# Analysis of Protein Pathway Networks Using Hybrid Properties

**DOI:** 10.3390/molecules15118177

**Published:** 2010-11-12

**Authors:** Lei Chen, Tao Huang, Xiao-He Shi, Yu-Dong Cai, Kuo-Chen Chou

**Affiliations:** 1 College of Information Engineering, Shanghai Maritime University, Shanghai 201306, China; E-Mail: chen_lei1@163.com (L.C.); 2 Centre for Computational Systems Biology, Fudan University, Shanghai 200433, China; 3 Key Laboratory of Systems Biology, Shanghai Institutes for Biological Sciences, Chinese Academy of Sciences, Shanghai 200031, China; E-Mail: tohuangtao@126.com (T.H.); 4 Shanghai Center for Bioinformation Technology, Shanghai 200235, China; 5 Institute of Health Sciences, Shanghai Institutes for Biological Sciences, Chinese Academy of Sciences and Shanghai Jiao Tong University School of Medicine, Shanghai 200025, China; E-Mail: xiaoheshi@163.com (X-H.S.); 6 Institute of Systems Biology, Shanghai University, Shanghai 200444, China; 7 Gordon Life Science Institute, San Diego, California 92130, USA; E-Mail: kcchou@gordonlifescience.org (K-C.C.)

**Keywords:** protein-forming system, regulatory pathway, minimum redundancy maximum relevance, gene ontology, biological graphic feature

## Abstract

Given a protein-forming system, *i.e.*, a system consisting of certain number of different proteins, can it form a biologically meaningful pathway? This is a fundamental problem in systems biology and proteomics. During the past decade, a vast amount of information on different organisms, at both the genetic and metabolic levels, has been accumulated and systematically stored in various specific databases, such as KEGG, ENZYME, BRENDA, EcoCyc and MetaCyc. These data have made it feasible to address such an essential problem. In this paper, we have analyzed known regulatory pathways in humans by extracting different (biological and graphic) features from each of the 17,069 protein-formed systems, of which 169 are positive pathways, *i.e.*, known regulatory pathways taken from KEGG; while 16,900 were negative, *i.e.*, not formed as a biologically meaningful pathway. Each of these protein-forming systems was represented by 352 features, of which 88 are graph features and 264 biological features. To analyze these features, the “Minimum Redundancy Maximum Relevance” and the “Incremental Feature Selection” techniques were utilized to select a set of 22 optimal features to query whether a protein-forming system is able to form a biologically meaningful pathway or not. It was found through cross-validation that the overall success rate thus obtained in identifying the positive pathways was 79.88%. It is anticipated that, this novel approach and encouraging result, although preliminary yet, may stimulate extensive investigations into this important topic.

## 1. Introduction

During the past decade, the continuous development of high-throughput experimental technologies has increased the sizes of large-scale datasets, including both metagenomes and personal genomes, which necessitate renewed efforts to develop computational technologies for better biological interpretation of all this data. A vast amount of information about different organisms, both on the genetic and metabolic levels, has been accumulated and systematically stored in specific databases that are available on various websites including KEGG [1,2], ENZYME [3], BRENDA [4,5], and EcoCyc and MetaCyc [6]. 

KEGG (Kyoto Encyclopedia of Genes and Genomes) [1,2,7] is a widely used knowledge database for the systematic analysis of gene functions in terms of the interactions between genes and molecules; it consists of graphical diagrams of biochemical pathways, including most of the known metabolic pathways and some of the known regulatory pathways. Nowadays, KEGG PATHWAY is supplemented with a new global map of metabolic pathways, which is essentially a combined map of about 120 existing pathway maps. KEGG BRITE is an ontology database, which represents functional hierarchies of various biological objects, including molecules, cells, organisms, diseases and drugs, as well as relationships among them [8,9]. In these databases, experimental knowledge is organized and diagramed as smaller networks, and web interfaces and visualization tools have been developed to overview and analyze computationally generated global networks [10,11,12].

Many studies from various research laboratories around the world have indicated that mathematical analysis, computational modeling, and the introduction of novel physical concepts to solve important problems in biology and medicine, such as protein structural class prediction [13,14], modeling of 3D structures of targeted proteins for drug design [15,16,17,18], diffusion-controlled reaction simulation [19,20,21,22], cellular responding kinetics [23,24], bio-macromolecular internal collective motion simulation [25,26,27], identification of proteases and their types [28,29], membrane protein type prediction [30,31], protein cleavage site prediction [32,33], and signal peptide prediction [34,35], can provide very useful and timely information and insights for both basic research and drug development. Encouraged by these promising outcomes, the present study was initiated to address a fundamental problem in system biology and proteomics.

For most pathways stored in the KEGG server, it is barely possible to acquire their graph characteristics by manual query execution. The present study was devoted to the development of a new approach to address this problem that maybe of use for in-depth study of the various pathway network systems.

## 2. Materials and Methods

### 2.1. Materials

The data of regulatory pathways was collected from the public available database KEGG (ftp://ftp.genome.jp/pub/kegg/xml). Those pathways without GO information or biological properties were removed. Pathways involving less than three proteins were also excluded. As a result, 169 regulatory pathways, or protein-forming systems, were obtained and they are termed as “positive pathways”. The 169 positive pathways as well as the protein codes contained in each of such pathways are given in Online Supporting Information S1.

The negative pathways data was generated by the following two routes: first, proteins were randomly picked as the nodes of a graph, followed by the creation of some arcs between these proteins in a random manner. The number of arcs in each pathway was assigned according to the size distribution of the arcs in the positive pathways. Second, about half of proteins were replaced by other proteins in each positive pathway, and the arcs between the proteins, including both the original and the replaced ones, left unchanged. Since positive pathways are very rare in comparison with the vast majority of negative pathways, in this study the number of negative pathways thus generated was 100 times as big as that of the positive ones. The 16,900 negative pathways thus obtained are given in Online Supporting Information S2.

### 2.2. Features

The use of graphic approaches to study biological systems can provide useful intuitive insights, as indicated by many previous studies on a variety of important biological topics, such as enzyme-catalyzed reactions [36,37,38,39,40], protein folding kinetics [41], inhibition of HIV-1 reverse transcriptase [42,43,44], inhibition kinetics of processive nucleic acid polymerases and nucleases [45], and drug metabolism systems [46]. Recently, graphical methods have also been utilized to deal with various biological and medical related problems [47,48,49,50]. 

In this study, both graphic features and biological properties were used to code each pathway. We downloaded the human KGML (KEGG XML) files from KEGG FTP site (ftp://ftp.genome.jp/pub/ kegg/xml) and parsed them into graphs using KEGGgraph [51], an interface between KEGG pathway and graph objects in R. The vertices in graphs parsed from KGML files are proteins and the arcs indicate the relations between the protein vertices. Each graph is a directed graph or digraph [39,41], since the relation between two proteins is directional, *i.e.* one protein **P**_1_ can regulate another protein **P**_2_ while **P**_2_ cannot always regulate **P**_1_. In this study, 88 graph features were extracted from each directed graph that represents a pathway, and 264 features of biological properties were derived from biochemical properties and physicochemical properties, including amino acid compositions, hydrophobicity, normalized van der Waals volume, polarity, polarizability, solvent accessibility and secondary structure. Thus, we have a total of (88 + 264) = 352 features altogether. For the codes of the 352 features and how they were used to quantitatively define each of the 169 positive pathways, see Online Supporting Information S3. Similarly, we can also uniquely define each of the 16,900 negative pathways in a 352-D (dimensional) space as done for the 169 positive pathways. Here, the detailed results for the 16,900 negative pathways are not shown because the corresponding file is too large to be submitted. However, it is available upon request. 

Actually, many graph features were derived in [52,53,54], where the features were extracted from an undirected graph. In this study, every pathway can be deemed as a directed graph, where vertices denote proteins and arcs denote relations. The arcs are weighted by the likelihood that they may interact with each other, as will be further explained in Section 2.3. The 352 features were divided into the following groups.

(1) Graph size and graph density. Suppose the graph of a pathway is formulated by *G* = (*V*, *E*) where *V* represent the vertices and *E* the arcs. The size of the graph is the number of proteins in the pathway. Suppose |*E*|_max_ = |*V*|^2^ is the theoretical maximum number of possible arcs in *G*. The graph density is defined as |*E*| divided by |*E*|_max_ [52]. 

(2) Degree statistics. The in-degree (out-degree) of a vertex is defined as the number of in-neighbors (out-neighbors) of the vertex. Considered in this study were the mean in-degree, variance of in-degree, median in-degree, maximum in-degree, mean out-degree, variance of out-degree, median out-degree and maximum out-degree as features [53].

(3) Edge weight statistics. Let *G* = (*V*, *w*(*E*)) be a weighted pathway graph where each arc is weighted by a weight *w* in the range of [0,1]. It is possible when *w*(*e*) = 0 for some arc *e*∈*E*; we extracted features in two cases: (a) all arcs in graph were considered including those with zero weights, and that mean and variance of those weights being taken as the features; (b) arcs with non-zero weights were considered so as to take mean and variance of the non-zero weights as features [52].

(4) Topological change. Let *G* = (*V*, *w*(*E*)) be a weighted pathway graph. This group of features was to measure the topological changes when different cutoffs of the weights were applied to the graph. The weight cutoffs included 0.1, 0.2, 0.3, 0.4, 0.5, 0.6, 0.7 and 0.8. Let *G_i_* = (*V*, *E_i_*) (*i* = 1,2,3,4,5,6,7,8) be the graph that only includes arcs with weights higher than *i*/10 remained; *i.e. E_i_* ={*e* | *w*(*e* ) > *i*/10}. Topology changes are measured as *T_i _* = (|*E_i_*|-|*E_i+1_*|)/|*E_i_*| for *i* = 1,2,3,4,5,6,7 (*T_i_* = 0 if |*E_i_*| = 0). 

(5) Degree correlation. Let *G* = (*V*, *E*) be a pathway graph with *V* = {*v*_1_,*v*_2_,…,*v_n_*}. For each vertex *v_i_*, denote its in-neighbors as *V ^′^_i_* = {*v_i_*_1 _,*v_i_*_2 _,…,*v_ik _*} and out-neighbors as *V ^″^_i_* = {*v_j_*_1_,*v_j_*_2 _,…,*v_jl _*}. Let *H ^′^_i_ = * (*V ^′^_i_ , E^′^_i_*) and *H ^″^_i_ = * (*V ^″^_i_, E ^″^_i_*) be two subgraphs of *G* induced by *V ^′^_i_* and *V ^″^_i_*, respectively. Define *D ^′^_i_* = |*E ^′^_i_*| / *k* (*D ^′^_i_* = 0 if *k* = 0) and *D ^″^_i_* = |*E ^″^_i_*| / *l* (*D ^″^_i_* = 0 if *l* = 0). Take the mean, variance and maximum of *D*^′^_1_,…,*D^′^_n_* and *D*^〞^_1_,…,*D^〞^_n_*, respectively, as features in this group [54].

(6) Clustering. Let *G* = (*V*, *E*) be a pathway graph with *V* = {*v*_1_,*v*_2_,…,*v_n_*}. For each vertex *v_i_*, let its in-neighbors be *V ^′^_i_* = {*v_i_*_1_,*v_i_*_2_,…,*v_ik _*} and out-neighbors be *V ^″^_i_* = {*v_j_*_1_,*v_j_*_2_,…,*v_jl _*}. Let *H ^′^_i_ = * (*V ^′^_i_ , E ^′^_i_*) and *H ^″^_i_ = * (*V ^″^_i_, E ^″^_i_*) be two subgraphs of *G* induced by *V ^′^_i_* and *V ^″^_i_*, respectively. Define *C ^′^_i_* = |*E ^′^_i_*| / *k* ^2^ (*C ^′^_i_* = 0 if *k* = 0) and *C ^″^_i_* = |*E ^″^_i_*| / *l* ^2^ (*C ^″^_i_* = 0 if *l* = 0). Take the mean, variance and maximum of *C*^′^_1_,…,*C^′^_n_* and *C*^〞^_1_,…,*C^〞^_n_*, respectively, as features in this group [53].

(7) Topological. Let *G* = (*V*, *E*) be a pathway graph with *V* = {*v*_1_,*v*_2_,…,*v_n_*}. For each pair of vertices *v_i_*, *v_j_*(*i*≠*j*), denote *n^1^_ij_* as the number of both in-neighbor of *v_i_* and in-neighbor of *v_j_*, *n^2^_ij_* as the number of both in-neighbor of *v_i_* and out-neighbor of *v_j_*, *n^3^_ij_* as the number of both out-neighbor of *v_i_* and in-neighbor of *v_j_* and *n^4^_ij_* as the number of both out-neighbor of *v_i_* and out-neighbor of *v_j_*. For each vertex *v*_i_, denote *n^1^_i_* and *n^2^_i_* as the number of in-neighbors and out-neighbors of *v_i_*. Let *T ^1^_ij_* = *n^1^_ij_*/*n^1^_i_* (*T ^1^_ij_* = 0 if *n^1^_i_* = 0), *T ^2^_ij_* = *n^2^_ij_*/*n^1^_i_* (*T ^2^_ij_* = 0 if *n^1^_i_* = 0), *T ^3^_ij_* = *n^3^_ij_*/*n^2^_i_* (*T ^3^_ij_* = 0 if *n^2^_i_* = 0), and *T ^4^_ij_* = *n^4^_ij_* /*n^2^_i_* (*T ^4^_ij_* = 0 if *n^2^_i_* = 0). For each vertex *v_i_*, let *T ^i^_i_* be the mean of *T ^k^_il_*,…,*T ^k^_in_* for *k* = 1,2,3,4. Features in this group are defined as the mean, variance and maximum of *T ^k^_l_*,…,*T ^k^_n_* for *k* = 1,2,3,4 [54].

(8) Singular values. Let *G* = (*V*, *E*) be a pathway graph and *A* be its adjacent matrix. Take the first three largest singular values as the features [52].

(9) Local density change. Let *G* = (*V*, *E*) be a pathway graph with *V* = {*v*_1_,*v*_2_,…,*v_n_*}. This group of features was to measure the similarity of the in-neighbors and out-neighbors of a protein in the pathway. For each vertex *v_i_*, suppose *V ^′^_i_* = {*v_i_*_1 _,*v_i_*_2_,…,*v_ik _*} and *V ^″^_i_* = {*v_j_*_1_,*v_j_*_2_,…,*v_jl _*} be the in-neighbors and out-neighbors of *v_i_*, respectively. We only show how to gain features from the in-neighbors of each vertex under different cutoffs, which included 0, 0.1, 0.2, 0.3, 0.4, 0.5, 0.6, 0.7, 0.8 and 0.9. Construct a weighted undirected complete graph *K ^′^_i_* with vertex *v_i_*_1 _,*v_i_*_2_,…,*v_ik _* and the weight of each pair of vertices is the likelihood of the corresponding proteins (see Section 2.3). Suppose the cutoff is *w*, which may be 0, 0.1, 0.2, 0.3, 0.4, 0.5, 0.6, 0.7, 0.8 or 0.9. Extract a spanning subgraph *G^′^_i_*(*w*)of *K ^′^_i_* with edges whose weights are greater than *w*. Compute *L^′^_i_*(*w*) = 2|*E*(*G^′^_i_*(*w*))|/(*k*(*k*-1)) (*L^′^_i_*(*w*) = 0 if *k* ≤ 1). Take the mean and maximum of *L^′^_1_*(*w*),*L^′^_2j_*(*w*),…,*L^′^_n_*(*w*) as features under cutoff *w*. 

The above features are for the pathway graph representation. The following are for the biochemical properties and physicochemical properties, where biochemical properties include amino acid compositions and secondary structure, while physicochemical properties include hydrophobicity, normalized van der Waals volume, polarity, polarizability and solvent accessibility. These properties have been widely applied in the field of computational biology [55,56,57,58,59,60,61,62,63]. Suppose a pathway consists of *n* proteins, the mean and maximum values of biological properties of the *n* proteins are taken as the features.

(10) Hydrophobicity, normalized van der Waals volume, polarity and polarizability: 42 features can be extracted from each of these physicochemical properties [64,65]. Here we will only describe how to obtain features from the hydrophobicity property, as features from other properties can be obtained in a similar way. Each amino acid is assigned into one of the three categories, polar (P), neutral (N) and hydrophobic (H). For a given protein sequence, we use P, N or H to substitute each amino acid in the sequence, and the resulting sequence is called a protein pseudo-sequence. Composition (C) is defined as the percentage of P, N and H in the whole pseudo-sequence. Transition (T) is defined as the changing frequency between any two characters (such as P and N, P and H, N and H). Distribution (D) is defined as the sequence segment (in percentage) of the pseudo-sequence that is needed to contain the first, 25%, 50%, 75% and the last of the Ps, Ns and Hs, respectively. In conclusion, there are three, three, and 15 properties for (C), (T) and (D), respectively. Totally 21 × 2 = 42 features are obtained.

(11) Solvent accessibility: each amino acid can be predicted by ACCpro [66] as hidden (H) or exposed (E) to solvent. Then the protein sequence is coded with letters H and E. Use composition (C) for H, transition (T) between H and E, and five distributions (D) for H in this property, resulting in totally 7 × 2 = 14 features. 

(12) Secondary structure: each amino acid in the protein sequence is substituted by one of three letters like hydrophobicity property. For details, please see [67,68]. 21 × 2 = 42 features can be derived from this property.

(13) Amino acid compositions: the percentage of each amino acid in the whole sequence. Totally, 20 × 2 = 40 features about amino acid composition are extracted. 

**Table 1:** Amount of properties in feature group 10–13.

Properties	C	T	D	Total
**Hydrophobicity**	3	3	15	21
**Normalized van der Waals volume**	3	3	15	21
**Polarity**	3	3	15	21
**Polarizability**	3	3	15	21
**Secondary structure**	3	3	15	21
**Solvent accessibility**	1	1	5	7
**Amino acid composition**	20	---	---	20
**Total**	---	---	---	132

**Table 2:** The distribution of 352 features.

Group ID	Group Name	Number of features
1	Graph size and graph density	2
2	Degree statistic	8
3	Edge weight statistics	4
4	Topological change	7
5	Degree correlation	6
6	Clustering	6
7	Topological	12
8	Singular values	3
9	Local density change	40
10	Hydrophobicity, normalized van der Waals volume, polarity and polarizability	4 × 2 × 21 = 168
11	Solvent accessibility	7 × 2 = 14
12	Secondary structure	2 × 21 = 42
13	Amino acid compositions	2 × 20 = 40

Shown in Table 1 are the numbers of the properties in the above feature group 10–13. Before taking the mean and maximum values of properties in these groups, the following conversion was taken to adjust their values according to a standard scale:

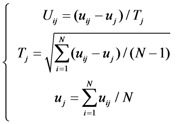
(1)
where *T_j_* is the standard deviation of the *j*-th feature and *u_j_* the mean value of the *j*-th feature. The total number of features is

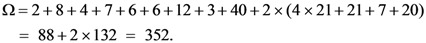
(2)


As for the detailed distribution of the 352 features, see Table 2.

### 2.3. Gene ontology

As mentioned above, some features need the arc weight to indicate how likely it is that an interaction may happen between two proteins. In order to generate the edge weight of two interacting proteins, we used gene ontology consortium (GO) [69] to represent each protein. “Ontology” is a specification of a conceptualization and refers to the subject of existence. GO is established by the following three criteria: molecular function, biological process, and cellular component. GO consortium is considered to be a very powerful and helpful vehicle for investigating protein-protein interactions [70], because these three criteria reflect the attribute of gene, gene product, gene-product groups and core features reflecting the subcellular localization [71,72]. The steps of using GO (gene ontology) encoding are described as following:

(1) By using Uniprot2GO mapping provided by GOA Uniprot 34.0 on November 21st 2005 (http://www.ebi.ac.uk/GOA/) [69] which contains 9525 GO items, the functional annotations of proteins provided by GO were obtained.

(2) Each protein can be represented in a 9,525-dimensional vector using each of the 9525 GO items as the vector base, e.g., if a given protein hits a GO item which is the *i*-th entry of the 9525 GO items, then the *i*-th component of the 9,525-dimensional vector is set to be 1, otherwise 0.

(3) Thus, each protein sample can be formulated as a 9,525-D vector:

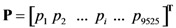
(3)
where *p_i_* = 1 if the sample hit the *i*-th GO item; otherwise, *p_i_* = 0. The interaction between **P***_i_* and **P***_j_*, *i.e.*, the weight of arc between the two proteins, is computed by the following formula:

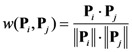
(4)
where **P***_i_·***P***_j_* is dot product of **P***_i_* and **P***_j_*, || **P***_i_* || and || **P***_j_* || are their modulus. 

### 2.4. Minimum redundancy maximum relevance (mRMR)

Feature selection can reduce the feature dimensions so as to improve the efficiency of a learning machine. The concrete procedure can be realized by utilizing the mRMR approach, which was first proposed by Peng [73]. This is because it can balance the minimum redundancy and the maximum relevance. The maximum relevance would guarantee selection of those features contributing most to the classification, while the minimum redundancy would guarantee exclusion of those already been covered by the selected features. During the selecting process, one feature at a time was selected by mRMR into the selected list. In each round, a feature with maximum relevance and minimum redundancy was selected. As a result, we obtained a complete list of the selected features with some order. When computing the redundancy and relevance, the mutual information (MI) was adopted, as defined below:

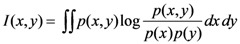
(5)
where *x* and *y* are two random variables; *p*(*x*,*y*) is the joint probabilistic distribution of *x* and *y*; while *p*(*x*) and *p*(*y*) the marginal probabilities of *x* and *y*, respectively. 

Let Ω denote the whole feature set. The selected feature set with *m* features is denoted by Ω*_s_*, and the rest of *n* features is denoted by Ω*_r_*. The relevance of a feature *f* and the target variable *h* can be computed as *I*(*f*, *h*), the redundancy between a feature *f* and the selected Ω*_s_* is computed as:

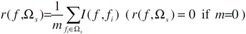
(6)


For each feature *f* in Ω*_r_*, compute the following equation:

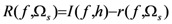
(7)


To maximize the relevance and minimize redundancy, select a feature *f* ^′^*∈*Ω*_r_* such that:

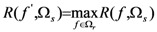
(8)


Then take *f* ^′ ^into Ω*_s_* and remove *f* ^′^from Ω*_r_*. For the rest features, in each round the most relevant and least redundant feature is removed from Ω*_r_* and put into Ω*_s_*, until all features are in Ω*_s_*. Thus, for a feature pool Ω with *N*(*N* = *n*+*m*) features, mRMR program will execute *N* rounds and provide an ordered feature list:

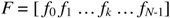
(9)
where *k* denotes the round at which the feature is selected.

### 2.5. Nearest neighbor algorithm

In this study, the NN (nearest neighbor) algorithm [74] was adopted to predict the class of pathway (positive or negative). The “nearness” is defined by the Euclidian distance:

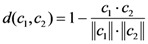
(10)
Where *c*_1_*c*_2_ is dot product of two vectors *c*_1_ and *c*_2_, || *c*_1_|| and || *c*_2_|| are the modulus of vector *c*_1_ and *c*_2_, respectively. The smaller the *d*(*c*_1_, *c*_2_), the nearer the two vectors are [75]. 

In the NN algorithm, suppose there are *m* training pathways, each of them is either positive or negative, and a query protein system needs to be determined as forming either a positive or negative pathway. The distances between each of the *m* pathways and the new pathway are computed, and the nearest neighbor of the new pathway is found. If the nearest neighbor is positive or negative, then the query protein system is assigned to be with positive or negative pathway, respectively. 

### 2.6. Jackknife cross-validation

The prediction model was examined by the jackknife test. In statistical prediction, the following three cross-validation methods are often used to examine a predictor for its accuracy: independent dataset test, subsampling (K-fold cross-validation) test, and the jackknife test [14]. However, as elucidated by [76] and demonstrated by Eq. (50) in [75], among the three cross-validation methods, the jackknife test is deemed the most objective that can always yield a unique result for a given benchmark dataset, and hence has been increasingly used and widely recognized by investigators to examine the accuracy of various predictors (see, e.g., [77,88]). Accordingly, in this study the jackknife test was adopted to examine the quality of our prediction method as well. During the jackknifing process, each of the statistical samples in the benchmark dataset was in turn singled out as the prediction target and the rest of the samples were used to train the prediction model. 

### 2.7. Incremental feature selection (IFS)

From mRMR, we obtained an ordered feature list *F* = [ *f*_0_*f*_1_ … *f_k_* … *f_N_*_-1_]. Let *F_i_* = {*f*_0_, *f*_1_ … *f_i_*} (0 ≤ *I* ≤ *N*-1) be the *i*-th feature set taken from *F*. For every *i* (0 ≤ *i* ≤ *N*-1), we executed NN algorithm with the features in *F_i_* and obtained an accuracy of correctly predicting the positive pathways, evaluated by jackknife cross-validation. As a result, a curve named IFS curve, with identification accuracy as its y-axis and the index *i* of *F_i_* as its x-axis, was obtained.

## 3. Results and Discussion

### 3.1. Results of mRMR

The mRMR program was downloaded from http://research.janelia.org/peng/proj/mRMR/. It was run with default parameters. The following two feature lists were obtained through the mRMR program: (1) MaxRel features list; (2) mRMR features list (see Online Supporting Information S4).

**Figure 1:**
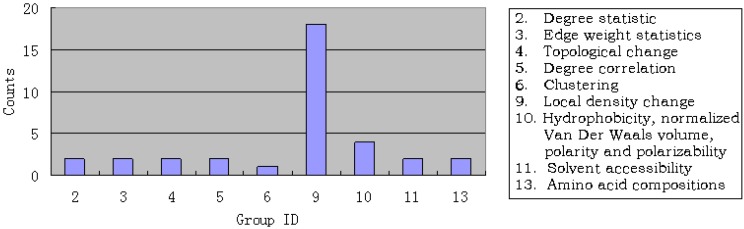
Illustration to show the distribution of features. See the text in Section 3.1 for further explanation.

For the MaxRel feature list, we investigated the most relevant 10% of the features (35 in total). Shown in Figure 1 is the distribution of these features. It is straightforward to see that 27 (77.1%) features come from pathway graph, indicating that among the adopted features, graph features contribute most to the forming of regulatory pathways. Of the 27 features, 18 (51.43%) were from the 9-th feature group, which reflects the essence of the similarity concerned, implying that similar proteins can be regulated by the same protein. 

### 3.2. Results of IFS

**Figure 2:**
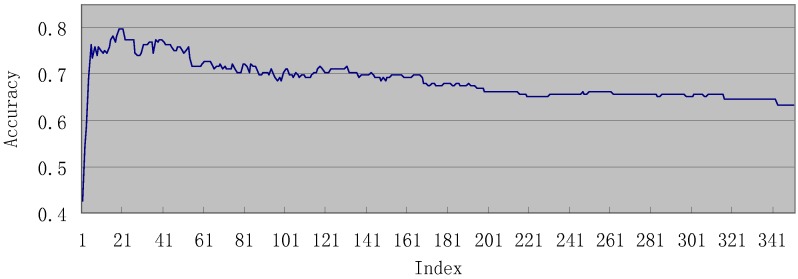
The IFS (incremental feature selection) curve. See the text in Section 3.2 for further explanation.

Shown in Figure 2 is the IFS (incremental feature selection) curve. The highest accuracy of IFS for the positives is 79.88% using 22 features (see Online Supporting Information S4). When using these optimized 22 features, the accuracy of negative pathways and total accuracy were 99.69% and 99.49%, respectively. The detailed IFS data can be found in Online Supporting Information S5.

**Figure 3:**
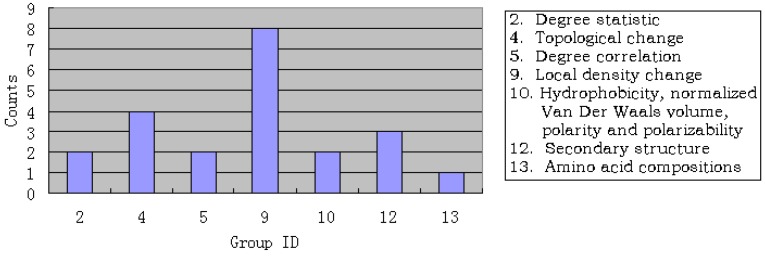
Distribution of the optimized 22 features. See the text in Section 3.2 for further explanation.

Shown in Figure 3 is the distribution of the optimized 22 features. It is again straightforward to see that 16 (72.72%) features were from the pathway graph, among which 8 (36.36%) features were from the 9-th feature group, reaching the same conclusion as that in Section 3.1. 

### 3.3. Analysis of the important features

In this work, we present a novel KEGG pathway network analysis method based on hybrid properties, the graph properties and biochemical and physicochemical properties. It was found that the features contributing most in forming pathways were the “out_local_density” and “in_local_density”, both of which were involved with the change of the number of the edges when different weight cutoffs were applied to the graph. Therefore, more edges might remain in the positive graph when higher weight cutoffs were applied. The other graph feature with more contribution to the pathway is the “topological mean”, reflecting various proteins topologies in the regulatory pathway. For a non-broken graph, linear graph (proteins in the graph form a linear path) has a minimum topological mean, while a complete graph has a maximum topological mean. A densely-connected graph always has higher topological mean, indicating a higher likelihood to form a regulatory pathway. The “in_degree_variance”, “out_degree_variance”, and “out_degree_correlation_max” represent the difference of similarity between each of the protein pairs. Most of the forefront features with the dominant contribution are graph features, indicating that graph features are the most important ones. The biochemical and physicochemical properties, including “polarity_composition_P_max”, “secondary_structure_distribution_P-1.0_mean”, “secondary_structure_distribution_P-1.0_max”, “secondary_structure_distribution_P-0.0_max”, “polarizability_distribution_N-1.0_max”, and “AA_composition_ C_mean” also had considerable contributions in determining the regulatory networks. The distribution of the polarity of proteins structures had strong impact on the conformation of proteins, and hence their interactions as well as their binding sites. 

## 4. Conclusions

We analyzed 352 features extracted from each of the generated positive pathways and negative pathways. Of the 352 features, 88 were graph ones, meaning that each pathway was treated as a graph; and 264 were derived from protein biological properties. The mRMR (minimum redundancy maximum relevance) and IFS (incremental feature selection) techniques were employed to analyze these features. Nearest neighbor algorithm and jackknife test were used to evaluate the accuracy of our model in searching for the positive pathways. As a result, 22 features were found to be the important features for the classification. These findings might be of use for stimulating further studies on such an important and challenging topic.

## Supplementary Materials

Supplementary File 1

Supplementary File 2

Supplementary File 3

Supplementary File 4

Supplementary File 5
