# Supplementary material for: Analysis of Protein Pathway Networks Using Hybrid Properties
Source: Molecules. 2010 Nov 12;15(11):8177–92. doi: 10.3390/molecules15118177 (PMC6259184; doi:10.3390/molecules15118177)
Supplement: Supplementary File 1 [file molecules-15-08177-s001.pdf]

Online Supporting Information S1: Proteins in each positive regulatory pathway

Index	Proteins
Positive_1	DLD, GCK, GPI, HK1, HK2, HK3, ACSS1, ACSS2, ADH1A, ADH1B, ADH4, ADH5, ADH6, ADH7, AKR1A1, ALDH1A3, ALDH1B1, ALDH2, ALDH3A1, ALDH3A2, ALDH3B1, ALDH3B2, ALDH7A1, ALDH9A1, ALDOA, ALDOB, ALDOC, BPGM, DLAT, ENO1, ENO2, ENO3, FBP1, FBP2, G6PC, G6PC2, GALM, GAPDH, LDHA, LDHAL6A, LDHAL6B, LDHB, LDHC, PCK1, PCK2, PDHA1, PDHA2, PDHB, PFKL, PFKM, PFKP, PGAM1, PGAM2, PGAM4, PGK1, PGK2, PGM1, PGM3, PKLR, PKM2, TPI1
Positive_2	CS, DLD, FH, PC, ACLY, ACO1, ACO2, DLAT, DLST, IDH1, IDH2, IDH3A, IDH3B, IDH3G, MDH1, MDH2, OGDH, OGDHL, PCK1, PCK2, PDHA1, PDHA2, PDHB, SDHA, SDHB, SDHC, SDHD, SUCLA2, SUCLG1, SUCLG2
Positive_3	GPI, PGD, RPE, TKT, ALDOA, ALDOB, ALDOC, DERA, FBP1, FBP2, G6PD, H6PD, PFKL, PFKM, PFKP, PGLS, PGM1, PGM3, PRPS1, PRPS2, RBKS, RPIA, TALDO1, TKTL1, TKTL2
Positive_4	RPE, AKR1B1, DCXR, GUSB, UGDH, UGP2, UGT1A10, UGT2A1, UGT2A3, UGT2B10, UGT2B11, UGT2B17, UGT2B28, UGT2B4, UGT2B7, XYLB
Positive_5	FUK, GCK, HK1, HK2, HK3, KHK, MPI, AKR1B1, ALDOA, ALDOB, ALDOC, FBP1, FBP2, FPGT, GMDS, GMPPA, GMPPB, MTMR1, MTMR2, MTMR6, PFKFB1, PFKFB2, PFKFB3, PFKFB4, PFKL, PFKM, PFKP, PGM2, PHPT1, PMM1, PMM2, SORD, TPI1, TSTA3, UGCGL1, UGCGL2
Positive_6	GAA, GCK, GLA, HK1, HK2, HK3, LCT, AKR1B1, B4GALT1, B4GALT2, G6PC, G6PC2, GALE, GALK1, GALK2, GALT, GANC, GLB1, LALBA, MGAM, PGM1, PGM3, UGP2
Positive_7	ACACA, ACACB, FASN, MCAT, OLAH, OXSM
Positive_8	ACAA2, ECHS1, HADH, HADHA, HADHB, HSD17B4, MECR, PPT1, HSD17B10
Positive_9	ACAA1, ACAA2, ACADL, ACADM, ACADS, ACADSB, ACADVL, ACAT1, ACAT2, ACOX1, ACOX3, ACSL1, ACSL3, ACSL4, ACSL5, ACSL6, ADH1A, ADH1B, ADH4, ADH5, ADH6, ADH7, ALDH1A3, ALDH1B1, ALDH2, ALDH3A1, ALDH3A2, ALDH7A1, ALDH9A1, CPT1A, CPT1B, CPT1C, CPT2, CYP4A11, CYP4A22, ECHS1, EHHADH, GCDH, HADH, HADHA, HADHB, HSD17B4, HSD17B10
Positive_10	ACAT1, ACAT2, BDH1, BDH2, HMGCL, HMGCS1, HMGCS2, OXCT1, OXCT2
Positive_11	EBP, LSS, MVD, MVK, DHCR24, DHCR7, FDFT1, FDPS, GGCX, GGPS1, HMGCR, HSD17B7, IDI1, IDI2, NQO1, NSDHL, PMVK, SC4MOL, SC5DL, SQLE, TM7SF2, VKORC1
Positive_12	CEL, ACAA1, ACAA2, ACAD9, ADH1A, ADH1B, ADH4, ADH5, ADH6, ADH7, AKR1B10, AKR1C4, AKR1D1, ALDH1A3, ALDH1B1, ALDH2, ALDH3A1, ALDH3A2, ALDH7A1, ALDH9A1, BAAT, CYP27A1, CYP7A1, HADHB, HSD3B7, LIPA, SLC27A5, SOAT1, SOAT2, SRD5A1, SRD5A2
Positive_13	COQ3, COQ5, COQ6, COQ7
Positive_14	AKR1C4, AKR1D1, CYP11A1, CYP11B1, CYP11B2, CYP17A1, CYP21A2, HSD11B1, HSD11B2, HSD3B1, HSD3B2
Positive_15	STS, AKR1C4, AKR1D1, ARSD, ARSE, CYP11B1, CYP11B2, CYP19A1, HEMK1, HSD11B1, HSD11B2, HSD17B1, HSD17B2, HSD17B3, HSD17B7, HSD17B8, HSD3B1, HSD3B2, LCMT1, LCMT2, METTL2B, METTL6, SRD5A1, SRD5A2, SULT1E1, SULT2A1, SULT2B1, TRMT11, UGT1A10, UGT2A1, UGT2A3, UGT2B10, UGT2B11, UGT2B17, UGT2B28, UGT2B4, UGT2B7, WBSCR22, HSD17B12
Positive_16	ATP12A, ATP4A, ATP4B, ATP5A1, ATP5B, ATP5C1, ATP5D, ATP5E, ATP5F1, ATP5G1, ATP5G2, ATP5G3, ATP5H, ATP5I, ATP5J, ATP5J2, ATP5L, ATP5O, ATP6AP1, ATP6V0B, ATP6V0C, ATP6V1A, ATP6V1D, ATP6V1F, ATP6V1H, PPA1, PPA2, TCIRG1, ATP6V0A1, ATP6V0A2, ATP6V0A4, ATP6V0D1, ATP6V0D2, ATP6V0E1, ATP6V1B1, ATP6V1B2, ATP6V1C1, ATP6V1C2, ATP6V1E1, ATP6V1E2, ATP6V1G1, ATP6V1G2, ATP6V1G3
Positive_17	ADC, ASL, OTC, SMS, SRM, ABP1, ACY1, AGMAT, ALDH1A3, ALDH1B1, ALDH2, ALDH3A1, ALDH3A2, ALDH7A1, ALDH9A1, AMD1, ARG1, ARG2, ASS1, CPS1, GATM, MAOA, MAOB, NAGS, ODC1, SAT1, SAT2, ALDH18A1

Positive_18	ADA, ADK, AK1, AK2, AK5, AK7, DCK, GDA, XDH, ADCY1, ADCY10, ADCY2, ADCY3, ADCY4, ADCY5, ADCY6, ADCY7, ADCY8, ADCY9, ADSL, ADSS, ADSSL1, AK3L1, AMPD1, AMPD2, AMPD3, APRT, ATIC, CANT1, DGUOK, ENPP1, ENPP3, ENTPD1, ENTPD2, ENTPD3, ENTPD4, ENTPD5, ENTPD6, ENTPD8, GART, GMPR, GMPR2, GMPS, GUCY1A2, GUCY1A3, GUCY1B3, GUCY2C, GUCY2D, GUCY2F, GUK1, HPRT1, IMPDH1, IMPDH2, ITPA, NME2, NME4, NME6, NME7, NPR1, NPR2, NT5C, NT5C1A, NT5C1B, NT5C2, NT5C3, NT5E, NT5M, NUDT2, NUDT5, NUDT9, PAICS, PAPSS1, PAPSS2, PDE10A, PDE11A, PDE1A, PDE1B, PDE1C, PDE2A, PDE3A, PDE3B, PDE4A, PDE4B, PDE4C, PDE4D, PDE5A, PDE6D, PDE6G, PDE6H, PDE7A, PDE7B, PDE8A, PDE8B, PDE9A, PFAS, PKLR, PKM2, PNPT1, POLA1, POLA2, POLD1, POLD2, POLD3, POLD4, POLE, POLE2, POLE3, POLE4, POLR1A, POLR1B, POLR1C, POLR1D, POLR1E, POLR2A, POLR2B, POLR2C, POLR2D, POLR2E, POLR2F, POLR2G, POLR2H, POLR2I, POLR2J, POLR2K, POLR2L, POLR3A, POLR3B, POLR3D, POLR3G, POLR3H, POLR3K, PPAT, PRIM1, PRIM2, PRPS1, PRPS2, RRM1, RRM2, RRM2B, TYMP, ZNRD1
Positive_19	XDH, CYP1A2, CYP2A13, CYP2A6, CYP2A7, NAT1, NAT2
Positive_20	AK3, CAD, CDA, DCK, DUT, TK1, TK2, CANT1, CMPK1, CMPK2, CTPS, CTPS2, DCTD, DHODH, DPYD, DPYS, DTYMK, ENTPD1, ENTPD3, ENTPD4, ENTPD5, ENTPD6, ENTPD8, ITPA, NME2, NME4, NME6, NME7, NT5C, NT5C1A, NT5C1B, NT5C2, NT5C3, NT5E, NT5M, NUDT2, PNPT1, POLA1, POLA2, POLD1, POLD2, POLD3, POLD4, POLE, POLE2, POLE3, POLE4, POLR1A, POLR1B, POLR1C, POLR1D, POLR1E, POLR2A, POLR2B, POLR2C, POLR2D, POLR2E, POLR2F, POLR2G, POLR2H, POLR2I, POLR2J, POLR2K, POLR2L, POLR3A, POLR3B, POLR3D, POLR3G, POLR3H, POLR3K, PRIM1, PRIM2, RRM1, RRM2, RRM2B, TYMP, TYMS, UCK1, UCK2, UCKL1, UMPS, UPB1, UPP1, UPP2, UPRT, ZNRD1
Positive_21	CAD, GLS, GPT, ABAT, ALDH4A1, ALDH5A1, CPS1, EARS2, EPRS, GAD1, GAD2, GFPT1, GFPT2, GLS2, GLUD1, GLUD2, GLUL, GMPS, GOT1, GOT2, GPT2, NADSYN1, PPAT, QARS
Positive_22	ASL, CAD, DDO, GPT, PC, AARS, AARS2, ABAT, ACY3, ADSL, ADSS, ADSSL1, AGXT, AGXT2, ASNS, ASPA, ASRGL1, ASS1, DARS, DARS2, GAD1, GAD2, GOT1, GOT2, GPT2, NARS, NARS2
Positive_23	AMT, CBS, CTH, DAO, DLD, SDS, AGXT, AGXT2, AKR1B10, ALAS1, ALAS2, AOC2, AOC3, BHMT, CHDH, CHKA, CHKB, DMGDH, GAMT, GARS, GATM, GCAT, GLDC, GLYCTK, GNMT, HSD3B7, MAOA, MAOB, PEMT, PHGDH, PIPOX, PISD, PSAT1, PSPH, SARDH, SARS, SARS2, SHMT1, SHMT2
Positive_24	CBS, CTH, MTR, SRM, TAT, AHCY, AHCYL1, AHCYL2, AMD1, BHMT, DNMT1, DNMT3A, DNMT3B, DNMT3L, MARS, MARS2, MAT1A, MAT2B, MTAP, MTFMT, TRDMT1
Positive_25	CTH, CARS, CARS2, CDO1, GOT1, GOT2, LDHA, LDHAL6A, LDHAL6B, LDHB, LDHC, MPST
Positive_26	AUH, DBT, DLD, IVD, MUT, ABAT, ACAA1, ACAA2, ACAD8, ACADM, ACADS, ACADSB, ACAT1, ACAT2, ALDH1A3, ALDH1B1, ALDH2, ALDH3A1, ALDH3A2, ALDH6A1, ALDH7A1, ALDH9A1, AOX1, BCAT1, BCAT2, BCKDHA, BCKDHB, ECHS1, EHHADH, HADH, HADHA, HADHB, HIBADH, HIBCH, HMGCL, HMGCS1, HMGCS2, HSD17B4, MCCC1, MCCC2, MCEE, OXCT1, OXCT2, PCCA, PCCB, HSD17B10
Positive_27	BCAT1, BCAT2, IARS, IARS2, LARS, LARS2, VARS
Positive_28	AASDH, AASS, ACAT1, ACAT2, ALDH1A3, ALDH1B1, ALDH2, ALDH3A1, ALDH3A2, ALDH7A1, ALDH9A1, BBOX1, DLST, DOT1L, ECHS1, EHHADH, EHMT1, EHMT2, GCDH, HADH, HADHA, HSD17B4, NSD1, OGDH, OGDHL, PLOD1, PLOD2, PLOD3, SETD1A, SETD1B, SETD2, SETD7, SETD8, SETDB1, SETDB2, SETMAR, SPCS1, SPCS3, SUV39H1, SUV39H2, TMLHE, WHSC1, WHSC1L1, AASDHPPT, HSD17B10, SUV420H1, SUV420H2
Positive_29	ASL, CKB, CKM, OAT, OTC, ALDH4A1, ARG1, ARG2, ASS1, CKMT2, CPS1, EPRS, GAMT, GATM, GLUD1, GLUD2, GOT1, GOT2, LAP3, NOS1, NOS3, P4HA1, P4HA2, P4HA3, PARS2, PRODH, PRODH2, PYCR1, PYCR2, PYCRL, RARS, RARS2
Positive_30	DDC, HAL, HDC, ABP1, ALDH1A3, ALDH1B1, ALDH2, ALDH3A1, ALDH3A2, ALDH3B1, ALDH3B2, ALDH7A1, ALDH9A1, AMDHD1, CNDP1, FTCD, HARS, HARS2, HEMK1, HNMT, LCMT1, LCMT2, MAOA, MAOB, METTL2B, METTL6, TRMT11, UROC1, WBSCR22
Positive_31	DBH, DCT, DDC, FAH, HGD, HPD, MIF, TAT, TH, TPO, TYR, ADH1A, ADH1B, ADH4, ADH5, ADH6, ADH7, ALDH1A3, ALDH3A1, ALDH3B1, ALDH3B2, AOC2, AOC3, COMT, GOT1, GOT2, GSTZ1, MAOA, MAOB, PNMT, TYRP1
Positive_32	DDC, HPD, LPO, MIF, TAT, ALDH1A3, ALDH3A1, ALDH3B1, ALDH3B2, AOC2, AOC3, GOT1, GOT2, MAOA, MAOB, PRDX6

Positive_33	ACP1, ACP2, ACP5, ACP6, ACPP, ACPT, ALPI, ALPL, ALPP, ALPPL2, CYP3A4, CYP3A43, CYP3A5, CYP3A7, PON1, PON2, PON3
Positive_34	CAT, DDC, KMO, AADAT, AANAT, ABP1, ACAT1, ACAT2, ACMSD, AFMID, ALDH1A3, ALDH1B1, ALDH2, ALDH3A1, ALDH3A2, ALDH7A1, ALDH9A1, AOX1, ASMT, CYP1A1, CYP1A2, CYP1B1, ECHS1, EHHADH, GCDH, HAAO, HADH, HADHA, HEMK1, HSD17B4, INMT, KYNU, LCMT1, LCMT2, MAOA, MAOB, METTL2B, METTL6, NFX1, OGDH, OGDHL, TDO2, TPH1, TPH2, TRMT11, WARS, WARS2, WBSCR22, HSD17B10
Positive_35	PAH, TAT, FARS2, FARSA, FARSB, GOT1, GOT2, YARS, YARS2
Positive_36	ABAT, ACADM, ALDH1A3, ALDH1B1, ALDH2, ALDH3A1, ALDH3A2, ALDH7A1, ALDH9A1, AOC2, AOC3, CNDP1, DPYD, DPYS, ECHS1, EHHADH, GAD1, GAD2, HADHA, HIBCH, UPB1
Positive_37	ADO, BAAT, CDO1, CSAD, GAD1, GAD2, GGT1, GGT5, GGT6, GGT7
Positive_38	CHPT1, PCYT1A, PCYT1B
Positive_39	CBS, CTH, AHCY, AHCYL1, AHCYL2, HEMK1, LCMT1, LCMT2, MARS, MARS2, MAT1A, MAT2B, METTL2B, METTL6, PAPSS1, PAPSS2, SCLY, SEPHS1, TRMT11, WBSCR22
Positive_40	GLS, GLS2, GLUD1, GLUD2
Positive_41	GSR, GSS, SMS, SRM, ANPEP, GCLC, GCLM, GGT1, GGT5, GGT6, GGT7, GPX5, GPX7, GSTA1, GSTA2, GSTA3, GSTA4, GSTA5, GSTK1, GSTM1, GSTM2, GSTM3, GSTM4, GSTM5, GSTO1, GSTO2, GSTP1, GSTT1, GSTZ1, LAP3, MGST1, MGST2, MGST3, ODC1, OPLAH, TXNDC12
Positive_42	AGL, GAA, GBA, GCK, GPI, HK1, HK2, HK3, SI, AMY2A, AMY2B, ENPP1, ENPP3, G6PC, G6PC2, GANC, GBA3, GBE1, GUSB, GYS1, GYS2, LYZL1, MGAM, PGM1, PGM3, PYGB, PYGL, PYGM, UGDH, UGP2, UGT1A10, UGT2A1, UGT2A3, UGT2B10, UGT2B11, UGT2B17, UGT2B28, UGT2B4, UGT2B7, UXS1
Positive_43	ALG1, ALG10, ALG10B, ALG11, ALG12, ALG13, ALG2, ALG3, ALG5, ALG6, ALG8, ALG9, B4GALT1, B4GALT2, B4GALT3, DAD1, DDOST, DHDDS, DOLPP1, DPAGT1, DPM1, DPM2, DPM3, FUT8, GANAB, MAN1A1, MAN1A2, MAN1B1, MAN1C1, MAN2A1, MGAT1, MGAT2, MGAT3, MGAT4A, MGAT4B, MGAT5, MGAT5B, RPN1, RPN2, ST6GAL1, STT3B
Positive_44	OGT, B4GALT5, C1GALT1, GALNT1, GALNT10, GALNT11, GALNT12, GALNT13, GALNT14, GALNT2, GALNT3, GALNT5, GALNT6, GALNT7, GALNT8, GALNT9, GALNTL1, GALNTL2, GALNTL4, GALNTL5, GCNT1, GCNT3, GCNT4, ST3GAL1, ST3GAL2, WBSCR17, C1GALT1C1, ST6GALNAC1
Positive_45	GALE, GALT, TGDS, UGDH, UGP2, UXS1
Positive_46	GCK, GNE, HK1, HK2, HK3, NPL, AMDHD2, CHIA, CHIT1, CMAS, CTBS, GFPT1, GFPT2, GNPDA1, GNPDA2, GNPNAT1, HEXA, HEXB, MTMR1, MTMR2, MTMR6, NAGK, NANP, NANS, PHPT1, RENBP, UAP1
Positive_47	GNS, IDS, LCT, ARSB, GALNS, GLB1, GUSB, HEXA, HEXB, HGSNAT, HPSE, HPSE2, HYAL1, HYAL2, IDUA, NAGLU, SPAM1
Positive_48	B3GALT6, B3GAT1, B3GAT2, B3GAT3, B4GALT7, CHPF, CHSY1, CHSY3, XYLT1, XYLT2, CSGALNACT1, CSGALNACT2
Positive_49	EXT1, EXT2, EXTL1, EXTL2, EXTL3
Positive_50	AGK, CEL, GK, GK2, LPL, AGPAT1, AGPAT2, AGPAT3, AGPAT4, AGPAT6, AKR1A1, AKR1B1, ALDH1A3, ALDH1B1, ALDH2, ALDH3A1, ALDH3A2, ALDH7A1, ALDH9A1, DGAT1, DGAT2, DGAT2L4, DGKA, DGKB, DGKD, DGKE, DGKG, DGKH, DGKI, DGKQ, DGKZ, GLYCTK, GPAM, LIPA, LIPC, LIPF, LIPG, MGLL, PNLIP, PNPLA3, PPAP2A, PPAP2B, PPAP2C, PNLIPRP1, PNLIPRP2
Positive_51	IMPA1, IMPA2, INPP1, INPP4A, INPP4B, INPP5A, INPP5B, INPP5E, INPPL1, IPMK, IPPK, ISYNA1, ITPK1, ITPKA, ITPKB, MINPP1, MIOX, OCRL, PI4KA, PI4KB, PIK3C3, PIK3CA, PIK3CB, PIK3CD, PIK3CG, PIP4K2A, PIP4K2B, PIP4K2C, PIP5K1A, PIP5K1B, PIP5K1C, PIP5K3, PLCB1, PLCB2, PLCB3, PLCB4, PLCD1, PLCD3, PLCD4, PLCE1, PLCG1, PLCG2, PLCZ1, PTEN, PTPMT1, SYNJ1, SYNJ2
Positive_52	DPM2, GPAA1, GPLD1, PGAP1, PIGA, PIGB, PIGC, PIGF, PIGH, PIGK, PIGL, PIGM, PIGN, PIGO, PIGP, PIGQ, PIGS, PIGT, PIGU, PIGV, PIGW, PIGX
Positive_53	ACHE, AGPAT1, AGPAT2, AGPAT3, AGPAT4, AGPAT6, ARD1A, CDIPT, CDS1, CDS2, CHAT, CHKA, CHKB, CHPT1, CRLS1, DGKA, DGKB, DGKD, DGKE, DGKG, DGKH, DGKI, DGKQ, DGKZ, ETNK1, ETNK2, GDE1, GNPAT, GPAM, GPD1, GPD1L, GPD2, LCAT, LYPLA1, LYPLA2, NAT5, NAT6, PCYT1A, PCYT1B, PCYT2, PEMT, PGS1, PISD, PLA2G10, PLA2G1B, PLA2G2A, PLA2G2D, PLA2G2E, PLA2G2F, PLA2G3, PLA2G4A, PLA2G5, PLA2G6, PLD1, PLD2, PNPLA3, PPAP2A, PPAP2B, PPAP2C, PTDSS1, PTDSS2, PHOSPHO1, PLA2G12A, PLA2G12B

Positive_54	AGPAT1, AGPAT2, AGPAT3, AGPAT4, AGPAT6, CHPT1, ENPP2, ENPP6, PAFAH2, PLA2G10, PLA2G1B, PLA2G2A, PLA2G2D, PLA2G2E, PLA2G2F, PLA2G3, PLA2G4A, PLA2G5, PLA2G6, PLD1, PLD2, PPAP2A, PPAP2B, PPAP2C, PAFAH1B1, PAFAH1B2, PAFAH1B3, PLA2G12A, PLA2G12B
Positive_55	AKR1C3, ALOX12, ALOX12B, ALOX15, ALOX15B, ALOX5, CBR1, CBR3, CYP2B6, CYP2C18, CYP2C19, CYP2C8, CYP2C9, CYP2E1, CYP2J2, CYP2U1, CYP4A11, CYP4A22, CYP4F2, CYP4F3, EPHX2, GGT1, GGT5, GGT6, GGT7, GPX5, GPX7, LTA4H, LTC4S, PLA2G10, PLA2G1B, PLA2G2A, PLA2G2D, PLA2G2E, PLA2G2F, PLA2G3, PLA2G4A, PLA2G5, PLA2G6, PTGDS, PTGES, PTGES2, PTGIS, PTGS1, PTGS2, TBXAS1, PLA2G12A, PLA2G12B
Positive_56	ALOX15, ALOX5, CYP1A2, CYP2C18, CYP2C19, CYP2C8, CYP2C9, CYP2E1, CYP2J2, CYP3A4, CYP3A43, CYP3A5, CYP3A7, PLA2G10, PLA2G1B, PLA2G2A, PLA2G2D, PLA2G2E, PLA2G2F, PLA2G3, PLA2G4A, PLA2G5, PLA2G6, PLA2G12A, PLA2G12B
Positive_57	FADS2, PLA2G10, PLA2G1B, PLA2G2A, PLA2G2D, PLA2G2E, PLA2G2F, PLA2G3, PLA2G4A, PLA2G5, PLA2G6, PLA2G12A, PLA2G12B
Positive_58	GBA, GLA, LCT, ARSA, ARSD, ARSE, ASAH1, B4GALT6, CERK, DEGS1, DEGS2, ENPP7, GAL3ST1, GALC, GLB1, KDSR, NEU1, NEU2, NEU3, NEU4, PPAP2A, PPAP2B, PPAP2C, SGMS1, SGMS2, SGPP1, SGPP2, SMPD1, SMPD2, SMPD3, SMPD4, SPHK1, SPHK2, SPTLC1, SPTLC2, UGCG, UGT8
Positive_59	ABO, B3GALT1, B3GALT2, B3GALT5, B3GNT5, FUT1, FUT2, FUT3, ST3GAL3, ST3GAL4
Positive_60	ABO, B3GNT2, B3GNT3, B3GNT4, B3GNT5, B4GALT1, B4GALT2, B4GALT3, B4GALT4, FUT1, FUT2, FUT3, FUT4, FUT5, FUT6, FUT7, FUT9, GCNT2, ST3GAL6, ST8SIA1
Positive_61	GLA, A4GALT, B3GALT5, FUT1, FUT2, GBGT1, HEXA, HEXB, NAGA, ST3GAL1, ST3GAL2, ST8SIA1, B3GALNT1
Positive_62	LCT, B3GALT4, GLB1, HEXA, HEXB, SLC33A1, ST3GAL1, ST3GAL2, ST3GAL5, ST8SIA1, ST8SIA5, B4GALNT1, ST6GALNAC3, ST6GALNAC4, ST6GALNAC5, ST6GALNAC6
Positive_63	DLD, ME1, ME2, ME3, PC, ACACA, ACACB, ACAT1, ACAT2, ACOT12, ACSS1, ACSS2, ACYP1, ACYP2, AKR1B1, ALDH1A3, ALDH1B1, ALDH2, ALDH3A1, ALDH3A2, ALDH7A1, ALDH9A1, DLAT, GLO1, GRHPR, HAGH, HAGHL, LDHA, LDHAL6A, LDHAL6B, LDHB, LDHC, LDHD, MDH1, MDH2, PCK1, PCK2, PDHA1, PDHA2, PDHB, PKLR, PKM2
Positive_64	ARD1A, NAT5, NAT6, PNPLA3
Positive_65	AKR1B10, EPHX2, HSD3B7
Positive_66	CS, HYI, PGP, ACO1, ACO2, AFMID, GLYCTK, GRHPR, HAO1, HAO2, MDH1, MDH2, MTHFD1, MTHFD1L, MTHFD2
Positive_67	ARD1A, ECHS1, EHHADH, GCDH, HADHA, NAT5, NAT6, PNPLA3
Positive_68	MUT, ACACA, ACACB, ACADM, ACAT1, ACAT2, ACSS1, ACSS2, ACSS3, ALDH6A1, ECHS1, EHHADH, HADHA, HIBCH, MCEE, MLYCD, PCCA, PCCB, SUCLA2, SUCLG1, SUCLG2
Positive_69	ADH1A, ADH1B, ADH4, ADH5, ADH6, ADH7, ALDH1A3, ALDH1B1, ALDH2, ALDH3A1, ALDH3A2, ALDH7A1, ALDH9A1
Positive_70	FAH, HGD, GSTZ1
Positive_71	AACS, ABAT, ACADS, ACAT1, ACAT2, ACSM1, ACSM2A, ACSM3, ACSM4, ACSM5, ALDH5A1, BDH1, BDH2, ECHS1, EHHADH, GAD1, GAD2, HADH, HADHA, HMGCL, HMGCS1, HMGCS2, HSD17B4, OXCT1, OXCT2, HSD17B10
Positive_72	AMT, MTR, ALDH1L1, ATIC, DHFR, FTCD, GART, MTFMT, MTHFD1, MTHFD1L, MTHFD2, MTHFR, MTHFS, SHMT1, SHMT2, TYMS
Positive_73	MTHFR, SHMT1, SHMT2
Positive_74	FH, ACLY, ACO1, ACO2, ACSS1, ACSS2, IDH1, IDH2, MDH1, MDH2
Positive_75	MTMR1, MTMR2, MTMR6, PHPT1, THTPA, TPK1
Positive_76	RFK, ACP1, ACP2, ACP5, ACP6, ACPP, ACPT, ENPP1, ENPP3, FLAD1
Positive_77	AOX1, PDXK, PDXP, PNPO
Positive_78	NNT, AOX1, BST1, C9orf95, CD38, ENPP1, ENPP3, NADK, NADSYN1, NAMPT, NMNAT1, NMNAT2, NMNAT3, NNMT, NT5C, NT5C1A, NT5C1B, NT5C2, NT5C3, NT5E, NT5M, NUDT12, QPRT
Positive_79	COASY, DPYD, DPYS, ENPP1, ENPP3, PANK1, PANK2, PANK3, PANK4, PPCDC, PPCS, UPB1, VNN1
Positive_80	BTD, HLCS, SPCS1, SPCS3

Positive_81	GGH,PTS,ALPI,ALPL,ALPP,ALPPL2,ASCC3,ATP13A2,DDX18,DDX19A,DDX23,DDX4,DDX41,DDX47,DDX50,DDX51,DDX52,DDX54,DDX55,DDX56,DHFR,DHX58,ENTPD7,FPGS,GCH1,IFIH1,MOV10L1,NUDT5,NUDT8,SKIV2L2,SMARCA2,SMARCA5
Positive_82	ADH1A,ADH1B,ADH4,ADH5,ADH6,ADH7,ALDH1A1,ALDH1A2,BCMO1,CYP1A1,CYP1A2,CYP26A1,CYP26B1,CYP26C1,CYP2A13,CYP2A6,CYP2A7,CYP2B6,CYP2C18,CYP2C19,CYP2C8,CYP2C9,CYP3A4,CYP3A43,CYP3A5,CYP3A7,CYP4A11,CYP4A22,DGAT1,DGAT2,DGAT2L4,DHRS3,DHRS4,DHRS4L2,DHRS9,LRAT,PNPLA4,RDH10,RDH11,RDH12,RDH16,RDH5,RDH8,RETSAT,RPE65,UGT1A10,UGT2A1,UGT2A3,UGT2B10,UGT2B11,UGT2B17,UGT2B28,UGT2B4,UGT2B7
Positive_83	CP,ALAD,ALAS1,ALAS2,BLVRA,BLVRB,COX10,COX15,CPOX,FECH,FTH1,FTMT,GUSB,HCCS,HMBS,HMOX1,HMOX2,PPOX,UGT1A10,UGT2A1,UGT2A3,UGT2B10,UGT2B11,UGT2B17,UGT2B28,UGT2B4,UGT2B7,UROD,UROS
Positive_84	FDFT1,FDPS,GGPS1,IDI1,IDI2,SQLE
Positive_85	ACOT11,ARD1A,CYP2C19,CYP2C9,NAT5,NAT6,PNPLA3,YOD1
Positive_86	AMT,CTH,GLS,HAL,ASNS,ASRGL1,CPS1,GLS2,GLUD1,GLUD2,GLUL
Positive_87	BPNT1,CHST11,CHST12,CHST13,PAPSS1,PAPSS2,SULT1A1,SULT1A2,SULT1A4,SULT1E1,SULT2A1,SULT2B1,SUOX
Positive_88	ECHS1,EHHADH,HADH,HADHA,HSD17B4,HSD17B10
Positive_89	DDC,TAT,TYR,GOT1,GOT2
Positive_90	AADAC,CES1,CES7,DDHD1,LIPA,PLA1A,PRDX6
Positive_91	MARS,MARS2,MTFMT
Positive_92	ADH1A,ADH1B,ADH4,ADH5,ADH6,ADH7,AKR1C1,AKR1C2,AKR1C3,AKR1C4,ALDH1A3,ALDH3A1,ALDH3B1,ALDH3B2,CYP1A1,CYP1A2,CYP1B1,CYP2B6,CYP2C18,CYP2C19,CYP2C8,CYP2C9,CYP2E1,CYP2F1,CYP2S1,CYP3A4,CYP3A43,CYP3A5,CYP3A7,DHDH,EPHX1,GSTA1,GSTA2,GSTA3,GSTA4,GSTA5,GSTK1,GSTM1,GSTM2,GSTM3,GSTM4,GSTM5,GSTO1,GSTO2,GSTP1,GSTT1,GSTZ1,MGST1,MGST2,MGST3,UGT1A10,UGT2A1,UGT2A3,UGT2B10,UGT2B11,UGT2B17,UGT2B28,UGT2B4,UGT2B7
Positive_93	ADH1A,ADH1B,ADH4,ADH5,ADH6,ADH7,ALDH1A3,ALDH3A1,ALDH3B1,ALDH3B2,AOX1,CYP1A2,CYP2A13,CYP2A6,CYP2A7,CYP2B6,CYP2C18,CYP2C19,CYP2C8,CYP2C9,CYP2D6,CYP2E1,CYP3A4,CYP3A43,CYP3A5,CYP3A7,FMO1,FMO2,FMO3,FMO4,FMO5,GSTA1,GSTA2,GSTA3,GSTA4,GSTA5,GSTK1,GSTM1,GSTM2,GSTM3,GSTM4,GSTM5,GSTO1,GSTO2,GSTP1,GSTT1,GSTZ1,MAOA,MAOB,MGST1,MGST2,MGST3,UGT1A10,UGT2A1,UGT2A3,UGT2B10,UGT2B11,UGT2B17,UGT2B28,UGT2B4,UGT2B7
Positive_94	CDA,TK1,TK2,XDH,CES1,CES2,CES7,CYP2A13,CYP2A6,CYP2A7,CYP3A4,CYP3A43,CYP3A5,CYP3A7,DPYD,DPYS,GMPS,GUSB,HPRT1,IMPDH1,IMPDH2,ITPA,LIPA,TPMT,TYMP,UCK1,UCK2,UCKL1,UGT1A10,UGT2A1,UGT2A3,UGT2B10,UGT2B11,UGT2B17,UGT2B28,UGT2B4,UGT2B7,UMPS,UPB1,UPP1,UPP2
Positive_95	DBI,GK,GK2,ILK,LPL,ME1,SCD,UBC,ACAA1,ACADL,ACADM,ACOX1,ACOX2,ACOX3,ACSL1,ACSL3,ACSL4,ACSL5,ACSL6,ADIPOQ,ANGPTL4,APOA1,APOA2,APOA5,APOC3,AQP7,CD36,CPT1A,CPT1B,CPT1C,CPT2,CYP27A1,CYP4A11,CYP4A22,CYP7A1,CYP8B1,EHHADH,FABP1,FABP3,FABP4,FADS2,HMGCS2,MMP1,NR1H3,OLR1,PCK1,PCK2,PDPK1,PLIN,PLTP,PPARA,PPARD,PPARG,RXRA,RXRB,RXRG,SCD5,SCP2,SLC27A1,SLC27A4,SORBS1,UCP1

Positive_96	CRK, EGF, FAS, FOS, JUN, MAX, MOS, MYC, NF1, NGF, NLK, SRF, TNF, ACVR1B, ACVR1C, AKT1, AKT2, AKT3, ARRB1, ARRB2, ATF2, ATF4, BDNF, BRAF, CACNA1A, CACNA1B, CACNA1C, CACNA1D, CACNA1E, CACNA1F, CACNA1G, CACNA1H, CACNA1I, CACNA1S, CACNB1, CACNB2, CACNB3, CACNB4, CACNG1, CACNG2, CACNG3, CACNG4, CACNG5, CACNG6, CACNG7, CACNG8, CASP3, CD14, CDC25B, CDC42, CHUK, CRKL, DAXX, DDIT3, DUSP1, DUSP10, DUSP14, DUSP16, DUSP2, DUSP3, DUSP4, DUSP5, DUSP6, DUSP7, DUSP8, DUSP9, ECSIT, EGFR, ELK1, ELK4, EVI1, FASLG, FGF1, FGF10, FGF11, FGF12, FGF13, FGF14, FGF17, FGF18, FGF19, FGF2, FGF20, FGF21, FGF22, FGF23, FGF3, FGF4, FGF5, FGF6, FGF7, FGF8, FGF9, FGFR1, FGFR2, FGFR3, FGFR4, GADD45A, GADD45B, GADD45G, GNA12, GNG12, GRB2, HRAS, HSPA1B, HSPA2, HSPA8, HSPB1, IKBKB, IKBKG, IL1A, IL1B, IL1R1, IL1R2, JUND, KRAS, MAP2K1, MAP2K2, MAP2K3, MAP2K4, MAP2K5, MAP2K6, MAP2K7, MAP3K1, MAP3K11, MAP3K12, MAP3K13, MAP3K14, MAP3K2, MAP3K3, MAP3K4, MAP3K5, MAP3K6, MAP3K7, MAP3K8, MAP4K1, MAP4K2, MAP4K3, MAP4K4, MAPK1, MAPK10, MAPK11, MAPK12, MAPK13, MAPK14, MAPK3, MAPK7, MAPK8, MAPK9, MAPT, MEF2C, MKNK1, MKNK2, MRAS, NFATC2, NFATC4, NFKB1, NFKB2, NR4A1, NRAS, NTF3, NTF4, NTRK1, NTRK2, PAK1, PAK2, PDGFA, PDGFB, PDGFRA, PDGFRB, PLA2G10, PLA2
Positive_97	BAD, BTC, CBL, CRK, EGF, JUN, MYC, SRC, ABL1, ABL2, AKT1, AKT2, AKT3, ARAF, AREG, BRAF, CAMK2A, CAMK2B, CAMK2D, CAMK2G, CBLB, CBLC, CDKN1A, CDKN1B, CRKL, EGFR, ELK1, ERBB2, ERBB3, ERBB4, EREG, FRAP1, GAB1, GRB2, GSK3B, HBEGF, HRAS, KRAS, MAP2K1, MAP2K2, MAP2K4, MAP2K7, MAPK1, MAPK10, MAPK3, MAPK8, MAPK9, NCK1, NCK2, NRAS, NRG1, NRG2, NRG3, NRG4, PAK1, PAK2, PAK3, PAK4, PAK6, PAK7, PIK3CA, PIK3CB, PIK3CD, PIK3CG, PIK3R1, PIK3R2, PIK3R3, PIK3R5, PLCG1, PLCG2, PRKCA, PRKCB, PRKCG, PTK2, RAF1, RPS6KB1, RPS6KB2, SHC1, SHC2, SHC3, SHC4, SOS1, SOS2, STAT5A, STAT5B, TGFA, EIF4EBP1
Positive_98	F2R, PLN, ADCY1, ADCY2, ADCY3, ADCY4, ADCY7, ADCY8, ADCY9, ADORA2A, ADORA2B, ADRA1A, ADRA1B, ADRA1D, ADRB1, ADRB2, ADRB3, AGTR1, ATP2A1, ATP2A2, ATP2A3, AVPR1A, AVPR1B, BDKRB1, BDKRB2, CALML3, CALML5, CALML6, CAMK2A, CAMK2B, CAMK2D, CAMK2G, CAMK4, CCKAR, CCKBR, CHRM1, CHRM2, CHRM3, CHRM5, CYSLTR1, CYSLTR2, DRD1, DRD5, EDNRA, EDNRB, EGFR, ERBB2, ERBB3, ERBB4, GNA11, GNA14, GNA15, GNAL, GNAQ, GNAS, GRM1, GRM5, GRPR, HRH1, HRH2, HTR2A, HTR2B, HTR2C, HTR4, HTR5A, HTR6, HTR7, LHCGR, LTB4R2, MYLK, MYLK2, MYLK3, NOS1, NOS3, NTSR1, OXTR, PDE1A, PDE1B, PDE1C, PDGFRA, PDGFRB, PHKA1, PHKA2, PHKB, PHKG1, PHKG2, PLCB1, PLCB2, PLCB3, PLCB4, PLCD1, PLCD3, PLCD4, PLCE1, PLCG1, PLCG2, PLCZ1, PPP3CA, PPP3CB, PPP3CC, PPP3R1, PPP3R2, PRKACA, PRKACB, PRKACG, PRKCA, PRKCB, PRKCG, PRKX, PRKY, PTAFR, PTGER1, PTGER3, PTGFR, TACR1, TACR2, TACR3, TBXA2R, TRHR
Positive_99	AMH, EDA, EGF, EPO, FAS, GH1, GH2, GHR, HGF, IL2, IL3, IL4, IL5, IL6, IL7, IL8, IL9, KDR, KIT, LEP, LIF, LTB, MET, MPL, OSM, PRL, TNF, TPO, ACVR1, ACVR1B, ACVR2A, ACVR2B, ACVRL1, AMHR2, BMP2, BMP7, BMPR1A, BMPR1B, BMPR2, CCL1, CCL11, CCL13, CCL14, CCL15, CCL16, CCL17, CCL19, CCL2, CCL20, CCL21, CCL22, CCL23, CCL24, CCL25, CCL26, CCL27, CCL28, CCL3, CCL4, CCL5, CCL7, CCL8, CCR1, CCR10, CCR2, CCR3, CCR4, CCR5, CCR6, CCR7, CCR8, CCR9, CD27, CD40, CD40LG, CD70, CLCF1, CNTF, CNTFR, CRLF2, CSF1, CSF1R, CSF2, CSF2RA, CSF2RB, CSF3, CSF3R, CTF1, CX3CL1, CX3CR1, CXCL1, CXCL10, CXCL11, CXCL12, CXCL13, CXCL16, CXCL2, CXCL3, CXCL5, CXCL6, CXCL9, CXCR3, CXCR4, CXCR5, CXCR6, EDA2R, EDAR, EGFR, EPOR, FASLG, FIGF, FLT1, FLT3, FLT3LG, FLT4, GDF5, IFNA10, IFNA13, IFNA14, IFNA16, IFNA17, IFNA2, IFNA21, IFNA4, IFNA5, IFNA6, IFNA7, IFNA8, IFNAR1, IFNAR2, IFNB1, IFNG, IFNGR1, IFNGR2, IFNK, IFNW1, IL10, IL10RA, IL10RB, IL11, IL11RA, IL12A, IL12B, IL12RB1, IL12RB2, IL13, IL13RA1, IL15, IL15RA, IL17A, IL17B, IL17RA, IL17RB, IL18, IL18R1, IL18RAP, IL19, IL1A, IL1B, IL1R1, IL1R2, IL1RAP, IL20, IL20RA, IL21, IL21R, IL22, IL22RA1, IL22RA2, IL23A, IL23R, IL24, IL25, IL28A, IL28B, IL28RA, IL29, IL2RA, IL2RB, IL2RG, IL3RA, IL4R, IL5RA, IL6R, IL6ST
Positive_100	CALML3, CALML5, CALML6, CDIPT, CDS1, CDS2, DGKA, DGKB, DGKD, DGKE, DGKG, DGKH, DGKI, DGKQ, DGKZ, IMPA1, IMPA2, INPP1, INPP4A, INPP4B, INPP5A, INPP5B, INPP5E, INPPL1, ITPKA, ITPKB, OCRL, PI4KA, PI4KB, PIK3C2A, PIK3C2B, PIK3C2G, PIK3C3, PIK3CA, PIK3CB, PIK3CD, PIK3CG, PIK3R1, PIK3R2, PIK3R3, PIK3R5, PIP4K2A, PIP4K2B, PIP4K2C, PIP5K1A, PIP5K1B, PIP5K1C, PIP5K3, PLCB1, PLCB2, PLCB3, PLCB4, PLCD1, PLCD3, PLCD4, PLCE1, PLCG1, PLCG2, PLCZ1, PTEN, PTPMT1, SYNJ1, SYNJ2

Positive_101	CGA, F2, F2R, GH1, GH2, GHR, LEP, LHB, PLG, PRL, CSH1, CTSG, F2RL1, F2RL2, F2RL3, FSHB, FSHR, GZMA, LEPR, LHCGR, PARD3, PRLR, PRSS1, PRSS2, PRSS3, TSHB, TSHR
Positive_102	ATM, RB1, SFN, ABL1, ANAPC1, ANAPC10, ANAPC11, ANAPC2, ANAPC4, ANAPC5, ANAPC7, BUB1, BUB1B, BUB3, CCNA1, CCNA2, CCNB1, CCNB2, CCNB3, CCND1, CCND2, CCND3, CCNE1, CCNE2, CCNH, CDC14A, CDC14B, CDC16, CDC2, CDC20, CDC23, CDC25A, CDC25B, CDC25C, CDC26, CDC27, CDC45L, CDC6, CDC7, CDK2, CDK4, CDK6, CDK7, CDKN1A, CDKN1B, CDKN1C, CDKN2A, CDKN2B, CDKN2C, CDKN2D, CHEK1, CHEK2, CREBBP, CUL1, DBF4, E2F1, E2F2, E2F3, EP300, ESPL1, FZR1, GADD45A, GADD45B, GADD45G, GSK3B, HDAC1, HDAC2, MAD1L1, MAD2L1, MAD2L2, MCM2, MCM3, MCM4, MCM5, MCM6, MCM7, MDM2, ORC1L, ORC2L, ORC3L, ORC4L, ORC5L, ORC6L, PCNA, PKMYT1, PLK1, PTTG1, RBL1, RBL2, RBX1, SKP1, SKP2, SMAD2, SMAD3, SMAD4, SMC1A, SMC1B, TFDP1, TGFB1, TGFB2, TGFB3, TP53, WEE1, YWHAB, YWHAE, YWHAG, YWHAH, YWHAQ, YWHAZ
Positive_103	ATM, BAX, BID, FAS, SFN, APAF1, BAI1, BBC3, CASP3, CASP8, CASP9, CCNB1, CCNB2, CCNB3, CCND1, CCND2, CCND3, CCNE1, CCNE2, CCNG1, CCNG2, CD82, CDC2, CDK2, CDK4, CDK6, CDKN1A, CDKN2A, CHEK1, CHEK2, CYCS, DDB2, EI24, GADD45A, GADD45B, GADD45G, GTSE1, IGF1, IGFBP3, LRDD, MDM2, MDM4, PERP, PMAIP1, PPM1D, PTEN, RCHY1, RFWD2, RPRM, RRM2, RRM2B, SESN1, SESN2, SESN3, SHISA5, SIAH1, STEAP3, THBS1, TP53, TP53I3, TP73, TSC2, ZMAT3, SERPINB5, SERPINE1, TNFRSF10B
Positive_104	BET1, BET1L, GOSR2, SNAP23, SNAP25, STX10, STX12, STX16, STX1A, STX1B, STX2, STX3, STX4, STX5, STX6, STX7, STX8, TSNARE1, VAMP1, VAMP2, VAMP3, VAMP4, VTI1A, VTI1B, YKT6
Positive_105	ATG12, ATG3, ATG7, GABARAP, PIK3C3, PIK3R4, GABARAPL1, GABARAPL2
Positive_106	INS, PGF, AKT1, AKT2, AKT3, BRAF, DDIT4, EIF4B, EIF4E, EIF4E2, FIGF, FRAP1, HIF1A, IGF1, MAPK1, MAPK3, PDPK1, PIK3CA, PIK3CB, PIK3CD, PIK3CG, PIK3R1, PIK3R2, PIK3R3, PIK3R5, PRKAA1, PRKAA2, RHEB, RPS6, RPS6KA1, RPS6KA2, RPS6KA3, RPS6KA6, RPS6KB1, RPS6KB2, STK11, TSC2, ULK1, ULK2, ULK3, VEGFA, VEGFB, VEGFC, EIF4EBP1
Positive_107	ATM, BAD, BAX, BID, FAS, IL3, NGF, TNF, AKT1, AKT2, AKT3, APAF1, BCL2, BCL2L1, BIRC2, BIRC3, CASP10, CASP3, CASP6, CASP7, CASP8, CASP9, CFLAR, CHUK, CSF2RB, CYCS, DFFA, DFFB, FADD, FASLG, IKBKB, IKBKG, IL1A, IL1B, IL1R1, IL1RAP, IL3RA, IRAK1, IRAK2, IRAK3, IRAK4, MAP3K14, MYD88, NFKB1, NFKBIA, NTRK1, PIK3CA, PIK3CB, PIK3CD, PIK3CG, PIK3R1, PIK3R2, PIK3R3, PIK3R5, PPP3CA, PPP3CB, PPP3CC, PPP3R1, PPP3R2, PRKACA, PRKACB, PRKACG, PRKAR1A, PRKAR1B, PRKAR2A, PRKAR2B, PRKX, PRKY, RELA, RIPK1, TNFSF10, TP53, TRADD, TRAF2, XIAP, TNFRSF10A, TNFRSF10B, TNFRSF10C, TNFRSF10D, TNFRSF1A
Positive_108	JUN, MYC, NLK, APC2, AXIN1, AXIN2, BTRC, CAMK2A, CAMK2B, CAMK2D, CAMK2G, CCND1, CCND2, CCND3, CER1, CHD8, CREBBP, CSNK1A1, CSNK1E, CSNK2A1, CSNK2A2, CSNK2B, CTBP1, CTBP2, CTNNB1, CXXC4, DAAM1, DAAM2, DKK1, DKK2, DKK4, DVL1, DVL2, DVL3, EP300, FBXW11, FOSL1, FRAT1, FRAT2, FZD1, FZD10, FZD2, FZD3, FZD4, FZD5, FZD6, FZD7, FZD8, FZD9, GSK3B, LEF1, LRP5, LRP6, MAP3K7, MAPK10, MAPK8, MAPK9, MMP7, NFAT5, NFATC1, NFATC2, NFATC3, NFATC4, NKD1, NKD2, PLCB1, PLCB2, PLCB3, PLCB4, PORCN, PPARD, PPP2CA, PPP2CB, PPP2R1A, PPP2R1B, PPP2R5A, PPP2R5B, PPP2R5C, PPP2R5D, PPP2R5E, PPP3CA, PPP3CB, PPP3CC, PPP3R1, PPP3R2, PRKACA, PRKACB, PRKACG, PRKCA, PRKCB, PRKCG, PRKX, PRKY, PSEN1, RAC1, RAC2, RAC3, RHOA, ROCK1, ROCK2, RUVBL1, SENP2, SFRP1, SFRP2, SFRP4, SFRP5, SIAH1, SMAD4, SOX17, TBL1X, TBL1XR1, TBL1Y, TCF7, TCF7L1, TCF7L2, TP53, VANGL1, VANGL2, WIF1, WNT1, WNT10A, WNT10B, WNT11, WNT16, WNT2, WNT2B, WNT3, WNT3A, WNT4, WNT5A, WNT5B, WNT6, WNT7A, WNT7B, WNT8A, WNT8B, WNT9A, WNT9B, CSNK1A1L, CTNNBIP1, PRICKLE1, PRICKLE2
Positive_109	ADAM17, APH1A, CREBBP, CTBP1, CTBP2, DLL1, DLL3, DLL4, DTX1, DTX2, DTX3, DTX3L, DTX4, DVL1, DVL2, DVL3, EP300, HDAC1, HDAC2, HES1, JAG1, JAG2, KAT2A, KAT2B, LFNG, MAML1, MAML2, MAML3, MFNG, NCOR2, NCSTN, NOTCH1, NOTCH2, NOTCH3, NUMB, NUMBL, PSEN1, PSEN2, PSENEN, PTCRA, RBPJ, RBPJL, RFNG, SNW1

Positive_110	DHH, IHH, SHH, SMO, BMP2, BMP4, BMP5, BMP6, BMP7, BMP8A, BMP8B, BTRC, CSNK1A1, CSNK1D, CSNK1E, CSNK1G1, CSNK1G2, CSNK1G3, FBXW11, GAS1, GLI1, GLI2, GLI3, GSK3B, HHIP, PRKACA, PRKACB, PRKACG, PRKX, PRKY, PTCH1, PTCH2, RAB23, STK36, SUFU, WNT1, WNT10A, WNT10B, WNT11, WNT16, WNT2, WNT2B, WNT3, WNT3A, WNT4, WNT5A, WNT5B, WNT6, WNT7A, WNT7B, WNT8A, WNT8B, WNT9A, WNT9B, ZIC2, CSNK1A1L
Positive_111	AMH, DCN, FST, ID1, ID2, ID3, ID4, MYC, NOG, SP1, TNF, ACVR1, ACVR1B, ACVR1C, ACVR2A, ACVR2B, ACVRL1, AMHR2, BMP2, BMP4, BMP5, BMP6, BMP7, BMP8A, BMP8B, BMPR1A, BMPR1B, BMPR2, CDKN2B, CHRD, COMP, CREBBP, CUL1, E2F4, E2F5, EP300, GDF5, GDF6, GDF7, IFNG, INHBA, INHBB, INHBC, INHBE, LEFTY1, LEFTY2, LTBP1, MAPK1, MAPK3, NODAL, PITX2, PPP2CA, PPP2CB, PPP2R1A, PPP2R1B, RBL1, RBL2, RBX1, RHOA, ROCK1, ROCK2, RPS6KB1, RPS6KB2, SKP1, SMAD1, SMAD2, SMAD3, SMAD4, SMAD5, SMAD6, SMAD7, SMAD9, SMURF1, SMURF2, TFDP1, TGFB1, TGFB2, TGFB3, TGFBR1, TGFBR2, THBS1, THBS2, THBS3, THBS4, ZFYVE16, ZFYVE9
Positive_112	DCC, FES, FYN, ABL1, ABLIM1, ABLIM2, ABLIM3, CDC42, CDK5, CFL1, CFL2, CXCL12, CXCR4, DPYSL2, EFNA1, EFNA2, EFNA3, EFNA4, EFNA5, EFNB1, EFNB2, EFNB3, EPHA1, EPHA2, EPHA3, EPHA4, EPHA5, EPHA6, EPHA7, EPHA8, EPHB1, EPHB2, EPHB3, EPHB4, EPHB6, GNAI1, GNAI2, GNAI3, GSK3B, HRAS, ITGB1, KRAS, L1CAM, LIMK1, LIMK2, LRRC4C, MAPK1, MAPK3, NCK1, NCK2, NFAT5, NFATC1, NFATC2, NFATC3, NFATC4, NGEF, NRAS, NRP1, NTN1, NTNG1, PAK1, PAK2, PAK3, PAK4, PAK6, PAK7, PLXNA1, PLXNA2, PLXNA3, PLXNB1, PLXNB2, PLXNB3, PLXNC1, PPP3CA, PPP3CB, PPP3CC, PPP3R1, PPP3R2, PTK2, RAC1, RAC2, RAC3, RASA1, RGS3, RHOA, RHOD, RND1, ROBO1, ROBO2, ROBO3, ROCK1, ROCK2, SEMA3A, SEMA3B, SEMA3C, SEMA3D, SEMA3E, SEMA3F, SEMA3G, SEMA4A, SEMA4B, SEMA4C, SEMA4D, SEMA4F, SEMA4G, SEMA7A, SLIT1, SLIT2, SRGAP1, SRGAP2, SRGAP3, UNC5A, UNC5B, UNC5C, UNC5D, ARHGEF12
Positive_113	BAD, KDR, PXN, SRC, AKT1, AKT2, AKT3, CASP9, CDC42, HRAS, HSPB1, KRAS, MAP2K1, MAP2K2, MAPK1, MAPK11, MAPK12, MAPK13, MAPK14, MAPK3, NFAT5, NFATC1, NFATC2, NFATC3, NFATC4, NOS3, NRAS, PIK3CA, PIK3CB, PIK3CD, PIK3CG, PIK3R1, PIK3R2, PIK3R3, PIK3R5, PLA2G10, PLA2G1B, PLA2G2A, PLA2G2D, PLA2G2E, PLA2G2F, PLA2G3, PLA2G4A, PLA2G5, PLA2G6, PLCG1, PLCG2, PPP3CA, PPP3CB, PPP3CC, PPP3R1, PPP3R2, PRKCA, PRKCB, PRKCG, PTGS2, PTK2, RAC1, RAC2, RAC3, RAF1, SH2D2A, SHC2, SPHK1, SPHK2, VEGFA, MAPKAPK2, MAPKAPK3, PLA2G12A, PLA2G12B
Positive_114	BAD, CRK, EGF, FN1, FYN, HGF, ILK, JUN, KDR, MET, PGF, PXN, SRC, TNC, TNN, TNR, VCL, VTN, ZYX, ACTB, ACTG1, ACTN1, ACTN2, ACTN4, AKT1, AKT2, AKT3, ARHGAP5, BCAR1, BCL2, BIRC2, BIRC3, BRAF, CAPN2, CAV1, CAV2, CAV3, CCND1, CCND2, CCND3, CDC42, CHAD, COL11A1, COL1A1, COL1A2, COL2A1, COL3A1, COL4A1, COL4A2, COL4A4, COL4A6, COL5A1, COL5A2, COL5A3, COL6A1, COL6A2, COMP, CRKL, CTNNB1, DIAPH1, DOCK1, EGFR, ELK1, ERBB2, FARP2, FIGF, FLT1, FLT4, GRB2, GRLF1, GSK3B, HRAS, IBSP, IGF1, IGF1R, ITGA1, ITGA10, ITGA11, ITGA2, ITGA2B, ITGA3, ITGA4, ITGA5, ITGA6, ITGA7, ITGA8, ITGA9, ITGAV, ITGB1, ITGB3, ITGB4, ITGB5, ITGB6, ITGB7, ITGB8, LAMA3, LAMA4, LAMB1, LAMB2, LAMB3, LAMB4, LAMC1, LAMC2, LAMC3, MAP2K1, MAPK1, MAPK10, MAPK3, MAPK8, MAPK9, MYL2, MYL5, MYL7, MYL9, MYLK, MYLK2, MYLK3, PAK1, PAK2, PAK3, PAK4, PAK6, PAK7, PARVA, PARVB, PARVG, PDGFA, PDGFB, PDGFC, PDGFD, PDGFRA, PDGFRB, PDPK1, PIK3CA, PIK3CB, PIK3CD, PIK3CG, PIK3R1, PIK3R2, PIK3R3, PIK3R5, PIP5K1C, PPP1CA, PPP1CB, PPP1CC, PRKCA, PRKCB, PRKCG, PTEN, PTK2, RAC1, RAC2, RAC3, RAF1, RAP1A, RAP1B, RAPGEF1, RHOA, ROCK1, ROCK2, SHC1, SHC2, SHC3, SHC4, SOS1, SOS2, SPP1, THBS1, THBS2, THBS3, THBS4, TLN1, TLN2, TNXB, VASP, VAV1, VAV2, VAV3, VEGFA, VEGFB, VEGFC, XIAP, PPP1R12A
Positive_115	FN1, GP6, TNC, TNN, TNR, VTN, AGRN, CD36, CD44, CD47, CHAD, COL11A1, COL1A1, COL1A2, COL2A1, COL3A1, COL4A1, COL4A2, COL4A4, COL4A6, COL5A1, COL5A2, COL5A3, COL6A1, COL6A2, COMP, DAG1, IBSP, ITGA1, ITGA10, ITGA11, ITGA2, ITGA2B, ITGA3, ITGA4, ITGA5, ITGA6, ITGA7, ITGA8, ITGA9, ITGAV, ITGB1, ITGB3, ITGB4, ITGB5, ITGB6, ITGB7, ITGB8, LAMA3, LAMA4, LAMB1, LAMB2, LAMB3, LAMB4, LAMC1, LAMC2, LAMC3, SDC1, SDC2, SDC3, SDC4, SPP1, SV2A, SV2B, SV2C, THBS1, THBS2, THBS3, THBS4, TNXB

Positive_116	CD2,CD4,CD6,MAG,MPZ,PVR,SPN,ALCAM,CADM1,CADM3,CD22,CD226,CD274,CD28,CD34,CD40,CD40LG,CD58,CD80,CD86,CD8A,CD8B,CD99,CDH1,CDH15,CDH2,CDH3,CDH4,CDH5,CLDN1,CLDN10,CLDN11,CLDN14,CLDN15,CLDN16,CLDN17,CLDN18,CLDN19,CLDN2,CLDN20,CLDN22,CLDN3,CLDN4,CLDN5,CLDN6,CLDN7,CLDN8,CLDN9,CNTN1,CNTN2,CNTNAP1,CNTNAP2,CTLA4,ESAM,F11R,GLG1,HLA-C,HLA-DMA,HLA-DOA,HLA-DOB,HLA-DRA,HLA-F,HLA-G,ICAM1,ICAM2,ICAM3,ICOS,ICOSLG,ITGA4,ITGA9,ITGAL,ITGAM,ITGB1,ITGB2,ITGB7,JAM2,JAM3,L1CAM,MADCAM1,MPZL1,NCAM1,NCAM2,NEGR1,NFASC,NLGN1,NLGN2,NLGN3,NRCAM,NRXN1,NRXN2,NRXN3,OCLN,PDCD1,PECAM1,PTPRC,PTPRM,PVRL1,PVRL2,PVRL3,SELE,SELL,SELP,SELPLG,SIGLEC1,VCAM1,HLA-DPA1,HLA-DPB1,HLA-DQA2,HLA-DQB1,HLA-DRB3,HLA-DRB4,HLA-DRB5,PDCD1LG2
Positive_117	FER,FYN,MET,NLK,SRC,VCL,WAS,ACP1,ACTB,ACTG1,ACTN1,ACTN2,ACTN4,ACVR1B,ACVR1C,BAIAP2,CDC42,CDH1,CSNK2A1,CSNK2A2,CSNK2B,CTNNA1,CTNNA2,CTNNA3,CTNNB1,CTNND1,EGFR,ERBB2,FARP2,FGFR1,IGF1R,IQGAP1,LEF1,LMO7,MAP3K7,MAPK1,MAPK3,MLLT4,PARD3,PTPN1,PTPN6,PTPRB,PTPRF,PTPRJ,PTPRM,PVRL1,PVRL2,PVRL3,PVRL4,RAC1,RAC2,RAC3,RHOA,SMAD2,SMAD3,SMAD4,SNAI1,SNAI2,SORBS1,SSX2IP,TCF7,TCF7L1,TCF7L2,TGFBR1,TGFBR2,TJP1,WASF1,WASF2,WASF3,WASL,YES1
Positive_118	CGN,ACTB,ACTG1,ACTN1,ACTN2,ACTN4,AKT1,AKT2,AKT3,CASK,CDC42,CDK4,CLDN1,CLDN10,CLDN11,CLDN14,CLDN15,CLDN16,CLDN17,CLDN18,CLDN19,CLDN2,CLDN20,CLDN22,CLDN3,CLDN4,CLDN5,CLDN6,CLDN7,CLDN8,CLDN9,CRB3,CSDA,CSNK2A1,CSNK2A2,CSNK2B,CTNNA1,CTNNA2,CTNNA3,CTNNB1,CTTN,EPB41,EPB41L1,EPB41L2,EPB41L3,F11R,HCLS1,HRAS,IGSF5,INADL,JAM2,JAM3,KRAS,LLGL1,LLGL2,MAGI1,MAGI2,MAGI3,MLLT4,MPDZ,MPP5,MRAS,MYH1,MYH10,MYH11,MYH13,MYH14,MYH15,MYH2,MYH3,MYH4,MYH6,MYH7,MYH7B,MYH8,MYH9,MYL2,MYL5,MYL7,MYL9,NRAS,OCLN,PARD3,PARD6A,PARD6B,PARD6G,PPP2CA,PPP2CB,PPP2R1A,PPP2R1B,PPP2R2A,PPP2R2B,PPP2R2C,PPP2R2D,PRKCA,PRKCB,PRKCD,PRKCE,PRKCG,PRKCH,PRKCI,PRKCQ,PRKCZ,PTEN,RHOA,RRAS,RRAS2,SPTAN1,TJP1,TJP2,TJP3
Positive_119	EGF,SRC,ADCY1,ADCY2,ADCY3,ADCY4,ADCY5,ADCY6,ADCY7,ADCY8,ADCY9,ADRB1,CDC2,CSNK1D,DRD1,DRD2,EGFR,GJA1,GJD2,GNA11,GNAI1,GNAI2,GNAI3,GNAQ,GNAS,GRB2,GRM1,GRM5,GUCY1A2,GUCY1A3,GUCY1B3,GUCY2C,GUCY2D,GUCY2F,HRAS,HTR2A,HTR2B,HTR2C,KRAS,LPAR1,MAP2K1,MAP2K2,MAP2K5,MAP3K2,MAPK1,MAPK3,MAPK7,NPR1,NPR2,NRAS,PDGFA,PDGFB,PDGFC,PDGFD,PDGFRA,PDGFRB,PLCB1,PLCB2,PLCB3,PLCB4,PRKACA,PRKACB,PRKACG,PRKCA,PRKCB,PRKCG,PRKG1,PRKG2,PRKX,PRKY,RAF1,SOS1,SOS2,TJP1,TUBA1A,TUBA1B,TUBA1C,TUBA3C,TUBA3E,TUBA4A,TUBA8,TUBAL3,TUBB,TUBB1,TUBB2A,TUBB2B,TUBB2C,TUBB3,TUBB4,TUBB4Q,TUBB6
Positive_120	A2M,C1S,C2,C3,C4B,C5,C6,C7,C8A,C8B,C8G,C9,CFD,CFH,CFI,CR1,CR2,F10,F11,F12,F2,F2R,F3,F5,F7,F8,F9,FGA,FGB,FGG,PLG,BDKRB1,BDKRB2,C1QA,C1QB,C1QC,C3AR1,C4BPA,C4BPB,C5AR1,CD46,CD55,CD59,F13A1,F13B,KLKB1,KNG1,MASP1,MASP2,MBL2,PLAT,PLAU,PLAUR,PROC,PROS1,TFPI,THBD,SERPINA1,SERPINA5,SERPINC1,SERPIND1,SERPINE1,SERPINF2
Positive_121	CD4,CD74,CD8A,CD8B,CIITA,CREB1,CTSB,CTSL1,CTSS,HLA-C,HLA-DMA,HLA-DOA,HLA-DOB,HLA-DRA,HLA-F,HLA-G,IFNA10,IFNA13,IFNA14,IFNA16,IFNA17,IFNA2,IFNA21,IFNA4,IFNA5,IFNA6,IFNA7,IFNA8,KIR2DL1,KIR2DL2,KIR2DL3,KIR2DL4,KIR2DS4,KIR2DS5,KIR3DL1,KIR3DL2,KIR3DL3,KLRC1,KLRC2,KLRC3,KLRC4,KLRD1,LGMN,NFYA,NFYB,NFYC,PSME1,PSME2,PSME3,RFX5,RFXANK,RFXAP,HLA-DPA1,HLA-DPB1,HLA-DQA2,HLA-DQB1,HLA-DRB3,HLA-DRB4,HLA-DRB5,KIR2DL5A
Positive_122	TNF,CASP8,CD14,CD40,CD80,CD86,CHUK,CXCL10,CXCL11,FADD,IFNA10,IFNA13,IFNA14,IFNA16,IFNA17,IFNA2,IFNA21,IFNA4,IFNA5,IFNA6,IFNA7,IFNA8,IFNB1,IKBKB,IKBKE,IKBKG,IRAK1,IRAK4,IRF3,IRF5,IRF7,MAP3K7,MYD88,NFKB1,NFKBIA,RAC1,RIPK1,STAT1,TBK1,TICAM1,TICAM2,TIRAP,TLR2,TLR3,TLR4,TLR5,TLR9,TOLLIP,TRAF3,TRAF6

Positive_123	CBL, GHR, MPL, MYC, AKT1, AKT2, AKT3, BCL2L1, CBLB, CBLC, CCND1, CCND2, CCND3, CISH, CNTFR, CREBBP, CRLF2, CSF2RA, CSF2RB, CSF3R, EP300, EPOR, GRB2, IFNAR1, IFNAR2, IFNGR1, IFNGR2, IL10RA, IL10RB, IL11RA, IL12RB1, IL12RB2, IL13RA1, IL13RA2, IL15RA, IL20RA, IL20RB, IL21R, IL22RA1, IL22RA2, IL23R, IL28RA, IL2RA, IL2RB, IL2RG, IL3RA, IL4R, IL5RA, IL6R, IL6ST, IL7R, IL9R, IRF9, JAK1, JAK2, JAK3, LEPR, LIFR, OSMR, PIAS1, PIAS2, PIAS3, PIAS4, PIK3CA, PIK3CB, PIK3CD, PIK3CG, PIK3R1, PIK3R2, PIK3R3, PIK3R5, PIM1, PRLR, PTPN11, PTPN6, SOCS1, SOCS2, SOCS3, SOCS4, SOCS5, SOCS7, SOS1, SOS2, SPRED1, SPRED2, SPRY1, SPRY2, SPRY3, SPRY4, STAM, STAM2, STAT1, STAT2, STAT3, STAT4, STAT5A, STAT5B, STAT6, TYK2
Positive_124	BID, FAS, FYN, LAT, LCK, SYK, TNF, ARAF, BRAF, CASP3, CD244, CD247, CD48, CSF2, FASLG, FCER1G, FCGR3A, FCGR3B, GRB2, GZMB, HCST, HLA-C, HLA-G, HRAS, ICAM1, ICAM2, IFNA10, IFNA13, IFNA14, IFNA16, IFNA17, IFNA2, IFNA21, IFNA4, IFNA5, IFNA6, IFNA7, IFNA8, IFNAR1, IFNAR2, IFNB1, IFNG, IFNGR1, IFNGR2, ITGAL, ITGB2, KIR2DL1, KIR2DL2, KIR2DL3, KIR2DL4, KIR2DS4, KIR2DS5, KIR3DL1, KIR3DL2, KLRC1, KLRC2, KLRC3, KLRD1, KLRK1, KRAS, LCP2, MAP2K1, MAP2K2, MAPK1, MAPK3, MICA, NCR1, NCR2, NCR3, NFAT5, NFATC1, NFATC2, NFATC3, NFATC4, NRAS, PAK1, PIK3CA, PIK3CB, PIK3CD, PIK3CG, PIK3R1, PIK3R2, PIK3R3, PIK3R5, PLCG1, PLCG2, PPP3CA, PPP3CB, PPP3CC, PPP3R1, PPP3R2, PRKCA, PRKCB, PRKCG, PTK2B, PTPN11, PTPN6, RAC1, RAC2, RAC3, RAF1, SH2D1A, SH2D1B, SH3BP2, SHC1, SHC2, SHC3, SHC4, SOS1, SOS2, TNFSF10, TYROBP, ULBP1, ULBP2, ULBP3, VAV1, VAV2, VAV3, ZAP70, KIR2DL5A, TNFRSF10A, TNFRSF10B, TNFRSF10C, TNFRSF10D
Positive_125	CBL, CD4, FOS, FYN, ITK, JUN, LAT, LCK, TEC, AKT1, AKT2, AKT3, BCL10, CARD11, CBLB, CBLC, CD247, CD28, CD3E, CD40LG, CD8A, CD8B, CDC42, CHUK, CTLA4, GRAP2, GRB2, HRAS, ICOS, IKBKB, IKBKG, KRAS, LCP2, MALT1, MAP3K14, MAP3K8, NCK1, NCK2, NFAT5, NFATC1, NFATC2, NFATC3, NFATC4, NFKB1, NFKBIA, NFKBIB, NFKBIE, NRAS, PAK1, PAK2, PAK3, PAK4, PAK6, PAK7, PDCD1, PDK1, PIK3CA, PIK3CB, PIK3CD, PIK3CG, PIK3R1, PIK3R2, PIK3R3, PIK3R5, PLCG1, PPP3CA, PPP3CB, PPP3CC, PPP3R1, PPP3R2, PRKCQ, PTPN6, PTPRC, RASGRP1, RELA, RHOA, SOS1, SOS2, VAV1, VAV2, VAV3, ZAP70
Positive_126	BTK, FOS, JUN, LYN, SYK, AKT1, AKT2, AKT3, BCL10, BLNK, CARD11, CD19, CD22, CD72, CD79A, CD79B, CHUK, GSK3B, HRAS, IKBKB, IKBKG, KRAS, LILRB3, MALT1, NFAT5, NFATC1, NFATC2, NFATC3, NFATC4, NFKB1, NFKBIA, NFKBIB, NFKBIE, NRAS, PIK3CA, PIK3CB, PIK3CD, PIK3CG, PIK3R1, PIK3R2, PIK3R3, PIK3R5, PLCG2, PPP3CA, PPP3CB, PPP3CC, PPP3R1, PPP3R2, PRKCB, PTPN6, RAC1, RAC2, RAC3, RASGRP3, RELA, VAV1, VAV2, VAV3
Positive_127	BTK, LAT, LYN, SYK, AKT1, AKT2, AKT3, FCER1G, GRB2, HRAS, KRAS, LCP2, MAP2K1, MAP2K2, MAP2K3, MAP2K4, MAP2K6, MAP2K7, MAPK1, MAPK10, MAPK11, MAPK12, MAPK13, MAPK14, MAPK3, MAPK8, MAPK9, MS4A2, NRAS, PDK1, PIK3CA, PIK3CB, PIK3CD, PIK3CG, PIK3R1, PIK3R2, PIK3R3, PIK3R5, PLA2G10, PLA2G1B, PLA2G2A, PLA2G2D, PLA2G2E, PLA2G2F, PLA2G3, PLA2G4A, PLA2G5, PLA2G6, PLCG1, PLCG2, PRKCA, PRKCB, PRKCD, PRKCE, RAC1, RAC2, RAC3, RAF1, SOS1, SOS2, VAV1, VAV2, VAV3, PLA2G12A, PLA2G12B
Positive_128	EZR, ITK, MSN, PXN, TXK, ACTB, ACTG1, ARHGAP5, BCAR1, CD99, CDC42, CDH5, CLDN1, CLDN10, CLDN11, CLDN14, CLDN15, CLDN16, CLDN17, CLDN18, CLDN19, CLDN2, CLDN20, CLDN22, CLDN3, CLDN4, CLDN5, CLDN6, CLDN7, CLDN8, CLDN9, CTNNA1, CTNNA2, CTNNA3, CTNNB1, CTNND1, CXCL12, CXCR4, CYBA, CYBB, ESAM, F11R, GNAI1, GNAI2, GNAI3, GRLF1, ICAM1, ITGA4, ITGAL, ITGAM, ITGB1, ITGB2, JAM2, JAM3, MAPK11, MAPK12, MAPK13, MAPK14, MLLT4, MMP2, MMP9, MYL2, MYL5, MYL7, MYL9, NCF1, NCF2, NCF4, NOX1, NOX3, OCLN, PECAM1, PIK3CA, PIK3CB, PIK3CD, PIK3CG, PIK3R1, PIK3R2, PIK3R3, PIK3R5, PLCG1, PLCG2, PRKCA, PRKCB, PRKCG, PTK2, PTK2B, RAC1, RAC2, RAP1A, RAP1B, RAPGEF3, RAPGEF4, RASSF5, RHOA, ROCK1, ROCK2, SIPA1, THY1, VAV1, VAV2, VAV3, VCAM1
Positive_129	ARNTL, CLOCK, CRY1, CRY2, CSNK1D, CSNK1E, NPAS2, NR1D1, PER1, PER2, PER3
Positive_130	ADCY1, ADCY8, ARAF, ATF4, BRAF, CACNA1C, CALML3, CALML5, CALML6, CAMK2A, CAMK2B, CAMK2D, CAMK2G, CAMK4, CREBBP, EP300, GNAQ, GRIA1, GRIA2, GRIN1, GRIN2A, GRIN2B, GRIN2C, GRIN2D, GRM1, GRM5, HRAS, KRAS, MAP2K1, MAP2K2, MAPK1, MAPK3, NRAS, PLCB1, PLCB2, PLCB3, PLCB4, PPP1CA, PPP1CB, PPP1CC, PPP1R1A, PPP3CA, PPP3CB, PPP3CC, PPP3R1, PPP3R2, PRKACA, PRKACB, PRKACG, PRKCA, PRKCB, PRKCG, PRKX, PRKY, RAF1, RAP1A, RAP1B, RAPGEF3, RPS6KA1, RPS6KA2, RPS6KA3, RPS6KA6, PPP1R12A

Positive_131	CRH, LYN, ARAF, BRAF, C7orf16, CACNA1A, CRHR1, GNA11, GNA12, GNA13, GNAI1, GNAI2, GNAI3, GNAO1, GNAQ, GNAS, GNAZ, GRIA1, GRIA2, GRIA3, GRM1, GRM5, GUCY1A2, GUCY1A3, GUCY1B3, GUCY2C, GUCY2D, GUCY2F, HRAS, IGF1, IGF1R, KRAS, MAP2K1, MAP2K2, MAPK1, MAPK3, NOS1, NOS3, NPR1, NPR2, NRAS, PLA2G10, PLA2G1B, PLA2G2A, PLA2G2D, PLA2G2E, PLA2G2F, PLA2G3, PLA2G4A, PLA2G5, PLA2G6, PLCB1, PLCB2, PLCB3, PLCB4, PPP2CA, PPP2CB, PPP2R1A, PPP2R1B, PRKCA, PRKCB, PRKCG, PRKG1, PRKG2, RAF1, PLA2G12A, PLA2G12B
Positive_132	PDC, ADCY3, ADRBK2, ARRB2, CALML3, CALML5, CALML6, CAMK2A, CAMK2B, CAMK2D, CAMK2G, CLCA2, CLCA4, CNGA3, CNGA4, CNGB1, GNAL, GUCA1A, GUCA1B, GUCA1C, OR10A2, OR10A3, OR10A4, OR10A5, OR10A6, OR10A7, OR10AD1, OR10AG1, OR10C1, OR10G2, OR10G3, OR10G4, OR10G7, OR10G8, OR10G9, OR10H1, OR10H2, OR10H3, OR10H4, OR10H5, OR10J1, OR10J3, OR10J5, OR10K1, OR10K2, OR10P1, OR10Q1, OR10R2, OR10S1, OR10T2, OR10V1, OR10X1, OR10Z1, OR11A1, OR11G2, OR11H1, OR11H4, OR11H6, OR11L1, OR12D3, OR13A1, OR13C2, OR13C3, OR13C4, OR13C5, OR13C8, OR13C9, OR13D1, OR13F1, OR13G1, OR13H1, OR13J1, OR14A16, OR14C36, OR14I1, OR14J1, OR1A1, OR1A2, OR1B1, OR1C1, OR1D2, OR1D5, OR1E1, OR1E2, OR1F1, OR1G1, OR1I1, OR1J1, OR1J2, OR1J4, OR1K1, OR1L1, OR1L3, OR1L4, OR1L6, OR1L8, OR1M1, OR1N1, OR1N2, OR1Q1, OR1S1, OR1S2, OR2A12, OR2A14, OR2A2, OR2A25, OR2A4, OR2A42, OR2A5, OR2AE1, OR2AG1, OR2AG2, OR2AT4, OR2B11, OR2B2, OR2B6, OR2C1, OR2C3, OR2D2, OR2D3, OR2F1, OR2F2, OR2G2, OR2G3, OR2G6, OR2H1, OR2H2, OR2K2, OR2L13, OR2L2, OR2L3, OR2L8, OR2M2, OR2M3, OR2M4, OR2M5, OR2M7, OR2S2, OR2T1, OR2T10, OR2T11, OR2T12, OR2T2, OR2T29, OR2T3, OR2T33, OR2T34, OR2T4, OR2T5, OR2T6, OR2T8, OR2V2, OR2W1, OR2Y1, OR2Z1, OR3A1, OR3A2, OR3A3, OR4A15, OR4A16, OR4A
Positive_133	ADCY4, ADCY6, ADCY8, GNAS, GNAT3, GNB1, GNB3, GNG13, GNG3, KCNB1, PDE1A, PLCB2, PRKACA, PRKACB, PRKACG, PRKX, PRKY, TAS1R1, TAS1R2, TAS1R3, TAS2R1, TAS2R10, TAS2R13, TAS2R14, TAS2R16, TAS2R3, TAS2R38, TAS2R4, TAS2R40, TAS2R41, TAS2R42, TAS2R48, TAS2R49, TAS2R5, TAS2R50, TAS2R60, TAS2R7, TAS2R8, TAS2R9
Positive_134	CRK, CSK, EZR, F2R, GSN, MOS, MSN, PXN, RDX, VCL, WAS, ABI2, ACTB, ACTG1, ACTN1, ACTN2, ACTN4, APC2, ARAF, ARHGEF1, ARHGEF4, ARHGEF6, ARHGEF7, ARPC1A, ARPC1B, ARPC2, ARPC3, ARPC4, ARPC5, ARPC5L, BAIAP2, BCAR1, BDKRB1, BDKRB2, BRAF, C3orf10, CD14, CDC42, CFL1, CFL2, CHRM1, CHRM2, CHRM3, CHRM5, CRKL, CYFIP1, CYFIP2, DIAPH1, DIAPH2, DIAPH3, DOCK1, EGFR, ENAH, FGD1, FGD3, FGFR1, FGFR2, FGFR3, FGFR4, GIT1, GNA12, GNA13, GNG12, GRLF1, HRAS, INSRR, IQGAP1, IQGAP2, IQGAP3, ITGA1, ITGA10, ITGA11, ITGA2, ITGA2B, ITGA3, ITGA4, ITGA5, ITGA6, ITGA7, ITGA8, ITGA9, ITGAD, ITGAE, ITGAL, ITGAM, ITGAV, ITGAX, ITGB1, ITGB2, ITGB3, ITGB4, ITGB5, ITGB6, ITGB7, ITGB8, KRAS, LIMK1, LIMK2, MAP2K1, MAP2K2, MAPK1, MAPK3, MRAS, MYH10, MYH14, MYH9, MYL2, MYL5, MYL7, MYL9, MYLK, MYLK2, MYLK3, NCKAP1, NCKAP1L, NRAS, PAK1, PAK2, PAK3, PAK4, PAK6, PAK7, PDGFRA, PDGFRB, PFN1, PFN2, PFN3, PFN4, PIK3CA, PIK3CB, PIK3CD, PIK3CG, PIK3R1, PIK3R2, PIK3R3, PIK3R5, PIP4K2A, PIP4K2B, PIP4K2C, PIP5K1A, PIP5K1B, PIP5K1C, PIP5K3, PPP1CA, PPP1CB, PPP1CC, PTK2, RAC1, RAC2, RAC3, RAF1, RHOA, ROCK1, ROCK2, RRAS, RRAS2, SCIN, SLC9A1, SOS1, SOS2, SSH1, SSH2, SSH3, TIAM1, TIAM2, VAV1, VAV2, VAV3, WASF1, WASF2, WASL, ARHGEF12, PPP1R12A
Positive_135	BAD, CBL, CRK, INS, ACACA, ACACB, AKT1, AKT2, AKT3, ARAF, BRAF, CALML3, CALML5, CALML6, CBLB, CBLC, CRKL, EIF4E, EIF4E2, ELK1, EXOC7, FASN, FBP1, FBP2, FLOT1, FLOT2, FOXO1, FRAP1, G6PC, G6PC2, GRB2, GSK3B, GYS1, GYS2, HRAS, IKBKB, INSR, IRS1, IRS2, IRS4, KRAS, LIPE, MAP2K1, MAP2K2, MAPK1, MAPK10, MAPK3, MAPK8, MAPK9, MKNK1, MKNK2, NRAS, PCK1, PCK2, PDE3A, PDE3B, PDPK1, PFKL, PFKM, PFKP, PHKA1, PHKA2, PHKB, PHKG1, PHKG2, PIK3CA, PIK3CB, PIK3CD, PIK3CG, PIK3R1, PIK3R2, PIK3R3, PIK3R5, PKLR, PKM2, PPP1CA, PPP1CB, PPP1CC, PPP1R3A, PPP1R3B, PPP1R3C, PPP1R3D, PRKAA1, PRKAA2, PRKAB1, PRKAB2, PRKACA, PRKACB, PRKACG, PRKAG1, PRKAG2, PRKAG3, PRKAR1A, PRKAR1B, PRKAR2A, PRKAR2B, PRKCI, PRKCZ, PRKX, PRKY, PTPN1, PTPRF, PYGB, PYGL, PYGM, RAF1, RAPGEF1, RHEB, RHOQ, RPS6, RPS6KB1, RPS6KB2, SHC1, SHC2, SHC3, SHC4, SLC2A4, SOCS1, SOCS2, SOCS3, SOCS4, SORBS1, SOS1, SOS2, SREBF1, TRIP10, TSC1, TSC2, EIF4EBP1, PPARGC1A

Positive_136	JUN, SRC, ADCY1, ADCY2, ADCY3, ADCY4, ADCY5, ADCY6, ADCY7, ADCY8, ADCY9, ATF4, CALML3, CALML5, CALML6, CAMK2A, CAMK2B, CAMK2D, CAMK2G, CDC42, EGFR, ELK1, GNA11, GNAQ, GNAS, GNRH1, GNRH2, GNRHR, GRB2, HBEGF, HRAS, KRAS, MAP2K1, MAP2K2, MAP2K3, MAP2K4, MAP2K6, MAP2K7, MAP3K1, MAP3K2, MAP3K3, MAP3K4, MAPK1, MAPK10, MAPK11, MAPK12, MAPK13, MAPK14, MAPK3, MAPK7, MAPK8, MAPK9, MMP14, MMP2, NRAS, PLA2G10, PLA2G1B, PLA2G2A, PLA2G2D, PLA2G2E, PLA2G2F, PLA2G3, PLA2G4A, PLA2G5, PLA2G6, PLCB1, PLCB2, PLCB3, PLCB4, PLD1, PLD2, PRKACA, PRKACB, PRKACG, PRKCA, PRKCB, PRKCD, PRKX, PRKY, PTK2B, RAF1, SOS1, SOS2, PLA2G12A, PLA2G12B
Positive_137	DCT, KIT, TYR, ADCY1, ADCY2, ADCY3, ADCY4, ADCY5, ADCY6, ADCY7, ADCY8, ADCY9, CALML3, CALML5, CALML6, CAMK2A, CAMK2B, CAMK2D, CAMK2G, CREB1, CREB3, CREB3L1, CREB3L2, CREB3L3, CREB3L4, CREBBP, CTNNB1, DVL1, DVL2, DVL3, EDN1, EDNRB, EP300, FZD1, FZD10, FZD2, FZD3, FZD4, FZD5, FZD6, FZD7, FZD8, FZD9, GNAI1, GNAI2, GNAI3, GNAO1, GNAQ, GNAS, GSK3B, HRAS, KITLG, KRAS, LEF1, MAP2K1, MAP2K2, MAPK1, MAPK3, MITF, NRAS, PLCB1, PLCB2, PLCB3, PLCB4, PRKACA, PRKACB, PRKACG, PRKCA, PRKCB, PRKCG, PRKX, PRKY, RAF1, TCF7, TCF7L1, TCF7L2, TYRP1, WNT1, WNT10A, WNT10B, WNT11, WNT16, WNT2, WNT2B, WNT3, WNT3A, WNT4, WNT5A, WNT5B, WNT6, WNT7A, WNT7B, WNT8A, WNT8B, WNT9A, WNT9B
Positive_138	LEP, NPY, TNF, ACACB, ACSL1, ACSL3, ACSL4, ACSL5, ACSL6, ADIPOQ, ADIPOR1, ADIPOR2, AGRP, CAMKK1, CAMKK2, CHUK, CPT1A, CPT1B, CPT1C, FRAP1, G6PC, G6PC2, IKBKB, IKBKG, IRS1, IRS2, IRS4, JAK2, LEPR, MAPK10, MAPK8, MAPK9, NFKB1, NFKBIA, NFKBIB, NFKBIE, PCK1, PCK2, POMC, PPARA, PRKAA1, PRKAA2, PRKAB1, PRKAB2, PRKAG1, PRKAG2, PRKAG3, PRKCQ, PTPN11, RELA, SLC2A1, SLC2A4, SOCS3, STAT3, STK11, TRADD, TRAF2, PPARGC1A, TNFRSF1A, TNFRSF1B
Positive_139	INS, TNF, ABCC8, ADIPOQ, CACNA1A, CACNA1B, CACNA1C, CACNA1D, CACNA1E, CACNA1G, FRAP1, IKBKB, INSR, IRS1, IRS2, IRS4, KCNJ11, MAFA, MAPK1, MAPK10, MAPK3, MAPK8, MAPK9, PDX1, PIK3CA, PIK3CB, PIK3CD, PIK3CG, PIK3R1, PIK3R2, PIK3R3, PIK3R5, PRKCD, PRKCE, PRKCZ, SLC2A4, SOCS1, SOCS2, SOCS3, SOCS4
Positive_140	FAS, CD28, CD80, CD86, FASLG
Positive_141	INS, FOXA2, FOXA3, HES1, HHEX, HNF1A, HNF1B, HNF4A, HNF4G, IAPP, MAFA, MNX1, NEUROD1, NEUROG3, NKX2-2, NKX6-1, NR5A2, ONECUT1, PAX4, PAX6, PDX1, PKLR, SLC2A2
Positive_142	APP, APBB1, APOE, BACE1, BACE2, GAPDH, GSK3B, MAPT, NAE1
Positive_143	UBB, APAF1, CASP3, CASP9, CYCS, HTRA2, PPID, SLC25A4, SLC25A5, SLC25A6, UBA1, UBA7, UBE2L3, UBE2L6, VDAC1, VDAC2, VDAC3, SLC25A31
Positive_144	BAD, BAX, TNF, ALS2, APAF1, BCL2, BCL2L1, CASP3, CASP9, CYCS, DAXX, DERL1, MAP2K3, MAP2K6, MAP3K5, MAPK11, MAPK12, MAPK13, MAPK14, NEFH, NEFL, NEFM, PPP3CA, PPP3CB, PPP3CC, PPP3R1, PPP3R2, PRPH, PRPH2, RAB5A, RAC1, SOD1, TOMM40, TOMM40L, TNFRSF1A, TNFRSF1B
Positive_145	CASP3, CASP8, DCTN1, HAP1, HIP1, IFT57
Positive_146	INS, ATN1, BAIAP2, GAPDH, INSR, ITCH, MAGI1, MAGI2, RERE, WWP1, WWP2
Positive_147	APLP1, BCL2, GFAP, LAMC1, NFE2L2, PRNP, RPSA
Positive_148	ACTA1, ACTA2, ACTB, ACTC1, ACTG1, ACTG2, ADCY3, ADCY9, CFTR, ERO1L, GNAS, KCNQ1, PDIA4, PLCG1, PLCG2, PRKACA, PRKACB, PRKACG, PRKCA, PRKCB, PRKCG, PRKX, PRKY, SLC12A2
Positive_149	CSK, IL8, JUN, LYN, SRC, ADAM10, ADAM17, CCL5, CHUK, CXCL1, EGFR, F11R, GIT1, HBEGF, IGSF5, IKBKB, IKBKG, IL8RA, IL8RB, JAM2, JAM3, MAP2K4, MAP3K14, MAPK10, MAPK11, MAPK12, MAPK13, MAPK14, MAPK8, MAPK9, NFKB1, NFKBIA, NOD1, PAK1, PTPRZ1, RELA, TJP1
Positive_150	WAS, ACTB, ACTG1, ARHGEF2, ARPC5, ARPC5L, CD14, RHOA, ROCK1, ROCK2, TLR4, TUBA1A, TUBA1B, TUBA1C, TUBA3C, TUBA3E, TUBA4A, TUBA8, TUBAL3, TUBB, TUBB1, TUBB2A, TUBB2B, TUBB2C, TUBB3, TUBB4, TUBB4Q, TUBB6, WASL
Positive_151	WAS, ACTB, ACTG1, ARHGEF2, ARPC5, ARPC5L, CD14, RHOA, ROCK1, ROCK2, TLR4, TUBA1A, TUBA1B, TUBA1C, TUBA3C, TUBA3E, TUBA4A, TUBA8, TUBAL3, TUBB, TUBB1, TUBB2A, TUBB2B, TUBB2C, TUBB3, TUBB4, TUBB4Q, TUBB6, WASL

Positive_152	BAD,BAX,DCC,FOS,JUN,MYC,ACVR1B,ACVR1C,AKT1,AKT2,AKT3,APPL1,ARAF,BCL2,BIRC5,BRAF,CASP3,CASP9,CCND1,CTNNB1,GSK3B,KRAS,LEF1,MAP2K1,MAPK1,MAPK10,MAPK3,MAPK8,MAPK9,PIK3CA,PIK3CB,PIK3CD,PIK3CG,PIK3R1,PIK3R2,PIK3R3,PIK3R5,RAC1,RAC2,RAC3,RAF1,RALGDS,SMAD2,SMAD3,SMAD4,TCF7,TCF7L1,TCF7L2,TGFBR1,TGFBR2
Positive_153	CRK,HGF,JUN,MET,PGF,AKT1,AKT2,AKT3,ARAF,ARNT,ARNT2,BRAF,CDC42,CREBBP,CRKL,EGLN1,EGLN3,EP300,EPAS1,ETS1,FIGF,GAB1,GRB2,HIF1A,MAP2K1,MAP2K2,MAPK1,MAPK3,PAK1,PAK2,PAK3,PAK4,PAK6,PAK7,PDGFB,PIK3CA,PIK3CB,PIK3CD,PIK3CG,PIK3R1,PIK3R2,PIK3R3,PIK3R5,PTPN11,RAC1,RAF1,RAP1A,RAP1B,RAPGEF1,SLC2A1,SOS1,SOS2,TGFA,TGFB1,TGFB2,TGFB3,VEGFA,VEGFB,VEGFC
Positive_154	BAD,EGF,PGF,RB1,ACVR1B,ACVR1C,AKT1,AKT2,AKT3,ARAF,ARHGEF6,BCL2L1,BRAF,CASP9,CCND1,CDC42,CDK4,CDK6,CDKN2A,CHUK,E2F1,E2F2,E2F3,EGFR,ERBB2,FIGF,IKBKB,IKBKG,JAK1,KRAS,MAP2K1,MAPK1,MAPK10,MAPK3,MAPK8,MAPK9,NFKB1,PIK3CA,PIK3CB,PIK3CD,PIK3CG,PIK3R1,PIK3R2,PIK3R3,PIK3R5,PLD1,RAC1,RAC2,RAC3,RAF1,RALA,RALB,RALBP1,RALGDS,RELA,SMAD2,SMAD3,SMAD4,STAT1,STAT3,TGFA,TGFB1,TGFB2,TGFB3,TGFBR1,TGFBR2,VEGFA,VEGFB,VEGFC
Positive_155	BAD,EGF,ILK,MYC,AKT1,AKT2,AKT3,APC2,ARAF,AXIN1,AXIN2,BRAF,CASP9,CCND1,CTNNB1,EGFR,ELK1,FOXO3,GRB2,GSK3B,HRAS,KRAS,LEF1,MAP2K1,MAP2K2,MAPK1,MAPK3,NRAS,PDPK1,PIK3CA,PIK3CB,PIK3CD,PIK3CG,PIK3R1,PIK3R2,PIK3R3,PIK3R5,RAF1,SOS1,SOS2,TCF7,TCF7L1,TCF7L2
Positive_156	EGF,RB1,AKT1,AKT2,AKT3,ARAF,BRAF,CALML3,CALML5,CALML6,CAMK2A,CAMK2B,CAMK2D,CAMK2G,CCND1,CDK4,CDK6,CDKN1A,CDKN2A,E2F1,E2F2,E2F3,EGFR,FRAP1,GRB2,HRAS,IGF1,IGF1R,KRAS,MAP2K1,MAP2K2,MAPK1,MAPK3,MDM2,NRAS,PDGFA,PDGFB,PDGFRA,PDGFRB,PIK3CA,PIK3CB,PIK3CD,PIK3CG,PIK3R1,PIK3R2,PIK3R3,PIK3R5,PLCG1,PLCG2,PRKCA,PRKCB,PRKCG,RAF1,SHC1,SHC2,SHC3,SHC4,SOS1,SOS2,TGFA,TP53
Positive_157	AR,BAD,EGF,INS,RB1,AKT1,AKT2,AKT3,ARAF,ATF4,BCL2,BRAF,CASP9,CCND1,CCNE1,CCNE2,CDK2,CDKN1A,CDKN1B,CHUK,CREB1,CREB3,CREB3L1,CREB3L2,CREB3L3,CREB3L4,CREB5,CREBBP,CTNNB1,E2F1,E2F2,E2F3,EGFR,EP300,ERBB2,FGFR1,FGFR2,FOXO1,FRAP1,GRB2,GSK3B,HRAS,HSP90B1,IGF1,IGF1R,IKBKB,IKBKG,INSRR,KLK3,KRAS,LEF1,MAP2K1,MAP2K2,MAPK1,MAPK3,MDM2,NFKB1,NFKBIA,NKX3-1,NRAS,PDGFA,PDGFB,PDGFC,PDGFD,PDGFRA,PDGFRB,PDPK1,PIK3CA,PIK3CB,PIK3CD,PIK3CG,PIK3R1,PIK3R2,PIK3R3,PIK3R5,RAF1,RELA,SOS1,SOS2,TCF7,TCF7L1,TCF7L2,TGFA,TP53,HSP90AA1,HSP90AB1
Positive_158	MYC,RET,TFG,TPR,BRAF,CCDC6,CCND1,CDH1,CTNNB1,HRAS,KRAS,LEF1,MAP2K1,MAP2K2,MAPK1,MAPK3,NCOA4,NRAS,NTRK1,PAX8,PPARG,RXRA,RXRB,RXRG,TCF7,TCF7L1,TCF7L2,TPM3
Positive_159	SHH,BMP2,BMP4,CTNNB1,DVL1,DVL2,DVL3,FZD1,FZD10,FZD2,FZD3,FZD4,FZD5,FZD6,FZD7,FZD8,FZD9,GLI1,GLI2,GLI3,GSK3B,HHIP,LEF1,PTCH1,PTCH2,TCF7,TCF7L1,TCF7L2,WNT1,WNT10A,WNT10B,WNT11,WNT16,WNT2,WNT2B,WNT3,WNT3A,WNT4,WNT5A,WNT5B,WNT6,WNT7A,WNT7B,WNT8A,WNT8B,WNT9A,WNT9B
Positive_160	BAD,EGF,HGF,MET,RB1,AKT1,AKT2,AKT3,ARAF,BRAF,CCND1,CDK4,CDK6,CDKN1A,CDKN2A,E2F1,E2F2,E2F3,EGFR,FGF1,FGF10,FGF11,FGF12,FGF13,FGF14,FGF17,FGF18,FGF19,FGF2,FGF20,FGF21,FGF22,FGF23,FGF3,FGF4,FGF5,FGF6,FGF7,FGF8,FGF9,FGFR1,HRAS,IGF1,IGF1R,KRAS,MAP2K1,MAP2K2,MAPK1,MAPK3,MDM2,NRAS,PDGFA,PDGFB,PDGFC,PDGFD,PDGFRA,PDGFRB,PIK3CA,PIK3CB,PIK3CD,PIK3CG,PIK3R1,PIK3R2,PIK3R3,PIK3R5,RAF1,TP53
Positive_161	EGF,RB1,ARAF,BRAF,CCND1,CDK4,CDKN1A,CDKN2A,DAPK1,DAPK2,DAPK3,E2F1,E2F2,E2F3,EGFR,ERBB2,FGFR3,HRAS,KRAS,MAP2K1,MAP2K2,MAPK1,MAPK3,MDM2,NRAS,RAF1,RASSF1,RPS6KA5,TP53

Positive_162	BAD, BCR, CBL, CRK, MYC, RB1, ABL1, ACVR1B, ACVR1C, AKT1, AKT2, AKT3, ARAF, BCL2L1, BRAF, CBLB, CBLC, CCND1, CDK4, CDK6, CDKN1A, CDKN1B, CDKN2A, CHUK, CRKL, CTBP1, CTBP2, E2F1, E2F2, E2F3, EVI1, GRB2, HDAC1, HDAC2, HRAS, IKBKB, IKBKG, KRAS, MAP2K1, MAP2K2, MAPK1, MAPK3, MDM2, NFKB1, NFKBIA, NRAS, PIK3CA, PIK3CB, PIK3CD, PIK3CG, PIK3R1, PIK3R2, PIK3R3, PIK3R5, RAF1, RELA, RUNX1, SHC1, SHC2, SHC3, SHC4, SMAD3, SMAD4, SOS1, SOS2, STAT5A, STAT5B, TGFB1, TGFB2, TGFB3, TGFBR1, TGFBR2, TP53
Positive_163	BAD, JUP, KIT, MYC, PML, AKT1, AKT2, AKT3, ARAF, BRAF, CCNA1, CCND1, CEBPA, CHUK, FLT3, FRAP1, GRB2, HRAS, IKBKB, IKBKG, KRAS, LEF1, MAP2K1, MAP2K2, MAPK1, MAPK3, NFKB1, NRAS, PIK3CA, PIK3CB, PIK3CD, PIK3CG, PIK3R1, PIK3R2, PIK3R3, PIK3R5, PIM1, PIM2, PPARD, RAF1, RARA, RELA, RPS6KB1, RPS6KB2, RUNX1, RUNX1T1, SOS1, SOS2, SPI1, STAT3, STAT5A, STAT5B, TCF7, TCF7L1, TCF7L2, ZBTB16, EIF4EBP1
Positive_164	FN1, MAX, MYC, RB1, AKT1, AKT2, AKT3, APAF1, BCL2L1, BIRC2, BIRC3, CASP9, CCND1, CCNE1, CCNE2, CDK2, CDK4, CDK6, CDKN1B, CDKN2B, CHUK, CKS1B, COL4A1, COL4A2, COL4A4, COL4A6, CYCS, E2F1, E2F2, E2F3, IKBKB, IKBKG, ITGA2, ITGA2B, ITGA3, ITGA6, ITGAV, ITGB1, LAMA3, LAMA4, LAMB1, LAMB2, LAMB3, LAMB4, LAMC1, LAMC2, LAMC3, NFKB1, NFKBIA, NOS1, NOS3, PIAS1, PIAS2, PIAS3, PIAS4, PIK3CA, PIK3CB, PIK3CD, PIK3CG, PIK3R1, PIK3R2, PIK3R3, PIK3R5, PTGS2, PTK2, RELA, SKP2, TRAF1, TRAF2, TRAF3, TRAF4, TRAF5, TRAF6, XIAP
Positive_165	BAD, EGF, RB1, AKT1, AKT2, AKT3, ARAF, BRAF, CASP9, CCND1, CDK4, CDK6, CDKN2A, E2F1, E2F2, E2F3, EGFR, ERBB2, FOXO3, GRB2, HRAS, KRAS, MAP2K1, MAP2K2, MAPK1, MAPK3, NRAS, PDPK1, PIK3CA, PIK3CB, PIK3CD, PIK3CG, PIK3R1, PIK3R2, PIK3R3, PIK3R5, PLCG1, PLCG2, PRKCA, PRKCB, PRKCG, RAF1, RASSF1, RASSF5, SOS1, SOS2, STK4, TGFA
Positive_166	FAS, CD28, CD40, CD40LG, CD80, CD86, FASLG
Positive_167	C1S, C2, C3, C4B, C1QA, C1QB, C1QC, CD28, CD40, CD40LG, CD80, CD86
Positive_168	FAS, CD28, CD40, CD40LG, CD80, CD86, FASLG
Positive_169	FAS, CD28, CD80, CD86, FASLG, HLA-C, HLA-F, HLA-G, KIR2DL1, KIR2DL2, KIR2DL3, KIR3DL1, KIR3DL2, KLRC1, KLRD1, KIR2DL5A
