# Supplementary material for: Analysis of Protein Pathway Networks Using Hybrid Properties
Source: Molecules. 2010 Nov 12;15(11):8177–92. doi: 10.3390/molecules15118177 (PMC6259184; doi:10.3390/molecules15118177)
Supplement: Supplementary File 3 [file molecules-15-08177-s003.pdf]

Online Supporting Information S3: The vector representation of each positive regulatory pathway

Index graph_size,
graph_density,
in_degree_max,
in_degree_mean,
in_degree_median,
in_degree_variance,
out_degree_max,
out_degree_mean,
out_degree_median,
out_degree_variance,
weight_edge_mean(with_missing_edge),
weight_edge_variance(with_missing_edge),
weight_edge_mean(without_missing_edge),
weight_edge_variance(without_missing_edge),
topological_change_0.1_0.2,
topological_change_0.2_0.3,
topological_change_0.3_0.4,
topological_change_0.4_0.5,
topological_change_0.5_0.6,
topological_change_0.6_0.7,
topological_change_0.7_0.8,
in_degree_correlation_mean,
in_degree_correlation_max,
in_degree_correlation_variance,
out_degree_correlation_mean,
out_degree_correlation_max,
out_degree_correlation_variance,
in_clustering_mean,
in_clustering_max,
in_clustering_variance,
out_clustering_mean,
out_clustering_max,
out_clustering_variance,
in_in_topological_mean,
in_in_topological_max,
in_in_topological_variance,
in_out_topological_mean,

in_out_topological_max,
in_out_topological_variance,
out_in_topological_mean,
out_in_topological_max,
out_in_topological_variance,
out_out_topological_mean,
out_out_topological_max,
out_out_topological_variance,
first_singular_values,
second_singular_values,
third_singular_values,
in_local_density_0_max,
in_locta_density_0_mean,
in_local_density_0.1_max,
in_locta_density_0.1_mean,
in_local_density_0.2_max,
in_locta_density_0.2_mean,
in_local_density_0.3_max,
in_locta_density_0.3_mean,
in_local_density_0.4_max,
in_locta_density_0.4_mean,
in_local_density_0.5_max,
in_locta_density_0.5_mean,
in_local_density_0.6_max,
in_locta_density_0.6_mean,
in_local_density_0.7_max,
in_locta_density_0.7_mean,
in_local_density_0.8_max,
in_locta_density_0.8_mean,
in_local_density_0.9_max,
in_locta_density_0.9_mean,
out_local_density_0_max,
out_locta_density_0_mean,
out_local_density_0.1_max,
out_locta_density_0.1_mean,
out_local_density_0.2_max,
out_locta_density_0.2_mean,
out_local_density_0.3_max,
out_locta_density_0.3_mean,
out_local_density_0.4_max,
out_locta_density_0.4_mean,
out_local_density_0.5_max,
out_locta_density_0.5_mean,
out_local_density_0.6_max,

out_locta_density_0.6_mean,
out_local_density_0.7_max,
out_locta_density_0.7_mean,
out_local_density_0.8_max,
out_locta_density_0.8_mean,
out_local_density_0.9_max,
out_locta_density_0.9_mean,
hydrophobicity_composition_P_max,
hydrophobicity_composition_P_mean,
hydrophobicity_composition_N_max,
hydrophobicity_composition_N_mean,
hydrophobicity_composition_H_max,
hydrophobicity_composition_H_mean,
hydrophobicity_transition_PN_max,
hydrophobicity_transition_PN_mean,
hydrophobicity_transition_PH_max,
hydrophobicity_transition_PH_mean,
hydrophobicity_transition_NH_max,
hydrophobicity_transition_NH_mean,
hydrophobicity_distribution_P-0.0_max,
hydrophobicity_distribution_P-0.0_mean,
hydrophobicity_distribution_P-0.25_max,
hydrophobicity_distribution_P-0.25_mean,
hydrophobicity_distribution_P-0.5_max,
hydrophobicity_distribution_P-0.5_mean,
hydrophobicity_distribution_P-0.75_max,
hydrophobicity_distribution_P-0.75_mean,
hydrophobicity_distribution_P-1.0_max,
hydrophobicity_distribution_P-1.0_mean,
hydrophobicity_distribution_N-0.0_max,
hydrophobicity_distribution_N-0.0_mean,
hydrophobicity_distribution_N-0.25_max,
hydrophobicity_distribution_N-0.25_mean,
hydrophobicity_distribution_N-0.5_max,
hydrophobicity_distribution_N-0.5_mean,
hydrophobicity_distribution_N-0.75_max,
hydrophobicity_distribution_N-0.75_mean,
hydrophobicity_distribution_N-1.0_max,
hydrophobicity_distribution_N-1.0_mean,
hydrophobicity_distribution_H-0.0_max,
hydrophobicity_distribution_H-0.0_mean,
hydrophobicity_distribution_H-0.25_max,
hydrophobicity_distribution_H-0.25_mean,
hydrophobicity_distribution_H-0.5_max,

hydrophobicity_distribution_H-0.5_mean,
hydrophobicity_distribution_H-0.75_max,
hydrophobicity_distribution_H-0.75_mean,
hydrophobicity_distribution_H-1.0_max,
hydrophobicity_distribution_H-1.0_mean,
secondary_structure_composition_P_max,
secondary_structure_composition_P_mean,
secondary_structure_composition_N_max,
secondary_structure_composition_N_mean,
secondary_structure_composition_H_max,
secondary_structure_composition_H_mean,
secondary_structure_transition_PN_max,
secondary_structure_transition_PN_mean,
secondary_structure_transition_PH_max,
secondary_structure_transition_PH_mean,
secondary_structure_transition_NH_max,
secondary_structure_transition_NH_mean,
secondary_structure_distribution_P-0.0_max,
secondary_structure_distribution_P-0.0_mean,
secondary_structure_distribution_P-0.25_max,
secondary_structure_distribution_P-0.25_mean,
secondary_structure_distribution_P-0.5_max,
secondary_structure_distribution_P-0.5_mean,
secondary_structure_distribution_P-0.75_max,
secondary_structure_distribution_P-0.75_mean,
secondary_structure_distribution_P-1.0_max,
secondary_structure_distribution_P-1.0_mean,
secondary_structure_distribution_N-0.0_max,
secondary_structure_distribution_N-0.0_mean,
secondary_structure_distribution_N-0.25_max,
secondary_structure_distribution_N-0.25_mean,
secondary_structure_distribution_N-0.5_max,
secondary_structure_distribution_N-0.5_mean,
secondary_structure_distribution_N-0.75_max,
secondary_structure_distribution_N-0.75_mean,
secondary_structure_distribution_N-1.0_max,
secondary_structure_distribution_N-1.0_mean,
secondary_structure_distribution_H-0.0_max,
secondary_structure_distribution_H-0.0_mean,
secondary_structure_distribution_H-0.25_max,
secondary_structure_distribution_H-0.25_mean,
secondary_structure_distribution_H-0.5_max,
secondary_structure_distribution_H-0.5_mean,
secondary_structure_distribution_H-0.75_max,

secondary_structure_distribution_H-0.75_mean,
secondary_structure_distribution_H-1.0_max,
secondary_structure_distribution_H-1.0_mean,
solvent_accessibility_composition_H_max,
solvent_accessibility_composition_H_mean,
solvent_accessibility_transition_HE_max,
solvent_accessibility_transition_HE_mean,
solvent_accessibility_distribution_H-0.0_max,
solvent_accessibility_distribution_H-0.0_mean,
solvent_accessibility_distribution_H-0.25_max,
solvent_accessibility_distribution_H-0.25_mean,
solvent_accessibility_distribution_H-0.5_max,
solvent_accessibility_distribution_H-0.5_mean,
solvent_accessibility_distribution_H-0.75_max,
solvent_accessibility_distribution_H-0.75_mean,
solvent_accessibility_distribution_H-1.0_max,
solvent_accessibility_distribution_H-1.0_mean,
VanDerWaal_composition_P_max,
VanDerWaal_composition_P_mean,
VanDerWaal_composition_N_max,
VanDerWaal_composition_N_mean,
VanDerWaal_composition_H_max,
VanDerWaal_composition_H_mean,
VanDerWaal_transition_PN_max,
VanDerWaal_transition_PN_mean,
VanDerWaal_transition_PH_max,
VanDerWaal_transition_PH_mean,
VanDerWaal_transition_NH_max,
VanDerWaal_transition_NH_mean,
VanDerWaal_distribution_P-0.0_max,
VanDerWaal_distribution_P-0.0_mean,
VanDerWaal_distribution_P-0.25_max,
VanDerWaal_distribution_P-0.25_mean,
VanDerWaal_distribution_P-0.5_max,
VanDerWaal_distribution_P-0.5_mean,
VanDerWaal_distribution_P-0.75_max,
VanDerWaal_distribution_P-0.75_mean,
VanDerWaal_distribution_P-1.0_max,
VanDerWaal_distribution_P-1.0_mean,
VanDerWaal_distribution_N-0.0_max,
VanDerWaal_distribution_N-0.0_mean,
VanDerWaal_distribution_N-0.25_max,
VanDerWaal_distribution_N-0.25_mean,
VanDerWaal_distribution_N-0.5_max,

VanDerWaal_distribution_N-0.5_mean,
VanDerWaal_distribution_N-0.75_max,
VanDerWaal_distribution_N-0.75_mean,
VanDerWaal_distribution_N-1.0_max,
VanDerWaal_distribution_N-1.0_mean,
VanDerWaal_distribution_H-0.0_max,
VanDerWaal_distribution_H-0.0_mean,
VanDerWaal_distribution_H-0.25_max,
VanDerWaal_distribution_H-0.25_mean,
VanDerWaal_distribution_H-0.5_max,
VanDerWaal_distribution_H-0.5_mean,
VanDerWaal_distribution_H-0.75_max,
VanDerWaal_distribution_H-0.75_mean,
VanDerWaal_distribution_H-1.0_max,
VanDerWaal_distribution_H-1.0_mean,
polarity_composition_P_max,
polarity_composition_P_mean,
polarity_composition_N_max,
polarity_composition_N_mean,
polarity_composition_H_max,
polarity_composition_H_mean,
polarity_transition_PN_max,
polarity_transition_PN_mean,
polarity_transition_PH_max,
polarity_transition_PH_mean,
polarity_transition_NH_max,
polarity_transition_NH_mean,
polarity_distribution_P-0.0_max,
polarity_distribution_P-0.0_mean,
polarity_distribution_P-0.25_max,
polarity_distribution_P-0.25_mean,
polarity_distribution_P-0.5_max,
polarity_distribution_P-0.5_mean,
polarity_distribution_P-0.75_max,
polarity_distribution_P-0.75_mean,
polarity_distribution_P-1.0_max,
polarity_distribution_P-1.0_mean,
polarity_distribution_N-0.0_max,
polarity_distribution_N-0.0_mean,
polarity_distribution_N-0.25_max,
polarity_distribution_N-0.25_mean,
polarity_distribution_N-0.5_max,
polarity_distribution_N-0.5_mean,
polarity_distribution_N-0.75_max,

polarity_distribution_N-0.75_mean,
polarity_distribution_N-1.0_max,
polarity_distribution_N-1.0_mean,
polarity_distribution_H-0.0_max,
polarity_distribution_H-0.0_mean,
polarity_distribution_H-0.25_max,
polarity_distribution_H-0.25_mean,
polarity_distribution_H-0.5_max,
polarity_distribution_H-0.5_mean,
polarity_distribution_H-0.75_max,
polarity_distribution_H-0.75_mean,
polarity_distribution_H-1.0_max,
polarity_distribution_H-1.0_mean,
polarizability_composition_P_max,
polarizability_composition_P_mean,
polarizability_composition_N_max,
polarizability_composition_N_mean,
polarizability_composition_H_max,
polarizability_composition_H_mean,
polarizability_transition_PN_max,
polarizability_transition_PN_mean,
polarizability_transition_PH_max,
polarizability_transition_PH_mean,
polarizability_transition_NH_max,
polarizability_transition_NH_mean,
polarizability_distribution_P-0.0_max,
polarizability_distribution_P-0.0_mean,
polarizability_distribution_P-0.25_max,
polarizability_distribution_P-0.25_mean,
polarizability_distribution_P-0.5_max,
polarizability_distribution_P-0.5_mean,
polarizability_distribution_P-0.75_max,
polarizability_distribution_P-0.75_mean,
polarizability_distribution_P-1.0_max,
polarizability_distribution_P-1.0_mean,
polarizability_distribution_N-0.0_max,
polarizability_distribution_N-0.0_mean,
polarizability_distribution_N-0.25_max,
polarizability_distribution_N-0.25_mean,
polarizability_distribution_N-0.5_max,
polarizability_distribution_N-0.5_mean,
polarizability_distribution_N-0.75_max,
polarizability_distribution_N-0.75_mean,
polarizability_distribution_N-1.0_max,

polarizability_distribution_N-1.0_mean,
polarizability_distribution_H-0.0_max,
polarizability_distribution_H-0.0_mean,
polarizability_distribution_H-0.25_max,
polarizability_distribution_H-0.25_mean,
polarizability_distribution_H-0.5_max,
polarizability_distribution_H-0.5_mean,
polarizability_distribution_H-0.75_max,
polarizability_distribution_H-0.75_mean,
polarizability_distribution_H-1.0_max,
polarizability_distribution_H-1.0_mean,
AA_composition_R_max,
AA_composition_R_mean,
AA_composition_K_max,
AA_composition_K_mean,
AA_composition_E_max,
AA_composition_E_mean,
AA_composition_D_max,
AA_composition_D_mean,
AA_composition_Q_max,
AA_composition_Q_mean,
AA_composition_N_max,
AA_composition_N_mean,
AA_composition_G_max,
AA_composition_G_mean,
AA_composition_A_max,
AA_composition_A_mean,
AA_composition_S_max,
AA_composition_S_mean,
AA_composition_T_max,
AA_composition_T_mean,
AA_composition_P_max,
AA_composition_P_mean,
AA_composition_H_max,
AA_composition_H_mean,
AA_composition_Y_max,
AA_composition_Y_mean,
AA_composition_C_max,
AA_composition_C_mean,
AA_composition_V_max,
AA_composition_V_mean,
AA_composition_L_max,
AA_composition_L_mean,
AA_composition_I_max,

AA_composition_I_mean,
AA_composition_M_max,
AA_composition_M_mean,
AA_composition_F_max,
AA_composition_F_mean,
AA_composition_W_max,
AA_composition_W_mean

Positive_1 61.000000,0.062618,11.000000,3.819672,3.000000,9.416940,9.000000,3.819672,3.000000,10.116940,
0.486452,0.024906,0.486452,0.024906,0.030043,0.057522,0.276995,0.396104,0.408602,0.436364,
0.870968,0.353257,1.571429,0.253310,0.277049,2.333333,0.312692,0.080789,0.250000,0.012571,
0.045659,0.388889,0.008787,0.071885,0.133333,0.002083,0.038378,0.166667,0.001744,0.044290,
0.133333,0.001714,0.078229,0.166667,0.002941,8.623475,5.708297,5.114596,1.000000,0.770492,
1.000000,0.770492,1.000000,0.770194,1.000000,0.766617,1.000000,0.690063,1.000000,0.538167,
1.000000,0.382178,1.000000,0.311316,1.000000,0.170405,0.666667,0.119304,1.000000,0.704918,
1.000000,0.704918,1.000000,0.699688,1.000000,0.671468,1.000000,0.617590,1.000000,0.453578,
1.000000,0.396500,1.000000,0.311684,1.000000,0.255998,1.000000,0.115561,0.382239,0.297323,
0.446677,0.377283,0.436975,0.325394,0.271095,0.213854,0.259825,0.201869,0.339888,0.247693,
0.036923,0.011125,0.293179,0.230737,0.582888,0.496531,0.798258,0.743098,1.000000,0.994760,
0.032468,0.007041,0.350649,0.257766,0.662338,0.502340,0.826923,0.744524,1.000000,0.994703,
0.006494,0.002453,0.286252,0.243898,0.574961,0.489792,0.789799,0.741433,1.000000,0.994435,
0.580499,0.415704,0.406433,0.185815,0.728291,0.398481,0.164223,0.085062,0.109515,0.076515,
0.013369,0.005162,0.006494,0.002453,0.564935,0.245657,0.662338,0.486521,0.845475,0.734817,
1.000000,0.999853,0.525974,0.062915,0.545455,0.238069,0.642857,0.472566,0.928571,0.724351,
0.998221,0.936831,0.181287,0.044168,0.554869,0.274556,0.701700,0.499729,0.883152,0.753023,
1.000000,0.969895,0.747899,0.627127,0.418269,0.346320,0.032544,0.011597,0.343195,0.266635,
0.561205,0.502179,0.805255,0.744579,1.000000,0.987585,0.474498,0.399545,0.451737,0.366175,
0.318182,0.234280,0.354545,0.292168,0.235925,0.185190,0.217054,0.167510,0.022680,0.006371,
0.357143,0.255247,0.675325,0.500286,0.799451,0.740157,1.000000,0.995180,0.036145,0.011288,
0.281567,0.239316,0.586614,0.494346,0.800525,0.742970,1.000000,0.996094,0.006494,0.002453,
0.310256,0.238905,0.588757,0.499064,0.834225,0.748355,1.000000,0.995728,0.481793,0.351889,
0.418856,0.329173,0.413127,0.318938,0.286863,0.235359,0.290698,0.231621,0.242370,0.199450,
0.006494,0.002453,0.281567,0.248522,0.562597,0.492481,0.785162,0.741507,1.000000,0.995440,
0.051948,0.007360,0.396104,0.253748,0.694805,0.501895,0.826923,0.741337,1.000000,0.994344,
0.027692,0.010340,0.293179,0.233180,0.578804,0.498268,0.797436,0.743857,1.000000,0.994886,
0.378947,0.332085,0.509653,0.433635,0.318182,0.234280,0.356037,0.283968,0.193798,0.155731,
0.248366,0.196969,0.022680,0.006931,0.350649,0.251012,0.694805,0.497870,0.837662,0.743283,
1.000000,0.994818,0.025974,0.010032,0.312253,0.249123,0.586614,0.495087,0.820059,0.742793,
1.000000,0.996506,0.006494,0.002453,0.310256,0.238905,0.588757,0.499064,0.834225,0.748355,
1.000000,0.995728,0.076655,0.045783,0.103118,0.065679,0.104247,0.064019,0.071006,0.049111,

0.063922,0.034846,0.057915,0.037885,0.113949,0.086835,0.120879,0.084540,0.090361,0.058481,
0.082902,0.053118,0.106646,0.046199,0.051948,0.021615,0.053097,0.026495,0.043478,0.021261,
0.113772,0.079676,0.142857,0.089314,0.096386,0.060435,0.049462,0.024811,0.077922,0.036726,
0.038961,0.013172

Positive_2 30.000000,0.111111,7.000000,3.333333,3.000000,5.057471,6.000000,3.333333,3.000000,2.919540,
0.618338,0.029317,0.618338,0.029317,0.000000,0.010000,0.101010,0.123596,0.371795,0.387755,
0.500000,0.749524,3.000000,1.049182,0.877778,3.000000,1.300868,0.145392,0.600000,0.038418,
0.190556,0.750000,0.066893,0.098183,0.172414,0.002208,0.069622,0.137931,0.001863,0.075613,
0.172414,0.001843,0.120115,0.206897,0.003872,5.099020,4.640324,3.806286,1.000000,0.733333,
1.000000,0.733333,1.000000,0.733333,1.000000,0.730159,1.000000,0.610794,1.000000,0.564286,
1.000000,0.388254,1.000000,0.302540,0.666667,0.150794,0.200000,0.059206,1.000000,0.833333,
1.000000,0.833333,1.000000,0.833333,1.000000,0.817778,1.000000,0.773333,1.000000,0.721111,
1.000000,0.658889,1.000000,0.596667,1.000000,0.460000,1.000000,0.180000,0.349537,0.289648,
0.480000,0.396265,0.377358,0.314087,0.259861,0.220917,0.248260,0.190137,0.398734,0.253074,
0.046667,0.015026,0.295082,0.244217,0.561538,0.498412,0.810651,0.747386,1.000000,0.994924,
0.015385,0.005928,0.340000,0.246418,0.553333,0.483143,0.791383,0.736258,1.000000,0.993385,
0.006667,0.002433,0.293737,0.248372,0.574961,0.503148,0.809935,0.749132,1.000000,0.997079,
0.580499,0.432275,0.245580,0.164641,0.620172,0.403084,0.127953,0.075270,0.101266,0.077553,
0.011893,0.003870,0.006667,0.002433,0.333333,0.227860,0.600000,0.462143,0.802721,0.729018,
1.000000,1.000000,0.433255,0.096618,0.648712,0.280105,0.859485,0.478633,0.906323,0.664353,
0.999151,0.877998,0.058957,0.013764,0.554869,0.273522,0.701700,0.522223,0.866559,0.768851,
0.997788,0.982473,0.792453,0.614424,0.441558,0.337274,0.027322,0.009178,0.343931,0.270949,
0.596026,0.517025,0.818985,0.741021,0.999151,0.990282,0.474498,0.409185,0.425926,0.350952,
0.293333,0.239863,0.354545,0.290075,0.234409,0.193249,0.205811,0.170123,0.015385,0.006241,
0.320000,0.245024,0.533333,0.487609,0.777778,0.732713,1.000000,0.994642,0.026667,0.009820,
0.300578,0.247512,0.560706,0.498996,0.794872,0.747769,1.000000,0.996947,0.006667,0.002433,
0.307692,0.238404,0.557377,0.499552,0.819527,0.751468,1.000000,0.995170,0.408805,0.341825,
0.418856,0.342822,0.362319,0.315353,0.329114,0.236804,0.273438,0.227126,0.243011,0.206485,
0.006667,0.002433,0.288312,0.248169,0.596026,0.504862,0.805031,0.748123,1.000000,0.997230,
0.015385,0.006293,0.320000,0.240040,0.532880,0.476481,0.802721,0.731205,1.000000,0.993142,
0.046667,0.014869,0.296407,0.242907,0.556410,0.499804,0.798817,0.748244,1.000000,0.994955,
0.375723,0.336799,0.474537,0.423338,0.293333,0.239863,0.356037,0.283979,0.206452,0.163391,
0.255034,0.199981,0.015385,0.006614,0.313333,0.241070,0.559748,0.490145,0.788804,0.737006,
1.000000,0.994145,0.026667,0.009234,0.306358,0.252635,0.560241,0.494695,0.792308,0.739979,
1.000000,0.997390,0.006667,0.002433,0.307692,0.238404,0.557377,0.499552,0.819527,0.751468,
1.000000,0.995170,0.074941,0.049578,0.092814,0.061945,0.083601,0.057758,0.070796,0.046639,
0.053996,0.034811,0.066158,0.038918,0.127168,0.083671,0.144654,0.094218,0.106667,0.060060,
0.072643,0.052210,0.106646,0.052662,0.066667,0.025706,0.050000,0.027738,0.050000,0.019723,
0.099338,0.073213,0.169811,0.087249,0.083815,0.059005,0.041558,0.028818,0.051485,0.035167,
0.031447,0.010912

Positive_3 25.000000,0.198400,13.000000,4.960000,6.000000,10.790000,14.000000,4.960000,3.000000,20.123333,
0.458484,0.020328,0.458484,0.020328,0.064516,0.086207,0.216981,0.445783,0.630435,0.647059,
0.833333,1.406722,4.769231,1.797928,1.159524,3.857143,1.628504,0.218842,0.500000,0.034382,
0.171835,0.551020,0.024720,0.282770,0.468750,0.027318,0.183018,0.312500,0.009756,0.166210,

0.541667,0.023287,0.228353,0.395833,0.017610,7.940723,4.812392,3.802585,1.000000,0.800000,
1.000000,0.800000,1.000000,0.737949,1.000000,0.653875,1.000000,0.567707,1.000000,0.350410,
1.000000,0.216059,1.000000,0.136505,0.178571,0.073363,0.071429,0.021062,1.000000,0.800000,
1.000000,0.800000,1.000000,0.779048,1.000000,0.750330,1.000000,0.679963,1.000000,0.541612,
1.000000,0.218718,1.000000,0.153040,1.000000,0.094103,0.055556,0.012527,0.348624,0.300124,
0.437984,0.387690,0.349057,0.312186,0.271095,0.225126,0.223975,0.185318,0.287938,0.243069,
0.062016,0.012625,0.344961,0.244808,0.531008,0.480748,0.798450,0.739461,1.000000,0.991386,
0.012862,0.006104,0.324176,0.245511,0.607143,0.501283,0.826923,0.746647,1.000000,0.996927,
0.005618,0.002466,0.360129,0.249204,0.591640,0.500054,0.803859,0.738864,1.000000,0.994533,
0.511254,0.403907,0.258007,0.172554,0.587537,0.423540,0.112903,0.073309,0.116071,0.080301,
0.009677,0.003567,0.005618,0.002466,0.294564,0.232477,0.563798,0.497745,0.796703,0.741911,
1.000000,1.000000,0.301887,0.062100,0.498392,0.276736,0.630225,0.492361,0.839228,0.666510,
0.998221,0.921867,0.196141,0.033703,0.403727,0.235960,0.621118,0.488813,0.854037,0.755137,
0.997472,0.973366,0.690209,0.624821,0.441640,0.344595,0.038576,0.013465,0.343195,0.262930,
0.550179,0.501695,0.772189,0.727974,0.997472,0.987456,0.461240,0.408292,0.398754,0.360365,
0.286239,0.231343,0.346304,0.295768,0.223529,0.182478,0.238971,0.168067,0.033708,0.006804,
0.321429,0.244243,0.565934,0.495196,0.799451,0.735038,1.000000,0.997432,0.031008,0.012652,
0.347267,0.248016,0.538760,0.491891,0.793624,0.746808,1.000000,0.995460,0.005618,0.002466,
0.310256,0.250925,0.636656,0.514271,0.839228,0.748596,1.000000,0.995927,0.364780,0.339642,
0.403101,0.336603,0.372477,0.323755,0.291829,0.229935,0.265537,0.217694,0.242370,0.209068,
0.005618,0.002466,0.369775,0.255888,0.594855,0.505028,0.803859,0.743686,1.000000,0.996494,
0.033708,0.007002,0.315934,0.241328,0.620879,0.494685,0.826923,0.737999,1.000000,0.996524,
0.062016,0.010335,0.372093,0.246497,0.554264,0.483034,0.806202,0.745494,1.000000,0.993764,
0.385093,0.348090,0.476744,0.420567,0.286239,0.231343,0.350282,0.293707,0.188797,0.154962,
0.273897,0.195583,0.033708,0.009204,0.307692,0.236313,0.543956,0.488219,0.826923,0.733443,
1.000000,0.994881,0.028090,0.009977,0.311897,0.255480,0.542636,0.493622,0.820059,0.746116,
1.000000,0.996614,0.005618,0.002466,0.310256,0.250925,0.636656,0.514271,0.839228,0.748596,
1.000000,0.995927,0.078899,0.049760,0.089021,0.058881,0.077151,0.060027,0.071006,0.053253,
0.065740,0.040860,0.051447,0.037343,0.118971,0.079529,0.139535,0.099374,0.080537,0.062995,
0.077640,0.052938,0.093023,0.041766,0.044248,0.023631,0.053097,0.027456,0.028302,0.018436,
0.093458,0.073347,0.143411,0.087497,0.100629,0.061290,0.067416,0.025567,0.053381,0.035849,
0.022472,0.010199

Positive_4 16.000000,0.082031,10.000000,1.312500,1.000000,5.562500,9.000000,1.312500,1.000000,4.362500,
0.271329,0.019283,0.271329,0.019283,0.400000,0.250000,0.555556,0.750000,1.000000,0.000000,
0.000000,0.000000,0.000000,0.000000,0.000000,0.000000,0.000000,0.000000,0.000000,0.000000,
0.000000,0.000000,0.000000,0.300000,0.533333,0.074667,0.003750,0.060000,0.000225,0.004167,
0.066667,0.000278,0.375000,0.600000,0.090000,3.162278,3.000000,1.000000,1.000000,0.062500,
1.000000,0.062500,1.000000,0.062500,0.977778,0.061111,0.800000,0.050000,0.755556,0.047222,
0.600000,0.037500,0.488889,0.030556,0.466667,0.029167,0.288889,0.018056,1.000000,0.062500,
1.000000,0.062500,1.000000,0.062500,1.000000,0.062500,1.000000,0.062500,0.944444,0.059028,
0.750000,0.046875,0.611111,0.038194,0.583333,0.036458,0.361111,0.022569,0.342052,0.287799,
0.423507,0.352045,0.398866,0.360156,0.237323,0.198144,0.262097,0.211889,0.292181,0.251978,
0.039326,0.009054,0.273279,0.243956,0.540707,0.506367,0.804916,0.754996,1.000000,0.997395,
0.024590,0.007779,0.313093,0.266929,0.537879,0.508687,0.779851,0.740069,1.000000,0.993705,

0.005618,0.002334,0.261569,0.233965,0.539235,0.476998,0.772636,0.738941,1.000000,0.988685,
0.519201,0.401387,0.273425,0.165362,0.500000,0.433251,0.126984,0.069073,0.102881,0.077717,
0.010081,0.001570,0.005618,0.002334,0.335443,0.286725,0.560127,0.497036,0.813291,0.722044,
1.000000,1.000000,0.197183,0.048380,0.476861,0.179452,0.649899,0.474390,0.905433,0.665113,
0.993964,0.842237,0.119403,0.027352,0.330224,0.232237,0.658986,0.481862,0.848315,0.798593,
0.996269,0.971808,0.672131,0.636274,0.429379,0.342358,0.020492,0.009806,0.286885,0.261633,
0.549180,0.492205,0.757296,0.723007,1.000000,0.983770,0.434701,0.360288,0.420523,0.368189,
0.317580,0.271523,0.325103,0.266316,0.225379,0.201600,0.222011,0.193936,0.033708,0.008804,
0.292220,0.259081,0.534091,0.506933,0.770624,0.745320,1.000000,0.996506,0.028090,0.009411,
0.258065,0.227556,0.511066,0.479235,0.747191,0.727108,1.000000,0.987835,0.005618,0.002334,
0.287879,0.249243,0.610656,0.512363,0.819887,0.765776,1.000000,0.997734,0.427221,0.389497,
0.364754,0.295238,0.364185,0.315265,0.275720,0.231382,0.294355,0.247184,0.226337,0.186650,
0.005618,0.002334,0.277666,0.238299,0.515091,0.472035,0.768612,0.736530,1.000000,0.988685,
0.033708,0.009184,0.309298,0.257576,0.537736,0.507754,0.779851,0.732055,1.000000,0.992311,
0.012658,0.007299,0.279352,0.246271,0.548387,0.521046,0.798771,0.763391,1.000000,0.997755,
0.360656,0.291091,0.490506,0.437387,0.317580,0.271523,0.350282,0.257110,0.189300,0.159192,
0.268939,0.236343,0.033708,0.009184,0.296015,0.246224,0.563567,0.505528,0.776660,0.749063,
1.000000,0.994862,0.028090,0.009411,0.251509,0.236988,0.509054,0.487519,0.751866,0.728118,
1.000000,0.990273,0.005618,0.002334,0.287879,0.249243,0.610656,0.512363,0.819887,0.765776,
1.000000,0.997734,0.061475,0.040981,0.080483,0.065799,0.072785,0.054625,0.060729,0.047301,
0.057836,0.037051,0.068410,0.042042,0.090164,0.060707,0.106557,0.066270,0.091418,0.067789,
0.065574,0.049023,0.063291,0.051449,0.039400,0.027466,0.056836,0.029341,0.024291,0.017749,
0.131148,0.079982,0.120075,0.097846,0.082996,0.056643,0.067416,0.032918,0.077505,0.054177,
0.030246,0.020841

Positive_5 36.000000,0.125000,12.000000,4.500000,4.500000,9.800000,13.000000,4.500000,3.000000,15.114286,
0.440187,0.031259,0.440187,0.031259,0.055556,0.189542,0.298387,0.298851,0.360656,0.743590,
0.800000,0.751852,2.666667,0.762314,0.573168,2.666667,0.837240,0.128302,0.296296,0.013973,
0.074609,0.500000,0.013549,0.168492,0.342857,0.013851,0.095370,0.182857,0.004737,0.104768,
0.371429,0.012710,0.137869,0.314286,0.007996,8.538681,5.388625,3.726778,1.000000,0.805556,
1.000000,0.805556,1.000000,0.775926,1.000000,0.589689,1.000000,0.490542,0.857143,0.391991,
0.642857,0.272655,0.500000,0.208995,0.333333,0.139177,0.333333,0.084103,1.000000,0.861111,
1.000000,0.861111,1.000000,0.859329,1.000000,0.789520,1.000000,0.707594,1.000000,0.597704,
1.000000,0.440659,1.000000,0.406283,1.000000,0.371978,1.000000,0.331259,0.369919,0.314281,
0.435484,0.368292,0.377261,0.317427,0.264463,0.221882,0.259825,0.203813,0.276573,0.233898,
0.048780,0.011548,0.289744,0.247109,0.572581,0.491349,0.809140,0.740211,1.000000,0.994161,
0.033557,0.006232,0.324176,0.241222,0.607143,0.502420,0.826923,0.751953,1.000000,0.996955,
0.008065,0.002326,0.295117,0.246836,0.562406,0.495102,0.787686,0.743201,1.000000,0.992541,
0.580645,0.430045,0.335917,0.183166,0.464286,0.386788,0.155440,0.078010,0.105882,0.076437,
0.011236,0.004189,0.008065,0.002326,0.335443,0.236963,0.661290,0.508254,0.875000,0.764861,
1.000000,1.000000,0.163399,0.043351,0.602564,0.245403,0.666667,0.468021,0.879433,0.681035,
0.998445,0.925396,0.140940,0.038673,0.434174,0.270424,0.640860,0.486622,0.869128,0.723609,
0.998917,0.935396,0.664541,0.590912,0.442553,0.363812,0.048387,0.013416,0.343195,0.270905,
0.564516,0.508479,0.791398,0.738346,1.000000,0.990116,0.451613,0.385487,0.416021,0.369672,
0.286634,0.244840,0.344353,0.281640,0.220408,0.180346,0.246809,0.189272,0.026846,0.005852,

0.321429,0.239203,0.565934,0.494790,0.809615,0.747375,1.000000,0.996064,0.048387,0.012869,
0.297240,0.248172,0.539850,0.496154,0.793510,0.735587,1.000000,0.995723,0.008065,0.002326,
0.330645,0.248913,0.588757,0.498670,0.817949,0.746904,1.000000,0.994501,0.395349,0.350461,
0.365385,0.310340,0.382114,0.339198,0.261708,0.216377,0.304255,0.243739,0.250674,0.200354,
0.008065,0.002326,0.291729,0.251925,0.559398,0.498233,0.780451,0.741134,1.000000,0.994197,
0.033557,0.006367,0.315934,0.228134,0.620879,0.500173,0.842308,0.748770,1.000000,0.995747,
0.048780,0.010285,0.314516,0.251582,0.564516,0.493099,0.803763,0.742837,1.000000,0.995163,
0.365462,0.318724,0.509044,0.436436,0.286634,0.244840,0.346774,0.278741,0.186136,0.149082,
0.277628,0.220536,0.033557,0.006837,0.307692,0.231856,0.550000,0.486370,0.826923,0.738859,
1.000000,0.994750,0.024194,0.008696,0.299363,0.250976,0.550000,0.500965,0.820059,0.744966,
1.000000,0.996757,0.008065,0.002326,0.330645,0.248913,0.588757,0.498670,0.817949,0.746904,
1.000000,0.994501,0.076759,0.054165,0.085366,0.059828,0.103226,0.068581,0.080645,0.053337,
0.063922,0.041208,0.053430,0.037162,0.105128,0.075330,0.120879,0.076535,0.101089,0.063553,
0.070513,0.049969,0.078571,0.044954,0.056452,0.024918,0.061571,0.033034,0.040323,0.021810,
0.104027,0.075167,0.138376,0.093580,0.069921,0.053974,0.049462,0.023391,0.076336,0.038866,
0.021218,0.010639

Positive_6 23.000000,0.130435,9.000000,3.000000,3.000000,6.909091,9.000000,3.000000,2.000000,5.727273,
0.374599,0.030897,0.374599,0.030897,0.057971,0.384615,0.500000,0.250000,0.466667,0.375000,
0.600000,0.581643,1.888889,0.507942,0.602053,3.000000,0.749642,0.143033,0.500000,0.033255,
0.159784,0.600000,0.034269,0.158893,0.318182,0.015184,0.150285,0.409091,0.020043,0.161739,
0.318182,0.013885,0.178910,0.363636,0.008063,5.215355,3.833512,2.885756,1.000000,0.652174,
1.000000,0.652174,1.000000,0.644928,1.000000,0.528502,1.000000,0.289855,1.000000,0.181159,
0.666667,0.115459,0.666667,0.096618,0.333333,0.036232,0.166667,0.016908,1.000000,0.739130,
1.000000,0.739130,1.000000,0.734300,1.000000,0.606970,1.000000,0.373430,1.000000,0.294928,
1.000000,0.287819,1.000000,0.255556,1.000000,0.052657,0.000000,0.000000,0.346237,0.291781,
0.438025,0.375628,0.436975,0.332591,0.264550,0.206150,0.262097,0.201782,0.339888,0.250873,
0.014177,0.007326,0.408602,0.247619,0.604839,0.503365,0.825269,0.735661,1.000000,0.994694,
0.042254,0.008850,0.350649,0.260317,0.662338,0.490453,0.806452,0.744024,1.000000,0.993856,
0.007042,0.002307,0.308707,0.242440,0.539235,0.492811,0.772636,0.738709,1.000000,0.997278,
0.618090,0.435187,0.300875,0.182475,0.728291,0.382338,0.126984,0.080685,0.134752,0.067540,
0.011527,0.003912,0.007042,0.002307,0.564935,0.263064,0.662338,0.488009,0.813291,0.731790,
1.000000,1.000000,0.525974,0.095540,0.545455,0.281021,0.769231,0.504081,0.928571,0.722190,
0.999461,0.938163,0.079114,0.019792,0.421225,0.249850,0.640860,0.482104,0.822319,0.717513,
0.998917,0.942651,0.747899,0.609541,0.425532,0.349579,0.028169,0.012312,0.343008,0.263469,
0.554090,0.495378,0.791398,0.734328,1.000000,0.990320,0.436224,0.388919,0.420523,0.362699,
0.318182,0.248382,0.331887,0.279338,0.227414,0.187561,0.217742,0.179818,0.042254,0.007515,
0.357143,0.260913,0.675325,0.493639,0.805369,0.752200,1.000000,0.996766,0.035211,0.008694,
0.283920,0.233002,0.566434,0.495715,0.793067,0.731493,1.000000,0.997032,0.007042,0.002307,
0.419598,0.254130,0.605528,0.501999,0.798995,0.738701,1.000000,0.993156,0.481793,0.367057,
0.382653,0.314107,0.367742,0.318836,0.278090,0.232765,0.294355,0.241217,0.232804,0.184661,
0.007042,0.002307,0.329815,0.250701,0.525126,0.493079,0.768612,0.738004,1.000000,0.997278,
0.051948,0.009697,0.396104,0.258358,0.694805,0.490117,0.808602,0.748815,1.000000,0.992712,
0.014085,0.006947,0.408602,0.246552,0.591549,0.505998,0.819892,0.740891,1.000000,0.994846,
0.362947,0.316320,0.490506,0.435298,0.318182,0.248382,0.338395,0.272886,0.195742,0.152747,

0.266667,0.214631,0.077465,0.010334,0.350649,0.258462,0.694805,0.500866,0.837662,0.759031,
1.000000,0.993506,0.035211,0.008290,0.258794,0.237273,0.525210,0.490804,0.782563,0.734538,
1.000000,0.998512,0.007042,0.002307,0.419598,0.254130,0.605528,0.501999,0.798995,0.738701,
1.000000,0.993156,0.091398,0.051333,0.084507,0.047804,0.103226,0.057688,0.084507,0.053789,
0.068602,0.043384,0.068410,0.037783,0.106322,0.075899,0.122449,0.069675,0.084034,0.065552,
0.071082,0.051405,0.089710,0.051576,0.051948,0.027055,0.048744,0.034466,0.056338,0.021023,
0.098039,0.069033,0.142857,0.104467,0.098592,0.050344,0.049462,0.025585,0.077922,0.045275,
0.039627,0.016864

Positive_7 6.000000,0.472222,4.000000,2.833333,2.500000,0.966667,5.000000,2.833333,2.500000,1.366667,
0.705073,0.028795,0.705073,0.028795,0.000000,0.000000,0.000000,0.117647,0.133333,0.461538,
0.142857,1.416667,2.250000,0.416667,1.555556,2.000000,0.251852,0.493056,0.562500,0.007060,
0.612963,1.000000,0.092860,0.461111,0.600000,0.014407,0.366667,0.500000,0.014667,0.377778,
0.600000,0.024296,0.438889,0.600000,0.027741,3.211529,2.022378,1.345525,1.000000,1.000000,
1.000000,1.000000,1.000000,1.000000,1.000000,1.000000,1.000000,1.000000,1.000000,0.888889,
1.000000,0.777778,0.333333,0.166667,0.166667,0.055556,0.000000,0.000000,1.000000,1.000000,
1.000000,1.000000,1.000000,1.000000,1.000000,1.000000,1.000000,1.000000,1.000000,0.911111,
1.000000,0.788889,1.000000,0.477778,1.000000,0.422222,0.000000,0.000000,0.320755,0.283739,
0.450980,0.395040,0.334033,0.321222,0.224891,0.205285,0.230479,0.189642,0.290503,0.258097,
0.022222,0.008377,0.260852,0.245722,0.507763,0.488972,0.769016,0.726713,0.999602,0.993929,
0.015094,0.006381,0.291939,0.238604,0.531590,0.494418,0.764706,0.745057,1.000000,0.994675,
0.005556,0.002122,0.322222,0.259769,0.544444,0.495710,0.766667,0.746254,1.000000,0.999044,
0.483019,0.417571,0.204784,0.158968,0.611111,0.423461,0.078603,0.064152,0.111732,0.078022,
0.006105,0.003319,0.005556,0.002122,0.266667,0.214878,0.513219,0.458708,0.740741,0.715640,
1.000000,1.000000,0.288889,0.078506,0.448802,0.293384,0.623094,0.504972,0.886710,0.765610,
0.999204,0.945765,0.147170,0.031920,0.332075,0.269617,0.592453,0.512693,0.796097,0.758300,
0.997063,0.971384,0.664488,0.613474,0.377834,0.341448,0.056604,0.014640,0.315904,0.249756,
0.526851,0.498096,0.764443,0.747066,0.998805,0.991438,0.459695,0.403066,0.391523,0.365389,
0.256604,0.231544,0.311554,0.288866,0.208333,0.184249,0.203191,0.168298,0.015094,0.006245,
0.281046,0.236675,0.520697,0.486671,0.762527,0.745977,1.000000,0.994675,0.016667,0.005813,
0.338889,0.267333,0.566667,0.491546,0.766667,0.754745,1.000000,0.998135,0.005556,0.002122,
0.278275,0.223485,0.538242,0.497220,0.751017,0.718436,1.000000,0.993828,0.367604,0.347523,
0.405556,0.340492,0.358491,0.311985,0.281659,0.236125,0.275819,0.228868,0.224891,0.198956,
0.005556,0.002122,0.322222,0.268144,0.538889,0.502483,0.777358,0.750434,1.000000,0.999044,
0.015094,0.006381,0.281046,0.228402,0.525054,0.476687,0.762527,0.742343,1.000000,0.994675,
0.022222,0.008377,0.262843,0.246144,0.507763,0.493072,0.766627,0.730056,0.999602,0.993929,
0.387800,0.327901,0.456604,0.440555,0.256604,0.231544,0.310757,0.278958,0.170455,0.153812,
0.222502,0.198735,0.015094,0.006381,0.287582,0.244983,0.518519,0.491834,0.783333,0.759770,
1.000000,0.993749,0.016667,0.005813,0.277778,0.252560,0.538889,0.487167,0.756272,0.746414,
1.000000,0.998961,0.005556,0.002122,0.278275,0.223485,0.538242,0.497220,0.751017,0.718436,
1.000000,0.993828,0.077778,0.056262,0.067925,0.042068,0.083333,0.069173,0.053714,0.043246,
0.049383,0.039554,0.049057,0.033436,0.100000,0.077036,0.144444,0.090330,0.079251,0.072894,
0.058824,0.044394,0.066667,0.055838,0.037736,0.028246,0.033571,0.026302,0.030189,0.019328,
0.105556,0.075982,0.125846,0.100006,0.062946,0.047240,0.032312,0.023533,0.056604,0.043352,
0.015094,0.011782

Positive_8 9.000000,0.259259,4.000000,2.333333,2.000000,1.750000,6.000000,2.333333,2.000000,2.750000,
0.600670,0.028819,0.600670,0.028819,0.000000,0.047619,0.000000,0.200000,0.437500,0.444444,
0.600000,0.703704,1.333333,0.290123,0.444444,2.000000,0.527778,0.271605,0.500000,0.051076,
0.120370,0.500000,0.036651,0.277778,0.500000,0.042101,0.252315,0.375000,0.010872,0.266204,
0.625000,0.025149,0.265046,0.375000,0.008711,3.313764,2.313271,1.732051,1.000000,0.777778,
1.000000,0.777778,1.000000,0.777778,1.000000,0.703704,1.000000,0.703704,1.000000,0.629630,
1.000000,0.481481,1.000000,0.407407,0.000000,0.000000,0.000000,0.000000,1.000000,0.666667,
1.000000,0.666667,1.000000,0.666667,1.000000,0.666667,1.000000,0.666667,1.000000,0.633333,
1.000000,0.525926,1.000000,0.151852,0.066667,0.007407,0.000000,0.000000,0.317241,0.291001,
0.426160,0.388658,0.356209,0.320340,0.238095,0.210345,0.222973,0.195365,0.278689,0.256462,
0.071895,0.022434,0.333333,0.279734,0.584967,0.517330,0.800000,0.751228,1.000000,0.993078,
0.010101,0.005972,0.275862,0.225585,0.544081,0.484710,0.763224,0.734094,1.000000,0.996674,
0.003968,0.002726,0.257937,0.234560,0.514946,0.481246,0.777778,0.741136,1.000000,0.993607,
0.436141,0.396950,0.232337,0.166018,0.516383,0.437032,0.104730,0.074736,0.121311,0.089621,
0.017677,0.006234,0.003968,0.002726,0.290761,0.220683,0.547619,0.492367,0.801587,0.729778,
1.000000,1.000000,0.124138,0.050767,0.359477,0.227891,0.589674,0.448588,0.798913,0.669551,
0.996633,0.935566,0.073048,0.022774,0.329966,0.267780,0.627586,0.495394,0.820690,0.763999,
0.996732,0.970461,0.697733,0.629432,0.396166,0.344789,0.019841,0.009122,0.324138,0.259727,
0.555172,0.493332,0.773300,0.738896,0.998689,0.993889,0.452381,0.415300,0.400673,0.360558,
0.257962,0.224142,0.390438,0.303993,0.260042,0.185562,0.196721,0.157600,0.010101,0.005972,
0.272414,0.226638,0.516373,0.476029,0.754915,0.732068,1.000000,0.996063,0.031746,0.012354,
0.269841,0.246249,0.519841,0.486821,0.777778,0.743784,1.000000,0.994547,0.003968,0.002726,
0.343434,0.273336,0.592593,0.521579,0.831034,0.766159,1.000000,0.991451,0.385621,0.341008,
0.396825,0.352237,0.324138,0.306755,0.283298,0.251089,0.239865,0.218331,0.220408,0.198078,
0.003968,0.002726,0.261146,0.240154,0.544586,0.493351,0.777778,0.743971,1.000000,0.995518,
0.010101,0.005972,0.258621,0.214208,0.544081,0.477790,0.760705,0.724219,1.000000,0.994531,
0.071895,0.022434,0.333333,0.282414,0.584967,0.515330,0.786207,0.749350,1.000000,0.994893,
0.388889,0.352791,0.478114,0.423067,0.257962,0.224142,0.362550,0.297579,0.213531,0.159759,
0.219672,0.183403,0.010101,0.005972,0.303448,0.229453,0.531034,0.479133,0.774510,0.729883,
1.000000,0.993244,0.019841,0.009791,0.277174,0.245806,0.547619,0.486569,0.797619,0.740971,
1.000000,0.995691,0.003968,0.002726,0.343434,0.273336,0.592593,0.521579,0.831034,0.766159,
1.000000,0.991451,0.047138,0.040634,0.098726,0.070549,0.068966,0.054175,0.050955,0.046967,
0.067340,0.042997,0.047619,0.035680,0.119048,0.087804,0.134921,0.103065,0.076433,0.064764,
0.076433,0.050190,0.063973,0.046413,0.026144,0.015754,0.029412,0.020667,0.027586,0.016096,
0.095238,0.077565,0.114478,0.096357,0.062500,0.053784,0.040084,0.028063,0.054140,0.040762,
0.019608,0.007714

Positive_9 43.000000,0.137912,18.000000,5.930233,6.000000,19.447398,17.000000,5.930233,7.000000,19.209302,
0.536509,0.016068,0.536509,0.016068,0.000000,0.023529,0.120482,0.296804,0.487013,0.708861,
0.826087,1.505565,7.083333,3.464457,1.348086,6.000000,3.232996,0.196576,0.777778,0.055940,
0.141182,0.479339,0.032827,0.182743,0.333333,0.010868,0.090700,0.230159,0.005407,0.073330,
0.265873,0.007391,0.171217,0.357143,0.009623,9.792693,6.480741,6.370851,1.000000,0.744186,
1.000000,0.744186,1.000000,0.744186,1.000000,0.744186,1.000000,0.717307,1.000000,0.600837,
1.000000,0.445305,0.523810,0.229947,0.523810,0.137932,0.190476,0.035175,1.000000,0.860465,
1.000000,0.860465,1.000000,0.860465,1.000000,0.860465,1.000000,0.812457,1.000000,0.715707,

1.000000,0.569781,1.000000,0.408258,1.000000,0.316549,0.047619,0.007135,0.334917,0.288680,
0.432024,0.386255,0.361413,0.325065,0.273810,0.211035,0.231608,0.193963,0.325758,0.256191,
0.023810,0.010065,0.332494,0.246911,0.582888,0.512902,0.800000,0.746269,1.000000,0.996980,
0.027190,0.006099,0.297927,0.246188,0.544081,0.490378,0.787629,0.742131,1.000000,0.995702,
0.003968,0.002085,0.276744,0.241496,0.537405,0.489236,0.784543,0.741899,1.000000,0.994906,
0.472000,0.397534,0.259358,0.166750,0.601527,0.435716,0.107239,0.071280,0.114187,0.076528,
0.017677,0.006636,0.003968,0.002085,0.353201,0.222842,0.598234,0.477703,0.848485,0.722657,
1.000000,0.999139,0.399543,0.090570,0.465753,0.284369,0.778420,0.493548,0.939577,0.703906,
0.998145,0.946887,0.155440,0.035149,0.500000,0.279334,0.665761,0.519782,0.883152,0.756464,
1.000000,0.972606,0.701389,0.641123,0.400000,0.341273,0.021333,0.008318,0.324138,0.269233,
0.567696,0.503639,0.785498,0.750214,1.000000,0.992385,0.480363,0.405677,0.418557,0.356826,
0.289683,0.237497,0.390438,0.285961,0.260042,0.197557,0.216216,0.169075,0.027190,0.006132,
0.306843,0.251577,0.544041,0.492760,0.779381,0.734943,1.000000,0.996122,0.031746,0.008394,
0.273342,0.239500,0.541985,0.491479,0.799729,0.746307,1.000000,0.995207,0.003968,0.002085,
0.321429,0.246644,0.582888,0.508724,0.834225,0.766439,1.000000,0.996455,0.391753,0.350889,
0.407855,0.339657,0.346793,0.309454,0.313131,0.245902,0.260331,0.222933,0.240476,0.198873,
0.003968,0.002085,0.281395,0.245950,0.544586,0.491354,0.783721,0.746061,1.000000,0.996424,
0.027190,0.006163,0.286976,0.240794,0.574181,0.490540,0.785567,0.732415,1.000000,0.994386,
0.023810,0.010065,0.332494,0.248454,0.578804,0.511537,0.789398,0.749373,1.000000,0.997031,
0.401813,0.336447,0.482474,0.426056,0.289683,0.237497,0.362550,0.277210,0.213531,0.165172,
0.260618,0.201460,0.027190,0.006465,0.310881,0.256122,0.553191,0.497353,0.786269,0.737198,
1.000000,0.995279,0.019841,0.007920,0.285256,0.240233,0.547619,0.486320,0.797619,0.741770,
1.000000,0.995807,0.003968,0.002085,0.321429,0.246644,0.582888,0.508724,0.834225,0.766439,
1.000000,0.996455,0.073059,0.046401,0.098726,0.065834,0.081395,0.059814,0.057751,0.044520,
0.061657,0.038315,0.051389,0.033796,0.119048,0.086018,0.150485,0.089957,0.092172,0.062393,
0.082902,0.053559,0.065007,0.047730,0.049242,0.020774,0.043651,0.025825,0.043478,0.021500,
0.104278,0.072452,0.144509,0.094165,0.081579,0.058283,0.040084,0.025860,0.059343,0.041876,
0.025253,0.010927

Positive_10 9.000000,0.296296,7.000000,2.666667,2.000000,7.250000,6.000000,2.666667,2.000000,4.750000,
0.524828,0.014401,0.524828,0.014401,0.000000,0.000000,0.166667,0.350000,0.615385,0.200000,
1.000000,0.465608,1.428571,0.377425,0.503704,1.600000,0.576790,0.094734,0.222222,0.012662,
0.095802,0.320000,0.021447,0.382275,0.625000,0.060895,0.113757,0.250000,0.010775,0.074074,
0.250000,0.012924,0.464815,0.750000,0.097236,4.306952,1.857462,1.414214,1.000000,0.555556,
1.000000,0.555556,1.000000,0.555556,1.000000,0.555556,1.000000,0.417989,1.000000,0.365079,
1.000000,0.248677,0.142857,0.031746,0.000000,0.000000,0.000000,0.000000,1.000000,0.777778,
1.000000,0.777778,1.000000,0.777778,1.000000,0.777778,1.000000,0.770370,1.000000,0.666667,
1.000000,0.592593,1.000000,0.577778,0.000000,0.000000,0.000000,0.000000,0.322449,0.285105,
0.413098,0.394882,0.338462,0.320013,0.227360,0.198248,0.237705,0.194640,0.325758,0.267541,
0.021077,0.011881,0.332494,0.262714,0.529981,0.495143,0.773694,0.737956,1.000000,0.995834,
0.011811,0.006519,0.299213,0.242232,0.555102,0.501017,0.795918,0.753505,1.000000,0.994200,
0.004082,0.002520,0.277551,0.235212,0.507692,0.486306,0.784543,0.738310,1.000000,0.996911,
0.526923,0.437869,0.253385,0.185037,0.502041,0.377094,0.127168,0.080826,0.099415,0.080261,
0.012626,0.006182,0.004082,0.002520,0.261538,0.216629,0.548077,0.494071,0.805769,0.741373,
1.000000,1.000000,0.106299,0.066532,0.367505,0.283198,0.583138,0.460085,0.803150,0.739867,

0.997658,0.982712,0.075567,0.027065,0.352770,0.250271,0.603499,0.474406,0.782201,0.721088,
0.996154,0.962627,0.687657,0.649331,0.395062,0.333658,0.020151,0.010142,0.293878,0.265320,
0.556851,0.496028,0.781341,0.746765,0.996154,0.993274,0.448363,0.418494,0.391837,0.356614,
0.274052,0.224892,0.356061,0.296589,0.248538,0.192483,0.181118,0.159027,0.011811,0.006519,
0.275591,0.236421,0.559184,0.495755,0.759615,0.744627,1.000000,0.993702,0.024615,0.009758,
0.300292,0.239115,0.506297,0.479134,0.768262,0.730335,1.000000,0.999033,0.004082,0.002520,
0.334623,0.279855,0.555118,0.506781,0.798834,0.736226,1.000000,0.995631,0.364431,0.348338,
0.385390,0.351056,0.330612,0.300606,0.314815,0.257915,0.245902,0.218729,0.208092,0.189878,
0.004082,0.002520,0.279883,0.241419,0.524781,0.490230,0.772834,0.740996,1.000000,0.996911,
0.011811,0.006519,0.275591,0.227692,0.555102,0.491899,0.771654,0.742788,1.000000,0.992477,
0.021077,0.011881,0.334623,0.277744,0.539370,0.498345,0.782692,0.746686,1.000000,0.996048,
0.380353,0.356398,0.440816,0.418710,0.274052,0.224892,0.336420,0.290287,0.222222,0.162254,
0.211240,0.189256,0.011811,0.006733,0.281496,0.235537,0.542857,0.496859,0.775385,0.745727,
0.998077,0.991930,0.024615,0.009117,0.288630,0.240657,0.502041,0.483081,0.758186,0.734431,
1.000000,0.999462,0.004082,0.002520,0.334623,0.279855,0.555118,0.506781,0.798834,0.736226,
1.000000,0.995631,0.067698,0.047055,0.073077,0.062177,0.069231,0.057379,0.071154,0.047716,
0.048980,0.032679,0.050000,0.038099,0.108312,0.087399,0.119438,0.096619,0.093294,0.069908,
0.066929,0.054757,0.049563,0.042374,0.020151,0.015501,0.048077,0.028325,0.024615,0.019723,
0.103275,0.082782,0.106299,0.089557,0.075567,0.056119,0.039813,0.031755,0.041339,0.031689,
0.014577,0.008389

Positive_11 22.000000,0.082645,6.000000,1.818182,1.000000,3.108225,4.000000,1.818182,2.000000,1.679654,
0.622192,0.025785,0.622192,0.025785,0.000000,0.050000,0.026316,0.108108,0.363636,0.571429,
0.555556,0.549242,3.000000,0.977949,0.742424,3.000000,1.202982,0.149937,1.000000,0.067996,
0.232323,0.888889,0.102921,0.054654,0.142857,0.002267,0.069444,0.238095,0.004664,0.066378,
0.174603,0.003867,0.079004,0.206349,0.005642,3.997612,2.374325,2.009050,1.000000,0.409091,
1.000000,0.409091,1.000000,0.409091,1.000000,0.409091,1.000000,0.348485,1.000000,0.295455,
1.000000,0.207576,1.000000,0.133333,1.000000,0.118182,0.166667,0.013636,1.000000,0.545455,
1.000000,0.545455,1.000000,0.545455,1.000000,0.500000,1.000000,0.500000,1.000000,0.409091,
1.000000,0.303030,1.000000,0.151515,0.333333,0.075758,0.000000,0.000000,0.375000,0.283273,
0.427500,0.359581,0.441718,0.357146,0.247350,0.199483,0.240838,0.184215,0.340528,0.266443,
0.073620,0.014866,0.312500,0.248492,0.648148,0.508382,0.913580,0.766491,1.000000,0.995478,
0.026756,0.009749,0.352423,0.250358,0.567568,0.501729,0.805755,0.745612,1.000000,0.994501,
0.006173,0.003070,0.300613,0.233640,0.564417,0.478653,0.780488,0.719727,1.000000,0.988498,
0.502203,0.383345,0.218992,0.112438,0.697842,0.504217,0.117216,0.046797,0.111801,0.082117,
0.018519,0.006049,0.006173,0.003070,0.329955,0.226259,0.629630,0.481848,0.852843,0.737531,
1.000000,1.000000,0.966555,0.159257,0.966555,0.300436,0.969900,0.423847,0.973244,0.608746,
0.991579,0.825868,0.161616,0.046635,0.409692,0.266767,0.655052,0.515638,0.846690,0.746948,
0.998258,0.970139,0.839506,0.650753,0.418848,0.298992,0.036667,0.015332,0.301053,0.260626,
0.552928,0.491698,0.785714,0.710392,0.997067,0.986298,0.457500,0.354031,0.433333,0.376360,
0.382716,0.269609,0.364557,0.268485,0.246835,0.186037,0.266019,0.195185,0.023460,0.008619,
0.312775,0.236330,0.563063,0.484446,0.805556,0.739300,1.000000,0.989321,0.067485,0.013624,
0.331288,0.251611,0.583333,0.498967,0.833333,0.734333,1.000000,0.994312,0.006173,0.003070,
0.344498,0.248532,0.593301,0.508708,0.820574,0.752739,1.000000,0.995671,0.478528,0.395984,
0.382500,0.289433,0.414097,0.314583,0.308642,0.235117,0.297659,0.228556,0.212014,0.179789,

0.006173,0.003070,0.286957,0.242632,0.564417,0.487161,0.796247,0.728941,1.000000,0.988715,
0.026756,0.009749,0.343612,0.241311,0.567568,0.485814,0.819398,0.733819,1.000000,0.986467,
0.073620,0.014866,0.306220,0.252593,0.586420,0.512701,0.871166,0.765676,1.000000,0.996582,
0.367500,0.287523,0.489437,0.442869,0.382716,0.269609,0.356962,0.260672,0.222222,0.152500,
0.305369,0.228722,0.055556,0.011228,0.345679,0.234503,0.586420,0.482577,0.819398,0.736344,
1.000000,0.987341,0.049080,0.010822,0.300613,0.249556,0.558047,0.497997,0.807292,0.738227,
1.000000,0.995143,0.006173,0.003070,0.344498,0.248532,0.593301,0.508708,0.820574,0.752739,
1.000000,0.995671,0.093750,0.055470,0.087591,0.049063,0.096916,0.058057,0.062500,0.044601,
0.067708,0.040542,0.063380,0.035540,0.086124,0.064951,0.115000,0.071418,0.104294,0.059035,
0.064516,0.047518,0.076555,0.046511,0.086420,0.031310,0.066890,0.038838,0.042945,0.019997,
0.095652,0.066048,0.186603,0.121407,0.080268,0.054767,0.037162,0.021197,0.100334,0.051534,
0.047826,0.022196

Positive_12 31.000000,0.086368,7.000000,2.677419,2.000000,7.559140,11.000000,2.677419,1.000000,12.025806,
0.442057,0.010724,0.442057,0.010724,0.000000,0.048193,0.354430,0.607843,0.600000,0.875000,
1.000000,0.072581,0.750000,0.038306,0.032991,0.272727,0.007605,0.030242,0.250000,0.006477,
0.006848,0.062500,0.000363,0.118548,0.333333,0.011824,0.029928,0.200000,0.002061,0.028055,
0.366667,0.005186,0.060313,0.195833,0.005908,7.399138,3.502325,2.548182,1.000000,0.516129,
1.000000,0.516129,1.000000,0.516129,1.000000,0.516129,1.000000,0.446237,1.000000,0.413978,
1.000000,0.291398,0.466667,0.138710,0.333333,0.100000,0.066667,0.012903,1.000000,0.387097,
1.000000,0.387097,1.000000,0.387097,1.000000,0.370926,1.000000,0.316129,1.000000,0.179570,
1.000000,0.116101,0.333333,0.048429,0.333333,0.016716,0.166667,0.005376,0.344937,0.276365,
0.511905,0.385610,0.416364,0.338025,0.256410,0.202893,0.251553,0.191971,0.335968,0.262551,
0.049603,0.011796,0.337681,0.233041,0.611594,0.504307,0.800613,0.736589,1.000000,0.994069,
0.031496,0.008969,0.316327,0.246214,0.623016,0.490878,0.828042,0.741228,1.000000,0.996139,
0.005102,0.002470,0.328185,0.251855,0.568966,0.493286,0.791411,0.743523,1.000000,0.994829,
0.617725,0.417876,0.259358,0.166985,0.712727,0.415139,0.126984,0.074297,0.103015,0.074451,
0.017677,0.006828,0.005102,0.002470,0.369565,0.231805,0.661376,0.493138,0.845475,0.748667,
1.000000,1.000000,0.706564,0.090750,0.710425,0.226571,0.777778,0.489079,0.980695,0.722227,
0.998677,0.965583,0.186603,0.058335,0.500000,0.300798,0.665761,0.506719,0.883152,0.737077,
0.995215,0.954964,0.806950,0.644806,0.406154,0.334651,0.051020,0.012095,0.341818,0.268004,
0.565134,0.498847,0.785498,0.745512,1.000000,0.992492,0.538360,0.399122,0.418557,0.356310,
0.338182,0.244568,0.327273,0.276748,0.260042,0.201174,0.236794,0.172605,0.027190,0.008290,
0.318783,0.245892,0.632275,0.484253,0.838624,0.731861,1.000000,0.993908,0.030612,0.010533,
0.300613,0.238195,0.548023,0.486722,0.791411,0.733614,1.000000,0.996097,0.005102,0.002470,
0.362205,0.258711,0.594488,0.519606,0.862205,0.777522,1.000000,0.995483,0.469091,0.368101,
0.465608,0.333060,0.376582,0.298839,0.324111,0.248813,0.310559,0.223167,0.246154,0.187663,
0.005102,0.002470,0.329091,0.256657,0.578182,0.497374,0.782383,0.747171,1.000000,0.997007,
0.031496,0.008969,0.321429,0.236703,0.654762,0.481765,0.842593,0.730834,1.000000,0.992723,
0.049603,0.011796,0.336232,0.235265,0.604348,0.506854,0.800613,0.743069,1.000000,0.994513,
0.402116,0.320933,0.482474,0.434499,0.338182,0.244568,0.323232,0.267394,0.213531,0.160989,
0.290698,0.212791,0.031496,0.008852,0.311224,0.245871,0.572751,0.485247,0.808201,0.732341,
1.000000,0.989331,0.030612,0.010130,0.294479,0.241802,0.553672,0.486627,0.777778,0.734586,
1.000000,0.997230,0.005102,0.002470,0.362205,0.258711,0.594488,0.519606,0.862205,0.777522,
1.000000,0.995483,0.074713,0.045985,0.098101,0.060353,0.085443,0.057877,0.065015,0.042398,

0.065913,0.035488,0.057644,0.034265,0.109462,0.082745,0.120846,0.085681,0.075188,0.055298,
0.082902,0.054811,0.128307,0.054524,0.045918,0.022474,0.062992,0.030076,0.043478,0.023665,
0.117347,0.076684,0.149275,0.097315,0.081579,0.054681,0.040084,0.023592,0.105455,0.048368,
0.035088,0.013720

Positive_13 4.000000,0.187500,1.000000,0.750000,1.000000,0.250000,1.000000,0.750000,1.000000,0.250000,
0.752219,0.007178,0.752219,0.007178,0.000000,0.000000,0.000000,0.000000,0.000000,0.333333,
0.500000,0.000000,0.000000,0.000000,0.000000,0.000000,0.000000,0.000000,0.000000,0.000000,
0.000000,0.000000,0.000000,0.000000,0.000000,0.000000,0.166667,0.333333,0.037037,0.166667,
0.333333,0.037037,0.000000,0.000000,0.000000,1.000000,1.000000,1.000000,0.000000,0.000000,
0.000000,0.000000,0.000000,0.000000,0.000000,0.000000,0.000000,0.000000,0.000000,0.000000,
0.000000,0.000000,0.000000,0.000000,0.000000,0.000000,0.000000,0.000000,0.000000,0.000000,
0.000000,0.000000,0.000000,0.000000,0.000000,0.000000,0.000000,0.000000,0.000000,0.000000,
0.000000,0.000000,0.000000,0.000000,0.000000,0.000000,0.000000,0.000000,0.330275,0.289227,
0.442308,0.386793,0.336043,0.323980,0.217391,0.194576,0.222222,0.198428,0.301927,0.263176,
0.050691,0.029665,0.290520,0.270850,0.517615,0.491893,0.764977,0.739996,1.000000,0.998084,
0.009217,0.006934,0.230352,0.199044,0.517094,0.472126,0.775068,0.746100,0.997863,0.990328,
0.004608,0.003128,0.327189,0.274696,0.519878,0.499152,0.769585,0.741590,1.000000,0.997042,
0.435897,0.382016,0.175214,0.110640,0.737327,0.507344,0.068522,0.040950,0.116848,0.091687,
0.008565,0.005713,0.004608,0.003128,0.179487,0.151293,0.422018,0.357711,0.691057,0.624185,
1.000000,1.000000,0.094851,0.040282,0.406504,0.251890,0.599388,0.387522,0.813008,0.517394,
0.990826,0.657598,0.048930,0.025421,0.331797,0.305980,0.702991,0.577377,0.882479,0.805056,
0.995726,0.987240,0.620795,0.598706,0.385870,0.352877,0.018970,0.011561,0.281106,0.267575,
0.523504,0.510455,0.773504,0.731234,0.995726,0.991827,0.448718,0.388860,0.382114,0.357869,
0.275229,0.253272,0.301630,0.265234,0.205567,0.194398,0.193252,0.175952,0.009217,0.006934,
0.238482,0.199318,0.502137,0.460505,0.745726,0.713283,0.997863,0.988974,0.055300,0.028119,
0.377880,0.324786,0.594470,0.546272,0.778802,0.763855,1.000000,0.995440,0.004608,0.003128,
0.238532,0.209929,0.452599,0.423074,0.747967,0.731888,1.000000,0.998084,0.368564,0.355760,
0.378205,0.325120,0.354740,0.319120,0.282655,0.253946,0.269939,0.240232,0.190217,0.166341,
0.004608,0.003128,0.304147,0.259203,0.525994,0.499612,0.779817,0.746768,1.000000,0.997042,
0.009217,0.006934,0.222222,0.182915,0.527778,0.449368,0.767094,0.719524,0.997863,0.988974,
0.050691,0.029665,0.290520,0.280878,0.534562,0.506168,0.778802,0.752604,1.000000,0.998084,
0.397436,0.335522,0.455285,0.411206,0.275229,0.253272,0.293478,0.276289,0.177914,0.169577,
0.211656,0.200772,0.009217,0.006934,0.235772,0.202462,0.510684,0.457830,0.754274,0.711510,
0.997863,0.988974,0.018970,0.013928,0.364055,0.316826,0.589862,0.541341,0.778802,0.756745,
1.000000,0.995440,0.004608,0.003128,0.238532,0.209929,0.452599,0.423074,0.747967,0.731888,
1.000000,0.998084,0.092166,0.060407,0.062331,0.050323,0.073733,0.062180,0.059829,0.047692,
0.061162,0.037955,0.040650,0.030670,0.085627,0.075370,0.119816,0.091824,0.100427,0.075689,
0.065041,0.044948,0.048780,0.037290,0.038462,0.029894,0.036697,0.031779,0.024390,0.016048,
0.083333,0.074107,0.108974,0.105568,0.055300,0.047390,0.036866,0.024453,0.045872,0.034870,
0.024465,0.021546

Positive_14 11.000000,0.553719,9.000000,6.090909,6.000000,4.290909,9.000000,6.090909,5.000000,3.890909,
0.612833,0.019850,0.612833,0.019850,0.000000,0.000000,0.014925,0.303030,0.304348,0.500000,
0.500000,3.635354,5.888889,2.268588,3.725685,5.250000,1.603985,0.584450,0.722222,0.009401,
0.608034,0.720000,0.007144,0.566465,0.633333,0.003017,0.561061,0.616667,0.004807,0.570620,

0.620000,0.002021,0.570620,0.640000,0.004525,6.709978,2.854364,2.366341,1.000000,1.000000,
1.000000,1.000000,1.000000,1.000000,1.000000,1.000000,1.000000,0.935859,1.000000,0.616667,
0.833333,0.472727,0.500000,0.262626,0.333333,0.155556,0.166667,0.072727,1.000000,1.000000,
1.000000,1.000000,1.000000,1.000000,1.000000,1.000000,1.000000,0.966667,1.000000,0.710029,
1.000000,0.548773,0.600000,0.309019,0.333333,0.174964,0.166667,0.084704,0.343653,0.294754,
0.419753,0.358297,0.367677,0.346949,0.221538,0.200753,0.251553,0.209230,0.282178,0.254041,
0.045576,0.016982,0.288889,0.247972,0.533742,0.495170,0.818493,0.751660,1.000000,0.998903,
0.012346,0.007919,0.330645,0.238999,0.565657,0.495242,0.787654,0.732347,1.000000,0.995541,
0.003425,0.002483,0.300613,0.247725,0.539877,0.490123,0.791411,0.750587,0.998012,0.989400,
0.457055,0.411635,0.164087,0.115089,0.547945,0.473276,0.099379,0.056769,0.094059,0.083451,
0.004950,0.002328,0.003425,0.002483,0.318519,0.248031,0.624691,0.541190,0.906173,0.794731,
1.000000,1.000000,0.207407,0.060162,0.274074,0.181469,0.775432,0.452941,0.913386,0.702009,
0.996024,0.926442,0.110429,0.034869,0.318618,0.263429,0.552279,0.500762,0.796247,0.706434,
0.990157,0.950513,0.654321,0.616683,0.406154,0.342562,0.024768,0.009206,0.252485,0.237505,
0.513699,0.491331,0.751491,0.730680,0.998012,0.989638,0.439506,0.350486,0.404040,0.388708,
0.291747,0.260806,0.293522,0.270884,0.206989,0.186490,0.235060,0.200383,0.012346,0.007080,
0.280822,0.242163,0.555556,0.491732,0.773737,0.724415,1.000000,0.995355,0.027397,0.010975,
0.309055,0.260254,0.546012,0.503727,0.791411,0.748334,1.000000,0.998143,0.003425,0.002483,
0.273006,0.235442,0.603217,0.508455,0.820375,0.762474,1.000000,0.994184,0.414384,0.381597,
0.380247,0.295825,0.368421,0.322577,0.280323,0.237885,0.310559,0.249649,0.198020,0.178649,
0.003425,0.002483,0.288344,0.246662,0.525912,0.496853,0.775348,0.752322,1.000000,0.991641,
0.012346,0.007919,0.309140,0.238329,0.577778,0.490258,0.773737,0.722773,1.000000,0.992991,
0.045576,0.016982,0.303704,0.250094,0.533742,0.494516,0.801370,0.751200,1.000000,0.998903,
0.338272,0.278355,0.496970,0.460839,0.291747,0.260806,0.289604,0.251610,0.171821,0.144134,
0.282869,0.242738,0.019753,0.007754,0.304175,0.252659,0.553535,0.494189,0.757576,0.717227,
1.000000,0.995355,0.027397,0.009642,0.295276,0.255938,0.546012,0.499908,0.777397,0.746880,
1.000000,0.998143,0.003425,0.002483,0.273006,0.235442,0.603217,0.508455,0.820375,0.762474,
1.000000,0.994184,0.081511,0.057666,0.077399,0.054692,0.076687,0.060310,0.065015,0.040432,
0.055666,0.041228,0.055215,0.040427,0.079012,0.057782,0.113580,0.073234,0.082192,0.061662,
0.056452,0.045245,0.079012,0.057903,0.037773,0.027823,0.051370,0.034648,0.022222,0.014228,
0.085616,0.068784,0.169697,0.128999,0.070866,0.048960,0.054795,0.025621,0.059501,0.043302,
0.022222,0.017054

Positive_15 39.000000,0.211703,16.000000,8.256410,9.000000,18.458839,23.000000,8.256410,3.000000,83.037787,
0.421033,0.032167,0.421033,0.032167,0.124224,0.209220,0.242152,0.360947,0.314815,0.729730,
1.000000,1.315828,6.428571,4.600309,1.233822,3.750000,1.727962,0.103247,0.459184,0.026620,
0.114584,0.468750,0.026096,0.383903,0.578947,0.036072,0.181859,0.421053,0.008036,0.109382,
0.434211,0.019030,0.159055,0.394737,0.018559,14.770270,7.625650,5.243895,1.000000,0.948718,
1.000000,0.948718,1.000000,0.943646,1.000000,0.910923,1.000000,0.882403,1.000000,0.740583,
1.000000,0.619358,1.000000,0.481435,1.000000,0.407627,1.000000,0.258945,1.000000,0.666667,
1.000000,0.664842,1.000000,0.600993,1.000000,0.564153,1.000000,0.501402,1.000000,0.326730,
1.000000,0.262742,1.000000,0.218293,1.000000,0.174269,1.000000,0.140932,0.383803,0.284339,
0.450253,0.363009,0.398866,0.352652,0.275000,0.197792,0.262799,0.195614,0.335968,0.253539,
0.045576,0.012476,0.357853,0.252184,0.588336,0.510238,0.832740,0.758354,1.000000,0.995501,
0.031496,0.007882,0.330645,0.245148,0.593548,0.499784,0.826220,0.739458,1.000000,0.994852,

0.003937,0.002610,0.328185,0.250140,0.558201,0.479972,0.791411,0.733315,1.000000,0.988080,
0.699831,0.419622,0.249271,0.131681,0.652510,0.448697,0.103650,0.054199,0.123239,0.080696,
0.020478,0.003623,0.003937,0.002610,0.339093,0.265879,0.624691,0.510608,0.906173,0.758469,
1.000000,1.000000,0.706564,0.100710,0.710425,0.233131,0.798942,0.481871,0.980695,0.676770,
0.996024,0.885426,0.110429,0.026184,0.334646,0.235368,0.571930,0.480730,0.836812,0.737553,
0.995627,0.953156,0.806950,0.619754,0.417266,0.324261,0.042705,0.012775,0.388889,0.250501,
0.587302,0.488501,0.783069,0.715350,0.998012,0.984465,0.478927,0.364747,0.412903,0.362873,
0.350340,0.272381,0.361538,0.261874,0.260745,0.204053,0.269625,0.193557,0.023460,0.007517,
0.302817,0.235698,0.554286,0.486995,0.828571,0.735725,1.000000,0.995599,0.032028,0.012983,
0.336918,0.250498,0.566308,0.489877,0.791411,0.731932,1.000000,0.992001,0.003937,0.002610,
0.362205,0.256604,0.603217,0.515447,0.862205,0.774684,1.000000,0.994547,0.440945,0.386911,
0.409962,0.301518,0.401408,0.311571,0.324111,0.233010,0.320819,0.233564,0.257143,0.183112,
0.003937,0.002610,0.326772,0.250841,0.560847,0.483375,0.779528,0.736370,1.000000,0.989095,
0.031496,0.008056,0.309298,0.235906,0.585714,0.490338,0.837143,0.728298,1.000000,0.993687,
0.045576,0.011938,0.351421,0.256695,0.586563,0.519037,0.832740,0.764874,1.000000,0.995648,
0.417625,0.291198,0.479876,0.436422,0.350340,0.272381,0.384615,0.249137,0.207254,0.160595,
0.324232,0.237016,0.032590,0.009063,0.304175,0.225807,0.563567,0.485608,0.823171,0.729149,
1.000000,0.991516,0.028470,0.011555,0.304659,0.253038,0.546012,0.492624,0.777397,0.734973,
1.000000,0.994281,0.003937,0.002610,0.362205,0.256604,0.603217,0.515447,0.862205,0.774684,
1.000000,0.994547,0.096085,0.051586,0.102041,0.057769,0.098639,0.059743,0.073944,0.043107,
0.058201,0.035761,0.055215,0.036373,0.112985,0.064537,0.130268,0.068863,0.097633,0.067856,
0.064516,0.046835,0.108571,0.053427,0.053963,0.027232,0.062992,0.034259,0.043011,0.020122,
0.103448,0.067071,0.167901,0.112785,0.087475,0.051140,0.054795,0.028534,0.082677,0.054007,
0.042105,0.018993

Positive_16 43.000000,0.044348,41.000000,1.906977,0.000000,76.324474,2.000000,1.906977,2.000000,0.181617,
0.371759,0.006331,0.371759,0.006331,0.048780,0.141026,0.507463,0.939394,1.000000,0.000000,
0.000000,0.000000,0.000000,0.000000,0.000000,0.000000,0.000000,0.000000,0.000000,0.000000,
0.000000,0.000000,0.000000,0.001107,0.023810,0.000026,0.000000,0.000000,0.000000,0.000000,
0.000000,0.000000,0.908084,0.952381,0.041183,9.055385,0.000000,0.000000,1.000000,0.046512,
1.000000,0.046512,1.000000,0.046512,1.000000,0.046512,0.974390,0.045320,0.807317,0.037550,
0.568293,0.026432,0.369512,0.017187,0.218293,0.010153,0.080488,0.003744,1.000000,0.953488,
1.000000,0.953488,1.000000,0.953488,1.000000,0.953488,1.000000,0.953488,1.000000,0.953488,
1.000000,0.953488,1.000000,0.953488,1.000000,0.953488,0.000000,0.000000,0.508475,0.314361,
0.471831,0.353060,0.530864,0.332580,0.333333,0.205864,0.310345,0.201914,0.425000,0.239674,
0.370370,0.032712,0.521739,0.260127,0.753086,0.495520,0.851852,0.729169,1.000000,0.986466,
0.092593,0.014075,0.352273,0.233815,0.646018,0.486238,0.802381,0.711276,1.000000,0.985455,
0.019608,0.005199,0.347458,0.245945,0.686441,0.501057,0.866197,0.749813,1.000000,0.989870,
0.647059,0.333249,0.303571,0.102843,0.813559,0.563908,0.156250,0.046249,0.112500,0.070838,
0.034483,0.005083,0.019608,0.005199,0.566372,0.274380,0.906780,0.497261,0.949153,0.724598,
1.000000,1.000000,0.974576,0.195534,0.974576,0.302556,0.974576,0.414958,0.974576,0.536570,
0.997101,0.732909,0.574394,0.058974,0.640138,0.303999,0.773810,0.519728,0.886905,0.735300,
0.998192,0.932503,0.832258,0.547382,0.520000,0.354625,0.078431,0.025768,0.353982,0.252278,
0.631068,0.504859,0.838983,0.724378,0.996759,0.977580,0.490446,0.355972,0.482301,0.389467,
0.409091,0.254561,0.363057,0.278420,0.235294,0.172570,0.317757,0.202199,0.092593,0.014067,

0.343137,0.231534,0.607843,0.475207,0.813149,0.705107,1.000000,0.984047,0.074074,0.020371,
0.387097,0.255240,0.619048,0.500189,0.832258,0.753208,1.000000,0.991599,0.019608,0.005199,
0.329545,0.236486,0.607330,0.490559,0.863636,0.751697,1.000000,0.990100,0.580247,0.367419,
0.452229,0.302428,0.525424,0.330153,0.388535,0.232464,0.355140,0.236682,0.290598,0.182036,
0.019608,0.005199,0.372881,0.255187,0.686441,0.512325,0.866197,0.749375,1.000000,0.991192,
0.092593,0.014142,0.343137,0.219511,0.646018,0.470835,0.813149,0.699497,1.000000,0.980457,
0.117647,0.025225,0.521739,0.263048,0.753086,0.501216,0.886364,0.741110,1.000000,0.987659,
0.436620,0.302722,0.518519,0.442717,0.409091,0.254561,0.356688,0.269919,0.218391,0.146282,
0.345794,0.228487,0.115942,0.016631,0.337580,0.226408,0.607843,0.480822,0.819048,0.700635,
1.000000,0.978888,0.074074,0.019447,0.374194,0.253484,0.587045,0.494716,0.823529,0.752106,
1.000000,0.993227,0.019608,0.005199,0.329545,0.236486,0.607330,0.490559,0.863636,0.751697,
1.000000,0.990100,0.127119,0.053298,0.156863,0.066962,0.144068,0.068290,0.096886,0.040829,
0.169492,0.046965,0.066456,0.038016,0.139241,0.061634,0.196429,0.090800,0.146497,0.064001,
0.067961,0.045458,0.086420,0.040535,0.034940,0.015792,0.102273,0.034840,0.037037,0.012715,
0.126214,0.072113,0.142169,0.099649,0.139241,0.064433,0.067797,0.029552,0.092593,0.044749,
0.061728,0.009369

Positive_17 28.000000,0.088010,10.000000,2.464286,1.000000,7.961640,6.000000,2.464286,2.000000,3.665344,
0.437608,0.024789,0.437608,0.024789,0.072464,0.140625,0.236364,0.428571,0.708333,0.714286,
0.000000,0.239881,1.250000,0.158937,0.184524,1.500000,0.157077,0.070764,0.500000,0.016204,
0.038889,0.250000,0.006243,0.074361,0.185185,0.004372,0.035758,0.191358,0.002769,0.042328,
0.185185,0.001930,0.120040,0.250000,0.009932,5.415130,3.849579,3.183179,1.000000,0.464286,
1.000000,0.464286,1.000000,0.457143,1.000000,0.439683,1.000000,0.365760,1.000000,0.286224,
1.000000,0.200397,0.333333,0.023696,0.000000,0.000000,0.000000,0.000000,1.000000,0.571429,
1.000000,0.571429,1.000000,0.547619,1.000000,0.526190,1.000000,0.458333,1.000000,0.332143,
1.000000,0.192857,1.000000,0.123810,1.000000,0.123810,0.000000,0.000000,0.336066,0.299795,
0.434088,0.377077,0.360870,0.323128,0.251185,0.212567,0.235616,0.201403,0.311526,0.248385,
0.029412,0.010153,0.267157,0.229834,0.580645,0.512596,0.824561,0.742611,1.000000,0.996417,
0.033898,0.009317,0.316384,0.249145,0.546638,0.488151,0.787629,0.747174,1.000000,0.997141,
0.005882,0.002547,0.307116,0.255017,0.576832,0.497237,0.797814,0.739440,1.000000,0.991630,
0.595206,0.437990,0.295606,0.198310,0.650862,0.363701,0.127962,0.082033,0.090652,0.071525,
0.018692,0.005914,0.005882,0.002547,0.353201,0.233489,0.647826,0.505676,0.845475,0.769732,
1.000000,1.000000,0.331897,0.085240,0.440341,0.284215,0.616477,0.517358,0.820116,0.728076,
0.998145,0.934627,0.251656,0.042638,0.386364,0.236680,0.629139,0.471649,0.846591,0.712340,
0.996680,0.957672,0.684720,0.626384,0.412776,0.366696,0.046358,0.013030,0.320312,0.277569,
0.530055,0.505284,0.778409,0.741676,1.000000,0.990190,0.468750,0.392432,0.418557,0.361396,
0.327485,0.246172,0.345794,0.280549,0.226667,0.191343,0.214112,0.176150,0.033898,0.009064,
0.316384,0.249361,0.531073,0.489737,0.779381,0.744892,1.000000,0.996932,0.029240,0.010381,
0.306818,0.244215,0.543011,0.496028,0.793785,0.742617,1.000000,0.995009,0.005882,0.002547,
0.298246,0.241375,0.580574,0.506618,0.825843,0.745608,1.000000,0.992462,0.415205,0.356605,
0.386364,0.321105,0.356796,0.322290,0.283224,0.228736,0.288235,0.238589,0.232295,0.196748,
0.005882,0.002547,0.323864,0.260708,0.568306,0.502564,0.797814,0.743732,1.000000,0.992117,
0.033898,0.009317,0.305085,0.240049,0.536723,0.485119,0.801923,0.746600,1.000000,0.996678,
0.029412,0.010153,0.271638,0.234414,0.575290,0.507696,0.817204,0.741402,1.000000,0.996496,
0.361423,0.320466,0.482474,0.433362,0.327485,0.246172,0.352025,0.276469,0.203024,0.158513,

	0.244701,0.208981,0.033898,0.009758,0.316384,0.245810,0.519774,0.487555,0.784615,0.739415,<br>1.000000,0.995225,0.029240,0.010174,0.278689,0.245372,0.560109,0.493611,0.792277,0.742594,<br>1.000000,0.996979,0.005882,0.002547,0.298246,0.241375,0.580574,0.506618,0.825843,0.745608,<br>1.000000,0.992462,0.073034,0.050930,0.080097,0.057685,0.111111,0.066568,0.081967,0.051953,<br>0.076159,0.039099,0.048473,0.033560,0.111801,0.077790,0.106383,0.076799,0.099251,0.061555,<br>0.075269,0.052369,0.085227,0.052592,0.047936,0.022495,0.058480,0.033477,0.039130,0.019374,<br>0.090686,0.073636,0.138418,0.093340,0.080412,0.055193,0.048387,0.025051,0.069892,0.042091,<br>0.026005,0.014442
Positive_18	141.000000,0.190131,97.000000,26.808511,27.000000,424.698784,106.000000,26.808511,30.000000,380.698784,<br>0.325625,0.013801,0.325625,0.013801,0.118631,0.390545,0.567194,0.660959,0.764310,0.628571,<br>0.576923,9.243610,26.727273,34.977839,8.788357,31.830189,45.643628,0.360998,1.000000,0.046380,<br>0.286974,0.666667,0.027413,0.291661,0.500000,0.014252,0.260992,0.407540,0.013355,0.226100,<br>0.344286,0.008574,0.289976,0.685714,0.016381,40.195127,25.339666,23.048501,1.000000,0.971631,<br>1.000000,0.970280,1.000000,0.905702,1.000000,0.739714,1.000000,0.555981,1.000000,0.436686,<br>1.000000,0.338928,1.000000,0.258711,0.333333,0.105674,0.166667,0.026503,1.000000,0.921986,<br>1.000000,0.920817,1.000000,0.868239,1.000000,0.682228,1.000000,0.462089,1.000000,0.306399,<br>1.000000,0.212527,1.000000,0.152763,0.333333,0.072549,0.136364,0.019018,0.530612,0.323542,<br>0.467513,0.359439,0.402985,0.317019,0.310811,0.222280,0.302013,0.204712,0.295455,0.225870,<br>0.111111,0.013722,0.408163,0.255987,0.687075,0.498072,0.850340,0.745708,1.000000,0.993358,<br>0.090226,0.009018,0.406667,0.236462,0.718274,0.492105,0.870558,0.740003,1.000000,0.993563,<br>0.017241,0.003001,0.518072,0.250257,0.722892,0.495088,0.831325,0.733951,1.000000,0.992526,<br>0.819277,0.453413,0.494186,0.159788,0.676056,0.386798,0.152047,0.062641,0.158940,0.075598,<br>0.023256,0.004238,0.017241,0.003001,0.387718,0.229403,0.693878,0.480338,0.925373,0.743183,<br>1.000000,1.000000,0.933162,0.094627,0.935733,0.261685,0.951157,0.472682,0.987603,0.692856,<br>0.999148,0.898565,0.698795,0.048582,0.722892,0.268306,0.826667,0.499457,0.933333,0.715410,<br>0.998258,0.923910,0.668550,0.543815,0.479452,0.359893,0.258621,0.020144,0.566265,0.266661,<br>0.722892,0.511068,0.826633,0.733921,1.000000,0.987152,0.512821,0.379730,0.445367,0.369684,<br>0.362069,0.250585,0.361314,0.274054,0.280702,0.185859,0.269690,0.183955,0.059701,0.008295,<br>0.332024,0.231599,0.656853,0.487404,0.853807,0.738224,1.000000,0.994895,0.119658,0.013686,<br>0.351051,0.255598,0.674699,0.502018,0.879518,0.744683,1.000000,0.995291,0.017241,0.003001,<br>0.401575,0.252620,0.637931,0.494584,0.824708,0.737824,1.000000,0.990497,0.462687,0.347355,<br>0.435897,0.303649,0.530612,0.348996,0.279352,0.208350,0.348993,0.242915,0.279817,0.203826,<br>0.017241,0.003001,0.518072,0.256866,0.722892,0.500113,0.839080,0.738796,1.000000,0.993405,<br>0.090226,0.009254,0.353333,0.224099,0.728426,0.480857,0.869036,0.731163,1.000000,0.991850,<br>0.111111,0.013355,0.408163,0.258014,0.687075,0.500432,0.850340,0.746783,1.000000,0.994654,<br>0.435897,0.307672,0.512821,0.441742,0.362069,0.250585,0.387931,0.271193,0.212500,0.150366,<br>0.324582,0.219447,0.134328,0.010032,0.344000,0.234026,0.651961,0.487993,0.833503,0.740188,<br>1.000000,0.993256,0.102564,0.011369,0.317757,0.249232,0.602410,0.498450,0.850340,0.743326,<br>1.000000,0.996268,0.017241,0.003001,0.401575,0.252620,0.637931,0.494584,0.824708,0.737824,<br>1.000000,0.990497,0.120690,0.056679,0.126609,0.059062,0.176871,0.075468,0.120000,0.055001,<br>0.080952,0.041285,0.061224,0.036047,0.118257,0.065285,0.222222,0.071204,0.114721,0.064800,<br>0.084337,0.051383,0.120482,0.050977,0.062222,0.025454,0.060000,0.030336,0.092593,0.021081,<br>0.104651,0.065290,0.179104,0.098107,0.102564,0.053486,0.051724,0.025557,0.088319,0.042785,<br>0.037383,0.010713

Positive_19 7.000000,0.265306,4.000000,1.857143,2.000000,3.476190,3.000000,1.857143,2.000000,0.809524,
0.364422,0.000646,0.364422,0.000646,0.000000,0.000000,0.923077,1.000000,0.000000,0.000000,
0.000000,0.190476,1.333333,0.253968,0.238095,0.666667,0.091270,0.063492,0.444444,0.028219,
0.103175,0.250000,0.016645,0.115079,0.222222,0.011877,0.162698,0.555556,0.062803,0.206349,
0.333333,0.017196,0.349206,0.500000,0.036486,2.901241,2.060856,0.579374,1.000000,0.571429,
1.000000,0.571429,1.000000,0.571429,1.000000,0.571429,1.000000,0.476190,1.000000,0.476190,
1.000000,0.476190,1.000000,0.476190,1.000000,0.476190,1.000000,0.476190,1.000000,0.857143,
1.000000,0.857143,1.000000,0.857143,1.000000,0.857143,1.000000,0.761905,1.000000,0.476190,
1.000000,0.476190,1.000000,0.476190,1.000000,0.476190,1.000000,0.476190,0.334483,0.313501,
0.390848,0.342036,0.358300,0.344463,0.225152,0.210114,0.235294,0.201851,0.262976,0.249613,
0.050607,0.025472,0.295547,0.261939,0.538462,0.527846,0.761628,0.747793,1.000000,0.995459,
0.017241,0.008297,0.296552,0.252577,0.520630,0.467809,0.780195,0.742630,0.997976,0.997272,
0.003448,0.002237,0.237931,0.227603,0.505814,0.478809,0.784884,0.750965,1.000000,0.997988,
0.510345,0.434291,0.268966,0.150857,0.536437,0.414851,0.096886,0.066899,0.081136,0.069191,
0.005825,0.003322,0.003448,0.002237,0.331034,0.242352,0.544574,0.517235,0.843023,0.789849,
1.000000,1.000000,0.296552,0.124065,0.393103,0.244176,0.765182,0.672742,0.844130,0.811731,
0.997976,0.992566,0.019505,0.010837,0.302326,0.225607,0.578395,0.420688,0.786197,0.678112,
0.987997,0.947239,0.627907,0.610861,0.373225,0.352076,0.012146,0.009345,0.269317,0.248657,
0.555889,0.479796,0.762191,0.733339,0.998500,0.995249,0.417854,0.352341,0.420690,0.370581,
0.311741,0.277077,0.303303,0.263457,0.225152,0.196736,0.223124,0.201516,0.006897,0.005341,
0.286207,0.249310,0.498875,0.472543,0.770443,0.753446,0.997976,0.997272,0.010345,0.006165,
0.269380,0.231680,0.511628,0.491282,0.767442,0.715518,1.000000,0.998265,0.003448,0.002237,
0.287449,0.265095,0.566802,0.545040,0.793522,0.759276,1.000000,0.994136,0.400000,0.373418,
0.345086,0.290223,0.362069,0.336359,0.244745,0.224578,0.294118,0.237748,0.207207,0.185221,
0.003448,0.002237,0.255172,0.233321,0.505814,0.476251,0.775194,0.743692,1.000000,0.997988,
0.017241,0.008297,0.300000,0.253074,0.511628,0.461455,0.777944,0.748494,0.997976,0.997272,
0.050607,0.025472,0.313953,0.263390,0.538462,0.528694,0.779310,0.747108,1.000000,0.995459,
0.332333,0.285346,0.482759,0.437577,0.311741,0.277077,0.296547,0.246194,0.184584,0.159320,
0.257606,0.238932,0.006897,0.005341,0.279310,0.253958,0.511628,0.444766,0.774944,0.739852,
0.997749,0.994669,0.010345,0.006165,0.253876,0.224943,0.506897,0.494788,0.769380,0.736102,
1.000000,0.999133,0.003448,0.002237,0.287449,0.265095,0.566802,0.545040,0.793522,0.759276,
1.000000,0.994136,0.070850,0.059210,0.064516,0.056931,0.075862,0.064791,0.058621,0.047294,
0.051724,0.045417,0.051724,0.039858,0.081020,0.067412,0.074269,0.044393,0.077519,0.063120,
0.079310,0.063127,0.067829,0.052171,0.027586,0.022857,0.048276,0.028956,0.030008,0.014825,
0.070518,0.060476,0.118217,0.105231,0.075862,0.054808,0.040486,0.029375,0.093117,0.070509,
0.017442,0.009240

Positive_20 86.000000,0.130070,57.000000,11.186047,4.500000,172.200274,41.000000,11.186047,13.000000,77.564979,
0.345336,0.019468,0.345336,0.019468,0.146722,0.318293,0.431127,0.600629,0.716535,0.583333,
0.533333,2.184205,11.020000,8.269711,3.407346,7.827586,6.064696,0.146104,0.471074,0.030312,
0.284459,0.500000,0.028663,0.262919,0.407843,0.015922,0.267059,0.469281,0.019851,0.164869,
0.334454,0.016711,0.297341,0.509244,0.027414,20.912637,13.185781,9.851594,1.000000,0.941860,
1.000000,0.941688,1.000000,0.907929,1.000000,0.747353,1.000000,0.546030,1.000000,0.473823,
1.000000,0.398983,0.771739,0.287377,0.431159,0.096019,0.119565,0.014015,1.000000,0.883721,
1.000000,0.879037,1.000000,0.814385,1.000000,0.703695,1.000000,0.469389,1.000000,0.277127,

0.511364,0.167430,0.410985,0.102846,0.229167,0.043825,0.062500,0.006792,0.530612,0.328721,
0.496032,0.359027,0.402985,0.312252,0.310811,0.226700,0.295302,0.204804,0.296552,0.220135,
0.111111,0.018552,0.408163,0.254661,0.687075,0.496197,0.850340,0.745846,1.000000,0.993183,
0.090226,0.011223,0.406667,0.239802,0.718274,0.495181,0.870558,0.737081,1.000000,0.991968,
0.017241,0.003800,0.418803,0.246072,0.622047,0.487203,0.826633,0.727627,1.000000,0.989793,
0.701005,0.469856,0.494186,0.183239,0.676056,0.346905,0.152047,0.073100,0.158940,0.073331,
0.021459,0.004297,0.017241,0.003800,0.387255,0.224638,0.693878,0.481780,0.925373,0.741564,
1.000000,1.000000,0.933162,0.110641,0.935733,0.269020,0.951157,0.497277,0.956298,0.712141,
0.999148,0.914612,0.350427,0.057503,0.580000,0.267884,0.826667,0.470219,0.933333,0.687759,
0.998652,0.894164,0.684878,0.531282,0.479452,0.363231,0.258621,0.025186,0.480315,0.263672,
0.639344,0.503348,0.826633,0.729943,0.999281,0.984813,0.512821,0.383292,0.441077,0.365382,
0.362069,0.251326,0.361314,0.268939,0.280702,0.189483,0.269690,0.181234,0.059701,0.010290,
0.332024,0.234923,0.656853,0.491297,0.853807,0.736325,1.000000,0.994646,0.119658,0.017717,
0.384365,0.251961,0.626984,0.490165,0.863946,0.738468,1.000000,0.993237,0.017241,0.003800,
0.401575,0.252023,0.637931,0.498338,0.800000,0.739555,1.000000,0.988372,0.462687,0.344148,
0.448413,0.304164,0.530612,0.351689,0.296552,0.205268,0.348993,0.241454,0.270270,0.206945,
0.017241,0.003800,0.418803,0.253557,0.622047,0.493519,0.826633,0.733336,1.000000,0.990521,
0.090226,0.011729,0.353333,0.227951,0.728426,0.484340,0.869036,0.723545,1.000000,0.990343,
0.111111,0.018147,0.408163,0.253149,0.687075,0.498574,0.850340,0.748331,1.000000,0.994678,
0.435897,0.306011,0.512821,0.442663,0.362069,0.251326,0.387931,0.267740,0.212500,0.150504,
0.324582,0.220213,0.134328,0.011946,0.344000,0.237599,0.651961,0.489863,0.833503,0.734508,
1.000000,0.992803,0.102564,0.015046,0.317757,0.244281,0.570033,0.489990,0.850340,0.739429,
1.000000,0.994835,0.017241,0.003800,0.401575,0.252023,0.637931,0.498338,0.800000,0.739555,
1.000000,0.988372,0.120690,0.059898,0.126609,0.059770,0.176871,0.076770,0.120000,0.056265,
0.080952,0.042712,0.068783,0.033306,0.126984,0.067251,0.222222,0.071253,0.114721,0.061185,
0.082707,0.050057,0.112150,0.054417,0.048000,0.022968,0.060000,0.031896,0.092593,0.022864,
0.104651,0.066051,0.179104,0.093750,0.102564,0.052793,0.051724,0.024444,0.088319,0.042108,
0.037383,0.010243

Positive_21 24.000000,0.204861,15.000000,4.916667,3.500000,25.210145,8.000000,4.916667,5.500000,8.166667,
0.480847,0.016868,0.480847,0.016868,0.042373,0.026549,0.190909,0.460674,0.500000,0.791667,
0.800000,1.075018,4.461538,1.872488,1.542262,2.875000,1.464292,0.130641,0.395062,0.022055,
0.232607,0.408163,0.024956,0.165012,0.304348,0.016029,0.135862,0.550725,0.026547,0.125878,
0.195652,0.005710,0.322024,0.532609,0.028251,8.282596,5.208579,2.703087,1.000000,0.583333,
1.000000,0.583333,1.000000,0.552086,1.000000,0.536065,1.000000,0.422848,1.000000,0.230723,
0.333333,0.120472,0.333333,0.064057,0.333333,0.052864,0.333333,0.028439,1.000000,0.833333,
1.000000,0.833333,1.000000,0.833333,1.000000,0.833333,1.000000,0.713988,1.000000,0.525298,
1.000000,0.368056,1.000000,0.141171,1.000000,0.112798,0.035714,0.001488,0.359788,0.306981,
0.418692,0.377875,0.335777,0.315145,0.266129,0.220735,0.218346,0.200373,0.270234,0.238654,
0.026810,0.011340,0.295508,0.245362,0.562617,0.503401,0.804185,0.754362,1.000000,0.997076,
0.013540,0.004349,0.281870,0.232965,0.512748,0.479876,0.769120,0.728183,1.000000,0.997123,
0.002681,0.001679,0.319312,0.263952,0.560229,0.514887,0.773148,0.751522,1.000000,0.997491,
0.587131,0.432090,0.233068,0.154899,0.519713,0.413011,0.098837,0.066255,0.096506,0.077047,
0.013410,0.004694,0.002681,0.001679,0.299355,0.213647,0.547416,0.470893,0.812379,0.725610,
1.000000,1.000000,0.415825,0.083902,0.574074,0.298664,0.780645,0.476687,0.870968,0.683082,

0.998677,0.925183,0.056093,0.019361,0.453083,0.282210,0.665392,0.530141,0.827916,0.759386,
0.998652,0.983234,0.684588,0.619840,0.416185,0.342135,0.018145,0.007017,0.326599,0.276270,
0.558710,0.510492,0.776455,0.739891,0.998652,0.994381,0.444860,0.391434,0.401530,0.358466,
0.281501,0.250100,0.312950,0.273173,0.231183,0.192826,0.220307,0.176336,0.013540,0.004824,
0.285924,0.231121,0.521994,0.477548,0.769120,0.726645,1.000000,0.997170,0.024129,0.009378,
0.317757,0.257980,0.586998,0.510859,0.794393,0.758468,1.000000,0.997875,0.002681,0.001679,
0.316785,0.256638,0.557920,0.506129,0.837110,0.753717,1.000000,0.996208,0.372521,0.345955,
0.383178,0.321151,0.380291,0.332894,0.260300,0.224735,0.266176,0.239013,0.241935,0.202722,
0.002681,0.001679,0.304933,0.266683,0.543319,0.513844,0.783178,0.754502,1.000000,0.998426,
0.013540,0.004947,0.284703,0.227430,0.525794,0.474512,0.770563,0.723142,1.000000,0.996686,
0.023932,0.010826,0.307329,0.246779,0.562617,0.503437,0.805680,0.754468,1.000000,0.997076,
0.368224,0.319741,0.474187,0.430160,0.281501,0.250100,0.316479,0.271780,0.188811,0.158818,
0.254789,0.210344,0.013540,0.005187,0.286119,0.226371,0.534000,0.474730,0.774799,0.723999,
1.000000,0.996705,0.024129,0.007452,0.291589,0.256395,0.544933,0.506951,0.786916,0.756278,
1.000000,0.997875,0.002681,0.001679,0.316785,0.256638,0.557920,0.506129,0.837110,0.753717,
1.000000,0.996208,0.080306,0.055370,0.097884,0.058832,0.087302,0.065893,0.066079,0.049604,
0.065010,0.038781,0.060533,0.038501,0.097196,0.076343,0.130841,0.081293,0.086379,0.062790,
0.062049,0.049711,0.066532,0.051015,0.038206,0.025913,0.044917,0.030810,0.032578,0.020679,
0.087041,0.070353,0.141491,0.092193,0.077922,0.052745,0.042735,0.025272,0.060000,0.042889,
0.021792,0.011014

Positive_22 27.000000,0.150892,12.000000,4.074074,2.000000,15.148148,12.000000,4.074074,2.000000,17.609687,
0.387225,0.013667,0.387225,0.013667,0.036364,0.207547,0.464286,0.577778,0.736842,0.400000,
1.000000,1.122949,5.083333,2.972490,1.091035,5.083333,2.971837,0.129185,0.423611,0.030769,
0.116381,0.423611,0.027493,0.234482,0.423077,0.016841,0.086149,0.269231,0.008041,0.067628,
0.461538,0.011827,0.162960,0.423077,0.022099,8.454681,3.137622,2.828427,1.000000,0.629630,
1.000000,0.629630,1.000000,0.629630,1.000000,0.552782,1.000000,0.407739,1.000000,0.197229,
1.000000,0.161210,1.000000,0.139594,1.000000,0.139594,0.333333,0.021840,1.000000,0.666667,
1.000000,0.666667,1.000000,0.657309,1.000000,0.573435,1.000000,0.378521,1.000000,0.298311,
1.000000,0.228927,1.000000,0.198752,1.000000,0.192603,0.022222,0.002768,0.361277,0.306736,
0.435737,0.373607,0.350195,0.319657,0.239460,0.216822,0.237395,0.207645,0.290448,0.236329,
0.025559,0.013028,0.295720,0.250592,0.564202,0.493838,0.811284,0.745563,1.000000,0.994598,
0.009740,0.004848,0.285429,0.238761,0.545455,0.494207,0.795349,0.739409,1.000000,0.996442,
0.003247,0.002034,0.285470,0.254036,0.547046,0.502811,0.778509,0.749191,1.000000,0.995681,
0.504702,0.417973,0.249322,0.168675,0.661157,0.413353,0.100977,0.068179,0.101786,0.079116,
0.018908,0.004991,0.003247,0.002034,0.301095,0.213705,0.547416,0.463872,0.794157,0.714518,
1.000000,1.000000,0.415825,0.082986,0.574074,0.284466,0.783058,0.519386,0.987603,0.753405,
0.999151,0.953335,0.096491,0.032476,0.418895,0.276037,0.626478,0.514600,0.821281,0.751615,
0.998652,0.986106,0.684825,0.617829,0.399123,0.348526,0.019960,0.009782,0.359489,0.270841,
0.563869,0.506440,0.799270,0.731557,0.999151,0.993017,0.470779,0.386387,0.392544,0.363458,
0.297683,0.250156,0.328252,0.270743,0.235867,0.191020,0.234000,0.182282,0.009740,0.004743,
0.281022,0.232735,0.542208,0.485832,0.795527,0.733144,1.000000,0.996881,0.035124,0.012519,
0.317073,0.257201,0.558266,0.504388,0.796053,0.752811,1.000000,0.996380,0.003247,0.002034,
0.316785,0.254224,0.581633,0.501047,0.808511,0.744070,1.000000,0.993368,0.381462,0.349574,
0.386364,0.316252,0.389222,0.334174,0.265107,0.218399,0.291667,0.247385,0.227508,0.202105,

0.003247,0.002034,0.290146,0.256728,0.551422,0.505531,0.786496,0.749135,1.000000,0.996093,
0.009740,0.004963,0.283422,0.230940,0.542208,0.489323,0.792415,0.734175,1.000000,0.995674,
0.025078,0.012320,0.307329,0.249407,0.541880,0.495581,0.789883,0.747585,1.000000,0.995419,
0.415584,0.314910,0.472813,0.434935,0.297683,0.250156,0.329268,0.267447,0.203024,0.154887,
0.280000,0.218415,0.029221,0.005878,0.299611,0.232012,0.555195,0.482286,0.784496,0.728959,
1.000000,0.995246,0.020661,0.008642,0.300813,0.253402,0.555785,0.502274,0.785088,0.753956,
1.000000,0.997615,0.003247,0.002034,0.316785,0.254224,0.581633,0.501047,0.808511,0.744070,
1.000000,0.993368,0.075127,0.056344,0.080097,0.055033,0.089416,0.066141,0.077479,0.050930,
0.061929,0.043327,0.060533,0.034962,0.129870,0.077981,0.110390,0.078500,0.077586,0.057171,
0.067751,0.050328,0.081505,0.052272,0.044728,0.027438,0.049911,0.029917,0.033074,0.019205,
0.097403,0.070894,0.135204,0.097561,0.086262,0.050574,0.042735,0.025736,0.069182,0.044336,
0.027100,0.011351

Positive_23 39.000000,0.094675,11.000000,3.692308,2.000000,13.218623,15.000000,3.692308,2.000000,16.060729,
0.485470,0.019145,0.485470,0.019145,0.013889,0.007042,0.234043,0.490741,0.654545,0.421053,
0.454545,1.219533,4.909091,3.062718,1.204851,4.727273,2.703907,0.164788,0.530612,0.038485,
0.161450,0.520000,0.045137,0.115339,0.368421,0.012553,0.082874,0.236842,0.005565,0.085851,
0.289474,0.006681,0.096568,0.250000,0.008609,8.720494,5.023735,3.471436,1.000000,0.666667,
1.000000,0.666667,1.000000,0.666667,1.000000,0.666667,1.000000,0.521384,1.000000,0.315140,
1.000000,0.133777,1.000000,0.064874,1.000000,0.035470,0.000000,0.000000,1.000000,0.538462,
1.000000,0.538462,1.000000,0.538462,1.000000,0.537973,1.000000,0.423251,1.000000,0.263844,
0.190476,0.033973,0.066667,0.016213,0.047619,0.004373,0.000000,0.000000,0.383268,0.288395,
0.441176,0.392584,0.391960,0.319020,0.263069,0.210320,0.249513,0.191858,0.404040,0.255600,
0.059322,0.011053,0.338983,0.248944,0.564202,0.485147,0.811284,0.747215,1.000000,0.995295,
0.025292,0.008062,0.354086,0.242122,0.566148,0.499042,0.787645,0.741565,1.000000,0.997195,
0.005102,0.002371,0.306173,0.252985,0.576832,0.498351,0.796690,0.741867,1.000000,0.992646,
0.596330,0.446384,0.305556,0.176173,0.653266,0.377443,0.127962,0.075343,0.111111,0.077982,
0.014218,0.003841,0.005102,0.002371,0.339768,0.231547,0.615764,0.496308,0.813291,0.743897,
1.000000,1.000000,0.405333,0.089251,0.597333,0.329613,0.752000,0.522825,0.881356,0.711781,
0.997821,0.898741,0.162963,0.035179,0.412658,0.227163,0.663580,0.464076,0.875274,0.706068,
0.998039,0.963827,0.708543,0.613056,0.473214,0.349314,0.051020,0.011337,0.344358,0.269321,
0.553611,0.509795,0.779961,0.736832,0.997821,0.989059,0.435946,0.394987,0.395556,0.356944,
0.309333,0.248068,0.345865,0.274358,0.238095,0.195346,0.226121,0.177549,0.020173,0.007198,
0.311224,0.237661,0.558974,0.491225,0.793436,0.739305,1.000000,0.996245,0.038136,0.009910,
0.341463,0.254793,0.559322,0.494966,0.804878,0.742321,1.000000,0.994594,0.005102,0.002371,
0.374179,0.251079,0.590810,0.501669,0.813559,0.748277,1.000000,0.994710,0.447236,0.350136,
0.386233,0.331110,0.402724,0.318754,0.363636,0.235069,0.298413,0.232623,0.258010,0.198601,
0.005102,0.002371,0.317287,0.253260,0.569873,0.502663,0.783646,0.742486,1.000000,0.993259,
0.025292,0.008367,0.317121,0.235055,0.560859,0.488179,0.801923,0.740635,1.000000,0.995635,
0.059322,0.010692,0.338983,0.250320,0.555799,0.491660,0.789883,0.748192,1.000000,0.997197,
0.372849,0.319805,0.470460,0.432127,0.309333,0.248068,0.343985,0.276352,0.201745,0.157580,
0.282540,0.215314,0.021277,0.008050,0.311224,0.240012,0.562667,0.487592,0.784615,0.738429,
1.000000,0.994878,0.030612,0.008304,0.329268,0.249304,0.550505,0.496390,0.804878,0.745995,
1.000000,0.995512,0.005102,0.002371,0.374179,0.251079,0.590810,0.501669,0.813559,0.748277,
1.000000,0.994710,0.088803,0.055664,0.098101,0.051668,0.098522,0.063486,0.074576,0.044973,

0.061776,0.041148,0.055556,0.031456,0.113949,0.081164,0.141491,0.083182,0.085938,0.060725,
0.070352,0.049761,0.083151,0.056277,0.045918,0.030359,0.055276,0.031116,0.033074,0.018905,
0.117347,0.070487,0.160804,0.102290,0.074656,0.048077,0.037221,0.024721,0.066456,0.040343,
0.029661,0.014197

Positive_24 21.000000,0.149660,6.000000,3.142857,3.000000,5.328571,7.000000,3.142857,4.000000,6.828571,
0.383529,0.015762,0.383529,0.015762,0.045455,0.174603,0.480769,0.703704,0.625000,0.333333,
0.000000,0.373016,1.333333,0.163624,0.295238,2.000000,0.323844,0.095899,0.250000,0.010492,
0.052812,0.333333,0.009485,0.159325,0.275000,0.011288,0.085119,0.183333,0.004779,0.100102,
0.350000,0.015297,0.112948,0.250000,0.011913,5.536712,4.383051,2.531444,1.000000,0.714286,
1.000000,0.714286,1.000000,0.714286,1.000000,0.644444,1.000000,0.420635,1.000000,0.242857,
1.000000,0.147619,1.000000,0.077778,1.000000,0.061905,0.000000,0.000000,1.000000,0.571429,
1.000000,0.571429,1.000000,0.571429,1.000000,0.520635,1.000000,0.456009,1.000000,0.303175,
0.800000,0.245578,0.600000,0.173696,0.200000,0.057596,0.000000,0.000000,0.386029,0.314089,
0.421585,0.369881,0.350384,0.316030,0.286327,0.226854,0.258974,0.199092,0.280353,0.237484,
0.038869,0.009298,0.284238,0.245696,0.580645,0.502821,0.822581,0.749843,1.000000,0.995918,
0.025707,0.006975,0.275449,0.223593,0.529114,0.471273,0.774834,0.735215,1.000000,0.996629,
0.005376,0.002225,0.395082,0.283252,0.610746,0.519571,0.811254,0.750308,1.000000,0.990359,
0.629156,0.492161,0.278146,0.166238,0.478921,0.341601,0.118919,0.070720,0.096408,0.075407,
0.011038,0.004316,0.005376,0.002225,0.310345,0.219264,0.615764,0.483724,0.793103,0.734975,
1.000000,1.000000,0.206140,0.050399,0.437500,0.257524,0.645244,0.465953,0.814882,0.664452,
0.982005,0.893443,0.251656,0.070061,0.547703,0.300901,0.731449,0.523105,0.890026,0.776951,
0.998162,0.971351,0.671378,0.570146,0.400000,0.348171,0.046358,0.014950,0.386885,0.281966,
0.611842,0.513945,0.822368,0.736934,0.998904,0.990086,0.436066,0.396935,0.445013,0.358919,
0.274194,0.244146,0.326733,0.284239,0.237589,0.185863,0.209877,0.174283,0.020566,0.006507,
0.299401,0.223441,0.499387,0.463444,0.769461,0.720369,1.000000,0.995296,0.035336,0.010366,
0.327869,0.271602,0.573271,0.514789,0.805654,0.757554,1.000000,0.993527,0.005376,0.002225,
0.361757,0.255027,0.571856,0.521449,0.805419,0.762825,1.000000,0.994901,0.388747,0.346239,
0.352459,0.316660,0.405025,0.337101,0.256071,0.217551,0.315385,0.238221,0.264255,0.210458,
0.005376,0.002225,0.401639,0.284585,0.607456,0.522254,0.800439,0.753559,1.000000,0.992503,
0.025707,0.006975,0.296407,0.214560,0.521519,0.458861,0.769461,0.723456,1.000000,0.993622,
0.038869,0.009298,0.284238,0.245886,0.550926,0.501735,0.817204,0.750674,1.000000,0.997715,
0.350820,0.313514,0.519182,0.442340,0.274194,0.244146,0.326733,0.280980,0.189189,0.146498,
0.252577,0.213648,0.028133,0.008404,0.323353,0.223188,0.514139,0.460682,0.770370,0.718117,
1.000000,0.992509,0.035336,0.008051,0.304918,0.265406,0.570926,0.509960,0.775330,0.750551,
1.000000,0.995713,0.005376,0.002225,0.361757,0.255027,0.571856,0.521449,0.805419,0.762825,
1.000000,0.994901,0.082063,0.053754,0.083946,0.060475,0.098522,0.067610,0.078481,0.054041,
0.076159,0.043608,0.053892,0.034601,0.091133,0.070400,0.100000,0.071606,0.096774,0.065349,
0.091873,0.052118,0.079692,0.057187,0.044444,0.023012,0.043011,0.030208,0.043928,0.026234,
0.101266,0.071131,0.132992,0.090557,0.077739,0.051412,0.048387,0.024458,0.069892,0.037763,
0.026981,0.014475

Positive_25 12.000000,0.229167,5.000000,2.750000,3.000000,3.113636,7.000000,2.750000,3.000000,7.477273,
0.390648,0.009409,0.390648,0.009409,0.000000,0.000000,0.636364,0.666667,0.500000,1.000000,
0.000000,0.611111,1.600000,0.289158,0.535714,1.750000,0.633813,0.159259,0.320000,0.014559,
0.121173,0.437500,0.035354,0.324747,0.484848,0.031565,0.190404,0.333333,0.019911,0.161346,

0.477273,0.043770,0.157107,0.363636,0.020877,4.401688,3.150031,1.529893,1.000000,0.750000,
1.000000,0.750000,1.000000,0.750000,1.000000,0.716667,1.000000,0.658333,1.000000,0.427778,
1.000000,0.411111,1.000000,0.108333,1.000000,0.108333,0.000000,0.000000,1.000000,0.583333,
1.000000,0.583333,1.000000,0.583333,1.000000,0.569444,1.000000,0.432540,1.000000,0.349206,
1.000000,0.321429,0.666667,0.150794,0.666667,0.150794,0.666667,0.150794,0.365824,0.302883,
0.424242,0.367625,0.376506,0.329492,0.270270,0.203172,0.234234,0.207351,0.294737,0.258630,
0.023622,0.013739,0.296099,0.249244,0.560284,0.518867,0.820698,0.759886,1.000000,0.995215,
0.020000,0.007488,0.290000,0.234302,0.550000,0.491054,0.800000,0.729098,1.000000,0.985625,
0.005000,0.002768,0.306173,0.240278,0.532558,0.491180,0.771084,0.746325,1.000000,0.991927,
0.535000,0.419088,0.240000,0.159562,0.516245,0.421351,0.111486,0.075139,0.093240,0.077705,
0.010135,0.006876,0.005000,0.002768,0.390000,0.204838,0.580000,0.455683,0.815000,0.686739,
1.000000,1.000000,0.295000,0.074877,0.375000,0.250166,0.655000,0.510852,0.842520,0.740496,
0.986532,0.931936,0.162963,0.037142,0.351706,0.287369,0.656028,0.530848,0.817088,0.722046,
0.997674,0.952103,0.683735,0.599887,0.397590,0.344344,0.035000,0.013170,0.293023,0.261347,
0.539535,0.497065,0.756024,0.720868,0.997674,0.989624,0.434343,0.381243,0.440120,0.376248,
0.285000,0.242508,0.344411,0.290706,0.241206,0.180736,0.211480,0.187330,0.020000,0.007282,
0.270000,0.227080,0.540000,0.483218,0.800000,0.727622,1.000000,0.989596,0.016835,0.010541,
0.303704,0.253901,0.553086,0.500676,0.800525,0.763991,1.000000,0.994573,0.005000,0.002768,
0.299401,0.264089,0.545181,0.508918,0.753012,0.730325,1.000000,0.992878,0.397590,0.354874,
0.356902,0.312098,0.400000,0.333028,0.284211,0.237409,0.271357,0.243244,0.253378,0.195123,
0.005000,0.002768,0.296296,0.249633,0.532558,0.486695,0.781145,0.742382,1.000000,0.991927,
0.020000,0.007488,0.262048,0.223040,0.550000,0.481028,0.815000,0.727638,1.000000,0.985344,
0.023622,0.013158,0.296099,0.255743,0.546099,0.519710,0.808664,0.754448,1.000000,0.997628,
0.361702,0.319808,0.488024,0.437683,0.285000,0.242508,0.326733,0.286158,0.206039,0.154458,
0.246231,0.213608,0.020000,0.007282,0.256659,0.228505,0.539157,0.486271,0.810000,0.733203,
1.000000,0.989596,0.013468,0.009655,0.291358,0.246645,0.545679,0.494884,0.792169,0.756426,
1.000000,0.995807,0.005000,0.002768,0.299401,0.264089,0.545181,0.508918,0.753012,0.730325,
1.000000,0.992878,0.080808,0.044605,0.084337,0.066426,0.084236,0.062245,0.061372,0.051856,
0.048135,0.036616,0.070000,0.041135,0.090426,0.073713,0.102326,0.072766,0.090361,0.073580,
0.065000,0.047894,0.074074,0.044146,0.055000,0.030145,0.031288,0.025382,0.024691,0.017289,
0.113772,0.074682,0.114458,0.099332,0.096386,0.062238,0.035000,0.023181,0.055690,0.035423,
0.023569,0.017346

Positive_26 46.000000,0.094991,17.000000,4.369565,4.000000,10.638164,19.000000,4.369565,3.000000,13.438164,
0.508409,0.015679,0.508409,0.015679,0.004975,0.040000,0.156250,0.339506,0.588785,0.704545,
0.846154,0.814774,3.692308,0.754377,0.796519,3.736842,0.633860,0.143254,0.360000,0.015864,
0.151740,0.360000,0.018301,0.128224,0.266667,0.007627,0.081888,0.168889,0.003469,0.083812,
0.251852,0.003778,0.140496,0.355556,0.008595,7.547469,5.659017,5.408676,1.000000,0.847826,
1.000000,0.847826,1.000000,0.847023,1.000000,0.826892,1.000000,0.748531,1.000000,0.674693,
1.000000,0.442467,1.000000,0.159001,0.200000,0.033267,0.000000,0.000000,1.000000,0.782609,
1.000000,0.782609,1.000000,0.781296,1.000000,0.759094,1.000000,0.630389,1.000000,0.481209,
1.000000,0.355583,1.000000,0.135678,1.000000,0.079391,0.000000,0.000000,0.337079,0.293474,
0.432024,0.388952,0.369898,0.317575,0.273810,0.208536,0.239085,0.193213,0.325758,0.255521,
0.061947,0.013441,0.332494,0.246433,0.575290,0.510127,0.815730,0.748975,1.000000,0.997434,
0.027190,0.006646,0.299213,0.241937,0.544081,0.487756,0.787629,0.740746,1.000000,0.996211,

0.005682,0.002267,0.293367,0.244961,0.543367,0.489636,0.784543,0.739573,1.000000,0.994160,
0.526923,0.406446,0.305699,0.183608,0.570071,0.409947,0.137143,0.079965,0.114187,0.078718,
0.017677,0.006067,0.005682,0.002267,0.353201,0.209465,0.598234,0.488441,0.845475,0.724182,
1.000000,0.999195,0.375904,0.082551,0.462963,0.294373,0.612789,0.496002,0.939577,0.711574,
0.998626,0.938512,0.132812,0.019780,0.407738,0.256950,0.660241,0.500796,0.839286,0.742200,
1.000000,0.970126,0.701531,0.644312,0.399225,0.341220,0.022727,0.007776,0.324138,0.269260,
0.578838,0.503295,0.819502,0.749567,1.000000,0.993286,0.480363,0.407581,0.418557,0.359512,
0.278652,0.232907,0.390438,0.288384,0.260042,0.191054,0.216851,0.168211,0.027190,0.006501,
0.306843,0.243300,0.532308,0.487509,0.779381,0.737901,1.000000,0.995712,0.031746,0.009606,
0.311224,0.249761,0.580723,0.499117,0.797590,0.741063,1.000000,0.996772,0.005682,0.002267,
0.334623,0.245870,0.580574,0.502326,0.831034,0.757256,1.000000,0.993922,0.395408,0.343891,
0.407855,0.343730,0.366292,0.312379,0.314815,0.245683,0.267532,0.221670,0.240476,0.195820,
0.005682,0.002267,0.303571,0.248985,0.544586,0.494268,0.777778,0.744175,1.000000,0.996028,
0.027190,0.006646,0.290456,0.235824,0.544081,0.483436,0.796680,0.732484,1.000000,0.994327,
0.047198,0.013120,0.334623,0.250031,0.590909,0.510819,0.820225,0.751095,1.000000,0.997626,
0.401813,0.342135,0.482474,0.424959,0.278652,0.232907,0.362550,0.283467,0.213531,0.160367,
0.241713,0.198898,0.027190,0.006623,0.303448,0.244844,0.534000,0.489420,0.796680,0.739213,
1.000000,0.994119,0.024615,0.008925,0.313776,0.248852,0.573980,0.494665,0.797619,0.739477,
1.000000,0.997204,0.005682,0.002267,0.334623,0.245870,0.580574,0.502326,0.831034,0.757256,
1.000000,0.993922,0.078652,0.048655,0.098726,0.063068,0.080311,0.061041,0.071154,0.045287,
0.065169,0.038494,0.051136,0.036929,0.119048,0.087955,0.153392,0.097988,0.092262,0.060970,
0.076433,0.049935,0.071429,0.046882,0.039773,0.018906,0.051685,0.026316,0.036145,0.018564,
0.107955,0.074420,0.130102,0.090190,0.080412,0.058438,0.050595,0.027717,0.060000,0.038872,
0.028061,0.009373

Positive_27 7.000000,0.204082,3.000000,1.428571,2.000000,1.952381,2.000000,1.428571,2.000000,0.952381,
0.452130,0.000939,0.452130,0.000939,0.000000,0.000000,0.000000,1.000000,0.000000,0.000000,
0.000000,0.000000,0.000000,0.000000,0.000000,0.000000,0.000000,0.000000,0.000000,0.000000,
0.000000,0.000000,0.000000,0.095238,0.166667,0.007937,0.142857,0.500000,0.059524,0.142857,
0.333333,0.031746,0.190476,0.333333,0.022487,2.449490,2.000000,0.000000,1.000000,0.571429,
1.000000,0.571429,1.000000,0.571429,1.000000,0.571429,1.000000,0.571429,1.000000,0.571429,
1.000000,0.571429,1.000000,0.285714,1.000000,0.285714,0.000000,0.000000,1.000000,0.714286,
1.000000,0.714286,1.000000,0.714286,1.000000,0.714286,1.000000,0.714286,1.000000,0.714286,
1.000000,0.714286,1.000000,0.285714,1.000000,0.285714,0.000000,0.000000,0.346088,0.305159,
0.394778,0.363713,0.369898,0.331128,0.236400,0.209470,0.230638,0.205784,0.276053,0.245030,
0.020408,0.006834,0.285601,0.247993,0.517787,0.491308,0.795918,0.758460,1.000000,0.994768,
0.012953,0.004609,0.273533,0.233943,0.500000,0.482369,0.767829,0.727807,1.000000,0.997324,
0.002591,0.001381,0.293367,0.270638,0.522152,0.509549,0.769657,0.753372,1.000000,0.998769,
0.489480,0.450667,0.305699,0.189386,0.460443,0.359947,0.122078,0.084711,0.088032,0.073947,
0.007792,0.005131,0.002591,0.001381,0.249604,0.203225,0.490491,0.455462,0.774960,0.698421,
1.000000,1.000000,0.099490,0.041019,0.299842,0.265290,0.533163,0.465189,0.900510,0.722015,
0.999150,0.985296,0.116580,0.023817,0.352332,0.283834,0.631423,0.593302,0.818383,0.778184,
0.999209,0.975680,0.609694,0.586304,0.379574,0.358238,0.010363,0.005385,0.279272,0.256281,
0.515544,0.491079,0.742347,0.721838,0.999150,0.996908,0.412975,0.377198,0.398574,0.366681,
0.288265,0.256122,0.306413,0.269451,0.201774,0.188952,0.236596,0.195203,0.007772,0.003868,

	0.282392,0.239258,0.507772,0.481385,0.771791,0.731593,1.000000,0.997974,0.015544,0.006789,
	0.311224,0.282717,0.573980,0.531466,0.798450,0.771370,1.000000,0.998248,0.002591,0.001381,
	0.257120,0.218348,0.494898,0.461418,0.785714,0.721148,1.000000,0.996675,0.395408,0.363714,
	0.347310,0.308956,0.367347,0.327330,0.248337,0.223740,0.270638,0.243514,0.215628,0.190475,
	0.002591,0.001381,0.293367,0.265955,0.522152,0.509778,0.763834,0.748790,1.000000,0.998769,
	0.012953,0.004609,0.284607,0.232977,0.497409,0.478715,0.797147,0.733693,1.000000,0.996595,
	0.020408,0.006608,0.285601,0.243828,0.508893,0.490802,0.803571,0.758187,1.000000,0.995618,
	0.318653,0.297412,0.474490,0.446467,0.288265,0.256122,0.318290,0.261992,0.175166,0.151959,
	0.276596,0.232196,0.010301,0.004661,0.275748,0.245346,0.512953,0.483468,0.773376,0.730020,
	1.000000,0.995788,0.015306,0.006049,0.298469,0.267894,0.538265,0.519837,0.791805,0.766595,
	1.000000,0.998361,0.002591,0.001381,0.257120,0.218348,0.494898,0.461418,0.785714,0.721148,
	1.000000,0.996675,0.069620,0.050593,0.094388,0.063538,0.079082,0.068452,0.057823,0.049418,
	0.054264,0.041612,0.041997,0.031547,0.085492,0.067825,0.098101,0.066126,0.077075,0.059922,
	0.067358,0.054120,0.079114,0.060962,0.032115,0.022171,0.041204,0.032586,0.025907,0.018824,
	0.081633,0.072814,0.130102,0.106427,0.053360,0.045829,0.034014,0.025158,0.045959,0.038645,
	0.029900,0.023430
Positive_28	47.000000,0.057945,18.000000,2.723404,2.000000,17.247919,18.000000,2.723404,3.000000,13.856614,
	0.393010,0.017616,0.393010,0.017616,0.000000,0.226562,0.585859,0.439024,0.565217,0.600000,
	0.500000,0.148936,1.000000,0.129510,0.103343,1.857143,0.129283,0.046099,0.333333,0.012623,
	0.021248,0.333333,0.005223,0.180311,0.369565,0.024304,0.017422,0.065217,0.000259,0.014858,
	0.391304,0.003489,0.152306,0.369565,0.030299,7.348469,6.000000,4.027032,1.000000,0.617021,
	1.000000,0.617021,1.000000,0.617021,1.000000,0.617021,1.000000,0.617021,1.000000,0.616604,
	1.000000,0.582395,1.000000,0.468919,1.000000,0.416354,1.000000,0.397997,1.000000,0.617021,
	1.000000,0.617021,1.000000,0.617021,1.000000,0.609929,1.000000,0.582371,1.000000,0.569732,
	1.000000,0.537672,1.000000,0.505859,1.000000,0.421777,1.000000,0.154221,0.396947,0.319844,
	0.489162,0.383967,0.377778,0.296188,0.324786,0.233065,0.282383,0.192858,0.327381,0.228314,
	0.023810,0.008000,0.332494,0.249694,0.575290,0.504421,0.911243,0.745426,1.000000,0.998317,
	0.022680,0.005909,0.303101,0.240829,0.598569,0.484726,0.855556,0.749210,1.000000,0.996532,
	0.005917,0.001970,0.369800,0.254028,0.619318,0.503272,0.838313,0.745048,1.000000,0.993567,
	0.747270,0.510017,0.434426,0.177697,0.534247,0.312286,0.164384,0.078384,0.114187,0.067387,
	0.013274,0.005540,0.025714,0.002456,0.378788,0.244669,0.646064,0.502626,0.845475,0.752830,
	1.000000,1.000000,0.433255,0.079435,0.648712,0.291657,0.859485,0.529050,0.941238,0.748070,
	0.998145,0.956860,0.573770,0.034938,0.693989,0.247842,0.797814,0.459114,0.969945,0.668214,
	0.998352,0.954798,0.689498,0.547997,0.414508,0.355209,0.053254,0.009259,0.401387,0.271639,
	0.627841,0.524008,0.836555,0.760928,1.000000,0.991570,0.507020,0.405383,0.418557,0.350146,
	0.300971,0.244471,0.390438,0.277014,0.259259,0.193001,0.243506,0.171874,0.028571,0.006570,
	0.334286,0.241744,0.604424,0.487944,0.809061,0.744502,1.000000,0.996250,0.082840,0.009667,
	0.325444,0.252093,0.564327,0.499820,0.793204,0.738203,1.000000,0.996825,0.005917,0.001970,
	0.479290,0.248785,0.674556,0.507499,0.831034,0.757219,1.000000,0.995365,0.405556,0.325229,
	0.445811,0.332798,0.422392,0.341973,0.313131,0.214544,0.343066,0.226270,0.316239,0.213244,
	0.005917,0.001970,0.376860,0.255683,0.630682,0.507017,0.838313,0.747484,1.000000,0.994491,
	0.028571,0.006732,0.325309,0.232358,0.615485,0.479725,0.816667,0.738619,1.000000,0.995238,
	0.023810,0.007850,0.332494,0.251133,0.575290,0.507059,0.911243,0.746359,1.000000,0.998425,
	0.388889,0.324243,0.482474,0.431286,0.300971,0.244471,0.362550,0.273183,0.212291,0.157790,

0.285714,0.207085,0.028571,0.006949,0.332524,0.236561,0.612232,0.486961,0.822857,0.739968,
1.000000,0.995105,0.053254,0.008086,0.302285,0.255520,0.549180,0.499307,0.797619,0.743515,
1.000000,0.997370,0.005917,0.001970,0.479290,0.248785,0.674556,0.507499,0.831034,0.757219,
1.000000,0.995365,0.121212,0.055873,0.105114,0.064760,0.103105,0.069056,0.080103,0.050575,
0.071318,0.039788,0.078880,0.039793,0.119048,0.074621,0.134921,0.077285,0.122855,0.069720,
0.077778,0.052041,0.158606,0.059130,0.049881,0.022129,0.062842,0.029040,0.052015,0.022010,
0.103275,0.066282,0.122222,0.087101,0.080412,0.048126,0.039813,0.021056,0.077778,0.039957,
0.029126,0.011657

Positive_29 32.000000,0.104492,9.000000,3.343750,2.500000,8.490927,8.000000,3.343750,3.500000,5.652218,
0.429654,0.030515,0.429654,0.030515,0.028037,0.259615,0.259740,0.473684,0.466667,0.500000,
0.750000,0.679067,3.888889,1.422390,1.065402,3.142857,1.421531,0.110954,0.500000,0.030441,
0.261532,1.000000,0.108927,0.107599,0.225806,0.006043,0.082613,0.193548,0.004462,0.091141,
0.225806,0.003425,0.127224,0.258065,0.006372,5.778923,5.368018,4.154236,1.000000,0.656250,
1.000000,0.656250,1.000000,0.653274,1.000000,0.579787,1.000000,0.528100,1.000000,0.445362,
1.000000,0.410615,1.000000,0.330580,1.000000,0.159747,1.000000,0.108681,1.000000,0.718750,
1.000000,0.717634,1.000000,0.715402,1.000000,0.633854,1.000000,0.536384,1.000000,0.328571,
1.000000,0.188318,1.000000,0.103051,1.000000,0.100818,1.000000,0.071205,0.366667,0.305307,
0.454545,0.384001,0.340909,0.310692,0.258912,0.221157,0.259484,0.197885,0.311526,0.243136,
0.059322,0.013166,0.338983,0.247801,0.564972,0.498350,0.802508,0.744716,1.000000,0.997801,
0.033898,0.006724,0.316384,0.240901,0.539185,0.488186,0.792135,0.741745,1.000000,0.996545,
0.004237,0.002155,0.300847,0.253315,0.576832,0.505636,0.796690,0.748200,1.000000,0.994202,
0.490237,0.412919,0.233068,0.147407,0.650862,0.439674,0.127962,0.068019,0.110638,0.083314,
0.018692,0.004093,0.004237,0.002155,0.497186,0.239845,0.673546,0.494111,0.868750,0.740597,
1.000000,1.000000,0.588235,0.135243,0.639706,0.305840,0.786765,0.517482,0.904412,0.710358,
0.998677,0.908773,0.096667,0.025337,0.371269,0.246680,0.653846,0.492766,0.818996,0.733313,
0.997674,0.961596,0.684588,0.594133,0.410765,0.370559,0.038136,0.008397,0.314351,0.267166,
0.576108,0.502270,0.784200,0.740397,1.000000,0.992556,0.467085,0.396107,0.406574,0.357689,
0.290780,0.246204,0.345794,0.275184,0.226107,0.195637,0.224880,0.177409,0.033898,0.007031,
0.316384,0.239211,0.534375,0.488657,0.772983,0.737647,1.000000,0.997446,0.038136,0.009549,
0.338983,0.252649,0.574032,0.504527,0.803150,0.755051,1.000000,0.994924,0.004237,0.002155,
0.319697,0.243578,0.565299,0.499657,0.803468,0.738400,1.000000,0.995839,0.374242,0.340661,
0.405594,0.327806,0.398950,0.331534,0.282132,0.227514,0.303490,0.237136,0.232295,0.202772,
0.004237,0.002155,0.300847,0.255905,0.559322,0.506256,0.783333,0.748965,1.000000,0.994388,
0.033898,0.007530,0.305085,0.232142,0.552434,0.483276,0.794007,0.737854,1.000000,0.996337,
0.059322,0.012948,0.338983,0.248696,0.552239,0.498275,0.783599,0.744243,1.000000,0.997976,
0.404389,0.328376,0.460821,0.425420,0.290780,0.246204,0.352025,0.280521,0.203024,0.163812,
0.255981,0.209234,0.033898,0.007942,0.316384,0.240974,0.548951,0.485249,0.776224,0.736933,
1.000000,0.995810,0.024845,0.007476,0.309322,0.248730,0.560364,0.500727,0.792373,0.752840,
1.000000,0.995863,0.004237,0.002155,0.319697,0.243578,0.565299,0.499657,0.803468,0.738400,
1.000000,0.995839,0.085000,0.056335,0.097884,0.059019,0.088180,0.065108,0.081365,0.052254,
0.065744,0.038116,0.060533,0.034475,0.111801,0.075943,0.136364,0.084155,0.090395,0.063118,
0.068323,0.052906,0.088944,0.051684,0.041995,0.026227,0.051471,0.029969,0.037895,0.016047,
0.094406,0.068437,0.147388,0.102306,0.073286,0.049247,0.033898,0.023482,0.055690,0.037990,
0.029661,0.013183

Positive_30 29.000000,0.095125,9.000000,2.758621,2.000000,7.189655,12.000000,2.758621,1.000000,16.046798,
0.305185,0.018581,0.305185,0.018581,0.237500,0.393443,0.540541,0.764706,0.500000,0.000000,
0.500000,0.637931,2.166667,0.881010,0.321839,3.583333,0.691703,0.116284,0.361111,0.026009,
0.029334,0.298611,0.005132,0.172017,0.392857,0.025136,0.033251,0.083333,0.001253,0.038752,
0.428571,0.006562,0.111700,0.285714,0.014251,7.193131,3.275628,2.888211,1.000000,0.551724,
1.000000,0.551724,1.000000,0.535249,1.000000,0.478161,1.000000,0.311686,1.000000,0.222797,
1.000000,0.214559,0.333333,0.068391,0.333333,0.066475,0.333333,0.046935,1.000000,0.275862,
1.000000,0.275862,1.000000,0.238334,0.904762,0.200537,0.619048,0.132587,0.428571,0.076967,
0.190476,0.032721,0.190476,0.031676,0.100000,0.020331,0.100000,0.013092,0.387560,0.310521,
0.434088,0.365113,0.356701,0.324366,0.275000,0.212242,0.262590,0.201745,0.276952,0.237481,
0.117647,0.012582,0.333333,0.241550,0.576512,0.511424,0.832740,0.744086,1.000000,0.994302,
0.039216,0.008524,0.306338,0.246219,0.598086,0.488656,0.797403,0.741263,1.000000,0.994732,
0.019608,0.002854,0.309859,0.248619,0.558201,0.486730,0.766764,0.729718,1.000000,0.993694,
0.595206,0.424380,0.295606,0.173474,0.607843,0.402146,0.103650,0.073962,0.160000,0.079783,
0.017986,0.005523,0.019608,0.002854,0.353201,0.240875,0.617225,0.518239,0.882353,0.771278,
1.000000,1.000000,0.509804,0.137689,0.626984,0.316878,0.798942,0.522205,0.915344,0.716011,
0.998145,0.932225,0.186603,0.034278,0.362292,0.216406,0.678373,0.449549,0.839187,0.701509,
0.997041,0.957668,0.684720,0.602416,0.480000,0.358283,0.098039,0.015352,0.388889,0.272899,
0.587302,0.495526,0.783069,0.733445,1.000000,0.983356,0.422741,0.378423,0.422397,0.368065,
0.392157,0.253512,0.324074,0.269438,0.260000,0.187822,0.243816,0.182235,0.039216,0.008204,
0.306843,0.246019,0.598086,0.486403,0.827751,0.743106,1.000000,0.997119,0.156863,0.014487,
0.336918,0.249914,0.588235,0.495006,0.806250,0.741694,1.000000,0.994454,0.019608,0.002854,
0.291866,0.243293,0.595833,0.507183,0.861210,0.758118,1.000000,0.991156,0.391753,0.355537,
0.362974,0.310087,0.406699,0.334376,0.267658,0.216731,0.302158,0.238578,0.257143,0.192274,
0.019608,0.002854,0.316901,0.249448,0.560847,0.490418,0.784314,0.736200,1.000000,0.994378,
0.039216,0.008524,0.299848,0.238064,0.617225,0.484491,0.837321,0.738840,1.000000,0.992687,
0.117647,0.012177,0.333333,0.245051,0.593750,0.513666,0.832740,0.745843,1.000000,0.994514,
0.352031,0.305307,0.485714,0.441181,0.392157,0.253512,0.312634,0.258848,0.240000,0.155291,
0.282686,0.214766,0.039216,0.008900,0.306213,0.239460,0.593301,0.482714,0.827751,0.736321,
1.000000,0.990941,0.156863,0.013561,0.450980,0.253499,0.647059,0.497111,0.843137,0.742661,
1.000000,0.997907,0.019608,0.002854,0.291866,0.243293,0.595833,0.507183,0.861210,0.758118,
1.000000,0.991156,0.096085,0.059979,0.090909,0.054751,0.100478,0.073012,0.073944,0.046246,
0.066986,0.042770,0.052910,0.033763,0.109462,0.074258,0.121996,0.074324,0.117647,0.063106,
0.067308,0.047373,0.082557,0.051026,0.058824,0.023855,0.049676,0.031172,0.058824,0.022090,
0.088975,0.070188,0.130178,0.098012,0.080412,0.050322,0.078431,0.025653,0.078431,0.044352,
0.035503,0.013749

Positive_31 31.000000,0.154006,14.000000,4.774194,4.000000,15.313978,14.000000,4.774194,3.000000,18.513978,
0.373856,0.017697,0.373856,0.017697,0.040541,0.281690,0.490196,0.596154,0.380952,0.615385,
0.600000,1.495079,5.230769,1.682031,1.348576,3.636364,1.531383,0.253059,0.562500,0.038410,
0.229225,0.444444,0.028820,0.216289,0.400000,0.022291,0.166832,0.433333,0.021279,0.176878,
0.411111,0.019897,0.210724,0.422222,0.019889,8.401308,4.720270,4.304932,1.000000,0.741935,
1.000000,0.741935,1.000000,0.711903,1.000000,0.518094,1.000000,0.382681,1.000000,0.265450,
1.000000,0.215759,0.333333,0.084044,0.066667,0.020820,0.066667,0.017452,1.000000,0.709677,
1.000000,0.709677,1.000000,0.684488,1.000000,0.544030,1.000000,0.424390,1.000000,0.295859,

1.000000,0.243473,0.333333,0.092989,0.072727,0.013516,0.021978,0.004350,0.348601,0.284261,
0.437746,0.386579,0.387454,0.329160,0.252252,0.210952,0.256250,0.191403,0.324561,0.262909,
0.060870,0.012943,0.309963,0.243664,0.582888,0.501487,0.806250,0.738981,1.000000,0.992254,
0.031056,0.006896,0.297927,0.245431,0.546067,0.491678,0.826568,0.747320,1.000000,0.996504,
0.008696,0.002582,0.333333,0.246294,0.556738,0.497561,0.783688,0.742068,1.000000,0.994746,
0.631285,0.463466,0.305556,0.193201,0.614907,0.343333,0.122807,0.079662,0.118750,0.071780,
0.023923,0.007018,0.008696,0.002582,0.353201,0.226380,0.621118,0.478708,0.869565,0.735534,
1.000000,1.000000,0.409594,0.091908,0.549645,0.267877,0.755319,0.471355,0.886525,0.681573,
0.997409,0.926555,0.155440,0.057833,0.761798,0.283089,0.851685,0.505059,0.937079,0.751067,
0.997674,0.966462,0.683761,0.619498,0.456140,0.355203,0.046099,0.014145,0.320312,0.268458,
0.569416,0.506068,0.769231,0.739305,0.997674,0.990573,0.465789,0.405693,0.440994,0.357061,
0.290076,0.237246,0.350000,0.285000,0.239234,0.192874,0.247449,0.167779,0.031056,0.006844,
0.306843,0.244231,0.542435,0.490309,0.804428,0.736494,1.000000,0.996613,0.043478,0.012532,
0.312977,0.243895,0.577608,0.496528,0.806250,0.745754,1.000000,0.995187,0.008696,0.002582,
0.302583,0.253227,0.595833,0.510212,0.834225,0.761849,1.000000,0.993851,0.416974,0.358858,
0.371053,0.332406,0.381679,0.308736,0.289474,0.245599,0.288265,0.228832,0.234234,0.193688,
0.008696,0.002582,0.312057,0.251115,0.549645,0.503251,0.787234,0.746460,1.000000,0.995176,
0.031056,0.007152,0.286976,0.236824,0.541573,0.486985,0.804428,0.739649,1.000000,0.996258,
0.060870,0.012633,0.289583,0.244160,0.593750,0.501029,0.806250,0.742279,1.000000,0.993005,
0.378947,0.318639,0.540373,0.444115,0.290076,0.237246,0.350000,0.275701,0.188811,0.153133,
0.272959,0.207520,0.069565,0.009083,0.343173,0.253471,0.549815,0.496498,0.797753,0.742846,
1.000000,0.996160,0.028369,0.010052,0.307888,0.238877,0.552163,0.484706,0.797917,0.742073,
1.000000,0.995984,0.008696,0.002582,0.302583,0.253227,0.595833,0.510212,0.834225,0.761849,
1.000000,0.993851,0.076459,0.047936,0.086957,0.053003,0.086519,0.059345,0.062731,0.047277,
0.080745,0.039059,0.069565,0.037641,0.106952,0.079058,0.114688,0.078005,0.096408,0.062450,
0.082902,0.051849,0.083532,0.061044,0.042328,0.024474,0.047438,0.029698,0.043478,0.026010,
0.104278,0.072840,0.154982,0.095604,0.081579,0.052571,0.040573,0.023216,0.061657,0.044688,
0.026465,0.014230

Positive_32 16.000000,0.148438,5.000000,2.375000,2.000000,4.650000,9.000000,2.375000,3.000000,6.516667,
0.318261,0.014029,0.318261,0.014029,0.131579,0.454545,0.388889,0.636364,0.750000,1.000000,
0.000000,0.140625,0.750000,0.091406,0.088542,0.750000,0.058767,0.035156,0.187500,0.005713,
0.016348,0.187500,0.002424,0.173958,0.366667,0.022332,0.018750,0.100000,0.001625,0.013542,
0.150000,0.001601,0.121991,0.266667,0.014042,4.664016,3.290284,2.068175,1.000000,0.625000,
1.000000,0.625000,1.000000,0.575000,1.000000,0.575000,1.000000,0.291667,1.000000,0.200000,
0.666667,0.129167,0.100000,0.025000,0.100000,0.025000,0.100000,0.025000,1.000000,0.562500,
1.000000,0.562500,1.000000,0.487847,0.833333,0.338542,0.666667,0.284722,0.500000,0.126736,
0.500000,0.079861,0.000000,0.000000,0.000000,0.000000,0.000000,0.000000,0.348601,0.294115,
0.437746,0.381311,0.350220,0.324574,0.245614,0.212756,0.229592,0.190386,0.324561,0.258456,
0.060870,0.013214,0.290179,0.245183,0.582609,0.515130,0.806250,0.748826,1.000000,0.993603,
0.017391,0.007265,0.282561,0.239857,0.532692,0.478713,0.797403,0.741558,1.000000,0.996404,
0.008696,0.002593,0.306167,0.247211,0.540730,0.495674,0.767442,0.737824,1.000000,0.994613,
0.596330,0.440025,0.305556,0.193595,0.510417,0.366380,0.122807,0.082712,0.093946,0.072719,
0.017544,0.006182,0.008696,0.002593,0.353201,0.227623,0.598234,0.495427,0.845475,0.745980,
1.000000,1.000000,0.297917,0.104845,0.495833,0.312036,0.637500,0.511384,0.858333,0.708062,

0.988281,0.933489,0.132812,0.046578,0.301708,0.212359,0.648855,0.462460,0.825427,0.694741,
0.997674,0.964683,0.683761,0.621236,0.456140,0.358018,0.026786,0.012728,0.320312,0.266691,
0.539535,0.507590,0.769231,0.739154,0.997674,0.993303,0.426087,0.389496,0.394805,0.364054,
0.290076,0.246450,0.328477,0.279198,0.226107,0.186291,0.247449,0.185410,0.017391,0.007340,
0.306843,0.241378,0.519231,0.478682,0.782051,0.733701,1.000000,0.996735,0.043478,0.012070,
0.312977,0.254169,0.577608,0.500865,0.806250,0.747495,1.000000,0.995089,0.008696,0.002593,
0.299157,0.254453,0.595833,0.527081,0.833766,0.759964,1.000000,0.994621,0.383260,0.357518,
0.365217,0.324458,0.381679,0.318024,0.289474,0.238465,0.288265,0.228779,0.228070,0.192108,
0.008696,0.002593,0.303965,0.251892,0.537445,0.505953,0.762500,0.742405,1.000000,0.995171,
0.017391,0.007936,0.286976,0.233432,0.530769,0.473593,0.801923,0.735529,1.000000,0.996046,
0.060870,0.012613,0.289583,0.245974,0.593750,0.516313,0.806250,0.749879,1.000000,0.994320,
0.339535,0.308377,0.485714,0.445173,0.290076,0.246450,0.312634,0.272762,0.188811,0.153124,
0.272959,0.218577,0.069565,0.010968,0.295806,0.239444,0.518764,0.477089,0.787013,0.736457,
1.000000,0.995532,0.027990,0.008443,0.307888,0.250186,0.552163,0.490075,0.797917,0.744557,
1.000000,0.996071,0.008696,0.002593,0.299157,0.254453,0.595833,0.527081,0.833766,0.759964,
1.000000,0.994621,0.068820,0.052450,0.083969,0.052963,0.083969,0.061760,0.071429,0.046046,
0.059740,0.040325,0.069565,0.040571,0.083879,0.074212,0.106250,0.077878,0.086957,0.060737,
0.067308,0.049504,0.084821,0.062126,0.042328,0.023909,0.047438,0.032944,0.037445,0.018992,
0.083333,0.071753,0.121693,0.098568,0.070640,0.051077,0.034783,0.023679,0.061599,0.045397,
0.022770,0.015108

Positive_33 17.000000,0.145329,10.000000,2.470588,0.000000,14.514706,4.000000,2.470588,3.000000,2.139706,
0.364581,0.013956,0.364581,0.013956,0.095238,0.210526,0.533333,0.714286,0.500000,0.500000,
1.000000,0.000000,0.000000,0.000000,0.000000,0.000000,0.000000,0.000000,0.000000,0.000000,
0.000000,0.000000,0.000000,0.066176,0.187500,0.007066,0.147059,0.625000,0.074678,0.147059,
0.250000,0.016085,0.352941,0.562500,0.068331,5.477226,3.464102,0.000000,1.000000,0.411765,
1.000000,0.411765,1.000000,0.411765,1.000000,0.411765,1.000000,0.392157,1.000000,0.329412,
0.666667,0.207843,0.155556,0.027451,0.088889,0.015686,0.022222,0.003922,1.000000,0.764706,
1.000000,0.764706,1.000000,0.764706,1.000000,0.764706,1.000000,0.764706,1.000000,0.764706,
1.000000,0.568627,1.000000,0.176471,1.000000,0.176471,0.500000,0.088235,0.310748,0.283942,
0.460094,0.385998,0.382937,0.330060,0.263862,0.216160,0.232604,0.182446,0.327059,0.259805,
0.058685,0.015299,0.316103,0.264706,0.532803,0.474880,0.763077,0.700953,1.000000,0.983018,
0.017857,0.008153,0.330357,0.256515,0.556923,0.517602,0.812500,0.772237,1.000000,0.995406,
0.008929,0.002912,0.242254,0.207219,0.562500,0.483651,0.776786,0.734599,1.000000,0.992871,
0.519774,0.466722,0.361582,0.179441,0.500000,0.353837,0.141643,0.081092,0.091816,0.073168,
0.009940,0.004456,0.008929,0.002912,0.303571,0.250901,0.565737,0.509819,0.840637,0.782320,
1.000000,1.000000,0.200000,0.084650,0.427692,0.251830,0.738342,0.501972,0.898462,0.786606,
0.991549,0.945132,0.178571,0.024063,0.377934,0.197288,0.544393,0.399998,0.758929,0.595915,
0.996183,0.915321,0.673239,0.608185,0.382716,0.328540,0.062500,0.012562,0.313559,0.246448,
0.536723,0.487336,0.765537,0.733485,0.998092,0.987412,0.458647,0.385717,0.429379,0.368142,
0.287500,0.246141,0.360360,0.297264,0.257862,0.189544,0.226640,0.177212,0.017857,0.006558,
0.285714,0.248199,0.551643,0.505278,0.800469,0.767265,1.000000,0.996056,0.081250,0.013647,
0.294235,0.235628,0.536723,0.489951,0.758929,0.728928,1.000000,0.994407,0.008929,0.002912,
0.315493,0.255880,0.535385,0.491871,0.809231,0.711835,1.000000,0.980122,0.412698,0.365673,
0.401408,0.322842,0.336449,0.311485,0.315294,0.243459,0.266289,0.218009,0.244821,0.200269,

0.008929,0.002912,0.247887,0.222318,0.526761,0.491633,0.773364,0.739783,1.000000,0.992871,
0.017857,0.008153,0.285714,0.238651,0.546948,0.498644,0.821429,0.773194,1.000000,0.994410,
0.030534,0.013089,0.306773,0.272117,0.533865,0.484160,0.763077,0.708725,1.000000,0.984021,
0.390977,0.308143,0.494350,0.445716,0.287500,0.246141,0.331445,0.284297,0.220126,0.153418,
0.272366,0.213338,0.017857,0.006558,0.285714,0.245561,0.546948,0.499712,0.812500,0.742740,
1.000000,0.993613,0.081250,0.013495,0.282383,0.236266,0.531073,0.494846,0.781250,0.756156,
1.000000,0.997841,0.008929,0.002912,0.315493,0.255880,0.535385,0.491871,0.809231,0.711835,
1.000000,0.980122,0.093750,0.048437,0.083499,0.050939,0.077720,0.057905,0.062147,0.049451,
0.063084,0.037891,0.064972,0.039320,0.095865,0.068636,0.121212,0.071108,0.089286,0.062573,
0.067669,0.056376,0.105634,0.064149,0.042373,0.027543,0.049223,0.035613,0.044643,0.013425,
0.096045,0.070390,0.166667,0.118913,0.067460,0.043723,0.037773,0.025990,0.065476,0.045151,
0.031250,0.012467

Positive_34 49.000000,0.075385,16.000000,3.693878,3.000000,14.883503,11.000000,3.693878,4.000000,8.050170,
0.351640,0.031723,0.351640,0.031723,0.215909,0.275362,0.300000,0.414286,0.536585,0.684211,
0.666667,0.516950,3.000000,0.442754,0.661158,2.200000,0.691727,0.129668,0.500000,0.023862,
0.146076,0.440000,0.031957,0.095437,0.187500,0.004684,0.080098,0.243056,0.006413,0.085427,
0.208333,0.003302,0.126701,0.291667,0.010808,7.412473,5.204242,4.912959,1.000000,0.795918,
1.000000,0.741497,1.000000,0.702054,1.000000,0.581904,1.000000,0.439434,1.000000,0.308618,
1.000000,0.249926,1.000000,0.148726,1.000000,0.037547,0.333333,0.015372,1.000000,0.693878,
1.000000,0.670330,1.000000,0.588867,1.000000,0.481982,1.000000,0.350203,1.000000,0.263022,
1.000000,0.194679,1.000000,0.080411,1.000000,0.043258,1.000000,0.025331,0.383803,0.305130,
0.434088,0.370912,0.368313,0.323958,0.275000,0.208639,0.262590,0.199180,0.378641,0.247803,
0.023810,0.010100,0.332494,0.244848,0.576512,0.499946,0.832740,0.746612,1.000000,0.996659,
0.024775,0.007648,0.331081,0.238608,0.604895,0.487215,0.839901,0.742012,1.000000,0.996024,
0.004831,0.002364,0.344643,0.259356,0.566540,0.500892,0.792969,0.742944,1.000000,0.994413,
0.774107,0.434455,0.307692,0.158558,0.593596,0.406987,0.115789,0.067426,0.114187,0.078620,
0.017986,0.005337,0.004831,0.002364,0.353201,0.223001,0.598272,0.496453,0.847656,0.739463,
1.000000,1.000000,0.433255,0.116614,0.648712,0.306167,0.859485,0.530109,0.915344,0.715858,
0.998145,0.916283,0.202899,0.030411,0.516461,0.246810,0.822321,0.489045,0.893750,0.729417,
0.996700,0.960380,0.690702,0.604956,0.446828,0.354951,0.042705,0.011370,0.388889,0.277055,
0.587302,0.507221,0.787500,0.742204,1.000000,0.991116,0.462500,0.383806,0.418557,0.362566,
0.312808,0.253628,0.390438,0.274077,0.247761,0.193398,0.274074,0.181205,0.022680,0.007189,
0.346154,0.240078,0.608392,0.488471,0.839901,0.739929,1.000000,0.996089,0.038023,0.009793,
0.336918,0.252053,0.613861,0.494750,0.806250,0.746047,1.000000,0.995413,0.004831,0.002364,
0.321429,0.247654,0.595833,0.510438,0.861210,0.754330,1.000000,0.995324,0.403292,0.353502,
0.396825,0.315779,0.401478,0.330718,0.313131,0.227880,0.302158,0.236598,0.257143,0.193829,
0.004831,0.002364,0.339286,0.260905,0.560847,0.504514,0.779297,0.745473,1.000000,0.994991,
0.027027,0.007946,0.346154,0.229156,0.615385,0.481945,0.832512,0.736962,1.000000,0.995268,
0.023810,0.009834,0.332494,0.248526,0.593750,0.501748,0.832740,0.747282,1.000000,0.996704,
0.388889,0.310471,0.482474,0.435901,0.312808,0.253628,0.362550,0.264720,0.210526,0.157255,
0.296296,0.217348,0.022680,0.007566,0.388112,0.235775,0.646853,0.486008,0.839161,0.739594,
1.000000,0.994609,0.038023,0.008873,0.307985,0.252043,0.603960,0.496226,0.797917,0.742450,
1.000000,0.997629,0.004831,0.002364,0.321429,0.247654,0.595833,0.510438,0.861210,0.754330,
1.000000,0.995324,0.096085,0.054925,0.098726,0.059117,0.103448,0.067871,0.082803,0.047389,

0.062937,0.040681,0.062619,0.035147,0.119048,0.073167,0.134921,0.076561,0.097633,0.064691,
0.076433,0.048662,0.083036,0.052698,0.062802,0.025589,0.049676,0.029544,0.089286,0.020637,
0.103275,0.070156,0.135266,0.097502,0.080412,0.051209,0.043210,0.024689,0.073944,0.046555,
0.035503,0.013210

Positive_35 9.000000,0.296296,6.000000,2.666667,1.000000,4.750000,5.000000,2.666667,3.000000,2.750000,
0.378288,0.003013,0.378288,0.003013,0.000000,0.000000,0.708333,0.857143,1.000000,0.000000,
0.000000,0.342593,0.833333,0.167438,0.438889,1.200000,0.200278,0.076389,0.222222,0.008825,
0.121574,0.240000,0.013485,0.369213,0.500000,0.006920,0.318287,0.500000,0.018531,0.176620,
0.406250,0.015084,0.335417,0.541667,0.058307,3.936968,2.223087,1.577214,1.000000,0.444444,
1.000000,0.444444,1.000000,0.444444,1.000000,0.444444,0.666667,0.240741,0.666667,0.240741,
0.666667,0.240741,0.666667,0.240741,0.533333,0.174074,0.166667,0.033333,1.000000,0.777778,
1.000000,0.777778,1.000000,0.777778,1.000000,0.777778,1.000000,0.455556,1.000000,0.444444,
1.000000,0.433333,0.500000,0.066667,0.500000,0.055556,0.166667,0.018519,0.354167,0.316009,
0.393023,0.364871,0.350220,0.319119,0.258383,0.224329,0.250474,0.213282,0.283814,0.247270,
0.021792,0.012662,0.293501,0.250658,0.545073,0.497631,0.773836,0.746586,1.000000,0.997719,
0.006652,0.004721,0.292020,0.235523,0.541596,0.477804,0.764007,0.731679,1.000000,0.993736,
0.002421,0.002115,0.306167,0.254539,0.565410,0.518607,0.775591,0.756554,1.000000,0.995425,
0.478936,0.428182,0.224109,0.161199,0.482180,0.410619,0.091837,0.072458,0.096639,0.077147,
0.011038,0.004298,0.002421,0.002115,0.405512,0.235477,0.568898,0.460407,0.807087,0.722921,
1.000000,0.999369,0.139535,0.051954,0.443182,0.309355,0.742424,0.560422,0.922432,0.775065,
1.000000,0.960091,0.099558,0.036399,0.360619,0.251418,0.606112,0.485017,0.797674,0.725713,
0.998302,0.976371,0.646512,0.570720,0.390533,0.371852,0.013258,0.008066,0.330709,0.279281,
0.604331,0.519153,0.799213,0.733687,1.000000,0.993447,0.404651,0.367773,0.399621,0.370399,
0.303769,0.261828,0.315673,0.269493,0.226107,0.191833,0.246679,0.203914,0.006652,0.004477,
0.280136,0.236500,0.541596,0.487705,0.764007,0.729045,1.000000,0.993795,0.015152,0.010276,
0.308370,0.255255,0.539535,0.513763,0.788372,0.768565,1.000000,0.997116,0.002421,0.002115,
0.307087,0.253620,0.527559,0.489937,0.749446,0.726518,1.000000,0.995772,0.383260,0.352126,
0.339535,0.302987,0.381375,0.344887,0.256071,0.224235,0.282732,0.255795,0.244576,0.200813,
0.002421,0.002115,0.303965,0.260495,0.556541,0.515462,0.783465,0.755004,1.000000,0.995425,
0.006652,0.004721,0.288625,0.224419,0.556876,0.476940,0.769100,0.731529,1.000000,0.992572,
0.021792,0.011887,0.285115,0.249303,0.542977,0.495989,0.779070,0.746434,1.000000,0.997719,
0.339535,0.299627,0.480176,0.438545,0.303769,0.261828,0.300221,0.264354,0.188811,0.156975,
0.284630,0.238773,0.006652,0.004665,0.271647,0.233179,0.529711,0.470325,0.747029,0.715786,
1.000000,0.993607,0.011062,0.007493,0.303965,0.256447,0.543237,0.515547,0.793307,0.771411,
1.000000,0.997361,0.002421,0.002115,0.307087,0.253620,0.527559,0.489937,0.749446,0.726518,
1.000000,0.995772,0.072835,0.053667,0.115530,0.064806,0.096591,0.069847,0.057725,0.049562,
0.057650,0.040481,0.060533,0.037646,0.092243,0.068043,0.102326,0.073815,0.072835,0.061042,
0.066214,0.047165,0.070485,0.052922,0.062084,0.028878,0.048673,0.033007,0.037445,0.015224,
0.081439,0.064975,0.136268,0.104209,0.088285,0.053241,0.033040,0.020584,0.059735,0.048488,
0.021792,0.012398

Positive_36 21.000000,0.149660,11.000000,3.142857,1.000000,10.228571,11.000000,3.142857,2.000000,10.928571,
0.355067,0.018797,0.355067,0.018797,0.060606,0.370968,0.435897,0.545455,0.600000,0.500000,
0.500000,0.385714,3.000000,0.614286,0.468254,3.000000,0.585053,0.050606,0.272727,0.005915,
0.113957,0.272727,0.015388,0.206169,0.500000,0.035090,0.054221,0.500000,0.013796,0.045587,

0.154545,0.001834,0.238612,0.475000,0.036911,6.447624,3.798958,1.732051,1.000000,0.476190,
1.000000,0.476190,1.000000,0.472342,1.000000,0.295382,1.000000,0.235498,1.000000,0.178403,
1.000000,0.096344,0.333333,0.054978,0.100000,0.035257,0.000000,0.000000,1.000000,0.666667,
1.000000,0.666667,1.000000,0.662290,1.000000,0.632516,1.000000,0.576555,1.000000,0.146774,
1.000000,0.104810,0.333333,0.032832,0.018182,0.000866,0.000000,0.000000,0.334917,0.301639,
0.437746,0.377580,0.356701,0.320781,0.273810,0.212990,0.231169,0.196370,0.299338,0.246987,
0.023932,0.009739,0.283237,0.234935,0.575290,0.519694,0.800000,0.757598,1.000000,0.997625,
0.022680,0.006177,0.312500,0.251194,0.557292,0.489931,0.787629,0.742515,1.000000,0.996335,
0.003448,0.001928,0.285470,0.248831,0.520202,0.486506,0.769231,0.736177,1.000000,0.994536,
0.596330,0.424920,0.305556,0.176173,0.570071,0.398907,0.099609,0.074836,0.114187,0.075481,
0.013274,0.004102,0.003448,0.001928,0.353201,0.234882,0.610790,0.511084,0.845475,0.762653,
1.000000,1.000000,0.415825,0.105279,0.574074,0.311488,0.688552,0.495990,0.868687,0.717192,
0.998145,0.937267,0.159923,0.025030,0.342967,0.214225,0.641330,0.449238,0.820690,0.687401,
0.996633,0.970017,0.684878,0.638211,0.399225,0.346793,0.015625,0.008195,0.326599,0.281730,
0.567696,0.510798,0.783848,0.746855,1.000000,0.993852,0.440000,0.394139,0.418557,0.360666,
0.277202,0.245195,0.328477,0.277143,0.223938,0.193298,0.210744,0.172712,0.022680,0.006461,
0.306843,0.245801,0.528646,0.488807,0.779381,0.740646,1.000000,0.996239,0.026128,0.009643,
0.264957,0.241655,0.536680,0.493347,0.781197,0.739509,1.000000,0.997598,0.003448,0.001928,
0.305671,0.256568,0.580574,0.517511,0.831034,0.767101,1.000000,0.997237,0.391753,0.349351,
0.361730,0.327645,0.362694,0.323004,0.281250,0.232603,0.267532,0.228479,0.240476,0.197620,
0.003448,0.001928,0.289562,0.255035,0.525097,0.491571,0.769231,0.742983,1.000000,0.995428,
0.022680,0.006555,0.289062,0.238339,0.541667,0.482773,0.785567,0.735319,1.000000,0.994382,
0.023932,0.009613,0.279383,0.237239,0.575290,0.521629,0.786207,0.756510,1.000000,0.998543,
0.369942,0.322774,0.482474,0.432031,0.277202,0.245195,0.305960,0.270763,0.195238,0.161348,
0.239669,0.204662,0.022680,0.006638,0.317708,0.252735,0.544271,0.498774,0.786458,0.742859,
1.000000,0.995482,0.019002,0.007338,0.262548,0.237781,0.528958,0.484394,0.786341,0.738214,
1.000000,0.998139,0.003448,0.001928,0.305671,0.256568,0.580574,0.517511,0.831034,0.767101,
1.000000,0.997237,0.062500,0.050349,0.093054,0.061294,0.085938,0.065606,0.067437,0.046919,
0.050773,0.039756,0.054688,0.037715,0.109462,0.081579,0.131034,0.087376,0.075397,0.056559,
0.067437,0.050341,0.079365,0.051790,0.042328,0.021365,0.039735,0.028570,0.035122,0.019574,
0.088975,0.070828,0.121693,0.090409,0.080412,0.056352,0.042735,0.025076,0.061599,0.045776,
0.024119,0.012766

Positive_37 10.000000,0.250000,7.000000,2.500000,2.500000,3.833333,5.000000,2.500000,2.000000,3.833333,
0.312305,0.006061,0.312305,0.006061,0.080000,0.434783,0.615385,1.000000,0.000000,0.000000,
0.000000,0.171429,1.714286,0.293878,0.240000,0.800000,0.149333,0.024490,0.244898,0.005998,
0.048000,0.160000,0.005973,0.263492,0.444444,0.031141,0.117460,0.285714,0.015688,0.137778,
0.555556,0.050129,0.382222,0.666667,0.070211,4.000000,2.449490,1.732051,1.000000,0.800000,
1.000000,0.800000,1.000000,0.800000,1.000000,0.766667,1.000000,0.752381,1.000000,0.466667,
0.333333,0.166667,0.333333,0.166667,0.333333,0.157143,0.047619,0.004762,1.000000,0.500000,
1.000000,0.500000,1.000000,0.500000,1.000000,0.500000,1.000000,0.440000,0.600000,0.246667,
0.600000,0.246667,0.600000,0.246667,0.400000,0.186667,0.100000,0.030000,0.345000,0.277673,
0.525355,0.412316,0.350000,0.310010,0.256506,0.220882,0.215852,0.175012,0.292683,0.239377,
0.023932,0.010605,0.316865,0.231034,0.555556,0.508623,0.786325,0.758725,1.000000,0.995271,
0.020000,0.008036,0.290000,0.251632,0.550000,0.497197,0.800000,0.735597,1.000000,0.995422,

0.005000,0.002341,0.285470,0.244752,0.530211,0.486721,0.769231,0.738435,1.000000,0.993960,
0.607407,0.487719,0.240000,0.153926,0.490385,0.358355,0.100503,0.064298,0.094435,0.077798,
0.010050,0.002299,0.005000,0.002341,0.390000,0.237442,0.584327,0.505147,0.815000,0.755834,
1.000000,1.000000,0.415825,0.156394,0.574074,0.406014,0.737160,0.621457,0.913897,0.802307,
0.998489,0.946433,0.186603,0.043487,0.459330,0.237136,0.590909,0.395246,0.827751,0.603828,
0.996633,0.908990,0.659051,0.608817,0.359712,0.328681,0.035000,0.014095,0.326599,0.274767,
0.546828,0.504162,0.768882,0.741865,0.996593,0.993824,0.527383,0.419841,0.394737,0.358622,
0.285000,0.221538,0.329352,0.283976,0.241206,0.181692,0.206030,0.159861,0.020000,0.008036,
0.294118,0.231789,0.540000,0.495660,0.800000,0.740344,1.000000,0.996139,0.023932,0.012079,
0.299094,0.236530,0.542296,0.493309,0.781197,0.738979,1.000000,0.997469,0.005000,0.002341,
0.304274,0.267801,0.563462,0.497779,0.807302,0.757204,1.000000,0.987308,0.375000,0.335710,
0.478702,0.355929,0.400000,0.308361,0.270325,0.224683,0.271357,0.210471,0.246231,0.213955,
0.005000,0.002341,0.303704,0.251068,0.530211,0.486686,0.769231,0.735561,1.000000,0.998130,
0.020000,0.008036,0.290061,0.230751,0.550000,0.488884,0.815000,0.728912,1.000000,0.995076,
0.023932,0.010605,0.296422,0.243963,0.553846,0.513762,0.821501,0.765141,1.000000,0.995271,
0.411765,0.338296,0.461722,0.440167,0.285000,0.221538,0.353659,0.282664,0.201005,0.144970,
0.246231,0.196582,0.020000,0.008777,0.290061,0.219900,0.536028,0.488410,0.810000,0.732342,
0.998316,0.994287,0.015332,0.007912,0.285498,0.244222,0.551852,0.504614,0.772727,0.746056,
1.000000,0.998386,0.005000,0.002341,0.304274,0.267801,0.563462,0.497779,0.807302,0.757204,
1.000000,0.987308,0.077778,0.052773,0.070707,0.039321,0.080000,0.061181,0.066667,0.046046,
0.057922,0.044199,0.070000,0.034154,0.103918,0.081448,0.133874,0.092031,0.087613,0.067663,
0.065000,0.051108,0.125926,0.063679,0.055000,0.030688,0.035897,0.025700,0.025641,0.017866,
0.086116,0.066275,0.152130,0.112557,0.056239,0.040257,0.042735,0.023093,0.050505,0.037756,
0.020513,0.012207

Positive_38 3.000000,0.222222,2.000000,0.666667,0.000000,1.333333,1.000000,0.666667,1.000000,0.333333,
0.597124,0.003253,0.597124,0.003253,0.000000,0.000000,0.000000,0.000000,0.500000,1.000000,
0.000000,0.000000,0.000000,0.000000,0.000000,0.000000,0.000000,0.000000,0.000000,0.000000,
0.000000,0.000000,0.000000,0.000000,0.000000,0.000000,0.000000,0.000000,0.000000,0.000000,
0.000000,0.000000,0.333333,0.500000,0.083333,1.414214,0.000000,0.000000,1.000000,0.333333,
1.000000,0.333333,1.000000,0.333333,1.000000,0.333333,1.000000,0.333333,1.000000,0.333333,
1.000000,0.333333,1.000000,0.333333,0.000000,0.000000,0.000000,0.000000,0.000000,0.000000,
0.000000,0.000000,0.000000,0.000000,0.000000,0.000000,0.000000,0.000000,0.000000,0.000000,
0.000000,0.000000,0.000000,0.000000,0.000000,0.000000,0.000000,0.000000,0.373297,0.301719,
0.392954,0.385051,0.426108,0.313231,0.252717,0.206745,0.213115,0.182181,0.328395,0.231164,
0.029557,0.017992,0.314363,0.281298,0.574526,0.552790,0.832512,0.761198,1.000000,1.000000,
0.008174,0.006173,0.242507,0.217387,0.482289,0.460719,0.831978,0.774031,0.987685,0.986837,
0.002725,0.002633,0.278325,0.266689,0.524631,0.473792,0.748768,0.726042,0.997537,0.986503,
0.493188,0.422236,0.102981,0.085646,0.650246,0.492118,0.051630,0.038103,0.086420,0.070577,
0.012346,0.009565,0.002725,0.002633,0.219212,0.157303,0.445813,0.419427,0.864499,0.835441,
1.000000,1.000000,0.166213,0.086029,0.546798,0.308215,0.770936,0.453546,0.940887,0.638874,
0.980926,0.976398,0.233062,0.141004,0.392371,0.342973,0.623978,0.579965,0.739837,0.725660,
0.956640,0.911782,0.810345,0.530996,0.402174,0.325760,0.013624,0.011439,0.273399,0.261419,
0.500000,0.454702,0.731527,0.682252,0.983651,0.979935,0.411924,0.393627,0.374384,0.338564,
0.288828,0.267809,0.259259,0.239886,0.215847,0.194370,0.213115,0.200536,0.005450,0.005265,

0.216749,0.203594,0.476839,0.451431,0.834688,0.768925,1.000000,0.998193,0.034483,0.017837,
0.275204,0.251606,0.546798,0.523737,0.790640,0.738188,0.997290,0.996546,0.002725,0.002633,
0.327913,0.304959,0.531165,0.525411,0.778325,0.748576,1.000000,0.991913,0.467980,0.347120,
0.338753,0.323192,0.400545,0.329687,0.274074,0.210329,0.251366,0.219710,0.228261,0.181079,
0.002725,0.002633,0.276423,0.268092,0.524631,0.475604,0.748768,0.704313,0.997537,0.986503,
0.008174,0.006173,0.196185,0.191847,0.485014,0.449560,0.864499,0.787560,0.986450,0.986016,
0.029557,0.017992,0.285714,0.275466,0.596059,0.554660,0.834975,0.760213,1.000000,1.000000,
0.325203,0.316213,0.440887,0.415979,0.288828,0.267809,0.271739,0.266813,0.180328,0.164171,
0.248634,0.230736,0.013550,0.007975,0.226158,0.223434,0.485014,0.464420,0.807588,0.750809,
1.000000,0.998193,0.027094,0.014471,0.228883,0.213338,0.536946,0.489670,0.768473,0.740765,
0.997290,0.996546,0.002725,0.002633,0.327913,0.304959,0.531165,0.525411,0.778325,0.748576,
1.000000,0.991913,0.073569,0.055317,0.079019,0.058693,0.092643,0.071365,0.062670,0.051437,
0.037940,0.035861,0.032698,0.029046,0.071429,0.053713,0.083744,0.075021,0.097561,0.085220,
0.062331,0.050821,0.073171,0.058417,0.029557,0.027968,0.041872,0.033890,0.027094,0.018998,
0.076355,0.068945,0.133005,0.080553,0.071429,0.052795,0.032020,0.026069,0.061576,0.051323,
0.024631,0.014548

Positive_39 20.000000,0.122500,7.000000,2.450000,2.000000,4.471053,9.000000,2.450000,2.000000,6.260526,
0.371611,0.046023,0.371611,0.046023,0.142857,0.452381,0.260870,0.470588,0.222222,0.285714,
0.400000,0.200000,2.000000,0.378947,0.200000,2.000000,0.378947,0.100000,1.000000,0.094737,
0.100000,1.000000,0.094737,0.210526,0.421053,0.039364,0.044737,0.368421,0.007661,0.046491,
0.122807,0.001208,0.142105,0.315789,0.018108,4.582576,4.242641,2.000000,1.000000,0.800000,
1.000000,0.800000,1.000000,0.800000,1.000000,0.785714,1.000000,0.292857,1.000000,0.197619,
1.000000,0.161905,1.000000,0.145238,0.095238,0.014286,0.047619,0.007143,1.000000,0.550000,
1.000000,0.550000,1.000000,0.538889,1.000000,0.527778,1.000000,0.494444,1.000000,0.361111,
1.000000,0.347222,1.000000,0.230556,0.083333,0.008333,0.027778,0.002778,0.383803,0.313664,
0.424719,0.367515,0.347670,0.318820,0.275000,0.216479,0.262590,0.197041,0.279703,0.240333,
0.018519,0.008958,0.289941,0.245228,0.576512,0.499099,0.832740,0.741536,1.000000,0.994064,
0.014815,0.006512,0.306338,0.224421,0.566901,0.481191,0.798077,0.742308,1.000000,0.997633,
0.003584,0.002318,0.395082,0.280001,0.593443,0.513438,0.782214,0.745089,1.000000,0.988920,
0.524781,0.454178,0.249271,0.166314,0.478921,0.379507,0.103650,0.067568,0.104317,0.081234,
0.017986,0.004260,0.003584,0.002318,0.339093,0.232607,0.598272,0.485373,0.792657,0.740633,
1.000000,1.000000,0.343195,0.089256,0.626984,0.323027,0.798942,0.527644,0.915344,0.724672,
0.992832,0.916766,0.162963,0.046922,0.390164,0.242406,0.610455,0.482041,0.810036,0.722853,
0.996627,0.959829,0.656790,0.578433,0.417266,0.352657,0.042705,0.014564,0.388889,0.277919,
0.587302,0.516850,0.783069,0.732336,0.996721,0.988572,0.436066,0.386166,0.397778,0.364233,
0.299296,0.249601,0.326733,0.278162,0.228571,0.185682,0.243816,0.179909,0.014793,0.006389,
0.302817,0.225611,0.511879,0.471540,0.778061,0.732359,1.000000,0.996023,0.032028,0.009775,
0.337079,0.274073,0.566308,0.504926,0.791289,0.746321,1.000000,0.989542,0.003584,0.002318,
0.328889,0.245669,0.571856,0.518334,0.861210,0.757390,1.000000,0.992904,0.379928,0.348453,
0.366292,0.310212,0.401408,0.341335,0.248082,0.214255,0.302158,0.236810,0.257143,0.203527,
0.003584,0.002318,0.401639,0.278836,0.606557,0.516695,0.782214,0.747379,1.000000,0.991322,
0.014815,0.006512,0.296407,0.216815,0.521519,0.471318,0.793367,0.733099,1.000000,0.995544,
0.018519,0.008958,0.289941,0.247796,0.565089,0.500852,0.832740,0.741946,1.000000,0.995774,
0.350820,0.309729,0.483333,0.440670,0.299296,0.249601,0.326733,0.273658,0.185279,0.148740,

0.282686,0.216852,0.014793,0.006560,0.323353,0.220575,0.511879,0.469101,0.770370,0.726952,
1.000000,0.993394,0.028470,0.008520,0.312360,0.269070,0.547541,0.503066,0.773770,0.746396,
1.000000,0.994449,0.003584,0.002318,0.328889,0.245669,0.571856,0.518334,0.861210,0.757390,
1.000000,0.992904,0.096085,0.058968,0.084507,0.054426,0.093190,0.069047,0.078481,0.052442,
0.063333,0.043246,0.053892,0.035536,0.088968,0.070708,0.105618,0.074123,0.097633,0.061302,
0.068878,0.051154,0.079882,0.052924,0.044444,0.027671,0.049676,0.029632,0.043011,0.023513,
0.101266,0.071444,0.130178,0.097749,0.074074,0.047211,0.045918,0.024233,0.073944,0.039223,
0.035503,0.015447

Positive_40 4.000000,0.250000,2.000000,1.000000,1.000000,1.333333,2.000000,1.000000,1.000000,1.333333,
0.746298,0.005759,0.746298,0.005759,0.000000,0.000000,0.000000,0.000000,0.000000,0.250000,
0.666667,0.000000,0.000000,0.000000,0.000000,0.000000,0.000000,0.000000,0.000000,0.000000,
0.000000,0.000000,0.000000,0.166667,0.333333,0.037037,0.000000,0.000000,0.000000,0.000000,
0.000000,0.000000,0.166667,0.333333,0.037037,2.000000,0.000000,0.000000,1.000000,0.500000,
1.000000,0.500000,1.000000,0.500000,1.000000,0.500000,1.000000,0.500000,1.000000,0.500000,
1.000000,0.500000,1.000000,0.500000,0.000000,0.000000,0.000000,0.000000,1.000000,0.500000,
1.000000,0.500000,1.000000,0.500000,1.000000,0.500000,1.000000,0.500000,1.000000,0.500000,
1.000000,0.500000,1.000000,0.500000,1.000000,0.500000,0.000000,0.000000,0.301075,0.297618,
0.413978,0.402903,0.315615,0.299478,0.235189,0.228733,0.186715,0.183884,0.262895,0.245668,
0.005376,0.004640,0.279522,0.243732,0.518685,0.494814,0.804185,0.771448,0.995516,0.992257,
0.008969,0.005280,0.254480,0.219758,0.485663,0.469856,0.736559,0.722097,1.000000,0.998422,
0.001792,0.001685,0.297459,0.281229,0.538206,0.529309,0.759857,0.749167,1.000000,0.999104,
0.490284,0.412696,0.123656,0.096086,0.519713,0.491218,0.061041,0.045493,0.096506,0.092214,
0.008982,0.003910,0.001792,0.001685,0.274194,0.227493,0.539427,0.515591,0.787375,0.744132,
1.000000,1.000000,0.322259,0.195047,0.397608,0.305918,0.586379,0.440316,0.711510,0.647995,
0.761649,0.748277,0.010463,0.006102,0.303438,0.230162,0.527653,0.500040,0.818996,0.789624,
0.998339,0.992862,0.684588,0.638499,0.369838,0.344648,0.005979,0.004532,0.299003,0.280388,
0.545590,0.509234,0.756272,0.744634,0.998339,0.997194,0.417040,0.410370,0.340532,0.331324,
0.261649,0.258306,0.302829,0.277350,0.213645,0.206233,0.174147,0.171986,0.010753,0.008864,
0.245520,0.218336,0.480066,0.462258,0.722591,0.715082,1.000000,0.998422,0.011628,0.008882,
0.288490,0.268136,0.525090,0.522532,0.774086,0.768142,1.000000,0.997312,0.001792,0.001685,
0.298954,0.253561,0.536622,0.507144,0.788530,0.770160,0.998339,0.996446,0.343854,0.331708,
0.349462,0.339685,0.331541,0.328607,0.239601,0.229441,0.233393,0.231221,0.211849,0.205626,
0.001792,0.001685,0.304933,0.291250,0.538206,0.532148,0.770609,0.758654,1.000000,0.999104,
0.010753,0.008864,0.250896,0.213300,0.478495,0.455286,0.725806,0.707128,1.000000,0.998422,
0.005376,0.004640,0.292975,0.241346,0.524664,0.493118,0.805680,0.769745,0.995516,0.992257,
0.356631,0.344948,0.411061,0.396746,0.261649,0.258306,0.284526,0.280704,0.188510,0.172954,
0.221557,0.205264,0.010753,0.008864,0.245520,0.221200,0.498339,0.474521,0.734219,0.725382,
1.000000,0.998422,0.011628,0.008882,0.284053,0.258794,0.512545,0.509366,0.759136,0.752078,
1.000000,0.997312,0.001792,0.001685,0.298954,0.253561,0.536622,0.507144,0.788530,0.770160,
0.998339,0.996446,0.062724,0.055289,0.062724,0.060322,0.066308,0.059946,0.056801,0.053060,
0.036545,0.029305,0.041854,0.039696,0.094982,0.088493,0.094982,0.086865,0.086379,0.071565,
0.048387,0.044964,0.059791,0.047797,0.038206,0.030989,0.037634,0.032230,0.026578,0.017626,
0.070254,0.065471,0.101329,0.087108,0.068100,0.049797,0.033223,0.027802,0.049327,0.044154,
0.008969,0.007520

Positive_41 36.000000,0.236883,24.000000,8.527778,6.000000,50.770635,28.000000,8.527778,7.000000,72.542063,
0.339236,0.009776,0.339236,0.009776,0.072368,0.234043,0.583333,0.833333,0.933333,1.000000,
0.000000,1.595073,7.291667,3.523934,1.476865,3.821429,1.269246,0.186111,0.303819,0.013979,
0.160703,0.244898,0.011264,0.528751,0.771429,0.075082,0.339853,0.600000,0.027349,0.312270,
0.771429,0.038743,0.396254,0.608163,0.062819,13.139063,10.774565,2.442686,1.000000,0.916667,
1.000000,0.916365,1.000000,0.894525,1.000000,0.821592,1.000000,0.608575,1.000000,0.449264,
1.000000,0.370373,1.000000,0.359909,0.666667,0.255452,0.214286,0.087963,1.000000,0.777778,
1.000000,0.777338,1.000000,0.716526,1.000000,0.410381,1.000000,0.349701,1.000000,0.295240,
1.000000,0.269079,1.000000,0.251427,0.666667,0.186069,0.236842,0.081106,0.373333,0.306692,
0.525355,0.360525,0.394558,0.332783,0.269006,0.213503,0.308036,0.210319,0.356164,0.241906,
0.106952,0.020654,0.366279,0.247314,0.587209,0.491135,0.870748,0.733275,1.000000,0.994676,
0.032258,0.009856,0.289474,0.222357,0.606452,0.480637,0.792793,0.741356,1.000000,0.989710,
0.006803,0.003802,0.308642,0.248817,0.571038,0.499648,0.797814,0.744314,1.000000,0.993123,
0.520362,0.391956,0.278146,0.125566,0.795918,0.482478,0.103647,0.054834,0.118750,0.081726,
0.010753,0.001917,0.006803,0.003802,0.323210,0.218664,0.621118,0.454429,0.927632,0.771137,
1.000000,1.000000,0.393548,0.049318,0.453152,0.165621,0.737160,0.331172,0.913897,0.497387,
0.998489,0.676467,0.251656,0.052901,0.457084,0.311406,0.705274,0.512706,0.883721,0.735413,
0.998447,0.958324,0.743421,0.587323,0.442396,0.352752,0.051613,0.018479,0.312925,0.255468,
0.580645,0.487725,0.792035,0.730552,1.000000,0.987837,0.527383,0.368701,0.445946,0.375114,
0.330275,0.256186,0.350000,0.268455,0.244240,0.191484,0.257919,0.189787,0.031056,0.009498,
0.302632,0.226212,0.579646,0.490297,0.796053,0.746734,1.000000,0.991637,0.040146,0.016266,
0.304598,0.256570,0.554160,0.492552,0.796791,0.732153,1.000000,0.992817,0.006803,0.003802,
0.326531,0.234815,0.563063,0.492467,0.807302,0.745543,1.000000,0.994508,0.455782,0.370338,
0.478702,0.301486,0.386667,0.328176,0.317881,0.223528,0.339286,0.246794,0.245614,0.194584,
0.006803,0.003802,0.311475,0.246737,0.568306,0.498389,0.797814,0.740122,1.000000,0.994368,
0.032258,0.009856,0.316742,0.217725,0.597345,0.488861,0.806306,0.752649,1.000000,0.987538,
0.106952,0.020654,0.300885,0.246258,0.569767,0.489320,0.870748,0.731508,1.000000,0.995224,
0.411765,0.297410,0.540373,0.446404,0.330275,0.256186,0.353659,0.260989,0.214286,0.153362,
0.317972,0.227909,0.037267,0.011339,0.298137,0.213337,0.606195,0.475048,0.782895,0.727241,
1.000000,0.988798,0.040146,0.013949,0.319767,0.262325,0.560109,0.501888,0.796791,0.742014,
1.000000,0.995213,0.006803,0.003802,0.326531,0.234815,0.563063,0.492467,0.807302,0.745543,
1.000000,0.994508,0.070968,0.049016,0.108108,0.063853,0.104651,0.066986,0.081967,0.050540,
0.080745,0.040322,0.073423,0.035974,0.118789,0.065436,0.133874,0.077000,0.102190,0.061467,
0.068323,0.042967,0.095335,0.054616,0.042596,0.021485,0.065789,0.037555,0.045267,0.016675,
0.096257,0.061046,0.152381,0.118256,0.080745,0.052529,0.048673,0.026276,0.074830,0.045131,
0.026738,0.012871

Positive_42 40.000000,0.088125,12.000000,3.525000,2.000000,9.999359,12.000000,3.525000,1.000000,13.691667,
0.409328,0.031961,0.409328,0.031961,0.107143,0.160000,0.333333,0.457143,0.657895,0.461538,
0.142857,0.819643,3.000000,0.752613,0.633750,3.583333,0.865825,0.233684,1.000000,0.062789,
0.103315,0.469388,0.022003,0.181063,0.282051,0.006506,0.059356,0.128205,0.001359,0.031357,
0.134615,0.001833,0.136969,0.235897,0.006366,6.963727,4.647430,4.368974,1.000000,0.900000,
1.000000,0.900000,1.000000,0.867289,1.000000,0.593344,1.000000,0.527462,1.000000,0.332242,
1.000000,0.298025,1.000000,0.278887,1.000000,0.110498,1.000000,0.089277,1.000000,0.450000,
1.000000,0.449444,1.000000,0.429242,1.000000,0.393146,1.000000,0.316017,0.800000,0.207056,

0.600000,0.105281,0.488889,0.074910,0.466667,0.029268,0.288889,0.020682,0.355107,0.299634,
0.438025,0.360915,0.436975,0.339452,0.271095,0.208164,0.262097,0.208568,0.339888,0.245437,
0.036247,0.006633,0.329897,0.242073,0.603093,0.501079,0.829897,0.753614,1.000000,0.997458,
0.032468,0.006934,0.350649,0.262660,0.662338,0.505245,0.816825,0.742206,1.000000,0.996471,
0.006494,0.001801,0.280627,0.240857,0.539235,0.486832,0.772636,0.738221,1.000000,0.994105,
0.622703,0.427204,0.300875,0.168659,0.728291,0.404136,0.150980,0.071217,0.109515,0.071051,
0.010081,0.003596,0.006494,0.001801,0.564935,0.262621,0.662338,0.504837,0.869742,0.731636,
1.000000,1.000000,0.525974,0.073114,0.545455,0.235414,0.698630,0.487238,0.928571,0.701566,
0.999461,0.906588,0.118557,0.018807,0.421225,0.253970,0.658986,0.480158,0.836812,0.751530,
0.998917,0.957205,0.747899,0.626771,0.419355,0.334186,0.030928,0.008589,0.319088,0.266627,
0.550179,0.496737,0.791398,0.732423,1.000000,0.988862,0.448454,0.368915,0.420523,0.359087,
0.318182,0.271998,0.331887,0.266397,0.252496,0.198744,0.227111,0.194784,0.017613,0.005859,
0.357143,0.252934,0.675325,0.503906,0.805970,0.749574,1.000000,0.997432,0.036247,0.007299,
0.299459,0.234984,0.552521,0.494086,0.793067,0.736826,1.000000,0.994990,0.006494,0.001801,
0.340206,0.255132,0.578947,0.497831,0.824742,0.745241,1.000000,0.996108,0.481793,0.375637,
0.373950,0.296335,0.382423,0.328028,0.278090,0.224088,0.309156,0.250123,0.242370,0.188713,
0.006494,0.001801,0.288660,0.244580,0.527985,0.483456,0.768612,0.736107,1.000000,0.994248,
0.051948,0.007421,0.396104,0.253280,0.694805,0.504462,0.824966,0.740606,1.000000,0.995869,
0.036247,0.006382,0.329897,0.247200,0.628866,0.509530,0.829897,0.757206,1.000000,0.997768,
0.376289,0.302247,0.469048,0.425755,0.318182,0.271998,0.338395,0.258937,0.209700,0.161848,
0.268939,0.231680,0.022267,0.006146,0.350649,0.247835,0.694805,0.504599,0.837662,0.750336,
1.000000,0.997005,0.025974,0.006198,0.270789,0.239543,0.550107,0.496258,0.782563,0.739927,
1.000000,0.995505,0.006494,0.001801,0.340206,0.255132,0.578947,0.497831,0.824742,0.745241,
1.000000,0.996108,0.074822,0.048518,0.080483,0.058015,0.103226,0.057539,0.068702,0.053600,
0.063922,0.036841,0.084149,0.045120,0.099804,0.068631,0.091008,0.063166,0.092784,0.064317,
0.079912,0.052533,0.085084,0.047689,0.051948,0.028394,0.062678,0.036185,0.056701,0.018979,
0.098039,0.069747,0.142857,0.094884,0.082996,0.054955,0.049462,0.027213,0.077922,0.053344,
0.038961,0.020329

Positive_43 41.000000,0.045211,5.000000,1.853659,1.000000,2.328049,10.000000,1.853659,1.000000,5.028049,
0.662078,0.024640,0.662078,0.024640,0.000000,0.000000,0.039474,0.164384,0.196721,0.306122,
0.617647,0.125610,1.000000,0.077640,0.093438,1.000000,0.066230,0.039654,0.500000,0.010516,
0.026384,0.500000,0.008033,0.068577,0.158333,0.003728,0.016768,0.050000,0.000327,0.016770,
0.100000,0.000778,0.034463,0.100000,0.001183,4.520621,3.517433,3.454260,1.000000,0.463415,
1.000000,0.463415,1.000000,0.463415,1.000000,0.463415,1.000000,0.422764,1.000000,0.377236,
1.000000,0.367480,1.000000,0.163415,1.000000,0.110569,1.000000,0.041463,1.000000,0.365854,
1.000000,0.365854,1.000000,0.365854,1.000000,0.365854,1.000000,0.361789,1.000000,0.334495,
1.000000,0.296516,1.000000,0.216260,1.000000,0.192954,1.000000,0.134727,0.368263,0.267749,
0.447837,0.369556,0.485207,0.362696,0.264051,0.190314,0.255255,0.180127,0.404959,0.270390,
0.097345,0.017681,0.416667,0.270095,0.803279,0.528226,0.928571,0.768931,1.000000,0.995078,
0.042424,0.008772,0.304212,0.228884,0.545082,0.480114,0.796438,0.735579,1.000000,0.994786,
0.011905,0.002769,0.307692,0.244585,0.555556,0.490315,0.796296,0.730823,1.000000,0.993038,
0.618090,0.409867,0.290254,0.126966,0.805310,0.463166,0.107692,0.053364,0.120482,0.071054,
0.017568,0.005560,0.011905,0.002769,0.459016,0.222084,0.741408,0.507944,0.858607,0.749297,
1.000000,1.000000,0.729508,0.156488,0.729508,0.349029,0.883721,0.590402,0.972387,0.753747,

0.999126,0.905168,0.143590,0.029224,0.711697,0.226120,0.803954,0.477377,0.897858,0.705546,
0.998353,0.951864,0.804734,0.620930,0.439024,0.306518,0.095238,0.015128,0.389127,0.273036,
0.589413,0.502289,0.795567,0.727435,0.999126,0.987292,0.419847,0.361738,0.424020,0.366695,
0.334038,0.271568,0.363636,0.261236,0.221939,0.190422,0.250000,0.198925,0.031320,0.009142,
0.286585,0.223531,0.543897,0.475012,0.796438,0.736775,1.000000,0.995132,0.083333,0.013101,
0.285714,0.248315,0.550481,0.495447,0.806373,0.744889,1.000000,0.994401,0.011905,0.002769,
0.419598,0.275325,0.605528,0.522479,0.845238,0.751344,1.000000,0.994652,0.526427,0.401862,
0.368852,0.302542,0.395210,0.295596,0.404959,0.245497,0.279279,0.222733,0.229226,0.169872,
0.011905,0.002769,0.314070,0.254548,0.555556,0.496073,0.796296,0.735043,1.000000,0.993312,
0.042424,0.009825,0.288618,0.213076,0.546039,0.467548,0.816794,0.732512,1.000000,0.993720,
0.097345,0.017402,0.483607,0.280221,0.803279,0.531617,0.916667,0.763937,1.000000,0.995797,
0.360656,0.290165,0.500000,0.438268,0.334038,0.271568,0.347107,0.252788,0.188854,0.152496,
0.288136,0.236850,0.042424,0.010309,0.326053,0.220550,0.559796,0.475394,0.820359,0.735514,
1.000000,0.994888,0.083333,0.011569,0.284779,0.244716,0.549261,0.491283,0.786469,0.743606,
1.000000,0.995485,0.011905,0.002769,0.419598,0.275325,0.605528,0.522479,0.845238,0.751344,
1.000000,0.994652,0.094148,0.055045,0.091133,0.050158,0.090352,0.050884,0.065678,0.041556,
0.068862,0.037412,0.048598,0.032695,0.094148,0.064494,0.122951,0.067587,0.106195,0.068401,
0.084553,0.048127,0.104326,0.053933,0.042122,0.027847,0.062193,0.039167,0.041420,0.017640,
0.119048,0.068711,0.221311,0.126937,0.088462,0.050057,0.030864,0.021233,0.122622,0.059504,
0.032787,0.018614

Positive_44 28.000000,0.127551,23.000000,3.571429,2.000000,30.698413,23.000000,3.571429,2.000000,31.291005,
0.531460,0.038534,0.531460,0.038534,0.010000,0.040404,0.389474,0.172414,0.208333,0.473684,
0.250000,0.579969,1.869565,0.197124,0.271118,2.565217,0.480622,0.202235,0.250000,0.009002,
0.029676,0.320000,0.007366,0.689383,0.814815,0.075053,0.128077,0.444444,0.012472,0.116985,
0.271605,0.004064,0.596135,0.814815,0.111760,7.505716,5.521252,3.343651,1.000000,0.857143,
1.000000,0.857143,1.000000,0.857143,1.000000,0.821429,1.000000,0.782609,1.000000,0.764469,
1.000000,0.755717,1.000000,0.739342,1.000000,0.731437,0.241107,0.035079,1.000000,0.892857,
1.000000,0.892857,1.000000,0.892575,1.000000,0.891869,1.000000,0.838142,1.000000,0.148617,
1.000000,0.143958,1.000000,0.132242,1.000000,0.124760,1.000000,0.109373,0.353261,0.327077,
0.396667,0.345486,0.359813,0.327437,0.292154,0.211754,0.264637,0.220437,0.268734,0.222932,
0.018248,0.006850,0.311429,0.247272,0.541237,0.489210,0.786082,0.750240,1.000000,0.996974,
0.025114,0.008894,0.323529,0.249502,0.573003,0.511388,0.796143,0.750589,1.000000,0.995964,
0.003650,0.001994,0.340000,0.246752,0.586170,0.485832,0.788298,0.733651,1.000000,0.993900,
0.640000,0.505350,0.288321,0.181232,0.585086,0.313419,0.117216,0.076673,0.105263,0.064480,
0.009050,0.003141,0.003650,0.001994,0.409178,0.250521,0.640535,0.531441,0.802528,0.771775,
1.000000,1.000000,0.531549,0.159397,0.608987,0.406880,0.696941,0.588884,0.885106,0.809097,
0.998392,0.971410,0.204380,0.015084,0.249619,0.172664,0.558333,0.367695,0.846501,0.628899,
0.996705,0.868664,0.641509,0.554329,0.400000,0.354599,0.018779,0.008184,0.418333,0.286657,
0.643333,0.522725,0.820000,0.728446,0.998392,0.993495,0.397849,0.352803,0.398744,0.363402,
0.322857,0.283795,0.303349,0.245559,0.220630,0.197493,0.252212,0.209160,0.018265,0.008813,
0.286976,0.251305,0.550314,0.503020,0.794362,0.751920,1.000000,0.995634,0.022375,0.008436,
0.297143,0.244050,0.531915,0.481233,0.781991,0.746812,1.000000,0.997530,0.003650,0.001994,
0.305785,0.243510,0.552496,0.504359,0.806667,0.736544,1.000000,0.994043,0.408879,0.364684,
0.338333,0.276801,0.379787,0.358515,0.242894,0.198079,0.314159,0.266046,0.258765,0.190017,

0.003650,0.001994,0.371667,0.252946,0.605000,0.497188,0.798333,0.741210,1.000000,0.994489,
0.034247,0.010260,0.300221,0.240650,0.573951,0.500068,0.785294,0.737008,1.000000,0.994955,
0.018248,0.006737,0.311429,0.248269,0.541237,0.493100,0.787120,0.755319,1.000000,0.997482,
0.318996,0.279359,0.477759,0.436847,0.322857,0.283795,0.290909,0.236095,0.186978,0.158908,
0.287293,0.247745,0.037559,0.011328,0.288014,0.246876,0.544118,0.497400,0.802941,0.745459,
1.000000,0.995252,0.019284,0.007860,0.288571,0.247979,0.544025,0.489926,0.786730,0.748605,
1.000000,0.997693,0.003650,0.001994,0.305785,0.243510,0.552496,0.504359,0.806667,0.736544,
1.000000,0.994043,0.091211,0.064020,0.087234,0.061784,0.092943,0.062635,0.068841,0.053633,
0.067293,0.039668,0.062141,0.045337,0.085814,0.060609,0.105163,0.057642,0.077263,0.063119,
0.080000,0.044355,0.063927,0.051076,0.048571,0.031438,0.063361,0.037247,0.036496,0.022369,
0.087894,0.065887,0.131868,0.098714,0.075055,0.051161,0.053459,0.024847,0.067010,0.043264,
0.036496,0.021194

Positive_45 6.000000,0.222222,3.000000,1.333333,1.000000,1.066667,3.000000,1.333333,1.500000,1.466667,
0.504917,0.033611,0.504917,0.033611,0.000000,0.125000,0.142857,0.500000,0.000000,0.333333,
1.000000,0.250000,1.000000,0.175000,0.250000,1.000000,0.175000,0.097222,0.333333,0.023380,
0.097222,0.333333,0.023380,0.194444,0.400000,0.026852,0.177778,0.400000,0.024296,0.177778,
0.600000,0.056296,0.116667,0.300000,0.017667,2.276388,1.185926,1.000000,1.000000,0.333333,
1.000000,0.333333,1.000000,0.333333,1.000000,0.333333,1.000000,0.333333,0.333333,0.055556,
0.333333,0.055556,0.000000,0.000000,0.000000,0.000000,0.000000,0.000000,1.000000,0.500000,
1.000000,0.500000,1.000000,0.500000,1.000000,0.500000,1.000000,0.333333,1.000000,0.333333,
1.000000,0.333333,1.000000,0.277778,0.000000,0.000000,0.000000,0.000000,0.342052,0.322468,
0.411609,0.364501,0.328571,0.313031,0.264550,0.226192,0.262097,0.215285,0.236311,0.222097,
0.020000,0.009695,0.275862,0.236659,0.574286,0.517468,0.811429,0.757417,0.997988,0.993951,
0.022267,0.008362,0.311905,0.265860,0.511905,0.488786,0.788918,0.736632,1.000000,0.998849,
0.002874,0.002464,0.308707,0.237822,0.539235,0.481865,0.772636,0.730715,1.000000,0.993799,
0.498681,0.445390,0.221328,0.171770,0.442857,0.382840,0.092219,0.075041,0.085193,0.070707,
0.011527,0.007103,0.002874,0.002464,0.323810,0.250321,0.548851,0.490446,0.797571,0.766056,
1.000000,1.000000,0.197183,0.078254,0.476861,0.272905,0.649899,0.506137,0.905433,0.726818,
0.993964,0.913247,0.080000,0.031956,0.398417,0.256598,0.551451,0.456786,0.782857,0.709132,
0.992084,0.956118,0.657143,0.596893,0.419355,0.362890,0.026385,0.013779,0.343008,0.277062,
0.554090,0.495478,0.778364,0.714985,0.995976,0.985260,0.395778,0.360820,0.420523,0.381718,
0.288571,0.257462,0.304260,0.277817,0.203704,0.179279,0.240688,0.203642,0.016194,0.007350,
0.278736,0.246146,0.543103,0.487011,0.775862,0.736295,1.000000,0.998514,0.023747,0.011540,
0.257545,0.236687,0.537143,0.500695,0.762857,0.738000,1.000000,0.995236,0.002874,0.002464,
0.300792,0.250114,0.578947,0.509788,0.814286,0.754762,1.000000,0.993759,0.380000,0.350647,
0.335092,0.299190,0.366667,0.350163,0.224784,0.201544,0.294355,0.262937,0.232804,0.204652,
0.002874,0.002464,0.329815,0.249986,0.515091,0.488396,0.768612,0.733893,1.000000,0.993799,
0.022267,0.008362,0.276190,0.243233,0.521127,0.488881,0.775862,0.742854,1.000000,0.996502,
0.020000,0.009695,0.279352,0.244406,0.571429,0.517832,0.805714,0.758319,1.000000,0.994683,
0.330460,0.292545,0.469048,0.449993,0.288571,0.257462,0.304260,0.256617,0.152738,0.140912,
0.300860,0.242010,0.022267,0.008362,0.257085,0.245081,0.517241,0.481268,0.776660,0.735844,
1.000000,0.994108,0.021108,0.010148,0.257143,0.245458,0.537143,0.496966,0.764286,0.742432,
1.000000,0.995236,0.002874,0.002464,0.300792,0.250114,0.578947,0.509788,0.814286,0.754762,
1.000000,0.993759,0.068602,0.049737,0.080483,0.064298,0.082857,0.069860,0.060729,0.047373,

0.068602,0.041048,0.068410,0.050151,0.106322,0.067232,0.097625,0.069214,0.072435,0.061460,
0.058350,0.047266,0.089710,0.054018,0.044855,0.027695,0.051429,0.037616,0.024291,0.014257,
0.085714,0.075760,0.108652,0.090610,0.082996,0.054288,0.033333,0.023754,0.054286,0.039080,
0.031662,0.015282

Positive_46 27.000000,0.109739,10.000000,2.962963,3.000000,5.037037,8.000000,2.962963,3.000000,7.575499,
0.370128,0.019167,0.370128,0.019167,0.062500,0.266667,0.527273,0.692308,0.500000,0.500000,
0.000000,0.612346,1.700000,0.341957,0.460758,1.571429,0.394455,0.152901,0.333333,0.021026,
0.080979,0.312500,0.012661,0.147388,0.269231,0.009555,0.052944,0.153846,0.002410,0.053359,
0.269231,0.004202,0.131563,0.346154,0.012550,4.831741,4.754799,3.464102,1.000000,0.740741,
1.000000,0.740741,1.000000,0.674486,1.000000,0.567490,1.000000,0.448971,1.000000,0.304527,
1.000000,0.256790,1.000000,0.166255,0.333333,0.054733,0.166667,0.011111,1.000000,0.555556,
1.000000,0.555556,1.000000,0.536861,1.000000,0.450088,1.000000,0.240917,1.000000,0.197531,
1.000000,0.169136,1.000000,0.153263,1.000000,0.135979,0.333333,0.061376,0.352475,0.307991,
0.461957,0.368061,0.353261,0.323948,0.252604,0.215115,0.259825,0.210516,0.282132,0.237862,
0.040323,0.009957,0.325540,0.235718,0.570144,0.482878,0.800000,0.735401,1.000000,0.995795,
0.032609,0.008960,0.304348,0.245575,0.562061,0.495713,0.806452,0.744907,1.000000,0.995618,
0.008065,0.002512,0.297235,0.250057,0.566820,0.512627,0.779944,0.749148,1.000000,0.993216,
0.580645,0.439237,0.266304,0.175542,0.545667,0.385220,0.111872,0.073212,0.097166,0.074561,
0.011737,0.005263,0.008152,0.002714,0.334239,0.232213,0.661290,0.502178,0.856250,0.760173,
1.000000,1.000000,0.211957,0.060601,0.489130,0.212057,0.620968,0.447339,0.826613,0.650224,
0.998445,0.924109,0.122093,0.033471,0.415038,0.290438,0.640860,0.500982,0.845622,0.731812,
0.998917,0.940278,0.662791,0.592860,0.453441,0.357337,0.048387,0.010924,0.332815,0.271978,
0.564516,0.512815,0.791398,0.740894,0.998672,0.989599,0.462415,0.384946,0.419802,0.360438,
0.297424,0.254616,0.331887,0.275978,0.237089,0.186907,0.233871,0.185888,0.034602,0.008183,
0.320652,0.251498,0.580645,0.503786,0.807065,0.745495,1.000000,0.995366,0.027174,0.009431,
0.304348,0.247085,0.545961,0.497217,0.784962,0.741958,1.000000,0.995733,0.008065,0.002512,
0.330645,0.239830,0.590116,0.479977,0.820652,0.733077,1.000000,0.994477,0.387524,0.357516,
0.388587,0.306621,0.381643,0.335864,0.263323,0.219305,0.286026,0.253148,0.248858,0.193749,
0.008065,0.002512,0.313364,0.252451,0.566820,0.505900,0.782609,0.747376,1.000000,0.995188,
0.043478,0.009798,0.312500,0.247051,0.580645,0.493778,0.808602,0.741995,1.000000,0.993752,
0.040323,0.009634,0.314516,0.240507,0.561151,0.482786,0.800000,0.734148,1.000000,0.996463,
0.378132,0.318612,0.477228,0.426772,0.297424,0.254616,0.338395,0.273685,0.187793,0.154589,
0.272581,0.218206,0.034602,0.009385,0.309783,0.248659,0.557065,0.496687,0.807065,0.731504,
1.000000,0.993015,0.024194,0.007647,0.290398,0.248221,0.548387,0.504843,0.806867,0.752689,
1.000000,0.996672,0.008065,0.002512,0.330645,0.239830,0.590116,0.479977,0.820652,0.733077,
1.000000,0.994477,0.077283,0.051389,0.086957,0.058322,0.103226,0.066013,0.080645,0.055598,
0.064378,0.041275,0.065217,0.035394,0.119186,0.077104,0.109339,0.071570,0.101089,0.064584,
0.081522,0.049755,0.062950,0.043607,0.056452,0.027873,0.057143,0.033568,0.040323,0.022727,
0.096425,0.070229,0.120260,0.095921,0.077025,0.051605,0.049462,0.026278,0.068750,0.043732,
0.030445,0.013455

Positive_47 17.000000,0.076125,3.000000,1.294118,1.000000,1.095588,4.000000,1.294118,1.000000,1.845588,
0.526006,0.045361,0.526006,0.045361,0.045455,0.142857,0.222222,0.214286,0.181818,0.333333,
0.666667,0.166667,0.666667,0.072917,0.102941,1.000000,0.086397,0.070261,0.250000,0.012646,
0.025735,0.250000,0.005400,0.065564,0.187500,0.005517,0.037990,0.125000,0.001898,0.050551,

0.250000,0.006944,0.037684,0.125000,0.002108,2.689994,2.557612,1.662508,1.000000,0.470588,
1.000000,0.470588,1.000000,0.470588,1.000000,0.431373,1.000000,0.392157,1.000000,0.333333,
1.000000,0.333333,1.000000,0.294118,1.000000,0.117647,0.000000,0.000000,1.000000,0.352941,
1.000000,0.352941,1.000000,0.343137,1.000000,0.333333,1.000000,0.245098,1.000000,0.176471,
1.000000,0.156863,1.000000,0.127451,1.000000,0.127451,0.000000,0.000000,0.309122,0.274466,
0.441041,0.399699,0.401575,0.325835,0.236887,0.213951,0.215686,0.184065,0.293375,0.260301,
0.039080,0.008561,0.325540,0.272502,0.570144,0.502433,0.808883,0.755755,1.000000,0.991928,
0.010135,0.005125,0.293944,0.236507,0.568710,0.492506,0.784355,0.740307,1.000000,0.997370,
0.002299,0.001706,0.283465,0.233671,0.533784,0.494393,0.774803,0.740647,1.000000,0.998045,
0.689091,0.519377,0.273425,0.153547,0.537008,0.327076,0.118462,0.064854,0.083558,0.058363,
0.006135,0.003396,0.002299,0.001706,0.345643,0.239110,0.553914,0.491186,0.797637,0.754806,
1.000000,1.000000,0.111511,0.059515,0.395100,0.223940,0.702910,0.507652,0.868300,0.738505,
0.998425,0.944643,0.040541,0.016230,0.347826,0.255464,0.658986,0.496628,0.804916,0.681438,
0.996364,0.941029,0.681890,0.622890,0.372549,0.333409,0.016575,0.007358,0.314961,0.247151,
0.570079,0.496538,0.796850,0.728051,0.998464,0.994933,0.441379,0.399085,0.390551,0.347089,
0.273425,0.253826,0.324885,0.276232,0.237327,0.205394,0.223350,0.173905,0.009208,0.004920,
0.316100,0.232270,0.547569,0.497300,0.786693,0.743673,1.000000,0.996285,0.019027,0.008755,
0.272016,0.244423,0.532609,0.499291,0.779693,0.741858,1.000000,0.996301,0.002299,0.001706,
0.314748,0.268549,0.554531,0.491629,0.798425,0.755231,1.000000,0.997565,0.439370,0.367909,
0.373660,0.331744,0.337838,0.300346,0.268139,0.244606,0.255499,0.225325,0.223502,0.191658,
0.002299,0.001706,0.288189,0.242677,0.532283,0.496183,0.779528,0.742231,1.000000,0.998244,
0.010135,0.005125,0.327917,0.221178,0.568710,0.491988,0.790698,0.734176,1.000000,0.995605,
0.017241,0.006803,0.311151,0.273499,0.561151,0.501092,0.798771,0.757388,1.000000,0.993431,
0.348587,0.314976,0.462992,0.431198,0.273425,0.253826,0.327189,0.275301,0.195742,0.162974,
0.257191,0.216325,0.012681,0.006304,0.323486,0.238482,0.568710,0.494187,0.786469,0.732370,
1.000000,0.994826,0.012598,0.006389,0.267833,0.239556,0.524372,0.497533,0.796935,0.752411,
1.000000,0.997316,0.002299,0.001706,0.314748,0.268549,0.554531,0.491629,0.798425,0.755231,
1.000000,0.997565,0.082452,0.055020,0.071823,0.040482,0.068053,0.044263,0.070909,0.051601,
0.064368,0.041586,0.063406,0.041514,0.088123,0.070016,0.127860,0.072872,0.083550,0.068226,
0.063406,0.052260,0.094545,0.068370,0.038055,0.025880,0.056836,0.042074,0.027397,0.015739,
0.080338,0.063937,0.154696,0.116157,0.069291,0.039631,0.028986,0.016727,0.068053,0.047981,
0.040169,0.025662

Positive_48 12.000000,0.187500,4.000000,2.250000,2.500000,1.477273,5.000000,2.250000,1.000000,3.477273,
0.744807,0.036946,0.744807,0.036946,0.000000,0.000000,0.000000,0.037037,0.269231,0.368421,
0.250000,1.250000,3.000000,2.386364,0.750000,3.000000,1.840909,0.416667,1.000000,0.265152,
0.150000,0.600000,0.073636,0.196970,0.363636,0.026797,0.174242,0.272727,0.012710,0.112121,
0.327273,0.018502,0.166667,0.272727,0.008765,3.872983,2.449490,2.000000,1.000000,0.833333,
1.000000,0.833333,1.000000,0.833333,1.000000,0.833333,1.000000,0.833333,1.000000,0.833333,
1.000000,0.833333,1.000000,0.805556,1.000000,0.541667,1.000000,0.527778,1.000000,0.416667,
1.000000,0.416667,1.000000,0.416667,1.000000,0.416667,1.000000,0.416667,1.000000,0.391667,
1.000000,0.266667,1.000000,0.266667,1.000000,0.241667,0.666667,0.186111,0.357143,0.314562,
0.434839,0.381730,0.326568,0.303708,0.270186,0.238808,0.218456,0.179184,0.279141,0.232244,
0.015291,0.007069,0.325228,0.271336,0.541796,0.503349,0.805970,0.754393,1.000000,0.994866,
0.032836,0.009220,0.247761,0.217264,0.535168,0.471835,0.782288,0.736115,1.000000,0.996666,

0.003096,0.002064,0.359750,0.259543,0.573696,0.510190,0.785193,0.749145,1.000000,0.996009,
0.570386,0.507207,0.192661,0.156603,0.415414,0.336190,0.074534,0.060715,0.077844,0.063911,
0.009009,0.004369,0.003096,0.002064,0.313433,0.238544,0.580547,0.505124,0.811940,0.770716,
1.000000,1.000000,0.248120,0.140587,0.429889,0.352546,0.648526,0.585626,0.899083,0.769488,
0.996259,0.925918,0.015291,0.008312,0.339355,0.209902,0.498866,0.417303,0.744526,0.688945,
0.996310,0.957719,0.623100,0.554723,0.371608,0.343440,0.024465,0.010774,0.347237,0.289508,
0.571956,0.526760,0.789668,0.746050,0.996942,0.990207,0.428387,0.380788,0.411439,0.348366,
0.305810,0.270846,0.284431,0.251660,0.231707,0.203915,0.207241,0.175786,0.032836,0.009220,
0.247761,0.213663,0.525373,0.474506,0.769327,0.748325,1.000000,0.996666,0.030581,0.010867,
0.329932,0.268974,0.544218,0.499819,0.790419,0.744614,1.000000,0.994379,0.003096,0.002064,
0.327068,0.281559,0.554511,0.515386,0.798872,0.740384,1.000000,0.991637,0.367159,0.336310,
0.371613,0.320021,0.387218,0.343669,0.230061,0.205263,0.267420,0.228563,0.254658,0.215857,
0.003096,0.002064,0.364964,0.266408,0.572562,0.513897,0.783447,0.747695,1.000000,0.996009,
0.032836,0.009220,0.247761,0.199373,0.516763,0.453833,0.776879,0.733142,1.000000,0.996666,
0.015291,0.007069,0.334347,0.280114,0.541033,0.501536,0.811940,0.756880,1.000000,0.994866,
0.349544,0.297148,0.489552,0.432005,0.305810,0.270846,0.306202,0.252368,0.210366,0.162444,
0.246246,0.217256,0.032836,0.010934,0.301493,0.223124,0.538226,0.482521,0.795107,0.747320,
1.000000,0.996159,0.015480,0.007220,0.289277,0.244085,0.524505,0.491280,0.790274,0.750543,
1.000000,0.997181,0.003096,0.002064,0.327068,0.281559,0.554511,0.515386,0.798872,0.740384,
1.000000,0.991637,0.136778,0.084540,0.073308,0.044300,0.103321,0.063997,0.054711,0.046795,
0.056716,0.045023,0.045113,0.029907,0.081633,0.071487,0.151976,0.076278,0.074830,0.055438,
0.083832,0.047152,0.101493,0.069667,0.045872,0.029107,0.042394,0.032602,0.024465,0.013972,
0.095808,0.067920,0.140299,0.111183,0.048753,0.030336,0.033666,0.019454,0.061162,0.042314,
0.030395,0.018529

Positive_49 5.000000,0.400000,3.000000,2.000000,2.000000,1.500000,2.000000,2.000000,2.000000,0.000000,
0.557331,0.002502,0.557331,0.002502,0.000000,0.000000,0.000000,0.000000,0.600000,1.000000,
0.000000,0.000000,0.000000,0.000000,0.000000,0.000000,0.000000,0.000000,0.000000,0.000000,
0.000000,0.000000,0.000000,0.200000,0.250000,0.012500,0.266667,0.500000,0.050000,0.300000,
0.500000,0.012500,0.400000,0.500000,0.018750,2.449490,2.000000,0.000000,1.000000,0.800000,
1.000000,0.800000,1.000000,0.800000,1.000000,0.800000,1.000000,0.800000,1.000000,0.800000,
1.000000,0.800000,1.000000,0.666667,1.000000,0.533333,1.000000,0.400000,1.000000,1.000000,
1.000000,1.000000,1.000000,1.000000,1.000000,1.000000,1.000000,1.000000,1.000000,1.000000,
1.000000,1.000000,1.000000,1.000000,1.000000,0.600000,1.000000,0.600000,0.309166,0.292063,
0.442308,0.369060,0.372727,0.338876,0.232215,0.210734,0.221918,0.192288,0.256296,0.239153,
0.008705,0.005176,0.275758,0.247653,0.522572,0.477281,0.755130,0.746298,0.998660,0.997301,
0.015152,0.007620,0.312121,0.268334,0.569697,0.519636,0.772727,0.749316,1.000000,0.994388,
0.003030,0.001661,0.277480,0.226390,0.526810,0.488466,0.768226,0.740591,1.000000,0.999112,
0.545576,0.515598,0.155951,0.141785,0.415152,0.342617,0.072603,0.065977,0.077037,0.070724,
0.007625,0.005169,0.003030,0.001661,0.315560,0.263279,0.498368,0.447243,0.733333,0.719298,
1.000000,1.000000,0.130178,0.039638,0.284848,0.259185,0.505362,0.469601,0.793939,0.747940,
0.983678,0.973406,0.042424,0.016444,0.383037,0.271810,0.630303,0.557116,0.838577,0.796060,
0.995896,0.991586,0.618331,0.600861,0.383442,0.342679,0.009383,0.007491,0.298150,0.272022,
0.533333,0.521855,0.766756,0.754654,0.996736,0.994410,0.455621,0.374111,0.396970,0.362387,
0.301609,0.263502,0.298630,0.267272,0.210738,0.190552,0.243161,0.196072,0.009091,0.005313,

0.272727,0.252941,0.542424,0.495826,0.747989,0.731825,1.000000,0.994147,0.018182,0.006835,
0.260054,0.244775,0.518767,0.479068,0.732318,0.717550,1.000000,0.999408,0.003030,0.001661,
0.266594,0.242466,0.558215,0.528097,0.829162,0.803764,0.998912,0.996971,0.409091,0.376566,
0.397929,0.305080,0.341823,0.318354,0.251852,0.227329,0.268493,0.233808,0.217778,0.187665,
0.003030,0.001661,0.272118,0.236524,0.537534,0.500868,0.775843,0.754171,1.000000,0.999112,
0.030303,0.010650,0.312121,0.268574,0.557576,0.508756,0.741124,0.722759,1.000000,0.991567,
0.008705,0.005176,0.275758,0.249345,0.521204,0.484036,0.769697,0.757936,0.998660,0.997301,
0.335799,0.289111,0.467852,0.447387,0.301609,0.263502,0.291852,0.254683,0.165101,0.143077,
0.282675,0.243548,0.033333,0.011256,0.312121,0.262798,0.563636,0.501962,0.757373,0.734613,
0.998632,0.992528,0.009091,0.005017,0.256032,0.245592,0.516086,0.478726,0.733406,0.718465,
1.000000,1.000000,0.003030,0.001661,0.266594,0.242466,0.558215,0.528097,0.829162,0.803764,
0.998912,0.996971,0.069527,0.058960,0.061662,0.047624,0.068553,0.050741,0.060192,0.050648,
0.053254,0.043971,0.060606,0.040120,0.066568,0.053025,0.094675,0.066245,0.085799,0.073977,
0.051143,0.045216,0.100592,0.066618,0.034853,0.026291,0.048257,0.037690,0.020520,0.018383,
0.090287,0.069802,0.122781,0.106249,0.072727,0.051505,0.045455,0.025192,0.053619,0.047719,
0.028107,0.020028

Positive_50 45.000000,0.206420,18.000000,9.288889,12.000000,49.528283,24.000000,9.288889,2.000000,92.710101,
0.345833,0.034253,0.345833,0.034253,0.201923,0.370482,0.349282,0.470588,0.472222,0.421053,
0.409091,4.773244,11.400000,22.851901,3.237482,10.708333,22.404243,0.325427,0.900000,0.114091,
0.149474,0.489796,0.045144,0.302253,0.505682,0.031992,0.172611,0.338636,0.010111,0.107414,
0.410606,0.018997,0.189722,0.386364,0.020694,17.674978,7.258250,3.932658,1.000000,0.733333,
1.000000,0.733333,1.000000,0.706765,1.000000,0.570976,1.000000,0.473638,1.000000,0.301594,
1.000000,0.137789,1.000000,0.085893,1.000000,0.052716,1.000000,0.025872,1.000000,0.733333,
1.000000,0.729075,1.000000,0.625047,1.000000,0.539314,1.000000,0.338825,1.000000,0.291991,
1.000000,0.207352,1.000000,0.167129,1.000000,0.106022,1.000000,0.067012,0.329915,0.283352,
0.511905,0.379126,0.412234,0.337522,0.243137,0.208000,0.243869,0.187469,0.325301,0.255910,
0.057789,0.011008,0.339223,0.251111,0.612613,0.517819,0.855856,0.756308,1.000000,0.994873,
0.062500,0.009011,0.318021,0.252709,0.623016,0.500025,0.828042,0.750378,1.000000,0.996460,
0.004065,0.002151,0.307377,0.237862,0.571721,0.476160,0.772541,0.728306,1.000000,0.990307,
0.617725,0.448381,0.298925,0.158093,0.688525,0.393526,0.148148,0.066884,0.108312,0.071616,
0.013274,0.005284,0.004065,0.002151,0.378092,0.253146,0.661376,0.524709,0.902778,0.772298,
1.000000,1.000000,0.468310,0.094141,0.502193,0.271219,0.764454,0.507349,0.860814,0.711375,
0.998677,0.909879,0.181518,0.033321,0.368421,0.236005,0.627863,0.448832,0.833969,0.684314,
0.997279,0.934419,0.733607,0.616210,0.403175,0.320750,0.046205,0.010745,0.320312,0.264752,
0.559426,0.490299,0.778203,0.725973,1.000000,0.987146,0.538360,0.391990,0.418557,0.357590,
0.309309,0.250420,0.335145,0.277353,0.262048,0.193314,0.221978,0.178180,0.038194,0.008160,
0.342756,0.251699,0.632275,0.497762,0.838624,0.747423,1.000000,0.995544,0.057377,0.009052,
0.336518,0.243198,0.551155,0.489306,0.804805,0.740257,1.000000,0.995092,0.004065,0.002151,
0.364754,0.246675,0.612705,0.501633,0.808247,0.745288,1.000000,0.990812,0.449468,0.369662,
0.465608,0.321792,0.358209,0.308545,0.301205,0.235570,0.286104,0.223230,0.232759,0.193410,
0.004065,0.002151,0.319672,0.242996,0.577869,0.481890,0.780738,0.730730,1.000000,0.992622,
0.062500,0.009518,0.332155,0.248954,0.654762,0.498245,0.842593,0.753035,1.000000,0.994521,
0.045226,0.010523,0.329268,0.251155,0.597173,0.515649,0.804805,0.754426,1.000000,0.995385,
0.402116,0.313111,0.490506,0.436469,0.309309,0.250420,0.312261,0.266599,0.210843,0.154159,

0.266667,0.217335,0.038194,0.009183,0.374558,0.251563,0.613821,0.496806,0.851590,0.751773,
1.000000,0.993645,0.032787,0.008077,0.287801,0.242861,0.547855,0.493625,0.786432,0.740634,
1.000000,0.997489,0.004065,0.002151,0.364754,0.246675,0.612705,0.501633,0.808247,0.745288,
1.000000,0.990812,0.084154,0.050438,0.082090,0.056181,0.076172,0.056531,0.064815,0.045746,
0.061185,0.036990,0.070352,0.037466,0.109462,0.074947,0.141491,0.071944,0.106029,0.067026,
0.082938,0.053448,0.128307,0.054428,0.042904,0.025193,0.069444,0.032141,0.049383,0.024451,
0.105691,0.072486,0.154876,0.102254,0.080412,0.051863,0.040650,0.022820,0.078078,0.046003,
0.035088,0.017644

Positive_51 47.000000,0.204165,25.000000,9.595745,7.000000,73.159112,22.000000,9.595745,12.000000,56.246068,
0.377895,0.017891,0.377895,0.017891,0.039911,0.260970,0.496875,0.590062,0.606061,0.730769,
0.285714,2.084853,7.400000,6.680376,2.605845,7.045455,4.904555,0.163099,1.000000,0.034074,
0.285498,1.000000,0.083469,0.261484,0.400362,0.017240,0.077702,0.215415,0.005948,0.085805,
0.254476,0.006799,0.281249,0.521739,0.032059,16.661075,9.854703,4.833216,1.000000,0.851064,
1.000000,0.851064,1.000000,0.803416,1.000000,0.714744,1.000000,0.586254,1.000000,0.479975,
1.000000,0.376615,1.000000,0.298727,0.333333,0.078283,0.333333,0.031404,1.000000,0.808511,
1.000000,0.808511,1.000000,0.781915,1.000000,0.659280,1.000000,0.455731,1.000000,0.365336,
1.000000,0.299978,0.400000,0.097979,0.314286,0.069548,0.166667,0.020737,0.391447,0.331340,
0.453416,0.362044,0.362360,0.306616,0.257908,0.224752,0.258185,0.208534,0.274648,0.216281,
0.064133,0.008910,0.350000,0.253831,0.568710,0.504412,0.826160,0.743723,1.000000,0.996910,
0.014035,0.004674,0.348371,0.244250,0.614749,0.493569,0.833333,0.750356,1.000000,0.997222,
0.004975,0.001617,0.385093,0.250825,0.591615,0.495895,0.802603,0.733127,1.000000,0.995073,
0.677590,0.502238,0.259928,0.158897,0.546838,0.338865,0.101449,0.059972,0.100000,0.068803,
0.013333,0.005184,0.004975,0.001617,0.375000,0.251736,0.664439,0.500826,0.885305,0.746426,
1.000000,1.000000,0.307453,0.062521,0.627635,0.274645,0.833882,0.493532,0.918534,0.720207,
0.998355,0.934499,0.201923,0.030174,0.503831,0.266412,0.692827,0.491047,0.891827,0.739808,
0.998410,0.965409,0.638989,0.512554,0.409639,0.362624,0.076355,0.010499,0.347222,0.265372,
0.580745,0.501329,0.801932,0.739769,0.999047,0.993674,0.492236,0.379293,0.410655,0.364460,
0.322816,0.256247,0.334171,0.270161,0.264085,0.183288,0.238443,0.186359,0.014035,0.004564,
0.333333,0.245041,0.612206,0.494609,0.838164,0.745411,1.000000,0.997952,0.029851,0.007786,
0.320000,0.256093,0.562500,0.504551,0.810096,0.747581,1.000000,0.997538,0.004975,0.001617,
0.366594,0.246379,0.630021,0.492755,0.812112,0.733955,1.000000,0.995002,0.398876,0.338602,
0.424947,0.304358,0.409548,0.357040,0.239437,0.195883,0.305893,0.248580,0.245503,0.202609,
0.004975,0.001617,0.402174,0.255753,0.593168,0.498362,0.803140,0.734360,1.000000,0.995703,
0.014035,0.004732,0.359903,0.238978,0.652893,0.493119,0.840580,0.744617,1.000000,0.996308,
0.064133,0.008910,0.350000,0.255178,0.582452,0.503849,0.819409,0.746108,1.000000,0.997373,
0.378882,0.304512,0.493896,0.439241,0.322816,0.256247,0.318818,0.266824,0.204225,0.149538,
0.284672,0.220109,0.014035,0.005179,0.346232,0.247987,0.608696,0.495512,0.828502,0.743514,
1.000000,0.997288,0.029851,0.006260,0.312000,0.250503,0.544994,0.502638,0.780000,0.749673,
1.000000,0.997726,0.004975,0.001617,0.366594,0.246379,0.630021,0.492755,0.812112,0.733955,
1.000000,0.995002,0.101952,0.056467,0.100329,0.063517,0.106329,0.072465,0.101754,0.056068,
0.073491,0.045917,0.057072,0.036906,0.100423,0.058648,0.131944,0.063523,0.121118,0.076631,
0.074627,0.049641,0.095137,0.055914,0.041872,0.025700,0.057072,0.031986,0.034623,0.018867,
0.104693,0.062123,0.136015,0.100639,0.075812,0.046410,0.043321,0.024633,0.070388,0.043352,
0.021053,0.010593

Positive_52 22.000000,0.066116,6.000000,1.454545,1.000000,2.735931,8.000000,1.454545,1.000000,3.593074,
0.814111,0.037734,0.814111,0.037734,0.062500,0.000000,0.000000,0.000000,0.000000,0.000000,
0.300000,0.011364,0.250000,0.002841,0.005682,0.125000,0.000710,0.002841,0.062500,0.000178,
0.000710,0.015625,0.000011,0.138203,0.333333,0.019956,0.025216,0.142857,0.002543,0.013258,
0.095238,0.000627,0.067911,0.214286,0.006827,3.531718,2.950285,1.970134,1.000000,0.272727,
1.000000,0.265152,1.000000,0.250000,1.000000,0.250000,1.000000,0.250000,1.000000,0.250000,
1.000000,0.250000,1.000000,0.250000,1.000000,0.243939,1.000000,0.159091,1.000000,0.454545,
1.000000,0.452922,1.000000,0.443182,1.000000,0.443182,1.000000,0.443182,1.000000,0.443182,
1.000000,0.443182,1.000000,0.441558,1.000000,0.372835,1.000000,0.188312,0.308861,0.224153,
0.429762,0.371123,0.485057,0.404723,0.219124,0.160815,0.217593,0.153574,0.371560,0.312382,
0.071429,0.016417,0.416667,0.231494,0.759494,0.483852,0.928571,0.742155,1.000000,0.994176,
0.050228,0.010406,0.306397,0.246237,0.548736,0.491342,0.783410,0.733955,1.000000,0.993499,
0.011905,0.003012,0.334049,0.254966,0.576799,0.507392,0.815668,0.751429,1.000000,0.990039,
0.572619,0.367193,0.294931,0.108951,0.750000,0.523856,0.097222,0.041441,0.120482,0.073393,
0.021390,0.005172,0.011905,0.003012,0.348810,0.208856,0.680365,0.460405,0.850258,0.715562,
1.000000,1.000000,0.436170,0.134974,0.580122,0.271712,0.940639,0.447517,0.972414,0.597658,
0.998016,0.804899,0.101240,0.023977,0.439313,0.264587,0.741935,0.519144,0.925606,0.749290,
0.998163,0.973287,0.813793,0.673279,0.462963,0.265620,0.095238,0.017549,0.321429,0.259784,
0.557166,0.502182,0.784219,0.733134,0.998016,0.987796,0.423810,0.356121,0.433180,0.384532,
0.328520,0.259348,0.351613,0.285345,0.210753,0.178277,0.246445,0.195540,0.023810,0.008013,
0.323232,0.238353,0.553571,0.477611,0.777381,0.731830,1.000000,0.993626,0.083333,0.013699,
0.324468,0.259641,0.555785,0.507632,0.796690,0.751460,1.000000,0.995272,0.011905,0.003012,
0.321429,0.230002,0.581756,0.487123,0.845238,0.751406,1.000000,0.994675,0.547126,0.446544,
0.377410,0.301804,0.339241,0.251652,0.361446,0.280095,0.282407,0.195946,0.207171,0.148062,
0.011905,0.003012,0.328679,0.257496,0.574871,0.511727,0.811060,0.750750,1.000000,0.990956,
0.054795,0.010816,0.353535,0.240138,0.561644,0.480728,0.783410,0.731428,1.000000,0.992360,
0.071429,0.015666,0.416667,0.229460,0.740506,0.483128,0.916667,0.741815,1.000000,0.994902,
0.367857,0.287757,0.525346,0.452896,0.328520,0.259348,0.341935,0.267616,0.185936,0.145899,
0.285714,0.227918,0.031646,0.009146,0.350168,0.237470,0.560714,0.462651,0.786905,0.719042,
1.000000,0.992756,0.083333,0.012359,0.322314,0.259958,0.545455,0.509998,0.796690,0.752772,
1.000000,0.997252,0.011905,0.003012,0.321429,0.230002,0.581756,0.487123,0.845238,0.751406,
1.000000,0.994675,0.074010,0.044608,0.068330,0.041540,0.078341,0.041681,0.060759,0.033941,
0.045089,0.033064,0.045662,0.029319,0.104762,0.053829,0.112948,0.072265,0.101064,0.074896,
0.077855,0.052826,0.081315,0.047989,0.046931,0.027498,0.070922,0.041821,0.050691,0.020375,
0.119048,0.076298,0.193237,0.145324,0.095238,0.058847,0.037975,0.023166,0.103448,0.060460,
0.041096,0.020254

Positive_53 64.000000,0.085205,27.000000,5.453125,4.000000,41.172371,29.000000,5.453125,4.000000,31.331101,
0.392806,0.019918,0.392806,0.019918,0.045977,0.219880,0.459459,0.457143,0.552632,0.676471,
0.818182,0.890757,4.750000,1.457331,1.037941,4.750000,1.460411,0.115287,0.320000,0.018004,
0.144691,0.500000,0.019938,0.206934,0.444444,0.027647,0.073327,0.222222,0.004876,0.066384,
0.136508,0.002797,0.162327,0.298413,0.012958,11.977153,7.944316,7.091620,1.000000,0.640625,
1.000000,0.639259,1.000000,0.631604,1.000000,0.489404,1.000000,0.382979,1.000000,0.340465,
1.000000,0.250609,1.000000,0.160513,0.500000,0.083146,0.153846,0.041467,1.000000,0.781250,
1.000000,0.781173,1.000000,0.752383,1.000000,0.641067,1.000000,0.514410,1.000000,0.410385,

1.000000,0.253633,1.000000,0.176170,0.333333,0.079615,0.166667,0.014274,0.374468,0.281377,
0.457792,0.378250,0.426108,0.340373,0.290598,0.208611,0.243869,0.181710,0.404040,0.259293,
0.193939,0.014237,0.472727,0.270315,0.666667,0.521126,0.832512,0.763715,1.000000,0.993133,
0.062500,0.010734,0.318021,0.240879,0.601626,0.491660,0.831978,0.746978,1.000000,0.994098,
0.007246,0.003026,0.317829,0.232334,0.569767,0.473928,0.782946,0.722138,1.000000,0.988953,
0.626623,0.440746,0.235955,0.115070,0.772277,0.444184,0.085561,0.047780,0.129252,0.076750,
0.020408,0.005089,0.007246,0.003026,0.378092,0.229573,0.710638,0.497043,0.902778,0.756441,
1.000000,1.000000,0.673267,0.140243,0.706117,0.311948,0.837766,0.483958,0.980198,0.653643,
0.997585,0.848388,0.233062,0.040280,0.440415,0.257638,0.670954,0.503110,0.875274,0.748660,
0.998201,0.962178,0.810345,0.586684,0.448980,0.333848,0.056872,0.014021,0.347072,0.249527,
0.582902,0.479036,0.779070,0.710509,0.998639,0.980616,0.473934,0.394031,0.413105,0.346553,
0.331924,0.259416,0.336245,0.268701,0.299270,0.199994,0.238961,0.174710,0.047297,0.009818,
0.342756,0.238972,0.608511,0.489120,0.859574,0.747126,1.000000,0.996658,0.042254,0.012279,
0.341232,0.242778,0.630332,0.485575,0.826087,0.738384,1.000000,0.990743,0.007246,0.003026,
0.374179,0.255884,0.590810,0.502187,0.827586,0.743374,1.000000,0.987831,0.467980,0.377275,
0.415584,0.313714,0.412766,0.309011,0.363636,0.232959,0.316883,0.225065,0.273504,0.188199,
0.007246,0.003026,0.321705,0.237970,0.589147,0.480560,0.782946,0.724804,1.000000,0.990231,
0.062500,0.011158,0.332155,0.228302,0.613821,0.484040,0.864499,0.744857,1.000000,0.993420,
0.193939,0.014237,0.460606,0.270134,0.636364,0.520628,0.862579,0.763962,1.000000,0.993840,
0.350318,0.304004,0.539394,0.436580,0.331924,0.259416,0.332248,0.266441,0.211679,0.153164,
0.298701,0.221540,0.047297,0.011277,0.374558,0.238231,0.638298,0.487895,0.876596,0.741679,
1.000000,0.993587,0.037915,0.010495,0.383886,0.238593,0.639810,0.491602,0.838863,0.743133,
1.000000,0.995387,0.007246,0.003026,0.374179,0.255884,0.590810,0.502187,0.827586,0.743374,
1.000000,0.987831,0.093633,0.056394,0.097222,0.051354,0.101124,0.055704,0.068085,0.046629,
0.076621,0.036967,0.060811,0.034328,0.108696,0.069810,0.123762,0.069775,0.106029,0.067562,
0.077922,0.050229,0.152597,0.056338,0.042667,0.027634,0.074257,0.036902,0.105634,0.033689,
0.105691,0.065046,0.164319,0.108353,0.089109,0.046154,0.043290,0.025087,0.094382,0.046084,
0.047228,0.015960

Positive_54 29.000000,0.155767,19.000000,4.517241,3.000000,20.401478,13.000000,4.517241,2.000000,24.901478,
0.412368,0.014866,0.412368,0.014866,0.083969,0.100000,0.333333,0.555556,0.718750,1.000000,
0.000000,0.118945,2.526316,0.243716,0.159151,0.923077,0.125922,0.007033,0.132964,0.000760,
0.012242,0.071006,0.000745,0.211041,0.418367,0.035444,0.013402,0.230769,0.002606,0.016294,
0.071429,0.000866,0.295756,0.535714,0.049291,9.344600,5.251530,3.800519,1.000000,0.655172,
1.000000,0.654971,1.000000,0.652349,1.000000,0.546172,1.000000,0.419088,0.705128,0.254315,
0.679487,0.095651,0.538462,0.086755,0.371795,0.018668,0.179487,0.009012,1.000000,0.758621,
1.000000,0.758621,1.000000,0.756410,1.000000,0.743389,0.933333,0.287533,0.866667,0.273563,
0.803030,0.213914,0.636364,0.178105,0.439394,0.128888,0.212121,0.052472,0.344978,0.281139,
0.417062,0.371447,0.426108,0.347414,0.263158,0.212118,0.232850,0.179226,0.328395,0.255500,
0.193939,0.018124,0.472727,0.282329,0.666667,0.534418,0.832512,0.757918,1.000000,0.986745,
0.062500,0.014230,0.318021,0.259548,0.601626,0.494329,0.825203,0.741587,1.000000,0.993808,
0.007246,0.003712,0.290244,0.208913,0.553846,0.456656,0.792683,0.721412,1.000000,0.989373,
0.612978,0.455179,0.495122,0.111688,0.650246,0.433133,0.176039,0.051391,0.129252,0.073498,
0.020408,0.004742,0.007246,0.003712,0.378092,0.270229,0.639576,0.517522,0.902778,0.772634,
1.000000,1.000000,0.611111,0.171355,0.706117,0.332698,0.837766,0.509042,0.940887,0.669814,

0.997104,0.833479,0.142857,0.028883,0.327974,0.215031,0.670954,0.472443,0.881818,0.720374,
0.997727,0.913517,0.810345,0.596679,0.448980,0.335318,0.056872,0.015509,0.303030,0.229953,
0.542471,0.453570,0.760618,0.692647,0.998069,0.975204,0.473934,0.392376,0.436681,0.338833,
0.319444,0.268791,0.304878,0.255116,0.299270,0.212790,0.223684,0.174025,0.047297,0.012933,
0.342756,0.258894,0.601626,0.500269,0.833922,0.748308,1.000000,0.996499,0.042254,0.014568,
0.341232,0.219553,0.630332,0.466354,0.826087,0.733689,1.000000,0.986837,0.007246,0.003712,
0.345455,0.256787,0.586207,0.498802,0.827586,0.737524,1.000000,0.982207,0.467980,0.388410,
0.359528,0.300537,0.384279,0.311053,0.285714,0.230582,0.285990,0.227894,0.260870,0.183372,
0.007246,0.003712,0.280488,0.217989,0.528205,0.468094,0.787805,0.726616,1.000000,0.991784,
0.062500,0.014784,0.332155,0.244568,0.613821,0.485183,0.833333,0.739577,1.000000,0.992614,
0.193939,0.018124,0.460606,0.286208,0.636364,0.534645,0.834975,0.759724,1.000000,0.989469,
0.340278,0.297437,0.539394,0.433773,0.319444,0.268791,0.324468,0.255223,0.211679,0.157952,
0.267544,0.228863,0.047297,0.013505,0.374558,0.250952,0.613821,0.488962,0.851590,0.733239,
1.000000,0.993302,0.037915,0.013582,0.383886,0.230528,0.639810,0.493364,0.838863,0.744723,
1.000000,0.996784,0.007246,0.003712,0.345455,0.256787,0.586207,0.498802,0.827586,0.737524,
1.000000,0.982207,0.092338,0.054975,0.097222,0.055487,0.078772,0.049822,0.067568,0.047088,
0.076621,0.036534,0.060811,0.037233,0.108696,0.073771,0.089431,0.062219,0.092308,0.063656,
0.076389,0.050702,0.084848,0.050189,0.069264,0.029913,0.072464,0.040996,0.105634,0.044751,
0.105691,0.064522,0.151515,0.107422,0.080386,0.043300,0.040650,0.024570,0.071809,0.045097,
0.036232,0.017752

Positive_55 48.000000,0.210938,30.000000,10.125000,11.500000,74.750000,28.000000,10.125000,4.000000,107.941489,
0.454635,0.046820,0.454635,0.046820,0.014644,0.259023,0.361032,0.300448,0.333333,0.240385,
0.177215,4.578706,12.724138,18.363084,3.873924,11.538462,18.152219,0.299696,0.531250,0.051580,
0.302622,0.500000,0.038213,0.311311,0.570213,0.041921,0.188238,0.638298,0.020014,0.173181,
0.574468,0.014898,0.312634,0.601064,0.046578,19.128341,6.672224,4.236773,1.000000,0.750000,
1.000000,0.748488,1.000000,0.746828,1.000000,0.613261,1.000000,0.555265,1.000000,0.508151,
1.000000,0.442544,1.000000,0.407875,1.000000,0.337974,0.600000,0.166208,1.000000,0.812500,
1.000000,0.810130,1.000000,0.792304,1.000000,0.711106,1.000000,0.596853,1.000000,0.536051,
1.000000,0.372637,1.000000,0.236033,0.892857,0.158313,0.535714,0.051353,0.357401,0.288515,
0.525355,0.371985,0.401316,0.339500,0.262887,0.207420,0.242754,0.190183,0.355705,0.253312,
0.193939,0.023650,0.472727,0.279423,0.666667,0.519379,0.821053,0.751231,1.000000,0.991543,
0.047297,0.009557,0.295775,0.241579,0.589796,0.496923,0.819905,0.740794,1.000000,0.993428,
0.007246,0.003145,0.279601,0.217817,0.553846,0.466326,0.780000,0.732583,1.000000,0.994697,
0.573460,0.427390,0.368421,0.107220,0.800000,0.465391,0.099379,0.052570,0.129252,0.083509,
0.020408,0.003698,0.007246,0.003145,0.347826,0.242100,0.642000,0.506378,0.887324,0.776388,
1.000000,1.000000,0.611111,0.112816,0.706117,0.241520,0.837766,0.518539,0.940385,0.678628,
0.998489,0.892371,0.249258,0.034835,0.540098,0.263375,0.692308,0.495696,0.857895,0.714632,
0.996011,0.950349,0.733333,0.595257,0.448980,0.351659,0.056872,0.014001,0.311178,0.237757,
0.572368,0.475400,0.769737,0.716519,0.998363,0.985701,0.527383,0.390497,0.404082,0.352267,
0.319444,0.257236,0.329352,0.264420,0.299270,0.204058,0.254545,0.173045,0.047297,0.008893,
0.340278,0.247171,0.549801,0.498078,0.800407,0.747828,1.000000,0.995984,0.042254,0.013516,
0.341232,0.234070,0.630332,0.480490,0.826087,0.738122,1.000000,0.992121,0.007246,0.003145,
0.345455,0.253239,0.586207,0.498285,0.827586,0.741184,1.000000,0.989473,0.446667,0.373100,
0.478702,0.310046,0.377709,0.316854,0.322148,0.234239,0.285714,0.232518,0.238095,0.187874,

0.007246,0.003145,0.291777,0.224180,0.546053,0.474671,0.775401,0.732154,1.000000,0.996339,
0.047297,0.009557,0.316742,0.230637,0.574181,0.489793,0.829384,0.743304,1.000000,0.992532,
0.193939,0.018305,0.460606,0.279287,0.636364,0.518037,0.821501,0.753197,1.000000,0.991956,
0.411765,0.296634,0.539394,0.446130,0.319444,0.257236,0.353659,0.266629,0.216931,0.155862,
0.304545,0.221241,0.047297,0.009700,0.291667,0.234015,0.589796,0.488273,0.799669,0.725432,
1.000000,0.992243,0.037915,0.011580,0.383886,0.244791,0.639810,0.496747,0.838863,0.751367,
1.000000,0.997663,0.007246,0.003145,0.345455,0.253239,0.586207,0.498285,0.827586,0.741184,
1.000000,0.989473,0.092338,0.056102,0.104072,0.052518,0.078772,0.055538,0.072202,0.047124,
0.076621,0.042283,0.061224,0.034950,0.108696,0.067906,0.186667,0.071189,0.094737,0.060980,
0.089474,0.049434,0.095335,0.060536,0.046243,0.028339,0.072464,0.033600,0.105634,0.033327,
0.096257,0.061483,0.213333,0.115745,0.073469,0.042268,0.045045,0.023361,0.079430,0.047571,
0.036232,0.015744

Positive_56 25.000000,0.286400,24.000000,7.160000,12.000000,47.640000,14.000000,7.160000,1.000000,43.973333,
0.353984,0.013929,0.353984,0.013929,0.017544,0.279762,0.520661,0.672414,0.947368,0.000000,
0.000000,0.698333,6.458333,1.650069,0.822637,1.785714,0.792096,0.047431,0.269097,0.003591,
0.058963,0.127551,0.004045,0.290764,0.541667,0.072003,0.029097,0.269097,0.002865,0.034277,
0.074405,0.001375,0.695943,0.958333,0.075495,12.925463,3.328127,0.925191,1.000000,0.560000,
1.000000,0.558841,1.000000,0.558406,1.000000,0.551159,1.000000,0.483412,1.000000,0.457971,
1.000000,0.455652,1.000000,0.454058,0.945455,0.427739,0.618182,0.278957,1.000000,0.480000,
1.000000,0.480000,1.000000,0.480000,0.935897,0.448425,0.794872,0.365421,0.730769,0.314505,
0.679487,0.288278,0.538462,0.229451,0.371795,0.159927,0.179487,0.074872,0.330861,0.298868,
0.417062,0.354566,0.382937,0.346567,0.262887,0.205992,0.232604,0.198727,0.321168,0.246409,
0.193939,0.020015,0.472727,0.296093,0.666667,0.537945,0.817568,0.758732,1.000000,0.988451,
0.047297,0.011308,0.295775,0.240007,0.589796,0.491372,0.819905,0.733690,1.000000,0.995381,
0.007246,0.003432,0.275964,0.204865,0.553846,0.454992,0.784884,0.728956,1.000000,0.994957,
0.573460,0.438299,0.178905,0.086346,0.541833,0.475355,0.066845,0.047335,0.129252,0.086569,
0.020408,0.004938,0.007246,0.003432,0.347826,0.254890,0.579568,0.515192,0.887324,0.790403,
1.000000,1.000000,0.611111,0.147546,0.706117,0.262300,0.837766,0.561296,0.867021,0.705766,
0.997959,0.917678,0.249258,0.030147,0.406528,0.251214,0.661538,0.486884,0.798220,0.704747,
0.996011,0.940211,0.659363,0.582009,0.448980,0.372765,0.056872,0.012497,0.311178,0.222283,
0.542296,0.451439,0.753846,0.699549,0.997033,0.983762,0.473934,0.387904,0.404082,0.345819,
0.319444,0.266277,0.304878,0.256254,0.299270,0.211782,0.226640,0.176030,0.047297,0.010348,
0.340278,0.256022,0.549801,0.496750,0.772908,0.740966,1.000000,0.997433,0.042254,0.013130,
0.341232,0.227376,0.630332,0.470272,0.826087,0.735923,1.000000,0.988390,0.007246,0.003432,
0.345455,0.248471,0.586207,0.502485,0.827586,0.748852,0.998665,0.983807,0.427536,0.383333,
0.359528,0.292315,0.356083,0.324352,0.284672,0.228282,0.284722,0.244527,0.230360,0.179376,
0.007246,0.003432,0.274882,0.212321,0.528205,0.467628,0.775362,0.732046,1.000000,0.994957,
0.047297,0.011308,0.280585,0.228580,0.563798,0.477168,0.829384,0.733664,1.000000,0.995381,
0.193939,0.019856,0.460606,0.297205,0.636364,0.535055,0.817568,0.757798,1.000000,0.988451,
0.340278,0.283349,0.539394,0.450373,0.319444,0.266277,0.324468,0.255846,0.211679,0.154053,
0.272366,0.233759,0.047297,0.011142,0.291667,0.237401,0.589796,0.478557,0.783673,0.705811,
1.000000,0.991461,0.037915,0.011760,0.383886,0.243446,0.639810,0.499589,0.838863,0.755105,
1.000000,0.998451,0.007246,0.003432,0.345455,0.248471,0.586207,0.502485,0.827586,0.748852,
0.998665,0.983807,0.092338,0.051873,0.097222,0.063877,0.078772,0.053855,0.067568,0.049602,

0.076621,0.037888,0.061224,0.041773,0.108696,0.068222,0.081081,0.053491,0.092308,0.059252,
0.076389,0.052781,0.084848,0.058568,0.036511,0.025484,0.072464,0.036766,0.105634,0.045986,
0.079523,0.057628,0.151515,0.108405,0.073469,0.046270,0.037773,0.025054,0.071713,0.049735,
0.036232,0.013489

Positive_57 13.000000,0.071006,12.000000,0.923077,0.000000,11.076923,1.000000,0.923077,1.000000,0.076923,
0.376453,0.008485,0.376453,0.008485,0.000000,0.083333,0.636364,0.500000,1.000000,0.000000,
0.000000,0.000000,0.000000,0.000000,0.000000,0.000000,0.000000,0.000000,0.000000,0.000000,
0.000000,0.000000,0.000000,0.000000,0.000000,0.000000,0.000000,0.000000,0.000000,0.000000,
0.000000,0.000000,0.846154,0.916667,0.064637,3.464102,0.000000,0.000000,1.000000,0.076923,
1.000000,0.076923,1.000000,0.076923,0.939394,0.072261,0.833333,0.064103,0.833333,0.064103,
0.803030,0.061772,0.636364,0.048951,0.439394,0.033800,0.212121,0.016317,0.000000,0.000000,
0.000000,0.000000,0.000000,0.000000,0.000000,0.000000,0.000000,0.000000,0.000000,0.000000,
0.000000,0.000000,0.000000,0.000000,0.000000,0.000000,0.000000,0.000000,0.325768,0.289006,
0.417062,0.379196,0.375758,0.331799,0.262887,0.225727,0.213049,0.181460,0.321168,0.243855,
0.193939,0.026453,0.472727,0.302139,0.666667,0.568402,0.817568,0.783851,1.000000,0.985387,
0.047297,0.016079,0.295775,0.252922,0.520270,0.464652,0.819905,0.711927,1.000000,0.992561,
0.007246,0.004845,0.306306,0.189674,0.553846,0.445686,0.769231,0.716315,1.000000,0.992560,
0.573460,0.472334,0.178905,0.074788,0.545045,0.452877,0.066845,0.040915,0.129252,0.089894,
0.020408,0.005239,0.007246,0.004845,0.347826,0.273569,0.579568,0.500731,0.887324,0.757033,
1.000000,1.000000,0.611111,0.215490,0.706117,0.360055,0.837766,0.530652,0.867021,0.641529,
0.973298,0.839092,0.066351,0.029223,0.331081,0.216814,0.661538,0.485578,0.791722,0.748777,
0.996011,0.944668,0.700450,0.544127,0.448980,0.388913,0.056872,0.020076,0.340090,0.209110,
0.538288,0.428329,0.753846,0.668816,0.996011,0.973654,0.473934,0.421137,0.378989,0.311066,
0.342342,0.267798,0.304878,0.245531,0.299270,0.232806,0.261851,0.156049,0.047297,0.016079,
0.340278,0.269094,0.541667,0.481212,0.760563,0.725098,1.000000,0.995988,0.042254,0.019531,
0.341232,0.199671,0.630332,0.451213,0.826087,0.740734,1.000000,0.979323,0.007246,0.004845,
0.345455,0.258730,0.586207,0.512432,0.827586,0.745083,1.000000,0.978663,0.427536,0.376914,
0.359528,0.302640,0.351135,0.320445,0.284672,0.223159,0.284722,0.241154,0.230360,0.190337,
0.007246,0.004845,0.317568,0.208369,0.576577,0.466662,0.775362,0.721956,1.000000,0.992560,
0.047297,0.016079,0.280585,0.224347,0.541667,0.447625,0.829384,0.705889,1.000000,0.992041,
0.193939,0.026453,0.460606,0.303056,0.636364,0.558121,0.817568,0.777956,1.000000,0.985387,
0.340278,0.298626,0.539394,0.433576,0.342342,0.267798,0.324468,0.262076,0.211679,0.162626,
0.291196,0.226229,0.047297,0.016079,0.291667,0.233622,0.509309,0.446622,0.731383,0.683067,
1.000000,0.991398,0.037915,0.017981,0.383886,0.231361,0.639810,0.506941,0.838863,0.763619,
1.000000,0.997905,0.007246,0.004845,0.345455,0.258730,0.586207,0.512432,0.827586,0.745083,
1.000000,0.978663,0.092338,0.051335,0.097222,0.062096,0.078772,0.045994,0.067568,0.048224,
0.076621,0.040287,0.060811,0.041070,0.108696,0.080998,0.081081,0.060047,0.092308,0.059630,
0.076389,0.049727,0.084848,0.052238,0.067568,0.031440,0.072464,0.045115,0.105634,0.070272,
0.073138,0.048513,0.151515,0.101249,0.072072,0.033953,0.037383,0.021824,0.074324,0.039080,
0.038288,0.016908

Positive_58 37.000000,0.170197,24.000000,6.297297,4.000000,41.770270,18.000000,6.297297,5.000000,26.103604,
0.430202,0.016664,0.430202,0.016664,0.021459,0.131579,0.363636,0.436508,0.605634,0.892857,
0.333333,1.749936,6.722222,3.305738,1.916808,6.000000,4.187874,0.222756,0.500000,0.028126,
0.195688,0.420118,0.025933,0.223692,0.416667,0.017740,0.106470,0.500000,0.011216,0.098761,

0.250000,0.005610,0.276846,0.522222,0.023106,11.492379,5.378864,4.459493,1.000000,0.783784,
1.000000,0.783784,1.000000,0.775708,1.000000,0.631244,1.000000,0.542127,1.000000,0.391019,
1.000000,0.283328,1.000000,0.124254,1.000000,0.097066,0.035714,0.000965,1.000000,0.783784,
1.000000,0.783784,1.000000,0.749349,1.000000,0.652468,1.000000,0.473386,1.000000,0.262713,
1.000000,0.134174,0.333333,0.028651,0.333333,0.026854,0.000000,0.000000,0.315011,0.258192,
0.502016,0.401940,0.408629,0.339868,0.274406,0.203516,0.221963,0.166402,0.318021,0.272499,
0.078313,0.010473,0.328244,0.239183,0.563667,0.487543,0.808050,0.748212,1.000000,0.993613,
0.062500,0.008552,0.293944,0.247819,0.562691,0.499211,0.778169,0.742779,1.000000,0.996180,
0.003521,0.002223,0.360544,0.246975,0.571429,0.493738,0.809524,0.740715,1.000000,0.990848,
0.699831,0.484137,0.363158,0.154527,0.591371,0.361336,0.125253,0.061247,0.099379,0.060226,
0.015748,0.004818,0.003521,0.002223,0.346749,0.246483,0.681115,0.492756,0.902778,0.750621,
1.000000,1.000000,0.468310,0.084447,0.611708,0.266064,0.769231,0.516153,0.966102,0.764439,
0.997080,0.907440,0.314516,0.031540,0.806452,0.238043,0.877016,0.471193,0.971053,0.697577,
0.997817,0.944449,0.761610,0.638837,0.404199,0.296120,0.030733,0.010182,0.351474,0.262068,
0.571429,0.497370,0.795107,0.730939,0.998134,0.988187,0.524194,0.397569,0.418605,0.341833,
0.346749,0.260598,0.310873,0.270224,0.248485,0.208780,0.235437,0.171727,0.038194,0.007746,
0.316100,0.241890,0.562648,0.490100,0.775717,0.738364,1.000000,0.996653,0.030354,0.010021,
0.314092,0.247172,0.566434,0.499078,0.797040,0.748689,1.000000,0.994780,0.003521,0.002223,
0.299320,0.249482,0.562701,0.498329,0.802218,0.744409,1.000000,0.992946,0.455206,0.380509,
0.457661,0.329968,0.342932,0.289524,0.300395,0.252151,0.270341,0.208553,0.271768,0.186928,
0.003521,0.002223,0.374150,0.251244,0.571429,0.496256,0.809524,0.743712,1.000000,0.993191,
0.062500,0.009342,0.327917,0.238279,0.607565,0.487948,0.794760,0.734897,1.000000,0.994618,
0.078313,0.010060,0.334351,0.248724,0.567468,0.494751,0.808050,0.752722,1.000000,0.994424,
0.386588,0.311560,0.486258,0.427843,0.346749,0.260598,0.333992,0.265797,0.213720,0.165436,
0.303398,0.215071,0.038194,0.009344,0.323486,0.245021,0.576832,0.488207,0.786145,0.734897,
1.000000,0.994288,0.024648,0.008117,0.295416,0.245492,0.550403,0.499086,0.796943,0.753395,
1.000000,0.997500,0.003521,0.002223,0.299320,0.249482,0.562701,0.498329,0.802218,0.744409,
1.000000,0.992946,0.084677,0.056085,0.072089,0.039705,0.093023,0.048777,0.069930,0.045574,
0.086842,0.034497,0.062827,0.033554,0.114919,0.076066,0.108481,0.074410,0.093976,0.065613,
0.077121,0.049897,0.120795,0.063982,0.055728,0.031332,0.069444,0.040640,0.045553,0.022028,
0.105263,0.064591,0.151376,0.112318,0.089783,0.048095,0.043147,0.022779,0.071207,0.048980,
0.041162,0.021077

Positive_59 10.000000,0.340000,8.000000,3.400000,3.000000,9.377778,6.000000,3.400000,3.500000,4.933333,
0.759206,0.006104,0.759206,0.006104,0.000000,0.000000,0.000000,0.000000,0.000000,0.205882,
0.666667,0.891071,2.375000,1.077413,1.166667,2.250000,0.739198,0.184662,0.750000,0.059135,
0.268056,0.562500,0.041187,0.304365,0.555556,0.051147,0.141071,0.444444,0.026107,0.210185,
0.333333,0.008993,0.540741,0.777778,0.053102,5.107399,2.013503,1.709456,1.000000,0.700000,
1.000000,0.700000,1.000000,0.700000,1.000000,0.700000,1.000000,0.700000,1.000000,0.700000,
1.000000,0.700000,1.000000,0.675000,1.000000,0.430952,0.666667,0.205952,1.000000,0.700000,
1.000000,0.700000,1.000000,0.700000,1.000000,0.700000,1.000000,0.700000,1.000000,0.700000,
1.000000,0.680000,1.000000,0.570000,0.666667,0.316667,0.066667,0.020000,0.305687,0.283005,
0.409972,0.368817,0.380645,0.348178,0.266667,0.212962,0.220065,0.188400,0.324561,0.260520,
0.021333,0.011384,0.372603,0.293045,0.562327,0.508923,0.797784,0.735091,0.997260,0.991535,
0.020408,0.010994,0.268222,0.242924,0.584656,0.505712,0.793313,0.753687,1.000000,0.997657,

0.003226,0.002827,0.242331,0.199917,0.498480,0.471614,0.770968,0.732952,1.000000,0.997234,
0.590047,0.518404,0.201058,0.150659,0.396501,0.330937,0.090652,0.066242,0.093567,0.067664,
0.007958,0.002860,0.003226,0.002827,0.328000,0.225026,0.574194,0.481294,0.822581,0.732562,
1.000000,1.000000,0.267773,0.103446,0.443128,0.333811,0.600000,0.526343,0.925926,0.705169,
0.997354,0.937410,0.116564,0.028909,0.565097,0.266763,0.725762,0.507337,0.872576,0.773567,
0.994460,0.965077,0.653061,0.606509,0.402266,0.353909,0.030471,0.014706,0.300948,0.256177,
0.540284,0.500361,0.775076,0.743595,0.997230,0.990129,0.412742,0.359625,0.370056,0.341549,
0.322086,0.298826,0.282967,0.245787,0.263889,0.214452,0.237530,0.209947,0.021327,0.010954,
0.291005,0.240761,0.574074,0.499180,0.798834,0.747327,1.000000,0.995516,0.025806,0.010415,
0.274882,0.244323,0.519355,0.480094,0.780645,0.743100,1.000000,0.995165,0.003226,0.002827,
0.304709,0.249743,0.576177,0.515896,0.788344,0.733768,1.000000,0.993655,0.422581,0.391891,
0.336986,0.294794,0.348341,0.313314,0.285714,0.231388,0.275862,0.242712,0.238889,0.183705,
0.003226,0.002827,0.258294,0.215217,0.530806,0.483294,0.761290,0.737499,1.000000,0.997234,
0.028436,0.011942,0.280423,0.230767,0.584656,0.489208,0.793003,0.742858,1.000000,0.993675,
0.021333,0.011384,0.342466,0.291309,0.562327,0.511646,0.797784,0.751159,1.000000,0.992300,
0.317808,0.281302,0.449153,0.419872,0.322086,0.298826,0.262032,0.229665,0.186111,0.162469,
0.291785,0.261931,0.028436,0.012248,0.280423,0.250041,0.591837,0.505400,0.798834,0.750756,
1.000000,0.990738,0.015337,0.008848,0.281991,0.233036,0.535484,0.476234,0.800000,0.743436,
1.000000,0.996883,0.003226,0.002827,0.304709,0.249743,0.576177,0.515896,0.788344,0.733768,
1.000000,0.993655,0.080332,0.062469,0.082067,0.053143,0.056497,0.045732,0.060847,0.046212,
0.050265,0.038052,0.066351,0.037398,0.072000,0.054535,0.084932,0.063915,0.083102,0.063522,
0.064417,0.053118,0.094183,0.059704,0.043732,0.030310,0.068783,0.043713,0.029032,0.018619,
0.101695,0.062359,0.120548,0.107028,0.073620,0.050981,0.036723,0.028072,0.083871,0.056813,
0.047091,0.024307

Positive_60 20.000000,0.380000,15.000000,7.600000,8.000000,22.884211,13.000000,7.600000,8.000000,21.936842,
0.654798,0.026122,0.654798,0.026122,0.000000,0.000000,0.039474,0.082192,0.335821,0.426966,
0.411765,3.328135,7.000000,6.912614,3.467960,7.076923,5.743178,0.336349,0.600000,0.046554,
0.406441,0.687500,0.029383,0.459067,0.614035,0.031359,0.288705,0.532895,0.020286,0.305112,
0.513158,0.013221,0.528266,0.736842,0.011122,10.210561,4.391076,2.788874,1.000000,0.900000,
1.000000,0.900000,1.000000,0.900000,1.000000,0.900000,1.000000,0.898095,1.000000,0.884744,
1.000000,0.712321,1.000000,0.576185,1.000000,0.438417,1.000000,0.338641,1.000000,0.950000,
1.000000,0.950000,1.000000,0.950000,1.000000,0.950000,1.000000,0.859091,1.000000,0.732840,
1.000000,0.576562,1.000000,0.403061,1.000000,0.317498,0.333333,0.101232,0.337209,0.276736,
0.483019,0.390779,0.375706,0.332485,0.266667,0.215905,0.233236,0.177957,0.324561,0.263738,
0.016854,0.009177,0.408602,0.307230,0.604839,0.528323,0.825269,0.762630,1.000000,0.992708,
0.022901,0.009940,0.268222,0.233741,0.584656,0.484495,0.796438,0.737576,1.000000,0.997174,
0.003021,0.002689,0.322642,0.222929,0.556604,0.478606,0.796226,0.733345,1.000000,0.994456,
0.624528,0.537019,0.201058,0.151176,0.396501,0.311805,0.102426,0.067681,0.093567,0.064637,
0.011268,0.003115,0.003021,0.002689,0.320225,0.224746,0.554707,0.465476,0.793651,0.716804,
1.000000,1.000000,0.252688,0.118148,0.522613,0.354103,0.633166,0.526479,0.925926,0.719183,
0.997354,0.914263,0.045226,0.024332,0.565460,0.254374,0.732620,0.541731,0.888679,0.780496,
0.996226,0.965335,0.653061,0.588764,0.418367,0.362991,0.044974,0.016254,0.326633,0.265519,
0.561713,0.501558,0.765743,0.734930,0.997500,0.990740,0.469811,0.380912,0.395349,0.331685,
0.311047,0.287404,0.297229,0.247296,0.270950,0.216863,0.267677,0.195456,0.024194,0.010335,

0.291005,0.226891,0.574074,0.475711,0.798834,0.736542,1.000000,0.996491,0.028090,0.010550,
0.330189,0.262058,0.577358,0.498206,0.783019,0.741200,1.000000,0.991915,0.003021,0.002689,
0.419598,0.278198,0.605528,0.528766,0.810160,0.758523,1.000000,0.993346,0.419940,0.373979,
0.416981,0.314593,0.375000,0.311428,0.285714,0.230797,0.297376,0.231058,0.240223,0.190834,
0.003021,0.002689,0.335849,0.235925,0.592453,0.494060,0.796226,0.739089,1.000000,0.994590,
0.024194,0.010746,0.280423,0.214510,0.584656,0.461447,0.816794,0.727235,1.000000,0.995685,
0.016854,0.009177,0.408602,0.304092,0.586022,0.528766,0.819892,0.761994,1.000000,0.993236,
0.352830,0.287906,0.473118,0.424690,0.311047,0.287404,0.288265,0.243755,0.198324,0.163923,
0.308081,0.248397,0.029570,0.012305,0.280423,0.238248,0.591837,0.500697,0.801527,0.742729,
1.000000,0.994605,0.022284,0.009031,0.313208,0.240251,0.516616,0.476077,0.778090,0.736371,
1.000000,0.994436,0.003021,0.002689,0.419598,0.278198,0.605528,0.528766,0.810160,0.758523,
1.000000,0.993346,0.114035,0.069553,0.075567,0.040336,0.069767,0.042300,0.060847,0.046512,
0.056452,0.039392,0.069638,0.038644,0.094148,0.059616,0.122642,0.069937,0.086351,0.064827,
0.064140,0.047015,0.104326,0.073199,0.043732,0.034692,0.068783,0.041493,0.028090,0.019807,
0.101695,0.062003,0.142857,0.108871,0.071429,0.040475,0.042135,0.023152,0.069486,0.052374,
0.049057,0.025803

Positive_61 13.000000,0.159763,4.000000,2.076923,2.000000,1.910256,9.000000,2.076923,2.000000,5.910256,
0.583335,0.043985,0.583335,0.043985,0.000000,0.074074,0.200000,0.250000,0.066667,0.428571,
0.125000,0.230769,0.750000,0.086984,0.085470,1.111111,0.094967,0.070513,0.222222,0.008157,
0.009497,0.123457,0.001172,0.310897,0.666667,0.055155,0.059295,0.250000,0.006564,0.044160,
0.138889,0.003371,0.125356,0.250000,0.008939,3.636115,2.472967,2.182652,1.000000,0.615385,
1.000000,0.615385,1.000000,0.615385,1.000000,0.564103,1.000000,0.474359,1.000000,0.410256,
1.000000,0.397436,1.000000,0.333333,1.000000,0.333333,1.000000,0.141026,1.000000,0.538462,
1.000000,0.538462,1.000000,0.538462,1.000000,0.467949,1.000000,0.405983,1.000000,0.241453,
1.000000,0.235043,0.666667,0.170940,0.333333,0.113248,0.027778,0.002137,0.307692,0.275700,
0.408163,0.370259,0.395770,0.354041,0.224432,0.193505,0.229226,0.196290,0.324561,0.270027,
0.036254,0.012291,0.372603,0.279451,0.570144,0.516941,0.798271,0.744762,1.000000,0.995244,
0.020408,0.009448,0.323529,0.249109,0.561765,0.495660,0.788235,0.754524,1.000000,0.997402,
0.003226,0.002667,0.251418,0.207984,0.508516,0.475517,0.776435,0.732560,1.000000,0.994591,
0.542135,0.486187,0.221411,0.162663,0.396501,0.351150,0.104878,0.074502,0.093567,0.076406,
0.011268,0.003803,0.003226,0.002667,0.320225,0.233049,0.574194,0.493138,0.822581,0.750427,
1.000000,1.000000,0.414706,0.122605,0.502857,0.311719,0.776156,0.529842,0.892944,0.717273,
0.991429,0.936868,0.029155,0.015178,0.362606,0.241508,0.570888,0.457215,0.821530,0.742083,
0.994334,0.973783,0.653061,0.617874,0.383481,0.351974,0.021918,0.011765,0.274286,0.248295,
0.548571,0.495540,0.764706,0.732874,0.994521,0.990690,0.408219,0.367168,0.380665,0.344356,
0.335347,0.288476,0.303468,0.254586,0.228169,0.207540,0.263636,0.199232,0.020408,0.010198,
0.285294,0.246542,0.562682,0.493706,0.798834,0.743264,1.000000,0.995430,0.028090,0.012678,
0.297143,0.245674,0.566910,0.496038,0.780645,0.738797,1.000000,0.995477,0.003226,0.002667,
0.314748,0.250164,0.563739,0.498849,0.771583,0.733680,1.000000,0.992612,0.465257,0.394904,
0.336986,0.298701,0.335294,0.306395,0.285714,0.244213,0.284848,0.242439,0.215909,0.177624,
0.003226,0.002667,0.255618,0.216240,0.516854,0.484418,0.766854,0.736996,1.000000,0.995276,
0.020408,0.010557,0.285294,0.240267,0.538235,0.486304,0.793003,0.740897,1.000000,0.994383,
0.036254,0.012069,0.342466,0.280854,0.566572,0.515704,0.789625,0.748537,1.000000,0.996398,
0.317808,0.287687,0.444234,0.423836,0.335347,0.288476,0.303738,0.242079,0.186246,0.163812,

0.303030,0.242959,0.026239,0.011902,0.342939,0.265580,0.593660,0.508447,0.802941,0.744268,
1.000000,0.993531,0.017291,0.009079,0.288571,0.233023,0.535484,0.482757,0.800000,0.739181,
1.000000,0.997022,0.003226,0.002667,0.314748,0.250164,0.563739,0.498849,0.771583,0.733680,
1.000000,0.992612,0.069164,0.057487,0.070968,0.043032,0.068053,0.047836,0.072993,0.048286,
0.053613,0.039488,0.052941,0.039570,0.076923,0.061613,0.084932,0.061662,0.078652,0.062645,
0.076471,0.053481,0.084986,0.059300,0.048571,0.030695,0.069486,0.040863,0.030303,0.020180,
0.077143,0.062187,0.130312,0.109066,0.061224,0.046208,0.051095,0.030468,0.083871,0.058752,
0.039627,0.027180

Positive_62 16.000000,0.195312,7.000000,3.125000,3.000000,5.450000,9.000000,3.125000,3.000000,4.650000,
0.673036,0.077047,0.673036,0.077047,0.020000,0.122449,0.162791,0.138889,0.000000,0.064516,
0.103448,0.553571,1.333333,0.306236,0.451389,1.555556,0.346759,0.167623,0.500000,0.039359,
0.113233,0.444444,0.022131,0.245288,0.533333,0.022691,0.163373,0.377778,0.015365,0.164583,
0.311111,0.006736,0.264815,0.400000,0.013383,4.559862,3.301348,2.650372,1.000000,0.750000,
1.000000,0.750000,1.000000,0.750000,1.000000,0.683333,1.000000,0.411905,1.000000,0.394048,
1.000000,0.394048,1.000000,0.381548,1.000000,0.360714,1.000000,0.162500,1.000000,0.750000,
1.000000,0.750000,1.000000,0.750000,1.000000,0.700000,1.000000,0.675000,1.000000,0.629861,
1.000000,0.629861,1.000000,0.556250,1.000000,0.405208,1.000000,0.269444,0.318452,0.278613,
0.420420,0.386586,0.391621,0.334801,0.226895,0.198822,0.243655,0.188577,0.310219,0.249658,
0.022785,0.010394,0.377483,0.269456,0.596026,0.502868,0.798013,0.737391,1.000000,0.993381,
0.031746,0.008190,0.323529,0.250128,0.577049,0.499692,0.800532,0.756886,1.000000,0.996559,
0.003311,0.002400,0.291667,0.224050,0.550595,0.484124,0.768617,0.739324,1.000000,0.995526,
0.556869,0.502295,0.233383,0.149125,0.617486,0.348580,0.096154,0.066207,0.093909,0.075102,
0.011278,0.003738,0.003311,0.002400,0.345643,0.253720,0.574074,0.489607,0.797637,0.751347,
1.000000,1.000000,0.414706,0.137577,0.502857,0.330847,0.848816,0.524210,0.865209,0.720825,
0.992714,0.957212,0.090090,0.024164,0.480480,0.203454,0.618619,0.458388,0.825516,0.734332,
0.995569,0.969555,0.723133,0.595128,0.424812,0.343720,0.023179,0.009686,0.333333,0.270455,
0.555851,0.516500,0.771107,0.751216,0.997340,0.990945,0.427767,0.380357,0.375661,0.341774,
0.331148,0.277869,0.308394,0.253597,0.239203,0.207257,0.218289,0.180602,0.031746,0.008065,
0.316100,0.243404,0.537666,0.477057,0.788235,0.742428,1.000000,0.997232,0.028090,0.012559,
0.297143,0.241393,0.528090,0.480870,0.778090,0.720859,1.000000,0.994864,0.003311,0.002400,
0.390728,0.281973,0.613508,0.526605,0.827815,0.772362,1.000000,0.993283,0.431694,0.376684,
0.370370,0.315126,0.366071,0.308190,0.299270,0.235582,0.314667,0.227698,0.211319,0.183389,
0.003311,0.002400,0.294643,0.232255,0.553571,0.501079,0.766854,0.743010,1.000000,0.995705,
0.031746,0.008356,0.327917,0.235978,0.540620,0.477745,0.787234,0.733068,1.000000,0.995740,
0.022785,0.010281,0.377483,0.271632,0.596026,0.513677,0.827815,0.759606,1.000000,0.995308,
0.341463,0.298854,0.476190,0.423277,0.331148,0.277869,0.300752,0.248031,0.195742,0.165269,
0.272000,0.222590,0.031746,0.008448,0.323486,0.252874,0.544118,0.483044,0.802941,0.740205,
1.000000,0.994845,0.015957,0.008017,0.288571,0.232348,0.531876,0.477123,0.778090,0.730618,
1.000000,0.997308,0.003311,0.002400,0.390728,0.281973,0.613508,0.526605,0.827815,0.772362,
1.000000,0.993283,0.096096,0.061988,0.081013,0.046344,0.068053,0.048237,0.060197,0.044060,
0.077381,0.040409,0.053191,0.037575,0.092593,0.066622,0.089947,0.060620,0.099099,0.071134,
0.076471,0.056417,0.081081,0.060332,0.048571,0.029577,0.068852,0.041883,0.039735,0.021171,
0.082552,0.069413,0.156085,0.104060,0.065574,0.042080,0.042135,0.023458,0.069149,0.052945,
0.034392,0.021674

Positive_63 42.000000,0.109410,14.000000,4.595238,3.000000,12.685830,17.000000,4.595238,3.000000,27.710221,
0.468231,0.031446,0.468231,0.031446,0.031088,0.122995,0.292683,0.370690,0.410959,0.418605,
0.840000,1.839966,3.785714,1.592402,0.903183,4.769231,2.304699,0.415923,1.000000,0.120033,
0.090731,0.444444,0.016541,0.235785,0.390244,0.014942,0.060308,0.170732,0.001181,0.037986,
0.190244,0.003053,0.138734,0.317073,0.012148,8.840355,7.267615,3.789717,1.000000,0.880952,
1.000000,0.880952,1.000000,0.858370,1.000000,0.827301,1.000000,0.647259,1.000000,0.435621,
1.000000,0.277978,1.000000,0.250297,1.000000,0.063946,0.109890,0.007849,1.000000,0.714286,
1.000000,0.713239,1.000000,0.690935,1.000000,0.666857,1.000000,0.576613,1.000000,0.535948,
1.000000,0.476562,1.000000,0.433092,1.000000,0.390370,1.000000,0.303566,0.383838,0.298766,
0.446677,0.376559,0.376506,0.324675,0.316327,0.211641,0.265306,0.200591,0.325758,0.253678,
0.050505,0.011232,0.332494,0.240883,0.592308,0.513987,0.834615,0.755307,1.000000,0.995673,
0.026923,0.008334,0.316436,0.248887,0.545181,0.496076,0.792683,0.740582,1.000000,0.991777,
0.010101,0.002645,0.290480,0.242210,0.574961,0.478825,0.789799,0.741763,1.000000,0.996078,
0.580499,0.420804,0.414141,0.199674,0.487113,0.379522,0.127953,0.084150,0.105079,0.072842,
0.013274,0.005448,0.010101,0.002645,0.353201,0.230197,0.598234,0.484830,0.845475,0.735757,
1.000000,1.000000,0.215035,0.063966,0.395543,0.268749,0.618384,0.511831,0.897284,0.736309,
0.999151,0.920799,0.232323,0.040140,0.554869,0.269568,0.734615,0.501459,0.866559,0.735482,
0.997097,0.956047,0.710306,0.621057,0.530612,0.354900,0.070707,0.012901,0.320312,0.263282,
0.561205,0.496236,0.805255,0.737927,1.000000,0.990756,0.474498,0.396103,0.440120,0.369929,
0.282828,0.233968,0.360856,0.298181,0.244898,0.183626,0.211480,0.172213,0.026923,0.008278,
0.306843,0.248175,0.539384,0.491656,0.779381,0.736687,1.000000,0.992660,0.050505,0.009461,
0.286424,0.240201,0.552268,0.492444,0.800525,0.748714,1.000000,0.996381,0.010101,0.002645,
0.319899,0.247118,0.585859,0.505542,0.819527,0.749895,1.000000,0.996176,0.397590,0.350977,
0.418856,0.328374,0.414141,0.320649,0.313131,0.241106,0.316327,0.231104,0.285714,0.199818,
0.010101,0.002645,0.296989,0.245706,0.562597,0.480883,0.785162,0.742895,1.000000,0.996804,
0.026923,0.008334,0.316436,0.247016,0.570205,0.490824,0.802721,0.732904,1.000000,0.990568,
0.050505,0.011232,0.332494,0.242905,0.575290,0.514992,0.798817,0.751705,1.000000,0.995726,
0.380353,0.326291,0.490506,0.439741,0.282828,0.233968,0.356037,0.289318,0.234694,0.154641,
0.266667,0.201198,0.039007,0.009861,0.301370,0.244245,0.539384,0.491352,0.783505,0.736763,
1.000000,0.991936,0.050505,0.009177,0.289694,0.244235,0.551532,0.492544,0.792308,0.745383,
1.000000,0.997294,0.010101,0.002645,0.319899,0.247118,0.585859,0.505542,0.819527,0.749895,
1.000000,0.996176,0.076655,0.048692,0.101010,0.063571,0.092199,0.064828,0.097826,0.048459,
0.080808,0.035889,0.050898,0.037327,0.113949,0.081339,0.119438,0.082726,0.141414,0.060338,
0.088462,0.053430,0.106646,0.050541,0.046154,0.021883,0.046392,0.026302,0.033505,0.019270,
0.113772,0.081614,0.127660,0.089427,0.096386,0.060844,0.039813,0.025206,0.054159,0.035202,
0.026114,0.013111

Positive_64 4.000000,1.000000,4.000000,4.000000,4.000000,0.000000,4.000000,4.000000,4.000000,0.000000,
0.604163,0.076866,0.604163,0.076866,0.000000,0.125000,0.285714,0.000000,0.400000,0.000000,
0.333333,4.000000,4.000000,0.000000,4.000000,4.000000,0.000000,1.000000,1.000000,0.000000,
1.000000,1.000000,0.000000,1.000000,1.000000,0.000000,1.000000,1.000000,0.000000,1.000000,
1.000000,0.000000,1.000000,1.000000,0.000000,4.000000,0.000000,0.000000,1.000000,1.000000,
1.000000,1.000000,1.000000,1.000000,0.833333,0.833333,0.500000,0.500000,0.500000,0.500000,
0.166667,0.166667,0.166667,0.166667,0.000000,0.000000,0.000000,0.000000,1.000000,1.000000,
1.000000,1.000000,1.000000,1.000000,0.833333,0.833333,0.500000,0.500000,0.500000,0.500000,

0.166667,0.166667,0.166667,0.166667,0.000000,0.000000,0.000000,0.000000,0.374468,0.294394,
0.457792,0.402372,0.370062,0.303234,0.290598,0.242804,0.197740,0.183795,0.314583,0.258927,
0.028090,0.012333,0.292135,0.262921,0.573034,0.530932,0.820225,0.753471,1.000000,0.994957,
0.025532,0.014290,0.293617,0.256288,0.531915,0.494121,0.804255,0.777182,1.000000,0.987369,
0.005618,0.003800,0.272727,0.219460,0.490260,0.455984,0.740125,0.676851,1.000000,0.986893,
0.626623,0.486938,0.235955,0.167934,0.430353,0.345126,0.074919,0.056312,0.097917,0.081781,
0.006250,0.002377,0.005618,0.003800,0.328482,0.269043,0.710638,0.589889,0.868085,0.828704,
1.000000,1.000000,0.178723,0.056007,0.292135,0.259701,0.470779,0.382416,0.626623,0.535598,
0.990260,0.805385,0.100649,0.053590,0.353896,0.205760,0.574675,0.501902,0.754678,0.688709,
0.993763,0.977326,0.627859,0.517232,0.403909,0.360317,0.022472,0.016214,0.282468,0.233846,
0.509740,0.443282,0.717256,0.664368,0.994382,0.984759,0.464286,0.408903,0.365169,0.351640,
0.280899,0.239457,0.316384,0.312320,0.204167,0.187962,0.196581,0.161536,0.025532,0.014810,
0.310638,0.250236,0.608511,0.509848,0.859574,0.803678,1.000000,0.995860,0.022472,0.011968,
0.252809,0.237473,0.528090,0.491290,0.735967,0.709850,1.000000,0.993617,0.005618,0.003800,
0.353896,0.232012,0.581169,0.479636,0.727273,0.694172,0.995842,0.979676,0.390852,0.338018,
0.415584,0.340120,0.412766,0.321861,0.285417,0.233966,0.256410,0.226899,0.273504,0.235034,
0.005618,0.003800,0.272727,0.216967,0.493506,0.453678,0.735967,0.677473,1.000000,0.986893,
0.025532,0.015330,0.323404,0.269306,0.612766,0.525768,0.855319,0.800570,1.000000,0.987369,
0.028090,0.012333,0.292135,0.256976,0.567416,0.509672,0.820225,0.729706,1.000000,0.994957,
0.331915,0.311997,0.509740,0.448546,0.280899,0.239457,0.332248,0.306091,0.163842,0.151110,
0.222222,0.198388,0.025532,0.014810,0.361702,0.270600,0.638298,0.509207,0.876596,0.781159,
1.000000,0.995860,0.022472,0.011968,0.266112,0.232764,0.528090,0.492184,0.772727,0.748208,
1.000000,0.993617,0.005618,0.003800,0.353896,0.232012,0.581169,0.479636,0.727273,0.694172,
0.995842,0.979676,0.074675,0.061462,0.068085,0.042410,0.101124,0.077125,0.068085,0.050772,
0.045455,0.029261,0.055319,0.033364,0.067416,0.060303,0.085106,0.074389,0.106029,0.079038,
0.077922,0.047494,0.152597,0.078896,0.038298,0.027468,0.061798,0.034784,0.037422,0.018010,
0.068607,0.058947,0.149351,0.117363,0.050562,0.035580,0.038298,0.029109,0.050562,0.034572,
0.016632,0.009654

Positive_65 3.000000,0.222222,2.000000,0.666667,0.000000,1.333333,1.000000,0.666667,1.000000,0.333333,
0.325656,0.000487,0.325656,0.000487,0.000000,0.000000,1.000000,0.000000,0.000000,0.000000,
0.000000,0.000000,0.000000,0.000000,0.000000,0.000000,0.000000,0.000000,0.000000,0.000000,
0.000000,0.000000,0.000000,0.000000,0.000000,0.000000,0.000000,0.000000,0.000000,0.000000,
0.000000,0.000000,0.333333,0.500000,0.083333,1.414214,0.000000,0.000000,1.000000,0.333333,
1.000000,0.333333,1.000000,0.333333,1.000000,0.333333,1.000000,0.333333,1.000000,0.333333,
0.000000,0.000000,0.000000,0.000000,0.000000,0.000000,0.000000,0.000000,0.000000,0.000000,
0.000000,0.000000,0.000000,0.000000,0.000000,0.000000,0.000000,0.000000,0.000000,0.000000,
0.000000,0.000000,0.000000,0.000000,0.000000,0.000000,0.000000,0.000000,0.344937,0.300313,
0.433673,0.366267,0.356757,0.333420,0.256410,0.222845,0.244444,0.211995,0.260317,0.245524,
0.018987,0.013833,0.246835,0.228384,0.517117,0.504242,0.792793,0.758107,0.998198,0.996644,
0.010204,0.006712,0.316327,0.278573,0.550633,0.520978,0.770408,0.731235,1.000000,0.998799,
0.005102,0.003356,0.252252,0.229525,0.488288,0.461738,0.750000,0.720938,1.000000,0.987277,
0.489796,0.466823,0.178571,0.151144,0.425225,0.382033,0.126984,0.079086,0.095238,0.082945,
0.001805,0.000602,0.005102,0.003356,0.335443,0.309458,0.560127,0.535459,0.813291,0.788061,
1.000000,1.000000,0.056122,0.024728,0.324324,0.218093,0.454054,0.377929,0.637755,0.605476,

0.954082,0.907165,0.091837,0.068395,0.255102,0.213205,0.525510,0.458161,0.756329,0.715740,
0.980180,0.943320,0.625225,0.594334,0.400000,0.363454,0.051020,0.024083,0.266667,0.242187,
0.520408,0.502884,0.770408,0.736202,0.987342,0.982972,0.413265,0.371183,0.367568,0.365634,
0.303797,0.263183,0.290614,0.252346,0.238095,0.205284,0.219048,0.198238,0.010204,0.006712,
0.311224,0.272390,0.510204,0.491690,0.780612,0.734490,1.000000,0.996689,0.030612,0.017280,
0.255856,0.226507,0.506306,0.477647,0.751351,0.711980,0.996835,0.995443,0.005102,0.003356,
0.261261,0.242904,0.533333,0.504201,0.809009,0.781747,1.000000,0.991497,0.380180,0.360280,
0.362245,0.307547,0.376582,0.332174,0.222222,0.212943,0.298413,0.254490,0.246154,0.207496,
0.005102,0.003356,0.261261,0.231473,0.500901,0.476146,0.740506,0.718671,1.000000,0.991497,
0.010204,0.006712,0.311224,0.261256,0.510204,0.494400,0.780612,0.733436,1.000000,0.996689,
0.018987,0.013833,0.246835,0.235888,0.526126,0.508881,0.800000,0.765784,0.998198,0.996644,
0.326531,0.291746,0.455856,0.445072,0.303797,0.263183,0.276923,0.259519,0.174603,0.154390,
0.282540,0.249131,0.010204,0.006712,0.311224,0.257037,0.479592,0.455907,0.739796,0.725105,
0.996396,0.993288,0.030612,0.017280,0.270270,0.248490,0.528481,0.509794,0.749550,0.742875,
1.000000,0.997143,0.005102,0.003356,0.261261,0.242904,0.533333,0.504201,0.809009,0.781747,
1.000000,0.991497,0.056122,0.045926,0.098101,0.063325,0.085443,0.075215,0.060127,0.044161,
0.051020,0.041223,0.034234,0.030463,0.102041,0.068601,0.091837,0.077389,0.057658,0.047593,
0.066327,0.054002,0.064865,0.059962,0.045918,0.031861,0.031646,0.026860,0.025510,0.019476,
0.117347,0.084545,0.111712,0.092929,0.060127,0.041259,0.045045,0.029202,0.066456,0.045670,
0.021622,0.020339

Positive_66 15.000000,0.084444,4.000000,1.266667,1.000000,2.066667,3.000000,1.266667,1.000000,1.066667,
0.588769,0.072416,0.588769,0.072416,0.052632,0.166667,0.066667,0.071429,0.153846,0.363636,
0.428571,0.466667,2.500000,0.945238,0.622222,2.666667,1.172487,0.150000,1.000000,0.103125,
0.251852,1.000000,0.187772,0.039683,0.142857,0.002544,0.056349,0.214286,0.005430,0.068254,
0.214286,0.006144,0.084127,0.214286,0.006954,3.020448,1.847759,1.414214,1.000000,0.333333,
1.000000,0.333333,1.000000,0.333333,1.000000,0.333333,1.000000,0.333333,1.000000,0.244444,
1.000000,0.222222,1.000000,0.200000,1.000000,0.111111,0.333333,0.022222,1.000000,0.400000,
1.000000,0.400000,1.000000,0.400000,1.000000,0.333333,1.000000,0.333333,1.000000,0.333333,
1.000000,0.333333,1.000000,0.288889,0.333333,0.044444,0.000000,0.000000,0.318627,0.294458,
0.426386,0.382174,0.350427,0.323368,0.270936,0.219549,0.226601,0.197877,0.284866,0.254399,
0.044118,0.013565,0.293413,0.229471,0.561538,0.502896,0.815182,0.753107,1.000000,0.997516,
0.016502,0.006638,0.342742,0.252992,0.528684,0.484322,0.792683,0.731720,1.000000,0.995546,
0.004902,0.002564,0.299065,0.252975,0.557632,0.497746,0.783784,0.752401,1.000000,0.993075,
0.552419,0.440620,0.203846,0.147675,0.620172,0.411705,0.115533,0.073273,0.106250,0.086133,
0.007702,0.002879,0.004902,0.002564,0.284589,0.236584,0.573077,0.487861,0.766730,0.734751,
1.000000,1.000000,0.180258,0.067393,0.387097,0.260662,0.600806,0.489133,0.843645,0.701324,
0.997955,0.941654,0.051829,0.021257,0.356436,0.262398,0.584046,0.498366,0.838284,0.764528,
0.997750,0.980105,0.658120,0.609555,0.394286,0.363546,0.024194,0.011124,0.316832,0.271518,
0.560748,0.507068,0.778203,0.743975,0.997955,0.990036,0.473520,0.406278,0.403226,0.377088,
0.253219,0.216634,0.360856,0.314556,0.234409,0.178045,0.192412,0.163361,0.012097,0.006198,
0.290323,0.247247,0.531549,0.484535,0.778203,0.736033,1.000000,0.995546,0.024194,0.009750,
0.330484,0.251395,0.613861,0.507004,0.798680,0.748887,1.000000,0.997384,0.004902,0.002564,
0.307692,0.237996,0.550898,0.506312,0.819527,0.746597,1.000000,0.991565,0.366337,0.345406,
0.393491,0.338131,0.344872,0.316462,0.278932,0.241385,0.275862,0.224526,0.261084,0.213503,

0.004902,0.002564,0.305296,0.251720,0.554517,0.496496,0.778970,0.752341,1.000000,0.993075,
0.016502,0.006638,0.342742,0.252093,0.530934,0.482378,0.792683,0.732254,1.000000,0.995546,
0.044118,0.013565,0.296407,0.230453,0.556410,0.507685,0.815182,0.750266,1.000000,0.997516,
0.389408,0.337624,0.480392,0.445742,0.253219,0.216634,0.356250,0.303447,0.206452,0.150281,
0.216749,0.191124,0.036585,0.009557,0.298387,0.244140,0.531549,0.487926,0.778203,0.731957,
1.000000,0.994654,0.015576,0.008341,0.316239,0.250543,0.603960,0.499742,0.792079,0.743698,
1.000000,0.997639,0.004902,0.002564,0.307692,0.237996,0.550898,0.506312,0.819527,0.746597,
1.000000,0.991565,0.073529,0.052326,0.092814,0.055004,0.073529,0.057307,0.068862,0.053053,
0.078431,0.040318,0.052419,0.036451,0.112745,0.084615,0.141491,0.094032,0.074850,0.052795,
0.071006,0.053130,0.068627,0.053560,0.034615,0.022004,0.038627,0.022039,0.028490,0.015094,
0.104839,0.080081,0.154876,0.106729,0.084677,0.056202,0.036290,0.022308,0.056106,0.032201,
0.019802,0.010753

Positive_67 8.000000,0.250000,4.000000,2.000000,3.000000,2.857143,4.000000,2.000000,1.000000,2.857143,
0.319744,0.003433,0.319744,0.003433,0.062500,0.200000,0.833333,1.000000,0.000000,0.000000,
0.000000,0.000000,0.000000,0.000000,0.000000,0.000000,0.000000,0.000000,0.000000,0.000000,
0.000000,0.000000,0.000000,0.214286,0.428571,0.052478,0.053571,0.428571,0.022959,0.053571,
0.142857,0.005466,0.321429,0.428571,0.021866,3.464102,2.000000,0.000000,1.000000,0.625000,
1.000000,0.625000,1.000000,0.625000,1.000000,0.604167,1.000000,0.562500,1.000000,0.562500,
1.000000,0.520833,0.333333,0.187500,0.000000,0.000000,0.000000,0.000000,1.000000,0.375000,
1.000000,0.375000,1.000000,0.375000,0.833333,0.312500,0.500000,0.187500,0.500000,0.187500,
0.166667,0.062500,0.166667,0.062500,0.000000,0.000000,0.000000,0.000000,0.374468,0.298689,
0.457792,0.391533,0.370062,0.309778,0.290598,0.228306,0.204724,0.186309,0.314583,0.258495,
0.028090,0.010801,0.292135,0.255493,0.573034,0.519890,0.820225,0.761167,1.000000,0.997306,
0.025532,0.009415,0.293617,0.256496,0.531915,0.490976,0.804255,0.755543,1.000000,0.990734,
0.005618,0.002953,0.272727,0.225513,0.520548,0.476077,0.744292,0.703432,1.000000,0.990684,
0.626623,0.428287,0.235955,0.144115,0.534247,0.427598,0.079585,0.057333,0.114187,0.086571,
0.006250,0.002867,0.005618,0.002953,0.328482,0.240642,0.710638,0.538712,0.868085,0.778657,
1.000000,1.000000,0.399543,0.097905,0.465753,0.234721,0.506849,0.361292,0.647444,0.556166,
0.990260,0.812375,0.100649,0.033336,0.353896,0.239054,0.655251,0.534063,0.820690,0.735672,
0.993763,0.977015,0.689498,0.576174,0.403909,0.346273,0.022472,0.010845,0.324138,0.249137,
0.555172,0.480319,0.771689,0.708415,0.998689,0.988571,0.464286,0.405621,0.366972,0.357108,
0.280899,0.237271,0.316384,0.303825,0.235698,0.195766,0.196581,0.160643,0.025532,0.009675,
0.310638,0.251250,0.608511,0.502277,0.859574,0.768971,1.000000,0.995885,0.022472,0.009410,
0.252809,0.232461,0.528090,0.489973,0.757991,0.722686,1.000000,0.995096,0.005618,0.002953,
0.353896,0.249545,0.581169,0.495204,0.831034,0.737310,1.000000,0.988639,0.390852,0.339615,
0.415584,0.340006,0.412766,0.320379,0.285417,0.243405,0.256410,0.221813,0.273504,0.218229,
0.005618,0.002953,0.272727,0.227462,0.518672,0.476784,0.748362,0.705376,1.000000,0.990848,
0.025532,0.009935,0.323404,0.257428,0.612766,0.506908,0.855319,0.763441,1.000000,0.989915,
0.028090,0.010801,0.292135,0.252858,0.567416,0.509102,0.820225,0.750077,1.000000,0.997306,
0.348276,0.324280,0.509740,0.438449,0.280899,0.237271,0.332248,0.297103,0.210526,0.161324,
0.222222,0.195084,0.025532,0.009675,0.361702,0.263643,0.638298,0.504138,0.876596,0.759041,
1.000000,0.995721,0.022472,0.009410,0.266112,0.229474,0.528090,0.488973,0.772727,0.744453,
1.000000,0.995096,0.005618,0.002953,0.353896,0.249545,0.581169,0.495204,0.831034,0.737310,
1.000000,0.988639,0.074675,0.057876,0.093054,0.055701,0.101124,0.070490,0.068085,0.046562,

0.045455,0.035827,0.055319,0.032232,0.098174,0.074024,0.131034,0.085001,0.106029,0.071048,
0.077922,0.047645,0.152597,0.062288,0.038298,0.021690,0.061798,0.029837,0.037422,0.019053,
0.083879,0.062640,0.149351,0.110070,0.060288,0.045848,0.038298,0.028075,0.050562,0.035430,
0.016632,0.008663

Positive_68 21.000000,0.083900,6.000000,1.761905,2.000000,2.390476,6.000000,1.761905,1.000000,3.390476,
0.517160,0.022596,0.517160,0.022596,0.000000,0.027027,0.222222,0.250000,0.523810,0.500000,
0.600000,0.253968,2.166667,0.345470,0.253968,2.166667,0.345470,0.057540,0.361111,0.015278,
0.057540,0.361111,0.015278,0.093651,0.183333,0.003761,0.046032,0.150000,0.003294,0.042857,
0.183333,0.004238,0.065079,0.250000,0.007461,3.958183,2.449490,2.449490,1.000000,0.571429,
1.000000,0.571429,1.000000,0.571429,1.000000,0.571429,1.000000,0.565079,1.000000,0.492063,
1.000000,0.231746,1.000000,0.190476,0.333333,0.038095,0.000000,0.000000,1.000000,0.476190,
1.000000,0.476190,1.000000,0.476190,1.000000,0.476190,1.000000,0.438095,1.000000,0.387302,
1.000000,0.346032,0.666667,0.174603,0.666667,0.085714,0.000000,0.000000,0.349537,0.297014,
0.453757,0.383187,0.340909,0.319799,0.273810,0.214231,0.248260,0.195140,0.325758,0.249005,
0.028902,0.012005,0.332494,0.257715,0.569948,0.511953,0.800000,0.749872,1.000000,0.998110,
0.011364,0.004995,0.316436,0.233440,0.543779,0.481943,0.762523,0.737284,1.000000,0.994207,
0.005682,0.002036,0.306288,0.256969,0.555076,0.498100,0.809935,0.746794,1.000000,0.996021,
0.505831,0.400082,0.303571,0.186194,0.570071,0.413724,0.137143,0.078461,0.114187,0.081233,
0.012626,0.005055,0.005682,0.002036,0.291859,0.216074,0.558783,0.484181,0.796680,0.730205,
1.000000,1.000000,0.395538,0.077940,0.507099,0.300282,0.589286,0.492554,0.874239,0.712666,
0.998626,0.934650,0.075567,0.013946,0.317333,0.244931,0.641330,0.489955,0.820690,0.750549,
0.997097,0.971463,0.687657,0.624464,0.441558,0.355203,0.022727,0.007416,0.343931,0.270223,
0.567696,0.511814,0.799189,0.749995,0.998689,0.993302,0.448363,0.398666,0.425926,0.368426,
0.277202,0.232908,0.356061,0.291883,0.221429,0.181149,0.205285,0.172812,0.011364,0.004957,
0.293395,0.235095,0.513057,0.488523,0.760989,0.736669,1.000000,0.994479,0.026128,0.009817,
0.300578,0.261097,0.539554,0.510070,0.800292,0.753542,1.000000,0.997938,0.005682,0.002036,
0.319899,0.244499,0.568182,0.492670,0.831034,0.746168,1.000000,0.992778,0.377880,0.344688,
0.407514,0.337114,0.362694,0.318198,0.313131,0.235404,0.275819,0.224202,0.241299,0.201866,
0.005682,0.002036,0.306288,0.257906,0.535637,0.498445,0.801296,0.747716,1.000000,0.996423,
0.011364,0.004995,0.316436,0.226370,0.543779,0.478466,0.771978,0.731963,1.000000,0.992828,
0.028902,0.012005,0.332494,0.259578,0.590909,0.512958,0.795356,0.751328,1.000000,0.998110,
0.380353,0.333832,0.477273,0.433261,0.277202,0.232908,0.328283,0.284419,0.195238,0.152418,
0.233766,0.201544,0.011364,0.005065,0.303448,0.235316,0.542117,0.491062,0.768049,0.739096,
1.000000,0.993295,0.022727,0.008966,0.306358,0.257657,0.532468,0.501680,0.771058,0.748319,
1.000000,0.998352,0.005682,0.002036,0.319899,0.244499,0.568182,0.492670,0.831034,0.746168,
1.000000,0.992778,0.073022,0.048810,0.093054,0.064313,0.080311,0.062932,0.059369,0.044509,
0.053996,0.039756,0.062500,0.036695,0.127168,0.085971,0.131034,0.096083,0.072802,0.059015,
0.072254,0.048253,0.067055,0.047792,0.039773,0.021184,0.039939,0.024889,0.030722,0.017043,
0.107955,0.078552,0.135903,0.089065,0.083815,0.061427,0.039813,0.027576,0.051813,0.036417,
0.026114,0.009721

Positive_69 13.000000,0.248521,6.000000,3.230769,6.000000,9.692308,7.000000,3.230769,0.000000,13.192308,
0.394250,0.006004,0.394250,0.006004,0.000000,0.071429,0.538462,0.777778,0.750000,1.000000,
0.000000,0.000000,0.000000,0.000000,0.000000,0.000000,0.000000,0.000000,0.000000,0.000000,
0.000000,0.000000,0.000000,0.269231,0.500000,0.067308,0.000000,0.000000,0.000000,0.000000,

0.000000,0.000000,0.192308,0.416667,0.046741,6.480741,0.000000,0.000000,1.000000,0.538462,
1.000000,0.538462,1.000000,0.538462,1.000000,0.538462,1.000000,0.538462,1.000000,0.538462,
0.866667,0.466667,0.466667,0.251282,0.266667,0.143590,0.066667,0.035897,1.000000,0.461538,
1.000000,0.461538,1.000000,0.461538,1.000000,0.461538,0.761905,0.351648,0.571429,0.263736,
0.333333,0.153846,0.095238,0.043956,0.000000,0.000000,0.000000,0.000000,0.322266,0.283557,
0.400000,0.380179,0.361413,0.336264,0.227006,0.201901,0.231608,0.205919,0.296791,0.258379,
0.016304,0.009432,0.272000,0.227274,0.582888,0.515579,0.768340,0.734911,1.000000,0.994145,
0.022680,0.007981,0.297927,0.253519,0.517526,0.488096,0.787629,0.746481,1.000000,0.996005,
0.002717,0.002294,0.275391,0.251658,0.536269,0.498701,0.782383,0.749310,1.000000,0.996316,
0.472000,0.425273,0.259358,0.220494,0.434879,0.354233,0.107239,0.094050,0.080429,0.069750,
0.013369,0.009353,0.002717,0.002294,0.353201,0.233858,0.598234,0.469485,0.845475,0.744687,
1.000000,1.000000,0.211921,0.069245,0.357616,0.234028,0.560297,0.473452,0.820116,0.749401,
0.998145,0.981991,0.155440,0.074082,0.500000,0.334736,0.665761,0.524704,0.883152,0.754837,
0.993814,0.970170,0.684720,0.645218,0.400000,0.373610,0.021333,0.011978,0.320312,0.279983,
0.519531,0.504704,0.775629,0.755123,1.000000,0.992940,0.465789,0.415372,0.418557,0.359439,
0.251546,0.225189,0.310992,0.289655,0.235925,0.200463,0.210744,0.154738,0.022680,0.007981,
0.306843,0.258363,0.518764,0.488863,0.779381,0.734216,1.000000,0.995190,0.018667,0.010316,
0.261719,0.230512,0.538043,0.488780,0.771739,0.738347,1.000000,0.995944,0.002717,0.002294,
0.302139,0.249831,0.582888,0.523699,0.834225,0.795136,1.000000,0.998756,0.391753,0.358455,
0.371053,0.341617,0.338491,0.299928,0.286863,0.250114,0.260331,0.230327,0.213307,0.189709,
0.002717,0.002294,0.281250,0.257779,0.538860,0.498019,0.782383,0.752598,1.000000,0.997109,
0.022680,0.007981,0.286976,0.246315,0.515789,0.489063,0.785567,0.738015,1.000000,0.995190,
0.016304,0.009432,0.266667,0.227057,0.578804,0.510686,0.771739,0.741529,1.000000,0.994315,
0.378947,0.336270,0.482474,0.438542,0.251546,0.225189,0.302452,0.274628,0.193798,0.162880,
0.239669,0.192322,0.022680,0.008267,0.310881,0.267162,0.531579,0.501756,0.783505,0.743550,
1.000000,0.994739,0.018667,0.010316,0.262548,0.234882,0.528958,0.475362,0.750000,0.727573,
1.000000,0.996094,0.002717,0.002294,0.302139,0.249831,0.582888,0.523699,0.834225,0.795136,
1.000000,0.998756,0.056093,0.037411,0.086957,0.073347,0.076172,0.063269,0.054159,0.044044,
0.050773,0.030813,0.044527,0.034673,0.109462,0.093220,0.106383,0.085138,0.067935,0.054350,
0.082902,0.059517,0.058594,0.049391,0.022075,0.016371,0.039735,0.022191,0.043478,0.029711,
0.104278,0.085779,0.111340,0.080321,0.081579,0.064584,0.030888,0.021686,0.054159,0.044001,
0.024119,0.010182

Positive_70 3.000000,0.222222,1.000000,0.666667,1.000000,0.333333,1.000000,0.666667,1.000000,0.333333,
0.653163,0.002277,0.653163,0.002277,0.000000,0.000000,0.000000,0.000000,0.000000,1.000000,
0.000000,0.000000,0.000000,0.000000,0.000000,0.000000,0.000000,0.000000,0.000000,0.000000,
0.000000,0.000000,0.000000,0.000000,0.000000,0.000000,0.166667,0.500000,0.083333,0.166667,
0.500000,0.083333,0.000000,0.000000,0.000000,1.000000,1.000000,0.000000,0.000000,0.000000,
0.000000,0.000000,0.000000,0.000000,0.000000,0.000000,0.000000,0.000000,0.000000,0.000000,
0.000000,0.000000,0.000000,0.000000,0.000000,0.000000,0.000000,0.000000,0.000000,0.000000,
0.000000,0.000000,0.000000,0.000000,0.000000,0.000000,0.000000,0.000000,0.000000,0.000000,
0.000000,0.000000,0.000000,0.000000,0.000000,0.000000,0.000000,0.000000,0.310559,0.289952,
0.403341,0.380154,0.347826,0.329893,0.252252,0.221056,0.256250,0.202142,0.299043,0.273930,
0.019093,0.012752,0.231504,0.224185,0.474940,0.453449,0.739857,0.737190,1.000000,0.991565,
0.031056,0.013441,0.272076,0.254760,0.546067,0.520134,0.788820,0.774118,1.000000,0.999251,

0.006211,0.003615,0.280899,0.257125,0.496894,0.486817,0.735084,0.713616,0.995227,0.986029,
0.622472,0.471873,0.284010,0.198520,0.614907,0.329607,0.119617,0.087489,0.118750,0.075239,
0.023923,0.011559,0.006211,0.003615,0.284010,0.223095,0.621118,0.523307,0.869565,0.797607,
1.000000,1.000000,0.037267,0.018508,0.350562,0.223630,0.491647,0.338540,0.749403,0.478787,
0.985680,0.838044,0.105590,0.071652,0.761798,0.422687,0.851685,0.556762,0.937079,0.761591,
0.987578,0.943911,0.676404,0.634566,0.363636,0.330972,0.024845,0.012869,0.296629,0.265214,
0.514607,0.479703,0.749403,0.727378,0.990453,0.984437,0.422434,0.401188,0.440994,0.364555,
0.269663,0.234257,0.350000,0.311787,0.239234,0.190987,0.171171,0.157296,0.031056,0.013441,
0.267081,0.254486,0.534161,0.516607,0.791011,0.770996,1.000000,0.999251,0.018634,0.011641,
0.229213,0.221742,0.505967,0.465113,0.758950,0.703241,1.000000,0.994268,0.006211,0.003615,
0.301124,0.243469,0.566292,0.484040,0.768539,0.739792,0.993789,0.987071,0.366460,0.359135,
0.346062,0.327488,0.323596,0.313378,0.287081,0.261272,0.268750,0.234694,0.234234,0.209468,
0.006211,0.003615,0.294382,0.249666,0.505967,0.493838,0.749403,0.718389,0.995227,0.986029,
0.031056,0.013441,0.267303,0.254709,0.541573,0.518636,0.797753,0.775793,1.000000,0.999251,
0.019093,0.012752,0.231504,0.227181,0.470167,0.460847,0.752809,0.742820,1.000000,0.991565,
0.322196,0.299443,0.540373,0.466301,0.269663,0.234257,0.350000,0.291473,0.174641,0.134021,
0.227477,0.214263,0.037267,0.015511,0.298137,0.265076,0.541573,0.509089,0.797753,0.764635,
1.000000,0.997753,0.018634,0.011641,0.229117,0.222067,0.515513,0.479396,0.749403,0.721250,
1.000000,0.995064,0.006211,0.003615,0.301124,0.243469,0.566292,0.484040,0.768539,0.739792,
0.993789,0.987071,0.049689,0.045115,0.056180,0.048335,0.062112,0.051364,0.059666,0.050861,
0.080745,0.050177,0.060674,0.044101,0.090692,0.070941,0.074534,0.064162,0.069663,0.062855,
0.068323,0.050624,0.083532,0.078906,0.029213,0.023425,0.040449,0.029241,0.026966,0.022839,
0.068323,0.061343,0.118012,0.095925,0.080745,0.061645,0.040573,0.031367,0.060674,0.043142,
0.017978,0.013632

Positive_71 26.000000,0.121302,11.000000,3.153846,2.000000,7.975385,9.000000,3.153846,3.000000,8.615385,
0.524177,0.016418,0.524177,0.016418,0.000000,0.012195,0.160494,0.308824,0.553191,0.619048,
0.875000,0.791825,4.363636,1.228015,0.858974,3.111111,1.089131,0.132143,0.396694,0.022959,
0.161681,0.375000,0.025313,0.144509,0.270000,0.007153,0.102602,0.320000,0.009402,0.099744,
0.310000,0.009474,0.114872,0.280000,0.011830,6.556685,3.465098,2.889085,1.000000,0.576923,
1.000000,0.576923,1.000000,0.576923,1.000000,0.576224,1.000000,0.476590,1.000000,0.401332,
1.000000,0.154612,0.333333,0.084665,0.000000,0.000000,0.000000,0.000000,1.000000,0.615385,
1.000000,0.615385,1.000000,0.615385,1.000000,0.612179,1.000000,0.537821,1.000000,0.455128,
1.000000,0.330128,1.000000,0.167308,1.000000,0.051282,1.000000,0.038462,0.323232,0.292047,
0.427184,0.387268,0.339688,0.320685,0.268166,0.213406,0.237705,0.195005,0.325758,0.248301,
0.023932,0.012082,0.332494,0.248859,0.568221,0.514024,0.800000,0.759172,1.000000,0.997261,
0.015598,0.005888,0.303754,0.244531,0.555102,0.490674,0.795918,0.739738,1.000000,0.995796,
0.004082,0.002198,0.285470,0.241328,0.520202,0.492026,0.784543,0.735455,1.000000,0.996295,
0.526923,0.422419,0.253385,0.173954,0.567961,0.403627,0.127168,0.073058,0.114187,0.080234,
0.013841,0.006178,0.004082,0.002198,0.324090,0.229582,0.573402,0.496225,0.805769,0.731125,
1.000000,1.000000,0.415825,0.097780,0.574074,0.298081,0.688552,0.500789,0.868687,0.730139,
0.997658,0.947844,0.075567,0.018577,0.352770,0.240680,0.650485,0.467376,0.820690,0.717931,
0.996633,0.977674,0.687657,0.636646,0.396166,0.331124,0.020151,0.008503,0.326599,0.273657,
0.556851,0.506098,0.781341,0.741527,0.998689,0.991073,0.452381,0.408344,0.391837,0.352160,
0.276451,0.239496,0.390438,0.280931,0.248538,0.194783,0.188870,0.163365,0.015598,0.006487,

0.287695,0.239546,0.559184,0.485369,0.759615,0.734655,1.000000,0.995750,0.031746,0.010470,
0.317757,0.245074,0.573832,0.497826,0.794393,0.743245,1.000000,0.997358,0.004082,0.002198,
0.334623,0.258872,0.560166,0.505934,0.831034,0.755473,1.000000,0.996535,0.373512,0.347289,
0.396825,0.342559,0.352000,0.310152,0.314815,0.238645,0.251712,0.223293,0.240484,0.198825,
0.004082,0.002198,0.289562,0.245895,0.544586,0.499164,0.783178,0.741784,1.000000,0.996993,
0.015598,0.006487,0.284229,0.232875,0.555102,0.481819,0.771654,0.727697,1.000000,0.994457,
0.023932,0.011882,0.334623,0.254831,0.562617,0.512091,0.788396,0.760521,1.000000,0.997334,
0.388889,0.342284,0.452381,0.418221,0.276451,0.239496,0.362550,0.277589,0.222222,0.162486,
0.226252,0.195663,0.020725,0.007159,0.303448,0.238914,0.542857,0.482952,0.775385,0.732104,
1.000000,0.994323,0.024615,0.008358,0.291589,0.245774,0.547619,0.500473,0.797619,0.744210,
1.000000,0.997659,0.004082,0.002198,0.334623,0.258872,0.560166,0.505934,0.831034,0.755473,
1.000000,0.996535,0.067698,0.047943,0.098726,0.065951,0.077381,0.059589,0.071154,0.046696,
0.051587,0.036080,0.050000,0.035789,0.119048,0.083488,0.150485,0.091557,0.093294,0.067250,
0.076433,0.053293,0.069085,0.046971,0.032000,0.018105,0.048077,0.026604,0.028037,0.019090,
0.103275,0.074551,0.107884,0.090856,0.075567,0.055295,0.042735,0.028185,0.060000,0.039232,
0.029463,0.013476

Positive_72 16.000000,0.457031,15.000000,7.312500,8.000000,25.429167,13.000000,7.312500,8.000000,12.629167,
0.483070,0.029172,0.483070,0.029172,0.000000,0.128205,0.186275,0.445783,0.565217,0.500000,
0.300000,3.760629,6.933333,5.130643,4.458093,6.923077,3.015828,0.490392,0.812500,0.047557,
0.655361,1.000000,0.028538,0.515387,0.777778,0.057525,0.356862,0.616667,0.028265,0.435003,
0.600000,0.005810,0.684436,0.933333,0.013304,9.389693,3.268152,2.760409,1.000000,0.875000,
1.000000,0.875000,1.000000,0.871537,1.000000,0.756345,0.714286,0.437527,0.500000,0.292118,
0.166667,0.055010,0.054545,0.017808,0.018182,0.005736,0.000000,0.000000,1.000000,1.000000,
1.000000,1.000000,1.000000,0.998397,1.000000,0.967803,1.000000,0.892797,1.000000,0.563266,
1.000000,0.322065,1.000000,0.176439,0.166667,0.033051,0.000000,0.000000,0.374384,0.313144,
0.407867,0.368663,0.338710,0.318193,0.234184,0.215112,0.242574,0.208770,0.293532,0.241823,
0.049261,0.012191,0.277027,0.237128,0.559122,0.493843,0.807487,0.762602,1.000000,0.996842,
0.025707,0.007312,0.342742,0.245717,0.535980,0.494414,0.768379,0.731913,1.000000,0.995526,
0.005348,0.002291,0.283537,0.252239,0.565495,0.496180,0.772866,0.743276,1.000000,0.992131,
0.565553,0.446241,0.336898,0.181030,0.560074,0.372729,0.107527,0.077772,0.107356,0.077908,
0.012821,0.004476,0.005348,0.002291,0.374384,0.232946,0.556650,0.481298,0.866995,0.740129,
1.000000,1.000000,0.251232,0.067599,0.394089,0.298295,0.664537,0.532553,0.851117,0.736602,
0.998891,0.959994,0.155080,0.035437,0.362292,0.240327,0.678373,0.493003,0.839187,0.719997,
0.996303,0.945937,0.640203,0.586196,0.419355,0.373299,0.029557,0.010251,0.294207,0.264342,
0.561576,0.505962,0.771341,0.732390,1.000000,0.993004,0.420792,0.388671,0.403226,0.375022,
0.283422,0.236307,0.345178,0.291698,0.230616,0.184624,0.263441,0.180195,0.020566,0.007238,
0.290323,0.239712,0.551724,0.490700,0.763975,0.730435,1.000000,0.996675,0.029557,0.008797,
0.309451,0.248811,0.571646,0.497409,0.803354,0.752093,1.000000,0.996505,0.005348,0.002291,
0.298986,0.251034,0.532806,0.500824,0.804627,0.755786,1.000000,0.993538,0.363636,0.344138,
0.355446,0.320309,0.394089,0.335553,0.273632,0.228429,0.279570,0.242046,0.223769,0.201881,
0.005348,0.002291,0.289634,0.257285,0.568690,0.502563,0.774390,0.748775,1.000000,0.993011,
0.025707,0.007560,0.342742,0.239344,0.538462,0.489142,0.761905,0.723010,1.000000,0.991409,
0.049261,0.012191,0.294118,0.237453,0.565878,0.494430,0.798722,0.759067,1.000000,0.997388,
0.356436,0.317804,0.478149,0.445888,0.283422,0.236307,0.340102,0.278324,0.184891,0.148177,

0.295699,0.216642,0.025707,0.008211,0.298387,0.241759,0.527094,0.493203,0.769960,0.728601,
1.000000,0.995752,0.029557,0.007268,0.300493,0.245458,0.549521,0.493430,0.798030,0.752681,
1.000000,0.996825,0.005348,0.002291,0.298986,0.251034,0.532806,0.500824,0.804627,0.755786,
1.000000,0.993538,0.081349,0.057076,0.090909,0.060440,0.096037,0.068014,0.067093,0.051894,
0.059126,0.039669,0.053476,0.036050,0.097030,0.077919,0.121996,0.082038,0.083744,0.057354,
0.067380,0.048599,0.079692,0.054399,0.033126,0.022409,0.039337,0.025945,0.024814,0.016468,
0.104839,0.074070,0.120148,0.102147,0.084677,0.055071,0.037433,0.024809,0.048780,0.035053,
0.025707,0.010574

Positive_73 3.000000,0.222222,2.000000,0.666667,0.000000,1.333333,1.000000,0.666667,1.000000,0.333333,
0.444844,0.000064,0.444844,0.000064,0.000000,0.000000,0.000000,1.000000,0.000000,0.000000,
0.000000,0.000000,0.000000,0.000000,0.000000,0.000000,0.000000,0.000000,0.000000,0.000000,
0.000000,0.000000,0.000000,0.000000,0.000000,0.000000,0.000000,0.000000,0.000000,0.000000,
0.000000,0.000000,0.333333,0.500000,0.083333,1.414214,0.000000,0.000000,1.000000,0.333333,
1.000000,0.333333,1.000000,0.333333,1.000000,0.333333,1.000000,0.333333,1.000000,0.333333,
0.000000,0.000000,0.000000,0.000000,0.000000,0.000000,0.000000,0.000000,0.000000,0.000000,
0.000000,0.000000,0.000000,0.000000,0.000000,0.000000,0.000000,0.000000,0.000000,0.000000,
0.000000,0.000000,0.000000,0.000000,0.000000,0.000000,0.000000,0.000000,0.327744,0.313124,
0.407867,0.386165,0.312500,0.300710,0.226640,0.224718,0.214712,0.206989,0.242739,0.224796,
0.021825,0.012940,0.253049,0.224734,0.524390,0.492631,0.789683,0.771481,0.998016,0.997632,
0.007622,0.005905,0.277778,0.251559,0.496032,0.488822,0.728778,0.715979,1.000000,0.998620,
0.002070,0.001859,0.283537,0.267476,0.513458,0.501994,0.772866,0.752072,1.000000,0.991410,
0.500000,0.439326,0.144817,0.120535,0.498965,0.440139,0.068465,0.065200,0.107356,0.089519,
0.003976,0.002526,0.002070,0.001859,0.245427,0.230618,0.539634,0.491357,0.739329,0.708371,
1.000000,1.000000,0.140873,0.110064,0.347826,0.321307,0.559451,0.505813,0.785061,0.694974,
0.948171,0.875926,0.062500,0.032221,0.240079,0.229946,0.613095,0.546848,0.787698,0.758298,
0.987805,0.983771,0.625000,0.592164,0.366412,0.360370,0.021341,0.010507,0.294207,0.274282,
0.504573,0.488297,0.771341,0.729221,0.994048,0.992897,0.408730,0.394803,0.375000,0.355418,
0.253049,0.249780,0.262595,0.258801,0.230616,0.200077,0.189313,0.177669,0.009921,0.007228,
0.259921,0.233010,0.507937,0.484880,0.763975,0.737430,1.000000,0.997987,0.010352,0.005790,
0.309451,0.254433,0.571646,0.521035,0.803354,0.768187,0.998016,0.996252,0.002070,0.001859,
0.251984,0.245206,0.509146,0.490971,0.730159,0.706470,1.000000,0.997459,0.344512,0.335075,
0.335404,0.325708,0.349085,0.339217,0.236515,0.218353,0.247328,0.243570,0.216700,0.208200,
0.002070,0.001859,0.289634,0.261975,0.512195,0.491642,0.774390,0.739008,1.000000,0.991410,
0.009921,0.007228,0.267857,0.237256,0.521739,0.498358,0.761905,0.733788,1.000000,0.995974,
0.021825,0.012940,0.250000,0.229123,0.510671,0.495563,0.783730,0.762307,1.000000,0.998294,
0.345238,0.324624,0.455793,0.425597,0.253049,0.249780,0.280318,0.268512,0.184891,0.158281,
0.227481,0.219464,0.009921,0.007228,0.261905,0.232185,0.509921,0.475887,0.761905,0.736894,
0.998476,0.997479,0.008282,0.005100,0.294207,0.252773,0.547256,0.510202,0.797256,0.766183,
1.000000,0.997958,0.002070,0.001859,0.251984,0.245206,0.509146,0.490971,0.730159,0.706470,
1.000000,0.997459,0.081349,0.062988,0.062112,0.052531,0.096037,0.071235,0.057540,0.051313,
0.041667,0.034768,0.045732,0.040290,0.084886,0.075632,0.107143,0.085897,0.068598,0.063411,
0.051760,0.048370,0.064024,0.052397,0.033126,0.026092,0.039337,0.034365,0.020704,0.017782,
0.062112,0.057515,0.111111,0.105262,0.051829,0.046348,0.021825,0.020274,0.047256,0.041373,
0.024390,0.012156

Positive_74 10.000000,0.120000,4.000000,1.200000,1.000000,1.733333,2.000000,1.200000,1.500000,0.844444,
0.506196,0.019043,0.506196,0.019043,0.000000,0.000000,0.333333,0.250000,0.333333,1.000000,
0.000000,0.000000,0.000000,0.000000,0.000000,0.000000,0.000000,0.000000,0.000000,0.000000,
0.000000,0.000000,0.000000,0.061111,0.111111,0.003052,0.100000,0.444444,0.034156,0.088889,
0.222222,0.007682,0.133333,0.333333,0.018656,2.288246,2.000000,1.414214,1.000000,0.400000,
1.000000,0.400000,1.000000,0.400000,1.000000,0.400000,1.000000,0.400000,1.000000,0.366667,
1.000000,0.350000,1.000000,0.333333,1.000000,0.216667,0.000000,0.000000,1.000000,0.500000,
1.000000,0.500000,1.000000,0.500000,1.000000,0.500000,1.000000,0.300000,1.000000,0.300000,
1.000000,0.300000,1.000000,0.300000,1.000000,0.200000,0.000000,0.000000,0.340580,0.294106,
0.433962,0.391800,0.338323,0.314093,0.249395,0.224489,0.210654,0.190795,0.284866,0.245760,
0.020710,0.008787,0.293413,0.244028,0.561538,0.505970,0.810651,0.768483,1.000000,0.997898,
0.009217,0.004679,0.316436,0.258561,0.543779,0.495652,0.761530,0.737084,1.000000,0.996477,
0.002994,0.001884,0.275148,0.241808,0.532243,0.487505,0.782925,0.742372,1.000000,0.995758,
0.465893,0.405908,0.218126,0.176911,0.582353,0.417182,0.115533,0.078403,0.092338,0.077648,
0.009231,0.005341,0.002994,0.001884,0.291859,0.234621,0.573077,0.461768,0.741546,0.721523,
1.000000,1.000000,0.130624,0.053811,0.362845,0.259931,0.557329,0.488193,0.843645,0.704128,
0.993642,0.931571,0.044910,0.013374,0.328402,0.251441,0.608696,0.511800,0.838496,0.773040,
0.997788,0.993881,0.668627,0.631469,0.379822,0.343933,0.014970,0.008198,0.285922,0.262462,
0.547684,0.507355,0.778383,0.741866,0.998183,0.992396,0.455621,0.408739,0.379078,0.348799,
0.285398,0.242462,0.353116,0.288015,0.223947,0.197404,0.205811,0.173228,0.009217,0.005071,
0.293395,0.251979,0.513057,0.490243,0.760369,0.735578,1.000000,0.996477,0.012077,0.007768,
0.290196,0.238725,0.524336,0.498123,0.776549,0.754223,1.000000,0.996711,0.002994,0.001884,
0.307692,0.247993,0.550898,0.505896,0.819527,0.757467,1.000000,0.997059,0.377880,0.344524,
0.393491,0.337764,0.362319,0.317712,0.278932,0.236669,0.256739,0.223209,0.225519,0.205157,
0.002994,0.001884,0.281567,0.240083,0.522252,0.488974,0.775658,0.741228,1.000000,0.995758,
0.009217,0.005071,0.316436,0.254733,0.543779,0.493961,0.761530,0.733040,1.000000,0.996477,
0.020710,0.008787,0.296407,0.248944,0.556410,0.511809,0.798817,0.771986,1.000000,0.997989,
0.372781,0.341248,0.452193,0.416290,0.285398,0.242462,0.329377,0.282406,0.193705,0.166854,
0.228381,0.203778,0.009217,0.005071,0.289089,0.251833,0.509662,0.500694,0.768049,0.739984,
1.000000,0.996281,0.012077,0.007383,0.272549,0.237104,0.513170,0.486198,0.756863,0.736535,
1.000000,0.997104,0.002994,0.001884,0.307692,0.247993,0.550898,0.505896,0.819527,0.757467,
1.000000,0.997059,0.059507,0.042108,0.092814,0.069282,0.070048,0.056143,0.070796,0.054462,
0.043478,0.033951,0.058824,0.038160,0.096154,0.083723,0.127451,0.088605,0.074850,0.055571,
0.071006,0.058886,0.062992,0.050979,0.034615,0.023605,0.041063,0.030431,0.030722,0.016512,
0.095808,0.075814,0.095808,0.082314,0.074359,0.062416,0.041176,0.026264,0.049494,0.037866,
0.026114,0.012906

Positive_75 6.000000,0.166667,1.000000,1.000000,1.000000,0.000000,6.000000,1.000000,0.000000,6.000000,
0.345656,0.103959,0.345656,0.103959,0.333333,0.750000,0.000000,0.000000,0.000000,0.000000,
0.000000,0.000000,0.000000,0.000000,0.166667,1.000000,0.166667,0.000000,0.000000,0.000000,
0.027778,0.166667,0.004630,1.000000,1.000000,0.000000,0.166667,0.200000,0.006667,0.027778,
0.166667,0.004630,0.000000,0.000000,0.000000,2.449490,0.000000,0.000000,0.000000,0.000000,
0.000000,0.000000,0.000000,0.000000,0.000000,0.000000,0.000000,0.000000,0.000000,0.000000,
0.000000,0.000000,0.000000,0.000000,0.000000,0.000000,0.000000,0.000000,1.000000,0.166667,
1.000000,0.166667,0.866667,0.144444,0.666667,0.111111,0.533333,0.088889,0.466667,0.077778,

0.400000,0.066667,0.200000,0.033333,0.066667,0.011111,0.000000,0.000000,0.342995,0.312001,
0.435484,0.377792,0.353909,0.310207,0.242991,0.227769,0.214876,0.204558,0.252066,0.228756,
0.040323,0.011822,0.275188,0.253739,0.543478,0.489733,0.813043,0.732518,1.000000,0.991074,
0.016129,0.009040,0.274194,0.231655,0.563786,0.485866,0.806584,0.753616,1.000000,0.996274,
0.008065,0.003533,0.286159,0.244898,0.562406,0.521436,0.774436,0.745875,1.000000,0.996560,
0.580645,0.487166,0.341564,0.200480,0.380032,0.312354,0.148760,0.079773,0.083871,0.066899,
0.026201,0.007744,0.008065,0.003533,0.282258,0.225948,0.661290,0.514804,0.854839,0.800849,
1.000000,1.000000,0.126316,0.055595,0.370370,0.208058,0.699588,0.379281,0.855967,0.581835,
0.998445,0.918720,0.085346,0.031129,0.415038,0.337767,0.621053,0.516568,0.780715,0.658131,
0.967794,0.823814,0.584541,0.553678,0.427948,0.366070,0.048387,0.019034,0.332815,0.275201,
0.564516,0.524504,0.782258,0.726526,0.996779,0.993267,0.451613,0.386702,0.400000,0.360457,
0.286634,0.252840,0.293388,0.270276,0.200000,0.176608,0.233871,0.199527,0.016461,0.010030,
0.267490,0.226862,0.530864,0.476927,0.790123,0.730923,1.000000,0.993855,0.024194,0.011891,
0.292379,0.252267,0.539850,0.506700,0.784962,0.739856,1.000000,0.996562,0.008065,0.003533,
0.330645,0.257936,0.547368,0.489905,0.766129,0.750782,0.996779,0.990620,0.382716,0.347100,
0.330645,0.307198,0.381643,0.345703,0.223140,0.205104,0.276423,0.258644,0.219626,0.200237,
0.008065,0.003533,0.291729,0.244131,0.559398,0.511344,0.780451,0.742389,1.000000,0.996560,
0.016461,0.010531,0.267490,0.227599,0.567901,0.478956,0.810700,0.743988,1.000000,0.993586,
0.040323,0.011822,0.314516,0.263586,0.543478,0.499644,0.771337,0.728862,1.000000,0.994806,
0.354839,0.312776,0.473913,0.434383,0.286634,0.252840,0.292683,0.261432,0.162602,0.144756,
0.272581,0.231379,0.016461,0.010030,0.292181,0.225902,0.534979,0.472524,0.814815,0.705800,
1.000000,0.993586,0.024194,0.009636,0.278261,0.251208,0.524194,0.511565,0.776050,0.752195,
1.000000,0.997904,0.008065,0.003533,0.330645,0.257936,0.547368,0.489905,0.766129,0.750782,
0.996779,0.990620,0.069565,0.055756,0.070853,0.053331,0.117391,0.072350,0.080645,0.056619,
0.048309,0.040366,0.051530,0.033580,0.096774,0.067004,0.091304,0.068238,0.101089,0.072493,
0.090535,0.048422,0.060870,0.051040,0.056452,0.033702,0.048387,0.036892,0.040323,0.022886,
0.078261,0.066524,0.134783,0.097126,0.065844,0.050513,0.032258,0.021514,0.051530,0.035766,
0.018045,0.015880

Positive_76 10.000000,0.210000,7.000000,2.100000,2.000000,3.655556,7.000000,2.100000,1.000000,6.766667,
0.294349,0.011058,0.294349,0.011058,0.190476,0.411765,0.500000,1.000000,0.000000,0.000000,
0.000000,0.000000,0.000000,0.000000,0.000000,0.000000,0.000000,0.000000,0.000000,0.000000,
0.000000,0.000000,0.000000,0.466667,0.666667,0.103704,0.022222,0.222222,0.004938,0.022222,
0.111111,0.002195,0.488889,0.666667,0.082853,3.741657,2.645751,0.000000,1.000000,0.800000,
1.000000,0.800000,1.000000,0.795238,1.000000,0.785714,1.000000,0.771429,0.428571,0.042857,
0.190476,0.019048,0.047619,0.004762,0.000000,0.000000,0.000000,0.000000,1.000000,0.200000,
1.000000,0.200000,0.952381,0.190476,0.857143,0.171429,0.714286,0.142857,0.428571,0.085714,
0.190476,0.038095,0.047619,0.009524,0.000000,0.000000,0.000000,0.000000,0.320988,0.287462,
0.460094,0.390361,0.348214,0.322178,0.245283,0.221097,0.236025,0.182498,0.327059,0.265279,
0.058685,0.017262,0.306250,0.255816,0.592593,0.487346,0.827160,0.713163,1.000000,0.990643,
0.017857,0.008980,0.330357,0.263241,0.556923,0.507332,0.812500,0.755906,1.000000,0.995063,
0.008929,0.003563,0.257297,0.209423,0.562500,0.486098,0.776786,0.739778,1.000000,0.989693,
0.622703,0.491306,0.283951,0.157260,0.461140,0.351434,0.093168,0.061482,0.085714,0.073796,
0.009009,0.003772,0.008929,0.003563,0.303571,0.236062,0.562500,0.486959,0.839864,0.766106,
1.000000,1.000000,0.200000,0.080540,0.427692,0.238375,0.738342,0.476347,0.898462,0.741633,

0.985981,0.926858,0.178571,0.040499,0.377934,0.253168,0.777778,0.521431,0.870370,0.725451,
0.993186,0.957598,0.609813,0.568678,0.409938,0.361168,0.062500,0.017564,0.289730,0.240786,
0.516757,0.477554,0.756571,0.721861,0.995676,0.979935,0.446009,0.388569,0.383178,0.353532,
0.302469,0.257899,0.360360,0.291375,0.257862,0.201451,0.213115,0.170614,0.017857,0.007949,
0.285714,0.237015,0.551643,0.487849,0.800469,0.747719,1.000000,0.995029,0.081250,0.021536,
0.299459,0.250688,0.623457,0.508211,0.783951,0.740501,1.000000,0.994014,0.008929,0.003563,
0.298462,0.245061,0.535385,0.488783,0.809231,0.719766,1.000000,0.986607,0.380829,0.360172,
0.401408,0.322661,0.358025,0.317166,0.315294,0.246816,0.273292,0.223645,0.226415,0.204054,
0.008929,0.003563,0.277838,0.230117,0.517857,0.488867,0.773364,0.739421,1.000000,0.989693,
0.017857,0.008980,0.285714,0.238377,0.546948,0.480914,0.821429,0.749521,1.000000,0.994446,
0.026786,0.013506,0.306250,0.264009,0.586420,0.494110,0.827160,0.721291,1.000000,0.991878,
0.339286,0.297264,0.471963,0.444836,0.302469,0.257899,0.327059,0.267141,0.220126,0.158472,
0.250585,0.213592,0.024691,0.009184,0.285714,0.234126,0.546948,0.481998,0.812500,0.739180,
1.000000,0.994179,0.081250,0.018190,0.282383,0.242610,0.549383,0.506159,0.781250,0.754390,
1.000000,0.995860,0.008929,0.003563,0.298462,0.245061,0.535385,0.488783,0.809231,0.719766,
1.000000,0.986607,0.093750,0.058936,0.080247,0.044836,0.077720,0.059076,0.058462,0.043777,
0.063084,0.044028,0.058286,0.036808,0.091549,0.070248,0.119718,0.064680,0.089286,0.066433,
0.062500,0.052126,0.105634,0.069174,0.037037,0.029705,0.049223,0.037995,0.044643,0.022131,
0.080357,0.062016,0.166667,0.112708,0.080247,0.038894,0.037037,0.025180,0.052308,0.044309,
0.031250,0.016939

Positive_77 4.000000,0.437500,3.000000,1.750000,1.500000,0.916667,2.000000,1.750000,2.000000,0.250000,
0.371348,0.011430,0.371348,0.011430,0.000000,0.285714,0.200000,1.000000,0.000000,0.000000,
0.000000,0.166667,0.666667,0.111111,0.250000,0.500000,0.083333,0.055556,0.222222,0.012346,
0.125000,0.250000,0.020833,0.305556,0.333333,0.003086,0.472222,0.666667,0.052469,0.416667,
0.666667,0.027778,0.416667,0.666667,0.083333,2.135779,1.414214,0.662153,1.000000,0.500000,
1.000000,0.500000,1.000000,0.500000,1.000000,0.500000,1.000000,0.500000,0.000000,0.000000,
0.000000,0.000000,0.000000,0.000000,0.000000,0.000000,0.000000,0.000000,1.000000,0.750000,
1.000000,0.750000,1.000000,0.750000,1.000000,0.750000,1.000000,0.250000,0.000000,0.000000,
0.000000,0.000000,0.000000,0.000000,0.000000,0.000000,0.000000,0.000000,0.337165,0.307304,
0.429054,0.376902,0.342949,0.315794,0.257692,0.231542,0.241158,0.195284,0.264024,0.239878,
0.022989,0.010257,0.302682,0.250236,0.547893,0.497015,0.750958,0.730849,1.000000,0.993361,
0.028846,0.011564,0.297297,0.245972,0.522436,0.503208,0.768311,0.748792,1.000000,0.995677,
0.003831,0.002790,0.256410,0.242962,0.496622,0.483861,0.763514,0.742221,1.000000,0.996395,
0.473094,0.443322,0.287356,0.213140,0.397436,0.343538,0.088462,0.078527,0.094915,0.070684,
0.005984,0.002458,0.003831,0.002790,0.263514,0.224003,0.486486,0.462075,0.812261,0.745118,
1.000000,1.000000,0.070946,0.028642,0.363985,0.246524,0.535127,0.473273,0.823755,0.736992,
0.996795,0.979837,0.080460,0.044520,0.304487,0.259913,0.532138,0.515620,0.817308,0.749569,
0.996622,0.922989,0.652466,0.578020,0.423077,0.363743,0.016026,0.011566,0.297297,0.240860,
0.557432,0.493370,0.766284,0.753478,0.998505,0.991702,0.472973,0.396522,0.426282,0.357369,
0.298851,0.246109,0.311892,0.273730,0.253846,0.197596,0.202572,0.178626,0.016026,0.007985,
0.293919,0.250524,0.554487,0.487378,0.767564,0.728717,1.000000,0.998211,0.019157,0.011736,
0.302682,0.251822,0.578544,0.509658,0.790541,0.754406,1.000000,0.997240,0.003831,0.002790,
0.250374,0.234172,0.513453,0.492469,0.747126,0.733643,0.997010,0.974181,0.375000,0.344125,
0.391892,0.323258,0.360153,0.332617,0.243082,0.214446,0.279743,0.226957,0.250847,0.223798,

0.003831,0.002790,0.249042,0.245682,0.506757,0.486762,0.770270,0.744669,1.000000,0.996395,
0.028846,0.011564,0.277027,0.240868,0.573718,0.489700,0.774290,0.728536,1.000000,0.995677,
0.022989,0.010257,0.295019,0.249208,0.563218,0.509167,0.756757,0.747966,1.000000,0.993361,
0.391892,0.315967,0.487179,0.437924,0.298851,0.246109,0.322034,0.275424,0.196154,0.159691,
0.246154,0.216532,0.028846,0.011190,0.277027,0.244817,0.538462,0.485940,0.774290,0.729484,
1.000000,0.995945,0.013514,0.008976,0.287356,0.247930,0.559387,0.503909,0.758621,0.753485,
1.000000,0.999156,0.003831,0.002790,0.250374,0.234172,0.513453,0.492469,0.747126,0.733643,
0.997010,0.974181,0.104730,0.071371,0.062033,0.042670,0.091954,0.073524,0.057692,0.050552,
0.057692,0.039556,0.041667,0.029632,0.104730,0.081888,0.141892,0.082598,0.062033,0.057882,
0.047833,0.043047,0.072797,0.057842,0.029148,0.025313,0.034483,0.028330,0.031390,0.022712,
0.112179,0.074131,0.138514,0.105469,0.066517,0.035057,0.038462,0.027734,0.053640,0.038026,
0.026820,0.012666

Positive_78 23.000000,0.319471,15.000000,7.347826,7.000000,17.782609,18.000000,7.347826,5.000000,31.964427,
0.414262,0.028225,0.414262,0.028225,0.065476,0.242038,0.310924,0.414634,0.583333,0.350000,
0.846154,2.515729,5.866667,3.138024,2.111994,6.000000,3.958713,0.292287,0.888889,0.043032,
0.240177,0.666667,0.030955,0.483577,0.772727,0.030502,0.265310,0.530303,0.010096,0.255121,
0.590909,0.027642,0.374779,0.545455,0.030517,10.313173,5.909609,3.691236,1.000000,0.869565,
1.000000,0.867893,1.000000,0.741780,1.000000,0.604947,1.000000,0.442562,1.000000,0.317271,
1.000000,0.283581,1.000000,0.259795,0.200000,0.085873,0.133333,0.052454,1.000000,0.826087,
1.000000,0.826087,1.000000,0.780245,1.000000,0.635273,1.000000,0.559481,1.000000,0.492822,
1.000000,0.441297,1.000000,0.260965,1.000000,0.164197,0.666667,0.104209,0.372760,0.311524,
0.444444,0.365861,0.360000,0.322615,0.305755,0.227188,0.287162,0.200683,0.296774,0.227322,
0.014925,0.007806,0.320000,0.247308,0.575758,0.495961,0.796667,0.749020,1.000000,0.995685,
0.021930,0.008160,0.288372,0.240478,0.568182,0.493117,0.795349,0.742787,1.000000,0.995738,
0.005714,0.002748,0.307065,0.245039,0.600000,0.503124,0.801639,0.736775,1.000000,0.987301,
0.622703,0.488822,0.253363,0.157108,0.500000,0.354070,0.094595,0.067135,0.107143,0.079095,
0.011982,0.003504,0.005714,0.002748,0.358306,0.244628,0.550000,0.488301,0.841355,0.751219,
1.000000,1.000000,0.233766,0.074857,0.560000,0.273485,0.750000,0.543326,0.835031,0.721203,
0.988571,0.948281,0.122807,0.040872,0.434426,0.256836,0.714286,0.489612,0.874286,0.709954,
0.998258,0.968971,0.676796,0.568311,0.422907,0.364373,0.032258,0.013657,0.357377,0.257316,
0.639344,0.504384,0.806557,0.732853,0.998505,0.984244,0.471380,0.383358,0.441077,0.366512,
0.304813,0.250130,0.324324,0.268736,0.257143,0.187943,0.212838,0.179137,0.032558,0.008837,
0.315682,0.237617,0.568182,0.488342,0.795349,0.740636,1.000000,0.995470,0.028571,0.010006,
0.302632,0.258920,0.549189,0.511789,0.800000,0.746700,1.000000,0.995555,0.005714,0.002748,
0.294054,0.231888,0.531148,0.475358,0.837110,0.743360,1.000000,0.989504,0.397306,0.356129,
0.410774,0.305428,0.401434,0.338443,0.299539,0.209866,0.334459,0.243274,0.266355,0.203440,
0.005714,0.002748,0.342623,0.251269,0.600000,0.500556,0.796721,0.737358,1.000000,0.987537,
0.032558,0.009171,0.321792,0.228777,0.587121,0.480960,0.804651,0.738572,1.000000,0.994395,
0.014925,0.007806,0.308244,0.251925,0.579125,0.501506,0.787293,0.752471,1.000000,0.996332,
0.390572,0.305971,0.484848,0.443899,0.304813,0.250130,0.334459,0.263455,0.212500,0.154073,
0.256131,0.213006,0.032558,0.009271,0.331976,0.238562,0.554348,0.491346,0.823256,0.747284,
1.000000,0.992582,0.028571,0.009248,0.303030,0.257441,0.531429,0.506164,0.782857,0.744710,
1.000000,0.996699,0.005714,0.002748,0.294054,0.231888,0.531148,0.475358,0.837110,0.743360,
1.000000,0.989504,0.114428,0.051556,0.095723,0.061171,0.091429,0.066323,0.079602,0.053850,

0.062857,0.040611,0.058286,0.038013,0.107735,0.064641,0.171717,0.070430,0.109836,0.068139,
0.065378,0.048911,0.076087,0.053307,0.055814,0.026919,0.055258,0.033514,0.053333,0.024079,
0.101010,0.069097,0.132576,0.099664,0.081433,0.052804,0.040000,0.022527,0.061404,0.039517,
0.030702,0.014925

Positive_79 13.000000,0.242604,6.000000,3.153846,4.000000,4.307692,6.000000,3.153846,4.000000,3.974359,
0.389211,0.072394,0.389211,0.072394,0.292683,0.413793,0.058824,0.000000,0.625000,0.166667,
0.000000,0.635897,2.333333,0.564623,0.720513,2.166667,0.526303,0.129316,0.388889,0.018854,
0.160342,0.361111,0.021059,0.210897,0.333333,0.017614,0.246368,0.500000,0.025625,0.198504,
0.366667,0.018075,0.204915,0.354167,0.023607,4.612389,3.617126,1.723403,1.000000,0.615385,
1.000000,0.564103,1.000000,0.353846,1.000000,0.323077,1.000000,0.317949,1.000000,0.261538,
1.000000,0.205128,1.000000,0.205128,1.000000,0.205128,1.000000,0.200000,1.000000,0.692308,
1.000000,0.641026,1.000000,0.512821,1.000000,0.358974,1.000000,0.358974,1.000000,0.333333,
1.000000,0.220513,1.000000,0.220513,1.000000,0.220513,1.000000,0.215385,0.326087,0.296013,
0.412331,0.370226,0.377451,0.333761,0.240876,0.202256,0.242812,0.204095,0.298103,0.259515,
0.072464,0.013227,0.369565,0.252639,0.615942,0.503775,0.818841,0.748085,1.000000,0.995323,
0.021739,0.008370,0.312500,0.246181,0.557292,0.488677,0.778646,0.730926,1.000000,0.995782,
0.007246,0.002644,0.267516,0.243529,0.530401,0.487296,0.794595,0.749496,1.000000,0.991784,
0.622703,0.461790,0.297101,0.188075,0.462366,0.350135,0.109489,0.076833,0.108374,0.076579,
0.009585,0.003997,0.007246,0.002644,0.307292,0.229292,0.610790,0.481616,0.816956,0.717380,
1.000000,1.000000,0.157838,0.050951,0.384016,0.253180,0.590643,0.446449,0.804657,0.710401,
0.994792,0.944925,0.159923,0.050780,0.394904,0.268775,0.697452,0.520141,0.891304,0.753649,
0.996454,0.976235,0.684878,0.600656,0.433393,0.349192,0.034314,0.011083,0.322581,0.275695,
0.582803,0.508327,0.797297,0.739471,0.998227,0.991407,0.440000,0.389648,0.442029,0.363656,
0.292994,0.246696,0.315271,0.276201,0.223938,0.195537,0.198187,0.175454,0.036232,0.009194,
0.289062,0.238512,0.528646,0.485905,0.778646,0.731550,1.000000,0.997501,0.028986,0.009710,
0.318841,0.253883,0.586957,0.507996,0.799283,0.763914,1.000000,0.996177,0.007246,0.002644,
0.307992,0.252204,0.547206,0.485876,0.788054,0.732706,1.000000,0.994726,0.397849,0.366322,
0.356098,0.313428,0.347826,0.320251,0.284173,0.239613,0.274760,0.237327,0.239382,0.185544,
0.007246,0.002644,0.277838,0.248316,0.526520,0.486161,0.789809,0.750683,1.000000,0.991784,
0.036232,0.009485,0.289062,0.240513,0.541667,0.490007,0.791423,0.730842,1.000000,0.995782,
0.072464,0.013227,0.369565,0.256638,0.615942,0.502600,0.826087,0.749110,1.000000,0.995619,
0.369942,0.314599,0.492754,0.438706,0.292994,0.246696,0.297852,0.264878,0.191120,0.160085,
0.234987,0.210906,0.036232,0.010224,0.317708,0.241831,0.544271,0.495748,0.786458,0.740578,
1.000000,0.994624,0.028986,0.008363,0.289855,0.245836,0.550725,0.498330,0.788530,0.756647,
1.000000,0.996973,0.007246,0.002644,0.307992,0.252204,0.547206,0.485876,0.788054,0.732706,
1.000000,0.994726,0.062500,0.044907,0.082803,0.060307,0.094203,0.064637,0.067437,0.052112,
0.053191,0.031878,0.060510,0.042171,0.100358,0.069475,0.102941,0.074568,0.079922,0.065888,
0.076023,0.052556,0.069714,0.050941,0.034682,0.024238,0.044324,0.032561,0.041081,0.024108,
0.102941,0.070410,0.136525,0.101519,0.094203,0.053041,0.047771,0.024677,0.060932,0.047612,
0.024510,0.012393

Positive_80 4.000000,0.562500,4.000000,2.250000,2.000000,1.583333,4.000000,2.250000,2.000000,2.250000,
0.516543,0.049307,0.516543,0.049307,0.000000,0.222222,0.285714,0.000000,0.800000,0.000000,
0.000000,1.312500,2.250000,0.890625,0.979167,2.250000,1.335069,0.515625,0.750000,0.125977,
0.279514,0.562500,0.104179,0.770833,1.000000,0.061921,0.687500,1.000000,0.057292,0.743056,

1.000000,0.091242,0.743056,1.000000,0.091242,2.700478,1.130641,0.655035,1.000000,0.750000,
1.000000,0.750000,1.000000,0.750000,1.000000,0.666667,0.500000,0.125000,0.500000,0.125000,
0.166667,0.041667,0.166667,0.041667,0.166667,0.041667,0.166667,0.041667,1.000000,0.500000,
1.000000,0.500000,1.000000,0.500000,1.000000,0.416667,1.000000,0.375000,1.000000,0.375000,
0.333333,0.125000,0.333333,0.125000,0.333333,0.125000,0.333333,0.125000,0.312672,0.273000,
0.479290,0.390982,0.377778,0.336018,0.234483,0.207544,0.202759,0.169059,0.327381,0.291733,
0.017751,0.011127,0.322222,0.265485,0.533333,0.490729,0.911243,0.760364,1.000000,0.997222,
0.016667,0.010112,0.244936,0.206960,0.548803,0.476643,0.855556,0.754051,1.000000,0.993148,
0.005917,0.003673,0.349112,0.279934,0.568047,0.516110,0.761708,0.734025,0.994490,0.980870,
0.579882,0.521842,0.366667,0.225298,0.390533,0.252860,0.111732,0.081216,0.067586,0.047329,
0.004138,0.001034,0.005917,0.003673,0.316667,0.233004,0.555556,0.469330,0.804734,0.740711,
1.000000,1.000000,0.254438,0.130338,0.388889,0.340730,0.607438,0.514270,0.836096,0.675649,
0.990792,0.950006,0.112426,0.052976,0.508876,0.258414,0.609467,0.381167,0.845730,0.544673,
0.988981,0.828475,0.615101,0.535290,0.391061,0.356545,0.053254,0.022432,0.360947,0.259006,
0.579882,0.522913,0.757576,0.730706,0.994475,0.978209,0.485207,0.400559,0.418733,0.358622,
0.265193,0.240819,0.308966,0.278348,0.240223,0.193946,0.206704,0.182024,0.016667,0.009079,
0.261510,0.202855,0.517495,0.453120,0.794444,0.727338,1.000000,0.993140,0.082840,0.026939,
0.325444,0.271245,0.544379,0.521910,0.758953,0.741127,1.000000,0.992146,0.005917,0.003673,
0.479290,0.306984,0.674556,0.529771,0.828402,0.762203,1.000000,0.996581,0.405556,0.368985,
0.431953,0.332519,0.341598,0.298496,0.303571,0.255684,0.262069,0.212056,0.202759,0.189023,
0.005917,0.003673,0.360947,0.285326,0.591716,0.528503,0.776860,0.741957,1.000000,0.986886,
0.016667,0.011490,0.246777,0.194305,0.535912,0.460378,0.816667,0.728112,0.995868,0.990378,
0.017751,0.009286,0.313609,0.261797,0.544379,0.499775,0.911243,0.762086,1.000000,0.997222,
0.349112,0.317604,0.488981,0.441576,0.265193,0.240819,0.292414,0.267792,0.212291,0.162936,
0.234637,0.213036,0.016667,0.009079,0.239411,0.186456,0.532228,0.429449,0.771639,0.714556,
1.000000,0.990385,0.053254,0.019542,0.295858,0.268692,0.538889,0.511964,0.783333,0.759314,
1.000000,0.992491,0.005917,0.003673,0.479290,0.306984,0.674556,0.529771,0.828402,0.762203,
1.000000,0.996581,0.049587,0.044452,0.061111,0.049047,0.077135,0.050856,0.066667,0.049580,
0.053254,0.036315,0.077778,0.042750,0.082840,0.070690,0.094675,0.065376,0.089532,0.074040,
0.077778,0.057920,0.112426,0.064494,0.044199,0.025497,0.044199,0.032966,0.029466,0.018461,
0.088889,0.070520,0.122222,0.110524,0.050964,0.047657,0.029586,0.024102,0.077778,0.051206,
0.017751,0.013549

Positive_81 32.000000,0.147461,28.000000,4.718750,0.000000,93.886089,6.000000,4.718750,6.000000,4.918347,
0.274447,0.009001,0.274447,0.009001,0.170068,0.606557,0.625000,0.777778,0.750000,1.000000,
0.000000,0.283854,4.250000,0.650251,1.134375,1.500000,0.377813,0.025360,0.500000,0.008350,
0.200312,0.250000,0.009810,0.032342,0.161290,0.003767,0.009157,0.137097,0.000677,0.036593,
0.048387,0.000393,0.678730,0.870968,0.067740,11.953315,1.830102,1.732051,1.000000,0.218750,
1.000000,0.217679,1.000000,0.209229,1.000000,0.184539,1.000000,0.156647,0.592885,0.099442,
0.375494,0.063109,0.189723,0.032132,0.059289,0.010217,0.007937,0.001401,1.000000,0.812500,
1.000000,0.812500,1.000000,0.812500,1.000000,0.764583,1.000000,0.385417,1.000000,0.385417,
0.600000,0.354167,0.600000,0.306250,0.400000,0.204167,0.100000,0.051042,0.473171,0.335782,
0.453008,0.362203,0.368511,0.302015,0.298165,0.235624,0.250000,0.191471,0.299830,0.217119,
0.069182,0.011160,0.310000,0.236846,0.626984,0.497284,0.840000,0.742663,1.000000,0.992095,
0.021429,0.005948,0.312195,0.234641,0.570732,0.484291,0.817505,0.738201,1.000000,0.996247,

0.007143,0.002139,0.452000,0.277043,0.625610,0.507785,0.816000,0.740408,1.000000,0.993948,
0.632597,0.461773,0.336898,0.166114,0.529468,0.372113,0.107527,0.063047,0.098901,0.077601,
0.015773,0.005483,0.007143,0.002139,0.288641,0.210265,0.595978,0.466781,0.812925,0.745656,
1.000000,1.000000,0.289024,0.078558,0.609756,0.328293,0.767805,0.530075,0.916098,0.718980,
0.998780,0.935925,0.479452,0.051080,0.525114,0.270066,0.817352,0.524765,0.894977,0.738166,
0.998081,0.970191,0.660377,0.517564,0.419355,0.341326,0.068966,0.010366,0.512195,0.284985,
0.642683,0.505625,0.819512,0.726763,0.998638,0.988301,0.464286,0.378854,0.427317,0.371535,
0.319512,0.249611,0.352518,0.273823,0.248996,0.187421,0.263441,0.185151,0.021429,0.005890,
0.296089,0.229337,0.543762,0.480827,0.804469,0.734397,1.000000,0.997911,0.027586,0.007960,
0.356354,0.259682,0.607143,0.508788,0.813793,0.745527,1.000000,0.993745,0.007143,0.002139,
0.380220,0.253846,0.605119,0.504247,0.833638,0.747547,1.000000,0.991351,0.396596,0.328913,
0.392045,0.311325,0.492683,0.359762,0.291312,0.199644,0.298611,0.226792,0.279817,0.217386,
0.007143,0.002139,0.436000,0.281425,0.628049,0.512206,0.808000,0.743494,1.000000,0.994331,
0.021429,0.006417,0.309125,0.224942,0.569878,0.472432,0.817505,0.732520,1.000000,0.994312,
0.069182,0.010974,0.312088,0.246840,0.606576,0.503279,0.835000,0.746668,1.000000,0.992768,
0.390977,0.309776,0.484255,0.440614,0.319512,0.249611,0.330935,0.265363,0.207880,0.156586,
0.295699,0.215985,0.028571,0.006156,0.286778,0.228732,0.540027,0.482460,0.785617,0.731856,
1.000000,0.996748,0.027586,0.006586,0.342541,0.257522,0.592857,0.502346,0.806897,0.747845,
1.000000,0.995944,0.007143,0.002139,0.380220,0.253846,0.605119,0.504247,0.833638,0.747547,
1.000000,0.991351,0.121951,0.062480,0.111940,0.065947,0.109589,0.073742,0.076829,0.052016,
0.082596,0.045948,0.075862,0.035649,0.111878,0.068941,0.121212,0.074890,0.107191,0.062833,
0.073059,0.051095,0.092857,0.053566,0.041985,0.023980,0.055172,0.026898,0.028571,0.015513,
0.124138,0.067868,0.148080,0.101863,0.069028,0.046465,0.041379,0.024682,0.066038,0.036655,
0.018622,0.008969

Positive_82 54.000000,0.239712,21.000000,12.944444,15.000000,46.091195,45.000000,12.944444,7.000000,144.506289,
0.603175,0.056304,0.603175,0.056304,0.004292,0.103448,0.195513,0.133466,0.147126,0.371968,
0.197425,5.808709,13.523810,24.814293,4.070958,14.892857,24.781242,0.364827,0.643991,0.059260,
0.190322,0.531888,0.042288,0.450186,0.641509,0.033874,0.228786,0.358491,0.006231,0.181981,
0.528302,0.012896,0.239632,0.339623,0.017308,20.412013,11.551205,7.758521,1.000000,0.962963,
1.000000,0.962963,1.000000,0.958781,1.000000,0.908803,0.895238,0.758387,0.814286,0.577576,
0.804762,0.514707,0.669118,0.325365,0.669118,0.302514,0.470588,0.189109,1.000000,0.814815,
1.000000,0.814815,1.000000,0.800526,1.000000,0.702456,1.000000,0.566740,1.000000,0.449857,
1.000000,0.402392,0.551471,0.247189,0.415344,0.138157,0.273846,0.074738,0.323887,0.280722,
0.429119,0.358772,0.398866,0.360506,0.234432,0.191096,0.232604,0.198528,0.325301,0.262118,
0.072785,0.017648,0.316103,0.263983,0.612613,0.508680,0.855856,0.743231,1.000000,0.992584,
0.032258,0.008474,0.313093,0.246194,0.589796,0.504395,0.785489,0.741034,1.000000,0.994601,
0.004348,0.002391,0.307377,0.229112,0.571721,0.478856,0.804348,0.746317,1.000000,0.993852,
0.606947,0.398014,0.307130,0.139042,0.688525,0.462944,0.120301,0.060113,0.119355,0.081336,
0.013369,0.004218,0.004348,0.002391,0.395257,0.256060,0.643678,0.515403,0.858934,0.766283,
1.000000,1.000000,0.264264,0.073780,0.446640,0.189506,0.778420,0.539543,0.939453,0.724221,
0.997976,0.935391,0.155440,0.032648,0.500000,0.277267,0.704348,0.491896,0.912249,0.732132,
0.993443,0.956929,0.733607,0.638985,0.400000,0.331300,0.046763,0.010365,0.320144,0.251991,
0.582734,0.490972,0.817391,0.736757,1.000000,0.988910,0.465789,0.372305,0.405145,0.364969,
0.317580,0.262726,0.354839,0.268499,0.262048,0.203210,0.226640,0.183710,0.032258,0.008437,

0.292220,0.245110,0.556291,0.500407,0.808044,0.745142,1.000000,0.995730,0.057377,0.009819,
0.294235,0.239837,0.546547,0.488984,0.804805,0.727152,1.000000,0.993455,0.004348,0.002391,
0.369775,0.251889,0.612705,0.506359,0.834225,0.767247,1.000000,0.993662,0.432432,0.387113,
0.381295,0.307226,0.345521,0.305661,0.303030,0.243310,0.262357,0.230989,0.215873,0.179935,
0.004348,0.002391,0.319672,0.231887,0.577869,0.479797,0.791304,0.745336,1.000000,0.993986,
0.032258,0.009017,0.309298,0.237868,0.589404,0.497972,0.800000,0.739503,1.000000,0.994450,
0.072785,0.017406,0.316406,0.264771,0.578804,0.511552,0.804805,0.746122,1.000000,0.992721,
0.384892,0.299430,0.491837,0.437844,0.317580,0.262726,0.361011,0.256794,0.210843,0.163928,
0.272366,0.222992,0.062893,0.010631,0.310881,0.239399,0.589796,0.492550,0.808044,0.732490,
1.000000,0.993233,0.032787,0.008853,0.297414,0.244827,0.549407,0.493341,0.782609,0.738174,
1.000000,0.994481,0.004348,0.002391,0.369775,0.251889,0.612705,0.506359,0.834225,0.767247,
1.000000,0.993662,0.091954,0.049210,0.090909,0.063599,0.073314,0.054489,0.063985,0.044079,
0.061657,0.032975,0.061914,0.036371,0.106952,0.069095,0.120690,0.067039,0.083004,0.064423,
0.082902,0.054794,0.069583,0.051875,0.050314,0.024939,0.053016,0.026607,0.043478,0.021000,
0.107759,0.075578,0.157233,0.109246,0.081579,0.056310,0.047170,0.027589,0.093117,0.056304,
0.032787,0.014479

Positive_83 29.000000,0.054697,6.000000,1.586207,1.000000,2.394089,9.000000,1.586207,1.000000,7.036946,
0.417737,0.031599,0.417737,0.031599,0.021739,0.244444,0.529412,0.312500,0.090909,0.400000,
0.833333,0.005747,0.166667,0.000958,0.017241,0.500000,0.008621,0.000958,0.027778,0.000027,
0.008621,0.250000,0.002155,0.094007,0.285714,0.017273,0.020525,0.047619,0.000485,0.029557,
0.321429,0.006817,0.048030,0.178571,0.003920,5.196152,2.524338,2.000000,1.000000,0.448276,
1.000000,0.448276,1.000000,0.445977,1.000000,0.434483,1.000000,0.360920,1.000000,0.149425,
0.666667,0.133333,0.333333,0.013793,0.000000,0.000000,0.000000,0.000000,1.000000,0.241379,
1.000000,0.241379,1.000000,0.241379,1.000000,0.241379,1.000000,0.241379,1.000000,0.235632,
1.000000,0.215517,0.611111,0.063218,0.583333,0.060345,0.361111,0.037356,0.398907,0.294176,
0.451456,0.374002,0.398866,0.331821,0.234146,0.199370,0.247253,0.193096,0.337104,0.253419,
0.048507,0.011381,0.334711,0.252194,0.572687,0.490009,0.804916,0.745505,1.000000,0.992714,
0.033962,0.008087,0.313093,0.247418,0.600000,0.496156,0.803797,0.740125,1.000000,0.993552,
0.005464,0.002578,0.317164,0.245586,0.570896,0.496992,0.802239,0.746558,1.000000,0.985743,
0.615672,0.408507,0.337089,0.139394,0.683060,0.452099,0.118462,0.054901,0.111498,0.078032,
0.007752,0.001781,0.005464,0.002578,0.330171,0.228212,0.590278,0.455431,0.843750,0.710380,
1.000000,1.000000,0.337629,0.071972,0.440299,0.214445,0.829897,0.465741,0.966495,0.618880,
0.996875,0.760923,0.081967,0.025396,0.485075,0.279833,0.667401,0.511513,0.868243,0.789447,
0.996970,0.979955,0.688144,0.599369,0.450549,0.347756,0.082090,0.014720,0.447761,0.274719,
0.630597,0.508843,0.809701,0.734371,0.998464,0.985345,0.461165,0.376316,0.435811,0.357795,
0.317580,0.265889,0.371212,0.264263,0.237082,0.197639,0.269231,0.192024,0.030189,0.007957,
0.297968,0.240206,0.548611,0.495833,0.819672,0.745319,1.000000,0.994343,0.043716,0.009582,
0.351240,0.244343,0.586777,0.493759,0.797521,0.738590,1.000000,0.989180,0.005464,0.002578,
0.388060,0.264788,0.640777,0.497330,0.825243,0.751331,1.000000,0.993261,0.427221,0.363845,
0.404612,0.312042,0.453552,0.324113,0.305430,0.230474,0.307692,0.229467,0.233193,0.190257,
0.005464,0.002578,0.317164,0.250841,0.604478,0.495903,0.813433,0.746122,1.000000,0.988597,
0.033962,0.008087,0.309298,0.232910,0.581132,0.488710,0.837736,0.731008,1.000000,0.992562,
0.048507,0.010992,0.338843,0.257776,0.572687,0.503355,0.798771,0.754318,1.000000,0.993294,
0.393204,0.302785,0.483108,0.431326,0.317580,0.265889,0.393939,0.260777,0.188450,0.157983,

0.280220,0.231679,0.030189,0.008881,0.302083,0.233907,0.563567,0.497199,0.819672,0.743970,
1.000000,0.993979,0.043716,0.009348,0.286052,0.244475,0.572104,0.495288,0.792188,0.737445,
1.000000,0.991962,0.005464,0.002578,0.388060,0.264788,0.640777,0.497330,0.825243,0.751331,
1.000000,0.993261,0.082645,0.051306,0.082278,0.058624,0.118243,0.062098,0.081967,0.046070,
0.062500,0.041001,0.065574,0.035079,0.114537,0.064678,0.121212,0.076933,0.094340,0.067578,
0.087379,0.047527,0.089552,0.055327,0.054645,0.029936,0.060094,0.032024,0.030837,0.018204,
0.121359,0.067637,0.146907,0.106658,0.066414,0.045323,0.047170,0.028829,0.077505,0.047509,
0.044776,0.017661

Positive_84 6.000000,0.305556,4.000000,1.833333,1.500000,3.366667,3.000000,1.833333,2.000000,1.366667,
0.720302,0.030664,0.720302,0.030664,0.000000,0.000000,0.000000,0.000000,0.272727,0.375000,
0.200000,1.000000,2.000000,1.200000,1.333333,2.000000,1.066667,0.333333,1.000000,0.166667,
0.555556,1.000000,0.207407,0.166667,0.400000,0.034667,0.300000,0.800000,0.088000,0.311111,
0.600000,0.072296,0.355556,0.600000,0.079407,3.020448,1.000000,0.936426,1.000000,0.500000,
1.000000,0.500000,1.000000,0.500000,1.000000,0.500000,1.000000,0.500000,1.000000,0.500000,
1.000000,0.388889,1.000000,0.277778,1.000000,0.277778,0.166667,0.055556,1.000000,0.666667,
1.000000,0.666667,1.000000,0.666667,1.000000,0.666667,1.000000,0.666667,1.000000,0.666667,
1.000000,0.555556,1.000000,0.444444,1.000000,0.444444,0.000000,0.000000,0.374449,0.337072,
0.372822,0.324568,0.353659,0.338360,0.247350,0.227703,0.227425,0.203968,0.278846,0.240183,
0.026132,0.012218,0.292254,0.249064,0.546667,0.492742,0.786667,0.731048,1.000000,0.997118,
0.019185,0.010902,0.352423,0.255271,0.544365,0.518777,0.805755,0.746669,1.000000,0.991322,
0.004405,0.002964,0.240418,0.228262,0.519824,0.482847,0.780488,0.740978,1.000000,0.992945,
0.502203,0.377621,0.180617,0.093508,0.697842,0.528872,0.084806,0.039638,0.110368,0.090893,
0.010033,0.004169,0.004405,0.002964,0.316901,0.241590,0.514085,0.474609,0.791367,0.731733,
1.000000,1.000000,0.549161,0.185423,0.549161,0.276222,0.750600,0.376852,0.752998,0.558826,
0.989437,0.749574,0.030837,0.014467,0.409692,0.270857,0.655052,0.535553,0.846690,0.781844,
0.998258,0.976774,0.649165,0.605275,0.389423,0.347615,0.036667,0.014368,0.279236,0.248848,
0.541812,0.484082,0.785714,0.713173,0.996516,0.989089,0.367596,0.326730,0.433333,0.405761,
0.273381,0.267509,0.302885,0.268512,0.183946,0.170576,0.230088,0.206422,0.014388,0.010103,
0.312775,0.241848,0.510000,0.481931,0.774580,0.730504,0.995204,0.978091,0.017621,0.010427,
0.278169,0.247461,0.524648,0.497648,0.763723,0.744584,1.000000,0.995561,0.004405,0.002964,
0.273519,0.240650,0.577093,0.521694,0.820423,0.766319,1.000000,0.997044,0.391408,0.377567,
0.311847,0.257146,0.414097,0.365287,0.240838,0.200872,0.297659,0.254561,0.212014,0.196274,
0.004405,0.002964,0.264916,0.246241,0.541850,0.505027,0.794425,0.752042,1.000000,0.993342,
0.019185,0.010902,0.343612,0.240975,0.544365,0.497677,0.796163,0.727970,0.995204,0.968115,
0.026132,0.012218,0.288732,0.247180,0.546667,0.497015,0.786667,0.733968,1.000000,0.998679,
0.306620,0.266191,0.489437,0.466300,0.273381,0.267509,0.278846,0.261165,0.157191,0.136306,
0.265487,0.240692,0.019185,0.011698,0.280000,0.233252,0.513333,0.473901,0.774580,0.717167,
0.995204,0.977357,0.017621,0.010029,0.267606,0.244592,0.529976,0.498051,0.763723,0.745234,
1.000000,0.995561,0.004405,0.002964,0.273519,0.240650,0.577093,0.521694,0.820423,0.766319,
1.000000,0.997044,0.083700,0.058845,0.080000,0.061291,0.096916,0.077253,0.061674,0.051224,
0.062053,0.046379,0.063380,0.042080,0.073171,0.051148,0.085919,0.065556,0.081882,0.056058,
0.053333,0.042205,0.048780,0.042178,0.039648,0.028215,0.057279,0.039208,0.031690,0.018361,
0.080139,0.063153,0.133333,0.115524,0.073944,0.061371,0.035971,0.020867,0.059233,0.042063,
0.026432,0.017021

Positive_85 8.000000,0.187500,2.000000,1.500000,2.000000,0.857143,4.000000,1.500000,1.000000,3.142857,
0.579938,0.095485,0.579938,0.095485,0.000000,0.000000,0.500000,0.333333,0.000000,0.000000,
0.000000,0.500000,2.000000,0.857143,0.500000,2.000000,0.857143,0.250000,1.000000,0.214286,
0.250000,1.000000,0.214286,0.250000,0.428571,0.039359,0.035714,0.142857,0.004373,0.035714,
0.142857,0.004373,0.071429,0.142857,0.005831,2.828427,2.000000,0.000000,1.000000,0.750000,
1.000000,0.750000,1.000000,0.750000,1.000000,0.750000,1.000000,0.750000,1.000000,0.750000,
1.000000,0.250000,1.000000,0.250000,1.000000,0.250000,1.000000,0.250000,1.000000,0.500000,
1.000000,0.500000,1.000000,0.500000,1.000000,0.458333,1.000000,0.375000,1.000000,0.375000,
1.000000,0.291667,1.000000,0.291667,1.000000,0.250000,1.000000,0.250000,0.374468,0.304470,
0.457792,0.374222,0.377551,0.321308,0.290598,0.225857,0.229039,0.193888,0.314583,0.243391,
0.028090,0.009960,0.310345,0.274926,0.591954,0.527878,0.820225,0.742107,1.000000,0.993852,
0.025532,0.010989,0.293617,0.234768,0.571429,0.503789,0.804255,0.763390,1.000000,0.992456,
0.005618,0.002975,0.281609,0.231737,0.522989,0.470039,0.759184,0.711558,1.000000,0.992829,
0.626623,0.470584,0.316310,0.164748,0.534694,0.364668,0.085809,0.059019,0.097917,0.072618,
0.014409,0.004012,0.005618,0.002975,0.328482,0.245601,0.710638,0.545836,0.868085,0.814256,
1.000000,1.000000,0.178723,0.072572,0.292135,0.219226,0.763265,0.499790,0.842857,0.667097,
0.997959,0.890784,0.198276,0.054963,0.514368,0.271523,0.600575,0.495049,0.841954,0.684046,
0.993763,0.969272,0.630612,0.547311,0.403909,0.357478,0.034483,0.014360,0.282468,0.238736,
0.537356,0.465613,0.758621,0.703216,0.995918,0.989664,0.464286,0.393109,0.404082,0.362226,
0.280899,0.244665,0.316384,0.289218,0.204167,0.187800,0.200409,0.166375,0.025532,0.010739,
0.310638,0.236848,0.608511,0.503815,0.859574,0.778791,1.000000,0.996701,0.034483,0.013002,
0.307471,0.244370,0.534483,0.493653,0.764368,0.719790,1.000000,0.996397,0.005618,0.002975,
0.353896,0.240233,0.581169,0.493326,0.767347,0.725868,0.995842,0.986406,0.400000,0.351249,
0.415584,0.318867,0.412766,0.329884,0.285417,0.221620,0.259714,0.230889,0.273504,0.215262,
0.005618,0.002975,0.304598,0.234600,0.531609,0.477242,0.759184,0.713409,1.000000,0.992829,
0.025532,0.011508,0.323404,0.234910,0.612766,0.503501,0.855319,0.773480,1.000000,0.992456,
0.028090,0.009960,0.298851,0.271900,0.591954,0.519034,0.820225,0.735181,1.000000,0.993852,
0.362069,0.303090,0.509740,0.452245,0.280899,0.244665,0.332248,0.284677,0.167147,0.151320,
0.243354,0.202855,0.025532,0.010739,0.361702,0.241700,0.638298,0.504354,0.876596,0.766667,
1.000000,0.991089,0.022472,0.009618,0.287356,0.241699,0.528090,0.491854,0.772727,0.742958,
1.000000,0.996397,0.005618,0.002975,0.353896,0.240233,0.581169,0.493326,0.767347,0.725868,
0.995842,0.986406,0.080725,0.062764,0.071429,0.047916,0.101124,0.070000,0.068966,0.052076,
0.048851,0.033950,0.055319,0.037763,0.091954,0.062727,0.085106,0.065706,0.106029,0.070665,
0.077922,0.051915,0.152597,0.067854,0.038298,0.025414,0.061798,0.029940,0.037422,0.022165,
0.085667,0.064364,0.149351,0.108016,0.073469,0.048133,0.038298,0.026533,0.067347,0.042038,
0.018122,0.010060

Positive_86 11.000000,0.355372,7.000000,3.909091,5.000000,5.290909,9.000000,3.909091,3.000000,9.690909,
0.572288,0.012108,0.572288,0.012108,0.000000,0.000000,0.093023,0.153846,0.363636,0.714286,
0.833333,1.268831,2.428571,0.749666,0.940115,3.222222,1.374633,0.266413,0.360000,0.019112,
0.135465,0.358025,0.025131,0.512165,0.800000,0.045823,0.169740,0.420000,0.019509,0.131313,
0.466667,0.030582,0.321717,0.533333,0.049447,5.492929,3.057385,1.432804,1.000000,0.818182,
1.000000,0.818182,1.000000,0.818182,1.000000,0.818182,1.000000,0.818182,1.000000,0.818182,
1.000000,0.596104,0.500000,0.133333,0.200000,0.035498,0.000000,0.000000,1.000000,0.727273,
1.000000,0.727273,1.000000,0.727273,1.000000,0.727273,1.000000,0.708297,1.000000,0.438456,

0.733333,0.225397,0.400000,0.087807,0.133333,0.037951,0.000000,0.000000,0.324397,0.296533,
0.422078,0.393623,0.337469,0.309844,0.266129,0.219424,0.214618,0.192711,0.293532,0.252818,
0.026810,0.007994,0.279522,0.246104,0.525469,0.496701,0.804185,0.752851,1.000000,0.993928,
0.014815,0.006426,0.296804,0.236691,0.545455,0.496946,0.764706,0.739804,1.000000,0.998072,
0.003247,0.001962,0.306173,0.261221,0.538206,0.499535,0.759857,0.739114,1.000000,0.994721,
0.587131,0.440487,0.240260,0.149411,0.523592,0.410101,0.100977,0.067924,0.101786,0.082186,
0.009772,0.004010,0.003247,0.001962,0.274194,0.216647,0.539427,0.498988,0.790885,0.757679,
1.000000,1.000000,0.322259,0.086817,0.397608,0.240194,0.597403,0.408429,0.860390,0.653167,
0.982175,0.881375,0.162963,0.038459,0.453083,0.285897,0.614973,0.521194,0.818996,0.755310,
0.998339,0.962925,0.684588,0.645417,0.378109,0.334925,0.012407,0.006878,0.300268,0.273028,
0.545590,0.506704,0.756272,0.729571,0.998339,0.989637,0.470779,0.405468,0.381142,0.345749,
0.297683,0.248783,0.326733,0.288038,0.231183,0.194441,0.226786,0.178861,0.012346,0.007343,
0.272451,0.237704,0.542208,0.488464,0.758025,0.734111,1.000000,0.997618,0.024129,0.008573,
0.303704,0.257164,0.553086,0.502011,0.775309,0.744983,1.000000,0.995286,0.003247,0.001962,
0.298954,0.244478,0.536622,0.504402,0.793226,0.762788,1.000000,0.993151,0.381462,0.339858,
0.386364,0.333312,0.353887,0.326829,0.273632,0.233753,0.262500,0.234366,0.241935,0.204544,
0.003247,0.001962,0.304933,0.263369,0.538206,0.502561,0.770609,0.743177,1.000000,0.995511,
0.014815,0.007729,0.299848,0.235994,0.542208,0.487274,0.761141,0.734503,1.000000,0.997341,
0.021448,0.007506,0.292975,0.243355,0.538874,0.500370,0.805680,0.755817,1.000000,0.995948,
0.415584,0.336894,0.450533,0.414323,0.297683,0.248783,0.326733,0.284833,0.188510,0.158399,
0.271429,0.214903,0.029221,0.010053,0.267884,0.236504,0.555195,0.491952,0.782468,0.741385,
1.000000,0.996540,0.024129,0.007635,0.291358,0.255334,0.545679,0.497493,0.761787,0.741153,
1.000000,0.996928,0.003247,0.001962,0.298954,0.244478,0.536622,0.504402,0.793226,0.762788,
1.000000,0.993151,0.069479,0.051491,0.074675,0.060044,0.080670,0.062749,0.064935,0.052306,
0.044444,0.031347,0.053619,0.038595,0.129870,0.084814,0.110390,0.083832,0.086379,0.064487,
0.061688,0.051456,0.061662,0.048724,0.044444,0.030296,0.049911,0.030015,0.029491,0.019850,
0.099256,0.072445,0.119107,0.091060,0.068100,0.049551,0.037221,0.027585,0.057041,0.039667,
0.018767,0.009685

Positive_87 13.000000,0.207101,13.000000,2.692308,0.000000,27.064103,3.000000,2.692308,3.000000,0.230769,
0.340458,0.051621,0.340458,0.051621,0.200000,0.500000,0.357143,0.444444,0.200000,0.000000,
0.500000,0.414201,2.692308,1.022227,2.000000,2.000000,0.000000,0.031862,0.207101,0.006049,
0.769231,1.000000,0.025641,0.034517,0.166667,0.004339,0.032544,0.211538,0.006311,0.153846,
0.166667,0.000979,0.923077,1.000000,0.002849,5.727585,1.481476,0.000000,1.000000,0.230769,
1.000000,0.230769,1.000000,0.216963,1.000000,0.179487,0.777778,0.126890,0.500000,0.077909,
0.388889,0.059500,0.277778,0.043064,0.194444,0.028764,0.111111,0.016437,1.000000,1.000000,
1.000000,1.000000,1.000000,1.000000,1.000000,1.000000,1.000000,0.538462,1.000000,0.538462,
1.000000,0.538462,1.000000,0.538462,0.000000,0.000000,0.000000,0.000000,0.367347,0.318119,
0.417143,0.363961,0.368421,0.317920,0.257880,0.219239,0.262799,0.204632,0.265306,0.229518,
0.028571,0.009730,0.288571,0.262785,0.589831,0.522580,0.797143,0.754304,1.000000,0.995955,
0.027119,0.010926,0.272727,0.239271,0.554286,0.497050,0.805714,0.752248,1.000000,0.993984,
0.003509,0.002802,0.258481,0.229650,0.528986,0.470315,0.771261,0.717953,1.000000,0.992151,
0.559633,0.475474,0.249541,0.123634,0.480702,0.400892,0.086397,0.060706,0.123239,0.085864,
0.020478,0.005788,0.003509,0.002802,0.291743,0.255674,0.620000,0.500660,0.862857,0.780432,
1.000000,1.000000,0.287390,0.073232,0.433028,0.244233,0.677064,0.421227,0.847706,0.581591,

0.997067,0.881508,0.088571,0.042921,0.315254,0.243842,0.571930,0.493775,0.798701,0.716948,
0.991922,0.962001,0.586538,0.552268,0.421769,0.372354,0.030508,0.019296,0.324675,0.244022,
0.536349,0.483513,0.778675,0.709101,0.996610,0.988403,0.431429,0.362776,0.389610,0.344346,
0.350340,0.292878,0.327206,0.242932,0.260745,0.211250,0.269625,0.206099,0.020339,0.010165,
0.254286,0.225013,0.554286,0.468235,0.828571,0.760418,1.000000,0.994655,0.029326,0.012643,
0.292271,0.241548,0.562802,0.471927,0.792271,0.721793,1.000000,0.997311,0.003509,0.002802,
0.359322,0.272188,0.583051,0.538702,0.809295,0.757684,0.998397,0.993165,0.400000,0.358523,
0.362857,0.294405,0.397959,0.347072,0.244300,0.206909,0.320819,0.251917,0.243553,0.198476,
0.003509,0.002802,0.266862,0.233694,0.545894,0.481665,0.774194,0.721407,1.000000,0.992151,
0.027119,0.011209,0.268707,0.228236,0.585714,0.477502,0.837143,0.766383,1.000000,0.993540,
0.022857,0.009149,0.300000,0.272644,0.582456,0.524213,0.789474,0.751360,1.000000,0.996237,
0.347403,0.282754,0.453211,0.424368,0.350340,0.292878,0.319218,0.236152,0.200573,0.165149,
0.324232,0.252199,0.020339,0.010507,0.268571,0.216069,0.524771,0.481842,0.820339,0.770949,
1.000000,0.991875,0.021053,0.009760,0.277778,0.241456,0.529825,0.463346,0.792271,0.728274,
1.000000,0.999162,0.003509,0.002802,0.359322,0.272188,0.583051,0.538702,0.809295,0.757684,
0.998397,0.993165,0.152493,0.062198,0.102041,0.062869,0.098639,0.071423,0.062802,0.053407,
0.047706,0.036898,0.051136,0.031324,0.081169,0.055024,0.137830,0.064702,0.097143,0.062194,
0.062500,0.047427,0.108571,0.065059,0.036932,0.028953,0.059659,0.040603,0.026393,0.014963,
0.093578,0.065793,0.149560,0.097501,0.074675,0.041407,0.047458,0.029316,0.077193,0.048917,
0.042105,0.020023

Positive_88 6.000000,0.333333,4.000000,2.000000,2.000000,4.800000,3.000000,2.000000,3.000000,2.400000,
0.656990,0.017920,0.656990,0.017920,0.000000,0.000000,0.000000,0.083333,0.272727,0.625000,
0.666667,0.500000,1.000000,0.300000,0.666667,1.000000,0.266667,0.125000,0.250000,0.018750,
0.222222,0.333333,0.029630,0.200000,0.400000,0.048000,0.091667,0.200000,0.010417,0.122222,
0.200000,0.009630,0.400000,0.600000,0.096000,3.464102,0.000000,0.000000,1.000000,0.500000,
1.000000,0.500000,1.000000,0.500000,1.000000,0.500000,1.000000,0.500000,1.000000,0.500000,
0.500000,0.250000,0.166667,0.083333,0.000000,0.000000,0.000000,0.000000,1.000000,0.666667,
1.000000,0.666667,1.000000,0.666667,1.000000,0.666667,1.000000,0.666667,1.000000,0.666667,
1.000000,0.666667,0.333333,0.222222,0.000000,0.000000,0.000000,0.000000,0.317241,0.296098,
0.416667,0.388440,0.326343,0.315462,0.238095,0.213486,0.204724,0.196069,0.263158,0.250587,
0.023810,0.013169,0.279161,0.261737,0.537931,0.504992,0.800000,0.764016,1.000000,0.998882,
0.007937,0.005103,0.275862,0.231472,0.497925,0.487412,0.759336,0.736750,1.000000,0.995463,
0.003968,0.002442,0.257937,0.239798,0.514946,0.497305,0.777778,0.733810,1.000000,0.994393,
0.436141,0.385720,0.232337,0.146507,0.516383,0.467773,0.088435,0.067656,0.114187,0.091461,
0.006390,0.003221,0.003968,0.002442,0.290761,0.240237,0.558783,0.510658,0.801587,0.748786,
1.000000,1.000000,0.124138,0.052432,0.324728,0.171841,0.589674,0.375216,0.798913,0.622357,
0.993631,0.885510,0.035961,0.015685,0.305374,0.262195,0.627586,0.503030,0.820690,0.762985,
0.995924,0.970403,0.662069,0.610086,0.396166,0.348874,0.019841,0.008009,0.324138,0.266261,
0.555172,0.520205,0.768966,0.740542,0.998689,0.992849,0.452381,0.412217,0.388889,0.360342,
0.257962,0.227441,0.390438,0.302611,0.212245,0.186987,0.178231,0.159359,0.007937,0.005103,
0.272414,0.231926,0.500655,0.482653,0.754915,0.736148,1.000000,0.994547,0.031746,0.011974,
0.269841,0.243067,0.519841,0.491506,0.777778,0.736409,1.000000,0.996154,0.003968,0.002442,
0.321429,0.273704,0.560166,0.515578,0.831034,0.783656,1.000000,0.996852,0.349934,0.336580,
0.396825,0.352006,0.324138,0.311414,0.270916,0.241734,0.234014,0.222160,0.220408,0.201427,

0.003968,0.002442,0.261146,0.247162,0.544586,0.509650,0.777778,0.740624,1.000000,0.996204,
0.007937,0.005103,0.258621,0.220139,0.503448,0.485052,0.750983,0.724338,1.000000,0.992248,
0.023810,0.013169,0.279161,0.261707,0.517241,0.502878,0.786207,0.761050,1.000000,0.998882,
0.388889,0.352776,0.452381,0.419784,0.257962,0.227441,0.362550,0.291415,0.189116,0.159590,
0.201361,0.186755,0.007937,0.005103,0.303448,0.224111,0.531034,0.472974,0.753604,0.716219,
0.997283,0.991022,0.019841,0.009763,0.277174,0.249935,0.547619,0.503976,0.797619,0.746510,
1.000000,0.996815,0.003968,0.002442,0.321429,0.273704,0.560166,0.515578,0.831034,0.783656,
1.000000,0.996852,0.059474,0.043107,0.098726,0.076090,0.068966,0.059091,0.050955,0.045250,
0.051587,0.038554,0.047619,0.034004,0.119048,0.090254,0.134921,0.103698,0.076433,0.061933,
0.076433,0.051641,0.065007,0.044480,0.025478,0.015317,0.029046,0.021118,0.027586,0.014961,
0.095238,0.079040,0.107884,0.093767,0.062500,0.055885,0.031034,0.023574,0.054140,0.042228,
0.013587,0.006008

Positive_89 5.000000,0.160000,1.000000,0.800000,1.000000,0.200000,4.000000,0.800000,0.000000,3.200000,
0.366712,0.002339,0.366712,0.002339,0.000000,0.000000,0.500000,1.000000,0.000000,0.000000,
0.000000,0.000000,0.000000,0.000000,0.000000,0.000000,0.000000,0.000000,0.000000,0.000000,
0.000000,0.000000,0.000000,0.600000,0.750000,0.112500,0.000000,0.000000,0.000000,0.000000,
0.000000,0.000000,0.000000,0.000000,0.000000,2.000000,0.000000,0.000000,0.000000,0.000000,
0.000000,0.000000,0.000000,0.000000,0.000000,0.000000,0.000000,0.000000,0.000000,0.000000,
0.000000,0.000000,0.000000,0.000000,0.000000,0.000000,0.000000,0.000000,1.000000,0.200000,
1.000000,0.200000,1.000000,0.200000,1.000000,0.200000,0.500000,0.100000,0.500000,0.100000,
0.500000,0.100000,0.000000,0.000000,0.000000,0.000000,0.000000,0.000000,0.309927,0.292544,
0.393023,0.378847,0.350220,0.328608,0.230583,0.211970,0.225470,0.196497,0.280353,0.258641,
0.026465,0.015087,0.281250,0.252574,0.552083,0.516411,0.806250,0.764190,1.000000,0.998866,
0.007561,0.005983,0.266541,0.233409,0.533081,0.464439,0.748184,0.721987,0.998110,0.993811,
0.002421,0.002185,0.306167,0.259600,0.537445,0.513503,0.767442,0.755677,1.000000,0.995621,
0.604915,0.437629,0.174009,0.123234,0.510417,0.439137,0.081678,0.054839,0.093946,0.083914,
0.011038,0.004528,0.002421,0.002185,0.213611,0.178267,0.523629,0.467743,0.710417,0.677163,
1.000000,1.000000,0.297917,0.126889,0.495833,0.341744,0.637500,0.467616,0.858333,0.668447,
0.951163,0.921241,0.083700,0.028839,0.283721,0.263468,0.602326,0.509323,0.882798,0.799970,
0.997674,0.990662,0.677083,0.635868,0.384106,0.352167,0.012500,0.007929,0.293023,0.276686,
0.539535,0.519363,0.741860,0.728365,0.997674,0.992743,0.404651,0.393480,0.372247,0.347940,
0.275000,0.258581,0.315673,0.270396,0.226107,0.185179,0.211165,0.187631,0.007561,0.005542,
0.226872,0.217096,0.487713,0.463358,0.728814,0.700361,0.997797,0.995635,0.014528,0.008534,
0.308370,0.277110,0.539535,0.527851,0.806250,0.779005,1.000000,0.997748,0.002421,0.002185,
0.288136,0.252832,0.595833,0.523736,0.766667,0.745794,1.000000,0.998237,0.383260,0.360721,
0.339535,0.320202,0.331719,0.319078,0.256071,0.234908,0.263048,0.235717,0.207459,0.188091,
0.002421,0.002185,0.303965,0.263105,0.537445,0.515316,0.762500,0.756007,1.000000,0.995621,
0.007561,0.005983,0.249395,0.215803,0.504726,0.448266,0.745763,0.698762,0.997674,0.993433,
0.026465,0.013691,0.289583,0.260681,0.593750,0.527887,0.806250,0.770368,1.000000,0.999622,
0.339535,0.312241,0.480176,0.429178,0.275000,0.258581,0.300221,0.266712,0.188811,0.154123,
0.230583,0.218687,0.007561,0.005542,0.262760,0.235093,0.510397,0.464197,0.745763,0.704933,
0.997797,0.995635,0.007264,0.005759,0.303965,0.252739,0.518750,0.508399,0.797917,0.769594,
1.000000,0.998189,0.002421,0.002185,0.288136,0.252832,0.595833,0.523736,0.766667,0.745794,
1.000000,0.998237,0.060417,0.052894,0.067442,0.051098,0.068750,0.059532,0.056711,0.049169,

0.043478,0.035892,0.060533,0.043960,0.083721,0.068874,0.106250,0.082738,0.096408,0.069723,
0.053269,0.041736,0.070485,0.057130,0.033333,0.026533,0.043478,0.032113,0.037445,0.024109,
0.075061,0.061452,0.103970,0.093933,0.068282,0.053171,0.033040,0.027016,0.058601,0.050067,
0.026465,0.018860

Positive_90 7.000000,0.244898,3.000000,1.714286,3.000000,2.571429,4.000000,1.714286,0.000000,4.571429,
0.462792,0.013353,0.462792,0.013353,0.000000,0.000000,0.333333,0.375000,0.800000,1.000000,
0.000000,0.000000,0.000000,0.000000,0.000000,0.000000,0.000000,0.000000,0.000000,0.000000,
0.000000,0.000000,0.000000,0.285714,0.500000,0.071429,0.000000,0.000000,0.000000,0.000000,
0.000000,0.000000,0.142857,0.333333,0.031746,3.464102,0.000000,0.000000,1.000000,0.571429,
1.000000,0.571429,1.000000,0.571429,1.000000,0.571429,0.666667,0.380952,0.333333,0.190476,
0.000000,0.000000,0.000000,0.000000,0.000000,0.000000,0.000000,0.000000,1.000000,0.428571,
1.000000,0.428571,1.000000,0.428571,0.833333,0.357143,0.833333,0.357143,0.166667,0.071429,
0.000000,0.000000,0.000000,0.000000,0.000000,0.000000,0.000000,0.000000,0.316964,0.278884,
0.424312,0.383568,0.366228,0.337548,0.242250,0.202277,0.216080,0.182979,0.279121,0.257098,
0.040179,0.012053,0.328042,0.277897,0.587302,0.519637,0.824561,0.772618,1.000000,0.995184,
0.017544,0.007373,0.265664,0.223079,0.478697,0.460077,0.785550,0.739300,1.000000,0.995231,
0.004464,0.002331,0.313073,0.243804,0.549312,0.503931,0.751304,0.730029,1.000000,0.994244,
0.678899,0.503947,0.271930,0.169457,0.436090,0.326596,0.112108,0.073090,0.103015,0.071994,
0.010989,0.003430,0.004464,0.002331,0.325893,0.231576,0.591479,0.513568,0.812500,0.773380,
1.000000,1.000000,0.122807,0.052396,0.353070,0.203930,0.747807,0.422279,0.875000,0.665780,
0.997807,0.967849,0.212156,0.052979,0.486239,0.320900,0.581422,0.479487,0.749559,0.702278,
0.994987,0.931237,0.686067,0.619514,0.358744,0.329524,0.026786,0.009685,0.280702,0.255588,
0.528670,0.481894,0.761468,0.721731,0.998261,0.994689,0.428899,0.390953,0.385965,0.359029,
0.280702,0.250018,0.309187,0.278817,0.215247,0.179926,0.226131,0.190339,0.017544,0.007536,
0.283208,0.226160,0.481203,0.462144,0.762615,0.731067,1.000000,0.987522,0.022321,0.011756,
0.307339,0.261501,0.528670,0.504018,0.765351,0.740155,1.000000,0.997375,0.004464,0.002331,
0.302752,0.272624,0.574956,0.534387,0.795414,0.765831,0.997494,0.992280,0.406015,0.369001,
0.353211,0.323934,0.345183,0.307064,0.254417,0.232002,0.278894,0.225868,0.210103,0.184682,
0.004464,0.002331,0.307339,0.251069,0.552752,0.510741,0.764411,0.737034,1.000000,0.996594,
0.017544,0.007536,0.265664,0.216832,0.495536,0.453868,0.793578,0.732226,1.000000,0.987209,
0.040179,0.012053,0.322751,0.281039,0.571429,0.531481,0.811287,0.766028,1.000000,0.995184,
0.350917,0.314074,0.467105,0.435907,0.280702,0.250018,0.287912,0.265516,0.183417,0.148112,
0.243719,0.222153,0.017544,0.008965,0.285714,0.239887,0.481203,0.448794,0.779817,0.727197,
0.998261,0.984525,0.015038,0.008276,0.290179,0.244707,0.519495,0.507803,0.760965,0.741448,
1.000000,1.000000,0.004464,0.002331,0.302752,0.272624,0.574956,0.534387,0.795414,0.765831,
0.997494,0.992280,0.060780,0.041040,0.080357,0.056783,0.081422,0.056285,0.071429,0.052380,
0.039474,0.030886,0.057644,0.041511,0.091743,0.073649,0.085217,0.064897,0.094037,0.070923,
0.062500,0.052225,0.084821,0.062240,0.037844,0.028180,0.052632,0.031453,0.035088,0.014639,
0.080201,0.069413,0.132832,0.111873,0.065789,0.049061,0.028219,0.020340,0.067826,0.052169,
0.035088,0.020053

Positive_91 3.000000,0.222222,2.000000,0.666667,0.000000,1.333333,1.000000,0.666667,1.000000,0.333333,
0.564054,0.004195,0.564054,0.004195,0.000000,0.000000,0.000000,0.000000,0.500000,1.000000,
0.000000,0.000000,0.000000,0.000000,0.000000,0.000000,0.000000,0.000000,0.000000,0.000000,
0.000000,0.000000,0.000000,0.000000,0.000000,0.000000,0.000000,0.000000,0.000000,0.000000,

0.000000,0.000000,0.333333,0.500000,0.083333,1.414214,0.000000,0.000000,1.000000,0.333333,
1.000000,0.333333,1.000000,0.333333,1.000000,0.333333,1.000000,0.333333,1.000000,0.333333,
1.000000,0.333333,1.000000,0.333333,1.000000,0.333333,0.000000,0.000000,0.000000,0.000000,
0.000000,0.000000,0.000000,0.000000,0.000000,0.000000,0.000000,0.000000,0.000000,0.000000,
0.000000,0.000000,0.000000,0.000000,0.000000,0.000000,0.000000,0.000000,0.317778,0.294462,
0.421585,0.382324,0.326478,0.323213,0.224694,0.218810,0.206186,0.189180,0.278716,0.258317,
0.005141,0.004141,0.278889,0.240423,0.513333,0.506038,0.774444,0.762520,1.000000,0.999438,
0.025707,0.013040,0.257069,0.233937,0.493573,0.488992,0.750000,0.737851,1.000000,0.993377,
0.002571,0.001789,0.276560,0.265494,0.526138,0.499809,0.758853,0.741960,0.997429,0.990777,
0.565553,0.447764,0.195373,0.154015,0.478921,0.398221,0.074742,0.061114,0.084459,0.078787,
0.007732,0.004445,0.002571,0.001789,0.293423,0.244668,0.489039,0.482418,0.763496,0.737205,
1.000000,1.000000,0.125964,0.067462,0.342222,0.272044,0.645244,0.483073,0.807198,0.626597,
0.982005,0.926884,0.033419,0.017079,0.262211,0.239843,0.610455,0.554102,0.784148,0.732041,
0.996627,0.994453,0.586118,0.555698,0.387097,0.371997,0.007712,0.004806,0.241147,0.233219,
0.519393,0.489777,0.712222,0.707133,0.994859,0.993253,0.423272,0.391891,0.397778,0.367339,
0.252951,0.240769,0.315907,0.296384,0.184122,0.177246,0.190722,0.177722,0.020566,0.011326,
0.239075,0.223428,0.485666,0.482383,0.740000,0.733956,1.000000,0.996805,0.007712,0.004806,
0.290488,0.271992,0.534704,0.506472,0.764444,0.757952,1.000000,0.994226,0.002571,0.001789,
0.328889,0.249350,0.541111,0.501532,0.804627,0.761657,1.000000,0.995154,0.357504,0.355568,
0.349073,0.327174,0.334444,0.317258,0.239865,0.231567,0.239691,0.225562,0.212458,0.209931,
0.002571,0.001789,0.277635,0.264732,0.526138,0.504697,0.757167,0.741376,0.997429,0.990777,
0.025707,0.013040,0.244216,0.220096,0.493573,0.486744,0.762222,0.734775,1.000000,0.993377,
0.005141,0.004141,0.281111,0.241164,0.511111,0.494240,0.770000,0.761039,1.000000,0.999438,
0.328836,0.299332,0.483333,0.459898,0.252951,0.240769,0.297297,0.285944,0.140203,0.129382,
0.252577,0.225587,0.025707,0.013040,0.257069,0.222119,0.514139,0.492052,0.741111,0.736924,
1.000000,0.993377,0.007712,0.004806,0.300169,0.270771,0.526138,0.502220,0.763912,0.754017,
1.000000,0.994967,0.002571,0.001789,0.328889,0.249350,0.541111,0.501532,0.804627,0.761657,
1.000000,0.995154,0.070826,0.064152,0.064267,0.049139,0.068889,0.056697,0.047778,0.041948,
0.063333,0.053748,0.034444,0.028777,0.070826,0.065772,0.077778,0.074063,0.075885,0.062527,
0.058889,0.055023,0.079692,0.069789,0.033727,0.022796,0.038786,0.032355,0.026981,0.022770,
0.079692,0.072203,0.121111,0.115146,0.051414,0.040768,0.020566,0.011875,0.045531,0.035852,
0.026981,0.024600

Positive_92 59.000000,0.278656,34.000000,16.440678,16.000000,197.767972,44.000000,16.440678,20.000000,154.457627,
0.510383,0.047751,0.510383,0.047751,0.002062,0.107438,0.270833,0.393651,0.366492,0.314050,
0.114458,10.169730,22.911765,116.989482,12.180405,23.846154,119.237633,0.365563,0.997500,0.130407,
0.495742,0.997500,0.205569,0.419093,0.741379,0.056051,0.306774,0.585345,0.028766,0.246807,
0.455570,0.047122,0.351832,0.568966,0.042521,27.624742,10.662900,7.310495,1.000000,0.847458,
1.000000,0.847458,1.000000,0.842373,1.000000,0.797235,1.000000,0.705227,1.000000,0.592549,
1.000000,0.511388,1.000000,0.444536,1.000000,0.429186,1.000000,0.358735,1.000000,0.813559,
1.000000,0.813559,1.000000,0.809801,1.000000,0.775400,1.000000,0.694602,1.000000,0.531780,
0.866667,0.388071,0.466667,0.260056,0.336842,0.205117,0.236842,0.115769,0.373333,0.300204,
0.473684,0.344811,0.398866,0.354985,0.263158,0.201382,0.308036,0.215442,0.356164,0.249285,
0.057143,0.014394,0.316103,0.256161,0.582888,0.500151,0.870748,0.735791,1.000000,0.994670,
0.032258,0.008373,0.313093,0.233303,0.606452,0.491940,0.797403,0.742218,1.000000,0.993058,

0.006803,0.003091,0.308642,0.241125,0.559671,0.489540,0.792969,0.746278,1.000000,0.992524,
0.472000,0.386055,0.259358,0.122981,0.795918,0.490964,0.107239,0.057488,0.118750,0.082830,
0.013369,0.003167,0.006803,0.003091,0.353201,0.239217,0.636905,0.476117,0.927632,0.772782,
1.000000,1.000000,0.393548,0.056107,0.393548,0.137522,0.763747,0.415607,0.843177,0.585030,
0.997963,0.777465,0.155440,0.051068,0.500000,0.308087,0.665761,0.511330,0.883152,0.745934,
0.995833,0.961089,0.743421,0.613431,0.442396,0.351607,0.051613,0.014938,0.320312,0.255241,
0.580645,0.482233,0.792035,0.728332,1.000000,0.988522,0.465789,0.357321,0.445946,0.372424,
0.330275,0.270255,0.350000,0.266621,0.244240,0.202481,0.257919,0.195090,0.031056,0.007379,
0.306843,0.237879,0.579646,0.497240,0.800407,0.746675,1.000000,0.993188,0.037037,0.013339,
0.296296,0.245770,0.538043,0.490338,0.795918,0.731382,1.000000,0.992798,0.006803,0.003091,
0.326531,0.245968,0.582888,0.498720,0.834225,0.762829,1.000000,0.996176,0.455782,0.388631,
0.371053,0.288697,0.386667,0.322672,0.317881,0.232033,0.339286,0.250192,0.235294,0.182877,
0.006803,0.003091,0.304527,0.240280,0.543210,0.487375,0.782383,0.743046,1.000000,0.993784,
0.032258,0.008373,0.309298,0.227832,0.597345,0.493300,0.806306,0.746634,1.000000,0.991548,
0.057143,0.014394,0.316406,0.256163,0.578804,0.503447,0.870748,0.738725,1.000000,0.995106,
0.378947,0.281194,0.540373,0.448551,0.330275,0.270255,0.350000,0.249460,0.214286,0.157170,
0.317972,0.240400,0.037267,0.008726,0.310881,0.230123,0.606195,0.489558,0.787013,0.732778,
1.000000,0.989453,0.037037,0.011607,0.303226,0.251282,0.541916,0.493899,0.775510,0.736959,
1.000000,0.994451,0.006803,0.003091,0.326531,0.245968,0.582888,0.498720,0.834225,0.762829,
1.000000,0.996176,0.081031,0.046982,0.108108,0.070212,0.099585,0.061394,0.077982,0.048722,
0.080745,0.035128,0.061224,0.037766,0.118421,0.061882,0.108844,0.063916,0.080078,0.059421,
0.082902,0.047253,0.074534,0.056225,0.039474,0.022468,0.065789,0.033647,0.045267,0.019903,
0.104790,0.066385,0.162698,0.114285,0.081579,0.057466,0.048673,0.028625,0.079430,0.055312,
0.030246,0.013009

Positive_93 61.000000,0.119860,25.000000,7.311475,1.000000,97.451366,19.000000,7.311475,10.000000,12.384699,
0.544631,0.060005,0.544631,0.060005,0.006726,0.146727,0.253968,0.241135,0.271028,0.141026,
0.320896,1.365564,5.333333,3.679197,2.564970,5.263158,3.906293,0.099952,0.592593,0.029536,
0.288535,0.480000,0.035429,0.108401,0.300000,0.010912,0.128927,0.383333,0.022577,0.112060,
0.196667,0.003269,0.319333,0.400000,0.013882,14.628739,11.279106,6.543980,1.000000,0.459016,
1.000000,0.459016,1.000000,0.457850,1.000000,0.436310,1.000000,0.405158,1.000000,0.354522,
1.000000,0.292472,1.000000,0.249944,1.000000,0.223932,1.000000,0.163419,1.000000,0.934426,
1.000000,0.934426,1.000000,0.934426,1.000000,0.919912,1.000000,0.830045,1.000000,0.685610,
1.000000,0.576647,1.000000,0.495600,1.000000,0.459707,0.781818,0.294937,0.373333,0.299222,
0.473684,0.344663,0.398866,0.356115,0.263158,0.200953,0.308036,0.212855,0.356164,0.249785,
0.057143,0.015722,0.316103,0.254100,0.582888,0.497908,0.870748,0.729713,1.000000,0.993434,
0.032258,0.007724,0.313093,0.237645,0.606452,0.495166,0.797403,0.747034,1.000000,0.993270,
0.006803,0.002937,0.308642,0.239051,0.559671,0.489047,0.786008,0.746155,1.000000,0.994211,
0.498938,0.391314,0.259358,0.129179,0.795918,0.479507,0.107239,0.059025,0.118750,0.080327,
0.013369,0.003416,0.006803,0.002937,0.353201,0.234944,0.621118,0.469964,0.927632,0.767528,
1.000000,1.000000,0.393548,0.051744,0.393548,0.142198,0.770624,0.413223,0.935614,0.588828,
0.997976,0.788378,0.155440,0.045203,0.500000,0.320074,0.712946,0.526908,0.883152,0.752738,
0.998124,0.963463,0.743421,0.619518,0.442396,0.349483,0.051613,0.013816,0.320312,0.256032,
0.580645,0.482318,0.792035,0.730444,1.000000,0.989048,0.465789,0.357785,0.445946,0.369304,
0.330275,0.272911,0.350000,0.266973,0.244240,0.201778,0.257919,0.197632,0.031056,0.006975,

0.306843,0.239723,0.579646,0.498430,0.800407,0.752485,1.000000,0.993608,0.037037,0.012357,
0.296296,0.242920,0.547771,0.488853,0.795918,0.724956,1.000000,0.992975,0.006803,0.002937,
0.326531,0.246404,0.582888,0.500379,0.834225,0.760600,1.000000,0.996195,0.455782,0.389148,
0.371053,0.290056,0.386667,0.320796,0.317881,0.232648,0.339286,0.246710,0.235294,0.182700,
0.006803,0.002937,0.304527,0.237838,0.543210,0.486152,0.787594,0.743116,1.000000,0.994315,
0.032258,0.007724,0.309298,0.233002,0.597345,0.498716,0.806306,0.753571,1.000000,0.992353,
0.057143,0.015722,0.313953,0.255285,0.578804,0.500688,0.870748,0.733283,1.000000,0.993856,
0.378947,0.282571,0.540373,0.444517,0.330275,0.272911,0.350000,0.250334,0.214286,0.157789,
0.317972,0.241621,0.037267,0.008213,0.310881,0.234090,0.606195,0.489156,0.787013,0.739207,
1.000000,0.989976,0.037037,0.010834,0.303226,0.246299,0.547771,0.492777,0.775510,0.734293,
1.000000,0.994670,0.006803,0.002937,0.326531,0.246404,0.582888,0.500379,0.834225,0.760600,
1.000000,0.996195,0.076459,0.046592,0.108108,0.070863,0.099585,0.062208,0.077982,0.047931,
0.080745,0.034367,0.061224,0.037261,0.118421,0.064092,0.108844,0.061958,0.084926,0.059125,
0.082902,0.049466,0.076459,0.055415,0.039474,0.021575,0.065789,0.033033,0.045267,0.019799,
0.104278,0.065911,0.152381,0.109779,0.081579,0.059777,0.048673,0.029990,0.093117,0.057513,
0.030246,0.013346

Positive_94 41.000000,0.079120,13.000000,3.243902,3.000000,7.089024,12.000000,3.243902,2.000000,11.789024,
0.396435,0.021770,0.396435,0.021770,0.082707,0.180328,0.360000,0.453125,0.857143,0.600000,
0.000000,0.715697,3.076923,0.818453,0.678221,2.600000,0.714997,0.149418,0.500000,0.025686,
0.142159,0.520000,0.030157,0.131646,0.275000,0.009350,0.089019,0.200000,0.003583,0.071328,
0.293750,0.007895,0.130470,0.300000,0.010791,6.907973,5.073455,3.944509,1.000000,0.707317,
1.000000,0.707317,1.000000,0.701644,1.000000,0.658791,1.000000,0.541178,1.000000,0.361360,
1.000000,0.255897,1.000000,0.253708,1.000000,0.179353,0.500000,0.109082,1.000000,0.536585,
1.000000,0.536585,1.000000,0.533259,1.000000,0.484257,1.000000,0.355543,0.763636,0.208955,
0.509091,0.115952,0.500000,0.106640,0.500000,0.086524,0.236364,0.046600,0.360153,0.296027,
0.431535,0.359661,0.398866,0.344312,0.276923,0.205832,0.241803,0.197657,0.296552,0.242513,
0.050607,0.014268,0.328042,0.260381,0.622793,0.511390,0.834671,0.755684,1.000000,0.996114,
0.017544,0.006572,0.313093,0.240357,0.582474,0.493193,0.783333,0.742642,1.000000,0.996205,
0.006849,0.002449,0.351792,0.245104,0.579805,0.483267,0.782847,0.732007,1.000000,0.988866,
0.519201,0.432673,0.273425,0.164094,0.536437,0.403233,0.119816,0.070958,0.103015,0.075566,
0.016393,0.003138,0.006849,0.002449,0.330171,0.237439,0.610790,0.486539,0.840637,0.751683,
1.000000,1.000000,0.205479,0.065277,0.380634,0.229978,0.765182,0.493379,0.873444,0.715454,
0.998557,0.912428,0.202555,0.042910,0.442997,0.277944,0.741294,0.506977,0.902062,0.748962,
0.994992,0.969923,0.686067,0.612402,0.426230,0.344718,0.044776,0.012496,0.331551,0.260057,
0.591743,0.493701,0.816514,0.728935,0.998500,0.987514,0.481328,0.377738,0.406130,0.360263,
0.317580,0.261999,0.332640,0.271994,0.240000,0.200819,0.226640,0.183005,0.017544,0.006036,
0.292220,0.238516,0.572165,0.488307,0.800995,0.740470,1.000000,0.996044,0.049270,0.012229,
0.384365,0.248592,0.609121,0.491536,0.801303,0.728207,1.000000,0.992059,0.006849,0.002449,
0.333333,0.260497,0.597111,0.514447,0.824708,0.769593,1.000000,0.993767,0.427221,0.373391,
0.417012,0.306348,0.393035,0.320261,0.296552,0.228395,0.271889,0.229567,0.253846,0.192457,
0.006849,0.002449,0.361564,0.247134,0.599349,0.486106,0.782847,0.736730,1.000000,0.990355,
0.017544,0.006572,0.309298,0.232440,0.577320,0.482472,0.800766,0.731908,1.000000,0.994832,
0.050607,0.014268,0.322751,0.260873,0.608347,0.516354,0.831461,0.761676,1.000000,0.996566,
0.396266,0.302247,0.474227,0.435754,0.317580,0.261999,0.353430,0.261419,0.198157,0.161177,

0.272366,0.222647,0.017544,0.006115,0.317708,0.238392,0.577320,0.485973,0.791045,0.727870,
1.000000,0.994108,0.034826,0.010554,0.315961,0.239158,0.570033,0.493215,0.798046,0.734643,
1.000000,0.994186,0.006849,0.002449,0.333333,0.260497,0.597111,0.514447,0.824708,0.769593,
1.000000,0.993767,0.107492,0.048805,0.085714,0.063402,0.085938,0.059003,0.096330,0.051256,
0.076628,0.039004,0.057644,0.034557,0.118257,0.067873,0.136929,0.067787,0.078799,0.063066,
0.072018,0.052264,0.078652,0.055357,0.044776,0.024234,0.056836,0.029079,0.061644,0.020134,
0.091743,0.073355,0.145228,0.099502,0.077922,0.054841,0.047170,0.029241,0.093117,0.054115,
0.035088,0.013124

Positive_95 62.000000,0.064776,6.000000,4.016129,4.000000,2.180063,56.000000,4.016129,0.000000,178.507932,
0.283397,0.009411,0.283397,0.009411,0.120482,0.662100,0.797297,0.600000,0.333333,0.250000,
0.333333,0.000000,0.000000,0.000000,0.000000,0.000000,0.000000,0.000000,0.000000,0.000000,
0.000000,0.000000,0.000000,0.698374,0.827869,0.055731,0.000000,0.000000,0.000000,0.000000,
0.000000,0.000000,0.005771,0.066745,0.000317,14.635548,4.117144,3.324097,1.000000,0.903226,
1.000000,0.903226,1.000000,0.903226,1.000000,0.903226,1.000000,0.903226,1.000000,0.903226,
1.000000,0.627419,0.500000,0.380645,0.333333,0.253763,0.166667,0.126882,1.000000,0.096774,
0.971429,0.093078,0.881935,0.083034,0.603129,0.051265,0.467994,0.034850,0.266003,0.018088,
0.122333,0.008429,0.087912,0.004481,0.038407,0.002579,0.014778,0.000882,0.413793,0.303004,
0.459016,0.378835,0.385965,0.318161,0.290698,0.217306,0.285714,0.196156,0.381232,0.244538,
0.069672,0.016600,0.393996,0.261584,0.575428,0.506032,0.803030,0.752517,1.000000,0.994236,
0.050000,0.008335,0.299213,0.238336,0.582418,0.481712,0.809524,0.737791,1.000000,0.996308,
0.011494,0.002758,0.345216,0.239426,0.652908,0.493973,0.825516,0.738556,1.000000,0.992323,
0.801084,0.432066,0.598425,0.141165,0.906367,0.426770,0.174603,0.056598,0.141414,0.071351,
0.026316,0.003399,0.011494,0.002758,0.676768,0.234463,0.838384,0.492810,0.919192,0.728990,
1.000000,1.000000,0.545788,0.121700,0.787879,0.282150,0.793939,0.479359,0.939577,0.656356,
0.998452,0.814198,0.339093,0.053578,0.580087,0.261401,0.723514,0.470939,0.908898,0.683204,
0.997294,0.914661,0.765472,0.573956,0.641221,0.351005,0.068966,0.012058,0.340090,0.253663,
0.602459,0.502002,0.795082,0.735302,1.000000,0.986267,0.504690,0.388306,0.448819,0.356060,
0.344828,0.255634,0.354545,0.274183,0.255814,0.196850,0.261851,0.181172,0.050000,0.007849,
0.286713,0.235691,0.556777,0.484675,0.795082,0.734028,1.000000,0.996711,0.062992,0.012226,
0.369330,0.250497,0.636023,0.499517,0.827392,0.742750,1.000000,0.995013,0.011494,0.002758,
0.385246,0.254614,0.628998,0.498803,0.835821,0.751491,1.000000,0.991549,0.418129,0.348638,
0.423372,0.322325,0.436782,0.329037,0.351906,0.227988,0.321429,0.232634,0.267442,0.203289,
0.011494,0.002758,0.354597,0.244907,0.651032,0.497886,0.827392,0.742906,1.000000,0.993803,
0.050000,0.008449,0.293706,0.229866,0.582418,0.478956,0.805861,0.731244,1.000000,0.993994,
0.065574,0.015519,0.393996,0.262839,0.577869,0.505841,0.803030,0.751021,1.000000,0.994816,
0.424242,0.318682,0.490000,0.425684,0.344828,0.255634,0.347727,0.268425,0.232558,0.164144,
0.291196,0.213878,0.101010,0.009735,0.309168,0.235929,0.553191,0.482758,0.803030,0.732671,
1.000000,0.996009,0.062992,0.010026,0.315789,0.242679,0.573099,0.500493,0.813417,0.746053,
1.000000,0.996065,0.011494,0.002758,0.385246,0.254614,0.628998,0.498803,0.835821,0.751491,
1.000000,0.991549,0.077519,0.051833,0.149425,0.062020,0.126437,0.063051,0.083333,0.048303,
0.101093,0.044624,0.065678,0.033174,0.159836,0.072331,0.151515,0.075871,0.130031,0.067582,
0.142857,0.054596,0.121951,0.051946,0.067568,0.026033,0.064067,0.030477,0.040598,0.017678,
0.128788,0.062768,0.153558,0.103436,0.094488,0.049008,0.045977,0.026394,0.076759,0.044187,
0.038288,0.014690

Positive_96 252.000000,0.013527,26.000000,3.408730,0.500000,37.445820,23.000000,3.408730,2.000000,12.139047,
0.385413,0.023111,0.385413,0.023111,0.062937,0.279851,0.442142,0.526316,0.437908,0.500000,
0.581395,0.026167,2.000000,0.029228,0.028888,1.125000,0.023527,0.005171,0.222222,0.001007,
0.005736,0.250000,0.000964,0.015070,0.087649,0.000641,0.014306,0.091633,0.000647,0.011464,
0.091633,0.000282,0.035297,0.099602,0.001242,12.489996,12.171508,9.536059,1.000000,0.456349,
1.000000,0.455925,1.000000,0.451814,1.000000,0.429278,1.000000,0.388032,1.000000,0.332116,
1.000000,0.292691,1.000000,0.242028,1.000000,0.071693,1.000000,0.032067,1.000000,0.626984,
1.000000,0.626984,1.000000,0.623760,1.000000,0.617174,1.000000,0.585732,1.000000,0.527573,
1.000000,0.463785,1.000000,0.317190,1.000000,0.152135,1.000000,0.049999,0.536913,0.316544,
0.564961,0.382386,0.409910,0.301070,0.333333,0.227497,0.331361,0.190202,0.369492,0.222845,
0.193939,0.014730,0.472727,0.260712,0.697406,0.510024,0.846154,0.750733,1.000000,0.991505,
0.047297,0.007988,0.347732,0.239911,0.631250,0.495916,0.846962,0.745654,1.000000,0.995189,
0.013889,0.002774,0.363409,0.237664,0.641350,0.482114,0.834320,0.722528,1.000000,0.993329,
0.873711,0.534615,0.449749,0.156235,0.731006,0.309150,0.194444,0.065594,0.129252,0.066358,
0.043478,0.005527,0.070866,0.003024,0.401575,0.245976,0.743750,0.510373,0.911704,0.764300,
1.000000,1.000000,0.958333,0.099622,0.958333,0.279089,0.958333,0.453827,0.973019,0.613871,
0.999083,0.861227,0.619355,0.051239,0.860812,0.271598,0.880155,0.489079,0.978093,0.702092,
0.998890,0.933095,0.649412,0.499622,0.500000,0.353800,0.152778,0.017243,0.420048,0.251789,
0.632911,0.489312,0.868557,0.714059,1.000000,0.985976,0.559055,0.401603,0.468750,0.351822,
0.352941,0.246575,0.392405,0.268586,0.299270,0.188608,0.263514,0.175593,0.062992,0.008236,
0.345588,0.239686,0.622817,0.493982,0.842520,0.747908,1.000000,0.996075,0.128440,0.012751,
0.367893,0.246676,0.652174,0.490868,0.853026,0.731244,1.000000,0.994136,0.013889,0.002774,
0.407767,0.252664,0.697816,0.503473,0.877660,0.742792,1.000000,0.989605,0.441441,0.331346,
0.533465,0.326143,0.563758,0.342511,0.328814,0.203774,0.349112,0.227848,0.314465,0.208305,
0.013889,0.002774,0.375527,0.242150,0.670139,0.485884,0.841019,0.722822,1.000000,0.993841,
0.062992,0.008733,0.366589,0.234676,0.675000,0.491745,0.854464,0.744879,1.000000,0.994123,
0.193939,0.013911,0.460606,0.261335,0.703170,0.509897,0.841499,0.750896,1.000000,0.992826,
0.484252,0.314917,0.539394,0.438508,0.352941,0.246575,0.373026,0.271492,0.235669,0.149518,
0.293651,0.214683,0.062992,0.008906,0.347732,0.234601,0.612340,0.485615,0.839196,0.737207,
1.000000,0.993656,0.125000,0.010819,0.383886,0.248449,0.639810,0.497883,0.838863,0.743667,
1.000000,0.996500,0.013889,0.002774,0.407767,0.252664,0.697816,0.503473,0.877660,0.742792,
1.000000,0.989605,0.182353,0.060044,0.154362,0.057960,0.201342,0.068086,0.111765,0.051834,
0.114990,0.042357,0.073593,0.036263,0.171765,0.066838,0.178674,0.067285,0.152985,0.078387,
0.116142,0.050574,0.152490,0.063060,0.077799,0.025968,0.072464,0.030276,0.105634,0.023626,
0.099476,0.062035,0.165333,0.097010,0.097222,0.046072,0.048880,0.023686,0.078341,0.037983,
0.037037,0.010659

Positive_97 87.000000,0.028273,8.000000,2.459770,2.000000,3.809409,17.000000,2.459770,2.000000,10.344293,
0.410806,0.027717,0.410806,0.027717,0.028169,0.275362,0.406667,0.370787,0.446429,0.516129,
0.466667,0.022989,2.000000,0.045977,0.012843,0.307692,0.003506,0.002874,0.250000,0.000718,
0.000908,0.023669,0.000018,0.064417,0.186047,0.004235,0.035001,0.093023,0.000880,0.036827,
0.197674,0.003165,0.029718,0.081395,0.000882,6.928392,4.898979,3.922114,1.000000,0.678161,
1.000000,0.678161,1.000000,0.653941,1.000000,0.641817,1.000000,0.614778,1.000000,0.493350,
1.000000,0.395375,1.000000,0.323618,1.000000,0.260783,0.333333,0.038396,1.000000,0.597701,
1.000000,0.596893,1.000000,0.594999,1.000000,0.578347,1.000000,0.529185,1.000000,0.435382,

1.000000,0.380100,1.000000,0.278537,1.000000,0.126836,0.333333,0.048518,0.431718,0.322972,
0.518692,0.386728,0.402516,0.290300,0.335025,0.234738,0.259053,0.190573,0.291139,0.209982,
0.095238,0.011665,0.432203,0.251883,0.658610,0.503040,0.872881,0.747252,1.000000,0.994498,
0.029762,0.006771,0.313084,0.243912,0.600900,0.491148,0.846962,0.747488,1.000000,0.995859,
0.008696,0.002428,0.401015,0.249237,0.653130,0.496197,0.848341,0.734260,1.000000,0.991495,
0.810748,0.567159,0.377880,0.146608,0.660259,0.286233,0.194444,0.065786,0.102041,0.063134,
0.016541,0.005514,0.008696,0.002428,0.472292,0.256640,0.709677,0.505665,0.877834,0.758149,
1.000000,1.000000,0.451923,0.086999,0.592045,0.233703,0.869863,0.406002,0.961538,0.621082,
0.994709,0.875213,0.474576,0.057208,0.860812,0.333228,0.868268,0.533555,0.958213,0.717456,
0.998286,0.943331,0.619835,0.471637,0.432671,0.365930,0.103659,0.016327,0.420048,0.259538,
0.644670,0.498617,0.848341,0.724771,0.998823,0.987224,0.567797,0.405479,0.434562,0.347066,
0.313364,0.247455,0.333333,0.261826,0.238494,0.188522,0.251656,0.173541,0.029762,0.006367,
0.329975,0.242821,0.583646,0.489245,0.838710,0.745109,1.000000,0.996160,0.076271,0.013849,
0.409483,0.251384,0.614213,0.500630,0.837644,0.738690,1.000000,0.995888,0.008696,0.002428,
0.396450,0.252522,0.631420,0.497358,0.877660,0.742348,1.000000,0.991347,0.433884,0.321263,
0.491525,0.329268,0.455947,0.349469,0.246835,0.190683,0.306407,0.228898,0.299492,0.213122,
0.008696,0.002428,0.404399,0.252437,0.651438,0.495762,0.848341,0.732491,1.000000,0.991844,
0.029762,0.006956,0.352853,0.243578,0.631658,0.488022,0.854464,0.746844,1.000000,0.995001,
0.077694,0.011273,0.432203,0.254182,0.643505,0.502141,0.863905,0.747177,1.000000,0.995562,
0.428571,0.313618,0.496855,0.438928,0.313364,0.247455,0.333333,0.265744,0.191617,0.145982,
0.289130,0.216081,0.059524,0.007497,0.304425,0.234794,0.611111,0.483741,0.826790,0.740132,
1.000000,0.993775,0.059406,0.010630,0.305970,0.253919,0.575800,0.503148,0.804598,0.745968,
1.000000,0.997521,0.008696,0.002428,0.396450,0.252522,0.631420,0.497358,0.877660,0.742348,
1.000000,0.991347,0.121951,0.059299,0.127962,0.060579,0.102083,0.069620,0.085106,0.053243,
0.095299,0.044052,0.068282,0.036180,0.121951,0.066297,0.138365,0.064674,0.160714,0.078703,
0.101695,0.050702,0.144860,0.068893,0.060870,0.026497,0.060738,0.030963,0.094340,0.022969,
0.119497,0.060972,0.153846,0.090445,0.077778,0.045797,0.045455,0.023858,0.078341,0.035515,
0.029762,0.010745

Positive_98 119.000000,0.026693,38.000000,3.176471,0.000000,56.231306,11.000000,3.176471,3.000000,3.994018,
0.328095,0.010469,0.328095,0.010469,0.075269,0.316860,0.668085,0.807692,0.866667,0.500000,
0.000000,0.000000,0.000000,0.000000,0.000000,0.000000,0.000000,0.000000,0.000000,0.000000,
0.000000,0.000000,0.000000,0.014007,0.084746,0.000626,0.030129,0.322034,0.004774,0.033905,
0.093220,0.000834,0.143062,0.313559,0.014746,12.339438,6.708204,5.959061,1.000000,0.327731,
1.000000,0.322449,1.000000,0.318007,1.000000,0.305483,1.000000,0.268919,1.000000,0.238579,
1.000000,0.159064,1.000000,0.088546,0.666667,0.053985,0.238095,0.015879,1.000000,0.840336,
1.000000,0.840336,1.000000,0.838044,1.000000,0.827502,1.000000,0.779068,1.000000,0.771632,
1.000000,0.570155,1.000000,0.245225,1.000000,0.146371,0.238095,0.016683,0.429530,0.287094,
0.492537,0.361881,0.538462,0.351025,0.324138,0.200844,0.331361,0.183540,0.353474,0.251597,
0.038462,0.009025,0.384783,0.261036,0.679300,0.522510,0.874036,0.767783,1.000000,0.992781,
0.115385,0.007176,0.319176,0.239731,0.590678,0.490845,0.822041,0.744312,1.000000,0.990972,
0.019231,0.002200,0.538462,0.248651,0.730769,0.484757,0.865385,0.727944,1.000000,0.996098,
0.656327,0.442415,0.361024,0.098973,0.826923,0.458613,0.129639,0.043247,0.112360,0.065771,
0.021008,0.006014,0.019231,0.002200,0.428571,0.254069,0.765743,0.523422,0.921053,0.789931,
1.000000,0.999194,0.919626,0.152950,0.919626,0.313562,0.954262,0.494808,0.984340,0.701112,

0.998355,0.891292,0.174497,0.044166,0.814525,0.265693,0.858412,0.490666,0.895973,0.718131,
1.000000,0.945778,0.725904,0.558602,0.500000,0.296877,0.122905,0.015533,0.496644,0.256336,
0.666107,0.473340,0.848049,0.725109,1.000000,0.988952,0.502488,0.379688,0.557692,0.370360,
0.319088,0.249952,0.362538,0.275497,0.235119,0.183706,0.294118,0.183968,0.153846,0.006915,
0.303732,0.238430,0.600515,0.492543,0.821803,0.747729,1.000000,0.993749,0.038462,0.008613,
0.365385,0.240216,0.596154,0.491501,0.807692,0.736746,1.000000,0.997801,0.019231,0.002200,
0.386364,0.264920,0.642247,0.508349,0.821734,0.744539,1.000000,0.991573,0.557692,0.382136,
0.465174,0.309234,0.436242,0.308630,0.334152,0.229781,0.349112,0.218389,0.303448,0.183270,
0.019231,0.002200,0.459732,0.251929,0.711538,0.486332,0.846154,0.728516,1.000000,0.996463,
0.153846,0.007517,0.352853,0.233517,0.629661,0.491830,0.832653,0.742863,1.000000,0.988226,
0.038462,0.008816,0.400504,0.264420,0.661808,0.522585,0.856041,0.767219,1.000000,0.993189,
0.405473,0.303393,0.634615,0.446655,0.319088,0.249952,0.346437,0.267262,0.200000,0.146546,
0.333333,0.221128,0.153846,0.007338,0.304094,0.230674,0.574742,0.487130,0.845209,0.742120,
1.000000,0.989439,0.038462,0.007235,0.403846,0.244948,0.634615,0.494507,0.807692,0.744737,
1.000000,0.998151,0.019231,0.002200,0.386364,0.264920,0.642247,0.508349,0.821734,0.744539,
1.000000,0.991573,0.094527,0.055140,0.100329,0.051955,0.134228,0.058703,0.114094,0.044768,
0.096154,0.037257,0.075107,0.039271,0.110140,0.058167,0.161692,0.073752,0.124706,0.072421,
0.083051,0.054284,0.105392,0.050610,0.043321,0.021536,0.058968,0.031110,0.057692,0.025685,
0.105651,0.069895,0.192308,0.107555,0.153846,0.057680,0.060773,0.026221,0.090652,0.048635,
0.035885,0.015355

Positive_99 244.000000,0.004115,15.000000,1.004098,0.000000,3.748954,5.000000,1.004098,1.000000,1.320971,
0.351900,0.022125,0.351900,0.022125,0.139241,0.210784,0.440994,0.488889,0.695652,0.857143,
0.500000,0.000000,0.000000,0.000000,0.000000,0.000000,0.000000,0.000000,0.000000,0.000000,
0.000000,0.000000,0.000000,0.002113,0.016461,0.000015,0.000000,0.000000,0.000000,0.000000,
0.000000,0.000000,0.007708,0.057613,0.000213,5.477226,4.871954,3.487565,1.000000,0.204918,
1.000000,0.204918,1.000000,0.200273,1.000000,0.179176,1.000000,0.140788,1.000000,0.116130,
1.000000,0.084368,1.000000,0.063564,1.000000,0.031792,0.580952,0.011934,1.000000,0.315574,
1.000000,0.315574,1.000000,0.314208,1.000000,0.314208,1.000000,0.312022,1.000000,0.281557,
1.000000,0.269809,1.000000,0.212842,1.000000,0.128825,1.000000,0.014071,0.409639,0.289047,
0.550562,0.373977,0.468468,0.336976,0.338346,0.207769,0.325243,0.184006,0.349398,0.247869,
0.336134,0.027454,0.527473,0.293234,0.747253,0.534022,0.885965,0.762042,1.000000,0.989531,
0.065327,0.014232,0.359206,0.231258,0.681159,0.482076,0.850877,0.732604,1.000000,0.991020,
0.010989,0.004260,0.361650,0.209900,0.593830,0.465135,0.826196,0.716621,1.000000,0.987788,
0.783080,0.503871,0.449749,0.158405,0.734463,0.337725,0.133501,0.062474,0.124294,0.056235,
0.026163,0.005399,0.010989,0.004260,0.433071,0.237331,0.755906,0.481464,0.929972,0.748756,
1.000000,1.000000,0.968992,0.176355,0.968992,0.300581,0.976744,0.439921,0.984496,0.588023,
0.998004,0.790998,0.676923,0.039539,0.938224,0.243892,0.957529,0.478103,0.972973,0.723337,
0.998561,0.927271,0.687500,0.487053,0.517073,0.349044,0.067194,0.018222,0.338078,0.218364,
0.660668,0.469969,0.869018,0.723119,0.999082,0.976432,0.576087,0.399030,0.475410,0.366442,
0.347826,0.234528,0.380531,0.282008,0.271739,0.175209,0.322581,0.170536,0.061404,0.013790,
0.338164,0.229308,0.652174,0.475658,0.840580,0.730709,1.000000,0.992666,0.089286,0.017275,
0.336842,0.240568,0.681319,0.498439,0.858696,0.732001,1.000000,0.991444,0.010989,0.004260,
0.458333,0.267872,0.712000,0.508491,0.885417,0.747445,1.000000,0.985509,0.523944,0.363445,
0.510870,0.323387,0.429952,0.313168,0.346260,0.226552,0.383495,0.218067,0.315789,0.194119,

0.010989,0.004260,0.439320,0.213626,0.645244,0.468633,0.863980,0.718836,1.000000,0.988761,
0.065327,0.015406,0.368231,0.224957,0.700483,0.473538,0.862360,0.728460,1.000000,0.989429,
0.244898,0.024270,0.517544,0.293682,0.747253,0.533317,0.885965,0.760730,1.000000,0.991006,
0.415730,0.302097,0.566667,0.463375,0.347826,0.234528,0.374486,0.281440,0.218085,0.134795,
0.344086,0.210950,0.075342,0.015972,0.342995,0.228772,0.676329,0.476904,0.879227,0.728617,
1.000000,0.989649,0.070707,0.014936,0.299145,0.238473,0.596958,0.491456,0.795455,0.733914,
1.000000,0.994627,0.010989,0.004260,0.458333,0.267872,0.712000,0.508491,0.885417,0.747445,
1.000000,0.985509,0.123016,0.054615,0.192000,0.054025,0.096618,0.057561,0.103627,0.039715,
0.097087,0.046328,0.103226,0.036802,0.168033,0.055105,0.175000,0.067334,0.138577,0.081471,
0.149123,0.058472,0.152174,0.061005,0.094488,0.024121,0.069444,0.026469,0.099585,0.035928,
0.129032,0.063501,0.226131,0.115889,0.112245,0.046360,0.063492,0.022416,0.095775,0.037851,
0.037175,0.015031

Positive_100 63.000000,0.336609,46.000000,21.206349,15.000000,193.521249,52.000000,21.206349,14.000000,268.456733,
0.420184,0.027641,0.423353,0.026507,0.018154,0.181818,0.382298,0.606707,0.453488,0.255319,
0.238095,13.369528,23.673913,56.111015,12.603629,22.730769,64.038784,0.618077,1.000000,0.071409,
0.592894,1.000000,0.119811,0.535488,0.822581,0.041977,0.490710,0.741935,0.022715,0.484604,
0.822581,0.063302,0.463567,0.725806,0.047808,30.196521,13.864423,9.262951,1.000000,0.963850,
1.000000,0.963120,1.000000,0.943952,1.000000,0.826707,1.000000,0.659847,1.000000,0.475874,
1.000000,0.402029,1.000000,0.339583,1.000000,0.261209,1.000000,0.102515,1.000000,0.948651,
1.000000,0.948232,1.000000,0.934107,1.000000,0.814988,1.000000,0.644480,1.000000,0.482023,
1.000000,0.395744,1.000000,0.323176,1.000000,0.242954,1.000000,0.079743,0.431718,0.331066,
0.453416,0.360347,0.422535,0.308586,0.324138,0.225289,0.258185,0.206070,0.344340,0.219524,
0.064133,0.008646,0.350000,0.255808,0.597527,0.508888,0.826160,0.749823,1.000000,0.997657,
0.013699,0.004496,0.348371,0.236485,0.614749,0.485499,0.819957,0.747521,1.000000,0.996665,
0.006849,0.001731,0.385093,0.252911,0.591615,0.497554,0.802603,0.735533,1.000000,0.993595,
0.677590,0.481102,0.342541,0.151895,0.727700,0.367003,0.101449,0.057210,0.100000,0.068854,
0.020690,0.005388,0.006849,0.001731,0.375000,0.232984,0.664439,0.490526,0.859060,0.742613,
1.000000,0.998478,0.659436,0.082595,0.659436,0.299396,0.833882,0.515854,0.916350,0.738390,
0.998355,0.928508,0.106888,0.024053,0.503831,0.268640,0.692827,0.491040,0.891827,0.733168,
1.000000,0.965894,0.793427,0.514424,0.500000,0.362966,0.076355,0.010727,0.347222,0.267097,
0.580745,0.505991,0.801932,0.742471,0.999047,0.992741,0.492236,0.381035,0.410655,0.363826,
0.322816,0.255138,0.337931,0.269015,0.231144,0.184189,0.251656,0.185333,0.017354,0.004647,
0.333333,0.233835,0.612206,0.485968,0.808933,0.743590,1.000000,0.997365,0.029851,0.007985,
0.336518,0.261015,0.557047,0.508092,0.800434,0.749757,1.000000,0.996875,0.006849,0.001731,
0.366594,0.249881,0.630021,0.498356,0.812112,0.735867,1.000000,0.995004,0.465169,0.339315,
0.424947,0.304061,0.455947,0.356624,0.278302,0.197298,0.305893,0.245727,0.303448,0.204394,
0.006849,0.001731,0.402174,0.256852,0.593168,0.498823,0.803140,0.736131,1.000000,0.994100,
0.017354,0.004787,0.335840,0.229423,0.652893,0.484977,0.826303,0.744373,1.000000,0.996104,
0.064133,0.008646,0.350000,0.255752,0.597527,0.507873,0.819409,0.750516,1.000000,0.998022,
0.404110,0.306969,0.493896,0.437893,0.322816,0.255138,0.318818,0.263908,0.200000,0.150958,
0.289130,0.218565,0.017354,0.005198,0.325815,0.236455,0.581454,0.490230,0.821340,0.746374,
1.000000,0.996747,0.029851,0.006645,0.312000,0.252371,0.544994,0.500671,0.787671,0.748759,
1.000000,0.997200,0.006849,0.001731,0.366594,0.249881,0.630021,0.498356,0.812112,0.735867,
1.000000,0.995004,0.101952,0.056622,0.100329,0.062243,0.134228,0.073816,0.114094,0.056757,

0.073491,0.044958,0.068282,0.036669,0.100849,0.061127,0.143836,0.064490,0.121118,0.074587,
0.074627,0.050008,0.096154,0.053849,0.041872,0.025558,0.060738,0.030729,0.049383,0.020218,
0.104693,0.060241,0.164319,0.100662,0.083146,0.047479,0.060773,0.025551,0.094382,0.042922,
0.021164,0.011513

Positive_101 27.000000,0.063100,7.000000,1.703704,0.000000,7.293447,5.000000,1.703704,1.000000,4.370370,
0.280531,0.021413,0.280531,0.021413,0.377778,0.321429,0.631579,0.428571,0.500000,0.500000,
1.000000,0.000000,0.000000,0.000000,0.000000,0.000000,0.000000,0.000000,0.000000,0.000000,
0.000000,0.000000,0.000000,0.032764,0.153846,0.003608,0.000000,0.000000,0.000000,0.000000,
0.000000,0.000000,0.074074,0.230769,0.009550,5.916080,2.000000,1.732051,1.000000,0.333333,
1.000000,0.333333,1.000000,0.333333,1.000000,0.315697,1.000000,0.253968,1.000000,0.201058,
1.000000,0.116402,1.000000,0.045855,0.047619,0.008818,0.000000,0.000000,1.000000,0.296296,
1.000000,0.296296,1.000000,0.296296,1.000000,0.244444,1.000000,0.166667,1.000000,0.140741,
1.000000,0.114815,0.333333,0.064198,0.100000,0.025926,0.000000,0.000000,0.394543,0.271526,
0.480519,0.382433,0.430481,0.346041,0.263469,0.200406,0.221229,0.175632,0.394161,0.263454,
0.123188,0.018892,0.334802,0.262133,0.577093,0.492350,0.793451,0.737640,1.000000,0.988911,
0.042553,0.012667,0.379845,0.247452,0.643411,0.500219,0.790588,0.750920,1.000000,0.994750,
0.008621,0.003593,0.294118,0.219291,0.558901,0.480055,0.801235,0.726976,1.000000,0.994687,
0.711652,0.515394,0.366071,0.175963,0.628342,0.308643,0.169291,0.071990,0.092593,0.048309,
0.018072,0.004400,0.008621,0.003593,0.390071,0.245391,0.627907,0.483716,0.876574,0.754228,
1.000000,1.000000,0.589862,0.113390,0.589862,0.273265,0.793103,0.455293,0.853448,0.592660,
0.992908,0.822351,0.179221,0.021490,0.485988,0.183203,0.774336,0.430661,0.958015,0.687866,
0.998765,0.875440,0.707809,0.553421,0.424779,0.347660,0.034483,0.014038,0.311874,0.232075,
0.599678,0.476759,0.797531,0.723213,0.998392,0.983399,0.477922,0.403447,0.431718,0.358744,
0.284483,0.237810,0.343373,0.275653,0.313869,0.206382,0.200524,0.166417,0.042553,0.012607,
0.333333,0.242848,0.604651,0.510168,0.813953,0.750012,1.000000,0.995559,0.086207,0.017516,
0.282116,0.236156,0.555066,0.465423,0.801235,0.712833,1.000000,0.989856,0.008621,0.003593,
0.353247,0.245922,0.601286,0.493973,0.799035,0.749896,1.000000,0.990244,0.497326,0.385878,
0.418182,0.318168,0.417404,0.295954,0.386861,0.246590,0.274860,0.215862,0.255351,0.185543,
0.008621,0.003593,0.315508,0.227390,0.558901,0.485903,0.786420,0.737406,1.000000,0.995726,
0.068966,0.015632,0.341085,0.233136,0.635659,0.494698,0.798561,0.737062,1.000000,0.991927,
0.123188,0.014674,0.325991,0.262563,0.571788,0.495366,0.808511,0.742172,1.000000,0.989447,
0.389610,0.308708,0.539007,0.453482,0.284483,0.237810,0.349398,0.268179,0.226277,0.156446,
0.249330,0.216353,0.042553,0.015729,0.341085,0.240999,0.643411,0.517184,0.829457,0.751763,
1.000000,0.992070,0.086207,0.015619,0.282116,0.240935,0.537445,0.477893,0.771605,0.722585,
1.000000,0.995832,0.008621,0.003593,0.353247,0.245922,0.601286,0.493973,0.799035,0.749896,
1.000000,0.990244,0.133333,0.046631,0.072519,0.048342,0.081994,0.049315,0.073009,0.044510,
0.071856,0.039290,0.072874,0.043437,0.097166,0.059605,0.140260,0.062183,0.124706,0.082682,
0.100775,0.059729,0.113475,0.053970,0.043103,0.024428,0.079710,0.039837,0.124031,0.040768,
0.095718,0.062159,0.200000,0.110905,0.089209,0.053637,0.043478,0.022336,0.080214,0.041721,
0.029940,0.014514

Positive_102 109.000000,0.048817,18.000000,5.321101,3.000000,37.460754,17.000000,5.321101,4.000000,24.201495,
0.596617,0.046572,0.596617,0.046572,0.005190,0.045217,0.173042,0.270925,0.184290,0.244444,
0.279412,1.686697,11.222222,14.636696,1.928595,11.235294,17.082590,0.103477,0.916667,0.051868,
0.140933,0.916667,0.081970,0.040151,0.145062,0.001814,0.035879,0.155864,0.001854,0.043481,

0.154321,0.001714,0.077718,0.157407,0.002384,15.829760,7.348469,6.958415,1.000000,0.568807,
1.000000,0.568807,1.000000,0.563612,1.000000,0.533381,1.000000,0.469629,1.000000,0.351826,
1.000000,0.239374,1.000000,0.160491,0.893939,0.096056,0.333333,0.031602,1.000000,0.798165,
1.000000,0.798165,1.000000,0.798165,1.000000,0.791364,1.000000,0.740486,1.000000,0.625639,
1.000000,0.481505,1.000000,0.377230,0.893939,0.204174,0.333333,0.085261,0.494118,0.339208,
0.550633,0.361833,0.398104,0.298959,0.335025,0.226123,0.290098,0.209717,0.300236,0.213767,
0.063348,0.011413,0.376344,0.243364,0.626984,0.484613,0.855670,0.746142,1.000000,0.994869,
0.070588,0.009739,0.482353,0.243657,0.618750,0.502674,0.853081,0.749391,1.000000,0.994190,
0.011765,0.002756,0.350701,0.253471,0.618812,0.493495,0.819048,0.731948,1.000000,0.989643,
0.876582,0.486467,0.487730,0.111646,0.850975,0.401888,0.172308,0.047070,0.121212,0.074866,
0.016304,0.003797,0.042654,0.003104,0.747911,0.253156,0.828691,0.506961,0.937326,0.754751,
1.000000,1.000000,0.969355,0.105828,0.969355,0.254828,0.972581,0.413531,0.976303,0.584042,
0.998841,0.791982,0.305556,0.040273,0.598142,0.268517,0.774809,0.497743,0.900763,0.703374,
0.998752,0.930775,0.620805,0.481459,0.576923,0.365507,0.115942,0.017320,0.497685,0.269073,
0.643519,0.506332,0.870474,0.730956,0.999528,0.986186,0.588608,0.385266,0.468750,0.376474,
0.294606,0.238260,0.392405,0.282243,0.224900,0.171291,0.256494,0.177617,0.070588,0.009040,
0.411765,0.244720,0.626298,0.504436,0.838863,0.749890,1.000000,0.995752,0.064815,0.010787,
0.397849,0.254252,0.643564,0.491191,0.799026,0.739209,1.000000,0.995107,0.011765,0.002756,
0.350000,0.241877,0.666667,0.482252,0.871951,0.734185,1.000000,0.986018,0.421801,0.327509,
0.537975,0.309386,0.506083,0.363106,0.266667,0.193918,0.329975,0.246556,0.299492,0.206781,
0.011765,0.002756,0.370370,0.257297,0.608911,0.495489,0.833692,0.732764,1.000000,0.989988,
0.070588,0.011420,0.482353,0.238583,0.636042,0.498948,0.857820,0.747592,1.000000,0.993314,
0.055556,0.010719,0.376344,0.247430,0.626984,0.488553,0.848797,0.746091,1.000000,0.995590,
0.423077,0.308704,0.535545,0.453036,0.294606,0.238260,0.431746,0.279053,0.193548,0.138401,
0.295238,0.210507,0.082353,0.010527,0.411765,0.243449,0.660900,0.505316,0.853659,0.745413,
1.000000,0.994153,0.064815,0.009601,0.361111,0.251475,0.648515,0.493976,0.821782,0.743223,
1.000000,0.996621,0.011765,0.002756,0.350000,0.241877,0.666667,0.482252,0.871951,0.734185,
1.000000,0.986018,0.121951,0.056897,0.126521,0.062280,0.142061,0.078100,0.118750,0.055121,
0.115162,0.048139,0.095238,0.038672,0.123188,0.053607,0.202532,0.073074,0.134809,0.076323,
0.091892,0.050579,0.174051,0.055802,0.054348,0.023897,0.065327,0.028550,0.074074,0.020760,
0.123223,0.059881,0.165094,0.106794,0.082927,0.044887,0.050808,0.024037,0.062069,0.032662,
0.055556,0.009937

Positive_103 66.000000,0.018365,5.000000,1.212121,1.000000,0.969697,40.000000,1.212121,0.000000,25.400466,
0.367667,0.021382,0.367667,0.021382,0.077922,0.211268,0.517857,0.555556,0.583333,0.400000,
1.000000,0.008081,0.333333,0.002258,0.015152,0.500000,0.007459,0.002290,0.111111,0.000209,
0.007576,0.250000,0.001865,0.373831,0.600000,0.076311,0.049852,0.076923,0.001101,0.038735,
0.615385,0.018962,0.008636,0.061538,0.000314,6.328771,4.000000,2.828427,1.000000,0.136364,
1.000000,0.136364,1.000000,0.136364,1.000000,0.119697,1.000000,0.112121,1.000000,0.051515,
1.000000,0.038384,0.166667,0.010101,0.166667,0.010101,0.000000,0.000000,1.000000,0.121212,
1.000000,0.121057,1.000000,0.118357,1.000000,0.113986,1.000000,0.098046,1.000000,0.082348,
0.500000,0.034654,0.166667,0.012421,0.071429,0.001141,0.035714,0.000561,0.388889,0.317782,
0.545833,0.364138,0.440415,0.318080,0.309677,0.217049,0.302721,0.201205,0.344969,0.224301,
0.074074,0.012943,0.450382,0.249832,0.660131,0.493789,0.836601,0.748626,1.000000,0.991189,
0.041026,0.010989,0.366379,0.240729,0.650000,0.497001,0.853081,0.739823,1.000000,0.995089,

0.018519,0.003487,0.425926,0.253759,0.666667,0.489700,0.833333,0.730882,1.000000,0.990841,
0.825440,0.495545,0.419204,0.114594,0.782443,0.389861,0.145540,0.049234,0.116129,0.074215,
0.018519,0.003841,0.042654,0.004062,0.457516,0.241059,0.653226,0.485585,0.927481,0.747675,
1.000000,1.000000,0.951501,0.166584,0.951501,0.317877,0.958716,0.479533,0.976303,0.666856,
0.998347,0.816837,0.511450,0.053174,0.587786,0.277790,0.761364,0.482968,0.913495,0.693940,
0.998340,0.907943,0.673575,0.505705,0.461538,0.355610,0.314815,0.024374,0.481481,0.275999,
0.666667,0.508744,0.862366,0.736688,0.998586,0.986676,0.558333,0.396048,0.468750,0.363523,
0.342857,0.240429,0.392405,0.280926,0.236842,0.178898,0.242363,0.171539,0.037037,0.010398,
0.334052,0.238051,0.626298,0.487531,0.838863,0.732786,1.000000,0.995217,0.166667,0.014434,
0.388889,0.249223,0.603834,0.489805,0.800000,0.733496,1.000000,0.991315,0.018519,0.003487,
0.432990,0.254610,0.687285,0.513854,0.871951,0.755541,1.000000,0.988948,0.492228,0.344532,
0.475000,0.313979,0.420635,0.341489,0.326360,0.206201,0.354582,0.235740,0.296774,0.201393,
0.018519,0.003487,0.425926,0.259415,0.666667,0.498545,0.833692,0.737611,1.000000,0.991579,
0.042654,0.012246,0.360190,0.229169,0.620833,0.489521,0.857820,0.733395,1.000000,0.993565,
0.074074,0.012778,0.450382,0.252944,0.660131,0.495265,0.836601,0.751576,1.000000,0.992092,
0.423077,0.308204,0.531250,0.451367,0.342857,0.240429,0.388889,0.277368,0.202899,0.141027,
0.280778,0.209410,0.055556,0.011974,0.377163,0.233085,0.660900,0.485783,0.821340,0.732067,
1.000000,0.991443,0.068702,0.011737,0.333333,0.245563,0.561983,0.490948,0.789256,0.736600,
1.000000,0.994741,0.018519,0.003487,0.432990,0.254610,0.687285,0.513854,0.871951,0.755541,
1.000000,0.988948,0.121951,0.057471,0.171429,0.056925,0.133065,0.071178,0.111966,0.052407,
0.091603,0.043211,0.069231,0.036590,0.123810,0.059075,0.185897,0.072343,0.138614,0.074493,
0.083582,0.049886,0.179167,0.058182,0.055556,0.023707,0.058333,0.026452,0.111111,0.029663,
0.137615,0.061785,0.168659,0.106070,0.088083,0.044688,0.050808,0.025698,0.088319,0.037942,
0.038168,0.012233

Positive_104 25.000000,0.068800,4.000000,1.720000,1.000000,2.543333,12.000000,1.720000,0.000000,10.876667,
0.511087,0.024574,0.511087,0.024574,0.000000,0.023256,0.285714,0.366667,0.315789,0.615385,
0.600000,0.000000,0.000000,0.000000,0.000000,0.000000,0.000000,0.000000,0.000000,0.000000,
0.000000,0.000000,0.000000,0.202917,0.458333,0.026621,0.000000,0.000000,0.000000,0.000000,
0.000000,0.000000,0.022083,0.125000,0.001864,5.430312,2.604090,2.000000,1.000000,0.440000,
1.000000,0.440000,1.000000,0.440000,1.000000,0.440000,1.000000,0.393333,1.000000,0.346667,
1.000000,0.220000,1.000000,0.126667,1.000000,0.080000,0.000000,0.000000,1.000000,0.280000,
1.000000,0.280000,1.000000,0.280000,1.000000,0.279394,1.000000,0.256554,1.000000,0.183775,
1.000000,0.140346,1.000000,0.095048,0.060606,0.002424,0.000000,0.000000,0.480583,0.390028,
0.401559,0.301183,0.370000,0.308789,0.262238,0.216237,0.274306,0.229355,0.290909,0.166333,
0.137931,0.026719,0.327586,0.243955,0.545809,0.462440,0.781991,0.673876,1.000000,0.949803,
0.034602,0.014829,0.315972,0.211061,0.552083,0.453827,0.777778,0.691883,1.000000,0.994432,
0.010000,0.004987,0.432203,0.290538,0.771186,0.595121,0.889831,0.832082,1.000000,0.990091,
0.440678,0.252471,0.161616,0.018456,0.850000,0.729072,0.050761,0.008509,0.087838,0.061156,
0.036364,0.002769,0.010000,0.004987,0.451456,0.147741,0.558252,0.354859,0.636620,0.509255,
1.000000,1.000000,0.578544,0.114855,0.578544,0.116382,0.877119,0.169863,0.974576,0.216393,
0.983051,0.250655,0.271186,0.068618,0.484848,0.309151,0.696970,0.537975,0.851852,0.792968,
0.997183,0.985850,0.464646,0.360839,0.493506,0.430345,0.275862,0.039666,0.387931,0.261413,
0.593870,0.510658,0.797170,0.740904,0.991525,0.961575,0.441441,0.341963,0.501684,0.422060,
0.288136,0.235977,0.342857,0.257803,0.281818,0.153285,0.270142,0.188472,0.033898,0.012061,

0.308057,0.201545,0.537736,0.448818,0.753472,0.685371,1.000000,0.990351,0.137931,0.036015,
0.358674,0.269749,0.596491,0.514779,0.850000,0.772358,1.000000,0.987698,0.010000,0.004987,
0.457627,0.256816,0.737288,0.502797,0.847458,0.752182,1.000000,0.980337,0.389831,0.324997,
0.372320,0.269697,0.490291,0.405306,0.281818,0.154992,0.313589,0.253740,0.252381,0.196214,
0.010000,0.004987,0.423729,0.289685,0.771186,0.590824,0.906780,0.831805,1.000000,0.990804,
0.034602,0.015031,0.313589,0.207510,0.553398,0.454945,0.766990,0.679435,1.000000,0.990351,
0.137931,0.026719,0.327586,0.246735,0.543860,0.465318,0.781991,0.678054,1.000000,0.950485,
0.423423,0.305180,0.529661,0.458843,0.288136,0.235977,0.319048,0.263412,0.281818,0.142497,
0.279621,0.199260,0.078014,0.015178,0.264808,0.211164,0.548822,0.480868,0.766990,0.695817,
1.000000,0.990351,0.077586,0.025463,0.297980,0.250629,0.576471,0.498333,0.850000,0.761902,
1.000000,0.987698,0.010000,0.004987,0.457627,0.256816,0.737288,0.502797,0.847458,0.752182,
1.000000,0.980337,0.115207,0.071875,0.096886,0.063478,0.121528,0.087483,0.099099,0.062082,
0.122605,0.064656,0.080569,0.040453,0.101695,0.047834,0.137931,0.069511,0.108108,0.074243,
0.080569,0.051510,0.076271,0.026599,0.037736,0.015277,0.050847,0.016209,0.028436,0.010184,
0.110000,0.061915,0.129310,0.097206,0.101045,0.070346,0.063107,0.034609,0.050847,0.026629,
0.040000,0.007899

Positive_105 8.000000,0.109375,5.000000,0.875000,0.000000,2.982143,2.000000,0.875000,1.000000,0.410714,
0.494237,0.072852,0.494237,0.072852,0.000000,0.285714,0.200000,0.250000,0.333333,0.500000,
0.000000,0.125000,1.000000,0.125000,0.125000,1.000000,0.125000,0.025000,0.200000,0.005000,
0.062500,0.500000,0.031250,0.021429,0.142857,0.002507,0.103571,0.714286,0.062493,0.151786,
0.285714,0.021046,0.321429,0.571429,0.080175,2.288246,1.000000,0.874032,1.000000,0.125000,
1.000000,0.125000,1.000000,0.125000,0.500000,0.062500,0.400000,0.050000,0.300000,0.037500,
0.200000,0.025000,0.000000,0.000000,0.000000,0.000000,0.000000,0.000000,1.000000,0.125000,
1.000000,0.125000,1.000000,0.125000,1.000000,0.125000,1.000000,0.125000,1.000000,0.125000,
0.000000,0.000000,0.000000,0.000000,0.000000,0.000000,0.000000,0.000000,0.393162,0.337982,
0.411765,0.349508,0.350427,0.312509,0.252688,0.224221,0.238149,0.191144,0.251852,0.224323,
0.021390,0.011873,0.273885,0.222475,0.534759,0.452224,0.735207,0.689164,1.000000,0.986417,
0.076923,0.025258,0.307692,0.254585,0.589744,0.524489,0.793078,0.768095,1.000000,0.995237,
0.008547,0.004690,0.282051,0.249793,0.566845,0.516568,0.821656,0.733032,1.000000,0.995752,
0.604278,0.490826,0.222222,0.187256,0.448703,0.321919,0.103448,0.078551,0.092652,0.063463,
0.017241,0.008065,0.008547,0.004690,0.341880,0.273486,0.598291,0.518695,0.782544,0.727134,
1.000000,1.000000,0.239316,0.126455,0.617834,0.368693,0.801915,0.514182,0.899853,0.744300,
0.999264,0.953835,0.069519,0.034407,0.405862,0.192569,0.583991,0.352402,0.859873,0.660598,
0.997745,0.882730,0.600884,0.505172,0.399549,0.380779,0.042781,0.025654,0.350427,0.293213,
0.636943,0.538748,0.821656,0.762593,0.999264,0.981958,0.443850,0.354830,0.388535,0.359186,
0.376068,0.285983,0.303704,0.272860,0.194074,0.169155,0.258621,0.197518,0.085470,0.032737,
0.316239,0.269151,0.558910,0.494268,0.790133,0.743718,1.000000,0.994955,0.059829,0.022712,
0.284104,0.267826,0.545455,0.510901,0.759358,0.729523,1.000000,0.986185,0.008547,0.004690,
0.232484,0.180840,0.589172,0.473895,0.796178,0.715877,1.000000,0.996085,0.393162,0.358628,
0.374332,0.278184,0.418803,0.363187,0.248889,0.195267,0.278781,0.238444,0.236559,0.185612,
0.008547,0.004690,0.282051,0.250279,0.582888,0.521316,0.805732,0.749783,1.000000,0.996821,
0.085470,0.037011,0.316239,0.268998,0.615385,0.528683,0.793078,0.743191,1.000000,0.994955,
0.021390,0.011873,0.273885,0.217879,0.529412,0.456240,0.754438,0.701214,1.000000,0.986815,
0.336898,0.281280,0.458599,0.432736,0.376068,0.285983,0.293333,0.262446,0.163793,0.135515,

0.301724,0.231159,0.136752,0.039147,0.367521,0.297764,0.641026,0.535804,0.790869,0.749186,
1.000000,0.994955,0.059829,0.022342,0.278467,0.260855,0.514092,0.488190,0.757962,0.734654,
1.000000,0.986185,0.008547,0.004690,0.232484,0.180840,0.589172,0.473895,0.796178,0.715877,
1.000000,0.996085,0.068376,0.051141,0.111111,0.081536,0.127389,0.078705,0.076923,0.061914,
0.050810,0.039164,0.040501,0.025521,0.066568,0.050097,0.084320,0.059507,0.090909,0.059847,
0.089172,0.049915,0.080214,0.058818,0.038217,0.025205,0.085470,0.046119,0.026738,0.014732,
0.094017,0.072682,0.124260,0.090873,0.076923,0.052240,0.034188,0.023447,0.076923,0.047652,
0.017094,0.010883

Positive_106 44.000000,0.040806,12.000000,1.795455,1.500000,3.933932,14.000000,1.795455,1.000000,10.026956,
0.354819,0.029824,0.354819,0.029824,0.179487,0.328125,0.372093,0.481481,0.357143,0.555556,
0.750000,0.000000,0.000000,0.000000,0.000000,0.000000,0.000000,0.000000,0.000000,0.000000,
0.000000,0.000000,0.000000,0.153101,0.302326,0.015153,0.033915,0.279070,0.002792,0.030867,
0.127907,0.001295,0.097939,0.255814,0.011474,5.291503,3.525370,3.333132,1.000000,0.500000,
1.000000,0.500000,1.000000,0.499188,1.000000,0.485365,1.000000,0.482955,1.000000,0.149129,
1.000000,0.075758,1.000000,0.058933,0.151515,0.005879,0.060606,0.002189,1.000000,0.272228,
1.000000,0.272228,1.000000,0.270718,1.000000,0.216728,1.000000,0.204196,1.000000,0.177528,
1.000000,0.092016,1.000000,0.051174,1.000000,0.039591,1.000000,0.032806,0.459902,0.328472,
0.516949,0.373683,0.372727,0.297845,0.340984,0.227904,0.259053,0.200753,0.302752,0.209758,
0.076271,0.013401,0.432203,0.258297,0.664706,0.514982,0.872881,0.754578,1.000000,0.995188,
0.028571,0.007434,0.360277,0.247868,0.681159,0.493319,0.850242,0.753652,1.000000,0.994061,
0.009091,0.002572,0.412245,0.239906,0.620408,0.473723,0.816190,0.714627,1.000000,0.991541,
0.815057,0.526555,0.329412,0.149015,0.660259,0.324430,0.088757,0.058984,0.119266,0.071372,
0.016949,0.005934,0.009091,0.002572,0.457516,0.257823,0.647059,0.509487,0.882353,0.764420,
1.000000,1.000000,0.500000,0.100708,0.646552,0.238969,0.843468,0.389466,0.905172,0.614951,
0.995781,0.863841,0.474576,0.047360,0.578313,0.297398,0.822394,0.506540,0.932367,0.699095,
0.998340,0.958969,0.558650,0.476033,0.446602,0.365418,0.059322,0.015318,0.388186,0.248424,
0.594937,0.470452,0.783178,0.698434,0.998823,0.988439,0.567797,0.394546,0.418182,0.346997,
0.341365,0.258456,0.320574,0.254592,0.295082,0.194693,0.251656,0.186790,0.059322,0.008624,
0.374134,0.250235,0.652174,0.500757,0.840580,0.755620,1.000000,0.995524,0.076271,0.011868,
0.396552,0.243335,0.615385,0.490497,0.833333,0.730095,1.000000,0.994675,0.009091,0.002572,
0.359223,0.241629,0.670588,0.494894,0.835294,0.733779,1.000000,0.992946,0.409091,0.328916,
0.491525,0.315955,0.466448,0.355129,0.247706,0.189154,0.306407,0.239616,0.295082,0.208256,
0.009091,0.002572,0.439320,0.244290,0.604082,0.475071,0.816190,0.714380,1.000000,0.992528,
0.059322,0.009078,0.394919,0.244775,0.700483,0.496202,0.859903,0.757697,1.000000,0.991875,
0.076271,0.012262,0.432203,0.258350,0.664706,0.513448,0.847458,0.751682,1.000000,0.995637,
0.423895,0.308772,0.527273,0.432772,0.341365,0.258456,0.337662,0.260006,0.247541,0.151757,
0.289941,0.229726,0.088235,0.011233,0.374134,0.245026,0.676329,0.495645,0.879227,0.751465,
1.000000,0.990458,0.059322,0.009693,0.357759,0.250296,0.617021,0.501103,0.800000,0.739549,
1.000000,0.996736,0.009091,0.002572,0.359223,0.241629,0.670588,0.494894,0.835294,0.733779,
1.000000,0.992946,0.128514,0.064866,0.144578,0.062178,0.102083,0.070856,0.126023,0.054744,
0.063636,0.043780,0.068282,0.032047,0.109091,0.062831,0.140097,0.063972,0.168103,0.076467,
0.101695,0.050759,0.130435,0.061927,0.042373,0.026658,0.060738,0.031070,0.090692,0.023847,
0.100000,0.056704,0.181818,0.100064,0.092391,0.043546,0.039216,0.025486,0.058824,0.036652,
0.033755,0.011545

Positive_107 80.000000,0.026094,17.000000,2.087500,1.000000,7.447943,9.000000,2.087500,1.000000,4.207437,
0.429680,0.032776,0.429680,0.032776,0.067073,0.183007,0.344000,0.329268,0.418182,0.531250,
0.666667,0.023125,0.750000,0.014300,0.028125,0.750000,0.020560,0.004885,0.187500,0.000691,
0.007031,0.187500,0.001285,0.028812,0.101266,0.001149,0.015476,0.113924,0.000692,0.021157,
0.113924,0.000619,0.070388,0.202532,0.004774,5.036796,4.898979,4.501809,1.000000,0.475000,
1.000000,0.474632,1.000000,0.471081,1.000000,0.443748,1.000000,0.394252,1.000000,0.312747,
1.000000,0.256750,1.000000,0.210191,1.000000,0.091091,0.333333,0.014738,1.000000,0.400000,
1.000000,0.400000,1.000000,0.398859,1.000000,0.376558,1.000000,0.352093,1.000000,0.242312,
1.000000,0.207282,1.000000,0.126190,1.000000,0.080804,0.333333,0.036558,0.431718,0.332660,
0.517857,0.358212,0.364162,0.309127,0.270833,0.220231,0.331361,0.206119,0.292490,0.216100,
0.070539,0.011886,0.384342,0.254310,0.597527,0.496784,0.828571,0.746551,1.000000,0.991987,
0.041026,0.007985,0.366379,0.239322,0.625483,0.492113,0.819957,0.740111,1.000000,0.994806,
0.009524,0.002709,0.405858,0.249023,0.640167,0.501765,0.864706,0.744883,1.000000,0.992460,
0.737452,0.499021,0.359788,0.146758,0.731006,0.354220,0.119863,0.064384,0.104430,0.070078,
0.017751,0.005615,0.009524,0.002709,0.368750,0.228168,0.672365,0.464936,0.927885,0.738844,
1.000000,1.000000,0.876147,0.123285,0.885321,0.307514,0.958716,0.469168,0.974763,0.630391,
0.998382,0.852398,0.288256,0.039576,0.938224,0.304886,0.957529,0.539508,0.972973,0.728914,
0.998131,0.938871,0.601140,0.481334,0.500000,0.379328,0.122905,0.019591,0.389892,0.268435,
0.602510,0.512282,0.864706,0.741482,1.000000,0.988297,0.540541,0.384450,0.460526,0.367280,
0.342857,0.248270,0.351515,0.265868,0.238494,0.182387,0.257143,0.185278,0.029762,0.007363,
0.334052,0.238088,0.586207,0.488587,0.798265,0.739788,1.000000,0.994931,0.046025,0.010922,
0.372385,0.253252,0.624595,0.501263,0.801980,0.745592,1.000000,0.995893,0.009524,0.002709,
0.340967,0.246694,0.618123,0.498399,0.844660,0.735131,1.000000,0.989932,0.398876,0.337748,
0.470238,0.305393,0.455947,0.356859,0.255599,0.196567,0.349112,0.242113,0.295833,0.203111,
0.009524,0.002709,0.389121,0.252940,0.631799,0.505406,0.828452,0.741432,1.000000,0.992876,
0.041026,0.008169,0.355603,0.234577,0.633205,0.488404,0.824219,0.740970,1.000000,0.993211,
0.070539,0.011639,0.384342,0.254797,0.597527,0.492129,0.811705,0.742112,1.000000,0.992944,
0.428571,0.304778,0.552632,0.446952,0.342857,0.248270,0.366667,0.266144,0.210084,0.146928,
0.289130,0.220737,0.059524,0.008795,0.330961,0.241469,0.606178,0.499210,0.818359,0.748167,
1.000000,0.994130,0.046025,0.009893,0.320819,0.244993,0.579288,0.492504,0.796117,0.740813,
1.000000,0.996452,0.009524,0.002709,0.340967,0.246694,0.618123,0.498399,0.844660,0.735131,
1.000000,0.989932,0.097633,0.058025,0.171429,0.059680,0.125257,0.075514,0.111765,0.055495,
0.114990,0.045550,0.085526,0.038396,0.123810,0.060565,0.125000,0.065083,0.160714,0.073574,
0.131274,0.050062,0.138790,0.056109,0.043321,0.024199,0.060738,0.028621,0.065934,0.023562,
0.091228,0.060012,0.184211,0.101811,0.087873,0.045997,0.044610,0.024014,0.074349,0.040968,
0.032110,0.012763

Positive_108 142.000000,0.037245,25.000000,5.288732,4.000000,46.064979,29.000000,5.288732,3.000000,37.767106,
0.443197,0.018250,0.443197,0.018250,0.022758,0.149315,0.265700,0.412281,0.630597,0.898990,
1.000000,0.247748,3.040000,0.624960,0.248341,6.551724,1.193905,0.013179,0.240000,0.001741,
0.024866,0.250000,0.004825,0.056697,0.168186,0.003094,0.033241,0.177305,0.002255,0.031103,
0.085106,0.000961,0.062687,0.170213,0.003936,16.489030,10.540962,9.484597,1.000000,0.676056,
1.000000,0.674988,1.000000,0.671156,1.000000,0.632951,1.000000,0.587666,1.000000,0.515013,
1.000000,0.354065,1.000000,0.223373,1.000000,0.117015,1.000000,0.059672,1.000000,0.661972,
1.000000,0.661044,1.000000,0.649841,1.000000,0.570084,1.000000,0.519301,1.000000,0.432376,

1.000000,0.369604,1.000000,0.226997,0.700000,0.124271,0.339181,0.063602,0.462334,0.311727,
0.537344,0.385961,0.437778,0.302311,0.306667,0.220046,0.331361,0.190382,0.374165,0.225262,
0.098214,0.012123,0.371585,0.251418,0.658610,0.508057,0.835260,0.751113,1.000000,0.994444,
0.061728,0.007022,0.353488,0.244582,0.616236,0.494252,0.861893,0.748273,1.000000,0.993308,
0.012346,0.002419,0.362429,0.240688,0.576970,0.480341,0.812274,0.725302,1.000000,0.994251,
0.829091,0.527268,0.467433,0.122848,0.765705,0.349884,0.180422,0.053206,0.156463,0.066654,
0.016541,0.003642,0.042654,0.002686,0.403387,0.246691,0.703704,0.496824,0.891165,0.756804,
1.000000,1.000000,0.978261,0.123396,0.978261,0.321171,0.980676,0.510576,0.991914,0.678337,
0.998815,0.884275,0.902786,0.053496,0.960322,0.272479,0.963467,0.492782,0.973363,0.677776,
0.999132,0.889481,0.788889,0.508626,0.500000,0.354742,0.122905,0.015388,0.389892,0.250754,
0.643463,0.478921,0.813243,0.703626,1.000000,0.988568,0.545643,0.407620,0.440932,0.340287,
0.326409,0.252093,0.343333,0.263861,0.279070,0.191444,0.265774,0.175447,0.061728,0.007491,
0.370370,0.244458,0.634686,0.492073,0.872123,0.748305,1.000000,0.995289,0.037190,0.011441,
0.351044,0.236269,0.603834,0.485135,0.828194,0.730941,1.000000,0.995875,0.012346,0.002419,
0.432331,0.262396,0.676768,0.512040,0.850318,0.743632,1.000000,0.992004,0.480000,0.331208,
0.494813,0.327771,0.483013,0.341021,0.327394,0.204953,0.349112,0.227465,0.297778,0.204148,
0.012346,0.002419,0.362429,0.243677,0.573487,0.481749,0.800464,0.727421,1.000000,0.994343,
0.061728,0.008459,0.360190,0.237676,0.682864,0.489337,0.892583,0.744749,1.000000,0.991473,
0.098214,0.011514,0.383459,0.255081,0.643505,0.510737,0.834586,0.753816,1.000000,0.995197,
0.412017,0.313112,0.528814,0.434794,0.326409,0.252093,0.366379,0.267467,0.209302,0.148324,
0.300191,0.218566,0.061728,0.009205,0.382716,0.241839,0.660900,0.490826,0.859335,0.741686,
1.000000,0.993028,0.034682,0.009082,0.324478,0.238402,0.556364,0.489601,0.791781,0.742762,
1.000000,0.997296,0.012346,0.002419,0.432331,0.262396,0.676768,0.512040,0.850318,0.743632,
1.000000,0.992004,0.115591,0.061220,0.117834,0.057068,0.116691,0.065234,0.111765,0.048540,
0.138889,0.045058,0.062257,0.034607,0.142651,0.068258,0.163090,0.072996,0.142322,0.074519,
0.080569,0.048800,0.152490,0.063199,0.077799,0.029293,0.065714,0.028896,0.093750,0.031308,
0.093750,0.058961,0.168889,0.093694,0.087719,0.042732,0.061728,0.025034,0.071225,0.037684,
0.033333,0.012898

Positive_109 44.000000,0.055785,19.000000,2.454545,0.000000,30.811839,7.000000,2.454545,3.000000,2.765328,
0.473552,0.020814,0.473552,0.020814,0.027778,0.038095,0.277228,0.479452,0.473684,0.400000,
0.916667,0.000000,0.000000,0.000000,0.000000,0.000000,0.000000,0.000000,0.000000,0.000000,
0.000000,0.000000,0.000000,0.024313,0.139535,0.002062,0.078013,0.441860,0.025328,0.090683,
0.162791,0.004681,0.279221,0.418605,0.028369,7.549834,5.881614,3.225928,1.000000,0.295455,
1.000000,0.295455,1.000000,0.294657,1.000000,0.281761,1.000000,0.250877,1.000000,0.223810,
1.000000,0.212167,1.000000,0.156482,1.000000,0.113078,0.028571,0.002894,1.000000,0.818182,
1.000000,0.818182,1.000000,0.781385,1.000000,0.781385,1.000000,0.778139,1.000000,0.761905,
1.000000,0.720779,1.000000,0.573593,1.000000,0.429654,0.142857,0.009740,0.451493,0.289022,
0.540925,0.428775,0.455446,0.282203,0.291589,0.219308,0.221875,0.159751,0.378788,0.236306,
0.135231,0.012986,0.345196,0.243162,0.609966,0.487854,0.894340,0.731940,1.000000,0.995065,
0.069307,0.005927,0.405941,0.256733,0.600000,0.507670,0.851485,0.763072,1.000000,0.996385,
0.009901,0.001839,0.323661,0.242023,0.553191,0.487118,0.779443,0.724188,1.000000,0.992934,
0.801957,0.614960,0.336000,0.122477,0.742574,0.262563,0.130261,0.055879,0.095066,0.054799,
0.013423,0.003958,0.009901,0.001839,0.357143,0.249899,0.642857,0.513904,0.928302,0.769548,
1.000000,1.000000,0.910891,0.112108,0.910891,0.287213,0.920792,0.496616,0.954717,0.684151,

0.999595,0.927049,0.610959,0.063026,0.711762,0.252380,0.877668,0.496311,0.898194,0.710822,
0.999217,0.949051,0.762264,0.488597,0.480172,0.371857,0.053571,0.010607,0.386677,0.259889,
0.641003,0.509824,0.814286,0.743221,0.999609,0.992004,0.577768,0.454333,0.419700,0.322779,
0.326733,0.222887,0.336910,0.274167,0.234438,0.190512,0.240000,0.148734,0.069307,0.005566,
0.405941,0.250992,0.603960,0.501999,0.861386,0.751811,1.000000,0.997123,0.024631,0.009512,
0.345361,0.245750,0.573883,0.497565,0.809061,0.738773,1.000000,0.997164,0.009901,0.001839,
0.327177,0.238569,0.611779,0.490377,0.801683,0.732324,1.000000,0.991922,0.514851,0.309097,
0.508897,0.375684,0.470149,0.315218,0.359848,0.222664,0.270270,0.191633,0.265339,0.211496,
0.009901,0.001839,0.330357,0.244340,0.554361,0.489122,0.779443,0.727108,1.000000,0.993099,
0.069307,0.005973,0.405941,0.252478,0.607143,0.506256,0.861386,0.761119,1.000000,0.995757,
0.135231,0.012925,0.348754,0.243479,0.596463,0.491085,0.848797,0.733635,1.000000,0.995084,
0.416370,0.336025,0.505190,0.441087,0.326733,0.222887,0.336638,0.284876,0.201869,0.142838,
0.280000,0.196408,0.069307,0.006128,0.405941,0.249324,0.603960,0.500829,0.861386,0.751835,
1.000000,0.996108,0.024169,0.007491,0.310292,0.247338,0.577997,0.499691,0.791541,0.744353,
1.000000,0.998132,0.009901,0.001839,0.327177,0.238569,0.611779,0.490377,0.801683,0.732324,
1.000000,0.991922,0.098881,0.058327,0.098881,0.043014,0.099585,0.055590,0.078883,0.045269,
0.167820,0.049537,0.081281,0.037285,0.126214,0.080317,0.137931,0.077825,0.131285,0.079414,
0.074733,0.053200,0.135714,0.084928,0.044289,0.026197,0.059406,0.026894,0.107553,0.033380,
0.098113,0.056711,0.158879,0.086800,0.069663,0.036856,0.048882,0.021755,0.108911,0.034733,
0.059406,0.011968

Positive_110 56.000000,0.046237,16.000000,2.589286,3.000000,12.719156,28.000000,2.589286,0.000000,39.119156,
0.256340,0.005450,0.256340,0.005450,0.158621,0.713115,0.742857,0.888889,1.000000,0.000000,
0.000000,0.000000,0.000000,0.000000,0.000000,0.000000,0.000000,0.000000,0.000000,0.000000,
0.000000,0.000000,0.000000,0.245733,0.490909,0.057860,0.143460,0.290909,0.020973,0.147078,
0.509091,0.053437,0.087152,0.272727,0.014672,9.173368,6.928203,2.449490,1.000000,0.607143,
1.000000,0.606250,1.000000,0.602679,1.000000,0.595706,1.000000,0.591412,0.758333,0.366305,
0.666667,0.358397,0.458333,0.024554,0.300000,0.016071,0.150000,0.008036,1.000000,0.392857,
1.000000,0.392857,1.000000,0.392149,1.000000,0.382370,1.000000,0.348923,0.666667,0.227324,
0.666667,0.214002,0.261905,0.014031,0.214286,0.011480,0.161376,0.008645,0.388186,0.304455,
0.580827,0.394628,0.353283,0.300917,0.282609,0.222425,0.262857,0.187716,0.302829,0.232008,
0.078378,0.012284,0.350725,0.265495,0.591549,0.506869,0.807263,0.739050,1.000000,0.994537,
0.037975,0.007999,0.322511,0.244212,0.599567,0.491379,0.827004,0.750172,1.000000,0.995601,
0.004219,0.002370,0.339350,0.232286,0.563177,0.478942,0.812274,0.737042,1.000000,0.993813,
0.820886,0.528749,0.346221,0.158457,0.533080,0.312794,0.126761,0.062915,0.098935,0.061809,
0.014493,0.004969,0.009886,0.002533,0.383133,0.273673,0.681928,0.538747,0.880342,0.778072,
1.000000,1.000000,0.449315,0.171323,0.797260,0.429104,0.925659,0.577608,0.959233,0.713375,
0.998155,0.899032,0.207229,0.041674,0.391061,0.216559,0.643836,0.383531,0.898990,0.615943,
0.999369,0.838310,0.648379,0.524777,0.436911,0.335939,0.122905,0.016597,0.389892,0.247688,
0.582911,0.475005,0.866541,0.702930,1.000000,0.989103,0.565217,0.416097,0.405063,0.321090,
0.326409,0.262812,0.319635,0.250070,0.250696,0.205694,0.257143,0.180290,0.042980,0.008072,
0.313853,0.250121,0.577922,0.505959,0.836466,0.765763,1.000000,0.996573,0.027714,0.009190,
0.332645,0.229643,0.524366,0.473898,0.777577,0.725144,1.000000,0.992081,0.004219,0.002370,
0.321300,0.258621,0.583548,0.495263,0.809117,0.729139,1.000000,0.996778,0.378229,0.330618,
0.483083,0.333231,0.396624,0.336150,0.281198,0.204003,0.305714,0.231295,0.265358,0.205449,

0.004219,0.002370,0.361011,0.235451,0.567251,0.483581,0.814815,0.741606,1.000000,0.993813,
0.048711,0.009106,0.322511,0.230953,0.610390,0.486707,0.853383,0.746046,1.000000,0.993451,
0.078378,0.011789,0.339130,0.268998,0.597183,0.508727,0.796089,0.742296,1.000000,0.995971,
0.434783,0.325345,0.495817,0.411843,0.326409,0.262812,0.320362,0.262618,0.203343,0.162069,
0.285714,0.223915,0.048711,0.009204,0.324675,0.250241,0.580087,0.496289,0.866541,0.744690,
1.000000,0.994095,0.027397,0.007460,0.300752,0.230217,0.544160,0.490152,0.795455,0.752435,
1.000000,0.995874,0.004219,0.002370,0.321300,0.258621,0.583548,0.495263,0.809117,0.729139,
1.000000,0.996778,0.115591,0.074208,0.099715,0.050883,0.080579,0.057672,0.069832,0.046165,
0.074074,0.039703,0.063877,0.035824,0.156522,0.080004,0.133459,0.077446,0.136076,0.073804,
0.073903,0.047926,0.134720,0.054052,0.077068,0.031695,0.053412,0.029701,0.073446,0.036701,
0.088608,0.057629,0.168821,0.094781,0.070111,0.035483,0.041543,0.021394,0.071225,0.039298,
0.033333,0.015632

Positive_111 86.000000,0.031368,14.000000,2.697674,2.000000,9.342818,13.000000,2.697674,2.000000,7.625171,
0.403533,0.030275,0.403533,0.030275,0.087719,0.230769,0.368750,0.326733,0.485294,0.542857,
0.875000,0.139867,1.428571,0.092805,0.083721,0.900000,0.056124,0.038071,0.222222,0.006509,
0.013488,0.160000,0.001509,0.045618,0.117647,0.002070,0.027790,0.137255,0.001903,0.030812,
0.117647,0.001607,0.059238,0.152941,0.002492,6.006132,5.833815,5.636865,1.000000,0.593023,
1.000000,0.593023,1.000000,0.566524,1.000000,0.524790,1.000000,0.486052,1.000000,0.288996,
1.000000,0.267788,1.000000,0.169825,1.000000,0.052548,1.000000,0.038816,1.000000,0.639535,
1.000000,0.639535,1.000000,0.631008,1.000000,0.597941,1.000000,0.562328,1.000000,0.503832,
1.000000,0.341307,1.000000,0.173747,1.000000,0.049116,1.000000,0.028957,0.462334,0.316819,
0.509317,0.381409,0.365025,0.301772,0.311966,0.230033,0.276730,0.195550,0.293233,0.223417,
0.065574,0.014315,0.374194,0.260090,0.596916,0.501648,0.825991,0.739134,1.000000,0.993821,
0.050725,0.008168,0.341404,0.235020,0.593315,0.488165,0.820388,0.746222,1.000000,0.995836,
0.009259,0.002556,0.368545,0.244830,0.591549,0.498118,0.822355,0.750175,1.000000,0.995046,
0.856734,0.567523,0.313305,0.157504,0.765705,0.274973,0.112717,0.063818,0.156463,0.057524,
0.017928,0.004711,0.009259,0.002556,0.416465,0.238822,0.714286,0.494137,0.891566,0.741310,
1.000000,1.000000,0.907563,0.123727,0.907563,0.282334,0.907563,0.471835,0.981366,0.672335,
0.998051,0.864486,0.493617,0.040189,0.585205,0.224314,0.841410,0.434354,0.927313,0.653879,
0.999350,0.920725,0.593897,0.494394,0.508380,0.350875,0.115942,0.012600,0.365957,0.257757,
0.627477,0.509702,0.822222,0.750493,0.999037,0.990201,0.552795,0.412988,0.440932,0.356563,
0.331325,0.230449,0.364221,0.286980,0.269231,0.176926,0.237251,0.165719,0.050725,0.007565,
0.334140,0.233660,0.582090,0.490997,0.831707,0.745387,1.000000,0.997107,0.064815,0.012151,
0.385093,0.257985,0.589202,0.495169,0.828194,0.735857,1.000000,0.993017,0.009259,0.002556,
0.411311,0.255743,0.791774,0.504629,0.859897,0.749391,1.000000,0.992346,0.392079,0.327866,
0.471111,0.327611,0.483013,0.344523,0.263158,0.201361,0.327103,0.231018,0.282051,0.212569,
0.009259,0.002556,0.373239,0.252558,0.612676,0.502424,0.828889,0.753631,1.000000,0.995663,
0.050725,0.008697,0.365617,0.228310,0.604478,0.474946,0.849398,0.736287,1.000000,0.994258,
0.065574,0.012388,0.380645,0.263370,0.603524,0.504505,0.824074,0.740033,1.000000,0.995259,
0.440000,0.314988,0.537815,0.454563,0.331325,0.230449,0.364221,0.284225,0.217949,0.136156,
0.275766,0.206488,0.050725,0.008963,0.338824,0.234549,0.605327,0.487525,0.853659,0.731093,
1.000000,0.994210,0.064815,0.010979,0.385093,0.254583,0.577640,0.495658,0.824444,0.748245,
1.000000,0.997032,0.009259,0.002556,0.411311,0.255743,0.791774,0.504629,0.859897,0.749391,
1.000000,0.992346,0.115942,0.060670,0.126506,0.049501,0.116691,0.062717,0.129458,0.054168,

0.115162,0.048119,0.075209,0.041644,0.125964,0.068224,0.180124,0.069505,0.151235,0.074505,
0.087404,0.048585,0.124224,0.066791,0.056373,0.027704,0.052778,0.026094,0.119874,0.031209,
0.088285,0.062117,0.169643,0.099975,0.077778,0.041990,0.048882,0.020891,0.066265,0.031803,
0.055556,0.013786

Positive_112 116.000000,0.023038,18.000000,2.672414,2.000000,8.465667,10.000000,2.672414,2.000000,6.726537,
0.386453,0.022736,0.386453,0.022736,0.058442,0.282759,0.432692,0.508475,0.413793,0.558824,
0.933333,0.035783,2.222222,0.053030,0.052586,0.800000,0.035863,0.004825,0.187500,0.000753,
0.007716,0.187500,0.000871,0.023960,0.078261,0.000653,0.017734,0.156522,0.000923,0.015710,
0.078261,0.000328,0.031127,0.104348,0.000899,7.338314,5.774229,5.633950,1.000000,0.534483,
1.000000,0.534483,1.000000,0.534483,1.000000,0.496229,1.000000,0.431335,1.000000,0.388736,
1.000000,0.263203,1.000000,0.175536,1.000000,0.109497,1.000000,0.075869,1.000000,0.603448,
1.000000,0.603038,1.000000,0.591851,1.000000,0.530788,1.000000,0.431609,1.000000,0.380213,
1.000000,0.293979,1.000000,0.186939,1.000000,0.105679,1.000000,0.067577,0.462334,0.311914,
0.537344,0.386200,0.419355,0.301886,0.275830,0.228550,0.331361,0.190109,0.323944,0.222937,
0.096639,0.010752,0.440860,0.256434,0.688172,0.506166,0.827957,0.749465,1.000000,0.991859,
0.032258,0.005934,0.367470,0.247494,0.621395,0.492367,0.828652,0.742581,1.000000,0.994585,
0.010753,0.002087,0.401015,0.240621,0.653130,0.492340,0.819193,0.735404,1.000000,0.995631,
0.829091,0.552497,0.406523,0.201734,0.679775,0.245769,0.138535,0.078874,0.101695,0.053586,
0.013060,0.004128,0.010753,0.002087,0.493917,0.250065,0.700465,0.488389,0.926966,0.746626,
1.000000,1.000000,0.642395,0.059012,0.690939,0.240202,0.724919,0.438861,0.875723,0.615432,
0.997442,0.890729,0.251397,0.037061,0.760194,0.345635,0.929577,0.585638,0.988943,0.812469,
0.998975,0.961851,0.640449,0.499184,0.510471,0.363947,0.088235,0.010864,0.351145,0.239956,
0.644670,0.487463,0.818448,0.725615,1.000000,0.991540,0.545643,0.408631,0.451613,0.350332,
0.304516,0.241037,0.342243,0.274620,0.278607,0.187755,0.237251,0.170887,0.037671,0.006292,
0.335484,0.248493,0.626047,0.487600,0.823034,0.742924,1.000000,0.995062,0.034682,0.009485,
0.321865,0.248393,0.614213,0.502734,0.802632,0.745199,1.000000,0.996453,0.010753,0.002087,
0.373950,0.245208,0.611399,0.498137,0.877660,0.741427,1.000000,0.992297,0.463483,0.335666,
0.494813,0.327756,0.483013,0.336579,0.278873,0.207711,0.349112,0.227141,0.261372,0.210050,
0.010753,0.002087,0.404399,0.241599,0.651438,0.490118,0.813875,0.730534,1.000000,0.996052,
0.037671,0.006401,0.359589,0.248122,0.648372,0.492537,0.862360,0.745527,1.000000,0.993274,
0.096639,0.010096,0.408602,0.258434,0.666667,0.506822,0.818533,0.751742,1.000000,0.992712,
0.374481,0.317726,0.548387,0.441237,0.304516,0.241037,0.337184,0.274551,0.200000,0.147178,
0.278351,0.211465,0.044776,0.006740,0.337500,0.245299,0.610938,0.485734,0.862360,0.740124,
1.000000,0.993015,0.034682,0.007665,0.305761,0.250232,0.575800,0.501014,0.807229,0.747493,
1.000000,0.997419,0.010753,0.002087,0.373950,0.245208,0.611399,0.498137,0.877660,0.741427,
1.000000,0.992297,0.097594,0.057347,0.150602,0.056317,0.116691,0.064476,0.111765,0.054084,
0.138889,0.041999,0.086022,0.037691,0.164706,0.066642,0.117172,0.065638,0.142322,0.076895,
0.093750,0.054467,0.152490,0.064114,0.063415,0.024665,0.062958,0.033779,0.074212,0.026792,
0.119048,0.066956,0.159204,0.092979,0.101124,0.046232,0.041176,0.020755,0.070588,0.036326,
0.026316,0.011847

Positive_113 70.000000,0.044490,8.000000,3.114286,2.000000,4.160663,22.000000,3.114286,1.500000,20.827329,
0.390504,0.025930,0.390504,0.025930,0.059908,0.308824,0.439716,0.316456,0.574074,0.565217,
0.500000,0.066667,0.666667,0.030918,0.008929,0.625000,0.005580,0.031746,0.250000,0.006940,
0.000558,0.039062,0.000022,0.137598,0.304348,0.010920,0.026749,0.115942,0.000522,0.019998,

0.231884,0.001717,0.032748,0.101449,0.001186,7.753300,6.928203,5.000000,1.000000,0.785714,
1.000000,0.785714,1.000000,0.782653,1.000000,0.720476,1.000000,0.634286,1.000000,0.552517,
1.000000,0.514286,1.000000,0.293605,1.000000,0.211088,1.000000,0.034014,1.000000,0.500000,
1.000000,0.499757,1.000000,0.496293,1.000000,0.438768,1.000000,0.364164,1.000000,0.307432,
1.000000,0.271546,1.000000,0.224075,1.000000,0.147438,1.000000,0.085088,0.431718,0.316518,
0.537344,0.378432,0.375758,0.305049,0.317010,0.230441,0.331361,0.194461,0.321168,0.221588,
0.193939,0.012559,0.472727,0.266670,0.666667,0.523033,0.817568,0.756950,1.000000,0.991525,
0.047297,0.008265,0.317308,0.234081,0.579327,0.481155,0.819957,0.738654,1.000000,0.992944,
0.007246,0.002900,0.361650,0.241019,0.619048,0.487203,0.818951,0.732879,1.000000,0.992785,
0.829091,0.523099,0.282448,0.143559,0.540541,0.333342,0.099631,0.060733,0.129252,0.074659,
0.020408,0.004722,0.007246,0.002900,0.357955,0.238557,0.607069,0.490186,0.887324,0.758835,
1.000000,1.000000,0.611111,0.101520,0.706117,0.275957,0.837766,0.430726,0.918960,0.602470,
0.996313,0.843183,0.177112,0.052666,0.649705,0.319590,0.810211,0.540940,0.973363,0.747160,
0.998890,0.956782,0.619205,0.505539,0.500000,0.379299,0.088235,0.016144,0.370270,0.249062,
0.578009,0.477047,0.819712,0.705982,1.000000,0.984690,0.545643,0.402079,0.412145,0.344355,
0.319444,0.253566,0.342784,0.264284,0.299270,0.202116,0.251656,0.173712,0.047297,0.008228,
0.340278,0.239289,0.564885,0.485426,0.799669,0.745411,1.000000,0.995294,0.042254,0.011998,
0.348797,0.247640,0.630332,0.492182,0.826087,0.741216,1.000000,0.993459,0.007246,0.002900,
0.361702,0.253418,0.671743,0.500693,0.877660,0.737047,1.000000,0.988004,0.427536,0.340353,
0.494813,0.316720,0.455947,0.342927,0.294118,0.207326,0.349112,0.237117,0.322165,0.205308,
0.007246,0.002900,0.439320,0.246275,0.619048,0.491523,0.818951,0.731421,1.000000,0.993159,
0.047297,0.008495,0.304012,0.224355,0.579327,0.478418,0.829384,0.741012,1.000000,0.991880,
0.193939,0.012545,0.460606,0.267078,0.636364,0.517645,0.817568,0.753314,1.000000,0.991897,
0.428571,0.306474,0.539394,0.439960,0.319444,0.253566,0.348039,0.268908,0.211679,0.155001,
0.289130,0.220827,0.059524,0.009137,0.302439,0.235920,0.556190,0.477037,0.818359,0.731978,
1.000000,0.991208,0.037915,0.009913,0.383886,0.249320,0.639810,0.503126,0.838863,0.750128,
1.000000,0.997624,0.007246,0.002900,0.361702,0.253418,0.671743,0.500693,0.877660,0.737047,
1.000000,0.988004,0.101942,0.056972,0.111702,0.059351,0.102083,0.066015,0.111765,0.055192,
0.138889,0.044434,0.068282,0.034554,0.113095,0.068830,0.121993,0.062524,0.160714,0.068925,
0.083770,0.051003,0.152490,0.065439,0.047753,0.026409,0.072464,0.035304,0.105634,0.030166,
0.099476,0.059880,0.151515,0.096070,0.077778,0.043402,0.041667,0.024255,0.070588,0.038930,
0.036232,0.012347

Positive_114 183.000000,0.046254,47.000000,8.464481,2.000000,200.667687,23.000000,8.464481,6.000000,60.788567,
0.335164,0.011982,0.335164,0.011982,0.073092,0.360084,0.633588,0.672619,0.645455,0.615385,
0.600000,0.117500,1.600000,0.104612,0.132100,2.000000,0.108020,0.012487,0.250000,0.001317,
0.016605,0.250000,0.001863,0.029985,0.105915,0.001166,0.025745,0.258242,0.002743,0.021045,
0.087912,0.000456,0.086346,0.252747,0.009879,30.680457,11.659655,10.392305,1.000000,0.562842,
1.000000,0.562234,1.000000,0.541279,1.000000,0.494922,1.000000,0.427219,1.000000,0.324697,
1.000000,0.215216,1.000000,0.129867,1.000000,0.044293,0.333333,0.015777,1.000000,0.737705,
1.000000,0.736353,1.000000,0.711163,1.000000,0.665663,1.000000,0.602698,1.000000,0.531742,
1.000000,0.421781,1.000000,0.277591,1.000000,0.114410,0.333333,0.044041,0.462334,0.323036,
0.642565,0.387453,0.456954,0.289511,0.376582,0.235721,0.278912,0.192396,0.327684,0.214667,
0.054893,0.008226,0.411765,0.254012,0.664706,0.509846,0.841176,0.749474,1.000000,0.995889,
0.029762,0.005704,0.345972,0.243754,0.681159,0.485614,0.853081,0.738478,1.000000,0.994874,

0.006623,0.001978,0.454698,0.242328,0.653130,0.500055,0.888813,0.746297,1.000000,0.992887,
0.929322,0.559245,0.451807,0.167540,0.737336,0.273214,0.194444,0.066928,0.116959,0.055212,
0.026163,0.004044,0.042654,0.002185,0.457516,0.239317,0.709677,0.485229,0.891165,0.731424,
1.000000,1.000000,0.909953,0.113262,0.909953,0.297091,0.983114,0.509243,0.990619,0.690259,
0.999230,0.903185,0.896028,0.055790,0.923481,0.315325,0.953289,0.548691,0.981426,0.743437,
0.999213,0.956404,0.619835,0.470196,0.612507,0.403201,0.092715,0.011131,0.496644,0.247533,
0.666107,0.492545,0.866387,0.733610,1.000000,0.990728,0.681011,0.419527,0.456193,0.347308,
0.313953,0.233166,0.342408,0.275189,0.263158,0.188232,0.251656,0.163665,0.059322,0.006517,
0.338164,0.245344,0.652174,0.489024,0.840580,0.737893,1.000000,0.996061,0.093023,0.010004,
0.372385,0.250731,0.624595,0.504291,0.853142,0.746160,1.000000,0.996047,0.006623,0.001978,
0.368201,0.244051,0.671743,0.506070,0.870398,0.758690,1.000000,0.993381,0.496689,0.318332,
0.623634,0.335461,0.483013,0.346206,0.298137,0.199903,0.311475,0.227737,0.327759,0.216884,
0.006623,0.001978,0.459732,0.242663,0.657787,0.499488,0.896999,0.748720,1.000000,0.993383,
0.059322,0.007188,0.360190,0.241804,0.700483,0.485793,0.859903,0.735019,1.000000,0.994188,
0.046377,0.007569,0.392157,0.254474,0.664706,0.509914,0.841176,0.751396,1.000000,0.996357,
0.478825,0.323979,0.528814,0.442856,0.313953,0.233166,0.452603,0.286254,0.251462,0.148344,
0.289941,0.203553,0.088235,0.007912,0.377163,0.243048,0.676329,0.486060,0.879227,0.734635,
1.000000,0.994227,0.087209,0.008423,0.333333,0.252167,0.586420,0.501167,0.797753,0.744946,
1.000000,0.997348,0.006623,0.001978,0.368201,0.244051,0.671743,0.506070,0.870398,0.758690,
1.000000,0.993381,0.117647,0.054876,0.109827,0.057622,0.189274,0.069739,0.156667,0.057348,
0.085324,0.045358,0.075710,0.038094,0.286399,0.079783,0.157812,0.066163,0.160714,0.069474,
0.108280,0.051211,0.204142,0.068831,0.053333,0.023170,0.072555,0.028822,0.090692,0.026717,
0.105960,0.060539,0.175487,0.089404,0.106742,0.044174,0.058140,0.021731,0.084337,0.036349,
0.029762,0.010597

Positive_115 70.000000,0.096939,36.000000,6.785714,0.000000,103.214286,16.000000,6.785714,4.500000,47.996894,
0.283882,0.008607,0.283882,0.008607,0.178723,0.520725,0.697297,0.857143,0.750000,1.000000,
0.000000,0.000000,0.000000,0.000000,0.000000,0.000000,0.000000,0.000000,0.000000,0.000000,
0.000000,0.000000,0.000000,0.082147,0.217391,0.008617,0.000000,0.000000,0.000000,0.000000,
0.000000,0.000000,0.161065,0.420290,0.023394,17.569017,9.352838,6.646140,1.000000,0.428571,
1.000000,0.428571,1.000000,0.428043,1.000000,0.399136,1.000000,0.347705,0.890110,0.229459,
0.681319,0.158504,0.600000,0.093049,0.400000,0.045991,0.333333,0.016008,1.000000,0.528571,
1.000000,0.528571,1.000000,0.528571,1.000000,0.507755,1.000000,0.447988,1.000000,0.371789,
0.666667,0.225995,0.400000,0.147930,0.333333,0.098076,0.066667,0.028182,0.419558,0.298169,
0.642565,0.430481,0.426282,0.271350,0.376582,0.244306,0.254190,0.163893,0.311897,0.224626,
0.060897,0.006182,0.354029,0.250446,0.620261,0.514887,0.834553,0.753693,1.000000,0.997159,
0.019900,0.003855,0.331230,0.252287,0.586751,0.483984,0.803513,0.732213,1.000000,0.997780,
0.005051,0.001217,0.334250,0.223211,0.645975,0.498361,0.888813,0.768303,1.000000,0.996253,
0.929322,0.648183,0.411465,0.187651,0.642857,0.164166,0.154762,0.072088,0.075758,0.030249,
0.019293,0.002288,0.005051,0.001217,0.333682,0.242830,0.589077,0.478957,0.830128,0.704848,
1.000000,1.000000,0.874583,0.107742,0.907272,0.355712,0.948632,0.569908,0.965310,0.735716,
0.999230,0.935290,0.896028,0.070459,0.923481,0.244744,0.953289,0.574716,0.981426,0.802496,
0.999019,0.965647,0.778917,0.472873,0.612507,0.421091,0.020362,0.005029,0.325409,0.232611,
0.634473,0.478093,0.866387,0.746981,0.999444,0.994528,0.681011,0.472799,0.429487,0.325168,
0.294290,0.202033,0.342408,0.296497,0.245283,0.184107,0.209497,0.129181,0.019900,0.003639,

0.321767,0.249993,0.573604,0.484307,0.810585,0.722447,1.000000,0.998053,0.067873,0.007143,
0.322705,0.247254,0.614046,0.513079,0.853142,0.768610,1.000000,0.996888,0.005051,0.001217,
0.400000,0.243204,0.651741,0.515531,0.893548,0.782323,1.000000,0.996234,0.458333,0.297713,
0.623634,0.383983,0.460000,0.318304,0.295820,0.214722,0.293296,0.194516,0.327759,0.232166,
0.005051,0.001217,0.328841,0.224101,0.657787,0.504539,0.896999,0.776098,1.000000,0.997293,
0.019900,0.004029,0.340694,0.251712,0.588913,0.481459,0.804061,0.727533,1.000000,0.997311,
0.060897,0.006079,0.326288,0.251642,0.604102,0.515731,0.834553,0.756393,1.000000,0.997522,
0.478825,0.362863,0.491228,0.435104,0.294290,0.202033,0.452603,0.324259,0.191460,0.144934,
0.259777,0.168354,0.025070,0.004337,0.331230,0.253727,0.561514,0.485928,0.793872,0.728842,
1.000000,0.997181,0.029851,0.004622,0.302415,0.247216,0.576336,0.499645,0.796915,0.748814,
1.000000,0.998011,0.005051,0.001217,0.400000,0.243204,0.651741,0.515531,0.893548,0.782323,
1.000000,0.996234,0.089590,0.050549,0.074627,0.044195,0.189274,0.062447,0.156667,0.058388,
0.085324,0.043911,0.075710,0.038679,0.286399,0.113616,0.133484,0.066884,0.133333,0.069921,
0.128959,0.054054,0.204142,0.079508,0.053333,0.020135,0.072555,0.026363,0.074873,0.030428,
0.096154,0.059371,0.175487,0.081402,0.096154,0.039357,0.049780,0.015913,0.078167,0.034754,
0.027419,0.010124

Positive_116 113.000000,0.037434,19.000000,4.230088,1.000000,46.375158,19.000000,4.230088,1.000000,45.321587,
0.786866,0.039041,0.786866,0.039041,0.000000,0.023013,0.029979,0.059603,0.079812,0.181122,
0.161994,3.342035,19.000000,50.246454,3.333333,19.000000,50.287202,0.232824,1.000000,0.169483,
0.223943,1.000000,0.165427,0.031359,0.160714,0.003468,0.030189,0.160714,0.003518,0.030308,
0.160714,0.003513,0.040435,0.160714,0.003602,19.000000,3.316625,3.131508,1.000000,0.407080,
1.000000,0.407080,1.000000,0.407080,1.000000,0.407080,1.000000,0.389381,1.000000,0.346903,
1.000000,0.292044,1.000000,0.209717,1.000000,0.157629,0.333333,0.080826,1.000000,0.407080,
1.000000,0.407080,1.000000,0.407080,1.000000,0.404130,1.000000,0.373156,1.000000,0.333333,
1.000000,0.277778,1.000000,0.227630,1.000000,0.135693,1.000000,0.079646,0.379884,0.271775,
0.580000,0.403600,0.439614,0.324625,0.321429,0.216409,0.268719,0.165345,0.405286,0.258943,
0.085106,0.016430,0.509579,0.264226,0.697318,0.499382,0.896552,0.745111,1.000000,0.993335,
0.032967,0.008471,0.373219,0.255605,0.623932,0.512314,0.857778,0.758736,1.000000,0.996419,
0.005917,0.002707,0.353135,0.220150,0.656716,0.478805,0.814545,0.719759,1.000000,0.994919,
0.880597,0.498423,0.548023,0.272452,0.640898,0.229125,0.175637,0.098487,0.108985,0.034664,
0.030303,0.004165,0.005917,0.002707,0.390805,0.260259,0.808696,0.540330,0.919431,0.785807,
1.000000,1.000000,0.245211,0.069708,0.620690,0.237989,0.878109,0.424819,0.883085,0.652893,
0.999354,0.912525,0.898799,0.045786,0.912521,0.278732,0.953458,0.582577,0.988462,0.747877,
0.998409,0.889288,0.669856,0.501130,0.564860,0.352539,0.036789,0.011447,0.316832,0.217779,
0.820896,0.470830,0.878109,0.711008,0.999233,0.984201,0.600000,0.422274,0.417582,0.359027,
0.291457,0.218699,0.403990,0.303412,0.243762,0.175151,0.238462,0.152299,0.032967,0.007887,
0.335570,0.254914,0.579487,0.504996,0.819905,0.752852,1.000000,0.994325,0.041420,0.012001,
0.335249,0.243828,0.615385,0.487929,0.834320,0.729653,1.000000,0.996024,0.005917,0.002707,
0.338957,0.235579,0.669154,0.490359,0.852843,0.747095,1.000000,0.992831,0.478261,0.356828,
0.567500,0.351018,0.407315,0.292154,0.387665,0.244422,0.317804,0.199074,0.315476,0.200176,
0.005917,0.002707,0.363036,0.225972,0.656716,0.482296,0.814545,0.727154,1.000000,0.995323,
0.032967,0.008634,0.334483,0.250316,0.621083,0.507441,0.836364,0.755430,1.000000,0.992599,
0.082677,0.015872,0.463602,0.263986,0.693487,0.501870,0.896552,0.749114,1.000000,0.994116,
0.502500,0.335801,0.537906,0.445500,0.291457,0.218699,0.416459,0.298340,0.205374,0.139879,

0.268293,0.187571,0.047619,0.009619,0.321608,0.250605,0.592308,0.494906,0.810078,0.745805,
1.000000,0.993707,0.041420,0.010970,0.356000,0.249804,0.564103,0.497360,0.796000,0.739514,
1.000000,0.997569,0.005917,0.002707,0.338957,0.235579,0.669154,0.490359,0.852843,0.747095,
1.000000,0.992831,0.090551,0.048135,0.111111,0.042985,0.090551,0.055536,0.094675,0.043745,
0.074236,0.042081,0.072682,0.039293,0.142012,0.075055,0.149254,0.073330,0.145000,0.077547,
0.159204,0.066124,0.123037,0.058962,0.042308,0.020378,0.084291,0.032204,0.083032,0.027511,
0.112583,0.076517,0.184332,0.098624,0.082192,0.046976,0.061611,0.021645,0.080460,0.037439,
0.040909,0.015913

Positive_117 71.000000,0.037691,25.000000,2.676056,2.000000,14.479276,9.000000,2.676056,2.000000,6.250704,
0.406671,0.033311,0.406671,0.033311,0.047368,0.248619,0.455882,0.418919,0.465116,0.391304,
0.428571,0.577637,4.000000,1.507309,0.480047,4.000000,1.083624,0.118539,1.000000,0.068927,
0.093212,0.750000,0.035500,0.037726,0.114286,0.001513,0.033606,0.157143,0.001715,0.039272,
0.128571,0.001610,0.126730,0.342857,0.014603,6.658437,5.215842,4.880755,1.000000,0.521127,
1.000000,0.521127,1.000000,0.519671,1.000000,0.480673,1.000000,0.418457,1.000000,0.336097,
1.000000,0.263567,1.000000,0.157230,0.333333,0.036255,0.166667,0.010896,1.000000,0.549296,
1.000000,0.549296,1.000000,0.530203,1.000000,0.448357,1.000000,0.375196,1.000000,0.230595,
1.000000,0.201252,1.000000,0.152191,0.100000,0.009703,0.100000,0.008920,0.433225,0.322678,
0.532313,0.392204,0.354455,0.285118,0.302805,0.233437,0.260918,0.185125,0.277567,0.218260,
0.033333,0.009107,0.371585,0.256990,0.628272,0.503308,0.857923,0.748548,1.000000,0.995461,
0.029885,0.005659,0.416335,0.254083,0.616466,0.496970,0.861893,0.751168,1.000000,0.994481,
0.008929,0.002056,0.362429,0.236140,0.578358,0.482972,0.798715,0.731968,1.000000,0.996941,
0.803279,0.529677,0.423006,0.149463,0.737336,0.320859,0.152091,0.061392,0.112821,0.062237,
0.015571,0.004204,0.008929,0.002056,0.544231,0.254285,0.676923,0.509842,0.891165,0.762674,
1.000000,1.000000,0.905598,0.107307,0.905598,0.222100,0.983114,0.453492,0.991914,0.640874,
0.999394,0.888802,0.251397,0.040070,0.836569,0.318117,0.884876,0.567947,0.964334,0.734735,
0.998561,0.951632,0.618438,0.469870,0.494382,0.374195,0.070896,0.012443,0.361186,0.248135,
0.591331,0.486334,0.810384,0.713393,0.998646,0.989670,0.533865,0.406968,0.434307,0.354781,
0.325714,0.238252,0.360360,0.281321,0.299625,0.185075,0.240688,0.168187,0.025287,0.005459,
0.352415,0.247372,0.618474,0.498402,0.872123,0.751794,1.000000,0.996328,0.035857,0.009529,
0.351044,0.242950,0.574627,0.485574,0.810017,0.731657,1.000000,0.996425,0.008929,0.002056,
0.362791,0.255916,0.643939,0.500461,0.832884,0.740037,1.000000,0.993428,0.392079,0.317314,
0.470120,0.333510,0.454397,0.349177,0.267016,0.205115,0.338109,0.224814,0.288973,0.212308,
0.008929,0.002056,0.362429,0.239055,0.589552,0.484321,0.783726,0.731685,1.000000,0.997190,
0.029885,0.005978,0.431727,0.250028,0.682864,0.498438,0.892583,0.750928,1.000000,0.993632,
0.033333,0.009034,0.369403,0.258956,0.617801,0.502275,0.852459,0.746972,1.000000,0.995978,
0.398887,0.315611,0.524150,0.446137,0.325714,0.238252,0.368030,0.277632,0.232210,0.145160,
0.288018,0.208103,0.031873,0.006860,0.338824,0.240825,0.601023,0.489706,0.859335,0.748660,
1.000000,0.994276,0.030556,0.007634,0.324478,0.247976,0.563433,0.498347,0.781421,0.742307,
1.000000,0.997441,0.008929,0.002056,0.362791,0.255916,0.643939,0.500461,0.832884,0.740037,
1.000000,0.993428,0.081121,0.054629,0.102190,0.059157,0.104235,0.068972,0.077720,0.053531,
0.076503,0.047259,0.062180,0.039130,0.125498,0.061054,0.124765,0.070321,0.141791,0.075591,
0.100186,0.055113,0.196787,0.071430,0.077799,0.026499,0.065714,0.032195,0.052239,0.019927,
0.099476,0.061302,0.120359,0.089651,0.077778,0.048467,0.046512,0.023587,0.051163,0.030175,
0.023555,0.012009

Positive_118 111.000000,0.059573,32.000000,6.612613,3.000000,70.439476,24.000000,6.612613,2.000000,77.294021,
0.633423,0.072650,0.637768,0.070374,0.057024,0.079646,0.083333,0.092657,0.188825,0.258907,
0.201923,4.163037,19.000000,56.601994,3.748989,19.125000,50.526009,0.222805,1.000000,0.154748,
0.205651,1.000000,0.101785,0.085795,0.209091,0.007414,0.072391,0.281818,0.006397,0.049301,
0.171212,0.003961,0.111292,0.281818,0.005878,21.415869,9.346314,7.665759,1.000000,0.575036,
1.000000,0.569036,1.000000,0.535213,1.000000,0.464445,1.000000,0.400663,1.000000,0.367400,
1.000000,0.336680,1.000000,0.245636,0.666667,0.146287,0.333333,0.069773,1.000000,0.540541,
1.000000,0.538138,1.000000,0.499804,1.000000,0.452671,1.000000,0.430045,1.000000,0.373525,
1.000000,0.266501,1.000000,0.136228,0.402174,0.084177,0.333333,0.040379,0.482959,0.334931,
0.521505,0.359820,0.439614,0.305248,0.339623,0.226633,0.279177,0.195387,0.405286,0.216407,
0.050459,0.011690,0.509579,0.264300,0.700000,0.514505,0.896552,0.757127,1.000000,0.992954,
0.039370,0.006778,0.353488,0.239966,0.613636,0.494047,0.861893,0.756832,1.000000,0.993753,
0.008333,0.002575,0.353135,0.236520,0.561056,0.467453,0.825103,0.707418,1.000000,0.994621,
0.844086,0.438193,0.451807,0.136644,0.817955,0.425163,0.149296,0.054642,0.156463,0.061725,
0.019737,0.005024,0.070866,0.003143,0.401575,0.234749,0.808696,0.494829,0.919431,0.733424,
1.000000,1.000000,0.905598,0.093696,0.905598,0.204857,0.917695,0.380913,0.962963,0.591147,
0.999510,0.832804,0.273927,0.035006,0.795302,0.301713,0.825503,0.512203,0.883459,0.709666,
0.999193,0.931616,0.669856,0.491966,0.509693,0.363638,0.119266,0.015997,0.390788,0.237551,
0.578009,0.463479,0.860082,0.698839,0.999510,0.988210,0.508065,0.384992,0.460432,0.368801,
0.325714,0.246206,0.365741,0.276186,0.264117,0.188265,0.261905,0.177658,0.062992,0.006561,
0.335570,0.245555,0.601023,0.497992,0.872123,0.757170,1.000000,0.994054,0.128440,0.014628,
0.344037,0.248310,0.576132,0.490761,0.823009,0.729275,1.000000,0.997948,0.008333,0.002575,
0.370192,0.240943,0.675000,0.494141,0.877660,0.740871,1.000000,0.992947,0.478261,0.334694,
0.467742,0.308877,0.496457,0.356428,0.387665,0.204891,0.338109,0.230725,0.305299,0.204688,
0.008333,0.002575,0.363036,0.239030,0.577558,0.468315,0.823045,0.712611,1.000000,0.995027,
0.062992,0.007102,0.340541,0.237005,0.682864,0.496344,0.892583,0.754915,1.000000,0.990305,
0.050459,0.011483,0.463602,0.262264,0.700000,0.513206,0.896552,0.757970,1.000000,0.993472,
0.421053,0.318847,0.508333,0.434946,0.325714,0.246206,0.350490,0.273775,0.253188,0.156712,
0.309220,0.209212,0.062992,0.006793,0.304348,0.238922,0.601023,0.494464,0.859335,0.748055,
1.000000,0.991967,0.087209,0.012753,0.337449,0.254580,0.619342,0.499403,0.816667,0.740754,
1.000000,0.998246,0.008333,0.002575,0.370192,0.240943,0.675000,0.494141,0.877660,0.740871,
1.000000,0.992947,0.110215,0.056758,0.111702,0.063264,0.146780,0.076502,0.105134,0.056161,
0.098919,0.046410,0.069977,0.035837,0.122605,0.065563,0.137097,0.077072,0.123894,0.066878,
0.096234,0.053173,0.118280,0.046191,0.040921,0.021497,0.084291,0.029446,0.062802,0.019954,
0.111111,0.064825,0.184332,0.095086,0.082192,0.050142,0.061611,0.026092,0.084337,0.037985,
0.040909,0.011164

Positive_119 91.000000,0.028861,18.000000,2.626374,1.000000,11.169963,20.000000,2.626374,2.000000,9.125519,
0.327897,0.019483,0.327897,0.019483,0.123404,0.393204,0.616000,0.520833,0.521739,0.545455,
0.600000,0.065324,2.000000,0.126584,0.356960,2.000000,0.572278,0.013469,1.000000,0.011211,
0.166688,1.000000,0.134632,0.063026,0.211111,0.006318,0.053643,0.200000,0.005676,0.044145,
0.222222,0.002350,0.060169,0.188889,0.004051,6.708204,6.655445,6.063457,1.000000,0.472527,
1.000000,0.471917,1.000000,0.469556,1.000000,0.445033,1.000000,0.413043,1.000000,0.324048,
1.000000,0.284695,1.000000,0.184950,0.555556,0.070430,0.333333,0.021625,1.000000,0.714286,
1.000000,0.714286,1.000000,0.708793,1.000000,0.694577,1.000000,0.521852,1.000000,0.495711,

1.000000,0.407300,1.000000,0.322631,1.000000,0.071115,0.357895,0.038781,0.408629,0.314564,
0.459559,0.360579,0.466877,0.324858,0.302805,0.219331,0.262857,0.202673,0.354430,0.228869,
0.046377,0.008827,0.366812,0.254751,0.620087,0.507037,0.849257,0.765456,1.000000,0.994350,
0.024896,0.007003,0.302799,0.238423,0.633508,0.493407,0.846962,0.743437,1.000000,0.996459,
0.005319,0.002049,0.350746,0.247641,0.578358,0.494449,0.812274,0.723354,1.000000,0.994852,
0.751144,0.474677,0.377880,0.149034,0.722397,0.376288,0.194444,0.061484,0.111732,0.067626,
0.026163,0.007370,0.005319,0.002049,0.391598,0.252321,0.709677,0.503313,0.880342,0.764981,
1.000000,1.000000,0.436019,0.072751,0.785863,0.247619,0.954262,0.447109,0.968815,0.661351,
0.998284,0.874082,0.308756,0.036210,0.860812,0.300022,0.868268,0.534657,0.883408,0.748433,
0.997758,0.959775,0.703470,0.551009,0.430233,0.344818,0.122905,0.012736,0.389892,0.247441,
0.597738,0.487406,0.844244,0.711799,1.000000,0.988416,0.490566,0.378506,0.443231,0.367115,
0.319088,0.254379,0.348101,0.270691,0.235119,0.185850,0.257143,0.185537,0.024324,0.007027,
0.345588,0.240915,0.638743,0.495177,0.838710,0.745655,1.000000,0.995630,0.033333,0.007990,
0.312450,0.242137,0.574627,0.487707,0.786062,0.737032,1.000000,0.996793,0.005319,0.002049,
0.451104,0.263293,0.618297,0.512116,0.877660,0.749283,1.000000,0.993709,0.492114,0.358744,
0.419118,0.302012,0.426396,0.339244,0.313291,0.209372,0.306407,0.244169,0.282198,0.196070,
0.005319,0.002049,0.361011,0.250420,0.589552,0.495317,0.779783,0.723367,1.000000,0.996481,
0.024896,0.007859,0.352941,0.233869,0.651832,0.493661,0.854464,0.743317,1.000000,0.994265,
0.046377,0.008597,0.368973,0.253557,0.613537,0.504141,0.845011,0.763005,1.000000,0.994843,
0.358491,0.305299,0.524017,0.440322,0.319088,0.254379,0.335443,0.268685,0.199275,0.150135,
0.285714,0.221251,0.024896,0.007743,0.296572,0.230526,0.623037,0.480916,0.825996,0.739839,
1.000000,0.994667,0.030556,0.006820,0.305970,0.247475,0.563433,0.497182,0.785388,0.744342,
1.000000,0.997465,0.005319,0.002049,0.451104,0.263293,0.618297,0.512116,0.877660,0.749283,
1.000000,0.993709,0.113744,0.057052,0.111702,0.055819,0.106329,0.069354,0.085106,0.053656,
0.058347,0.039235,0.072052,0.039448,0.087035,0.063534,0.138365,0.067246,0.112527,0.067408,
0.076923,0.053454,0.136029,0.050369,0.043321,0.024681,0.052778,0.033887,0.053853,0.022839,
0.100437,0.068004,0.173502,0.095160,0.096070,0.055915,0.052198,0.026429,0.078341,0.045644,
0.023041,0.010868

Positive_120 63.000000,0.022172,9.000000,1.396825,1.000000,3.856119,9.000000,1.396825,1.000000,2.404506,
0.540985,0.055117,0.540985,0.055117,0.045977,0.108434,0.202703,0.169492,0.346939,0.250000,
0.375000,0.060607,1.000000,0.040065,0.070547,1.250000,0.051818,0.016280,0.333333,0.003507,
0.019155,0.312500,0.003869,0.031754,0.129032,0.001603,0.030217,0.145161,0.002123,0.026242,
0.145161,0.001057,0.052547,0.129032,0.002481,3.388422,3.128079,3.040113,1.000000,0.238095,
1.000000,0.238095,1.000000,0.228269,1.000000,0.173280,1.000000,0.104056,1.000000,0.093222,
1.000000,0.068833,1.000000,0.051436,0.607143,0.026732,0.500000,0.014248,1.000000,0.269841,
1.000000,0.269841,1.000000,0.264550,1.000000,0.244268,1.000000,0.171958,1.000000,0.137566,
1.000000,0.111993,1.000000,0.073633,1.000000,0.062610,1.000000,0.056437,0.366599,0.298080,
0.485714,0.384007,0.457801,0.317913,0.278689,0.221175,0.248996,0.187854,0.320917,0.238159,
0.103960,0.015463,0.367188,0.262865,0.636829,0.501999,0.838875,0.747289,1.000000,0.992587,
0.032020,0.007808,0.348730,0.246483,0.572178,0.490422,0.816273,0.743072,1.000000,0.996901,
0.007812,0.002168,0.294118,0.228133,0.620408,0.498257,0.844068,0.747575,1.000000,0.996339,
0.731225,0.566788,0.381579,0.250400,0.671429,0.182812,0.163158,0.104771,0.072289,0.036380,
0.023622,0.003328,0.007812,0.002168,0.389016,0.236142,0.615561,0.477510,0.917143,0.743925,
1.000000,1.000000,0.532258,0.096801,0.656126,0.356770,0.800512,0.556640,0.935484,0.746533,

0.999101,0.954479,0.990923,0.084439,0.990923,0.278062,0.992436,0.443397,0.993949,0.676436,
0.998765,0.862282,0.716714,0.510486,0.589641,0.394110,0.039062,0.008623,0.346939,0.256265,
0.616327,0.510370,0.868526,0.755088,0.998853,0.990420,0.542609,0.414361,0.433428,0.347873,
0.281059,0.237766,0.348178,0.281665,0.236496,0.197411,0.216867,0.159919,0.023715,0.008253,
0.324480,0.242121,0.562929,0.481379,0.805415,0.740421,1.000000,0.997469,0.035714,0.008746,
0.320479,0.248576,0.608871,0.507889,0.827471,0.747956,1.000000,0.997071,0.007812,0.002168,
0.375494,0.254964,0.604743,0.505425,0.824847,0.741280,1.000000,0.993528,0.480818,0.351896,
0.420408,0.326801,0.384929,0.321303,0.318052,0.223830,0.287599,0.225381,0.275304,0.204803,
0.007812,0.002168,0.298824,0.233511,0.624490,0.501409,0.816949,0.745798,1.000000,0.996396,
0.036021,0.009029,0.346420,0.242031,0.569794,0.485707,0.820312,0.743606,1.000000,0.996375,
0.103960,0.013732,0.347826,0.262139,0.636829,0.502602,0.838875,0.745477,1.000000,0.995099,
0.404157,0.316200,0.507082,0.446035,0.281059,0.237766,0.400810,0.276748,0.181193,0.151434,
0.256522,0.205896,0.036021,0.009360,0.337182,0.248228,0.572082,0.486717,0.834646,0.746549,
1.000000,0.995155,0.023438,0.007388,0.297746,0.242807,0.564516,0.495633,0.800670,0.741759,
1.000000,0.998663,0.007812,0.002168,0.375494,0.254964,0.604743,0.505425,0.824847,0.741280,
1.000000,0.993528,0.083004,0.047698,0.081340,0.054729,0.097368,0.062175,0.073227,0.047153,
0.084158,0.041746,0.083665,0.044579,0.167347,0.075893,0.118812,0.056855,0.124706,0.076007,
0.115486,0.060292,0.122449,0.057754,0.043902,0.023223,0.065053,0.033983,0.096085,0.040408,
0.101695,0.064677,0.152975,0.090194,0.074169,0.044502,0.044776,0.018749,0.090652,0.043282,
0.032538,0.016101

Positive_121 60.000000,0.066944,12.000000,4.016667,3.000000,22.050565,18.000000,4.016667,2.000000,28.118362,
0.624375,0.079884,0.624375,0.079884,0.073059,0.039409,0.035897,0.069149,0.108571,0.282051,
0.294643,2.936111,11.000000,16.561292,2.437500,11.000000,19.060646,0.451296,1.000000,0.222725,
0.239236,1.000000,0.175914,0.126766,0.288136,0.013280,0.055353,0.170904,0.004279,0.047976,
0.305085,0.006932,0.085734,0.186441,0.007456,11.492921,7.422924,6.000000,1.000000,0.550000,
1.000000,0.550000,1.000000,0.547778,1.000000,0.528889,1.000000,0.513889,1.000000,0.490556,
1.000000,0.470000,0.833333,0.247778,0.833333,0.215556,0.833333,0.161667,1.000000,0.500000,
1.000000,0.500000,1.000000,0.500000,1.000000,0.497727,1.000000,0.496895,1.000000,0.491573,
1.000000,0.473860,1.000000,0.389989,1.000000,0.340069,1.000000,0.257325,0.437751,0.304142,
0.502924,0.373967,0.386973,0.321890,0.304348,0.222167,0.288538,0.173791,0.323077,0.253939,
0.094737,0.036139,0.378676,0.270648,0.695473,0.503915,0.870732,0.746064,1.000000,0.990624,
0.037975,0.012064,0.322368,0.238935,0.590551,0.497121,0.790795,0.735033,1.000000,0.988045,
0.006329,0.003896,0.405063,0.221816,0.595588,0.483543,0.845588,0.739072,1.000000,0.991966,
0.617647,0.448362,0.475610,0.199017,0.687831,0.352621,0.189602,0.078364,0.150943,0.059866,
0.018519,0.002867,0.006329,0.003896,0.356287,0.231916,0.588235,0.477247,0.840090,0.739788,
1.000000,1.000000,0.518750,0.066404,0.601208,0.194646,0.806647,0.367265,0.917874,0.500582,
0.996979,0.678498,0.260870,0.032603,0.796053,0.257846,0.875546,0.530685,0.947368,0.764370,
0.995575,0.925492,0.609195,0.513834,0.453782,0.344353,0.056250,0.016107,0.343750,0.224299,
0.559078,0.471605,0.800000,0.728319,0.999115,0.979092,0.511029,0.385359,0.472622,0.373774,
0.299094,0.240867,0.390173,0.290189,0.247706,0.179243,0.254032,0.170799,0.037975,0.011231,
0.335347,0.237845,0.557927,0.483023,0.798450,0.724649,1.000000,0.991352,0.068750,0.015184,
0.336842,0.252904,0.676471,0.510297,0.875000,0.744222,1.000000,0.993131,0.006329,0.003896,
0.723343,0.249134,0.835735,0.486427,0.887608,0.744854,1.000000,0.989556,0.409962,0.350435,
0.444853,0.318958,0.453815,0.330607,0.311538,0.233836,0.332016,0.208450,0.282927,0.202203,

0.006329,0.003896,0.386076,0.228058,0.595588,0.490038,0.834559,0.746413,1.000000,0.992582,
0.037975,0.012529,0.335526,0.235141,0.575188,0.490377,0.814961,0.728671,1.000000,0.984644,
0.094737,0.035758,0.378676,0.266873,0.610294,0.497562,0.851220,0.747237,1.000000,0.991759,
0.430147,0.308039,0.530259,0.451094,0.299094,0.240867,0.365031,0.292949,0.218085,0.142910,
0.282258,0.207132,0.047619,0.013236,0.329305,0.235535,0.581590,0.488553,0.810078,0.721802,
1.000000,0.988935,0.067633,0.013972,0.356000,0.251654,0.573529,0.504217,0.852941,0.740247,
1.000000,0.994726,0.006329,0.003896,0.723343,0.249134,0.835735,0.486427,0.887608,0.744854,
1.000000,0.989556,0.090551,0.054531,0.120482,0.047089,0.116466,0.066881,0.073077,0.043266,
0.206587,0.056782,0.072961,0.035593,0.125000,0.062093,0.125000,0.064301,0.128000,0.082391,
0.100917,0.055987,0.105519,0.054184,0.057737,0.026465,0.060423,0.028545,0.055866,0.023136,
0.109890,0.066178,0.148673,0.104505,0.086614,0.043835,0.068750,0.027067,0.080460,0.042138,
0.035398,0.015031

Positive_122 50.000000,0.025200,5.000000,1.260000,1.000000,0.971837,13.000000,1.260000,1.000000,4.645306,
0.376167,0.021857,0.376167,0.021857,0.047619,0.383333,0.378378,0.478261,0.583333,0.400000,
0.666667,0.027000,0.600000,0.012981,0.035000,0.750000,0.020434,0.008650,0.250000,0.001573,
0.013750,0.250000,0.003076,0.086728,0.244898,0.009128,0.041020,0.102041,0.001042,0.027857,
0.193878,0.002408,0.014589,0.081633,0.000477,3.625922,2.709723,2.587208,1.000000,0.220000,
1.000000,0.220000,1.000000,0.220000,1.000000,0.198000,1.000000,0.188667,1.000000,0.110667,
1.000000,0.097333,1.000000,0.043333,1.000000,0.023333,0.000000,0.000000,1.000000,0.300000,
1.000000,0.300000,1.000000,0.273333,1.000000,0.255333,1.000000,0.185333,1.000000,0.126513,
1.000000,0.119590,1.000000,0.106769,1.000000,0.042103,0.705128,0.014103,0.429158,0.323975,
0.487360,0.340968,0.393617,0.335057,0.261194,0.198772,0.295699,0.214205,0.319149,0.249259,
0.094737,0.029730,0.468085,0.282993,0.712766,0.525137,0.851064,0.768355,1.000000,0.995366,
0.040816,0.008593,0.366379,0.227566,0.600000,0.480796,0.820000,0.733121,1.000000,0.984239,
0.010638,0.003434,0.398190,0.228529,0.588235,0.476772,0.809145,0.726347,1.000000,0.988797,
0.765343,0.466276,0.364583,0.103063,0.731006,0.430661,0.113402,0.042795,0.105820,0.074220,
0.018315,0.003954,0.010638,0.003434,0.442667,0.221391,0.645333,0.434314,0.927885,0.724844,
1.000000,1.000000,0.452586,0.074134,0.783451,0.217909,0.914826,0.332384,0.974763,0.457772,
0.997669,0.615439,0.272340,0.029032,0.749226,0.275602,0.871528,0.568214,0.920139,0.776989,
0.997449,0.956663,0.574132,0.495700,0.501750,0.396443,0.053191,0.015573,0.434389,0.233849,
0.647059,0.488030,0.846154,0.745978,0.998628,0.984557,0.524887,0.368489,0.454545,0.392543,
0.299465,0.238968,0.355615,0.282925,0.223404,0.172367,0.322581,0.188549,0.040816,0.008410,
0.334052,0.225707,0.589333,0.472990,0.808000,0.720939,1.000000,0.990674,0.042105,0.013467,
0.407240,0.256456,0.588235,0.511789,0.813121,0.733623,1.000000,0.993346,0.010638,0.003434,
0.438776,0.250733,0.683673,0.504676,0.846154,0.769886,1.000000,0.991351,0.427807,0.359826,
0.446629,0.293826,0.449692,0.346348,0.294118,0.226912,0.360215,0.247693,0.258621,0.181470,
0.010638,0.003434,0.402715,0.231042,0.606335,0.484268,0.809145,0.736909,1.000000,0.988797,
0.040816,0.008593,0.358667,0.223563,0.616000,0.475046,0.817333,0.725703,1.000000,0.983924,
0.094737,0.029183,0.468085,0.279490,0.712766,0.518206,0.851064,0.761197,1.000000,0.995432,
0.393665,0.288587,0.551020,0.472445,0.299465,0.238968,0.352590,0.281060,0.218085,0.139625,
0.344086,0.221290,0.043321,0.009202,0.303249,0.224921,0.570423,0.479916,0.797333,0.715696,
1.000000,0.988002,0.037037,0.012048,0.302128,0.246947,0.563830,0.500905,0.784672,0.732533,
1.000000,0.995869,0.010638,0.003434,0.438776,0.250733,0.683673,0.504676,0.846154,0.769886,
1.000000,0.991351,0.091346,0.054639,0.138298,0.054983,0.125257,0.072025,0.073276,0.046530,

0.114990,0.056776,0.085177,0.039022,0.100703,0.048728,0.114667,0.062466,0.144796,0.081752,
0.079861,0.049111,0.136236,0.051768,0.045977,0.022373,0.053476,0.024769,0.083032,0.028133,
0.095745,0.056269,0.186047,0.121017,0.112245,0.047434,0.063492,0.027578,0.074074,0.042216,
0.032787,0.012410

Positive_123 99.000000,0.088562,53.000000,8.767677,10.000000,99.772006,45.000000,8.767677,4.000000,162.772006,
0.313037,0.015130,0.313037,0.015130,0.109524,0.417112,0.610092,0.629412,0.603175,0.560000,
0.818182,0.312560,7.735849,2.343896,0.368126,3.644444,1.218404,0.005897,0.145959,0.000834,
0.008181,0.080988,0.000602,0.231584,0.448980,0.035544,0.095868,0.540816,0.021429,0.105535,
0.250464,0.006396,0.267185,0.530612,0.055173,21.745511,12.211993,9.991085,1.000000,0.838384,
1.000000,0.837592,1.000000,0.791973,1.000000,0.698814,1.000000,0.551981,1.000000,0.389770,
1.000000,0.288792,1.000000,0.208197,1.000000,0.169461,0.166667,0.026975,1.000000,0.828283,
1.000000,0.828283,1.000000,0.814009,1.000000,0.770513,1.000000,0.732395,1.000000,0.609856,
1.000000,0.550301,0.666667,0.371889,0.500000,0.275551,0.333333,0.111359,0.431718,0.307163,
0.550562,0.386516,0.398714,0.306321,0.285024,0.224701,0.278912,0.190104,0.309973,0.232490,
0.076923,0.012572,0.315113,0.250956,0.597527,0.492122,0.825991,0.736881,1.000000,0.994471,
0.026432,0.006613,0.345972,0.254997,0.601604,0.505260,0.853081,0.757581,1.000000,0.996502,
0.005882,0.002107,0.428571,0.236905,0.650794,0.490907,0.864706,0.737528,1.000000,0.995580,
0.727273,0.564655,0.391978,0.184226,0.606780,0.251119,0.194444,0.070927,0.102041,0.050381,
0.017751,0.003716,0.042654,0.002490,0.479339,0.278402,0.728453,0.528301,0.877834,0.767636,
1.000000,1.000000,0.909953,0.106594,0.909953,0.277253,0.955017,0.449569,0.976303,0.629974,
0.997576,0.928472,0.676923,0.042202,0.760766,0.252567,0.940860,0.545895,0.967742,0.733349,
0.999133,0.954282,0.578199,0.476495,0.459854,0.373647,0.050167,0.009799,0.350746,0.248513,
0.638095,0.503070,0.864706,0.744256,0.999133,0.989848,0.546816,0.406428,0.448000,0.359757,
0.313364,0.233814,0.348794,0.277160,0.265700,0.180689,0.251656,0.171572,0.027027,0.006664,
0.331754,0.257227,0.626298,0.511292,0.838863,0.761678,1.000000,0.996863,0.044944,0.008052,
0.407583,0.242162,0.672986,0.493249,0.838863,0.737884,1.000000,0.995211,0.005882,0.002107,
0.432014,0.239742,0.697074,0.480268,0.829412,0.712748,1.000000,0.992282,0.434084,0.336833,
0.498127,0.329863,0.455947,0.333303,0.287411,0.210818,0.305893,0.230463,0.257962,0.205544,
0.005882,0.002107,0.431746,0.239497,0.653968,0.493848,0.833735,0.736688,1.000000,0.995800,
0.042654,0.008099,0.360190,0.256258,0.631658,0.504234,0.857820,0.757570,1.000000,0.995692,
0.076923,0.012159,0.315113,0.251550,0.603524,0.492416,0.823789,0.735910,1.000000,0.995224,
0.415730,0.305721,0.531140,0.460465,0.313364,0.233814,0.363462,0.275081,0.186192,0.136805,
0.297710,0.215455,0.040678,0.008206,0.377163,0.250326,0.660900,0.501695,0.841398,0.753279,
1.000000,0.995307,0.020356,0.006468,0.328244,0.248856,0.596958,0.503034,0.829384,0.750229,
1.000000,0.997362,0.005882,0.002107,0.432014,0.239742,0.697074,0.480268,0.829412,0.712748,
1.000000,0.992282,0.137441,0.051547,0.090121,0.051230,0.115254,0.068246,0.090090,0.049246,
0.115162,0.049964,0.074722,0.036931,0.092417,0.055661,0.123223,0.058589,0.141707,0.086291,
0.108614,0.055935,0.151463,0.073388,0.045699,0.026141,0.063158,0.030512,0.093750,0.027319,
0.094070,0.059518,0.155112,0.101390,0.090476,0.043708,0.048882,0.020707,0.078341,0.036013,
0.037175,0.017666

Positive_124 124.000000,0.023348,15.000000,2.895161,2.000000,13.753147,14.000000,2.895161,2.000000,7.639326,
0.359159,0.017811,0.359159,0.017811,0.086592,0.269113,0.569038,0.495146,0.653846,0.611111,
0.571429,0.051613,2.000000,0.095309,0.106452,1.500000,0.146625,0.004247,0.166667,0.000661,
0.018065,0.250000,0.004204,0.031565,0.105691,0.001080,0.015377,0.113821,0.000358,0.016250,

0.065041,0.000218,0.040800,0.105691,0.001493,8.155230,7.483315,5.874382,1.000000,0.575038,
1.000000,0.574654,1.000000,0.528470,1.000000,0.455000,1.000000,0.414335,1.000000,0.348307,
1.000000,0.252610,1.000000,0.190616,1.000000,0.141769,0.604396,0.030309,1.000000,0.661290,
1.000000,0.660936,1.000000,0.654479,1.000000,0.611701,1.000000,0.566989,1.000000,0.475070,
1.000000,0.417205,1.000000,0.318016,1.000000,0.128771,0.333333,0.009151,0.431718,0.310760,
0.537344,0.375018,0.383721,0.314222,0.308642,0.221732,0.331361,0.186305,0.351648,0.233245,
0.206522,0.027484,0.420601,0.275361,0.815217,0.522930,0.880435,0.750009,1.000000,0.989526,
0.041026,0.009331,0.332083,0.233671,0.627907,0.491239,0.846962,0.743870,1.000000,0.990325,
0.011628,0.003328,0.344017,0.224553,0.614828,0.477549,0.834897,0.733342,1.000000,0.991053,
0.829091,0.509812,0.475610,0.182130,0.689840,0.308058,0.194444,0.074850,0.105820,0.059395,
0.015038,0.003637,0.011628,0.003328,0.593023,0.237802,0.755814,0.485009,0.891566,0.750379,
1.000000,1.000000,0.833021,0.081825,0.864916,0.247494,0.889306,0.401963,0.958724,0.570207,
0.996441,0.790037,0.308756,0.043957,0.938224,0.279474,0.957529,0.542378,0.973363,0.731673,
0.998890,0.929834,0.597826,0.490398,0.500000,0.362516,0.088235,0.015851,0.357500,0.228678,
0.632911,0.479608,0.840525,0.725245,1.000000,0.984770,0.545643,0.391924,0.476744,0.361830,
0.331325,0.246247,0.366071,0.272725,0.247967,0.187886,0.322581,0.174665,0.054348,0.009058,
0.319079,0.232260,0.616279,0.483884,0.838710,0.739849,1.000000,0.992906,0.042169,0.013500,
0.348797,0.250190,0.583815,0.502246,0.832618,0.737117,1.000000,0.994325,0.011628,0.003328,
0.424779,0.251592,0.637168,0.498560,0.877660,0.743194,1.000000,0.989510,0.453488,0.346250,
0.494813,0.317259,0.455947,0.336492,0.296703,0.215439,0.360215,0.223820,0.273973,0.202159,
0.011628,0.003328,0.344017,0.229468,0.605787,0.485021,0.861163,0.737436,1.000000,0.992240,
0.054348,0.009739,0.335835,0.229698,0.654897,0.487871,0.854464,0.743407,1.000000,0.988097,
0.162791,0.025409,0.404908,0.272426,0.793478,0.516098,0.869565,0.748893,1.000000,0.991324,
0.402174,0.305620,0.546512,0.448133,0.331325,0.246247,0.392857,0.274120,0.218085,0.148747,
0.344086,0.213805,0.056940,0.010198,0.360515,0.235502,0.627907,0.490618,0.822086,0.739072,
1.000000,0.988957,0.042169,0.011867,0.314917,0.244821,0.562212,0.495917,0.788760,0.738228,
1.000000,0.996061,0.011628,0.003328,0.424779,0.251592,0.637168,0.498560,0.877660,0.743194,
1.000000,0.989510,0.092308,0.054199,0.126506,0.055997,0.098712,0.066293,0.111765,0.049667,
0.138889,0.047669,0.072961,0.036936,0.119565,0.060401,0.130435,0.060587,0.142322,0.081681,
0.131274,0.053285,0.152490,0.061306,0.053498,0.025731,0.072000,0.032028,0.074122,0.024998,
0.104478,0.061477,0.197674,0.102658,0.085837,0.046797,0.063492,0.024775,0.078341,0.040107,
0.041667,0.013411

Positive_125 82.000000,0.028108,10.000000,2.304878,2.000000,5.424420,9.000000,2.304878,2.000000,4.510840,
0.363482,0.021559,0.363482,0.021559,0.095745,0.276471,0.536585,0.473684,0.600000,0.416667,
0.571429,0.053659,0.888889,0.040066,0.107927,0.800000,0.064453,0.007407,0.120000,0.000715,
0.027022,0.187500,0.003871,0.033522,0.098765,0.001040,0.012316,0.111111,0.000368,0.011891,
0.048011,0.000202,0.032146,0.111111,0.001046,5.856646,5.000000,4.221123,1.000000,0.573171,
1.000000,0.573171,1.000000,0.568835,1.000000,0.515912,1.000000,0.371012,1.000000,0.281707,
1.000000,0.226055,1.000000,0.172435,1.000000,0.149206,1.000000,0.062698,1.000000,0.573171,
1.000000,0.573171,1.000000,0.566783,1.000000,0.541086,1.000000,0.476500,1.000000,0.341812,
1.000000,0.279752,1.000000,0.171893,0.700000,0.075494,0.333333,0.026229,0.431718,0.329337,
0.537344,0.375637,0.417085,0.295026,0.308642,0.233981,0.331361,0.193794,0.311966,0.213454,
0.085106,0.009919,0.420601,0.256871,0.658610,0.496968,0.850575,0.739771,1.000000,0.993822,
0.031818,0.007554,0.341421,0.250130,0.625285,0.495500,0.846962,0.752675,1.000000,0.994157,

0.006135,0.002431,0.448000,0.240369,0.653130,0.494419,0.834897,0.734933,1.000000,0.994301,
0.829091,0.541412,0.377880,0.158513,0.731006,0.300075,0.194444,0.067495,0.108197,0.061194,
0.030303,0.004416,0.006135,0.002431,0.515021,0.249889,0.709677,0.496982,0.911704,0.754962,
1.000000,1.000000,0.833021,0.093913,0.864916,0.276738,0.914826,0.451584,0.974763,0.641816,
0.996904,0.891744,0.308756,0.055727,0.656388,0.316277,0.875546,0.550484,0.973363,0.734978,
0.998890,0.951254,0.582569,0.477353,0.500000,0.370353,0.088235,0.012156,0.351145,0.253049,
0.644670,0.503796,0.840525,0.737990,1.000000,0.990733,0.545643,0.392478,0.445585,0.359393,
0.316957,0.248128,0.331579,0.268147,0.238494,0.185989,0.251656,0.180815,0.031818,0.007713,
0.341421,0.247221,0.593394,0.492606,0.838710,0.752824,1.000000,0.994441,0.036810,0.009018,
0.412000,0.250770,0.614213,0.502298,0.832618,0.735168,1.000000,0.995223,0.006135,0.002431,
0.361702,0.244607,0.631420,0.488814,0.877660,0.733177,1.000000,0.991791,0.467337,0.327703,
0.494813,0.318221,0.455947,0.354076,0.269129,0.196059,0.349112,0.233151,0.291803,0.212859,
0.006135,0.002431,0.438000,0.244988,0.651438,0.496618,0.861163,0.736409,1.000000,0.995107,
0.031818,0.007893,0.395147,0.248264,0.654897,0.494639,0.854464,0.753703,1.000000,0.992242,
0.072340,0.009702,0.404908,0.258359,0.643505,0.497526,0.833333,0.738269,1.000000,0.994336,
0.415789,0.304509,0.529774,0.447362,0.316957,0.248128,0.338182,0.266104,0.203704,0.144714,
0.309220,0.222090,0.047619,0.008723,0.360515,0.246809,0.584392,0.488844,0.822086,0.749575,
1.000000,0.992666,0.036810,0.007994,0.336000,0.250863,0.575800,0.502991,0.788760,0.741843,
1.000000,0.996783,0.006135,0.002431,0.361702,0.244607,0.631420,0.488814,0.877660,0.733177,
1.000000,0.991791,0.087986,0.057086,0.111702,0.060544,0.125257,0.072940,0.111765,0.053651,
0.138889,0.047059,0.068282,0.038059,0.113071,0.062919,0.133987,0.062627,0.144737,0.075378,
0.083770,0.049936,0.152490,0.067362,0.057576,0.024739,0.072727,0.032677,0.055276,0.020607,
0.099476,0.059183,0.145110,0.098336,0.066479,0.043816,0.041176,0.024362,0.078341,0.037442,
0.031250,0.011279

Positive_126 58.000000,0.040428,8.000000,2.344828,1.000000,4.756201,11.000000,2.344828,3.000000,4.335148,
0.365773,0.019798,0.365773,0.019798,0.059259,0.314961,0.528736,0.487805,0.619048,0.500000,
0.500000,0.003448,0.200000,0.000690,0.008621,0.500000,0.004310,0.000690,0.040000,0.000028,
0.004310,0.250000,0.001078,0.062986,0.175439,0.003439,0.020891,0.140351,0.001088,0.021917,
0.070175,0.000734,0.041571,0.122807,0.001859,5.000000,4.898979,4.429915,1.000000,0.448276,
1.000000,0.448276,1.000000,0.444704,1.000000,0.391092,1.000000,0.322126,1.000000,0.199220,
0.666667,0.147701,0.400000,0.066256,0.333333,0.034852,0.333333,0.022537,1.000000,0.655172,
1.000000,0.655172,1.000000,0.652508,1.000000,0.614316,1.000000,0.541432,1.000000,0.350522,
1.000000,0.318809,1.000000,0.201881,0.700000,0.113009,0.333333,0.052038,0.431718,0.332472,
0.537344,0.372806,0.364162,0.294722,0.282407,0.232182,0.331361,0.197897,0.253298,0.211900,
0.039823,0.010199,0.389381,0.265964,0.658610,0.509452,0.821752,0.743675,1.000000,0.993485,
0.018476,0.006489,0.341421,0.240522,0.625285,0.491856,0.819957,0.752556,1.000000,0.993854,
0.005882,0.002344,0.448000,0.241577,0.626000,0.490474,0.824000,0.733771,1.000000,0.991843,
0.829091,0.523837,0.459268,0.160586,0.731006,0.315576,0.153664,0.067867,0.108197,0.064612,
0.011834,0.003968,0.005882,0.002344,0.515021,0.238881,0.686695,0.491240,0.911704,0.766288,
1.000000,1.000000,0.777778,0.067028,0.833718,0.275686,0.914826,0.443838,0.974763,0.629939,
0.997691,0.909045,0.192105,0.047467,0.541752,0.315034,0.810211,0.532519,0.973363,0.724145,
0.998890,0.929285,0.576419,0.489616,0.500000,0.382985,0.088235,0.012360,0.341176,0.252822,
0.632794,0.494919,0.842956,0.729484,1.000000,0.991711,0.545643,0.393055,0.445585,0.362372,
0.308977,0.244573,0.329114,0.272996,0.238494,0.185105,0.251656,0.180732,0.015625,0.006369,

0.341421,0.239747,0.593394,0.490533,0.811159,0.754868,1.000000,0.995627,0.034682,0.009432,
0.412000,0.258563,0.600000,0.501350,0.798000,0.736001,1.000000,0.995944,0.005882,0.002344,
0.361702,0.247748,0.631420,0.491510,0.877660,0.728968,1.000000,0.991396,0.398876,0.327527,
0.494813,0.315593,0.455947,0.356879,0.269129,0.199782,0.349112,0.238088,0.291803,0.210475,
0.005882,0.002344,0.438000,0.243029,0.604000,0.490965,0.831409,0.733561,1.000000,0.992384,
0.018476,0.006863,0.395147,0.240644,0.654897,0.490291,0.845266,0.753182,1.000000,0.991813,
0.039823,0.009937,0.350975,0.265051,0.643505,0.506931,0.818731,0.741796,1.000000,0.993932,
0.415789,0.308216,0.529774,0.447210,0.308977,0.244573,0.342222,0.272822,0.187755,0.145688,
0.289130,0.220148,0.025180,0.007448,0.335355,0.245021,0.593525,0.498208,0.826790,0.757980,
1.000000,0.992635,0.034682,0.007720,0.336000,0.250680,0.562000,0.494456,0.788760,0.738476,
1.000000,0.997186,0.005882,0.002344,0.361702,0.247748,0.631420,0.491510,0.877660,0.728968,
1.000000,0.991396,0.084967,0.055676,0.111702,0.060253,0.125257,0.074564,0.111765,0.056344,
0.138889,0.049216,0.068282,0.036419,0.113071,0.063412,0.133987,0.061854,0.144737,0.074388,
0.081545,0.052217,0.152490,0.063721,0.047753,0.024407,0.060738,0.032806,0.036458,0.021117,
0.093750,0.058925,0.145110,0.099418,0.066479,0.043829,0.041176,0.023975,0.070588,0.035920,
0.030576,0.011537

Positive_127 65.000000,0.040473,11.000000,2.630769,2.000000,7.705288,16.000000,2.630769,2.000000,11.486538,
0.366940,0.022678,0.366940,0.022678,0.070175,0.352201,0.456311,0.500000,0.464286,0.466667,
0.625000,0.078322,1.545455,0.084033,0.059203,1.312500,0.034537,0.019708,0.250000,0.004129,
0.007247,0.082031,0.000321,0.101985,0.234375,0.006368,0.047034,0.171875,0.002815,0.025699,
0.187500,0.001534,0.035249,0.156250,0.003141,7.888240,4.898979,4.167875,1.000000,0.676923,
1.000000,0.676923,1.000000,0.672310,1.000000,0.594545,1.000000,0.579262,1.000000,0.562526,
1.000000,0.511857,1.000000,0.378355,1.000000,0.166101,0.333333,0.007504,1.000000,0.507692,
1.000000,0.507564,1.000000,0.503205,1.000000,0.485736,1.000000,0.388333,1.000000,0.346099,
1.000000,0.293408,1.000000,0.218353,1.000000,0.100919,0.333333,0.037424,0.431718,0.327050,
0.502146,0.356417,0.392562,0.316533,0.300752,0.228263,0.261117,0.206583,0.321168,0.217876,
0.193939,0.016879,0.472727,0.264586,0.686047,0.518309,0.848837,0.757453,1.000000,0.991616,
0.047297,0.008814,0.332083,0.240164,0.627907,0.493560,0.846962,0.746124,1.000000,0.993267,
0.011628,0.003094,0.363409,0.232562,0.558824,0.474710,0.834897,0.724157,1.000000,0.993126,
0.778612,0.493018,0.377880,0.148691,0.627907,0.358291,0.194444,0.061462,0.129252,0.076150,
0.020408,0.004706,0.011628,0.003094,0.593023,0.254222,0.755814,0.511035,0.887324,0.769709,
1.000000,1.000000,0.833021,0.105772,0.864916,0.254646,0.889306,0.395713,0.958724,0.578698,
0.995413,0.810698,0.308756,0.063701,0.541752,0.319111,0.760243,0.519835,0.890744,0.742774,
0.998131,0.954494,0.627049,0.509037,0.448980,0.370054,0.068862,0.017351,0.420048,0.252261,
0.580448,0.483254,0.840525,0.706734,0.998419,0.985272,0.519313,0.378998,0.476744,0.354885,
0.319444,0.266117,0.316872,0.255791,0.299270,0.203399,0.251656,0.186073,0.047297,0.008934,
0.340278,0.242227,0.616279,0.495385,0.838710,0.749119,1.000000,0.995582,0.060150,0.013716,
0.341232,0.234851,0.630332,0.482001,0.832618,0.734329,1.000000,0.993063,0.011628,0.003094,
0.406977,0.260425,0.593023,0.504598,0.877660,0.741216,1.000000,0.987829,0.453488,0.356540,
0.433476,0.291041,0.455947,0.352419,0.284672,0.201713,0.306407,0.252997,0.259398,0.195717,
0.011628,0.003094,0.368421,0.238493,0.575985,0.484094,0.861163,0.728176,1.000000,0.993953,
0.047297,0.009061,0.335835,0.227621,0.654897,0.489518,0.854464,0.746052,1.000000,0.992625,
0.193939,0.016354,0.460606,0.264939,0.686047,0.514954,0.860465,0.754292,1.000000,0.992032,
0.377682,0.291308,0.546512,0.442576,0.319444,0.266117,0.324468,0.255792,0.211679,0.155802,

0.289130,0.233669,0.047297,0.009722,0.360515,0.235765,0.627907,0.484440,0.819205,0.735716,
1.000000,0.991015,0.037915,0.010646,0.383886,0.243219,0.639810,0.496119,0.838863,0.746752,
1.000000,0.997446,0.011628,0.003094,0.406977,0.260425,0.593023,0.504598,0.877660,0.741216,
1.000000,0.987829,0.092338,0.054922,0.111702,0.067634,0.102083,0.067376,0.085106,0.057631,
0.076621,0.042624,0.068282,0.036863,0.108696,0.062446,0.098712,0.058946,0.120172,0.063517,
0.076389,0.048768,0.151970,0.057364,0.036111,0.025369,0.072464,0.040007,0.105634,0.030326,
0.093750,0.060581,0.197674,0.098250,0.090164,0.049192,0.045455,0.026523,0.078341,0.039692,
0.036232,0.011969

Positive_128 103.000000,0.065793,21.000000,6.776699,4.000000,56.900628,21.000000,6.776699,3.000000,59.194746,
0.647173,0.074719,0.648101,0.074224,0.032999,0.139466,0.122414,0.090373,0.088553,0.172986,
0.232092,4.297087,21.000000,72.154403,4.315534,21.000000,72.018335,0.206990,1.000000,0.162868,
0.215615,1.000000,0.163166,0.076623,0.196078,0.005176,0.057932,0.196078,0.005302,0.057003,
0.196078,0.005335,0.065753,0.196078,0.004908,21.000000,6.480741,6.335602,1.000000,0.766990,
1.000000,0.766990,1.000000,0.729868,1.000000,0.601496,1.000000,0.525180,1.000000,0.486771,
1.000000,0.423328,1.000000,0.305428,1.000000,0.168306,0.271429,0.057504,1.000000,0.776699,
1.000000,0.776699,1.000000,0.772992,1.000000,0.667329,1.000000,0.579247,1.000000,0.523020,
1.000000,0.411136,1.000000,0.316255,0.580952,0.169102,0.271429,0.063811,0.481802,0.308550,
0.481609,0.366835,0.439614,0.324615,0.321429,0.213880,0.293478,0.187271,0.405286,0.238228,
0.033816,0.012849,0.509579,0.262546,0.697318,0.517082,0.896552,0.759991,1.000000,0.993167,
0.032258,0.007198,0.313636,0.243955,0.625285,0.496305,0.857778,0.752905,1.000000,0.993059,
0.010753,0.002753,0.353135,0.233227,0.605753,0.479259,0.826087,0.717847,1.000000,0.995718,
0.724196,0.449359,0.535859,0.156345,0.734078,0.394296,0.160075,0.060590,0.116959,0.064667,
0.019737,0.005689,0.010753,0.002753,0.415385,0.255863,0.808696,0.526469,0.926966,0.783311,
1.000000,1.000000,0.697143,0.087945,0.697143,0.243478,0.879558,0.426083,0.902857,0.664553,
0.999394,0.867819,0.273927,0.037736,0.795302,0.278001,0.921053,0.514842,0.962209,0.732395,
0.998131,0.930949,0.689474,0.520307,0.509091,0.358472,0.036789,0.013656,0.331395,0.234364,
0.580645,0.471903,0.797909,0.705486,0.998419,0.987394,0.532544,0.389735,0.451613,0.365401,
0.313953,0.244864,0.365741,0.280107,0.263158,0.185544,0.261538,0.177268,0.054348,0.007897,
0.335570,0.247726,0.593394,0.492405,0.823034,0.749049,1.000000,0.993344,0.093023,0.014070,
0.342105,0.247935,0.615385,0.492267,0.834320,0.733185,1.000000,0.996747,0.010753,0.002753,
0.354839,0.238732,0.671743,0.502384,0.852843,0.751571,1.000000,0.992625,0.478261,0.357871,
0.435897,0.313902,0.497400,0.328227,0.387665,0.224231,0.336957,0.223758,0.315476,0.192636,
0.010753,0.002753,0.363036,0.235543,0.617597,0.480917,0.818951,0.723813,1.000000,0.995938,
0.054348,0.008136,0.313636,0.239076,0.654897,0.492463,0.862360,0.746761,1.000000,0.989975,
0.033816,0.012687,0.463602,0.262099,0.693487,0.517171,0.896552,0.760469,1.000000,0.993521,
0.437870,0.314048,0.548387,0.441087,0.313953,0.244864,0.370180,0.275023,0.251462,0.150295,
0.289130,0.212516,0.054348,0.008493,0.317949,0.244117,0.592308,0.486474,0.862360,0.741337,
1.000000,0.991292,0.087209,0.011834,0.314917,0.251979,0.584615,0.497161,0.795031,0.738978,
1.000000,0.997519,0.010753,0.002753,0.354839,0.238732,0.671743,0.502384,0.852843,0.751571,
1.000000,0.992625,0.093301,0.051763,0.109827,0.060282,0.136915,0.064986,0.104651,0.051064,
0.081522,0.043364,0.086022,0.037091,0.142012,0.065805,0.135314,0.075006,0.130435,0.067623,
0.099379,0.054550,0.122989,0.050918,0.044420,0.019677,0.084291,0.033257,0.074122,0.024769,
0.118280,0.068481,0.184332,0.100850,0.101124,0.050630,0.061611,0.026068,0.084337,0.040121,
0.040909,0.013697

Positive_129 11.000000,0.735537,10.000000,8.090909,8.000000,4.890909,10.000000,8.090909,10.000000,8.890909,
0.657499,0.033682,0.657499,0.033682,0.000000,0.000000,0.101124,0.112500,0.225352,0.290909,
0.564103,6.965909,8.500000,6.100284,6.545455,8.500000,8.656061,0.819602,1.000000,0.080945,
0.730303,0.850000,0.063749,0.798864,0.900000,0.013214,0.779708,0.842857,0.001896,0.754848,
0.810000,0.007450,0.760909,0.783333,0.000522,8.873233,3.073592,0.904858,1.000000,1.000000,
1.000000,1.000000,1.000000,1.000000,1.000000,1.000000,1.000000,0.881674,1.000000,0.748918,
1.000000,0.585859,1.000000,0.491342,1.000000,0.281025,0.333333,0.101732,1.000000,0.909091,
1.000000,0.909091,1.000000,0.909091,1.000000,0.909091,1.000000,0.810101,1.000000,0.739394,
1.000000,0.626263,0.866667,0.490909,0.400000,0.222222,0.066667,0.060606,0.338942,0.305953,
0.518605,0.424204,0.315700,0.269843,0.254237,0.229407,0.216867,0.174521,0.251282,0.217553,
0.013158,0.005851,0.294788,0.235537,0.531250,0.479706,0.787597,0.739269,1.000000,0.998667,
0.014458,0.005797,0.331311,0.277783,0.571200,0.537739,0.812048,0.773844,1.000000,0.998820,
0.002410,0.001494,0.285016,0.228467,0.583062,0.446205,0.811075,0.700464,1.000000,0.995591,
0.693798,0.616099,0.151442,0.108290,0.421502,0.275611,0.072289,0.045914,0.105983,0.065454,
0.004831,0.001789,0.002410,0.001494,0.383133,0.294996,0.681928,0.561139,0.856655,0.782099,
1.000000,1.000000,0.252800,0.073581,0.401600,0.191360,0.618421,0.321234,0.806189,0.547368,
0.997502,0.883207,0.244300,0.069465,0.480456,0.211077,0.763844,0.436052,0.887622,0.681332,
0.997610,0.930440,0.564846,0.470701,0.393162,0.326486,0.030340,0.008964,0.286400,0.248358,
0.540800,0.457824,0.775244,0.688627,0.996800,0.990671,0.541860,0.432718,0.390071,0.328556,
0.306024,0.238726,0.287179,0.264973,0.209040,0.184612,0.236145,0.166643,0.014458,0.005025,
0.303081,0.265013,0.568675,0.534216,0.816000,0.775419,1.000000,0.998239,0.024436,0.006158,
0.294788,0.240936,0.579196,0.486980,0.822476,0.745679,1.000000,0.997428,0.002410,0.001494,
0.299674,0.231554,0.539088,0.453239,0.770358,0.679880,1.000000,0.993278,0.349829,0.298176,
0.475194,0.368407,0.367612,0.333417,0.226754,0.201591,0.274699,0.210666,0.235503,0.213492,
0.002410,0.001494,0.270358,0.229233,0.563518,0.451855,0.807818,0.697856,1.000000,0.995591,
0.014458,0.005797,0.342233,0.281637,0.595337,0.552471,0.831325,0.784865,1.000000,0.998163,
0.013158,0.005851,0.298046,0.238391,0.540717,0.479237,0.767442,0.735434,1.000000,0.998667,
0.404651,0.336902,0.472087,0.424372,0.306024,0.238726,0.338247,0.281122,0.169082,0.144769,
0.267470,0.206486,0.014458,0.005347,0.303081,0.252395,0.551807,0.522625,0.810096,0.779286,
1.000000,0.996600,0.006515,0.003980,0.288274,0.254302,0.570922,0.502874,0.781324,0.753861,
1.000000,0.998118,0.002410,0.001494,0.299674,0.231554,0.539088,0.453239,0.770358,0.679880,
1.000000,0.993278,0.098795,0.062163,0.064904,0.048525,0.077068,0.060540,0.073600,0.045113,
0.120567,0.053693,0.060261,0.035917,0.087597,0.065713,0.088372,0.064041,0.133550,0.107834,
0.076832,0.054199,0.116279,0.076619,0.035461,0.027464,0.043373,0.028333,0.025498,0.019197,
0.053191,0.047998,0.108010,0.090647,0.057600,0.039761,0.031325,0.024348,0.045113,0.037472,
0.029010,0.010420

Positive_130 63.000000,0.061728,11.000000,3.888889,3.000000,6.293907,16.000000,3.888889,3.000000,11.229391,
0.372976,0.032914,0.372976,0.032914,0.118644,0.302885,0.365517,0.445652,0.431373,0.517241,
0.500000,0.000000,0.000000,0.000000,0.000000,0.000000,0.000000,0.000000,0.000000,0.000000,
0.000000,0.000000,0.000000,0.093310,0.241935,0.006099,0.076822,0.177419,0.001812,0.066415,
0.258065,0.003969,0.072782,0.161290,0.001959,7.291503,7.097903,5.000000,1.000000,0.841270,
1.000000,0.840917,1.000000,0.815470,1.000000,0.763114,1.000000,0.677249,1.000000,0.639962,
1.000000,0.446107,1.000000,0.378415,1.000000,0.197863,1.000000,0.040028,1.000000,0.761905,
1.000000,0.760714,1.000000,0.748791,1.000000,0.690212,1.000000,0.597468,1.000000,0.583825,

1.000000,0.440703,1.000000,0.322317,1.000000,0.108409,0.500000,0.035317,0.429530,0.332501,
0.488024,0.358649,0.369080,0.308850,0.324138,0.229153,0.331361,0.207441,0.285714,0.212695,
0.034682,0.009771,0.313349,0.250188,0.601093,0.502290,0.846154,0.755919,1.000000,0.993575,
0.035088,0.006175,0.303883,0.239102,0.608187,0.497185,0.819572,0.751470,1.000000,0.994012,
0.006849,0.002377,0.339350,0.241251,0.563177,0.481644,0.812274,0.713970,1.000000,0.995520,
0.783626,0.496324,0.342541,0.136097,0.616438,0.367579,0.098361,0.060050,0.111732,0.072239,
0.020690,0.006041,0.006849,0.002377,0.375335,0.252822,0.672365,0.515330,0.880342,0.770980,
1.000000,0.998478,0.313592,0.072084,0.651934,0.225281,0.836296,0.414774,0.922825,0.601858,
0.999065,0.850958,0.134503,0.039927,0.503715,0.274483,0.814815,0.536360,0.907767,0.732248,
1.000000,0.942289,0.610711,0.512024,0.500000,0.359573,0.122905,0.019316,0.389892,0.252060,
0.581227,0.481510,0.786787,0.701575,1.000000,0.988903,0.495509,0.382053,0.434783,0.361961,
0.319088,0.255986,0.337931,0.262515,0.235119,0.190633,0.257143,0.186125,0.054348,0.007165,
0.302294,0.242424,0.608187,0.500873,0.814407,0.749304,1.000000,0.994233,0.034682,0.010168,
0.317919,0.240828,0.583815,0.487991,0.794521,0.733820,1.000000,0.997435,0.006849,0.002377,
0.361702,0.252483,0.595442,0.495982,0.877660,0.743007,1.000000,0.991581,0.399464,0.339384,
0.434880,0.303973,0.436242,0.356643,0.268669,0.195686,0.349112,0.245288,0.305882,0.205471,
0.006849,0.002377,0.361011,0.244337,0.572254,0.482262,0.780528,0.712089,1.000000,0.995797,
0.054348,0.007372,0.352853,0.238002,0.629661,0.500871,0.832817,0.754342,1.000000,0.992339,
0.034682,0.009771,0.313349,0.249903,0.599727,0.501606,0.837607,0.754177,1.000000,0.994515,
0.404110,0.309693,0.495029,0.434321,0.319088,0.255986,0.314286,0.263804,0.200000,0.156757,
0.285714,0.220001,0.054348,0.007595,0.296524,0.235640,0.614035,0.500194,0.818359,0.743121,
1.000000,0.992498,0.034682,0.008785,0.299145,0.244907,0.543478,0.491213,0.787671,0.743473,
1.000000,0.997710,0.006849,0.002377,0.361702,0.252483,0.595442,0.495982,0.877660,0.743007,
1.000000,0.991581,0.077096,0.054302,0.111702,0.065206,0.134228,0.072444,0.114094,0.059361,
0.115162,0.044019,0.057377,0.037169,0.102545,0.067574,0.143836,0.065666,0.116505,0.066780,
0.099415,0.050310,0.117647,0.053642,0.044420,0.024142,0.052778,0.030534,0.042813,0.018718,
0.092391,0.063070,0.137595,0.093168,0.077778,0.052091,0.060773,0.027353,0.071225,0.044286,
0.023520,0.010163

Positive_131 67.000000,0.069949,21.000000,4.686567,3.000000,24.248756,12.000000,4.686567,3.000000,15.521483,
0.298793,0.016444,0.298793,0.016444,0.205788,0.497976,0.540323,0.543860,0.692308,0.750000,
0.500000,0.039801,1.333333,0.052264,0.034115,0.571429,0.018608,0.006633,0.222222,0.001452,
0.004874,0.081633,0.000380,0.086085,0.166667,0.004311,0.031841,0.318182,0.002241,0.046146,
0.181818,0.002270,0.134794,0.303030,0.012103,10.954451,8.195171,5.841619,1.000000,0.776119,
1.000000,0.775693,1.000000,0.751031,1.000000,0.729353,1.000000,0.610306,1.000000,0.374911,
1.000000,0.308955,1.000000,0.215068,1.000000,0.091471,1.000000,0.078394,1.000000,0.835821,
1.000000,0.835821,1.000000,0.828358,1.000000,0.811714,1.000000,0.788391,1.000000,0.751873,
1.000000,0.538181,1.000000,0.346484,0.500000,0.086793,0.212121,0.035776,0.438710,0.325357,
0.418644,0.354280,0.414458,0.320363,0.275510,0.226548,0.259053,0.203678,0.321168,0.221227,
0.193939,0.013447,0.472727,0.260947,0.666667,0.510149,0.836601,0.757074,1.000000,0.991051,
0.047297,0.007590,0.335484,0.243183,0.590678,0.484233,0.819905,0.740619,1.000000,0.994976,
0.007246,0.002671,0.321038,0.232837,0.566929,0.491409,0.777228,0.725351,1.000000,0.996344,
0.716129,0.462858,0.248677,0.129937,0.765705,0.407205,0.116327,0.056212,0.156463,0.080612,
0.020408,0.004990,0.007246,0.002671,0.457516,0.257805,0.647059,0.495317,0.887324,0.757011,
1.000000,1.000000,0.611111,0.102768,0.706117,0.296519,0.837766,0.476686,0.916129,0.636222,

0.998881,0.867109,0.090323,0.028225,0.541752,0.254261,0.700073,0.514070,0.887755,0.746594,
0.997468,0.952469,0.657831,0.529071,0.480000,0.373483,0.056872,0.012839,0.357500,0.245924,
0.590551,0.490266,0.787097,0.712856,0.998605,0.987540,0.473934,0.383536,0.440932,0.355944,
0.319444,0.260521,0.343333,0.262238,0.299270,0.197288,0.249364,0.182966,0.047297,0.007348,
0.340278,0.245247,0.567797,0.487420,0.814407,0.740167,1.000000,0.994916,0.042254,0.011968,
0.341232,0.235589,0.630332,0.486482,0.826087,0.737851,1.000000,0.994033,0.007246,0.002671,
0.361702,0.259121,0.586207,0.509621,0.877660,0.751085,1.000000,0.990806,0.460241,0.355660,
0.387755,0.295390,0.477419,0.348950,0.284672,0.202425,0.306407,0.247992,0.256410,0.198921,
0.007246,0.002671,0.332487,0.241131,0.556430,0.495145,0.780528,0.728066,1.000000,0.996837,
0.047297,0.007798,0.317152,0.233724,0.629661,0.480530,0.832203,0.736892,1.000000,0.992464,
0.193939,0.013375,0.460606,0.261273,0.660131,0.508595,0.836601,0.756678,1.000000,0.991589,
0.354237,0.302594,0.539394,0.436886,0.319444,0.260521,0.325000,0.265416,0.211679,0.155528,
0.279898,0.224727,0.047297,0.007952,0.311224,0.237294,0.567797,0.477136,0.825806,0.726733,
1.000000,0.992870,0.037915,0.010299,0.383886,0.242053,0.639810,0.497340,0.838863,0.747438,
1.000000,0.997872,0.007246,0.002671,0.361702,0.259121,0.586207,0.509621,0.877660,0.751085,
1.000000,0.990806,0.104987,0.058863,0.122581,0.063003,0.106329,0.065728,0.116129,0.056877,
0.081633,0.042129,0.062180,0.038756,0.108696,0.066200,0.112245,0.063706,0.101852,0.065010,
0.076389,0.050802,0.127551,0.049674,0.038710,0.023593,0.072464,0.035297,0.105634,0.031268,
0.089947,0.060434,0.168367,0.097554,0.087324,0.051342,0.039216,0.025577,0.070866,0.043411,
0.036232,0.010777

Positive_132 355.000000,0.008260,327.000000,2.932394,2.000000,297.724230,327.000000,2.932394,1.000000,597.396546,
0.420636,0.018534,0.420636,0.018534,0.017375,0.299607,0.126227,0.531300,0.034247,1.000000,
0.000000,0.000000,0.000000,0.000000,0.000000,0.000000,0.000000,0.000000,0.000000,0.000000,
0.000000,0.000000,0.000000,0.849322,0.920904,0.060015,0.001470,0.156309,0.000130,0.005727,
0.022599,0.000006,0.848910,0.920904,0.060707,25.573424,18.083141,4.898979,1.000000,0.966197,
1.000000,0.966197,1.000000,0.966197,1.000000,0.043783,1.000000,0.038632,1.000000,0.038632,
1.000000,0.028974,0.999869,0.020174,0.992964,0.003575,0.787678,0.002594,1.000000,0.028169,
1.000000,0.027264,1.000000,0.026660,1.000000,0.020282,1.000000,0.017626,1.000000,0.014125,
1.000000,0.012072,0.999869,0.011267,0.992964,0.007787,0.787678,0.004941,0.429530,0.184676,
0.453125,0.364559,0.521595,0.450765,0.325503,0.129892,0.301508,0.138526,0.427215,0.339171,
0.060897,0.011082,0.421053,0.247322,0.730650,0.530714,0.934985,0.818643,1.000000,0.994067,
0.040000,0.010825,0.345048,0.259483,0.621359,0.503238,0.815534,0.754585,1.000000,0.993169,
0.006849,0.003127,0.361789,0.229930,0.569106,0.475529,0.822742,0.700839,1.000000,0.993062,
0.745819,0.310029,0.408313,0.099602,0.685246,0.590369,0.102972,0.041209,0.125628,0.083155,
0.035948,0.015994,0.006849,0.003127,0.375000,0.233802,0.672365,0.540778,0.896936,0.792872,
1.000000,0.999730,0.459350,0.036654,0.724852,0.081118,0.790274,0.382463,0.965517,0.802966,
0.996951,0.896626,0.128492,0.057317,0.391061,0.268947,0.597765,0.487084,0.846154,0.709904,
1.000000,0.979843,0.780822,0.726201,0.500000,0.213029,0.122905,0.016895,0.389892,0.265396,
0.601626,0.476570,0.856187,0.708667,1.000000,0.980049,0.458194,0.357040,0.440252,0.384778,
0.319088,0.258182,0.371795,0.277147,0.250000,0.184709,0.268421,0.192381,0.035230,0.010597,
0.334365,0.265220,0.608414,0.504028,0.793750,0.742598,1.000000,0.993290,0.048338,0.010609,
0.306859,0.214763,0.570435,0.487312,0.813008,0.719730,1.000000,0.993425,0.006849,0.003127,
0.331250,0.250829,0.584848,0.490445,0.843478,0.773066,1.000000,0.994094,0.559105,0.491729,
0.418060,0.296380,0.436242,0.211891,0.373041,0.301392,0.331658,0.174888,0.335570,0.127881,

0.006849,0.003127,0.365854,0.234444,0.569106,0.475820,0.822742,0.707668,1.000000,0.993371,
0.068571,0.011598,0.352853,0.255248,0.653722,0.503633,0.815625,0.750848,1.000000,0.991504,
0.045317,0.010700,0.402477,0.248570,0.712074,0.529232,0.904321,0.801144,1.000000,0.994856,
0.404110,0.283621,0.548495,0.458197,0.319088,0.258182,0.362179,0.266558,0.200000,0.146402,
0.300654,0.230688,0.056911,0.011756,0.341676,0.254510,0.634304,0.498022,0.810897,0.741532,
1.000000,0.990697,0.028846,0.009856,0.300813,0.231647,0.565000,0.494574,0.798722,0.721808,
1.000000,0.994673,0.006849,0.003127,0.331250,0.250829,0.584848,0.490445,0.843478,0.773066,
1.000000,0.994094,0.073826,0.034551,0.099715,0.035928,0.143813,0.027499,0.114094,0.028145,
0.068493,0.024712,0.071770,0.033841,0.089041,0.046467,0.143836,0.063367,0.130573,0.081654,
0.099678,0.063989,0.150502,0.040904,0.062893,0.027215,0.068852,0.040964,0.054313,0.032515,
0.116505,0.077892,0.188854,0.139341,0.149682,0.081494,0.071839,0.040194,0.112179,0.071801,
0.025237,0.007528

Positive_133 39.000000,0.040105,22.000000,1.564103,0.000000,13.883941,5.000000,1.564103,1.000000,2.041835,
0.392210,0.017133,0.392210,0.017133,0.034483,0.178571,0.391304,0.357143,1.000000,0.000000,
0.000000,0.000000,0.000000,0.000000,0.000000,0.000000,0.000000,0.000000,0.000000,0.000000,
0.000000,0.000000,0.000000,0.022329,0.105263,0.001621,0.030364,0.578947,0.009496,0.026316,
0.098684,0.000743,0.323887,0.552632,0.059818,4.711479,4.000000,3.872983,1.000000,0.307692,
1.000000,0.307692,1.000000,0.307692,1.000000,0.307692,1.000000,0.307692,1.000000,0.307692,
1.000000,0.273504,1.000000,0.228283,1.000000,0.198490,1.000000,0.089488,1.000000,0.230769,
1.000000,0.230769,1.000000,0.230769,1.000000,0.230769,1.000000,0.179487,1.000000,0.128205,
1.000000,0.128205,0.600000,0.097436,0.500000,0.066667,0.200000,0.032479,0.417910,0.251818,
0.409037,0.335367,0.538462,0.412815,0.241730,0.164747,0.318182,0.187708,0.337539,0.269438,
0.070234,0.017823,0.313349,0.242290,0.557003,0.503782,0.826160,0.739508,1.000000,0.991173,
0.104478,0.011409,0.334448,0.250211,0.606061,0.510628,0.815851,0.743379,1.000000,0.992476,
0.014925,0.003231,0.339350,0.244907,0.567797,0.482201,0.812274,0.737869,1.000000,0.996235,
0.558275,0.305231,0.485294,0.088674,0.849498,0.606094,0.171091,0.035307,0.112360,0.064076,
0.015152,0.003823,0.014925,0.003231,0.597015,0.328340,0.719064,0.591926,0.896440,0.816750,
1.000000,1.000000,0.960000,0.430749,0.960000,0.520705,0.960000,0.672591,0.973333,0.794237,
0.997059,0.908749,0.133333,0.022872,0.391061,0.227797,0.692827,0.455090,0.858228,0.665150,
0.997622,0.917297,0.800687,0.666050,0.484848,0.266626,0.173333,0.024215,0.389892,0.258495,
0.581227,0.479783,0.791176,0.732814,1.000000,0.988045,0.476471,0.338148,0.448161,0.386115,
0.319088,0.275737,0.318449,0.256049,0.230088,0.180805,0.281046,0.223411,0.074627,0.010270,
0.313433,0.241073,0.600233,0.513956,0.808858,0.747050,1.000000,0.993384,0.053333,0.010940,
0.306859,0.229736,0.531646,0.471132,0.779747,0.727407,1.000000,0.994504,0.014925,0.003231,
0.352564,0.266132,0.555838,0.501946,0.866667,0.752648,1.000000,0.992241,0.565217,0.442450,
0.347059,0.280417,0.426396,0.277133,0.293375,0.240239,0.348485,0.222117,0.229730,0.154835,
0.014925,0.003231,0.361011,0.249532,0.563177,0.483967,0.779783,0.739391,1.000000,0.996235,
0.104478,0.011581,0.334448,0.238969,0.628205,0.505663,0.815851,0.741213,1.000000,0.990165,
0.070234,0.016247,0.355049,0.254308,0.566775,0.507103,0.824104,0.741991,1.000000,0.991173,
0.420588,0.280098,0.522388,0.444165,0.319088,0.275737,0.303173,0.248464,0.212389,0.150231,
0.318182,0.253985,0.074627,0.011280,0.316151,0.228085,0.586247,0.500490,0.807692,0.739719,
1.000000,0.989186,0.053333,0.009636,0.281375,0.236877,0.522782,0.481712,0.769620,0.739473,
1.000000,0.997364,0.014925,0.003231,0.352564,0.266132,0.555838,0.501946,0.866667,0.752648,
1.000000,0.992241,0.073604,0.045599,0.134328,0.056235,0.119403,0.043961,0.088235,0.035180,

0.053299,0.030503,0.065147,0.040342,0.080986,0.049323,0.106667,0.064198,0.116719,0.078208,
0.082353,0.053189,0.089552,0.035499,0.043321,0.025314,0.050167,0.029636,0.053333,0.022551,
0.090301,0.065195,0.200637,0.130343,0.136808,0.075772,0.059701,0.029893,0.107023,0.066978,
0.045603,0.022082

Positive_134 173.000000,0.023088,26.000000,3.994220,3.000000,26.552292,18.000000,3.994220,2.000000,16.470897,
0.364275,0.021551,0.364275,0.021551,0.076246,0.288889,0.537946,0.502415,0.514563,0.440000,
0.607143,0.015414,0.666667,0.010097,0.010276,0.888889,0.009081,0.001285,0.055556,0.000070,
0.000571,0.049383,0.000028,0.029499,0.098837,0.000915,0.016232,0.151163,0.000480,0.015674,
0.104651,0.000459,0.041130,0.145349,0.002270,11.401754,10.631004,7.694423,1.000000,0.601156,
1.000000,0.599650,1.000000,0.596322,1.000000,0.548547,1.000000,0.451735,1.000000,0.404278,
1.000000,0.321826,1.000000,0.213560,1.000000,0.079620,1.000000,0.019613,1.000000,0.669745,
1.000000,0.661268,1.000000,0.621568,1.000000,0.537224,1.000000,0.445170,1.000000,0.391899,
1.000000,0.324920,1.000000,0.171721,1.000000,0.101838,1.000000,0.046334,0.481990,0.333322,
0.501992,0.364410,0.457801,0.302268,0.292871,0.225236,0.311688,0.199089,0.305128,0.215737,
0.064133,0.011152,0.384783,0.259504,0.679755,0.506352,0.838875,0.747850,1.000000,0.994702,
0.102564,0.006451,0.450085,0.242735,0.625285,0.487993,0.846962,0.744515,1.000000,0.993900,
0.013889,0.002302,0.454698,0.242706,0.653130,0.487761,0.823529,0.736232,1.000000,0.993781,
0.724196,0.489947,0.451807,0.163405,0.764796,0.346649,0.157182,0.062058,0.116959,0.062275,
0.016043,0.004092,0.089744,0.003111,0.544231,0.240513,0.709677,0.497112,0.933333,0.748060,
1.000000,0.999968,0.958333,0.102919,0.958333,0.279479,0.983114,0.483876,0.990619,0.673242,
0.998902,0.900625,0.230337,0.038355,0.814525,0.288716,0.953289,0.516053,0.975676,0.753767,
1.000000,0.959695,0.716714,0.477391,0.509091,0.368205,0.152778,0.014275,0.496644,0.246002,
0.666107,0.473241,0.839056,0.720076,0.999082,0.988763,0.533865,0.388405,0.456984,0.367761,
0.423077,0.243834,0.355615,0.273034,0.263158,0.182540,0.261905,0.179194,0.102564,0.006561,
0.456853,0.242297,0.618474,0.490261,0.842520,0.744995,1.000000,0.995804,0.128440,0.013357,
0.359060,0.252955,0.614213,0.495457,0.807198,0.738318,1.000000,0.995971,0.013889,0.002302,
0.370192,0.248533,0.671743,0.498133,0.877660,0.744895,1.000000,0.993425,0.480818,0.331700,
0.470120,0.312463,0.502573,0.355837,0.294872,0.199572,0.376623,0.234319,0.267823,0.205178,
0.013889,0.002302,0.459732,0.243981,0.651438,0.485489,0.818951,0.735349,1.000000,0.994239,
0.102564,0.006767,0.465313,0.239101,0.654897,0.489080,0.854464,0.742865,1.000000,0.993250,
0.064133,0.010549,0.384783,0.260058,0.662577,0.506383,0.838875,0.748409,1.000000,0.995197,
0.401650,0.309682,0.524150,0.446483,0.423077,0.243834,0.361842,0.271763,0.251462,0.148685,
0.293651,0.213049,0.102564,0.007451,0.319231,0.236066,0.644670,0.484809,0.819757,0.739196,
1.000000,0.993723,0.125000,0.010919,0.316514,0.253176,0.576923,0.502957,0.816514,0.745651,
1.000000,0.996765,0.013889,0.002302,0.370192,0.248533,0.671743,0.498133,0.877660,0.744895,
1.000000,0.993425,0.109983,0.056412,0.150602,0.063405,0.146780,0.073926,0.104651,0.055378,
0.094488,0.045374,0.075107,0.038827,0.138686,0.062239,0.130265,0.067767,0.132128,0.073164,
0.087379,0.051135,0.196787,0.058160,0.041872,0.022515,0.062016,0.029432,0.074873,0.020563,
0.109489,0.063593,0.165333,0.096960,0.106667,0.049081,0.089744,0.023904,0.115385,0.037957,
0.035135,0.010211

Positive_135 130.000000,0.021834,12.000000,2.838462,2.000000,9.206261,15.000000,2.838462,2.000000,10.911688,
0.408645,0.028078,0.411995,0.026925,0.061453,0.217262,0.285171,0.404255,0.580357,0.702128,
0.785714,0.034286,1.600000,0.028311,0.043269,0.875000,0.023913,0.009865,0.250000,0.002077,
0.009976,0.187500,0.001234,0.032802,0.108527,0.001109,0.018999,0.093023,0.000476,0.014208,

0.077519,0.000470,0.029527,0.085271,0.000721,8.391025,7.158403,5.659033,1.000000,0.576923,
1.000000,0.576154,1.000000,0.566031,1.000000,0.522934,1.000000,0.475766,1.000000,0.342994,
1.000000,0.222060,1.000000,0.167781,1.000000,0.098168,0.333333,0.045087,1.000000,0.607692,
1.000000,0.604957,1.000000,0.563529,1.000000,0.511099,1.000000,0.452637,1.000000,0.397875,
1.000000,0.269316,1.000000,0.210354,1.000000,0.107650,1.000000,0.031673,0.445783,0.322119,
0.588191,0.376812,0.436975,0.301069,0.331242,0.230356,0.262857,0.198290,0.348425,0.218244,
0.076271,0.009970,0.432203,0.250169,0.618644,0.500358,0.872881,0.747717,1.000000,0.994693,
0.032468,0.006755,0.350649,0.239141,0.662338,0.491598,0.846962,0.748674,1.000000,0.995265,
0.009091,0.002425,0.412245,0.255445,0.620408,0.494291,0.822901,0.734889,1.000000,0.992820,
0.825954,0.507958,0.377880,0.148004,0.741228,0.344038,0.194444,0.060396,0.119266,0.067424,
0.020690,0.005368,0.009091,0.002425,0.564935,0.246193,0.709677,0.504432,0.896956,0.760328,
1.000000,0.999262,0.525974,0.096067,0.902752,0.274233,0.930275,0.435259,0.970760,0.640345,
0.999204,0.885038,0.474576,0.054000,0.738971,0.283679,0.822758,0.504466,0.907308,0.709130,
1.000000,0.948324,0.747899,0.510891,0.500000,0.361756,0.122905,0.016763,0.389892,0.266956,
0.594937,0.493827,0.815421,0.725688,1.000000,0.988905,0.587444,0.394476,0.439252,0.355567,
0.341365,0.249957,0.354545,0.264155,0.264085,0.187780,0.257143,0.182425,0.029762,0.006400,
0.357143,0.237007,0.675325,0.492962,0.838710,0.746879,1.000000,0.995674,0.076271,0.010969,
0.436190,0.250598,0.672986,0.496138,0.845714,0.741386,1.000000,0.996588,0.009091,0.002425,
0.389724,0.255062,0.665414,0.498626,0.877660,0.738228,1.000000,0.991618,0.481793,0.332266,
0.535127,0.320380,0.457831,0.347353,0.340551,0.198514,0.309156,0.237156,0.303448,0.208966,
0.009091,0.002425,0.411429,0.258896,0.619048,0.494949,0.818519,0.735458,1.000000,0.993387,
0.051948,0.007029,0.396104,0.233334,0.694805,0.491052,0.854464,0.749933,1.000000,0.994093,
0.076271,0.009389,0.432203,0.251308,0.618644,0.499716,0.847458,0.747492,1.000000,0.995174,
0.438714,0.314504,0.527273,0.435539,0.341365,0.249957,0.352558,0.266263,0.211268,0.152278,
0.289130,0.217926,0.059524,0.007271,0.350649,0.231048,0.694805,0.487267,0.853261,0.743728,
1.000000,0.992966,0.059406,0.009145,0.333333,0.250190,0.584762,0.500500,0.829384,0.746626,
1.000000,0.997489,0.009091,0.002425,0.389724,0.255062,0.665414,0.498626,0.877660,0.738228,
1.000000,0.991618,0.137441,0.059559,0.144578,0.057833,0.134228,0.070658,0.114094,0.055717,
0.084309,0.043277,0.068282,0.035076,0.118087,0.066909,0.143836,0.068213,0.160714,0.074648,
0.101695,0.049018,0.144860,0.061593,0.051948,0.025234,0.060738,0.031197,0.054545,0.018379,
0.108761,0.062606,0.181818,0.095385,0.092391,0.048566,0.060773,0.024497,0.078341,0.039794,
0.038961,0.011843

Positive_136 85.000000,0.030588,9.000000,2.600000,2.000000,3.980952,23.000000,2.600000,1.000000,19.052381,
0.386795,0.031007,0.386795,0.031007,0.082569,0.305000,0.431655,0.405063,0.255319,0.457143,
0.736842,0.000000,0.000000,0.000000,0.000000,0.000000,0.000000,0.000000,0.000000,0.000000,
0.000000,0.000000,0.000000,0.093067,0.261905,0.010515,0.027906,0.107143,0.000429,0.023601,
0.273810,0.003376,0.023729,0.095238,0.000892,8.317451,6.708204,3.464102,1.000000,0.682353,
1.000000,0.682353,1.000000,0.682353,1.000000,0.663529,1.000000,0.620000,1.000000,0.532549,
1.000000,0.415294,1.000000,0.332484,1.000000,0.209150,0.333333,0.021765,1.000000,0.447059,
1.000000,0.447059,1.000000,0.445385,1.000000,0.429161,1.000000,0.419754,1.000000,0.382978,
1.000000,0.349034,1.000000,0.246422,1.000000,0.109684,0.333333,0.046855,0.429530,0.322009,
0.541667,0.363348,0.435976,0.314644,0.324138,0.226984,0.262857,0.203793,0.327217,0.220295,
0.193939,0.013642,0.472727,0.260332,0.666667,0.510623,0.846154,0.761436,1.000000,0.992831,
0.047297,0.007615,0.313084,0.235693,0.600900,0.487383,0.846962,0.735864,1.000000,0.993889,

0.010870,0.002901,0.363409,0.239381,0.578358,0.485502,0.812274,0.725215,1.000000,0.996081,
0.810748,0.484086,0.377880,0.130225,0.631098,0.385689,0.194444,0.054278,0.129252,0.073626,
0.020690,0.005861,0.010870,0.002901,0.391598,0.259670,0.709677,0.508750,0.887324,0.759682,
1.000000,0.998872,0.611111,0.107143,0.706117,0.253987,0.837766,0.410394,0.981707,0.602753,
0.997104,0.809815,0.308756,0.050361,0.589552,0.275108,0.814815,0.527600,0.897436,0.746142,
1.000000,0.949112,0.685976,0.519799,0.500000,0.363795,0.122905,0.020203,0.420048,0.253343,
0.603834,0.484095,0.815421,0.703379,1.000000,0.985984,0.525000,0.385228,0.423913,0.354220,
0.319444,0.260552,0.337931,0.258577,0.299270,0.197041,0.257143,0.182932,0.047297,0.007548,
0.345588,0.241057,0.583646,0.493262,0.838710,0.738361,1.000000,0.993961,0.066667,0.014186,
0.341232,0.240130,0.630332,0.490186,0.826087,0.745014,1.000000,0.994710,0.010870,0.002901,
0.379808,0.251500,0.631420,0.494596,0.877660,0.745377,1.000000,0.990081,0.466463,0.349006,
0.483645,0.303159,0.436242,0.347835,0.287462,0.202248,0.306407,0.246435,0.303448,0.200467,
0.010870,0.002901,0.368421,0.244663,0.591346,0.487523,0.807692,0.725776,1.000000,0.996269,
0.047297,0.007727,0.352941,0.226836,0.631658,0.486046,0.854464,0.734291,1.000000,0.992130,
0.193939,0.013231,0.460606,0.259991,0.643505,0.507809,0.837607,0.759617,1.000000,0.993491,
0.416667,0.303153,0.539394,0.436295,0.319444,0.260552,0.332553,0.262146,0.211679,0.155803,
0.285714,0.224170,0.097826,0.008583,0.296524,0.229936,0.567402,0.478858,0.819205,0.724750,
1.000000,0.992001,0.066667,0.011036,0.383886,0.249647,0.639810,0.504127,0.838863,0.753068,
1.000000,0.997833,0.010870,0.002901,0.379808,0.251500,0.631420,0.494596,0.877660,0.745377,
1.000000,0.990081,0.092338,0.055491,0.111702,0.064374,0.134228,0.067652,0.114094,0.055304,
0.078550,0.042671,0.060914,0.036517,0.108696,0.066075,0.143836,0.066334,0.119709,0.065305,
0.083770,0.050136,0.144860,0.055309,0.050000,0.025826,0.072464,0.034363,0.105634,0.026765,
0.099476,0.060152,0.157846,0.098947,0.077778,0.048282,0.060773,0.026808,0.085366,0.041876,
0.036232,0.011814

Positive_137 96.000000,0.040799,19.000000,3.916667,1.500000,32.245614,10.000000,3.916667,2.000000,14.077193,
0.426689,0.018661,0.426689,0.018661,0.040214,0.148045,0.314754,0.454545,0.640351,0.853659,
1.000000,0.000000,0.000000,0.000000,0.000000,0.000000,0.000000,0.000000,0.000000,0.000000,
0.000000,0.000000,0.000000,0.038306,0.094737,0.001158,0.025251,0.200000,0.001552,0.025213,
0.084211,0.000439,0.075856,0.189474,0.005416,13.784049,6.324555,4.898979,1.000000,0.500000,
1.000000,0.500000,1.000000,0.500000,1.000000,0.488745,1.000000,0.462480,1.000000,0.405554,
1.000000,0.330777,0.833333,0.201759,0.500000,0.108685,0.339181,0.062612,1.000000,0.593750,
1.000000,0.593750,1.000000,0.588889,1.000000,0.575893,1.000000,0.559524,1.000000,0.429572,
1.000000,0.374521,1.000000,0.248686,0.666667,0.126496,0.357143,0.060590,0.429530,0.309318,
0.532313,0.379985,0.407240,0.310696,0.324138,0.219370,0.262857,0.189043,0.336824,0.231243,
0.078378,0.011363,0.419811,0.255141,0.603774,0.505556,0.840979,0.751846,1.000000,0.994631,
0.018868,0.006186,0.302799,0.240941,0.603774,0.482709,0.827327,0.746397,1.000000,0.995107,
0.006849,0.002313,0.339350,0.240643,0.576507,0.494571,0.812274,0.728276,1.000000,0.995508,
0.803279,0.495922,0.342541,0.114533,0.616438,0.389545,0.105641,0.046257,0.112821,0.069041,
0.020690,0.004335,0.006849,0.002313,0.387718,0.256103,0.672365,0.498007,0.891165,0.767274,
1.000000,0.999001,0.748634,0.127317,0.797260,0.356060,0.925659,0.540624,0.991914,0.725783,
0.998073,0.914173,0.128492,0.025698,0.558402,0.285065,0.856269,0.500889,0.917431,0.695814,
1.000000,0.908327,0.676471,0.525238,0.500000,0.347214,0.122905,0.014292,0.389892,0.250280,
0.584906,0.489353,0.816712,0.706325,1.000000,0.991029,0.509511,0.405576,0.423181,0.341564,
0.319088,0.252860,0.343558,0.262522,0.255924,0.193925,0.257143,0.176926,0.042980,0.006657,

0.306849,0.241567,0.579515,0.486356,0.821002,0.748522,1.000000,0.995711,0.033333,0.010294,
0.314534,0.235274,0.562384,0.490369,0.805861,0.732720,1.000000,0.995432,0.006849,0.002313,
0.362369,0.265077,0.737003,0.515608,0.880734,0.746115,1.000000,0.993997,0.438914,0.340122,
0.467687,0.324180,0.436242,0.335699,0.314136,0.210533,0.306407,0.226933,0.303448,0.201784,
0.006849,0.002313,0.361011,0.244091,0.574148,0.495507,0.794344,0.731472,1.000000,0.996036,
0.048711,0.007236,0.352853,0.234734,0.608059,0.478141,0.845266,0.740199,1.000000,0.993423,
0.078378,0.011119,0.372642,0.256393,0.613208,0.505889,0.840979,0.752810,1.000000,0.995377,
0.404110,0.317946,0.522911,0.429194,0.319088,0.252860,0.365031,0.267727,0.203343,0.153339,
0.293839,0.217512,0.048711,0.007763,0.315864,0.241157,0.592068,0.485920,0.842776,0.739377,
1.000000,0.993993,0.030556,0.008056,0.300555,0.235025,0.544160,0.490539,0.793296,0.746174,
1.000000,0.997004,0.006849,0.002313,0.362369,0.265077,0.737003,0.515608,0.880734,0.746115,
1.000000,0.993997,0.115591,0.060671,0.111702,0.055891,0.134228,0.064989,0.114094,0.049552,
0.115162,0.042260,0.073986,0.035955,0.142651,0.070702,0.143836,0.073090,0.131285,0.072317,
0.100917,0.052285,0.136612,0.055786,0.059299,0.026381,0.052778,0.029425,0.073446,0.031844,
0.089947,0.059722,0.172507,0.093983,0.077778,0.044655,0.060773,0.025856,0.071225,0.040794,
0.033333,0.013843

Positive_138 60.000000,0.054722,8.000000,3.283333,3.000000,5.698023,13.000000,3.283333,3.000000,11.664124,
0.365491,0.020915,0.365491,0.020915,0.076923,0.305556,0.528000,0.423729,0.500000,0.823529,
0.666667,0.008333,0.500000,0.004167,0.003333,0.200000,0.000667,0.004167,0.250000,0.001042,
0.000667,0.040000,0.000027,0.078406,0.203390,0.002679,0.032420,0.067797,0.000524,0.030255,
0.152542,0.002285,0.036720,0.118644,0.001805,7.937254,5.494611,5.451175,1.000000,0.733333,
1.000000,0.733333,1.000000,0.733333,1.000000,0.600000,1.000000,0.494563,1.000000,0.402738,
1.000000,0.270159,1.000000,0.187341,0.333333,0.043452,0.333333,0.024008,1.000000,0.566667,
1.000000,0.560185,1.000000,0.508526,1.000000,0.432671,1.000000,0.370282,1.000000,0.273469,
1.000000,0.187011,1.000000,0.139436,1.000000,0.075794,1.000000,0.038571,0.429158,0.298981,
0.588191,0.386674,0.436975,0.314344,0.331242,0.224928,0.255319,0.184140,0.349398,0.238545,
0.143939,0.014869,0.363636,0.248705,0.587629,0.491766,0.802083,0.743368,1.000000,0.994219,
0.032468,0.007200,0.360277,0.244920,0.662338,0.495341,0.808843,0.740316,1.000000,0.996844,
0.010309,0.002423,0.448000,0.250685,0.626000,0.483816,0.824000,0.735494,1.000000,0.994763,
0.798206,0.512988,0.366071,0.126013,0.731006,0.360999,0.130726,0.054497,0.183206,0.066996,
0.018450,0.004970,0.010309,0.002423,0.564935,0.242305,0.662338,0.478268,0.911704,0.747150,
1.000000,1.000000,0.825758,0.115281,0.825758,0.292232,0.914826,0.440535,0.974763,0.639442,
0.998505,0.859809,0.310476,0.051877,0.738971,0.274574,0.817582,0.506951,0.952009,0.726134,
0.998211,0.947214,0.756098,0.537374,0.492000,0.345234,0.072165,0.013693,0.388119,0.258575,
0.602459,0.495367,0.795082,0.734247,0.998823,0.986799,0.587444,0.401097,0.445585,0.357222,
0.318182,0.241681,0.375000,0.277629,0.225847,0.181382,0.243972,0.175076,0.030928,0.006723,
0.374134,0.246091,0.675325,0.494472,0.815345,0.743369,1.000000,0.997346,0.044944,0.009550,
0.436190,0.253617,0.640000,0.496759,0.845714,0.747455,1.000000,0.994939,0.010309,0.002423,
0.389724,0.241517,0.665414,0.487460,0.848485,0.730229,1.000000,0.989102,0.481793,0.344906,
0.535127,0.329412,0.449692,0.325682,0.340551,0.215903,0.302128,0.221358,0.294856,0.206512,
0.010309,0.002423,0.438000,0.254402,0.604000,0.493904,0.820000,0.738380,1.000000,0.995251,
0.051948,0.007920,0.396104,0.242722,0.694805,0.490131,0.853261,0.740020,1.000000,0.996260,
0.143939,0.012536,0.393939,0.251710,0.587629,0.494475,0.797428,0.742463,1.000000,0.994219,
0.438714,0.313272,0.529774,0.445047,0.318182,0.241681,0.406250,0.280167,0.192162,0.141749,

0.309220,0.214709,0.030928,0.007622,0.374134,0.239296,0.694805,0.493372,0.853261,0.745002,
1.000000,0.995917,0.035928,0.007990,0.336000,0.253871,0.584762,0.497426,0.798095,0.744954,
1.000000,0.996829,0.010309,0.002423,0.389724,0.241517,0.665414,0.487460,0.848485,0.730229,
1.000000,0.989102,0.092949,0.055116,0.088060,0.052220,0.125257,0.065355,0.076441,0.048123,
0.114990,0.045087,0.058304,0.033080,0.159836,0.070950,0.133987,0.068924,0.146538,0.076412,
0.084599,0.048863,0.133034,0.064263,0.051948,0.026701,0.057377,0.030562,0.083333,0.023562,
0.108761,0.061203,0.164948,0.105561,0.075203,0.046936,0.045455,0.025412,0.082902,0.038654,
0.038961,0.013015

Positive_139 40.000000,0.078750,15.000000,3.150000,2.000000,17.823077,8.000000,3.150000,3.000000,3.925641,
0.339082,0.014646,0.339082,0.014646,0.096774,0.321429,0.605263,0.466667,0.750000,1.000000,
0.000000,0.030000,0.400000,0.011385,0.105000,1.200000,0.083308,0.002000,0.026667,0.000051,
0.024750,0.240000,0.004454,0.080513,0.179487,0.004699,0.094252,0.384615,0.022725,0.101282,
0.205128,0.006156,0.213675,0.358974,0.012484,6.804244,4.974151,4.898979,1.000000,0.550000,
1.000000,0.550000,1.000000,0.535476,1.000000,0.494325,1.000000,0.475060,0.666667,0.229980,
0.571429,0.071944,0.428571,0.036905,0.214286,0.013631,0.071429,0.004782,1.000000,0.725000,
1.000000,0.725000,1.000000,0.722321,1.000000,0.615417,1.000000,0.524583,1.000000,0.394702,
1.000000,0.100000,0.733333,0.062738,0.400000,0.030536,0.133333,0.011845,0.431718,0.308014,
0.588191,0.382844,0.398821,0.309142,0.289809,0.219638,0.259053,0.187983,0.348425,0.225146,
0.069672,0.012013,0.409091,0.264670,0.705382,0.513056,0.815864,0.755241,1.000000,0.995906,
0.020492,0.005485,0.294686,0.238692,0.560000,0.487324,0.825994,0.748903,1.000000,0.995919,
0.009091,0.002089,0.383886,0.239991,0.606635,0.492496,0.810427,0.732861,1.000000,0.994649,
0.830389,0.516640,0.313305,0.126847,0.693517,0.356512,0.119342,0.052397,0.119266,0.067724,
0.012853,0.003791,0.009091,0.002089,0.357955,0.244080,0.643741,0.485225,0.863436,0.759363,
1.000000,1.000000,0.438163,0.076117,0.643443,0.278107,0.843468,0.423841,0.922825,0.640465,
0.999083,0.858443,0.264463,0.046365,0.558304,0.296382,0.713881,0.505230,0.891010,0.705669,
0.998735,0.936764,0.728880,0.523831,0.432671,0.346274,0.044818,0.012038,0.349633,0.255699,
0.602459,0.495520,0.795082,0.721951,0.998823,0.992217,0.587444,0.391325,0.439153,0.358221,
0.305857,0.250454,0.349138,0.262935,0.234074,0.183530,0.251656,0.181619,0.020492,0.005680,
0.303103,0.235678,0.543590,0.484636,0.819581,0.751997,1.000000,0.996446,0.037190,0.009301,
0.407583,0.257741,0.672986,0.508468,0.838863,0.746321,1.000000,0.997506,0.009091,0.002089,
0.419263,0.251015,0.586066,0.504157,0.817289,0.729014,1.000000,0.991241,0.433884,0.342189,
0.535127,0.321518,0.455947,0.336293,0.340551,0.202796,0.306407,0.227172,0.279459,0.198430,
0.009091,0.002089,0.379147,0.245529,0.606635,0.497848,0.810427,0.733928,1.000000,0.995178,
0.020492,0.005841,0.307876,0.231995,0.547677,0.477923,0.829842,0.747998,1.000000,0.992990,
0.065574,0.011156,0.416431,0.263905,0.620397,0.509083,0.794271,0.752859,1.000000,0.995940,
0.438714,0.307300,0.527273,0.442246,0.305857,0.250454,0.336207,0.266892,0.176955,0.145495,
0.289130,0.219654,0.020492,0.006114,0.294007,0.233063,0.543590,0.483738,0.810571,0.747603,
1.000000,0.993017,0.030556,0.007748,0.301653,0.251414,0.582938,0.503846,0.829384,0.751851,
1.000000,0.998425,0.009091,0.002089,0.419263,0.251015,0.586066,0.504157,0.817289,0.729014,
1.000000,0.991241,0.137441,0.059937,0.087278,0.052353,0.094714,0.065566,0.105134,0.049251,
0.080688,0.044377,0.068282,0.036530,0.161473,0.069463,0.123675,0.070497,0.146538,0.072241,
0.080808,0.045848,0.169611,0.063469,0.090652,0.028278,0.060738,0.033047,0.054545,0.020556,
0.090909,0.058369,0.181818,0.104958,0.082051,0.048420,0.045455,0.024690,0.068047,0.040019,
0.020202,0.012130

Positive_140 5.000000,0.120000,2.000000,0.600000,0.000000,0.800000,1.000000,0.600000,1.000000,0.300000,
0.553909,0.012718,0.553909,0.012718,0.000000,0.000000,0.000000,0.666667,0.000000,1.000000,
0.000000,0.000000,0.000000,0.000000,0.000000,0.000000,0.000000,0.000000,0.000000,0.000000,
0.000000,0.000000,0.000000,0.000000,0.000000,0.000000,0.000000,0.000000,0.000000,0.000000,
0.000000,0.000000,0.100000,0.250000,0.018750,1.414214,1.000000,0.000000,1.000000,0.200000,
1.000000,0.200000,1.000000,0.200000,1.000000,0.200000,1.000000,0.200000,1.000000,0.200000,
1.000000,0.200000,1.000000,0.200000,1.000000,0.200000,0.000000,0.000000,0.000000,0.000000,
0.000000,0.000000,0.000000,0.000000,0.000000,0.000000,0.000000,0.000000,0.000000,0.000000,
0.000000,0.000000,0.000000,0.000000,0.000000,0.000000,0.000000,0.000000,0.379104,0.300741,
0.448399,0.363546,0.354545,0.335713,0.254491,0.208931,0.242515,0.188530,0.267857,0.244953,
0.050746,0.020868,0.384342,0.286183,0.570149,0.516919,0.829721,0.758865,0.996441,0.993342,
0.031818,0.013629,0.281818,0.223290,0.554545,0.486528,0.786364,0.720376,1.000000,0.995450,
0.004545,0.003531,0.309609,0.236869,0.530249,0.492569,0.790036,0.739016,1.000000,0.995455,
0.583630,0.514359,0.364583,0.261382,0.298507,0.224258,0.114155,0.089231,0.074850,0.043547,
0.008982,0.002710,0.004545,0.003531,0.277273,0.214646,0.554545,0.476688,0.845455,0.713568,
1.000000,1.000000,0.128472,0.081678,0.626335,0.334115,0.704626,0.454412,0.882562,0.633485,
0.996441,0.808988,0.288256,0.074229,0.749226,0.357490,0.871528,0.707980,0.920139,0.804588,
0.986364,0.933700,0.545455,0.477069,0.419162,0.342370,0.018182,0.014316,0.338078,0.238754,
0.601423,0.492811,0.793594,0.721909,0.992883,0.978334,0.402135,0.367692,0.402985,0.374618,
0.286364,0.257690,0.326347,0.263109,0.232877,0.184743,0.212544,0.183738,0.031818,0.013629,
0.277273,0.238605,0.550000,0.490231,0.759091,0.710537,1.000000,0.994645,0.024306,0.011154,
0.327402,0.235528,0.558209,0.485706,0.794030,0.719248,1.000000,0.978275,0.004545,0.003531,
0.313167,0.267658,0.562278,0.512287,0.831818,0.787125,1.000000,0.992811,0.427273,0.373142,
0.373665,0.298819,0.411940,0.328039,0.236934,0.217918,0.269461,0.230689,0.239521,0.189230,
0.004545,0.003531,0.309609,0.246017,0.590747,0.503280,0.793594,0.739398,1.000000,0.998182,
0.031818,0.013629,0.277273,0.220957,0.590909,0.493982,0.786364,0.709412,1.000000,0.994026,
0.050746,0.019479,0.384342,0.281601,0.526690,0.498666,0.826625,0.763843,0.996441,0.993342,
0.277612,0.264619,0.501493,0.477691,0.286364,0.257690,0.302395,0.254391,0.164384,0.133092,
0.268293,0.235390,0.056940,0.022170,0.330961,0.250859,0.551601,0.495676,0.807829,0.734432,
1.000000,0.991943,0.024306,0.011154,0.253472,0.223808,0.524306,0.474797,0.774306,0.725639,
1.000000,0.992108,0.004545,0.003531,0.313167,0.267658,0.562278,0.512287,0.831818,0.787125,
1.000000,0.992811,0.062500,0.046693,0.095522,0.067513,0.077612,0.057601,0.050746,0.035517,
0.046440,0.038321,0.065672,0.055096,0.067616,0.048488,0.045455,0.035275,0.108359,0.084453,
0.083582,0.060886,0.138790,0.069716,0.032836,0.027298,0.072727,0.037429,0.062687,0.033356,
0.095455,0.072021,0.124555,0.103352,0.074303,0.048227,0.042705,0.025334,0.050000,0.039802,
0.017361,0.013621

Positive_141 23.000000,0.056711,6.000000,1.304348,1.000000,2.039526,4.000000,1.304348,1.000000,1.948617,
0.568640,0.050076,0.568640,0.050076,0.066667,0.142857,0.125000,0.000000,0.047619,0.400000,
0.750000,0.000000,0.000000,0.000000,0.000000,0.000000,0.000000,0.000000,0.000000,0.000000,
0.000000,0.000000,0.000000,0.069170,0.136364,0.002391,0.058136,0.272727,0.004528,0.043808,
0.181818,0.003402,0.060935,0.227273,0.007192,2.964141,2.239035,2.000000,1.000000,0.260870,
1.000000,0.260870,1.000000,0.260870,1.000000,0.260870,1.000000,0.260870,1.000000,0.247826,
1.000000,0.179710,1.000000,0.098551,0.066667,0.002899,0.000000,0.000000,1.000000,0.391304,
1.000000,0.391304,1.000000,0.347826,1.000000,0.231884,1.000000,0.173913,1.000000,0.173913,

1.000000,0.144928,1.000000,0.065217,1.000000,0.043478,0.000000,0.000000,0.345506,0.279277,
0.588556,0.463187,0.410305,0.257536,0.290441,0.208782,0.227477,0.153732,0.349904,0.225414,
0.056180,0.014086,0.588556,0.313875,0.760218,0.518784,0.866485,0.712363,1.000000,0.994515,
0.022472,0.008605,0.370787,0.245654,0.620787,0.492880,0.833708,0.738189,1.000000,0.994870,
0.011236,0.003313,0.359813,0.237064,0.600733,0.497845,0.803738,0.735721,1.000000,0.992142,
0.848812,0.654127,0.182927,0.036635,0.675573,0.309238,0.082024,0.022518,0.119266,0.065350,
0.009174,0.002521,0.011236,0.003440,0.382022,0.249872,0.674157,0.504243,0.847191,0.755945,
1.000000,1.000000,0.782143,0.176881,0.789286,0.254305,0.825000,0.384145,0.985423,0.600319,
0.996516,0.771029,0.345794,0.078642,0.649462,0.336616,0.750538,0.492975,0.868817,0.643725,
0.994382,0.862695,0.757634,0.512619,0.416901,0.297875,0.053571,0.017577,0.443925,0.263710,
0.648876,0.497517,0.845506,0.724148,0.998258,0.988012,0.580381,0.454871,0.460674,0.321876,
0.273118,0.223253,0.336508,0.270229,0.240260,0.180299,0.227273,0.141903,0.022472,0.008665,
0.303371,0.231143,0.596429,0.483106,0.812721,0.742931,1.000000,0.995713,0.033708,0.010680,
0.370370,0.270485,0.657224,0.521837,0.833787,0.745380,1.000000,0.996035,0.011236,0.003313,
0.444142,0.280308,0.648501,0.490855,0.801527,0.704356,1.000000,0.991506,0.442748,0.284387,
0.547684,0.404108,0.384831,0.311505,0.328872,0.219851,0.277328,0.182679,0.262911,0.207887,
0.011236,0.003313,0.448598,0.241833,0.616822,0.493720,0.808411,0.740618,1.000000,0.992142,
0.022472,0.008860,0.362360,0.239935,0.607143,0.485298,0.841772,0.735897,1.000000,0.991737,
0.056180,0.013147,0.585831,0.301635,0.754768,0.511376,0.866485,0.718109,1.000000,0.994515,
0.473815,0.350720,0.527273,0.426027,0.273118,0.223253,0.366197,0.291985,0.187793,0.140421,
0.238636,0.181781,0.025926,0.009303,0.303371,0.229131,0.561014,0.474581,0.825926,0.739193,
1.000000,0.983984,0.033708,0.010011,0.360691,0.246586,0.566085,0.510647,0.797980,0.755264,
1.000000,0.996937,0.011236,0.003313,0.444142,0.280308,0.648501,0.490855,0.801527,0.704356,
1.000000,0.991506,0.107477,0.053772,0.067416,0.046209,0.101124,0.058159,0.062921,0.033955,
0.098743,0.051913,0.101124,0.035269,0.161473,0.082448,0.179551,0.092750,0.163488,0.093496,
0.080824,0.048071,0.169611,0.087343,0.090652,0.032228,0.057143,0.026851,0.054545,0.016807,
0.099303,0.046487,0.181818,0.098119,0.087786,0.031929,0.066955,0.025191,0.080153,0.029492,
0.020408,0.009510

Positive_142 9.000000,0.086420,3.000000,0.777778,1.000000,0.944444,3.000000,0.777778,1.000000,0.944444,
0.252724,0.003991,0.252724,0.003991,0.285714,0.600000,1.000000,0.000000,0.000000,0.000000,
0.000000,0.000000,0.000000,0.000000,0.000000,0.000000,0.000000,0.000000,0.000000,0.000000,
0.000000,0.000000,0.000000,0.083333,0.250000,0.015625,0.125000,0.375000,0.035156,0.125000,
0.375000,0.035156,0.083333,0.250000,0.015625,1.732051,1.732051,1.000000,1.000000,0.111111,
1.000000,0.111111,1.000000,0.111111,1.000000,0.111111,0.333333,0.037037,0.000000,0.000000,
0.000000,0.000000,0.000000,0.000000,0.000000,0.000000,0.000000,0.000000,1.000000,0.111111,
1.000000,0.111111,0.666667,0.074074,0.333333,0.037037,0.000000,0.000000,0.000000,0.000000,
0.000000,0.000000,0.000000,0.000000,0.000000,0.000000,0.000000,0.000000,0.413249,0.315929,
0.514175,0.399508,0.341317,0.284563,0.312258,0.241426,0.284810,0.189976,0.272727,0.210339,
0.020779,0.009242,0.288312,0.251150,0.540541,0.497549,0.764179,0.732399,1.000000,0.997700,
0.018927,0.005816,0.287066,0.247323,0.558891,0.491940,0.806005,0.742518,1.000000,0.996099,
0.003155,0.002030,0.350704,0.254642,0.621127,0.511537,0.829897,0.757891,1.000000,0.986176,
0.873711,0.486645,0.311377,0.160945,0.782334,0.352409,0.114120,0.065463,0.096774,0.069136,
0.009873,0.004763,0.003155,0.002030,0.308712,0.235272,0.535797,0.440167,0.826790,0.716105,
1.000000,0.999005,0.128169,0.048786,0.784794,0.274153,0.828608,0.411225,0.871134,0.544457,

0.998106,0.834755,0.048499,0.014828,0.387991,0.240849,0.880155,0.601689,0.978093,0.819253,
1.000000,0.977001,0.644776,0.477660,0.481013,0.364196,0.023095,0.011607,0.325352,0.242423,
0.630155,0.512205,0.868557,0.750682,0.997403,0.988491,0.541237,0.418510,0.441640,0.361302,
0.244776,0.220187,0.339915,0.288918,0.203593,0.176031,0.221519,0.164788,0.018927,0.005605,
0.292537,0.248135,0.563510,0.493838,0.810624,0.745509,1.000000,0.994396,0.027714,0.009010,
0.291506,0.243374,0.541558,0.493682,0.795103,0.741022,1.000000,0.997059,0.003155,0.002030,
0.342254,0.265482,0.576056,0.510007,0.795455,0.748534,1.000000,0.994367,0.381238,0.310668,
0.480670,0.352202,0.419558,0.337130,0.266925,0.195928,0.303797,0.220105,0.303226,0.227255,
0.003155,0.002030,0.359155,0.258592,0.621127,0.510937,0.831186,0.755121,1.000000,0.986176,
0.018927,0.005816,0.292537,0.239961,0.598152,0.495947,0.845266,0.739178,1.000000,0.994045,
0.020779,0.009242,0.284416,0.251797,0.540541,0.495737,0.767164,0.736448,1.000000,0.998677,
0.411082,0.342624,0.473186,0.437189,0.244776,0.220187,0.339915,0.286363,0.191617,0.146424,
0.231499,0.194395,0.018927,0.005750,0.287066,0.238626,0.558891,0.492366,0.826790,0.748177,
1.000000,0.994239,0.011940,0.007214,0.258661,0.241561,0.532819,0.492871,0.777992,0.743830,
1.000000,0.997842,0.003155,0.002030,0.342254,0.265482,0.576056,0.510007,0.795455,0.748534,
1.000000,0.994367,0.107256,0.053800,0.083763,0.051293,0.126183,0.076977,0.064935,0.051259,
0.100946,0.047256,0.058712,0.035344,0.106959,0.074557,0.123028,0.088344,0.103093,0.071595,
0.073903,0.056868,0.125000,0.060838,0.032468,0.021202,0.041570,0.026105,0.023377,0.015049,
0.095522,0.069509,0.129338,0.088769,0.077652,0.043448,0.031169,0.021120,0.048263,0.033295,
0.025237,0.013373

Positive_143 18.000000,0.052469,9.000000,0.944444,0.000000,5.584967,1.000000,0.944444,1.000000,0.055556,
0.465823,0.040681,0.465823,0.040681,0.000000,0.235294,0.230769,0.400000,0.500000,0.333333,
0.500000,0.055556,1.000000,0.055556,0.000000,0.000000,0.000000,0.011111,0.200000,0.002222,
0.000000,0.000000,0.000000,0.000000,0.000000,0.000000,0.023856,0.264706,0.004464,0.049020,
0.058824,0.000509,0.307190,0.470588,0.034014,3.000000,2.236068,1.414214,1.000000,0.166667,
1.000000,0.166667,1.000000,0.166667,1.000000,0.139198,1.000000,0.137654,0.638889,0.046605,
0.444444,0.035802,0.222222,0.017901,0.111111,0.011728,0.100000,0.007099,0.000000,0.000000,
0.000000,0.000000,0.000000,0.000000,0.000000,0.000000,0.000000,0.000000,0.000000,0.000000,
0.000000,0.000000,0.000000,0.000000,0.000000,0.000000,0.000000,0.000000,0.415584,0.317956,
0.451965,0.371628,0.350084,0.310417,0.292683,0.232277,0.282895,0.209288,0.271335,0.224461,
0.039216,0.014698,0.318777,0.237147,0.559459,0.486147,0.828571,0.754529,1.000000,0.995071,
0.030568,0.009880,0.317308,0.249711,0.579327,0.492978,0.773032,0.732827,1.000000,0.995881,
0.009524,0.003572,0.288210,0.237201,0.552381,0.498295,0.790614,0.745157,1.000000,0.991282,
0.571429,0.422153,0.552817,0.207775,0.721477,0.370072,0.157895,0.080207,0.114650,0.068068,
0.026316,0.002717,0.009524,0.003572,0.289782,0.233534,0.585427,0.481279,0.809524,0.710945,
1.000000,1.000000,0.838926,0.143238,0.838926,0.282008,0.838926,0.414607,0.843384,0.538213,
0.999055,0.717888,0.205776,0.043231,0.613514,0.204824,0.732432,0.440687,0.870130,0.647574,
0.996644,0.848754,0.765101,0.572003,0.470990,0.342777,0.066667,0.024199,0.346570,0.280982,
0.559567,0.510901,0.819712,0.738940,0.999055,0.987690,0.482993,0.395101,0.445415,0.344748,
0.342857,0.260151,0.323851,0.265821,0.254417,0.205059,0.250000,0.175429,0.030568,0.009649,
0.300481,0.238742,0.550481,0.493009,0.768007,0.729600,1.000000,0.994530,0.045752,0.018451,
0.358079,0.249700,0.595238,0.506026,0.816594,0.742489,1.000000,0.992937,0.009524,0.003572,
0.298658,0.243798,0.549149,0.483487,0.837662,0.758188,1.000000,0.990070,0.389262,0.341720,
0.421397,0.321412,0.428571,0.336868,0.275618,0.214856,0.315789,0.247184,0.284553,0.213009,

0.009524,0.003572,0.294760,0.251032,0.561905,0.508174,0.790614,0.748082,1.000000,0.992042,
0.030568,0.010409,0.302885,0.238920,0.579327,0.487773,0.778894,0.731963,1.000000,0.990956,
0.039216,0.013543,0.323144,0.240294,0.552402,0.487991,0.805195,0.747534,1.000000,0.996074,
0.428571,0.332606,0.542484,0.407243,0.342857,0.260151,0.361050,0.264546,0.236749,0.172158,
0.269231,0.208330,0.030568,0.010217,0.281879,0.237988,0.551089,0.497757,0.810458,0.737571,
1.000000,0.994123,0.045752,0.016539,0.312227,0.245773,0.561905,0.496417,0.801310,0.737461,
1.000000,0.994997,0.009524,0.003572,0.298658,0.243798,0.549149,0.483487,0.837662,0.758188,
1.000000,0.990070,0.096070,0.048660,0.171429,0.078618,0.090909,0.054766,0.072202,0.055572,
0.078603,0.039698,0.071895,0.040642,0.123810,0.083737,0.114094,0.076275,0.093863,0.057899,
0.106007,0.059123,0.117647,0.044378,0.041502,0.018913,0.047619,0.031303,0.032663,0.018117,
0.096070,0.062326,0.135375,0.091632,0.091703,0.055684,0.038095,0.021392,0.079365,0.048640,
0.021776,0.012625

Positive_144 36.000000,0.040123,7.000000,1.444444,1.000000,2.939683,10.000000,1.444444,1.000000,3.796825,
0.370136,0.037637,0.370136,0.037637,0.183673,0.350000,0.230769,0.150000,0.588235,0.571429,
1.000000,0.000000,0.000000,0.000000,0.000000,0.000000,0.000000,0.000000,0.000000,0.000000,
0.000000,0.000000,0.000000,0.084127,0.257143,0.011099,0.013539,0.057143,0.000369,0.016746,
0.114286,0.000808,0.040159,0.152381,0.003310,4.323241,3.051161,2.828427,1.000000,0.277778,
1.000000,0.277778,1.000000,0.265079,1.000000,0.230952,1.000000,0.224603,1.000000,0.164418,
1.000000,0.155159,0.400000,0.034788,0.133333,0.010053,0.066667,0.005026,1.000000,0.333333,
1.000000,0.330864,1.000000,0.326543,1.000000,0.317901,1.000000,0.310494,1.000000,0.225926,
1.000000,0.141975,1.000000,0.058642,0.333333,0.019753,0.166667,0.009259,0.492593,0.326178,
0.517857,0.376007,0.411255,0.297814,0.373896,0.228966,0.331361,0.195439,0.326087,0.217094,
0.034682,0.013650,0.350649,0.255108,0.604167,0.506624,0.839827,0.757054,1.000000,0.993935,
0.029762,0.007956,0.317308,0.226329,0.606936,0.492185,0.812500,0.743760,1.000000,0.992279,
0.009524,0.003367,0.405858,0.241850,0.640167,0.485685,0.864706,0.725703,1.000000,0.987643,
0.783080,0.503257,0.438312,0.143375,0.617021,0.353367,0.130719,0.059195,0.097493,0.064564,
0.017751,0.004191,0.055556,0.004859,0.494220,0.238386,0.822511,0.494553,0.909091,0.752011,
1.000000,1.000000,0.876147,0.142093,0.885321,0.287453,0.958716,0.467011,0.972477,0.582025,
0.997487,0.776555,0.370130,0.090939,0.636905,0.293064,0.817582,0.513042,0.896703,0.703282,
0.997817,0.900795,0.753247,0.486332,0.500000,0.360600,0.097674,0.026601,0.380734,0.259642,
0.602510,0.479302,0.864706,0.713903,1.000000,0.969185,0.568330,0.397998,0.435185,0.357674,
0.342857,0.244327,0.377215,0.273848,0.237488,0.186525,0.240385,0.174358,0.029762,0.007602,
0.300481,0.233963,0.560694,0.490844,0.798165,0.743021,1.000000,0.993395,0.046512,0.017389,
0.372385,0.256481,0.615063,0.502945,0.800000,0.743234,1.000000,0.995334,0.009524,0.003367,
0.339450,0.232799,0.588235,0.487234,0.829412,0.730857,1.000000,0.984651,0.471861,0.325231,
0.470716,0.325618,0.503704,0.349151,0.296203,0.203064,0.349112,0.228435,0.368008,0.212718,
0.009524,0.003367,0.389121,0.243483,0.631799,0.488919,0.828452,0.720786,1.000000,0.988216,
0.029762,0.007956,0.302885,0.221361,0.606936,0.492559,0.832370,0.748747,1.000000,0.989464,
0.034682,0.012946,0.350649,0.258388,0.597403,0.501952,0.839827,0.753724,1.000000,0.994962,
0.428571,0.324788,0.494444,0.430885,0.342857,0.244327,0.346405,0.274261,0.211973,0.156463,
0.269231,0.204420,0.059524,0.008429,0.337662,0.235844,0.577922,0.494085,0.831169,0.746863,
1.000000,0.991294,0.046025,0.015329,0.308511,0.249781,0.564854,0.500947,0.795349,0.740203,
1.000000,0.995756,0.009524,0.003367,0.339450,0.232799,0.588235,0.487234,0.829412,0.730857,
1.000000,0.984651,0.102128,0.053274,0.171429,0.062981,0.255556,0.080240,0.111765,0.053985,

0.065957,0.040642,0.074419,0.035056,0.162338,0.071034,0.141176,0.072528,0.160714,0.076559,
0.084599,0.050682,0.097614,0.054815,0.051948,0.022973,0.060606,0.027416,0.065934,0.018395,
0.096296,0.063024,0.128755,0.094228,0.076190,0.044484,0.044025,0.024374,0.086580,0.041051,
0.043290,0.012257

Positive_145 6.000000,0.111111,1.000000,0.666667,1.000000,0.266667,1.000000,0.666667,1.000000,0.266667,
0.565139,0.014739,0.565139,0.014739,0.000000,0.000000,0.000000,0.500000,0.500000,0.000000,
1.000000,0.000000,0.000000,0.000000,0.000000,0.000000,0.000000,0.000000,0.000000,0.000000,
0.000000,0.000000,0.000000,0.000000,0.000000,0.000000,0.066667,0.200000,0.010667,0.066667,
0.200000,0.010667,0.000000,0.000000,0.000000,1.000000,1.000000,1.000000,0.000000,0.000000,
0.000000,0.000000,0.000000,0.000000,0.000000,0.000000,0.000000,0.000000,0.000000,0.000000,
0.000000,0.000000,0.000000,0.000000,0.000000,0.000000,0.000000,0.000000,0.000000,0.000000,
0.000000,0.000000,0.000000,0.000000,0.000000,0.000000,0.000000,0.000000,0.000000,0.000000,
0.000000,0.000000,0.000000,0.000000,0.000000,0.000000,0.000000,0.000000,0.387931,0.374970,
0.366321,0.336608,0.325431,0.288422,0.247664,0.232828,0.254860,0.232606,0.199275,0.182988,
0.032634,0.008635,0.350498,0.275399,0.543189,0.473577,0.755245,0.731627,1.000000,0.994262,
0.014440,0.006521,0.366379,0.224885,0.577586,0.496040,0.782921,0.759102,1.000000,0.998605,
0.003610,0.001920,0.307309,0.258969,0.539867,0.498427,0.790614,0.744161,0.999202,0.996278,
0.537906,0.383734,0.212996,0.083368,0.745419,0.532897,0.115942,0.044058,0.086449,0.065716,
0.007246,0.003828,0.003610,0.001920,0.325431,0.171647,0.532328,0.432593,0.833998,0.733559,
1.000000,1.000000,0.452586,0.114322,0.581897,0.202484,0.683190,0.309980,0.971269,0.605704,
0.988827,0.912591,0.205776,0.068800,0.385382,0.293634,0.638695,0.512588,0.815884,0.725522,
0.998404,0.964969,0.504310,0.426453,0.453668,0.410142,0.046931,0.013566,0.346570,0.266576,
0.561852,0.515114,0.772563,0.745999,0.998404,0.995581,0.387869,0.363624,0.424242,0.398561,
0.292419,0.237815,0.272201,0.253517,0.231884,0.167557,0.241901,0.198898,0.014440,0.005320,
0.334052,0.231226,0.581897,0.496000,0.784483,0.746261,1.000000,0.994252,0.012931,0.008968,
0.373754,0.269962,0.549834,0.489358,0.755245,0.730156,1.000000,0.990093,0.003610,0.001920,
0.285199,0.227009,0.544850,0.488273,0.808664,0.744234,1.000000,0.998363,0.364224,0.314926,
0.330407,0.289245,0.410256,0.395829,0.192029,0.165505,0.298056,0.268026,0.219634,0.212997,
0.003610,0.001920,0.312292,0.259385,0.546512,0.501527,0.790614,0.743603,1.000000,0.997268,
0.014440,0.006521,0.355603,0.222795,0.584378,0.487075,0.799569,0.763232,1.000000,0.993616,
0.032634,0.008635,0.360465,0.278153,0.547786,0.477810,0.755245,0.733052,1.000000,0.997270,
0.332003,0.305408,0.490033,0.456776,0.292419,0.237815,0.286741,0.253248,0.202899,0.142852,
0.280778,0.223603,0.014440,0.006428,0.297975,0.232809,0.564127,0.498801,0.777242,0.755238,
1.000000,0.993975,0.012931,0.008138,0.320598,0.262041,0.524917,0.492746,0.746209,0.729275,
1.000000,0.994523,0.003610,0.001920,0.285199,0.227009,0.544850,0.488273,0.808664,0.744234,
1.000000,0.998363,0.079734,0.058698,0.079422,0.063946,0.146179,0.109996,0.073276,0.055348,
0.067837,0.052369,0.054152,0.034612,0.059801,0.051590,0.104549,0.072148,0.093863,0.074903,
0.067599,0.051420,0.058140,0.039185,0.028881,0.020859,0.038793,0.026503,0.028881,0.019030,
0.062937,0.050239,0.119713,0.106290,0.073276,0.045056,0.036101,0.028782,0.054152,0.032564,
0.009967,0.006462

Positive_146 11.000000,0.082645,8.000000,0.909091,0.000000,5.690909,1.000000,0.909091,1.000000,0.090909,
0.342138,0.005774,0.342138,0.005774,0.000000,0.400000,0.500000,1.000000,0.000000,0.000000,
0.000000,0.000000,0.000000,0.000000,0.000000,0.000000,0.000000,0.000000,0.000000,0.000000,
0.000000,0.000000,0.000000,0.000000,0.000000,0.000000,0.010227,0.100000,0.000901,0.018182,

0.100000,0.001636,0.509091,0.700000,0.106909,2.828427,1.000000,1.000000,1.000000,0.090909,
1.000000,0.090909,0.821429,0.074675,0.357143,0.032468,0.178571,0.016234,0.107143,0.009740,
0.071429,0.006494,0.071429,0.006494,0.000000,0.000000,0.000000,0.000000,0.000000,0.000000,
0.000000,0.000000,0.000000,0.000000,0.000000,0.000000,0.000000,0.000000,0.000000,0.000000,
0.000000,0.000000,0.000000,0.000000,0.000000,0.000000,0.000000,0.000000,0.353579,0.312275,
0.602521,0.422114,0.372727,0.265611,0.292871,0.236483,0.218351,0.173662,0.302752,0.212008,
0.054545,0.009784,0.409091,0.241900,0.610924,0.495387,0.800000,0.757564,1.000000,0.998828,
0.018182,0.004256,0.327928,0.270703,0.585586,0.495207,0.792440,0.724090,1.000000,0.996467,
0.009091,0.002008,0.313225,0.234645,0.652941,0.507600,0.836134,0.753376,1.000000,0.995878,
0.789076,0.591936,0.268657,0.129911,0.518182,0.278154,0.110778,0.059459,0.119266,0.059655,
0.009174,0.002749,0.009091,0.002008,0.544231,0.287975,0.676923,0.495231,0.853846,0.700650,
1.000000,0.999186,0.726923,0.158608,0.757692,0.269333,0.798077,0.453617,0.946154,0.640579,
0.998077,0.930430,0.038806,0.020554,0.668908,0.348305,0.755965,0.555215,0.888235,0.752428,
1.000000,0.973511,0.644776,0.437001,0.431599,0.357931,0.027273,0.014861,0.435294,0.270670,
0.652101,0.516786,0.847382,0.757896,0.996746,0.993786,0.557143,0.427606,0.418182,0.339460,
0.258621,0.232935,0.293578,0.261276,0.217726,0.184138,0.204833,0.165815,0.018182,0.004256,
0.350000,0.263730,0.584615,0.490823,0.795876,0.719396,1.000000,0.997187,0.027273,0.009800,
0.283193,0.225206,0.620168,0.496695,0.813445,0.742928,1.000000,0.998454,0.009091,0.002008,
0.408894,0.272070,0.610924,0.509125,0.800766,0.751902,1.000000,0.996618,0.409091,0.296800,
0.528571,0.364237,0.373102,0.338962,0.247706,0.194299,0.246225,0.208139,0.267823,0.221964,
0.009091,0.002008,0.349188,0.250465,0.635294,0.504824,0.828571,0.758287,1.000000,0.995878,
0.018182,0.004256,0.355769,0.272727,0.585586,0.485836,0.824055,0.704480,1.000000,0.990518,
0.054545,0.009784,0.336364,0.242151,0.615126,0.497626,0.792437,0.760624,1.000000,0.999483,
0.376119,0.327044,0.527273,0.440021,0.258621,0.232935,0.311927,0.273793,0.191617,0.146148,
0.233888,0.203805,0.018182,0.004583,0.319231,0.247265,0.588462,0.483839,0.804124,0.719382,
1.000000,0.990423,0.027273,0.009142,0.274585,0.241536,0.563636,0.497998,0.790909,0.740395,
1.000000,0.998645,0.009091,0.002008,0.408894,0.272070,0.610924,0.509125,0.800766,0.751902,
1.000000,0.996618,0.068966,0.055842,0.080769,0.053169,0.081345,0.067596,0.059701,0.046217,
0.063636,0.047336,0.060738,0.042115,0.109091,0.074755,0.096154,0.068225,0.128571,0.082282,
0.080260,0.055566,0.187395,0.083410,0.048739,0.026687,0.036903,0.031190,0.054545,0.017151,
0.095522,0.054837,0.181818,0.091179,0.065672,0.036397,0.029851,0.020084,0.048724,0.033205,
0.023202,0.012759

Positive_147 7.000000,0.122449,5.000000,0.857143,0.000000,3.476190,1.000000,0.857143,1.000000,0.142857,
0.287491,0.006034,0.287491,0.006034,0.166667,0.400000,0.666667,1.000000,0.000000,0.000000,
0.000000,0.000000,0.000000,0.000000,0.000000,0.000000,0.000000,0.000000,0.000000,0.000000,
0.000000,0.000000,0.000000,0.000000,0.000000,0.000000,0.119048,0.833333,0.099206,0.119048,
0.166667,0.006614,0.476190,0.666667,0.105820,2.236068,1.000000,0.000000,1.000000,0.142857,
1.000000,0.142857,0.600000,0.085714,0.400000,0.057143,0.200000,0.028571,0.100000,0.014286,
0.000000,0.000000,0.000000,0.000000,0.000000,0.000000,0.000000,0.000000,0.000000,0.000000,
0.000000,0.000000,0.000000,0.000000,0.000000,0.000000,0.000000,0.000000,0.000000,0.000000,
0.000000,0.000000,0.000000,0.000000,0.000000,0.000000,0.000000,0.000000,0.431555,0.328122,
0.486166,0.400979,0.301255,0.270898,0.248837,0.222399,0.281395,0.191061,0.277311,0.209179,
0.025105,0.011710,0.307220,0.261991,0.620261,0.538413,0.814792,0.754706,1.000000,0.984275,
0.016241,0.007972,0.308475,0.230350,0.531323,0.454211,0.796610,0.715910,1.000000,0.998222,

0.004184,0.002523,0.405858,0.250522,0.640167,0.513990,0.845850,0.755596,0.998135,0.992654,
0.628099,0.504365,0.108475,0.056876,0.654292,0.438758,0.061224,0.033357,0.071429,0.058997,
0.009302,0.002199,0.004184,0.002523,0.342373,0.211379,0.654237,0.432943,0.902552,0.683605,
1.000000,1.000000,0.509881,0.122125,0.513834,0.165964,0.644269,0.357442,0.966942,0.610677,
0.983471,0.754052,0.120650,0.034421,0.695463,0.402073,0.799254,0.623433,0.894344,0.768947,
0.993856,0.935637,0.711462,0.484978,0.488806,0.389967,0.037657,0.013564,0.313808,0.238501,
0.602510,0.470168,0.774059,0.693834,0.998135,0.985354,0.456067,0.413744,0.436195,0.354001,
0.284585,0.232255,0.316225,0.268284,0.246032,0.180662,0.220930,0.163816,0.016241,0.007027,
0.308475,0.231132,0.511864,0.440145,0.813559,0.710633,1.000000,0.997860,0.046025,0.015688,
0.372385,0.298473,0.679842,0.555696,0.837945,0.773664,1.000000,0.991316,0.004184,0.002523,
0.272727,0.218329,0.604959,0.510524,0.831405,0.737480,1.000000,0.996381,0.334728,0.298827,
0.397490,0.346197,0.450116,0.354976,0.247899,0.191905,0.323256,0.231455,0.228234,0.208253,
0.004184,0.002523,0.389121,0.254867,0.631799,0.513903,0.845850,0.755259,0.998135,0.992654,
0.016241,0.007972,0.311864,0.225558,0.515254,0.432996,0.813559,0.702455,1.000000,0.997624,
0.020339,0.009917,0.304348,0.262865,0.604102,0.538540,0.809198,0.751443,1.000000,0.984275,
0.368201,0.331836,0.488479,0.435909,0.284585,0.232255,0.311258,0.275699,0.210084,0.145008,
0.230159,0.199469,0.016241,0.007027,0.271074,0.233331,0.501160,0.460496,0.810169,0.715180,
1.000000,0.995749,0.046025,0.013755,0.301255,0.265939,0.628458,0.526431,0.830040,0.761359,
1.000000,0.993258,0.004184,0.002523,0.272727,0.218329,0.604959,0.510524,0.831405,0.737480,
1.000000,0.996381,0.111369,0.064592,0.064463,0.039135,0.127610,0.081780,0.074380,0.049865,
0.070661,0.054143,0.062150,0.038607,0.177866,0.077310,0.125424,0.086780,0.110744,0.065223,
0.081356,0.052659,0.093702,0.064226,0.039526,0.026854,0.051383,0.027929,0.062150,0.017683,
0.066946,0.055306,0.129930,0.089134,0.050847,0.035030,0.047431,0.025962,0.050209,0.031614,
0.035573,0.016169

Positive_148 24.000000,0.098958,8.000000,2.375000,2.000000,4.853261,8.000000,2.375000,0.500000,9.548913,
0.281494,0.013668,0.281494,0.013668,0.137255,0.386364,0.777778,0.666667,1.000000,0.000000,
0.000000,0.218750,1.375000,0.168818,0.148065,2.125000,0.262186,0.066406,0.250000,0.011554,
0.019571,0.265625,0.004479,0.187500,0.304348,0.018732,0.013134,0.086957,0.000562,0.008864,
0.119565,0.000917,0.087118,0.304348,0.012957,4.898979,4.733601,2.926968,1.000000,0.666667,
1.000000,0.648810,1.000000,0.630952,1.000000,0.622024,1.000000,0.619048,1.000000,0.616071,
1.000000,0.182540,0.600000,0.042857,0.200000,0.014286,0.200000,0.014286,1.000000,0.458333,
1.000000,0.453869,1.000000,0.430556,1.000000,0.236111,1.000000,0.216270,1.000000,0.193948,
1.000000,0.185020,1.000000,0.162202,1.000000,0.130456,0.095238,0.011409,0.408629,0.318754,
0.417160,0.360830,0.375000,0.320416,0.257764,0.211119,0.262857,0.201831,0.262032,0.230117,
0.015228,0.006899,0.350592,0.248056,0.636964,0.506142,0.820957,0.756716,0.999324,0.995917,
0.018568,0.007386,0.279202,0.230684,0.524217,0.481494,0.802030,0.735846,1.000000,0.994041,
0.003610,0.001987,0.339350,0.258972,0.563177,0.507284,0.812274,0.741658,1.000000,0.998988,
0.590774,0.453224,0.203101,0.150783,0.552027,0.395993,0.093583,0.067437,0.111702,0.084459,
0.014493,0.006362,0.003610,0.001987,0.375000,0.244041,0.672365,0.507277,0.880342,0.764943,
1.000000,1.000000,0.264865,0.067625,0.535533,0.222835,0.678218,0.400686,0.907343,0.576784,
0.999175,0.896302,0.128492,0.021033,0.503715,0.304103,0.671620,0.529700,0.827586,0.757058,
0.998450,0.973979,0.636139,0.549818,0.402036,0.348728,0.122905,0.025657,0.389892,0.272680,
0.581227,0.498573,0.780237,0.733353,1.000000,0.994563,0.418317,0.373784,0.409459,0.353311,
0.319088,0.272905,0.293333,0.254680,0.235119,0.194930,0.257143,0.206133,0.016000,0.004841,

0.287162,0.219706,0.564103,0.487784,0.786802,0.741859,1.000000,0.994023,0.017831,0.008572,
0.313531,0.265676,0.541667,0.501360,0.771452,0.737626,1.000000,0.994567,0.003610,0.001987,
0.337984,0.250856,0.599835,0.507995,0.797834,0.754785,1.000000,0.998215,0.402027,0.356860,
0.365385,0.299449,0.426396,0.343691,0.254011,0.209010,0.305714,0.241862,0.221705,0.186734,
0.003610,0.001987,0.361011,0.265420,0.563177,0.509575,0.779783,0.739099,1.000000,0.999056,
0.018568,0.007386,0.284459,0.226326,0.552707,0.481671,0.789096,0.736733,1.000000,0.988385,
0.015228,0.006868,0.357988,0.245323,0.640264,0.499370,0.818482,0.754865,1.000000,0.996095,
0.362211,0.305826,0.465812,0.421269,0.319088,0.272905,0.299465,0.259660,0.199275,0.157353,
0.285714,0.243710,0.016000,0.006390,0.286486,0.219160,0.552707,0.487708,0.789174,0.740443,
1.000000,0.989736,0.017094,0.006366,0.299505,0.258465,0.530435,0.495255,0.773913,0.741990,
1.000000,0.997226,0.003610,0.001987,0.337984,0.250856,0.599835,0.507995,0.797834,0.754785,
1.000000,0.998215,0.073964,0.052892,0.100775,0.065258,0.096154,0.072747,0.085271,0.057046,
0.053299,0.036069,0.060914,0.034743,0.084983,0.066230,0.103960,0.067873,0.095344,0.063188,
0.071618,0.051489,0.080344,0.050669,0.043321,0.024937,0.045584,0.036444,0.032051,0.017289,
0.085664,0.062392,0.123649,0.088147,0.080405,0.059214,0.045333,0.027923,0.071225,0.051450,
0.022346,0.014001

Positive_149 37.000000,0.034332,7.000000,1.270270,1.000000,2.813814,3.000000,1.270270,1.000000,0.869369,
0.394224,0.038410,0.394224,0.038410,0.085106,0.372093,0.444444,0.200000,0.333333,0.250000,
0.666667,0.000000,0.000000,0.000000,0.000000,0.000000,0.000000,0.000000,0.000000,0.000000,
0.000000,0.000000,0.000000,0.018447,0.055556,0.000493,0.024024,0.194444,0.001979,0.024024,
0.083333,0.000679,0.048549,0.166667,0.003441,3.825343,2.000000,2.000000,1.000000,0.270270,
1.000000,0.270270,1.000000,0.270270,1.000000,0.259974,1.000000,0.200772,1.000000,0.180824,
1.000000,0.178250,1.000000,0.108108,0.333333,0.018662,0.333333,0.011583,1.000000,0.378378,
1.000000,0.378378,1.000000,0.378378,1.000000,0.378378,1.000000,0.378378,1.000000,0.342342,
1.000000,0.297297,1.000000,0.135135,0.333333,0.009009,0.333333,0.009009,0.429158,0.316547,
0.461538,0.371393,0.454286,0.312061,0.302805,0.225943,0.255144,0.198490,0.292479,0.218893,
0.095238,0.012811,0.527473,0.262285,0.747253,0.513567,0.846154,0.759642,1.000000,0.993538,
0.043956,0.008237,0.321767,0.236057,0.591652,0.493390,0.808333,0.734979,1.000000,0.995826,
0.010989,0.002836,0.363409,0.245392,0.614828,0.485612,0.807692,0.734466,1.000000,0.993727,
0.751144,0.511432,0.401338,0.156161,0.731006,0.332407,0.147651,0.069059,0.104430,0.063245,
0.018868,0.006316,0.010989,0.002836,0.313131,0.231084,0.615245,0.478690,0.925000,0.753276,
1.000000,1.000000,0.502778,0.109378,0.581731,0.219047,0.914826,0.351132,0.974763,0.542634,
0.998284,0.804445,0.264463,0.068048,0.795302,0.334296,0.825503,0.579675,0.890110,0.794943,
0.997521,0.962049,0.680000,0.499953,0.438172,0.367674,0.041322,0.015688,0.408521,0.252203,
0.632911,0.483284,0.802885,0.711249,0.999136,0.985422,0.479129,0.389532,0.445585,0.368490,
0.304110,0.241978,0.329114,0.273007,0.236735,0.182557,0.232527,0.178565,0.043956,0.007665,
0.335570,0.240769,0.586207,0.490061,0.794444,0.737174,1.000000,0.996055,0.060150,0.015335,
0.318296,0.250551,0.681319,0.503362,0.824176,0.753200,1.000000,0.997385,0.010989,0.002836,
0.406593,0.241809,0.631420,0.484640,0.852843,0.734676,1.000000,0.990095,0.491429,0.344069,
0.404834,0.315006,0.449692,0.340926,0.280802,0.203911,0.295265,0.237166,0.282198,0.206824,
0.010989,0.002836,0.368421,0.249686,0.605787,0.487546,0.807692,0.733319,1.000000,0.994168,
0.043956,0.008237,0.312303,0.231524,0.613430,0.484238,0.819444,0.737410,1.000000,0.994900,
0.077694,0.011889,0.505495,0.264703,0.747253,0.511866,0.846154,0.753953,1.000000,0.994685,
0.367171,0.304843,0.529774,0.453178,0.304110,0.241978,0.338182,0.268429,0.187755,0.142785,

0.306122,0.218337,0.043956,0.007966,0.301342,0.231138,0.584392,0.475169,0.813889,0.732544,
1.000000,0.994800,0.056075,0.011979,0.305970,0.255859,0.563433,0.510403,0.794486,0.755646,
1.000000,0.997901,0.010989,0.002836,0.406593,0.241809,0.631420,0.484640,0.852843,0.734676,
1.000000,0.990095,0.087324,0.054633,0.101010,0.062008,0.125257,0.067072,0.078883,0.049147,
0.114990,0.045390,0.065421,0.038297,0.092879,0.058423,0.153846,0.070859,0.124838,0.074864,
0.090301,0.051551,0.132486,0.059309,0.038770,0.024379,0.054945,0.032008,0.056511,0.025379,
0.096990,0.066150,0.180556,0.104657,0.069444,0.046924,0.045455,0.024313,0.068571,0.034297,
0.022113,0.010341

Positive_150 29.000000,0.034483,17.000000,1.000000,0.000000,10.000000,2.000000,1.000000,1.000000,0.357143,
0.395757,0.004247,0.395757,0.004247,0.000000,0.034483,0.785714,0.666667,0.500000,1.000000,
0.000000,0.000000,0.000000,0.000000,0.000000,0.000000,0.000000,0.000000,0.000000,0.000000,
0.000000,0.000000,0.000000,0.007389,0.035714,0.000217,0.028325,0.607143,0.012790,0.028325,
0.071429,0.000490,0.339901,0.571429,0.078774,4.123106,2.000000,2.000000,1.000000,0.172414,
1.000000,0.172414,1.000000,0.172414,1.000000,0.172414,1.000000,0.172414,1.000000,0.103448,
1.000000,0.103448,1.000000,0.101927,1.000000,0.092799,0.500000,0.017241,1.000000,0.172414,
1.000000,0.172414,1.000000,0.172414,1.000000,0.172414,1.000000,0.172414,1.000000,0.172414,
1.000000,0.172414,1.000000,0.172414,1.000000,0.068966,0.000000,0.000000,0.462334,0.318636,
0.501992,0.373976,0.466877,0.307388,0.260000,0.220826,0.284010,0.204878,0.354430,0.226457,
0.025907,0.007192,0.322767,0.243915,0.564767,0.508350,0.820399,0.763233,1.000000,0.994718,
0.016129,0.011085,0.338614,0.244435,0.598020,0.487601,0.810931,0.737511,1.000000,0.995352,
0.006623,0.002516,0.313725,0.249853,0.556291,0.488945,0.823529,0.729645,1.000000,0.991140,
0.707171,0.464760,0.233161,0.147500,0.722397,0.387740,0.093583,0.063687,0.109626,0.076985,
0.020316,0.010697,0.006623,0.002516,0.358566,0.197770,0.613546,0.439407,0.871037,0.723922,
1.000000,1.000000,0.113565,0.026733,0.464509,0.145150,0.928105,0.391559,0.941176,0.630390,
0.995825,0.856317,0.178808,0.038187,0.427928,0.341272,0.709178,0.589815,0.883408,0.804442,
0.997758,0.971962,0.703470,0.570665,0.494033,0.374897,0.065359,0.014836,0.398693,0.247759,
0.629139,0.472753,0.814570,0.715713,0.997333,0.979482,0.533865,0.398581,0.442193,0.366617,
0.275480,0.234803,0.355615,0.290418,0.223958,0.175061,0.237251,0.169702,0.022173,0.011191,
0.336634,0.240757,0.605941,0.481235,0.806499,0.737296,1.000000,0.996910,0.035857,0.009870,
0.321270,0.252234,0.535948,0.487951,0.801980,0.741272,1.000000,0.996093,0.006623,0.002516,
0.451104,0.244304,0.618297,0.521551,0.797927,0.744557,1.000000,0.991465,0.492114,0.337802,
0.470120,0.320181,0.483013,0.342017,0.313291,0.211360,0.325776,0.244778,0.237200,0.200523,
0.006623,0.002516,0.313725,0.250039,0.575163,0.486452,0.779180,0.725337,1.000000,0.995660,
0.022173,0.012787,0.350495,0.245703,0.625498,0.492283,0.810931,0.740747,1.000000,0.993575,
0.025907,0.007192,0.320532,0.239618,0.564767,0.501658,0.822616,0.754861,1.000000,0.994871,
0.384106,0.324796,0.501577,0.440401,0.275480,0.234803,0.361842,0.294867,0.187500,0.140854,
0.260274,0.203908,0.022173,0.012419,0.274667,0.230638,0.536534,0.467739,0.805941,0.738550,
1.000000,0.995628,0.026490,0.008704,0.305761,0.256849,0.550495,0.502307,0.776025,0.739146,
1.000000,0.997096,0.006623,0.002516,0.451104,0.244304,0.618297,0.521551,0.797927,0.744557,
1.000000,0.991465,0.098121,0.052818,0.103746,0.047249,0.116691,0.076198,0.086093,0.057514,
0.065562,0.043069,0.065554,0.041787,0.125498,0.070827,0.114667,0.077052,0.096544,0.062343,
0.073733,0.057060,0.181275,0.052899,0.035874,0.023382,0.042411,0.030414,0.044164,0.020885,
0.094637,0.069066,0.173502,0.088769,0.077333,0.047727,0.045333,0.028847,0.069130,0.042929,
0.013825,0.009165

Positive_151 29.000000,0.034483,17.000000,1.000000,0.000000,10.000000,2.000000,1.000000,1.000000,0.357143,
0.395757,0.004247,0.395757,0.004247,0.000000,0.034483,0.785714,0.666667,0.500000,1.000000,
0.000000,0.000000,0.000000,0.000000,0.000000,0.000000,0.000000,0.000000,0.000000,0.000000,
0.000000,0.000000,0.000000,0.007389,0.035714,0.000217,0.028325,0.607143,0.012790,0.028325,
0.071429,0.000490,0.339901,0.571429,0.078774,4.123106,2.000000,2.000000,1.000000,0.172414,
1.000000,0.172414,1.000000,0.172414,1.000000,0.172414,1.000000,0.172414,1.000000,0.103448,
1.000000,0.103448,1.000000,0.101927,1.000000,0.092799,0.500000,0.017241,1.000000,0.172414,
1.000000,0.172414,1.000000,0.172414,1.000000,0.172414,1.000000,0.172414,1.000000,0.172414,
1.000000,0.172414,1.000000,0.172414,1.000000,0.068966,0.000000,0.000000,0.462334,0.318636,
0.501992,0.373976,0.466877,0.307388,0.260000,0.220826,0.284010,0.204878,0.354430,0.226457,
0.025907,0.007192,0.322767,0.243915,0.564767,0.508350,0.820399,0.763233,1.000000,0.994718,
0.016129,0.011085,0.338614,0.244435,0.598020,0.487601,0.810931,0.737511,1.000000,0.995352,
0.006623,0.002516,0.313725,0.249853,0.556291,0.488945,0.823529,0.729645,1.000000,0.991140,
0.707171,0.464760,0.233161,0.147500,0.722397,0.387740,0.093583,0.063687,0.109626,0.076985,
0.020316,0.010697,0.006623,0.002516,0.358566,0.197770,0.613546,0.439407,0.871037,0.723922,
1.000000,1.000000,0.113565,0.026733,0.464509,0.145150,0.928105,0.391559,0.941176,0.630390,
0.995825,0.856317,0.178808,0.038187,0.427928,0.341272,0.709178,0.589815,0.883408,0.804442,
0.997758,0.971962,0.703470,0.570665,0.494033,0.374897,0.065359,0.014836,0.398693,0.247759,
0.629139,0.472753,0.814570,0.715713,0.997333,0.979482,0.533865,0.398581,0.442193,0.366617,
0.275480,0.234803,0.355615,0.290418,0.223958,0.175061,0.237251,0.169702,0.022173,0.011191,
0.336634,0.240757,0.605941,0.481235,0.806499,0.737296,1.000000,0.996910,0.035857,0.009870,
0.321270,0.252234,0.535948,0.487951,0.801980,0.741272,1.000000,0.996093,0.006623,0.002516,
0.451104,0.244304,0.618297,0.521551,0.797927,0.744557,1.000000,0.991465,0.492114,0.337802,
0.470120,0.320181,0.483013,0.342017,0.313291,0.211360,0.325776,0.244778,0.237200,0.200523,
0.006623,0.002516,0.313725,0.250039,0.575163,0.486452,0.779180,0.725337,1.000000,0.995660,
0.022173,0.012787,0.350495,0.245703,0.625498,0.492283,0.810931,0.740747,1.000000,0.993575,
0.025907,0.007192,0.320532,0.239618,0.564767,0.501658,0.822616,0.754861,1.000000,0.994871,
0.384106,0.324796,0.501577,0.440401,0.275480,0.234803,0.361842,0.294867,0.187500,0.140854,
0.260274,0.203908,0.022173,0.012419,0.274667,0.230638,0.536534,0.467739,0.805941,0.738550,
1.000000,0.995628,0.026490,0.008704,0.305761,0.256849,0.550495,0.502307,0.776025,0.739146,
1.000000,0.997096,0.006623,0.002516,0.451104,0.244304,0.618297,0.521551,0.797927,0.744557,
1.000000,0.991465,0.098121,0.052818,0.103746,0.047249,0.116691,0.076198,0.086093,0.057514,
0.065562,0.043069,0.065554,0.041787,0.125498,0.070827,0.114667,0.077052,0.096544,0.062343,
0.073733,0.057060,0.181275,0.052899,0.035874,0.023382,0.042411,0.030414,0.044164,0.020885,
0.094637,0.069066,0.173502,0.088769,0.077333,0.047727,0.045333,0.028847,0.069130,0.042929,
0.013825,0.009165

Positive_152 50.000000,0.041600,8.000000,2.080000,1.000000,4.483265,12.000000,2.080000,2.000000,3.462857,
0.392876,0.021296,0.392876,0.021296,0.048077,0.262626,0.438356,0.390244,0.600000,0.700000,
1.000000,0.011429,0.571429,0.006531,0.026667,0.333333,0.008345,0.001633,0.081633,0.000133,
0.008889,0.111111,0.000927,0.074242,0.224490,0.007613,0.024796,0.163265,0.001441,0.024422,
0.081633,0.000650,0.054626,0.142857,0.002740,4.898979,3.751583,3.464102,1.000000,0.380000,
1.000000,0.380000,1.000000,0.374524,1.000000,0.352333,1.000000,0.324333,1.000000,0.274905,
1.000000,0.230571,1.000000,0.138381,0.333333,0.036429,0.333333,0.018810,1.000000,0.580000,
1.000000,0.579697,1.000000,0.579394,1.000000,0.550303,1.000000,0.453030,1.000000,0.318485,

1.000000,0.268788,1.000000,0.146061,0.333333,0.074242,0.333333,0.073636,0.431718,0.317232,
0.532313,0.381880,0.392562,0.300887,0.272727,0.219946,0.278912,0.195442,0.308824,0.217856,
0.080292,0.011332,0.371585,0.252334,0.658610,0.507862,0.825991,0.741844,1.000000,0.990285,
0.029762,0.007444,0.320000,0.242561,0.579327,0.490429,0.819957,0.746398,1.000000,0.994897,
0.007299,0.002581,0.405858,0.249532,0.640167,0.497358,0.828452,0.741745,1.000000,0.992692,
0.803279,0.530372,0.345543,0.135722,0.606780,0.333906,0.116183,0.054977,0.112821,0.069499,
0.013910,0.004506,0.007299,0.002581,0.431594,0.239575,0.607069,0.488082,0.891165,0.750479,
1.000000,1.000000,0.876147,0.118640,0.885321,0.241006,0.958716,0.424402,0.991914,0.619603,
0.997305,0.844598,0.264463,0.054244,0.760194,0.332967,0.847270,0.576260,0.988943,0.760144,
0.998131,0.957674,0.619835,0.502848,0.455882,0.359832,0.055046,0.015873,0.380734,0.266601,
0.602510,0.497796,0.821101,0.718794,0.998131,0.987216,0.528947,0.398224,0.433898,0.351827,
0.308977,0.249949,0.328054,0.264998,0.238494,0.187796,0.251656,0.176367,0.029762,0.007357,
0.320000,0.236277,0.564885,0.488024,0.810624,0.747612,1.000000,0.996002,0.046025,0.011784,
0.372385,0.248527,0.615063,0.493262,0.828194,0.735809,1.000000,0.995459,0.007299,0.002581,
0.361702,0.259333,0.631420,0.512599,0.877660,0.743284,1.000000,0.990996,0.433884,0.332773,
0.470238,0.322499,0.455947,0.344728,0.279412,0.204202,0.306407,0.233582,0.263473,0.199745,
0.007299,0.002581,0.389121,0.250385,0.631799,0.498030,0.828452,0.740243,1.000000,0.993272,
0.040678,0.008540,0.331765,0.236030,0.598152,0.487050,0.845266,0.747416,1.000000,0.994168,
0.080292,0.010749,0.342105,0.256533,0.643505,0.506940,0.823789,0.738609,1.000000,0.991332,
0.428571,0.308271,0.528814,0.441780,0.308977,0.249949,0.328494,0.266951,0.210084,0.145754,
0.289130,0.218409,0.059524,0.009353,0.338824,0.232493,0.579661,0.489615,0.826790,0.745532,
1.000000,0.993891,0.046025,0.010041,0.301653,0.247082,0.564854,0.492691,0.781421,0.741403,
1.000000,0.996125,0.007299,0.002581,0.361702,0.259333,0.631420,0.512599,0.877660,0.743284,
1.000000,0.990996,0.095238,0.056090,0.111702,0.059610,0.115254,0.066852,0.085106,0.053007,
0.078550,0.045499,0.068282,0.036175,0.113095,0.059700,0.112971,0.067178,0.160714,0.076112,
0.074946,0.052275,0.136612,0.067234,0.059299,0.027496,0.060738,0.031886,0.051095,0.022719,
0.093750,0.059820,0.136015,0.095967,0.077778,0.047514,0.045455,0.026198,0.065693,0.035669,
0.043796,0.013000
Positive_153 59.000000,0.030738,8.000000,1.813559,2.000000,3.568089,12.000000,1.813559,2.000000,7.016365,
0.424627,0.024695,0.424627,0.024695,0.056075,0.188119,0.365854,0.365385,0.575758,0.714286,
0.250000,0.008475,0.500000,0.004237,0.008475,0.500000,0.004237,0.004237,0.250000,0.001059,
0.004237,0.250000,0.001059,0.083577,0.189655,0.006435,0.029710,0.103448,0.001449,0.027323,
0.189655,0.003429,0.031147,0.120690,0.001956,4.898979,4.690416,3.478364,1.000000,0.508475,
1.000000,0.508475,1.000000,0.489709,1.000000,0.476554,1.000000,0.467514,1.000000,0.439911,
1.000000,0.300000,1.000000,0.080952,0.107143,0.007708,0.035714,0.001816,1.000000,0.508218,
1.000000,0.508218,1.000000,0.504982,1.000000,0.496713,1.000000,0.484746,1.000000,0.381510,
1.000000,0.324294,1.000000,0.151412,1.000000,0.068053,0.333333,0.047714,0.431718,0.327035,
0.516908,0.375832,0.420732,0.297133,0.303529,0.232254,0.278689,0.197113,0.334012,0.212539,
0.063348,0.011238,0.335294,0.250831,0.664706,0.513818,0.841176,0.744117,1.000000,0.994846,
0.024896,0.006599,0.314010,0.244589,0.681159,0.484366,0.850242,0.743711,1.000000,0.994936,
0.006289,0.002366,0.401015,0.246177,0.653130,0.503459,0.819193,0.736455,1.000000,0.993258,
0.798271,0.544265,0.377880,0.165160,0.715447,0.290575,0.194444,0.066783,0.102041,0.064534,
0.015038,0.005128,0.006289,0.002366,0.391598,0.259101,0.709677,0.504169,0.882353,0.752969,
1.000000,1.000000,0.432927,0.084092,0.680751,0.266829,0.788636,0.440464,0.923077,0.639319,

0.997738,0.886095,0.308756,0.039880,0.656388,0.272506,0.782477,0.488903,0.958213,0.719345,
0.998752,0.963363,0.756098,0.478594,0.446602,0.362296,0.049296,0.015368,0.429577,0.248269,
0.644670,0.497871,0.808252,0.719833,0.998757,0.988783,0.555556,0.397117,0.434783,0.354238,
0.313364,0.248646,0.331579,0.267165,0.250344,0.189902,0.251656,0.178075,0.059322,0.009061,
0.338164,0.244872,0.652174,0.487756,0.840580,0.746623,1.000000,0.996334,0.062706,0.010496,
0.345070,0.247300,0.614213,0.500141,0.797583,0.729483,1.000000,0.995070,0.006289,0.002366,
0.359223,0.244348,0.670588,0.507398,0.838174,0.750718,1.000000,0.992082,0.447154,0.327666,
0.473430,0.319547,0.455947,0.352787,0.317719,0.194578,0.311475,0.233668,0.280000,0.212318,
0.006289,0.002366,0.439320,0.250672,0.651438,0.501275,0.813875,0.732878,1.000000,0.993759,
0.059322,0.009364,0.318391,0.237778,0.700483,0.480656,0.859903,0.740908,1.000000,0.994675,
0.044025,0.009179,0.352941,0.253532,0.664706,0.513145,0.841176,0.744151,1.000000,0.995428,
0.386473,0.302323,0.500000,0.449031,0.313364,0.248646,0.330097,0.263467,0.193182,0.144374,
0.289941,0.223603,0.088235,0.011596,0.342995,0.233832,0.676329,0.478942,0.879227,0.732961,
1.000000,0.993591,0.059406,0.009232,0.302876,0.254068,0.575800,0.504409,0.785814,0.743167,
1.000000,0.996816,0.006289,0.002366,0.359223,0.244348,0.670588,0.507398,0.838174,0.750718,
1.000000,0.992082,0.117647,0.059143,0.093511,0.061378,0.102083,0.068500,0.082949,0.052189,
0.115162,0.048122,0.068282,0.037703,0.110329,0.060800,0.140097,0.062241,0.134006,0.075759,
0.083770,0.051334,0.130435,0.069412,0.056604,0.025752,0.060738,0.030533,0.094340,0.025381,
0.119497,0.062531,0.136015,0.092061,0.077778,0.045321,0.048882,0.024974,0.078341,0.035858,
0.026099,0.011007

Positive_154 69.000000,0.031086,9.000000,2.144928,2.000000,3.331628,12.000000,2.144928,1.000000,7.331628,
0.383033,0.024570,0.383033,0.024570,0.081081,0.242647,0.524272,0.428571,0.428571,0.500000,
0.500000,0.138889,1.500000,0.091027,0.127174,0.900000,0.081055,0.034722,0.250000,0.004708,
0.031120,0.250000,0.005232,0.056177,0.161765,0.002755,0.020016,0.073529,0.000505,0.023415,
0.147059,0.001140,0.025532,0.117647,0.001173,5.336526,5.198776,4.472136,1.000000,0.550725,
1.000000,0.550725,1.000000,0.547826,1.000000,0.461353,1.000000,0.442995,1.000000,0.234783,
1.000000,0.197746,1.000000,0.102415,1.000000,0.067633,0.333333,0.027375,1.000000,0.492754,
1.000000,0.492534,1.000000,0.491268,1.000000,0.451381,1.000000,0.407809,1.000000,0.349627,
1.000000,0.306782,1.000000,0.174060,1.000000,0.043895,0.333333,0.007224,0.451908,0.327427,
0.517857,0.363753,0.402516,0.308820,0.309677,0.223665,0.278912,0.207237,0.291139,0.211362,
0.063348,0.012278,0.376344,0.260951,0.664706,0.510901,0.841176,0.750352,1.000000,0.993729,
0.029762,0.007067,0.334667,0.236163,0.681159,0.488353,0.850242,0.744555,1.000000,0.993630,
0.006410,0.002575,0.361650,0.243343,0.619048,0.492155,0.864706,0.731882,1.000000,0.993731,
0.732143,0.507632,0.329412,0.157374,0.731006,0.334993,0.111940,0.062303,0.116129,0.067862,
0.017751,0.005518,0.006410,0.002575,0.442667,0.250801,0.647059,0.500846,0.911704,0.772271,
1.000000,1.000000,0.764706,0.098994,0.764706,0.255543,0.938983,0.411788,0.942373,0.584180,
0.997738,0.829313,0.264463,0.038869,0.860812,0.306571,0.868268,0.525218,0.932367,0.726485,
0.999133,0.961771,0.619835,0.494539,0.446602,0.371637,0.063107,0.014511,0.335878,0.253122,
0.594118,0.491703,0.864706,0.718050,0.999133,0.988786,0.555556,0.388413,0.448000,0.365377,
0.308977,0.246210,0.332308,0.270701,0.240876,0.182473,0.251656,0.181478,0.059322,0.008284,
0.338164,0.237785,0.652174,0.492285,0.840580,0.751598,1.000000,0.996427,0.037190,0.009798,
0.397849,0.247571,0.600000,0.488321,0.786260,0.727268,1.000000,0.995062,0.006410,0.002575,
0.361702,0.257615,0.670588,0.506612,0.877660,0.740881,1.000000,0.990277,0.433884,0.338953,
0.473430,0.307845,0.476336,0.353202,0.267016,0.195633,0.306407,0.244933,0.296774,0.200589,

0.006410,0.002575,0.439320,0.246461,0.619048,0.492826,0.800915,0.730723,1.000000,0.994099,
0.059322,0.009042,0.358667,0.229260,0.700483,0.485853,0.859903,0.746234,1.000000,0.993473,
0.044025,0.010459,0.376344,0.263464,0.664706,0.510219,0.841176,0.747049,1.000000,0.994152,
0.428571,0.299706,0.529774,0.454084,0.308977,0.246210,0.387615,0.268077,0.209756,0.141321,
0.289941,0.222631,0.088235,0.011109,0.342995,0.234359,0.676329,0.490322,0.879227,0.742329,
1.000000,0.992848,0.030556,0.008373,0.329771,0.248952,0.587097,0.492968,0.787097,0.739824,
1.000000,0.996745,0.006410,0.002575,0.361702,0.257615,0.670588,0.506612,0.877660,0.740881,
1.000000,0.990277,0.117647,0.058604,0.111702,0.061095,0.155725,0.072987,0.085106,0.052667,
0.114990,0.046246,0.068282,0.035829,0.113095,0.058075,0.185897,0.066669,0.160714,0.071258,
0.083770,0.051037,0.132486,0.060806,0.056604,0.025775,0.060738,0.030132,0.094340,0.027901,
0.119497,0.063972,0.136015,0.099384,0.077778,0.046960,0.045455,0.024343,0.056250,0.034008,
0.029762,0.012253

Positive_155 43.000000,0.046512,8.000000,2.000000,2.000000,3.047619,9.000000,2.000000,2.000000,2.238095,
0.436454,0.024152,0.436454,0.024152,0.000000,0.197674,0.333333,0.478261,0.458333,0.538462,
0.666667,0.000000,0.000000,0.000000,0.000000,0.000000,0.000000,0.000000,0.000000,0.000000,
0.000000,0.000000,0.000000,0.065338,0.190476,0.004673,0.046512,0.190476,0.002347,0.045404,
0.214286,0.001885,0.058693,0.166667,0.003330,4.000000,3.000000,3.000000,1.000000,0.511628,
1.000000,0.511628,1.000000,0.509967,1.000000,0.500000,1.000000,0.488372,1.000000,0.400332,
1.000000,0.348837,1.000000,0.203765,1.000000,0.121262,0.333333,0.048173,1.000000,0.697674,
1.000000,0.697674,1.000000,0.697028,1.000000,0.692506,1.000000,0.551034,1.000000,0.496770,
1.000000,0.447028,1.000000,0.178295,1.000000,0.056202,0.333333,0.031654,0.431718,0.328454,
0.532313,0.384818,0.362360,0.286728,0.275534,0.232618,0.278912,0.192685,0.247098,0.204759,
0.033333,0.007989,0.371585,0.240817,0.597527,0.498226,0.825991,0.740707,1.000000,0.993639,
0.029762,0.006470,0.326965,0.251658,0.600900,0.500015,0.846962,0.756419,1.000000,0.995599,
0.005952,0.002154,0.309973,0.243706,0.619048,0.492765,0.799304,0.734655,1.000000,0.992092,
0.843982,0.554730,0.377880,0.128318,0.606780,0.316953,0.194444,0.054981,0.112821,0.066983,
0.015038,0.004035,0.005952,0.002154,0.403387,0.250246,0.709677,0.509512,0.891165,0.769910,
1.000000,1.000000,0.762712,0.112200,0.762712,0.245231,0.938983,0.454945,0.991914,0.680634,
0.997680,0.893049,0.308756,0.047136,0.860812,0.318506,0.868268,0.540796,0.927313,0.714785,
0.999132,0.949790,0.618438,0.488435,0.432671,0.357433,0.045822,0.011820,0.361186,0.258326,
0.566122,0.488935,0.819712,0.716581,0.998263,0.990283,0.527489,0.399918,0.433898,0.350279,
0.313364,0.249803,0.314286,0.260079,0.238494,0.188212,0.251656,0.172423,0.029762,0.006423,
0.329570,0.245668,0.583646,0.500429,0.838710,0.757322,1.000000,0.997142,0.033333,0.008909,
0.323210,0.234979,0.567608,0.488789,0.828194,0.728851,1.000000,0.996498,0.005952,0.002154,
0.361702,0.260776,0.627753,0.503933,0.877660,0.740959,1.000000,0.992646,0.398876,0.317972,
0.483645,0.325479,0.455947,0.356550,0.228027,0.189552,0.306407,0.231738,0.266033,0.213221,
0.005952,0.002154,0.299191,0.244572,0.619048,0.489119,0.800464,0.733618,1.000000,0.992682,
0.040678,0.007744,0.342597,0.247335,0.631658,0.502053,0.854464,0.758915,1.000000,0.995436,
0.033333,0.007989,0.322404,0.245824,0.603524,0.499415,0.823789,0.738287,1.000000,0.994473,
0.442793,0.310784,0.528814,0.439413,0.313364,0.249803,0.332553,0.264798,0.191617,0.146524,
0.289130,0.214111,0.059524,0.008730,0.305085,0.238746,0.579661,0.493781,0.826790,0.754666,
1.000000,0.995672,0.030556,0.007313,0.297180,0.241648,0.562212,0.494930,0.781421,0.742434,
1.000000,0.997211,0.005952,0.002154,0.361702,0.260776,0.627753,0.503933,0.877660,0.740959,
1.000000,0.992646,0.095238,0.059077,0.111702,0.060028,0.115254,0.071161,0.085106,0.055026,

0.076503,0.047704,0.068282,0.035459,0.113095,0.063213,0.130265,0.065207,0.163447,0.078910,
0.073903,0.048428,0.144860,0.069721,0.059299,0.028096,0.060738,0.031244,0.053853,0.019413,
0.089947,0.057020,0.136015,0.093856,0.077778,0.045080,0.044248,0.025415,0.078341,0.034856,
0.029762,0.011088

Positive_156 61.000000,0.047568,9.000000,2.901639,2.000000,8.356831,14.000000,2.901639,1.000000,15.123497,
0.350878,0.019276,0.350878,0.019276,0.069767,0.318750,0.486239,0.553571,0.600000,0.800000,
0.500000,0.000000,0.000000,0.000000,0.000000,0.000000,0.000000,0.000000,0.000000,0.000000,
0.000000,0.000000,0.000000,0.067280,0.216667,0.006623,0.032206,0.150000,0.001537,0.061415,
0.233333,0.006671,0.044572,0.133333,0.002317,8.823575,5.196152,3.874777,1.000000,0.606557,
1.000000,0.606557,1.000000,0.605191,1.000000,0.553279,1.000000,0.487575,1.000000,0.385311,
1.000000,0.240632,1.000000,0.148712,1.000000,0.063687,0.333333,0.026802,1.000000,0.491803,
1.000000,0.489468,1.000000,0.487133,1.000000,0.470785,1.000000,0.436525,1.000000,0.342728,
1.000000,0.264957,1.000000,0.172829,1.000000,0.099554,0.333333,0.058445,0.431718,0.331131,
0.487973,0.367621,0.402516,0.301248,0.324138,0.236916,0.278912,0.203741,0.291139,0.212432,
0.044025,0.010808,0.411765,0.265246,0.660131,0.511679,0.836601,0.753111,1.000000,0.993918,
0.024896,0.006813,0.302799,0.237957,0.600900,0.488152,0.846962,0.750648,1.000000,0.995123,
0.006849,0.002529,0.346237,0.241508,0.565591,0.485771,0.800915,0.729643,1.000000,0.994116,
0.768293,0.522407,0.377880,0.153278,0.660259,0.324315,0.194444,0.062478,0.116129,0.067593,
0.020690,0.005773,0.006849,0.002529,0.457516,0.254643,0.709677,0.517556,0.877719,0.778984,
1.000000,0.998428,0.762712,0.100878,0.762712,0.261788,0.938983,0.439436,0.942373,0.655059,
0.994709,0.879255,0.308756,0.038184,0.860812,0.305069,0.868268,0.514303,0.875725,0.696676,
1.000000,0.946623,0.597360,0.486913,0.500000,0.377344,0.103659,0.016091,0.357500,0.250531,
0.578009,0.488542,0.786787,0.719681,0.999082,0.990233,0.506873,0.393742,0.433898,0.357107,
0.313364,0.249151,0.337931,0.267372,0.238494,0.187576,0.251656,0.178543,0.023729,0.006998,
0.301802,0.236517,0.583646,0.490963,0.838710,0.748127,1.000000,0.995600,0.033333,0.010418,
0.397849,0.251342,0.591398,0.494525,0.826599,0.735018,1.000000,0.995557,0.006849,0.002529,
0.361702,0.260875,0.601896,0.504784,0.877660,0.744752,1.000000,0.991154,0.408805,0.330907,
0.434708,0.311400,0.455947,0.357692,0.246835,0.192011,0.306407,0.243760,0.303448,0.213307,
0.006849,0.002529,0.322581,0.244318,0.556989,0.487401,0.800915,0.729640,1.000000,0.994545,
0.040678,0.007577,0.352853,0.236005,0.631658,0.485370,0.854464,0.748186,1.000000,0.994618,
0.044025,0.010522,0.392157,0.266863,0.660131,0.510976,0.836601,0.753326,1.000000,0.994723,
0.423077,0.309460,0.528814,0.441389,0.313364,0.249151,0.387615,0.268247,0.200000,0.149298,
0.289130,0.216821,0.040678,0.008361,0.305085,0.235594,0.579661,0.486864,0.819205,0.741509,
1.000000,0.993328,0.030556,0.008658,0.329032,0.246858,0.587097,0.496327,0.787671,0.742956,
1.000000,0.997239,0.006849,0.002529,0.361702,0.260875,0.601896,0.504784,0.877660,0.744752,
1.000000,0.991154,0.121951,0.060298,0.111702,0.059669,0.134228,0.075221,0.114094,0.058661,
0.068493,0.042876,0.068282,0.034405,0.121951,0.063029,0.185897,0.067094,0.134809,0.069340,
0.070539,0.051336,0.123570,0.060601,0.056604,0.026561,0.060738,0.029659,0.094340,0.023681,
0.119497,0.061611,0.136015,0.096410,0.077778,0.046584,0.060773,0.025560,0.078341,0.036986,
0.023041,0.010418

Positive_157 86.000000,0.039886,10.000000,3.430233,3.000000,10.718605,11.000000,3.430233,2.000000,10.789193,
0.392018,0.020929,0.392018,0.020929,0.034130,0.236749,0.458333,0.478632,0.590164,0.640000,
0.444444,0.039535,1.000000,0.032772,0.081395,1.000000,0.058003,0.004729,0.166667,0.000530,
0.023256,0.500000,0.005703,0.050733,0.117647,0.001996,0.042189,0.117647,0.001912,0.033725,

0.129412,0.001695,0.051870,0.105882,0.001305,8.485281,8.000000,6.057376,1.000000,0.581395,
1.000000,0.581395,1.000000,0.570238,1.000000,0.532641,1.000000,0.484690,1.000000,0.303055,
1.000000,0.260105,1.000000,0.185308,1.000000,0.122462,0.333333,0.062689,1.000000,0.627907,
1.000000,0.627907,1.000000,0.625323,1.000000,0.591167,1.000000,0.545537,1.000000,0.403826,
1.000000,0.352145,1.000000,0.268900,1.000000,0.172887,0.333333,0.036822,0.431718,0.325898,
0.532313,0.381659,0.402516,0.292444,0.339056,0.228965,0.278912,0.192820,0.302752,0.212419,
0.100840,0.011117,0.411765,0.259316,0.660131,0.509865,0.846154,0.745471,1.000000,0.993527,
0.036913,0.007345,0.321767,0.242187,0.603774,0.488824,0.846962,0.751382,1.000000,0.996125,
0.009091,0.002410,0.405858,0.245250,0.647826,0.494475,0.841880,0.732230,1.000000,0.993235,
0.825954,0.551622,0.377880,0.128721,0.731006,0.319656,0.194444,0.056890,0.119266,0.064242,
0.026163,0.004452,0.009091,0.002410,0.457516,0.241949,0.709677,0.489463,0.911704,0.765106,
1.000000,1.000000,0.952991,0.108482,0.952991,0.246028,0.952991,0.424390,0.991914,0.634620,
0.998073,0.880353,0.308756,0.038319,0.860812,0.329258,0.868268,0.554246,0.917431,0.721411,
0.998752,0.952250,0.620805,0.473825,0.438172,0.364138,0.103659,0.013210,0.403465,0.251382,
0.645299,0.486455,0.819712,0.711618,0.999082,0.989373,0.523664,0.402280,0.445585,0.360049,
0.313364,0.237671,0.343558,0.272161,0.238494,0.177948,0.251656,0.170436,0.036913,0.007603,
0.325503,0.236827,0.590604,0.491835,0.838710,0.752628,1.000000,0.996716,0.046025,0.009505,
0.397849,0.249307,0.615063,0.494587,0.791328,0.730629,1.000000,0.995016,0.009091,0.002410,
0.395652,0.266861,0.737003,0.508622,0.880734,0.739607,1.000000,0.992895,0.409091,0.320871,
0.470238,0.326157,0.455947,0.352972,0.248945,0.194536,0.306407,0.229724,0.330472,0.212108,
0.009091,0.002410,0.435897,0.249195,0.688034,0.496531,0.897436,0.731292,1.000000,0.993655,
0.040678,0.008203,0.348485,0.236408,0.631658,0.487045,0.854464,0.752213,1.000000,0.994731,
0.100840,0.010736,0.392157,0.260539,0.660131,0.507088,0.840979,0.745467,1.000000,0.994098,
0.428571,0.310101,0.529774,0.452228,0.313364,0.237671,0.387615,0.275550,0.210084,0.137008,
0.289130,0.211377,0.059524,0.009934,0.308725,0.233049,0.611111,0.488977,0.826790,0.749259,
1.000000,0.994148,0.046025,0.008011,0.329032,0.244795,0.587097,0.495699,0.790909,0.739797,
1.000000,0.996779,0.009091,0.002410,0.395652,0.266861,0.737003,0.508622,0.880734,0.739607,
1.000000,0.992895,0.121951,0.056676,0.111702,0.057704,0.132597,0.073698,0.085470,0.053293,
0.115162,0.050265,0.068282,0.034260,0.121951,0.061098,0.138365,0.065483,0.160714,0.078130,
0.100917,0.052096,0.136612,0.069349,0.102981,0.027074,0.060738,0.028427,0.094340,0.022830,
0.119497,0.058445,0.181818,0.100382,0.077778,0.042997,0.070461,0.024796,0.078341,0.031935,
0.037815,0.011058

Positive_158 28.000000,0.062500,7.000000,1.750000,1.000000,4.787037,5.000000,1.750000,2.000000,1.898148,
0.431708,0.027368,0.431708,0.027368,0.040816,0.234043,0.333333,0.333333,0.437500,0.777778,
1.000000,0.000000,0.000000,0.000000,0.000000,0.000000,0.000000,0.000000,0.000000,0.000000,
0.000000,0.000000,0.000000,0.050265,0.148148,0.002968,0.022487,0.259259,0.003083,0.021958,
0.074074,0.000799,0.084656,0.222222,0.008013,4.582576,2.828427,2.449490,1.000000,0.392857,
1.000000,0.392857,1.000000,0.382653,1.000000,0.341837,1.000000,0.306122,1.000000,0.282313,
1.000000,0.134354,0.333333,0.039116,0.333333,0.011905,0.333333,0.011905,1.000000,0.607143,
1.000000,0.607143,1.000000,0.600000,1.000000,0.592857,1.000000,0.450000,1.000000,0.363095,
1.000000,0.272619,1.000000,0.229762,0.666667,0.130952,0.333333,0.107143,0.540323,0.331548,
0.532313,0.391064,0.359322,0.277387,0.290727,0.226791,0.279352,0.191239,0.292490,0.204309,
0.033333,0.010114,0.393996,0.258173,0.596916,0.507416,0.825991,0.740786,1.000000,0.991992,
0.026432,0.006180,0.405000,0.238802,0.719409,0.497088,0.858650,0.749366,1.000000,0.995374,

0.005319,0.002432,0.345216,0.251537,0.652908,0.485836,0.825516,0.726327,1.000000,0.994533,
0.803279,0.541727,0.341270,0.092665,0.963710,0.365608,0.114642,0.039974,0.112821,0.063236,
0.015038,0.003079,0.005319,0.002432,0.770631,0.238220,0.844689,0.509832,0.920017,0.744594,
1.000000,1.000000,0.784810,0.194223,0.791790,0.267715,0.938983,0.443620,0.991914,0.616600,
0.997305,0.802132,0.339093,0.073608,0.580087,0.316805,0.841410,0.555742,0.927313,0.735235,
0.997368,0.945645,0.618438,0.481050,0.510121,0.349372,0.045822,0.014796,0.361186,0.247009,
0.591111,0.479966,0.810834,0.715365,1.000000,0.986461,0.511111,0.407494,0.504032,0.361073,
0.292553,0.231433,0.329381,0.274721,0.231092,0.177263,0.233983,0.161450,0.023729,0.006170,
0.355000,0.239723,0.672996,0.501412,0.856540,0.750413,1.000000,0.996700,0.033333,0.011142,
0.369330,0.248642,0.636023,0.487817,0.828194,0.726242,1.000000,0.996404,0.005319,0.002432,
0.361702,0.269424,0.627753,0.508884,0.877660,0.749381,1.000000,0.992772,0.380556,0.306900,
0.467687,0.335623,0.548387,0.357477,0.267261,0.197940,0.321429,0.226131,0.258824,0.209136,
0.005319,0.002432,0.354597,0.251393,0.651032,0.486400,0.827392,0.734594,1.000000,0.996028,
0.040678,0.007640,0.395000,0.234585,0.719409,0.497966,0.869198,0.747619,1.000000,0.994048,
0.033333,0.010034,0.393996,0.260782,0.603524,0.505412,0.823789,0.736713,1.000000,0.993265,
0.397778,0.316882,0.528814,0.451684,0.292553,0.231433,0.318841,0.280672,0.187166,0.137357,
0.275766,0.201357,0.040678,0.008179,0.331111,0.228838,0.628862,0.491682,0.832840,0.744591,
1.000000,0.995451,0.030556,0.009628,0.291405,0.245707,0.572327,0.495792,0.813417,0.741164,
1.000000,0.997004,0.005319,0.002432,0.361702,0.269424,0.627753,0.508884,0.877660,0.749381,
1.000000,0.992772,0.080645,0.054307,0.111702,0.060098,0.217742,0.075222,0.085106,0.052892,
0.137500,0.053976,0.056689,0.035054,0.112570,0.063621,0.112903,0.068916,0.146667,0.080642,
0.090703,0.050812,0.136612,0.071632,0.059299,0.025929,0.052778,0.029512,0.044068,0.018979,
0.089947,0.056267,0.126316,0.094636,0.077778,0.045919,0.040678,0.026084,0.054507,0.028855,
0.022801,0.006647

Positive_159 47.000000,0.140335,19.000000,6.595745,3.000000,46.289547,27.000000,6.595745,3.000000,46.811286,
0.438476,0.022392,0.438476,0.022392,0.038710,0.208054,0.173729,0.410256,0.652174,0.925000,
0.000000,1.707447,3.000000,2.218259,0.191489,3.000000,0.549491,0.565160,1.000000,0.245747,
0.007092,0.111111,0.000754,0.371647,0.565217,0.052168,0.083834,0.413043,0.008333,0.048001,
0.215781,0.002737,0.206804,0.391304,0.028212,13.784049,9.002082,5.477226,1.000000,0.872340,
1.000000,0.872340,1.000000,0.872340,1.000000,0.871096,1.000000,0.849944,1.000000,0.614744,
1.000000,0.597885,0.666667,0.159512,0.456140,0.121165,0.339181,0.087769,1.000000,0.702128,
1.000000,0.702128,1.000000,0.699400,1.000000,0.683943,1.000000,0.669940,1.000000,0.640244,
1.000000,0.586598,0.666667,0.342153,0.333333,0.192908,0.333333,0.135370,0.331476,0.278652,
0.534358,0.418591,0.400372,0.302757,0.260925,0.211890,0.245714,0.168068,0.336824,0.248559,
0.078378,0.011177,0.371585,0.249303,0.597598,0.496934,0.816712,0.737500,1.000000,0.996333,
0.014327,0.006154,0.322511,0.257646,0.599567,0.489722,0.827327,0.752843,1.000000,0.995960,
0.002865,0.002079,0.328530,0.231052,0.576507,0.488701,0.796569,0.733940,1.000000,0.994258,
0.820886,0.542298,0.285354,0.106291,0.573624,0.351411,0.088608,0.044221,0.112821,0.061597,
0.008677,0.002628,0.002865,0.002079,0.363889,0.261175,0.650142,0.501421,0.891165,0.764221,
1.000000,1.000000,0.748634,0.186744,0.797260,0.452799,0.925659,0.677421,0.991914,0.813227,
0.997549,0.966888,0.127215,0.023479,0.467213,0.248283,0.778976,0.429506,0.846361,0.624768,
0.999369,0.864124,0.648379,0.535020,0.418100,0.331228,0.045822,0.009420,0.361186,0.248233,
0.582911,0.484488,0.806373,0.698916,0.999369,0.991867,0.530741,0.441608,0.395647,0.308073,
0.291667,0.250319,0.314286,0.258465,0.250696,0.206258,0.218391,0.160008,0.042980,0.006968,

0.313853,0.258317,0.577922,0.494634,0.820113,0.763404,1.000000,0.998009,0.027714,0.008758,
0.280380,0.218048,0.546110,0.478696,0.777577,0.720855,1.000000,0.993942,0.002865,0.002079,
0.362369,0.264496,0.625337,0.509056,0.832884,0.736049,1.000000,0.995275,0.433892,0.330882,
0.467687,0.359372,0.379902,0.309746,0.314136,0.226482,0.281609,0.204344,0.265358,0.204444,
0.002865,0.002079,0.312210,0.232878,0.573487,0.491917,0.801471,0.739610,1.000000,0.994258,
0.048711,0.007243,0.322511,0.249713,0.610390,0.482546,0.845266,0.745799,1.000000,0.994759,
0.078378,0.010694,0.327635,0.253356,0.605105,0.500583,0.819820,0.740574,1.000000,0.996791,
0.402597,0.334564,0.465503,0.415116,0.291667,0.250319,0.320362,0.271394,0.203343,0.155651,
0.267241,0.210616,0.048711,0.008772,0.324675,0.262265,0.592068,0.494732,0.842776,0.746882,
1.000000,0.995549,0.014946,0.006582,0.289116,0.220074,0.544160,0.486705,0.795455,0.748533,
1.000000,0.997243,0.002865,0.002079,0.362369,0.264496,0.625337,0.509056,0.832884,0.736049,
1.000000,0.995275,0.115591,0.069668,0.073239,0.044504,0.072776,0.054344,0.064516,0.039485,
0.076503,0.039106,0.054131,0.031545,0.142651,0.083426,0.123377,0.079706,0.136076,0.080577,
0.073903,0.051370,0.136612,0.064294,0.059299,0.031094,0.042042,0.028125,0.073446,0.042750,
0.085470,0.057418,0.152951,0.091048,0.068901,0.034612,0.041308,0.023151,0.066202,0.037054,
0.033333,0.016724

Positive_160 67.000000,0.061261,28.000000,4.104478,1.000000,61.882858,11.000000,4.104478,3.000000,6.549525,
0.342414,0.013083,0.342414,0.013083,0.029091,0.378277,0.650602,0.568966,0.560000,0.727273,
0.666667,0.000000,0.000000,0.000000,0.000000,0.000000,0.000000,0.000000,0.000000,0.000000,
0.000000,0.000000,0.000000,0.037313,0.151515,0.003106,0.041987,0.424242,0.008239,0.041651,
0.166667,0.001864,0.203999,0.409091,0.031385,12.961481,5.744563,4.898979,1.000000,0.477612,
1.000000,0.477612,1.000000,0.475065,1.000000,0.448887,1.000000,0.408659,1.000000,0.298353,
1.000000,0.194338,1.000000,0.122649,0.333333,0.068100,0.333333,0.062533,1.000000,0.805970,
1.000000,0.805156,1.000000,0.804342,1.000000,0.798643,1.000000,0.787155,1.000000,0.547354,
1.000000,0.426594,1.000000,0.190412,0.333333,0.072094,0.333333,0.070466,0.431718,0.322238,
0.517857,0.382564,0.362360,0.295198,0.331776,0.240761,0.278912,0.190532,0.311628,0.216759,
0.126214,0.018448,0.418269,0.273688,0.660131,0.520879,0.836601,0.752142,1.000000,0.989477,
0.047059,0.009241,0.307692,0.239203,0.570048,0.490836,0.819957,0.752354,1.000000,0.996456,
0.006536,0.003231,0.346237,0.227568,0.631944,0.477905,0.800915,0.712918,1.000000,0.989080,
0.768293,0.558827,0.364706,0.203098,0.606780,0.238075,0.168831,0.085043,0.116129,0.059143,
0.043478,0.005224,0.006536,0.003231,0.457516,0.247280,0.647059,0.512169,0.858974,0.779038,
1.000000,1.000000,0.762712,0.110101,0.762712,0.282272,0.938983,0.441950,0.942373,0.617411,
0.995192,0.873252,0.619355,0.047548,0.860812,0.269351,0.868268,0.450545,0.902158,0.660297,
0.998561,0.906545,0.631068,0.502480,0.432671,0.354121,0.103659,0.018040,0.357500,0.247441,
0.557756,0.480618,0.786720,0.701559,0.999082,0.983428,0.517857,0.394181,0.433898,0.347265,
0.352941,0.258553,0.332308,0.260142,0.285714,0.195338,0.251656,0.182629,0.047059,0.010153,
0.293289,0.232575,0.564885,0.482191,0.829146,0.749543,1.000000,0.996943,0.053140,0.014020,
0.397849,0.242398,0.649306,0.482935,0.784946,0.717489,1.000000,0.991491,0.006536,0.003231,
0.407767,0.271485,0.660194,0.523289,0.877660,0.751994,1.000000,0.988415,0.398876,0.329597,
0.470238,0.321638,0.455947,0.348764,0.294686,0.195528,0.306407,0.231643,0.313589,0.218453,
0.006536,0.003231,0.364583,0.237423,0.670139,0.485209,0.815972,0.714494,1.000000,0.990244,
0.047059,0.010675,0.305085,0.231536,0.574163,0.483253,0.854271,0.750649,1.000000,0.995965,
0.126214,0.017009,0.392157,0.273645,0.660131,0.519897,0.836601,0.754324,1.000000,0.991926,
0.428571,0.310779,0.528814,0.430668,0.352941,0.258553,0.387615,0.260233,0.233645,0.156175,

0.289130,0.221792,0.059524,0.011695,0.305085,0.235029,0.579661,0.474259,0.839196,0.737497,
1.000000,0.994683,0.030556,0.011726,0.329032,0.237705,0.587097,0.488732,0.805970,0.732698,
1.000000,0.994592,0.006536,0.003231,0.407767,0.271485,0.660194,0.523289,0.877660,0.751994,
1.000000,0.988415,0.182353,0.069092,0.111702,0.058419,0.115254,0.067469,0.085106,0.049540,
0.087963,0.041817,0.072115,0.035900,0.163194,0.071710,0.185897,0.061996,0.160714,0.075787,
0.077419,0.051747,0.124402,0.060400,0.047809,0.026527,0.060738,0.034399,0.065359,0.023003,
0.090047,0.060938,0.138756,0.098162,0.077778,0.042979,0.046392,0.023618,0.059701,0.035690,
0.029762,0.010808

Positive_161 29.000000,0.054697,5.000000,1.586207,1.000000,2.679803,3.000000,1.586207,2.000000,1.036946,
0.510005,0.023958,0.510005,0.023958,0.000000,0.108696,0.170732,0.323529,0.391304,0.785714,
0.666667,0.000000,0.000000,0.000000,0.000000,0.000000,0.000000,0.000000,0.000000,0.000000,
0.000000,0.000000,0.000000,0.028325,0.071429,0.000923,0.026108,0.178571,0.002005,0.030788,
0.107143,0.001250,0.071018,0.142857,0.003406,3.162278,3.000000,2.449490,1.000000,0.482759,
1.000000,0.482759,1.000000,0.482759,1.000000,0.379310,1.000000,0.372414,1.000000,0.324138,
1.000000,0.206897,1.000000,0.186207,0.333333,0.058621,0.333333,0.044828,1.000000,0.551724,
1.000000,0.551724,1.000000,0.551724,1.000000,0.551724,1.000000,0.540230,1.000000,0.471264,
1.000000,0.448276,1.000000,0.298851,0.333333,0.022989,0.333333,0.022989,0.407489,0.326472,
0.467949,0.374562,0.359322,0.298966,0.309677,0.232783,0.278912,0.203042,0.254085,0.208379,
0.038902,0.011787,0.376344,0.261987,0.580153,0.509018,0.820339,0.753985,1.000000,0.991545,
0.019231,0.006916,0.302799,0.230995,0.564885,0.492582,0.822041,0.747901,1.000000,0.995782,
0.006410,0.002624,0.346237,0.248214,0.565591,0.483874,0.800915,0.725312,1.000000,0.993008,
0.768293,0.548268,0.248677,0.138070,0.606780,0.313662,0.111940,0.060928,0.116129,0.069759,
0.015038,0.005505,0.006410,0.002624,0.323171,0.246795,0.594966,0.522350,0.858974,0.776913,
1.000000,1.000000,0.762712,0.099135,0.762712,0.225531,0.938983,0.413106,0.942373,0.561515,
0.998601,0.827513,0.181141,0.042976,0.860812,0.310643,0.868268,0.543865,0.902941,0.738877,
0.997521,0.944335,0.597360,0.483566,0.424779,0.369716,0.103659,0.020048,0.357500,0.258411,
0.557756,0.491872,0.786720,0.711975,0.998367,0.988682,0.500000,0.403857,0.433898,0.360263,
0.292553,0.235880,0.320565,0.274682,0.212389,0.177688,0.233983,0.170017,0.023729,0.007543,
0.293289,0.235857,0.564885,0.493263,0.804898,0.748766,1.000000,0.997488,0.033333,0.010818,
0.397849,0.252477,0.591398,0.486466,0.794118,0.727802,1.000000,0.993864,0.006410,0.002624,
0.361702,0.264468,0.592875,0.506058,0.877660,0.741198,1.000000,0.989162,0.380556,0.326800,
0.423077,0.321925,0.429515,0.351274,0.238411,0.193158,0.306407,0.238784,0.296774,0.209996,
0.006410,0.002624,0.322581,0.251887,0.556989,0.484864,0.800915,0.726645,1.000000,0.993391,
0.040678,0.008127,0.305085,0.226708,0.572519,0.492978,0.832653,0.747210,1.000000,0.995692,
0.038902,0.011787,0.376344,0.266427,0.580153,0.508392,0.813559,0.752573,1.000000,0.992825,
0.423077,0.317869,0.528814,0.446251,0.292553,0.235880,0.387615,0.281046,0.193548,0.144291,
0.275766,0.203414,0.040678,0.009301,0.305085,0.229590,0.579661,0.492806,0.801633,0.740602,
1.000000,0.994710,0.030556,0.009078,0.329032,0.249036,0.587097,0.492979,0.787097,0.740238,
1.000000,0.996650,0.006410,0.002624,0.361702,0.264468,0.592875,0.506058,0.877660,0.741198,
1.000000,0.989162,0.121951,0.061932,0.111702,0.056277,0.125551,0.072790,0.085106,0.058336,
0.064073,0.043432,0.054545,0.033705,0.121951,0.066883,0.185897,0.068269,0.134809,0.073776,
0.069149,0.050606,0.123570,0.062392,0.039900,0.024802,0.052778,0.027834,0.053853,0.023596,
0.089947,0.062437,0.131183,0.100249,0.077778,0.047650,0.040678,0.023844,0.054054,0.032595,
0.022370,0.008596

Positive_162 73.000000,0.034340,8.000000,2.506849,2.000000,3.808980,13.000000,2.506849,1.000000,7.975647,
0.389412,0.019859,0.391552,0.019126,0.043956,0.218391,0.492647,0.565217,0.466667,0.562500,
0.571429,0.000000,0.000000,0.000000,0.000000,0.000000,0.000000,0.000000,0.000000,0.000000,
0.000000,0.000000,0.000000,0.056507,0.166667,0.003573,0.022076,0.111111,0.000993,0.019458,
0.083333,0.000752,0.035578,0.097222,0.001008,6.324555,5.099020,4.898979,1.000000,0.753425,
1.000000,0.753425,1.000000,0.739628,1.000000,0.682877,1.000000,0.416667,1.000000,0.259198,
1.000000,0.176712,1.000000,0.127984,1.000000,0.092531,0.333333,0.045760,1.000000,0.493151,
1.000000,0.493151,1.000000,0.489300,1.000000,0.457622,1.000000,0.425940,1.000000,0.295677,
1.000000,0.271619,1.000000,0.172914,1.000000,0.085315,0.333333,0.048460,0.431718,0.329063,
0.536424,0.381489,0.362360,0.289448,0.335025,0.235439,0.278912,0.198399,0.280374,0.211508,
0.063348,0.010042,0.376344,0.255380,0.609966,0.506052,0.855670,0.748457,1.000000,0.994932,
0.029762,0.006995,0.321767,0.245891,0.600900,0.496340,0.846962,0.751061,1.000000,0.995905,
0.006410,0.002372,0.379229,0.240666,0.619048,0.484266,0.864706,0.730544,1.000000,0.992312,
0.874172,0.548844,0.377880,0.133781,0.731006,0.317375,0.194444,0.058121,0.116129,0.065150,
0.017751,0.005339,0.006410,0.002372,0.472292,0.258239,0.709677,0.515115,0.911704,0.767529,
1.000000,1.000000,0.764706,0.115105,0.764706,0.255569,0.938983,0.436222,0.974763,0.619579,
0.997738,0.849676,0.308756,0.042891,0.636905,0.282445,0.841410,0.489693,0.927313,0.691961,
0.998230,0.939811,0.597360,0.481601,0.470377,0.370146,0.103659,0.013715,0.357500,0.257472,
0.595594,0.491130,0.864706,0.725523,0.998426,0.989703,0.523179,0.401193,0.445585,0.356402,
0.313364,0.242405,0.333333,0.272643,0.238494,0.180714,0.251656,0.175883,0.029762,0.006874,
0.329975,0.244473,0.586207,0.498767,0.838710,0.754305,1.000000,0.996986,0.062706,0.009389,
0.397849,0.250542,0.591398,0.491565,0.828194,0.736486,1.000000,0.995224,0.006410,0.002372,
0.361702,0.252550,0.627753,0.492370,0.877660,0.732392,1.000000,0.991188,0.398876,0.319279,
0.476821,0.324692,0.455947,0.356029,0.254425,0.192977,0.306407,0.237691,0.299492,0.213327,
0.006410,0.002372,0.357986,0.243955,0.619048,0.486650,0.806452,0.728752,1.000000,0.992751,
0.040678,0.008020,0.348485,0.243692,0.631658,0.495026,0.854464,0.752753,1.000000,0.995715,
0.038902,0.009236,0.376344,0.258274,0.603524,0.505670,0.848797,0.746453,1.000000,0.995539,
0.428571,0.312353,0.529774,0.445242,0.313364,0.242405,0.387615,0.274860,0.193548,0.141841,
0.289130,0.214756,0.059524,0.008519,0.338824,0.244443,0.611111,0.497370,0.819205,0.749406,
1.000000,0.994784,0.059406,0.008310,0.329032,0.246991,0.587097,0.496134,0.787097,0.744325,
1.000000,0.997271,0.006410,0.002372,0.361702,0.252550,0.627753,0.492370,0.877660,0.732392,
1.000000,0.991188,0.121951,0.061310,0.111702,0.055335,0.125257,0.073319,0.085106,0.055522,
0.114990,0.047329,0.068282,0.036248,0.121951,0.063215,0.185897,0.068087,0.160714,0.076600,
0.072848,0.048930,0.132486,0.067860,0.054348,0.026966,0.060738,0.029832,0.045608,0.020979,
0.089947,0.058185,0.145110,0.096523,0.077778,0.044798,0.040678,0.023788,0.078341,0.033833,
0.029762,0.011341

Positive_163 57.000000,0.046784,8.000000,2.666667,3.000000,4.083333,14.000000,2.666667,2.000000,10.869048,
0.388293,0.028587,0.388293,0.028587,0.105263,0.279412,0.387755,0.416667,0.400000,0.619048,
0.625000,0.000000,0.000000,0.000000,0.000000,0.000000,0.000000,0.000000,0.000000,0.000000,
0.000000,0.000000,0.000000,0.083876,0.214286,0.005611,0.044987,0.142857,0.001889,0.032070,
0.196429,0.002036,0.036559,0.125000,0.001778,7.004373,4.898979,3.872983,1.000000,0.666667,
1.000000,0.666667,1.000000,0.663033,1.000000,0.606725,1.000000,0.550585,1.000000,0.393358,
1.000000,0.339766,1.000000,0.259231,1.000000,0.134294,0.333333,0.074394,1.000000,0.649123,
1.000000,0.648166,1.000000,0.621524,1.000000,0.570528,1.000000,0.538398,1.000000,0.365682,

1.000000,0.320672,1.000000,0.247990,1.000000,0.080729,0.333333,0.060354,0.431718,0.328432,
0.541899,0.381156,0.362360,0.290412,0.273148,0.227885,0.278912,0.198628,0.272138,0.205764,
0.076271,0.009032,0.432203,0.251409,0.709497,0.505482,0.872881,0.743573,1.000000,0.992774,
0.029762,0.006956,0.304636,0.243011,0.600900,0.494525,0.846962,0.756235,1.000000,0.995536,
0.008475,0.002254,0.309973,0.246356,0.619048,0.491693,0.821229,0.732226,1.000000,0.993271,
0.874172,0.529111,0.377880,0.121718,0.731006,0.349172,0.194444,0.052330,0.107527,0.066880,
0.015038,0.004150,0.008475,0.002254,0.472292,0.238408,0.709677,0.500623,0.911704,0.765029,
1.000000,1.000000,0.762712,0.116371,0.762712,0.227897,0.938983,0.394435,0.991914,0.610064,
0.997305,0.828384,0.474576,0.061923,0.661631,0.337882,0.841410,0.550720,0.927313,0.732093,
0.998131,0.945984,0.598658,0.482956,0.458333,0.369341,0.090032,0.014271,0.424569,0.264664,
0.625000,0.491971,0.788793,0.717004,0.998823,0.989895,0.567797,0.394650,0.445585,0.357216,
0.313364,0.248133,0.314286,0.261840,0.238494,0.185072,0.251656,0.178836,0.029762,0.006988,
0.329975,0.240653,0.586207,0.497539,0.838710,0.757633,1.000000,0.996807,0.076271,0.009353,
0.323210,0.241490,0.578212,0.488078,0.857542,0.730300,1.000000,0.995189,0.008475,0.002254,
0.361702,0.262314,0.634686,0.511076,0.877660,0.738253,1.000000,0.993341,0.398876,0.321293,
0.491525,0.321911,0.455947,0.356795,0.254425,0.191312,0.306407,0.238412,0.275316,0.207381,
0.008475,0.002254,0.303855,0.246799,0.619048,0.492532,0.796089,0.732388,1.000000,0.993873,
0.040678,0.007917,0.331234,0.239346,0.631658,0.495077,0.854464,0.759136,1.000000,0.994473,
0.076271,0.009032,0.432203,0.254222,0.618644,0.502495,0.847458,0.740542,1.000000,0.994162,
0.428571,0.305031,0.529774,0.446836,0.313364,0.248133,0.338182,0.267135,0.191617,0.143207,
0.289130,0.220701,0.059524,0.009168,0.309637,0.240208,0.584392,0.496077,0.819205,0.753213,
1.000000,0.994408,0.059322,0.007862,0.297180,0.241369,0.562212,0.489912,0.784566,0.739447,
1.000000,0.997545,0.008475,0.002254,0.361702,0.262314,0.634686,0.511076,0.877660,0.738253,
1.000000,0.993341,0.095238,0.057775,0.111702,0.058414,0.125257,0.073404,0.085106,0.053382,
0.114990,0.051389,0.068282,0.034068,0.113095,0.061588,0.131285,0.065072,0.160714,0.075581,
0.101695,0.049407,0.153631,0.070262,0.059299,0.028363,0.060738,0.030882,0.044068,0.019357,
0.089947,0.055904,0.136015,0.097245,0.077778,0.045207,0.040678,0.026071,0.078341,0.035846,
0.029762,0.010782

Positive_164 74.000000,0.037071,14.000000,2.743243,2.000000,15.261940,14.000000,2.743243,1.000000,9.289337,
0.378600,0.018640,0.378600,0.018640,0.039409,0.282051,0.500000,0.542857,0.500000,0.500000,
0.750000,0.000000,0.000000,0.000000,0.000000,0.000000,0.000000,0.000000,0.000000,0.000000,
0.000000,0.000000,0.000000,0.060916,0.178082,0.004334,0.023033,0.191781,0.001360,0.024462,
0.191781,0.001296,0.069298,0.178082,0.004287,9.165151,5.338726,4.898979,1.000000,0.513514,
1.000000,0.513514,1.000000,0.512066,1.000000,0.502487,1.000000,0.458992,1.000000,0.374949,
1.000000,0.346299,1.000000,0.306917,0.500000,0.033423,0.333333,0.019381,1.000000,0.445946,
1.000000,0.445946,1.000000,0.443978,1.000000,0.430465,1.000000,0.416135,1.000000,0.301245,
1.000000,0.243248,1.000000,0.160474,0.333333,0.092612,0.333333,0.050097,0.462500,0.326176,
0.615385,0.378867,0.363208,0.294957,0.335025,0.228579,0.278912,0.200239,0.300236,0.218992,
0.050633,0.008036,0.376344,0.252621,0.620261,0.505037,0.829642,0.747045,1.000000,0.997026,
0.050725,0.006425,0.321767,0.239685,0.631250,0.488144,0.829308,0.744594,1.000000,0.996899,
0.012658,0.002032,0.346237,0.249704,0.565591,0.497808,0.864706,0.743165,1.000000,0.992793,
0.929322,0.547011,0.411465,0.136386,0.731006,0.316602,0.149108,0.057781,0.116788,0.061640,
0.017751,0.003550,0.012658,0.002032,0.357955,0.224076,0.743750,0.471853,0.911704,0.738885,
1.000000,1.000000,0.870327,0.106929,0.895920,0.312735,0.938983,0.471373,0.974763,0.655206,

0.998832,0.860578,0.896028,0.067353,0.923481,0.338620,0.952687,0.563592,0.981426,0.734936,
0.998880,0.959194,0.620805,0.471338,0.612507,0.410562,0.115942,0.012406,0.403465,0.258748,
0.641827,0.498728,0.866387,0.742998,0.999444,0.991101,0.643787,0.407963,0.445585,0.357185,
0.405063,0.234852,0.337187,0.275396,0.238494,0.182580,0.256410,0.169724,0.050725,0.006514,
0.325503,0.238097,0.590604,0.489879,0.815686,0.743519,1.000000,0.997780,0.063291,0.008910,
0.397849,0.265570,0.624595,0.514755,0.828194,0.751239,1.000000,0.995200,0.012658,0.002032,
0.311828,0.237321,0.627753,0.483734,0.838826,0.731919,1.000000,0.992308,0.398876,0.322981,
0.584781,0.325704,0.500000,0.351315,0.285799,0.203372,0.305893,0.237046,0.314465,0.210463,
0.012658,0.002032,0.351220,0.249022,0.573014,0.497116,0.857988,0.741808,1.000000,0.993129,
0.050725,0.007319,0.367089,0.241963,0.675000,0.491141,0.831707,0.748582,1.000000,0.996625,
0.038902,0.007771,0.376344,0.253188,0.604102,0.502904,0.823789,0.746174,1.000000,0.997097,
0.439252,0.312997,0.529774,0.452151,0.405063,0.234852,0.445444,0.284755,0.201923,0.140138,
0.346154,0.212166,0.050725,0.007497,0.319149,0.245787,0.628366,0.502189,0.837255,0.748141,
1.000000,0.992874,0.063291,0.007637,0.329032,0.253525,0.587097,0.501011,0.796117,0.744085,
1.000000,0.996734,0.012658,0.002032,0.311828,0.237321,0.627753,0.483734,0.838826,0.731919,
1.000000,0.992308,0.115942,0.056064,0.171429,0.057608,0.125257,0.071519,0.093750,0.054104,
0.114990,0.049876,0.072682,0.037005,0.286399,0.073019,0.144928,0.063379,0.141707,0.073402,
0.108280,0.049094,0.204142,0.066810,0.075949,0.025140,0.063291,0.028024,0.073254,0.028156,
0.089109,0.056290,0.165094,0.100194,0.076733,0.042301,0.050633,0.022132,0.062914,0.035358,
0.025316,0.010526

Positive_165 48.000000,0.053819,9.000000,2.583333,2.000000,6.248227,12.000000,2.583333,2.500000,4.460993,
0.433601,0.026688,0.433601,0.026688,0.032258,0.208333,0.305263,0.439394,0.540541,0.529412,
0.375000,0.065972,0.888889,0.051308,0.131944,0.750000,0.080329,0.011381,0.250000,0.001820,
0.033565,0.187500,0.005096,0.067967,0.234043,0.005532,0.043624,0.191489,0.002363,0.043667,
0.148936,0.001866,0.073312,0.170213,0.003217,5.856646,4.709537,3.795791,1.000000,0.604167,
1.000000,0.604167,1.000000,0.601687,1.000000,0.505903,1.000000,0.462847,1.000000,0.295635,
1.000000,0.270486,1.000000,0.174603,1.000000,0.120635,0.333333,0.028869,1.000000,0.666667,
1.000000,0.666351,1.000000,0.666035,1.000000,0.659542,1.000000,0.594291,1.000000,0.404356,
1.000000,0.366793,1.000000,0.281611,1.000000,0.106106,0.333333,0.050911,0.431718,0.331650,
0.517857,0.364468,0.402516,0.303882,0.309677,0.230250,0.278912,0.207624,0.291139,0.206940,
0.044025,0.009997,0.376344,0.259292,0.599589,0.501943,0.820339,0.748043,1.000000,0.993219,
0.029762,0.006532,0.317308,0.234218,0.600900,0.488704,0.846962,0.750528,1.000000,0.995453,
0.006410,0.002328,0.346237,0.249127,0.619048,0.500278,0.800915,0.733990,1.000000,0.993664,
0.843982,0.525895,0.377880,0.147641,0.606780,0.326464,0.194444,0.063490,0.116129,0.070369,
0.015038,0.005113,0.006410,0.002328,0.391598,0.247729,0.709677,0.509872,0.877719,0.772392,
1.000000,1.000000,0.762712,0.093761,0.762712,0.252384,0.938983,0.413956,0.968796,0.618655,
0.994709,0.860219,0.308756,0.038596,0.860812,0.331154,0.868268,0.545027,0.913876,0.737830,
0.998131,0.968167,0.597360,0.493367,0.432671,0.371131,0.044818,0.013890,0.357500,0.258804,
0.578009,0.503503,0.819712,0.727515,0.998419,0.989731,0.527489,0.389916,0.433898,0.359452,
0.313364,0.250632,0.332308,0.263056,0.238494,0.187859,0.251656,0.182149,0.029762,0.006947,
0.300481,0.233806,0.583646,0.488967,0.838710,0.751617,1.000000,0.996578,0.033333,0.009411,
0.397849,0.252295,0.591398,0.498246,0.818182,0.733049,1.000000,0.996355,0.006410,0.002328,
0.361702,0.261234,0.583333,0.501880,0.877660,0.738333,1.000000,0.990224,0.408805,0.334131,
0.470238,0.308371,0.455947,0.357497,0.246835,0.189767,0.306407,0.246312,0.296774,0.207110,

0.006410,0.002328,0.327751,0.252583,0.619048,0.501522,0.800915,0.735334,1.000000,0.994209,
0.040678,0.007503,0.313578,0.229740,0.631658,0.486554,0.854464,0.750425,1.000000,0.995399,
0.044025,0.009997,0.376344,0.260182,0.597527,0.501359,0.813559,0.746565,1.000000,0.994043,
0.442793,0.304714,0.528814,0.444654,0.313364,0.250632,0.387615,0.264681,0.193548,0.148132,
0.289130,0.221876,0.059524,0.008948,0.305085,0.233161,0.579661,0.489794,0.819205,0.746261,
1.000000,0.995134,0.030556,0.007764,0.329032,0.247330,0.587097,0.496828,0.787097,0.743674,
1.000000,0.997267,0.006410,0.002328,0.361702,0.261234,0.583333,0.501880,0.877660,0.738333,
1.000000,0.990224,0.108974,0.060281,0.111702,0.059676,0.115254,0.072344,0.085106,0.058863,
0.065476,0.045982,0.068282,0.034503,0.113095,0.062062,0.185897,0.064272,0.163447,0.070664,
0.069149,0.048852,0.123570,0.062521,0.056604,0.025848,0.060738,0.030249,0.094340,0.022681,
0.119497,0.060717,0.136015,0.098382,0.077778,0.047524,0.041068,0.024730,0.078341,0.038581,
0.029762,0.011268

Positive_166 7.000000,0.081633,2.000000,0.571429,0.000000,0.619048,1.000000,0.571429,1.000000,0.285714,
0.558911,0.008579,0.558911,0.008579,0.000000,0.000000,0.000000,0.500000,0.500000,1.000000,
0.000000,0.000000,0.000000,0.000000,0.000000,0.000000,0.000000,0.000000,0.000000,0.000000,
0.000000,0.000000,0.000000,0.000000,0.000000,0.000000,0.000000,0.000000,0.000000,0.000000,
0.000000,0.000000,0.047619,0.166667,0.006614,1.414214,1.000000,1.000000,1.000000,0.142857,
1.000000,0.142857,1.000000,0.142857,1.000000,0.142857,1.000000,0.142857,1.000000,0.142857,
1.000000,0.142857,1.000000,0.142857,1.000000,0.142857,0.000000,0.000000,0.000000,0.000000,
0.000000,0.000000,0.000000,0.000000,0.000000,0.000000,0.000000,0.000000,0.000000,0.000000,
0.000000,0.000000,0.000000,0.000000,0.000000,0.000000,0.000000,0.000000,0.379104,0.301924,
0.448399,0.363826,0.354545,0.334250,0.254491,0.209318,0.242515,0.183812,0.267857,0.245718,
0.050746,0.018095,0.384342,0.280926,0.570149,0.509437,0.830325,0.751524,1.000000,0.994697,
0.031818,0.014503,0.283525,0.238676,0.563218,0.497603,0.808429,0.734737,1.000000,0.991982,
0.004545,0.003585,0.309609,0.224409,0.530249,0.482793,0.790036,0.736168,1.000000,0.994690,
0.765343,0.533658,0.364583,0.249476,0.306513,0.216865,0.114155,0.089441,0.074850,0.041807,
0.011538,0.005137,0.004545,0.003585,0.287356,0.228407,0.554545,0.484039,0.845455,0.728369,
1.000000,1.000000,0.245211,0.098013,0.626335,0.374255,0.735632,0.498263,0.882562,0.675694,
0.996441,0.861468,0.288256,0.065452,0.749226,0.281790,0.871528,0.640560,0.920139,0.731818,
0.986364,0.931540,0.545455,0.465681,0.419162,0.343563,0.019157,0.013994,0.338078,0.234902,
0.601423,0.505122,0.793594,0.730969,0.992883,0.980335,0.440433,0.377006,0.402985,0.377565,
0.286364,0.245428,0.326347,0.273530,0.232877,0.176290,0.238462,0.188599,0.031818,0.014503,
0.283525,0.244458,0.563218,0.497669,0.808429,0.719974,1.000000,0.991407,0.024306,0.010094,
0.327402,0.236271,0.558209,0.489490,0.794030,0.722184,1.000000,0.984482,0.004545,0.003585,
0.313167,0.257295,0.562278,0.492561,0.831818,0.762616,1.000000,0.993802,0.427273,0.366396,
0.373665,0.304256,0.411940,0.329348,0.236934,0.216836,0.269461,0.222399,0.239521,0.196345,
0.004545,0.003585,0.309609,0.228753,0.590747,0.493822,0.793594,0.734251,1.000000,0.996638,
0.031818,0.014503,0.287356,0.237557,0.590909,0.506085,0.816092,0.730064,1.000000,0.990965,
0.050746,0.017103,0.384342,0.277622,0.526690,0.490790,0.826625,0.756879,1.000000,0.994697,
0.310345,0.273576,0.537906,0.480996,0.286364,0.245428,0.302395,0.263130,0.164384,0.128885,
0.268293,0.236004,0.056940,0.024214,0.330961,0.263010,0.551601,0.501885,0.807829,0.736978,
1.000000,0.989477,0.024306,0.010094,0.264368,0.228058,0.524306,0.477088,0.774306,0.731739,
1.000000,0.994363,0.004545,0.003585,0.313167,0.257295,0.562278,0.492561,0.831818,0.762616,
1.000000,0.993802,0.062500,0.044014,0.095522,0.064717,0.079422,0.062342,0.050746,0.034326,

0.068592,0.045929,0.065672,0.050596,0.067616,0.051643,0.061303,0.040143,0.108359,0.082775,
0.083582,0.064689,0.138790,0.065006,0.032836,0.027425,0.072727,0.032145,0.083032,0.038424,
0.095455,0.070453,0.124555,0.099400,0.074303,0.048846,0.042705,0.022990,0.057471,0.041798,
0.017361,0.012340

Positive_167 12.000000,0.090278,4.000000,1.083333,0.000000,2.446970,2.000000,1.083333,1.000000,0.628788,
0.727518,0.021742,0.727518,0.021742,0.000000,0.000000,0.000000,0.153846,0.181818,0.111111,
0.250000,0.000000,0.000000,0.000000,0.000000,0.000000,0.000000,0.000000,0.000000,0.000000,
0.000000,0.000000,0.000000,0.015152,0.090909,0.001252,0.030303,0.363636,0.011019,0.030303,
0.090909,0.002004,0.121212,0.272727,0.014025,2.828427,1.414214,1.414214,1.000000,0.333333,
1.000000,0.333333,1.000000,0.333333,1.000000,0.333333,1.000000,0.333333,1.000000,0.333333,
1.000000,0.333333,1.000000,0.333333,1.000000,0.277778,0.500000,0.083333,1.000000,0.333333,
1.000000,0.333333,1.000000,0.333333,1.000000,0.333333,1.000000,0.333333,1.000000,0.333333,
1.000000,0.333333,1.000000,0.333333,1.000000,0.333333,0.000000,0.000000,0.336140,0.291749,
0.485714,0.390422,0.354545,0.317828,0.278689,0.223756,0.211063,0.174000,0.264493,0.240942,
0.025266,0.014154,0.347826,0.278387,0.565217,0.511011,0.830325,0.758465,1.000000,0.993047,
0.031818,0.012462,0.283525,0.244196,0.563218,0.481248,0.808429,0.724742,1.000000,0.995406,
0.004545,0.002886,0.260638,0.207267,0.620408,0.503213,0.824490,0.752343,1.000000,0.993353,
0.765343,0.565878,0.364583,0.291032,0.306513,0.143090,0.138282,0.101358,0.044750,0.026324,
0.011538,0.002877,0.004545,0.002886,0.287356,0.229365,0.554545,0.469049,0.845455,0.715949,
1.000000,1.000000,0.486166,0.170842,0.656126,0.364317,0.735632,0.524148,0.877470,0.724970,
0.996169,0.935807,0.688953,0.073255,0.860465,0.267887,0.970930,0.511983,0.979651,0.566968,
0.997133,0.734633,0.551020,0.501195,0.459016,0.381713,0.020408,0.010282,0.346939,0.258460,
0.616327,0.521118,0.795918,0.747384,0.998853,0.989165,0.474308,0.406911,0.397474,0.358923,
0.286364,0.234166,0.326087,0.277045,0.232877,0.190266,0.238462,0.176063,0.031818,0.011176,
0.283525,0.245810,0.563218,0.485185,0.808429,0.727603,1.000000,0.996415,0.024306,0.009667,
0.320479,0.232415,0.573123,0.502922,0.820408,0.740574,1.000000,0.988116,0.004545,0.002886,
0.375494,0.264115,0.604743,0.508214,0.831818,0.762337,1.000000,0.994954,0.427273,0.350420,
0.420408,0.336182,0.353578,0.313398,0.245902,0.222738,0.248908,0.209463,0.274590,0.211867,
0.004545,0.002886,0.269388,0.212535,0.624490,0.508366,0.790514,0.745362,1.000000,0.994489,
0.031818,0.012462,0.287356,0.242398,0.590909,0.483563,0.816092,0.722880,1.000000,0.995406,
0.025266,0.012774,0.347826,0.275344,0.569170,0.507087,0.826625,0.761287,1.000000,0.993047,
0.355731,0.308904,0.537906,0.456931,0.286364,0.234166,0.344262,0.286553,0.170635,0.147591,
0.268293,0.218738,0.043321,0.014547,0.303249,0.249743,0.540230,0.486487,0.800766,0.732960,
1.000000,0.994476,0.024306,0.009134,0.264368,0.230025,0.526531,0.486262,0.774306,0.736768,
1.000000,0.995241,0.004545,0.002886,0.375494,0.264115,0.604743,0.508214,0.831818,0.762337,
1.000000,0.994954,0.062500,0.045890,0.077399,0.055963,0.079422,0.057382,0.059593,0.041774,
0.068592,0.045846,0.062500,0.044896,0.167347,0.086614,0.079128,0.049446,0.108359,0.074116,
0.079861,0.056954,0.122449,0.069052,0.032491,0.021649,0.072727,0.032591,0.083032,0.028956,
0.095455,0.071291,0.114106,0.091129,0.074303,0.048380,0.039526,0.023078,0.057471,0.043343,
0.020349,0.011651

Positive_168 7.000000,0.081633,2.000000,0.571429,0.000000,0.619048,1.000000,0.571429,1.000000,0.285714,
0.558911,0.008579,0.558911,0.008579,0.000000,0.000000,0.000000,0.500000,0.500000,1.000000,
0.000000,0.000000,0.000000,0.000000,0.000000,0.000000,0.000000,0.000000,0.000000,0.000000,
0.000000,0.000000,0.000000,0.000000,0.000000,0.000000,0.000000,0.000000,0.000000,0.000000,

0.000000,0.000000,0.047619,0.166667,0.006614,1.414214,1.000000,1.000000,1.000000,0.142857,
1.000000,0.142857,1.000000,0.142857,1.000000,0.142857,1.000000,0.142857,1.000000,0.142857,
1.000000,0.142857,1.000000,0.142857,1.000000,0.142857,0.000000,0.000000,0.000000,0.000000,
0.000000,0.000000,0.000000,0.000000,0.000000,0.000000,0.000000,0.000000,0.000000,0.000000,
0.000000,0.000000,0.000000,0.000000,0.000000,0.000000,0.000000,0.000000,0.379104,0.301924,
0.448399,0.363826,0.354545,0.334250,0.254491,0.209318,0.242515,0.183812,0.267857,0.245718,
0.050746,0.018095,0.384342,0.280926,0.570149,0.509437,0.830325,0.751524,1.000000,0.994697,
0.031818,0.014503,0.283525,0.238676,0.563218,0.497603,0.808429,0.734737,1.000000,0.991982,
0.004545,0.003585,0.309609,0.224409,0.530249,0.482793,0.790036,0.736168,1.000000,0.994690,
0.765343,0.533658,0.364583,0.249476,0.306513,0.216865,0.114155,0.089441,0.074850,0.041807,
0.011538,0.005137,0.004545,0.003585,0.287356,0.228407,0.554545,0.484039,0.845455,0.728369,
1.000000,1.000000,0.245211,0.098013,0.626335,0.374255,0.735632,0.498263,0.882562,0.675694,
0.996441,0.861468,0.288256,0.065452,0.749226,0.281790,0.871528,0.640560,0.920139,0.731818,
0.986364,0.931540,0.545455,0.465681,0.419162,0.343563,0.019157,0.013994,0.338078,0.234902,
0.601423,0.505122,0.793594,0.730969,0.992883,0.980335,0.440433,0.377006,0.402985,0.377565,
0.286364,0.245428,0.326347,0.273530,0.232877,0.176290,0.238462,0.188599,0.031818,0.014503,
0.283525,0.244458,0.563218,0.497669,0.808429,0.719974,1.000000,0.991407,0.024306,0.010094,
0.327402,0.236271,0.558209,0.489490,0.794030,0.722184,1.000000,0.984482,0.004545,0.003585,
0.313167,0.257295,0.562278,0.492561,0.831818,0.762616,1.000000,0.993802,0.427273,0.366396,
0.373665,0.304256,0.411940,0.329348,0.236934,0.216836,0.269461,0.222399,0.239521,0.196345,
0.004545,0.003585,0.309609,0.228753,0.590747,0.493822,0.793594,0.734251,1.000000,0.996638,
0.031818,0.014503,0.287356,0.237557,0.590909,0.506085,0.816092,0.730064,1.000000,0.990965,
0.050746,0.017103,0.384342,0.277622,0.526690,0.490790,0.826625,0.756879,1.000000,0.994697,
0.310345,0.273576,0.537906,0.480996,0.286364,0.245428,0.302395,0.263130,0.164384,0.128885,
0.268293,0.236004,0.056940,0.024214,0.330961,0.263010,0.551601,0.501885,0.807829,0.736978,
1.000000,0.989477,0.024306,0.010094,0.264368,0.228058,0.524306,0.477088,0.774306,0.731739,
1.000000,0.994363,0.004545,0.003585,0.313167,0.257295,0.562278,0.492561,0.831818,0.762616,
1.000000,0.993802,0.062500,0.044014,0.095522,0.064717,0.079422,0.062342,0.050746,0.034326,
0.068592,0.045929,0.065672,0.050596,0.067616,0.051643,0.061303,0.040143,0.108359,0.082775,
0.083582,0.064689,0.138790,0.065006,0.032836,0.027425,0.072727,0.032145,0.083032,0.038424,
0.095455,0.070453,0.124555,0.099400,0.074303,0.048846,0.042705,0.022990,0.057471,0.041798,
0.017361,0.012340
Positive_169 16.000000,0.105469,3.000000,1.687500,2.500000,2.095833,8.000000,1.687500,0.000000,9.962500,
0.600426,0.011915,0.600426,0.011915,0.000000,0.000000,0.037037,0.230769,0.350000,0.461538,
1.000000,0.000000,0.000000,0.000000,0.000000,0.000000,0.000000,0.000000,0.000000,0.000000,
0.000000,0.000000,0.000000,0.233333,0.466667,0.058074,0.000000,0.000000,0.000000,0.000000,
0.000000,0.000000,0.033333,0.133333,0.002963,4.898979,1.414214,1.000000,1.000000,0.562500,
1.000000,0.562500,1.000000,0.562500,1.000000,0.562500,1.000000,0.562500,1.000000,0.562500,
1.000000,0.562500,1.000000,0.229167,1.000000,0.229167,0.333333,0.166667,1.000000,0.187500,
1.000000,0.187500,1.000000,0.187500,1.000000,0.187500,1.000000,0.187500,1.000000,0.187500,
0.892857,0.167411,0.678571,0.127232,0.214286,0.040179,0.107143,0.020089,0.379104,0.282357,
0.493333,0.405780,0.354545,0.311863,0.304348,0.234661,0.242515,0.146367,0.287462,0.243314,
0.069959,0.029650,0.384342,0.278298,0.695473,0.538742,0.844595,0.771576,1.000000,0.991368,
0.031818,0.010713,0.293103,0.242476,0.554545,0.492049,0.786364,0.725230,1.000000,0.993772,

0.005587,0.003395,0.326180,0.211028,0.559172,0.471796,0.808743,0.737587,1.000000,0.995522,
0.583630,0.490208,0.475610,0.306442,0.350394,0.203350,0.189602,0.113416,0.074850,0.036858,
0.008982,0.002273,0.005587,0.003395,0.301724,0.243269,0.583333,0.521895,0.845455,0.775812,
1.000000,1.000000,0.368715,0.084424,0.626335,0.289148,0.704626,0.469010,0.882562,0.643618,
0.996441,0.910256,0.288256,0.039707,0.749226,0.270713,0.871528,0.566589,0.925333,0.697339,
0.988739,0.878735,0.591463,0.500208,0.419162,0.321380,0.025751,0.012748,0.338078,0.216080,
0.601423,0.462015,0.793594,0.696168,0.997126,0.980518,0.496000,0.408332,0.403433,0.345436,
0.286364,0.246232,0.326347,0.275860,0.247706,0.199754,0.212544,0.160484,0.031818,0.009909,
0.304527,0.252290,0.557927,0.498289,0.773224,0.730470,1.000000,0.994249,0.024306,0.010913,
0.327402,0.243488,0.582677,0.506273,0.794030,0.745845,1.000000,0.989878,0.005587,0.003395,
0.313167,0.228450,0.562278,0.467811,0.831818,0.731892,1.000000,0.988711,0.427273,0.345881,
0.429333,0.338842,0.411940,0.315276,0.278287,0.224987,0.269461,0.189576,0.273973,0.217718,
0.005587,0.003395,0.321888,0.221102,0.590747,0.478392,0.793594,0.737029,1.000000,0.996374,
0.031818,0.010713,0.307471,0.239466,0.590909,0.496661,0.806011,0.732150,1.000000,0.989006,
0.069959,0.029216,0.384342,0.271614,0.608000,0.518111,0.826816,0.770804,1.000000,0.992201,
0.381333,0.312070,0.501493,0.441698,0.286364,0.246232,0.328530,0.283855,0.173913,0.146677,
0.268293,0.213561,0.056940,0.012578,0.330961,0.253907,0.557927,0.498677,0.807829,0.732431,
1.000000,0.988813,0.024306,0.010175,0.279528,0.244759,0.559172,0.499633,0.788288,0.750566,
1.000000,0.995760,0.005587,0.003395,0.313167,0.228450,0.562278,0.467811,0.831818,0.731892,
1.000000,0.988711,0.090551,0.053906,0.095522,0.047051,0.079882,0.057327,0.057377,0.040782,
0.062842,0.041989,0.072961,0.041302,0.088415,0.062174,0.106299,0.055817,0.128000,0.093205,
0.083582,0.060092,0.138790,0.067554,0.053498,0.032920,0.072727,0.034018,0.062687,0.028708,
0.095455,0.064960,0.124555,0.097023,0.085837,0.042835,0.042705,0.023257,0.051829,0.037090,
0.033520,0.017991
