# Supplementary material for: Analysis of Protein Pathway Networks Using Hybrid Properties
Source: Molecules. 2010 Nov 12;15(11):8177–92. doi: 10.3390/molecules15118177 (PMC6259184; doi:10.3390/molecules15118177)
Supplement: Supplementary File 4 [file molecules-15-08177-s004.pdf]

Online Supporting Information S4: Two lists obtained by mRMR

(1) MaxRel features list

Order	Feature name
1	out_local_density_0.6_mean
2	in_local_density_0.6_mean
3	in_local_density_0.5_mean
4	out_local_density_0.7_mean
5	out_local_density_0.5_mean
6	in_local_density_0.7_mean
7	in_local_density_0.4_mean
8	topological_change_0.1_0.2
9	out_local_density_0.4_mean
10	out_local_density_0.8_mean
11	topological_change_0.2_0.3
12	polarity_composition_P_max
13	hydrophobicity_composition_H_max
14	in_local_density_0.8_mean
15	solvent_accessibility_composition_H_max
16	in_local_density_0.3_mean
17	weight_edge_mean(with_missing_edge)
18	weight_edge_mean(without_missing_edge)
19	out_local_density_0.3_mean
20	out_local_density_0.7_max
21	hydrophobicity_transition_NH_max
22	in_local_density_0.7_max
23	in_local_density_0.6_max
24	polarity_transition_PN_max
25	out_local_density_0.6_max
26	solvent_accessibility_composition_H_mean
27	AA_composition_I_max
28	out_degree_correlation_max
29	out_local_density_0.9_mean
30	in_degree_correlation_max
31	in_degree_variance
32	AA_composition_C_mean
33	out_local_density_0.8_max
34	out_degree_variance
35	out_clustering_mean
36	in_local_density_0.9_mean
37	hydrophobicity_distribution_P-0.75_max
38	in_clustering_mean
39	topological_change_0.3_0.4
40	topological_change_0.7_0.8

41	in_local_density_0.5_max
42	in_local_density_0.8_max
43	out_local_density_0.5_max
44	AA_composition_S_mean
45	AA_composition_L_mean
46	AA_composition_K_mean
47	hydrophobicity_transition_PH_mean
48	topological_change_0.6_0.7
49	in_clustering_max
50	AA_composition_F_max
51	second_singular_values
52	polarity_transition_PH_mean
53	out_out_topological_variance
54	secondary_structure_composition_P_max
55	out_clustering_max
56	secondary_structure_transition_PH_mean
57	hydrophobicity_transition_PN_mean
58	AA_composition_L_max
59	AA_composition_D_mean
60	secondary_structure_composition_P_mean
61	AA_composition_V_mean
62	polarizability_transition_PH_mean
63	secondary_structure_composition_H_max
64	in_in_topological_variance
65	AA_composition_F_mean
66	polarity_composition_H_mean
67	in_degree_correlation_variance
68	secondary_structure_distribution_H-0.5_max
69	in_local_density_0_mean
70	AA_composition_E_mean
71	secondary_structure_transition_NH_max
72	hydrophobicity_composition_P_mean
73	secondary_structure_transition_NH_mean
74	out_out_topological_max
75	polarity_transition_PN_mean
76	polarity_composition_P_mean
77	solvent_accessibility_transition_HE_mean
78	out_local_density_0_mean
79	solvent_accessibility_transition_HE_max
80	hydrophobicity_composition_H_mean
81	AA_composition_S_max
82	out_degree_correlation_variance
83	hydrophobicity_transition_NH_mean
84	polarity_transition_NH_mean

85	VanDerWaal_transition_PN_mean
86	in_degree_max
87	polarity_distribution_H-0.75_max
88	AA_composition_A_mean
89	secondary_structure_composition_N_max
90	out_degree_max
91	AA_composition_W_mean
92	polarity_distribution_P-0.75_max
93	AA_composition_C_max
94	VanDerWaal_composition_P_max
95	first_singular_values
96	in_in_topological_max
97	polarizability_composition_H_mean
98	VanDerWaal_composition_H_mean
99	secondary_structure_distribution_H-0.75_max
100	secondary_structure_distribution_N-1.0_mean
101	solvent_accessibility_distribution_H-1.0_mean
102	secondary_structure_transition_PN_max
103	out_local_density_0.1_mean
104	in_in_topological_mean
105	VanDerWaal_transition_PH_mean
106	polarity_composition_N_max
107	solvent_accessibility_distribution_H-0.25_mean
108	out_degree_median
109	solvent_accessibility_distribution_H-0.75_mean
110	in_local_density_0.1_mean
111	secondary_structure_transition_PN_mean
112	AA_composition_G_mean
113	hydrophobicity_composition_P_max
114	hydrophobicity_distribution_H-0.0_mean
115	VanDerWaal_distribution_H-0.0_mean
116	polarizability_distribution_H-0.0_mean
117	polarity_distribution_P-0.0_mean
118	solvent_accessibility_distribution_H-0.5_mean
119	in_degree_median
120	weight_edge_variance(with_missing_edge)
121	hydrophobicity_composition_N_max
122	polarity_transition_NH_max
123	out_out_topological_mean
124	polarizability_transition_PN_mean
125	third_singular_values
126	AA_composition_P_max
127	weight_edge_variance(without_missing_edge)
128	AA_composition_T_mean

129	AA_composition_M_max
130	AA_composition_P_mean
131	polarity_composition_H_max
132	secondary_structure_distribution_N-0.75_mean
133	secondary_structure_composition_H_mean
134	AA_composition_I_mean
135	hydrophobicity_distribution_N-0.0_mean
136	hydrophobicity_distribution_P-0.5_max
137	solvent_accessibility_distribution_H-0.75_max
138	polarity_composition_N_mean
139	polarizability_composition_N_mean
140	secondary_structure_distribution_H-0.25_max
141	secondary_structure_distribution_P-0.0_mean
142	hydrophobicity_distribution_H-0.25_mean
143	AA_composition_E_max
144	AA_composition_R_mean
145	out_in_topological_mean
146	in_out_topological_mean
147	polarizability_transition_NH_mean
148	out_local_density_0.2_mean
149	VanDerWaal_composition_N_max
150	secondary_structure_distribution_P-0.5_max
151	secondary_structure_composition_N_mean
152	secondary_structure_distribution_P-0.75_max
153	AA_composition_Q_mean
154	in_local_density_0.4_max
155	polarizability_composition_P_mean
156	in_out_topological_max
157	VanDerWaal_composition_P_mean
158	secondary_structure_distribution_N-0.75_max
159	hydrophobicity_distribution_H-0.75_mean
160	hydrophobicity_distribution_H-0.75_max
161	topological_change_0.5_0.6
162	secondary_structure_distribution_N-0.5_mean
163	polarizability_composition_N_max
164	VanDerWaal_transition_NH_mean
165	solvent_accessibility_distribution_H-0.0_mean
166	in_local_density_0.2_mean
167	hydrophobicity_composition_N_mean
168	secondary_structure_distribution_H-0.0_mean
169	secondary_structure_distribution_P-0.75_mean
170	AA_composition_T_max
171	polarity_distribution_P-0.0_max
172	hydrophobicity_distribution_H-0.0_max

173	polarizability_distribution_H-0.0_max
174	VanDerWaal_distribution_H-0.0_max
175	polarity_distribution_H-0.5_max
176	polarizability_composition_P_max
177	secondary_structure_distribution_H-1.0_mean
178	AA_composition_Y_mean
179	AA_composition_H_mean
180	AA_composition_W_max
181	VanDerWaal_distribution_P-0.0_mean
182	polarizability_transition_PN_max
183	secondary_structure_distribution_N-0.25_max
184	polarizability_distribution_H-1.0_mean
185	VanDerWaal_distribution_H-1.0_mean
186	AA_composition_R_max
187	secondary_structure_distribution_N-0.0_max
188	in_local_density_0.9_max
189	secondary_structure_distribution_N-0.5_max
190	polarity_distribution_N-0.0_mean
191	VanDerWaal_distribution_P-0.75_max
192	polarity_distribution_H-0.25_mean
193	polarizability_distribution_P-0.0_mean
194	polarizability_distribution_N-0.0_mean
195	hydrophobicity_transition_PN_max
196	polarity_distribution_H-0.25_max
197	hydrophobicity_distribution_P-0.25_max
198	polarity_distribution_P-0.25_mean
199	polarity_distribution_N-0.75_max
200	polarizability_composition_H_max
201	VanDerWaal_composition_H_max
202	out_local_density_0.9_max
203	polarity_distribution_H-1.0_mean
204	VanDerWaal_transition_NH_max
205	in_out_topological_variance
206	polarity_transition_PH_max
207	polarity_distribution_P-0.5_max
208	polarizability_distribution_N-0.75_mean
209	polarity_distribution_N-0.5_max
210	hydrophobicity_distribution_P-0.25_mean
211	AA_composition_D_max
212	polarizability_distribution_P-0.5_max
213	solvent_accessibility_distribution_H-0.5_max
214	polarity_distribution_H-0.0_max
215	AA_composition_G_max
216	VanDerWaal_distribution_N-0.5_max

217	out_local_density_0.4_max
218	AA_composition_Q_max
219	VanDerWaal_distribution_N-0.25_mean
220	hydrophobicity_distribution_N-0.75_max
221	polarizability_distribution_N-0.25_mean
222	VanDerWaal_distribution_H-0.75_mean
223	polarizability_distribution_H-0.75_mean
224	polarity_distribution_N-0.0_max
225	polarity_distribution_P-0.75_mean
226	secondary_structure_distribution_H-0.25_mean
227	polarity_distribution_H-0.0_mean
228	hydrophobicity_distribution_P-1.0_mean
229	out_local_density_0_max
230	polarizability_distribution_P-0.75_max
231	hydrophobicity_distribution_H-0.5_max
232	secondary_structure_distribution_H-0.5_mean
233	secondary_structure_distribution_H-0.75_mean
234	hydrophobicity_distribution_P-0.75_mean
235	VanDerWaal_distribution_P-0.75_mean
236	hydrophobicity_transition_PH_max
237	VanDerWaal_distribution_P-0.5_max
238	AA_composition_K_max
239	hydrophobicity_distribution_P-0.0_mean
240	polarity_distribution_N-0.25_max
241	out_local_density_0.1_max
242	topological_change_0.4_0.5
243	polarity_distribution_P-1.0_mean
244	hydrophobicity_distribution_N-0.5_max
245	polarizability_distribution_P-0.25_mean
246	hydrophobicity_distribution_H-0.25_max
247	secondary_structure_distribution_N-0.25_mean
248	polarity_distribution_H-0.75_mean
249	hydrophobicity_distribution_N-0.0_max
250	VanDerWaal_composition_N_mean
251	VanDerWaal_distribution_H-0.5_max
252	polarizability_distribution_H-0.5_max
253	VanDerWaal_distribution_N-0.75_mean
254	polarity_distribution_P-0.5_mean
255	secondary_structure_distribution_N-0.0_mean
256	out_degree_mean
257	in_degree_mean
258	secondary_structure_distribution_H-0.0_max
259	AA_composition_V_max
260	VanDerWaal_distribution_N-1.0_mean

261	AA_composition_Y_max
262	VanDerWaal_distribution_H-0.25_max
263	polarizability_distribution_H-0.25_max
264	VanDerWaal_distribution_N-0.75_max
265	VanDerWaal_distribution_P-0.0_max
266	polarizability_transition_NH_max
267	VanDerWaal_transition_PN_max
268	VanDerWaal_distribution_N-0.0_mean
269	out_in_topological_max
270	solvent_accessibility_distribution_H-0.0_max
271	VanDerWaal_distribution_H-0.75_max
272	polarizability_distribution_H-0.75_max
273	hydrophobicity_distribution_H-1.0_mean
274	secondary_structure_distribution_P-0.5_mean
275	VanDerWaal_distribution_P-0.25_mean
276	hydrophobicity_distribution_N-0.25_mean
277	secondary_structure_distribution_P-0.25_max
278	solvent_accessibility_distribution_H-1.0_max
279	polarity_distribution_P-0.25_max
280	hydrophobicity_distribution_H-0.5_mean
281	AA_composition_N_mean
282	polarizability_distribution_P-0.0_max
283	AA_composition_N_max
284	polarizability_distribution_P-0.75_mean
285	AA_composition_H_max
286	solvent_accessibility_distribution_H-0.25_max
287	out_in_topological_variance
288	secondary_structure_distribution_N-1.0_max
289	polarizability_distribution_N-0.75_max
290	hydrophobicity_distribution_P-0.0_max
291	secondary_structure_distribution_P-0.25_mean
292	VanDerWaal_distribution_N-0.25_max
293	in_degree_correlation_mean
294	polarizability_distribution_N-0.5_max
295	polarizability_distribution_H-0.25_mean
296	VanDerWaal_distribution_H-0.25_mean
297	polarizability_distribution_N-1.0_mean
298	polarity_distribution_N-0.25_mean
299	hydrophobicity_distribution_P-0.5_mean
300	VanDerWaal_distribution_P-0.25_max
301	polarity_distribution_H-0.5_mean
302	AA_composition_M_mean
303	out_degree_correlation_mean
304	VanDerWaal_distribution_H-0.5_mean

305	polarizability_distribution_H-0.5_mean
306	hydrophobicity_distribution_N-0.25_max
307	VanDerWaal_distribution_N-0.5_mean
308	polarity_distribution_N-0.75_mean
309	polarizability_distribution_P-1.0_max
310	polarizability_distribution_P-0.5_mean
311	hydrophobicity_distribution_N-0.75_mean
312	out_local_density_0.2_max
313	secondary_structure_distribution_P-1.0_mean
314	in_clustering_variance
315	polarizability_distribution_N-0.0_max
316	graph_density
317	AA_composition_A_max
318	VanDerWaal_distribution_N-0.0_max
319	secondary_structure_distribution_H-1.0_max
320	in_local_density_0.3_max
321	polarizability_distribution_N-0.5_mean
322	polarizability_distribution_N-0.25_max
323	secondary_structure_transition_PH_max
324	polarizability_transition_PH_max
325	hydrophobicity_distribution_H-1.0_max
326	polarity_distribution_N-1.0_mean
327	VanDerWaal_distribution_P-1.0_mean
328	hydrophobicity_distribution_P-1.0_max
329	VanDerWaal_distribution_H-1.0_max
330	polarizability_distribution_H-1.0_max
331	polarizability_distribution_P-0.25_max
332	VanDerWaal_transition_PH_max
333	out_clustering_variance
334	in_local_density_0_max
335	hydrophobicity_distribution_N-0.5_mean
336	VanDerWaal_distribution_P-0.5_mean
337	in_local_density_0.1_max
338	polarity_distribution_H-1.0_max
339	polarity_distribution_N-1.0_max
340	VanDerWaal_distribution_P-1.0_max
341	hydrophobicity_distribution_N-1.0_max
342	polarity_distribution_P-1.0_max
343	polarizability_distribution_P-1.0_mean
344	hydrophobicity_distribution_N-1.0_mean
345	polarity_distribution_N-0.5_mean
346	VanDerWaal_distribution_N-1.0_max
347	out_local_density_0.3_max
348	in_local_density_0.2_max

349	graph_size
350	polarizability_distribution_N-1.0_max
351	secondary_structure_distribution_P-0.0_max

(2) mRMR features list

Order	Feature name
1	out_local_density_0.6_mean
2	polarity_composition_P_max
3	in_local_density_0.7_max
4	in_local_density_0.5_mean
5	topological_change_0.6_0.7
6	topological_change_0.1_0.2
7	in_degree_variance
8	topological_change_0.7_0.8
9	out_local_density_0.8_mean
10	secondary_structure_distribution_P-1.0_mean
11	topological_change_0.2_0.3
12	in_local_density_0.6_mean
13	out_degree_variance
14	secondary_structure_distribution_P-1.0_max
15	out_local_density_0.5_mean
16	out_degree_correlation_max
17	out_local_density_0.7_max
18	polarizability_distribution_N-1.0_max
19	in_local_density_0.7_mean
20	AA_composition_C_mean
21	in_degree_correlation_variance
22	secondary_structure_distribution_P-0.0_max
23	out_local_density_0.7_mean
24	AA_composition_S_mean
25	out_degree_correlation_variance
26	VanDerWaal_distribution_P-1.0_max
27	polarity_distribution_P-1.0_max
28	in_local_density_0.8_mean
29	AA_composition_K_mean
30	polarity_distribution_H-1.0_max
31	polarizability_transition_PH_mean
32	in_clustering_max
33	hydrophobicity_distribution_N-1.0_max
34	solvent_accessibility_composition_H_max
35	out_local_density_0.9_mean
36	VanDerWaal_distribution_N-1.0_max
37	secondary_structure_transition_PH_mean

38	in_local_density_0.3_mean
39	hydrophobicity_distribution_H-1.0_max
40	in_local_density_0.9_mean
41	out_local_density_0_max
42	solvent_accessibility_distribution_H-1.0_mean
43	in_local_density_0.6_max
44	AA_composition_V_mean
45	VanDerWaal_distribution_H-1.0_max
46	polarizability_distribution_P-1.0_max
47	out_local_density_0.6_max
48	hydrophobicity_distribution_P-1.0_max
49	solvent_accessibility_distribution_H-0.5_mean
50	secondary_structure_distribution_H-0.0_mean
51	in_local_density_0_max
52	solvent_accessibility_composition_H_mean
53	out_degree_correlation_mean
54	out_out_topological_variance
55	AA_composition_W_mean
56	out_local_density_0.8_max
57	out_local_density_0.1_max
58	hydrophobicity_transition_PH_mean
59	polarity_distribution_N-1.0_max
60	in_local_density_0.8_max
61	VanDerWaal_transition_PN_mean
62	out_clustering_max
63	AA_composition_L_mean
64	in_local_density_0.1_max
65	secondary_structure_distribution_P-0.0_mean
66	out_local_density_0.3_mean
67	AA_composition_A_mean
68	AA_composition_H_mean
69	polarizability_distribution_H-1.0_max
70	VanDerWaal_distribution_H-0.5_mean
71	in_in_topological_variance
72	AA_composition_G_mean
73	secondary_structure_distribution_P-0.75_mean
74	in_degree_correlation_mean
75	secondary_structure_distribution_N-1.0_max
76	AA_composition_T_mean
77	polarity_distribution_H-1.0_mean
78	out_local_density_0.9_max
79	secondary_structure_distribution_N-0.5_mean
80	in_local_density_0.9_max
81	in_local_density_0.4_mean

82	secondary_structure_distribution_H-1.0_mean
83	polarizability_distribution_N-0.75_mean
84	solvent_accessibility_distribution_H-0.0_max
85	solvent_accessibility_distribution_H-0.25_mean
86	AA_composition_D_mean
87	out_local_density_0.2_max
88	polarizability_distribution_H-1.0_mean
89	polarizability_distribution_P-1.0_mean
90	in_degree_max
91	weight_edge_mean(with_missing_edge)
92	hydrophobicity_distribution_P-0.0_mean
93	secondary_structure_transition_PN_mean
94	out_clustering_mean
95	polarizability_distribution_N-0.25_mean
96	secondary_structure_distribution_H-0.5_mean
97	AA_composition_Q_mean
98	solvent_accessibility_distribution_H-0.75_mean
99	AA_composition_P_mean
100	secondary_structure_distribution_P-0.25_mean
101	hydrophobicity_composition_H_max
102	polarity_distribution_H-0.25_mean
103	AA_composition_N_mean
104	hydrophobicity_distribution_N-0.0_mean
105	in_local_density_0.2_max
106	AA_composition_R_mean
107	out_local_density_0.4_mean
108	VanDerWaal_distribution_P-0.75_mean
109	out_degree_max
110	polarizability_distribution_N-1.0_mean
111	polarizability_distribution_H-0.25_mean
112	hydrophobicity_distribution_N-1.0_mean
113	VanDerWaal_transition_PH_mean
114	polarity_distribution_H-0.75_mean
115	in_clustering_mean
116	polarity_distribution_P-1.0_mean
117	secondary_structure_transition_NH_mean
118	secondary_structure_distribution_H-0.25_mean
119	AA_composition_Y_mean
120	polarizability_distribution_P-0.25_mean
121	in_degree_correlation_max
122	polarizability_composition_N_mean
123	VanDerWaal_distribution_H-0.75_mean
124	solvent_accessibility_distribution_H-0.0_mean
125	VanDerWaal_distribution_N-0.5_mean

126	polarizability_transition_PN_mean
127	secondary_structure_distribution_N-0.25_mean
128	AA_composition_E_mean
129	out_local_density_0.3_max
130	VanDerWaal_distribution_P-1.0_mean
131	hydrophobicity_distribution_P-1.0_mean
132	hydrophobicity_distribution_H-0.75_mean
133	secondary_structure_composition_P_mean
134	secondary_structure_distribution_N-0.75_mean
135	AA_composition_G_max
136	secondary_structure_distribution_H-0.75_mean
137	in_local_density_0.3_max
138	AA_composition_F_mean
139	VanDerWaal_distribution_N-1.0_mean
140	polarity_transition_PH_mean
141	hydrophobicity_distribution_H-0.25_mean
142	secondary_structure_distribution_N-0.0_mean
143	graph_size
144	weight_edge_mean(without_missing_edge)
145	polarizability_distribution_P-0.0_mean
146	secondary_structure_distribution_P-0.5_mean
147	secondary_structure_distribution_H-1.0_max
148	polarity_distribution_H-0.5_mean
149	polarizability_distribution_N-0.0_mean
150	polarizability_distribution_N-0.5_mean
151	topological_change_0.3_0.4
152	secondary_structure_composition_N_mean
153	polarizability_transition_NH_mean
154	solvent_accessibility_distribution_H-1.0_max
155	polarity_distribution_N-1.0_mean
156	solvent_accessibility_transition_HE_mean
157	polarizability_distribution_P-0.5_mean
158	hydrophobicity_distribution_H-1.0_mean
159	polarity_distribution_H-0.0_mean
160	secondary_structure_distribution_N-1.0_mean
161	hydrophobicity_distribution_N-0.75_mean
162	out_in_topological_mean
163	AA_composition_I_mean
164	VanDerWaal_distribution_H-1.0_mean
165	hydrophobicity_distribution_P-0.25_mean
166	AA_composition_M_mean
167	VanDerWaal_distribution_N-0.75_mean
168	hydrophobicity_transition_PN_mean
169	in_clustering_variance

170	polarity_distribution_N-0.25_mean
171	polarizability_composition_H_mean
172	VanDerWaal_composition_N_mean
173	hydrophobicity_transition_NH_max
174	AA_composition_Q_max
175	VanDerWaal_distribution_P-0.0_mean
176	out_clustering_variance
177	polarity_distribution_P-0.5_mean
178	VanDerWaal_distribution_N-0.0_mean
179	VanDerWaal_distribution_H-0.25_mean
180	hydrophobicity_distribution_P-0.75_mean
181	VanDerWaal_distribution_N-0.25_mean
182	polarizability_distribution_P-0.75_mean
183	secondary_structure_distribution_H-0.0_max
184	polarizability_distribution_H-0.75_mean
185	hydrophobicity_distribution_P-0.0_max
186	polarizability_composition_P_mean
187	in_out_topological_variance
188	secondary_structure_composition_H_max
189	polarity_transition_PN_mean
190	in_local_density_0.4_max
191	polarity_distribution_P-0.25_mean
192	polarizability_distribution_H-0.5_mean
193	secondary_structure_distribution_N-0.75_max
194	in_in_topological_mean
195	hydrophobicity_distribution_N-0.5_mean
196	polarity_distribution_N-0.0_mean
197	VanDerWaal_transition_NH_mean
198	out_local_density_0.4_max
199	polarity_distribution_P-0.75_mean
200	in_out_topological_mean
201	hydrophobicity_composition_P_mean
202	out_in_topological_variance
203	AA_composition_L_max
204	hydrophobicity_distribution_P-0.5_mean
205	hydrophobicity_composition_N_mean
206	polarity_distribution_N-0.75_mean
207	AA_composition_I_max
208	secondary_structure_composition_H_mean
209	VanDerWaal_distribution_P-0.25_mean
210	out_out_topological_mean
211	polarity_transition_PN_max
212	VanDerWaal_composition_H_mean
213	hydrophobicity_distribution_H-0.5_mean

214	out_local_density_0.2_mean
215	polarity_transition_NH_mean
216	secondary_structure_composition_N_max
217	secondary_structure_distribution_N-0.5_max
218	hydrophobicity_distribution_H-0.0_mean
219	AA_composition_H_max
220	VanDerWaal_distribution_P-0.5_mean
221	hydrophobicity_distribution_N-0.25_mean
222	AA_composition_R_max
223	first_singular_values
224	AA_composition_W_max
225	hydrophobicity_transition_NH_mean
226	polarity_transition_NH_max
227	secondary_structure_distribution_H-0.75_max
228	polarity_composition_N_mean
229	in_local_density_0.2_mean
230	VanDerWaal_distribution_N-0.0_max
231	solvent_accessibility_distribution_H-0.5_max
232	secondary_structure_transition_PH_max
233	polarity_composition_H_mean
234	polarity_distribution_N-0.5_mean
235	hydrophobicity_distribution_P-0.75_max
236	AA_composition_S_max
237	AA_composition_E_max
238	VanDerWaal_distribution_P-0.25_max
239	polarizability_distribution_N-0.75_max
240	AA_composition_C_max
241	AA_composition_Y_max
242	AA_composition_T_max
243	VanDerWaal_distribution_H-0.0_mean
244	in_degree_median
245	AA_composition_D_max
246	solvent_accessibility_distribution_H-0.75_max
247	VanDerWaal_composition_N_max
248	hydrophobicity_composition_H_mean
249	polarizability_composition_H_max
250	VanDerWaal_composition_P_mean
251	secondary_structure_transition_PN_max
252	VanDerWaal_distribution_H-0.25_max
253	AA_composition_K_max
254	VanDerWaal_distribution_P-0.0_max
255	AA_composition_F_max
256	polarizability_distribution_P-0.5_max
257	polarizability_distribution_N-0.25_max

258	AA_composition_N_max
259	out_degree_median
260	polarizability_distribution_N-0.0_max
261	secondary_structure_distribution_H-0.5_max
262	AA_composition_A_max
263	polarizability_distribution_P-0.75_max
264	polarity_composition_P_mean
265	polarizability_distribution_H-0.0_mean
266	polarizability_composition_N_max
267	polarity_distribution_N-0.25_max
268	VanDerWaal_transition_PH_max
269	VanDerWaal_distribution_H-0.5_max
270	AA_composition_V_max
271	polarizability_transition_NH_max
272	polarity_distribution_H-0.75_max
273	polarity_distribution_P-0.75_max
274	secondary_structure_distribution_P-0.25_max
275	VanDerWaal_distribution_N-0.5_max
276	hydrophobicity_distribution_N-0.5_max
277	out_degree_mean
278	polarizability_transition_PH_max
279	solvent_accessibility_distribution_H-0.25_max
280	solvent_accessibility_transition_HE_max
281	VanDerWaal_distribution_P-0.75_max
282	AA_composition_M_max
283	polarity_distribution_P-0.0_mean
284	hydrophobicity_distribution_P-0.5_max
285	VanDerWaal_transition_NH_max
286	out_local_density_0.1_mean
287	VanDerWaal_transition_PN_max
288	hydrophobicity_distribution_H-0.5_max
289	secondary_structure_transition_NH_max
290	polarizability_distribution_N-0.5_max
291	hydrophobicity_composition_P_max
292	VanDerWaal_distribution_H-0.75_max
293	hydrophobicity_transition_PN_max
294	polarizability_distribution_P-0.0_max
295	VanDerWaal_distribution_N-0.25_max
296	polarity_transition_PH_max
297	VanDerWaal_distribution_P-0.5_max
298	in_local_density_0.1_mean
299	hydrophobicity_distribution_N-0.25_max
300	secondary_structure_distribution_N-0.25_max
301	secondary_structure_composition_P_max

302	hydrophobicity_distribution_H-0.25_max
303	polarity_distribution_H-0.0_max
304	secondary_structure_distribution_P-0.5_max
305	secondary_structure_distribution_H-0.25_max
306	in_degree_mean
307	VanDerWaal_composition_H_max
308	topological_change_0.4_0.5
309	out_out_topological_max
310	polarizability_distribution_H-0.25_max
311	polarizability_distribution_P-0.25_max
312	polarizability_composition_P_max
313	secondary_structure_distribution_P-0.75_max
314	polarity_distribution_H-0.5_max
315	polarity_distribution_N-0.75_max
316	hydrophobicity_distribution_N-0.0_max
317	VanDerWaal_distribution_N-0.75_max
318	in_in_topological_max
319	hydrophobicity_distribution_P-0.25_max
320	out_local_density_0_mean
321	polarizability_distribution_H-0.5_max
322	polarity_composition_H_max
323	VanDerWaal_composition_P_max
324	polarity_distribution_N-0.5_max
325	polarity_distribution_P-0.25_max
326	polarity_distribution_N-0.0_max
327	hydrophobicity_transition_PH_max
328	hydrophobicity_distribution_N-0.75_max
329	secondary_structure_distribution_N-0.0_max
330	polarizability_distribution_H-0.75_max
331	in_local_density_0_mean
332	AA_composition_P_max
333	polarity_distribution_P-0.5_max
334	topological_change_0.5_0.6
335	polarity_distribution_P-0.0_max
336	hydrophobicity_distribution_H-0.75_max
337	polarity_distribution_H-0.25_max
338	weight_edge_variance(with_missing_edge)
339	hydrophobicity_composition_N_max
340	polarizability_transition_PN_max
341	in_out_topological_max
342	out_local_density_0.5_max
343	weight_edge_variance(without_missing_edge)
344	hydrophobicity_distribution_H-0.0_max
345	in_local_density_0.5_max

346	polarity_composition_N_max
347	polarizability_distribution_H-0.0_max
348	VanDerWaal_distribution_H-0.0_max
349	out_in_topological_max
350	second_singular_values
351	graph_density
352	third_singular_values
