# Supplementary material for: Analysis of Protein Pathway Networks Using Hybrid Properties
Source: Molecules. 2010 Nov 12;15(11):8177–92. doi: 10.3390/molecules15118177 (PMC6259184; doi:10.3390/molecules15118177)
Supplement: Supplementary File 2 [file molecules-15-08177-s002.pdf]

Online Supporting Information S2: Proteins in each negative pathway

Index	Proteins
Negative_1	ADO,ACTA1,DEGS2,EPHA3,EPHB4,EPHX2,EPOR,EREG,FTH1,GAD1,HTR6,IGF1R,KIR2DL4,NCR3,NME7,NOTCH1,OR10S1,OR2T33,OR56B4,OR7A10,OR8G1,PDGFC,PLCZ1,PROC,PRPS2,PTAFR,SGPP2,STMN1,VDAC3,ATP6V0A1,MAPKAPK2
Negative_2	DCC,IDS,VTN,ACTN2,AKR1B10,CACNA1A,CHIA,DAAM2,FUT5,GCLM,GNAZ,ITPA,NEU4,NTF3,OR10A3,PAPSS1,PARD3,PLOD1,RGS3,SCLY,SHC1,TNFRSF4,TP53
Negative_3	DAO,CACNA1D,HMGCS2,LAMB4,OR56A3,PRKCQ,SLC25A5
Negative_4	IL5,LHB,PGD,ADCY3,ALDH1A3,ATP13A2,BUB3,CD244,CYFIP2,EPHX2,FCER1G,FGD1,FGF4,FZD9,HSD17B7,IL6R,ITGAV,LEFTY1,LIPG,MAN1C1,MPDZ,PGM1,PGM3,PIGM,PLD1,PPP3CC,TBXAS1,TKTL2,TPH2,YWHAQ,PPP1R12A
Negative_5	HK2,MOS,TKT,TNN,B3GALT4,B3GAT3,CASP7,CDH1,CYFIP1,EFNA5,EXTL1,FCGR3B,FGF20,GSTA5,GUK1,HSD3B7,ITGB4,MCM6,MYH3,NOD1,OR10H1,OR1C1,OR1E1,OR4C11,OR56A3,PPA1,PRKAA1,PRKAB2,RDH5,SLC27A1,SLC2A4,SMPD2,STK36,THBS1,SERPINC1
Negative_6	TNR,ATP5A1,CNGB1,CX3CL1,DEGS1,DNMT3B,EFNB2,FMO2,GUCY1B3,JAG2,LARS2,NUMB,PCCB,PGAM1,PLA2G1B,PLOD2,PRDX6,PRPS1,RFXANK
Negative_7	FER,MVD,PAH,ACTC1,ADCY4,ADCY8,CBR3,CLDN16,CPT1A,DDOST,DDX56,DKK1,EFNB1,EPHA8,FCGR3A,GLS2,GSTM1,GZMB,HADHA,IL13RA2,KIR2DS4,KLRK1,LAMB4,LGMN,MAGI1,NUDT2,OR13A1,OR1I1,OR4D11,OR4X2,OR6K2,OR8B4,OXCT1,PIK3R4,PPM1A,PRKAG3,SELP,SPHK2,SUCLG1,TAS1R2,TAS1R3,THY1,TUBA1C,ZIC2,AASDHPPT,SERPIND1
Negative_8	MTR,ACAT2,ADCY2,ATP5D,BMPR1A,CACNA1E,CD38,CYP2A7,DDIT4,EXTL1,FCER1G,FGD3,FZD5,ITGAM,MAPK8,NR4A1,OR10V1,OR4F17,OR52D1,OR8J3,PLD1,PPA1,PSEN2,SKP1,TACR3,VNN1,CTNNBIP1
Negative_9	APAF1,APOA1,CARD11,CCDC6,CSF3R,CYP4F2,DAPK1,FLOT1,GSTM1,IL22,KLRK1,LARS,NGEF,OR7A17,PFN3,PIGL,POLR2E,PPCS,RAF1,SEMA3C,SULT1A4,TAS1R1,TCF7L1,TRMT11,XIAP,TNFRSF6B
Negative_10	LPL,ADORA2A,ATP6V1H,B3GALT1,CLTCL1,CNGA4,DTX3L,IL23R,IRF5,NEU2,OR4M1,RDH12,SEMA4F,STAT5A,THBS4
Negative_11	AGL,CBS,FUK,ACSS1,ALAS2,ASAH1,ATP2A2,CD14,CD44,CLDN17,CYP1A1,DDX23,DNMT1,EFNA4,ENPP7,EPOR,EXTL1,FLT1,GFPT1,GRIA1,HAO1,HTR2A,IFNA4,LEFTY2,MTHFD2,NCR2,OR10A2,OR1L6,OR2L3,OR51M1,OR5B12,OR5M3,OR6C1,ORC3L,PLA2G2E,PLCB2,PTCH1,TAS1R2,ULK2,ATP6V0A2,CACNA2D3
Negative_12	BID,BPNT1,CACNA1D,CXCL16,ENPP7,EPHA1,FUT4,GMPS,GRIN2C,IBSP,IL22,IL2RA,LLGL2,MAPK13,MTFMT,MTHFR,NOS3,OR56A1,PAK2,PIGU,PLCZ1,POLR3B,PTPN6,RAPGEF4,TUBA8,TUBB1,TNFRSF12A
Negative_13	EGF,LTB,ACAT2,ADCY10,CCL26,CCNB3,CLDN1,DGAT2L4,DVL1,FBP1,IFNK,ITGAE,LAMA4,MCCC2,NEU3,NKD1,NLGN1,NT5C2,OR10H4,OR1L4,OR2A42,OR2AE1,OR2Z1,OR4K1,OR51F2,OR5AK2,OR5V1,OR9G4,PDE1C,PIAS2,PPAP2C,PPP1R1A,RBL1,ROBO1,SEMA4C,THBD,TUBA1B,B4GALNT1,HSD17B10,TNFRSF18

Negative_14	MOS, OSM, WAS, AASDH, B3GALT2, B4GALT1, CDKN2A, CLDN19, DDX50, ENTPD3, F2RL1, GALNTL4, GNA13, HIF1A, IL12RB2, NAE1, NKD1, OR1J1, OR2M4, OR9I1, PLOD1, PLXNB1, SDHC, SH3BP2, STX6, TOLLIP, TUBB2B
Negative_15	ADK, CFH, DAO, F9, MUT, ADCY5, ADH1A, ALDH1L1, CARS2, CD14, CD79A, CTBS, CYP2A7, DGKH, DUSP10, DUSP16, DUSP5, DVL2, FGF21, GALM, GNAI1, GSTA2, HADHA, IDH1, IFNK, LDHD, LFNG, LTA4H, MASP1, OR10G8, OR51G2, PAX4, PIM1, RND1, TKTL2, TLR9, TNFRSF17
Negative_16	FUK, GH1, SPN, ACSM4, CACNB1, CACNB2, CCDC6, CCR4, CDK2, CKS1B, EHHADH, FADS2, GALNT12, GRIA1, HCLS1, HIF1A, HMGCS1, HMGCS2, IL3RA, ITPA, KCNB1, MAP2K6, MAPK8, MTHFR, MYH2, NTF4, OR10A5, OR1C1, OR1J2, OR2T10, OR52L1, OR5AK2, PDE8A, PIGM, PIK3CA, PIP5K3, PLCG1, POLE, SHC1, SKIV2L2, SV2B, TJP1, TKTL1, TUBA3C, UROC1, HLA-DPA1
Negative_17	CALML6, CDKN1B, EFNA3, OR2T4
Negative_18	ME3, ACOX3, ADORA2B, AKR1C2, ALDH9A1, ATP5F1, B3GAT3, BAIAP2, BMP8A, CXCL3, CYP1B1, DHRS4L2, HADHB, IDH1, IRS1, KLRC4, MAN1A1, MAP3K4, NKX6-1, OR1Q1, OR2B6, OR2H1, OR7G3, PDE1A, PECAM1, PGAM4, PIGX, PPP2CB, PPT1, RHEB, SLC2A4, SV2C, SYNJ1, TAP1, UGCGL1, WNT7B
Negative_19	CAT, CDA, LEP, PTS, B3GNT4, BLVRB, CLDN20, COASY, COL4A2, CTNNA2, EFNA1, FGF13, GALE, GBA3, GCNT3, GSTM4, HSD17B3, IL12RB1, IL2RB, IPPK, LRDD, MYH9, NOX1, NTF3, OR14C36, OR2T2, OR4K13, OR4N4, OR56A1, PDE3B, PIP4K2A, PTGFR, QARS, SPHK2, STK3, TP73, TUBA8, TUBB2A
Negative_20	AR, C8B, CTH, IL9, ALDH1A1, ALOX5, BCL10, CACNB4, CDC25C, FOXO1, GALT, GRIN2C, GSTT1, HAP1, IDI2, IL20, NRXN1, OR10C1, OR3A1, OR4K5, OR51Q1, OR7G2, OR8D1, ORC4L, PER3, PIAS4, SPRY4, UGCGL1, YWHAZ, ATP6V1B2
Negative_21	FMO3, GSK3B, HLA-F, NPAS2, NTSR1, OR1I1, OR1L1, OR1Q1, OR52M1, P2RX4, POLD4, POLE4, STX5, TNFRSF12A
Negative_22	PC, AASDH, ACP6, ALDH7A1, ASRGL1, CCR3, CDC16, CDH2, CNGB1, COL4A6, DRD1, EFNB2, EIF4E2, FARS2, FBP1, GLI3, GNAI2, GRIN2C, GUCY1B3, HAO1, HNF1B, OR6C6, OR7G1, PIK3R5, PPP3CC, PRNP, RASSF5, SLC9A1, ST3GAL5, TGFBR1, WNT9A, XCL1, YARS
Negative_23	ADA, C8G, IL8, AGPAT2, AMD1, CCKAR, COMP, DAPK2, EPHA2, GYS1, OR10J5, OR10K2, OR1L6, OR51T1, OR6B3, PARD6A, PDE2A, PPARA, RAPGEF2, RHOQ, SETDB1, SMPD4
Negative_24	AGK, FH, GPT, IL4, ARRB2, ATP6AP1, B3GALT6, CASP10, CCL24, CFLAR, CYP2A6, EPHA6, FGF1, FLT4, GALC, HBEGF, HSPA8, ITGAX, KNG1, MAP3K14, MAPK1, MGST2, MTFMT, NT5C1A, OR2A25, OR2L8, OR51G2, PKMYT1, POLD4, POLR3D, PPP1R3C, PRNP, PTGS1, RAC3, RASGRP2, RPIA, RPS6KB2, SLC33A1, ST3GAL3, STAT5B, TAS1R3, TLN2, ULK1
Negative_25	CKM, PDC, ALG5, ALPL, ANAPC4, ARSD, ATP5O, BDH2, BMP5, CAV2, CES1, CXCL11, DHRS4, GALE, GAMT, HSD11B2, IFNA16, IMPDH1, IRAK3, MAP2K1, MAPK1, NADSYN1, OR13F1, OR3A1, OR52A5, OR5AK2, OR5P2, PDGFD, PIGT, PIPOX, PLAT, PNPLA4, PPM1B, PPP1R3C, RND1, RRM1, SEMA4A, TPH2, USE1, WNT5A, CACNA2D2, GABARAPL2, PRICKLE2
Negative_26	ADIPOR1, ALOX12, ALOX5, ATP5J, BCL2, CALML5, CD36, CFLAR, CLDN14, CREB3L1, CXCL9, CYP19A1, DPYD, ECSIT, EGFR, EPHA6, GALNT6, GBGT1, GCLM, HCLS1, KIR2DS4, KLKB1, LDHB, LHCGR, LIPC, LMO7, MAP2K3, MPST, NCF1, NRXN2, NT5C2, OGDHL, OR1B1, OR2T34, OR2W1, OR6A2, OR8B4, PIK3CG, PKMYT1, RDH8, SLC9A1, SMAD2, SMAD7, SPCS1, TAS2R42, TUBB2A, TUBB3, UCKL1, HLA-DQB1

Negative_27	IHH,ACAT2,BTRC,CDC6,CKS1B,CNTF,CREBBP,DRD1,GAPDH,GBA3,GIT1,GSTM1,ITGA11,JAK2,MOV10L1,NCOA4,ONECUT1,OR10C1,OR10S1,OR2T2,OR4K2,OR4M1,OR6C2,OR8G1,P2RX7,PFKFB3,PGAP1,PLXNC1,PPARA,PRKAA2,PRNP,PTPN11,SETD1A,SMARCA5,STK36,TGFB3,VAV3,XCL1,PDCD1LG2,PHOSPHO1
Negative_28	DCK,ID3,IVD,AKT3,APAF1,ARPC1A,B3GALT5,BIRC2,CACNB3,CREB3,CRKL,CX3CL1,CYP3A7,DTX4,FRAT2,GCLM,GMPS,GNPAT,GSTO1,GZMB,HNF1A,MAPK12,NAGS,NEUROG3,NUDT2,OR10G2,OR1A1,OR4N5,OR51A4,OR51G2,OR9Q1,OXCT2,PI4KB,PTGS2,RFXAP,RPS6KB1,RUNX1,SHC1,SLC9A1,SPHK1,SRD5A1,TNFSF18,UGT2A1,WNT16,WNT3A
Negative_29	AARS2,ACSM1,C3AR1,OR5D13,SGMS2,TNFRSF14
Negative_30	C3AR1,PIP5K1A,UGCGL2
Negative_31	C4B,ACACA,ADCY2,ADIPOQ,CCL11,CD34,DDX54,DTX3,DTX4,ELK1,HAGHL,HEMK1,INPP5B,ITGB1,JAM2,LIAS,MAPK13,MAT2B,MPDZ,MTFMT,OR5AR1,OR6C75,OR6K2,OR7G1,OXTR,PDE2A,PHGDH,PIGO,PYCR2,RALA,RRM1,SETDB2,SH2D1B,SKP1,STAT2
Negative_32	CCL20,FUT6,ITGAE,OR2B6,OR5T3,PEMT,PPA1,PPP1CB,GABARAPL2
Negative_33	ME3,TNC,BACE2,C1QA,CASP8,CCNB1,CLDN7,COQ7,CRKL,CYP2A7,CYP2C9,CYP3A5,CYSLTR2,DAD1,DVL3,GALK1,GNPNAT1,HLA-DRA,IFNA10,INHBE,MAPT,MTHFD1,MYH6,OR1B1,OR2T2,OR4C16,OR5D16,PDE4B,PDGFA,PIK3R5,PRKAA2,RALA,RAPGEF1,SESN2,SLIT1,STX2,TGFB3,TJP2,UGT2B10,WHSC1,WNT7A
Negative_34	F11,FAH,FUK,ACAA2,ACTB,ACVR1C,ADCY6,AGRP,ATP5E,BLNK,CCNB1,CNTNAP1,DAPK3,DHRS4,ENTPD4,EPHA6,GNPAT,GZMA,HLA-DRA,HNF4G,ITGB3,MMP9,NCOR1,OR11H4,OR2V2,PANK2,PIGK,POLE3,POLR3D,PPP2R1B,PPP2R5E,PTK2B,RAP1A,TACR1,TRADD,UGP2,UNC5D,WARS,EIF4EBP1,KIR2DL5A
Negative_35	F11,JUN,SFN,SMO,CACNA1F,CREBBP,GAD2,OR13H1,OR8A1,PARS2,PIP5K1C,POLE4,POLR2L,POLR3B,SESN1,UGP2
Negative_36	C8G,F10,FOS,ACTN2,ARG1,C4BPA,CACNB2,CCNG2,CD226,CD3E,CD99,CHD8,CPT1C,CSF1R,CSF2,CYP21A2,DERL1,DGKB,DUSP3,EI24,FGF9,IDI2,IFNA16,IL10RA,ITGA8,KLRD1,MAD2L1,MTHFD1L,MYH11,NRXN3,NT5M,OR1M1,OR52H1,PAK2,RASA1,RDH10,SETDB1,STX5,TICAM1,TOMM40L,UGCGL1,UNC5B,YES1,ZMAT3
Negative_37	MVD,CX3CR1,METTL6,OR4D5,RBKS,RPRM,RRAS2,SLC25A5
Negative_38	LAT,ADCY3,ARPC5L,ATG12,CD46,CDKN2D,COL1A1,DEGS1,DUSP5,HRAS,IKBKE,LDHD,MAP4K2,NFYC,PLXNB3,RBL1,SPRED2,SSH3,TJP2,VAMP1
Negative_39	CS,LTB,AARS,ABLIM2,ACOX3,AGPAT1,AGRN,ALDH1A3,ALG6,AMPD3,BBOX1,CAV2,CREBBP,CYP11B2,CYP2F1,EDAR,GNAI2,HPRT1,HSD17B4,IKBKE,LDHAL6B,MYH8,MYL5,NCKAP1,NT5C1A,OR2T11,OR51B4,OR52W1,OR6X1,OR8H3,PCYT1B,PDE3B,PIGV,PLOD3,POLR2K,POLR3G,PPP1R1A,PPP2R2D,PRKACB,RBPJL,RPS6KB1,STK11,SUCLA2,TJP1,TUBB1,TUBB4,UGDH,VCAM1,SERPINC1
Negative_40	ADC,ACSL5,ACTN2,ATP5E,CHST11,EXOC7,FOXO1,FZD4,FZD7,HADHB,HIF1A,ITGAE,MARS,OR14I1,OR4K17,PDE8A,PLCE1,PMM2,SNW1,SYNJ1,WNT4,CTNNBIP1
Negative_41	CFI,CGN,FOS,MVD,RFK,ACPP,ADRA1A,ARHGEF6,CCKAR,CCR8,CD72,DUSP1,EPHA7,EXT1,FGF2,G6PD,GSTA4,HPSE,HSD11B2,HSD3B2,IFNA10,IL10,IPMK,LLGL1,MARS2,MDH2,MYH14,NCOA4,NFATC2,NRG2,OR10AG1,OR52E2,OR5L2,OR8J3,PDHA2,PPAP2A,PTPRB,RALGDS,RFX5,RPS6KA3,SDC4,TJP3,TRAF3,UNC5D,TNFRSF10B

Negative_42	C3,F12,AARS,ADCY4,ADH5,ADORA2A,ADRA1D,ALDOA,ALG3,CNGB1,CNTFR,CXCL5,CXCR4,DGKA,DGUOK,EFNB3,F2RL2,FASLG,GDF6,GMPR,GMPS,GNAQ,IFNA14,IL23R,ITGAE,MAP3K14,MMP9,NAGLU,NEFH,OCRL,OR10G4,OR6S1,PCCB,PRKAA2,PSEN2,RFX5,SC4MOL,UGCG,SUV420H1
Negative_43	AK1,BAD,AADAC,ACLY,CLDN11,DDB2,GPT2,MYH10,PFKFB4,SPTLC2,TLR9
Negative_44	ABO,LYN,SFN,SPN,ACTG2,ADH5,ALOX15B,BACE1,CDC2,CNTF,DDX23,IL19,INPP5E,KIR2DL2,KIR3DL2,MAP3K3,NEU4,NKX2-2,NTRK1,OR2Y1,OR8B4,PDE9A,RALGDS,RASGRP1,ROBO1,ST8SIA5,TAS2R3,TLR2,TNFRSF4,TUBB4,UCP1,VEGFB
Negative_45	AK1,GSR,NLK,SYK,UBC,ACSM1,ALDH5A1,ALPPL2,B3GALT1,B4GALT1,C1QB,CCNG1,CCR6,CD28,CD46,CDC25A,CTNNA1,DAPK2,DDX55,DUSP10,DUSP3,EPHA1,ETNK2,FABP4,FLOT2,HTR5A,IFNA7,IL1B,MAFA,MDM2,NRXN2,OR4D1,PHKA1,PLA2G6,PON3,RDH5,RFWD2,SIPA1,SMAD7,SPRY2,SRGAP3,TLR9,TSTA3,PAFAH1B3
Negative_46	APOA1,CD19,OR10P1,RAC1
Negative_47	PGD,TXK,ACSM4,ARHGEF2,ARPC5L,CACNA1A,CAMKK2,COL4A2,CSAD,E2F3,EFNA1,GNAQ,GNPDA2,HLA-DRA,ITGB1,OR1C1,OR1E2,PIK3R5,TUBB2C,ATP6V1G1,CACNA2D1
Negative_48	LTB,ACLY,B3GALT4,CREB3L2,CYP2F1,DHRS3,EPB41,GUCY2F,HSPA1B,IL12B,LIPC,MTMR6,NFYB,OR1B1,OR1D5,OR4A16,PIGW,SARDH,UGCGL2,YKT6,B3GALNT1
Negative_49	AK3,MAX,ADCY8,BCL2L1,BST1,CXCR5,CYP2J2,IL1R2,INPP5E,LAMB2,MBL2,MYH15,MYL9,NARS2,OR13C3,RASGRF2,RASGRP1,SHC3,SPRY3,GABARAPL2,SERPINF2
Negative_50	ID2,ACMSD,CAMKK1,CCL28,CDC45L,CYCS,DAAM2,DCTN1,DOT1L,DUSP2,ENPP3,GDF6,IL12A,LAMB4,NAMPT,NEUROD1,OR4D5,PLA2G2D,PLXNA3,POLR2D,PPAP2B,PRKAB2,PRKACA,PRKAR1B,PTPRJ,RDH8,SETD1B,TNFSF14,TUBB2B,VASP
Negative_51	GLS,ALDH4A1,ALOX15,ATP2A3,CPT2,CYBA,ELK4,FABP4,HADHB,IL8RB,IMPA1,ITGA6,NLGN3,NT5M,OR2L3,OR52R1,OR6B2,P2RX6,PGK2,PRKACB,PRODH2,YARS2,TNFRSF6B
Negative_52	CD4,CGN,FAS,PRL,ALDH4A1,ANAPC1,APOA1,ASS1,C5AR1,CPT1C,CXCL2,DKK4,EFNA5,FZD10,NAGA,OR52E2,OR52W1,OR5M8,OR7D4,PDE2A,PON1,PYCR1,RELA,RPSA,TAS2R16,TRMT11,UPRT,VAV3,VEGFB,ZFYVE16,ATP6V1G3
Negative_53	IL9,ADCY1,AKR1C4,ATP2A2,BAAT,CLDN9,FMO1,GNAZ,IL10,IL2RG,IMPDH2,NME7,OR1L6,OR1N1,OR4F15,OR6C2,PLXNA3,PPOX,RDH10,ROBO3,SEPHS1,ST8SIA1,TUBB1,UGP2,HSD17B12,PLA2G12B
Negative_54	EZR,CSNK1G3,GLUL,PIGL,SEMA3D,STX18
Negative_55	ADH4,CSNK2B,DTX1,KNG1,PIK3C2G,TYMP
Negative_56	F5,SMO,CCDC6,CSF2,DFFA,HAGH,IL15,LRRC4C,MAOB,NCF4,OR10AD1,OR2K2,OR2Z1,PHPT1,PPARG,RAB23,RAP1B,SC5DL,ATP6V1B1,SERPINC1,SERPINE1
Negative_57	FES,MPZ,VCL,ABCC8,ALOX15,ASCC3,ATP5I,BMPR2,CPS1,CYP27A1,DHFR,DTX1,EFNA5,GALNT1,GCLC,GFAP,GNA12,GSTA3,HLA-F,HNF1A,IFNK,IRS4,NFE2L2,NUDT5,OR2M5,OR5B12,OR5D13,OR5R1,OR8B4,PIGT,SOCS3,TAP2,TNFRSF18
Negative_58	HPD,AK3L1,ANAPC4,ATP2A1,DNMT3L,DVL2,ENO3,GALNS,GLB1,GLI1,IFNW1,IL6ST,LARS2,MYL2,OR5B21,RRM1,SDHB,TKTL2,TUBA1C,TUBB3
Negative_59	OGT,ARRB2,ATF4,CACNG4,DGKB,ENPP7,FZD1,IL11,LCMT2,MAD1L1,OR13G1,OR2L13,OR2M3,PFKFB1,RPS6KA5,SPP1,TRAF6,YWHAQ

Negative_60	IL9,ALPP,APLP1,ARRB2,ATIC,CSNK2B,CYBA,CYP2E1,ENTPD4,HSD17B8,ITGA2B,MAP3K4,MGST1,OR2AG1,PAK2,POLR2I,POLR2J,POLR3K,PTPRB,SIGLEC1,SYNJ2,TACR2,TBL1XR1,TDO2,TUBA8,UROC1,VAMP1,ST6GALNAC4
Negative_61	GLO1,HLCS,ORC2L,PAK1,PPP1CA,PYCR1,STX8
Negative_62	CD2,HK2,MPZ,ACP5,ACTN2,AGPAT2,AKT3,ALDH9A1,ATP5G1,B3GNT4,CD3E,CDK5,CLDN11,COQ3,CREB3,F11R,FABP1,GMPPA,GNPNAT1,HSD17B1,IQGAP3,ITGAD,LAMA4,LAMB1,LPAR1,MAD1L1,MAP3K4,MCM3,MCM5,MGAT1,MMP7,OR13J1,OR2K2,OR52K2,OR52R1,OR6K6,OR9A2,P2RX2,PDPK1,POLE4,PTGDS,RRM1,SCLY,SETD7,SMC1A,SULT1A4,YOD1,TNFRSF10B
Negative_63	AK1,DCN,MAG,ATP2A1,B4GALT3,CALML6,CCNB2,CD226,CDKN1B,CLDN22,CSF2RA,CYP2A13,DAD1,DGKB,DGUOK,GANC,GNAI3,GNAQ,GNMT,GSTM2,H6PD,HNF1B,IKBKG,IL2RB,LFNG,MECR,MTMR1,OR7A10,SH3BP2,WNT8A,PLA2G12A
Negative_64	BAD,ADCY10,ARPC4,ARRB1,CUL1,CYP11B2,CYP2D6,DDOST,E2F4,IDH3G,IFNA7,LAMA4,LIMK1,NFASC,NT5C2,OR10J3,OR2A14,OR51B5,OR52J3,OR5AR1,PGM2,ROCK1,TP73,WNT9A,AASDHPPT,ATP6V0E1,TNFRSF12A
Negative_65	ATP5J,FGF2,KIR2DL2,PDGFD,TRAF2
Negative_66	EBP,AACS,CAV3,CCND1,DNMT1,HSD17B4,IL28A,KIR2DL4,NNMT,OR6S1,PDE7A,PFKFB3,PHGDH,PLIN,PTDSS1,RDH16,SGMS1,SPCS3
Negative_67	ATM,GDA,JUP,ADAM17,APPL1,BDH2,COMP,CYP2S1,DGKG,DVL3,EFNB1,HEMK1,IQGAP1,LAMB3,NMNAT2,OCRL,OGDHL,OR1B1,OR6B2,OR9Q1,ORC1L,PDE6G,PDE8A,PRKACA,RIPK1,THY1
Negative_68	EGF,IL5,PTS,TXK,VTN,ABL2,ACADVL,ALG1,BIRC2,CHRM5,CNDP1,CRHR1,DDIT4,DDX18,DGKH,EIF4B,ENO3,FGF1,GRIN1,GRIN2C,HCCS,HLA-DOB,HSD17B8,HTR2A,KCNJ11,LIAS,MMP14,NFKBIB,NGEF,NLGN1,OR3A1,ORC1L,PDE10A,PIGP,PRKCB,PTGIS,RPS6KA5,SDHB,SEMA4C,SMAD9,SPRED2,SQLE,SRD5A1,SSH1,SUCLG2,TGFA,HLA-DPA1
Negative_69	ADC,EGF,ADCY3,ADCY8,AGPAT2,ALPP,AMY2B,APBB1,ARNT2,CACNG8,CCL3,CD44,CD82,CLDN11,CNTNAP2,DAXX,DERA,DGKB,DLL3,DOLPP1,DUSP16,ENAH,GAD2,GFPT2,HLA-DOB,HSD17B2,IL18R1,ITGAE,NCF4,NCKAP1,OR10H2,OR1L8,PDE4A,PIP4K2C,POLR2H,RALB,SOD1,SORBS1,TLR3,USE1,WNT6,XCR1,ZNRD1
Negative_70	CFH,NGF,PLG,TH,ABAT,ACP6,ACTG2,AQP7,BCL10,C1QA,CASP7,CCL11,CD244,DFFB,DHX58,DPYSL2,EIF4E,EPHA3,FARSB,FOXO3,GART,GCDH,GCNT4,GLG1,GPAM,GUCA1A,HLCS,KCNQ1,MAP2K2,MAPK12,MTMR2,NCK2,NFYB,NME6,NOX1,OR4S2,PIK3R5,PIM2,PKMYT1,PSPH,SKP1,SPI1,TAS2R48,TPH1,VAV1,CACNA2D4,PPP1R12A,TNFRSF18,TNFSF13B
Negative_71	AUH,OGT,PTS,SRF,ALDH3A2,ARAF,FGF9,GARS,INPP4B,IRAK3,LAMB1,NADSYN1,ORC5L,OSMR,PGAM1,PRKAR1B,ROCK2,TIRAP,TRIP10,SUV420H1
Negative_72	ADK,DBT,IL2,XDH,ACTG1,ARNT2,ATP5L,BET1L,CYP2A13,CYP2D6,EFNA1,EPHA5,IL3RA,IL7R,NAT6,NODAL,OR52H1,OR5D14,PARD6A,PHKG1,POLE,POLR2E,SEMA4G
Negative_73	LPL,BACE2,BIRC5,CCL20,COMP,GJD2,GRIN2D,IL28A,IL8RB,LCP2,LPAR1,NANP,NT5C3,PPP2R5A,TRADD
Negative_74	C8G,FAS,HK2,ACSS1,ALAS1,BMP6,CACNG7,CDS1,CLDN8,CSF2RB,CTNNA2,CX3CR1,GALNTL2,GPX7,GRIN2B,IL1R1,ITGB4,ITPKA,KDSR,MAN2A1,MEF2C,NFATC4,NLGN1,NRG4,OR10A2,OR9G4,PFKP,PLOD3,PTK2B,RDH16,SEMA4B,THBS4
Negative_75	ME1,CAV2,CCR8,FOXA3,IFNA17,IL2RG,LGMN,OR2T4,OR51M1,PAICS,PLXNB3,SC4MOL,SEMA4C,SESN3,TAS1R2,ULK3
Negative_76	ADCY6,ADCY9,AHCYL1,AKR1D1,AMY2A,BIRC3,C4BPA,CDH1,CPOX,CYP1A2,DRD1,DUSP14,FGF18,FTCD,GRIN2A,KCNJ11,KIR3DL1,MLYCD,MTMR6,OR13A1,PIP5K3,PRKAB1,SEMA3B,SOCS1

Negative_77	UBB,ACSL4,CSF1,CYP2A13,EPHB1,FGF10,HCST,IKBKB,IL28A,ITGB1,KLRC4,LAMB4,MAP3K6,OR5A2,OR5F1,OR8J3,PDX1,PGLS,PLXNA2,SLC25A4,TJP1,TNFRSF1A
Negative_78	DDC,F2,GSN,AANAT,ACSL4,ACSM3,AGPAT3,APOA5,ASPA,CFL1,CHRD,CSDA,CYCS,DDX47,FGF1,FOXO1,GALT,HAO2,HEXB,HLA-G,IFNGR2,IL2RA,IMPDH2,LCP2,LDHAL6A,NR5A2,OR4X1,OR5M8,OR6C75,OR9G1,PDE4B,PDE6G,PECAM1,PEMT,PIGH,PLAUR,POLR2I,PPCDC,PRKAR1A,RDH12,SORD,STX18,SUFU,TAS2R9,TRAF2,WNT1
Negative_79	C9,CKB,DCC,GP5,MET,OGT,ACVR2B,ADCY5,ADH7,ADRA1A,ALPL,ANAPC4,ATP5L,BACE2,BIRC3,BST1,CDH15,CDK7,CHPF,COL11A1,DGKZ,FRAP1,GALNT3,IL13RA1,IL7R,KIR3DL3,LLGL1,MCM2,MTMR6,NAMPT,OR2A2,OR4A15,OR4K15,OR4K2,OR51B2,OR52H1,PARVB,PCNA,PDGFB,PDHA2,PIGV,PTPRC,RPS6KA2,SEMA3G,SEPHS1,TGFB3,TJP2,WNT3A,ATP6V1G2
Negative_80	ATP2A1,ATP6AP1,BBC3,BCL10,CCL23,CLDN15,CNTNAP1,ERBB4,EXT1,FZD6,NEFH,PFKFB1,PTGER3,PYGB,RXRA
Negative_81	MAT2B,MCAT,SULT1A1,TAS2R7
Negative_82	CAT,HYI,OTC,PTS,ADCY6,AMPD1,ARPC3,CARS2,CXCL2,CXCR5,DGKI,GNPDA1,IQGAP1,SELP,SRGAP3,UCKL1,UPP2,WWP1,SERPINE1,TNFRSF10A
Negative_83	SRM,ACVR2A,ATP5D,B3GALT1,C1QC,C3AR1,CACNG3,CD27,CLTA,CNTF,CNTNAP2,CTSB,DEGS1,DHRS9,GLDC,IL15RA,IL28A,MGAT5,MYH2,OR10J5,OR13G1,OR2A2,OR2C3,OR4K17,OR51S1,OR6C2,OR8H3,OXCT2,PCYT1A,PERP,PLA2G5,PLCD1,PRPS2,RRM1,SEMA3G,SHMT1,STX6,CACNA2D1,MAPKAPK3
Negative_84	ABLIM1,ARPC5,ATP5A1,ATP5E,CNTNAP1,CYCS,ERBB2,HNF4G,ITGA3,MAP4K1,MCCC1,OR10H1,OR2G6,POLR2K,PTAFR,RALA,TSC2,XCL2,PNLIPRP2
Negative_85	GP9,AARS2,ACTN1,ASS1,ATP5F1,BMP5,C4BPB,CCNB1,CD22,CDC42,CES2,COASY,COL6A2,CXCL9,CYP27A1,CYP2C9,EFNA1,EGLN3,EPHA6,FGF2,FZD3,GALNT5,GGT7,GNB1,HLA-DOA,IKBKB,IL22RA2,KAT2B,LTA4H,NFKBIB,NTN1,OR4E2,OR5B3,P2RX1,PAFAH2,PAK6,PCK2,PERP,PLCG2,PPID,SEMA4D,SESN3,SGPP1,SOCS7,SPHK2,SPTLC2,STAT2,VARS
Negative_86	ME2,ACAA2,APOE,CARD11,CMBL,DCXR,GNG12,KITLG,MCM5,NOTCH3,PIGL,POMC,PPP1R3D,PRKX,PTK2B,RASGRP4,SHC3,SMARCA5,SOAT2,STX7,TAS2R1,CACNA2D4,SERPIND1
Negative_87	MVD,BMP2,CCL5,CCR5,DERA,ENTPD5,F13A1,FARP2,GLB1,GNRHR,GSTM1,IFNA4,IFNAR2,MGLL,MYL7,NFX1,NOX1,OR2F1,OR4C13,OR52N2,OR5A2,OR5K1,PIK3C2A,PMVK,POLR2D,PPOX,PRIM1,PRKAA1,PRKG1,RARS2,RGS3,SPRY3,ST8SIA5,TNXB
Negative_88	CDO1,DGKD,LTC4S,SGPP1,TNFRSF9
Negative_89	CR2,MOS,ABLIM3,ADSSL1,ALPP,ATP5H,BCAT2,BTRC,CCR6,CCR9,CDH1,CYP2E1,DDX51,DFFB,DGKA,DGKD,DLAT,FPGT,GALNT2,GJD2,GLI2,GPAM,HEMK1,HMGCL,IL9R,IMPA2,MGLL,NFKBIA,OR5AR1,PANK1,PDGFRB,PIK3R1,PLAUR,PLXNB3,RASGRP3,ROBO1,SPRY4,SV2A,TGFB3,TIAM1
Negative_90	C8B,CDA,GPT,LAT,LEP,AADAT,ACSL1,ARPC5L,BBC3,C3AR1,CHRD,CIITA,CKMT2,COL4A2,CPT1A,CRHR1,CSNK1A1,CYP2U1,CYP8B1,EGLN1,GALM,GALNS,GDF7,GZMB,HGSNAT,ITGA6,JAK1,KLKB1,MAP3K13,MGST3,MRAS,MS4A2,MTHFD1,MYL9,NFATC2,OR2B6,OR8I2,PDCD1,PIGO,PIP4K2B,PLCB3,PPP2R5B,PRKCA,PYGB,PYGM,SARS,TUBA3C,YOD1,SERPINB5

Negative_91	GNS,NGF,AANAT,AARS,ABL2,ACVR1,ACVRL1,ALG12,AMY2B,ARSE,ATP4A,CACNG7,CFTR,CHEK1,CSDA,CSNK2B,DAPK3,DLL1,EFNA1,ETNK1,GMPS,GRIN1,IL19,NADK,NFKBIA,OR11H1,OR14C36,OR1A1,OR2T12,OR4K1,OR4S2,OR51A4,OR51I1,OR52E2,OR56B4,PDGFB,PER1,PIAS1,PIGU,PNPLA4,POLR2G,PPP2R5A,RASA1,RUNX1T1,TAS2R48,TDO2,TFPI,MAPKAPK5,PAFAH1B3
Negative_92	ID3,TAT,TNC,ACTG1,ADSS,ALDH2,BNIP1,CYP11B1,CYP4A11,DDX47,DUSP7,DUSP9,E2F5,EDNRB,ENTPD2,FGF22,GAL3ST1,GALNT9,GNA12,GNPNAT1,GSTM4,GUCA1B,HPRT1,INPP5A,JAK2,LDHC,LIPA,LRDD,MAPK10,MGAT1,NR4A1,NRP1,OR10T2,OR2M5,OR4N4,OR51T1,OR56B4,OR6M1,PFAS,PLXNA1,PPP1R3D,PTPRJ,RASGRP2,SNAP23,UGCGL2,ULK1,UPB1,ZIC2
Negative_93	AR,CKM,HYI,STS,ACO2,ADCY9,ADH1B,ALAS2,ATP5C1,ATP6V1D,AXIN2,B3GAT3,BBC3,CCND1,CHRM3,CLDN5,COMT,CSF2RA,CYP3A7,DBF4,DDX19A,EIF4E2,ETS1,EVI1,FBXW11,GLG1,GNMT,GRIN2A,GSTA1,INHBA,IRF3,MAN1A2,MEF2C,NAGS,OR10G9,OR11A1,OR2W1,OR4K1,OR5I1,OXCT1,SCD5,SDHA,TNFRSF4,UCKL1,WEE1,SUV420H2
Negative_94	CGN,GCK,GSN,TPR,ACAD8,AOC3,CNTFR,CNTNAP1,FGFR4,FZD1,HNF4A,HSD17B3,IL29,MTHFS,MYH9,OR10H1,OR1K1,OR4K15,OR4M1,OR5D16,OR6A2,OR6C3,OR8B4,OR8D1,PLAUR,PSEN1,SCP2,TMLHE,UCK2,WNT7A,WWP2,XCL2,ZNRD1,MAPK8IP1
Negative_95	ADC,CBS,TK1,ACADVL,ALDH1A2,APAF1,C5AR1,CCNG1,CDC25A,CLDN1,CTF1,CYFIP1,CYP3A7,FBP2,GBE1,GCNT2,GRM1,IARS2,IL28A,NEFH,NUDT2,OR2A2,OR2B2,OR4K15,OR5I1,OR6F1,OR9Q1,PIP5K3,PSENEN,PTPN1,RXRA,SARDH,SPTLC1,SSH2,TDO2,TLR2,TUBB,ATP6V1G3,HLA-DQB1
Negative_96	IL4,LCK,LTB,ADSS,ADSSL1,ALG5,ASNS,C3AR1,C5AR1,CCL25,CTNNA3,EFNB1,EXTL2,FABP3,FARSA,FGF10,FRAT2,GIT1,GPX7,GRB2,HNF1B,IL28B,ITGAE,LCAT,MAP3K6,MAPK3,NKD1,NKX3-1,NR4A1,OR10Z1,OR1D5,OR2B6,OR2T33,OR52A5,OR9Q2,PCYT2,PDE11A,PIK3C2A,PON3,PRKACA,PRKCQ,SARS2,SHMT2,SLIT1,SPCS1,SRGAP2,SSH2,TAS2R1,WASF2
Negative_97	BTK,GDA,PLG,TK1,AKR1C3,ALG1,ATP5D,CD28,CSNK1G2,EXTL3,FCGR3A,GANAB,OR2L3,OR9G1,PTGER3,SLC25A5,B3GALNT1,PRICKLE1
Negative_98	ME1,ALDH9A1,DPYSL2,FABP1,GARS,LIPE,OR13H1,OR1I1,OR5AC2,SERPINA1
Negative_99	AK3,TPR,ADRB1,AGPAT4,ATP5F1,B3GNT5,CASP6,CDH5,CDKN1A,CSF2RB,FPGT,FRAP1,GALNTL4,HLA-C,IL17B,IL19,LRP5,MAP4K2,METTL6,MYH4,OR2H1,OR6K6,P2RX1,PDHA1,POLE2,PPP1R1A,PPP1R3C,PTCH1,PTK2,RPIA,SLC25A6,STAT4,STAT5A,TRAF3,VAV2
Negative_100	C1S,EGF,GBA,MAG,RDX,TNR,AARS2,ABAT,ABL2,ALAS2,CCL7,CER1,COQ5,CPOX,CYFIP1,CYP19A1,DHCR24,DUSP14,GGT6,GGT7,GUK1,GZMB,HLA-DOB,IL20RB,IL8RB,LRRC4C,MAD2L1,MAP2K4,MARS,MGAT4A,MINPP1,NLGN2,NT5E,OR2M4,OR9K2,PDE3B,PDGFRA,PHGDH,PLA2G3,PPAT,SRD5A2,THBS2,XIAP,SERPINA1
Negative_101	LPL,LTB,NOG,ABL2,ACSM5,ACVRL1,AMDHD2,B4GALT4,BMP8B,CCL2,CCL27,CDIPT,CFLAR,CLDN17,CREB3L3,CTNNA3,EFNB1,FZD6,FZR1,GALNT13,H6PD,HAO2,IDI2,LIMK2,LTBR,MAOB,MGAT1,MLYCD,MPZL1,MYH10,NRXN1,NUDT12,OR6C2,PCNA,PLA2G3,PMVK,RPN2,SETDB1,ST6GAL1,SUV39H1,TAS2R49,TLR4
Negative_102	HGF,HK1,ACAA1,ACSM2A,ACVRL1,ATP5J,CACNG1,CFTR,CSAD,DHDDS,EIF4B,FARSB,FCGR3A,HSD17B3,IL20,IRS1,ITPKB,JAM2,KIR2DL1,KLRD1,NCOR1,NFKB2,NKD2,NLGN1,OR2M4,SLC2A1,SLC33A1,ATP6V0A2

Negative_103	GNE, PRL, ACAA2, CACNA1S, CDK7, CES7, CHST11, DGKG, ETS1, FTH1, FZD6, GUCA1B, HIP1, IFNA21, IL22RA2, NFATC1, OCRL, OR2T6, PARD6A, PIAS1, POLR2H, POLR3A, PYCR1, RPS6KB1, SGMS2, ST6GAL1, UBE2L6, WASF3, XYLT1
Negative_104	GSN, ATP5H, ATP6V0B, CD19, CERK, ELK4, ESPL1, GRPR, GSTM4, OR2S2, OR6C75, PIGO, PTGIS, PTGS1, SDC4, PLA2G12B
Negative_105	CR2, DCT, GBA, ADRB3, CCL5, CHKA, CHST12, CLDN14, CTBP2, CXCL2, CXXC4, CYP11B2, DHODH, EFNA4, HSD11B2, IFNA14, IQGAP3, MKNK1, OR10R2, OR2G3, OR2T8, POLR1C, POLR2D, PPP2R2C, RUNX1, SARS, SMC1A, SSX2IP, SUCLG1, TDO2, TGFBR1, TICAM1, UROC1, YKT6, GABARAPL2
Negative_106	TEC, ACADM, ACYP2, ATP2A1, CACNG5, CBR3, CDS2, CFTR, CTSS, DHRS4L2, FGF19, IARS2, ICOS, LPAR1, NME4, OR13H1, OR2T33, OR4C16, PDE7B, POLD1, POLR2K, PRKAR1B, PTGS2, RAB5A, TSC2, UCP1, UPP2, WNT2B
Negative_107	ALDOC, BIRC2, C9orf95, CDC25C, FZD7, OR56A4, PAX6, PRKACG
Negative_108	ACYP2, ATP6V0C, B3GALT4, CACNA1S, CD72, CREB3, CYP2C8, ENPP1, FGF18, GLI1, GNAS, GPD1L, HAGH, HRH1, HSD17B7, IL8RA, INPP5E, ITGA8, JAM2, MAD2L2, MAN2A1, OR11G2, OR2M7, PANK3, PDE5A, POLR1C, RBPJ, SLC33A1, SPAM1, STAT3, TAS2R49, TGDS, TRAF1, TUBA8, WNT8B
Negative_109	C5AR1, CD19, CD8A, COL2A1, CYP3A7, GAMT, MAP2K5, OR2T2, OR4S1, OR51A4, OR5L2, P4HA1, PMAIP1, PON1, PTCH2, SGMS2, SLC25A6, TNFSF10, TUBB2A, YARS, GABARAPL1
Negative_110	DUT, ID2, ACSM5, ALDH2, ATP5J2, B3GALT1, B4GALT6, CCL24, CCR4, CDC42, CYCS, CYP1B1, DTX3L, EDN1, GDF5, GFAP, GMPR2, GPX7, GSTM4, HSPB1, IL1RAP, IL2RG, IPPK, ITGA8, MAD2L1, MAN2A1, MGAT5, NMNAT3, NOX3, OR4F17, OR51I2, OR5F1, OR8B8, PGAM2, PHGDH, PLCG1, POLR2I, PPA2, SETMAR, SMARCA5, SOCS7, SPTLC2, TYROBP, YWHAZ
Negative_111	CP, HPD, ALOX15, CARS2, CUL1, EPHB2, F13B, GAMT, GNG3, MGAT1, MMP9, OR12D3, OR52E6, OR8B8, ORC5L, PLTP, RBPJ, SLC33A1, SMURF2, TBXA2R, TUBA1C, CSGALNACT1
Negative_112	BACE2, CNTN1, MAOB, NTF4
Negative_113	LEP, ATP6V1H, BCL10, CACNB4, CCL19, CCR1, CDKN2A, CHST12, CXCL12, CXCR3, DUSP3, ENTPD1, GRPR, GUCA1A, HMGCL, MAP3K11, MAP3K2, MCCC1, MGAT5, NRXN2, OR6M1, PDHA1, PIK3R1, PPP2R5D, PSEN1, RAD51, SEMA4F, TFDP1, TICAM2, ULK2, WARS, WBSCR17
Negative_114	PML, STS, ABLIM3, ARSB, ASNS, CANT1, CCL13, CHKA, CREB1, CX3CL1, CXCL12, GALNTL4, GBE1, GUCY2C, LAMB1, OR13D1, PFKFB1, RDH16, RPN2, SRD5A1, HLA-DRB3
Negative_115	BAX, BTK, CTH, FUK, GDA, SYK, ARHGEF4, CDH1, CLDN11, CLDN22, CYFIP1, DHCR24, DUSP10, FZD6, IGSF5, MAGI2, PGAM2, PHKG2, PIP5K1B, PKMYT1, RARS2, SAT1, SEMA3A, SREBF1, SRGAP1, TAS2R8, TICAM1, ZIC2, CACNA2D3
Negative_116	ID3, PLN, CMPK1, CNTNAP1, CSNK1G2, EPB41L3, EREG, GRB2, IFNB1, MGST3, NAT5, OR1J2, OR2T34, OR52N4, PLA2G1B, PMM1, POLA1, PRKAG1, RPS6KA6, SCIN, TCIRG1, THBS3, TNFSF12, TRIP10, UGT2B28
Negative_117	ACP5, ACY1, ALG10, ALG8, CCL24, CHUK, COL5A2, CREB3L2, CTBP2, GNAI3, GUCY2C, KIR2DL2, LAMB3, MAPK8, MINPP1, OR10G7, OR2D3, OR5L1, OR8B4, PLCE1, PPP5C, PSPH, RFWD2, ZNRD1
Negative_118	CR1, EGF, ACSM5, ATP5G2, CDK2, DDX19A, FABP1, IFNA17, IL18RAP, IL21, LTA4H, MAPK11, OR4F17, PDGFA, PDHB, PPAP2B, SCIN, SKP2, TUBA1C, TNFRSF11A
Negative_119	ADA, CADM3, COL4A6, CYP17A1, CYP19A1, ESAM, GALNT12, GRIN1, HSD3B7, OR2T12, OR51B6, OR6N1, OR9Q1, PARS2, RRM2B, SLC2A1, SPTAN1
Negative_120	GBA, ILK, VTN, AASDH, ADCY3, ALDH1A2, ATP5D, CCNG1, CCR4, CMPK2, DUSP1, EPHB6, FDFT1, FUT8, GSTM5, IFNK, MAP3K3, NAGA, OR4M1, OR5K1, PDXK, RBPJL, RPIA, SARS, SLC2A4, ZFYVE16, TNFRSF18

Negative_121	MYC, ACTN4, ACVR2B, ADCY2, ANAPC5, BMP8B, C5AR1, COL5A2, COQ7, DTX3L, E2F5, EFNB2, FZD5, GNAI2, IL9R, KNG1, MAP3K14, MCCC1, NOD1, NT5C1B, NTF4, OR4A5, OR52E8, PER2, PITX2, PRDX6, RAP1B, SIAH1, TNFSF11, VAV2, PNLIPRP2, TNFRSF18
Negative_122	MIF, ACADM, AKR1C1, ATP2A3, CDIPT, CES7, CHIT1, CHRNA7, CXCL16, DHDH, EPOR, FGF22, FGFR3, GAMT, GCNT4, GMPPB, IFIH1, IL1B, LDHB, MYLK3, NARS2, NCAM1, ODC1, OR2T34, OR5I1, P2RX6, PAK4, PAK7, PANK2, PDE1B, PER1, PLA2G2F, PLCD3, PLCZ1, RDH8, SELE, SEMA3E, SH2D2A, SMAD9, STK36, UCP1, WNT3, PPP1R12A, TNFRSF10B
Negative_123	TNC, ADCY2, ADRBK2, ARG2, ARNT, ARPC3, BMPR1B, BRAF, CCR10, CD80, CFL2, CYP2D6, DHCR7, DPM3, EXT1, FGF13, HLCS, IRAK4, IRS2, KIR3DL3, KITLG, LDHAL6A, MCAT, NAT1, OR1A1, OR1J1, OR2D2, OR4K1, ORC6L, PCNA, PCYT2, PDE6H, PFKFB1, PIPOX, RAP1B, SHISA5, TACR2, ALDH18A1
Negative_124	CFH, ACTB, AFMID, AHCY, ALDH3A1, ALG8, CACNA1I, CLTCL1, COL5A1, CYP2D6, DIAPH2, EFNB2, ENTPD8, FUT6, GALK1, GDE1, GUCA1C, KCNJ11, LAMB2, MAN1A1, MAOB, MAP2K6, MAPK11, METTL2B, NAGA, NFASC, OR10G9, OR1E1, OR4E2, OR4K15, OR6B3, OR8S1, PCK2, PIGA, PRKAR2B, PRKX, SC4MOL, SNAP23, SRD5A2, TNFRSF4, TP73, TNFRSF12A
Negative_125	ADH7, APC2, DGKB, ENTPD2, IFNA16, IL17RA, LCAT, LYPLA1, MYH13, OR1J4, RBL1, RXRG, WASF2, TNFRSF1B
Negative_126	GCK, GSS, RDX, SHH, ALPPL2, ATP2A3, ATP6V1F, CD40LG, DCTN1, DGKH, GALNTL1, GBA3, HMGCR, MAML3, MTHFD1L, MTHFS, NKX2-2, OLR1, OR10X1, OR1G1, OR51D1, PPP1CC, PSME3, PTGES, PYGM, SHISA5, SMAD6, SOS2, TOLLIP, TPH2, TSC2, WNT6, GABARAPL2
Negative_127	IL2, IL5, LCT, AASS, ANPEP, CSAD, CYP26C1, DKK2, EFNA2, IKBKB, NAGS, OR2T10, OR5AR1, OR5R1, OR7C2, OR8D4, PIK3CB, TGDS
Negative_128	IL8, TYR, ADIPOR2, AGRP, ATP13A2, CCR9, CDC26, CXCL16, DDX56, FABP4, FUT6, GP1BA, HSD11B2, IBSP, MAP2K6, MGST2, NAT2, NEFM, OR10V1, OR10Z1, OR1I1, OR2L13, OR51D1, OR6B3, PAK4, PIGH, PIK3CB, PPCS, PPP2CB, PYGB, RAPGEF3, RDH8, TLR3, XCL1, ATP6V1B1, CTNNBIP1
Negative_129	GLA, OAT, PXN, AKR1D1, ATP6V1F, CASP7, CD22, CEBPA, CTNNA2, DARS, DDIT3, DRD5, MDH2, NMNAT1, OR10H1, OR2K2, OR5A2, OR8H3, PI4KB, PRLR, TAS2R16, SERPINF2
Negative_130	CD6, UBC, ACVR1, ADH7, ALS2, ATG12, CCL22, CCND3, CD44, CD72, CDC42, CNTFR, CRY2, DNMT1, DTX1, ENTPD2, HADHB, HES1, HPSE, IL20, IL23R, JAK2, LCMT2, MAPT, MGAT3, MKNK2, MYH10, MYL2, NADK, OR10H4, PLXNC1, PROS1, RDH10, RPRM, SGMS2, SMPD4, STX3, SV2A, TGFB2, TOLLIP, TUBA1A, TUBB2C, ZIC2, HLA-DQA2
Negative_131	DCN, PGP, GMPR, MLYCD, NTF4, OR2M3, OR8B4, PDGFA, SETMAR, SH2D1B, STAT6, TUBB2C
Negative_132	IVD, ATP6V1A, CLDN7, CRLS1, OXSM
Negative_133	AKR1C2, CLTB, CMAS, GP1BA, IDH2, IFIH1, IFNW1, MYL2, NFE2L2, OGDH, PLA2G3, SLIT1, TRPM5
Negative_134	MET, OGT, AVPR1A, CYP2A6, DAAM1, FLT3, GSTZ1, IRS1, MAP3K1, NLGN1, OR4C46, OR51A2, OR5P3, PMAIP1, PON2, PPP2CB, PTPRR, SGPP1, ULK1, TNFRSF1B
Negative_135	BTK, GSR, ALG3, ANAPC10, ARPC1B, ATP2A3, ATP5I, B3GNT2, CADM1, CHKA, CHST13, CNGB1, CNTN2, CRKL, DPYS, FBP2, GPD2, GRIN2B, IL15, INPPL1, MYH10, NRXN2, OR13C9, OR2S2, PRKAA2, RAPGEF1, ROCK1, SDC3, SLC25A6, SOCS7, ST6GAL1, STK11, TSLP, VCAM1, ATP6V0D2
Negative_136	ME1, ACMSD, ALOX12B, ARG1, BIRC5, CACNA1D, CDH4, CHKA, COX15, F13A1, GDF6, GGT5, HADHA, HPRT1, IDH3B, MAGI3, OR1J1, OR2T12, OR51L1, PAICS, PDE6G, PHGDH, PNPO, PPP3CA, ROBO1, SOCS7, ULBP1, VKORC1, ST6GALNAC1

Negative_137	AK1,APP,DBH,ADRBK2,AK3L1,ATP6V0B,CANT1,CCL25,CD34,CHSY3,CNGA4,CREB5,CYSLTR1,DGKE,DGKZ,DPYS,EPHA7,ETNK1,FZD7,G6PD,HLCS,HPRT1,IL22RA1,IMPDH2,JAK1,KITLG,LAMB1,LCMT1,LGMN,MMP7,OPLAH,OR1F1,OR2D3,OR4C16,OR5AP2,P4HA3,PCCB,PHKG1,PSME1,SHISA5,SIGLEC1,STX12,TGFB2,ZBTB16
Negative_138	DCC,F5,GNE,TXK,ADORA2B,AFMID,ASRGL1,ATP5O,BBOX1,CACNA1H,CD80,CLDN4,CYP4F3,DDX50,EPHB1,FGF14,IGF1,LAP3,MBL2,MICA,NR4A1,OR10AD1,OR13C3,OR4P4,OR52K1,OR6C1,PPP2R5B,PPP3R2,RAPGEF3,SAT1,STK11,TBL1XR1,TICAM2,TKTL2,TUBB3,VTI1A,WNT6,WWP1,ZMAT3
Negative_139	AGTR1,APOE,B3GALT5,CACNA1E,CCL7,CD247,CD40LG,CD8A,CPT1C,CRLF2,CXCL11,CYP3A7,DUSP16,DUSP2,ERBB3,FDFT1,FIGF,FOXO3,FSHB,GABARAP,GALC,GIT1,IL18R1,IL20RB,IL22,IRAK4,JAG2,KIR2DS5,LAMA4,LAMC1,NEFL,OR10K2,OR13C4,OR1L8,OR51B2,OR6N1,PPM1A,PPP2R2C,SRGAP3,SUV39H2,TPK1,ULBP2,UROS
Negative_140	GH2,NF1,AASDH,ARNT2,B4GALT1,CACNA1H,CCL23,CCNB1,CD3E,CD59,CFLAR,CSF3R,CYP2C19,CYP2U1,DDHD1,DLL4,DUSP2,E2F2,EFNA3,GADD45A,GALNT9,GRIA2,GRM1,IKBKG,IL18RAP,ITPKB,JAK3,JAM2,MAPK12,MCM7,MGAT5B,NAE1,NLGN3,NOTCH2,OR1N1,OR5M8,PDE1B,PER1,PLA2G6,RCHY1,RPSA,SCP2,SIAH1,SLC25A6,SLC33A1,SMAD2,TNFRSF6B
Negative_141	JUN,ALPI,CD58,CD8B,CDS2,CSF1,EDAR,HPRT1,HSD3B7,P4HA3,SOD1,WHSC1
Negative_142	AUH,ADAM10,C1QC,DGAT2,HARS,IFNA5,NEFH,NUDT8,OGDHL,PAK7,SMAD3,TBL1XR1
Negative_143	FAH,OAT,ALDH3B2,ALOX12B,AREG,ARSE,BCL10,CCL4,CHST11,CPT1C,CREB3,CYP3A5,EI24,ELK4,EREG,FZD1,GMPR2,GUCY1B3,HSD3B1,IDI2,IL25,IL2RA,ITGB3,LAMC1,LRRC4C,MCCC1,MDM2,MNX1,NARS,NGEF,PDE4B,PIGL,PIGN,PPP2R1A,PRKAG1,PRPS1,RAPGEF2,RBKS,RPS6KB2,TUBAL3,UGCG
Negative_144	C1S,ADCY3,APOC3,CD38,CDK7,CHST11,CIITA,F2RL1,GTSE1,HTRA2,OR8U1,TAS2R10,ALDH18A1
Negative_145	HGD,MET,CAMK2G,CCL5,CDKN2D,CSF1R,FUT3,IL22RA2,METTL6,MYH13,NRG4,NT5C1B,NTN1,NTRK2,OR10V1,OR6C4,PLA2G3,PPBP,RALB
Negative_146	ACPP,ALAS2,CARS2,CCR9,CHUK,CTF1,E2F4,GUCY2D,HLA-F,IFNGR1,NCSTN,NKD2,OR13C5,OR6C4,OR8J1,PITX2,POLR2J,PTK2,SIPA1,SSH2,STX4,TGFBR2,TRHR
Negative_147	C6,EDA,IL6,AVPR1B,CREB3L2,CTLA4,E2F3,HCST,IKBKB,ITGA8,JAG1,MNX1,OR4K17,PNMT,SPTAN1
Negative_148	ADH7,CACNB4,CHRM3,CLDN10,COQ7,CRHR1,EHHADH,FMO5,GLYCTK,HMOX1,HTR5A,LLGL1,MAGI1,OR10Z1,PFAS,PPARD,PRKAG2,SUV39H2,SV2C,TAS2R7
Negative_149	ASL,C6,MOS,CCR4,CYCS,ERBB2,GBE1,HSD17B7,NARS2,OR52B4,PANK4,PCNA,PIP5K1A,SPRY4,THY1,VTI1A
Negative_150	ARPC1A,CTNNA3,F2RL1,GNAI3
Negative_151	CKM,IL4,ACSL1,ACSM5,BST1,CACNB3,CCR9,CD22,CDC14B,CLDN18,E2F5,FOXO1,HADHB,HMGCR,IFNA4,IGFBP3,LRRC4C,NADK,NFATC3,NFYC,NLGN3,NOX3,OR11L1,OR13D1,OR13J1,OR52H1,PGM1,PNPLA4,SCLY,VAMP2,YWHAH,TNFRSF13B
Negative_152	INS,AARS2,ALDOC,CCNE2,CHRNA7,CXCR6,ENTPD2,LILRB3,MAD1L1,MASP1,OR6C4,OR7D2,PPP1R1A,PRKAA2,PVRL2,SDC4,VARS,WWP2
Negative_153	DDO,ADCY6,CD40,FGF18,FLOT1,FUT4,HLA-DOB,MTMR6,NFX1,SDC3,SKP2,TUBA1C
Negative_154	BUB1B,EPHA4,GNA13,LIPE,MPST

Negative_155	ADK,PC,ADH1A,AKR1C4,CAMKK1,CD22,CHRNA7,COL4A2,CREB3L1,CTBP1,CYP26C1,GALNT2,GSTO2,KYNU,OR2A5,OR2L8,OXSM,POLR3G,PPAP2B,PPAP2C,PRKY,RPS6KA5,SHMT1,SULT1A4,UGT2B10,TNFRSF10D
Negative_156	BTD,CD3E,CXCL5,DHCR7,DIAPH3,FGF14,GALNTL5,GNAL,ITGAM,LAMC1,LIFR,MAPK9,MTMR1,RAD51,TAS2R4,TAS2R5,TICAM2
Negative_157	F9,NGF,ABLIM2,ACO1,ACPP,CD8B,CDIPT,CLDN11,FLT1,GNPNAT1,KARS,MAOA,NSDHL,OR11G2,OR56B4,PDE10A,PLA2G3,PLXNC1,SEMA4D,TUBB3,ZAP70,HSD17B12,TNFRSF10A,TNFRSF17
Negative_158	C7,DBI,ADH1B,AFMID,ALOX15,CCL4,CYP2C9,DTYMK,GGT5,IFIH1,IMPA1,IRAK2,ITGA6,OR1E1,OR1I1,POLR2A,PRKG1
Negative_159	FGF11,HLA-DMA,SMURF2
Negative_160	DBI,RET,ADH5,AHCYL1,ALG9,APC2,B3GAT3,CBR3,CCND2,CREB3L3,CSNK1G2,DGKD,DTX3L,FMO1,GNRH1,LAP3,MAD2L1,MAN1B1,MAP2K1,MYH11,NFKB1,NT5M,NTNG1,OGDHL,OR2A5,OR2T1,OR4D9,OR4K2,PDE3A,PDGFA,POLR3A,SLC27A1,SMPD3,SPAM1,SULT2A1,SUV39H1,THBS2,TIAM1,TUBB6,UNC5C
Negative_161	KIT,ACAA2,ACO2,B4GALT4,BPNT1,C3orf10,CACNG4,DHRS4L2,EPHB3,FLOT2,GLUL,IDH3B,IQGAP3,LRRC4C,NFYC,OR1D2,PHKA2,TGDS,TLN1
Negative_162	C1S,EBP,F7,SRF,ACOT11,ADCY8,ALOX15,ATP13A2,BCKDHB,CACNA1B,CD80,CYP1A1,DUSP10,FDPS,FLOT2,GCLM,IL10RA,IQGAP1,KDSR,LAMA4,MGST2,MLLT4,MTMR6,NKD2,OR10J5,OR4F15,OR7C1,PIK3C3,PIK3CA,PRDX6,RDH10,SCLY,SH2D2A,SIGLEC1,SLC2A4,VKORC1
Negative_163	CBL,AASS,ACSL6,ADAM10,ADH1A,AMPD2,ARRB1,CD59,CLDN17,CYP8B1,EARS2,ENTPD6,ERO1L,IL12A,OR10J3,OR10Q1,OR13C2,OR4S1,P2RX1,SCD5,SEMA7A,SHISA5,SOCS7,STK11,STX16,VNN1,WASF1,WNT7A
Negative_164	TPR,XDH,AADAC,ANAPC10,ANAPC5,CDC16,CDC7,DHX58,DUSP2,DUSP3,FMO2,GRPR,HHIP,KLK3,MAP3K3,MMP9,MYH2,MYL5,OR10A2,OR1E2,OR2T2,OR6B3,PIGB,PLOD3,PLXNC1,PPP1R3A,PRSS2,PTPN7,PTPRM,RAC3,SARS,SH2D2A,STAT5A,STX16,SYNJ2,TJP3,TPK1
Negative_165	FGB,ACP6,ADH5,BDH2,CTSL1,GGT6,GLG1,GNAI1,GNG13,GRB2,ITGAV,LALBA,MAP4K1,NCAM2,OR6N1,PCCB,PPM1A,RAPGEF4,SEMA3C,PAFAH1B1
Negative_166	PDC,ADIPOR2,AGXT2,COL5A2,CSF1R,CYP2F1,DLL1,GNAS,HAGHL,HNF4A,ICAM2,LIPC,MTHFD1,NEFH,PIP4K2A,PPA1,PTK2B,RASGRF1,SKIV2L2
Negative_167	ADSSL1,ASPA,ITGAD,MAP2K2
Negative_168	CRH,GNS,ID4,LSS,ALG10,ALG9,ANAPC10,ATP5F1,B3GALT5,CNGB1,DAXX,ETNK2,GNAO1,GUCY2C,IL5RA,ITGA1,MGST3,MYL7,NME2,NRG2,OR52M1,OR5B17,PCNA,PRKAR2A,SETD7,TJP1,ULBP2,UNC5A
Negative_169	NF1,ATP6AP1,CD46,GANAB,GNG3,HLA-F,INPP4A,KLKB1,MAFA,OR6F1,OR9I1,PHGDH,PPP1CB,PPP2R1A,PRKG2,PTGER1,SMAD6,STX1B,STX4,TACR1,HLA-DQA2
Negative_170	IL2,MAG,MVK,ATG12,BMP7,CDC26,CSF3R,CTLA4,CTPS2,CYP26C1,DARS2,EPB41L2,GZMB,HPRT1,IFNA7,IL2RA,MAPK12,NCOA4,OR2T3,OR4A16,OR5A2,OR7G3,OR8B4,PAK3,PDHA1,PIK3R2,SPP1,TJP1,UNC5C,EIF4EBP1,PRICKLE2
Negative_171	CFH,AACS,ADSS,ARNTL,COQ6,EHMT2,EIF4E,EPB41L2,EXT1,FGF7,FZD3,HDAC2,LDHB,MADCAM1,MGST3,NRP1,OR10G3,OR13C2,OR4C11,OR56B4,OR6F1,POLD2,PPP1R1A,RASGRF1,SMPD1,SOD1,TBXA2R,TUBA1C
Negative_172	LHB,RB1,ALAS1,ATF2,BMP8A,CDKN2A,CERK,CHIT1,CXCL13,ELK4,EPB41L1,F13B,FLOT2,G6PD,GSTA5,GSTZ1,HAO2,HSD17B2,IL12A,KAT2A,NR4A1,OR10Q1,OR14C36,OR5B17,OR6N2,ORC3L,PARD3,PDE4B,PITX2,PLA2G10,PPP2R1B,PRKCZ,PRLR,RPS6KA5,RPS6KA6,TBL1Y,TJP3,UGT8,WARS2

Negative_173	CD6, LYN, MPL, ADCY9, AGPAT1, AKR1C1, ALG12, ARHGAP5, ARHGEF2, ATP5F1, BUB1B, CASK, CCR2, CDKN2C, EPHA6, EXTL3, FGD3, G6PC2, HSPA1B, HTR4, IL18, IMPDH2, INMT, NCKAP1L, OR1C1, OR2S2, PLCD3, POLR1C, PRKCH, PRPS2, RELB, ROCK1, SLIT2, TPI1, EIF4EBP1, TNFRSF11B
Negative_174	C4B, EGF, FAS, GP6, ACSL3, ACTN2, AKR1C1, AKR1C4, ANAPC2, ATP5G3, CHD8, DUSP4, DUSP8, E2F2, EFNA5, FBP1, FZD3, HLA-DMA, IFIH1, IMPDH1, INADL, LMO7, MAGI1, NCK2, OCRL, OR52M1, PDXP, POLR1B, POLR2G, PRNP, PTK2, PVRL4, SELE, SH3BP2, SHC3, SSH3, TPM3, TUBB3
Negative_175	ANAPC2, COL1A2, CTNNA2, DHRS9, GMPS, IL11RA
Negative_176	C8A, ACACB, B3GAT2, CBLB, COL4A2, DGKH, DGKI, FLOT2, GNAS, HDAC1, IFNA17, IFNA4, IL23A, MEF2C, MMP7, NAGK, NKX6-1, OR13C9, OR14I1, OR1D5, OR2AE1, OR2G6, OR2L2, OR5F1, P2RX6, P4HA1, PFKP, PIP5K1A, PLXNA1, POLR3H, PON1, TAS2R16, TSC1, WASL, ZFYVE16, CACNA2D3
Negative_177	CKMT2, CLTCL1, CYP2U1, DHDDS, DHRS4, DPM2, EHMT1, MYH8, OR10G7, OR10J5, PNMT, PSPH, SRGAP1
Negative_178	CYP19A1, IL1B, LFNG, MAP3K8, OR52M1, OR5AN1, SRD5A1, UGDH
Negative_179	CCL13, FUT1, PGAM1, SPHK2
Negative_180	FUK, GLA, SMO, ACADM, AKR1B1, ARAF, BACE1, BPGM, CAMK2G, CEBPA, CLTCL1, DPYD, DUSP6, EPHA8, FRAT2, FUT1, GDE1, HNF4A, IFNA16, ITGA11, KLRC4, MAPK3, NCF4, NCOR2, NOTCH1, OR2D3, OR2M2, OR4M2, PAK1, PDE8B, PIGX, PLIN, PLXNA2, POLD1, PPP2R5E, PRNP, SELP, SPRY3, STX4, THY1, TNFSF4, TUBA4A, ATP6V1G2, PLA2G12B, SUV420H2
Negative_181	CAD, CFI, LEP, RFK, B4GALT1, BMP5, CACNB4, CHKA, CYP2D6, DHFR, FGF13, FTMT, MARS2, MASP1, MYLK, NAT2, NCK2, OR5AC2, OR6A2, OR6C70, OR6C75, PHKG2, PPP2R2B, SPP1, TLN2, UGCGL2
Negative_182	F11, FES, HYI, ACOX3, ACSS3, ADCY4, ARHGEF6, ARRB1, CACNA1I, CHAD, CREB5, DDX19A, EDNRA, EHMT2, ENTPD5, ENTPD7, GMPS, GNG12, HPRT1, HTR7, IL1A, ITGA1, MAP3K12, MAP4K2, MYH8, NT5C1B, OR10J5, OR11G2, OR5C1, PIM1, PRKCZ, PTCRA, PTTG1, RASSF1, SORD, STAT3, STEAP3, TALDO1, TCF7L1, TNXB, UBE2K, UGT2B10, ULBP2, UXS1, SERPINC1
Negative_183	CD2, DHH, OTC, PAH, ACSM5, ASNS, BBOX1, BLVRB, CD8B, CDH2, CDH4, CNDP1, DDX51, DGAT1, DPAGT1, EPHA2, GOSR2, GOT1, GP1BA, HLA-DOB, HSD17B3, IDI1, NT5C1B, OR13C9, OR13H1, OR51T1, OR5T1, PIK3CA, PIK3R1, PTPRZ1, RAB5A, RRM2B, SESN2, SMURF1, STX18, TBXA2R, TLN2, WNT10A, YES1
Negative_184	EZR, PDC, ADRB2, ARPC4, CCR2, CD14, CD3E, CDH15, EGLN1, FADS2, GGT1, GSTM5, GSTZ1, HAP1, HIF1A, HRH1, IL15, KITLG, LDHAL6B, NAMPT, OR2D2, OR4F4, OR51B5, OR5T3, OR6K2, PLCG2, PRKY, QPRT, RETSAT, SGPP2, STX5, TM7SF2, TP53I3, UNC5D, WNT3, ZFYVE9, PNLIPRP2, SERPINA1
Negative_185	AR, SPN, ACOT12, ACP6, CCL3, CDC16, CDC25B, ECSIT, EFNA3, ENPP1, FGF10, GNAO1, GNPDA2, HARS2, HLCS, IFT57, IL29, INHBB, ITGA10, JAK1, LAMA4, LIAS, MTHFD1L, NARS2, OR6K2, PAK4, PLXNA2, SHC2, ST8SIA1, STAT3, TOMM40L, TRAF5, CACNA2D4
Negative_186	AUH, C6, GP5, ANAPC10, ATP5I, BMP5, CD46, CHAD, CHRNA7, DGUOK, DKK2, FGD3, GMPR, HSD11B2, IDI2, IKBKG, ITPKB, MPZL1, MYL5, NMNAT3, OR13C2, OR52B4, OR9A4, PAK2, PCYT1B, PIGS, PPP2R1B, RASGRP3, SMC1A, TAS2R48, THBS4, TUBB3, SERPIND1, ST6GALNAC1
Negative_187	BMP8A, FTMT, GSTM5, GYS1, LYPLA2, OR10A3
Negative_188	F5, ADIPOR1, AKT2, ATP6V1D, BUB3, CAMK2D, CD70, CLDN22, CREB3L3, CSNK1G2, DDX51, DGKE, EHMT1, FABP1, FGF23, GART, H6PD, IFNA7, KIR2DS5, KITLG, LEFTY2, MYH6, NR5A2, OR10H3, OR1A2, OR4C13, OR4C46, OR52B6, OR8B8, OR9Q2, PDGFRA, SPRED2, ST3GAL2, UGCG, WEE1

Negative_189	HYI, ABL1, ADRB1, CACNG6, CCL14, COQ7, CSNK2B, CTNND1, GNAZ, GRAP2, PFKFB2, POLR1A, RASGRP3, SFRP2, YARS2
Negative_190	NPY, AGPAT6, ALOX12B, ATP6V1F, B4GALT2, CBR3, CHD8, CLDN4, COL5A1, CSNK1E, CXCL6, ENTPD5, ETS1, GDF5, GIT1, GNA12, HSPA1B, KIR2DL2, MGST3, MOV10L1, MTHFR, OR10Q1, OR1C1, OR4P4, PDCD1, PPARG, PSPH, RFXAP, STK11, TAS2R13, WNT4
Negative_191	HK2, MYC, SDS, AMPD1, BLNK, BMP8B, CBR1, CBR3, DMGDH, F2RL2, FCGR2B, GBA3, GDF5, HADHB, ICOSLG, IL20, MMP1, MMP7, NCR3, OR10A4, OR56A1, OR5D16, PANK4, PDK1, PIM1, PTDSS2, QPRT, RASGRP2, SFRP4, SUOX, TAS2R48, TNFSF9, TPH2, USE1, YWHAB
Negative_192	ILK, KIT, RET, ACTA2, AK3L1, APOC3, ATN1, B4GALT6, CACNG2, CAMK2B, CCL19, CNDP1, CRY1, CSNK1A1, DDX54, DDX55, FGF4, GRM5, GSTM3, IL25, ITGB4, KARS, KLK3, LDHC, MAP2K6, NT5C1B, OPLAH, OR10T2, OR13J1, OR3A1, P2RX6, PAK3, PFKL, PIGF, PLCB4, PTCH2, RPS6, RRM1, SEMA7A, SLIT2, SUOX, TAS2R16, TNFSF4, ATP6V1E2, TNFRSF12A
Negative_193	UBB, AKR1C1, ALDH6A1, ALDH7A1, BMP8A, CACNB3, CD40LG, CYP2B6, DHCR7, DUSP1, ELK4, FGF14, GCLM, GMPPB, GSTO1, HTR5A, IL21R, MASP1, MGAT3, NAGLU, NANS, OR51E1, ORC1L, PAK3, PLIN, PRKACG, SMARCA5
Negative_194	C4B, HYI, MVK, ABLIM2, ACSL4, ACSL5, ACYP2, AKR1D1, ALG5, AMHR2, AOC3, APOC3, ATP6V1H, BCAR1, BMPR2, CSDA, CXCL10, EXT1, FGF1, FZD8, GLI3, GLS2, GSTK1, GSTZ1, GYS2, IFNAR2, IFNK, KNG1, MYH9, NCAM2, NLGN1, OR5R1, OR8D2, PGAM4, PKM2, PLCB3, PTPN7, RRAS2, SFRP2, SPHK1, STAT3, STX3, TAS2R49, THY1, TJP3, TRAF6, ATP6V1G3, MAPK8IP3, ST6GALNAC6
Negative_195	GNAQ, GOT2, OR13C2, PKMYT1, PLCG1, POLE3, TSHR
Negative_196	ACAD8, CD36, GRIN2C, OR4P4, PERP, TAS2R49
Negative_197	BTK, DLD, LTB, MPL, ABLIM2, ADCY6, BNIP1, CACNA1G, CD19, CHRM2, CTNND1, DTX3L, EPHA2, FPGT, FZD3, GALK1, GCNT4, HES1, IFNA2, ITPKB, JAK3, LDHD, LYZL1, MAML2, MAOA, MAP3K6, ODC1, OR10H1, OR2B11, OR2T4, OR4A15, OR56A3, OR6S1, OR8D4, ORC3L, PFKM, PLA2G2D, PRKACB, PTPRM, RENBP, SMAD1, SPTLC2, SQLE, TUBA1A, VTI1B, WNT6, WNT8B, CACNA2D1, TNFRSF10A
Negative_198	ACSL3, AMY2A, CLDN1, CX3CL1, CXCL10, DGKH, E2F4, GALNT7, GDF7, OR1A2, OR52M1, PDE7A, PHKA2, PI4KA, PPP5C, SH2D1B
Negative_199	ADRB1, CTSL1, IL25, OR10G7, OR5T3, PPAP2B, SREBF1, SLC25A31
Negative_200	IL8, ADCY6, ALDH2, ATP4A, DAPK1, DKK2, GALNT11, HSD3B7, KNG1, OR10K1, OR12D3, OR3A1, OR6K2, PTGES, ROCK2, TRPM5, TUBB, WNT3
Negative_201	ACSM4, ACVRL1, ALDOC, BMPR1A, CHEK1, CSF2RB, CXCL9, CYP2C18, DHDDS, DUSP6, F2RL1, FUT2, HEMK1, IL8RB, MLLT4, OR10V1, POLR3H, RAP1A, SMURF2
Negative_202	AK7, SYK, ACSL4, B3GALT1, BMP2, CAMKK2, CCL20, COMP, EFNB1, ENPP6, GNA11, GOSR2, HMGCR, IRF7, NAT5, NCK1, NRCAM, NUDT2, OR52M1, OXTR, PLAUR, POLR1C, PRKCG, RARS2, RBX1, SLC2A4, TLR9, WNT7B, WNT9A
Negative_203	CFI, ADAM10, ALG8, ANAPC10, ATP4A, CACNA1S, CCR5, CHRD, CLCA4, DERL1, ECHS1, FGF6, FLT3, GBE1, GMPS, GNAI3, GRIN2B, IPPK, MTHFR, MYH7B, NAT1, NRG2, OR2G6, OR2T33, PRSS1, RBKS, RCHY1, RPS6, RPS6KA2, RUNX1T1, SELP, SYNJ2, TCIRG1, TUBB2A, VAV2, CACNA2D2
Negative_204	APOA2, APPL1, ASIP, CLDN2, CYP4A22, DDX54, DFFA, GSTZ1, LEF1, MARS, NR1D1, P4HA3, PAK1, POLE3, RBL1, RPRM
Negative_205	FOS, GK2, ACHE, ALG10, CLDN4, KIR2DS4, MTHFR, MTHFS, NEU2, NME4, OLR1, OR2T3, OR4C16, PLIN, SMAD7, SPRY2, TUBA8, TNFRSF1A
Negative_206	CGA, MYC, TPO, ACAA2, ADIPOQ, ALG10, APOA5, BCL2, C1QB, CCL17, CCL2, CD79B, CHRM5, CKS1B, CYP7A1, DHDDS, DUSP2, EGLN1, EPHA3, FABP1, FOXA3, FUT5, FZD2, GGT5, GSTA5, HES1, HNF4G, ISYNA1, ITCH, KLK3, L1CAM, LDHD, MAPK3, MAT1A, OR1Q1, OR2C3, OR7A17, PDE8A, PDK1, PPP2R5D, RAF1, RPN1, RPS6KA1, SARS2, SEMA7A, TBK1, WASF2

Negative_207	C3, AKR1D1, CALML5, EPAS1, GSTA1, IL12RB2, INPP5B, LIFR, OR2T8, OR51E1, OR51V1, OR5AP2, PERP, SKP1, STMN1, TYROBP, XIAP
Negative_208	AGXT2, ARPC3, CDH5, CMAS, COL2A1, EPAS1, MARS2, PKMYT1, PLA2G4A, PLD1, RAB23, SRGAP1, TUBB6
Negative_209	ZYX, AKR1C2, GSTM5, HAO1, HMOX1, HNMT, PEMT, SHC1, ST3GAL2
Negative_210	NLK, CYP19A1, FABP3, MAPK11, PTK2, ST6GALNAC1
Negative_211	DCC, IDS, TFG, ADCY5, CD36, DGKB, EFNA1, ENPP3, FDFT1, IL1A, MBL2, OR5AU1, ROBO1, HLA-DRB4
Negative_212	LAT, LCT, ACADSB, ACOT12, ACYP2, AKR1C4, ALDH2, ALOX15B, ALOX5, ATF4, CACNG1, CD27, CNTN2, CTSS, CXCL11, EFNB3, EREG, FLOT1, GAL3ST1, GALNT5, GATM, GLUL, GRIN2A, GUK1, IRS2, MAOA, MAP2K7, MYLK, OR2A42, OR2AG2, OR52K2, OR6K2, PDE8B, PHKA1, PHKG2, PLA2G2F, PPP1R3A, PRODH, SAT1, SELP, SMAD2, SSH3, SSX2IP, SULT1A4, TNFSF8
Negative_213	ENPP1, NTSR1, OR2D2, SLC2A1
Negative_214	AKR1C3, ALDH2, AMDHD1, BCKDHB, CCR2, CD44, CDC14A, CNTN1, CTNNB1, DUSP1, ERBB3, FDFT1, FGFR4, FZR1, GALNT9, GPLD1, GUCY1B3, HTR2B, LIMK1, NAT6, OR2B11, OR8B12, PTGIS, SPI1, STAT6, PRICKLE1, TNFRSF10B, TNFRSF12A
Negative_215	AK5, PGF, ACADM, ANGPTL4, ATG7, C9orf95, CDK2, DLL3, EGLN1, EXTL2, FGF9, FMO5, IDH3G, ITGA4, KIR2DS5, LCAT, MAP2K6, NEFM, NRXN3, OR13A1, OR52N4, OR52W1, OR6C6, OR8J1, PMVK, POLD3, POLR2F, PPP1R3C, SMAD7, SUCLG1, TLR2, TSLP, TUBA4A
Negative_216	ALCAM, B3GALT2, CDKN1C, CDKN2B, CYP26A1, GDE1, GRHPR, MMP7, OR1K1, OR51M1, OR8H2, PCYT2, PPP2R1A, PTCH2, SC4MOL, SPCS1, SUV39H2, TRHR
Negative_217	ALAS2, MAP4K4, OR4K15, OR5P3, RRM2
Negative_218	BCMO1, CACNB4, ENPP7, FLT4, HDAC2, IFNA5, IL13RA1, IL18, MECR, NME7, TPK1, TUBB6, WARS2, HLA-DQB1, PRICKLE1
Negative_219	ACSL1, ACSM5, CARS2, CCL5, CDC14B, CYFIP1, CYP2J2, DPM2, ENPP6, FARSA, IFNGR1, ITGB5, LTB4R2, NFKB1, PRKG1, TAS1R2, TM7SF2, VDAC1
Negative_220	C8A, AKR1B10, ALDH5A1, AMDHD1, CACNA1S, CNTNAP2, DPM3, DUSP2, FUT9, FZD1, GAB1, GALNS, GGPS1, INSRR, MAP3K5, MDH1, MYH2, NLGN1, NOTCH1, NUDT5, OR10Z1, PIAS2, PLCE1, PPAP2A, PTGIS, RPS6KA5, SGMS1, TAS1R3, TNFSF14, ULBP1
Negative_221	EPO, ACO1, ALDH4A1, DFFA, OXSM, SPHK1
Negative_222	DBT, ID2, SDS, ACSL6, ACSS1, ARSB, BPGM, CD79B, CHEK2, CTNNA2, GRIN1, GUCA1A, GUCY2F, IFNGR1, ITGB2, KIR2DS4, KIR2DS5, KLRC4, MAGI1, MCM3, MFNG, MGST2, NEFM, OR11A1, OR13H1, OR1K1, OR4C12, OR51B4, OR52R1, PIGC, PTGS1, RAB5A, RAC1, RASA1, RHOD, RPS6KB1, SOD1, TAS2R48, TCF7L2, ZFYVE9, ATP6V1C1
Negative_223	CKM, IL8, JUP, PGF, RB1, ACADS, AGRP, ALG8, ALOX5, ALPP, ASAH1, B3GALT5, B4GALT5, BMP6, CAMK2A, CD58, CLDN11, CSNK1A1, DDX41, DGKH, DLST, HSP90B1, IL2RG, IRS4, ISYNA1, ITCH, ITGA11, MAP2K1, NCAM2, NEUROD1, NFKB2, NUMB, OR1L1, OR52E6, OR5M3, OR8I2, OR8U1, P2RX2, PGK2, PIGU, SEMA3G, TLN1, UNC5C, VAV1, WNT3, ATP6V0D1, SERPINE1
Negative_224	ID1, CREB3L3, EXTL3, F2RL3, FGF2, IKBKG, IL1R2, IL7R, ITGA6, LRP5, MAD2L1, NKD1, OR11G2, OR6C6, OR7G1, PLCE1, PPAT, PPP2R5D, SELL, WNT9A
Negative_225	AR, CKM, MIF, ALCAM, ATP2A1, ATP6V0B, B3GNT3, C3orf10, CACNG2, CACNG4, CDK6, CHIT1, COX10, CRLF2, CYCS, F13B, GBE1, GP1BA, IFNB1, IL20RA, MFNG, NFYB, NR5A2, NT5C2, OR10H3, OR13A1, OR2Z1, PIK3R1, PLA2G4A, PTGIS, SH3BP2, SLC27A1, SSH1, TUBA1A, USE1, WHSC1L1, SUV420H2
Negative_226	DBI, GPI, ME3, SI, ACAD8, AGTR1, ALDH3B1, ALDOA, CALML3, CASP6, CCL2, CD22, CHEK2, COL11A1, CSDA, CYP3A43, CYP8B1, FGFR3, FRAP1, GP1BB, IL11RA, IL15RA, IL5RA, IQGAP2, ITGA3, MAP4K3, MTMR1, OR10Q1, OR8D1, OR8D2, PPAP2A, PTPRR, SDHB, SSH1, THBS3, WARS, WNT10B

Negative_227	APP, FGB, GP6, GPI, PDC, PRL, AK3L1, AMY2A, ARSB, CPT1B, CSF2RA, GAD1, GDF7, GMPS, GNAL, GPX5, GPX7, HSPA8, IQGAP2, LYZL1, MAFA, MAP4K4, MGAT3, NEFM, NR4A1, OR13G1, OR3A2, OR6S1, PIAS1, PIGA, POLD1, PPCS, PRKCB, ROBO3, SETDB1, TXNDC12, USE1, WASF2
Negative_228	MAX, ABCC8, ARRB1, BMPR1A, CBR1, CDH1, CHRM5, CMBL, ESPL1, HMOX1, HTR6, IL4R, KIR2DL4, LAMC1, MDH1, MGAT4B, OR10C1, OR2S2, OR4C16, PLXNC1, PPP1CB, PTGER3, TIAM2, VANGL1, HLA-DPA1
Negative_229	ARPC5L, CBR3, CDC2, CNGB1, FARP2, FECH, FGF10, FUT8, GNB3, HTR7, KLRC1, NCOR1, NTN1, OR10H1, OR1S2, OR51I2, OR51T1, PAX6, PDGFB, POLR1D, PRKCQ, PTGES2, STK4, TSLP
Negative_230	AMT, GK, MPZ, PXN, ACSM1, ACVRL1, ADSSL1, AOC3, ARHGEF7, ASPA, BMP8A, CCR10, CLCA4, CPT1B, FGF1, GNRH1, GUCY1A2, ITGAX, MAML1, MAPK12, MPP5, NEU1, NEU3, NFASC, OR10A2, OR51S1, OR7A5, PDE10A, PDGFRA, PGM3, PIGC, PIK3CG, PPM1A, PPP1R1A, PPP3R2, PRKCQ, PRSS1, PRSS3, RDH5, SHC4, SV2A, UBA1, UGT2B4, WASF3, YARS
Negative_231	IL5, BAI1, CDC26, CHRM5, GALNT12, GNMT, GSTO1, IFIH1, LRRC4C, OR2M5, OR4K17, OXCT1, PHKB, PPP1R3D, PSEN2, ROBO1, SPRED1, SUCLA2, UNC5D, YWHAB
Negative_232	BAD, C8B, ACHE, ACVR2B, ADCY4, APC2, ARNT, ATP5G1, B4GALT4, BCKDHB, BDH2, CAV1, CIITA, EDA2R, ENPP1, ENPP7, ERBB4, GAD1, GRIN2A, HPSE, HSPA1B, HTR6, IFNAR2, IL24, MTHFD1, OR10A2, OR10H2, OR1L6, OR4K14, OR7A10, PAK7, PISD, SMAD5, TCF7, TRAF1, TUBB6, VAMP1
Negative_233	MSN, ACADS, ACOX1, B4GALT2, CACNG7, CCL24, CCL27, CDC2, CHUK, CYP26C1, CYP2B6, CYP3A5, DHRS4, GNAT3, GRIN2C, HAGH, IRS1, L1CAM, MAP3K6, OR4D5, OR5P2, PIGM, PLA2G10, PLA2G5, PPARD, PPP3R1, PRSS3, RASSF1, SOCS4, TPH1, WNT7A, PPARGC1A
Negative_234	NPY, A4GALT, B4GALT1, CARS, CCND2, FGD3, FGF13, OR2M4, OR5AR1, OR9Q1, POLD4, PPAP2A, SEMA7A, SENP2, TFDP1, TGFBR2, CTNNBIP1, HSD17B12
Negative_235	F9, SHH, SMS, ADRBK2, ARSB, CD19, CLDN17, CPT1B, CTPS2, CYFIP1, DCTN1, ECHS1, EFNA2, ENPP7, FGF20, G6PC2, GALNTL5, GGT7, HMGCS1, HTR7, IL20RB, IQGAP2, IRF3, KLRC1, MGST1, MYH3, NKX6-1, NPAS2, NR1H3, OR52D1, OR6Y1, OR9G1, RAP1A, RPS6KA3, STX18, SULT2B1, TGFB2, UPP1, WNT1, PAFAH1B1
Negative_236	CP, FAH, GLS, IL5, SDS, TPO, ADAM10, ALDOC, APOE, CNGA3, CRB3, DUSP1, E2F4, GNA12, GNAO1, GNRHR, GYS1, KIR2DL3, LAMA3, LFNG, LIMK1, NEFL, NME7, NTRK1, OR2H1, OR6T1, OR8H2, PAK2, PLA2G6, PLXNA3, POLR1B, POLR1D, PTK2B, RRAS2, TAS2R8, TBL1Y, TMLHE, TYK2, UGDH, VAMP2, YWHAZ, CTNNBIP1, TNFRSF1B
Negative_237	C3, CR1, NLK, ASCC3, CAV2, CCNG2, CDK7, COASY, CYP2A6, EPB41L1, EPHB6, GCNT3, GNA13, HTR2B, ICOS, ITGA11, LAMB2, NCOA4, NCOR2, NPAS2, OGDHL, ONECUT1, OR13C3, OR4X2, OR6B2, PIGO, PLCD3, QPRT, RARS, SLIT1, SULT1A4, TAS2R9
Negative_238	SDS, ARHGEF7, ASS1, BET1, CACNA1D, CKMT2, ECHS1, KNG1, LAMB4, PDK1, PITX2, PSPH, TAS2R1, TLN1, VAMP2, SERPINA5, ST6GALNAC5
Negative_239	DBI, GGH, GSR, ID2, IL5, PGP, A4GALT, AGPAT3, B4GALT1, CACNB1, CD55, CDC16, CSF2RA, CSNK1G1, CYP1B1, DIAPH1, EPHB1, ERBB4, GRIN1, GRPR, GSTK1, IFNA14, IFNB1, IQGAP3, LEF1, MAPK7, NKX3-1, NTRK2, OR6C70, OR7A5, PIGO, POLE, POLR2G, RASGRP2, SDHD, SH2D1B, THBS2
Negative_240	CHD8, IL18RAP, LAMB1, MAP2K4, OR12D3, OR4K5, OR8K1, PAK4, RASGRP4, PAFAH1B1
Negative_241	HK3, ATG7, CYP2A7, DGKQ, EFNA5, GART, MAP3K4, MYH4, NFX1, OR4M2, PDE4D, PERP, PIAS4, RENBP, RPS6, SMAD1, STX8, TALDO1, TAS2R60, TM7SF2, UXS1

Negative_242	CCNB3,MAP3K2,OR51E1,OR51Q1,OR8B4,SDC3,TAS2R49
Negative_243	EDA,FAS,SP1,SYK,TNR,B4GALT1,CACNA1S,CCNE1,CCNE2,CD74,CSF2RB,CYCS,CYP27A1,ENPP7,GLI1,GMPPA,GRPR,GUK1,HCLS1,IL19,INHBB,JAM3,LIPT1,OR10A5,OR10G8,OR2T6,P2RX4,PDE6G,PIGF,PIGH,PTPRM,RAB23,SEMA4D,TBL1XR1,VTI1B,WARS
Negative_244	C1S,C9,FN1,RFK,ACP5,ACSL5,ALG12,AMPD3,ASIP,CCR8,CDC20,CSNK2A2,DRD1,F13B,GNB3,GNRH2,HIBADH,HNF4A,IL23A,MADCAM1,NT5C1A,OGDHL,OR2B6,OR2L8,OR4D6,PAK6,PPP1CA,RALBP1,SUFU,THBS3
Negative_245	PXN,RPE,ACTC1,APOA5,AREG,DGAT2L4,HMBS,OR1M1,PTPRM,TOMM40,VCAM1,WBSCR22
Negative_246	PC,ABI2,ADCY8,ATIC,ATP5A1,C3AR1,CASP10,CCNG2,CYSLTR1,EFNA3,FSHB,FUT8,GCNT2,GRIA2,HSPA8,IFNA21,IL1R1,IL1R2,LAMC1,LRAT,MAP2K3,MECR,MTHFD2,NR1H3,OR1N1,OR2AG1,OR4E2,OR51Q1,OR5D13,OR7C1,PDHA1,PLA2G2D,PPP1R3D,PPP2R5D,PRKCD,PTGS1,SDHA,STX10,STX7,SV2B,TAS2R40,TAS2R50,TMLHE,TRIP10,TYROBP,UCK2
Negative_247	EDA,NPY,ACVR1B,ALDH1A1,CHRM3,CPT1B,GSTK1,OR4C16,SAT2
Negative_248	ALDH1A3,COMP,DCXR,DHCR24,DUSP3,HIP1,IDH3A,IMPDH1,LIAS,MGAT5,MGAT5B,NR1D1,NUDT9,OR10A3,PORCN,RUNX1T1,STX5,YARS2
Negative_249	CCKBR,CSAD,GADD45G,GNA11,GRM5,IL2RA,MGAT5B,PANK2,PTGES,SSH1,TACR3,TOMM40L,UGP2
Negative_250	ANAPC7,BAIAP2,BCMO1,CASP6,CTNNA1,CYP11A1,DPYSL2,DTX3L,ENAH,ENPP6,EPHB2,EXOC7,EXTL2,FARP2,GNB3,METTL6,NEFL,OR10H5,OR51F1,PGM1,SOCS5,STEAP3,SULT2A1,TYMP,UGT2B11,VEGFC,TNFRSF14
Negative_251	CFD,EGF,GP5,GP9,MET,ACOT12,ADCY1,ADRB1,ALDH2,ARPC4,ASAH1,ATP5G2,ATP5G3,CDH3,CHIA,CHRM3,CLDN2,DHX58,EPHB3,FZD6,GAS1,HADH,IL10,KLK3,LAMB1,LDHB,MAP4K1,MTMR1,MYH13,MYH8,OLAH,OR10R2,OR10T2,P4HA3,PDE6G,PFKP,PIGB,PLOD3,SH2D1B,SLC27A5,ST3GAL2,UGT2A1,ATP6V0A1,B3GALNT1
Negative_252	FST,IL6,ACY1,ALDH6A1,APOA2,ATG3,BLVRB,CCR5,CD244,CYBB,DNMT1,DVL2,EP300,FGFR2,GAL3ST1,GRIA3,HARS,HTR4,IFNG,MAP3K4,MGST3,MYD88,MYH6,OR4B1,OR4F6,OR5L1,OR6B2,OR9A2,PIGH,PSEN2,PTDSS1,RAD51,RDH16,STK3,STX16,TACR1,TACR2,ATP6V1C1,HSD17B12,SERPING1
Negative_253	ACMSD,ARG1,ARNT2,DAG1,EDA2R,GALE,HADHA,INHBE,IQGAP3,LAMB1,NCF2,OR2H2,RRAS,UGDH,WNT7A,ZNRD1
Negative_254	BAX,CFI,RFK,WAS,ACLY,ACTN1,ADH7,ASS1,B3GAT1,B4GALT4,CACNG7,CACNG8,CCL21,CD48,CD74,CRB3,DIAPH1,E2F3,EIF4E2,FPGS,FZD7,GLI2,GNA13,GRM1,IFIH1,INPP4A,ITGB3,ITGB5,LAMB1,LLGL2,MAN1C1,MAP3K13,MYLK2,NFYB,OR11H6,OR8S1,PIAS4,PIK3CG,POLE4,PRKCQ,SEMA7A,SETD1B,SFRP1,TAS2R7,TOMM40L,VEGFB,WNT9A,ZFYVE16,HLA-DRB4
Negative_255	FAS,GGH,HGF,COL3A1,CYP2S1,DDX19A,FGF11,FGF5,FOXA2,GLO1,GRIN1,GSTO1,HLA-DRA,HLA-F,IL6R,INPPL1,ITGA10,MKNK1,MS4A2,MYH13,OCRL,OR2M2,PFN2,PIK3R3,POLR2B,PPAP2A,PPAT,ROBO2,TSLP,WASF3,PHOSPHO1
Negative_256	KMO,AADAC,ADRA1B,BIRC5,CCL5,CSF2RB,CYP3A5,DDX23,DPM1,ENPP3,KIR2DL3,LAMB3,UROD,TNFRSF11A
Negative_257	AUH,GP5,GSN,IL7,AARS,ACSS2,B3GALT2,B4GALT6,CACNA1B,CD3E,CHRD,CRHR1,DBF4,EFNA5,ENPP1,FLT4,FUT4,GUCY1A2,HLA-DOA,HPRT1,HSPA1B,IFNA2,IFNA8,IKBKB,MAFA,MEF2C,MYH8,NAGA,NFATC3,OR2G6,OR2L3,OR4D9,OR6C74,OR8D2,PARVB,PGAP1,PLA2G10,PPP3CB,PRKAA2,PRKAB2,RETSAT,SHC2,SMAD1,SUCLA2,TBL1Y
Negative_258	OSM,FGF5,IL6ST,KARS,OR8J1,RENBP,SCD5,STX7,TLR4

Negative_259	CR2, GPT, CD72, CDH2, EREG, FMO1, ITPA, KIR2DL4, OR3A1, OR4X1, OR51A7, OR5J2, PIAS4, PIK3CG, PRKACB, RRAS, TGFB3, TUBAL3
Negative_260	AMY2A, CD79B, CDC14B, DDX4, GZMA, HEMK1, IRF3, LCMT1, OR12D3, OR2AG2, OR6K3, POLR1A, RHOQ, RPIA, SC5DL, SDHD, SOCS3, TAS2R8, TBL1XR1, WNT8B
Negative_261	PLN, SDS, ABL1, AKT3, ATP2A3, CCL11, CCL16, DIAPH3, E2F2, EPHA2, ERO1L, GRIN2D, GSTO1, INPP1, MAPT, NCR3, NUDT8, OR10A2, OR1S1, OR2T29, OR52E2, OR6C75, P2RX6, PDPK1, PIK3CA, PLAT, SENP2, VANGL1, HLA-DPA1
Negative_262	LAT, ACSS2, ACTN4, CCL13, FZD6, GALNT12, PGM3, PIGN, PIK3R1, PLA2G1B, RASGRP2, SULT2B1, TAS2R3, THBS2, ULK2
Negative_263	IL7, LPL, ATP6V1F, COL2A1, DARS, DCXR, DGUOK, DKK1, GALC, GBE1, HIP1, IL11, IL1RAP, NRCAM, OR10H2, OR2L13, OR4M1, OR8D4, ORC2L, PGK1, POLR1C, RAC1, SEMA4C, VKORC1
Negative_264	APAF1, BDH2, BMPR1B, CACNA1S, CCNE1, CD86, CDC7, CDO1, CHKA, CHRM1, CSNK1G3, GRIA2, GUCY2C, HAO2, HDAC1, HLA-G, HMGCL, IRF5, IRF9, MMP7, NAMPT, OR13D1, OR1F1, PHKG1, PPT1, PRIM1, RDH8, SCLY, SHISA5, STK11, SULT1A4, VEGFC, XYLT1, ST6GALNAC4, TNFRSF10C
Negative_265	AUH, CFD, CKB, ATP2A1, BCAT2, BLVRA, CACNA1G, CACNG4, CASP6, CYP2C9, DCTN1, DGUOK, DOT1L, EPHA8, EPHB1, FLAD1, G6PD, MAP3K5, MCM7, NCKAP1, OR13C2, OR2B2, PDE6H, PLCD3, SLC2A2, SMAD3, SMURF2, UGT8, MAP3K7IP1
Negative_266	C1S, DHH, FGB, ABL2, ALAS2, ALOX12B, BBOX1, CANT1, CD72, CREB3, CXCL12, IL12RB1, MAD2L2, PAK7, PCK1, PPM1A, ST3GAL3, SV2C, TAS2R38
Negative_267	C8A, SRM, ACOX2, ATP6V1D, CER1, CLDN8, CLTB, COL5A1, CXCR5, CYP4F3, DDX51, FIGF, FOXO1, FUT5, GFPT1, ICOSLG, IKBKB, MYH11, NCF2, OR10AG1, OR5AP2, OR6C4, OR7D4, ORC1L, PCCA, PFKFB2, PGAM1, PITX2, PLA2G2F, PLAU, PPM1B, PSENEN, SHC4, SV2A, TAS2R10, TRHR, TSNARE1, USE1, ATP6V1B1, ATP6V1B2, ATP6V1C2, SLC25A31
Negative_268	FUK, HPD, ABCC8, ACTA1, ADH4, ATG12, BTRC, CLTCL1, CREB5, CXCL16, CYP1B1, CYP2C9, CYP8B1, DDX55, DPM1, EPHA8, EXTL3, FMO5, FZD3, GBA3, GLI1, HHEX, IFNA13, IL18RAP, MAPK7, OR4K1, OR52N1, OR5AU1, OR6C3, PNPLA3, RAF1, SMAD5, SPRY3, SV2A, XYLB
Negative_269	AK7, ACY3, B4GALT6, CTNNA3, GALNTL1, MAP2K6, MYLK, NNMT, OR10AD1, OR1G1, PLA2G2E, POLR2G, RPE65, SAT1, UGT2A1, ST6GALNAC4
Negative_270	ACADSB, ACTN4, BET1L, CMBL, CXCR5, GUCY1A3, IL23R, IRF3, MAML2, MYL7, OR2M4, PVRL2, ROCK2, SLC12A2, YWHAE
Negative_271	LEP, NPL, XDH, ACAD8, AGTR1, AMDHD1, APOA5, CARS, CAV2, CD274, CDKN1A, DDX23, DPYS, DUSP10, EFNB3, ENPP2, FSHR, GRIA2, HMGCR, IQGAP3, ITGB1, MGST2, OR11H6, OR1J1, OR9A2, PFKFB4, PRIM1, RETSAT, RFXAP, SAT1, SETDB2, TAS2R41, TGFB3, UGT8, HLA-DQB1
Negative_272	EDA, MET, PAH, AASDH, ATF4, CANT1, CXCL3, ELK4, IFNA2, KITLG, LDHC, LIMK1, METTL2B, MYH7, OR10AD1, OR13C3, OR4X1, OR5AK2, PDE6H, PHKA2, POLD3, PRKCD, RRM2, SHMT2, SMPD4, SUCLA2, WNT4, ATP6V0E1
Negative_273	PGP, CES2, CNTFR, GNA13, GSTO1, IFNB1, ISYNA1, OR11A1, OR5T2, PDE1C, PDGFA, PNPLA3, PPARD, RAPGEF1, TAS2R16, TCF7L1, TP53, HSD17B12
Negative_274	PTS, ADRB3, BDKRB1, CLCF1, CLTCL1, DAPK3, DDX4, HDAC1, HSD17B8, MYD88, OR10AD1, PHKB, PIGF, POLR2K, POLR3K, PTCH1, SDHC, SGMS2, SH2D1B, TGDS, TNFSF8, WNT2, ATP6V0A4, ATP6V1C1
Negative_275	CSK, GH2, HK1, PGP, ACVR2A, BMP8B, CDC42, CHIT1, COL1A1, DNMT3B, ENTPD6, GSTM1, GSTO1, HCST, HLA-DOB, IL10RA, INPP5E, MAPT, MS4A2, MYH11, PGAP1, PLTP, UGT2A3

Negative_276	ASL, ADSS, AMDHD1, APOA2, ATP5C1, BAIAP2, CCR3, CD46, CHKB, CSNK1G2, CTNNB1, EFNB2, GABARAP, GCNT1, IFNA7, IL12B, ITGAV, LAMA3, NAT1, NR4A1, OR52L1, OR5AR1, OR6T1, OR8K1, PCK2, RPIA, SRD5A2, SV2C, TLR3, TLR4, VNN1, PAFAH1B2, SUV420H1
Negative_277	FAH, B3GNT4, CXCR3, FZD1, GGT6, OR10H2, OR5AC2
Negative_278	GSS, CACNB4, CYBA, GIT1, KIR3DL2, LEFTY2, PDXP, PGK2, TBXA2R, UROS, CACNA2D2
Negative_279	IL6, CCL15, GNRH1, PFN3, VAMP3
Negative_280	ADRA1B, APOA1, CLDN7, CNGA4, FOXA2, IKBKB, RPS6, SERPINE1, TNFRSF10C
Negative_281	AGK, RFK, ALG5, C1GALT1, CBR1, CCNB2, CCNG1, CD40, CDC14B, CHIA, CLDN17, CSF2, CX3CR1, CYP2D6, EPHB4, ERBB4, LDHAL6B, MAT2B, NFE2L2, NOS1, NT5C1A, OR51F2, PIM1, PRODH2, RND1, SEMA3C, SFRP1, SLC25A6, SULT2A1, TPM3
Negative_282	CBL, DCN, HDC, AARS2, ACAD8, AMY2A, ASPA, BCAT1, CYP3A43, GNA12, HES1, HSD11B1, HYAL1, LAMC3, NFKBIA, OR9A4, PHKA1, PTK2, SFRP5, STX6, TLN2, TNFSF18, UGP2, VANGL1, ZAP70
Negative_283	AK5, MPI, AADAC, ACOX2, ACP2, AMY2A, APOA2, CDKN2A, DDX23, ENTPD6, ETS1, EVI1, GJA1, GNRH1, IL8RA, INPP5A, KLRC4, LIFR, LIPA, LTBP1, NRG1, NRG2, NUMB, OR1L6, OR2M2, ORC3L, PARVB, PDGFRB, SGPP2, SH3BP2, SMARCA5, SPRED2, SQLE, SV2B, TPM3, TRHR, UGDH, WNT10A, WNT2, WNT5A, HLA-DPB1
Negative_284	CFI, ACADL, AK3L1, CD8B, CPT1B, CTBP1, ETNK2, FIGF, GAD1, GALNT1, GCLC, GUCA1A, HIBCH, IDI2, OR2D2, PANK4, PER2, PFN4, POLE4, PRKCB, PRKCQ, SETD1B, SETD7
Negative_285	G6PC2, RRM1, SPCS1, UGT2A3
Negative_286	ADO, SMO, ACSM3, AREG, BLNK, CCR7, CD19, CHRNA7, CSNK2A1, CYFIP2, GAMT, MADCAM1, NAGLU, NEU1, PLAUR, SV2B, TAS2R8
Negative_287	GP6, ACAD8, ALOX5, AQP7, ARNT, CACNG3, CDH2, DHDDS, DPYS, ETS1, GNAO1, GUCY1A3, HRH2, ITGA2B, ITGAE, MAGI3, MYL5, NODAL, OR1L3, OR2T3, OR6A2, PERP, STK3, TLR4, CSGALNACT2, SERPINA5
Negative_288	IL4, CCR1, CD79A, DOLPP1, EDN1, FGD1, GNA14, IFNA6, L1CAM, OR5AP2, OR5AS1, OR5M8, RBL1, XYLB, SUV420H1
Negative_289	AARS2, B3GALT6, CDKN2C, GSTA5, PLCD3, PPCS, PPP1R1A
Negative_290	DCT, ID2, MAG, CCDC6, DLL3, INPP4A, IPMK, ITPK1, LLGL1, PISD, PYGB, TGFBR2
Negative_291	F5, LCK, PDC, ADCY7, ADRB3, B3GALT1, BAAT, BDH1, BMPR1B, CCDC6, CDC42, CLCF1, CNGB1, CREB3L1, CREB5, E2F1, EFNA5, EIF4E, EXTL3, GPX7, HAGH, HGSNAT, IRAK2, KIR2DL2, KLRK1, LRDD, MGAT3, OR4C16, OR6C6, P2RX5, PIGA, PLXNB2, PTPMT1, PTPN7, RAD51, SETD7, SH2D1B, STX1B, SULT1A1, TRADD, UCK2, C1GALT1C1
Negative_292	CP, IL4, JUP, LPL, TNN, WAS, ADRA1A, APH1A, CYP2S1, EFNA5, ENTPD3, FARP2, FGF22, GADD45B, GNAZ, GYS1, HSPB1, IBSP, IL17A, IL6R, KIR2DL4, KNG1, MAGI3, MALT1, MAPK1, MDH1, MYL9, NFKB1, OR10A5, OR13J1, OR52I1, OR5B12, PIM2, PLCB2, PLXNB2, PRPH2, RAC1, SAT1, SELPLG, SLC2A1, STT3B, TOLLIP, UGCGL1, WNT3, YARS, ALDH18A1, HLA-DPA1
Negative_293	BAD, FST, AKT2, EFNA5, EI24, FDPS, GRPR, GSTA3, GSTP1, HYAL1, IKBKB, IL28A, MGAT3, MPDZ, NR1H3, NR4A1, NRG2, OR13A1, OR2F1, OR5T3, OR6B3, OR7C1, OR8G1, PFKFB1, PPP3CC, PTPRZ1, RAP1A, RDH16, STX2, TNFRSF8, TSHR
Negative_294	BTK, DBT, DCN, FN1, ABL1, ADH1B, CCKAR, CCND1, CCR1, CNGB1, DGKG, EGFR, EPHX2, FZR1, GYS2, HCLS1, IFNGR2, KDSR, MALT1, MAPK10, MPZL1, NAT1, NAT2, OR11L1, OR52N1, OR56A4, OR8H1, PDHA1, PPP1R1A, PPP2R1B, SHMT1, TBK1, TOLLIP

Negative_295	CD6, CP, FOS, LCK, NPL, SMS, TAT, TNN, ACACA, ACSS3, B3GNT4, BDKRB2, BIRC3, CCL27, CD36, CDO1, CSF2RB, DTX4, EPHA3, ETNK2, FARSB, FPGS, GRM5, IL12RB2, LHCGR, MAP2K4, MITF, NEFL, OR10A5, OR10G9, OR13C4, OR4K2, PIK3R2, PRKAG1, PTPN1, PVRL3, RPRM, SEMA4G, SPRED2, TAP1, TAS1R2, TAS2R1, ZFYVE16, ATP6V0D1
Negative_296	FGB, GPI, ALAS2, BIRC5, BLVRB, CAPN2, CAV1, CCL15, DCTN1, GALNTL1, HSD17B4, KIR2DL4, KYNU, MAP3K12, MYL7, NAMPT, NEUROD1, OR10Z1, OR5AK2, OR8D4, PAX4, PIAS4, PTK2, PTK2B, PYGB, RDH5, RPS6KA1, SHC3, SOCS2, TAS2R3, WASF3, WWP2, MAPKAPK5, SERPINB5
Negative_297	DBI, TAT, CCL27, ELK4, OR10J1, OR5H6, SHISA5
Negative_298	DUT, AKT3, ATP5G2, FOSL1, MFNG, MKNK1, PIGW, RASSF5, TCF7L2
Negative_299	DCT, ABL2, ALG12, CACNA1C, CHST11, CSNK1D, GALT, HMGCL, ITGAM, MYD88, NCOA4, OR4F15, P2RX7, PIAS2, PIK3C3, PLAU, RPS6KA4, TACR3
Negative_300	KDR, LHB, BCMO1, FARP2, LILRB3, OR52I2
Negative_301	FGA, ACOT11, AGPAT2, AKR1B1, CCNB3, CD28, CDH2, CMAS, COL5A2, COQ7, DDX54, DNMT3B, EGLN1, ENTPD1, ETNK1, FIGF, GNA11, GPT2, GRAP2, GSTO2, HMOX1, HSPA8, KIR2DL1, LTC4S, MAPK8, OR10C1, OR2A5, OR8I2, PIP4K2A, PIP4K2C, PON3, SH2D1A, TAS1R3, THBS4, TSTA3, UNC5D
Negative_302	F2, LEP, MOS, MVK, TK1, ACPP, ADAM17, ADCY1, B3GAT1, BUB1, COQ5, CPT2, EXT1, GLUL, GPAM, HCLS1, LARS2, MAOB, MYH7B, NCAM2, OR10J5, PLXNA3, PTK2B, TM7SF2, SERPING1
Negative_303	CFI, EPO, GGH, PXN, ACACA, ACSL3, ACTB, ATP5O, CDC45L, CFLAR, CHRM5, CXCL9, CYP2A6, DAAM1, FMO1, GARS, GFPT2, GRIN2A, GSTM5, HAO2, IL20RB, IRS1, ITGA1, ITGB6, LTC4S, NLGN2, NRAS, OR4C13, OR4K14, OR51F2, OR52A1, OR5F1, PRKCB, PTCRA, SPRED1, TUBAL3, VANGL2, HLA-DPA1, TNFRSF13C
Negative_304	ACO2, ALG5, CAV2, CCL20, CCNA1, CREB1, DDHD1, ETS1, GSTM5, HLA-F, LCMT2, MAP4K4, NADK, OR10X1, OR1B1, ORC1L, PVRL4, SEMA3F, SLC25A4, SYNJ2, WHSC1L1, PDCD1LG2
Negative_305	PTS, TNR, WAS, ARPC2, CACNA1E, CACNA1S, CD28, CHRM5, GALNT8, KRAS, MGAT4B, MKNK2, NRXN2, NTN1, OR1N2, PRKAB1, PRSS2, PSPH, PTPRZ1, RARS, RDH16, RHOD, STAM, TUBB2A
Negative_306	BBC3, CNTF, FGF11, FGF19, GALNT9, GNAL, GSTO2, ITGA6, OR2L13, OR4D5, OXCT1, PHKG2, PPBP, RALB, ZFYVE16
Negative_307	AK2, ACADSB, ADCY2, AGPAT2, ALDH4A1, ALOX5, ATF4, CDC25A, CNTN1, COL1A1, COL6A1, ENO2, EPRS, FGF21, FLT1, GGT5, GLUL, GNAI3, GNPAT, GSTA4, ICAM3, KNG1, LDHD, METTL6, NGEF, NKX2-2, NQO1, NRXN3, OR10H2, OR56A1, OR5M8, OR6B3, OR6C75, PLAT, PPP2R1B, RASGRF2, SCD5, SETD8, SOCS7, ST8SIA5, STK11, TNXB, ATP6V1G1, PAFAH1B1
Negative_308	GHR, GLS, GPT, NF1, AGPAT3, ATP6V1H, CACNB2, CCL15, CHRM2, FGF21, FGFR1, GOSR2, HAP1, IFNA16, IL12B, KIR3DL1, LARS, NCF2, NCK1, RXRB, SLC9A1, UGT2B7, ARHGEF12
Negative_309	ACAD9, ACSM2A, ACYP1, ALDH5A1, APOE, ASPA, BMP4, BMP8A, CNTN2, COL4A1, CYP2A7, F2RL2, GLO1, HSD17B3, IL23A, NME2, OR6N1, PRKACA, RPS6KB2, TUBA3C, ZBTB16, SLC25A31
Negative_310	CDA, CGA, CRH, DAO, LAT, ABI2, ACYP1, ADRB1, AFMID, AGPAT3, ATP5A1, ATP5I, BCAT2, CCNG2, CDC20, CES2, CLCA4, CLDN9, CTSB, CTTN, CYP11B2, E2F4, ENPP3, GSTP1, HTR2A, LDHC, MAD2L1, NCOR2, NUMBL, OR13C8, OR1E2, OR1L1, OR2L3, OR52N1, OR5R1, OR8B12, PIK3R4, PRKCA, PTK2, SOCS5, SOCS7, ST8SIA1, TAS2R5, THBD, TPK1, TRADD, ATP6V1B2
Negative_311	SRC, ACVR2B, CACNG3, CLDN6, DHDH, EFNA3, FARP2, GMPPA, GNRH1, LALBA, MAD2L1, OR2A5, OR2T8, OR4A16, RHOD, SPTLC2

Negative_312	FOS,NOG,AXIN1,CACNB1,CASP9,CLDN18,COQ7,FRAP1,GNA11,GRIN1,IDH1,KLRD1,PITX2,POLR2L,SLC9A1,THBS1,TRHR
Negative_313	LHB,BAIAP2,C4BPB,CACNA1S,CARS,CCL26,CCND1,CD44,CDC25B,CDS1,COL2A1,DUSP16,DUSP5,FCGR2B,FGFR2,GNAI2,GSTO1,ITGA11,LDHA,MAP3K7,MAP4K2,OR2B11,OR3A3,OR4B1,PARVA,SARDH,SKIV2L2,SPCS1,STAM,TP53I3,UPP1,XYLB
Negative_314	IL7,ILK,SDS,TNC,ACVR2A,ATP5I,CDC26,CTBP1,DHODH,EPHA2,FCGR3A,FECH,FMO3,FUT8,GADD45A,GLG1,GNA11,GNAI2,GNAO1,GP1BB,GRIN2A,HCST,KRAS,NAT2,OR13A1,OR1N1,OR2H1,OR2M2,OR2Z1,OR4D1,OR6K2,PARVA,PFKL,PIM2,PNLIP,PRKX,PTGER1,PTGS1,PTPRF,SC5DL,STAT6,TYMP,TYRP1,UCK1,XIAP
Negative_315	AGL,IDS,PGF,A4GALT,ALCAM,ALDH1A2,APOA5,CD47,COMT,CPOX,CYP8B1,DAXX,EDAR,GRIA3,ICAM3,IFNA6,IL20,ITGAE,JAK1,MKNK2,NFATC2,OR4N4,OR51E2,OR8D1,OXCT2,PDGFC,PIAS2,PIK3C2G,PPP2CA,PRKCI,RPS6KA3,SGPP1,ST3GAL1,TPH1,XYLT1,XYLT2
Negative_316	ALDH7A1,GRM1,PIK3R1
Negative_317	C6,CSK,DBT,XDH,ACVR1B,AOC2,B4GALT2,BACE1,CD38,CHRM5,CSNK1G1,CUL1,DARS2,DGKG,DPYD,F2RL1,F2RL2,GNA13,GPD1,HGSNAT,HLA-DRA,IL12RB2,IL7R,ITCH,MAML3,MAOA,MASP2,NCAM1,OR10Z1,OR2M2,OR4D6,OR51B2,OR6B3,OR7A10,PGS1,PIPOX,PLA2G1B,PPP1R3D,PRKAR1A,RASGRP2,RFXAP,SPRY3,TNFSF11,TP53I3,TYK2,UBE2K,ATP6V1C1,MAP3K7IP1
Negative_318	MET,A4GALT,AARS2,CERK,CHUK,COQ5,DLAT,FGF1,GART,GMPPA,GRB2,HNF4G,LAMA4,LRRC4C,MAP3K14,OR10Q1,OR2A12,PDE8A,PFKL,PIGQ,PLCB3,PPM1B,PRKG2,PRNP,SDC3,SEMA4D,SMAD9,SMURF1,SULT1A1,TPK1,TRAF2,WWP2,ZAP70,SERPINC1
Negative_319	GCK,SMO,ALDH2,ATP4A,CACNA1B,CACNG5,DUSP4,ENPP3,ENTPD5,EXOC7,IL21R,LIPA,MAML3,MAN1A1,MGAT2,NCOR1,OR13G1,OR2AG2,OR6C74,OR8H2,PFKFB1,PIGO,POLE2,PPARA,RAC3,SGPP2,TGFB3,UGT2B17,WNT9B
Negative_320	CR2,ADCY1,ARG1,CASP7,CBLC,CSNK2A1,CXCL13,CYCS,CYP3A5,EPAS1,FZD5,IDH3A,ITGB8,LIPE,MAP3K3,NEU2,NGEF,OR13D1,OR2T34,OR2T4,PIP5K1B,POLR2D,PRKCG,RDH11,SEMA3F,TGFBR1,TSC1,UGT2B10
Negative_321	BAD,DCT,GK2,GP9,ALOX12,BLNK,COL6A1,CSF3,CTPS,CYP1B1,GTSE1,GUCY2F,LAMB2,MYL7,NAGK,OR13F1,OR1J4,OR1N1,PIK3CA,POLE,RAB23,SUOX,TAS2R3,TLR9,TPK1,UAP1,YKT6
Negative_322	ACSM2A,CD47,ETNK2,HAGHL,HMGCR,NFAT5,OR11H6,OR2A2,OR4M1,OR5M8,PGM1,SNAP23
Negative_323	GLS,HDC,KIT,MYC,ACADS,ACTN1,ADH4,ADSL,ALCAM,ALDH1L1,CACNA1B,CACNB1,CD44,CNTNAP2,EFNA3,EPHA3,IFNA17,MAP2K2,NARS2,NTF4,OR1K1,OR1S2,PLA2G2E,PLCZ1,PLOD1,POLR2F,PPARD,RBPJ,SRD5A1,TPMT,WIF1,CACNA2D3
Negative_324	BID,F3,ACAD8,ALDH7A1,DDX47,DGKG,NKD2,RDH12,RPS6KA2
Negative_325	F2R,UBB,ACTA2,ARPC5,CBLB,CCL22,CCNE1,CDC14B,CDK7,CER1,CSNK2A1,DDX50,DNMT3A,ENPP3,GATM,HSD3B1,IFNGR2,IQGAP3,JAG2,MAP3K14,MGST3,MICA,MYH11,NAGK,NQO1,OR5L2,PANK1,PGLS,PIGF,PMM2,PNPT1,RARS2,RASGRF2,RDH12,SUFU,SULT1A4,SV2A,TP73,TPH2,UGT1A10
Negative_326	AASDH,B3GNT2,BACE1,CACNG2,CCL19,CCNH,CD70,CDC2,CHRM5,CNGB1,CNTNAP1,CTNNA1,EPHA8,FDFT1,FZR1,GANAB,GDF5,GRIN2A,HRH2,LAMA4,MAD1L1,MDH1,MYL5,NCK1,NT5C,NT5M,OR2C3,OR2G2,OR2Y1,OR52K1,OR9Q2,PDXK,SLC2A1,SOAT2,STX12,TGFB3,PLA2G12B,PNLIPRP2
Negative_327	CACNG7,CREB1,CSF1,CYP3A4,GSTP1,OR9I1,PPOX,PPP1CA,PTTG1
Negative_328	AUH,MUT,SDS,CASP8,CDKN1C,LIPF,MAP3K1,OR1N2,OR51A2,OR52I1,OR9K2,PAPSS1,TAS2R16,VAMP2,SERPINF2
Negative_329	GDA,NLK,CYP4F2,CYSLTR2,GSTA2,OR5C1,SLIT1,TIAM2,WWP1

Negative_330	BAX,FGA,ITK,LTB,PRL,ACSM3,ATP5D,ATP6V1F,CCL3,CD40,CDC25C,CSF3R,CYP3A5,FOXO1,GAB1,ITGB2,LEFTY2,LHCGR,MAP3K11,MGAT1,MYH9,NTRK1,PIK3R3,SLC2A4,SMAD3,SOS2,TJP1
Negative_331	FER,MSN,TPR,ACSM5,ANAPC10,CRLS1,CSF1R,CTNNA3,FADD,KRAS,LLGL2,LTC4S,OR52L1,PRKCA,SOCS7,SSH2,WNT10A,SERPING1
Negative_332	BTC,SRF,APC2,APH1A,ARSE,BAAT,CAMK2A,CCR10,CHRNA7,EPHA1,GNPDA1,GSTA5,IFNGR2,IL17RB,IL21,IPMK,JAG2,MITF,MYL9,OR12D3,OR51B4,OR51V1,OR5T3,PIGA,PMAIP1,PRKAB2,PTGS2,PYCRL,ROCK2,SC4MOL,SESN2,SLIT2,SNW1,SORBS1,SUCLG1,WASF3
Negative_333	CD2,CCL22,CDH3,CES7,CHIA,CLCA2,CSF3R,DCTD,GSTM3,IFNA14,LARS2,LCMT2,MGAT4A,OR13G1,PGM2,RERE,SEMA3D,SSH2,STX2,SUV39H2,TBXAS1,UROS,VTI1B
Negative_334	CFI,CGA,FAS,ACVRL1,ALDH3B1,ANAPC2,BIRC5,CDIPT,CYP3A43,DRD5,EFNB2,GNMT,GSTO1,HRAS,IL17RA,IL20RA,MAOB,MYH11,NKD2,OR10X1,OR56A1,OR5K2,OR6A2,OR7A17,PDE1A,PFKP,PGK1,PHGDH,PRKACA,QPRT,RRM2,SDC1,SEMA7A,SH2D1A,STX2,TNFRSF9,TRAF1,TRAF6,SERPINC1
Negative_335	CKM,GHR,VCL,AADAC,ACADVL,ALG8,AMDHD2,BMPR1A,BST1,CD226,CHAT,CISH,CLDN16,CNGB1,COL6A1,CTNND1,DARS2,DHCR7,ECHS1,FGF11,GAMT,GFPT1,IPMK,MDH1,NR1H3,OR13A1,OR14A16,OR1M1,OR52R1,PIK3C2A,POLE,PPP1CC,PPP2R2D,RASA1,RRM1,RXRB,SYNJ2,TICAM1
Negative_336	IL5,MSN,ANAPC11,CCL11,CD46,ECHS1,ENTPD8,FGF1,FUT6,GATM,GRIN1,GSTM1,HLA-DOA,KCNB1,NEGR1,NFAT5,OR1N2,OR4D6,OR51I1,OR6M1,PHGDH,PPP1CC,PRKAG3,RAB23,ROCK1,VAV1,ZIC2,MAP3K7IP2
Negative_337	SYK,ADCY7,CACNG1,CACNG7,CREB3,CREBBP,CYP3A43,EPHB3,FARSB,GLDC,GLUD1,GNPAT,HNF4A,HSD11B2,HSD17B3,IL2RB,IRF5,KIR2DS5,MAPT,NT5E,NTF3,OR5AC2,PCCB,PDE7A,POLR1C,PVRL2,RASGRP3,SHC4,TJP2,PAFAH1B3,PNLIPRP2
Negative_338	SRF,TH,CCNA2,CD48,ERBB4,HMOX1,LAMC1,PRKCQ,SNAP23,SNAP25,TUBA3E
Negative_339	ID3,WAS,ATP5E,BAI1,CACNG4,CD19,CD58,CNTF,CYFIP1,DHCR7,EPAS1,FGD3,GALNT14,GLG1,GRB2,GRIN2B,GRM5,GSTO1,HEXA,HNMT,IL13RA1,IL1RAP,MAP2K3,MYH8,NFATC4,NFYB,NRXN2,NT5M,NUDT2,ONECUT1,OR2Y1,OR56A3,OR8B12,PARVG,PIAS2,PIGF,PIK3CD,PRIM2,PTK2B,PTPMT1,RUVBL1,SRD5A1,TLR5,TSLP,TUBB1,TYMP,WNT5A,ZIC2,MAPK8IP3
Negative_340	LAT,CXCL2,FCGR2B,ITGB7,OCLN,OR2V2,OR4E2,OR6C1,OR7A5,PAFAH2,PSME3
Negative_341	DHH,ABLIM1,CAV3,CNTNAP1,CSNK2A1,GCH1,GMDS,GNAI3,OR4P4,PTPN7,SHMT1,TUBA1C,VARS
Negative_342	ZYX,ABL2,GRAP2,IRF3,MAML1,OR10V1,OXSM,POLD3,UNC5C,PAFAH1B1
Negative_343	AK1,CTH,DCT,AGRN,ALG6,ARG1,ARHGEF1,ARHGEF6,ARNTL,ATP5C1,BAAT,CACNG6,CCL3,CHKA,CREBBP,DARS2,EVI1,FLT4,FZD1,GAPDH,HTR2B,HTRA2,KAT2B,MAP3K13,MMP7,MTFMT,NADK,NEUROD1,NODAL,OR2K2,OR2T8,OXCT1,PDGFC,PPP1CC,PRKAR1B,PRKAR2A,RPS6KA3,SELPLG,SH2D2A,STX5
Negative_344	ABO,FAH,GBA,HDC,PVR,ALDH3B2,CDC14B,CHPF,CHRNA7,CHSY3,DARS,DDX52,DTX2,DTYMK,EHMT2,EI24,EIF4B,GSTO2,GSTT1,GUSB,HNF4A,HYAL2,IFNGR1,IL17RA,IL7R,NRG1,NRG2,OR2A25,OR51A4,OR6C4,OR6N1,PFKFB3,PIAS2,PIGN,PIK3R1,PLXNA1,PTDSS1,RALBP1,SETMAR,SOCS4,ST3GAL6,TNFSF9,VCAM1,PRICKLE2,TNFRSF18

Negative_345	CAT,GK2,PGF,SFN,ADCY1,ADRA1D,AMDHD2,BACE1,CCNB3,CCR9,CHDH,CXCL1,DPYD,EIF4E2,ENO1,ETNK2,FADS2,FMO5,GNAZ,HTRA2,ICOSLG,IL1R2,IL2RB,IRS1,LCMT1,MADCAM1,MAP3K3,MAPK11,MCCC2,MYL2,NCK2,NFKBIB,OR4K17,PDE1C,PDE2A,PDE8B,SIGLEC1,STX6,TAS1R3,THBD,TIAM1,TUBA1B,UGT8,ULBP1,UNC5D,ZFYVE9,TNFRSF10A
Negative_346	BCR,ADORA2B,ALDH6A1,BHMT,CD14,CLDN11,COL1A1,CSF2,CXCL5,CYFIP1,DHRS9,DPM3,DUSP3,EFNA3,ENPP3,ENPP7,FARS2,GADD45A,GOT2,HAO1,HIP1,KLRC3,MAP2K5,MCCC1,MGLL,MPST,OLAH,ONECUT1,OR10AD1,OR1E1,OR1L1,OR2G3,ORC5L,OXTR,PDHA1,PNPO,PRSS1,RASGRF2,TOLLIP,VEGFC
Negative_347	C7,ALG6,CAMKK1,CLTA,CRY2,DDX54,ENTPD4,FZD9,GNAZ,GNPAT,GSTM5,HYAL1,ITGB8,LYZL1,MAGI3,MEF2C,OR10A3,OR1L4,OR51Q1,PPA1,PPT1,PRKAA2,PRKCE,RASA1,SHC2,SPP1,TNFRSF9,TOMM40L,XYLT2,MAPKAPK5
Negative_348	CFD,FN1,ARHGEF2,ATP5G3,ATP6V0B,CD19,CDC6,CMPK1,CRHR1,CSF3,EFNA3,EPHB3,IFNA17,IGFBP3,IRAK4,IRF9,ITGAD,MYH6,NFYC,NRXN1,OR10H2,OR2T33,OR51G1,OR5T2,PCK2,PNPLA3,POLA2,PROC,PRSS3,SAT1,TSC2,TYROBP,VAMP2,SERPINB5
Negative_349	CKB,CSK,RPE,ACPT,ADRB3,B3GALT6,B3GAT3,CACNG8,CCND2,CHKB,CLDN6,CSNK1A1,CYP4F2,ERBB4,GRIN2C,GRIN2D,HIBADH,HTR6,IGF1R,IL22,MAP3K2,MAP3K3,MITF,NKX6-1,NOS3,NRXN3,OR5AC2,OR8S1,PDIA4,PLCB2,PLCB3,PNLIP,POLR2J,PPP2R5E,PSME3,ROBO3,SESN1,SQLE,SRGAP2,SV2C,YWHAQ,TNFRSF13B
Negative_350	CSK,GSS,PTS,PXN,AGMAT,AKR1A1,ALDOC,ALS2,CAMK2G,CCL20,CREB3,ELK4,FOSL1,GALK1,GGT7,GNA13,GSTZ1,IFIH1,IFNGR2,IFT57,KIR3DL3,LIPG,MYH6,NFKB2,NUMBL,OR11L1,OR4D9,OR4K14,OR4K2,P2RX7,PDE10A,PDIA4,PMAIP1,SREBF1,STX1A,STX1B,STX5,PAFAH1B1,TNFRSF12A,TNFSF13B
Negative_351	NOG,ACTN4,ARPC2,CDC16,CRB3,GPT2,GUCA1B,HSD3B2,ICAM2,IL13,LEFTY2,NCF2,NEFL,NRP1,OR2G6,OR6X1,TLN2,WBSCR22,PRICKLE2
Negative_352	SRF,ALG3,ATP5B,BDNF,CAMK2D,CDH3,CHUK,CLOCK,GNA15,IRF7,JAK3,MYH7B,NFKBIE,NKD1,NTF3,OR2T12,OR4K5,PDGFRA,PLA1A,PPP1R3B,PYGB,ROBO3,RPRM,SDC4,TJP2,ATP6V0E1,MAPKAPK5,SLC25A31
Negative_353	PVR,SRM,ACPT,ACSL4,ACSM5,ALPI,ANAPC11,APOA2,B4GALT5,B4GALT7,BIRC5,BUB3,CACNA1C,CYP19A1,DGKE,EXTL1,FARSB,FGF10,HSD3B7,IFNA6,IKBKB,IL1B,ITGB4,LAMC2,LYPLA2,MDH1,NCF2,NSD1,OR13J1,OR5AC2,OR5B21,OR8D1,PAX4,PIP5K3,PPP2R2D,PTPRC,RALB,RHOD,SARDH,SIAH1,SULT2B1,THBD,WHSC1
Negative_354	DAO,PXN,SMS,UBB,BMP6,CSNK1D,ENO3,FPGS,FUT2,GSTT1,IL13,MAGI2,MGAM,MGAT5B,RENBP
Negative_355	AK2,GSS,IVD,OAT,OSM,SRC,ACSL3,AGRP,ATG12,CACNA1D,CACNG4,CBLC,CPT1B,CYSLTR2,DCTN1,ENO2,GAB1,GALNS,GLG1,GNRHR,JUND,LIAS,LRDD,MMP9,NAE1,NRP1,OR10H3,OR10R2,OR1N1,OR51T1,OR52B4,OR56A1,PIP4K2B,RFX5,SENP2,SOAT2,SORD,SSX2IP,STAM2,SUV39H1,TCF7,TIRAP,TRMT11,HLA-DPB1,PAFAH1B2
Negative_356	AMT,PTS,TNN,AADAC,ACAA1,ACSS2,ADCY6,AVPR1A,CD19,CD27,CHRM5,F11R,FABP3,FPGS,GGT1,KARS,MAN2A1,NCR1,NLGN3,NME6,NTRK2,OR4K5,OR5D16,PECAM1,PGLS,POLR1A,PPM1A,SUV39H2,WNT2B,ATP6V0A1
Negative_357	BAD,AHCY,BACE2,CACNA1F,CHEK2,CKMT2,EP300,GALNTL4,IARS2,IL7R,MAP3K5,MCM7,NANS,OR4C16,PDE7A,PDHA1,PRKAR1A,PTGER3,SMAD5

Negative_358	GBA, HYI, MVD, CACNB1, CACNG8, CCL14, CCNB1, CD80, CDH3, CLDN8, CYFIP2, DERA, ENO1, FZD7, GAPDH, GNAS, HES1, HSD17B3, HSD17B4, KLKB1, KLRC1, MAP3K1, MYH11, NCSTN, NME7, NOTCH3, OLR1, OR2L8, ORC3L, PAFAH2, PERP, PLAT, PPP3R2, PRKY, SETD8, SGPP2, SHC3, SMPD4, SYNJ1, TMLHE, TNFSF8, TP73, TSLP, TYROBP, WNT9A, PLA2G12A, ST6GALNAC3
Negative_359	ADA, CTH, FER, ACAT2, AGMAT, ALG13, BMP2, CBR3, CSF3R, CTF1, CYSLTR1, DPM3, DVL1, EARS2, FGF23, GOT1, HHIP, HTR5A, IL17B, ITGA9, MAP3K7, MEF2C, MFNG, NCK2, NFATC2, OPLAH, OR2A25, OR8G1, OR8H1, OR8I2, PCYT2, PDE10A, PFN3, PIK3R2, PIP4K2B, RUVBL1, SAT2, SUOX, C1GALT1C1, HLA-DRB5, PPP1R12A
Negative_360	ALAS2, AMD1, CLDN14, COQ3, CSNK1G1, CXCL6, DGKE, HMGCR, ICAM2, MGLL, OR1G1, OR4K13, OR6B2, OR8J3, OXCT1, PARD3, PHGDH, PLAU, PRSS2, SMPD4, MAPK8IP1, TNFRSF1B
Negative_361	AGK, DBT, DDC, GSR, NPY, CD44, CLOCK, CYP2C18, DGKG, FGFR4, GLYCTK, GNA13, GTSE1, HNF4A, MAML1, MCCC1, OR10G3, OR9I1, PLD2, TCIRG1, TMLHE, ATP6V1E2, TNFRSF10D
Negative_362	ADAM17, COL6A2, FUT8, NOD1, ULBP1
Negative_363	CGA, F11, PML, PRL, TNN, ALG8, ATP6V1A, CCL28, CD274, CD44, CYFIP1, DCXR, DEGS2, GALNT7, GNRH2, HADH, IL10RB, KAT2A, MAOB, MYLK, NME2, OR56A4, OR8B12, ORC6L, P2RX4, PAK7, PIGU, PLCD3, PPP2R5B, PRKACA, PSEN2, PTPRM, RXRG, SDC3, SMPD3, HSD17B10, SERPINA1
Negative_364	ATM, DBH, IL8, MVD, TKT, ACP6, ACSM5, ADH5, ADRB3, CANT1, CCNB2, CHAT, CNTNAP1, ENTPD1, GAB1, GAMT, HADHB, IPMK, MAP2K2, MTHFD1, NAGA, NKX3-1, NTSR1, PDHA2, PLA2G2D, POLR2D, RFWD2, RXRA, TRAF2, UGT2B10, ATP6V0A4
Negative_365	FAS, GSN, ZYX, ADIPOR2, ADSL, ALDH1A2, C1QA, C5AR1, CD74, CDKN1A, CLTA, CLTCL1, CSNK1G3, CYP1A1, CYP2J2, DIAPH1, EXTL1, FECH, FGF23, FUT4, FZD1, IARS, LAMB4, METTL6, MYH10, MYH11, NFASC, OR10A2, OR10X1, OR2T12, OR2T8, OR4S1, OR51F1, OR52N4, OR6T1, OR8D2, PDE8A, PEMT, PPM1D, PPP2R2B, PRKCG, RDH10, SGMS2, SNAP23, ST3GAL6, SV2C, TACR1, TAS2R16, ST6GALNAC5
Negative_366	LTB, ATP5L, CACNG8, CASP10, CD55, CYBB, DARS, DFFA, FABP4, GALNT3, GALNT8, HGSNAT, IL19, IL25, MAD1L1, MAP2K5, MYH9, NFKBIE, OPLAH, OR2A12, OR2A25, OR2T4, OR6B3, OR6C75, PDGFD, PIM1, PRKCI, ROBO3, RRM2B, SLIT2, UBA7, WASF3, HLA-DPB1
Negative_367	ADORA2B, CERK, COL6A2, DNMT3A, MYH8, PLK1, SETMAR, STX7, UPB1, WASL
Negative_368	F10, KIT, ME1, NGF, TFG, ACADL, ACTC1, ADRB3, BCL2L1, BUB1, CD8A, DDX52, DHCR24, EPRS, GMPPB, HEXB, HSD17B7, ITGA5, MAP3K5, MYH15, NTNG1, OR7C2, PDE3B, PDE4D, PDHA2, PLAUR, PLCD4, PPCDC, PRODH, PTDSS1, PYCR2, RAPGEF4, RPRM, STK3, TACR1, TJP1, TOLLIP, TUBA1B, UCK2, ULBP1, WHSC1L1, YOD1
Negative_369	CNGA3, FMO5, FOSL1, IMPDH2
Negative_370	PC, APOC3, ASNS, C1QB, CAMK4, CD55, CLDN6, CNTF, CRKL, CSDA, CYP2S1, DDHD1, FGF18, FPGT, GNB3, GPX5, GUCY2C, ITGAM, OR10H4, OR13C5, OR1L6, OR2A2, OR5T1, OR9A4, PLCD1, PLCD4, PLCZ1, PRKACB, SDHB, SELPLG, SFRP4, SMARCA5, SPTLC1, THBS4, TRDMT1
Negative_371	DCN, ACVR2B, AKR1C4, AOC3, ARPC5, ASAH1, B3GALT1, CACNG5, CDC2, CHIA, CLTCL1, COQ3, CTPS2, DMGDH, EPHA7, GGCX, GNB3, HSPA8, IKBKB, IL11, MAP3K14, NUDT8, OR10Z1, OR2M5, OR4C13, OR4D9, OR51A7, PAK7, PDE4D, PHKG1, PMAIP1, POLR2I, PPP5C, PRKACG, RHOA, SENP2, ARHGEF12, ATP6V1G3, ST6GALNAC6, TNFRSF10A

Negative_372	F2R, GHR, ACSM4, ACSM5, ADAM10, ARSA, CPS1, CPT1C, CX3CL1, DRD1, EGFR, ETS1, GALNT9, GANAB, GGT1, GNA13, GNMT, HSD17B7, HSD3B7, KRAS, NAGA, NR1H3, NRG3, OR10A7, OR10K2, OR11A1, OR14I1, OR4K17, OR51V1, ORC2L, POLR1D, PORCN, PTGER3, PYGB, RAC3, RHOA, SDC1, SMURF1, STX1A, SULT1A2, SYNJ2, TFPI, TNFSF4, TRADD, TUBB4, UGCG
Negative_373	C3, ALG12, ARD1A, CCR8, CDKN2B, DHRS9, F13B, GAD2, GMPR, HRAS, ICOS, INPPL1, NR4A1, OR2G6, OR6C76, ORC2L, PFKFB3, RDH5, RPE65, RPS6KA1, SLC25A5, SPCS3, ST3GAL1, STT3B, TP73, TRIP10, UBE2L3
Negative_374	CRH, MPI, NF1, ADCY9, ATP6V1F, BCL10, CCR8, CLDN20, CSH1, CTBS, CUL1, GAD1, GALNT3, GCNT2, GCNT3, IL23A, IL24, ITGA9, MAP4K1, NCR1, NUDT12, OR10A3, OR14A16, OR2S2, ORC2L, PAK4, PDGFRB, PHKA1, PKMYT1, PSME3, RAF1, SCLY, TNFSF8, TNXB, UGCGL2, WNT10A, PLA2G12A
Negative_375	CFD, DDC, F9, FGG, IHH, ACMSD, ACSS2, ALPI, CDC20, CES2, CLTCL1, CREB1, DDX19A, ELK1, G6PC2, GNPAT, GOSR2, GYS1, HLCS, HSD17B3, LCP2, LTC4S, NKD1, NR1H3, OR13J1, OR1C1, OR1K1, OR4N4, PCCB, PDE9A, PLCD1, PPA1, PRKAG3, PRKCB, RPS6KA4, RUNX1T1, SDC1, SEMA7A, SNAP25, SOAT2, SV2B, TAS2R50, TAS2R9, TNFSF14, WWP2, ATP6V0D2, CSNK1A1L, TNFRSF17
Negative_376	CAD, GBA, GK2, ME3, TNR, AARS, ALAD, ANAPC2, CBLC, CDKN2D, CLDN10, CLDN18, COQ6, CTSB, CX3CR1, CXXC4, DAAM1, FRAT2, FZD10, FZR1, GLDC, IARS2, IFNA8, KARS, KIR3DL1, LARS, MYH8, NOS1, NR1D1, OR51F1, OR52A1, OR56A4, OR8D4, PCYT1B, PIK3C2G, PPP1R3C, PRDX6, PRPH, SETD1B, SETD7, SOAT1, SULT1A2, TUBA1B, VASP, WNT5B, YKT6, PPP1R12A
Negative_377	CGA, FAH, TH, AADAC, ATP5J2, B3GALT4, BCKDHB, C4BPA, CD44, CDC45L, CEBPA, CLDN3, CLOCK, CYP1A1, EFNA3, GALNT5, GNA11, GSTM5, HCCS, IGF1R, JAK2, LDHAL6B, LRP6, MYD88, MYLK3, NANP, NFYC, NSDHL, OR1D2, OR2A5, OR4D11, OR52R1, OR8D2, PDE4C, PIGT, PIP4K2B, PPID, SH2D2A, SOS2, TCF7L2, TSC2, VAMP4, YWHAE
Negative_378	BET1L, C7orf16, CLDN4, IQGAP1, OR51A4, PIP5K1B, HSP90AA1
Negative_379	ME3, ADIPOR1, APAF1, APOA1, ATG3, BIRC2, BMP8B, CCL8, CES7, CMBL, COL3A1, CSF1, CYP11B2, DCTD, DDX50, GALM, GRAP2, GRM5, HES1, INSR, KRAS, LAMC1, LDHAL6A, NT5C2, OR1J4, OR2C3, OR52N5, OSMR, PHKB, PRKCE, RDH8, SDC4, TAS2R42, TJP3, ULK1, VEGFB, ZNRD1
Negative_380	AK7, C6, GH1, ALG10, AMDHD1, CDC14A, DOCK1, EARS2, IL20RB, MMP14, OR8K5, THY1, WEE1, WNT10B
Negative_381	ADA, APP, BAD, F5, GHR, OSM, ACSL3, ADRB2, AQP7, ATP13A2, CBLC, CES2, CHIT1, CYP2A6, DGKB, DTX3L, GARS, GDE1, IARS, IDH3A, IL12RB2, LALBA, MGAT1, OR4A16, OR6A2, PARVA, PHKA1, PIP5K1B, POLA2, POLE4, PRKCE, RAB5A, RND1, SMAD6, SPRED2, ST3GAL3, SV2C, TBL1XR1, TJP2
Negative_382	FGA, HAL, PAH, VCL, ATG7, CBR1, CPS1, CYP11B1, DDOST, EPAS1, FASLG, GAB1, GLYCTK, GUSB, MAML2, NEU2, OR4S1, PLAT, PMM1, PPP3R2, RAB5A, SCD5, SPTLC1, STX2, UBA1, ST6GALNAC5
Negative_383	MOV10L1, NME7, PDE8B, RDH12
Negative_384	FES, IL3, ACSL3, ACSS3, ADH5, ADRA1D, ALDH7A1, ALDOB, BACE1, CADM3, CALML5, COL6A1, HAGHL, IFNA5, IKBKE, ITGA9, ITGB8, MAPK3, NT5E, OR10A5, OR4F15, OR5A1, SHC2, MAPKAPK3
Negative_385	MPL, ACSS1, ADCY9, CACNB4, CXCL1, CYFIP1, GMPS, GUCY1A3, MCM5, MDM4, OR5AC2, POLR2C, PPP1R1A, RAD51, SELL, SESN3, THBS1
Negative_386	ACVRL1, APLP1, GALNT3, GNG12, IDH3G, PPCDC, UGT2B28
Negative_387	GSS, ACAT2, CHRM1, CLDN5, GNB1, IGF1, MYL2, POLA2, PPP1R3C, SLIT1, TAS2R13
Negative_388	AHCYL2, ALDH2, APC2, ATG12, B3GALT6, CCNB2, CTBP2, CYBA, DAPK3, DLL3, EPHA7, GALNTL5, GNA14, IFNGR2, LAMB1, MGAT1, NEGR1, OR6B3, OR7A17, PYGL, SMAD1, SMPD4, SPTAN1, SRD5A2

Negative_389	ACADM, APPL1, CLOCK, CX3CL1, CYP3A7, DERL1, DIAPH3, ESPL1, GNAO1, HNF4G, IL19, IL20RA, IL3RA, ITGA8, ITGAD, LCAT, MAP2K5, NRXN1, OR3A3, OR52D1, OR5T3, OR8D4, PGAP1, PITX2, PKMYT1, PRDX6, PTPN6, RFX5, RPS6KB2, SAT1, SMURF2, SSH3, TAS2R3, TYMS, UGT2B11, ULBP3, UPP1
Negative_390	AGPAT3, CHST12, CXCL16, GAS1, HNF4G, LAMC2, MAPK10, MFNG, OR9Q1, PGM1, PIK3C2B
Negative_391	GGH, GSS, RDX, CACNB1, CACNG1, CAV1, CCR2, COL6A1, CXCL1, CYP1B1, DDIT3, DHRS9, EPAS1, EPHA5, G6PC, GLUL, GSTM5, GYS2, HARS, IDH1, JAG2, MAP2K5, MAPK13, MBL2, MCM2, MKNK2, MYH4, OR10Q1, OR2S2, OR2T3, OR4K5, OR8J3, OR9I1, P2RX2, PIK3R3, SEMA3C, SFRP1, SMARCA5, SYNJ2, TAS1R1, TIAM1, TRPC1, UBE2L6, UGP2, UPP1, VEGFA, ZAP70, ATP6V1C1, B3GALNT1
Negative_392	GHR, LCT, MUT, ACAD9, ALPL, BLNK, CYBB, DUSP6, FZD3, IFIH1, IL1R2, OR2D2, OR4C15, PIK3CD, RBX1, RPN2, SEMA3F, SRGAP2, TBL1Y, SUV420H1
Negative_393	ALCAM, DKK2, DOCK1, KLRC3, NOTCH2, NOX1, PCYT1B
Negative_394	CR1, ACP2, CACNG4, CASK, CAV1, CCL14, COQ3, DDX52, EDN1, EREG, ICAM1, MYH10, NEGR1, NUDT9, OR2AG1, OR6S1, SERPINB5
Negative_395	LIF, NLK, NOG, PTS, ABLIM1, ALDH3B1, ARD1A, ATIC, ATP2A1, BDNF, CD79B, CDC25A, CSNK1G1, CXCR3, DAG1, EIF4B, FPGS, GNA11, HSD17B8, IKBKE, IL1B, IL21R, MYH13, NCOR1, NPR1, OR11G2, OR3A2, OR4C15, OR5V1, POLE4, PPOX, STAT4, SUV39H2, TBK1, UGT1A10, PPP1R12A, SUV420H1
Negative_396	IL4, LHB, PGP, SDS, ABL2, ACP6, AGPAT6, BBOX1, CACNG5, CD55, CHKB, CRLF2, CYP3A5, CYP8B1, DDX55, ECHS1, FABP4, FASLG, FPGT, GAD1, GNAI2, GSTO1, HARS2, ICAM2, IFNA10, IL24, IPPK, MAP3K13, MAP3K2, MYH3, OR10H1, OR10S1, OR14A16, OR2T12, OR51A2, OR5D16, ORC3L, PAPSS2, PDE1C, PIK3CA, PIM1, POLR2K, PRODH2, SLC12A2, SPRED1, SRGAP1, ST6GALNAC3
Negative_397	FABP1, LLGL2, SPCS1
Negative_398	ABAT, ACTN1, AKR1C4, ALG13, AMY2A, ATP5J2, ATP5O, BCMO1, CACNA1E, CACNG1, CACNG7, CDC20, CHRM2, CLDN2, COASY, CYP1B1, DEGS1, FARSB, GLO1, GNA14, GRIA2, IL17B, IL20RB, JAG1, MINPP1, MMP1, OR13C5, OR2A4, OR6N1, OR6T1, OR8J1, PDE4B, PGM2, PHKB, PIK3C2B, POLE, POLR3B, PTPRM, SPRY2, STX1A, TRPC1, ATP6V1G3, PPARGC1A, TNFRSF17
Negative_399	OGT, AKR1B1, ALG1, ATP5G3, CD55, CHRM1, ENTPD8, EXTL3, FABP4, FRAP1, FTCD, FUT2, GFPT2, GP1BB, HSPA8, HTR6, IQGAP1, MAPK10, MICA, MYH7B, NADSYN1, NCK2, NMNAT1, NRG2, OR1F1, OR5B12, OR5C1, OR5H6, OR6K3, OR9A2, PFAS, PLCD4, PPP1CA, PPP2R5D, PYGM, RFWD2, RGS3, SDC1, STX7, SULT2B1, THY1, TLR3, TUBB2C, KIR2DL5A
Negative_400	HYI, WAS, FGFR3, IL20, ITGA5, OR4K1, PLXNA3, STK3
Negative_401	AACS, ANAPC7, B3GNT2, BST1, GRIA2, HSD3B2, IL10RA, IL21R, MAOA, NCAM2, OR8K5, PON2, PPBP, PTPRM, SEMA3D
Negative_402	CDH1, DAD1, NRXN3, OR1M1, PPP1CC, PPP2R2A, RBKS, RFWD2
Negative_403	CS, F7, ALOX15, B4GALT7, CASP10, CD19, DAPK2, FCGR3B, HAGH, HTR4, NOX3, OR10H1, OR51S1, OR52D1, OR8A1, PNPO, RDH5, SEPHS1, SLC33A1, ST3GAL3, ULK3, WASL, XIAP, CACNA2D2
Negative_404	AR, IDS, LEP, TNC, ALDH1L1, ARHGEF2, B4GALT6, BCL10, CARS, CASK, CLCA2, CLDN1, CLDN4, CMPK2, CXCL13, DKK1, DMGDH, ENO2, ENTPD7, FECH, GUCY2D, HTR5A, INPP4A, ITGB1, MDH2, NCAM1, NSDHL, OR10K2, OR1S1, OR2G2, OR2M7, OR52N5, OR5T1, OR6M1, ORC6L, P2RX2, PIP4K2A, PLXNC1, PPP2CA, PTPRC, RASA2, RELA, RRM2, STX6, TUBA8, UGT2B4, UROC1
Negative_405	LIF, SCD, SRF, TFG, CACNB2, CCL21, CD48, CFTR, CSF3, CYP2A13, DLL1, FGF11, GNA11, GNA12, GUK1, HAO2, HSD17B8, INHBB, IPMK, LAMB4, LDHB, LIPA, MCEE, NLGN3, NUMB, OR10Q1, OR2A14, OR7D2, PDGFC, PI4KA, PIK3C2A, PLEKHA8, POLR2F, PRLR, PTDSS2, QPRT, RAPGEF1, RRM2, RRM2B, SARDH, SORD, SRGAP2, SUCLG1, TUBA1C, PPP1R12A, ST6GALNAC1, TNFRSF1A
Negative_406	ACSL6, CXCL11, DUSP3, LRP5, OR1A2, SDC4, ULK1, SUV420H1

Negative_407	CD2,CFD,FES,AASDH,ABLIM3,ATP4A,B3GAT1,BHMT,BMPR1A,CCL25,DAAM2,DDX51,DEGS2,DFFB,EXTL2,GAD2,GALNTL5,LTA4H,MAP3K14,MCEE,NKX6-1,OR2F2,OR52B6,OR7G1,PLCB3,PPOX,PTPN5,PYGB,RPE65,SUV39H1,VAMP1,ATP6V0A2
Negative_408	AK2,APP,CD4,ACVR2A,ARSB,ATP4A,ATP5A1,ATP5I,ATP5L,CACNA1H,CD82,CHPT1,CLTC,CTSG,CYCS,CYP1A1,DHRS3,ERBB4,FARS2,GSTK1,HLA-F,ITPA,LEFTY2,MADCAM1,MAML3,NCF4,OR10A3,OR10K1,OR4C13,OR4C6,PANK4,PON2,PPP2CB,PPP2R5A,PRKAA2,RAC2,RELA,RPN1,SDC2,SDHA,SELL,SMPD3,SPRED2,SUV39H2,TCIRG1,WARS,WNT7B,ST6GALNAC6
Negative_409	HDC,ACSL5,APBB1,ATIC,CASK,CDKN2C,DEGS2,FABP1,FGF7,ITGB8,LRP5,MAPK3,NTRK2,OR5H6,PLA2G2E,POLR2L,SPRED1,TICAM1,CTNNBIP1
Negative_410	BAX,CSK,ID3,CCL28,COQ6,CTNNA1,EP300,EXT2,FGF20,GUK1,GYS1,IFNAR2,IGF1R,ITPA,MINPP1,NEU2,ODC1,OR2D3,OR4K2,PRKAA1,RALB,RASGRP2,RPSA,SETD8,TAS2R5,HLA-DPB1,ST6GALNAC4
Negative_411	C8B,GLS,IL3,ADCY2,ALG12,ALG9,BCKDHB,BHMT,CDK5,DHCR24,EPAS1,FASLG,FGF12,FUT4,GALM,GANC,HRAS,IL17B,LAMB3,LARS,LLGL1,METTL6,NLGN3,NRP1,NTSR1,OR11H4,OR13C5,OR2C3,OR2F1,OR8D4,PAX6,PIGN,PLXNA2,PTGER3,RBL1,RHOD,SLC9A1,SMAD7,STAT1,STX1B,STX3,TAS2R1,TGFA,TNFRSF9,UGDH,SERPING1,SUV420H1
Negative_412	GPI,MDH2,NCR1,OR2A42,OR52W1,SERPINA1
Negative_413	ACVR2A,ADSSL1,ATG12,BAAT,CCL1,CDH15,GRIA2,HNF4A,MARS2,MLLT4,NCOR2,NSDHL,OLR1,OR13C8,OR2L3,OR4B1,SFRP2,STAT5A,TAS1R3,WNT1
Negative_414	XDH,CACNB3,CALML5,CAPN2,CCL17,CNDP1,CREB3L4,CYP3A7,EPHA8,EPHB2,IL5RA,MCEE,OR1J2,OR6C3,POLD2,WNT10B,WNT4,SUV420H2
Negative_415	CD6,FN1,ACSS2,ALG8,ATP2A1,CACNA1D,CACNG6,CCNG1,CD8A,CES7,COL5A3,CTNNB1,DPM2,DUSP1,EXTL1,FSHR,GCAT,GSTO2,HPRT1,HSD3B1,INPP5A,MAPK10,MGAT5B,OGDH,OR2AG1,OR4F15,OR51Q1,OR5AU1,OR8B12,PAK4,PDE1B,PDGFD,PIGA,POLR2I,RAF1,RBPJL,ROCK2,STAT1,STK3,UNC5B,VAV2,HLA-DRB3,ST6GALNAC4
Negative_416	ALPI,ARPC1A,BMP6,CNTNAP1,CREBBP,EPHA3,GALNTL2,HSD11B2,ICOSLG,IFNA8,IFNG,IL10RA,IRAK1,IRS1,LAMC1,MAN1C1,NARS,NRG1,NRXN3,OR10H1,OR1D2,OR1J1,OR2B2,OR51L1,PDPK1,POLR1A,POLR3G,PRLR,SPP1,TCF7L1,SERPINF2
Negative_417	AMH,AMT,SRC,ADAM10,AGXT2,ALG12,ALPPL2,CD22,CSNK1E,DDX55,EGLN3,EXOC7,FABP4,FARSB,FGF5,FPGT,FZR1,GALNT2,GALT,GGT7,GLUL,GNA12,HAO1,HMGCS1,HMGCS2,IL8RA,LDHC,MASP1,MDM2,MPDZ,NCR2,NFATC1,NPR1,OR1A1,OR2D3,OR2T6,OR4K2,PAK7,PDE7A,PLXNC1,RARA,RASA2,SETD8,SLC2A1,SPRY4,TBL1Y,TRAF6
Negative_418	SFN,AARS,LFNG,MDM4,NEUROD1,OR52L1,PPP2R5B,PTTG1,SETMAR,SMAD5,STAM2,ATP6V0A4,HSD17B10
Negative_419	FH,AMPD2,APPL1,AXIN2,BCL2,BCMO1,CCL13,CYP27A1,GRB2,MAD2L1,NCOR1,OGDHL,OR3A1,OR4D9,OR5T3,PIK3CA,PTDSS2,SFRP1,SHMT2,SSX2IP,PAFAH1B1
Negative_420	ALG3,ALG6,CASK,CLDN1,CLTC,COMP,ERO1L,IFNA16,OR10H1,OR11H4,OR6X1,PFKFB4,PIGW,PRNP,SLIT2,WASF2
Negative_421	ACTG1,ALDH6A1,ALG9,ARAF,ARG1,ATIC,ATP4A,BCAT1,CCL17,CCL8,CHPF,COL2A1,COMP,CSF3,CSNK1D,DLAT,GNB3,GYS1,HPRT1,IPPK,IRF3,MAP3K7,MAT2B,MCM2,MYD88,MYH11,NAGS,NFAT5,OR10G8,OR1L6,OR51B4,OR5A2,PARVB,PIK3R1,POLR3B,PRKAB2,PRODH2,RPN1,SPCS1,TAS2R13,THBD,TRAF3,WHSC1,MAPK8IP3

Negative_422	HGF, ILK, RDX, ACAT1, ACSL6, ATP6V0B, CD244, CLCA2, CSNK2A2, CTNNB1, CYP11A1, DKK1, FGF17, FZD8, GNA12, IRAK3, ITGB1, MAPK3, MYH8, NFKB2, NT5C1A, OR1C1, OR2A2, OR4K2, OR4Q3, OR52N2, OR5B17, OR6T1, PARD6G, PGK2, PIPOX, POLR3B, PPP2R2A, PPP2R2B, PRKAG3, PVRL4, SMAD4, SMURF2, SNAI1, TAS2R7, TBK1, TCIRG1, TRAF3, UGT1A10, WNT8B, GABARAPL1
Negative_423	CEL, GGH, BCAT1, GALC, GANAB, GYS1, IL6R, LIFR, LIPE, MAGI2, MAOA, MDH2, MGLL, NEU2, OR2AE1, OR2T8, OR5D18, ORC3L, PLXNB2, SOAT1, ST3GAL6, TPM3, VEGFB, WHSC1, WNT5A
Negative_424	CACNA1A, CACNG6, GSTO1, ITGB7, OR13G1, SELPLG
Negative_425	CAD, ME2, ADH7, AMY2A, AOC3, ARG2, B3GAT3, B4GALT2, BACE2, CD80, CNTN2, COL5A1, DDIT3, DTX4, E2F5, EARS2, EGFR, G6PC, GRLF1, GTSE1, GUCA1B, GUK1, HSD17B1, HTR6, IL15RA, IL1R2, MAP3K8, OR2AG2, OR4L1, OR6S1, PDE1C, PFN3, POLR2B, POLR2K, PRKAG3, RFXAP, SSH1, TRAF6, UGT1A10, WASF2, WNT8A, PDCD1LG2, PRICKLE2, SUV420H1, TNFRSF1A
Negative_426	EGF, ATP5J2, CADM3, CDKN1B, CERK, DVL3, ENTPD4, EREG, FASN, GMPS, LAMA4, LDHAL6A, MYH9, MYL2, OR10G4, PDHA1, RCHY1, SH2D1B, SLC2A4, SQLE, SSH2, TAS2R8, TRAF3, CTNNBIP1
Negative_427	GCK, UBB, ACP5, CADM1, CLDN4, DHRS4L2, DHRS9, DIAPH3, GSTM2, GSTM5, HEXA, HNF4G, HRAS, INMT, LHCGR, MYH13, OR10AD1, OR11H6, OR11L1, OR6C2, P2RX1, PFN2, PIGB, POLD4, PPP1R3A, PRKAG3, PTPRR, PYCR1, ROBO2, SLC27A5
Negative_428	CD2, CKB, ID2, KIT, ADH4, ADH5, ALCAM, AMY2A, ATP6V0B, CCL14, CSF2RB, DGAT1, EI24, EPHB4, FGF2, IDH1, KAT2A, MAGI2, MINPP1, NFYB, OR2B6, OR2T3, OR5K1, PFN1, PIK3C2G, PIP5K1A, PLXNC1, PNPLA4, POLR1D, PPM1A, PTCH2, SETDB2, SSH1, ST8SIA1, TAS1R1, TAS2R10, WBSCR17, HLA-DRB4
Negative_429	C8B, ILK, SCD, ADRB3, AKR1C3, AMD1, ARSE, CD8B, CHAD, CHRM3, CLTB, CREB5, CSNK1G2, CTNNA1, DIAPH1, FARSB, GALNT8, GGPS1, HIF1A, HPSE2, IDI1, IFNA16, IL11, ITGB1, MALT1, NT5C1A, NTNG1, OGDH, OR6N2, OR8D2, PDE6D, PIAS1, PIGF, PIM1, POLR1A, RBL1, RND1, ST3GAL4, STMN1, TAS2R14, TP53I3, TUBA8, UPP1, WNT2, ATP6V1E1, TNFRSF1A
Negative_430	MPDZ, NCKAP1L, OR4Q3
Negative_431	FN1, ATP5G1, BAIAP2, CCL20, CUL1, ECHS1, ELK1, ENPP7, NKX6-1, OR6C4, OR7G2, PLOD1, PPP2R2A, SMAD4, STK11, SUCLG1, UGT8, WNT16
Negative_432	SMS, TKT, ALPPL2, ARG2, CAV1, CD274, CD8B, CLDN2, CNDP1, CSNK1G1, DIAPH2, EFNA1, ERBB4, FUT7, GATM, GLB1, GSTK1, HSD3B1, KAT2A, LEFTY2, MYH9, OR13C9, OR13J1, OR2T8, OR5AK2, OR5D13, OR8B8, ORC2L, PCK2, PDE4D, PIK3R1, PLAU, PTGDS, PTGS1, SDHB, SLC25A4, UBE2K, UXS1, PLA2G12A
Negative_433	AGL, GNE, ALDH1A3, ALDH6A1, AMY2A, APH1A, CACNB4, CCND1, CHDH, EPAS1, FDPS, ITGA10, MPST, NCK2, NFATC1, NFYA, OR2Z1, OR8H3, P4HA2, PLA1A, PON1, PYGM, RUNX1T1, SNAI1, TUBB2B, TYRP1, UGCG, VASP, PLA2G12A
Negative_434	AMH, TK1, ADSSL1, ARHGEF1, CACNG7, CCL2, CD14, CDK2, CLDN16, CTSB, CXCL13, DDHD1, GALNTL1, GP1BB, HHIP, IFNGR1, IL12RB1, IL1B, LIPE, LTBP1, NEU4, NUDT2, OR4M1, OR5D16, PFN2, PPP5C, SUFU
Negative_435	GSN, ALDOC, APC2, ATP5I, ATP6V1F, CCL23, CES1, EFNA4, FTH1, HDAC2, HMGCS1, MCCC2, PFKFB1, PIM2, PRSS3, CACNA2D1
Negative_436	ALPL, CCL11, IL21, OR1G1, ORC4L, POLE4, WNT5B, HLA-DRB4
Negative_437	AK1, GPI, AGPAT6, BAAT, BPNT1, CACNB4, CASK, CREB3L1, CRLS1, CSDA, CSNK2A1, CTBP1, ECHS1, FDPS, HAP1, HSPA2, IGF1R, ITGA3, LAP3, MECR, NEUROG3, OLAH, OR13C4, OR1J2, OR1L3, OR4F4, OR52N2, OR5D16, OR5H2, OR6T1, PKLR, SEMA3A, WWP1
Negative_438	ADORA2A, AGPAT1, ATP6V1D, CACNB2, CD8B, CDH1, FCER1G, FZD2, GUCA1A, NMNAT2, NOTCH2, OR10H4, OR4D11, OR52E8, OR56A3, OR6K2, PCYT1A, POLD1, POLR2B, PTPRB, RHOD, SNAI2, TJP2

Negative_439	C4B,DUT,ACADSB,AGRP,ALG1,ARPC2,ARPC3,ATP5E,BBOX1,BCAT1,BPGM,CCL25,COL4A4,CYP1A2,DGKH,DHCR24,DHX58,DNMT3B,ECHS1,ENTPD4,EPB41L2,FGF22,FRAT1,GPAM,GRB2,HTRA2,INPP5A,ITGB1,MMP2,NAE1,OR10G7,OR10K2,OR13F1,OR2M4,OR6C6,OR7D2,POLE2,PVRL4,RBL1,RBL2,SMARCA2,SMPD4,SSX2IP,ST3GAL4,TUBA4A,WBSCR17,WNT6,YARS2
Negative_440	AKR1B1,ARG1,CNGB1,FGD3,KIR2DL1,OR2T29,PLA2G4A,SMARCA5
Negative_441	AK2,AK5,TPR,ACSL5,BLNK,COL5A1,CX3CR1,IQGAP3,MAT1A,OR8J1,POLD3,SFRP5,STAT2,UBE2L6,UGT8,WNT3A
Negative_442	SP1,ADH1A,ARPC2,ATG3,BLNK,COASY,CXCL13,CXCL2,DGAT2L4,DPAGT1,DTX2,DTX3,EFNA5,FARSA,GARS,GNA11,GNB3,HPRT1,HSD11B2,IL4R,KCNQ1,KYNU,MAML3,MDM4,MGST1,OR10A2,OR10A4,OR2A14,OR4K17,OR7G1,OR9Q2,PGK2,PIGX,PPP2R2A,RFXAP,RPIA,SHC3,SLC25A6,TJP2,VDAC2
Negative_443	AGL,ACAA1,ACTA2,AK3L1,ALDH6A1,APBB1,APOC3,ATP5O,CNTNAP2,CTNND1,CXCL13,CYFIP2,DOCK1,EDN1,GNAO1,GP1BB,GSTM2,HTR2A,ICOSLG,IGF1R,IL18RAP,ITGB4,LEF1,MAP2K1,MAP4K2,NCOA4,OR3A2,PARD6B,PER2,PYGB,RASA1,SOCS7,STAT1,STK4
Negative_444	EPO,STS,ABL1,AGXT2,BRAF,CD79A,CFTR,COL2A1,CYP26C1,DDX52,ETS1,FGFR1,GSTM4,HIP1,IQGAP2,LEFTY2,MYH11,OR10G8,OR4B1,PARS2,PLCB4,PRIM2,PYGB,RAC1,TUBA1C,TUBB6,UGT2B11,WNT11,XYLT1,YES1,TNFRSF11B
Negative_445	DBT,GSS,SMO,ABLIM1,ADRA1B,ASPA,CNTNAP1,DDHD1,FZD4,MTHFD1,NT5E,OR51G2,PARS2,PER2,SREBF1,WASL
Negative_446	CR2,ADCY5,ADCY8,APAF1,APOE,B4GALT1,B4GALT3,CACNA1B,CACNB1,CADM3,CD3E,CHIT1,CYP19A1,CYP1A1,FGFR4,GADD45B,GGCX,GSTM5,HAGHL,HLA-DOA,HPSE,HSD17B8,KNG1,LRP6,OR2AT4,OR2V2,OR52R1,OR5D18,OR9G4,PCNA,PLA2G2F,PLXNC1,PMM2,POLR3H,SEMA7A,SPP1,TAS2R41,TBL1XR1,TNFSF10,TPH2,AASDHPPT,ARHGEF12
Negative_447	EPHA4,FGF9,IARS2,OR2T33,PANK2,PIK3R3,RAB5A,RAD51,RXRG,SEMA4A,TRHR
Negative_448	CTH,DCN,MVK,SCD,AGTR1,AKR1A1,ALG13,ALG2,APLP1,ATP6V0C,BBOX1,CDC14A,CDC7,CDH5,DKK4,ECSIT,FZR1,GCNT3,GNAZ,INHBA,JAG1,KLRD1,LDHAL6B,MAPK13,MTHFD1,NADSYN1,NUDT9,OR4C12,OR51L1,OR5AC2,OR5M9,PDE1A,PDK1,PIAS2,PIPOX,POLR3K,PRKACA,RERE,RHOQ,SLC27A4,SOAT2
Negative_449	AK5,CFI,LSS,ATP5G3,BACE2,BRAF,CLTCL1,CYP1A2,CYSLTR2,ENAH,FGFR2,FSHR,IL6ST,KCNB1,NAT5,NR1D1,NTF3,OR11H1,P2RX2,P2RX3,P2RX6,PER3,PIGL,PIK3CG,SETDB1,SMAD7,SPCS1,TCF7,TRAF5,UMPS,TNFRSF12A
Negative_450	DBT,PLG,ACP5,B3GAT1,CACNG2,CD40,CDH4,DARS2,DRD1,EHMT2,IL20,IL25,KIR2DL4,NEU4,OR2G3,OR52E6,OR5AR1,ORC3L,POLR2E,TGFBR2,SERPINE1
Negative_451	EHHADH,EXTL1,GNAL,GNB3,HAGH,OR4K15,PCYT1B,SUFU,C1GALT1C1
Negative_452	CEBPA,DMGDH,FCER1G,ITGB5,JUND,KIR3DL1,MYH8,NT5C2,PPP3R2,TLR2,PAFAH1B1
Negative_453	CGA,HK1,NPL,ALG11,APOE,ARHGEF4,ATP5J,BLVRB,CCNB1,CCND2,CD46,CLTB,CPT1C,CYP2U1,EPRS,FOXO1,FUT1,HMOX1,MCM3,NR5A2,OR10H1,OR10H3,OR1K1,OR2Y1,OR5H6,OR7D2,PARVB,PIGU,PKLR,POLR2I,PRKAR2B,RASGRP4,SENP2,TAS2R48,TAS2R9,TUBB3,ULK3,CSGALNACT1,TNFRSF6B
Negative_454	ADC,SMS,ACOX2,ATN1,CALML6,CD40,GSTA4,LAP3,OR4D5,OR5J2,OR5L1,OR6C70,OR6M1,P2RX5,PPP1R3C,ST3GAL6,SULT2A1,TRDMT1,VCAM1,WNT10B

Negative_455	ABLIM2,ACO1,ADH5,BLNK,C1GALT1,CAMK2D,DKK4,FLOT2,FUT9,GBA3,MTHFD1,OR1J2,PLAT,WNT6,XYLB,HLA-DQB1,TNFRSF14
Negative_456	GSR,IL5,ANAPC10,ATP12A,AVPR1A,BIRC3,CMPK2,FCGR2B,GCLC,GNMT,IL11,NUDT12,OR1F1,OR6N1,PFN4,POLR2L,SLC25A5,SLC33A1,TAS2R38,TAS2R9,TOMM40L,TUBAL3
Negative_457	ATP5C1,IRF3,MYL7,PIK3C2B,TNXB,WNT8B
Negative_458	GH2,ADRA1A,GALNT8,MAP2K4,MASP2,OR13C4,OR2K2,OR51F1,PFKFB2,SAT2,SC5DL,SSX2IP,STAM2
Negative_459	CKB,IL3,IL5,AANAT,AARS2,AKR1D1,ALCAM,AMY2A,ARPC5L,BCAR1,CFTR,CYP2C8,GGCX,LFNG,OR10H5,OR51A7,OR52B6,OR6Y1,PAPSS2,PDHA1,PRKAR2B,SC5DL,SULT1E1,TRHR,TRPC1,TNFRSF11A
Negative_460	B4GALT4,COL1A1,CYFIP2,DAPK3,GALNT2,GP1BB,IFNG,IL1RAP,IL22RA1,LYPLA2,MGAT4A,PDGFRB,STAT3,VAV3,HLA-DQB1
Negative_461	F2R,ACTN1,ITGAM,MYH14,OR2T8
Negative_462	DDC,XDH,APOA2,ARNT,B3GAT1,CCNB1,CHPF,CHSY1,CRLF2,FPGT,GNA14,HLA-DOB,HSD17B1,JAM3,KITLG,LDHAL6B,LYPLA1,MCM5,MKNK2,NFE2L2,NUDT2,OR10S1,OR2F2,OR4A15,OR4Q3,OR6Q1,OR7C2,PDE4B,PLAT,PLCG2,PLXNA3,PPP2R2C,PSME2,PYGL,TJP2,SLC25A31
Negative_463	C8A,DCK,FOS,NOG,ADCY3,ADH4,ALDH1L1,ALDH3B1,ARHGEF6,ATP6V1D,CALML3,CCKAR,CSNK1E,DGKH,DUSP4,ENPP7,EPHX1,ERBB2,ETNK2,HMGCR,ITGA11,KIR2DL1,KIR3DL1,MAD2L2,MAN1A1,MAT1A,NAT6,NRG2,NRXN2,OR14C36,OR4F17,OR52M1,OR6K2,PGAP1,PIP5K1B,PRKCB,PSME3,PTDSS1,RDH11,SMPD2,TALDO1,WASL
Negative_464	C1S,AKR1C3,ATP2A2,BDH1,BMP6,CXCL13,GNG13,IARS2,PLA2G4A,PRKCD,SH2D1B,SOS2,TALDO1,TRIP10
Negative_465	GPT,RB1,ACSM4,C4BPA,CD3E,CHAT,CLDN19,CLDN9,CNGA4,COQ3,CSF3,CXCL6,DDX47,ETNK1,HMBS,HTR2A,IL17B,IL1R2,INADL,IRS1,ITGAV,LRP5,MAPT,NAMPT,OR5T3,ORC1L,PDE7B,POLR2B,PPARA,PPP2R2D,PRKX,RRM2,SDC1,ST3GAL5,TRAF5,WNT6,TNFRSF10C
Negative_466	DLD,ABLIM3,ACMSD,ADH6,ALDOA,ARHGEF4,ATP5E,BDH1,BMP8A,C1GALT1,CACNA1C,CD19,CD226,DUSP3,DUSP7,FABP4,GART,GUCY1A3,ICAM2,IDH2,KITLG,MTAP,NAGS,NANP,NARS2,OR10AG1,OR10G2,OR13C4,OR2B2,OR2C3,OR52E8,OR52I2,OR52N5,OR5B17,OR5T2,PDGFA,PDGFRA,PIGV,RHEB,SFRP5,STAT1,STAT2,STK4,TAS2R9,VTI1A,WNT7B
Negative_467	GP5,MET,CACNA1S,CACNB1,CDC16,CHST13,CRY2,CSAD,DERA,FGF21,HADH,HSD17B1,IL1RAP,ITGA3,NFATC1,NFYB,NT5C2,OR10G9,OR10Q1,OR4N4,OR5M9,OR9I1,PIGX,PPP2CB,SOCS3,STX10,UGP2,WNT5A,CACNA2D2,HSD17B12
Negative_468	UBC,ABP1,ADRB3,ALS2,AXIN2,CPT1A,CXCL16,DHRS3,EGFR,HTR2A,IL25,METTL6,OR1L4,OR4K15,OR5AC2,OR7G2,RAB5A,RPN2,SLC12A2,STEAP3,TKTL2,TP73
Negative_469	DUT,FAH,MUT,OSM,ADSL,ARPC2,CLOCK,INPP5E,KIR3DL1,MFNG,NFKB2,ODC1,OR4P4,OR8J1,POLE4,POLR2F,PTGER1,RPN2,TUBB2B,UNC5A
Negative_470	OSM,PVR,ACO2,ANAPC10,AQP7,B3GAT3,CD226,CTLA4,CXCL11,FGFR4,GALNT12,GDE1,GJD2,IL17RB,ITGA5,ITGAL,LLGL2,MTFMT,NOD1,NQO1,OGDHL,OR2F2,OR51G2,PANK3,PCNA,PPP3CB,PROC,PSPH,ROCK1,SPTAN1,TRAF4,TUBB1,ATP6V0A1,SUV420H2
Negative_471	GSS,IVD,ME2,ALDH3A1,ANAPC4,APOE,CDIPT,CHUK,CIITA,CRLF2,CXCL1,CYP3A43,EARS2,EPHX1,FCER1G,GALM,GMPR2,HMOX1,HRH2,HTR7,IL18,IL22RA1,IL28A,ITGA1,ITGAD,KAT2B,KLRC4,LAMB2,MAPK7,MKNK1,NKD1,NME4,NUMBL,OR13G1,OR2L13,PGAM4,PIGH,PIP5K1B,PRPS2,SEMA4F,SMAD4,SOS2,SULT1A4,TYMS,VDAC2,XCL2,GABARAPL1

Negative_472	FGB,HAL,A4GALT,CD27,CXCL9,FZD10,GAL3ST1,GMPPA,MAPK11,NRG3,OR2C1,SRGAP2,TAS2R7,TAS2R9,TLN2,WNT1
Negative_473	CFD,DBT,F10,BET1,CACNG3,CMPK2,EDAR,EFNB1,FADS2,FUT5,HTR2C,IDH2,IFNAR1,KLRC4,NFASC,NPR2,OR1S1,OR2T10,P4HA3,PANK2,PER2,PIGK,STK3,TUBA1B,PNLIPRP2,ST6GALNAC5
Negative_474	ACSL4,EPHA4,HEMK1,OR1L6,PAX4,SORD,TICAM1,TUBA3C
Negative_475	C8G,ALDH5A1,CACNG2,CDK7,CLDN17,CPT1A,CYP3A43,DDX18,G6PC,GLI2,GNPAT,HHEX,HNF4A,ITGA5,NODAL,OGDH,OR11H6,OR13D1,OR1E2,OR2A2,OR4K17,OR52A1,OR5M9,P2RX6,PGLS,PLXNA2,PPP2R5C,PRPH2,SRGAP1,STK3,ULK3
Negative_476	GK2,HYI,ALDH3A2,ARSD,ATP5G2,B3GNT3,B4GALT7,CASP3,CCL8,CDC42,CDKN1B,COL3A1,EPHA2,EPHX1,FASLG,FGF1,FGF22,GCH1,GFPT2,GNPAT,GRIN2A,HEMK1,HYAL1,IKBKB,LDHB,MLLT4,MYH13,OR10A2,OR51T1,OR5AP2,OR8U1,OXTR,PARVG,PCK2,PIAS4,PIP5K1C,PPP2CA,PRKAG1,PRODH,SHC3,SPRED2,TLR3,TNFSF11,UGT2A3,VAV3,WASF2,ATP6V0A1
Negative_477	FGG,BAI1,CAMK2B,CD40,EPHA1,FSHR,GLS2,GNA14,GRHPR,HAGH,ITGAD,MS4A2,NR1H3,OR1L8,OR2H2,OR52H1,OR8H3,OR8J3,PFKFB3,PRSS2,RXRA,THBS2
Negative_478	HYI,PGP,ABLIM2,ACTN2,ACY1,ADRA1B,AGPAT4,B3GNT5,CALML6,CCL27,CREB3L4,CSF1R,CSNK2A1,CTBP1,CYP2C18,CYP2D6,DDIT4,EPHA8,FGF23,GNA11,GSTZ1,HMGCS1,KITLG,LEF1,MCCC1,MFNG,NARS2,OR1E1,OR2AG1,OR2L3,OR4C12,OR5AC2,PARD6A,PER1,PFKFB3,PIGP,PIP5K3,PLAT,PLOD3,POMC,RXRA,TRAF2,TRAF4,UROS
Negative_479	ARPC1B,ATP5H,BMP8B,BMPR1A,CAMK2D,CD70,CDKN2B,CLDN7,CNTN2,COL5A1,COMT,EHMT2,FGF1,G6PC,GCLM,HTR5A,KYNU,LRP6,MKNK1,NEU3,OR13C3,OR1J2,OR1L6,OR2T12,OR4K13,OR52N2,OR5T2,OR6C3,OXTR,PAFAH2,PAK4,PANK1,PDE4A,SNW1,SOX17,STX4,SUCLA2,TFDP1,TFPI,TLN1,TUBA1B,TYMS,UBA1,UGT8,VAMP4,TNFRSF10C
Negative_480	SRC,ADCY3,ADCY7,AKT2,ASIP,ATP6V1D,CCKBR,CCL15,CHAD,CHRM2,CYP11B1,DVL3,EPHA5,EPRS,FECH,FGD3,FGF23,GNAZ,HMGCR,IL28A,KIR2DL2,MAN1A2,MAP4K3,NAGLU,NARS2,NFE2L2,NFKBIA,NLGN3,NOX3,OR5V1,PCYT2,PDIA4,PFAS,PLXNB1,POLR2D,PVRL3,SGMS1,VAMP3,VTI1A,WASF3,ATP6V0D1,HSD17B10,ST6GALNAC5
Negative_481	HDC,MVD,AMHR2,B4GALT1,CADM3,CAPN2,CCL8,CHIA,CHSY1,DPYSL2,EPHA4,FDPS,FZD5,GDF5,GSTO2,HAGH,HAP1,HIBADH,IL17RB,ITPKA,LIPG,MPP5,NAT1,NCR2,NOTCH3,NTNG1,OR11H6,OR4C12,P2RX3,P2RX7,PARVG,PDIA4,PFN4,PLA2G5,PLEKHA8,POLA2,PSEN1,PSME2,PTGES,SEMA4A,SETMAR,SLC33A1,SRD5A1,STAT6,UPB1,UROS,WNT9A,CACNA2D1
Negative_482	IL8,SDS,TK2,VCL,AACS,ANAPC10,ARPC5L,ATP6V1A,BCL2L1,BIRC3,CCL19,CDC7,CSNK1G2,CXCL5,CYP3A4,DAD1,DGUOK,DHCR24,EPHB4,FGF23,GANC,GGT5,GNRH1,IDI1,IL10RB,KAT2A,MAP3K14,MAP4K4,METTL2B,NCR2,OR51V1,OR8H2,ORC3L,PDE7A,PI4KA,PLA2G2A,POLR1D,POLR2G,PPM1A,PRLR,PSEN1,PTCH2,QARS,SDHA,SIAH1,ST3GAL4,YOD1,CACNA2D3
Negative_483	GSR,MET,ASAH1,ATP6V1D,ATP6V1F,CREB3L2,CSDA,CSNK1E,ERO1L,IDH2,IFNA10,ITGA11,LARS2,MEF2C,NFKBIA,OR52K2,OR8B8,PDK1,PLEKHA8,RAP1B,RARS2,SIAH1,TM7SF2,WEE1,ST6GALNAC3
Negative_484	ID4,AKR1C2,AMY2B,ATP6V1A,BDNF,CDC26,CRLS1,CYP2C9,EI24,FZR1,GALC,GCNT2,GRIN2B,IL2RA,IL5RA,KLRC1,P2RX5,RBL1,STT3B,TYROBP,WARS2,XCR1
Negative_485	CGA,DCC,F8,ACP2,ALDH4A1,C1QB,CER1,CTBP2,DLL4,FDPS,FUT4,GALK1,GNA13,GYS1,HMOX2,METTL2B,NMNAT2,NTF3,OR13C8,OR1J1,OR4K5,OR52E4,OR7A10,ORC6L,PDX1,PLOD3,PNPO,PTGIS,TAS2R49,TNFRSF10C

Negative_486	LAT, OAT, TFG, AFMID, BNIP1, CARS, CCL20, CD79B, CDC14A, CDS1, CXCL6, DAAM1, DHRS3, DRD2, FPGT, GDF7, GPAA1, GSTM2, HADHB, IGF1, ITGA11, KLKB1, NEU2, NT5E, OR10K1, OR6C74, OR9G1, PAFAH2, PLA2G5, PLOD2, RHEB, ROCK1, SHC3, TSHR, VARS, ATP6V1E2
Negative_487	CTH, HPD, UBB, AGPAT4, AGRP, CACNG8, CDC25C, CES7, CHKB, COL11A1, COQ3, CPT2, CXCL11, EHHADH, GLG1, GLO1, IDH3A, IL22RA2, JAM2, KITLG, LIMK1, NRG3, OR2T4, OR51F1, OR51L1, OR5B17, OR6C6, POLR3B, POLR3G, PPP2R5B, PRIM1, ST8SIA1, TALDO1, TAS2R7, TGFB1, TGFB3, VEGFC, ATP6V1G2, PHOSPHO1
Negative_488	ACSS2, GNPDA2, GRIA3, MGST3, SH2D2A, TAS2R38, ST6GALNAC5
Negative_489	ATM, ME3, CD40LG, CDKN1C, CNTN1, CSF3, DLST, DUSP4, EDNRB, GBE1, GCNT3, GFAP, IL25, INHBB, ITGAM, LAMC2, LRP5, MECR, NFATC1, OR1L6, OR51E2, PNPT1, PRKG2, SCP2, SMPD2, SOS1, XYLT2
Negative_490	AK5, ABLIM1, FARP2, PDHA2, PFN1
Negative_491	DDX55, IL13RA2, TAS2R5, TBK1
Negative_492	DBT, KHK, ARHGEF2, C4BPA, CBLB, CBLC, DMGDH, FOXO3, FZD2, GLUD1, H6PD, HAGH, HSD3B7, MTMR1, NOX3, NTSR1, OR4D9, PDE1C, PECAM1, POLE, PVRL4, RALB, VANGL2, VNN1
Negative_493	GRLF1, PFN1, UNC5A
Negative_494	DDB2, IFNA10, IL2RB, MMP14
Negative_495	CAT, F10, XDH, ACP2, AFMID, APBB1, ATP5G1, B3GNT2, BMP5, BRAF, CDC23, ENPP7, GSTM4, HCLS1, HTR5A, ICAM3, KCNB1, KIR3DL1, MAP2K1, MYD88, OR4N5, OR52R1, OR7A17, PHKG2, PPP1R1A, SH2D2A, SMAD1, STMN1
Negative_496	CSK, DCN, F12, HAL, ALG9, ATP4A, B4GALT4, CTNNA1, DHRS9, DUSP4, EIF4E, HNF4G, IFNAR2, LARS, NFKB2, NKD2, OR1J1, OR2V2, OR5B17, PPP1R3D, PTTG1, RAPGEF1, ST8SIA5, STAT5B, TAS2R7, THTPA, USE1, VAV2
Negative_497	DBI, TK2, APRT, ARPC4, NUMB, OR14I1
Negative_498	GSN, OSM, ANAPC1, ARHGEF2, ATIC, ENTPD8, GRIN2D, HMOX1, KIR3DL2, NT5C1A, OR8G1, PDE1A, PTGES2, RAC1, ARHGEF12, MAPK8IP3
Negative_499	NOG, PRL, ACOT12, AMDHD1, BLVRB, CACNA1H, CACNG6, CDC16, CDH1, CLCA4, DGKB, EPHA3, F2RL2, FARP2, G6PC, GPLD1, LARS2, MAPT, MMP7, MS4A2, NTSR1, OR56B4, OR8H1, PARVB, PFKFB2, PIK3C2G, PPARA, PPOX, PPP1R1A, SETDB2, STAT2, STMN1, STX5, WNT10B, YWHAB, CACNA2D2, HLA-DQB1
Negative_500	DCN, F3, GP9, PGF, ALDH6A1, ALG2, AQP7, B3GNT3, BRAF, CACNG7, CCNB1, CSF2RB, DGKA, EGLN1, EXT1, FOXA2, GAS1, HAP1, HLA-DOA, ITGAV, ITPKA, JAM3, NTN1, POLR1C, PSME3, SH3BP2, SKP1, SMAD3, TRHR, UNC5B
Negative_501	ARPC2, B3GALT4, BLNK, CDKN1B, IFT57, ITGA7, KIR2DL2, LEFTY1, LILRB3, OPLAH, OR1L4, POLR3G, SMPD3, SSH1
Negative_502	CBS, ACTB, ACY1, ARHGEF2, CD99, CYP3A43, DDB2, DHDH, ITGB4, OR10J1, OR10J3, OR10K1, OR6N2, PCK1, SMURF2, TNFSF12, UGT2B4, ATP6V1B2, PLA2G12B
Negative_503	ATM, FUK, AKR1A1, B3GALT1, B3GAT2, BIRC5, CCND1, CYCS, CYP2J2, DHODH, DLAT, FLAD1, GALNT1, GNA12, GRIN2D, GSTM2, HCST, HRH1, HTR7, ITGAV, LDHAL6A, LTC4S, MAN1C1, MCM2, NLGN3, NMNAT1, OR4A5, PDCD1, PRKCB, RASSF1, SMPD1, ST6GAL1, STK3, UCK1
Negative_504	GBA, HGD, PGF, ADH7, B3GAT3, BBOX1, CCNA1, CDC25A, CFL2, CHRD, DDIT3, DGKD, E2F1, HSD17B2, IDH2, MKNK1, NFKBIA, OR13A1, OR4C6, OR4X2, OR5T2, OR8K3, RAC3, SARS2, STX2, TLR2, VDAC2, GABARAPL2, TNFRSF10B
Negative_505	SYK, BCAT2, C3orf10, CPT1B, DHODH, DVL1, EXTL3, FLT4, GALT, GARS, IMPA2, ITPKB, LAP3, LYPLA1, MPZL1, PLEKHA8, PLOD2, POMC, PTPN5, RETSAT, SSH2, ULK2

Negative_506	DBT, AADAT, ATG12, CCKAR, CKMT2, CYBA, GSTK1, IFNA7, IL1A, IL6ST, NAT5, NCR1, OR4L1, OR52I2, OR6C70, OR6C74, P2RX3, PCCB, PGK1, PIGU, PIK3CB, PLA2G1B, PLXNB1, PTGES2, RALBP1, SMAD5, SMARCA2, SPRY3, TUBA3E
Negative_507	ADO, CR1, ID3, WAS, ACAD9, ARPC3, CDKN1A, CDKN2B, IL12B, IL17RA, IL18, IRAK2, KLRC2, MASP1, MGAT4B, MMP2, MMP9, NARS, NLGN2, OGDH, OR10J1, OR10R2, OR6N1, OR8K5, PIP4K2B, PRKY, RAB5A, SLC2A2, TSHR, UGT2B11, VANGL2, XYLB, HSD17B12
Negative_508	C7, CS, DHH, FER, PGD, RDX, ADH4, ALG5, ANAPC4, CACNG3, CD8B, E2F5, MCM2, MPDZ, NKD2, OR4B1, OR4X2, OR8G1, PFKL, PPAT, PYGL, WNT7A, ZFYVE16, TNFRSF13B
Negative_509	LTB, VTN, ACTN1, ACVR2A, ADH6, AGMAT, AGXT, AMY2A, ATG3, B4GALT2, B4GALT6, CD19, CD82, CDH4, CNTNAP1, CYP11A1, CYP3A7, DHCR24, DKK1, DPYS, ERBB2, ETS1, FDPS, GSTA4, HSD11B1, IDH1, IL15RA, LARS, MAML3, NMNAT1, NPR2, NUMB, P2RX2, PGM3, PLXNC1, PMM2, PNLIP, POLE2, PRKCH, PVRL2, RELB, ROCK2, ST8SIA1, THTPA, TUBAL3, TUBB4, WARS, CTNNBIP1
Negative_510	ADCY6, ADRBK2, ATP12A, AVPR1B, CCND1, CD59, CD80, COL1A1, ECSIT, EDNRB, FGFR2, HLA-C, ITPA, LDHA, LDHAL6A, LIPT1, LYZL1, NEFH, NFKB1, NLGN3, OR10A6, OR3A2, OR51G2, OR6C1, PDE11A, PLCD1, POLR2E, POLR3G, PPP3CC, RARS, SFRP5, SOCS7, STAT5A
Negative_511	VCL, ACSL1, AKR1C1, EIF4B, FMO5, IL13RA1, IRS4, MYH6, MYL5, OR10A5, OR13G1, OR2S2, OR4K14, OR5A2, ORC4L, PIAS3, PRSS3, SEMA3E, TGFB3, TP73, SERPINF2
Negative_512	GSN, PML, AADAC, ACMSD, ALOX12, ALOX12B, B3GALT2, CASP3, CCNB3, CFLAR, CNGA3, CRLF2, DDHD1, DPYSL2, GALNTL2, GRIN2B, IFNA5, IGF1R, IL19, ITGAD, LARS, MAP3K8, MGAM, MPP5, NFKBIE, OR2T33, OR51B5, P4HA2, PLAU, PPP2CA, PSEN1, PTK2B, PTPRJ, PYCRL, RAPGEF4, RHOA, ROBO1, SEMA4D, SPHK1, TUBA1A
Negative_513	ADK, BCR, CBL, CBS, CR1, CS, AADAT, AASS, ADIPOQ, ADSS, ALDH1L1, ALDOB, AMPD2, ANAPC5, ASIP, ATG7, BMPR1B, CALML6, CCNG1, FGF23, FZR1, GANAB, GMPPA, GRIN2B, GSTA1, INHBE, MGAT4B, OR10H5, OR4M1, PDHA2, PIGU, POLA1, POLR2E, PRLR, PRSS2, PTPN11, SUV39H1, TP53, YKT6, HLA-DQB1, SERPINC1, TNFRSF11B, TNFRSF12A
Negative_514	LPL, ACSM2A, ALOX12, ANAPC5, CCND3, CD247, CLOCK, CREB3L1, CXCL11, CXCR6, CYP4F3, DPM1, FABP4, FARSA, FGF19, GBGT1, HSP90B1, IFNGR2, ITGA2, MAP3K7, MTHFR, OR6K6, OR6Y1, ORC6L, PAK1, PNPT1, PPP2R2C, PSME1, RASGRP1, RASGRP2, SAT2, SCLY, SEPHS1, SMC1B, SPCS1, STK3, TBXA2R, TPH1, UGT2B17, VTI1A, YES1, PAFAH1B3
Negative_515	ADK, DDO, TBXAS1, TRPC1
Negative_516	AK7, HDC, MVD, AHCY, ALPL, ARSB, B3GALT6, CACNA1S, CACNB3, CACNG1, CD58, CDC26, CYP11B2, EIF4E, FLOT1, HEXB, IKBKE, IL28RA, LLGL2, MECR, OR1A1, OR4S2, PDE1C, PDE8A, POLR3D, PRKACA, PTAFR, PTEN, RAPGEF2, WNT5A, CSNK1A1L
Negative_517	BCR, TEC, ACOT12, APOA2, ARD1A, BCL2, C4BPB, CLDN18, CNTN1, CYP1A2, CYP2A13, DGUOK, DVL2, GALNT6, GGT1, GPD1, IL15, INPP4B, INPPL1, IPPK, JAK2, MTHFD1L, OR10A2, OR14J1, OR4D11, POLR3B, RETSAT, ST8SIA1, VARS, PLA2G12B, TNFRSF1B
Negative_518	AK5, BID, C1S, ACAD9, ACSL3, AOC2, ATP5G3, CYP2B6, ENTPD4, NFKB2, NFYA, OR2A42, OR4N5, OR56A1, PDE6G, PIP4K2B, PPP2R2B, PRKAG2, RAC2, RFX5, SGMS2, SLC12A2, TLN1, UNC5A, ZIC2

Negative_519	DBI, AARS, ABLIM2, ADCY2, AGPAT3, ARHGEF2, ATP5I, CACNA1A, CCL17, CCR5, CDH2, CDKN1A, CTSS, DDX54, DGKD, DHDH, EFNB3, FLAD1, GALK1, GATM, GCNT4, HSD3B2, ITPA, MDM4, NEGR1, NKD1, OR10K1, OR2A42, OR2H1, OR2T10, OR51I2, OR5A2, OR5AR1, PRKAA1, PTAFR, PTPRM, RRAS, SNAP23, TAS2R14, TICAM1, TPH1, TUBB6
Negative_520	DLD, LEP, NPY, TK1, ACADS, ACYP1, CDKN2A, COL2A1, CSF1R, CYSLTR1, DNMT1, EIF4E, FGF21, FRAP1, GCAT, GNG12, GRHPR, IL6ST, IPPK, MYH10, NAE1, NANP, NEU4, NFKBIB, OR10G7, OR2A5, OR4N2, PCYT1A, PIP5K1B, PIPOX, PLOD1, POMC, PPP2R2D, RAPGEF1, SLC27A5, SOAT2, TUBA3E, ULK1, VEGFC, WWP2, CTNNBIP1, GABARAPL1, KIR2DL5A, PHOSPHO1
Negative_521	ACAA1, DDX52, FUT7, MYH6, NCK2
Negative_522	SMO, ATP5D, ATP5J, CBR3, COQ6, CSNK1E, ETNK2, FLT1, GAD1, GNAQ, HBEGF, JUND, MCM5, PDE6G, PFN3, PRLR, SARDH, SDC4, SELPLG, SEMA4D, TALDO1, UGT2B10, WNT1
Negative_523	CACNG5, CCNE2, DHDH, EPHB2, MTHFR, NME4, OR52M1, OR5J2, PHKG1, PIK3C2A, PIPOX, SLC27A1, TRAF4
Negative_524	EBP, APAF1, BMP8A, DHRS4L2, EPRS, FGFR4, GOT1, HLA-F, IL17RA, LGMN, LHCGR, LTBR, MAPK11, OR4C12, PIK3CG, SDHD
Negative_525	FST, LPL, ME1, MET, ACTN2, ALDOA, APOA1, BIRC2, BMPR1B, CD47, CSNK1A1, EXTL1, GALNTL4, GLUD1, HCLS1, HIP1, HNF4A, IL12B, MCM3, MIOX, MYH10, MYH2, OR5J2, OR7A5, ORC2L, POLR3K, TAS2R7, TLN2, TYROBP, YWHAB
Negative_526	ASMT, BIRC2, CLDN3, CNTF, DOCK1, GALNT7, GIT1, IDH2, KIR2DL3, KIR2DS5, NANS, NEU3, PIAS2, PIAS4, PNPT1, PPP2CA, PRNP, SMURF2, SOCS4, SPHK1, STAT4, SULT2A1, TIRAP, TLR2, ZFYVE16
Negative_527	CCR1, PIK3C3, PTPRJ, TNFRSF14
Negative_528	FER, GHR, ALDH4A1, ALG11, CDH2, CLTCL1, CSNK1G2, GLI1, HMOX1, INMT, NKX3-1, OR4X1, OR6C1, ROBO2, SRGAP2
Negative_529	IL9, APOA2, CHIT1, GSTZ1, IFNAR1, IMPDH2, OR51F1, OR52B4, OR52R1, OR5M3, OR5P3, PDGFRB, PER2, RPRM, TDO2
Negative_530	BTC, F2R, AKR1C4, ARG2, ARRB2, CCKBR, CDKN1C, CYP2C18, DOT1L, EXTL2, FUT4, GALNT5, GALNT6, GALT, GFPT1, GMPR2, GNB3, GSTM1, HEXA, HMGCR, IL22RA1, ITGB8, KARS, MFNG, MKNK1, NEFM, NRG1, NRG3, NUDT8, OR10J1, OR2T33, OR6K3, P4HA2, PARD6G, PIAS1, PIGO, POLE3, PTEN, RBPJ, SC5DL, SH2D2A, SMPD1, SNW1, TGFB1, TKTL2, VANGL2, ATP6V1E2, PRICKLE1, ST6GALNAC6
Negative_531	EZR, LCT, MIF, TYR, AKR1D1, ALDH1A3, AMY2A, BMPR1A, BUB3, CCNE2, CHRD, CHSY1, CLDN16, CLDN2, DERL1, ENPP3, GFPT1, GRHPR, HSD3B7, IKBKG, IL6ST, LDHAL6A, MAP2K5, MAP3K12, OR1L8, OR56B4, OR5B21, OR5T1, PSEN1, PTGIS, QARS, QPRT, RALA, RASGRP1, RELA, SEMA3C, VEGFC, ARHGEF12, SUV420H1, TNFRSF1B
Negative_532	COQ5, GNAI1, NEU1, OR4C15, PLA2G2F, TAS2R9
Negative_533	GBA, SRF, APLP1, CLDN16, COL4A6, FZR1, GCDH, GNRH2, MCM2, NR5A2, PERP, PLA1A, PLA2G5, PRKAB1, RPS6KA3, SHMT1, TJP2, TPMT, TUBB6, UGT2A3
Negative_534	AGRN, AGTR1, AKR1C3, CSNK1G2, DGKZ, FGFR3, HPSE, IRAK1, METTL2B, NRXN1, NT5C1A, OR4L1, OR7C2, OR8J3, OR9Q1, PCYT1A, POLE3
Negative_535	CR2, ADCY10, ATP2A1, ATP5J, BMP8A, CDKN2C, CLDN2, DDIT4, GRHPR, IRS4, MAPT, MYH1, NAT5, NPR2, POLR1E, RARA, SMPD4, SULT1A2, UGT8, XCL2
Negative_536	EXT1, ITGB7, PIK3C3
Negative_537	AR, HDC, MVK, ADH4, ANAPC1, BIRC3, CRLS1, FZD10, GNA14, GSTP1, HMOX1, INPPL1, LCMT1, OR13J1, OR1E2, PIK3CB, PLXNA2, PRKAR1B, PSEN2, TLR2, TUBB3, B3GALNT1, MAPK8IP3

Negative_538	DCC, TPO, ZYX, ACTB, B3GALT1, CCR10, CD47, CHAD, CLDN15, CSDA, DAXX, DTX2, FZD7, GANC, GDF7, GRLF1, GSTA4, IL1A, MCM4, MGST1, MLLT4, NUDT9, OR4C11, PFKFB2, PNLIP, POLR2K, PPP1R3B, PPP2R5D, PROS1, SLC2A1, ST8SIA5, TAS2R10, TICAM1, TNFRSF9, XYLT2, PRICKLE1
Negative_539	AUH, F8, ILK, IVD, PGF, SPN, ADRA1D, AK3L1, ANAPC10, AQP7, CD72, CHIT1, DAPK2, EIF4B, FCGR2B, FGFR3, FUT2, HDAC2, HNMT, IL28B, IMPA1, LALBA, MAOB, MAP3K5, MAPK3, NUDT2, OR10A4, OR4C16, OR52J3, OR8H3, PDPK1, PERP, PHKA2, PLCD4, POLD3, PPAP2C, PSPH, RASGRP2, SARS2, SEMA3F, SEMA7A, SUFU, UROC1, SERPINF2, TNFRSF13C
Negative_540	F9, FGG, ADRA1D, AKT1, ATP5J, BDH2, CHAD, CLDN6, CYP4A11, DHFR, E2F1, EPOR, EREG, ETNK2, GNPDA1, GRIA1, MLLT4, NFYC, OR52N1, PFN1, PIGB, PTGDS, PTGES2, PTPMT1, SPTLC2, STMN1, STX5, UAP1, UGT2B4, YARS, HSD17B10, TNFRSF10B
Negative_541	CYP1B1, ERBB2, FARS2, OR10Z1, PFKFB4
Negative_542	GAA, RPE, ACY3, ALPI, AMPD2, C3AR1, CBR3, CCNE1, COASY, COL1A2, CPT1B, EFNB2, EPHB1, KAT2B, KIR2DL3, LAMB2, LIAS, MGAT5B, NFATC1, NOTCH2, OR4N5, OR56A1, OR5AC2, P4HA1, PIK3C2G, PPP2CA, PRPH2, TICAM1, TUBB2B, ARHGEF12, CACNA2D3
Negative_543	ARPC2, CSF3, GUK1, HLA-C, IL1RAP, IL23A, OR52E6, PDE1A
Negative_544	SMS, ALG9, CD46, CD80, DDX55, DPM1, EPHB6, GLYCTK, HIP1, IL5RA, IL7R, INHBB, ITGB6, KARS, MAGI2, MAP4K3, MEF2C, MGST1, MPST, NAGS, OLAH, OR1D5, OR2K2, OR4C12, OR4C15, P4HA3, PIAS3, PIGV, PPCS, RFXAP, SLC27A1, SMAD6, SOCS5, TAS1R3, TSLP, TUBB4, TYK2
Negative_545	AHCY, CACNA1D, CD36, CD55, CHIT1, CSNK1G2, CTNND1, CYBB, CYP2F1, DAPK3, EIF4B, ELK1, EPB41L2, FUT2, G6PC2, GADD45G, GDF5, GDF6, GNAZ, GNRH2, GZMB, HSD3B7, ICAM3, IL17B, IRF9, MAP2K5, MNX1, NR1H3, NUDT8, OR11L1, OR13F1, OR1B1, OR5L1, OR5M9, OR9Q1, ORC6L, P2RX1, PDE7A, PTPN5, RGS3, SELP, TKTL1, TP53, TXNDC12, MAPKAPK5
Negative_546	GDA, KHK, ACHE, ALDH1L1, C1QB, CCR9, CNTNAP1, CYBB, DEGS1, DLL3, IL1RAP, IL22RA2, ISYNA1, ITGB4, MYH15, NCKAP1L, OR10K1, OR2A2, OR4M2, OR6B2, OR8B8, PFAS, PIGT, PON2, PSEN2, RDH16, SARS, SDHA, SPRED1, TP73, TUBB3, HLA-DQB1
Negative_547	FGB, STS, CDH3, CDKN2C, EPHA6, MARS2, OR2L13, OR4M1, OR5B17, POLR1C, TAS2R7, VAMP1, WNT7A, PRICKLE1
Negative_548	CD2, GBA, LCK, MVK, AADAC, ACSM5, ASIP, BCAR1, BMPR2, CEBPA, CLDN16, CREB3L1, DAPK3, GPD1L, GSTM5, GUCA1C, ITPKB, MDM4, OGDH, OR2AE1, OR2K2, OR51B4, OR52B4, OR52N4, OR5P2, PLA2G4A, PLXNC1, SOAT1, SREBF1, STAT2, TYMS, UGDH, HSD17B10
Negative_549	GPI, UBB, ACVR1B, ARNTL, DARS2, FABP1, IDI1, LCMT2, NFKB1, PAX6, PDXP, RALA, SC4MOL, SHC1, SRGAP3
Negative_550	C6, PGD, ANAPC11, ASNS, BMP4, CCND2, CRLS1, CYP2C9, DERA, FZD5, G6PC2, GAL3ST1, HLA-DOB, NCK1, NPR1, OR10H3, SETD2, SOCS3, VAMP4, VEGFB, WEE1
Negative_551	C8A, GNS, MOS, AGPAT4, BDH1, CASP9, CCL14, CCL21, CCR6, CD74, CDC25C, CLTA, COL6A1, CREB3L4, DUSP7, FGF13, GARS, GATM, GFAP, GNPNAT1, HAGHL, IFNA16, IRS4, ITGA6, KIR2DL4, LAMC1, MAP3K4, NUDT8, OR1J2, OR4M1, OR9Q2, ORC2L, PLOD2, PSEN2, RELA, RXRB, SLIT1, STAT5A, TRAF3, TXNDC12, ULK3, CACNA2D4
Negative_552	DBT, ILK, ACADVL, ACTA2, ATP4B, BCAT2, CASP8, CAV3, CCL28, CLDN10, CTTN, FUT4, HDAC2, HTR2C, MGAT5, OR2H2, PPP3CA, PRKCD, SLIT2, TAS2R16, TOLLIP, UCP1, SERPINE1
Negative_553	FUT7, GNAO1, GRIN2A, GSTO1, MAD1L1, P4HA3, TCF7, WBSCR22

Negative_554	ACADL,ADH5,ALAD,BST1,CARD11,CNTFR,CYP26C1,FGF12,GBA3,ICAM3,IL18R1,KDSR,MAP3K12,MOV10L1,NT5C1B,OR2M7,PDGFA,POLR2E,PRKCA,PRKCH,TAP2,TAS1R1,TPK1,ATP6V0D1,HLA-DRB4
Negative_555	ATM,C9,MVK,ACLY,ACSL6,ADIPOR2,APOA5,ARHGEF7,BET1L,CHIT1,CLDN15,FGFR1,GGCX,GSTM5,IDI2,IFNAR1,IL10,IL4R,ITGB7,LIPC,LMO7,LTC4S,MGST1,NR1H3,OR10J1,OR51A4,OR52J3,OR5L2,PDE4D,PDE9A,PHGDH,PLCB2,PPP2R5D,PTPN7,RAB23,SOCS7,TAS2R60,TGFB1,TRAF5,TSC1,TUBA3E,TUBA8,ULK3,ATP6V0E1,ATP6V1C2,MAP3K7IP1,TNFRSF10D
Negative_556	BTC,AHCYL1,ATP2A3,BCAT1,CSF3,CYFIP2,DNMT3B,EDA2R,EFNB3,EHMT2,FARS2,FARSA,FCER1G,FGF21,FLT1,G6PD,GALNT5,GBGT1,GNRH2,HGSNAT,HHEX,HMBS,HPRT1,HYAL2,IL13RA1,LILRB3,MDH2,NCOR1,OR1I1,PDHA2,PFKFB2,PKM2,PLCB2,PPP2R2B,PTGIS,RASGRP1,ROBO2,RPS6KA4,SUCLA2,TUBA1B,WASF3
Negative_557	CFH,AKR1C1,C3AR1,CCL3,CLTA,CYP11B2,EPB41L2,FTH1,GPX5,GRLF1,IL1RAP,INADL,ITGAM,KLRC2,OR10H4,OR4F17,OR52N2,OR5A2,PARS2,PGM2,YWHAZ
Negative_558	CR1,CRK,FAS,GK,KIT,MUT,ACTN2,AGTR1,AOC2,ATG3,CD48,CXCR5,CXCR6,CYP3A4,DDB2,DGAT1,DHRS3,EPHA7,EPRS,FECH,FGF22,GATM,GSTT1,HRH1,HTR2A,HTR7,IL12B,LAMB3,MCM4,MYL9,NCF1,NRXN2,OR2W1,OR52D1,OR52N2,OR8D2,PDCD1,PIGW,PLD2,PPP3CC,RARA,SH2D1A,TAS2R7,TUBB2B,YWHAH
Negative_559	DBI,GH1,LSS,TKT,A4GALT,ADH5,ADRA1A,ATP6V1F,BDKRB1,CD274,CHIA,CLDN18,CNGA4,CREB3,DDX41,GBE1,GLYCTK,GYS2,IFIH1,JAK2,KIR2DS5,MGAT4A,MKNK2,NCKAP1,OR1J2,OR5V1,OR6C75,OR8K5,OR8S1,PAK6,PIAS4,PNLIP,POMC,SEMA3E,SHMT1,SLC25A5,STX8,TRAF1,ATP6V0D2,PPARGC1A,SERPINC1,TNFRSF10D
Negative_560	A2M,AUH,C4B,MYC,ADCY1,AGPAT2,B3GALT2,BLVRB,BST1,EIF4B,EPHA1,FGFR3,GALNT13,GGT1,GNRHR,HMGCR,HNF1B,IL12RB1,LAMA4,LCP2,MAD2L2,MAP2K4,MAPK3,METTL6,MTFMT,MYH4,NRXN1,OCRL,OR2F1,OR51B5,OR5AU1,PANK2,PDE6D,PDE9A,POLR2L,PRKCE,RDH5,RHOD,SLC27A1,SPI1,TRMT11,UGT2B11,ULK3,KIR2DL5A
Negative_561	DCK,MOS,PLG,ALOX15,ATP5J,ATP6AP1,BDKRB2,BIRC2,CD44,CDC7,CDH5,CISH,CKMT2,COASY,COMP,CREB3,DHDDS,DTX3,ETNK2,FZR1,GCNT3,GUK1,HLA-DOB,HTR4,IFNA5,IGF1R,IRAK3,ITGB4,MAD2L1,MAFA,MBL2,MYLK,NAT2,NCAM1,OR2G2,OR5D14,OR7A17,OR9K2,PERP,POLR2D,PROC,PTGER1,ROCK2,STX6,TAS2R1,UBE2L6,UCK2
Negative_562	KDR,MPZ,ADCY8,ADRB3,BBC3,CASP8,CDK7,CSNK1A1,CSNK2B,CYFIP2,DLL1,DVL1,FZD1,GJA1,HCST,IL11,IL18RAP,IL21,MECR,MGAT2,NAGA,NEFH,OR2T6,PFKP,POLE3,PPP2R1B,QPRT,RASA2,SHMT2,SPI1,ST3GAL4,STK3,STX4,TUBB4,UBA7,YWHAZ,PLA2G12B
Negative_563	APP,FES,MPZ,AOC3,CSNK1A1,CXCL13,CXCL16,DAPK1,DAXX,DLL1,FGFR2,GGT5,GLYCTK,IL22RA1,ITGA3,ITGA5,KLRC1,NFKB1,NLGN2,NRP1,OR10Q1,OR1J1,OR4A15,OR4K13,OR51M1,OR5A1,PPAT,RASGRP3,RPN2,RPS6KA2,SELPLG,SHISA5,THBD,TRDMT1,ULBP3,GABARAPL1,HLA-DRB4
Negative_564	EZR,HAL,PRL,UBB,ACPP,CKS1B,CREB3,DGKA,DGKG,DPYD,EFNA4,EIF4E2,ENO3,GNB1,GNRH1,GOSR2,GOT2,GRIN2A,HLA-DOB,HNF4A,HSD17B4,IGF1R,IL21,MAP3K6,MGST3,OR14C36,OR1A1,OR1L1,OR2B6,OR4K14,ORC3L,PIK3C2B,PLCD3,PPAP2C,PPBP,PRIM2,PROS1,RBX1,RPS6KA6,SUV39H1,VAMP2,PAFAH1B2,SLC25A31
Negative_565	B4GALT4,CDKN1C,DDIT3,GMPPA,GSTM5,KIR2DL3,LCMT2,MAOB,MGAT4B,OR14I1,PCYT1A,PPM1B,SOAT2,STAT5A
Negative_566	C4B,WAS,ARHGEF4,B3GALT2,DLST,GAPDH,GDF7,IL28A,MAPK1,MDM2,MECR,MTHFS,NRXN3,OR52N1,PFN3,RUVBL1

Negative_567	CDA, GPI, AASS, ACYP2, ARNTL, ATIC, CDK5, CXCL1, CYP1A1, FDPS, GDE1, HMBS, IL7R, LIPT1, LTB4R2, OR10H4, OR4Q3, ORC4L, POLE3, POLR3G, PPP2R2B, PTPRZ1, RPS6KA4, SETDB1, TAS2R14, WNT9A, MAPKAPK2
Negative_568	AK2, BAD, FYN, GCK, HGF, MOS, PAH, PML, ACSM5, ALG6, ATP5B, ATP5J, BIRC3, CLTCL1, CYP17A1, DAPK3, ETNK2, GLUD1, HDAC2, LIAS, MAP3K14, NAGA, NFATC2, NPAS2, NTF4, OPLAH, OR2B6, OR2T8, OR56B4, OR6T1, P2RX1, PFN2, PKLR, PORCN, PRSS3, SPTLC1, STAT6, TNFSF9, TUBB6, UPRT, ATP6V1C2
Negative_569	F12, TFG, ACOX1, ACSL3, BRAF, CX3CL1, F2RL3, GSTM4, GTSE1, HSD3B1, IL21, IL9R, LTBR, MAGI3, MALT1, ONECUT1, OR10J3, RPN2, SETD7, UNC5B
Negative_570	TK1, ATP2A1, CD82, DPYS, ENO2, EPHB4, FBXW11, FCGR2B, FMO3, GALNT2, GSTK1, ICAM3, NCAM1, NRXN1, PIK3R3, PLXNA2, CACNA2D1
Negative_571	DBI, AADAC, ABAT, ABL1, ACOX3, ALG3, BBOX1, CDC25C, CDS1, CREB3L4, DOLPP1, EXT2, GALNT2, GALNTL4, GUSB, HSD3B1, IFNA8, IMPA1, JAK3, MAP3K7, OR10H5, OR11H6, OR1G1, OR1N2, OR2A2, OR52A5, PDE4B, PGM1, PIGF, PIK3CG, PLA2G2D, POLR2F, PPARD, RFXANK, RPS6KB1, SEMA3B, SLC2A1, TP73, TPH2, UGCGL2, ULBP3, VCAM1
Negative_572	IDS, RB1, ALDH1A3, CACNB3, EFNB3, FGFR1, GNA15, HSD3B2, INHBE, MAP3K6, OR4C46, OR5V1, OXCT1, PANK4, POLR2B, PRKX, PTTG1, SMAD6, SULT2B1, ATP6V1E1, HLA-DRB3
Negative_573	CBS, MVD, AGTR1, CDC42, CTNNA1, DUSP14, EDA2R, ENPP7, FGF11, ITGAE, ITGB8, JUND, MDM4, NFKBIB, NR4A1, OR52E2, PROS1, TUBB2C, PDCD1LG2
Negative_574	GLS, PLG, RET, AGRN, ARPC5L, DHFR, GCNT2, HSD3B7, IFNA10, LTBR, OR10A4, OR2T12, OR2T5, PCNA, PDE4A, PIGT, PPP2CB, PPP2R2B, SPTLC1, TBL1X, TBL1XR1, THTPA, TM7SF2, VARS, ATP6V0A4, B3GALNT1, PRICKLE2
Negative_575	DUT, FGA, LEP, TXK, WAS, ZYX, ADRA1B, APOA2, BET1, BNIP1, CACNA1S, CBR3, CCL1, CD14, CNTNAP2, CSNK1G1, CXCL13, CXCL5, CYP1B1, ERO1L, GRIA3, GSTP1, GUCY1A3, HSPA1B, IL6ST, INHBE, IPPK, LDHB, MAP3K12, MTHFR, NEU4, OR4F4, OR51F2, OR51Q1, OR52A1, OR52R1, PAX4, PGS1, PIK3CA, PPP2R5B, PRKAG1, PTAFR, PTPN5, RRM2B, TGFBR2, CTNNBIP1, HLA-DRB4
Negative_576	BTC, ALDH9A1, ALG11, APOA5, CDIPT, CISH, CLDN16, CNTNAP1, CPT1C, CYP4A11, EFNA5, GNAO1, GNRH1, GRIA3, HEXA, HLA-DOA, IFNGR1, LARS, NFKBIA, NMNAT1, NR4A1, OR10H5, OR2M3, OR51S1, PDE4D, PDHA1, PLTP, PSEN2, SPRED1, TAS1R3, UPP1, ST6GALNAC4
Negative_577	C8A, F11, SCD, COL6A2, DCTN1, GSTZ1, HSD17B7, IGF1, IL10RA, IL7R, INHBE, INPP5A, ITPK1, LIPT1, NAGLU, NME2, OR13J1, OR51I1, OR52R1, PPM1A, RND1, SGMS2, SORD, VNN1, YARS, CSNK1A1L, SERPINE1
Negative_578	SRC, GALNT7, WBSCR22
Negative_579	F9, TKT, AHCY, CDIPT, DUSP2, GALNT5, GNA11, GNG12, IL1A, KITLG, OR13H1, PER1, SMARCA5, TNFRSF13C
Negative_580	AVPR1A, CPT1B, FZD2, GNPAT, IRF5, OR2AE1, OR6C76, OR8D1, RALGDS, RPS6, SRGAP1
Negative_581	TKT, ACAT2, ATP2A3, BDH1, BUB1B, CAV1, CCL13, CYP8B1, FTMT, GLUD2, MOV10L1, NFKBIA, NUDT5, OR5A1, PFKFB4, PGAM2, PHKA1, PPM1A, PTEN, SFRP5, SHC2, SLC25A4, TACR3, UCKL1, XCL1, ST6GALNAC6
Negative_582	MPL, AACS, ACSM2A, ARPC4, AVPR1B, BDKRB1, BLVRA, CXCL5, FADS2, GADD45A, HEXA, HNF4A, IFNA14, IL8RB, LDHAL6B, MAGI3, MAOB, MCM4, MITF, MYL7, NT5C1A, OR13C2, OR14C36, OR2Y1, OR4F15, PI4KA, PIGX, PPARG, ROCK2, SMAD1, SOCS7, SRGAP2, TNFRSF10B
Negative_583	GP5, MIF, ADIPOR2, APOA2, ARD1A, ASAH1, BMP7, BMPR2, BNIP1, CAMK4, CCL17, CD38, CHIA, CLTC, COL3A1, FOXA3, GCNT1, GSTM3, HTR2C, ICOS, IL13RA2, IL24, LEFTY1, MAPT, MDM4, NAT6, OR10A5, OR4C15, OR5C1, OR5V1, PIP5K1B, PNLIP, PPP3CC, PRIM2, PRPS1, RAC1, SNAI1, STEAP3, UGT2B7, ATP6V1G1, SERPINE1, TNFRSF11B

Negative_584	ABLIM1, ADH7, ATG7, INPP5A, KLRC4, LPAR1, NEGR1, NRG3, OR13C5, OR1J1, PAK3, PARD6B, PRKAB2, THBS1, ATP6V1G1
Negative_585	CFH, EBP, GH1, PVR, ADCY1, ADH7, ALDH1A1, ARPC5, ARSD, BPGM, CCL13, CDC16, CDKN2A, COL4A1, CYP21A2, DERA, DLL4, E2F1, EPHX1, FGF20, GNPNAT1, HRH2, HSD11B2, HSPA1B, IDUA, IFNA21, KNG1, LAMB3, LLGL2, MCM7, MITF, NOS3, OCLN, OR10H2, OR4A16, OR52N5, PARS2, PIGK, PLCG1, SFRP5, SPRY2, STK4, TAS2R50, TNFSF12, TRPC1, UBA1, CSNK1A1L, PRICKLE2
Negative_586	C9, CCR5, DUSP1, DVL2, EGLN3, ESAM, NMNAT2, OR11H4, OR1L4, PDE8A, PIM1, RERE, SPTAN1, TOMM40, TUBB2C, UBE2K, WNT8A, XIAP, YKT6
Negative_587	CBS, OCRL, OR2D3, TNFRSF10D, TNFRSF11A
Negative_588	AVPR1B, CDS2, DPYS, FGF14, GMPR, GNG12, OR4N5, PAX8, RALBP1
Negative_589	GDA, GP6, ZYX, ACADSB, ACSM3, ADH5, AKT1, B4GALT6, BIRC5, BST1, CD36, CD55, CLDN19, CLDN2, CLDN5, DDX51, EHMT2, FPGS, GALNTL2, LEFTY2, MAP4K4, MYL2, NEUROD1, OR10Z1, OR13C5, OR14I1, OR1L6, OR7C1, OR8K5, PDE5A, PGAP1, PKMYT1, POLR3G, RARS, RELB, SDHC, SHC3, SLC25A4, ST3GAL1, SULT1A4, SV2A, UBE2L3, VDAC1, VTI1B, ZIC2, GABARAPL2, SUV420H2
Negative_590	BID, CEL, FGA, A4GALT, ACVR2A, ACVRL1, ADAM10, ADCY4, ALS2, B3GAT2, BMP2, CCL15, CCL16, CCR5, CD244, CDS2, CHST11, CYP2S1, DDX52, E2F1, EDNRB, ENTPD8, ERBB3, GAMT, GBE1, GCDH, GUCA1C, IDH1, IDI2, MAGI1, MYH14, MYH2, NFYA, NFYC, OR10H5, OR13G1, OR14A16, OR1D2, OR4K5, PISD, POLR3B, PPP3CA, RAPGEF1, RFXANK, RPS6KA4, SFRP5, STX8, TJP3
Negative_591	FUK, GNS, PML, SPN, ADCY9, ALS2, APRT, AREG, CDKN2C, CER1, CSNK1G2, CTBP1, EHMT1, ENO2, EPB41L1, FZD10, GMPR, GUCY1A3, ITPKB, LAMB2, MAPK1, OR8S1, OXTR, PHKA1, POLR1E, POLR2K, PPM1D, RARA, SCD5, TPH2, UGT2A1
Negative_592	CP, FGB, GH2, RPE, TK1, ABP1, ACMSD, ACVRL1, ADAM10, ADCY9, ADRA1D, C1GALT1, CASP6, CFL2, CX3CL1, CYP21A2, DTYMK, EXT1, GAMT, GSTM4, IL10RB, INHBE, MYLK3, NFATC1, ORC2L, PDXP, PGM1, PLCE1, SGMS1, SMAD6, TGFB1, UNC5D, UPRT, VTI1A
Negative_593	DBH, ID4, TNN, BMP6, CACNA1G, CD44, CD47, CNGA4, CYP1B1, DDB2, DIAPH3, DLL3, F2RL2, FOXA2, FSHR, GNAI2, IFNA17, IL5RA, INPP5E, IRF7, JAK3, KIR2DL3, LLGL2, MAD2L1, MCM3, METTL6, MITF, OR13F1, OR2T1, POLR2C, POLR2G, PRKG1, PTGER1, TAS2R4, TAS2R49, TCF7L2, HLA-DPB1, PNLIPRP1, TNFRSF10C
Negative_594	FADD, INSRR, MYL7, OR10G9, STAT5B, WASL
Negative_595	BTD, C4B, CBL, KIT, AGTR1, CAMK4, CLDN19, GJD2, HYAL1, IRF5, MTMR2, NANS, NAT1, OR10A3, OR4B1, OR51G2, OR6C3, OR8J1, PIAS4, PNPT1, PPP1R3C, PTK2B, RAC1, RAPGEF4, RFNG, ROCK2, RPS6KA4, TAS2R14, UGT1A10, VEGFC, HLA-DQA2
Negative_596	BAX, ME1, MVD, ALDH6A1, B3GALT1, CD40LG, MALT1, NTN1, NUDT2, NUMB, ODC1, OR5A1, PPP3R1, PRODH2, PTDSS2, RALGDS, TSHR
Negative_597	ARNT2, DHRS4, HARS2, KAT2B, MNX1, OR51A7, OR51B6, YARS
Negative_598	C6, C9, CS, EBP, FST, ACAA1, ACSL3, AKR1C3, ARHGEF6, ATP6V1A, CBLC, CD28, CDC27, CDS1, CHSY1, COL4A2, CSF1, DDX54, DHRS4, DOT1L, F2RL2, FTCD, FUT4, GFPT1, GLB1, H6PD, IMPDH2, MYL2, NFYA, NME7, NMNAT3, OR10Z1, OR2A25, OR2H2, OR4D9, OR51I1, OR5M8, POLD2, POLR1D, PON2, PPM1B, PPP3CC, PRPH2, RFXANK, SFRP5, STX8, SULT1A1, WNT10B, TNFRSF12A
Negative_599	MIF, PC, ACOX2, ACSL1, ATF2, CARD11, CASP10, CCND1, CCND3, CHAD, DNMT3B, FPGS, FZD3, GAS1, LIPG, MAP2K4, MARS2, MTHFD1L, OR14A16, OR1G1, OR4K1, OR51F2, OR56A1, OR5H2, OR7C2, OR8I2, OR8K3, PISD, PRIM2, PYGB, RXRG, STAM2, STK36, THY1, TRAF1, TRAF2, WNT16, ZFYVE9
Negative_600	C8A, ACOT11, ADH5, AOX1, BMP8B, CACNG1, CACNG8, CLDN17, CYP2C9, DLL4, DUSP4, ESPL1, FADS2, GNPAT, IDH3A, IL18RAP, ITGA2B, LLGL2, MMP7, NME2, OR11H1, OR2A14, OR51E2, OR52I1, OR6B2, PRKCI, RPS6KA2, RRM2, ST3GAL1, PAFAH1B2

Negative_601	BAX,DUT,F2R,F7,GK2,ALG10,APC2,B4GALT3,B4GALT4,BPGM,CASK,CCL22,CCR9,CDK5,CLDN14,COQ6,CREB3L1,CSNK1E,DHODH,EDN1,EFNA1,GNA13,GOT1,GPD1L,IFNA6,IL2RG,INHBA,ITGA7,MAP3K5,MARS2,NMNAT2,NQO1,OR10X1,OR14J1,OR4D1,OR5A1,OR5M3,OR9K2,PIK3CB,PIK3R1,PPM1D,SLC12A2,SUV39H2,SYNJ1,THBD,TSHR,UNC5A,VDAC2,CSGALNACT1
Negative_602	FGD3,GRIN2B,OR52I2,OR9A4,PRLR,RARS2
Negative_603	ADC,C9,FES,KMO,ABI2,ARSB,CACNA1I,CDKN2B,CXCL10,DAXX,DTX1,DVL2,FARSB,GADD45G,GUCA1C,IL25,MECR,MYL9,NAT6,OR2AT4,OR5L1,PIK3C2B,PLCD3,RASA2,RASSF1,TBL1Y,TRAF5,SERPINB5
Negative_604	AARS2,ATP5F1,DDX41,MTMR2,NCOA4,OR4F4,PTCH2,SEMA4A,SLIT2,SUV420H1
Negative_605	CBS,DCN,CACNA1F,CD80,CRLF2,EXTL3,HLCS,IL28B,IL29,MAPK11,MYH4,NAGA,OR4F15,PDE8B,PDGFRB,PFKFB3,PIGU,PROC,SC4MOL,SORBS1,ALDH18A1,ATP6V1E1
Negative_606	C7,IVD,NOG,PLG,SRM,AKR1B10,APOA5,ATP4A,ATP5L,CXCR5,CYBA,CYP26A1,CYP3A4,DFFA,DHRS4,DHX58,FCGR3A,FOXA2,FSHR,GALC,GALNT1,GALNT14,GZMA,HIF1A,HSPA1B,MAP2K6,MCM7,OR11H1,OR4C15,OR52N4,OR5I1,PRODH2,PVRL2,RHOQ,SLC12A2,SPRED1,SV2B,TICAM2,TJP3,TSC1,UPB1,ZBTB16
Negative_607	CFH,ZYX,AFMID,ALOX12B,C1QA,CACNA1C,CCL21,CDC42,CSDA,CSNK2B,GUCY2F,IGF1,LPAR1,LYPLA2,MAD2L1,OR2L8,OR52A1,OR52B4,OR5M3,OR7G3,PI4KA,SIPA1,SLC33A1,SNW1,SOCS2,ZFYVE9
Negative_608	ALAS2,CD44,CDC20,CDH5,EPHB1,EPRS,GALNTL5,NTN1,OR1K1,OR52E4,OR5B3,OR5D16,PIP5K1C,UGT2B28
Negative_609	MPI,TNC,AKR1C3,CD82,DTX3,ENTPD3,HYAL1,MDM4,OR4Q3,PGK2,PRKCB,PSAT1,RPS6KB1,SDC4
Negative_610	A2M,MTR,PGF,TNF,WAS,ADH6,ANAPC4,CACNA1D,CACNB4,CFL2,CLCA2,CLDN20,CMAS,CPT2,CYP11A1,EFNA1,EFNB3,FASLG,FGF22,FLT3LG,GNAO1,GNAQ,HIBADH,HIF1A,ITGA8,LAMC3,MMP14,MYH11,NPR1,OR10K2,OR4D5,OR6Y1,OR8B8,PAK2,PARS2,PLCD3,PNMT,POLR2K,PTGDS,RFWD2,SLC25A6,SPCS3,VEGFC,ATP6V1B1,TNFRSF1A
Negative_611	ADH6,MPZL1,OR52N5,UNC5A
Negative_612	FGG,TPO,ACADS,CD79B,DRD2,IFNG,IL23A,OR1K1,OR51A2,OR7G3,PAK2,PDE6G,PEMT,PER1,PI4KA,RASGRF1,RASGRP3,SETDB2,ST3GAL3
Negative_613	C8G,CCR10,DGKI,EPRS,F2RL1,GNRHR,GPD2,OR6A2,TGFB3
Negative_614	CFI,DLD,PVR,ALG10,CACNA1G,CACNG8,COQ5,GALT,GNG13,H6PD,JAM3,MMP14,NFKBIA,OR52E4,PARD6B,SDHB,SMC1B,SOCS4,VAMP1
Negative_615	TNC,ALG11,CDC14B,COL6A2,CTNNA2,CTSB,ENO3,FZD8,HMOX1,IFNA10,IL13,IL21R,NTF3,OR14I1,OR6C4,RXRB,SORD,UGT2B17,ATP6V1C1
Negative_616	CR2,SHH,ACP5,ACSM1,ADRB1,ADSS,APC2,CD226,CDC23,CDC42,CLDN4,COMT,CPOX,CXCL11,CYP3A7,DARS,DDX18,DPYS,DRD2,FGF13,FOXA2,GALNT7,GLUD2,GPX7,GSTM4,GSTT1,IL1A,INMT,MCM5,OR10AG1,OR10C1,OR2G6,ORC2L,ORC4L,PCCB,PDE8A,RASGRP2,TGFB3,TNFSF8,UROC1,HLA-DPB1
Negative_617	C8G,IL5,ACACB,AGPAT4,AKR1D1,ATP6V0C,CTNNA2,DAPK2,DDX4,EARS2,EPB41,EXTL1,G6PD,GMDS,GZMA,ITGAE,JAK2,KLK3,MDM2,MMP9,MYL7,NAE1,OR10J3,OR51A4,PGM3,PRLR,SMAD1,WNT2B,PPARGC1A,SERPIND1
Negative_618	C5,GDA,CBLB,CYP2C9,DTYMK,FOXA2,G6PD,GBA3,HAGHL,IL10RA,LEFTY1,MDH1,P4HA2,SOCS1,UBA1,ARHGEF12
Negative_619	F11,FGB,IHH,INS,ASRGL1,BCKDHB,BMPR2,C3AR1,CAMK4,CCL22,CPT2,DAPK2,DBF4,GPX7,GUCA1B,IL12RB1,JAM2,KLRK1,LCMT2,MYH13,PAK4,PARS2,PDE9A,PERP,PIK3R1,PTPN11,PTPRM,SFRP5,TAP1,TOMM40,VAMP2,XIAP,PPP1R12A

Negative_620	AK1,C8A,CD4,GHR,PDC,ACSL1,AGPAT4,AHCYL1,ARG2,B3GAT1,BLVRA,CCNG2,CHSY3,CKS1B,CLDN11,CNDP1,COL3A1,CSF3,GALC,HNF4A,IAPP,IGFBP3,IL1B,ITGA2B,NOTCH2,NRCAM,OPLAH,OR2M2,OR4B1,OR51T1,OR5AP2,ORC2L,PDE7A,PDGFB,PLXNC1,POLR3H,PPM1A,PPP2CB,PRPH,RXRA,SCLY,SOCS3,THY1,TPK1,ZIC2,CSNK1A1L
Negative_621	ATG12,FZD8,IRAK4,LCMT2,PKLR,POLR2B,PRKG2,SETD2,TAS2R50
Negative_622	BTC,GNE,AARS2,AKR1A1,AMPD1,B3GAT3,BCKDHB,BPNT1,CANT1,CD99,COQ3,CPT1C,CREB3L1,E2F1,EHMT1,FMO1,GADD45B,GP1BB,GRIA2,IGSF5,IL23R,IMPDH2,KDSR,MIOX,OR1E2,OR1L6,OR2B11,OR2T6,OR52A5,OR6C3,OR8G1,PHKA2,PLAU,RUNX1T1,UGT2B28,UGT8,XCL1,TNFRSF10B
Negative_623	CAT,DHDH,LAMC2,OR6C76,OR8B8,PRNP,RUNX1,SKP1,SMAD3,SUCLA2,PNLIPRP2
Negative_624	AGPAT6,CFTR,ENO1,HPRT1,IL18RAP,OR10K1,OR1K1,P4HA3,PFKM,RRAS,TAP1
Negative_625	PTS,AGXT,APOC3,CAMK2B,GSTT1,OR8B8,PIGT,PIM1,PLXNA2,PPP2R5B,SLC25A6,WNT3A
Negative_626	GNS,LAT,TEC,ACO2,CADM3,CALML6,CCR3,CD22,CD28,CHPF,COL4A6,CSAD,DDHD1,EXTL3,F2RL1,FASN,FBXW11,FDFT1,FZD1,GUCA1C,HARS2,IGSF5,IL12A,IL2RA,ITPK1,KAT2B,KLK3,NCSTN,NRP1,NSD1,NUDT5,OR11L1,OR12D3,OR4A5,OR4C12,PFN1,PLCG1,PRKACB,PSEN2,RASSF5,RDH16,SPHK1,STX1A,TBL1X,VAMP2,WNT5A,ATP6V1E1,SERPING1
Negative_627	AK7,F10,LPL,UBC,ACP5,ADCY8,AGPAT1,AKR1D1,CCNB3,CCR7,CD247,CDC45L,CDKN2A,COL11A1,CXCL1,DCXR,EVI1,FLOT2,GBE1,GFPT1,GRM5,GTSE1,HIF1A,IL17RA,IQGAP2,ITGAE,LALBA,LCMT1,LIPT1,MAP2K3,MAPK13,MYL9,OR2C1,OR4F5,OR9Q2,PI4KB,POLA1,PPP2R5D,RAPGEF4,SGMS2,SOCS3,ST8SIA5,STX1A,STX1B,VAMP3,WHSC1,YWHAB,TNFRSF13C
Negative_628	LSS,C1GALT1,CCL27,FZR1,GLI1,HNF1A,LCMT2,OR4S2,PDGFB,PIK3R5,PVRL4,SULT1A1,TNXB
Negative_629	C1S,CBS,MAG,PAH,RET,ACSS3,ADIPOR1,ARPC1B,B3GNT5,C3orf10,CACNA1H,CARS2,CCL26,CD28,CDKN1C,CLDN3,CREBBP,CTSL1,DFFB,DIAPH2,E2F1,FARP2,FTH1,GADD45A,GBA3,IL6R,LIMK2,MAP3K2,NANP,OR2A5,OR52N2,P2RX4,PPCS,PRKAA1,PRSS3,RAPGEF4,RCHY1,SHC1,SMAD6,SMPD2,SNAI2,TAS2R3,TUBAL3,UBE2L6,VANGL1,WNT16,WNT2,PNLIPRP2
Negative_630	GPT,ACVR1B,AHCYL1,ARPC5L,BCL2,CD59,COL2A1,DLL1,DOCK1,HTR5A,IL23R,MGAT4B,OR10G7,OR1A2,OR2A25,OR4K2,OR4S2,OR56A1,OR5I1,OR5K2,ORC6L,PDGFB,PHKA1,PIK3R4,PROC,ATP6V1G1,KIR2DL5A,TNFRSF10B
Negative_631	ADRA1A,ATP5C1,B3GNT5,CCL25,CCND3,CCR10,CDC25A,CNGA3,DCXR,DDX52,DHRS4,DVL2,FARP2,GAB1,GALK1,GALNT8,GNA11,GNG3,HLA-DOB,IFNA10,IFNA8,IRAK3,KARS,MAP2K1,MBL2,NCOR2,NLGN2,OR4C13,OR4D9,OR51V1,OR6C4,OR6C6,OR6F1,OR8D4,PARS2,PFKP,PTGDS,RELA,SMAD2,TAS2R5,WNT11
Negative_632	CRK,SI,SYK,TYR,B4GALT7,BDKRB2,CDC16,CHD8,CLDN20,CYP8B1,DDX56,FGF22,FUT4,GADD45A,GNG12,HTR2A,ITGA9,ITGB6,KLKB1,LTBP1,MGAT4B,MGST1,MYH7B,OR4X1,PIGO,PPP1CB,PTPN1,RHOA,SETD1B,ST3GAL5,SUCLA2,TAS2R8,TBL1XR1,TLN2,ATP6V0D2,TNFRSF14
Negative_633	LPL,MAG,AMDHD2,B3GALT5,CHKB,COL2A1,CSNK2A2,GUCY2F,IL9R,ITGA2,NFKBIE,NOX1,OR8G1,SULT1A4,ATP6V0A4
Negative_634	CD79A,DUSP14,FADS2,INPP4B,IRS4,JUND,KIR2DS4,MKNK1,PAX8,PIGQ,PPARD,RDH11,SEMA4C,XCR1,ATP6V1C1,HLA-DQA2
Negative_635	APOA1,CNTF,CXCL10,EP300,GZMA,HADHB,KARS,MDM4,MTHFD1,NRAS,OR2M2,OR6C3,PTPRJ,RASGRP1,SYNJ2,UPB1
Negative_636	AK1,HYI,ID4,ATP6V0B,CYP2S1,FTCD,LDHA,MS4A2,OR2T3,OR4K1,OR5AC2,PFKL,PNPO,PRPS2,RARS2,RPS6KB2,TUBB4Q,WASF1,SERPINE1

Negative_637	CS, ALDH1A1, B3GAT1, FOSL1, GMPS, INSR, ITGB6, OR2A4, PRKCI, THBS1, PDCD1LG2
Negative_638	HYI, KMO, ATP5A1, BCKDHA, FOSL1, GALNT11, NRG4, RASA2, CSNK1A1L
Negative_639	FGG, FYN, HK1, ACYP2, ATG7, ATP12A, ATP5H, B4GALT2, BAAT, BDH2, CACNG5, CCL16, COASY, CRLS1, EPHA4, GANAB, GCLM, IRAK4, KIR2DL3, LEFTY1, LRP5, MAML1, MGAT5, MGST3, MYH15, OR4C46, OR4F15, OR6C4, OR6C6, ORC3L, PIP5K3, PIPOX, PPP5C, PRPS2, RALBP1, SUFU, TAS2R41, THBS1, VDAC3, WNT2, WWP2, KIR2DL5A, SLC25A31
Negative_640	AK1, GPT, MPI, AARS2, ADCY7, ATF4, B4GALT1, CAMKK2, CHKB, COL1A2, CTBP1, DGAT1, DUSP8, FGD3, GDF7, GMPR2, GMPS, GRIN2B, GSTO2, HEXA, HSD3B2, IDH3G, IRF7, ITGA11, ITGA3, MAD2L1, MBL2, MDH2, NAGS, NFKB2, NRG3, OR10J3, OR2T8, PAPSS1, PRLR, PSME3, ROBO2, SDHA, SEPHS1, SGPP1, SLC25A4, THBS4, TUBA3C, UBA7, CACNA2D3
Negative_641	ME2, ACAA1, ACADVL, ACO2, ACTN2, ADCY2, ALG5, ALPP, AMD1, APOA5, BDKRB1, BMP7, CHDH, COL5A2, DDIT4, GAD2, GALK2, GGT7, GLUL, HBEGF, HPSE2, IFIH1, IL12A, IL20, IL23R, IRF3, ITPKB, KYNU, MITF, NMNAT3, ONECUT1, OR4C11, OR5M9, PCYT2, PLA2G2D, RDH11, RPIA, SMPD4, SULT1A2, TAS2R9, TBXAS1, THY1, VAV2, MAPKAPK2
Negative_642	GSS, ME2, PGD, ADCY7, ALDH9A1, ENTPD1, EXT1, HARS, IL1R1, IL22, INPP5A, ITPKB, LIPF, MAP4K4, NTF4, OR8H1, OR9G1, OXTR, SFRP5, STX1B, TAS2R42, TCF7L1, ZIC2
Negative_643	DERA, NOX1, TUBA4A, TXNDC12
Negative_644	C8A, TPO, AKT1, ALDH2, ALG12, ARHGEF4, ARPC1B, BCMO1, BMP6, CTTN, FARS2, FARSB, GJD2, GLG1, GSTM2, HMOX1, IL1R2, IRF5, KIR2DL1, MFNG, MGAT4A, MYH15, OR13G1, P4HA3, PDXP, PIGQ, PLCD3, RAPGEF2, SC5DL
Negative_645	F2R, PGD, ACTB, ADCY3, AHCY, CCNG2, CHSY1, EREG, F2RL3, GALNT3, HMGCS2, OR10H2, OR52K1, OR6T1, PDE8B, PIGX, PRIM1, PRKAR2B, SH2D1B, SMPD4, TSC1, UBE2K, XYLT2
Negative_646	MPZ, BLVRB, CCR5, CHIT1, CYP3A4, DDX55, GFAP, HRAS, LIAS, OR51B4, OR51B6, OR56A4, POLR1A
Negative_647	GSS, ANAPC1, AOC3, ARAF, ATG7, BLVRB, CAMK4, CCL26, CD47, DNMT3A, E2F2, EDAR, FGF18, GDE1, GNAI3, GRIA3, HRAS, IFNA8, IL2RA, JAG1, LAMA3, MAD2L2, NADK, NEU3, ODC1, OR4P4, PER2, QPRT, RAC2, SNW1, ST3GAL5, UNC5A, YKT6, CSNK1A1L
Negative_648	HK2, LPO, PLN, ACAA1, AGMAT, ARSD, AVPR1B, B3GALT1, CACNG6, CCL20, CDK5, CHDH, COX10, CXCL10, DPM3, DTYMK, GPX7, GSTT1, IFNA5, IFNB1, IL18R1, INPP4B, ITGAM, KIR2DL1, MMP14, OR1C1, OR8H3, PFN1, POLR2H, PTGS2, RFXAP, SLIT2, SRGAP3, TSC2, TUBA3C, TUBA3E, VAV3, ARHGEF12, ATP6V0D2
Negative_649	EDA, GSS, AGPAT3, AGPAT6, CACNG2, DHDDS, DOLPP1, E2F3, EFNB1, ENTPD7, FTMT, GALNT5, GANAB, GAS1, GUSB, IBSP, IFIH1, IGSF5, IRS4, KDSR, LDHC, MAP3K13, MKNK1, NT5C2, OR2A4, OR2H2, OR2T6, OR4C11, OR6X1, PDE5A, PGM1, PLD1, POLR3A, PYGB, SELPLG, SESN2, TFDP1, TRMT11, TSTA3, TUBB2C, ATP6V1E2, ST6GALNAC3, TNFRSF11B
Negative_650	ACADVL, CREB3L2, IL17RB, IL18, OR4C11, OR51D1, PRKCD, PTGER3, SGPP1, SMAD2, STAT4, SUV39H1, TUBB, UGCGL2, PPARGC1A
Negative_651	F8, IL9, TK2, CACNG4, COL4A4, COMT, CPOX, CREB5, DKK4, DPM3, DPYD, EFNA2, HHEX, IFNGR1, IL12RB1, IL15RA, JUND, KRAS, LDHA, LIPA, NAGA, NCKAP1L, NFKBIE, NTNG1, OR10H2, OR2AG2, OR5K2, OR5T2, OR8A1, PIGN, PIP5K3, PPP2R2C, SEMA4F, SETD1B, TAS2R49, TNFSF14, UPP1, VEGFA
Negative_652	DAPK1, KAT2B, LEF1, RRAS, SLC27A4
Negative_653	FOS, IL4, ANAPC1, CD27, COL1A1, CSF3R, FMO2, GANC, GP1BB, GSTM2, LYPLA1, MALT1, OR5P3, PIGN, RPS6KA1, TAS2R4, TUBAL3, TYMP, UBE2L6, ULBP3

Negative_654	AK3,ASL,BLVRA,FZD4,GFAP,IPMK,OR13D1,OR51S1,RASGRF1,SLC25A31,ST6GALNAC5
Negative_655	ID1,CSF2RB,GGT5,ITGB5,TBL1X
Negative_656	XDH,ACO2,ACOT11,CBR3,CCL8,CHST12,CXCR3,DTX3,EPB41L2,FGF9,FOXA3,FZD4,GRB2,LLGL2,MYH3,NR1D1,OR4C12,PAK4,PIM1,POLR3A,PTGES2,PVRL4,SDC4,SEMA4B,SFRP1,SFRP2,TUBA1C,TYK2
Negative_657	CRK,B3GALT4,ELK1,F11R,GUCY1B3,NKD2,WNT5B,HLA-DRB5
Negative_658	ADA,DCC,GAA,ME1,ADRB2,ARSB,ATP6V0B,CYP2C19,CYP3A5,DUSP3,DVL3,ENTPD1,EPHB2,FARP2,FCGR3A,FGF21,GALNTL1,GPD2,GRPR,KDSR,KIR2DS5,MAP3K8,NAMPT,OR1C1,OR4L1,OR4X1,OR51B5,OR5AU1,OR7D4,PAK1,PARS2,PDCD1,PDHB,PNMT,PRKACG,PRKCB,PRNP,PYGM,RBX1,RPE65,SEMA3D,TRAF3,TSLP,UBA1,WASF2,ZMAT3,ST6GALNAC6
Negative_659	F10,AANAT,ACMSD,ASPA,CBR3,CCR10,CCR3,CD38,CXCR5,DDX4,DDX47,FLAD1,FLT3,FRAP1,FZD4,GRAP2,HGSNAT,IL12A,LAMB4,MMP2,MYH10,NCAM2,NRG4,OR4X1,OR6N1,ORC6L,PCCA,PGM1,PIGX,PLA2G5,POLE2,PON1,PSENEN,QPRT,SFRP4,SGPP1,TUBB2C,ATP6V1B2
Negative_660	SPN,TXK,ACSM2A,ALOX5,ATP5O,ATP6V1F,CCR1,CDKN1A,CXCR4,GCNT4,GGT1,GPT2,GUCY1A2,IFNA2,IL22,KLRC3,LDHD,MAP3K5,MYL7,NFATC4,NRXN3,OR1F1,OR4M1,OR51A2,OR51A7,OR52A1,PFAS,PIGF,RASA2,WHSC1L1,MAPK8IP1,PDCD1LG2
Negative_661	BTD,CFD,CFI,HYI,OTC,AK3L1,ALOX15,AMD1,ANPEP,ATP4B,B3GNT2,C4BPA,CACNG6,CAMK2A,COQ7,CTNNB1,CYP17A1,DCTN1,EIF4E,ENPP3,EPHA7,F13A1,F2RL3,FIGF,FUT8,GOT2,IMPDH1,INMT,ITGA10,MGAT4A,MYH11,NARS,OR10G4,OR4K5,OR6B2,OR6S1,OR8G1,PIGA,POLD2,POLR2G,PPP1CC,PTCRA,VASP,ZFYVE9,ATP6V1E2
Negative_662	BTC,ACP6,ADAM10,ANAPC5,ARRB2,ARSA,ATP5G3,ATP5O,BUB1,CACNA1I,CHST12,DTX4,GADD45G,GALK2,GSK3B,GSTM5,IL8RB,IPMK,NR5A2,OR7G2,PDIA4,PPP2R2C,PTPRB,RAD51,SETD7,SETDB2,SMAD4,TSLP,ULK1,YWHAB,CSGALNACT1
Negative_663	C2,CD6,FES,JUN,LCK,ACOT12,ADRB2,AGXT2,CACNB3,CDK6,COQ3,DLL1,EDN1,FGF22,HSPA8,IFNA13,ITGA4,LRRC4C,NFAT5,ONECUT1,OR1B1,PDE1A,PDHA1,PDPK1,PDX1,PON3,RALBP1,RARA,RFNG,RFWD2,RPS6KA1,SCP2,SLC9A1,STX1A,TACR3,TAS2R38,TRPM5,TUBA4A,TUBAL3,UBE2K,UGCGL1,VEGFB,WASL,WNT2,XCL1,ZFYVE9,CACNA2D1,TNFRSF10B,TNFRSF13B
Negative_664	AGXT2,ARNTL,CCL1,CHUK,CMPK2,EARS2,EHHADH,GADD45G,LIPC,MAPK14,MCAT,MNX1,MYH1,NEFM,OR10G4,OR10Z1,OR3A3,PIGV,PIK3C3,SELP,SFRP4,STAM2,SUCLA2,TBL1Y,TNFRSF10B
Negative_665	CAT,EBP,HYI,KHK,SCD,ACAD8,ACADSB,AKR1C3,ALG5,AMD1,ANAPC4,AOC2,APLP1,AREG,ARNT,BIRC2,CES7,COX10,ENO2,FGF17,GALK1,GLUD2,HCLS1,IL12RB2,INPP4B,LRP5,LTBP1,MLLT4,MYLK,NRXN2,OR13C3,OR6C70,OR6F1,POLE2,POLR2L,PSAT1,PTPN11,RELA,ST3GAL6,STK3,SYNJ1,TRMT11,UPP2
Negative_666	CCL16,CDK2,CREBBP,CYP3A7,LCP2,OR2A4,OR52N1,RETSAT,SELP,TNFSF11,WBSCR22,PPP1R12A
Negative_667	BAD,OTC,CD3E,CLDN10,COQ5,CXCR4,DGKG,DHDDS,F13B,FIGF,GLI3,HDAC2,IARS,IL13RA2,IL17A,OR10H1,OR52A1,PDIA4,PIP5K1C,PPP1CB
Negative_668	ACOX1,ACOX2,ACVRL1,AHCYL2,COQ7,CTSL1,EFNA4,EHMT2,FASN,FGF9,GALNT13,GSTA1,PDGFB,PFKFB4,SYNJ2,TLR4,TNFRSF9
Negative_669	CDC26,PRKCI,TNFRSF18
Negative_670	MUT,SFN,TKT,BCL2,CXCL12,E2F5,ESPL1,GUCA1B,HAP1,MAPK14,NRG2,OR10C1,OR6K6,PGS1,PIP4K2A,PON3,PYGM,QARS,RAC3,RASA2,SGMS2

Negative_671	CAD,ACAT1,ATP6AP1,CCL16,CCL26,CD48,CTSS,CTTN,E2F4,GALNT9,GUK1,IL12A,LDHA,NEUROD1,NFASC,NFE2L2,OR4N4,OR5AP2,PGAM1,PIGT,STK11
Negative_672	CBS,TNN,ACTA1,AGXT2,ALOX12,CBR3,CCKBR,CD46,CHEK1,CXCR5,CYP27A1,CYP8B1,ETNK1,GNAS,GSTK1,GSTM2,IGF1,INPP4A,JUND,LMO7,MAOA,MPDZ,OR3A2,OR4C15,OR4L1,OR6N2,P2RX4,PHGDH,PI4KB,POLR3A,PON3,PPP2R2B,PRODH,RBKS,RUVBL1,SULT2A1,TAS2R3,CACNA2D1
Negative_673	C2,IL6,JUN,NOG,CASP3,CSDA,CTPS2,ENPP7,EPHA8,EXT2,GALNTL1,GSTA4,NRXN2,OR51A2,OR5A2,OR7G3,ORC4L,PDE7B,PIGA,PLCB4,PNPO,POLD3,PROS1,PRPS2,SEMA3F,SMAD7,SRGAP3,STK4,UGT2A3,WNT3,ZFYVE16,PRICKLE1
Negative_674	JUN,OSM,PAH,PLG,AGMAT,ALDH3A2,ALG5,CCL16,COL6A1,CYP1A1,DDX41,EPHA6,FCGR3B,FZD1,G6PC2,GCAT,HARS2,IL17RB,MAPK12,MECR,MTHFR,NFAT5,OR10H4,OR2A12,OR2L13,OR2T10,OR56B4,OR5L1,P2RX3,PAK2,PFKFB4,PIGH,PLA2G2F,RASGRP4,ROCK1,RRM2B,UGCGL1,ULBP2,XYLB,MAPKAPK2
Negative_675	AUH,ILK,ITK,PC,ALG8,ATP5J,CAMK2A,CD28,CHEK2,CREB1,CRHR1,CSNK1G2,CXCL6,CYP3A5,DAG1,DPYD,EPHB4,F11R,GADD45A,GOT2,GRIA3,GUCA1B,HDAC2,IFNG,IL12RB1,ITGB6,MAPK14,MPZL1,NADK,OR10J5,OR13C9,OR1L8,OR2AG2,OR5A2,PCK2,PCYT1B,PMAIP1,RPS6KA2,SARS2,STMN1,STX18,TAS2R13,TPMT,WASL,PDCD1LG2
Negative_676	C1S,CR1,HDC,IHH,LIF,ACACA,ACOX2,ARNTL,C3orf10,CACNB3,CCNA2,CCR4,CTSG,CXCR4,DAD1,EFNA2,FIGF,FUT7,IL29,ITGB4,LIPA,MICA,MOV10L1,OR51S1,OR52L1,PARS2,PCNA,PGM1,PNMT,SH2D1B,TDO2,WNT3,TNFRSF1B
Negative_677	RET,CYP7A1,OR51B2,TUBB4
Negative_678	BID,CKM,ADSS,ALPI,AOX1,BCKDHA,CAMK2A,CD36,CHIT1,CMPK2,CRLS1,CTBP1,CYP2C8,DGKE,DOCK1,DPM1,EXT1,GALNT8,GNG12,GRIN2C,IFNGR2,ITGA9,ITGB6,LRDD,MAP2K6,OR2F2,OR2T29,OR51B6,OR51E2,PFN2,PIGT,PIGW,PPA1,SETDB2,SMC1A,TKTL1,TLR3,TPK1,TUBB4
Negative_679	BTK,NF1,TK2,ACP6,ACSM2A,ALAD,ASS1,CDKN1C,CRHR1,CXCL16,EFNB2,EPB41,FARP2,GCLM,GMPR,GNAT3,GRLF1,IFNA14,INPP5E,MAML3,NKX3-1,OR2T34,OR2Z1,PLA2G4A,POLR2H,PPP1R3D,PPP2R2A,PTGES,RBL1,ROCK1,RXRG,THBS4,YARS2
Negative_680	CBS,AARS,ADCY6,ALG5,CLDN7,CSF2RA,EFNA3,EPOR,GALNT7,MAD2L1,MAGI2,METTL6,OR8G1,PDGFA,PIP4K2C,PSME2,ZMAT3,ATP6V1E1
Negative_681	JUN,AVPR1A,CLDN1,CTNNA3,CYP7A1,FSHR,FZD4,G6PC,GJD2,JAG1,OR1F1,PTPN11,RDH5,STEAP3,ULK1,WARS
Negative_682	FGB,GPI,ACAT1,ANAPC7,ASIP,BDH2,CASP3,CD79B,CDC6,CES2,CRLF2,CSDA,EGFR,EVI1,GPT2,HTR2B,ICOS,IRS1,ITGB1,NTRK1,OR14I1,OR2M7,PAK2,PFKP,PLCZ1,POLR2K,PPP2R5C,PPT1,PRKAB2,PYCR1,RUNX1,SLC2A2,SOAT1,SPAM1,SUV39H2,THBS2,TJP3,TRAF3
Negative_683	FUK,NOG,CCDC6,CDH1,CDS1,CRHR1,CSF2RA,DPAGT1,EGLN1,EPHB4,EXTL1,FARSA,GMPPA,ITGB3,KIR2DS4,LIAS,NRG4,OR11H4,OR51Q1,OR8H2,OXCT1,PDE8B,POLR1D,PPA2,RPIA,SHISA5,SOD1,TPH2,YWHAH
Negative_684	PRL,ACSM2A,B3GAT2,CCL11,CCNB2,CYBA,DHRS9,FLOT1,HMBS,IFNW1,IL8RA,IRAK2,IRF3,LAMB3,LEPR,MAPK7,OR10A4,OR52A5,PDGFRA,PFKFB4,PHKA2,PIAS2,POLR2H,RHOA,RIPK1,SC5DL,SDC1,SPRY4,SRGAP3,TBXA2R,UCP1,YARS
Negative_685	EPO,FES,HK2,ADH1A,ADH6,APRT,ATP6V1A,BHMT,CACNB1,COL4A2,CRY1,CTSB,DAG1,DKK4,EPHB1,GLYCTK,IFNW1,IL6ST,KIR2DS4,LEPR,NGEF,NME2,OCRL,OR2V2,OR4D6,OR7A5,OR8K3,PDGFC,PIGH,PLEKHA8,PLXNA1,PPP1CC,ST3GAL6,UAP1,VANGL1

Negative_686	LPL,ACSL1,ALDH3B1,BIRC2,CASP6,CCL1,CMPK2,CYSLTR2,DGAT2L4,DIAPH2,DKK1,DPYD,GGCX,GNAT3,GSTA5,NUDT2,OR2L13,OR4K2,OR51V1,OR52N2,PKM2,PLXNA3,PSENEN,PTGS2,PTPN6,RAC2,SKP1,SNAI1,SPRED1,SPRY2,TP53I3,WNT10B
Negative_687	ASMT,DAPK1,HMGCS1,OR10A6,OR4X2,PARS2,POLR2J,SRGAP3,TNFRSF8
Negative_688	DAO,GLA,IL2,TNC,CBLC,ENTPD1,GALNS,HSD17B4,KNG1,LAMB4,MAPK11,MYH11,NCR2,PCYT1A,PIPOX,PROS1,SFRP4,TAS2R8,ZNRD1,ALDH18A1
Negative_689	ATP5G3,FGF14,OR5P2
Negative_690	ME2,AQP7,C9orf95,CIITA,COX10,CXCR6,CYP1A1,DERA,DHODH,EPB41,FUT4,HSPA2,LRRC4C,MAML1,MAP3K1,MCM6,NANS,OR51M1,OR52W1,OR5I1,PIK3CD,PLCB4,RFX5,SEMA7A,SESN3,SFRP5,TLR3,TRAF1,VAMP2,HSD17B10,TNFRSF12A
Negative_691	MPZ,CSF3,DKK4,FADD,CTNNBIP1
Negative_692	EZR,ID3,JUP,ME3,TK2,AKT1,ARHGEF1,BMPR2,CACNG6,GRHPR,IFNAR1,IL23A,KLRC1,METTL6,MYH15,OR10H2,OR10H4,OR52R1,OR5D14,PGLS,PIAS3,PTCRA,PTPMT1,TLR4,ST6GALNAC4
Negative_693	ALAS1,CD59,CDK4,CLDN9,COMP,CTBP2,DUSP4,ELK1,HMOX2,HSPA1B,IFNAR2,LIPG,MDH2,MYH11,NRG2,OR10AG1,OR1L8,OR2W1,OR5B12,PLA2G10,PLCB3,TGFBR1,TNFSF9,TUBB3,ULK1,VNN1,SERPIND1
Negative_694	TNR,ALDH3B1,CCL20,CCL24,CCL7,CCR9,CTNNA1,CXCL11,DLST,DUSP7,EDNRA,EPHA3,FGF2,H6PD,HYAL1,LIPC,OR10G7,OR11L1,OR2T8,OR8S1,RALA,SELL,SGPP1,SKP1,TNFRSF4,TPH2,TUBA1A,C1GALT1C1,HSD17B10,SUV420H2,TNFRSF10A,TNFRSF12A,TNFRSF18
Negative_695	GH2,ALDH1A1,ALDOB,ANAPC5,CCND3,CD74,CDC45L,CPT2,DTX3L,ERBB2,FUT5,GSTA2,HHIP,IL5RA,MYH10,OR1L4,OR4F17,PAX8,PGS1,PLXNB1,PPP5C,SDC2,SLC25A5,STK11,TAS2R48,TLR3,TRPM5,ULBP2
Negative_696	ABAT,ACAA1,ACLY,ADCY7,ADRA1B,B3GNT4,CACNB3,CCL11,CSDA,CSF3R,IL2RA,NOTCH3,OR51A4,OR5AR1,OR6F1,OR8G1,RFX5,SETD2,STX1B,SV2B,TGFB3,TRAF1,TSHB,TNFRSF12A
Negative_697	CD4,CRH,F2R,FAH,AGXT,ALDOB,ALPI,ASNS,ATP5I,CD14,CDK6,CHRM2,COL1A1,DPM3,DRD2,ENTPD8,FARSB,FCGR3B,FGF10,FUT1,GFAP,GNG12,GYS2,HLA-F,IKBKB,IL8RB,ITGA7,MYH3,NFX1,NUDT12,OR4L1,OR51B4,OR52J3,OR52R1,ORC6L,PFKL,PNLIP,PPP2R2A,PPP2R2C,PYGL,RETSAT,SEMA3A,SEMA4C,SKP2,SPRY4,STAT1,TAS2R49,THTPA,WNT4
Negative_698	RDX,CD28,CDC7,TAS2R10
Negative_699	VCL,A4GALT,ACSL3,ALDOC,ANAPC5,C4BPA,CDC23,CLDN20,DUSP9,FMO5,MYL2,NEFL,OR10G3,OR4C46,OR5D18,OR6C74,P2RX3,PISD,RPS6KA5,SUCLA2
Negative_700	FGB,ALDH5A1,BCL2L1,CLDN7,CREB5,CYP2S1,DPM1,DUSP3,E2F2,F13B,FLAD1,GAPDH,GARS,GFPT2,GNB1,GPD2,IL18,IL2RG,IL6R,MICA,MPST,NPR1,NRAS,OR4K1,OR8H1,PCCA,PECAM1,PFKFB1,PFKFB4,PFN2,POLR3B,PPP2R5A,PRKCG,SEMA3A,SETD1B,SPRY4,SULT1A2,TACR1,UCP1,WARS,WNT6
Negative_701	DDB2,ENTPD8,ERBB4,FBXW11,GALNT1,OR52A1,OR5D16,PDXP,RASGRP4,RHEB,RPS6KA4,SLC2A2,THBS4,VANGL1
Negative_702	CD4,TPR,ACPT,ADRA1A,ANGPTL4,ATN1,CDH5,CKMT2,CSH1,CSNK1D,DDX18,DDX19A,DGAT2L4,DUSP1,EPAS1,EPHB6,GMPR2,HPRT1,HSPA1B,IKBKE,ITGB8,KLRC3,MAP4K3,MCAT,MCM4,MPST,MTHFR,MYH14,OR10A5,OR2M4,OR4M1,OR52K1,OR56A1,P2RX2,POLD3,PPP3R2,PTCH1,PTPRC,SUV39H2,TALDO1,TAS2R8,TUBB4Q,WNT9B

Negative_703	ACP5, ACTB, ARPC1A, BDH2, CACNA1G, CDC26, CES2, CXCR5, ENTPD6, ERBB4, EXTL3, GGT1, GLS2, GNG13, HSD3B1, HSPB1, IMPDH2, INADL, INMT, KIR2DS4, LPAR1, MAT2B, NAGS, OCRL, OR5B3, OR7A10, OR7G3, PIK3R4, RAPGEF3, RASGRP1, SAT1, SGPP1, SIAH1, STAT5B, SYNJ1, TAS1R2, TNFSF9, TNFRSF10B
Negative_704	TNR, ARHGEF4, ATP2A3, CACNB1, COMP, DHRS9, ELK1, IL22RA1, ITCH, NOX3, OR2A25, PPP1R1A, PRKCQ, PSAT1, PTPRJ, TAS2R8, UGT2B7
Negative_705	PXN, CCKAR, CDKN2D, CHST11, DAAM1, DDX18, DKK1, FGD3, FLOT1, HTRA2, MCM6, MGAT5, OR13C8, OR5H6, PPP2R1B, PPP5C, SAT1, SFRP1, SMARCA5, SULT1E1, WEE1, WIF1, PAFAH1B3
Negative_706	ASL, ABL2, ADCY7, ADORA2B, AOC3, CAV1, CLOCK, CYP1A1, ITGA7, NTN1, NUDT2, OR8B12, PAK7, PCK1, RAD51, STX6, ATP6V0D2
Negative_707	BCR, C7, GNS, ID2, LAT, TH, ACP5, ACTC1, ACY3, ADCY6, APH1A, ARHGEF1, BCL2, BPGM, CCL11, CKS1B, CNDP1, CNTNAP2, CTSG, CYSLTR2, DLAT, DRD5, DTX1, ENPP2, ENPP6, FLAD1, GNB1, HAO1, HHEX, IL1A, JAM3, MCM5, NFATC2, PARVB, PNPLA3, TNFSF14, WNT6, XIAP, ATP6V1C1, SERPINA1
Negative_708	AK2, BCR, OGT, AARS, ADSSL1, AGPAT2, AGRP, CD14, CD226, CMAS, CSNK1D, DAPK2, DDX55, DGKH, DIAPH3, ELK1, EPB41, GALNT10, GLG1, GRIN2C, HMOX1, IRAK4, MAN2A1, NME2, NRP1, OR11H4, OR12D3, OR2AE1, OR3A2, OR4S1, ORC1L, P2RX2, PARD6A, PDIA4, PFN3, PLCE1, PLEKHA8, PMAIP1, PPA1, PPP1R1A, PSEN2, SEMA4F, VEGFC, ZMAT3, ATP6V0A2, HSD17B10
Negative_709	GP5, ID1, RFK, TK1, ACAA1, ALDH2, ALG6, ARPC2, ARSD, CD226, CD40LG, CD8A, DIAPH1, DLST, EDA2R, ENTPD2, GBE1, GGPS1, GNA13, HHIP, IDH3B, IRS4, LAMA4, LCMT1, LDHC, MAGI2, MAML2, MAP2K4, MDM4, NPR1, NQO1, OR2T29, OR4S2, OR51Q1, OR52B6, OR7A5, P2RX2, PDXP, POLD1, PPP1R3C, PPP2R5B, RALB, RRM1, SDHA, TRDMT1, TUBB4Q, VARS, VNN1
Negative_710	SFN, TNF, ALG3, ALPL, ARAF, ARPC3, BTRC, CD19, CD38, CLDN16, CPT1B, DHRS3, DUSP3, ENO3, ENPP7, GP1BB, GPX7, H6PD, HAAO, HSD17B2, IL12B, LAMB1, MCEE, MPDZ, OR2M3, OR5T3, OR7D4, PARD6A, PDE8A, PER2, PLCB3, PLXNB1, PPBP, PTGS1, SFRP5, ULBP3
Negative_711	ATP5G1, CDC23, CDH2, IFNA10, MYH14, OR4K17, PLD2, SC4MOL, VAMP4, KIR2DL5A
Negative_712	ADORA2B, ALPI, ANAPC4, BACE2, BNIP1, BST1, CCNE1, CSNK1E, CYP19A1, DGKG, EFNA3, FGF10, FMO1, GLG1, GMPPA, GRIN2C, HAO2, HPSE, HRH1, IPMK, ITGA1, ITGA3, KIR2DS5, LIPE, LRDD, NFE2L2, OR2T5, OR4F17, OR5K1, SEMA4B, SOAT2, ST3GAL1, STX6, TCF7L1, TIAM2, ZIC2, CSNK1A1L
Negative_713	CRH, AADAC, ACSS2, ACVR2B, B3GALT4, B3GNT5, BCKDHA, CACNG7, CADM3, CDK2, DUSP7, EFNA1, EP300, FARSA, FMO5, FPGT, GCDH, GNA12, GNG12, GNG3, GSTM1, HMOX2, HRAS, HSPA2, IFNA16, MAPK11, MAPK13, MARS2, MOV10L1, NCKAP1L, OR1K1, OR4D1, OR4K13, OR51L1, PAICS, PIK3R4, PON3, PRKACB, PRKCZ, ROBO3, SESN2, TAS2R10, TLR9, TOLLIP, CACNA2D4
Negative_714	CP, KDR, LPL, ME3, ACAD8, ACSM5, ADCY3, ADCY5, ADCY9, ALDOC, ARNT2, BCKDHB, BMP4, CDH5, CDK2, CYP26C1, DGAT2, DRD1, E2F4, ENTPD5, EPHB2, EXT1, F2RL2, GAD2, GLUL, GRPR, GSTM2, GSTM5, GSTZ1, GUCA1A, HDAC1, MYH13, MYL2, OR6C6, OR8H1, PDXP, PERP, PON3, PPP1R1A, PPP2R2C, PPT1, PTPMT1, TNFSF10, UNC5D, PPARGC1A, SLC25A31, TNFRSF6B
Negative_715	F2, RB1, TK1, ACPT, ADCY4, CD19, CSNK2A2, FTH1, HSPA1B, LIAS, MMP7, OR5AC2, PANK1, PFKM, SH3BP2, TRAF5
Negative_716	CSK, HK2, AKR1D1, ALPL, ANGPTL4, B4GALT1, CCL11, CDH3, CYP1B1, FGF18, GCAT, GRLF1, HBEGF, INPPL1, MAPK1, NT5C2, PIK3CG, PPA1, RUNX1T1, TAS2R9, TCF7, YARS2

Negative_717	FAH, GH2, MPZ, PLG, ACTN2, ATG12, ATP6V1F, BCKDHB, CCNE1, CD247, CD38, CDKN1C, CSNK1G1, CTNNA2, CYP2C18, CYP4A22, DFFA, DLL1, EPHA8, FGD1, LAMA4, MAFA, MAPK13, MCM2, METTL2B, NME6, NRXN3, OR10H4, OR11G2, OR51B5, OR5B3, OR8A1, P2RX4, PIK3C2A, PMAIP1, PPP2R5B, RASA2, RGS3, SMC1A, SNAI1, UGCGL1, ULBP1, XYLT2, ATP6V1C2
Negative_718	C8A, F5, LPL, PGF, ACSS2, ADH5, ARPC5L, CD27, DTYMK, FCER1G, GLS2, GLUL, GPX7, GSTA4, HPSE2, HRH1, IFNA8, IL22, LRDD, MAP2K7, MYL2, NOS3, NR4A1, OR2A4, OR2Y1, OR56B4, OR5D16, OSMR, PDHA1, PGM2, PLCB2, PPP2R5E, PRKAG1, STT3B, THTPA, TKTL2, UPB1, WARS
Negative_719	AK5, BNIP1, CMPK1, CSNK1G3, EPB41L3, FGF5, FGFR1, GUCY2C, HDAC1, IL12RB1, NAT1, OR5H6, PIGW, PLA2G6, PRKCB, PYGB, RXRA, SEMA4B, ST3GAL2, STT3B, B3GALNT1
Negative_720	GPT, SCD, COL11A1, GARS, PLA2G1B, POLA1, HLA-DPA1
Negative_721	GH1, BBOX1, GAS1, PLXNC1
Negative_722	MPZL1, NT5C1A, OR11H6, OR13D1, OR7C1, PDGFD, PRKAR1A, SERPINB5
Negative_723	CAMK2A, CCNH, DLL1, GRHPR, HRAS, ICAM3, IL18RAP, NOTCH1, NSD1, OR2B2, OR7G1, PGAM4, SLC27A5, ULK3
Negative_724	CFH, ABL1, ACTN1, APLP1, B3GALT2, B3GNT5, BAIAP2, CHPT1, CHRM5, CMAS, COX10, EDAR, FBP1, G6PD, GALE, GUCA1B, NTRK1, OGDH, OR1L1, OR2A12, OR2H1, OR4C12, OR4N4, OR52I1, PGK2, PHKA2, PHPT1, PIK3R5, PLA2G6, PMAIP1, PRKCD, PRODH2, PTPRR, RBL1, SDC1, SMARCA5, SNAP25, STEAP3, SULT1A1, UBA1, VTI1B, YKT6
Negative_725	AASS, EXTL2, GSTZ1, INPP1, MAP2K5, OR13C8, PRKAR1A, RBPJ, SLC9A1, WWP1
Negative_726	RFK, ACAD8, ATIC, CHST12, EFNB1, KLRC4, MCM7, OR52B6
Negative_727	SYK, ATP5C1, BHMT, C1QC, CCR10, CD36, IL6ST, IRF3, LIPF, MPP5, MTMR1, OR13D1, OR2L8, OR51G2, OR51S1, OR52A1, PIGN, POLR2F, YWHAH, PAFAH1B2, SERPINA1, SERPING1
Negative_728	KMO, LTB, SCD, ADH1A, AKR1C2, AVPR1B, CCR1, CYP4A22, DCXR, DOT1L, EDNRA, FGF10, FMO1, G6PC2, GLB1, HSD3B2, IL21R, IRAK4, ITGAV, LIPA, MYH13, OR10A6, OR2T12, OR51I1, OXSM, PDE1C, RPIA, SLC12A2, SPP1, SQLE, TP53I3, UGT2B17, WASF3
Negative_729	AGRP, ALS2, BMP8A, CCL16, CD34, CDK6, COL4A4, COL6A2, DVL2, EI24, GABARAP, GSTM2, GUCA1A, HAGHL, IAPP, IARS2, IL22, IL3RA, ITGA9, KLRC3, MAFA, MAPK10, MCAT, MCEE, MECR, NEU4, NUDT2, OR52W1, OR5T1, OR7C1, OR8J1, PCK1, PDE5A, PPP2CB, PPP2R2A, PTGS2, RDH11, SLC25A4, TDO2, TLR2, TNFSF4, TUBA3C, TUBB4, UPP1, UPRT, PPP1R12A, TNFRSF6B
Negative_730	C5, MAG, ME2, PLN, CASP3, DGKA, DGKH, DOCK1, DPM3, FOXO1, LRRC4C, MAP2K1, MPZL1, PDXK, PIK3CG, PTGDS, SRGAP2, TNFSF11
Negative_731	ILK, ATP13A2, CXCL9, DNMT3B, GNRH2, HMGCS1, LAP3, LPAR1, P2RX5, PIGB, PLA2G2D, SLC12A2, ST8SIA5, STK4, TUBA3C, WNT9A
Negative_732	GSS, PVR, ACY1, AGPAT2, AGTR1, ALS2, APOC3, ARPC5, CCNA1, DDX4, DERA, FZD3, GNRH2, GRPR, HHEX, IL1R2, LIPA, MAP2K5, MAP3K11, MAP3K2, METTL2B, NR4A1, OR10J1, OR5F1, PANK3, PDIA4, PISD, PPP2R5D, PVRL4, SC5DL, SMARCA5, TOLLIP, TPI1, UCK2, WASF1
Negative_733	DAPK2, DUSP6, EDNRB, HCCS, PRKCE, YES1
Negative_734	BAX, CR1, GP6, LYN, AHCYL1, AKR1B1, AKR1C1, ARHGEF7, ATG7, ATP13A2, CNTFR, COX10, CYP1B1, FGF12, FZD1, GNG3, ITGA4, MAP2K3, METTL6, MGAT1, MGAT5B, NFYA, NUDT2, OR1A2, OR2B11, OR4C11, OR6T1, OR8D4, PDE4B, PI4KA, POLE4, POLR2F, PTTG1, RPS6KA5, TNFSF9, TOLLIP, XCR1, ZFYVE16, ATP6V0D1

Negative_735	TNR,ACADS,CAV1,CCNB2,CD22,CD58,CD82,CDH4,CHST12,COQ5,CRY1,CTSB,FUT3,GART,IL28B,KIR2DL3,KLRC1,KLRC3,LCMT2,LHCGR,LIMK1,MAP4K2,MNX1,MPZL1,NUMB,OR10AD1,OR10R2,OR13G1,OR1E1,OR4C16,OR51T1,PDX1,PKMYT1,SDHB,SEMA4A,SKIV2L2,TIRAP,TLR5,UNC5D,UXS1,CACNA2D3
Negative_736	C6,CSK,TPR,APC2,BCAT1,C3orf10,CISH,CSNK1D,EIF4E,HEXA,IFNA14,IL28A,KCNQ1,LDHD,MAPK12,MKNK2,NAGK,NFASC,NFYC,NME4,NOD1,NSD1,NUDT8,OR51A2,PDE10A,PIAS2,PRKCQ,SEMA3B,TRAF4,UBE2L3,UCK2,UGT2B4,VAV1,VEGFA
Negative_737	F10,OTC,ABL2,CALML6,CD3E,CD74,CES1,CSF3,CSNK2A1,DDX23,DGKZ,DRD1,GALNT5,GRIN2B,HARS,HSD3B7,IL17B,INPP5E,LAMC3,LEPR,LRP5,MAN1B1,MECR,NCAM1,NCF4,NGEF,NT5M,ONECUT1,OR1A1,OR2F1,OR4K5,OR52A1,PFN3,PIGK,PLIN,PLXNA2,PYGM,RDH5,RRM2,SCP2,SLC25A6,SLC2A4,TAS2R3,TUBB,TYROBP,XYLB,XYLT1
Negative_738	ALDH3B1,APOE,CCR7,GALNT10,ITGA1,LTBR,PLA2G2E,PPP2R5C,RAC1,SARS2,SEMA4G,SLC27A4,SOAT1
Negative_739	EZR,IL7,ACAA1,ADRA1D,ALOX5,CDH5,CFLAR,DLAT,E2F5,ENTPD4,GALK2,GFPT1,GNAL,HAGHL,INPP4B,MAPK12,MAPK13,MAT1A,MEF2C,MGAT1,MMP2,NAGA,OR1B1,OR2W1,PANK2,PDE1C,PDHA1,PER3,PIGS,PKMYT1,PLA2G2D,POLD4,PRKAR1A,PRKCD,SETD7,TUBB4Q,UGCGL2,VCAM1,WNT9B,ATP6V1B1
Negative_740	C1S,PLN,AMPD3,CACNA1H,CHUK,CYP1A2,E2F2,IL1B,JAK2,MGAT2,NEU2,OR2Y1,PDE4B,PIAS4,PIGV,PKMYT1,PLD1,RBX1,STEAP3,PLA2G12B
Negative_741	GP5,ACADL,CARS,CCL2,FADD,FGF13,FSHR,GALNT3,GLS2,HARS2,LCP2,LEFTY2,LIPF,MAOA,MINPP1,NCK2,OR1G1,OR2T5,OR52I2,OR5AP2,PITX2,PLCG2,PPP2R2D,PPP2R5E,PPT1,SMURF1,TJP2
Negative_742	MET,NLK,ADAM10,ADIPOQ,ARNT2,ASCC3,ATP5D,C9orf95,CCR7,CLDN17,CMPK2,COL5A3,CSNK2B,DAPK1,DGKD,DLAT,DLL3,FUT8,G6PC,GNG12,GSTA2,HSPA2,LIPT1,NPR1,OR4D2,OR52K1,OR5M3,PAK4,PHKG1,PIGF,PPAP2B,PPP3R2,PRKAG1,PTPN1,SDHA,SELL,SELP,SEMA4D,STK4,UGT2B28,UGT2B4,WEE1,XYLB,ZAP70,PRICKLE1,TNFRSF13B,TNFRSF14
Negative_743	DUT,ALDH1A1,ALDH3B1,ALDOC,ALOX15,CACNA1I,CAMKK2,CCR1,CDC25C,CDK4,CHRNA7,CNGB1,COQ5,DDX41,GNAL,HSD17B8,IFNA14,IL10RB,MAP2K7,MDH2,NRG2,NUDT9,OR5M9,PFKM,PIK3CD,PTK2,RRAS,SARS,SLC27A1,SMURF1,TAS2R40,TCIRG1,TUBB,WNT8A,B3GALNT1,HSP90AB1
Negative_744	CRK,DBI,IL4,SP1,ATP2A2,B3GALT2,CD3E,CHRM2,DNMT3A,DNMT3B,DUSP16,EPHB2,GRIN2A,HSPA1B,IFNA21,IL4R,IRS1,MAP2K1,METTL2B,MTMR6,NAT5,NAT6,OR13H1,OR13J1,OR2T8,OR5AR1,P2RX5,PARD3,PDE7A,PHKG1,PIGX,RARS2,SAT1,SMARCA5,SPTLC1,TBL1XR1,TRDMT1,TYROBP,ATP6V0A1
Negative_745	FGG,ARHGAP5,CCL5,CHPF,ETNK2,FPGT,HEXB,HSD11B1,JAK2,PTK2,RFXAP
Negative_746	HK3,SHH,ADCY7,AKR1D1,CALML5,CBR1,CDKN1C,DDX19A,DHRS3,DPYS,ENO1,GCH1,GFPT1,GRHPR,GYS1,HTR2C,JAM2,MLYCD,OR1L8,OR2A42,PIGF,PTGFR,RARS,SLC27A4,SV2C,TACR2,HSP90AA1
Negative_747	DCT,ID1,INS,IVD,TK2,WAS,ALDH1L1,ARPC3,ATP6V1H,CCL20,CCNG2,CNTF,CYP4A22,GALNTL5,GCNT2,KCNB1,KLRD1,NKX6-1,OR4C46,OR9K2,PGK1,PIGH,PRKAA2
Negative_748	ARNTL,FGF6,GALK1,LTBP1,NLGN1,RHOQ
Negative_749	BCR,GSN,ME3,ACADVL,ADH4,AMY2B,ASIP,CALML3,CALML6,CHUK,CMBL,CNTNAP1,CYP2C18,CYP2E1,FPGT,GNAT3,GNG3,GP1BA,IL24,ITGAV,MAGI2,MYLK3,NAGA,NCK2,NCR2,NMNAT3,NOTCH1,OR51L1,OR5B17,OR5K2,OR6C70,PDXP,POLE3,PPP1R3C,PRPH2,PTK2,SEMA3E,SREBF1,STX2

Negative_750	CTH, DDC, FH, FN1, IL6, ACOX3, ACY1, ACYP2, ADCY4, ATP5A1, BMP8B, CACNG7, CHST12, CPT1B, CSF2RA, DKK4, DUSP4, FSHR, FZD8, G6PD, GUCY2C, IDH3G, IKBKB, IL1RAP, ITGAD, JAK2, MITF, MTHFD1L, MYH11, NKX6-1, OR5AN1, PAPSS2, PIAS4, PPP3CC, PPP3R2, RASA1, SETD7, SMAD5, STEAP3, TAS2R14, TNFRSF8, TRAF2, TUBB3, WWP2, ZFYVE9, SUV420H2
Negative_751	A2M, LCT, ACMSD, ADCY4, AGPAT3, ALOX15B, AMPD2, ATP5I, CHKA, CLCF1, CRLF2, CSF1R, CTPS, FGF19, GRAP2, GRB2, HIBCH, HYAL1, IDH2, IFNA16, JAG2, NAT1, OR10G7, OR11H6, OR1B1, OR2M3, OR3A1, OR5P3, OR6N2, OR8S1, PMVK, PRODH2, RAP1A, SC4MOL, SELPLG, SOCS7, ST3GAL6, STX3, TAS1R1, TAS2R60, TXNDC12
Negative_752	CACNA1D, CYP3A5, EDN1, IL6ST, INPP5B, NTF4, OR5B12, PLOD2, SARS
Negative_753	ALDH1B1, FUT3, LALBA, OR2T29, OR51B2, OR8J3, PFN3, POLE4, PRKCQ, SNAP23, UBE2L3, VTI1B
Negative_754	CD4, F10, ALAD, BCKDHA, CCNG2, CD34, CDKN2C, DIAPH1, FGF17, FGF18, GSTM3, JAG2, MAP3K13, MCM4, NKD1, NRXN3, NT5C2, NTF4, OR10G7, OR10H4, OR4D9, OR52N1, OR5A1, OR6F1, PLXNB3, RALA, SOCS5, TM7SF2
Negative_755	AK2, EGF, GHR, MIF, AASDH, ACY3, ALDOC, ARPC1B, ASIP, ATP6V1A, ATP6V1F, C3AR1, COX15, CSNK2B, DDX52, ENPP2, FOSL1, FOXO1, GJA1, HCST, HSD17B2, MAP3K7, MINPP1, ONECUT1, OR2D3, OR2T11, OR2T29, OR4P4, OR5A2, OR6K2, PIGP, PPARD, PRKY, RRM1, SARDH, STAT2, TUBA1C, TUBA3C, TUBA8, VAV3, WASF2, PAFAH1B1, PDCD1LG2, PNLIPRP2
Negative_756	DCT, ALOX15B, ATP5J, DGKE, DRD5, EHMT1, FCGR3B, GLI1, LIPF, NAGLU, OR6N1, PTEN, RBL1, SDHC, SIPA1, TALDO1
Negative_757	HPD, KIT, MPZ, AHCYL2, CADM1, COL4A6, COQ5, CXCR5, CYSLTR2, DUSP3, EPB41L3, FLOT1, GALNT1, GPD2, IL19, IL29, KRAS, LIFR, LTA4H, MAFA, NCSTN, NEGR1, NME6, NT5C2, OR13C8, OR2H1, OR4A5, OR51V1, OR5D18, OR5R1, OR5T3, OR9G1, PCNA, SEMA7A, SHMT2, SORD, SRGAP3, ST3GAL2, SUV39H2, TNFSF9, UNC5D, VAMP1, VAV3, WARS, WASL, XYLT1
Negative_758	CCNG2, CREB3, DHCR24, EDA2R, GGCX, GLUD2, ITGAD, OR4A5, PIK3C3, POLA2, SHMT1
Negative_759	CAD, CGN, GK, HGD, ALDH5A1, ASAH1, CAMK2G, CHKA, CYP4A22, CYSLTR1, DGKA, DUSP9, GRM1, IL12RB2, ITGB5, NNMT, OR5D13, ORC5L, PAK7, PFKFB3, PFN3, SHC2, STX12, TAS2R3
Negative_760	SFN, ACYP1, ADORA2A, ARPC3, CADM3, CASP7, CDC45L, CTSL1, CYBB, CYP1B1, DFFA, DHX58, ERBB4, FGF4, GALNT3, GIT1, IL15RA, IL1B, MYH14, NFAT5, NRG3, OR10J5, OR56A3, PFKFB4, PIGL, PTPRM, RFX5, SMAD5, TLR2, ULK3, WNT7B
Negative_761	EPO, AADAC, AASDH, ACSL6, ACY3, AKR1B10, CD86, CLCF1, ENPP7, GAL3ST1, GCH1, HTR4, LDHD, NRCAM, PDHA2, PIM1, RFXANK, SEMA4C, SMC1B, SMURF1, UBE2L6
Negative_762	A2M, C4B, FH, ITK, LIF, SRM, ARSE, CBR3, DTYMK, E2F4, ENTPD5, INPP5E, MCM4, NFATC2, NRG3, OR10AD1, OR10G2, OR4S2, OR5A2, PLA1A, RBL1, RFNG, TLR5, TLR9, ZMAT3, ZNRD1
Negative_763	CS, TNF, BBC3, CCR6, CFLAR, CRKL, CSH1, DERL1, ENTPD5, EPHA4, GAS1, GCLC, GLI3, GPAA1, HAP1, HEXA, IKBKE, NLGN1, NME2, OR13H1, OR6N2, OR8H2, PDE4B, PHKB, PLIN, PRKAR1B, PRPH2, PSME3, PYCR2, RPS6KB1, SMPD1, SV2A, TAS2R41, TIAM2, TNFSF14, TP53, HLA-DQA2
Negative_764	F12, MAX, ACVRL1, AMPD3, B3GALT1, CACNA1I, CD38, CD55, CDC14B, DVL3, EFNA3, GNA12, HIF1A, MDH2, MTHFD2, NFKB2, OR13F1, OR2B6, OR4A5, P2RX4, PFN1, PTPRM, STX2, TGFB1, HSP90AB1
Negative_765	DPYD, IFNA10, OR1F1, UGT2B10, UGT2B28

Negative_766	DHH,LPO,SDS,TH,ACOX1,ACVR2A,ADCY2,ALOX12,COQ6,DBF4,DPYS,DUSP14,ENTPD3,FZD6,FZD7,GP1BA,GRIA2,IGF1,IL3RA,IL6R,IQGAP1,MGST1,NCAM1,OR11L1,OR5P2,OR6N2,P2RX1,PFKFB4,PGLS,PHKG1,PRKG1,PRKX,PRODH2,RPRM,SDC1,SDC3,SETD1A,SV2A,TAS2R1,TAS2R60,TNFSF14,TRHR,UCK2,VAMP2,WNT2,ATP6V0E1,CTNNBIP1,PNLIPRP1
Negative_767	CSK,NCF2,OR9G4
Negative_768	ARRB2,CCR2,DAPK3,GNPDA1,HSD17B7,KRAS,PIGK,PPP1R3B,TAS2R60,TLR5,TRAF6,MAPKAPK2
Negative_769	ALDH3B1,BDNF,CARS,CD44,CLDN10,CRKL,CXCL5,EIF4E2,ENPP2,GGT1,IARS,IFNA16,IFNA21,IFNGR1,IL7R,LDHC,LRAT,OR5T3,OR6C76,PON2,SEMA4A,SETD1B,SPHK2,HLA-DRB5
Negative_770	F9,ACOT11,CSH1,FGF3,FLOT2,GALNTL2,GNG3,MAOA,NEUROG3,OR52M1,PANK3,PIGP,PLEKHA8,PRKAA2,SMC1A,ATP6V1B2
Negative_771	F10,AMPD3,APOE,ATP6AP1,CCKAR,CDKN1B,CHST12,CLDN14,CXCL11,FDPS,HMGCS2,ITGAL,KRAS,MAP4K4,NCF4,ONECUT1,OR2A4,OR4K14,OR52W1,PAK3,POMC,PPAP2B,SETD2,TIAM1,TKTL2,VAMP2,VEGFA
Negative_772	TNC,DGAT2,OR4D11,PLD1,PRKAA2,VDAC2
Negative_773	GCNT4,HAGHL,IFNA16,PAK6,PARS2,PIGS,PLIN
Negative_774	ADCY10,AKR1B10,ALDH1A1,BAIAP2,BMP8A,CMBL,CSNK2B,CYP19A1,DPYD,DTX2,ERBB4,FBP1,GALNT5,HEXB,ITGA7,LYZL1,MAP3K6,NAE1,PHPT1,POLA2,RASSF5,SMC1A,SMPD4,SOAT2,SULT2A1,TPK1,YWHAZ,PLA2G12B
Negative_775	AK2,CD6,CS,ACAA2,B3GAT3,CLCA4,DGAT1,FIGF,GNMT,GOSR2,IFIH1,ITGB4,KLRC4,NR4A1,PFKP,TUBA8,VEGFA
Negative_776	CDA,TFG,ASPA,CCL19,IL12RB1,PDE8B,PIGO
Negative_777	DBI,F2,COL5A1,CSF3R,DDX23,DEGS2,IL25,LEPR,OR10A6,OR2G3,OR8J3,PFKFB1,PLCB4,PRNP,SEMA4G
Negative_778	F2,PRL,ACTA2,ACTG2,ADSS,AHCYL2,ALS2,B4GALT5,BCL10,CACNG1,CAV3,CCL15,CCNA1,CTF1,DHDH,FECH,GALNT13,GP1BA,IGSF5,IL21,MARS,MKNK2,MRAS,MTMR6,NAGLU,NFYA,NKX6-1,ODC1,OR4A16,OR51D1,OR6C4,PARVG,PCK1,PIK3CG,PLXNB1,POLA2,PRNP,PSME1,PTK2B,SOCS7,TLN2,TNFSF12,TP53I3,UCKL1,UGDH,ULBP1,ST6GALNAC3
Negative_779	EDA,KDR,XDH,ABCC8,ACTB,ADORA2B,ADRA1B,ALDH9A1,ALPPL2,AMPD3,ANPEP,AXIN2,BDH2,BPGM,CACNA1B,CMPK2,CSH1,CYP2J2,DDX41,DGKD,DUSP7,ENTPD5,EXOC7,FZD9,IGF1R,IL24,LAP3,MDH1,MYH4,NR1D1,NUDT12,OR1A1,OR52H1,OR5R1,PDGFB,RFWD2,RFXANK,RFXAP,ROBO2,SEMA3G,SFRP1,SLC27A4,SMURF2,TLR3,TNFRSF9,UROS,XCL1,MAPK8IP1
Negative_780	ATM,BAX,UBB,AARS,ACVR1B,ADRA1B,AKR1A1,ATP5F1,BMP8B,CACNB3,CHAT,EPB41L1,ETNK2,FZD10,GALK2,GCNT3,GLUD2,GRIA2,HGSNAT,HIP1,ICAM2,INMT,KAT2A,LEFTY2,LRP5,MMP14,NSDHL,OR2T4,P4HA2,RBL2,SHMT1,STAT6,STX5,SULT1A4,TNFSF10,UCK1,UPB1,MAPK8IP3
Negative_781	CAD,IL3,APLP1,APPL1,ATP6AP1,BBC3,C3AR1,CASP7,CCL3,FZD9,GADD45G,H6PD,IL10RB,LIMK2,MAGI1,MAP3K11,MAP3K2,MGAT4A,MYH3,NFAT5,OR11H6,OR4C13,OR7A10,PCCB,PHKA1,PLCB1,POLR2K,POLR3D,PTCH1,PTTG1,RALA,SPRY4,TAS2R40,WWP1
Negative_782	AK5,BTD,CD4,GSN,MTR,SRM,ACAA2,ACPT,AGTR1,ARG2,B4GALT5,BBOX1,CDC14A,CHDH,CLDN9,CRY1,CTSL1,CYP2S1,DDX4,FGF12,FOXA3,FOXO1,GGT7,GLI3,GRIN2D,IFNW1,IL2RB,INSR,IRAK1,NANP,NKX2-2,OR10AG1,OR4A5,OR9Q1,PCYT2,PHKG2,PRKAA2,RHEB,VANGL2,MAPKAPK3
Negative_783	IL6,APC2,BIRC3,CD70,CD8B,CDH3,DARS,GMPPA,GPAM,ICAM1,OR52I2,OR5AC2,OR5M9,OR6B3,PDGFB,POLR3K,PRPH2,RALB,RARS2,TPI1,TPK1,ULBP3,EIF4EBP1,TNFRSF6B

Negative_784	AMT,C6,FUK,LCK,TNF,ACOX1,BAI1,CACNG5,CAPN2,CASP6,CCL27,CCNA1,CHST13,CLDN1,CYP2F1,DHX58,EDN1,GNG3,GUCY2D,LYZL1,MGAT4A,MIOX,NTF3,OR10A4,OR2A2,OR52N5,PDHA2,POLR1C,PRKCE,RAP1B,SC4MOL,SDC4,SNAP23,SOCS5,STK11,TAS2R38,TGFB1,TKTL2,TPI1,TRPC1,VAMP2,TNFSF13B
Negative_785	ATP4A,MGAT2,OR10V1,OR11A1,OR5AK2,PIK3R3
Negative_786	AMY2B,IGFBP3,IL18,KLRC3,NR1H3,OR2T1,OR6C2,PIGL,PLA2G2F,TLN1,TRDMT1,VASP
Negative_787	CBL,KDR,ACSL3,ADSSL1,AHCYL2,ANAPC1,ANAPC2,APAF1,ATP4A,ATP4B,CCNA1,CCNB2,CCR5,CD19,CER1,CHRM5,CYP19A1,CYP2F1,DGKG,ECSIT,ESPL1,FABP3,FBP1,GAD2,GFPT1,GGPS1,IMPA2,ITGB2,NMNAT3,OR10AG1,OR4C13,OR4F4,PGM2,PLXNB3,POLR3B,PRSS2,PSEN1,RAP1B,RRAS,SCP2,SLC25A6,SLC27A4,SSH3,TAS1R1,TOMM40L,TPH2,TPI1,PLA2G12B
Negative_788	AR,DUT,JUP,MVK,TNF,ADCY7,ADH6,ADRB2,ALOX12,C5AR1,CAMKK1,CCL20,CCL22,CDH3,CLDN10,CLDN17,DAPK3,DHODH,FBXW11,GALNT13,GLG1,GLYCTK,GMPR,GNPNAT1,HSD3B1,IL2RA,IRF5,LAMB3,LRRC4C,OR1B1,OR4C11,OR4K15,OR5AK2,OXSM,PAK6,PDGFA,RAF1,RHOA,SC4MOL,SEMA3D,SKIV2L2,TRAF4,TRPC1,VANGL1
Negative_789	KMO,AANAT,CACNA1H,CALML5,DARS2,FGF14,FGF8,OR5T3,OR8B12,PPP2CA,PSME3,RALA,SSH1,TNFSF12,TUBA4A,UGT2B28
Negative_790	FUK,ALG2,B3GNT5,CES7,CYFIP2,DERA,GLI1,GSTA2,HADH,HSD17B7,LTC4S,MYD88,OR2L8,PRKAG2,SMURF1,UROC1
Negative_791	GP5,SRC,CHRM3,CTBS,DDX19A,DGKI,F2RL2,NLGN2,OR10A2,POLR1D,POLR2J,RXRB
Negative_792	AKR1B10,ATP2A2,CASK,CCND1,CLDN15,COQ5,CXCL2,CYCS,HAGH,HRH1,MAP2K7,NAT2,PIGB,PLCB3,PRKACB,PRKCD,RFXANK,SLC27A5,SPTLC1,WASL
Negative_793	VTN,ALDH3B2,ALG5,CACNG3,FADS2,GALNT10,GLI2,GNAL,GNAZ,IDH3G,IQGAP1,NT5C2,OR2D2,OR4D9,OR8B8,PFN3,PIK3C2A,POLA2,POLD1,SFRP5,SPRED2,UPB1,VKORC1
Negative_794	OR8G1,PISD,PMM2
Negative_795	ITK,XDH,ALG3,CACNG4,CAV1,CCL13,CCL19,CLDN8,DARS,EDN1,EIF4B,ENPP2,FMO4,GCLC,GUCA1A,MAPK10,MAPK13,OR10J3,OR10P1,PDE1C,PFKM,PIGB,POLR2K,PRIM1,PTPN11,TAS1R1
Negative_796	FES,ARPC5,BMPR1B,CACNA1G,CDC26,CPS1,CREB3L4,CYBA,DCTD,GOT1,HSP90B1,INPP4A,KLRC1,NADSYN1,NFATC3,NPR1,OR51E1,PCCA,PDE8B,PI4KA,PRPS2,PTAFR,PTPN11,SOAT2,SPP1,SUV39H2,VEGFC,WNT6,ST6GALNAC6
Negative_797	CFD,EGF,FGG,GDA,LSS,ACOX3,ALDH1A1,ALS2,ANAPC10,CCL25,CCNE1,CYP2J2,DDHD1,G6PC,GALNT6,GAPDH,GGT5,GNA12,GUK1,IFNA13,IFNGR1,IL10,ITGB1,KIR3DL2,MYH3,NFATC1,OR13C8,OR4F15,PDE1C,PMM1,POLR3B,PPP2CA,TCF7,TMLHE,TSHR,TYMS,WNT8A,SLC25A31,TNFRSF10C
Negative_798	ADAM10,ADCY7,AKT3,DDIT3,FLAD1,FMO4,GUCY2F,IFNAR2,ITGA1,MAGI3,MDH1,MLYCD,MTAP,P2RX4,PDE3B,PLCB1,PLCZ1,PPP3CB,RFNG,VEGFC
Negative_799	AK3L1,ATG3,BDKRB2,MAP2K5,MMP1,NOTCH2,OR52B6,RASSF5,RHOA,TNFRSF10C
Negative_800	ACOX1,ANAPC11,ATP2A2,CHRD,EXT1,FGF6,GAD2,GADD45B,GATM,OR2T1,OR52R1,PARVG,PIAS4,PIP4K2A,PIP4K2B,PRPH,SLC27A5,SOCS4,WNT5B
Negative_801	C6,F10,GPI,NOG,PGD,AMPD2,ASRGL1,CLTCL1,CPS1,CREB3,CYP7A1,DFFA,DKK1,EDA2R,FMO1,HSD11B2,IFNG,IL9R,INPP5A,INPPL1,LAMA3,NAT5,NTRK2,OR2H2,PIGH,PIPOX,PPP2R2B,PPP2R5C,RXRB,SGPP2,SH3BP2,HSD17B12

Negative_802	LTB,ACO1,AKT1,BMP8A,CPOX,DRD5,EFNA5,IL12RB2,IQGAP3,LRP6,MAML3,MYH11,NCF2,NFYA,OR5AP2,OR5P3,PCK1,SELL,SUV39H2,YES1
Negative_803	CGN,PAH,ADRBK2,AHCY,APOA1,CASP6,CCR4,CDC45L,DDX54,DTX2,EHMT2,FGF17,GADD45B,GALNT1,HLA-DMA,ICAM3,MKNK1,MTHFD1,PDK1,PROS1,SDC2,STX1B,TP53,TRDMT1,TUBB3,YES1,YWHAB
Negative_804	AK2,CD6,GSN,TFG,ABL2,ADRA1B,BMP2,CCR6,CHEK2,COX15,CREBBP,CXCL6,DAXX,DTX2,GAL3ST1,GLUL,GUCY2C,HIBCH,HSD17B1,HSPB1,INPP4A,ITPKB,LAMC1,MLYCD,NAGS,NCF1,NUMB,OR10P1,OR13J1,OR2T10,OR4S2,OR5I1,PCYT1A,PLA2G3,PLOD2,PYCR1,RXRA,STX10,SULT2A1,TIAM2,WNT7A,WWP2,TNFRSF17
Negative_805	ANAPC10,ETNK2,HSPB1
Negative_806	AK1,BID,FAH,ALDH2,ATP2A2,IL20RB,KRAS,MMP14,OR1K1,OR5B3,OR5M9,OR5V1,OR8H3,PGK1,RALGDS,SC5DL,SMC1B,STEAP3,WNT4,HSD17B12
Negative_807	B3GNT3,BMPR2,CCL19,CDC45L,DGKA,DHDH,ERBB4,OR10R2,OR7D2,QARS,ROBO3,SLC27A5,ULBP2,WBSCR17
Negative_808	KLK3,SNAP23,TNFRSF6B
Negative_809	MVD,ALDH1A2,ALG12,AMY2B,CCL22,CMPK2,COQ7,CTSG,DBF4,DEGS2,HPSE,IL24,IL2RG,INHBA,LTBP1,NMNAT3,NRG4,NTNG1,OR10A3,OR10G7,OR4D2,OR7D4,PDE2A,PPOX,RAF1,RAPGEF3,RUNX1T1,SPAM1,STX8,UGCG,ST6GALNAC4
Negative_810	C8G,ALCAM,C3AR1,CCL25,CLDN3,DARS,DFFB,EGLN3,HTR5A,IL10RA,IL23R,IRF7,MAP2K7,MMP9,NAT2,OR11G2,OR4X2,OR51F2,OR51T1,PDGFA,PIGB,PPID,PRPS2,PTAFR,PTCRA,RELA,SHC4,ST3GAL6,TUBB2A,UBA7
Negative_811	MTR,ARSE,CCNG1,IFNB1,MYH15
Negative_812	AMT,ABL1,CD38,CD40LG,CLDN3,CSNK1D,CYP2B6,GYS1,HYAL2,IFNA10,IMPDH1,LIAS,OR13C4,OR5M9,PDCD1,PSAT1,TKTL1
Negative_813	F2R,GGH,GSR,MET,ABLIM3,ACTN2,AGTR1,BMP5,CASK,CASP6,CCL1,CLCA2,COQ3,DVL2,FOXO1,NRG1,OR13C9,OR5V1,OR6Y1,PIGM,PSME1,RDH5
Negative_814	GK2,IL5,ADIPOR2,ANAPC5,ASRGL1,B4GALT3,CYP1A1,ENTPD6,EXTL2,HADHB,INSRR,ITGB2,LHCGR,MAPK1,MGAT5B,MYH9,NNMT,TRHR,UNC5A,YARS2,PNLIPRP2
Negative_815	MYC,ACSL1,AKR1C2,ASIP,CACNA1G,CACNA1S,COL1A1,COL4A4,DMGDH,ENTPD6,FUT6,GFPT2,GRAP2,GRIA2,IFNG,LIPG,MAP3K2,MMP1,MRAS,NCKAP1,NUDT2,OR1K1,OR2Z1,OR4N2,PLAUR,PPP3R1,PRIM2,PRKCD,PRKG1,RAB23,RXRA,SPP1,XYLT2,TNFRSF13C
Negative_816	CBL,CD2,APRT,ARNTL,ARPC5L,CCL20,CHEK2,CHST11,DLST,EIF4B,FCGR3A,FZD10,GLI2,GMPPA,GNAO1,GNAT3,GSTA2,HHIP,HNMT,HSD17B7,IL11RA,LAMB4,LTA4H,MAPK14,MARS,MYH2,MYH8,NEGR1,NME4,OR5P2,P4HA3,PDE6D,PPP1R3A,RFWD2,SOCS7,STX6,PNLIPRP1,ST6GALNAC6
Negative_817	DLD,UBB,ACAT1,ACLY,ALPP,B3GAT2,C4BPA,CCL16,ENO3,EVI1,FBXW11,GALNTL1,GNPAT,GRAP2,KCNQ1,KIR3DL2,MICA,OR10J5,OR10T2,OR2L13,OR5D13,POLD3,PON1,PPP2R5C,PRKG2,SDHB,SHISA5,SLC25A5,TJP2,TRAF4,UBE2K
Negative_818	ALG11,CD58,EREG,GLB1,GNG12,HNF1B,IL18RAP,IL2RG,MYH2,OR10J3,OR1G1,OR52R1,PGS1,PRNP,ROBO2,RPE65,UBA1,ULBP1,SERPINC1
Negative_819	AANAT,ABI2,ACTN4,ACVR2A,ADCY5,ADCY8,CYP2C8,EFNB2,EGFR,GLUL,GNAZ,GZMB,HEMK1,IMPA2,LDHD,LIMK1,NANS,NEU4,NME2,NOX1,OR10G4,OR10H2,OR10K2,OR2S2,OR51L1,OR8S1,P2RX6,PIK3R5,POLR2E,RASGRP4,SOCS2,STX10,TP53I3,WNT11
Negative_820	AMPD2,OR4X1,OR5P2,PLAUR,RAB5A,RPRM
Negative_821	SRC,ASPA,CXCL12,GNRH1,GPD1L,NCR1,OR11L1,PPARG,PTPRC,ST8SIA5,UCK1,PLA2G12B

Negative_822	KMO, MVD, ACADSB, ALG12, ASPA, ATP6V1H, B3GNT2, CHKB, COX10, CSNK1D, DDHD1, DNMT3B, EXTL3, F13A1, FGF8, FGFR4, GALNS, GUCA1A, HAO2, HAP1, HTR2B, IL29, IQGAP1, KIR3DL2, MAP2K7, MCM7, MTHFR, NOX3, OR10G7, OR1S2, OR4F5, OR51F1, OR52B6, OR6B2, OR7C2, OR9A2, PDGFA, PPP3R1, PSME2, SELP, TRPC1, UNC5D, ATP6V0A1, EIF4EBP1, ST6GALNAC6
Negative_823	CD2, CFH, IL7, ACSL5, ACVRL1, ADRA1D, ARSB, BCAR1, BMP4, BMPR1B, CAMK2B, CD48, CDC14B, CXCR4, CYP4A22, DGKB, E2F4, FMO4, GNAQ, HARS2, IKBKG, IL24, ITGA10, KRAS, LRDD, NME6, OLAH, OR2A25, OR4F17, OR51E2, PFN2, PIAS1, PIGX, POLR2B, PPP1CA, SDC2, SOCS5, SRGAP3, TM7SF2, PNLIPRP2, SERPING1
Negative_824	GBA, GH1, PML, ACAA1, ATP6V0C, BCAT2, C4BPB, CCL28, CPT1A, CYP11B1, CYP2B6, CYP4F2, DARS2, ENTPD1, EPHA6, GAD2, GPAM, GRIA2, H6PD, INPP4B, INPP5B, MAGI3, OR12D3, OR52N5, OR5T3, OR6Y1, OR7G3, PAK4, PER1, POLR2F, POLR2H, SPRED2, STAT1, VCAM1, YWHAH, ATP6V0A1
Negative_825	TPR, ARAF, ARPC1A, CD48, CD99, CHRNA7, CHST11, COL4A1, CYP26C1, CYP2S1, FZD3, GALNS, NUMBL, OR11A1, OR51I2, OR51M1, OR5AP2, PCK1, POLR1D, PPOX, RHEB, RPN1, SENP2, SQLE, SUOX, TNXB, SERPIND1
Negative_826	MVD, CCL2, CSF3R, OR2M4, OR8K3, PDE3B, PTPN1, SNAP23
Negative_827	DUSP9, GLI1, IFNAR1, OR51M1, PNPT1, PRKAG1, SEMA3E, STX3, WNT5B, PRICKLE2
Negative_828	ITK, SRM, AGPAT1, CD36, COL5A2, GGT5, HEMK1, HTR2B, IL2RG, LMO7, MS4A2, NMNAT3, OR10G2, OR2H2, OR2T8, OR56B4, OR5L2, OR7D2, OR8D4, PARS2, PRNP, PYGM
Negative_829	GP5, HGD, CHST13, CNTN1, GALNTL5, GIT1, GNPAT, INHBC, MAOB, NRXN1, OR10K2, OR1L1, PIGN, POLR1A, PON1, SLC9A1, SMAD3, TFDP1, VAV2
Negative_830	AK2, SMO, AARS, ACSL4, ACTC1, ALDH5A1, CDK5, ICAM3, KLRK1, MTHFD1L, NFKB2, OR10Z1, PLOD3, PYCR1, SLC27A4, SOAT2, TAS2R10, TBL1X, TRAF4, UBA1, VNN1, SERPIND1
Negative_831	AK3, CTH, ID1, ADORA2A, CNTFR, DGKA, EIF4E2, PIK3R5, SPP1, TAS1R3, TGDS, VAMP3
Negative_832	ME1, OTC, ABP1, ADIPOR1, AXIN1, CHAT, CHST13, COL3A1, CXCR6, CYP2E1, DAPK2, DGKB, DHFR, DHRS4L2, EPHA2, ERBB2, F2RL2, GART, GNG12, GPLD1, GRHPR, GSTK1, HAO2, IFNA10, MAN1C1, MAPK7, NEFL, NOS1, OR10A5, OR10G2, OR10J3, OR2A2, OR52E8, RDH10, RUVBL1, SETD2, TNFSF9, TNFRSF13C
Negative_833	CLDN5, SHMT1, SLC2A2, UPRT
Negative_834	CR1, ALG5, CADM1, CCNB2, CPOX, CYP3A7, DGKH, DUSP7, GAD1, GGCX, GLI1, GUCY2F, IL15, NPR1, NQO1, OR6C2, OR9Q1, POLR3G, STK36, TUBB6, TNFRSF1A
Negative_835	AK7, ACVR2B, ATP5D, BIRC3, CD80, CSNK1G1, DAD1, EIF4E, FGF17, FGFR4, GRPR, IL2RA, MYLK, NAMPT, OR2T34, OR4N5, OR52B4, OR5P2, OR6C74, OR6K6, PFKFB2, PNPT1, POLR1E, PVRL1, RASA1, RBPJ, TAS2R49, TPK1, TUBB6, UGT2B10, SERPINA1, TNFRSF18
Negative_836	C2, KIT, LCK, ACP6, ALDOA, ATP5G3, CCL21, CCR7, CTLA4, DPM2, DUSP2, DUSP7, EPB41L1, FLT4, GALK2, GALNT5, GANAB, GNAL, IFNA6, IL22RA2, LAMB4, MAP2K1, OR11H1, OR4L1, OR4M2, OR4Q3, OR52K2, PDGFRB, PIGA, POLR2C, PPAP2A, PPM1D, RAC3, ROCK1, RPIA, STAT4, TGFA, UGT8, UMPS, UPP2, VANGL1, XCL1
Negative_837	ID4, ACSM5, AGPAT3, AGXT, ALDH2, ALDOA, ATG7, CD27, CD47, CDC45L, CLDN7, CNTN1, CXCR5, DHODH, DLL3, DMGDH, EFNA2, ETS1, FGF21, FIGF, FLT4, GLUL, HMBS, ICOS, IKBKB, LAMA4, LAMB2, MASP1, MYH1, NCF4, OR10AG1, OR10P1, OR2V2, OXTR, POLD1, PTEN, RRM2, STEAP3, STK36, TAS2R42, TAS2R49, TUBA4A, VDAC2, WNT10B, WNT11, WWP1, SUV420H2

Negative_838	AGXT,AMDHD2,CAMKK1,CRHR1,CTSB,EIF4E2,FGF13,FUT1,INHBB,NRG3,OR10G8,OR1L6,OR7D2,PDK1,PRKAG3,PYCR1,RARS2,TAS2R38,TNFSF10,UAP1
Negative_839	CD2,CSK,IL4,TYR,ACSM1,ADSSL1,GALE,GSTK1,GUCA1B,GZMA,IGF1R,IL22RA2,ITPA,LGMN,MAPK9,MDH2,MYH7,NAGLU,NFAT5,NRXN1,NT5C,OR10S1,OR2AG2,OR2T3,OR51E1,OR51Q1,OR52J3,PRKAG2,PTTG1,SDHC,SEMA4D,SEMA4F,SLC27A5,STT3B,TP53,TRADD,TRHR,CSGALNACT1
Negative_840	SRC,ACP1,CCKAR,FGF13,GAS1,IL13RA2,RPS6KA1,SPP1,TBL1X
Negative_841	UBB,CTSB,GALNT3,GCLC,ITGB8,KLRC2,OR10H2,OR5P2,PCCA,PCCB,SETD1B
Negative_842	CAMK2D,CLDN17,ITPA
Negative_843	AMT,DBI,NF1,AASDH,ACHE,AMY2A,CAMK2B,CD274,CDO1,E2F3,F2RL1,GIT1,GZMB,HSPA1B,HTR4,KAT2B,LARS2,MAPK9,MCAT,MFNG,NAT6,OR13C9,OR2T12,OR5I1,OR5P3,OR7G2,PIGK,POLD3,PPP1CB,PSME2,PYGL,RRAS2,SDC3,STX4,TSC1,TUBA3E,XYLT1,SERPINA1
Negative_844	IL6,BPNT1,GCNT1,GRIN2D,OR5D16,PRKCB,RPS6KB1,SC4MOL,SEMA3G,SGPP1,SOCS4,STX16,UGT2B4
Negative_845	CLDN9,CTNNA3,ENTPD3,GGT7,GJA1,GNAI1,HLA-F,OR4D11,PANK2,PGM3,QARS,SDC3,SIAH1,TAS2R38
Negative_846	C8A,TFG,ACTC1,AHCYL2,BCL2L1,BDNF,CACNA1A,CACNB1,CCDC6,CCR9,CLDN10,COASY,COL4A4,CPT2,CYCS,DAAM2,DBF4,DHRS9,DRD5,ENTPD5,GABARAP,GANAB,GNPNAT1,HHIP,ICOS,ITGA11,MAD1L1,OR10G3,OR10H4,OR2L13,OR4N2,OR6C76,OR7A10,PCYT2,PDE2A,PISD,PNPLA3,PPP1CB,PPP1R3B,PPP1R3C,PSME3,PYGL,RPS6KA3,ST6GAL1,TDO2,ATP6V1G2,CACNA2D1,SERPINA1,SERPINE1
Negative_847	OAT,STS,ACTN2,ACVR2A,CHKA,CHRNA7,DGKZ,DGUOK,FGF7,GNG13,HDAC2,IRF5,JAG2,MYH9,NAT2,OR1J2,OR52E4,OR5P3,OR8B12,PGS1,PIGB,PIGV,PIK3C2G,PLA2G6,POLR1B,PPP2R2D,PRKCA,SEMA4B,SLC25A6,SPP1,STAT5A,TM7SF2,ULK3
Negative_848	NPL,AASDH,AXIN2,CACNB4,CBLC,CLCA2,FLT3,GNPNAT1,GPLD1,HNF4A,HSD11B1,ICOS,IDI1,IL12RB1,ISYNA1,KLRC2,NME6,OR14C36,PRKX,SPRY1,TAS2R5
Negative_849	LAT,ME2,CD27,CYP2E1,DUSP2,GALE,GNPDA1,HNF4G,IL17A,KAT2A,MAPK11,OR10G9,POLR1E,PPP3CB,RDH16,SMURF2,TGFBR2,UGCG,XYLT2,ST6GALNAC6
Negative_850	GSN,CACNG5,CHAD,CXCR4,IL10RA,OR52D1,RRM2B,WNT8B
Negative_851	ABO,ALDH9A1,ANGPTL4,ARSB,CACNB4,CCL11,COL4A4,CTSL1,CYP1A2,CYP2S1,CYP3A7,CYP7A1,DPYS,FADS2,FGF18,FGF5,GSTO1,IL12RB2,IL18,IL24,ITGAM,MKNK1,NEFL,NKD1,NKX3-1,NSD1,NUMB,OR10X1,OR13H1,PDE7A,PDIA4,PECAM1,PIK3R5,PIP4K2A,PSME2,SELE,TSNARE1,UCKL1,UGT2B17,VAV2,SERPINE1
Negative_852	AGRP,ANAPC11,ATP5G3,CCL13,COL1A2,CYSLTR1,DDX51,FGF3,HHEX,ITGAE,PARVB,PIK3CA,PMAIP1,POLR1C,RBX1,RUVBL1,SPTLC1,TAP2,TJP2
Negative_853	MTR,ABLIM1,ACSL1,AGRP,ANGPTL4,APOC3,CAV1,CD72,EPHA6,FADS2,G6PC2,GAD1,GSK3B,HRAS,IL13RA1,MIOX,OGDHL,OR52K2,OR5AK2,OR5AR1,PCK2,PDE4D,PGAM1,PLOD3,PTPRF,SMAD3,TRMT11,TUBA8,TYMP,UBE2L3,VAMP1
Negative_854	AUH,C7,B3GALT4,B3GALT6,B3GNT5,COL6A1,CREBBP,CYP2C9,DKK4,EXT1,HCLS1,HLA-C,IFNAR1,IL1RAP,IL22RA1,ITGB2,KNG1,NEU2,OR2G3,PPM1A,SDC4,SH2D1B,SMAD2,ATP6V1G3

Negative_855	MIF, ACACB, ALG10B, ARPC1A, ATIC, CACNA1A, CCL27, CCL3, CNTN1, CREB3L4, DDIT4, DEGS2, DGKQ, EFNB1, EPHX2, HSD17B3, IDI1, IL2RA, JAM2, LAMC1, MAP4K3, MCM7, OR10K2, OR13C3, OR5AS1, OR5K1, PCNA, PDGFC, PER1, PFAS, PGAM4, PLCD4, PRSS1, PYCRL, RPS6KA2, SEMA4F, TAS1R3, TJP3, TSTA3, ATP6V0E1
Negative_856	IL6, BCKDHB, CDKN2D, CER1, CHKA, GALNT5, GSTK1, GUCY1B3, HGSNAT, NAT5, PDE1B, PITX2, PPAP2C, PRKACB, PTGES2, SHC3, SOAT2, ULK2, ATP6V1E2
Negative_857	CEL, PTS, AHCYL2, AKR1B1, CALML6, CD58, COL6A1, CRB3, CXCL1, CYP26B1, EFNA1, EPHB3, HARS2, HCCS, IL19, LTBR, MAN1A2, MASP2, MMP14, OR10A7, OR14J1, OR2L3, OR4K15, OR4S1, OR52E2, OR5K1, PANK3, PGM3, PIP5K1C, POLR1E, PPM1A, RAF1, RARS2, RXRG, SPHK1, STAT6, SUCLA2, TRAF6, CTNNBIP1, PLA2G12A
Negative_858	ACSM2A, ADCY7, BLVRB, COL3A1, CSF1, CYP2B6, GALNT6, GJD2, GNA15, INHBA, MAPK8, NFAT5, NMNAT2, OLAH, OR10G3, OR10Z1, OR2A12, OR2T12, PER3, PTGES2, TGFB3, TUBA8, TUBB2A, UCK2, VCAM1, HLA-DQA2
Negative_859	CNTN1, CYP2C9, OR8D2, PIGA
Negative_860	C1S, C3, KIT, MPI, RFK, AFMID, APH1A, ARHGEF1, ARSA, ARSD, BIRC3, CACNA1E, CANT1, CD79B, CHEK1, CPOX, EFNB3, GRIN2C, HBEGF, IL20RA, INSRR, KLRC4, LAMC2, MCAT, OGDH, OR10J1, OR2Z1, OR6C2, OR6C4, ORC3L, PDE4C, PDE8A, PIP5K1A, PLXNA2, POLA1, POLR2K, PRKG2, RFX5, SETD1B, SH2D1A, STX5, TLN2, TUBA4A, YOD1, SERPINC1, ST6GALNAC5, TNFRSF12A
Negative_861	AARS2, ACSS2, ACTN1, ADRA1B, AKT3, ALDH6A1, AMHR2, ATP5E, BACE1, CACNG4, CAMK2D, CCNB2, CDKN2B, CISH, CTNND1, CUL1, CYP3A7, EDNRA, ENAH, FRAT2, GARS, GATM, GBE1, GCNT2, GSTM2, MAGI1, MPST, MTMR1, NFATC1, NFATC2, OR5A2, OR8B4, OR8I2, PDE7A, PFKFB3, PHKG2, PIGV, PTGS2, SOS2, WASF3, WNT6, ZAP70, CSNK1A1L, SERPING1
Negative_862	CFI, TK1, ACP6, APOA1, ATG7, BDKRB2, BIRC2, BTRC, CAV1, CMAS, COMT, CSH1, DARS2, DEGS1, DUSP6, E2F1, F2RL3, GALNTL4, GBE1, GLDC, GNB1, HLA-DMA, HMOX1, HTRA2, LCMT2, NADSYN1, NME4, OR51B2, OR5AS1, OR5T3, OR7G1, PDHA2, PDK1, PTGER3, PTGIS, PYCRL, RARA, SDC3, SFRP5, SV2B, TALDO1, CSNK1A1L
Negative_863	CGN, EZR, OGT, UBB, ALOX12B, ATP6V1A, BDNF, CCR2, DTYMK, EXTL1, GDF6, KIR2DL2, LIPA, MAPK14, NAMPT, NAT2, NCF2, P2RX5, PDHB, PGM1, PI4KA, PLCD3, RGS3, SELP, SEMA4A, TGFBR1, UBE2K
Negative_864	CFH, ACYP1, ALDH2, AMDHD2, AVPR1A, C5AR1, CDO1, CLDN2, CLTB, COL1A2, CUL1, EPB41L2, GRIN1, IRAK4, LDHB, METTL2B, MGAT4A, NCK1, OR10A2, OR4L1, P2RX4, PDE7A, PIGF, PLA2G2A, PLA2G6, POLR2F, SSH2, SV2C, TFPI, TRIP10, WNT16
Negative_865	ARPC1B, CES1, CYP4A11, DGKZ, IFIH1, IL23A, MGST2, MMP14, OR2AG1, P4HA1, ST3GAL4
Negative_866	LARS2, SYNJ2, ZAP70
Negative_867	AK1, ANAPC7, CCNE1, CYP4A22, ENO1, FADS2, GADD45A, GOSR2, ICOS, IFNA2, INADL, INHBA, JUND, LCMT2, MAP2K4, MAPK1, OR10A7, OR2M3, OR51E1, OR5H2, OR6B2, OR6T1, OSMR, PIAS4, PIGS, PIGW, PISD, POLR2H, PPT1, PRKACB, RAB5A, SETD7, SLC2A1, STAT5A, TAS2R1, WNT7A, YWHAH, ZNRD1, SERPINC1
Negative_868	ME2, ANAPC11, BLVRA, CDH15, CHRNA7, CTBP1, GCLC, OR1J2, PFAS, PIGM, PTPRB, TCF7L1
Negative_869	ACPP, ACSM1, AGRN, ALDH7A1, ASS1, CALML3, CHRM3, CHST11, CPT1C, CRLS1, CYP2C8, CYSLTR2, DDX18, DKK1, DUSP6, FMO2, GALNT13, GMPR2, GTSE1, JAK2, MAPK7, MPP5, MPST, NRXN2, OR1L4, OR56A1, OR5A1, PHKG2, PIGW, PPP2R2D, SDC4, SKP1, SMAD6, SYNJ2, TAS2R16, TMLHE, TRAF4, ULBP1, VARS, WBSCR22, WNT4

Negative_870	CKM, AARS2, ACSL1, AGPAT2, ATP5O, AXIN2, B4GALT2, CCR3, GGT7, GNAZ, GPAA1, IRAK1, ITGB6, MCM7, NEU2, OGDH, OR6B3, PIP4K2C, POLR2G, RAF1, SARS2, SNAI2, SUCLG1
Negative_871	LYN, ATP13A2, CLDN16, EPB41L1, HSP90B1, MAD2L2, MARS2, OCRL, P2RX1, PAK6, PPP1CC, PTEN, PYGL, RAB5A, RBX1, RFXAP, SREBF1, TBK1, MAPK8IP3
Negative_872	GBA, ME1, TFG, ACAA1, ACOX3, ADCY1, BPNT1, CCKBR, CCL1, COL5A1, DLAT, EHHADH, GALNT6, GGT5, HADH, IFNAR2, INSRR, IRAK4, LEF1, MADCAM1, MYH4, MYLK2, OR4F6, OR6C4, PFKFB3, PIK3C2B, PLA2G1B, PLAT, PLCB3, PPAT, PVRL4, RASGRP4, RPE65
Negative_873	GK, PRL, ADIPOR2, ALPP, ARG1, CD72, CTSS, CYP4F2, DPM2, ENPP3, GCNT3, GLI3, GNAZ, GSTA4, ICAM2, IL20RA, LRDD, MAP4K2, MARS2, MTHFD1L, NCOR1, OCRL, OR10A7, OR10H1, OR5P2, PDE10A, PIAS3, PLIN, SMURF2, STK11, SUCLA2, TAS2R10, TLN2, TLR2, VDAC1, ATP6V1C2, PPARGC1A
Negative_874	AADAT, ACHE, ADH1B, ADRBK2, ASRGL1, CHKA, CHRM3, CHSY3, CLCF1, COQ6, CTBP2, CXCL1, CYSLTR2, EPHA8, FLT3, FMO4, GFPT2, GMPPA, GRIA1, GSTA3, GSTA5, IFNA17, IL1B, IL21R, IRAK3, MCM2, MGAT5B, NFYC, NOD1, OR11G2, OR2T6, OR51I1, OR51I2, OR5P3, OR6N1, PARVA, PIGU, PIP5K1A, PPP1R3A, PPP2R5C, PRNP, RPS6KB2, SAT2, SRGAP1, STX1B, TRPM5, C1GALT1C1
Negative_875	RPE, ACOT11, ACOX1, ADRB3, AKR1A1, AKR1B1, ALG10, CCND1, CDK4, CKMT2, CYP2C19, DHRS3, EPB41L3, ETNK2, FZD8, GDF6, GGT5, GMPR, GP1BA, IL10, IRF9, KAT2B, NCKAP1L, NEU2, NR5A2, OR13G1, OR2F2, OR2G6, OR56A4, OR5B21, OR5F1, OR6T1, PDE6G, PIGO, RPIA, SORBS1, ST3GAL5, STEAP3, TUBB2C, ZNRD1
Negative_876	SP1, AGMAT, ARPC1B, ARRB2, CAMK4, CBLB, COL1A2, IL18, IL6ST, LLGL2, NCKAP1L, NEUROG3, PIAS1, PNPT1, PRKY, SEMA4G, UNC5B, ATP6V0A1
Negative_877	CRK, CKMT2, OR13J1, PFKM, PRKCH, VAMP2
Negative_878	ACVR1, BPNT1, GUCY2F, ITGA8, NME7, NODAL, NT5C, NT5C2, OR2T34, OR8U1, PIK3CG, PLAUR, POLR2L, RPS6KA5, TACR1, VEGFA, PAFAH1B1
Negative_879	ACAT2, ALDH3A2, ANAPC11, CCR3, FRAT1, GALT, KIR3DL3, PPA2, PPP2R5D, RPS6KB2, STEAP3, WNT11, ATP6V1C1
Negative_880	DAO, FST, ADORA2B, AGPAT6, BTRC, DDX47, MDM2, NFKBIA, OR4K17, PYCR2, TRADD, TRAF3
Negative_881	AMH, GSN, HK2, KMO, LSS, ACSL4, AGRN, AHCY, ALDH1A1, ALG5, ATP5D, BBC3, CCR7, CTPS, DPYSL2, DUSP6, EPHA2, FABP3, GNA14, IGSF5, IMPA2, MKNK2, NCF1, OR4C15, OR51A2, OR51L1, OR7G1, OR8D2, PIGH, PIGT, PIGV, PLA2G1B, PPP2R2B, PYCR2, RBX1, RPS6KA6, SETD2, SOS1, VAV1, MAP3K7IP1, ST6GALNAC5, TNFRSF17
Negative_882	AK5, GLS, MPL, ACSS2, AOC2, ATP5C1, ATP6V0C, CADM3, CCR10, CCR5, CD274, CXCL11, CYP17A1, DOLPP1, FGF23, GLI2, HIP1, IFIH1, ITGA5, JAM3, KLRK1, MAP3K3, NME7, OR11H6, OR4D2, OR51I2, P2RX1, PDE8A, PFKFB4, PIK3C3, PNPO, PTPMT1, PTPN6, RCHY1, SOD1, SULT1A1, TBXA2R, TUBA1B, ULBP3
Negative_883	ADAM10, ADCY7, ATP6V0C, CCR9, CUL1, DRD5, FGF4, FGF5, FGF7, METTL6, NOD1, NRCAM, OR5AR1, OR5D14, OR6C2, SETDB2
Negative_884	ADC, AK3, GK, ADCY5, AKT1, ALDOC, CHAT, CRHR1, DLST, EPB41L1, GNRHR, PSEN1, SESN1, VNN1
Negative_885	AADAT, ACVR2A, ALOX15B, ATP5C1, CCL23, CLDN19, CYP4F3, E2F1, EGFR, EHHADH, ENTPD7, EPRS, FUT1, FUT2, FZD9, HIBCH, IL15, JAM2, MAD2L2, MTMR1, NKD1, NOS3, OPLAH, OR13G1, OR5M8, OR6C1, PDHA1, PHKB, PIGU, POLR2I, PPARG, RAB23, RAF1, RDH16, SETDB1, SIAH1, TUBB, UPRT, XCR1, MAPK8IP2
Negative_886	LHB, TYR, ABL2, ACP2, ACSM1, AMY2A, ANAPC2, ATP5G3, ATP5O, B3GNT4, BPNT1, CACNB4, CLDN7, DHFR, FOXO1, GADD45A, GLI3, HCST, IKBKE, IL15RA, IPPK, MCM4, MTHFR, MYH1, OR7A10, PDE1C, PIGB, PNPLA4, POLR2J, SIGLEC1, SPTLC2, ST3GAL4, STX12, SV2A, XYLB, ATP6V1B1, PLA2G12A

Negative_887	CCL2,MMP2,OR51B5,PIK3C3,RAB23,TAS1R1
Negative_888	DCT,DLD,AGXT2,AKT3,ANAPC2,BLVRA,CCNA1,DIAPH1,EIF4E,EPHX1,FASLG,FRAT2,FUT6,GUSB,HCCS,LAMA4,NAT1,NUMB,OR51A2,OR52A5,OR52R1,OR5J2,PDE1A,POLR3K,PRKACA,RFXAP,SEMA3D,SETD8,SHMT2,TUBA1A
Negative_889	OR1D5,PIGM,PTK2B
Negative_890	ADRBK2,ALPP,BTRC,CHRD,CHRM3,DHRS3,FGF22,GAPDH,GSTO1,OR1J1,OR52N5,OR6N1,PPP1R3D,STX1A,TJP3,TOMM40,TSC2,VEGFC
Negative_891	CSK,PTS,ACVRL1,BAAT,BUB1,CANT1,CAPN2,CHDH,CXCL16,CXCL2,CXCL5,CYBB,CYP2F1,CYP2J2,GNAS,HADHB,HMGCS1,ITGA10,JAM2,KLRC2,LAMA3,LDHB,NME2,OGDHL,OR1L6,PARD6B,PGM1,PPA2,RENBP,SLC2A1,TCIRG1,TGFBR2,HLA-DRB5
Negative_892	CBL,MPL,ARG2,ATP5G2,B3GALT2,CACNA1I,CDC42,CXCL11,DTX3,ENPP1,FGD3,GALNT12,GGT7,GSTM5,IL8RA,INHBC,INPP4A,INPP4B,ITGA5,LAMC3,NFATC3,OPLAH,OR10J1,OR2C3,OR4D1,OR52M1,ORC3L,PGM1,PIGW,PON2,PPP2R2D,RALA,SLIT2,SPCS3,TM7SF2,UPP2,EIF4EBP1,MAPK8IP2,TNFRSF12A
Negative_893	CFH,ACTC1,ACYP1,ALDOC,ALG1,BPNT1,CCND1,CD80,CFLAR,CHST11,CLTCL1,CSAD,CSNK1G3,DGUOK,DUSP7,FGF17,FGF7,GDF7,HNF4A,IFNB1,IFNG,IL23R,KLRC4,LIAS,NCF2,NCOA4,NCOR1,NT5C2,OR2G6,OR5L1,OR7A17,PGAM4,PHPT1,POLR2F,PON1,PTDSS1,RASGRF1,RHOD,RPS6KA2,SMARCA2,SMPD3,STMN1,TMLHE,TUBB2B,PRICKLE1
Negative_894	AK1,CGA,GSN,RFK,ACADM,ACTN2,ANAPC2,ASIP,ATG3,ATP6V1F,BBOX1,BCMO1,CCL4,CD22,CD38,CSF2,DDHD1,DDX23,EGFR,ENTPD4,EPHB1,GABARAP,GALNT2,GMPS,GOSR2,GPD1L,GRIN2A,ITGB7,ITGB8,LIAS,MGAT2,OR10G2,OR14I1,OR52A5,OR5B3,OR5M8,PLXNB1,SRD5A1,STAM2,TIAM2,TUBAL3,TYROBP,UGT2B11,ATP6V0E1
Negative_895	IL6,RB1,AKR1B1,ALG10B,BCAT2,BHMT,CCR10,CYP1A2,EARS2,FGF21,GLDC,KIR3DL2,MAP3K14,OR1L8,OR5B21,PIGF,PLA2G3,PLOD3,PRKG1,RBPJL,RDH5,SKIV2L2,STX7,TAS2R41,TLN2,TPH2,TYROBP
Negative_896	AKR1A1,ALDH5A1,CAPN2,DTYMK,FRAP1,GCH1,GRIN1,IMPDH1,MAGI2,MAOB,OR4C15,OR6K3,PFKFB3,RCHY1,SGPP1,STAT3,YWHAH
Negative_897	AK3,MOS,CHRM5,DCTD,SLC25A31
Negative_898	ADRBK2,CDH4,EPHA5,INPP5A,LDHD,OR13G1,OR1B1,OR4F4,PDE7A,PIK3C3,PIM1,PLA2G2A,PLXNC1,POLE,RBX1,RRAS2,SPTAN1
Negative_899	DBI,ACVR1B,ARNTL,AVPR1B,BDH2,C4BPA,CCNB2,CRKL,CTNNA3,DDB2,DDX50,DGKG,DOT1L,DPYS,FGF10,GALE,HAO2,IFIH1,INHBE,IPPK,KLRD1,MOV10L1,NRG1,NUMBL,OR1K1,OR1N2,OR2B11,OR51Q1,OR52N5,OR5AS1,OR6C74,PARD3,PDE7B,PDGFA,PIK3CD,PIK3R1,PTGES2,RELB,SETD8,SHMT2,SPTLC2,TLR4,TM7SF2,TMLHE,WEE1,GABARAPL1,TNFSF13B
Negative_900	GK2,HAL,APPL1,CDS1,CYP4F2,DGKG,HHIP,LIAS,OR56A1,OR5AK2,OR8I2,OR8S1,PARVG,POLR1D,POLR2E,PTGS2,PTTG1,PVRL4,RAP1A,SGPP2,SHC2,TACR3,UGT2A3
Negative_901	ADC,F7,GP9,OTC,TFG,AASS,ACAA1,ACP1,ACP5,AGPAT4,AMY2A,ANAPC7,ATP2A3,B3GNT3,CANT1,CNTFR,DARS2,DERA,DGKZ,FUT3,GALNT6,GLUD2,HDAC2,HYAL2,IFNGR2,IRS2,KDSR,NODAL,OR2M4,OR56A3,POLD4,PPP3CB,PSME2,PTCH1,RALA,RPS6KA4,RRAS2,SRGAP2,STAT1,STAT6,TGFA,CSGALNACT1,CSNK1A1L,SERPING1,TNFRSF10C
Negative_902	F10,INS,ACYP1,AGXT2,ANAPC4,ATP5H,ATP5J2,CHSY1,COL6A1,CYP2U1,DNMT3B,GJA1,HAAO,HEMK1,HIBCH,KLRK1,NUDT5,OR2A4,PAK7,PAX4,PDE6H,PIGS,PIM1,PLA2G2D,PLA2G6,PPP1R1A,PPP2CB,PRKAB1,RBL1,SDHB,SIGLEC1,ST3GAL1,SULT1E1,TYK2,ST6GALNAC3
Negative_903	TNR,CBLC,CRB3,CTF1,EVI1,FARSB,GOT2,INHBB,IRS1,MAP3K4,MAPK12,NAT6,OR10P1,OR9Q1,OXTR,PAK6,TP53,ATP6V1C2

Negative_904	AGL,CP,F10,MUT,ALDH5A1,ARPC1A,ASS1,ATP5H,B4GALT3,BCAT1,CCNB1,CNGA4,CRLS1,CYSLTR1,DIAPH2,DRD2,EFNB1,EPHA7,GALNT9,GNAS,GOT2,IL17RB,IRAK2,ITGA10,ITGA9,LEFTY1,MAGI1,MGST2,NME4,OR11A1,OR2AG2,OR2F2,OR4M2,OR5A2,OR7D2,ORC1L,PDE1A,PRSS2,RAC1,SETD1B,TRAF3,YWHAG,ZAP70,ZFYVE16,HLA-DQA2,MAPKAPK2
Negative_905	EPO,ADH1A,BCMO1,BMP8B,CCL21,CD55,CYBB,IQGAP1,MYH11,NRP1,OR10A3,PVRL1,SMAD7,VANGL1
Negative_906	GLS,ACADSB,ACOX2,ADRA1B,AGXT2,ATP6V1A,BACE1,CEBPA,CYP27A1,DAAM1,DAG1,DAXX,ENTPD2,EPB41L2,FGF1,GSTM2,IL22,OR10X1,OR4C16,OR4F6,OR51F1,OR5T3,PAK2,PNMT,PTDSS1,RAPGEF3,RXRA,SLC25A6,THBD,UGT8,VTI1A,CSGALNACT2
Negative_907	F2,F2R,LPL,ACAD9,B3GALT6,BAAT,CYP1A2,ENO2,GALNT7,GNAS,IL18RAP,IL28B,KIR2DL2,LAMA4,MAN1A2,NOTCH1,NT5E,OR4D1,OR5K2,ORC2L,PPP1R3D,PRKACA,RAB23,SOCS5,ST6GAL1,STMN1,TAS1R3,VAMP1,WNT10B
Negative_908	CCNE2,CLCA4,COL5A3,CYP2D6,DNMT3A,GLB1,GRAP2,IQGAP1,LEFTY2,MMP9,MTHFD2,MYH7B,NCOR2,OR2F1,OR2F2,OR2M7,OR2T2,OR2T33,OR9I1,ORC1L,PIGT,PIK3CB,PLXNC1,POLD4,POLR2I,PPP1R1A,PRPS2,SFRP2,SMAD9,TRHR,UBE2K,UROD,ZBTB16
Negative_909	AK2,FUK,TPO,ABCC8,ALAS2,ARPC2,CER1,FGF9,GAPDH,HAO1,IL1B,LALBA,PIGC,PRKACB,PTGDS,RAP1A,UGT2B4
Negative_910	ACP1,ATP2A3,CACNG7
Negative_911	AMH,GHR,MUT,ACVRL1,C7orf16,CDS1,CREB3L2,DAG1,DGAT2L4,ENPP3,FARS2,FGF22,FTCD,G6PC,GNAI2,IARS,IL12A,IL1RAP,ITGA6,KARS,KLRC3,LIPG,NUMB,OR10G3,OR10Q1,OR1A1,ORC5L,PIGL,PIP5K1B,POLR2E,SARS2,TDO2,TGFB1,THBS2,TNFRSF9,TUBB4,UBE2L3,WNT7B,MAP3K7IP1
Negative_912	CEL,FER,GCK,GSS,ARPC5L,BHMT,CD244,CD40LG,CD80,CDKN2A,CHAT,CHRM3,CLDN18,COL5A2,CTBP1,DKK1,EDA2R,FGF23,GMPS,GSTM4,HSD17B2,HSD3B2,IFNW1,KNG1,LIPF,LRDD,MAP2K4,MIOX,MYLK2,NEU1,NRG4,OR10G8,PCNA,PFN1,PIGP,PLA2G2F,POLR3H,PSPH,RAC1,RFNG,WNT9A,PDCD1LG2
Negative_913	CAT,EBP,EZR,GPT,GSS,NPY,SP1,AGPAT4,ALDH6A1,ALOX5,ATG12,B3GAT3,BET1L,BIRC2,CAV3,CDKN2B,CHIT1,CNDP1,CSH1,DUSP8,GALE,H6PD,IL21R,OR10G3,OR10H4,OR4C16,OR5B21,PDHB,POLE4,PSME3,PTPN1,RBPJ,RRM2,SH2D1A,UGP2,WNT9A,TNFRSF17
Negative_914	ADH5,ALDH2,ASCC3,EI24,MAP4K1,NT5C1B,PARS2,SAT1,TMLHE,MAPK8IP1
Negative_915	KIT,ACACB,ALDH7A1,APC2,BUB3,CYP3A5,DAXX,DCTN1,DRD5,DVL3,EDN1,ETS1,FGFR3,FUT3,GNAS,HLA-DOA,MAD2L1,MAP3K14,MCCC1,NME6,NRG1,OCRL,OR11H1,OR2B6,POLR2I,PRKAB2,ROCK2,SEMA4D,ST3GAL2,SUFU,SUV39H2,TRIP10,UPRT,ALDH18A1
Negative_916	ID2,INS,MOS,ALG3,ATP5L,COQ5,CREB1,CSF1R,E2F3,FGF14,FUT1,IDUA,MAP2K2,MKNK1,OR1L4,OR4B1,PTGFR,RAPGEF1
Negative_917	ITGA11,LIPG,VDAC2
Negative_918	DCN,ABCC8,ADCY9,ATP6V1H,BPGM,CD226,COMT,CXCL2,FUT1,GP1BA,GUK1,IDI2,IL12B,KIR2DL4,OR2B6,PDGFRA,SDC1,TP53
Negative_919	B4GALT7,NAGK,NTF3,OR2T29,SETDB2
Negative_920	CTH,SI,ACSL1,ALDH1A1,ANPEP,ATP5O,CALML3,CAMK2A,CAMKK2,CD3E,CYFIP2,CYP2A6,CYP2U1,DAG1,DDIT3,EFNB3,FZD5,GALNT13,GAS1,JAM3,LDHAL6B,LIPT1,MIOX,OR1L8,OR4C11,PANK2,PDE4D,PER1,PIGN,POLR1E,POLR3B,RALB,RDH8,RFNG,SGMS1,SMPD4,WNT10A,XCL1,MAPK8IP3

Negative_921	EBP, ACTG2, APC2, BTRC, CCNA2, CDC23, CDKN1A, CSNK1G2, EPHA6, IL12A, PDE3A, ST3GAL1
Negative_922	AVPR1B, CDC6, CSNK1A1, CYCS, DAPK2, FGF1, IL1RAP, LDHB, NAE1, TXNDC12, UXS1, HSP90AB1
Negative_923	AUH, ACACA, AKR1C3, CCL22, EREG, HAAO, OR4Q3, OR8D2, RPSA, SLC27A1, TYMP, PDCD1LG2
Negative_924	CP, TPO, ACOX1, ALPP, APRT, ATP4A, B4GALT2, C1QA, CACNG8, CCDC6, CCL21, CCR2, CLDN8, CRKL, CTBP1, CYP2C8, CYP2J2, CYP8B1, FCGR2B, FUT8, GCNT1, GNA11, IRF3, ITGAX, LTA4H, MAPK13, MCCC1, MTMR2, MYH7B, OR8K1, PCYT2, PITX2, PKMYT1, PRNP, PSME2, PTCH2, ROBO2, SIGLEC1, TGFB2, TGFBR1, XIAP, ZNRD1, C1GALT1C1, HLA-DQA2
Negative_925	BTC, FH, DUSP3, F2RL1, FMO1, MTHFD1L, NMNAT3, PAK3, SLC25A6, TAS2R13, HSD17B12
Negative_926	PDC, TNN, ACHE, ACSL6, AHCY, AKR1B1, APOA1, CALML6, CD247, CDC7, CHEK2, CHKB, CHPT1, CISH, COL11A1, COQ3, DDX50, EDA2R, FGD1, FGF22, FUT8, IL6ST, ITGAM, MFNG, NEU2, OR10A6, OR10X1, OR1S1, PIAS4, PPP1R3C, PRPH, ROCK1, SMPD1, STX1B, TSC1, WNT10A, WNT2B, GABARAPL2, HLA-DQB1
Negative_927	IHH, IVD, LAT, ANAPC10, APC2, B3GAT2, CLDN15, CSF2RA, E2F1, EGFR, EPHX1, EREG, F2RL1, GALNT9, GBGT1, GCLC, IL11RA, ITGB1, LARS2, MAPK11, MCM4, MYH7, NAT5, NKX2-2, OR2T6, OR4F6, OR52I2, P2RX3, P2RX7, PEMT, POLE4, RALA, RFX5, ROCK1, SPRY4, SSH1, SYNJ1, TAS2R5, TRADD, UGT2B4
Negative_928	ACACA, HSD17B8, KIR3DL3, NT5C, OR4D9, PTCRA, TAS2R13, TNXB, WNT7B
Negative_929	FES, CDC20, CXCR3, DOT1L, HNF1A, NODAL, PLOD2, PPP2R2D, RCHY1, VEGFB
Negative_930	AARS, ACP6, ALOX5, CDS1, CHRD, DEGS1, ENTPD3, FASLG, GALNT8, HSPA8, IFNAR1, ISYNA1, MARS, NUDT2, OR10V1, OR1L1, OR4C13, PDCD1, PDPK1, PHPT1, PLA2G10, PMVK, POLR1D, PON1, RAF1, SETD8, SSH3, SULT2B1, TFDP1, TNFSF4, TRAF5, TUBB, UROD, KIR2DL5A
Negative_931	COL1A2, CSF3R, EFNA2, GALNT10, GNPNAT1, MYH9
Negative_932	SDS, INADL, LAMB1, LAMB3
Negative_933	CS, TFG, ALG2, AMDHD1, APLP1, ASAH1, CARS2, CCL26, CTSB, EARS2, FGF23, GNAI3, IL25, IL4R, KIR2DS4, MAP3K11, NCOR2, OR10J3, OR1E2, OR5B21, OR8D1, PDE10A, PGM1, PNMT, PRKCD, PYCRL, RBPJ, SCD5, SOX17, STX3, TAS2R9, TUBB3, TNFRSF10B
Negative_934	SYK, CASP9, CCND3, CES1, CLDN20, CSF1R, CTLA4, EFNA2, ENO1, EPHX1, GOSR2, GSTM1, HTR4, LFNG, MCCC2, MPP5, OR52E2, OR56A3, PLCD3, PPCS, RBPJL, RPS6KB2, RUNX1, SGMS2, TCIRG1, TYMS
Negative_935	CP, ABAT, ACSS2, ACVR1, AHCYL1, ALDOB, BUB1B, CLCA4, CPS1, CXCR4, CYP3A7, DGKI, GLI3, GUCY1B3, HARS2, NFKBIE, NT5C1A, NUDT8, OR10R2, OR10S1, OR2T11, OR2Y1, PLCE1, ROCK1, TACR2, TLN2
Negative_936	CES1, EFNA5, IQGAP3, OR2T33, ROCK2, THBS2, SERPINF2
Negative_937	AFMID, CACNA1E, CDC7, DKK1, EFNB1, ENPP7, IFNG, LIPT1, NR1D1, PMVK, RAB5A, RBPJ, SMURF2, TJP3
Negative_938	KDR, FBP1, FZD3, IL3RA, MAP3K6, PPA2, SKP2, SORD, SUFU
Negative_939	ACADM, ALAS1, TNFSF4, TSC1, UGT2B11
Negative_940	IVD, CCL26, CYP27A1, EFNA2, GMPPA, IL6ST, MAP4K4, MAPK12, NEU3, NR5A2, OR4D6, PDE6H, PLXNB1, TSLP, HLA-DRB3
Negative_941	ACOX3, CES2, DDX51, FCGR2B, FZD2, GMPPA, IL22RA1, MYH10, OR9G4, PLXNA3, SARS2
Negative_942	GP9, ABLIM2, ADCY8, BCAR1, BDH1, CACNB4, CASP9, CDO1, CRY1, CSNK1A1, EIF4B, ENAH, GALK2, GRB2, GSTA5, HLA-DOA, HSD17B1, ICAM3, IFNGR2, IGF1, IL12RB1, ITGA3, MPP5, NAT6, NSDHL, OR52A1, OR9G1, ORC6L, PKMYT1, PRKAR2A, RGS3, SDC2, SMAD3, SMARCA2, SMC1B, SPRY3, TAS1R3, UBE2K, VTI1B, WNT9B, CSGALNACT2, HLA-DPA1

Negative_943	AK1,ADRB1,ALAD,AMD1,ATP5E,CD38,CNTN1,CTNNA2,EPHA5,F13A1,GLG1,GRHPR,GSTM4,HAAO,HYAL2,IRS4,LIPE,MAP3K13,MAPK8,MARS,NOX3,NT5C,OR2B2,PLA2G2D,PRKCA,PTPRZ1,PYGL,RENBP,RXRG,SETDB1,TCIRG1,TYK2,VCAM1,KIR2DL5A,PLA2G12B,TNFRSF10B
Negative_944	GGH,GK,PLG,ACOX1,ADCY7,ALOX15,APOA1,B4GALT7,BDH1,CD99,CDKN1B,CLTB,DAPK3,DIAPH3,DLL1,EPAS1,GALNT14,GNAI2,HSPA2,HTR6,IL28A,NCF1,NKD1,OCRL,OR2L8,OR52W1,OR5AK2,OR9A2,POLR2E,PRKCH,PTGER1,RASGRP3,RRM2,SGMS1,SHMT1,SMPD2,SPHK1,SULT1A2,TAP2,TSHR,SERPIND1,SERPINE1
Negative_945	FES,NPL,ACOT12,ADRB2,AGXT2,AMDHD1,ASIP,BMPR2,CCNH,DIAPH1,DRD5,ECSIT,EPHA3,HAGHL,IL10RB,JAM3,KNG1,MAN2A1,NCK1,NKD1,OR52I1,OR7G2,PAK6,PGS1,PRKAB1,PRSS1,RBPJ,RELB,SPP1,SUCLG1,TGFB2,MAP3K7IP1
Negative_946	RET,TPR,ALG12,ATG12,CALML6,DPM1,GOT2,HTR7,IQGAP3,NCOR2,NT5C3,PROS1,ROBO3,SESN1,SOCS4,WWP1
Negative_947	MUT,OSM,ACHE,ALDH1A3,CES2,CLCA2,CLDN22,CYSLTR2,DOCK1,EARS2,IL12RB2,LAMB1,LFNG,MAP3K6,NRG3,NT5M,OR1M1,OR2T2,OR3A1,OR6C1,OR8D1,OR8H2,OR9Q2,PLA2G10,PLD1,POLR3G,PPP2R2C,PRKAR2A,RFXANK,RPS6KA1,SKP2,SNAI2,SPRY1,STK3,UBE2L6,UCKL1,VCAM1,YWHAZ
Negative_948	CS,ITK,MYC,ADORA2B,ARHGEF4,BDNF,CCR5,CDS2,CHST12,CNTFR,CTBP2,CTNND1,CXCR4,CYP17A1,CYP2A7,DDX55,EFNA4,EHHADH,F13A1,FGF11,FGF6,GNA15,GPLD1,GUCA1B,MAP2K3,MGLL,NARS2,NCOR2,NLGN3,OGDHL,OR11L1,OR14I1,OR7D4,OR8H1,PCYT1B,POLR2B,PTCH2,RASGRP2,RASSF1,SARS2,STX18,TAS2R16,TAS2R48,TNFSF14,VNN1
Negative_949	LIF,NF1,TNN,CACNA1I,CDC2,CDKN2C,CTBS,DUSP14,EXOC7,FLAD1,GRIA3,GUCY2D,HSD17B4,IDI2,IRS2,KLRC1,MYH2,NGEF,NTRK1,OR10H5,OR1D5,OR1S1,OR51E1,OR5B21,OR5D18,OSMR,PPP1CA,PYCR1,SRGAP3,STX10,TAS2R8,UBE2K,UXS1,VANGL2,YARS,SERPING1
Negative_950	ADA,ADK,HK1,MVD,TH,ABLIM3,ADCY1,AKT2,ALOX12B,ARSD,ASS1,BMPR1B,CAMKK1,CASP3,CTPS2,CYP3A5,DEGS1,FUT7,HMOX2,HPSE2,MKNK1,OR56A4,OR8D2,PLIN,RRAS2,SMURF2,SUV39H1,SV2B,TNFSF18,TP53
Negative_951	CRH,TYR,ACADM,ARPC2,BCKDHB,BMP7,CACNB1,CCL21,CCNA2,CERK,CPT1C,DHDH,EDNRA,ENPP1,FZD2,HADHA,HARS,HMOX2,IL10RA,IL18RAP,INHBC,JAG1,LARS2,MAP3K8,MAPK3,MDM2,OR10A3,OR2AE1,OR2M5,OR4A16,OR52H1,P4HA2,PIK3CA,PIK3R4,SOCS1,STK4,UGT1A10,UGT2B10,UNC5A,WNT3
Negative_952	BCR,C3,DCT,HDC,NGF,ACP6,ATIC,B3GNT2,CAPN2,CCR1,CD244,COL3A1,CYP19A1,ENPP6,FTH1,GAD1,HCLS1,IL5RA,IRF5,ITGB3,LCMT2,MCM7,OR13J1,OR1B1,OR2M4,OR51B2,OR7A17,P2RX6,PHKA2,PIGB,PIK3R5,PLD2,POLE3,POLR3D,PRKCA,RBL2,SEMA4G,SIGLEC1,SMC1B,SOD1,SPAM1,STEAP3,UBE2L3,HLA-DPB1,PNLIPRP1
Negative_953	AMT,AREG,ARG1,CCL27,CYP2S1,DUSP4,EIF4E,ELK1,ERBB2,HMGCS2,IAPP,IL2RG,JAM3,KDSR,MCM6,MTAP,OR10AD1,OR4A5,OR52W1,OR5D18,OR7G3,PRSS3,SH2D2A,TAS2R7,TNFSF12,TOMM40L,WNT6,MAPK8IP2,ST6GALNAC6
Negative_954	CR1,F11,MVK,RB1,TK2,ACP6,ACSL4,ADCY9,AKT3,ALDH1A2,AMD1,AMPD3,ANAPC4,ARSA,ATP4B,C3AR1,CARS2,CCR5,CHST12,CLTA,COL4A6,CSNK1G1,CYP2J2,CYP2U1,DDX47,H6PD,HLA-G,ITGA5,ITGB4,MAP4K1,MTHFR,NOX1,NRXN2,OR2C3,OR2M4,OR4K17,OR51B6,OR51M1,PANK3,PFKP,PIK3R5,PMAIP1,PNPLA4,PPARD,RBX1,RPS6KA1,SMAD7,ATP6V0A2
Negative_955	ADO,BAD,C3,ASMT,CACNG7,CCNH,CD72,CDC26,CDKN1C,CLDN5,CREB3L1,EPHA1,ERBB3,FUT8,GAMT,GPX5,IFNAR2,IGF1R,NODAL,OR2T4,P2RX1,SLC2A2,WNT16

Negative_956	CLCA2, CPT1C, CTSS, FTMT, GNA11, OR13F1, PTPN7
Negative_957	TKT, ADSSL1, CDH2, CLDN5, EXTL1, FLT1, GALNT5, PDE5A, PIGW, TICAM2, WASF3
Negative_958	AR, GDA, RDX, TKT, ACTG1, ALPP, ARHGEF1, BCKDHB, CCL5, CCNB1, CYBB, DUSP1, E2F3, HAO2, IDH1, IFNGR1, IL17RB, IL20RA, ITGA11, MAP3K2, MCM7, OR51B2, ORC1L, PI4KA, PLXNC1, PPA1, PPP2R2C, RAC2, SC4MOL, SEMA4F, SETDB1, SH2D1A, SNAP23, ST3GAL4, TAS2R40, THBD, TUBB2B, TNFRSF10C
Negative_959	C5, CCL25, CREB3, CXCL10, DDX23, DHCR24, EIF4B, FCER1G, IL21, IRF3, ITGA4, KITLG, KLRC3, LEF1, LIPG, MCM2, OR5B12, PAK6, POLD2, PYCR2, RBX1, SEMA4A, SFRP4, STAT3, STMN1, TAS2R5, YARS2, ATP6V1E2
Negative_960	AGL, CSK, ID4, ACOX2, ADRB1, CAMKK2, CD48, CDS1, CHIA, DARS2, ENTPD3, GSTM4, GSTZ1, HAAO, HMBS, HYAL1, IL6ST, IRF5, MGAT1, MTMR1, NTSR1, OR10Z1, OR4B1, PDPK1, PPP2R5E, SUOX, TAS2R40, TUBB4, PNLIPRP1, TNFRSF10A
Negative_961	CBS, LPO, NF1, PGP, ALG9, ARHGEF6, CD226, CUL1, DUSP3, ENPP2, GZMA, PAX6, PI4KB, PRSS2
Negative_962	ABO, LCK, TNN, AANAT, CD74, CDS1, FABP4, GAL3ST1, GGT7, IRF7, MAP3K8, OR11L1, OR51V1, PLXNA1, PPP1CA, RALBP1, TSHR
Negative_963	LSS, ACSM1, C9orf95, COL4A6, CRLF2, CYBB, DDX50, FUT3, GPLD1, IBSP, INPP5A, IRF9, ITGA3, NFKBIE, NUDT8, OR5V1, PVRL4, RASGRP3, SELL, SFRP2, SKIV2L2, SLC2A2
Negative_964	CLDN3, PIP5K1B, STAT6
Negative_965	ALDH3B2, CISH, CRHR1, FZD6, GYS2, HADH, IL20, OR2A25, PIGW, PLAU, PLCB4, QPRT, SQLE, SV2B, TSHR, TYRP1, ATP6V0D1, HLA-DQA2
Negative_966	CDK5, ENO1, HADHB, NEGR1, OR3A1, P2RX2, PAICS, PIP5K3, VEGFC
Negative_967	ALS2, BIRC5, CKMT2, CSNK2A2, FUT3, GNAS, HMGCS2, IFNA13, IL21R, IMPDH1, NAGA, NMNAT3, ONECUT1, OR8I2, OXTR, PDE6G, PDGFB, PIAS3, POLR2F, PVRL2, ROCK2, SHC3, SUFU, TSLP, HLA-DPB1
Negative_968	CS, F5, SDS, ATP5E, CACNG8, CTF1, DVL2, HNF4G, HSPA2, IFNA4, KCNB1, MAML2, MCM6, OR10R2, OR11H6, PHKB, PPP1R1A, PPP1R3C, PPP2R1B, STAM2, STT3B, STX1A, UNC5A
Negative_969	DCN, ITK, LIF, RFK, SRM, STS, ABLIM2, ACADM, ACTN4, ADH5, AGPAT1, ANAPC2, ARPC5, AXIN1, BMPR2, CCDC6, CCR8, CSNK1E, CTNND1, CYP11B2, DAPK2, DUSP7, EPHA7, FGF17, FGF22, FSHR, FUT4, GUSB, HLA-G, INPPL1, MYH7, MYLK2, NCF1, NCK2, OR10G7, OR52B6, OR6C1, OR6N2, PDGFD, PLA2G2F, PTDSS1, TGFBR2, UNC5C
Negative_970	FOS, GSS, HK2, MPZ, ATP5G2, AXIN1, CPOX, CSNK1G1, CYP2C19, CYP2S1, DPYS, EPHA3, GNRH1, HTR4, KLRC3, MAML3, MEF2C, MTMR6, NME4, NODAL, NUDT5, OR1S2, OR2A5, OR2B11, OR4M1, P2RX7, PDGFRA, PLCB4, PPP1CB, PTGER3, RRAS, SEMA3F, SREBF1, MAPK8IP3
Negative_971	FAH, FST, JUN, LPO, TNC, ABCC8, ALAD, ALG1, ALG8, ARHGEF2, ATP6V1H, CARS2, CCL4, CES1, COL6A2, CYFIP2, DHCR7, EGLN1, FADD, FUT4, FUT9, IFNB1, IL29, IPPK, ITGAX, OR10H2, OR1I1, OR4D9, OR52I2, PCYT1B, PDE3B, PRKAG2, PSPH, PTDSS2, RBL2, RPN2, SELL, SLC25A4, SMC1A, STX12, UROD, WNT7B
Negative_972	GSR, NPY, ACOT12, ALDH1A1, ALG12, BAIAP2, CCL26, CD79B, CSF2, DERL1, DGAT1, DGKE, DNMT3A, DTX3, ELK1, GALNT13, GUCY1B3, HLA-DOB, IL17A, INPP5B, IRF5, OR10J1, OR2A25, OR2M3, OR4C15, OR51E1, OR5H2, OR5P3, OR6K3, OR7A5, PLA2G2F, PLAT, POLE3, SPRY1, STAT6, SUCLG1, SULT1A1, YWHAB, YWHAE, HLA-DQB1, ST6GALNAC1
Negative_973	ATM, BTD, CRY1, DHRS4, G6PC, INHBA, NUDT12, RAPGEF2, SDC1, TCIRG1, YWHAG

Negative_974	C9, F2, MPI, ACSS1, AGPAT2, ALDH6A1, B3GNT5, CBLC, CFLAR, CREB3L4, CRY1, CXCL2, EPHB4, ETNK2, FGD3, FRAT1, IFNA8, JAK1, NCOR2, NOD1, ONECUT1, OR1L4, OR51E1, OXCT2, PARD6A, PAX6, PIK3R5, POLD3, PRKAR1B, PRKCG, PRPH, PSME1, RASGRP4, RDH10, SESN3, TAS2R3, TJP2, TLN2, TRPM5, TUBB2C, UPP1, WIF1, WNT10A, ZFYVE9, ATP6V1G2, PNLIPRP2, SERPINB5
Negative_975	GP5, ID2, ACTB, DUSP5, MAP3K8, MAT1A, NFKBIA, POLE, PRDX6, RASGRP2, RUNX1T1
Negative_976	APAF1, ELK1, GADD45A, GLI2, GNPDA1, MAP3K12, MAP3K2, MAP3K4, MECR, MMP14, MMP2, MYH11, NARS2, OR5M9, PIAS4, PLA2G10, PLA2G6, PMVK, PSEN1, ROCK1, RRM1, SHMT2, ST8SIA1, TRAF2, MAPKAPK2
Negative_977	OGT, CCR3, CNGA4, FRAT2, IFNW1, OR2T3, OR6A2, RAB23
Negative_978	MTR, AADAT, ACADM, BACE2, CBLB, CBR1, ETS1, NCAM1, NFAT5, NR5A2, NUDT12, PDGFB, PLAT, PLAUR, RAP1B, RFNG, TNXB
Negative_979	CAD, GK, AXIN1, BCMO1, F2RL3, G6PC, GALNTL5, GZMA, HMGCL, IL23A, ITGA7, ITGAL, NFKBIE, OR2K2, OR2W1, OR6T1, OR8D2, PARS2, PARVA, PDE4A, POLD3, PPT1, PTPMT1, PVRL3, TGFBR2, WASF2, PPARGC1A
Negative_980	F11, HPD, LCT, PAH, WAS, ALG5, APRT, BMPR1A, C3orf10, CACNA1I, CD3E, CDC42, CXCL1, CYP11B1, CYP4A22, ECHS1, EPHB6, FTCD, INSRR, JAM3, LTB4R2, OR4F6, OR4X2, OR6Y1, P2RX1, PDE4C, PPP1R1A, PRKACA, RELB, ROCK2, RPE65, RPS6, SH3BP2, VTI1B, ATP6V0D1, TNFRSF17
Negative_981	KHK, SPN, ACVR2A, ALDOC, CAMKK1, CASP10, COL2A1, COL5A2, CYP3A4, DDX19A, DGKZ, DVL3, EPB41, FGF21, GCLC, GRIN1, IFNA21, IFNA6, IFNAR2, MAP3K8, NODAL, OR13J1, OR1G1, OR4C46, PDXP, PIGT, PPP1CC, PRKCG, RALBP1, SIAH1, SQLE, STX4, TNFSF11, TOLLIP, WNT16, SERPING1
Negative_982	ACHE, ACTN2, ACVR1, ADSS, ALG3, ANPEP, BCKDHA, CLDN17, CSNK2A1, EPB41L2, EPHX1, FGF13, FMO5, FRAT2, GUCY1A2, HADH, INSR, MAP3K14, MYH7B, NAGA, OR1L6, OR2T1, OR9Q1, P4HA3, PIGO, PLCD3, PLOD3, POLR2A, SH2D1B, SPAM1, C1GALT1C1
Negative_983	CR1, FGA, GP9, JUN, KIT, LCK, NNT, B3GALT4, B3GAT2, CHPT1, CHUK, CYP27A1, EPHA8, GGT1, GSTP1, ITGAM, KCNJ11, NFKBIB, OR13C3, OR1J4, OR2D2, OR2M3, OR6Q1, PDGFC, PIK3CD, PIPOX, POLR2D, PPP2R2D, QPRT, SELL, SKP1, SOAT2, TBL1X, TGFBR1, WNT16, PAFAH1B1, PRICKLE2, SERPING1
Negative_984	F5, CASP8, CES2, CSF1R, DDX52, DDX56, DKK2, FGD3, GRM5, GSTM5, HRH2, MFNG, MLYCD, MMP2, MYH4, NANS, OR10A6, OR1L6, OR4S1, OR5D13, PDK1, PPM1A, PPT1, PRODH, PTAFR, STX1B, TRIP10, TRPM5, TUBA1B, YWHAB
Negative_985	HK3, AKR1A1, B4GALT7, CCL19, CPT2, DGKA, EPRS, FUT3, GALNTL1, HTR2C, IDH3B, IL3RA, ITGA2, MAP2K7, OR1D2, OR4X2, P4HA3, PLA2G2A, PSEN2, SLC2A4
Negative_986	ADCY9, CDK7, CLTB, EFNA5, GALNT6, HTR5A, IFNA4, MAPK1, NCOA4, OR13C4, OR4C15, PPP1R1A, RDH11, SMPD2, TLN1, UGCGL1
Negative_987	C4B, LIF, SI, ALDH3A1, ANAPC5, CALML3, CKMT2, FZD5, GLI2, HMOX1, IQGAP2, LHCGR, MAN2A1, OR1D2, OR4D1, OR4M1, STK3, VAMP1, CACNA2D2
Negative_988	ACAT2, BTRC, CCL2, GRIN2C, HYAL2, ICOSLG, IQGAP2, LCMT2, MAPK8, OR5M9, PRKAG1, TNFRSF4
Negative_989	AMT, PGF, ACAD8, BMP4, CACNA1F, CCL27, CCR6, CHST12, CHSY1, COASY, DGAT2, DGAT2L4, DTX1, E2F2, GPD2, GRIN2C, GSTM5, IKBKB, JAG1, KCNQ1, LYPLA1, MAD2L2, MYLK, NAGLU, OR13G1, OR51B6, OR6K2, PAX4, POLR1A, POLR2E, SEMA4A, SMARCA5, XCR1, YWHAZ
Negative_990	SCD, ACSM3, ACSS3, ACVRL1, ATP5G3, BCL10, BMPR1A, BUB3, CD244, CHRNA7, CLDN10, CSNK1E, CYP21A2, DDX41, DIAPH1, DUSP3, ESAM, FGF17, GOT1, GRAP2, IL17B, IL6ST, KLKB1, MAP4K3, MYLK2, NKD2, NKX3-1, NRAS, NT5C, OR52E8, OR52N2, OR5R1, PAK2, PLAU, PRKCA, PRKCE, SOAT1, TPMT, UPRT, WNT10B, PRICKLE2
Negative_991	ATP6AP1, CCND1, FUT8, OR5D16, POLE

Negative_992	AK1,GH1,SMS,ALOX15B,ALOX5,ARHGEF2,BIRC3,BUB1B,CCL19,CCL23,CD36,CD55,CDKN2B,CES1,CLDN1,COL11A1,CXCL6,CYFIP1,CYP2A13,DPAGT1,FUT1,GALNT2,GFPT2,GJD2,IBSP,IL18,JAM2,MTHFD1,MTHFR,NOTCH2,OR10P1,OR2B2,PTAFR,RASGRP1,RFXAP,SETD8,SLC25A6,SOAT2,SUCLA2,VAMP2,VASP,PHOSPHO1
Negative_993	ACOX3,ALG6,CDH15,CTSS,LDHD,OR3A1,ZIC2
Negative_994	DCK,F2R,SI,ACP5,APH1A,ATP6V1A,CCKAR,CCL16,CFL2,CHSY3,CLCA2,COL11A1,CTTN,EPHB6,FGF9,GZMB,MAP4K4,MAPK11,MCCC2,MGST3,NCF2,NCR3,NTSR1,OR10G9,OR10H3,OR52K2,OR52N2,PCK2,PIK3R4,PPBP,RASA1,RPS6KA1,SCIN,SDC1,TACR2,TCIRG1,TNFSF11,TNFSF14,XYLB,HLA-DQA2,MAPKAPK2
Negative_995	BAX,CGN,FER,GPT,IL2,LHB,ACY1,AGTR1,B3GALT2,BDKRB2,CLDN1,CSF1R,CXCL11,DDOST,FUT7,GMPPB,GNAI1,GUCA1A,IL29,MECR,MYH11,NAT5,OR8J3,PFKP,PIGN,POLA1,RAC1,SIAH1,SULT1A2,TBL1X,TOMM40,UXS1,VEGFA,YWHAE,YWHAZ
Negative_996	CGA,HK3,PML,ACSM2A,ADCY5,BACE1,CCR9,CD8B,COL5A2,DDX18,DGKE,EPHB4,GNB1,HLA-F,HSD17B4,IL6R,MAOB,NT5M,NTN1,OR14C36,OR4F17,OR51B2,OR51F1,OR52D1,OR56A4,OR8H1,PIK3R1,POLR1B,POLR2H,PRKCH,PSEN2,PYGM,RELB,SLC27A1,TUBA1B,YKT6,YWHAQ,HLA-DRB3,PRICKLE2
Negative_997	GSR,RDX,AGTR1,ALDH2,ATG7,ATP6AP1,CASP6,CCR7,CYP27A1,DDX47,ENTPD7,EPB41L1,EPHB4,RELA,RHOQ,SCP2,SEMA4B,UGCG,TNFRSF13C
Negative_998	LCK,ACSL5,DAPK3,DDX50,ENPP1,FZD10,INHBB,ITGB8,LAP3,OR2M3,OR52L1,PCCB,PRNP,TAS2R50,TYROBP,VAMP1,HSP90AB1
Negative_999	BAD,C8B,GP5,KDR,MIF,PVR,AADAT,ACOX2,ACSM3,ADH4,AHCYL1,BCKDHB,COL4A1,CREB5,DDX50,DHCR7,DTYMK,EFNA3,EFNA4,EPHA5,FBXW11,GMPPA,GNPDA1,H6PD,LAMB4,LCMT1,LDHA,MAPK3,MCM5,OR10AG1,OR52L1,PCNA,PDXK,POLR2J,SOS2,TRAF6,TRPC1,ULK1,VAV1,PPP1R12A
Negative_1000	CR1,GP5,IL5,SYK,AADAC,CACNA1H,CHSY3,DIAPH3,DTX3,DUSP10,EXTL2,FGF3,IL11RA,LLGL1,MAPK9,OR10A6,OR2M7,OR51B4,OR51B6,OR56A1,OR5B21,OR6C70,OR8B12,PIGB,PYCR2,RALB,SC5DL,STK4,TP53
Negative_1001	FN1,ACSL3,ACTB,AKR1D1,ARNT,ARPC3,BCL2,BCMO1,CARS2,CNGA3,CSF2RA,CSNK2A1,DRD2,HSD17B1,IFNAR1,ITGA3,KIR2DS4,LRDD,NUDT2,OR12D3,OR4L1,OR4N5,OR51B6,SSH3,WARS,WNT3,YWHAB
Negative_1002	ALDH4A1,ALPI,B3GAT1,CACNB2,CCL23,CDKN1C,CLDN7,CTLA4,CYP2U1,GUCY1B3,ICAM3,IDH3A,KLRK1,MAD2L2,MAOB,NFASC,NRXN2,NTNG1,OR10H3,OR51Q1,OR9K2,PARD6G,PLOD1,PYCRL,RAPGEF2,RASSF5,ROCK2,SETD2,SMC1B,SOCS4,SOX17,TCF7L2,THY1,TLR4,ZIC2,ATP6V0E1,B4GALNT1,TNFRSF10C
Negative_1003	CRK,EZR,GLA,TNC,AMHR2,B4GALT1,BST1,CASP7,CCL26,CMAS,CRKL,DHRS4,DOLPP1,FGF14,GDE1,IL7R,MGST2,PAK2,POLR3D,PRSS3,PTCH1,SMURF1,TCF7,TCIRG1
Negative_1004	ID4,NGF,ACOX1,ADRB2,ANAPC5,AOC3,GALNT7,IFNGR1,KYNU,MAD1L1,MAPT,MAT1A,NODAL,PDGFB,PRODH,TNXB,UGT1A10,PRICKLE1
Negative_1005	HK1,ALG5,CD28,CXXC4,DDX23,ENPP1,GAB1,GART,NTSR1,OR51B4,OR52E6,PIK3CD,PLA2G6,PRKACG,PSME2,RGS3,SC4MOL,ULBP1,PHOSPHO1
Negative_1006	F2R,ID2,MVD,TNF,ALDH3B2,ALPPL2,AMHR2,ARPC4,B3GALT2,CD8A,DDX18,DIAPH2,DLAT,DPYD,EREG,HAP1,HSP90B1,IL13RA1,KCNQ1,LTBR,OR51I1,OR52W1,OR5D18,PDE4D,PFKL,PIK3CD,PTCH1,RASGRP4,RASSF5,TRMT11,MAPK8IP1,ST6GALNAC6
Negative_1007	AOC2,B4GALT1,CNTNAP1,CYP26C1,DAG1,DGAT2,DTX2,GRIA2,HIF1A,IFNGR1,IL12A,IL25,ITGA10,KDSR,MS4A2,NUDT5,NUDT8,OR51S1,OR7D2,OR7G1,PI4KB,PIGL,PKM2,SLC27A1,SMURF1,SNAP25,SOCS5,TLR9,TNFSF9,HSD17B10,HSD17B12

Negative_1008	SDS, ABL2, ACVR1B, ADRA1B, APOA5, APOE, BTRC, CAV2, CCND1, CES2, CLTC, DDX18, IDUA, LCMT2, MYLK, OR13C8, OR2M5, OR2T11, OR9Q2, POLD1, RXRA, SEMA3B, SLC25A4, STAT3, STX4, UGDH, MAP3K7IP2
Negative_1009	AK2, ACADVL, ACTA2, ADRB2, ASCC3, ATP5L, AVPR1B, CREB3L3, DAD1, EXOC7, FGF9, GLG1, GSTA3, HPSE2, IL18R1, LAMB1, MAN2A1, MEF2C, MGST1, MTHFR, NCAM2, NME7, OR10K1, OR14C36, OR4C12, OR8D4, PCCB, PER2, PPP2R5C, PRODH2, PTGER3, SORD, TACR3, TRDMT1, UGCG, UPP2, XCL2, CSGALNACT1
Negative_1010	IL2, TYR, ARSA, ATP5J2, BBOX1, BST1, CACNA1G, CBLC, CCL26, CLDN10, COL5A2, DDX23, E2F1, ENTPD5, EXOC7, FUT8, GAL3ST1, GSTK1, GUCY2F, JAM2, KLRC2, LARS2, NMNAT2, NRXN2, OR2M2, OR2T2, OR51V1, OR5K2, OR5R1, OR8G1, PAX8, PDIA4, POLR3K, PRKAB1, PTGER1, PYGB, RPE65, STX1A, TAS2R48, TFDP1, TMLHE, UGCG, VDAC1, ATP6V1B1
Negative_1011	AGL, KDR, ACP6, ANAPC1, APAF1, BET1L, C7orf16, CSNK2A1, CUL1, CYP17A1, DLAT, DVL2, EFNA4, ERBB4, GARS, GBGT1, GUCY1A2, GUCY2C, HMGCS1, INMT, LIMK2, LTC4S, MALT1, MDH2, MYH15, MYL5, NUDT5, NUMB, OR1C1, OR2A25, OR4K15, OR6M1, PDGFA, PDPK1, PHKG1, POLR2I, PTGES2, PTPN5, RFWD2, TACR2, TAS2R4, TUBA3E, USE1, ATP6V0D1
Negative_1012	ACSL4, ALDOB, BET1L, CHIA, ITGB5, PIGT, SLC12A2, TAS2R5, TP53I3, VARS
Negative_1013	C2, GSTA1, PTDSS2, SRGAP1
Negative_1014	F2, SFN, SYK, B4GALT7, CES2, CKMT2, CYSLTR2, DHDH, E2F3, EFNB3, ETNK1, FASLG, FSHR, GALNT8, GLDC, GLS2, GNAI3, GNAZ, HMGCS2, IL23A, IRF3, MAP2K1, MAP3K11, MAPT, MTAP, NAGS, NT5C2, OR10Z1, OR4N2, OR52E8, OR52W1, OR7A17, PDHA2, PFKFB3, PPP2CA, PTK2B, RHEB, SORBS1, SRD5A2
Negative_1015	FOS, CHST12, COL1A2, CYP2C19, EFNA4, FGF1, GZMA, JAM3, OR6N2, PHKA1, PRDX6, RBX1, STX3
Negative_1016	CEL, CKM, F2, MET, PTS, RFK, AKR1C1, APBB1, APH1A, CD79B, CREB1, EFNA1, ENO1, EPHA3, OR11L1, OR1N2, OR2M3, OR7A5, PIK3R4, PLA2G4A, PLK1, POLR2F, RASGRF1, SOAT1, STX4, TGFBR1, TMLHE, TNFSF10, TNFSF9
Negative_1017	BAD, C8A, MOS, ABAT, ATP5A1, CACNA1I, CALML3, CD38, CDC25C, CER1, CHRD, CREB3, CYP2F1, FZD10, G6PC2, GAL3ST1, GGT1, GNA12, GP1BA, GSTA3, GSTO1, HADHB, IL11RA, LYZL1, MAP3K2, NT5C, OR13A1, OR1B1, OR52D1, OR5L2, OR7C2, PHKB, PLA2G5, PRKCG, PSME1, RASA2, SELL, SLC33A1, SPTAN1, STX7, SV2A, TSHB, TYK2, VAV3, YWHAH
Negative_1018	HGF, ACOX3, ACVR1B, ADIPOR1, CHPT1, GNRH2, HMGCS1, IKBKB, LYPLA1, MIOX, RPN2
Negative_1019	MUT, AGPAT4, ALDOC, ATIC, BMP2, CNGA3, CREB3L1, ECSIT, FLT3, GDF6, GTSE1, HMGCL, HSPA2, ITGB2, KIR2DS5, MGAT4A, NCOR1, OR10C1, OR11H4, OR2C3, OR2L13, OR4N2, OR51L1, OR7G2, PARD6B, PGK1, PIK3CD, PRKCZ, SMPD4, UBA1, WBSCR22
Negative_1020	BAD, ACAA1, ACACB, ARPC4, ARSD, ATP5J2, CCR4, CDH1, CDKN2C, CX3CR1, CXCL2, DDX50, EDN1, EFNA1, FGFR3, FUT2, FZD5, FZD8, GDF6, GMDS, GPT2, GRIN2C, HSD11B1, IARS2, IL7R, ITGA11, NKD2, OR1K1, OR2A14, OR4P4, OR52B4, PFAS, POLR2F, SIAH1, SMC1B, SORD, ST3GAL4, STAM2, STX12, TDO2, UNC5D, YARS, MAPK8IP3
Negative_1021	C3, KHK, VTN, ACTG2, ANAPC11, ASNS, ATP6V1F, B3GNT4, BMPR1B, CCR2, CHPF, CXCL6, DAAM2, DGKD, EIF4B, GNAI1, GNAL, GNAT3, GNRH1, GRIA2, IFNAR2, IL1B, KIR2DS5, LYPLA1, MYH14, MYLK3, OR3A1, OR6M1, PDGFRB, PGS1, POLR3D, PRKCG, PRKCH, SEMA3B, SMAD2, SPHK2, STX12, SV2A, TRMT11, UNC5C, UPRT, WASL, YOD1, YWHAH, ATP6V1E1, TNFSF13B
Negative_1022	ABO, EZR, AKR1B1, ALDOC, ALG11, CCL3, CLDN6, CREB3L1, CYP2F1, DDX18, DVL1, EDNRB, ENPP1, GAD2, HCLS1, ICAM2, IL1R2, IMPDH2, MAPK8, OR1M1, OR4K1, OR51F2, OR51L1, PIK3C2G, PTPRC, RAPGEF3, SEMA3E, SH3BP2, SLC2A2, STX16, TAP2, VAMP2, VAMP3, KIR2DL5A

Negative_1023	CCL3,CYP2S1,EVI1,GADD45A,GAS1,HSD17B2,LEFTY2,NODAL,OR1S2,OR51T1,OR52E2,PARVA,PDE3B,PSME1,RGS3,SLC2A1,SULT2A1,TCF7L1,WNT10B,EIF4EBP1,PAFAH1B2
Negative_1024	ATM,SHH,ATP5C1,CCL17,CDKN2D,DGKG,FTCD,FTMT,IGF1R,IRF5,ITGA1,ITPK1,MAPK1,MDH1,NLGN2,OR2T4,OR8D2,ORC4L,PNPT1,PON2,PPARA,SARS2,SEMA4F,WIF1,XCL1,EIF4EBP1
Negative_1025	EGF,GLS,IHH,ACTC1,ADH1A,AGRN,ALDH7A1,C1QB,CADM1,CASP10,CD74,DHDH,EXTL2,FGF14,GPX5,GUCA1C,HDAC2,IAPP,ITGB1,ITPA,LEPR,MYH7,NANS,NFATC4,OR4C46,OR5B3,PDK1,PFKFB2,POLR3G,PSME1,PTCRA,PTEN,SOCS3,ST8SIA5,STX3,TAS2R9,TJP1,WIF1,WNT5B,WWP2,CACNA2D2,PAFAH1B2
Negative_1026	F10,AGRP,APH1A,ASPA,CACNA1C,CD3E,DGKH,FLAD1,GAL3ST1,GMPR2,GUCA1C,IFNK,IRS4,ITGA11,ITGA9,KLRC2,MAPK14,MYH14,NAT6,NCOR2,NPR1,OR2F1,OR2S2,OR4A16,OR4N5,OR52I1,PIGB,PPP1R1A,PPP3CB,PYCRL,RRAS2,SPTLC2,STMN1,TSNARE1,VDAC3
Negative_1027	ADK,PVR,APBB1,AXIN2,CACNA1C,CCND3,CD8B,GAB1,HLA-C,HMOX2,IDH3B,IL7R,LAMB2,OR2A25,OR6X1,OR8D1,OSMR,PDE7B,PDHA2,PIGC,PPP2R2A,PTPRB,RASGRP3,TICAM1,TRMT11,SUV420H1
Negative_1028	CDA,DDC,GLS,HK2,MTR,MUT,PGD,VCL,CBR1,CBR3,CLCA2,CLDN10,COL4A1,CXCL5,CYP1A2,DKK1,DPYS,EARS2,EDN1,EHMT2,ENTPD5,FCGR3A,GSTK1,IL1R1,INADL,ITGAE,ITGB2,MARS,NCAM1,NFATC4,NT5C,NT5E,NUDT2,OR10A5,OR4D6,OR52K2,OR6X1,PIAS4,PROC,RFXAP,SEMA3B,STAM,TACR2,TNFSF8,TRAF2,ATP6V1C2,SERPINC1
Negative_1029	CR1,FES,GAA,SMO,ACY1,AKT2,CALML5,CAMK2D,DGKB,EPHB4,GOT2,GRIN2D,GUCA1C,LAMC1,MGAT1,OR5P3,OR6C6,OR8U1,POLR2A,RBPJ,MAPK8IP1
Negative_1030	ABO,CRK,ALPL,C1GALT1,CCL25,CHD8,CXCL1,DUSP1,E2F4,EGLN1,GLI1,HHEX,IL20RA,KLKB1,LDHC,NEU2,PDE1C,PITX2,TAS2R60
Negative_1031	CS,ACAD9,ACADSB,ALDH3B2,ALDH7A1,ALOX15,ANAPC10,ASS1,BCKDHB,BDKRB2,BLVRB,CHRM5,CHST11,CSDA,CYCS,CYP4F2,DGAT2L4,DGKQ,EFNA3,EFNB1,ENTPD7,FIGF,FMO4,GAL3ST1,GALNT7,HDAC1,HLCS,IFNA5,IFNGR2,IL2RG,MYH3,MYL2,NKD1,NT5C1B,OR9G1,PI4KB,PLD2,PTPRR,PYCR2,PYGM,RAB5A,RPS6KA4,SELE,SHC1,TYROBP,YWHAE
Negative_1032	AVPR1A,DTX3,IRF9,JUND,LYZL1,NUDT2,OR5AN1,P4HA3,PCYT1B,PHKA1,PIK3C2B,PPARG,SOAT1,TPI1,UGT2A1,B4GALNT1
Negative_1033	ABO,C8G,IL4,ATP6V1A,BMPR2,CNTNAP1,FLOT2,GNRH1,HARS2,KLRC2,LRP5,OR1L1,OR5AR1,PDGFRB,PFKFB1,PNPO,POLD4,PPP2R5A,SCLY,SOX17,TAS1R1,TGFB1,ZAP70,HLA-DRB5,HSP90AA1
Negative_1034	TNF,ADAM10,CCR1,CSNK1G1,CYP2C18,EREG,GTSE1,IL23A,NT5C3,OR1N1,OR4D1,OR5B3,PGAM1,PIK3CB,SETD1A,ST6GAL1,TAS2R5,WNT3,YKT6,ATP6V1G1
Negative_1035	CFH,FYN,LPO,ACOT11,CCR6,CHRD,CNGA3,CTSS,ENO3,FLOT1,HLA-G,IFNK,IL13,ITGAX,KCNQ1,MASP1,MECR,NAT1,NCR2,NEU4,NFYC,OR2AG2,OR8B12,PARVB,PIK3CB,PPP1R3D,RALGDS,STAT2,SULT1E1,THY1,TYROBP,UBE2K
Negative_1036	GK2,GNE,LHB,ACACA,ALDH1B1,ARHGAP5,CADM1,CCL3,CDC14B,CES7,CPT1A,CXCL13,DGKD,ENPP6,FBP2,FGF11,GBE1,GCAT,HYAL1,IFIH1,IKBKE,IL20RB,IL22,IRF7,MGAT4A,MTMR2,NEUROD1,NT5C1B,OR2H1,OR4C11,OR5P3,OR6T1,OR7C2,PDPK1,PFAS,POLR2K,PPP3CB,PRKCG,PRPH2,PRPS2,RALA,SHC3,SMAD1,SPRY1,STK11,TFDP1

Negative_1037	HAL,ID1,LAT,LPO,AANAT,ABI2,ABLIM3,ACO2,ALDH3A2,ALPL,ANAPC4,ATP4B,CCNG1,CDC7,DGAT2,DOT1L,GALNT2,GANC,IL2RG,INPPL1,ITGAM,LCMT1,MYH11,NKX6-1,OR2AE1,OR51S1,OR6C76,OR8H3,POLR1B,POLR1C,PSEN1,PTGFR,RELB,SCIN,STX10,TAP2,TBK1,TGDS,THBD,TIAM1,TICAM2,UNC5C,UPRT,PDCD1LG2,PRICKLE1
Negative_1038	FOS,OAT,ACTN1,ALDH4A1,B4GALT1,BET1,CLCA4,COL5A3,CYFIP1,EFNA4,EPB41,GMPPA,GNA12,GUCY2D,HRH2,HSD3B1,ICOSLG,IRAK2,MAD2L2,MAFA,NAGLU,OR2T1,OR52B6,OR5P3,POLD2,RFX5,SETD1B,SOCS3,SOD1,STX1B,SULT2A1,TAS2R4,TGFB3,TYMS
Negative_1039	ACOX3,BACE2,BDNF,DDX18,FOXA2,GRIA2,MASP1,NCF4,ODC1,OR2T5,OR4F5,OR5C1,PER3,PGK2,PHKG1,PLCD4,PPP2R2D,PTPN7,RPIA,SMURF2,ST3GAL6,TAS2R16,TIAM2,TUBB2C
Negative_1040	ARPC1B,EHMT2,GLI1,MS4A2,OR10R2,OR1Q1,PLD1,PLXNB3,PPAP2C
Negative_1041	CRH,CES7,CSNK1E,OR4Q3,OR7D2,PNPLA3,SEMA4C,SETDB1
Negative_1042	DBT,GLS,DIAPH1,DMGDH,NEFH,PARD6B,POLR3A,PPCDC
Negative_1043	GP5,ILK,ADRB2,BCL2,BDH2,CASP6,CDH3,CRLF2,CYP1A1,FZD5,GLI2,GPLD1,ITGA2B,NOX3,NRG2,ORC4L,PIPOX,PTPN7,SH3BP2,STX12,UPRT,VCAM1,XCL1,ATP6V1B2,PLA2G12B,PRICKLE2,SERPINE1
Negative_1044	GP9,BST1,CDC25C,DDX23,GIT1,GLB1,GRB2
Negative_1045	AGK,EBP,CD70,CLCF1,INADL,MDM4,MIOX,OR1E2,PCYT2,RAC3,RASGRP1
Negative_1046	ACHE,ACVR1B,ADIPOR1,ALDH3B2,ALG2,ANGPTL4,CACNA1D,CCNB3,CD36,CDC14A,CES1,CYP2F1,DPYS,DVL1,FUT5,IDH3B,ITGA8,LRDD,NR1D1,OCLN,OR51F2,OR52I2,OR5C1,POLA1,PPP2R2B,RALGDS,SHMT1,SNAI1,STX10,WARS,TNFRSF10A,TNFRSF17
Negative_1047	FGF8,FGFR1,GNAZ,NCR3,PRKG2
Negative_1048	ARG2,DGUOK,OR4C15,OR6B2,PDE11A,ST3GAL1,SUCLA2,THBS2,TRAF5,ZAP70,TNFRSF10A
Negative_1049	C3,GPI,ADCY10,CAMKK2,CD244,CDH3,CNGB1,FGF11,FUT9,GCNT1,HMGCR,IL22,IL2RG,IMPDH1,INSRR,JAM3,LPAR1,NRP1,OR10H5,OR2T1,RAPGEF4,TAS2R41,ULBP3,VEGFA,WBSCR17
Negative_1050	EGF,HDC,ACMSD,ADCY6,BCMO1,CD28,CDC42,CLDN2,CLDN9,CNTN2,CYP2C9,DGKZ,DTX3,ENTPD8,EPHB2,GALNTL5,GRHPR,IL22RA2,MAP3K5,MAP4K4,OR10G2,OR52B4,OR5K2,OR7A5,OSMR,PCK1,PDE4B,PIGQ,PLAUR,POLD3,PPA1,RASA1,RFXANK,RPS6KA1,SARS2,SLC25A5,SLC2A4,SLC33A1,TAS1R2,TAS2R40,TGFA,TPM3,VDAC2,YARS2,ATP6V1E1
Negative_1051	CRH,DUT,RET,ADH1A,AGXT,AHCY,ALG6,ANAPC2,APH1A,ATIC,B3GALT4,C5AR1,CACNG4,CCNA1,CES7,COQ5,CXCR3,DNMT3A,DPYSL2,EGLN1,EPB41L2,GALNTL2,GGCX,GNAI1,GUCY2D,HES1,IGFBP3,IL20RB,LDHC,MAFA,MAP2K7,NADSYN1,NANP,OR10G8,OR11H6,OR14C36,OR1J4,ORC6L,P2RX5,PDK1,PGAP1,PHKA2,PLCD1,PPAT,RALBP1,TAS2R49,TGFB2,UXS1,SERPINA5
Negative_1052	ACSS3,ATP5J2,CCL3,DERL1,DGKQ,DNMT1,ICOS,IFNK,IGF1R,LARS2,MAOB,NFAT5,OPLAH,OR52B6,SUCLG1,SUV39H2,TYMP,C1GALT1C1
Negative_1053	ACAA1,ADRB2,B4GALT3,CCL4,CRY2,CTSS,CYP2C18,CYP4F2,EIF4E,OR10K1,OR3A2,PHKA1,PPP2R1A,SESN2,YOD1
Negative_1054	RET,HIBCH,OR5D13
Negative_1055	FGB,CD34,IL17RB,OR10J5,SNAI2,SOAT2,UGT1A10
Negative_1056	ACTB,ARNT2,B3GALT4,BAIAP2,CCR10,CD14,CLDN15,DIAPH2,DUSP16,ENO3,GALNT13,GALNT8,HADHA,HBEGF,IL10,ITGA9,MAGI3,NCOR1,NRG4,NT5C2,OR13A1,PGM2,PHKA1,PNPLA3,POLR1C,PPP2R5E,PPP3CB,PTGES2,PTK2,TLN1,TUBA3C,UXS1
Negative_1057	AGL,F3,CCDC6,CCR2,CDC45L,CDKN2D,EGLN1,EPAS1,HMOX2,NOTCH2,PGM1,PPP3CA,RGS3,THBS2,UGT2B28,MAPKAPK5

Negative_1058	ACADS, B4GALT1, B4GALT4, CCL25, CCL26, CD3E, DFFA, GLUL, HHEX, IL18, OR5AN1, OR5T3, PDGFD, PLA1A, PNPLA3, PPAP2C, PVRL2, RAP1B, THBS4
Negative_1059	CDA, ACOX3, ANAPC10, ASNS, CACNG1, CCL15, CLDN6, CSF1R, FGD3, FIGF, FZD5, GFPT2, GP1BA, IL1RAP, INPP1, ITGB2, MAD1L1, MAOA, MAPK13, MGAT1, MMP1, NNMT, OR2A12, PGK2, POLE3, POLR3B, QARS, SELP, TAS2R4, WWP1
Negative_1060	SMS, AMDHD1, EPOR, GART, GRIN2D, MAN1B1, OR10J1, OR13C2, OR2S2, OR2V2, PIGU, SEMA3C, TNFSF9, SERPINF2
Negative_1061	GK, BCL2, CAV2, CYCS, DTX4, FMO2, FOXA3, IRF3, NFKBIB, OR2L13, PFKFB3
Negative_1062	C8A, FGB, LPO, ABCC8, AREG, ARPC1B, CASP7, CD47, CD48, CDK7, DGKB, ENO3, GCDH, GDF5, IDH3G, IL25, ITGA6, LHCGR, NR1D1, OR2G3, PDX1, PNPO, POLR1E, PRKAG3, PTPN7, PYCRL, SMC1B, SNW1, TACR1, TP73, TSC2, UNC5A, WBSCR22, XCL1
Negative_1063	CGN, CR1, FUK, ME1, ACSS3, ADRA1A, ATP6V1F, ATP6V1H, CCNB2, CDC23, GNG13, HSPA2, IGF1R, IL23R, MYH13, NUDT8, OR14J1, OR5H2, OR6C4, OR9Q2, PFKFB2, PPAP2A, RXRB, SOCS4, ULK3
Negative_1064	HK1, ME1, ACADM, ACP5, ACVR1C, ALDH2, CCR9, CREB3L4, CSF2RA, CYP11B2, FZD7, GRIA2, IL10, LFNG, MAD2L2, OR2C1, OR2M4, PAFAH2, PCK2, RAPGEF3, ROBO1, SLC27A4, SREBF1, SRGAP2, TAP1, ATP6V1G1
Negative_1065	SP1, TPO, ALDOA, IDI2, KLKB1, PIP5K3, POLR2K, ST3GAL5
Negative_1066	BHMT, CHDH, OR8U1, PIK3R4, PLA2G4A, HLA-DQA2
Negative_1067	CIITA, CYFIP1, GARS, IL21R, NFX1, SV2C, TNXB, UNC5B
Negative_1068	ACTA2, ACTB, ALG5, BPGM, CAMKK1, CSF2, DIAPH3, FLOT2, FOXA3, GLYCTK, GNAS, IFNA16, IL5RA, LMO7, LRP6, MAP2K6, NAT5, NFX1, NME2, OR4C15, PCCB, PFKM, POLE4, SDC2, UNC5D, ATP6V1G2, MAPKAPK3
Negative_1069	CEL, HPD, ALS2, B4GALT7, CREB3L3, CYP19A1, ECHS1, EIF4B, FGD3, GNG3, HTR2A, ICOS, KIR2DS5, NFATC4, OR51S1, OR52R1, OR8D1, PPP1R3A, SPI1, TOLLIP, UROS
Negative_1070	CDA, ACADM, ACTN4, ALDH2, APOA1, ASMT, ATIC, BLVRB, CCNA2, CCNH, DDX51, DFFB, DHRS4L2, DVL2, GRB2, HARS2, HMOX1, IL21, IRAK3, ITCH, ITGA6, KIR3DL2, MAT2B, NCF1, NFE2L2, OR10C1, OR11A1, OR4P4, OR5AP2, PIGF, PLCD4, POLR2B, PPP2R5C, PRODH, PROS1, PTCH2, RASA2, SMURF2, SOS1, SV2A, TYRP1, UBE2L6, UGT2B11, VASP, YWHAE, SERPINB5
Negative_1071	GNRH2, LAMB1, PMAIP1, SEMA3E
Negative_1072	ID4, ADSSL1, CD59, CDH15, CRKL, CTF1, CXXC4, DIAPH3, EPHA2, FGF20, FUT7, GPAM, GRIN2D, HIF1A, HTR5A, MAP3K6, NTRK2, OR10X1, OR2B6, OR5A2, PIGX, PIP5K1A, POLE3, PPP2CA, TACR1, TP73, UGT2B4, KIR2DL5A
Negative_1073	AR, MET, APOA2, ARPC1B, BMP8A, CACNA1E, CLDN7, CRY2, EPHA6, EPRS, FMO1, FMO4, GMPR, LALBA, NEFL, OR1S2, OR2T6, OR52K1, OR5A2, PDCD1, PLA2G10, PPP2R2B, PTEN, PTGIS, PTGS2, RPS6KB1, SKP2, ST8SIA5, TFPI, TNFSF10, UBA1, WASF3, SERPINC1
Negative_1074	CRK, PVR, SRF, ATN1, ATP5A1, B3GNT2, C5AR1, CANT1, CYP11A1, DDHD1, ENTPD6, GNA13, GNB1, GRLF1, HTR2A, IMPA2, ITPKB, MAN2A1, MAP3K14, MAT1A, NKX3-1, NRG4, OR10Z1, OR1M1, OR2T8, OR4N2, OR5AU1, PARD6B, POLE, PPP3CA, PVRL1, RELB, SPRY3, ST3GAL1, VARS, ATP6V0A1
Negative_1075	F2R, UBB, AHCYL2, BACE2, GLI3, OR52K1, PCNA
Negative_1076	CKB, DHH, STS, ABI2, CLDN5, COASY, GDF5, LTB4R2, MAN1A2, OR4F15, OR4F5, OR7D4, PIGL, TGFA, UROS
Negative_1077	SRC, FRAT1, JAK3, OR52W1, PANK4, SEMA3A, SOS2
Negative_1078	DRD5, EFNB3, GALNT7, HGSNAT, VARS
Negative_1079	ADSS, ALPL, ARAF, CACNG5, CDKN2C, CHEK2, CHIT1, FASN, FOSL1, GRB2, INPP4B, NOS3, NUDT2, OR4N2, OR5A2, PPOX, PPP1R3A, RBPJL, TNFSF10
Negative_1080	ACP6, CTLA4, CXCR6, GARS, NAT6, WNT16

Negative_1081	BTC,CRH,CS,DBT,GDA,LIF,NPL,TAT,ACHE,ADORA2B,BAI1,CAMK2D,CHRNA7,CHUK,CLDN6,CXCL6,DPM1,GSTO2,GUCY1B3,HIBCH,MPZL1,MTHFS,OR10A6,PLXNB1,POLR3H,PRKY,SC4MOL,SUV39H1,TCF7,TGDS,TRHR,WIF1
Negative_1082	AK3L1,ALDH9A1,OR4D1,RGS3,RPE65
Negative_1083	INS,KDR,ACACA,AKR1B1,C1QC,CD48,CDC25C,DIAPH3,DUSP10,FCGR3A,FGF20,FLT3,G6PC,GNA15,HSP90B1,LAMC3,NCKAP1L,NCOA4,PAPSS1,PERP,PKMYT1,PLA2G4A,PLXNA3,POLR2F,PVRL4,RETSAT,SKP1,SKP2,SOAT1,TNFRSF9,TUBB
Negative_1084	CEL,ACADL,ACP6,ACSL4,ALG3,BMP8A,CACNA1C,CD34,CDK6,CYSLTR1,DAXX,ENTPD7,F13B,FDFT1,FOXA2,GCLM,GSTM3,GTSE1,HIP1,HPSE2,HSPA8,ISYNA1,MAPK13,MDH2,MGAT1,MGST2,OGDHL,OR10G3,OR13C2,OR52M1,PRODH2,PTPRB,PTPRR,RBPJL,SULT2A1,TAS2R40
Negative_1085	GK,LAT,LPO,AANAT,ATP6V0C,AXIN1,CDK7,CSNK1D,DLL4,DTX3L,FRAT1,GRIA2,HSPB1,IL21R,LCAT,MAML1,MDM2,NANS,NEU2,NRG3,NT5C1B,NUDT2,OR1N2,OR8K5,PPP2R5E,PTDSS2,RASGRF1,SCIN,SYNJ2,TNFSF11,TNFSF18,WNT3A
Negative_1086	MUT,ALG12,ATP5C1,C1QC,CAV1,CYFIP1,DUSP1,FGF18,GALNT13,GATM,GCLC,GMPPA,MADCAM1,NFX1,NKD1,OR1S2,OR2M4,PPA2,PRKCA,RDH11,SLC25A4,SOX17,SPP1,SPRED1,TBL1Y,HLA-DQB1
Negative_1087	NF1,ACP1,ACVR1,ARHGEF7,BBC3,CD40LG,CD80,CFL1,CHDH,CX3CR1,CYP1A2,DDX52,DGKG,DNMT3A,DUSP3,EPHA8,EPHB4,FABP3,FBP2,FGF20,FUT5,GJD2,GUCY2F,HLA-DOA,IGF1,IL10RB,IL12A,IL24,NAT5,PIK3C2B,PLAU,PRPH,PSEN2,RRAS,SELPLG,SFRP4,SUCLG1,TAP2,TGDS,TMLHE,YARS,SERPIND1
Negative_1088	DDO,FST,MOS,TNR,ACSL1,ADIPOR1,ALDH7A1,AMPD3,CAMKK2,CIITA,CLDN2,CNTNAP2,DUSP16,EDA2R,ENPP7,EPHB3,FGF11,FRAT2,FZD2,GSTO2,HSD17B4,HSD3B7,IL15RA,IRF5,MAP2K6,MECR,MGLL,MKNK1,MTMR1,MYLK,OR10H5,OR1L4,OR2M3,OR56A1,OR9K2,PIK3C3,PIK3R5,POLD3,PRKCE,SORD,TGFBR2,TRAF4,UBE2L3,UNC5C,USE1,VAMP3,PRICKLE2
Negative_1089	BTC,C7,CBL,EPO,MSN,SDS,UBB,ACSL6,AKT1,ALDH1A1,ALDOB,B4GALT7,BRAF,CNDP1,CNGB1,COL5A1,CREB5,CSNK1G2,DGKH,E2F2,ELK1,GANC,IFNG,IL2RG,ITGA3,MCM4,MDM2,METTL2B,NANP,NT5C3,OR10AD1,OR52E6,P2RX2,PDGFB,PDPK1,PIK3CB,PSENEN,RAPGEF1,RBL1,RPE65,TBK1,TLR3,WNT8A
Negative_1090	CASK,CD19,CD36,CLDN1,EFNA5,IL9R,IMPDH1,LEF1,NUDT2,OR51I2,PCYT1A,PFN2,PIGH,PTPN11,PVRL2,WNT11
Negative_1091	GH1,MVK,TK1,XDH,ACADVL,ALOX15B,ARPC3,C3AR1,CACNG8,CADM3,CCR1,CXCL6,DDIT3,DKK2,DNMT3A,EHHADH,EHMT1,ELK4,GNRHR,GSTM4,IL18RAP,KLRD1,MAGI2,MCM5,MGST2,NFKB2,NT5E,OR10H3,OR10K2,OR51V1,OR5R1,ORC3L,PAK7,PLA2G4A,PLXNC1,PVRL4,QARS,RRM1,SMAD3,SORD,UBE2L3,HLA-DRB3
Negative_1092	ASIP,CNTNAP1,CYP26A1,GUCY2F,NPR1,NRXN1,NT5C3,OR5H2,PAK6
Negative_1093	ME3,RPE,ACSL6,ALDH1A1,CAMK2G,CD80,CDH3,CDK7,CDKN1A,COQ5,CYFIP1,ENTPD7,EXTL1,GLI2,GOT1,GPD1L,HSD3B7,HYAL1,NMNAT1,PI4KA,PKMYT1,PRKAR2A,PRKG2,RASSF5,SEMA3E,TAS2R40,TRADD,TRHR,UBE2L3,UGT2B17,YWHAG,YWHAQ,HLA-DRB3
Negative_1094	ADRB3,ALDH1B1,AMPD1,APAF1,B4GALT3,CHIA,CKMT2,CLTC,CRY1,CTNNA3,DCTD,DDIT3,EXTL2,G6PC,IFNA16,IRAK3,MAN2A1,NR1H3,NUDT2,OR10P1,OR2AE1,OR5M8,OR6F1,PTGES,SAT1,SESN3,TRIP10,TYROBP,WNT2B,CACNA2D1
Negative_1095	ACAT1,ACOT12,ACTC1,AHCY,ARSB,DAPK3,DHCR24,FUT4,HNF4G,IL28RA,LTBP1,MAOA,MYH15,OR11H6,OR2S2,OR2T1,OR52A5,OR8H2,PAK1,PPP1R3B,PVRL1,SDHA,SETDB1,TRAF6,WNT9A

Negative_1096	CGA,CKM,FGB,OSM,SHH,ACSL5,ATG12,CCL5,CDKN1C,CYP2A13,CYP3A43,DVL1,ETS1,FZR1,GBGT1,IL13RA2,MAD2L2,MMP2,MTHFS,NAT5,NCOR2,OR10G3,OR11H6,OR5P2,PIK3CA,PIK3CG,PIM2,PRKY,PRSS3,SDHC,SHC2,SRD5A2,TAS2R40,TIAM1,TPK1,TRAF4,UBE2K,ATP6V1G2
Negative_1097	IL6,LPO,TYR,ABCC8,ABP1,ALDH4A1,ATP5H,AVPR1B,BCAT2,BPNT1,CHAD,CYP2A7,ECSIT,EDAR,GLDC,GUCA1C,GUCY2C,HLA-C,IL12A,ITGAE,MAPK3,OR13C2,OR2F2,PLCB3,POLR2B,POLR2H,PRNP,RPS6KA1,RPSA,SREBF1,SUCLG2,VKORC1,XIAP,YARS2,TNFRSF1A
Negative_1098	CACNA1A,FGD3,GNRHR,IRF9,ITGB8,SGPP2,SPRED1,TICAM1,TJP2
Negative_1099	CR2,F2,ALDH2,CDC25A,CLCA4,CMBL,CNTN1,CYP2C18,DGKB,ENTPD1,EPHB2,GNPNAT1,GRPR,GUCY1B3,HSD3B2,IL17A,LDHC,LTB4R2,NCOR1,NME2,OR10H2,OR56A3,POLR2A,TFPI,TLR3,TRAF5,TUBA8,TYMS,ATP6V1E1
Negative_1100	IL3,ACTC1,AFMID,ALDH3B1,ALOX15,APAF1,BST1,CD47,CDKN2D,CHIT1,CXCR3,DEGS2,DPYSL2,EFNB3,EPHB1,FZD3,GUSB,HLA-DOA,ICAM3,IL15RA,IL21R,KARS,LILRB3,MAML2,MTMR6,MYL7,NCR3,OR1F1,OR2T1,OR6T1,OR9Q2,PDE2A,PLA2G2D,PLD1,POLR3D,PPM1B,RALB,RPS6KA6,SDHB,SOCS1,ST3GAL4,TSC1,TUBA3C,SUV420H1
Negative_1101	MPL,TK2,AARS,ADRA1B,ALG3,ALG9,ARRB2,CD19,CD247,CDKN1A,DLL4,ENTPD8,GRIA3,LALBA,LLGL1,OR4B1,OR6Q1,PIGF,PPP2R5B,RPS6,SMPD4,TUBB4Q,UGT1A10,VEGFB,VTI1B,SERPINA5,TNFRSF13C
Negative_1102	ADO,APOC3,CDC20,CHEK1,DGAT2L4,DGKQ,FSHR,INPP5B,IQGAP3,KCNQ1,LDHAL6A,OR10A4,OR2AG1,OR5A1,PDE6H,PIK3R3,PLCZ1,PTAFR,RBPJL,ROCK1,TXNDC12,HSD17B12
Negative_1103	IVD,ADCY5,B3GNT2,CMAS,OR6C1,OXCT1,PLTP,RDH5,STAT1
Negative_1104	JUP,PVR,APOE,APPL1,ARHGEF2,BMP7,CCR9,CERK,CPS1,CSNK1E,CTBS,DGKB,DPM1,GAS1,GSTM4,HAP1,IL18R1,LAMC1,LDHC,MAN2A1,OR2H1,OR52D1,PAK2,PAX6,PFAS,PFN1,POLD1,POLR2B,PPP1R1A,PPT1,SC5DL,SETMAR,SMPD2,WNT11,WNT9A
Negative_1105	B3GNT3,BHMT,CCL22,CCND3,CLDN4,CSF3R,CSNK1G3,GALNT3,NSDHL,OR5V1,SOCS1,WBSCR22
Negative_1106	FMO2,ITPK1,MYLK2,OR4S1,PAK4,TACR1,HLA-DRB4
Negative_1107	AK2,GNRH1,MITF,OR52N2,PRKAG2,PROC,RRM2,SSH1
Negative_1108	C7,F3,LPO,MOS,OGT,ACSS3,AHCYL1,ALG6,ARPC1B,ATP6V1D,CCND1,CFLAR,CLDN4,CLTB,DDX47,DERA,DKK1,DKK4,EFNA5,ENTPD8,FGF10,FGF11,GDE1,GLI1,GPT2,GUSB,HPSE,ICOS,ITGAL,KIR2DL3,KIR3DL3,MAD2L2,MAP3K4,NEFL,NRXN3,OR3A3,OR5K2,PDIA4,PRKG1,RAC3,RRAS,SKP2,SMURF2,SOS2,STAM,SERPINA5
Negative_1109	ID4,PVR,SPN,SRC,ALDH1A1,ALG12,ATP6V1F,BRAF,CDC14B,DDOST,DOCK1,GALNTL2,HSD17B8,HSPA1B,IDH3A,LAMA3,LPAR1,NADK,NCK2,NEFH,NUMBL,OCRL,OR13C8,OR1L1,OR2T29,OR4K1,OR51I2,OR52D1,PCK2,PDE6H,PIGT,PRKAA2,QARS,SCP2,TCF7,TNFSF9,TRADD,TRDMT1
Negative_1110	ASS1,CASK,CCNA2,CCNB3,CD80,CER1,GLI1,IMPDH1,MCCC1,MKNK1,OXCT1,SEMA4A,SLIT2,SUCLG1,TNFSF18,UGT2B28,EIF4EBP1,ST6GALNAC4
Negative_1111	MIF,CD74,CDC2,CHKB,CHRM2,CTSG,CYP1B1,CYP21A2,CYP2C19,DEGS2,EDNRA,ENTPD8,FABP3,FGF9,GARS,GGPS1,GLUD1,GPLD1,GRIN2D,HES1,HGSNAT,HMOX2,IMPDH1,ITGA2,KLRD1,LAMC1,MAP2K4,MAP2K5,MLLT4,MYH11,NFAT5,OR10AG1,PLXNA2,POLE4,POLR2L,PPA1,PPP1R1A,PTGES2,RAC2,RND1,RPIA,SELL,SGMS2,SUCLG1,TNFSF18,TNFRSF13C
Negative_1112	GH2,TH,ABL2,ASCC3,ASPA,C1QB,CSNK1G1,CXCL10,DGKI,DPYSL2,EHHADH,FARP2,FSHB,FUT9,GSTA3,NCR2,OR51E2,OR52I2,OR52N4,OR8H3,PLA2G2D,STAT3,TBXA2R,TCF7
Negative_1113	AOC3,APAF1,CHSY3,GARS,HSD3B7,MAPK11,PIK3CB,PLA2G3,PPP1R3D,ST8SIA1,TAS2R7

Negative_1114	APP, FAS, GK, GLA, AARS2, AHCYL2, ALOX12, ALS2, ANPEP, AVPR1A, BUB1B, C3orf10, CALML5, CD79B, CLDN18, CLDN5, CXCR6, DHCR24, DHFR, EDA2R, FTH1, GRPR, HADH, HAGH, IFNA14, IRAK2, MAP2K4, MAPK7, OR4M1, OR8K5, PCYT1A, PIK3C2A, PLA2G2D, PPM1A, PRKCE, PTPN6, RENBP, SAT2, SLC27A5, TRPM5, UGT2B4, WARS, PNLIPRP2
Negative_1115	GAA, PGP, SPN, CDC2, DERL1, DPYSL2, GALNT7, HRH2, HSD3B7, IFNA10, KIR2DL1, KYNU, OR4D11, OR5D14, MAPK8IP2
Negative_1116	FGB, GP6, CRLF2, CSF2RA, GOT1, GOT2, MAP3K5, MTMR1, OR2T12, OR5T3, PI4KB, PPAP2A, VCAM1, WIF1, ATP6V0A4, GABARAPL2
Negative_1117	CFI, F11, ABAT, BLVRB, CACNA1I, CAMK2B, CDK6, ENAH, FOXA3, IAPP, IARS, MAP3K12, MTMR6, MYH8, PAX8, PPP2R5B, SDHC, SHMT1, TPH2, TUBB3, VKORC1
Negative_1118	ALOX5, FUT2, OR10H1, SOCS3, YARS, ATP6V1G3
Negative_1119	BID, HAL, ACOX1, ACPT, AGPAT3, ALOX12, B3GAT2, BAAT, BCAR1, BMP7, CARD11, CCL17, CCL3, CDC42, CLDN3, CYP3A4, EI24, FADD, FECH, FGF19, FOXA2, FUT9, GGT5, GUCY2C, GYS1, HEXA, HSD17B4, MAFA, MYD88, OR10K1, OR10V1, OR1D2, OR5D13, OR6C76, PAK3, PDK1, PLCD4, RALA, RDH8, RELA, RHOQ, STX1B, TAS2R50, UMPS, UPRT, XIAP
Negative_1120	IL8, TH, TK2, VCL, ACAT2, AGPAT1, AKR1B10, CACNA1D, CALML3, CCR4, CD14, ENPP3, FGD1, FMO3, GSTA1, HTR2B, IL12RB1, IL25, LIPT1, MAP4K3, MGAT4A, NCF1, NEFH, OR10H5, OR13C5, OR14J1, OR1A2, OR4B1, OR52K2, OR5AR1, OR8H1, OXCT2, PARD6B, PIK3R2, PLA2G2D, POLR3A, PPP2R5A, PRKAA1, RRM2, SESN3, SMAD5, SMURF1, TAS2R60, ULK2, XCR1, ARHGEF12, CACNA2D1, MAPK8IP2
Negative_1121	AK1, GPT, LCT, OAT, PTS, A4GALT, ABLIM3, ADAM10, ATP4A, CACNA1D, CMBL, COL5A2, CPT2, CXCL13, DOCK1, ENTPD5, EPHA3, GPT2, HCST, HNMT, IFNAR1, LALBA, LRRC4C, MAN1B1, NTN1, OR1D5, OR4E2, OR5R1, OR6M1, ORC4L, PDGFA, PLCD3, POLR2H, QARS, RDH5, RERE, SMC1A, ST3GAL5, SULT1A4, TAS2R50, XCR1, ATP6V1E1, HLA-DRB3
Negative_1122	GP9, UBC, ALG9, CDIPT, CYP3A5, EP300, GGT1, NAT5, NCR2, OR10X1, OR2AE1, PIP5K1B, PTTG1, RBKS
Negative_1123	GH1, ALPPL2, APBB1, APOE, CAMK2G, CDC25C, COMT, CTNNA2, DUSP10, DUSP8, GTSE1, MPP5, PDE8A, PHKB, PMVK, PON1, PPT1, PRKAB1, ST3GAL2, TAS2R10, TLR5, UGT2B17
Negative_1124	ACAD8, C3orf10, CMPK2, DMGDH, MAP3K4, OR2M7, OR51S1, OR6C6, PDE6H, POMC, PROS1
Negative_1125	C4BPA, CACNA1B, COL6A2, CSF1R, DCTN1, ICAM3, IL12B, ITGA1, PRKAG3, CSGALNACT2, SLC25A31
Negative_1126	ARSD, CACNA1H, CASP10, CRY1, CTSS, CYSLTR2, HLA-F, IL20RB, IL22RA2, OR2M7, OR5H2, POLD3, SQLE, TBK1, WNT1, MAPKAPK5
Negative_1127	F11, FES, TK2, AMHR2, ARHGEF6, B4GALT6, CCL8, CCNH, FUT4, IFNA4, IL11, LAMA3, LIPC, NANS, NCSTN, PIK3CG, SLIT2, TACR2, TAP2, TLN2, TYMP, CSNK1A1L, HSP90AB1
Negative_1128	AFMID, ASMT, DNMT3A, EREG, GALNT8, HRH1, IL13, INPP5B, IQGAP3, NT5E, OR2T12, PDE6G, TAS1R3, TCF7L2, WNT6, XCL1
Negative_1129	HGF, TNN, ABL2, ACTC1, BBOX1, CACNA1A, CCNA2, COQ5, CPS1, CREBBP, DGAT2L4, DHRS4L2, GMPR2, HCST, KIR2DS5, LRRC4C, OR10A6, OR10T2, OR4F5, OR51A2, OR6K2, POLR3G, RHOA, RPS6KA4, SLIT1, SOCS1, TNFSF10
Negative_1130	FES, AKR1C3, CADM1, CEBPA, CMBL, DAXX, DFFA, FMO2, MGAT1, OR2T34, OR51A4, PLTP, TUBB2C, HLA-DRB5
Negative_1131	ILK, PRL, ADCY5, AGRP, AKR1D1, ANPEP, B3GAT3, CCL26, CLCA4, CLDN18, DNMT1, DUSP4, FARSB, GALE, GALNT10, GPT2, IKBKB, ITGA4, ITGB7, KLKB1, MGAT4B, OR13C5, OR1E1, OR1M1, OR4C11, OR5I1, PARVG, POLR3B, PSME2, ROCK1, SGPP1, SOCS1, SOS1, UPP2, CTNNBIP1

Negative_1132	MYC, ADCY4, AKR1D1, ALDH2, ALG10B, LDHAL6A, MITF, NOX1, OR6B2, PIM1, PRPS2, RPN1, SGPP2, STX2, UGT2B4, VDAC1, CACNA2D2
Negative_1133	IL2, SRF, ACYP1, CACNG5, CDS2, ENPP1, EREG, F2RL2, FGD1, FGF2, GALE, GRIN1, HRH1, IL2RG, IL4R, MAP4K2, MCM2, NT5C1A, OR10K1, OR13C2, OR4D1, OR5A2, OR5D14, OR6B3, PANK1, PTGS2, RHEB, SDHD, SESN1, SMAD5, SPTLC2, TNFRSF4, TRAF4
Negative_1134	CP, FST, IL3, ALG10, CACNA1A, CACNG6, CACNG8, CD47, CYP2C9, DAAM2, DOCK1, DTX2, ENO2, FLT3LG, FUT5, GUCY1B3, KIR2DS4, KLKB1, MTFMT, NAT1, NEU3, NEUROD1, NFKBIA, NKD1, OR1S1, OR2AT4, OR2T12, OR51B6, OR5AR1, PHKA1, PIGT, PIK3R3, PIPOX, PRKCG, PTGES2, PTGS1, PTPN1, RDH16, SNAP23, ST3GAL3, STAT5B, TAP2, TGFBR2, TLN2, TSTA3, VAV2, VTI1A, YARS2
Negative_1135	ACP1, ADAM10, AMY2A, CLTCL1, COL1A2, DAPK3, DLL1, EXTL2, F13A1, ITGA4, NCF1, PRKCB, RASGRF2, UROD
Negative_1136	CRH, DDC, LTB, ATP5L, CDKN1C, DHRS4L2, EIF4E, EPHB6, KCNJ11, MAPK10, OR1L8, PDE5A, PDE8A, PPAP2C, PTCH2, RASSF5, SSH2, TAS2R14, VAMP2
Negative_1137	EGF, ATP6V1D, CACNA1A, CD38, CD79A, FUT4, IL24, IRS1, LIPT1, OR1L3, OR56A3, UROD, SUV420H2
Negative_1138	CRK, IHH, SCD, BACE2, CDK2, ELK4, FADS2, GP1BA, HMGCR, OR4X1, OR8D1, OR9A4
Negative_1139	GLUD2, MECR, RASGRF1, SELL
Negative_1140	CEL, SPN, ADSS, ALPI, AMDHD1, BMP7, CBLB, CDK2, CLDN19, DGAT2, DNMT3L, EDNRB, GALNT5, GYS2, HSD11B2, L1CAM, MGLL, MLLT4, MYH6, NSDHL, OR1L6, OR4X2, OR5AC2, OR7D4, PGM3, PPA2, PRKAB1, PSME1, RASGRP1, SARDH, SAT1, SHMT1, SOAT1, TAS2R10, TP73, VDAC3, ST6GALNAC1, ST6GALNAC6, TNFRSF11B
Negative_1141	FGA, GDA, ACAA2, AFMID, DDX50, DEGS2, DHFR, DHRS3, DTX1, DUSP16, E2F5, ESPL1, GJA1, GPX5, GRLF1, HLA-G, IL1RAP, LIFR, MAGI1, MAGI2, NTSR1, OR10C1, OR12D3, OR4N4, PANK1, PDK1, PER1, PFKFB1, PIGO, PPCS, RENBP, SMPD4, SULT2A1, TGFB1, TYROBP, WNT9B, GABARAPL1, HLA-DRB5
Negative_1142	GSR, EPHB1, HARS2, LEFTY1, SMAD4
Negative_1143	VTN, ATP5F1, CCR2, CHST13, DKK2, IL2RB, MAP2K1, MTMR2, MYL9, RBPJL, SOCS7, SPRY2, UCK2
Negative_1144	C5, TKT, ADRB2, AMHR2, AMY2A, APOC3, ATP5G2, BCL2, CCL23, CD80, CYP17A1, DGAT1, DHX58, DLL1, FOXO1, FUT6, GOSR2, GPAM, HTR7, IRF5, ISYNA1, MARS2, NFASC, NFKB1, NFKBIB, NFYC, OR4K2, OR5I1, PCCA, PDGFB, PHGDH, PLA2G5, PLIN, PPOX, RASSF5, SCIN, SGPP2, SLC25A6, SLC27A5, SMPD1, SULT2A1, UNC5D, YWHAE
Negative_1145	OSM, SP1, ACAD8, AFMID, CDK2, F11R, GSTK1, OR52A5, RGS3, TRHR, TXNDC12, YWHAG, PDCD1LG2
Negative_1146	F5, AXIN1, NT5C3, PLAUR, SMARCA5, UGT2B4
Negative_1147	ENTPD6, GALNTL4, PNPO
Negative_1148	AGL, BTK, IL7, MET, TK2, ARSE, ATP5J, CCNE1, CD74, CDC2, EPB41L2, IFNA8, ITGA4, LAMA3, MMP2, NEFH, NMNAT1, OR52E4, PPOX, PTAFR, SMAD6, TSTA3, VAV3, VEGFC, ZFYVE9, TNFRSF11A
Negative_1149	ACHE, ALG9, ATP12A, CACNA1G, CCL28, CLTB, COL2A1, CSF2RA, EPB41L1, GANC, GRB2, GSTA4, GSTM4, HCCS, IDH3G, MGST1, MYH7, NRG1, OR13D1, OR1S2, OR3A2, OR4L1, OR52B4, OR5AS1, PANK1, PANK3, PFN4, PRLR, PSME3, SELL, SHISA5, SIGLEC1, SNAP25, ST3GAL1, SV2A, TIRAP, TSNARE1, SLC25A31, TNFRSF13C
Negative_1150	C8G, MVD, TFG, ACSM4, ALDH3B2, B3GALT5, CAMK2B, CBLB, CHPT1, GUCY2C, IRF5, KAT2B, MFNG, NMNAT1, PRKAA2, SMARCA2, STAM2, STK11, TAS2R60, TRAF1, YWHAH, CTNNBIP1

Negative_1151	AK3,CFI,TFG,TK2,VCL,ACAA1,ACSM1,ADCY1,ALG10,ATP5A1,BMP8A,C4BPB,CD80,CLDN18,COQ5,CRY2,CSF2RB,DGAT1,ENO3,EPHB3,FGF20,FMO1,G6PC,G6PC2,GSTA4,HLA-DRA,HMBS,HNF1B,INPP4B,INPP5B,ITGA8,OR10T2,OR13C8,OR1N2,OR2F2,OR2W1,PLA2G5,PMAIP1,PPP1CA,PPP2R2C,RDH5,SELL,SETD7,SOAT2,STAT6,TKTL1,TPMT,ATP6V0A2
Negative_1152	UBC,AHCYL2,ALG6,ANAPC10,CACNA1C,CAV2,CFL1,CTNNA1,CXCL13,GGPS1,HMGCS1,IGSF5,LILRB3,MAP3K1,NCK1,OR4F17,OR4N2,OR52E2,OR6Y1,PARD6B,PFKFB1,PIGH,PLXNB1,PRKAG2,RBKS,RPN1,RPS6KA6,TAS2R8,WNT9A
Negative_1153	ID4,ACAD8,ACTN4,ALDH3B1,GALT,GSTM4,GYS1,HRH1,HSPA1B,IKBKB,LYPLA1,NT5C2,OR51L1,PTCH2,SHMT1,TRAF1,XCL2
Negative_1154	CFD,EGF,OGT,TAT,ACVR2B,B4GALT2,BCAT2,CBLB,CHRD,FADD,MTHFD2,POLR2G,PPARD,PRKCI,RIPK1,SEMA3F,SLC2A4,TACR1,UGT2A1
Negative_1155	ABO,ACLY,AGPAT1,CASP3,COL11A1,DHCR24,GSTK1,HLA-G,IFNA5,KCNQ1,NFATC4,OR8U1,RBL1,SMC1B,WNT3A,WNT6
Negative_1156	LYN,SDS,BRAF,CCL26,CCNG1,DGKI,GLO1,NCKAP1L,OR5M3,RHEB,VAV2
Negative_1157	LHB,ARRB1,CACNA1F,CNGA4,CREB3,CYP17A1,DLST,GALK1,GALNT1,GANC,GNRH1,GSTA5,IFNA21,INSR,KIR2DL4,LRP5,OR12D3,OR2A12,OR4K2,OR6C76,OR7G2,PDGFB,PRSS1,PTCH2,RAPGEF1,RASA1,SEMA3C,SMAD3,SRGAP3,ST3GAL3,STAT6,TIRAP,TSHR,UBE2L6,WNT3A,YWHAE,CSNK1A1L
Negative_1158	MSN,PAH,ADCY1,ALDH9A1,ASAH1,ATP2A3,ATP4A,CCL1,CD8B,CXCR5,DPM2,EFNA5,F2RL3,FGF21,FLT3,HEXB,HPSE2,IARS,IFNA21,ITGA3,LRRC4C,MAP3K2,NSDHL,NT5C,OR10J1,OR2L3,OR5B12,OR5H6,OR8K3,PDE1C,PGK2,PTEN,RASSF1,SGPP2,SLC27A1,SPRY3,SRD5A2,TAS2R13,TPM3,TUBA1B,VTI1B,XCL2,ATP6V0D1
Negative_1159	AGPAT2,ARRB1,CLDN4,CYP1B1,GALNT8,GDE1,ITPA,KIR3DL3,LCMT1,NUDT2,SUCLG2
Negative_1160	CFD,APAF1,ATF4,CYP3A7,FUT2,NEFM,NFYA,OR52I1,OR5P3,TAS2R3,TNFSF8,ATP6V0E1,TNFRSF10B
Negative_1161	HK3,AASS,ACOX3,BAAT,CCL11,CHST12,FARS2,FIGF,GSTK1,IBSP,IGF1R,INHBA,MBL2,MTMR1,MYH15,OR10A4,OR5A1,PIP5K1B,PPP1CC,RDH10,SSH3,TGDS,THY1,TOLLIP,UNC5A,ST6GALNAC3
Negative_1162	AHCY,FGF2,GRIA3,MAP3K13,MAP4K2,NEUROD1,OR2AE1,ORC4L,PDE5A,PHKA2,PTGER1,TRDMT1
Negative_1163	AK3,FES,ARNT2,ARRB1,ATIC,CACNA1G,CD55,CDIPT,CPT1C,CYP2C9,DAPK2,DHRS4,EDA2R,EFNA2,ELK4,FGF18,HTR2B,INADL,KCNJ11,MCM6,MCM7,NUDT9,OR10A2,OR2A5,PLD2,PLEKHA8,POLR1E,PPP1R3B,PRODH,RBX1,SDC4,SESN1,SETD7,SLC2A4,TGFB2,TUBB1,VAV2,CTNNBIP1,GABARAPL1,MAPK8IP2,ST6GALNAC1
Negative_1164	LYN,ABL1,ACSL5,AGPAT6,ALAS1,BBC3,CCNH,CHST11,CRLS1,DDX23,DGKE,DUSP3,FOXA2,GUK1,HSPA1B,IPMK,IRAK4,LEPR,MAOB,MAPK7,MTAP,NTF3,OR2T33,OR52B6,OR8S1,PAPSS1,PGAM1,PHGDH,PIP5K1C,POLR1A,PTPN11,SMC1A,STMN1,SUV39H1,TIAM2,UBE2L3,UGT2B28
Negative_1165	FER,ACTN2,AKR1A1,C5AR1,CASP10,CDKN1B,DUSP3,EDAR,GNRH1,MPZL1,NLGN3,NME2,NRCAM,OR2H1,OR52B4,PGM2,PIGB,PIP5K1B,PLCZ1,VAMP3,SERPINF2
Negative_1166	F10,HDC,ACTN4,ATF4,ATP2A2,CACNA1A,CASP3,CHPF,CRY1,CSDA,ECHS1,EREG,EVI1,GALNT2,ITPK1,LIPG,MTMR6,NSDHL,OR12D3,OR5M9,OR6K3,PRNP,RDH8,SH2D2A,SHMT2,SPRED1,STAT6,TDO2,UNC5B,VTI1A,YKT6,SERPIND1

Negative_1167	GHR,APLP1,ATP6V1A,BACE1,BAIAP2,BCL2L1,CCNE1,CD34,CKMT2,CSNK1G2,CTF1,DUSP1,EPHB1,FSHB,GDF5,GYS1,IL21,INHBB,LAMB1,MAP2K2,MGAT2,MTHFS,MYL9,OR4C12,OR52N4,OR8K3,PDK1,RASGRP1,SHC2,SNAI1,SNW1,TNFSF4,TSHB,UPP1
Negative_1168	FOS,BMP2,CDS1,EPOR,GLUD1,NAE1,NTRK1,PARD6A,PER3,POLE3,SFRP5,SH2D2A,TGFA
Negative_1169	AGK,CAT,GH2,MUT,TPO,VTN,ACOX1,ACSM3,CCNA2,CLDN18,CYP3A4,DGAT2L4,DKK2,GALNT12,GRB2,GRM1,H6PD,HIF1A,IKBKB,KCNJ11,MAP3K6,MAPK10,MEF2C,MGAT4B,MKNK1,NLGN3,OR1L6,OR51T1,OR5F1,OR6T1,PHKG1,PLAUR,PPP2R1A,RALBP1,RBX1,RIPK1,RPN2,SMC1A,STAT1,TUBB2B,TYROBP,WNT3A
Negative_1170	CDA,GSS,RFK,APLP1,ARAF,ARHGEF2,ARHGEF4,BCL10,CARD11,CCR9,CLOCK,CYP27A1,DPM3,FARP2,FOSL1,FRAT1,GAD2,GSTA5,GUCY2C,GYS1,HAP1,HSD17B3,IFNAR2,INHBE,LIPE,NAMPT,NUMB,OR2A42,OR2H1,OR52A1,ORC4L,PDX1,PECAM1,PIGC,PROC,RALB,RBKS,SEMA4D,SHC3,STMN1,TAS2R38,TAS2R7,TGFBR1,TNXB,UGT1A10,UGT2B11,ULBP2,YWHAB
Negative_1171	EBP,GK,GSR,GSS,APAF1,CCR3,CD14,CMPK1,CTTN,DARS,DIAPH1,DOT1L,ELK1,EPHA8,FRAP1,ICOS,ITGA6,ITGA7,ITGA8,KCNJ11,MCAT,NFATC4,ODC1,OR2L3,OR4K1,OR4K13,OR4Q3,OR5L1,PAX6,PLXNA2,PTCRA,PYCRL,SNW1,SULT1E1,SV2C,TACR2,TGFB3,UBE2L3,PNLIPRP2
Negative_1172	CDC6,CDH4,CDKN2A,IKBKG,INHBE,ITGA11,LTBP1,NSDHL,OR8G1,PARD6B,PCYT1B,PGM2,POLR2I,PPAP2B,PRKCE,SGPP1,STAT3,VEGFC,ST6GALNAC5
Negative_1173	CXCL13,EXT1,FBXW11,G6PC2,GNAI3,IFT57,IRAK1,OR10G3,OR2A12,PDE8B,POLR2C,PYCR1,SOAT2
Negative_1174	ACSL4,AMDHD1,ANAPC2,BCKDHB,CARS2,COASY,CYP4A22,EHMT2,ENTPD1,G6PC,GLI1,MCM4,MYH10,OR10H5,OR51E2,OR6C3,OR8H3,PNMT,POLR2D,PTPN7,SLC25A4,TJP1,TRAF4,XIAP,MAPKAPK5,PAFAH1B1,SERPINF2
Negative_1175	AGL,C3,HK2,OSM,SDS,ABCC8,ADRBK2,ALPP,CACNB2,CD46,CD86,CLCF1,CSF2,CTPS,DHDH,ECSIT,EDN1,FMO4,GLDC,GPX5,GRIA1,GSTM5,IFNAR2,IL13,INPP5E,ITGB6,MAPK10,MAPK13,MCM5,MYLK,OR13G1,OR2K2,OR4F15,OR4F5,PLAT,ROBO2,RPRM,SOAT2,STX4,UPP2,WNT9A,YARS2,TNFRSF11A
Negative_1176	GLA,HPD,PGP,TK1,AKR1C3,ARPC5,ARPC5L,ATP5J,C4BPB,CCNB1,CISH,CYP1A1,E2F1,FGD1,HSD17B8,HSD3B2,IFNB1,IL18RAP,MAML2,MDM4,MGAT1,MGAT5B,NARS2,OR14I1,OR2M4,OR4C13,OR51M1,OR5A1,OR6K2,PPID,PRKAR1A,SDHA,SMPD2,TNFRSF9,TSNARE1,UCKL1
Negative_1177	DAO,GBA,OTC,TPR,AGPAT6,AKR1B1,ALG11,AMPD1,ATP5J2,ATP6V1A,BAAT,CCKAR,CCNA1,CRKL,DRD2,G6PC,HBEGF,HCCS,IPMK,ITGA6,ITGB1,MAP4K2,MITF,MYH2,NME2,OR51Q1,OR6C75,PIK3C2B,POLE4,RASSF1,SARDH,TAS2R49,TBXA2R,UBE2K,WNT7A,HLA-DQA2,TNFRSF13C
Negative_1178	C6,MET,PXN,SPN,ALAS1,ESPL1,FCER1G,FGF17,FUT8,KIR3DL2,MAP4K4,NPR2,OCLN,OR4A16,OR51D1,PAFAH2,RFWD2,SOCS7,VAMP3
Negative_1179	AKR1A1,ATP6V1H,CAMK4,GSK3B,HES1,ITPA,ITPK1,OR2M3,PLA2G2D,PRKCI,SLC25A5,TNFRSF13B
Negative_1180	INS,CACNG7,CD8B,CSF3R,CYP2C8,HSD17B7,LAP3,OR2F2,OR6K6,OR8D2,PER3,PLCG1,PLK1,POLE,PPP2R2D,SFRP1,SOCS5,TAS2R49
Negative_1181	ACP1,ALDH5A1,CD38,CLTA,CYP26B1,DTX4,FGF21,GNG13,ICOS,INPP5E,LDHD,MYH1,NAGK,PARVG,PRPS1,PTCH2,RARA,UBE2K,WNT3
Negative_1182	CFH,FGA,LAT,ACAA2,ADRB1,ALOX15B,ANPEP,BAIAP2,CACNB1,CD40,CDO1,CHPF,CLDN6,CSNK1G1,CTNNA2,DNMT1,FABP4,GRHPR,HSD17B3,INSR,NANS,NR4A1,NTNG1,NUDT2,OR1L6,OR9A2,PDE1C,RIPK1,ROCK2,RPS6KA1,SPHK2,STAM,STK4,SULT2A1,VARS

Negative_1183	GAA,ME3,WAS,B4GALT3,CD72,COL1A2,CTSL1,CXCL12,DGAT1,DUSP5,ERBB3,GAPDH,GLDC,HEXB,IL15,KAT2A,KCNB1,LARS2,LIFR,OR11A1,OR51S1,OR52N2,OR6B2,OR8K5,PDE1C,QPRT,RASA1,RERE,RPS6KA5,SETD2,SETDB1,TSC2,UGT2B11,HLA-DQB1,ST6GALNAC3
Negative_1184	GSR,IDS,LYN,PML,ADH1B,ANAPC10,ARD1A,ATP5G3,CD59,CD82,CEBPA,CTNNA2,EFNA2,GGPS1,GUCA1B,HRH2,IL2RB,ITGB8,MAN1C1,MAP4K4,MASP1,NCAM2,OR10G4,OR1E2,OR2A5,OR2S2,OR2V2,OR6C3,PIP5K1A,POLR1C,PTCRA,TAS2R38,TGFB2,TSLP,ULK1
Negative_1185	ILK,AADAC,ACOX1,APLP1,ARPC1A,B3GAT2,BMP6,CDC23,CDC27,CDKN2A,CLTCL1,CRHR1,CTSB,CUL1,CYSLTR2,DDX41,ESAM,FGF19,FGF4,FZD8,GUCY2D,HNF1A,IFNA7,IL17RB,IL20,IRF9,ITPK1,MAP4K2,MAP4K4,MAPK10,MCM3,MMP7,MYH2,NPR1,NT5C1B,OR10G4,OR10G8,OR1J4,OR2L8,OR5AR1,OR8D1,P2RX4,PDE1C,RASGRP4,SGMS1,SLC27A4,SPCS1,TSC1,UNC5B
Negative_1186	CR1,GCK,RET,ARHGEF2,BET1,CADM3,CARD11,CXCR6,DDX4,DRD1,ENTPD4,ERBB4,FTMT,FUT8,HADHA,IKBKE,IRS1,LAMC1,LAMC3,LIFR,NAGS,OR1B1,PDK1,PER2,PIK3R2,PPP1CC,PRLR,SKIV2L2,SORBS1,VANGL1
Negative_1187	ALDH1A1,CBLB,CSF1,HPSE,INPP1,ITGB5,ITGB6,LIMK2,MAML3,NCAM2,OR5M9,OR8U1,POLE3,RALB,STAM,TNFRSF10A
Negative_1188	UBC,CREB3L1,MAP3K4,OR2H2,SEMA3E
Negative_1189	CDA,CKM,JUN,ADCY1,ALOX12,ATP5B,ATP5E,CACNG3,CXCR5,ENTPD1,FGF20,GNA15,GOSR2,IL10RB,LDHAL6B,LTC4S,MAML3,OR1A1,OR4S2,PTPN7,SMAD7,B3GALNT1
Negative_1190	ABLIM3,CRY1,FGF6,HLA-G,IL18R1,MAP4K3,NPR1,OR2M3,OR4X1,OR51F1,OR8B4,PPP3R2,SMARCA2,SNW1,SORD,SUCLG1,TAS2R14
Negative_1191	F10,ALOX12,BBC3,CLDN17,DERA,DHRS4L2,EPB41L1,FABP3,FGF2,GARS,GLS2,GRIN2B,GUCY2F,KYNU,LDHD,LIPC,MAP3K13,MAPK11,MCM7,NFE2L2,OR4D6,OR5D18,PDE4B,PLXNB1,POLR1A,PRKAR2A,PRKG2,PRPS2,PSME3,RBL2,TSC2,XYLT1,YWHAQ,HLA-DPB1,HLA-DRB5
Negative_1192	C4B,ARPC1B,B4GALT6,CHRD,CYBB,FABP1,FUT7,GCH1,ICOSLG,KIR2DL1,LAMA3,LRDD,NFKB1,PDE11A,POLE3,STAT3
Negative_1193	F8,RPE,TPO,XDH,AQP7,BAAT,BCMO1,CHIT1,CNGA4,CTPS2,CYP7A1,DVL3,E2F4,ENTPD4,FBXW11,FGF12,IRAK2,LLGL1,MPST,OR1F1,OR2W1,PFAS,PIAS4,PLA2G2D,PLA2G4A,PPAP2B,PRKAG2,ROBO1,SEMA4F,SMURF2,ST3GAL3,STX1B,UGT2B10,WNT6,ST6GALNAC3
Negative_1194	HYI,CASP10,CCR10,CHRM5,EPOR,EXTL1,FZD5,GNPAT,GRIN2D,IFNA21,MPDZ,NARS,NKX3-1,OR5T3,PTGES,RASGRP2,ROCK1,SDHB,SESN2,WNT5A
Negative_1195	AK3,AMH,FST,FYN,ALDH3B1,ATP5L,CXCL10,DARS,EHMT2,ESAM,F2RL1,GALE,MAN1B1,MAP4K4,MEF2C,MRAS,MYL7,NFKBIE,OR10H1,OR10J3,OR1L4,OR5AK2,OR6C6,OR6K2,OR6Y1,PGAM4,SHISA5,STK11,STX6,SYNJ1,TPH2,UGT1A10,YOD1,B3GALNT1,PNLIPRP2
Negative_1196	DBH,MAX,AACS,B4GALT5,BACE1,CCL27,CDH2,CDKN2D,CRHR1,CYP1B1,EVI1,FBP2,FLAD1,GGT5,GPT2,GYS2,IL2RB,MARS2,MDM2,MYH6,OR4N5,OR4P4,OR6C70,P4HA2,PRKAA2,RHOQ,SEMA4D,SPRY3,YOD1,ATP6V0D1
Negative_1197	GH2,TYR,ADSSL1,ALPP,ASIP,ASNS,C3AR1,CACNB3,CACNG1,CCL24,CDC25C,CHRM1,CLDN10,CLDN14,COL4A6,CUL1,DCTN1,ENAH,EXTL3,GAB1,GALNS,GIT1,GOT2,HAAO,HCCS,HSD11B2,ITPKA,MAP2K6,MTHFD2,NFKBIE,NRG4,OR52L1,OR6N2,OR6T1,OR7A5,OR8A1,ORC6L,PARVA,PDK1,PLD1,RDH5,SETD7,SMPD2,TBL1X,TM7SF2,TSHB,TYMS,WNT7B,ATP6V1B2
Negative_1198	CD48,GALK2,IFNB1,IL2RA,YWHAE
Negative_1199	ACTC1,CDH15,CXCL11,CYP1B1,DLL4,PPP3CC,PRPH2

Negative_1200	IL5, ABCC8, ARPC1A, BCAT1, CCNH, CNTF, DUSP9, EDNRA, FASLG, HSD17B1, IFNB1, IGF1, LCMT1, MGAT1, NOX3, NUDT5, OR4M2, OR8S1, OR9A4, PCYT1A, POLE2, PROC, RDH8, SLC2A2, TAS2R3, TCF7L1, TSTA3, VNN1, TNFRSF10A
Negative_1201	MPI, TNF, ACAA1, ALDH3B2, ANAPC4, BIRC5, CAV2, CDC7, EHMT2, GADD45A, GALK1, GIT1, GPAA1, GSTK1, HLA-DOB, IL24, KLKB1, MAP3K7, MGAT2, NFKBIB, PFKFB4, PRKAB1, PRKCH, PSEN2, RHOQ, SPRY2, THBD, YARS
Negative_1202	ADK, GBA, IL9, SRM, TNF, ACVR2A, AFMID, ATP4B, ATP5D, B4GALT2, GCNT1, ICAM1, MAP3K1, OR10A7, OR52N4, PLA2G2A, PPP1R3C, PPP2R5E, SELL, SUCLA2, TNFSF12, TRDMT1
Negative_1203	ACOT11, CCR1, OR2L8, UCK2
Negative_1204	ADA, CEL, ID3, PC, ACTB, ADCY8, ATP5F1, BMP2, CAMK2B, CDC26, CRHR1, CSNK2A2, DPAGT1, ENTPD4, ENTPD7, FGF6, GADD45G, GGCX, IL20RB, MAP2K7, MPP5, OGDHL, PPP2R1B, PSPH, SOCS1, TGFBR1, VASP
Negative_1205	ADH7, CANT1, CBLC, CCL14, CD22, CDC27, CDC6, CERK, CHSY1, FOSL1, GNB3, GPD2, IGFBP3, IL21, MTMR2, MYL5, OR2T11, P2RX2, PHKA1, PNPLA4, PTK2B, PYGL, RBPJ, STX5, TNFSF11
Negative_1206	HK2, DDB2, MYLK2, NEGR1, OR14A16, PLAU, POLE4, PPP3CB, PTGDS, CACNA2D4
Negative_1207	GK, AMY2B, CAMK2G, CHEK1, COMP, DPYD, ENTPD5, IL22RA2, OR13C9, OR13H1, OR52N4, PTGDS, PTTG1, SPRED1, TGDS, TMLHE, UBA7, WNT2B
Negative_1208	CCL20, CES2, CSNK1G3, DGAT2, EPRS, IL10, NOS1, OR10Q1, OR1K1, PRKACG, SEMA3E
Negative_1209	FGB, AVPR1B, CYP2A7, DLST, E2F4, GALM, GANAB, HSD3B2, IL2RG, KNG1, LEFTY1, MAP4K3, NMNAT3, OR1F1, OR2F1, OR2K2, OR2L3, OR51T1, PPP1R3B, PRODH
Negative_1210	ABO, CFI, DBI, F3, FER, NLK, ACOX1, ACY1, ASAH1, B4GALT7, ESAM, FTCD, GUCA1B, HAGH, INPP4A, PLA2G10, POLR1A, PPP3R1, PSENEN, PTGS2, RPN1, ST6GAL1, STAM
Negative_1211	CKM, HDC, MVD, CDC14B, CDKN1A, CREB3, DARS2, DVL3, GLG1, GNG13, GUCY1B3, MTMR6, NFYA, NUMBL, PRKCI, RAPGEF1, SEMA4A, WNT10A, XCR1, PNLIPRP2
Negative_1212	C1S, EGF, ID1, TKT, TYR, ACOT12, AOC2, BUB3, CD8B, CDKN2B, CTLA4, CX3CL1, DRD5, EFNA2, FABP3, FECH, GCLC, GCNT4, GMPPB, GMPS, HADHB, HSPA1B, IL8RA, LAMC3, LARS2, MAP3K1, MAP3K6, MASP2, MGAT5B, MLYCD, OCLN, OR2M7, OR2T8, OR4D6, OR5D18, PGAM2, PHKA1, PTTG1, SARS2, SGPP1, SLC25A5, SMPD3, STAT6, VAMP2, ATP6V1G1, CACNA2D2
Negative_1213	DBI, GH2, PGD, TPO, ARPC5, CCR1, COMP, CPT2, CTNND1, DARS2, DLL3, EPHA5, F13B, FRAT2, GALNS, GCH1, MMP1, OR4K5, PFN3, SNW1, TRAF2
Negative_1214	GK, ACVR1B, ADORA2B, ALG6, ARRB2, CACNG3, CCKAR, CCL22, CCR2, CD226, CDH3, CSF1, DDX47, DFFA, DRD5, EFNA4, EPHX1, FLOT2, FLT4, GALK2, GLI3, IL8RA, ITGB5, ITGB6, ITGB7, MAP3K6, NFAT5, NME4, OR10G4, OR2T3, OR4C15, OR51A7, OR51B5, OR51M1, PAX8, PIGA, PIGW, PTGES2, SARS, TM7SF2
Negative_1215	AGL, AK5, C2, TAT, ACADSB, FOSL1, FRAP1, IQGAP2, MIOX, NCAM2, OR4D6, OR6F1, PGAM4, PIK3CA, PPOX, PYGB, SHC1, SLC27A5, SLIT1, TRMT11
Negative_1216	GDA, PGP, ACTG2, ADRB1, ATP6V1D, CAV2, ENPP2, HMBS, HTRA2, IFNA17, IFNB1, KARS, KLRC1, MAP3K5, MGAT3, MGST1, OR2T1, OR6C4, PDE11A, SH2D1A, TNFSF4, TNFSF8, TRAF2
Negative_1217	CCL11, CNDP1, ERBB4, GAD2, GALNT13, GNPDA2, MAGI1, OR2S2, OR51A4, RASGRP4, SUOX, WNT3

Negative_1218	ATM,BAX,CBL,ID3,ABLIM2,ATP4B,ATP5F1,B3GAT3,C1GALT1,CALML5,CCNH,CLDN14,DNMT3B,EFNB2,EFNB3,ENTPD5,EPHA1,FGF17,FGF18,GALK1,GSTM1,IFNA17,ITGA8,NRXN2,NT5C,OGDH,OR1N1,OR2M4,OR6S1,OR9A2,ORC3L,OXCT2,PDE10A,PYCR1,RDH12,SPTLC1,ST3GAL2,ST3GAL5,TCF7L1,THBS4,WWP1,XYLT1,YWHAQ,ALDH18A1
Negative_1219	LDHD,MPZL1,NKX2-2,OR9I1,SEMA4C,SHC2
Negative_1220	DAO,JUN,ACTB,ALG1,ATP6V0B,BCMO1,C3orf10,CASP6,CLDN22,CLDN4,CLOCK,FZD4,GSTM3,IL28A,INPP5E,LEPR,NEFM,NOX1,OR4K15,PCCB,POLR2K,SC4MOL,SHC3,SLC27A1,TBL1X
Negative_1221	PC,ACTG1,ALDH1A2,ALDH3B1,AMPD2,ATP12A,BACE1,CAMK2D,CCR2,CYP2C8,DHCR7,DIAPH2,EDAR,GRIN2C,IFNGR1,INPP5E,LAMC2,MAP3K8,MGAT3,MYH2,NFKBIE,OR10J1,OR1N1,OR4L1,OR51E2,OR7D2,PCK2,PLA1A,PPP3R1,PRKACA,PSME3,PTEN,SKIV2L2,THBS4,TUBA3C,WNT8B,TNFRSF13C
Negative_1222	AR,ADCY9,ARPC1A,CACNA1H,CCNA1,CCR2,CYP2S1,DUSP10,EFNB1,ETNK1,GCDH,GNRH1,GUK1,IL1R2,KIR2DS4,MDM2,MICA,MLYCD,NAGK,NEFM,NOTCH1,OR10C1,OR10K2,OR10V1,PHKG2,PLAU,PLXNA1,PRPH2,SH3BP2,STEAP3,UROD,VAV3,XYLB,PRICKLE2
Negative_1223	MAG,ACSS2,AKR1B1,AKR1C3,ALG10B,ARHGEF7,CADM3,CCL3,CHUK,DDX56,DGKD,GAB1,GALNTL1,HGSNAT,HTR6,IRAK1,KYNU,LLGL2,OR5B17,POLR2C,PON3,PTK2,ROCK2,RPE65,SDHD,SRGAP1,STMN1,TPH1,TYMP,WNT4
Negative_1224	C1S,ZYX,ACPP,AKT3,ALPI,ARHGAP5,BUB1B,CADM3,CCNB1,CXCR5,CYP1B1,DEGS2,DGKB,EDA2R,EPB41L3,EPHB2,GLYCTK,KLKB1,LTBR,NMNAT2,OR10G9,OR6N1,OXCT2,PHKG2,PIP4K2B,POLA2,POLR3D,PTPRM,RDH16,THBS1,UGT2A3,UGT2B28,UROC1,WNT9B,TNFRSF11B
Negative_1225	FGG,KDR,PGD,SMO,ARPC2,ATP5I,CASK,CHUK,CLDN20,CREB3L3,CTNND1,DPM1,IFNA2,IL17B,IL22RA1,KLRD1,LCMT2,MAGI1,MAP3K8,METTL6,NAT5,NODAL,NQO1,NTF3,OR10AG1,OR1N2,OR3A3,OR5M9,PDXP,PMAIP1,PPP2R5A,SEMA7A,SSX2IP,TNFRSF4,ATP6V0A1
Negative_1226	KRAS,OCRL,WNT3A
Negative_1227	BAX,F3,FGB,GBA,GCK,AGXT,AKR1D1,B3GNT3,BMPR1B,CCR1,CCR9,CD3E,CDC14A,CDK6,CXXC4,FGF9,FUT3,GLI3,ICAM2,LDHD,MADCAM1,MAML3,NFATC4,NLGN3,NRXN3,OR10T2,OR4C46,OR52K2,OR5A1,OR5AU1,P2RX5,PAX4,PCCA,PIAS2,PON3,PTCH2,QARS,STAT5A,TACR2,TLN1,TYMP,UGDH
Negative_1228	GGH,MUT,PML,ALDH1B1,ATP5O,BET1,MPP5,OR52A1,OR8J1,SFRP2,SHC3,UGT1A10,ULBP2
Negative_1229	EZR,TYR,ADRA1A,AGPAT2,AOC2,AOX1,CCR7,CD19,CD40,CDKN2B,CHKB,CREB3L4,CSAD,IFNB1,ITPKB,LDHA,ORC5L,PARS2,PFKFB1,PHKA1,PIGT,SLC27A5
Negative_1230	GNAT3,ITGAD,TYMS
Negative_1231	C8G,FES,HK3,PML,ACPT,ANAPC7,ATP5L,BCL10,CACNB1,CCR10,COL5A2,CYP1A1,GRHPR,GZMB,MAPK3,NAE1,NFYA,NLGN3,OR10R2,OR51E2,OR8J3,ORC2L,PGS1,PNLIP,POLD3,POLR2J,PPA1,PPP2R2A,RUNX1T1,SH2D1A,SRGAP1,TAS2R9,TMLHE,ULK1,ULK2,YWHAH
Negative_1232	BMP5,CDIPT,DHFR,DUSP9,FOXA3,GAD1,HARS2,HIP1,IFIH1,PPP1CA,RAPGEF4
Negative_1233	FUK,ID1,RFK,ADCY9,ALDH2,APOC3,ATG12,CDC6,DHODH,DRD2,EFNB1,IDUA,MYH4,NOTCH1,OR1I1,OR2M3,PAPSS1,PIGW,PPP5C,PPT1,SETDB2,CSNK1A1L,TNFRSF1A
Negative_1234	DCN,APOA1,CCL2,CHKB,COL1A2,COMP,CYP19A1,CYP26B1,DAXX,DNMT1,HES1,ITGB6,MTMR6,NEGR1,NR1D1,NR5A2,NUDT12,OR6K3,OR8B8,PORCN,PPP1CA,PTCH1,ROCK1,SPHK1,SPTLC1,UNC5B,VAMP2,VTI1B,WBSCR22

Negative_1235	ADK,DLD,GGH,GK,ID3,SDS,TK2,AARS2,ACO2,AGTR1,AKT1,ALDH1A1,AMHR2,BNIP1,CCNG1,CD226,CHST13,CYP1A2,DPYD,DUSP16,EPHA4,FZD1,GAMT,GCLC,GSTM4,GUCA1C,HLA-F,HNF4G,HTR4,JAG1,KIR2DL2,KIR3DL2,LRP5,MTHFR,OR10A5,OR1E2,OR2B2,OR51D1,P2RX5,PIK3CA,PSEN1,PTPN11,RUVBL1,SETD8,SETMAR,SH2D2A,SULT1A2,TYMP
Negative_1236	ABO,ADCY9,AKR1B1,ARSE,GNRH2,ICOSLG,PLCG2,POLA2,WNT11,ALDH18A1,ATP6V1E2
Negative_1237	CFI,GSR,ALDH6A1,APLP1,B3GNT2,BIRC3,CACNB1,CES1,CYP11B1,FGF20,GABARAP,GSTM3,HARS2,IRF3,MYH14,OR51B4,OR6C70,P2RX3,PRKAG2,PTGFR,STK36,UROC1,PLA2G12B,SERPINF2
Negative_1238	ADK,GHR,MUT,MVK,NNT,ACYP1,ANAPC4,ARPC3,ATP6V1H,AXIN2,C3orf10,C5AR1,CADM3,CAMK4,CD47,CDK4,CREB1,CXCR3,DDX41,DPAGT1,ERBB4,FMO3,GANAB,GAPDH,GGT6,GLI2,HLA-DMA,IL8RB,MAP3K2,MCM6,OR13C2,OR2T8,OR5A2,OR5P3,OR8D1,OR9I1,PIP4K2B,PIP5K1C,POLR2D,PVRL3,SDC1,SETD2,SLC25A4,SMURF1,STAM2,STX1A,TALDO1,UROS,TNFRSF13B
Negative_1239	BTK,ACSM2A,ANAPC11,CCNA2,CCND2,CDC16,CDKN2D,CHPF,CLDN3,CYFIP2,DGAT1,ENO3,FPGT,FRAT2,FZD4,GALK2,LDHA,MAP3K8,NOX3,OR1E1,OR4X1,OR6K6,OR6N1,PIGO,PTPRJ,PTPRR,SCLY,SELPLG,SIPA1,SOCS1,SPTLC2,TP73,TUBB4Q,UCK1,PDCD1LG2
Negative_1240	LYN,AASDH,ACY3,ADRB1,CD55,DDX19A,ENTPD8,FCER1G,IL11RA,IL8RB,MS4A2,OR1I1,PDHB,PHGDH,PIK3R5,PRKCD,RHOQ,SLC2A4
Negative_1241	ADH1B,AGPAT1,AMDHD1,AVPR1A,CACNA1S,CACNB4,CDC14B,CFLAR,CLCF1,COL11A1,CYP8B1,ENO2,EPHB3,ITGA6,MAP2K3,NKX3-1,OR1I1,OR2T6,OR4K15,OR52N4,PIAS4,RAP1A,SLC9A1,SOD1,SULT1A2,TBK1,TLR4,UXS1,VANGL2,WBSCR17,GABARAPL1
Negative_1242	DBI,DHH,CCNG1,CTF1,CYP2C8,DAAM1,DVL1,GCAT,GNB1,NTNG1,OR2M2,OR4A16,PIK3CD,PPCDC,PPP1R3A,TM7SF2,SERPINE1
Negative_1243	INS,AMY2A,AVPR1B,CD46,CTNNA3,CYP3A7,DARS2,DRD2,GART,GMPS,GRB2,HDAC2,LFNG,LHCGR,LRP5,LTBP1,MAPK1,OR1B1,OR1E1,OR52W1,PAK6,PFN1,PLIN,POLR2J,PROC,STX4,TAP1,TRAF1,YARS,TNFRSF10B
Negative_1244	ADCY9,COL6A1,ENPP6,HLA-DOB,MGAT1,MGAT2,NR5A2,OPLAH,OR6B3,OR7C1,ORC2L,PPM1A,PTGS1
Negative_1245	ADK,GSN,IDS,BTRC,CDK4,COL11A1,FGF19,FSHB,GNAZ,HLA-F,HSPA8,IL1R2,IL22,NEGR1,NOTCH1,OR10Z1,PDE8A,PLCZ1,POLE3,PSME1,TAS2R48,TBK1,TIAM1
Negative_1246	VCL,CHST11,CXCL9,ERBB3,EXOC7,GSTA4,HCST,IFNA10,MAP3K8,MMP14,NME2,PHPT1,PLCB1,POLR1C,TJP3,UXS1
Negative_1247	ID3,STS,CACNG2,CASP3,CD86,HDAC1,LAMB2,MAD1L1,TUBA3E,UGT2B7
Negative_1248	ITK,PVR,ANPEP,CCL16,CD3E,CFTR,COL4A4,CTNND1,GBE1,GNA11,LAMB3,MAML1,NMNAT3,OR11H1,OR2L8,OR4S2,OR4X1,PGAM4,PGK2,PIK3R4,PRPS2,RHEB,SEMA3C,SRD5A1,TNFSF18
Negative_1249	NKD2,OR56A3,TYROBP,CSGALNACT1
Negative_1250	FH,PDC,SFN,ASRGL1,B3GNT4,BCL2L1,CBR1,CYFIP2,DDX18,EPHA4,EPHA5,GADD45G,GCLM,GNA12,HMGCS1,HSD17B2,IFNW1,MAP4K4,MCM5,MDH1,MGAT5,NCK1,NUDT12,NUDT5,OGDH,PDE8A,PDGFA,PPM1D,PRKCZ,PTPN5,STK4,VEGFC,ATP6V0A4
Negative_1251	MIF,MPL,ACTB,B3GAT3,BCL2,BLVRA,BMPR1A,CACNA1B,CCR1,CDH5,CDK5,CTNNB1,DUSP14,EFNB1,ENTPD7,ENTPD8,EPHA1,HSD17B8,HSPA8,IRAK1,IRS2,LTA4H,MGAT4A,NPR1,NTRK1,NUDT9,OR10G7,OR10H4,OR4C11,OR51F2,OR52K1,PAK2,PARVA,PHKG1,POLR3G,RELA,SEMA7A,TLR3,TNFSF11,UROC1,VAV2
Negative_1252	B3GALT6,MGST1,PGK2,POLD3,THBD,TM7SF2

Negative_1253	F3, F9, WAS, ABCC8, ACSM2A, AMDHD2, ANAPC2, DDX50, ENAH, EPB41L2, EPHA1, FARP2, FZD4, GSTM2, HGSNAT, IFNA4, IL12A, IL1R2, IRF9, JAG1, KIR2DS5, LTA4H, MAP4K4, NLGN1, NOX1, OR10Q1, OR5D18, PIK3R4, PLA2G2E, POLR1A, PTEN, SMPD4, UGT2B7, WARS, CTNNBIP1, PRICKLE2, TNFRSF12A
Negative_1254	CD6, CDA, GK, HAL, ALDH2, ALOX12B, AMDHD2, APRT, CHEK2, CLDN8, DKK4, FLT4, G6PC2, GUCY2C, HAGH, HARS2, IL12A, IL28A, MPP5, OR2A12, OR4F15, OR7D4, PLIN, PRKAB1, PRKG1, ZFYVE16, HLA-DRB5
Negative_1255	AK5, IL4, ACP5, ADIPOQ, AKR1C1, ALDH1A2, ARHGEF7, BDH1, BIRC2, C1QA, CD22, CLCA2, CPT1C, CSF2, CSNK2A2, CXCL16, DBF4, DUSP2, EIF4B, GCAT, GLI1, GNAI3, GPAM, GUK1, HSPA1B, KLRD1, OR5A2, OR5AU1, OR9A2, P2RX7, PRKCI, PRPH2, SARDH, SCLY, SEMA4G, SGPP1, SHC3, SIGLEC1, SKIV2L2, SPTLC1
Negative_1256	AK3, FAS, IL8, NPL, ADAM17, ADH7, AQP7, ASMT, ATP5G1, B3GALT5, CCL8, CHKB, CYP4F3, DAAM1, DUSP14, FGF18, FOSL1, GLUD2, GSTM5, HIBCH, HMGCS2, HSPB1, IL2RB, KNG1, LDHA, LILRB3, METTL6, NAT6, NFYB, NUDT9, OR8B8, PGM2, PRKAR2A, RASA2, SGPP2, UGCGL1, SERPINC1, SERPINF2, ST6GALNAC3
Negative_1257	AUH, C4B, FH, HK1, LEP, ACADVL, ACTB, ALDOC, BDH2, CAMK2B, CCL1, CCR5, CHRM3, COL5A2, CTNNA3, CTNNB1, DGKD, DNMT1, GALNT12, GJD2, GRHPR, IFNA6, IL6R, MYL5, NCAM2, NCOR1, NT5C2, NUDT8, OR51T1, OR8J3, PAK1, PIP4K2C, PPARA, PSME2, SLC2A4, SMARCA5, STX18, TCF7L2, TRMT11, VTI1B
Negative_1258	C6, CFH, IL9, LPL, TAT, ALDOC, AQP7, ARPC3, BUB1B, COL5A2, DIAPH2, DOLPP1, FABP3, HTR4, LRDD, NFATC1, OR51S1, OR7G1, PCK2, PIK3R5, PIM2, PTK2, RAC3, TAS2R10, TCF7L2, TUBA1A, VEGFB, ZFYVE9
Negative_1259	EPHB4, HTR2B, LPAR1, NEFL, OR9G4, PCNA, VAMP4
Negative_1260	LTB, CCNG2, CXCL10, HARS2, MYL7, MYLK, PARS2, PPAP2B, PPCDC, TLR4
Negative_1261	MPL, SRC, ADORA2A, NFATC2, OR51V1, PDXP, PLXNA3, PTK2B, TUBA3C
Negative_1262	DLD, IL7, NOG, RET, SRC, ADH1A, AMHR2, ARPC3, CDH15, CDH5, CHPT1, DAAM2, DGAT1, DOT1L, FABP3, FUT9, GLUD1, IL28A, INSRR, KIR3DL2, KLRC1, LDHD, MGAM, MINPP1, NME2, NTRK2, OR10H3, OR10H4, OR10J5, OR2Y1, OR51M1, OR7A5, OXCT1, PDE11A, PIK3R4, PTDSS2, RARA, RRAS, SMPD3, STMN1, VDAC2, WASF3, WNT7A, SLC25A31
Negative_1263	EFNA2, GDF6, GNAL, LAMB1, NKD2
Negative_1264	CD2, ARSA, FBP2, JAM3, MECR, PAK4, ATP6V0A4, MAP3K7IP1
Negative_1265	ABO, F5, F8, IL2, MET, AHCYL2, ARHGEF4, CACNB3, CERK, DEGS2, DVL3, EPHA8, FRAP1, FZD6, GFPT1, GRIA2, IGFBP3, IKBKE, IL24, L1CAM, MTAP, NADSYN1, NODAL, NUDT9, OR10P1, OR2S2, OR2T11, OR5P3, PARD6A, PDE6D, PIGW, PLA2G2E, PLXNB3, POLR1D, PTPRM, RPS6KA1, SOS2, SREBF1, UPP2, WNT2B, HSP90AB1, ST6GALNAC4
Negative_1266	CCL28, CTSB, HDAC2, LAMC3, MAD2L1, MAPK14, OR10A2, OR4C6, PIAS4, POMC, PSME1, RAPGEF4, UXS1
Negative_1267	DCT, ALG11, BDKRB1, COL6A2, CYFIP2, KIR3DL3, ST8SIA1
Negative_1268	ACADSB, AGRN, AMY2A, CBLC, CCNE2, CCR2, CD34, CLDN8, CLTB, CTBP2, CYP27A1, EI24, ERBB2, GALNT6, GALNTL4, GNA12, GNG13, GUCY1A2, HAGHL, HEXA, HHIP, ITGA9, MAP3K14, OR13J1, OR1D5, OR8U1, PLXNB1, SLIT1, SMPD3, TAP2, UPRT, ZIC2, HLA-DPB1
Negative_1269	PDC, PLG, BMPR1B, CAPN2, DGKG, EXTL3, GRM5, METTL6, OR2M4, OR4C15, OR5H2, ORC5L, RPSA, SHC1, SHC2, TAS1R2, ULBP2
Negative_1270	CKM, DDO, FGA, MPL, PXN, RPE, AGPAT2, ANAPC5, ASS1, CACNA1I, CAMK2G, CD34, CD40, CYP2D6, DHRS9, ENPP3, FGF3, FGF8, FMO5, HSD3B2, IL24, IL6R, LARS, LARS2, MOV10L1, NFYA, OR2A14, OR2T11, OR9A4, ORC3L, PIGB, POLR2C, PRIM2, PRPS2, SARS, SLC27A4, SPHK2, SPRY4, STX5, TAS2R49, TPMT, TNFRSF10A

Negative_1271	BTK,ACP1,ACSL1,AHCYL1,AKR1B1,ARHGEF4,CARS2,CAV1,CCL3,CYP26A1,CYP4A22,EPRS,GALNT13,HSD17B8,INSRR,NOD1,NRXN3,PAK3,PDE8B,POLR2F,PRNP,SDC4,SNAI2,TAS2R9,TSHR,CACNA2D2
Negative_1272	CYP26A1,GALNT9,HIBADH,MGST1,OR4K15,PDHB
Negative_1273	IVD,MUT,AMPD2,CXCL6,DDIT4,DDX54,DGKZ,EP300,FUT3,FZD3,HLA-DOB,IRF5,KIR2DL2,MDM4,NAE1,OCRL,OR11H6,OR1I1,OR4C15,OR51B2,RXRB,SETDB2,SGPP1,SORBS1,ZAP70,ATP6V1C2
Negative_1274	FPGT,IDH3A,IDI2,NAE1,PDE6D,PLD1
Negative_1275	F12,IHH,SRC,TNF,TPO,ACADM,ACSS3,ADRB3,CCDC6,CDC14B,CLCA4,CSNK2A2,CX3CR1,DKK1,FZD5,FZD7,ITGA8,ITGB4,KAT2B,OCLN,OR14I1,OR9K2,P2RX1,PRSS1,PTGES2,RASGRP4,SCIN,SOCS3,SRGAP3,SV2B,TGFB1,TSTA3,XIAP,ATP6V0A2,HSD17B12
Negative_1276	ABO,MAX,MPI,ACADL,ALPI,ARPC4,ARSD,ATP5J,C7orf16,CAMK2D,CCL15,CD86,CYP1A2,CYP2A13,CYP2F1,DGKH,DMGDH,EPHA1,FGF23,GRHPR,GRPR,HHEX,IL28A,IL5RA,LAMA4,LAMC1,NKX2-2,OR2M2,OR4D9,OR4L1,OR6C70,OR6T1,ORC5L,PFAS,PHKA2,PIGO,PIP4K2A,PLCB3,PLXNA1,PLXNA3,SC4MOL,SH3BP2,SMAD1,TAS2R14,TCF7L2,TRDMT1,UNC5C
Negative_1277	OAT,CD3E,DNMT3B,EPHX1,FUT8,HSD11B2,ICOS,KLK3,MAPK11,NUDT12,OR2AT4,PDE7B,PRKCI,RXRB,SLIT1,UBE2L6,UGCG,PAFAH1B1
Negative_1278	ABL2,AGMAT,CYP2C19,HRAS,ITGA5,PER1,PIK3CB,POLR3A,TAP1,UBA7,UGT2B11,CACNA2D1
Negative_1279	IL5,NPL,AASDH,ABP1,ACSS1,ACYP2,AKR1B10,ANAPC2,C9orf95,CACNB2,CACNG4,CCL28,COL5A3,CSNK2A2,DARS2,DEGS1,EFNA1,EPB41,GDF6,GIT1,GPX5,IGSF5,IL5RA,IMPA2,MAP2K5,MYH7B,NOS3,NR1H3,OR11H6,OR12D3,OR2H1,OR52N5,OR5A1,OR5K1,PCYT2,PLA2G1B,PTPN6,SMAD5,TAS2R10,TNFSF12,TUBB2B,VTI1A,ATP6V1G2,CSNK1A1L,TNFRSF1B
Negative_1280	CS,AK3L1,ALG12,CHRM1,CXCL10,GALC,GCDH,HARS2,HDAC2,IFNA5,NCR3,OR1G1,OR1M1,OR2W1,OR4K2,PCYT1B,PFN4,PTPRC,TNFRSF9,VAMP4
Negative_1281	CKB,ACSM5,ADRA1A,AMD1,AOC3,ARNT2,C7orf16,CACNG4,CAMK4,CD244,CDC7,CHUK,CLDN2,CSF1,CYP1A2,DUSP2,ECSIT,EDN1,FTH1,GADD45G,GNAQ,HNF4G,HTR4,IDH3G,IL1RAP,KLRC4,LYPLA1,MTHFD2,MYH3,NPAS2,NSDHL,OR13C4,OR13C9,OR2A14,OR2T29,OR3A2,OR52K2,OR5P3,PARD6G,PFAS,RAD51,RCHY1,RPS6KA1,SYNJ1,UBE2L6,WNT9A,ATP6V1B2,TNFRSF11B
Negative_1282	FGA,GP6,PDC,ACACA,ACSS3,AMHR2,AMY2A,C3AR1,CACNA1A,CCNG2,CCR6,CD19,CDH5,CER1,CRY2,CTLA4,CXCL3,CYP26A1,DDX23,DLL1,DTX3L,EPHA2,GCNT2,GSTM2,HSPA2,HSPA8,IFNA21,INADL,KCNB1,KLRC2,KLRC3,KLRK1,MMP2,OR11L1,OR4N4,OR52N2,PFN2,PLAT,PROS1,RDH10,SGPP2,STAT6,STEAP3,TRADD,USE1,PDCD1LG2,ST6GALNAC1
Negative_1283	C5,CFD,DLD,IDS,OSM,AANAT,ADCY4,AHCYL1,CASP6,CAV2,CLDN17,CSNK2A2,EXTL1,GMPS,IARS2,IL11,ITGA2,ITGB3,MAD2L2,NLGN2,NTSR1,OR14C36,PANK1,PDX1,POLR2B,PTGDS,PVRL2,RRAS,SDHC,SEMA4B,UNC5D,WASF3,MAP3K7IP2
Negative_1284	ADA,AQP7,DDX55,DNMT3B,ETS1,IL10,MADCAM1,MASP1,OR13H1,OR4A5,OR6C6,PAPSS1,RUNX1,SOS2,TUBA3E
Negative_1285	TK2,ALDH7A1,CADM3,CCL2,CLDN20,DDX4,FMO5,GRIN1,GUCA1B,INPP4B,MAPK1,MTMR2,OGDHL,OR2A42,PIK3CB
Negative_1286	C2,CAT,HDC,AADAC,ACO1,ALOX15,AMPD1,ATP5A1,ATP5I,CCR8,CYP2C19,CYSLTR2,DKK4,DUSP5,EPHA3,GJA1,GLI2,KLRD1,NCOA4,NNMT,NTRK2,OR1L1,OR2C3,OR5H2,PANK3,PDPK1,PIK3R2,PYCR2,SGPP1,SMPD3,SOCS7,THBS2,TUBB1,YWHAZ,HSD17B12,PPP1R12A,SERPINB5,ST6GALNAC6

Negative_1287	LIF,OSM,ACOX1,ADCY7,ARNT,ARSD,CBR3,CCND1,CD19,CD27,CDK5,DUSP2,ERBB2,FGD3,FLAD1,FZD5,GGPS1,GMPPB,GMPR,GNRHR,GPD1,IL12A,NCF4,NKD2,OR10H1,OR13D1,OR52N1,PDE7A,PLA2G10,PLCB1,PLCD3,POLD2,PTAFR,RFXANK,SKP1,SLIT2,SMPD2,SUCLG1
Negative_1288	GPI,GSS,ACAA1,AGXT,ALG10,CD82,CDC20,CPOX,FMO5,GANC,GAS1,GSTO2,JAK2,KARS,MOV10L1,PIGQ,ROCK1,THY1,WBSCR22
Negative_1289	AR,AMPD2,COL4A2,CTTN,CYP2F1,DRD2,DUSP9,EPOR,GNA14,GSK3B,GUCY1B3,ICAM2,LIPF,MYH7B,OR1L8,OR1N1,OR5H6,PIK3R2,PSME3,RASA1,TXNDC12,VAMP3,VEGFA,YWHAG,GABARAPL2
Negative_1290	LCMT2,OR5V1,OR7C2,POLR1B,TIAM1,TMLHE
Negative_1291	MTR,ADCY1,AGPAT3,ALDH3A2,ARAF,ARHGAP5,CACNG3,CD34,CDH2,DHDDS,EFNB3,FUT9,IFNA7,IFNK,ITPK1,MAP3K11,MAPK14,MEF2C,MGAT5,MTHFS,OR10A7,PIPOX,PNPLA4,PPP2R1A,RPS6KB2,RRAS,SFRP4,SORBS1,STAT4,TAS2R5,TUBB4Q,ULK1,VAMP4,VANGL1,WBSCR22,MAPKAPK2,TNFRSF11B
Negative_1292	ASL,TH,CCND3,CD38,CREB3,DHRS3,ENTPD7,EPB41,EXT2,GALNT5,GGT6,GPAA1,HRAS,IL12B,JAG2,NAT6,NFATC4,OR10V1,OR2T11,PIP5K1B,RAD51,RASGRF1,RUVBL1,UGCGL1,UGCGL2,VARS,MAPK8IP2
Negative_1293	FAH,ID1,RPE,ALDH1A3,ANAPC5,CADM1,CAMK2B,CDKN2B,CDO1,DKK4,ENPP6,FARSA,FIGF,GAD2,GUCY2F,GZMB,HLCS,HYAL1,MAPK13,MYH10,OR1G1,OR52B6,PERP,PPP1R3B,SESN3,STAT4,TAS2R10,UXS1,VKORC1
Negative_1294	PC,ALS2,CACNB3,CCL19,CES2,FARS2,FZD4,HTR5A,ITGB2,LDHAL6B,PIAS4,PIGN,PIPOX,PLCG2,PLXNB2,PPARA,RBL2,RPS6,SC4MOL,SDHB,SESN2,SLC33A1,SRGAP3,TAS2R49,TNFSF10,TRAF5,UROS,WASF3,WNT10A,YWHAZ,MAPK8IP3
Negative_1295	CS,DUT,LIF,SPN,ADSL,B4GALT3,BUB1B,CACNG6,COL1A1,FBP1,GLG1,GYS2,HEXA,IFNA4,ITGA2,ITGA3,ITPA,NFYC,NRXN3,OR1N1,OR2C1,OXTR,PCNA,PLA2G2A,PPP2R5D,PRDX6,ST3GAL5,TUBA8,XCL1
Negative_1296	SMO,DCXR,EXTL1,FGF4,IL22,MAP2K7,PDE10A,RAD51,SFRP5,TFPI,TKTL1
Negative_1297	JUN,AANAT,ALAD,ALDH1L1,ARHGEF7,CCND3,CCNH,CLCF1,DGAT1,ENTPD2,EPHA1,FABP1,FARSB,FGF13,GALNT3,GPX7,IL1A,INHBA,KIR2DL3,LCMT1,LTB4R2,MYH1,NSDHL,OLAH,OR2T2,OR4P4,OR52N1,ORC2L,P4HA2,PCYT2,PIGA,PMAIP1,POLR2I,PPT1,SCD5,SLC27A1,SOAT1,ST3GAL5,WNT7A
Negative_1298	ADK,C8B,CEL,EZR,SRF,APLP1,ARPC4,ATIC,CCDC6,CNTF,CXCL9,DGKD,FECH,FUT2,IL1B,IQGAP3,MECR,NAGK,NME7,OR10H3,OR1I1,OR4D5,OR4X1,OR51T1,OR5AP2,ORC6L,PAK7,POLE3,PTGS1,PTTG1,SETD1B,SLC25A4,SOAT1,TGFB3,HSP90AA1
Negative_1299	ABLIM1,AREG,B3GAT2,BCAT1,BMP8A,C4BPA,CAMK2D,CSNK2A1,CYP4A22,DAAM1,DUSP9,GALNS,IL17A,MLLT4,OR2AG1,OR2T12,OR5T3,OR8B12,ORC4L,ORC5L,PFKFB1,PIGO,PLA2G2F,PLCB3,PPOX,PRKCE,RAC3,SELP,SRD5A2,TNFRSF4,UGT2B4,SLC25A31
Negative_1300	ADC,EBP,MAX,ALDH1A3,ALDH3B2,ARRB1,CCNA1,CTNNB1,DFFA,EGLN3,FOSL1,GNMT,HAGH,HLA-C,IL11RA,OR2G6,OR4Q3,OR7C2,OXSM,PDGFB,PLCZ1,PPP5C,PTPN5,SLC27A4,SOAT1,SORD,SPTLC2,SV2B,TPK1,VAV1
Negative_1301	CFD,FGG,A4GALT,ADH4,ARG2,BPNT1,CDC6,CLDN9,DGKI,EIF4E2,EPHA7,FZD2,GNAO1,GNG3,GRIN1,HSD17B7,IFNA2,INMT,KLKB1,LPAR1,MTMR6,OR10A4,OR10G2,OR10J5,OR12D3,OR4F17,OR8B12,PDE3B,PDK1,POLR2H,RXRB,SV2A,TAS2R48,TAS2R60,TPI1,TRPM5,WNT2B,XIAP,TNFRSF6B

Negative_1302	BTK, C2, CGA, EPO, TEC, AGMAT, ARNTL, ATP4B, AXIN1, B3GAT1, B4GALT5, CCL14, CCNA1, CDS1, CDS2, COL4A6, CSNK1E, DHDDS, DPM1, EREG, FGF12, FTMT, FZD3, GPX5, HNF1B, NAT5, NUDT8, OR5B3, OR5M8, OR9Q1, PDK1, PIGS, PLIN, PNPLA4, POLD1, PRKAA1, RELA, SDHB, SGPP1, TAS1R2, TCF7L2, TM7SF2
Negative_1303	GSR, MET, ARHGEF7, CREB1, EIF4E2, NME7, PRKAB1, QARS, SRGAP2, ST3GAL1, SULT1A4
Negative_1304	NPY, CD79B, DHODH, EFNB2, GNAI2, IFNA16, NRAS, OR1A1, PARD6B, PRKAB1, RENBP, TGDS, WASL, WNT3A
Negative_1305	ACAT2, ARHGEF2, COL5A1, DVL3, ERBB4, IL23R, MAD2L1, OR2AG2, OR4S1, OR5L2, OR6T1, OR7D2, PAX6, PIAS4, PTGFR, SLC25A5, SOAT1, SPTLC1
Negative_1306	FUK, ABLIM1, AGRP, CDKN2B, FLOT1, KLRK1, LLGL2, MKNK1, MTHFD1L, OR5P2, PIK3R2
Negative_1307	C8B, CGA, CKM, F3, MPZ, SPN, ACSS2, ACVR1, AKR1C4, ANGPTL4, ARNTL, BET1, CAMK2A, DKK2, FUT5, FZD3, GCDH, HBEGF, IFNGR1, IL7R, IMPA1, JAK2, KAT2A, NEGR1, NRG1, NUDT2, OR10K1, OR10Q1, OR13J1, OR1A2, OR1G1, OR4F6, OR5AN1, POLE3, POLR2F, PRKAB1, PTPRM, RALBP1, RXRB, SARS, SIGLEC1, SORD, STMN1, SV2B, TAS2R14, TGFBR1, TRAF4, ATP6V0A2
Negative_1308	ADC, ME2, ADSSL1, AMY2B, CACNG5, CCL24, CD34, CDKN2D, CYP2C18, EFNA5, ENTPD4, FABP1, HHEX, HSD3B1, IMPA2, LARS2, LPAR1, MYH11, NAT6, NFKB1, NOS1, NUMBL, OR52A5, PARVA, PIGQ, PIK3C3, PKMYT1, PPARG, PYCRL, SAT2, SDC2, SESN2, SIPA1, SMURF2, SPI1, TSLP, WNT11, PAFAH1B1, SERPINA1, SERPIND1
Negative_1309	ALAD, ALOX15, ATP6V1F, CDK7, IPPK, LGMN, MAPK11, MLYCD, NAGA
Negative_1310	CAPN2, CARD11, CDH4, CTTN, FABP3, GALK1, GOSR2, GP1BA, HNF4A, IRF7, ITGA2B, ITGB5, MAGI2, MAPK12, MYL7, NKD2, NKX2-2, OR10T2, OR2W1, OR6C70, PAFAH2, PCYT2, PFN4, PRKAB1, PRKACA, PTK2B, PTPRZ1, SDHA, SLC27A4, SMARCA5, STK11, TLR9, TPK1, WNT1, HLA-DRB5
Negative_1311	SMS, CAMK2A, CYP27A1, JUND, NAT6, OR2AG1, OR9A2, ORC3L, PIGV, PPAP2C, THBD, TYK2
Negative_1312	DCC, XDH, ADH1B, BDNF, CYCS, EPHX2, FGFR3, MAP3K14, NAT1, PPP2R5C, PPP3CB, PRKAG2, RRAS
Negative_1313	ALG12, CACNB1, KNG1, OR2T33, PER2, RUNX1
Negative_1314	ADSSL1, B4GALT2, CD80, CMPK1, DDX47, G6PC2, GOT2, GPX5, MAGI2, MAP2K6, PLCD1, RFXANK, SFRP2, SPI1, TUBA1A, WARS2, WNT3
Negative_1315	AHCY, CDH2, CDH3, CYP8B1, HMGCL, MAP3K4, MKNK2, PTGES, ST3GAL2
Negative_1316	ALDH7A1, ARNT2, CACNG3, CNTFR, CYBA, DVL3, HMGCS2, ITCH, ITGB4, JUND, KIR3DL2, MGAT1, MLYCD, OR51L1, OR8J1, OXCT2, PFKFB1, PIGU, PLCB2, PNPT1, PPARG, PPP2CB, PSAT1, RBPJ, RDH12, STX18, TSHB, WNT1, HLA-DPA1
Negative_1317	CKB, F7, AACS, AGMAT, CASP3, DDHD1, DIAPH2, FUT3, GNAQ, HCST, HSD17B1, IARS2, IL17RB, LDHC, LTA4H, MAGI1, MAP3K6, MYLK2, NOTCH1, OR1J2, OR2T6, OR51D1, OR5D18, OR5R1, PARVA, PERP, PKMYT1, PMVK, POLE3, RPS6KA1, RPS6KA2, SMC1A, SUV39H1, TNFSF14, TOLLIP, VARS, MAPK8IP3
Negative_1318	AACS, ABCC8, ACP5, ATF4, DGKA, DPM2, EGLN1, EPHA8, IL12RB1, IL19, ITGB5, LIPT1, MDH1, NOS1, OPLAH, OR4K13, OR4K15, OR7A5, PLCD1, POLR2L, PRKAG1, RUNX1, SETDB2, SV2A, XCL1, ZBTB16
Negative_1319	ABO, CFH, ANAPC1, CBR3, CCR1, HTR2C, ITGA3, MAPK12, MMP2, NAGS, NCAM2, NME2, NPAS2, NUDT8, OR10AD1, OR2T1, OR4M1, PLAT, POLR2F, PPP1R3C, RELB, TGFBR2
Negative_1320	AGL, C6, ADORA2B, AMPD1, ATG7, B3GALT4, C4BPB, CDC27, CHAD, CTSG, DAPK3, DKK4, ENTPD6, EXOC7, ICAM2, KLRD1, NAGK, NFYA, OR52W1, OR5T3, OR8U1, PAX6, POLE4, POLR1E, SHC2, STX1A, TUBA4A, UBA7
Negative_1321	B3GNT3, B4GALT7, FZD8, GCNT2, MMP2, PLCB4, SEMA3G

Negative_1322	MPL,CD40LG,CLDN4,CREB3L4,FGF1,FGFR4,GNG13,IFIH1,LAMB3,LRRC4C,NFKBIB,NME2,NRXN1,NUMB,OR10K2,OR4F17,OR56A3,OR8D2,OR9G1,PIP5K3,SLIT1,TICAM2,TKTL1,ZBTB16
Negative_1323	RET,ALCAM,ARG1,CLDN1,CSNK1G1,CXCL10,DDX56,EARS2,FLT1,GOT1,HRH2,IL10RB,LDHA,NRG3,OR56A4,ORC3L,PIGV,RAPGEF4,RRM1,SPTAN1,STK3,TOMM40L,TYK2,WNT9B,ATP6V1G3
Negative_1324	AGRN,ATP5G1,BAI1,NCOR2,OR10A6,OR5B12,PRKAG3,SOCS1,TBL1XR1,TNFSF11,YARS,HLA-DRB5
Negative_1325	F8,SP1,AKR1C3,ALG6,ARSD,ATP5B,BCMO1,CACNA1H,CDC14A,CDK5,CXCL9,CXXC4,CYP11B1,CYP21A2,DHRS4,F2RL1,FGF11,FMO5,GLYCTK,GSTA4,IARS,IRF3,LAMB4,LCP2,NKD1,NME7,OR10C1,OR5P2,OR6A2,PERP,POLR2G,RFX5,SV2A
Negative_1326	AR,TFG,ACO2,ARRB1,DDOST,HEMK1,HLCS,HMGCS1,HSD3B7,IFNGR2,IL1RAP,INHBC,LTBR,MAGI3,OR52W1,PARVA,PCYT1B,PI4KB,PIGB,RBKS,SIGLEC1,UROC1
Negative_1327	AR,CREBBP,FABP3,IL2RG,MAP4K1,PVRL3,RAB5A
Negative_1328	CKM,F5,MAX,PDC,AGRP,CCR10,CD27,CD58,CDC25B,CER1,CMPK1,CTSB,CYP1A2,CYP2D6,DAAM2,DGKI,GNAL,HLCS,IBSP,IFNB1,MAN1A2,MAP2K1,MDM4,NAT5,NEU3,NFYC,OR51G1,PFN2,PHKA2,PLCZ1,PPP2R2D,PSME1,PTPN1,RARS2,RELB,SIGLEC1,SLC25A6,ST6GAL1,VEGFB,WNT7B,C1GALT1C1,HLA-DPA1,PPARGC1A
Negative_1329	CR2,GSS,SRC,ACSL6,ADIPOR1,ALDH3A1,ALG11,ARD1A,ARPC1B,CCL25,CCL28,CDC20,CLDN16,COL1A1,CYP2U1,EFNA4,FARSA,GSTO1,GUSB,HLA-DRA,INPP5A,LIAS,MADCAM1,MGAT1,MTAP,NCF1,NKD1,NME6,OR11H1,OR2H2,OR5A1,OR5AR1,OR6B3,ORC6L,PAK3,PDXP,PLK1,RAC3,RFNG,RGS3,RPS6KA6,SHC2,SLC2A2,ST3GAL4,C1GALT1C1,CSGALNACT1,PHOSPHO1,SERPINA5
Negative_1330	CYP3A4,FTCD,GADD45G,HSD17B3,IFNW1,IL20RB,METTL2B,OR10J5,PFKFB2,POLE3,RRAS,SELL
Negative_1331	C4B,CD2,TAT,AKR1C4,ATP5E,CACNG2,CD79A,CHEK2,CXCL11,DCTD,DLL3,DLL4,E2F2,ETNK2,EXTL3,FUT8,GP1BA,HHIP,IQGAP2,LYPLA1,MAPK9,MYH7B,NTRK2,OR2T34,OR4C46,OR4F17,OR4K2,OR4M2,OR5C1,PDE4B,RALA,TFDP1,TRHR,SERPINC1,ST6GALNAC4
Negative_1332	SYK,GALNT8,OR5B21,OR6C74,QARS,STAT5B,TPM3,WNT5B,YWHAH,B3GALNT1,GABARAPL1
Negative_1333	EPO,AKR1B10,CD226,HSPB1,NUDT8,OR13C4,OR2F2,OR8G1,PIAS1,RAPGEF4,SCD5,TCF7L1,WNT9B
Negative_1334	AK3,CD2,GDA,NPY,ADCY1,ADRA1D,BCAT1,CACNG4,DUSP6,E2F1,ENPP2,HRH1,HSP90B1,IL3RA,KLRC4,MAP3K11,NEUROG3,OR4F5,PFKFB4,PTGER1,RDH8,SDC3,TACR3,TUBB2A
Negative_1335	AMT,OSM,ACVRL1,FBXW11,IQGAP2,LALBA,LAMC3,MDM2,MNX1,NAT5,NCF4,PDHA1,PRKAR1A,SFRP2,TMLHE,TYK2
Negative_1336	JUP,CD40LG,CTTN,CYSLTR1,IFNAR1,MMP14,OR2F1,OR56A1,PRKAR1B,TPH1
Negative_1337	F9,ACAD9,AKT1,BCKDHA,CDC6,COL4A6,CSF1R,CSNK2B,CYP1A1,DOCK1,EDA2R,EPHB4,FGF1,GNA14,GUCA1A,HLA-C,HMGCL,HSPA8,LAMB3,LGMN,NEU2,OPLAH,OR1L4,OR4A15,OR52H1,OR6C75,PDE2A,PI4KA,PIGU,PNPO,PRSS1,PTCRA,PTPN6,RASGRP3,RPN2,RUNX1T1,SMAD5,TAS2R4,UBA1,WNT6,ZBTB16
Negative_1338	ADK,F9,AARS,ACAT1,ACAT2,ACVR2A,ACYP2,APOA5,CCL23,CD36,CD38,CHAD,CTSB,DDHD1,DGKI,DHRS4,E2F2,ENTPD6,FARSB,LDHD,MICA,MPST,NCOR1,NSDHL,ONECUT1,OR2L3,OR6N2,PYGM,SETD2,THBS3,TNFSF14,TYK2,VDAC3,CACNA2D2,TNFRSF13B

Negative_1339	C9, SCD, ACYP1, BAAT, CCR10, CNGA4, DHX58, ECHS1, GNG3, GP1BA, GRAP2, LAMB3, LIPA, MCCC2, MGST2, MTHFD2, OR1E2, OR5L1, PDGFB, PGM3, POLE, PRODH, PSME3, RDH5, SH2D1A, TAS2R4, TGFB3, TOMM40
Negative_1340	C8G, C9, DDO, F9, MUT, ABAT, AOC2, CACNA1I, CDH15, CDK2, CHAT, CHRD, CHSY3, CSNK1D, CX3CR1, CYP2U1, FGF2, FGF9, FUT2, GALNT5, GNAI3, IDH3G, IDI1, JAK2, MYH7, NRXN3, OR1J1, OR52M1, OR8I2, OR8J3, PLA2G3, POLR2C, PPP2CB, PRKAG1, RAPGEF4, RASA2, SH3BP2, SKP2, TJP3, TXNDC12, XYLB, KIR2DL5A, MAPKAPK2, ST6GALNAC5
Negative_1341	ACTA1, DDX47, GSTK1, PLCG2, PTPRB, TGFB2, MAPK8IP3
Negative_1342	DAO, MIF, MPL, SHH, ATP12A, CLDN1, COL11A1, CXCL12, DGKG, FZD7, HRAS, IKBKE, INSR, IQGAP1, ITGB5, LRAT, NOTCH3, OR5T2, OR7D2, OR8I2, PFKFB4, PLCD4, POLR2B, RALGDS, ST3GAL3, STX7, TCF7, TUBB2C, PAFAH1B1, PLA2G12A
Negative_1343	APP, ACY1, ALDH1B1, ANAPC2, ARG2, ATP5J2, CADM1, DHCR7, DHRS3, DUSP4, EPAS1, EXTL1, GALNT1, INPPL1, KIR3DL1, MAP3K2, NEUROD1, OR10G9, OR10H2, OR51I2, OR7C1, PCCA, PDGFD, PFKFB2, PLCD1, POLR2A, POLR2G, PPT1, RAP1B, SDC4, SGMS2, SGPP1, ST3GAL5, TRPC1, UGP2, ULK1, PNLIPRP1
Negative_1344	PGP, ALG13, CCL7, CUL1, EDN1, HADH, ITGAD, MS4A2, OR2F1, OR2H2
Negative_1345	FGA, GK2, ACSS1, AKR1C1, ALDH4A1, ATP6V0B, CHPT1, COX15, F13B, FGF20, FOSL1, FRAT1, HAAO, IFNG, IL13RA1, ITGA4, MCAT, NAT2, OR51E2, OR5AK2, OR8G1, OSMR, PER3, PIGV, PKM2, POLR1B, RAB23, RAPGEF3, RPRM, RUNX1, STAT2, TALDO1, UGT2A3, TNFRSF1A
Negative_1346	CFD, ACOX1, AKR1C3, AMPD3, APOE, CTPS2, DAD1, FGF2, GALC, MALT1, OR10J3, OR1F1, OR2M5, OR52I2, OR52N4, STAT2, SUFU, SUV39H2, TUBB2C, WASL
Negative_1347	C9, EGF, IDS, ILK, MPL, OSM, ATP5G3, B3GNT5, CCR6, CD22, CDH4, CERK, CHAD, CTSL1, CUL1, DAAM2, DDIT3, FGF6, GAD1, GMPPB, GNAS, GUCY2D, HSD11B1, HSD3B7, LDHA, MAP3K12, MARS2, OR10AG1, OR10T2, OR5D13, OR6C6, PDE5A, PDGFRA, PIM2, PPBP, PPCS, RAP1A, RASA2, SOCS4, TCF7L2, TDO2, TPMT, UAP1, VAMP4, YWHAE, ATP6V1G2, TNFRSF18, TNFRSF1B
Negative_1348	FES, LYN, ABP1, AGXT, ALG9, CNDP1, CTNNA2, CYP3A7, ENTPD1, GRIN2C, GSTA4, KAT2B, LPAR1, NEU4, OR4B1, OR52N2, OR8G1, PIAS4, PLEKHA8, POLR1B, POLR2F, PPP3CB, PRKACA, PRKAR2B, SEMA3D, SOCS3, TCF7, SERPINF2
Negative_1349	ARPC3, GALNTL1, INPP5B, OR2B11, PLA2G2D, PLCD3, ST3GAL2, YWHAG
Negative_1350	DUSP9, KIR2DL3, MGST3, OR8G1
Negative_1351	CGA, FER, ID2, ADCY10, ALG10, CLCA4, CXCL1, DNMT1, EGFR, HAO1, HLA-DOA, IGSF5, MCM4, MLYCD, NCOR1, OR1I1, OR5K1, PCCA, PEMT, PYGM, RAB5A, SULT2B1, ZBTB16
Negative_1352	CACNA1G, DTX3, EIF4E, ENO3, MYH10, OR5H6, POLR2F, SARDH, SMPD2, TYROBP, MAPK8IP2
Negative_1353	AKR1C3, BET1L, C3AR1, CCR4, CD80, CDKN2C, DEGS2, GALNT12, GCNT1, GDF5, GDF6, HTR5A, LIFR, MYL7, OR10H4, OR51B6, P2RX1, PANK3, PDGFC, PGK2, PYCR1, SDC1, TYK2, USE1
Negative_1354	ADRB3, FARP2, GOSR2, IL20RA, MMP2, MTMR2, POLR1D, PTTG1, UNC5C
Negative_1355	MOS, PGF, PML, AKT3, ATG7, BDNF, BIRC3, BUB1B, CACNB3, COASY, FZD6, GRIA2, GSTO1, INSR, OR13C5, OR8H3, P4HA3, PCYT1B, RASGRP1, RBL2, SHC3, SLC9A1, TICAM2, TLR3, WBSCR22
Negative_1356	FH, SP1, ARHGEF2, CCR2, CLTB, DUSP5, GALNT6, GLS2, HADHA, IL8RB, IL9R, MDH1, NRP1, OR2A14, OR2T11, OR2T29, OR51A2, OR51A4, OR51B5, PGAM1, PKLR, PLXNB3, PRKCZ, RARA, RUNX1, SEMA3G, ST3GAL3, STAT6, STX8, TBL1X, TBL1XR1, TCIRG1, TGFBR2, UPP1
Negative_1357	SI, TNN, EP300, EPB41L2, GCLC, OR1S2, OR7C1, PANK2, PAX4, PVRL3, SRGAP3, TLR9, UNC5B
Negative_1358	INSR, OR14J1, PFN3, ULK3

Negative_1359	LEP, AKR1D1, ALG5, ALPI, ARHGEF7, ASNS, CCR3, CTSB, EIF4B, ESPL1, GCNT2, GNPDA1, HMGCR, LAMC3, LHCGR, MICA, NOTCH2, OR2L2, OR2T11, OR51S1, OR52B4, PLA2G4A, POLR3D, PRKAB2, RPS6KA1, SAT1, STAM2, STX16, TGFB2, TUBA3C
Negative_1360	PLN, TNF, ACADVL, ALG13, ALOX12B, AOC3, ATP12A, BMP8B, CCNB2, CLDN1, CNTN2, CRLF2, CSDA, G6PC, GLO1, GNMT, HADHB, HPSE2, IPPK, KLRC2, LEFTY1, LIMK2, OR2M5, OR4F6, OR52E8, OR5D16, OR8K3, PEMT, POLR2A, PPARG, PRIM1, PSPH, RPS6KA6, SLC12A2, SLC25A5, SSX2IP, TAS2R9, TICAM2, UGT2B4, YWHAZ, ATP6V1C2, ATP6V1G2, MAPKAPK5, PHOSPHO1
Negative_1361	IVD, PRL, TPR, ACADS, ADCY9, ALG3, ANAPC1, ASS1, ATP5B, ATP6V0C, CCL26, CD19, COL1A2, DDX19A, EDA2R, EFNB2, F13A1, FUT2, GCH1, GNA15, IGF1R, MPP5, NMNAT3, OR4C13, OR4D11, P4HA1, PARD6G, PCYT1A, PGM3, PPP3CA, PTDSS1, RPS6KB2, SPHK1, SUV39H1
Negative_1362	GAA, IL8, ADAM17, ADH1A, ALDH3A2, B3GNT2, BCKDHB, CACNB3, CASK, CDC25A, CDC7, CDKN1A, CDKN2C, CLDN22, CTNNB1, EPB41L2, FUT1, GATM, GNB1, GNG13, GRHPR, GRIN1, HTR4, HTR6, IDUA, IL19, MAP3K11, OR10A6, OR4C13, OR4L1, OR4S2, OR51E1, OR56A4, OR5H2, OR6Y1, PAK6, PIAS4, PIGC, PIK3C3, PRIM1, PRKY, SPRY3, YWHAZ, CACNA2D3, PRICKLE2
Negative_1363	TK2, ACMSD, AKR1C4, ALDH1A3, ATF2, ATP4A, BBOX1, BET1, C9orf95, CACNG8, CNGA4, CYP11A1, DDIT3, DLAT, DNMT3A, DTX2, ENTPD4, GNA12, GNPAT, GUCY1B3, HCCS, HEXA, HHIP, ICAM1, INHBC, IRF3, MARS2, MFNG, NCKAP1L, NRG4, OR13G1, OR4D9, OR52N1, OR5A2, OSMR, PIK3C2A, PMM2, PNPLA3, RAB5A, ROBO3, SSH1, STAT3, SUOX, SV2B, TSHB, UCK2, TNFRSF1B
Negative_1364	ACSM3, ADH7, ALDH6A1, CX3CR1, DGAT1, DGKG, DLL1, DPM3, EPB41L2, FZD9, GALNT3, GGT6, HSD17B3, IFNA21, ITPA, LDHA, LIAS, LRP6, MASP2, OR1A2, OR2T2, OR52E8, OR6F1, PGAP1, PGM2, PGS1, PHKB, PRKY, SHC4, UGT2B17, WNT7B, XYLB
Negative_1365	APP, ASL, GLS, INS, TNF, ACTG1, ATP5I, BMP5, BUB1, CD55, CLOCK, DHRS4L2, EPHB4, GAB1, GALNT6, MAGI3, MCEE, MS4A2, NODAL, OR2M4, OR4B1, OR5R1, OR9Q2, PDK1, PON3, RAPGEF4, SETMAR, C1GALT1C1, TNFRSF10D
Negative_1366	C2, IDS, ACACA, ANAPC2, APLP1, BIRC3, C7orf16, CASP9, CD58, CDC7, CDK4, CFL2, CSDA, CXCR4, CYP2A6, DBF4, FGF8, GLI2, GNG12, GRLF1, HSD17B4, IFNA13, ITGA8, KLK3, MGST1, NCKAP1L, NCR2, NKD2, OR1L3, OR2A2, OR51F2, OR51G2, OR8D2, PCYT2, PIGQ, PLXNB2, POLR3B, PSPH, PTPRR, STX5, STX7, TACR1, TBXA2R, UGP2
Negative_1367	AK5, C1S, ITK, ACSM1, EGFR, GLI2, LMO7, NEU1, PDE6H, PIAS3, TRDMT1, VAMP4, ZFYVE9
Negative_1368	FER, CDS2, GARS, OR2B11
Negative_1369	DBT, ACACB, BBC3, FGF20, FGFR3, FPGS, FTMT, FUT9, GALNT3, GSTM4, NEFH, NFKB2, PDGFRA, PPP2CA, PTTG1, TBL1X, AASDHPPT, HSD17B10
Negative_1370	CSK, AMDHD2, ATG7, B3GAT2, BPGM, C4BPA, CAV1, CCR10, CD40, CHDH, CSF1, DUSP4, ENAH, EPHX1, F2RL1, FARSA, FGF1, FGF13, FUT9, GALNT3, GAPDH, GMPR2, HLA-G, HSD11B2, IDH3B, IKBKB, KCNJ11, NFX1, OCLN, OGDHL, OR2A4, OR2T5, OR4D1, OR51G1, OR5L1, P2RX4, PHPT1, PNPLA3, POLE4, PSAT1, PSME1, RBPJL, SMAD3, SPRY3, TOMM40L, TUBB4Q
Negative_1371	MPZ, NLK, UBB, ACSM5, C3AR1, DHRS4, GCNT3, GRHPR, GUCY1A2, IL17RA, ITGAE, LALBA, MAP2K4, MMP9, MYH7, NAE1, NAT6, OR4K5, OR7C1, OR8H3, PDE3A, PIM2, PLCD1, PRKAB1, RENBP, SDHD, SETD1A, SMAD1, SRD5A2, SSH1, THBS4, TRAF3, UGCGL2, UGT2B7, VNN1, ATP6V0D2, HLA-DQA2

Negative_1372	CS,GCK,ADH1B,ARSE,B4GALT5,BDKRB1,CADM1,COQ5,CREB3L1,CREB3L3,DFFA,E2F4,FUT2,FZD5,GNAZ,IL17B,MICA,NSD1,OR11A1,OR14J1,OR2M5,OR6C74,OR6K3,PDCD1,PGM3,PHPT1,PLA2G2F,POLR3G,PRKAR2A,PTEN,RFNG,RRAS,SH3BP2,SYNJ2,TAS2R4,TKTL2,UBE2L6,ATP6V1C2,MAP3K7IP1
Negative_1373	DDO,NOG,ACACA,AGPAT3,CD72,CREB3L2,CTNNA2,CXCL2,CYP4F3,DDX18,DOCK1,GALNT7,HSD17B1,MASP1,NCOR1,OR4D6,OR4K13,OR5T3,PFAS,PMM2,PRIM2,RASSF1,STAM,SUFU,TUBB3,WNT9B
Negative_1374	BTD,C4B,LAT,PTS,ADH4,ALG5,CNGA4,CREB1,CX3CL1,EGFR,EPHA6,F2RL3,GAD1,GLDC,GSTA3,HLA-DMA,HSPB1,IL5RA,IMPDH2,INHBC,IQGAP1,JAG2,LCMT2,PAK4,PDE6H,PECAM1,PFN3,PIAS1,POLE4,RASGRP1,RASGRP3,SMPD4,SPHK2,SSH3,SUV39H1,TAS2R1,TDO2,UGT2B17,VKORC1
Negative_1375	FGB,KIT,CHD8,CHKB,CHSY3,FGF11,GLG1,GSTM2,IL22,KIR2DS5,MAP2K5,MPST,NRCAM,PTCRA,RENBP
Negative_1376	CAT,MIF,MPI,A4GALT,AMPD1,B3GAT2,B4GALT5,BBC3,CAMKK1,CARS2,CXCL2,DCTN1,DTX3,ENO2,FASN,FOSL1,GGT5,GJA1,IFNA14,ITGA2,LEFTY2,LIPA,LLGL1,LMO7,MLYCD,MYH4,OR10AG1,OR10X1,OR1K1,OR4X2,OR52I2,PCYT1A,PDK1,PRKACA,RFWD2,RPSA,SV2C,UGT2B7,UPP2,VAV2,XCL1,TNFRSF10B
Negative_1377	C1S,CDH4,CLDN14,COL3A1,CSNK1E,DKK4,EVI1,FGF14,GALK2,GDF6,HCLS1,IFIH1,KIR2DS5,KIR3DL3,MAPK1,MGAT4A,OR13G1,OR2G2,OR4C11,OR4S1,OR51A2,OR5D18,OR5J2,OR9G1,PIM1,QARS,RBX1,SDHC,SMAD7,SULT1A2,TUBB2A,TUBB2B,SERPINB5
Negative_1378	F12,FAH,GGH,GLS,LHB,MPL,NLK,ACACA,ALDH2,AMY2B,ARPC1A,ASS1,ATP5J,COL1A1,COL2A1,DEGS2,DGAT2L4,DHFR,DNMT3A,FGF8,GANC,HBEGF,HLA-C,HMOX2,HSP90B1,IFT57,INSR,ITGA11,ITGB4,LCMT1,MAN1A1,MAP3K6,MLLT4,MYH6,NCOA4,NFKB2,OR8B4,OR8D2,OR9Q2,PCNA,PTK2B,PTPMT1,RARS2,SOCS3
Negative_1379	MPI,ACOT12,ALDH6A1,AMDHD2,CXCL6,GYS2,IFNA13,JAM3,MGAT4A,NOS3,NRXN1,ODC1,OR2M2,OR5F1,OR7C1,PER2,PFAS,PISD,PLOD3,PPP3R2,RALBP1,SC5DL,TBL1X,TIAM2,TRAF3,UNC5D,MAP3K7IP1
Negative_1380	COQ5,DKK4,EPHB6,F11R,FLT1,FTMT,FUT5,FZD1,GALNT13,GPAM,HIBCH,MRAS,NAGLU,NLGN3,NTF3,OR5AS1,OR6C76,PAK7,RPS6KA4,SETD1B,ST3GAL6,TAS2R49,TCIRG1,TFDP1,TNFRSF8,UCKL1,ATP6V1G2,PNLIPRP1
Negative_1381	AR,SRC,ACAA2,ALDH1A3,BDH2,CDKN1B,GGT7,LYPLA1,MAGI1,OR11G2,OR13C3,OR6C3,P2RX4,PLA2G2A,SLC27A5
Negative_1382	F7,MVD,ACPT,ALOX15,BACE1,BET1L,CPS1,CYP11B1,DFFA,EPHA2,EXTL1,F2RL3,FGF1,FMO1,GAD1,GALNT6,GCNT2,GDF7,GGCX,HSD11B1,IL18R1,KAT2B,KIR2DL2,NCF4,NCR3,NEU3,NUDT5,OCRL,OR4X2,OR52E2,OR5K1,OR6M1,PFKFB3,PGAM2,PIAS4,RAP1B,SEPHS1,THBS1,TPI1,TSC2,UBA1,SERPINA1,TNFRSF13B,TNFRSF13C,TNFRSF1A
Negative_1383	ACP6,EPB41L1,EXTL3,LRP6,PAK2,SETD7,ST8SIA1,UGT2B4,PPP1R12A,TNFRSF10B
Negative_1384	CSNK1G3,CTBP1,NAT2,NME2,ROBO2,TIRAP
Negative_1385	CRH,FES,LYN,MAX,AADAC,ALDH1A1,ARNT2,B3GALT1,BMP2,CD55,CMPK1,CSF3,CYP4F2,EPB41L3,FGFR4,FUT5,IFNA16,INADL,JAK3,LDHA,MAN1C1,MGAT4A,NMNAT3,OR1B1,OR1L3,OR2AG1,OR51G2,OR51S1,OR52L1,P2RX5,PDHB,PIGM,PLCB2,PLCD4,POLD2,PPP1R3C,PTGIS,PVRL4,RASGRP4,SDC3,SETMAR,SMC1B,SPHK1,ST3GAL3,TJP1,TRIP10,UGT2A3
Negative_1386	CKM,DCT,FN1,HK2,NGF,PLN,TKT,COQ5,CYP1A1,DHRS3,HNF1B,IFNA4,IFNK,IL8RB,LCP2,MAPK8,NFATC1,OR13C3,PANK3,PIGT,PSAT1,SELE,STEAP3,TNFSF11,USE1

Negative_1387	ALAS2,ANAPC2,ATP2A1,CDC25C,ENPP2,ETS1,GIT1,GPX5,NCKAP1L,NFYC,OXCT2,RARS
Negative_1388	HK3,PVR,ARPC3,CLDN11,CLDN3,CYP1B1,ENTPD5,NODAL,OR4P4,OR52K2,P2RX6,SLC2A1,STMN1,TAS2R10,TGFBR1,TRPM5
Negative_1389	AK7,RB1,CACNA1F,CLDN3,KCNJ11,MITF,NARS2,NOS1,OR4B1,OR8J1,PGAM2,PKM2,PRKAR1B,SHC1
Negative_1390	F5,IL3,OTC,ACSL6,ADH4,ALG11,BLNK,BMP4,BNIP1,COL4A2,CREB3L3,CTF1,CXCL13,CYP11B1,CYP3A4,DHCR24,EFNB1,EFNB2,EIF4E2,ENPP2,FGF10,FZD7,GFAP,GPX5,GUCA1C,HARS,MLLT4,NEFL,OR10T2,OR2A12,OR2H1,OR51V1,PPP1R3A,PPT1,RAPGEF2,SIPA1,SV2C,TSTA3,WIF1,WNT8B,CSGALNACT2,TNFRSF14,TNFRSF1A
Negative_1391	AKR1B10,DGAT2,DPM1,GNA14,OLAH,OR5D13,PAX6,PITX2,SESN2,TBL1Y
Negative_1392	PAH,ACVR1,ALDH3B1,BBOX1,BIRC3,C1QB,CAMK4,CCL1,CCNA2,CLDN1,CLDN16,CLDN5,COX15,CYP2C18,DRD1,EP300,FLT3LG,GADD45B,GCLC,GUK1,IFNGR1,IL9R,IRF3,LAMB3,MKNK2,OCLN,OR1C1,OR5P2,OR5T1,PDK1,PIK3CG,PKLR,PLXNA1,PLXNB3,PPP1R1A,PRNP,RAC2,RDH12,SEMA3B,SHISA5,SMC1B,SMURF1,ST3GAL4,TGFBR2,THBS2,UGCGL1,UGT2B4,WNT10B,TNFRSF14
Negative_1393	F11,ANAPC1,CCL2,CLCF1,CTF1,FZD3,IFNA6,IL28RA,MAML2,OR9G1,PPP2R2B,SDHA,SEMA4A,SNAI1,THBS2
Negative_1394	ALOX5,CARS,CDC2,CLDN22,GSTM1,NAT1,NFYC,NOX3,OR8K5,PARS2,PIGB,PIK3R1,ATP6V1G3,ST6GALNAC5,SUV420H2
Negative_1395	NPY,ADORA2B,ADRB3,AMPD1,CCL2,CLTB,CNTN2,COL1A1,DLAT,EFNB2,FGF17,GNRH2,HMBS,HSD17B8,HSD3B7,IL1R1,IL6ST,INHBB,MECR,NUMB,OR10Q1,OR11H1,OR6C6,OR6C76,OR6S1,OR7G1,PIP5K1C,PLA2G10,PPP2R5D,PRKAA2,PRKAB2,QARS,RDH10,SFRP5,SMC1A,TUBA8,TNFRSF11B
Negative_1396	CLCF1,CYP26A1,FASN,OR8A1,OR9I1,P2RX2,PCCA,UGT2A1
Negative_1397	ABLIM3,ATP2A2,F13B,GRAP2,HYAL1,IL11,IL17B,NSD1,OR4F4,OR51T1,OR8B4,PFKFB2,PTCRA,PTK2B,THBS1,TPH1
Negative_1398	HK2,UBC,ACPT,ERBB4,MAPT,MGAT2,NFKBIA,NLGN2,OR5AS1,THBD
Negative_1399	CKB,ACSM5,ACTB,ACVR2B,ANAPC10,CACNB1,GNPDA2,IAPP,IL10,IRS1,OR9Q1,PDE6D,PDE9A,PHKG2,RAF1,UBE2L3
Negative_1400	C8G,KMO,MTR,ALDH3B2,ALS2,APOC3,CLDN7,CNGA3,DIAPH3,DPM2,DUSP14,EPHA8,FGF6,FLT4,GALK1,GANAB,GPD1L,HNMT,ICAM3,IFNA5,IL6ST,IRAK1,KLRC1,LCP2,LIAS,MNX1,MTAP,NSD1,NTN1,OR10A7,OR11H4,OR2F1,OR5AP2,OR5J2,PDE4B,PGM2,POLA1,PRKACB,PRPH,RAP1B,SC4MOL,SC5DL,SRD5A1,UNC5C,WASF1,SUV420H1
Negative_1401	F3,FGB,GK2,ACOT11,ATP5C1,CASP6,CAV2,CBLC,CCNE1,CCR2,CD27,CHSY1,DTX2,EPHA1,FLT1,FZD9,GGCX,GRAP2,HCST,HTR7,IDH3G,IL10RA,LEFTY2,MCM6,NTNG1,OR10J3,OR1Q1,OR2T12,OR4F5,OR8B8,PAX8,PFKL,PON3,PRKAB1,PRKCD,RCHY1,SCIN,SPRED1,SPTAN1,STX16,TAS2R42,TAS2R50,WWP1,YOD1,HLA-DPA1
Negative_1402	GBE1,HNF1B,SGPP1,SNAP23
Negative_1403	AK3,BTK,ACACB,DDB2,GALK1,GNAI2,GNB1,GUCY2F,LLGL2,MYH1,NT5C,OR4N2,TGFB3,SUV420H1
Negative_1404	AK5,ACSL1,ACTC1,ANAPC5,ATP5L,CCKBR,COL4A4,F11R,FGF1,GLUL,GUCY2C,IARS2,IDH3A,IFNAR1,IGF1R,IRAK2,MAPK11,MCEE,MMP9,MYH1,NKD1,NMNAT3,OR2M5,OR4M1,OR9A4,ORC2L,PAK4,PARD6G,PAX8,PLXNA1,PNPLA3,PORCN,PPAP2C,PRKG2,PROC,RAP1A,SELE,TSHB,HLA-DRB5
Negative_1405	AGK,OR5B21,OR8B12,SARS,TFPI,UCK2

Negative_1406	CD2,NOG,TFG,ACY3,AKT3,AMPD2,ANAPC1,ATG12,ATP5A1,ATP5J2,BIRC5,CAMK2G,CCL16,CHRM5,CYP3A4,DDX52,DEGS2,DLAT,DVL3,ENO1,EPHB2,FUT4,GNAT3,ICAM3,IL13RA2,MASP1,MAT1A,OR4Q3,ORC6L,P2RX1,PLA2G3,PMM2,PTPN7,SNAP23,SOS2,SPP1,TAS1R1,TAS2R49,TGFBR2,TRAF6,TNFRSF10B
Negative_1407	ADO,FER,AMY2B,ARNTL,ARPC2,CCL14,CCL24,CDO1,CFL2,CLOCK,CTPS,CYP3A7,EFNB3,EIF4E2,ENTPD4,FGF22,GALNT5,GPD1L,GSTK1,IL13RA1,LEF1,NGEF,NR5A2,NTRK1,OR2AG1,OR4K1,OR51E2,OR9K2,PAK2,POLR3H,PON3,PPID,PPP2R2C,PSME1,PTGES,PTPRC,RND1,TACR3,TBXA2R,THBS1,TLR5,TMLHE,TSC1,TUBA1B,WNT5A,HLA-DRB4
Negative_1408	FOS,MET,AGPAT6,AKR1C1,ALG1,CANT1,CCNH,CLDN6,DGKQ,DOLPP1,FECH,FGFR3,GALM,GALNT10,GNA13,GNRHR,HLA-DRA,HSPB1,IGF1,IL5RA,IL7R,ITCH,ITGA11,MGAT5B,MYH3,NFAT5,OR2L13,OR51T1,OR5K1,OR8H2,PDIA4,PDK1,PEMT,PFN1,PFN4,PIAS2,PLK1,PTPN6,SCD5,SKP1,SOX17,SRD5A2,STAT5A,TNFSF11,UNC5B,HLA-DQA2
Negative_1409	C1S,SRC,ADRB3,AMHR2,ATP4A,BMP4,CSNK2A2,CYP2D6,DFFB,DGKA,DUSP6,DUSP9,ECHS1,EI24,FCGR2B,FGF23,FGF8,FOSL1,GGT1,GLI2,GUSB,IFNA17,LAMC1,LFNG,MGST3,NKX2-2,OR14A16,OR1L8,PDE4C,PIGM,PIK3C3,PIP5K3,PTPN6,RFX5,SELE,ST3GAL3,TKTL2,WEE1,XYLT1,ATP6V1G3,PPARGC1A
Negative_1410	AUH,IL5,NF1,FLT4,GLS2,NLGN2,NRG1,OR10R2,PLOD3,SKP1,TJP1,TUBB4Q,VAMP3,ZFYVE9
Negative_1411	ALG9,APBB1,BIRC2,CLDN6,CYBB,CYP2S1,DIAPH1,DUSP3,E2F2,GFAP,GNA14,HSPA1B,IL1RAP,ITGAX,LDHD,MTFMT,OR4M1,OR52L1,PIK3R1,POLA2,STAT6,TPI1,TYRP1,ZFYVE9,TNFRSF17
Negative_1412	EBP,SRM,ABI2,BDNF,C3orf10,CLDN18,COL1A2,ENTPD4,HDAC2,IRF5,NUDT2,OR4M1,PIAS1,PTCH2,ROCK2,SEPHS1,XCR1
Negative_1413	C1S,BMP4,BST1,FGF17,NANS,NCAM1,OR52N4,SV2A,TGFBR1,WNT10B,TNFRSF10D
Negative_1414	PDC,SRM,ADCY10,ALG10,ALG12,ARPC2,CAPN2,CASP6,CDH4,CHIT1,CLDN9,CTBP2,FGF3,GNG13,GYS1,INHBE,MAOA,MAP2K3,NUMBL,OR1C1,OR2A42,ORC1L,PER2,PLXNC1,PTPMT1,TYMS,VAV3,VTI1B,ZFYVE16
Negative_1415	AK3,GLA,GSS,LAT,PLN,ALG6,ATP5G2,ATP6V0C,C1GALT1,CDC25A,CFTR,DDX52,DEGS2,E2F1,EARS2,ENO2,ENPP2,ENTPD2,FGD1,FOXO1,GNB1,GOT1,HADHA,HLA-DRA,IRF7,JAK3,NEFH,OR4A5,OR51E1,OR5A1,OR5T2,OR5T3,ORC1L,PANK1,PANK3,PCYT1A,PDE1A,PMM2,POLR1C,POLR3B,RAD51,RASGRP1,RFX5,RPS6KA2,RXRG,STAM,TCF7L2,TLN2,WEE1
Negative_1416	F3,ACP5,BCMO1,BMP8A,CACNB1,CDK7,DDX55,EVI1,FGF23,GNG12,HIF1A,KAT2B,MAN1A2,MTHFD2,NME2,OR5AR1,PARD6A,RALB,RRM1,SETD2,SPRY4,TAS2R8,UBE2L3,UROD,ZFYVE9
Negative_1417	A2M,MPZ,ATIC,CHRNA7,GLO1,GMDS,HSD17B4,IFNA17,IL1A,INADL,JUND,NEUROD1,OR1D2,OR2AT4,OR4M2,PLD2,PPP2R1A,PSME2,ULBP2,ALDH18A1
Negative_1418	IL3,OSM,ACAT1,CASP10,CCL25,CTSL1,CYP2C19,DPYD,DVL3,EPHB3,FGFR4,GCNT4,GNAT3,GNG3,IFNA14,IFNA7,ITPKA,KCNJ11,MYLK3,NAT5,NMNAT2,OR1E2,OR4D2,OR52E8,PKM2,RALB,RASSF1,SH2D2A,SNW1,TAS2R4,XCL1
Negative_1419	GSTA1,NCF4,OR5L2,OR7A17,STX10
Negative_1420	LCK,ACTA2,BCL2,BLVRB,C1GALT1,CDK2,CDS2,CKMT2,CYP17A1,DGKZ,DHRS4L2,ECSIT,FCGR3B,FMO4,GNA12,GPT2,GRIN2D,HGSNAT,IKBKE,INHBE,LCMT2,MYH13,NME7,OR10C1,OR52E6,OR6C4,ORC6L,PER1,POLR2A,PRKAG2,RARS2,SLC2A1,SMAD3,SRGAP1,STAM2,SUCLG2,TLN1,UMPS,WNT9B,SERPINF2

Negative_1421	CAT, SMS, TXK, ACAA1, ALOX12, ARPC3, CD74, CDC2, CHRM2, CLDN2, CYP11A1, CYP1B1, DNMT3L, DPM1, DVL3, FGF8, FPGT, GRIN2C, GRM1, HTR2B, IL28RA, MAP3K2, NAT1, NCF4, OR51F2, OR9K2, PAPSS1, PDE8A, PDGFRA, POMC, PPP2R1B, RELA, SEMA4G, TBXAS1, TGFBR2, TNFSF8, VANGL2, VAV2, WHSC1
Negative_1422	ID4, TK2, AK3L1, ARNTL, DCXR, DDX47, GCLC, HPSE, IGF1R, IL2RG, OR6M1, PI4KB, PIK3C2B, RND1, SCP2, TUBB2C, UPP2
Negative_1423	AADAC, ADRBK2, AKR1C1, ALOX12B, CNGB1, CRB3, DFFA, OR13C4, SDHA
Negative_1424	F10, GSS, ID4, ASCC3, B3GALT2, CALML6, CES7, CHRM5, CXCL6, DVL2, EPOR, FGF8, FUT2, GALNT11, GSTA4, GSTA5, IFNA7, IL28B, JAM2, LEPR, LTBP1, METTL2B, NME6, NPAS2, NPR1, NRP1, NRXN1, OR10V1, OR2M4, OR2Z1, OR4K2, OR5F1, PAK6, PDE8A, PFKP, PRKAR1A, PTCRA, RARS2, RASGRF2, SIPA1, THBS2, VDAC3, B4GALNT1, HSD17B10
Negative_1425	NNT, AGXT2, AVPR1B, CDK7, CHEK2, CREB3L4, CTNNA3, FMO5, GADD45A, KIR3DL2, MARS2, MYH6, OR1N2, OR2M3, OR4F6, OR6F1, OR8K3, POLD4, PSME1, RUNX1, SPTAN1, STMN1, TAP2, TAS2R49, TNFSF9, TPH2, UGDH
Negative_1426	HDC, TH, ACAA1, ACTB, AGXT2, ALDH4A1, ASPA, EGLN3, ENAH, EPHA5, EPHX2, FLT1, GNAS, GSTM5, HLA-C, MAP4K4, MAT1A, OR1L3, OR52B6, PFN1, PGM2, POLR2K, PRKAG1, RALBP1, SIAH1, TBK1, THBS3, TNFSF10, TRMT11, VAMP4, WASF2
Negative_1427	TH, ACAT1, ACO2, ARPC4, ARRB2, AVPR1B, C3AR1, CCNA2, CTLA4, EGLN1, ENPP3, FARSA, FBXW11, FLT3, GALT, ITGA2, NEUROD1, NME4, NMNAT1, OR10G9, OR4C46, OR51B5, OR8H1, PLA1A, POLD2, POLR1B, PVRL1, SIGLEC1, ST6GAL1, STEAP3, STX2, TRPM5, TUBA3E, TYMS, WWP2
Negative_1428	ALG6, CD274, HPSE, ITGA6, LILRB3, NFKBIE, PDE8A, TNFSF12
Negative_1429	MAG, ADRA1D, ADSL, ARPC3, CCL14, COL5A2, CSNK2A2, ETNK1, ITCH, ITGB6, JUND, MMP14, NRG1, OR14C36, PGM2, RAC1, SETD2, TAP1, UBE2L3, TNFRSF1A
Negative_1430	ALDH4A1, ASCC3, CYP11B2, DDX23, EPHB3, GNRHR, GRIN2D, HSPA1B, MPST, NODAL, OR11H6, OR1L8, OR5K2, OR6C6, OR9A2, PIAS2, PRDX6, PRPH2, RBX1, SGPP1, SHC1, STMN1, TAS2R1, TBK1, TNFSF18, VEGFC, WWP1, ATP6V0A1
Negative_1431	IL3, MIF, OGT, ACTN2, ARHGEF6, ATP2A1, CACNA1E, CAPN2, CDKN2A, CPS1, CSNK1G1, CYP2C9, DPYD, DTX1, DUSP4, ETS1, FGFR4, GUCY2C, IDH1, IL12A, IL15RA, LRDD, MAP2K2, MAP3K12, MAPK11, MYL9, NCR1, NEU2, NR1D1, OR14C36, OR2A14, OR52N5, PDE8B, PIAS1, PIGP, PLCZ1, POLD1, PRKX, PRSS2, RDH11, RDH16, RENBP, SCD5, SMC1B, SOD1, STAM, THTPA, UGT2A3, MAPKAPK3
Negative_1432	ADA, AR, BID, CGA, PAH, SDS, STS, AADAC, ADH1A, AKR1B10, APOA2, ATP5G1, BMP2, CCNG2, CCR4, CDS2, CREB3, ENO1, EPHB2, FBP2, FLOT1, HSD17B8, IL15RA, LRP5, MCCC1, NKD1, OR2M3, OR4F4, OR6K3, PCYT1A, PMM2, RASSF1, SEMA3B, SHC3, SLC2A4, TAS2R60, TM7SF2, UCP1, VANGL1, VAV1, SUV420H2
Negative_1433	INSR, OR4K14, POLR2F, SHC4, STAT4
Negative_1434	AASDH, DBF4, OR5L2, PDE6H, PFN3, PIK3R2, TAS1R2, TNFSF14
Negative_1435	BID, TPO, CCL25, CFTR, DGAT2, DHRS4, F2RL3, GLG1, GNAZ, HNF4A, IRS4, MAP4K3, MYL5, NRG1, OR1M1, OR51I2, ORC4L, PIGM, POLR3K, PRLR, PTGES, PTPRR, SLC2A2, WNT3
Negative_1436	ALPL, ANAPC5, APOA5, CCND2, DAAM2, FRAP1, HTR2C, MITF, OR51I1, PFKFB1, PIK3C3, PLA2G2D, PPAT, PPP2CB, PRIM2, RFXANK, THY1, TYK2, WNT3, PRICKLE1, TNFRSF13B
Negative_1437	DCC, CAMKK1, CCR1, CXCR3, DLL4, IQGAP2, NR1D1, OR4X2, OR5AC2, OR6C76, OR8H2, PIGQ, POLR1D, POLR2H, PRKAB1, TMLHE, TNFSF12
Negative_1438	CS, GDA, LSS, ANAPC5, ARD1A, CD19, FUT6, HEMK1, LDHA, MCCC1, NRG4, NT5M, OR10H4, OR11A1, OR52D1, OR52E8, PIK3C2G, PLCB4, POLR2C, SEMA4C, SGPP1, SNAI1, SPRED1, TGFB2, YWHAG, YWHAZ, GABARAPL2

Negative_1439	CD2, CKM, SP1, ACPP, C7orf16, CLOCK, CRLS1, DHRS4, DTX4, DUSP10, EP300, FGF23, GPD1L, HMBS, IKBKE, IL24, IL2RB, IPMK, OGDHL, OR13H1, OR2L8, OR5D14, OR5D18, OR8J3, POLR3K, THY1, TRAF3, TUBA1A, VDAC2
Negative_1440	APP, OGT, ABLIM3, ACOT12, ACSS2, ADCY5, ALG11, ARD1A, ARG2, ATP4A, BPNT1, CACNA1B, CACNA1E, CAPN2, CCNG1, CD247, CD80, CHEK2, COQ5, CRLF2, CYP4A22, ENTPD3, FUT4, GALNT8, GLI1, GPD1, GRIA2, ITGA4, MGST1, NTF4, OR13D1, OR1I1, OR2M2, OR5AK2, PHGDH, PIGA, PPP3CA, PTPMT1, RPS6KA4, RUNX1, SELE, SOX17, TAS2R50, TGFB1, THBS2, TSHB, TSLP, TUBB6, UGT2B28
Negative_1441	MVD, CARS, CRLS1, GFPT2, KDSR, MYL5, NQO1, PARD6G, PDE8A, PLCB4, PRKACB, SH2D1B, SLC25A5, SMAD1
Negative_1442	ADK, CCDC6, CCL7, CLDN20, IQGAP3, KYNU, MFNG, OR1L4, OR5V1, SERPINA1
Negative_1443	F10, AARS, AKR1D1, BRAF, COL6A2, GALNT8, GRHPR, IL3RA, LDHA, PDE8A, PFKFB3, PFN2, SLIT1, TKTL2, TUBAL3, VEGFA, WNT10B
Negative_1444	CAD, ACACA, AGXT, AKR1C3, ALG9, CCL21, CCL26, CCNB1, CDKN2D, CYFIP2, CYP3A5, DDX4, DNMT3L, FLOT2, FMO4, GNAQ, HHIP, IAPP, IL18R1, KLKB1, KLRD1, NFKBIE, OR13F1, OR1S1, OR4F15, OR5B21, PAK4, POLR2J, POLR3K, RELA, SARDH, SLC25A6, SOS1, TOMM40, ULBP1, XYLT1, ATP6V1B2, CSNK1A1L, PPP1R12A, SLC25A31, SUV420H1
Negative_1445	AK2, ATM, MET, CREB1, CREB3L3, DEGS1, FOSL1, GSTT1, MCCC1, NCK2, OR4D9, STAT5A, HSD17B10
Negative_1446	GLA, VCL, ACADS, ACVR1B, ALDH1L1, AOC3, ATP2A1, CACNA1G, CADM1, CHRM5, CTSG, CXCR3, CYP26C1, DUSP8, F2RL2, GSTO1, GUCA1A, HNF4A, LAP3, MAPK3, MAPK9, MMP7, OR10A3, OR10AG1, OR10H4, OR11H6, OR51B5, OR5AS1, PANK4, PIGT, POLR2I, PTPRZ1, QPRT, SRGAP3, STX10, UNC5A, WBSCR22, AASDHPPT
Negative_1447	STS, AADAT, BDH1, DBF4, HRH1, MCCC1, NME4, NRXN3, OR2Z1, PHGDH, PIGS, PTPRR, SMAD6, SRGAP1, CSGALNACT2
Negative_1448	CD2, CD6, FYN, ADSS, ALCAM, BMP7, CADM3, CDH5, CHRD, CRLF2, EXTL3, FLOT1, GALNS, GPLD1, GUCY1A3, HAGHL, IL15, NOD1, OR10G3, OR56A1, PDIA4, PIP4K2A, PLA2G10, POLA1, SMARCA5, TICAM2, VEGFC
Negative_1449	CAD, ALDH4A1, AMY2A, B3GNT5, CAMKK2, CCNB1, CCND1, COASY, DCXR, DDB2, IGF1, NCF1, NFATC1, NLGN2, NRCAM, OR2B2, PTPRB, SUOX, TACR3, TAS2R38, THBS1, UMPS
Negative_1450	DDO, F9, ID3, GALNTL5, GRIA1, IFNA2, OR2T4, PRKAG2, HLA-DRB5
Negative_1451	FN1, FYN, IL5, SMO, ADAM17, CYP1B1, CYP2F1, DIAPH1, GGT5, IGFBP3, IL1RAP, IL28B, MCM6, OR1J2, OR5T2, POLR1E, SLC2A4, TIAM1
Negative_1452	ABI2, ALDH3A1, ASS1, ATP5I, CACNA1F, CDC23, CDH15, COMP, DTX4, FGF21, FGF4, GFPT2, GSTZ1, IKBKB, IL20RB, POLR2H, PPP5C, SDC1, SERPINF2
Negative_1453	CCL22, GUSB, ITGA11, KIR2DL3, OR13D1, POLD3, RCHY1, ROBO1
Negative_1454	GBA, GLS, ACAA1, ACSS3, ARHGEF4, ATP12A, ATP5G2, BCAR1, C4BPA, C5AR1, CBLB, CDK4, CLCA4, CLDN11, CLDN18, CSF3R, CXCR6, EHMT1, FGF4, HLA-G, HMGCS2, HTR7, IL12A, KLRC3, MAP4K3, NFATC2, OR13G1, OR6K3, PAK4, PDE4D, PDE6D, PDPK1, PIP5K3, POLD1, PRKAB2, SGPP1, SMAD5, SPCS3, TGDS, VAV3, WASF3, ATP6V0D1
Negative_1455	PLXNA2, QPRT, TAS2R10, UROS
Negative_1456	F2R, ADH6, ADSL, AGPAT3, APLP1, ATF4, BTRC, CCL8, CCNE2, CD70, CYP21A2, EPHB3, FGF14, GGCX, IQGAP1, IRS2, MAOA, NEU1, OR10H3, OR2A12, OR5J2, PIK3R5, PIP4K2B, PPP1CB, PTGS2, RPSA, SDHD, STEAP3, TKTL1, TPH1, TPMT, UCK2, SERPINE1, ST6GALNAC3

Negative_1457	ASL, DCK, FUK, OAT, VCL, AADAC, ACOX2, ALOX12B, BMP2, CDC14B, CDC25B, COMT, CPS1, CRY2, DOT1L, DRD5, EFNA4, ENAH, GANAB, GJA1, GPLD1, GUCY2D, IL17B, LIMK2, MAP4K4, MEF2C, NCAM1, NEU3, NME7, NR1H3, OR13C3, OR4A5, PIPOX, PLAUR, PPAT, RAB5A, RDH5, RFXAP, RND1, SLC2A1, SORBS1, TCF7L2, TGFB2, TOMM40, UPP2, WBSCR22, WNT3A, WNT5B
Negative_1458	EGF, TNR, ACAA1, ACSM1, ALDOC, BCKDHA, CCL23, CCNA2, CD72, CD79A, CEBPA, CLDN14, CLDN15, CSH1, CYBB, EIF4E2, GCDH, GLI2, GLUD2, HMBS, HYAL1, IAPP, IGF1, IRAK3, ITGB8, MAP3K1, MDM2, NAT1, OR10A2, OR10C1, OR51I1, OR6C75, OR9Q2, PDCD1, PGK1, PIGK, PPP2R2B, PSEN1, SMAD5, UGCGL2, ATP6V1G1
Negative_1459	ASL, GPT, HPD, CCNA1, DCTN1, DDX4, DTX4, GALE, GALNT12, GNPDA2, HHIP, IL5RA, IRAK4, ITGA10, ITGA8, LLGL2, MAPK12, MARS2, NLGN3, OR11H6, OR1M1, OR2A12, OR2Z1, OR5A1, OR5J2, PAX4, PNMT, RRM1, SMAD9, TUBB6, UBE2L6, MAPKAPK5
Negative_1460	ADA, ALG6, ARHGEF1, FGF19, G6PC, NEFM
Negative_1461	IHH, TNN, ACHE, ADCY5, AMPD2, B3GAT2, CCL1, CD34, CTBP1, DAAM2, ETS1, EXOC7, FGF17, GALNT13, GJA1, HTR2B, MYL5, OR11A1, OR2M2, OR5D14, OR6C74, PANK3, PARD6A, PIK3CG, PLA2G4A, PMM1, PRKCB, PSPH, RBPJ, SPRED2, TPM3, TRAF2, UCK1, VAMP1, WHSC1L1
Negative_1462	DUT, RPE, ARG2, ARSD, CCR8, CD80, GALNTL1, GGPS1, GOSR2, GSTM2, GUCY1B3, HMOX2, HNF1A, ICAM1, IDH3B, INPP5A, INPPL1, MAP2K2, NKD2, OR2B6, OR5R1, OR8H1, OR9Q1, P2RX3, POLR3K, RPE65, RRAS, SCD5, SDC2, SPTLC1, ZFYVE16
Negative_1463	GP9, OSM, SP1, ACTN4, AMY2A, C3orf10, CCKAR, CDH1, CKS1B, DGKA, DLL3, ENTPD7, FGF7, GDF6, HGSNAT, IDH3G, IFNA21, IL18R1, INPP4A, ITGA2B, NAT2, NCAM1, NCOA4, NKX6-1, OR10G4, OR51V1, OR5T3, OR8B12, OR9Q1, PHKA1, PLXNA2, POLA1, PPP2R2A, PTPRZ1, SEMA7A, TNFSF18, TRDMT1, TUBB6, TYRP1, ATP6V0A2
Negative_1464	MAG, AANAT, ACVR1B, ADH7, AMY2A, FPGS, FZD8, HDAC1, IFNGR2, LHCGR, MYL7, OR13G1, OR1M1, OR4D5, OR52B6, PRKG2, PTGER3, RPN1, RPS6KB2, TALDO1, UROD, ZNRD1
Negative_1465	ACLY, ALS2, ARHGEF1, ATP4B, BAIAP2, CHST13, COL3A1, COL5A3, DUSP1, IGSF5, OR51G1, OR5T2, OR6K3, OR6K6, PHPT1, PPAT, SEMA3C, STX4, TCF7L1, VDAC3
Negative_1466	TH, ADIPOR1, CACNA1I, CCND2, CNTNAP1, CSNK1D, DFFA, MAPK8, NCF1, OR13D1, OR1D5, OR4E2, PCCA, PPA1, PRPH2, ST3GAL2, STAT5A
Negative_1467	ASAH1, COL4A2, EFNA1, ENO3, IL7R, PCNA, PIPOX, PPARGC1A
Negative_1468	FOS, INS, ABL1, CD48, CDC2, CSNK1G2, DPM2, ENPP2, GAS1, IRF5, NOX3, OR6F1, PLA2G5, PRDX6, PRPS2, SELE, TGFB2, WNT2, ZFYVE9, HSP90AB1, MAPKAPK2
Negative_1469	DCC, PC, AARS, ACAT1, ALAS1, ALOX15B, AXIN1, B4GALT6, C9orf95, CBLC, COL4A6, CSAD, CTPS, CXCL5, EHMT1, FGF12, GALM, GALNTL5, GLI2, GSTM3, HADH, IPPK, IRAK3, LAMC3, LDHD, LIPC, MCM6, MLYCD, MS4A2, MYLK2, NME2, NRCAM, PCK1, PDE6H, PDE8A, PIP5K1B, PPAP2C, PRSS1, RETSAT, SPRED2, ST3GAL4, TNFSF11, TP53, TUBA8, ULK2, VDAC3, VNN1, YWHAQ, ZMAT3
Negative_1470	LCK, NPL, AK3L1, ANAPC5, CHKB, CNTNAP2, ENO1, FGF22, GRAP2, IDH3G, IRF3, LIPT1, MAPK9, NOX1, OR1F1, OR1L3, OR52J3, OR6N1, OR8D1, PCCA, POLD1, PPP2CB, PPP2R1B, PRKAR2A, PRKG1, PVRL1, RALBP1, RENBP, SMC1A, SSX2IP, TNFRSF8, UCP1, ULK1, VANGL1, XIAP, HLA-DRB3
Negative_1471	CCL13, GRIN2C, HSD3B7, SAT1
Negative_1472	GAA, SYK, ACP5, AGRP, ALOX12, ALOX15B, ATP5I, BDKRB2, CCL11, CDK5, CLDN9, DKK1, EPAS1, EPHX2, FBP2, HMGCS1, HSD17B4, KIR3DL2, LDHA, LEFTY2, NFX1, OR1K1, OR2T29, OR51M1, ORC4L, PDE3A, PGLS, PGM1, PI4KB, PON3, PTGES2, QARS, RAP1A, RFNG, TRAF5, UCP1

Negative_1473	APP,C8A,CD4,ATP5O,CACNG1,CAMKK1,CCNB1,CCR1,CDKN1B,CHST11,CLCA4,COQ5,COX10,CPS1,CXCR5,CYP1B1,EHMT1,ENPP3,EPHA6,GLUD2,GRM5,HHEX,HLCS,IFNA5,IL7R,IRF9,MAP4K2,MCM2,MPZL1,NAE1,NR1H3,NSD1,OR10A6,OR13C3,OR13C5,OR1N2,OR51E1,OR5A2,PIK3R2,PVRL2,RRM2B,SLIT1,UGT2B4,WNT5B,SERPIND1
Negative_1474	GH2,GSN,MAG,ABL1,ACACA,ADH1A,ALCAM,AMPD1,ATP5O,CACNA1S,CANT1,CD34,CXCL16,DHRS4,EPHA2,FZD4,GAD1,GALNT3,GPX5,ICOSLG,ITPA,KLRC4,LRDD,MGAT1,NCOR1,NFKBIA,NTN1,NUDT9,OR5H6,PKM2,POLE3,PRNP,PTCH2,RRAS,SDC1,SETD1B,SPTAN1,STAT3,TAS1R3,TPM3,HLA-DPA1
Negative_1475	CRK,DLD,ACSM3,ARD1A,BLVRA,CYP11B1,DUSP8,ECHS1,GFAP,GPX7,GRIN1,GSTA1,IL18,IRF7,ITGB7,L1CAM,MYH11,NANP,OR10AD1,OR1C1,OR2AT4,PANK4,PCNA,PI4KA,PIK3CG,PRKAB2,RBL1,SDC1,SFRP4,SPRY2,SSH1,TLN1,TUBB2A,YWHAB,SERPINF2,TNFRSF6B
Negative_1476	AK1,C5,ACP5,CREB1,CXCR4,CYP26B1,DDOST,FZD1,IARS,IMPA2,IRF5,ITGA1,ITGAV,JAG2,MAT1A,MNX1,NFX1,NOD1,OR10H2,PIGQ,PON3,PRKAA2,RPIA,TIAM2,UGT8,WEE1,ATP6V1G3,PPP1R12A,PRICKLE2
Negative_1477	CD6,IDS,KHK,MYC,PLG,PXN,ACTN1,AKR1A1,ARD1A,CALML3,CSF3R,DDX4,FCGR3A,GANAB,GOT1,IL10,L1CAM,OR10A7,OR1N2,PIK3R4,PIP4K2A,PRKAB1,PTAFR,PTPRF,RAPGEF2,RDH11,RPS6KA3,SOD1,CTNNBIP1,TNFRSF17
Negative_1478	OSM,RB1,KIR3DL2,MAP3K3,SLIT1
Negative_1479	GPT,IDS,OGT,APC2,BMP2,CCNB1,CD44,COL6A1,DNMT3A,DUSP14,ESAM,GCDH,GRIA2,HLA-DMA,IL17B,KLRD1,MAP3K14,NCF1,OR10A7,OR11G2,OR12D3,OR13C4,OR14A16,OR2B2,OR4A15,OR7C1,PDE3A,PDGFD,PEMT,PLA2G2E,PLOD2,POLR1B,RASGRF2,TGFB1,WASL,CSGALNACT2,PRICKLE1
Negative_1480	HK3,B4GALT2,CACNG6,CSNK1G3,DHDDS,MAP4K4,NPR2,OR2A4,OR9Q1,POLA1,PPP2CA,PRIM2,PRKAG3,TFPI
Negative_1481	BID,CBS,CDA,AARS2,CCR5,CDK6,CYP3A4,DLL3,DTYMK,EDA2R,ENPP3,GALC,GSTT1,ITGA3,LARS2,LHCGR,MAPK12,MYH10,OR13J1,OR2T3,OR5T2,OR9G1,PIK3CG,PTGS1,ST3GAL3,STX5,SUV39H2
Negative_1482	ACVR2A,APOE,C3orf10,CCL28,CD70,CDC27,CLDN4,CSNK2B,CYFIP1,EDNRB,FDPS,GALNT7,HPSE2,IFNAR1,IFNGR1,INSRR,ITPA,NT5E,ODC1,OR10H3,OR10Q1,OR2A4,OR3A2,PDE7A,PLEKHA8,POLR3B,PYCR2,RASGRP3,SV2B,TBL1XR1
Negative_1483	ADC,FH,LCK,OSM,PVR,AADAC,ABL2,ACSS1,AGPAT1,AHCYL2,ALG1,APC2,ATP13A2,B3GAT1,B3GAT3,BCL2,CACNA1E,CACNG3,CAMK2D,CLDN19,COL1A2,COX10,DNMT3L,EARS2,EGLN3,EHMT1,FLOT2,FOXA2,GALNS,GNA11,GNA12,MINPP1,NCKAP1L,NEFL,NNMT,OR10A7,OR4A5,OR4F17,PDE2A,PEMT,PIK3R4,PPP2R2A,RAPGEF2,RXRB,STX10,STX1A,SLC25A31
Negative_1484	JUN,ME1,ACADM,ADSL,ARRB2,ARSA,CD19,CLDN6,ENPP7,ERBB2,FUT7,GADD45A,HHIP,IRF3,LEFTY1,LTB4R2,NCF2,OR10S1,PGM2,PLA2G6,PPAP2B,PTK2,PTPN7,SELL,SGPP2,SPRY1
Negative_1485	AK7,APP,DAO,DBH,AKR1C1,ALDH6A1,CCL1,CDC14A,CDC45L,CMPK2,CNGB1,COL5A2,CPT2,CRLF2,DAPK3,DKK2,ETS1,FGF9,FRAT1,G6PC,GSTM5,HSD17B7,INPP5E,IRF5,IRF9,LIPC,NEFH,NFYC,OR2S2,OR52W1,OR5M3,OR6C1,PARD6B,PARVA,PPP1R1A,PPP2R5C,PPP2R5D,PRKCE,ROCK2,SHC4,SOCS3,ST8SIA5,TPI1,YWHAB
Negative_1486	ACYP1,DAAM1,DAPK2,GSTA5,PAX8,PLIN
Negative_1487	ATF4,NRXN2,RRAS2

Negative_1488	DUT, PTS, UBC, AGMAT, ALDOB, ARHGAP5, CAMK2A, CCL14, CCND2, CHRD, DGKG, F2RL3, FGF5, GALNT2, IL2RA, MAD1L1, NAT2, OR13C8, OR51F2, OR5T3, PDE1B, PIGA, PLD1, POLE, POLR2C, PRKG2, SEMA3E, SETD2, TBL1Y, UBA7, UCK1, UPRT, VCAM1, YWHAB
Negative_1489	CDA, FOS, IL3, NLK, AANAT, ACTA2, ALG13, APC2, BAAT, CBLB, CCL25, CRB3, DMGDH, EPB41L1, FMO1, FUT1, GNAZ, HSD3B7, HTRA2, IL5RA, ITGAE, KIR3DL3, LRP6, NKD2, NR1H3, OR10K2, OR11L1, OR8I2, OXTR, PDE3B, PHKB, PRLR, PYCR2, SMAD6, SPCS1, SPRY4, SPTAN1, SRGAP2, UGT2B28, B3GALNT1, PAFAH1B3, SERPIND1
Negative_1490	ACO2, EDNRB, GAB1, GCDH, GUCY1B3, OR2K2, POLR3G, TAS1R1
Negative_1491	ADO, FUK, GCK, INS, ABP1, ACACA, ALDH1A3, APOA2, CASP10, CCR3, CISH, CLDN11, COL6A2, COQ3, DTX4, DVL1, GALNT14, GRIN1, INHBB, MADCAM1, MYH7B, NAGLU, NME4, OR2A25, OR4D2, OR51F2, OR52E4, OR5AU1, OR5K1, OR8H1, OR9Q1, PDK1, PIGB, PIK3R3, PLAUR, PMAIP1, QPRT, RPIA, SNAI1, ST6GAL1, TAS1R3, TNFSF9, TPI1, UGDH, YES1, YWHAQ, TNFRSF13B
Negative_1492	BTD, CRK, FAH, PGP, XDH, ACACA, ATP5C1, CCNB1, CHSY3, CLDN4, DUSP1, ENTPD5, EPHA4, EPHA7, IFNA14, IFNA4, IFNAR2, IL20RB, LYZL1, MMP7, NGEF, NT5C2, NTNG1, OR11A1, OR1D2, OR2D2, OR4D6, OR4M2, OR52E6, OR6F1, OR8K3, PDE4C, PFKFB4, PNPLA4, PPP2R5B, PRNP, PRPH, SORD, TNFRSF4, TRPC1
Negative_1493	DDO, EZR, INS, SRF, ALOX15B, APOE, AQP7, ARHGEF1, ARHGEF2, ARHGEF6, ATP5I, CACNA1E, CCL5, CDC16, CDKN2D, DDX41, DGAT1, DHX58, DLL3, E2F3, ESAM, GP1BA, GPX5, HEXB, IL1RAP, LDHC, LIMK1, LRP6, OR13C5, OR1G1, PAK3, PFN1, POLR3H, RFWD2, SOS2, TLR4, WNT11, B3GALNT1, GABARAPL1
Negative_1494	C4B, GNS, AGTR1, BMP6, CASP3, CCR8, CHD8, DDHD1, ENO2, FTH1, GALM, GCLM, HSD17B4, IL17RB, IL21, MAP2K7, NRG1, OR8J3, PLCD1, SGMS1, SRD5A2, SULT1A2, THBS2, HLA-DPB1, PNLIPRP1
Negative_1495	ATP2A1, B3GNT5, CNTN1, ENTPD4, FOXO1, HSD17B1, IL10, KAT2A, LTBR, MYL9, NRG3, STT3B, STX10, ATP6V1C2, SERPINA5
Negative_1496	DBH, ACADSB, ACTB, ADSL, ATP6V0C, B4GALT5, CCND1, CD274, CYP4F2, DTX3, FBP2, LIPG, NPR1, OR11A1, OR2Z1, OR6N1, PDCD1, PGM2, POLR2B, PRKAA1, TJP1, TJP2, ATP6V1E1
Negative_1497	HDC, ACAA2, AMY2B, BMP4, CD3E, DLL4, DTX2, DUSP4, FLT3LG, FRAT2, GALNT1, GRIN2D, IL21, JAG1, KIR2DL1, MAP2K5, MTHFD2, PLOD2, PLXNA3, PLXNB1, POLR1D, POMC, QARS, TSTA3
Negative_1498	ID1, B3GALT4, CD27, CNTNAP2, CREB3L2, CTBP2, CYP4A11, EFNA2, GPAM, MINPP1, NAT6, NFKB2, OR10A4, OR14J1, OR52M1, OR6K3, OR7C1, P2RX7, SCD5
Negative_1499	ADA, APP, DBH, EZR, ALS2, CACNG7, CD72, CES2, CHPF, CNTNAP1, CRLF2, DMGDH, DNMT3L, EPHA4, FARSA, GLO1, GP1BB, GZMA, IFNB1, NPR2, OR10G8, OR11L1, OR6K2, OR6M1, OR9A2, PPP2R5E, SEMA3E, SESN3, SOS1, TAS1R1, TPH1, TPH2, WNT9A, ATP6V1E1, ATP6V1E2
Negative_1500	F8, FES, NPL, AKR1C2, CACNB2, CDC14B, MLLT4, OR10Q1, OR2T6, OR2W1, OR5AK2, RALGDS, STX16
Negative_1501	C2, NF1, ACSM2A, BUB3, CDIPT, EFNB1, NFKB2, OR8A1, PLCB1, POLR1E, RARA, STK11
Negative_1502	CFL2, E2F2, HNF1A, OR10C1, PPP2R2D, TSLP, UGT2B4
Negative_1503	FST, APC2, OR5T2, PDE1B, SC4MOL, XYLB, TNFRSF11B
Negative_1504	AMY2B, CLDN7, IL4R, LIPC, MAML2, NT5C1B, OR52E6, PLA2G2F, RRAS2, SLC2A1, TGFB2, TUBA8

Negative_1505	EBP, FUK, LPO, PDC, ABLIM2, ACSM3, AGXT, CDC26, CHPT1, CYP1A1, CYP27A1, CYP8B1, FZD5, GLG1, GNA11, GRIN1, HLCS, HPRT1, HTRA2, IAPP, IBSP, IL11RA, IPPK, NFX1, NOX1, OR6C76, OR9I1, P2RX5, PARD6G, PLCB3, POLR2L, PTAFR, PTK2B, PTPRB, RDH5, SCP2, SDHB, SETD1B, SMAD2, TAS1R1, TAS2R41, TGFB2, TNFSF10, TUBA1B, VANGL1, VNN1, WNT1, WNT7A, MAPKAPK2
Negative_1506	GPT, RFK, ABP1, ARPC1B, ATP5A1, CD40LG, CSF2RA, ENPP6, FABP3, FGFR3, FLOT2, FMO1, FOXO3, GSTT1, MAP2K2, MAP2K7, MDM4, OR10H3, P4HA1, PECAM1, PER2, PFAS, PPP1CA, PPP3R1, PPP3R2, PRKCI, SNAI1, WHSC1L1, ARHGEF12, KIR2DL5A, ST6GALNAC1
Negative_1507	AASDH, AKR1C1, ALOX15, ATP5B, BAIAP2, CCL17, CD46, CDC14B, CDC25A, CLTC, CSNK2B, CTBP2, DHCR24, DUSP8, DVL1, EGLN3, EPHB6, FLT1, GNAO1, IFNA2, IL17RA, ITGAE, ITPA, JAK1, LAMC3, MGST2, MYH7, NCF1, NCF4, NTNG1, OR1Q1, OR2T2, OR52E6, PANK2, PAPSS2, PIAS1, PLCD4, PSENEN, RAPGEF4, RRM1, SDC2, SOCS2, TNFSF11, TNFSF18
Negative_1508	ARHGEF4, ATP5A1, ATP6AP1, AVPR1B, BCL10, BHMT, CADM1, CALML5, CARS, CYP26A1, DAD1, DHDH, EXTL1, HADH, HDAC1, HLA-G, IL23A, JAG2, KIR2DS4, KLKB1, MAP3K3, MGAT4B, MYLK2, NAGLU, OR4C16, OR52E4, OR5D18, PMM2, POLR2H, PPP3R2, RFNG, RPSA, SSX2IP, STX4, TGFBR2, VARS, WNT7B, SERPINE1
Negative_1509	FST, IL2, PTS, VCL, ACSM4, ALG11, APOA1, BMP7, BUB3, CCL3, CD72, CLDN5, COL2A1, DUSP9, FUT7, GLI1, GLUD2, HMGCL, IDH3G, ITGA11, ITGB1, KLRC2, LDHB, MAP3K12, MCM3, MCM7, OR10H2, OR2A4, OR7G2, PDCD1, PHKB, PIK3CA, PIK3R2, PPCS, PRKAB2, RASGRF2, RRAS2, SMAD7, SULT2B1, TLR9, TNFSF18, VDAC3, WNT5A, WWP1, HLA-DPB1
Negative_1510	F12, GLS, ACAT2, CCNB1, CD19, CDKN1B, CDKN1C, CDKN2D, CYP4F2, DAG1, DDX55, DGKE, DHDH, DHRS4L2, EHMT1, FGFR4, FLOT1, GADD45B, GFPT2, HAP1, ITGA9, ITGAL, ODC1, OR10X1, OR5AU1, P2RX6, PITX2, POLR2D, PPAP2B, PRKCZ, PSME2, PTEN, RBL2, UNC5D
Negative_1511	RET, ARHGEF1, ATP13A2, CFTR, CHRM1, CRKL, CRY2, DAAM2, DARS2, ENPP6, ENTPD5, FECH, FLT3, FMO3, FZD2, ITGA6, ITGB2, MAP3K13, MAP4K3, MDM2, OR1F1, OR52A5, PGM2, POLE4, PRKAG2, PTPMT1, PTPRM, RPS6KA6, SEMA3E, SEMA4F, SMPD3, WNT9A
Negative_1512	BUB1B, MTHFD2, SEMA3E
Negative_1513	AGL, BAX, APLP1, CARS2, CDKN1A, CHRM2, CLDN15, CYP3A4, DDHD1, FUT6, GSTM4, IPMK, JAG1, LIFR, MAP3K7, NME7, OR13C3, OR5AU1, OR5L2, OR6C4, PFKFB2, PIAS2, PPA2, PPID, PTGES2, QARS, RAPGEF1, RPN2, TUBA3E, UGT2B4, ZNRD1
Negative_1514	IL22, KCNQ1, MYH13, POLR3K, TCF7L1
Negative_1515	ADORA2B, AKR1B10, BTRC, CAV3, CDKN2D, DDX18, FADD, FARS2, GDF5, GLB1, IL17B, IL1A, IRAK1, ITGAX, LCMT2, NAMPT, NTF4, OR13C3, OR2T10, OR4B1, OR4C12, OR52A5, OR5A2, OR5M3, PANK4, RAB23, SEMA4D, SETD2, SPRED2, SPTLC2, STX16, SULT2A1, SV2C, TACR1, TUBB2A, TUBB4, UAP1, UGT2A1, YWHAZ, MAPKAPK2, TNFRSF10A, TNFRSF12A
Negative_1516	CS, GH1, KMO, ACADSB, AGRN, ATG7, ATP5A1, BDKRB2, CD8A, FZD3, GSTM4, LEPR, MGAT4B, OR51A7, PIP5K1A, RERE, SNW1, UGT2B17, WWP2, ST6GALNAC1
Negative_1517	BLVRA, BMP4, CADM3, CAMK2D, CNTNAP2, COL6A1, DGUOK, FGF4, GCLC, GLI2, GNB1, IRAK2, MAPT, NCOR1, OR2T8, OR6B2, OR6K2, RDH5, ROBO2, SHISA5, VAV2, WBSCR22
Negative_1518	DAO, FZD5, KIR2DS5, PRKAA2, ST6GALNAC5
Negative_1519	ID4, LPO, PTS, TNF, ABLIM1, ACSS3, ALPI, ATP6AP1, CSNK1D, CXCR3, DAAM2, E2F3, EREG, F2RL2, FUT1, ITGA2B, ITGA5, KLKB1, MAML3, MAPK3, NTF4, OCRL, OR9I1, PDGFD, PTPRZ1, RASA2, RRAS, SH2D1B, SLC2A4, SMAD6, STK4, TAS2R7, TNFSF11, UROD

Negative_1520	A2M,ILK,KMO,B4GALT1,BCKDHA,CACNG5,CPT1A,E2F5,HSD3B2,IFNK,IL22RA1,NODAL,OGDHL,OR10Q1,OR2L2,PON1,TBXA2R
Negative_1521	APP,ATM,ID4,KIT,WAS,ACADVL,ADAM10,AKR1C1,CCR3,CD86,CHAD,CHIA,CYP11B1,CYP2S1,DGKI,DNMT1,FIGF,GALE,GLO1,HDAC2,HSPB1,IFNA5,IFNA6,IL20,INADL,INHBC,MGST1,MPDZ,NEU1,NRG1,OR1L4,OR4D2,OR4S1,PFKFB3,PIGT,PIP5K1C,PLK1,PLXNA3,PRKX,PSEN2,RAC3,RPS6KA6,SNAI2,TACR3,TAS2R8,TRAF6,TYRP1,HLA-DPA1,MAPKAPK2
Negative_1522	HK3,ACSM3,COQ5,CSAD,GFPT2,MAP3K1,OR10H4,OR2AG1,OR56B4,PTK2,PTPN7,SGMS1,STX18,SYNJ1,TCF7L1,TPH2,WNT7A,TNFSF13B
Negative_1523	ACO2,AKR1A1,ALOX12,ATF2,BLVRB,CCNE2,CDKN2D,CSNK1A1,CSNK2A2,CYP2C9,CYP2J2,DAPK1,DGAT2,EIF4B,GNAS,GPT2,GRIN1,HLCS,ICAM3,IL4R,LLGL2,LYZL1,MAP2K1,MAPT,NKX3-1,NOD1,NQO1,OR2W1,OR5D16,OR6M1,OR7C2,OR8U1,ORC1L,PDE10A,PIP5K3,PRDX6,PSEN2,SPAM1,ST3GAL5,TBXA2R,TGFB3,UGT2B7,ATP6V0D1
Negative_1524	ADH6,CACNG3,CDC7,CXCR6,DAPK2,DAXX,DDX51,DFFB,DGKA,EFNA4,ERBB3,GNB1,GRLF1,HCCS,ITGA7,LHCGR,NFE2L2,OR10R2,OR1C1,OR2T3,OR5D14,PARD6B,PDPK1,PIK3R5,PRKACG,PRKCE,PTAFR,PTPN6,RRM1,SETDB2
Negative_1525	AGL,FGG,ITK,KHK,UBC,ARG2,ASS1,ATF4,B3GALT5,CD47,CXCL13,DGKH,EDN1,FIGF,GALNT2,ICAM3,IL18R1,INPPL1,KDSR,LTBR,MCEE,MYH7B,NCAM2,NTSR1,OR5AC2,OR5T1,PDE9A,PGK1,PIM1,PLXNB2,PRKAB1,SMAD9,SMPD2,SPTLC1,THBS1,CACNA2D1,MAPKAPK5,TNFRSF1B
Negative_1526	ADORA2A,AGPAT1,AOX1,BMP6,CD226,CD27,DCXR,DDIT3,FABP3,FSHB,GDE1,GUCA1B,HNF1B,HSD11B1,IFNG,ITPK1,ITPKA,JAG1,KAT2B,LILRB3,MTFMT,NKD1,NR5A2,OR10A5,OR4F4,OR51F2,OR51T1,OR6C6,OR8U1,PNMT,PPP3CA,SEMA4G,SETD2,ST8SIA5,TSHB,TSNARE1,CACNA2D3,PAFAH1B1
Negative_1527	FZD3,GAL3ST1,GCDH,HMBS,IBSP,IL20,LLGL1,MAP4K3,OR10A4,OR1S1,OR4A16,SEMA3E,TOMM40L,HSD17B10
Negative_1528	OGT,C1QC,CREB1,CYP2A7,DDX23,DUSP9,GCAT,GSTP1,GUCA1C,HSD3B2,NME6,OR8D2,PARD6G,SC4MOL,TAS2R48,YARS,ST6GALNAC1
Negative_1529	ADA,CR2,FH,HGD,MUT,NNT,ACAT2,AGRP,ALDH3B2,ARAF,B3GALT1,BAIAP2,CAMK2B,DBF4,DGKE,FTMT,GPD2,GRIN2A,HARS2,HSD11B1,IL15RA,JAK3,MYH9,MYL9,NCR3,NT5C1B,OR1L1,OR2G2,OR2L2,OR4F4,OR56A3,OR5AK2,OR9I1,PAX4,PLCD1,PMM1,POLR2K,PPCS,PRKCB,PTAFR,PTCRA,PYCRL,RALA,RPS6KB1,ULBP1,PPARGC1A
Negative_1530	AUH,PAH,ACSS3,ADRB3,ALPL,ATP13A2,ATP4B,CD38,CHAD,CHDH,GSTA3,HAAO,HSD3B7,KCNB1,LEF1,MAP4K1,MGAM,NEUROG3,OR13C3,OR13F1,OR3A3,OR51L1,OR52K2,P2RX7,PPM1B,PVRL4,RASA1,SOAT1,STAT5B,SULT1A4,SV2A,THBD,UCK2,UMPS,VDAC3,ZFYVE9,C1GALT1C1
Negative_1531	ASL,CCL15,CCL20,COL5A1,DLAT,ENAH,FASN,GCAT,GPX7,IL11,IMPA1,IRAK3,IRF9,NANP,OGDH,PTGER3,QPRT,SDHB,XYLT1,ZMAT3
Negative_1532	KMO,LTB,NNT,BDH2,C3orf10,CD70,CXCL12,FGF8,FRAP1,GMPS,GRIA1,HSD17B3,HTR4,IL10RB,IRF5,KCNB1,MDH1,MPDZ,MYH7B,NCR1,NFKBIA,NMNAT3,OR1I1,OR6X1,ORC2L,PAK6,PDE8A,PFKFB4,PRKG1,RASA2,RELA,ROBO3,SDC2,SGPP1,SH2D2A,SMARCA2,SPAM1,TYMS,VAMP4,PLA2G12A
Negative_1533	TKT,ACACA,APOC3,CDK4,CREB3,ENTPD4,FGF20,FOXO3,GALNT9,ITPA,OPLAH,OR51G1,OR5D13,PLXNA1,RPS6KB2,WHSC1L1
Negative_1534	C7,NF1,AOX1,BMP8A,CHIA,CRLS1,CTSB,DCXR,DDX51,DUSP1,DUSP2,FPGT,HNF4G,IDI1,KLRC4,KLRD1,NADSYN1,NUDT8,OR2AE1,OR4D9,OR51B4,OR51B6,OR52N4,OR5B21,OR5L2,P4HA2,PDE6G,PDE7B,PLA2G6,POLR1D,PTGER3,SEMA4A,SSH1,TAS2R5,TCIRG1,UGT1A10,UROC1

Negative_1535	EBP,KIT,ANAPC2,APBB1,ARSE,ATF2,ATN1,ATP5D,CDK2,COQ5,COX10,CSNK2B,HLA-F,INPP1,NNMT,NPR1,OR2H1,OR52N1,OR5F1,OR5T2,OR8B8,PCYT1A,PIAS2,PTDSS2,SESN1,TMLHE
Negative_1536	ADCY6,DDIT4,GFPT1,ITGAE,NCR1,SOCS7
Negative_1537	PVR,ACVR2B,ALG8,ARPC3,CD99,CSNK1D,CSNK1G3,CXCL6,CYFIP2,CYP2A7,DERA,F13B,GPD1,GRB2,HMBS,HRH2,IL24,KIR2DL3,LDHAL6B,MTFMT,P2RX7,PKM2,POLR2K,SEMA4G,SSX2IP,TAS2R9,VAV2,VTI1A
Negative_1538	GSN,CAMK2G,FLT1,GFAP,GIT1,HTR2C,OR2V2,OR5AS1,OR6N2,PI4KB,PIAS4,PTPRC,RELA,SOX17
Negative_1539	IL9,ARG1,VAMP3
Negative_1540	ADO,FN1,OAT,ADCY10,ALDOB,FOXA3,FTH1,ITGB3,OR1B1,OR52L1,OR5L1,PHKA1,SDHC
Negative_1541	GNG12,OR4M1,PITX2,POLR3G,SLC27A5
Negative_1542	CSNK2A1,CXCL6,MAP4K4
Negative_1543	GSN,HK1,ACP1,ACSS2,AGTR1,AHCYL1,BMPR1B,CAV1,CCND2,CD226,CDKN2B,CYP26B1,FADD,FADS2,GIT1,GRIN1,HRH2,ITGA9,LCAT,MARS,NEGR1,NEU1,NKX3-1,OR2K2,OR4C11,OR52A1,OR52D1,OR5AS1,PER3,PIGL,PIK3R3,PRKAB2,PVRL4,PYCRL,SARS2,TAS2R3,CACNA2D4,GABARAPL1,HSD17B10,TNFRSF10B
Negative_1544	HPD,PRL,ADIPOR2,ARHGEF1,ATP6V1F,BMP5,CALML5,CAMK2A,CDC2,CHD8,CKS1B,COL3A1,CTBP2,DUSP6,EIF4B,FMO2,GANAB,GRIA3,GZMA,HPSE,IFNK,MAN1B1,NCR2,OR2A25,PHKA1,PIGK,PLXNA2,PTGFR,SETD8,VASP
Negative_1545	AK3,GPT,ACLY,ADORA2B,ADRA1B,AKR1B1,ALG6,CAMK2B,CCL15,CDH2,CDKN2C,CPT1C,CYP2B6,DDX52,DHX58,DOLPP1,DPM1,DRD1,ECHS1,EI24,EPOR,EXT2,GUCA1B,IL11,INSRR,IQGAP3,LAMB4,MS4A2,NFYC,NLGN1,OR10H2,OR13C2,OR4E2,OR6C3,OR7G1,P4HA1,PDE7A,PPCS,PRKAR2B,PTDSS2,RASGRF2,UBE2K,WBSCR22,WNT7B,ST6GALNAC1
Negative_1546	C8G,CBS,AANAT,ADCY4,ADRB3,AMY2B,ASRGL1,CCND3,CD247,CD27,CHRM3,CPT1A,ETS1,FGF22,GSTM4,GUK1,HSD17B8,IDUA,LYPLA1,MAP2K5,NANP,OR1B1,PDGFRB,PFN2,PIGB,PLXNB3,PRPH,RXRB,STX16,UGT2B28,WNT9A
Negative_1547	FYN,ITK,TEC,ALAS2,CACNG6,CD82,CDK4,COL1A1,DDX52,DGKQ,EPRS,FLT3,GALNT1,GSTZ1,HIBCH,HMGCR,HSD17B4,MAN2A1,MAPK1,MCM2,NME4,NMNAT3,NTF3,OLR1,OR13C5,OXCT1,PARS2,PDGFRA,PIGL,POLR3A,PRKCI,PRKCZ,SUCLG1,TCF7L1,CACNA2D2,ST6GALNAC6
Negative_1548	CGN,ID4,ACTA2,CASP10,CBLC,CCL28,CCR9,CHD8,CHRNA7,CNTN1,COL4A6,COL6A1,CTBS,CYP2A7,DGKD,FRAP1,GNAI3,HMGCR,IL2RG,LFNG,MASP2,MMP14,NAT6,NCSTN,NMNAT2,OR1C1,OR2Y1,OR4A15,OR51A7,OR51S1,OR52K2,OR6K3,OR8H1,OR8S1,OXTR,PDE2A,PHKG1,POLD3,RBL1,RPS6,SESN1,SIPA1,TCIRG1,TGDS,TNFSF18,UNC5B,TNFRSF10C
Negative_1549	ME2,TPR,CCL16,CHPT1,CHRD,CLDN2,COL3A1,COL4A6,CREB3L2,CX3CL1,CYP26C1,DHDDS,DOLPP1,ENTPD2,EPHX2,GGCX,GP1BA,HLA-G,JAK1,MDM2,OR1G1,OR2H2,OR51A2,OR5T3,PDE4C,PLK1,PPP2R1A,PRKACA,SMAD7,STT3B,TRAF1,XYLB,XYLT1,ATP6V1C1,SERPINB5
Negative_1550	ACO2,AMY2A,DNMT3L,FPGT,GAS1,ITGB5,JUND,NEUROD1,OR4C13,PPA1,SDC2
Negative_1551	MTR,B3GAT2,FARSB,MAP3K11,MTHFR,PAPSS1,PAX6,PLEKHA8,RAPGEF3,RFWD2,SOCS2
Negative_1552	CGN,IHH,LSS,MIF,AASDH,ALG1,ALPP,AOC2,CNTNAP2,EXTL1,EXTL3,GNA12,HMGCS1,LIPG,MAML3,NTRK2,NUDT9,OR10K2,OR2AE1,OR2F1,OR2G2,OR52L1,PDXK,PGAM2,PLCD3,PPP2R5E,RFX5,SEMA7A,SFRP1,SH2D2A,SMPD2,SNAI2,SUV39H1,TAS2R41,TRAF2,YWHAG

Negative_1553	EPHA7,HBEGF,IRS2,NFKB2,OR10S1
Negative_1554	TNF,ALOX12B,CD79B,DTX3L,GDF7,OR1J2,OR52M1,PRSS3,RASGRP1,SOX17,VARS,WNT8B
Negative_1555	C4B,FGA,ILK,ABP1,ACPT,BACE1,CD40LG,CD86,CYP2C8,DHODH,DPYS,DUSP14,EHMT1,GALNTL2,GSTA3,GSTM3,HLA-F,IL1A,LTBP1,MCCC2,NCF2,NFKB1,NTRK2,OR2B11,OR4K15,OR5K1,OR8B12,PIP5K1C,PLA2G2E,PRKCB,PSME1,PTPRF,PVRL3,RAPGEF3,STAT3,TNFSF8,ULBP1,XYLT2,CACNA2D2
Negative_1556	BTC,MPL,RFK,SI,SRF,ASS1,CCL14,CSAD,CYP11B1,CYP1A2,DRD1,FBXW11,GLG1,HLCS,HNF1B,IL22,IL28B,IMPA1,MAP2K3,MTFMT,OR2A5,PDHA1,PNMT,TGFB2,PRICKLE2
Negative_1557	DBH,IL5,OGT,TK1,ACO1,ADCY5,BCAT1,CCL19,DDX18,DGKE,DHRS9,DPM3,ELK1,EXT2,FGFR3,FPGS,FUT2,GALNT2,GSTO1,IDH2,IKBKE,IL18,LIPA,MGAT4B,OLAH,OR13G1,OR2L3,OR3A1,ORC4L,PDE6G,PHKA2,RIPK1,SGPP2,SIPA1,SLC25A6,SORD,TAS2R1,TGFB2,TNFSF11,TNFSF18,CACNA2D3
Negative_1558	AK1,AASS,ADCY10,CDC7,CLDN20,CLDN3,CTNNB1,CXCR3,CYP2C19,EHHADH,EPHB6,GCLM,HAAO,IL29,IMPA2,ITGB2,MAP3K4,MGAT3,NAT5,NT5C,OR1L4,OR52I1,OR8B4,OR8J1,PER2,PFN2,PIK3R2,RALB,RASGRP2,RFX5,TNFSF14,TRHR,WNT8B,ATP6V0A1
Negative_1559	IHH,APOA2,BCL2,GABARAP,GALNTL4,GPD2,GUCA1B,ICAM2,IL20RB,IL3RA,IL5RA,OR1D5,OR4K17,PIGS,PIK3R5,PRIM1,ROBO2,TPI1,WNT2,TNFRSF17
Negative_1560	CAD,ACTN4,ALG10,ALG3,BCL2,BMP8B,BRAF,CCL16,CCNH,CDC42,CLDN4,CLTA,COQ5,DHRS9,E2F3,ENPP6,FZD1,GSTO2,HMGCS2,IARS2,IL20,IL4R,IL8RA,ITGAX,ITPA,LIMK2,MCM7,MLYCD,MYH7B,NSDHL,OR4C6,OR52M1,OR5AC2,OR7D2,PARD6B,POLR2E,SC5DL,SEMA3E,SEPHS1,SETMAR,SH2D1A,SHMT1,STAT5A,TAS2R1,THBS4,WARS2,WNT2B,B4GALNT1,C1GALT1C1
Negative_1561	DCC,GLS,ME1,TK1,CDC45L,CYP4F3,EPAS1,EPHA2,FZD1,GPX5,GSTM3,GSTZ1,ITGB3,LTBP1,MTFMT,NME7,OR52D1,OR56A4,PRKG1,RAC3,RAPGEF1,SOS1,TNFSF10,TUBA4A,TUBB6,UGDH,PRICKLE2
Negative_1562	ADA,FGA,FGB,PLN,UBB,VCL,ARG1,C1QC,CES7,CHEK1,CHRM2,CMPK2,CYSLTR2,DDX41,DHODH,DLST,DPM1,DRD5,DUSP14,DUSP16,EFNA2,FGF7,FGFR3,GNRHR,HLA-C,NCR2,OR1E1,OR51G1,OR52I1,PGM1,PGM3,PIGH,PLAT,PLCG2,PPM1A,PPP3R1,PRKAB2,RBKS,SKIV2L2,SOCS5,SPCS1,SV2B,TNXB,ULBP1,ATP6V1C2
Negative_1563	CDA,LEP,PRL,ADCY5,ALPL,CACNG3,CD14,CYSLTR1,DDX55,ENTPD4,FZD5,GGT6,GMPR2,MGAT1,NKX6-1,NRXN3,OR51B5,OR52H1,OR52K1,OR8I2,PAK1,PAK6,PPT1,PYGL,SPRED2,THBD,UGCGL1,UROS,ATP6V0A4
Negative_1564	ADH1A,ARG2,ARHGEF2,ARRB1,ATP2A2,BCL2,CCL21,CCL7,CDC7,COMP,E2F5,FARSA,GALNT5,GLUL,GNMT,HIP1,HLA-G,HSPA8,LAP3,LLGL2,LRRC4C,NFYC,OR1A1,OR1L6,OR2A14,OR51B6,OR5B12,OR8B8,PAK3,PDE4C,PDGFRA,PIP5K1C,POLD1,PPAT,PPP1R3B,PPP2R2C,RAPGEF4,RBL2,SETD1A,SLC27A4,ST8SIA1,STAT1,TRAF2,UGP2,YWHAH,SERPING1
Negative_1565	MOS,ABLIM2,ADSSL1,ALG2,APRT,ATF2,ATP2A1,B4GALT4,CCL28,CCNB3,CD72,CSF2RA,CXCR6,FUT4,IL2RG,LIPF,MAPK9,MCCC2,MYL2,OR10R2,OR4D2,OR6B2,PAPSS1,PDE7A,PIGN,PLA2G1B,ROCK2,SETD1A,STAT5A,TUBA1C,VAMP4,PPP1R12A
Negative_1566	CARD11,CREB5,MAPK14,OR10H1,SCLY

Negative_1567	CGA, GH2, LAT, ME1, ACVR2A, ACYP2, ARHGEF1, C4BPA, CDC14A, CFL2, CHRM2, CMPK1, CNTF, CNTFR, EFNB3, EIF4E, ENPP6, ETS1, EXTL2, FGD1, FGFR1, FUT4, GRB2, HNF4G, HYAL2, IL12RB1, IRF3, ITGA2, LAP3, MTMR6, OR10R2, OR4D6, OR4N4, OR6Y1, PAX4, PDCD1, PGS1, PIM2, PLCB3, PPP1R3B, RXRG, SPRY2, SUV39H1, CACNA2D4, HSD17B10, SERPINA1, SERPING1
Negative_1568	ABO, EGF, ACP1, AKT1, ANPEP, CACNA1C, DUSP8, MYH3, PDHA2, PKMYT1, PTEN, TAS2R42
Negative_1569	GBA, CYP2B6, CYP4F2, DOT1L, ENTPD4, ENTPD8, FUT5, GALNT8, GMPPB, GRM1, ITGB2, ITPKA, MTAP, NCR1, OR4N2, OR6X1, PDIA4, PLCB4, PLCE1, PPAP2A, PPP2R5A, PRSS1, TAS2R8, THBS2, TNFSF11, TUBA1C, TNFRSF10D
Negative_1570	CDA, ADCY1, CLDN6, COQ6, CSF3, GRIN1, MYL2, NEGR1, PTGES, RAB5A, SUOX, MAP3K7IP1
Negative_1571	CTH, GLA, ATP5O, BET1, FUT3, HPRT1, IL20RB, KLRD1, MAP3K14, MLLT4, NAGLU, OR51S1, PTPN1, RPS6KB2, SEMA4G, SNAP23, SUCLG1, UAP1, WEE1, PPP1R12A
Negative_1572	C7, F11, ACADSB, ACAT2, ADH4, ATP5F1, AVPR1A, B3GALT4, BIRC3, CACNG3, CCL26, CD40LG, COL1A2, CYP4A22, DLL4, DNMT3B, ENTPD5, GLYCTK, HLA-DOA, IL1B, INPP5E, LDHAL6A, LTA4H, MASP2, NAGK, NARS2, NAT1, NFYC, OR10K1, OR2G6, OR6F1, PDCD1, PDGFA, PIGX, POLA1, POLR3A, POLR3H, PPA2, RUNX1, SCD5, TNXB, TRAF1, TUBAL3, XYLT1, ARHGEF12
Negative_1573	ADK, IL2, ACAD8, AK3L1, CYP2F1, DAPK3, DHDDS, EPHX1, FGF1, HYAL1, MAT2B, NOS1, OR13C2, OR1Q1, OR2M4, OR5M9, OR6C3, OR9Q2, PLEKHA8, RAPGEF4, RHOQ, RRM2, SDC4, SRD5A1, STAT1, SUCLA2, TOMM40, VEGFC, WASF1
Negative_1574	CRK, ID3, STS, ACSS2, ATG12, B3GNT4, CCKBR, DFFA, GNA14, GRLF1, HDAC2, IL15, IL19, LARS, NRG3, OR12D3, OR4E2, PAPSS1, POLR1D, PPP2R2B, PRODH, RAD51, TALDO1, SERPINF2
Negative_1575	IL5, SRM, ADCY4, ARSE, ATP6V1A, CASP10, CCNE2, CD27, CD34, CYP2C19, EHMT2, GALNT7, GMDS, GNAO1, GSTA2, IFNA21, IFNG, IL22RA2, IRAK4, LLGL1, NFATC4, OR2AT4, OR2F2, OR2L8, OR4C11, OR4D6, OR52N5, PRKCH, PTGER3, RDH11, SIPA1, UBE2L3, VAMP1
Negative_1576	F2R, INS, RB1, ADORA2B, BLVRB, CASP7, CCNA2, EARS2, GNRH2, HIF1A, MAP3K8, OR10H4, OR52K2, PPP1R3D, PPP2R2A, PPP3R1, PTGIS, SELL, HSD17B12
Negative_1577	FST, ACAA2, AKR1C4, ALDH5A1, B3GAT2, C3AR1, COL3A1, DPM2, GYS2, IFNA4, IL21R, MCAT, NODAL, OR4F17, OR51T1, OR52R1, OR5AC2, OR5R1, PCYT2, PLA2G6, POLA2, PRKCH, RRM1, SESN1, SLIT1, SREBF1, TAS2R16, HSP90AB1
Negative_1578	C8B, FOS, GCK, TH, ACTN1, AGRN, ATIC, BIRC3, BLVRA, CALML5, CCL3, CCR5, CDH5, DFFA, DKK1, DPM1, DRD1, EPHA3, EXTL2, GALM, GCLM, GSTM5, HNF1B, IDH3A, IL11RA, IL22, KAT2A, MAGI2, NUDT5, OR10P1, OR2F1, OR52I2, OR6B3, OR8I2, PLXNA1, POLR2L, PSME2, RASGRF1, RRM1, SARDH, SEMA3A, SETDB1, SLC2A4, SULT1A1, SV2B, UGT2B7
Negative_1579	MAG, ADCY3, ALDH1A1, ASRGL1, BTRC, CAMK2A, CCL23, CCR3, CD44, CDC20, CES7, CSNK2A2, EFNB3, F13A1, FGF22, FGF8, FTCD, GALM, GALNT1, GSTA2, IFNA7, IL18RAP, ITGA4, JAM3, LTBP1, OR6Q1, PFKFB1, PLCB2, RBPJL, SARDH, SHMT1, SKIV2L2, SMC1B, TAS2R40, TPMT, UGT2B10, WHSC1L1, B4GALNT1
Negative_1580	B4GALT4, CALML3, EFNB1, HDAC1, ITGA9, OR52H1, RETSAT
Negative_1581	C9, CD2, SRM, ARHGEF7, C1QB, CARS2, CCND3, CD19, CD36, CD72, CLDN17, CSAD, ENO2, FCGR2B, GNA15, GPD1L, GRAP2, MECR, OR10G8, OR2D3, OR2T10, PARD6G, PGK1, PHPT1, POLR2A, PRIM1, PRKG1, PRPS1, RPS6KA6, SH2D1B, SLC27A1, SLC2A1, SOCS5, SPP1, SREBF1, STAT5B, VEGFC, YOD1
Negative_1582	B4GALT4, CLDN5, CSNK2A1, LIMK2, NANS, OR4A5, OR52E4, PDE1A, PFKFB4, PPBP, WEE1

Negative_1583	OSM, ACADSB, ACYP1, AGPAT2, ALAS2, BMP6, CCL21, CLDN20, COL1A1, CPT1C, CTNNA3, CYBA, EFNB3, ENPP7, EPHA4, EXT2, FPGS, GALNT12, GBA3, GMPR2, GNB1, GNPAT, GRIN1, GSTA4, IFNA2, IL1R2, KLRK1, LAMA4, LEFTY2, MAPK3, MAPK9, MDH2, OR10Q1, OR13C4, OR2T3, OR5AR1, OR8H3, PAX6, PCNA, PDE4C, PGLS, PLXNB1, RPS6KA3, TP73, VAV2, CACNA2D4, SERPIND1, TNFSF13B
Negative_1584	BAX, MUT, ABCC8, ALDH5A1, CD28, CDC25C, CSF2, DKK4, DUSP9, GZMB, HAGHL, OR10T2, PHKA1, PHKB, PNMT, PTCH2, RPS6, STK4, UROD
Negative_1585	DBH, DDC, GLS, GSN, ARAF, ARPC3, ARRB2, ATP5J, CD70, CHKB, COL4A6, ERBB2, F13B, FGF7, FPGS, GALNTL1, GMPR2, HSD17B4, IAPP, IFNAR2, IL19, IL1R1, IL1R2, ITGAV, MAGI3, MAPK10, MLYCD, MYL9, OR10Z1, OR2T10, OR5T1, OR8K5, PKM2, PNPLA4, PPID, PPP3R1, PRDX6, PRKAR2B, PTGIS, SDHB, SEMA4A, SEMA4B, SPRED1, TUBB4, ZMAT3, PNLIPRP2, SERPINA1
Negative_1586	HAL, PLG, ACSL5, ACVR2A, ACY3, ADIPOR1, ANAPC1, BMP2, CHRM5, CLDN2, CMPK1, CPT2, CREB3L3, DUSP3, ENTPD6, FABP3, GCDH, GMPPB, GPAA1, HAO1, HTR2B, IAPP, IL29, ITGB2, KNG1, LAP3, LDHA, MAD2L1, OR10G7, OR10H3, OR52I2, PFKM, PGK2, PPM1D, PPP3CB, RDH11, RHOD, SAT2, ST3GAL5, TAS2R7, UBA1, XCL1, YWHAG, MAP3K7IP1
Negative_1587	CRK, DDC, HDC, WAS, ADRA1D, ARG1, BMPR1A, CCL2, CCL4, CD247, CLDN6, CSDA, DKK1, EFNA5, F2RL1, FARS2, HPRT1, HPSE, IFNA8, IGSF5, JAM2, KAT2A, MAD2L1, MCEE, NAT6, OR10C1, OR10T2, OR1D2, OR2AT4, OR2Y1, OR6A2, PDE1A, PGAP1, PNPT1, RHOQ, RRAS, SLC27A1, SMC1B, TBXA2R, TUBA1B, ZAP70, ATP6V0D2
Negative_1588	SP1, ANAPC7, APOA1, BCMO1, CDC14A, DNMT3B, F2RL1, FGF23, ITGA7, MCM3, NUMB, OR10H2, PCYT1A, SMPD2
Negative_1589	AMT, LSS, ADSSL1, ALPPL2, APRT, BET1, CHKA, DERA, DLL1, DPAGT1, ETS1, FRAT2, FSHR, GNPDA1, GRM5, GRPR, MAP3K8, NCKAP1, OR2Z1, OR52J3, RASGRF2, SMPD2, SNAP23, TUBB4, UPRT, USE1, WNT16, CSGALNACT2
Negative_1590	CKB, ME3, ACACA, ACAT2, ACVR2A, ALG2, CD244, CDK5, CDKN1C, CHSY1, CRY1, DBF4, FPGS, GGT6, GLI2, GNPAT, GNRH2, HARS, ICAM1, MAN1A2, NEFM, NEGR1, OR10G8, OR10K1, OR2S2, OR4S2, OR4X2, OR51L1, PDE4A, PFN4, PHGDH, PIK3CG, PLOD1, POMC, PPAP2C, PPP2R1B, RASA2, RASGRP4, RDH10, RPRM, RPS6KA3, STX1B, UCP1, WBSCR17, ZAP70, EIF4EBP1, TNFRSF1A
Negative_1591	ACP2, CCL23, CD38, CERK, CHAT, DLST, DUSP3, MAT1A, NME4, NR1H3, OR51E2, PFKL, POLA1, PYCR1, SQLE, TAS2R1, TRPM5, WWP2
Negative_1592	AMT, ADCY6, GART, GRIN2D, HSD11B1, OR4X1, OR5B12, PNPO
Negative_1593	LHB, TAT, CACNG2, CHRM5, CLDN19, CNTNAP1, CXCR4, EXT1, HSD3B7, INPP4B, KIR2DL3, KLRD1, MAPK1, NAMPT, OR51I1, PON1, PPA2, RFNG, SUV39H2, TNFRSF12A
Negative_1594	IDS, ME3, AADAC, B3GALT6, BUB1, CALML3, DCTD, DERL1, DUSP16, EFNA4, ENTPD3, EPOR, EXTL2, F2RL2, GALNT14, GFPT2, IL12B, MDM4, NTRK1, OR4K5, PANK1, PDGFB, PIGU, PLCZ1, PMAIP1, PPAP2C, PPP2R2C, PRKG2, RAC1, RASA1, STX18, TAS2R14, TJP1, TLR5, TUBB6, ULK1, VKORC1, ZFYVE9, HSP90AA1, TNFRSF1A
Negative_1595	CDA, OGT, ACADM, ACSL4, ALDOC, C5AR1, CREB3L3, GCLM, NTF4, OR4F5, OR4X2, PCCA, SPTLC2, TGFB2, TLN2
Negative_1596	EBP, EGF, GHR, GP6, AARS, ADRA1B, CDH4, CRB3, CTNNB1, CTPS2, DDX51, DOT1L, FGF7, GRIA1, IFNAR2, IL28RA, LCP2, NAMPT, NQO1, OR51B4, PIGP, PLOD2, PVRL3, SEMA3F, TNFSF14, TUBB4Q, SERPINC1, SUV420H1
Negative_1597	BTD, C6, HYI, MAX, PGP, SI, ADORA2B, ALDH3A1, AMDHD1, ATG12, BCAR1, CCL27, CD55, CFLAR, CHUK, DAPK1, DLL1, DNMT3L, FOSL1, GPLD1, IL23R, LIPA, MAP3K11, METTL2B, MGAT5B, NAT2, OR10A2, OR10J1, OR1L6, OR51B5, OR51D1, PAK6, PNPLA4, PSENEN, SKIV2L2, SLC27A4, TSC1, UCKL1, WNT8B, MAPK8IP2, SERPIND1, ST6GALNAC5

Negative_1598	C5, OTC, ACY3, ADORA2B, ALG9, ATP6V0C, CYP19A1, DDX50, EFNA3, ENAH, FRAT2, GALNT13, LAP3, LIPA, LRRC4C, NCF2, NCF4, NME4, OGDHL, OR10H5, OR10J1, OR10Z1, OR11G2, OR1G1, OR1J1, OR2C1, OR2T34, OR4A5, OR5A1, OR5AS1, PLA2G5, PPM1B, RARS, RDH16, RPS6KA3, SETD8, SPCS3, TRAF4, UCP1, UXS1, WBSCR22, ATP6V1B2
Negative_1599	AR, F9, MPZ, PGP, ACMSD, ADCY10, BMP4, CACNG6, CD48, CLDN9, DUSP1, EFNA1, EIF4E2, EXOC7, EXTL2, GALNTL5, GGT7, ITGA7, MS4A2, MYH10, OR10H1, OR2L13, OR2V2, OR4D1, OR51D1, OR8G1, PECAM1, PLAUR, PLTP, POLR2F, PRLR, PSENEN, RRAS2, RXRB, SESN1, SETD7, SMPD3, SOS2, SRGAP3, STX1B, TICAM2, TRAF2, TRPM5, VCAM1, WNT8B, YWHAG
Negative_1600	CNGA3, DAG1, TSHR
Negative_1601	JUP, SDS, ACSS3, ADCY4, CYP2E1, DHRS9, DVL3, FASLG, GLDC, MGAM, NCF4, OR2S2, OR5J2, PAICS, PPARA, PRKCG, PYGM, SMARCA2, TBL1Y, VEGFA
Negative_1602	CDA, GP5, ALDH9A1, AREG, ARHGEF2, ATN1, ATP5C1, CCNA2, CDC45L, EXTL1, FLT1, HMOX1, HTR2B, KLRC2, MAP2K6, NUDT8, OR6T1, STT3B, SYNJ1, THBS2, WBSCR22, SERPINA1
Negative_1603	F5, GHR, MTR, ARHGEF6, ATP4B, AVPR1B, CACNB1, CD58, CSNK2A2, ERBB2, FGFR1, FPGS, GFPT1, GPD1, GSTM1, HAGH, IFNA7, LAMA4, LIMK1, MAD1L1, NOTCH1, OCRL, OR6F1, PIGH, PRKAR1B, RFWD2, SEMA3A, SMURF1, STK11, TAS1R2, TBXAS1, TPK1, TSNARE1, UNC5D, WEE1
Negative_1604	ACAD8, ALOX12B, ANAPC2, C5AR1, CACNA1H, CHPF, CHRM5, CLDN22, CLDN4, CSNK2B, CTBP2, DCXR, FCGR2B, FMO4, GAD2, GGPS1, GNG13, LAMB3, ODC1, OR4C16, PCK2, PIGH, RDH8, SLC2A1, TIAM2, TSC1, UNC5D, VARS, SERPINB5
Negative_1605	BTK, C8A, CR1, MAG, ACYP1, B3GALT5, BTRC, C1QC, CACNG8, CLDN6, CSF3, E2F3, EGFR, FADS2, FGF4, FGF8, GAL3ST1, LAP3, MAP3K14, MKNK2, MPDZ, MYH6, OR1J4, OR2H2, OR2T1, OR3A1, OR52R1, PAK6, PANK2, RARA, RRAS2, SDC4, SEMA3F, SLC12A2, SOX17, TIAM1, TRADD, TSHB
Negative_1606	F8, GK2, PRL, CARD11, GGPS1, MAP3K8, OR2T33, OR52E6, PANK3, PLCB1, POLR2L, RASA2, RPS6KB1, TBL1Y
Negative_1607	PVR, ACOT11, ACSL3, ACTG1, AKR1C3, ALOX15B, APRT, ARSD, CCL24, CXCL10, DNMT3B, GMDS, HTR5A, IFNA13, ITGA1, LYPLA2, MAOA, NAMPT, OR3A2, OR9Q2, PGM3, PMAIP1, PMM1, POLR3K, QPRT, SNAP23, STAM, SV2A, TAS1R2, TJP1, TNFRSF8, TYMP
Negative_1608	FUT6, PRKAA1, TNFRSF9, TRAF2
Negative_1609	GLA, GP9, BCL2, BMP4, CASK, CDC16, FGF2, FZD5, HDAC2, MAP4K1, MAPK11, NMNAT1, OR51G1, PAK2, PPP3CA, RERE, THBS3, TUBA1C
Negative_1610	HGD, IL6, LEP, OAT, ACAT2, ATP4A, CHPT1, GMDS, KIR2DL4, NUDT5, OR10A4, OR5D13, ORC5L, PNPO, SULT2B1, TGFB1, UGP2, ZAP70
Negative_1611	OR4K1, PIK3CA, PLA2G12A
Negative_1612	AR, SMO, ALDH4A1, ALPL, ARPC5L, BIRC3, CADM1, CASP6, COASY, FGF18, FGF5, GALNTL1, GNAS, GRHPR, HLA-F, IRS1, NCK1, OR2A14, OR52I2, OR8J3, PDE1B, PPP5C, RASGRF1, SHC2, TKTL2, UGT2A3, WASF2
Negative_1613	MSN, PAH, ABCC8, ARHGEF6, CLDN11, CREB3L4, CSAD, CYP26C1, DAXX, EIF4B, ENTPD2, GSTT1, HMOX1, IQGAP1, MAP3K2, OR4S1, OR52B6, OR8J3, PARD6A, PRKAG2, QPRT, UGCG
Negative_1614	BTK, EZR, LHB, NOG, OTC, ACVR1C, AGRP, BMPR1B, C4BPB, CCNA2, CDC23, CSF2RB, DNMT3B, DOLPP1, DOT1L, FCGR2B, FUT4, ICOS, IL13, IL21, IL23A, IMPDH1, INPP4A, ITGA5, KLK3, MAD1L1, MAPK11, NME7, NUDT2, OR10J5, OR2A2, OR51A7, OR52H1, PEMT, PLOD1, POLE2, RAC1, RBL1, RBPJL, RHOD, TAS1R2, TPH1, VEGFC, ST6GALNAC5
Negative_1615	ABAT, ACY1, ALCAM, ARHGEF6, CCNB3, CLDN18, CSF2RA, CYP1A2, CYP2D6, GRIN2D, HSD17B7, MECR, METTL6, MYL5, OR51B5, OR5R1, P4HA3, RBPJL, RCHY1, SFRP1, TAP2, TRAF6

Negative_1616	ATM, PDC, ADRB3, AGPAT4, B3GNT2, CADM1, CYSLTR1, E2F5, GPD1, HBEGF, IKBKE, IL7R, ITGB4, ITPK1, MCM5, MCM7, NAGLU, OR10J5, OR13A1, OR2AG2, OR4M2, POLR2D, RRAS, SEMA4F, THBS3, UGT1A10, UMPS, VASP
Negative_1617	CGN, FOS, KMO, ACTN4, ADRA1B, BDKRB2, C1QC, CAPN2, CCR2, CMAS, CNTFR, CSDA, CSF1, CXCL3, CYP19A1, CYP2B6, CYP2D6, FGF5, GLUL, GNPAT, GSTA5, GUCY2C, HMOX1, HSD17B1, ICOSLG, IFNA4, IRF7, KLKB1, LIMK1, MAP2K6, MIOX, NOX1, NSD1, OCLN, OR56A1, OR7C1, PAK6, PARD3, PDPK1, PIK3CB, PLCB1, SNAI2, ST3GAL4, TUBB2B, UROD, YWHAB, CSGALNACT2, PPP1R12A
Negative_1618	BTD, IL12RB2, PDGFB
Negative_1619	FH, EREG, IL18RAP, LCMT2, NRXN1, PLA2G4A
Negative_1620	A2M, LHB, ABI2, ACVR1B, ADIPOR1, ATP5G3, CCKAR, DUSP1, DUSP10, ENO3, GDF6, GNMT, IRAK2, LRP5, MNX1, MYH13, OR4K17, PLA2G10, POLR3A, SHC1, SLC2A2, TRIP10, VEGFC, WNT1, ARHGEF12, CACNA2D1
Negative_1621	AK5, AMT, CKB, MPL, ABAT, ACTA1, APOE, BCKDHB, CCR6, CHSY3, CLDN15, CNGA3, CXCR3, CYP2J2, E2F3, ENO1, FABP4, GALC, GAS1, GDE1, GNB3, GUCY1B3, IFNW1, KLRK1, LDHD, MCM6, MYLK2, NAMPT, NAT1, NOTCH3, OR52E4, OR5A1, PAPSS1, PIK3R5, PRKAG2, SDC2, SEMA4D, TAS2R8, TOMM40, TYMP
Negative_1622	AK7, FST, GAA, ANAPC2, APH1A, CACNA1D, CACNA1E, CCL16, CCR1, CD80, CHEK1, CLDN8, COQ6, F2RL1, GDF6, GLUD1, HSPA2, HYAL2, IDH3G, IFNAR1, IFNGR2, IL3RA, LCMT2, MMP1, OR10Z1, OR11H4, OR2M7, OR2T5, OR5D16, OR6C2, OR6C74, PARS2, PARVB, PIAS3, PPP2R5B, PPP3R2, PRKAB2, PTPRB, RAC3, RPSA, SARS2, SOS2, TLR3, TUBA1B, TUBB2C, ZFYVE16, ATP6V0A1, ATP6V1C2, ST6GALNAC6
Negative_1623	GH1, ITK, LSS, ACADSB, ACAT2, ACTC1, ARSD, ATP5J2, B4GALT4, C9orf95, CACNB2, CDIPT, CTSG, CTSS, CYP3A43, EPHA3, EXTL2, FGF14, GP1BB, HADHA, HCST, HNF4G, HSD17B2, HSPB1, HTRA2, LYZL1, MCM6, NEFM, NFYC, NTRK2, OR10G4, OR11H1, OR1J1, OR1S1, PKMYT1, PNPLA3, PON3, RASGRP1, RPS6KA1, SPRY2, TGFBR1, TLR9, TNFSF8, UNC5C, HLA-DRB3, TNFRSF1A
Negative_1624	ACAD9, ALG1, CAV1, CCNH, FABP3, IFNK, MYH6, NFE2L2, OR11L1, OR13G1, PER3, POLD2, SCLY, VAMP3, TNFRSF11A, TNFRSF6B
Negative_1625	ABLIM3, ALG12, APOC3, CCL13, CCL5, CDKN2B, CSF2RA, FOXA2, HHIP, IL11, IL12RB2, KITLG, NFATC3, OR1S2, OR3A2, OR4K17, OR5J2, OR6T1, OR7G3, P2RX3, PDHA2, PIK3CA, PIP5K1A, PPP2R5D, RBPJ, SFRP5, STX1A, TAS2R14, WHSC1L1, PAFAH1B3
Negative_1626	CDKN2D, CHIA, CNTNAP2, IGF1, MICA, NOX3, OR4X2, OR6C2, PRIM1, SKP2, TNFSF8
Negative_1627	GP6, JUP, OGT, TAT, ACAT2, APOA5, CAV3, CCL19, CCNB1, CD99, CER1, CPT1B, ENTPD6, F11R, F13A1, FLAD1, GPD1, GRB2, HTR2A, IARS2, LAMB1, LCMT2, MAD2L1, MPDZ, NCR3, NT5C2, OR8G1, OR8H3, OR8J1, PAK3, PRPS2, PSEN1, RPS6KA3, RPS6KA6, TAS2R60, TCIRG1, TLR2, UPRT, WNT11, WNT5B, ZIC2, HSD17B10
Negative_1628	DGKB, PPARA, PTCRA, STX16
Negative_1629	F11, ACSS3, ANPEP, CCKBR, CCL7, CD86, DDX19A, GLUL, MGAT4B, NOTCH3, OR4K1, OR8D1, PLAT, SETD2, TBK1
Negative_1630	SPN, CYP2B6, EXT1, FARS2, ITGA2
Negative_1631	GSS, IL8, SP1, ADCY7, AGPAT6, AMHR2, CSAD, CSNK1D, DDX19A, E2F1, EPHA8, EREG, FZD9, GANC, GPLD1, IL13, ITPKB, KLRK1, MAP3K8, MAPK1, MLLT4, MYL2, MYL9, OR4K13, OR4S1, OR6N1, ORC5L, PRPH2, PSENEN, PTGFR, SETD1A, SLC27A4, TCF7L1, TNFSF18, TSHR, B3GALNT1, CACNA2D3
Negative_1632	F2, ID1, ANPEP, CDKN2C, GJD2, HPSE2, LIPT1, NFKB1, OR1L1, TBL1Y, TUBA1B

Negative_1633	CRK, AARS, ACSM1, ACTN4, CAMK2G, CDKN2C, CREB3L3, CRKL, DKK2, EDA2R, FZD3, GALNT8, GIT1, IL18R1, MCAT, NFATC2, OR13C5, OR2G3, OR6T1, PFKFB4, PIP4K2C, POLR3K, PPCDC, PRKAR2A, PTPMT1, PTPN6, PTPRM, RAP1A, SPCS3, STX1A, SV2B, TNFRSF4, VAMP3, WNT4, CSNK1A1L
Negative_1634	CD2, CFI, DAO, ACSL6, ALS2, CCNA1, CHRNA7, CRLS1, CYP2D6, DHDDS, ENPP6, EXTL1, FOXA3, GUK1, MYLK, NANP, NCKAP1L, OR4K15, PPP2CA, PRKAG3, RASA1, SLC25A4, SPP1, SPTLC1, TNFSF4, ATP6V0D1, ST6GALNAC3
Negative_1635	AK3, LCK, PVR, TPR, ACO1, CASK, CDKN1B, CXXC4, DDX54, EPOR, HES1, HSPA1B, INHBE, LIFR, MAP3K7, OR4K15, PDGFC
Negative_1636	C6, ME1, ARPC5L, B3GNT5, CACNG1, CDC25C, CNGB1, COQ3, DHRS3, EXTL1, FGF23, GGPS1, GLDC, HDAC1, LDHD, OR1A1, OR4K14, OR51G1, PCYT1A, PHKA1, POLA1, PPP1R3A, RPS6, RUNX1T1, SMAD7, TAP1, YOD1, ATP6V0D2, SUV420H1
Negative_1637	C8A, NOG, AACS, ADH5, APC2, CACNG2, CAMK2A, CRKL, DDIT4, DPYS, FGFR4, HIF1A, HSD17B2, KLRC1, LDHB, LIPF, LIPG, MFNG, NCOR1, OR10K2, OR3A1, OR4D5, OR52N1, OR6M1, PARVG, PDPK1, PLOD1, PPP1R1A, PRKAR2B, PTGES, SSH1, THY1, TM7SF2, UPB1, WNT16
Negative_1638	F7, ITK, PGD, ALAD, ANAPC4, BLVRA, BTRC, CSF2RB, DRD5, DUSP7, EFNA5, FGF8, FMO5, GALNTL5, NOS3, OR52R1, PFKFB1, PGS1, PLA2G2D, PRKAG1, SNAP23, ST3GAL3, TAS2R41
Negative_1639	AHCYL1, ALDH7A1, ANAPC10, BET1L, COQ3, E2F4, MYH14, OR13C5, OR51A4, OR5C1, OR8J3, PFN3, POLE2, PPAT, PPP3CB, PTGIS, SETD1A
Negative_1640	ALG10B, ATP5C1, ATP6V0B, BAAT, CCL25, DDX18, FADD, FLOT2, GOSR2, HRAS, HSD17B4, IL7R, ITGA7, LILRB3, MPDZ, MYH8, OR10AG1, OR6M1, OR7G2, PFKP, PPP3R1, SDHC, SGMS2, TRDMT1, XCL1
Negative_1641	HGF, KDR, MSN, NNT, AASS, ACACA, ADCY10, ADCY7, AKT2, ALDH6A1, ASPA, BCAT1, CAPN2, CD55, CD58, CHAT, CHRM5, CHST13, CNGA3, COASY, DLL1, ERBB4, GALNT9, GPD2, GSTO2, HHIP, IL11, IL1B, IMPA2, ITGAD, LTA4H, MICA, MITF, OR12D3, OR4D5, OR4D6, OR5M8, OR6C70, PHKB, POLR3H, PRKCQ, PTGIS, STX10, UBE2L3, ULBP2, UROD, VAV3, CSNK1A1L, HLA-DQB1
Negative_1642	GP9, TXK, ACADVL, ACOT12, ALG2, ARHGEF1, BMP4, BMP7, CHST11, DARS, DUSP6, ENTPD6, GRIN2B, HSD3B7, IL2RA, IL3RA, IQGAP2, IQGAP3, LAMC1, MAFA, MCM7, MYH11, NCAM1, NFYC, OR6T1, P2RX1, PIAS2, PLA2G6, PLXNA3, PSEN1, RGS3, RRM2B, SLIT1, TAS2R42, UGT2B28, VNN1, ATP6V0A4
Negative_1643	BCKDHB, BDH1, CASP6, CHIT1, CYP27A1, ERBB4, GAL3ST1, GFPT1, HES1, IL8RB, MAML3, OR10C1, OR11A1, OR2A42, OR51F2, P2RX4, PARD3, PDE3A, PIK3CA, PRKY, RPE65, SEMA3G, SEPHS1, SGMS2, SOCS1, SPAM1, SPRED2, STX6, TAS2R41, TMLHE, WNT6
Negative_1644	DBT, FN1, NPY, AADAC, ABLIM1, ACO2, ACSM2A, ANAPC2, ARHGEF1, BBC3, CTSL1, CX3CR1, DHDH, ENO1, GSTP1, IFNA17, MGST2, OR4F4, OR6C1, OR9A4, PPP3R1, PRKAR2A, PTGDS, SPHK1, VANGL2
Negative_1645	C6, GPT, HAL, HK3, LCT, ACAD9, ACOX1, CACNA1B, DDX18, FOXA3, GNPDA2, HMOX1, OR10A7, OR5H2, PIGX, PTGER1, SOCS4, STK11, TGFB1, TRAF5
Negative_1646	C8A, CFH, DAO, AARS2, ACPP, ACSS1, CCND3, CD3E, CD79B, CPT1C, CXCL16, FCGR3A, GDF7, GRIA3, HEMK1, HRH2, ITGA9, MAPK12, MITF, MKNK1, MMP14, NR4A1, OR4B1, OR4M1, OR5D14, OR5D18, OR5P3, OR8S1, OXCT1, OXSM, PIGH, PNLIP, POLA1, PPA2, PRKCI, SPTLC2, TSNARE1, TYRP1
Negative_1647	FN1, DEGS1, ERBB2, LEF1, ATP6V1G1
Negative_1648	CS, MPL, NPL, AKR1C3, C1QC, CDH1, CDH2, CREB3, FGF5, FUT5, GCAT, GSTA3, IFNK, LEF1, MCM6, NCAM1, NME7, OR1J1, OR2A14, OR5AU1, OR5R1, OXSM, PAFAH2, PDE8B, PSME3, PYCRL, RAF1, SETD7, STAT1, STAT5A, SULT1A1, UGT2B17, ZFYVE9

Negative_1649	FAH,GP9,AMY2A,ANAPC4,AVPR1A,B3GALT2,ENTPD2,IL25,ITGB5,KLRC3,MAP4K1,OR10A6,OR10AG1,OR4A15,OR51E1,PFKM,PIGC,PIK3R1,RRM2B,STX6,TUBA1B
Negative_1650	ALOX12,HMGCS1,PON2,UGCGL1
Negative_1651	MOS,COQ5,CYFIP1,EGLN3,ITGB7,LIFR,NME2,NRXN2,OR10X1,PPP1R3C
Negative_1652	TFG,CACNA1F,DDX23,ETNK1,FGF4,HHIP,MAGI2,MAP3K4,NR5A2,OR13C9,OR1N1,OR8K3,PAK7,RALB,SLC33A1,SPHK2,SERPINA5
Negative_1653	GBA,AKR1D1,AREG,ARPC3,ATP5E,C3AR1,CTLA4,CXCL9,GLS2,GPD2,HADH,HSD11B2,MAPK13,NT5C2,OR4M2,PRSS1,PTEN,PTGES,SCP2,SULT1A4,XCR1,ATP6V1C2
Negative_1654	GPI,LIF,B3GNT2,BLVRA,CAPN2,CD19,CHST12,DNMT3L,DTX4,EFNA4,EPHX1,FGF3,FPGT,IRS4,JUND,LIMK2,MYH4,OR9I1,P4HA1,PLXNB3,RERE,SC4MOL,SHISA5,SKP1,SLC2A2,SMURF2
Negative_1655	CGN,DCT,FGG,MAG,ACOT11,CAMK2G,CDKN2B,CLDN9,CMAS,ETNK2,FCGR3A,HLA-<br>C,IFNA4,LIPC,NCAM2,OR4D11,PDE6H,PER1,POLR2E,PTPN6,SEMA4B,STX10,TBXAS1,TNXB,WASL
Negative_1656	ABO,CDC7,PFN1,STT3B,YWHAB
Negative_1657	MSN,ACSL6,ALOX15,ANAPC1,CCL25,CCL5,CDC7,CLCA4,COL6A2,CXCL10,EXT2,GDF5,GLUD2,IARS,LCAT,MGAT5B,MYH13,OR2A12,OR2K2,OR5H6,OR6C3,PAX8,PLIN,RAPGEF3,SMAD2,STAT3,STX8,TBK1,TUBA3E,ATP6V0D2,PPP1R12A
Negative_1658	OTC,TNC,WAS,CES1,CHKA,CLDN15,CYP4A22,CYP7A1,EDN1,EPHA4,FLT1,GIT1,GRM5,HEMK1,HSP90B1,IL6R,INMT,NAGLU,NAT5,NFAT5,OR14C36,OR1A1,OR2T29,OR3A2,OXTR,PFKFB3,PLAUR,PNPLA3,POLR2F,POLR2G,PTGS2,RAD51,SKP2,SULT2A1,SULT2B1,TIRAP,TLN1,TNFSF4,ULK3,PNLIPRP1,TNFRSF10D
Negative_1659	CDC25A,DRD1,GNG13,ITPA,OR52I1,PLXNA2
Negative_1660	F12,GBA,WAS,ALG2,B4GALT7,CCDC6,CD8B,CDH1,CDK7,CKS1B,COX10,CPT1B,CYP4A22,EI24,FUT6,FZD2,GALC,GNRHR,GUCY1A3,IL23A,IL2RB,MAT1A,NT5C3,OR2F2,OR2T29,OR2Y1,OR4C12,OR52E8,OR5T3,OR6C70,OR6N1,OR7A17,PCYT1A,PGLS,PIK3R1,PRKAB2,PRKCA,PSAT1,SAT1,SELP,SSH1,SUV39H1,TGFB1,TUBA1C,VEGFA,YOD1
Negative_1661	PGF,TPO,ABL2,ABLIM1,ASAH1,ATP5I,CPT1B,DBF4,FGF7,GMDS,GNAT3,GOSR2,GSK3B,HIBADH,HTR6,IFNA17,IQGAP2,LDHB,MMP9,NAMPT,OR11H1,OR2M2,OR5D16,PARVA,PIK3R4,PTGDS,PTGER3,PTK2,SHISA5,SPP1,TM7SF2,UGT2B7,ATP6V1G3,ST6GALNAC3,TNFRSF18
Negative_1662	OTC,ACO1,ACSL1,ARSD,ASCC3,ATP5I,CADM1,CCL20,CCL22,CCL4,CDC26,CDC45L,CLCF1,COL1A1,COX10,CYP2A6,CYP3A7,DDX4,EXT1,GALNT5,GLUD1,GMPPA,GNPAT,HSP90B1,IL25,MAPK14,NT5E,OR2L13,OR2T29,PLA2G2E,PMM2,POLR2I,PPP1CA,PPP2R2B,PTEN,RAC3,RASSF5,RDH12,RFX5,SFRP5,SIGLEC1,TGFB3,TGFBR2,TJP1,TKTL1,TMLHE,TRIP10,UGT2B28,XYLT2
Negative_1663	ITK,ADRA1B,AGTR1,ALG1,APC2,APOA1,C5AR1,CHAT,CHST11,CSNK2A2,EXT2,FRAP1,GNA12,HTR2A,IFT57,IL29,ITGA7,MTMR2,NOTCH2,OR2T5,OR4K17,POLD2,SNW1,ST3GAL3,UCK2,WNT4,SERPING1
Negative_1664	ATM,BTK,GP6,OGT,AKR1C2,ALAS2,AXIN1,AXIN2,CASK,CD8A,CNTNAP2,COX10,CXCR3,CYP11B1,CYP3A7,DHRS4,FGF9,HHEX,HMOX1,HRH1,IL10RB,INSR,IQGAP2,KLRC1,MAN1B1,MAP3K5,MAP3K7,MGST1,MYH6,MYH7B,OR1J1,OR51E1,OR6Q1,ORC1L,PKM2,PPARG,ROCK2,SRD5A2,TUBA1C,TUBB,UGCGL2,UROS,ZBTB16,ZIC2
Negative_1665	ATM,ACTG1,ASS1,B3GNT3,BIRC3,CDC27,CNTFR,GCDH,GDF6,GNRH2,GUCY1A3,HSD17B4,LAMB2,LIPF,OLAH,OR4A15,OR4N5,OR52D1,PRIM2,PSEN1,SH3BP2,SUCLA2,VAMP3,CSNK1A1L

Negative_1666	CBL, F5, ACACB, APRT, ARPC2, ATP2A1, DBF4, DDX4, GALK1, GLI2, GNRH2, IDH3G, MAOB, OR52B4, OR6X1, SUCLG1, TAS2R48, PDCD1LG2
Negative_1667	AR, EBP, ADH6, ATP13A2, CCNA1, CDC25A, CDIPT, CLDN10, CLDN9, CPT1C, CYCS, EIF4E2, FGF13, GAL3ST1, GALNT7, GLI1, GLI2, HCCS, HMBS, HMGCL, HNF4A, IDI1, LIPE, METTL2B, MYH13, NTRK2, OR11H4, OR56A3, PDE4C, PFKP, PHGDH, PLOD3, POLR2L, POLR3D, PORCN, PRKAA1, PTPRC, RARA, SAT2, SMURF2, SNAI1, SNW1, SPRED1, SUCLG2, TUBAL3, CTNNBIP1
Negative_1668	APOC3, CCND3, COL6A1, COL6A2, DGKE, HSD17B2, IRAK4, MYH4, NOX1, OR10S1, PRKAG2, RALBP1, RFXAP, UCK1, MAPKAPK5, PHOSPHO1
Negative_1669	CFD, ARPC1A, ARPC2, BDH1, CCR2, CCR5, CYP4A22, DDIT4, FUT7, HRH2, IL8RA, INADL, OR10A2, OR1C1, OR5T1, PRLR, PTTG1, SEMA3F
Negative_1670	FYN, HK3, SCD, ACAA1, ALDH3B1, C7orf16, CASP8, CLDN2, CNDP1, CYP1A2, EPHB4, ESPL1, GGPS1, GOT1, IDH1, IKBKB, ITGA5, KIR2DL3, KITLG, MAP3K2, NOS1, NRG2, OR10A6, OR10AD1, OR10H5, OR1L6, OR1Q1, OR2A42, OR51B6, OR52H1, PDHA1, PGLS, PIGS, PLD2, PPP1R1A, PROS1, SRGAP2, ATP6V0E1, MAPK8IP3
Negative_1671	FOS, GK2, ARNTL, DDX50, DDX56, FGF2, HNF4A, L1CAM, NME2, OR5M9, PAK6, PIGA, POLR1D, SMC1B, SOX17, TAS2R1, TAS2R42, TKTL2
Negative_1672	CBS, GBA, GLS, LTB, NLK, STS, AASDH, AGRP, AMDHD1, B3GALT5, BCL2L1, CAPN2, CCKAR, CCL24, CCR7, CDC23, CLTCL1, CXCR6, CYP19A1, DCXR, ENO3, EPHB4, FGF9, GALNT1, GNA15, GNPDA1, GSTM1, IL13RA2, IL1R2, INHBB, KLRC4, MAGI2, NARS2, OR2W1, OR5C1, OR6K3, PDE4A, PLTP, POLR2F, PPM1B, PTCH1, SMAD3, SMAD4, SPRY2, SUCLG1, VAV3, ATP6V0D2
Negative_1673	C7, COL5A1, CTNND1, CYP2D6, IKBKE, OR1D5, PDE6D, PFKFB2, PPM1A, PPP1CC
Negative_1674	EBP, SRC, ALDH1A3, ALOX5, ATP13A2, CAMKK2, COL3A1, CXCL13, DOLPP1, DTYMK, ETNK2, INADL, MAP2K4, MAP3K12, MYH1, NARS2, OR2A12, OR2M2, OXCT2, POLR2I, PPID, PRKG1, PYCR1, RERE, SKP1, SOCS3, SSH1, TRAF4, WNT3, XYLT2, ATP6V0A2, TNFRSF13C
Negative_1675	CAD, CD2, ID4, ALDH1L1, APRT, ARRB2, AXIN2, CD244, CDKN2C, CER1, CNTF, CNTNAP2, CSH1, CXCL16, CYP26B1, CYP8B1, CYSLTR1, EDN1, ETS1, FLT3LG, GIT1, GP1BB, GRIN2C, HSD11B2, HTRA2, IFNG, IL24, IRF9, KYNU, NR1D1, OR2AG2, OR2B2, OR2T11, OR4N2, OR6C70, PDGFA, POLR2K, PPAT, PPP1CB, PPP2CB, PTEN, SAT1, SCP2, TAS2R41, TGFB1, VANGL2, VAV2, WASF2, MAPKAPK3
Negative_1676	C1S, GLA, LHB, ALG6, DDHD1, DDOST, DDX19A, ENPP1, EPRS, GJA1, GUCA1B, HSD17B2, IL20, MAOA, MDM4, MYL9, NEGR1, NT5M, OR4N4, OR5M9, OR8K1, OXSM, PLA1A, PPARA, PTDSS1, RALGDS, SAT2, SETD8, THBS3, TRPC1, SUV420H2
Negative_1677	MUT, WAS, AARS, ACTN1, AMHR2, APH1A, ATP6AP1, CHUK, CLDN2, CLDN22, CPOX, CREB3, CXCL3, DPM1, DUSP5, EREG, EXTL3, F13A1, F2RL2, FABP1, GAS1, GNAT3, GPD1, GRIN2B, GTSE1, LALBA, LPAR1, MGAT1, MPZL1, MTAP, OR13H1, OR4N2, OR5AS1, OR5AU1, PANK1, PIGB, POLR1A, PPP1R3C, PRPH2, RCHY1, RPSA, SOCS4, SOS1, STX1B, SUCLG1, THBS1, TXNDC12, UCK2, ULBP2
Negative_1678	IL3, TXK, ACOT11, ALDH1A1, ARRB1, ASAH1, AXIN1, CACNB3, CD244, CLDN11, CSNK2A2, DAD1, DGKE, FMO4, GCH1, GNAS, GNPDA1, GOT2, HSD17B8, IFNA4, IFNGR2, MAP2K4, MAPK14, MDH2, MTMR2, NOX3, NT5E, OR56A3, OR5A2, OR5T1, OR6K6, ORC6L, PANK3, PLXNC1, POLE3, PPARG, RIPK1, SSH1, STAM2, TMLHE, TYMS, WASL, YES1, ALDH18A1
Negative_1679	IL5, MAG, PGD, RPE, STS, ACVR1B, ACY3, ALG12, AMD1, CD274, CD48, CD79B, COL3A1, CYP2B6, DUSP5, EPHA5, FGF14, GALNTL2, IFNB1, IL21R, INSR, LYPLA1, NEFH, NMNAT2, NRXN2, OR2T6, OR52J3, OR7G2, PAX4, PLD1, PPBP, PPP2R5B, PPP2R5D, PRIM1, SEMA3B, SESN3, SKP1, TCF7, THTPA, HLA-DRB5, SUV420H1, TNFRSF14

Negative_1680	ACSM2A,ACTC1,AGPAT2,AGXT,AMDHD2,ANAPC1,ATP5E,B3GNT4,BDH1,CACNA1D,CCR8,CDH2,CTBS,FGF10,FMO3,FTH1,GLI2,HAP1,IFNW1,ITGA4,ITGA9,MS4A2,NAT1,NFAT5,OR10G7,OR13C8,OR1L4,OR4N4,OR52N4,OR8H2,PAX8,PHKG1,POLE3,PPP2CA,PPP2R2D,RASGRP2,RDH10,RELA,SPP1,SUCLA2,TRPM5,CSGALNACT2,MAP3K7IP1
Negative_1681	DAO,HGF,IL4,LCK,ADRB1,ADSL,BMP5,C9orf95,CACNG7,CD3E,CD44,CD47,CMPK1,CTTN,EGLN3,ENTPD7,GPD1,GSTA1,GSTM1,HMOX2,IFNAR2,OR5I1,OR5V1,PGM3,PIK3C2G,PLCB1,PPP1R3D,PSAT1,PTGER3,RENBP,SMARCA2,STX5,TAP1,THBS3,TJP2,VEGFA,ST6GALNAC6
Negative_1682	CAPN2,NAE1,QPRT
Negative_1683	GDA,GLS,LYN,OGT,VTN,AKR1C3,ATP13A2,B3GALT4,BBOX1,BMP4,CYP2A7,ECSIT,ENTPD1,FBP2,FGFR1,FLT1,GFAP,GRLF1,IRF3,ITPKA,LTBR,MTFMT,NCOR2,NME4,NT5C,NTF3,OR2T12,OR51D1,OR51E1,OR5M8,OR6C2,OR6N2,PDE2A,SC4MOL,SMAD6,STAT1,TAS2R4,TNFSF4,UGT2B10,C1GALT1C1
Negative_1684	C4BPA,GJA1,PLCD3
Negative_1685	CD2,HGF,CCL7,COASY,ETS1,FOXA3,HLCS,OR5R1,PANK1,PTPRB,HLA-DRB3
Negative_1686	DBH,LIF,MAX,PRL,RB1,ACPP,ACVRL1,ATP5D,CCL16,CD19,CDKN1A,CHEK1,CTNND1,DMGDH,FRAT2,GLS2,GUSB,HPSE,IFNK,INHBC,ITPK1,JAG2,MAP3K2,MMP9,MYH13,NAT2,NT5C1A,OR2T1,OR5B3,PFAS,PIP4K2C,PLA1A,PON1,PORCN,PPBP,PRKG1,RASGRP2,RASGRP4,SC4MOL,SOCS7,SREBF1,TALDO1,TAS2R8,TJP1,UGCGL1,WASF1,CSNK1A1L,SERPINA5,TNFRSF1A
Negative_1687	GNE,KIT,AADAT,ACOX2,ACSL3,ACTN2,ADCY6,AMDHD1,AVPR1A,BPGM,COASY,DVL1,DVL2,ENO3,FADS2,FRAT1,GALE,GALNT10,GMPPA,HSD3B7,IL10,IL24,IRAK4,LAMA3,LRDD,MYL7,NEGR1,NQO1,NR5A2,NTF4,OR2T2,OR4F6,OR51A4,OR5M8,POLD3,PSME2,PSME3,RELB,SSX2IP,ST3GAL2,TBL1XR1,TP53I3,HSD17B10
Negative_1688	SI,ACAT2,ADCY9,AGPAT1,AHCYL2,ALDH1A3,B3GAT1,B3GNT5,BNIP1,CAV1,CCL26,CDKN2A,CSF2RB,CTSB,CYP21A2,CYP3A7,DEGS2,EPHA7,ERO1L,EXTL1,GRM5,HMGCL,IAPP,ICOSLG,INHBA,LIPE,MAP3K13,MARS2,NEU1,NFYB,OGDHL,OR1S2,OR2B11,OR2T34,OR51F2,OR52K1,OR5T2,P2RX5,PGM1,PTDSS1,PTGFR,PYCRL,SDHA,ST3GAL2,UGCG,UGT2B7,WNT7A,ATP6V1G3,ST6GALNAC5
Negative_1689	OAT,CCL13,CD3E,CSNK2B,DIAPH2,FOXA3,GALNTL1,IFNK,IMPA2,MAT2B,NCOA4,OR52A1,PGM1,POLR2A,RERE,RFNG
Negative_1690	GSN,BLVRA,DLL1,DUSP14,MAD1L1,MMP7,OR4C16,SMAD2,SULT1A2,TGFBR2
Negative_1691	ALG9,CCL25,CCR7,CYP2C9,CYP3A7,FGF21,GARS,GSTA1,OR1J2,OR2S2,OR5K2,OR8U1,PGM1,RASGRP1,SLC27A1,TBL1Y
Negative_1692	CAD,CD2,MYC,TNF,CCL15,CCR2,CLTA,DIAPH2,FLT1,GALC,HADHA,IL18R1,LARS2,MYL9,OR10A4,OR11H1,OR2A25,OR2H2,OR8S1,PCYT1A,RENBP,SEMA3E,TGFBR1,TRIP10
Negative_1693	CFI,CRH,CRK,PXN,TNC,ACLY,ACSL4,ARHGEF1,ATP13A2,CACNA1I,CCR6,CLCF1,CLDN20,CRY1,CYP1A1,DPM2,FTCD,GADD45B,HAGHL,HYAL1,IRF5,JAG2,KLRK1,LIMK2,LIPF,NRCAM,OR10G8,OR3A2,P2RX3,PHKG2,POLR3A,PPP2R5C,SDHC,TGFB3,TUBA1A,UGT2B4,WNT16,TNFRSF11A
Negative_1694	GP6,GSR,MPI,OGT,SFN,ADRA1B,AGPAT1,ALG1,ALOX12B,ATP5H,CALML3,CDC14B,COQ5,CYCS,DUSP6,EDAR,FBP1,HLA-F,IFNA13,IFNA21,ITGB1,JAK2,JAM2,MEF2C,MGAT3,OR14C36,OR2T5,OR4M2,OR51V1,OR5B21,OR7A10,P2RX2,PDCD1,PDGFRB,PERP,PIP5K1A,PON2,PPM1A,PTGES2,RDH10,RPSA,SMURF1,SPHK2,SPP1,THBS4,UPP2,KIR2DL5A,PHOSPHO1

Negative_1695	LSS,PAH,PXN,TKT,AKR1C4,ASS1,ATP5F1,CADM3,CCDC6,CES7,CREB1,CYP3A43,EFNB3,ETS1,FASN,FGF7,FRAP1,FZD7,GABARAP,GALNT12,GLS2,GOT1,HHEX,IFNGR1,IL1B,IL3RA,KAT2A,OR10V1,OR1D2,OR6C6,PAK6,PDHA2,PIP4K2B,PLOD1,POLD3,POLR2L,PRKG1,TAS2R10,HSD17B10,SERPINC1
Negative_1696	EPO,MSN,TFG,AMDHD1,ATN1,BHMT,BMP7,CBR1,CCL1,CNDP1,CYP2C9,GLO1,GRIN2A,IFNA17,ITPKA,LEFTY2,MAP2K2,MGAT5,MGST2,MTHFR,NANS,OR4D2,OR51L1,OR7A5,TLR9,ZBTB16
Negative_1697	C1S,CTH,ACTN4,ADH6,ASMT,DGKZ,HPRT1,IL20RA,ITGB6,LAMA3,OR5B21,OR7C1,PLD1,PLK1,POLD2,PPP2R5B,PRKG1,PSEN2,SARDH,SIGLEC1,SMAD6,ST3GAL6,THBS3,UGCGL2,UGT2A1,WNT8B,GABARAPL2,TNFRSF11B
Negative_1698	AGK,TNF,UBB,ADCY8,CD47,CYP4A11,FARSB,FGF21,GALNTL1,GARS,GRAP2,MAP2K4,NR5A2,OR10J5,OR52M1,POLE3,RBPJ,SUFU,TAS1R1,TUBA1A,WASF1,CACNA2D2,HLA-DRB3
Negative_1699	CDC14A,FASLG,FGF5,IL21R,MTHFS,MYH11,OR10J5,RRM2,SPRY3,THBS3,AASDHPPT
Negative_1700	CFI,GP6,HGF,IL7,LTB,AACS,ACMSD,ADCY9,ALAS1,AMY2A,ANGPTL4,ARHGEF1,ARNT,B3GNT5,BACE1,CAMKK2,CD40,CYP3A7,CYP4F2,DHDH,DHRS9,DRD5,ENPP1,EXT2,FGF18,FZD7,FZD9,GNAT3,IL19,NR5A2,OR6K3,OR6Q1,OR8J3,OR9Q2,PLD2,PPP2R5D,PTPMT1,RAC2,SCIN,SKP1,STX1B,TIAM1,UGCGL1
Negative_1701	F10,ABLIM2,ENTPD3,IDH3B,IGF1R,IKBKG,ITGA4,JAG1,NT5C2,P2RX6,PCNA,PDE2A,PIK3R5,PPT1,TAS2R49,TBL1XR1,TUBAL3
Negative_1702	FYN,RFK,ACVR1C,ADCY8,AHCYL1,ARSB,ASCC3,CDC25B,CEBPA,CHEK1,GIT1,GNMT,HIF1A,IGSF5,IL2RG,IPPK,ITGA2B,METTL2B,MYH2,MYH9,NPR2,NRXN1,OR2B2,PLCG1,POMC,PYCR1,RAF1,ROBO2,SEMA4D,SETD8,STAT3,TNFSF4,ARHGEF12,MAPK8IP2
Negative_1703	C6,CKB,RPE,ACSL1,ASIP,BMP2,CACNG7,CADM3,CALML3,CCNE1,CD3E,CSF2RB,CYP2S1,DPM2,ENPP7,FGF13,G6PC,GALK1,GZMB,IL12RB2,IL17B,IL7R,ISYNA1,MAP3K4,MAPK8,MGAT5,MGST2,MTHFR,NME2,OR10H4,OR3A3,OR4C6,OR5H6,PLK1,POLR1A,POLR3G,PPP1CA,PPP5C,PRPS2,RENBP,SEMA3C,SIGLEC1,SLC25A4,SORBS1,ST3GAL5,STX4,TICAM1,VDAC2,ATP6V0A2
Negative_1704	BAD,BTC,PRL,WAS,ADRA1B,AGPAT1,ALG10,BACE2,CDH4,CFLAR,CLDN18,CSF1,CTBP2,CYSLTR2,DARS2,DGKA,E2F5,IDH3A,IFNA14,IKBKB,IL20RA,IRS1,PRKY,PVRL1,RUNX1T1,SEMA4D,SMAD6,TGFBR2,THBD,THBS1,TNFSF10,VAV2,PAFAH1B3
Negative_1705	AMT,ATM,OAT,AMDHD2,AOC3,ATG3,B3GALT6,B4GALT6,CD14,CD8A,CDKN2D,CPT1A,CYP4F2,DAAM2,EPHA6,FARSA,FTMT,GNG13,HGSNAT,IDUA,IFNG,IL21,ITGA5,LRP6,MCM5,METTL2B,MGAT2,NME7,OR2L3,OR2Z1,OR4C13,OR6N1,PTGIS,PTGS2,SETDB1,SMAD7,TAP1,TBK1,KIR2DL5A
Negative_1706	UBC,ABL2,C9orf95,CAV3,CCR6,CMPK2,COQ7,CSNK2A1,DKK4,DNMT3B,EHMT2,FLT3LG,FZD3,GAB1,GGT1,GRLF1,GUSB,ICOSLG,IL17RA,LIPF,LYPLA2,OR10J1,OR10V1,OR1J1,OR2D3,OR5K2,PHKA1,PLD2,PRPS2,PSENEN,TGFB3,TNFRSF9,VDAC3,ATP6V1C1,HLA-DPB1,HLA-DQB1
Negative_1707	AR,BAX,XDH,ADSL,AKR1C4,APH1A,ATP5B,B3GALT6,BLVRA,CCND1,CYP3A5,CYP4A11,FMO1,GALNT7,GAS1,GNPDA2,HEXA,IL15,INHBE,KLKB1,KLRC1,KLRC4,LARS,MMP1,OR1M1,OR2L3,OR2M7,OR4L1,OR6F1,OR8K5,PLA2G3,PRODH2,PTK2B,SDHA,SEMA3E,SH2D1A,STAM2,SULT2B1,TUBB6,TYROBP,UBA7,UPB1,VARS,VTI1A,YOD1
Negative_1708	APP,BCR,F2R,GPI,PGP,AGRP,CCNE1,CHEK1,CHRM5,COQ6,DIAPH1,DVL3,GAD1,GNRH2,GSTM4,ICOSLG,IL10RA,JAK1,MAP3K8,NCKAP1,OR5B12,ORC1L,PANK2,PDE9A,PLA2G2D,POLR2B,RHEB,SETMAR,SNW1,TAS2R41,TUBA8,TUBB4

Negative_1709	ABO, PXN, AKT3, ARG1, CYP2F1, DARS, DNMT1, MAP3K13, OR10H5, OR2T11, OR8I2, PIGS, POLR2H, SFRP2
Negative_1710	GLA, JUP, ACSM1, ACYP2, CASP7, CCKAR, CCNA2, CCND2, CHST12, CIITA, CLTB, CUL1, CYP2A6, CYP4F3, DAPK2, DIAPH2, DRD2, EHMT2, GART, GAS1, GJA1, HNF1A, HSD3B1, IL1R1, IRS4, ITGB7, NAE1, NAGA, OR13G1, OR2A14, OR2Y1, PLXNA1, RASSF5, SOS1, SPCS3, TRAF3, TSHR, SERPINB5, ST6GALNAC3
Negative_1711	SMO, TK1, ACYP2, ASCC3, CAMK2G, CD82, CHD8, GBA3, PIP5K1C, PLEKHA8, PLXNB1, PRKG1, SEMA3E, SETDB2, SHC3, SNAP23, SQLE, SRGAP3, TRPC1, WWP2
Negative_1712	F10, ALDH9A1, CCL19, CCNE1, CLCA4, CNTNAP2, CYP2F1, FBXW11, GNAI1, MARS2, MGAT2, OLR1, OR5F1, OR6C1, PFN2, PIK3CD, PIP5K1B, PPM1D, PSEN1, SHC1, SYNJ2, TACR2, TGFBR2
Negative_1713	ADA, C5, PGF, PRL, STS, ACSL1, ADRBK2, AMD1, CACNA1F, CCL28, CCL3, CYP2A13, EDA2R, ESAM, FADS2, GDE1, GSTK1, INHBA, IRF5, MAPT, NAT1, OR4D2, OR4M2, OR52N4, OR6C4, PAK3, PDE10A, PDGFD, POLR2E, PTPN11, PVRL1, PVRL2, SGPP2, SNAP25, UGCGL2, VAV1, WNT10A, WNT16, WNT5B, XYLT2
Negative_1714	LPO, ABI2, ACSL6, ALDOC, ALG11, ATP5O, CD38, CEBPA, CHIT1, CLDN22, COL3A1, CREB3L1, CYSLTR1, DFFA, DUSP1, EI24, GALNT14, GLUL, GSTZ1, IL1R2, MPZL1, NRXN2, OR2A4, OR2L3, OR2T29, OR56A4, PI4KA, POLR3B, RAF1, RRM2, SOCS2, UPB1
Negative_1715	FES, MIF, TNN, ADORA2B, ARHGAP5, ARPC1B, CCL2, EFNA3, EFNA4, GNAI1, IL17RB, IPPK, LYZL1, NRP1, OR5H2, OR8J3, ROCK2, SMURF1, ULK1, ULK3
Negative_1716	C5, C9, DCK, MET, SP1, A4GALT, ATP5B, B4GALT2, BCKDHB, BMP8A, CACNA1E, CHDH, DGKA, DHDH, EDN1, EIF4B, ELK4, ENTPD8, FGF20, GALNTL1, GDF6, GNAZ, GUK1, IRAK3, KIR2DL1, LEPR, LIFR, LIPE, LRP5, NPAS2, OR1N1, OR2M4, PLA1A, PLCB1, PRIM1, PRKCI, PRLR, SCP2, SLC25A5, TYROBP, UBE2K, UGT2B28, WHSC1, TNFRSF10B
Negative_1717	AKT3, CD38, CSF1, DUSP4, DVL1, EI24, GSTZ1, HEMK1, ITGAD, MTHFD1L, NFKBIA, NLGN2, OR51L1, ORC5L, PSME1, RAB5A, RRM1, SOCS2, SRGAP3, SV2A, TNFRSF13C
Negative_1718	MUT, ADCY5, ALDH1B1, ARPC2, ATP6AP1, CCR7, CDC27, CDO1, CERK, CTF1, CYP2D6, CYP4A22, DDIT3, DERL1, F2RL1, FGF3, FGF8, FUT7, FZD1, GNAI2, IFNA10, IL21, MAN2A1, MGST1, MMP9, NCKAP1L, OR13C4, OR2A14, OR52K2, OR5C1, OR5I1, PARD6B, PFKFB4, PGS1, PHGDH, PIM1, PLA2G5, PLCB3, PLCG1, PRKCQ, RERE, SEMA4D, TAS2R40, UAP1, WNT7B, XCR1, YWHAG, TNFRSF10B
Negative_1719	ALG12, ATP6V1D, CRB3, EIF4E, HSD17B2, SESN1
Negative_1720	MTR, CD38, CSNK2A2, ITGB5, MAML3
Negative_1721	ABO, GPI, HDC, NNT, PLG, ACAA1, AKR1A1, ATP5L, B4GALT3, BMP8A, CACNA1E, CACNA1S, CDO1, COL11A1, COL1A2, CREB5, DDX47, FBP2, FLOT2, FMO4, GAD2, GALNT7, GRIA3, HSD17B4, MAPK10, MCAT, OR1C1, OR2C1, OR56A1, PANK4, PAPSS1, PCK2, PIK3CA, PPARG, PPP2R2A, PSME2, PTGS2, PTK2, RAPGEF1, SMPD1, TBL1X, TNFRSF4, TRAF6, UGT2B7, VKORC1, PAFAH1B2, PHOSPHO1
Negative_1722	AK1, BCR, ID3, LCK, TPR, AK3L1, ATP5G1, BIRC3, CACNA1H, CARS, CCL27, CCR5, CLDN9, CXCR3, DERA, DGKI, DHDDS, DUSP2, EPHB3, EXT1, FGF6, GNA11, HTR5A, HTR6, IDH3G, IFNW1, IL24, MYH7B, OR11L1, OR52N1, ORC2L, P2RX6, PIGO, RHOQ, RND1, RPS6KB1, SOCS7, ST3GAL6, TOLLIP, TPH1, ULBP2, WHSC1L1, ZMAT3
Negative_1723	ARNT, ATP2A1, CHIT1, FPGT, GNAZ, IFNA7, OR10A4, OR10G2, OR13G1, OR7A5, OR7D4, OR8K3, PAK6, PDE6H, PKMYT1, PLK1, RXRG, SOCS1, TUBA1A, TYMS, UCK1, VEGFB, WNT7B
Negative_1724	LYN, ADIPOR2, B4GALT7, CAMK2D, GCH1, GNG12, NKX3-1, OR5P2, PARD6B, PIGX, PIK3C2G, RASGRF1, UBA1, UGT1A10, TNFRSF6B

Negative_1725	CP,F3,OTC,CACNA1C,CCR2,CD79B,CSNK2A1,CXCL3,DDX54,DKK1,ERBB2,ERBB3,IKBKG,JAG1,LLGL1,MMP14,MYH7,OR3A2,OR4C12,OR5T1,P2RX4,PDGFB,PMVK,ROCK2,SDC2,SOD1,TUBA8,WNT6,YWHAE
Negative_1726	C8G,FAS,FGA,OTC,AHCY,BMP2,CACNA1H,ENTPD4,IMPDH1,LARS2,OR2M5,PANK4,ULK2
Negative_1727	DBT,KIT,ADIPOR2,CLDN18,DAXX,DOT1L,EPHB1,GALE,IL12B,IL18,IL18RAP,MAN1C1,MAPK14,MTFMT,NADSYN1,NKX6-1,OR51B2,OR51B4,P2RX4,PDE4D,PIGA,PLOD1,POLR2C,PRKCB,RPN1,SMURF1,SPI1,STAT1,TAS2R13
Negative_1728	BCR,DHH,F7,GAA,GP5,PGD,ABL2,ARHGEF4,ATP4A,CRHR1,CYP26A1,GADD45A,HSD17B8,IFNG,IL13,KIR2DL1,LAMC1,MAGI2,MAPK3,NAGA,NCF1,NOTCH1,OR6Q1,PANK2,PERP,PPP2R2B,PROC,RDH8,SH2D2A,STAT5A,TACR1,UGT2B17,HSD17B10
Negative_1729	OGT,ACP5,ALAD,CHSY3,DHCR7,FGF10,MAP3K5,MLLT4,NCR1,OR4A15,PLCG1,PTGS1,UGCG,TNFRSF10D
Negative_1730	MPI,ACSS3,ATN1,B4GALT2,CACNA1D,DNMT3B,EXOC7,FLT3,GPX7,HAP1,IQGAP1,JUND,LALBA,MAP3K5,MECR,NCKAP1L,OR51I2,OR52B4,OR52B6,OR8D2,PAX4,PHKG2,PIGM,PTPRJ,RALBP1,RPS6KA6,SEMA3D,SESN2,TJP2,TUBB4
Negative_1731	ADCY1,AKR1B1,AKR1D1,ARG2,ARHGEF6,ENO1,FLOT2,FUT4,ITGA6,ITGB1,LCAT,MPP5,NEUROD1,NKX2-2,NTRK1,OR13H1,OR1L1,P2RX1,P4HA3,PDE11A,PIGF,PPP3R1,TAS2R9,TGFBR1,THY1,TRADD,WASF1,WNT5A
Negative_1732	ID4,AMDHD2,ATP2A1,BCAT1,BDKRB1,CCL19,CDC42,CTNNA3,FABP4,GMPPB,IL19,ITPKB,LAP3,MAP2K5,MCAT,OR1J1,OR2L3,PFKFB1,PLCZ1,PPM1B,PPP2R2B,PRKCG,SAT2,SGMS2,TLR3,TSHR,TUBB6,MAP3K7IP1
Negative_1733	CKB,IL9,ACAA2,ACY3,AXIN1,BMPR1B,CDKN2C,CES2,CLDN11,CLDN20,ECHS1,ENTPD3,FSHB,GCNT3,GNAQ,GNG3,ITGA2,ITGAE,ITPKB,MCM3,NODAL,OR11L1,OR2M7,PI4KA,PIGV,POLR2E,PRKAB2,PRKCE,PVRL4,RAC1,STX8,CSGALNACT2
Negative_1734	CAT,ACOX2,ADSL,AKR1D1,CDIPT,CDKN2A,CSF2RB,HYAL2,MAP3K2,NCSTN,NT5C1A,NTSR1,OR4D1,OR5AC2,OR5AS1,PARD6A,PPP1CC,PPP2R2B,PVRL3,SETD8,SHC2,SUOX,TAS2R13,TP53I3,VAV2,WNT6,WNT7A
Negative_1735	IL7,MVK,ABLIM2,ACADM,ACP6,AKR1C3,AKR1D1,CAMK4,CD47,COL4A1,EPB41L2,GNMT,HAGHL,HLA-DRA,HSD17B1,IFNA7,IFT57,IL29,NCKAP1L,OR1A1,OR2T34,OR4C13,OR6T1,OSMR,P2RX7,PARVB,PIP5K1C,PPP3R2,TAS2R9,TIAM2,TYMP,WNT10A,GABARAPL2
Negative_1736	ADORA2B,ANAPC11,ARHGEF1,ARSA,CACNA1H,DAG1,DTX3,EPRS,FGF6,GGT5,GNPDA2,IGF1R,LIPC,MYH1,NMNAT2,OR13A1,OR51E1,OR6T1,ORC1L,PDE4A,RERE,RPS6KA5,SDHC,SLC2A4,SMC1A,ST3GAL3,SUOX,TFPI,TM7SF2,TNFSF12,TUBB,TUBB3
Negative_1737	DBI,HGD,PLG,ABLIM3,ACADSB,ALPL,C5AR1,CAMK2B,CAMKK1,CDK6,CLDN2,FSHR,FZD1,GUCA1B,HARS2,ICAM2,ISYNA1,MTMR2,NOTCH3,OR51F2,OR5H6,PHKG1,PLCG2,POLR1E,PPCDC,PPP3CB,SDC2,SRGAP3,STAT2,TNFSF18,VAV1
Negative_1738	ACO2,CASP6,DAPK3,E2F4,IL8RA,MGAT3,OR8D2,PPP2R5E
Negative_1739	ID2,IL2,APOA5,ATG7,ATP4B,BCL10,CTPS2,DAPK3,ENPP6,GALK1,GLI3,GLYCTK,HTR2A,MAGI1,MAP2K1,MYH8,OR5B3,OR5D18,PPAP2A,PRKG2,STX6,TJP2,TLR3,TPMT,WNT8B
Negative_1740	ALDH1A2,ASIP,EREG,HLCS,MAN1A2,MINPP1,NRG3,PRKAR2B,RCHY1,UGCGL1,PAFAH1B3
Negative_1741	ADCY6,ARNTL,ATP2A3,CCNH,CLDN8,GGT5,MAP2K4,NTRK2,OLAH,OR13C9,OR2T10,OR4A16,PER2,PMAIP1,PRKAB2,RUVBL1,STAT1

Negative_1742	DAO,GNE,SHH,UBC,ACTA2,AGXT,AKR1C4,ATP5D,ATP5I,B3GALT4,BCAT1,CCL16,CCNB1,CDC16,COL4A4,COL6A1,CPOX,CTSL1,DPYSL2,FZD4,GALNT8,GCH1,HTR2C,LIPF,LRP5,MAD1L1,MAOB,MPDZ,OR1C1,OR4D9,OR52K1,OR52N2,OR6C6,OR8J1,PANK4,PHGDH,PLXNA1,RCHY1,SMAD9,SMARCA5,SNAP25,STX8,TAS2R49,TAS2R60,TCF7,TNFSF4,UGCGL1
Negative_1743	DAO,LPL,AGTR1,ARPC3,ATP6V1A,CDIPT,CDKN2C,CLCA4,COL5A2,CSAD,DDX23,ERBB3,FUT7,FZD7,GCLC,GGPS1,GLO1,GRIN2D,GSTM3,HGSNAT,IL17RA,ITGB2,MAGI2,MICA,NODAL,OR4N4,OR6C76,PROS1,PTCH2,RALBP1,RASGRP1,ROBO2,RXRA,SFRP5,TRAF1,TSHB,UGCGL1,YES1,MAP3K7IP2,SERPINF2
Negative_1744	ALG6,ARPC5,CLDN11,GAL3ST1,KDSR,MAN2A1,PGLS,RAC3,RDH5,ST6GAL1
Negative_1745	CAT,DAO,HK1,HYI,INS,OGT,ACOX3,CDKN1B,CPT1A,CYP2C8,CYP3A4,DUSP1,FGF9,FUT5,IL13,IL20,KLRC2,LTA4H,MAT2B,MTMR2,OR10A5,OR1L8,OR2A14,OR2D3,OR52E6,OR5AC2,OR5AS1,PER3,PFKFB3,PGM1,PTCH1,SPRED2,SULT1E1,UBE2L3,WASF2,SUV420H1
Negative_1746	AMH,GP6,MPI,ACAA2,AMDHD1,ARPC3,ATF4,ATP4A,BDH1,BIRC3,CSF2RB,CTTN,DHDH,ERBB3,GRAP2,GRIN1,HLA-DOA,IFNA5,MAFA,NKD1,OR1I1,OR2A4,OR51A7,OR6F1,OR8B12,PDE4B,PIM1,POLR2D,PPT1,PRKACB,PTGES2,PYCRL,SEMA3G,SIAH1,TLR5,WASF1,WNT10B,SERPINA5,SERPIND1
Negative_1747	ABO,PGF,TNR,AANAT,ACP6,ADAM10,CDH2,CXCL13,CYP4A22,CYSLTR1,DUSP3,EI24,EIF4E,EXOC7,FGF20,GJD2,GNAI1,GNAL,GSTA2,HNF1A,HSD17B8,IFNA2,IRAK4,KIR2DS5,LIFR,LILRB3,LTBP1,MAML3,MDM2,NAE1,NCAM1,NME2,NTRK1,OR13A1,OR13G1,OR52N2,OR5M3,OR8G1,PDE10A,PDE7A,PHKB,POLR3D,PPA1,PRKACB,PRKCG,ROCK2,SARS2,SOS2,ST3GAL6
Negative_1748	AR,C8A,DCK,GAA,IL8,PTS,ALG3,ANAPC7,ANPEP,ARPC1A,BBOX1,CACNB3,ELK4,ENPP2,EPHB4,GALNT5,GCNT2,HLA-C,ITGA2B,LAMB1,LAMB4,MAML1,MAPK11,MCM4,OR13H1,OR52E4,PLXNB3,POLR3D,PSME3,RCHY1,RHEB,SPRY2,VCAM1,XYLT2
Negative_1749	CFLAR,CYP3A4,DGKQ,PIP5K1A,PNPT1,UROC1
Negative_1750	ADH5,ADSS,CAMK2B,CARS2,CYP1A2,DPYD,DUSP16,EPHB4,HNF4G,MLLT4,MLYCD,OR4D5,OR51F1,OR52K1,OR6K3,OR6X1,P4HA1,PCYT1A,PIGF,PPOX,PRKCB,PTPN1,SDC4,SOS1,TCF7,TICAM2,WNT2,TNFRSF10B
Negative_1751	CD2,FGB,GBA,PML,BPGM,CDC7,CTSL1,CYP3A7,DDX56,DPM1,F13B,FMO3,FZD4,GAD2,GALNTL1,GNAI1,GNAI3,GNAL,GSTM2,HARS2,ITGA11,KCNB1,KCNQ1,LCMT2,LDHB,MAP3K13,NEFM,NFKBIB,NT5E,NTRK2,OR10A4,OR1N2,OR2H1,OR51B4,PIK3R3,POLR2B,RAB23,SYNJ1,YWHAH
Negative_1752	C7,ACP1,CD44,CD79B,FZD4,PARVB,PFKFB4,WBSCR22
Negative_1753	ADA,AK1,GNE,ADRA1D,BMPR2,COASY,GLUD2,GRB2,GUCY1B3,MGST3,MYLK,NNMT,OR2L2,PLA2G1B,SIAH1,STX1A,UPP2,VDAC2,ATP6V0A4,B3GALNT1,HLA-DPB1
Negative_1754	CBS,CFD,SYK,TPO,ADSSL1,B3GALT4,C3orf10,CDK2,CYP2C18,EHMT2,MITF,MMP14,NFATC4,OR10C1,OR4N5,PIP5K1B,PLA2G2D,PRKAA1,RPS6KA5,SLC27A4,SMC1B,STK36,STT3B,TALDO1,TNFSF14,TPK1,TRAF1,VKORC1,WNT10A,ST6GALNAC4
Negative_1755	CCL15,CCL21,CNGB1,CTNND1,HARS,MAPK11,MAPK8,MDM2,PIGM,THBD,TIAM2,UGCG,WNT11,TNFSF13B
Negative_1756	ADO,ADCY9,BNIP1,CCL15,COL2A1,IDH3A,IFNA21,NAGA,NOTCH3,PLA2G5,PPM1A,RXRA
Negative_1757	AKR1B10,CXCR4,CYP3A5,DARS,GBA3,GNAS,LEF1,LIPT1,NKD2,OR2AT4,OR4X2,OR6C76,PPP1R3C,PSEN1,SETD2,SLC9A1,WEE1
Negative_1758	B4GALT6,CCL26,EHMT1,FGF17,HPSE2,NPR1,OR2T34

Negative_1759	C6,HK2,TFG,CAMKK2,CCL3,CD36,CD79B,CDC27,CLDN20,COL4A1,CYP11A1,EDNRA,ENTPD1,EPHA8,GLYCTK,GRPR,HHEX,HLA-DOB,IRF3,LAMB1,NCOA4,NLGN2,OR10G7,OR10S1,OR11H6,OR4C12,PDGFD,PLA2G4A,PMM1,PON2,PRNP,RALGDS,SOCS7,SORD,STX10,TACR3,TAS2R38,TLR2,YWHAQ
Negative_1760	DCK,CLDN2,ESPL1,EXTL1,INHBE,MAP3K4,OR14J1,OR4E2,OR5A2,OR5M9,PHKG2,PIK3R1,PKLR,SSH3,TAS2R42,WASL,C1GALT1C1
Negative_1761	F2,LEP,AK3L1,BBOX1,CASP9,CDC16,CISH,CLDN8,CMPK2,DLL4,DTX1,DUSP7,ECSIT,FTMT,FUT2,GNPDA2,GPX7,GRLF1,GRPR,INPP4B,KRAS,NPR1,OR1L6,OR4K5,OR5AS1,PDGFB,PDK1,PIGW,PISD,PPOX,SLC2A1,SORD,SOS2,SYNJ2,TAS2R4,TRPM5,XYLT1,ZFYVE16,ZMAT3
Negative_1762	IDS,LTB,ACO2,ACSM1,ACTN2,ARG2,ATIC,CARS2,CCL22,CHRNA7,CSF1,DDX18,DGKQ,DIAPH2,DTX3L,EIF4E2,GALNT6,HEXA,MAN1A1,OR1L3,OR52K2,OR56B4,PLCG1,PTPN1,TAS2R3,ZMAT3,CSGALNACT1
Negative_1763	BTC,C7,FGA,LEP,LHB,TNN,ANGPTL4,BCL10,BST1,C1QB,CDH5,CHKA,CHRM1,CXCL9,CYP3A5,CYP4A22,ENTPD8,EPB41,FARP2,FGF7,FSHB,GNAO1,IL4R,ITGAE,MAP3K12,MDH1,MEF2C,OR2A2,OR2D3,OR52A5,PDE4A,PDIA4,PLCB4,PLIN,PRKAR1A,RASSF5,RPS6KA3,SFRP5,SMC1B,ST6GAL1,TYROBP,VANGL1
Negative_1764	DDC,GNE,GPI,LHB,PVR,RET,ACAT2,ALDH3B1,ARPC5,CALML3,CCL25,CD46,CDH1,CDH4,CDKN2A,CSNK1E,CTSB,DGKH,DLL4,DPM2,FTMT,GSTM2,HAO2,IAPP,ICAM3,IRAK1,KLRC2,MGST3,MYH8,NEGR1,OR10G7,OR2F2,OR51E2,OR51M1,OR6C3,OR7G3,P2RX3,PIGO,PMVK,POLR3D,RPS6KA5,ST6GAL1,TCIRG1,TRDMT1,UROD
Negative_1765	PC,ALOX15,CD14,OR51Q1,PER1,PRDX6,SEMA4B,SPP1
Negative_1766	GALNTL1,MAN1A1,RBPJ,SDC2
Negative_1767	CKB,PTS,CBR3,CCL5,CCNA1,CDC6,CRB3,CYP2C18,G6PC,NFE2L2,OXCT1,POLD1,PSEN2,PYCR2,SDC4
Negative_1768	DCN,GGH,GNE,KMO,AKR1B1,ARNT2,CD44,CD59,CDS2,CSDA,DIAPH2,FZR1,LEFTY1,NRG4,OR10K2,OR51F1,PERP,PLA2G1B,PMM2,TBK1,TFPI,TYMS,WIF1
Negative_1769	MSN,ADRA1A,ARPC5,B3GNT4,CCR3,COL5A3,CYCS,DLST,GAMT,GPT2,HSD11B2,KIR3DL3,LAMA4,LCP2,MAD1L1,MAML2,MAP4K4,OR51A2,OR51F1,ORC2L,PAPSS2,PRKY,SLC25A4,STAT2,TAS2R1,TCF7L1,TP53I3,TYRP1,WARS2
Negative_1770	C1S,PC,ACAA2,ALG5,BET1L,CD59,CPT1B,CSF3,EVI1,HARS2,HNF1A,IFNW1,OR1L4,OR4F6,OR51M1,OR9I1,PISD,RAP1B,ST6GALNAC5
Negative_1771	AKT1,ATN1,ATP5C1,CCL1,CDH4,CRY2,DAD1,EPB41,GADD45G,GUSB,HSD11B2,IL4R,NME6,OR2T12,OR56B4,ORC5L,PHKG1,PIK3C2B,PPP3CC,TAS1R3,ST6GALNAC1
Negative_1772	CHAD,COQ5,FBP2,G6PC,GOT1,PIK3R5,TLN2,VANGL2
Negative_1773	BTK,C5,OAT,PVR,AGPAT3,COX15,FMO2,GALNT12,GGPS1,GLB1,HIF1A,HMGCL,MMP2,NAT5,OR2T34,OR52R1,OR5K1,PON1,PPM1B,RPS6KB2,SETDB2,SMAD9,SMURF2,TSC2
Negative_1774	KHK,SMS,ADRB2,AVPR1A,B3GNT4,CARS2,CCR9,DDX23,DUSP3,EFNB2,FGF22,GNAO1,GPLD1,GPX7,GRIN2B,HADHB,HDAC2,IFNAR2,IL10RB,IL19,IL29,ITGA11,ITGA8,LALBA,MAPK7,MYD88,MYH15,MYLK3,NME4,OR1E1,OR2A5,OR51A4,OR51V1,OR52N4,OR5B12,OR5D16,OR7D2,PLCG2,PPP3R2,TAS2R14,TRIP10,TRMT11,TUBAL3,TYRP1,WNT7A

Negative_1775	CBS, F9, GP9, ID2, MSN, XDH, ABLIM1, ACPT, ARNT, ATF4, BACE1, CACNA1C, CACNG1, CD40LG, COQ3, COX10, CTPS2, CXCL10, CYP2A7, CYP2B6, DDX47, ELK1, GMPR, HGSNAT, HLA-DMA, HSD17B8, INHBA, INHBC, KIR2DS5, LAP3, LARS2, MRAS, NR4A1, NT5C1B, OR14C36, OR2H1, OR4K17, OR9Q1, PCYT1A, PIK3C2B, RBPJ, SMARCA2, SULT2B1, TAS2R7, ATP6V1C1, GABARAPL2, TNFRSF13B
Negative_1776	F2, ADSSL1, CACNA1G, CACNB4, CALML5, CAMKK2, CHST11, DAD1, DFFB, DUSP9, EPHB3, FLOT1, FZR1, GSTM3, IFNAR1, IL21R, MDH2, MDM4, MGAT3, MMP1, NCF1, NCKAP1L, NR4A1, NSD1, NT5E, OR2T4, OR4F17, OR52L1, OR5F1, PDE5A, PIP4K2A, POLE2, PRKG2, RRAS2, SEPHS1, STX6, SULT1E1, UGT1A10, TNFRSF17
Negative_1777	SPN, TAT, ACVRL1, ADIPOR1, ALDOB, ATP6V0C, CCR5, CDC27, COMT, CYP4F3, CYSLTR1, DEGS2, DPM2, F13A1, FUT7, GCNT2, HAP1, HMOX1, HTR4, LEF1, MMP14, NOTCH2, NRG4, OR8D1, PDPK1, POLR3G, RDH12, RENBP, RPRM, RUNX1, SEMA7A, SNAP23, SOS1, TFDP1, UGT2B11, ATP6V1B1
Negative_1778	FGG, GH1, ACAA1, AGPAT6, ALG3, ATG3, CCL22, CNGB1, DOCK1, FECH, GADD45G, IMPA1, MCCC2, MKNK1, MYH15, NCF2, NCK1, OR2T11, PGAM1, PNLIP, PNMT, POLD3, POLR1C, PTPN7, RAP1B, SSH1, TAS2R41
Negative_1779	RDX, CAMKK1, CCL21, FRAT2, HSD17B3, MAP3K8, OR4D1, OR7C2, PFKFB2, PTPN1, PYGB, TSHB
Negative_1780	MASP2, OR5D14, PFKFB4, VAMP2
Negative_1781	ACAA2, ALDH1A2, ALDH9A1, ALOX5, ANAPC1, CASK, CRLF2, EFNA3, NTRK1, OR1J1, PITX2, RUNX1, SESN2, TNFSF9, TRAF5, UGT2A1, ULK1, YWHAE, SERPINE1
Negative_1782	AK5, GBA, ATP13A2, B3GALT1, CCNA1, CD46, CDC25C, CHKA, COL2A1, CRB3, CSF2RB, DERA, ECSIT, OR2T1, OR51B2, PIGV, PPARG, PPP2R2D, PRKCE, RASGRP2, SFRP5, SPRED1, SUCLG1, SV2B, TBXA2R, TNFRSF10A
Negative_1783	AK2, ACOT12, ALOX15, APPL1, B3GAT3, BUB1B, CACNG4, CLDN4, CLDN8, CMPK2, CRLS1, CYP2A7, E2F3, F13B, FBXW11, FGF1, FUT4, GCAT, GRPR, GZMB, HAP1, HLA-C, IDH3B, IL12A, MMP14, OR1L6, OR2M4, ORC1L, PDGFRA, PDIA4, PFKFB3, PLA1A, PRKAB2, RUNX1, SEMA4F, STX10, UGT8, ULK1, HLA-DPB1, SERPINA5
Negative_1784	C6, LTB, MSN, ALDH3B1, ARPC4, B3GALT2, CACNA1C, CCR6, CD99, CYP2A13, DEGS1, DERA, DRD2, FUT4, GADD45B, HAGHL, HTR2B, IL7R, MFNG, NAT6, NCK2, NCSTN, NFASC, NUMB, OR10A6, OR51B2, PFN4, PLA2G2F, PTPRF, RAPGEF2, RIPK1, ROCK2, SETD2, SMURF2, STX5, TAS2R42, THY1, TSLP, ULK3, UPRT, WASF2, TNFSF13B
Negative_1785	LCT, TK1, TK2, ABL1, ADCY7, ALAD, APC2, CD226, CDC45L, CLOCK, DDX51, GADD45B, KLK3, KLRC2, MAN1A2, MAPK3, MCCC1, NCSTN, RASGRP3, SCIN, SKIV2L2, TRPM5, TYMS, UNC5A, SLC25A31
Negative_1786	APP, LEP, ACOX1, AHCY, ARPC1B, B3GALT6, BCMO1, BDH2, CLDN6, CXCR4, CYP11B2, FASLG, FPGT, G6PD, GLI2, HLCS, IDH3A, ITGAM, JAM3, LEFTY1, NAT5, OR2T11, OR5AR1, OXCT2, PPP3R1, RPS6KA2, SENP2, TP53, CACNA2D2
Negative_1787	EPO, HDC, IL6, TK2, BMPR1B, CACNG4, COQ7, CYP2C18, DAPK1, GALNT1, GGT5, GP1BA, GPT2, GRLF1, HSP90B1, IFIH1, ITGA2, MAP2K3, MYH1, NTRK1, OR10H4, PARD6B, PARVB, PFAS, PHKA2, PLOD3, SARS, SEPHS1, STX4, SULT2A1, VDAC1
Negative_1788	CBL, CGN, GNE, HK3, ACTG2, ALOX15, AREG, CCKAR, CCR3, CD80, CRY2, DEGS2, DHCR7, E2F2, EHHADH, EPHA7, FARP2, FASLG, FZR1, G6PC, GADD45G, GALK1, GJD2, HNF1B, IGFBP3, IL13RA1, ITGB2, LAMB3, LCAT, MAPK1, NCKAP1L, NCSTN, NFKBIB, OR14C36, OR5T1, OR7C1, ORC4L, PDE7B, PFAS, PIGV, PRIM1, PRKAB1, RRM1, TMLHE, TRAF6, UCKL1, VAV1, ATP6V0E1, ATP6V1E1
Negative_1789	C2, B3GNT3, CASP7, DDB2, DDX50, FGF12, HSPA2, IFNA16, OR52E8, OR56B4

Negative_1790	EPO, ACADSB, ACP6, ACTN2, ARPC5, DAG1, FUT6, FZD5, HEMK1, ICAM1, ICAM2, LAMB1, LIMK1, NMNAT3, OR4M1, OR6C2, PDXP, PLEKHA8, POLD3, POLE4, PROS1, PTTG1, RASSF1, RFNG, ROBO2, SMAD4, STAM, SERPINF2, SLC25A31
Negative_1791	GH1, CXCL1, OR6A2, PDE3B
Negative_1792	DDO, TKT, CCL13, CHPT1, DHFR, ENPP3, ERO1L, FZD2, IL3RA, NR1H3, PFN3, PLAUR, POLR2D, SCP2, SFRP2, SPCS3, TUBAL3
Negative_1793	MAG, ACADM, ACO1, ACSM5, ACVRL1, AKT2, COL5A2, DAG1, DAXX, ENO2, FARSB, FLAD1, GAD2, GUCY2F, HLA-DOB, INPP4A, IRF9, NCAM1, NFKBIB, NUDT12, OR1D2, OR1S1, OR4N2, OR5J2, OR9K2, PAX8, PDIA4, PIP5K1A, PSEN2, PYCR1, RBL2, RBPJ, TPK1, TPMT, UGT2B17, UGT2B7, UPP2, ATP6V1E1
Negative_1794	AKR1D1, CACNA1B, RXRA, TLN1
Negative_1795	HGF, LCK, LTB, NLK, ASAH1, B3GALT6, B3GNT5, BET1L, C1QC, CDKN2C, CSF1R, CXCL3, DAD1, EARS2, ESAM, FDPS, ITCH, LIMK1, MNX1, NPR1, OR1L4, OR1L8, OR2L13, OR6C74, PDGFA, PFN4, PLA2G4A, POLD2, SOX17, WNT16
Negative_1796	CAMK2G, CRLF2, DNMT1, ELK4, GRPR, GSTT1, IL12A, IL4R, IMPA2, LALBA, NR1H3, OR10G8, OR2B2, OR5D13, PER3, PIAS4, PIM2, PMVK, SDC3, SMPD4, UROC1, MAPK8IP2
Negative_1797	DAO, IL6, IL7, MTR, SRC, ADCY2, ADRA1A, ADRB1, AKR1C4, BLVRA, BMPR1B, CCL11, CXXC4, ERO1L, GCH1, GMDS, GP1BA, HAO2, MAOA, MCM3, MTHFR, NCKAP1L, OR2G3, OR2L13, OR51M1, OR52E6, OR5AK2, OR6M1, OR7A5, P4HA3, PARD3, PARVA, PDE8A, PIGO, POLR1A, PPARD, PYGL, RAC3, RASGRF1, RDH10, SDHA, SLC25A4, TCF7L2, TSNARE1, WNT6, YARS2, B4GALNT1, PLA2G12B
Negative_1798	DDC, ATP6V0C, ATP6V1H, BCAR1, BLVRB, CREBBP, EIF4B, FUT9, GGT6, GNPNAT1, HLA-DOA, IL21, IL25, JUND, MAP2K2, MKNK2, MLLT4, MYH15, NKX6-1, NT5M, OLAH, OR5B12, PDE2A, PHKA1, PIK3CD, PIP4K2C, PIP5K1C, PPM1A, PTGDS, SMARCA5, THTPA, UMPS, VKORC1, ZFYVE9, PHOSPHO1
Negative_1799	DDO, GSN, ALAS2, ARAF, ATP5G2, CD82, CLTA, CYP26A1, DIAPH2, ENPP7, ERBB2, FBXW11, FGF17, GCNT4, GPT2, HSD17B4, HTR2C, IPPK, LARS2, MAP4K4, NAGK, NKD1, NKX3-1, NME7, NRCAM, OR2B11, OR4S1, OR5AP2, OR5P2, OR8D4, PDGFC, PFN3, PIGT, PITX2, PPM1B, RAF1, RELB, SMAD6, SPAM1, SRGAP2, STT3B, SUV39H2, TUBA3C, TUBB, ATP6V1B2, SERPINF2, TNFRSF10B, TNFRSF12A
Negative_1800	DAO, F2, CDKN1B, HTR2C, RUNX1
Negative_1801	EDA, IL7, ZYX, ARNT, GGT1, IFNA4, KLRK1, RAC3, RAPGEF1, RPS6KA4, SDC2
Negative_1802	DCT, GAA, NLK, ABLIM3, ACADSB, ACSM2A, ACSS2, ADRA1B, B4GALT7, CACNG6, CHSY1, CREB3, CYP11A1, DDX4, DGAT2, DPM3, DVL3, GOT2, GSTP1, GUCY1A2, IDI1, IL25, MAFA, MAP3K14, NFASC, NRG3, OR51B2, OR8H3, PDHB, PMM2, PRKACG, RASGRP4, TCIRG1, TJP2, UGT2B10, UGT2B17, WARS2, YOD1
Negative_1803	TFG, XDH, AADAC, ADCY3, ADORA2B, BST1, CD244, CHAD, CHKA, CHPT1, CKMT2, CLTA, CTSL1, DARS2, DDHD1, DDX52, DHRS4L2, DLST, FBP2, FGF8, GALNTL2, GPX5, HARS2, HNF1B, ITGA2B, KDSR, MAP3K4, MCM6, NCF2, ODC1, OR1G1, OR2T33, OR4F4, OR4K1, PAK4, PMAIP1, PPAP2C, PRPS1, SELE, SLC2A2, SOCS5, TJP1, TPH1, TUBB3, UNC5A, VDAC1, VTI1A
Negative_1804	SFN, ADRB1, ADSSL1, APPL1, CLDN18, FMO4, GRIN1, GUCA1B, HADHB, IDH3B, IL2RA, JUND, MAP4K4, NPR1, OR1L3, OR5H2, OR6K3, OR9G4, PKMYT1, PPP2R5B, PYCRL, STX6, TCF7, WBSCR17, MAPKAPK2
Negative_1805	ALG3, OR2L2, TRAF2
Negative_1806	ALDH1B1, ATP13A2, CD40LG, CREB3L2, CSF3, CTNNA3, ENTPD5, FGF17, GOSR2, KLKB1, OR10H4, OR4K2, OR6C70, OR9G1, PPOX, PPP1CC, SUFU, SERPINB5
Negative_1807	EPHA3, MAPK10, MRAS, PPM1D, PPP1CC, RFX5

Negative_1808	ZYX, ACSS2, AGXT2, ALDH9A1, ATP6V0C, CCR1, CREB1, CTSB, CX3CR1, FBP2, HTR6, ICOS, JUND, OR2A25, OR2T12, OR5AU1, OR6N1, OXCT1, PGAM2, PIK3CA, PLAT, POLD1, POLR3A, PTPRR, SEMA4G, STK36, TAS1R3, TAS2R14, UGCGL2, VEGFA, WARS2, WHSC1, WNT8B, HLA-DRB3, PAFAH1B1
Negative_1809	ILK, SHH, SP1, AFMID, ATP5A1, CCR6, DDIT4, ENTPD7, FGF4, GPD1, HTR6, MARS2, MKNK2, NFKBIB, NKD2, OR4C46, PLEKHA8, PRKAG3, YOD1, ATP6V0D1
Negative_1810	ATP6V0C, CYP26B1, DHCR24, DLAT, OR5P2, OXCT1, PER2, WEE1, ATP6V1B2, ATP6V1C1
Negative_1811	CRH, CS, PGD, TYR, ACSS2, ALDH3B1, AVPR1A, BACE2, CDC2, CDC25B, CFL2, COL1A2, CSF3R, DCTD, ENO3, GDE1, GNA15, GPD2, GZMB, HADHA, IDI1, IFNA2, IPMK, KCNQ1, LALBA, MAP3K7, NAE1, NLGN1, NTRK2, ONECUT1, OR2M7, OR2T5, OR4L1, OR51B2, OR52E6, ORC2L, PAPSS1, PDE10A, PIGX, PLCB4, PPP3R1, PTGER1, SEMA3D, TIAM1, TNFSF11, TUBB6, WNT2B, ARHGEF12, ST6GALNAC5
Negative_1812	GSR, TNF, ABCC8, ALG10, ARNTL, ARSB, B3GAT1, BCKDHA, BMPR1B, CACNA1E, CACNG7, CCL21, CHRD, CNTF, CYP2F1, DDX54, DVL3, EDNRB, EPHA2, FGF6, GAD1, IL28RA, LAMB1, MIOX, MKNK2, NCR3, NNMT, NR5A2, OR2T11, OR4C16, OR4K1, OR7G1, OR8D4, P2RX3, PDE3A, PDE9A, POLA2, PPARA, PTGER3, RALGDS, RPS6KA3, SIPA1, SMURF1, SORD, TAS2R60, TLR4, UGCGL2, WNT7B, YARS2
Negative_1813	STS, ALDH6A1, GADD45G, GUCY2C, JAM3, MAP3K4, MAP3K7, OR10G7, OR10Z1, OR52N4, SETD1B, SKP1, TACR3, TGDS
Negative_1814	KHK, LSS, NOG, ACSL4, ACVR2B, AKR1C1, ARG2, ASRGL1, BNIP1, CAPN2, CNTF, CPOX, CYP17A1, DHCR7, ENTPD1, FLOT2, GNRH1, GRPR, GSTO1, HPSE2, IAPP, IFNA8, IPMK, JAG2, MMP9, MNX1, NCF2, NRG3, OR2B2, OR2Z1, OR4C11, OR52A5, OR52K2, OR5B12, PFN4, PIK3CA, PLA2G6, POLR3B, PPT1, PRKAR2B, RAPGEF1, RDH5, SIPA1, SMARCA2, STAM2, TBL1X, TMLHE, PPP1R12A
Negative_1815	BST1, CLDN17, COMP, NCOR1, OR9K2, PIK3C3, PRKCQ, RARS, SLC2A4, TAS2R7, ULBP2
Negative_1816	EPO, FH, MPL, MUT, SP1, AARS2, ADSS, ALAS2, ALDH1A1, C3AR1, CCL15, CFL2, COL5A1, CXCL5, DDB2, DDX19A, GP1BA, HHIP, HPSE, IL23A, MGAT4B, NEFM, OR52J3, OR6C76, PLIN, PNPO, POLR2F, PRKAR2A, PTAFR, PTK2B, RAF1, TCIRG1, TUBB4, ARHGEF12, ST6GALNAC3
Negative_1817	ADK, GNS, HGF, AGPAT2, ALDH1A2, ALPP, ATP2A1, CD247, CES2, CTPS, EHMT2, EXT1, FGF23, FZD10, IFNA8, ITGA11, LILRB3, LIMK1, LRP5, MAP2K6, MAP3K1, MYH11, MYH2, NCR1, NR1H3, OGDH, OR2B2, OR52L1, OR9G4, PGM2, PIGC, PIK3C3, PTPRB, RPS6KA6, SEMA3D, SEMA4C, SLC25A5, SULT2B1, TAS2R48, UBA1, ULBP3, VDAC2, VEGFB, WBSCR22, ATP6V1B2, CACNA2D1
Negative_1818	MYC, ADH1B, AGRN, ARSE, CCNG1, CERK, MAP3K5, OR51M1, PIGF, PRKG2, SMPD1, UNC5B
Negative_1819	AMT, SMS, ADRB2, EXT2, FARS2, HEMK1, OR13G1, OR1G1, PLCE1, PSME3, TRIP10
Negative_1820	HGD, RB1, TEC, ABL1, ACSL4, ATP4A, BST1, CCKAR, CCL15, CCR2, CD19, CD244, CDC7, CHAD, CYP2S1, DPAGT1, ESAM, FUT9, GMPPB, HTR2A, ITPK1, LIMK2, MAPK7, MASP1, MCM3, MIOX, MPDZ, NR4A1, NT5E, OR2L8, OR4F6, OR5AN1, PHKG2, PLA2G4A, PRKCQ, PRKCZ, PSME2, PTGER1, SLC2A1, SLIT2, TAS2R4, TAS2R41, TSC1, ULBP3, ULK1, UROC1, WASF2, SERPINA5
Negative_1821	AMH, GHR, MAX, PGP, TNF, ADAM10, ALDH1A3, APOC3, ARRB1, CCDC6, CD80, CHEK1, CHRD, CLOCK, CSF2RB, CYP3A4, DHRS3, EPHB1, FSHR, GLDC, ICOS, IFNA21, IL11, IL12RB2, LCAT, MAP3K12, NFATC1, NFATC4, NRG3, NTSR1, OR2M7, OR7D4, PARD6A, PEMT, PIP4K2A, POLD2, POLR2E, PPP2CA, PRKCD, SEMA4A, SEMA4F, TOMM40L, UBE2K, UGT1A10, VAMP1, WBSCR22, WNT11, ATP6V1G2, HLA-DQB1
Negative_1822	PXN, ACP5, CDH2, CDH4, FBP1

Negative_1823	CACNA1F,GUCY2C,PTAFR,RPS6KA2,TAS2R3
Negative_1824	NNT,ADCY1,AGTR1,AKR1C2,APPL1,APRT,ATP5C1,BMP7,CPS1,EGLN1,FARSB,HRAS,METTL2B,MTHFD1L,MTMR2,OR1B1,OR1L8,OR2A12,OR5L2,PAICS,PDE2A,PDGFRB,PGAM4,SEMA4D,TDO2,TNFRSF12A
Negative_1825	FST,AMD1,CADM1,DUSP4,EPHA5,IL12RB2,IL3RA,OR10H2,P2RX3,PIGW,PRPS1,RGS3,SOX17,TOMM40L
Negative_1826	FGB,ARHGEF6,CD40,CDS2,CNTN1,CYP2D6,FLT3,GNAO1,INADL,LILRB3,MYL7,NANP,NCK1,NEU1,PLA2G5,PTPRB,SEMA4D,SUV39H1,ZMAT3
Negative_1827	DLD,HK2,MPI,PTS,AGPAT4,APOA1,CLDN18,CPT1C,CYP2C9,ENTPD2,ITGA2B,MAN1A2,MAP3K11,MTMR1,NEFH,NMNAT2,PIGK,PIPOX,PLA2G2F,PLCZ1,PRKACB,PRKAR1A,SDC1,ST3GAL5,TAS2R40,TRPC1,UGT1A10,UGT2B7,PLA2G12B,SERPINA5,SERPING1
Negative_1828	CRH,DBI,FUK,HGF,SFN,AMD1,ATIC,BTRC,CACNG8,DIAPH2,GNRH1,HLA-DOA,ITGAD,MGAT5,NCF1,OR3A1,OR4M1,OR5I1,PDE6D,PFKL,PLA2G2F,PYGB,SEMA4A,STX5
Negative_1829	CR1,IVD,SP1,TH,BCL10,C1GALT1,C1QA,CCDC6,CNGB1,CSDA,CTSB,FGF8,GALNT13,GLB1,GLO1,GNA12,GNG12,IFNA17,IGFBP3,IL17RA,IL8RB,ITGA8,MYH4,OR10K1,PGAM2,PIGU,PIP4K2B,PLCB4,STAM,TNFSF18,TPH1
Negative_1830	C4B,CBL,ITK,ABCC8,ACACA,ACTG2,ACTN2,ACVR1C,ARRB1,ASPA,BACE1,CBLC,CD59,CDK6,CDK7,CDKN1A,CHEK2,CLTB,CNTNAP1,CREB3L3,CRHR1,CXCR6,CYP11A1,CYP26A1,DHDH,DOLPP1,EPHX2,HCCS,IFT57,INSRR,MAP3K4,MASP1,MYH1,NFKBIB,NRP1,NUDT8,OR10G3,OR2L13,OR52D1,PER2,PHKG1,PMAIP1,PORCN,PTPMT1,SMURF2,TNFSF8,ZFYVE16,ATP6V0D2
Negative_1831	DCK,MUT,APC2,ATP12A,ATP5C1,CUL1,PDE4A,PPP3R2,PRKAR2B,PTPN6,SPRY4,STAM,SULT1A1,YES1
Negative_1832	ACAA1,ADCY8,ARPC1B,ARSD,ASMT,B3GALT2,B4GALT7,CACNG4,CACNG6,CCL20,NTNG1,OR2T11,OR52A5,RAC1,SPHK1,UXS1,SERPINA5,SERPINB5
Negative_1833	ATM,HYI,ME3,MVK,PLN,PTS,RFK,TFG,ACP5,ADIPOR1,AKR1C3,AMPD1,ANAPC7,CLTC,CPT1C,DHFR,FGF7,GSTP1,HCCS,KIR3DL2,MMP2,NFX1,OR10A7,OR6M1,OR8H3,PCK2,PHPT1,PLA1A,PNPT1,POLR3G,PPARA,RAF1,SLC9A1,SPP1,ST8SIA1,SV2A,TUBB4,UNC5B,VTI1A,HLA-DRB3
Negative_1834	ATM,GSR,IHH,SHH,AKT3,ATG12,BDKRB2,CD80,CLDN14,CLDN8,CTF1,DKK1,DNMT3A,DPM1,FADS2,GRHPR,HNF4A,HSD3B2,IL22,ITPK1,MFNG,MNX1,NADK,OR8I2,ORC4L,PCCA,POLR1B,POLR2G,STX16,TNFRSF9,TPH2,UROC1,WNT8A
Negative_1835	BTD,ACVR1B,ACYP1,ALG12,ALG5,APOC3,ATP5H,C3orf10,CCL4,CD14,CHKA,DDX51,DGAT2,DOLPP1,DPM1,DUSP2,E2F2,ENTPD2,FGF22,GABARAP,GALNS,IL29,LAMC3,NFKBIA,OLR1,OR2AE1,OR2M4,OR2T34,OR2T6,OR4X2,OR51B2,OR5L1,OR6N1,PDHB,PPP2R2A,PPP3CC,PSME1,RAPGEF1,RPIA,RPS6KA3,SORD,STAT3,TGFB3,TPM3,TUBB6,VEGFB,AASDHPPT,ST6GALNAC5
Negative_1836	CGA,ADSSL1,ARPC3,GALNT6,GALNTL4,GRIA1,IL8RA,LILRB3,NCOR1,OR1L1,OR52E2,OR52L1,PANK3,PIP5K1A,VAMP3,ATP6V0A2
Negative_1837	DCK,IL9,WAS,AHCY,ARHGEF6,AXIN2,CAMK2G,CASP6,CDC16,CDKN2B,CREBBP,CSNK1G1,EFNA1,FCER1G,FZD8,IDH1,IL8RB,ITGA10,KLRC2,MYH2,NCOR1,NR1D1,NR1H3,OR10A7,OR4C15,OR5D13,P4HA2,PDE4D,PIGA,RAPGEF1,ROBO3,SCP2,SLC27A4,SLC2A2,SYNJ2,TAS2R9,THBS1,TIRAP,TLR4,TUBB2B,UGCG
Negative_1838	GLS,MTR,TK2,ACVR1C,AMHR2,DHRS3,DIAPH3,DKK4,FGF23,GRB2,KIR3DL2,LMO7,MAML1,MTMR2,OR1A2,OR52N1,SRD5A2,ST8SIA1,SYNJ2
Negative_1839	ETNK1,HRAS,OR52N5,SETMAR,SPCS1
Negative_1840	ACP6,ATP5G1,NT5C2,RPS6KB2
Negative_1841	PDHA1,THY1,TRPC1

Negative_1842	AK7, MAG, SMO, ACO2, ADAM10, ALDH1A1, CACNG4, CALML6, DKK1, DLST, DUSP1, FIGF, FRAT1, HADHA, HSPA1B, LAMB3, LTC4S, MCM6, NADSYN1, NOTCH2, NT5C2, RASGRP3, SLC27A1, TUBA1A, UGT2B17, ATP6V0A4
Negative_1843	ADA, ATP5G2, BMP2, COL3A1, CRKL, CTNNA1, CYP4A22, DLAT, FADS2, IRF9, MAP3K11, MAPK10, OR13C5, OR2H2, P2RX2, PMAIP1, PPA1, ST3GAL6, TUBA8, VDAC2, WNT7B
Negative_1844	CD6, CFI, CD274, CDKN2A, CEBPA, CYP2A7, GABARAP, NAGA, OR4C46, RGS3, STX5, TNFRSF13B
Negative_1845	CRK, AACS, ACSM1, ACSS1, AKT1, ALCAM, ASPA, ATP5H, B3GNT2, CLDN17, DNMT3L, EXTL2, FOXO1, FUT6, GCNT3, GUCY2C, HEXA, IDH2, IL8RB, IQGAP3, ITGAV, MTMR2, MYH6, NAT5, NCOR1, NTRK2, OR10P1, OR1N2, OR4A15, OR4C16, OR4L1, OR4X1, OR9K2, P2RX1, PHKG1, POLA2, PPARA, PPP3CA, PRKAR2B, SMPD2, ATP6V0A4, C1GALT1C1
Negative_1846	MOS, AARS, ADRA1D, AHCY, APBB1, C1QA, CACNA1A, CARS2, CCL17, CCR1, COQ5, DCXR, DHRS4L2, DTYMK, EXOC7, FGF20, FGF23, FGF9, GADD45B, HEXB, IL24, JAG1, NAT2, OR14C36, OR2T1, PIAS1, PPP5C, PRKG2, RBX1, RXRA, SETDB1, TICAM2
Negative_1847	ADA, ACACA, ACOX2, ACSM4, BNIP1, CARS2, CCL11, CCL5, CDC25B, CKS1B, CLDN17, COL6A2, CSNK1E, DARS, EPHA3, GCNT3, HNF1B, IBSP, LRAT, NAT6, NMNAT2, NUDT12, OR10A6, OR10J3, OR3A3, OR52K2, OR6C3, PDHA1, POMC, SC4MOL, SDC4, TLR3, TP53, TPH1, UPB1
Negative_1848	DBI, GAA, CACNA1D, GLI2, HMOX2, SULT1E1
Negative_1849	KDR, LIF, STS, ACMSD, ACSM4, ADCY10, ADRA1B, AGPAT6, AKR1B10, AKT1, C3AR1, C3orf10, CCL16, CD274, CKMT2, CSNK2A2, CTNNA2, DAG1, ENPP7, FLT3LG, GALNT2, GALT, HMGCS1, HNMT, IFNA2, IL12B, IRS1, LCP2, LIPA, MDH1, NAGLU, NEFH, OR2M3, OR4A15, OR4D2, PEMT, PIGV, PLXNB2, PPP1R3D, PPP3CC, PRKCQ, PRLR, SOS2, TACR3, TAS2R5, TP53I3, WNT4, PDCD1LG2, ST6GALNAC5
Negative_1850	BAD, GDA, IL6, ALOX12B, ATP5C1, CASP7, CD72, CYP2F1, DEGS1, GALC, GFPT1, GSK3B, HCLS1, IBSP, IRAK2, ITPKA, MARS, MCM5, NEFM, OR52K2, ORC6L, PDE4C, PDE8B, PIGC, PRKAR2A, PTGDS, SC5DL, SDC4, TNFRSF11B
Negative_1851	CGA, DCT, ACAD8, ADCY8, CACNG4, CCR8, CDC14B, CDO1, CNTF, DDX41, DHRS4L2, E2F2, EXTL3, FARS2, GALC, GRM5, HYAL2, IFNA5, IQGAP1, ITPKB, LCAT, LYPLA1, MTFMT, OPLAH, OR10A2, OR52E4, PGAM4, PTGES2, RBL1, TGFBR1, TRDMT1, TUBA1C, UNC5B, HLA-DQA2, HLA-DRB5, SERPINA1
Negative_1852	CD4, AGRN, B4GALT1, CCNA1, CMBL, CSNK2A2, CYP2U1, DAAM1, DDX54, DERL1, DGKD, EHMT1, EPOR, HSPA8, LAMB3, MCM2, NAGS, NCSTN, NUMB, OR5J2, PLOD1, ROCK2, SARDH, SMARCA2, STX5, TALDO1, UGT2B17, WASF2, ZFYVE16, HSD17B12
Negative_1853	FGB, GLA, ADH1A, ALDH4A1, B4GALT4, CSF2, CYP26A1, EIF4B, GALNT9, JAG2, KIR2DL2, LYPLA2, OR10C1, PANK1, PDGFRA, PRKAG3, SETD7, SRGAP2, UGT2B11, VANGL1
Negative_1854	AK3, HK2, NLK, RET, ABL2, ACVR1B, ADCY4, ATP5B, B3GNT3, BRAF, CACNG8, CAV1, CES2, DTX4, EARS2, FOSL1, IGF1, IMPDH1, IMPDH2, ITGB8, MYH11, MYH4, NLGN1, NMNAT3, NOS3, OR10J3, OR11G2, OR4D1, PDIA4, PRKCG, SHMT1, SLC9A1, TAS2R50, THBS2, TRAF6, TRPM5, YARS, AASDHPPT
Negative_1855	ACSL1, ACTB, ASMT, CCR10, DDIT3, MGAT3, MYH2, NT5C1B, OR6X1, PANK2, POLR2K, RPRM, SH3BP2, UXS1, TNFRSF18
Negative_1856	AGXT, ALG6, CACNB2, CD58, CLDN14, EPHB6, GRM1, INSRR, ITPK1, MINPP1, NNMT, SFRP1, PLA2G12A

Negative_1857	BCR, GP5, NNT, SRF, ADRB3, ALCAM, ANAPC10, ASPA, BCAT2, CASP9, CCL5, CLDN9, CYP3A4, DAD1, DERL1, DFFA, DGAT2, DPM1, ELK1, ENTPD8, EPHB1, EPHB6, EXTL2, FARS2, GNG13, HAO2, HDAC1, IRF9, LGMN, MAP3K3, OR10G4, OR10R2, OR1S1, OR2B2, OR5AK2, OR5L2, OR8B4, PFKFB3, PGAM1, PKLR, PLD2, PORCN, PPP3R2, SNAP23, STX2, SUFU, TAS2R5, UGDH, SUV420H2
Negative_1858	ATF4, B4GALT6, DRD2, GALNS, ITGA6, MRAS, NMNAT2, OR10X1, PAK6, PER1, PLOD3, PLXNC1, PPM1A, XYLT1
Negative_1859	F11, FGG, NPY, PC, PGF, PLG, ACP6, ACSL3, ADCY7, AKR1C3, B3GNT5, CD72, CD8B, CER1, CHSY3, CLDN14, CXCL6, CYP2E1, DFFA, GALNT7, GMPR2, IL23R, MADCAM1, MTHFD1L, MYH8, OR2F1, OR4F6, OR6C76, ORC1L, ORC6L, PDGFA, PKM2, PRKAA2, PRPH, ROCK2, RPS6KA4, SELPLG, SEPHS1, SH3BP2, SLC9A1, SPTLC1, TAS2R16, TGFA, WNT16
Negative_1860	ACTG1, ACY3, ARHGEF7, GMDS, ITGAD, PRKG2, RASGRP2, UPP1
Negative_1861	NOG, ADIPOR2, ADORA2A, ANAPC10, CCNG2, CCR4, CDK2, CLDN1, CLDN22, DDX47, EPB41L2, FSHR, GIT1, GJD2, GRM5, HAO2, HSPA1B, IL2RB, LAMC2, LMO7, MGAT4B, NFKBIB, NRXN3, OR1S2, OR2T1, OR51B4, OR51S1, OR56A1, PARD6G, PGAM2, PPP1R1A, SCIN, ULK3, ATP6V0E1
Negative_1862	ADC, BAX, F10, LIF, UBB, AASDH, ALDH5A1, ANAPC5, B4GALT6, CCL17, CCL19, CCR1, CNTFR, DAAM2, EDNRA, EPHA6, GAB1, GLDC, GUK1, LDHB, MMP7, NAT2, OR10G9, OR13A1, P2RX1, PLD1, PNMT, POLR1D, PPBP, RAC3, RASGRP1, RASGRP4, SDHC, SEPHS1, SULT2B1, TAS2R9, TGDS, UGCG, TNFRSF12A
Negative_1863	ARPC2, DLL1, ERBB2, OR10H5, OR2T4, OR8H2, SETD8
Negative_1864	ADK, BAD, GK2, MTR, ACO1, ADCY9, ATP4B, CACNA1A, CACNA1D, CACNA1S, CANT1, CASP9, CCL23, CD40LG, CDK2, CHST13, CRY1, CXCR3, DGKH, DOCK1, FBXW11, FSHB, HAO1, HHIP, HTR4, IMPDH2, ISYNA1, ITPK1, MAP3K4, NAT1, NCKAP1L, NRXN2, OR13G1, OR4E2, OR52L1, PAPSS1, PERP, PIAS1, PPAP2C, SLC27A1, SMAD3, SORD, TAS2R38, TLN2, YWHAH, ZBTB16, ZFYVE16, PNLIPRP1
Negative_1865	IL8, ACTC1, ACVR1B, ADCY9, ADH7, AK3L1, ATP5I, CD47, CYP2C18, EPB41L3, FMO1, GALNT2, GOSR2, IL17A, ITGB2, KIR3DL1, L1CAM, MYH3, NUMB, OR10T2, OR13C3, OR4N4, OR51F2, OR8B12, OR8D1, OR8D2, PPARG, PRSS2, PSME3, RPS6KA1, RRM2B, SDHA, UGCG, ATP6V1C1, HLA-DRB4
Negative_1866	CKB, F9, PAH, ADCY1, ADH7, ALDH9A1, ALG12, ATP6V1D, CCL4, CDK6, CKS1B, CLDN3, CRLF2, ELK4, FASN, FZD4, IFNA4, IL1A, IQGAP2, IRAK4, ITGAX, KITLG, LFNG, MCM7, OR10H4, OR51G2, PLA2G10, PLD1, PLTP, PPBP, PTGES2, RBL2, RND1, SPI1, TICAM2, TYROBP, UGCG, SERPINA1
Negative_1867	C4BPB, CACNA1E, DHRS4L2, DUSP5, KAT2A, LIFR, MAPK13, OR4S1, PARD6B, PDE11A, PFKFB3, PHPT1, PLA2G3, PNLIP, PPA2, PRKAG3, PTDSS1, RHOD, RUVBL1, SEMA3B, STAT5A, STMN1, TAS2R48, TNFSF10, TUBA1C, HSD17B12
Negative_1868	ADA, CLDN20, EPHA2, GZMB, NOX3, OR51I2, OR52E4, PDPK1, POLR1C, POLR3D, PYGB, QPRT, RHOA, RPS6KA3, SESN1, TPK1, ATP6V1C2
Negative_1869	DDHD1, JUND, MOV10L1, NEU3
Negative_1870	ALDH3A1, CCL27, E2F4, FTH1, MYH9, PI4KB, PPP2R5B, ST3GAL6
Negative_1871	ATP4A, MYH15, OR4M2, PMM2, TAS1R1
Negative_1872	ATP5J2, CCNH, CER1, EPHA8, NCR1, OR4D11, PRKCI, SMURF1, VAMP1
Negative_1873	CR2, ARHGEF1, BCAT2, BMP4, DHFR, EPHX2, GNPNAT1, GPX7, HBEGF, MAP4K4, NEU2, NQO1, OR2AE1, OR5J2, OR6C3, OR8H1, ORC5L, PMM2, PSEN1, RBX1, SELP
Negative_1874	ADO, APP, F5, GSR, ID2, UBC, ACTN1, ACTN2, ALDH1A2, ALOX15B, ATP2A1, ATP6V1H, CD8B, CLDN7, COL11A1, COL1A2, COL5A3, CYP1B1, DUSP8, DVL1, EREG, GALNT8, GPT2, HADHA, IL8RB, MAPK9, MCEE, NOTCH2, NUDT2, OR51B5, OR51T1, OR5T1, OR6C3, POLD2, PON2, PPP2R2B, PYGM, SESN2, SPRED2, TP53I3, TUBA4A, UGT2A1, ULBP2

Negative_1875	ADA,NLK,PGP,ACAT1,ADH7,ATP6AP1,B3GNT2,CCL21,CLDN19,COQ6,CYP8B1,ERBB2,FABP4,GALNS,GALNTL5,GNA15,GSTA3,IDH1,IL23R,ITGA6,ITGB6,LAP3,MAP2K5,NCKAP1,NEFM,NLGN1,NRAS,NRG1,OR10C1,OR10T2,OR3A1,OR5L1,OR6F1,OR7A10,OR8J3,PCYT2,PIK3C2G,PIK3CD,PITX2,POLE3,PPP2CA,PTEN,ST3GAL1,TAS2R13,YWHAQ,ATP6V1C1,PPARGC1A
Negative_1876	TYR,ACAA1,GALK2,IMPA1,TJP3
Negative_1877	DBI,ID4,MSN,ARHGAP5,AXIN1,CDKN2D,EREG,HEXB,IL17RB,MAN1A1,MPST,NFASC,NFAT5,NR1H3,OR52E2,OR6C74,PDIA4,PHPT1,POLR2F,POLR3D,PPP2CA,SMAD1,STK36,ULK2,YWHAZ
Negative_1878	IL4,ACTN2,ADSS,AQP7,ARHGEF2,CD55,CDO1,CNTNAP1,DPYSL2,FGF22,FOXO1,FUT2,FUT8,GALNT12,GBE1,LIPC,MYH1,OR13A1,OR2L8,OR4F4,OR5D14,OR7C1,PDE1C,PLCD3,RPIA,SESN2,SPTLC2,TGFBR2,TPI1,UBE2K,WNT3
Negative_1879	C8B,FGB,GSN,ACAD8,APOE,CACNG2,CCNB2,CD247,CHRM3,COL1A2,CYBA,CYP2C9,DCTN1,DOCK1,DTX1,E2F4,ENTPD7,FGF2,GNMT,GNRHR,HHEX,HNMT,HSD11B2,IDH1,IFIH1,IL28RA,LIPC,MAGI2,NKD2,NKX3-1,NR5A2,OR13G1,OR1D5,OR52N2,PFKM,PIAS4,PMVK,POLR2I,PPA2,PRKAG2,RAC1,RAP1B,RPIA,SHC4,SNAP25,TSC1,YARS,PAFAH1B3,TNFRSF10D
Negative_1880	CCNE2,NADK,PFN4,TFPI,TOMM40,ZFYVE9
Negative_1881	FGG,ATP5F1,ATP5G2,B3GALT5,BACE1,CAMKK1,CDK4,COL2A1,GNRHR,HCLS1,HTR6,MCM7,OR52A5,OR5P3,OR6A2,OR9G4,POLR2E,PTGES,RHOA,RUNX1T1,SELPLG,UGT2A3,VDAC1,TNFRSF10A
Negative_1882	C6,DCC,AASS,ACOT12,ACPT,ACSM2A,ACVR2A,ADCY8,ALG9,ARPC1B,B3GALT1,B4GALT2,CAMKK2,DDX56,EDAR,FCER1G,HARS,HTR7,IFNA7,IL10RA,ITGAL,LLGL1,MYLK3,NMNAT2,NT5M,OR10P1,OR2L8,OR5L2,PAPSS2,PAX4,PDE1C,PDE9A,PLA1A,POLR3A,POMC,PVRL4,SEMA3G,STEAP3,STK4,TUBA1A,UBA7,YES1
Negative_1883	TPO,C1QB,COL3A1,CSF1,CYP2C18,DDIT3,GALNT9,GSK3B,IFNGR2,ITGA6,ITGB4,LCP2,LFNG,NCK2,NOTCH1,NRG1,OR2Y1,ORC3L,PRKY,PRSS1,RFX5,RPN2,TOMM40,VEGFA
Negative_1884	CES2,MNX1,SETD8
Negative_1885	CCL14,CYP2C9,MAML2,OR7A10,GABARAPL2
Negative_1886	ACO1,ADCY5,ALDH6A1,ANAPC11,ATP13A2,CCNB2,CLDN5,CTBS,CYFIP2,DGKH,FMO4,GDF6,GPX7,GRIN2C,INPP5B,NOS1,OR10R2,OR4F6,OR52A5,OR56A1,OR5M3,OR5R1,OR9Q1,PARD6A,PDE10A,PHKA2,PLCG2,POLR2B,PPP2R2B,PRKAR1B,RAC1,TPK1,UGCGL2,UGT2B17,ULK2,HLA-DRB5
Negative_1887	TPR,ACP5,ADRB1,AKR1C1,ATF4,ATP5E,BIRC2,CD82,CNTNAP2,DDX41,HTR2B,IFNA14,KLRD1,LTA4H,MCCC1,OR13A1,OR5T3,PIM2,POLR3A,SAT1
Negative_1888	IL7,ALG12,ANAPC11,ARHGAP5,ARNT,ARPC1A,ATP6V1F,CHEK1,COL5A2,DGUOK,DHFR,DUSP4,FMO4,GGT5,GLB1,GLI3,GSTO2,H6PD,IL23A,LRP5,NCAM1,NFX1,NR1H3,NT5C,OR2Y1,OR51S1,OR5V1,OR6N1,OR9A2,ORC5L,PI4KA,PLD1,PRKG2,PTCRA,RAPGEF1,SEMA4A,SMAD5,SPHK2,SREBF1,TCF7,TUBB2B,TUBB4,YOD1,GABARAPL1,HSP90AB1,SERPINA5
Negative_1889	CGA,CKB,GSN,PC,ADCY4,B3GNT2,CACNG7,CD40LG,CD79B,CHUK,GLO1,GLS2,GSK3B,HDAC2,LYPLA1,NRG1,PDHB,PKM2,PRNP,SARS,TAS2R41,TUBB6,TYRP1,CACNA2D2
Negative_1890	ACAD8,ACO2,BDNF,CCL27,EPHA4,EXTL2,IL20RB,NFE2L2,OLR1,OR2W1,OR4N5,PMM2,PRPS1,PTGES,RASA1,RUVBL1,TAS2R3,TRAF4
Negative_1891	DHH,LSS,AADAT,ADRB1,ALDH5A1,ARNT2,ASIP,BLVRA,BUB1B,CASP7,CD244,CTNNA3,EIF4B,FUT3,GADD45G,GALNTL4,HNF1A,IFNA14,IL17RA,IPPK,NTNG1,OR6C70,OXTR,PDE3A,PIK3C2G,PMM1,PRIM2,SPTLC1,ST3GAL5,STX2,TP73,TPI1,TRAF3,WNT4

Negative_1892	CD44,CHSY3,COL5A1,ENPP1,EXT2,HAGHL,JAK1,MMP9,POLD2,SPCS1,SPRY3,TSNARE1,VDAC2
Negative_1893	HGF,ACLY,AKR1C2,ALDH6A1,ALS2,C1QC,CACNA1E,CALML6,CD22,CD38,CES7,CHRM3,DDX23,DUSP10,ESPL1,FGFR2,IKBKB,IL10RA,INPP1,IRS1,JAG1,LYPLA2,OR13A1,OR2A42,OR4S2,OR7G3,OR8S1,P2RX5,PDXP,PLCD4,PLCE1,PNMT,PRKAA1,PTGIS,RXRB,SIPA1,SKP1,ST3GAL4,TRAF6,TYMP,WBSCR17,ATP6V1E2,TNFRSF18,TNFRSF1B
Negative_1894	DDC,F5,GNS,ME3,ABCC8,ACSL6,ADCY1,ALDH2,ATP5A1,CDC25C,CDC27,CREB3L2,CSNK2B,CXCL13,DGKZ,GALM,GNAT3,GSTT1,HARS2,HNMT,IDH2,IDH3B,IGSF5,IL8RB,ITGAD,LCP2,OR1A1,OR52N4,PDE4D,PFKFB2,SPRED1,SSH1,UBE2K,VDAC3
Negative_1895	BTC,PDC,ADSL,ALPL,B3GALT2,CDKN2C,CHEK1,DTX3,HAO1,HSD17B7,LYZL1,MYH13,NKX2-2,OR2A12,OR51A2,PPP2R1A,PYCR2,RELA,SESN1,TAS2R41,TAS2R42
Negative_1896	BID,LHB,MVK,AGXT,CDKN1C,COL4A6,CXCL5,GALT,IFNA4,IL15RA,KAT2A,KITLG,NPR1,NRCAM,OR4C12,P4HA2,PDE3A,PIGU,PLCE1,RAP1B,RDH11,SOCS7,SPHK2,UGT2B7,VANGL2,WNT2B,MAPKAPK5
Negative_1897	GGH,TYR,ABLIM2,CDH2,CFLAR,FZD4,GAD2,HTR6,IFNA5,IL9R,MAP3K5,MPDZ,NT5E,OR10K2,OR10T2,PDE5A,PIGO,PIGX,PRPH2,PTPRZ1,SMAD5,TUBB3,VEGFC,YOD1
Negative_1898	ARPC4,CCNG2,DGUOK,FGF5,FUT3,GLI2,GNG13,HAO2,HIBCH,IARS2,IKBKE,KLRD1,NCOR2,NT5C,PAICS,PLA2G4A,POLR3A,PRKAR2B,ROCK1,SCIN,SELE,TMLHE,TUBA1A,UXS1,SERPINB5
Negative_1899	ALG1,CNGA4,CXCL3,GNG3,NKD2,PIGX,PYCR2,STX12,TAS2R8,CACNA2D1
Negative_1900	FGB,LIF,ACAD8,ALG12,CYFIP2,CYP4F3,F2RL3,GLB1,IGSF5,IL13RA1,MGAT5,OR10A6,OR1L1,OR2T34,OR6B2,PIGU,PIGV,POLR2C,PRKACA,PRNP,PRSS1,PTGFR,RAF1,RPS6KA3,RRM1,STEAP3,SYNJ1,TAS2R13,TUBB2B,UCKL1,UGCGL1,VCAM1,ATP6V1C1
Negative_1901	FH,SDS,ACHE,ACPT,ADRA1D,BDKRB2,CCNB3,CXCR5,CYP2C8,CYP2E1,CYP4F2,FDFT1,FECH,FUT2,HHEX,IRS1,JAK3,MAD2L2,MDM4,OR7C1,OR9G1,PPM1D,PRLR,STX16,TAS2R60
Negative_1902	ACACA,ALDH5A1,AQP7,CLDN2,G6PC2,GUCY2F,HADHA,IQGAP1,MAP3K3,NCF1,PLK1,PTGS2,SELL,SEMA3D,WNT2B,KIR2DL5A,TNFRSF11A
Negative_1903	OTC,CXCL10,DERL1,DUSP1,HPRT1,IFNA8,OR4K14,OR52K1,OR7G1,SRD5A1,WNT2B
Negative_1904	ATM,DDC,DDO,GLS,LYN,ACP6,ALG11,ATP6V1D,BBOX1,CTBS,DGUOK,DOLPP1,DTX3,DTYMK,DUSP5,ENPP3,F13A1,GRIN2C,HAGHL,HSD17B1,IFNA5,ITGA3,ITGA5,MAP3K12,NEFH,OR4K17,PDGFA,POLR2L,PPM1A,PRKCH,PTDSS1,SMPD4,STMN1,STX7,SV2C,TICAM2,TYRP1,UGP2,PNLIPRP1
Negative_1905	C9,F8,CAMK2D,DUSP16,GALNT7,GSTM5,OR52B6,PRKCH,PRSS3,SKIV2L2,STAT3,ATP6V0E1
Negative_1906	PVR,SFN,ADSSL1,ALG5,APAF1,B3GNT4,B4GALT5,CASP6,CCL17,CDKN2A,CFL1,COL5A1,CYSLTR2,DKK2,DLST,DUSP6,EHMT1,EREG,GAD1,GALNTL2,GRLF1,HADHA,HIBCH,IL1A,LIFR,MTHFD1L,NTF3,OR1S1,OR2Z1,OR51B2,OR51I1,OR6B3,OR6S1,PLA1A,PLA2G4A,POLR3K,PPP1R3B,TUBAL3
Negative_1907	AKT3,FGF17,ITGB6,OR2G2,TPK1
Negative_1908	LTB,AACS,ALOX15,BUB1,CHKB,CLDN15,CYP11B1,CYP17A1,DAD1,DCXR,DDX23,DPAGT1,DTX1,E2F2,FIGF,FMO4,GALNT14,GALNTL4,IARS2,IFNA7,IL6R,MCCC1,MGAM,MS4A2,MYH14,OGDH,OR4F17,OR51E2,PAK6,PIGN,PORCN,PPP2R5D,PTCH1,STAT6,TAS2R5,TCF7L2,TUBB
Negative_1909	ACVR1,ADCY2,CD8B,CLDN18,HIP1,IRS2,MCM3,MDH1,OR1A2,OR51L1,OR5B12,PDGFC,PDXP,RDH11,SDHB,SPCS1,TAS2R49,TUBB2B
Negative_1910	ENPP1,IL12A,MAPT,MAPK8IP2

Negative_1911	TNN,AADAC,CACNG5,EREG,LAMC2,MYH8,OR5T2,PLA2G2D,PLAU,PLOD3,TCF7L1,TYK2
Negative_1912	DBI,EZR,ACSL4,ARPC1B,ATP4A,CHRNA7,CRLS1,CYP11A1,DGAT2,DHFR,HMGCS2,HTR2B,IL12A,IL1B,MYL9,NCOA4,NOX1,OR10V1,OR52N4,PFKM,PSENEN,SUCLG2,SUV39H1,TCF7L2,TNFRSF8,WNT11,HLA-DRB5
Negative_1913	ACP1,C1QB,GPAM,KLRK1,NRAS,OR1N2,PLOD1,PTPRM,TNFRSF13B
Negative_1914	CEL,ACOX1,ACSS2,AK3L1,ANAPC4,AOC3,B3GAT1,CLTCL1,DLL4,ESPL1,GALNT7,GBA3,GLO1,GPD2,GSTK1,GUCA1C,ICAM1,INPP5E,KDSR,MAP3K3,MCM4,MPDZ,MTFMT,NEUROG3,NR1D1,OR10A7,OR2AG2,OR52E8,OR9A4,PCYT1B,PHKG2,PLA1A,PPAP2B,RARS,RHOA,SETD2,TLR4,TNFSF10,XIAP,ATP6V0A1
Negative_1915	INMT,OR52R1,OR9G4,PPP2CA
Negative_1916	BAX,PGD,ACHE,ACOX1,CNTN1,DAPK3,HSD17B7,IGSF5,KLRC2,MAPK10,MAPK14,MCM4,OR10S1,OR2M4,WIF1,CACNA2D1
Negative_1917	HPD,SP1,ADCY9,ADH7,ARHGEF4,CD79B,CXCL9,CYP3A43,DEGS1,EHMT1,EPHB6,FGF8,FOSL1,HSD11B2,IL29,LDHB,OR2A5,OR4S1,PLA2G2A,TXNDC12,UROC1,TNFRSF10D
Negative_1918	ADA,CAT,CSK,GGH,ASRGL1,CACNG8,CSF2RA,CYP2C8,DCXR,GCNT4,GUCY1B3,HSPA8,HTR7,IL15,LRAT,MAFA,MASP2,OR6X1,OR9A2,PI4KB,PPP3CA,SDC2,SNAI2,TUBAL3,UGT2A1,UGT2B28
Negative_1919	KMO,SRM,ABLIM3,ACOX1,ADH4,AKR1B10,ATP4A,ATP5H,CAMK2B,CDH4,CHSY3,CLDN10,CTNNA2,CYP2U1,DEGS1,DHX58,ECSIT,FGF6,GNRH1,GUCA1B,HSD17B2,JAM3,MAP3K6,METTL6,MTHFD1L,OR1D2,OR6N2,PIGO,PTDSS1,PTGFR,PVRL4,RPSA,SC5DL,SDHC,SETD2,SOCS1,TAS2R40,UAP1,UGT2B4,EIF4EBP1,HLA-DQB1,SERPINA1,SUV420H2,TNFRSF1B
Negative_1920	ACY1,APOC3,BET1,CCR1,CDK2,CSNK1G3,DDX41,EIF4E,GNG12,OR2T1,OR51V1,OR6C3,TAS1R2,UGCGL2,WNT8B
Negative_1921	FGG,AADAT,BIRC3,CDKN2A,COASY,CSH1,DUSP7,GANC,GATM,GRHPR,GRIN2D,GUCY1A3,HCLS1,IRAK1,ITPK1,LEFTY1,OR51G1,OR52R1,TAS2R50,TRAF4,TRHR,WNT11,ZBTB16
Negative_1922	ACSL1,ALDH1B1,AMPD2,ARPC5,ATP13A2,BCMO1,BTRC,BUB3,C4BPA,DGKA,DNMT3A,EPHA6,FGF5,IL23R,ITPKA,MAP2K4,MTAP,NEU2,NEU3,OR2T11,OR4X2,PKLR,POLR1D,SULT1A4,TRIP10,TUBB,ATP6V1G3,CACNA2D2
Negative_1923	C2,CTH,FYN,GH1,KMO,ACSS2,AK3L1,B3GALT5,BET1L,CD27,CD74,CSF2,CSNK2B,DLST,ENTPD6,EPHA1,IL18,MAPK8,MMP2,MYLK,NCF2,OR4D5,OR4N4,OR51I2,PAK4,PDE4D,PLA2G3,PPP3CB,RUNX1,SETDB2,SIGLEC1,ST3GAL1,TCF7L2,TJP2,PPARGC1A
Negative_1924	HGF,ACP2,ALAS2,ARHGEF6,CD59,CREB3L2,GRAP2,GUCY1A3,HLA-DOB,IFNA17,LFNG,LIAS,MAP4K1,NCOR2,NRAS,OR52K2,PDE4C,PDE6G,PGK2,PI4KA,PIP4K2A,PNPLA3,RASGRP4,HSD17B10
Negative_1925	DHH,AANAT,ACVR2A,CASP8,CCR1,CDH2,EP300,EPHB4,FGD3,G6PC,GNAQ,IDH2,IFNA5,IL1R1,IRF7,JUND,KIR2DS4,LCP2,MAD1L1,MGAT1,NKD1,OR13A1,PARD6G,PLAT,PLXNA1,PMM1,SGMS2,SOX17,STK11,TACR1,UBE2K,WNT5A,CSGALNACT1
Negative_1926	F8,ME1,TKT,ACP1,ACTB,ACY1,ALAS2,ALG1,CALML6,CAMKK1,CCL23,CDH3,FUT5,HLA-G,JAM3,MYH11,NCOR2,OR10A4,OR10S1,OR52I1,OR6A2,PIM1,PLCB2,PNPLA3,PPARD,PRNP,RFXANK,SOD1,XYLT1
Negative_1927	ALAS1,ARHGEF2,NOS1,OR6K6
Negative_1928	CD4,ACTN4,GANAB,GCNT4,KRAS,LRP6,OR5M9,WNT11,MAPKAPK2
Negative_1929	C8A,FGB,ADSSL1,AHCYL1,ASRGL1,CD8A,CDC14A,CLCA2,DIAPH3,FIGF,GALNT1,GMPPB,HMGCS1,NLGN1,NQO1,OR51V1,ORC3L,PDHA2,PGS1,PKM2,PRKAB1,PRKAB2,SEMA3A,TUBA3C,UCKL1,WARS2,WNT8B
Negative_1930	CCL5,EFNB3,IDI2,LEPR,OR3A3,RRAS,UGT1A10,YWHAZ

Negative_1931	RB1, ADCY7, AHCY, AHCYL1, ALDH1L1, BCL2, CDC27, CDKN1A, CLDN17, CMBL, CSF1, CSF3, CXCL16, DNMT1, EFNA1, EIF4E, FMO1, GLI1, GNAI2, GSTM4, HSD17B1, HSPA2, HSPA8, IFNA17, IFNB1, IL11, LAMB4, MAP2K2, OR51D1, OR56B4, PIGK, PIK3CB, PIK3R1, PMVK, PTGDS, STX12, TNFSF18, SERPINA5
Negative_1932	LCT, ALDH5A1, BACE1, CLTC, EFNA4, ENTPD1, GALNT3, GFAP
Negative_1933	C7, CBL, LCT, ACSS3, ARHGEF7, ATP5L, BIRC2, C4BPA, CCR1, CNTN1, CREB3, CSNK2A2, EFNA2, ETS1, GCNT2, GPT2, IL17A, INPP5A, INPPL1, ITCH, KIR2DL3, LYZL1, NAE1, NAGLU, NARS, NAT5, NCF4, NCOR1, OR11H4, OR1N2, OR52E8, PARVA, PGM3, PRKX, RPS6KB1, SFRP4, SLC2A1, SPCS3, SV2A, TUBA3C, VAV1
Negative_1934	C5, DCN, GCK, IHH, KHK, AADAC, ACO1, ACVR1C, ADH6, CCL2, CTSS, CYP26C1, DAAM2, DGKB, DUSP3, ERBB4, FGF14, FGF22, GPAA1, HSD3B7, IDUA, ITGB6, LIPG, MAN2A1, MAP3K13, MCCC1, MGAT4B, MKNK1, NARS, OR10G3, OR13C3, OR4F17, PIAS2, PIGC, PPAP2C, PPP2R5D, PTPMT1, PVRL1, RAC3, RDH5, RELA, SOX17, WNT4, YWHAH, SUV420H2, TNFRSF17
Negative_1935	GSS, LSS, NF1, PML, TYR, ACAD8, ACSM1, AGPAT1, BBOX1, CAMK2A, CASP9, CCL13, CHIT1, CXCL13, DLST, FLAD1, GAB1, GRLF1, HADHA, HSD11B1, IL15, IL21R, MAD2L2, NAGK, OR4D1, OR51B6, OR52B4, OR8U1, PCNA, PIK3R2, PRKAG3, PRPH, SELP, SLC25A5, SMPD2, SQLE, TAS1R2, TCF7L2, TM7SF2, TYROBP, YWHAH, ZFYVE16, HSP90AA1
Negative_1936	APP, HYI, ADCY7, ALOX12B, B4GALT1, BCAT1, BMP7, CAMK4, CCND1, CD36, CDH4, CEBPA, COL3A1, CREB5, CSF1, CXCL13, DCTD, DHRS3, EFNB3, FGF1, FTCD, GALNT6, GRPR, GUCY2D, IDH2, MMP7, MPST, MTHFR, NOTCH2, NT5C1A, OR52I1, OR52L1, OR5D16, OXCT2, PHGDH, PIGB, POLD4, PSENEN, ROBO2, SELP, SLC25A5, SMAD6, SNAP25, SUCLG1, TAS2R10, TYMS, WHSC1, YWHAQ
Negative_1937	EBP, TAT, ATG7, FGF21, HEXA, HMGCS1, HTR4, METTL6, OR2A4, OR4D5, PIAS4, PIP5K1A, PRSS3, PYGB, ATP6V1G2
Negative_1938	CRH, GNS, SI, ADIPOQ, CACNA1B, CSF3, CYP7A1, DHRS3, FGF20, FZD9, GAMT, GSTA4, HNF4G, IMPDH2, IRS4, ITGB8, LIPT1, MAP2K6, MKNK1, MS4A2, MYL5, NCKAP1L, NR4A1, OR10C1, OR10H4, OR1L3, OR4E2, OR51F2, OR5B3, OR6X1, PDGFRA, PEMT, PFAS, PFN2, PPP1R3B, RDH5, RETSAT, SARS2, SCD5, STAT5B, TACR2, TAS2R40, TAS2R7, TLR3, UCK1, ATP6V1G3, PRICKLE1, SERPINF2
Negative_1939	SDS, ARPC3, ARSA, B3GNT4, CD86, CX3CL1, FSHB, GCLM, ITGA9, MAP3K14, NCSTN, NKX3-1, NOD1, NSD1, OR10S1, OR1L3, OR5L2, PLEKHA8, PMAIP1, POLR3K, PTGFR, PTK2B, RPIA, SOAT1, STAT4, THY1, TYMS
Negative_1940	BLVRB, CDKN1A, GNB3, MAML2, NEUROG3, OR5B17, PSME2, RPN1, SERPIND1
Negative_1941	C1S, CDA, CGA, EZR, ID3, ACY1, ARHGEF7, ARPC5L, CACNA1S, CCNE1, CDC26, CLCA4, CXCL1, DARS2, DFFA, DLAT, DVL2, FGF18, GANAB, GCLM, GLDC, GSTM1, HADH, IL6R, INPP1, JAK1, KAT2A, MAGI2, NEU2, NRAS, OR11L1, OR52I1, OR5M3, OR5M9, OR6N1, OR8D4, PAK6, PDE4A, PDE8A, PLK1, RHOA, TLN2, UCK1, UGDH, UGT1A10, YOD1, MAPKAPK2, PDCD1LG2
Negative_1942	C7orf16, COL11A1, DMGDH, DUSP2, EHMT2, FASLG, NUDT5, OR1J1, OR4K15, OR52I1, OR6C76, OR7D2, OR7G3, OR8D4, PPP2R5B, PTK2B, SDHB, TAS1R3
Negative_1943	STS, ABP1, ALDOA, B3GAT3, BRAF, CACNB4, CARD11, CSH1, CTBP1, CTF1, DGKZ, DPM3, DRD1, DUSP14, FABP4, FGFR4, FMO4, GALNTL5, GNA15, GRIN2D, GSTO1, GUCY2D, HPSE2, HSPA1B, IL22RA1, ITGB8, MAML1, MAP2K1, NCAM2, NCR2, NRXN1, OR51A7, OR5AU1, PDGFC, PKMYT1, POLA1, PPP1CC, RBX1, SULT1E1, TIAM2, TRAF4, TYK2, ULK1, VAV3, YWHAZ
Negative_1944	IL7, AARS2, ADCY9, APAF1, CDIPT, GCNT3, GLI1, GUCY2D, IFNA13, INPP4A, LDHA, NEU2, NTF4, OR2F2, TGFBR1, TXNDC12, ULBP1, YWHAQ
Negative_1945	AK1, CBL, LTB, AOC2, APRT, BNIP1, MAP2K3, MYL9, OR13F1, OR2A25, OR51B6, PLA2G3, PMM2, RPS6KA1, SLC2A4, SMPD3, TNFSF4, MAPKAPK2

Negative_1946	AARS2,ACADVL,AFMID,ATP5D,CD34,CDS2,CYP2C19,DUSP4,E2F1,GALNT2,GALNTL1,HSD11B2,IFNA16,LIPC,MGAT3,MYD88,OGDH,OR4F15,OR5AS1,OR7A10,PDX1,PTGER1,PVRL3,WNT9B,MAP3K7IP1
Negative_1947	F3,GH2,ACSS1,CADM3,CKMT2,DDX47,EPHX1,GYS2,HNF4A,OR3A2,OR8B12,OR8S1,PKMYT1,PLXNA3,PPBP,SRGAP2,STT3B,SYNJ1,TCF7,TNFSF4,VEGFB
Negative_1948	DAO,GDA,MVD,ACADL,AMD1,ATN1,B4GALT7,CACNA1B,CDKN1A,CXCL16,DTX3L,HEXA,IL5RA,KIR2DL1,LDHD,MNX1,NCAM1,NFASC,NPAS2,PAPSS2,PPAP2B,PPP1R3A,PPP3R1,PRKX,QARS,SEMA4D,SMURF2,STX7,TAS2R38,TAS2R5,YARS
Negative_1949	LSS,SFN,ARPC5,BUB1,CYBA,DPYSL2,FRAT1,HMBS,MAT1A,NUDT12,OR10C1,OR52K1,OR6C6,ORC5L,PIK3R2,PIM2,POLR2F,SEMA3E,TUBA1B,WNT3,WNT8A,B4GALNT1
Negative_1950	AK3,CACNA1G,IARS2,OR1L3,PRPH,ATP6V0E1
Negative_1951	ADO,ATP2A3,B3GALT5,DDX51,GSTK1,HAO2,IL29,IRF3,LRRC4C,MTFMT,OR13J1,OR2T4,OR51Q1,PORCN,SEMA4A,TALDO1,TNFSF9
Negative_1952	CR2,FYN,IL5,TNN,TPR,ALG8,ATP5C1,BIRC5,CAMK2B,CHEK2,COL1A2,CREB3,CXXC4,DPM2,ELK1,EPHB3,FARS2,GALNTL5,HAAO,IFNGR2,IL13,IL20RB,KLRC4,LTA4H,MAML2,NRAS,OR10G3,OR2T11,OR4Q3,ORC3L,PDGFRB,PLA2G5,PRODH,RALGDS,RXRA,SGMS2,ST3GAL2,TUBB2B,TUBB6,UROD,WBSCR22,CACNA2D4,MAPKAPK3,PRICKLE2,SERPINF2
Negative_1953	CFH,ID1,AKR1B1,ATF2,AXIN1,CMPK2,CREB3,DAPK2,DUSP8,ENTPD4,GLUD2,GRM1,ICAM3,IQGAP3,ITPKA,MALT1,MAN1A1,MAPK7,NAGA,NAMPT,NLGN1,OR10H4,OR1J1,OR2L2,OR4A5,OR5H2,PCYT1A,PLA2G6,POLR3K,PTPN1,RAC2,SC4MOL,SEMA4G,SHMT2,TAS1R3,TP53I3,VAV2,MAPK8IP3
Negative_1954	HDC,OSM,TH,BCKDHB,BST1,CKS1B,CMPK2,CREB3L3,DIAPH3,EPHB1,ESAM,FGF10,FGFR4,FLOT2,GGPS1,GRIN2B,ICAM3,KIR2DL2,LIAS,MAP4K2,OR2M7,OR52I1,OR6C1,OR8B12,P2RX1,PLXNA2,PON2,PPP1R3D,PPP2R1A,RFNG,RFXAP,RUNX1T1,SDHC,SOS1,VAV1,WNT11
Negative_1955	RET,CACNG5,EPHX1,FZD3,GALE,GLUL,GNB1,HAAO,HAGH,MARS,MYH11,OR1J4,PLIN,PRKAG1,SMC1B,TAS2R13,TAS2R49
Negative_1956	F2R,C3AR1,CADM3,CCNE1,CD47,CDH5,DVL1,E2F4,EDA2R,ETNK2,GALNT13,GART,GNRHR,GPD1L,GRIA3,IL23A,INMT,IRF5,MAOA,MCEE,MDM4,OR14C36,OR2H2,OR51B2,OR52N5,OR7A5,PARD3,POLR2F,SEMA4C,SEMA4F,SFRP4,SLC25A4,SUV39H1,TAS2R1,TAS2R40,THBS2
Negative_1957	ADO,DCC,GPI,HGF,KDR,TK1,ABL1,ACAD9,AKR1C4,APOE,ARHGEF2,B4GALT1,BCAT2,CTNNA3,HNMT,IL12RB1,IL12RB2,LYPLA2,MASP1,MLYCD,NCR1,OR13D1,OR13F1,OR2T10,OR4A16,OR4F5,OR7G1,OR8S1,PAK7,PARD6G,PDE1A,PFKFB3,PFN4,PTGDS,PTK2,TGFA,VAMP3,VANGL1,VNN1
Negative_1958	PGP,ADCY8,CALML3,CHST13,RASSF1,SPI1,VAV1
Negative_1959	GSN,MPZ,ALDH4A1,AOC3,ARSA,BCL2,CDKN2C,CNTF,CSNK1A1,DDX51,HTR2B,NFYA,NME2,NRXN1,NTRK2,NUDT5,OR4A16,ORC5L,PDHB,TDO2,TLR3,WNT8A,YKT6,HSD17B12
Negative_1960	C8B,OTC,ACPT,ATP6AP1,C1QA,CCL20,DGUOK,DVL2,GPT2,IAPP,IARS,IL4R,JAK3,LIMK2,MTMR1,OR1E1,OR6N1,PIK3CD,PRKCI,PRKCQ,YWHAH,ZMAT3,TNFRSF1B
Negative_1961	DCT,GSN,ACAT1,AHCYL1,AMY2B,AOC3,ARSE,ATP5J,B3GNT5,CACNG6,CES1,CSF2RB,CYP2D6,DRD1,DTX3L,DUSP4,EFNA2,GMDS,GRIN2C,IFIH1,IL23A,INPP4A,IRF3,MMP9,NGEF,OR2T3,OR8G1,POLR2H,POLR2J,PPP2R5D,RAB5A,SARS,SELPLG,SEMA3B,SORBS1,SPRED2,STK36,B3GALNT1
Negative_1962	CR1,LAT,ACTA1,C1GALT1,CD44,CLDN18,CTSG,CXCL5,CYP2C18,DNMT3B,FADD,FMO4,GALNT3,GNRH2,GUCY1A2,GUCY2F,HAAO,INHBB,MAP3K12,MAPK14,MCCC1,MYH7,NFYC,PNPT1,PPP2R5D,PRKCD,RUNX1T1,SHISA5,STMN1,TBL1X,TJP3,VTI1B,TNFRSF1B

Negative_1963	F12,JUN,AKR1B1,ALOX15B,CADM3,CDC25C,COL4A4,CSNK2B,CYP11B2,EFNA2,EPHA6,F13A1,GDF7,GRIN2B,GUCY1B3,HTR2A,IFNW1,KARS,MAN1A2,MAP4K1,MAPK11,MEF2C,NME4,NUDT2,ONECUT1,OR2F1,OR2T6,OR4A15,OR4D11,OR4E2,PIP4K2B,PLOD3,POLR2H,PPM1B,PPP3CC,RBL1,RPS6KA1,RPSA,SETD8,SMC1A,SPHK2,ATP6V1G2,ST6GALNAC6
Negative_1964	APP,GH2,HPD,LYN,ME2,ALDH1L1,ALDH7A1,ALG8,CCKBR,CCL8,CHD8,CLDN19,CLDN4,CLDN9,CLOCK,CSF3,CSNK1G3,CTNNA3,DPYD,FZR1,GABARAP,GALNS,HLA-DOB,IL2RG,MALT1,MAP2K5,MAP2K7,MAP3K13,MMP9,NCOA4,NEU3,OR1J2,OR52H1,OR56A4,OR5T2,OR8K5,PARS2,PLCB3,PRLR,PTPRR,RBPJ,ROBO1,SMURF1,TAS2R38,TFDP1,TICAM1,TLR3,TUBB4Q,HSP90AB1
Negative_1965	GP6,ITK,CREB3,EIF4E2,MAP3K14
Negative_1966	C1S,CFI,ACTN2,AVPR1A,BPNT1,C3AR1,CCL24,CLCA4,CYP4F3,DPYD,DUSP2,DUSP3,EFNA5,ETNK1,FSHB,GNAT3,HLA-F,HYAL1,MAP4K3,MYH6,NEFH,OR10Q1,OR4C13,OR51I1,OR52B4,PFKFB2,PFKL,POLD2,PPP2CB,PTGS2,PTPN6,SPAM1,TNFSF18,WNT1,TNFRSF11B
Negative_1967	BID,FAH,GDA,HK3,IL2,RPE,TPR,ACY1,CACNA1H,CES2,CXCL10,CXCR5,CYP3A43,DHODH,ELK4,EPHX1,GALNT13,HBEGF,HIF1A,IL12RB1,IL20RB,KIR2DL1,MFNG,OR3A3,OR4B1,OR7G2,OR8K1,PEMT,RHEB,SLC25A4,SLC27A4,TSHR,VAV1,PAFAH1B2
Negative_1968	ARD1A,CDK6,CNTNAP2,DLL4,EPHA3,FGF2,HGSNAT,INHBB,LEFTY2,TLR2,TUBB6
Negative_1969	CBL,GNS,MOS,STS,ABLIM2,ACPT,B4GALT6,CALML3,CHAD,CXCR5,CYP8B1,DDX54,FADS2,GLI3,GNG12,HBEGF,HCCS,INPP1,MKNK2,NCR2,NFKB1,NR1H3,OR11G2,OR4A5,OR51B2,OR8B4,PCYT1B,RBL2,SMPD3,STK11,STX3,SUCLA2,TAS2R42,THBS1,VAV2,YKT6,ATP6V1E2,PDCD1LG2
Negative_1970	BTC,DDO,SI,APOE,ARNTL,CLDN17,DLL3,F13B,FMO5,GUCY1A2,IDI1,JAK3,NME7,NMNAT3,SDC1,SETDB1,ST8SIA1,TACR1,TNXB,ATP6V1B1,MAPK8IP1,SERPINA1
Negative_1971	AKR1D1,GALNT12,HAP1,HARS2,IL10RA,IL8RA,NOS3,PARVG,PPP1R3C,SV2C,TACR3
Negative_1972	BAD,EZR,ALDH9A1,ALG11,AOX1,BIRC2,CREB3L1,DDX55,GNAZ,IFNG,LYPLA2,MTMR6,NUMB,OR52B6,SRD5A1,TIRAP,UXS1
Negative_1973	CACNB2,CCNB3,CISH,CNTFR,FBXW11,FLT3,GALK2,IFNGR1,ITPKB,KLRK1,LAMC3,OR2T8,PLD2,QPRT,SFRP5,SV2C,UGCG,UGT2A1
Negative_1974	BTC,CR1,DBT,FGB,ADCY8,ANAPC4,APOA2,CCL5,CDH3,COL5A2,CYP11A1,DOLPP1,EPHB4,FRAP1,GALNS,GCAT,GGT1,GPX5,GRIN2D,HEXA,KCNB1,LMO7,MYH8,MYL5,OR4X1,OR52K1,PCYT1B,PDE4C,PDE7A,PDIA4,PIGA,PLD2,POLD2,PROS1,PYCRL,RAB23,TIAM1,YOD1,CACNA2D4,PNLIPRP1
Negative_1975	GNS,PDE7B,PIK3C2G,PVRL4
Negative_1976	AGPAT4,ARPC3,CACNA1A,CD48,CD79B,COMP,DLL4,ERBB3,GNAO1,H6PD,HMGCR,IL18,LDHAL6B,MAP4K1,OR2H1,PAFAH2,PIGC,RBX1,SMURF1,THBS1,TRAF1,WNT5A,WWP2
Negative_1977	MUT,ATP5O,CCR7,GBE1,IL1A,OR4D6,PRIM2,RPS6KA4,STK3,TYROBP,WIF1,CSGALNACT2
Negative_1978	CR1,TNC,ABLIM3,ACADSB,ALAS2,CYFIP1,EDNRA,FUT2,HAAO,KLRC2,MASP2,MGAT3,MLYCD,OR10R2,OR5C1,PPCDC,PRKCZ,SIPA1,ST3GAL1,UMPS
Negative_1979	ACPT,ALAS1,FOXA3,GALNT1,IL10,NFYC
Negative_1980	PAH,AGRP,ALG11,CD99,DAG1,FOXA2,GP1BA,GZMA,MGAT2,NADK,NAGLU,NAT6,NQO1,NTRK1,OR2A42,OR2M3,OR4K15,OR8I2,PIGB,SELE,TRPC1,VANGL2,MAPK8IP2

Negative_1981	TNF, ACADL, ADRA1D, CASP10, CCKBR, CSAD, CTNNA3, CYFIP2, DHCR24, E2F2, EHMT2, ENTPD4, ENTPD8, EPHX1, FPGS, FUT7, IFNA13, IL5RA, IRF3, LIPT1, MAP2K5, NAGLU, OGDH, OR10X1, OR1L1, OR2A42, OR4P4, OR6T1, PANK1, PANK4, PFKFB4, PNPO, PRPS2, PVRL1, RALA, SGPP1, SH2D2A, STX16, SULT2A1, TAS2R1, TIRAP, VEGFA, VTI1A, HLA-DRB4
Negative_1982	DAO, KDR, OSM, TXK, ACACA, AOC2, ATN1, CAV2, CDH15, CDKN2A, CDS1, CFL1, COL6A2, CYP2B6, DLL1, ENAH, ETS1, GAD1, GPX7, HAP1, HEXA, HMOX1, HTR2B, IFNA6, IMPA2, ITCH, ITGA2B, KDSR, LRP6, NAGLU, NCOA4, OR2C1, OR4D9, PISD, PPP1R3B, RARS, RDH10, SULT1A2, ST6GALNAC6
Negative_1983	KHK, ALDH1A3, APAF1, DDHD1, DRD2, GJD2, MAP3K3, OR5P3, PKLR, POLR3G, PPP3CC, SUV39H2, PAFAH1B2
Negative_1984	AK2, WAS, AMPD1, ATP5C1, BDH2, CCNA1, CLTA, CTSL1, EARS2, ENTPD5, FGF3, GALM, GNAO1, IL6ST, LAMA4, LEFTY2, MAML2, NEU1, NFATC3, OR2A2, OR4C15, OR4D11, OR6N1, PCCB, PDHA2, PHKB, PIGK, PRODH, SEMA3G, SESN3, SMAD5, TAS2R1, TLR2, TMLHE, WNT16, SERPINE1
Negative_1985	AGK, DAO, EZR, GH2, ACVR1C, ADSL, ATP6AP1, AVPR1A, B4GALT1, BPNT1, CPT2, FMO2, FRAT1, GMDS, GNA15, NAT6, NFKB1, OR12D3, OR2C3, OR6C74, PDCD1, PDXP, PIGA, PON2, PPA1, PYGM, RPS6KA3, SDC3, SEMA4C, SUFU, TSNARE1, YWHAG, CSNK1A1L, SUV420H1, TNFRSF10A
Negative_1986	FOS, UBB, AASDH, ACADSB, ACVR2A, ALDH1L1, ARG1, BPNT1, CCL2, CRLF2, CTTN, CYFIP2, DDIT3, FZD8, GAL3ST1, GRIN2A, GSK3B, HRAS, HSPA2, INSR, MPZL1, NRP1, OR10A3, OR2L2, OR2W1, OR6X1, OR7G2, PDE3A, PKMYT1, POLR2H, POLR3H, PON3, PPAP2C, RDH8, ROBO1, SEMA4A, SGPP2, TAS2R4, UBA1, CACNA2D2, CACNA2D4, MAP3K7IP2
Negative_1987	GP5, IL3, ACOX1, ALDH1A3, ATP5J, CLDN8, GAL3ST1, GPAM, GPX5, GRIA2, GSTP1, HAP1, MMP9, NR1D1, PIGT, PIGX, PLXNB2, ARHGEF12
Negative_1988	CDA, LCT, RPE, ADRA1D, AGXT, ALPPL2, ATIC, BACE1, CCL17, CCNG1, CDH1, CNGB1, CREB5, CSF2RA, CXCR5, DTX3, FMO3, GIT1, HEMK1, HSPA2, HTR2A, IFNA6, IL20RA, MFNG, MGLL, OR1L3, OR2T4, OR5B17, OR9Q2, P2RX4, PAK7, PHGDH, PITX2, PLCD3, PNLIP, PPP1R3C, PRKY, RALB, SLC27A1, SMAD7, SPRY2, SPTAN1, TAS2R60, TBXAS1, VAMP2, WASF2, PHOSPHO1, PRICKLE2, TNFRSF10D
Negative_1989	CFI, FES, ADCY8, ALDH7A1, CNTN1, CREB1, CYP26C1, DDIT3, DEGS1, DGKQ, ECSIT, FABP1, GNA11, IFNA13, IL4R, LDHC, MCAT, MMP14, NEFL, NFYB, OR2T2, OR4B1, OR52R1, OR5A1, PIP5K1B, POLE, PPAT, PPP3R1, PTPN7, RASA2, RPSA, SH3BP2, SNW1, VANGL2, ATP6V1G2
Negative_1990	C6, NGF, ZYX, ACSL4, ACVRL1, B3GAT1, B3GNT4, BHMT, BMP8B, CD226, CD274, CYP2B6, DDX23, DERL1, DUSP2, EPAS1, FSHR, GLB1, GNG3, GUK1, IARS, IDH1, INPP5B, IRS1, LGMN, NOTCH1, NOTCH2, OR2T12, OR4A5, PANK4, PLXNB1, PRNP, RASSF5, SCD5, SFRP5, SSX2IP
Negative_1991	ACSM1, CACNG3, CAMKK2, CYP2C8, DDX50, FTMT, GALK2, GCNT2, GFAP, GGT6, HRH2, IBSP, IL25, IRF3, KLRC1, MGST2, NFKBIB, OR2M3, OR51A4, OXSM, PKM2, PLCB4, PLXNB1, POMC, PPM1B, RALGDS, RDH16, SEMA3G, SLC2A4, TUBA1A
Negative_1992	FGG, APLP1, APOA5, ARPC5L, BMP6, CD274, CHEK1, CRY1, CSF1, CSNK1G1, CYP3A7, DAD1, IFIH1, LMO7, MYH13, NEFH, OR2T2, OR5K2, POLD2, POLR3G, PRKCB, RAPGEF3, RFNG, WNT2B, EIF4EBP1
Negative_1993	AGL, CFD, DCK, FH, FOS, ACVR2B, ALDH4A1, CBLC, CDC23, COX15, CYP2C18, DGKG, FGF11, FGFR2, IFNA2, JAK3, KARS, LLGL2, MNX1, NKX2-2, OR2M3, OR52L1, OR6N2, PARD6A, POLR1D, PRKAB1, PTCH1, SRGAP2, TBXA2R, TUBB2B, TUBB4, UROC1
Negative_1994	DGAT1, DGKA, DTX1, FECH, FZD10, GALNT13, GLS2, IGF1R, IL1A, KCNQ1, NCK2, NRXN2, OR2Z1, SEMA3F, SMARCA2, WNT5B, SUV420H1

Negative_1995	CHRNA7,DGKH,DIAPH3,FBXW11,FPGT,FZD10,FZD8,INPP5E,NRXN1,OR2A2,OR4K5,OR52E6,OR8D1,OR8K3,PPM1A,PRKAA2,SULT2A1,TNFRSF10C,TNFRSF1B
Negative_1996	CDA,GH1,OSM,RFK,SRM,TNN,ALDH4A1,ASRGL1,B3GALT4,CACNA1E,CLDN7,CNGB1,CYP2C9,DKK1,E2F5,ENO3,FASLG,FASN,FLT3LG,GPAM,GPX7,H6PD,LCAT,MAPK10,MITF,MYH14,OR10H4,OR2A12,OR3A3,OR4D1,OR4K17,OR4L1,OR52E6,OR56A3,OR8J3,PITX2,PMAIP1,STAT4,TAP1,TSC2,UGT2A1,ULBP2,UMPS,UPB1,VEGFC,SERPING1,TNFRSF11A
Negative_1997	BTD,F2,GLS,ACVR2A,BMP5,CCR8,CDH15,CFTR,CMPK2,COQ3,CPOX,CYP19A1,DGUOK,DUSP16,ECHS1,HLA-DRA,INHBB,LIAS,MYL2,OR10G2,OR2M7,OR6M1,P2RX1,PAX4,PCNA,PIK3R1,PPP1R3B,RHOD,ROBO3,RPS6KA4,SPRY4,STAT5A,ZNRD1,SERPINC1
Negative_1998	DBI,KMO,MAG,ME1,XDH,ACVR1B,ADCY3,ADH4,APOA2,CASP9,CCR4,CD86,DGKZ,DUSP2,EPHA5,ESAM,FBP2,FGF18,FUT6,GALNT11,GNAI2,GSTA4,HTRA2,IFNA6,MAP3K12,OR10K1,OR5AN1,OR5AU1,OR5K1,OR5V1,OR6K3,PCYT1B,PGK2,PGM3,PIGX,PTPN11,PVRL1,SETD7,SHC4,TGFB2,VDAC2,ATP6V1B2,MAPK8IP1
Negative_1999	VTN,B3GALT2,B3GAT3,BMP4,CHEK1,CLDN3,CNTNAP2,CPOX,CSH1,CTBP1,EFNA1,EP300,EPB41L1,FBXW11,GMPPB,GNAI1,MAN1B1,NCAM1,NCF1,NME6,OR10G8,OR2T29,OR2T3,OR4C11,OR52N4,OR5AS1,PDE1A,PLCG2,PPP2CA,PRKY,SMURF2,STK11,TAS2R50,PPP1R12A
Negative_2000	BID,GNS,HYI,ILK,MIF,AKR1B1,AKR1C1,ALDH9A1,AMHR2,ATP6V1F,B3GALT6,CACNA1B,CACNB1,CCR8,CDH4,CLDN9,CLTB,CYP4F2,GNAI3,HSD3B1,HSD3B7,IRF5,ITGA5,KIR2DS5,MDH1,MYD88,MYL2,NAGK,NT5E,OR10H2,OR2G2,OR4K2,OR52B4,OR6C76,OR7A5,PGK1,PIK3R4,POLE2,PTPRM,PYGB,QARS,RUNX1T1,SCP2,SMPD1,TCIRG1,ATP6V0E1
Negative_2001	EGLN1,OR6B3,OR6K2,OR6N2,RARA,SCLY,TRAF5
Negative_2002	CAT,IL4,TEC,ADH7,ALG3,AMDHD2,ATF4,CDC14A,CIITA,DGKE,FOXO3,FRAP1,FRAT1,GNPDA2,HNF4A,HTRA2,KLK3,MAPK14,MGST3,OR4C13,OR51G2,PIK3CB,PLXNC1,PPP2R2A,RARA,RRM1,SH2D1A,SPHK1,STAM2,TRAF6,YWHAE,TNFSF13B
Negative_2003	IL4,VCL,AOC3,ATP6V1F,CCL3,CD22,CD55,CDKN2C,CMAS,DUSP14,FRAP1,GMPPB,HSD11B1,HSD17B4,MOV10L1,OR2A4,OR6T1,SMAD4,SPRED2,SV2B,TPM3,UGDH,UROS,VKORC1,GABARAPL2,TNFRSF1A
Negative_2004	BAX,ACOX1,BUB1B,CD55,E2F4,HAAO,IL10RA,IL18R1,ITGB4,LIPT1,LYZL1,NNMT,OR1L6,OR2K2,OR2L13,OR6A2,P2RX2,RASA2,SMAD2
Negative_2005	MVD,C1GALT1,CNTN2,FBP2,FOSL1,HTR6,PIGC,PPM1A,RALGDS,SELPLG,SETDB2,WHSC1,ATP6V0D1,HSD17B12
Negative_2006	CAT,PC,ACSL1,ACYP2,ARPC1A,BUB1B,CD28,CLDN6,CYP21A2,EHMT2,FARP2,FBXW11,IFNK,LIMK1,MAGI2,NFE2L2,OR11L1,OR2G6,OR2M4,OR6T1,PAK2,PDE1C,PIP4K2B,PLA2G2F,PLCZ1,RBPJ,RPN2,RXRB,SFRP2,ST3GAL6,TAS1R3,TGFB3,UPB1,PDCD1LG2
Negative_2007	GCNT3,P2RX6,PAK7
Negative_2008	ACACA,ACSM5,ALG2,CRKL,CXCR5,DDX51,ETS1,EVI1,FOXO1,GALE,GTSE1,HTR5A,ICAM1,IFNA14,IL8RB,IRF9,LCMT1,MAP2K3,MAT2B,MECR,MINPP1,MYH11,NR1H3,NR4A1,OR10A6,OR2A12,OR2T5,OR2T6,OR4A15,PDGFA,PISD,POLR2F,PTAFR,RBX1,RFX5,SLC25A4,TCIRG1,WNT9A,HLA-DRB4
Negative_2009	C2,ACTG1,FASLG,GANC,IFNA2,ITGB4,ITGB5,OR4D11,OR51B2,PCYT2,HLA-DRB3
Negative_2010	ASL,KHK,MPI,TPR,ADRA1A,ADSSL1,ALG11,B3GALT6,BMPR1B,CCNA1,CCND3,CD38,CSNK2A1,DAAM2,DAG1,FGF19,FRAP1,GALNT5,IFNG,KARS,LCMT1,NEFH,NFX1,OR51B2,OR5T3,OR8H1,OR8J1,OXCT2,PLA2G10,SHISA5,SLC33A1,ST3GAL3,SULT1A2,TP53I3,TRDMT1,VTI1A,ZNRD1,ST6GALNAC6

Negative_2011	APP,CFD,NPY,A4GALT,ABLIM2,ACP1,ATP2A1,DEGS2,FUT2,LEF1,NLGN1,PDE3B,RBPJL,SORBS1,TDO2,UPP2
Negative_2012	F10,NLK,SYK,UBC,ACTG1,ADCY10,ALAD,ALDH3A1,ALPL,B3GALT5,BMP4,CCR8,CPT2,CYP7A1,ENTPD4,FMO2,GPD2,HDAC1,HMGCS2,HSPB1,IL6R,IMPDH2,ITGA2,LIPE,MYH6,NTNG1,OR12D3,OR1E2,OR4C15,OR8B8,P2RX5,PDE2A,PRDX6,PRIM1,PRPH,RHOA,SHC1,SMPD4,ATP6V1B1
Negative_2013	HGD,TH,ADCY3,ATP2A2,BHMT,CD48,CD55,CDKN2A,CSDA,CYBB,OR10T2,OR4K2,OR56A4,ORC3L,PANK2,PLCZ1,POLR1A,SPI1,TAS2R41,ZBTB16,SUV420H1
Negative_2014	AK7,MVD,OSM,PTS,ATP5H,BLVRA,CCR2,CHUK,COL2A1,CSNK1E,DMGDH,EPOR,ITGA2B,LDHB,METTL2B,MGAT5B,NRAS,NTF4,NTSR1,OR51A2,OR6Q1,PIAS1,SLC25A5,TAS2R9,UNC5C,YWHAG
Negative_2015	EBP,GBA,GK2,PLN,ADCY1,ARPC3,ATP2A1,CD38,CES1,CLTC,CNTNAP1,COL3A1,CPT2,CYP1A2,CYP2C19,CYP2C9,EPHX2,FGF2,FRAT1,FZD4,GBGT1,GNA13,GNAL,GNAO1,GRIN2B,HTR2C,IRF9,MICA,MTHFD1,NCAM2,OR2A25,OR4P4,OR51B2,OR51F2,PAPSS1,PARD6B,PLIN,POLD4,PPP1CA,RASGRP4,RPS6,SCP2,TNFSF8,WHSC1,ATP6V0A2
Negative_2016	F9,KIT,ME2,NNT,ABAT,ACY1,AKR1C4,ALDH1L1,ANAPC1,B4GALT7,BAAT,BACE2,CLDN2,COL4A6,CXCL13,DUSP14,EP300,GLUD1,GUK1,IL17B,IL22RA1,INPP5B,IRAK3,LAMC3,MAP3K6,MYH11,NKX6-1,NUMBL,OR14J1,OR2AE1,OR4C6,OR4X1,OR7D2,PPP2R1B,PRKX,SKIV2L2,SLC27A4,SLIT2,SNAP25,TNFSF14,ATP6V1G2,CSGALNACT1,TNFRSF10B
Negative_2017	ATP6AP1,B4GALT7,GSTM1,LLGL1,LRDD,PPARD,TRADD,ATP6V1C2
Negative_2018	F9,SFN,SYK,TPR,AANAT,ALDH1A1,ALDH3B1,ALPPL2,CAMK2A,CHRD,CSNK2A2,CYP4A22,DRD5,DVL2,FGF4,GLUL,HMGCS1,IFNA21,IL15RA,INPP5A,KCNQ1,LDHAL6A,NRXN1,OGDH,OR1Q1,PIAS2,PLA2G4A,PPP2R1A,TJP2,TRIP10,WNT7B,ATP6V0D2
Negative_2019	GSN,SFN,ACADSB,ACSM2A,ACTC1,ATP5H,C4BPB,CDH1,CDKN2A,COQ3,CTPS2,CTTN,CXCL9,EFNB2,ENTPD6,FOXA2,GUCY2F,HAO2,IGSF5,ITGAL,JAK2,KYNU,MAPK11,NANP,NFATC1,NPAS2,NT5C2,OR14J1,OR2M2,OR8H1,PANK4,PARS2,PCCB,PIGU,POLR2K,PRKACG,SHC1,SIGLEC1,TAS2R16,TBXA2R,TNXB,TUBB2A,WNT11,HLA-DRB3
Negative_2020	BAX,ZYX,AARS,AASS,ACP2,ATF2,ATP2A1,ATP5J,B3GALT1,BMP6,CDC14B,CHKB,CREB1,EPHA6,F13A1,FASLG,FLOT2,GANC,GLYCTK,GPT2,GSTA5,GUCY1A3,HEMK1,HSD3B2,IDH3A,IL24,LAMC2,MCM2,MYH9,NEU3,NOTCH3,OR1B1,OR51L1,OR5K1,OSMR,PAK3,PPP2R2C,RASGRP4,RDH11,SLC27A5,VTI1A,WNT2,YWHAZ
Negative_2021	MVK,SPN,ZYX,AMPD1,ATF4,C1QA,CACNG2,CD8B,CD99,COL1A2,CREBBP,CXCL13,CYP17A1,DDX47,ENO3,GCLM,IARS2,IL1RAP,IL22RA1,LIAS,LTC4S,MAP2K3,MCM4,MYD88,NLGN1,NT5C1A,OR10H5,PAK2,PCNA,PDHA2,PECAM1,PLD1,POLR2F,RXRG,SDC3,SDHC,TDO2,VCAM1,WNT3,WWP1,YWHAH,SUV420H1
Negative_2022	ACSS2,ALDH6A1,CFL1,CHPT1,CYP3A7,DNMT3A,FPGS,GABARAP,GCAT,GRIA1,HGSNAT,IL11,IL12RB2,MCM7,MECR,MYH7,OR5L1,OR7C2,OR7G2,PCYT1A,PDE8A,PPARG,PPOX,PPP1CB,PTGFR,SEPHS1,SETD1B,TJP1,UPP2,UPRT,WNT4,TNFRSF17
Negative_2023	CAD,DBH,GK,NNT,STS,ACADSB,ARPC2,CACNG5,CLDN11,CYP4F2,EPAS1,EPHA1,IL28A,ITGA6,MAP2K7,NCOR1,NMNAT1,OR4E2,OR6C74,RBX1,RRAS
Negative_2024	ICAM1,ICOS,MAOB,TAS2R13

Negative_2025	ACVR2B,AMD1,ATP5G1,ATP5J,CCND2,CMBL,COQ6,CXXC4,EFNB2,EIF4E2,FUT2,GALNT1,GNA14,HGSNAT,IFNA10,IFNA21,IL18R1,KCNB1,KDSR,LIFR,MAPK7,MCM7,MTHFD1L,NARS2,NEFL,NLGN1,OR11G2,OR1D2,OR2A25,OR51F1,OR56A3,PAFAH2,PFKP,PLCB2,PPARD,PYGM,RAPGEF2,RHOD,SDHD,SHC4,SSH1,TALDO1,TLR5,UNC5D,YWHAH,ATP6V0D2,PPP1R12A,PRICKLE2
Negative_2026	ACAD8,B3GNT4,CDK2,CHRM3,ENO2,GALNT14,GRLF1,GSTT1,IL13RA1,ITGAE,MGST1,MPST,MYH14,NCK2,NCKAP1L,NFYC,OR2L2,OR4K15,OR52R1,PAPSS2,PPARD,RELB,RPS6KB2,SEMA3D,SGMS2,SMAD4,TAS2R9,TDO2,TRAF4,WASF1,ATP6V0A1,HLA-DPA1,TNFSF13B
Negative_2027	F5,PAH,ACO1,ADH7,CCL17,CXCL12,ENO1,ESAM,FASLG,GAL3ST1,GART,GDF7,GNA12,HAGHL,HES1,IL22RA2,KCNQ1,LMO7,MCEE,MCM7,MGLL,MITF,MTHFD2,NEU4,OR2B2,OR4K15,OR51V1,PGAM4,PHGDH,PLA2G3,PLCZ1,PPA1,PPP2CB,RELB,RFXAP,SEMA3F,SESN2,SRD5A1,SULT1A1,TACR1,TUBA8,UGT2B11,WBSCR17,WNT16,HLA-DPA1,PNLIPRP2
Negative_2028	C2,ME3,ACSS1,ADCY10,APOA5,ATG12,BCKDHA,CCL2,CCL7,CCR3,CISH,CLDN4,DDX47,DLST,DVL1,FCER1G,GAMT,GGT6,GJD2,ICAM2,IL19,INPP5A,MAGI1,OCLN,OR13D1,OR14I1,OR2G3,OR4A15,PAX6,PLCB2,POMC,PON1,PPP1R1A,PROS1,PTPN1,SOD1,ST3GAL4,TAS2R42,TAS2R48,TCF7,UCK2,UGT8,VANGL2,PNLIPRP1,SERPIND1,TNFRSF10A,TNFRSF13C
Negative_2029	FYN,IL4,MPL,AADAC,ALG11,CACNG2,CASP10,CD79A,CDH5,COL4A4,CX3CR1,CXCL11,DGKD,DPM3,GART,GPD1L,GRIA1,HLA-C,HNF1B,IFNA8,IL12B,IL19,KARS,KIR2DL2,MASP2,MCCC1,MICA,NTF3,NTN1,OR10AG1,OR2A25,OR4F6,OR4N4,OR8K3,PAK2,PFKL,PGS1,PRODH2,PSME1,PSME3,RAPGEF2,TUBA4A,VAMP3,WASL,YOD1,YWHAQ,KIR2DL5A
Negative_2030	DBF4,DRD5,GADD45A,HLA-C,HSD17B2,KLRD1,NUDT12,OR51I1,SULT2B1,TCIRG1,TNFRSF12A
Negative_2031	CAT,MSN,APOE,ATP5E,BET1L,CHST12,DEGS1,FGF19,GBGT1,GMPR,GSTM3,HLA-DRA,MAP3K8,MKNK1,OR10A3,OR1A2,OR2H1,OR2T10,OR52M1,OR7C1,OR8G1,PIP4K2B,QARS,VANGL1,GABARAPL1,TNFRSF10D,TNFRSF12A
Negative_2032	B3GAT1,B3GNT5,BLVRB,BMPR1B,CARD11,CSF1,GALNT8,OR2G2,OR52B4,PLOD1,POLA2,PVRL1,SNAI1,SYNJ2,ZFYVE16
Negative_2033	DCN,ATP5F1,CCL16,IFNA21,MAPK7,OR6K3,PAK2,PLXNA1,XCL2,HLA-DQB1
Negative_2034	ADA,AMH,APP,DBT,LIF,OAT,ACAA1,AGMAT,ARNT,BAI1,CALML5,CBLC,CD86,CSNK2A2,DVL2,EIF4E,GADD45G,GLI2,GOT1,HNF4G,IPMK,ITGAX,LAP3,MGAM,MYH13,NCSTN,NFKBIA,OR11H1,OR13A1,P2RX6,PARVG,PGK2,PHKB,PIGV,POLD4,PRPS1,RASA1,RBKS,RELB,RFXAP,RGS3,RPS6KA2,SEMA3A,SPCS1,SPRY2,TIAM1,HLA-DQB1
Negative_2035	AARS2,ALAS2,BMPR1B,EFNB1,MYH10,OR10T2,OR2B11,OR4K5,PARD3,TNFRSF11B
Negative_2036	BCR,CAT,DDO,F9,FER,HYI,MSN,ALG11,ARNT2,CD22,COX15,CTLA4,EPB41,GALE,GNMT,GSTT1,GZMA,HTR2B,IFNW1,IL22,JAK2,NME2,OR2B2,OR56A4,OR6C6,PANK4,PPAP2B,RDH10,RPS6KA6,SARS2,TAS2R13,THBS3,TLN2,TPM3,TUBB4Q,ST6GALNAC3
Negative_2037	F3,IL6,ACAT1,BMP5,CD8A,CHRD,CNTN1,CREB1,CRHR1,CSDA,DDX4,DUSP7,EDAR,FRAT2,GALNTL4,GCNT4,HSPA8,KLRC4,KYNU,LLGL1,MAD2L2,OR1N2,OR2T12,OR2T2,OR2T29,OR5AN1,PLCE1,PRKAG1,WNT8A
Negative_2038	PXN,SRF,TNN,ACOT11,ACTG1,ACTG2,ADORA2B,ATP4B,BIRC3,BUB1B,CALML6,CCNA2,CUL1,EGFR,F13A1,GARS,GNA14,GSTK1,HAGH,HMGCS1,IFNA14,IL18RAP,KLKB1,LAMC2,NME4,OR11L1,OR2AE1,OR2AG2,OR2G2,P2RX1,PIP4K2C,POLE2,POLR2J,PON2,PPAP2C,PPP2R1A,PTPN5,RENBP,RHOQ,SELL,TUBAL3,UAP1,UCP1,VNN1,HLA-DPA1

Negative_2039	MPL, AGPAT4, AMD1, ANAPC11, CRB3, DDIT3, DGKE, ENTPD7, GLG1, PTGES, PTGES2
Negative_2040	SMS, ACVR1C, IL17RB, LAMC3, LDHC, OR13C2, PIGK
Negative_2041	APP, NPY, APOA2, B3GNT3, CACNA1C, CCL26, FGF1, GNAI1, HMGCR, IFNA13, OR10T2, PGK1, RND1, SHISA5, SPHK2, SUFU, WNT4
Negative_2042	CKB, ALDH1L1, ARPC1B, ATP4B, DAPK2, FARP2, FPGS, GALNT6, GGT6, HSPA8, IL11RA, IL1R2, IQGAP3, NT5E, OLAH, OR10G8, OR52H1, OR52I2, P4HA1, PAK1, PCYT1A, PMM1, PRKCB, ST8SIA1, VANGL1, WASL, ATP6V0D1, ATP6V1G3
Negative_2043	CCL15, FGF5, LRP6, PAK3, PFN3, PLCB4, SEMA3F
Negative_2044	A2M, DUT, F2, MAX, ACYP1, AKR1B1, ANAPC2, APRT, AQP7, C1QA, CDK6, COL4A2, COMT, CXCL13, DIAPH3, EPHB4, FSHB, G6PD, GALNT6, GNPDA1, HAO2, HSD17B8, IFNA7, IL17RA, IRAK2, MYH8, NANP, OR10H2, OR1J2, OR5C1, OR8H2, PFN2, PGAM2, PI4KB, PIK3C2B, PIP5K1A, PLCD3, PLXNA2, SDHB, SEMA4A, SGMS2, SPRED1, STT3B, STX3, UCKL1, UGT8, MAPKAPK3, ST6GALNAC4
Negative_2045	F2R, GK2, MTR, ALDH3B2, AMDHD2, AMHR2, ATP5H, BDNF, BLVRA, CCL22, CCL27, CCND3, CIITA, EFNB2, EXTL1, GJD2, GLB1, IFNK, IL2RA, INPP4A, JUND, LAMA3, MGAM, NLGN2, OR2AG2, OR4N5, OR52E8, OR6C70, PDE5A, PIP4K2B, POLR2J, SC4MOL, SDHA, SRGAP3, STAT6, HLA-DPB1, ST6GALNAC5
Negative_2046	TNC, VCL, AXIN2, CCL23, CRY1, DVL3, PDE5A
Negative_2047	DAO, EDA, LEP, SI, ACOT12, ADRB3, AKR1B1, B4GALT7, CTLA4, EXT1, GNAI3, HTR2B, ITGA6, LIPA, MYH6, NANP, NEU4, OR13C5, OR4A5, OR52E8, OR5D14, PAK6, POLR3D, THBS3, WNT6
Negative_2048	EPHB1, GALK2, GLUD2, KCNB1, MAP2K4, OR6B2, PIP4K2A, SULT1A1, TUBB4, VANGL2, ATP6V1C2
Negative_2049	MYC, ACAT1, ACP5, ATG3, ATP5B, CASP10, CASP7, CLDN16, DLL3, FSHR, GNA15, GPX7, INHBE, LIFR, MYL2, NFYA, PPP2CA, PRDX6, PRKCA, RXRA, TAS2R48, TNFRSF4
Negative_2050	C2, DRD5, DUSP8, IFNAR2, NME4, OR5I1
Negative_2051	CR2, LPO, UBB, ACAT1, ADH4, ARPC3, CCND3, CCNE1, CDC14B, CYBA, DGAT2L4, DHDH, DUSP6, EGLN3, FZD5, HDAC2, HMGCS1, MAP3K8, NFKB1, OR1S1, OR2AG1, OR4M2, OR51A2, OR5C1, PGLS, POLR2L, PRKAR2B, PRSS1, PTDSS1, STAM2, STX8, UGT2A3, PLA2G12A, SUV420H1
Negative_2052	C7, HGF, AMY2A, ARPC4, ATP5E, BMP8A, BNIP1, C5AR1, CREB3L2, CYCS, DDIT4, DDX4, DDX55, FBXW11, GNAS, GSTA2, HAP1, IFNA16, IL17RA, ITGA11, LDHC, MAP3K14, NKX3-1, NTF3, OR51E1, OR6K2, OR9G4, PAK3, PAX6, PLOD2, POLE4, POLR3B, PRKAA1, RAD51, STAM, TUBAL3, ATP6V0A1
Negative_2053	ADC, KHK, LYN, ANPEP, APBB1, ARG1, BLNK, CCL7, CXCL11, FUT6, GIT1, GNA11, HARS2, HYAL1, IL9R, KLRC2, LCMT1, LDHD, LEFTY1, LIPA, MAGI2, MASP1, NOX3, OR13A1, OR1L8, OR2T8, OR4K13, OR5P2, OR8S1, PIGA, PIK3C3, PIP5K1A, PPCS, PPP3R1, RASSF1, RBL1, RRAS2, STX1A, STX6, VNN1
Negative_2054	AUH, TNC, ACSS2, ARNT, CCNE2, CHIT1, EGLN1, EPHA7, FBXW11, GALC, GCNT2, GP1BB, HIP1, ICAM1, IFNA7, ITPA, NFKB2, NME6, OR11H6, OR1N1, OR4C16, OR4F4, OR51S1, OR56A3, PPP2CB, PRSS2, RRAS2, SELL, SLC27A4, SMAD1, SV2B, TSHB, TYRP1, XYLT2, ATP6V1B1, EIF4EBP1, MAPKAPK3, ST6GALNAC6
Negative_2055	BDKRB2, CXCL1, IL20, MYL2, OR2T12, PYGM, RASGRP3, STK3
Negative_2056	FAH, KMO, PGF, TYR, ABLIM3, ALOX15, BACE1, CACNA1G, CCKAR, CCL27, CHEK2, COL4A1, COMT, CSF3R, CXCL16, CYP1A1, DHODH, DHRS9, GADD45B, GALNTL5, GRHPR, H6PD, IL17RA, IL9R, LRP6, MYH8, NR5A2, OR10H4, OR2K2, OR2T10, OR4A15, OR4C16, OR8H2, PANK3, PGAP1, POLR3D, PSEN2, RBL2, RDH12, SDHB, SFRP1, SMARCA5, SOCS5, TLR4, UNC5B, WWP2, EIF4EBP1, HLA-DRB3, PDCD1LG2

Negative_2057	BAX, DCN, AMHR2, BCKDHB, CCNE2, CSDA, DTX3, EPHB3, IRS1, OR52H1, SEMA3G
Negative_2058	DCC, LCK, TFG, XDH, AGPAT1, AK3L1, ANAPC11, CACNA1I, COMP, CYP2B6, DAPK1, EIF4E2, FASN, GCH1, GLI2, GSTK1, ICAM3, ICOSLG, IFT57, KCNJ11, LIAS, NFYB, ONECUT1, OR5AR1, OR9A2, PPP2CB, RPS6KA2, RPS6KA5, RXRG, SPRY1, TLN2, EIF4EBP1, ST6GALNAC6
Negative_2059	AK3, GK, IL3, TPR, ACO2, ADCY1, AGRN, ARHGEF1, CDKN1C, CLDN14, CLTB, CLTCL1, CNTNAP1, CYP17A1, DDX23, DPM2, DTYMK, ENTPD6, GNAT3, GSTA2, HNMT, HSD17B8, IL28RA, IQGAP3, KIR2DL4, MAD2L1, MAGI2, MAPK10, MKNK1, NEU3, OR51B2, OR5AR1, OR9G4, OXSM, PHKA1, PIP4K2A, PISD, PNPO, POLR1D, PSME1, RUNX1, SEMA4D, SOCS5, SV2B, ATP6V1E1
Negative_2060	BCR, CEL, CGA, IDS, IVD, STS, TPO, ARSB, ATP4B, B4GALT2, B4GALT5, CCND2, CCNG1, CYSLTR1, G6PC, GCLC, GLDC, HAAO, IL12A, IMPA2, ITGA3, MAP3K6, MICA, NOS1, OR10A2, OR10G4, OR10H5, OR1M1, OR3A1, OR9A4, ORC2L, PDE5A, PDXK, PIAS1, PIK3R1, PRKAG3, PSAT1, PTDSS1, PTGES2, RHOQ, RPRM, SEMA4C, STX5, TALDO1, TAS2R60, TPM3, VAMP1, VANGL2, MAPKAPK5
Negative_2061	ADH1A, ALOX15B, B3GNT3, BAAT, COL3A1, COQ3, CYP2A7, GUCY2C, ITGA7, ITGAE, LRP5, MAD2L2, MAP4K2, METTL2B, MYL9, NEGR1, OR51B2, PIAS2, POLA1, RDH11, SPRY4, SUCLA2, TRAF5, TNFRSF12A
Negative_2062	ALDH9A1, BCL2, CDH2, CYP2S1, EHHADH, FGF5, GP1BA, IL10, IL15RA, KCNJ11, LIPA, MAP3K5, PIPOX, SV2A, TNFRSF8, WNT16, MAPK8IP3
Negative_2063	CD28, CDC20, CSDA, EPHB1, IL8RB, LCMT2, NUDT9, TP53I3, TPI1, WNT6
Negative_2064	SRC, ARRB1, DPAGT1, ENTPD8, HSD11B2, IBSP, ITGA7, PDHA1, PLXNA2, SORD, THBS3
Negative_2065	AMH, FST, GGH, LCK, RB1, SI, AASDH, ACO2, ACTN1, ARPC1B, BLVRB, C1QA, CACNA1C, CD247, CD8B, CDO1, CTSB, CXCR4, CYP26C1, CYP4A22, DDIT4, EIF4E2, EPHB1, IKBKE, ITGB3, LEFTY1, MAP3K5, MAPK12, MOV10L1, MYH1, NAT1, OR10J5, OR2G6, OR4K17, OR51B2, OR6Q1, OR8B8, OR8K1, PAK2, PPP2R5B, PRPH, PVRL1, RPS6, RPS6KA5, SEMA4B, SOAT2, SSH3, TSHR, ULBP1
Negative_2066	LEP, CMBL, EGFR, GSTM1, MAPK11, NLGN1, PI4KA, RFWD2, SOCS7
Negative_2067	HK1, ME2, NLK, ALPL, BDNF, CACNG3, CD40LG, CREB3L4, DHDDS, EI24, ENTPD1, HLA-<br>C, OR10Z1, PIP5K1B, PLCB2, PRKCG, PTCH1, RETSAT, TNFSF4, XYLB, ARHGEF12
Negative_2068	F9, GNE, ID4, ATP2A1, ATP2A2, BCL2, CASP6, CCL2, CES7, CHSY1, CLDN4, COL3A1, CRLF2, DKK4, DPM2, EPHA3, ERBB4, GBE1, GLG1, HIF1A, LDHD, MAD1L1, MAP3K14, OR13C2, OR1L8, OR52B4, OR5D13, PLOD2, POLR3G, PPP2R2D, SELP, SOCS3, SUCLG2, TGFB1, VASP, CSGALNACT2
Negative_2069	HK2, ACSM4, ARSD, BPNT1, DGAT2, GRLF1, IRF9, MIOX, OR1C1, OR2M7, PIK3CB, PRKAR2A, PTGER3, PTGS2, SESN1, STX10, TGFB2, ATP6V0A4, TNFRSF10B
Negative_2070	PDC, ACOX3, AGPAT1, ATN1, CCNB2, CMPK1, CREBBP, CSF2RB, CXCL9, DGAT2, DVL2, EIF4E2, ENTPD4, ERBB4, EXT1, GALE, IGFBP3, IL9R, JAG2, MCM2, NAT2, NUDT12, OR2A12, OR4B1, OR5D13, OR9G4, OR9I1, PAX6, PIGA, PIPOX, PPP2R2C, PPP2R5A, PTGES, PTGES2, RGS3, SLC27A5, SLC2A1, SRGAP1, SSX2IP, TLR9, TNFRSF4, UGT8, GABARAPL2
Negative_2071	ALAS1, AMPD2, ATF2, ATP2A3, CACNA1I, CDC25A, CMPK1, G6PC2, GMPR2, GNA11, GSK3B, IL23R, JAM2, MAP2K6, OR10AG1, OR5K1, PCYT2, PPM1A, PTAFR, RPS6KA4, MAPK8IP3
Negative_2072	DAO, EPO, ALG3, AMY2A, APOA5, B4GALT1, CACNA1A, CD226, CDKN1A, CYP11A1, F2RL2, FGF20, GNAI3, MAP3K14, MAPK10, MTHFS, NRP1, OR2M4, OR51F2, OR52R1, OR56A4, PIGL, PIM1, PIPOX, PRPS1, PTDSS2, RHOD, SEMA7A, STAT5A, SULT1A2, HLA-DRB5, SUV420H1

Negative_2073	ADRA1A, ATG12, CBLC, CD28, CNTN1, IL20RB, IRF3, MAP3K12, NTF3, PDE8A, SESN2, SETDB1, PAFAH1B3
Negative_2074	FES, AKT3, APOA1, BMPR2, CACNB4, CCKAR, CDC25A, CXCL2, CXCR6, DDX50, DEGS2, DUSP5, ENO1, GPT2, HARS, HCLS1, HDAC1, KRAS, MYH1, NEU3, NLGN3, NSDHL, NT5C3, OR1K1, OR5AP2, OR5K2, P2RX1, PIGM, PROC, PSPH, PTGES2, SSH2, SUCLG1, VAV2, PNLIPRP2
Negative_2075	GSN, HK2, ACVR1, ADH7, ALDH3A2, BMP2, CFTR, DHCR7, EDNRB, ENPP1, GRIN2D, IL18RAP, IL8RA, INPP5E, JAK2, LTC4S, NRCAM, OR1C1, OR2G6, OR51A4, OR5C1, OR8D2, OR8U1, PGAM2, POLE4, PRSS2, PTAFR, RARS, RPE65, ST3GAL3, ST3GAL4, SV2C, TBXA2R, TLR2, TUBA1A, ULK3, UPB1, VAV2
Negative_2076	TNN, CCR8, CD58, CYSLTR2, DGKQ, EDA2R, EPHB3, GADD45B, GNG12, GPX5, OR2A5, OR4Q3, OR6K6, OR8J3, PTPMT1, TLN1
Negative_2077	DAO, ALDH3A2, ALPPL2, AMHR2, ANAPC4, APRT, CACNB3, CHRM3, CLTC, DTX1, DUSP6, FASN, FBP1, IFNB1, ITGA3, KCNQ1, MAML2, OR13C5, OR2A12, OR56B4, PCNA, PDX1, PFKM, PFN3, PLCB2, POLR2G, PPP2R5D, PTGS1, PTK2B, PTPN11, RAPGEF3, SFRP4, TKTL2, TRAF2, UGT2B17, WNT10A, ALDH18A1, HSD17B12
Negative_2078	MOS, PGP, TFG, ANAPC10, BCKDHA, CASP3, CCL8, CSNK2A2, E2F3, GCAT, GSTA3, IDH3G, IKBKG, LCMT1, LGMN, LRP5, MAP3K1, MYL5, NCF1, OR1J2, OR5AP2, OXCT2, PHGDH, PPAT, PYCR1, SDC1, SLC9A1, TAP1, TSNARE1, UGT2A3, UGT2B7, VEGFB, YWHAQ, MAPK8IP2
Negative_2079	DUT, TXK, ALOX12B, ARPC1B, CASK, CDC6, EPHA1, JAG1, JUND, MNX1, MYH9, NFKBIE, OR1N1, OR2Z1, OR4D11, PIK3R2, POLR2H, RARS2, STAT2, TXNDC12
Negative_2080	BTK, PGD, STS, ACACA, ADAM10, ADCY4, B3GAT1, CAV1, CCL21, CMPK1, COL1A1, CYP17A1, DAPK1, ENPP1, FDPS, FUT5, GSTA1, HPSE, IFNW1, IL17RB, LAMB3, LMO7, MAPK3, NEUROG3, OR5P3, OR8J1, PFKFB1, PPP2R5B, RFXANK, ROBO1, ST6GAL1, WWP2, ZMAT3
Negative_2081	ALOX12, CAV2, FBP2, MAP2K3, MICA
Negative_2082	C8G, FST, LSS, ACTG1, AKR1C4, ALDH6A1, ALG13, CCL27, CD3E, DGKE, DKK2, DLAT, EDA2R, IFNAR2, KIR3DL3, LAMA4, MAD2L1, MAP3K4, MAP3K7, NEFM, NME7, PDGFC, PIK3C2A, PORCN, PRKAR2A, PTGER3, RPE65, SMC1B, TNFSF12, TNFRSF10D
Negative_2083	C1S, FH, LTB, MVD, ALPPL2, AMDHD2, APBB1, B4GALT1, BDKRB1, CAMK2B, CASP3, CBR3, COL3A1, COL5A2, DGAT2L4, EPHB2, FDFT1, FGF21, GNAQ, GNMT, GZMA, HGSNAT, HIBCH, IFNGR1, MAN1C1, MCM4, OR10G9, OR14J1, OR1S2, OR4C46, OR4K5, OR51D1, OR51V1, OR52A1, OR52R1, OR52W1, OR8D4, PPAP2A, RPE65, SFRP5, SULT2B1, TRIP10, TSHR, VDAC1, WNT6, MAPK8IP3, PLA2G12A
Negative_2084	NPL, ACAA2, CD8B, DDHD1, FSHB, FTMT, MAN1A1, OR56A1, OR5R1, PIK3R3, RFWD2, TNFSF12
Negative_2085	CR2, TNC, BCAT2, CCND3, CSNK1G1, DTX2, EI24, ENPP7, EVI1, FGFR1, FUT8, GGT6, IGF1, IL10RA, IL2RB, IL6R, IMPDH1, KLRC2, LTA4H, MAPK12, MCCC1, NR1H3, OR1C1, OR2G3, OR4C13, OR4N4, OR51L1, OR6C75, PARS2, PDE8B, PLA2G2F, POLR2K, RBPJL, RUVBL1, SDC4, SEMA4G, SV2C, THBS3, TLR4, TYMS, VEGFC, WWP1, XYLT1, XYLT2, ALDH18A1
Negative_2086	ABO, BTD, CBL, ATP5G3, BCL2L1, CHRM3, CNTN1, CSF3R, DPM1, EPHB2, FASLG, GFPT1, GFPT2, HSPA2, IL21R, IL24, IL28RA, INHBB, LHCGR, MAP3K14, MAPK1, MAPK12, OR11A1, OR4P4, OR52K2, OR7A10, OR8J3, PFKFB1, PNPLA4, PPAT, RBX1, SEMA3E, SLC27A4, ST3GAL2, STK11, THBS1, TNFRSF9
Negative_2087	ABO, C8G, ABI2, ACAT2, ACOT11, ACTG2, AHCYL1, ATN1, CCNE2, CDC25C, CDK2, COL2A1, CSNK1A1, DPM1, DUSP8, EPHA2, F2RL2, FARP2, GRIA3, HSPB1, HTR4, OR13D1, OR2M3, OR51A7, OR52A5, OR5AP2, OR6M1, P2RX7, PDGFB, PLAU, PLTP, PNLIP, PPAT, PRKAR1A, PRKCI, PRPH, SC4MOL, SH3BP2, SHMT2, SLC27A1, SPRY2, THBD, ULK1, VAV1, VEGFB, TNFRSF10D

Negative_2088	OGT,GPT2,P4HA1,PIK3R2
Negative_2089	MVK,AGRP,ALDH7A1,CAV2,CCNH,ENPP1,GBA3,MGAT4B,NOD1,OR4A15,ORC3L,P4HA1,PDK1,PMM2,PPM1B,SHMT1,YARS
Negative_2090	LPL,ACAA2,ACSM4,AMPD1,BDKRB1,CDH3,CNDP1,COQ5,DAAM1,EARS2,FABP1,FRAT2,FZD10,GYS1,HLA-DOA,IDH2,MCAT,MTAP,MYL7,OR10K2,OR4C12,OR52E2,OSMR,PDE2A,PPP3R1,PRKAG1,PRKCI,PTCH2,RCHY1,RDH8,SDHD,SKP1,SOCS4,STK4,STX3,TBL1X,TNFSF4,TNXB,TP73,TUBA8,UNC5A,YES1,ATP6V0A1,ATP6V0D1
Negative_2091	EGF,EPO,F10,ATG12,ATP5G1,CAV3,CD47,CYP2A13,CYSLTR2,DGKB,DPYSL2,FARSA,FTMT,FZD8,GMPPB,GOT1,GPD1L,GRIN2B,HARS,IDH3G,IFNA10,IL15,IL2RB,KIR3DL3,NAGS,OR4C11,OR8B12,OR8D2,OXCT1,PDE6D,PLCB2,SULT1A2,TAP2,TAS2R7,TSLP,UGCGL1,WASF3,HLA-DRB3
Negative_2092	GZMB,KLRC1,MEF2C,PFKFB4,PLOD2,TPMT
Negative_2093	AMH,GCK,ACP6,ACSS3,AQP7,ARHGEF7,CACNB2,CREB3,CSF2,CTNNA3,EXTL2,FIGF,FLT3,GCNT2,IL1A,ITGA10,NME2,OR51F2,OR5V1,OR6N1,P2RX5,PER2,PLK1,POLR1E,PTPN6,QPRT,SDC2,THTPA,TP73,ATP6V0A1,ATP6V0D1,HSP90AA1,TNFRSF10C
Negative_2094	CS,ME1,MPI,ACSM2A,ACTN2,ALG10,ATP2A1,CD55,COL6A2,CUL1,CYP2E1,DMGDH,FGF18,FMO4,FPGS,HIF1A,IL20,LCMT2,MAD2L2,MS4A2,NCOR1,NFKB1,ODC1,OR10G2,OR13C8,OR4F6,PAFAH2,POLR2J,RDH11,SDHA,SMPD1,TLR2,UPP1,VAV3
Negative_2095	F11,ID2,PGD,A4GALT,AHCY,B3GNT4,C1QA,CAMK2G,CARS2,CCND3,CER1,CKS1B,CLDN1,FECH,FZD5,GRIA1,GUCA1A,HCCS,HIP1,HLA-DMA,HSD17B8,ITGA8,LIMK1,LRP6,MADCAM1,OR7C1,PKLR,PLTP,PTPRZ1,SELP,SMC1A,TXNDC12,UGT2B28,VCAM1,WEE1
Negative_2096	BBC3,CLDN4,DNMT1,KCNJ11,MTMR1,OR4P4
Negative_2097	SFN,ABI2,ADH1A,ADIPOR1,B4GALT5,CDKN2A,CES2,CYP3A7,GFPT2,GMPS,GNAQ,GNRH1,GSTM2,MASP2,NEU2,NMNAT1,NTF4,OR2F2,OR51M1,OR5D13,PDE8B,PLCG1,PRODH,TIAM1
Negative_2098	AK7,MAG,AARS2,ALAS2,ATP6V1D,BMP4,COQ6,CSF2,CYP19A1,CYP8B1,DDIT4,DGKB,ENTPD2,ENTPD6,EPHA3,FMO4,GALNTL4,GNA15,GRHPR,IL25,INPP4B,ITPKA,KIR3DL2,MAPK3,MTHFR,NUDT8,OR13D1,OR1E2,OR2T33,OR52A1,OR9A4,PIAS4,POLE2,SARDH,SENP2,SETD1B,SRD5A2,STK4,TAS2R14,TFDP1
Negative_2099	AK2,IL6,MVK,SRM,ACAD9,ACO1,ADH7,AMPD1,AOX1,ARHGEF6,C9orf95,CAV1,CCL22,CCR1,CCR4,DDX55,DKK2,EPHA2,FGF8,HSD3B7,IGF1,IL9R,IRS2,ITGAE,LAP3,MAP3K2,MBL2,MNX1,OR2T1,OR4K13,PLA2G3,PLOD2,PVRL4,PYGM,RAPGEF3,STAT5B,WNT8A,ATP6V1G1,CSGALNACT1
Negative_2100	CP,ALG10,ALOX12,CASP3,CCL15,CD38,CNTFR,DTX4,ENPP2,EPHA8,FUT6,GALNT2,IL21,IRAK4,ITGA8,MAP3K6,NMNAT2,OR5M9,OR6K6,OR7A5,PDE6D,PPM1B,SETD7,SKIV2L2,SOD1,VAV2,MAPK8IP2
Negative_2101	CSK,KHK,ACAT1,ACVRL1,ADCY5,B3GNT4,CAMK4,DDX18,EFNA4,EPHA4,ETNK1,F2RL1,FGFR1,G6PD,GLS2,HDAC2,HMBS,HNF1A,IFIH1,MADCAM1,MAP3K1,MCM4,MLLT4,NFKB1,NSD1,ODC1,OR56B4,PIK3CB,PLD1,PLXNB3,RIPK1,ROBO3,TACR1,TLR5,WNT16,XYLT1
Negative_2102	CAT,ACVR1B,AGMAT,AGPAT3,CCL23,HSD3B1,MAFA,OR13F1,OR2G2,PIGH,PLXNA1,SPRY3,SRGAP2
Negative_2103	CD6,ADCY9,AKT3,BCAR1,COMT,CX3CL1,CYFIP1,DLAT,EPB41L1,FDFT1,GLG1,GSTZ1,GUCY2F,IDH2,LCP2,LDHA,LIMK2,MAGI2,MAML3,MPP5,MYLK3,OR10T2,PIGX,PLA2G4A,PLA2G5,PMM2,POLR2E,PVRL2,RAB5A,RARS,RPN2,RRM2,SGPP1,STX3,STX8,TBL1XR1,TUBA8,UBE2L6,TNFRSF13B

Negative_2104	AASS,ADH7,ALPI,AOC2,BCMO1,CAMK2D,CDKN2C,CMPK1,CMPK2,DDX54,DLL4,DRD5,DTX3L,EPAS1,EPRS,G6PC2,GNA12,GNA15,GSTA3,GUCY1A3,LEPR,MTHFD1L,NLGN1,OR8I2,PLXNC1,POLR2L,PPP2R1A,PRKCD,PSEN1,SV2A,HSP90AA1
Negative_2105	C6,AARS2,BIRC5,CARS,CCL22,CDC42,CHAT,CSDA,EPHA4,LLGL2,NCR3,NT5C1A,OR1K1,OR4C15,OR51A4,POLR2F,PSENEN,RPS6,SH2D1A,SLC33A1,UGP2
Negative_2106	COL5A1,FGF21,IKBKE,MAN2A1,POLR1E,SENP2
Negative_2107	ACTN4,ATG12,BCMO1,GADD45B,ITGA8,ITPK1,JAK3,MGAT2,OR2H2,OR5AN1,PIGA,YOD1
Negative_2108	PML,ALG5,TUBA8,TYMP
Negative_2109	PDC,B3GAT3,EPHB1,NAT5,OR13A1,OR51E1,OR5P3,SCLY,SPTLC2,UGT2B4
Negative_2110	KCNJ11,OR2Y1,OR8K1
Negative_2111	ABO,CRHR1,CRLS1,CUL1,DGKQ,INHBB,LIPA,MAPK9,ORC2L,PNPO,PPAP2A,PPP3R2,PTDSS2,YWHAE
Negative_2112	ADO,AK3,APP,GK2,NPL,SYK,ACAD9,ALDH3B1,ALG6,AREG,CAMK2G,CD80,CLDN4,CLTB,CRB3,CTF1,CYP2D6,DHRS3,DNMT3L,F2RL2,FMO3,FUT1,GALNT3,GPAA1,HIBCH,IFNA6,IL23R,MAOA,MAP4K3,MAPK3,MFNG,NFKB1,NRG1,OR10G4,OR3A3,OR5M8,OR6B2,OR9A2,PPP1R3C,PTGS2,RFXANK,RXRB,SARDH,TSC2,WNT3,TNFRSF10C,TNFRSF14
Negative_2113	IL3,ABAT,BET1L,COQ5,CXCR4,DHCR24,FGFR4,FZD8,LCMT1,MAP2K3,OR10A2,OR4Q3,PLA2G6,RPS6KA5,SDHA,SMAD5,UNC5D
Negative_2114	SMS,AGPAT2,CDK6,CRB3,DHCR7,ENTPD2,FUT4,GGT6,HADH,IBSP,LEFTY2,MRAS,NFATC2,NGEF,NME2,OR56A4,OR5H2,ORC5L,P2RX7,PRKAG1,PYCR1,ROCK1,SEMA3C,SETDB2,SPHK1,TGFA,ATP6V0E1,ST6GALNAC3
Negative_2115	DDC,KHK,MVD,AASDH,APAF1,CBR3,CEBPA,CNDP1,IL1RAP,LGMN,MAP2K7,NEUROD1,OR2AE1,OR5A1,PIK3CD,PNPT1,POLR3A,RAB5A,RELA,SDHB,ST3GAL5,STX3
Negative_2116	GNE,GP9,ACOX3,ACSL6,AGPAT3,ARPC2,ATG12,CAMK2D,CCL15,CD14,CDC45L,CLDN7,CREB5,CTPS,CYP11A1,DPM1,FGD3,FZR1,HAGHL,HIF1A,IKBKG,IL1R1,IL20RA,INPP5A,JAM3,MGAT4B,MMP1,NFATC1,NTF4,OR10K1,OR2B2,OR2L8,OR2M3,OR4D2,OR51F1,PIGB,PIM2,PLOD1,PRSS3,PTAFR,RAC1,RRAS2,STX8,TUBA3E,ULK2,UROD,WNT7A
Negative_2117	IL6,TH,AASS,ACADSB,CASP6,CDH5,DHX58,HAP1,ICAM2,IL29,IL4R,INPP1,IRS4,MGAM,NCR1,NFKBIE,OR52R1,PLA2G2D,POLR2B,SOCS3,VKORC1,SERPIND1
Negative_2118	ACADM,BDKRB2,COL4A2,GNAZ,ICAM1,IL6ST,PANK1,PDE3A,PPP2R2A,SHC3,HLA-DRB4
Negative_2119	ATP5G3,ATP6V1D,BBC3,BTRC,CCL22,CDC45L,CLTB,CRY2,DGKG,DTX1,ENAH,FTH1,GALC,GALK1,GALM,GNAS,GYS1,HIP1,HNF1B,HTR5A,JAK1,LAMB3,LDHC,MAPK12,NADK,NKX6-1,OLAH,OR13J1,OR3A3,OR4D2,OR4P4,OR52D1,OR56B4,OR8H3,OR9A2,OSMR,PARD3,PHKB,PKM2,SFRP4,TMLHE,UXS1,SERPINE1,TNFRSF17
Negative_2120	AMH,TNR,BMP8B,CAMK4,CD58,CD80,CSF2,CYP26C1,DTX4,EGLN1,GALK2,GIT1,GMPR2,GRIA3,GUCY1A2,HMOX2,IFNA7,IL18R1,ITGA8,LALBA,LAMB3,MAP4K3,MPZL1,MYH14,NR1H3,OR1E1,OR1F1,OR4L1,OR5M9,OR7A10,PIK3R3,PNLIP,POLR1D,PROS1,PTK2,RAC1,RRM1,SLC2A1,SLC2A4,SUCLA2,TGFB3,TNFRSF9,VDAC1,SERPINA5,SERPINF2
Negative_2121	F12,F8,ITK,LPO,ACACB,ACYP1,ADAM17,ADIPOQ,ARHGEF6,ASAH1,B4GALT6,BMP5,CACNG6,CASP10,DERA,FMO3,G6PC2,KLRC1,MGAT1,NEFL,NODAL,NPR2,NRG1,NRP1,OR10H3,OR1L8,OR2B11,OR4C13,OR52E6,OR56A1,OR5A2,OR7G1,PDE1C,PGAM2,PTCH1,RDH11,RXRG,SDHA,SEMA3E,SMAD6,SPRY1,TSLP,UNC5D,ATP6V0A1

Negative_2122	BTC, CP, GH2, ACTC1, ADSS, AOC3, BCL2, BMP6, BMPR2, DGAT2, DUSP7, ENPP3, ETS1, EXT2, GLUD1, GNAO1, GNAZ, HLA-F, IL1B, KAT2A, MAGI1, MFNG, MYH15, NCF1, NEGR1, OR10A6, OR52W1, OR56A3, PIAS2, PIGT, PIK3CB, PPARA, PRKX, PTCRA, RDH16, SKP2, SOCS3, TALDO1, TJP3, UBE2L3, XIAP, YWHAH, B4GALNT1
Negative_2123	ALDH1B1, ATF4, B3GALT2, DAAM2, GFAP, INSR, MLLT4, OR13C8
Negative_2124	ARSA, AVPR1A, CAMKK1, CYP19A1, DIAPH3, F13A1, GALNTL5, GLG1, GRPR, INHBA, ITGB1, LIPC, LRP6, MINPP1, MYH7B, NFATC2, NRG4, OR8J1, PIGT, POLR2D, TLR2, TUBA1B, TYRP1, UROD, VEGFC
Negative_2125	CGN, EGF, FGA, LCK, ACHE, ACP6, ADCY7, BAI1, BDKRB1, C4BPA, CCNA1, CDC7, EPOR, FRAT2, GADD45A, GSTT1, HEMK1, IDI2, MAP2K4, MPP5, NEU3, NUMBL, OR10J1, OR12D3, OR2T33, PAK1, SELPLG, SETD2, STEAP3, STX18, TUBB, TYMS, UGT2B17, UPP2, MAP3K7IP1
Negative_2126	EGF, ACADVL, DOT1L, FGF5, OR56A1, OXTR, UGT8
Negative_2127	AK5, GDA, ADH1A, ANAPC7, BMP7, CLCA2, DPYS, EREG, FABP4, INHBA, PLOD1, PPP2R2C, ROBO2, SLIT2, TBL1X, HLA-DQA2
Negative_2128	ATP4A, GPAM, NME6, PRIM1, RAPGEF4
Negative_2129	DPM3, FMO5, OR51E1, PFKP, PLA2G6
Negative_2130	GH1, RB1, BDH2, SMAD2
Negative_2131	LCT, ATP6V0C, CADM1, MAOB, OR52A1, PLCG1, PRKCE, SPP1, SSH3
Negative_2132	ACSL5, CALML3, CNTN1, EPHA4, EPRS, F13A1, FCGR3A, ITGA1, MCM5, PNPO, PTCH2, PTGER3, SPHK2
Negative_2133	ADC, F11, ASCC3, CAMK2B, CPS1, DDX19A, DUSP6, EPHA2, GFPT2, GUCY1A2, HPRT1, IDI1, IL10RA, IRAK2, OR2T29, OR4B1, PTCH1, TNFSF8, WNT7A, TNFRSF12A
Negative_2134	JUN, NPL, ACOT11, ACTG2, ADH5, ALG12, ALOX12B, CCKAR, CHAD, CYP2A13, CYP2C9, DDB2, DTX3L, EGLN3, HDAC1, ICAM3, IFNA17, IL10, IL17A, ITGA6, JAK1, KIR2DL3, MINPP1, MYH4, NCAM2, NFAT5, NQO1, OR1M1, OR51T1, PIK3R4, PLA2G10, PLXNA1, PMAIP1, POLD3, PON1, PPP1CA, PRPH2, RARS2, RPS6KA5, RRAS2, SETMAR, SMAD6, STK4, THBS4, UGCGL1, WARS2
Negative_2135	DFFB, DHFR, FDPS, GLG1, ROCK2
Negative_2136	CFI, F7, GGH, GSS, PVR, TK2, ACSM4, ADAM10, ADRB3, ADRBK2, AFMID, ATP5O, BMP6, CASK, CD79B, CER1, CIITA, DNMT3B, DUSP16, DUSP7, EPHB6, FGF20, FGF22, GPAA1, IL8RB, KIR2DL3, LRP6, NAMPT, OR11H4, OR2T11, OR52E2, ORC6L, PDE4A, PLCD3, PNLIP, PPAP2A, PRKAB2, PTPRM, RAB23, RELA, RXRA, TAS2R16, TAS2R40, TAS2R41, THBS3, TNFSF11, TNFRSF1B
Negative_2137	ACO1, ALG8, BCL10, CCND2, DAAM1, EPHA1, EPHA7, GAD1, LIMK1, LRP6, NEFH, OR4S2, SMAD7, UGT2B7
Negative_2138	DHH, CCR7, CNTN1, FASN, GART, JAG2, OR4K14, OR5M9, RALGDS, RASA1, RERE
Negative_2139	ADA, AK1, CR2, IL7, AARS2, ACAA1, ACAT2, ACSL6, ADCY2, ADCY3, AK3L1, CHD8, CPT2, CYP11B2, DDHD1, EDNRA, EPHA6, F2RL2, FRAT1, GLI1, GUCY2F, HLCS, IAPP, IL19, IL22, IMPA2, INADL, KLK3, OR10AG1, OR6N2, OR8H2, PDE6H, PPP1CB, RALB, RPS6KA3, SEMA3A, SLC25A6, SPRED1, TUBA3C, TUBB3, UPRT, ATP6V1E1
Negative_2140	CFD, MTR, MVK, TAT, AKR1A1, BPGM, CALML3, CD14, CDK6, CDKN1A, DDX50, GCNT4, GNG13, GSTM4, GUCY2C, GZMB, HPSE2, HRH1, IFNA6, IL2RA, IL6ST, ITPA, MAP4K3, OR1D2, OR2D2, OR6C74, OR7D4, PGK1, POLA1, PRKACB, PTPRF, RAB23, RFXANK, SMAD9, SOS2, STAT6, STEAP3, SUV39H1, TACR2, TGFB3, TPH2, WASF2, WBSCR17, XYLB, CACNA2D3, PAFAH1B3
Negative_2141	ARD1A, ARHGEF4, COQ7, CYP4F2, DDIT4, DPM3, E2F3, ENTPD3, FGFR1, MAP3K8, OR51T1, PGM1, PIGL, PRPS2, PTPN6, SH2D1A, SKIV2L2, TAS1R1, UCK1
Negative_2142	ACACA, AK3L1, C9orf95, EIF4B, ENPP2, FTMT, FZD3, HSD3B7, MYH3, POLD4, PRKCA, TRDMT1

Negative_2143	FAH, ARG1, ARPC1B, CES1, MGAT4B, NME6, OR2G6, OR56A1, PANK3, PPAP2A, SV2C
Negative_2144	ADK, BID, GPT, PVR, AFMID, AGMAT, AGPAT4, ALG6, APOA1, C4BPB, CACNG7, CDC6, CLDN3, CYFIP2, CYP26A1, CYP4A11, DDX18, DIAPH3, ENTPD3, GFPT2, GNRHR, KLRC1, LTBP1, OR5B3, PER1, PPP1R1A, PPP1R3A, PRKCI, PSME2, PTGS1, PYCRL, RALA, SDHC, SMAD7, TUBB1, USE1, B3GALNT1, ST6GALNAC3
Negative_2145	AGK, BCR, HK1, WAS, ADRA1D, AOC2, ATP4B, B3GALT2, BIRC2, DGAT2, FGF14, FRAT2, FZD6, HMGCL, IARS, ICAM2, IL17A, ITGB6, KAT2B, MAPK1, MYLK3, NAGLU, PAX8, PIK3CG, SFRP4, SSH2, UGDH, ALDH18A1, PPP1R12A
Negative_2146	HGF, KDR, TFG, CASP8, DIAPH1, FUT5, GLB1, GMPR, OR13C2, OR5D13, SNAP25, PNLIPRP2
Negative_2147	F12, GSR, ADH4, ASRGL1, CACNA1C, CCND3, CD19, CYP1B1, CYP3A5, CYP4A11, GNRHR, HLA-G, IAPP, ICOSLG, ITGA10, LIPT1, MAML1, MAP4K1, MCM5, NCOR1, NUDT2, OR2A2, OR52N4, OR5L2, OR8J3, PLCZ1, PTCH1, RAP1B, RPS6KA1, TAS2R48, TNFSF18, UGT8
Negative_2148	CRK, DCC, AANAT, ACTG1, ALDH4A1, ALG10, AOC2, APRT, ATP5G3, CACNG8, CXCL13, CYP11B2, DDIT4, DDX54, DPYD, EI24, EXT1, GSTO1, HTR4, IFNA5, LAMA4, MTHFS, NANP, NEFH, OR2F1, OR51G2, OR52A5, OR9G4, PDE5A, PDGFA, PGAM1, PGAM2, PGLS, PKLR, POLA1, POLE, PSME1, RAC1, ST8SIA1, SULT2B1, TAS2R9, TCF7L2
Negative_2149	GCK, GDA, TNF, AKT2, ALAS1, B4GALT1, BPGM, CDO1, CLTCL1, CREB3, CSDA, CYP3A43, DUSP3, DUSP9, EPHA2, EPHB3, FGD3, FGF2, FZD6, GANAB, GNG3, GRIA3, GRM1, HGSNAT, IL12RB2, KIR3DL2, LTBP1, MAP3K1, NEFH, OR13F1, OR4A15, OR4K1, OR56A4, OR5M8, OR7C1, PANK3, PI4KB, RHOA, RUNX1T1, SC4MOL, ST8SIA5, STX12, THBS4, TICAM1, SUV420H2, TNFRSF6B
Negative_2150	MAX, RPE, ACTC1, CHKA, CSNK2A1, CTSL1, CXXC4, DDX54, GLB1, GNRHR, ICAM3, IL10RA, KIR3DL3, LRDD, MAD1L1, MKNK1, OR2T8, OR2W1, OR4C16, OR4K14, OR52D1, OR56B4, PAX4, PSME3, SHISA5, SRGAP2, TMLHE, VTI1B, XYLT2, MAPK8IP3, TNFRSF11B
Negative_2151	AKR1C4, ARHGAP5, BAIAP2, CADM3, CAMKK1, CHD8, DTX1, HSD3B1, IL20, KIR3DL3, MCM6, OR2T4, OR51I2, OR56A4, PANK1, PIGW, RETSAT, WNT5A, CSGALNACT2
Negative_2152	CR2, TXK, AASS, ACSM3, AMPD1, APPL1, CACNA1S, CRHR1, FGF3, FMO3, HRAS, HSP90B1, KIR2DL2, NARS, OR2T33, OR5T2, PRPS2, RXRA, TUBB4, TNFRSF1B
Negative_2153	DHH, FN1, FUK, AADAT, ABP1, ADIPOR2, AGPAT4, ALDH2, ARRB2, B3GNT3, CXCL11, CYP21A2, CYP26C1, DDX51, DHDDS, GMPPA, HAGHL, IL12B, ITGA10, LILRB3, LRDD, LYPLA1, MTHFR, NRP1, NUDT9, OR11L1, PDCD1, PFKL, PIPOX, PPP2R1B, PPP2R5A, PRKAR2A, PTEN, PTPRM, ROCK1, SHC3, SLC25A5, SNAI2, STAT5B, TRAF1, WWP2
Negative_2154	AGL, F7, LIF, AKR1B1, APH1A, ATP6V1A, C3orf10, DAPK1, FOXA3, FZD2, GADD45B, GRM1, LIPG, NCF2, NPR1, OR10A2, OR1J1, OR4C12, OR52E8, OR6B2, P2RX6, PIGT, PLA2G2D, PPP1CC, PRKAA2, PRKY, RCHY1, RENBP, SELP, SEMA3B, SEMA4F, TSLP, UGT2B28, UGT2B4, TNFRSF11B
Negative_2155	CCL2, LCAT, LCMT2, LIPA, OR11G2, PPP2R5E, SOS2, VAMP1, ATP6V1G1
Negative_2156	COL11A1, EPHB3, HMGCS1, MAP4K2, NOD1, PRPH2, SHC4, TGDS
Negative_2157	ACHE, ADRB3, AK3L1, APC2, GLI3, P2RX2, SNAP25
Negative_2158	UBB, AASDH, BCAT1, CBR3, CREB1, DDX55, DRD1, EFNB3, FZD5, GALC, GALNT10, NME2, OR2T12, OR6C4, OR7G1, OR8H3, PHKG2, SDC1, TJP3, TYROBP, ST6GALNAC3
Negative_2159	IL3, OGT, TK1, DBF4, DDX54, GRPR, HNF1A, OR13J1, OR1K1, OR8A1, OR9I1, PFKFB1

Negative_2160	CBL, GCK, ME2, AARS2, ADRA1A, APOA2, ATP6V1H, BBOX1, C3AR1, CACNB3, CACNG6, CAMK4, CDC20, CDK5, CLDN9, CTSB, CYP2D6, EREG, FGF11, FSHR, GADD45G, GUK1, HIBCH, HTR2B, MAT2B, MGAM, MMP9, MTFMT, NADK, OR10A3, OR7A10, PCCA, PCYT1B, PIM2, PORCN, SDC4, SIPA1, TAS1R1, TAS2R1, TAS2R49, THBS4
Negative_2161	DAO, FAH, GPI, ADRB1, ALDH5A1, ATP6AP1, BMPR2, CD79B, CLDN16, CTLA4, CYP1A2, CYP4F2, DHFR, DRD5, EFNA2, ENTPD6, GP1BA, IL13, IL6R, ITGA3, KNG1, LFNG, LIMK2, MYH7B, NLGN3, NOTCH1, OR1L1, OR2K2, OR52I1, PDXK, PORCN, PPARD, SAT2, SEPHS1, SOCS7, SPHK1, SULT2B1, WNT1
Negative_2162	PDC, ACTB, AKT2, ALAS2, ANAPC1, ASMT, ATP5L, C3orf10, CCKAR, CCNB3, CDC16, CRLF2, CTLA4, CYP2S1, CYP4A22, DAXX, EDAR, EFNB2, EPHB2, F13B, GALK1, GNRH2, IMPDH2, INSRR, ITGA2B, KLKB1, MPZL1, OR1N2, OR4E2, OR51G2, OR7A10, PCCB, PGAP1, POLR2J, RELB, SRGAP1, UGDH, ULBP2, WNT7A, ATP6V0E1, CSGALNACT2, MAPK8IP3, SUV420H1
Negative_2163	ABO, AK1, C1S, FUK, GHR, GK, TEC, ARHGEF1, ARPC4, BDH1, CCNA1, CDC25B, CHRD, CLDN15, CMAS, COL5A1, CXCL9, DLAT, EFNA1, ENTPD6, GALNT14, GAPDH, GRLF1, HIF1A, IDI2, IL13RA1, IL18R1, ITGA9, OR51E1, PDPK1, PTAFR, PTDSS2, RALGDS, RENBP, RIPK1, SAT1, SC5DL, SETD1A, SLIT2, TOMM40L, WASF1, ATP6V1E2
Negative_2164	HGD, IHH, IL6, ADCY2, ALDOC, ANGPTL4, CDC25A, CDH1, CXCR6, CYP2U1, DHRS3, DLL3, DNMT3L, ERO1L, FUT7, GSTM3, GSTO1, HCST, IARS2, IFNK, ITCH, MAGI2, MMP9, MPST, NMNAT1, ONECUT1, OR1S2, OR2G6, OR8J1, PAPSS1, PDE8A, PLA1A, PRKAA2, PTCH2, SOS1, SQLE, STAT4, STX1A, TNFRSF9, TNFSF18, UGT2A1, VTI1A, TNFRSF1A
Negative_2165	CAT, PAH, ATP5D, BMPR1B, IFNB1, LDHD, OR2Y1, PVRL1, QPRT, ROCK2, SETD8
Negative_2166	ADA, AGL, FAS, MOS, PGD, ACADS, ADCY10, ADCY3, AKT2, AMDHD2, AMHR2, ARG2, ATP5G2, BIRC2, BPGM, CACNB1, CALML6, CBLB, CD19, CD40, CD74, CHKA, CISH, COL4A1, CREB3L3, CYSLTR1, DPM3, DTX3L, GAS1, HMOX2, IRF5, ITCH, KIR2DL4, MAN1A1, OR1F1, OR52N5, OR5L1, PGM2, PIGO, PRDX6, PRSS1, SELP, TBXA2R, TRHR, TSTA3, UPP1, ZIC2, KIR2DL5A, SUV420H1
Negative_2167	MVK, BET1L, C3AR1, EPHB1, FCER1G, KLRC1, MPZL1, NADK, PLOD1, RHOA, SLC25A4, SMAD2, SPRED1, SUOX
Negative_2168	ALG11, ARHGEF7, CAPN2, CPOX, CYP2J2, DGKA, DGKQ, MAP3K1, MDM4, PPP1R3D
Negative_2169	FN1, IL6, LSS, ALDOC, ALG10B, CACNB3, DAPK1, DAXX, DVL1, ENO2, FZD7, GALNT9, HAAO, HSD17B3, LDHD, LGMN, MICA, MTHFD1, NCR3, NODAL, NRG3, ODC1, OR8U1, PCYT1A, POLR2F, PPM1A, PPP2R5C, RASGRP2, UGP2, VAMP4, VDAC1
Negative_2170	CFH, MTR, ALDH2, C4BPA, CAMK4, CHST12, DAG1, EPHA7, HCLS1, MAP3K4, MFNG, NKX3-1, OR2L8, OR7D4, PECAM1, SESN2, SHMT2, STX2, TYROBP, SERPINF2
Negative_2171	APP, IL9, AARS2, ADH6, AMD1, ARPC5L, BST1, CTNNA2, CYP2C18, DHCR24, LAMB4, MTAP, NRAS, OR11G2, OR2D2, OR2M7, OR52K2, OR5C1, OR5D18, OR6C6, PGM1, PIK3R2, PPM1D, SELP, SHMT1, TAS2R49, UBE2K, HSP90AB1
Negative_2172	BMP8B, CPT2, GALM, NOS1, PLA2G6, PPP2R2C, RAPGEF3, RPS6KA2
Negative_2173	AACS, AADAT, ACVR1B, ADAM10, CAMKK2, CCNG1, CCR5, CDIPT, CDK6, CYBA, CYP2A7, DGKG, GDF7, GP1BA, HES1, IQGAP2, MAP3K2, MAPK9, NRG2, NT5C, OR10A2, OR4A5, OR4M1, OR4X2, OR56A3, OR7D4, PI4KA, PIGO, PPT1, PTPRZ1, SEMA4B, ST3GAL4, TRAF3, TUBB2B, WNT3
Negative_2174	F10, ME2, PAH, ATP12A, EVI1, FGF14, GYS2, HLCS, MDM4, OR11H1, OR6C2, OR8J1, PER3, SH3BP2, TGFB1, MAPKAPK5, TNFRSF11B

Negative_2175	RET,ADRB2,AFMID,ATIC,ATP5L,CAMK4,CHEK1,CREB3L1,CXCR5,CYP11A1,DLL4,EXTL1,FPGT,G6PD,GALM,GARS,GGCX,HAP1,IL17B,LAMB1,MAD2L2,NKX3-1,NR1D1,PDE4B,PNPLA4,PRKACG,PVRL1,RASA2,SETD7,TIAM1,TJP3,CSGALNACT1,ST6GALNAC5,TNFRSF1A
Negative_2176	ALDOA,CCR5,CYP4F3,DKK1,PLXNA1,PTPRF,PTPRR,SPCS1,SERPINA1
Negative_2177	DBT,GSR,ADIPOR1,ANAPC7,B3GALT1,B4GALT6,BDH1,CHD8,COQ6,CSDA,CSH1,CTSS,DLL3,EPHB3,ERO1L,GALNT12,GOSR2,HNF4A,HPSE,IL28B,IL2RG,INSRR,MASP2,NCK1,NKX3-1,OR10J5,OR11H4,OR2C3,OR6S1,OR8K1,OR8K5,PANK3,PCK1,PIGB,POLR1A,POLR3B,PPAT,PTPN7,RASGRP2,SMAD7,TAS2R13,TSC2,WNT2B,YWHAH,HSD17B12,PAFAH1B1
Negative_2178	AMT,CGN,ACOT12,ACTN1,AKR1C4,APOC3,APRT,ARRB1,ATP5F1,CADM3,DAD1,GMPPB,GUCY1A2,IL29,ITGA5,OR10A6,OR13C3,OR1L6,OR4A16,OR4N2,OR51I1,OR52J3,P2RX1,PDE3A,PER1,PGS1,PIAS4,POMC,PTK2,SC5DL,SDHA,SQLE,STX12,TGFB3,TNFSF9,TPH1,UGP2,YWHAZ,ATP6V0D2
Negative_2179	LEP,ABL2,ALDOC,ATP2A1,BUB3,CD59,CDC6,CSF2RA,CX3CL1,CYBB,CYP21A2,CYP27A1,FCGR2B,GNAI1,GNAQ,HSP90B1,LAMB1,LAMC2,LTBR,MASP1,MS4A2,NFATC3,OR10K2,OR51L1,OR6X1,PDGFC,PDGFD,PGS1,PIGP,PLA2G10,PLAUR,PLD2,POLR1B,PPOX,PTGER1,SOCS1,SRGAP1,VEGFB,YWHAB,ATP6V0D1,SERPINA5
Negative_2180	ALDH3B1,HADHB,UNC5C
Negative_2181	GSR,LIF,ALDH1L1,CD8A,CDK7,CLDN10,CSNK2B,DPM3,ENTPD6,GALM,GMDS,GNPDA2,MAFA,NAGK,NCR1,OR6C75,OR6K6,PECAM1,PTGES2,PTK2,TGFB3,WASF3,WHSC1L1,XYLB,YWHAZ
Negative_2182	C2,ACSL4,BMP7,CPT1C,DFFA,DVL2,EFNB1,EVI1,GALNTL1,GPAA1,NARS2,PARVA,RENBP,TRIP10,C1GALT1C1,CSGALNACT2,SLC25A31
Negative_2183	LEP,LPO,ABI2,ACSL5,ALG5,ARPC5,CAMKK2,CCND3,CCR3,CLTA,CMPK2,CNDP1,CYP2C19,DNMT3L,GAPDH,GCNT1,GLI3,H6PD,HHEX,INHBA,LIFR,LRP6,MAP3K2,MAPK13,MIOX,NKD2,NOS1,OR1N1,OR2B6,OR5P3,PGM2,PKM2,PLCB4,POMC,PPP2CB,ROCK1,RPE65,SETD1B,TAS2R14,TCIRG1,WHSC1,HSP90AA1
Negative_2184	GAA,IL9,AMD1,BDNF,CD59,CXCL2,FASLG,GNB1,MAPK7,OR2T1,OR51M1,PDE4D,PLA2G2A,PLIN,PMM1,PPCDC,PRPH2,QARS,SESN3,WNT16,XCL2
Negative_2185	F7,GLS,LPO,SHH,ACTA2,ACTG2,AKR1C3,ALDH7A1,ARPC2,ATP5J2,CCL23,CD79B,CRKL,CTLA4,EHMT2,ENTPD8,ETNK2,GSTA4,HTR4,IAPP,IL8RA,IRAK1,KIR2DL1,MAD2L2,OR5AC2,OR5M8,OR6B3,PARD3,PDXP,PIK3R1,PPBP,PPID,RENBP,RFNG,SPCS3,SPRY2,XYLB,ATP6V1B1,SERPINA5
Negative_2186	GP5,MAG,AKR1C2,AKR1C3,DAAM1,FGF8,FPGS,GFPT2,HSD17B3,KIR3DL3,NME7,NT5E,OR10A4,OR7A10,PAK2,PPP1R3C,SOS2,TUBA1C,YARS2
Negative_2187	IL3,ADRB1,FGF19,NGEF,PEMT,PPP2R5A,UXS1,MAPK8IP3
Negative_2188	AGL,DCK,LSS,ARHGEF7,CCL1,CDK4,CDK5,CDKN1C,DGKG,EARS2,EFNA4,FCGR3A,FGF20,FUT8,FZD9,ITGB1,NAGS,NCAM1,NEU1,NFKBIB,NRXN1,OR52J3,OXTR,PDHA1,PLCB3,PLCE1,PLEKHA8,PON2,PPBP,TAP2,TGDS
Negative_2189	ANAPC10,ATP6V0B,CASP6,CBR1,CCNA2,CCR5,CD14,CLTC,CTSL1,DGKD,F13B,IDH3A,IL6R,IRF9,MAP3K14,MAPK13,OR1L6,OR2A14,OR2T12,OR51B4,OR52W1,PARD6G,PDE8A,PPP2R2B,PPP2R2D,PYCRL,RND1,TP53
Negative_2190	TEC,ACPT,AKR1D1,ANAPC11,CBLB,CBR3,DCXR,GSTO2,ICAM3,IGSF5,IL2RB,IRAK3,MAPK9,NRAS,OR11G2,OR2Z1,OR5AK2,PDE10A,PTPN6,SV2B,TLR2,GABARAPL1,SERPINA1
Negative_2191	CGA,GNAS,NANP,OR13C8,OR4X2,TAS2R4

Negative_2192	F12, PTS, ACPT, AGXT, AKT2, AMY2A, ARHGEF1, CARS2, CDH4, COX15, CYP27A1, DPM1, ENPP7, ERBB2, FZD9, GALNT14, GNAI1, HAO2, IRF5, JAK2, KLKB1, LAMC2, MTAP, OR13C2, OR4N2, OR51F1, OR52K2, OR5L1, OR5R1, PRKCG, TRPC1, VEGFA
Negative_2193	FAH, HK3, AK3L1, ARD1A, BMP8A, CCNB3, CCNE1, CLDN14, CLDN7, CREB3L1, CYBB, FZR1, GLDC, GRB2, HTR5A, KLRD1, MAP3K8, MARS, NR1D1, NT5C1B, OR1S1, OR4F5, OR4N4, OR52K2, OR5L2, OXCT1, PIK3R5, POLR1C, PRKX, RDH12, RELB, RXRB, SETMAR, SNAP25, TACR3, TBL1XR1, VDAC1, VDAC2, WNT7B, ATP6V0A4
Negative_2194	CGA, GP6, NPL, ADCY6, AGXT, AHCYL2, ALG5, BPNT1, BST1, CD48, CISH, CPT1A, CSNK1G2, CYP4A22, FZD5, GBA3, GLDC, IFNA4, IL17RB, IRF5, LRRC4C, MYLK, NAT6, NKD1, OR10A6, OR5P3, OR6C6, ORC5L, P2RX4, PDGFD, PI4KA, PIAS1, PIGM, PLCE1, PRKAB2, PRKCI, PRSS2, RALA, RAPGEF4, RND1, STX12, THBS3, TPI1, UNC5D, ATP6V1E1, SERPINC1
Negative_2195	B4GALT4, CACNG2, CCR9, CD226, DUSP9, ENPP3, EPHA5, GGT7, GSTA3, IARS2, JAM3, LAMA3, NCF1, OR10H1, OR2T11, PANK1, PDE9A, PLXNB1, POLA1, POLR2D, POLR2G, POLR2K, PORCN, RDH10, SLC27A1, SOCS4, SPTLC1, TUBA3C, VAV3, WNT6
Negative_2196	ATP5G3, BCAT2, FABP1, HCST, PFN4, RBKS, STAM2
Negative_2197	CKM, HPD, SI, ATP6AP1, BACE2, CACNG4, CLDN20, CRKL, DIAPH2, EPHA4, FADS2, FASN, HIP1, KLRK1, LALBA, MYH1, NPR1, OR2M3, OR2Z1, OR52B4, OR6C70, ROBO2, RPS6KB2, SLC27A4, TBL1X, TLN1
Negative_2198	ABO, DBT, HGF, PGP, ACP1, AGXT, AHCYL1, ARPC2, CCL28, CDK7, DHCR7, IL20RA, IQGAP2, KARS, LRRC4C, MAGI1, MCM2, NANP, NOX3, OR10S1, ORC6L, PANK1, PHKG1, PRKACA, SELPLG, SNAP25, SOS1, ST8SIA1, TFPI, TNFSF11, WASF2
Negative_2199	CAD, F2R, ARPC5, ARSE, BAAT, CACNA1I, CDK4, CHIA, CSF2, FABP1, GNAL, GPD2, GRIA1, IFNGR1, KLKB1, MYH7B, NMNAT1, OR4N2, OR9Q2, POLR2A, PPID, PPP3R2, PRKCI, RASGRP3, RDH8, SEMA4C, SENP2, SMAD9, TPMT, WHSC1L1
Negative_2200	IHH, AKR1C4, ATP5J2, DDX52, GOT1, MGAT1, OR4F15, OR5AS1, YWHAE
Negative_2201	CBL, F2RL1, MAN1B1, OR2A2, OR8B4, PHKA2, PIK3CG, PRKCG, SDC1
Negative_2202	C8A, HPD, AHCYL1, ARNT2, CACNB4, CCKBR, CCL22, CD247, CDH3, COMT, CTNNA3, CXCR5, DDX50, ELK4, HEXA, IKBKG, JAG1, LAMC1, PDE4A, SIGLEC1
Negative_2203	SMO, ACTC1, ALDH1A1, APAF1, ASPA, CCL24, CD72, DHCR7, HEMK1, ITGB2, MCAT, NRG2, OR9A2, PANK4, PAX6, PIM1, PPP1R3D, SOAT1, TAS2R9, VNN1, WNT5A
Negative_2204	CR1, AGPAT1, ALOX12, AMY2B, AXIN1, CCKAR, CDC20, CDH2, CYP8B1, DDX51, DOCK1, DUSP3, ENTPD4, FMO5, GFPT2, IDUA, IFT57, IL15, IL23A, IL2RG, LTA4H, MAP3K6, MAPT, MARS2, MKNK1, NNMT, NTF3, NTSR1, OLAH, OR1A1, OR1D5, OR51F2, OR5AR1, PFKM, PIK3CG, PPP1CB, RAC3, SENP2, SMAD7, STK36, TAS2R50, TBXAS1, TLR4, TNFSF8, TPMT, UGT2B17, WIF1, TNFSF13B
Negative_2205	BID, ACSL4, ACY1, CCL26, CCNG2, CDC6, CFL2, CPT1C, CYP2U1, E2F3, FGF18, FPGT, FZD9, GALNT2, GRIN2B, IL7R, ITGA6, LAMC3, LGMN, MAOB, MTMR6, OR1D2, OR52L1, OR9G1, P2RX2, PIK3R2, PLCD1, PPARA, PPP2CA, RBL1, SRD5A1, TBK1, THY1, TIRAP, TRDMT1, UGT2B7, TNFRSF17
Negative_2206	DCK, FER, FGB, IL2, TAT, AASDH, AHCY, ALDH3B1, APOE, B3GNT3, BBOX1, BUB1, CCND1, CHPF, CLDN20, COL2A1, COQ5, COX15, CYP26A1, DHDDS, DPAGT1, GNPDA1, HAGHL, HRAS, IL18R1, IL19, IL28A, ITGAE, KCNB1, LAMC3, LIFR, MAML3, MAP3K11, MTHFD1L, MYH13, OR10A3, OR2B2, OR51B4, OR51T1, PGAP1, PIK3C2A, PRKCG, RDH16, RHEB, SETDB2, SHC2, TLR4, TRPC1, WNT7B
Negative_2207	AK7, KIT, ALDH1L1, ALDH2, ASAH1, ATP5D, ATP5J2, B4GALT6, CADM1, CCND2, CCNE2, CSNK1D, CSNK1E, CYCS, CYP2U1, EHMT1, FGD1, GJA1, HCLS1, HNF1A, HSP90B1, IFNG, KLKB1, LCMT2, MAP3K5, OR10A3, OR2H2, OR2T3, PAICS, PFKFB2, PRKY, RPS6KB1, SEMA4A, ST3GAL1, TRAF5, ZNRD1, PPP1R12A

Negative_2208	LAT, ADCY3, BCAT1, BNIP1, CRY2, CYSLTR2, DARS2, DUSP5, EARS2, ENPP3, IL1B, LMO7, MAP2K7, OR10G3, OR2L13, OR2T29, P4HA1, PRKAA1, PTPN1, STX3, STX5, WNT8B
Negative_2209	NGF, ARPC4, CNGA3, HIP1, IDH3B, MYH7, NSD1, OR11H4, OR1M1, PPP2R2C, SMAD3, SMURF2, STAT6, CSGALNACT1, PHOSPHO1
Negative_2210	GHR, KDR, TK2, ALDH2, ALG11, AMDHD2, ANAPC2, BMP4, CAMK2A, CAV3, CCND2, CCNH, CD27, CD72, CDKN1B, CLDN15, CTNNB1, CX3CR1, DMGDH, EPHB3, EVI1, FUT8, HEXB, ITGAX, LEF1, LIMK2, MGAT5, NRXN3, NTRK2, NUDT2, OR11A1, OR11G2, OR1C1, OR9G1, PARD6A, PARVB, PAX6, PDE3A, PNLIP, SKP2, SOCS3, SOS2, ST3GAL6, SUFU, TAS2R8, VAMP1, AASDHPPT, PHOSPHO1, ST6GALNAC6
Negative_2211	C9, HDC, HGD, ADCY2, APOA2, ASRGL1, B4GALT5, BCAR1, BMP7, CACNA1S, CDH3, CLDN4, COL3A1, CYP11B1, CYSLTR1, ELK1, FABP4, GNAZ, GP1BA, GRB2, IFNA5, LIPG, MDH1, OR10A2, OR13G1, OR3A2, OR4D5, OR4M2, OR5D13, OR6T1, OR7D4, OR8D2, PAX8, PDX1, PGM1, PIK3R4, POLR2L, PPP1R3A, PTPN6, RARS, RPN2, SC4MOL, SDC2, SPRED2, TNFSF10, TRAF1, TSHR, WNT8B
Negative_2212	ACAT2, APC2, ARNTL, BMP4, BMP8B, CCR7, G6PC2, MAML3, MASP1, METTL6, OR13C5, OR2D2, OR2M3, PANK3, SDHC, SRGAP2, TUBA1C, UBE2L6, UNC5B, WNT16, PHOSPHO1, PNLIPRP1
Negative_2213	AARS2, BCAT1, CACNA1B, FGF17, FGF19, GNRH1, IL5RA, INHBA, ITGA4, MMP9, MYH15, MYLK2, NEU2, NLGN3, PLCB1, PMVK, POLR3H, PTGER3
Negative_2214	APP, CAD, CS, CTH, FER, FGA, PLG, TPO, AKR1D1, B3GALT5, BACE2, CACNG2, CASP10, CCL19, CCR9, CTSL1, CYP7A1, DAPK2, DUSP6, FOXO1, GNA15, GRAP2, HPSE, HTR7, IL15RA, INPP4B, NFKB1, OR10Q1, OR13C5, OR2T34, OR51I1, OR6C1, OR6X1, OR7A17, OXCT1, POLD2, PPP5C, PYGB, SARS, SETD1A, SLC25A6, SOX17, STAT3, TAS1R1
Negative_2215	ATP5B, DGKI, LIMK2, LTA4H, OR10A5, OR5T2, P4HA3, RBPJL, TAS1R1, UNC5C, CTNNBIP1
Negative_2216	IL6, CACNG8, IMPA1, ITGAE, ITPA, MYH7, NRCAM, P4HA3, TGFB3, TUBB6
Negative_2217	CTNND1, DGAT1, GDF6, OR6C3, OR6C4
Negative_2218	MAX, ALOX15B, GSTA2, KARS, LTB4R2, NFATC2, OR14C36, PTAFR
Negative_2219	CD6, ADRB3, AGTR1, ALDH3A1, CACNB1, CD27, DDX55, EVI1, GBE1, GIT1, HHIP, IFNA6, JAK3, MCM5, MTHFR, NLGN2, NME6, NMNAT3, OR52N1, OR9K2, PDE4B, PMM2, SULT1A1, TAS2R8, TUBB4Q, WNT4
Negative_2220	ACACA, APH1A, BMP8B, CDH1, CDIPT, CHST12, CTSL1, CTTN, DDX54, DGKG, DIAPH2, EPHB1, HDAC2, IL12A, NCK1, OR6M1, PARS2, PDIA4, PHKB, TALDO1, TSNARE1, UGT2B10, ULK2, XIAP
Negative_2221	MVK, ACOT11, ACPP, ACSM5, ALDH2, ARNTL, ASAH1, AVPR1B, BCMO1, CACNG1, CACNG5, CD226, CD28, CMPK2, CSDA, DHRS4L2, ERBB3, FOXA3, GNA13, GNPNAT1, GPD1, GPD1L, GPT2, HNMT, HSD17B2, IDH3B, IQGAP2, LPAR1, NFAT5, NQO1, NTF3, OR1L4, OR2C1, OR2G6, OR4A15, OR5AN1, OR6C2, OR6Y1, PAK1, PERP, PIAS2, PIP5K1A, PORCN, SLC12A2, SORBS1, SPAM1, TNFSF12, VAMP3, B4GALNT1
Negative_2222	ESAM, GALNT14, MNX1, PANK1, ZBTB16, PPP1R12A
Negative_2223	L1CAM, OR2T29, PLCE1, PTPRJ
Negative_2224	ACADM, ARPC3, DNMT1, TACR1, USE1
Negative_2225	ID3, CTSS, DGAT2, GGCX, GSTM5, IL22RA1, JUND, MYH10, NKX6-1, OR4C11, PIGU, PLCD4, PLXNB1, PRKCG, RAP1A, VEGFA
Negative_2226	GPI, RDX, SRM, TK2, ACTA1, ACTB, ALDH3A1, ARPC1A, ATP5C1, CHRNA7, CSH1, CSNK1G3, CTF1, CYP11B2, CYP2C19, FCGR3B, ITGB4, MCAT, NT5C2, OR14A16, OR2F2, OR5V1, OR8D4, PANK1, PDE7A, PDXK, PPARA, SNAP25, WNT3A, ZNRD1, HLA-DPB1, PDCD1LG2
Negative_2227	ASPA, DAPK2, PDE4B, PHKA2, SUCLG1

Negative_2228	CP, F9, LIF, AARS, DCXR, DEGS2, IFNAR2, INPP4A, NEU1, OR4C11, OR51F1, OR6C76, OR8S1, PRKAR1B, SH2D1A, SREBF1, SRGAP3, ST8SIA5, SUFU, WASF1, CSNK1A1L
Negative_2229	F11, FOS, ADH1A, FSHB, GOT2, HADHB, IFNK, INSRR, MASP1, NFATC3, NRCAM, OR1L3, OR4K5, OR8J3, PARD6G, PDE3A, SLC27A4, SLIT2, WNT1
Negative_2230	ZYX, ADCY8, ADRBK2, APOE, DDIT3, ENPP1, FARSA, FGFR3, GCNT2, GLYCTK, HMOX1, HNF1A, IDI2, IFNAR2, IKBKB, JAM2, KCNQ1, MAP3K11, MAP3K8, NEUROD1, NFATC1, NMNAT3, OR4A16, PIK3CB, PPP1R3C, PYGM, RPS6KA2, SFRP1, SLC27A1, SMAD4, SMPD1, SPRY3, SULT2B1, THBS3, TUBB6, UGT2B28, WNT10A, SLC25A31
Negative_2231	AMH, NNT, TEC, ACVR2A, ASRGL1, AXIN2, CD36, DAPK1, FGFR3, FZD1, IKBKB, IL1R2, INPP1, JAM2, MAOB, MCM5, MGST1, MINPP1, NAT1, NFYA, OR14C36, OR2H1, OR2T5, OR5T1, OR8K3, PIK3C2G, PKLR, PPP5C, SPRY1, TCIRG1, TUBB, WASF2, YOD1, TNFRSF11A
Negative_2232	JUN, BCL10, ITGA2B, NT5C1A, OR14C36, OR2F2, PDGFD, PLAT, POLE4, PRKCZ, PTGFR, STX16, WASF2, ZMAT3
Negative_2233	PTS, ABL2, ACSM4, ALAD, CDC27, FMO4, FPGT, HMOX2, OR5AS1, OR5M9, OR5P3, PLA2G5, PRPH, PTAFR, SOS1
Negative_2234	PC, ATG7, ATP13A2, CAPN2, CCL1, CLDN1, CYP7A1, DAG1, DHRS4, DUSP8, GUCY2C, HEXB, MAGI3, NCR1, NGEF, OR13H1, OR51T1, OR5M9, PIK3C3, PROC, RASGRF1, WNT1, SERPINB5, TNFRSF13B
Negative_2235	HYI, NPY, TKT, ACVR1B, B3GALT2, BPGM, CHRD, CNGA4, COL1A1, CPOX, CRLF2, CYP4F2, DDX54, ENAH, GALNT1, GALNT11, GNAO1, GPX7, GTSE1, GYS1, HNF4A, IL28B, IRF5, ITGA4, ITGB1, KLRC4, LIPC, LTB4R2, MAPT, NADK, NCOR2, NTF3, OR4C11, OR9K2, PHKB, PIAS4, PIGV, POLR3B, RARA, RDH16, RPN2, SLC12A2, TNFSF11, TRIP10, VKORC1, WASF3, TNFRSF10A, TNFRSF10C
Negative_2236	ATM, CFI, DLD, LIF, SYK, ABAT, ABLIM2, ADCY6, ARG1, ARNT2, ATF4, CACNA1D, CDC16, CDC45L, CMAS, CYCS, CYP11B2, EFNA5, ENTPD8, EPOR, F2RL3, FBP1, GNAO1, GNG12, IL17B, IL17RA, IQGAP3, KCNQ1, KIR2DS4, KIR3DL1, MAP3K2, METTL2B, MGAT5, MYD88, NMNAT3, OR10J5, OR13G1, OR51A7, PIAS1, PLXNC1, POLR3A, PON1, RPS6KA1, SELPLG, TCIRG1, TKTL2, UGT2B7, CACNA2D4, TNFRSF13C
Negative_2237	AMH, IL5, TKT, ACO1, AGMAT, ATG3, CLDN16, COX15, FASLG, HSPA8, IFNA10, MAP3K6, NFE2L2, OR10G9, OR2T34, OR2T5, OR5K2, OR6C3, PIGM, SELL, UNC5A
Negative_2238	LCT, TEC, AADAC, AGPAT4, CDC7, CYP8B1, FGF4, IL10, ITGA11, LRAT, MGAT2, NEU3, NOX3, ODC1, OLR1, OR1J2, OR52E8, PDE7B, PGM3, PRKCA, SCP2, SEMA4D, TLR9, UNC5A, WNT8A, HSP90AB1
Negative_2239	OTC, ABL2, ADH4, CMAS, GAMT, MCM7, MEF2C, ST3GAL5, STAT6
Negative_2240	ADAM17, CACNA1D, CYP3A5, NCR2, ZNRD1
Negative_2241	HDC, MPZ, TPO, ADRB3, ARG2, B3GALT6, CACNG2, CDC23, DDX19A, EPRS, GCNT2, GLI2, GNA12, GPLD1, HSPB1, ITGA7, MAP3K14, MTHFR, MTMR6, NME2, OR11G2, OR14A16, OR2M4, OR2W1, OR52H1, PAK1, PAK3, PDGFRB, POLR2H, PPP1R3D, RAP1A, RDH10, SSX2IP, SULT1E1, UBA1, VANGL2, WEE1
Negative_2242	BAD, CKB, IL6, SP1, ADCY7, ATP6AP1, CCL7, CLCF1, DDIT4, E2F5, EFNB1, ENPP6, F2RL3, FIGF, GMPR, GNAI2, GNAL, GNPNAT1, HADH, HCLS1, HDAC2, MALT1, MAPK8, NCOR1, NQO1, NRG4, NT5C3, NUDT9, OR1S2, OR7C1, PARVB, PDE6H, PGK1, PLOD1, PNLIP, POLR3H, PVRL1, RALGDS, RFXAP, STX12, TKTL1, UNC5D, UXS1, CACNA2D4, TNFRSF6B
Negative_2243	ALCAM, OR12D3, POLE3, WWP1
Negative_2244	C6, DDC, SHH, APOA5, AQP7, ATG7, BET1L, CDC42, CDH5, CEBPA, CSF1, CXCR5, DDX23, DKK4, FGF5, HGSNAT, IFNA4, NFATC4, NME7, OR10H5, OR9K2, OXCT2, PAK7, PDE8B, PDHB, POLE3, PPCS, PRKCG, PRODH, PSME1, PTGDS, RFWD2, STX4, ZMAT3, PPP1R12A, ST6GALNAC5

Negative_2245	SRM, TNN, ADRBK2, ALCAM, APC2, ATF2, ATG7, BIRC5, CREB3L2, CTNNA2, CTSS, DDIT4, FGF7, FRAT2, GYS1, IQGAP1, ITGAM, KAT2B, MGST1, NODAL, OR13C2, OR2AT4, OR4A16, OR8B4, PARD6G, PIK3C2A, PIP5K1B, PLA2G4A, PLAT, PPP1R3C, PPP2R1B, PRKAG2, RPS6KA6, TUBB2C, UGT2B17, ATP6V0A2
Negative_2246	ADH5, ADRA1A, GPD2, UROC1, TNFRSF12A
Negative_2247	IL4, AANAT, AFMID, ARPC2, ARPC5, ATP5G2, BMP8A, BUB1, BUB3, CREB5, EHMT2, FLT4, GALNT12, GRM5, GUK1, IL15, ITGA10, JAM3, LAP3, MGAM, MGAT1, MGST2, MITF, OR11L1, OR2M3, OR51I1, OR5H2, OR9I1, PROS1, RAP1A, SHC1, SKP1, TGDS, TUBB1, ATP6V0E1, TNFRSF14
Negative_2248	EZR, CHRM1, CMPK2, FUT5, GGT5, IDH3G, LLGL2, OR10AD1, OR4P4, OR6N2, OR7G1, PDX1, PFN3, ROCK2, YWHAB
Negative_2249	GCK, GH1, ME3, UBB, ADRA1A, ALOX12, ALPP, AMD1, CADM3, CD36, CDS2, CSNK2A2, CTF1, CYBB, CYP1A2, CYP2C8, CYP4F2, DHDDS, DPM1, EXTL3, FADD, FLT3, GALNT7, GATM, GPD1, HAO2, HEXB, HLA-G, INPP5B, LILRB3, LIPE, MAPK11, MGST2, MYH2, NUMB, OR10S1, OR13F1, OR4C46, OR56A3, PAK7, PLCD4, PLXNB3, POLR2J, PSEN2, PTDSS1, SEMA4G, WIF1, EIF4EBP1
Negative_2250	AK3, GCK, PGF, ACVRL1, AGXT2, ALDOB, AOX1, ARSD, CD22, CYP7A1, ENPP6, FGF14, GDF7, GUCY1A2, HLA-G, ICOS, IL10, IL11RA, IL19, JAG1, LDHAL6A, MADCAM1, MYL7, NEFL, NEU2, OR10G2, OR4X2, OR51E2, OR6S1, OR8A1, PIK3R1, PIP4K2B, PPP2CB, PSEN1, PTGS2, PTK2, PTPN7, SHMT2, SLC27A5, SPHK2, TLN1, TPM3, TRDMT1, TUBB2A, WNT6, MAP3K7IP2
Negative_2251	ADC, CRK, ACAT1, ACVR2A, BDH2, BPNT1, CD79B, CDC25C, CES7, CYFIP2, DFFB, EIF4B, FABP4, FBP2, FGF6, GNAI1, HPRT1, IFNA21, IKBKE, INHBA, KAT2A, LIPA, LPAR1, MAPK7, MGAT2, NTN1, OR51A4, OR8D1, PDE9A, PRKY, PTGES, RBL2, SDHC, ST3GAL3, TACR3, TICAM1, TLN1, TYMS, UGT8, PPP1R12A
Negative_2252	FES, INS, LCT, APC2, ATP2A2, CAMK2B, CCL3, CD46, CHST11, COL11A1, CSNK1A1, DGKG, DOT1L, EPHA3, FUT2, GALNT14, GIT1, GNAQ, GNRH1, MPZL1, OR2C1, OR6C1, PHGDH, PYCRL, SAT1, ST3GAL1, ATP6V0A1
Negative_2253	CDA, KDR, ACSS3, AGPAT2, AKT3, AMDHD1, AOC2, ARHGEF6, CACNA1G, CD3E, CD86, CDC45L, CKS1B, CSNK1A1, CYP2S1, DGKZ, GJA1, HDAC2, IGFBP3, MGAM, NT5C1B, OR10K1, OR2G6, OR5AP2, PIAS1, PTPN5, RAP1A, RFXAP, SEMA7A, TICAM1, TLR4, TOMM40, VARS, HLA-DRB3, MAPK8IP1
Negative_2254	BTC, ALPL, COL11A1, GCNT1, GNAZ, GSTO1, IARS2, IRS1, ITGA8, MAPK12, OR1N2, OR4A15, OR4K1, OR5T3, PAK2, PAX6, PDE8B, PIK3R5, PLOD1, PNMT, POLR1E, PRKCG, RALB, SAT1, SMAD7, STMN1, TAS2R38
Negative_2255	NF1, ABP1, ACO2, AQP7, CHSY1, CSF3, ERBB3, GNRH1, HMGCS1, IPPK, MAGI2, OR13C8, OR2L8, OR4K1, OR52L1, P2RX2, PIP5K1A, PRIM1, PRKY, PTGES2, RPS6, UGCGL1, ULK1
Negative_2256	BAX, CEL, CP, GGH, JUN, ABP1, ACTA1, ALDH7A1, C9orf95, CACNA1F, CNDP1, CXCL9, DCTN1, DLAT, DNMT3A, DUSP4, FPGS, GNPNAT1, GPD1L, HSD17B7, IFIH1, IL28RA, IL8RA, ITGA7, MYH4, NCOA4, OLR1, OR51L1, OR5H2, OR8H3, PIAS4, PLCG1, PVRL4, RFNG, SLC12A2, TRADD, CACNA2D1, GABARAPL2
Negative_2257	AGL, ACAD8, CES1, EDN1, GNAI1, MAP4K3, PLCG2, PTEN, SKIV2L2, TLR5
Negative_2258	AASDH, ACAT1, COQ7, DPM3, FDFT1, FGF4, FMO3, INPP5A, LAMA4, NFASC, OR1N2, OR2H2, OR9G1, SMC1B, SOS1
Negative_2259	C7, DBT, ALDOC, ARHGEF2, B3GNT2, BBOX1, BCKDHB, BIRC2, CD8A, CRLS1, GSTO2, GUCY1A3, HCST, IDH3B, IL2RB, IL2RG, LLGL1, NCF2, NEU4, OR2A2, OR4D9, OR6C1, PCCB, PDXK, PIGC, PRPS2, RUNX1, RUVBL1, SORBS1, TPH1, TRADD, TSHR, UCK2, PAFAH1B3, TNFRSF1A

Negative_2260	SI,ACAT1,ALDH9A1,AMD1,ATP12A,B4GALT4,CCL19,CD80,CRLF2,DMGDH,KCNJ11,KLK3,LARS,LIPC,LLGL2,MAPK11,NLGN1,OR1E2,OR2T12,OR51A4,OR8D4,P4HA2,PDE8A,PECAM1,PSEN1,RALA,RALB,RDH16,SESN1,ST8SIA1,SUCLG2,TAP2,TPH2,UGCGL1,ZNRD1,PPP1R12A,SERPINE1
Negative_2261	C8A,PC,TNC,ABL1,ACPP,ACTN2,BMP2,CACNA1C,CALML5,CBR3,CDK7,COQ7,DDX52,DFFB,DUSP4,ETS1,GALNT6,GNA14,IRF9,ITGA2B,JAG2,KIR3DL3,MAN1C1,MTHFD1L,OR2C1,OR2T1,OR4N2,OR51A7,PANK4,PIK3R3,PIP5K1A,PLXNB2,PPP1R3A,RFXAP,SETDB1
Negative_2262	CS,APAF1,BACE1,BTRC,EXTL2,MYLK,NRXN3,PITX2,PPP2R5A,PRLR,SLC27A4,STAT5A,YKT6
Negative_2263	GCK,GLA,AGTR1,CLDN17,EDAR,FGF11,FGF17,FLT4,FZD3,GJA1,GLB1,IL4R,LYZL1,MAN1B1,MICA,MTFMT,NAGS,NCAM2,OR13C3,OR2Y1,OR52N2,OR52N4,PARD6G,PDE10A,PIGA,SNAI2,SNAP25,TNXB,GABARAPL2
Negative_2264	ALPP,BCMO1,CD47,CDC27,CYFIP2,ELK4,OR2K2,PEMT,PLCB1,POLA2,PRKAA2,PRSS1,SEMA3D,SMPD1,WNT3
Negative_2265	BTK,CSK,EPO,SHH,SRF,TK1,AHCYL2,ALDH3A2,B3GALT2,BLNK,CCL22,CEBPA,CFTR,CSNK2A1,DGKH,DRD1,DTX2,FGF20,GALNT14,GALNT6,GCDH,GNAO1,IL13RA1,IL4R,IL8RA,MAP2K5,NSD1,NT5C3,OR11G2,OR4C6,OR7C2,PANK1,PIGL,PLA2G2D,PPARA,PPP3R1,PRPS1,PYGL,RHOQ,SDC1,SIPA1,SMC1B,ST8SIA5,STK4,SULT1A2,TGFB2,TOMM40L,TPH1,XYLT1
Negative_2266	ACPP,AGXT2,CTBP1,CYP11A1,ENO1,ENTPD1,F11R,FCGR3A,FGF21,GADD45A,IGF1,LTC4S,MGLL,NOS1,NT5C3,OR2A14,OR2G6,PIGU,PLCE1,PRKAA1,PTDSS2,PTPN6,RPS6KA1,STK4,TIAM1,TNFSF8,TUBB3,WNT1,SERPINF2
Negative_2267	ADK,AR,KDR,ABLIM2,ADCY4,AGRN,ANPEP,CACNA1A,CCL23,CCNH,CHUK,COL4A1,CTPS,CXCR6,DAAM1,DEGS1,FIGF,FMO4,GAPDH,GSK3B,HAGH,IRAK4,ITGB5,MTAP,OR2Y1,OR4F5,OR4K2,OXTR,PGAM1,PGAM4,PI4KB,POLR3K,PPAP2A,PRKAR1B,PRKCA,PTGER3,SCLY,SDHC,SEMA4A,SEMA4D,SRD5A1,TAS2R7
Negative_2268	C4B,CCL3,CDKN1B,CREBBP,EFNA4,ENTPD2,EPHA2,FTMT,GUCY1B3,ITCH,LAMB3,MITF,OR11G2,PDCD1,PGK2,PGM1,PIK3C2A,PNPO,POLR1C,PSME2,RDH11,RPSA,SPI1,TIRAP,TLN1,TNFSF4
Negative_2269	CR2,ADAM17,ADIPOR2,ADRA1D,ALDH3A1,CCR10,CHPF,CYP1A1,DNMT1,DOT1L,GPT2,NFKBIE,OR1E2,PDK1,PFKFB2,PIGX,PNLIP,SV2B,PAFAH1B1,ST6GALNAC1
Negative_2270	ACP1,AKR1C2,CASP6,CDC25B,DIAPH1,DUSP10,DUSP2,EDAR,GPD1L,ICAM1,ITGAM,ITGAX,MAPK7,MYH8,NEGR1,NUMB,OR11G2,OR6F1,PFKFB2,PIK3CD,PPP2R2D,PRSS1,SMAD7,TPH1,ATP6V0E1,ATP6V1E1
Negative_2271	ADC,PDC,ACAA2,AGPAT3,ANPEP,CCNA2,CMPK1,CSAD,IGF1R,IL18R1,INPP1,LLGL2,NFX1,NFYB
Negative_2272	DUT,NPL,PRL,SP1,ACPT,ACSS1,ACTC1,BLVRA,CACNB1,CDH4,CHUK,CLDN11,COX10,CYFIP1,DLL1,DOLPP1,DVL1,FOXO1,FZD2,GLI2,GRM1,HSP90B1,IARS,IDH3G,ITGA9,JAK2,KLRC4,MAPK1,MAPK13,OR10H4,OR52D1,OR52K2,OR52L1,P2RX3,POLR2L,PPOX,PTTG1,PVRL3,RUNX1,TAS2R5,TNXB,PPARGC1A
Negative_2273	FST,AANAT,ACAT2,ALDOC,CNGB1,CNTN1,DUSP10,F2RL3,FGF22,HYAL2,ICAM3,MAN1C1,MDH1,NUDT5,OR10AG1,OR2G6,OR2V2,OR52I2,P4HA3,PARVB,PFN1,PPOX,PTPRZ1,RPS6KA3,SNAP23,STX4
Negative_2274	ATM,CCL15,FZD1,GNRH2,OR5AU1,PTTG1,UGT2A1
Negative_2275	ADSL,AGPAT4,AGXT2,CMBL,CYP1A2,GSTA5,GUCY2D,HSD11B1,LLGL1,LRP6,OR2D2,OR6B3,PDE6H,PLOD3,ROCK1,TAP2,TBL1X,TGFBR1,TUBB1,WBSCR22,ST6GALNAC5

Negative_2276	F11, LPO, MPL, ABI2, ACVR1C, ACVRL1, ALOX15B, CD79B, CDH4, CLTA, CXCL2, DCXR, DDX41, DLL4, ELK1, EPB41L3, EXTL1, FGF19, FIGF, GAL3ST1, GAMT, GNPAT, HPRT1, INPP1, IRAK3, KARS, LARS2, MAT1A, MCCC1, NFKB2, OR10A5, OR12D3, OR13G1, OR4F4, OR4K2, PFKFB2, PHGDH, PIP4K2C, POLR2C, POLR2I, PPP1R3A, PROC, RALB, SIGLEC1, SLC25A6, SMAD3, TYMS, GABARAPL2, TNFRSF13B
Negative_2277	KIT, SRM, ZYX, AARS2, ACPP, ACTN1, APOA1, CACNA1C, CLDN11, CNDP1, DNMT1, EFNB2, ERBB3, FARSB, GALNT8, GSTM3, HMOX1, IFNAR1, KLRC3, NRG3, NTNG1, PDIA4, PFKFB1, PIGA, POLR2J, PPA2, PPARG, PSME2, RARA, RPE65, RXRG, ST8SIA1, SUCLA2, UNC5A
Negative_2278	C4B, CGN, F11, HAL, ACTG2, ARPC3, BDH1, C9orf95, CALML5, CDKN2C, COL5A2, CYP2E1, DGAT2L4, DHRS9, DNMT1, EPHA1, EPHA6, FSHR, HARS2, HSD17B3, MAP2K2, MMP2, MYD88, NCF4, NUDT5, OR10G9, OR1D2, OR4F6, PORCN, PPAP2B, PTPRB, SMARCA5, TAS1R2, TUBAL3, UPRT
Negative_2279	FH, IL9, TK2, ALG12, CACNB3, CPT1A, CPT2, CX3CL1, DDHD1, FRAT1, FSHB, GLI3, HTRA2, IL12RB2, IL20RB, ITPK1, JAM2, MAGI2, MMP1, MYH4, OR10S1, OR2T33, OR6Y1, OR8G1, PLCZ1, PPM1A, PTPN6, RAPGEF3, ROCK2, SETD1A, SETDB1, SULT1A2, TIRAP, TRAF3, VAV2
Negative_2280	DDO, MTR, PGD, AADAT, ARHGEF6, CBR3, CD244, CD8B, CDC27, COL1A1, CYP2C8, DARS, ELK4, GCLC, GOT2, GUK1, LYPLA2, MAP3K1, MYH7B, OGDH, OR1L1, OR2A12, OR51F2, OR5P2, OR6N1, OR7C1, PARVG, PDE8B, RARS2, RASA1, RASSF1, SOCS3, UPP1, WARS2, WNT8B, ATP6V1E1, PDCD1LG2
Negative_2281	C7, CRH, DHH, HYI, IDS, NPL, AARS2, ALG6, BMPR1A, CD36, CHEK1, CLTCL1, CMPK1, CXCL2, CYP4A22, DGKG, EDN1, FGF9, GCLC, GLUD2, HLA-G, HNF4A, HTR6, IFNA10, INPP5E, KLRD1, LALBA, LDHD, MITF, MPZL1, MYD88, NAT2, NKX6-1, OR1F1, OR51B5, OR5P3, SCD5, SLC9A1, SNAI1, TAS2R16, TBK1, WHSC1, MAP3K7IP2
Negative_2282	CD6, ABCC8, CCL3, CDC25B, CLDN22, COL1A2, FGF1, GPT2, IFNA16, IRS1, MTHFD1, OR56A4, POLD1, PPP2R5B, PRKCI, PTGES2, RAB5A, RXRB, SESN1
Negative_2283	HK1, LPO, NOG, ADCY5, BET1, CCR8, CYP2C18, DPYS, FRAT2, ITGAL, MYLK2, NAGK, NARS, OR4A5, OR4F4, OR51G1, OR51V1, OR56A1, PCK2, PRKAA2, PRKG1, ZFYVE16, TNFRSF10B
Negative_2284	GLA, ALAS2, OR5M3, RXRB, THBS4
Negative_2285	CRH, ACO1, ADRA1A, B4GALT2, BCL10, CD48, COQ5, CUL1, E2F1, EPHA2, EXT1, GALNT5, GCH1, GCNT3, HNF1A, HRH2, HSD3B1, IFNA10, IL10RA, MAP2K5, MAP3K11, MAP3K7, MAPK13, MYH9, NADK, OR10AD1, OR2T11, OR52H1, OR6B2, PI4KB, PPP2R5C, PRIM2, SH2D1B, ST8SIA1, STAT2, STX4, TAS2R8, ULBP3, VAMP3
Negative_2286	FES, INS, LCK, AASS, ADRB3, BIRC5, BPNT1, COMP, CXCL10, DTX3L, FUT9, INPP1, INPP4B, IRAK1, JUND, MMP14, NFKB2, NMNAT1, NPAS2, NT5E, OR2A42, OR2B11, RHOD, SMAD5, TRPC1, TUBA1B, UCP1
Negative_2287	GLS, ACSL3, AKR1B10, AKR1C1, ATP5J, CD19, CYP3A5, ELK4, FBXW11, FUT3, GNAI3, HIBCH, IL13, KLKB1, LIPE, MAPK14, MFNG, NR1H3, NUDT9, OR10G9, OR2B6, OR2F2, OR2T2, OR4A15, OR5T2, PCYT1B, PDE9A, POLR2J, PRKAA2, RASGRF2, RBPJ, SUFU, TBXA2R, XIAP, ATP6V1G1, ST6GALNAC3
Negative_2288	CCR3, CD36, DGKQ, IL22RA2, NADK, NOS1, OR4F6, OR4S2, PEMT, PYCR2, RAC3, RAP1A, SCP2, SPRY4, TPM3, TRPC1, WNT4
Negative_2289	BCR, CDA, CRK, CTH, PML, AKR1A1, APRT, ARPC5, BBOX1, BUB1B, CCL23, CCR7, CD40, CERK, CLDN1, CNTNAP2, COL5A3, CTSL1, CYP26B1, CYP3A7, DUSP8, ENTPD5, FBXW11, FGF1, FZD8, GALT, GRAP2, HSD3B7, IKBKB, IL10RA, IL13RA1, IL18RAP, MAOB, MAP4K3, MKNK1, MYL9, OR10V1, OR4C12, PIP5K1A, PLCZ1, PLOD1, POLD3, PPM1B, PROC, SEMA3D, SLC2A1, SMAD4, YES1, ALDH18A1

Negative_2290	IL8, AMHR2, CDS2, CHD8, CLDN8, CNTN1, CXCL1, DKK1, DVL3, EFNB1, EPHA2, IFNA21, MAPK13, NPR2, OR2T11, PIK3CD, PPP2R5D, TJP1, TPM3, UAP1
Negative_2291	NPY, ADRA1B, ARPC2, B4GALT5, C3AR1, CDKN1B, DGKD, DGKH, DHCR7, ELK1, FUT7, GCNT1, HTR2B, IFNG, IFNK, IL13, LAMB3, LCP2, OR10R2, OR2AG1, OR8I2, PI4KA, PRKAB1, RRAS, SMURF1, TIAM1, UXS1
Negative_2292	CEL, DLD, GSR, TEC, ABL1, ARD1A, ARHGEF6, ARSD, ATP5G3, CCL23, CDC25C, CMPK2, CREB3, CSF3, CYP27A1, EXOC7, FARSB, FZD10, FZD5, FZD9, GMDS, IL21, IL6R, JUND, KAT2B, LFNG, MAD1L1, MLLT4, NME2, NOTCH1, OR10X1, OR2C3, OR51A4, OR5T1, OR6Q1, OR8D4, PLXNA1, PYGB, RENBP, SCP2, SDC4, SLC33A1, SMAD4, TAS2R10, TUBA1A, UBE2L6, VTI1A, SERPINC1
Negative_2293	AK3, C8B, EPO, ADCY6, ARNT2, B4GALT4, BET1, C4BPB, CCL2, CD48, CD82, CLDN22, ENPP7, FGFR2, GALNTL4, GRM1, GSTA3, GSTO1, IARS, MYL9, OR2A2, OR5D14, PLK1, POLR2H, PPT1, RALA, SLC27A1, SPI1, THBS3, TLN2, TRADD, UROS, ZMAT3, SERPING1
Negative_2294	CFI, CCL27, CXCL11, FGF5, PPP2R1B, SEMA4C
Negative_2295	ACP2, ATF4, ATP5O, CCR1, CYP3A43, DAXX, ENTPD1, FGF6, FLT1, FPGS, GCAT, GPLD1, GRIA3, GSTM3, INSR, ITGB8, LDHAL6A, LIMK1, LRDD, MAD2L1, OR52B4, PIP5K3, PRKY, STAT4, TICAM1
Negative_2296	LHB, ADH6, ALDH3B2, AMY2A, B3GAT3, CALML6, CSF3, CTBS, CYP2B6, DAPK1, DERL1, GANC, GRB2, GRIA2, GSTA4, IFNG, IL1A, INPP4A, JAK3, KAT2B, NUMB, OR13A1, OR2B2, OR2M7, OR4B1, PAPSS1, PGAP1, PLA2G5, PRDX6, PRKACB, RAP1A, SARS, SELL, TRDMT1, TUBB2C, WNT3A, WNT7A, PPARGC1A
Negative_2297	DBI, LEP, TFG, TKT, CCL4, CD8B, CDC7, CNGA3, COMP, CTPS2, INPP4A, IRS2, OR4C12, OR51A7, OR5J2, OR6C2, OR6F1, PARVA, PDE2A, PDGFA, RELB, UCK1, ULBP3, UNC5D, UROS, B3GALNT1
Negative_2298	AK2, FN1, HGF, TK2, ACVR1, AK3L1, CCND3, CHIA, CREB3, DIAPH2, FCGR2B, GLI1, HSD3B2, IDH3G, MYH6, NTRK2, OR13G1, OR52E8, OR5T1, P2RX4, PPA1, PRKAB2, SGMS1, TAP1, TRMT11
Negative_2299	AKT1, CREB5, CYP3A4, FZD6, GUCY1B3, ITCH, KLRC4, PER2, PRKAR1B, YWHAE
Negative_2300	ADRB3, CLTC, EPHA5, PRKAB1
Negative_2301	GP5, MVD, CACNA1G, CADM3, CHRM5, CLDN22, CLDN6, CRHR1, CYBA, CYP4F2, DTYMK, GNB3, H6PD, HSD3B1, IL21R, IRF3, LIFR, MTHFS, OR2T3, OR51A2, OR52K2, OR8J1, PANK2, PLXNB2, PMAIP1, POLD3, PPP2CB, PPT1, RRM2, RUNX1T1, SEMA4A, TLN1, UNC5A
Negative_2302	FUK, ID4, KDR, ACSM1, ALAS1, ALDH3B2, AMDHD2, AMPD3, APPL1, ATP5L, ATP6V1H, CLTCL1, CYP2U1, DNMT1, EFNA5, FUT4, GCDH, IDH3B, ITGB1, KLRC3, LAMB4, LYZL1, MAD1L1, NEUROD1, NR4A1, OR10A7, OR2A2, OR51D1, OR51V1, OR8B8, PARVG, PFAS, PPARD, PPP1R3C, RAC3, SAT1, SMAD4, SMURF1, STAT4, SV2B, TKTL2, TLN2, WARS, WNT4
Negative_2303	MVD, ASCC3, CACNG6, CHRM3, CRB3, IL17B, KIR2DL4, OR52N1, PHKA1, TPMT, WASF1, TNFRSF10C
Negative_2304	EZR, TFG, ACSL3, ADCY6, ADH7, CCL28, CIITA, CXCL9, DHRS4L2, FLT4, GAB1, GLYCTK, GSTZ1, GUCY2C, IL2RG, MGAT5B, OR52E4, OR6Y1, PFKFB4, PLA2G1B, PLCG2, PLOD3, PTEN, RASA2, SCIN, SEMA4D, SLC27A5, SOX17, TCF7, UGP2, ATP6V1G3
Negative_2305	AUH, C5, C7, CAD, CR2, SRC, AACS, ABL2, ABLIM1, ACSS1, CDKN2A, CDKN2D, CYP1A2, DHDH, DRD5, DTX3L, EDN1, FARSB, GAPDH, GFPT1, GOT2, GYS1, IFNA6, IL21R, MAP3K12, MCM5, MYLK, OR13G1, OR4S1, OR5AN1, OR6K6, OR7G2, PAK7, PCNA, PIP4K2A, PPP2R5B, PRKCI, STK3, TGFA, TUBA8, TNFRSF10C
Negative_2306	AKR1A1, AMHR2, APOA1, BAIAP2, BDNF, BUB1B, CD70, GNRH1, GRIA3, HAAO, HAGHL, HTR5A, IL9R, LEPR, LFNG, OR2T4, OR51B2, PAK3, PANK1, PDE8A, PRIM2, TAS2R13
Negative_2307	ALPL, CDIPT, G6PC2, LCAT, OR51B4, RPIA, STX2, TJP3

Negative_2308	CHPT1,COL4A2,GCDH,HRH1,OR6A2,PPP1R3C,SHMT2
Negative_2309	C2,IHH,TYR,ADCY3,ARG1,ASAH1,AXIN2,BBC3,CACNG7,CAMK2G,CD3E,CD46,CD74,CERK,CXCL11,EFNA2,FBXW11,FUT4,GADD45A,GNAZ,GNG3,HIBADH,IFNAR1,MAN1B1,MAP4K1,MEF2C,NUMB,OR2W1,OR5L1,OR5M3,OR6Q1,OR7A5,PDE3A,POLD3,PPM1A,PRNP,RFX5,RHOQ,SETD7,SPCS3,SPHK1,TALDO1,VDAC1,WNT2B
Negative_2310	TNN,ACAD9,CFTR,IBSP,ICOSLG,IL22,LRP5,MAN1A1,MAP2K7,MAP3K11,MGST3,OR10A7,PLA2G1B,PPCS,PTGIS,STX10,VEGFB
Negative_2311	ARHGEF7,ARPC3,ATP5E,BDH2,CDC16,CDC25B,CNTFR,COX10,CTNNB1,DFFB,DGKB,DGKD,ESAM,FGF3,FSHR,FZD3,GALNT9,H6PD,IKBKG,INPP4A,ITGA9,KARS,LAMA3,LILRB3,MAOB,MICA,OR11A1,OR11H4,OR1L6,OR2C1,OR52A5,PDE6D,PER2,PIGP,PPARD,PPM1B,PPP1CB,PRDX6,PRKCG,RAC3,RELB,ROBO1,SHMT1,SMPD3,TLR5,UPP1,WASF3
Negative_2312	TNR,ACP1,ACTN1,CES1,EI24,FMO3,FZD7,GMPPA,INHBC,MGAT1,MOV10L1,MYH13,PIGF,PIK3R2,RALB,TACR2,ALDH18A1
Negative_2313	MVD,SRM,AARS,ACY3,ADH4,ARPC5,ATP5L,CACNB4,CCND1,CCR6,CD40LG,CYP26B1,DAPK3,EDNRB,GUCY1A3,IFNA2,IL12RB2,IL17RB,IL1R1,MCM4,NFKBIA,OR51A7,OR52D1,PCNA,RARS2,RPS6,SCLY,SESN3,STX18,TSNARE1,TYMS,UGT2B4,ZNRD1
Negative_2314	F5,OSM,ABCC8,ACACB,ACLY,ARD1A,ATP6AP1,CCNG1,CD38,CLDN1,CXCL1,CYP2C8,FGF4,GALM,GGT1,GNA15,GSTO1,HADH,IFNA8,IL19,IL20,KLRC3,MAD2L2,MAP3K5,MMP7,MPST,NCOR1,OR1S1,OR2K2,OR4B1,OR8B8,ORC2L,PFKL,PNPLA3,PPP1R1A,PPP2R5B,PTPRB,SCLY,SETD1B,STT3B,TAS2R50,ULK2,PDCD1LG2,PRICKLE1
Negative_2315	CKM,NGF,DDIT3,EP300,EPB41L1,HMBS,LIMK2,OR2M3,PLA2G2D,PRKCZ,SMAD5,MAPK8IP3,TNFRSF1A
Negative_2316	ALOX15,B4GALT4,CCNG1,CTSB,DKK2,FGF19,HMOX2,IDH3B,IL13,IL23A,ITGB4,MAP3K4,PAK3,PTPN7,SOAT2,TXNDC12
Negative_2317	BAD,LIF,ALDH1L1,ANAPC10,ARPC2,C5AR1,CLOCK,CXCL13,DAPK2,DGKQ,FRAT1,GCNT1,GSTM2,IL18,IL19,IRAK1,NEU2,OR4N2,PCK2,PFKFB4,PPP1CC,STAM2,STX4,TNFSF10,TSC1,TUBA3C
Negative_2318	A2M,C8A,GGH,ME1,XDH,AKR1A1,ALOX15,ATP12A,BCAT2,CD46,CHST13,CLDN18,CTPS2,DDX18,FECH,FMO1,FZR1,GDF6,HSP90B1,MCAT,NOTCH1,OR2T3,OR4S2,ORC4L,PARD6A,PEMT,PLA2G5,PSME3,SKIV2L2,SLIT2,SPRY1,VAV3,YOD1
Negative_2319	ARG1,CCR4,CDC6,CYP1B1,ENPP3,EXT1,FARSB,GUCA1A,JAK3,JAM2,MAP4K2,NKX2-2,OR14I1,OR4X1,PIK3C2B,PIP5K1B,PRODH,QPRT,RHOD,STX7,THTPA,TUBA4A,ALDH18A1
Negative_2320	CEL,HK2,MET,TNF,AANAT,ACAD9,ACADVL,ASAH1,ATP5C1,BAAT,BCKDHA,CAMK2D,CD22,CSAD,CYBA,DDHD1,DNMT1,FBP1,GALNT5,GALNT6,GP1BA,GUK1,IFNA13,IFNG,ITGB1,ITGB5,LAMA3,MLYCD,OR4D5,OR4M2,OR6C74,OR6K6,OR9K2,PAPSS1,PCNA,PEMT,PIAS2,PIP5K1C,PLA2G10,PMM1,SEMA4A,SNW1,SV2B,TM7SF2,ATP6V0A2
Negative_2321	CGA,GDA,HPD,ID1,AADAC,ADH1B,ALG10,CCNG2,CMAS,DARS,E2F5,GGT7,GSTT1,HEMK1,IBSP,IDI1,ITGAM,MAT2B,MGAT2,NOTCH2,OR52H1,PTPN7,PTPRZ1,SEMA4G,UCP1,VANGL2,TNFRSF10D
Negative_2322	BAD,PML,ALDH1A3,CARS2,CCND1,CD36,CHKB,CHRD,CHSY3,CLDN2,CNGB1,COQ3,EFNA2,GLI3,GSTA1,HTR6,IL22,MYH10,MYH7,NKD1,OR52I1,OR5B12,OR5H6,OR6F1,OR7D2,OR8H1,PARS2,PER2,PLEKHA8,SETD7,SH2D2A,SMPD1,SOCS3,YWHAQ
Negative_2323	MPI,ABL1,FARSB,FGF8,IFNA7,RBL1,SPHK1,STAM

Negative_2324	PGP, ACSL5, ACVR2B, ADH1A, BMP8A, CCL3, CCNE2, CSNK1G2, CYP3A5, DUSP14, EDNRA, FGF23, GFPT1, IL22RA1, LAMB4, LARS2, MARS2, MDM2, NFYB, NOTCH3, NSDHL, OR4S1, OR5M9, OR6C6, OR6C74, PLA2G2D, PLCB3, PMAIP1, PPA1, PROC, PSME1, SELP, SHC1, TBL1Y, VDAC3, MAPKAPK3
Negative_2325	AGL, GK, ACAT2, AGRN, BAAT, BMPR1A, BTRC, CHRM3, CRB3, CREB3, CTNNA2, CYP19A1, CYP3A7, EXTL1, FADD, FOXO3, FZD7, GCNT2, GSTM5, HPRT1, JAK3, MAP2K1, MAP3K11, MCCC2, MPP5, OR2T2, OR52E8, OR52I2, PDE1C, PIGA, PIK3R4, POLD2, SEMA3B, SLC27A1, MAPK8IP1, TNFRSF1A, TNFRSF6B
Negative_2326	F2R, NOG, ADH1A, ADIPOR2, ATP6AP1, BUB1, COL5A1, CTSG, CYP2C18, DARS, DGKD, GLYCTK, GSTO1, HEXA, IRS1, MKNK1, MYD88, MYH6, OR4N4, OR52A5, OR52M1, OR6C74, OR7D4, PDE1C, PITX2, PRKX, RAB5A, RDH8, SNAP25, STAT1, SV2B, UCK2, PPARGC1A, SERPINA1
Negative_2327	ACOT12, DHRS3, DUSP4, GALNT6, GRHPR, GSTT1, LAMB1, MAP2K2, NRXN2, OR10A2, OR52E4, OR6C1, OR6K6, PLAU, PRIM1, PSEN1, PTDSS1, SPRY1, THY1, TNFSF11, TPMT, TUBA3E, YWHAQ, PHOSPHO1, PPP1R12A
Negative_2328	SMS, ATP5J, CD247, CYP19A1, GNAO1, GSTA1, IRAK1, OGDH, OR2G3, OR2L8, PRKAR2A, PTCH2, SEMA4F, WNT8A, CSGALNACT2, MAP3K7IP2
Negative_2329	GNS, SFN, ALG10, AMDHD2, AXIN2, BET1L, CD274, CXCR3, DDX4, DRD2, DUSP1, GALNT12, GLG1, GMPR2, GNAT3, GUCY2F, IL23R, IL9R, MAML3, MTMR1, NME7, NSD1, OR6S1, OR7A5, PIK3R4, PLA2G5, PLAT, PYCRL, RFXAP, UNC5A, ZAP70
Negative_2330	AQP7, COL3A1, HAO2, IL28RA, PAFAH2, PKMYT1, PRKCE, YKT6
Negative_2331	AGL, BTD, AMPD2, CD274, CD40, CTBP1, ENTPD5, FADD, GNMT, HAO1, IFNA8, ISYNA1, MAGI3, MYL5, P2RX5, PLCZ1, PRSS2, VAV3, ZIC2
Negative_2332	CRK, ID3, TEC, B4GALT6, GLI3, GSTM4, HSP90B1, MYLK, NMNAT3, SEMA3D
Negative_2333	MET, PGF, ADCY9, ARSA, ATP2A3, BLVRA, BLVRB, BNIP1, C4BPB, CASP9, CCDC6, CD59, CFTR, CNTN2, COL4A2, CTBS, ECSIT, EIF4E2, EXTL3, F2RL3, FLT3, IGF1R, IL18, IL22, ITGA7, ITGAV, MGAT4A, MGST3, MTHFD1L, NLGN2, OR1J2, OR2T4, OR4M1, OR51G2, OR6C76, P4HA2, PIP4K2B, POLA2, RPN1, SLC27A5, STMN1, TNFSF12, TNFSF14, TRAF5, UAP1, UGCG, HSD17B12, ST6GALNAC1, TNFSF13B
Negative_2334	AK5, TEC, CCR1, COX15, DDX54, FGF19, FZR1, IL28A, MAP3K4, OR10H1, OR2T8, PER2, PNPLA4, SARS, SLC27A5, SPHK1, THBS4, TSHR, ATP6V0A4
Negative_2335	F10, HK2, ACSS2, ADRB2, CACNG6, GRB2, IFNG
Negative_2336	CR1, ACADSB, ALDH3A2, ATP6V0C, AXIN1, CAMK2A, CBR3, CD79B, COL3A1, CSDA, CTBS, CTTN, DHCR7, DIAPH2, DVL2, FLT3LG, FZD6, FZD7, HNMT, IL23A, LCP2, LIPC, MAP2K4, MAP3K12, MECR, OR2G2, OR4C11, OR4X1, OR52K1, OR5M3, OR9K2, PAK7, PECAM1, PITX2, PPP3R1, PTPN1, RDH16, RPRM, SELL, SEMA3G, SMC1A, SNAI1, SRGAP2, ST3GAL6, TICAM1, TKTL1, TNFRSF4, TPH1
Negative_2337	CRK, FGG, ID1, JUN, STS, ACACA, ADCY9, ADH5, ALG8, AOC3, BMP5, CACNG5, CCL13, CD34, CDH1, CREB3, CRHR1, EARS2, FOXA2, GAL3ST1, GNA15, IL12RB2, IRF5, ITGB5, MAOA, MASP2, MTHFD1L, NCOR2, OR10AD1, OR14J1, OR4A16, OR4N2, OR8I2, PIK3C2B, PPP2R5D, PRSS2, PTCH1, PTGIS, PYCRL, SREBF1, TAS2R49, TOMM40, WNT9A, ATP6V0A2
Negative_2338	A2M, DCT, F2, IL9, ACHE, APPL1, ATP4B, BMPR1B, CCL21, CDC6, CRLS1, E2F2, EFNA4, ELK4, GSTO1, GZMA, HCCS, HSP90B1, HTRA2, LYPLA2, NANS, NAT5, NCR1, NT5C3, OR1F1, OR51B5, OR51E2, OR5A2, PDE9A, POLR2C, PRKAB1, RAF1, RALB, RENBP, RRAS, SPHK1, WNT16, ALDH18A1
Negative_2339	ALOX15, DHRS4L2, FGF17, IQGAP1, NRG1, PPA1
Negative_2340	GLA, CAMK2D, COL1A1, CXCL1, DAPK2, DHRS3, FGF17, GAD1, GCNT2, MAFA, NCR3, NUDT5, OR10K1, OR1J2, OR4D1, PARS2, PDE3A, PRKAB2, PTGES, PVRL1, SEMA3A, SETDB1, ST6GAL1, THBS2, WIF1, CSNK1A1L
Negative_2341	CXCL1, DDX51, EFNB2, GALC, GNB3, GRIA1, KIR3DL3, OR1J1, OXTR, PAX4, SDC2, TAS2R5, THBS2, UNC5C, AASDHPPT

Negative_2342	AK1, FGG, MIF, ACPP, ACSM3, ADSL, AGRN, AMY2B, B3GALT6, BCAT1, CDK7, CSNK2A1, CTNNB1, ERBB3, FCGR3B, FLOT1, GALNT14, GBGT1, GFAP, GFPT1, GLO1, HARS2, HIF1A, IFT57, IL12A, ITGA10, ITGB3, LIMK2, NKX2-2, NSD1, OR10G7, OXCT2, PDE5A, PHKG1, PIK3C2G, PTGES2, RAB23, RASA1, RPS6KA5, SEMA3B, STX3, THY1, TUBA4A, HLA-DQA2, HSD17B12
Negative_2343	F8, FER, RPE, ACTG1, ACVR1B, AOC2, ATP5C1, ATP5F1, B3GNT2, BAI1, BMP7, C1QA, CCL16, CNTNAP2, DDX54, FDFT1, H6PD, HEXA, ICAM1, IL22RA1, MAML2, NAT5, NRG1, NRG4, NT5C2, OR4Q3, OR51B2, OR51E2, OR6C75, OR8J3, OXSM, PIK3R4, PLA1A, PPP1CB, PRPS2, TAS2R4, TGFBR2, THTPA, UROD, WARS, WARS2, ATP6V1G3
Negative_2344	MPI, MPZ, AGPAT3, CCR4, COL3A1, GLDC, GLO1, HTRA2, IL18, OR10C1, OR6B3, OR8I2, POLA1, PRKCH, GABARAPL2
Negative_2345	ASL, DDO, F12, AGPAT3, ASS1, BCMO1, BMP6, CTSL1, DUSP2, GAPDH, GIT1, GPT2, GRIA3, HTR2A, IRF3, MTHFR, OR10A3, OR10A4, OR10H3, OR52E8, OR5F1, PPP2R1A, PTPRM, WEE1, SERPINC1
Negative_2346	ABL2, ALG13, ARRB2, C7orf16, CCNB3, CD79A, CHIA, CLDN17, EPB41L2, FASLG, GMPS, GRAP2, MAGI2, NFATC2, OR2M3, PLCD3, PMM2, PRKCE, ROCK1, SULT2A1, THBS2, WASF1, XYLT1, MAPK8IP3
Negative_2347	ALDOC, ANAPC1, CACNA1H, CD247, F13A1, FADD, FUT9, GAPDH, GBE1, HSPA2, NMNAT3, OR1L3, PIGW, TAS2R8, TCIRG1
Negative_2348	GSR, MUT, ACTA1, AGXT2, BCMO1, BMP8A, CDC16, CHSY3, CTBP2, DHCR7, FMO4, FSHB, FZD1, GALK2, GLUD2, GMPR2, HYAL2, IL28RA, IL8RA, JAK3, NGEF, NUMBL, OR10S1, OR11G2, OR13A1, OR6C70, OR6T1, PIGM, PLA2G2F, PLA2G3, PLD1, RND1, SCIN, SEMA4B, SHISA5, SMAD7, SNW1, STAT6, ATP6V0D1, PPP1R12A
Negative_2349	ALPL, ATP6V0C, CCL14, CXCL2, DUSP2, DUSP3, GDF6, HGSNAT, HIBCH, IKBKE, ITGA10, MAPK14, MLLT4, OR1J4, OR51B5, OR5A2, P2RX1, PGAM2, PIK3R3, PLTP, PRKY, SETD8, SLC12A2, TACR2, TDO2, TICAM2, CSNK1A1L
Negative_2350	CACNB1, OR5AK2, OR5B17, PLXNB1, POLR2L, TLR2, VANGL1
Negative_2351	CTNNA2, HSPA2, JAM3
Negative_2352	CP, HGD, ABLIM3, B3GALT4, B3GNT4, CCL7, CTNNA2, CYFIP1, KLRC1, LYPLA2, MIOX, MMP9, OR10G7, PCYT2, PLXNB3, SPI1, TLR9
Negative_2353	FOS, TPR, ABLIM1, ADCY5, BET1, CASP8, CCND1, CCR4, CLDN1, CMAS, CXCR4, EDN1, EPHA4, FMO3, FRAT1, FUT7, GMPPA, GP1BA, HAO1, HARS, HIF1A, HSD3B2, IDUA, IL18R1, IL21R, IQGAP3, KCNJ11, KIR2DS4, LIMK2, LIPE, LRDD, NRXN3, OR2A14, OR5P2, OR9A2, PCCA, PHKG1, PHKG2, PPP2R5B, PTPRJ, RETSAT, RPS6KA4, SLIT2, SOCS3, CSGALNACT1
Negative_2354	GCK, NOG, SHH, ZYX, A4GALT, ADCY1, ADRB2, ADSL, ATP5F1, ATP5J, AXIN1, BST1, CACNG5, CCNE2, CKMT2, CLTA, DDX18, DDX19A, ENPP6, FASLG, GCNT4, GNB1, GOT2, GSTK1, HSD3B1, HSPB1, ITGA6, MDH2, MMP9, MYH2, NT5E, OR2B2, OR5H2, PDGFC, PDGFRA, PIK3R5, PKMYT1, PMM1, PNPT1, RRM2B, SGMS2, SMPD2, SOS1, UCK1, WWP1
Negative_2355	ADO, GLA, IL5, MSN, PGD, ACAA1, ATP2A1, BDKRB1, CAPN2, CASP6, CCR10, CSF2RB, DDOST, EFNA3, FIGF, GGT7, GNB3, GPLD1, IL15, IL3RA, JAG1, MCM6, NMNAT2, OR2M5, PAFAH2, PAK7, PHKA2, PISD, PLA2G1B, PPP1CA, PRSS2, QARS, SH2D1A, TACR3, UGT2B7, VANGL1, WNT8B, ZAP70, ALDH18A1
Negative_2356	BID, ADCY10, ARNT, B3GNT2, OR8J1, PLXNB1, PRKAG1
Negative_2357	ACMSD, AHCY, APLP1, ARHGEF2, ATP5E, AXIN1, BUB1B, CDC20, CNTF, CRY1, CYP19A1, FGF2, GLUD2, GMPR, GSTA2, HCLS1, HLA-DRA, HPRT1, IFNG, IKBKG, NAGA, OR4K15, OR52A1, OR6C74, RARA, RELA, SDC2, SGPP2, SH2D1A, SMC1B, ST3GAL3, YWHAZ, CTNNBIP1, PRICKLE2
Negative_2358	CRK, BET1L, SQLE

Negative_2359	ACAA1,AKR1C3,AMY2A,CALML6,CCL21,CCL4,CDC42,CDC45L,CNGA4,CYP3A7,DUSP2,EGLN3,EHMT1,FCGR3B,FMO4,FUT4,HES1,HSD17B3,HTR2A,IFNW1,IKBKE,NOD1,NOS3,OGDH,OR4K17,OR51D1,OR8K1,PPP2R2B,PRSS3,PTCRA,SFRP5,SLC2A1,TAS2R8,TGDS,VTI1B,WWP1,ST6GALNAC3,SUV420H2
Negative_2360	C8A,HGD,IL2,ACSS3,ADCY6,AQP7,CCL2,CDK2,COASY,COL2A1,CSDA,CYSLTR1,E2F2,EXTL3,FGF3,FGF4,GCH1,GMPPB,HGSNAT,HSD3B1,IFNAR2,ITPK1,KAT2B,MAP2K2,NCOR1,NFATC4,NTF3,OR10H3,OR2M4,OR4C11,OR5L2,OR6C1,OR6S1,PAK6,PAPSS2,PARS2,PIK3C2B,SPTLC2,SV2A,TUBB6,YWHAZ,PPP1R12A
Negative_2361	ALPPL2,APH1A,ATP5J,CDC25A,CSNK1E,GOT1,GUCY1A2,HSD3B1,ITGA11,NUDT2,OR1L8,OR6B2,PLA2G10,RAPGEF3
Negative_2362	AK5,ACOT12,AGPAT4,APAF1,CCL22,CD82,DAPK1,GARS,OR4M2,OR7G2,OR8H3,PPP2CB,RASGRP4,SMARCA2,TNFRSF1A
Negative_2363	ABI2,HAGH,MIOX,NCAM2,OR52H1,PLEKHA8
Negative_2364	CKM,GPT,NGF,SRC,TNF,ABCC8,ATP2A3,BAI1,CER1,CNGA3,CSNK1D,DEGS1,EIF4E2,FLT3,GLG1,GNAS,ITGA2B,LAMB2,LDHAL6A,MAN1C1,MCCC2,OR10A7,OR13C5,OR5A1,OR9Q1,OXCT1,POLD1,POLE3,PPP2R5B,PRKCE,SHC4,SHISA5,SLC2A4,SPHK2,STAT3,SV2B,TAS2R8,TCF7L1,TRPM5,WARS2
Negative_2365	AKT1,CACNG7,DGKA,DOLPP1,EREG,EXT2,FUT5,GALNT3,GRIN1,ITPKB,JAK2,MGAT3,MYH6,NFATC2,NR5A2,OR4X1,SPRED1,TDO2,TLN1,UGCGL1,UROS,YWHAQ
Negative_2366	ACLY,BCAR1,CCNG1,ENTPD6,ITGB5,MYL7,OR8U1,SIPA1,TSC2,TYMS
Negative_2367	C4BPB,CTNNA2,CYP27A1,ENPP7,ETNK1,LLGL2,NOS1,OR5C1,RPS6KA2
Negative_2368	GP6,SYK,AGXT2,ALOX5,ARPC3,CD74,CHRM5,FABP1,FMO3,GSTM4,HLCS,NEFL,NFATC4,OR2B11,OR6S1,POLR3G,PPA2,PVRL3,RASGRP4,SETD7,SOCS5,SPRY1,TGFB2,TLR3,UMPS,SERPINA5,ST6GALNAC4
Negative_2369	BAX,TK2,AACS,ABLIM1,ATP6V0C,BDH1,BDKRB1,CRLS1,CXCL2,DCTD,DGUOK,DNMT3L,EGFR,FGFR1,GPLD1,GRIN2B,HLA-G,HSPA8,IFT57,IQGAP1,IRAK2,MCM3,MPDZ,OR10R2,OR3A2,OR6Y1,PIK3CG,PIK3R3,PON3,PRKACB,RXRG,SGMS1,SH2D1A,SOD1,SOS1,SOX17,SRGAP3,SSX2IP,ST3GAL3,SULT2B1,TAS2R3,TPI1,TRAF3,TRPM5,PDCD1LG2
Negative_2370	CP,EPO,IL9,MPL,ABL1,ACADSB,C7orf16,CD22,CD44,CERK,CTSL1,DUSP14,EPHB6,GLUL,GSTM1,H6PD,HTR2A,KNG1,LRRC4C,MAP4K1,OR10G2,OR5AP2,OR7A10,OXCT1,PDIA4,POLR2C,PTPN6,TAS2R48,TPM3,TYMP,SUV420H1,TNFRSF13C
Negative_2371	FST,IL2,ACTA1,ADIPOQ,ATP5G3,CCKAR,CCL17,EIF4E,IFNA13,KLK3,LAP3,MAP3K14,MFNG,OR2A14,P4HA2,PIGF,PPP1CC,RRM2B,TRAF5,WASF2
Negative_2372	DBH,ID1,LSS,ACHE,ALPI,CACNG1,CCL2,CD46,CD47,CD79B,CLDN18,CLDN4,DDX55,DFFB,GMPR,GRIA1,HNMT,IRS1,MAP2K1,NLGN2,OR2F1,OR51B5,OR7D4,PIM1,PLEKHA8,POLD4,ST3GAL2,ST6GAL1,TAS1R3,TBXAS1,TFDP1,UBE2K,UNC5C,CSGALNACT2,PRICKLE2
Negative_2373	ALDH3A2,CCNB1,GDF6,GLI3,GUCY1A3,MITF,TNFRSF9,UBA7,ULBP2
Negative_2374	ZYX,FUT8,HSD17B2,MAGI3,NEFL,NRP1,OR8H2,PDIA4,PRKAR1A,RPS6KA3,UROS
Negative_2375	C1QA,CNTN2,NUDT12,SENP2,TIRAP
Negative_2376	F3,AKR1D1,CDC26,CMAS,CSNK1A1,FUT1,IFNA16,IL28RA,MAGI1,OR1M1,OR1N2,OR5P3,PGS1,POLR2D,SPHK2,TAS2R40,TLN2,TLR9,MAPK8IP2

Negative_2377	AR, ABCC8, ACVR2B, APLP1, BAI1, CCL25, CCNE2, CD36, CISH, CSNK2A1, EHMT1, ENTPD8, FOXA3, GAD1, GALNT8, GDF5, HSPB1, IL17A, IL22, ITPKA, LDHB, MAN2A1, MASP1, MYH14, NFYC, OR2B11, OR4C16, OR6M1, PAK3, PDE3A, PIK3CD, PLA2G3, SLC25A5, SMAD7, SULT1A1, TAS2R4, TCF7, TSC2, UBA7, ARHGEF12, PPARGC1A, TNFRSF17
Negative_2378	FGB, ACOX1, ANAPC5, ARNT2, COL4A2, IL10, KIR2DL4, NFATC3, PGS1, PIGH, RFX5, SELP
Negative_2379	ADK, AGL, AK3, B4GALT6, DUSP2, FUT8, GRLF1, GSTT1, HLA-DOB, IL13, ITGAL, LEFTY2, NRG2, OR51V1, OR52E4, PDE6H, PDXP, PIGB, PLA2G3, POLR3B, PPP1R3B, PTGS1, PTTG1, RRAS, SLC2A2, SNAP25, TAP1, TJP3, ATP6V0A4
Negative_2380	ANAPC10, ARSD, BCL2L1, CACNB4, CDKN2C, CMPK1, GALNT11, HNF4G, IFNGR2, JAM3, LAMC3, MAGI3, PLXNB3, PTK2B, PYGL, SGPP1, MAPKAPK2
Negative_2381	GDA, GGH, LEP, APOE, ARHGEF2, DGAT1, DPYD, FOXA3, ITGA10, LMO7, OR13C8, OR5F1, OR6K6, PIP5K1A, RPN1, ATP6V1G3, MAPK8IP1, MAPKAPK3, TNFRSF18
Negative_2382	AMH, CFD, IL3, LSS, PLG, ALAS2, ALG12, AXIN1, BDH2, CCDC6, CCL16, CCNB1, CRB3, CYP11B2, EPHB4, FUT9, GSTA4, HTR2A, IRF3, MAD1L1, MAD2L2, METTL2B, MGAT5B, MYL7, NOX3, OR14J1, OR2F1, OR5A1, PLK1, PRKAR2A, PRKCE, PTGER3, PTPRF, RHEB, SH2D1B, SREBF1, ST3GAL1, TAS2R48
Negative_2383	FN1, ACHE, ADH1B, COX10, DUSP2, FZD2, HCCS, HLA-DOA, LPAR1, NAE1, OR2G3, PPP2R5D, SMURF2, TUBA3E, TUBB
Negative_2384	CRLF2, GAL3ST1, NKD1
Negative_2385	CRH, ACADM, ADCY4, ADORA2A, AGXT2, ATP5B, C1QC, CCNB3, EPHB6, FADS2, FPGT, FRAP1, GPAA1, IFNA8, IFNAR2, LEFTY2, LGMN, MCCC2, NTNG1, OR2K2, OR4K13, OR4K2, OR51E2, OR52I1, OR7G3, P2RX6, PARD6A, PTEN, PTGES2, STAM2, TNFSF8, ULK1, B4GALNT1, CACNA2D2, ST6GALNAC6
Negative_2386	BAI1, CARD11, CD8B, IL10RB, ITGA9, PAX4, RUNX1T1, SMARCA2, STMN1
Negative_2387	AKT2, AOC3, B4GALT7, OR10A5, OR52I1, OR52N1, PFKP, PPP2R1B, PYGB, RFX5, RPRM, RUNX1, XYLT2
Negative_2388	ADC, LPO, ADCY1, ALPP, APPL1, BMPR1B, CACNB1, CDC14B, DHRS4L2, DUSP8, EGLN1, FCER1G, FUT7, GMPS, GOSR2, GSTM2, GUK1, IDH3A, MAN1B1, OR11H1, OR1S1, OR4F6, OR51L1, OR52R1, OR8I2, PDE5A, PFKFB1, PGAM1, PLAUR, PPAP2C, PRKCB, PRKX, PTGER1, SEMA3G, SETD1B, SUFU, TJP3, TNFSF8, CSNK1A1L
Negative_2389	LEF1, LRP6, MAP2K7, OR2H2, OR5A1, OR9K2, PIGL, SELE, HLA-DQA2
Negative_2390	HDC, LAT, LCK, TK1, ABL2, ACTN1, ADSL, ARNTL, ASCC3, ATP6V1D, BDKRB1, CACNA1B, CDS1, CHPT1, CMAS, CNTNAP2, CREB3L4, CSDA, CXCR4, ENTPD3, EXTL3, FGF4, GALNT11, GRHPR, HSPA1B, ICOS, IRF3, LARS, LTBR, NEU2, OR10G7, OR52E4, P2RX7, PARVG, PLK1, POLE, PPP1R1A, PRKAB1, PTPN1, SLC2A4, ZNRD1, PAFAH1B1
Negative_2391	TK1, TKT, ATIC, ATP5G2, CALML3, CASP7, CCNE2, COQ3, GPX5, IFNA17, IL17B, IL9R, LTC4S, OR1C1, OR52A1, OR5H2, PPAT, PPP2R2A, SGPP1, SKP2, SULT1E1, TNFRSF13B
Negative_2392	AK7, GBA, ARHGEF7, ARPC3, ASS1, ATP12A, BACE1, CALML3, COL2A1, DAD1, DTX3L, FCGR3A, FUT4, GNA13, IFNA17, IL10RA, IL17B, IRS1, LAMC2, NCAM2, NEFH, NEFM, NMNAT2, OR7C2, OR8B4, ORC3L, PAX6, PLA2G2E, PLCB2, PLK1, PON3, PSAT1, PVRL1, PVRL4, RPE65, SDC4, SETMAR, SNAI2, SOCS2, SUV39H1, SUV39H2, TUBA1C
Negative_2393	DHH, ABL2, CLDN5, CSF2RB, IFNA8, IL17RB, MAP2K4, MPZL1, PLAU, QPRT, SETDB1, SOS2

Negative_2394	ALG8, ALPP, CACNB1, CDK2, CXCR4, CYP2C8, EGLN3, EI24, FRAT1, GART, GPD2, IL12RB1, IL15RA, IL20RB, ITGB1, JUND, LIPF, NFKBIB, OR7G2, OXSM, PDHA1, POLR3D, PPA2, PPP2R5E, PTPN6, RDH16, RFX5, STX2, TSHB, VEGFC, ST6GALNAC5, TNFRSF13C
Negative_2395	CGN, NPL, PGF, CCL1, CDC2, F2RL2, FGF21, GSTM5, IL22, IL9R, OR10AD1, PFKFB1, SLIT1, TOLLIP, WNT3, ST6GALNAC6
Negative_2396	IL9, ABP1, AGRN, CD82, CLDN8, CLTCL1, GCNT3, GNAS, GNG13, IARS, INPP5E, MAP3K1, MMP14, NCF4, PARD6G, PDGFRB, PDHB, PDX1, PROS1, RXRG, SORBS1, SPTLC1, TCF7L2, THBS2, TLR5, TUBB2C, UNC5C
Negative_2397	ITGA9, LEFTY2, PTDSS1, ST3GAL4
Negative_2398	AKR1C2, ARHGEF2, B3GAT2, CACNA1H, CLDN22, COASY, CREB1, CSF1R, DDX4, DGKI, GAD1, GUCY2F, OR10G8, OR10K1, OR13J1, OR1C1, PFN2, PLA2G5, RERE, RPS6, SLC27A4, STX3, SUFU, TAS2R50, TBL1X, TGFBR2, TOMM40, HSP90AA1
Negative_2399	AGL, DBT, ALDH5A1, CHEK1, CXXC4, DUSP2, FCGR3B, IMPDH1, INPP5B, LTBR, OR3A2, OR4F6, PNPLA3, PPP1R3B, TAS1R1, TFDP1
Negative_2400	CD6, GSS, LCT, ABAT, ACACB, ALDH3A2, AMD1, B3GNT3, CCL24, CD70, EPHA5, FABP1, GADD45A, GMPPB, GNA14, IDUA, MAP3K1, MARS2, MPZL1, OGDH, PIK3R5, SLC27A4, SV2A, UBE2L3, UBE2L6, VEGFC
Negative_2401	A2M, C4B, SMO, ADH4, BDKRB1, BIRC3, BUB1, CDKN1C, CKS1B, CTTN, DAPK2, DGKI, ENTPD6, FGF22, FMO3, GFAP, GGT1, GSTA4, KLRC4, MYL5, NARS, NOTCH1, OR1E2, OR2AT4, OR4C11, OR4C13, OR5AU1, PAPSS1, PER2, PRKCH, PRKG2, PROS1, RAB5A, RASSF1, SHISA5, SPCS3, STAM2, SULT1A1, TGFA, TPH2, TUBA1A, TUBA8, ST6GALNAC5
Negative_2402	KIT, CX3CR1, IRS1, MYL5, OR10Z1, OXSM, PRKACB, PRKAG2, SETD2, THBS4, TRMT11, WNT7A, YARS2
Negative_2403	C8A, ACAA2, ACPP, AKR1C1, ALDOC, CASP8, F2RL1, GRIN2B, IL6ST, ITGB7, MGAT4B, OLR1, OR6C4, PDGFB, RBL1, TSLP, WASF1
Negative_2404	CD4, F9, ADCY4, ALCAM, BDKRB1, CD19, CHRM2, CYP2U1, DCTN1, ENO2, GRIA2, MAP3K6, MTMR2, OR4C46, OR4M1, PCCA, RARS2, TYK2, UCP1, PRICKLE2
Negative_2405	ACSS2, CD34, DVL3, PNLIP
Negative_2406	BCR, EGF, LHB, TEC, AADAT, ACY1, ALDOC, ATP5G1, CCNG2, CD86, CDH1, COL4A2, CYFIP1, DDX41, EPHA4, F13A1, F13B, FCGR2B, FZD3, FZD5, GALNT12, GNAO1, HMGCL, HTR2A, IL10RB, ITPKA, MBL2, MTHFD1, NFATC3, OR52B6, OR52W1, PDE3A, PER1, PLXNA1, PPARA, PRKAB1, RETSAT, SPTLC1, SUCLG1, TAS1R3, TNFSF10
Negative_2407	LPL, UBC, APOA2, C1QB, CACNA1B, CCR5, CMPK2, COL4A1, CSNK1G3, EPAS1, FZD4, GJA1, GPLD1, LAMC2, LTBR, MCM5, NT5C, OR14J1, OR1L8, OR2AG1, OR4K2, OR5F1, PIGM, PIGS, PLA1A, PLCE1, PON1, SHISA5, SPTAN1, SSX2IP, STX12, UCK1
Negative_2408	ADH6, ARSE, CALML6, CCL11, CYP11B1, DARS2, DCTN1, ENTPD2, ESPL1, FGF7, MDH2, NTNG1, PARD6B, PLCG1, PPA2, TRAF2, WNT16
Negative_2409	ACAA1, ACSM3, B3GAT2, BAIAP2, CACNG8, CCL25, DGKI, DOT1L, HAGHL, IDI2, LYZL1, MTHFD1, NFKB2, OR4C12, OR51F1, OXSM, PDGFC, PIK3C2A, RHOA, VAMP3, SERPINC1, TNFRSF1B
Negative_2410	ACP1, BNIP1, FMO1, HRH1, IL22RA2, LAMA4, MAPT, NUDT8, OCLN, OR10V1, OR2T5, PAK6, PPP2R2A, RASGRP2, STAT3, STX2, TPH1, TYRP1, WEE1
Negative_2411	CCL15, CHSY1, DHDH, SPCS1, STK36
Negative_2412	ADA, ADCY2, ADH6, CASP10, CDIPT, CIITA, CXCR3, DEGS1, EPHA1, EXTL3, FPGS, FZD1, FZD3, GNAI2, HNF1B, IL10RB, IL15, IQGAP2, ISYNA1, KITLG, LAMC1, MAPK9, NCKAP1, NCR3, NFATC3, OR10H1, OR1G1, OR4P4, OR51E2, OR51V1, OR6C6, PDE4C, POLR3B, PPP2R1A, PPP2R5B, PRKAA2, PRSS1, PRSS3, RDH16, RDH5, TAS2R1, TNFSF4, WNT9A, YWHAB, PNLIPRP1

Negative_2413	AK2,F11,HAL,IL2,ACTG2,ADAM10,B4GALT7,CASP6,CD8B,CYP3A4,ESPL1,ETNK2,FGF1,GALNTL5,GBE1,IFNGR1,JAM2,LCAT,MAP3K4,METTL2B,NCOA4,OR4N4,OR7G1,PFN2,POLR2I,RAB5A,RASA2,RBX1,UROD,UXS1,WNT8A,PPARGC1A,SUV420H2
Negative_2414	ADC,ATP13A2,DEGS1,GJA1,HLA-DMA,HSD3B1,IRF9,LIPG,OR1J2,OR2T1,OR4C6,PDE7A,PDE8B,PIGX,PKM2,POLR3D,SH2D1A,SLC2A2,SMAD6,SMC1A,SPRED1,TCIRG1,UGT2B28,PAFAH1B2,PRICKLE2,SERPINF2
Negative_2415	CGN,GCK,ACHE,APOA5,ARHGEF7,CACNG7,COX10,FGF23,FGF9,HBEGF,HNF1B,IFNA21,IFNA5,INPP4A,KYNU,LIPC,LTBP1,MAGI2,MAN1A2,MS4A2,NCAM1,NT5C,OR2G2,OR4K1,OR52L1,OR6A2,PDE5A,PIAS1,PRKAB2,PRKCB,RELA,SHC2,SOCS7,SULT1A4,TAS2R3,TFDP1,TIAM1,UGT1A10
Negative_2416	GPI,PGP,TNC,AARS,ACP2,ACTC1,CLDN10,CXCL16,GAD1,GYS2,NUDT9,OR51I2,PIAS3,PLK1,POLR2E,PPCDC,QPRT,RAB5A,RASGRP3,SRGAP2,TAS2R50,UGT8
Negative_2417	AK2,GSN,IVD,AGXT,ALDOB,CACNG6,CAMKK2,CCR7,GALNT10,HSPA8,IL7R,KRAS,LRP5,MAPK13,MASP1,MCEE,NLGN3,OR4S1,OR51Q1,PAICS,PEMT,PRKCD,RAF1,RBPJL,SELP,SH2D1A,SMAD1,SORBS1,TBL1Y,TGDS,TUBAL3,WARS,ATP6V1G2,PDCD1LG2
Negative_2418	LTB,AGPAT1,ALDH3B2,APOE,ATP5G2,BCMO1,CAMK2B,CCL11,CDC25C,CHUK,DGAT2,ENO2,FMO4,FUT8,FZD3,GALE,GSTM5,HCLS1,HIBCH,IARS,INPP5E,KCNB1,MAP3K12,OR11H4,OR2AT4,OR2T8,OR4P4,PDGFRB,PFN3,PGAP1,PIGP,PLAU,PPP3CA,PTGS2,RRM2B,RUVBL1,SOCS2,TAS2R3,TUBA4A
Negative_2419	ABL1,AGXT,AMY2A,ASPA,ATF4,ATP5H,CCL24,CCR6,CD36,CHRM1,CYP26C1,DLL3,FLAD1,GALNT9,GBGT1,HLA-DOA,ITGB6,MAPK8,NEUROD1,NKX3-1,NME7,OR12D3,OR2T29,OR8D2,P2RX2,PAK3,PGAM2,PIK3R1,PLA2G5,PPID,SKP2,SPTAN1,STK3,STX4,TACR1,TAS2R10,TAS2R60,TJP2,TM7SF2,TPK1,UPP1,VDAC2,HLA-DQA2,MAPK8IP2,MAPKAPK2
Negative_2420	GNS,GP6,CD27,CPT1A,IBSP
Negative_2421	ITK,AACS,ACO1,ACVR1C,AKR1C3,B4GALT1,CAPN2,CCL1,CCL3,CFLAR,CHRM5,COL4A4,CYP2D6,FGF22,HSPB1,IL1R2,INPP1,NCAM1,NFKBIE,NNMT,OR52E2,OR5H2,OR9I1,OXCT2,PDXK,PIGH,POLR3K,PRKCD,RUVBL1,SFRP1,STK3,TAS1R1,TRIP10,UGT2B10,MAPKAPK2,ST6GALNAC4,TNFRSF11A
Negative_2422	LPL,STS,ACOT12,ADRB3,B3GALT6,CACNA1H,CCR4,CD226,CSF1R,DDHD1,E2F4,GNPDA1,HAGHL,IL13,INPPL1,ITGAE,NMNAT3,NR1H3,OR10H4,OR2A42,OR2AG2,OR4C12,OR52A1,OR9Q2,PDE4B,PDIA4,PHKA1,PKM2,PRDX6,PTPN11,TICAM2,TLR2,UCP1,ATP6V0A2
Negative_2423	AKR1B10,CHD8,GAD2,OR4C6,PLCZ1,PYGB,TAS2R38,WIF1,TNFRSF6B
Negative_2424	SCD,ACSL6,ADH7,ADSSL1,BDH1,C7orf16,CREB3L1,DRD1,ERO1L,GALNT11,OR12D3,OR1D2,PHGDH,PRKACB,PTCH1,SHISA5,SLC27A4,SPRED1,STEAP3,TAS2R49,THBS3,TP73,UROC1
Negative_2425	AK3,GP5,AKR1B1,ALG12,AOX1,BTRC,CCL13,CLTB,CNTN1,DERL1,EFNA4,ETS1,GRIA2,HRH2,ICAM2,MAOB,OR52L1,PYCR2,TGFB3,CSGALNACT2
Negative_2426	C8G,CTH,F2R,SRC,TAT,TKT,ADCY4,ADORA2A,ALDH9A1,APC2,BET1L,CACNA1H,CAMK2A,CDC14A,CFLAR,CREB3L3,DDHD1,EDNRB,FZR1,GADD45G,GART,HSD3B1,IFNB1,LCP2,LTB4R2,MAT2B,NFATC2,OR11H6,OR2M4,OR2V2,OR4P4,OR9K2,PDHA2,PIK3C3,POLA1,POLE2,PPAP2C,PPP2R1A,SSX2IP,TAS1R1,TMLHE,TRDMT1,WHSC1L1,WNT2,CACNA2D3,MAPKAPK3
Negative_2427	GDA,BCKDHB,BDH1,CDC14A,CXCL10,EFNA1,FABP4,FMO3,GALM,NRXN1,OR10J3,SPTAN1
Negative_2428	DLD,BMPR1B,DDX51,EFNA1,GADD45A,HADHB,MKNK2,NEU1,NFATC4,PLA2G5,RGS3,SHC1,TXNDC12

Negative_2429	GP6, MET, ALDH3B1, AMY2A, CDC45L, CHRD, CX3CR1, DDX4, GARS, GLUD1, IL20RB, NKX6-1, OR10J1, RASGRF1, RBPJ, RPS6KA2, RRM1, SETD1B, ST3GAL2, STX10, SUV39H1, PHOSPHO1
Negative_2430	CACNA1B, GAL3ST1, OR6C6, RBKS, SRGAP2, TPMT, TYMS
Negative_2431	IL2, SDS, ADH7, ALG13, ALG2, B3GALT4, CCNE1, CDO1, COL4A4, DVL3, GBGT1, GGCX, GNA12, GNA13, INPP4A, IRS4, MAP3K14, MAP3K6, MAP3K7, MTFMT, NT5C1A, OR10G3, OR1J2, OR2F1, OR2T10, OR4K17, OR52L1, OXSM, POLE, POLR1A, PPP2R5D, SGPP1, TPH1, TSC1, WASL, HSP90AA1
Negative_2432	AKT3, B3GAT1, GAB1, GNPDA1, KIR2DL4, NCOR1, SRGAP3, STAT3, STX3, TUBB2A, WNT2B
Negative_2433	FGB, LAT, SHH, TK1, ALDH5A1, ALPI, ARHGEF4, BDH2, C4BPB, CD79B, CDKN1C, COL5A3, DGKA, ENPP3, EPHB2, FGFR3, GGT7, GSTK1, HAGH, HIBCH, ICAM3, IL13, IL5RA, KIR3DL3, LDHAL6B, LTA4H, MARS, MTHFD2, MYH2, MYH9, NAT6, NKD2, NME4, OR2C3, OR2T2, OR6K6, OR9G1, PARS2, PDE11A, PIGP, PIGQ, SIAH1, SLC27A1, SPTLC2, ST6GAL1, SUCLG1, TRAF2, TUBA1A, WWP1
Negative_2434	F12, MUT, AKT3, ALPPL2, FLT3, GALNT9, GFPT1, LAMB1, OR8S1, PAPSS2, RRM1, SV2B, TP73
Negative_2435	AGRP, HARS2, MAP3K5, MGAT4B, NKX6-1, OR8J3, PANK2, PYGM, SPAM1, HLA-DPA1, SERPINA5
Negative_2436	CS, TNN, ALDH1A2, CSDA, EDA2R, GALNS, GRIN1, PDE1A, SDC4, UGT2A3
Negative_2437	ACSS1, ADH6, ARNTL, ARPC1A, B3GALT6, CAMK2B, FMO3, GCNT3, HDAC1, HLA-G, KIR3DL2, NAGA, OR10Q1, OR2L8, OR2T33, OR4N2, OR5K2, OR7G2, PAK3, PAK6, PKM2, POLA2, POLR3K, PPP2R1A, RARA, RRAS2, SARS2, STK3, STX2, TICAM1
Negative_2438	ACLY, ACOX1, ACPP, AMD1, AMDHD1, ATP2A2, CAMK2D, COQ6, CYBA, DHDH, ECSIT, FPGT, GUCY2D, IL18, IL28A, ITGA7, ITGB4, MAP3K4, NFATC2, NT5C, OR10A6, PDXP, PIAS2, PLAU, PMM2, RDH8, SETD2, SETD7, SUCLG1, TM7SF2, TP53I3, TRAF3, TUBB, UGCGL2, WNT2B, YKT6
Negative_2439	CD2, EBP, F8, ID4, RET, APOE, ARHGEF6, CDC20, CX3CR1, CXCR6, DDX18, DGKQ, F11R, INHBC, INPP1, ITGA10, MTMR2, MYH7B, NCF4, NEFH, NKX2-2, NME7, OR12D3, OR13C3, OR52N5, OR5K2, PISD, PLCD1, PPAP2C, PPP1R1A, PSME1, PTCH1, RBL2, RPS6KA5, SOCS7, SQLE, STX10, STX1A, TACR1, WBSCR17, WNT10B, TNFRSF13C
Negative_2440	C5, SRM, TEC, TNF, BTRC, CCL26, CCNG2, CSH1, CXCL16, DDHD1, DDX41, DDX47, DNMT3B, GPD1L, GYS1, IFNA21, MAPK14, MAT2B, MGAT4A, NMNAT2, OR10X1, OR11H6, OR2G6, OR2T34, OR52I2, OR6N1, OR6Q1, OR8H1, OXSM, PANK3, PDE4D, PGK2, PLCG2, POLR3A, PTDSS1, RARS, RASGRP3, RND1, SMAD7, SPTAN1, ST3GAL3, TIAM2, TP73, TUBA1B, TUBB2B, YARS, TNFRSF10A
Negative_2441	DDC, IL3, IL7, ACSL4, AHCYL1, ALOX12, ALOX5, ARG1, CACNA1F, CADM3, CD19, CHAD, CHRNA7, CREB3L4, CYP2S1, DDX23, DVL3, EARS2, GFPT2, GOT1, GSTA1, HBEGF, HLA-G, HNF1A, HNMT, MAP4K1, MAPT, MPDZ, NAGLU, OR2L2, OR2W1, OR4A5, PLCB4, POLR3G, PTK2, SDHA, SFRP4, TAS2R41, TUBA3C, PNLIPRP2
Negative_2442	ALDH2, ANAPC2, ATP5C1, B4GALT6, CCR2, CHEK2, ENPP7, F13A1, FGF2, GALNT5, HCST, ITGAD, ITGB1, LIPE, LIPF, MAP3K6, MGLL, MTMR6, NCKAP1L, NT5C1A, OR2D2, OR4A5, OR52J3, OR8I2, ORC6L, PAX8, PHPT1, PIM1, PON1, PPP2R1A, RAC2, RPRM, RPS6KA6, SULT1E1, TAS2R50, TCF7, THBS3, UNC5A
Negative_2443	C2, AVPR1B, CSF3R, DDX56, GSTM4, IL17A, MAP3K4, OR5L1, PI4KB, STX7
Negative_2444	C8G, TNN, ALAS1, APC2, APPL1, ATP6AP1, CASP8, CCDC6, CCND2, CYP3A4, GCNT1, GDF6, HARS, IL11, IL29, KYNU, LTBP1, OR1E2, OR4F5, OR6C74, PDIA4, PON3, PPP2R1A, RASGRP2, RFX5, TALDO1, ULBP3

Negative_2445	B3GAT2, CAPN2, CHST11, COX10, DLL3, FARS2, GRIN1, IFNW1, PRKAG3, RHEB, SETDB1, TP73, UGP2
Negative_2446	ACSS2, ARNT, ATP5G2, CHIA, ENTPD3, EPHB1, HMGCR, HTR6, IL1R2, LARS2, LIPG, MAP2K5, OR51L1, RPSA, STX5
Negative_2447	DLD, GSN, LPO, ADCY3, ADRA1A, ALDH1A1, APAF1, DBF4, HIBCH, IMPA1, MAD2L1, OR11H1, OR6Q1, PDE6D, PIAS1, PTDSS2, SELP, TUBA1B
Negative_2448	CBS, EDA, LPO, MAX, ACO2, APBB1, DFFA, DHRS4L2, FGF21, GALNT11, IRAK2, LHCGR, LILRB3, MYD88, MYH10, OR13C8, OR4S1, PDE3A, PLCG1, PLTP, RASA2, SSH3, STAM2, SUCLG2, TACR2, WARS, YARS, ATP6V1G1
Negative_2449	BTK, AGMAT, APOA2, ATP2A2, ATP5L, BMP4, CLDN3, COX15, CPOX, CRLS1, DDX18, DKK4, FZD5, GCLC, GNB3, HAGHL, IL22, ITGA6, JAK2, KDSR, KRAS, LARS2, LIPE, MAP3K12, MTHFR, NEU1, OR10V1, OR2D3, OR51B5, PHKG1, PPP1CC, PPP3R1, RXRG, SEMA3B, TAS2R10, THBS1, TKTL2, UCP1, VKORC1, TNFRSF13C
Negative_2450	ATG7, ATP5H, FGF4, FIGF, OGDH, OR8B8, POLR2B, YWHAE, HSD17B12
Negative_2451	C2, C5, CAD, CDA, IL3, IL9, ADCY7, ALS2, ANAPC7, ATP5O, BMP4, CCL13, COL5A1, CXCL12, CXCR4, CYP11A1, GLI3, HARS, IL23A, IL8RB, MAP2K2, MFNG, NADSYN1, NRG4, OR1D2, OR1D5, OR2T3, OR51F1, PCK1, PDE1B, PDE6D, PER3, PFKFB3, PGS1, PIGB, PRKAR1B, PRKCH, SARS, SLC25A4, TAS2R40, TAS2R49, WASF3, HSP90AB1, MAP3K7IP2
Negative_2452	HGF, ID4, MUT, NNT, ABCC8, ACAD9, AGMAT, ALPL, AMDHD1, ANAPC7, ARHGEF2, ATP5E, BNIP1, CACNB4, COQ3, CXCL9, DCTN1, FPGS, GNA13, GNMT, HES1, KIR2DL1, MALT1, MAML2, MMP2, NUDT2, OR2H1, OR2T6, OR3A1, OR51I1, OR5V1, OR6M1, PDGFRA, PLOD3, PRPH, PRPS1, PTGES2, PYCRL, RAPGEF2, SPCS3, STAT5A, STX4, SULT2A1, TGDS, TGFB2, HLA-DPA1, SERPINE1, SERPING1
Negative_2453	CD79A, HCST, MAPK7, NCAM1, OR51T1, TPI1
Negative_2454	FST, IL3, OSM, RDX, ACTN1, ALG6, ATP5F1, BTRC, CDC25C, CSNK1E, DUSP5, E2F1, GCDH, GCNT4, GNAI2, GNAT3, MYL9, NTN1, OR2A2, OR4E2, PLCB1, PPP2CB, SLC25A5, STX2, TAS2R50, TNFSF14, TUBA3E, UGT2B7
Negative_2455	SMS, ARPC5L, CACNA1D, CXCR6, MAP2K5, NOTCH1, NQO1, OGDH, OR52B6, PARD3, PKM2, PRKACA, PRKCH, RDH5
Negative_2456	OTC, SP1, CD72, CPT1A, CXCL6, KYNU, MYLK, OR2T10, RELA, TUBB2A, TYROBP, YES1
Negative_2457	CEL, FZD1, IFNA17, OR6K2, PAK4, PIGL, CTNNBIP1
Negative_2458	AK1, AGPAT2, B3GNT3, IFNA13, IL9R
Negative_2459	BPNT1, CAV3, CREB3L2, CXCL3, ECSIT, KIR2DL1, METTL6, OR9A2, PAX6, PDE1C, PI4KA, SEMA4C, SNAI1, TRAF3, TNFRSF10B
Negative_2460	EZR, ATP6V0B, BCL2, BMPR2, CDC23, CLDN5, COX15, CSNK1D, CYP11B1, CYP2C8, DHCR7, IARS2, IFNA7, IMPDH1, IRAK2, JAG1, MAPK1, NUDT12, OR10A7, OR13A1, OR2AG1, OR2T33, OR2T34, OR4C15, OR4P4, OR52A1, ORC3L, PCK2, PGAM2, PLAU, WARS2, PAFAH1B2
Negative_2461	AK5, AASDH, ACTG1, ALOX15B, APC2, ARHGAP5, C9orf95, CHEK1, CLCA2, CLDN18, CXCL9, DDB2, EPHB4, FCGR3B, FGF12, FGF13, GADD45B, GALE, HRH1, IL13, IL22RA2, IMPA1, LIPG, MGST1, OR10X1, OR2A4, OR2M4, OR2T10, OR51L1, OR52N5, OR5D13, P4HA1, PPARG, PPP3CA, PRIM1, PRKCE, SLC33A1, TSC2
Negative_2462	TAT, AADAC, ACLY, CCL20, CCNG2, CSNK2B, FTH1, GSTA3, GYS1, NEU4, SMAD4
Negative_2463	AGRN, GMDS, IL17RB, PPP2R1A
Negative_2464	A2M, C9, F10, GGH, TPO, ALG11, B4GALT6, CACNG4, CLDN19, DHDDS, DIAPH2, DNMT3L, EI24, EIF4B, EPAS1, FGF11, FZD8, GALC, GUCA1A, IL1R1, ITGA2B, METTL2B, OR12D3, OR52E2, OR52I1, OR52K1, PIGC, PLAT, POLA2, POLR2C, PRKCZ, RASGRP3, SKIV2L2, SPRY3, SERPINA1

Negative_2465	AR,BID,CD4,CRH,MAX,APOA5,ATP5G2,B3GALT4,BST1,C3AR1,CHPT1,CLDN14,CNDP1,CYP2J2,DGKA,DPYD,DUSP4,GLUD2,GPLD1,HPSE2,LAMC3,MAPK7,NOTCH2,NT5C,NTRK1,OR2V2,OR52N5,PDE3A,PER1,PGAM4,PLCB1,PON2,PPT1,PTPN5,TNFSF12,TUBB2C,UNC5B,ATP6V1G3,PPARGC1A,ST6GALNAC6,TNFSF13B
Negative_2466	MYH7B,SH3BP2,SLC27A4
Negative_2467	TPO,ACVR1B,CAMK4,CD247,NCR2,OR6Q1,AASDHPPT
Negative_2468	TNC,ACHE,ADORA2B,B4GALT2,BRAF,COQ3,CXCR5,CYP2D6,DHFR,EPHB3,EXT2,FARP2,FCGR2B,FGF12,GLS2,HEMK1,IKBKG,LLGL2,MCM3,NFE2L2,NOS1,NTF4,OR4D6,OR5B21,OR5V1,PAFAH2,PDE11A,PDGFC,PDGFD,PLAT,PPP2R5C,PPP3CB,SETDB2,TDO2,TUBA1A,UXS1,WNT2B,PRICKLE1,TNFRSF13C
Negative_2469	FGF3,KAT2B,NME4,OR2T5,SEMA3B,SMAD9,STAT3,WNT6
Negative_2470	CGN,AANAT,AGPAT6,APOA2,ATP4A,BLVRB,CACNA1A,CASP10,CCR2,DDX50,EFNA2,F13B,GRIN2A,GUCY1A2,HRAS,IL1A,ITGB1,LDHA,MTHFS,OR10V1,OR2T33,OR6N2,ORC1L,PMM2,PRKAR2B,RIPK1,RPS6KA3,SH2D1B,SMC1B,SULT1E1,TALDO1,TLR2,MAPK8IP1,PDCD1LG2
Negative_2471	EDA,ACTG1,AGPAT1,ALOX15,ARG2,ATP6V1A,BCKDHB,CTNNB1,FARSA,IFNA2,IFNA6,IKBKG,IL15,MYL7,OR2K2,OR51V1,OR5T1,PAK1,PDE7A,PGK1,PHKB,PHKG1,PLCD4,RBL2,RPE65,SREBF1,TAS1R1,UCP1
Negative_2472	GPT,ADCY1,ADRB2,CDO1,CES1,CHEK2,DDHD1,DPYD,ENTPD2,IL17RA,IRF9,KLRC2,LAMC3,MAP2K3,MCM5,MKNK2,MYH15,NAE1,NPR2,OR10G3,OR14C36,OR1E2,OR4D5,OR52E6,OR5M8,OR7G1,PLXNA1,POLE2,POLR1B,PPP5C,SNAI2,STX7,TYROBP,XYLT1,ARHGEF12,KIR2DL5A,PNLIPRP1,PPARGC1A
Negative_2473	CKM,CRK,RDX,BTRC,CALML6,CASP8,CD14,CDC26,CHAT,CHST11,CLDN7,CPOX,CSNK2A1,CTNNA3,CX3CL1,DGKA,DGKH,DGKQ,DUSP1,ENAH,GALE,GALNS,GALT,GGT1,IARS,IFNA7,INSR,ITGA1,MGST3,NRXN3,OR13G1,OR4S1,PDE3B,PDXP,PLCB4,RBL2,RBX1,RPE65,SOAT2,STX12,TMLHE,TRMT11,CACNA2D2,HLA-DRB4,HSD17B10,TNFRSF11A
Negative_2474	LCK,PLG,ARPC2,ATP2A1,CTNNB1,CX3CR1,CYP2C19,ENTPD4,EPHA6,FBP2,FGFR1,FZD4,GGT5,GJA1,HAGH,HSD3B1,IFNA14,IGF1,INPP5B,KITLG,KNG1,MGST3,NLGN2,OR1L3,OR1L8,OR2T2,OR52L1,PCCB,PSAT1,RDH5,STAT1,STK4,TNFRSF8,UGCGL1,PHOSPHO1
Negative_2475	ATF4,BCL10,COQ6,EPHA6,GUCY2C,HIF1A,TSTA3
Negative_2476	CCL24,OR52B4,SFRP4
Negative_2477	WAS,ADRA1B,ALPP,BRAF,CACNA1B,CDKN1B,CLTCL1,DVL1,EPB41L3,FPGT,IDI1,IFNA14,IL5RA,IMPDH1,INADL,KLRC2,LRRC4C,MAP3K14,MAPK9,MCM5,OR10G7,OR2T11,OR4F5,PANK4,PAPSS1,PARD6B,PCCB,PFN4,PGAM4,POLR1C,PPCDC,PSEN2,PVRL4,RBKS,SEMA3F,TOLLIP,VANGL2,B4GALNT1,CACNA2D3
Negative_2478	DDO,PAH,ACAA1,ACSS2,ALAD,APPL1,ARRB1,BST1,CD79A,CLTB,CNGA3,COX10,FGF20,FGF5,GNAI1,GYS2,ITGA3,LIPC,MADCAM1,NRXN1,OR52A1,OR6C3,PFKFB1,PPARA,PPP1R3A,RCHY1,RELB,RHOA,SCD5,SELP,SULT1A4,THBS2,THBS4,TPH1,TUBB4,UROS,VDAC1,VKORC1,WASF1,ZIC2,ATP6V0E1,ATP6V1C2,B3GALNT1,ST6GALNAC5
Negative_2479	C7,SRC,ADCY8,ANGPTL4,B3GNT5,CNTF,CRB3,DDIT3,HPRT1,IGF1R,LIPF,MYH7B,NOS3,OR4K15,PCNA,RALBP1,ST3GAL3,WARS,YWHAE,SERPIND1,TNFRSF14
Negative_2480	CUL1,LIPF,MAP2K7,POLA2
Negative_2481	C5,DCN,ARPC5L,ARRB2,B4GALT2,C1QB,CACNG2,CCL21,CD8B,CHST12,CREB3,DGAT1,DPM1,FGF9,GUCY1B3,GYS1,IFNW1,MFNG,NFATC1,NFYA,OR4D9,OR52E4,OR52E8,PLXNB3,PTGIS,RPRM,SLC25A4,ST8SIA5,STX2,TAS1R3,TDO2,TFDP1,THY1,TKTL2,PLA2G12A,PPP1R12A,SERPIND1

Negative_2482	GPT, OGT, TFG, TNF, AGTR1, AHCYL2, ALDH2, ALDH3B1, BLVRB, CAMK2A, CDC26, CHRD, CHRM1, CXCL5, CXCL6, CYP3A7, ECHS1, FTMT, GADD45A, GBA3, GFAP, GGT1, GNAT3, GNPDA1, IL20RB, IRAK2, JAG1, JAK2, JUND, MAP4K1, MIOX, NCR1, OR2M7, OR52R1, OR5F1, PARVA, PIP4K2A, PSME1, RDH5, SLC2A1, TICAM2, TUBB2B, TYMS, VEGFC, PDCD1LG2
Negative_2483	FST, CCL4, CHRM2, EGFR, OR11L1, OR2H2, OR4D1
Negative_2484	HDC, IL3, ACSL3, ALS2, APBB1, CASP9, CCNE2, CHRM5, DAPK2, DRD1, EHMT2, EIF4E, GAD2, GGT5, GUCY2C, ITGA11, MAPT, MYL2, NR5A2, OR10T2, OR2A25, OR4D5, OR5A1, OR8H1, PDE4B, PDXK, PPP2R2B, PROS1, SHISA5, TIAM1, UGT2B28, ATP6V1E2
Negative_2485	CR2, EDA, GP6, TFG, XDH, ACVRL1, ADH1A, ARHGEF4, BIRC5, CCL28, CCR4, CFL2, CIITA, CREB3L3, CSNK1E, CTNNA1, CYP2C19, DFFA, DOT1L, EPHA1, GAB1, GAS1, GRAP2, GSTK1, HTRA2, MAGI3, NEFH, OR13F1, OR2T11, OR4D2, OR7G1, OR8S1, P2RX6, PHKG2, PRIM2, PRKCB, PRKG1, RDH5, TBXAS1, TGFB2, TRAF3
Negative_2486	GLS, ACADVL, ACSL1, AGXT2, ALDH3A1, AMPD3, APOC3, ATP5J, CARD11, CDC16, DUSP14, EFNB1, FGD1, GALC, GALNTL5, GGPS1, GGT1, GNPNAT1, HAGHL, HRH2, IFNW1, INSRR, MICA, OR11H1, OR2M7, OR51F2, PGAM2, PPP1R3B, PYCRL, RARS2, TOMM40, VCAM1, WBSCR22
Negative_2487	KIT, AASS, ACSL3, ANAPC7, ARPC1B, ATP6V1F, B3GALT5, CYP19A1, DDOST, DDX19A, DTX2, ENTPD3, EXOC7, GARS, GNG13, H6PD, ICOS, NOTCH2, OR10C1, OR13C9, OR1L8, P2RX3, POLE4, SIPA1, TAS2R1, TGDS, TNFSF4, TUBB4, XYLT1
Negative_2488	SMS, ACOX1, ACTG1, BCMO1, CCL11, CTPS2, CXCL3, DUSP16, EHMT2, EPB41L3, FZD10, GNPDA1, GP1BB, HSPA2, HTR2C, KIR2DL3, MAGI3, MAP3K6, NANS, NCF1, NUDT12, OR2W1, PARVA, PDGFB, PIAS1, PIK3CA, PLA2G1B, PPAP2A, PPP1R3B, PTPN7, ROCK1, RPS6KB1, RUNX1, SLC12A2, STX10, ULK3, VNN1
Negative_2489	CBL, HYI, ADIPOR2, ALDOA, APOC3, CACNA1I, CCNB2, DDX54, EPHB3, GSTM5, PSEN1, SDC3, SETD2
Negative_2490	C9, CERK, DAPK3, DKK1, OR5P3, OR8J1, RASGRP3, WNT10B
Negative_2491	PML, ACYP1, ARPC1B, ATP5G1, ATP6V1A, CD274, CSF3, CXCL1, FDPS, GLS2, HADHB, IL1R2, IL22RA1, IQGAP2, KLKB1, NRG3, OR10S1, OR13F1, OR1F1, OR1L8, PDE3A, PFKFB3, PGAP1, PLAT, PTPMT1, RAF1, RDH11, SOCS4, SPTLC1, SV2B, TBXAS1, TYROBP
Negative_2492	ALG13, CNTN1, CNTNAP1, FBP1, GALNTL4, GBA3, GNAQ, HLA-G, HTR2C, IFNA4, IL12RB2, IL2RG, ITGA1, MAP3K13, NMNAT2, OR1A1, OR4D5, OR4F5, PDGFD, PIK3CG, PLXNB3, RAC2, SMAD9, TJP2, UGT8, WNT4, CACNA2D3, GABARAPL1
Negative_2493	F3, ACSM5, IGF1R, KLK3, NUDT9, OR9K2, SCLY, TALDO1
Negative_2494	CEL, HK1, HYI, TK1, TYR, UBC, ADH1B, AOC2, CACNB4, CCL11, CCL14, CCNA1, CD8B, CFL2, CREB5, CXCL10, CYP2A7, DAPK2, DDX50, EHMT1, ERO1L, FGF9, FZD1, GSTT1, HSD3B7, HTR4, KLRC3, LARS2, MAP4K1, OR10A2, OR11G2, OR52E6, OR6C6, OR6X1, PDGFC, PHKB, PRKCG, PTGS2, RRAS, SARDH, SOCS4, STX3, SULT1E1, THBS1, ULBP3, VANGL2, WNT5B, EIF4EBP1
Negative_2495	GNE, ADCY1, ARRB1, CXCL11, DUSP8, EHMT2, OXTR, TAS2R16, WHSC1
Negative_2496	ADRA1D, CYP21A2, FZD7, GNAI3, LYPLA2, SFRP1
Negative_2497	ADA, DAO, AKR1C1, AXIN2, GRHPR, GSTT1, IAPP, IL12A, IL3RA, KCNJ11, LCAT, LDHAL6B, LTBR, MAPK11, NLGN1, PFKFB1, PIK3R5, PPP1CA, PPP1R3A, PRKAB1, PRKAR1A, PVRL4, RAPGEF3, SDC3, STX12, SULT1A2, THBD, TNFRSF4, SERPINA1
Negative_2498	ACPT, APOA5, ATP5J2, CACNA1A, CD46, CFL2, CLCA4, DTX4, EPB41L3, FUT4, GALNTL4, GCH1, GLI2, GUCY1B3, IL18, IQGAP3, IRAK3, IRF7, MECR, NEU1, NOTCH3, NSD1, OR10P1, OR1L8, PFN1, PLCD1, PRKCQ, RPRM, RRM2B, STAT5B, STX10, TBK1

Negative_2499	C4B, CAD, JUP, MOS, OGT, ACVR1C, ATG7, ATP6V1H, CHAD, COL5A1, CYP2J2, DHRS4, EHMT2, FGF4, GMPR, GNG3, HSD3B1, IL20RA, ITGAM, MAD2L2, MAGI1, MGAT5B, NFATC2, OR10A2, OR2L3, OR4F6, OR51G1, OR52B4, OR5M3, OR9Q1, OXCT2, PIGW, PLXNB3, PRKAG1, PTEN, RAB5A, SDHA, SFRP1, SOCS2, TJP2, TUBB6
Negative_2500	ANPEP, KNG1, NRG4
Negative_2501	MVD, WAS, B3GALT5, CASP9, DGAT1, DUSP10, GUK1, ITPKB, SDHB, STAT4, YWHAZ
Negative_2502	AOX1, ARG2, CCL13, CCL15, CDH3, CRY1, CYP26A1, DBF4, DCTD, DUSP16, GLUD1, HIBCH, IDI2, IFNA13, IL28RA, OR2W1, OR7D4, PARD6A, PDE4D, PPP3CA, PTGFR, RAF1, TLN1, TRAF2, TUBAL3, YARS
Negative_2503	ABCC8, ADH4, AGTR1, AOX1, BMPR1A, CCNB1, DAAM2, GBA3, GPX7, NUMB, OR4A15, OR51A4, OR5K2, PSENEN, RALBP1, RFX5, SMAD7, STEAP3, TAS2R13, VDAC3, CACNA2D3
Negative_2504	DCN, NNT, SRC, ADIPOQ, ARPC5L, BST1, DUSP2, GLUD1, IL5RA, IPPK, MGST1, TAS2R38, TIRAP, HLA-DRB4
Negative_2505	C2, MOS, ATP5O, COQ7, CXCL3, ERBB3, GDF6, GLUD1, GSTM5, HCLS1, KIR3DL2, LGMN, NFKBIE, NLGN3, OR10K1, OR11H6, OR4K14, OR4N5, OR51M1, PNPLA4, PPP2R1A, SMPD2, TUBA8, UNC5C, ZBTB16, MAPK8IP1
Negative_2506	C8A, LCT, ADRBK2, ALG2, B4GALT5, BCL2, BHMT, CACNA1E, CRHR1, CXCL2, GAMT, GNPAT, HIF1A, IDUA, IGFBP3, IL22RA2, IL25, IL2RB, ITGA9, LEFTY1, LGMN, MYH4, OR2D3, OR4D1, ORC1L, OXTR, PDE9A, PGK1, PLCD3, RAB5A, RAC2, RAPGEF3, SKP2, SLC27A4, UGT2B28, AASDHPPT
Negative_2507	ACADM, ACPP, NSD1, OR1N2, PPP3R1, TAS1R1
Negative_2508	BID, F11, IDS, ACYP1, AGPAT4, ALG2, ARNTL, B3GALT4, CALML3, CD38, CD40LG, CD72, CERK, CSF2RA, DAPK1, DERA, DHCR7, DUSP6, EPHB2, EVI1, GALNT6, GUCY2C, IL6R, LAP3, MCM7, MTHFD1, MYL7, OLAH, OR10J1, OR3A3, OR4C16, OR7A10, PPP2R5E, RAB23, SLC25A4, STX1A, SULT2B1, SUV39H2, TGDS, TRHR, UROD, ATP6V1C2, SERPIND1, TNFRSF6B
Negative_2509	CGA, EPO, SFN, AARS2, ALDH1A3, ARNTL, ATP5G1, BTRC, CHDH, COX10, CRY1, CSNK1G1, CYP7A1, FMO2, FZD9, GNAZ, GNG12, IL6ST, ITGA3, KLRC1, KLRK1, LIMK2, MAT1A, OR2C1, OR2W1, OR4L1, OR5AC2, PDE11A, PIGA, PIGV, PTPRC, RARS, SEMA7A, SOS2, TCIRG1, UNC5A, ATP6V0A2, TNFRSF10A
Negative_2510	ATM, ACAT2, ACSS1, ARHGEF4, ARPC2, CDK4, DDX50, DUSP3, EXTL3, GART, GCNT4, HNMT, MICA, NAGLU, NUDT12, OR1Q1, PIM1, PLAUR, PTAFR, TAS2R9, WARS, SUV420H1
Negative_2511	FER, SMS, TKT, ACTG2, CCKBR, COQ5, EGFR, EGLN3, FDPS, OR10AD1, OR52A5, PDGFB, PIGA, PKMYT1, SOCS1
Negative_2512	CYP17A1, OR10Q1, SEMA4G
Negative_2513	DUT, FH, IL8, ACTN1, B3GALT1, BCKDHB, BRAF, BUB1B, C9orf95, CASP7, CCL17, CREB3L1, DGAT2, DUSP14, EPB41, F11R, GALNT9, GPAA1, IL15, IRAK1, IRS4, KIR2DL2, MPZL1, MYH15, NME6, NUDT5, OR1A2, OR4D9, OR5D16, OR8H2, OR8K5, PGLS, PIP4K2C, POLR3D, PRKCE, PTAFR, RASSF1, SDC4, SDHA, SESN3, SKP2, SPRY4, TBXA2R, XIAP, ATP6V1E1
Negative_2514	ABL1, ATP6V1F, CHDH, CPOX, CREB1, EPHA3, GADD45G, GDE1, HSD3B2, IFNGR2, ITGA6, KIR3DL1, LCAT, OR10R2, PIP5K1C, SNAI1, SRD5A2, VCAM1, WNT10B
Negative_2515	ADO, C1QB, CDH15, CREB3L2, DDX56, EPHB3, FGF5, GARS, HMOX1, IARS, IRAK1, NEUROD1, OR1L1
Negative_2516	PGD, ASNS, B3GNT3, BLVRA, CLDN2, CXCL6, ESPL1, IARS, PCK2, PIM1, ATP6V0A1
Negative_2517	ITGA10, MYL7, GABARAPL1

Negative_2518	SHH, ATP2A2, ATP6V0C, CACNG7, CDC6, COL1A2, DOCK1, EVI1, HCCS, HGSNAT, HSPB1, IFNAR1, MPDZ, NKX6-1, OR1S1, OR2V2, P2RX1, PTAFR, TGFBR1, WASF2
Negative_2519	MAG, SMO, ACADL, ATG3, CNGA3, DRD1, DUSP2, EREG, FLT3, FZD2, ICAM1, ITGA3, OR4F5, OR5J2, PIK3C2G, PITX2, PTPN6, PTPRM, SEMA3E, WNT1
Negative_2520	ACTN2, CASP9, CCL14, FGF11, FUT3, SDHC, HSD17B12
Negative_2521	BTK, FAH, GSR, SCD, SPN, AXIN2, BAAT, BCL10, CACNA1E, CD79B, CYP1A1, CYP26C1, FCER1G, FLT4, GALNT9, ISYNA1, MYH3, OR10T2, OR1J4, OR2T6, PANK1, PER3, PRKAR2A, PTPRZ1, SHC2, TALDO1, TAS2R16, TAS2R4, TNFSF13B
Negative_2522	BTK, ACSS2, ALG1, APPL1, BCL10, CADM3, CTNNA2, EFNB3, GMPR, GSK3B, HMOX1, LIPT1, MYH7, NFASC, NFKBIB, ODC1, OR2W1, OR4P4, OR52N1, PARD6A, PDHA2, SHC1, SPRED1, STK3, TAS2R8, TNFSF10, UPP2
Negative_2523	KHK, SMO, ALOX5, ATP12A, CADM3, CNTFR, DDX19A, DGKI, DPYSL2, EPB41L1, FGF9, FOXA3, GBA3, GDF7, HHIP, IL10RA, MYH7, NKX2-2, NLGN2, NUDT9, OR10A3, OR13A1, OR2Y1, PARD6A, PDGFA, PLA2G10, RAC1, RASGRP3, RASSF5, RPE65, SELPLG, SEMA4C, THBS2, UGT2B10, GABARAPL1
Negative_2524	CGA, FAS, IL2, ACY1, ALG10B, ARHGEF1, B4GALT7, CDK6, CNTNAP1, CXCL10, FARS2, FGF11, FUT7, FZD2, GNAI3, HIBCH, HIP1, HMGCS2, IL10RA, MYH13, NFKB2, OPLAH, OR6Y1, PGS1, PIAS3, PLXNB2, POLR1D, POLR3D, PPP2CB, RAB23, SLC12A2, STEAP3, TAS2R14, TM7SF2, TYMP, UBA7
Negative_2525	APP, ADCY4, B4GALT4, CD48, MCM4, OR2T11, OR4C11, OR52E2, OR56A3, OR5A1, SMARCA2, TPMT, UBA7, WNT2
Negative_2526	CRK, CCL7, FGF17, GGT1, OR10T2, PLAT, PRKAG2
Negative_2527	GSS, BCL2L1, CCL15, CDIPT, CSF2, CTBP1, CYP11B1, CYP2B6, E2F5, FGF20, LEF1, MEF2C, OR10G3, OR4X1, OR52D1, PDK1, PLTP, RAC1, SEMA4D, SOS1, TNFSF9, TP53I3, VEGFC
Negative_2528	MTR, ALAS2, ALG13, APLP1, ARPC5L, AXIN1, B4GALT1, CCNE2, CRHR1, CTNNA3, DERL1, DHRS4, DHRS4L2, DUSP7, EFNA1, EFNA3, FUT4, FUT8, FUT9, GAMT, GCLM, GLUD2, GNAS, HDAC1, ITGB3, MCM6, NEGR1, OR51D1, OR52R1, PANK2, PFKFB1, PFN1, PFN2, PGM3, PLA2G10, PNLIP, PPAP2B, PPP1R3A, RDH11, RUNX1, SFRP1, SULT1E1, TP53I3, VAV2, YWHAQ, PLA2G12A
Negative_2529	ALDH2, ATP6V0B, CCL25, CCND3, CD3E, CHRM2, CRLF2, DUSP5, EPHB4, HMOX1, HPSE, IL20, LAMB2, LDHAL6B, MTAP, OR52A5, OR5K1, OR6S1, P2RX6, PAK3, POLR3G, RAC1, SARS2, VNN1, WNT7B, YWHAZ, HLA-DRB3, HSD17B12
Negative_2530	ARNT2, B4GALT4, CACNG2, CCR2, CD58, CLDN20, CRB3, DGUOK, GCNT2, HTR4, IL8RA, L1CAM, MAPK3, MGLL, NANS, OR2K2, OR52A5, PCCB, PHKG1, POLD4, POLR1D, PSENEN, PSPH, RDH8, TPH2, UCKL1
Negative_2531	ADIPOR2, ALOX12B, APOA5, CLDN1, DCTD, IL12A, MAPK9, MYH14, NKD1, OR5AC2, OR6S1, OR8D2, POLE, POLR2A
Negative_2532	IL9, INS, SFN, SRF, VCL, ABAT, ARHGAP5, CACNG3, CD27, CD38, CD80, COL1A2, COL5A1, DPAGT1, ENPP3, FBXW11, FGF23, FLOT2, GNAL, HTR6, KAT2B, LAP3, LARS, MAP3K5, NPR1, OR2L3, OR4C15, OR8A1, ORC2L, PMVK, POLR2C, POLR3B, PTK2, PYGB, RAB5A, ROBO2, SUV39H1, USE1
Negative_2533	BTC, HAL, ACSM1, DMGDH, DTX1, GALNT9, GNG12, IDH3B, IRAK3, MAPK11, NEU4, OR11L1, PCYT2, PIGU, RDH16
Negative_2534	ID2, ARHGEF1, FUT1, HCCS, HNMT, KRAS, LAMB2, LEPR, NFE2L2, OR10G8, OR2L8, OR4K15, OXCT1, POLR3B, PTPRZ1, RPIA, SEMA4D, ST3GAL3, TUBA3E, SERPINB5
Negative_2535	RB1, CYP2C19, OR11H1, PTPN1, TNFRSF10D
Negative_2536	IL2, ANAPC2, BNIP1, DEGS1, DGAT1, OR4K17
Negative_2537	STS, CD28, DUSP14, ENO2, FRAT1, LAMB4, LIMK1, NR1D1, OR2T1, OR5B21, PIAS3, POLR1D, YWHAE, ZFYVE9, HLA-DPA1
Negative_2538	ACADVL, ACOX3, CHRD, DNMT3L, GARS, IL28A, IRAK1, KLK3, L1CAM, LFNG, MRAS, NCR3, PAX4, PIGM, PRKAR1B, PRODH, RARS, TGFBR2, TLN2, TNFSF9

Negative_2539	FAH,ADRB3,APH1A,APRT,CCNE1,GMPPA,GUCY1A2,IFNAR2,OR13A1,OR1A1,OR5B21,PLA2G2D,PPP2R5E,PTGDS,SC5DL,SHC4,SREBF1,SRGAP3,ST8SIA5,WNT2B,ATP6V0D1,MAP3K7IP2
Negative_2540	ADO,GK2,HK1,AGMAT,ALDH4A1,ALG13,ARAF,BACE1,CACNA1S,CASP9,CCR1,CD27,CFTR,CHUK,CSNK1D,CYFIP1,DDX47,DUSP10,E2F3,GPAM,HES1,ICOSLG,IL1R2,KIR2DL3,NADSYN1,OR10A5,OR10V1,OR4M1,PDE7A,PDXK,PRODH,PTDSS1,PYCRL,QPRT,SLC2A1,SMPD2,SPRED2,TCF7L1,TFDP1,WARS2,WNT11,ZIC2,ATP6V0A1,TNFRSF1A
Negative_2541	ACTN1,ALDOB,BCAT1,CLCA2,FOXA2,GCNT1,GLB1,GSTZ1,HNF1B,HSD17B2,HSD17B8,IL10,LAMB1,MALT1,OR1D2,PDE10A,PDE7A,PDE8A,PGS1,PLA2G2A,POLR1C,PRKACB,RFXAP,RPSA,SMAD6,SOCS4,SUV39H2
Negative_2542	AMT,DDC,A4GALT,ACPT,ACVR2A,ADRB2,ALDH4A1,ARRB1,BCL2,CCR7,CD44,CHRM1,CMPK2,CYP4A22,EI24,FGF14,GALNT12,GUCA1C,GUK1,MYH2,NAGLU,OR51D1,OR52K1,PDE4B,PIK3C2A,PITX2,POLR2D,PRSS1,RAF1,SHMT2,SRD5A2,TNFRSF17
Negative_2543	F12,ACOX1,ACSM2A,ATN1,CCL4,CD3E,CD58,CERK,CLDN5,CTPS2,CYBB,DPYSL2,E2F5,EFNA4,ENO1,EPHA3,EPHX2,FLAD1,FZD10,GJD2,GSTK1,HIF1A,IFIH1,INADL,INPPL1,KLRK1,NAT2,NR1H3,OR10V1,OR52J3,OR56B4,PIP4K2A,PIP5K3,PPP1R3B,PPP2CB,RAB23,RALBP1,SETDB1,ST3GAL3,STK36,TAS2R4,TCF7,TSNARE1
Negative_2544	FES,FUK,OAT,AFMID,ALG2,APOA5,CACNB3,CCNB2,CX3CL1,DUSP3,EFNA1,EPB41L2,EPHB3,ERO1L,FABP3,GMDS,LDHA,NCK2,NODAL,NOX1,OR1S1,OR6C76,PAK3,PFKFB2,PHGDH,PIGW,PLXNA3,PPP2R2D,PPP3CA,RAPGEF1,RENBP,SDHA,SMAD3,TSHB,UBE2L6,MAPKAPK3
Negative_2545	FH,AGTR1,GLUL,MYH3
Negative_2546	CDC20,CTSL1,CYP3A7,ENTPD7,IGFBP3,IMPA2,INPP5B,ITGB3,OR13D1,OR4K1,PFKFB2,PMM1,SLC25A4,SLC27A1,TNXB,TPI1,TNFSF13B
Negative_2547	ASMT,DDB2,HSPA1B,IFNGR2,LTA4H,MAN2A1,OR10C1,OR13C4,OR5F1,PAFAH2,PRKCQ,SEMA3B,SENP2,WASF2
Negative_2548	C1S,FGA,GGH,MYC,ACOX1,ALOX15,CDKN2A,CSH1,CTBP1,CYP2U1,DDX4,ELK4,ENTPD3,EPHA7,EPHX2,FZD7,GFPT2,GSTM3,GUCY2D,GUK1,HAO2,IL19,INHBB,JAG2,MAN1B1,MAP3K1,NME2,OR10H2,OR4D6,OR4K14,OR51G1,OR5A2,PDE6G,PLA1A,PLA2G10,POLR1D,PPP3CA,PVRL3,RAC1,SFRP1,SMAD2,SOCS7,STK11,TAS2R50,TKTL2,WNT10B
Negative_2549	ACSM5,FARSB,IARS,MAP3K2,OR52I1,PARVA
Negative_2550	INS,ACADSB,ACSS3,ALPP,BNIP1,CFL1,CHST11,CPT1C,EHMT1,FGF21,ITPA,MAP3K14,MLLT4,NCOR2,OR13C8,OR14C36,OR6Y1,PDE2A,PIGT,SUV39H1
Negative_2551	GRM1,OR5J2,PRKCD
Negative_2552	DCC,F8,HDC,ACTN2,BCL10,CHKA,FGFR1,IFNA17,IL18R1,IL7R,ITGB1,MTHFD1,NEFL,OR14I1,OR51G2,POLE,RAC2,RBL1,SRD5A1,SUOX,UGT2B7,WHSC1L1
Negative_2553	FST,AADAT,ACOX1,ALG10,B3GALT6,BNIP1,C9orf95,CASP10,DCTN1,DNMT3B,DUSP14,ETNK2,GCAT,HDAC1,HSPA2,IFNGR2,IL13RA1,NUDT2,OR4C46,OR5AR1,P2RX3,PGM1,PPAT,PPP1R3A,PRKACG,SMURF2,SOAT2,SPP1,TIAM2,UGT1A10,VAMP1,VTI1A,PHOSPHO1
Negative_2554	ADA,C9,EBP,GNS,LCT,LPL,PDC,ADIPOR1,AGRP,ALPPL2,AMPD3,BAIAP2,BBC3,CDKN2C,CTSS,CYFIP1,DAXX,DDX18,DDX54,DNMT3A,EDNRA,ENPP7,HLA-DMA,IDH1,IRS2,LYZL1,NCOA4,NLGN3,OR10H1,OR4F4,PDE4B,PIGN,PIK3C2G,PLK1,RAF1,RBX1,RIPK1,SELP,SMAD9,SPRED2,TAP1,WASF1,SERPINA1

Negative_2555	C3,GLA,OTC,ACVR1,BDH1,BHMT,CCNE1,CDH1,CHRM2,CSF2,CYP4A11,DLST,EPOR,FOXA2,IL12RB1,ITGA4,MGAT4A,OR2T33,OR4K14,PLCG1,PNMT,C1GALT1C1
Negative_2556	DGAT1,LPAR1,PIGQ,WNT9B
Negative_2557	BTC,MPI,ALOX12,CNTN1,CPOX,CRB3,DVL1,GNG13,IL19,IRS1,KLK3,NGEF,OR12D3,OR13C4,OR51B4,OR5B3,OR7A10,PAK2,PDE6H,PIGH,PIK3R2,PPM1A,RPS6KA5,SCP2,SEMA3C,TACR1,TAS2R16,TPK1,TUBA3C,UPP2
Negative_2558	KIT,AHCYL1,CD80,CNTF,CSF2RB,DHODH,EVI1,FRAT2,FZD4,HPRT1,ITGA10,ITGAL,SETD2
Negative_2559	LLGL1,OR11H4,PFKFB4,SESN1,WNT3,WNT5B
Negative_2560	ALDH3A2,VEGFC,WNT16
Negative_2561	CKB,AACS,ADCY5,AFMID,AOX1,ARPC5,CACNG3,CACNG6,CCL13,CCR2,CHSY1,CSF1R,DAG1,FGF10,GIT1,GMPR2,GRLF1,GSTT1,HIF1A,IFNK,IL28B,KLKB1,NAMPT,OR4B1,OR7D4,PECAM1,PPBP,PTGS1,RPS6KA2,SYNJ2,TYMS,TYROBP,CACNA2D4
Negative_2562	HAL,PDC,RFK,AANAT,ALOX15B,CDC23,CDS2,CHDH,CRY1,CXCL11,EDA2R,GSTM3,HAAO,HHIP,IL25,MTHFD2,NOS1,OR8D2,PEMT,PPID,PPP1CB,PPP2CB,PRSS3,SHC3,SOCS2,SPRY1,ST3GAL6,TIAM1,CTNNBIP1
Negative_2563	HGD,MOS,ATP6V1F,AXIN2,CACNG2,CAV1,CDC26,CDH15,CLDN14,CYP8B1,DDHD1,DEGS2,DTYMK,EHMT1,GALNT11,GALNT14,GMPPA,GMPR2,HEXB,HSD17B2,LLGL1,MMP7,MYH7B,NFYC,NMNAT3,NSDHL,OLAH,OR2T2,OXCT1,PAFAH2,PDE4A,PDPK1,PER1,PHKA1,PIK3R1,PIK3R3,PPP3CB,ROBO1,SMPD4,TRPC1,UNC5D,VEGFA,WNT9B
Negative_2564	AK5,FGG,NLK,ADORA2B,ALDH7A1,BCAT2,CACNG7,CASK,CCNG2,CHRM2,CLDN3,CXCL3,CYSLTR2,DAXX,EFNB2,FUT3,GALNT6,GNRH1,GPT2,IARS2,ICAM3,IGF1,IL2RG,IRS1,KCNJ11,MYH10,NEU4,NOX1,OR2AE1,OR2L2,PGAP1,PITX2,PKM2,PSME2,RFWD2,SDC2,SREBF1,TYMP,ATP6V1G2,CACNA2D2
Negative_2565	GMPPA,MGAT5B,OR10H4,OR13C8,OR5AR1,PNMT,STX1B
Negative_2566	MAX,MOS,AARS2,ACO1,ALDOC,ALOX5,AQP7,BMP6,CCL16,CD22,CDH2,CHIA,CLDN8,COL4A1,CXCL3,CYSLTR1,ENTPD3,EPHA1,EPHB4,FMO2,GAD1,IDH3G,IL19,IL2RB,IPPK,ITGA1,MAP4K2,NFKBIB,NFYB,OR2L13,OR2Y1,OR4K13,PAK3,RPN1,SENP2,SNAI1,SSX2IP,STK3,TAS2R4,TYRP1,WASF1
Negative_2567	AK3,CGA,IL3,ACOT12,ACSS1,ALDH1B1,ATP5H,B3GALT5,CASP3,CCL24,CD3E,CDH15,CLDN2,COL4A6,CYP2J2,FCGR3A,GRIN2D,IL17RB,IL1A,ITGA3,ITGB6,KLK3,LAMA3,MAN2A1,MAP3K12,NME6,OR10H3,OR2L2,OR4K15,OR51B2,OR5J2,PIGK,PLA2G3,PLD1,PMM1,PYCR1,RBPJ,RDH16,ROCK2,SORD,SPRY2,TAS2R42,TCF7,TNFSF9,ULK1
Negative_2568	ATM,BID,C4B,ID3,ASIP,B3GAT1,CD34,CNDP1,COQ7,DHRS4,DUSP14,DUSP9,ERBB3,GDE1,GNRHR,HRH1,HSD17B3,LAP3,MGAT4B,NOTCH2,OR10G7,OR4D2,OR7D4,PLTP,PLXNC1,SOS1,SULT2B1,TAS2R38,THBS1,TNFSF11,TOLLIP,TUBA1A
Negative_2569	APP,HGD,ID2,TH,ATP6V1D,CCNB2,COX10,CSNK1G1,CYP3A7,CYSLTR1,DUSP16,GALC,GCNT2,GLB1,GNRHR,IRF7,MAPK9,MCCC1,MYH9,NLGN1,OPLAH,OR51F2,OR6X1,P2RX7,PGM3,PHKA2,PIAS3,PLA2G5,PPID,PRKACA,SOCS3,TAS2R48,TNFSF14,UBE2L3
Negative_2570	CYP8B1,FGF13,FZD9,IL17B,KDSR,NFYB,NUMB,OR1L4,OR4K17,OR52N1,RBPJ,SOAT1,TAS2R8
Negative_2571	C6,SMS,ALDH3A1,B3GALT1,BAI1,BTRC,CASP7,CCNE1,CHST12,CREB3,CREB3L3,CYP11B1,ECHS1,FZD5,GMDS,GNMT,GPT2,IDI1,IL19,KIR2DL1,LCMT1,NFYA,NFYC,OR51L1,OR5K1,OR6T1,PCK1,PIM1,PRKX,PTGER1,RUVBL1,SPCS1,SQLE,SULT2B1,YWHAE,ZMAT3,ATP6V1G2

Negative_2572	MSN,ACSL5,BCL2,BMPR1B,CD48,ENPP3,GAL3ST1,ITGA4,OR10G2,OR10H1,OR10H2,PDE6G,SEMA7A,TSC1,CSNK1A1L
Negative_2573	CAD,FAS,GPI,ACHE,ALG6,BMPR1A,CHRD,CYP2C8,HYAL2,IRS1,ITGB3,LAMB3,NAT2,OR1I1,PLXNA1,PRKCE,PRKCI,PSAT1,SC4MOL,SETD1A,TJP1,TLN2
Negative_2574	GLS,AHCYL2,ATG3,CAV2,CCNA2,MYH13,OR4K14,OR5K2,POLR1A,PPARA,SLIT1,UCK1
Negative_2575	ACAA2,ACY1,APAF1,CD70,IL8RA,NTRK2,OR10AG1,OR2G3,PYGB,SETDB1,UCK2,ATP6V1G1,PPP1R12A
Negative_2576	A2M,AK7,DCC,DCK,EDA,OTC,RB1,AOC3,ARG1,BIRC5,CFTR,CHUK,CPOX,CTPS,DCTN1,DMGDH,FCGR3B,GLB1,GPD1,GSTA3,HHEX,HSD3B1,HTR2C,IL22,ITGB5,JUND,KIR3DL2,MECR,MGAT4B,NAT1,NCAM2,ODC1,OR2T2,OR51I2,PIAS4,PIM1,PKMYT1,RAD51,RUNX1,SPRY1,VARS,ATP6V1C1,MAP3K7IP1,TNFRSF1B
Negative_2577	HK3,IL8,ABLIM3,ACO1,ADH1B,ADIPOQ,B3GALT5,CASP9,CHKA,COL1A1,CTBP1,DKK1,DNMT3L,EHMT2,EPB41L2,FCER1G,GLG1,HARS2,IL20,ITGA2B,KITLG,MAP2K6,MTHFR,NAGA,NCSTN,NFATC3,NFKB1,NFKBIB,NOTCH1,OR13C4,OR1E1,PARD6A,PLAU,PLOD1,PLTP,POLR2F,PRKAR2B,SAT2,SPRED1,SPRED2,STAT1,STK4,THTPA,ULK3,YWHAH,ATP6V0D1,ATP6V1G1
Negative_2578	A2M,AK2,GDA,MVK,ACSL5,ATP6AP1,CANT1,CD27,CTSB,DDX4,DPM3,DUSP8,ENTPD4,FADS2,FCGR3A,FGF19,FSHR,GALNT1,GALNT11,IL28RA,IMPA2,MAML2,OR10J3,OR10S1,OR1L3,OR1N2,OR52E8,OR6C2,PIK3C2B,PIK3C2G,PLA2G2A,PLTP,PLXNA1,POLE,POLR3G,PPP2R2C,PTPMT1,RAPGEF2,SKP2,SLC2A1,SOCS5,SUOX,TAS2R10,VDAC3,WIF1,XYLT1
Negative_2579	CR1,ACO2,ACOT12,AKR1D1,ALG1,ALG5,ARPC5L,ARSA,B3GALT4,CSNK1G3,CTNNA3,CYBB,DDX41,GSTP1,ITPK1,JAK1,MASP1,MCM4,NFKBIB,OR10Q1,OR14C36,OR4C15,OR6C3,PDE8A,PIGK,PLCB3,PPARD,PPP2R5A,TAS2R7,ULK2,MAP3K7IP1,PDCD1LG2
Negative_2580	HK3,LPL,ALDH1A1,ARNTL,ATP5H,CBLB,CD22,CLDN18,CLDN22,CTLA4,CX3CR1,FARS2,FARSA,FMO4,GADD45B,GLG1,GLUD1,GRIN2C,HARS2,IDUA,IFNA4,KIR2DS5,MAD2L1,MMP2,OR2G6,OR52W1,OR8B8,PARD6G,PIGS,PIP4K2B,PIP4K2C,PLCD4,PLOD1,POLR1E,POLR2K,PPP1CC,ROBO2,SOCS1,SPRY1,STAT1,THY1,ST6GALNAC5
Negative_2581	CCNG2,DGKZ,EDNRB,GSTA4,IL12B,OR2T10,PDGFA,SOCS7,UCP1,HLA-DRB3
Negative_2582	AKT1,B3GALT5,CTSB,DAAM1,ESAM,NAGLU,NOD1,OR51S1,RFX5,TBK1,HLA-DQA2
Negative_2583	AR,ACSM3,APLP1,CDC7,CDKN2A,CYSLTR1,DHRS4,EPHA1,GAL3ST1,LRAT,MAP3K6,NAT6,NTN1,OR7G3,PMVK,RDH8,RERE,RFNG,SETD1B,TAS2R41,TNFSF11,USE1,PDCD1LG2
Negative_2584	F2,BNIP1,DPYSL2,GLUL,GUCY2F,MAP2K1,MYH4,PLA2G2A,PLOD3,ULK3
Negative_2585	AHCYL2,EDNRB,GAD1,IL1R2,LRAT,NTF3,PIK3CA,PRODH2,RFX5,ATP6V0D1,MAPKAPK2
Negative_2586	FAH,FUK,IL7,MET,AGPAT4,ARNT,CARS,CCNE1,CD55,CDH1,CEBPA,CSNK2B,DDX23,DKK2,FDPS,FGF20,GNAO1,HSPB1,INHBC,KRAS,MCM5,METTL6,MTHFD2,NEFH,NKD2,OR4N4,OR4S2,OR51S1,OR5AK2,OR5D18,OR5P3,OR5R1,OR5T2,OR6A2,OR7C1,P2RX2,PIGQ,PLD1,POLR3H,PPP2R5D,RPS6KA3,SLC25A5,STAM2
Negative_2587	CFH,CGA,KDR,MET,MPZ,MYC,ASCC3,ATP5J,B4GALT5,CASK,CD70,CDK2,CDK6,CTSB,ENPP6,ENTPD3,EP300,FOXA2,FZD9,GALK2,GALNT5,GCNT3,HAP1,IARS2,IL20RA,MAP4K1,OR13A1,OR2G2,PARD3,PLXNC1,PRKCA,PRPH,PTPN1,PTPRB,RPS6KA5,SEMA4G,SMURF2,SV2C,TUBB,CACNA2D4,TNFRSF14

Negative_2588	TEC, AACS, ACSM3, ADCY3, AMHR2, ASPA, CACNB2, CALML5, CD247, CD38, CFL2, CNGA3, CSAD, CYP4F2, DIAPH1, EFNB1, FGF8, FLT1, GALNT10, GRIA1, GSTA2, HMGCS2, IFNA7, IL10RA, KLRK1, LIPF, LRRC4C, NSDHL, OR10S1, OR2T29, OR51I2, OR51Q1, OR51T1, OR5I1, PCYT1B, PDE11A, PDE1A, PDGFRB, PI4KA, PRODH2, PTGS1, RPS6KA4, SELL, SOS2, STAT4, THBD, TLR9, TXNDC12
Negative_2589	LYN, CACNB2, CAMK4, CHST12, MDH1, OR11A1, OR2A4, TJP1, YARS2
Negative_2590	FGA, RFK, AANAT, ACSM5, AMPD3, ARRB2, AXIN2, BCL2, CCNA1, CDH2, CHRD, CLDN16, CYP21A2, CYP2A13, EIF4E2, GGT1, GMPR2, HCCS, INHBA, ITGAE, LAP3, LIMK2, MTHFD1, NAGLU, OR1E1, OR6C2, OR7C1, PDE10A, SEMA4B, SHISA5, SPRED1, STAT5A, TAS2R7, UPRT, B3GALNT1, PAFAH1B2
Negative_2591	ATM, AREG, CCL14, CDS2, FGF11, G6PC2, GALNT5, GGT6, HCCS, NCKAP1, NME2, OR10A5, OR10J1, OR2A4, OR5L2, PFKFB3, PFKM, SPRY4, ST8SIA5, TAS1R3, TLR9, VAV2
Negative_2592	CHRM1, CSF2, FGFR3, INPP1, MAPK7, OR4K14, OR8H3, SSH3, EIF4EBP1
Negative_2593	ADC, CKM, FN1, PAH, ACAD8, ACTG2, ADCY8, ADRB3, ATP5A1, CARD11, CBLB, CES1, CHKB, CYP2F1, DAPK1, DDX41, FGF18, FRAT1, GSTZ1, HEMK1, KAT2A, LDHA, MYH13, NADK, NRG3, OR2L2, OR2S2, OR3A3, OR7C2, P2RX1, PARVA, PCYT2, PPM1A, PPP1CB, PPP2R2C, PTGS2, SETDB2, SMAD5, SOCS1, TRAF2, MAPKAPK2, PHOSPHO1, SERPINA5
Negative_2594	ME1, MUT, OTC, AASS, ARAF, ATP5G1, BMP2, CACNG8, CCNB1, CLOCK, CYP2A7, DNMT3L, EPB41L2, FGF19, FGF3, GFPT1, GMDS, GUK1, HEXA, IFNA8, IGF1, JAG2, KIR2DS4, MAP3K3, MMP7, OR8H3, ORC5L, PRKCQ, RFWD2, SGPP1, TCF7L1
Negative_2595	LCK, TAT, ALDH3B1, BCL2, CCL20, COQ6, CSNK1G3, EPHX2, EVI1, F11R, GAPDH, GAS1, GGT6, GRIN2B, IL4R, INPP4A, MAGI3, MAT1A, MYH10, MYH15, OR5T2, OR9A4, PAK1, POLE3, POLR3H, PRKACG, PTPN5, PTPRM, RASA1, RDH11, SLC2A1, TPK1
Negative_2596	C1S, F9, ERBB3, IL15, RAC1, WNT1
Negative_2597	ADC, C4B, SDS, SP1, AACS, ACPT, ALDH3B1, ARPC4, ASPA, BPNT1, C1QB, CNTFR, CYCS, ERBB4, GADD45B, GRIN2D, HPSE, HPSE2, ICOS, IRS1, L1CAM, LDHD, MGAT4A, NFX1, NUDT2, OR2AE1, OR2M2, OR2Y1, OR4A16, OR52M1, PLA1A, PTPRF, RBL1, SETD2, SNAI2, MAPKAPK3
Negative_2598	F10, HPD, PML, UBC, ACSS3, ARRB1, GCLM, HRH1, IGFBP3, KIR2DL2, LAMA3, LTA4H, MASP2, OR1D2, OR2T10, OR52M1, OR5AU1, PAK7, PARD6G, PDX1, PLCG2, POLD2, RDH12, RFXANK, TRADD, TUBA1A, UGT2B10, UNC5A, VDAC2, MAPKAPK2, SERPINE1
Negative_2599	ASL, BAX, ACADSB, ACVR2B, APLP1, C5AR1, CD34, CLDN3, EPHB2, HEMK1, IDH3G, LAMB4, NFKBIE, OR4F5, OR51A4, PDE1A, PLK1, RAC3, RUNX1T1, SLC2A2, SNAI2, TKTL2, ULBP2, KIR2DL5A, MAP3K7IP2, MAPKAPK5
Negative_2600	ADK, SPN, ACACB, CIITA, HADHB, ICOS, IRAK3, MAT1A, OR13A1, OR2D3, OR4A15, PLA2G5, SELP, TOMM40, UGT2B17, ATP6V1C2, GABARAPL1
Negative_2601	FAS, LPL, CYBB, DAPK2, DDX47, FLOT1, GRM5, MAT2B, NOTCH2, NPR1, OCRL, OR2L2, OR2Z1, PPP1R1A, PTPRJ, RPIA, SCD5, SCIN, SETD8, SULT1A2, TCF7L1, TGFB1, UCP1, ST6GALNAC5
Negative_2602	CACNG1, GALNT2, IDH3G, IL10RA, ITGA10, OR10Z1, OR2Y1, PAX6, PLA2G2D, PRODH2, RHOQ
Negative_2603	IL9, CHRM5, CXCL11, FASLG, FGF6, GGCX, IFNA4, IRF3, MAP2K6, OR10J5, PSENEN, RENBP, ROBO2, TAS2R5
Negative_2604	CYP4F2, EI24, FSHB, IFNA14, PDE9A, PDGFRB
Negative_2605	CDC7, PIGQ, SULT2A1
Negative_2606	FYN, ABL2, ACSL5, ALG12, ATP5J, B3GNT5, CDH2, CES7, CLDN20, CPT1A, CYP2J2, DGAT2, DGKB, DTX4, FLOT2, GSTP1, HADH, ITGAL, LAMA4, LCP2, NAT5, NFATC2, NOTCH3, NTF4, OR2B6, OR2H1, OR6C1, OR9Q2, PPP3CA, SH2D1A, TJP1

Negative_2607	TAT, TYR, BCAT2, CHAT, GNB1, IDH2, IRS2, LIMK2, NCAM1, NCOR1
Negative_2608	C2, CBS, MET, SMS, VCL, WAS, ADRA1A, ALG3, AOX1, ARNT, C9orf95, CCR3, CFL2, CHST13, DKK2, DUSP1, EXT1, FGF8, HADHB, INPP5A, LDHAL6B, LLGL2, MPP5, OR13C4, OR1I1, OR2L8, PAX8, PKM2, PLAUR, PPM1A, PPM1D, PPP1CB, RDH5, SFRP5, TNFRSF4, TUBA1B, CSNK1A1L, PNLIPRP2
Negative_2609	MVK, ADH1B, ATP5C1, CCR10, CRKL, DKK1, DTX2, ENTPD1, FADS2, GRIN2C, HADH, HNMT, IGSF5, MCM3, MYH7, NKX2-2, NUDT9, OR1F1, OR2M4, OR52E4, POLR1B, PRKAG3, RASGRF2, TGFB2, XCL2
Negative_2610	MPI, AASDH, ADCY8, ADRB3, AHCYL2, CACNA1I, CAV3, CTSS, EFNB3, ENPP7, GSTA4, GUCA1A, HHIP, LAMB2, MAPT, MITF, NCF2, NEU1, NME6, OR4X1, OR51A4, POLD1, PRKAR2B, PRODH2, SLC27A4, SNAP25, SRD5A1, TAP2, TAS2R50, TSNARE1, VNN1, SERPIND1
Negative_2611	AGK, DBH, NF1, ACADSB, ACOT12, ADCY2, AKR1B10, AOX1, APH1A, BDH2, C3AR1, CACNG8, CCL28, CLDN5, CTSS, DHRS4L2, ENTPD7, FGF10, FGF9, GALNTL1, GCAT, GUK1, HMOX2, IBSP, IL23R, IL2RA, MAP3K7, NCF2, OR10G2, PARD6G, PI4KB, PRKCH, PTPRF, SLC2A2, TAS2R40, TBXA2R, UCK1, VAMP2, ATP6V0D1, HLA-DRB4
Negative_2612	DHH, ADORA2A, AGTR1, ATP5A1, CACNB4, CEBPA, CNDP1, DNMT3L, FTCD, GLB1, GSTM2, GUK1, MAT2B, RDH11, RPS6KA2
Negative_2613	AGL, C8G, CFI, PXN, ACTA1, ACTC1, ACY1, ADH1A, ARRB1, BAAT, CACNB3, CCR5, COMT, COQ6, CSH1, CYP2C18, CYP2S1, DOLPP1, FGF17, FPGT, GALNT9, GLUL, HADH, HSPA1B, HTRA2, IL15, LAMB2, MAP2K2, MARS, OR1Q1, OR4K1, OR52L1, OR8J3, PAK6, PPCDC, RXRA, SMAD6, STAT2, VAV2, ZFYVE9, ATP6V1B1, HLA-DPA1
Negative_2614	GGH, IL3, ACAD9, ACP6, ACVR2B, ADH6, CBR3, CCL26, CD55, CDC7, CNGA3, CRY2, CTBP2, EARS2, F2RL1, FTMT, GALNTL1, GCNT4, GNAI3, GNG3, GNRH1, GRIN2C, GSTO1, IL2RB, JAG2, LDHAL6A, LMO7, LTBR, MCCC1, MFNG, NCF1, NFKBIE, OR2T11, OR4K17, OR6C4, PIAS1, PITX2, PLA2G4A, PPCS, RAC1, SEMA4B, SLIT2, UGT2B7, PAFAH1B2, TNFRSF18
Negative_2615	CRK, ACAA1, ALDH3B2, BMP8B, CDS2, CES7, CHUK, CUL1, CX3CL1, CXCR3, DDHD1, DUSP16, EPHA4, FGFR3, GNPDA2, GRIN2B, HLA-C, HYAL2, ITGB1, LAMB3, MYD88, MYL2, NFATC1, OR2AG2, OR4C15, OR4F17, OR7A10, OR7C2, PDE10A, PPARD, PPP2R2B, PYGM, RALA, RARS2, RETSAT, RPS6KA1, SMPD2, SNAP23, STK4, STX16, TPI1, WNT9B
Negative_2616	MET, CACNA1E, CD34, CLCF1, DUSP14, GFPT1, GLUD1, IFNA21, KIR2DL3, NCR2, NPR2, OR10A3, OR10G3, PDE8A, PFN4, SDHC, TCIRG1, TFPI, UBE2L3, UGT1A10, WHSC1, TNFRSF1B
Negative_2617	ACOX2, ADH6, B3GALT6, CD40, CYP2D6, DAAM2, EGLN3, FLOT1, GGT1, GJA1, GPD1L, HTR7, IL17RA, MGAT2, MYH6, OR8H2, PDE10A, POLR1A, RALA, RARA, RPS6KA3, SLC2A1, SMC1A, SOCS7, SPTLC1, SUCLA2, TM7SF2, TPK1, TNFRSF1A
Negative_2618	LAT, ARPC5L, ARRB1, ATP2A3, ATP5G1, CACNA1A, CDC14A, CLDN2, CNTN2, CXCR6, CYBA, CYP2S1, EFNB2, FGF6, FOXA3, GZMA, HNF1A, HSD17B2, ISYNA1, MAP3K8, MGAT5B, MTMR6, NCR2, NEFL, NFASC, OR4M1, OR51F1, OR52B6, OR5T1, OR6Q1, OR6Y1, PIAS2, PIGL, PPP3R1, RALBP1, RBPJ, SEMA3C, STK11, THBS1, TJP1, UGP2, PPP1R12A
Negative_2619	MVD, CCL26, CLDN2, MAPK8IP3, SERPINE1
Negative_2620	CKB, ACSL6, ATF2, ATG7, ATP6V1A, CD14, COL4A4, CSNK1G1, CTSB, CYP3A7, DDX47, EFNA4, ENPP3, FSHR, GUCA1A, HSD3B1, HTR2A, IRS1, MAP3K5, MGAT5B, MICA, MLYCD, NTN1, OR2F2, OR4P4, OR7G3, PFN1, PIK3C2A, PLA2G3, PTPRB, RPS6KB2, SMAD3, SRD5A1, TNFSF4, VAMP3
Negative_2621	SDS, ALDH9A1, GALNT9, TAS2R41, TOLLIP
Negative_2622	ACO2, CRLF2, CYP3A4, FIGF, FZD3, NEFM, OR13C2, OR4Q3, RELB, SUCLA2, TAS1R2, TGFBR2, WBSCR22

Negative_2623	AUH,CBS,EGF,ID1,SMO,ACSL3,ALDH5A1,B3GNT5,BMP4,BUB1,CLDN1,CXCL11,CYP26B1,DCXR,HLA-F,HSD17B3,IL24,IMPA2,ISYNA1,NT5C,OR13C5,OR5AP2,OR6N1,OR9G1,PLA2G3,PTGS2,RPS6KB2,SMPD2,SSX2IP,STX1A,TAS2R1,TAS2R14,TRDMT1,UNC5D
Negative_2624	AK2,CR2,GAA,GSR,ACAA2,ACSM2A,AGPAT3,ARNTL,ATP6AP1,C1QA,CDO1,COL4A6,DDOST,GAD1,HSPA2,IDI2,IKBKE,ITGA8,ITGAM,LIFR,LYPLA1,MAPK1,NLGN3,NTRK1,OR5AS1,PIP5K3,PLD1,POLR1B,POLR2K,PRIM1,PTDSS1,PTGER3,RASGRP4,SHMT1,SPAM1,SUOX,UPP2
Negative_2625	CAD,GH2,IL5,LCK,ADCY9,ALDH3A2,ALOX15,ARAF,ARPC1B,BDKRB2,CCL28,DHRS4,EFNB1,FLT1,GNPNAT1,IRS2,JAM2,MAP3K14,MGLL,NOS1,NOX1,OR10A4,OR10Q1,OR52N4,OR6C1,OR7A17,PANK1,PDGFA,PPM1A,PRKCZ,PSAT1,SPRY1,UAP1,UGT2B7,ULBP3,YWHAB
Negative_2626	SCD,ADIPOR1,ARRB1,CCL15,CISH,MAPK12,NKX3-1,OR1C1,OR52N5,OR8U1,PPP1CC,PTEN,RRAS,SPTAN1,ZMAT3
Negative_2627	CP,ACTG2,CD82,CPOX,CREB3,DGKG,DHDDS,FGF20,GGT6,GLI1,GMPPA,HADHB,IDH3A,LTA4H,NRG1,OR2Z1,PI4KA,PIK3R5,SMAD3,TAS2R50,YARS2,CACNA2D4
Negative_2628	CSNK1G3,HADHA,OR10P1
Negative_2629	IVD,LTB,ARHGEF6,BCKDHB,CERK,CLDN20,CTF1,CYP3A4,DGKH,GADD45G,GLI3,HEXB,IL2RG,MDH1,MLLT4,MTHFD1L,OR56A3,OR5L2,PLCB4,PSME1,PTPN11,SC5DL,ST3GAL3,STAT6,TAS2R48,UPRT,UROC1,ATP6V0D1
Negative_2630	C2,CBS,IL7,ACOT11,ACVR1,ALG10B,ATP5L,B3GALT5,CACNA1H,CADM3,CALML5,CASP9,CD34,CLCF1,CXCL3,CYP26C1,DCTN1,DERA,FZD4,GALNT14,GSTA1,IL1R1,IQGAP2,MAP3K12,MYLK,NCSTN,OR10H5,PIGX,PLCD1,PLOD3,PON3,SIGLEC1,ST3GAL4,TP73,CACNA2D3,HLA-DPB1
Negative_2631	APP,IL2,PGF,ATP6V1D,DAPK3,DEGS2,FARSA,FPGS,G6PD,GNAL,GNG3,MDM2,OR2A14,OR6B2,PARVG,PPP2R2A,SOCS2,TNFRSF9
Negative_2632	ADRA1D,AMPD1,IFNA16,INHBB,NRXN1,PHGDH,PLAU,SMC1A,STX7
Negative_2633	GPT,INS,ACO2,ACP2,ACP6,ACSS1,AGXT,ASPA,BAIAP2,DLAT,ERO1L,FARSA,FGF21,GART,GLO1,HMGCS2,HSD17B2,IL10,IL20,IRS4,KLRC3,LIMK1,MAML1,MAPK9,MTMR2,NME2,OR10G7,OR14C36,OR2L3,OR51B4,OR5K2,OR6C74,P2RX6,P2RX7,PHKA1,PYGM,RUNX1,SLC27A5,SMAD1,SMC1B,STX1B,THBS3,TUBB6,UCKL1,UNC5C,WNT1,XYLT2,HLA-DQB1
Negative_2634	RPE,ABL1,ADCY7,ATP5H,B3GNT5,DUSP9,FMO3,GALM,GRPR,ITGA10,MECR,MTHFD1L,NFATC2,OR2Y1,OR52E2,OR5J2,PARVG,PER3,PON1,SOCS1,SUV39H1,ATP6V1G1,CACNA2D4,MAP3K7IP2
Negative_2635	ADK,C8B,CR2,ACO2,AMDHD2,AMPD3,ASRGL1,ASS1,ATP6V1A,B3GALT6,BMPR1A,CCL22,CCR8,CERK,DGKI,DTX4,DUSP8,EFNB2,EXOC7,GALNT11,KLRC4,NME4,NTN1,NTSR1,OR10H4,OR10J1,OR11G2,OR2B2,OR4K15,OR5M9,OXCT2,PARVA,POLD1,PRKCG,RRM2,SCD5,SNAI1,TACR2,TRAF3
Negative_2636	CR1,FES,SCD,SRF,ACSM5,AMDHD2,BCAT2,BHMT,CACNA1S,CDC25A,CDKN2B,CLOCK,CLTA,CSF3R,CYBA,CYCS,CYP2S1,DERL1,DVL1,EPHA4,GNPDA1,GPLD1,GRLF1,GSTA2,ITGB2,KIR3DL3,MAD2L1,MMP9,MYH6,NAE1,OR11L1,OR1B1,OR51V1,ORC5L,PAFAH2,PTPRB,SLC25A6,STAT4,TBL1X,YWHAE,TNFRSF13B
Negative_2637	BAD,ITK,ALDH1L1,AMHR2,APOA5,ATG3,ATP5L,CNTFR,CTNNB1,DUSP5,EHMT1,FABP1,GALNS,GCAT,HMGCL,HSPB1,HTR4,LAMC2,LIAS,LYPLA2,MCM3,OR13C9,OR2A42,OR2M3,OR7D2,OR8B4,PDE7A,PIK3CA,PTCH2,PTGFR,PTGIS,RAC1,SENP2,SOCS3,STEAP3,SUFU,TACR2,UGT2B17,UGT2B28,WNT16

Negative_2638	C4B, C6, EGF, FER, GAA, ADIPOQ, ALS2, CCND3, CCNH, CCR10, CD8A, COL6A2, CREBBP, CYFIP1, EPHA1, EPHA2, GAS1, GJD2, GNPDA1, ICOSLG, IFNA16, IFNA8, IL17RA, IL6R, MAP3K5, MAPK12, NKX6-1, OR2L3, OR5AK2, PCNA, PVRL3, SARS, SLC2A4, SMPD1, SPTLC1, ST8SIA5, TUBB, ARHGEF12, ATP6V1E1, SUV420H2
Negative_2639	ASL, C3, EGF, F10, MSN, ABAT, ABL2, ARPC5, BAAT, CACNA1G, CANT1, COX15, CYP3A5, DDX18, DUSP8, E2F1, EDNRA, ELK1, GLYCTK, GSTA1, ICAM2, IRF9, LAMB1, MARS2, MCM6, MTFMT, NFKBIA, NR5A2, NT5C1A, OR1B1, OR9G1, PDE6D, PDGFRB, PDIA4, POLR1E, PPP1R3A, RAPGEF3, RCHY1, RPIA, SELE, TAS2R10, TNFRSF8, UROD, VCAM1, ATP6V0E1
Negative_2640	CCNE1, CDK5, CLDN7, CXCL6, ENTPD2, ICAM2, LDHC, NT5E, WNT8A
Negative_2641	PGP, ACAA1, ADH1A, BLVRA, C9orf95, CCNB1, CCNH, COQ6, CSF2RB, CSNK1D, EPHA8, FLT3LG, FLT4, G6PC, GRLF1, HTRA2, IL10RA, IL25, IL8RA, KLRC2, LAMB3, MGAT1, MGAT5, MLYCD, NT5C2, OGDHL, OR1S2, PAPSS2, PDGFD, PIK3CB, PPID, PPP1R1A, PPP2R2B, RUVBL1, SDHB, SFRP1, SFRP4, SLC25A5, SOAT2, STK3, STX3, TAS2R13, ULK3, YARS2
Negative_2642	E2F3, EFNB1, FMO3, GALNT7, IL7R, MAPK14, MDH1, MGST2, MMP9, OR2B6, PIP4K2C, POLR2G, PPP1CB, PRODH, WHSC1L1, TNFRSF14
Negative_2643	AGL, CAT, ACADSB, ADCY6, ADSL, ALDH1B1, ALG11, APBB1, ARPC1A, CACNG4, CER1, COQ5, CREB3L4, CTNNB1, CTSL1, DDHD1, DGKE, EDA2R, EXT2, GNRH1, GPD1, HMGCS2, IL13RA2, KLRD1, LIPF, MAP3K8, MGAT2, MYLK3, OR11H1, OR1G1, OR1L4, OR4D6, PAK3, PIK3R2, PLA2G2F, PORCN, RAP1B, RBL1, ROCK1, SMAD1, STAT3, TAS2R38, UGT2B7, WASF3, WEE1, HSP90AB1
Negative_2644	ACO1, ACOX2, TLR2
Negative_2645	FER, HAL, IL3, PVR, ACVR1B, ANAPC2, BMP7, CCDC6, CDKN2D, CLDN16, CRB3, CXCL5, DFFB, GLUL, HTRA2, IFT57, IL11, IL22, IL2RB, IMPA2, IRAK1, IRF5, LLGL2, MYL5, NEFH, OR1L3, OR2L13, OR52W1, OR5P3, OR8K3, OR9A2, PAK7, PDE4B, PIP5K3, PORCN, PPARD, PPP1CC, PPP3CC, PTTG1, SDC3, SEMA7A, SENP2, SESN2, SMAD5, SOAT1, TDO2, VAV2
Negative_2646	C8A, ALG13, BDH1, CREBBP, FMO3, FRAT1, MYH11, NPR1, OR14C36, OR14I1, OR51B4, PDE2A, PLA2G4A, SARS, SH2D2A, HSP90AB1, PAFAH1B1
Negative_2647	CR1, F7, FAS, HAL, NLK, STS, ACP6, AGRP, ALDH3A2, AOX1, APOA5, AVPR1B, B3GNT3, BDKRB1, CACNA1E, CD3E, CDC42, CDK7, CDKN2C, CLDN4, CPS1, CYCS, DERA, EFNA5, EXTL3, FUT7, HARS, HLA-DOB, HRH2, IMPDH2, INSR, JAG1, LIFR, MAP2K1, MAP3K11, MAPK14, MCEE, MKNK2, NPAS2, OR14I1, OR52A5, OR6C3, OR8D4, PSENEN, PTGES2, PTGFR, SHISA5, SMAD7, TNFRSF12A
Negative_2648	HPD, ARSD, ATP5J2, CCL8, CSNK2A2, DHX58, DLAT, FLOT1, GATM, GNAI2, GUCY2F, HCCS, HSPA1B, IRAK1, ITGA11, METTL2B, NADSYN1, NT5E, NTSR1, OR1L3, OR2T2, OR4N5, OR6C1, PDGFB, PIGS, PRNP, SETDB1, SMURF1, TPI1, UNC5D, VAV3
Negative_2649	FADS2, MMP9, OR7C1, POLR2L, PTPRC
Negative_2650	DAPK2, FSHB, HCCS, OR10AG1, OR5A2, PDHB
Negative_2651	DAO, GP5, ACMSD, ACVR2B, AKR1C4, ATP5E, BST1, CNTN1, CSH1, E2F4, ENTPD5, FGF23, FGF9, GLUL, GRIN1, HSD17B8, LCMT2, MAP4K3, MASP2, MDM2, NCOR1, OR2C3, PGAP1, PLAU, PRKAB1, PRSS1, SEMA7A, ST8SIA5, SULT2A1, TUBAL3
Negative_2652	CBS, DLD, ABP1, ATP6AP1, BCMO1, BMP8B, CARD11, CCL26, CD46, COASY, ETNK2, FGF18, GRIA3, IL12RB2, LDHAL6B, NEU4, NFE2L2, NOX3, OR4D11, PDGFD, PLA2G2E, PRDX6, RALBP1, UNC5D
Negative_2653	BBC3, BLVRA, CALML3, CAMK2B, CCKAR, CD44, CNTNAP2, DUSP9, EPHB6, FGF9, ITGB8, LAMA4, MAD2L1, MAPK10, NCR3, OR11G2, OR2Z1, OR3A1, P4HA2, PDPK1, PERP, PLOD3, PRIM1, RFWD2, RPIA, SPRY3, TAS2R9, TNFSF8, TNFSF9, TNFRSF1B

Negative_2654	LTB, PAH, ANAPC7, AVPR1B, B3GALT5, CCR3, CD40LG, CDKN2B, DDOST, FMO3, GPX7, GRIA1, H6PD, HLA-DOA, INHBA, INSR, MCM2, MYLK2, NKX3-1, NOS3, OR10K1, OR7A10, OR8J3, ORC1L, PHKA2, PIK3R1, PRKAG1, PSME3, ROBO2, SH3BP2, ST3GAL2, PAFAH1B2, SERPINA5
Negative_2655	CAMK2B, MYH6, NEFM, STX4
Negative_2656	AK3, BAD, ABLIM1, ACACB, ANGPTL4, APOA2, ATP4A, ATP6V0B, BDNF, CAV3, CCL17, CCNE2, CDC25C, COL5A2, FSHR, FUT4, FUT9, GJD2, GLI3, IMPDH1, LAMB1, LAMB2, LEFTY1, LYZL1, MAP3K6, NUDT9, OR10G7, OR1S2, OR2T33, OR4F17, OR4K13, OR56B4, PCCB, PCK1, PDE6G, PPP2R5C, PYGL, RARS, RXRG, SNAP23, TBL1Y, TICAM2, TPI1, TUBA1C, WHSC1, HSD17B10
Negative_2657	AMH, CBL, IDS, LCT, ALOX15B, ALPP, BCL2L1, CACNG6, CARS, CCL14, CCL26, CD27, CNTN1, CXCL11, CYP1A1, CYP2A13, CYP2C9, EDN1, EGFR, EIF4E2, F13B, FECH, GNA15, GSTA1, HHIP, INPP4B, ITGA3, ITGA5, ITGB2, ITGB7, LCP2, LEFTY2, MYL9, OR5L2, PCYT1B, PLK1, PSENEN, PTDSS2, STAT5A, STX8, TAP1, TFDP1, THBS3, TIAM1, TUBAL3, VDAC3, TNFRSF10B
Negative_2658	GADD45G, GALK2, HCST, P2RX3
Negative_2659	BTC, C1S, CR2, AACS, AARS2, ADRA1A, ALG12, ANPEP, ARPC5L, CACNG7, CCKAR, CDH3, DDX52, DIAPH3, FARSA, GMDS, IL25, MPP5, OR4F5, PRKAB1, PRKCQ, RHOD, SETD1A, SMPD2, SPTLC2, STAT1, SUCLA2, UBE2L3, VAV3, WNT1, ATP6V1E1
Negative_2660	ACP1, CKMT2, CNGA3, CSNK1G3, DGAT1, DOCK1, DTX2, EIF4E, EREG, F2RL1, FOSL1, GSTM5, GZMA, KIR2DS4, MAP3K3, NAGK, NEUROD1, OR1E1, P2RX4, PIP5K1C, PLOD3, POLD3, PPP1CB, RIPK1, SLC2A1, SMAD7, SMURF1, SOX17, SPCS1, SPHK2, VANGL1, VDAC1, VDAC3, WNT10B
Negative_2661	ASL, FN1, MSN, NNT, ACAD9, BBC3, CEBPA, CLDN9, CSNK1D, CTBS, EPB41, EPB41L1, EPHA2, GATM, GGT6, GOSR2, GP1BB, GPT2, GRIA3, IFNW1, IL18RAP, MAPK1, MCM6, NT5E, OR56A4, OR5AU1, OR8K1, PIAS4, PPP3R1, PRKAR1B, PRKCQ, PRKG2, PRSS3, PTPRF, RPS6KA2, SELL, SKP1, SOAT2, SOX17, SPRED2, THBD, TYK2, WNT6, YKT6, MAPKAPK5
Negative_2662	A4GALT, EPHX2, ERO1L, GZMA, HTR2A, LCMT1, OR4E2, OR4X1, PRKAR2A, WNT9A
Negative_2663	ADH6, ARHGEF1, BACE2, CCL1, CSNK1G2, CYP2A7, EVI1, FBP2, IL12RB2, MAGI2, OR4B1, PIK3R3, PRKCD, SMAD7
Negative_2664	ALDH1A2, ALDH3A1, AREG, BPNT1, DBF4, HDAC2, OR8K5, PAX8, PDE7A, PKM2, ROBO2
Negative_2665	ME2, AKR1C1, CDC14A, CLDN3, CSNK2A1, DARS2, EFNA4, EPOR, FZD6, GNG12, GPT2, HIBADH, HSPA2, KIR2DS4, NAGS, NRP1, OR10AG1, OR2A12, OR5B3, OR5L1, PARD3, PLXNB2, PRKCD, ROBO1, SSX2IP, SV2C, UCK1, WNT4, WNT5B, XIAP, HSD17B12
Negative_2666	GP6, TNN, AASDH, ACSL6, ALPP, BAI1, CLDN18, DVL3, EPHB4, FGF7, GSTM3, IL10RB, INPP4B, NPR1, OR11G2, OR13C3, OR2T11, OR51A2, OR51B4, OR51B5, OR52E8, PARVG, RASGRP1, SDC4, SETD1B, TBK1, TIRAP, TRAF5, VEGFA, YWHAQ, SERPINF2, TNFRSF11A, TNFRSF17
Negative_2667	AR, ALOX5, CDC6, CSF2RA, DDX18, DGKG, OR2K2, ORC2L, PDE1B, PFAS
Negative_2668	CDA, SRC, VCL, ACVR1B, APLP1, CCNA2, CD55, CTBP1, FLT1, GJA1, GPD1L, HSD3B1, IL15, IL1A, INSRR, IRS2, MAP3K11, MPZL1, OR51Q1, OR52L1, OXSM, PIAS1, PIK3C2G, RAC2, WNT6, ATP6V0E1
Negative_2669	CACNB2, JAM3, OXCT1, SMAD5, TUBA3C
Negative_2670	AK3, ACOX2, ACPP, ACTA1, ADRA1B, ALDH1A3, B4GALT4, CSNK1G1, DRD2, GNA14, GPD1, GPLD1, GTSE1, GUCY1A3, ITGA2B, OR8J3, SHMT1, TUBB2A
Negative_2671	BCL2, EXOC7, PCK2, TFDP1

Negative_2672	ADC,CDA,ADSS,APPL1,CACNG6,CBLB,CLDN1,CPOX,CYP2C18,CYP4A11,DDX52,EPHB6,EPHX2,FOXO1,GAMT,IL19,IL4R,IPMK,MASP1,METTL6,NANP,NCSTN,NR1H3,NT5C1A,OR1J2,OR5B12,OR6C1,OR6N2,OR7G2,PDE9A,PDPK1,POLR2B,PPA1,RALB,RELA,SDC4,SELL,SMAD9,TJP2,CTNNBIP1,PNLIPRP2
Negative_2673	DBI,KDR,LAT,LPO,ACTN1,ALDH3B1,ALG1,ARG2,ARPC1B,ATP6V1A,BMPR1A,CCKBR,CLTB,CTBS,CXCR6,ELK1,GNAL,GRHPR,GSTK1,HIF1A,IL20RB,MGAT2,NCK1,NFE2L2,OR4N5,PPAP2C,PTPRR,SARS,SNAP25,TACR3
Negative_2674	SI,ADAM10,AKT1,BPGM,CDKN2C,CHST13,CSNK2A2,DHFR,EPHA1,FLOT1,FSHB,LLGL2,LTC4S,NFATC1,NTF3,OR3A2,OR51F1,OR52N5,OR8K3,PDE8B,PIAS1,POLR2L,RFXAP,SLC27A1,SMAD6,TNFSF8,UCK2
Negative_2675	EZR,ARSB,CACNG3,MAP2K4,OR1C1
Negative_2676	HDC,CCR4,CLDN8,DGUOK,FABP1,GAMT,GFAP,IL4R,NODAL,OR2D3,OR2T3,OR4N4,OR52K2,STT3B,THY1,TJP1,TUBB2A
Negative_2677	BID,DUT,VCL,ADIPOQ,DCTN1,EPHB2,FUT6,IL1R1,INPP5E,INPPL1,KITLG,NRG2,OR4X2,OR5AP2,OR6Y1,OR9G1,PDE3B,PDGFD,TSC2,XYLT1,PLA2G12A
Negative_2678	BCR,C4B,C9,DLD,F7,MPZ,PC,SDS,AASDH,ACTN2,ALG1,AMY2A,BMPR1A,CHIT1,CHRM3,DAPK1,DDIT4,ENO2,ERBB3,FBP2,FUT1,IPPK,IRAK4,LALBA,NEFM,NME7,NOX1,OR2S2,OR4B1,OR4C15,OR4K1,OR51B4,OR52A1,OR6S1,PPM1D,PPP3CC,ROBO3,SLIT1,STAM2,TLR9,TUBB4Q,WBSCR17,ATP6V1E2
Negative_2679	GP6,SI,ADH5,ADSS,ARPC4,CD44,CD8A,CDKN2C,CES2,DTX3L,GSTK1,HGSNAT,HNF1A,IFNA8,IFNGR1,KCNQ1,LAMA3,NCF1,OR10G9,OR2D3,P2RX4,PLAU,PPP2R1A,TRIP10,WNT7A,WNT8A,CSNK1A1L,HSP90AB1
Negative_2680	CRH,HK1,ACSS3,ALDOC,ALS2,BPGM,CD59,CSNK1A1,CTBS,FMO3,HAP1,NCR3,NFAT5,RELB,STAT5A
Negative_2681	LAT,ADCY9,ALDOA,CARS2,CD46,DPYSL2,E2F5,EPHA5,GLUD2,OR10A2,OR7G1,ORC6L,SEMA4G,STMN1,PLA2G12B
Negative_2682	GCDH,PIK3C3,TLN2,TPI1
Negative_2683	SRC,AOC3,CDC45L,CDH5,CHRM1,DDX4,DRD2,ECHS1,EFNA4,FDFT1,GLS2,HRH2,IL15,IL20RB,OR51B2,PDE6D,PFKFB1,PLCE1,POLE4,PRKAA1,PSME3,PTPRF,SRD5A1,ST8SIA5,TAS2R1,ZFYVE16
Negative_2684	KDR,AKR1D1,ARNTL,CRKL,FGF6,FLT4,FPGS,GADD45A,MAP3K3,NFKB1,OR1L6,OR2L2,OR2M4,OR4A16,OR51D1,OR5B21,SHC1,SKP1,STAT4,TFDP1
Negative_2685	ADH5,ALOX12B,B3GNT3,BLNK,CCNA1,CKMT2,CSNK1G1,CYP3A4,DAAM2,DAXX,DTX1,EPB41L1,EPHA4,FBP2,FLOT1,HARS2,HTR7,IL12B,IL3RA,IL8RA,JAK3,MAPK1,MOV10L1,OR3A2,OR4F6,OR4N2,PFN4,PLXNA3,PNPLA4,PTGER1,RPS6KA4,SGPP1,SLC12A2,SLC2A1,SSH1,SUCLA2,SUV39H1,TLR9,TNFSF18,WNT9B,SERPINB5,TNFRSF17
Negative_2686	APOE,FGF11,GLYCTK,MYH11,NRG1,OR2C3,OR6B2,OR6X1,OR8B4,PDE1B,PTCH1,SLC25A4,UNC5A,VARS
Negative_2687	GAA,ACADL,AGMAT,ALCAM,ALDH5A1,CYP26B1,CYP3A43,DUSP3,ENPP2,EPHB1,IFT57,IL12B,MAP3K8,MGAT4B,MGST1,OR10X1,OR1C1,OR4K5,ORC3L,PARD6B,PCK2,PRKCB,SOCS3,ST3GAL6,TSHR,UBA7,GABARAPL1,HLA-DRB3,PRICKLE2,SUV420H2
Negative_2688	SI,ACTA2,OR2Z1
Negative_2689	ACHE,ATP4A,CAV2,CD226,COX10,HEXA,MYH6,NFATC1,OR4F6,PLXNB2,PLXNB3,VAMP1,WNT1
Negative_2690	CSK,FGA,GH1,AARS2,AGRN,CDIPT,COL6A2,IDI2,IL10RB,LDHAL6B,MCM4,NFKBIA,NTRK2,OR5B12,OR6B2,ORC3L,STK11,TAP2,TJP2,TRDMT1
Negative_2691	NF1,NLK,RDX,TKT,ACVR2A,CDC7,DDX41,ERBB3,IFNA8,IFNB1,IL22RA2,KIR2DL2,OR51A2,PROC,RPN2,RPS6KA3,RPSA,TNFRSF12A

Negative_2692	ACAT1,CAMK2A,CCNG2,DGUOK,EP300,FGF3,GRM5,HTR7,IL24,MAP3K4,OR2T34
Negative_2693	WAS,APAF1,ARPC2,CBR3,COL1A2,CSNK2B,CXXC4,DAD1,ENPP3,ETNK2,FGF23,GNA13,GNPDA1,INHBA,JAK3,KCNQ1,LDHC,NAT5,NODAL,OR12D3,OR52D1,PCYT1A,PDXK,POLR2G,PTK2B,SESN1,SH2D1B,SMARCA5,TLR4,VTI1B,XYLB,ATP6V0A2,CSGALNACT1
Negative_2694	C7,ACAT1,CDC26,EDA2R,FGF4,MTHFR,MTMR2,NTSR1,PDE7A,PHKA1,POLR3B,B4GALNT1,TNFRSF13C
Negative_2695	PC,ALPPL2,ASCC3,ATP5D,CCKBR,CD72,CDH1,CLDN18,CSNK1G3,CSNK2A1,DPYSL2,ERBB4,FBXW11,GMPR,GPAA1,GSTM5,IRAK2,ITGB5,LYPLA1,MYH1,NRCAM,OR2AT4,OR56A3,OR5H6,PIGU,PLOD1,POLR2B,PPP2R5B,PRIM2,PRODH2,PTPN6,RHOQ,STX18,STX3,SUCLG1,WBSCR22,ATP6V1C1,CACNA2D1
Negative_2696	BCR,ID4,ABLIM3,ACY3,ADORA2A,ALDH1A3,CDH3,CYCS,DGUOK,DKK2,FGF17,GFPT1,GMPPB,GNAZ,GNPNAT1,HLA-F,IL1R2,IRAK2,ITGB5,MAP2K3,MCM3,NCOR2,OR2H2,OR2T6,OR52N5,OR6C6,POLR3K,PPT1,RBX1,SEMA7A,SGMS2,SOCS5,SPCS3,SULT1A1,VAV3
Negative_2697	BTC,GP9,APOA2,B3GALT6,C1QB,CACNG3,COL1A1,CYSLTR2,EPHA5,FADD,FGF14,GNPAT,IGFBP3,LCP2,NCSTN,NT5C1A,OR2G3,OR6X1,PDCD1,PIGF,POLE,POLE2,PPM1A,RAP1A,STAT5B,CSGALNACT2
Negative_2698	F10,ACSL1,ADRA1A,ALDH1A2,ARHGEF2,ATP5B,ATP5G3,B4GALT1,BPNT1,CD40,CDKN1B,CMPK2,CXXC4,EFNB2,FGF5,FGF7,GNA13,HTR5A,IRF7,MAP4K3,MECR,MYH15,NEFM,NR5A2,OR2S2,PMM2,POLE3,PVRL2,PYCR1,PYGM,RASSF5,SMAD3,TAS2R48,TBL1XR1,UCK2,YWHAE,ATP6V1G1
Negative_2699	AUH,ADCY3,ASS1,CRLS1,CSF3,FABP4,FDPS,FZD4,GUCA1A,HCST,IL23A,IL4R,PAK2,PNMT,PRKACA,RHEB,ZBTB16
Negative_2700	BTK,CCR4,HRH1,SC5DL,STK11,UNC5D,SERPINA5
Negative_2701	CKB,F8,ID4,AMHR2,ATP4A,BBC3,CD247,CD274,DGKZ,ENPP1,FGF2,GNRHR,HGSNAT,IL13,LDHA,MTFMT,NFKB2,OR2B6,OR2T10,OR4C13,OR4K17,PIK3C2A,PRKCE,RHOD,RND1,SGPP1,SMAD3,SOAT1,SPHK1,SRD5A1,TGFBR2,THBS4,TRPM5,VAV2,PPP1R12A
Negative_2702	FGG,CNTNAP1,HADHA,HLA-DMA,OR2M2,PPP1CB,TICAM1
Negative_2703	GNS,STS,ACSM5,ANPEP,AVPR1A,CACNA1C,CD79A,CTPS2,CTSB,CYP2A13,DGKZ,DUSP7,FSHR,GAL3ST1,GCH1,HGSNAT,HMGCL,INMT,OR6C4,OR7A5,PDE6H,PIGK,SPTLC1,TAS1R1,TCF7L1,VAMP2,ST6GALNAC5
Negative_2704	A2M,DDO,GPT,PGP,ACADL,ACTB,ALS2,AMDHD1,BMP5,CREBBP,CUL1,ECSIT,EIF4E2,ESAM,GCLC,GOT2,GPT2,IL12RB2,IL13RA2,ITGA7,JAG2,KAT2B,LDHB,MINPP1,MTHFR,MTMR1,NTF4,NTRK1,OR10H4,OR4F6,OR51A7,PDE4B,PDGFD,RBX1,SARS,SEMA4C,TAP1,TAS2R3,TNFRSF4,TNFSF12,TNFSF4,TYROBP,CTNNBIP1,TNFRSF10C
Negative_2705	BAD,ACP6,ALAS1,ALDH1A3,ARSB,ATP12A,ATP5C1,ATP5F1,CAMK2G,CD14,CHPF,CHRM5,EDAR,FBP2,GGCX,GP1BB,GUCA1C,IDH3G,IL15RA,KCNQ1,MOV10L1,MPST,MTHFS,NAT5,NT5C1B,OR5H6,OR8D2,PAICS,PCNA,PIGF,PPP1R3A,PRDX6,PRKCQ,PTGDS,SLC2A1,STAT2,TRAF5,UGT2B28,SERPINF2
Negative_2706	SFN,CDK2,GALNT9,HAAO,NEU1,NR1D1,PDE6H,PIAS4,POLR1D
Negative_2707	C8G,CR2,ADIPOR1,AKR1B1,BPGM,COX10,CPT2,EGLN1,F13A1,GRIA1,IGFBP3,IL11,MAP2K4,MYH1,NCKAP1,OR1D5,OR4C15,OR8S1,PDE5A,SMAD5,UGCGL2,CACNA2D3
Negative_2708	PGF,ADRA1D,KCNB1,NARS2,OR4F15,PTEN
Negative_2709	CDC26,CTSG,GALK1,GUSB,RXRB,ST6GAL1,WNT9B

Negative_2710	AK3, LHB, LPO, ACADVL, ATP5F1, CAMK4, CCR6, CD226, COL11A1, CTNNA1, CXXC4, ENTPD3, GBA3, GCNT4, GFPT2, GMPR, HAP1, HMGCR, IL1R2, INHBA, IRF7, LGMN, LRRC4C, MAPK11, MAPK9, OR13G1, OR2T2, OR56A3, POLA1, PTAFR, SFRP1, SFRP5, SGMS1, SLC2A1, SORBS1, SV2A, TNFRSF8, ZNRD1
Negative_2711	ANAPC10, CKS1B, CTF1, DUSP3, OR2T34, OR3A2
Negative_2712	C6, GNE, MAG, OSM, ADRA1A, ADRBK2, ALG13, ANAPC10, CACNB4, COMT, CXCL3, DDX41, DLL3, DTX4, EIF4E, ENPP7, FADS2, FUT2, GJA1, GPLD1, HMBS, HTR4, IL13RA2, MCM7, OR2Y1, OR4F5, OXCT1, PDE6G, PLAUR, PLD1, RALGDS, RPS6KA2, VEGFA, YWHAB, SERPIND1
Negative_2713	BCAT2, CACNG8, DEGS1, GLUD2, MADCAM1, OR4C46, OR8I2, PLAT, PLXNA2, PPP3R1, PTGS1, RPRM, RPS6KA4
Negative_2714	LIF, ACVR1, BET1, BMP6, CCNG2, CHAT, CSNK1E, CYCS, CYP2F1, DDX56, DHDDS, DOT1L, ENTPD7, ETNK2, FABP1, FMO4, FUT1, FUT4, GLI1, IFNB1, IL7R, ITGAD, ITGAX, MAPK14, MYL2, ODC1, OLR1, OR11L1, OR56A4, PDCD1, PLTP, PLXNA3, PRKG2, RAC2, SEMA3E, SFRP1, ST3GAL6, SUV39H1, THTPA, TP53, TYMP, XYLT1, GABARAPL1
Negative_2715	F10, TFG, APPL1, BCL2, CALML6, CCDC6, CCR9, CLDN18, CYP11B2, DIAPH3, FGF10, FZD4, GLI1, HCCS, HPSE, IL1R2, LIPE, MAML1, MAP3K6, MDM2, NRXN1, OR4A16, OR51G1, OR6C70, PARD6G, PDCD1, PIK3R4, POMC, PON3, PPP2CA, RPS6KA3, SHC4, SPTLC1, SULT1A2, TAS2R3, TGFB1, TUBAL3, WNT11, HLA-DPB1
Negative_2716	CLCA4, ENPP6, IL11, MAP2K1, MAPK11, OR2M2, OXCT2, POLE3, SESN2, STX5, TYRP1, ST6GALNAC5
Negative_2717	FST, GNS, LEP, PAH, SYK, ACSL1, ADRA1D, AFMID, AGPAT2, ALOX15, ARNT, ATP4A, BCAT1, BMP8B, CLDN14, CSNK1G1, CYP2J2, DOLPP1, DTX1, EDA2R, GALK2, GLI3, IAPP, IDUA, IRAK3, ITGB7, KIR3DL1, MAOB, MLYCD, NCAM1, OR1F1, OR2L8, OR52N4, PDHB, PLA1A, THY1, TNFSF8, ATP6V0A2, MAPK8IP2, PPARGC1A
Negative_2718	DUT, FES, KHK, ACPP, APOA5, BCL2, CCL13, CUL1, DIAPH3, DPYSL2, GNPAT, KLRD1, NME4, POLR3G, SDC4, SERPINA5
Negative_2719	ADA, ABL1, AMDHD1, APLP1, GFAP, HDAC1, HLA-G, ITGAD, LIAS, NUDT2, OR5B3, RRAS2, ULBP2, CACNA2D4
Negative_2720	CRK, GCK, PRL, ADSL, B4GALT1, BET1L, CCR3, CHRD, DLST, ENAH, OR2F1, OR8G1, OR8H2, OR8K3, PDXP, PKLR, POLD1, PPP2CA, PRSS3, PVRL3, SCIN, TAP2, TP53I3, ZMAT3
Negative_2721	INS, PVR, APOE, CNTN2, DERA, GAD1, GARS, INPP5A, MYD88, OR10A4, RAP1A, PHOSPHO1
Negative_2722	BCR, PRL, ADCY10, ATP6V1D, CACNA1C, CARD11, CCL2, CD274, DDX56, DPM2, EFNA3, EPHX2, FADS2, FTMT, GART, GPAM, IGF1, IL4R, KLRD1, MDH2, NFYB, NGEF, OR12D3, OR4S1, PROC, RUNX1, SEMA3B, SHC1, STX18, TAS2R7, PPARGC1A
Negative_2723	DCN, SRC, ACAD8, ADAM10, ALG10B, AMHR2, ATP5F1, CPOX, CYSLTR2, DDHD1, EFNB3, FUT8, FZD4, GGPS1, GLI1, GSTO2, IL1A, ITGB1, ITPKA, KAT2B, LDHAL6A, OR10R2, OR2L3, OR2T4, OR8D1, PIGV, SRGAP3, TLR3, UPP1, VDAC1, WNT3
Negative_2724	DBI, PRL, SCD, AHCY, ALG2, ANAPC7, ARHGEF4, BCL2L1, CD274, CD36, CDH15, CDH2, CLDN15, CSNK2A1, CXCL5, CYP2U1, EPHX1, FARS2, FGD1, FGF10, FLT1, GNAS, GNPDA1, IFNK, IL20RB, MCM6, NKX2-2, OR4X1, PIPOX, RDH16, RPS6KA3, SMAD2, SULT1A4, TAS2R40, THBS3, WNT10B
Negative_2725	C5, CSK, DBH, F8, HDC, TNN, AHCYL2, B3GALT2, BAAT, BHMT, CHRD, EIF4E2, ENTPD8, FRAT2, GNA12, IFNG, IL13, INHBB, L1CAM, LAMB3, LCMT2, MALT1, MYH10, OR10A7, OR13C4, OR52W1, PDE4C, PI4KB, PON2, TUBAL3, WASL, TNFRSF17

Negative_2726	F2, IL4, LIF, ACOT12, ANAPC11, BMP5, CCL22, CCL23, CD46, CLCA2, CRKL, DAPK3, GALNT1, GNAL, HNF4A, MAP2K6, MAPK14, MPST, NFYA, NME6, OR10H5, OR8U1, OR9G1, PDE8B, PNLIP, POLR2F, PTGFR, PTGS2, RASSF1, RXRG, SOCS4, WARS, XCR1, SERPINA1
Negative_2727	ACTG1, BET1L, C5AR1, CD247, CDH5, COL5A3, EFNB3, FABP1, G6PD, H6PD, HEXA, ITGAM, MAP3K3, NLGN2, OR2C1, PON2, SH2D1A, SMAD1, ST3GAL6, STX12, TNFRSF8, TRAF2, WNT4
Negative_2728	ACADVL, ACOX1, AMHR2, APOC3, BIRC2, CAMK2G, CLDN7, COL4A4, CRY2, CSNK2A1, DNMT1, DRD2, EARS2, ENO3, ETS1, FMO5, FUT6, GUCA1A, GUCY2C, HSD17B2, HSPA8, LAMC2, LEF1, MAN1B1, MAP2K4, OPLAH, OR10H2, OR10T2, OR1E2, OR2M5, OR4M1, OR4N5, OR52B4, PIGV, PNPLA3, POMC, PRSS1, PYGL, SKP1, SNAP23, SOCS4, SRD5A2, SULT1A1, TAP1, TAP2, TLN2, TSTA3, VAV1, VDAC3
Negative_2729	RPE, ALPPL2, CD55, OR10K1, OR2A2, OR8K3, THBS1, VAMP4
Negative_2730	F12, LIF, ADRB3, CCL25, CEBPA, GRLF1, HSD11B2, ITGAM, KIR3DL2, MITF, OR10C1, OR2W1, OR51S1, PLTP, POLR3G, RDH8
Negative_2731	AARS, ITGA5, OR2T2, OR8H2, ST6GALNAC3
Negative_2732	CRK, DGKD, ELK4, NRCAM, OR10G4
Negative_2733	F9, FOS, MUT, RPE, ACTC1, AOX1, ARHGEF2, B3GNT5, BLVRB, BMPR1A, CASP10, CREB3L4, DTX3, GALNTL1, GLUD2, GNB1, IBSP, ICOS, INPP5E, KITLG, LAMB1, LLGL2, NUDT9, OR13C2, OR2D3, OR2L8, OR6N2, PAX6, PDE8B, PDIA4, PFN4, PLA1A, PPP1R3C, PRKCA, PYCRL, RFXANK, RPS6KA4, RRM2B, SOAT1, SV2C, TAS2R13, THBS4
Negative_2734	ADIPOR2, BET1, METTL6, MYH13, NUMBL, OR51L1, PIP5K1B, PNPLA4, PPCS, SLC27A4, SLC9A1, ST8SIA1
Negative_2735	LPL, CLDN20, CTLA4, CTNNA3, CYSLTR2, DGAT1, DGKG, EP300, EXTL3, FARSA, GNRH2, GPD1L, GZMA, IL22RA1, LDHAL6B, MAP3K5, NEU4, OR10R2, OR1C1, OR2H2, OR6C6, OR7A10, OR8H1, PKMYT1, SMARCA5, TAS2R14, TPH1
Negative_2736	C8B, ACP6, ADCY7, BMP2, CD72, CTSS, DUSP9, ELK4, FABP3, FZD3, GLO1, GNAQ, GSTA1, HLA-DOA, INSRR, LIPA, MAN1A1, MAPK1, OR2Y1, OR4M2, OR51L1, OR8D1, OR9Q1, PIK3C2B, SELL, SPRED2, AASDHPPT, HSD17B10
Negative_2737	GP5, IL6, ADCY1, AKR1C1, ALDH1A2, ATP5J2, CHKB, DHCR7, DPM2, FGF2, GCAT, IL7R, LTBP1, MAD2L1, MAPK14, NEU2, NFAT5, OR7C1, PHKA1, PI4KB, PRKAR2A, PROS1, SEMA3B, UBE2K, UBE2L3, VEGFA, WBSCR22
Negative_2738	AMH, IL4, NPY, BCAT1, CACNA1G, CBR1, CCL25, CDK4, CLCA4, CLDN3, CYP2D6, EFNB3, FGFR1, GFPT1, GUCA1C, ICOS, LAMB2, LYZL1, MAP2K6, MCEE, MYH10, NCF2, NTNG1, OR10P1, OR8U1, PAPSS2, PDE3A, PDE4D, PLA2G6, PSEN1, PTPRZ1, SH3BP2, TAS2R49, TBL1XR1, VARS, CSGALNACT2
Negative_2739	GK2, ADIPOR1, APOA2, ATP5G1, B3GALT2, BCL10, CTPS, DGKH, FRAT1, GRIN2B, INSRR, JAK1, KIR2DL1, LEFTY1, MPST, NMNAT3, OR5K2, OR8K5, OR9A2, PERP, RDH10, TRAF6, TUBA3C, UGT2B4, UPB1, KIR2DL5A, SUV420H1
Negative_2740	C8B, CGA, CRK, LCT, ZYX, ADH1A, AGRN, ALDOC, ARRB2, ATF2, CDH15, CHKA, CSF2, DUSP4, EGLN1, ELK4, FGF22, GMPR, IL24, INPP4B, ITGA5, MAGI3, MYH1, NFYA, NRG2, OR52D1, PEMT, PFAS, PIGL, PIK3C2B, PLAT, PSME1, PVRL3, RASSF5, SOCS7, STAT4, TIAM1, VCAM1, WNT4
Negative_2741	C4B, FST, HPD, PDC, PLN, ADH5, ALOX15, B3GNT3, CD14, COL6A1, CSF3, CTBP1, FPGS, GCH1, GRIA3, GRM1, IFIH1, IL22RA1, IL28B, ITGA2, ITPKB, MAFA, MKNK2, OR4F6, PDX1, PFKFB2, PI4KA, PIGP, PLA2G2F, POLR2J, POLR3H, PPP2R1B, PPP2R2C, PTK2B, RALGDS, RDH8, RRAS2, SEMA7A, SLC2A4, SMAD4, SMC1A, TUBB2B, ULK1, UPP1
Negative_2742	CFI, DCN, SP1, ADSL, ANAPC4, CACNA1F, CCNG1, CD38, CREB3L4, ETS1, FBXW11, FGF14, GALNT13, HNF1B, IL7R, MDH2, OR10A7, OR10G7, POLR3B, PON1, PPCDC, SMAD7, ST3GAL6, TSC2, WASF2, ST6GALNAC6

Negative_2743	BAX,SMS,AHCYL1,ARG1,ARSD,ATP5G2,BIRC3,DAG1,DPM1,DUSP14,DUSP5,DVL1,DVL2,E2F5,EREG,GADD45B,GALNTL1,GMDS,GPLD1,GUCY1A2,GUCY2F,HLCS,IKBKB,IL6ST,ITGA11,NPR2,OR10H4,OR1N1,OR4E2,PAK1,PGAP1,PGS1,PLA2G2F,PNPLA4,POLD3,POLR2B,SPCS3,TFDP1,UCKL1,UGDH,GABARAPL1
Negative_2744	BMP8A,CACNG4,CRLS1,CTTN,CXCL13,DHCR7,FUT5,GLUD1,HAAO,HSD17B3,IFNW1,KIR3DL3,KLRK1,LAMB3,LCMT1,OR11H1,OR2T33,OR5V1,PDGFA,PLA2G1B,RHOD,THTPA,TJP1,VARS,WWP1
Negative_2745	GDA,ABL2,AKT2,ASCC3,CCNB1,CYP1B1,DDHD1,DUSP3,GRAP2,GUCY1B3,IL22RA2,ITGB6,NCKAP1L,OR10J3,OR3A1,OR6Q1,OR8U1,P2RX2,PGAM1,PLAU,PLCD3,PYCR2,SIGLEC1,SYNJ1,TAS2R60,TPM3
Negative_2746	IHH,ALG13,DHX58,IDH3A,LDHAL6B,NAMPT,NT5C1A,OR10C1,PDE4A,PGAM2,PLOD2,PPP2CB,RBL2,ROBO1,RPN1
Negative_2747	CDA,ACTN4,CBLC,DOT1L,ELK1,ENTPD3,IKBKB,IL2RB,ITGB7,MAP3K2,MAPK12,NAMPT,NTN1,OR4F4,OR8B4,PTPRZ1,SMAD5,TACR3,VNN1,XYLB
Negative_2748	C4B,CTH,ADSS,BCL10,C3AR1,CACNA1E,CCNB3,CLCA4,DDX19A,FASLG,GAPDH,MADCAM1,NAT1,NCOA4,OR4F5,OR51M1,PAFAH2,PDCD1,PLXNB1,RAD51,TNFRSF12A
Negative_2749	DDO,ID1,IL9,ACADSB,ARHGEF6,ATP5O,B3GNT4,CYP11B2,DOLPP1,DUSP2,EHHADH,FASN,FDPS,GMPPB,GNPDA2,GPT2,IDI1,IFNA10,IFNG,IL8RB,MMP9,NFKBIE,NR5A2,OR10G7,OR2AG2,OR51D1,OR8B12,PON3,PRPS1,PSME1,SHC3,SLIT2,SMURF1,TICAM1,TOMM40,HLA-DPA1,SERPINB5,SERPINC1
Negative_2750	AK2,ATM,HK1,ACAA2,CRKL,DNMT3L,DVL2,HCLS1,IFNA5,IL17A,MARS,OR14A16,PRLR,PTPN6,RAC2,RBX1,SPI1,TNFSF18
Negative_2751	LAT,LYN,B3GNT2,BET1,CD58,CISH,CNGB1,CTBS,ENTPD8,FZD10,GAPDH,KIR2DL3,MCM4,NKX3-1,NME4,OR51V1,OR5D16,PDCD1,PLCG2,PLXNB3,PRPS1,PSME2,RBPJL,SETD1B,SMURF1,SPRY1,TLR2,TSNARE1,PAFAH1B2
Negative_2752	C4B,GNS,ACVR2A,ADRA1D,AOC2,ARPC5L,B3GALT6,B3GNT5,C7orf16,CAMKK2,CAV1,CDC26,CNTN2,CXCL10,DFFB,ENPP2,FASN,FZD6,GATM,GLI3,HLA-DMA,HTR2B,MAP2K6,MAPK3,NADK,NR1D1,OR13C9,OR13D1,OR1J1,OR1K1,OR4D6,OR52A1,PAICS,PLXNB2,POLR2B,TAS1R2,TM7SF2,TNFSF10
Negative_2753	ADIPOR1,BMP4,CHD8,COMT,CRLS1,HTR4,IL17RB,KIR2DL1,LCMT2,MNX1,PI4KA,PIGH,PIK3R3,PROS1,RXRB,TRAF4,UGT2B11
Negative_2754	CR2,INS,MPZ,PGP,ACTB,ACVR1,ALDH9A1,CACNA1F,CCNA2,CCNG1,CCR8,CDC23,CLDN10,COL5A1,CYSLTR2,EDNRA,ENTPD2,GNA13,GNMT,GPD1L,GSTM3,IFNAR2,LYZL1,MMP1,OR14J1,OR2G2,OR2L8,OR2T33,ORC2L,PAK2,PCYT1B,PDGFRB,PGM3,PGS1,POLR3D,PPP2R5E,RASSF5,RBX1,SMARCA5,SOCS1,STX4,TLR2,TNFSF12,TUBB4Q,USE1,ZBTB16,ATP6V1G2
Negative_2755	NOG,ACAA2,ACAD8,ACP5,ALDH3A1,ALG11,APH1A,ATP5A1,BCKDHB,CCDC6,CD79A,CTF1,DUSP6,GALNT12,GNA11,GPD2,LPAR1,LYZL1,METTL6,NSD1,NUDT8,OR10R2,OR2A5,PPP2R1A,PVRL4,PYGL,QPRT,RAC3,RAP1B,RFNG,SMPD2,SOCS2,TSLP,ATP6V1C1
Negative_2756	CSK,DBT,FAS,ACPT,BLNK,BMP8A,CADM3,CCNB3,CD22,COMP,COQ3,CUL1,CYBA,CYP2S1,DRD5,EDA2R,ENPP7,GSTA2,GSTA4,HES1,HTR6,IRS1,ITGB2,LDHAL6B,MMP14,MTHFD1,MYL5,NCR1,OR2A12,OR2L3,OR51E2,OR52K1,OR52R1,PDHA2,PLCD1,POLR2G,PRKCH,PSEN2,PTAFR,PVRL3,SFRP4,SMAD6,ST3GAL3,STX3,STX5,TAS2R60,YARS
Negative_2757	CD80,CTNND1,EPHA5,FDPS,HES1,IL23A,JAG2,PI4KA,TOMM40L,B4GALNT1
Negative_2758	GH2,ALG3,BCAT1,FGF3,HSD17B7,OR52J3,PDE8A,POLR1C,RERE,SEMA3E

Negative_2759	NF1,ADH7,AKR1B1,ARHGEF7,BRAF,CACNA1I,CHEK2,CSNK1D,FABP3,FLAD1,GSTM3,HAO1,HPSE2,IL22RA1,MAGI2,MCM4,NAT6,NME7,NRAS,OR5M8,OR5T1,PDE4C,PLA2G6,PPA1,PTPRR,RASGRP3,STMN1,TXNDC12,VCAM1,XYLB,B4GALNT1,PNLIPRP1
Negative_2760	MPZ,AGMAT,COMP,GALNTL5,GNB1,IFNA16,OR6C74,OR7A10,PIGF,SETD8,TAS2R40
Negative_2761	FN1,IL7,TNR,ABLIM1,ACY3,ALAD,ARPC1B,ARPC4,ARSB,BET1L,CAMK2A,CDC25A,CXCL3,CYCS,DDIT4,DVL1,EPHA6,FADD,FGF6,FSHB,GBE1,GSTM3,HTR5A,IFNGR2,IL19,ITCH,MDM2,NCF1,OR10A7,OR10G9,OR13C9,OR51G2,OR5B17,OSMR,PDE6G,PGAP1,PIP5K1C,PPP1R3B,PPP3R1,PTTG1,SLC25A4,SMAD7,SUV39H1,TLR3,VKORC1,WNT8A,XCR1
Negative_2762	ACP2,GANAB,RPS6KA2,RUNX1T1,VNN1,PHOSPHO1
Negative_2763	DBH,AFMID,CCL14,CHIT1,CRY1,DGKE,DGUOK,GNPDA1,ITGA4,ITGB8,MAPK9,NCAM2,PLCB2,PLCE1,PLD1,RAPGEF1,SAT1,SMAD5,UGT2A3
Negative_2764	F7,GSS,MET,PAH,ADRB3,AMY2B,BHMT,CXCL13,DOLPP1,FCGR3A,GLUD1,HSD17B7,MAGI1,NANP,NOTCH3,OR7G3,PIGX,POLR3A,PTK2,PYCR1,TRAF3
Negative_2765	F12,FGB,RPE,ADCY1,ADH1B,ADIPOR2,AGPAT4,ALOX5,APH1A,CDC26,CDKN2D,COX10,CRHR1,EIF4E2,ENTPD5,F2RL1,FGF2,INPPL1,LIPG,MAML2,NTRK1,OR4F17,OR51F2,OR5L2,PEMT,PGM1,PIP5K1C,PON2,PRKX,PRLR,RND1,SLC33A1,TOMM40,UMPS,HLA-DQB1,HLA-DRB5,MAPKAPK5,TNFRSF11A
Negative_2766	ABLIM1,ACLY,ADSS,ALOX12,BDH1,C9orf95,CD27,CLDN20,CRLS1,DDIT3,DKK2,E2F4,EPHB2,EXOC7,FOSL1,FZD9,G6PC,GNAL,GRIA3,GUK1,IL18RAP,KAT2A,KLRC4,MAPK3,MTHFD1,NMNAT1,NOTCH3,ODC1,OR10X1,OR4K1,OR51V1,OR52E6,PFN4,PSEN1,SARDH,SETD2,SIAH1,STT3B,TSHR,UROS
Negative_2767	BAD,DBI,AKR1D1,ALDOB,ALG6,CACNA1S,CD44,CSH1,EDA2R,FBP2,GAB1,GUCA1B,NEU4,NNMT,PDE4D,PKM2,PPAP2C,PPP1CC,RPS6KA4,SSH2
Negative_2768	BAD,DERL1,FGF5,FUT5,HNF4G
Negative_2769	ABAT,ACOX2,CDC42,CHSY1,CLDN18,CYP21A2,CYP2S1,DDIT4,GP1BA,IFNA5,LIAS,LLGL2,MAP3K13,MCM7,MPZL1,NEU4,OR10G8,OR4D9,OR51L1,OR5H2,PMM1,POLR1B,PRKACG,RBPJL,ROBO2,RPS6KA1,RUVBL1,SHC4,SKIV2L2,SLC2A2,STX16,TBL1XR1,TUBA1C,UPB1,UROD,VARS,VNN1,CSNK1A1L,HSD17B10,MAPKAPK2,ST6GALNAC1
Negative_2770	DCC,ITK,AKR1D1,ATF2,ATP2A3,CDH5,CHSY1,CLDN22,CNDP1,CXCL2,DAG1,FUT5,GPX7,MAP2K6,NAT2,NEU2,NPAS2,OR5L2,OR5M9,OR7A5,OR7C1,OR7C2,PLCG2,PLXNB1,SKP1,SOS2,THBS2,VDAC3,WARS,WEE1,YES1
Negative_2771	CKB,LSS,ABCC8,ACTA2,AK3L1,AKR1B10,ALOX12,AMPD2,ATP5E,B3GALT5,CDKN2C,CTLA4,DUSP3,EPHA2,FGF1,FGFR1,GALNT13,GCLM,GSTM2,GUCA1C,HBEGF,HRH1,IPMK,KIR2DS4,KLK3,MAP3K12,MFNG,MTHFR,MYD88,MYH15,MYH9,OR5H6,P2RX7,PDGFRB,PIGL,PIP5K3,PLCB4,PLXNA1,RAP1A,SPRY2,TUBA3C,TYK2,UBE2K,UPP1
Negative_2772	C4B,CRK,FGA,PLG,ACY1,ALG9,ATP5J2,BCL2,CBLB,CTSS,EGLN1,ENTPD4,EPOR,F2RL2,FGF22,FUT1,GTSE1,GUCY1A3,MTHFD1L,NADK,OR2A25,OR4D6,OR51A7,OR5AS1,OR6T1,PPA2,PRKAR1A,SELPLG,SMAD9,SMC1A,SMPD1,TGFBR2,THTPA,VAV3,WNT11,XYLT1,SERPINB5,TNFRSF13B
Negative_2773	APP,GAA,ALPPL2,ATP5D,BMP6,GNPNAT1,HAGHL,INHBE,NCR1,PARD6B,PER1,PFKP,PSEN1,PTDSS2,SMAD6,SPAM1,TAS1R1,TUBB2C,WNT5A,ZAP70
Negative_2774	LHB,STS,AACS,ACAT1,ACSS2,ADCY10,ALG6,ARSB,ATP5G1,C1QC,CDH2,CNTF,COL2A1,CTNNA1,EFNB1,F13A1,F2RL2,GALNTL4,GBA3,GCLC,GLUL,GRM5,GSK3B,HDAC2,HTRA2,ITPA,KIR2DL1,LHCGR,LLGL2,MCM2,MYH7B,NEU2,OCLN,OR2H2,OR2L3,OR51F1,OR51S1,PFN1,PIGQ,PLA2G10,PLD1,POLR2E,PRKY,SHMT1,STX6,TAS2R48

Negative_2775	BIRC2,MYLK3,RDH16
Negative_2776	AR,DBT,EBP,FAH,SI,ADRB2,ALDH1A1,ALDH4A1,CAMK2D,CASP10,CLTA,CLTCL1,DUSP7,GANC,GP1BA,HLA-DRA,HPRT1,IL22RA2,INADL,IRS4,KRAS,MAP3K14,MAP3K2,MRAS,NCF2,NFATC3,NFKB1,OR13H1,OR1J4,OR2G2,OR2M3,OR4F5,OR51A4,P2RX1,PFKFB3,PLA1A,PPBP,PRPH2,SHISA5,SLC25A5,SPRY4,TALDO1,TCF7,TCF7L2,UGCGL1,UGDH,CACNA2D1
Negative_2777	EDA,ACACB,ADRB2,AK3L1,AXIN2,B3GALT5,C7orf16,CCNE1,CDKN2B,COL5A3,DGKQ,DLST,FBXW11,FCGR2B,GSTA5,IL20RA,ITGAD,LAP3,LARS2,LIFR,OR10AD1,OR5L2,OR8H2,OR8S1,PIAS1,PPARA,PROS1,PSENEN,PTCH2,SCIN,SKP1,SLC12A2,ST3GAL6,SYNJ1,ATP6V1B2
Negative_2778	LAT,UBB,ACADVL,ACOT11,BET1,BMP5,CACNG3,CCR5,CD44,COL1A2,CYP2S1,CYP3A4,DDX47,DGKB,FECH,GMPPB,GSTP1,HDAC2,IL15,IMPDH1,IRF3,MDH2,NCAM1,NRCAM,OR1A2,OR2AG1,P2RX3,PFKL,PIGX,PLA2G1B,PLA2G4A,PYGM,RAP1A,SCP2,TAS2R50,UCK1,VEGFB
Negative_2779	CTNNA1,CXXC4,DDX23,MYH13,ATP6V0A4
Negative_2780	ASAH1,BHMT,CDKN1B,DTYMK,FARP2,GALNT10,GCLM,GLDC,MYLK2,PRDX6,PTPRJ,RPS6KA6,SMC1A,STAT5A,TAS2R38,TUBA3C,ATP6V1G3
Negative_2781	CYP4A22,GRIA3,OR1S1,OR5T3
Negative_2782	BAD,BCR,LHB,ACADM,ADCY4,CADM3,CCKAR,E2F1,EDN1,FGF13,G6PD,GALK1,GGT6,GNAT3,GNPNAT1,GOSR2,HADH,IL13,ITGA4,LAMC2,LRP5,MAPK7,MGAT4A,MGAT4B,MYH1,NCR2,NFATC4,OR1J2,OR2C1,OR5P3,OR5T2,PAK6,PIGA,PIP5K1A,PLTP,PPA2,PPM1D,PPP2R2B,PRKAR1A,PRODH2,TAS2R1,TGFA,WNT9B,MAPKAPK5,SERPINA5
Negative_2783	PRL,ALDH3A2,ALDH7A1,B4GALT2,BUB3,CACNG7,CD14,CDH5,COL4A4,COL4A6,CSF2,CYP2E1,DHRS4L2,GALNT14,GLUD2,GNA15,IFNA13,INPP5E,KLRC1,LTBR,MCM2,METTL2B,MPST,OR2M2,PFN1,PIP4K2C,SPAM1,SPRY4,SULT2A1,SV2B,TAS2R13,TUBB2C
Negative_2784	EHHADH,GATM,LARS2,MCM7,NUDT5,PDE8B,PPCS,PRKAB1,SESN3,SORD,TIRAP
Negative_2785	LPO,APOA1,CD86,DGAT2L4,FBP1,FBP2,FMO5,IQGAP2,LTB4R2,OR52D1,PPP2R2B,SHC1,ST6GAL1,SUCLA2,TFDP1,TOLLIP,TYMP
Negative_2786	AK3,ACACA,ARPC5L,CAPN2,CCL3,CD40LG,CREB3L1,DPM1,DRD1,GNA11,GNPDA1,HLA-DMA,HLA-DRA,IRAK3,KIR2DS5,LDHAL6B,MCM4,NARS2,OR10G9,OR1L4,OR6B2,OR7G1,OR9G4,PAFAH2,PARD6A,PNPT1,POLD2,RAPGEF4,RASSF1,SOCS7,TNFSF8,VCAM1,YES1,HSP90AA1,SERPINA5
Negative_2787	BID,FGG,ADIPOR2,ADRB2,AMPD3,BDH1,BIRC2,BPNT1,CDC20,CSF3R,CTPS,CX3CR1,CXCL6,EFNA4,ESPL1,FCGR3B,GDF5,GRIA1,IDH3B,IL2RA,KCNB1,LALBA,MAD2L1,MAP3K11,NANS,NFYA,NMNAT3,OLR1,OR10Z1,OR2AG1,OR2AG2,OR51T1,OR8B8,P2RX6,PDGFD,PER2,PIK3R4,PRKCQ,SNAP25,STAT6,STX12,TNFSF9,WNT7A,YWHAH
Negative_2788	PGF,CD14,CDC27,CHUK,COL4A4,COL5A3,DGKZ,FCGR3B,GADD45A,IL2RA,NFATC2,NME7,OR4F17,OR56A4,PARD3,PFKFB3,PIGA,RRAS2,STAT4,ATP6V1G2,CACNA2D1,PDCD1LG2
Negative_2789	FN1,ACSM2A,ADCY4,ADSL,CALML6,CAMKK2,CD22,CDH1,CTNNA1,FCER1G,FLT3,GAS1,GCLC,GPD1,GUCY1A2,HAO1,HYAL2,IL21R,ITGB3,KIR2DL1,LDHB,MAOB,MMP14,NTF4,NUDT5,OR1I1,OR6C3,OR7A10,OR9I1,PARS2,PDE3B,PLA2G10,PLA2G2D,PLCG2,POLR2D,RASSF1,SHC3,SIGLEC1,UGT2B17,VAMP1,WWP2,XCL2,HSP90AB1
Negative_2790	ATP5A1,CACNG1,CSNK1E,DUSP8,IRAK1,LTA4H,MAN1A2,OR2T6,OR52E8,OR52I2,PPP3CB,SOD1,TGFB1,TUBAL3,PHOSPHO1

Negative_2791	CR1,DCK,LTB,RFK,AK3L1,AKR1C2,BMP4,CEBPA,CLDN2,CNGA3,CRKL,CYP26A1,CYP2U1,CYP4A11,CYP4F3,DVL2,ENTPD3,GALNT3,GALNT5,GPX5,GSTA2,HLA-F,HMBS,LILRB3,MAD2L2,NAT5,OR4X2,OR51A4,OR52A1,ORC2L,PIGF,RFXANK,RXRA,SMPD3,SORD,SPTLC1,TNFSF18,UBE2L3,PNLIPRP1
Negative_2792	AK1,ILK,AGPAT4,CD79B,CER1,CREB3,CREB3L2,CSNK1G1,DDX50,DKK2,DNMT1,F11R,FGF13,FLT3LG,HLA-DMA,NCKAP1L,NEFH,OR1E2,OR2L2,OR2M7,OR4K17,OR5K1,PLCB4,POLR1D,POLR2C,PORCN,PTGS1,PTPN5,RPS6KA5,SKIV2L2,SMAD1,TAS2R49,TCIRG1
Negative_2793	IVD,ADRA1A,ALDH1A3,ASIP,CAMK4,CFL2,CFLAR,CHKA,COQ3,DDOST,DDX23,DOCK1,ECHS1,FGF14,JAK1,KIR3DL2,LAMA3,LMO7,MAP3K14,NOS1,OR4K13,PDXK,PPCDC,PRKACA,PRKAR1A,RHOD,STK4,TCF7,TGDS,TXNDC12,PNLIPRP2
Negative_2794	ID3,ADCY9,CD38,CDK7,CPT1C,EFNA5,ENTPD2,EPB41L1,HDAC2,NT5C1A,SDC3,TLR2
Negative_2795	AK3,C4B,CTH,TNR,ADH4,ADRB3,AKR1A1,ALS2,ATP6V1A,B3GALT1,CBLB,CCNB3,CD79B,CHAD,CSNK1G2,CXCL3,GSK3B,HYAL1,MAP3K2,MRAS,MYH15,NMNAT1,OR10J1,OR10Z1,OR1I1,OR3A1,OR5D14,OR9Q2,PCNA,PCYT1A,PDE1B,PHKA1,PORCN,RRM2,SUV39H1,TUBA1C,EIF4EBP1
Negative_2796	HK2,IL5,ACP6,ACSL5,B4GALT2,CACNG3,CCL21,CCR3,CX3CR1,CYP1A1,DUSP5,EPB41L2,HNF1A,IRF9,MAPK12,NFAT5,NRXN2,OR4C12,OR6C4,OR6C76,PARVG,PFKFB2,PGK2,PIGH,POLR1C,PPP1R3A,RPS6KB2,STMN1,STX8,WNT5B,WNT9A,ZFYVE9,HSP90AB1,MAP3K7IP2
Negative_2797	GK2,MET,SRF,ACP1,ACP5,AGPAT2,ATP5J,CCL1,CDC42,CHKA,CNTF,CRLF2,CXXC4,DFFB,EGLN1,ENPP3,FGF9,FLT3,GLI2,GNAI3,GNRHR,HAGH,HAGHL,HAP1,IL1R1,IL9R,INHBA,KLRC2,LCMT2,LDHAL6A,LMO7,NADSYN1,NPR1,OR13G1,OR2T1,OR8H2,PAK3,PCYT1B,PLCE1,PTPN6,RAC1,SLC25A4,SMURF1,TCF7L1,TNFRSF9,WNT10B,SERPINA5,TNFRSF13C
Negative_2798	ACOX3,AKR1B1,ALG8,DDX55,FGF13,G6PD,H6PD,KNG1,NFKB2,ONECUT1,OR1L4,OR52L1,PHGDH,TLR9,TNFSF10,YWHAZ
Negative_2799	ALDH4A1,DAD1,DTX4,GALNT2,OGDH,OR4F15,ORC4L
Negative_2800	CR2,FH,GP6,MOS,PAH,ACAT2,AGXT,ALAS2,ATP5C1,BMP6,CCL19,CCL27,CD58,CLDN11,CLDN5,CRB3,DAAM2,DDX52,GNA14,GUK1,IL17B,INHBC,KIR2DL3,LEF1,MBL2,NEFL,NTNG1,OR7A10,PDE2A,PDE8A,PLCD4,RAP1A,ROBO3,SFRP4,SLC25A4,SNAP25,SPRED2,SSH2,TIAM2,TYMS,VKORC1,WNT7B,CACNA2D1
Negative_2801	CFH,GLS,MUT,TNC,ACADM,ACSM4,ADCY9,ASCC3,AXIN2,BMP4,CDIPT,CHRNA7,CLTA,CSF1,CTSG,DOT1L,GALNT7,GSTA3,GYS1,IL12RB1,ITPA,KLKB1,LDHA,OR1D5,OR1J4,OR52A5,PAX4,PIAS1,PIGT,PIGU,PLOD2,SUV39H1,TUBB6,MAPKAPK3
Negative_2802	C5,GK2,TK2,CACNB4,CASK,DDX41,EREG,FBXW11,FMO1,HTRA2,MDH1,NOX1,OR51B5,PAK2,PDE1C,PIK3R4,PRSS3,RDH10,SOCS2,STAT5A,TBL1Y,TUBA1B
Negative_2803	HGF,ACYP2,ARNTL,ASPA,BMPR1A,CDC2,CLDN3,CLTCL1,GCAT,HLA-DRA,IL1RAP,NADSYN1,OR2W1,OR52A1,OR5F1,OR8S1,PPP3R1,RARS2,RPS6KA1,UBE2K,WNT10B
Negative_2804	ADK,CD6,GK2,ABLIM2,ARHGEF4,BPNT1,CACNG8,CLTB,CNDP1,CNTN1,CPS1,CXXC4,CYP1B1,DAD1,DPM1,EIF4B,GARS,HTR7,IL22RA2,ITPKA,LRAT,MINPP1,NAGLU,NEFM,NFATC2,NRG2,NTRK2,OR13C2,OR4D6,OR6C6,PARVA,PAX6,PIGU,PISD,POLR2A,POLR2J,RASGRP2,RPN1,SFRP1,SKIV2L2,SLC25A5,UBE2K,VAMP1,WASF1,WNT5B,PLA2G12A

Negative_2805	ADCY1,ADSS,CCL7,DGKZ,DIAPH1,DTYMK,FECH,FUT8,HLA-G,IL6ST,ITGA8,MGAT5,OR10A2,OR1L3,PCYT1A,POLR2B,PTGS1,RPIA,SOCS1,SPI1,UBE2K
Negative_2806	CEL,GCK,IL8,OSM,ACOT12,ACTG1,AGRP,ALDH3A2,AMHR2,ATP5E,CASP8,CD8B,CDH4,CYP11B2,CYSLTR1,DNMT3L,DOCK1,E2F4,FGF20,FGF9,GALNT11,HSD3B1,IL1R1,KCNB1,NCAM1,NOX3,NTN1,OR10A7,OR13C2,OR1L4,OR1M1,OR4L1,ORC4L,PDXK,PLXNB1,PYCR1,SEMA3E,SULT2B1,SUOX,TGFB2,WNT16,CSGALNACT2,ST6GALNAC5,TNFRSF10A
Negative_2807	ADO,DLD,ACTA2,ACVR2B,ALCAM,AMY2B,C9orf95,CADM3,CAMK4,CCL26,CDIPT,CEBPA,CHUK,CNGA4,CYP2S1,DAG1,E2F2,E2F5,FZD1,GALNT10,HMOX2,HTR2C,IFNA10,IL12RB2,IRAK4,ITGB8,KAT2A,LDHAL6B,MGAT1,MTMR1,NCOR2,NT5C,OPLAH,OR51S1,OR5H2,OR6C74,PAFAH2,PDK1,PIK3CD,SMPD4,TAS2R38,TAS2R50,UGT2B17,PAFAH1B2
Negative_2808	IL6,ACP1,ACTA1,ACVR1,CCL20,CD22,CRKL,CYP11B1,CYP1B1,DGUOK,EDAR,EPHA7,FOXO1,ITGA9,LIPA,MAFA,MAPK3,NCKAP1,NME7,OR10A5,OR2T6,OR4K13,OR8G1,PIGN,PTDSS2,SCD5,TGFBR1,TYMS,WNT3,EIF4EBP1
Negative_2809	ASL,CCNE1,CLDN10,CYP2S1,E2F1,FTCD,GYS1,IL28B,JAM3,TMLHE
Negative_2810	AMT,DAO,BAIAP2,CXCL12,DAAM1,FZD3,MAP2K1,MYH1,OR2M2,OR2T11,OR4K2,PAPSS2,RDH11,RPRM,WNT6
Negative_2811	NF1,ACACA,ACADM,AGMAT,AGTR1,ATP5H,BLVRA,CALML5,CD72,CHDH,CHPT1,DUSP9,EPHA1,EXTL2,FGF5,FLT1,FSHR,FZD3,GUCY2F,HMGCR,IFNA7,IFT57,IKBKG,LCMT1,MYL2,OR10K2,OR4C15,OR52N2,PIK3C2B,SCIN,SEMA3B,SH3BP2,TAS1R1,TNFSF12,TP53I3,TRDMT1
Negative_2812	DBH,EBP,GCK,ACADM,ACMSD,ACVR1,APOA1,BACE1,CD14,CD99,CYP11B2,IL10RA,INPP4B,KIR2DL1,NPAS2,OR4M2,POLR2D,PRODH2,SELP,SKIV2L2,SRD5A1,ST3GAL5,STX7,TDO2,THBD,UCP1,HSD17B10,MAPKAPK3
Negative_2813	CD274,GFPT2,GNA12,IFNA14,NUDT5,OR10G7,PHOSPHO1
Negative_2814	OR51A4,PDK1,PFN2,TPH2
Negative_2815	CAT,NPY,ACACA,ACSL3,AKR1B1,ATP6V1A,BUB1B,CACNA1A,CCNG2,CERK,CHRM2,CHRNA7,CSF3R,CYP26B1,CYP4F3,DPYD,EPHA2,GBA3,IKBKG,IL7R,JAK3,LDHB,MAP3K3,NT5C2,OR11H4,OR1L4,OR2AG1,OR2T12,OR2T29,OR4C6,PCYT1A,PCYT1B,PFN4,SLIT2,TDO2,TLN1,TOLLIP
Negative_2816	LCK,MTR,MVK,TNR,ACSS3,ALDH9A1,ASCC3,C1QB,CACNA1F,CASP3,CCL20,CCR10,CD58,CKS1B,COL5A1,CSNK2B,DGKH,DKK4,E2F1,GLG1,HEXB,HMGCR,HSD3B2,IGFBP3,IL19,KCNJ11,LCP2,LIMK2,MAP2K3,MAPK14,MMP1,MYL7,OR8K3,ORC2L,PDK1,PIP4K2C,POLR1A,PTCRA,RAB23,SELPLG,SEMA4A,STAT6,SUV39H2,TIAM2,TSC2,ULBP1,VEGFC,WNT3
Negative_2817	C8G,CRK,F11,GNE,NPL,ACSS2,AMY2B,ARNTL,ATG7,BLNK,CXXC4,DHRS4,DNMT3A,GCLM,GDE1,GGT1,GNRHR,GRIN1,HNF1B,HSD11B1,IDI2,IFNA17,IFNA4,IL15RA,ITPK1,LDHA,LIFR,MAPK7,NCK2,NLGN3,NTN1,OR10H2,OR2K2,OR51E1,OR9I1,PAK2,PCCA,PGAM2,PPP2R5C,PRKAG3,PRKAR1B,STAM2,STAT3,THBS2,VANGL1,VTI1B,WNT10A,CACNA2D1,ST6GALNAC3
Negative_2818	CR2,A4GALT,AARS,ACOT12,AKR1D1,ALG12,ARRB2,B3GNT3,CRY1,DGAT2,EXTL1,FZD5,GOT1,HMOX1,IKBKE,ISYNA1,MAPK9,ODC1,OR12D3,OR8G1,PGAP1,PIGV,PLA2G2D,RND1,SDHD,SLC2A4,STAM,USE1,VANGL2
Negative_2819	EBP,IL4,ALDH1A3,ALOX15,ATIC,BCL2L1,CALML5,CALML6,CDC14B,CDC23,CLDN19,DDX19A,FUT5,GGT1,HARS2,HGSNAT,IFNA10,ITPKA,LYPLA2,MDM2,MTHFD1,NCR1,NCR3,NCSTN,OR4D1,PAX6,PLCB3,PNMT,PRKCE,RAPGEF2,RHOA,SMURF2,TRPC1
Negative_2820	CSK,ADCY7,ALOX12,AVPR1B,CACNG4,CHKB,DDX4,GAMT,IL12RB2,MAP3K2,NR1H3,OPLAH,OR5B12,PPARD,RASGRP2,TNFRSF9,TP53,TRPM5,UGCGL1

Negative_2821	PC, ABL2, ABP1, ACO1, ADIPOQ, ALOX15, ALPPL2, ASAH1, B3GALT5, BCL10, CAMK4, CDK5, CHAT, CHRM3, CNTNAP1, CYP7A1, DAPK1, DEGS1, EPHA3, FGF6, GFAP, HAGHL, IL28RA, IMPDH1, ITGAE, LIPG, MAGI2, MAN1A2, MAN1C1, MAOA, MGST2, MTMR1, NEU3, NT5C1B, OR10H4, OR2A25, OR4K2, OR51B2, OR5V1, OR7D2, PGM3, PLCZ1, PMAIP1, POLR3K, PPP5C, SELP, SHISA5
Negative_2822	RB1, BMP6, CCR7, CDK4, CHRM2, CSAD, CTPS, HMOX2, IPMK, OGDHL, OR10A3, PDE4D, PDXP, PIGV, PPAP2C, PPP1R3B, STAT6, SUOX, TRDMT1
Negative_2823	ABLIM1, AMDHD1, CCL22, CCL27, CDC25C, CXCR5, CYP2C8, DCXR, DNMT3L, EPAS1, FTH1, HSPA1B, IFNAR2, KLRD1, MAP2K2, OR4K14, PLCZ1, PLEKHA8, POLD3, PPP2CB, RAP1B, SORD, TOMM40L, UGT2B10, TNFRSF1B
Negative_2824	C6, APPL1, ATP5A1, ATP5J2, CBR1, CD99, CYP26B1, DLL1, ENAH, GART, GSTO2, HMGCS2, INPPL1, MAD2L2, OR11H6, OR5B17, PIP4K2C, ROBO1, WASF3, B4GALNT1
Negative_2825	GK2, ALDH3B2, BMP8B, GAS1, NOX3, NT5M, PRKAA1, SUOX
Negative_2826	ADRBK2, AKR1D1, BMPR2, CCL8, CD36, CSF1, DUSP1, GBA3, GPD2, ITGAL, KIR2DL1, MAML1, MAP3K4, MAPK7, OR5D13, OR6C76, PI4KA, POLR3D, PTK2, ST6GAL1, SUCLG1, TIRAP, MAPKAPK5, PHOSPHO1
Negative_2827	CCR4, CLTCL1, CPT1A, ENTPD4, FADS2, FZD6, GNAO1, GUCA1A, HSD17B8, HYAL2, IFNAR1, LIPF, MOV10L1, NOTCH2, NRXN3, OR2F2, PIK3C2B, PLIN, SEMA3E, SPHK1, STX1B, TALDO1, TAS2R40, TICAM1, TJP2
Negative_2828	CD4, GLA, AKT1, ALDH1A3, ASPA, ATP5O, BMP5, CASP9, CCL23, CD72, CLTCL1, CMPK1, CYP2J2, DAAM1, DGKE, DPM3, EXTL2, FGF13, FTCD, FZD1, HMGCS1, HMOX2, ITGB8, LEFTY2, MAP2K5, MMP14, MYH6, NCK1, NUDT8, OR10G3, OR2D3, OR4K13, PAICS, PMVK, PNLIP, POLR3A, PPM1B, TJP1, PAFAH1B3
Negative_2829	BAD, LTB, PTS, ACAA2, ADH7, COL1A1, DAAM1, DUSP8, F13B, G6PC, IL17B, OR8D2, PLK1, RBPJL, SAT1, SEMA4A
Negative_2830	A2M, CAT, CGA, GP6, SRM, ACADVL, ACY1, ARNT2, ARRB1, CASP6, CFTR, EFNA2, ENTPD7, GADD45G, KCNB1, LAMA4, LEFTY2, LIMK2, MGAM, MITF, MYH4, OR10A2, OR10A4, OR13D1, OR1A2, OR1F1, OR4M2, OR4X1, OR52W1, OR8B4, OXCT2, PIGT, PIP4K2A, PIP4K2B, PPP2CB, PVRL3, RASSF5, SOCS3, TAP2, TLR3, TUBA1B, TXNDC12, UGT2A3, WNT1, XCL1, ATP6V1C2, GABARAPL2
Negative_2831	FAH, FAS, HYI, WAS, ACVR1C, ASCC3, ATP2A2, CACNB1, CD19, CER1, CSF3R, DHCR24, GALNT10, GANAB, GDF6, GSK3B, HMGCR, HPSE, ICOS, IL2RA, IL2RG, ITGB1, NAMPT, OR56A3, OR5K1, PCYT1A, PPP1CB, ROBO1, SDHD, SEMA3A, SMAD9, TNFSF9, TSC2, UNC5B, YWHAB, TNFRSF14
Negative_2832	ABLIM1, ADIPOR2, ALAD, ALDH1A1, BCAT1, DAAM2, DARS, DARS2, DDX50, GLS2, MDH1, NME7, OR1J4, OR52K2, OR5B17, PDE6G, PIAS2, PIGL, PLA2G5, PLTP, PTCRA, RDH12, TCIRG1, TXNDC12, SLC25A31, TNFRSF13B
Negative_2833	COASY, MAGI2, PCYT1A, PPP5C
Negative_2834	GNS, OTC, TNC, ATP2A2, ATP6V0B, CCR8, DHRS4L2, FPGT, PPP1R3A, PRKCE, PTGS2
Negative_2835	CR2, ACLY, APOA2, ARG1, BBC3, BCL2, CCL13, CCND1, CCR9, CD22, CLDN15, GPT2, GSTZ1, ICAM1, INPPL1, MAN1A2, MAP4K2, MOV10L1, NTNG1, ONECUT1, OR10A4, OR10Z1, OR4K1, POLR2F, RAPGEF1, SDHC, ST3GAL6, TAS2R38, TCIRG1, TRAF2, TUBA1B, VAMP1, WNT7B
Negative_2836	ID1, AADAT, ACPT, ACYP1, ADCY10, ADORA2A, ALDH7A1, AREG, BIRC2, CHST11, CHST12, CLDN4, CRY1, CYBA, EPHA3, GNAT3, HMBS, IGSF5, ITPK1, LCMT1, MAPK12, NAGS, NCKAP1L, NFKBIA, OR10H2, OR2L8, OR2M2, OR4F15, OR4K13, PIP4K2B, PROC, PRPH2, RIPK1, SCLY, SEMA3G, TNFRSF8, UGT2B28, PAFAH1B3

Negative_2837	AGK,BID,CTH,F11,FYN,GHR,PTS,WAS,ARHGAP5,CDC2,CHSY1,COL4A1,CXXC4,CYP3A4,DFFB,ELK4,EPB41L1,FECH,GBGT1,GNA12,GUCA1C,GUCY2C,IL15,ITGB5,JAM2,LRAT,MAD2L2,NMNAT3,OR1C1,OR2T8,OR52N4,OR5L1,P2RX3,PDGFA,PDGFC,PDK1,PLCG1,PLXNB3,PPP2CA,SEPHS1,SETDB2,SSX2IP,SV2B,TCF7,UCP1,UGT2A3,CACNA2D4,TNFRSF13B
Negative_2838	ILK,MTR,PLN,AKR1B10,CAMK4,CCL19,CCNH,CES7,CNTNAP1,CSF1R,DERL1,DPYS,GALNT7,HNF1A,IL11,MYH14,OR56A4,OR9A4,PPP1R3A,RENBP,SETD1A,STAT5B,STX10,THBS4,WHSC1,WNT1,TNFRSF11A
Negative_2839	TXK,ADAM17,ADH4,ALPL,CDKN1B,CLDN17,COL4A1,CTLA4,DGKZ,DUSP3,FARSA,FUT8,GMPR,GNRH2,HRH2,IGSF5,LIAS,MYH4,OR6A2,PDHB,PECAM1,PHGDH,PPOX,SGPP2,PLA2G12A
Negative_2840	F2,CHRM5,CPT2,CREB3L1,CYP11A1,DCTN1,DIAPH2,ENTPD6,FSHR,GDE1,GLI3,GYS1,H6PD,HLA-C,IL29,KNG1,LILRB3,MKNK2,MTFMT,OR2L2,OR52D1,OR6C1,PDXP,PI4KB,POLR3A,PROC,RARA,SPCS3,TAS2R7,WNT9B,YWHAH,ATP6V1E1
Negative_2841	SCD,MAD2L2,MNX1
Negative_2842	MPL,CCNE1,ENTPD4,OR5P2,SEMA4C,TOLLIP,TRADD
Negative_2843	IL6,AADAT,ACYP1,AKR1C1,ALG10B,ALG12,ATP5C1,ATP5G2,CAMK2A,CCL25,CHEK1,CREB3L2,CYP2S1,ENO3,FTH1,GNB3,GNRH1,HNF1A,IARS2,INHBA,INHBC,INPP4B,ITGAM,KYNU,LDHAL6A,NCKAP1,NPR2,OR1N1,OR2AG2,OR52E6,OR5H6,PDXK,PLCZ1,PLOD2,RHEB,SETDB1,SH2D1B,TBXAS1,TJP2,TLN1,TPI1,TRADD,TRHR,WASL,CSGALNACT1
Negative_2844	C9,ACSM2A,CACNB2,CCL5,CMAS,COASY,DNMT3A,EDNRB,IL15RA,INHBC,MINPP1,MMP7,NCOA4,NOD1,NR1D1,OR2AG1,OR5H2,PARD3,PDE1C,PTPN1,STEAP3,EIF4EBP1
Negative_2845	AK3,IVD,ADH4,BLNK,CACNA1I,CALML6,CD274,CRHR1,CSF3,DAAM2,GALNT13,GALNT6,HRH2,IL12B,NR5A2,OR1L1,OR4K1,OR52W1,OR7A10,PAICS,PARVB,PCYT1B,PLA1A,PNPO,POLR3B,SELP,SLC27A5,SOAT2,SOS2,ULBP1,WARS,ZNRD1
Negative_2846	ADK,ALG3,ATP6V1H,C1QA,CACNA1E,DGKA,EIF4E,EPHX2,GALNT2,GNPNAT1,IRF5,ITGA11,ITGA8,LHCGR,NFKBIE,OR2K2,PAK3,PARVA,PPP2R2C,PPP3CA,PPP3R2,PRDX6,SH3BP2,SMPD3,SOD1,TCF7
Negative_2847	PGD,FABP4,PAX6,PCK1,SCD5,UMPS
Negative_2848	ACTA2,CTBP2,GALNT9,GUCY2C,HDAC2,HEXA,NCF2,OR1J2,OR4K2,P4HA2,PDE4B,PLA2G4A,TUBB4Q,UGT2B28,VTI1B,WASF3,PAFAH1B3
Negative_2849	CD6,PC,ACSL6,ACTN4,ADAM10,ADCY4,ANAPC2,AOC3,ASNS,DBF4,DRD5,EPHB1,FARS2,FLT3LG,IDUA,IFNA7,IL29,KYNU,NPAS2,OR4K15,OR6C3,PIK3CA,PSPH,RBPJL,SETD8,SGMS1,SLC27A4,STAM2,TUBA1A,WNT1,XYLB,CACNA2D2
Negative_2850	C7,DHH,GNS,PLG,ACPT,AGPAT3,ATP13A2,C1QA,CDC20,CDK7,CERK,CLTA,DAD1,DOT1L,FARS2,GP1BA,IL20RA,JAK1,OR1D5,OR1N2,ORC1L,PAFAH2,PAK7,PARVA,PIGL,PLA1A,PLA2G2F,PLCD4,PNMT,PPP1R3B,RARS2,RASGRP1,RPSA,SOCS2,SRGAP2,TCF7L1,TGFB3,TNFRSF1A
Negative_2851	ALAS2,ATG7,CREB1,DLL1,ENPP3,EPHB3,FUT2,GMPR,HIF1A,LRDD,LTBR,OR5P2,PLCB1,PRPS1,STX1A
Negative_2852	ADAM10,CCNG1,CLCA2,IKBKB,OR14A16,OR4S1,PCCB,RXRG,TLR4
Negative_2853	F9,HPD,ME1,MTR,ACMSD,ACSM2A,ALDH3A2,ARRB1,BUB3,CACNB1,CACNB4,CCNE1,CDC23,CDKN1B,COL11A1,CXCL11,CYP26B1,EPHB6,FGF17,FGF8,FMO5,GAD1,GCLM,GLI1,GUCY2C,HAO2,HMGCR,KLK3,LTB4R2,MGLL,MYLK3,NFYC,NOTCH3,OR6C70,ORC1L,PANK4,PAX4,PDE4D,PIGT,PIGU,PIK3C2A,PPP2R2B,PTGES,RASGRF1,SELP,SLIT1,WHSC1L1,WNT3A

Negative_2854	F12, ACVR2B, ALAS2, APC2, ATP5E, CLDN16, CYP11A1, EPHX1, GAD1, GALK2, GALNT11, HSD17B4, MAML2, MMP2, NFAT5, NUDT8, OR10H2, OR4K5, OR7D4, PDE11A, PGM3, PIK3C3, PRKAR2B, PRKCB, PVRL4, RFNG, SETD1A, TAS2R1, TMLHE, UBE2L3
Negative_2855	IL9, SCD, TFG, ACYP2, APBB1, CCR2, CMBL, DAD1, EPB41L1, FARSB, FASLG, FMO3, GPT2, GPX7, GRAP2, GRIA2, HADHB, IDI1, IL20RA, IRAK3, LDHB, LTB4R2, NOTCH1, NPR1, NTSR1, OR10G2, OR1A1, OR51B2, OR51I2, OR5T3, ORC6L, PGM3, PKLR, PKM2, PRKAR1A, PRODH, PVRL2, SETDB2, SMPD2, SPP1, VCAM1, PPP1R12A
Negative_2856	ADK, GPI, PC, TEC, ACADL, ALG13, AMY2A, ARPC3, ATP5A1, BMPR1A, CD99, CNTF, CXCR5, DBF4, DDX4, DGKB, FGF10, GLUD2, GPD2, GSK3B, GSTM5, HCST, HMGCR, ICAM2, KLRC4, MYH10, NT5C, OR13G1, OR2D2, OR4F17, OR5L1, PGAP1, PHKA1, PIK3CD, PRKAR2A, RBX1, SARS2, SOCS7, TAP2, TAS2R38, TBXA2R, TFDP1, THBD, TUBB, WNT7B, CSGALNACT2, SERPINC1
Negative_2857	AR, ID4, ALDH4A1, ATP6V0B, CACNA1H, CCL5, CFLAR, CLDN18, CXCL5, GMPPA, GNAI3, IL10RA, IL10RB, IL17A, NCF2, NEUROD1, OR1C1, OR4D5, PTGES2
Negative_2858	ME2, ACSL5, ADRA1B, AOC3, ATP13A2, BAIAP2, CCND2, CYP2E1, ERO1L, FCGR2B, GCNT4, NFYB, PI4KA, POLR2G, RRAS2, TAP2, TAS2R1, TBXA2R
Negative_2859	AK3, DCN, HGF, SRF, A4GALT, ADRA1A, ARPC1B, ATP6V0C, B3GAT2, BACE1, CCR6, CHKB, CSNK1G2, ENTPD2, GALNT1, GNA14, GNRHR, GRIA2, GZMA, IL22, MGLL, OLR1, OR1L4, OR6N1, PFN3, PLOD1, POLR2B, RFWD2, SCLY, SELL, SMARCA5, UGT2B17, VAMP3, VAV2
Negative_2860	BTK, AADAT, ACO1, ACTB, ANAPC7, CCND1, CDC26, CYP17A1, DAXX, EREG, FCER1G, GIT1, HSPA2, JAK2, MAP2K7, OR10G7, OR1E1, OR5AC2, OR6C4, PARD6G, RBPJL, TGFBR2, TRIP10
Negative_2861	KIT, ALOX15B, GPAM, ICAM3, LTC4S, PIK3R4, TPH1
Negative_2862	AK3, CEL, GAA, JUP, ALOX15, ATP5G2, CACNG4, CLDN18, CXCR5, DHRS3, DKK2, ELK1, FGF12, IFNA16, IL22RA1, ITGA11, MAN1C1, MTMR6, MYH9, NOD1, NUDT2, OR14A16, OR4D6, OR5AC2, OR6C74, OR9A2, PCYT1A, SV2C, ATP6V1E1
Negative_2863	PGP, AKR1C2, CDKN1C, COMT, INHBC, MAOB, NCKAP1, OR51A2, P4HA1, SLC25A4, SQLE, ATP6V1B1
Negative_2864	FOS, RDX, AHCYL2, ANGPTL4, ATG7, ATP5G2, CNDP1, CRY1, CTPS2, DUSP8, F2RL1, FUT8, HDAC2, HSD17B8, IDI1, IMPDH2, ITGAV, ITGB4, JAK2, LRP5, MCAT, MYH4, OR1Q1, OR2AT4, OR4C16, OR51E2, OR6S1, OR8K5, ORC4L, PLCB1, PLCD3, PRKAR2B, PRKCQ, PSME1, SLC2A2, SMPD3, SNAI1, STAT1, TBXAS1, VAMP4, HLA-DQB1, ST6GALNAC3
Negative_2865	MPZ, BCAT1, BDKRB2, C1QC, CARS2, CCDC6, CD244, CD70, CHRNA7, CYBA, CYP11B1, CYSLTR2, GOSR2, GSTM3, IDH1, IRAK2, IRS2, MYH3, NPR2, OR4Q3, PDE2A, PLTP, PPOX, PPP2CB, PRKCD, PYCR1, STK11, TCF7, MAP3K7IP1
Negative_2866	ABO, PML, ADIPOR2, AKT3, AOC3, ATP2A2, C5AR1, CACNG1, CCL19, CCNE1, CDC42, CXCR3, DGKH, FGF4, HARS2, HPSE, HSPA1B, KCNB1, MYH13, NCF1, NFYB, NR4A1, NRG3, OR8B4, PDE1B, PDGFC, PIGP, PIP4K2C, POLD2, PTPN11, SSH2, ST6GAL1, UGT2B28
Negative_2867	DBI, ADSSL1, ATP2A3, COL6A1, EVI1, FLAD1, GADD45A, MAP3K8, MAPT, MCAT, MYLK3, OR4D11, PLCB3, POLA2, PPP2R5C, PRDX6, SESN2, STAM2, STK11, STX4, TCF7L2, ULBP2, GABARAPL2
Negative_2868	BAAT, BPNT1, EPHA8, PDHB
Negative_2869	GLA, JUP, ATP5G3, NAT6, RXRA
Negative_2870	FOS, PGF, AADAC, ADIPOR1, B3GAT1, BCL2L1, CTLA4, CYSLTR2, DCTD, DDIT4, EIF4E, EP300, GALNT6, GSTZ1, HMOX2, HSD11B2, IL13, IL1RAP, MAPK12, OR10H1, OR10X1, OR1E1, OR4C46, OR52E6, PDE3B, PDE4C, PIGV, POLD3, PPARA, PPAT, PRKACG, SHC3, SOD1, SSH1, UGT2B28, VAV1, WWP1, MAPK8IP1
Negative_2871	GLS, AMDHD2, B4GALT7, BCKDHB, CACNG5, CD274, COL6A2, DRD2, ENTPD3, IDI2, KCNJ11, NOTCH3, OR5A2, OR6N1, PPP3R2, RFWD2, STEAP3, TJP2

Negative_2872	AGK,AUH,DCN,F11,GP5,NOG,ACSL5,ATP5O,CCL4,CD80,CNTF,EPHB3,GNRHR,GSTK1,GSTT1,GZMA,KLRC2,MAP3K11,NKD1,OR1C1,OR2B11,OR2F2,OR51F2,OR52N4,POLE3,PTGIS,SOCS1,SPI1,SSH2,STX16,TAS2R8,VNN1
Negative_2873	LPO,CACNG7,CCL13,CD79B,CMPK1,COL3A1,CXCL12,FLOT1,GAB1,GRIA2,HTR6,IRF9,ITGAX,KIR2DL2,MITF,MYH7B,NLGN1,OR10X1,OR1N2,ORC4L,OXSM,PCNA,PIP5K1A,PIPOX,SEPHS1
Negative_2874	BRAF,C5AR1,CLDN16,COL1A1,DGKA,HDAC1,IL1R2,MAPK1,NME6,NT5C2,OR1B1,OR4F17,OR4K2,PDE1A,PGM1,RPRM,UCP1,UGP2,VAV2
Negative_2875	FGB,GLA,HGF,TK1,BMP5,C3AR1,CDKN2B,CNTNAP2,CYP2B6,DGKB,DUSP16,ENTPD1,FARP2,FOXA2,GALM,GALNTL4,ICAM2,IFIH1,IFNA10,LEF1,MAGI1,MTAP,NAGLU,NKD2,NUDT5,OR1S1,OR4K17,OR52B4,OR6C3,PRSS2,RIPK1,SOD1,TNFRSF13C
Negative_2876	MPI,SI,AADAC,ACAA1,ACSS1,ADCY5,ADRA1D,ALDOB,ALG8,ARNTL,AVPR1A,BHMT,CACNA1E,CAV2,CSH1,DHFR,EFNB1,FARP2,FBP2,FLT3,GCNT2,GSTM5,GSTP1,GYS1,HPSE2,HTRA2,IL10RB,INPP1,INPPL1,MDM2,OR10Q1,OR1B1,OR2M4,PIGH,PLXNB3,PRKACB,PRKG2,SKP2,TAS2R7,TSNARE1,WNT1,WNT2,CACNA2D3,PLA2G12A,PLA2G12B
Negative_2877	CNTNAP1,OR1M1,PPP2R5C
Negative_2878	DUT,HK3,ACO2,AKT2,C3AR1,CUL1,FMO3,MAPK13,POLD3,PSEN2,ROBO2,HLA-DRB5
Negative_2879	C5,MVD,CACNG4,CSNK1A1,IL10RB,MAP2K7,PCYT1B,TXNDC12,UNC5C
Negative_2880	DBH,ALDH5A1,ASAH1,BIRC3,CCL27,CLDN17,CRLF2,CYP4A22,DDX47,DNMT1,ENO2,ETNK1,HARS2,IL13RA2,IL23A,ITGA11,METTL2B,MYLK,NAT2,NFKB2,OR10A2,OR10G8,OR5R1,OR6K3,OR9A2,PIK3R1,PPP1R3C,PTEN,RIPK1,SIAH1,STAM2,STEAP3,STX1A,HSD17B12
Negative_2881	F7,E2F4,GJA1,GLB1,GMPS,ITGA4,LCMT1,MYH6,OR2T12,PAPSS1,PARVA,PLA2G4A,PRPH2,SMPD2,TLR2,WNT16,HSD17B10
Negative_2882	NOG,ACADS,ACSM2A,AGMAT,ARHGEF6,ARNTL,AVPR1B,C4BPB,CHST11,CNGA4,CXCL1,FMO4,GALNT5,GUCY2F,IQGAP1,LAMB1,LAMC3,NEU2,NLGN2,NME6,NR1D1,OR2AG2,OR4C13,OR4D9,OR51Q1,PDE7B,PIGM,PIK3CB,PNPLA3,POLR3G,UROS
Negative_2883	DCK,NPY,TKT,BCMO1,CMPK2,DTX1,ENTPD8,EPRS,FGFR1,GALNT6,GGPS1,GSTM5,IL12B,KIR3DL3,LAP3,MGST1,NANP,NCOR1,OR2C1,OR3A2,OR5H2,PCNA,PDE4C,PIAS2,PIGM,POLE,POLR2E,PPCDC,PRKAB2,RASGRF2,TRPC1,UXS1,VEGFB,CACNA2D2,CSGALNACT2,GABARAPL1
Negative_2884	GDA,HCCS,KITLG
Negative_2885	GP9,ADCY1,ALDH1L1,ASMT,CDC42,CSF2,EDA2R,GALNT12,GALNT14,GMPR,HPSE2,IPPK,ITGA1,ITGB5,OR2A2,OR51I2,OR52A1,OR52A5,OR8J3,OR8K5,PON3,PPARA,PPP1R3D,PRKCZ,SOX17,STX4,VANGL2,ATP6V1B2
Negative_2886	CMPK1,GLG1,KIR2DL2,LEF1,MAP4K2,MAT1A,OR7A10,RFNG,SOAT2,UGT8
Negative_2887	LCK,PTS,AGXT2,AREG,ATG12,BMP2,CCL25,CNTF,CPOX,DGUOK,DHRS4,GCLM,GIT1,ITGA8,JAK2,KLKB1,MCM6,MS4A2,MYH6,NEU1,OR10AG1,OR10J5,P2RX5,PAPSS2,PLCB3,PLCE1,SLC25A6,SMARCA5,STMN1,TRAF4,VDAC2,YES1,CSGALNACT2
Negative_2888	C4B,F9,IL3,SMO,TNF,ACADVL,ACTC1,B3GALT2,CCKAR,CCL3,CTNNB1,CTTN,CXCL16,EFNB3,IKBKB,IL23A,IL7R,IRAK1,LEFTY1,MAPK7,MKNK2,NKX6-1,OR10S1,OR4C6,PPAP2C,RHOA,TOMM40L,TUBB2A,VAMP4,WWP1
Negative_2889	GP9,OTC,XDH,APOC3,CAV1,CD22,EPHX1,FGF18,FTH1,GAD1,OR10G7,OR1K1,OR2D3,OR2L2,OR4F15,OR5AU1,PDE9A,PTGIS,PTPRB,RAF1,SEMA4D,TAS1R3,TIRAP,TNFSF11,UGT2A1,CSNK1A1L,SERPINF2
Negative_2890	AMY2A,CNDP1,INPP5A,NOS3,OR1J1,PTK2,SELP
Negative_2891	PML,ADSSL1,ATG7,ATP5O,CCL28,F2RL1,FGF6,GOT2,MNX1,OR13F1,OR2Y1,OR4C6,POLE,PTDSS2,SLC2A4,TBXAS1

Negative_2892	ACOX2, ATP5H, EPHA7, GALE, GNG3, GRIN2B, NADSYN1, NAT5, P2RX3, PPP5C, PSPH, SARDH, SOCS2, ST3GAL4, TRAF1
Negative_2893	CGA, PML, ACADSB, AOX1, ATP2A1, ATP5J2, CACNA1D, CCR7, CDKN1A, CLDN10, CLDN4, CREBBP, DGKZ, DHRS4L2, DPM1, FABP4, GADD45A, GCNT2, GFPT1, GRPR, IL23R, MPDZ, MTHFR, NAT1, NMNAT3, OR10H2, OR52E6, OR56A3, PDE9A, PIP5K1B, PTEN, PTPN11, SH2D2A, SOCS1, TAS2R9, TGDS, THTPA, TNFSF12, XCL2, TNFRSF1B
Negative_2894	GH2, KMO, TK1, BHMT, BMP6, CCL7, COMT, CREB1, FASLG, FGF18, FZD10, GALE, GALNT7, GGT6, IKBKG, LEFTY1, MYH7B, MYLK3, OR10A6, OR1D5, OR2M3, OR2V2, OR52A1, OR5P3, OR8K5, RFX5, SETD2, SULT1A2, TRHR, TYRP1, UGT8, VDAC1, WNT9B, ZBTB16, CACNA2D2
Negative_2895	DLD, GK, ACAA1, ACOX2, CSH1, ERBB3, GALE, GMPPB, KLRC1, NTNG1, OR14A16, OR52B4, PAICS, PIK3C3, TICAM1
Negative_2896	CKM, TNC, ARNT2, EHHADH, IL1B, IRF5, OR10A4, OR2T2, PPP2R1B, PPT1, RND1
Negative_2897	CFI, F12, ANAPC11, ARSE, CD14, CDC27, CDK4, CHKA, CLDN17, CSF3R, DDX55, GSTA1, KAT2A, LTBP1, MYH14, NFAT5, NFKBIA, NUMBL, OR4D2, OR5K1, OR7D2, PDE4D, PRDX6, RCHY1, RDH11, RPIA, TCF7L2, TCIRG1, TUBA1A, TYMP, UGT2B17, VAMP4, YWHAZ
Negative_2898	ACHE, AGPAT2, DGUOK, IL22RA2, INSR, KLK3, NPAS2, NRG2, NRG4, OR2A5, OR4D1, ORC2L, PLXNA3, PORCN, SMPD1, UPP1, SERPINF2, TNFRSF1A
Negative_2899	EGF, JUN, COL3A1, OR4F5
Negative_2900	SPN, ABL1, ACYP1, ADH4, ADRB1, ADRBK2, AMPD3, ATP5G1, CBR1, CDKN1C, CFLAR, CKMT2, CNTNAP1, CREB3L2, CSNK1D, DHCR24, DTX4, DUSP16, EXT1, F2RL3, FCER1G, FGF11, GJD2, GRB2, IL18, MAPK11, MTHFD1L, MYH2, NARS2, NEU1, OR14J1, OR2A12, OR51Q1, OR52N4, OR52W1, PANK1, PLXNA3, PRKAG1, TRDMT1, UXS1, VAV1
Negative_2901	SFN, ALG3, APLP1, CDC20, CSF2, CTF1, DTX3L, DUSP8, FSHB, FZD5, GALNT2, IFNGR2, IGF1R, IMPDH2, LIMK1, MICA, MYD88, NEUROD1, NKX3-1, OR10G9, OR8K1, PIK3CB, PPM1A, PTPRZ1, RALGDS, STX12, TACR2, TUBA3E
Negative_2902	B4GALT7, BDH1, CNGA3, CPT1C, CYP1A1, DIAPH3, FGFR4, GADD45A, GNAZ, HADHB, KCNB1, LAMB4, NFYC, OR2T1, OR4D6, OR51A7, PGAM2, PRKAG3, SMPD1, TNFRSF4, HLA-DRB3, HSD17B12
Negative_2903	AR, ALAS2, ARAF, AVPR1B, CACNG7, COL6A1, DNMT3L, DTX3L, FZD9, GIT1, IL6R, INHBC, MAN1A1, NOX1, OR5B21, OR6C70, PANK3, TRAF4
Negative_2904	CCL5, CD99, CDC26, DAG1, FGF10, FGF12, FLT1, FZD9, GNB3, HMBS, IBSP, IL9R, IRAK3, MAN2A1, MAP3K7, NCR2, OR4F5, OR7G3, PLA2G2E, PPP1R3D, PPP2CB, SGPP2, SYNJ1, TAS2R50, TM7SF2, AASDHPPT
Negative_2905	FOS, ALPL, BHMT, CDC6, COL3A1, DHFR, DPYSL2, FZD10, IFNA8, IL11, ITGAL, LDHC, MAGI1, NAT2, PDE1B, PGM2, PIGW, PKM2, RBKS, SUCLG1, TIAM1, TLR9, ZBTB16, ATP6V1G3
Negative_2906	TPR, ARNT, BCMO1, CMAS, CPT1C, DTX3, EARS2, ENPP7, ITGA6, LAMB2, NFASC, OR52R1, PRKG2, RRAS, SRGAP1
Negative_2907	ALG10, GCNT3, GNA12, GPAA1, NPR1
Negative_2908	A2M, DBH, AADAC, AGRN, APLP1, CALML5, CD79A, CER1, CRLS1, CTNNA2, CYP21A2, DEGS2, DGKZ, DVL2, FRAP1, GLUD2, LPAR1, MAGI2, MAGI3, MAOB, MIOX, NCAM1, OR13C4, OR2D2, OR51L1, OR52K1, OR8H3, PFN3, POLR2B, POLR2H, PPAP2A, RARA, RXRB, SEPHS1, SMURF1, THBS1, TRHR, TSNARE1, VCAM1, VDAC3, WHSC1L1, WNT5B, SUV420H2
Negative_2909	AGL, KHK, ALDH3B1, BCL2L1, CSNK1E, CYBB, DERA, DOCK1, FLT3LG, FZD1, GGT6, HEXA, IL4R, MYH3, NCAM1, OR4K5, OR52E4, OR56A4, PGAM4, RDH11, SELPLG
Negative_2910	CKB, DVL3, EPOR, OR5B3, UNC5D, YKT6

Negative_2911	ATG3,SOCS2,PRICKLE2,TNFRSF13C
Negative_2912	ME1,TXK,ACSS1,APC2,ARPC1B,CARS,CD79A,CLDN4,CLDN7,CTF1,CYP1A1,DNMT3A,EPHB1,FGF2,HTR6,IBSP,IL11RA,NKX2-2,NTNG1,OGDH,OR10G2,OR2M7,OR2T11,OR3A2,OR4F15,OR51I1,PAX4,PDE3B,POLR1A,PON2,PROC,RBPJ,SLC33A1,TAP1,TKTL1,TRAF4,TYMP,CSNK1A1L,HSD17B12
Negative_2913	LPO,ARNT,BCKDHB,CLDN1,EPHB2,GRIN2C,MKNK1,OR51S1,OR5M8,STT3B
Negative_2914	MAOB,OR10P1,OR52N4,SIGLEC1
Negative_2915	IDS,ADCY3,ADH4,ADH6,ALDH1B1,ALG8,B4GALT3,BLNK,CD59,CLTCL1,CNTN2,CYP4A22,DKK4,EPHA1,EPHB4,GALNTL1,GSTA5,GUCY1B3,IL17B,IPMK,MARS2,NTSR1,OR6T1,PGK2,PIK3C3,POLD3,RENBP,RPSA,SEMA3D,SEMA7A,SIAH1,SMAD7,SMURF1,TAS2R9,TNFSF10,ULBP1,ULK3,SERPINA1
Negative_2916	GOT2,PIAS2,RAC3,TAP1,TBL1XR1,TNFSF11
Negative_2917	F3,ADSS,ALDH9A1,BMP8A,CYBA,EARS2,ENTPD6,GPAM,GSTK1,HCST,INADL,IPPK,MLYCD,NRXN3,OR2T10,OR5D16,PDE2A,PTTG1,PYGB,SETD8,TAS2R40,TUBA3C,TUBB4,TUBB4Q,VDAC2
Negative_2918	KHK,MTR,AACS,ABCC8,ACTA1,AKR1C2,ALG8,ANAPC10,APOA2,ARAF,BDH1,CACNG8,CCL1,CCL8,CLDN8,CNDP1,DUSP7,EPHA2,ERBB3,FGF4,GPLD1,HIF1A,IFNA17,LYPLA1,OR10K2,OR5T3,OXTR,PFKFB3,PIAS3,PLOD2,PMM1,RASGRP4,STAT3,THBS2,TRAF3,VEGFB,SUV420H1
Negative_2919	ARAF,CCL13,CHST11,FDPS,HHIP,INPP1,PFKFB3,PIM1,PLXNC1,TICAM1,TNFRSF6B
Negative_2920	CTNNB1,FGF10,FSHB,GCDH,IL4R,MAP3K13,MYL5,NRG3,NTSR1,OR10J5,OR13D1,PDXP,PIGC,PTPRF,PTPRM
Negative_2921	CACNG1,DPM2,HTR6
Negative_2922	PML,ATP2A1,BLVRA,CYP19A1,DFFB,DGUOK,FGF23,LRP5,POLR1B,PRKG2,PTGES2,SEMA7A,TLR3,ATP6V0D2
Negative_2923	DBH,AFMID,CCNA1,CTNNA3,EDNRB,EFNB2,ENTPD1,FADD,FMO3,FZD4,GRLF1,HAGH,ITGA1,LAMB4,METTL6,MICA,NPR2,NUDT8,OR10G2,OR5AR1,OR6K6,OR8H3,PAPSS1,PRDX6,PROS1,PYGB,RAP1B,RFXANK,SOCS2,SPP1,TNFSF9,WASF2
Negative_2924	CSF2RA,OR4D2,OR5L2,OR6C70,VAMP4
Negative_2925	FGG,FUK,C1QC,CCDC6,CCL27,CD44,CYP11B1,DUSP14,DVL1,ENPP7,G6PC,GCDH,IFNGR2,INHBA,LIMK1,MYH2,NODAL,OR10K2,OR11L1,OR8K5,PKLR,PLA2G1B,PRKX,RAPGEF1,RAPGEF4,SNW1,SOCS1,TNFRSF10C
Negative_2926	LYN,ACADS,ADIPOQ,CCL17,CDS2,CSF3,CYP19A1,ENPP3,FARSB,FLAD1,G6PD,GALNT14,GLI1,GSTM1,ITGB5,OR2AT4,OR4M1,OR52D1,OR8D1,PLA2G5,PPT1,SYNJ1,TCIRG1,TGFBR1,TYK2,UROD
Negative_2927	EZR,SRC,ACSM2A,ALDH1A1,AMY2A,BPGM,CCL3,CCNB2,CDC14B,CDH15,CDK2,CHAD,CHEK2,CNTNAP1,CREB3,CTSG,CTSL1,DTX2,FBXW11,FCGR3B,FMO3,GGT5,HIF1A,IFNA14,IL25,LCMT1,MPST,NGEF,NUDT2,OGDH,OR10G9,OR3A1,OR4M2,OR6C4,P4HA3,PCK2,PCYT2,PLA2G10,PLA2G2D,PTGFR,RDH16,RERE,SELP,SEMA4G,TYMP,B4GALNT1
Negative_2928	GDA,HK3,ANGPTL4,C4BPB,CFLAR,CPOX,CREB3L1,F2RL1,GAD2,GUCY2D,GZMA,HDAC1,IFNA13,IFNA14,JUND,MAOB,MS4A2,MTHFD1L,OR10A7,OXSM,PAPSS2,PDIA4,PIK3R4,PITX2,POLD1,POLR2A,POMC,PYCRL,PYGB,RUNX1T1,SLC25A4,STX10,WNT16,WNT4
Negative_2929	C8B,CBL,TK2,ACACB,ACTN2,ALOX12B,CACNA1E,CXCL2,CYP26C1,CYP3A5,DIAPH2,DPYS,EPB41L3,GCNT2,GTSE1,HNMT,IQGAP1,ITPKA,LIPE,OR1L8,OR2AE1,OR4A15,OR5M9,PDE1C,PFKFB2,PFN1,PIGO,PPP3R1,PRKAA2,RASGRP1,SDHA,SLC2A2,SMPD1,SPRED2,SSH1,TBL1X,UNC5C,VAMP2,ZNRD1,ARHGEF12,CSNK1A1L,SUV420H1

Negative_2930	BAX,DCK,ACP2,ACSM2A,ADCY7,ADSSL1,ALPP,ASCC3,ATG7,AXIN2,CCL2,CD59,CTNNA3,CYP2S1,DDX51,FGF12,HIBADH,HTR2B,LDHAL6A,MAN1B1,MASP1,MGAT3,NRXN3,OR10G8,OR6C75,PGK1,PLA2G2F,PSEN2,RDH5,SPHK1,ST8SIA5,TNFSF14,TOMM40,TUBAL3,VAV2,VEGFA,ATP6V1B1,B3GALNT1,SERPIND1
Negative_2931	DCN,INS,A4GALT,CD70,EPB41,EPHB2,FGF3,IFNA21,JAM2,LIPC,MAP2K4,MDM2,OR2G3,OR2L2,OR4D6,OR52E4,PLAT,RASSF5,SHC1,SPRY3,TAS2R14,UAP1,YWHAB,CSGALNACT1
Negative_2932	PML,SMS,OR10T2
Negative_2933	AR,UBC,ACPP,ALDH9A1,ATP6V1A,CLDN1,HHIP,HMBS,IRS1,METTL2B,NFKB1,OR2T29,OR4C13,PLXNA2,PMVK,SCIN,SUOX,TLR9,TSC1
Negative_2934	F8,FGG,B3GALT6,CCNE1,DDB2,DUSP1,GNAL,HEXB,HLA-C,HSD17B3,NAGS,OR5A1,POLR1D,SKP2,SOS1,ST3GAL5,TAS2R48,TRAF4,TRHR,USE1,HLA-DRB3
Negative_2935	C8A,HYI,ITK,AK3L1,BCMO1,CCL17,CDK7,DFFA,DLAT,DPAGT1,ENPP6,GRIA2,GSTA1,GUCA1A,HPSE2,HSD17B2,IFNA21,KDSR,MTAP,MYH13,OR13C4,OR2S2,OR4M2,OR8D2,PIM1,PLD2,PPCS,PRKCG,PSEN2,RUVBL1,SEPHS1,SLIT2,THBS4,TUBB,TYRP1,ULBP2,XYLT1,PNLIPRP2
Negative_2936	DCT,EPO,ALDH1B1,ALOX12,APPL1,ARHGAP5,ARNTL,BACE1,CD8B,CREB5,CSF2,GNAQ,HLA-F,IFNA6,IGFBP3,LAP3,LDHC,LRDD,MAP3K11,METTL6,NRCAM,OR2A5,OR2AT4,OR5V1,PCYT2,PDE2A,PIP4K2A,POLR3G,SLC25A6,STX10,UGT2B10,UROS,ALDH18A1
Negative_2937	ACTN1,OPLAH,PGAP1,RASGRP1,STX5,TNXB,ATP6V1B1
Negative_2938	ACVR1B,ADSS,ATP5E,DHFR,GRLF1,KIR2DL4,NQO1,OR11A1,OR4C15,OR4C46,SMAD5,SPCS3,TACR2,TPH1
Negative_2939	SDS,BIRC2,CYP2F1,DEGS2,EDNRB,GALNT7,HYAL2,IL23R,OR4A16,PDE11A,RFWD2,UGT2A3,MAP3K7IP2
Negative_2940	GH2,ANPEP,ARRB2,BMPR1A,CCR4,CLDN1,CTSG,EPB41L1,FGF11,GALM,GALNT3,GRIN2B,GSTO2,H6PD,HNF4A,IL17RB,KAT2B,NCR3,NEFL,OR52E2,OR52I2,OR6C2,PAICS,PRODH2,PTPN5,RALBP1,TAS2R3,TP53,TRDMT1,WNT7A,YARS
Negative_2941	AK5,C7,FUK,GH2,OSM,APOA2,APPL1,ASAH1,ATP12A,ATP6V0C,CCR4,CDKN1A,DAAM1,EFNA3,EPHA3,ETNK1,F2RL1,FGF11,GCLC,IKBKE,IL1B,IL21R,MCM3,MMP7,MYL9,NR4A1,NUDT9,OR13J1,OR4D6,PGAM4,PLA2G10,PORCN,PPID,SEMA4C,STAM2,TLR4,TSLP,TUBB3,UGT2B28,WNT5A,XIAP,PHOSPHO1
Negative_2942	LCT,BBOX1,COL1A2,CXCL13,CYP4F3,DDX41,DUSP6,FOXO3,KRAS,LALBA,OLR1,OPLAH,PER3,TSC2,SLC25A31
Negative_2943	C1S,NNT,SYK,ALCAM,ATP5F1,BCAT1,C1QA,C1QC,CAMK2B,CASP7,CCNB3,CDH2,CLCA4,COL4A2,EFNA4,FIGF,FUT4,FZD5,HLA-DMA,ITGB5,LCMT2,MAPK11,MASP2,MGAT5,NEUROG3,NFYB,OR10K1,OR4C15,OR4K1,OR4K15,OR51M1,OR5AK2,PDE5A,PIGO,PIK3C2B,RPS6KA4,SEMA4C,SHMT2,THY1,TNFSF12,TRDMT1,UGCG,PAFAH1B1
Negative_2944	CDH3,FGD1,OR4D6,RFWD2,VEGFB,YWHAQ
Negative_2945	GSR,ACVR2B,CD27,DAXX,EARS2,ENPP7,FMO5,GANAB,IL1A,IL3RA,ITGB1,MAN1B1,OR1L4,OR5H6,P4HA3,PAK4,PARS2,PCK1,PIGV,PLCD3,PRPH2,PVRL4,RRM2,RUNX1,SAT1,SMAD3,TRAF3,UCK2,YWHAG
Negative_2946	C4B,CR2,PDC,TNR,ABP1,ACVRL1,ADORA2B,ALAS1,ATP5E,ATP6V1F,CD59,COL4A4,COL4A6,CSNK1G3,EHMT2,GALNT3,HPSE,ICOS,ITGA1,MCCC2,MCM5,MPST,NSD1,OR2T33,OR2T5,OR4F4,OR4K14,OR4X2,OR5I1,OR8J3,PGM3,PHGDH,PPM1D,PPP1R3D,PPP2R5B,RBL1,SELP,SLC33A1,SNAP23,SPRY4,TAS2R16,THBS4,TUBB3,TYMS,UGT2B10,UPB1,WNT6

Negative_2947	ITK,KIT,TEC,ADH5,ARHGEF2,CDC25B,CDC7,CLDN2,CYP4F2,DAPK2,G6PC,GALNT1,GANC,GBE1,HLA-C,HSD11B1,INPP4B,IQGAP3,MAN1A2,MMP7,MYH14,OR10G7,OR2T33,OR8S1,PDGFC,PFN3,PISD,RFX5,RRAS,SDC3,SMAD3,SNAP23,TPMT,WNT7B
Negative_2948	IL8,BBOX1,CD247,CD36,CLDN16,COQ7,CYSLTR1,DPYD,ELK1,GADD45B,GGCX,GLO1,HAP1,NKX6-1,OR10H3,OR13J1,OR2A4,OR51B4,PANK1,RAB23,SYNJ2
Negative_2949	GK,KMO,LHB,AK3L1,B4GALT3,CCKBR,CFL1,CHST11,DNMT1,EDA2R,EIF4B,FARSA,FDFT1,NME7,OR1F1,OR3A1,OR4B1,PPA2,SDHA,SMPD4
Negative_2950	ACTN4,ADCY10,ALDH3A1,ATP6V0C,CD59,DAPK1,GALNTL1,IMPA1,KIR2DL4,LPAR1,LRRC4C,MAN1B1,NEU2,NGEF,OR10G9,OR6B2,PAPSS2,PFKFB2,POLR3H,SUFU,WNT1
Negative_2951	AMT,CP,GGH,NOG,PGD,ARHGEF1,B4GALT7,BLVRA,CCL13,CDKN2D,CSF1,CSNK2B,DGKG,DKK1,DKK2,DOLPP1,EGLN1,EHHADH,FPGT,GNRH2,GSK3B,GUCA1C,GUCY1A3,IDUA,IFNGR2,LAMB4,MECR,MYH9,NAT2,OR14C36,OR1J4,OR2AG2,OR4M2,OR5AN1,OR6Y1,OR8J1,PERP,PRKCI,PVRL4,RXRB,SHMT2,SIGLEC1,ULK2,UNC5A,VAMP1,WNT7B,PPP1R12A,SERPING1
Negative_2952	GSS,ADCY2,ANAPC2,AOX1,CNGA4,EIF4E,ENPP1,GNAQ,HSD17B7,MAP3K4,MCEE,NMNAT2,OR51E1,PDE9A,PIK3R3,PRKCI,RFXANK,SESN3,TRPM5,YES1,ATP6V1B2,GABARAPL2
Negative_2953	SRM,ACVRL1,CCNG2,CD40LG,CDO1,CERK,FASN,FCGR2B,GRLF1,IL9R,OR2L2,OR2T1,OR4S1,RBPJL,SMAD1,WNT2B,TNFRSF17
Negative_2954	FGB,PRL,ABCC8,ABL2,ACADS,COL5A2,CREB3L3,DDHD1,DRD2,DUSP8,F2RL2,FGF5,HLCS,HMGCS1,IFNA13,IRF9,MAD2L2,MAPK3,MAPK7,MGLL,NAT6,OR10V1,OR4P4,OR6K6,ORC4L,OSMR,PAX4,PLD2,RDH5,SPRY1,VAMP2,B3GALNT1
Negative_2955	FAH,RFK,ARSB,ATP6V1H,CCDC6,CYP1A2,EPB41L3,FLT3,FOXO3,HIBCH,IDH3G,IFNAR2,IFNW1,IL17RA,MAP2K5,NLGN2,NT5C2,NT5E,OR2T8,OR51A4,OR52L1,P4HA2,PLA2G3,PRKCB,RAB23,TCF7L1,TGFB1,UBA7,WNT5A
Negative_2956	GH2,ALDH3B1,ARPC5,CCNB3,CCR5,CDKN2C,CHRD,CTNNA2,DAAM1,FZD3,GPAM,OR51B2,OR8U1,PIAS1,PPP2R5B,RPIA,RXRA,SETDB2,TGFBR2,SERPINC1
Negative_2957	TEC,BMP5,CCNA1,CXCL3,DPM2,DUSP7,DUSP8,FSHR,GNRHR,LFNG,LTBP1,MKNK1,OR1A1,OR2T5,UNC5A,HLA-DQA2
Negative_2958	C3,DLD,SHH,TFG,ACSL1,ACTC1,AFMID,ATG12,ATP5J2,BTRC,CHPT1,CTNNB1,DDHD1,EPB41L3,FGF7,GCDH,GSTA3,HMGCR,IL12RB1,IRS2,ITPK1,JUND,LAMB3,MAP4K1,NANS,NPAS2,OR11L1,OR1N2,OR2T1,OR4K5,OR6C1,PCNA,PIAS4,PPP2R5E,PPP3CB,PYCR1,RRM2B,STAT6,UGT2B4
Negative_2959	ABO,ALG6,APC2,DCTN1,FGF3,LCAT,LRAT,OGDHL,OR1N1,OR4K13,PDK1,WNT8A
Negative_2960	ADC,CAD,MVD,SRC,AARS,BCAT2,CYP26B1,DDX19A,EFNB2,FGF18,FIGF,GALNT12,GNG13,KLRK1,LARS2,LCAT,LCP2,MAP3K8,NAT2,NUDT8,OR11H4,OR13C3,OR51A7,OR51B4,OR5B21,PPP3CC,RDH12,UGT2A1,WIF1,PPP1R12A
Negative_2961	C1S,HGF,ABLIM1,ACOX3,ADORA2A,ALG3,ANAPC1,BCAR1,CD40LG,CHST13,FUT4,HSD17B8,IFNG,ITGB1,LCMT1,MARS2,MYH7B,OR3A1,OR4K14,OR5T3,OR6N1,OR6Q1,PFKFB1,PIK3CG,PIP5K1B,PRKAB1,PROC,PTGER3,RHOQ,STX10,HLA-DPA1,TNFRSF13C
Negative_2962	CACNG8,CCR1,DAD1,DAPK3,DHCR7,EDAR,EDNRB,ENO1,EPHA1,GADD45A,HADHA,HIBADH,HLA-DMA,LIFR,MASP1,MTHFD1L,NFASC,ONECUT1,OR4N4,OR52D1,OR5AK2,PDE6D,PNMT,ROBO2,RPS6,TP53I3,WNT7B
Negative_2963	DCC,MPL,ABI2,ASIP,GLS2,GNB1,IFNA7,IL6ST,KLK3,MASP1,MRAS,MYL9,OR13G1,OR2F2,OR51B2,PDE1A,TAS2R1,TFDP1

Negative_2964	FST, BUB1, DTX3L, HAO2, HSD3B1, JAM3, LYZL1, NFAT5, NLGN1, PIGP, PLCD1, RXRG, SMAD1, STAT5A, TJP3
Negative_2965	CSNK2A1, ELK1, EPHA2, FGF22, LAMB2, MCCC2, TYMP
Negative_2966	EZR, ACTN1, ACY1, IL11, SETD2
Negative_2967	ACSM1, ALS2, ASNS, ENO2, EP300, FZD2, GMPPB, GNA12, IFNA17, IL5RA, INPP5B, MAP3K1, MAPK10, MAPK11, METTL2B, OR2G6, PDHB, PLOD1, POLR2F, PPP1CA, PRPH2, XCL2
Negative_2968	AARS, ALDOB, DUSP14, PANK2, PGAP1, TMLHE, YARS
Negative_2969	DBT, DCN, EZR, FYN, SYK, ALDH1L1, ALG11, ARPC4, B3GAT2, BCKDHA, BDH2, BLVRA, CASP3, CASP7, CKS1B, COX10, CXCL2, DDHD1, DKK4, EI24, EPB41L3, FOSL1, GSTA2, GSTO1, HDAC2, HTR2C, IFNA17, IL20RA, MAP3K11, MYH13, NEUROG3, NKX3-1, NME6, NOTCH2, OR13C4, PPBP, PPP2R2B, PRKCA, PSME2, PTAFR, PTPRZ1, SPCS1, SPHK1, TNFSF11, TPH2
Negative_2970	CBL, GHR, JUP, ACAA1, ADORA2A, AKR1B10, ALAD, BCKDHB, CACNA1S, CSNK1E, CYP3A4, DAAM2, FGD1, GLUD1, GNAZ, ITGAD, JAM2, MTHFD1, MTHFR, NKD2, OR10G8, OR2AE1, OR3A2, OR52N2, PDIA4, PLXNB2, PPP1CA, SMAD9, SPRED1, SRD5A1, ST8SIA1, TFDP1, TLR9, UXS1, VAMP4, VAV2, VCAM1, WARS2, WHSC1L1, TNFRSF14
Negative_2971	LSS, PML, ACY1, BAAT, C3AR1, CALML3, CALML6, CD48, DRD2, GALNT3, LAMC3, LIPF, OR11G2, OR2A4, OR4D2, OR4X1, UBE2L3
Negative_2972	AMH, FAH, ID2, LCK, ME1, ACAD8, ADORA2A, ADRA1D, ATP5G2, CDH1, CMPK1, CSH1, CYFIP1, CYP2A7, EPHB1, FGF17, GALNT9, GGCX, HTR5A, IL17RB, IL18RAP, IMPDH2, ITGB1, MAP3K1, MAP4K4, MDH2, MLYCD, MTHFR, NEFH, OLAH, OR14I1, OR2B2, OR4K13, OR5M8, OR6C75, PHKA2, POLR2I, PORCN, PRPH, RELA, SEPHS1, SETD7, TAS2R1, TFDP1, TGFB2, UBA1, WASF2, SERPIND1
Negative_2973	SRM, TPR, CCL23, CLOCK, GOT2, HNMT, POLD4, SLC27A4, TLR4, UGCGL1, XYLT2
Negative_2974	PML, BUB1B, CACNG5, CFTR, COL1A2, DCXR, FIGF, GSTO2, HNF1A, IFNA14, LRP6, NAGA, NFATC2, PGM3, RPS6KB1
Negative_2975	ALG8, CLCA4, DCXR, GGT5, OR2AG1, OR2F1, OR51I1, PPM1D
Negative_2976	AK5, C9, CS, TNR, VCL, ARSE, B3GALT1, CHKA, CTTN, DVL2, EFNA5, IPMK, MTHFS, NAGA, OR10AG1, OR10G2, OR10G8, OR2T12, PARD6B, PGM2, RBL2, SCIN, SOS1, SUCLG2, TRAF4
Negative_2977	BST1, CYP17A1, ECHS1, NME6, RPS6KA4, TP53I3, WNT6
Negative_2978	NPY, ACSM2A, CBLC, CCL7, CCNE1, CCNH, CDC25B, CHEK1, CLDN4, ENTPD7, FGF21, GNRH2, HCCS, IRS1, ITPKA, MAP2K2, NAMPT, NUMBL, OR10G9, OR2AG1, OR51Q1, OR8K5, PIK3R3, RBX1, RFXANK, RPSA, SH2D2A, STAT3, STX8, UGT2A1, WWP1, TNFRSF13B
Negative_2979	GCK, ADORA2A, ALOX15B, C1QB, CACNG1, CARD11, CCR6, CXCL3, CYP2D6, DDX19A, DGKQ, DHRS4, EPAS1, EPHB3, FARSA, FGF23, FZD3, GNPDA2, IL10RB, LAMC1, MAP4K4, NCSTN, OR11H6, OR6C75, OR6X1, PAPSS1, PIGV, PIK3C2A, PIP5K1A, PLA2G1B, PLIN, POLA1, POLR3B, PPARG, PPP3R2, RAB23, RASA2, SARS2, SENP2, SMURF1, SOCS7, TGFB1, TSTA3, WARS2, XCR1, YWHAG, CTNNBIP1, MAP3K7IP1, PAFAH1B3
Negative_2980	DCT, ACSS1, ACSS3, ACTN2, AK3L1, ALAS2, B3GALT4, BCKDHB, CAMK2A, CCL11, CCR2, CDK5, CIITA, COL4A2, CRLF2, CUL1, CYP2S1, DAAM1, DGKD, DHRS3, EXTL1, GAL3ST1, GART, GNAQ, GUCY1A2, IL18R1, ITGA2B, ITPA, LDHD, MOV10L1, MYH1, NCF4, NKX3-1, NUDT12, OR10V1, OXCT1, PNPT1, PRDX6, PTGS2, RDH12, RIPK1, SCP2, SEMA3B, SIGLEC1, SMAD3, TRAF1

Negative_2981	KMO,ACACA,ALDH3A2,ATP5A1,C4BPA,CAMK2G,CCL7,CYP2B6,FADD,FOXA3,GLI1,GNAI3,GPX7,HARS2,IFNA17,IQGAP2,IRF9,LTBP1,MAP2K2,MAP4K1,NEFH,NEUROD1,OR1L3,OR1Q1,OR4K15,OR51D1,OR9A4,PDE9A,PER3,PI4KA,PLCB4,POLD2,SEMA4C,ST3GAL4,SUOX,SV2A,TAP1,VAV2,MAPKAPK3
Negative_2982	INS,LPO,MVK,OGT,ALDH3B1,BDKRB1,CARS,CHDH,DLL4,DOCK1,DRD1,FUT8,GUCA1C,IDH3A,IDH3B,IFT57,LIPE,NAT2,OGDHL,OR1D5,OR5I1,PDE7A,PDK1,PEMT,PER1,PTPMT1,SC4MOL,TBXA2R,WNT16,YOD1,HSD17B12
Negative_2983	AR,FES,ID2,ACAD9,ACO2,ALG3,ANPEP,BIRC5,C9orf95,COL4A1,COL4A2,CSF1,ECHS1,GLUL,IRF5,LARS2,LDHC,MAP3K6,MAPK3,NFKBIB,OR10AG1,OR2T10,OR2T11,OR4N5,PDE4C,PDK1,PFKL,PIK3R4,PLA2G5,PLCG2,PRKY,PSME1,SDC3,TAS2R42,TICAM1,TUBB,ATP6V0A1,HLA-DQA2,HLA-DRB3,PHOSPHO1,PLA2G12B
Negative_2984	F7,LPL,NNT,OSM,ACADM,ACVR2B,ANGPTL4,APOA1,ARD1A,ATP5F1,C1QB,CDKN2C,CMBL,CREB5,DGKZ,DUSP16,ENO1,HMGCL,KIR2DS5,MYH6,NUDT8,OR10A7,OR52H1,OR8D2,PAICS,PDCD1,PDE11A,PRKCE,PTK2B,SH2D1A,STK36,TAS2R49,TLR2,TNFSF9,WNT16,WNT8A,XYLT2
Negative_2985	BID,AMPD3,CHIA,CXCR3,DCTN1,PDE8B,PIGB,PPP2R2D,SLC2A4,HSD17B10
Negative_2986	FYN,JUP,LPL,PRL,ACSM4,APOC3,B3GAT2,CBLC,CD244,CDH1,CDK5,CLCA2,CYFIP2,FZD10,GAD2,GFPT2,GRM1,HSD3B2,IL10RB,ITGAE,KLRC4,LDHB,MCM2,NKX2-2,OR7A5,OR9G4,PANK4,PDE4B,PDGFA,PPARD,SEMA4C,SUV39H1,TSHB,UNC5D,VNN1,YKT6,ATP6V1C1
Negative_2987	PXN,TNR,ALG8,ARD1A,ARHGEF7,ATP6V0B,B3GAT1,B4GALT3,BET1,C1QA,CDC25B,CHSY1,COL6A1,DIAPH1,DNMT3B,EPHB1,FGF2,FOXA2,FZD10,GCDH,GMPPB,GNB3,HIF1A,IL13RA2,IRF7,LTBR,MBL2,MGAT2,MGAT4B,MKNK1,NAT5,OR2AG2,OR2T4,OR2W1,OR51M1,OR5F1,PARS2,PLXNC1,POLR2F,ROCK2,SESN1,ST3GAL5,STK3,UBE2L6,XCL2,SLC25A31
Negative_2988	CCKAR,CD46,FZD7,GNAI2,RAP1A,RBKS,TYRP1,UNC5A
Negative_2989	ABLIM1,ACSL3,ACTN4,APH1A,CCL24,COL5A2,CTBP1,CTPS2,GLI2,GMPPB,IRAK3,LFNG,LHCGR,LIPE,MMP2,OR12D3,OR2T6,OR4C15,OR52E6,PCCB,PDE2A,PFKFB3,PHKA2,PIP5K1B,SOCS2,SPAM1,SV2A,TRPM5,TNFRSF14
Negative_2990	GK,SP1,ALDOA,APOC3,CACNA1E,CCL27,CD99,CDC42,CDH2,CXCL16,CYP2J2,DHX58,DLL3,DUSP10,DUSP7,EPHA1,FDFT1,FGF11,GALNS,GGPS1,GOT1,GPD2,HLA-DOA,IFNB1,IKBKG,IL13,IPPK,JAK1,LARS2,LRAT,MYH15,OR4A16,OR4S1,PDGFC,PIPOX,PLA1A,PRSS1,ROCK2,SFRP2,SLC2A4,SULT1E1,TAS2R42,VDAC1,HLA-DRB5,SLC25A31
Negative_2991	CRK,TXK,AANAT,ACOT11,ADCY3,AHCY,CACNB2,CCL23,CLDN7,CYCS,DPM1,DUSP8,EPHB3,ESPL1,FGF3,HSD3B7,HTR4,IL11,IL1R2,IRF9,IRS1,JAM2,LCMT2,MMP9,NCK2,OR10H3,OR2B11,OR4L1,OR51A7,PLA2G2F,RHEB,SLC27A4,ST3GAL6,TFPI
Negative_2992	AK1,BCR,CSK,LYN,AANAT,APOA2,CLDN22,CREBBP,CYP3A43,DCTN1,DUSP14,EFNA1,EFNA5,ERO1L,FGF19,FGFR1,FLOT1,FUT9,GRM1,HTR5A,ITPK1,L1CAM,LIPE,NARS2,NEUROD1,NTRK2,OR2D2,OR2T33,OR51I1,OR6C3,OR8G1,PGM1,PIGV,PON1,PPP2R5A,PPP2R5B,RDH8,SMAD4,SOCS3,SULT1E1
Negative_2993	C2,DDC,LYN,AASDH,BCAR1,CISH,DAG1,DLL3,MPST,OR5M9,PLAUR,SELE
Negative_2994	EBP,MTR,HSD11B2,HSD3B1,MDM4,NME4,OR52K1,STAT6,SUCLG1,UBE2L3,UCK1

Negative_2995	GH1, XDH, ADIPOQ, ALDH3B1, CASK, CHRM3, CXCL10, DNMT3A, DTX2, EARS2, EDNRB, F11R, HLA-DOA, HSPB1, IL22RA1, ITPK1, MARS2, NFKBIA, NT5C, OR2T5, PCYT2, PGAM2, RAF1, RUVBL1, ST6GALNAC4
Negative_2996	BTC, PC, SPN, ADAM10, ATP5D, CCL8, CDH2, CSH1, CSNK1G2, DGAT2L4, EPB41L1, GADD45A, GGT6, ITGA2, ITGA5, LAMC2, LEFTY1, OR10C1, OR8B8, PIP4K2B, PLCG2, SH2D2A, SLC2A4
Negative_2997	ARNTL, HSP90B1, PKLR, SERPING1
Negative_2998	C3, GDA, GNE, KDR, TYR, ADRA1D, AGPAT4, ATN1, B3GAT2, CCR1, CHEK1, COL6A1, DHCR7, EPHB1, FOXO3, GALNT8, GDF7, HARS2, LDHC, NEU4, OR13C4, OR14J1, OR2L2, OR4F4, OR5M8, PANK1, PGM1, PPA2, PPARG, PRLR, RBX1, RPS6KA5, ST8SIA1, THBS3, UBE2L6, MAPK8IP2, ST6GALNAC3
Negative_2999	LCT, ACPP, ADH6, B3GALT2, BNIP1, CD70, CD80, CDS1, CHRM2, CIITA, CYP11A1, DUSP8, E2F3, GP1BA, IL12RB2, IL13RA2, IL22, KLRD1, NOS3, OLAH, OR10A4, OR2F1, OR8J3, OR8S1, PFKL, POLD2, PPAP2A, PPP1CA, PTGES2, SESN2, SOCS4, ST3GAL6, STAT4, THBS2, TRHR, WNT2B, ATP6V0D1, ATP6V1C1
Negative_3000	LPO, ACOT11, AKR1C4, CACNG6, CACNG7, CDIPT, CFTR, CHST12, DARS2, ENPP1, HLA-G, IARS, MKNK2, OR1E1, OR2W1, OR7A17, SV2C, CACNA2D3, SERPIND1
Negative_3001	IL3, B3GNT4, IL17RB, NEFH, OR52W1, OR6K3, OR9Q1, PKLR, POLD4, ST8SIA5, TRIP10
Negative_3002	PGF, ALAD, AOC2, ARHGEF4, BCL2, BHMT, C1GALT1, CREBBP, CSF3R, CTF1, CYP3A4, ERBB4, GSTM5, GSTO1, HYAL2, KAT2B, NADSYN1, NEFM, OR4N5, OR52K1, PAPSS2, PCCA, PIM2, PRIM2, PTGFR, SGPP1, SOD1, SRD5A1, TALDO1, VNN1, WASL, TNFRSF17
Negative_3003	ANAPC7, CPT2, SLC9A1, SSH3, THBD, TRIP10
Negative_3004	DBT, DDO, GLA, A4GALT, ACSM2A, AGXT, B3GAT2, CAV3, CIITA, CXCR6, DARS2, EDNRA, G6PD, GADD45A, GLI3, GSTZ1, IL29, ITPK1, MYL9, OLR1, OR1C1, OR2Z1, OR51I2, OR5AC2, OR6C75, OR8H2, OXCT1, SETD2, ST8SIA5, TGFBR2, TLR4, TRMT11, VEGFB, TNFRSF18
Negative_3005	ACSM3, BPNT1, CNGA3, FABP1, GARS, ITGB6, MAD2L1, MAN1A2, MAP4K2, MDH1, OCLN, OR1J2, OR1J4, OR51A2, OR5D13, OR8K3, PDE10A, PLA2G6, PLXNC1, RFXANK, RFXAP, SEMA4F, STK3
Negative_3006	CGA, CRK, DBT, CCL15, ERBB4, FGF19, MAT1A, MYH11, NKD2, PIGN, PRKCE, THBS3, TNFRSF8
Negative_3007	AGL, EGF, ACACA, ACSL6, ADIPOR2, AKT3, CAPN2, CCND2, EPHB2, FMO1, GNA12, GRIN1, HLCS, INSRR, MAT1A, NAT1, OR10H3, OR1N2, OR2A12, PDGFD, PIAS4, PIGW, PIK3C2G, PIP5K1B, RFX5, TLR9, UPP1, UROD, VTI1B, WNT7A
Negative_3008	FAH, PTS, A4GALT, ATP5A1, BIRC2, CMPK1, DPYD, DRD1, F13B, FLOT1, GALNT3, GMPS, GNMT, GUCY2F, HIP1, HSPA2, IFNA10, IL10, IL20, IL28A, KIR2DS5, P2RX7, PON1, PRKCH, RFNG, RPS6KA4, SAT1, TPMT, TUBA1B, UROS
Negative_3009	CAT, GGH, HK3, NOG, PVR, SRF, ADCY6, C9orf95, CD36, CLDN19, CMBL, CNGA4, DDOST, DEGS2, DUSP8, FBP1, FGF17, FUT6, GAPDH, IARS, IDH1, KAT2A, KAT2B, NRXN2, OR1N1, OR1S2, OR2K2, OR4A5, OR8H3, P4HA2, PDE1B, PEMT, PIGO, POLD3, PTCH1, RELA, SCP2, SENP2, SIGLEC1, SLC27A1, SLIT2, SPRED1, STX18, THTPA, YWHAQ, MAPKAPK5, ST6GALNAC3, TNFRSF13B
Negative_3010	ATM, FGG, GGH, ALPL, ATP5G2, B4GALT1, CAMKK2, CCL7, CD55, CDH4, CYBA, CYP17A1, DFFA, DHCR7, ETNK1, GALE, GDF5, GNAT3, GNPAT, HLA-C, IKBKE, IL7R, MAML3, MAPK12, MCM2, MYH7B, NCAM1, OR10G7, OR10G8, OR1J4, OR2M7, OR4C6, OR7G1, PDE4A, PDE8B, PGAM1, PPID, PRKCA, PSME1, PTPRF, SETD2, SUCLG1, SUCLG2, TUBB4Q, UGT8
Negative_3011	CR2, HDC, HK2, AGMAT, CAV1, IDH2, LIPT1, LTC4S, OR11L1, OR2AG1, OR2M7, OR2S2, PGM1, SETD1A, SOCS1, WASF3

Negative_3012	CCNG1,CD3E,CHRM2,COL5A1,CREB5,DEGS2,FZD9,ITGA9,JAK3,NNMT,OR6C2,OR7G3,PARD3,PDGFRA,PIGQ,PIK3CD,PMM2,PPM1D,RHEB,SMPD1,THTPA,YWHAE
Negative_3013	ALDH3B2,ATP6V1A,EXTL3,FIGF,FLAD1,LRP6,NCK2,SLIT1,TAS2R7,ATP6V0A4
Negative_3014	GAA,ME3,MET,AKR1C4,BIRC3,CCL25,CD80,COQ7,CREB5,CXCL12,DKK4,GLUD2,GNAQ,GZMA,HSD17B8,NT5M,PPID,RFXANK,SPI1,TAS2R8,YWHAE
Negative_3015	PRL,CASP3,CCL26,CDKN2D,CXCL12,CXCR6,ENTPD6,FDFT1,ITGB7,OCRL,OR10H2,OR2Y1,OR51Q1,OR6X1,PCK2,ROBO3,UGP2,VAMP2
Negative_3016	AGK,AUH,FES,ID2,TFG,ACP6,ACSM5,AFMID,ALDH3B1,ARHGEF7,B4GALT3,CADM3,CASP8,CMAS,CSNK1G2,DOCK1,ERBB3,ERBB4,EXTL2,FABP4,FZR1,GART,GNAS,IRF9,LRDD,MAP4K4,NEUROD1,NOS1,OR2AG1,OR3A2,OR4D6,OR4M1,OR6C3,PARD3,PDE1B,PLOD2,PLTP,PPP3R2,PRKAB1,RBL1,STAT3,TPH1,TYRP1,VEGFB,WNT9A
Negative_3017	F2R,FGA,AGPAT1,ARG2,C4BPB,DAPK2,DTX3,FGF3,GCNT3,GNB3,GRB2,GSTM3,ITGB7,OR4L1,PDGFB,PTPN6
Negative_3018	CBS,IHH,TH,ALPI,B3GALT4,DRD2,GALNT9,GNA12,GPLD1,GZMB,HSPA8,IGF1R,OR1J1,OR2M5,ULBP2,WNT10A
Negative_3019	CD2,NGF,ABP1,ALPP,ALPPL2,ATP6V1A,BUB1B,CCL15,CCR5,CER1,CFL2,DARS,DHX58,DLL1,DRD1,GLI2,GNPDA2,GSTA2,HMBS,IL11RA,MAP3K3,MDM2,MYLK,NTF3,OR2T1,OR51F2,OR51V1,P2RX2,PANK2,PARD3,PDGFA,PIGK,PPP2R1A,RASA2,RETSAT,SLC33A1,STX12,STX6,TLN1,TP73,TPH1,TYK2,UCP1,UGT2B17
Negative_3020	ACP6,ALPPL2,CYP2C8,DAD1,FGF12,HAP1,NFATC4,PIGN,SEMA3G,ST6GALNAC5
Negative_3021	DCK,NNT,RPE,ADH1B,ADH5,AVPR1B,B4GALT1,C7orf16,CD79B,CD99,CER1,CHAT,CYP2F1,EVI1,FGF20,FGF8,FTMT,FZD8,GPAM,GPX5,HTR2B,ITGAD,KIR2DS4,KIR3DL2,MAGI3,MGAT5,NFASC,NGEF,OR4A15,OR7D2,PIGM,PRIM2,PRKAR1A,SDC2,SDC3,SIAH1,SKIV2L2,SLC25A6,SQLE,STEAP3,GABARAPL1,TNFRSF1B
Negative_3022	BAX,NNT,ACAT1,ACSL6,ARNT2,CD3E,CTNND1,CXCL6,DARS2,DDX55,DTX4,INPP4B,KLRD1,MGAT5B,MMP2,NANS,NFASC,NT5C1A,NUDT5,OR11H1,OR2L3,PIGT,PITX2,PMM1,PNLIP,PRKCD,PSME1,SGMS2,SOAT2,WNT10B,ATP6V1B1
Negative_3023	IL7,ILK,ACY1,ALDH1A2,AMY2A,ARPC3,B3GAT2,C3orf10,CES7,CYP11A1,EXTL2,IFNK,ITGAX,ITPKB,JAG1,PAFAH2,PCK2,PIGQ,PSAT1,PSPH,SLC2A1
Negative_3024	CBL,EDA,GAA,GHR,ABLIM1,ATP2A2,CASP9,CBLC,CMAS,DGKB,ENTPD7,EPB41,EPHA1,FABP1,FGF11,GATM,GUSB,IL12RB2,ITGB3,LEF1,MAP3K8,MAPK1,MCM2,MMP9,NR1D1,NTN1,OR2AG1,OR52I1,OR5AK2,PAK2,PIAS4,PLAT,PLCZ1,PRODH2,RXRB,SHC1,SRGAP3,WASF1,ATP6V1C1,PPARGC1A
Negative_3025	AMT,C1S,GP9,HGF,IL3,PTS,ABL2,ACADSB,ALOX15B,ARSE,ATP5H,BET1L,CAMK2A,CHKA,GMPPB,HCST,HIF1A,ITGA9,KLRD1,MAD1L1,MAPK14,OR11L1,OR2M3,OR4A16,ORC5L,PGK2,PIK3R3,QARS,RDH10,RFNG,SMC1B,TAS2R48,TP53I3,TYROBP,UGT2A3,VTI1A,WWP2,ATP6V1C1
Negative_3026	HAL,ACAD9,ARHGEF7,ARNT2,CD44,E2F4,HNF1B,HTR6,KARS,LAMC2,MAPT,MLYCD,MYLK2,NARS,OR10A4,OR14J1,OR1S1,OR4E2,OR52I2,PAPSS1,PPARG,QPRT,RAD51,RAF1,SPRY3,STX10,VDAC2,VDAC3,WNT16,ZIC2,CACNA2D3
Negative_3027	MPZ,ADSSL1,ANAPC5,ANAPC7,B3GNT3,CLDN17,CYP1A1,DDX55,GJD2,GLDC,GNPDA1,HSD17B8,MYL2,NOX1,OXTR,PPP2R5E,SEMA4G,SMAD5,SMAD9,SPRY2,SUV39H2,VAMP4,CACNA2D1
Negative_3028	BID,ID4,ACACB,ACTN1,CCL16,CHDH,CYP2S1,FZD5,FZD8,HLA-G,LILRB3,NT5M,OR2T10,PRKAG3,SCD5,STX6,TP53I3,ULK3

Negative_3029	FGB,IHH,CD38,COL4A1,CSNK1A1,DHDDS,GSTK1,IL17A,MAP3K12,MAP3K4,MGST3,NTNG1,OR1N2,P4HA1,PAICS,PDHA2,PIGW,PPCS,PPID,PSME1,RUVBL1,SETD8,UCKL1
Negative_3030	DUT,HGF,ME2,ATP5L,BMP6,CCNB1,CLCF1,CYP19A1,GGT1,GPD2,GRIA1,INADL,ITGA9,ITPK1,LEF1,MAP2K3,MYH13,NFX1,NME7,OR1E2,OR2B2,OR4F5,OR51D1,OR6Y1,PARD6A,PDGFA,PTPRC,SNAI1,STX7,TBXA2R
Negative_3031	C4B,LCK,ME3,AKT2,ALG8,ATP5J2,BET1L,FBXW11,FDPS,GNPDA1,IDH2,IFNA17,IL13,IL15RA,MAGI3,NMNAT1,NR1H3,NTRK2,OR10A6,OR2C3,PCYT2,PIGV,PLCD3,RASGRF1,SNAP23,TICAM1,TSC2,WEE1
Negative_3032	CAT,FST,HAL,KIT,ABLIM2,ACADM,ADCY10,AOC2,BHMT,C7orf16,CD58,DIAPH1,FGF2,FIGF,FUT1,HEMK1,HMOX2,LIPT1,LTBP1,MAP2K2,OR2W1,OR6N1,OR8J1,ORC2L,PAK7,PDPK1,PHKG1,PIGM,PIM1,POLR1D,PTPMT1,SCP2,SHC3,TUBB6,ZBTB16
Negative_3033	C5,C8B,CS,FGA,PC,PVR,ASNS,CARD11,CASP3,CD22,CTSS,EDN1,EXT2,FASN,HSPB1,INMT,MAPK3,NRG4,OR2M5,ORC2L,PARD6G,PDE4D,PDGFB,PRKAA2,PSME2,RFXAP,TJP3,VKORC1,PAFAH1B1
Negative_3034	C7,AASS,ALDH3B1,ARHGEF7,CACNG3,CLDN20,FUT8,ICOSLG,IDH1,MECR,NCOA4,NUDT8,OR14A16,OR1N1,OR5AC2,OR6B2,PIP5K1B,PMM2,RENBP,SELE,TUBB4,UCP1,SERPINA1,TNFRSF10A
Negative_3035	EGF,C4BPB,DDX52,EPHA6,FGF6,GMPR2,GNA15,GUCA1B,JAK2,LCAT,MGAT5B,OR11H6,PDIA4,RAC1,RPS6KA5,SDHA,ST3GAL6,TNXB,TOMM40,TRAF2,TYK2,ST6GALNAC6
Negative_3036	WAS,XDH,ADH1B,AMHR2,ARD1A,ARHGEF2,BCKDHA,CCKBR,CDK4,DDB2,DERA,DUSP4,GDF6,GRIN2D,HAAO,HPRT1,IL23A,MCM3,OR10K1,P4HA1,PRKY,PSME3,RPS6KA5,TACR3
Negative_3037	CDC14A,CISH,ITGA2B,PDCD1,PIK3C2B
Negative_3038	CBS,GNS,NPY,SHH,ABLIM2,ACACB,ACTA1,ACYP2,ALAS2,ALCAM,BCKDHA,CASP3,CCL13,CDC42,CES2,CLDN9,COL1A1,CTPS2,CXCL3,FPGS,GCNT3,HAO1,MTHFS,NKD1,NOTCH2,OR10H3,OR1S2,OR4N2,OR52I1,PDCD1,PIM2,PPP2CA,PTGFR,PTGS2,QARS,RAC3,SEMA4C,SNW1,STX2,TAS1R2,TSC2,VAV3,WNT16,HLA-DPA1
Negative_3039	CBS,HGD,NPL,ACAA1,ADH7,ARPC5,ASRGL1,ATP4B,BMP5,BNIP1,CD14,CD72,CDC20,CDKN2D,CFL2,CHRM1,CYP2C8,ENTPD8,F11R,FGD1,FGFR2,GNAT3,HDAC1,IAPP,LDHC,LIMK2,MCM3,NCOR1,OR10J5,OR2W1,OR2Y1,OR4E2,OR4K1,OR52E8,OR52J3,OR8J3,PFKFB1,PFKFB3,PPP2CA,RASSF1,SH3BP2,SMAD7,SPHK2,STAT5A,TUBA3C,UGT2B28,YOD1
Negative_3040	BTC,CCR10,CD38,COL4A6,GALNT5,MAPK14,POLR2D,SDC4,SRGAP3
Negative_3041	CTH,BET1L,CEBPA,CSF3,ELK1,F2RL3,FZD8,GABARAP,GADD45A,GUCY1A2,IFNW1,IL21R,INPPL1,MAP3K5,MMP14,OLAH,OR5AU1,UBE2L6
Negative_3042	ATP5L,DIAPH2,GSTA1,GUCY2D,ITGB4,LTBR,OR4C15,OR52N1,PROC,RUVBL1,SOD1
Negative_3043	CS,MYC,ADAM10,ARRB1,ATP5J,CACNA1I,CD14,CDH3,CISH,COL1A1,CXCR6,CYP2C18,DTX1,EFNB3,EHMT1,GCDH,GGT7,IARS2,IL12A,IL6R,LAMA3,LAMC3,MARS2,MYLK,NFX1,NTRK1,OR2H1,OR2T11,OR52B6,PIGP,PNPT1,RRM2,SDC2,SNAI1,SRD5A1,TACR1,TMLHE,UCP1,UGT1A10,ULK2
Negative_3044	C8G,EPO,IVD,PGD,PTS,AMPD1,ARPC5L,B3GNT5,CACNA1E,CDH1,CNGA3,CNTF,CPT1C,CRLF2,DGUOK,ERBB3,HLA-DRA,NOS3,OR10X1,OR1L3,OR1S2,OR4K5,OR5M3,OR5T1,OR7A10,OR7A5,PCNA,PDE6H,PDXK,PFN4,PIGT,PTK2B,RDH11,RPS6,RPS6KA6,SH2D1B,SSH3,TPMT,UGT8,VAMP2
Negative_3045	IL4,ALDH1A3,ALOX12B,BCKDHB,CAV2,CCDC6,CSNK1E,CTF1,DTX3,EPHA4,FGFR3,IMPA1,OR8J3,PDE10A,PDGFRA,PIGW,RALB,SEMA4B,STX18

Negative_3046	C2,HYI,MVK,SDS,ALPPL2,ATP4A,BBOX1,CRY2,CYSLTR1,EPHB2,FOXO3,GOT2,IL20RB,IL5RA,ITGB4,ITPA,NRG2,OR13C2,OR2L3,PIGS,PTPN7,RAPGEF3,ST3GAL6,THY1,MAPKAPK3
Negative_3047	NNT,B4GALT2,COL1A1,F13A1,G6PD,GMPS,LALBA,MAFA,MALT1,OR10K1,OR1S1,OR2T12,OR7C1,PIGP,SNAP25
Negative_3048	C8A,RET,E2F5,EDN1,NCKAP1,OR4A5,OR6C76,PPBP,PPM1B,TXNDC12
Negative_3049	GPI,HDC,ZYX,AOC3,APOA1,ARRB2,BACE2,C9orf95,CASK,CDK5,CSF3,CYP4A22,DHRS4,FDFT1,FGF19,GALNT6,GNG12,KNG1,MAP3K3,MASP2,METTL6,OR1E1,OR4A5,OR4C12,OR8K1,RFXAP,RHEB,ROCK1,SMPD1,TMLHE,UGT8,VAMP4,ZMAT3,GABARAPL1,SERPINF2,TNFRSF13C
Negative_3050	IVD,ACTN2,ACYP1,ADCY3,ANPEP,ARRB2,ATP5B,CDK4,CLTA,EPHB3,HAO1,HAP1,IDH2,IDI1,IL28B,IL8RA,IRAK4,KIR2DL4,KLRC1,LHCGR,METTL2B,MPDZ,NPR1,NRG4,OR4D11,PAK4,PDGFRB,PGAM1,PHKG2,PIGL,POLR2F,PPP1CA,PRPH2,THBS3,TSTA3,TYMP,TYMS,VEGFC,TNFRSF11B
Negative_3051	UBB,HPSE,IL13,NOS3,PRKAR1A
Negative_3052	CTH,RET,SP1,ACAA2,ADH5,ARHGAP5,B3GNT4,CANT1,CSF2RB,CTPS,CYP26C1,DLL1,FARSA,IFNA8,IL13,IL15RA,KCNJ11,MAOA,MFNG,OR10H3,OR8U1,PIGM,PSAT1,RPS6KB2,SEMA3F,SMAD1,SUCLG2,TUBB2A,ULBP2
Negative_3053	ID4,CACNG8,EDA2R,NR1D1,OR11H1,PDE1B,PDGFC,PIP5K1B,PTGS2,WNT1,CACNA2D4
Negative_3054	PDC,CNTF,CRY1,DDX55,GUCA1B,IFNK,IPMK,IRS1,LAMC3,NAMPT,NUDT2,OR4C6,PCCB,PGM3,SC4MOL,TBK1,TCF7L1,TNFRSF10A
Negative_3055	C7,CSK,GBA,GP9,ALCAM,DOLPP1,F13B,OR2B11,OR2Z1,OR9G1,PIK3C2G,RAD51,RAPGEF1,SLC27A4,THBS2,TNFRSF8,UCKL1,TNFRSF13C
Negative_3056	CRH,KDR,UBB,ALG5,AMPD1,ATP5C1,CAMKK1,CASP8,CCL11,CCL28,CNGA4,COQ6,CRLF2,FZD4,GALNTL5,GSTK1,HSD17B2,MLYCD,NADSYN1,NQO1,NTRK2,OR10AD1,PER1,PHKG2,PLCB3,PLXNB2,POLR2K,PTPN6,PVRL3,PYCR2,SEMA7A,STAT4,SYNJ1,TSC1,YARS2
Negative_3057	ITK,AADAC,ACSL6,ADCY3,ADCY6,ADIPOQ,AGTR1,ALAD,ALG8,CDKN1C,CHRM1,COQ7,DDX50,DVL3,FSHR,GMDS,IDH3B,IL12B,IL18R1,NCF1,NRP1,NUMB,P2RX2,PAK3,SELE,SETD1A,SKP1,SUCLG1,TACR1,TPH2,B3GALNT1
Negative_3058	CR2,ID2,SDS,ALDH6A1,ATP6V1H,CHST13,CLCA4,COL4A2,DERA,HSD3B1,IRAK2,KNG1,LAMA3,LDHC,MCM7,METTL2B,MYLK2,OR10A6,OR10X1,OR51A4,PRPH,RAB23,SOAT1,TBL1XR1,TCF7L2,VCAM1,PHOSPHO1
Negative_3059	AK1,TEC,ACTN4,ALAS1,ATP5G3,CCL1,CD59,FGF23,FRAP1,GNPDA1,IL22RA2,LDHC,MAP2K6,MCM5,MTAP,MTMR6,NOD1,OR4K14,PAK7,PIP5K3,STEAP3,UBE2K
Negative_3060	ARRB2,CKS1B,CXCL12,FZD10,LAMA4,OR6C6,PDE5A,PPP2R1A,PTGES
Negative_3061	FOS,IL3,LCT,SRF,ADRB3,ALG11,ATP5C1,B4GALT3,CALML3,CCL25,CD36,CD59,CLDN1,FGF13,FLAD1,FZD4,GCLC,HRH2,IDH3B,IL15RA,IMPA1,ITPK1,JAK1,JAM2,KDSR,KIR3DL3,LDHAL6A,LRP6,MAP3K12,OR10G4,OR2G6,OR5J2,OR7G1,OR8D1,PFN4,PLA2G3,PLCD4,PLXNA2,POLR2H,PPM1B,PPP1R3C,RRM2B,TAP1,TMLHE,ULK2,WASF3,HSP90AB1
Negative_3062	DHH,F10,FGB,TYR,ACTA1,AKT1,ALDH3B2,APRT,ARHGEF6,ARPC3,BDH1,CALML3,CAMK2D,CD38,CLDN22,CPS1,CXCL13,FGF21,FGFR1,FTCD,GALC,GALNT13,GCLC,HMGCL,HMOX1,IL29,IL4R,KIR2DS4,NAT5,OCLN,OR13D1,OR2AG2,OR4C46,OR4S2,OR52B6,OR52R1,OR9K2,POLD2,PTK2,RASGRP1,SLIT1,SULT2A1,TCF7L2,ALDH18A1,ATP6V0D1,CACNA2D3,ST6GALNAC5
Negative_3063	DAO,GK2,ITK,MVD,PTS,AFMID,ARNT,COL6A1,CSF2RB,DDIT3,HBEGF,IL22RA1,MCEE,OR13C9,POLR2I,SHISA5,TSHR

Negative_3064	AGL,F11,KHK,MYC,ACVR2B,BUB1B,COQ6,CX3CL1,GBE1,INHBB,LIPC,LPAR1,OR4P4,P2RX7,PRKCE,SCIN,SELE,SIPA1,TAS1R1,UROC1,CSNK1A1L,HLA-DRB3,TNFRSF13B
Negative_3065	FN1,ILK,TEC,ALG13,CDK6,CLDN7,DDIT3,DDX55,DOCK1,EP300,FGF5,FGFR3,GALNT6,GYS1,IDI2,INPP5B,JAG1,MAD2L2,MYLK,NCSTN,OR52N2,OR5AK2,PARD3,PFN3,PGAM4,PLXNC1,QPRT,SH2D1B,SIGLEC1,STEAP3,UGDH,HLA-DPA1,KIR2DL5A
Negative_3066	CAT,CFD,MPL,TKT,ARSD,ASNS,CACNA1C,CUL1,DCXR,GCLM,IL21R,ITGA8,MAP2K7,MAPK8,MYLK,OR2M7,OR6S1,P4HA1,RALGDS,RENBP,SARS,SEMA3F,SFRP4,TNFSF18,TUBB2A,VDAC1,ZIC2,ATP6V0D1
Negative_3067	F5,XDH,ATP6V1D,CLDN16,CNTNAP1,CPT2,CREB3,DUSP9,EGLN3,EXTL2,FCGR3A,FOXA2,FZD10,GALNTL2,GSTT1,HRH2,IL18RAP,IL6R,IRS1,LIFR,MGAT2,NEFL,NRG1,NTF4,OR4N2,OR5B12,OR5B3,PRKCA,SRD5A2,TAS2R7,TPH1,TXNDC12,ATP6V1C1
Negative_3068	AGL,MUT,SYK,ABP1,ACOX2,ACTB,ALDH6A1,APOA1,CACNA1E,CACNA1I,CCL26,COL5A3,CSF3R,CYP1A1,DGKZ,DHX58,EXT1,GNB3,GRIA3,GRIN1,HPRT1,INPPL1,IRF3,ISYNA1,ITGB2,LAMA4,MAP2K6,NGEF,OR13D1,OR2AG2,OR2B2,OR9G4,PDE11A,PIGP,POLE3,PRKAB1,SESN2,SETDB2,TIAM1,TRAF6,UBE2L3,UGDH,YWHAE,TNFRSF14
Negative_3069	F3,AGPAT6,APC2,ARRB2,ATP5G1,ATP6V1D,BPNT1,CDC25B,CEBPA,CLTC,CSNK2A2,DHCR7,DIAPH1,ERBB4,FASN,FMO2,GNRHR,HAO1,HGSNAT,ITPK1,LEFTY2,MAP4K2,MRAS,NKD2,NSD1,NUDT2,OR1L1,OR7D4,PCYT1A,PIK3CG,PLOD2,POLR2E,PPAP2C,PRKCB,PRKG1,PTGES,PTPRJ,ROBO1,SAT2,TIRAP,TP53,TYRP1,UGT2B17,WNT1,ZFYVE16
Negative_3070	ABI2,ALOX5,AOX1,BMP6,COL4A2,CPS1,ENO1,ESPL1,FUT4,IGF1,KIR2DL4,LAMC1,MAP3K13,MTMR1,NAGK,OLAH,OR10J3,OR2K2,OR5H6,OR8A1,PMM1,PTGER1,RND1,SAT2,SCD5,SDC4,SEMA3F,TP53I3,TRDMT1,VAV2,WNT2,ATP6V1C1
Negative_3071	CCR9,EXOC7,MFNG,UPP1
Negative_3072	ADRB3,ALCAM,CCL1,ERO1L,NUDT8,OR3A2,PCK2,PLCD3,PLXNC1,PPP1R3D,SAT2,SEMA7A,WBSCR22
Negative_3073	ADK,DCK,DHH,ACOT11,ACSL1,ADAM10,ALAD,ARD1A,ATP5G1,CHPF,CHST13,CMPK2,CSAD,CYBA,ENPP2,GAS1,GNA15,GSTA4,KCNB1,KLRC4,LRAT,MAPK3,MAPK8,OR51S1,OR6T1,PAPSS1,PARVG,PFKM,PISD,PPARG,PRKAR1A,RPS6KA3,THBS2,ATP6V1B2,ATP6V1G1
Negative_3074	ACPT,ANAPC1,INPP4A,ITGAX,MGAT2,NCAM2,PRKCD,TUBA8,WARS2,TNFRSF11A
Negative_3075	BID,BTC,CD4,IHH,ACTC1,AGRN,ARSE,ATP5E,B3GALT5,CCL20,CDC25A,CES1,CYP4F2,DGKB,F11R,FBP2,FCER1G,GALNT13,GLI1,GNAS,GRIA3,GSTA4,IDH2,IDUA,LALBA,LDHD,LMO7,NCF1,OR2L13,OR4D9,OR52K1,OR7G1,ORC3L,PIGO,PPP2R2B,PSME1,SLC25A5,TACR1,TAS2R40,TNFSF14,ATP6V0A1,TNFRSF11B
Negative_3076	SMS,TNR,TPO,AKR1C4,ARSA,CACNA1E,CDC14A,CXCR3,EPHA8,FASLG,MAGI2,OR52N2,PFKFB4,PPP2R1B,SELE,SLC33A1,TAS2R38,TAS2R41,UGT8,VDAC2,WNT2B
Negative_3077	SHH,ASAH1,AVPR1A,CADM3,CAMK2A,CLDN2,GCLM,HRH1,INPP5A,LRAT,MTHFR,OR14I1,OR52N2,OR7A17,SEMA3F,SSH2,SV2B
Negative_3078	IL9,AASDH,ALOX15B,ALPI,ATP6V1F,C1GALT1,CCL7,CD28,CDKN1A,CTSG,CYP3A5,DKK2,DUSP6,ESPL1,FABP1,GLB1,HNF4A,ICAM1,INPP5B,JAM3,LCMT1,LCP2,MAML2,MAN1A2,MAPT,MAT2B,MFNG,OR2K2,OR5AK2,OR5V1,OR6T1,PIP4K2A,PLA2G2F,PPARG,PRIM2,RFWD2,ROBO3,SCD5,SEMA4G,SOS1,STX16,TAS2R49,TBL1X,TGFBR1,TRPM5,TYROBP,TNFRSF6B

Negative_3079	IL6,ALG3,ARPC1B,ATP5A1,B3GALT2,C3orf10,CASP9,CCNA1,CDH1,CHSY1,CNTNAP2,COX15,CTNND1,CYP2C18,GALNT5,IL6ST,MYH13,NRXN1,OPLAH,OR10G2,OR2A42,OR2AG2,OR4D1,OR56A3,OR5AN1,OR5AP2,PDE1C,PDE4D,PDHB,PIK3R3,PLCZ1,PPAP2A,PPARD,PPP1CA,PTPN5,RARS2,RHOA,RRAS2,SELPLG,SLIT1,SLIT2,STK11,TAS2R16,TAS2R60,THBD,TICAM1,VANGL1,VDAC3,PRICKLE1
Negative_3080	C4B,TYR,ATG12,C1GALT1,FMO4,GALE,IMPA1,LAP3,LDHB,LDHC,LEF1,MAP3K6,NEU3,NRG2,OR52B4,OSMR,PARD6G,PDHB,PER2,POLR2F,STMN1,SV2C,WNT16
Negative_3081	DHH,MTR,ALPI,ATP6AP1,B3GAT3,C7orf16,CD72,CHIA,CXCL6,DGKH,EGFR,EXT1,FLT1,FRAP1,GADD45G,GPX5,IL10,IL12B,LIMK2,MAP3K13,NCF1,NMNAT3,NRG2,OR2H1,OR4D1,PIGB,PPP2R2A,SAT2,SETD8,SH2D1B,SLC27A5,SLC33A1,TFPI,TJP2,TNFSF10,TOLLIP,ATP6V1G1,PRICKLE1
Negative_3082	CR1,PGF,ADCY9,CACNB2,CAV3,CLTCL1,CSNK1D,DHX58,DIAPH1,ENPP6,FASLG,FPGT,FTMT,IFNG,KLRC2,LIPT1,OR13C8,OR13F1,OR51V1,OR52K1,OR5B21,OR6S1,PAK4,PLCE1,PPP2R5C,SETD1A,SMPD3,SOCS1,SORD,SSH3,SUCLG1,TAS2R38,TLN2,ST6GALNAC3
Negative_3083	F3,GP5,ID4,SHH,AREG,ARPC2,BMPR1B,BNIP1,FCGR3B,HTR4,IFNGR2,MAPK1,MARS,NARS2,OR1K1,OR2B6,OR2D3,OR2L2,OR2T29,OR3A3,OR4A15,OR4D6,OR52E4,OR52N1,OR5A2,OR5AS1,PDE1A,PDXK,PERP,PPP1R3C,PRKACA,PTGES2,RRAS,SCD5,SCIN,SEMA3B,TBL1Y,TICAM1,TOLLIP,TUBB4Q,UAP1,WARS,WNT6,ZNRD1,SERPINB5
Negative_3084	DDC,MYC,ALPL,ATP5J2,CDC27,CLDN6,COL4A2,CX3CL1,DTX2,FPGS,FZD6,GDE1,GJD2,GNAI1,GRIN2A,HSPA8,IL24,IL6R,IL6ST,ITGB8,JUND,LIMK1,NODAL,OR2T33,OR2T8,OR56A1,OR56A3,OR6C70,OR8D4,PARD3,PIGH,PIGT,PLCG2,PPP1CA,PPP2R2C,PSAT1,QARS,SCD5,SH2D2A,SLC25A5,SOCS7,SSH2,TIRAP,TUBA4A,UGCGL1,UGT2B7,WNT3A,PAFAH1B2,ST6GALNAC6
Negative_3085	HK2,NPY,TK2,ACADS,ANAPC7,ARD1A,BCL2L1,CACNA1A,CCL21,CD14,CD27,CTNND1,CYP17A1,DDX23,DDX52,DTYMK,EPB41L2,ERBB2,ERBB3,FRAT1,G6PC2,GALNT1,GSTA3,HGSNAT,IL23R,INPP1,MYH14,MYL7,NAT5,NFATC2,OR11H4,OR14I1,OR1J4,OR5B17,OR5C1,OR5P3,PDGFB,PIK3C2G,PLCG1,PMM2,PPP2R1B,RAPGEF3,SNAI1,UBE2K,VAMP4,VANGL2,ATP6V0E1,HSP90AB1,PLA2G12B
Negative_3086	ACOX1,BPNT1,CCND2,GALNT1,GOT2,MAML2,NR1H3,OR10A4,OR9A4,P2RX4,PAK3,PIP5K1B,PTPN5,SHMT2,SRD5A2,TCIRG1,TNFRSF4,TUBA3C,ATP6V0D2
Negative_3087	AGL,AMH,C8G,EDA,GPI,PDC,TKT,AOX1,ARSA,ARSE,CBLB,CD14,CLDN9,CLTB,COL11A1,CRHR1,CYP2A6,DHDH,DTX2,FASLG,FUT7,FZD10,FZD3,GALNTL1,HAGH,IARS,IBSP,ICOS,MGAT3,MGAT5,MYLK,NFATC2,NGEF,NT5C1B,OR1E2,OR51I1,OR51L1,OR52K2,PDE3B,PLCB2,PLCB4,RXRA,SMC1A,ST3GAL4,TAS2R5,VAMP4,WNT10A
Negative_3088	ACHE,BLVRA,GRIN1,MCM6,NOS1,PMM1
Negative_3089	ADK,DBI,SHH,ADORA2A,AQP7,ARPC1A,ATP5H,BCKDHA,BCL2L1,CCR3,CISH,EXTL2,FUT9,GSTP1,HLA-G,LRAT,MYH8,NFATC3,OR1E2,OR2T2,OR5C1,OXCT2,PIGV,PKMYT1,PPM1A,TKTL1,UBE2K,UGT2B7
Negative_3090	APP,CFD,CP,LHB,OTC,AADAT,ACP1,ADCY10,ALDH1A1,AOC3,BCL2L1,CACNG5,CCL1,CCNB1,CD40,CDC42,CYP27A1,CYP2A6,DOLPP1,ENTPD5,FADD,FARSB,FECH,GBGT1,GUCY1A2,GZMB,HTRA2,IMPDH1,INPPL1,JAG1,LCMT1,MDM2,MYLK2,NFAT5,OR11A1,OR13J1,OR1S1,OR5AN1,PDGFRA,PDHB,PIGP,PISD,PPT1,SEMA3D,SOCS5,TJP1,VANGL1,WWP1
Negative_3091	C8A,OSM,BPGM,CDC16,CNTN2,CREB3L3,GALNS,LLGL1,MAP3K14,PTPN1,TM7SF2,TUBB1,ZAP70,HLA-DQA2

Negative_3092	ADCY6,ADCY8,ADCY9,ADRA1D,ALDOC,ALPI,ARHGAP5,BCL2,BMP8A,CCL16,CTPS,CYP2D6,DHRS3,DUSP7,ERO1L,EXOC7,FGF22,GADD45G,IL23A,KIR2DL4,MARS,MYL7,NAT6,NCR1,NEU1,NLGN2,NR1D1,NUDT8,OR13C5,PARD6G,PDGFD,PKLR,POLR2D,PTK2,RPIA,TLN1,TRIP10,VDAC1
Negative_3093	AGPAT1,CFTR,CYP2E1,CYP4A22,GUSB,OR8H1,SEMA3G,SIAH1,TSHB
Negative_3094	DCK,AARS2,AQP7,ASIP,BMP8A,CAV2,CHRD,CKS1B,CLOCK,COL4A6,CYP2C19,DDX50,DPYD,DUSP1,EPB41,EPHB6,F13B,FUT4,GNAO1,GSTO2,IRAK2,ITGA2B,JAG1,L1CAM,LCMT2,MYH10,OR13G1,OR51B6,OR52K1,OR52N1,PFN4,PLCD1,PLCZ1,PPP2R1A,PPT1,PRKCA,RAC1,RENBP,TLR4,TP53,TUBA8,CSGALNACT2,PLA2G12B
Negative_3095	FES,MSN,RFK,ACHE,ALDH4A1,ASAH1,ASPA,CD80,CDC25C,CXCL9,DDX54,DLL4,DVL2,F2RL1,HCLS1,HLA-DOA,HSD17B8,MPST,OR1N1,PARVA,PCYT1A,PLCZ1,POLR2D,RASA1,RPS6KB2,ST8SIA1,SULT1A4,TICAM2
Negative_3096	HYI,CAMK2G,CD38,CDKN2D,CRLF2,CRY2,DAG1,DUSP4,ENPP6,EPRS,EREG,FOSL1,FRAT2,GARS,GRIA1,HEXB,IFNA10,IL1R2,KITLG,KLRK1,L1CAM,MYLK,PDE5A,PIGH,RPS6,SHISA5,UNC5C,XCR1,SERPINA5
Negative_3097	ILK,MPI,ABCC8,ALDH3A1,BAI1,DLL1,INPP5B,MARS2,PGS1,RHOQ,SEMA4F,TYMP
Negative_3098	AK1,GNS,ADCY10,BBC3,CACNA1E,CCR3,CCR5,CLTC,DLL4,DPYSL2,GRLF1,HNF4A,HTR2B,IFNA4,ITGA8,ITGB1,LAMA3,LIMK1,MYLK,NCAM2,OR1D5,PIM2,PTPRR,RAP1A,RBX1,THBS4,TNXB,CACNA2D1,CSGALNACT2,PAFAH1B3
Negative_3099	F7,FOS,ADH1A,AGXT2,ANGPTL4,ATG7,ATP5D,CACNA1B,CARS,CLDN6,COL1A1,DFFA,EIF4E2,EPHA5,FARS2,FGF23,GLUL,GMDS,GUCA1A,HSD11B2,IL20RA,MGAT3,MGAT4A,NCF2,PCYT1A,PFN4,PIP4K2C,PIP5K1A,PKMYT1,POLR2E,PRKACA,PRKAR2A,PVRL1,SHISA5,SOCS4,STAT6,UCP1,YWHAB
Negative_3100	IL2,ALG10B,B3GALT1,CTPS2,FBP2,GAPDH,NCR3,NNMT,PIK3R5,PPP1R3A,PTPN7,SNAI1,PRICKLE1,ST6GALNAC3
Negative_3101	HYI,B4GALT1,CHAD,CHKB,CKS1B,CLDN2,CYP26C1,EFNA3,FDPS,GOT1,ICAM1,IGSF5,IL17RA,IQGAP2,MKNK1,NCF4,NUDT9,OR4S2,OR52E6,PANK4,PAPSS2,PIGU,PON3,PPARG,SLC25A4,SMAD2,TP53I3,TUBB2A,CACNA2D4,SUV420H2
Negative_3102	CFI,DCT,MET,ACSS3,ALDOB,AMDHD1,ANGPTL4,ARPC4,ATP5H,BDNF,CAMK4,CREB3,CXCR4,FBP1,FGF1,FLAD1,NUDT8,OR13C4,OR14A16,OR5R1,PDE4C,PDE6H,PIK3CG,PRKCB,RAF1,RAP1A,ST3GAL5,STX3,SUCLG1,ATP6V0E1,SERPINA1
Negative_3103	ILK,PLN,ALPPL2,ANGPTL4,CARD11,CCL1,CCNG1,CHRD,CLDN19,CYSLTR1,EXOC7,FARSB,FGFR4,GRIN2B,GSTA1,GSTA3,IAPP,ITGA3,MAFA,NGEF,OR13G1,OR52R1,PRKCD
Negative_3104	AVPR1B,CDC26,CRY1,FUT9,GALM,PLEKHA8,PRIM1,SHC3,ST6GALNAC5
Negative_3105	APP,CSK,EDA,MPI,TFG,ALDH3A1,ASCC3,BMP4,CLDN20,COASY,CYP2E1,DHDDS,FCGR2B,GALK1,GSTA5,GUK1,IGFBP3,IL22RA2,ITGA2B,ITGA4,MAP4K3,NSDHL,OR10K1,OR13C3,OR2G2,OR4M2,OR52N1,PDXP,PGM1,PRPS2,RELA,SETD1A,SMAD1,SULT1A1,TPH1,WNT11,CTNNBIP1,HLA-DPB1
Negative_3106	A2M,CR1,DLD,MPI,ADRB1,APOC3,CD79A,CHST13,CMPK2,CNTN1,CXCL13,CYSLTR2,DPYSL2,EPHB2,FARS2,FBXW11,FGF6,GALNT3,GAMT,GLI2,GNRHR,GRAP2,GSTZ1,IDH3B,IL24,MAP2K4,MAP3K14,METTL6,NEFM,NMNAT1,NSD1,OR2S2,OR4S1,OR6C1,PGK2,POLR3H,PTDSS2,RAP1A,SAT1,SEMA3F,SEMA4D,SETD1A,SFRP1,TNFSF18,WNT8B

Negative_3107	CD2, CEL, CFD, CRH, FOS, MYC, TK2, ADIPOR1, ALPPL2, AOX1, ARPC3, ATP6V1H, BCAT2, CACNB2, CCL8, CDK4, CES7, CHIT1, CNTNAP2, COASY, CX3CL1, CXCL6, CYP17A1, DERA, EDAR, EIF4B, FLT1, GALNT14, GSTM3, IFNA16, MADCAM1, OR13D1, OR52D1, OR6K6, OR8A1, OSMR, PCK2, PDE6H, PLA2G1B, POLR2D, PPOX, PRKAR2A, RAB5A, SDHA, SLC9A1, UGT1A10, MAPK8IP1
Negative_3108	HK2, ACADL, ACTG1, ADH1A, ARD1A, ATP6V1A, BIRC3, CADM3, CAPN2, CCL11, CCR4, DFFA, DUSP10, FPGT, GRIN2A, HADH, HLA-F, HMBS, IL1A, IL1R2, ITGA10, MAP3K14, MFNG, MICA, MMP2, NFE2L2, NKD1, OR5B21, OR5T3, PERP, PNMT, RPS6KB2, SLC27A5, SMC1A, TRAF5, TYMS, ATP6V0D1, TNFRSF14
Negative_3109	GP6, ADCY3, ALDH1A3, ARHGEF1, AVPR1B, BDH2, CYP2C18, DERL1, DMGDH, EGLN1, FGF21, GLI2, GNA12, HARS, HSD17B1, IFNK, IL21R, IL6R, IL8RB, KIR3DL2, LARS2, MCM4, OR10Z1, OR4N2, PDGFRB, PFAS, PIGM, PKLR, PKM2, POLR3B, RFX5, RPS6KB2, STAM, STX3, SULT2B1, YWHAG
Negative_3110	C4B, PLG, ACP5, ADRA1B, ARSB, B3GALT5, CTBP2, ERBB3, FCGR3A, GNAI1, HAAO, INPPL1, IRAK3, KIR3DL3, MALT1, NUMB, OR10K2, OR1C1, OR2L3, OR52B6, P2RX1, PCCB, PGM1, PMM2, PPP1CC, PPP2R1B, PRPH, RALA, SETD2, TRAF2, TRAF6, UGDH, SLC25A31
Negative_3111	AR, AUH, BAD, BCR, ACSS1, ACTN2, ALG2, CACNB2, CAPN2, CDC14A, CLDN1, CLDN17, DARS2, DCTD, DHRS9, DUSP6, EFNA3, F2RL3, FGFR1, GCNT2, GGT1, GSTP1, HAO2, IKBKG, LIMK2, MAML2, MAN1A2, MKNK2, MPDZ, NFE2L2, NTF4, OR10AG1, OR1A1, OR51A7, OR52E6, PCK1, PIP5K1C, RRM2B, SARDH, SLC25A4, SOAT2, SPI1, SPRED2, TAS2R48, TAS2R50, VCAM1, XCL2
Negative_3112	CGA, FUK, GLA, ABLIM3, ACADVL, ACSS2, ARPC5L, ATP6V0C, C1QA, CAPN2, CDH1, CNTN1, CYBA, DGKA, DUSP5, ENPP2, FGF5, FGFR1, GDF6, IFNA2, IL13, MTFMT, NT5C1A, OR2F1, OR51E2, PFN1, PGAM1, POLR2J, PPP1R3A, PPP2R5B, PTGES2, RIPK1, RPS6KA1, RXRB, TNFRSF9, XYLB, PAFAH1B1
Negative_3113	DHH, F7, GP5, ME3, PLN, ACSL5, ADIPOQ, AMPD2, APOA1, COL4A6, CSNK1G2, EIF4E, GLI2, GSTA2, GSTZ1, HNF4A, KLRC2, MAP4K1, NTF3, OR10G4, OR1C1, OR1I1, OR52A1, OR5D14, OR5L1, OR5P2, OR5T1, PGS1, PIAS4, PLA2G5, SOCS4, SOX17, ST3GAL4, TBL1XR1, CACNA2D4, PLA2G12A
Negative_3114	MIF, TNF, AMY2A, ATP2A3, CCL28, CD38, CYP3A43, DUSP7, F11R, F13B, FGFR1, GRIN2B, IFNA2, JAG1, LIPF, LYPLA2, MGAT4B, MGST3, OR11H4, OR1C1, OR2AE1, OR2B2, OR4E2, OR5J2, ORC5L, PAK7, PCCA, POLD2, PSME2, PVRL2, RAC3, SULT1A1, TGDS, TUBA3E
Negative_3115	CD6, ACAD8, ACTN2, AKT3, B3GALT6, BLVRB, CARS, CD36, CDK5, CDS2, CMBL, CTPS, EXOC7, F2RL2, FDPS, GALM, GNPAT, HTR2A, ICAM2, LARS, NTF4, OR6C74, PAK6, PNPT1, PRODH2, PTPRC, RARA, SAT1, THBS3, TOLLIP, TUBB, UGCG, UPRT, WARS2, YWHAG
Negative_3116	AMT, ASL, DDC, GCK, IL9, MPL, SDS, ARHGEF2, CCL23, CHRD, COL1A1, CYP4A22, DAD1, ENPP3, FGF10, FGF12, FLT1, FSHR, GAD2, GALNTL4, GFPT2, GRIN2B, ITGA5, LIPT1, MCM2, MCM7, MPST, NME4, OCLN, OR10A2, PIK3R3, POLD2, PTTG1, PVRL1, QARS, RASA2, RHOQ, SMPD1, SOCS5, SPP1, STX7, PNLIPRP2
Negative_3117	MTR, ACACA, ACOX3, ANAPC10, ATP5A1, CCL2, CCNA2, CHSY3, CYP21A2, EFNA1, GLI3, GRIN2D, IL25, IL8RB, MAPK9, MCM2, MKNK2, MTHFD1L, NRXN3, OR52K1, OR6C75, PAX4, PDE1B, PHKG1, PIGA, PLCE1, SELP, TAS2R49, TSLP, WASF3, XCL2, MAPK8IP3
Negative_3118	C8G, DDC, DDO, NGF, ACADM, ALDH5A1, ARD1A, COL11A1, CTBP2, DGAT1, EPHB1, FARS2, FLAD1, FSHR, FTCD, GMPS, GP1BA, KIR2DL4, MTAP, MYH13, MYH7B, NEU1, NUMB, OR10A7, OR10G9, OR13D1, OR2M4, OR52R1, OR8B4, OR8I2, PIGH, POLR1C, PPP1CA, PPP2CB, SMC1B, TNFSF9, YKT6, YWHAG, TNFRSF13C
Negative_3119	AGTR1, ALDOB, FRAT2, FTMT, GPD1, HSPB1, ITGA6, PGK2

Negative_3120	AUH,CFD,DLD,FGG,IL2,ALDH3B1,AOC3,BLVRB,CCL17,CCR5,CES7,CXXC4,DARS2,FLT1,GSTM4,IFNA13,ITGB2,KCNJ11,LDHAL6B,LIAS,OR10G2,OR4K5,OR51B5,PDGFRB,PLXNB3,PRIM2,PRKAR1B,RBL2,RETSAT,RXRA,SCIN,SHC3,SMARCA5,SRGAP3,SUV39H1,WBSCR22
Negative_3121	HGF,ACADVL,ACSL1,BAIAP2,CAMK2B,CBR1,CCR2,COL11A1,CSNK1D,GGT1,GOSR2,GPAA1,GSTA5,HADHA,HIF1A,INMT,IPMK,LLGL2,MALT1,NOTCH1,PLA2G2F,PRKACB,PTPN7,ROBO1,SGMS1,THBS4
Negative_3122	MYC,ABLIM1,AOX1,ARSD,ATP5L,B3GNT4,CCL21,CD226,CD27,CDC20,CDC23,CXCR3,DAAM1,DHFR,EPHA2,FUT4,GCNT1,GLB1,GOSR2,GRIA3,IDUA,IL22,INADL,IPMK,ITGA11,KIR3DL1,LEFTY1,MAN1A2,MGST2,MYH1,OR2W1,OR5D18,OR8I2,P4HA3,PER2,PGK1,PIGS,ST6GAL1,WHSC1L1,PPP1R12A,SERPINC1
Negative_3123	ADC,GCK,IL2,ACTA2,ATP5O,BACE1,CCL11,CCL3,CD22,CXCR5,DHRS9,DPAGT1,ENO1,FIGF,GMPPB,GNMT,GUSB,HNF1B,HSPA8,IAPP,ITGB6,L1CAM,MAP3K1,MAPK11,MRAS,NFKBIA,NT5M,OGDHL,OR4N2,OR5K2,OR6C3,OR6C75,PDHA2,PGM1,POLD3,POMC,PRIM2,PRKAR2B,PSME2,PTGDS,PTGS2,QARS,SEMA3G,SOCS1,TJP2,TUBB1,TUBB2A,WIF1
Negative_3124	GH1,PVR,ENPP3,MTMR6,PDGFRB,TKTL1,VDAC2,ATP6V0A2
Negative_3125	ACADS,CACNG8,DDX56,DIAPH1,DLL4,DRD1,FECH,INSR,MGAM,MGAT4A,MPZL1,OR10J5,OR10Z1,OR2A2,SGMS2,STX3,TBL1Y,ULK1,VDAC2
Negative_3126	GHR,LCT,ACAA2,ACAT2,ACP5,ADCY1,ARSA,CD38,CES2,COQ3,CRLS1,CYP27A1,HSD11B1,IL9R,MAML3,MAP3K7,MAPK7,MKNK1,NCKAP1,OR1I1,OR1J4,OXTR,PMAIP1,PPARD,PYGB,RALBP1,SDHC,SETD1B,STEAP3,TPH2,YARS2,YWHAH,HLA-DRB5
Negative_3127	ADO,AMDHD2,ATP6V0B,CHST11,CMAS,COL4A1,DUSP8,GGPS1,GMPPA,HSD3B2,MAD1L1,MAP3K3,PDGFRA,SULT2B1,TAP2
Negative_3128	AK7,ADCY9,ADIPOR1,ALG13,ALPPL2,ARAF,BAAT,CDC25C,EFNB1,FOXO1,GALNT13,LIPE,MLLT4,NFKBIE,OPLAH,OR2K2,OR51Q1,OR5P2,PIK3R2,PYCRL,TGDS,TP53
Negative_3129	BCR,GSN,IL7,LPL,STS,TNR,ABAT,ALDH7A1,ATP6V1D,AXIN1,C1QC,CCR6,CD82,CLDN4,CXCL13,GRIN2D,JAM2,LALBA,LEFTY2,MLLT4,OR10Q1,OR2A12,PIK3R2,RHEB,STEAP3,ZFYVE16,PPP1R12A,ST6GALNAC5
Negative_3130	ATM,B3GNT4,CDC14A,CDC20,CDC25A,CFTR,CYFIP1,DHRS9,GSTA4,OR56A3,PIGQ,VKORC1,PRICKLE1
Negative_3131	F3,NOG,SP1,ADCY2,ATP2A1,CAMK2B,CCL13,CDH3,CYP4A22,DDX18,EPHB1,GCLC,GRB2,HSD3B2,MADCAM1,MAN2A1,MCM3,NAGLU,NAGS,OR1B1,OR52A5,PLCD1,PLXNA1,PPP2R5A,PRIM2,RDH11,SETMAR,VEGFC
Negative_3132	CFH,CKB,MOS,ARG2,ATP5J,B4GALT7,DTYMK,ETS1,GZMA,IL22RA2,NEU1,OR2L2,OR8K5,PLA2G2A,PPP2CA,PRKCZ,RAP1B,SLC2A1,SRD5A2,SSH3,TNFSF18,VEGFA
Negative_3133	STS,BNIP1,BUB3,CAMK2G,CCL25,CTBP2,CYP4F2,DDX47,DUSP14,ENTPD7,FGFR3,FZD7,GALK1,GCLM,HAAO,HAO1,HLA-DOB,HSP90B1,IFNGR2,IL25,ISYNA1,LAMC3,LARS2,LYZL1,MAP3K5,MPDZ,OR14J1,OR5AS1,OR7G3,PDE1B,RRM1,SEMA7A,SESN2,SRD5A2,SUV39H2,TYMS,ULK1,WNT9A,MAP3K7IP1
Negative_3134	FGG,SCD,VTN,ACVR1C,ADIPOR1,AQP7,ATP6AP1,BCL2L1,BET1,BLNK,CASP7,CCL23,CDKN1C,CNGA3,DPYSL2,DUSP2,EDA2R,ENPP1,H6PD,HHEX,IL5RA,MMP9,NFKB1,OR4Q3,OR51E2,OR5M9,OR6K3,OR6N1,P2RX1,PAK3,PHKG2,PLA1A,PPP2R2C,PRNP,PVRL2,RAB23,SARS,STX1B,TCIRG1,TGFBR1,TNFRSF8,UGT2B17,CACNA2D4,PRICKLE1
Negative_3135	OSM,CALML5,CDK6,CYP2B6,FGF20,IL1A,ITGB4,MAPK1,NCK2,OR13C3,PIK3R5,POLR2D,POMC,SMC1A,VAV3
Negative_3136	C5,GH2,IL5,LIF,CACNA1E,CACNA1G,DHRS4L2,GALNT6,HSD17B2,HTR2C,INSRR,OR51M1,OR8S1,ORC2L,PNLIP,POLE4,SNAI1

Negative_3137	AK5, TNF, VCL, ACTN4, APRT, CDC14B, CLTCL1, CXCL9, DAD1, DGKQ, GALNT6, GJA1, GNAI2, HEXA, HTR5A, IL6R, ITGAD, MTHFD1, OR9G4, POLR3D, RPS6KA4, SLC2A1, SNAI1
Negative_3138	BCKDHB, IL11RA, OR4D1, PNLIP, PRKAR1B, RAP1B, TYK2
Negative_3139	CD4, GK, GSR, RFK, ACAA2, ACAT2, ACVR1, ATP2A3, ATP5F1, CD27, CLDN22, CXCL3, CYP2D6, F13B, GFPT2, GRHPR, GSTM5, LAMA4, MYH10, MYH4, NNMT, NOX3, OR5D14, PECAM1, PER1, SCLY, SDC4, SRGAP3, STX16, TAS2R40, TGDS, TIRAP, TM7SF2, UGDH, USE1, WASF1, B3GALNT1
Negative_3140	AK1, LCT, NPL, SCD, AXIN2, BACE1, CACNA1B, CASP6, CCR9, CD3E, CDKN2B, CSNK1G1, CYP11A1, CYP26B1, DRD5, ETNK1, FMO4, FZD1, HSD17B3, IL1R2, IMPA1, ITGB3, KIR2DS5, KITLG, NAGK, NRG3, OR52R1, PIAS3, PSEN1, PTPN6, SEMA3E, SHMT1, TAS2R42, TFPI, THBS3, TNFSF10, TPH1, VEGFB, WNT16, WNT2, SUV420H1
Negative_3141	STS, BET1L, CALML6, CAMK2B, CCNE1, CRB3, E2F1, FADD, FGF13, FOXO3, HSD17B3, HTR7, IFNA13, IL10RB, IL22RA2, LEFTY2, MAP3K2, MAP3K5, MYLK2, NUMB, OR10H1, OR1K1, OR2Z1, OR4K17, OR5H2, OR5H6, OSMR, POLR3K, RAPGEF3, SDHA, SEPHS1, SMC1A, SMPD2, SPRY1, STAM2, SUOX, TAP1, VDAC2, MAPK8IP2
Negative_3142	IL4, TNN, ABLIM2, AGRP, ANAPC4, ASRGL1, BUB1, CES7, CNTNAP2, CYP2C18, DVL3, FZD6, GAS1, GGT5, GIT1, GRIN2D, HMGCS1, IAPP, IDH3B, IFNA2, IL10, IL23A, MALT1, MAML1, MAP3K3, NEFL, NFATC1, NTF3, OR13C8, OR2D3, OR4F15, OR8K1, OR9Q1, PDGFC, PERP, PIGC, POLR2L, PPA2, PPP2R2A, PPP2R5A, PPP3CA, RALBP1, SEMA4F, SLC2A1, STX3, TBXA2R, YKT6
Negative_3143	ACOT12, ACSL6, BMPR1B, CEBPA, CXCL1, DKK2, ENO3, FGF6, GRHPR, HEMK1, ICAM1, MAOB, MAPK3, MASP2, OR1F1, OR51G1, OR6C70, ORC5L, OSMR, PIP5K1A, ROCK2, SOAT1, SOD1, SSH2, STAT3, TGDS, VAMP1, VDAC2, WNT1, XYLB
Negative_3144	RFK, SMS, A4GALT, AADAC, ATIC, ATP5G1, B3GAT3, BCL10, CACNA1B, CAV3, CD40LG, CDH4, CMAS, CNDP1, CSAD, CTTN, CXXC4, DIAPH3, DLAT, ENTPD1, FGF6, HADHB, HSD3B2, IGF1R, IL25, ITGAV, KIR3DL2, LDHD, MALT1, MAML3, MIOX, NFKBIB, OR2G6, OR4A16, OR6C4, OR7D2, OR8A1, OR8D2, PTCH1, RASSF1, RBL1, SNAI1, SORD, ST3GAL4, TRMT11, WNT5B, WNT6, ARHGEF12, TNFRSF10B
Negative_3145	SHH, ISYNA1, METTL2B, PANK3, PGM1, PLXNA2, SH2D1B, ULBP3, XIAP, TNFRSF10B
Negative_3146	MVK, ALG5, ALPI, CDC26, CHKA, CHRM2, CMAS, CSF2, CUL1, CXCL12, IL1B, MTAP, OR2T1, OR8S1, PFKFB3, PLCB2, PRKCB, RAC3, SEMA3E, WHSC1, HSD17B12, MAP3K7IP2
Negative_3147	LCK, SRF, ACAD8, ACOT12, AXIN1, B4GALT7, CALML6, CD99, CMPK2, NCR3, OR1N2, PPP2R2B, STAT5B, TAS2R4, UCK1, UROC1, VEGFB, PNLIPRP1
Negative_3148	BTD, DCN, ALDH1L1, CCND2, CFLAR, EPHB6, HGSNAT, KIR3DL2, OR10S1, OR5A1, PIP4K2A, RAP1A, RASGRP2, RASSF5, SEMA3C, SMURF1, SRGAP1, STX16, STX7, TDO2, TGFA, TMLHE, CSGALNACT1
Negative_3149	ACSS2, ACVR1B, ALDH1A2, ALG11, ASRGL1, ATG7, BET1L, CCL3, CCNH, CYP2C8, DDX55, DHRS4L2, DVL2, FGF12, GMPPB, GNRH2, GPD1, HNF4G, IDH1, IGF1R, IL1A, IL6R, IL8RA, ISYNA1, LILRB3, MAML1, MPDZ, MYH10, NT5C1B, OR1S2, OR2B2, OR7A10, PDX1, PTGER3, SLC25A6, STX1B, TAP2, ZFYVE16, ZFYVE9, PDCD1LG2, TNFRSF13B
Negative_3150	CRK, IL7, CCL5, CCNB1, CDC45L, CDIPT, CYBA, DTX3, GALNT2, HAGH, INPP5A, ITGAV, MAPK11, OR1S1, OR4S2, OR51I2, OR5M3, POLA1, PRKAR2B, SCD5, SMAD1, SSX2IP, SULT1E1, TAS2R42
Negative_3151	C1S, F11, XDH, ALDH1L1, CACNA1F, CCL4, CDIPT, CSF3R, DNMT3A, DUSP16, F2RL1, F2RL3, FTCD, GLUL, HARS, HEXB, IKBKB, IPPK, KLRD1, MAP2K3, MAP3K4, MLLT4, MYL5, NOD1, OR8I2, PDE10A, PER1, PLCE1, POLR3D, PRKAB2, SC5DL, SREBF1, TACR1, XCL2

Negative_3152	AASDH, ACVR2B, ASCC3, CD99, DPM3, GGT6, MAD2L2, NFYC, UGT2B11
Negative_3153	DBT, GSN, KHK, ACTA1, ASMT, B3GALT4, BCL2L1, CACNA1E, CDC2, CDC42, CHRD, CLTC, CXCR6, DVL1, FABP3, FLT3, GLI3, HMBS, IFNA16, LARS2, MAN1A1, MMP7, MTMR1, NOS3, NR1D1, NRG4, NUDT5, OR10H5, OR10R2, OR52N2, PCK1, PDE10A, PDE5A, PLCB4, POLD4, ROCK1, SDC2, SENP2, SOCS1, TAS2R38, TAS2R40, KIR2DL5A
Negative_3154	AGK, STS, ADH1A, AGPAT1, AHCYL2, ALG1, ALOX15B, APOE, ATP2A1, CAMK2G, COL6A2, CTSS, DDX4, DGKE, DRD1, DTX1, ERBB2, FGFR4, GNG13, GUCY2F, HHIP, LTBR, MAN1A2, MPST, OLAH, OR2A2, OR4C6, OR56A3, P2RX3, PDGFB, ST3GAL5, XYLB
Negative_3155	C4B, CAT, BCKDHA, CACNA1G, CDC25C, CYP2C9, DHCR7, DTYMK, ELK4, GAMT, GARS, GNG12, HTR2A, IFNGR2, JAG2, OR6C1, P4HA1, PDHA2, PIK3R2, RAB23, RBKS, TNFRSF9
Negative_3156	C5, CAT, HK3, ID2, UBB, ADAM17, AKR1A1, AKR1C1, CMPK1, CNTFR, CRLS1, FIGF, FPGT, GALNT2, HTR6, IL17RB, LIPT1, MAPK8, NAGK, OR10AG1, OR1M1, OR52D1, PRKAA2, RCHY1, SESN1, SGPP1, TJP2, WARS
Negative_3157	C5, A4GALT, ADCY7, CAMK2B, CDK7, CSF2, DLL1, EPB41L1, IFNAR2, MAPK1, NADK, OR5C1, RAPGEF4, SAT1, TUBA1A, VAMP4
Negative_3158	DBH, MIF, A4GALT, AGPAT6, CARD11, CAV1, COL4A2, CXCL11, DGKA, DIAPH2, DVL2, GRIA1, IL18, LARS2, OR10G7, OR2B2, OR2G6, OR4C6, OR4E2, OR6N1, OR8A1, PIP4K2A, PLCD3, PNPT1, PRPH2, PTDSS2, PTGFR, STX7, MAP3K7IP2, PPARGC1A
Negative_3159	PC, AARS2, AASS, AKR1D1, ALG10, ANAPC10, CCL19, CYP2B6, CYP3A5, ENTPD2, GUCA1A, IFNA7, LARS, MAP3K13, MYL9, NAT2, NLGN2, NOX1, POLR1A, PPBP, TAS2R16, TYMP, UCP1, UNC5A, USE1, WNT10A, AASDHPPT, ATP6V0A4, B4GALNT1
Negative_3160	CRK, SRC, ALDH1A2, BCKDHA, CCND1, CDO1, CES1, CHPF, CTLA4, CXCL13, EFNA4, FECH, FGF20, IKBKE, IL10, ITGA11, LDHC, LTBP1, MYL7, MYLK3, PPA1, QPRT, TLR9, UGT2A1, ST6GALNAC3
Negative_3161	EPO, SRM, ACACA, ACTB, ADCY8, ATP13A2, B4GALT6, CCKAR, COQ5, CSNK2A2, DHFR, DUSP8, ENAH, GALC, GJD2, IL7R, LLGL1, MAFA, MYLK3, NCF1, NCSTN, OR6B3, OR6Y1, PAX6, PPARG, PRKAB2, RBPJL, ROCK2, TAS2R8, TBXAS1, THY1, UBA1, SERPINE1
Negative_3162	MPZ, ACTN1, ALG12, APOA5, ASAH1, B3GNT3, BDNF, CNTF, CYP8B1, FDPS, FGD1, FOXA3, GALNT13, GFAP, ICAM1, ICAM2, IL8RB, INSRR, KIR2DS4, MAOB, MPST, MYH10, NEU2, NKX2-2, NRXN1, OR13G1, OR1S1, OR2A25, OR2F2, OR5J2, OR7G2, PHGDH, PLA1A, PLCE1, PPP1R3B, PVRL2, SEMA3A, ST3GAL4, THBD, TOMM40L, TRHR, WNT10B, WNT11, YOD1, ZMAT3
Negative_3163	ABO, AUH, C2, DPYSL2, FZD2, GGT7, GMPR, MTFMT, SFRP4
Negative_3164	CD6, IDS, TK1, ACSL4, ALDH1A3, ATP6V0B, CCL2, CCL25, CES2, CKS1B, CYCS, ENTPD2, ENTPD6, EPOR, GUCY2C, H6PD, HGSNAT, IL22, ITGA4, LYPLA1, NT5C3, NTF3, OR2A25, OR5A2, OR5AP2, OR6X1, PAK4, PDGFRB, PLOD2, POLR2K, PRKCE, SEMA3E, SOD1, STAT5B, UCK2
Negative_3165	RB1, ADCY10, ALDH1L1, ARPC5L, ATP6V0B, C4BPB, CCNG1, COL1A1, DHRS4, EGLN3, ENPP7, EPOR, FMO4, FTH1, FZD1, IQGAP1, LIPC, MAPT, OR1E2, PIP4K2A, PRKCQ, SGMS2, SOAT2, SOCS2, SORBS1, TNFRSF13C
Negative_3166	GDA, LTB, NGF, NPL, ABP1, DGKG, EP300, EPAS1, FCER1G, FTH1, IL20RA, IL5RA, INHBE, NARS, OR2V2, OR51S1, OR8D2, OR8I2, ORC3L, TKTL2
Negative_3167	ADIPOR2, AOC2, ARHGEF1, ARPC5L, AVPR1B, B4GALT7, CCND2, CDH15, CLDN15, EFNA5, EIF4B, GALK2, GLUD2, GNAZ, IFT57, IKBKB, IRS2, ITGB7, NLGN2, ONECUT1, OR1S1, OR51A4, PI4KA, POLR2B, POLR3A, PPOX, PRKCQ, PTTG1, SFRP1, TRAF6, TSTA3, TYROBP, PHOSPHO1
Negative_3168	EPO, FUT7, PIP4K2B

Negative_3169	PRL,ADSSL1,BMP5,CARS,CLDN19,CMAS,CREBBP,CYP2C19,DUSP5,FDFT1,FZD4,ITGA7,MYH7,OR7C2,PDE2A,PGLS,PNLIP,PRKAR1B,RPE65,SEMA3C,VANGL1,PPARGC1A
Negative_3170	ABL2,ACSM4,ACVR2B,ATF4,CALML5,CD79A,DGAT1,ENPP3,EPHA5,LTBR,MMP14,NAGLU,NEU1,OR1M1,OR2A4,OR4A16,OR4K2,SOAT1,TAS2R8,TLR4,WNT3
Negative_3171	EFNB2,GGT7,GNAQ,OR13C4,OR4K13,P2RX1,PARVA,PPP2R5A,PTPN5,STAT3
Negative_3172	CD4,CKM,F11,MOS,ACSS1,CD70,COX15,DHRS3,ENO2,FTCD,GALNT7,GANC,GCLC,GDF6,GRB2,HDAC2,HLA-G,HSD3B7,HTRA2,ITGAD,KLRC1,LAMC3,LDHAL6A,LLGL2,MCM7,NOTCH1,OLR1,OR10P1,OR14J1,OR4B1,P2RX5,PFKFB4,PIGB,PROS1,SFRP5,SNAI1,TGDS,UGT2B17
Negative_3173	BCR,PXN,ACAA2,ACSM5,ALAS1,ATP5G2,CHKA,CLDN10,CYP2A13,EXTL2,GLUD1,GNAZ,GRIA2,GUCY1A2,IDH3A,IFNG,KLRC4,MARS2,MCEE,NCOA4,NKD2,NR1H3,OR51Q1,PNPO,PPP2R5D,SEMA4A,SNAP23,TLN1,UGT2B17
Negative_3174	EPO,GSR,NPL,ARSE,ATP5G3,CD40LG,CDC16,CRKL,DHRS4L2,DPM3,GFAP,HMGCR,MAP4K2,NAGK,NEU4,OR51T1,PIGV,PIK3C2B,POLR2F,PPAP2B,PPP2R5E,PRKCI,PRPH,PYCR1,RDH10,RHOQ,SDC4,SLC33A1,SPAM1,TGFB2,TNFSF8,WARS2,WNT5A,PAFAH1B1
Negative_3175	ABL1,ACVRL1,ARSB,ATG3,CLOCK,DGAT2L4,ENTPD3,EPHA2,FDFT1,HLA-F,HMOX2,KCNJ11,MAML1,OR10Q1,OR51M1,OR5AR1,OR8D1,OR8H2,PDX1,SENP2,SETD2,TUBB3,XCL2
Negative_3176	AUH,TEC,TNN,ALCAM,ARPC2,BCL10,CAMK2G,CES7,CLDN8,CTBP1,CTTN,CXCL9,DPM3,EGLN1,ENTPD3,EPHA5,ESPL1,EXT2,GNG13,GUCY1B3,HEXA,HLCS,HSD11B1,IFNG,LAMB3,MAP3K8,MAP4K2,MCCC1,MCM4,MDM2,NT5C2,PNPT1,PTPN7,SH2D1A,SHISA5,SIGLEC1,STX4,TLN2,TNFRSF9,TPH1,CACNA2D3,PAFAH1B1,PLA2G12B
Negative_3177	A2M,CBR3,GFPT1,GNAI1,ITPK1,PGAM1,PIGM,POLR3A,SAT1,CACNA2D3
Negative_3178	C1S,C8B,UBB,ACADVL,ACSM4,B4GALT1,BBC3,CDC27,CHRM5,COL5A2,DGKA,DUSP3,EPHX2,GADD45B,GNAI2,HLA-DMA,HPSE2,IL1R2,LEPR,MGAT2,MTMR1,OR13C5,OR1C1,OR1L1,OR2M4,OR52W1,P2RX6,PIGN,PLCG1,PON1,PTCH1,SELP,TFPI,TIRAP,VCAM1
Negative_3179	FUK,ADH6,ALDH7A1,ALG10,ALG9,AMDHD1,AMPD2,AMY2B,APOC3,BNIP1,CD72,CDK2,COL5A1,CXCL10,CXCR4,CYP11B1,DAAM2,EDA2R,GAMT,GUCA1A,IBSP,ITPK1,LRP6,MAN1B1,MAP2K5,MCCC1,MDH2,NRG1,ODC1,OR10G7,OR10G9,OR2AT4,OR8D2,OR8U1,PIAS2,POLR3B,PTGIS,RAF1,RARS2,SC5DL,SPCS1,SPHK1,TAS2R48,VAMP4,WBSCR17,XYLT2,YKT6,PNLIPRP1
Negative_3180	C8G,DBI,ABLIM2,BDNF,CALML5,CCL25,CTBP2,CTNNB1,CXCL16,CYP2C18,CYP8B1,DLAT,FCGR2B,FZD5,GALNT11,GJD2,GUCA1A,GUCA1B,IL20RB,ITCH,MAP3K3,MCM2,MLLT4,NFASC,OR2A4,PIK3CG,PLXNB1,SPP1,STK36,STK4,SULT1E1,TBK1,TNXB,TRDMT1,TUBA1B
Negative_3181	ACOX1,ALDOB,CD247,CLOCK,EDAR,EIF4E2,GAD2,IQGAP1,NME7,OR10A4,PRKAG3,PSENEN,RDH11,SETD1B,TGFBR2,TPI1,WNT9A
Negative_3182	GPI,HYI,C4BPA,CACNB2,DARS2,DIAPH3,ENO2,FMO2,NMNAT1,OR1L4,TACR3
Negative_3183	CACNB2,CACNG3,EIF4B,ERBB4,GPLD1,PIK3CD,PLD1,POLD1,POLR1B
Negative_3184	F9,IL2,ACTA2,ATP4A,B4GALT1,CXCR3,EXTL2,FCGR2B,GARS,IL18RAP,OR13C8,OR13G1,OR52A1,OR52B6,OR5L1,PAPSS2,PFKM,PIAS3,PPP2R2B,ATP6V0D2
Negative_3185	CS,FGA,KHK,VCL,ACO2,ATP5J,BCL2,CARS,CSNK2A2,DHDH,ELK1,FADD,FGF11,GNRHR,IAPP,IFNA10,MAP3K7,MARS,NLGN3,NMNAT2,OR1A1,PLTP,RARS2,SOX17,TNFSF18,WNT9B,MAP3K7IP1

Negative_3186	CSK,HYI,PLN,ADCY5,AMDHD1,ATP5E,CACNG8,CASP8,DIAPH2,DTYMK,GCNT3,HSD3B2,IL10,L1CAM,LIPT1,LLGL1,MARS,NME6,NRAS,OR14A16,OR2L3,PANK3,PDGFB,PIM2,PKM2,PLCD3,PRKAA2,PRPH,QARS,RALBP1,RPS6,SETMAR,TYK2
Negative_3187	EPO,FYN,MPL,PAH,ACSM3,ARPC2,ARSA,CCDC6,CCNB2,CHRM3,CMPK2,COL1A1,CSF3R,CXCL5,CXCR6,ENTPD3,FGF9,GNA12,GRLF1,HLA-F,HSP90B1,IFNA14,LAMC1,MTHFD1L,MTMR1,MYL7,NFX1,NKX2-2,OR14I1,OR1A1,OR2A12,OR56A1,PGM3,PLA2G2F,PPP3CB,PRKCH,PRPH,PTDSS1,PYCRL,RDH12,RUNX1T1,SARS2,SEMA7A,SEPHS1,SNAI1,SOCS5,TNFSF10,XYLT1
Negative_3188	DAO,F5,SRC,ACO1,ACSS3,AGPAT6,ALDH7A1,BAAT,CACNG7,CLDN16,CLDN7,CXCL1,ENTPD7,FUT6,GALNT2,GYS2,HRAS,HSD3B1,ITGA6,MAD2L1,OLAH,OR4D5,OR51I1,OR8B12,PSME2,PYGB,SUV39H2,SV2A,TAS2R38,TBXA2R,VAMP4
Negative_3189	CCL2,CCR8,INHBA,POLD3,SUV39H1,TAS1R1
Negative_3190	IVD,MIF,ATP5D,CACNA1E,FLT3LG,HMBS,HRH2,INPP5A,MMP7,NOS1,OR10A2,OR10H2,OR51E1,OR52I1,PNPO,CSGALNACT2
Negative_3191	AK2,EDA,F7,FGG,FH,TK1,ACSM2A,ACSS3,AKR1B1,ALG6,ASNS,CARS,CBLB,CRB3,CRHR1,CYP1B1,DAAM1,DLST,DVL1,FCER1G,GCDH,GSTM4,GUCY1A3,GUCY2D,IDH3A,IGF1R,KIR2DS4,MALT1,OR10G2,OR11H4,OR2F2,OR2T10,PPID,PPP3CC,SLC25A5,SMC1A,STX7,SUV420H2
Negative_3192	DDO,EGF,ID1,ME3,AASS,ACPP,ADCY1,ADCY8,ALG12,ARSD,ATP4B,CD22,CLDN8,COL6A1,DHDDS,DHRS4L2,FLAD1,GTSE1,HNF4G,HTR2A,HTR2C,IFNG,IL22RA1,IRF5,MASP2,MITF,NRXN2,OR51E2,OR52J3,OR8I2,P4HA1,PARVB,PDE4B,PLA2G6,PLD1,PPP1R3B,PSME2,RPN2,SLC12A2,SMURF1,SQLE,TAS2R4,TGFBR1,TICAM2,TRPC1,YWHAZ,ATP6V0D1,CTNNBIP1,HLA-DRB4
Negative_3193	ALDH1L1,APRT,ARG2,DPM3,EPHA6,GOT1,OR2A4,PIGB,PRKCZ,PTPN1,STK11,PRICKLE1
Negative_3194	C9,F7,FOS,GP6,ADSS,ADSSL1,ALG1,ALG11,ATP5L,CDK5,CLDN5,CLDN6,CLTC,CMBL,CSNK2A2,EPHB6,EPOR,GUCY2D,IGF1R,MAGI3,MYH2,NFATC1,NODAL,NRAS,NRXN1,OR1D5,OR2G6,OR8D1,PERP,PIGH,POLR1A,RUNX1T1,SULT2B1,XYLT2
Negative_3195	MUT,ACSL1,CLDN14,CREB3,CXCL11,FADS2,GSTP1,HEMK1,HMGCL,IL28A,NFE2L2,OR13C2,OR4Q3,OR5T3,OR8B12,USE1
Negative_3196	PARVA,PI4KB,PSEN1,UGT2A1
Negative_3197	DBI,FGG,GPI,UBB,ADIPOQ,ATP5B,AVPR1B,BDNF,CACNA1F,CREB3L4,CRHR1,CYFIP2,ENTPD8,FLT3,FRAT1,FZD4,GADD45G,GFAP,GRIA2,HMGCL,IFNA14,IFNA6,IKBKG,IRS4,JAM2,KIR2DL2,LDHC,LEF1,MAPK12,OR52B6,PARS2,PFKL,PIGS,PPARG,PPOX,PPP1CB,PTPN11,PTPN6,RPS6KB1,STX1A,TAS1R2,TP53I3,TUBB6
Negative_3198	BID,CAD,CEL,AADAT,ACPT,ACSM2A,CSF1R,DUSP14,FOXA3,HSD17B8,IFNA8,INADL,LLGL2,MAP3K6,NUMB,OR10V1,OR1J4,OR2A12,OR6T1,PCNA,POLR2I,RPS6,RRM2B,TOMM40L
Negative_3199	FUK,GNS,NPY,ADRA1A,CD8B,CDC27,CLDN22,FUT4,HTR5A,JAG1,LAMB1,LDHA,MMP14,NTN1,OR10R2,OR2AE1,OR4D2,OR56B4
Negative_3200	ADO,GSS,ACYP1,APOE,CACNA1F,CDC45L,CMAS,CSF3,ESPL1,GATM,GNA15,HTR7,KAT2B,NR1D1,OR4D6,OR51G1,OR51I1,PGS1,PIGS,ROBO2,SCIN,SOS1,ATP6V1E1
Negative_3201	APP,ATM,FYN,ID2,IL9,ABLIM2,ADSS,AKT2,ARNT,ARSE,BET1,CDKN2B,CKMT2,CTSL1,CXCR6,CYP11B1,DGKA,EDAR,EIF4B,FRAT2,GRIN2B,HSD17B1,IL6R,IQGAP1,MPST,NARS,OR4X2,PLCZ1,POLR2C,RGS3,SETD7,SHMT1,SLC25A4,SLIT1,SOX17,ST3GAL3,TPH2,UBE2K,UBE2L3,UXS1,XYLT2
Negative_3202	KRAS,TAS2R16,TGDS

Negative_3203	ABP1,CACNA1C,CCKBR,GAPDH,HEMK1,LDHAL6A,NCK1,OR2T8,PDE4C,RAP1B,RAPGEF1,SERPIND1
Negative_3204	CKM,RPE,ACVRL1,ALAS1,ATP13A2,CDC23,CTTN,CXCL13,CYP2A7,GFPT1,HGSNAT,IL28B,KIR2DL3,OR4K14,PIK3R2
Negative_3205	C8B,CFD,PAH,UBB,CLDN20,ERBB2,FMO1,OR1L4,OR6C4,OR6N1,OXCT1,ST3GAL2,TDO2,TM7SF2,TNFRSF4,TP53I3,YKT6
Negative_3206	MVK,ACTG1,ADSS,AQP7,CDKN1C,DCXR,DERA,DGAT2L4,DNMT3L,GNPDA2,HTR4,IFNK,IQGAP3,LAMC3,LRDD,LRP5,MAPK14,MCCC1,OR2T34,OR4L1,PDHA2,PLCB2,PVRL1,WHSC1
Negative_3207	C8G,FER,LHB,ACSL1,ADORA2A,AKR1C2,AREG,ARNT2,BMP8A,CDKN2B,CHRM2,CLDN18,CSNK1G1,EPHA1,F2RL1,FZD8,GZMB,HLA-DMA,ITGAD,KARS,OR13C8,PDE1B,PDE6D,PIK3CA,PRODH2,RAB5A,RFNG,SGPP1,TBL1XR1,WNT7B
Negative_3208	EPO,FGA,ZYX,B3GALT4,BACE1,CAMKK2,CCNE1,CCR8,CHD8,CYP3A43,DIAPH2,GCNT1,HLA-G,HPSE,IFNA21,MINPP1,NEGR1,NT5C,NTRK1,OR4M1,OR6B2,PCK1,PDE9A,PGLS,POLA1,RELA,RFXANK,SEMA3A,SPRED2,CSGALNACT1,SLC25A31
Negative_3209	CKB,ABAT,ALAS2,CTBP1,CTLA4,DFFB,DGAT1,DGKE,FLT1,FUT2,FZD2,GARS,GLDC,IGF1R,IL20,ITGB7,LAP3,OR10Q1,OR1S2,OR2AT4,OR2G3,OR5K1,PDE5A,PPP2CB,PSEN1,TALDO1,UAP1
Negative_3210	GLS,ABI2,AMPD3,ANAPC11,B3GALT6,CD79B,CSNK1D,DDB2,EPHA6,GBGT1,HSD17B2,JAK1,LEPR,PPAP2C,SOAT1,SPHK1,TMLHE
Negative_3211	IL5,ME2,ALDOB,AOC2,ASRGL1,ATF4,ATP5B,BLVRB,CCL27,CLCF1,COX10,CSF2RB,CYP27A1,DFFA,FGF5,FOXA3,FZD6,GMPR,HMBS,ICAM1,IL28RA,ITGB4,KDSR,MGLL,NADSYN1,OR10J5,OR2A5,OR51Q1,OR5AR1,PKMYT1,POLR2A,POLR2K,POMC,PRIM1,RCHY1,ROBO1,RRAS2,SMAD3,SOCS5,SOCS7,TAS2R4,WBSCR22,ZNRD1,MAPK8IP1
Negative_3212	LCK,SRC,C4BPA,DDX50,DUSP1,EARS2,FGF22,FGF8,FPGT,GNRHR,GPAA1,GRIA1,GRM1,GSTA4,GSTO1,GUCY1B3,IL2RB,MAPK12,NLGN1,OR11L1,OR1D5,OR2L8,OR2M3,OR4C13,OR4C15,OR6C3,PHKB,PLXNC1,POLD1,POLR2H,PTEN,PTPRC,PYGM,RAB5A,SGMS2,SULT1A2,TACR2,TUBB,ATP6V1G2,SERPINB5,TNFRSF18
Negative_3213	KDR,ACHE,ACOX3,CACNG4,CASP10,CDH2,CES2,CLOCK,DGAT1,DLL4,FRAT1,GABARAP,HAAO,IDH3A,INSR,LAMB4,MAPK1,NCR3,NUMBL,OR2A42,OR4K1,OR7A17,PAPSS1,PDE7A,PHKA1,PIPOX,PLCD4,SDC1,SETD7,SUFU,TUBB1,WNT5B
Negative_3214	DCC,GNE,ACTG2,ALDH1B1,ATP5B,ATP5G1,BBOX1,BCAT2,CAPN2,CCNE1,CCR1,CHRD,DRD5,GPX7,HIBADH,MYH6,NCK2,NKD1,OR13C9,OR1E1,OR2M7,OR4A16,OR4D6,OR51S1,OR52E2,PARS2,PLA2G2F,POLR2K,SC5DL,TRAF5,VTI1B,ATP6V0A4,EIF4EBP1,PLA2G12A
Negative_3215	C1S,DCN,STS,TAT,AARS2,ACSS2,ADCY3,ADH7,ALDH3B1,APOE,CHD8,CLDN7,CRHR1,CSAD,CTNNA2,DGAT2,ENO2,FZD4,HAO2,HSD11B1,IL1B,IL22,JAK3,NFATC3,NTF4,OR10V1,OR2A14,OR4F15,OR51E2,OR5F1,OR6C6,PDGFC,PIK3R4,POLE2,PRKAR1A,PRKCE,PSME1,PTPRF,RRAS2,SPRY3,TRAF3,TRMT11,TUBA3E,WNT5B,SERPINA5,TNFRSF18
Negative_3216	BTC,ADCY9,ATP4A,BACE1,CCL15,CDKN2D,COQ3,CTNNA1,CYP21A2,FGF19,FMO3,FMO4,FPGS,FRAT2,FSHR,GALM,GALNT14,GNAI3,GNAS,HSD17B3,IL20RA,KLRC4,LALBA,MAPK3,MAPK7,MFNG,NANS,NCOA4,OR13C4,PDE6D,PPP2R2C,PSME3,PYGL,RPSA,RRM2B,SEMA4B,SIPA1,SMPD1,SMPD3,TNFSF4,WEE1,WNT9B,WWP1,ST6GALNAC4
Negative_3217	ADK,LTB,PLG,SDS,ZYX,ATP6V0C,B3GAT3,B4GALT3,C3AR1,CALML5,CASP10,CCL15,CNGB1,CRLS1,CYP27A1,DDX51,FTMT,GNAQ,GSTA3,IL11,LILRB3,OR10A4,OR10AG1,OR1B1,OR1F1,OR51E2,OR5B12,OR5H2,RAB23,SEMA4C,TCIRG1,TGFB1,B4GALNT1,MAPK8IP2

Negative_3218	DAG1,E2F5,EHMT2,GCLC,GJD2,ITGB2,LAMC1,MAGI3,OR5M3,RELA,SAT1,TLR2
Negative_3219	ATM,DAO,NLK,ACVR1C,AGPAT4,CAMK4,CXCL6,DCXR,ETNK2,GGT5,GNA13,GNAZ,IL15,NT5C,OR10T2,PDE2A,PPAP2A,SDC4,SELE,WNT3,PAFAH1B3
Negative_3220	BAX,DCC,FOS,NLK,ACADSB,AKR1C2,ALDH7A1,C1QA,CES7,CHKA,CLDN18,CMPK2,DLL4,EPHA6,FGD3,FZD9,GNPDA2,IRS1,LIPE,MAP3K14,MGAT4B,MINPP1,MMP14,NEUROG3,NME7,OR1M1,OR4S1,OR52K1,OR6K2,PDXP,PLA2G2E,PLA2G2F,PLAUR,PTPRZ1,SLC25A6,SLC27A5,SPTLC1,STAT2,SULT1A2,TLR3,VDAC3,VEGFC,ZNRD1,CSGALNACT1,CSNK1A1L,KIR2DL5A,TNFRSF13B
Negative_3221	C6,CAD,CR1,MPL,ADH4,B4GALT7,CNTN2,COL4A1,CREB3L3,CSNK2A1,CXXC4,DDIT3,DLAT,ETNK1,GBA3,GCAT,GOT2,GSTM4,HNF4G,IL20RA,IL22RA2,ITPKB,JAK1,LIPT1,NFKB2,NFKBIA,OPLAH,OR10H3,OR2L2,OR2T5,OR6X1,PIP5K1A,PLA2G2E,PNPLA3,PTPRC,SMAD5,SPP1,STX1B,TALDO1,TAS2R4,TPH2,UGCG,VASP,YOD1,ATP6V0D1,CACNA2D2,PHOSPHO1
Negative_3222	ALDH3B2,ANGPTL4,GNG3,IFNW1,OR2Y1,STK4,TYK2
Negative_3223	DBI,FH,HK3,ACAD8,ADCY6,ARHGEF4,CD70,CHRM3,FGF20,GADD45G,GALNTL4,LAMA3,MAPT,MTHFD2,MYLK2,OR1K1,OR5AS1,PCYT1B,PLCE1,SIGLEC1,TAP1,TP73
Negative_3224	BTC,CGN,EPO,MPL,ALAD,CARS,CD8A,CLCF1,CSNK1G3,CXCL12,FTMT,ITGB6,NCF2,NCK2,NFATC3,OR10S1,PCCA,PGS1,PHKA1,PIK3R5,PLCB4,POLR2K,PPP1CB,RASGRP4,SENP2,SOCS7,STAM2,TNFRSF14
Negative_3225	F2,ADRA1D,CNTFR,JAG2,MDH2,PHKG2,PLCZ1,SCIN,SMARCA5,VCAM1
Negative_3226	AR,DDO,JUP,MTR,TNR,ADRBK2,BST1,CHDH,COL3A1,DDX19A,EFNB1,EIF4B,GANAB,HSPA2,HTR2C,IFIH1,IFNA4,IRAK4,ITCH,ITGA7,MGAT5,MMP7,MYH10,NAE1,NAT5,NCF4,NFKBIA,OPLAH,OR5AU1,OR7G2,OR7G3,PAX4,PCYT1A,PLA2G1B,PLCB4,POLR3B,PRKACG,RDH5,SFRP1,SIPA1,STX5,TGFB2,THBS4,TJP2,TNFRSF9
Negative_3227	CAT,IL3,CACNG1,CCL15,CERK,EDNRB,HTR6,IL25,ITGB3,KIR2DL3,KIR3DL1,LIPE,NMNAT3,OR5AR1,PDGFB,PHPT1,UGT2B10,UNC5D,YOD1
Negative_3228	ADC,HK2,LCT,ADAM17,BMP2,CRLF2,DDX47,EHMT2,FGF12,G6PD,KLRC1,MDM4,OR52E8,PRKACB,PTGS2,PVRL4,SEPHS1,ST8SIA1,STAT5A,TAS2R7,TYMS,VAV1,XYLT1,YWHAE,HSP90AA1,SERPINC1
Negative_3229	CSK,ADRB1,CCKBR,CYP26A1,DAPK1,FMO4,GNRHR,IFNA14,IFNGR1,IL15,MAT1A,NCR3,NOTCH2,OR1L1,PARD6B,PDE1C,PKMYT1,PLA2G5,PLCB3,PPP2R1B,PPP3R2,PYGL,RDH11,SEMA3A,SMURF1,TRAF1,VAMP3
Negative_3230	VTN,AGRN,ALPPL2,BAI1,CDC27,CYP3A7,EFNB3,ENTPD8,FGFR3,FPGT,GPX7,HPSE2,IRF5,NCSTN,NEGR1,OR51B2,OR52B6,OR5M9,P4HA1,PLCB2,PMM2,POMC,TNFRSF8,TPM3,WNT5A,YWHAH
Negative_3231	CCL24,ENTPD7,KLRD1
Negative_3232	BTK,CD6,EDA,F9,FGG,LEP,ABLIM1,ACADSB,ADCY8,ARAF,CARD11,CCL25,CCR1,CHSY1,CTBP1,CTSS,CYP4A22,DCXR,DNMT3B,EDNRB,ENO3,ERBB2,GNB3,GNMT,GSTM5,GUCA1A,HIF1A,IRF5,ITGB3,KNG1,LAMA4,LDHAL6B,MYL7,OR1D2,OR5D13,OR5F1,PDE3A,PIGL,PIGX,PLA2G2A,PLA2G5,ROCK1,SESN1,SMARCA2,SUV39H1,UGCG,UGP2,SERPINF2
Negative_3233	IL5,PLG,ACP1,AMDHD1,CACNA1D,CFLAR,CTBP2,CXCR6,DUSP1,EARS2,EI24,GRM1,GSK3B,HNMT,IL20RA,IRF9,OR10K2,OR52K2,P2RX5,PIM2,PLOD2,PNPLA4,RASGRP1,SV2C
Negative_3234	CDA,FGFR3,GDF5,NUDT9,PLCZ1,PPP2R2B,UGT2A1
Negative_3235	CYP21A2,EFNA1,FGF7,KIR2DL2,OR4D2,PLIN,SPI1,TGDS
Negative_3236	CFH,VCL,CTNNB1,CYP2J2,DDX47,EPB41,FUT1,GCLM,GLG1,GPX5,IFNA7,LAMB2,MAP3K2,NRG1,OR2T1,OR4K13,OR5K1,OR8B8,PON2,SV2A,TAS2R1,TAS2R10
Negative_3237	BCAR1,CACNG4,IFNGR1

Negative_3238	ADA, AGK, DUT, EDA, MYC, ACSS2, ADH4, ALDH3A2, ASPA, ATP5E, ATP5F1, CCKBR, CSNK2B, CXCL10, EFNA5, EHMT1, ENTPD7, F13B, GAD2, GFAP, GSTK1, GSTM4, GTSE1, HMGCR, IL10RB, KIR2DL1, LTBR, MAP3K7, MDH1, MMP7, NAE1, NCKAP1L, NLGN1, NRAS, OR4F15, OR51G1, PDE1A, PEMT, SFRP4, SPP1, SPRY2, STAT4, TCF7, TICAM2, TJP2, TNFRSF8, TNFSF14, TRHR, WIF1
Negative_3239	LCK, MPI, TYR, AKR1A1, ALPP, AXIN1, B3GNT2, CX3CR1, CYP2C8, DAPK3, FARSA, FGF11, GNAO1, HLA-C, MAD2L2, MPZL1, OR10J3, OR10P1, OR13C4, OR14I1, OR2A5, OR2L2, PAICS, PAX6, PFKP, PYGB, PYGL, RALBP1, SEMA3B, SEMA4B, SLC25A5, SMPD2, TLR3, TRPM5, TUBA1A, GABARAPL1, ST6GALNAC6
Negative_3240	AMPD1, ANAPC1, DHODH, ERBB2, GUSB, KLRC4, METTL2B, NCK2, PDXK, POMC, RAD51, SLC27A1, TNFSF14, XYLB, ALDH18A1
Negative_3241	ACADS, CCL17, CHRM2, CHUK, CLTCL1, COL1A2, CSF2RB, CTTN, CXCR5, EDN1, EXTL1, FOXA3, GLDC, GNPDA2, GYS1, IRF7, METTL2B, OR2A14, OR2T29, OR8H3, SOCS3, TGFB1, TIRAP, TP53
Negative_3242	NOG, RPE, ALG9, ARHGEF1, BACE1, C9orf95, CCL13, CD82, CDC16, CDC26, CHEK2, HTR2A, IL10RA, IL28A, MIOX, NEU3, NFKBIE, OR2M5, OR4A15, OR52L1, PGAM1, PIGT, SAT2, SHMT2, TKTL2, CSGALNACT2
Negative_3243	DBT, MIF, ACAT2, ARG2, CACNA1I, CCNB3, CHIA, DGKG, DHRS4, DKK4, DNMT3L, FZD8, GALNTL2, GLUL, GMPR, GNAI2, IFNW1, IL13RA1, MAGI3, MAPK11, OR1A2, OR4D9, OR4F5, OR52B4, OR5B21, OR8S1, PDGFC, PGK1, PLCG1, RBL1, RDH11, ROBO3, SEMA7A, SOCS4, XCL1, YARS2, ATP6V0E1, TNFRSF10A
Negative_3244	ATM, SP1, ACADS, ALOX12B, AMDHD1, AMPD2, CCNH, CCR8, CD38, CDH2, CDS2, CFTR, CSF3R, DAPK3, DKK4, EPAS1, FUT6, GNG12, GRIA1, HTR7, IFNAR1, IL28A, JAM3, KLKB1, MAP3K3, MARS, MGAT4B, MTAP, NAMPT, NEFL, NEUROD1, NT5C, OR1E2, OR2T3, OR4K2, OR9A2, PAK3, PDGFB, PPP1R3A, RALBP1, RAPGEF3, SMAD1, SOD1, TUBB2A, WEE1
Negative_3245	PVR, CHD8, COL3A1, CXCR4, DPM3, IAPP, MYH11, OR10G2, OR14J1, OR2AE1, PIGB, RND1, TIRAP, YOD1, PHOSPHO1
Negative_3246	CD2, HPD, ACY3, ADH4, AK3L1, ARSD, B3GALT4, B4GALT7, CASP3, CBLC, CCKBR, DAPK3, DEGS2, E2F1, EPOR, FARP2, FCGR3A, FZD2, GMDS, GTSE1, HSD11B1, IDH3G, IL20RB, ITPKA, MTAP, NAGLU, NPR2, NT5M, NTSR1, OR11H4, OR1F1, OR2T34, OR4C15, OR51B6, OR6K6, OR8J1, PRKCH, PTGS2, RAF1, SAT1, SLC9A1, SLIT1, SMARCA2, STAT6, SUCLG1, TRPC1, UROS, WNT2
Negative_3247	LYN, ADRB1, BIRC5, CDC42, HYAL2, NCOR1, NKD1, NPR1
Negative_3248	ALDH1A2, CCNG1, CDKN1B, CHD8, CLDN4, CSF2RB, CXCR4, CYP3A43, F2RL3, IL20, INPP4A, KIR3DL1, L1CAM, MAP3K12, OR2T11, OR2T29, OR4N2, OR51G1, PECAM1, PYCR1, SHMT2, SNAI2, TALDO1, TAS2R8, TUBA1A, PRICKLE1, ST6GALNAC6
Negative_3249	ANAPC10, C7orf16, CD40LG, CTSS, CYFIP1, ENTPD1, SV2B
Negative_3250	IL8, RFK, ABP1, ACO2, ALCAM, ATP6V1A, EGFR, EPAS1, GPD1L, HRH1, MAP4K4, NMNAT3, RARS, RDH5, SHC2, SMURF1, TNFRSF1A
Negative_3251	ABO, C5, F8, MPZ, SYK, ADCY5, ARRB1, BUB3, C7orf16, CACNA1D, COL1A2, CSF3R, CXXC4, CYP4A22, DAG1, DDIT4, EPHA4, F2RL1, FGF7, FGFR1, GBGT1, GNPNAT1, IL12RB2, IL15, IL28RA, ITPKB, MAGI3, MYL7, OR1I1, OR4L1, OR52H1, PANK1, PANK4, PDE1B, PDHA1, PIP4K2C, PLXNA3, PTEN, RRAS2, SHC1, STK4, TMLHE, TPMT, VNN1, PAFAH1B3
Negative_3252	CCL25, CMPK1, F2RL2, GNG3, IDI1, INPPL1, MYH13, OR2T11, PRKCH, ULK3
Negative_3253	CCL23, GADD45B, NFKBIB, OR2G2, OR6N2, UGT2B17, ZAP70
Negative_3254	ACO1, ADCY7, AKR1C1, ARHGEF6, B3GALT1, CACNB3, CACNG7, F13B, GNRHR, GSTA4, HTR4, IL10, INHBE, MALT1, MMP2, MYH6, NCR2, NT5C3, OLAH, OR10K2, OR2T5, OR8B4, PAICS, PARD6G, PHPT1, SDC3, UPB1, ZNRD1, ATP6V1E1, TNFRSF1A

Negative_3255	EBP,HDC,APC2,CD58,EFNB3,FZD3,GNAO1,HAGH,HTR2A,LDHD,MYH1,NEU2,OR10H4,PANK4,PIK3CG,RBX1,TAS2R5
Negative_3256	GLA,ARG2,CD47,CPT2,DKK1,FDPS,HSD3B7,LAMB4,LTB4R2,OR2T1,OR4L1,PFKFB2,SETD2
Negative_3257	GPT,MVD,CD36,DUSP6,FUT6,GRIN2A,HSD11B1,IL21R,KLRC4,MAPK1,NANS,OR1L8,PDPK1,PISD,PLAUR,POLR1B,PPP3R1,SEMA3A,SEMA4F,SETD7,SLC27A4,SSX2IP,TPM3,CACNA2D3
Negative_3258	PDC,SCD,ATG3,CBR1,CDC42,CSH1,DUSP9,EPAS1,GSK3B,GSTO1,GSTZ1,IFNW1,IL20RB,INPP5E,MOV10L1,MTMR6,NT5C3,OR1K1,OR8S1,PPP2R2B,PRKCB,RAF1,RAP1B,RASGRP4,RFWD2,RPN2,SMAD2,TCF7,TCIRG1,TPM3,TNFRSF13C
Negative_3259	C8A,DCC,GNE,VCL,A4GALT,APH1A,ATP5C1,CACNA1I,CYP3A4,ETNK2,GART,GMPPB,HSD11B1,ITGA9,MAGI1,OR52D1,PIK3C2B,PIM2,PLA2G2F,PRKCZ,RAF1,RDH8,SMAD4,STX8
Negative_3260	ID1,ADCY6,AMDHD1,CACNA1A,CLDN6,FGFR3,GNAL,HPSE2,OR10H3,OR10X1,PTPRM,RPSA,SYNJ2,TNFSF8,ATP6V1G2,MAPK8IP2
Negative_3261	DCC,GK,LIF,ADCY2,CHKB,DTX3,EDAR,IL1A,ISYNA1,MAT2B,OR6S1,PCYT2,PLXNA2,PPP2R1A,HLA-DRB4
Negative_3262	CR1,F11,LTB,ME2,ASMT,BBOX1,BLVRB,BMP6,CACNA1B,CANT1,CMAS,COL11A1,CSNK1E,CXCL1,CYP19A1,DPM1,EDNRA,ENPP6,FGFR3,HNF4A,IL22,LDHB,LIMK2,OR2G3,OR8J3,PCYT2,PIGC,PSEN1,RCHY1,RGS3,SESN3,SH2D1B,ST3GAL6,STK36,STX8,TIAM1,TPH1,TUBA3C,PNLIPRP1,PPP1R12A
Negative_3263	EPO,EFNA2,HSPA8,MARS,RXRA,TAP2
Negative_3264	C5,GDA,GNS,AASDH,ALG1,ALG12,ARRB2,CAMKK1,CTSL1,DAPK2,DPYSL2,FGF14,GADD45A,GNB1,LRP6,NTRK2,OR4Q3,OR4X1,OR5K2,RASGRP1,TRMT11,UGT2B7,VKORC1,MAPK8IP1,SERPINE1
Negative_3265	CKB,ID1,NLK,ADAM17,ALDOA,B3GAT1,CDC25C,CLDN9,CSH1,FGF13,FGFR4,GGT1,GNAI3,IL28B,ITGB1,NARS2,PHPT1,PIK3C2G,PIPOX,POLR1A,RALGDS,SMAD5,SORD,STX2,TNFRSF9,UBA7,UGT2B17,YWHAH,ARHGEF12,CSGALNACT2,SUV420H2
Negative_3266	KIT,XDH,BIRC3,CCNE2,CD19,CD79A,COL3A1,CXCL12,CYP2U1,DAAM2,DARS,GBGT1,GPT2,HADH,IFNA17,ITGAV,LAMB2,LRP6,MGAT5,MYH8,NAGA,NFKB2,OR6C70,OR8I2,PDE1B,PFKFB1,RASSF5,RPS6KA1,SEMA3G
Negative_3267	DBI,DHH,FYN,HAL,LIF,AGPAT1,ALCAM,ALG5,C7orf16,CACNB4,CD19,CSNK1A1,CSNK1D,CTNNA1,EARS2,EFNA5,EPHB3,FGF2,FMO3,GDF6,GOSR2,GSTA4,IFNA13,IL10RB,INSR,LEFTY1,LTC4S,MAML1,MGAT5B,OR51B2,OR51G2,OR5B12,PANK1,PCNA,PGAP1,PHKG2,PIK3C2A,PPARD,PPP1R1A,SORD,TGFA,TGFBR2,THBS4,TRAF3,VAV1,WNT11,YES1
Negative_3268	ALPI,APH1A,CACNA1C,CHRM3,DGAT1,IL6ST,NAGA,NCF1,OR3A2,OR5L2,OXTR,POLR2D,SERPINF2
Negative_3269	GK,ALDH3A1,ATP5G2,CHRM5,CLDN8,CYP2S1,DDB2,ENO3,EXTL1,FGF3,HIBADH,HLA-DOA,HLA-G,INHBB,ITGA2B,LTBR,MYH3,OR10AD1,OR2A4,OR4B1,OR7C2,OR8B8,ORC3L,P2RX5,PAX6,PIK3R2,PTPRR,RALGDS,SETD8,SFRP4,SKP2,SRGAP1,TSC2,SERPINC1
Negative_3270	ASL,GCK,MET,ACAA1,ACY1,ADRA1B,ASIP,CCL3,CCND2,CD28,CDC20,CDH4,CNTNAP2,CYP2B6,DRD2,DVL1,FSHR,GALNT14,HBEGF,IARS,IL10RA,ITPK1,LEFTY1,OR10A2,OR10G9,OR13J1,OR4X2,OR51B5,OR6C74,OR6N2,PARVB,PDE1A,PDIA4,PLXNA1,POLR3H,PTCH2,RND1,SH2D2A,TOMM40,UGCGL1,UGCGL2,WNT6,SERPINA1,TNFRSF14,TNFRSF1B,TNFSF13B

Negative_3271	DBH,FGG,RPE,ACAA2,ADCY1,ATP2A1,B4GALT4,CACNA1S,CACNG8,CALML3,CASP9,CCL1,CCR4,CD274,CYP7A1,DCTN1,DGAT2L4,FGF10,GCNT1,GJD2,GNMT,IL10RB,KIR2DL3,MAP4K2,MAPK8,MLYCD,NCK2,NKX2-2,OR1F1,OR1S2,OXCT2,PFKFB1,PRKY,PRODH,RDH8,RPIA,RPS6KA1,SMAD4,SUV39H1,ULBP1,WASF3,WNT5B,AASDHPPT,ATP6V0A2,KIR2DL5A,ST6GALNAC4
Negative_3272	CR1,GK2,MAG,ACADSB,ACP6,ACPT,ALG3,CES1,CYP4A11,DHDH,DPM2,DTX2,GLUD2,GRIA1,OR10G4,PDE6D,PDGFC,PFAS,PIK3C2B,POLR3H,PPBP,UAP1,UBE2L3,ZNRD1
Negative_3273	JUP,APRT,CREB3L2,FGD3,FMO4,GFPT1,GSTO1,HSPB1,INPP5E,NNMT,NTF4,OR2T11,OR5V1,OXSM,PAK7,PERP,PLXNB1,POLD3,RPS6KA6,SETD2,TNFRSF8,TNFRSF10D
Negative_3274	ME2,ADRA1A,AGXT,CXCR6,EHMT2,EPHA1,EPHA5,GSTA4,IARS2,OR8B8,ORC4L,PCCA,PIAS2,PRDX6,SEMA3F,WNT6
Negative_3275	AGTR1,PDIA4,TIAM2,UGDH
Negative_3276	MVD,SHH,ADH1B,AGPAT4,B3GALT1,CACNB3,CCL17,CD46,CD48,CEBPA,CYP17A1,CYP3A5,DDX47,FZD10,FZD7,GCNT2,GMPS,GNA11,GSTM4,HSD3B7,MAP3K12,MAP3K8,MYH7B,NOTCH1,OR2T5,OR4D11,OR4M2,OR51V1,P4HA2,PDGFB,PIK3R2,PLA2G2D,POLR3B,PPP2R5B,PRKAA1,PTK2B,SMURF2,TBL1XR1,TNFSF8,TRAF5,VAMP2
Negative_3277	C8G,ARHGEF7,ATP6V0C,B3GALT6,C1QA,CACNG5,CARS,CCL13,CHRD,DLL3,E2F2,FUT4,GOT1,IKBKB,LAMC2,MAGI3,NTRK2,OR5D14,PIGS,PIK3R5,PLCZ1,POLD3,PRKAR2B,RPIA,STX12,STX1A,TJP2,TYMS,WNT2B,YWHAG,HLA-DRB4,PPP1R12A,ST6GALNAC1
Negative_3278	CDIPT,EGFR,MAPK11,OR13C9,OR2T10,P4HA1,UROD
Negative_3279	GPT,RB1,UBC,VCL,AASDH,ACPT,ACVR1B,ASIP,ATP6V1F,CACNG3,CLDN6,CYP2S1,EHHADH,EXOC7,GALK2,HPRT1,HTR2C,IFNA14,INPP4B,LDHD,LIPT1,MAP3K3,NGEF,NLGN1,OR2M7,OR2Y1,OR5R1,PLXNC1,PRKY,SIPA1,TAS2R48,UAP1,VANGL1,PDCD1LG2
Negative_3280	CEL,ADCY9,ADIPOR2,AMPD3,CCL7,CD27,CSAD,CYP4A11,CYP7A1,DCTD,FUT9,GZMB,HTR5A,ITGAL,KIR2DL1,KIR2DL3,LIMK1,MMP14,OR10S1,OR2A14,OR2C3,OR4S1,OR52J3,OR8D4,OR8U1,ORC3L,RELB,SULT1A2,WIF1
Negative_3281	CS,CD79A,CLTB,CNTFR,DHDDS,GFAP,IRS1,ITGB4,NPAS2,OR5B12,PAPSS2,PARS2,PGAM2,PRKAR2A,RRM2B,TUBB2C,ST6GALNAC1
Negative_3282	LCT,LYN,NLK,ADIPOR2,BCKDHA,CSF2RB,CYP26C1,DHFR,DOT1L,EREG,FZD9,FZR1,GLI3,GNG3,IKBKE,IL22,IL25,MAOB,NFX1,NRG1,OR10AG1,OR1J1,OR5AK2,OR5AN1,OR6A2,OR6N1,PER1,PIK3R3,RBKS,SFRP2,SGMS2,SPHK2,ATP6V0D1,PHOSPHO1
Negative_3283	LIF,C3AR1,CCL15,CD22,CNTN1,DHRS3,IGF1,IL11RA,LARS,MAD2L1,MYD88,OR2L3,OR3A1,PIGW,POLE4,SIPA1,SLC25A6,STT3B,SULT2A1,SUOX,ATP6V1C2
Negative_3284	CHEK2,EDNRB,EGFR,GRAP2,GRLF1,IL28B,LAMA3,MYH8,OR51B4,PTPRR,TFDP1
Negative_3285	NPL,CARS,CDS2,CLDN20,CTLA4,GBA3,IL10,INMT,OR11H1,OR1L6,PDGFRA,AASDHPPT
Negative_3286	CTH,ACAA1,ADH1B,AKR1B1,ATP5J,CBLB,DAPK2,DCTN1,DLL1,DTX4,GALNT11,GMDS,IL20RA,KITLG,MADCAM1,MYH9,MYL2,OR1G1,OR4E2,OR51M1,OR6Q1,PFKFB2,RASSF5,STX1B,STX3,TLR3,VKORC1,WASF3,TNFRSF13C
Negative_3287	CLDN3,CLTA,CREB3L4,GMPS,MGAT3,MTHFD1L,NFKBIB,SH2D2A
Negative_3288	ACSS3,C3orf10,CARD11,CDC2,CLDN4,CYFIP2,LCAT,MGAT5,MYH6,OR5D13,OXTR,ATP6V1C2
Negative_3289	GPI,AKR1C3,BDH2,CHAD,CLDN3,CSF3,CTNNA1,FMO4,IL17B,ITGA3,ITGA5,MARS,OR11G2,OR1J2,PANK2,PRLR,PYCR2,UCP1,UGCGL2,ULBP2

Negative_3290	EPO, GLS, ADCY1, B3GAT3, BAAT, BBC3, CACNG1, CACNG2, CCL25, CD8A, CIITA, CMBL, CYSLTR1, DUSP16, EFNB3, EGFR, FMO5, FUT1, GALE, GDF6, GNPDA1, GSTO2, HAO2, HPSE2, HRAS, HTR7, INPP5A, IRS4, OR10V1, OR13H1, OR9Q2, PHKA2, PIK3C2G, PLD2, PLXNA2, POLR3B, RAPGEF4, SETDB1, THBS1, ULK3, CSNK1A1L
Negative_3291	HK2, ADRA1B, BIRC3, DDHD1, FGFR2, HIF1A, HSD17B7, IDH3G, ITGA1, MAPK3, NEUROD1, OSMR, PISD, PLCD1, PPP3CA, TNFSF4, VTI1A
Negative_3292	C3, FUK, LYN, ACVRL1, AGPAT4, AHCYL2, ARHGEF2, B3GAT2, CDH4, CHEK2, EXOC7, FGD3, GALNTL2, GNPNAT1, IARS2, IL13, ITGAD, LIPG, LMO7, MAPK12, NLGN1, NOTCH3, OCLN, OR11A1, OR4A15, OR4N5, OR8K5, PDPK1, PLOD2, PYCR1, SKP2, SLC2A1, SMAD7, SSH2, STX2, SUOX, TFPI, TICAM2, TLR9, UGCG, VASP
Negative_3293	HDC, ACPP, ALG2, AOC2, CACNB3, CCL8, CD22, CDK6, CTF1, DEGS2, DUSP1, EGFR, ENPP6, EPB41L1, FCGR3B, FGFR4, GDF6, GFAP, GZMB, IFNA6, IFNG, IKBKB, IL12RB2, ITPA, KIR2DS5, LTB4R2, MAP3K5, NME6, OR8S1, PER1, PLAUR, PRIM2, PRPS2, QPRT, SMPD2, SNW1, TAS2R5, TBL1X, TRDMT1, ULK3, XYLT1, ATP6V0A2, GABARAPL2, TNFRSF10D
Negative_3294	C1S, LCK, LIF, SHH, AKR1C3, AKT2, APBB1, ARNTL, ATG7, CASP9, CDC6, CISH, CSAD, EFNA3, FGF14, FGF19, IL1B, IL2RB, LTBR, MAP3K8, MGAT4B, MTMR1, MYLK2, NADK, NTNG1, OR4A15, OR4C15, OR5AP2, PLCB3, QARS, TAS2R3, TNFSF18, TSHB, CSGALNACT1, PAFAH1B3, SERPINC1
Negative_3295	B4GALT6, CCR2, DKK2, GCNT1, ICOSLG, ITGA4, KLRK1, MAP3K3, OR1K1, OR4A15, PIGO, PRSS3, RASGRP4, RPS6KA6, TUBA1A
Negative_3296	CDA, RFK, ACVR1B, ASAH1, CASP9, CDH4, DDX47, DGKI, GCNT4, GSTM1, HPSE, IL1R1, MAN1B1, MAP3K13, MMP1, NADSYN1, NEGR1, OR1L4, OR7C1, PLCE1, PTPRF, SGPP2, TAS1R3, THBD, XYLT2
Negative_3297	CAT, GSN, ATP6V1A, AVPR1B, AXIN2, CCL19, CD19, CHST11, CSNK1A1, GAD2, HGSNAT, IL11, IPPK, KIR3DL2, LRAT, MYH4, OR10Z1, OR6Y1, PAK3, PAPSS1, PGM1, PIGK, POLD2, RIPK1, ROCK2, SMPD3, SSH3, TSLP, TUBB2C
Negative_3298	C8G, CMPK1, DDX55, GJD2, LAMA3, OR2A42, PIP5K1C, TGDS
Negative_3299	AHCYL2, DBF4, DDX19A, DDX23, E2F4, GAL3ST1, GFPT2, HMGCS1, IFIH1, IFNK, IL6ST, INPPL1, IQGAP2, MDM2, MTMR2, OR52E2, PECAM1, RAP1B
Negative_3300	DBT, BCAT1, BCKDHA, BUB1B, C3orf10, CACNB3, CD47, CER1, FGF3, FUT1, GARS, GLUD1, GSTM4, ITGA8, ITGB5, MCM5, OR2A2, OR2G6, OR51F1, OR9G1, PFKM, PGM2, PIGC, PIGU, PIK3C2G, PORCN, PPBP, PRKCD, PTCH1, PTPRM, PTPRR, SRGAP1, TSHR, TYMP, XCL1, SERPINF2
Negative_3301	F12, TYR, ADIPOR2, B3GAT3, CCR1, CD14, CD40, CD99, CDC14A, DGAT2L4, DHX58, EFNA3, FLT3LG, FMO2, FMO3, FOSL1, FSHB, GALNT1, GAPDH, GIT1, GNAT3, GSTA5, GSTZ1, HNF4A, HSPA1B, ITGAM, LAMB4, MAOA, MPST, NTRK1, OR1C1, OR5D13, OR6N2, P4HA1, PAK2, PDE7A, PIP4K2B, PSENEN, RXRA, SMAD5, SPHK1, TGFBR2, UCKL1, UPP2, TNFRSF6B
Negative_3302	DBH, ME1, ACTB, ADCY2, ADRA1B, BMP4, CCR6, DPYD, FUT8, IKBKE, LCAT, MAP3K3, NCSTN, PLXNB3, PRKX, PTPN6, RUVBL1
Negative_3303	AK5, ACTA1, CACNA1G, MAD2L2, MGST1, NFX1, OR2A12, OR5D18, SULT2A1
Negative_3304	TPR, ACTG2, AKR1B1, ALG6, B4GALT7, BUB1B, C3AR1, CACNA1F, CNGA4, COX15, CSNK1D, CXCL1, DARS, DGAT1, EGFR, ERBB4, GMPPA, GNAI2, HADHB, HSPB1, IL17RA, ITGA9, MAP4K4, MGST3, MYH4, NEGR1, NOX1, OCRL, OLR1, OR2Y1, OR6C70, PNMT, PTAFR, SGPP2, SLC2A4, SOCS4
Negative_3305	HDC, HK3, ALDH3B2, ALG5, ARHGEF6, ASMT, ATF2, CACNG8, CDC25B, CYP2E1, NRG3, OR14A16, OR4E2, OR6C74, PTGES2, RFNG, SLC33A1, ATP6V1G3, MAPKAPK2
Negative_3306	ACVR2A, ADRA1D, ALDH3A1, CACNG8, CYP27A1, GNPNAT1, GRIN2C, INHBC, ITGA1, L1CAM, LLGL2, MGAT5B, NFKBIA, PFKL, PIGS, RRAS2, SREBF1, TUBB4Q, VDAC1, WNT2

Negative_3307	MPZ,COL4A2,DKK1,DUSP14,GLYCTK,IL19,ITGA11,KARS,MAP3K3,NT5M,OR51S1,OR8H3,PDE4B,PNLIP,SNAP25
Negative_3308	CEL,IVD,ACSS1,APRT,CAV3,COL6A1,CRLS1,EFNA1,GALNT1,GBGT1,MYLK3,NLGN1,NT5C3,OXSM,POLR2L,PSAT1
Negative_3309	PDC,ATP6V1D,CCL5,CFL2,DBF4,EXTL3,GALE,GNPDA2,HEXA,HLCS,NCF2,NNMT,PRPH,SMARCA5,ST6GAL1,VAV3,WARS
Negative_3310	AK3,DCK,GNE,LCT,SRM,ACAA2,ARHGEF6,ARHGEF7,CCR8,COL4A1,DEGS2,DHODH,DIAPH1,EDNRA,EPRS,GAB1,GBE1,GDF7,GNA13,GSTO1,ICAM1,IRAK4,MAN1C1,MAP3K12,NAGS,OR10A4,OR1I1,OR2L8,PIGN,POLR2H,SDC4,WARS
Negative_3311	ANAPC7,CARD11,CBLB,CCNH,CYP2C18,FARP2,FGF11,GGCX,ITGAD,LAMB4,MMP2,NARS2,NEU1,RPN2,UGT2B4
Negative_3312	ADO,ACVR1C,ACY1,AK3L1,ANAPC11,ARD1A,ASMT,CAMK4,CLDN1,DEGS2,FABP3,FGF1,GP1BA,HSD3B1,IAPP,IRF3,LAMC1,NFATC1,NR4A1,OR2A42,OR2F1,OR52A1,OR5AU1,OR5D18,PARVB,PGM3,PLA2G4A,PPP3CA,PRKCB,RASA1,SMAD7,STK3,SYNJ2,TRAF1,ATP6V1G3
Negative_3313	CD4,CFH,GBA,ACOX3,AOC3,BUB1,CD79A,E2F4,FGF6,GNG13,ICOS,ITPK1,LPAR1,OR5M3,PGAM2,POLR3B,RASA2,WBSCR17,WNT1,TNFRSF14
Negative_3314	HAL,AADAC,AMDHD1,CXCL6,GNA15,GUCY2F,HMBS,HMGCL,OR14A16,PDGFRB,PGK1,PGS1,PSPH,STX4,UCK2
Negative_3315	CS,TK2,ALG10B,ARHGEF4,ATF2,BIRC2,CDKN2C,DBF4,FCGR2B,GNA13,HGSNAT,ITGAM,ITGB4,KCNJ11,MAGI1,MAPK14,MASP1,OR2B11,OR6N2,P2RX5,PLCD4,RASGRF1,RPS6KA2,SMAD2,ST3GAL3,TCIRG1,VEGFB
Negative_3316	EDA,ADCY6,BMP6,EPHA8,FGF7,OR2H2,PDE8B,PFKFB3,PGM3,PON3,PTPRR,RXRB,SETDB1,SMAD5,SUV39H2,TPM3
Negative_3317	LYN,ACADSB,ACTC1,CASK,FABP3,FADD,FZD8,HHEX,IL6ST,MCM4,MTAP,MYH14,NKX3-1,OR10K1,OR2AG1,OR51E1,OR51L1,OR52A1,ORC2L,PDIA4,PIK3C2G,POLR2H,POLR3D,PON3,RARS2,RXRB,SDHC,SNAP25,SOCS4,SPRY4,SSH3,STX16,TRMT11,UROS,TNFRSF13C
Negative_3318	CGN,EBP,FOS,GH1,SMO,ADH4,ATG12,CMAS,CTF1,DAG1,FGF22,GRIN2D,HADH,IL17B,MAP2K3,MCM6,PON2,PPP2R2A,PRPH,SETD8,SGPP1,SUV39H2,TAS1R1,TAS2R9,TJP3,TXNDC12,UGT2B28,PHOSPHO1
Negative_3319	ID2,PSEN1,SEMA3D,TAS2R60,TIRAP
Negative_3320	CD19,CDKN2D,CLDN3,COQ3,CYBB,DARS,EPB41L3,KIR3DL1,MAPK9,NUDT5,P2RX3,PAX8,PFAS,PLD1,TKTL2,TUBB,TYRP1,YWHAE
Negative_3321	ATP5L,B3GAT2,CACNG3,CD8A,CDH4,CLDN5,CRB3,CSF2RB,DARS,DPYSL2,ERBB2,FZD1,GNA13,GTSE1,HSD17B8,IL15RA,IL22RA1,INHBB,INHBC,ITGA8,LRP5,MAPK7,MGAT4B,MTHFS,MYH3,NOD1,OLR1,OR10A5,OR11A1,OR52B6,PAFAH2,PIP5K1C,PLIN,PPID,PPP1CA,PTPN5,SCD5,TRPM5,YOD1,MAP3K7IP1,PPP1R12A
Negative_3322	ADAM10,ALDOA,ATG12,CLDN3,CNDP1,DNMT3L,E2F1,GP1BA,GRIA3,HTR6,INSRR,IRAK3,OR4K5,OR6A2,PIGK,PLD1,PLXNA1,POLR2D,PRSS3,ROCK1,SDHC,TAS2R38,UGCGL1,ULK2,ATP6V1G2
Negative_3323	AGMAT,ALPP,CACNB3,CD40LG,CDH3,CLTB,CREB3L1,CYP3A5,DAD1,EHHADH,ENO3,FUT6,GNAT3,HCCS,IL12A,MITF,MS4A2,OR2T34,OR4K17,OR8B8,PAPSS1,PFAS,POLR2F,PPP2R2B,STX3,TPI1,ZMAT3,TNFRSF13B
Negative_3324	C5,DCK,CSF3R,PGLS,PIM1,TBL1XR1
Negative_3325	ADH4,CCL22,CYFIP1,DAAM1,DARS,EARS2,EXOC7,FARSA,GUCA1A,IL1B,ITGA2B,ITGAX,MAP2K7,MAP3K6,OR1S2,OR2M4,OR5AS1,OR5B12,OR9A4,PAK2,PPP3R1,RBX1,SETDB2,SMPD1,THBS1,TNFRSF14
Negative_3326	F2,AGMAT,CLDN22,FGF19,FRAT2,GALNT12,GNA13,HADH,HHIP,INPP4B,MCEE,NOTCH2,OR51A4,OR6N2,PDHA1,PGK2,PRKAG3,TAS2R13,UNC5B

Negative_3327	LHB, ACVR1C, ADRB3, APBB1, CAMK2G, CISH, CPT1B, DCXR, GRAP2, HIBCH, HLA-DOA, KIR3DL3, NFKBIA, OR1E1, PIGL, PLA2G2A, TJP2, WNT11
Negative_3328	EPO, ITK, NF1, SP1, ADH5, ADORA2B, ARPC1B, CYP3A7, IL6R, MTHFD1, MYH7, NRP1, OR2M3, OR52E6, PIAS2, PLA2G2D, PLOD3, PRKACB, RASSF1, RGS3, CSGALNACT1
Negative_3329	VCL, CAV2, FOXO1, OR4F17, PTPRM
Negative_3330	ALDH4A1, ALS2, ASRGL1, CACNA1C, CCR2, CCR9, ERO1L, EXTL3, GGCX, IFNAR1, NAE1, OR2Z1, PLOD3, STX6, WNT5A
Negative_3331	ARPC5, BMP8B, CCL17, DFFB, EPHX2, IL25, MAP2K4, OR11H1, OR5B12, PAK1, RXRA, SETD8, SMAD5
Negative_3332	BTD, CFH, ACSS1, ATP5C1, BPNT1, CACNA1D, CHPF, CLTCL1, CSAD, CYBA, FABP3, FGF11, FGF2, IFNW1, KARS, NADK, OR13D1, OR51Q1, OR51S1, OR8H2, PIK3CG, PIP4K2C, PRSS1, RPS6, SEMA3C, SESN2, STAT2, TUBB4Q, UBA7, ULBP2, WNT9B
Negative_3333	GK2, PLN, ABP1, ACVR2A, ADRA1A, CCL26, CD27, CFL2, CSDA, CYP4A11, CYSLTR2, EPHB6, GALNT6, HBEGF, HHIP, HSD3B7, ITGB8, MAML1, OR52K1, OR52W1, OR6Y1, PDCD1, ROCK1, SLC33A1, SMAD9, TAS2R1, UCKL1
Negative_3334	ACAT1, ADCY1, ALOX12, ARAF, BDH2, BET1L, CLDN18, CREB3L1, CXCL11, EFNA1, FDFT1, GCDH, GPT2, GUCA1A, IL9R, IMPDH1, KCNQ1, MAFA, METTL6, OR2T4, OR6C74, PFKFB3, PIGN, RRM2B, SHC3, SOCS3, SPRED2, TBL1Y, VCAM1, ZBTB16
Negative_3335	F7, IL9, VTN, AKR1A1, ATP6V1A, DAAM1, DARS2, DUSP3, FZD3, GALNT2, GLI3, GPD2, GSTO2, HAGHL, HNMT, IL7R, MALT1, MAT2B, MIOX, NCR3, NEU2, OR2G2, PCYT1B, PDE7A, PIP4K2C, PLA1A, POLA1, POLR2C, PON2, PPBP, PSME2, RAF1, RASSF1, RUVBL1, SMAD4, SMURF2, TMLHE, TUBB2A, TYK2, VAMP1, HSD17B12
Negative_3336	HPD, MVD, RFK, ACADL, B3GAT1, C1QA, CCNB3, CDS1, CMPK1, DDX52, EPHB4, FARSB, FGF20, FUT9, GALNT9, GCH1, GNA11, GNA12, HGSNAT, JAM2, OR2L2, OR2M3, OR51A2, OR5A1, OR6C74, POLR2E, RBPJL, SH3BP2, TYMS, AASDHPPT
Negative_3337	C8B, ACVR1B, AGPAT3, CYP2U1, MDM2, MYH15, OR6C76, PLCG2, UPP2
Negative_3338	APP, MOS, ADH6, ATP12A, BRAF, CCL16, CLDN15, DDX19A, DUSP4, DVL1, EI24, FGFR4, GNAI1, IDI2, NMNAT2, NUDT2, OR51I2, OR6K3, PRKACG, RASGRP4, UBA1, USE1
Negative_3339	ARPC5, CHPF, DPYS, EFNA2, IL18RAP, LRP5, MAP3K4, MEF2C, PIGC, PPARA, PPOX, PPP1R3C, PTCH2, SULT2A1, ULK3, SERPINB5
Negative_3340	CGN, GAA, AK3L1, CAMKK2, EPB41L3, HMOX2, OR10V1, OR4C15, OR5B21, PAK7, POLR2L, PPP2CA, PRKAR2A, TMLHE, HLA-DQA2, MAPKAPK2
Negative_3341	LIF, CHEK2, CNGA3, CPOX, DCTD, DRD2, FABP1, G6PC, GRIN2D, GSTM5, GUSB, IL4R, ITGA9, KYNU, OR4K14, OR5B12, OR5L1, OR5R1, OR6C2, PPAP2C, RGS3, RPE65, SEMA4A, SUOX, WNT2B, ARHGEF12, TNFRSF13C
Negative_3342	A4GALT, ALOX12, AMD1, CCL19, CD70, CYP1A2, DUSP14, GSTO2, IFNA10, IL22RA1, KDSR, NFX1, NMNAT3, OR52E8, PRKCG, PVRL2, SMPD4, SOCS2, STX2, TNFRSF4, USE1, WBSCR22
Negative_3343	CD2, CS, NPL, ALDH1L1, ARSB, ASIP, ATP5C1, CDO1, CLCF1, DUSP2, EVI1, GAL3ST1, GCLM, GLB1, HAO2, IDH3A, ITPA, MAP3K11, MCM3, NCF4, NEU2, NEU3, OCLN, OR1A2, OR1L8, OR2A14, OR5H6, ORC6L, P4HA1, PRKAA2, RARS2, SDHC, SV2B, THY1, TSC1, TUBB1, UPP2, XCL1, MAPKAPK3
Negative_3344	CD8B, FOXO3, OR3A1, OR5L2, OR6F1, PRICKLE1
Negative_3345	GSS, ALG10B, BMP2, CMPK2, FTMT, FZD1, GNA15, HLA-DOB, MTHFR, OR2G2, OR5A1, PAICS, PTK2, SETMAR, SH2D2A

Negative_3346	CAD,ID4,KHK,AASDH,ADCY10,ADSS,AHCYL1,ALDH9A1,ATG3,ATP5J,CCDC6,CDH15,CSAD,DGAT2L4,DPM3,EPB41L3,FDFT1,FGF19,FIGF,GABARAP,GOT2,HSD11B2,ISYNA1,MLLT4,MYH13,NAGS,NCK1,OR13D1,OR51L1,OR9Q1,PARVB,PIGF,PLTP,PTPRB,PYCRL,RIPK1,SMPD2,SREBF1,TJP1,TXNDC12,UROS,ZMAT3,HLA-DQB1
Negative_3347	AKR1C3,CDS1,CEBPA,CYP2C19,DDX55,DHX58,GRIN2D,OR10S1,OR2A5,OR52H1,ORC5L,PLXNA3,PRKY,SV2B,TM7SF2
Negative_3348	VCL,AACS,ACTB,B4GALT2,BCAT2,COL1A2,IL22RA2,INHBE,INPPL1,NAT1,OR4C46,OR5R1,OR5T1,PCCA,RPN2,TAS2R42,WNT7B
Negative_3349	FST,GSN,NPL,PAH,ACAA1,ACAT1,ACTC1,ADSL,ARPC1A,BAIAP2,BNIP1,CCL23,CD27,CHPT1,CYP26C1,DHCR7,DIAPH2,EDNRA,EVI1,FCER1G,IFNA13,IL28A,KNG1,LAMC2,LTBR,MAGI3,MYD88,NQO1,OR1B1,OR4X1,OR51E1,OR5AS1,PIGB,PIP5K1B,PRSS2,RPS6KA1,SETMAR,SSH3,SSX2IP,SUCLA2,SULT1A2,TBK1,TICAM1,TSHR,UNC5B,VAMP1,SERPINA5
Negative_3350	BUB3,CCL2,CDC25B,CSF3,CSNK1E,CXXC4,CYP21A2,CYP2U1,DOCK1,EFNB2,FGF3,GCNT1,GGT5,HMGCR,HSD11B2,HSD3B7,IL20RA,ITGB7,KIR3DL2,MARS,MIOX,NCOA4,NEFL,OR10G4,OR3A3,OR4P4,PHKG1,PLA1A,PLXNA2,PNMT,PPP2CB,RAF1,RPS6KB2,SETMAR,SGPP2,SH3BP2,SKP2,SLC25A4,SNW1,SPCS3,STAM,STX1B,TUBA4A,UAP1,ZBTB16,ZIC2
Negative_3351	ACSL4,ADRBK2,ARRB2,BMP6,CACNA1A,CES1,CNGA4,CYP3A43,DDHD1,FTMT,GALNT12,GALNT2,GBA3,GNB3,HAO2,HSD11B2,IMPA2,IRS4,MGAT2,NNMT,OR4K15,OR4X2,OR5D13,OR6N2,ORC2L,P2RX3,PFKP,PGS1,PIK3C3,PSENEN,PTPRJ,PYCRL,ROBO1,SAT1,SHMT2,SOCS7,TKTL2,UCK1,VAV1,WWP1,ATP6V1B2
Negative_3352	ACVR1,ADIPOQ,ATP5J2,BDNF,CFLAR,CHAD,CTNNB1,CXCL16,MYH14,NMNAT2,OR6Q1,PIGQ,PIK3CD,SESN3,TRAF1,UGP2,HLA-DRB5
Negative_3353	FN1,ATP12A,CLDN14,CLDN3,CYP11A1,FGD1,GNMT,IL12B,MARS,OR52R1,PRKCD,SDC3,YKT6
Negative_3354	ME2,PXN,BET1L,CD226,CD36,CLDN1,EXOC7,GALNT2,GLI3,GYS1,HSD11B1,IBSP,LAMA4,MAGI2,MAN1B1,MAOA,MAT2B,MPZL1,NUMBL,OR2D3,OR2T33,OR4D5,OR52W1,OR9Q2,ORC4L,ORC5L,PLD2,PNMT,POLR3A,SELE,SGMS2,SPRY3,SQLE,UBA1,CACNA2D3,TNFRSF1A,TNFRSF6B
Negative_3355	CR2,PML,ALG13,AMPD1,ATP6V0C,BMP4,CCL14,CHKA,CHRD,DDX54,EPHB3,GALNT14,IARS,IL12B,ITGB5,NOS1,PDPK1,PIGF,PKMYT1,SEMA3E,TNFSF14,TUBA1A,ZMAT3
Negative_3356	C4B,F3,GSS,ATP5A1,ATP5J,ATP6V1D,AVPR1B,CCNA1,CDC25A,CHIA,DDX18,DERL1,EPAS1,FRAP1,GAB1,GARS,GMPS,GOSR2,GOT2,HYAL2,IL5RA,KAT2A,MAOB,MAP4K2,MIOX,MYL5,NKX2-2,OR13C9,OR4C15,OR4K15,OR51M1,PFAS,PFKM,PGS1,RBL2,SEMA3F,SLC12A2,SMAD2,STX1B,SUV39H1,TJP3,TLR5,UBE2L3,VANGL1,WNT2,MAP3K7IP2
Negative_3357	CBS,FAS,KIT,AASS,ACAT2,ALDH3B2,BCL2,BDNF,BLVRB,CTBP2,CTBS,ERBB2,FGF13,GBA3,GSTA1,IFNA8,INHBC,ITGB6,OR1A2,OR4C46,PIGH,PIK3R2,SMAD3,TCIRG1,CTNNBIP1
Negative_3358	AGL,ABLIM1,ASS1,EDNRB,FABP4,IFIH1,LCP2,MAN1A1,MCEE,OR6C74,SMURF2,TNFRSF10A
Negative_3359	AMH,CKB,ILK,LSS,ME1,MVK,NLK,VTN,ATP4B,BCAR1,CAV3,CCL3,CDKN1C,CHRM3,CHRM5,DDX47,DUSP10,DVL1,EGFR,FGFR1,FUT1,GPLD1,GRAP2,IL28B,ISYNA1,KIR2DL1,OR10P1,OR13C4,OR13J1,OR2B2,OR2S2,OR4X2,OR51M1,OXSM,PFKFB3,PPAP2C,RHEB,SEMA3D,SESN1,SMURF2,SRGAP2,TAS1R2,TAS2R42,THY1,UGT2B10,WHSC1L1

Negative_3360	GSN, SRM, TFG, ACTA1, APOA1, ARPC1B, ARPC4, BMP8A, CANT1, CCL20, CCNA1, CDC14A, CES1, CYFIP2, DHDDS, EFNB3, MAPK14, MPDZ, MTAP, ONECUT1, OR11H4, OR2A42, PARD3, PFKFB2, PFN2, PPAP2B, PPP1CA, PSPH, PTPRC, PTPRM, TCF7L1, THBS4, WNT7A, YWHAB, ZIC2, HSP90AA1, PNLIPRP1
Negative_3361	MUT, ADCY4, BLVRB, FMO4, IL15, ITGAM, KAT2B, MNX1, NT5C1B, PARD6A, PDGFRA, PLCB3, PLOD3, PTDSS1, SLC2A4, SRGAP1, WHSC1L1, YARS, MAPKAPK2
Negative_3362	F11, JUP, ACO1, CLDN3, DUSP5, GARS, OR10J1, OR8K1, PISD, PPOX
Negative_3363	ADCY10, ADCY3, B3GALT2, B4GALT1, B4GALT5, CCL21, CDKN2B, CSF3R, FGF9, GCNT3, GGT1, GMPPB, HDAC1, ITGA9, L1CAM, NT5E, OR2H2, OR4C6, OR51B5, OR5M8, OR7D2, PAICS, PANK3, PDE6D, PLAT, PLCD3, SMPD3, SSX2IP, SYNJ1, THBD, TNFSF14, VTI1B, PLA2G12B
Negative_3364	PCYT1B, PLAUR, ROBO3, VAV1
Negative_3365	PDC, ABL2, ACYP2, ALDH2, ATP5C1, CACNB4, CDK4, CERK, CHST12, COQ6, CXCR4, CYP26A1, CYSLTR1, EGLN3, FLOT2, FPGS, GALNT14, GSTM4, GYS1, IQGAP1, MECR, MYH8, NADSYN1, NKX6-1, OR1B1, OR2A2, OR4C11, OR52B6, PDE6D, PDGFA, PDK1, PPA1, PPP3CA, RCHY1, SNAP25, ST3GAL1, TCF7L2, TOLLIP, TRAF4, WIF1, XIAP, YWHAH, PPP1R12A
Negative_3366	ID3, SHH, ACY1, BCKDHA, CARS2, CCNE2, CCR9, DHFR, FCGR3A, FGF11, FGFR4, GALM, GCLC, IFNA8, IL18R1, IL1R2, INADL, NFASC, NR4A1, OR5K2, OR6X1, PDE7B, PMVK, POLD3, PRIM1, PRPH, PTEN, SPI1, ST8SIA1, TUBB4Q, VAMP3
Negative_3367	RPE, TNR, ACMSD, ARRB2, CCL15, CCR1, DUSP3, ENTPD6, FABP3, IL21, KLRC4, LCMT2, NCAM2, NR1H3, OR2T12, OR56A4, PARVA, PER3, SDHD, SETD1A, UGT2B17, WNT10A
Negative_3368	ACTG2, CCNG1, DDX23, FMO4, G6PC2, GLB1, GNA14, HAO2, IRF7, KIR3DL3, LRP5, ONECUT1, OR10A5, OR2G6, PCYT1A, PLCB4, PPP5C, PRSS1, SESN1, SMAD9, SPRED1, ZFYVE16, AASDHPPT, TNFRSF11B
Negative_3369	GBA, IHH, ABAT, ABLIM2, ACAD9, APOA5, CARD11, CCL7, CD44, CHD8, DDX54, EPB41L2, FGF13, FLAD1, FLOT1, GALNT5, GALNT7, GBA3, IQGAP2, MAP2K1, OR10A3, OR1A1, OR2AG1, OR51A2, OR8J3, OR8K3, PPM1D, PRKAG1, RASSF5, ST3GAL5, TBK1, TRPM5, UGCG, VAV3, VEGFA, WBSCR22, PNLIPRP2
Negative_3370	ATP6V1F, CACNA1E, CACNB2, CAMK2B, CDH15, CFLAR, CHRM5, CLDN8, CLTA, CYP4A22, DERA, EDAR, EPHA6, GNAI2, IFNA7, LEF1, MAP2K3, NCOA4, OR6C2, OR8D2, P2RX4, PDE4A, PIK3R5, PNPLA3, RPS6KA2, SCD5, SMC1B, TPH2, TUBB3, UPB1, XIAP
Negative_3371	HK1, TKT, AXIN1, BDH2, BMP8B, CPS1, DLAT, DUSP16, EDN1, ERBB3, GALNT6, HBEGF, NFKBIE, OR2T3, OR5L1, PARS2, PPCDC, SLC12A2, TNFSF8, TUBB4, UROD, WWP1, HLA-DQB1
Negative_3372	C5, MPL, SMS, ACTG1, ATP5D, ATP5L, BUB1, CAMKK2, CD8B, CFLAR, CHEK2, COL3A1, CPT2, DDIT3, DDX19A, EFNA3, F2RL2, FMO5, HRH1, IFNG, IL22RA2, KLRD1, MAP3K1, MTAP, NNMT, NOTCH1, OR56A4, OR5AN1, P2RX1, P2RX2, PDE8A, PLD2, PON3, PRKAR1B, PYCR1, SMURF2, SOCS2, SQLE, UCP1, CACNA2D4, TNFRSF11B
Negative_3373	AK5, PLG, ACSM5, ADH7, APPL1, BDH1, BET1L, BIRC5, CCR4, CD19, CTBP1, DAPK1, DGAT1, DIAPH3, ENPP3, FMO4, GMPPB, GSTK1, IL1R1, INSRR, ITGA1, LTB4R2, MICA, MTHFR, OR10A5, OR10Q1, OR2T2, OR52N2, OR5V1, PDXK, PIK3CA, PIK3R2, PLCZ1, POLR3B, PSME2, RAF1, ROBO2, SDC2, SNAP25, SULT1E1, TJP3, TUBB4Q, WBSCR22, XYLT1, ZIC2, HSD17B12
Negative_3374	DDC, XDH, ACTC1, AGPAT1, AGXT2, ATG12, ATP5I, BMP2, CDC45L, CSF1R, CXCL3, FARP2, GNPDA2, LIPF, MAFA, MAP2K1, NOS1, NR1H3, NT5E, OR11G2, OR4A5, PIP5K1B, PLCG2, PRLR, PTEN, RBKS, RDH16, SOCS2, STX3, ZFYVE9, B4GALNT1, CSGALNACT1, MAPKAPK3, PNLIPRP2

Negative_3375	AKR1C3,ALG8,ANPEP,CCNB3,CDC14A,DDIT4,EGLN1,FECH,INHBC,OR13H1,OR51F1,PDE8A,PLD2,PNLIP,POLR1E,RELA,RFXANK,SETD7,TGFBR1,UCK1,UGT2B28
Negative_3376	F8,LPL,ASCC3,CD44,CLDN8,CYP27A1,CYP4F2,DPYS,EPB41L3,GBA3,GNB3,GUCY2F,HSD11B1,IFNA7,IL10RA,IL12B,IL1R2,MAP2K4,MAPK11,MIOX,OR1J4,OR52I1,OR52I2,PAFAH2,PDE3A,PIP5K1B,PLXNB2,POLR2F,PPARA,PTGS1,RDH5,SGMS1,SUCLA2,TAS2R49,TNXB,TUBB2C,TUBB4Q,TXNDC12,ZBTB16,PLA2G12B
Negative_3377	ACVR1B,ADORA2A,B4GALT4,CREB3L2,CSNK1G2,DAPK1,DGAT1,DGKZ,DNMT3A,DTYMK,EPHB1,FGD1,FLT3,GAMT,GBA3,GNG3,IDI1,IL17RA,IQGAP3,NFKBIB,OR10J3,OR2T2,OR4C6,PHKG2,PKM2,SDC1,SPRY1,TGDS,TRAF1,TSC1
Negative_3378	RET,ADCY6,ADRB3,AK3L1,ASPA,C4BPA,CDH5,COL4A2,CXCL2,F13B,FGFR2,GAS1,IFNAR2,ITPKB,MAP3K7,MARS2,OR2T6,PDCD1,PPAP2C,PRKAA2,RASSF5,RBKS,RBX1,RDH5,TIAM1,UPRT,VASP,WNT5B,ZAP70
Negative_3379	DBH,ACO2,CTNNA3,CYP2U1,DAPK2,FLT3,GAD1,GATM,GSTM4,MTAP,OR10X1,OR1L8,OR2B6,OR9A2,PTCH1,ROCK2,SKIV2L2,SREBF1,WHSC1
Negative_3380	FST,LEP,SHH,TKT,ARHGEF7,ARNT,ATP4B,CACNA1I,CDKN2D,CXCL12,CYP2A13,CYP3A5,EIF4B,GCLM,GNRH1,ICOSLG,IL9R,IPPK,LCP2,MAN1C1,MECR,MPZL1,MYH11,MYH3,NRAS,NRG2,OLAH,OR52N4,POLE,ROCK2,RUNX1,SOCS3,SULT1A4,MAPK8IP3
Negative_3381	CFI,F2R,APRT,ENPP3,F13A1,ITGA9,MAP2K5,RAPGEF3,STX5,TAS1R1,UGT2B10,HSD17B12
Negative_3382	A2M,CS,FAS,IL2,MUT,PGP,SMS,ABL2,ACTA2,ACVR1C,ALCAM,APOC3,BUB1,CACNB3,CAMK2G,CD99,CDS2,CLDN5,CLTCL1,DGKE,EDA2R,FARSB,FASLG,GALNT2,GBE1,HSPA1B,IL11RA,INHBE,LIPF,NR4A1,NUDT5,OR4K15,OR4X1,OR5P3,OR9G4,PIK3R1,PLAU,PPID,RPS6KB2,SQLE,STAM2,TOLLIP,ATP6V1E1,CSGALNACT1,TNFRSF1B
Negative_3383	AMT,DDC,F8,ATG12,AVPR1B,CDH15,CRLF2,GOSR2,IL12B,LIPT1,OR13H1,OR56A1,PFAS,PFKFB1,PPP2R2D,PTPN6,RPS6KA3,TOMM40L
Negative_3384	FAH,HGF,MPI,ALG2,ARG2,B4GALT2,CD274,CDH15,CHRD,CYP2F1,FUT3,MYLK2,NFKBIE,ONECUT1,OR4K13,OR56A3,OR8J3,PANK4,SORBS1,TAS2R38,TNFRSF9,UGCGL1,YWHAQ
Negative_3385	LPO,ACSM5,ATG3,BHMT,FGF8,IL17RB,LLGL1,MAD2L2,NFATC4,OR13C8,PER2,PLOD3,TNFSF12
Negative_3386	BAX,ABI2,ACP1,ALDH1A1,ARHGAP5,B3GNT5,BMP7,CYP2E1,FPGS,FZD9,GNG12,IFNA10,OR10H4,OR2A12,OR5T3,OXCT1,PAK7,PCYT1A,PI4KB,POLR3D,PTGS1,RBKS,WNT3A,ZMAT3,CACNA2D3
Negative_3387	CS,F8,LTB,ADH7,ALPL,B3GAT1,BMP4,BMP8A,CCR7,COL1A1,CREBBP,CX3CR1,DVL3,EPB41L2,FUT2,GALT,GLI1,GUCA1C,HIBADH,HSP90B1,IL10,LMO7,LPAR1,MASP2,MYL2,NME7,OR10A6,OR13D1,OR5T1,ORC1L,OXCT1,PDE6H,PPP1R3A,PPP2R2C,RRAS,RRAS2,SESN3,SHC3,SLC27A1,SLC2A1,SMAD1,TLR2,WNT3,XIAP,XYLT2,YOD1,ST6GALNAC6
Negative_3388	TFG,ACP5,ALDH7A1,FZD5,GPLD1,MYLK,OR10J1
Negative_3389	ADO,AMH,DLD,ID4,XDH,AADAC,ACACB,AHCY,ALDH4A1,ARPC3,CBR3,CDK2,CDO1,CHAD,COL4A4,DCXR,DPM1,ENTPD6,FGF3,FZD8,GCDH,GNAQ,HHIP,IL17RB,INADL,ITGA9,LRAT,MTFMT,NCKAP1,NFYC,NT5C2,OR10A2,OR2A5,OR2B2,OR3A2,OR4D2,OR4F15,OR52N2,PAFAH2,PIP4K2B,PRKAR2B,PTGS1,PTK2B,SOX17,TNFSF11,ATP6V1G1,HLA-DRB5
Negative_3390	BTD,IL6,TNF,ANAPC7,ARPC3,ATP5B,BCKDHA,DDIT3,DIAPH2,DTX2,ERBB2,GUCY2F,ITGA4,NEGR1,OR11G2,OR13G1,OXTR,PDE6D,PRPS2,RPS6KA5,STAM,ATP6V1E2,TNFRSF10B

Negative_3391	ADK, GNS, IL9, ACTA2, ATP2A2, BCAR1, CACNG4, CCL8, CD28, CDH2, CRLS1, FIGF, FZD2, GLUL, GRIA1, GUCY1A2, HEXA, IQGAP3, ITGA9, OR2M4, OR6N1, PDE3A, PIGV, PMVK, PTGER1, TLR2, WNT1, TNFRSF6B
Negative_3392	CBS, HGF, ACOX2, ALG9, ALPP, AMDHD2, BMP5, CCL14, CCR5, CXCR4, DBF4, EIF4E2, FMO3, GNAT3, HAO1, HCCS, MAPK1, MAPK13, OR10J5, OR14I1, OR2G2, OR6C75, PARVB, PDE5A, PROC, PYCR2, RXRA, SETD1B, SRD5A1, ST3GAL1, SULT2A1, TGFB2
Negative_3393	C4B, CRK, LCK, SP1, ALPI, APH1A, ATN1, B4GALT5, CAV2, CEBPA, CLTA, COMT, CRLS1, CSNK1A1, CTSS, DCTN1, DDX4, DHCR7, EPHX1, FGF10, GALNT3, KLK3, MAGI2, MCM7, MDM4, NRCAM, OR10G3, OR10G8, OR11G2, OR2G2, OR4N5, OR52I2, OR8K5, OR8U1, PDPK1, PIP5K1C, PNPLA3, POLR1D, POMC, PRIM2, PVRL3, RBKS, SDC4, SLC25A5, SMAD6, TAP2, TAS2R5, ATP6V1E1
Negative_3394	ID2, ANAPC4, ARHGEF6, CADM1, CD86, CLDN22, CXCR3, DHCR7, DUSP6, FASN, FTH1, GARS, GFAP, GMPR2, GSTA4, HNF4A, IL9R, KRAS, MAPK3, MGAT4B, NME2, NOTCH3, OR11L1, OR51Q1, OR5T1, OR7A5, PGAM4, PIGF, PPM1B, PRSS3, RDH12, RGS3, STK4, THBS3, THTPA, TIRAP, VEGFC, WASF2, ZFYVE16
Negative_3395	GNS, LCK, NPL, ACAT1, ACTN2, AGXT, ALOX5, AMHR2, APOA2, BMP7, CHEK2, CHKA, CLDN3, DIAPH3, FASN, GALNT6, GALT, GJA1, GP1BA, GRIA1, HCST, HMGCL, IL28B, LAMC2, NEFM, NEUROG3, NFATC4, NFYA, NUMBL, OR13H1, OR2T1, OR4N5, PDGFD, PHKG1, SPRED2, SUCLG2, TDO2, UGT2A3, WNT9A
Negative_3396	CCR5, CHRD, CYP2C8, DPYD, GSK3B, ITGAM, OR2F2, OR51G2, SMARCA2, SOAT2, ST6GALNAC6
Negative_3397	F8, LHB, B4GALT2, BBC3, BCMO1, CACNG5, CCL7, CLTC, CREB3L3, EIF4E, HDAC2, HSD11B1, LRRC4C, NCOR1, PDHA1, PDXK, PPAP2C, RPS6KA3, RPS6KA4, TYRP1, UGT2B4
Negative_3398	CD4, HAL, ACTA2, CAMKK2, CD28, CD72, CLTC, CPT1C, CRLF2, CSF2, DLST, ENPP1, GALNTL4, ITGB4, KLRC4, NRG3, NT5M, OR10H3, OR13J1, OR5K1, OR9A2, OR9I1, PEMT, PER1, PIGL, PIP4K2A, PPP1CA, TCF7L1, TSNARE1
Negative_3399	PXN, PLA2G1B, RPN2
Negative_3400	CS, FAS, GNE, KHK, LAT, MVK, PLN, XDH, ACADL, ACOX1, ATP4A, ATP5J, CCL22, CREB3L4, EARS2, IDI2, IFNA13, IL11, INPP5A, MAML2, MMP7, NAT6, OR2T29, OR4S2, OR51A7, OR6C74, OR6C75, OR6K6, OR9A2, PIK3C3, PLXNB1, PPP1R1A, PTPRM, SELP, SPHK1, ST3GAL3, STK11, TACR3, TUBA1B
Negative_3401	IL4, PGP, CASP10, GADD45B, IARS2, IL11RA, OR4K1, PARD6A, RASA2, SEMA3B
Negative_3402	AR, CAD, TFG, TNF, ADRB3, APOE, BIRC5, BPGM, CRB3, CYP27A1, DTX3, EFNB1, EFNB2, EHMT2, EPOR, FGFR1, FUT3, GGT6, GNG13, GUK1, HSD11B1, IRS4, MAP3K11, MYL2, NGEF, OR13C3, OR4P4, OR51F2, OR6C3, OR6T1, OR9K2, OXCT2, PDCD1, PDE3B, PGAM2, PLA2G6, PLCZ1, PLOD2, PLXNC1, POLD1, POLD3, PRKAA1, PRKAR1A, PTGER3, PTGS2, USE1, WNT7A, YWHAG, TNFRSF18
Negative_3403	CSK, GK2, WAS, ALPP, ATN1, CCL28, CCR8, CD19, CSAD, CYP11B2, DTX4, DUSP1, ETNK1, FLOT2, GAB1, GALNTL4, GNAI1, HSD17B1, HTR2A, IMPA2, KCNB1, MAP4K4, NAMPT, NOTCH1, NRXN2, NTN1, NUDT9, OR2T29, PARD6A, PCYT1A, PLAT, PLAUR, PPAP2A, PPAT, PVRL4, RALA, SMC1B, SMURF1, STX2, TSHR, VDAC1, ATP6V0D1
Negative_3404	C8G, CBL, GK2, INS, ADIPOR1, ALCAM, ALOX12, AVPR1B, BAAT, BDKRB1, CBR3, CDC16, COL6A2, DGAT2, DIAPH1, DPYS, DTX4, ENTPD6, FADS2, FGF8, FLT3LG, FUT1, GALNT9, GMPPA, KAT2B, KCNJ11, MASP1, NANP, OR10G9, OR2C1, OR4D2, OXSM, PTGDS, RBPJ, SFRP1, SMARCA5, SPHK1, STAT1, THTPA, TOLLIP, MAPK8IP1, PHOSPHO1
Negative_3405	GK, LHB, ANAPC11, CD70, CD8A, CYP1A2, DDOST, DUSP3, EFNA1, ENPP6, EXTL2, GRIN1, IPPK, OR10AG1, PIK3CG, POLR2E, SHMT2, SOS2, TNFRSF18

Negative_3406	ACOX2, ACPT, ALG3, CACNA1E, CDH4, CYSLTR2, EDA2R, EFNA3, EI24, HAP1, HSPA1B, LCMT1, LEF1, MOV10L1, OCRL, PPP3R1, ROBO2, RPS6KA3, SNAP23, TPMT, VANGL2
Negative_3407	GPT, ACHE, ACPT, CCL24, CCNE2, CCR10, CKMT2, CNGA3, ECSIT, EPAS1, HARS, ICOSLG, ITGB1, MAN1C1, OR1L6, OR52I2, PDE7A, PMAIP1, PPP3R2, PRKAR2B, PYGB, SAT1, SEMA3F, SPTAN1, SRD5A1, STX1B, STX2, TUBB2A, VAMP3
Negative_3408	BAX, MPI, RPE, ADH7, ADRB2, ALCAM, ASNS, BPGM, CPOX, CYP3A4, ENO3, ENTPD6, FABP3, GPX5, HLA-DOB, IDI1, IFNA4, INPP5B, IRS4, KNG1, LGMN, LTBR, MCEE, NCAM2, NOTCH3, NRG2, OLR1, OR51B4, OR52E4, OR6Q1, OR8D4, OXTR, PECAM1, PLCG1, PLTP, PPARG, PRKG1, QARS, RPS6KA3, SELL, SKIV2L2, SYNJ2, TAS1R3, TIRAP, TLR5, TYK2, UGT1A10, UMPS, WARS
Negative_3409	PRL, ACADM, ALDH3B1, ALDOA, ANAPC1, BCL10, CACNG8, CBR1, CD47, CDC25C, CDH2, CDH4, CHAT, CHUK, CYSLTR1, DGKI, DPM2, DPYSL2, DTX4, E2F5, ENO2, GRIA2, GSTM4, HPSE2, HTR6, IARS, ICAM3, INHBC, IRF5, LIFR, MAML3, MYH10, OR11G2, ORC6L, PAK6, PDGFRB, PPP2CB, RPS6KB2, SESN2, SETDB1, SLC33A1, TNFRSF4, UNC5A
Negative_3410	SHH, AKR1C3, ALOX12, ARHGEF6, AVPR1B, CCNB3, CD46, DOLPP1, ENTPD2, ERO1L, ESPL1, F13B, MAP4K3, NT5C2, OR1G1, OR4P4, P2RX3, PIK3C2A, PTGER3, PTPN11, RPN2, RXRG, SC5DL, SEMA4C, SH2D1A, SHISA5, SLIT1, TBXA2R, TGDS, TUBA3C, VDAC1, YWHAH
Negative_3411	DDC, F12, FER, GGH, HK2, SCD, ACO2, ACSM4, ACYP1, ADCY9, ALDH3A2, ANAPC2, ASIP, ATP5F1, CD82, CNDP1, DDX56, DUSP9, DVL2, ENTPD7, EPRS, FLAD1, FZD7, GBGT1, GCNT4, IFNGR1, IL17RA, INPPL1, KLRC3, MAP3K3, MAPT, METTL2B, MTHFR, OR10A6, OR10T2, OR4C11, OR6K2, PERP, PIGA, PIGX, RAC3, SELP, SOCS4, THBD, TRAF2, ATP6V1E2, TNFRSF11B
Negative_3412	GH2, HK2, ID1, LCT, ATG7, CCR1, CDH1, CHKB, CLDN15, CNTN2, CYP3A43, CYP3A7, ENO1, FASLG, FMO1, GAB1, GDF6, HBEGF, IFNGR2, LEF1, MAD1L1, MAPT, MFNG, MMP1, NMNAT2, NT5M, NTRK1, OR2A2, OR2B6, OR2H1, OR4C16, OR4N2, OR4P4, OR51A7, PLCG2, POLD2, RERE, SLC2A2, SSX2IP, STAM, TBK1, TBXAS1, TGFA, TNFSF18, ZFYVE16, HSD17B12
Negative_3413	CCR7, CHST13, GUCY1A2, OR2H1, OR5H2, PCK1, PLCB4, RASGRP3, SLC12A2
Negative_3414	KDR, ME1, MYC, ACOT11, ACSM3, AGRN, ATP6V0B, CLDN4, COQ7, CSAD, FGF13, FGFR2, GALNTL4, GCH1, GOT1, HSD11B1, IL7R, KDSR, MAFA, MAP4K1, NFATC4, OR2T12, OR2T33, OR4C11, OR6B2, PITX2, PPP2R1B, RAPGEF1, SESN3, SLIT2, TNFSF18, TRAF6, TUBB6, UGP2, YWHAZ, HLA-DPB1
Negative_3415	B3GAT1, CTSL1, ELK4, GAMT, GPAM, OR1J4, OR2G6, OR4F17, OR52E8, OR8H2, PIGA, PPAT, UGCGL1
Negative_3416	CFI, FES, GK, OTC, ASRGL1, ATG3, C1QC, CHPF, CNGA3, CPT1A, CREB3, DHX58, DLL1, EPAS1, FGF4, FUT2, JAM2, MAN1A1, MAP3K13, MFNG, NCF1, NFATC1, OGDHL, OR1D2, OR8K5, PDE8B, PLXNC1, PRKCZ, RDH8, RFXANK, SETD1B, SPAM1, SUOX, PNLIPRP2, TNFRSF10C
Negative_3417	IL5, OGT, ANAPC5, ATIC, CASK, CCR3, CLDN19, CLTC, CYP1B1, GAD1, GBA3, GNB1, IL13RA1, IL21, JAK1, LDHD, MAN1A2, MCEE, MYL2, NAT5, OR3A3, OR52E2, OR6C74, OR9I1, PDE10A, PDE2A, PLA2G6, PLCG1, PPP3CC, RDH8, SELPLG, SRD5A2, TSTA3, UGCGL2, TNFRSF13B
Negative_3418	IDS, ABI2, ACAT1, ADSL, AMPD2, BMP5, BUB1B, CASP6, CASP8, CCL25, CDS2, CNTN2, CXCL9, EIF4E, EPHA5, ERO1L, FPGS, GMPS, GP1BA, HDAC2, HSPB1, HTRA2, IL6ST, ITGB1, MAD1L1, OR10R2, OR51D1, OR56A4, PARD3, PIK3R4, SC5DL, SEMA3G, SESN3, SORD, THTPA, TNFSF8, WNT1, CTNNBIP1
Negative_3419	AK7, NOG, SYK, ACADVL, AMHR2, CCND2, DERA, GGT7, HSPA8, OGDHL, OR4B1, P4HA2, SNW1, SERPINC1

Negative_3420	ADK, ATM, CAT, CD6, ACVR1C, ADIPOQ, ALDH1A2, ANPEP, ARPC1B, ATN1, CCNB1, CCR9, CDK5, DDIT3, DVL1, FDFT1, FZD7, GLG1, GNA13, GNPDA1, IARS2, IDUA, IL28B, LEFTY1, LYPLA1, MAN2A1, MAP4K1, MCM3, OR10A6, OR4E2, OR51B2, OR5AS1, PDXK, PGM2, PIAS1, PIK3R2, PRKAR1A, PTEN, PTPN1, RFWD2, SDHC, SETD1A, STAT3, STX2, ULBP1, ULK1
Negative_3421	AGK, C1S, CD2, CGN, ACACA, ACADSB, ALG8, BUB3, CACNG3, CLDN3, CNGA3, COL5A1, COX10, CREBBP, CTNNB1, CXCL3, CYFIP2, ENTPD5, GALNT8, GMPR2, GPX5, IGF1, MYH9, NCF4, NOX1, NSD1, NSDHL, NT5C1B, OR10A2, OR10G7, OR10J3, OR52B6, OR52W1, OR6A2, P2RX5, PCNA, PGM3, PIK3C3, PORCN, RFWD2, RXRA, SEMA4G, SETD1A, SLC9A1, TAS2R9, TJP3, HLA-DQA2, PPP1R12A, TNFRSF13C
Negative_3422	AGL, C2, HK3, RPE, ADH1A, AGXT, ALDH3A1, ALG2, BCKDHA, C7orf16, C9orf95, CCKAR, CLCA2, CLDN9, CRY1, DHCR7, ENO3, ENTPD2, GAD2, GALNT10, GSTA2, HTR6, IFNA10, IFNA13, IL15RA, KLRC1, KYNU, LCMT1, NAE1, NEU3, OR2T10, OR5F1, OR5T1, PDGFRB, PERP, PIGF, PITX2, PTAFR, SHMT1, SLIT1, SPHK2, SRGAP2, TAS2R5, UPP2, VARS, YES1, MAPK8IP3
Negative_3423	PTS, CD99, ETS1, FZD3, OR4F17, OR6C1, PAK2, PPP1R3D, TAS1R2
Negative_3424	AKR1D1, DDX55, HTR2C, HTR6, NKX2-2, PDE1A
Negative_3425	C8G, TNF, AFMID, CCNG2, CLDN9, CPT2, CYP2A7, DDB2, ECHS1, EPB41L1, GALNT5, GNA12, GNRH2, GRIN2A, GRM1, LIMK1, MCCC1, MYH8, OR2A42, OR7C1, OR8K1, ORC6L, PIP5K1B, PLCB2, PLEKHA8, POLR1C, PTK2B, SUV39H2, TAS2R5, TRPC1, ZAP70
Negative_3426	GH2, TXK, ACSL3, CCL1, CLDN16, ERBB2, IRS1, NR1D1, PDGFD, PGM1, PIP5K1C, STAT5B, SULT1A4, WNT9A
Negative_3427	F5, NOG, PGP, ADH4, ASMT, ATP5H, BCL10, CACNG2, CAV1, CES1, CHSY3, CLTB, CYP27A1, CYP3A7, DDX4, FUT4, GATM, GNA13, GPLD1, HTR2B, IL7R, MLLT4, NAGA, NARS2, NFATC3, OR13D1, OR56B4, OR9Q2, ORC3L, P4HA1, PANK3, SPTLC1, TGDS, TLR9, TUBA1A, WHSC1L1, WNT11, WNT2B, TNFRSF17
Negative_3428	LPL, LPO, ACP2, AGXT, CACNG5, CLDN18, CLDN22, CLDN7, COL5A1, ENPP7, IFNA16, ITGA3, OR52E4, OR5F1, OR8H1, PDE1B, PIGQ, PLOD1, RPS6KA1, ULBP3, XCL1, ATP6V1E2, HSP90AB1
Negative_3429	ALG13, CCNA1, DARS, DGKG, DHRS4, FARSB, GGT7, GIT1, GUCY1A3, INMT, OR14I1, PLCB4, PRKAG2, TAS2R49, TLN1, TLR3, UGT2A3, WBSCR22, PDCD1LG2
Negative_3430	BMPR1B, CSNK2A2, DDX56, DFFB, GART, GMPR2, GSK3B, IDH3G, KDSR, MAGI1, NRG2, OR10P1, OR10T2, OR11A1, OR51G2, OR52I2, OR7A5, P2RX1, P2RX6, PDK1, PRKCE, SEMA4A, SULT2B1, TLR9, TNFRSF4, TNFSF4, TYRP1, CSGALNACT2
Negative_3431	FGB, FH, ADRA1A, ALG10B, ANAPC10, ARNTL, BCL10, BMPR1B, BNIP1, CACNG8, CCNG2, CLDN1, CMPK1, COL6A2, CREB3, CREB3L3, DPYSL2, ENTPD7, FCGR2B, FGF20, FPGT, GAD2, GMPS, GUCY2D, IL20RA, INHBC, INSR, IRAK3, MCM4, MTFMT, MTHFD2, NSDHL, OR2L2, OR4N5, OR52K2, OR5AN1, PDE7A, PFKFB1, PRKACB, RASA2, SESN1, STAT3, SULT1E1, TUBB2A, UGT2B7, ATP6V1E2
Negative_3432	CD2, CKM, MET, ANAPC11, ANPEP, CD247, CDC7, DDHD1, DHRS3, EDAR, EFNB3, EGFR, EPHA7, FASN, FGF5, FSHB, FUT3, FZD4, GNRH2, ITGA1, JAK2, LIPC, MAP2K6, OR2D2, OR2M2, OR4D11, OR51M1, PIGN, PLA2G1B, PRSS1, RRAS2, SCP2, SMAD5, SPRY2, TGFB2, TYROBP, WNT5A
Negative_3433	LSS, DLAT, RAPGEF3
Negative_3434	ABP1, ALAS2, CFL2, CSNK1D, EFNA4, EPHA2, GGT6, GLI1, IFNA2, MAP3K1, MAP3K12, MAP3K4, NT5E, OR2F2, OR51S1, OR52L1, OR56B4, OR5B3, OR5M3, PLCG2, SPRY3, STX3, TUBA3C, HLA-DRB5

Negative_3435	CBS, AMHR2, ARPC5L, BBC3, BST1, CACNA1B, CD274, CHSY1, CLDN1, CTBP2, DAPK1, EDA2R, EPB41L2, FSHR, GALNT13, GANC, GAS1, GCNT1, HLA-DOB, IKBKE, NCF1, NEU3, OR5B21, OR6K3, OR8G1, PARS2, PGK2, PIGX, PLTP, POLE, POLR2J, PPP1CA, RERE, ROBO2, SDC2, SEMA4C, SEMA4G, SLC2A1, STAT6, TP53, TUBB1, UCK2, WEE1, WNT10A, CACNA2D1, SUV420H1
Negative_3436	BCR, DBI, ACSL1, ATG3, BLVRB, CAMK2B, CBR1, CD40, CD48, CD86, CDC25A, COL6A2, CSF2RA, CTSL1, CYSLTR1, DGKA, DHRS3, ECSIT, ESAM, GDF5, GNB3, HLA-G, ICAM2, ITGB6, LCMT2, OR10G3, OR1I1, OR1L3, OR4L1, OR4Q3, OR51E2, OR51M1, PAX4, PFKFB3, PON2, PPM1B, PTTG1, PYCR2, PYGL, SOD1, SRGAP3, WIF1, WNT7B, TNFRSF13B
Negative_3437	RPE, ACTB, ALDH1A1, B3GNT5, CKS1B, CNTFR, CYSLTR1, GALK2, GRIN2D, GSTA1, HLA-C, ITGB3, ITPKA, MCM3, METTL6, MLLT4, MTHFS, NME4, NPAS2, OR4C11, OR5V1, OR7A10, OR7G2, PGK1, PRKCG, PTPN1, TM7SF2, TSTA3, VAV3, VTI1B, XYLT1, YWHAQ
Negative_3438	AMPD1, CAMK2B, CLDN1, CPS1, CSNK2B, DRD5, FGF4, GMDS, IL20RB, PRKG1, RBX1, TUBB2A, TNFRSF10B
Negative_3439	PLG, ADCY7, ATP6V1H, BCL2, BMPR2, CHSY3, CRY1, CYP1A2, EPHB3, EREG, FCGR3B, FOSL1, GALNT9, GCDH, GMPPB, IFNA4, IFT57, IL12B, MAN1A1, MAPK14, MGST2, OR10AG1, OR4K15, OR51M1, PCYT1A, PDHA1, PI4KB, PIM1, PIP4K2A, POLR2J, YARS2, ATP6V1G3
Negative_3440	C9, AASS, B4GALT3, CD82, CDKN1C, FGF3, FLAD1, HLA-C, IL25, ITGA9, NRCAM, OR5T3, PARD3, PIP4K2B, PLA2G2E, PLXNA2, PPARD, PTPN6, SLIT1, SMURF2, TIAM1, TRAF2, YWHAQ, ATP6V1E1
Negative_3441	INS, ADRB2, ARHGEF2, BCAT1, CCNA2, CD14, CFL2, CHIT1, F2RL2, FLOT2, FUT9, GBE1, LAMB3, MAP4K2, MGAM, MGST1, MKNK2, MPST, NEGR1, OR10G8, OR13C9, OR1L3, OR2AG2, OR2M4, OR51I2, OR7G1, PLCB4, PLCG1, PLXNC1, POLE2, RERE, RHOQ, RPE65, RPN2, SLC27A1, SMPD1, SORBS1, STAT1, SUV39H2, TBXA2R, TGFB3, TNFRSF18, TNFRSF6B
Negative_3442	HK3, ACVR2A, AK3L1, ALAS1, CD44, CHPF, CSAD, CYP7A1, DGKH, ENTPD5, EXTL3, LYPLA2, MCM3, MEF2C, OR52K2, ORC2L, PIK3CD, PPP2R5C, SOD1, SV2B, UNC5B, WASF3, MAP3K7IP2
Negative_3443	F11, GHR, HK2, HK3, ABI2, ACOT12, ARRB2, COQ3, CPT1A, CYBB, DHFR, DNMT3B, EPHX1, F2RL1, FASLG, GAPDH, GOSR2, GSTA2, INADL, LAMC3, LTA4H, MAOA, NEFH, NFASC, OR1A2, OR4D6, OR56A1, OR6S1, OR8K3, OR9A2, PDE4A, PIK3R2, PPM1A, PPP1CB, PRKAR2B, RFXANK, SEMA4C, SETMAR, STT3B, STX3, VAMP3, VDAC1, VTI1A, ZMAT3, HSP90AA1, SERPINE1
Negative_3444	CBS, CGN, DBI, ALDOB, ALG13, ALPL, BMP7, CTNNA2, EVI1, GADD45G, GATM, GJA1, GMPPB, IPPK, ITGA4, LIMK1, MYH7B, MYL5, OR1S1, OR52K1, OR8I2, OR8K5, PDGFD, PIK3CA, PPP3CB, RELA, STAT4, TAP2, TJP1, WBSCR22, XCL2
Negative_3445	CGA, MPI, MSN, TFG, ACADM, ALG10, APOA2, ARPC1B, CFLAR, CLDN22, CXCL9, CYP3A5, DAAM2, DEGS2, DNMT1, ENO2, EPHB1, FSHB, GNAO1, HEXA, IDH3G, KARS, LDHD, MAGI3, MYL7, NOTCH1, OCLN, OR1L4, OR2L2, OR52I2, OR52K2, OR6K6, PDK1, PIAS2, PLCB1, PNMT, POLR1D, PPP1R1A, PRIM1, RXRG, TAS2R14, TICAM1, TRAF3, UCKL1, VANGL2, ATP6V0D1, ATP6V1C1, GABARAPL1, SERPINC1
Negative_3446	IL6, ITK, SP1, ACTN1, ADRA1A, ANAPC1, ATP5J, BLVRA, CDC27, CFLAR, CHKB, CSF1, CSNK1G2, DLL1, DMGDH, EPHA7, EXTL1, GNPNAT1, HLA-DOB, HMGCS1, IL1B, IL2RA, ITGA7, MAP3K11, MAP4K3, MS4A2, NAT1, OR10H3, OR13C9, OR13D1, PDE6H, PPAP2B, PTDSS1, RAF1, SEMA4F, SKP2, SMARCA2, SRD5A1, SRD5A2, UMPS, WHSC1L1, WNT2, WNT8B, XCL1, YWHAQ, ATP6V1C2, HLA-DPA1, TNFRSF13C

Negative_3447	ME3,ADCY10,ATP5F1,FGD3,FUT8,GSTM3,HCST,INMT,LILRB3,MAP3K13,MAP3K7,MAPK13,NT5C1A,OR1L4,OR2G6,OR52B4,WBSCR22,YOD1
Negative_3448	STS,TK1,ADCY8,ALDH1L1,ALDH7A1,BMPR1A,CLDN16,CYBB,CYP2A7,DHODH,FMO5,GLI3,HCST,IL17B,INPP5E,NGEF,OR2L8,OR51A7,OR5H2,OR7A17,PDHA2,PER1,PFKFB3,RGS3,STK4,TPI1,WWP1
Negative_3449	CSK,PLG,TAT,TNN,ASAH1,BUB1,CCL1,CD59,CDKN1B,CSF2RA,DLL4,DTX3L,EPHA2,EPHB1,EPHB2,FBP1,FGF23,FLAD1,FZD5,GRIN1,HLA-G,HSP90B1,LALBA,NCKAP1,NFX1,OR1A1,OR2D3,OR52J3,PIGA,PPP2R5A,RASSF1,SOCS7,TCF7,TNFSF12,UBA7,UPP1,YOD1
Negative_3450	BTC,CKM,UBB,ACMSD,ALPL,APOA1,BUB3,C3AR1,CACNG1,CANT1,CASP10,CBLB,CDKN2A,CDO1,CHAT,CLDN17,CREB3L4,DKK2,DNMT3B,EXTL2,HARS,INPP5B,LARS2,MAP2K1,MGAT1,NAT2,NT5C1A,NTSR1,OR10G2,OR10K1,OR1L4,OR51T1,PDE2A,PIGV,PPP1R1A,PRKY,RUNX1,SFRP5,SMPD4,SOCS1,TGFB1,TSHB,WIF1,WNT10B,WNT3,ZFYVE16
Negative_3451	BID,CRH,NPY,SDS,TNF,AGPAT6,ASS1,CYP21A2,DDIT3,DNMT3L,ECSIT,FGD1,GALNT1,LILRB3,MAN1C1,OR10H1,OR10K1,OR9Q2,PLA2G10,PPM1B,PPP2R2C,PTGDS,SEMA3C,TOMM40
Negative_3452	LYN,ACP2,AMPD3,CACNB3,CHRM5,CLDN2,CYP1B1,GGT5,GRIN2A,GSTA5,HSPB1,LYPLA1,MNX1,MPDZ,PDE7B,PIGS,PLA2G2D,SC4MOL,STX4,TXNDC12,CACNA2D4
Negative_3453	LPL,B3GNT4,BCKDHB,CACNG4,CAPN2,CD58,CDC42,CHSY3,COL1A1,CTLA4,DERL1,FBP2,FBXW11,GUCY2F,ITGB8,MAD2L1,MAP2K1,NMNAT2,NPR2,PCCA,PDE4B,POLR2K,PRNP,SOS1,STAT6,SULT1A4,TAS2R8,ATP6V1G2
Negative_3454	C8G,GNE,UBB,AASS,ADRB2,ALDH4A1,B3GALT2,CCNE1,CD22,CES2,CHPF,COMT,CXCL1,CYP11A1,DARS2,DHDH,DTX3L,ENO1,GLI1,GNG12,GNPNAT1,GRPR,ITGAM,ITGB2,KIR2DL3,KLK3,LRP5,MAPK1,MMP9,NFYC,OR5M8,PLCZ1,POLE3,PPARA,PPAT,PSEN2,PTCRA,PTGS1,RASGRP4,RXRB,RXRG,SLC25A6,SLC9A1,SRGAP3,TNFRSF13B
Negative_3455	GPT,LYN,TH,ACLY,ACMSD,B3GAT3,B4GALT1,BMP2,CAMKK2,CFTR,CLCA4,CLDN1,DHRS4L2,DPM2,FGF7,FGF9,GAD1,GJA1,HPSE,ICAM2,IFNW1,IL1RAP,ITGB6,LAMB1,MAP4K3,NCAM1,OR10G3,OR51F2,OR52W1,PDE9A,PHPT1,PLAU,POLR2J,POLR3H,PPP2R2A,PRKCB,RPSA,SESN1,SFRP5,SLC2A2,STK11,WWP2,XYLB
Negative_3456	ADK,AR,TPO,ALDH4A1,ALG13,ATP12A,CDC42,COL2A1,CYSLTR2,DAAM2,DDX52,FGFR1,GALNT3,GP1BA,IL2RB,JAM3,MCM7,NFX1,NOTCH1,OR52L1,PRKAB2,PROC,SAT1,SEMA4F,SH2D1A,TUBAL3,UROS,ATP6V1G2,PLA2G12B
Negative_3457	TKT,TXK,ACTN2,ADORA2B,ALDH1A2,ARHGEF1,ASIP,C7orf16,CCL21,CCL4,CD82,CLDN4,DGAT2L4,DUSP3,EFNB3,FGD1,FSHB,GCLM,GNAQ,HCST,HTR7,INADL,ITGB8,LCAT,MAN1A1,MTHFD1,NKX2-2,OR10A6,OR10T2,OR4P4,P2RX5,PDGFD,PIAS2,PPARD,PYGB,RBPJL,RGS3,RPRM,SARDH,SELL,SHMT1,SNAI1,SV2C,TSHR,UBE2L3,ATP6V0E1
Negative_3458	BAD,C2,CSK,FGG,WAS,ABI2,ATP4B,ATP5L,C1GALT1,CCL14,CDC20,CTNND1,DDX56,EPB41,FECH,FGF9,GBE1,GCLM,GRLF1,HIBADH,IQGAP2,KIR2DL2,LAMA4,MMP2,NCAM2,NPR2,OR2T12,OR5P2,OXCT2,POLR3B,PPP1R3C,RDH16,SIPA1,SMPD2,STEAP3,THBD,UCP1,UPB1,YWHAQ,HLA-DQB1,HSD17B10
Negative_3459	ALDH1B1,ALPL,AQP7,BDH1,EPRS,ETS1,FUT6,GAS1,MYH2,PROC,CACNA2D2,SERPIND1
Negative_3460	UBC,B4GALT7,CSF2RA,DNMT1,H6PD,LILRB3,MICA,NFKBIA,OR52A1,PDXP,TLR5

Negative_3461	BCR,CAD,PAH,ACOX2,CBR1,CNTN1,COL5A3,COMT,CRLS1,DDX50,DHCR7,DIAPH2,DKK1,E2F5,EFNA4,GNG12,HTR2A,IFNA16,IL28RA,JAK2,LDHA,MAP2K5,MMP1,MMP9,MPP5,MYL9,OR10H1,OR12D3,OR4C15,OR4D6,OR6C74,P2RX1,PCYT2,PDIA4,PLXNB1,POLR2K,PTPN11,RASGRP1,RDH16,RPN2,SETD7,TAP2,TAS2R7,TRDMT1,TRPC1,UROD,ATP6V1G1,C1GALT1C1,HLA-DRB4
Negative_3462	CTSL1,CYSLTR2,EFNB1,FZD5,HAAO,MAP3K14,METTL2B,NANS,NCOR2,NFATC2,NRCAM,PARD6B,SMAD6,STT3B,TUBB1
Negative_3463	TFG,TNN,AADAT,ACADSB,ACSL4,ADIPOQ,AMDHD1,ANPEP,B4GALT2,CACNG5,CASP9,CCL16,CCL27,CCR8,CES1,CLTA,COL1A2,DNMT3B,ESAM,FOXA3,GLUD2,GNA11,IFNA7,IL17B,MAP2K3,MPZL1,NTF4,OR13C2,OR2A2,PDE7B,PKLR,PRODH2,PTPRZ1,PVRL4,RRM2B,SHC2,SLC2A2,TAS2R14,TUBB
Negative_3464	ABO,CDC23,CLDN18,HIBADH,LCMT1,OR10A4,OR2M3,OR6T1,PDE6H,PDE8A,PRKAA2,PTCH1,TBXAS1,TCF7L1,TUBB2B
Negative_3465	ACTG1,CDH4,CHIT1,DRD5,GLO1,HLA-DOB,INHBA,OR4K2,OR6C75,PANK2,PIK3R1,PLA2G1B,RENBP,SV2C,TAS2R1,WHSC1,WNT11,YOD1,PAFAH1B2,SERPINE1,SUV420H2
Negative_3466	CBS,GLS,AKR1A1,ARSB,CAPN2,CDIPT,CLTA,COQ7,CTLA4,CYP1B1,EDAR,ENO2,FLT3LG,FUT6,GALNTL2,GRIN2A,KIR2DL4,LEFTY1,NRCAM,OR1S2,OR51F1,OR6T1,PLCB3,PLXNB1,SCD5,WASF2
Negative_3467	PGF,CAMK2B,GNAS,OCRL,OR3A3,OR4S1,OR6F1,SESN2,TGFBR1,MAPKAPK2
Negative_3468	C4B,LTB,SMS,ACADL,ADRB2,ADRB3,AKR1A1,ASCC3,CCL4,CREB3L1,CXXC4,DLAT,EDN1,EPHA8,FZR1,GALNT11,GLO1,GSTA3,HLA-F,IMPDH1,KLRC2,OR2T33,OR8J1,PDK1,PLAU,PPM1A,SMAD5,SSH1,ATP6V0A1,ATP6V1E1,MAPKAPK3
Negative_3469	PGP,AASS,ACAA1,AKR1B10,CADM1,CCL26,CRY2,DAXX,F11R,HADHA,NAGS,NCR3,NSD1,OR10A5,OR5L2,PDHA1,PLXNB2,PMAIP1,PPP2R2B,RAP1B,TPH2,CSGALNACT2
Negative_3470	ALG12,CAMK2A,CYP11A1,DPM2,OR8H1,UMPS
Negative_3471	GCK,NPY,CYSLTR2,E2F5,EFNA5,GMDS,IKBKG,OXSM,P2RX1,PHPT1,PPP1R3D,PRKACB,SLC25A6,THBS4,TRAF5,PAFAH1B1
Negative_3472	ATM,GNS,HK1,MPL,SHH,ACADS,ACTN1,ADH7,AGMAT,AOX1,CBR3,CCL16,CHST12,CPS1,CYP1A2,DUSP16,ESAM,FGF12,FUT5,GCNT4,GOT1,HLA-C,ICOSLG,IFIH1,IL17B,IL18R1,ITGA2B,ITGAE,LAMC1,MIOX,MMP2,NR4A1,NUDT5,OR10H2,OR10K1,OR5A2,OR5H2,PIP4K2A,PRSS2,PTGER3,RPS6KA2,SCD5,SOAT2,SOCS2,SUCLA2,SULT2A1,ULBP2,ATP6V1E2,TNFRSF11A
Negative_3473	LPO,CD44,CLDN20,CSNK1E,DDX47,HAO2,OR5P2,OR5T2,PARD6A,PTPRZ1,RASSF5,SDHD,SUFU,TUBA1B,UGT2B4,ST6GALNAC4,TNFRSF11B
Negative_3474	GNG13,OR10A7,OR1S2,P2RX1,PANK1,SPTLC2
Negative_3475	FAH,GSN,HGD,HYI,ANAPC5,C7orf16,CDC25C,CHPF,CLDN19,COL4A1,COL4A4,CXCL11,DAXX,DVL1,ENTPD5,FGF2,HDAC1,HMOX1,IFNA14,IL20RA,LAMC3,MADCAM1,MGST2,NRP1,OR2T4,OR5H2,OR8J3,PDK1,PFKFB4,PIK3C2B,PLA2G6,PLXNB3,POLR1E,RHOQ,RUVBL1,SLC25A4,SLC25A5,SULT2A1,SYNJ2,TYMS,VARS
Negative_3476	ASL,C6,HGF,LAT,SPN,AKT1,CD34,CLDN18,CXCR6,DDX19A,HMGCR,MLLT4,OR1L1,OSMR,PPA1,PPP1R3C,PYCRL,RPN1,ST3GAL1,UBE2L3,SERPINA5
Negative_3477	EDA,F12,OTC,TNN,VTN,ACSS2,AKT1,ALOX12,C3AR1,CCL28,CDH1,COL3A1,CYP2D6,ELK1,EP300,EVI1,FABP4,FGF23,GUCA1C,IL12RB1,INHBA,LAMC1,OR1J2,OSMR,PER3,PGK1,PI4KA,POLR2L,PSAT1,RAC1,RARS2,ROBO3,RXRB,SHMT1,SMPD4,STAT6,STMN1,UGT8,ATP6V0A2,PPP1R12A

Negative_3478	C3orf10, CHPT1, CYP2A6, IQGAP2, MCM3, OR11H1, OR52H1, OXCT2, SOAT1, STX5
Negative_3479	CPT1A, FGD3, GSTM4, ITCH, MTFMT, NEUROD1, OR9Q1, RHOA, TGFB1, ATP6V0A2
Negative_3480	CCL5, CLDN17, MITF, HSD17B12, ST6GALNAC4
Negative_3481	DBI, TNF, ADSL, ALDH3B2, ALPP, C1QC, CLDN1, CYP4A11, GPD2, MAP3K6, MAPK10, MGAT5B, NADK, NCOR1, OR1S1, OR4S1, OR51M1, PANK4, PDE7A, SNAI1, TAS2R1, UBE2K, WNT8A, SERPING1
Negative_3482	IDS, PTS, AKR1B1, AXIN2, CRHR1, DHRS9, EXT2, GNG3, HEMK1, HEXB, IFNB1, JAG1, LRAT, LTC4S, LYPLA2, NLGN3, OR10G9, OR1J1, OR6C76, OR6Q1, RARS, SEMA4C, SPRY3, UPRT, VASP, WBSCR22, HLA-DRB5, SUV420H2
Negative_3483	ASL, F9, BIRC2, COL6A2, CTBS, STX3, TLR3, MAPKAPK3
Negative_3484	F9, SRM, ALG1, ATP5H, CCKAR, CRLF2, EREG, FUT7, FZD4, GAS1, GSTM2, IFNW1, LDHAL6A, MAPK3, NCKAP1L, NFKB1, NRAS, PCK1, PIK3R3, POLR3B, PRKCZ, SETD1A, SIPA1, SNAI1, SOCS3, VARS, TNFRSF10D
Negative_3485	C7, CFH, MET, ACAT1, ALG11, AMD1, B3GALT4, BMP8A, CD58, CD86, CDC25A, CDS2, CYP2A7, DHDH, DUSP16, FBXW11, FCER1G, GALNTL4, GIT1, GSTP1, ITGA11, MCCC1, NKD1, OR10V1, OR1E2, OR52N4, OR8U1, PAX8, PDE10A, PIGN, PRKAG2, SEMA4G, SRGAP3, TAS2R4, TCF7L1, UGDH, USE1, WASL, WNT2B
Negative_3486	CP, FER, IL2, ALOX5, ANAPC2, APOC3, BACE1, BUB1B, CCL27, CD247, CDC25A, CDK2, CES1, CMPK1, CYFIP1, DAPK3, DDIT4, EXTL1, GLYCTK, INPP1, ITGA9, NFATC1, OR2A25, OR5I1, OR6Y1, PMM1, PPP3CC, PRKACA, PTTG1, SDHC, SULT1A2, TBXA2R, THBS3, TXNDC12, UPB1, XCL2, TNFRSF12A, TNFSF13B
Negative_3487	ABL1, ACOT12, ACTN2, CCL24, CCR5, CDC7, DHCR7, ERBB2, GPX5, GPX7, GUCY1A2, HRH1, KIR2DL2, LRP6, MGST3, OR6C76, PARVB, PCK2, PDE2A, SLIT2, SNAI1, TNFSF10, TUBB, XCR1
Negative_3488	CDA, CS, UBB, AGTR1, ALAD, ALCAM, ARSA, ATN1, C3AR1, C3orf10, CCL7, CMAS, CREB3L1, EFNA5, IDI2, IL10, NKX2-2, OR6Q1, PIGC, POLR2J, RBX1, STK11, TNFSF8, TYMP, ATP6V0E1, HSD17B10
Negative_3489	GPT, SRF, ACSL1, ARSB, ATP6V1D, BLNK, BMP5, CACNA1H, CAMK2A, CASP6, CCL25, EPHB1, FARS2, FPGT, GADD45A, HHIP, HSD17B1, IFNB1, MTHFR, OR10Z1, OR6F1, OXCT2, PIK3C2B, PIK3CB, RASGRP2, RPS6KA3, TBXAS1, WARS2, SERPIND1
Negative_3490	AK3, ABLIM1, ACADS, BMP8B, CDC6, CHPT1, COL6A2, CYP21A2, DAPK1, ENTPD1, FRAP1, GRIN1, GSTA2, ITGB8, MOV10L1, OR10AD1, PAK7, PGAM4, POLE2, PPA2, TDO2, CSNK1A1L, HSD17B10
Negative_3491	MTR, ALDH3B2, ATG12, CACNA1S, CDKN2A, CLDN1, DHCR7, DHDDS, EHHADH, EPHA2, FGF4, GLO1, GUCA1C, LCAT, MDM4, METTL2B, MGAT1, MYL2, NAMPT, NQO1, NTNG1, NUDT12, OR10Q1, OR2A5, OR8D2, PDIA4, PKM2, POLR3A, PTDSS2, RHEB, RRM1, ST3GAL1, TGFB3, TNXB, TOLLIP, UCKL1, VTI1B, CSNK1A1L, TNFRSF13B, TNFRSF1A
Negative_3492	CAMKK1, DNMT3L, DPM3, IGF1R, NT5C3, OR1K1, OR2T3, OR7C1, PAK4, PIGL, PLCZ1, POLD2, PPP3CC, PRKAB1, SOS2
Negative_3493	FUK, ACAA2, ACO2, APOA5, ATP5A1, CHSY3, CPT2, DDX19A, DUSP3, DVL3, GNB1, GRM1, IL1R2, IL3RA, LEFTY2, MAGI2, MAP3K3, MYH10, OR10X1, PGK1, PLCD3, PMM2, PSME3, SETD7, SLC25A4, SLIT1, SMPD2, TAS2R48, TLR5, TNFSF9, TSC1, UBE2K, UGCG, HSD17B12
Negative_3494	HK3, IDS, SI, SRM, ACTN2, AGMAT, AGXT, AMDHD1, ARPC5L, AVPR1A, BMP7, BST1, CHRD, CXCR4, EFNA3, HHEX, IDH3B, IL10RA, IL10RB, KIR2DL1, KIR2DS5, MAP3K3, MGST2, MYH2, MYH3, OR1K1, OR2A4, OR2T2, OR4F15, OR5F1, OR9K2, PHKA1, PRSS2, PTPN5, RAPGEF2, RPS6KB2, RRAS2, SNAP25, SPHK1, TACR2, ULBP2, ATP6V1E2

Negative_3495	MAX,ACMSD,ACTN2,ACVR2B,ALG9,BMP8A,C1GALT1,CARD11,CCNB1,CD226,CDK4,CLDN3,CNGA4,DDX50,DRD5,GMPPA,HIP1,HMGCS2,IFNB1,KCNQ1,L1CAM,MASP1,NCKAP1,OR52A5,PDE7B,PPCDC,PSPH,SFRP2,SMAD4,SULT1A2,ULK2
Negative_3496	SYK,ENPP2,KITLG,OR10R2,OR5P3,OR7G2,ST8SIA1,TAS2R50
Negative_3497	AR,CCNE1,CD27,ENTPD3,GNMT,IFNA16,LAP3,METTL6,MTHFD1,OR52B6,OR56B4,PDE2A,PIPOX,STX1A,TPI1
Negative_3498	DAO,FES,IL2,ALOX12B,ARHGEF2,CCL7,CD19,CNGA4,ENO2,EXTL3,GFAP,GNAL,IL10,LRDD,NOX1,OR4A5,OR56A4,PAK4,PAX4,RASGRP4,SOAT2,SPTLC1,ST8SIA5,TAS2R7,UGT2B11
Negative_3499	AUH,DBT,VTN,ABLIM3,CDK7,DUSP2,ENAH,EPHB4,IDH3A,IRS1,ITGAL,OR4B1,PPM1B,TLR9
Negative_3500	CFI,ACACB,ACOX1,ACSL4,ACYP1,ADIPOQ,AGPAT2,AK3L1,AKR1C2,ARHGEF2,AXIN1,CACNA1B,CBR1,CCL21,CDC20,DNMT1,DPYS,EPHB6,F2RL3,FGF12,FLOT1,FOXO1,FZD5,GSTM1,INSRR,ITGA2B,KITLG,KLRK1,LDHA,MGST2,NCOR1,OR1L1,OR52M1,OR6N2,OR7A5,PIM1,POLR1C,SOAT2,TAS2R14,TFPI,WASF3,WIF1,MAPK8IP3,ST6GALNAC4
Negative_3501	MPZ,ABLIM3,ALDOA,CACNA1C,CACNG4,COX10,DHODH,DIAPH3,E2F1,ENPP6,IL21,LAMB2,MPDZ,NAT6,NQO1,NT5C1B,OR14C36,RALGDS,RARS2,TRDMT1,UGCGL1
Negative_3502	ASL,CS,MSN,ABLIM1,ACADSB,ACSL4,CCL1,CREB3,CSF3,CYP2C8,DAAM2,DDX55,DPM2,ENAH,FZD8,GARS,GSTA1,GSTT1,H6PD,HBEGF,HMGCR,HMGCS2,HRAS,IFNGR1,IGSF5,ITGA3,ITPA,LTC4S,MAP3K5,NCAM2,OR1E2,OR2M3,OR51A2,PDGFD,SHC4,SOCS4,SPRY4,ST8SIA1,STX1A,TOMM40,TSC1,TSHB,WHSC1
Negative_3503	CSK,CRKL,NLGN3,OR11L1,OR4F15,PPAP2B,RHEB,RND1
Negative_3504	BAD,CEL,ADCY9,ASPA,BCL2L1,BIRC3,BMPR2,CCL5,CDC42,CRY2,CYP11B1,CYP17A1,DDX54,FLT3LG,GGCX,GGT5,GPD1L,GUCA1C,JAK1,MMP1,MPDZ,NAT6,NRP1,OR2A4,OR2D2,OR6C4,OR9K2,PDGFA,SCLY,SDHA,STK11,VANGL1,WARS2,WNT11
Negative_3505	ACAA2,APOA5,APOE,ATP5G2,BIRC2,CACNA1G,CDC25C,CPT1B,CREB3,GPAM,HAGH,IL22RA1,LIPA,NOX1,OR7G1,OR8H1,PLXNB2,PON2,PPAT,PPP3CB,PSME3,UBE2K
Negative_3506	A2M,AK3,FES,ALAS1,ASAH1,BMP6,CSNK1E,CYP1A1,DDIT4,E2F1,FASLG,GCLM,GNA12,HLA-C,ITPKA,MKNK2,MPP5,OR4C6,PHPT1,PPP2R2D,PRDX6,RPS6,SC5DL,ST6GALNAC5
Negative_3507	AK1,GSR,LSS,APRT,ATP5G3,CHST13,CXCR6,DAPK1,DDX51,FGF21,GPD1,GRHPR,HCST,NFX1,NT5C,OR2T29,OR5AC2,PCYT2,PI4KB,PIAS2,PKMYT1,PLA2G2D,PLA2G2F,PMVK,PPP2R5D,PRNP,PVRL3,RIPK1,RPS6KA4,SEMA4D,SHC2,SULT2A1,TBXAS1,UCK1,UCP1
Negative_3508	CAD,IL6,ADH6,AGPAT2,B4GALT1,CDKN2C,DUSP1,FMO1,KIR2DL1,LAMC3,MASP2,MCCC1,NRXN3,OR10S1,OR51B6,PLA2G1B,PPP3CB,RENBP,STK3,STX7,TAS1R3,UROC1,WNT8A,PPP1R12A,TNFRSF11A
Negative_3509	OTC,SPN,AARS,AGMAT,ATIC,CCL26,CD55,CNDP1,CXCL16,DOT1L,EPHX1,GALNT1,HADH,NTRK1,PHPT1,PLCG1,PNPLA4,POLR2D,PRKCA,RPS6KB2,SLIT2,SUCLG2,SULT1A1
Negative_3510	CTH,NNT,ACSL1,CHKB,CSF3R,CYSLTR2,MCM7,OR8A1,P2RX5,PPP3R2,PRKAB1,PTPRB,TAS2R38
Negative_3511	NFKBIB,PLXNC1,TUBB6

Negative_3512	ADA, CFH, CSK, F2, FAS, ME3, PTS, SDS, ALDH9A1, ARPC2, CES2, CLDN20, CLOCK, CLTC, CXCL10, DDX41, DHDDS, DIAPH3, DLL1, ECHS1, ENTPD2, GBE1, GLUL, GNB3, HCST, IL18RAP, IL8RA, MAML1, MAPK12, MAPK13, NFKBIE, OR1A1, OR1F1, OR56A3, PARD3, PARD6G, PDE2A, PHPT1, PLXNB1, POLR2G, POLR2K, PPP3CB, UGT2A3, UNC5C, VTI1B, ZAP70
Negative_3513	FES, GP9, LCT, ADCY8, ALAS2, ALCAM, ATP4B, ATP5G2, CDK6, CRY2, CXCL3, EFNB1, GALT, GNAI1, HEXA, IQGAP2, ITGB5, LEF1, NOX3, NRAS, OR14C36, OR51I1, OR6K2, PDE1A, PDGFB, PIGB, PRKCG, PRLR, RASSF1, RBX1, SKP1, UPP2, UROD, VAV2, WNT10B, PHOSPHO1, TNFRSF10C
Negative_3514	DHH, GNS, AREG, CKS1B, CSNK2B, DDX55, GRAP2, IL21R, MAP3K2, MCM5, OR51G1, ORC6L, RPSA, TACR2
Negative_3515	CRK, MTR, AGPAT6, ATP6V1A, CCR6, CXCR4, CYP2J2, DMGDH, DPYS, EHMT1, FUT1, GANC, HDAC1, HTR2B, ICAM3, IFNA5, IGSF5, IL20, INPP5B, INSRR, ITCH, KLRC4, MAP2K5, MAP4K3, MCEE, NCKAP1L, NRG2, NT5E, OR10G8, OR2A2, OR2L2, OR4X2, OR7A5, P2RX2, POLR1C, PTEN, PTGER3, PYGM, RARA, SEMA4D, SEPHS1, UAP1, UGT2B7, UPP2, VAMP4, PHOSPHO1
Negative_3516	AMY2B, BMP6, CYP3A5, IFNGR1, ITGB3, KIR3DL2, MAGI2, NOX3, OR5B3, PPID, RPS6KB1, TGFA, TPMT, VEGFA
Negative_3517	BMP8B, CLDN6, MAP3K7
Negative_3518	CCL23, CD59, DUSP5, DVL3, E2F3, GLI1, GNRH1, MAOB, MGAT4B, NCAM2, NRG3, NTF3, OR2L2, OR4K2, OR5I1, OR5L2, PDE1C, PHKA1, PIGQ, PLAUR, POLR2B, PPP3CA, ROBO2, SDHA, SPI1, SQLE, SREBF1, SRGAP3, TYK2, UGP2, WHSC1L1, PRICKLE1
Negative_3519	CBR1, CFTR, CYP2C18, DRD1, ETNK1, GNA15, ITGB8, LAMC3, MCM4, MMP9, MYH11, NARS2, OR2A2, OR51F2, PDE4B, PIP5K1A, PTPN5, RAPGEF3, VNN1
Negative_3520	ATP5I, BPNT1, FARS2, YKT6
Negative_3521	F8, PGF, ADRA1A, ALG5, CCR7, CD36, CD40LG, CD48, CDH1, CDKN2D, COQ3, CSNK1G1, DGKB, E2F5, EPHA3, ERBB3, F13B, GALNT13, GCNT2, HTR2B, IL13, IRS2, ITGAM, KNG1, LEFTY2, LIAS, MARS, NRCAM, OR12D3, OR5AK2, OR8B8, OR8D1, PDE11A, PDE7B, PLOD3, PLXNB1, SLIT2, STX12, TAS2R60, UNC5D, VAMP3, VTI1B, PRICKLE2
Negative_3522	INS, FOXO3, MAP3K12, MKNK1, NCSTN, PHKA1, SLC2A2, SMARCA2, TBXA2R, TUBA3C
Negative_3523	ACSL4, ASAH1, CCR4, DARS2, DLL3, FZR1, NEGR1, OR1L6
Negative_3524	OGDH, OR4Q3, RARS2, RPS6
Negative_3525	ILK, TNN, ACAT2, ACHE, ACSL5, AGTR1, APH1A, ATP5G3, CCL5, CDIPT, CHIT1, DLL3, DRD1, EDNRB, HSD17B3, IL6R, IMPDH1, LCP2, MAML2, METTL2B, METTL6, NPR2, OR10A4, OR11A1, OR6K2, OR7C1, OR8B8, P2RX6, PDK1, PER2, PGK2, PIGC, RAF1, SETMAR, STAT1, STAT2, SUV39H1, TAS2R5, TJP1, TYMS, HLA-DRB3
Negative_3526	PDC, ATIC, ATP4A, BIRC5, CD70, CD80, CEBPA, CPT1C, CXXC4, CYP17A1, FGF2, FOXO3, GCNT2, GSTM2, IGSF5, IL17A, LAP3, MCM3, MITF, NLGN1, NRP1, NRXN1, OR10G4, OR10H1, OR1L6, OR2T33, OR2T5, OR4P4, OR6B2, PIK3C2B, SDHB, SHC3, SIAH1, SMARCA2, TAS1R3, TLR5, TNFSF12, UGP2, ULK1
Negative_3527	CD2, AKR1B1, ARRB1, ARRB2, ATP5H, BDH2, BUB1B, CACNB1, COL4A6, DGKG, DHDDS, ENTPD5, OR2C1, PLCD4, PTPRJ, RASGRP4, ROBO1, TLR9, VDAC2, WASF3
Negative_3528	ACTN4, AKR1C3, ATP5I, ATP6AP1, B4GALT5, C1QA, CD14, CD27, CD79B, CYP27A1, CYP2D6, DHODH, DTX3L, ENPP2, ENPP6, FABP3, FASN, FMO1, FMO5, FPGT, GALE, GRAP2, HSD3B7, IGFBP3, IL20RB, IL21R, ITGA3, JAM2, MAP4K2, MGAT4A, MTHFR, MYH9, NCF4, NTF4, OR1A2, OR1J2, OR4K15, OR52I2, OR6Q1, PIGP, SDC3, UGT2B10, WNT7B, HLA-DPB1, ST6GALNAC5

Negative_3529	C5,F2R,HK2,JUP,BET1L,CD19,CDH1,CES1,CNTF,DPAGT1,ENO3,EXT2,FGF13,GSTO2,HSD17B1,LYPLA2,MOV10L1,MTMR2,MYL5,MYL9,NEU4,OR13C4,OR13C5,OR1J2,OR1S2,OR51G1,OR52E8,OR6K6,OR8D4,PFKP,PORCN,PPP2R1B,PPP2R5A,PPP3CA,PRKAR2B,SPAM1,UPP2
Negative_3530	LRAT,OR14A16,POLR2C,SELL
Negative_3531	IL6,ACOT11,CACNG2,EHHADH,FUT5,GALC,GRIN2D,INPP5A,KYNU,LRDD,MYH1,NFKBIA,OR2T29,OR2T33,OR5D13,OR5K2,OR9A4,OXCT1,PRKAR1A,SLC2A4,TNXB,UCKL1,UGT2B11,WNT3
Negative_3532	ADIPOR2,ALG5,CD28,CDC42,HSPB1,NRG1,NT5C1A,TAS2R60
Negative_3533	CRH,OAT,ALDH1L1,B3GAT2,CD79B,CTNND1,DGKG,FGF6,GRPR,IDI1,ITCH,MGAT4B,NLGN3,OR2AG1,OR52E8,OR5D13,PDCD1,PROC,SCLY
Negative_3534	BCR,ACADS,ATP5H,BIRC2,CTTN,GMDS,GZMA,IL12B,KAT2B,MAP4K2,NCSTN,OR13D1,OR9G4,ST3GAL2,STX10,TBXAS1,TLR3
Negative_3535	ACP1,ADRBK2,ANAPC10,AVPR1A,C1QC,CACNG4,CCR10,CDC20,CDH3,COX10,CPS1,CYP4F3,DGAT1,GMDS,HLA-DRA,HLA-F,IL20RA,IRS1,MAGI3,MCM4,MRAS,NCKAP1,NSD1,OR10G2,OR11H4,OR2A12,OR51B6,OR52A1,ORC1L,PECAM1,PIGN,PIK3CB,PNPO,PRKAB2,RAC2,RAP1A,SGMS2,SHC1,ST3GAL3,SULT1E1,UGT2B11,YWHAZ,MAPKAPK3,ST6GALNAC3
Negative_3536	ACSM2A,ADRB3,ANAPC4,CACNA1D,CACNG5,CHSY3,CYBA,CYCS,CYP3A7,DBF4,EPOR,G6PC2,HCCS,METTL6,OR1J2,OR56B4,PAK7,PIK3R5,PLTP,PRKAR2A,PVRL3,RPS6KA6,SDC1,SEMA3F,SMAD6,SRGAP2,ST3GAL6,STAT3,TAS2R16,TRMT11,UCKL1,ULBP1,ZIC2
Negative_3537	DBH,LSS,ME2,OSM,RET,SHH,AANAT,ACSL6,ACTB,ADAM10,CAMKK1,CAV1,CD27,CSF2,CTF1,CX3CR1,CXCL2,CYP4F2,DGUOK,EFNA4,FGF8,GRIN1,HLA-DMA,IL13RA2,ITGA3,KIR3DL2,MAGI3,MASP1,NFYA,OR2T6,PANK3,PHKA1,PLXNC1,PPCS,PTPRM,RAF1,STK36,TAS2R1,TJP3,TNFSF10,TRPC1,TUBA1C,UPP2,VARS,WWP1,ZAP70
Negative_3538	EZR,FER,MAG,ACP6,ALCAM,ARRB1,C3orf10,DEGS1,EDA2R,FASN,FGFR1,FLAD1,FTMT,GRAP2,HMGCR,HMOX2,HSPA8,ITPK1,MMP14,NCK2,OR51S1,PDE8A,POLR2A,PRKG1,PYCRL,SAT1,SEMA3D,SKIV2L2,SMAD1,SNAI1,STMN1,TICAM2,TP53,HSD17B12
Negative_3539	DCC,GNS,LAT,ALG13,ARD1A,ATP5O,CD48,CD86,CDH5,CLCA4,CNGB1,DCTN1,DGAT2L4,HLA-DOA,IL21R,ITGA9,KARS,KCNB1,MGAT4B,NKX2-2,OR2A4,OR4C13,PPP2R2A,PPP3CA,PRKCI,PSME3,RDH5,STK36,SULT2B1,UGCGL1,WNT3,PHOSPHO1
Negative_3540	TYR,ACSM3,ADH4,AGRP,AKR1C1,ATG12,B4GALT5,C1QA,CACNA1B,CAMK2B,CCNE2,CD70,CDC2,CLDN19,CSH1,CXCR5,CYP4F2,DAPK2,DOCK1,DUSP4,E2F1,EPHA3,G6PD,ITGB1,LARS2,MAP3K6,MDM2,MYH14,NLGN1,OR2M5,OR6B3,OR8D1,ORC4L,PDX1,PHKG2,PORCN,SH3BP2,SSH2,TNFRSF10B
Negative_3541	OR10C1,OR13A1,OR4E2,PDE7A,PFN4
Negative_3542	ACSL4,ARNT2,CHUK,CLTB,EPHA1,EXTL3,GSTZ1,IDH3A,IL1R2,JAM2,NCAM1,NCOA4,NFATC4,NUDT12,PARD6G,PDXK,PIP5K1C,PITX2,POLR1B,PTPN7,RBKS,SEMA3A,SEMA4B,SLC2A1,SRGAP1,SSX2IP,STAT1,TAS2R13,TAS2R4,TBXAS1,TLR3,TUBB4,UROD,VAMP4,HLA-DPB1
Negative_3543	DUT,NF1,AMHR2,BBOX1,BMP8A,CCL15,CD40LG,CDH15,CDKN2A,CLDN1,COL6A2,CPT1C,CSNK1G2,CYP2A13,GMPR,GSTM2,HLA-C,ICOSLG,IFNA4,IL22RA2,IRS2,KLRK1,LIFR,MAP3K11,MGAT5,NEU3,NRXN3,OR10H1,OR10Q1,OR1L1,OR7D4,PCK2,PDE4D,PRODH,PTAFR,ZNRD1,PAFAH1B1

Negative_3544	LCK,LEP,LSS,ABLIM3,BMPR1A,CACNA1B,CCNA2,CD48,CLDN5,CNGB1,COL4A2,COQ6,CREB3L2,CXCR3,EPHX2,EXOC7,FLT4,GNRH1,HMGCS2,IL1A,NRG2,OR2T34,OR4D9,OR51S1,ORC6L,P4HA1,PECAM1,PLAT,POLD1,PPM1B,PRKAG2,PTPRM,RUNX1,SULT1E1,TBK1,TNFSF12,WNT5A,ATP6V0A4,PAFAH1B3,SERPIND1,TNFRSF10B,TNFRSF10D
Negative_3545	C4B,SMS,ACACB,CAV3,CTNNA1,EPHB1,FGD3,GCNT2,GNG3,IL28RA,MAPK14,MGST1,OR1A1,OR51Q1,OR5F1,PDIA4,PTGES2,PLA2G12B
Negative_3546	GMPR,GNAQ,HES1,LIMK1,MGAT3,NCAM1,PFAS,PHGDH
Negative_3547	GLS,HAL,HGD,MVD,RDX,TKT,ACAA2,ALOX15B,AOC2,ARPC5,BPGM,CAV1,CCL15,CRLF2,CYP2A7,DTX3L,ENPP1,EPHA4,EPHX1,FLOT1,FLT3,FOXO3,GALNT14,GFAP,GLI3,GRIN2B,HIF1A,IBSP,IDI1,IL7R,INSR,KYNU,MAPK12,NEUROD1,OR2T1,OR8I2,PIP5K1C,ROCK1,SEMA4D,SLC27A1,STAT3,TAS2R42,TUBB4,WNT7A,MAP3K7IP2
Negative_3548	IL9,MPL,SI,VTN,ADRA1A,AKT1,ALG12,ARG2,ARRB2,AVPR1A,BAI1,CACNA1E,CACNG3,CADM1,CERK,COASY,CREB3L2,CREB5,CSNK1A1,DRD1,DRD5,GLUD1,GOT2,HDAC1,HNF1A,IFNA5,IL22,ITGB7,LTBR,OR4N5,OR51I1,OR5R1,PIK3CB,PIP5K3,POLD1,POLR2J,PTPN1,RFXAP,STAT5B,TBK1,ULBP1,WNT3,ATP6V1B1
Negative_3549	FST,PAH,AGTR1,ALG5,B3GNT3,B4GALT2,B4GALT7,BCMO1,CACNG6,CASP6,CCL19,CHRD,E2F5,ETNK2,FMO3,FRAT2,HAGHL,HPSE,MMP9,MYH9,NNMT,OR4F4,OR4K14,OR5V1,PLXNA3,PMVK,POLR3D,SEMA3D,TGFA,TJP2,TLR2,UGT2B7,VAMP3,WNT7B,MAPKAPK2,PPARGC1A
Negative_3550	IMPDH1,LDHB,MAP3K6,PRPS2,TAS2R4,YWHAE
Negative_3551	PTS,SI,B3GNT3,BAAT,CDK4,GBA3,GGCX,GNA13,GUCA1C,IL1RAP,OR52L1,P2RX1,PLAUR,POLD1,PRDX6,PRPS2,PTCH1,SEMA4G,SMPD1,ST3GAL4,STAT4,TUBB4Q,YWHAE,ZIC2
Negative_3552	ADH6,IDUA,IL18R1,PMM1,STX6
Negative_3553	AKT1,ALG10B,CNGA3,GABARAP,IDH2,IL1A,INMT,OR51E2,PARVB,TLR5
Negative_3554	F12,AKR1C2,APBB1,GMPPB,MAGI2,SEMA4D
Negative_3555	RB1,ASMT,CDC6,CDK4,DBF4,EPHB3,FDPS,FZR1,GNRH2,GUCA1B,HTR2C,IFNB1,IL1R2,OR9I1,PCK2,PTPMT1,SMPD1,STX2,TPMT
Negative_3556	CRH,IL2,ACSL3,ADCY3,ALG10,ARHGEF7,CRLF2,CXCR3,CYP4A11,GALNT2,GRIN2C,GSTM3,IDH1,OR10A3,OR2V2,OR51B5,OR5AS1,OR9Q2,ORC2L,PDE3A,PFN3,SEMA3F,SEMA4B,SH2D2A,SMARCA2,TGFA,TPM3,USE1
Negative_3557	GPT,SI,TFG,ACADL,ACMSD,ACSM2A,ALDH1B1,ALOX12,APLP1,C1GALT1,CD72,CDC20,CYP26A1,CYP26B1,DPM3,GPD1,HMGCS1,IDH2,IFNA4,NOS3,NOX1,NR5A2,OR2B6,OR2L8,OR4A15,OR4C15,OR7G2,PANK4,PDE1B,PHPT1,SOAT1,SORD,SPAM1,ATP6V1G1
Negative_3558	DAO,HDC,SMS,ACSL3,ADIPOR1,BRAF,CALML5,FGF14,FRAP1,FZD5,HAO1,HLA-DOB,IRAK4,LHCGR,MLYCD,NTF3,OR8J3,PAK1,PDE1B,PDE7B,PIAS2,RUNX1,SORBS1,ST3GAL1,UCK2,PAFAH1B2
Negative_3559	CASP7,FOXA2,GALNT8,GUCA1A,OR9Q1,CSNK1A1L
Negative_3560	A2M,BTC,DDC,F2,KMO,NPY,ACTN4,CASK,CHUK,CNGB1,CPT1C,CSF2,CSNK1E,CYFIP1,DCXR,DDX52,DKK4,DPM1,EPHA8,FGF7,FSHB,FZD7,HSD17B1,LYZL1,MAOA,MGST1,MKNK1,MMP7,MYD88,MYH13,NAGS,OR14J1,OR52B4,OR56A1,OR5C1,OR6Y1,PFN1,PHPT1,PLCB1,PRPS1,SOCS4,THTPA,WNT7B,YES1,MAPK8IP1
Negative_3561	GGH,MTR,AGPAT2,AKR1C1,ALG9,CADM1,CLDN20,DDX41,EGFR,HMOX1,MMP2,MYL9,NAE1,NME2,NMNAT3,NOTCH1,NRG4,OR10S1,OR4E2,OR4N2,PGK1,PIGQ,PKMYT1,POLR3H,PPP3CB,PPP5C,RBKS,SHC4,STAM2,TPMT,TUBB2B
Negative_3562	ADSS,ALG6,ANAPC7,GUCY2C,KRAS,OR1D5,PIK3R5,PTPMT1,SMARCA5,UCKL1,ULBP3

Negative_3563	GSN, ID1, TYR, BCAT2, CACNB4, CD44, CD8A, CDH1, CDKN1B, COMT, CRY2, CYP1A1, DGKQ, DNMT1, EPHA4, EPHB1, FABP4, FTCD, GAL3ST1, GBA3, GCNT2, GJD2, GNG12, IFNA2, IL1B, LAMA3, LEFTY1, MCEE, NR4A1, OR10G7, OR1E2, OR1M1, OR5M3, P2RX4, PFKFB1, PFN4, PLA2G1B, POLR2D, PPP1R3A, RASGRP1, RFXANK, ROBO1, SDC4, SULT2B1, TUBA4A, TYROBP, VEGFC, ATP6V1G3
Negative_3564	ABO, CP, DCT, F8, IL8, KDR, PXN, SRF, ALCAM, ALG2, ARG2, ATP5O, B4GALT3, BIRC2, CHAT, COX10, DDOST, DDX41, DLL4, ELK4, ENTPD3, FGF22, GAD2, GNAQ, HBEGF, LRAT, NOS1, NRCAM, OR2T33, OR4D1, OR7A17, PPAT, PPBP, PPP3R1, PSAT1, PTAFR, PTK2B, SLC25A4, ST3GAL2, ZBTB16, SLC25A31
Negative_3565	CTH, HGD, HK2, PAH, ACO2, ADRB2, AMDHD1, ANAPC5, BMP6, BNIP1, CBR3, CCND2, CCR6, CSF2, CXCR6, DGAT2L4, ERBB2, FUT2, GCNT3, IPPK, LDHAL6A, LDHAL6B, LDHC, MGAT5B, OR2C3, OR2D2, OR4C6, OR5A2, OR5L2, OR6C76, OR9K2, PAK3, PAPSS2, PFAS, PIGW, PLOD1, PPP3CC, PYGL, RASA1, RUVBL1, SMARCA2, SMPD3, ST8SIA1
Negative_3566	AUH, CCL2, IRF7, MOV10L1, PDGFA, PISD, TACR3, PDCD1LG2
Negative_3567	GK2, DAD1, GZMB, IFNA8, MYH7, NRAS, OR2T29, OR4D5, OR6K6, OR8D2, PIAS1, PRODH2, SOCS4
Negative_3568	MOS, ACTN2, ADRBK2, ALDH6A1, ALG6, AMDHD1, ARHGEF4, BBC3, CADM3, CFTR, CHIT1, CKMT2, CLDN8, CTSL1, DAPK2, DHCR7, EGLN1, GAD1, GNPNAT1, GPX5, IDH3A, IL18, JAK1, MAGI1, MTFMT, OR13C3, OR4K17, OR4Q3, OR52E4, OR5D18, OR5L1, OR8J1, PDE11A, PIK3C3, PRKCA, PTK2, PVRL1, SDHD, SEMA4B, SV2A, TAS1R2, TOLLIP, TPH1, UCK1, WASF2, ATP6V1G1, TNFRSF14
Negative_3569	BAD, CD6, GSR, ACP6, ALDH6A1, BUB3, CCL14, CCL3, CTBP2, DCTN1, EFNA2, EPHA3, FLT3LG, GALK2, GNRHR, IGSF5, IRS1, OR2D3, OR5T1, PLA2G2A, PLA2G4A, PRKACB, PTPRR, PVRL1, PYGB, SCIN, SULT2A1, THTPA, TSHR, PHOSPHO1
Negative_3570	ACADM, CHPT1, CYCS, FOSL1, HEXB, MAPK7, MGAT5B, OR2Z1, RPS6KB2, TRPC1
Negative_3571	ALPI, ATP5D, CCR8, CD70, CD8A, CLDN4, CREB3L3, FGF2, FGF9, FOXA3, FPGT, GADD45B, GALNT6, GLUD2, GNG12, GPT2, HARS2, HPSE2, HSD11B1, IDH2, IPMK, MAML3, MAP3K1, MKNK2, OR2F1, OR2M5, OR2T2, OR5T1, OR6X1, OXSM, PDXP, PECAM1, PIK3R3, PTPN1, RAP1B, RXRB, SEMA7A, SMAD7, SREBF1, TKTL1, TPH2, UGT2B10, WNT10B
Negative_3572	CTPS, PDHB, UGT2B28
Negative_3573	SMO, ATP6V0C, AVPR1A, C4BPB, CACNA1D, CASP8, CCL8, CCR8, CD27, CDC23, CTBP1, CXCL12, DDX19A, DDX52, DIAPH2, ETNK2, FGF7, FOXO3, GMPS, GSTM3, GUCA1C, IFNA13, MAP2K1, MKNK2, NRG3, NT5C, NUDT9, OR2A25, OR2T6, OR5P3, OR6Y1, ORC2L, PCYT2, PFKM, PIGF, POLR1A, PRPH, PTGS1, RAC3, SH2D1A, SMARCA2, STAT1, TACR3, UCK1, TNFRSF1B
Negative_3574	FYN, IL5, SCD, SRM, ASNS, ATP5B, CAPN2, CCNH, CD59, CDK5, COL4A4, CYFIP1, GADD45B, GALC, GLUD1, GSTT1, HLA-DOA, ITGA3, ITGB3, JAM2, MAP2K7, NCAM1, NR5A2, OR3A1, OR4C11, OR52W1, PCCB, PDHA2, PIK3C2B, PLCG1, POLD3, POLR2K, PTGES, RDH8, SMARCA2, SMURF1, SPRY3, TAS2R7, TCF7L1, TRHR, WWP1, ATP6V1C2, CTNNBIP1, SERPINB5
Negative_3575	AK5, ACO1, ATP5D, LIPG, OR4F17, OR51B4, PPP2R5B, TJP3
Negative_3576	AK1, CKM, FAH, ACSL3, ADH7, ANAPC7, B3GALT5, BCKDHB, BCL2L1, CACNA1C, CBR3, CCNE2, CLDN2, CRLS1, FLOT1, FUT2, HAP1, HMGCS2, IKBKG, IL12RB1, IL21R, IL28RA, LAP3, LTC4S, NFX1, OR1E1, OR2T10, OR2T33, OR4K1, OR8K3, OR8K5, OXTR, PDPK1, PLA2G1B, PLA2G4A, PTGES, PTPN1, PVRL2, RND1, SOAT2, STX5, TAS2R4, TUBA1A, ULBP2, SERPINE1

Negative_3577	ID2,INS,ALG12,ALPI,APBB1,ATP5G3,CARS,CLDN18,CREB5,CSAD,CYP11B1,DOLPP1,EDAR,FGF19,GAS1,HSD11B1,IL11RA,INPP5E,KCNB1,LYPLA2,LYZL1,NRAS,OR13C4,OR1F1,OR4C6,OR5AS1,OR5D16,OR6T1,POLR1D,SLC2A4,SERPINE1
Negative_3578	C6,DBH,ADRBK2,AKR1B10,ALDOB,ALG13,ARHGEF6,BDKRB1,CCL21,CCR2,CHAD,COL4A6,CRKL,CYP26A1,CYP2A13,EPRS,FGF21,GLUD1,GOT1,GPAA1,GPX7,GSTA3,ICOSLG,IDUA,IL2RG,IRAK4,NOS3,OR10H4,OR2C3,OR4L1,OXSM,PAK2,PPAP2B,PPM1D,PTGIS,RFX5,RFXANK,ROCK1,RPS6KA3,RPS6KA5,SNAP25,THTPA,HLA-DRB4
Negative_3579	IHH,ACAA2,FGF13,OR4D6
Negative_3580	DCC,CD27,IFNA21,NPR1,OR2A42,PPARD
Negative_3581	AK5,DLD,GAA,LCT,CXCL6,DUSP10,FUT9,GRIA1,OR5D13,OR5P3,PER3,PFN1,PHPT1,PTGDS,SNAP25,SV2B,UGCG,ULBP1,UNC5A,AASDHPPT
Negative_3582	CAT,NF1,SPN,TH,ADCY5,ADCY7,AMDHD2,ARPC1B,ATP2A3,CD55,CDKN2A,CYFIP1,DGKH,DHDDS,FABP3,GSTZ1,INPP5B,ITGB8,KIR2DL3,LHCGR,NCF4,NRG2,OR4C16,OR56A1,OR8H1,PANK2,PHPT1,PYCR1,SDC1,SULT1E1,SV2A,TGFBR2
Negative_3583	PXN,WAS,CCDC6,COL5A1,GSTP1,HHIP,IFNA14,MAPK10,MOV10L1,OR3A2,OR4N4,SKP2,SMAD5,UPP2,TNFRSF10C
Negative_3584	DBH,OGT,AARS2,ABAT,ALAS2,ATP5F1,CACNA1A,CACNA1I,CACNG3,CCL27,CCL8,CDK2,CMAS,DUSP3,FSHR,GFPT1,GNB1,KLRD1,LHCGR,MAPK7,MITF,NEFH,OGDHL,OR13C4,OR51B2,OR6N1,ORC5L,PGM1,PLA2G2A,PLAT,PLTP,PRKCG,PRKCI,SC4MOL,SLC25A5,SLC2A4,SMPD1,SORBS1,TUBB6,UBA7,UGT2A3,VKORC1,KIR2DL5A,MAPK8IP2
Negative_3585	CGN,ALDH3A1,ATIC,B4GALT6,BBOX1,BIRC3,BMPR1A,CACNA1S,DCTD,DGUOK,FMO3,GNB1,INPP4A,MYH9,NCOR2,ODC1,OR4L1,OR5AK2,OR9G1,PDE4C,PDE7B,PFKFB2,PIP4K2A,PLA2G2D,POLR2H,PRKCA,PSME2,PTGES,RPE65,SDHB,SLC2A4,THTPA,UGT2B10,CSNK1A1L
Negative_3586	HGD,NPY,ACO2,ADH1A,BAIAP2,BMP2,CD48,COL6A2,CRY1,CTSG,CYP2C19,CYP2C9,DRD5,HAGH,HEXB,IFNB1,INPP1,KLRC3,LRP6,MTHFD2,MTMR1,OR10G7,OR1A2,OR1D2,OR2T4,OR7A5,RDH16,SETDB2,UGT2A1,SLC25A31
Negative_3587	CD2,CSK,DDO,IL2,ACACA,ACO1,ACYP2,ALG10B,AMPD1,AMPD3,ARD1A,CBLB,CCL13,CCNB2,CCNE1,CNDP1,COL5A1,COMT,CSF1,CXCL12,EP300,FARP2,FGF7,HMGCS1,IL21R,ITPA,MTHFD2,NSDHL,OR13C5,OR51L1,OR5AR1,PAX8,PLCD3,PLOD2,POLR3D,PTPN1,RCHY1,SELL,SPRY2,SPTLC2,TAS2R7,TPMT,TRAF4
Negative_3588	MVK,TFG,ARHGEF4,CYFIP1,GBGT1,ITGA6,MGAT5B,NAGK,NOTCH2,PFN3,PLD2,TRAF4
Negative_3589	ATN1,ATP12A,ATP5G1,CPS1,LIPE,NAMPT,NKX2-2,RPS6KA5
Negative_3590	GLS,NOG,PC,STS,TK2,AARS,ABCC8,ACMSD,ACSL4,BCMO1,CACNG2,CCR10,CDC26,CDC45L,CHAT,CLCA2,CNTN2,EXT1,FECH,FPGS,GRIA3,HIP1,IDH3B,KIR3DL3,MAPK10,MYH9,OR10A2,OR2H2,OR4N5,OR6X1,OR8K3,PARVA,PGAP1,PPP2R5A,PRKY,PYCR1,RRM2,SDHD,SLC27A4,SPI1,SPTLC1,UNC5A,VKORC1,C1GALT1C1
Negative_3591	ACYP1,B3GALT4,BCL2L1,GFPT2,MLLT4,NCF4,OR5J2,PKMYT1,TOMM40L,UGP2
Negative_3592	FOS,ACP6,CACNB4,CAV2,CNTN2,CREB5,CTLA4,CYP2C18,EPHA2,GALNT12,GUSB,MAD1L1,OCLN,OR2D2,OR2T33,PPT1,RRM2B,UGT1A10
Negative_3593	CD58,NEU1,POLR3A
Negative_3594	C5,ACSS2,ARNT,CCR3,CDC14B,CHDH,CNGA4,COL4A1,CYP26B1,CYP2S1,DIAPH2,DKK2,DNMT1,HSPA2,NARS2,OR4F17,P2RX1,PDE11A,PRODH2,PTGIS,TAS2R50,TUBB3

Negative_3595	BAX,KHK,ACADSB,ARRB1,B3GALT2,CASP3,CASP7,CCL22,CHUK,CKS1B,DHDDS,EPHA7,FUT6,GPD2,GRIN2D,GRM1,GSTP1,HSP90B1,HSPA2,IL11,ITGB4,ITPK1,MAOA,MARS,MMP1,MYH15,NAMPT,NKD2,NLGN1,NLGN3,NME4,NOTCH2,OR1L6,OR2A42,OR52N1,OR5AK2,OR5R1,PARVG,PGK1,PRKAR2B,PVRL1,RCHY1,RRM2B,RXRB,SDHA,SETD7,SMAD2,ST6GALNAC3
Negative_3596	CFLAR,GBGT1,GPD1L,GSTM4,P4HA3,PRNP,TNFRSF11B
Negative_3597	BTC,PRL,AANAT,ADH1B,ADRA1A,ANGPTL4,CDKN2A,COL5A2,DUSP2,DVL2,GGT6,GSTT1,IDUA,IFNA14,ITGAL,LALBA,MCEE,MCM6,NRG4,OR2G3,PAX8,PERP,PIP5K1B,PLXNA1,POLA1,PPAP2B,PRKAA2,PRKG2,RPS6KB1,STAT2,TGFB2,TLN2,WNT9A
Negative_3598	BTK,FGB,PGF,ACSM1,ARSD,CCL21,CSAD,CSH1,CYP19A1,DDIT4,DGKB,GGPS1,GNA13,ITGAM,LGMN,NEUROG3,NUDT9,OR10J3,OR13J1,OR3A2,OR51B6,PANK3,PDGFRA,PKLR,PTGER3,RAC2,RASSF1,STK4,ULBP2,VTI1A,WASF2
Negative_3599	CACNA1I,IL17RA,OR10H3,OR7D2
Negative_3600	CD2,IL5,ACP1,ANAPC11,ASIP,BMPR1B,CALML6,CCL28,COL4A6,COQ5,CTNNA2,ERBB4,GFAP,IQGAP2,MYH13,OR2A14,OR2T29,OR51B5,OR5AU1,OR5B3,OR5D13,OR8B12,OR9A2,PNMT,PPP2R5D,PRKAG1,SEMA4C,SH3BP2,ST3GAL3,UROS,ZBTB16,ZNRD1
Negative_3601	LPL,MPI,NGF,NLK,ABI2,ACADVL,ALDOC,BPGM,CACNG2,CLDN18,CSF1R,DKK4,DTX4,DUSP1,FUT4,GARS,GLI1,GOT1,GPD2,GUCY1B3,HMGCR,HSD17B1,MYH13,NFYB,NFYC,NR5A2,NUDT12,OR10A5,OR13J1,OR51E1,OR8S1,PAICS,PDE6G,PER2,PGM2,PIGV,PIK3C2G,PIP4K2B,POLR2K,PRLR,PTPN6,RFNG,RXRG,SOCS1,SRD5A2,UGT2B11,UGT2B7
Negative_3602	SFN,ABL2,ACLY,ADH6,CDIPT,CNTN2,DTX3,F2RL2,FOXA3,GALNT6,IFNA8,KIR2DL1,MTHFD1,OR52D1,OR52E6,SENP2,SGMS1,HLA-DRB4
Negative_3603	OR10G4,OR2B2,TNFRSF10B
Negative_3604	ASL,GK,GPI,SDS,AHCYL2,APOA2,ATP6V0C,BIRC2,CACNG6,CCL1,CCL21,CHIT1,CNGA4,CYP11A1,DUSP5,EARS2,EFNA3,ESAM,FSHR,GNPDA2,GSTM3,GUCA1B,HCCS,IFNA10,IL1R1,ITGAX,LAMB1,MGAM,MYD88,NCAM1,NMNAT2,OR10A4,OR52N1,OR52N4,OR56B4,OR5AP2,OR5L2,OR7G1,PARS2,PLAU,POLR2D,PPARD,SKIV2L2,SLC33A1,TSLP,VEGFA
Negative_3605	ACTG2,ACYP1,E2F5,ENO1,FASLG,MOV10L1,NUDT8,OR52R1,OR9I1,TUBB,PNLIPRP1
Negative_3606	DBI,PGF,TEC,ARSB,CD8A,DDX23,DUSP14,EGFR,ESAM,FOXO1,HEXA,HRH2,HSD17B1,IL1R1,IQGAP3,LALBA,MASP2,MKNK1,NUMB,OR3A3,OR51D1,OR9G4,PHGDH,PKMYT1,SMAD6,SRD5A1,ATP6V1B2
Negative_3607	AGK,GSN,ACTG1,AKR1C2,ARRB2,BPGM,CD244,CRLF2,CXCR6,DIAPH2,DPAGT1,EPB41L1,FADS2,GRLF1,HAGH,IL15,MGAT5B,NAGA,NAGS,PFAS,PIGP,PLAUR,PON3,PVRL4,RENBP,SQLE,STX12,TJP3,UAP1,UBE2K,VNN1,WNT11,B3GALNT1,SERPINB5
Negative_3608	IDS,NPL,ADCY10,ALOX12,ALOX15,ATP2A2,BMPR1B,CCNE2,CYCS,EFNB2,FLAD1,GLUL,GSTM3,IL1A,LIMK2,MAP2K1,MYH2,NUDT8,OGDHL,OR8H1,OR8K5,PIM2,PVRL2,RALBP1,ROBO3,SORD,SULT1A1,TAS1R1,TSTA3,TUBA1C,XCR1,PLA2G12A,SERPINA5
Negative_3609	ANAPC7,ATF2,ATN1,ATP5I,B3GALT1,BLNK,CCNA2,CCNH,CCR4,CCR6,CHRNA7,CYFIP1,CYP7A1,EPHX2,FDPS,GADD45A,GCH1,GNPNAT1,GRAP2,INPPL1,KCNQ1,LAMC1,LILRB3,MAPK14,MAPK7,MNX1,NAT1,OR10T2,OR2A2,OR56A3,OR5D18,OR6C2,PARD6G,PCYT1B,PIPOX,PLCE1,PRKAR2A,PRKCA,RASA1,RXRA,SLC2A1,SMAD6,STX16,SUCLG2,TAS2R13,WNT16,WNT5A,CSGALNACT1,SERPINC1

Negative_3610	CFI,MPI,MSN,ADH6,ALG13,B3GNT4,CCNE2,CCR6,CHAD,COL4A1,CXCL11,DUSP10,GAD2,GOT1,HMBS,IL17B,IL1RAP,MYL2,NAE1,OR5D13,OR5L1,P4HA2,PARD3,PARS2,PDE8A,PDGFC,POLR2I,PRKCG,SDC1,SHMT2,STAM,TAS2R40,UNC5B,VKORC1,WNT5A,SERPING1
Negative_3611	PGP,ABCC8,ARPC2,ATP5B,B3GNT3,BCAT1,C3AR1,CACNA1C,CHEK1,CHST11,ERBB2,FARS2,FBP2,FSHR,GUCY2F,HDAC2,HLA-G,OR7A10,PCYT1A,PFKFB3,PPT1,PTPRJ,PVRL3,ST3GAL5,TUBB2C,VEGFB
Negative_3612	NGF,ALDOA,COL6A1,FUT8,IRAK2,OR4Q3,TAS2R60
Negative_3613	BMP4,CLOCK,CYP3A43,FOXO3,GGCX,OR3A3,OSMR
Negative_3614	CD2,DCK,FGA,FST,ACOT12,ACOX1,ADORA2B,ATP5A1,CCR1,CD79B,CHRNA7,DRD1,EDA2R,EDN1,EPHA2,FRAT2,FUT6,GNPNAT1,GNRHR,GOSR2,HTR2A,IDH2,ITGB3,LIPC,MALT1,MMP2,NCK1,OR13A1,OR1J4,OR2H2,OR8K3,PAK4,PDE3B,PGAM2,PTPRF,SELL,SH3BP2,SLC2A2,SMC1B,SOCS5,ST3GAL4,YWHAH
Negative_3615	HK3,AARS,ADCY8,B3GALT4,CCR1,CDC20,CRY1,CXCR4,EDN1,EREG,GSTA3,HHEX,ITGA6,NEU4,NME6,OR51I2,OR5V1,ORC4L,PFKL,PIAS2,PIAS3,PLA2G4A,RPSA,SESN2,SLC12A2,SRD5A2,STX4,TYMP,VDAC1,ZAP70,SUV420H1
Negative_3616	TFG,TKT,ADRA1B,AGXT2,AKT1,ANAPC4,ARRB1,BMPR1A,CAV3,CNTN1,CYBA,CYP3A5,DLL4,DPM1,EGFR,ENTPD2,EPB41,FGF13,FUT9,GADD45B,GOT2,GP1BB,GUCY2F,GUK1,IL28A,LDHD,MECR,OR13H1,OR1L3,OR51S1,PLOD2,PPP1R3A,PTPRM,RALA,SDHA,SGPP2,SPP1,STAT5A,UNC5B,WNT7B,TNFRSF6B
Negative_3617	IL7,NPY,ABI2,ALDH3B2,ASCC3,CARS,CD22,EPHA3,EXT2,GAD2,IL28B,ISYNA1,MNX1,NFYA,OR1A1,OR5AN1,OR5B17,PIK3CB,PLA2G3,PRKY,RASA1,TNFRSF1A
Negative_3618	C4B,DDO,GCK,ACYP2,ALDH2,ANAPC2,CCR8,CPT1B,CXCL12,FGF13,FLOT2,GZMA,IDI2,ITGA1,MAP4K1,MFNG,OR2T33,OR3A2,OR5B17,OR8D1,P2RX4,PDGFC,PIGQ,PTGS1,RASGRP4,SDC1,SDC2,SPRY2,STT3B,TACR1,YARS,CSGALNACT1,TNFRSF11B
Negative_3619	F7,IL7,SPN,ACADSB,ACO2,ACVR1,ASNS,CADM1,CALML5,CAPN2,COL6A1,CSF2RA,CXCL2,GGT7,GLUL,GRIA1,HAGH,HAGHL,HCLS1,HLA-C,IKBKB,IL13,IRAK4,LEFTY2,MBL2,MCEE,MDH2,MKNK2,MPDZ,MRAS,NEU1,OR13H1,OR4P4,PDE8B,PIP4K2B,PMM2,RAPGEF3,YOD1
Negative_3620	FER,GSN,HDC,SFN,ZYX,ACVR1C,ADH4,ADORA2B,AKR1C4,ALDH1A2,ALPL,ANAPC4,ARSA,CACNA1B,COL1A2,COL5A2,CYP26B1,FARSB,FGF4,FLT1,GRIA2,NOS3,NRG4,PCK2,PDHA2,PTPN7,TNFRSF9,TUBB4,WNT10B,ZFYVE9,PAFAH1B3,TNFRSF10C
Negative_3621	CGA,DBT,HK2,ABLIM1,ACAD9,ACADSB,ADH6,ADRB2,ALG8,ARHGEF6,CCL7,CNTF,FARS2,GNPDA2,IL18RAP,INADL,IQGAP2,KIR2DL4,LYPLA1,MAP3K14,OR1F1,OR2M7,OR6B2,OR6K6,PDE6D,PIGK,PLA2G1B,PPP5C,RAPGEF4,SHC4,TUBA1A,ULBP1,WNT2,HSP90AB1,PPP1R12A,TNFRSF18
Negative_3622	AGK,AMH,C1S,HK1,ID1,AASDH,ACOT11,B4GALT7,CACNA1F,CCL7,CHRM3,CYP4F3,DOT1L,DPYD,DUSP5,GDF7,ICAM3,ITPKB,MYD88,MYL2,NCR2,NFATC2,NLGN1,NSD1,OR13D1,OR1A1,OR4P4,OR52K1,OR6M1,OR8H2,PAK7,PGAM1,PIGT,PLA2G1B,PLD1,PMM2,PPT1,RRAS2,SH2D1A,TP73,ATP6V0A4
Negative_3623	AK2,AK5,FAH,SRC,ACPP,AGXT,ANAPC5,CDC2,CDC25A,DTX3,DUSP7,FABP3,G6PD,GDF7,ITGB1,KIR2DS4,LAMA3,LHCGR,LIPC,MCM4,NCOR2,NEU2,OR13C4,OR1F1,OR4C11,OR4D11,OR56A3,OR5M9,OR5R1,RAP1A,RPS6KA4,SFRP2,TLR2,ULK1

Negative_3624	DBI, MET, TPR, ADCY6, ATP5D, BACE1, CSNK1D, ECSIT, GALM, HNMT, IFNA4, IFNAR1, IL13, IL2RB, MAML3, MAP2K7, MKNK1, OR4F17, OSMR, PDGFB, POLR3H, PRKAA2, RND1, SCD5, STAT6, TSC1, TUBA1C
Negative_3625	C8G, CR1, ITK, OSM, AGPAT6, ALG13, ATP6V1F, BMP6, CCL25, CDK2, CRY2, DKK2, FMO2, GMPPB, HSD17B1, LDHC, NCKAP1, NCOR1, OR4D6, OR7D4, OR8D1, PLAUR, PLCD4, PRKACA, PRPH, PTGER1, RENBP, SGMS2, SKP2, STX7
Negative_3626	MET, CCR7, IRAK2, OR2A2, OR52N1, PFKFB4, PLXNA2, PPM1B, SEMA7A
Negative_3627	DBH, PML, TNN, ACPT, AHCYL2, ALPP, ARRB2, ATP5J, ATP6AP1, BMP7, CACNA1B, CASP3, CCL23, CCNA2, CLDN1, COMT, CYP21A2, DGKE, ENTPD3, EPHB2, FABP4, FPGT, GALNT5, GRM1, H6PD, IL11RA, METTL6, MPDZ, MTAP, NFYC, OR11A1, OR51A7, OR51I2, OR5AC2, OR5B17, OR5D18, OR5P3, PARD6B, PCCB, PIGN, PIGV, PRKAA1, XIAP
Negative_3628	GDA, ACSL1, ATIC, ATP2A1, CNDP1, CXCR6, GADD45G, HSPA2, NPR1, POLR3K, RFX5, ATP6V0D1
Negative_3629	AK3, FOS, GP9, LEP, AGPAT3, ANAPC5, ATG7, ATP12A, BAAT, CSF3R, CTBS, CYP2E1, DIAPH2, EGFR, ENO1, FZR1, GAS1, GGT1, IARS2, IL10, IMPA2, LIPA, MAP3K2, NCF2, OR1K1, OR4F17, OR51V1, OR6T1, OR6Y1, PGK2, PHKG1, PIM1, PLCG1, PRKG2, RDH5, ROBO1, SC5DL, SEMA3E, SETD8, SGPP1, SHC2, TICAM1, TUBA1A, UGT1A10, ATP6V1B2, ATP6V1C2, PRICKLE1
Negative_3630	GPI, LYN, SMS, TEC, APOA1, ARPC1A, BIRC2, CDKN1A, CSNK1G3, DPM3, DUSP4, EHHADH, EIF4E2, EPB41L1, FOXO1, GNG12, HTR5A, IDUA, IFNA10, IGF1, KCNJ11, MAD1L1, MCM7, MGLL, NFATC3, NFKBIB, NLGN2, OR10A2, OR5AP2, OR6C76, OR8S1, PLA2G4A, POLR3A, PPP3R1, PTPN11, PTPRZ1, RGS3, SEMA4D, SH2D1B, SH2D2A, SHC4, SOAT2, ST3GAL5, STX1B, TJP2, TRMT11, VDAC1, WEE1
Negative_3631	FUK, MTR, NNT, ACSM1, CACNG4, CCR10, CD22, CD86, CDH5, CLDN9, COL1A1, CREB3L3, CSF2, CSF3, CYP8B1, ERBB2, FGFR1, GUCY2C, GUK1, HMGCS2, NANP, NFKBIB, NTF4, OR11A1, OR2A12, OR3A2, OR6C1, PI4KB, PLCD1, PPAP2B, PPP2R2D, RALGDS, RERE, RRM2B, RXRA, TPM3, TRAF6, ULBP2, TNFRSF10D
Negative_3632	HK1, AK3L1, BST1, CCNE2, CDC23, CER1, GALT, GNAO1, IFNA13, KIR2DL3, NAE1, NT5E, OR2T3, OR52A5, OR7G2, PIGP, PLXNC1, PRKAR1A, STX12, TYROBP, XCR1, ZNRD1, HSP90AB1
Negative_3633	ACADM, ACTG1, ALG2, ATG3, ATP2A2, BACE1, BMP4, CBLB, CCL7, CFL2, CHRM3, DCTN1, ENTPD5, ESAM, GABARAP, GNAL, GRM1, HSPA8, HTR5A, IFT57, IL25, INHBE, KLRC2, MECR, MTAP, MTMR6, NEU2, NTSR1, OR10A6, OR2B2, OR2T12, OR4X2, OR52E8, OR5R1, PLA2G2D, POLR2K, PPP1CA, PRKCZ, PSPH, SNAI1, SPTLC2, STAT4, SV2A, TAS2R5, VAMP3
Negative_3634	CCL1, COX10, DDX50, EPHA5, FZD9, LAMC2, LLGL1, MAP3K7, MECR, OR2AG1, OR6K2, PIGC, SHC1, TRPC1, VEGFA
Negative_3635	BTD, LSS, SYK, ACMSD, ALG11, ARHGAP5, ASIP, ATP5H, CACNG8, CCNA1, FMO3, HAGH, HES1, IDI2, IFNGR2, LDHA, MAN1A2, MGAT4A, MNX1, MTFMT, NANP, NANS, NR1D1, OR10J3, OR2L3, OR5D13, PCYT1A, PLXNA1, PRIM2, PRODH, RPS6KA2, SDHD, SHC1, SUV39H2, TNXB, TRAF1, TRAF6
Negative_3636	JUP, PML, ABLIM1, ACADL, ACTA2, AGPAT1, ARHGEF7, BBOX1, CTPS, CTSG, CYP1B1, CYP2A6, ENPP3, ENTPD4, EPHB4, GADD45G, GAPDH, HLA-DRA, IL22RA2, MAP4K4, OR7A17, PIP4K2C, PTGDS, RAB23, RARS2, RASGRF2, RXRG, SUCLG1, TNFSF11, TNXB, XYLT2, HLA-DQB1
Negative_3637	ADO, GLS, IL6, NNT, ACACA, AGPAT3, ALDH3B2, ALDOB, BMP5, BTRC, CAMKK1, CAMKK2, CD48, CDC26, COL6A1, DERA, DOCK1, FECH, FUT6, GALNT5, GPT2, GSTA4, HSD11B2, KIR2DL1, LIPE, MIOX, NR1H3, OCRL, OR10A4, OR2H1, OR2T4, OR4S2, OR5R1, PDGFD, PDHA1, PHKG1, PIP5K1B, PLA2G1B, PLA2G2D, PPBP, PTPN1, RXRB, SC5DL, SDHA, SMARCA2, SOAT2

Negative_3638	TK1, ACSL4, ACSL6, ALDH3A1, ALDOA, AOC3, ARHGEF4, CCNG1, CLCA4, CNGA3, DAXX, DLL3, FASLG, LEFTY1, MTHFD1L, NCF1, OR52A5, PANK4, PDE1B, PPP1CC, PSME2, RPS6KA1, RPS6KA3, SEMA3A, SRD5A2, UPP1, ATP6V1B2
Negative_3639	AK7, FOS, ADH5, ALDH7A1, CACNA1D, CHRM5, CTNNA1, CXCR3, CYSLTR1, DGUOK, DKK2, EDAR, EIF4E2, F13B, FABP3, FGF1, IFNA16, IGFBP3, IL8RB, IRF3, MAOB, MYH4, NME7, OR4F17, OR4L1, PFN1, PGM1, PIK3R2, PLA2G10, PLXNB1, POLR2F, PPP1CB, PRPS1, RAP1A, RFNG, RFX5, RRM2, SMPD2, TNFSF12, TUBA3C, UGT2B10, ULK3, HLA-DQA2
Negative_3640	ACTA2, BCAT1, GALNTL4, GMPPA, PTPRR, RERE, TCIRG1
Negative_3641	FOS, IDS, LEP, ALPP, ATF2, CES2, COQ3, COX10, CXCR3, DHRS9, DUSP6, F2RL2, FZR1, HNF4A, IMPA2, NEU3, NOTCH3, NRG1, NUDT2, OR10G3, OR11H4, PDIA4, PIGV, PPP2R5B, PTGFR, SDHD, SELL, STAT1, UGP2, WNT10B, WWP2, ST6GALNAC1
Negative_3642	ASL, DAO, F2, F3, RDX, SPN, ADH5, AHCY, AKR1C4, ALDH4A1, ATP5G2, BLVRA, BTRC, CD226, CFL1, CFL2, CHPF, CYBA, FGD3, GAS1, HIBADH, HLA-F, HSPB1, LAMB1, MECR, MGAT5, NEU1, NR5A2, NTF4, OR5AS1, OR5T2, OR8B12, ORC3L, PIGS, PIK3C3, PON1, PRSS2, PTGIS, RAC3, RUNX1, SEMA4F, SH2D2A, STK4, STX12, STX4, TRPC1
Negative_3643	BAD, ALOX12B, APLP1, BDKRB2, CACNA1B, CHKB, CHST11, CISH, FGD3, IL22RA2, LIMK1, MAOA, MAP3K1, MYH2, NODAL, OR10G3, PDE3B, PIGW, PIK3R3, PIP5K1C, POLR2D, PPP2R2D, RALA, RBPJL, SLC2A4, SV2A, UCK1, VAMP2, ZMAT3, HSP90AB1
Negative_3644	ILK, ADSSL1, BET1, OR5I1, ORC1L, PTK2, TALDO1
Negative_3645	GSS, PAH, SMO, ACTB, BMP7, CCL4, CHIA, COL4A2, CYP2C19, EPRS, EXOC7, FGD3, FUT1, GLI2, GSTO2, HLA-C, IL21R, IL24, IRAK2, IRS2, MAP2K5, MTHFD2, NCOR2, OR10G2, OR13A1, OR4K2, OR52A5, OR6C76, OR6N1, PDE6D, PPBP, PPP1CA, TRADD, HSD17B10
Negative_3646	CR1, TK2, AANAT, AFMID, BCMO1, CSNK2B, DGKQ, DUSP16, EPHA3, FECH, GUCY1B3, HMGCS1, KCNQ1, MYL7, NANP, OR2AG1, OR6X1, PAK4, PDGFA, PDGFB, PER1, PLD1, POLR3D, POMC, PRKAG1, PRSS2, RDH12, RRM1, SETD7, SIGLEC1, SMAD3, SNAP23, SOCS1, TNFRSF4, TP73, YARS, PNLIPRP2
Negative_3647	F12, OSM, ALG11, ANAPC10, APOA1, APOA2, BLVRB, BST1, C3AR1, CACNA1D, CACNB4, CBLC, CDC14A, CPT1C, CYCS, CYP26A1, DPYS, ENPP6, FLOT1, IDH2, MAP3K1, MDH2, OR51E2, OR5AC2, OR5H6, OR5I1, OR7A17, OSMR, PFKFB3, PGS1, PIGP, PLD1, RARS2, RPS6KB1, SH2D1B, VAMP2, WNT2, MAPK8IP1, SERPINB5
Negative_3648	DCC, IDS, RB1, ACAT2, ALG2, AMHR2, BMP2, CAMK2A, CCNA2, CFL1, CHD8, CYSLTR2, DCXR, FTH1, FZD7, GBE1, GCH1, HIBADH, IL28A, IRS2, MAPK3, NCOR2, NUMB, OR2T29, OR4F4, OR4K15, OR6K3, OR6Y1, PAX6, PDE7B, PPAP2A, PRKCE, ROCK2, SMPD4, TRHR, UPB1, UROC1, SERPIND1
Negative_3649	AGK, AK5, ITK, LCT, TH, AGPAT4, AKR1C4, CACNA1A, CLDN10, CXCL1, FOXO3, FUT8, HIBCH, IL22RA2, LMO7, MAP2K7, MDH1, NCF1, OR4C16, OR5D14, PLCE1, PLXNB3, PRODH, PRPS1, PTCH1, PYCR1, SNW1, UGT2B11, HLA-DQB1, PAFAH1B3
Negative_3650	AR, CRH, HGF, TPR, AKT1, APOC3, BET1, BIRC2, BIRC5, BMP5, CDH3, COQ7, CYP7A1, ELK1, EPRS, FUT3, GALNT14, GLI3, GSTA2, IRS2, KDSR, KIR3DL1, NAE1, OR14I1, OR1L1, OR56A1, OR5D13, OR6T1, OR8B12, OR8K1, PAK1, PDE3A, PDIA4, PGK1, PLA2G2F, PRODH2, RFXANK, RPSA, SELE, TAS2R50, TFDP1, TNFSF12, TSTA3, TYRP1, VDAC2, WWP1
Negative_3651	DUSP3, EDNRA, FGF17, GPAA1, IL23A, OR11H6, OR4L1, OR5H6, POLR3B, PPARD, PTPRJ, RAB23, ULBP3
Negative_3652	AASS, ACSM3, BMP2, CAV3, CDC25C, CHSY1, CTTN, DDIT4, EDA2R, FDFT1, GADD45G, IRAK3, LAP3, LIPT1, OR4D9, OR8H3, ORC5L, P2RX6, PDHA1, PHPT1, PLXNB2, POLR2E, PON1, RAP1B, RARA, RPS6KA5, TXNDC12, ATP6V1G1

Negative_3653	AK1, ASL, AGRP, AKT2, B3GALT1, B4GALT2, CD27, CDKN2C, CFLAR, CPT2, FDPS, FGF2, FGFR1, GAB1, GLG1, HLA-DOB, HMGCS1, IDI1, IFNA8, ITGA9, MAP3K14, MGAM, MIOX, NFE2L2, OR52D1, OR6C4, PANK1, PIGA, PIGH, POLR1A, PON3, PRKX, PTTG1, SREBF1, STAM2, TAS1R2, TRADD, USE1, WIF1
Negative_3654	GHR, ADRBK2, BPGM, CYBA, CYP4F2, DFFB, DKK1, PPP2R5B, SDC2
Negative_3655	ID1, ZYX, B3GALT2, CARD11, CERK, CLDN14, CLDN5, COL2A1, CYP4A22, DPYD, GADD45A, GRIA3, INPP1, JAM2, JAM3, MDH1, NFKB1, OCRL, OR10J3, OR11H1, OR2L2, OR4D9, OR56A1, OR5B21, OR6M1, PARD6A, PARS2, PAX8, PLAT, PLCZ1, PLXNC1, PPP2R2D, PRKAG2, PYCR2, QPRT, C1GALT1C1, TNFRSF13C
Negative_3656	ACSS1, ADRA1D, ATP5L, CSF2, FGF21, LRAT, MCCC1, MCM2, OR4E2, PGM2, PLCB4, RPS6
Negative_3657	IL3, ACTA1, AGXT, AKT2, AOC2, BACE2, CACNG8, CD46, CDC23, CREB3L2, CXCL1, DDX50, DKK2, DOLPP1, ELK4, FBP1, FGF8, HIP1, JAM3, MAPK8, NEU1, NMNAT1, OR1F1, OR5L2, OR5T1, PSEN1, PTGIS, RALBP1, SH2D1A, SULT1A4, SV2C, TAS2R49, TLN2, TMLHE, TUBB4Q, UGT2B4, VDAC3, MAPKAPK2
Negative_3658	GSS, A4GALT, ACMSD, ACP6, AKR1B10, ARPC3, ARSD, B4GALT2, BCAT2, BDH2, CD8B, DAPK1, DARS2, DGUOK, DHCR24, EFNA4, ENTPD6, EXTL1, FASN, FZD3, GADD45A, GALNT10, GMPPB, GNAI3, HNF4G, HRAS, IFNA7, LCP2, MGAT4B, NAMPT, NFKBIE, OGDHL, OR10H1, OR13C9, OR1S2, OR4D2, OR4S1, PPP1R3C, PPP2R5A, RUNX1, SEMA3F, STK3, STX1B, STX2, SV2C, TMLHE, HSD17B10
Negative_3659	CACNG6, FGF18, ITPKB, LAMC1, NGEF, OR4F15, PPCS, TUBA1C, HLA-DRB3
Negative_3660	DDO, CLDN3, CYFIP1, GCH1, JAM2, LLGL2, MMP14, TFPI
Negative_3661	CFI, ABL2, ACADVL, ACSL1, ADH1B, ALDH1A1, CCR5, CDO1, DUSP6, EFNA2, GUCA1A, H6PD, MAP3K5, NRP1, OR7D4, PIGC, PLA2G2A, PPAT, PTPN11, RETSAT, RPIA, SEMA3F, SLC27A5, UCK1, YWHAH
Negative_3662	F9, ID2, MYC, ASRGL1, CACNG7, CPT1B, CREB3L4, CSF3R, DAXX, FARS2, FGF11, GMPR, IL17A, LFNG, OR14C36, OR2T1, OR52I1, P4HA3, PLOD2, PPP1R3B, PPP2CB, PRSS1, RAD51, RASA2, SPRY2, TCF7L2, UROC1, ATP6V0A1, TNFRSF10A
Negative_3663	ALAS2, APH1A, CCNB2, CCR6, FGF4, FRAT2, GDE1, MAML3, MAOA, OR51V1, OR7A17, PRKCD, PTCRA, TBL1XR1, WNT16
Negative_3664	DLD, TH, AKR1A1, ARNTL, ATIC, CD36, CFL2, CLDN17, DLL3, ENPP7, FGF14, GNAT3, HNF4A, KIR2DS5, KLRC2, MYH13, OLR1, OR3A2, P4HA2, RFX5, TGFB1
Negative_3665	CAT, ACADSB, CLTA, COL6A2, CSNK1G2, EPHB3, GJD2, MAD1L1, NOX1, NT5C2, OR5M8, PKMYT1, POLD2, SDC3, TGFA
Negative_3666	HGF, MPL, ABLIM1, ALDH1A1, ATP5G3, BIRC5, HSD11B2, MAOA, PIAS4, POLR2G, SCP2, VARS, VEGFC
Negative_3667	CFD, GALNT12, OR10A4, OR51S1, PTGS2
Negative_3668	ABO, UBC, ACAA1, AGRN, ALDH5A1, ANAPC7, ATG3, B3GALT4, BACE1, CSNK1G3, CSNK2A2, CXCR4, CYBB, CYSLTR2, DARS, DBF4, DTX3, FABP4, FLOT1, FZD2, GDE1, GNG13, MGAT4B, MGLL, MTHFD2, NMNAT1, OR2D2, OR2G6, OR5D14, OR6K3, P2RX4, PER2, PIP5K1B, PNPLA4, POLA1, PTPN5, RPS6KA4, SCP2, SMAD5, SPRY4, TAS2R3, VTI1A, WNT6
Negative_3669	CANT1, CCL7, CD14, CFL1, DLL4, EFNA2, FGF20, IL10RA, IL18R1, MAML1, MAN1C1, MAP2K5, MAP3K12, OR11G2, OR2AE1, PCNA, RAP1B, STT3B, VTI1A
Negative_3670	GH2, ACVRL1, BBC3, CSF1, CYP21A2, FARSB, FGD1, GALK2, IL23R, MTHFD1, OR10Q1, OR2H1, OR5R1, P2RX4, PER1, PMM2, POLA1, PRKAR1A, PRKCQ, PRKY, SESN1, SQLE, TAS2R4, TBXAS1, UGDH, UGT2B10, VCAM1, HLA-DPA1, TNFRSF14, TNFRSF1A
Negative_3671	COMP, DDOST, OR5A1, PPP2R2D
Negative_3672	AR, CACNB2, CDC2, HDAC1, IFNA2, IFNA6, LALBA, LIMK1, LIPC, MALT1, SFRP4, SH2D1B, MAPK8IP3

Negative_3673	ACADVL,CXCR4,PIGW,RPRM,SELPLG,ST3GAL2,HLA-DQB1,MAPKAPK3,MAPKAPK5
Negative_3674	AK3,HPD,ACMSD,ADAM17,APOE,ARSB,ATP5H,BCL10,BET1L,CARD11,CFLAR,CTNNA1,DCTN1,ENPP6,FGF19,GAL3ST1,GNAI2,GRM5,INHBA,NNMT,OR10H2,OR10H4,OR13D1,OR2B11,OR51B5,OR6C74,PLA2G6,POLR1C,RAC2,TPK1,WNT5B,TNFSF13B
Negative_3675	ACOT11,CCNH,CD99,KIR2DL3,NRP1,OR2V2,TKTL2,TUBAL3
Negative_3676	AASDH,AGMAT,CBR1,CREB5,CYP1B1,GADD45B,INHBB,MAPK1,NT5C3,RRAS2,STAT2
Negative_3677	ARNTL,CBR1,CXCL1,CXCL12,CYP2C19,DDX52,ENPP6,GPX5,IFNA13,INPP5E,MAN1A1,NT5C2,OR2A42,OR3A1,OR4C46,OR4K1,OR51S1,OR52B4,OR52E2,PHGDH,PLA2G2A,PPM1D,RDH16,SETD1B
Negative_3678	DUT,MVD,OGT,SRC,ACSL6,ALDH5A1,APPL1,ARHGEF7,ASNS,CCL3,CCNG2,CD244,CXCR6,CYP2S1,FIGF,GFPT1,GNPAT,GPD2,LAMA3,LGMN,MYH8,NADSYN1,OR1A1,OR2C1,OR2T5,PDHB,PIGK,PMM2,POLE4,PPP3R2,PTPRB,RPS6KA2,SARS2,SEMA7A,SGMS1,SOD1,TUBA1C,TUBA4A,VAMP2,CACNA2D1
Negative_3679	IL3,ADORA2B,AKT1,CARS2,CDC6,CLDN16,COQ3,DARS,DOT1L,DPYSL2,DUSP10,DVL3,EFNB3,FIGF,GALNT1,GRLF1,HRAS,IFNG,IL15RA,ITGAE,JAK3,KITLG,MRAS,MYH11,NT5C,OR11A1,OR52D1,OR52I1,OR8U1,PANK2,PDK1,PIAS3,PPP3CA,RAP1B,RDH8,SETD7,SHMT1,SPRED1,TGDS,TIRAP,TSC1,TUBB6,VAMP3,VAV1
Negative_3680	SMS,AARS,ACVR1B,ANAPC1,ARHGEF6,B4GALT5,BCL2,CADM3,CAV1,DDB2,DUSP16,EPHA3,EPHA4,FOXA2,FZD6,GGT1,GNA11,IL17A,KLRC1,MARS2,MDM2,MMP2,OR2AG2,OR2C1,OR2L2,OR6B3,PCK1,PDX1,POLR2D,PVRL4,RPS6KA1,RUNX1,SEMA3C,SFRP2,SUCLG2,TAS2R5,TAS2R7,WASF2,WNT6,XYLB
Negative_3681	F11,FOS,CD80,CREB3L4,CTSL1,FUT5,GCNT3,HNF1B,IBSP,ITGA10,KCNJ11,LRP6,MAPT,MCM7,MGST3,OR12D3,OR4D5,OR4X1,OR5M9,PHKB,SEMA3G,STAT6,TSTA3,PHOSPHO1
Negative_3682	GH2,SRC,ACADS,ADCY9,ADIPOQ,AREG,BAI1,CACNA1I,CAMK2G,CCL23,CDC26,CEBPA,CLCF1,CNGB1,FRAT1,FUT5,LARS,MAT2B,MINPP1,NCOR2,NTN1,OR11G2,OR2AG1,OR8G1,PAK1,PIP4K2C,PTCH2,SULT1A2,SULT1E1,TACR2,TCIRG1,WNT10A
Negative_3683	C8B,FGB,NF1,AXIN2,CCKBR,CES1,CLDN5,CLTA,CNTNAP1,CRY2,CTNNA1,CYCS,DUSP2,ETNK2,EXTL1,FUT9,GSTO1,HNMT,IL21R,IL8RB,ITGB4,LAMB4,MAGI2,MGST3,NFKB1,OR10J3,OR2T11,OR3A2,OR6C76,PDHB,PFAS,PHKG1,PLAT,RFWD2,RPN1,SGPP2,STX18,THBS2,WWP1,XYLT2,HLA-DQA2,MAPKAPK5
Negative_3684	GNS,MAX,WAS,CD28,ENO2,ENPP2,FADD,GSTA4,KDSR,MYH1,OR8D4,PDE1B,PDE1C,ST8SIA1,TXNDC12,UCP1,VAV1,CSGALNACT2,HLA-DRB5
Negative_3685	AK5,CFI,AMY2B,BBC3,CLDN10,GNA12,GNG12,ITGB4,MPST,NEFH,OR10K2,PARS2,PFKM,TUBB2C,UGT2B28,HSD17B12,PRICKLE1
Negative_3686	FER,HDC,MVD,ARPC1A,CASP9,CERK,COASY,CSH1,CYP26C1,DIAPH1,GNAI1,GZMB,OR4F15,PDE6H,PGM3,SNAP25,ST3GAL3,TRIP10
Negative_3687	SYK,ABCC8,ADCY6,ALG2,BACE1,C3AR1,C4BPA,CD70,CFLAR,CMBL,CPOX,CSNK1E,CTNNA1,DAD1,EFNB3,EGLN1,EPHB1,GGT7,HNF4G,JAM3,LRP5,MAFA,MTFMT,NCF2,OR2T10,OR51F2,OR52A1,OR5M9,PAX8,PDE4B,PDGFRA,PIGU,PPP2CB,PTCH2,ROBO2,RPSA,SMAD2,SMURF2,STK4,STX1A,STX4,TAS1R1,TOLLIP,VEGFA,HLA-DQB1,ST6GALNAC3

Negative_3688	EGF,INS,KMO,ADRA1B,ALDH1A2,ANAPC1,ARRB2,BAIAP2,CACNA1E,CHSY3,COL2A1,COL5A1,COL5A2,CREB3L4,CTSB,DHRS4,FABP1,FARSB,FGFR1,GADD45G,GAS1,IKBKB,KIR3DL2,LCMT1,LEF1,LILRB3,LRDD,MGST1,NAT2,NOTCH3,OR4E2,OR4K13,OR4K14,OR6N1,OR6T1,PAPSS1,PARS2,PLA2G3,PRKAR1B,RALA,RBL2,SELE,SNAI2,SV2C,TRAF3,TRPM5,WASF3,ZAP70,ZFYVE16
Negative_3689	ATIC,BST1,CSF3R,DOLPP1,FGF3,FLT3LG,IFNK,MAD2L2,OR5B17,RPN1,STAT6,TCF7L1
Negative_3690	B4GALT2,BST1,CSF1R,POLD4,RASGRP3,SLC2A4
Negative_3691	GNE,ABP1,BIRC5,CCKAR,CCL24,CD99,CES7,CREB3L2,CYBA,ENPP3,GSTM1,MYH13,NKX3-1,OR3A2,OR51A7,OR52N4,OR5D14,OR6C1,PDE7A,PIK3R4,PRKAA2,RDH8,RELA,SLC27A5,SOAT2,STX1A,TJP1,TRMT11,ULK3,UXS1,VAMP1,AASDHPPT
Negative_3692	ATP2A3,CBR3,DAAM2,DAXX,FZD10,GALNT14,GGT6,HIBADH,KLRD1,MGST3,OR2B6,PPP1CC,RELB,RPE65,TNFRSF12A
Negative_3693	ACSL4,ALDH4A1,BCKDHA,CXCL16,DARS2,EPHA1,GSTA1,GSTA2,ICAM1,JAM2,MYH11,OR4M2,OR56A1,OR5M8,OR9Q2,ORC3L,POLE,UGT2B28,CACNA2D3,CSGALNACT2
Negative_3694	CAV3,COASY,COL6A1,FZD7,HLA-DRA,INPP5B,IRF7,NKD2,OR8D2,PLCZ1,PLEKHA8,ULBP1
Negative_3695	APP,F2,GH2,KMO,ACAT2,AOX1,ARHGEF1,AXIN1,CAV1,CCL14,CD40LG,CDC26,CFTR,COQ6,CXCR3,CYP2S1,DGAT2L4,DGKZ,G6PC,GALNT12,GARS,GLB1,GNAS,GPX7,GSTA2,GSTM3,HAGHL,HSPA8,HTR7,IKBKG,LDHB,MAP4K2,MCM3,OR11A1,OR2T6,OR2T8,OR6S1,OR9Q2,PIM1,PRLR,SARS2,SGMS2,SOX17,SPRY1,STAM2,ZFYVE16,ATP6V1C2,SERPINF2
Negative_3696	ATP2A2,CACNA1I,CCL19,CCL27,CLDN17,CYP3A4,DPAGT1,EPHA4,GALNT6,KNG1,LAMB2,LILRB3,MAP3K12,MASP2,MPZL1,OGDHL,PDE6H,PPP3R2,UBE2L3
Negative_3697	LCT,ADCY10,ALDH3B1,ALDOA,AOC2,B3GNT2,CDH4,CDK5,CDS2,CHIA,DFFB,EPHA7,FGD3,GMPPB,GMPR,HADHB,OR11H6,OR51A4,OR5M9,OR5R1,OR7G3,PDE5A,PIK3C2B,PRIM1,PRPS2,RUNX1,SESN3,SETD2,SMAD5,TBL1X,ST6GALNAC4
Negative_3698	TNR,ACSM2A,ARRB2,B3GAT1,CCL13,CYP4A11,DHDDS,EPHA7,ERBB2,FBXW11,FSHR,GJA1,GSTA5,MAN1A1,OR10V1,OR4D1,PDE1C,POLR2D,RGS3,SGPP1,STK4,STX6,TGFB2,TNFSF8,WASF3,PPP1R12A
Negative_3699	AMY2B,CYP2C19,EARS2,EGLN1,OR6C1,PLXNA3,TAS2R5
Negative_3700	CGN,TNF,ADRB3,ALDH1L1,ATIC,B4GALT1,BMPR1A,CDKN1A,CDKN2B,CES2,CHPT1,DBF4,EFNB2,FGF12,IL12RB1,MAP3K13,MYLK2,NAGLU,NUDT12,OR2G2,OR2G6,OR2Z1,OR8J1,PI4KB,STK11
Negative_3701	ABO,CGN,F9,ALDH4A1,ATP5J2,B3GALT2,B3GAT1,BUB3,CCNE1,CD72,CDC23,COMT,DGKE,E2F4,GRHPR,ICOSLG,IRS4,LAMA4,MECR,NEFM,NTF3,OR5B12,PNPO,POLR2A,PTPRB,RND1,TACR1,TP53,TRDMT1,ULK3,WNT10B,CSNK1A1L
Negative_3702	ID3,ASIP,BPNT1,C1QC,CCR9,CMPK2,CRY2,CXCL6,EPHB4,FGFR1,FGFR3,GUCA1C,HPRT1,KIR2DL4,NCOR1,NMNAT1,OR4S1,OR6T1,OR7A5,OR7G3,OR8B8,ORC4L,P2RX5,PGK1,RAB5A,RBKS,RPN1,SORBS1,TACR1,TAS2R10,VAV3,TNFRSF10B
Negative_3703	CSK,ACP6,ASMT,BMP8B,CAV3,CD59,CD79A,CLDN1,CYP11B2,DKK4,DLL3,ENAH,FUT4,INMT,LALBA,MYH13,MYH9,NARS2,OR4L1,OR8K3,PDE7A,PIK3C2B,RUVBL1,YARS2,ATP6V1B1,SERPINF2,TNFRSF18

Negative_3704	C8G, CRH, DCN, F3, IL6, AKT1, ALPI, ASRGL1, B4GALT2, CACNB4, CD14, CDC27, CPT1B, CSDA, CXCL12, DDIT4, DNMT1, DVL2, FUT5, GATM, GSTA1, HLA-DMA, HSD3B7, ITGA10, KLRC1, MAP2K4, MCCC1, NFATC1, NODAL, NOX1, NTSR1, OR10P1, OR11H1, OR2C3, OR2H2, OR7G3, PARVA, PARVB, PLCD4, PLXNC1, PON1, RERE, SDHD, SGPP1, SLC27A5, SUCLA2, TGFB2, TP53, TPH1
Negative_3705	A2M, RET, TKT, ARHGEF6, ATG7, ATIC, BDNF, CCL20, DPYS, EPB41L2, EPOR, FZD9, GAPDH, GNAL, HMGCL, HYAL2, ICAM1, LDHB, MICA, NLGN2, PAK1, PGK1, POLR1C, POLR2G, PTPRB, PTPRF, PYGL, RND1, RPSA, RRM2, SEMA4G, TP53, TUBB4, WHSC1
Negative_3706	ADA, ADO, GP9, ATP5B, MGAT1, RPN2, STAT1, TNFRSF4
Negative_3707	ACTA2, CACNA1E, CADM1, DPM2, F13B, FLT3, HMOX2, IL22RA2, LAP3, LCMT2, MAP2K1, NRG4, OR14J1, OR6X1, PIK3R3, KIR2DL5A
Negative_3708	ACO2, ACTB, ANAPC11, APRT, B3GNT4, CHST12, CLDN20, CYP26B1, DOT1L, FGF13, FMO4, GSTK1, HTR2A, ITGA6, JAK2, MAP3K11, MAPK8, MINPP1, NCK1, OR52E8, POLE3, PTCH1, PVRL3, WNT3
Negative_3709	HGF, STS, ABP1, ARSD, CCL22, COL4A1, CXCL16, CYP2C9, DPM2, EPHA8, FUT7, FZD9, GNAS, HNF4G, IFNA16, IL9R, MAD2L1, OR11H6, OR5D16, PLA2G10, PLCG2, RDH8, SC5DL
Negative_3710	BTD, ID2, STS, ADH1A, C1GALT1, CACNA1G, CD38, CREB3L1, CYP4A11, DDX4, FBP2, FTH1, GALNT3, GP1BB, HTR5A, ICAM1, IL1RAP, MTHFS, NFYB, OR1A1, OR2S2, OR2T29, OR51B5, OR5K1, OR5L1, PCCA, PDE5A, PFAS, PIAS1, PIGS, PKLR, RELB, RPS6KA4, SFRP5, SLC2A4, SPRY3, STAM, SULT2B1, TAS2R41, UGDH, UGT2A1, UPRT, VAMP2, XYLB, ST6GALNAC4
Negative_3711	CTH, DHH, FOS, NF1, ALDH1B1, ATP2A1, ATP6AP1, B3GAT2, CDKN2A, COL5A1, CREB3L3, CYP19A1, DARS2, DDX19A, EREG, FARSA, FUT6, GAL3ST1, GALNS, GPLD1, IFNA7, INHBB, ITGA8, MAN1C1, MS4A2, NEU2, NFATC4, OR10J1, OR52K1, PCYT2, POLE3, POLE4, POLR1B, PPP3R2, PTPN7, RALA, ROCK1, SC4MOL, SEMA3E, SHC3, SPRY2, STX2, TCIRG1, TIAM2, TSHR, UBE2L3, UPB1, PAFAH1B2, TNFRSF13C
Negative_3712	GK, TK1, ADCY6, C1GALT1, C1QB, C3orf10, CCR10, CDC20, COQ5, CRHR1, CSAD, CYP2A6, ENPP2, F2RL3, FCGR3B, FLOT1, FOXO1, GALNS, GALNT8, GCNT4, GDF5, GNPAT, GSTM2, IBSP, MYLK, OR4S2, OR5D13, PARVB, PIK3R1, PIK3R4, PIP5K1C, PLOD2, PLXNA3, POLR3K, PTDSS2, PTPN5, SARS2, ULBP2, VAV1, WNT16, WNT3
Negative_3713	GK, GPI, ACO1, ACTN1, BBOX1, BDKRB2, BMP2, CCL19, CHDH, CHIA, CKS1B, CLCA4, CYP2C18, E2F2, EGLN3, ERBB2, FABP4, FGFR4, GNA13, HYAL1, IFNA7, INHBA, JAK2, KIR2DL4, LEFTY2, LRP5, MAP3K13, NEFL, OR10G4, OR13D1, OR2W1, OR51M1, ORC2L, PDE11A, PER3, PIK3R5, PIPOX, PLAUR, SOD1, STAM2, STX12, TBL1XR1, ZAP70, ARHGEF12, TNFRSF6B
Negative_3714	ALPPL2, ITGA10, OR11H6, PIK3CB
Negative_3715	CAT, CBS, FOS, IHH, PAH, SHH, TYR, ACP5, ALAS2, BCAR1, BCMO1, CACNB1, CCR6, CYP2U1, DRD1, EPHA3, FABP3, FBP1, FOXO1, GDF5, GGT6, HES1, IFNA16, IL12A, IL22RA1, MAFA, OR5D13, OR5K2, OR6C76, P2RX4, PDPK1, PERP, PHGDH, PLA2G1B, PPT1, RRM2B, RUNX1, SEMA4D, SH2D2A, SHMT2, TNFRSF4, UGCG, UROC1, YWHAQ, CACNA2D1, MAPKAPK3, SERPINC1, SERPINE1
Negative_3716	ATP6V1D, CANT1, CCL1, CDC25A, CLDN6, COASY, IL20, MAP4K1, OR2Z1, OR7C1, PIGP, PLCB2, UAP1, WNT11
Negative_3717	AK7, IL7, ABCC8, APOC3, ARPC1B, ARSE, B4GALT5, BCAR1, CACNA1B, CACNB4, CCR8, CHIT1, CHST13, CTNNA3, CTSB, CYP2S1, DDX18, DIAPH2, FGF21, GBE1, GPD1, GSTA2, HCCS, IL22, IL2RG, IRS1, ITGB7, KITLG, MYL9, OR13C3, OR2A5, OR51A2, OR7G1, OR8S1, PAK2, PAK3, PIGN, PLOD3, PLTP, PPARA, PPID, PROC, PVRL4, RASGRP1, TUBB3, ZFYVE9, SERPINE1

Negative_3718	OGT, TYR, ALG10, B3GAT1, BCKDHB, CACNG4, CERK, CHD8, CHIA, DDX50, DLST, GALM, GGPS1, IPMK, ITGB3, KLRC4, NR1D1, PRIM1, PRIM2, RRAS, SCP2, UGT2A1, SERPING1, SLC25A31
Negative_3719	ACVR1B, ALDH3B1, CD79B, FGF18, IFNA10, PIGP, UGT2B7, YARS
Negative_3720	MVK, ADSS, CDO1, DEGS1, OR11H1, PIK3C3, PRKCH, SRD5A1, UGCGL2
Negative_3721	LCT, AFMID, BRAF, CACNA1G, CXCR6, CYBB, CYP27A1, DGAT2L4, EXTL2, FABP1, FOXO3, FUT7, HMBS, IL1R1, ITGA10, ITGB2, NAT2, NFATC4, NFKB2, OR1D5, OR1M1, OR4K2, OR6Q1, P2RX1, PDE4A, PDHA1, PGM1, PIK3R2, PLA2G3, PMVK, PTGER1, ROBO3, RPS6KA3, SFRP2, SIGLEC1, SREBF1, STAM2, TAS2R14, WNT4, TNFRSF17, TNFRSF6B
Negative_3722	MVD, PXN, ABAT, ADRBK2, ARSA, ASIP, B4GALT4, CCR6, CDK5, CHPT1, DGAT2, GALNT10, KRAS, MADCAM1, MGAT3, OR2M3, OR2Z1, OR4M1, OR6K3, PCCA, PDIA4, PFN2, PHKG1, PMVK, POLR3G, RASGRP4, SDC4, SDHD, STX4, TAS2R9, TBK1
Negative_3723	ALDH7A1, DDX19A, FZD8, MIOX, UGT1A10, VEGFB
Negative_3724	FGA, MPZ, RET, TEC, ALOX15, ATP5C1, BDH2, CCL14, CLDN15, CPS1, CREB3L1, DAXX, EFNA2, EPB41L3, FMO3, GDF6, GLYCTK, IL8RA, ITGA6, ITPK1, MAP2K4, MCM4, NAT2, NEU4, NLGN2, NR4A1, OR10G4, OR11G2, OR5A2, ORC4L, PARVG, POLR3B, PRKCQ, PSME2, RRM1, SPHK1, STAT5B, SULT1A4, TBXAS1, TGDS, ULBP1, WNT5B, WNT6, YWHAB
Negative_3725	CBL, HAL, MET, ABL2, ACOT12, ACSS1, ACVR1B, ALDH2, ALG9, ATP4A, ATP5O, CDC42, CRB3, CSNK1D, CYP3A43, DDX54, EXTL2, FGF18, FGF19, FRAP1, GANC, GART, GNPDA1, GPD1L, HDAC2, IFNGR1, IL10RA, IL23A, ITGA1, MPP5, NRG4, OR11H4, OR13C4, OR2AG1, OR2C1, OR52J3, OR5F1, OR5T3, OXCT1, PDE4A, PIM2, PISD, PTPN6, RAB5A, RBX1, TBXA2R, ATP6V1G1, TNFRSF12A
Negative_3726	SRM, ASPA, BHMT, BLVRA, CCR4, CD58, CHRM3, CLDN7, CSNK1E, CYP2U1, CYP3A7, DDX50, ECSIT, ENTPD3, FZD4, GMPR, GNA12, HTR5A, ICOS, IFNA4, IL2RA, INSR, MASP2, MYL9, NEU1, NOS3, OR10G3, OR4K17, RALA, RAPGEF1, RARS, SCLY, SLC2A4, SOS1, SOS2, TUBA1B, WARS, SERPINE1
Negative_3727	CRH, IL6, TNR, ACAD9, ACMSD, ACSS1, ACY1, ADORA2B, ALDH3A1, AOC2, BIRC5, CHRM1, CLDN6, CYP26C1, DDIT3, DDX41, E2F1, EDNRB, FCGR3B, FTCD, FUT2, GALNT11, GALNTL5, GRIN2B, HAP1, IFNGR2, IL15, INPP4A, MYH3, NFYB, OR4F6, OR52E6, ORC5L, PANK2, PFN1, PIP4K2A, PLAU, POMC, PSAT1, RAD51, ST8SIA1, TAS2R3, TOMM40, TUBA8, TYK2, WNT1, HLA-DPA1
Negative_3728	ALG2, ANAPC1, CPT1A, CYP2S1, DTX3L, GSTZ1, MASP1, POLR2C, SHC4, STAM2
Negative_3729	ASL, ALDH3B1, DHRS4L2, DHX58, GMPR, GNRH2, HCLS1, JAM3, OR5A1, SH2D1A, UGT2B4, YARS2, TNFRSF13B
Negative_3730	AGL, ACAT1, ACSL4, ITGA3, MYH14, OR10G8, OR2T4, OR52W1, PANK4, SH3BP2, TNFRSF8, HLA-DPB1
Negative_3731	CKB, CTH, INS, ARPC3, CACNG5, CANT1, CCND1, CDC14A, CNTN2, DMGDH, DUSP10, ENO3, GGT7, IL10, IL8RB, IRAK4, MGST2, OR10A4, PDE1B, PLAT, PNPT1, RASGRF1, RBL2, RPS6, SFRP2, SMAD7, STX1B
Negative_3732	BTC, ADIPOQ, BCL2L1, GMDS, IL29, KYNU, MAP4K3, OR10A2, PDE4D, PGAM4, PGK1, POLR1A, POLR2A, ZFYVE16
Negative_3733	GP5, HPD, CPOX, CXCL1, ITGA5, ITGAM, LEPR, NAE1, NR1H3, OR9G4, PPM1A, PYCRL, SNAP23, STAT4, HLA-DQA2
Negative_3734	AMH, C6, DBI, IL3, ACVR2A, AHCY, ALDH3B1, ALDH5A1, CACNG7, CD86, CLCA2, DAAM1, DDX19A, DTX3L, E2F5, ESAM, GALK2, GNA13, GP1BB, GPAM, GYS2, IL17RB, MNX1, MYD88, NAGLU, NEUROG3, OR2D3, OR6Q1, PFKFB1, PGAM2, PGM3, PPP3R1, PRKCA, PRKCE, RELB, RHEB, RRM2, SEMA3B, SMC1A, SMPD2, SOX17, TPMT, TRDMT1, WNT7B, WWP1, PLA2G12B
Negative_3735	UBC, AHCYL2, ALDH3A1, ARHGAP5, CHPF, DGKE, F2RL3, ITGA6, ITPKA, MALT1, MAPK14, OR2G3, PCNA, PER3, PPP1R3A, PSME2, RRAS, UCK1, PDCD1LG2

Negative_3736	IARS2, OR51E1, TCF7L1, TJP1, WNT4
Negative_3737	FH, GH2, RPE, VCL, ADRA1A, ATP6V1F, BMPR2, CARS2, CD58, CDH3, EPB41L1, F2RL1, FGFR1, GNA12, HLA-DOA, HSD17B7, IL22RA1, IRF9, KLRC2, KRAS, MAPK14, MCM6, MTMR1, MYLK3, NEFL, NPR1, NT5C1A, OGDH, OR10G7, OR2A14, OR2H2, OR5D14, P2RX3, POLR2F, POLR2K, PPP2R2C, RALBP1, RPS6KA6, SMPD2, SOS2, SUV39H2, TAS1R1, THTPA, ZAP70, EIF4EBP1, MAP3K7IP2, PAFAH1B3
Negative_3738	SDS, ACTN1, ACVR1C, ALDH3B1, ALDH5A1, ARHGEF2, BLVRB, BPNT1, CCNA1, CCR6, CCR9, CHIT1, COX15, CSF2, CXCL10, CXCL11, CYP11B1, DGKQ, DLL3, GADD45G, GCNT3, GRIA1, HYAL2, IFNGR2, IL6R, IQGAP3, OR51A4, OR6M1, P2RX1, PDIA4, PHGDH, PHKG1, PPAP2C, PPP2CB, PRKCA, PRKCG, PYGB, RDH8, RPN2, TAS1R3, TFDP1, TUBB2A, WNT6, XCR1
Negative_3739	XDH, ACAA2, ALDH7A1, ARNT, CHD8, CPT1A, CSNK1G2, CTLA4, CXCL2, DARS2, DDIT3, DIAPH2, DRD5, GAS1, GCLC, HADH, HSD3B1, IGF1R, LMO7, MAP2K4, MGAT1, MYH4, NME4, OR10A2, ORC2L, PIGO, POLA2, PRPS2, SDC2, ST3GAL5, VAV2
Negative_3740	BID, EBP, TNR, ADCY6, AHCYL2, ALG6, ATP5F1, ATP6V1A, AXIN2, C4BPB, CACNA1D, CCL27, CLDN6, CREB3L1, CTBP1, CYP8B1, DCXR, DUSP5, ECSIT, ERBB4, GDF5, HAGH, HGSNAT, HSD17B2, HTR6, LCP2, LDHA, MINPP1, MIOX, MYH1, NRG1, OR10J5, OR51M1, OR6C74, OR9G4, ORC3L, PGK2, PNLIP, RAP1B, UPB1, UPRT, TNFRSF13B
Negative_3741	GCK, GDA, GPT, ACSL4, ADRB1, AOC2, AVPR1A, CNTF, E2F4, FLT1, GALNT2, HAAO, HIP1, IDUA, ITGA10, KARS, KLRC1, MS4A2, MTHFD1L, NCOR2, OR4F15, OR52I2, OR7A10, ORC4L, OXCT2, PAX8, PDE8B, PNMT, PPP2CB, PPT1, PSEN1, PTGES, SMPD3, TNFSF11, TRAF4, VAMP1, WNT16, WWP1
Negative_3742	CGA, CKM, LYN, SP1, ALAD, ASIP, ASMT, CDKN2D, CSF1R, CSNK1G2, DLL3, EPHA4, ERBB4, FDPS, FMO5, GGT7, GNPDA2, HTR2B, IL21R, NLGN3, NOS1, OR10H4, OR2T8, OR4F4, OR9Q1, P2RX4, PCCA, PYGL, ROBO2, SNAP25, SOCS3, STAT2, VAV2, WNT3, WNT9B, TNFRSF14
Negative_3743	CAD, F10, GAA, LSS, SHH, SPN, AGMAT, AKT1, ALDOA, CES2, CLDN16, CSNK1E, CYP2F1, DGAT2, EXT1, GLS2, GUCY1A2, HAO2, INMT, MAPK13, MYL2, OR13C3, OR2M7, OR2T1, OR51A2, OR5T1, OR6C70, OR9Q1, PPARD, PRKAA2, PSEN2, SMARCA2, SORD, TPM3, VAV2, WNT7A, WWP1, ATP6V1C1, KIR2DL5A
Negative_3744	FCER1G, HMGCL, IPMK, LIPG, MPZL1, OR4C11, PAK6, PARD6G, PPP2R5A, PYCR2, THTPA, UGT2B4, GABARAPL2
Negative_3745	CD274, CYP3A7, LDHD, MPZL1, OR5AR1, PRKAR2B, RPS6KB2
Negative_3746	ABLIM2, ACTG2, CACNA1F, CCR4, CD40, CDC2, CES7, CHIA, CTSB, CXCR4, ENPP1, EPHA1, FGF5, GALNT10, GBA3, GNAI2, IFNA14, IL17A, IL6ST, INHBE, ITGA8, ITGB2, LDHAL6A, MAP2K4, MAP2K7, MFNG, MTMR6, OR13C4, OR1I1, OR2M2, OR2M5, OR2T8, ORC3L, PDHA2, PLAUR, PSME2, RBKS, RELA, SFRP1, SHC2, TBL1X, TGFBR1, TRAF5, TUBB4, VAV2, VTI1A, WASF3, WNT11, CSGALNACT2
Negative_3747	C5, GOSR2, NOTCH2
Negative_3748	KMO, NOG, ACSM3, ATP6AP1, BET1L, CACNA1S, CHEK2, DLL3, FRAT2, FUT4, HAGH, KAT2A, KCNB1, MASP2, NT5C3, OR4F17, OR4N2, OR52K2, OR5AC2, PDE8A, PIK3R2, PLAU, RELB, SCLY, TACR1, UPP2, ATP6V0A4
Negative_3749	ALDH5A1, ARPC5, ATP5E, CFLAR, CSNK1G1, F13B, FLT3, GNA11, LGMN, NAT1, OR13C9, OR51I1, OR5B21, OR6C4, TNFRSF4, TUBA3C, YWHAB
Negative_3750	CD2, DHH, ID1, PRL, SDS, ADH1A, ADRB1, B4GALT1, BMP8B, CLTA, CYP1A2, IL20, MAOB, MAPK3, MTMR2, MYL7, NTN1, OR10R2, OR13D1, OR7G2, PDHA2, PPP3R2, PRIM2, PRSS3, THBS3, VEGFA, ST6GALNAC4

Negative_3751	EZR, ASCC3, BCL10, C5AR1, CHIT1, CHSY3, CLDN1, CREB5, CSF1, CYCS, CYP2J2, DARS, DPM1, DTX3L, EFNB2, FZD5, GALT, GNG12, IL1R1, IL9R, IRF7, KCNQ1, LALBA, LMO7, OR4Q3, OR5H2, PITX2, PLA2G2A, POMC, PTDSS1, RASGRP3, RASSF1, SEMA3E, SETDB1, SH2D1B, SPAM1, TNFRSF8, UGT2B28, VAMP1, WEE1, ZNRD1, ATP6V0D2, SERPINC1
Negative_3752	BID, GBA, UBB, AKR1C2, ALPPL2, ATP6V1H, CACNG1, CAV2, CCR3, CD19, CD79B, CYP19A1, DDX54, DUSP7, EP300, FGF6, GAD2, ITCH, ITGAV, KLRC4, LCMT1, LCMT2, MAP2K4, MAP3K2, OR1L8, PAK4, PIGA, PKM2, PLCG2, PPP2R1A, PPP3CA, PPT1, PRPS1, RALBP1, RBPJ, RHOA, ROCK2, SESN3, SFRP4, SSH2, SSX2IP, TLR4, UNC5A, VAMP1, WNT5A
Negative_3753	GGH, NGF, AKR1D1, APOC3, ATP4B, BIRC2, CACNA1H, CAMK2G, CDC16, CDK7, CDS1, CLCA4, CREB5, DEGS1, E2F5, FOXA3, GNAI2, GUCY1A3, HADHA, HMOX1, HSPA1B, IRS1, MAPK10, MARS, MASP2, MTAP, MYL2, MYLK, NOD1, OR4K2, OR9Q2, PDXP, PFN1, PLXNA2, PRDX6, PRIM1, PRSS2, PTGIS, RHOD, RRM1, SETMAR, TKTL1, TOMM40, ULK2, UPP2, WNT10A, XYLT2, ZIC2, ATP6V0D1
Negative_3754	GDA, SDS, SRC, ADCY5, AHCYL2, ALDOA, ANAPC5, BUB1, CD48, CRLF2, CYP2A7, DHRS9, F13A1, FASLG, GADD45A, GLUD1, GP1BB, HAO1, MTMR1, NFKBIB, OR1L4, OR5C1, OR8A1, OR8H2, PFN2, POLR3G, PTTG1, SDHA, TAS2R41, ATP6V1G1
Negative_3755	BTD, CGN, GCK, LTB, NNT, RB1, ALDH3B1, ARNTL, ARSD, ATP6V1F, C7orf16, CADM3, CCL24, CDH3, CLDN6, CYSLTR2, DHRS4L2, E2F3, FGF20, GNA11, GRM5, HNF4A, IARS, IMPDH1, NODAL, NT5M, NTRK1, OR10AG1, OR11L1, OR1J4, OR2A2, OR2A25, PCYT2, PIP5K1C, RUVBL1, SGPP1, SHC4, SORBS1, SPAM1, SSX2IP, STX1B, SUOX, WWP1, ATP6V0D2, KIR2DL5A
Negative_3756	F3, GPI, HGD, TYR, ACOT12, ACVRL1, ALDH1A3, ARD1A, ARPC4, CAMKK2, CCL13, CD82, CDH5, CEBPA, CYP2D6, DAG1, DGKE, DHFR, EIF4E2, ENO1, EPRS, FGD1, FLOT1, FUT8, GMPS, GNMT, ICOSLG, IQGAP1, IRS4, KLRD1, LARS2, OCLN, OR52B6, PIP4K2A, PISD, PPP1R3B, PRKACG, PROC, RARA, RBL2, SNAI1, SSH1, TAS2R41, TPM3, VAMP1, VAMP2, VCAM1
Negative_3757	C8B, PML, UBB, BUB1, CHIT1, CREB3L3, CXCL2, CYBA, EPOR, FRAP1, GLUL, IL10RA, MTMR1, NQO1, OR2T3, OR4K1, OR52N1, PHKG1, PLEKHA8, PON2, PTGIS, PTPRJ, SEMA4B, SMAD9, SNAI1, SRGAP2, SUV39H1, TPM3, TRPC1, VCAM1, PAFAH1B1
Negative_3758	AGK, GGH, IL6, IL8, KIT, NF1, VCL, ATP13A2, CHUK, CYP2F1, ENPP3, GLDC, HBEGF, HLA-G, MAP3K14, MMP9, OR10H2, OR2L3, OR52K1, OR5P3, OR7D2, PANK1, POMC, PTAFR, PTK2, SEMA4G, ST6GAL1, STAT4, TAS2R41, UNC5C, WARS
Negative_3759	CKB, ACSS3, AGPAT6, BCL2L1, CAMK2B, CD40, CD48, CD70, COL5A2, CSNK1D, CSNK2B, DUSP2, FCGR3A, GMPR, GNG13, GUCA1C, HEMK1, INMT, MTMR6, OR11L1, OR1Q1, OR5L1, PIM1, PMAIP1, PPP2R5B, PROS1, PTCH2, RASGRP2, SHC4, SMURF1, SNAP23, VAV3
Negative_3760	CTH, GGH, RET, SI, ZYX, ARSB, ATP4B, CCND1, CD19, CD22, CD55, CSNK1G1, CXCL6, DLL4, EPHB6, GCLM, GPD1L, HSD11B2, HTR2A, IL18R1, IL1R2, ITGB3, OR2A25, OR2L8, OR2T11, OR52D1, OR52N4, OR8K3, PDE10A, PIGB, PLOD3, PRKAB2, QARS, RPS6, SC4MOL, SH2D1A, TRDMT1, TSC1, UGT2B17, AASDHPPT
Negative_3761	GLA, LHB, NOG, ALAS2, ALOX12, AMD1, CACNA1E, CD14, CXCL13, DGAT2L4, ECSIT, FBXW11, FGF7, IFNA14, IL1R1, IL20RA, MCM7, METTL6, MMP1, MMP9, NRXN3, OR51F1, OR7D4, PCYT1A, PGAM4, POLR1B, PPAP2C, SFRP4, SOAT1, TAS2R60, TKTL2, TNFSF11, VTI1A, YWHAE

Negative_3762	GSR, ACTN4, ALDH1L1, APH1A, ARPC5, B3GNT5, BDH1, CACNA1G, CCR6, CNTFR, CYP2S1, DUSP6, GSTA5, HGSNAT, HNF1A, IL8RA, INHBA, LHCGR, LIPC, MKNK2, OR10K1, OR13H1, OR2T3, OR51F1, OR6C76, OR8J1, PIGF, PKLR, PLA2G10, PRKAA1, PVRL2, PVRL4, RUNX1, SETD1B, SLC27A1, SOCS3, TCF7L2, TRAF6, VTI1B
Negative_3763	ADH5, ATF4, CLDN6, CYP19A1, DHRS4, FGD1, GUCY1A2, IFNA17, IL15, LCMT2, LDHAL6B, LTC4S, OR51B6, PDXP, SPRED2, UNC5C, PLA2G12B
Negative_3764	BAX, ATP5G3, B4GALT7, BLVRB, CCNG2, CDH1, CSNK1G3, DDIT4, IL1A, IMPA1, NLGN2, OR10A3, OR5AS1, OR9I1, PCK1, PKM2, STK3
Negative_3765	C3, PGP, AASDH, ADH6, AGMAT, ALDOB, B4GALT5, BMPR2, C1QB, CAV1, CES2, CHRM1, COL5A3, DAAM2, DFFA, DRD1, DTX3, DUSP8, EP300, FGFR2, FLT4, GCNT3, GGT1, GNAQ, GUCA1C, IFNA2, IL17A, KDSR, MMP2, NME6, OR10K2, OR1K1, OR2M2, OR52D1, OR52R1, OR5V1, PARVG, PDCD1, PIK3CA, PIK3CD, PIP5K1B, PPCDC, PRKAB1, SEMA3C, SGMS1, SMURF1, ULK3, SERPINC1
Negative_3766	HPD, LYN, NNT, ALDH4A1, ARPC5, B4GALT5, CAV3, DRD5, EDNRA, EFNA1, ENTPD1, EPHX2, FLT3, GNRH1, HIF1A, HMGCS1, HTR2C, IL13, MAML2, NGEF, NSD1, OR10H5, OR12D3, OR4M1, OR7G3, PDE5A, PIGW, PLEKHA8, PTGER1, RBL2, ROCK2, RPIA, TRAF4, UAP1, UCP1, CACNA2D1, SERPINB5
Negative_3767	ANAPC5, BDKRB2, PIAS2, PTCRA, TAS2R3
Negative_3768	LHB, RPE, AGPAT1, ALG2, ARPC5, BAI1, BMPR1A, CD70, CLDN19, CSNK2A2, DDX54, FASLG, FLOT2, FZD5, GNB1, GNMT, H6PD, INPP5B, ITGB1, ITPKB, MTAP, OR10H4, OR5AU1, OR6M1, PLXNA3, PVRL2, RALA, TKTL2, UGT2B10, MAPKAPK2
Negative_3769	BTK, DBI, DHH, ALDH3A2, ARHGEF4, BDKRB2, CCND3, CPT1B, CYP2C9, FLAD1, FZD5, GUK1, HAP1, IL2RA, KITLG, LDHC, NCOR1, NOD1, OR8S1, PTCRA, SELL, THBD, TRAF5
Negative_3770	GK2, A4GALT, ARG2, CCL8, CDKN1B, COL1A2, COL4A1, COL5A2, CXCL3, DAPK3, FBP2, GBE1, GRB2, HEXB, INHBA, MCAT, MFNG, OR2A42, OR5A1, OR6C75, PHGDH, PPP2R1A, PRKAA1, SAT1, SH3BP2, SPRY3, TAS2R3, TAS2R5, WNT2B
Negative_3771	UBB, ALDH3A2, CCND3, CYP26C1, DIAPH2, GNPDA2, GRIN2D, ITGA6, ITGB7, KLRC2, LARS2, NEFH, OR5AR1, POLR2B, PPAP2A, PSAT1, RXRB, SHISA5, SPRY1, VNN1, ATP6V1E1, TNFRSF13C
Negative_3772	ADA, ARSA, EPHB6, F2RL3, GALNT1, GRM1, GSTA2, GSTO1, IRAK2, LFNG, MAGI1, NEFH, P2RX6, XYLB, TNFRSF6B
Negative_3773	ACY3, ARD1A, EGFR, UPB1
Negative_3774	NPL, ACSL3, ACVRL1, B3GAT3, BUB1, INPPL1, ITGA11, MRAS, NSDHL, NT5C
Negative_3775	CR2, ADAM10, BAI1, CDKN2B, CFL2, CYP2U1, MALT1, MAP4K3, MGST2, NANS, NFX1, OR10G4, P2RX2, PIK3C2B, POLD3, RAP1B, SGPP1, SMC1B, SOD1, ST3GAL4, GABARAPL1, MAP3K7IP1
Negative_3776	CD4, ACOT11, ATP5G2, CACNG8, CCR5, CD38, CD44, CREB5, CX3CL1, DDX50, F11R, F2RL2, FZD8, GALNT7, GFAP, IFNB1, IL15RA, IMPA2, JAM2, MAD2L1, NTRK1, OR12D3, OR2AT4, OR2C1, OR4S1, OR52R1, OR7G1, PARVA, PECAM1, PFN3, PHGDH, SIAH1, SMPD4, TACR3, TAS2R10, TYK2, UGT1A10, CACNA2D3
Negative_3777	BUB1B, C1GALT1, CLDN4, CTSB, CYSLTR2, DAG1, DDX51, DHODH, GRIN2C, HHEX, IDI2, MAP3K5, OR2F2, OR5K2, PDGFB, PIK3R1, PRNP, RALA, ROBO2, RUVBL1, SEMA4C, ATP6V1G3, MAPK8IP2, TNFRSF10B
Negative_3778	DDC, GBA, JUN, TFG, ALAD, ALDH1A2, ALPI, ARNTL, CD28, CLDN5, CSF2, CTBS, CUL1, CYP2J2, EFNA3, GNAS, KYNU, LEPR, LTBP1, LYPLA1, MAPK3, MYH7, NNMT, OR10AG1, OR2A12, OR2A25, OR52B4, OR7G1, OR8H2, P4HA1, PANK3, PLA2G2F, PPP2R1B, PRDX6, SEMA4G, STAT2, SUFU, ATP6V1G3, SERPING1, TNFRSF13C
Negative_3779	IL4, PLG, ADAM10, ATP6V1H, CDK2, CLCF1, DARS, FECH, ITGB8, MCM5, NEU4, PPP1CB, RAD51, TFDP1, TNXB, VAMP1, WBSCR22, WNT2B

Negative_3780	FGA, ADCY8, ADH1B, CD38, CDC16, CREB3L3, CSF1R, FZD3, GALK1, IFNA10, IRAK3, KCNB1, LIPF, MMP9, OR13C2, PARVB, PDE5A, PLA2G2D, SGMS1, SLC2A1, SOCS5, SOS2, STAM2, VTI1A, MAPKAPK2
Negative_3781	ACP2, ACSS2, ACVR1C, CCL13, CHAD, CLDN3, CYP1A2, DGAT2L4, EP300, FGF12, FZD2, GDF5, HMGCS2, IL20, MYH3, OR3A1, OR5B3, PPOX, PRKAR2B, RASGRP2, RPS6KB2, SH3BP2, UGT2A1, WHSC1
Negative_3782	GK2, AGXT2, AMY2A, ARNTL, ATP5G2, ATP6V0B, CCL26, CCL8, CSNK2B, CTNNA1, CTSB, HAP1, INHBB, JAG1, LIPT1, LYZL1, MYL9, NFKBIA, OR2M3, OR4C46, OR5A1, OR5AP2, PRNP, PTGS1, RPIA, TAS2R7
Negative_3783	C7, ACSL1, ACVR2B, C4BPB, CDKN1B, DCTN1, DGKE, FUT6, GALK1, GNA11, HSPA1B, IDH1, IL15, JUND, LMO7, LRDD, NOTCH3, NUDT8, RRM1, SAT2, SEMA3G, ST3GAL1, STK4, ULBP3, PHOSPHO1
Negative_3784	FGG, ACVR1C, AKR1C3, ALG6, ARNT2, ATP5G1, CD80, CDK5, FARSB, FGFR4, HYAL2, IL11RA, LIPT1, MRAS, OR11A1, OR7G3, PLA2G3, PLCB2, PPAT, RAC1, ROCK2, SC5DL, SMPD4, TRAF4, UBA1, ATP6V0D2
Negative_3785	F2R, AFMID, AVPR1B, CFLAR, FMO2, GCNT1, GLDC, IFNB1, ITGA2, ITGB5, KCNQ1, MAPT, OR13F1, OR52H1, PFKFB1, PFN1, PHKB, PLA2G2D, PLCD1, POLD3, SDHA, SETD1B, SV2C, TDO2
Negative_3786	IL6, OAT, ACSM4, APAF1, B4GALT7, CLDN20, CNGA4, ENPP6, IFIH1, IL1R1, KLKB1, MAPK8, MTAP, NPR1, OLAH, PPP1CC, PTK2B, RASGRP4, SPRED2
Negative_3787	MVK, ARG1, OR4X2, OR9I1, SPTAN1
Negative_3788	AR, AGPAT3, CES7, EIF4B, MTHFD1L, NLGN2, PIK3CG, PMVK, PRDX6, STX18, VAMP2
Negative_3789	ME3, PXN, ARRB1, CAMKK2, CASP3, CCNB3, CDC16, COL4A4, EI24, EIF4B, HMGCS2, HTRA2, IFNA13, IL6ST, IRS1, ITGA6, KAT2A, LTC4S, MIOX, NUMBL, OR11G2, OR52E6, PDE6H, PRNP, PTPRZ1, PVRL1, PRICKLE1
Negative_3790	PLG, ATP5I, CDC26, GALNTL4, OR6F1, TBL1X, TBXA2R, THBS1, UAP1
Negative_3791	NOG, ACOX1, ALDH3A1, ALDH6A1, APBB1, ARHGEF2, ASAH1, ATP2A1, B4GALT5, C7orf16, CCL16, CCL28, CDK6, DAPK2, GDF5, HSPA8, IL23R, LTB4R2, MYH6, NOX3, NRG2, NUDT12, OR5C1, OR6K2, P2RX5, P2RX7, PCYT2, PKLR, PLA2G10, POLR3A, PPP2R5D, PTPRZ1, SETD7, SOD1, STK36, WNT1, HLA-DPA1
Negative_3792	VTN, ACHE, ALOX12B, CCKBR, CD27, CES1, CLDN10, CYP2A13, ECSIT, EFNA1, ENPP1, EPHA5, EPHB3, FASN, FCER1G, FOXA3, GNPDA1, GUCY2D, HLA-DOA, IL21R, IQGAP1, ITGAV, NKX3-1, OR10J5, OR4X1, P4HA2, P4HA3, PDE2A, PGAM4, PTGDS, SEMA4B, SETDB1, TYMS, UROC1, VASP, ATP6V1C1, KIR2DL5A
Negative_3793	ADC, MPZ, ACADL, ADCY1, ALDH1A3, ATP5F1, CASP10, CCL5, CD72, CDC26, DRD5, FABP3, GSTZ1, ICOSLG, LDHC, MLLT4, NANP, NCR2, NTF4, OR3A2, OR4S1, OR51M1, OR56A1, P2RX3, PDE1C, PRKAA2, RBX1, SARDH, ST3GAL6, PLA2G12B
Negative_3794	ADAM17, APOA1, AVPR1A, CCL22, CKS1B, GPAM, IL11RA, IL1R1, IL28RA, IL3RA, KYNU, LIMK1, OR4A5, OR4E2, OR8K1, PIP5K1A, RPSA, SLC2A4, CSGALNACT1
Negative_3795	F8, MIF, TK2, ACYP2, AGPAT3, AVPR1B, B3GNT5, CANT1, CFL2, CRHR1, CYP2A6, DFFB, DUSP16, EXT1, EXTL1, HPSE, LAMA3, MAP2K2, MAPK1, MTHFS, OR10A6, OR4A16, OR5B3, OXTR, PAX6, PIK3R3, PLD2, PON3, PPP3CA, PRKCE, PRKCG, RASSF1, RDH16, RETSAT, STAT1, SULT1A1, VAV3, WNT5B
Negative_3796	DCN, C9orf95, GARS, GNAZ, HSD3B2, MAPK1, NAT1, NR1D1, OR6N2, PRKY, PTPRC, TAS2R40, VCAM1
Negative_3797	GK2, ATP13A2, BDKRB1, CKMT2, GJD2, GSTM3, IL1R2, MPP5, NEFH, PITX2, PLCB2, PLCB3, POLA1, PON2, PTAFR, STEAP3, UCK2
Negative_3798	CYP1B1, GSTA3, GUCY1A2, LDHAL6A, MECR, NUDT9, OR14C36, OR2C3, PDE9A, SFRP1, ST3GAL2, TP53I3

Negative_3799	BTD, GPI, MIF, ZYX, ADCY1, ADH1A, BMPR2, CD8B, CDC20, DVL1, EFNB3, FGD3, FMO3, GTSE1, HSPA1B, LIFR, OR7D2, PRKAG2, PRPS2, TAS1R3
Negative_3800	C9, AARS, ADCY9, ALDH6A1, ANAPC5, ATF4, BCMO1, CD59, COQ7, CTBP1, DGKG, DHDDS, ECHS1, EFNA1, GJD2, HMGCL, IFNA6, IL2RB, INPP4B, ITGB4, ITPA, ITPKB, MAP4K4, MCM7, MLLT4, MYH4, MYL7, NKX6-1, OPLAH, OR13C2, OR2A5, OR2L13, OR51A7, OR5L2, OR6N1, OXCT1, PMM1, PRPH, RPN2, SDC1, SOCS2, SRGAP2, ULK1, HLA-DPB1
Negative_3801	CKB, FES, GSR, ARHGEF2, CD44, CHPT1, EARS2, FGF8, GGT1, GLS2, GNRHR, IL6ST, IRS2, NKX2-2, OR1I1, OR4F4, OR8J1, PGK1, PIGX, PIK3R2, PIM1, PPP2R2C, PTCH2, PVRL3, SETMAR, SPAM1, STT3B, UGT2B28, ST6GALNAC1
Negative_3802	C2, F2, GDA, LTB, OSM, PTS, ACOT12, CNTNAP2, COL1A1, FDPS, FGF9, IDH3A, OR2M4, OR6C74, PPARD, PVRL1, RAPGEF1, SH3BP2, TUBA3E, CTNNBIP1
Negative_3803	AK7, DCC, LCK, TH, ACAT2, ACSL1, ACTN1, ADRA1A, ADRA1D, ALAD, ANAPC11, ARD1A, ARHGEF4, CCL17, CDK2, CFLAR, CLDN19, CXCL12, DNMT1, ENTPD7, FGF10, FGF23, FLOT2, GPLD1, IFNGR1, IKBKB, IL1R1, LDHD, MAML1, MASP2, MYLK, OR4D6, OR51A2, OR51M1, OR56B4, OR5D18, OR8H2, PAK6, PCCB, PDGFD, PER2, RETSAT, SFRP2, SSH2, SUCLA2, TAS2R50, TBL1Y, UGT2B28, UNC5B
Negative_3804	AK3, APP, ATP5B, C4BPA, CACNG6, CD28, ECHS1, FASN, FGF21, FGF4, FTH1, GUCY2C, HNF1A, HRAS, HTR4, LDHA, OR4F15, OR6Y1, PROS1, RFXANK, SFRP2, SHC4, MAPK8IP1, TNFRSF11B
Negative_3805	GNS, GSS, MVK, RB1, ACAD8, ARPC5, CCR4, CPOX, DERL1, EFNA5, FGF11, FMO5, GLI2, GPX5, HLA-DOA, IL18R1, KARS, LIPE, MGAT1, NAGLU, OR52A1, OR8D2, PARS2, PLOD3, PPP1R3D, RFXANK, ST3GAL2, UROC1, WNT2B, XIAP, YARS, ZFYVE9
Negative_3806	AK1, FGA, IL9, ACADSB, ACADVL, ASPA, ATF4, ATP4A, B3GNT2, BLVRB, CKMT2, COL11A1, COQ5, CSNK1G1, CXCL11, DIAPH3, ECSIT, EFNA3, FZR1, GFAP, GSTP1, GUCY2C, IL20, MGLL, NFKB2, OR2B11, OR4X2, OR5P3, OR7A10, PAK6, PDHB, PDK1, PGLS, PLCG2, PLXNA1, PNLIP, POLD1, POLR1B, RDH16, SEMA3F, SPRY2, TCF7, UGCG, ULK1, WASL, WNT5A, MAP3K7IP2
Negative_3807	TAT, ACADVL, ALAD, ARSB, B4GALT5, CCNB1, DPM2, FGF3, HRH1, IL19, ITGB4, LDHAL6B, MAP2K1, MAP3K1, MYH4, OR1N2, OR2Z1, OR4M1, OR52A5, PIK3C2B, PIK3C2G, PPA2, PRKACA, PRKX, SMPD3, SPAM1, ST8SIA1
Negative_3808	ACAA1, ADCY5, AMDHD2, CRKL, CRLF2, CRLS1, CUL1, EDN1, EPOR, HSD3B2, IDH1, ITGA7, ITGB6, LCAT, MAN1B1, OR6K6, PGK2, PGS1, SLC12A2, SLC2A4, TAS2R7, TCF7, TUBA3E, WNT4
Negative_3809	ADSS, AGPAT2, CCNA1, CDH3, CDKN1C, CHSY3, COL5A3, DPYD, DVL2, EXTL3, GRIA3, HAAO, LIFR, MPZL1, NPR2, OR6C1, OR8G1, POLR2B, PRODH, PTAFR, TP53, WNT10B, YWHAZ
Negative_3810	EBP, FGB, APH1A, ARHGEF6, CBR3, DDX23, ENO3, FGFR2, FLAD1, MAD1L1, MAP2K4, NMNAT2, NRG1, ONECUT1, OR10G2, OR4K5, OR52B4, OR5AU1, OR9G1, PAPSS1, PARVG, PPARD, PRPH, PTPMT1, TUBB4Q, UGT1A10
Negative_3811	CFH, CR1, DCN, GK, HGF, NGF, AANAT, B4GALT7, BCAT1, C9orf95, CCR9, CHST12, CLDN8, CYP2A6, DDX19A, EXTL2, GCAT, GGT1, GNA15, GNPDA1, GRIN2D, GUCY2C, GUK1, HSD17B8, LARS, LIPF, NFAT5, NOX1, OR6C75, PANK1, PDX1, PIP5K1B, PLCE1, PRKG1, TAS2R48, ULK2, WNT16
Negative_3812	ME2, ARPC2, ATF2, ATP5A1, ATP6V1F, BCKDHA, CCL14, CYP2C19, DDX56, FRAT2, FZD6, GANAB, HTRA2, IL10, IL28B, IL2RA, KLRC4, MAD1L1, MAD2L1, MADCAM1, NODAL, OR4Q3, OR52R1, PARVG, PCNA, PIP5K1C, TAS2R14

Negative_3813	ADK,ASL,CBS,FOS,ME2,BBOX1,BCL2L1,CDK4,DLL1,DUSP5,EPB41,EPB41L1,EPRS,FABP1,G6PC2,GAB1,HSD17B1,ICOSLG,IDUA,IL11RA,ITGA2,LRAT,MAML2,MAP3K4,MMP14,OR2D3,OR8H3,ORC6L,PER2,PGM3,PIGU,POLE,PRSS1,PTPN11,RALB,SELE,SEMA4B,SNAP23,ST3GAL6,TCF7,WNT8A,ZBTB16,SLC25A31,TNFRSF1B
Negative_3814	HYI,ACTN2,ATIC,ATP5J,CD247,CLDN7,EPHB3,GNPNAT1,HIBCH,IL21,MAML1,NFKB1,OR11A1,OR51B2,PFKFB4,PGK2,PKMYT1,RUNX1,TIAM2,TJP1,YWHAQ,CACNA2D3
Negative_3815	MTR,AGPAT3,FUT6,OR5L1,PANK1,PDE10A,SOCS1,VEGFA
Negative_3816	ALDH1A2,BMP8A,SLIT2,TM7SF2
Negative_3817	SDS,ALPPL2,CACNA1I,CACNG1,CAMKK2,CCL5,CHEK2,CIITA,DARS2,DDX55,DGKB,DHRS4,DOT1L,E2F2,EFNB2,ENPP3,EPHA2,EPHA3,FECH,FRAT2,HADH,IL22RA2,ITGA2B,LAMB4,MAP4K3,MARS,MTMR6,MYH4,NFKB1,ONECUT1,OR10H2,OR1F1,OR6N1,OR8J1,PFKFB4,PIK3R2,PRDX6,PTCH2,RASA2,SEMA3D,SHC2,SHISA5,UGDH,MAP3K7IP1
Negative_3818	ACTC1,ADCY7,ADRB3,CACNG2,CAMKK2,CXCL11,EIF4B,GAB1,IFNA2,IL1RAP,MPZL1,OR5J2,OR5K1,OXCT2,POLD3,POLR2J,RASA1,SMAD3,KIR2DL5A
Negative_3819	CRH,DCN,F2,INS,NGF,ACADM,ACPP,ACSL6,ALG13,AMDHD2,AMY2B,ANPEP,ATP5C1,CACNA1B,DNMT1,ENPP2,GPX5,HRH1,MDM4,NRXN2,OR10H1,OR10Q1,OR1J4,OR4C13,OR4D1,OR51B2,OR52D1,PGM2,PIP5K1C,PLA2G2A,POLE4,PPM1A,PPP2CA,RPS6KA6,SELP,SEMA3C,SETMAR,STAM2,STX12,STX4,TAS2R38,TGFA,TGFB1,TLN2,WNT6
Negative_3820	GPT2,OR13J1,SERPINE1
Negative_3821	MVK,ADAM17,ARHGEF7,CLDN18,CMPK1,CNDP1,CRHR1,DAAM1,DDX19A,DLL1,FUT1,FUT7,GSK3B,HYAL1,IL11,IL2RG,NOX3,P2RX3,P2RX6,PAPSS1,PCK2,PDE1A,PPP2CA,PYGB,SDC1,SMC1B,SPRED2,WNT5A,EIF4EBP1
Negative_3822	ADC,BTD,C6,C8B,EZR,MIF,AHCYL2,AKR1C1,ARPC5,B3GALT5,CACNA1G,CD8A,CSF2RB,CSNK1E,CYP3A5,DDX41,DDX56,EFNA2,GPD1,HSD3B7,HTR4,LALBA,LHCGR,MAP4K3,MGAT4B,NTRK2,NUDT2,OR1L4,OR1S2,OR2C3,OR4A5,OR52E8,PCYT1B,PLOD1,POLA2,POLE3,POLR2D,PPP1R3C,SETDB2,TAS2R9,TJP1,TKTL2,TNFRSF4,UCP1,VANGL2,WNT6,YWHAQ,ATP6V1C1,EIF4EBP1
Negative_3823	ABO,LYN,ACTG1,CDK4,CERK,CYFIP2,DGKE,GLB1,GNA12,HNF1B,IL21,IL25,KLRC1,MAN1A2,MAP3K11,NME7,OR52J3,OR6C70,PDGFD,PRKAG2,SOAT2,STAT6,TAS2R41,TBXA2R,THBS1,UNC5D,UPRT
Negative_3824	CR2,CRK,GGH,LIF,ATG7,CACNA1D,CDC25B,CDH3,CDK6,CTBP2,CYP7A1,DARS2,DIAPH2,FGF9,GGT6,GNPNAT1,GRIA2,GRPR,GSTM5,HLA-DOA,ITGA8,LALBA,LDHC,LIPE,MAT1A,MNX1,MTHFS,NT5M,OR13C4,OR4F17,OR4F5,OR4X1,PAFAH2,PDK1,PNMT,PNPLA4,PPA2,PTGER1,RXRA,SLC25A4,SOAT1,SPHK2,SV2C,TSC2,UGT2B28,ARHGEF12
Negative_3825	BAX,HGF,MSN,AGXT2,ARPC5L,BCAT1,CACNA1C,CALML5,CYP2U1,DDIT4,DFFB,DOCK1,FASLG,FLOT2,GSTA3,GUCA1C,KLKB1,KLRD1,LDHAL6A,NKX2-2,NME2,OR5AR1,PCYT1A,PGK1,PITX2,PLA2G4A,PNLIP,PPP3CA,PRKCI,PTDSS1,SDC1,SUCLG2,THY1,CACNA2D2
Negative_3826	F11,IHH,RB1,AGPAT2,AKR1C1,BACE1,BDH1,BPGM,CADM1,CFL1,CXCR5,CYP11B1,DPM2,EHMT2,ENPP3,FLT3LG,NEU4,OR4N4,OR52N5,OR7A5,PAK1,PIGA,PPARG,PTAFR,RDH16,RRAS2,SYNJ1,TRAF1,TSHB,TYK2,UPP2,YOD1

Negative_3827	GPI, LPL, ACSL4, ADAM17, ALDH5A1, C9orf95, CMBL, CREB3, DERA, DGKQ, GRM5, HSD3B7, IMPA1, INSR, ITGA2, KIR3DL2, KLRC2, LPAR1, MAP3K2, MCCC2, MGAT1, MIOX, NAT5, NKX2-2, OR3A1, OR4K2, PAICS, PCYT2, PDE1A, PDE6G, PIGF, PPP2R5B, PRKAR1A, PRSS3, RRAS2, SHC2, SLC2A1, SOS1, ST8SIA1, SV2A, TBL1Y, TDO2, TSHB, WNT10A
Negative_3828	AMHR2, DHCR24, GMPS, KAT2B, LIMK1, LIPG, NTRK2, OR4K5, TACR2, UCKL1
Negative_3829	ALDOC, CCR7, CD244, CNGB1, COASY, CYBB, DHDDS, IAPP, IFNA21, KAT2B, LAMA3, MBL2, NFKB2, OR10K1, OR6C75, PERP, PGAM4, PIM2, POLR1C, PVRL3, SMAD5, STX2, TYMP, UGT2B10, VNN1, WNT1, MAPKAPK3
Negative_3830	EZR, PTS, TXK, APOC3, ATP5O, CD48, CTTN, ERBB2, HSPA8, ITGB8, MTAP, NUMBL, OR51V1, OR8H3, PFAS, PIK3R5, RFX5, STK36, UGT2A3, UNC5B, WNT5B
Negative_3831	PDC, SRF, ADCY9, ADH7, AHCYL1, ALDH1A3, BLNK, BTRC, CCL17, CCL22, CD44, CPOX, CSF2RA, FGF3, FZD10, GSTP1, HTR2A, IDI2, IQGAP2, ITGA10, ITGA8, KCNJ11, MAGI1, NEFH, NUDT9, OCLN, OR2F1, OR5AU1, OR5L1, OXTR, PDE6G, PHKB, PIGB, RBPJ, RPS6KA6, SMAD3, SPRY3, SPTLC1, SUCLG2, TAS2R60, TBL1XR1, TNFSF18, TYK2, UCKL1, UGT2A3, XYLB, YWHAZ, TNFRSF11A
Negative_3832	FOS, CKMT2, CNTF, COQ5, GNAI1, OR2B11, OR5I1, PLA2G2A, SRGAP1, TIAM2, TXNDC12
Negative_3833	GMPR, GRPR, POLR1E, TAS2R1
Negative_3834	F12, NPL, ACTG1, ACYP2, CEBPA, DCXR, GJD2, GRHPR, PTGER3, WNT4
Negative_3835	CBL, ACY3, AGTR1, ALOX15, BIRC2, BLVRB, C1GALT1, C1QA, CASP10, DDX4, DGAT1, DOLPP1, ETNK2, FZD4, GALNT2, GATM, IFNA10, KLRC2, LILRB3, MYH13, MYL5, NAE1, OR1N2, OR2A4, OR2M5, OR2T4, OR51T1, PHKB, PRKY, PSAT1, QARS, SPHK1, STX2, TBK1, YKT6, ZIC2, HLA-DRB3
Negative_3836	GH2, HYI, ALG10, ARNT2, CNGA4, DLL3, MAML3, MAP4K3, NCK1, NTN1, P4HA3, PPP2R5A, TFPI
Negative_3837	ADCY4, CCND3, CD274, GPLD1, HAAO, OR52N2, PAPSS1, PFN3, RAC2, RPS6KA5, TOLLIP, WWP1
Negative_3838	MET, OTC, SRM, ACSL4, ACTN1, AKT2, CAMK2G, CDH5, DDIT4, EHMT1, EPB41, EXOC7, EXTL1, FGD3, GAB1, GALNT1, HEXA, IFNAR2, IL12A, INADL, KITLG, LAMB4, MAP2K7, MAP3K8, MGAT3, NLGN1, OR2A12, OR2L3, OR52E2, OR52K1, OR6C76, PIK3C3, PIK3R5, PIM1, SIAH1, TAS2R13, TRAF3, TRIP10, ZFYVE9, ATP6V1G2
Negative_3839	AADAC, AKR1C2, ANAPC10, BUB1B, CDH1, CES7, CHRM1, CLDN1, CMPK2, CREBBP, DARS, ENTPD2, EPB41, GRM1, HDAC2, IRAK4, KCNQ1, MAN1B1, MLYCD, NEFL, NEU4, OR2A2, OR3A1, OR51A7, OR52B4, OR5P2, OR8S1, PDHA1, PDHA2, PFKP, PGM2, PGM3, PHKB, PIGQ, PLA1A, PPAP2A, PPP3CA, PTPRM, RAF1, SH2D2A, SULT1A1, TLR4, UGCGL2, WNT11, ATP6V1B2, GABARAPL2, HLA-DRB3
Negative_3840	DDO, F2R, MAG, MPL, TYR, ABLIM2, ACSL5, ADH1A, AGTR1, APAF1, ARG1, CALML6, CAMKK2, DTX3L, E2F3, ERBB2, FGD1, FZD7, GALNT3, GALNT6, HSD11B2, HTR6, INADL, ITGB7, LRP6, MAN1A1, MGAT4A, NUDT8, OR10G4, OR2L13, OR3A2, OR4D11, OR51E2, PFKFB4, PGAM2, PHPT1, PLAU, RASSF1, RFNG, ROCK1, SEMA4F, SOAT2, TGFB2, TUBA8, UGT8, XIAP
Negative_3841	ACO1, AKR1C2, BMPR1B, CNTF, DDX41, GLI2, MBL2, MYD88, OR2A2, PIGL, PLXNB3, PTPN7, RAC3, RBPJ, UGCG, UGT2B7
Negative_3842	CKM, ILK, ACSM4, ARPC4, CACNB2, CD86, CEBPA, DAD1, DGKH, DUSP14, EHMT1, FGD3, GGPS1, GOSR2, ICAM2, IL1B, ISYNA1, MAPK13, MYD88, NT5E, OR2H1, OR7D4, PARVG, PIK3R2, PPA1, RPN2, TSTA3, ULK3, SERPINF2
Negative_3843	AMH, DUT, BAAT, CAV3, CDKN1A, CEBPA, DGAT2L4, ENTPD6, GPX5, IL12RB2, OR2T4, OR5L1, SHC4
Negative_3844	APP, IL4, ALG12, BCAT1, CHSY1, COL3A1, IDH3A, MAT2B, MDM4, MGAT2, OLAH, OR1E1, PCK2, PTCH1

Negative_3845	ACACA, APOA1, APOE, ARSE, B4GALT4, BBOX1, CPS1, CREB3L4, DBF4, EFNA1, ERBB2, FARSB, GFPT2, GRIN2A, HTR2A, KAT2A, MAML2, OR51I2, OR52N4, OR5T1, OR8A1, OR9Q2, PERP, PRKAB1, SEMA3G, SMAD7, SNAI2, TGFB3, TNFRSF6B
Negative_3846	EDA, KHK, SHH, TNN, ACSM5, ACVR1B, ADH1B, CACNB3, CCNG1, CDC14A, CES2, CYCS, CYP11B2, DBF4, GRIN2A, GRIN2C, IL6R, LCP2, NCK1, OR1D2, ORC1L, PFKFB1, PGS1, PIGH, PLA2G1B, POLE3, STX18, UGT2A1, WIF1
Negative_3847	CAT, F2, KIT, KMO, PC, RB1, AGMAT, AMDHD1, ANAPC1, ASIP, ATP5D, ATP6V1H, CACNA1G, CACNA1S, CCR6, CDC27, CYP11A1, CYP2A13, CYP2C8, DAD1, DBF4, ENPP3, EVI1, FABP1, GALE, GNG13, HSD17B4, IDH3B, ITGA10, MCM3, NR1D1, OR2T4, OR2T5, OR51B5, OR5J2, OR8B12, PIGQ, PLD2, POLE4, PORCN, PPP1CB, PTGS1, RCHY1, SLC2A4
Negative_3848	FER, HGF, NNT, ACPP, B3GALT2, CCNE2, COL5A1, DERL1, GALNT11, GOSR2, GUCY2C, IKBKE, ITGB1, LAMA4, LAMB4, MGLL, OR4D5, OR4X1, OR51F2, OR5A2, PLCE1, PPP1R3A, PVRL1, RPS6KB2, SARDH, SCLY, SMAD1, SNAI2, SUOX, UBE2L3, WNT5A
Negative_3849	DBT, RDX, TNC, AGPAT1, ALOX12, APLP1, B3GAT3, BST1, CD72, CD80, CD86, CHEK1, COL1A1, COL5A1, DDX54, EPAS1, ETNK2, FRAT2, GSTM1, HEMK1, HMOX2, IARS, IFNG, IL1B, IL21, IPPK, IRF7, NCOR2, NMNAT1, NUDT5, OR10A3, OR1A2, OR1J1, OR5B21, PARS2, PDGFA, PDGFB, PDX1, PGK1, PLCE1, POLA1, RAP1A, RARS, SLC9A1, TALDO1, THBS2, TSHR, HLA-DRB5
Negative_3850	AGPAT6, ASNS, ENTPD4, ESPL1, FADS2, IL23A, LLGL1, OR2F1, PRIM2, ULK1
Negative_3851	OGT, AGPAT4, ALOX15B, CCL14, CCR6, CLDN15, DPM3, GNAI3, INSRR, LYZL1, MAGI2, MAPK1, OR14J1, PCCB, PDXP, PIP4K2A, SPTLC2, WHSC1, WIF1, ST6GALNAC1
Negative_3852	AGL, CAD, DCK, FGB, ABL2, ALDH3B2, ANAPC10, CBLB, CCL13, CCNB2, CCR3, CSNK1E, DDX52, FLT3LG, GIT1, GSTZ1, IL5RA, KIR2DS4, MCM5, MCM7, MDM4, NFKBIB, NMNAT3, NTSR1, OR5J2, OR6Q1, P4HA1, PCYT2, PDE1C, PDE6G, PRKG1, SLC9A1
Negative_3853	ACP5, ADH6, ALAS2, ATIC, AVPR1A, CMPK1, CSF2RA, DTYMK, DUSP9, EPHB3, ERBB4, HYAL2, IFNA14, IL20RA, MAML3, MCM3, MNX1, MYH3, NRXN2, OR2A5, ORC4L, PRKAA1, SESN1, SMC1B, WIF1, CACNA2D2, PAFAH1B3
Negative_3854	CACNA1D, CCNG1, CSNK1E, CXCL13, CYP8B1, G6PC, GNRH2, IBSP, IFNAR2, IMPDH2, NEU4, PFN2, PKLR, PSME3, THBS4, TUBB2A, TNFRSF17
Negative_3855	ACVRL1, ADRB1, ALDH7A1, ARHGEF4, CDC25A, CDC6, CSH1, DHX58, DLAT, EPHB6, FARP2, FDPS, GNA11, GPD2, HMGCL, INPP4B, ITGA2, KARS, KDSR, MAP3K4, MCM4, MYLK2, OR10G7, OR2S2, OR7D2, OR8U1, PLK1, RELB, RPIA, SLC27A4, STX3, TRAF1, UCKL1, UGT2A3, PPARGC1A
Negative_3856	DUT, LPL, MUT, SFN, ADCY6, AKT1, ATP5H, CARS, CAV2, CYFIP1, CYP2E1, G6PC2, GALNT10, GCH1, GNMT, IL21R, LILRB3, LYPLA1, MAP2K4, MAPK1, MTHFD1L, OR10H5, OR1F1, OR2D3, OR2H1, OR6S1, PAK2, PPP1CC, PRKAR1B, RBX1, RCHY1, SCP2, SESN2, SLC33A1, TAS2R16
Negative_3857	IL7, AASDH, AKR1C2, CCKBR, CCR9, CD28, EHHADH, FGF6, GFPT1, IARS, IFNA5, IL11, KLK3, KLKB1, LDHD, MGAT1, NFKB1, OR4K2, OR52H1, OR52L1, OR5I1, OSMR, PDE8B, PRSS3, PTPRC, RBPJ, RFXANK, UNC5C
Negative_3858	GP5, IL9, SRF, ACTB, BCL2, BHMT, CAMK2A, CSH1, CTNNA1, E2F1, HAP1, IFNA2, KITLG, KLK3, NANP, NQO1, OR1A1, OR2T2, OR3A2, OR5L2, PAK6, PIGT, PLA2G10, POLD1, POLR1B, POLR2H, PRKY, PRPS1, RERE, RRAS2, SLC25A4, SPAM1, TACR1, TRAF6, TRDMT1, TRHR, UCP1, UGCG, ULBP1, VDAC2, ZAP70
Negative_3859	HK3, ARSE, BCKDHB, CDS1, CLTCL1, E2F1, GJD2, GMPR, KCNB1, OR51A2, OR51G1, OR5R1, PDGFD, PLD2, POLR2K, STK11, TMLHE, UNC5C, VTI1A

Negative_3860	CRK, ABI2, ABL1, ADH1A, AMPD1, BMP7, CADM1, CD38, CDK2, DTX3, ERBB2, EXOC7, GLUL, GNAL, GYS2, HTR2A, HYAL1, IFNAR2, IL11RA, IL25, INPP5E, ITGA5, ITGAD, LCMT1, LEFTY1, LYPLA2, MAPK11, MGST1, MYH3, NAT2, NRG4, OR10J3, OR14A16, OR56B4, OR8S1, P4HA1, PDE1A, PHKA1, PLCB4, RETSAT, SETDB1, SMURF1, SNAI1, TAS2R16, TM7SF2, TNFRSF1B
Negative_3861	C8A, ALAS1, ALG10B, ALG8, AREG, BCAT2, CAV3, CCNB3, GNA11, IFNB1, MAD1L1, NPR1, PVRL4, SLC25A5, SPTLC1, STX8, WNT5B
Negative_3862	BTK, TFG, ABLIM3, ADAM17, ASMT, B3GALT6, B4GALT4, CACNA1H, CALML6, CASP7, CCNE2, CCNH, CD59, CDC20, CHRM1, CSAD, DGKI, DPM3, DRD2, FLT4, FZD10, GAPDH, GSTK1, ICOS, ITGB3, MAPK14, MYH11, NOTCH3, OR2Y1, OR6C75, PTPRJ, RARS, RDH8, RUNX1, SIAH1, TCF7L2, TICAM2, UNC5A, WNT2B, YWHAZ, CSGALNACT1, SERPINB5
Negative_3863	FYN, GK, AKR1B10, BMP4, CDK4, COL4A1, FLAD1, LAMA4, PDE6D, PI4KB, RDH11, RELA, ST3GAL1, TUBA3C, HSP90AB1
Negative_3864	ABO, DDO, FAS, ID1, IDS, LIF, ACOT12, ARHGAP5, B4GALT6, CACNB2, CCL26, CD79B, COL4A1, CTBP1, DLST, DOT1L, E2F4, ESAM, ETS1, GBE1, GNG13, IFNGR1, MAPK10, MTHFD1, NME2, NR4A1, OR10G9, OR10V1, OR11A1, OR1E2, OR2C3, OR51T1, OR6F1, OR8B4, PAK2, PDE1A, PDE7A, PTCH2, RFX5, SETD1B, SFRP5, TIRAP, TUBA8, UCK1, UROC1, ATP6V1G2, ST6GALNAC1
Negative_3865	C8A, C8G, CKB, DLD, FGB, ACAT2, ACY3, ARHGAP5, ARPC3, BCAT2, CD59, CMPK2, CYP3A4, DDX51, DUSP4, E2F1, ELK4, ENTPD5, FZD10, GLDC, GNAZ, GSTA2, HCST, IL18, IL1RAP, IMPA2, KDSR, LAMB3, LCP2, LEF1, MAP3K8, NTF4, OR4C12, OR4P4, OR4X1, OR51B5, OR6B3, OR6C1, PIK3R5, PNMT, PPP1R3B, PPP1R3D, PTDSS1, PTK2B, RXRG, SGPP1, TUBA1A, XCR1, SERPINF2
Negative_3866	ALG2, CCNA1, CTTN, DDHD1, DHFR, ERBB3, MEF2C, NCR3, OCRL, OR10H4, PDE11A, POLR2E, RPS6KA5, SCD5, WNT5B
Negative_3867	FYN, TKT, CCNB1, FGF6, FZD6, GRIN2B, GSTA3, ITGAE, MYH1, NCF2, OR11G2, PPAP2B, C1GALT1C1
Negative_3868	F2R, ALOX15B, PDXK
Negative_3869	CD2, CKM, F2R, GPI, AARS2, ACSL5, ADORA2B, AFMID, AGPAT4, ATF4, C3AR1, CASP7, CTSL1, CYP27A1, CYP2E1, DDX56, DHDDS, DLL3, E2F5, FCGR3B, FGF20, GPD2, HAGHL, HSPA2, KLRK1, MTHFD2, NFYA, OR13C2, OR2A4, OR2T34, OR51A2, OR6N2, OR8A1, PDE6D, PHKA1, PHKA2, PKMYT1, PPP5C, PTGIS, PTPMT1, SGMS2, SOCS5, ST6GAL1, TAS1R2, THBS4, TYK2, VANGL1, WEE1, HLA-DPA1
Negative_3870	C9, CFD, FES, JUN, A4GALT, AGPAT3, ALDH3B2, CACNA1D, CDC45L, CSDA, CYP2C9, FUT2, GALNT12, GUCY1B3, INPP5A, ITGA5, ITGB2, NFATC2, OR13C3, PFKP, RBPJL, SPRY4, TCF7, ATP6V1E1
Negative_3871	AARS2, ADSS, CDKN1C, CMBL, CYP1A2, FASLG, IMPDH2, NRXN1, PRKAG2, VAMP3
Negative_3872	ACVR1C, AKR1D1, CIITA, CISH, DHDH, DPAGT1, ENO1, FABP1, GCLM, GDE1, GRIA2, HRH2, HSD3B2, IL17RB, ITGA4, MAD2L1, NEU1, OR2M4, OR6C1, OR8U1, PLA2G4A, PPP1CA, SARS2, SLC27A4, SMAD4, TAS1R2, ATP6V1E2, TNFRSF10C
Negative_3873	ADO, GP9, HPD, NF1, NNT, ACP1, ADORA2B, ATP6V1F, CACNA1B, CD244, CRY2, CSNK1D, CTPS2, CTSL1, DDX18, DPM2, DPYS, EFNA1, FGF17, GNAZ, GNG3, GNPDA1, GRIA3, GSK3B, GSTK1, GUCY2F, IFNA2, MDH1, MGAT5, NUDT9, OR4C16, OR52I1, OR5A2, OR6A2, OR6X1, PDGFRA, PIGC, PLXNA1, POLR3A, PPP3R1, PRIM2, PSEN2, RARS, SLC12A2, STK4, TGFB1
Negative_3874	ABP1, CDKN2D, CHRD, G6PC2, GALNTL1, INHBA, OR4K15, RPIA, RUNX1, SHC3, TAS2R42
Negative_3875	ABO, ITK, ABAT, ACSM3, ATP6V0B, B4GALT2, BIRC3, C1QB, FGF3, FZD8, GLUD2, IL24, KCNJ11, NAT2, OR14I1, OR4K1, OR4N2, OR6X1, PDPK1, PFKL, PGM1, POLR2H, PVRL3, SSH1, SULT1A2, WASF3

Negative_3876	GDA,GGH,ALCAM,CHRM5,CLCA2,CSAD,DTX4,FGFR2,GOT1,GRIN2A,GUCY2F,HRH2,IDUA,IFNK,IL6R,ITGB5,LIMK2,NFYA,NODAL,OR10G3,OR5B3,OR8I2,PARVB,PER1,PI4KB,PIK3CB,PIM1,PLCB4,PPBP,SORBS1,SSH1,THY1,ATP6V1G2,KIR2DL5A
Negative_3877	FGB,BHMT,GDF6,GSTZ1,LCMT2,RBKS,SEMA4C
Negative_3878	LSS,CACNG4,CANT1,DDX18,MDM4,POLR2D,PORCN,PTGER3
Negative_3879	ACSL6,AKR1B1,ALDH3A2,ARSA,DPM1,DPYD,EIF4E,FARSA,FRAT2,OR1A2,OR2B2,PRKCD,RALB,SULT1A4,TPK1,TRPM5,VEGFB,MAPKAPK3
Negative_3880	BAD,CAD,GBA,ADH4,ATP6V1F,BMPR1B,CD59,CDC25C,CDO1,CTBP1,CYP26A1,DARS2,DHRS4,IFNB1,IL11RA,INPP4A,INPP5E,PIGB,PRKAR1A,PTEN,RPS6KA5,SHC2,SOCS7,THTPA,TYROBP,WNT6
Negative_3881	DUT,EZR,RB1,VCL,ABLIM3,APH1A,CACNA1A,CCL14,CCL20,CLDN1,CSNK1G2,CSNK2B,CTSG,DLL4,ENPP2,GAS1,GRIN1,HLA-DMA,IDH1,IL3RA,KITLG,LAMC2,MAN1B1,MYH6,MYH7,NEFM,NT5M,OR5B17,OR5J2,PARD3,PDCD1,PIGL,PIGU,POLR2H,PPP2R5D,PRDX6,PRKCI,PTEN,RASGRF1,SH2D1B,SLC25A6,SMPD4,SOS2,STX5,TBK1,TBL1XR1,SLC25A31
Negative_3882	MVD,ADH1A,CD70,CDK7,CYP2C18,DPYS,DTYMK,GNG12,IFNA8,IL2RB,MASP2,NTNG1,OR2A14,OSMR,PIAS2,RGS3,SLIT2,TAP1,VEGFB
Negative_3883	CR1,HGF,ACTA2,ALDH3B2,ALG2,B3GNT5,BCMO1,BLVRA,BRAF,C4BPA,CASP10,CCL4,CSF1R,DAAM2,DAXX,DOLPP1,ENTPD1,FGF12,GADD45G,GDF6,GFPT2,GMPS,HADHA,HSD3B2,IFIH1,IFNB1,IL13RA1,IL1B,MAPK10,NPR2,OR13G1,OR1L3,OR4K1,OR52I1,PDGFRA,PGS1,PPP3R2,PTCH1,RASGRP4,ROBO3,SQLE,ST3GAL1,TPMT,UGT2B11,UNC5C
Negative_3884	CDA,TH,ACAD8,ARPC3,BMP4,CASP10,CHRM1,CHRM2,COL11A1,CSNK2A1,CTLA4,DRD2,ENTPD8,GNG13,GNRHR,JAG2,L1CAM,LHCGR,LIPA,MAPK3,MYLK3,NEFM,OLR1,OR2A25,OR4C13,OR6C4,OR8G1,PGM1,PIGA,PTGER3,SFRP5,SLC2A2,SYNJ1,TACR2,VKORC1,YES1,TNFRSF13C
Negative_3885	AK5,ASL,F5,GDA,GSR,MPZ,VCL,ACACA,ALG1,ARAF,CACNA1I,CCL13,CD59,CDH1,CLCA4,CLDN6,CYP8B1,EDNRB,ENTPD1,EPHA7,EPHX1,EXTL2,GOT2,HSPB1,IDUA,IL15,JAK1,KLRC1,LAMC1,NUDT12,OR1B1,OR1N2,OR2A4,OR2AG2,OR2T5,OR5I1,PAX6,PLXNC1,PSAT1,PSEN2,PTCH2,QPRT,RHEB,ROBO2,SLC27A5,WNT16,CTNNBIP1,MAPKAPK5
Negative_3886	CP,HGD,MOS,B4GALT6,CD19,FZD10,GALK1,ITGB5,KLK3,MTMR6,NSD1,NTRK2,PFAS,PFN4,PPP2R1A,SETD1A,ULBP1,WBSCR22
Negative_3887	AR,BTD,DDC,AANAT,B3GAT2,B4GALT3,CSNK1G1,EPHA5,IL10RB,IL1B,OR10S1,OR2T11,OR9G1,PARD6A,PMM2,POLR1C,PPM1A,PPP3CB,PRKAB1,PRPS2,RGS3,STK36,ULK3,TNFRSF17
Negative_3888	OGT,AKR1B10,B3GALT2,BIRC5,CD8A,CDIPT,CLDN6,DERL1,DHRS4,EPHB2,FGF22,GALNT2,GATM,HNMT,HSPB1,ICAM2,MYH7,NOD1,NPAS2,NRG3,OR10G3,OR4K17,OR8K3,OXCT1,PDHB,PFN3,PIGM,PPCS,RPS6KA2
Negative_3889	NPL,ABLIM2,ACADVL,AGTR1,AMY2B,APH1A,APOE,ATP4B,ATP5A1,BET1,CD19,CD36,CYP4A22,GAPDH,GNAZ,IL10RA,IL1RAP,MOV10L1,NLGN3,NMNAT2,OR13J1,OR2T4,OR4D5,OR4N2,OR52R1,OR8A1,PANK1,PFKFB3,PIP5K1A,PLXNC1,ROCK2,SDHA,SPRY3,TPH1,TRMT11,TUBA1B,VCAM1,WHSC1,YARS2,ATP6V0E1
Negative_3890	DAG1,DHODH,GPAM,IQGAP2,MTAP,NEU3,OR2M7,OR4N4,OR51A2,POLR3G,SOCS5,TALDO1,TGFB1
Negative_3891	AMH,VTN,ALG8,ANAPC1,ANAPC7,C4BPA,CCL20,CD19,CUL1,CXCL1,DDIT3,DDX47,DEGS2,ENO3,EPHB2,GAS1,GSTM1,HDAC2,IL24,LLGL2,LYPLA2,MTAP,NCR2,OR13C9,OR2M2,OR4P4,PPAP2A,PRKCB,PTGER1,RAB23,RASGRF1,SMC1A,TSLP,UGT8,VDAC3,WBSCR22,KIR2DL5A,ST6GALNAC4

Negative_3892	ADAM17,APC2,ATP5J,CLTA,CSNK1G2,CYP2J2,FGF7,HEXA,HSP90B1,IFNW1,MAPK3,OR10P1,OR13C8,OR9G1,PER1,PGLS,PHKA2,PIGM,POLA2,RHEB,RHOD,RPE65,TAP2
Negative_3893	TKT,AACS,ACAT1,ALCAM,CES2,CISH,CLTB,COL5A3,EPHA4,LDHAL6A,MAPK1,PHKB,WNT7B,CSGALNACT1,MAPKAPK3,TNFRSF10A
Negative_3894	BCR,HK1,TK1,ALG1,AOC2,ASAH1,ATP5G3,CCL28,CLTA,COL4A1,DFFB,FGD1,FGF14,GMDS,HIBCH,IL13RA1,JAG2,LDHB,MAP3K2,NLGN3,NRAS,NRXN1,OR10G7,OR1L1,OR4D2,OR51I2,OR56B4,OR5B12,P2RX6,PIK3CD,POLR2K,PPARD,PRKACG,PRKAG2,PRSS1,PRSS2,SOS2,STT3B,UGT1A10,ZIC2,MAP3K7IP1
Negative_3895	LAT,TPR,ACPP,ALOX12B,C3AR1,C5AR1,CAMK2B,CCKAR,CERK,CXCL3,CYP4A11,CYP4A22,DCTN1,FGFR4,GALNTL1,GCLC,MALT1,MICA,MTHFD2,MTMR6,NCOR2,NUDT12,OCLN,OR2G3,OR2T29,OR51B2,OR52B4,OR5L1,OR6C76,PDK1,PFKL,PIP5K1C,PPP1R3B,PRKAG3,RAB23,STX8,TAS2R49
Negative_3896	C2,DCN,FN1,IL6,OAT,CD27,CD3E,CISH,CXCR3,FARSB,GGT1,GNG3,GNRH2,HIF1A,HTR2C,IFNK,IL28B,KNG1,MALT1,NRG4,NTNG1,NUMB,OR7A17,PER2,PRSS3,PTCH2,RFXANK,TSTA3,AASDHPPT
Negative_3897	AUH,GH2,ACP6,ADRB1,ALDH3B2,AQP7,CCL4,CCR3,CHIT1,CYP8B1,DCTD,DDIT3,DDOST,E2F2,F2RL2,ICAM2,IL3RA,NOTCH1,OCLN,OR8K5,PARS2,PDGFC,PPP2R2B,PTGS1,TP53I3,TUBA4A
Negative_3898	GPI,JUP,MTR,AARS2,ADH1B,ALOX12B,ARNT,CADM1,CCR3,CD274,CLTA,CSNK2A2,DDX18,GCNT3,GSTA5,ICAM1,IDH1,IL11RA,IL1R2,INHBE,MAP4K1,MS4A2,NUDT9,OR10R2,OR4N4,OR5AP2,PAK6,PIGF,PIGO,PIP5K1A,POMC,PRKCZ,RRM1,SPP1,STAT5B,TLR4,TLR9,TSHR,SERPINC1
Negative_3899	SP1,AANAT,ALG10B,ARSD,CCL19,CYP26A1,DGAT2L4,DIAPH1,EDA2R,EPHX2,ESPL1,GP1BB,GRIN2D,GRM1,INHBB,IQGAP1,KCNQ1,MTHFS,OR4A5,OR52B4,OR52I2,PAICS,PARD6A,PHPT1,PIGW,PLCZ1,POLR1A,PTK2,RASA1,SH2D2A,TUBB4Q,UAP1,UGT2A3,WNT16
Negative_3900	LCK,AGPAT3,ATIC,CBR3,DDIT4,OR9G1
Negative_3901	CPT1A,CYP26C1,EXTL1,JAG2,MALT1,OR1J4,RIPK1
Negative_3902	CFH,GSN,ITK,ARRB1,CDK2,CYP8B1,EDAR,FGFR3,GGPS1,HYAL2,IL15,IL7R,MAN2A1,MAP3K12,NFYB,OR14J1,OR2C1,OR5C1,OR5M9,OR6B2,OR7D2,P2RX6,PDGFA,PERP,QPRT,SFRP1,SPAM1,SSH2,TRPM5,TNFRSF6B
Negative_3903	ACACA,ALDH1A3,ATP6V1A,CHPT1,GALK2,GLB1,ITGA1
Negative_3904	ADC,EDA,GP9,ME1,AGMAT,ALDOC,CAPN2,CCNB3,CCR10,CDKN1B,CNDP1,COMT,CYP2D6,DDX4,DDX55,DOCK1,DPYD,GMPS,KIR2DL3,MAP3K12,MAP4K4,NFYA,OR52N1,OR5D16,OR5F1,PIGV,PPM1B,SARS,SHC4,SIAH1,SMARCA2,SRGAP2,ST6GAL1,SV2B,WARS2,SUV420H1
Negative_3905	DCK,LPL,ABL2,ALCAM,ALDH3B1,ATP6V0B,BUB1B,CACNB1,CAMK2D,CDK2,CTNNA3,DIAPH2,DUSP2,DVL2,ENTPD4,EPHX1,FGF8,GLI2,GSTM1,HLA-G,HSD17B7,IARS2,IFNA21,IMPDH1,MAPK7,MDM4,MYH11,NADK,NANP,OR2M4,P2RX6,PARD6A,PECAM1,PFN3,PLAT,POLE,PPP2R2C,SMC1A,SMPD2,ST3GAL4,TNFSF10,TXNDC12,WNT5B,TNFRSF1A
Negative_3906	DAO,GP9,JUN,AKT3,ALCAM,ALPPL2,ANAPC2,APRT,AXIN2,CCND3,CES1,CXCL3,DHRS9,EXTL1,GNAI3,GNAQ,GNPDA2,IFNG,INPP1,KNG1,LDHAL6B,NFYA,OR10Q1,OR4C6,OR52J3,OR7D4,POLA1,PPBP,SFRP4,SHC2,SLIT2,SOAT2,SPHK2,SPTLC1,TACR2,TAS2R13,TAS2R42,WARS,SERPINA1
Negative_3907	ALOX15,ATP6V1F,BIRC3,NAT2
Negative_3908	CACNA1D,GRB2,KCNQ1,MCCC2,ORC4L
Negative_3909	DLD,FUK,ID3,TAT,ACSM2A,ANAPC11,APPL1,C9orf95,CHAD,COL2A1,CSF2RA,DDX47,EPHA8,FASN,GNAZ,ICAM1,IL13RA2,MKNK2,NAT1,NFYA,NOTCH2,NRG2,OR10A3,OR10H3,OR51B2,OR51E1,RASGRP1,RASGRP3,STX16,TUBA1B,TYK2,VDAC2,WNT10B

Negative_3910	EBP, LEP, LPL, TXK, ACSL6, ALG8, AOC2, BCMO1, CAMKK1, CD48, CDKN2A, CYP1A2, DHRS3, EXOC7, HSD3B7, HYAL2, IFNA17, LDHA, MAP2K4, MAP2K6, MFNG, OR11H4, OR13A1, OR4L1, OR4M2, OR5AR1, OR5H6, OR5T3, PIAS2, POLE, RAD51, SPRY4, TAS2R5, TGDS, TJP1, VDAC2, VEGFA, ZMAT3
Negative_3911	F3, GLA, ACLY, ADAM17, ADORA2A, BMP8A, CD226, CD38, CDC2, CLDN20, CNTFR, CYP11A1, DAPK1, DGKQ, DNMT3L, DRD1, EPOR, GALNT9, GCNT1, GRIN2B, HDAC2, ITGA3, LAMB2, MAPK9, OR52K2, PCYT2, PIK3CD, POLR2H, PON1, PRODH, PRSS3, RASSF1, RFX5, SCIN, SDC3, SMAD7, TYMS, UGDH, XYLT2
Negative_3912	GLS, LHB, MPL, ACADL, ACO2, ACYP2, AOC3, ASS1, BMP8A, C5AR1, CHIA, CHKB, CLDN20, DNMT3L, E2F2, E2F5, EPHA3, FGF1, GABARAP, GGCX, HNF4A, IKBKE, IRAK4, KIR2DS5, MGAT1, MYH6, NCF4, NME2, OR8B12, PDE7A, POLR2E, PRKAG3, SMPD1, STX10, TSTA3, WEE1, ATP6V1G2
Negative_3913	CD2, ABAT, APOC3, AXIN1, BDH1, BUB1B, EARS2, GCAT, HTR4, KNG1, OR8A1, PPP2R5E, RAC3, RUNX1, HLA-DRB4, PAFAH1B3, ST6GALNAC3
Negative_3914	C9, IDS, MAG, ACY1, ADCY6, ALPI, CDK6, CER1, CNDP1, COL4A1, CYP2B6, CYP3A5, DGKD, EGLN3, EPOR, FGF6, GSTM3, HSD11B2, HSPA1B, HSPA8, ICOS, IL10RA, IMPA2, LCAT, LTBP1, NCR1, NRXN3, PPOX, PPP2R1A, PROS1, PSPH, SDHB, TFPI, VKORC1, WARS2, HLA-DPB1, HLA-DRB4, TNFRSF11A
Negative_3915	TNF, AARS2, ACAD8, ATP4A, BAAT, CLDN14, CLDN16, CNTF, COMT, CTF1, CYP26C1, CYP2A7, CYP2B6, DGKZ, DRD1, EPHA7, FZD7, HSP90B1, IDH3B, IL25, ITGB5, KIR2DS4, LTC4S, MAN1A2, MAP2K6, MLYCD, OR1E2, OR2T29, OR51B4, OR6C1, OR8K3, PGAM4, PITX2, PLCD1, PRKY, PTCRA, PTPN1, PYCR1, RBL1, SEPHS1, ATP6V0A1, CACNA2D4, SERPINC1
Negative_3916	BTD, DHH, F8, PVR, ABL1, ABLIM3, ACSL6, ADCY5, ALDH1B1, ARPC1B, ARRB1, ATP5J2, B3GAT3, BCAR1, C5AR1, CACNG3, CFLAR, CKS1B, DHX58, DKK4, DMGDH, DUSP1, EDA2R, EPHB3, FLOT2, FZD9, GSTM3, GSTZ1, IL29, LIPF, MAP3K5, MGAT3, OR2G3, OR4X1, OR52I2, PPP1R3A, PVRL4, SGMS1, SPTAN1, TP53I3, UCP1, WNT3, WNT8B, SUV420H2
Negative_3917	ACP5, ADCY3, CXCL6, NAMPT, NMNAT1, OR6K2, PYGM, RELA, SEPHS1, UAP1
Negative_3918	CHPF, CIITA, CLDN4, GMPPA, NRG2, OR2T10, OR4Q3, PLCZ1, PNPLA3, PPP1R3D, PRKACG, SLC27A4, MAPK8IP1
Negative_3919	C1S, GK, IL7, LYN, ADRA1B, ATP4A, B3GAT1, BET1L, BUB1B, CDC27, DAAM2, EIF4E2, ENAH, GLI1, GSTO1, HARS2, ITGA6, LAMA4, LLGL2, NRXN2, OR2AG1, OR6B2, OR7C2, OR9I1, PIK3R5, PPP2R5C, PTGER3, RAF1, RXRA, SUCLG2, TAS2R13, TAS2R49, TLN1, WNT7A, YWHAG
Negative_3920	GIT1, HTRA2, IARS2, IFIH1, IGF1R, ISYNA1, MAD2L2, NOTCH2, NT5C, PDE7A, PLXNB1, PPT1, PTGES, TMLHE, TNFRSF18
Negative_3921	C9, ENTPD1, GNAL, POLR2K, TRPM5, WNT7A
Negative_3922	ACVR2B, ALOX15, AQP7, AXIN1, BLNK, CACNA1I, CD34, CER1, CLDN6, CLDN9, CNTF, CPS1, DDX56, EPHA2, EPHA4, FGF7, FGFR2, GMDS, IGFBP3, LDHD, NAT5, NT5C, OR5L2, PARD6A, PARVG, PDX1, PFN3, SESN3, SKP2, STAT5B, STAT6, TBL1X, TIRAP, TNFSF12, TUBB, UBE2L3, UMPS, VAMP4, ATP6V1C2, PAFAH1B1
Negative_3923	TNC, ADORA2A, ANPEP, APH1A, BMPR2, CAMKK1, CSNK1E, CX3CL1, CYP3A7, ETS1, FABP1, FMO5, GADD45B, GALNT9, HMOX2, IDH3B, LEPR, MAPT, MDM2, MEF2C, MGAT4B, NOTCH3, NUMB, OR4N5, PARD6G, PYCR2, SC5DL, TPMT
Negative_3924	CD2, CFI, PXN, SMO, ARHGEF4, CHEK2, CHKB, CKS1B, CTSG, DUSP16, IL29, MMP7, MTFMT, PDX1, PLCB2, PYCR1, RASGRP4, VANGL1, WNT3, WNT4
Negative_3925	CRK, DCK, IL9, OTC, ACY1, ADCY3, ALPP, ARSB, CBR1, CDC16, CPT1A, DUSP16, ELK4, EPHA3, EXT1, GALNT13, GAMT, GSTO2, HLA-DRA, HTR7, IFNGR1, ITGA9, JAG1, MAP4K2, MAPK1, MCCC1, OR2AG1, OR4F15, OR5M3, OR6N2, PAK4, PIAS1, PPP1CC, RASSF1, RFWD2, SQLE, STX16, TUBB2C, UCKL1, WNT7B, ATP6V0A2, PAFAH1B1

Negative_3926	MET,PLG,SPN,TK2,TNN,ALPP,ARG2,BMPR1A,CACNG8,CBR1,CCL8,CCND2,CCNH,EI24,EPHA4,EPHX1,FASLG,FTCD,IGF1,IL13RA2,IL15RA,IPPK,ITGB5,NT5C1B,OR13C2,OR1L1,OR7A17,PCYT2,PDE11A,PDK1,SUFU,TUBA1B
Negative_3927	AR,B3GALT1,CCNB2,CTSS,DARS2,EDA2R,ENTPD4,GDF7,HPSE,LIAS,MASP1,MGST2,NCF4,NKD2,PDK1,SDHD,SEMA3B,VDAC3
Negative_3928	FH,TK1,AVPR1B,C1QA,ENTPD3,FZD1,FZD9,GALNTL5,GPAM,GSTM2,GUSB,IRF5,MALT1,MAML3,MAP4K2,MKNK2,NKX6-1,NME6,NSDHL,OR10A2,OR4P4,OR51A4,OR5B3,OR6C3,PDE6D,PIK3CD,PIP4K2C,PTPMT1,RARS,RASSF5,TPMT,ATP6V1G1
Negative_3929	C4B,DBI,PRL,AKR1C2,ALDH1A3,APOE,CACNA1F,CNTN1,COL4A6,FZD10,GTSE1,HHIP,LGMN,OR5B21,PAK7,PFAS,RAP1A,SMURF1,ST6GAL1,TSHR,ST6GALNAC4
Negative_3930	GBA,ACSM1,ADCY5,CASP7,CDS2,CLCF1,DGKD,ERO1L,HAGH,HEXB,HTR2C,MYH4,NFASC,OR2AE1,OR4F4,OR52N1,PARD6A,PIK3R4,RIPK1,STX6,TAS2R4
Negative_3931	HYI,AADAC,ACOX1,AHCYL1,APBB1,CASP10,CCL3,CLDN8,CREB1,CYP2F1,DLL1,DPYSL2,FLOT2,GALT,GNAZ,GSTO1,HMGCS2,IFNA4,IRF3,MAOB,MGAT5B,MYL2,OR11L1,OR4F15,OR5P2,OR8B8,PER2,PHKA2,PIGK,PPAP2B,PYCR1,RUVBL1,SEMA4B,SOAT1,ST8SIA1,THBS2,TJP2,UGT2B17,WBSCR22,WNT1,WNT2,YWHAE,ATP6V0E1
Negative_3932	TNC,ACTA1,ACVR1,ADCY1,ADCY3,ALDH1L1,APC2,ATG3,B4GALT4,CYP7A1,DHRS9,EHHADH,ETS1,FCER1G,FZD4,GCLM,GIT1,GUCY1B3,LIPA,MS4A2,NPR1,NRAS,OR3A1,OR4B1,OR4K1,OR5T2,OR7C2,OR8D2,PANK3,PPM1A,PRKY,PRPH2,PTGES,RBL1,RBX1,SEMA4G,SOAT1,SPHK2,TLN2,WNT1,PPP1R12A
Negative_3933	CGN,F8,GP6,ME3,ABLIM2,AKR1D1,ALCAM,ANPEP,BIRC5,CARS,CLDN11,CNTN1,CPT1C,CXCR6,CYP27A1,FCER1G,GNG3,ICAM1,IDH3B,IL1A,LIPG,LYZL1,MAML3,MYLK2,OLR1,OR2A42,OR2A5,OR5B3,OR5D14,PIGK,PLA2G10,SOCS4,TNFSF10,ULK2,WEE1,CACNA2D1,PAFAH1B2,PLA2G12A
Negative_3934	HAL,ACSS3,CHAD,DEGS2,DFFB,DHCR7,GAD2,GLUD2,HADH,HYAL1,MAP3K13,NME7,NR5A2,OR10A5,OR4A16,POLE4,POLR2B,TUBB2B,UPB1
Negative_3935	GCK,ACOT12,ALG10B,BAI1,CD86,DHCR24,DPAGT1,ERBB4,GRM1,ITGA9,KLRK1,MAPK14,MAT2B,OR10AG1,OR51B4,POLE3,POLR3K,PPBP,PRKCB,SEMA3E,SRGAP3,ST3GAL2,WNT11
Negative_3936	C8G,MTR,AADAC,ACAD9,ASAH1,ATP5G3,BMP5,BMP8A,CLDN1,CTBS,DDIT4,DGKA,DHCR24,EDNRA,EIF4B,ESAM,FGF1,FLOT2,FTCD,GART,GCAT,GYS2,LIMK2,MAP4K3,MAPK12,METTL6,MITF,NCKAP1L,NFKBIE,OR2A25,OR7G2,PRKAR2A,PRSS3,RASA2,SDC1,SPAM1,SREBF1,STEAP3,SUV39H2,TPM3,TUBA1C,UGDH,VASP,SERPINB5
Negative_3937	CAT,CACNB4,CADM1,CCL24,CD244,CXXC4,CYP11A1,GNRH1,GSTA4,GUCA1C,MAN1A1,MAN1B1,P4HA2,PGAM4,PIK3R1,PLEKHA8,PLTP,PTPRR,RASA2
Negative_3938	ACACA,B3GALT6,CBLB,CCL16,CD79B,DHCR7,HSD11B1,IL17RA,INHBC,MALT1,NAT2,NME2,NMNAT3,NUDT8,OR6T1,PLCD4,UROD,VANGL1
Negative_3939	FAS,GSS,APAF1,CFL1,CHRM3,CHST11,CSNK1A1,DDX18,DUSP6,DUSP8,ETNK2,GANC,GPD1,GRIA3,IL17RA,OR52E8,PDE6D,SAT2,STX3,THBS2,TRADD,ATP6V0A1,TNFRSF10D
Negative_3940	GNRH2,IAPP,ITGA4,NFYC,SOCS3
Negative_3941	BCL10,CASP10,CHEK2,CHRM2,CREB3,GSTA1,IFNW1,IL2RB,INADL,NRXN1,OR2AE1,OR2AG2,OR2W1,OR51G1,OR5T2,OR6C76,P4HA1,PAK1,PCK1,PDHB,PFKFB4,PLA2G5,POLR2B,PTGS1,TRPC1,HSD17B12,MAPK8IP3

Negative_3942	BTD, LTB, SPN, ACO1, APPL1, AQP7, ARHGEF7, ATP5G3, BMP2, CCL16, CCNE2, CYP2J2, GADD45A, GOT2, GYS1, H6PD, HTR2C, IFNA2, ITGA4, ITGA6, MAP3K3, MGST1, MYH7, NOS3, OR1J4, OR1L4, OR51Q1, OR8K1, PDE1B, PDE8B, PKLR, RARA, RASSF5, RRM2, TAS2R7, UPRT, WNT3, ZMAT3, ATP6V1E1, SUV420H1
Negative_3943	GDF5, OR7G3, P2RX6, PIGV, ATP6V1E1
Negative_3944	ILK, ACP5, ACPT, ADIPOR1, ANAPC11, CNTN1, DDX23, EDAR, EXTL2, ICOS, IL2RG, KIR3DL2, MAP2K5, NCK1, NCOR1, OR1J4, OR2Y1, ORC2L, PIK3CB, PITX2, PPP1CC, SARDH, TNFRSF8, TP73, TNFSF13B
Negative_3945	BID, SRM, CACNA1D, CCNB3, CSF1R, CYP4F3, GRIA2, GSTK1, KLRK1, MPP5, OR5V1, OR6K6, PRKG1, SOCS2, TICAM1, HLA-DRB5, TNFRSF13C
Negative_3946	AK1, CSK, FST, AMDHD1, BAIAP2, C1QB, CYP26C1, CYP2B6, DAG1, DDX56, FARSB, GIT1, GJA1, GUCY2D, IDH1, IMPA1, JAM2, KLRC4, LAP3, LARS2, LIAS, MAD1L1, MAD2L1, MAP3K13, MGST1, MPDZ, MTHFR, MTMR2, MYLK2, NFYA, ONECUT1, OR2T1, OR5B12, PIGM, RETSAT, SCIN, SEMA4B, SHISA5, SMAD5, TAS2R4, TGDS, TLR3, TNFSF18, TRAF6, TYMS, WHSC1, WNT4, TNFRSF10A
Negative_3947	LPO, ME2, AFMID, ALDH1L1, AOX1, ARHGAP5, BAIAP2, CALML5, CLDN16, COL4A6, DGKD, EDAR, ENTPD6, F2RL2, HEXB, IFNA6, IL24, KLRK1, MAPK8, OR2F1, OR4L1, PCCB, PDE3B, PDHB, SQLE, TIRAP, TPK1
Negative_3948	HGF, ACTA2, CSNK1G1, FRAT2, OR51B4, RPE65, SMPD1, TNXB, WNT2
Negative_3949	TK1, ACHE, ALDH1B1, ARPC1A, ATP5B, BAAT, BDKRB1, COMP, CXCR5, EFNB2, GCDH, GGT7, GUSB, HIF1A, HLA-DMA, HNF1A, IDH1, ITGA2, ITGB1, MAPK10, MINPP1, NRAS, NRCAM, OR1E2, OR2A2, OR5A1, OR5L1, OR6C2, PANK1, PIP5K3, PLXNB1, PPOX, PRKX, PRPS1, PTK2, RASSF1, SETMAR, SOCS2, SPHK2, TGFB1, WEE1, C1GALT1C1, PNLIPRP2
Negative_3950	ATG3, DOT1L, IL18R1, KITLG, P2RX2, PTCH1, TAS2R40, WWP1, ARHGEF12, CSNK1A1L
Negative_3951	RET, TFG, UBB, AASS, ABLIM2, AFMID, ALS2, B3GALT1, CCL3, CHST11, CXCL2, CYP1A1, CYP26B1, CYP2C19, DNMT1, E2F3, EHMT2, ENTPD8, EPB41L2, FADS2, FGF2, GOSR2, GRIN2B, HCLS1, IDI2, INPP5B, IQGAP1, LMO7, MAP3K3, MYL9, OR10K2, OR4C16, PGS1, PHKB, POLR1C, POLR3A, SELL, SEMA4F, STX6, TYMS
Negative_3952	CHAD, COL1A1, GDE1, NFYA, OR13C2, PPP2R5E, VASP, VDAC2
Negative_3953	GHR, MIF, ACAT1, ALOX12, CAMK2A, CD8A, CMBL, CSF2RA, CYP27A1, EGLN3, ENTPD4, FCGR3A, FMO4, GNAI2, HARS2, MYH7B, NR5A2, OR10H3, OR8H1, PFN4, PI4KA, PRKAR2A, RERE, SSH2, TBXAS1, TPMT, TUBB2B, WNT4, ATP6V1G3
Negative_3954	SI, BUB1B, CYP2C9, GIT1, IL22RA2, MAP2K5, NFYB, OR1E1, MAPKAPK5
Negative_3955	DLD, ACAD9, ACVR1C, ARHGAP5, ARNT2, ATP6V1A, B3GNT3, CCL28, CD14, CD74, ENPP2, EPB41L3, ERBB3, FGF1, GFPT1, HMGCR, IPMK, ITGA7, KCNQ1, KLK3, KLRC1, LYZL1, MAP2K2, MRAS, NR5A2, OR14I1, OR1S1, OR2T10, OR4L1, OR5AN1, OR6K2, PLAUR, PLXNB2, PMM1, POLR1B, POLR2I, SARS, SELPLG, SH2D2A, SMARCA2, STX5, WNT8A
Negative_3956	CFI, ID4, ACTA2, ALAD, ATP5F1, CAMK2G, CCND2, CHRM2, CSF3R, CSNK2A2, HMOX1, NCOA4, OR4X2, OR7D4, PANK4, PTCRA, TAS2R14, TUBA4A, WASF2, XCL1
Negative_3957	MSN, ACSM2A, ADSSL1, BACE1, CHRM1, CHST12, COASY, DLL3, FMO3, GGT7, HSD17B2, IDH2, NQO1, OR51F2, OR52I1, OR5B21, OR9G4, PDHB, PFKFB3, PIK3C2G, PIP5K3, RCHY1, STEAP3, SULT2A1
Negative_3958	CBS, MSN, ASAH1, WNT5A
Negative_3959	ME2, ALDH3B1, ARD1A, CD74, MAT1A, NAT1, ONECUT1, PFKP, TNFSF13B
Negative_3960	ADK, CS, DBI, IHH, PRL, SRM, CCL13, CCL4, CCR4, CNTF, CSNK2A1, CXCL5, DEGS2, DHRS4L2, DPM3, ESAM, FGFR2, GLUD2, GPAA1, HYAL1, MAP3K6, MAP4K4, MGAT3, OR10G2, OR11A1, OR1S2, OR2AG1, PARD6A, PIGA, PLA2G6, PPAT, RND1, STX7, TAS1R2, THBS1, TUBB3, ULBP3, VTI1A, ATP6V1E2, PAFAH1B3

Negative_3961	ACADM,ATP5O,CCL8,CCNH,CDKN1A,CLTCL1,CREB1,CSNK1A1,CXCL13,GALNTL4,GPD1,HSD17B7,IL21,IL21R,IMPA1,LTBP1,NFKB2,OR10Q1,OXSM,PARD6G,PHGDH,PSEN2,RFX5,TGDS,TSTA3,HLA-DPB1
Negative_3962	ZYX,BMP8A,BMPR1B,CACNA1H,CHAT,CNGB1,CNTFR,CREB3L1,CXCR3,CYP3A7,DTX2,EPHA3,EPHA6,F2RL3,FLOT1,FUT8,GALNT8,GNAS,GNRH1,GSTA2,HSD3B2,LRRC4C,MAP4K1,NAGLU,OR51D1,OR52K2,PDE8A,PER2,PIK3CA,THBS2,TRADD,ATP6V1E2,CACNA2D4
Negative_3963	C8A,CRHR1,HDAC2,INMT,LCP2,MAPK3,PLXNC1,PPBP,PTPRF,RAB23,RFNG,TOLLIP
Negative_3964	EGF,GP6,TK2,AARS,ALDOC,ARG1,ARHGEF4,ATP4A,CD3E,CTSB,CYP7A1,IL21R,LMO7,MYH10,NARS,NME7,NOX3,OR4S2,OR8J3,PAFAH2,PGLS,PIAS1,PIK3R4,PLCB4,PON3,SEMA4D,SOCS7,TGDS,TLR3,TOMM40L,ATP6V1C1
Negative_3965	ACADL,ACOX1,ACTA1,ALG12,CASP10,CCND2,CDH3,CHKB,FMO1,FPGT,GMDS,HBEGF,HCLS1,IFNA4,LDHAL6A,LIMK1,MAP2K7,NUMB,OR10G2,OR2D2,OR51F2,PLEKHA8,PLXNB2,POLR2D,POLR2K,PTPN1,PTPRM,RRM2,SELE,SGMS2,TNXB,UNC5A,VDAC2,WASF1,TNFRSF14
Negative_3966	ACAA2,ADCY5,CD8A,CLDN9,COL6A2,CTSS,ENTPD6,GYS2,IDH3G,MAP4K3,MGAT2,NFKBIA,OR10S1,OR4K1,OR6K2,PNPLA3,RPS6,SETMAR,SLC12A2,SLC2A1,STAT4,ULK1,VDAC3,YWHAG
Negative_3967	MAX,MPI,SRM,ACHE,ACSL6,ADCY3,ALS2,ATP5G1,ATP5J,BACE1,BUB3,CD44,CLDN18,CLTC,CTF1,DAPK1,DDIT4,DDX47,DPYD,H6PD,IL28RA,MAPK3,MYL2,NUMBL,OGDHL,OR10A6,OR51G2,OR52E8,OR7A17,PCYT2,PHKA2,PIK3R1,PKM2,YWHAZ,TNFRSF13C
Negative_3968	AK2,FYN,NNT,AACS,AKR1B1,ALOX15B,ARSE,ATP5C1,BIRC2,BST1,CCL4,CCL7,CYP2A13,DUSP5,EDNRB,EPHA5,FZD6,HLA-F,IL22RA1,IRS2,LTBR,NGEF,NT5C1A,OPLAH,OR4C12,OR51A2,OR51S1,P2RX3,PDE6D,PLD2,SMAD4,SMAD9,STAT5B,TAS2R1,TRAF3,TUBB4Q,WNT11,TNFRSF13C
Negative_3969	JUP,IL20,LIMK1
Negative_3970	ATP5G3,CD47,FZD8,OR2D2,PROC,SDC3
Negative_3971	SP1,ACAA2,ADRA1B,APBB1,ATP6V0B,BCL10,CACNA1B,CCR8,CD14,CD44,CDKN2A,CLDN5,COL4A4,CSDA,DDX54,EPHX1,GNAI1,IKBKB,KIR2DL3,LDHA,MCM5,NAE1,NKD2,NMNAT3,OR2W1,OR4C12,OR4F5,OR4F6,OR5A2,OR5T1,PAK2,PAX4,PFN1,PLOD3,POLD3,POLR3K,PRKG2,SEMA4C,TICAM1,TNFSF10,TYRP1,UCK1,WNT5B,ATP6V1C2,CACNA2D4
Negative_3972	AK1,RB1,ADRA1A,AKT1,CHDH,COL4A6,CREB3L2,CX3CR1,CYSLTR2,DHRS4,DRD5,GALNT11,GANC,NARS2,NCOA4,OR1L8,OR6M1,OR9Q1,POLA2,PRIM1,SLC27A5,TIAM2
Negative_3973	ACSM4,DDX47,FGF5,OR10H3,OR4C12,OR4L1,OR52E4,OR5C1,RDH5
Negative_3974	CD2,GBA,TYR,ALCAM,ALDH1A3,CCNG1,CD55,CD79A,CLDN20,COL2A1,DDHD1,DDX41,FLT3,GNB1,HLA-DRA,IL6ST,KRAS,MAML1,MAN1A1,MPZL1,NFYB,NUDT8,OLR1,PDE3A,PIGB,POLE3,PPP3CC,PTGER1,PYCRL,RAPGEF1,SLC27A4,SORBS1,MAP3K7IP2
Negative_3975	ASL,EBP,MTR,SYK,ACACB,ADH4,ADIPOQ,ALG13,ARSA,B4GALT3,CDC42,CDH4,CLDN17,DNMT1,DUSP14,ENTPD3,EXT1,FMO2,GALE,GALK1,GALK2,GLUD1,GNAI3,GNPNAT1,IL1R2,INHBC,INPP1,IRF5,LRP5,MAFA,MGAT3,NUDT2,OR11G2,OR1I1,OR3A2,OR4K5,PANK3,PDPK1,POLR1D,PRKCE,RASGRP1,SMURF2,STAT2,TACR1,TAS2R5,TIAM1,TOMM40,WWP1

Negative_3976	C1S, GH2, LPO, TNN, ALPI, ATP5C1, CACNA1S, CCKBR, CLTA, CLTCL1, CMAS, DDIT3, DDX19A, DIAPH3, FMO5, FZD2, GALC, GBA3, GLYCTK, HEXB, HSD17B7, IL18R1, INPPL1, IQGAP2, NARS2, OR1M1, OR2T34, OR8H1, OR8K3, OR9A2, OSMR, PECAM1, PHKB, PNPO, PPA2, PPP1R1A, PPP1R3B, PRKAG3, PRNP, RERE, SULT2B1, SYNJ2, UBE2K, VTI1A, WASF3, YARS
Negative_3977	TK2, ADRA1D, ARSE, B4GALT7, CACNB1, CD58, CDC14A, DEGS2, GPT2, GRIN2A, HHEX, IFIH1, IL10RB, IRAK4, LAMB1, LILRB3, MIOX, MYH7B, MYLK, NCK2, NCKAP1L, NME2, OR2T1, OR5B21, PARD6G, PIGP, PTK2B, PTPMT1, PVRL2, PVRL4, RFXAP, UBA7, UCK2, B3GALNT1, TNFSF13B
Negative_3978	ESPL1, GLG1, OR9A4
Negative_3979	FYN, HYI, SCD, ACLY, ACOT11, AGPAT3, CDC2, CDC23, COASY, CSNK2A2, CYP2C19, DVL1, GALE, GARS, GMPPA, GP1BA, GPD2, HGSNAT, HNMT, HPSE2, INPP4A, ITPKA, MARS2, MGAM, MKNK2, OR4C6, OR4M2, P2RX2, POLA2, RPS6KA5, SMC1A, TFPI, THBD, TRAF4, TRPM5, TNFRSF10D
Negative_3980	IKBKB, OR5H6, OR5P3, PRKAR1B, SPRY3, UGT2B10, YWHAH
Negative_3981	AMT, ID3, ARPC2, C1QB, CD70, CHRNA7, CYP1B1, EIF4E, GDF5, GLI3, GUK1, HADHB, IFNA16, IRAK3, KLRC2, METTL2B, NR4A1, OR10G7, OR1L4, PAPSS1, PGK2, POLD2, PTPN5, RPE65, SLC2A2, TACR2, UGP2
Negative_3982	ATM, CBL, EDA, FST, TK2, AADAT, AMPD2, C5AR1, CHPT1, CYBB, CYP2C19, E2F5, FCER1G, FGFR3, FLT3LG, FOXA3, GGCX, GGT6, GZMA, HSD3B2, JAM3, KIR2DS5, NARS, OR6C2, OR8A1, PGAM4, PIK3C2G, PIK3CD, POLR2A, PRKCA, RRM1, SHC2, ATP6V0A1, HLA-DRB3, HLA-DRB5
Negative_3983	NLK, RB1, ADRB3, AGPAT2, AGXT, AK3L1, AKT2, ALDOA, ANAPC1, APOA2, B4GALT7, BCAR1, CDC2, CDKN1C, COX10, CYP2A7, DOT1L, G6PC, GNAL, H6PD, IDI2, IKBKB, IL15, IRS4, ITPKA, KLKB1, MCM5, MFNG, NCAM1, NFATC2, NFKBIA, NFKBIE, NOX1, OR10C1, OR8G1, PIGT, PIK3CG, PMM2, POLR3G, PON1, PRPH2, PTGES, RRM2, SMAD6, TLN2, WNT11, ATP6V1G2, HSP90AB1
Negative_3984	CR1, F2, GAA, MUT, ABLIM3, ATF4, CCDC6, CNDP1, CNGA3, COMP, CRB3, CTSB, CYBB, CYP27A1, CYP4F2, E2F1, ELK4, FARSA, FGF14, GALNT11, GALNT12, GALNT6, GLUD2, GUSB, HSD11B2, IL13RA1, MYH14, NAMPT, OR52N4, OR5B12, OR5P3, PANK4, PDE3A, PDGFRB, PI4KA, PIK3CD, POLR3D, PROS1, SLC2A2, SPTAN1, SRGAP2, TDO2, TRIP10, TYMP, WNT9B, EIF4EBP1
Negative_3985	ALDH4A1, CACNA1I, DHRS4L2, GALNT9, MYH4, NT5C, OR1A1
Negative_3986	LHB, ADH1A, ALG3, APOA2, ATP4A, BAIAP2, FCGR3B, GANC, ITGA1, PDGFC, RFNG, TAS1R1
Negative_3987	DDC, AMPD1, ANAPC4, ARNT, ATP5A1, B3GNT5, CDKN2A, DAXX, DUSP10, GNA13, GPX7, GSTT1, HTR2A, MAGI3, MGST1, MYH3, NARS2, OR13H1, OR14I1, OR4C13, OR51B6, OR52E8, OR5A2, PPP3CA, TJP3, TOMM40L, ATP6V1B2
Negative_3988	CFD, CR1, FAH, ACADVL, ATP5B, CLDN9, CYP11B1, DVL2, ENO2, FZD2, HLA-F, HTR2C, IFNB1, IL1RAP, IRS2, MAPK3, MGAT2, NFATC4, NRXN3, OR10H2, OR4C15, OR52M1, OR5B21, PERP, PIGS, PLK1, PTPN6, TUBA8, ATP6V0D1
Negative_3989	C8G, PLG, ACAD9, AMPD2, ATP5D, BET1, BET1L, CCDC6, CCL13, CCL2, CFTR, CLDN2, COL3A1, EFNA3, FLAD1, FMO2, GNRH1, ICAM3, IL1B, ITGB1, KIR2DL4, LTC4S, MAD2L2, METTL6, NEUROD1, OLR1, OR10P1, PGAP1, PLCE1, PLCG1, PPID, PPP2R2A, PRPS2, RETSAT, RRM2B, SDC2, SHMT1, STAT2, TAS2R41, UGP2, WHSC1, HLA-DRB3, HLA-DRB4
Negative_3990	APP, DAO, GNS, ACVR2B, ALPPL2, ATP6V1A, B3GNT4, CCR3, CYCS, CYP2C9, CYP4A22, CYSLTR2, DDX47, FBP1, GARS, GCLM, NANS, NRXN1, NUDT2, NUMB, OR4K2, OR8K3, PDE6D, POLR3H, PPP2R1A, PTPRM, RAC1, RXRG, SEMA3G, TAS1R1, TAS1R2, TKTL1, TUBB3, ZFYVE16, ATP6V1E2, CTNNBIP1, TNFRSF11A
Negative_3991	CAT, DAAM1, ITGB1, OR52M1, PRKAR2B, SREBF1, SSH2, TIRAP, TNFRSF1A

Negative_3992	BPGM, CACNA1A, CACNA1C, CACNB1, CD22, CD8B, CHD8, CKS1B, COL6A2, CSNK1G3, DLAT, E2F2, ENPP7, ERO1L, GAL3ST1, GCH1, GNRHR, HTR7, ITGA1, KCNQ1, MAGI2, NEFL, OR1G1, OR2L3, OR5AS1, OR6C2, PDE1A, PNPLA3, PRSS1, RHEB, RRM1, SULT1E1, SV2C, TUBB2C, VAMP2, WNT16
Negative_3993	CEL, EDA, HYI, SDS, ADAM17, AKR1B10, ALG9, AMD1, ATP4A, ATP5A1, ATP6V1H, CD59, CHRM5, CLOCK, CTNNA1, CYBA, CYP2J2, ELK4, FZR1, GDF7, GGT6, IFNA7, NCKAP1L, NFATC4, OR11H4, OR5K2, PCK2, PFKFB4, PKMYT1, PPCDC, RALBP1, TAS2R41, UNC5C
Negative_3994	APP, F12, GHR, GK, VCL, ADCY6, CER1, CHDH, DAAM2, FZD9, HAGHL, MCM2, MRAS, NEFH, NEU3, OR52K1, P4HA3, PFN1, PIGB, SESN2, SLC33A1, SMARCA2, TNFSF18, UAP1, VAMP4, B3GALNT1
Negative_3995	F12, INS, IVD, JUP, NF1, PVR, ALG9, ATP5D, ATP5O, CCDC6, CCR1, CD48, CD74, CES2, CSF3, CYP4A22, DDX52, DOCK1, E2F1, EGLN3, FARSB, FGF17, FLOT2, GNAL, HSD17B7, LRP6, MAP2K2, NANP, NCK2, NCR3, NOX1, OR10AG1, OR52E6, OR6C75, ORC1L, PAK6, POLA1, POLR3A, PPP1R3C, RHOD, SMPD2, SOD1, SSH3, TRIP10, VCAM1, VDAC1, XYLT2, ST6GALNAC6
Negative_3996	CCL26, GRHPR, IL1A, NT5C, OR51D1, PIP4K2B
Negative_3997	BCR, CD4, KHK, ME3, MYC, DBF4, DUSP4, E2F5, EHMT2, FADS2, FMO1, IL22RA1, IL23A, LCAT, LYPLA2, PARVB, PFN1, PIK3CA, POLR3B, PRKAB2, SDHA, SMAD6, SMURF1, TOMM40, UAP1
Negative_3998	IL6, ALDH3B1, CD47, CLTA, DEGS1, DTX2, EHMT2, IFNGR1, NTN1, NTRK2, PIGM, PIGW, PLXNB2, TBL1Y, UGT2B11
Negative_3999	SI, AGPAT2, AGXT2, BAAT, BCL2L1, CAPN2, CCR5, CXCL9, GRIN2A, IRF9, OR2B2, OR4E2, PHPT1, PVRL4, RAP1B, SIGLEC1, TSHR, WNT7A
Negative_4000	CBS, CS, ARG1, CDC7, CLCA2, DUSP2, FIGF, FUT7, GMPR, GNB1, HMOX2, ICAM3, IL10RA, IMPDH1, NKX3-1, NUDT5, OR10AD1, OR2A14, OR2K2, OR2M3, OR2T10, SEMA4F, SEMA7A, STX12, TGFB1
Negative_4001	CBS, CRK, LCT, ALG9, ATP6V1F, B3GALT5, B4GALT6, BIRC2, CACNB1, CAMK2G, CCND2, CCNH, CCR5, CCR6, CD70, CDC14A, CDO1, CYP2E1, DRD1, EI24, EPHA2, GUSB, HTR2A, IL28A, IL9R, INHBC, INMT, LAMC2, LIMK2, LTBR, OR10K1, OR11H4, OR13J1, OR1L8, OR8A1, OSMR, PDX1, PIGK, PPP3CC, PRKAB2, PRKCQ, PSENEN, SESN3, SH3BP2, STK3, THBS2, TSC2, VAMP1, TNFRSF17
Negative_4002	CD4, GP9, CFL1, CLDN19, DDX23, DUSP9, FABP3, FDPS, GSTP1, HSD17B2, IFNGR2, KLRC1, MDH1, NFKBIE, OR10AG1, OR4Q3, OR51B6, OXSM, PNPLA3, PRKAG3, RFXANK, SOCS3, SULT2A1
Negative_4003	EBP, ABLIM2, AKR1B1, ATF4, CAPN2, CCL16, COMT, CSNK2A2, DTX3L, FSHB, GALNT11, GRB2, IBSP, ITGB4, KLRK1, LYZL1, NSDHL, OR2L2, OR2M3, OR5D16, OR6C75, OR8K1, PDE7A, PIK3C2G, PPARG, PVRL2, RAB23, RAP1B, RASGRP3, RERE, RRM2, RXRG, SEMA4F, SEPHS1, SNAP25, UROS, ST6GALNAC4
Negative_4004	APP, ACLY, ALG8, BIRC2, DHRS4L2, GLS2, GUCY2D, IFNA16, IL10RA, NARS2, OPLAH, OR10A5, OR2AG1, OR4A5, OR4K17, OR51E1, OR52B4, PANK3, PARD6A, POLR3B, RFXAP, SCD5, SDHD, TBL1X, TUBA3E, UGT1A10, VAV1, XYLT2, ATP6V1C1
Negative_4005	C8B, AGPAT4, BMP6, CDC45L, CTSB, FUT9, GRIN2A, LAP3, LDHD, MAP3K6, MYH1, POLD3, PTK2, PVRL4, SUV39H1
Negative_4006	ADH1B, CHRM3, CLCA4, CNGB1, CRY2, GFPT1, IL20, NNMT, OR51G2, OR6K3, SEPHS1, SMAD3, TNFRSF11B
Negative_4007	AASS, CADM1, CDC25B, CXCL2, GNAI1, OR4F6, POMC, RRM2, SYNJ2
Negative_4008	GGH, MUT, TNC, ANAPC4, AQP7, CAMK2D, CARD11, CCL1, COL5A1, COX15, EPHA7, GALT, GLS2, GNAO1, GTSE1, IL10RB, INPP4B, INPP5E, IRS1, LTC4S, MMP7, OR13H1, OR2C3, OR7A5, OR9I1, PIGB, PPP1R3A, SPRY4, TRAF1, UNC5D, UXS1, B4GALNT1, TNFRSF17

Negative_4009	AK5,TNR,ALOX15B,AREG,BLNK,CDC23,CFTR,CNTF,CYP27A1,DGKZ,GADD45G,GNG3,GNMT,GRHPR,GSTA1,HES1,IDH2,ITGA11,LEFTY1,MCM5,MYL5,NRXN1,OR1L1,OR2T6,OR4F17,OR5AK2,OR9G4,PHKA2,POLR1B,RAC1,RERE,RHEB,RPS6KA3,SC5DL,SOS2,SPP1,SV2B,TLR9,WNT7B
Negative_4010	B3GALT4,CSF1R,DHDDS,IL17A,MAP3K2,PPARD,THBD
Negative_4011	BAX,ACVR1B,ARHGEF1,BLVRB,CCNB3,NUDT12,OR52I1,PAPSS2,PVRL4,TRAF5
Negative_4012	FYN,IVD,LIF,AGRN,ALG5,ASCC3,ATP6V0B,AXIN2,CACNA1F,CCL16,CCR8,CYP3A5,DKK4,F11R,FGF2,GLUL,GNB3,HSD3B1,IFNA4,IRAK1,KCNB1,LIMK2,MTAP,NAE1,NAGS,NCF4,OR5A2,ORC5L,PMM2,PSME3
Negative_4013	GSR,NLK,NPL,TYR,ABLIM3,ACACA,ACP6,COL4A6,FZD4,IFNA7,IFNB1,IL5RA,IMPA2,MCM6,MGLL,NFKB2,NOX3,NUDT12,OR12D3,OR4K2,OR8H3,PAPSS2,PLA2G2D,RPS6KA1,RPS6KA3,SEMA4D,ST3GAL2,STAT2,VAV3,WNT10B,PLA2G12B
Negative_4014	F7,LTB,PML,TNN,ACSM4,ALDH1A2,ANAPC11,ARPC5,ASRGL1,BAIAP2,CCL2,CCL25,CDC2,CHSY1,CSDA,CSF1R,CYP8B1,DDIT4,EDNRB,EIF4B,FZD1,GLUD1,HPSE2,IL10,IL15,OR12D3,OR4F15,PHKG1,POLR1E,PTCH2,RALBP1,RPRM,ST3GAL5,TAS2R48,YWHAH
Negative_4015	ACTN1,CD244,NT5C2,OR10G2
Negative_4016	AK7,CEL,DCN,AKR1C2,AKR1C3,ALDH9A1,ARSD,CAPN2,CCNB2,CREBBP,DDX4,DKK2,FLT3LG,GATM,MAD2L2,MCCC2,MYH6,NAE1,OR1D2,OR1J2,OR5B17,OR6C3,PLA2G4A,PLA2G5,POLD4,POLE2,PTPRB,SLC25A4,SLC2A2,ST3GAL4,TPI1,ULK3,WNT5B,ATP6V1G1
Negative_4017	C7,ALAS2,BCL10,C1QC,CES2,CLOCK,CYP4A11,DDX4,FZD10,GSTO2,NFX1,OR52L1,PDXK,PPP2R2B,TRPC1,WASL,YWHAE,CACNA2D3
Negative_4018	AK3,MSN,PML,ADCY6,ATP5L,C4BPB,CCR4,CHRM2,DEGS2,DVL1,IL13,NPAS2,PFKFB1,PRKACA,PRPS2,SDHD,THTPA
Negative_4019	BTD,SRM,XDH,ACHE,CLDN11,CYFIP1,DIAPH3,FCER1G,GDE1,GRIA2,MAN1C1,OR5D14,PFAS,POLR1A,RHOQ,SEMA4G,WNT8B,ZAP70,ATP6V1G1,CACNA2D3,TNFRSF11B
Negative_4020	AK2,FN1,GP6,PGP,ACVR1C,ALS2,ATP5G1,CCR3,COL4A4,EXTL2,FZD10,GALNT1,GAPDH,HAO1,IL28B,ITGA8,KCNJ11,MAP3K2,MAP3K5,METTL2B,MTHFD1,MYLK2,OR7A10,PIK3CG,PNPT1,PRODH2,PTCH2,RARS2,RASA1,TLN1,VAMP2,YARS2,ATP6V0A4,HLA-DQB1
Negative_4021	AK5,BTK,HGF,TKT,AKT1,ANAPC11,B3GNT2,BDKRB2,CACNB1,DIAPH1,DRD2,ENAH,FZD10,GSTO1,HSD3B2,MAP3K6,NEFH,OR1N1,OR7A17,PGK2,POLR2K,PRSS2,PTGES,RASSF5,SPRY3,THBS1,THBS3,ULK3,ZNRD1
Negative_4022	ADK,AMT,EGF,TNR,ZYX,ANAPC5,B4GALT4,CCL1,CFTR,ENPP1,FGF20,FUT8,GAMT,GUCY1A3,LYPLA2,MAP3K5,MGAT5B,OR1S2,OR4F6,OR51B6,OR8U1,PPARG,PRKACB,ST3GAL3,TAS2R10,TJP1,WNT7A
Negative_4023	F8,HDC,LAT,PC,PVR,B3GALT4,B3GAT2,BPGM,C4BPA,CLDN2,CPT1C,CYP1B1,DDX18,DGKQ,FGF2,FUT5,G6PC,GUCY2D,GZMB,HDAC2,HSP90B1,IL19,IRS1,LIPA,MTMR2,NTRK2,OGDHL,OR2B11,OR7G2,PARD6B,PLA2G2F,POLR2G,PRIM1,PRPS1,PYCRL,RASA1,SORD,TNFRSF10A
Negative_4024	TFG,ACP5,AGPAT1,CHRD,CXCR5,CYP2U1,CYP7A1,FMO1,IDI2,IFNK,INSRR,LARS,NKX2-2,OR12D3,OR1L6,OR4S1,OR51V1,PFN2,PIK3CG,POLR3A,TM7SF2,TSHB,UROC1
Negative_4025	CASP10,GALNT11,NPR2,OR2F2,OR2G3
Negative_4026	CGA,ACSS2,CERK,E2F5,NANP,OR51B6,OR51D1,PAK3
Negative_4027	C8G,ADRA1A,ARRB2,CCR2,CDC25B,CDC7,DGKG,FMO3,HHEX,IL11,INPP4B,ITGB3,MAPK11,OR52I2,PIK3R2,PPBP,TSHB

Negative_4028	ABL1,CCL13,CD14,CYP17A1,DHODH,EDNRB,FABP4,GGT6,GSTO1,IL13RA1,LIPA,MASP1,NCAM2,OR2T8,OR3A1,OR56A3,OR6N2,PIGL,PSME3,RPN1,STAT6,TNFSF14,VTI1A,XCR1
Negative_4029	APP,ARPC4,BCKDHB,BMP6,CHRM3,ENAH,GRIN2D,IFNA14,PHKG1,PMAIP1,SOS2,SPTLC1,WEE1
Negative_4030	FGG,AHCYL2,B3GNT3,CAMK2G,CCDC6,CD8A,CDC7,CHKA,CHKB,CSNK1D,DDX50,ENTPD8,FGF11,HTR2B,IL11,KIR3DL2,MAP3K14,MAT2B,NARS2,OR1L8,OR2Z1,OR6C3,OR9K2,P4HA3,PIP4K2A,PLA2G5,PLXNA1,PPP2R5E,PTCRA,RND1,SEMA3F,TAS2R4,TOMM40,TNFRSF11B
Negative_4031	GP6,MYC,ACAT2,ACSS1,ARHGEF2,ARRB2,CALML6,P2RX5,PDE10A,PLOD3,PPAT,TALDO1
Negative_4032	C9,ACOT12,ACOX1,B3GNT5,CFL1,DHCR24,EPHA5,GCAT,GNB3,GUSB,HAGHL,HTR7,IFNA13,LRRC4C,MTMR6,OR2K2,OR3A3,OR7C1,POLR2K,RAP1B,SLC33A1,SOCS7,ZMAT3,PAFAH1B1
Negative_4033	F11,TFG,ADH7,ARHGAP5,CACNG1,CPT2,CSNK1G3,CYP2A13,CYP2C18,DDOST,DHCR7,DTX4,EFNB1,FCGR3B,GRIA1,HBEGF,HHEX,IBSP,IDH3A,IDH3B,IL11RA,IL29,IL6ST,INHBB,MCM3,MTHFD1,NMNAT1,OR10G2,OR11H4,OR2T3,OR52I2,OR5AR1,PAPSS1,PLCE1,PLCG1,POLR3A,PPP2R2C,PROS1,RAPGEF2,RARS2,RPRM,RRM1,SFRP5,SMURF2,ST3GAL4,STMN1,TXNDC12,WARS2
Negative_4034	BTK,ACO2,ADORA2B,ALG3,EXT2,FZD8,GP1BB,IL12B,NEFH,OR2G3,OR52N2,OR6B2,OR7A17,PLXNA2,RRM2B,SHISA5,SERPIND1
Negative_4035	FGG,PGF,HHIP,IL18,OR51I1,PFKP,PSEN2,THBS4
Negative_4036	ADK,DCK,ALG8,ALOX15,ATP6V1A,BPNT1,CACNA1A,CALML6,CD70,CTBP1,DUSP6,ELK4,EPHB2,FUT1,IFNW1,IL6R,IL8RA,KIR2DL2,LAMA4,MYH11,OR2T5,OR51A7,OR51B6,OR52I1,OR5A1,OR9Q2,PDGFRA,PIP5K1B,POLR3D,PPP2R2B,PRSS2,RASA2,ROBO3,RPN2,SELE,TACR2,TBK1,HSP90AB1
Negative_4037	SPN,ACACB,ADCY8,APOA1,CCR10,CD46,DEGS2,ESAM,FGF21,GLUL,GRIA2,GUCY1B3,HPSE,ICAM2,IL20,IL22RA1,LRDD,MAML2,MTHFD1L,MYLK,NEFL,NLGN2,NPR2,NUMB,OR4C15,OR8B8,PDE6D,PPP2R5D,PROS1,PSEN1,PTGER1,RPS6KB2,SPRY1,SUCLA2,TACR1,TAS2R48,TNFSF8,TSHB,UROS,UXS1,VANGL1,WNT4,WNT7A,ZFYVE16
Negative_4038	ADK,TK2,ACTB,ADAM17,BBC3,CACNB4,CYCS,DOCK1,G6PC,G6PC2,GNAI3,GOT2,GUCY1B3,HTR2C,IRF5,ITCH,LCAT,LDHAL6A,LEFTY2,LTBR,MAP2K1,NFKBIE,OR13C3,OR2G2,PDE1C,PIK3CB,PTEN,SETD8,SMARCA2,TUBB2A
Negative_4039	DHH,FGB,SYK,ACAD9,AOC2,ASAH1,DOT1L,EFNA1,EHMT1,FDFT1,GRLF1,HMBS,KLRK1,LPAR1,MDM2,OR10Q1,OR1D5,OR2T6,OR8B8,OR8S1,PIGT,PLCD1,POLR2L,PPAP2C,PRKCB,PRODH,PROS1,SESN1,SORBS1,SRGAP1,ST3GAL6,TAS2R41,TLN2,VANGL2
Negative_4040	TNF,BUB3,CD226,CDIPT,CIITA,CNTN1,CSH1,CTSB,DAG1,GGCX,HAP1,MAGI3,OR2T29,OR8D1,P4HA3,PDE1A,PLD2,PMM2,PNLIP,POLR3A,POLR3H,PPID,QARS,SMAD7,TRAF4,TSHR
Negative_4041	CGA,ACVR2B,ALG13,OR52E4,PIM2,PPM1D,TAS2R16,UGT2A3,MAPK8IP3
Negative_4042	ADK,ADCY5,ARPC4,ATP6V1H,CBR1,CDC14A,CREB3,CRKL,DCTD,EDNRA,FOXA2,INHBC,ITGB5,MAN1B1,OR1J1,OR1M1,RENBP,SNAI2,TAS2R7,VAV1
Negative_4043	C9,HGF,LPO,ADH6,ATG7,CHST13,CMPK2,CYP2A13,CYP2F1,ETNK2,FBXW11,FUT1,GALNTL2,GRIN2D,HTR2B,NRXN2,OR4Q3,PDXK,PRKACB,PSME2,PTAFR,RRM1,TAS2R8,UGT2B11,UPP1,SERPINA1
Negative_4044	ABLIM2,ACPT,CCL27,CTNNA3,GNA12,GRM1,HMGCL,MGAT4B,OR2M7,OR2T8,OR52E2,OR52E4,PYCRL,SEMA4G,SH2D1A,SKP1,SMAD6,SULT2A1,TBL1X
Negative_4045	CACNA1H,CD44,CDKN1C,CSNK1D,CX3CL1,IL17RB,MAN2A1,OR5D16,OR6C70,OR8H1,PIGQ,SPAM1,ARHGEF12

Negative_4046	ACADM,ADH4,ALOX15B,ANAPC10,ARG2,ATF2,ATF4,BIRC5,CACNA1G,CDC26,CIITA,CRHR1,CTLA4,DUSP1,EPHA6,FGF7,FPGT,FUT7,FZD1,GPAM,GUCA1C,GYS2,GZMB,HSD17B4,HTRA2,IL5RA,INSRR,NCF1,OPLAH,OR11L1,OR4C6,OR4K14,OR51B6,PDE5A,PON1,PTGDS,RAB5A,RASGRF1,RGS3,ROBO1,SMARCA5,SSH1,ST3GAL5,WNT11,ZBTB16,C1GALT1C1,PLA2G12A
Negative_4047	GAA,APBB1,CD28,CLDN5,GFPT1,IFNA16,IRAK4,MCM4,OR5D18,OR6C75,PNMT,POLR2D,PPM1D,RFXANK,TMLHE,VAMP4,YWHAQ
Negative_4048	CFD,IL2,LSS,AGXT,BCKDHA,CACNA1B,CD47,CD59,CDK2,CLDN3,DGKE,DLL3,DOCK1,DUSP6,EGLN1,GPX5,LARS2,MAP2K6,OR10G7,OR52B4,P2RX2,PPA1,PRKCG,SEMA4C,TAS2R1,TAS2R14,TBL1XR1,TYMP,ATP6V0E1
Negative_4049	LEP,ACAA1,ACOX2,ALDH4A1,ARG2,COMP,CTNNA2,CXCL12,CXCL3,CYP2B6,DPYSL2,EREG,FUT4,FZD10,GPD1,HAO2,IFNGR2,IKBKE,IL18,ITGB6,KLRC2,LRP6,MYH7,OR10A2,OR4A5,OR52R1,OR6B2,PGAM2,PIK3CG,POLR3H,PRNP,PTGER1,PTGER3,PYCR2,ROCK1,TUBA3C,UROD,EIF4EBP1
Negative_4050	DCK,GP6,C1GALT1,CACNA1I,CCNA2,CD40LG,CDKN1A,CXCL2,CYSLTR1,FGD3,FGF14,GNRH2,GPAA1,GRIN2C,HLA-DRA,HRH2,KIR3DL3,NT5C1A,NUDT5,OR14J1,OR2A2,OR2T8,OR52I1,PCYT1A,PFKFB3,PIP4K2A,RUNX1T1,SCP2,SDC3,SMURF2,TSNARE1,VANGL2,HLA-DRB5
Negative_4051	IDS,BHMT,C9orf95,CADM3,CLDN6,COL4A6,DAD1,DDX55,DFFB,HAGH,INPP4A,LDHAL6B,NT5C1B,OR52K1,PDXP,POLR2D,PPID,PTPRB,RFXANK,SGPP1,SKIV2L2,TBK1,TGFB3,PRICKLE1,SLC25A31
Negative_4052	ADRB2,CYBA,FGF8,MAPK10
Negative_4053	KMO,TEC,ADCY1,ALDH2,APAF1,B4GALT3,BCL2L1,BMPR1B,CACNG8,CAMK2G,CASP6,CDK6,CRHR1,CSF1,CSH1,CYP4A22,FADD,FASN,GGCX,GGT7,GNB3,IDUA,IFNK,IKBKG,LRAT,MNX1,NOD1,NRG4,OR10A3,OR13C2,OR1L8,OR52A1,OR52N4,OR8H2,PIK3CG,PNPT1,PORCN,SOCS2,ST3GAL1,TAP1,YOD1,HSD17B12,TNFSF13B
Negative_4054	CKB,PLG,RET,WAS,ACP1,CFTR,CHAD,MAPK13,POLR2C,PPAP2A,PRKCE,PRPS1,PTPN11,SHC4,STX7,SUCLA2,SULT2A1,UGCGL1,UROC1,WASF1
Negative_4055	AKR1C2,OR11H4,OR52E2,ZBTB16
Negative_4056	FAS,ME1,ALG5,ASS1,ATP5L,B4GALT7,BCL2L1,BDH1,CLCA2,CNGB1,CYP4A22,FCGR3A,GALT,GDF7,GSTM3,GSTM4,IRS1,KRAS,LAMB3,MOV10L1,NCK2,NOTCH1,OR5D16,OR5F1,OR8H1,P2RX7,PDE4B,PDE6D,POLD4,PPM1D,PPP2R2A,PRKG1,RBKS,RPE65,SPP1,STAT5B,SULT1E1,TNFRSF8,TNFSF12,TUBB2A,VANGL1,WNT9A
Negative_4057	GSN,ABCC8,ACSS1,ALDH3B1,ARSA,BLVRB,CCL20,CD34,CD99,CMPK1,CREB3L4,DERA,DFFB,EGLN3,FLT1,GNA14,IL12A,MGAM,NAMPT,OR10T2,OR2M4,OR4L1,OR51G2,OR51S1,OR8K3,PARVG,PDE7A,PIK3C2A,POLE3,PRSS3,SRGAP1,ST3GAL2,TYMP,ATP6V1G1,SERPINB5
Negative_4058	GK,GNE,MAX,ACSL3,BMPR1A,CACNG7,CSNK1D,EHMT2,EP300,MAP3K8,NAGLU,PDPK1,PTPRZ1,RENBP,ROCK2,RPS6KA3,SRGAP3,SULT2B1,TBL1XR1,THBS2,UGT2B4,WNT8B
Negative_4059	RDX,ARHGEF4,CACNG1,CAMK4,CCR1,F13A1,GALNT6,GSTA1,HPSE2,IFT57,IL6ST,MAP3K6,MAP4K4,MDM2,MGLL,NCR3,OR2T4,PLA1A,POLD4,PSENEN,PTDSS2,PTGDS,PTGFR,RDH12,SEMA3F,TRDMT1
Negative_4060	NGF,AGRN,ALDH1A2,ALG9,APC2,C1QB,CACNA1I,CAV2,DMGDH,GCNT2,GOT2,GRM5,HMBS,IKBKG,IL10,LCMT1,MS4A2,NEFH,NUDT2,OR10H5,OR52A5,OR52E6,OR52N5,OR9A2,P2RX2,PIK3R5,PPP2R5C,PTCH1,SULT2A1,TAS2R16,TFPI,TNFSF10,VAV2,ATP6V1B1,MAPK8IP3
Negative_4061	TFG,ACSS1,ASPA,CDC25A,CDH2,CLCA4,CLDN17,CNTF,COL4A2,DDB2,DPM2,ECSIT,ENO2,EPRS,GCNT4,GNAQ,HHIP,MMP2,MTAP,OR8B4,PAICS,PGAM4,PRPH,PTPN7,RPS6KA4,SEMA3C,SH2D1A,SHMT1,STAT5A,TUBA3C,UPRT,VAV3,VTI1B

Negative_4062	CR2,ACO1,ACTA2,ACTC1,ATP6V1F,B3GALT1,CAMK2D,CBLB,CDKN1B,CHRD,CNGA4,DDB2,EPB41L1,GAMT,IFNK,IL20RB,IPPK,MAFA,OR1L1,OR6N1,OR8K5,PCK1,PLXNB1,PPAP2B,PYCR2,RBPJL,STX16,TAS2R5
Negative_4063	BCR,C8B,MPI,PML,ACMSD,ADCY4,B3GALT4,CCL4,CDIPT,CTBP1,DTX3,FADS2,FGF3,FGFR2,FTMT,GNA11,GNAI1,GNAI3,GNAT3,GNPDA2,HSD17B1,IL1A,KCNB1,KCNQ1,MAPK10,MGST3,MYL7,NCSTN,OLAH,OR10R2,OR14C36,OR2A14,OR51L1,OR52N4,ORC1L,P4HA1,PDE3B,PDGFRA,PNPO,SPHK1,TLN1,TUBA3E,UBA7,WARS2,TNFRSF10A
Negative_4064	CBS,ABL1,ACTG2,AVPR1A,CACNG4,CAV2,CCR3,CD28,COL6A2,CYP21A2,DGUOK,EFNA1,ENPP6,GLB1,GNA11,IFNA21,IPPK,IRS2,ITGB6,KIR2DS5,NRG4,PCYT1B,PLA2G10,PLCG2,PPM1A,PSEN1,RAP1B,ROBO1,SEMA4A,SNW1,SQLE,TAS2R13,TGFBR1,UCK2,UGT2B28,UMPS,SUV420H1
Negative_4065	GK2,SMS,B4GALT5,BLNK,CSAD,CYP2D6,DGAT2,EPHA2,NFATC1,OR2AT4,OR4F5,OR6C1,PDHA1,PTGS2,RAD51,ZBTB16
Negative_4066	GP6,HGF,BTRC,CSNK1G2,GLS2,GTSE1,NFX1,OR4C12,SELP,TAS2R16
Negative_4067	CR1,IHH,TK1,AACS,ACTG2,AHCYL1,APOA2,ATG7,CAMK4,CEBPA,CLDN17,COL3A1,COQ3,CREB3L3,DAPK3,EFNB3,FSHB,GALNTL4,GNB3,GSTO2,IRAK2,ITGA1,ITGA11,LAMC2,LCMT1,MYH10,OR11L1,OR6N1,OR7C1,PARD6B,PDE2A,POLR1E,SEMA7A,SETDB1,SMAD6,STAT2,UPRT,YARS2,CTNNBIP1
Negative_4068	CRK,GSS,RDX,VCL,ARSA,CD99,CLDN5,CLTCL1,COMP,CREB3L3,EARS2,FGF10,FZD10,FZD4,GADD45B,GPAM,IRF7,LARS2,MAML2,NTF4,OR10H3,OR2M7,OR2T2,OR4S1,OR5H2,OR6T1,PDE4B,PER3,PISD,PPARD,PTGS1,PTPN5,SOS2,TBXA2R,TCF7L1,TUBAL3,VCAM1,YWHAZ,HLA-DRB4
Negative_4069	ILK,ADRB1,AKR1C4,AKT1,ALDOA,ALG3,ALOX12,AMDHD1,C1QC,CACNA1S,CARS2,CCNA2,CD46,CHAT,CHST11,CHST12,DDB2,DUSP9,NCKAP1,NEFM,OR4C12,OR6N1,PDE2A,PER1,PGAP1,PLIN,PRPH2,PRSS1,RPN2,SARS,SFRP5,SMAD1,TAS1R2,TAS2R38,TPI1,UGT2B17,PRICKLE2
Negative_4070	APOE,CLDN3,CLDN4,CXCL2,EPHA6,NUDT2,OR5AN1,ULK3,ATP6V1C1,HLA-DPA1
Negative_4071	UBC,CD82,CSNK1G1,CTPS,FGF2,GBGT1,IL11,MCAT,POLR2F,PPT1,RAPGEF2,SHMT1,SPHK1,THBS4,EIF4EBP1
Negative_4072	F8,ACSS1,ACTA1,ALDH1B1,APOA5,ATG3,B3GAT3,CD79A,CNGA3,CNTNAP2,CRY2,CYP26C1,DPM2,EFNA4,FMO1,FPGS,GAB1,GAS1,GNAI1,LAP3,LTB4R2,MAP3K12,MAPK11,NUDT2,OR2T6,OR51F1,OR52N4,OR6X1,POLR1A,POLR3B,PRSS3,SHISA5,SLC25A5,ZAP70,HSD17B12
Negative_4073	GGH,ACVR2B,ALPPL2,BLVRA,C4BPB,CASP9,CBLC,CCL2,CYP2B6,CYP8B1,DDIT3,DDX47,DRD1,E2F1,FOXO3,GAPDH,IL12A,IL2RG,OR10J3,OR13D1,OR4K1,PMAIP1,POLD1,POLR1A,PTGER3,RAPGEF3,RPRM,STX2,TUBAL3
Negative_4074	MPL,ACAA1,CCNB2,CPT1B,CYP2S1,HCST,IDH3A,IFNA5,MINPP1,OR5I1,PIK3CG,PTAFR,YWHAZ,ATP6V1C1
Negative_4075	CKM,ID2,SMS,TEC,ADIPOQ,AGPAT6,BMP8B,CDC14A,CDKN2D,CHIT1,DPYD,GNG12,GRM5,HMGCS1,KLK3,MDH1,OR10V1,OR1S1,OR4L1,P2RX2,P2RX3,PER1,PLA2G6,PPP3R2,PRODH2,SDC3,SFRP1,WNT11
Negative_4076	MVK,ADCY2,ADCY7,AKR1A1,ARSB,CHIA,CTNNB1,FZD3,GALNT1,GNAZ,HRH2,ITGAV,NFKBIE,OR9I1,PAK4,PDE3A,SCIN,SLC33A1,SMAD5,THBS3,TLR9,TSHB,TSLP,TUBB2C,UNC5C
Negative_4077	FH,PGF,DAAM2,FGF3,GFPT1,HSD17B7,NCK2,NMNAT2,OR13C9,PAICS,PVRL3,SH3BP2,UROS,VDAC2,WNT8A
Negative_4078	AK1,ABLIM2,APAF1,APLP1,ARPC2,CHAD,CKS1B,CUL1,CXCL16,DTX3L,EHMT2,ENPP1,F2RL1,GNRH2,GPLD1,GPX7,IL12A,INHBB,ITGAD,LAMC1,NME6,OR2A12,OR2A2,OR2G6,OR2T33,OR5H6,OR6F1,OR7C1,OR9G4,PIGT,PIK3R3,PRKX,RENBP,SEMA4D,SIGLEC1,SPCS1,TSLP,UCKL1,ULK1,PPP1R12A,TNFRSF6B

Negative_4079	CAMK4,CSF2,DLL3,DOLPP1,ERBB3,IARS2,LIMK2,POLR1D,RPSA,VTI1A
Negative_4080	CRH,OAT,ADH5,AKR1C3,ALDH3A1,CALML6,CASP8,CHKB,CNTN2,COMP,GNA13,ITGA1,KAT2A,MNX1,NOS1,OR2D3,OR2G2,OR4F17,OR5L1,ORC6L,PDE8B,PIK3CA,PIK3R5,PPP3R1,PTGES,PTPN1,PTPRR,SEMA3F,SHC2,SORBS1,SYNJ1,WNT9B
Negative_4081	ACACB,BUB1B,DDX51,GALNT12,HNF1A,NFE2L2,OR6N1,STAT4,TNFRSF13C
Negative_4082	CLDN4,CUL1,HDAC1,MTMR1,PPCS,PRPH2,XCR1
Negative_4083	AK3,F11,FGG,MET,RB1,TEC,ACSM5,BCMO1,CKS1B,CYP11A1,DHDDS,E2F5,GAMT,GCLM,GDF7,GGPS1,IL13,IL20,LIPT1,MMP14,NAT5,NCK1,OR13C2,OR1D5,OR2A4,OR2T11,OR7A5,PAFAH2,PDE6G,POLR3B,PPP1R3B,PPP3CC,PTPRZ1,RCHY1,RXRA,SOX17,STAT4,XYLT2,PLA2G12B
Negative_4084	DIAPH1,DTX3L,GSTO1,MAN2A1,MAP3K4,PDGFA,PLAU,RFWD2
Negative_4085	EPO,F9,SHH,ATP5L,B4GALT4,BMPR1A,CCDC6,CCL11,CCR2,CD226,CNGA3,COX10,EDNRA,ENTPD6,EPHA3,ERBB3,EVI1,FUT7,G6PD,GNRH1,GSTO2,ICAM2,IFNA8,KARS,KCNB1,KLRC3,LDHD,MYLK2,NCKAP1L,NFKBIE,OR51E2,OR56B4,PDHA1,PPP1R3A,PRKY,RASGRP1,SELP,SGMS2,ULBP1,ZNRD1,MAPK8IP2
Negative_4086	ABL2,ACY1,CCR9,CDS2,CMBL,DAD1,DDX23,EPB41L2,EXTL3,FGF22,FGF3,GALNT7,GAMT,GLI1,GMDS,IFNK,IL12RB1,MAP3K3,MNX1,NEGR1,OR1A2,OR5V1,PRKAG1,RELB,SEMA3B,SNAI1,SUV39H2,WWP2,PHOSPHO1
Negative_4087	DUT,IL2,ACP1,APBB1,ATF2,CAV3,CD44,CDC25B,CDKN1B,CLDN5,COL4A1,CYP2B6,DGUOK,GOSR2,JAK1,LCP2,LRP6,MITF,MYH15,OR2K2,OR2L2,OR7A5,PIGB,PIM1,PVRL4,RHEB,SARS2,SC4MOL,SC5DL,SSH3,THBS1,TUBA8
Negative_4088	GH2,FSHR,HCCS,HTR4,IFNAR1,MAPT,NOTCH3,OR4D6,PAX6,PGK1,PITX2,SMAD5,SOD1,PNLIPRP1
Negative_4089	F3,OGT,SCD,ADH1B,AFMID,AGPAT4,AGRP,AMY2B,AQP7,ATP4A,BCKDHB,CAMK4,CCR1,CRY1,DDOST,DHODH,EARS2,EPHA8,GDF6,GSTZ1,GUCY2D,IL28B,MKNK2,MLYCD,OR10P1,OR2A42,OR4K2,POLD1,PRKCE,PTPN11,RAPGEF3,SHC4,SLC27A1,SOS1,ST3GAL4,TAS2R38,TLN1,TRAF4,UBA7,UGT2A1,UGT2B11,WBSCR22
Negative_4090	FH,GAA,AHCY,ALDH4A1,ATP5B,CCR8,CDH15,COQ7,CUL1,DAAM2,DDIT4,ENTPD6,FARSA,FLT3LG,GADD45B,GFPT2,GPAM,GRIA2,GSTO1,HMGCS1,HRH2,IL28A,MAP3K4,MARS,MYH10,NFYA,ODC1,OR5D18,OR5K2,OR7C1,OR7G1,RALA,SPCS1,SQLE,STAT5A,TGFA,USE1,VAV2
Negative_4091	BAX,CAD,B4GALT2,BDH2,CCL4,CHPT1,CXCL1,CYP2C19,DDX55,GYS2,IL18,IL2RA,ITGA8,KIR3DL2,OGDHL,OR1Q1,PARD6A,PPP1CB,PYGB,RALB,STAM2,STMN1,SUV39H2,TALDO1,TAS2R8,WNT16,XYLB,TNFRSF10B
Negative_4092	BTC,F12,ACAA1,CASP3,CASP7,FGFR2,FOXA2,GART,KLRK1,OR4D2,PAPSS1,PFN1
Negative_4093	AGK,C7,MPL,TEC,ADH4,AGXT,ANAPC5,BCAR1,CASP8,COL5A2,CSF2RB,DNMT3A,GALC,GDF5,GLI1,GNAO1,GNPDA2,GNPNAT1,IDH2,IFNA4,IL4R,IMPA1,ISYNA1,KCNJ11,NR5A2,OR13C5,OR14J1,OR2AG1,PGAM2,PIGH,PON2,PPP2R5A,PRKAR2B,SIAH1,SPTLC1,SULT2B1,VAV1,VEGFC,WNT8B,HSD17B12
Negative_4094	F10,B4GALT6,BDNF,BPNT1,CYFIP2,EPHA6,HSPA1B,IL2RB,MAP2K6,PRKAR1B,RND1,SMURF1,TNFRSF10B
Negative_4095	CGA,MVK,ACLY,ANAPC7,BNIP1,CACNA1D,CCKBR,CCNB3,CD74,CDKN2B,CHD8,CMPK1,CMPK2,COL5A2,CTF1,CXCR3,CXCR6,DDIT4,DGKA,DUSP16,FCGR3B,GADD45G,HLA-G,IL22,ITCH,LILRB3,MAFA,MYH1,NFATC4,OR10G8,OR1F1,OR2L3,OR4A15,OR56B4,OR6K6,OR8K5,POLR2F,POLR2H,PPP2R5A,PRKACG,SARS,SYNJ2,TP73,WBSCR17,CACNA2D3

Negative_4096	ADK, GCK, MPZ, SRC, AGXT2, CD44, CHRM1, COQ3, CREBBP, CRY2, CYP26B1, DHRS4, EPHB4, EPOR, GNAS, GP1BB, IFNA13, IGF1, IL17RA, LIPC, MAP2K2, MKNK1, MYH2, NFKB2, NFKBIE, NME7, NR1H3, NRXN3, OR11G2, PLXNB1, RPS6KA4, SPHK2, STAM2, STX6, TAP2, TNFRSF4, TRAF5, TUBB3, VCAM1, CSNK1A1L
Negative_4097	F7, KDR, TH, CCNG2, CXCL9, DDX19A, DHDDS, EFNB1, FZD7, LDHAL6B, MGST1, NLGN1, OR2AG2, OR5T2, PLAT, PRKAG3, PROS1, PRSS1, RAPGEF3
Negative_4098	AUH, FGA, ATP6V0B, CCL14, CCL19, CCR1, CD48, DNMT3B, FARSB, G6PD, GDF7, GSTA5, IMPDH1, ITGA2, ITGB8, MAML3, MGAT2, NME7, OR51B4, PEMT, PPP1R3C, SPRED2, SV2C, TCF7L2, THBS1, TYROBP, WNT6, ATP6V0D2
Negative_4099	DUT, ADCY9, ALG12, ATP5G1, BPGM, C3orf10, CLDN20, DCTD, DGKA, ICAM3, PTK2, SETDB2, USE1
Negative_4100	CYP1A2, EPRS, LIMK1, LLGL1, MGST1, NFX1, PTPRM, RARA, STAT4, MAPKAPK2, PDCD1LG2
Negative_4101	FH, GSS, PLG, ARAF, COX10, CXCL13, HMGCL, MAP3K7, MAP3K8, MAPK7, MCM5, MMP2, OR10H1, OR2A2, PLCB3, PLOD1, SREBF1, SUFU, WARS2, SUV420H1
Negative_4102	FYN, ACSL6, ALAD, FGD1, GRIN1, HTR5A, IFNAR2, IL20, IPPK, KLRK1, MCEE, NANP, PCYT1B, PIGA, PIGK, PPP2R2D, PPP2R5A, RPS6KB1, SPI1, SPRY1, XYLT1, PPARGC1A
Negative_4103	BTD, IHH, AXIN2, FTH1, GNPDA2, IDH3G, IFNB1, KITLG, LCP2, METTL6, PDIA4, PPAP2A, SEMA4G, UNC5D, XCR1, YWHAG
Negative_4104	APP, C6, CGA, AASS, ALOX12, ASMT, CACNB1, CCR5, CD36, CHSY3, COASY, DOLPP1, EDNRB, ENTPD2, EPOR, ERBB2, FCGR3B, FUT5, GGT5, GNG13, GRIA3, HAP1, HMGCL, HSD3B7, IFNA10, IFNG, IL25, IL6ST, IPPK, IRS2, LIPA, LIPT1, MAT2B, MEF2C, NEUROD1, NFKB2, OR2T6, OR5A2, OR5AP2, PER1, PER2, PGM1, PLCG1, PTGES, QPRT, RENBP, SULT1A2, TSC1
Negative_4105	C8B, CR2, F12, F9, GK2, AOC3, B3GNT4, BUB1, CBLC, CKS1B, CYFIP1, F11R, GAPDH, GCLC, GPAA1, HSD3B7, IL7R, MAPK1, MFNG, NRXN3, NTN1, OR13G1, OR14I1, OR2T2, OR5B3, PANK2, PLIN, PPARG, SHC2, SOCS7, SV2B, TGFBR2, ULK1, ZFYVE16
Negative_4106	PGD, ACSM3, ADRA1A, ADRB2, B3GNT4, BNIP1, CARD11, CD48, CLDN3, DLST, ETS1, GANC, GCH1, GFPT2, MAP2K5, MGAT5B, NRXN1, OR10H2, OR10J5, PCCB, PHKA2, PLAT, POLR1C, PPP1R3A, PTCH2, ROCK1, RPS6KB2, RXRA, SHC3, SLC27A5, SLIT2, SOCS2, STT3B, WARS2, WNT3A, XYLT2
Negative_4107	AK5, C5, ABP1, ATIC, CACNB4, CAMKK1, CDC25A, CYP2S1, DGKD, DPYSL2, EHMT2, ENTPD8, FECH, FPGS, FTMT, GAD1, GJD2, IL9R, INPPL1, ISYNA1, ITPKA, LLGL1, MAP2K7, MYH2, NOTCH3, OR13F1, OR2A14, OR2K2, PIK3CG, PIP5K1B, PLCB4, PLEKHA8, PMM2, POLR2J, PRKAA2, PRKCZ, RARA, RIPK1, SLC12A2, SULT1A1, TGFB1, ZNRD1, MAP3K7IP1, PAFAH1B3, PLA2G12B
Negative_4108	BTD, HPD, CES2, CLDN8, CSF3R, ENTPD5, GLG1, HYAL2, IL15RA, MAGI3, MYH9, OR6K2, PCYT2, PGAM4, SULT1A2, VDAC2
Negative_4109	GLA, E2F5, FPGT, GALNTL5, GLO1, IL5RA, NADK, PARS2, TIAM2, ATP6V1E1
Negative_4110	C2, FN1, GNS, HPD, STS, ADCY6, CASP3, CPT1B, DDB2, DKK2, GCAT, GGT6, GUCY2C, IL18, IL25, KCNJ11, LIPG, LTC4S, MAP2K1, MGST1, MKNK2, OR10H5, OR2T34, P2RX4, PPP3CC, PTK2, RELA, RFXAP, SARDH, SLIT2, SPRY3, STAT2, UCP1, UROC1, WNT2B, ATP6V1B2
Negative_4111	APP, ITK, SFN, ZYX, ATP5G1, CD74, CDK7, CHAT, CYP2A13, DCXR, DGKB, EDN1, FGF2, FTCD, LIPA, MAPK7, NSDHL, OLR1, OR2T34, OR3A1, OR6C6, OXCT2, PIGM, POLR3A, PRODH, STMN1, SYNJ2, TPI1, VCAM1, YES1
Negative_4112	HK3, LYN, APLP1, ARSB, BMP5, FMO1, GSTA2, PIGM, PIGQ, UGT2B11, VAV1, ATP6V0E1
Negative_4113	ATP5B, BMP5, CACNG7, CCNG1, CD86, CDK2, CYBA, F13A1, GAPDH, GNAI3, IL13RA1, INPP5E, ITGB1, MAP2K7, NQO1, OR4C13, OR5D16, P4HA2, POLR1C, PRPS1, SGMS1, SUCLG2, UGT2B4, AASDHPPT, HLA-DQB1

Negative_4114	F10, ACACA, ACACB, ACPP, ACY1, ADCY2, ADH1B, AOC2, BUB3, CCL16, CLDN19, CREB1, DKK2, GNRH1, GSTA5, HBEGF, HCCS, IDUA, IL6R, ITPK1, JAK2, MARS, OR2D2, OR2T33, OR4M1, OR6M1, OR7A10, OR8S1, PAK3, RAF1, ST8SIA1, SULT1A4, TLR4, TUBB2B, VAV3, WHSC1L1, ATP6V1B2
Negative_4115	ADC, APP, C8B, FGA, TAT, ABLIM1, AFMID, AK3L1, ANAPC4, CLTCL1, CPT1C, DDX23, DHX58, DLL1, DTX4, EFNB1, EIF4E2, EPHB3, EXT1, FGF21, HSPA2, IMPDH1, IQGAP1, IRS2, ITGA6, LRP6, MMP7, OR12D3, OR5I1, PLA2G5, PLCB2, PLOD1, PLOD2, POLE3, POLR3K, PPM1A, PTPRB, ROBO2, RRM2B, SCIN, SFRP5, SV2A, TAS1R3, MAPK8IP1, TNFRSF11A
Negative_4116	XDH, ALDH3A2, BMPR1B, CDC16, CRHR1, CTBP1, HMGCL, LAMC3, NFASC, NR1H3, OR6C74, PIM2, PTGER3, SLC25A5, SLC25A6, TNFSF11, TNFSF8, WNT7A, SERPINA5
Negative_4117	BAX, KMO, NLK, TNC, ADCY10, C5AR1, CACNA1G, CDH1, CER1, GALNT2, GSTO2, HPSE, IL17RA, IQGAP2, MAP3K6, NAMPT, NCK1, NPR2, ORC5L, PFKFB4, PI4KA, PIM1, PPP1CA, RPS6KA6, RPS6KB1, SIAH1, VAMP2, VNN1
Negative_4118	CEL, GSR, OSM, PLN, ACPT, ANAPC5, ATP12A, B3GALT4, B4GALT5, CERK, CNGB1, DDX52, DLL3, EP300, FGF22, GPLD1, HEXA, HSD17B3, IFIH1, IL7R, LYPLA1, MYH7, NT5C3, OR10K2, OR2A2, OR2L13, OR6C76, PANK3, PDE7B, PDPK1, PLA2G2A, PMM2, PON2, PPARD, RXRB, SETD7, UCK2, WASF2, YWHAE, CSNK1A1L, PPARGC1A, SERPINE1
Negative_4119	GH2, ME1, BDKRB2, CYBB, DAAM1, FMO2, HLA-DOB, KIR2DL1, MTAP, NTNG1, PDE1A, PTPRM, TDO2, TLR4, UBA7
Negative_4120	GNE, RPE, ALOX5, CD70, CLDN16, G6PC2, GADD45B, GAPDH, IFNAR1, IL12A, IL22RA2, ITGAV, JAK3, LIMK1, LYZL1, MCM4, OR2K2, ORC5L, PRKG2, STX12, USE1, WBSCR22, YARS2, PPARGC1A
Negative_4121	A2M, EDA, ABLIM1, ACSM1, ALDH3B1, ARPC4, CADM3, CD36, CD59, CXCL10, DGAT2, FZD10, GNRHR, IL11, IL12A, KYNU, LRDD, OR11L1, OR52A5, PIK3R5, PTPN1, RHOQ, SPTLC1, STX4, TAS2R14, TNFRSF18
Negative_4122	C4B, C8B, TK2, ADRB2, CANT1, CCL24, CD82, CHST11, CSF3R, F13A1, FOXA3, FZD8, HPRT1, INSRR, KLKB1, NANP, OR4S1, PDE5A, PDXK, PPP1CB, PPP2R5E, PRLR, SCP2, SETDB2, TNFSF8, TSHB, C1GALT1C1
Negative_4123	MPL, NF1, ACAA2, ACAD8, AOC3, ATP6V1H, CAMKK1, CCNA2, COASY, CYP26A1, DAD1, EFNA1, ENPP6, GNRH1, GSTA4, IL18RAP, ITGA6, JAM3, KLK3, LDHAL6B, LEPR, LMO7, MGAT1, MGAT5B, MTAP, NEU2, OR6C6, OR8B12, PFKFB2, PRKAB1, RAF1, RGS3, RXRB, SEMA3D, SHC1, SLC9A1, SPAM1, STK3, SV2A, TACR2, TLN1, TUBB4Q, UGT2B17, VDAC1, XCL1, ATP6V0A4, HLA-DQB1
Negative_4124	C8A, CEL, DBI, HPD, ARPC1A, BMP5, FUT5, GLB1, ICAM2, IFNAR2, LAMB2, MYH14, OR10H5, OR2L13, PAK2, PAX6, POLD3, PPCDC, RDH16, SESN3, SGMS2, SPRED2, TRHR, TXNDC12, UGT2B11, MAP3K7IP1
Negative_4125	BID, EDA, ACADM, AKT1, AMD1, ARPC5, CLDN2, CYBB, FGFR2, GALNTL2, GRIN2C, GSTO2, HAGHL, HRH2, KLRC1, LAMC1, LAMC3, MYH1, NEFL, NMNAT3, NPR2, OR1K1, OR1L4, OR2T2, OR4D6, ROCK2, RXRG, ST3GAL4, TBK1, ST6GALNAC5
Negative_4126	UBC, ALG5, ARAF, ATN1, CACNA1A, CDKN1B, DHRS4L2, IFNB1, IL24, IL5RA, ITGA4, NKX2-2, OR4B1, PARD6G, PDGFB, PPBP, PPP2R1B, RPS6KA5, ST3GAL5, STAT6, STX1B, YWHAB, SERPING1
Negative_4127	ILK, ACADM, AKT3, ATP5C1, ATP5E, BDKRB1, BUB1, CDC27, CYP2S1, EPAS1, ICOS, IL20RA, IRAK3, MYH6, NAT2, PAX4, PFKFB4, PLXNA3, ST6GALNAC5
Negative_4128	ACP2, ADCY8, CISH, CTNNB1, CYP3A5, GADD45G, GCNT1, ICAM2, NFATC2, OR1G1, PARD6B, PGLS, PLCD1, PYCRL, STX4, TNFSF10, TRIP10, VTI1B, ATP6V1B2, HLA-DQB1, SERPINC1
Negative_4129	PLG, ALCAM, CACNA1B, GLO1, NT5C, OR4B1, PLD2, SEMA4D, SOCS1, TRAF2, UGT2A3

Negative_4130	ADO, CTH, DCT, GLS, ABL2, AGMAT, AKT3, ASPA, BMP2, CDH2, CEBPA, CES7, CREB3L3, DLL1, FGFR4, FUT4, FZD10, G6PC2, GZMA, IQGAP2, LTBP1, MTHFR, NFX1, NFYA, NPR1, OR2F2, OR5B21, PCCB, PCK2, PDHA1, PIK3R4, PPP1R3C, PRKACG, PRODH, RPS6, RRM2, SEMA3E, TAS2R8, TRPC1, YWHAG, PLA2G12A, PNLIPRP1, SERPIND1
Negative_4131	ACTG2, ADRB3, BAIAP2, BTRC, BUB1, COL11A1, CYP2U1, DARS, ETNK2, FGF1, IKBKE, IL1B, OR13C3, OR13F1, OR3A1, OR5P3, PNPO, PPOX, PPP1R3B, PRSS3, RBL2, RDH10, SULT1A2, WNT5A, XCR1, B3GALNT1
Negative_4132	IL9, ACAA1, B4GALT5, BDH2, CCL25, CDKN2C, EIF4E2, GALM, HNF4A, NCK2, NTN1, OR10A6, OR2B6, OR4D1, OR4F5, OR8U1, PARD3, PLCG1, PLCG2, ROCK1, SMAD2, SMAD4
Negative_4133	MOS, ACTA2, ADIPOR2, ALPL, APOA1, BDKRB2, CYP2U1, GMPPA, GNG12, GNRHR, GP1BB, IARS, IL1R1, ITGA6, ITGA8, KLRC1, LYPLA1, NCAM1, NFKBIB, OR10G9, OR2H1, OR4D2, OR4F4, OR51B6, OR8J3, P2RX3, PIGX, PLTP, POLR2A, PPT1, PTGDS, RALGDS, RASSF5, SDC2, SETDB1, SNAP23, TAS1R3, WASF3
Negative_4134	ID3, ADRB2, FGF8, FGFR1, FZD8, HMGCR, IL13, NKD2, OR52M1, OR5M9, OR6Y1, PIK3R4
Negative_4135	AKR1A1, ARSD, ATP5G3, CARS2, CD74, DAPK3, EXOC7, GAPDH, GSK3B, INHBE, NCAM2, NEU1, OR1I1, OR5B21, P4HA2, PISD, PLCB3, PROS1, PRSS3, RAPGEF3, RPIA, RUVBL1, RXRB, SAT1, SLC27A1, SNW1, ST3GAL5, TCF7L2, TGFBR2, TUBA1B, TUBA8
Negative_4136	GAMT, KLRC4, MMP7, OR1E1, OR5R1, PAX8
Negative_4137	LYN, ADH1B, ALDH6A1, ALS2, ARPC4, ATP5F1, B3GAT2, CACNA1S, CARS2, CCL26, CD48, CDH1, CER1, CFLAR, CLDN11, DDX56, EGFR, ENTPD3, GFPT1, GNA14, GNPDA1, HTR6, IFNG, IL11, ITGA6, MPZL1, MTHFS, NR4A1, OR2A14, OR3A3, OR5AK2, PARD6G, PDGFA, PMM1, POLA1, PPP1R3C, PPP2R5B, RAC2, RALBP1, SEMA4B, TAP1, TGFB1, VARS, ATP6V1B2
Negative_4138	CDA, BDH1, CACNA1A, CCL28, CYP2A6, ERBB4, LAMA3, OR1N2, OR9A4, VDAC1, WNT3A
Negative_4139	BID, BTC, BTD, MUT, ADAM17, ATP5D, B4GALT7, BST1, E2F2, FABP1, FIGF, FMO3, FUT4, GSK3B, IL5RA, IL7R, KITLG, MAN1A1, MDH2, OR10A2, OR4A5, PAICS, PDE2A, PDHB, PLOD1, PPAP2B, RPS6KB2, SHC1, TCF7, TGFB3, TUBB2A
Negative_4140	CRH, FER, NF1, ACTG2, ANAPC1, ANPEP, CACNA1G, CCL5, CHRD, CREB3, CYP2A7, DFFB, DPM3, FLT3LG, FUT5, GALM, GP1BA, GUCY2F, IFNAR1, IL18, IL2RB, ITPA, JAM2, MAP2K5, MYH8, NAGLU, NPR1, NRAS, OR4K5, OR5L1, OXCT1, PIM2, PTDSS2, PVRL1, RAPGEF2, RBKS, SFRP2, SUOX, WHSC1L1, WWP2, ZNRD1, MAPKAPK2
Negative_4141	LSS, ALDOB, CFTR, GAD1, MOV10L1, NSD1, ATP6V0D1, HLA-DQB1
Negative_4142	GCK, LYN, PLG, PTS, ADRA1B, ALG9, B4GALT4, CCL8, CD8A, CYP3A7, EGLN1, FBP1, GALM, GRIN2B, IBSP, ICOSLG, IL1A, IL20RA, JAG1, JAG2, MDH2, MGST2, NAMPT, NFYC, NUMBL, OR1S1, PIGT, PIP5K1A, PLA2G10, POLE2, POLR2B, POLR2L, PSEN1, RARA, RPS6KB1, SEMA3C, SESN1, TAS2R1, TLR9, TNFSF4, TUBA1A, UGDH, CACNA2D2, SERPINA1
Negative_4143	LIF, ACTA1, AGRN, ALG9, ATP5O, BMP6, CACNB4, CDS2, CHAT, CYP2U1, EXT2, GSTZ1, IGSF5, ISYNA1, KCNJ11, KIR2DL3, NAGLU, OR52N2, OR5T1, POLR3K, PPP1R1A, SELL, SEMA3B, SLC25A6, YWHAQ
Negative_4144	ME3, SI, ADCY8, ADORA2B, ADRBK2, AKR1A1, ATP5H, BET1, CYP1A1, EPHA3, GNAO1, GRM1, HAP1, IFNA6, IL28RA, MAD2L1, NAE1, NAGA, ONECUT1, OR10A7, OR11H1, ORC5L, OXCT1, PARS2, PLA2G6, PLCG1, POLD3, TAS1R2, TPH2
Negative_4145	ADC, DBI, ACO1, AHCY, ATP5G2, B4GALT6, BMP6, CBR1, CHD8, DAPK1, DGKE, EPHB2, EXT1, FBP1, GUCY2D, HADH, HSPA8, IDH2, ITGA9, LLGL2, MAN1A2, NEFM, OR10AG1, OR1S1, OR2T4, OR4F4, OR8U1, PDK1, PFN2, PIK3CD, POLR2B, PPP1CC, RERE, SCIN, STAT1, TP73, HLA-DPB1, MAPK8IP1

Negative_4146	FGB,ALDH3A2,BDH1,CCNG1,CD244,FTH1,IL10,IL1RAP,IL22RA2,ITGB2,NOTCH2,OR7A5,PLXNB2,PNPLA3
Negative_4147	ABCC8,ALPPL2,ARPC1B,ATP5E,CSF2RB,CX3CL1,DAAM2,DPM1,GMPPB,IL1R1,IL22RA2,IL6R,IRF3,KIR2DS5,KLRC4,NCR2,NEUROG3,OR51A2,OR5D13,PLCD4,PPARG,TAS1R1,TSHB,ZFYVE9,TNFRSF10B,TNFRSF11A
Negative_4148	HGD,PLN,ADCY4,AKT1,CCKAR,CEBPA,CHD8,CHEK2,CHST12,CTTN,ENO1,GMPPB,GNA13,GNAI1,GNMT,HEMK1,HMOX1,MEF2C,NOTCH2,NT5C3,OR10H5,OR4P4,OR51E2,PFKFB4,PGAM1,POLR1A,PRKY,RAB23,TM7SF2,ATP6V0E1
Negative_4149	F10,F7,GDA,KMO,SDS,AGMAT,ANAPC4,CACNB4,CCNH,CSNK1G2,CXCL1,CYP2J2,CYP7A1,DNMT3L,FGF1,GSTA5,IFNA5,ITGB5,NT5E,OR10C1,OR11H1,OR4P4,OR52M1,ORC1L,PIGX,PIP4K2A,POLR3G,RFXAP,SMAD3,SV2B,SYNJ2,UBA7,MAPKAPK3
Negative_4150	CD6,FYN,CNTN2,CSNK1G3,DPM1,EPHA3,EPHA6,GLUD2,OR2H1,OR51S1,OR7A10,PRIM2,PYCR2,TAS2R50,TSC1,SERPINA5
Negative_4151	GSN,ACSM3,ALOX12B,B3GNT4,CPOX,CPT1C,DCTN1,GNA15,KCNB1,MYH8,OR6C75,PON3,SLC27A5,TXNDC12
Negative_4152	IL2,VTN,ADRA1A,AGPAT1,AMPD1,BMP8A,BUB1B,CCNB3,CLDN4,CSF3,ERBB3,GALNT13,GLI2,GMDS,GNAI1,LIPA,LRDD,SC5DL,SPI1,WNT11,CACNA2D1
Negative_4153	C7,CFH,AMPD3,ARPC1A,B3GALT2,B4GALT6,CD74,CLOCK,DGKI,FZD3,GAD1,GNB3,IFNGR2,IFNW1,ITGB5,ITGB6,LGMN,MKNK2,NAGLU,NCOR1,NSD1,OR7D2,PRKAB2,PRKY,RDH16,RFXANK,SOCS2,SULT2B1,TALDO1,TNFSF12,UMPS
Negative_4154	B3GALT4,B3GNT2,BRAF,CD34,CXCR4,DDHD1,EPHA6,ITPA,JAK1,KIR2DS5,MAN1C1,MMP7,NTF4,OGDH,OR10AD1,OR1N2,PTPN7,RAC1,SDC2,SOCS1,VASP,ZFYVE9
Negative_4155	DCN,LHB,PXN,AADAT,ACSS1,AKR1C4,CCL28,CHRD,DGKB,DHRS4L2,DTX3,DVL2,ENO2,IL28RA,INPP5B,MAN1C1,NT5C1A,OR4S2,OR51Q1,OR5B3,OR6C75,OR8S1,PRKCI,PTK2B,RALB,RASGRP2,RPS6KB1,SETMAR,STK36,UGT2A3,WARS,ATP6V1B2,ST6GALNAC1
Negative_4156	IL12A,MALT1,NEUROD1,NOTCH3,OR1I1,PPP1R3D,PPP2R5E,MAPKAPK2
Negative_4157	CD2,HPD,FGF21,H6PD,MGAT4B,OR6S1,UROS
Negative_4158	HYI,BCAR1,BMP4,CBLC,CD82,CHRM2,CLTCL1,CYP2C18,DTYMK,ERBB2,GALNT13,GNB3,GNG13,HDAC2,IL10,IL20,ITGA8,KCNQ1,LIPF,MDM4,OR13C5,OR2K2,OR4F5,OR5L1,OSMR,PDE1C,PTGER1,RFXAP,RXRB,SGMS2,SH2D1A,SHC2,SPCS3,TNFSF9,XCL2,MAPKAPK5
Negative_4159	ACSS3,CCNG2,DGKZ,ELK4,GANC,GLDC,HDAC2,IARS,IPMK,MRAS,OR10A3,OR10J3,OR10T2,PIK3C3,PLEKHA8,POLD1,PTCH2,QARS,TLN2
Negative_4160	C3,GSS,PGP,APH1A,ARSE,CDIPT,CDO1,CFL1,COL5A2,CYP19A1,DDHD1,DGKG,DGUOK,GANAB,GUSB,HMOX2,MTAP,MYLK2,NFYC,NTN1,OR13A1,OR13H1,OR1Q1,ORC2L,ORC3L,PDE10A,PER3,PGAM4,PKM2,PLA2G2D,PLA2G6,PNLIP,PPP2CB,RALA,SARDH,SETMAR,TNFSF18,CSNK1A1L,GABARAPL1
Negative_4161	ADCY8,ALG13,GGPS1,HADHA,KYNU,NNMT,OR2T4,PLA2G3,TGDS
Negative_4162	MET,PTS,ARPC1B,BDNF,C3AR1,DGKI,FLOT1,GNAT3,GRIA2,HBEGF,LMO7,MCM2,MTHFS,NCK2,NCSTN,NRAS,NT5C2,OR10H5,OR52D1,P2RX6
Negative_4163	IDS,ACVRL1,APC2,ATP6V1F,CLDN15,DGKA,EDNRB,FMO4,GRPR,IL28RA,ITGA2B,LEFTY1,MGAT2,NT5C1A,OLAH,PPM1A,PPP2R2D,SDC4,TRAF4,WNT10B
Negative_4164	BCR,LCT,PTS,ALOX12,ATP5D,CERK,CSDA,FABP3,FCGR3B,FSHR,GADD45A,GRIN2D,IL21R,ITCH,ITGA7,MASP2,NPR2,OR10A7,OR1J2,PIM1,PIP5K1B,PRKAR2A,RBL2,RPS6KA3,SLC27A1,SOS2,TLR4
Negative_4165	EPO,AKR1A1,B3GNT4,C4BPB,CDC25B,CYP2C9,EREG,HNF4A,IL6R,PYCR2,ST3GAL4,PRICKLE2

Negative_4166	ADA, F3, HGD, AASS, ACP6, ARHGEF7, ARRB1, BIRC5, CTSG, CYP2E1, GSTM5, HMOX2, ITGA1, ITGA9, KARS, LHCGR, MGAT4A, MGST3, MYH3, OR51D1, OR52J3, PIGM, PPP3R1, TFPI, UNC5A, UPRT, VEGFC, YWHAE, CTNNBIP1
Negative_4167	BAAT, BET1, CBR3, DDB2, DLL1, DUSP16, FGF8, FUT3, GNPAT, ITGAV, LPAR1, MGAT5B, MYH14, OR10H4, OR14C36, OR4A16, OR5C1, OR6M1, PLOD2, PRKCB, SGMS2, SPHK2, TAS2R8
Negative_4168	CARS, CTNNA2, LIMK2, OR2C3, OR6X1, PCYT2
Negative_4169	F5, GH2, SI, TYR, ACTB, AGXT2, ALAS1, ARHGEF2, ATP6V1D, CD74, CLDN19, CXCL2, DDB2, DGKH, ENAH, GRIN2B, HDAC2, HNF1B, IL20RA, ITGB2, MMP14, NCOR2, OLAH, OR1A1, OR1D5, OR2A4, OR5AK2, OR5P2, OR8U1, PDE9A, PIGQ, PIK3CB, POLE2, POLR2J, PPA1, RBL2, SELPLG, SEMA4F, SETD8, STX5, TJP1, TKTL1
Negative_4170	DBI, ACSM3, ALDOC, BCKDHA, BUB3, CD40, COL6A1, COX15, CSF1, CSNK1G2, DAD1, DDX54, DERA, DHFR, GALNS, GALNT6, GSTM5, HTRA2, IL18RAP, IL21, IL24, IRS1, MGAT3, MIOX, OR13C5, OR4B1, OR51L1, PIGN, PLAT, PORCN, PSENEN, PTDSS1, PTGS1, TRAF1, TRAF5, TUBAL3, VAMP1, WNT3A, PHOSPHO1, TNFRSF18
Negative_4171	APP, NPY, AKT1, ATP5D, CCL4, CCR4, CDKN1A, CNDP1, COL4A6, CXCR3, DLL1, IRF9, ITGA4, LTBR, NRP1, OCLN, OR6K3, OR6Q1, PECAM1, PMM1, SFRP1, SMPD1, SOAT1, SULT2A1, TNFRSF17
Negative_4172	CR2, DHH, GDA, ASIP, CCR8, FDPS, HGSNAT, HIF1A, ROCK2, SELP, TLN1
Negative_4173	GDA, TPO, CADM1, CCNA2, OR52A5, OR5H6, PIAS3, PIGX, UPRT, UROS
Negative_4174	AGL, BTK, C2, CP, PGP, ACSS3, ADH4, AGMAT, AKR1B10, ALG2, ALOX15, CCL25, CNTF, EFNA2, F2RL2, GCDH, GGT7, GIT1, GLG1, GSTA1, HIF1A, HLA-DMA, HSD17B7, HSD17B8, IDI2, IL12RB1, IL1A, INHBE, KLK3, LARS2, LCP2, MAPK14, MTFMT, NEFM, OR10A4, OR4D9, OR4M2, OR4N5, OR52K2, OR7A17, PDGFRB, POLE2, PYCR2, SIPA1, SMAD1, SSH1, TNFRSF17
Negative_4175	GH2, ALG1, AXIN2, CD70, CTSS, EXTL3, FASN, FLT4, FUT4, GNG13, HNF4G, ICOSLG, IL6R, MMP7, OR1J2, PCK1, PIGA, SETMAR, TAS2R4
Negative_4176	ACOX1, ANAPC10, CHPF, EPAS1, FGF11, IFNA8, KIR2DS4, NNMT
Negative_4177	CR1, LPO, ACOX1, CACNA1H, CCKAR, CLDN14, ENTPD6, ERBB4, INPPL1, MAN2A1, NAT1, NCSTN, OR4C13, OR9Q2, PHKG2, PIK3C3, PLAU, RPIA, SETD2, TAS1R2, ATP6V1C2
Negative_4178	CYP4A22, DNMT3B, IRS4, JAG1, NUDT5, OR5C1, OR5L1, SETD1A
Negative_4179	CD4, CFI, F9, AGPAT3, AK3L1, AKR1D1, ALG2, AQP7, ARD1A, ATN1, CDK7, CLCF1, CYP2A13, DARS2, DGKE, ENTPD6, ERBB3, FSHR, GGT7, GMDS, GSTO1, IFNA8, IQGAP3, ITGB7, LYZL1, MAP3K1, NPAS2, OR10K2, OR51S1, PNLIP, PPP2R2D, RFXANK, SFRP5, SOCS2, TDO2, TPH1, TRAF4, UBA7, VAMP3, HLA-DQA2
Negative_4180	CFH, GAA, AGPAT2, ANGPTL4, CLDN1, GALNT12, LAMC3, NRXN3, OR51D1, OR8H2, PLAT, POLR1D, PTDSS1, ROBO2, YKT6, TNFRSF12A, TNFRSF1B
Negative_4181	F8, GPAM, METTL6, OR52N4, OR6C76, SLC2A1, SULT2B1, PRICKLE1
Negative_4182	BAX, CDA, DCC, SI, ABLIM1, ADIPOQ, ARRB1, B4GALT6, CACNG8, CDC2, CFLAR, CHD8, DPYSL2, DUSP16, EGLN1, EI24, HEXA, IL13RA2, IL17A, IL22, IL28B, INHBE, KIR3DL1, LALBA, MAP3K2, NFKBIA, NMNAT1, NR1D1, NRCAM, OR4D6, OR51I2, OR51L1, PIGL, PPP2CA, PRIM2, RBL1, SPRED2, TAS2R16, TAS2R42, TPK1, TRDMT1, UGT2B17, MAPKAPK5, PRICKLE2
Negative_4183	CTBP1, OR56A1, RAB23
Negative_4184	CRK, DLD, IL2, MTR, SRF, ABAT, AKR1B10, ALDH3A1, ANAPC10, ATP5J2, ATP6V1H, CHEK2, DGKI, DNMT3L, EPB41L3, GLB1, GNG3, GZMB, HIP1, HMGCS2, HRH2, HSD17B3, HSD3B1, INADL, L1CAM, NCF4, NKX3-1, NQO1, OR2M3, OR4F6, OR4X1, OR7A10, PARVB, PCNA, PIGA, PLA2G4A, PRKACA, RERE, RPSA, SETD2, SRGAP3, STAT4, TNFRSF6B

Negative_4185	DBH,VTN,AMY2A,ANAPC5,CDC45L,CFL1,CNTN1,CTBP2,CXCL3,DDX55,ETNK1,HLA-C,HMGCS1,IL15RA,IL28A,INPP4B,IRF5,JAG2,KLRC1,MADCAM1,MICA,NUMBL,OR2A4,OR52A5,OR5F1,OSMR,PARD3,PLD2,PRKCI,RASGRP1,SPRED2,TCIRG1,WNT3,WWP2
Negative_4186	ACACA,ACTN4,ASS1,C1GALT1,CD36,DDX47,F2RL1,FGF8,FZD8,GMDS,KIR2DS5,MARS2,NFKB1,NFKBIE,NME7,OGDH,OR10A4,OR51L1,TSC2,B4GALNT1
Negative_4187	ALDH3B1,AREG,ARHGEF7,ESPL1,ETNK2,GGT5,IMPA2,OR2F1,SOCS2
Negative_4188	CD2,CFH,FAS,GK2,WAS,ADCY4,ADH1A,ALDH1B1,ALDH5A1,ALDH6A1,CCL2,CHEK1,CXCL10,CYP3A5,FGF2,FLOT1,GNAI2,GSTM1,GUSB,IL17RA,NT5C1B,OR3A1,OR4D1,OR52N5,OR56A1,OR6C74,OR8G1,PAK2,PNPLA4,POLR2D,PRSS2,PTPMT1,SFRP1,SRGAP2,STAT1,TNFRSF4,VAV1,YES1,PDCD1LG2,PNLIPRP2
Negative_4189	ANAPC10,FGF10,NFKBIA,OR1E1,PPP1R3B,RPS6KA3
Negative_4190	AKT2,ATP13A2,ATP2A1,BAI1,CCL21,E2F1,GLDC,HLCS,NADK,OR2D2,OR5AC2,PDHB,PLOD3,PPA1,PPP1CB,PTPN7,PVRL4,RHOA,SARDH,SEMA3F,SLC25A4,UGT1A10,VANGL1,WNT5A,ST6GALNAC6
Negative_4191	C8B,FER,TNC,ACTA1,ATP6V1H,CCNE2,CDC2,COQ3,CYP2A13,GYS2,HSD3B7,INHBB,ITGAE,LCAT,MDM4,NUDT5,OR1A2,OR2T29,OR8S1,PAK2,PANK2,PFKFB4,PIP5K1C,PPP2R2C,RFWD2,VAMP4,ATP6V0D1,MAPK8IP3,SERPINF2
Negative_4192	ACSS1,ADH5,CSNK1G3,EPHA5,EVI1,EXTL1,GDF6,GLI2,GOT2,IL24,MGST1,PFKM,RUNX1,TPMT,WNT7A,C1GALT1C1,MAPKAPK3,PNLIPRP1
Negative_4193	AK5,FAH,ACVR1,ADRB1,ARHGEF2,ATP13A2,CLDN5,CRHR1,DTX3,FGFR1,GSTM2,LAMB1,LTBR,MAP2K4,MPP5,NR4A1,OR10K1,OR2A25,OR2A4,OR2B11,OR52E6,PDE8B,PLAU,PTGER3,UCK1,VCAM1
Negative_4194	DLD,RFK,BMP4,CACNA1I,CCNG2,CREB5,DOLPP1,EPHA1,GFAP,GMPR,HCLS1,HTR2C,LDHAL6B,MAN1A2,MAP2K7,MCAT,MYH7B,OR2A5,OR5V1,PGAM1,PMVK,PPP1CC,ST8SIA5,VAV3,WBSCR22
Negative_4195	C7,ABL2,ARAF,BUB1B,CYP4F3,DAAM1,EHMT1,ENPP7,FBP2,GDF6,GZMA,HHIP,INPP4A,ITGA2,NCK1,NRXN3,OR2L2,PRKAR1B,RDH10,SCD5
Negative_4196	F10,SDS,AACS,ACHE,ACYP2,ATP5O,CD226,CHAD,CLDN20,CREB3L4,CTPS,CYP8B1,DLL4,EPHB3,FABP1,GRIN2D,H6PD,IRF3,MIOX,MPST,MYH6,NOX1,NRCAM,OR1G1,OR1J1,OR2T6,OR4M2,OR51E1,OR5L2,PLCB1,PORCN,PPT1,SDHC,STAT5B,TALDO1,TBXAS1,TICAM2,TM7SF2,TOMM40,TRADD
Negative_4197	AK2,C9,CBS,PC,SMO,AARS,ABLIM3,ACY1,ARPC1A,BLVRA,CAMK2D,CCL23,CDKN1C,CHRM1,CHST12,COL2A1,COL5A2,COMP,CTNNA3,DUSP5,ENO3,FRAT2,HTR2A,IRF9,MAML1,MKNK2,NARS,NCAM2,NEU1,NFKB2,OR1F1,OR1L1,OR52N5,OR52R1,OR56A1,PCK2,PPARG,PPP2R5E,PRKCD,RPSA,SDHB,SMC1A,SPRY3,STEAP3,TJP1,WNT6,WWP2,ATP6V1C2
Negative_4198	BTC,FN1,HK2,PVR,ABAT,ACOT12,ADRB3,ALPI,ATP5B,ATP6V1A,B3GALT1,CD74,CDH4,DDX23,DTX2,EFNB2,GALNT8,GLYCTK,HSPA1B,ITGA4,LCMT1,MCAT,MYH2,NADSYN1,OR10J1,OR51B2,OR5D18,POLD2,POLD4,PRKAA2,PTGER1,SDHA,SGPP2,SOCS4,SPRY3,ATP6V1G1,PHOSPHO1
Negative_4199	ACSM3,BET1,BMP7,CHPT1,CREB5,CUL1,CYP2A13,GNG3,INADL,KLKB1,LYZL1,MAN1A2,OR14I1,OR2AT4,OR2T10,OR4F15,OR51F2,PANK2,PDCD1,PIM1,RBPJ,SARS,SC5DL,SETD1A,TAS2R41
Negative_4200	CBS,ALG9,BCAT1,CREB1,HSP90B1,ODC1,SULT2A1,THY1,VDAC1,HSP90AB1
Negative_4201	ADCY4,ALDH5A1,ANAPC2,ARSE,CLDN2,EGLN1,GALNT2,GRIN2A,IRF9,ITGA10,NRG3,OR7A17,OR8U1,PLA2G3,RRM1,THBD,TRAF3,TUBB,VEGFC,WNT3,WNT7A

Negative_4202	AADAT,ALG11,B3GALT2,CCL17,CLDN15,FZD6,NLGN2,OR14I1,OR1J1,OR52E6,OR5K1,PTDSS1,TAS2R8,ULK2,ZBTB16,MAP3K7IP1
Negative_4203	CCDC6,NLGN1,PAFAH2
Negative_4204	C1S,LAT,LHB,ABCC8,AKR1B10,ALDH1A3,ALG10,ALG10B,BDH2,CASK,CLDN4,CREB3L3,CTSB,DKK2,FGD1,GADD45G,GALNT5,HBEGF,IFNA8,IL18R1,ITGA9,MTFMT,OR1C1,PARD6B,PIM1,PLA2G2A,PRODH2,RALA,SEMA3F,TRPC1,UPB1
Negative_4205	ADH5,AMD1,CACNA1F,CACNB3,CYP1A1,DHRS9,DUSP1,FARSA,FGF5,GGT1,GLUD2,GP1BB,GUCY1A3,IRF3,NARS2,OR13A1,PRSS2,PTAFR,RXRA,SGPP1,TUBB6,WNT8A,ZIC2,TNFRSF10A
Negative_4206	ITK,ABLIM1,AK3L1,AKR1C2,AKT2,CADM3,CCL4,CLDN6,DGKH,DLAT,EXTL1,GBGT1,GNPNAT1,GUCY1B3,IARS,IGF1R,IL13RA2,NFKB1,OR2Z1,OR4A16,OR8D4,PLXNB3,PTGIS,STAT1,TSC1,YWHAE
Negative_4207	AK5,F5,ACADM,ACSM2A,ALG11,ANAPC1,ARAF,BPGM,COL4A6,DAD1,DGKH,FUT8,GALM,GALNT2,GBGT1,ITGA3,ITGB7,LEF1,MCM2,NT5C1A,OR4P4,OR52N2,POLR2G,PYCRL,RARA,SSX2IP,TM7SF2,TNXB,VTI1B,ATP6V0A2
Negative_4208	AK3,ASL,CD6,CFI,MSN,OSM,ADCY4,ALG11,ALG6,ANAPC4,BAI1,CDK7,CNDP1,COL11A1,CSNK1G2,CYBB,CYP2C8,DAAM2,DCXR,DGKD,DLAT,EPHA5,ETNK1,FUT8,GALNT13,GALT,GGT5,GNAT3,GRM1,IL10RA,IL15RA,JAK1,METTL6,MTMR1,NANS,NLGN1,OR6T1,POLR1C,SETDB2,SFRP2,SLC25A5,SOCS4,STX2,SV2C,TLR2,TPH2,ST6GALNAC3
Negative_4209	RB1,ATP2A3,DHCR24,FOXA3,FUT6,NKD2,NOS1,NT5E,OR2G6,PGK2,PPP1R3B,RASSF1,SKP2,SPRY1,STAM,TUBA1A
Negative_4210	FH,BDH1,CADM1,CCR8,CES1,FMO5,IFNA2,IL20,MCCC1,MKNK2,NRXN1,OR2H1,PRKAB1,RHOD,SC5DL,TYMP
Negative_4211	AK5,MTR,NNT,ACSS2,AGPAT3,AMHR2,AOX1,AQP7,CASK,CPT2,CRKL,CSF1R,EXT1,F13B,FZD10,GPX5,GRIN1,GRM1,HHEX,KIR3DL1,MTMR1,NCKAP1L,NCOA4,OR13H1,OR1J4,OR52A5,OR8A1,PHKA2,PIP5K3,PKMYT1,PLA2G1B,PRKG1,PTCRA,RALA,RND1,RXRG,SORBS1,TPM3,UBA7,VASP,VTI1A,B3GALNT1
Negative_4212	NGF,ABI2,ACAT1,ADCY4,ALOX15B,AMPD3,ATP2A1,BDNF,BMP5,BUB1,CACNG7,CALML3,CCR10,CLCA2,CREBBP,EGLN1,EPHB6,EXTL3,FBP1,GSTM2,HAO2,IL1RAP,NFATC4,NOX1,NRXN2,NTF4,OR10A4,OR10K1,OR13C2,OR2F2,OR52N1,PHKB,PPP1R1A,TSNARE1,VDAC3
Negative_4213	PAH,AMY2B,FMO1,IL25,MGAT4A,NRCAM,OCRL,OR6Q1,OR7A5,PIP4K2A,PRKAR2A
Negative_4214	MIF,MOS,ACACA,BRAF,C1QA,CARD11,CCDC6,CCKAR,CNTNAP1,IL24,LDHC,MCAT,MCM5,MNX1,MYH4,NFYB,NKX6-1,NODAL,NR4A1,NTSR1,OR10H5,OR1N2,OR2G2,OR6B2,PEMT,PRKAG3,PSME2,SNAP23,TFDP1,TPI1,C1GALT1C1,MAPK8IP3,TNFRSF1A
Negative_4215	UBB,ACAT1,ALDH1A1,CASP3,CCNG2,CYP7A1,EP300,GAS1,H6PD,IFNA7,MAD2L1,NOTCH1,OR10P1,P2RX1,PANK4,PDE1A,ST3GAL3,TGFB2,TGFBR1,TSTA3,B3GALNT1,TNFRSF11A
Negative_4216	ACSS2,BST1,CFTR,CRB3,CRLF2,CSF3,FUT9,GSTA3,NOD1,PMM2,PRKCZ,RASGRP3,TBL1Y,GABARAPL2
Negative_4217	AK3,MSN,ACADS,ACOX3,APC2,B4GALT6,CAMK2G,DAAM1,DLAT,DTX2,EIF4E,FGD1,IL25,ITGB5,NFYC,OR1J4,OR4Q3,OR6C74,RBPJL,SNAP25,UGT2B11,WARS
Negative_4218	ATP5B,BMP2,COL4A2,CREBBP,GBA3,IL17B,MGAT1,OR2B11,OR2T10,OR6C70,OR9G1,PIGS,PNMT,PTGES,SPRY2,YWHAE,ATP6V0E1,SUV420H1
Negative_4219	FGA,PVR,RDX,ACO1,CER1,CHD8,CRB3,DAPK1,DARS2,DMGDH,DTX4,GNAT3,GRAP2,IL10RA,MAT1A,OR1D5,OR1G1,PIGM,PPAP2A,PRKAG3,PTPRF,RHOA,RHOD,SDC4,TKTL1,TSC2,UPB1

Negative_4220	DCK, SPN, ARD1A, CDKN2C, DGAT2, DTX2, DUSP4, ELK1, EXT2, GGT7, GPX7, GSTZ1, GUCA1A, HDAC1, IAPP, ITPA, KYNU, LARS, LCMT1, MAPK9, NANP, OR10A4, OR2G3, OXSM, PIK3C3, PIK3CA, PIP5K1A, PLAU, PPP1CB, RASGRP3, ROBO3, RPE65, SELP, SMARCA5, TRAF5, TRAF6, UGP2, ALDH18A1
Negative_4221	AGL, F3, F7, ACSM1, ACY3, ASRGL1, C3orf10, CASP10, CCR8, CHSY1, CREB3L1, DHX58, ETS1, FLOT2, FZD4, GALE, GALK2, GALNT5, GART, KCNB1, KIR3DL1, LCP2, OR6C75, P2RX1, RHOA, RXRA, SMAD7, SPHK2, UGT2B4
Negative_4222	ARG1, CCND2, CTSS, FGF12, FGF18, NT5C, PON1, TNFSF10
Negative_4223	GP5, TAT, TFG, ACP6, ACTB, AKR1C1, ALG6, B3GNT4, C3orf10, CD99, CDH3, CREB3, DDX56, FASLG, GAB1, HES1, IFNA16, IL10RA, IRAK1, IRAK4, NFASC, NFE2L2, NFX1, NPR2, NRXN2, OR13H1, OR4K5, OSMR, PFKFB1, PPA1, PRDX6, PRKAA1, PRKG2, SSH1, TACR2, TNFSF18, TSTA3, ULBP3, UNC5A, HLA-DRB4, MAPK8IP2, ST6GALNAC3, TNFRSF1A
Negative_4224	ADORA2A, AHCYL2, ALAS2, APOA1, ATP2A2, ATP5B, ATP5F1, CD47, CDKN2D, ENAH, EXTL2, FGF11, GALC, GLI3, HSPA1B, IFNA13, IRAK4, ITGA5, KIR2DS5, LAMC2, MCM2, NAMPT, NARS, NCOA4, NOX3, OR13G1, OR2AG2, OR4A5, OR51E2, OR51S1, OR52L1, OR6F1, PDE4B, PITX2, POLR2A, PTPRB, RAC1, RENBP, SEMA3G, SPRY4, TAS2R49, PPARGC1A, ST6GALNAC3
Negative_4225	A2M, GP6, BCAR1, CDC6, CFL2, CYP2C18, DGKG, ESAM, F2RL1, FGF22, ICOSLG, IRS4, KNG1, LIPA, MAP4K2, NAT2, PIGP, PLCB4, PRIM1, PTPRJ, RAPGEF1, RND1, SEMA4G, TBXA2R, TSC1, VKORC1, WNT5B, CACNA2D2
Negative_4226	CASP6, MGAT4B, OR2B11, PDGFA, PLA2G5, PLCD3
Negative_4227	DDC, RB1, RFK, ALAD, BDKRB1, CCL4, CCNE2, CHKB, CHRM5, CSNK2A1, CXCL9, CYP2A13, CYP3A43, CYP3A7, DDB2, ERBB3, GALM, GUCY2F, HSD3B1, IGF1R, INHBE, MAP2K5, MARS, MEF2C, MYH7B, NOS1, NRG2, OR13C8, PCCB, PIGM, PLCD1, PNPLA4, PPM1A, PRKAG1, PTPRJ, RRAS, SDHA, SPHK2, TRHR, XYLB, B4GALNT1, CACNA2D2
Negative_4228	C3, TFG, ACVR2A, CYP26B1, FGF17, GLG1, HHIP, MAP4K2, OR13C4, POLE, SESN3, UROD
Negative_4229	GHR, APC2, BLVRB, BTRC, CYP2D6, DUSP9, FGFR4, FIGF, GALNS, GALNT2, GMPPB, GNPDA1, IBSP, ICOS, IL1A, INSRR, JAG1, OR51E1, OR51F1, OR6M1, PPCS, RASA2, RASGRF2, RDH10, SDC2, TAP2, UGT2B7, WARS2
Negative_4230	F5, GSS, ACVR1B, ADCY3, C9orf95, CCL11, CCR3, CD226, CLDN15, COQ3, CXCL16, EIF4E2, EXOC7, FGF9, GNAL, HADHB, HAGH, IFNA6, JAK1, MYH10, NRXN3, OR10H3, OR14C36, OR1L8, OR1Q1, OR5P3, PFKFB4, PPM1D, PRKAB1, PTGFR, RPS6KB2, RRM2B, SPTLC2, STT3B, SYNJ2, THBS3, UCKL1, VNN1, YWHAG, CSNK1A1L, HLA-DPB1, SERPINB5
Negative_4231	ADSL, ARHGEF4, CPT1B, CYP2A6, EPHA6, FUT8, GBE1, GCNT3, HTR2B, ITGA8, NFKB1, PHPT1, PRKAB2, PRKAR2A, PRSS1, PSEN1, STAT6, STEAP3, ATP6V1C2, SLC25A31
Negative_4232	C9, ACSL1, ALDH1L1, ALG10, ATIC, ATP6V0C, BDH2, CALML5, EFNB2, ELK4, GALNT11, GPT2, IGF1R, MGAT4B, NRAS, NUMB, OR10A2, OR10P1, PDPK1, PIAS4, PIK3C3, PPP3CB, RIPK1, SDC2, SUV39H1, TNFSF8, ATP6V1C2
Negative_4233	C7, ABL1, ABP1, ACSM2A, ATG7, ATP6V1A, BUB3, CNTF, G6PC, GSTM1, HEXA, HLA-F, IFNB1, IL22RA1, LAMB3, LYPLA2, NCR3, NFYB, NNMT, OR10R2, OR2B2, OR52A5, PAK3, PFKFB1, PPARA, PPP2R5E, PRKAR1A, RAC2, SULT2B1, TUBB2A, VEGFC
Negative_4234	ATM, BAX, F10, ME3, TYR, ACSL6, BMP8B, C3orf10, CLTB, ELK1, G6PC, GNA11, IL13RA2, INHBB, ITGA11, ITGA4, LDHAL6B, OR11L1, OR1S1, OR4C15, OR4C6, OR51I1, OR7C1, PHPT1, POLA2, PPARG, PTGDS, RHEB, UCP1

Negative_4235	FN1, GAA, ABL2, AGXT2, AKT2, ALDH1A1, CDKN1A, COL2A1, CSNK1G3, CXCL16, DOCK1, DUSP16, ENO1, ENTPD5, FPGS, IFNA6, IPMK, KAT2B, MAP3K4, NT5C, OR52I1, OR5AK2, PIK3R5, POLA2, PPP3CC, SNAI2, SSH2, SULT1A2, TP53I3, UBE2L6, UGT8, MAPK8IP2
Negative_4236	GAA, ACOT12, ADRBK2, ALDH3B1, DTX1, INPP5A, MECR, NOX3, NRXN3, PFKP, POLR2E, RXRG, SEPHS1
Negative_4237	DBH, GBA, ID4, IL2, SI, AKR1C1, ATP5B, CLDN9, CSF3, FGF21, IDI2, MASP1, MCCC1, MCM6, OR52W1, PFKFB1, PLA2G5, PLCD1, PRSS2, RIPK1, SMAD3, SPCS3, STAT2, XCL2, MAP3K7IP2
Negative_4238	AGL, EPHA3, FMO1, GALNT3, GNAI1, IQGAP2, TBXA2R
Negative_4239	CR1, DLD, F5, GP5, SRF, ABAT, ALG11, ANAPC11, ATP5L, B4GALT7, CD58, COL6A1, CREB3L2, CYSLTR1, FMO5, HADHB, HSD17B3, IARS, IMPDH1, INMT, INPPL1, ITGB4, MGAT5, NNMT, NODAL, NQO1, ODC1, OR10J3, OR10P1, OR11H6, OR51G2, OR52I2, OR6K6, PGM3, PKLR, PLA2G5, POLR3A, RRM2, SDC1, SOCS7, TACR1, USE1, XCR1, TNFRSF17
Negative_4240	GP9, ALDH1L1, CALML6, CDC20, EHMT2, FRAP1, TM7SF2, WNT8B
Negative_4241	MVD, ADIPOQ, CDS2, GLI1, ICOS, OR2C1, OR52K1, PDK1, SDHD
Negative_4242	AUH, LTB, ATP6V0B, C4BPA, CDC23, CYP27A1, DAAM1, OR10C1, OR1N1, POLR2J, SSX2IP, UBA7, WNT10B
Negative_4243	ADO, CFH, MET, STS, ACPP, ARSE, COQ3, DERL1, DUSP9, GBGT1, GRIA3, GUCY2D, IL22RA1, JAG2, LEPR, LGMN, MAT2B, MCM7, MLYCD, MYH11, NADK, NKX3-1, NSDHL, NTN1, OR10S1, OR52K2, PAICS, PER2, POLE2, PPP1R3A, RHEB, RRM1, SC5DL, SHMT2, SMAD3, SNAI1, SPAM1, STX8, SULT2B1, TCF7L2, TIAM1, VAMP2, WNT16, ATP6V1G3, HSP90AB1, TNFRSF12A
Negative_4244	ANAPC2, ASS1, BACE1, C1QB, CCKBR, CCNB2, CDKN2A, COMP, CRB3, CSF1, EPHA2, ERO1L, FGF23, FGF4, GUCY1A2, IL6ST, LDHAL6A, MYL2, NANP, NSDHL, OR2AT4, OR5B12, PDE5A, POLR1D, POLR2F, SESN1, WNT3A, XCR1
Negative_4245	AUH, C2, DUT, TPO, AGPAT3, ALPPL2, AMPD1, ASMT, CMAS, COMP, DPM2, EPHA7, F2RL3, FABP1, FLOT2, FZD8, GANAB, GLI3, HLCS, HTR5A, IFNA6, ITGA9, LYPLA1, MAP3K3, MCAT, MKNK1, NT5C, OR51I1, OR5B21, PDE1A, PPCS, PPP1CB, PRPS1, RBX1, SFRP4, SMC1B, TAS2R14, THBS2, VASP, WIF1, ATP6V1G1, GABARAPL1
Negative_4246	ADA, HPD, ACVR1B, ADIPOR1, ARPC4, CES7, COQ6, DGKG, DHDH, DUSP1, E2F5, ENTPD3, GNAI1, GSTM2, GUCY1A3, HSD17B4, HTRA2, IDI2, IL13, LTBR, MAML2, MITF, MYH10, ONECUT1, OR4C12, PDE8A, PGS1, PIK3C3, QARS, RBL1, RFNG, SDC4, SELL, SEMA3E, SMARCA5, STX3, TGFB2, UGCGL2, WNT6
Negative_4247	FAS, GPT, IL4, ACSM2A, ARHGEF2, ATP2A2, CAMKK1, CBR1, CHUK, CISH, CYP4A11, DDX18, DDX19A, DMGDH, EFNA2, EPHA7, GCH1, GLUL, GSTT1, HEXB, IFT57, IL22, KIR2DL2, KLRC2, LAMA4, NODAL, OR2S2, OR4D1, OR52A5, OR52K1, OR5I1, PPP2R5D, PTPN5, PVRL1, RXRG, SDC2, SGPP2, SHC4, STX6, TBXAS1, ATP6V1E2, MAPK8IP2
Negative_4248	ACMSD, ALAS1, BLVRA, CASP10, CD55, CHPF, EHHADH, FIGF, GAB1, GSTO1, HAAO, HBEGF, HSD3B1, HSD3B7, IBSP, LAMA3, MGAT4B, NODAL, OR10J1, OR2T3, OR4Q3, OR52W1, PDE11A, PDE4A, PGM1, PGM2, PLCG2, RDH10, SULT1E1, TAS2R5, TGFB2, THTPA, TLR5, TNFRSF18
Negative_4249	DBI, AARS, ACTC1, AHCYL1, DDX4, DGUOK, ENPP7, GSTM1, HARS2, IL13, ITGA8, KIR2DL1, LIFR, LRRC4C, MKNK1, OR2T8, ORC6L, PMVK, PRKG1, SESN3, TSC2, UROD, WNT7A
Negative_4250	BAD, ALAD, ALDH3A1, ALG8, BLVRB, CCR8, CD3E, CSNK1G1, ETNK1, HCCS, MLLT4, MTMR6, MYH2, NADSYN1, NAT5, PLTP, PNMT, SOCS1, SULT1A1, MAP3K7IP1

Negative_4251	AGL, F11, JUP, ABLIM2, ACOX1, ACSS3, CHST11, CHST13, CLDN14, CRY2, EPHA8, EPHX2, FMO4, GCLC, GRIN2B, HADHB, HEXA, IL22RA2, IL29, IQGAP1, JUND, LIMK1, OR10A2, OR10R2, OR11H4, OR13C9, OR4K1, OR51F1, OR51M1, OR5K2, PHKG2, PIGL, PON3, PPP1R3D, PRKCE, PSME2, PTPRF, SARS, SHMT2, SMAD6, ST3GAL4, STX18, TUBB2C, WNT1, YES1
Negative_4252	NGF, RET, ADSSL1, ANAPC10, ANGPTL4, ARSD, BDH2, BMP4, CDC16, COL3A1, CPS1, CSF3R, DLL3, DPYD, GALNTL4, GCLM, GDF7, GIT1, HAGHL, HLA-C, IDUA, IKBKG, JAG2, KIR3DL1, KNG1, LDHB, MIOX, NTRK2, OR1J1, OR5H2, PIAS4, PLCB4, PPP2R1B, PPP2R5D, PSAT1, PVRL4, SHC1, ST8SIA1, SUFU, TNFSF4, TRPM5, UBE2L6, UROS, WNT1, ATP6V1B1
Negative_4253	CD4, FES, ARHGAP5, CNTN2, DARS2, DOT1L, GBE1, GLB1, HTR4, ICAM1, MCM2, MTHFD1L, MYH8, NRXN2, OR2A5, OR4K15, PLXNA1, TAS2R38, UCKL1, ATP6V0A2, TNFRSF11B
Negative_4254	ACACA, ADH7, ATP2A1, ATP5G2, B3GALT2, BCAT1, CD226, CDC25A, CLDN17, CLDN20, CNTFR, CSNK1G3, CXXC4, DAPK3, DPYD, DRD2, FDPS, GDE1, GJD2, GRM5, IARS, INPP5E, ITGAD, KNG1, LDHA, MAP4K2, MYH9, OR2H2, OR52B6, PFN4, PIK3R2, RAPGEF1, RASA2, SMURF1, TUBB2C, UGT2B4, UXS1, CSNK1A1L
Negative_4255	C4B, DBT, RET, AMPD1, CHRM1, CTBS, FGF7, FUT7, ITGB2, MAN2A1, OR4C11, OR51B2, OR51F2, OR6T1, OXCT1, PDE8A, PDXK, RAC3, UNC5D
Negative_4256	C8B, DLD, EGF, ACVRL1, ANAPC10, APPL1, BCL2L1, CDC27, CHKA, CLDN19, CLDN7, COL4A4, DBF4, GALNTL2, HAP1, ITGA1, LTBP1, LYPLA1, MAPK3, MGAT2, MYH1, NFYC, NUDT9, OR2AT4, OR4D6, ORC6L, P2RX2, PLXNC1, POLD3, PON1, PPARA, PPARG, PVRL2, PYGL, RASGRP1, SDC4, SYNJ2, TPMT, VAMP1, VASP
Negative_4257	CS, ACADS, ALCAM, B3GNT3, C1GALT1, CYP2F1, DIAPH3, DUSP2, F13A1, FCGR2B, IDUA, MMP1, NME6, RAP1A
Negative_4258	FYN, ACVR2B, ATP2A3, CISH, DGKA, H6PD, LPAR1, MAPK7, MTHFS, MYH11, MYL2, NLGN1, OR10G2, OR10J5, PIK3R2, RDH10, RPS6KA1, TGFB1, TUBB4Q, UCK2, HLA-DQA2
Negative_4259	AK5, C8A, CAD, CD6, CTH, INS, LPL, AGPAT6, AKR1B1, ALOX15B, BUB3, CACNG1, CDK6, CDK7, CTBP1, EREG, GALNT6, GNRHR, IDH2, IL11, JUND, LDHC, LIAS, MCM4, MGAT4B, NCR2, NEU1, NEU2, NKD1, NRG4, OR2A25, OR4D9, OR56A1, OR5F1, OR6C75, PDPK1, PRKCI, RALB, RAP1B, TAS1R2, ATP6V0D1
Negative_4260	AGL, CD6, CS, DUT, AACS, ACTB, ARPC4, B3GNT4, CCL2, CD58, CNTNAP2, CXCL16, CXCR6, DTYMK, EFNB2, F2RL3, FGF3, FGF9, GUK1, HMGCL, HMOX1, IBSP, IL12RB2, IL20RB, JAM3, NRAS, OR10A6, OR1J2, OR2A42, OR5B17, OR6C1, P2RX4, PFN3, PIK3R1, PLCG2, PNPO, RASSF1, RERE, RHOQ, SLC33A1, TJP2, TPM3, UGT2B10, ZFYVE9, TNFRSF18
Negative_4261	BCL2L1, CCKAR, ITGB6, MYH11, NCF2, NRG1, OR5K1, PARD6B, PYCR2, RFX5, STT3B, SYNJ1, UGDH, XYLB, ZMAT3
Negative_4262	CTH, SRF, TNF, ACADS, ALCAM, AMPD2, BCL2, BLNK, CASP7, CKS1B, COL4A6, CREB3L1, CYP2B6, DRD1, FLOT1, FZD7, GCNT4, HDAC2, ICAM3, IL17A, MAT1A, MPP5, MYH14, NAE1, NFATC4, NUMB, OR2A14, OR4A15, OR4K5, OR5K1, PTK2B, RASGRP1, ST8SIA1, TJP2, UGT2B7, VDAC2
Negative_4263	C9, GSN, ADRA1D, AGRP, BBC3, BLVRA, CACNG2, CNGB1, ENPP1, GNAQ, GRIN2B, INHBA, MAP2K5, MAP4K4, NANS, NEUROD1, OR8K1, OXCT2, POLR2E, RASGRP2, SMAD6, STK11, TRAF6, ATP6V0D2
Negative_4264	ALPPL2, ITGA9, NR1H3, OR8A1, PFKFB4
Negative_4265	DCK, FH, ADRBK2, ALDH4A1, APBB1, CAMKK1, CD55, CEBPA, CNTFR, COQ7, CYP4F3, DDB2, DHRS4L2, DNMT1, F2RL3, FCER1G, GPT2, GPX5, IBSP, IL13, IL6ST, IQGAP1, KIR2DL2, KNG1, NCKAP1L, OR1D2, OR4S2, OR4X2, OR6C2, OR8D1, OR8H3, POLR2K, PRKX, SEMA4C, SETD1B, SH2D1A, SORBS1, SPP1, ST3GAL4, TCIRG1, TJP3, TMLHE, TSC2, TSLP, VAMP2

Negative_4266	ASL, HK1, JUN, MAG, SMS, ALG2, ALG9, ALOX12B, APBB1, CACNG3, CBR1, COL5A2, CX3CR1, CXCL16, DPAGT1, DUSP16, EPHA2, EPHB4, GALNT12, GALNT13, GMPR, IRF3, LAMC2, MGAM, MNX1, MTMR2, NAT2, NSD1, OR13C5, OR1F1, OR2T4, OR5B17, OR6N1, PIK3C3, PLXNA1, PRODH2, PSME1, RPN2, SDC4, SOAT1, SUV39H1, TAS2R1, TCF7L1, TDO2, THBD, TOMM40L, ZFYVE9, ATP6V1G3, TNFRSF1A
Negative_4267	CAT, GH1, AARS, ACADM, ACY3, ADCY9, AKT3, ALDH3A2, ALOX5, BCAT2, BLNK, BUB1B, CCKBR, CCNB3, CHRM5, CMPK1, CXCL5, CYP8B1, DUSP7, FOXO3, GALK2, GALNT1, GBE1, GMPPA, GSTM3, HSD17B1, IL22RA2, MAT2B, MLLT4, MRAS, NAMPT, OR10H5, OR1I1, OR4X1, OR5AN1, PIK3R3, PPAP2B, PTAFR, PVRL2, RPS6KA5, SAT2, SELP, SEMA3C, UGCG, CACNA2D1, HLA-DRB3, TNFRSF10C
Negative_4268	MSN, NPY, SRF, BCKDHA, CCKBR, CLTA, CSF2, CXCL16, DVL3, FLT4, GMPR2, IDI2, IL22RA1, OGDH, OR10G9, OR10R2, OR1J2, OR2F2, PFKP, PPOX, RPS6KA2, SKIV2L2, UGT2B10, ATP6V1E2
Negative_4269	IL4, MPZ, UBB, ACLY, AKR1C4, CD80, FGF2, FLAD1, HMGCS1, IDH3G, IL1R1, IL20RB, LHCGR, MCM5, NME7, OR13C8, PARD6A, PDGFC, PFKFB4, PIK3C2B, POLR2F, PVRL3, TBL1X, THY1, VTI1B, PLA2G12B
Negative_4270	CAT, ABCC8, ACVR1C, AKR1B10, ALOX15B, ASMT, C1GALT1, CCL1, CCL3, CCNG2, CNGB1, DDX56, ENPP7, EPHA7, HLA-DRA, HNF4G, IAPP, ITGAE, MMP2, MYLK3, NSDHL, OCLN, OR10A3, OR5D16, ORC3L, PPP2R2D, PRKACB, QARS, SMAD7, STX6, SUCLG2, TPK1, XCL2, XCR1, YARS2, SLC25A31
Negative_4271	CR1, DBI, LPL, SP1, TNR, AGPAT6, BAAT, CAMK4, CCKAR, CFLAR, CHRM1, CSNK1D, DFFB, DHODH, DLL4, DRD1, DVL3, ENTPD8, ETNK2, FADD, FGD3, GBGT1, GPAA1, HLA-DOB, IGF1R, INPP5E, IRAK4, ITGAD, MAPK10, MDH1, OR10A2, OR4D5, OR4D6, OR5P3, OR7D2, PARVB, PNLIP, PRKCB, PTPRZ1, RAC3, RFXANK, SFRP4, TYK2, TNFSF13B
Negative_4272	IL6, ACTN1, CD274, CD28, CD8B, CMAS, CSNK1E, DGUOK, EARS2, FGF18, FGF23, GAD2, GLG1, IRAK4, KLRK1, MYH1, NAGK, NANS, NEU2, NGEF, NUDT5, OR5B3, OR8D1, PPP2R5D, RUVBL1, SGMS2, SSH2, STX7, SUV39H1, TRAF6, VTI1B, CSGALNACT2, MAPK8IP2
Negative_4273	BDNF, OR10A5, PCK2
Negative_4274	LPL, CCNH, CFLAR, CYP2C9, DBF4, FGF19, FLT4, FRAT1, GALNTL1, KAT2A, NCKAP1L, NCOR1, RUNX1, SRD5A2, WNT6
Negative_4275	HPD, JUN, LPL, MVD, ABCC8, ABL2, GLUD2, IKBKB, MAN1B1, NARS2, OR2AG1, OR2H1, OR4K5, PIK3R5, PORCN, RARA, TFPI
Negative_4276	DCT, AMY2B, B3GNT4, CREBBP, ITGB3, LAMC3, OR2S2, PGM3, PIK3C2G, PPP2R5D, PRKAR1A
Negative_4277	CXXC4, FUT1, FUT5, GOSR2, KARS, MYL5, NCF4, PDE6G, PPA2, SEMA3B, YARS2
Negative_4278	APP, ASL, ADH1B, AGTR1, ANAPC2, B4GALT4, C3AR1, CACNA1B, CD86, CFL2, CHST11, COL1A1, CYP4F2, DDX19A, G6PC2, GCLM, IFNA13, IL17B, IL6R, MAPK10, OR1D5, OR2Y1, OR3A3, PLAUR, PLD2, PPARG, RHOD, TAS1R2, TAS2R13, TNFRSF12A
Negative_4279	ADO, AGK, GLA, INS, ME2, ACY1, ATG7, CD79B, CFL2, CHKA, CLDN19, CTPS, CXCL6, ERBB4, F13A1, GPX5, GRB2, GRIA2, GSTA2, HSD17B7, ICOSLG, IKBKG, IL8RB, ITGB2, ITGB8, MMP7, NANS, NPR1, OR52I1, OR7C2, PFKP, PHGDH, PLCG1, PLTP, PTK2, PTK2B, PYGL, SCP2, ST3GAL3, SYNJ1, TCF7, TRAF4, WIF1, ATP6V0A1
Negative_4280	TAT, KDSR, THTPA
Negative_4281	ACHE, CLDN14, GLG1, IL1R1, ITGB3, TDO2, WHSC1L1, MAP3K7IP1, TNFRSF1A

Negative_4282	EPO,FH,ACSL6,ADCY4,ATP5G2,CACNA1B,CPT1B,DPM2,FASN,GALK1,GUCA1B,INPP5E,KYNU,MDM2,NME2,OR4K13,OR51A4,OR5AS1,OR7D4,PLD2,PNPLA4,PORCN,PPA1,PPP2R5A,PVRL1,RAC3,SEMA3A,SPAM1,SPRY3,UGCGL2,XYLT2
Negative_4283	AGL,EGF,AGXT2,ATP12A,CNTN2,FGF20,GCNT4,ICAM1,IFNA5,IFNB1,LCP2,NSD1,OR1E1,PIGA,PIM1,SHC3,SMURF1,SSH1,STX16,TAS2R1,TRIP10,TRMT11,VCAM1
Negative_4284	IDS,ADAM10,ALDH3B1,ATP6V1F,BDH2,ERBB2,HPRT1,LAMA3,OPLAH,RAC1,RAC2,SULT1A1,SYNJ2,TBL1X,TJP2,WASF1
Negative_4285	ADK,AK7,F12,ME1,SPN,XDH,AANAT,ACSM5,ATP6V0B,BDKRB1,CXCL11,CYP1A2,DHRS9,DRD1,EXT1,EXTL1,GBGT1,GNG12,GRIN1,IFIH1,IFNA21,IFNA5,IL3RA,INHBC,IRAK2,MAP2K3,MAPK11,MCM3,MGST2,OR4C11,OR4K5,PDE7A,PIAS4,PLK1,PPP2R5A,PRKAG2,PTK2B,PYCR1,SEMA4B,SULT1E1,TPK1,TUBAL3,UGT2B4,UGT8,PDCD1LG2
Negative_4286	C8B,KIT,TNC,ABLIM1,ADCY6,ALDH6A1,ALG5,APOE,ARSD,B3GAT3,CACNA1H,DAPK1,DDX23,DGKB,ERBB2,F2RL3,FARS2,FCGR2B,FDFT1,FGF6,GFPT2,GRIA1,KIR2DL3,MYH9,OR11A1,OR4S2,OR5R1,OR5T2,PCYT1A,PDE8A,PRKCH,RBKS,SMAD7,SMPD2,SNAP25,SOX17,THBS3,TICAM2,TNFRSF4,UPP2,ATP6V1C1,TNFRSF13B
Negative_4287	AMH,GSN,HGD,CCL23,CDK4,CLDN18,CLDN9,CMBL,CPS1,CSNK1A1,CXCR6,CYP11B2,CYP2S1,DPM2,EGLN3,GSTA5,IDH3A,IL8RB,INPPL1,LARS2,NMNAT1,NRG2,NRG4,NT5M,OR2AE1,OR2AG1,OR51A4,OR5L1,ORC5L,PDE4B,PDE8B,PHKG1,PMM1,POMC,PPP2R2B,PPP3CC,PPP3R2,PRKAA1,PTK2,RRM2,RUNX1T1,SETD2,SRGAP3,TCIRG1,TUBB6,UNC5C,VAV1,GABARAPL2,MAPK8IP1
Negative_4288	DHH,ADRA1B,CCL25,DAG1,DAPK2,DDOST,ELK1,EP300,EPHB2,ERBB3,HLA-G,ITGA1,NFYB,PFN3,POMC,PTPMT1,RASGRF2,RDH12,RETSAT,STAM,TAS2R16,XIAP,ATP6V0A4
Negative_4289	FH,IL5,PVR,TKT,ACACA,ARHGAP5,BDKRB2,CPT2,CRLF2,CSF3R,DAD1,GALK1,GJD2,GRHPR,HIF1A,HNF4G,MYH7,NFKB2,OPLAH,OR2G2,OR51F2,OR5B21,PFN1,PLA2G2D,PLD2,PNLIP,PPP5C,TRAF3,UBE2K,WNT1,ZAP70
Negative_4290	EZR,FGA,MVK,BDNF,CHAD,FGF22,GAD1,HSD17B7,OR1L1,PHKA1,PTPN7,RAC3,SKP1,VAMP1,PRICKLE1
Negative_4291	AGL,F7,GK2,HK2,HYI,OTC,SMO,AARS2,AKR1C3,ASNS,BMPR2,BUB1,CAMK2B,FABP1,GLI1,GNAI2,GRIN1,HAO1,HEXB,HLA-DOA,HMBS,IL1R2,IL22RA1,ITGA4,ITGB1,MAOB,MAP3K5,MCEE,MGLL,NTRK1,NUDT12,OR10A5,OR51A2,OR8K1,PANK3,PGK1,PGLS,PGS1,PIGO,PIK3R4,POLR2K,RDH5,SOAT2,STAT1,TLR9,TPH1,TUBB2C,ZFYVE16,SUV420H1
Negative_4292	GBA,ALOX5,ALPP,CRLF2,GALNS,GRIN2B,LEF1,OR4C12,PDGFD,PPP3R1
Negative_4293	C5,F2,IHH,SRC,ADAM10,AGXT,ALG1,ALG6,BUB3,CREBBP,DHDDS,DKK2,DPM2,GRIN2A,GSK3B,GSTA2,IFNK,JAM2,MAPK10,METTL6,MNX1,OR4F6,PAK1,PDPK1,PIM1,PRKAB2,PRKAG1,PSME2,SUV39H1,THBS1,PNLIPRP1,TNFRSF6B
Negative_4294	INS,ACTN4,AKT2,ASPA,ATG3,ATP5J,CNGB1,DUSP4,GRM5,HLCS,IFNA14,IFNA2,INPP5B,LAMC2,LDHC,MGAT1,NAGS,OR9G4,PGK1,PRKCE,RPS6KA5,SNAP25,SOS2,SPRY2,SULT1A4,TNFSF14,TSC2,TUBA3C
Negative_4295	HK2,MVD,B3GALT6,CD34,CDKN1B,CTTN,CXCL11,DBF4,GALNTL1,GNPNAT1,GRIN2C,HSD11B1,ITGA5,MAML1,NRG2,OR1E2,OR4K1,PON2,PTGFR,PTPN5,RALA,SARS2,SHC3,TAS2R10,THBS1,TNFSF10,TUBB2A,HLA-DPB1
Negative_4296	CYP3A4,FARSA,GNPNAT1,HMOX2,IFT57,MAOB,MITF,NCSTN,OR1J1,OR6C6,OR6N1,PLXNB1,POLR1D,RASA1,SEMA3C,SYNJ1,ST6GALNAC1,TNFRSF1B

Negative_4297	DBI, PXN, ACACB, ALG13, ATG12, CACNA1F, CACNG4, CDH1, CPS1, CUL1, CYP1A2, CYP2B6, DUSP3, FPGS, FUT5, GCDH, GIT1, GPAA1, GRIA2, IFNA21, KDSR, LIMK1, LTA4H, NMNAT3, OR10AD1, OR14J1, OR1L3, OR4A5, OR5AC2, PARS2, PARVG, PFN4, PRKX, PTGFR, RAF1, RHEB, RPS6KA1, SLC12A2, SNAP25, ST3GAL6, TAS1R2, TBK1, TRMT11, XIAP, HLA-DPB1, SERPINE1
Negative_4298	F5, INS, OSM, ALOX15, CREB3L4, CSF3R, DUSP9, GALNT2, GCNT3, HIBCH, MITF, OR11A1, OR4K14, OR52E8, OR6N1, OR9A2, P2RX6, PIK3R4, POLR2K, PRKCE, RPS6KA4, RRM2, SEMA3B, SMC1B, SOAT2, STX10, TAS2R50, TBK1, WBSCR17
Negative_4299	ADA, ATM, BAX, MET, CD8A, CLDN6, CYSLTR1, DUSP14, DUSP4, FTCD, HLA-DOB, KNG1, MAD2L1, MAN2A1, OR2B6, OR2M7, OR4D11, PDHA1, PGS1, POLR3H, PPP2R5D, SCP2, VAMP1, HSP90AA1
Negative_4300	C7, CFH, F11, NPL, AASDH, ACPP, ADIPOR2, ALG3, ATG3, CFTR, CHPT1, DTX2, ECHS1, FABP4, FGD1, FGF9, GNAT3, HMOX1, IFT57, IL6ST, MAP2K7, MAP3K14, OR10A2, OR2F1, OR2M2, OR4F17, OR56A1, OR5B3, OR6K2, ORC2L, PDXP, PPP1R1A, PPP2R2A, RAP1B, SLC9A1, SPRY2, VEGFC, YWHAQ, ATP6V0D2
Negative_4301	BAD, FES, IL8, SHH, SMO, TNC, ABLIM2, ACVR2A, AGPAT2, AGXT, ALPP, B4GALT3, BMP8B, CAPN2, CLDN18, CXCL12, DIAPH1, GNPAT, GYS2, HADHA, NAMPT, NCK1, OR4K2, OR5AS1, OR5T2, OR7C1, OR7G3, PCCA, PORCN, PRKG1, RAPGEF2, SULT1E1
Negative_4302	IL9, AGPAT6, CD274, COL1A1, EDN1, GALNT10, GALNTL5, GSTA3, GUCA1C, IL12RB1, INHBC, IPMK, IRS1, ITGA2B, MAP2K2, MKNK1, MYH7, OR13J1, OR4L1, OR5M9, OR8A1, SPAM1, STAT1
Negative_4303	BTC, HAL, LYN, NPL, ACTB, ADH4, ATP5A1, BHMT, C3orf10, CACNA1B, CCL5, CHRM3, CNTNAP1, CREBBP, CXCL10, EFNB3, ENTPD8, GFAP, GGT1, HADHB, IMPA2, KRAS, LAMC3, LEF1, MAOA, MCEE, NANS, NMNAT2, NOTCH2, NTSR1, OR1Q1, OR5P2, OR8I2, PI4KA, PLTP, PLXNB1, PYGM, RPS6KA4, SIAH1, SIGLEC1, SLC2A1, STMN1, TAS2R13, TLR2, TNFSF11, TRPM5, YWHAH
Negative_4304	ARSD, IFNA7, PTGER1
Negative_4305	DAO, FGA, GAA, HGD, ADRB3, AKR1C3, ANAPC1, CARS, CD36, CD48, CDIPT, CHST11, CNDP1, DCTN1, DUSP1, ERBB4, FGF20, GLDC, GNA14, HIF1A, HIP1, INPP4A, MAD2L2, MDM2, NCKAP1L, NRG2, OR10T2, OR11L1, OR2B6, OR4D11, OR52H1, PGAM4, PPOX, PPP1CB, PSAT1, PVRL3, RFXANK, ROBO3, SETD7, SLIT1, SRGAP1, STAT6, TUBA1B, WNT10B, GABARAPL1, TNFSF13B
Negative_4306	AMT, F10, ACPT, ACTA2, CSNK1G3, FZD8, GPD1L, HYAL2, OR5M8, PARD6B, PRNP, STMN1, YKT6
Negative_4307	AMPD1, GNG13, IL20RB, KIR2DL3, OR11H6, PDHA2, SDC4, SESN2, TYMP
Negative_4308	ADA, HGD, AADAC, ACAD9, ADH1A, AMD1, CHST11, CRKL, CTSG, CXCL16, IDH3A, IFNW1, IMPDH2, LLGL2, LRAT, NEFH, NR5A2, NRG2, OR10A5, OR2L3, PFKFB4, PIK3CA, PIM1, SGMS1
Negative_4309	COL4A2, E2F2, OR9A2, SSH3
Negative_4310	CD44, FOXO3, IGSF5, LEFTY1, OR2C1, OR52N5, RASGRF2, SEMA3F
Negative_4311	NPL, SCD, ADH1A, APC2, BMP5, CYSLTR2, ENTPD7, GNA13, MMP7, MPP5, OR4D2, PYCRL, SUCLG2, B4GALNT1
Negative_4312	AKR1A1, ATP5G2, CHST11, CYP2S1, DDX23, FOXA2, GABARAP, GDF7, GGT5, GRIN2C, GSTM5, HLA-C, HLA-DMA, LTC4S, MAP2K7, NT5M, OR10T2, OR1E1, OR2AT4, OR5M8, PIK3CB, SRGAP3, TACR3, WHSC1L1, SERPIND1
Negative_4313	GSS, AREG, ATF4, ATP4B, ATP5G3, BCL2, CDH1, DUSP6, EI24, FZD9, ITGAX, JAG1, MYL2, NKX2-2, OR2D3, PFN2, POLR2C, SARDH, SNAP25, UGT2B4

Negative_4314	GP5, IL7, SRM, TEC, ACSM5, AKR1C1, ALAS2, ANAPC2, ARHGEF7, CARS2, CCL1, CCL16, CCL21, CDK5, CFL1, CYP2C8, E2F1, FOSL1, HAGH, HPSE, IRAK4, KYNU, MAN2A1, MCEE, NEU1, OGDH, OR10J1, OR10S1, OR1L6, OR2A42, OR8J3, PI4KA, PNMT, PRKAR1A, PRKCA, PRPS1, ROBO2, SEMA3B, SLC27A1, SLIT2, ST3GAL4, STX3, TAS2R49, VANGL1
Negative_4315	CR1, APAF1, CCL8, CD274, CHPT1, CLDN20, CREB5, CTNNA1, DUSP9, EXOC7, GALNT7, GRPR, HIP1, MKNK2, MYH11, NCK2, OR2T11, OR52A5, OR5B17, PLA1A, PLXNB1, POLD2, POLR2D, POLR2F, PPP1CB, PRKY, SUCLG1, UROC1, HLA-DQA2, PRICKLE2
Negative_4316	CD4, DCC, CACNA1S, CADM3, CD74, DAD1, IL3RA, INSRR, ITGAV, OR10Q1, OR1A1, OR2A42, PFKFB1, PHGDH, PLA2G2A, POLD4, PYCRL, SDHA, SUOX, TAS2R8, TYRP1, GABARAPL2
Negative_4317	CKM, HK3, LIF, SRC, ACADVL, ALDH9A1, ARPC5L, BMPR2, CCL20, CDC14A, CDC2, CSF1, F11R, GRIN2D, GSK3B, ITGB1, LRRC4C, MMP9, OR2T8, OR4A5, OXCT1, PFAS, PIP5K3, PRKY, RALA, SFRP4, THTPA
Negative_4318	ALS2, BACE1, BACE2, BAIAP2, FADS2, HSPA8, NUDT2, OR1S1, PFN4, PPP3CC
Negative_4319	OAT, ALDH3B1, CAPN2, GLUL, NCOR1, SHC2, TAS1R3, TJP3
Negative_4320	BAX, TAT, AASS, ALDH4A1, BMP8B, CD247, CLTCL1, CYP2C9, DAPK3, DUSP7, GRIN2B, IFNA17, IRF5, LIMK2, LRAT, MASP1, MYL9, NCKAP1, OR3A2, ORC5L, PDE6G, PDX1, PFN4, PIK3CB, PRKX, RAB5A, RFNG, SNAI2, WNT2B, YWHAH
Negative_4321	IVD, LCK, PC, CDC25C, DFFA, DFFB, DUSP10, EDN1, EGLN3, ENTPD1, EREG, FUT8, G6PD, HIF1A, IL10, MDH2, PIAS4, SETDB1, THBD
Negative_4322	FES, GGH, IHH, MPZ, MVD, PVR, RFK, AKT1, AXIN2, BMP6, CACNA1S, CDC20, CTNND1, EFNB3, ENTPD5, FZD10, GNAL, GRIA2, HMGCS1, HNF4A, ITPK1, KAT2A, MAD2L2, MYH1, MYH15, OR11G2, OR51A4, PCCB, PDE10A, PDXK, PIGH, POLR1E, PTGS2, PTPN5, PYCR1, RASGRF2, SDC4, SETD7, SOS1, TAS2R5, MAPKAPK3, TNFRSF10C
Negative_4323	ARHGEF6, BAIAP2, BBC3, CACNA1E, CD46, CPOX, EPHB4, FARSB, NOX1, NSDHL, OR2F1, OR4F15, SHC4, TRAF2, WEE1
Negative_4324	IFNG, ITGB4, MCCC2, OR52A5, PKLR, RASSF5, TNFSF10, EIF4EBP1
Negative_4325	VTN, CCNB3, DEGS2, FUT2, PLAU, SHC2, STEAP3, VCAM1, SERPINC1
Negative_4326	HAL, CACNG3, CLDN19, DDX52, EARS2, ENO3, LDHAL6B, LEPR, NFKBIE, OR51L1, PAX6, PDHB, SMPD2, ST3GAL6, SV2A, TNFRSF6B
Negative_4327	AHCY, CACNA1H, CCL11, CCL8, CPOX, CYP7A1, DDX47, FLAD1, HTR2C, IL15, IL6ST, OR5AC2, P2RX1, PFAS, PKMYT1, PVRL4, QARS, TUBB
Negative_4328	C8A, F2R, FAS, FES, SI, SMO, ADH5, APOA2, ATP5J2, BMP7, CASP7, CD34, CD47, CFL1, CHIA, COQ7, COX15, CSDA, CTTN, DARS, DEGS2, FGF14, GSTO2, HMOX2, IL28A, IL8RB, LDHA, MKNK2, OR10G3, OR10H1, OR11H4, OR2A14, OR2L8, PDE4B, PHKA1, PLAUR, POLR2E, PPARD, PPAT, PSME1, RENBP, SKIV2L2, SLC2A1, TUBB1, UBE2L6, UNC5D, GABARAPL1
Negative_4329	C7, DBI, HK2, IHH, VTN, AANAT, ABP1, ACLY, ARNT2, ASAH1, B4GALT4, BLVRA, CCL20, CCNH, CER1, CHKA, CHUK, CLCA2, CTBS, GMPR2, HAP1, HTR2C, LPAR1, MAPT, MITF, OR2S2, OR4F6, OR4Q3, OR52J3, OR5D18, ORC4L, PAK2, PARD6A, PAX8, PER2, RASSF5, RENBP, SFRP4, TICAM2, YWHAZ
Negative_4330	GP5, ALDH1A2, BLVRA, CCNB2, CIITA, IL22, IL28A, OR10J1, OR2C1, OR56A1, QARS, RDH5, ROCK1, TPMT, UGCG, WASF2, ZFYVE9, ST6GALNAC1
Negative_4331	TK2, BDKRB1, CCL1, CRKL, CYP2D6, DGKQ, FASLG, HLA-G, OR2L13, PFN1, PLCZ1, ST6GAL1, TOMM40, WASF1, CACNA2D3, HSD17B12
Negative_4332	CR1, F10, F5, ACOX2, CDC42, CDS2, CSNK1E, CXCL9, CYP1A2, DLAT, FPGS, GALE, GAMT, GDE1, GIT1, GPX7, GSK3B, GSTZ1, GUCY2D, IDH3G, INHBE, LIPE, LTC4S, MAPK9, MGAT5B, NCR1, NEU2, OR10A5, OR4N5, OR52R1, OR9G1, OXTR, PANK2, PGAP1, PGLS, PI4KA, POLD2, PPAT, PRDX6, SOCS3, ST3GAL6, SUCLG2, ULK3, ST6GALNAC1, SUV420H2, TNFRSF10D
Negative_4333	OR5M3, OR6M1, P2RX4

Negative_4334	CERK,COQ5,F11R,NCOR2,PNPO,SMAD4,TRAF1,VAV2
Negative_4335	BTK,CAT,ACSM3,ADRA1A,AGRN,ATN1,CACNB2,CARD11,CD38,CD3E,CD8B,CDKN2C,CPT1C,CSNK2A1,DGKG,EPHB6,ERBB3,FGF2,FMO2,GLB1,GNA14,GP1BB,IFNA13,IFNA4,MAML1,NEU1,NFKBIA,OR52H1,OR5AK2,OR5C1,ORC4L,PPCDC,RALA,SEMA3B,SMPD1,SOCS7,TACR1,TSTA3,CSGALNACT1,HSP90AB1
Negative_4336	ATG7,FABP1,GADD45B,GNRH1,IARS2,OR5B21,OR7G1,SHC3,TAS2R49
Negative_4337	MVK,ACOX1,ACTA2,ADRA1A,ALG5,ATP13A2,BCAT2,CTF1,DAXX,EGLN1,FGF21,GALNT6,GALNTL1,GYS2,HIBCH,HSPA8,ITGAL,ITPKA,KIR3DL1,LDHAL6B,MAP2K7,MGAT5,MITF,MYH8,NSDHL,OR2T8,PDE11A,PGAM2,PLA2G4A,PLD2,SLC27A4,SPCS3,TCF7,TLR9,TNXB,TUBB2A,VAV3
Negative_4338	HK2,ID2,LSS,PAH,TFG,ABL2,ACADL,ACYP2,ALG10B,ALPP,AQP7,BBC3,BCKDHB,BST1,CCKAR,CFTR,DEGS2,DGKA,DGKB,DGUOK,GMPR,HAGH,HCST,HLA-DOB,HNF4A,HYAL2,LARS,MALT1,MAP4K1,NCOA4,NME4,OR10J3,OR11A1,OR51I2,OR52W1,OR56A1,PDE11A,PFKP,POLR2E,PPP1R3B,PSPH,PTGES2,SOCS2,THBS4,TNFSF8
Negative_4339	ALDH1L1,CDH4,CDKN1C,FGF13,HLA-G,MYLK,OR52M1,PLA2G2A,PROS1,PTPRR,TPK1,YWHAB
Negative_4340	LHB,MUT,ACSL4,AGRN,ALG3,ANAPC7,B3GALT4,CCL26,CCND3,CPS1,CREB3L3,CSF2RB,CYP2A13,GANAB,KARS,MCM6,OR1L8,OR51B4,ROBO3,SAT2,SELE,SETD1A,SLC9A1,SOAT2,ST3GAL6
Negative_4341	CASP6,CCL28,CISH,COL2A1,DCXR,DPM3,EPHA2,FTCD,GUCA1A,ITGA7,LIMK1,LMO7,MAPK14,MGAM,NEU4,OR10C1,OR14I1,OR8K5,SMAD4,TRAF3,WNT10B,ATP6V1B1,KIR2DL5A
Negative_4342	C3,DCK,F8,GNE,AKR1D1,AMDHD2,BDH1,BMP8B,CASP9,CDH2,CHRM3,DGKH,DPYD,DUSP9,FARP2,GLUD2,KCNJ11,MAPK3,MARS2,MDH2,NCOR1,NRG3,OR1A1,OR4D2,OR6S1,PCNA,PHPT1,PLCD1,PRODH2,RAB5A,SEMA4A,SLIT1,SOD1,TAS2R38,ATP6V1C2,CSNK1A1L
Negative_4343	BID,HGD,AKT3,BIRC3,HSPB1,INADL,IRAK1,KRAS,MPP5,NEU4,NKD2,OR10H4,OR13C8,OR2T5,PDPK1,POLR2H,PPP3CA,PRKAB2,STX7,TLN2
Negative_4344	LSS,PML,AADAC,ACOT12,BMP4,CDC2,CREB3,CTLA4,CTPS,DGAT2,DPYSL2,FGF1,FGF8,FZD6,GADD45B,GPX5,HMGCR,IFNGR2,IKBKG,MDM4,OR13C2,OR2T4,OR4A15,OR4F5,OR6Y1,PIGS,PPP2CA,SHMT2,SOCS4,SSH3,STX3,TRAF4,TNFRSF14
Negative_4345	AARS2,ASMT,ATP5J,B3GAT1,CD40LG,COL1A2,COL6A2,COQ5,CSF1R,DPYD,FGF1,HNF4A,LALBA,MCM4,OR6N2,OR7G2,OR8J1,ORC2L,OXCT1,PARS2,PI4KA,PLA1A,PLXNB2,RPN1,SETDB1,SFRP1,SPP1,TRPC1,UGT2B10,MAPKAPK3
Negative_4346	EZR,ALOX12,CALML5,FARP2,FGF1,FGF4,GGT7,GZMB,HDAC1,ITGAL,NODAL,PIAS1,RFWD2,SLC2A2
Negative_4347	C8B,F5,ALDOC,BIRC2,CASP7,CCL27,CCR8,CDC26,EPHA1,FUT8,HAGH,IL22,IL2RG,OR2AT4,OR2T29,PCK1,PIK3C3,SHC3,SPP1,SUCLG1,TAS2R13,TBL1X,UNC5A,MAPK8IP2,SERPINA5
Negative_4348	CEL,B3GALT1,BMPR2,CLDN7,DKK1,GSK3B,IL10,IL18RAP,IL22,LCMT1,MYH6,NR5A2,NRG3,OR10G2,RELB
Negative_4349	CTH,ME3,MTR,ABL2,AKT2,ARSA,CD46,CDC2,CFLAR,EARS2,FGF22,G6PC,GDF5,GLI3,GMDS,HADHB,HSD11B2,IFNGR1,IL17B,ITGB7,LTBR,MCM2,MMP9,MYH1,MYH11,NFASC,NFAT5,OCRL,OR10X1,OR11L1,OR4F6,OR5B12,P2RX4,PDE3B,PLOD2,POLR2H,PON2,PPP2R5D,RAF1,SELP,SHMT2,SLC2A1,STK4,SULT1A4,TAS2R50,UMPS,VASP

Negative_4350	C2,HYI,UBC,ADCY6,APLP1,ARHGEF6,C1QC,CCL8,CD79B,CSNK1G1,CXCL12,DHX58,DUSP4,EPHX1,FCGR2B,GALNS,GALNT3,GPX7,HAP1,ICAM3,IDI1,INPPL1,ITGAL,LAMC1,MAGI2,MAPK7,OR2T11,OR9Q1,PDE2A,PFKFB4,PIAS3,PPP2R1B,PYGM,SC5DL,SESN3,SMPD3,TGDS
Negative_4351	ABP1,ADCY10,DGUOK,HMOX2,ICOSLG,IL1R1,IRAK3,IRS2,MASP1,OR10S1,OR4K15,OR4K17,P2RX2,PPP1CC,PVRL4,SDC1,ULBP1,UROD,TNFRSF1B
Negative_4352	BDNF,CCR3,PRKCH,TAS2R3,TAS2R5
Negative_4353	AK1,DCN,GNE,IVD,LPO,LSS,SRF,ACAA1,ADAM10,ARRB1,ATP5C1,C1QB,CACNG1,CALML3,CCNE2,CDC14B,CDC20,CDK4,COQ6,CRKL,DDHD1,DDX56,DGAT2L4,EPHX1,ESAM,FDPS,FSHR,GALNT12,GCNT3,GNRHR,IFNA6,IL1R2,IL20RA,IMPA1,LCMT1,NCK2,NFATC2,NUMBL,OR2G2,OR4C46,OR4P4,OR51E2,PRPH,SSH2,TAS2R38
Negative_4354	DBI,F9,LIF,ZYX,ACTA1,CER1,DFFB,DTX1,DVL3,EPB41L1,FGF9,GIT1,GRB2,IL1R1,KCNQ1,MAT2B,MGAT3,NAGA,NCR3,NOD1,OGDHL,OR1A2,OR51B2,OXTR,PERP,PIK3C2G,PLCB4,SLC2A1,SREBF1,ST3GAL1,TIRAP,TNFSF4,TUBB2A,UMPS,UPP2
Negative_4355	APP,AR,GCK,NLK,ACVR2B,ALDH1A3,ATP2A1,ATP5D,ATP5G1,ATP6AP1,BCL10,BIRC3,CADM3,CNTN2,CSDA,CTNND1,DHX58,EP300,FMO4,GIT1,GLYCTK,GNAI3,GUK1,HHEX,HMOX1,IL1RAP,IQGAP3,KIR2DL2,LCP2,MAP3K5,OCLN,OR2B11,OR2D3,OR4K14,OR7A10,OXCT1,PRDX6,PRKCD,PSENEN,SMARCA5,TNFRSF9,TYRP1
Negative_4356	DDC,GPI,ID1,ACSL5,ACSM3,ARHGEF7,CHKB,CREB1,CYBA,DARS2,DGUOK,DKK4,DPM2,DUSP3,FSHR,GNRHR,GRIN2C,GUCY2F,IKBKB,IL8RA,IRAK3,ITGAV,LIPE,MCCC2,OR9G1,PDE1C,PFAS,PHKB,POLE,PRKCZ,PSME1,PYCRL,RASGRF2,RBL1,SDHB,STK3,STK36,TAS2R13,TAS2R60,TOMM40L,TSNARE1,TUBB,PNLIPRP2
Negative_4357	GALE,GSTM3,IFNA8,LIPC
Negative_4358	IL5,JUN,ACACA,ARRB1,CHAD,CLCF1,CTNNA2,OR8H1,STAT5B
Negative_4359	UBC,ENO2,LIPF,NEU4,OR6C70,PIGF,PLCG2
Negative_4360	SHH,TEC,ACADL,CMPK1,E2F4,FARSA,FGFR3,GJD2,GNA12,GNG12,GRIN1,GSTT1,HMGCL,HSPA8,KLRC4,NCSTN,PCK1,PSME1,PVRL4,RPE65,STX10,TIRAP,ULK1,WWP2
Negative_4361	CD6,IL9,AADAC,ACACB,ADRBK2,B4GALT6,CCL2,CHRD,FABP1,FUT2,GALE,IFNA17,MAPK10,NRAS,OR2L3,OR2Y1,OR51E2,OXCT2,PIGF,PKM2,PMVK,PVRL2,RGS3,SETMAR,SULT1A4,HLA-DRB5
Negative_4362	APP,DDC,PLG,ASS1,BCAT2,CAV2,CHDH,CLDN17,CLDN18,DGKB,EHHADH,ENTPD3,EPAS1,FZR1,GALNT5,GNPAT,GRIN2C,HDAC2,IDH3B,LAMC1,MEF2C,MINPP1,NEU1,OR1N1,OR2A12,OR2G6,OR51G1,OR5M3,OR6C1,OR6C2,OR6M1,PGM3,POLR2G,PTGES,PTK2,PTPRC,RASGRP3,ROBO3,SDHD,SUCLG1,TBL1XR1,TKTL2,TNFSF9,ULK3,VANGL1,WASL,YWHAZ
Negative_4363	ACSL4,ADCY1,ALDH4A1,ATF2,ATP5I,CAMK4,CCR4,CHSY1,CLCA4,CLDN9,DRD1,EPRS,EXTL1,FGF17,GALNTL5,GLG1,GLI2,GNPDA1,HSPB1,IARS2,KIR2DL4,MYH8,NR1H3,NTNG1,OR51A4,OR6Y1,OR9A2,PDE4A,POLR2B,RDH5,SFRP4,STX10,TUBA3C
Negative_4364	CBS,HK1,ATF4,CLDN10,CLDN15,CSNK1G2,CXCR4,DRD2,FUT6,GADD45B,IL22RA1,KLKB1,OR13D1,PHGDH,PIK3R4,POLE3,PRKAB1,TAS2R14,TJP3
Negative_4365	CKB,GSR,AGPAT3,ALDOC,APLP1,BRAF,CCR2,COX15,FZD4,FZD5,NEU4,NFAT5,OR13C4,OR5T1,POLA2,PTPMT1,SETD1A,SETD8,SPRY1,TAS2R10,TNXB,TUBA1B,WNT7A,KIR2DL5A
Negative_4366	CBL,OTC,AREG,C7orf16,CAV2,CCR2,CREB3L3,FOXA2,IL28B,MAPT,NFAT5,OR2M7,PCK2,WHSC1
Negative_4367	ASL,FGB,IL3,MVK,GALNT9,NRG2,OR1B1,OR9G4,ORC6L,PTGES,SDC4,SLC25A6,TAS1R1,TAS2R42,TLR4,UGCGL1,WASF2

Negative_4368	ADC,F9,SMO,ACTN1,ADCY7,AVPR1B,BAI1,CACNA1E,CACNA1G,CACNG2,CHDH,CHST11,CTBP1,EFNB1,EPHB2,FASLG,GART,GDF5,GLS2,HLA-DMA,HMOX1,IMPDH1,ITGB4,LRDD,MKNK1,NLGN2,NOX3,OR1L3,OR2A25,OR51A2,OR52W1,OR5AR1,OR5D16,OR8K3,OR8K5,PCCA,PDE1A,POLR2J,RAC3,RAPGEF4,RPIA,SARS,SHMT2,TNFRSF4,TRADD,C1GALT1C1
Negative_4369	AR,F2,APPL1,BET1,CARD11,CLDN14,EXTL1,GGCX,GSTM5,HARS,HSD11B1,HSD17B2,LIPF,MPP5,NFKBIA,OR4K17,ORC4L,P2RX5,PDE8A,PFKFB3,PPARA,PTCH2,TBL1Y,TSC2
Negative_4370	CDA,CS,F8,GK2,PML,ACSL3,AGPAT1,ARG1,ARNT2,CACNA1E,CDH5,CFLAR,COQ3,CSNK1G3,GNA12,GSTM5,IDH3A,IL2RA,ITGA2B,ITPK1,NTN1,OR1A1,OR1S1,OR2L13,OR4X2,PFAS,POLR1E,PTPRR,PTTG1,RALGDS,RASGRP2,SC5DL,WASF2
Negative_4371	AGMAT,ALG13,CCL11,CD99,CLDN19,DGKH,EDNRB,FGF9,G6PC,GOT1,GUCY1A3,IGF1R,INHBE,ITGB5,LRDD,MAPK14,NCF1,OR6Y1,PPP1CB,RALA,SLC27A5,SQLE,SV2C,TYRP1
Negative_4372	BID,ILK,APBB1,CDH3,CTSL1,HADHA,MAGI1,MARS,NKD1,NKX2-2,OR51E1,PAFAH1B2
Negative_4373	TPR,ABCC8,ARPC5L,CLOCK,FGF22,GSTK1,HMGCS2,ITGA3,NCOA4,OR10H5,PIGO,POLD1,RALB,RUNX1T1,SH3BP2,SOS1,WBSCR17,MAPKAPK3
Negative_4374	ATM,TPO,AADAT,ACVR2B,B3GALT4,BMPR2,CAMK2A,CHRM2,CTSS,CXCR3,DGKZ,EFNA2,FZD1,KLRC3,MAD2L2,NEFH,NUDT2,STK11,TUBB3
Negative_4375	BTK,EFNA3,MAPKAPK2
Negative_4376	ADK,ILK,MAG,TEC,ACAT2,ADRB3,ALDH3B1,ALG6,ARHGAP5,CACNA1A,CDC25A,CDH4,CPT1A,E2F4,EPRS,FTH1,FZR1,GOT1,GRIN2A,HTR2C,IRAK3,LAMB1,LDHAL6A,MGLL,NANS,NCR1,OR4F5,OR4N4,OR6C70,PCK1,PCK2,PLAU,PRKAG2,PVRL4,SETD7,SLC9A1,TP53I3,TYK2,PRICKLE2
Negative_4377	BTK,CSK,DHH,UBC,CAV2,CD34,CTNNB1,DDIT4,PDHA1,PLCD4,PTPN1,RND1,XCL2
Negative_4378	C6,CKM,SI,ARG2,ARHGEF2,ARNT2,CD3E,DAAM1,EPHB6,ERO1L,FZD6,GALT,GGPS1,GMPR,IL17B,IMPA2,LLGL1,MAP3K4,MAPK13,MTHFD1,NT5C2,OR2M4,P4HA2,PHKG1,PRODH2,PTGS1,PVRL3,RAPGEF4,SCD5,SKIV2L2,SMAD2,SOCS1,SPAM1,TACR1,PPARGC1A
Negative_4379	FGB,ABAT,ACADS,ANAPC10,CADM3,CAMKK1,CHKA,CLDN16,CYP2C8,FGF1,GALNT1,GSTK1,HNF1B,IKBKB,IL21,LAMC1,LRDD,LYPLA1,MAP3K5,NR1D1,NRXN1,OR2L3,OR2M3,PAICS,PCYT2,PPARA,RBKS,RPRM,SCD5,WNT8B,CSNK1A1L,SLC25A31
Negative_4380	ASL,BID,TPR,ACAA1,ACSM5,ANAPC4,AOC2,ATP5J,B3GALT4,B3GNT5,CDKN1C,ENTPD3,F13A1,FADS2,FCGR3B,FLT4,FTH1,FZD1,GALK2,HRH2,IFNAR1,IMPDH1,LIPA,MASP2,METTL2B,MYH7B,NAGLU,NEFM,OR13G1,PANK3,PDCD1,PNPLA4,POLR2F,PPA2,PPP2R1A,PPP2R2B,PPP2R2C,PRKACB,PRKAR2B,PTCH2,PTPN6,PTPRB,SOAT1,SORBS1,STAT5A,STEAP3,TGFB1,WNT8A,CSGALNACT2
Negative_4381	B4GALT5,GCH1,RAC3,SFRP1
Negative_4382	KHK,MTR,ACTN1,APRT,BBC3,CCR5,CXCL10,CYP1A1,DAG1,ENPP2,HIBADH,IL13,IQGAP2,ITGB8,KIR2DS4,MAP2K1,NFKBIE,OR10H4,OR52B6,OR5T2,OR7G2,PGAP1,SERPINA5
Negative_4383	RET,BHMT,BIRC2,CCKBR,CCR9,CSNK1A1,DNMT1,EXTL2,FGF18,FGF19,GNG13,HSD17B3,HYAL2,IL10RB,ITGA2,KITLG,LRRC4C,MYL5,NAGS,NFYB,NGEF,OR1C1,OR1D5,OR6K3,P4HA1,PARD6B,RASA1,SNAP25,TRAF1,ALDH18A1
Negative_4384	LCK,AKR1A1,ALPP,AMDHD1,BNIP1,C4BPA,CDS1,CNTN2,DDX23,EGLN3,ENAH,FGFR1,FTMT,FZD5,GAL3ST1,GMPS,GRM1,HGSNAT,HSD3B2,IL5RA,IRS4,MCM6,OR5K1,P2RX5,PAFAH2,PANK3,PI4KB,PIGO,PIP4K2A,PKM2,PLA2G4A,PMM2,PNPO,PPP3CC,PTEN,VDAC1

Negative_4385	PPAP2A,SPRY4,STT3B
Negative_4386	CBL,DUSP14,LALBA,RARA
Negative_4387	CBLC,GFAP,OR51E2,PAK7,TNFSF14
Negative_4388	F7,FGA,LPO,PTS,SI,TH,TNR,UBB,ABAT,ACSS2,ADRA1D,ALDH3A1,ARHGEF1,BIRC2,CTBP1,EPAS1,FGFR2,GLO1,IFNA17,IGFBP3,IL23R,ITPKA,KIR2DL2,LFNG,LIPG,LMO7,MYH4,NOS1,OR4X1,OR51I2,OR52W1,P4HA3,PDE1C,PDE6D,PFKFB3,PSME3,PTPRC,SDC2,SETD1A,SRGAP3,ST8SIA1,TIRAP,TPH1,TUBB1,YKT6,ST6GALNAC5
Negative_4389	EBP,LPL,CCNG1,CD28,CTSL1,CYP1B1,CYP2C18,CYP2F1,DGKH,EFNA3,ENPP3,IFNAR1,IL21,IL29,LAP3,LDHC,MS4A2,NCF1,PFKFB1,POLA2,POLR2K,PPARG,PTK2,SLC27A5,SMC1A,STX10,TPI1,MAPKAPK2
Negative_4390	GSS,PAH,AGPAT4,BDKRB2,DTX2,ENTPD7,GSTM5,HADH,HTR4,LCMT2,LRRC4C,MAFA,MAGI1,MAGI2,NFASC,OR2L13,OR4N4,PGK1,PIK3R5,PRKY,TAP2,TM7SF2,TYK2
Negative_4391	AK3,IDS,LIF,SRM,ACAT1,CD86,CIITA,COQ3,CYBA,ENPP6,ENPP7,FMO5,HADH,HAO1,IL18,MAP2K6,MAPK3,MPDZ,NFKB1,OR10C1,OR10G7,OR4F4,OR6C4,PIGS,PLCE1,POLR3H,RASGRP4,SEMA3G,TAS2R38,UGT1A10,USE1,ALDH18A1,HSP90AB1
Negative_4392	BID,CAD,FH,GK2,JUP,KIT,MSN,ALG9,ASS1,ATP6V1D,B3GAT3,B4GALT3,C3AR1,CCL14,CCL3,CCR8,COL1A2,FMO3,FUT1,FZD4,GLG1,GLUD1,GNG12,GSTP1,KAT2B,LEFTY1,NTN1,OR1J4,OR52B4,OR6F1,OR7A5,OR9G1,POLR1C,SESN2,SHMT2,STAT5A,SULT1A1,SULT1E1,TAS2R13,TUBA3C,UPP1,XCL1,ATP6V1C1
Negative_4393	CAD,FST,LSS,ACSL5,ACSM3,ACTN1,C4BPA,CD59,CSNK1G1,DGKQ,DLL3,DNMT3L,FGF4,FLT3LG,FUT4,GALT,GNAI3,GNG13,GOT1,IFNA7,IL17RB,ITGAD,MAFA,MAN1C1,NRCAM,OR4C6,OR56B4,PDGFB,PLD2,PNPLA4,PPP2CA,PSME1,RBX1,RRM1,SETD1A,SLC27A4,SPCS1,UGT2B7,PRICKLE2
Negative_4394	ADCY5,CACNA1B,CD74,EVI1,MRAS,P2RX5,RAP1A,RPS6KA1,SIGLEC1
Negative_4395	F7,ADRB2,CD82,CDK4,CREB3L1,CYP26A1,CYP2A13,DDX50,DGAT2,DGKD,DKK2,DOCK1,EPB41L2,GART,GFPT2,GNPDA2,GUCA1B,HIP1,IAPP,ICOSLG,MYL2,NEU4,NFATC3,NRG3,NSD1,OR10J3,OR2A42,OR2T4,OR4K13,OR5AK2,PGAM4,PIGF,PLA2G3,PNPO,PTDSS2,PTPRR,PYGB,RND1,RXRB,SDC4,SETD2,SFRP4,ST3GAL4,TAS1R2,TPMT,TRAF2,WASF3,WNT5B,TNFRSF14
Negative_4396	MIF,ATG7,CADM1,CXCL2,ETNK1,FCGR3B,FRAT1,GSTM5,HHEX,IMPDH2,METTL6,NADSYN1,OR2G2,PON1,SPI1,TUBB4Q,UBA1
Negative_4397	C4B,ACY3,ECHS1,IFNA6,NCR3,OR51I2,PDE1A,PIK3C2A,TIAM1,ATP6V1G3
Negative_4398	PLG,ADORA2B,BUB1,EIF4E2,GDF6,LIAS,OR2T1,PDXK,SCD5,SEMA3D,THBD
Negative_4399	CAV3,CCNB2,FGFR1,MCAT,OR10K1,PRKCG,PRNP,SCIN,SESN1,TAS2R10
Negative_4400	IL7,MPZ,ABL1,ACADM,ACSL3,ALOX15B,ANAPC2,BHMT,CCL22,CDC25B,CDH5,CLDN14,DPAGT1,DTX2,EFNB3,EP300,EPB41L2,GGCX,GNA15,GZMA,IRAK3,ITPA,LCMT2,MALT1,MCAT,MCM3,OR10P1,OR11G2,OR13F1,OR1I1,OR1S1,OR2AE1,OR5B12,OR6C6,OR6N1,PDE6H,RPN2,SDHC,SFRP4,SLC25A6,SOCS1,TNFSF14,TUBB3,UGT2B4,YES1,SERPINB5
Negative_4401	EPO,FH,ACSM1,AGRP,ALDH1A1,APOA5,ATP5F1,CCNA1,CCR8,CHSY1,CTSB,CYP27A1,CYSLTR1,FOXA2,GALNT8,GUCY2F,GZMA,INSRR,ITGA2B,MAFA,MAP3K14,MCCC2,MDH2,MECR,NFASC,NUMB,OR10G7,OR14A16,OR2B6,OR4C6,OR51B2,PARVG,POLR2H,PRKCA,RASGRP3,SPRY4,SSH1,SUV39H2,TAS2R50,TRAF5,UCP1,VAMP4,VEGFC,WNT9A,YWHAZ,HSD17B12,SERPIND1

Negative_4402	BCR,CR2,MPL,ABLIM1,ACSL5,ANAPC5,ARRB1,BUB1B,CALML3,CCNA1,CDKN1A,CNTFR,DLST,DTX3,E2F1,FABP3,FABP4,FARSA,HDAC1,IL7R,LDHC,LEFTY2,LIMK2,LTBP1,MGAT1,MOV10L1,MYH11,MYLK,NRAS,NRG1,OR4N2,OR4Q3,OR51M1,PLCG2,PPP2R5E,RASGRF2,SNAP25,SUCLA2,TAS2R3,TAS2R49,TBL1XR1
Negative_4403	AKR1D1,ALDH1A2,ATP6V0B,AVPR1A,BDKRB2,BMP5,CACNG7,EHMT2,ENPP3,GGT6,HRH2,ICOSLG,MAP3K11,NPAS2,OR6C75,OR8H2
Negative_4404	ADA,LEP,CARS,DGKI,GNAI3,IL21R,KAT2A,MAFA,MKNK2,NCF2,OR5AU1,PDGFRB,RFXANK,SAT1,SOAT2,TACR2,TRPC1,UGCG
Negative_4405	CAD,IDS,OTC,SMO,ACAT2,ACP2,ADCY8,ADIPOR1,ALG8,ALG9,ANAPC11,APRT,CARS,CHRM3,CXCL1,DHRS4L2,DIAPH2,DKK4,FMO2,FPGT,GJD2,GNA11,GUCY1A2,HARS2,HPSE,IL12B,ITPKA,KAT2A,KIR2DL4,LAMB1,LIPF,MGLL,MYH3,NKX6-1,OR1A2,OR2C3,OR52E8,OR52H1,OR6Q1,PPARG,PRKCG,PTK2,RAD51,RASSF5,RFXANK,SFRP1,SMURF1,SSH1
Negative_4406	DBH,GPT,ARPC2,CACNA1A,CALML5,CD36,DUSP2,ENTPD8,GNMT,GNRH2,GOT2,HARS2,IFNAR1,IL28B,JAK1,MAGI3,MAPK9,OR2L3,OR4F6,PANK2,PPP1CC,RALGDS,RASSF5,SEMA3G,STAT3,STK11,STX4,TGFBR1,TOMM40
Negative_4407	LCT,SP1,ALDH6A1,ASS1,BLVRB,C1QA,CD86,CHIA,CHKB,COL1A2,CTSG,CYP26C1,DPM1,DPYSL2,DRD1,EPHA2,EREG,G6PC2,ITGA5,MYH13,NEU4,NFATC2,NME7,OR11L1,OR1J2,OR4S2,OR6C6,OR6C75,OR6N1,ORC2L,PFN2,PGM2,PIK3CD,PLA2G5,PLCG2,PMM1,POLR2D,PRKAR1A,RAB5A,RUVBL1,SDC4,SH2D2A,SLC25A6,SOCS4,TCF7L2,TNFRSF9,HLA-DRB5
Negative_4408	ATP5J2,CD58,CLDN22,CREB3L4,CXCR6,CYP2J2,GPD1L,IFNA16,IFNW1,P2RX6,PIAS4,SRGAP3,STAT2,ZFYVE16
Negative_4409	PDC,TPR,ACVR1C,CACNB1,CMBL,GNAL,MAT2B,OR51S1,PDE4B,RAC3,RPSA,SOCS7,SERPING1
Negative_4410	DBI,HDC,ZYX,ATP5O,B3GALT4,C7orf16,CHEK1,CXCL13,CYP2S1,DUSP8,DVL3,ELK4,EREG,F2RL1,FARSA,FGD3,GGT5,GRIN1,HLCS,IL17RB,IL2RB,INADL,INPPL1,NME7,OR11H4,OR51L1,OR5T3,PDE6G,PFN3,PIGS,PRNP,RUVBL1,SEMA4G,SESN3,SHISA5,SMAD2,SMPD4,TSNARE1,CSGALNACT2
Negative_4411	FAH,ALG5,APC2,BMP8B,CACNG2,CRKL,EPHA4,HSD17B1,LIPT1,METTL2B,NKD1,NTRK2,OR4S2,OR5A2,OR6K3,PDIA4,PRKACB,PTEN,RFWD2,SUCLG1,TCF7L2,PRICKLE2
Negative_4412	IDS,PGF,ABLIM3,ACVRL1,ADCY5,AKT3,ASS1,AVPR1A,BCL10,CAMKK1,CBR3,CES7,CHUK,CLTC,DEGS2,DPYS,ETS1,FGFR4,GALNT14,GGT5,GLDC,HTR2C,IFNA6,MAML2,MYH10,NAGLU,OR1I1,OR2L13,OR4A15,PIM2,PKM2,PPP3CA,PRKAB1,PTGFR,PVRL3,PYGB,SLC25A5,TUBB6,UBE2L6,UGT8,ULK2,TNFRSF11A
Negative_4413	EPO,ARG2,CACNA1B,CARS2,CHSY1,CLDN6,CYBA,DDB2,ENTPD6,GDF5,GFPT1,GP1BB,GUK1,HHEX,ITPKA,MAT2B,MDH2,NEU1,NMNAT2,OR6N2,PIP4K2B,PTK2B,RASGRP1,TICAM2,VNN1
Negative_4414	DBI,TFG,AGTR1,AKR1B10,ATP2A2,CDK7,CLDN3,CPS1,CPT1A,CTF1,DUSP4,GALNT10,NPR2,OR1L4,OR5L2,OR8D4,PANK3,PIM2,PRSS1,SEMA4A,TAS2R9
Negative_4415	AK7,KMO,CYP26B1,IL12RB2,LAMB2,OR4C16,TRAF4
Negative_4416	RPE,ALDOA,ALG10B,ASS1,ITGA5,TAP2
Negative_4417	BTRC,PDHA2,PIP5K1C,QARS
Negative_4418	CAT,SI,AKT3,ALG11,ANPEP,B4GALT5,CTSB,DFFA,DFFB,FOXO3,GLS2,GMPR2,GPD1,GPD1L,MAN1A1,NME2,OR6C76,PGM1,PLAU,PRODH2,RFNG,SLC12A2,SMAD9,SMPD4,TKTL2,TLR9,TNFSF10,TPI1,TUBB2B,TYK2,UAP1
Negative_4419	AK1,BCR,IDS,ME2,NNT,ABL2,CAMKK1,CANT1,CLTC,CREB3L2,ENAH,GRLF1,GUCA1C,MAML3,OR8I2,PARVA,PRKAB2,PRKCH,TACR2,VEGFC

Negative_4420	ASS1,CD8A,CSF2RB,DERA,FPGT,GRLF1,LALBA,NR4A1,OR4C11,PFN2,PLXNB1,PPAP2B,PTCH1,SHC4,SLC2A4,SNAI2,SORD,ATP6V1C2
Negative_4421	DDO,FN1,LPL,ACOT12,ADH1A,ALDH1B1,ARRB1,CAV3,CD274,CNTN1,CYP2C8,GALNT10,GLDC,HPSE2,IL19,ISYNA1,MAP2K7,OR4F4,OR9G1,OXTR,PAK7,PARVA,PGM1,PPM1D,PSPH,RBL2,SMPD1,SMPD3,SPHK1,STAT5A,TRHR,TYROBP,UGCG,UMPS
Negative_4422	ATP5H,MCM3,NPR2
Negative_4423	FN1,ID2,MYC,SMS,AGMAT,ARPC5L,BDH1,CCR3,CD274,CLDN10,CPT1A,DGKE,DHRS4,GOSR2,KCNJ11,NCF4,OCLN,OR10Q1,OR5D16,OR5P3,PLA2G4A,PPP2R5A,PRKAB2,PRPH,RDH12,TLR9,VAV1,WNT11,GABARAPL1,HLA-DQA2
Negative_4424	CBL,IL6,ALDH3B2,ANAPC7,CYP2F1,DKK4,ETS1,GATM,GCNT1,GGCX,IFNA8,LDHAL6B,LRP5,OR14J1,OR52R1,OR6S1,OXCT2,PFKM,PIPOX,PLTP,STK4,TJP3,TRIP10,ATP6V0A4
Negative_4425	CGN,FUK,ARSA,CCL21,GNG13,ITGA7,MICA,OR13D1,PFKFB3,SEMA3C,TRDMT1,PDCD1LG2
Negative_4426	ACSS1,CDC42,CHRM2,CLDN6,DPYS,EDNRB,EFNA5,FLT3LG,GSTM4,HCLS1,L1CAM,MGST3,NOTCH3,NT5C1A,OR8S1,PLA2G2D,PRKAG2,RASSF5,SOX17,SPAM1,UCK1
Negative_4427	FN1,ABCC8,ATG7,ATP5F1,B3GNT4,BDH2,C3AR1,CALML3,CCL17,CCL22,CDH5,CER1,CHUK,COL3A1,DHDDS,EXTL1,FUT8,HEMK1,HMOX2,HRH1,HSD3B2,IDI1,INSR,JAG1,MEF2C,MKNK2,MS4A2,NCOR2,NGEF,NOX3,NT5C2,OR13D1,OR1D2,OR2D2,OR5P3,PHKG2,POLR3K,RASA2,SMAD4,SMURF2,SPI1,TLR9,TNFRSF4,TRPC1
Negative_4428	F2,GNS,SPN,ACSL3,ALCAM,ALDH3B1,ALDH6A1,ALPP,ARHGEF1,ASRGL1,B3GNT3,C1QB,CCL13,COL11A1,CPT1B,CPT1C,DKK1,GALNT11,HSD3B7,ICAM2,INPP4A,IRAK4,MAD2L1,OR1A1,OR4S1,OR51A4,OR51G1,PIGC,TGFBR1,CSGALNACT1,MAPKAPK3,ST6GALNAC4
Negative_4429	CRB3,DLL1,HCST,IL17RB,NOTCH1,NOTCH3,OR13C8,PEMT,SETDB2
Negative_4430	DHH,ACSS2,BMPR1B,CER1,CYP1B1,FGF20,GSTT1,MPP5
Negative_4431	NPL,PTS,ACSL4,APH1A,ARNT2,BUB1,CAMK2D,CDKN2B,CX3CL1,E2F1,OR52E6,PANK1,PNPT1,POLR2F,RPIA,TKTL2
Negative_4432	GHR,ENO2,EPOR,HSD11B2,OR4D2
Negative_4433	ADH7,BCL2,DLAT,ENPP2,EPHB3,EPOR,FZD5,GALK2,GNB3,IFNW1,NOD1,OR1D5,OR5M8,PFKFB1,PIK3C2B,PIP4K2B,SIGLEC1,UGT2B28
Negative_4434	IL8,AMHR2,CCND3,CDC14A,DTX1,PFKFB4,SPCS3,TIAM2,PLA2G12A
Negative_4435	ADC,ME3,ACVR1C,AKR1B10,ARNTL,ATP5A1,B3GALT1,B3GALT4,BMP5,CD38,CSF3,EXTL3,GSTA5,IL9R,NOS3,OR14J1,PLXNB1,PPP1R3B,PRKAA2,RAP1B,SMARCA5,TAS2R41,TP73,HSP90AA1
Negative_4436	CDA,ID2,SP1,ACADS,ACY1,AKR1C3,ALDH1A2,AMY2B,AREG,ARPC1B,ARPC4,CCL1,CCL7,CXCL5,DUSP5,EIF4E2,F2RL2,FLT4,GUCA1B,IL11RA,INSR,KDSR,LARS2,LIPG,MALT1,MGAT4B,MYH7B,NUDT8,OR2T12,OR5H6,OR6C4,OR6Q1,PDXK,PIGX,PIK3R3,PIM2,PRKCQ,SPRY2,STX7,SUOX,TCF7L1,TUBA4A,ARHGEF12
Negative_4437	C8A,CGA,DBT,F8,LCK,ABCC8,ACSL4,ADIPOR1,AGPAT4,ARPC1B,CACNG4,CDS2,CHRM5,CLTCL1,CYP2C8,DUSP10,DUSP14,ETS1,GLB1,GUCY2C,GYS1,HIBADH,HSPA2,IL15RA,IL23A,LGMN,NOS3,NPR1,OR2A4,OR2H2,OR4X1,OR51E2,OR8G1,PAX6,PISD,PTGIS,SNAP23,ST3GAL4,TNFSF8,TPK1,TUBA1B,UCP1,VCAM1,MAPKAPK3,SERPINF2,SERPING1
Negative_4438	GPT,OSM,AADAT,ALDH1A1,ALDH3A1,ALDH7A1,BCAT2,CADM3,CCL5,CYP1B1,FPGS,GYS1,HADHA,IFNGR1,IL22RA2,JAK1,LCP2,MKNK1,MTHFS,NFATC3,OR1A2,OR1J2,OR2Y1,OR5AR1,P4HA2,PER1,PIAS3,POLD1,PSME1,PSME2,PTCH2,PTCRA,PTEN,PTGER1,RALBP1,RAP1B,TRPM5,WASF1

Negative_4439	ATP12A,CD38,CNGA4,CXCR5,GAD1,GOT2,GUCY1A2,IFT57,IGSF5,JAK1,NEUROD1,PAK3,PFKL,PIP5K1C,PLD2,PNPO,PRLR,PRNP,TJP3,TNFSF8,TP53,TPH1,TRAF1,UROS,ATP6V1E1
Negative_4440	F12,NGF,ANAPC7,CD36,CD40LG,CLDN10,COQ5,CSF2RB,CSNK2A1,DDX18,DOCK1,FGF13,G6PC2,GUCA1A,IL23R,MAP3K6,MLLT4,MTHFD1L,NKX2-2,OCRL,OR10Z1,OR51A7,OR7C1,OR9K2,PDGFD,PFKFB3,PGK1,PHKA1,PPP3R1,PSENEN,PTGDS,TGDS,TGFB1,TGFBR1,PAFAH1B1
Negative_4441	ADO,F10,IL6,ACSL4,ALPP,APOA5,BCL2L1,BMP6,CCL15,CPS1,CRLS1,CSNK2B,DDX23,HLA-DRA,ITGAE,LAMC3,MTAP,NCF2,NFATC3,NGEF,NKD2,NQO1,OR10T2,OR2B2,OR51F2,OR5J2,PCNA,PLAU,PPAP2C,PPBP,PRKAB2,PRKACA,PRKACG,RGS3,SMARCA5,CACNA2D2,HLA-DQA2
Negative_4442	DHH,F2R,FAS,CDKN2B,CLCF1,DDX47,DPAGT1,ENPP2,ENTPD3,FZD9,GBA3,GMPPB,NAGS,ONECUT1,OR2A25,PAK6,PTPMT1,SELP,SKIV2L2,ST8SIA1,STX1A,TAS1R3
Negative_4443	AK5,IVD,ABL1,AHCYL2,ALOX5,ASRGL1,B3GAT1,BAAT,BAIAP2,CACNA1H,CALML6,CCL16,CCR1,CD34,CDH3,CDKN2D,CHIT1,CHPT1,CLDN18,CSF1,DLL3,DRD5,DUSP2,ENAH,FGD1,FRAT2,HYAL2,IMPDH2,LIFR,MCCC1,OR1D2,OR1N2,OR52M1,OR7A10,P2RX1,PLCB3,POLR3A,POMC,PPAP2A,PPP3R1,PVRL3,QARS,RPE65,SDHA,TJP3,UBA7,ATP6V1C1,SERPINF2
Negative_4444	F10,ME1,ADCY6,ADRBK2,ATP5A1,B4GALT2,CD244,CD38,CHSY1,DDX23,DHRS9,DVL2,FADD,G6PC2,GALNT3,GNAI3,HTR6,IFNW1,ITGA1,MAN1A2,OLAH,OR5AS1,OR5M3,PDGFA,RAC3,RASGRF2,RASSF5,RFX5,RPS6KA5,SH2D1B,SPRED2,SULT1E1,TXNDC12,UGDH,WNT7B,ATP6V1G2,ST6GALNAC6,SUV420H2
Negative_4445	CDH4,CSNK1G3,DUSP7,FMO2,HTR2A,NRXN2,PGAP1,PPARD,PRKCI
Negative_4446	ABAT,ACOX1,ADCY9,AGRN,ALDH4A1,ARPC1B,ASPA,ATF2,B3GAT3,B4GALT2,CDH5,CMPK1,CREB3L1,CREB5,CTSL1,CYFIP2,CYP1A1,FCGR3B,FGF20,GALNT14,GFPT1,GPLD1,KIR3DL1,LIFR,LIPF,MAGI2,MAN1A1,MGAT5,NMNAT1,NT5E,OR14A16,OR51M1,OR9I1,OXCT2,PDE4D,PECAM1,POLE3,PPT1,PRSS3,PTTG1,RERE,SOCS1,SPRY2,SULT2A1,SYNJ2,UCK1,UGT2B28,ZNRD1
Negative_4447	DAO,IL7,LEP,OGT,PRL,ACAA2,AHCYL2,ALG13,ANPEP,APLP1,CHRNA7,CYCS,DUSP8,EGLN1,FGF4,GRIN2D,ITPK1,MMP9,MTHFD1L,NAGA,NLGN3,NME7,OR10G4,OR10Z1,OR2A2,OR2T12,OR52E8,OR5J2,POLR2F,PTPMT1,RELB,SPTAN1,UBE2L6,UCK2
Negative_4448	BMPR1B,DHFR,EPHA5,GAD2,HLA-F,IL12RB1,INHBA,IRF3,NFATC2,OR52I1,OR9I1,POLA1
Negative_4449	GH2,IL8,ADIPOR1,ARRB1,ATG3,B4GALT1,CCL8,GANAB,GSTM3,IL20,LRRC4C,NRCAM,OR10K1,OR3A3,OXSM,PANK2,PIGM,PLK1,PPM1B,PPP5C,RDH16,RPS6KA6,RXRA,SHC2,SORD,STAT4,ST6GALNAC4
Negative_4450	FST,PGF,A4GALT,ABL1,ABLIM1,ALDH9A1,ALPI,ARHGEF4,ARNT,ATG7,CCR2,CD244,CLTC,CRKL,CXCL1,CYP19A1,DARS2,FBP1,FGFR4,FLAD1,GAL3ST1,GSTA2,IL12RB2,IRAK1,LPAR1,MAPK11,MTFMT,OR13H1,OR5R1,OR8H3,PDPK1,PGK1,PIP5K1A,PLAU,PNPO,PRKAG1,PTPRB,RAB5A,SEPHS1,SETD7,SMPD4,SSH1,SULT2B1,TAS2R8,TSC2,VASP,VAV2,TNFRSF1A
Negative_4451	GP5,OGT,B4GALT1,BBOX1,CACNB2,CCL25,CHEK2,CPOX,DUSP2,EPHA1,EPHA5,GFPT1,HSD3B7,HTR6,NSD1,ODC1,OR51T1,OR52W1,P2RX7,PARD6A,PIPOX,PTPRF,UGT2B10,UROC1,WIF1
Negative_4452	DVL3,GALNS,HNF1B,MDH2,POLE2,TGFA,WNT6

Negative_4453	CS,DBI,NPY,ACYP2,ALG9,ASPA,CAMK2G,CD79A,CDH4,CDKN1A,CFL1,CSNK2A1,CYBA,CYP1B1,DCXR,DDHD1,DHRS3,DTX3,FARS2,FRAT1,GNAI3,GSTA1,HMOX2,HTR2A,ICAM1,IL11,MAN1B1,MAN1C1,MYL9,NCF4,NEFM,NSD1,NUDT5,OR2B6,OR4N4,OR52R1,OR5I1,PDE10A,PPARG,RALBP1,RAP1A,RASGRF2,SGPP1,SOCS5,THTPA,TUBB4,UBA7,ARHGEF12,HLA-DPA1
Negative_4454	ASL,F11,GH1,GPI,PTS,ZYX,AASS,ADH7,ALG9,BCAT2,CAMK2G,CFLAR,DAAM2,GRIN1,ITGAL,LFNG,LIAS,OR10Q1,OR1N2,OR2A14,P2RX7,PFKM,PIP5K3,POLA2,PPP1R3B,PTGIS,RFWD2,SDC3,UROC1,WASF2
Negative_4455	F2R,MTR,RPE,ACVR2A,ADH6,AKR1C2,AMPD3,APOE,CASP10,CHIT1,COL6A1,CXCR4,DTYMK,EFNB2,ENAH,EXT2,FZD8,GUCY1B3,HMGCS1,LDHD,MAP3K13,MLLT4,MPP5,MYH4,OCRL,OR10A6,OR2T29,OR4F17,OR5B17,POLR2D,PPA1,PPP1R3C,PTK2B,SEMA3G,SHC4,TBL1XR1,TSHR,WNT1,ATP6V1B2,CSNK1A1L,MAPK8IP1
Negative_4456	HYI,CCKAR,CHEK1,CHRM3,CPT1C,CUL1,GALNT7,GUCY1A3,HARS2,OCRL,OR13G1,OR4L1,OR8K3,PARVB,PIK3R1,POLR3D,PPAP2A,PRKAR1A,RBPJL,SLC25A5,SRD5A2,WNT3A
Negative_4457	JUN,ME1,TNC,ACSL5,AKR1C4,ALDH5A1,APLP1,CACNA1B,CCNG1,CD86,CDKN2A,COQ3,CRY1,DDX51,DGKA,DGKB,GMPS,GPD2,GSTM2,IL1R2,LPAR1,MALT1,NUDT2,OR10G4,OR10J3,OR2M7,OR51I2,OR5AR1,OR8G1,ORC2L,PIK3R4,POLR2F,PRKAR1B,PTK2,ROCK2,RRAS2,SCP2,SGMS1,TBXA2R,UPP1,VKORC1,HSP90AA1,TNFRSF1B
Negative_4458	SRC,ARPC4,ATP6V1A,CLDN7,CRY2,CYP2A13,CYP3A7,DDX23,GGT1,GRHPR,GSTO1,GSTZ1,GUCY2D,HARS,ICOS,LIMK1,MKNK1,NEFM,NT5C1B,OCLN,OR2T10,OR4F6,OR51G2,PDE4C,PIGK,POLR2I,POLR3K,PPP2R2C,PSAT1,RASA1,SH2D2A,TCF7L1,VTI1B,WARS,ATP6V0A1,HSP90AB1
Negative_4459	AGK,IL5,AADAT,ABCC8,ACTG2,ATP6V1F,CDC25A,COQ6,CPT1C,DFFB,DGAT2L4,DUSP3,DUSP4,EPHX1,FUT8,KLKB1,MARS2,NCR3,OR6C74,PARS2,PDGFC,PPT1,RAP1B,RASGRP3,RFXANK,RHOQ,RUNX1T1,SUV39H2
Negative_4460	SRM,AGPAT6,CCNG2,CDS1,CHRNA7,CYFIP1,DGKA,DHFR,DMGDH,F13A1,GUCY2C,NMNAT3,OR6C4,PGLS,PPP2R1B,RALB,ULBP2,ATP6V1G3,CACNA2D2
Negative_4461	CAD,DCT,SRM,ACTA1,ADRA1B,BMP8B,CCNA1,FZD7,GNRH1,JAK3,KCNJ11,MARS,MASP1,MGAT2,OR10G2,OR1C1,OR2C3,PDE4B,PDE7A,PFKFB1,PFN3,PGAM1,PHKG2,PIK3R5,PPP2R2B,RBL1,RPS6KA1,SORBS1,TAP1,TBL1XR1,TUBB4Q,HSP90AB1
Negative_4462	CRH,ABLIM2,AKR1C3,ALDH6A1,ATP6V1A,BAIAP2,CALML3,CYP11B2,DPYSL2,ERBB2,GLI3,GRM5,HTR2A,IFNA8,MAP4K3,MTMR1,OR51B6,OXCT2,PFN4,PLCG2,POLR3K,PTPN11,RASSF5,UGT2B4,ULK3
Negative_4463	C6,IL8,ADAM17,CD247,CSNK1G3,CSNK2B,CTBP2,DDX50,FCGR3B,FMO5,GMPR,GNRH2,HMOX2,IRAK2,MGAT2,MYH1,OR12D3,PAK7,PFN1,PGM1,SELE,TAS2R1
Negative_4464	ADK,TPO,ATP6V0C,CD38,GUCY1B3,IDH1,IL6R,IMPDH1,MGAT4A,NEFH
Negative_4465	GLA,CD38,E2F5,EDA2R,FGF5,GUK1,IFNAR1,IL22RA2,IL24,PANK2,PDHA2,RDH16,SFRP1,THBS3
Negative_4466	HYI,DFFB,EFNA2,FGF11,FZR1,HPRT1,KARS,KIR2DL2,MAD2L1,MAPK3,MTHFD1,OR10P1,OR13C8,OR1L8,PAK4,PIK3C3,PRODH2,SDC1,SESN1,SLC25A5,SPRY2,TAS2R10,SERPINA1
Negative_4467	DAO,ATN1,B4GALT4,CCL5,CKS1B,FGF23,GAD1,NFAT5,NMNAT1,OR2M7,PHKA2,TFPI,UGT2A1
Negative_4468	KLRC1,MMP2,THY1
Negative_4469	ATM,ATP2A1,CACNG3,CBLC,CCL28,CNGA3,E2F3,IL13RA1,MAP3K11,OR12D3,OR5K1,PDE10A,PDX1,PDXP,PLAU,SIAH1,SIGLEC1,SYNJ1,TAS2R38,TNFSF9,UGCG,B4GALNT1,CTNNBIP1

Negative_4470	AMH,PGP,ADH7,BMPR1B,CCL11,CD19,CLDN16,CREB3L1,CRKL,CRY2,CTNND1,CX3CR1,DLL3,DPYS,EFNA4,ENTPD3,FLOT1,FUT5,GALC,GALNT3,GUCY1A3,IARS,IL1R1,LGMN,LIPF,LTA4H,OR4F17,OR4F4,OR5J2,OR7A17,OR8D4,P4HA3,PLCD3,POLD2,PPP2R2D,PVRL4,RASGRP2,SLC25A6,SREBF1,TGFB3,TRAF3,TRDMT1,UCP1,UNC5C,XCL1,SERPINB5
Negative_4471	ADIPOQ,AKT2,CD82,CTPS2,DTX4,EPHB4,GALNT9,GLDC,KLRK1,NMNAT2,OR10H3,OR4F5,OR52L1,OR5D18,PNPLA3,PPA1,PTGFR,RENBP,SUV420H2
Negative_4472	ACSM1,ADCY9,CHSY1,CLCF1,COL4A4,DEGS1,GNG13,OR1E2,OR52N4,POLR3D
Negative_4473	C5,ALDH4A1,ALG1,ALOX5,AMY2A,CDC42,CNGA4,CPT2,DNMT3A,DUSP6,DVL2,EFNB2,F2RL1,FTH1,G6PC2,GALNT6,GDE1,GRIN2C,NEFL,OR10T2,OR1J2,OR4A5,OR52B6,OR5D13,OR9G1,PAK3,PIGB,PKMYT1,PLCB2,PPM1A,PTPMT1,RELA,RERE,SDC3,SEMA7A,SPTLC2,SUV39H2,UCK2,VAMP1,WHSC1L1
Negative_4474	IL8,ADRA1A,AHCYL2,ALOX15,ANAPC11,BACE2,CACNA1D,CACNA1G,CCL17,CFL2,CLDN20,IL18,IL5RA,OR6T1,P2RX2,PPP2CA,SNAI2,TDO2,TRADD,UAP1,ULK2,VTI1A
Negative_4475	GH2,ANAPC1,ATF2,CACNA1S,CASP10,CLDN22,CLDN4,COX15,CTNNA1,CYP2B6,DNMT3B,GFPT1,HIBCH,LDHC,NCR1,OR2T3,OR2Y1,OR4D6,OR4D9,OR4P4,OR5V1,PIP5K1B,RRAS,STAT3,SYNJ1,TAS2R9,WHSC1L1,WNT10B,ATP6V1C1,MAPKAPK5,TNFRSF10C
Negative_4476	CD6,INS,NPL,ATP5F1,CD28,CD59,CLDN15,COL4A1,DEGS2,ERO1L,FUT8,G6PC2,IL5RA,ITCH,LEF1,LPAR1,NAT5,NFKBIA,NUMB,PDE6H,PIGO,RAP1A,RASGRF2,SETD2,SLIT1,SMC1B,SPI1,TAS2R42,TYROBP,VAV1,XCL1,PPP1R12A
Negative_4477	PML,AACS,ADH4,ALDH6A1,ANAPC10,CD55,CDC45L,CMBL,CRLF2,DAAM1,DUSP4,FLT4,IAPP,IFNAR2,IFNK,IL13RA1,MLYCD,OR10G7,OR1A2,PAK7,PDGFB,PRKG1,RFX5,SMAD5,TGFB2,UBE2K,XCR1
Negative_4478	ACAT1,ACOT11,AOC2,B3GNT5,CTNNB1,GPD1L,OR10A2,PRKCE,SMAD4,SMURF1
Negative_4479	CDA,ID1,OSM,PDC,UBB,ABLIM2,ACO2,ALG3,AREG,CD79A,CD8B,CNTNAP2,CXCL11,CYP3A43,E2F2,GGT7,GLO1,GUCA1C,HCST,IDH3G,IFNA21,IKBKE,IMPDH2,LRAT,MYH15,NFATC4,OR10P1,OR1D5,ORC5L,PDGFB,PDX1,PHKA2,PHKG1,PI4KB,PIK3CA,SOCS5,ST3GAL5,SULT1E1,WBSCR22,WNT9A,PRICKLE1
Negative_4480	AK2,GP6,ALG8,CBR1,CLDN5,FZD7,GALNTL1,IDH1,OR52I1,PIGW,PRPS1,TKTL1,WNT3
Negative_4481	MPZ,MUT,AXIN2,CREB5,DAPK2,DERA,EXT2,LARS2,MYH8,OR4D6,OR4S2,OR51B2,P2RX7,PI4KB,PKM2,RPS6,SHMT1,TNXB,TRIP10,VAV3,ATP6V0D1,GABARAPL1,MAPKAPK5
Negative_4482	ITK,TXK,ADRB3,ALAS1,AQP7,ARNT2,B3GNT2,CANT1,CD244,CDKN2D,CNTNAP2,CRHR1,DERA,DTX1,EARS2,FABP3,FARSB,FGD1,GCAT,GUCY2D,ITGB3,KAT2B,LPAR1,MAP4K2,MFNG,MKNK2,NOTCH3,NR1D1,NT5C1B,OR1D2,OR1I1,OR4C11,OR4X2,OR52E2,PDE3B,POLR1E,SMARCA5,SPP1,TBXAS1,TRAF1,TRPC1,TSTA3,UROS,WASL,WNT5B
Negative_4483	C8B,ACSL3,ADRA1D,BIRC2,CCND2,CDC27,CXCL2,EDAR,GALM,LIFR,NT5C1B,OR5K1,OR8U1,SFRP5,TAS2R14,TRDMT1,UPRT,VAMP2,MAP3K7IP2
Negative_4484	CDKN2D,CYP19A1,DUSP7,HTR5A,IRS4,RENBP,RFXAP,RPE65,SQLE
Negative_4485	AK7,GP9,NPY,PML,PRL,AADAC,AARS,ABAT,ACY1,ADH7,DUSP5,EPHA4,GCDH,HEXA,HSD17B1,HSD3B1,LEFTY2,MTFMT,MYH10,MYL7,NEGR1,NRG2,OR10H4,OR2A14,OR52I1,PPP1R3B,RHOD,SDC2,SH2D2A,TLR2,VTI1B,HLA-DRB5
Negative_4486	RPE,SHH,ACP1,APRT,CD27,CDC23,CFL1,CHRD,CKS1B,CLDN10,GNA14,OR10G4,OR1G1,PAX8,SOCS5,ST3GAL4,TDO2,TICAM2,VASP,TNFRSF17

Negative_4487	BTK, CKM, INS, MYC, ACY1, AMPD1, ARPC5L, CCL3, CD14, COL5A1, CYP2C9, CYP2D6, DGKI, DRD5, GNG12, HHEX, HIP1, ICOS, OR1Q1, OR6Q1, P2RX3, PANK2, RASGRF1, RPIA, SHC4, STAT1, XCL2, XYLT1, ST6GALNAC5
Negative_4488	VCL, APRT, ASS1, EPHB3, GALNTL4, HTR2A, LAMB4, OR4A5, OR5R1, PDE1B
Negative_4489	PDC, SMO, TPR, AGXT, CCNG1, CDKN2A, CHRM1, CLDN14, CTNNB1, CTSL1, CYP3A43, DDX4, DERA, DNMT3L, DUSP16, FGF12, GAL3ST1, GALT, HTRA2, IFNA6, IFNAR2, IRAK4, IRS4, ITGA2, KIR2DL4, LHCGR, LLGL1, MS4A2, NPR2, OR11H6, OR1N2, OR4D11, OR51B4, OR8D2, PDGFRA, PIK3R2, POLR2D, PRSS3, PTDSS2, RAPGEF4, THBS3, TRAF6, UNC5B, WNT7B, YKT6, AASDHPPT, HLA-DQB1
Negative_4490	AR, GGH, LPL, RET, AKR1A1, BPGM, BST1, C9orf95, CACNG2, CFLAR, CHSY3, DAPK2, DHCR24, GALK2, HSD17B4, IDH3A, JAK1, LFNG, OR10X1, PDX1, PIAS1, PPP2CA, SMAD6, STX12, SUV39H1, TBXAS1
Negative_4491	VTN, BMP8A, CXCL11, HES1, HGSNAT, IL25, MTHFR, NFKBIB, OR13A1, OR1L4, OR5H2, SLC25A4, TM7SF2, UGCG, VAMP1
Negative_4492	AMH, AMT, GLS, KDR, ACVR1B, ADRBK2, ALOX5, AQP7, B3GAT1, BLVRA, C4BPB, CACNA1A, CAMK4, CYP2C19, CYP3A7, DDOST, DDX54, DUSP9, GLS2, ITGA9, LRRC4C, MAP2K4, NCF4, NEU3, ODC1, OR2A42, OR4C11, OR51B5, PARS2, PER1, PLA2G10, PPP2R2A, PSME1, PTGS1, RASGRF1, SMPD1, SPTAN1, SULT2A1, ULBP3, WNT3, XCL1, PAFAH1B2
Negative_4493	C9, GBA, GDA, CDC2, CTNNA1, CYFIP2, DIAPH1, LMO7, OR10A2, OR10C1, OR10G9, OR13J1, OR2C3, OR4D1, OR7C2, PDE3A, PDX1, PLAU, PPARG, PTDSS2, PTGIS, PTGS2, RASSF5, TGFB1, TRPM5, TXNDC12, PLA2G12B, SERPINE1, TNFRSF13B
Negative_4494	F2R, MUT, AGXT, ALG1, ALG13, ATN1, ATP5G1, BIRC2, BMP2, CYP1A2, FADD, GNAL, IKBKG, LAMA3, MLYCD, NSDHL, ONECUT1, OR5L1, OR6C75, OR8D2, PDGFC, PIAS1, POLR3G, POLR3K, PPID, SEMA3B, SESN2, SHC3, SPRY2, ST8SIA1, VARS, ATP6V1C2
Negative_4495	AUH, IL5, MTR, AGRP, ALPPL2, AMHR2, APOC3, ASAH1, ATP5B, BUB3, CCNB2, CHST12, DDX18, DGAT1, ENO3, EREG, GRM1, GRM5, GSK3B, HNF4G, IL19, IMPDH2, ITGB6, KIR2DL2, MAPK10, MECR, NCOR2, NUDT9, OR10T2, OR2T12, OR52E8, OR5P3, POLR3B, PPP2R2C, PTPN11, PTPRC, STMN1, SULT1A1, HLA-DRB3
Negative_4496	UBB, CCR8, DHX58, GADD45A, ITCH, MALT1, NTF3, OGDHL, OR7C2, PTPRF, RFWD2, TAS2R48, CSNK1A1L
Negative_4497	CLDN8, CSNK1A1, FGF12, RALB, SPHK1
Negative_4498	ATP2A1, CD80, CD8A, CLDN14, COQ5, CXCL6, CXCR5, GCNT2, GPX7, GRAP2, GSTM3, IL8RA, JAM3, LAP3, MAML3, NADK, NEU4, OR2T3, OR51B4, PTCRA, SCP2, SELL, STX3, ZBTB16
Negative_4499	CD4, F2R, GPT, RPE, AGPAT4, ALDH5A1, ATP5F1, AXIN1, CACNG8, CCL21, CLDN20, COL5A1, CYBA, DDX47, DUSP8, EPHB4, HTRA2, IGF1R, LAMB2, MAML3, OCRL, OR5L2, P4HA2, PANK4, PDE8A, RAB5A, RAPGEF4, SOCS3, TLN1, TRAF2, ULBP3, WASF3
Negative_4500	AR, GK, GP9, AASDH, ACADM, ACOT11, ALDH1B1, ARPC4, B4GALT3, CALML5, CASK, CAV3, CCNE1, CD274, COL4A6, CSH1, EFNB2, ENO1, FGF1, FMO5, GCNT4, GLI2, IARS, IGFBP3, IL12RB2, LYPLA2, MCCC2, MTHFD1, NADSYN1, NAGLU, NCAM2, NME4, ODC1, OR10K2, OR2H1, P4HA2, PLEKHA8, RPSA, STAT5B, STX12, TSNARE1, YWHAB, TNFRSF10B
Negative_4501	MPL, AASS, ACVRL1, ADCY2, ATP5I, BPGM, CACNA1C, COASY, DDX50, GNG12, HCST, NFATC2, OR10A5, OR10R2, OR14J1, OR56A4, OR6X1, PCCB, PIGS, POLR2K, PRKG2, PYCR2, RPRM, STK11, THY1, TXNDC12, TYMP, WNT10A, XYLT1
Negative_4502	IHH, PRL, ADSL, BET1, C3orf10, CACNB1, CES1, CLDN19, COQ7, CSDA, CXCL2, FADS2, FRAT1, FZD7, GMPR, IFNK, MAP4K2, NT5C2, NT5C3, OR8B12, OR9A4, PAFAH2, PLA2G4A, POLA2, POLR2I, PPBP, PPM1B, PTPRF, SMARCA2, TAS2R41, TNFRSF13B

Negative_4503	SHH,AGXT,CCKAR,CHRNA7,CPT1B,CSH1,DUSP3,FTH1,GNRH1,HCST,HSD3B7,HTR6,IL28A,ITGA8,KIR3DL1,LDHAL6A,LRRC4C,NADK,OR10G4,OR1L1,OR4C46,OR5AC2,PMM2,PPM1A,PTPRB,PYCR1,RDH16,RPE65,SETD1B,STX6,THBS2,TKTL2,TNFSF18,TSTA3,UGT8
Negative_4504	B4GALT7,CD86,FARSA,ITPK1,PIGS,SOCS7,VASP
Negative_4505	CGN,GGH,JUN,LTB,MSN,OAT,SHH,ARPC3,ATP5J,BAAT,BCMO1,BNIP1,CACNA1I,CMBL,CSNK2A1,DGAT1,E2F2,EARS2,EP300,FBXW11,GUCA1A,HADHA,HDAC2,HPSE2,INSRR,MAOB,MYH7,NODAL,OR11H6,OR51B2,OR51B5,PDE11A,PIGB,PIP5K1A,RDH8,SPP1,TNFRSF4,TOLLIP,TUBB,UGT1A10,WNT9A,MAP3K7IP2
Negative_4506	FGG,ACHE,ATP5H,CYFIP2,FZD3,GNA12,HMGCL,HSP90B1,IL3RA,MGAT2,OR10AG1,OR13C8,PIGL,RUVBL1,TAS2R3,THBS3,WNT10B
Negative_4507	SI,VTN,ACPP,ALG1,BUB1,CALML3,CCNE1,CYP2C19,DHCR7,ENO1,FSHB,FZD6,G6PC,GADD45B,GADD45G,HLA-F,LIPC,MAML1,OR10J3,OR2T5,OR52I1,PANK2,PLXNB2,PRODH2,RDH5,SULT1A1,TNFSF11,WASF2
Negative_4508	AK5,IL9,AKR1C4,BAAT,CHRD,CLCA4,DHCR7,DIAPH3,ENTPD3,EPHA3,EPHA6,FOSL1,FTH1,GLUD1,HAP1,ICAM1,IFNA14,IL1R1,ISYNA1,JAK3,KCNQ1,MAPK14,MTHFD2,NCAM1,OR10A2,OR10AD1,OR1L3,OR2T1,OR51B4,OR6C3,OSMR,PSEN1,RASGRP2,SDC3,TAP1,TGFB1,WNT9B,ATP6V0A2,HSP90AA1,MAPK8IP3,PDCD1LG2
Negative_4509	AKR1C4,CCL8,GUCY2D,IFNGR1,IL18RAP,ITGB3,NCAM2,OR6X1,PI4KB,PLCB3,PLXNB3,POLR3D,PRKAB2
Negative_4510	GP6,MAG,AASS,ACVRL1,ADSL,ATP2A3,BCAT2,CD82,CMAS,COL1A1,DAPK3,DGKD,DHODH,EPAS1,GPT2,GPX7,HSPA1B,LFNG,OR10H4,PIGV,PPP2CA,RFX5,RRM2B,SOCS1,SPAM1,SQLE,TJP2,TNFSF14,UPP1,USE1,WNT16,WNT4,MAPK8IP1
Negative_4511	C8B,PGD,ADH5,AKR1B10,AMPD1,ASMT,C7orf16,CACNA1D,CCNB2,CLDN6,CXCL16,CYP2C18,FDFT1,FGF2,FGF4,GPD2,GPX7,HEXB,IFNB1,OR3A1,OR4C6,OR51A7,OR6C70,PEMT,PHGDH,SCLY,SREBF1,VAMP2
Negative_4512	AK2,AUH,KHK,ALAD,B4GALT2,BAAT,CDKN2B,CMAS,DCXR,DDX47,DIAPH3,DMGDH,DUSP7,G6PD,GNAI1,GSTM1,HBEGF,IL11RA,IL20RB,METTL6,NCR1,NEFM,NFATC2,NRCAM,OR2A4,OR56A4,PLCD4,PLCG1,RETSAT,RPS6KB2,SMPD1,SMURF1,VARS,WBSCR22,YARS2,PLA2G12A,SERPINC1
Negative_4513	CD6,ADH7,ALDH1A2,ALDH1L1,ALG12,CFL2,CISH,CLCA4,CXCL11,DDX54,EPHA5,FMO5,GOT2,GPD1,IMPA1,IRF9,LAMC3,LIPT1,MALT1,MAPK7,MGST1,MKNK1,MYH10,NUMBL,OR13C3,OR2M2,OR52B6,OR8U1,ORC4L,PDE3B,PDXP,RAP1B,RAPGEF2,STX4,TCF7L2,UPP1,WNT9A,ATP6V0D1,ATP6V1E2
Negative_4514	CDC14B,DAAM2,ENO1,FADD,GALK2,GLS2,GUCY1B3,IL7R,ITGA4,NTRK1,OLAH,OR4F4,OR4X1,OR9K2,PCYT2,PDE3B,PFN1,PIGQ,PLCB4,POLE4,SEMA7A,UGT2B4,WNT5A,HSP90AB1,SERPIND1,TNFRSF18
Negative_4515	CP,ACMSD,CD40,DLL4,EFNB3,GRLF1,IRF7,KLK3,NAMPT,OR12D3,PCK1,PIAS1,PTPN6,SLC27A5,TAS2R40,TUBB3,MAP3K7IP1
Negative_4516	CFI,CLDN9,CYCS,IL3RA,MAML1,MAP3K7IP1
Negative_4517	CEL,SDS,VTN,ADRBK2,AREG,CACNB4,ENTPD8,FZD5,HMOX2,HTR5A,IL9R,LARS2,MDH1,MGAT3,OR1S1,PIK3C2B,PRKCE,RPS6KA6,ST3GAL1,SULT2A1,TBXA2R,VAV1,WNT2
Negative_4518	CKM,DDO,DHH,LTB,ACSL1,CALML3,CCL3,CDC27,CPT1A,CREB1,EIF4B,FGF9,GSTT1,HTR6,ITPKB,LTA4H,MAP3K1,MAT1A,MAT2B,NRG1,OR2M2,OR2T11,OR4B1,OR52A1,PARVB,PGLS,PI4KB,PIK3CG,POLR2B,PPP1R3C,PTDSS1,TAS2R10,TBXAS1,WNT9B,CACNA2D1,GABARAPL1,HLA-DRB3,PAFAH1B1

Negative_4519	AK1,KDR,TPO,AGTR1,ATP5J,B3GALT6,CD3E,CDC25A,CLDN14,LCMT1,OR13G1,OR1N2,OR2A4,PLD2,PTPRB,SLC2A4,SMARCA2,STX2,TP53,WEE1
Negative_4520	AR,IDI1,ITGB4,PLK1
Negative_4521	ABO,TK2,TPO,ACSM3,ALG10B,CACNG4,CD19,CSNK2A1,CTSL1,CXCR5,CYP2A6,FUT4,GP1BB,ITGA2B,KIR2DL3,MAP2K7,MAPK12,MYL7,OR1F1,OR4C16,PFKFB2,PRIM1,RPN2,SEMA4A,SGPP1,SPCS3,UAP1,WNT16,PPP1R12A
Negative_4522	WAS,ALDH3B2,CLCF1,CSF1R,DIAPH1,FZR1,GNPNAT1,HAGHL,IL12B,IL1A,IL2RB,ITGAL,MGAT1,OR5H2,PAX4,PDCD1,PDE3A,PDK1,PIGH,PPCS,PRKAB2,PSME3,PTGS2,TUBA3C
Negative_4523	IL3,MSN,ARHGEF2,CCL20,CCR8,CDC6,CDIPT,CRLS1,DRD5,DUSP7,FCGR3B,FGF3,FMO5,HSP90B1,OR10G3,OR10K1,PAK2,PIK3CG,RALBP1,RPS6KA5,SHC2,SORBS1,SV2A,WNT5A,ATP6V1C1
Negative_4524	ASL,CFD,ADH5,BMP2,BMP5,CDK2,DDX51,DHRS9,DIAPH1,FCGR3B,FZD5,HARS2,KIR2DL1,LIFR,NKD1,OR2D2,OR5P3,ORC2L,PKLR,SLC27A5,TAS2R16,TRADD,TNFRSF10C
Negative_4525	CAT,DLD,ABCC8,ABLIM3,ACOT12,ACTA2,ANPEP,AOX1,AREG,BAI1,BPGM,BUB3,CD48,CLDN4,CLTB,DGKA,E2F4,EFNA5,EGLN1,EPB41L1,FGF20,FPGS,GALNT3,GALNTL5,GGT7,GNPNAT1,GSTP1,GUCY1A3,HNF1A,MAML2,OR2T11,OR51L1,OR5B17,OR8H2,PGAM4,PIGF,PLA2G2D,PPARG,PTGES2,PTK2,RBKS,SETD2,TRAF2,TUBA1A,ULBP1,TNFRSF14
Negative_4526	ABO,C6,MUT,ACTA1,ADORA2A,DPAGT1,ERBB3,IL20RA,ITGB2,MAP3K13,NUDT2,OR13C8,PFKFB2,PFKL,PLOD3,PNPT1,SMPD2,ST6GALNAC3
Negative_4527	ATP6AP1,CXCL1,FLT3LG,GRIA2,IFNW1,JAK2,KIR2DS5,PTPN7,SETD1A,SLC27A4,SMAD5,STX2
Negative_4528	CRK,ADH7,ATP5D,BMPR2,CACNG4,CAMK2B,CDC23,CES7,CREB3L2,CXCL12,DHRS4,EDNRB,EPHA7,FZD9,IL20,IL22,ITGA10,KARS,KLRC2,L1CAM,MGAT4A,NFYC,NRG2,OR52H1,OR6C2,PDGFRA,PGK2,PPP2R1A,PRKAG2,PTGES2,PTPRB,SMURF1,TIAM2,UCKL1,ATP6V0A4,ATP6V1C2
Negative_4529	CNTFR,DOT1L,RASGRP4
Negative_4530	ATM,DBH,SMS,ACACB,ADRA1A,AGPAT4,ALDH5A1,ARG1,CLDN15,CLDN18,DRD1,EFNB2,EGFR,ENTPD1,EPHA2,FRAP1,IARS,IRS4,MGAT5B,OR1A2,OR2T5,ORC1L,ORC3L,PDE7B,PRIM2,PRKCI,PRKCQ,PTCH2,RPS6KA6,TP73,TPI1,UPB1,WIF1,HLA-DQA2,MAPK8IP2
Negative_4531	BTK,F12,ATP5G2,C1QB,DBF4,EPHB1,FMO2,HLA-G,IL18,IL28A,MAPK3,MYH7B,OR10G9,OR10J1,POLR1B,POLR2K,PRKCD,RND1,TJP1,TNFSF10,TRADD,UGT2B7,GABARAPL1,ST6GALNAC4
Negative_4532	EBP,GP9,TK1,ACSL5,ADH7,ADSS,AGMAT,BLVRA,CACNG6,CCL16,CDKN1B,CREB3L3,CSNK1G2,DTX3,GALM,GDF6,GLI2,GRIN2D,GSTA1,IL23R,ITPKA,KLRC4,LIPF,LPAR1,NGEF,OR4N4,OR51F2,OR52B4,OR5F1,OR7A5,PARS2,SORBS1,SREBF1,SYNJ2,TAS2R41,TNFSF9,TRMT11,ULBP3
Negative_4533	ACVR1,BDH1,IL17RA,MAN1A2,NFKBIE,OR4A15,UNC5D,WNT7A
Negative_4534	CGA,DUT,FH,GCK,TNN,TPO,ADH5,AGTR1,ALG10B,BLNK,CCL22,CCR2,CD40LG,CDC6,CHPT1,COL2A1,CSF3R,CSNK2A1,CTPS,E2F5,GATM,GBE1,GIT1,GZMB,IDH3A,IL3RA,ITGA4,MTFMT,OR52D1,PDE8A,PIP4K2C,POLE,SFRP1,SPI1,SUV39H2,TACR3,UGT2B7,WASF3,TNFRSF17
Negative_4535	RPE,AGPAT6,GGT6,GZMB,OR7G2,P2RX6,PTGDS,PYCRL,TGFB3,WNT1,XYLT2
Negative_4536	RPE,ALDH1B1,CCL7,CD274,CNGB1,CTNND1,EXTL2,GNPAT,GRM1,HNMT,IL6R,OR52E2,PRKCI,TLN2,MAPK8IP1
Negative_4537	GLA,GNE,PGD,ATP5G2,ATP6V1H,BIRC5,CDC2,CMAS,COL11A1,COL5A2,COX15,CXXC4,DAG1,DUSP1,GNB3,HHIP,IL12RB1,IL12RB2,IL19,LRAT,MAPK7,MITF,MTMR1,NCOR2,NME6,OR6C3,PGAM4,PHGDH,POLR2C,PON1,STAM2,WWP2,SUV420H2

Negative_4538	ACHE,ACSL1,DKK4,ORC5L,PLA2G6,PPP2R1A,SC4MOL,AASDHPPT
Negative_4539	F2,SRF,AARS,ACTC1,AGTR1,CDKN1C,CREB3,DDX4,FABP3,FASLG,GLUD1,HLA-DRA,IDH3G,IGFBP3,IL23R,LILRB3,MLLT4,NFKBIA,OR13A1,OR2B6,OR4N5,P2RX3,PLK1,PPM1B,PRKCQ,RDH11,SOS2,SUCLG1,TAS1R3,TIAM1,TP53I3,XCR1,MAPK8IP1
Negative_4540	TXK,ADCY7,BCMO1,BMP7,CCNB3,CCNH,CD8B,CDC42,CPT2,CTF1,CUL1,CYP2A6,DLL3,EFNB1,ELK1,GDF5,GMPR2,HSD11B1,IL1B,KIR2DL3,MAP3K11,NAGK,OR6C1,OR8K1,PLA2G5,PPP2R1B,SH2D1B,SHC1,UCK1,MAPK8IP1,TNFRSF14
Negative_4541	ADH4,ATP4A,CACNA1I,CACNG8,CCR4,CEBPA,CHPT1,COMT,CRKL,CSNK1G3,CXCR5,CYP17A1,CYP2A7,DGAT2,DHRS9,DLST,DUSP1,DVL1,EPAS1,EPB41,FGF1,FGF10,FZD4,GRM5,HEXB,IKBKE,IMPDH1,JAK3,LIMK1,MDM2,MFNG,NFATC1,OR10A3,OR5H2,OR6C75,PERP,PIK3R1,POLR2D,SETD8,STX18,XYLT1,MAPK8IP3
Negative_4542	DBT,ADSS,ASS1,CLTC,DGAT1,DUSP8,GALNTL4,GUCY1A3,MYH7,NTSR1,OR2C1,OR8D2,OR8J1,OR9A4,PLAUR,PLOD1,RUVBL1,STAM,UGT2B10,ARHGEF12
Negative_4543	APP,ACSS2,B3GAT1,DPYD,FGF12,GRB2,HDAC2,HIBCH,IL18R1,KAT2B,KCNJ11,MAML3,NRP1,OR5A2,OR7C2,PRPH2,THBS1,THBS2
Negative_4544	AGL,ANAPC5,ARHGEF2,C3orf10,CASP7,CHEK1,CREB3L2,CYP21A2,GDF7,ITGA11,ITGA2B,MMP14,NARS2,NRXN1,OR2M7,OR4C12,OR6S1,SORD
Negative_4545	C3,FGG,SFN,TK2,ACMSD,ACPT,ATP5O,CACNG1,CBR3,CCL1,CDC45L,CDKN2C,CHKB,CPOX,DGKH,DHRS4,EPB41L1,GLG1,HLA-C,ITGA2,LYZL1,MAP3K11,NADSYN1,NARS,NAT5,OPLAH,OR51E1,OR51G2,OR8D1,PARD3,PIK3R3,PRKAR1A,PRKCA,RAF1,RALB,RBL2,RIPK1,SEMA4D,SLC2A2,SPRED2,SULT1A2,SULT1A4,TJP1,TSHB,UGT2B17,WEE1,YES1
Negative_4546	ITK,UBC,ACOX3,ADCY2,AOC3,APOC3,BBC3,CREB3L2,CXCL10,CYP26B1,FCGR3A,FGF18,GALM,GDF5,GSTM4,JAK2,NOS3,OR1E1,OR8K3,PCK1,PLA2G2A,PPP2R2C,PRKAB2,SEMA7A,WNT3A,SERPINA5
Negative_4547	GPI,HPD,ITK,B3GNT3,B4GALT4,FDPS,OR52N2,PDGFB
Negative_4548	ALG9,AMHR2,COL4A2,HLA-C,IKBKB,INPP5E,LTBP1,POLE4,RASA2,RASGRP3,SULT1E1,HLA-DQB1
Negative_4549	HGF,TNN,ACTN4,ADCY10,ADRB3,ALOX5,BPNT1,BRAF,C1GALT1,CACNB2,CALML3,CCNA2,CLCF1,CLDN15,CLTA,CRY1,DGUOK,DHDDS,EHHADH,EPHA8,FMO3,GCLM,HRH2,IFIH1,IFNK,MAP2K1,MAP3K11,MPDZ,NT5C1B,OR10H5,OR2D2,OR2Y1,OR7C2,OR8I2,PCYT2,PDE6G,POLE4,POLR1D,PRODH,PTGER3,STAT5A,SULT1A2,TAS2R38,TAS2R60,TP53I3,TRAF3,TUBB1,UROC1
Negative_4550	ID1,ADCY4,ARPC5L,B4GALT5,CACNA1B,CLCA4,FBP1,FCGR3A,FGF9,FOXO1,GNA13,KCNJ11,LEFTY2,MAD2L2,OR2T34,PFKFB3,PLCD1,POLE4,TBL1Y,TNFSF11
Negative_4551	MVD,CFTR,CLDN6,GABARAP,GALM,NLGN3
Negative_4552	AUH,ME2,SRC,ACOX1,ALG10,AOX1,APRT,ARRB1,BPNT1,CASP6,CCL16,CD59,COASY,CREB3L3,DPYD,EFNA3,ENO2,ENPP3,EXTL3,GCLC,HRH1,ICAM1,IFT57,IL7R,NT5C2,OR10AG1,OR8I2,PPM1A,PPP1CC,PPP2R5B,RAC1,SAT2,SEMA4C,SLC12A2,TGFB2,TGFBR1,TUBB2B,VTI1B,WBSCR22,TNFRSF6B
Negative_4553	C3,MPL,SDS,BCMO1,CDC7,CYP2F1,DTX3L,EFNA5,EPHA8,FGF22,GAL3ST1,GALE,GLI3,GNMT,HDAC2,HSPA1B,KITLG,LILRB3,MAGI2,MGAT5,OR5M9,PFAS,PLA2G6,POLR1A,PPP1R3B,PSEN2,UPB1,WASL,WHSC1,ATP6V0A1

Negative_4554	BACE2, BRAF, BUB1, CACNG4, CARS, CCL2, CDH5, CDKN2C, CFLAR, CHPF, CNTF, CRY2, CXCR5, CYP11B2, EFNA1, ENTPD7, FUT1, HARS, HTR5A, IL4R, ISYNA1, ITPKB, KIR2DL1, LTBR, NCR2, NEU3, NOTCH3, NRG2, OR2M2, OR5J2, OR6C70, OXSM, PIAS2, PIGB, PIGF, POLR2D, PPAT, PPP1R1A, PRSS2, PTPN1, SC4MOL, SDC1, SGPP1, SIGLEC1, STK4, UBE2L6, YWHAE
Negative_4555	ADC, ATP5O, CRKL, GYS2, MYH11, OR5H2, PMM1, POMC
Negative_4556	IL4, ACHE, ADCY4, FGD1, MPP5, NUDT5, NUMB, OR5P3, SPAM1, SULT1E1, TYMP, UPP2
Negative_4557	BCR, PLG, ACTB, AHCY, BLVRA, BMP5, CARS2, CAV2, CD38, CDKN2C, CSF2RB, CXCL13, DPM1, EDNRA, EREG, GMPR, HMGCR, IDI1, MAN1A2, MTMR2, NR1D1, OR1D2, OR6A2, OR6C6, ORC1L, PHPT1, PLCD1, PPA2, PRKACG, PRKCE, PTCH2, RCHY1, SGPP1, ST3GAL6, SUCLG1, TAS2R9, VANGL1, ST6GALNAC3, TNFRSF1A
Negative_4558	PXN, ACTA1, ALG12, ASCC3, CACNA1C, CCND1, CXCL16, DAAM1, DNMT3L, DUSP9, FCGR3A, GPD2, MPDZ, MPZL1, MYH15, OR13C5, OR51A4, OR51E2, OR56A1, PDE8A, RFXAP, SMURF2, STK4, VEGFA
Negative_4559	AGK, BAD, TFG, ACACB, ACMSD, AGMAT, AGXT2, ATIC, ATP5B, CHDH, CSF2RA, DGUOK, DPYSL2, FGF21, GDF5, GLI2, GNAL, HARS2, HMGCS2, HTR6, LDHAL6B, NEFL, OR2C3, OR51A7, OR56B4, OR6M1, P2RX5, PAPSS2, PDGFA, PGAP1, PIGM, PLCG1, PPP1R3A, PPP2R5A, PTGS1, SIPA1, TNFSF14, UGT2B7, VNN1, ATP6V0A4, SERPINF2
Negative_4560	CHST11, G6PC2, NCAM2, OR4K14
Negative_4561	CAD, ADCY10, ARNT, ECHS1, FMO2, GART, HBEGF, IFNAR2, IFNGR1, MICA, OR5C1, PFKFB3, RUNX1T1, TIRAP, TUBA8, WHSC1, TNFRSF12A
Negative_4562	DRD2, GALNT14, NOS1, NUDT5, SPRY4
Negative_4563	ALOX15B, ATP6V1D, MTAP, SMAD1, TNFRSF13B
Negative_4564	CKM, FUK, SYK, ADCY5, AGXT, B4GALT1, BPNT1, CCNG2, CHIT1, CHST11, CLDN1, COL1A2, COL6A2, CRB3, CTNNB1, CTTN, DOT1L, EHMT2, FZD9, GLS2, GLUD2, GNA12, GNG3, LIPT1, MAP3K12, OR2T2, OR51B5, PDE7A, PIK3CB, PNPLA3, POLR2J, PPP1R3A, PRKACG, PTCH1, PTPRZ1, RPS6KA6, SULT2B1, TRPM5, TSNARE1, WASF2, WNT5A, YWHAH, HLA-DPA1
Negative_4565	KHK, TYR, ACOT12, AGMAT, CAV2, CAV3, CCNH, CSNK1G3, DUSP3, FGF14, GSTM5, HEXA, HEXB, MGLL, NEFL, NME4, NNMT, OR10Z1, OR11H6, OR3A2, PARVA, PCCB, PFKM, PHKB, POLR2F, PTPRZ1, RASGRF2, RXRB, ST8SIA5, TRHR, UGP2
Negative_4566	ILK, SMO, ABLIM2, ACP1, ACSM5, ADCY3, AKT1, ARSA, CD14, CDH5, CDS1, CFL1, CHPF, CLDN22, CPT1A, CSNK1G3, EIF4E2, EPHA5, FCER1G, GALNT6, GCH1, GCNT4, GGT1, GSTM5, MLLT4, NKD2, OR13C8, OR2T6, OR4B1, OR4S1, OR52E2, OR7G2, PCNA, PIAS3, PLA2G2D, PTGIS, PVRL2, RASGRP4, RBL1, RCHY1, ROCK1, SH2D1B, STK36, VANGL1, WNT1, ATP6V1B2, MAPKAPK3
Negative_4567	C4B, C8B, PGF, ACSS2, AMPD2, APRT, ATG7, BST1, CD46, CFL2, CHEK2, CRHR1, MGAM, NFYA, NRAS, OR1C1, OR51V1, OR8D2, PDXP, PPP2R5E, VAV2
Negative_4568	C3, FST, ID3, ABAT, B3GNT5, BBC3, BCL2L1, CDC25C, CDH15, CSNK1D, CXCL5, EPRS, FGF1, FMO5, FZD4, GADD45G, GALNT12, GANAB, HEMK1, IL10RA, INHBC, ITGB4, MAP3K1, MAP3K3, MAPK10, MPP5, NADK, OR2T6, OR5K1, OXCT2, PFKFB3, PIGF, POLR2L, PPM1A, PRKAB1, PTK2, SGPP2, TBXA2R, TGFBR1, UGT2B17, WNT1, WNT3, TNFRSF1A
Negative_4569	CD4, FYN, ACYP1, B3GNT4, CDK6, CLDN7, DHRS4L2, DPYD, ENO3, EXTL2, GALNT11, GALNT2, GRM5, LTC4S, MAP4K4, OR10G3, OR10G7, OR13A1, OR4C15, OR4Q3, OR4S1, OR5H6, OR7D2, PDE1A, PIP5K1B, POLR3A, PRKAR1A, PSENEN, SEMA3E, SLC27A5, SOCS7, STAT6, STX4, TCF7L2, TIAM1, TNFRSF8, WNT5B, ST6GALNAC5
Negative_4570	NGF, AOX1, BMP8B, CTPS2, FGFR1, GADD45B, MYLK3, NR5A2, PHKA1, PLCD3, POLR3K, WNT2

Negative_4571	CBL,CGN,ACTN4,ADORA2A,ALOX5,CAMKK2,CASP6,CAV3,CCND1,CD48,CDC27,CLDN15,DPYS,DUSP8,FGFR2,HIBADH,HPSE2,JAM3,NUMBL,OR52N4,OR5AP2,PLA2G10,POLR1D,POLR3H,PPP2R2C,PTK2,RALB,SOCS1,TYK2,VAV2
Negative_4572	ABO,ADO,FGG,IL5,ABI2,AKR1C3,ALDOA,ALG10B,ALG13,ALPI,ALPL,BHMT,BPGM,CDH4,CFL2,CNTFR,CRY2,CTSB,DLL3,EGLN1,EXT2,GNG3,GPD1L,GUSB,IDUA,IL15RA,IL28B,IRS2,KARS,NME7,NOTCH3,NTNG1,OR2Y1,OR51L1,OR5A1,P2RX1,PAK2,PLCE1,PRODH,SC5DL,SELL,TAS2R13,WNT7A,TNFRSF12A
Negative_4573	C8A,AGTR1,ALAD,APC2,ATP5O,BCAT2,CANT1,CCL23,CLDN5,CYP3A43,DGKQ,DVL3,F2RL1,FLAD1,GRIA1,GSTM2,IPPK,LAMA4,MAP3K5,MTMR2,NCR1,NFAT5,NFKBIA,OR10A2,OR2B2,OR4A16,PFN1,PPARA,PPARD,PPP3R2,SEMA4D,SEPHS1,SMPD2,TBXAS1,TPMT,YWHAE,SERPINC1,TNFRSF6B
Negative_4574	LSS,RFK,ADH7,AGPAT2,ALDH3A2,ALG10B,BIRC5,BUB3,CASP7,CCR7,CD79A,CDC23,CDC26,CHUK,CRY2,CXCL13,CYP1B1,ELK1,ENTPD7,EXTL2,GLS2,IL1R2,IL20RB,IL6ST,NAMPT,NAT6,NRAS,OR13C8,OR52N1,OR8D4,OR8H3,P2RX6,PIGM,PIK3CD,PLA2G2F,PLOD1,POLD3,POLR2D,SELE,SPTAN1,STX7,TGFB2,TICAM2,WNT16,WNT7B
Negative_4575	GLS2,PRKCD,YWHAB
Negative_4576	IL7,ARPC2,ATP2A2,BCAT2,CREB3L4,DNMT3L,GALNTL5,METTL2B,PIK3R5,PRKCA,RHOQ,RND1,VAMP1,VARS
Negative_4577	ABL1,ACADS,ACHE,ACSM5,ACTC1,ACTN1,APRT,ATP4A,C5AR1,CAV2,CPT1C,CXCL11,CYP26A1,CYP2C9,FPGS,GYS1,IL13RA1,IL6ST,KIR2DL1,LAP3,LDHA,LIFR,LTBR,MAML2,MAP3K6,MYH15,NCOR1,OR10A4,OR2A42,OR2B11,OR4S2,OR8U1,PIGM,POLR2B,PPP3CB,PRDX6,ROBO1,SEMA3B,ST3GAL3,ST8SIA1,STK3,THBS3,UGT8,ULK2,HLA-DQB1,TNFRSF13C
Negative_4578	C7,IL4,SMO,ABLIM1,ACAT2,ALOX15,ANPEP,B4GALT7,BIRC5,CACNG8,CDK7,CREB5,DBF4,DHRS3,EIF4E,ETS1,FGF18,FLAD1,FZD3,GSTM4,IL1R2,ITCH,MAP3K4,NR5A2,OR13D1,OR2S2,OR52I2,OR5P2,OR6B2,OR6C70,OR6C76,POLR1A,PON1,RPS6KA5,SFRP1,SOCS3,SPTLC1,TBL1XR1,WNT10A,AASDHPPT,PNLIPRP2,SUV420H2
Negative_4579	BACE2,DUSP9,FABP1,FZD7,IL17RB,PANK3,PFKP,PNPLA4,SHC4,PPARGC1A
Negative_4580	CFI,ALS2,CYP26C1,GPT2,HRH1,LAMB3,NKX6-1,NRG2,STX7,TXNDC12,YARS
Negative_4581	ADA,AK5,CBS,NNT,SYK,TYR,ACAT2,ACSM3,ADCY3,ALDH9A1,ARD1A,AVPR1B,BDKRB1,C9orf95,CACNA1C,CAMK2A,CDC25C,CMBL,COL1A1,CTSS,CYP2B6,CYP3A4,EPHX2,GDF5,IFNA8,IL18,IL20RA,INHBC,JAG2,LARS2,LIPE,MAP2K3,MYH9,NPR1,NR1H3,OR10K2,OR2V2,OR4D5,OR4K5,OR5F1,OR6C3,PANK1,PFN1,PGK1,TUBB4,ULBP1
Negative_4582	F7,MET,PML,SP1,ADRB3,ARHGEF6,B3GAT3,CACNB2,CCL14,CD22,CDH5,CHRM2,CLDN22,CSNK1G3,ENO2,EPHA1,FGFR4,FUT4,FZD7,GJA1,HIBCH,IBSP,IL20RA,MDH1,MPZL1,MYH14,NADK,NRXN2,OR13C8,OR4B1,OR4F15,OR5M3,OR8K3,POLE4,PPAT,PTGES,RAD51,SMAD7,TKTL2,TUBA3C,WWP1,XCL1,SERPIND1
Negative_4583	ACLY,CDH5,CDKN2B,DPM1,MAN1C1,MRAS,STK11,TLN1,VANGL2
Negative_4584	GSS,ALG1,ALOX15B,BCAT1,C1QC,C5AR1,CDH15,CFLAR,CPS1,CXCL10,ENTPD2,EPB41L1,EPHB6,FZD8,GCNT3,GDF7,HMGCR,IGF1R,MGAT5B,NNMT,OR13C8,OR1E1,OR52J3,OXTR,PAX4,PFKL,PLA2G6,PYGB,RASGRP3,RELA,SARDH,SIPA1,SLC25A5,SPRY1,STK3,TAP1,TAS2R16,TLR3,VEGFA,B4GALNT1,SERPINA5,SUV420H2
Negative_4585	ADRA1D,CSF2,EPHA8,GLO1,GNAT3,KLRK1,LGMN,MYH8,PTEN,RHOA

Negative_4586	AGL,MUT,ACAT2,ADCY4,B3GNT4,B4GALT6,BUB1,CLDN1,FASLG,FGF11,GNA11,GNAL,HMGCS1,METTL2B,OR1A2,OXSM,POLD4,PPP1R3A,RASGRF2,SEMA4F,SLC25A4,TGFB1
Negative_4587	APOA5,BDKRB2,CDC42,CYP8B1,GZMB,PLCB2,TJP2,ZAP70
Negative_4588	DCC,TNN,TPR,VTN,ACACB,ACO2,CKMT2,COMP,GALNT2,GDF5,GSTT1,IFNG,KLRC1,MCM4,MYH8,NT5C2,OR4S1,OR52K1,OR6C1,OR9Q2,PANK2,PGLS,PLA1A,PLTP,POLR3K,PTGDS,RALA,SDC3,TAS2R50,TRAF2,VCAM1,VNN1,XYLT2,ZMAT3,HLA-DRB5,ST6GALNAC1
Negative_4589	IL6,ACYP1,CMBL,CTNNA1,CXCL10,EHMT1,ITGA5,LDHC,MAOB,OR9A2,PRKCE,TM7SF2,WNT9B
Negative_4590	CRK,LEP,TNC,ADRA1D,ATP6V0C,B4GALT1,CD40LG,CD70,ERO1L,GAMT,IL17RB,PGM1,PIK3R2,RALB,RPS6KB1,RRAS,RRAS2,SLC9A1,SMARCA2,TFDP1,TRPM5,TYMP,MAP3K7IP2
Negative_4591	PRL,ARD1A,ASIP,ATP6AP1,CD47,CD70,CHPT1,CLDN2,CNTNAP2,CSH1,CXCL10,DLL1,F2RL3,FDPS,GAPDH,GFAP,GNAI1,HSD3B2,ICOSLG,IFIH1,LDHA,MAP2K2,MYH15,NPR2,NRG3,NUDT9,OR1J2,OR2A4,OR2F1,OR2T3,OR4F4,PANK2,PDGFA,PIPOX,POLE4,PON2,PPP2R5B,RALB,RALGDS,RPE65,SHC4,STX16,TAS2R4,TYMP,GABARAPL2,MAP3K7IP2,TNFRSF12A
Negative_4592	RET,ALDH1B1,ARSE,CACNG7,CRY2,ENTPD7,FMO4,FRAP1,IQGAP3,LIPC,LTB4R2,MAP2K5,MIOX,MKNK1,NFATC2,OLR1,OR10G2,OR4S1,OR52K2,OR5M8,OR6N1,P2RX3,PANK1,PFN2,PIGV,POLR3K,PPAP2B,PRPS1,QARS,RRAS,TAS2R13,TICAM2,TUBAL3,WNT7A
Negative_4593	PVR,WAS,ADSSL1,ARRB2,CCL2,CXCL16,CXCR3,EGFR,EPOR,GLG1,GNAT3,HLA-F,LDHD,MAP2K7,MYL7,OR14A16,OR56A4,OR6K2,PFN1,PGK1,ROBO3,SDC1,SUV39H1,TKTL2,TLN2,TP73,TRADD,TSLP,UGCGL2,WNT10B,WNT2,AASDHPPT
Negative_4594	CACNG1,CAMK4,CCR7,COASY,LHCGR,OR10T2,OR1J1,OR5M3,OR8D4,PAK3,SEMA4B,WASF1
Negative_4595	NGF,ATP5E,COASY,DRD2,IL7R,MYH14,PIGX,RPS6KA3,TDO2,WNT10B
Negative_4596	CFI,FUK,AFMID,AKR1C2,C5AR1,CCNB2,CHSY1,CMBL,FMO5,GSTA3,MAPK1,MAPK3,NCOA4,OR1K1,OR2A2,OR52N1,PIGO,PPP1R3C,PRKAR2A,RAP1B,RARS2,SMAD5,SOAT1,TAS2R49,YWHAQ,ATP6V0A2
Negative_4597	BAD,CR2,LTB,ATN1,B3GNT5,B4GALT2,CASP9,DOT1L,HGSNAT,ITGAM,OR10A4,OR2T34,OR4F15,PLCG2,STK3,TGFB3
Negative_4598	ADRA1D,ATP5H,CAMKK1,CCKAR,CD72,CD82,CD8A,CDKN2B,CFL1,CYP2C18,E2F4,IMPDH1,KLRD1,NCF4,OR5D14,PERP,PPP2R1A,PRKAR1B,TFPI,TUBA1C
Negative_4599	CTF1,IPMK,OR2Z1,TBL1X,TPH2
Negative_4600	APP,ACAA1,ANAPC11,BMP5,CD58,CTBP2,ITGA9,PFN1,PPA2,PPP2R1A,RPIA,SMAD9,THTPA,TP53I3
Negative_4601	FOS,NPY,SCD,ALAS1,ALDH4A1,AMY2B,APPL1,ARRB2,ATP6V1H,BST1,CCL23,CD82,CFL1,COL1A2,COQ5,CXCL12,DHRS4,DHX58,DLST,EDN1,ENPP1,FUT7,G6PD,IDI2,IFNK,IL10RA,IQGAP1,MAPK11,MARS,MGAT5B,MYH13,NEUROD1,NFKB1,NRG4,OR11A1,OR4K1,OR6X1,OSMR,PIAS1,PIP4K2A,PLK1,POLA1,PPP2R2C,TAS2R5,WNT10A,CACNA2D4
Negative_4602	ITK,MAG,OTC,AARS,APC2,ARNT,CYP2F1,DUSP8,GSTM5,HHEX,IGF1,MAGI3,MTHFD1,NCR1,NOTCH1,OR1K1,PAPSS2,POLD1,POLR3D,RALB,RDH12,STK36,STX1A,WNT10B
Negative_4603	CD6,FST,GCK,IL9,LSS,TNN,ACSM4,ACVR2A,ALCAM,ALDH1B1,AQP7,BDH1,BLVRA,C9orf95,CCL26,CD99,CDC27,CDO1,CMPK2,CNTN1,CNTNAP2,CSNK1E,ENTPD8,FGF18,GALNT8,GNA13,HES1,IPMK,MADCAM1,MAP3K6,MAPT,MINPP1,MYH14,OR2T34,OR56B4,OR8S1,PCK2,PDE9A,PSEN2,RASSF1,RPS6KA5,SEMA4A,SESN3,SLIT2,TAS2R4,TLR2,UAP1,WASF2,WNT8A

Negative_4604	DAO, TNN, ARG2, ATP6V1D, CLDN9, CYP2A13, DUSP10, FLOT2, GNRH1, GRB2, GUK1, HAP1, HGSNAT, HLA-G, IFNA5, IPMK, OR1I1, ORC3L, PDE7B, PFKFB1, PIAS1, PMVK, PRPH2, SOCS2, STAM, STEAP3, TNFSF18, WNT3A, HSD17B10
Negative_4605	AGL, SP1, CDC27, CHRM5, CSNK1G2, DUSP5, EIF4E, FARSB, FGF18, GCNT3, GMPPA, HEXB, HMGCL, IDH3G, MAPK11, NCAM1, OR13H1, OR4F15, OR4X2, OR51Q1, OR8A1, PDGFA, PFN1, PGM1, PRIM2, TLR5, UGT2A3, VAMP3, XYLB, HLA-DRB4
Negative_4606	GNE, ATP6V1F, CARS2, HNF1B, IL13, KIR3DL3, OR1G1, SCIN
Negative_4607	CCR5, DHDDS, EPHB3, GAD2, OR6C2, RCHY1, PAFAH1B2
Negative_4608	NGF, OSM, ACYP2, ADAM10, AGMAT, CACNG5, CLDN15, GALT, IFNGR2, KIR3DL3, OR1J2, OR1M1, OR2K2, OR4D9, P2RX6, PIAS3, PSME3, RAPGEF3, TPM3
Negative_4609	AK1, BTC, SI, CSAD, DRD2, FECH, FGD1, IFNGR2, MMP14, NT5E, OR5D14, RBPJ, RPS6KA1, TRADD, UGT1A10, VEGFB
Negative_4610	DLD, ADRA1D, C4BPA, CD274, CD58, CHKB, FDPS, FMO3, GAPDH, GNAL, IFNA5, IL10RB, IL21R, IL24, MDM2, OR1B1, OR2B2, PAX4, PFN2, PIM2, PRDX6, UBE2K, UGT2B10
Negative_4611	C6, FH, GSS, ADAM10, AFMID, ALG10B, ARHGAP5, ASPA, ATP5J2, BMP2, CCR2, COMP, CPT1A, CPT1C, CYCS, DAAM1, EXTL1, FDFT1, GNAZ, GNRH2, GPAM, HSD17B7, INADL, LYPLA2, MGST2, NMNAT3, OR10A7, OR10H1, PDIA4, PFKFB1, PHPT1, PIAS4, PNMT, RBPJ, RDH8, SMURF1, SUCLA2, TAS1R1, TAS2R40, THY1, TJP2, TPM3, MAPKAPK5
Negative_4612	DDC, GBA, GNS, PLG, ANAPC4, ATP5J, AVPR1B, BLVRA, CMPK2, COL4A4, CSNK2A1, CTBP1, CYFIP1, CYP1A1, DHCR7, FDFT1, GALNT14, GNG13, GNRH1, HADH, HARS, IL18, ITPK1, LAMA4, LEF1, NFYB, NKX3-1, NME6, NTF3, PRKCZ, SDC4, SFRP1, SOCS2, TDO2, TLR4, TUBA4A, YOD1, ATP6V1C2, ST6GALNAC4
Negative_4613	IHH, ACSM5, APOA2, BLVRA, CCR9, DDX23, EXTL3, GABARAP, OR4C46, PRPS2, RAPGEF3, STK36, STX18
Negative_4614	AK1, GCK, GH1, IL9, ADH6, APH1A, ARG1, ARPC1B, CARD11, CBR3, CCL7, CLDN7, COX10, CREB3L4, CTNND1, CYP11B1, CYP2A6, DAPK2, DDOST, EPB41L3, ETNK2, FADS2, FDFT1, IL23R, JAK2, LDHAL6B, MAN1A1, NCAM2, NOD1, OR10G3, OR5L1, OR8D4, PDGFRB, PDHA2, PLA2G5, RPS6KA6, VASP, MAPKAPK5
Negative_4615	CFI, FAH, JUN, MOS, ALG11, ASIP, CARS2, CHEK2, CTLA4, CX3CL1, DDX4, DFFB, DHRS4L2, HSD3B2, IFNA2, IL20RA, KLK3, LIAS, MAD2L2, MAP3K1, MARS, NT5E, OR1D5, OR4P4, OR52K2, OR5D14, PER2, PFKFB3, PLK1, PLXNC1, POLA1, PRKCA, RALBP1, SETDB1, SIPA1, TGFBR2
Negative_4616	BTK, ACPT, CAMK2G, CDH2, DGKQ, EPHA2, MYLK2, OR2B6, OR2L8, PTEN
Negative_4617	IVD, AHCYL2, ALDH1A2, ASNS, BCMO1, CCL8, CD74, CHSY1, CPT1A, DDX23, DKK2, FGF9, GALNT7, GALNT9, IL2RG, IL9R, INSR, MAML1, NFKBIE, OR10K2, OR2C1, POLR3A, PTGES2, RAB23, SNAI2, SULT2A1, UGT2B10
Negative_4618	C2, ZYX, ALAS2, ATP5G3, BCKDHB, CACNA1H, CALML6, CD22, EREG, FGF12, GGT6, GRIA2, HDAC1, HTR5A, IGF1R, KCNQ1, NANP, OR4K14, OR51S1, OR52R1, P2RX2, PAK3, PDE5A, PEMT, PKLR, POLR2L, PRKCI, STAT4, STX4, TAS2R5, WHSC1, WIF1
Negative_4619	BAX, TK1, ADH1A, AMPD2, BMP4, C7orf16, CCL15, CD55, DARS2, DDX23, DGKE, EPHA7, G6PC2, G6PD, GSTM4, IL23R, KLKB1, MAP3K8, MDM2, OR14I1, OXTR, PRKAG3, PTGER1, SEMA4A, SETDB2, SH2D1B, STX3, SULT1A4, TSLP, UNC5A, WNT2
Negative_4620	APOE, CD8A, CMAS, EPHA2, EVI1, FMO3, FOXO1, GAD2, HSD3B7, IDUA, LDHC, LLGL2, MAP3K8, MAT2B, MCM5, NFKBIB, OR4A16, OSMR, PIGP, PRKCG, PRSS1, PRSS2, PSME2, SQLE, SUFU, TAP1, UCKL1
Negative_4621	C3, ID2, ACSS2, ARSB, CACNB4, CCR8, GALM, GPD2, IL23R, OR2A12

Negative_4622	APP,C9,RB1,ALDOC,ARHGEF1,B3GAT1,CASK,CHRM3,CHUK,CSF2RB,CSNK1G2,DARS2,DDIT3,DHRS3,DUSP3,FGF18,FLT1,FZD2,GRIN2D,GSTO1,GUCA1B,GUSB,HTR5A,IL21,IL22,ITGAL,ITGB2,MAPK12,MGAT4A,NUDT9,OLR1,OR4K15,OR52I2,OR5D16,OR6K2,PFKFB4,PHKG2,PRKAG2,SEMA4G,SPTLC2,ULBP2,WNT7B,ARHGEF12,ATP6V1E2,ST6GALNAC3
Negative_4623	KHK,KMO,ALPPL2,B4GALT1,CCND3,CD14,CX3CR1,CYP2C18,EHMT1,FZD9,IL17RB,IQGAP2,JAK2,LDHB,LFNG,MCCC1,NFYB,OR11L1,OR14C36,OR52N5,OR5AP2,OR5AS1,ORC5L,OXTR,PIGP,RASSF1,RFX5,SCD5,SMC1B,SMPD2,SULT2B1,TBL1Y,TYRP1,WARS2,TNFRSF10A
Negative_4624	BMPR1B,CDKN2C,F13A1,NNMT,NRAS
Negative_4625	ABCC8,ACSM1,ACTB,AMDHD2,ARPC5,CACNA1F,CD80,CER1,CHST13,CLTA,COQ7,CTSG,CXCL9,CXCR6,DDX4,DHDH,DUSP9,EIF4E,ENPP2,FMO1,GALNT11,GGT1,GPD2,HNF1A,IRS1,ITGB4,LYZL1,NAGK,NAGS,OR3A1,PIK3R3,PPA1,PPP2R2C,PTK2B,RAP1B,RBPJ,SDC4,SMPD3,STX5,TAS2R16,TRAF5,YWHAE,ATP6V1G2,TNFRSF13B
Negative_4626	TH,LLGL2,RASGRP2,TUBB4Q,YWHAH,ATP6V0A4
Negative_4627	GGH,GP9,PDHA1,SULT1A2,CSGALNACT1
Negative_4628	DCT,AFMID,ATG3,BRAF,CACNB4,CD8B,CIITA,CXCL2,DAG1,EDA2R,G6PD,GALNT2,GLYCTK,IDH3G,KIR2DS5,LAMC1,MTFMT,OR8J3,POLR2F,PPP2R1B,RASSF1,TUBB1,UGT2B17
Negative_4629	ADC,ALOX12,EHHADH,GALNT5,ICOSLG,LMO7,NPR2,OR2T1,OR4A16,POLR1C,PPT1,SCLY,STAT5A,TBL1X,TBXA2R
Negative_4630	EZR,GH2,PTS,ADSL,AKR1C1,ALPP,B3GALT4,CCL28,CDC42,CTF1,CYP4A22,DPM2,DUSP8,FLT3LG,GCNT4,HCLS1,IDI2,MDH2,NMNAT2,ODC1,OR2T33,OR51S1,OR7C2,OR7G2,OR9Q2,PAK7,PDE6H,PIGH,PIGX,PIM1,POLR2D,PRPH,RDH10,RRAS2,UBE2L3,XYLB
Negative_4631	GH2,ITK,LEP,ANAPC4,BACE2,BCAT2,CACNA1G,CCL4,CLDN9,CMBL,CNTFR,EXT1,GAL3ST1,GCDH,GGT5,HPRT1,IGSF5,IL17B,IRF3,MAOB,MAP4K4,MAPK8,MYH7,NAT1,NR1H3,OR4N4,OR5C1,OR6C6,PAK7,POLR2B,PPAT,PYCRL,RBX1,RPE65,SMURF1,TCIRG1
Negative_4632	CBL,AFMID,AKR1B10,AKR1C1,AMDHD2,ATP5I,ATP6V1A,CES2,CHST13,CNGB1,CREB5,DCXR,ENTPD7,EXOC7,GNRH1,HTR2B,IDUA,IL1B,IL21,L1CAM,MYH13,NMNAT2,OLAH,OLR1,OR13C8,OR13G1,OR2C3,OR2M4,PDE7A,PIGU,PLCB4,PRKCE,SGMS2,SYNJ1,TMLHE,UCKL1,CTNNBIP1,HLA-DQB1,TNFRSF11B
Negative_4633	AMH,HK1,ID3,ME3,ALG13,AMD1,ANGPTL4,ASCC3,CACNA1H,CXCL16,CYP19A1,DGAT2,EFNB2,FLOT2,GALNT3,LAMC1,LYPLA2,MAT2B,MMP1,MYH10,NKX3-1,NPR1,OR1E2,OR4C12,OR51Q1,PERP,PLA2G2E,TGFB2,TIAM2,TPI1,UBA7,WNT10B,YWHAH
Negative_4634	FGG,IL5,LHB,XDH,ARHGAP5,BLNK,BMP8A,CPT2,DARS2,DTX3L,EIF4E,FARP2,FGF1,FMO4,G6PD,GRIN2C,GZMB,HHEX,IL21R,ITGAL,JAG2,MAPK3,MCEE,NFKB2,NME7,NRG2,OGDHL,OR10A7,OR2T2,OR2T4,OR56A3,OR5B21,PIP4K2C,PLXNB1,PRKACA,RASGRF1,RASGRP1,RDH8,RUNX1T1,RUVBL1,SELE,SEMA3F,STX2,SYNJ2,TICAM1,VDAC3,ATP6V0A2
Negative_4635	CFI,ABCC8,BCAT2,CHRNA7,GGT5,HADHB,IFNA4,MMP9,PDE4D,PECAM1,PIK3C2B,PLD1,PPM1B,PPP2R5D
Negative_4636	PML,ABAT,ARPC5L,B3GAT3,CD55,CHEK1,CREB3L1,CYP1A2,HTR7,IDH1,JAG2,KIR3DL1,KLRC2,LGMN,LRAT,MAGI1,MAP4K4,NAE1,OR2A14,OR2A25,OR51F2,OR52J3,PAK6,PDE9A,PLCB1,PON1,PON2,PSAT1,SFRP5,SNAP25,TUBB3,ATP6V1B1,MAP3K7IP1,MAPKAPK2
Negative_4637	TK1,ANPEP,ATP4B,CACNG2,CDK6,CPS1,CYSLTR2,DDIT3,FMO2,FTCD,GMDS,GNPDA2,ITGAE,NLGN2,NT5C1B,OR10G3,OR11A1,OR52E4,P2RX6,PAK3,PER1,PIP4K2C,RFXAP,SELPLG,TBK1,UCK2,VNN1

Negative_4638	CKM,GLS,INS,NGF,TKT,ACAD9,ACOT11,ACOX1,ACSM1,ACY1,ADCY3,AK3L1,AKR1C3,ARSD,B3GNT5,CD8A,CLDN10,COQ6,DTX1,DTX3,EPHB3,FUT3,FUT8,GBE1,GNG3,HLA-C,HYAL1,IL9R,ITGA6,KIR2DL1,MRAS,MTHFD1L,NAT5,OR1E1,OR2AT4,OR2L2,PKLR,SDC3,SH2D1B,SULT1A1,TAS2R14,TPH2,UROC1,VCAM1,YARS,ARHGEF12,HLA-DQB1
Negative_4639	BTK,C3,GK2,SYK,AHCY,AMPD3,AOC2,APBB1,BCL10,CCL5,CCNB2,CD36,CFTR,CX3CL1,CXCR4,EPB41,F2RL1,GABARAP,GCLC,GIT1,HLCS,HNF4G,IL15RA,JAK1,LHCGR,LPAR1,MGST2,NME6,NOTCH1,NTRK1,OR10Q1,OR2D3,OR51F2,OR51S1,P2RX2,PPP2R5E,PPP3R1,RDH11,SOCS5,STAT2,STAT5A,STX18,TPI1,TUBB2B,UBA7,ATP6V0A1,PAFAH1B3
Negative_4640	A2M,LTB,ACPT,ACVR1,ADIPOR1,ARPC3,BCL2L1,CCNE2,CCR6,CD34,CDC42,CER1,CERK,CNTN1,DAAM1,E2F4,ENPP2,FECH,GALNT7,HTR2B,IBSP,IFNGR2,LLGL2,LRDD,NAGA,OR2G2,OR51A2,OR52E4,OR8H2,PDCD1,PIGQ,PMM1,POLR2D,PTK2,RALBP1,RARA,RASSF1,SMAD7,VEGFA,WARS2
Negative_4641	HYI,CCNB2,COL5A3,CSF1,F2RL2,FDPS,FSHR,IMPDH1,NARS2,SDHA,SRGAP1,WASF1
Negative_4642	BTD,ACHE,ADCY8,ALDH3B2,ATN1,CDC6,CYP1B1,ETNK1,FGF20,KAT2B,MAPK11,NAT2,NODAL,NRCAM,OR13C2,OR2T29,OR7C2,PAPSS2,PPM1A,PRKCA,SPRY1,ST3GAL4,YWHAH
Negative_4643	PLN,ABI2,DGKB,FZD9,GPX7,IFNA6,IL12B,KIR2DL3,LYZL1,OR52B6,OR5F1,OR8K3,TGFBR2,THBS1,TIAM1
Negative_4644	CAD,GPI,MUT,ACADVL,ASPA,ATP5C1,CCL22,CXCL12,CXCR5,ERO1L,FZD8,GLG1,GNAI1,GRPR,HEMK1,IRAK1,LAMB2,MDM2,MS4A2,MYLK3,OR10G8,OR4K17,OR51I2,OR7C1,OR7C2,OR8K1,PDE10A,PDE2A,PPT1,PRKCI,RBX1,SMAD9,SPRY2,TAS2R48,WNT16,WNT7A
Negative_4645	ADK,AK2,CDA,FGB,GGH,LCT,ABP1,ACTB,BST1,CSH1,CYP2C18,DARS,DHFR,DIAPH1,ERO1L,F2RL3,GALK2,GTSE1,IL22RA1,IL6ST,IL7R,KCNJ11,LAMB1,LDHC,MAP2K1,MAP3K1,MYH14,NPR1,OR10J5,OR2Y1,OR3A3,OR6T1,ORC6L,PARD3,PNLIP,PPP1CA,PPP2R1B,PRSS1,SORD,STAT2,TLR5,UROD,SERPIND1
Negative_4646	ABI2,ADH1B,ADH4,CAMK4,CCNE1,CDC6,EP300,FASN,HCST,INMT,MYH11,OPLAH,OR10X1,OR1K1,OR52W1,PIGP,PLCE1,PVRL4,SOS2,STK3,TAS1R3,TJP1,TSLP,VDAC2,XIAP
Negative_4647	LSS,LYN,MUT,A4GALT,ACSM1,ACY3,ARPC4,ASAH1,CACNA1E,CALML3,CCL2,CLDN8,CXCL2,DGKH,DGKZ,EPHA1,GALC,GRIA2,GRIN2C,GSTA1,HTR2C,LCP2,LGMN,LIMK2,LTBR,MTMR1,MYH10,NCR3,OR1E1,PDE3A,PIGA,POLR2D,RXRA,SMAD6,ST3GAL1,TNFSF18,TYROBP,WNT5A,SUV420H1
Negative_4648	DDC,AKT1,B3GNT4,CAMK2A,CLDN6,CLTA,DAPK1,FBP2,GNPNAT1,GPX5,GRHPR,IBSP,IMPA2,IPMK,MTFMT,MYD88,OR14C36,P2RX4,PRNP,PTAFR,RDH5,RPS6KB2,SETD1B,VARS,VDAC2
Negative_4649	FAH,HPD,ID2,KHK,ME3,ARHGEF2,ARPC1A,BBC3,CCL3,CERK,CPT1C,DKK1,EPHA1,FRAP1,GCLM,GGCX,GLUL,GNRHR,GRIN1,HRH1,IRF3,LTBP1,MAPK1,MCCC1,MGAT4B,MTFMT,MYLK2,NCSTN,NFX1,NOTCH3,OR10A7,OR4K14,OR5A1,OR6B2,OR6K2,OR8K3,SEMA3F,SPI1,SUCLG1,SULT1A1,TAS2R5,UCK1,VDAC1,WASF1,ATP6V0E1
Negative_4650	CHIT1,CLCA4,FCGR3B,OR13C9,OR8D1,PPA2,SEMA4B,SERPINE1,TNFRSF12A
Negative_4651	CFD,ADH5,B3GALT6,BDH1,CD55,CHRNA7,CPT1C,EHMT2,FGF12,FPGT,LDHC,MAN2A1,MYH8,NANS,OR10A5,OR2A14,OR51A7,OR5M8,OR9A2,PDE5A,PDE7B,PFAS,PKLR,POLR2L,PPARA,SCIN,STX1A,UAP1,HSP90AA1
Negative_4652	MPZ,SYK,ALDH6A1,ALOX12,CYP11B1,DDX52,DUSP7,EFNA2,GCNT1,GRIN1,IL20RB,MICA,NOX3,OR10K1,OR13J1,RASGRP3,SEMA4C,SETD8,SHC4,TAS2R40,TJP1,TUBB3,XYLB,B3GALNT1

Negative_4653	GPT,RDX,ACSM4,ATP6V0C,AVPR1B,CCR6,CKS1B,CLDN18,CXCL11,DAAM1,DUSP2,GNMT,GRIA3,GRIN1,GUCY2C,HSD3B1,IARS,IL28RA,IL5RA,MTHFD1L,NRCAM,NSDHL,OR4A15,OR6C70,PCK2,PGK2,PIK3CB,SHC4,STK3,STX6,WNT10B,WNT7B
Negative_4654	AMT,ME1,RDX,ACPT,ACTN1,ARAF,ARSA,CCKAR,CD48,CDH3,CDH5,CYP2S1,CYP3A5,DIAPH3,FASN,FGF21,GDF7,GFAP,GNAS,HEXA,IL23A,KIR2DL4,LAMB4,LILRB3,MAP3K1,MMP7,NEFH,OR13A1,OR14C36,OR7C2,PCNA,PRKAA1,PRKAR2B,PSPH,PTPN11,RARS2,RPS6KA5,RPS6KB2,SEMA3C,TACR3,TYMP,WHSC1,WNT5B,XYLT1
Negative_4655	C8A,BUB1,DHDDS,LAMB3,NFKBIB,OR11H1,OR4S1,OR8H1,PPAP2C,SDC2
Negative_4656	NPL,AASDH,ADCY8,ALDOA,AQP7,CLTCL1,CREB1,EDNRA,GAPDH,HAP1,HLA-DRA,HTR7,IL17A,LLGL1,LPAR1,NFYA,OR4D6,OR4F15,PANK4,PCNA,PIK3C2A,PRKCB,PTPN5,SFRP5,TLR9,ULK1,WHSC1,WNT7A,MAPKAPK2
Negative_4657	MAX,BIRC3,CCNA2,HLA-DRA,HPRT1,INHBB,LAMC2,LIMK1,OR13D1,OR1E2,OR5L1,OR5T1,ORC4L,PARVB,PIGV,TUBA1A,YWHAH
Negative_4658	BAX,C9,ABCC8,ALDH4A1,ALDOA,ASNS,B3GAT1,BPGM,BUB1B,CES2,DOLPP1,DRD2,FTH1,GAB1,GUCA1A,GUCA1C,IFT57,IL6R,LRP5,MYLK,OR51Q1,OR6M1,OR6X1,PDE1C,PIGB,PLXNA3,PNLIP,SDC1,SHMT2,TCF7L2,TGFA,UGT2B28,VDAC1,WNT4,WNT9A
Negative_4659	AGL,CDKN2B,CSNK1D,IL28RA,LRRC4C
Negative_4660	ZYX,AKR1B10,ARSD,CACNG6,CSNK2A1,NRG4,PARD6B,TUBA1C
Negative_4661	ATP5E,GRIA3,RAF1,TRAF3
Negative_4662	GALM,PRLR,PVRL1,UPP1
Negative_4663	ID2,LCT,CCNE2,CHST13,DDIT4,ICOSLG,ITGA9,ITPKA,LYPLA1,MAP2K7,MAPK12,MAPK13,OR4D11,SEMA4G
Negative_4664	APP,CTH,VTN,ACPT,CCL20,CCL28,DFFB,FABP4,FZD9,GAPDH,HLA-C,IRAK3,MYH2,OR1N1,OR2C3,PAX6,PIM2,PPP2R2D,TBL1Y,TRMT11,WNT7B
Negative_4665	ACOT12,ARNT2,CXCR6,DARS,MCCC2,PIGA,RND1,USE1,WARS2,ATP6V0E1
Negative_4666	ARNTL,AVPR1B,FZD2,FZD3,HEMK1,NFASC,OR10A5,PLXNB2,PTDSS2,RAPGEF2,SLC27A4,SULT1A2
Negative_4667	GP5,ID1,LHB,MVD,AHCYL1,ARHGEF4,AXIN2,BMP8A,BUB1,CCNB3,CD58,CHRM3,CLDN3,COL5A3,CTNNA2,DGKH,FGF19,GCNT4,GNPNAT1,HCST,HTR6,LEFTY1,LIPF,OR14J1,OR1K1,OR4C13,OR5B3,RARS,RHOD,RPS6KA1,SEMA4A,SORBS1,ST3GAL6,TAS2R3,TOMM40L,TRAF4,UGT1A10,VEGFC,CACNA2D2
Negative_4668	A2M,IL2,CD80,GFPT2,MMP14,NT5E,OR4C12,ORC2L,SLC27A1
Negative_4669	CACNG1,CDKN2D,EIF4E2,GNA13,IDI2,P2RX2,PPP1CA,RFNG,SMARCA2,STAT5A,TRPC1,MAP3K7IP2,ST6GALNAC4
Negative_4670	C2,GNS,ACO2,ADRA1D,ARHGEF4,CD79B,CLDN10,CLDN19,CLDN5,CSNK1G1,CYP2A7,DTX3L,DUSP2,EXOC7,FCER1G,GPLD1,IDI2,ITGA10,KARS,MITF,MTHFR,MYH7B,OR2K2,OR51F2,OR52D1,OR52K1,PDXP,PPP2R2C,PPP2R5E,PSENEN,PYGB,SEMA7A,SH2D1B,UNC5B,VDAC1,YWHAZ
Negative_4671	C3,GNS,HYI,ME1,NGF,ATP5C1,DARS,ECSIT,EXTL2,GNA12,GRIA2,IFNB1,OR2A4,OR2F1,PHKA1,PLD2,POLR2K,UGCG
Negative_4672	CTH,ACYP2,ADH4,ALDH1B1,CCR8,CHAD,CSF1R,DLL1,EPHX1,FGF13,FGF17,FGFR4,FRAT1,FUT6,FUT9,GFPT2,GLI2,GNA12,HPRT1,IL17A,IL5RA,LCAT,MAML1,MEF2C,NODAL,ODC1,OXTR,PDIA4,PIK3CG,PORCN,PPCDC,PRKCZ,PRKY,SNW1,SPHK1,TALDO1,TAP1,THBD,TUBB1,WNT16,ATP6V1E1

Negative_4673	KHK,AARS2,ABLIM2,ACO2,ADORA2A,ADRBK2,ALOX12B,ARSB,ATG3,BUB1B,CACNB4,COQ7,DNMT3A,ETNK2,FGF14,G6PC2,GRIA2,IL2RG,ITGAE,KLRD1,MAP2K2,MOV10L1,NEUROD1,OR1N2,PISD,PNPT1,QPRT,RASGRF2,SETD1A,SLC27A5,TAS2R38,UPP1,SERPIND1
Negative_4674	LEP,CARS,CHSY3,DTX2,EDNRA,EPHA5,OR10Q1,OR51F1,OR6C74,PCCB,PDXP,PRLR,PVRL1,SPRED2,TFPI,TUBAL3,UGT2B17
Negative_4675	ADA,CRH,GH2,CASP9,CLTCL1,GLI1,GSTA2,HMGCS2,OR10G2,OR12D3,PIK3C2A,PRDX6,PRSS2,SLC27A5,TMLHE,TNFSF11,TXNDC12,WNT4,SERPINC1
Negative_4676	ACOX2,ALG6,EARS2,MDH1
Negative_4677	ASL,GNE,IHH,TNF,AGMAT,ALDOC,ANAPC1,B3GNT2,BIRC5,BMP4,CACNG1,CSNK1G1,EFNB2,EPHX2,FUT2,GLS2,GNB3,HAGH,IFNGR1,IL5RA,LDHD,MAP3K7,NEU3,NRG4,OR5D16,OR8B4,OXTR,P4HA3,PLA2G3,PNPLA3,PPP2R5E,SDHB,SLC33A1,TLR3,WARS,MAPK8IP1,PAFAH1B1,ST6GALNAC6
Negative_4678	CPT1C,OR5AN1,PPP5C
Negative_4679	ADCY4,ARSE,ASAH1,CDS2,CHIT1,DAAM1,DGUOK,ITGAV,PORCN,PTCRA,TBL1X
Negative_4680	F5,ALG11,B3GAT3,B4GALT4,CCNB3,CDC14B,CLDN10,GRIN2B,HSD11B1,KIR3DL3,NCR1,OR12D3,OR5AP2,OR5H6,OR8U1,PGM2,RDH11,SDHD,SLC25A4,SPHK1,SRD5A2,STX1A,VANGL2,PPP1R12A,SLC25A31,ST6GALNAC3
Negative_4681	ANAPC1,CD34,CD55,CDS2,CLDN1,CLDN2,CYP2U1,CYP3A43,DVL1,FGF11,GLUL,GNRH1,IL6ST,MAP3K7,NEFH,OR10A7,OR51D1,PDE7A,PDHB,PIGP,PRDX6,PTGFR,PTPN5,PVRL2,SEMA3G,SGPP2,STX2,UGT8
Negative_4682	F2R,GGH,GH1,HPD,MVK,ZYX,ADCY2,ADSL,AKR1B10,AKR1C4,ALDH1L1,BDNF,BIRC2,BIRC5,CCNG2,CDC42,CDS2,CES7,CSNK1A1,DPYD,FZD9,GRLF1,GZMB,HAGHL,IFNG,IL17RA,KIR3DL1,MAOB,MAT2B,MDH1,NEGR1,NKX6-1,OR2G3,OR2V2,OR52D1,PARD3,STK36,TOMM40L,TRADD,TRAF4,TYK2,VARS,VASP,HSP90AB1
Negative_4683	GHR,DAG1,EPB41L3,FADS2,FARP2,GALNT11,LIAS,MAPK1,NCAM2,OR11G2,PTGES,RAPGEF3
Negative_4684	ARPC3,BET1,BMP6,COQ7,CSF1,DVL2,F13A1,ITGA5,MAN1C1,MAP3K12,NANS,OR2T34,OR5AS1,PCYT1B,POLR3K,PSEN1,RRAS2,RRM2B,SMAD9,PPARGC1A,TNFRSF1B
Negative_4685	IL2,CHST12,IFNA7,OR10Q1,SLC25A5,TNFSF4
Negative_4686	CSK,FAS,TFG,ABL2,ACO1,ATP2A3,CACNG2,CD27,CSNK1G3,CYP3A7,ENTPD3,ESPL1,FOSL1,GALNT5,KIR2DL1,LAMB4,LLGL2,LTC4S,MARS2,OR1C1,OR3A2,OR4D2,OR8G1,PLXNB2,PRKACG,RFX5,RRM2B,SHC4,STT3B,UGT2B11,TNFRSF18
Negative_4687	LPO,MPZ,NGF,ALPL,C1QC,CADM3,CSNK1A1,DOLPP1,GUCY1B3,IRF5,LFNG,NR4A1,NUMBL,ONECUT1,OR1L4,OR2AG2,OR2M3,OR7A5,ORC2L,PARVB,PLXNB2,PNMT,PRKCE,PTPRJ,SC4MOL,SPRY3,SULT1A1,VARS
Negative_4688	ITK,RB1,ANAPC4,ARNT,CD47,ENPP2,FMO5,GALNT11,GNRHR,GPAA1,HSD3B1,IDH3B,KCNB1,LDHA,MGST2,NARS,NKD2,OR10A2,OR10Z1,OR1S1,OR2T3,OR52M1,PISD,POLR3D,PPP3CC,PSME1,STX18,TAS1R3,TAS2R1,TYRP1,WBSCR22
Negative_4689	ADH1A,ARSB,BTRC,CYP7A1,DGAT1,ENTPD3,HMBS,IDH3G,LCAT,MAP3K14,MYH1,NFYC,NMNAT1,PRKAB1,RIPK1,SEMA3E,SORBS1,TACR1,TNFSF4,ZMAT3
Negative_4690	AMT,SP1,ALAS2,BCL2,C4BPB,CCR5,CDC26,COL6A1,COQ5,CXCL11,DAD1,EGLN1,EPHX2,FUT5,GADD45B,HADHA,ICAM2,ITGA2B,MADCAM1,MTHFS,NEFL,NFATC4,OR10G8,OR10T2,OR2M4,OR4C6,PCK2,PIP4K2C,PNPO,POLA1,POLR3G,PRODH,PTGS1,PYCR1,SCD5,STAT1,VAV3,VDAC3

Negative_4691	FN1,ID2,IHH,IL3,IL8,ABCC8,ACADVL,AMPD2,ATP6V0C,CCL20,CNGB1,CXCL16,DGKQ,DUSP14,F11R,F2RL2,FECH,FGF9,GSTO2,IFNGR1,IL10,MAD1L1,MAD2L2,MTAP,MYL9,NCOR2,OR10J3,OR1S2,OR2A14,OR51A4,OR52E2,OR52L1,OR6C4,OR8H3,PDGFC,PDXP,PFAS,POLD1,PRNP,RASGRP4,RFNG,SPCS3,SPRY4,TIAM1,UAP1,UGCG,CSGALNACT2,HLA-DQB1
Negative_4692	CD82,DUSP14,NUDT5,TRAF2
Negative_4693	BAX,AGTR1,EFNA1,ENTPD7,GRIN2C,HARS2,HLA-G,MMP7,NME2,NRXN2,PIAS2,RFXAP
Negative_4694	C2,GP9,TNF,ACP1,ATP6AP1,B3GNT2,CALML3,CBLC,CD79B,CDC20,COL5A1,CYP17A1,DBF4,DNMT1,FSHB,IL17RB,MYL9,NEUROD1,NFAT5,OCRL,OR13A1,OR14C36,OR2C3,OXSM,PDGFRA,PGAP1,PGK2,PIK3C2G,PLAT,POLA1,POLR3H,PRNP,PTCH1,RETSAT,SETDB1,ST8SIA1,TSC1,UGCG,VAV1,WNT6,PRICKLE1
Negative_4695	EDA,FAS,FH,GK,GLS,PGF,ADIPOR2,ATP2A1,CDH2,CDH5,CHKA,CHRM2,CKS1B,CSNK1G3,CYP26A1,DHDH,EPHX2,FGFR3,GSTM3,HLA-F,IL12RB1,JAG1,LLGL1,MAML2,MARS2,MGAT4A,OR1M1,OR6C4,ORC1L,PDE3B,PHKG2,PLA2G1B,PNPLA4,PPARD,PTPRZ1,QPRT,RASGRP3,RDH10,SETD8,SMURF1,SPI1,STMN1,SYNJ2,USE1,VANGL2,VASP,VDAC1,VDAC3
Negative_4696	C2,IL8,MVD,A4GALT,ADH7,AQP7,CASP6,CASP8,CTBS,DUSP2,FBP1,FCER1G,GALNT8,GLI1,GMPS,NFATC3,OR52M1,OR56B4,OR6N2,ORC1L,OXCT2,PLXNB1,PPP2R1B,RUNX1T1,SEMA3F,SFRP1,SQLE,STAT3,TUBB3,TYRP1,VNN1,WNT4,HSD17B12
Negative_4697	CD2,F8,PTS,SCD,ALDH7A1,AMPD2,ATP4B,CAMK2A,ETS1,GAD1,GSTO2,INHBA,LCMT2,MAP3K13,MAP3K4,NAT1,OR13J1,OR51B6,OR6C75,OR6F1,POLE4,PRKAG3,SELPLG,SETD7,YKT6,HSD17B10
Negative_4698	GP5,TXK,ARPC5L,CXCL3,HSD17B3,JAK2,KLK3,OR10G7,PON1,UGCGL2
Negative_4699	AK7,CFI,GNE,NGF,ACADS,ATF4,CACNB1,CASP9,CD36,CDKN1A,CHAD,CHKB,CPT2,DDX47,ETS1,FCER1G,FGFR3,GUSB,GYS1,HLA-DMA,HSD3B7,HTR2A,IL9R,LDHB,LDHC,LIPT1,MGAT4A,MMP7,MTHFD1L,OR1E1,OR52N5,OR6F1,OR6N2,OR8K5,OXSM,PAK1,PAX4,PCNA,PDE5A,PGAM1,QARS,RARS2,RHOD,SDC4,SGPP2,ATP6V1C2,MAPK8IP1,ST6GALNAC5
Negative_4700	CCNA1,CCNB1,CDC25C,CYP2C19,DMGDH,FTCD,GCH1,GNA13,HSPA2,IL18,OR2S2,OR4A16,SOCS1,TBK1,UCP1
Negative_4701	IHH,PLN,AFMID,AGRP,CARS,CDO1,CLOCK,ETNK1,GRIA3,HEXB,NCKAP1,OR2C3,OR2L3,OR52I1,OR5K2,OR5M8,OR7G1,PIGK,PLCD4,PRKCQ,PRPS1,RASA1,STX16
Negative_4702	ID2,RDX,SRM,TAT,VCL,ADRBK2,ALG3,B3GALT4,B3GAT1,CCL21,CDC2,CYBB,DPM2,DUSP16,FUT6,HADHA,KCNQ1,MAP4K3,NR1D1,OR52E6,OR56A1,PANK1,PDE11A,PDGFRB,PKMYT1,PPAP2A,PPP5C,SDHB,SMC1A,TYRP1,UBA1,WBSCR22
Negative_4703	AASS,ADAM10,ADCY2,ALPP,ATP5J,CAMK2A,CD70,DNMT3A,EI24,FGF1,GBA3,GCNT1,GUSB,ITGA10,NCF2,OR2T34,OR2T8,OR6N1,PRKAA1,TICAM2
Negative_4704	ADCY1,AGRN,AKT1,ALDH1A2,ANPEP,ASS1,ATP5J,BIRC5,BUB1,CACNG8,CD40LG,CERK,CSNK1D,EXT1,FGFR3,GAMT,HDAC2,IDH1,IL1R1,IL29,INSR,ITGB2,LPAR1,MAN1C1,OR4K17,OR5T2,OR9Q2,PCK2,PDE4A,PPA1,PPP2R5E,PRKAB2,PTGDS,RASGRP2,RFWD2,UCK2,UNC5D,MAPK8IP2
Negative_4705	JUN,AKR1C3,ALDH1A1,ALDH6A1,ASMT,CSF2RA,DDX52,DPM1,FGFR1,GGT6,GMPR,GPX5,IL22RA2,KIR2DS4,LIMK1,MFNG,OR2A25,OR4C15,OR4F6,PKM2,TLR2,TUBB6,MAPKAPK2
Negative_4706	SYK,AGPAT1,BCAR1,BST1,CACNG1,CPOX,CX3CL1,DERA,E2F1,EPRS,ERO1L,FGF18,GALNS,IPPK,ITGA3,KIR2DL4,NCK2,OR2T10,ORC2L,PAX4,PEMT,PIK3CB,PMAIP1,RPS6KA6,TRPM5,UGT2B28,UNC5B,UPP1,USE1,VANGL1

Negative_4707	ADCY1,BUB3,CDC25B,COL5A2,DOT1L,ENPP6,GAL3ST1,GOT2,GRIN2B,HDAC2,IMPA2,KIR2DL4,NCF1,NEUROG3,NT5C1B,NUMB,OR10G2,OR51A4,SESN1,SUV39H2,TAS2R14,TUBB2C
Negative_4708	CTH,F2,RPE,ATP5C1,CDK6,E2F2,FGF5,GALK1,IFT57,LAMC1,MAD1L1,OR2L2,OR51L1,PIK3C2A,ST3GAL5,USE1
Negative_4709	ACY1,ADIPOR1,APC2,CYP2C9,DAPK1,EPB41L3,GNAQ,GP1BB,IRAK4,NEU3,PLIN,POLR3G,SDHD,TAS2R4,TOLLIP
Negative_4710	EPO,CYP3A4,PAPSS2,PHKA2,PPA2,HLA-DRB3
Negative_4711	DBH,HK1,BMP7,CCL19,CCNA1,CTNND1,CYP2B6,ERO1L,F2RL3,FADS2,GNPNAT1,GZMB,KIR2DL2,NKD1,TAS2R1,TBXAS1,ULBP1
Negative_4712	CDC7,CXCR5,DPAGT1,FGF18,GLG1,IL23A,NCK2,PGAM1,PRPS1,SUCLA2,TAS2R16,TXNDC12,WNT7B,ZNRD1,PNLIPRP2
Negative_4713	ADO,GSN,JUP,PGD,SRC,TK1,TNF,ALG11,CAMKK1,CSNK2B,CXCL11,MAPK7,OR51A4,OR56A3,SFRP2,SMPD1,STAM2,TAS2R48,TYROBP,ATP6V1B1,MAPKAPK5
Negative_4714	C4B,ACSS2,ARPC1A,ATP5L,CFTR,CRB3,CTNNA3,FZD9,GNG12,LYZL1,PTGIS,PTPRJ
Negative_4715	C2,F10,GSR,HDC,NPL,AASS,ADIPOQ,ALOX12,ANAPC4,BIRC5,CCNB2,CLCA4,CMPK1,COQ7,FABP1,GSTM4,GSTO1,GUCY1A3,HDAC2,LARS2,MAP3K14,MGAT1,MIOX,MYH8,OR10Q1,OR2D3,OR52I1,PNPLA3,ULK1,YWHAG
Negative_4716	ALDH1B1,DIAPH2,ENO1,FADD,GDF5,MAML1,MGAT5B,PPAP2C,WNT16,YOD1
Negative_4717	ADA,BTC,LPO,NNT,AANAT,ACOX3,ACP5,ACTN1,ADRB2,ALDH1L1,BHMT,CACNB1,CCL8,CHAT,CTBP1,CYP1B1,EXT2,FLOT2,GAD1,HEXA,IFNG,ITGA2,ITGA4,MARS,MTHFD1L,NCOR1,NCR2,NMNAT2,NOS3,NRAS,OR10H2,OR12D3,OR2H2,OR2T6,OR3A1,OR4S2,OR51A7,OR7D2,PLTP,PLXNC1,PRKCI,PTDSS1,SELL,SLC2A1,SSH1,UXS1,SERPINA1,SERPINE1,TNFRSF13B
Negative_4718	ACOT12,BPGM,CCR7,CD80,CDC25A,CLDN18,CXCR5,FUT8,GGT1,GNG3,HIP1,KCNB1,KIR3DL3,LIPA,MAPK14,OR4C13,OR5AN1,PARVA,PIK3C2A,PRKAR1A,PRKY,RAP1B,SLC12A2,TAS2R1,TNFSF18,UCP1
Negative_4719	A2M,GSN,AGPAT2,BMP6,BMP8B,CNTN2,COL4A1,CXXC4,EXOC7,GAS1,GSTM2,IL9R,NTSR1,OR2F2,OR6M1,OR6N1,OR7A10,TAS2R41,UGT2B11
Negative_4720	GSS,SMO,TFG,TPO,ANAPC10,ATP5C1,CCKAR,CCNB1,CMPK2,CPT1A,CSF3,CXCL10,CYP2D6,FZD1,GNA15,GSTA3,IFNK,IL12RB1,MDH1,MOV10L1,NOTCH1,OR10G7,OR10J5,OR51S1,OR52A5,OR5M8,OR6C1,POLR3A,PPP2R5B,PTGES,RASGRF1,SC5DL,SEMA3E,SIAH1,SLC27A1,SMURF2,TUBB4Q,UGT2B7,CACNA2D2
Negative_4721	C6,AKT1,AKT2,CACNA1H,CD19,CERK,CLDN4,CSNK1G2,DTX4,FLT4,GABARAP,GFAP,GSTK1,HMGCS2,IDH3A,ITGA6,LAMB3,NEUROD1,NPR2,OR2A5,ORC4L,PARS2,PCCB,PDHB,PER3,PFKFB1,PTK2,PTPN7,SMAD3,USE1,WBSCR22,WNT7B
Negative_4722	CYP4A22,HMGCR,PCCA,ULK2
Negative_4723	ADA,MET,MSN,NLK,ANAPC11,CES7,CHIT1,COL5A3,CSNK2A1,CTPS2,CX3CL1,DFFB,DOT1L,ENTPD2,ERBB3,FBXW11,FOXA3,FZR1,GAD2,GNA15,GNG3,IRAK2,MAD1L1,MAP3K7,MEF2C,NAT6,NFYB,NLGN1,OR13J1,OR2AE1,OR52I1,OR6C2,OR8H3,P4HA1,PPA1,PPP1R3B,PRKACA,SLC25A4,SMAD4,SSH2,STX4,SUCLG1,TPI1,GABARAPL1
Negative_4724	ALG8,CX3CL1,PCCB,TNFRSF14
Negative_4725	EBP,RFK,ALDH1A1,B3GNT2,B4GALT5,B4GALT6,BMPR2,CDKN1B,CDKN2B,CDKN2C,CNTN1,COASY,COL4A6,CYP2A7,DCXR,DGKA,ENTPD1,GALE,GALNT10,GDF6,IDH3A,IFNB1,JAM2,KAT2A,KAT2B,KIR2DL2,MAT2B,MGAT3,NFKB1,NODAL,OR2T8,OR5P2,PIGK,PIGP,PRLR,PTPN6,RPS6KA5,SELL,SPTLC2,STX4,TPH2,WNT3A,SERPINE1

Negative_4726	CCL14, COL5A2, CX3CL1, EPHX1, FARSB, IFNA10, IL1A, LMO7, MKNK1, OR1M1, OR5D18, PRKG1, PTCH2, SMAD9
Negative_4727	CLTC, DHX58, NEFM
Negative_4728	C9, DBH, TNN, ALAS2, ANAPC7, CASP10, CPT2, CREB1, DDX55, DIAPH2, EPHA3, FSHB, GGT1, GNA14, HSD17B7, IL1RAP, NPR2, OR14J1, OR1N1, OR2B6, OR6K2, OR9A4, PDXP, PPP2CB, PTPN1, RARS2, STT3B, SV2A, UCP1
Negative_4729	CISH, CYP1B1, GALE, OR2M3, PRODH2, SHC2, TRDMT1, SERPINA1
Negative_4730	GDA, BMP4, CCNH, CDH3, ERBB3, GALNTL2, GRIN2C, GZMA, IFNA8, MTHFD1L, OR8A1, PPP2R2C, RPSA, SMAD3, TOMM40
Negative_4731	ACP5, BMP5, DGKB, FGF1, GDF5, GNAQ, IKBKG, MASP2, MDM2, ORC5L, PHKB, RASA1
Negative_4732	IL6, LIF, ACAA1, ACSS3, CCNB3, CD19, GALNT11, HCST, HIBADH, KYNU, OR2M2, OR9A2, PIK3R1, PRKCB, SLC27A5, SNAI1
Negative_4733	GPT, ACSL6, ADAM10, ALDH1A3, AREG, BCL2L1, CDH4, CMPK2, CTSS, F2RL2, FGF7, FMO1, G6PD, GZMA, HLA-DOB, ISYNA1, KAT2A, KCNJ11, LLGL1, MS4A2, NADK, NFX1, PDE4C, PPARD, PPP2R5A, RHOQ, RIPK1, SORD, TAS2R10, TAS2R40, ATP6V1G1, CACNA2D3, MAPKAPK5, SERPING1, TNFRSF17
Negative_4734	GLA, GNS, ID3, LIF, PDC, PTS, TNN, AGXT, ALDH3B1, B3GAT3, BCAR1, C5AR1, CACNA1S, CADM3, CDKN2C, CLOCK, COL1A2, COMT, CSAD, CSF2RA, DHRS3, FOXA3, GNAI3, IL18RAP, ITGAE, LAMB2, LMO7, MFNG, MINPP1, MLLT4, NFATC4, NKX3-1, OR4C46, OR52K1, OR52N1, PGAM1, POLR2D, PRKAG1, RRM2B, SC5DL, SPCS3, TKTL1, TUBB6, HLA-DPB1, KIR2DL5A
Negative_4735	F2R, PGF, ACP2, APOC3, CLDN9, DPYS, DUSP2, EPB41L2, MAP2K3, MPST, MYH14, NTRK1, OR56A3, OR6K6, OR6Q1, PIAS3, PIK3C2B, PLOD2, STX16
Negative_4736	JUN, ABAT, CALML6, CCL11, CCNE2, CYP19A1, CYP2C19, EPB41, FRAT2, FZD9, HSP90B1, LYPLA1, MAD1L1, NARS2, OR10H4, OR7D4, PIGO, PPOX, PSME3, SDC2, STAM2, TNFRSF4, ULBP1
Negative_4737	F2, GPI, ALG1, ANPEP, ARHGEF1, ARPC1B, CCL28, CDKN1A, CFL2, DDX23, FADD, FGFR2, FOXA3, GALK1, ICOS, ITGA2, JAK1, MAGI3, MASP1, NAGK, NNMT, NOS1, OR13A1, OR4A5, OR4D9, OR51G1, OR6K2, PARD6A, PDE1C, PDHA1, PI4KA, PLA2G1B, POLR3G, PPOX, RPS6KA3, UGT2B10, UNC5B, WNT5A, XYLT1, ZFYVE9, MAPKAPK3, ST6GALNAC5
Negative_4738	ADC, C6, F2R, LHB, MVD, PVR, SCD, WAS, ALDH2, ATP4B, CD274, DCTN1, DEGS2, DNMT3L, EPHB6, FGF9, FLOT2, FUT4, FUT8, GOT2, GPX7, IFNG, MCEE, MGAT1, MMP2, NGEF, NR5A2, P2RX1, PLA2G10, PLK1, POLR1B, POLR3H, RPS6KB1, SOCS4, TCF7, YOD1
Negative_4739	MOS, ACP1, ADCY9, ALG6, ATP6V0C, B3GAT3, CHKA, CPT1B, CXCL11, CXCL2, DOCK1, FOXO1, FZR1, GNAL, MCCC1, NFX1, OR10G2, OR10H2, OR2Y1, OR51A7, OR51T1, PIAS2, PISD, PPP3CB, PRKAB2, PRKY, PRSS1, RPN1, RPS6KB1, RRM2B, SUCLA2, MAPK8IP1
Negative_4740	AGK, FAS, ADCY9, ADSS, B4GALT3, C1GALT1, CACNG6, CCL17, CCL4, CDH3, CLCF1, CLDN22, COQ6, CYP1A1, CYP2A6, DAG1, FARS2, FGD3, FGF13, FGF8, GAD2, GALC, GALE, GALNT10, GATM, GYS1, IDH3A, IMPDH1, MBL2, PIGN, PTTG1, RAC1, RHOA, SFRP2, SGPP1, SMARCA2, SPTLC1, TCIRG1, TRDMT1, TRHR, UBA7, UBE2L3, UGT8
Negative_4741	FGA, GLS, AADAC, ACOT12, ACOX3, C3AR1, CASK, CER1, CHD8, CLDN9, CYFIP2, DNMT3L, EPHA3, GPAA1, GPD1L, GRIA3, GRIN1, GSTK1, GUCA1A, HAGH, HSD17B2, IL24, IRAK3, LRP5, MCM4, NCAM1, OR10A4, OR2T8, OR56A3, OR5D14, PDE6G, PDHB, PGAM4, PIGC, PLXNA3, POLR2I, RFNG, SDHD, SORD, STAT4, VEGFA, WNT16, YOD1, HLA-DRB3

Negative_4742	CP,MOS,ABLIM3,ACPT,ADH4,ALDH4A1,ALOX12B,BMP5,CLCF1,CXCL11,DKK4,DRD1,E2F2,GBGT1,GPAM,IFNG,LTBP1,MAP3K1,MAPK12,NFKBIB,NUDT8,OCRL,OR10R2,OR1J2,OR4C15,P2RX4,PAK7,PIK3C2A,STAT5A,TAS1R1,UGT2A3,UGT8,VASP
Negative_4743	AGXT,AKR1C2,AOC3,CCNA1,EGLN1,ELK4,EPHB3,FGF7,IL5RA,MAN1B1,MDH1,MTAP,NFATC4,OR52N4,PFN4,PRKAA1,PTEN,SELE,TAS2R16,TAS2R48
Negative_4744	GP6,IDS,AOC3,ATP5C1,ATP5G1,ATP6AP1,CISH,CYP11B1,CYP3A4,DGAT2,EARS2,GCNT2,HEMK1,ITGB6,LAMC3,LARS2,NADK,OR2G2,P2RX2,P2RX3,PGAM2,RAB23,UGCGL2,ATP6V1E2,GABARAPL1,TNFRSF10C
Negative_4745	CLTC,IARS,MARS2,NKD1,OR2A42,SMURF2
Negative_4746	CALML6,CCL27,CDC42,CFTR,CHSY3,COL4A2,DEGS1,DUSP14,GNA15,ITPKB,OR10G8,OR1M1,OR4C16,OR51A2,OR51I2,PDGFC,SLC25A4,TGFB2,TNFRSF8,VDAC3,ALDH18A1,EIF4EBP1,TNFRSF10B
Negative_4747	BID,ARSB,BIRC5,IFNA7,IL22RA1,SEMA3A,TUBB4
Negative_4748	DBT,ALG2,B4GALT2,BTRC,BUB1B,CACNA1I,CCDC6,COQ5,CYP2A13,DOCK1,DTX4,EXTL1,FZD3,GNA12,GNMT,GSTZ1,HPSE,LARS,MAT1A,OR2L13,OR2M7,OR51A2,OR51I1,PDE1C,PIGB,PIK3CG,PLA2G2E,PLCG2,PLIN,PTDSS2,PTPRJ,RFXAP,SOX17,STX1B,WWP1
Negative_4749	GSTM2,PTCRA,PTPN1
Negative_4750	F12,AASDH,ALDH5A1,ARPC1A,ENPP7,MAP2K4,OR4D1,PPARG,PRKCD,PRPS1,RDH11,SARDH,SESN1,TICAM2,TUBB2B
Negative_4751	ALOX5,ARD1A,COL2A1,GP1BB,INPP5B,NT5C,PLA2G5,PPM1B,SYNJ2,TAS2R10,TAS2R9,UBE2K,HLA-DRB5
Negative_4752	CGA,GLA,NOG,ACVR1,ALG8,B4GALT6,CACNA1D,CACNB3,CD44,CLCF1,DKK4,ENTPD4,FUT8,HHIP,IRS1,LAMC3,LDHAL6A,MGLL,MMP14,NAGK,OR13C3,OR2L2,OR51M1,OR8D4,OR9G4,PGAM1,PIGV,PRKAB2,RELA,SIGLEC1,THTPA,WNT16
Negative_4753	DCC,ACAT2,AKT3,ATP6AP1,B4GALT7,BUB3,CD72,CIITA,COX15,CXCL2,ESPL1,FMO4,GCNT4,HLCS,HTR5A,IDI1,IMPA1,ITGA8,NLGN1,NRG1,OR4K15,OR4Q3,OR51B5,OR6T1,PIGK,PPP1R3B,PRLR,RASGRP2
Negative_4754	F12,LPO,TK2,ACTN2,AMDHD2,AMY2A,ANAPC10,ASAH1,ATP2A2,ATP5J,CADM3,CALML5,CYP4A22,DDX51,EFNA2,ENTPD5,ETNK1,GADD45G,HSPA1B,ITGA11,ITGAX,LYPLA2,NEU3,NPR2,OR13C3,OR1E2,ORC6L,PDE3B,PGK2,PTGS1,RBKS,ROCK2,SLC2A1,SOS1,SOX17,SPTLC1,SSX2IP,STX16,TAS2R1,VAMP3,SERPINE1
Negative_4755	DUT,GSN,LAT,C4BPA,CALML5,COL6A1,DHDDS,DTX4,EFNA2,FRAT2,FZD5,GADD45A,HLA-DMA,HYAL2,IL1A,INHBE,KLRC4,LDHA,LEPR,LILRB3,LTA4H,MMP9,OR10G7,PLA2G2D,PRKX,PTCH1,SPTLC1,TAS2R14,ARHGEF12
Negative_4756	BTK,DBT,FGA,LCK,TPR,ADIPOR2,AMHR2,B4GALT6,CAMKK1,CCR5,CCR6,CD70,CHKB,CSNK1G3,CTSG,DTX1,ECSIT,EFNA5,EPB41L3,FARP2,FSHR,GAD2,GNG13,HCLS1,HES1,ICAM3,IFNB1,IMPA2,LRDD,MAP3K7,MYH13,NMNAT1,OR13J1,OR52A1,OR7A5,PLXNC1,PRKY,PSPH,PTDSS1,PTK2,PYCRL,STAT1,TGDS,THBD,YES1,ATP6V0D1,MAPKAPK3,SERPINC1
Negative_4757	FLAD1,IL11RA,MTFMT,SMAD3
Negative_4758	CFI,EZR,F2R,F5,LPL,AGTR1,ATP5H,B3GALT5,BMP2,BMP8B,BRAF,CAMK2,CD3E,CD8B,CDKN2B,CTSB,CYP2A6,DDX52,DOT1L,DUSP8,EPHX1,FLT1,HMOX1,IL11,IQGAP2,KCNB1,KITLG,NMNAT3,OR4A15,OR4C46,OR4D5,OR4K1,OR5C1,PARS2,PIGL,PLXNB2,PRKAB1,PYCR1,RASGRF1,SETD7,STAT4,TAP2,TGFB3,WARS2,TNFRSF10A
Negative_4759	B4GALT7,CD34,PPA2,RRM1,TUBB4Q,MAPK8IP3

Negative_4760	BAX, TNR, ALG1, B3GALT6, B4GALT3, CPT1A, CSNK1G3, CTF1, DUSP16, E2F5, ETS1, HIBCH, IL18RAP, IL4R, KRAS, LIPT1, MEF2C, MYH2, OR8K5, PAK4, PRKCG, RFWD2, SETD2, TJP2, TUBA8, UNC5D, PAFAH1B2
Negative_4761	BTC, HYI, ADSL, AKR1D1, DDX23, DUSP6, FZD10, GLUD1, IL22, LAMB1, LIPE, MAP3K1, MBL2, NCK1, OR1D5, OR1N1, OR2M7, OR4C12, PDHA1, PLXNB1, PPP3R1, SMPD1, SPCS3, TUBAL3, WNT8A, PDCD1LG2, ST6GALNAC5
Negative_4762	ALG9, CBR1, CCNG2, CD28, CD86, CDC14B, CDO1, CKMT2, CSNK1A1, CXCR5, IL2RA, IMPDH1, MGAT3, MTMR6, OR6B2, PDE3B, PDHB, PLXNC1, RUVBL1, SOCS1, TAS2R5, TCF7L1
Negative_4763	EDA, ARPC5, ASNS, CCNB1, CYCS, EPHX2, GNAT3, GPAA1, GPD1L, HHIP, INADL, ITGB8, MYL7, OR10H3, OR13C9, OR5B12, POLA2, POLR2I, STX8, TBXAS1, UGT2B10, ATP6V0D1, TNFRSF1A
Negative_4764	MYC, NPY, ADCY7, ALDH1B1, B4GALT7, BAI1, BST1, CCNG1, EPHX1, FMO2, GRM1, IFNA14, MMP7, NRAS, NUMBL, OR52A1, OR5AP2, OR7A10, PARVB, PDCD1, PDK1, PLA2G2D, PLXNB3, RHEB, STK3, SUCLG1, TAS2R60, XCL2
Negative_4765	C4B, RFK, SMO, AANAT, ACYP2, ARRB1, ATP6V1H, BCAR1, CNTN2, DDX56, EXT1, FOXO3, HLA-DMA, ICOS, IL28A, LILRB3, LIPT1, MFNG, MGAT1, NEFM, NUDT5, OR51A2, OR6K6, PANK4, PER1, PFKFB3, PIGX, PIK3C2A, PISD, PSENEN, RAB23, RFXANK, SKP1, ST3GAL6, TRAF4, TSHB, ULK1, VAV3, YWHAB, TNFRSF1A
Negative_4766	F5, GK, ADH6, APPL1, ARNT2, CCL8, E2F2, ENPP7, FBXW11, FZD1, GSTO1, HTR4, NCR2, OR10H1, OR8B8, PARS2, PIGA, PIGK, PORCN, PTPN5, SPRED1, TCF7L1, TNFSF10, UCK1, UNC5C
Negative_4767	AK2, FER, GH1, ACTB, ADORA2A, ARNT, CCR6, CTSS, DFFB, DNMT1, EFNA3, EPHX2, FZD5, GLG1, GNAI3, HSPB1, IL11, MAP2K7, NT5E, OR2A4, OR4F17, OR51M1, OR8B12, POLR2I, PPP1R3A, PPP3R1, PYGB, RASSF1, SLC2A4, STX18, TOMM40L, TRAF5, TRPM5, VEGFA, ALDH18A1
Negative_4768	DHH, FUK, ACSL5, ARPC1A, CAMK2G, CDKN1C, DVL2, ECHS1, EGFR, EIF4B, FADD, FARSB, FDFT1, IL11, IL13RA1, JAG1, NPR1, OR2H1, OR51A2, PANK4, PPP2R2C, PVRL1, ULK1, UNC5A
Negative_4769	ILK, BMP6, IDH3B
Negative_4770	F8, PXN, ARSA, BRAF, CDC2, GAPDH, NPR2, NR4A1, PPP1CB, TAS2R50
Negative_4771	ABO, TPO, AOC2, ARHGAP5, ARPC1A, ASS1, ATP13A2, BDNF, CD36, CERK, COL5A3, CRY1, CYP2S1, DNMT1, FGF4, GALC, GGPS1, GMPPB, GSTO1, HSPA2, IL1R2, ITPKA, LYPLA2, MAPK14, NEU2, OR1J1, OR2AG1, OR51B5, P2RX5, PAX8, PGAM1, PHKG1, PSEN2, SCP2, SESN1, SPHK2, SPTLC2, TNFSF10, TYRP1, YARS2, ATP6V1C1, TNFRSF13B
Negative_4772	APP, EGF, SI, CACNA1F, CCNG1, CSF1, DOT1L, GBE1, LIPE, OR1L3, OR8D4, PDE7A, PYCRL, SCIN, STX3, TRPM5, WHSC1
Negative_4773	EDA, GLS, ME1, OGT, STS, ARG2, C9orf95, CCNH, CES1, CKMT2, CSF2RA, CTSL1, CYP4A22, EP300, FZD9, FZR1, GSTM5, HNMT, HPSE, HTR2C, HTR5A, INPP5A, LGMN, MAD2L1, MAP2K2, MAP3K12, MAP3K6, MPDZ, MTAP, NMNAT1, OR1D2, OR1G1, OR2A42, OR52N4, OR5P3, ORC1L, PDGFB, PGS1, PLOD2, PRKCE, RUNX1T1, STAM, STX5, TIRAP, TRIP10, TUBB3
Negative_4774	MVD, CD36, DLL4, DVL1, FASLG, GAD1, HAP1, IBSP, ITGAV, MDM4, MTHFR, NEGR1, OR5AP2, OR6C6, PRNP, RPS6KB2, SEMA4C, SUOX, EIF4EBP1, PRICKLE2
Negative_4775	ATM, BCAT1, CBR1, CCND2, CDC16, CHAT, CLCA4, CRLF2, DAAM2, DCTD, HTRA2, IL21R, LARS, MAP4K3, MYH1, NKX6-1, OR9G4, SMPD4, SOAT2, ULK3, UPP1, VAV1, ZFYVE9
Negative_4776	CFH, IL4, LEP, LTB, ACADS, AXIN1, B4GALT7, BMPR2, C4BPA, CXCL5, CYP1B1, DUSP7, GALNS, GUCA1A, GUCA1B, HES1, HTR2C, IL1R2, IRF7, LAMB4, MICA, NAGK, NRG2, OR4D6, OR4F17, OR6C3, PAPSS2, PDE1A, PDE4A, PLEKHA8, PPP3CC, PRSS1, PTCH1, PYGB, RARA, RETSAT, UGCGL1
Negative_4777	GANC, OR4C11, PPP2CA, PPP2R5D, ST3GAL5, VDAC1

Negative_4778	GH2, LTB, ACAT2, ACP6, ACY3, ADRA1B, AGTR1, B3GALT2, CAMK2A, CCL3, CD55, CRB3, CSF2, CSNK1A1, CXCR3, CXCR5, CYFIP1, DGKB, DOLPP1, GJA1, GPD1, GSTA4, IL2RA, ITGA3, MTHFS, OR5M9, OR5P3, OR8B8, PAFAH2, PRPH2, RPN1, TNFSF8, TSHB, ZAP70, ATP6V1E1
Negative_4779	ATP5E, FGF5, HNF4G, HSPA2, POLR2F, SDC4, UNC5B
Negative_4780	MPI, MVK, NLK, PGD, ACVR2A, ALPI, ARHGEF6, ATP5F1, BLVRA, BUB3, CAV3, CCND2, CTSG, CXCL16, CXCR6, DHCR24, FUT8, GCLM, ICOS, IGF1, LDHA, LMO7, LRDD, NAE1, OR10A6, OR2A12, OR2T8, ORC3L, PEMT, PIGK, PLCG2, POLR3D, PRKCI, RCHY1, SETMAR, SFRP1, SIAH1, SPRY2, UNC5D, VAMP3, VNN1, YARS2, HLA-DPB1
Negative_4781	APP, BAX, ACAD9, B4GALT1, CDC14B, CER1, CHRM2, COL4A1, CXCR4, HNF1B, IL13RA1, IL1R2, MAGI2, MAP3K5, NFKBIE, OR1A2, OR4M1, OR4X2, PARVB, PCCA, PDE4A, PIP5K1B, PRKAG3, RXRB, SGMS1, THBS3, TUBB6
Negative_4782	CALML5, IFNA8, INADL
Negative_4783	DAO, ACY1, CTBP2, HNMT, MOV10L1, PTGIS, RUVBL1, TAP2, TBL1XR1
Negative_4784	UBC, ACO2, ACSM1, ATP5I, BST1, CLCA4, CYSLTR2, DAPK2, E2F4, FMO5, ITGB4, LGMN, NAT2, OCLN, PTEN, RPRM
Negative_4785	F10, F2, HPD, AMD1, ECHS1, ICAM3, ITGA3, OR7C2, PGM3
Negative_4786	FUT3, RALA, ST8SIA1
Negative_4787	ABL2, AKR1C4, AKT1, BAI1, CCR7, EVI1, FUT7, GOSR2, IFNA16, IL3RA, IL6ST, ITGA4, LPAR1, MCEE, OR3A2, OR7A10, PPCS, QARS, RPSA, RRM2, TBXA2R, ULK1, ATP6V0A1
Negative_4788	C8B, F9, ACTG2, AKR1C3, CD55, G6PC2, GGT6, GNAI3, LRRC4C, MYH10, OR52E8, OR8K1, RAPGEF3, SH2D1B, TNFSF10, TOMM40, MAPKAPK3
Negative_4789	WAS, ACO1, ATP5O, B3GALT5, BLVRA, EPAS1, FLAD1, GARS, GNAO1, IL6R, IRAK2, ITCH, ITGA2, KARS, LALBA, MAD1L1, MAD2L2, MCM2, NRXN1, OR2L8, OR4Q3, OR56B4, OR5AN1, OR5C1, OR6C3, PANK1, PLAUR, PLXNB2, RAB23, RAF1, SDC1, STX1B, TSC2, UGT2B11, WASL, SERPINE1, ST6GALNAC6, TNFRSF11A
Negative_4790	C7, ACTB, AGXT2, COX15, CUL1, DNMT3A, EGFR, FARS2, FUT1, HYAL2, IDH3G, IFNG, MAD2L1, MAML3, NKX3-1, NR1D1, NTN1, ORC4L, PDE8B, RENBP, RPS6KA5, SEMA4G, SULT1A2, SV2A, ALDH18A1
Negative_4791	ACTA2, ANAPC2, CDH15, ENPP1, ENTPD3, ERO1L, GGT5, GSTM4, ITGA4, NRG4, ORC3L, POLR2H, PTK2B, SHC3, SLC25A5, TGFBR2, WNT2
Negative_4792	HYI, ADRA1D, ALAS2, ALOX12, AREG, ATP5G3, B4GALT6, BIRC2, CASP10, COL4A6, CPOX, CUL1, CYP2A13, DARS2, DERL1, DPM1, EPB41, FABP1, FADD, FGF1, FTCD, FZD7, G6PC, GAD2, GUCA1C, IFNA21, IQGAP3, LIAS, MAN1A2, MINPP1, NTSR1, OR10R2, OR6M1, OR8B4, ORC3L, PAK2, PLAU, PLCZ1, PNPO, PRKAG1, SLC25A4, SLC2A2, STX12, XCL2, ATP6V0A2, TNFRSF14
Negative_4793	ABO, ASL, CGN, FUK, GDA, PTS, ACO1, ALG6, ATF2, AVPR1B, BLVRB, CCL21, CNTF, COMT, CRLF2, CYP2U1, EGLN3, ENO2, GNB1, HLA-DOB, IBSP, IFNA4, IFNB1, IRS2, MGAT3, MPP5, MTHFD1, MYH9, NAT6, OR13J1, OR1F1, OR2A25, OR4F5, OR51M1, PDHA2, PMM2, PRIM1, RPE65, STX3, SULT1A4, TOMM40, ULK1, WNT8B, HLA-DRB3
Negative_4794	FGB, TEC, ALAS1, BCAR1, BDKRB2, CACNG5, CCL1, CCR6, CDC25B, COX15, DDX50, DNMT3L, ENPP3, FOXO3, FSHR, GLS2, GOSR2, HSD17B3, IL11, IMPDH1, MAP4K3, MMP2, MTHFR, NRG1, NUDT5, OR2G3, OR6C70, ORC4L, PCCA, PRDX6, PRKAR2A, PRKCZ, PTGS2, RASGRP3, RDH16, SARS, STAT5B, TAP2, TAS1R1, THBS3, TPH1, WNT8B, ZNRD1, B4GALNT1
Negative_4795	F12, JUP, ADRB2, AKT1, ANAPC2, ARSE, ATP5L, BCAT1, BMP8B, CD3E, CDH1, CSF1R, CTSG, CYP1B1, E2F1, EXT1, F11R, FABP1, GGPS1, IL17B, KAT2B, LMO7, MASP2, MTHFD1L, NFYB, NUDT2, OGDH, OR2A4, OR5AR1, OR6B2, PAK6, PRKCI, SMAD7, SOCS1, STX3, SUCLG1, TAS1R3, THBS4

Negative_4796	IL7, LAT, ACSM4, ALDH3B2, ARNT, CBR3, CDH15, CHRNA7, CHUK, CLOCK, DAPK1, EFNB1, FZD10, HLA-F, ITGA9, MAN1B1, NLGN2, OR10J5, OR2C3, OR4D6, OR51G2, OR5B17, OR7G3, PARD6G, PIK3C2B, POLA2, POLR3A, PRKAB2, RASGRP2, SPRY1, STX4, THBS3, TKTL2, TPK1, B4GALNT1
Negative_4797	DUT, EPO, IL4, BHMT, C4BPA, CCL22, CX3CR1, CXCL6, CYP2A13, CYP2F1, CYP3A5, GALNT10, GCDH, IFNB1, MAP3K6, NCK2, OR10H1, OR13C9, OR51L1, OR51Q1, OR9G4, PAPSS1, PCK2, PDHA2, POLR2F, PON1, TAS2R41, UXS1, YWHAH
Negative_4798	PLG, ABI2, CNTNAP2, CRB3, CREB5, CYSLTR1, FARSB, FMO4, GALNS, GLS2, GRAP2, HTR7, IFT57, ITGA7, ITPA, KLRC1, MAP3K12, NT5C3, OR4C11, OR9G1, PARS2, PPP3CA, SELPLG, SEMA3B, SEMA4A, SSH2, THY1, PNLIPRP2
Negative_4799	APOE, CTF1, EIF4B, PPP3CA, SEMA4B
Negative_4800	IL9, ABP1, ACTA1, ADH5, AK3L1, ATG3, CACNB2, CNGA3, COL4A6, CSNK2A1, EGLN1, GOT1, HMOX1, MAPK7, NARS, OR2T2, OR5T2, TAS2R4, TAS2R8
Negative_4801	BAD, CFD, DCK, ITK, LAT, MPZ, ADCY2, ALDH2, B3GNT2, CES1, CHKB, COASY, CREBBP, CYP4F2, DPYS, ETS1, FMO5, GCNT4, GMPS, GNMT, GOT2, GRHPR, HMOX1, HNMT, HTRA2, ICOS, IDI1, JUND, KARS, MECR, MIOX, OR10A6, OR1L6, OR2F1, OR5H6, OR6K2, PDE6D, PGM2, PLTP, RFX5, SRGAP2, ST3GAL5, STK4, TAS1R1, TUBA1A, MAPK8IP1
Negative_4802	TNC, ADRB1, CDC14B, CDKN2B, CTSB, DUSP16, EPAS1, ESAM, FGF11, FLT1, GDE1, KLRC2, NAGK, NEFM, OR6K3, OR7G3, PIK3R1, PITX2, POLA1, PPP1R3D, PPP2R5E, PRKAR1A, PTGS1, RAP1B, SMAD9, SPTLC1, SSH1, UGT2B28, CACNA2D4, PPARGC1A, SERPINA5
Negative_4803	LYN, PGD, ADSSL1, CD40, CXCR5, DLL4, EFNA2, EFNA5, GFAP, GLUL, HADH, HSD11B1, OR10J3, OR11H4, OR4A15, PIK3R3, PPCDC, PRPS2, PTK2B, PVRL3, QARS, ROCK1, SMAD4, SMAD7, SSH1, STT3B, TACR2, TNXB, UMPS, PAFAH1B1
Negative_4804	ATP5H, COX10, DLAT, EPHB1, FZD6, IFNGR2, LAP3, OR4C15, OR7C1, TNXB, CSNK1A1L
Negative_4805	FGA, GNE, ID2, RDX, UBC, ABLIM3, ADCY1, ADCY3, BDH1, C7orf16, CNTFR, CYSLTR2, DGUOK, ESAM, GGCX, MAP3K4, OR14C36, OR51G2, OR5AP2, OR5T3, PARD6B, PTPRJ, PYCR1, RFWD2, TLR4, ATP6V1B2, CACNA2D4
Negative_4806	CEL, DGKH, LEPR, LRP6, MAPK10, NCOR1, OR4A5, OR6C70, RDH11, TCF7
Negative_4807	PAH, AHCYL2, AKT3, CCL26, CD22, CHEK1, COL6A2, DDIT4, FLT3, GCH1, GCNT1, GNAO1, GRIA1, HAO1, NAE1, ORC3L, OXCT1, PI4KA, PRKACG, SEMA4F, ST6GAL1, SYNJ1, TRDMT1, WBSCR22, WNT10B, WNT11
Negative_4808	AMPD3, ATP5B, DPM1, EFNA2, EFNA3, FADS2, FGF8, LCP2, MAN2A1, MGAT2, OR6N2, PAK1, PCK1, PRKCD, SOCS1, STX6, TNFRSF1B
Negative_4809	C7, FGG, ACP5, ASMT, CACNG7, CCR7, CDC16, CSF3, CYP2S1, FZR1, GALNT12, GNPDA2, IL22RA2, IRF5, ITGA1, NOX3, OR10H5, OR2M5, OR5AR1, OR5AS1, OR5L1, PDXK, PFN3, PLA2G1B, PRKACA, PVRL1, RDH11, SEMA4G, TACR1, TUBA1B, HSP90AA1
Negative_4810	ARG1, B3GAT1, CCL22, CCND2, CLDN5, COL1A1, DOCK1, ESPL1, GOT2, INHBC, MDH1, OR2L3, PDE7B, PFAS, PRKAG1, PVRL1, RFWD2, SETDB2, TGFB2
Negative_4811	TNR, ANAPC4, ATF2, ATP2A1, CCL21, CCNB1, CDS1, CHEK1, DUSP4, FPGT, FTMT, GABARAP, IL13RA2, IQGAP2, LDHAL6A, MLLT4, MMP9, MYH14, NCKAP1L, OR10A7, OR51D1, PDE4C, PFN1, PLA2G3, PTGES, ST3GAL6, TAS2R40, UCKL1, MAPK8IP1
Negative_4812	FER, AVPR1A, CERK, CLDN10, CLTC, FLT3, IFT57, NEU1, OR10H1, OR52A1, PDK1, PIGK, PPAP2A, PTTG1, WNT5B, ATP6V1G3
Negative_4813	CSK, B4GALT3, CCKAR, CSF2, EPHA1, LYZL1, MYH7B, NKX3-1, OR10P1, OR8H1, PDE4D, PDE6G, PMM2, PPT1, PSEN2, RRM1, TAP1, ULK1

Negative_4814	ADC, FAH, ID2, KMO, UBB, ALPI, APH1A, CASP8, CDH4, CHUK, CREB3L1, CSNK1G1, DAPK2, DDX52, DOCK1, EFNA1, GALC, GALNT7, GALNTL2, GCNT4, GPAA1, IBSP, INPP4A, ITGB1, NFATC3, NRG4, OR10G9, OR13C5, OR1E2, OR2A25, OR2A42, OR2T12, OR4C13, OR52E8, OR5D13, P4HA1, PIGP, PIK3R5, POLR1E, RCHY1, SFRP1, SLC9A1, SOD1, SPTLC1, STEAP3, TRIP10, UCKL1, UXS1, ATP6V1B2
Negative_4815	MSN, ARHGAP5, CCL1, CCL11, CHRM1, FGD3, GAD1, IDH3B, OR7C2, ORC2L, PHKG2, POLE, PTPN6, TP53, TRAF3, WASL, EIF4EBP1
Negative_4816	DLD, ADSL, ALDH1A1, CACNB2, CALML3, CALML6, CPT1A, CYP2C19, DUSP16, INMT, LALBA, MLYCD, MYH6, NCAM1, NTNG1, OR5V1, PLK1, POLR1D, RBL2, SFRP2, STX6, SUCLG1, TNFRSF8, CACNA2D3, HLA-DPB1
Negative_4817	CDA, IVD, KIT, LCT, ACSL6, ACVR2A, ADH4, AGPAT2, ALAS1, APOA1, ARPC1B, CD34, CDKN2D, CLDN4, DAPK2, DUSP3, EDNRB, FMO2, GCNT2, GNA12, GPX5, IFNA7, IL12RB1, IMPA2, MAGI2, MAP3K1, NAT1, NAT6, OR10T2, OR4A16, OR4C16, OR4D2, OR51Q1, PAX6, PDIA4, PLA2G2A, PLAT, PLCD1, PLXNC1, PNPLA4, PRKCI, PRLR, PTCH1, UNC5C, VAV3
Negative_4818	DCC, ACAA1, ADCY9, ALG10, AMDHD2, BCAR1, CAMK2A, CAMK2D, CHST13, CIITA, CSNK1G1, CYP27A1, DAD1, DDX41, EPHB6, FABP1, FGF12, FOXO1, GMPPA, GRB2, HSD17B1, IDH2, IL12RB1, ITGB6, MMP9, NFKB2, NOS3, OR11H1, OR2AG2, OR2K2, OR4C15, PCYT2, PDCD1, PGK2, PRSS2, PVRL2, SMARCA5, SMURF2, TAS1R1, WNT2, CACNA2D4
Negative_4819	FST, ACTN2, ACYP2, AMDHD1, ARPC1A, ASCC3, B3GNT5, B4GALT3, CACNG6, CCND3, CDK4, CHEK2, CYP1A1, DUSP1, ENTPD5, GALNT10, HSPA2, IFNA8, IL21R, IMPDH2, MAGI3, MAN1A1, MINPP1, MYH9, NFKB2, OR10AD1, OR14I1, OR1J1, OR2T5, PDGFB, PIK3C3, PRKCZ, ATP6V1E1, PNLIPRP2
Negative_4820	ADC, AK3, ALDH1A1, BLNK, BLVRB, C9orf95, CDK4, CHRD, COMT, DNMT3L, GALNTL4, GMPR, GNAZ, HLA-DOA, ICAM2, IQGAP1, ITGA2, ITGAV, LLGL1, MAD2L2, MAP4K4, MINPP1, OR4X1, OR7A17, PIGL, PON2, PRKX, PTPN11, PVRL2, SENP2, ULK2, VARS, WARS, XCR1, MAPKAPK3
Negative_4821	TXK, AGXT2, AHCYL2, CCL2, CERK, DDX18, DERA, EPHB4, FZD1, GALNT3, HMGCL, KIR2DL2, LAMB3, MGAT5B, OR2T33, OR52D1, OR52I1, OR6N1, OXSM, PTGES, QPRT, SHISA5, TP73
Negative_4822	C4B, GGH, GPT, IHH, PML, RDX, TXK, AK3L1, BDH2, CCR9, CDC7, CDS1, CHPT1, CREBBP, CSNK1G1, CYP3A43, DHDDS, ENO1, FOSL1, GAL3ST1, GNAI3, GPD2, HCST, HRH1, ITGB1, LIMK1, MAML2, MDH2, MGST3, MYLK, NCAM1, NCSTN, OR13A1, OR4A15, OR4C12, OR5K2, PARVA, PDE4B, PDGFRA, PERP, PHKB, PIM1, PNPLA4, POLR1A, PPAP2B, PRODH, PRODH2, RHEB, SRGAP2
Negative_4823	ABCC8, B3GNT2, CCR2, CD47, CREB5, EPHA1, IFT57, NCR2, NPR1, OR51B4, PER1, RDH11
Negative_4824	AK7, C8G, CRH, PTS, ADIPOR1, ASAH1, CASP8, CCDC6, CCR4, CSNK1E, DAG1, DLST, FGF13, GOT1, IL28A, KLRC1, LYPLA2, MADCAM1, NFKBIB, OR4S2, OR51B4, OR51F1, OR51S1, OR5D14, OR5M9, OR7C2, PDE2A, PDK1, PFAS, PLCB1, PPID, PRODH, ST3GAL5, THY1, VEGFC, YARS, MAPK8IP1, MAPKAPK2
Negative_4825	C5, CR1, NPL, OAT, ACP6, AGPAT3, CTBP1, CYP2F1, DAPK3, DDX56, HMGCL, IFNA5, IL10, INHBB, ITGAE, ITPKB, MAPK3, MGAM, MYLK, NFATC4, NOS1, NRG1, OR10X1, OR1S2, OR6K2, P4HA1, PIGC, PIK3CB, PIM2, PIP4K2C, PLCB4, POLD4, RAP1B, SEMA3D, SETD8, STAT6, UGT1A10, WASF1, YOD1, HLA-DPB1
Negative_4826	CD6, CKM, ME2, XDH, AKT3, ARHGEF4, ATP2A1, EFNB1, EPHA5, FABP1, FGF12, FSHR, HLA-DMA, MAML1, MCM2, NMNAT1, OR10G8, OR2C3, OR3A1, PIGT, PIGW, PIP4K2C, RBX1, SELPLG, SEMA3F, SMURF1, SOAT1, TNFRSF14

Negative_4827	GPT, IL2, AKR1B1, ALPPL2, ARSD, CACNB3, CADM1, CAMKK2, CASP10, CASP9, CLDN20, CPT1A, CREB3, CRY1, CTSS, EGFR, ENTPD7, EPB41, FUT5, GALNT1, GLI2, GMDS, GSTZ1, IL2RG, LAMA4, LIPC, MAP3K2, MASP2, MCM4, MITF, NFAT5, NFATC1, NPR2, OR4C11, PGK1, PIK3R1, PKLR, PLA2G6, PLEKHA8, SOCS3, STX3, UBE2K, MAPKAPK5, SUV420H2
Negative_4828	IVD, STS, AASDH, ACVRL1, AHCYL1, ALG12, APLP1, C9orf95, CDKN1A, CHST13, CNTN1, COL2A1, CTSB, EPAS1, FARS2, FDFT1, FGF22, FTCD, GP1BA, GSTM4, GSTO1, IL15RA, IL9R, KIR2DL1, LRP6, MDH2, MGAT5, OR1S1, OR52B4, OR5A1, PLA2G6, POLR2B, RBL1, RPS6KA2, TJP2, TNFRSF8, VEGFC
Negative_4829	SHH, ACYP2, ALOX12, ASIP, BAAT, CAMKK1, CCL16, CD8B, CD99, CDK7, CHEK2, CTNNA1, CYP1B1, DGAT1, DUSP7, GAB1, GMPR2, GPAM, GTSE1, INPP4B, LTBR, MAML1, MAP4K3, MPZL1, MYH14, NAMPT, NFKB1, NT5C1A, NUDT5, OR10A3, OR2B2, OR51G2, OR51S1, OR5AR1, OR6X1, OR8H3, PAK4, PCNA, PDE3B, PFN1, PPA1, RHOA, SETMAR, SPRY3, SV2C, TRPM5, AASDHPPT, ATP6V0A1, ATP6V1G2
Negative_4830	ACOX3, AKR1D1, ATP13A2, DDX54, GSTM3, ICAM3, ITPKA, NRCAM, OR4C13, OR6Y1, PAICS, PARD6G, PRKY, RAPGEF3, STEAP3, TAS2R14, TIAM2, TYROBP
Negative_4831	CAT, INS, SHH, TNR, ADCY5, AGXT, ALG12, CIITA, DAG1, GPAA1, GPLD1, IFNA8, LAMA4, LCP2, LIPA, OR10R2, OR2D2, OR51G2, OR52B4, OR52I2, OR5F1, PDE6D, PIP4K2A, POLR3G, PPAP2C, RASSF1, RBPJL, RIPK1, RXRG, SEMA7A, TIAM1, TKTL2, TRAF1, TSTA3, WNT1, ST6GALNAC1
Negative_4832	ME3, MYC, UBC, AADAT, ACOT11, CX3CR1, DDX41, EPAS1, F2RL2, GALE, GNA11, GSTA2, IL19, IL1A, IPPK, ITGAX, KLKB1, MARS2, MDH1, OR4N4, OR51L1, OR6C2, OR7C1, ORC6L, PIAS3, PKLR, POLR1A, PPP3CC, PRKG1, PTDSS1, PVRL2, RAC2, SELL, SLC27A5, SNAI1, SSH3, STAM, TAS2R41, TLR4, TUBA1C, TUBB6, WNT7B, ATP6V0D2, HSD17B12, MAPK8IP2, PPP1R12A, ST6GALNAC6
Negative_4833	CBL, ANAPC1, ASMT, B3GALT6, CHSY1, COL4A4, CYP8B1, DVL2, E2F1, FOXA2, GCNT4, MGAM, NEFH, OR11H6, OR2T29, OR6C6, PDHA2, PFKL, SETD1B, SPTLC1, TAS2R48, TNFRSF11A
Negative_4834	DBH, FER, GSS, NPY, ACAT2, ACOX2, ACSM2A, BCKDHB, CCNB1, CDKN2D, CHRM1, CSF2RA, CSF3R, DGKI, FMO3, HLA-DMA, HSD17B7, IL17B, JAK2, MAP2K1, MAP3K2, MYH14, NEFH, NTN1, OR10G2, OR4E2, OR4F4, OR4Q3, OR51E1, OR8D2, PDE6G, PGM3, POLA2, SOAT1, SOS1, SPAM1, SSX2IP, ST3GAL4, VEGFC, GABARAPL1, MAPKAPK2, SERPINC1
Negative_4835	OGT, TNR, ARNT, ATP5F1, B4GALT7, CPS1, FZD8, ITGAM, LGMN, PAPSS1, PPAT
Negative_4836	IL3, ABP1, ACSM5, ARRB1, CD14, CLDN4, COQ5, CYFIP2, GNRHR, GRIA3, JAM2, LIPC, MINPP1, NFE2L2, OR4E2, PIP4K2B, PLA2G10, POLE3, PPT1, SOCS1, ST3GAL5, THBS2, TIAM2, TPK1, TUBB, XCL2, EIF4EBP1, SERPINA1
Negative_4837	KHK, CD46, CHD8, DFFB, DVL3, EI24, FGF19, GNAS, IL29, NR1H3, OR11H4, OR13C9, OR9A2, PDE4B, PFKL, PNPLA4, PPP2R1B, PRKCI, RASGRP4, RPN2, SMC1A, TNFRSF10A
Negative_4838	BAD, RPE, ADCY4, AMPD1, ARHGEF4, BLVRA, CHIT1, E2F3, EXOC7, FCGR3B, GBE1, GLB1, IFNA5, IL12RB2, ITGAD, JAM2, LIPE, LYZL1, MAGI3, MAPK10, MAPK8, MKNK1, MYL2, NTNG1, OR10J1, OR1E1, OR1J2, RAB23, RERE, RPS6KA1, RRAS2, RUNX1T1, TAS2R14, UPB1, ATP6V0D1, ATP6V1G3
Negative_4839	ACY3, CAMK4, CDH5, COL4A2, CXCR6, FGF18, FRAT1, MAOA, MAP3K12, MAP3K14, OR10G7, OR2A2, SOAT1, TBL1XR1
Negative_4840	EBP, AGXT, ALDH9A1, ARNT, COX15, CYP21A2, EFNA5, ETNK1, FGF6, FZD7, HSD11B1, HSD3B1, HTR2A, INSRR, KLKB1, LTBP1, MGAT5, MMP14, MYD88, NT5C2, ONECUT1, OR5D14, OXSM, PGAM1, PMM2, PPP1CA, PPP2R5A, PROC, PYGL, SPRY1, STX18, SV2C, TGFB3, TSC2, USE1, VDAC3, WNT8B
Negative_4841	BAD, F2R, PDC, FGF18, IRF5, ITGA5, NPR2, OR8K5, PLA2G6, WASF3

Negative_4842	C1S,TKT,ACHE,ACO1,AGRP,AKR1A1,ALAS2,ARHGEF2,BLVRA,BMP5,CNTNAP2,CYP3A43,DRD1,ENPP1,HSD3B7,IARS2,IDI1,IL2RG,MAN1A1,NFATC4,NRP1,OR4C46,OR51E1,OR51T1,OR7A17,OR7G1,PFN3,PGAM4,POLR2G,POMC,PYCR2,SMAD5,STX6,TBXA2R,TYROBP,XCR1,ZMAT3,CACNA2D1,ST6GALNAC1
Negative_4843	C8B,DCC,MOS,MPI,ACPP,APOE,ATP13A2,CALML3,CCL21,CHRD,CTTN,CXCR5,CYBB,EFNA3,GALNT14,GMPPA,GNPDA2,KIR3DL2,LAMB3,MYLK3,NFATC2,PDX1,PHPT1,PKLR,POLR2B,PPP3R2,RPN2,STAM,SULT2B1,TNFRSF6B
Negative_4844	FGF7,GNMT,INPP5A,SEMA4C,WASF2
Negative_4845	FST,ACSL6,BIRC2,CDK2,CSNK2A2,INPP5A,ITGAE,LIPF,OR1K1,OR52E8,PIK3CD,RASGRP4,WASF2,WNT10B
Negative_4846	ALPL,GALC,OR2AT4
Negative_4847	CD80,DOCK1,IFT57,LEFTY2,NCR3,PRPH,TRAF4,WNT2B
Negative_4848	DDO,GPT,PTS,AACS,ACADVL,ADSS,ARPC1B,ARPC3,ATP6V0C,CD82,CD86,CXCR4,DLL1,DPYD,EGFR,EPHA3,FPGS,GNAS,GNG3,GSTO2,IRS4,MCM7,MYL9,OR4X2,PAK6,PPAT,PPP2R2C,PTPN5,RAC3,SELE,SEMA4F,ST6GAL1,THBD,YOD1,B3GALNT1
Negative_4849	F11,MVK,OSM,ACTG1,AGRP,APOA5,CACNG3,CCND2,CD44,CRY2,CSF3R,CTPS,DDOST,DUSP3,ENO1,ENTPD1,FCGR3B,GFAP,GJD2,GNAQ,GOT2,HNF4A,IL22RA2,IMPA1,ITGA8,MAP3K13,MAP3K5,MAP3K8,MAP4K3,MYD88,OR2A4,OR4N5,OR52N5,PIGQ,PIK3CA,PKMYT1,POLR2L,PPP1R3C,RASGRP1,RENBP,SQLE,STK36,TCF7L1,TDO2,TUBB,ULK1,UNC5A,WEE1
Negative_4850	CKB,F9,PC,ACADM,ACSM1,ADAM17,ALDOB,B3GNT5,BIRC2,CDKN1C,CHAT,CNTNAP2,CTNNB1,DDX51,GALNTL2,GNAQ,GUCY1B3,HNF1B,LRP5,MAPK9,OR10A3,OR10A5,OR1E2,OR4D11,OR52W1,PTPRB,RALB,SEMA3A,SETD1A,SOCS2,SORBS1,STX2,TYMS,WASL,YOD1,ATP6V0D2
Negative_4851	GLS,MSN,STS,TPR,ACAD9,AGRP,ATF2,BST1,CACNA1I,CNTN1,CRY2,DAG1,DARS,EHMT1,ESAM,GAL3ST1,GPX7,HTR7,KIR2DL3,KNG1,MAOA,MAOB,MAP2K6,MARS2,MTHFD2,NFKB1,OR5AR1,OR5T1,OR8D4,OR8S1,OXSM,PAK7,YWHAZ
Negative_4852	ACLY,ACSM5,BTRC,CDK4,CTF1,IDH1,STX10
Negative_4853	DCC,ATP5D,C3orf10,CCR7,CDC14A,CDK4,CLDN6,CSNK2B,CYP4F3,DDOST,DTYMK,GAL3ST1,HCST,HMBS,IMPDH1,KLRD1,KNG1,MAPK3,MARS2,MCAT,MYH4,OR51G1,PARD6A,PDE2A,PIPOX,PRKACB,RAD51,TBXA2R,TFPI,C1GALT1C1
Negative_4854	DDC,IL8,KHK,SRF,AASS,ACSS3,ALDH1A3,CCL20,CD40LG,CLCF1,CYSLTR2,FOXO3,FPGS,GADD45B,GCLC,GUCA1C,HTR2A,IKBKG,IL11,IL18,IMPDH1,INPP5B,MAP3K1,MAP3K3,MAP3K4,METTL6,MYD88,OR10R2,OR1D2,OR2M5,OR4P4,OR52I1,OR8H1,OR8J3,PAPSS1,PLCE1,PRKG2,PRPH2,RASGRP1,SLC2A2,ST3GAL6,TNFRSF8,TNFSF8,TSC1,PDCD1LG2
Negative_4855	SCD,ACVR1,AMDHD2,AXIN1,CACNB3,CXCL10,DHCR24,GPAA1,HLA-G,MAT1A,MYH8,OR51F1,OR52N2,SEMA3A,SMAD3,ST8SIA5,TJP2,XCL1,C1GALT1C1,SUV420H1
Negative_4856	FGA,FUK,HK3,PVR,B3GALT4,BMP7,CAV2,CHEK1,CLDN5,CSNK1G2,DDHD1,EPOR,FGF7,GLI2,GNMT,MAPK8,NKX2-2,OR14C36,OR2B2,OR2C1,OR5B3,PAX4,SOAT1,SPTAN1,SRD5A2,STX8,TACR1,VAV3
Negative_4857	IL9,OTC,BUB1B,CANT1,CLTCL1,CYP2C19,DHDH,ENPP3,GALNT13,GNAZ,INPP4B,IRAK2,KLRC3,LCP2,LYZL1,MAN1B1,MAPK3,MAPK8,OR2M4,OR5H6,PNPT1

Negative_4858	IL5, LAT, RET, ACOX1, APAF1, CYP2B6, DTX3, EFNA4, EFNA5, EPB41L1, FGF22, FUT2, FZD1, IFNA7, INPP4B, IRAK1, ITGA6, ITGAM, JAK3, MTMR6, MYH8, NRXN3, OR2L3, PIGK, PLAU, POLR3A, PPP1CA, PPP2R5B, RUNX1T1, ST3GAL4, TNFSF12, TP53, TUBB, VTI1B, SERPINF2
Negative_4859	CSK, PXN, ADORA2B, ATP5F1, CCKAR, CES7, CSNK2A1, CYP7A1, GCLM, GSTO1, HTR4, IL12A, ISYNA1, ITGAL, ITPK1, MYH8, OR13C2, OR13F1, OR52N2, OR8B4, PHKA2, PNPO, PTTG1, SETD1A, TBL1XR1, TPMT, TRAF3, TUBB2A
Negative_4860	INMT, POLR1D, SLC27A1, UROD
Negative_4861	DBH, FGB, AGPAT1, AKR1C4, AOX1, ARAF, CBR3, CXCL12, CXCL13, FZD4, GLUD1, GOSR2, GUSB, HMGCL, IL19, MARS, MTHFR, NQO1, OR10T2, OR13C8, OR2A14, OR2A2, OR4C6, PGAM4, PGM1, PLXNA1, POLR2G, PTGIS, SMAD2, STAT2, UGT1A10, VEGFA
Negative_4862	ABO, CSK, MAX, ALDH1L1, ALPPL2, ATP5I, ATP5J2, BBC3, CHAT, CRY2, CTNNB1, CYP26C1, CYP3A7, EPHB1, FLOT1, GNPAT, GRIA2, HAO1, IQGAP1, MAP4K3, MBL2, MCM3, NMNAT1, OR4M2, PDGFA, PIGB, PLAU, PPA1, TUBB2C, PAFAH1B1
Negative_4863	AK7, DCK, EZR, ACVR2A, ALDH1A3, ARRB2, ASCC3, CALML6, CASK, CCDC6, CCND3, CCR10, CD36, CD99, CRLF2, DARS, DLL1, ESPL1, FZR1, HNF4G, ITGAE, ITGB6, LLGL2, NTF3, OR1N2, OR4F6, PIP5K1A, PRLR, RBPJL, SORBS1, SPAM1, ST3GAL6, TAS1R2, ULK3, ZAP70
Negative_4864	C3, F7, ID3, MPZ, ACTG1, ADRA1B, B3GAT3, BUB3, CDC25A, CHD8, CREBBP, DLAT, EPRS, FOSL1, HTR7, IFIH1, NCR1, NKX2-2, OR2L8, OR51B2, OR51B5, OR52N1, OR6C76, PGM1, PI4KB, PON1, RPE65, SNW1, SUCLA2, SULT1E1, TPI1, TUBA8, UCKL1, TNFRSF17
Negative_4865	DLD, ACOT12, ATG7, BMPR2, CLDN4, CSNK2B, FPGS, GFPT1, HRH2, INPP4B, LAMB3, MAP3K14, OR2AT4, OR5C1, PARD6A, PISD, PRPS2, SETD7, STX10, TSHR
Negative_4866	GPT, IDS, MIF, ALPP, DPM2, EARS2, ECHS1, GNAQ, GRIA1, IL15, IL17B, LAMB3, MYH13, NOS1, NOX1, OR10J3, OR14I1, OR1I1, P2RX7, PDE3B, PDE6G, TSLP, VDAC2, ST6GALNAC4
Negative_4867	SCD, ACO1, APC2, ARPC3, CCL25, DDOST, FGF21, MTFMT, TUBA8, UROS, WNT3
Negative_4868	RB1, ACP6, ADRB2, BUB1, CCNA2, CHRM1, CYP11B2, DGAT1, GLG1, ICAM2, SUCLG1, SV2A, TUBA3E, TUBA4A
Negative_4869	CS, DCC, FUK, RB1, AK3L1, ANAPC4, ATP5F1, CCL7, CDC6, CDK2, CYP4A22, DAD1, DHRS9, EHMT2, EXOC7, FZD9, GADD45G, GALNT7, HSD11B2, IL3RA, INPP5B, JAM2, MMP7, OLAH, OR56A3, OR5P3, OXCT2, PANK1, POLR3A, PPP3CC, PTPRB, RPSA, SCLY, SCP2, SORBS1, TALDO1, TAS1R2, TNFRSF11B
Negative_4870	AGK, IL6, ACSM4, ALPI, ALPP, B3GNT4, C1GALT1, CACNA1G, CCL20, CCNB1, CDH3, COL4A1, EFNB1, ENPP2, GLUD2, HLA-DOA, IL20RA, LIMK2, MYH4, NAGA, OR1J1, OR51G2, OR52I1, P2RX3, PGAM1, PIK3R5, PPBP, PPCS, PTGER1, PTK2B, PYCRL, RPIA, SKP1, SKP2, TSNARE1, UGT2B7, ULBP2, ST6GALNAC1
Negative_4871	CR1, CSNK1A1, CXCR3, DGUOK, GALNTL4, GLUD1, GPD2, IAPP, MITF, MS4A2, OLAH, OR10G4, OR4K17, OR8G1, P4HA3, PRPH2, SLC27A4, STX8, WARS, WNT9A, ZBTB16, ALDH18A1
Negative_4872	ACYP2, BACE1, BAIAP2, CACNA1H, CKMT2, GGCX, IFNGR1, MAP3K13, MYD88, OR52D1, PFN3, CACNA2D4
Negative_4873	AK2, CS, ACTG2, ACYP2, ARSE, CLCA4, COL3A1, DTX4, FZD3, LRAT, OR4K14, PRKCH, RAC2, STX4, SULT1A4, TAS2R1, ULK3, XCL2
Negative_4874	CACNG3, DPYD, GRIA2, IL28A, INPP4B, MYH9, RDH10, SMPD1
Negative_4875	CD6, GHR, SHH, ACMSD, ACVR2B, ALPI, BDH1, CACNA1B, CACNB4, CCR6, CDC25B, CNGB1, CPOX, CYP2E1, DTX2, ERBB2, FABP4, FZD4, GNA14, GNPAT, GRM1, HAP1, LAMC3, LIMK1, MAML2, MAPK8, MYD88, PAX8, PIGL, PNMT, PPA1, RFXANK, SPCS3

Negative_4876	ACAT1,ALDH6A1,AVPR1B,CD72,CDH15,CDO1,CER1,CSF2RB,DDX41,ENTPD5,EPHB6,FTH1,GPD2,GZMA,HLA-G,IL13RA2,IMPDH2,MAP3K2,MOV10L1,NTSR1,OR14A16,OR2G3,OR2T34,OR2T5,OR4C6,OR4F17,PAX4,PCCB,PFKFB1,PIGN,PSEN1,PSPH,SEMA4D,SFRP2,SHISA5,ST6GAL1,TAS2R60,UAP1,UGDH,ATP6V1E2
Negative_4877	JUN,KLKB1,LAMB4,PLA2G4A,POLR2I
Negative_4878	C8G,CTH,FUK,GCK,IL4,ADIPOR2,ARNTL,B4GALT7,BAAT,CACNG5,CMPK2,CPOX,DDX52,DEGS2,GNA11,GNAQ,GSTK1,GSTZ1,GUK1,LYZL1,MAPK11,MAPK7,MGAT4A,NRG3,OR1J2,OR4D1,OR52N1,OR5J2,OR8D1,PIK3R3,PRKAR2A,PRKX,RALA,RPSA,SH2D2A,SMURF1,TIRAP,MAPK8IP2
Negative_4879	INS,AASS,ADCY10,ADSS,CCNE1,CDS1,CLDN9,CREBBP,CSF2RA,CSNK1D,CYBA,DUSP16,DUSP6,EPHB6,FOXA2,GADD45B,GTSE1,IL20RB,INPP4B,IQGAP2,ITGA2,KAT2B,MAN1B1,MAPK1,MINPP1,MMP14,MYH11,NAT2,OR10G8,OR51V1,OR5I1,OR5M9,PDE9A,PPP1R3C,PRNP,RAB5A,RFWD2,UBE2L6,ULBP1,WNT2,ATP6V1E1
Negative_4880	C1S,HGF,AASDH,ADIPOQ,ALG1,ARHGEF4,ATP4B,AXIN2,CD74,CDS1,CHRM3,CMBL,CSAD,CTNNA3,DDX47,FGF17,FOXO1,GNRH1,IL23A,INHBB,KIR2DL4,KLRC3,NLGN3,OR13C5,OR2M2,OR8K1,PRKCH,PSAT1,RASA2,RASSF5,RPRM,SAT1,SCIN,SETD2,THBS2,TLR9,TRAF1,UGCGL1,WBSCR22,PPP1R12A
Negative_4881	CARS,CCL3,FBP1,GCH1,HSD17B8,INHBA,KCNJ11,OR7G2,OR8H2,POLR1E,PPP2R5E,SCLY,UGT2B4,XYLT1
Negative_4882	DDO,ACP2,ACY1,ADCY3,B3GNT3,BMPR1B,CACNA1F,CCNA1,CD40,CD44,CDC14A,CFL2,CXCL13,CYP2C19,CYP3A43,DGAT2L4,F2RL3,GAS1,GBA3,GDF5,GSK3B,HIBCH,LYPLA1,MAD2L2,MAML2,MAT1A,MYLK2,NR1D1,OR1D2,OR4F17,OR5D18,OR8B8,PDE7A,PRKAG3,RAP1A,RASA1,SMPD4,SUV39H2,TRPM5,UGCG,ULBP2,UROD,UXS1,WNT5B,ALDH18A1,ATP6V1C1,MAPKAPK5
Negative_4883	DUT,JUN,OSM,ACSL5,ARHGAP5,ARPC1B,ASCC3,CCL5,CCNG2,CES7,CSF1R,DGKI,EPB41L3,FGD1,G6PC2,GALNT3,HDAC2,HMOX2,IFNGR1,KLRC1,NT5C,NTF4,OR13A1,OR3A1,OR6C6,PHKB,PPM1A,PRKACB,RDH16,RPSA,SETD2,SPRED2,SQLE,SSH2,STK11,TAS2R60,TUBB1,TUBB4,UGCG,WBSCR17,XCL1,SERPINE1
Negative_4884	AK5,APC2,APH1A,ATP5G3,CISH,FRAP1,GLB1,GNAI2,INMT,MYH7,PFKP,TRDMT1,MAP3K7IP2
Negative_4885	AGK,CRH,DDO,F5,FAS,GSS,CACNG4,CARS2,CCKBR,CD82,CRY1,CTSG,CYP2D6,DPAGT1,ECHS1,FASLG,FGF21,FUT4,GALNT2,GCDH,GNA15,GSTA3,IFT57,IL20RA,INPP5B,LARS2,NEUROD1,OR2F1,OR51I2,OR56A1,OR5D18,PGAM1,PLA2G2D,PPM1D,RUVBL1,SHC1,STX5,SULT2A1,TAS2R13,TSHB,WNT8A,ATP6V1G2,TNFRSF13B
Negative_4886	ADK,DLD,EZR,GLA,IL3,SI,ATP5O,CCND3,DTX1,ENO2,FGF12,FRAT2,FZD10,GARS,GLI2,GSTM1,IL28B,LYPLA2,MYL5,NPR2,NUDT12,OR11G2,OR5B12,OR5T2,PER1,PFKM,PIK3R2,PLAU,POLR3D,PRKACB,SDC2,STAT1,XCL2,PAFAH1B2,ST6GALNAC6
Negative_4887	HK1,MAG,ACTA1,B4GALT4,CACNA1C,CCNB3,CSDA,EGFR,FGF11,GGT7,GRIN2C,LCP2,NCR2,NFKB1,NOS1,OR10T2,OR8J3,PARVB,PLTP,SCP2,SETDB2,TALDO1,TKTL2,TPK1,VARS,WHSC1L1,CACNA2D1
Negative_4888	ALG3,BCL2,CDKN2C,CSNK2B,GMPR,HSPA1B,LGMN,SC4MOL,STX5,TNFSF4,UGT8
Negative_4889	BTC,C1S,PXN,ACP6,ALDH1A2,ATP5B,BDH2,BMP2,CHEK1,CLDN16,CLDN4,CLOCK,CSF2RA,CXCR5,DKK2,ELK1,EPB41,ETS1,FUT3,GGT6,IFNA2,ITGA9,MKNK2,MYD88,NCF4,OR10G7,OR1F1,OR2M4,OR4E2,OR5M9,PARD6A,PECAM1,PGK1,PIAS2,PIGV,PLCZ1,POLE,PRSS2,SKIV2L2,SLC9A1,SOCS4,SOS1,TAS2R60,THBS4,WASF3,MAPK8IP2

Negative_4890	A2M, ALOX12B, AMHR2, AMPD1, CAMK2A, DAAM2, DPM1, DVL1, GAS1, GMPR2, MCM4, MGST2, NAE1, NEU3, OR14I1, OR2AE1, OR2T8, P2RX4, PFKFB2, POLR2I, PRDX6, PRKACA, PRNP, RASA2, ROCK1, SDHC, SEMA3G, SGPP2, TAP2, TRDMT1, VDAC3, ATP6V1B1
Negative_4891	LEP, ACP2, ACVRL1, ALDH3B2, CD19, CLDN18, GFAP, ITPKB, KARS, MAP2K3, MGAT5, OR10J1, OR52W1, OR5B3, OR5C1, PMAIP1, PRIM1, PTGES
Negative_4892	GLA, ITK, ABP1, ACAD8, ACOX2, AHCY, ATN1, BIRC2, CACNG7, CADM3, CLTC, COL4A1, CTF1, CYSLTR1, DLL1, GAMT, GNB3, LPAR1, MAGI3, MAML1, MAP4K3, NCF4, NRG3, OR10G4, OR13C5, OR2B11, OR4M1, OR8D4, PANK3, PDE4B, PGK1, POLE3, PTK2, PTPRJ, RDH5, SORD, TACR1, TGFB1
Negative_4893	PGP, AVPR1B, BDNF, CTSB, FGFR3, HIBCH, IFNA4, OR4X1, OR5B21, SEMA4G, SPRED1, TUBA1B, CSGALNACT2
Negative_4894	CRH, IL4, SCD, SRF, SYK, TKT, ADAM17, ALG8, CDKN1C, CHAT, CRB3, CSNK1A1, CYBA, CYP11B1, DPM3, ERBB4, FADS2, GARS, GLB1, HDAC2, HMOX2, MAT1A, MGST2, NOX3, NT5C2, OLR1, PAK4, PIK3C2B, PLCB2, SHMT1, SOS1, STX12, TIAM1, WIF1
Negative_4895	C4B, GDA, ADCY4, ADCY7, ANAPC7, OR9I1, PAK4, PANK1, PARD6B
Negative_4896	APP, EBP, HK3, MPI, ALDH7A1, APOE, AREG, ARPC2, ATP5E, CACNG3, CAV2, CD28, CLDN22, COQ6, CXCL12, DAXX, DTX3, ENTPD2, GLI2, GMPPB, HTRA2, IL28RA, OGDHL, OR10A5, OR14I1, OR2W1, OR4Q3, PAICS, PIGC, PLCZ1, PNLIP, PPP1R1A, PRKG1, PYCRL, RPSA, SEMA3C, SHISA5, STX1A, SUV39H2, UGT2B28, UGT2B7, YARS2
Negative_4897	LCK, WAS, ATP12A, BCKDHA, C3orf10, CBLC, CYP1A2, DFFB, DHRS4L2, DTX3L, FLOT2, FOSL1, GRPR, GUCY1B3, ICAM1, IL5RA, INHBC, ITGB6, LTBP1, MAP3K1, MCCC1, NCR3, NFAT5, OR10C1, OR52K2, OR52L1, OR5T3, OR9K2, PPP2R5D, PTGER1, RASGRP1, RDH12, SELE, TAS2R50, TRAF4, YKT6, TNFRSF13C
Negative_4898	GPD1L, HNF1A, PAK6, POLR3D, PPP3R2
Negative_4899	AMH, F10, GSN, ADRA1D, AKR1C4, AQP7, ATF2, BLVRB, CACNA1S, CAMK2D, CLDN6, DHCR24, ECSIT, GLUD1, IFNAR1, ITGB8, MDM4, METTL2B, NADSYN1, NAGLU, NMNAT1, OR2A42, OR2M4, OR4S1, OR51B6, OR52A1, OR52N5, PLOD2, PNPO, POLA1, POLR1A, SCP2, THTPA, UGT2B17, VANGL1, WNT10B
Negative_4900	C7, ACAA2, ACOX2, AKT2, ARPC4, ATP5O, CACNG2, CBLB, CLDN5, CRLS1, ECHS1, EFNB1, ENTPD1, FMO3, GAL3ST1, HPSE, ICAM1, KIR3DL2, KYNU, MCM6, NCF4, NR4A1, NRG1, NTNG1, PCK2, PFKFB2, PRKAA1, PRKAR2A, PRSS3, STX5
Negative_4901	LEP, AKR1D1, APBB1, CDK5, CYP2D6, KIR2DL1, NFAT5, PFKL, PPP1CC, PTGFR, RDH11, RHEB, RXRB, SOD1, SOX17, ST3GAL2, STX1A, TAP2, TAS2R9, UBE2K
Negative_4902	LIPG, NFYB, PRKCQ
Negative_4903	RET, CES2, CHAT, E2F1, FZD10, GPX5, IL21, ITGB7, ITPKB, JAK3, LEFTY1, MARS, NME7, PTGIS
Negative_4904	DDC, NNT, ACAT2, CACNA1S, CD40, CDC6, CNDP1, CPT1A, CXCL2, CXCL5, DDOST, DDX47, ERBB4, FGF1, HSD17B7, IARS2, IL10, IQGAP3, LIMK2, LRRC4C, MBL2, OR10A7, OR11H6, OR4B1, OR4F15, PDGFRB, PIK3C3, PPAT, PPP2R1B, SEMA3E, SLC27A4, TACR3, TAS2R41, UPP1, VDAC2, ZFYVE9, PNLIPRP1, TNFRSF11B
Negative_4905	ABCC8, CAMK4, MYH15
Negative_4906	CD8A, CHRD, DDX41, GNRH2, OR1J2, OR9G1, PLD1
Negative_4907	FOS, GAA, ACSM2A, ALG6, ATP2A2, BCL2L1, CDC26, CHKB, COL4A1, COX15, CSNK1G2, EPHB6, FLAD1, HMOX2, LAMC3, LRRC4C, NEU3, NFYC, OR10H5, OR2C3, OR2T34, OR6N1, OSMR, PDE6H, PFKL, POLE, PYCR1, RPRM, RRM2B, SDHB, SELPLG, SMAD9, SULT2A1, SV2B, SYNJ2, TAS2R49, TCIRG1, WASF3

Negative_4908	PLN, ACACA, ACY3, AKT1, ALOX12, ANAPC4, ATP2A3, B3GALT4, BCKDHB, CASP3, CLDN2, CLDN3, CREB3L1, CSNK2A2, DUSP16, ESPL1, FGF21, GAB1, GPX7, GUCY2C, IDH3G, KIR2DL4, LYPLA2, MTHFD1, NCK2, NFATC1, OR52E4, PLA2G5, PROC, RAC3, SCD5, SEMA3F, SETD8, SMAD6, SULT1A2, SV2A, TJP2, TNFRSF9, TRAF4
Negative_4909	AK1, MSN, ACSM4, ALOX5, BDH2, CD28, CXCL5, DPM1, FZD2, LARS2, OR6A2, PPAP2C, PPP1CA, PTGDS, SHC4, ST3GAL3, UGP2, ULK3
Negative_4910	KHK, NGF, ASRGL1, BBC3, CCR8, CDK7, CHRD, CYP2D6, DHCR24, DTYMK, ELK4, FSHB, FZD3, GDF7, GSTO1, IFNA14, IL11, ITGAV, KYNU, LMO7, MAD2L1, NFATC1, NRG3, OR1J4, OR2H2, OR52A1, OR5M8, PCCA, PCCB, PDE4A, POLR2E, PPP2R5E, PTGS2, PTK2B, RBKS, SPCS3, STX12, TSNARE1, UGT2B17, WWP2
Negative_4911	ACVR2A, ALDH1B1, CACNG6, CD3E, CDC14A, CLDN19, CXCR3, ENTPD2, ENTPD6, ENTPD8, GMPPA, ITGA5, KITLG, OR10X1, OR2F2, OR6A2, PSME1, RUNX1T1, SARDH, STX8, TNFSF9, TPH1
Negative_4912	CSK, EGF, IL9, ABLIM3, ADCY3, ATP6V1H, CLDN17, COL3A1, CYP11B1, DDOST, DGAT2, DVL2, GALNTL4, KITLG, MAN1C1, OR4M2, ORC6L, P2RX7, PAK2, POLR3G, RUNX1, SFRP4, SHC4, SLC33A1, STAT4, XYLB
Negative_4913	NLK, ACSM5, ALG2, CCR4, CDKN2A, HMGCR, IL20RB, ITGAV, MYH10, NCK2, PIAS3, PPARG, PPAT, RRAS, SPRY4
Negative_4914	CRH, MAX, OTC, ACACA, B3GALT4, BBC3, CANT1, CHRNA7, CXCL2, DGKQ, DPM3, HPSE2, IL17A, ITGA4, NFATC4, OR52D1, STX18, TBL1XR1, TRIP10, VKORC1, VTI1B
Negative_4915	CAT, GK2, MPL, MYC, CYP11B2, DNMT1, E2F3, GALNT11, LTB4R2, MYH10, NEGR1, OR11H4, OSMR, PIGT, PLXNC1, PNLIP, PVRL3, RALGDS, RETSAT, ROCK1, SLC12A2, SRD5A1, UROC1
Negative_4916	ACOT11, CREB3L2, SAT1, SH3BP2
Negative_4917	WAS, ASCC3, CACNA1G, CHSY3, CSNK1A1, FRAT1, GNB1, KCNB1, MGAT1, NEU1, OR10A7, OR2F1, OR8A1, ORC1L, RASSF5, TNFRSF11B
Negative_4918	AADAT, EXOC7, KIR2DS5, NRCAM
Negative_4919	GP6, KHK, ALG9, ATP5B, BCMO1, BMP4, CALML6, CAMKK2, CCL8, CMPK2, CTNNB1, CYP2A6, DUSP10, ENPP6, FGF4, GLUL, KIR3DL3, LDHD, NRP1, OR2T1, OR3A3, OR4S1, OR6C75, PLCE1, PMAIP1, PPID, PPP2R5E, PTPRZ1, RXRG, SEMA3A, SREBF1, TFDP1, TFPI, TJP3, VCAM1, WHSC1
Negative_4920	IDS, MUT, PXN, SMO, TFG, TPR, ANAPC7, ATP5G2, CACNB2, CCL25, CMAS, CSNK2A2, CTSS, CYP3A4, CYSLTR2, DDX4, DOCK1, ERBB4, FTMT, GAPDH, GARS, GMPR2, GPAA1, IL25, MAOB, MASP1, MYLK, NPR2, OR2L2, OR5AR1, OR6C3, PRPH2, RPN1, RPS6KA6, RXRG, SCD5, SEMA7A, SFRP2, ST3GAL5, TUBB2A
Negative_4921	CAD, CD4, CR1, DBI, GH1, GH2, CACNG7, CALML3, CASP3, CDO1, CREB1, EXT1, GALT, GRPR, LCMT2, MYH14, NAT5, OGDHL, OR1L3, OR4K13, PIK3CD, PIK3R2, SETDB1, SMAD4
Negative_4922	EGF, PLN, ACAT1, ACOX3, ACP6, ADCY7, AHCYL1, C9orf95, CAMKK2, CHUK, CLTA, EI24, EPHA6, EPHX2, GALNT10, GARS, GNG13, IL20, INPP5B, LAMB3, LRP5, MYH10, NCAM1, NCKAP1, NCR2, OR1D2, OR2T10, OR51G2, P2RX2, PNPLA4, PRPS2, PYGL, RBL2, SMARCA5, SOX17, UGT2B28, VAV3, CSGALNACT2, TNFRSF10A
Negative_4923	C3, DDC, LCT, MVK, SRM, TFG, CDC25C, CDK4, COL5A1, CYSLTR2, DDX18, EPB41, GSTO1, GSTT1, IFNA5, LAMC3, OR52I1, OR5AU1, PDE4C, PIK3CA, PIK3R2, STAT6, STX1B
Negative_4924	AMDHD1, CXCR3, INPP1, OR10A5, OR2T12, PLEKHA8
Negative_4925	DBI, MVD, ACLY, ARHGEF4, ASNS, CHEK2, CLTB, CTF1, DEGS1, DGKE, EXOC7, FGF9, GAPDH, GLO1, HCCS, HLCS, HPSE2, IL12RB1, IL13RA2, JAK3, MAP4K1, OR4D2, OR8B4, OR8H3, OXTR, PERP, PPP2R1B, PTGS2, PTPN1, STMN1, ZFYVE9

Negative_4926	BTK, SI, ATP5C1, DHDH, GPAM, LEFTY1, LIPE, MKNK2, NEFH, NEU1, RPRM, SULT1E1, TAS1R1, TAS2R41, TUBB4, UAP1
Negative_4927	FGB, SRM, ARPC2, CAMK2G, CCND1, CLDN17, ENTPD4, FABP4, GSTA1, IL13, LDHB, MAD2L2, MAN1A1, NCOA4, OR1J4, OR2Z1, OR4A15, OR52N2, OR5B17, PDGFD, PISD, SGMS1, STX1B, TLR4, TRHR, ATP6V0A1
Negative_4928	ADRB2, ATP6V1A, BCKDHB, BPGM, C3orf10, CLDN7, DGKB, DLST, ENTPD1, GDF5, GNA11, HLA-DMA, IRF3, MYH15, OR4D11, OR51B6, OR8D2, PDXK, PHKG1, PIP4K2A, PLA2G2E, PLCB1, POLR2I, PPP2R5E, RPS6KA6, SEMA3F, SEMA4G, STX1B, TACR3, TAS1R2, TUBA1B, VEGFC, WNT10A, CACNA2D4
Negative_4929	BID, ALG11, AMD1, CDC16, GALNT10, GMPR2, OR4C12, ORC4L, PLA2G2E, SHISA5, ST3GAL2, EIF4EBP1
Negative_4930	ADC, GDA, GH2, AGMAT, ARSB, BDH1, CXCR5, CYP2E1, GMPPA, GNB1, GRIA3, HIF1A, HRH2, HSPA8, IL17B, IL24, PDE8A, PTK2, RAPGEF1, ST3GAL4, TPM3, TUBB2A
Negative_4931	AUH, C4B, DBT, LSS, ALPL, ARNT2, ATP5L, CCL23, CCR4, CIITA, CLCA2, CYP4A22, EDNRA, ENO1, ENO2, GSTO2, KIR2DL2, KIR2DS5, MARS2, NKX2-2, OR4F15, OR5T2, OR8B4, ORC2L, PDCD1, PDGFC, POLR1D, SH2D1B, SOAT1, SULT2B1, UBE2K, ZFYVE16, ATP6V1B1
Negative_4932	F7, OTC, ACSL3, ADRA1D, AHCY, ALG5, AVPR1A, CCL3, CHEK1, CPS1, CTPS2, CTTN, DHODH, EPHB1, EPHB2, F2RL2, FUT1, GBE1, GRAP2, HHIP, IL12A, ITGA2, LTBP1, METTL6, NOX1, OR11G2, OR2H2, OR2Z1, OR4F4, OR4X2, OR5A1, OR5AC2, OR6N2, PGS1, PRKCZ, RAD51, RAPGEF3, RHOA, RUNX1, SPHK2, STX10, TRAF3, VANGL2, ATP6V0A2, HLA-DQB1
Negative_4933	ADO, GP5, LIF, SMS, UBB, ACVR2A, ADCY1, AMHR2, ATP5G3, CANT1, CREB5, CSNK2A1, CXCL16, ESPL1, GMPPA, HLA-DRA, IFNGR1, IL12B, MAN2A1, MAP3K5, NLGN1, OR1I1, OR5AK2, OR6A2, OR6C74, PDE1B, PIGP, POLR2C, PPP3CB, RAD51, RBKS, RFNG, TNFSF9, TRAF6, TUBA1B, UXS1, VAMP3, VAV3, WNT11
Negative_4934	INS, ATP5G3, BAIAP2, DARS2, FABP3, GALNT6, IDI2, IL20RB, MYH14, OR10H2, OR8H2, PDE4D, PTGIS, RFX5, STAM2, YES1
Negative_4935	AK5, EBP, ADRB2, ASRGL1, BACE2, CD40, CFL2, CLDN9, CXCR6, EPHA4, FRAP1, GFPT1, GOT2, HDAC2, HLA-DOB, HSD3B2, IFNA6, IL1R1, IL20RA, ITGB7, MAGI2, MGAT2, MGST1, MKNK2, MYH7B, NFATC2, NRCAM, NT5C3, NT5E, OR1F1, OR1J4, OR2L8, OR4D9, OR6C75, OXTR, PCK1, PIK3CA, PIP4K2C, PPCDC, RASA2, SULT2B1, TAS2R14, TAS2R60, UGT2A3, VKORC1, PLA2G12A, ST6GALNAC6, TNFRSF1B
Negative_4936	SMS, ADSSL1, CDK5, CKMT2, CTSL1, DBF4, FOSL1, GNA14, MAOB, NCF4, OR4K1, OR8U1, PRKAG1, SC4MOL, STX12, VDAC3
Negative_4937	MAX, ALOX15B, ATP2A2, CACNG5, CADM1, CDC6, CXCL2, CYBB, FOSL1, G6PC, GALE, GALNT7, GLI3, IL8RB, ITGA3, MAT1A, MPST, OR13C8, PI4KB, PLA2G1B, PRKAR1A, PRPS1, RAB23, RRAS2, SPCS1, TAS2R42, TFPI, YWHAE, TNFRSF14
Negative_4938	C5, SI, ALS2, BDNF, BMP2, CHAT, CKS1B, GLS2, GSTM4, OR52E4, RPS6KA1, SRD5A1, SRGAP3, VDAC2, ATP6V0D1
Negative_4939	AUH, KIT, ADCY8, ATG12, CALML6, COL4A2, DDX23, EP300, FRAT1, HTR4, MCM2, PDE4C, POLE2, PPP2R5A, STK36, TCF7
Negative_4940	F11, ACTA2, ADCY9, ATG7, CYP2S1, FGF11, GALNTL5, GAPDH, KLKB1, MAD2L2, NEUROD1, OR2AG2, OR2H1, OR6B2, PARD6A, POLR2A, SORBS1, TSNARE1, UROD, WNT3A, MAP3K7IP2

Negative_4941	AMH,DLD,GGH,GLS,LAT,ACTB,ADCY5,ALAD,ANGPTL4,BDKRB2,CCND1,CHPF,COL5A1,CPT1A,CSF1R,DUSP14,ENPP7,EXOC7,EXT1,FGF17,FGF20,HARS2,HYAL2,IDH3B,MAOA,MCM2,MYH11,OR10T2,OR14I1,OR4D2,OR5M8,OR8H3,PIGA,PIP4K2B,PLD2,POLE,PPP2R5D,PRKAG3,SMURF1,SNAI2,SNAP25,SRGAP2,STX12,SUV420H1
Negative_4942	C4B,HYI,RDX,AARS2,AMDHD1,ARHGEF4,ATP6V1H,BLNK,BPNT1,CSF2,CYBA,DRD1,FBXW11,FTH1,FZD8,GCNT2,HBEGF,HSP90B1,IDH3B,INSR,JAK3,LTB4R2,MGAT2,MYH1,NCKAP1L,NMNAT3,OR11H1,OR2G6,OR51D1,OR51E1,OR7A5,OR8K1,ORC2L,PLK1,PRKAA2,PRKACA,PROC,PTGES,RARS,SMPD2,SYNJ1,WBSCR22,KIR2DL5A,PNLIPRP1
Negative_4943	SRF,SRM,ACPP,ATP6V1F,CKMT2,CYP4F2,GUCY2C,HLA-C,HNF4A,HSPA2,LTBP1,MIOX,OR2AG2,OR4F17,OR52K1,POMC,PTGES,RENBP,RUVBL1,SKP1,WHSC1L1,XYLB
Negative_4944	AGL,FES,FST,ID2,IL6,ALDH1A3,ANAPC2,ARNT,BNIP1,FGD3,FGF3,FZD4,GRIN2D,GUCA1C,HSD11B1,HSD17B8,HSPA1B,IFNA5,IPMK,ITGA6,JAM2,MGST3,NCR2,OR10A6,OR6B3,OXSM,PDIA4,PRDX6,PTPRC,RALA,RAP1B,SETDB2,SULT2A1,TLR3
Negative_4945	BAX,IL5,TNN,ALDH3B2,ARNT,BCKDHB,BIRC3,CACNA1A,CACNA1E,CCL13,CCL28,CCL4,CD8B,CDKN2A,CRB3,EIF4E,GPT2,IFNAR2,LAMC2,LEFTY1,MAP3K14,NOTCH1,OR4C16,OR51V1,PARD6A,PARS2,PARVA,PDE1C,PFKFB1,PGM2,PLCB4,PSAT1,RUVBL1,SCIN,SFRP1,SPP1,TIAM2,WWP1
Negative_4946	AMT,LCT,C4BPA,CACNA1I,CYP2D6,EXTL1,GALNT3,IFNGR2,LDHC,OR10H4,RFXAP,SOS1,UGT2B4
Negative_4947	EXOC7,FCER1G,GNAS,OR4Q3,OR8B4,TBK1
Negative_4948	BTD,F12,SRC,TNR,ACTG1,ADORA2A,BDKRB1,CREB3L1,DNMT3A,GPD2,GZMB,OR10K2,OR2D2,OR2M3,OR4D9,OR4P4,ORC4L,PPP3CC,RAC3,SETDB2,SLC33A1,SYNJ2,MAPKAPK2
Negative_4949	AGL,ABLIM3,ABP1,B4GALT2,CACNB2,CASP9,DKK2,DNMT1,DPYS,EPB41L3,GANAB,GBE1,IDH1,LCMT2,LEF1,MAP3K4,MGAT1,ODC1,OR13C9,OR51B6,OR7C1,PDE8A,PFKFB2,POLR2A,PRKAA1,SDC3,SOAT1,SYNJ1,VKORC1,HLA-DQB1
Negative_4950	AK7,LIF,CDS2,DOT1L,E2F4,FGF13,GALC,ICOSLG,NTRK2,OR10G3,OR4C13,OR4L1,OR51B4,OR8D4,OR8J3,PRKACB,SPCS3,TUBAL3
Negative_4951	BTD,F7,LIF,ABLIM3,ARRB2,B3GNT4,BCMO1,BDH2,CD46,CD59,COL4A2,CYP4F3,DEGS1,DNMT3B,ECHS1,FGF4,FLOT1,HDAC2,HNF4A,HSD17B3,IL21,MPST,NME6,OR10AD1,OR10G7,OR10S1,OR2L2,OR52B6,OR52N5,PDIA4,PPA1,PRKCH,PSAT1,RAPGEF2,RPS6KA5,SIAH1,SRGAP2,SSX2IP,TNFSF9,TUBA3C,ZNRD1,CACNA2D3,PRICKLE1
Negative_4952	ARHGAP5,CCNH,CD47,COQ7,FSHB,HAO2,IFT57,OR10H1,RAC2
Negative_4953	F2,RDX,TPO,AGMAT,CAMK2A,CAV3,CLDN11,CRY1,CX3CR1,FRAT2,FZD5,GSTA1,GUCY2F,HSD17B4,IKBKB,OR2T12,OR2T8,PORCN,PPA2,RBPJ,SARS,SMURF1,WNT10A,B3GALNT1,HLA-DRB5
Negative_4954	ACVR2A,AKR1B1,AKR1B10,B3GAT2,CCL27,FGF2,GZMA,KLRD1,OR10G7,OR2T8,YES1
Negative_4955	VCL,ACAA1,ACVR1C,ASRGL1,BCL10,C3AR1,CACNB2,CCR8,CD55,CYP1A2,CYP26C1,CYP3A7,DUSP6,EPB41L3,FLOT1,GBGT1,GCNT3,GDF7,HMBS,ICOSLG,IFNA10,ITGA7,JAG1,MGAM,MITF,OR13C4,OR2M5,OR2Y1,OR7A17,P4HA2,PDXK,PGAM2,PLXNB2,POLE,PPARA,PTAFR,SCP2,SETD1B,SETDB1,SMC1B,SV2A,UCK2,YKT6,ATP6V1G1
Negative_4956	CSK,WAS,ERBB3,GALNT11,GLB1,GOT2,MAP3K4,MGAT4A,NT5C2,OR52I1,OR6K3,PRPS2,PTGER1,RPS6KA6,SRGAP3,STK36

Negative_4957	ADC,F7,GK,ACADS,ALPPL2,AMDHD1,APOA5,BCL10,CREB3L3,CYP2A6,DDOST,DEGS2,DUSP1,ENPP3,EPHA3,GALNTL5,GBE1,GNB1,GP1BA,GRIN1,GSTM4,GUCY1A3,IL13,ITGAD,KIR3DL1,LLGL1,LRP6,MAP3K1,NFKBIB,OR2T3,OR4C13,PAK2,PIK3R5,PLAT,PNLIP,PRKAA1,PRKAR1B,SCIN,SELL,SHMT2,SKP1,SMAD3,SPTLC1,STAT2,SUCLA2,HLA-DPB1,HLA-DRB4,MAPKAPK3
Negative_4958	FN1,PTS,ACSL4,ITGB6,MAPK7,PDXK,SOAT1,SPTAN1
Negative_4959	AR,C5,CGN,DLD,F11,SMS,ACSS3,ADCY8,AGPAT3,ALG13,CAV3,CCNH,CD8B,CMBL,DTX1,EFNA1,EPHB6,F13A1,FLT4,GAD1,GALK2,GCNT1,GNA12,GSTM4,LMO7,MFNG,MGAM,NCF4,NOX1,OR10K2,OR13G1,OR14J1,OR1S1,OR51V1,OR5B21,PAICS,PAPSS2,PIP5K1C,POLR2F,POLR2K,PRKACA,PRKG1,PRPH,SFRP1,SUV39H1,TP73,VTI1A,HLA-DQA2
Negative_4960	DBH,LPO,NNT,TEC,ARD1A,B4GALT3,CDS1,CPT1C,DHRS4,DMGDH,E2F2,FARSA,FMO3,FSHR,GALNTL5,GGCX,GSTA3,GUCY2D,HEXA,HLA-G,HTR2A,HTR2C,LDHAL6B,LIFR,NQO1,OR51F1,OXCT1,PLA2G5,PPA1,PPAP2B,PPID,PPM1B,RFNG,STT3B,TYMS,WBSCR17,WNT2B,WNT4,PNLIPRP2,SERPINA1
Negative_4961	GLS,HTR2A,KIR2DL2,PRDX6
Negative_4962	HGD,ADRBK2,ADSS,ALOX5,ARPC1B,ARSE,CES2,CHKA,CLDN15,CPT1A,CXCL9,GSTT1,IDH3A,IKBKG,LGMN,MAGI2,NCAM2,OR10K2,OR1L6,OR2A25,PLD1,RASGRP2
Negative_4963	ACSL3,ATP4B,BUB3,CACNA1S,CLCA4,CLDN11,CYP2D6,KLRC1,OR2T12,SESN2,VAMP1,WNT10B,TNFRSF1A
Negative_4964	AR,EZR,ACSL4,ADRB3,ALG10,ANAPC2,APPL1,ATP6V0C,CCL22,CHST11,CLDN3,CPT2,CYBB,DARS,DARS2,GUCY1B3,HTR5A,IMPA2,ITGA10,MYH9,NLGN1,NR1D1,NTRK1,OR10A2,OR4C12,PAPSS2,POLR2L,PPP2R5A,SETD1B,SH2D2A,SPTLC1,STX10,STX3,TAS2R38,TBXAS1,TUBA3C,TUBB,WNT7B,PNLIPRP2
Negative_4965	CR1,PAH,ASMT,ATP13A2,CANT1,DEGS1,IFNB1,IL10RA,MGAM,MGAT4A,MTHFS,MYH9,NANP,OR10J5,OR13G1,POLR2L,PPP2R2D,PRKY,ROBO3,SUV420H1
Negative_4966	GGH,TYR,ALDH3B1,ALG2,CD40LG,COL1A2,CPS1,E2F5,EPB41L1,MTFMT,WNT10A
Negative_4967	GCK,TH,AKR1B1,BNIP1,CDKN2C,CNGB1,COMP,GALK2,GAPDH,GLDC,HMOX2,IFNAR2,MTHFS,OR2A5,OR6S1,RCHY1,SPP1,TYROBP
Negative_4968	IHH,CASP3,GNAQ,GRPR,MAPK1,CTNNBIP1
Negative_4969	C4B,PGD,ADSS,AKR1A1,CFL1,COL5A3,CXCR3,CYSLTR1,DNMT3A,GALK2,GGT1,GMPR2,HPRT1,IARS2,IFNA14,MAP4K3,MARS2,OR11H1,OR1L8,PHKG1,POLR1A,RASA1,SMAD4,SMAD5,STAT2,STK36,UGT2B28,VDAC3,WNT9B,WWP2
Negative_4970	CD2,F2,ACOX3,ACSL3,AKR1B1,ASAH1,CCR10,CRY2,CTLA4,DUSP8,EHHADH,FGF1,FGF19,FTCD,GDF6,GNPAT,GPD1L,IL13RA1,ITGA11,MAP3K4,MCM5,NFYB,OR2B11,OR4A5,OR4N5,PIPOX,PKM2,PLXNB2,RGS3,SDHD,SOS1,TP53,TSNARE1,TSTA3,UBE2L6,WASF1,YWHAH,PAFAH1B1,PRICKLE1
Negative_4971	ANAPC10,CSNK1D,DDX23,IL1A,ITGAX,OR5H2,PSPH,STX4
Negative_4972	AGPAT2,LIPA,PLXNB1
Negative_4973	CAT,LEP,ADIPOR2,ALDH3A2,CACNG1,CDC25B,DHX58,EGLN3,FSHR,GAS1,HIBCH,ITGB8,MCM7,NOTCH2,NR1D1,OR2C1,PLCB3,RARS2,VEGFC,ATP6V1C2,SERPINE1
Negative_4974	DBH,EPO,IHH,TK2,ALDOC,AMY2A,BET1L,CALML5,CDKN2A,CHST13,DGUOK,GALNT6,GOT1,IGSF5,IL7R,ITGA1,LILRB3,LIMK2,MDH2,NRG2,OR5AN1,PDE11A,PDGFRB,PPP1CB,PRKCA,RARS,RPRM,SCLY,UGDH,MAPK8IP1

Negative_4975	C8B, GP9, ACOT12, ACY1, ALCAM, ARRB2, CCR6, CD40, CDC45L, CHAD, CXCL5, CYP7A1, DHCR24, EPHA1, ERO1L, FGF23, GATM, GNG13, GPX7, GSTA1, GUCY1A2, HTR2A, IARS, IL23A, IL5RA, ITGB7, KLRC4, NKX3-1, OR1J4, OR4M2, OR52M1, OR5M8, OR8H1, PER3, PFKL, PLA2G10, PRKCB, PRKCQ, PRKX, RAPGEF2, ROCK2, SPTLC2, UAP1, UNC5C
Negative_4976	ALDH1A1, ALPI, BDKRB1, CYP3A5, DDX56, IL24, MAML1, OR6C6, OXTR, PARVG, PSME2, PYGM, SOD1, STAT1, SULT1A1, VDAC2
Negative_4977	BAX, OAT, PRL, TNR, ACSL3, AXIN1, CACNB1, CACNG6, CCR3, CDC25A, DPAGT1, ENPP6, EPB41L3, FMO4, GALNT6, HRAS, IFNGR1, IFNW1, IRS2, MBL2, OR10A7, OR2G3, OSMR, POLR2H, PPM1A, PPP2R5A, PRKACB, RASGRF2, RPS6, SPP1, STAT5A, TRHR, TUBA3E, TUBA8, TUBB2C, ATP6V1C2
Negative_4978	DDC, EBP, LTB, MUT, ALOX12, ALOX15B, ARHGAP5, CCR6, CLDN10, CREB1, DPYS, ENPP1, GALNT12, GALT, GLYCTK, GNA12, GRIN2B, GSTM2, HMOX1, HSD17B4, IL18, INSR, MAOB, MGAT1, MPST, OR13A1, OR2G2, OR51I2, OR52N5, PFN2, PIAS1, PPAP2A, RELA, RND1, RUNX1T1, SOS2, TFPI, WNT10A, ATP6V0A1, SERPIND1
Negative_4979	IL20, NTF3, POLR2F
Negative_4980	MVD, CD47, CYP2A13, DKK2, FARS2, GUCY2F, OR2L13, POLR3D, TOLLIP, XYLT1
Negative_4981	LCT, SPN, ACAA2, ACAT1, ACOT11, ARSB, CACNG6, CCNG1, CDIPT, CYP2C8, NCR3, NT5M, OR11H4, OR51T1, PRKY, PTPN1, SEMA3E, SETDB1, SOCS4, SOD1, SRD5A2, SYNJ1, TAS2R8, WNT3A, ATP6V1B2, SERPIND1
Negative_4982	KHK, CDC25A, G6PD, GUCY2F, KLRC4, TACR2, TAS1R3, TUBA1C
Negative_4983	BTC, ME3, AMD1, CCL2, GALNT3, IKBKE, IL22, NFKBIA, OR1M1, OR2AG2, PIAS3, RAC3, SPRY2, TBXAS1, XCL1, MAPKAPK5, SERPING1
Negative_4984	ACADM, ACVR2A, EPHB4, FUT3, INHBC, NODAL, OR2AG2, POMC, PRKG2, PSENEN
Negative_4985	OAT, COX10, DDIT4, HTR2C, IDH2, INHBE, IQGAP3, OR5P3
Negative_4986	CR2, LAT, OGT, ARHGEF1, CALML5, COL11A1, COX15, DHODH, FOXO3, GCNT3, GSTO1, IRAK1, LIPT1, MAPK14, OR10H5, OR10R2, OR2Z1, PFN4, PIK3R4, PPM1D, SUCLG1, TAS2R42, WNT5B, WNT9A
Negative_4987	CARD11, DNMT3L, GALNT14, GALNTL4, HARS2, IFNA5, IL25, IRS2, ITGA2B, L1CAM, NGEF, NKD2, NT5C1A, OR2G6, OR52E2, P4HA3, PEMT, PPCDC, PPP1CB, PRKAB2, PTPRF, PYGM, RDH12, SDHD, SYNJ1, ZAP70, PRICKLE1
Negative_4988	COL4A1, LEPR, OR11H1, OR6X1, PLA2G4A, PYCRL, RUNX1
Negative_4989	CTH, ALDH1A1, DDX50, FGF23, IL23R, NADK, OR10A3, PCYT2, ST3GAL5
Negative_4990	C2, LAT, ADH7, ALDOA, ALG10, ATP2A1, CHAT, COL5A1, CYP21A2, DPYSL2, DTYMK, GPT2, HAAO, IL2RB, ITGA3, ITGA4, LPAR1, MAP3K7, MAPK8, NAGLU, NFKB1, OR52E4, OR8S1, PHKG1, PIK3R1, PRDX6, RPS6KA5, SMAD3, STX18, STX7, TAS1R1, TGFB3, THBS3, WBSCR17, WHSC1L1, ARHGEF12, HLA-DRB4
Negative_4991	HDC, HK2, LIF, MPI, ACVR1C, ALDH4A1, ALDOA, AMD1, BET1L, CAMK2G, CHPF, CLCA2, CYP4A22, DHRS4L2, DIAPH2, DUSP7, EDNRB, ENTPD1, FOXO3, FZD10, IGFBP3, INADL, MDH1, NADK, NGEF, OR10A4, OR1A2, OR1J2, OR4F6, OR51Q1, OR6N2, PCCB, PMM2, PRKAA1, RPSA, RRM2B, SLC27A4, SMARCA2, SOAT2, SPRED1, ST3GAL4, TOMM40L, TNFRSF11A, TNFRSF1A
Negative_4992	F2R, IL2, ACP6, ARSE, BAAT, HMOX1, LYPLA1, NMNAT3, NOX3, OR51G2, PIP5K1A, SELP, ATP6V1G2
Negative_4993	PLG, TNN, ACAA2, ACTA1, ALDH2, ARPC3, CASP3, CHD8, CXCR3, CYP17A1, DFFA, DHDDS, DNMT1, EFNA4, EFNB2, GALNT1, GSK3B, GUK1, GYS2, H6PD, IL3RA, ITGA11, ITGB4, KAT2B, KLRC4, LAMC2, LEFTY1, MAT2B, METTL6, NTF4, NTRK1, NTRK2, OGDH, OR10Z1, OR4C46, OR52E4, OR6C76, PDCD1, PON2, PRKCI, PTTG1, RAB5A, RAPGEF1, RBPJL, SEMA3A, SKP1, TUBB4Q, VAV3, HLA-DRB4

Negative_4994	CD4,GBA,ME3,ATP5O,CDC25A,CYP2E1,FGF7,GART,GP1BA,HTR2A,OR2C3,PIGH,PRPH2,RFXANK,SEMA4A,WNT10A,MAPKAPK5
Negative_4995	C2,DCN,FOS,PGF,TYR,ZYX,ACP5,ARNTL,AXIN2,CDC45L,CPS1,CYBB,EFNB3,FGF9,FGFR3,GLDC,GNAL,IL13RA2,ITGA5,LLGL1,MAPK3,METTL2B,MYH2,OR2F1,OR6C2,OR6K6,OR7D4,PGK2,PI4KB,PIGU,PLA2G10,PLAUR,PNPLA3,PPP3CC,PRKY,PRSS2,RPRM,SGPP2,SOCS3,TLR4,ZIC2,SERPINA5,SERPINB5
Negative_4996	A4GALT,AKR1B10,AOX1,ARG1,B4GALT7,BMP5,BUB1B,CCL15,CCL24,CCNB1,ENO1,FBP1,FGF7,HIP1,ICOS,KARS,KLRD1,LFNG,LTC4S,MAT2B,MGAT5B,MIOX,MTHFR,OR2T33,OR51B5,OR5B21,OR5B3,OR7A17,PDE3A,PDE4C,PDGFA,PDGFB,PIP4K2C,PLA2G2D,PLCG1,PSME2,RDH8,STEAP3,STX10,TNXB,TP53,UROD,MAPKAPK2
Negative_4997	ITK,ASPA,COL5A3,CRKL,EGLN1,FOSL1,IDH3B,IL2RA,ITCH,PFKFB2,PHGDH,PIPOX,PPP5C,SEMA7A,SNAI1,UGT2B10,WNT5A,YWHAB,ZMAT3,ATP6V0E1,HLA-DRB4,HSP90AB1,SERPING1
Negative_4998	BAX,CD2,IL9,TPR,VTN,APC2,CAV2,CCNB2,CD3E,CHDH,CHRD,CSF2RA,DLL4,DVL2,FGF19,FMO2,GNB3,IL17A,IL23A,IMPDH2,LAMB4,LEFTY2,LRAT,NCOR1,NUDT2,OR10G7,OR12D3,PDGFB,PPBP,PRKCG,PRPS1,PTGS2,PYCR2,SKIV2L2,STX1A,UBA1,USE1,VNN1,SERPINA1
Negative_4999	SHH,ACSL1,CDC16,FRAT1,GGPS1,IFNA6,MEF2C,OR12D3,SC5DL,SV2A
Negative_5000	DPAGT1,ENPP7,GCLC,IDH3B,MYH3,PHKA2,PRKCE,SDC4,SPRED2
Negative_5001	BCR,ALOX5,BET1,BLVRB,CIITA,CLDN1,CLDN18,DOT1L,MAPT,MCM3,MGAT3,NCKAP1L,OR10J5,OR2M5,OR5M8,PDE3A,PDE8B,PRKAB2,SLC9A1,TUBB,UCK1,TNFRSF6B
Negative_5002	TNR,ACSL4,AVPR1A,CCL22,CLCF1,CLDN14,CPT2,CYP2A13,DDOST,E2F3,GPLD1,GSTA4,H6PD,HSD11B1,IBSP,KIR2DL2,KLRK1,MCEE,MKNK1,NT5C,OR10X1,OR5T2,PLCZ1,POLR1D,PTEN,PYCR1,SETD2,TNFSF12,WASF1
Negative_5003	DCT,F3,AACS,ASNS,CD38,EHHADH,FDPS,GNPAT,GPD1,LEFTY2,MAP3K13,MTHFD1,OR2T1,OR2T33,OR4K1,OR4K14,OR4S1,OR7G3,PFKFB2,PGAP1,PLXNB1,POLR2F,PPID,SGPP1,STAT1,UCP1
Negative_5004	BLVRA,CBLB,FGF20,GALNT2,IRAK1,OR5AS1,ZNRD1
Negative_5005	FST,ALDH1A3,AOX1,ATP5G1,ATP5O,C3AR1,C9orf95,CCKAR,CNTF,COQ5,DDX4,FBP2,FSHR,GBA3,HLA-G,HMGCS2,IRS2,ITGA1,MAML1,MPST,NUDT9,OR10G9,OR13C3,OR51V1,OR56A1,OR5C1,OR6B3,PDGFRA,PIK3CG,PPP1CB,PPP1R3D,PSENEN,RELB,SLC2A4,TBL1XR1,TKTL1,TNFRSF11A
Negative_5006	CFD,GP5,ACTC1,ALDOC,ALG1,AOC3,ATP5G3,CAMKK2,CCL21,CD55,CDH1,CIITA,COL5A2,CYP21A2,CYP2D6,ESPL1,FABP3,GALNTL2,HADHA,HLA-C,HRH1,HSD17B7,INHBA,JAM2,LRDD,MRAS,NFATC4,OR13A1,OR1J4,OR6C2,PLXNB3,PVRL1,SHISA5,SOCS4,TBK1,UNC5D,AASDHPPT
Negative_5007	AKR1D1,BHMT,C4BPB,E2F5,NFATC2,OR1L3,OR5I1,OR6S1,PFKFB2,PPP2R5A,PVRL1,SGPP2
Negative_5008	CCR4,CXCL12,IL8RB,OR13G1,OR1D2,PIGS,YARS
Negative_5009	A2M,EBP,ACSL4,ASRGL1,CAMK2G,EPHB6,IFNB1,IL23R,JAG1,LFNG,MYH6,OR2M5,OR51S1,OR8J1,PIGN,POLR3D,RBKS,RELB,RUVBL1,SEMA4C,SV2A,TUBA4A,UBA7,ULK2,WBSCR17,YWHAB,B4GALNT1
Negative_5010	C8B,CS,HGD,LCT,OGT,ACY1,ADCY7,ALDH1A2,ARNT2,ATP5L,ATP6AP1,BMP6,BMPR1A,CACNB1,CBLB,CLDN2,COASY,CRY1,CTNND1,CYP3A4,DCXR,DNMT3B,EDN1,EXT2,FRAT2,IL10,IL13RA1,IL22,KIR2DL3,LEF1,MASP1,MYL7,NEUROD1,NOTCH3,OR51A2,OR5B21,OR9G4,PLTP,POLE2,POLR1E,PRKCQ,PRSS2,SIPA1,TUBB1
Negative_5011	TYR,ATP4B,CHST12,HSD17B3,IQGAP1,OR1L8,PRKAR2A,SUV39H1
Negative_5012	LSS,MTR,ADCY8,ATP5B,BUB1B,CFL1,CNDP1,CTBP1,PHKG2,SSH3,SUCLA2,TUBB4Q

Negative_5013	CAD,GLS,MIF,ZYX,ABL1,C3AR1,CACNA1A,CCL24,CDC25A,CDKN1B,CHAT,CYBA,DCTD,ENTPD6,EPRS,EXT1,FGF11,GGT6,GLI2,GPD1L,GSTM1,IFNA5,KIR2DL3,LEPR,MAP3K3,MLLT4,NFATC4,NFYC,NTSR1,OR11H1,OR13C3,OR2L13,OR4K14,OR4K2,OR52N5,OR8K1,PFKFB1,PIGC,PIGO,POLR2C,PRIM2,QARS,SARS2,SEMA3D,SEPHS1,VAMP4,ARHGEF12,MAP3K7IP2
Negative_5014	BAD,C2,C5,FOS,FUK,GDA,HPD,MPI,PAH,CACNG2,CCNG1,CD244,CTNNA1,CYP3A43,DDX18,DRD1,GAB1,GCLM,IFNA2,IL17B,LTC4S,MCM6,MMP1,MRAS,OR2A12,OR2W1,OR4A16,OR6A2,PLCB3,POLR3G,RETSAT,SFRP5,SKP2,SLC12A2,UGT2B28,HLA-DQB1,ST6GALNAC1
Negative_5015	ATM,FES,HAL,IHH,ACAD9,ALG6,ASAH1,AVPR1A,BCL2,CCL21,CD55,CDC7,CLOCK,DUSP8,FASN,FPGS,GAMT,GARS,GLS2,GNRH1,GSTM3,GUCY2F,IFNW1,IL3RA,LDHD,NEFM,NPR1,NT5E,OR10H1,OR13F1,OR1C1,OR4D6,OR51B5,OR52E4,OR5D13,PDE4B,PFN4,PLA2G2A,RARS,ROBO3,SESN1,SMAD4,SOX17,SULT1A1,TXNDC12,UGT2B17,VAV1,WNT3A
Negative_5016	ALDH6A1,DKK2,IL11,IL18,NAMPT,TGDS,TNFRSF6B
Negative_5017	MPI,CD36,CDC23,COX15,DLL3,DUSP1,EGFR,GDE1,IL11,IRAK4,OR51A7,PCCA,PDE1C,PDE4C,PTPRC,SMAD5,TCF7,ULK1
Negative_5018	C5,ALDH3A1,BCAR1,CHST12,CLDN11,COL6A1,CXCL9,CYP2A13,EGLN1,FBP1,GLB1,GP1BB,IGFBP3,ITPKB,LAMC2,LIAS,MS4A2,NFE2L2,OGDHL,OR10AD1,OR2T11,OR4C12,OR4K1,OR52J3,P2RX3,PANK1,PDE1A,PDXK,POLD4,POLR3D,PRKCD,PTPN6,RALGDS,RAP1B,RBPJL,SEMA3F,SH2D1A,SPHK1,STAT4,SUCLA2,TYMS,UMPS,WARS,MAPKAPK2,MAPKAPK3,TNFRSF17
Negative_5019	JUP,TPO,ACTA2,ACY3,ADCY9,B4GALT7,BLNK,CALML3,CCL28,CCR6,CHRM3,CISH,CRY1,DPM1,GRLF1,IMPDH1,KLRC1,LARS,LIPF,NLGN2,NOS1,OR13H1,PARVB,PLAU,PLCB1,PPP2R2D,PRKAA1,RARA,SMAD3,SMC1A,ST6GAL1,TACR3,TNFSF10,TUBB3,PLA2G12B
Negative_5020	F8,FH,ACSM4,ARNTL,B3GALT6,CTPS2,CYP2J2,DGAT2L4,DUSP14,E2F2,E2F3,EPHA2,ESPL1,GSTM3,IL15RA,IL20RB,IL22RA2,MYLK3,NARS2,NFYA,OCLN,OR52B6,PIPOX,TGFB3,TPH1,MAPK8IP1
Negative_5021	JUN,CCL25,CD70,CDH1,DDX41,FABP3,HSD11B2,KCNB1,NEU2,OR10J5,OR4A16,OR56A4,PDGFRB,PTCRA,TCF7L2,UAP1,WNT3,ATP6V0D1
Negative_5022	AADAT,AGXT,AQP7,ATP6AP1,CHKA,CPOX,ITGA8,KCNQ1,NT5M,OR11H4,PDE1B,PFKL,RASGRF2,RENBP,SDHB,WNT2B,WNT9B
Negative_5023	AK3,CR2,LCK,MPI,ACADM,AKR1C3,ANAPC1,ASS1,ATP6V1D,CCL11,CD72,CHST13,DRD5,DTX2,EPHB3,FGFR1,KIR2DS4,NRAS,OR10K1,OR4F6,OR5V1,PLCB2,RFXAP,SESN2,SETMAR,SPAM1,CACNA2D3,SERPINF2
Negative_5024	C5,ACAD9,CSAD,CSDA,CYP2E1,DAAM1,GADD45G,GCDH,IL6ST,MINPP1,NARS,OR2T5,OR5M3,PSME2,SGPP1,WNT3A,YWHAQ,SUV420H1
Negative_5025	CD6,GSS,ACAD8,CD14,CDH5,FIGF,FZD1,GRM1,IARS,IL2RB,ITGA4,KLKB1,MADCAM1,OR4C16,OR52I2,SHC3,THBS4,VEGFC
Negative_5026	ADCY2,ITGA8,OXSM,PIAS1,STX12,TACR1,XYLB
Negative_5027	FST,SRM,ACOX2,ADIPOR2,ARPC1B,BMPR2,CCL23,CD46,CD82,CNDP1,CYP11B1,DLAT,ERO1L,EVI1,F2RL1,FGF9,FOXO3,FSHB,FUT6,GANC,ITPKA,LAMB4,LDHA,LDHD,MAML3,MAPK9,OR11A1,OR4C46,PDGFB,PDXP,POLR2H,RALA,SHC2,SQLE,TPH2,TSLP,HSD17B12,PRICKLE2
Negative_5028	AGXT,B4GALT2,CCL8,CLDN11,DGKI,DPYS,FZD7,GNPDA2,GSTM3,HAAO,MS4A2,NEU1,OR6B3,OR8U1,PARD6B,PIGW,PMAIP1,RPSA,WNT5B,MAP3K7IP2
Negative_5029	AGPAT6,GPAM,PIGS
Negative_5030	AANAT,ADIPOR2,ADORA2A,AHCY,AOC2,CASP9,CLTCL1,ENTPD4,FGF19,HLCS,IL1A,LAMA4,MAN1A2,MAP3K6,NCOR2,NCR3,NEU4,OR11G2,OR13J1,OR1E2,OR5A1,OXTR,PRKG2,SEMA3E,SLC33A1,UCP1,WNT1

Negative_5031	KDR, LCK, ACADL, AFMID, ALG1, ALG11, ATP6V0C, CAMK2D, CTSS, EIF4E2, GPD1, GUK1, HTRA2, IFIH1, ITPA, KAT2B, LTA4H, MAFA, MCM7, NCK1, OGDH, OR13F1, OR2L2, PDE7A, PDGFRA, PFKL, PLCG1, PLOD1, PPP2R5A, PRODH2, SDC3, SPTAN1, ST3GAL1, STAT5B, TNFSF9, WNT3, TNFRSF11A
Negative_5032	F10, FH, BLVRB, BMP5, CHAT, CLDN7, CNGB1, COMT, CRLS1, CRY2, GNG3, GRLF1, IFNA13, IFNA21, IGF1, ITGAL, LTA4H, MAN1A1, MCM3, OR2B6, OR4K15, OR52K1, OR5D18, PON1, PSAT1, RALGDS, RXRB, TAP2, TAS2R13, TRAF4, UROC1
Negative_5033	ALG8, ALS2, C3AR1, CASP6, CHKA, CLTB, DNMT3A, EPHA3, ERBB2, ETS1, GADD45G, GFPT2, GRIA1, GSTO1, HYAL1, IDI1, ITGA3, ITGA6, NAGK, NT5M, OR13C5, OR13G1, OR4D1, OR5I1, PAPSS1, PCYT1A, PLA2G2E, PPP2R5B, SOCS5, SPRED1, SQLE, STX16, STX1B, TAS1R2, TCF7L1, TDO2, TSLP, UBE2K, UGT2B10, YOD1, YWHAG, ST6GALNAC4
Negative_5034	AUH, ALPL, AMHR2, CAV1, CTBS, DHODH, DVL2, FOXA2, GCNT4, IKBKG, INPP5A, IPPK, LRRC4C, MAP2K5, MCM2, OR10G4, OR13C3, OR2W1, OR9I1, PER2, PFKFB4, PROC, SEMA7A, SH2D1B, SKP2, SMC1B, SOAT1, THBD
Negative_5035	NOG, ARPC3, GSTA3, HLCS, KCNB1, KLRD1, MAP3K8, MMP1, NQO1, OR51A2, PLXNB2, RPRM, UCK1
Negative_5036	DHH, ADIPOQ, CNTNAP1, EFNA4, GALK1, GALNT1, GSTK1, HLCS, LYPLA2, MPZL1, NR1H3, OR5T3, PGAM2, SEMA3F, SEMA4A, SETD7, SPRY3, STX10, VCAM1
Negative_5037	LHB, OAT, TFG, TNF, ACTG1, AGRP, ALDH4A1, ALDOB, AXIN2, C4BPB, CACNB2, CHIT1, CLCA4, CYP2F1, DHCR7, DPM2, DVL1, EI24, GBGT1, IARS, IFNA16, KCNB1, KCNQ1, MAPK10, MAPK3, NEUROG3, NUMB, OR10G3, OR13G1, OR1E1, OR4K5, OR51G1, PIGT, PPP1R1A, PRKCE, PSME3, PTPRM, RFNG, SH2D2A, SUOX, SUV39H2, TAS2R9, TGFBR1, ULBP3, ST6GALNAC6, TNFRSF10A
Negative_5038	ADO, ABP1, ACSM5, CCND3, CXCL2, DDIT3, DNMT3B, ETS1, FGD3, GALK2, HADHA, IGF1, MADCAM1, OR2S2, OR2T3, OR4K14, PGLS, PON2, PRKCH, PRLR, QPRT, RARS2, SC5DL, SCLY, SMPD1, SNAP25, TGFBR2, WNT11, WNT2B
Negative_5039	AMH, CD4, IL3, MIF, ACAD9, B3GAT1, CAMK2B, CASP8, CMPK1, CPS1, CXCR5, GALNT10, GALNT13, GALNT14, HPSE, LIAS, MAGI3, MGAT3, NRG1, NSDHL, NUDT5, OR13G1, OR4C6, OR6F1, OR7G3, PARD6G, POLR1B, PPP2R5C, RPS6KA6, SETD1B, SV2B, TAS2R3, TGFB2, TNFSF9, TNXB, TSNARE1, UAP1, HLA-DPB1
Negative_5040	F7, BDH1, COL4A6, CYP17A1, EXT1, GALNT5, IFNA10, MAP4K4, NKD2, NUMB, OR10H3, OR1C1, OR4L1, PAICS, PIP4K2C, POLD2, PVRL1, RBL1, SGMS1, TAS2R4, TP73
Negative_5041	HDC, TAT, VCL, ALDH9A1, ARNT2, BMP6, CASK, CCL19, CCL3, CCR1, CMPK1, EVI1, FZD8, GAD1, GLG1, GLUD2, HAO1, LAP3, MGST2, OR52E2, OR5K1, OR9Q2, PIP5K1B, PPP2CB, PPP2R1A, PRIM2, PTGES2, RBX1, SHC2, TNFSF4
Negative_5042	ID2, ME3, RDX, ACSM4, ACVR2B, ALAS1, BMPR1A, CD27, COL1A2, CREB3, DHFR, FCGR3B, FZR1, GALC, GTSE1, HSD17B2, IQGAP2, ITGAL, KIR2DS5, MAN2A1, MAP4K2, MAPK12, MYH8, OR11H1, OR51V1, OR6K6, OR6N2, PDE9A, PER2, PHKB, PIGH, POLE, POLR1E, PRIM1, PRKCB, PRKCH, PRLR, SENP2, SOCS7
Negative_5043	DCN, ME1, ACOX3, B3GALT5, C1QA, CLDN22, CLDN9, CYP2C19, DNMT3L, FZD3, JAK2, KNG1, MYH3, NCF4, NCR1, NT5C, OR51B4, OR5D14, PCNA, PIK3R1, PIP4K2B, PORCN, RASGRP2, ROBO1, RPRM, SAT2, SDHB, TAS2R5, TBXAS1, TGFBR1, TYRP1, UBE2K, VARS, CSGALNACT1, HLA-DRB3, HSD17B10, PRICKLE1
Negative_5044	HAL, RB1, ACACB, ACADL, ALOX12B, CCL25, CD8B, CHKB, COX10, CXCR4, CYP2C9, HTR2B, IRS4, PLOD1, PRKAA1, PYCR1, SELP, TBL1XR1, TLR2, TUBB6, WNT5B, YWHAB
Negative_5045	GRIN1, PARD6B, PGM1, PTPRM, STT3B
Negative_5046	CD2, IL8, LCT, ME2, MPZ, ACYP2, CHPT1, CLTB, DHDDS, EI24, ENTPD6, FMO4, FOXA3, GRM1, HSPB1, IL25, LAP3, LDHC, LRAT, MAP4K2, MCCC1, MLYCD, NFE2L2, NUDT9, OR10H4, OR2Y1, OR52R1, P2RX3, PDE3B, PDPK1, PFN2, POLE4, PPP1R3B, PPP2R2A, RPSA, TBXAS1, TLR5, VEGFC, SUV420H2

Negative_5047	CD14,HAGHL,MAP2K3,PLXNA2,VANGL2
Negative_5048	AMDHD1,HEMK1,OR7D4
Negative_5049	AGRN,BDNF,FZD9,GAB1,NUDT12,OR52E6,TGFBR1
Negative_5050	CAT,CKM,SYK,ACADSB,CACNG2,CD19,CDK7,CFTR,CLDN19,CSNK1D,CTSB,CXCL1,CYP11B1,E2F4,EPHA1,GALT,HSD17B1,MAP3K13,NFKB2,NRXN1,OR2A5,OR4K2,OR5D16,OR6C4,OR8D4,PPID,PPM1D,PPP2R2B,PRPH2,PYGB,SNW1,SPRY3,TRAF5,UNC5B,VAMP2,XYLB,ATP6V0D1,CSGALNACT1
Negative_5051	LCK,GMPR,PCYT1B,RELB,SIGLEC1,SORBS1
Negative_5052	KDR,AADAT,ADRA1B,AKR1B1,AOX1,ARG1,ATP6V1D,BMP4,CASP9,CHDH,CSNK2B,CYP19A1,CYP2C19,DDB2,DGAT1,DUSP8,FGF2,FLOT2,GALNT8,GLUD2,H6PD,HLA-F,HMBS,IBSP,INPP5B,KAT2A,OR10V1,OR5D16,P2RX6,PIGM,PIPOX,PLOD3,SH2D1B,SMAD1,SMAD7,STAT5A,TRPC1,UGCGL1,UNC5C,UROS,WBSCR22,ATP6V0A4,CACNA2D4
Negative_5053	CES7,EPHB1,FADD,OR56B4,XYLB
Negative_5054	DDC,VCL,ABL2,APOA1,CBR3,CDC23,CDKN2C,CYP17A1,CYP2B6,DPAGT1,EFNB2,FGF12,GDE1,GSTP1,GUCA1A,GUCY1A2,INPPL1,KLRC3,MAML2,NPR1,NRXN2,OR1L3,OR2M4,OR3A2,OR51S1,OR5AS1,OR6A2,OR8H1,PIK3CA,PLA2G1B,PLCD1,PNLIP,RALBP1,SETD7,SH3BP2,TPH1,UCP1,ZMAT3,PAFAH1B1
Negative_5055	CHIA,GSTA2,IL10,IL21R,NEU4,PANK3,PLA1A
Negative_5056	AUH,FOS,FYN,GPI,GSS,ADORA2B,ADRB1,AKR1B1,BDH1,CAV2,CCNE2,CD40,CLTC,CSNK1A1,CYP4F2,DNMT3B,GMDS,HES1,IL24,INPP4A,IPMK,ITGA7,MGAT5,NOS3,NRG1,NT5C3,OR10J3,OR10K2,OR51G1,OR5H2,PANK4,PERP,PHKG2,PIK3R3,PLOD2,RELB,RUNX1,SEMA3C,SEMA4B,SLIT1,SOCS4,ST6GAL1,TIAM2,HLA-DRB4,SLC25A31
Negative_5057	SYK,AGPAT2,CRLF2,MTHFR,NR5A2,OR2T4,OR51G2,PTPMT1,TAS2R4
Negative_5058	EPO,ABL2,ACY1,ADIPOR1,AK3L1,ALOX15B,AMD1,CFL2,LIPT1,MDH2,OR14J1,OR1D5,OR2C1,OR2T29,OR52J3,OR52N1,PLA2G2A,RPRM,TNFRSF13C
Negative_5059	BMP8A,CAV1,CDH5,CDIPT,CXCL11,CXCL2,CXCL9,DHX58,DPYD,FBXW11,FCGR3A,GALNT3,HTR6,HTRA2,IFNA17,MAPK10,NFASC,NRXN1,OR6A2,SMAD7,STMN1,TAS2R9,TCF7L1,PNLIPRP1
Negative_5060	AGL,AUH,NPY,CACNA1C,CALML6,CSNK2B,EPHA2,FZD3,HSD3B2,OR6C1,TDO2
Negative_5061	TPO,DDIT3,GMPPA,GSTM5,HSD11B2,LTBR,NCF4,NCK2,NPR1,OR2C3,ATP6V1G3
Negative_5062	KDR,ACY3,AKT3,ARPC1A,CACNA1H,COL2A1,CTSS,DDX47,GSTA3,IL1R2,ITGAE,ITPK1,KIR2DS5,LEFTY1,NEU2,NLGN2,OR1I1,OR2C1,OR6C1,PHKA2,PPM1D,PTGES,RAF1,RAPGEF1,SORBS1,TUBA1B,WIF1,WNT3
Negative_5063	MPZ,SMO,ADH4,ASS1,ATP5G3,CCL13,CD40LG,CD58,CDK7,CYP3A5,EXTL2,GNAQ,GNRHR,ICAM1,IL2RG,KLRC4,LARS2,LIPE,MADCAM1,MAP2K2,MAP3K12,OR2H2,OR4N5,OR51A2,PAK1,RHOQ,SEMA3G,SLC2A2,TACR1,TMLHE,TUBB3
Negative_5064	GNA11,GYS2,HLA-DMA,SSH1
Negative_5065	AARS2,ALDH1A3,ARSD,ATP5E,B3GAT1,CPT1C,CYP2B6,DDX55,DHRS3,EGFR,GFPT1,GLUL,HIF1A,IRAK1,LAMC2,MAP3K3,MGAT4A,MYH2,NEFH,OR2T10,OR2T34,OR4K15,OR7G1,ORC1L,P2RX6,PPP1R3D,PTPN1,RDH8,STAT6,ZAP70,HSD17B12
Negative_5066	GDA,TYR,ACP1,ACSS3,ACTA1,ATP5G3,BMPR2,CADM3,CD58,CRY1,DKK4,DPYSL2,EP300,FSHB,G6PD,HLA-DOB,HSD3B7,IL13RA1,MAP2K6,MAPK9,NGEF,NKX3-1,OR10C1,OR1S1,OR52E6,OR5C1,P2RX5,PANK1,PDE2A,PLCB3,PPA2,PSME1,PTCH1,RAB5A,RAP1A,SEMA3G,SOCS5,PNLIPRP1

Negative_5067	LIF,ACOX1,ALG12,APOA1,BUB3,CD226,CD274,CD79B,CFTR,CHIA,COL5A2,CPT1C,CRHR1,ENTPD7,FBP1,FGD1,LEF1,MMP14,OR2T1,OR2W1,OR4D1,OR8D4,PAK7,PTCH1,PTPRB,SCLY,SETD1A,SHC4,SIPA1,TAS2R40
Negative_5068	GNA14,OR6C76,OR6K6,PARD3,SPRY4
Negative_5069	CTH,MSN,SMO,TNF,AADAC,ACSM3,ADCY8,ALG3,ANPEP,AOC2,ATP5D,CD58,CXCR5,FGFR1,GALC,GRIN2D,IDI1,IKBKG,IL1RAP,IL24,INPP4A,MAP4K4,MTAP,NUMBL,OR13C5,OR13F1,PAX4,PDE6H,PPAP2A,PTGS2,TAS2R5,TRMT11,TSNARE1,VDAC3,WARS2,ATP6V1G3
Negative_5070	GH1,BAI1,CCL27,CDC42,DPAGT1,DTX3L,DUSP8,GALNT5,GCLM,GNA15,GSTK1,IL15RA,IL6R,NCOR2,OR10C1,OR2L3,OR56A1,PDE9A,PLIN,PMVK,POLR2D,PPP1CB,RDH5,SOCS5,TNXB,ATP6V0A1,HLA-DQB1,MAPKAPK5,SERPINC1
Negative_5071	CD2,EPO,F12,GBA,OAT,ALDH1B1,CYP2A7,DAD1,DGKD,DGKG,DVL3,EFNA4,GPD1L,HADHB,IMPDH2,NOD1,OR4S2,OR6C75,PDE5A,PHKG1,PRSS1,PTGS1,RPS6,TAS2R9,TNFSF10
Negative_5072	DCT,EDA,GP6,NGF,ABI2,ACPT,ALG11,ASPA,BMPR1A,CACNA1S,CAMKK1,CDS2,CHAD,CHKB,CRKL,CXCL13,CXCR4,DGKH,EPHA5,G6PC,GCNT1,GSTK1,HRH2,IFNA7,IL15,ITGA9,MAD2L1,MYLK2,NAT5,OR13J1,OR1C1,OR1E1,OR4C16,OR9G4,PDPK1,PLCB1,PLK1,PRLR,RALB,RASGRF2,RASGRP1,SDC4,SRGAP2,THBS1,TICAM1
Negative_5073	DBI,LAT,AASDH,ACAT1,ALPPL2,APOC3,ASMT,BCAT1,CCL17,CCL27,CD40LG,CDC7,CDO1,CFLAR,CLDN10,COQ5,CTPS2,CTSG,DAD1,DVL1,FZD8,GADD45A,GALNT7,GNG13,GYS2,HNF1A,LDHB,NFASC,NFATC2,OR11L1,OR1E2,OR2T3,OR4F5,OR51E2,OR5B3,PDX1,PFKFB2,PGAM2,PLA2G10,PNMT,PPP2R2D,SESN2,SPHK1,ST3GAL3,TPMT,ATP6V0A2,CACNA2D2,CACNA2D4
Negative_5074	ACOT12,ALOX15,ANAPC4,CCND3,CD247,CDC25C,CHPF,COL6A1,DGUOK,EDAR,FCER1G,GALNT12,GNPDA2,HLA-G,IRF9,KNG1,LYPLA1,MAN1B1,OR10K1,OR5B17,OR5L1,PCYT1A,POLR2J,PVRL3,RDH12,SLC9A1,TGFBR2,ATP6V1E2
Negative_5075	CYP4F3,GRB2,HIP1,IL12RB2,IL2RB
Negative_5076	GLS,HAL,IL5,ACADM,ACLY,CADM3,CAMKK1,CD27,CMPK1,CYP26B1,DDX55,DLL4,DVL1,FUT3,GBA3,GUCY2F,HAGHL,HLA-DOA,IL10,IL10RB,LRP6,MAN1B1,NAT1,NFYB,OR11G2,OR2B2,OR56A1,OR56A4,OR5P3,OR8K5,PER2,POLE2,PPP3R2,RAPGEF2,RPN1,TRDMT1,TSTA3
Negative_5077	GLS,ACVR1C,ACYP1,AMY2B,CCL7,COX10,CSAD,DAPK1,DGKQ,DGUOK,EXOC7,GDF7,GMPPB,GRIA2,IL17B,MAP3K12,MAPK9,MGAM,NARS2,NAT1,NCOR1,OR1Q1,OR2T4,OR2W1,OR7G2,OR8I2,POLR1B,RASSF1,SKP2,STX12,SUV39H1
Negative_5078	CASP8,DOT1L,EDNRA,ENAH,ETNK1,EXOC7,IFNA10,NAGLU,OR52D1,PPP1CC,PYCR2,TOMM40
Negative_5079	AARS,CFL1,FGF22,HRAS,NCOR2,OR11H4,OR6K3,OR6N1,SERPINB5
Negative_5080	GP5,PRL,ACAD9,ACHE,ACOX1,ACPP,ATP6V1F,CCR3,CD72,CDH5,CSNK1D,CXCL11,DDX50,ERBB3,GDE1,GRIN2D,HRH2,IFNA14,IFNAR1,ITGAL,LFNG,NEGR1,NTF4,OR4C16,OR52L1,OR56A3,OR5I1,OR5P3,ORC6L,PIGQ,PSAT1,SC5DL,SLC27A5,TRDMT1,UBA7,WASL,HSD17B12,HSP90AA1
Negative_5081	UBC,ALDH2,CACNG1,CCL8,CCNE1,CD40,COQ7,CREB5,CYP19A1,DUSP10,FUT9,GALNT10,HSPA8,ICOSLG,MOV10L1,NARS,NFAT5,NFATC4,NFX1,OR4A16,OR5M9,OR6X1,OR9Q2,PAICS,POLD4,PRPS2,PRSS1,RBL1,RDH16,SDC2,SELP,SEMA3D,SPHK2,SUCLG2,TRDMT1,TRPM5,TSNARE1,UCK1,WNT1,WNT5B,ZMAT3,CSNK1A1L,PNLIPRP1

Negative_5082	AR, BID, C8G, NF1, SI, ACACB, ADCY8, AGMAT, ALDH1B1, ALDH7A1, CDK4, CDK6, COL2A1, DHX58, EXT1, GCDH, GP1BB, IRAK4, MAML1, MCCC2, NTF4, OLR1, OR10A6, OR1L1, OR4F5, PIP5K1B, POLR3B, PPCDC, PRSS3, RPS6KB1, ST3GAL2, STK4, TAS2R40, TNFSF11, UNC5B, CACNA2D1, ST6GALNAC3
Negative_5083	C3, CD40, CYP11B2, FBP2, FGF2, NOTCH1, PIGL, SUCLA2, TNFRSF10B
Negative_5084	CAD, CR1, ILK, UBB, ADCY4, ALDH5A1, B4GALT2, CAMK2D, CCR10, CPT1A, CYP4A22, DGKB, DKK2, DKK4, EPHB4, FRAT2, GGPS1, GPX5, GSTM4, IFNK, IKBKG, IL5RA, ITGA2, KIR3DL2, LEPR, LIPA, MAFA, NFKBIB, NOX3, NT5C1A, OR10H4, OR13H1, OR6K3, PARVA, PDE8B, PPT1, PRKAB1, SEMA3F, SHC1, TGFB2, ULK1, UNC5A, CACNA2D3, CTNNBIP1, PLA2G12B, SERPINA5
Negative_5085	ABO, ADA, AGK, AMT, DUT, GNE, GP6, IDS, AACS, ARNT2, DNMT3B, FGF3, G6PC, G6PD, GALNS, GDF5, HAGH, HLA-G, ICOS, IMPDH1, NKX3-1, NRAS, NUDT5, OR4C46, OR51A4, OR8H2, PAK2, PDCD1, PDX1, POLR2I, POLR3G, PRKACB, PRKAR2B, PYGB, SEMA4F, SH2D1A, UPP1, TNFRSF11A
Negative_5086	CRK, TPO, ADCY1, ALG13, CCR4, CD72, CDC6, CTPS, CTPS2, DRD1, DUSP8, EPHA7, EPHX2, FGF18, GAD1, GMPPB, IFNB1, ITGA8, KIR2DL2, MAP3K11, NTRK2, OR14A16, OR1I1, OR4A15, OR5T1, PDE4C, SARS2, SETD2, SNAP25, SULT1E1, SV2A, TACR3, TLR4, TNFRSF9, TYMP, UGT2A1, UNC5D, UPB1, ZFYVE9
Negative_5087	ANGPTL4, ATG12, BLNK, CD55, CHDH, CHEK2, CRY1, CSAD, CSNK1D, CTSB, DDX4, F2RL1, GPAA1, IAPP, ITGA2, ITGA4, LAMA4, OR2AT4, OR8G1, OR9K2, PLAUR, PLK1, RAC1, RFX5, RPN1, SEMA4D, SMAD2, STX10, TUBA1A, UCP1, XCL2, YWHAH
Negative_5088	BTK, FH, GH2, AACS, ABL2, ADCY7, ADORA2A, ADRB3, ATP4A, B4GALT6, CCNB3, CD80, CDKN1A, CLDN19, COL6A2, CXCL3, DARS, DUSP16, E2F5, FGF12, FRAP1, GPT2, GUCY1A2, GUCY2D, IGF1, IL25, ITGB6, LEFTY2, MAML2, MAP3K14, NAGS, NCK1, NCR1, NCR3, OR10G9, OR1A2, OR2D2, OR2T5, OR51B5, OR51G2, OR52R1, PDGFRB, PFKFB3, PPP2R1B, RAP1B, SMURF1, TRAF1, TYMP
Negative_5089	CS, LCK, B4GALT5, C3AR1, CHD8, DAAM2, EPHA2, GCH1, GUCY2C, IFNA16, IFNW1, MMP9, MTHFD1L, OR11H4, PNPLA4, PRDX6, TLR4, UGDH, VNN1, WNT11, ZBTB16
Negative_5090	AK2, BTK, GP9, GSN, WAS, ADAM17, ADCY5, CMPK1, CYP27A1, EPHX2, FZD8, GAD2, GSTA5, HGSNAT, HPSE2, IL24, IQGAP1, MAML3, MASP1, MYD88, OR14A16, OR4X2, OR5A2, OR5R1, OR6C1, OR8B8, OR9G1, OR9G4, P2RX1, PDE1A, PDGFRA, PIAS2, PLA2G5, PPCS, TAS2R42, THBS3, TLN2, VAV3
Negative_5091	HAL, ABCC8, ADH1B, CHIA, CXCL6, DNMT3A, FGF23, IL1B, P2RX1, PPARD, PRKAB1, RRM2B, SMARCA5
Negative_5092	CAT, GPT, IVD, AACS, ADCY6, ALDH9A1, B4GALT7, CTF1, DCTD, EXTL2, GALNT5, GALT, GPX7, GUSB, HAP1, IL17A, INSR, ITGA11, MCM3, MYH9, NFATC3, NFX1, OR1Q1, OR2T8, OR4E2, OR7G2, OR8D2, PANK3, PIK3R2, PPP3CB, PTDSS2, PTGS2, SEMA3F, SMURF2, TAS2R3, TNFSF4, WARS2, HLA-DPA1
Negative_5093	AMT, C9, CRH, LIF, ALDH1L1, BCL2L1, BLNK, CCL26, CHAT, CREB1, CSF2RB, CSNK1G2, CTNNA1, DRD1, DVL2, E2F5, ELK4, ENTPD2, FLOT2, GLUD1, GNAQ, IL15RA, MECR, MYH8, NAMPT, OR2T11, OR4P4, OR52N2, PLCD1, PPP1CA, PRODH, RAP1B, RHOD, SC4MOL, SLC27A1, SLC27A4, SOCS1, SUV39H1, WNT8A, TNFRSF13B
Negative_5094	TH, ACAT1, AKR1C2, CALML3, CFL2, CLDN11, DCXR, GALNTL1, GNG13, GOT2, ITPK1, KITLG, KLRC3, MYH15, NME4, OR1L3, OR52E8, OR8U1, PANK3, PCK2, PDX1, PFN3, TAS2R38, XYLT1, CACNA2D2
Negative_5095	DAO, GPI, SCD, AACS, ALG1, ANPEP, ARNTL, B4GALT2, B4GALT6, BLVRA, BMP7, CACNA1B, CCKBR, COL5A2, FUT9, GBE1, GPT2, GRIA2, GRLF1, HGSNAT, HSPA2, IFNA4, IL10RB, KLRK1, LAMB4, LHCGR, NKX2-2, NTF4, OR51G1, PISD, SAT1

Negative_5096	APP,LPO,ACAT1,ACTG1,ACY3,AHCYL2,ALPL,ARRB1,AXIN1,BET1,CCR10,CHAT,CSNK1E,CYP3A5,DGAT1,FRAT1,GALT,GSTT1,LIPA,NAT1,NCF2,NPAS2,NUDT2,OR51B2,PPM1D,RASGRP3,SETD1A,SH2D1A,TACR1,TCF7L2,TICAM1,TRADD,UGCGL1,UGT2B28,UGT2B7,PAFAH1B1,PAFAH1B2,ST6GALNAC1
Negative_5097	FST,C5AR1,CMPK2,OR1C1,OR51Q1,P4HA3,PPAP2C,SCD5,UBE2L3,ATP6V0A2
Negative_5098	CSK,FGA,ABLIM1,ADCY4,ALDH7A1,CCL27,COL5A3,DLL4,ENAH,GNA11,HLA-G,KRAS,LAMB2,LPAR1,MGAT1,MYLK,NAGK,NSD1,OR13C4,PDE4A,PFN1,PIP4K2A,PLA2G1B,PLCB4,PPAP2A,RXRG,TUBA4A,UCP1,B4GALNT1,MAPK8IP3
Negative_5099	AR,BAD,CAT,GP5,ACSL5,CCR1,CSF2RB,CXCL6,DTX4,EHMT1,GSTA5,HLA-DOA,ITGB4,MAP2K5,MAPT,MGAT1,NCR3,NNMT,OR5I1,OR5K2,PDE9A,PECAM1,PTDSS1,STX18,STX1B,TGDS,HSD17B12
Negative_5100	IL4,INS,ABLIM1,ACYP1,ADH1A,ANAPC2,ATP5G3,BUB3,CDC25C,CRY1,ERBB3,FMO1,HADHB,KLRD1,LMO7,MTAP,MTHFR,NFATC3,NTNG1,OR13C3,OR2L8,OR4C15,PPP3CC,PRKAR1A,RDH8,RHOA,RPN1
Negative_5101	DDO,PLN,CD99,CYP2B6,MPST,OR3A2,OR56B4,PIAS1,SULT2B1,TRPM5,UGT1A10,CACNA2D1
Negative_5102	BTC,BTD,AKT2,BMP6,CBLC,CD82,CSAD,EIF4B,ENPP2,GABARAP,GCNT2,GNMT,GRIN2A,GSTA4,HMGCL,HRH1,HSD17B1,HSP90B1,IL20,ITGA7,MAOB,NKX2-2,OR10G3,OR1A1,OR2T12,PANK4,RENBP,SENP2,TNFSF8,PPP1R12A
Negative_5103	DMGDH,ITGB6,OR2M2,PIGW
Negative_5104	MTR,ACADSB,ADSS,AFMID,AGPAT1,AKR1C1,BCAT2,BDKRB2,CACNA1E,CCL22,CCL5,CCNG2,CYP3A5,DNMT3B,DVL3,ENTPD6,FGF13,GYS2,HSD17B7,HTRA2,LIPC,MBL2,MTMR1,OR2L2,OR2T33,OR8B8,OR8G1,PAICS,PIP5K1C,PKMYT1,PLCG1,PTPN1,RUVBL1,SC4MOL,SMAD2,SRGAP2,ST3GAL4,STEAP3,TGFB1,THY1,TJP2,TP53I3,UROD,SLC25A31
Negative_5105	XDH,ASS1,CXCR6,FTMT,GNPNAT1,HSD11B2,ITGA7,MLYCD,NFATC3,OR5AC2,OR5L2,RDH10,SLC27A5,SMC1B,TUBA3C,ATP6V1G1,PAFAH1B1
Negative_5106	CRH,ACTC1,ATF2,ATP5E,CARS,CCND3,CDC23,CLDN8,CNTN2,CSAD,CYP4F3,DDX4,DGUOK,GNAT3,GUCY1A3,HARS2,HNMT,IL22RA1,ITGA2B,ITGB5,LTBP1,MAP2K7,OR14J1,OR1D2,OR4F6,OR8D1,PIK3R1,POLA2,RALBP1,SEMA3E,SMAD9,SMPD1,SRD5A1,STAM2,STX4,TUBB2A,UNC5C,VDAC1,TNFRSF17
Negative_5107	F10,HK3,PAH,ADIPOR1,B4GALT2,CCL3,CCNA1,CRLF2,EPB41,FGF10,GADD45G,MTMR2,NEFL,NUDT8,OR1A2,OR1M1,OR51F1,OR52A5,PDE4A,PGAM2,PIGK,SLC27A1,SMC1A,HLA-DPA1,TNFRSF10D
Negative_5108	CSK,LTB,ACSL5,ADCY3,ALDH3A2,ALOX15B,CACNA1E,CFLAR,CSNK1G1,CXCL12,DGKH,DTX1,EPHA7,GALNT7,GALNTL4,GLI1,GNA14,HSD17B4,HSPA1B,IFNAR1,IL17RB,INMT,ITGAL,KLRK1,OR11A1,OR11H6,OR4D5,OR52E2,OR52I2,OR5D13,OR6C74,PLK1,PMM1,PPP1R3B,PPP2R2C,PRKAA2,PTGFR,SEMA3A,SEMA3F,SEMA4G,SNAP25,SPRY2,STX18,STX4,TUBB2A,UNC5D,WNT5B,SERPINA1,SERPINB5
Negative_5109	HSPA2,NCOR2,OR6K3,PGAM4,TUBB6
Negative_5110	GK2,HGD,ACADS,ALOX15B,CACNA1F,CAV3,CD79B,CDC42,CHEK1,CLDN9,CYP11A1,CYP2A6,CYP2C8,DUSP16,EGFR,FMO2,GALNT13,GANC,HLA-C,HLCS,HTR2A,IL22,IL6ST,IRS2,KIR3DL2,LMO7,MAN1C1,NEFH,NGEF,NMNAT3,OR6C2,PIGB,PIK3R3,PIM1,QPRT,RBL2,RPN1,STK36,SUFU,TAS2R4,TRMT11,TYMS,VDAC3,VEGFB,YWHAB
Negative_5111	BMP6,COASY,ENTPD2,MAP2K4,NCOR2,PIGF,TRAF3

Negative_5112	ATM,KMO,ACP5,ATP5E,CCL1,CCL11,CCR5,CLDN8,CNTFR,CPOX,CSF2RA,CTNNA1,DAXX,DLST,E2F5,EPAS1,ETS1,IDH3B,ITCH,JAK1,KARS,MYH1,MYLK3,NFATC3,NMNAT3,OR10H1,OR2T12,OR4C6,P2RX3,PCNA,PDE3A,PPAP2A,SARS2,SGPP1,SPRY3,XYLB,SERPINC1
Negative_5113	TFG,ADH1B,AGPAT2,ATP5O,GATM,HHIP,NRXN2,OR10H2,OR2T5,OR51M1,PDX1,PLD1,SUV39H2,TMLHE,CACNA2D4
Negative_5114	ADK,C3,CSK,MUT,BCKDHB,CASK,CD86,CDKN2D,CYBA,FGF2,GADD45B,GRIA3,IFNA14,IL7R,NCF2,NT5C,OR1J2,OR5AN1,OR6A2,OR8H2,PAK1,PDE8B,PLCB4,POLR2E,POMC,PRODH2,SETD1B,SNAI2,ST8SIA1,TGFB1,TLR9
Negative_5115	ADK,FGB,FYN,GDA,PDC,AGXT,ALDH6A1,AMD1,BAI1,CCR4,CNGA4,CNTN1,DCTN1,DHRS3,GANC,GMPS,GNAI1,GNPDA1,HCCS,HMOX2,IL17RA,INHBA,INHBB,ITGA9,MAPT,MPP5,NANP,NUDT8,OR10AD1,OR1E1,OR1F1,OR2T6,OR4E2,OR5AP2,OR6Y1,P2RX5,P2RX6,RHOQ,SGMS2,SLC27A1,SLC33A1,STAT1,STEAP3,TRAF2,ATP6V1G1,CSGALNACT1
Negative_5116	BID,FH,NPL,SMO,ADRB2,ALDH1B1,BMP8B,CACNA1C,CCL7,CCNB3,COQ6,CSNK1G1,DHX58,FGF22,FGF3,GUCY1B3,HCCS,HEMK1,IFNA4,IL12A,IL22RA1,LAMA3,LIPE,LIPF,MAT1A,NAGK,NOD1,NSDHL,OR4B1,OR52H1,OR5D18,OR6C75,PAK7,PDE3A,PGAM1,PI4KB,PIP5K1A,PISD,RBPJ,RFNG,RPS6KB2,RRM2,SEMA3A,SEMA4D,SLC2A1,SMAD4,STAT6,THBS3,WNT10B
Negative_5117	ATP2A2,BMPR2,C9orf95,CASP3,CDC20,CXCR4,CYP11A1,DPM1,ENO1,FCGR3A,GIT1,HLA-F,MYLK2,NTRK1,OR1L3,OR5J2,OR6B2,OR8I2,PAFAH2,PDGFA,PER2,PPBP,RAPGEF4,RASGRP4,RPE65,RXRB,SSX2IP,STK4,TCF7L2,VAV3,WASF2,HLA-DPA1
Negative_5118	BID,GSN,MPZ,AARS2,BDKRB1,CASP6,CD80,CDC6,CHST13,CSF1R,ENPP2,ETS1,FGF13,FOXA3,GGCX,GGPS1,GNAI2,HSD3B7,IL15,IL3RA,IMPDH2,IRAK2,ITGB5,MCM7,MMP7,MYH11,OLR1,OR10AG1,OR4C13,OR4D1,P2RX1,PNMT,PRKCA,PRKCH,PTK2B,SDHB,SETD1A,SSH2,STT3B,TNFRSF8,TRMT11,VAV2,ZNRD1,ALDH18A1,CACNA2D4,PNLIPRP1
Negative_5119	APP,KDR,ALG12,CCNA2,CCR5,CHSY3,CSF1R,CXCL12,DKK4,GLO1,HADHB,IRAK3,LCMT2,MGAT3,OR1C1,OR4A5,OR56A1,ORC1L,PARD3,PLA2G2D,PNPLA3,PORCN,PRKAB1,PRODH2,PTTG1,RASA2,RETSAT,SMAD3,SORD,SPCS1,SULT1A4,TNFSF14,ZBTB16,PRICKLE1,TNFRSF14
Negative_5120	C2,CBS,ACSL5,B3GNT4,CFTR,CHEK2,CLDN1,CNDP1,CYP11A1,IFNA2,IL28B,INADL,MPST,OR52A5,OR5B21,PIK3R1,PLCD1,PMVK,RAPGEF2,SEMA4B,SOCS4,THBS3,TYRP1,UBE2L3,WNT8A,ATP6V0D1,SERPINC1
Negative_5121	A2M,ACVR2A,ADH1B,ADRA1D,AGPAT1,ANAPC2,CACNG7,CCL23,CHPF,CPT1C,CYP2S1,DHDDS,DHDH,EI24,ENTPD8,EPB41L1,HAO1,IL13,IL1R2,MAD2L2,MADCAM1,NCAM1,OR5AP2,OR5L1,OR8B4,OR8K1,PDE1B,PIGK,PLAUR,PPP1CB,PTPRM,PVRL3,SARS,SPP1,SYNJ2,TAS2R38,UGT2B28,VAV2,ATP6V0D1
Negative_5122	B4GALT7,CCR8,EXTL1,GP1BB,GRIA3,KCNB1,LAP3,NOS3,NTRK2,PLA2G1B,PPCS,TGFBR1
Negative_5123	CFD,ALDH1A1,ATP5G1,B3GALT6,CACNA1A,CHKA,EFNB1,FARS2,HPRT1,LIFR,MAGI1,MMP9,OR1L8,OR1N1,OR1Q1,OR2G6,OR2L3,PFKM,PFN3,RUVBL1,SGMS2,SULT1A2,TAS2R7,WNT3
Negative_5124	GDA,HK1,CCKBR,CSF2,GALNT1,GGCX,GUCA1B,GUCY1A3,HGSNAT,HSD17B7,IFNA17,ITGA11,ITGB1,KCNB1,LAMA4,MDH2,NUDT9,OR10AD1,POLR1B,PPP1R1A,SV2C,TUBA1A,UGCGL1,MAPKAPK3
Negative_5125	ACSL3,ADSL,AGXT2,ALCAM,ALDH3A1,ATP5G2,CCR9,CFL1,CLCA4,COQ3,DGAT2,DLAT,EPHX1,FARSA,GADD45A,GGT7,GRM1,GSTM4,IFNW1,JAG1,KIR2DL3,MAPK14,MCM4,OR13C2,OR14I1,OR4L1,OR5C1,PAK6,PIAS4,PRKACG,PVRL1,SNAI2,ST3GAL4,ST8SIA5,YKT6
Negative_5126	EBP,OR5M8,PPP2R5D,PSME3,RFX5

Negative_5127	ADIPOR2, CLDN6, DIAPH2, HTRA2, OR10J5, PLCB2, YARS
Negative_5128	C8B, DBI, IL8, LPO, ACVR1, ADCY10, BCAT2, CDC25A, CRLF2, DUSP8, FARP2, FOXA2, OR2L8, OR2M3, OR4D2, OR6B2, PLCE1, PON2, RASGRP4, SOAT1, UPRT
Negative_5129	ACVR1B, ARD1A, CD46, CD80, CXCL12, DIAPH1, FBXW11, IL28A, ITGB3, KIR2DL3, MAN2A1, MTHFD2, NARS, NCKAP1L, NCOR1, NOS3, OR2T10, OR9G1, PAX4, PIP5K1A, PRNP, RARS2, RELA, TLN1
Negative_5130	F12, FOS, ADCY4, ASCC3, ATF2, B3GALT2, CCL7, CD22, CTNNB1, DDIT4, DGKE, DGKH, ELK4, ENTPD2, FBXW11, GLYCTK, GUCA1B, HNF1A, IL8RA, OR9I1, OR9Q2, ORC2L, P2RX4, PIAS2, PLA2G1B, SELP, TUBA1B, TUBB2B, WNT16
Negative_5131	CBS, FYN, ME2, ADORA2B, ADRA1D, ADSS, AKR1A1, AKT2, AXIN2, CD38, DVL2, ENO3, ENPP1, FLT3LG, HMGCR, HRH2, IL25, KLRK1, MAN1A2, NFE2L2, OR5A1, PARD6G, PDE7B, PRKAA1, PYCR1, SMAD5, TALDO1, TLN1, WASL, WNT2B, SUV420H2
Negative_5132	ACTA2, AMD1, ATP12A, CHSY1, CREB3L1, CYP11B1, DNMT1, IL4R, MIOX, MMP9, OR52E6, POLR1B, RDH10, ST3GAL5
Negative_5133	EPOR, MAPK9, VDAC3
Negative_5134	SPN, ATP5A1, B3GALT1, CCDC6, CCL19, CD28, GSTA4, JAK3, LIPE, NCR2, NR4A1, OR52N5, OR5M8, PLCE1, SHISA5, SUOX, THTPA, WNT6, XIAP, MAPKAPK2
Negative_5135	EGF, LYN, MOS, PDC, PRL, RDX, ABI2, ACHE, AHCYL2, ALOX15, ARSD, CAPN2, CCDC6, CDH5, CDS2, DUSP4, DVL3, FOXO3, GAMT, GDF6, GSTA3, HADHA, HAO1, HLA-C, HMGCR, IL18RAP, INPPL1, ITGA1, LYPLA2, NFYB, NKX3-1, OR2W1, OR52B4, ORC1L, PARD6B, PDE5A, PON1, PPM1A, PSENEN, SDC4, SLC2A1, SLC2A2, TAS2R60, TNFSF12, TRAF5, XCL1, ATP6V1C1
Negative_5136	ADK, DLD, HPD, RFK, ACSM2A, ACSS3, AGPAT6, ALG3, AMPD2, C5AR1, CNTN2, CX3CL1, CXCR4, DTX1, ECSIT, F2RL3, GSTM3, IDH3G, IL23R, IPMK, KCNQ1, LEPR, OR10A5, OR10T2, OR13D1, OR4N4, PDE7A, PI4KA, PIAS2, PPP3CA, RPSA, SDC3, SKP2, SPRED2, TGFB2, THBD, TUBB4, UGCGL2
Negative_5137	ADO, ADCY3, ALDH9A1, ANAPC2, B4GALT6, C3AR1, CACNA1C, CCL11, CCL17, CCNE1, CD244, CFL2, DIAPH1, EDA2R, EFNA3, EFNB2, GPD1, ITGAV, KLRC4, MAP2K3, MAT2B, MDM2, NCR2, NPR1, NR4A1, OR10A2, OR2T34, OR51S1, OR52N2, OR5K1, OR8D4, PAICS, PGM3, PRPS1, SLC2A1, ST3GAL2, TAS2R49, HSD17B10
Negative_5138	PDC, SCD, CCNA2, EPB41, ERO1L, FGF2, GSTO2, MAPK1, NT5C, OR14I1, OR4D6, OR51G2, OR5D14, MAPKAPK2
Negative_5139	OAT, IFNAR1, MAP2K4
Negative_5140	GP5, MIF, ABAT, AKR1C4, ALG11, B3GALT1, CCR4, CHSY1, COL4A4, CTBP1, FASLG, IRAK4, KIR2DL4, LIAS, MAP3K1, MECR, NADK, NFATC3, OPLAH, OR10V1, OR14A16, OR2AG2, OR4C6, OR51G2, OR6C2, PDE2A, PDE9A, PGM2, PI4KA, PLXNB2, PRKAB2, RBL2, SEMA3G, SLC25A6, TAS2R42, TOMM40, ULK3, YKT6, CACNA2D2, PAFAH1B3
Negative_5141	CP, AHCY, B3GAT2, B3GAT3, IMPA2, MCM5, TUBB4, VTI1B
Negative_5142	GBE1, PDCD1, PPT1, STT3B
Negative_5143	GNS, MTR, AGPAT4, BCL2, CLDN16, CRY2, ENTPD5, ENTPD6, MAPK8, MECR, NARS, NCF1, OLR1, OR4K15, POLR2H, SEMA3F, SESN3, SMC1B, THBS2, TSHR, TUBA3E
Negative_5144	CFI, PGF, TH, AKR1B1, BAI1, BLVRB, BRAF, CD14, CDH2, CHST11, CLTA, CTBP1, CYP2C9, E2F4, GARS, GP1BB, GYS2, KLK3, KLRD1, MAPK7, MAT1A, NRP1, OR10T2, OR6K2, OR6T1, PDE1A, PHGDH, PLXNA3, PPP5C, PSENEN, RASSF5, STX2, TBL1XR1, TMLHE, YWHAQ
Negative_5145	HDC, KMO, AGTR1, ALDH3A1, ALS2, AMD1, APC2, AREG, ARG1, ARPC5, ATP5J, BCL2L1, CAMK2A, CCL23, CDH15, DGKH, FCER1G, GSTM1, HPRT1, IFT57, METTL6, MGAT2, OR8A1, PIGX, PITX2, PNPO, SCLY, UGP2, WNT3, WNT5B

Negative_5146	B3GNT5, CACNG3, CLDN17, GALNT3, GNPDA2, INSR, LGMN, LRRC4C, MYH6, NCF4, NFATC3, NT5C3, OR2T2, OR51F1, PLCB1, PNPT1, PPT1, RALB, SEMA3G, TYMP, WNT8B, SERPINE1
Negative_5147	SMO, ACSL4, ACSL6, ADCY3, ATP5G2, CACNA1A, CMBL, COQ6, DAPK3, DKK2, DUSP5, FGD1, IL12B, IL13RA1, IL13RA2, KIR2DL4, LRDD, MTAP, MYH10, OR52L1, PDCD1, PDE1A, PRKAG1, RASGRP4, RFXAP, SUOX, ATP6V0A1
Negative_5148	DAO, GSR, TPR, ADORA2A, AGRP, ASIP, BCKDHA, BCL10, BMP2, CALML6, CCL2, CDKN1B, DOT1L, FMO3, GCLC, GCNT2, HHEX, IFNA21, LIAS, MYH13, MYLK3, NAGLU, ODC1, OR10R2, OR2D3, OR5M9, OR6K6, PGAM2, PIGQ, PIM1, PTGES, PTGIS, RAB23, RFNG, RUVBL1, RXRB, SMPD3, SNAI1, TAP2, TJP2, TPMT, WBSCR17, EIF4EBP1
Negative_5149	ATM, IHH, OAT, TNR, ACOT12, B3GALT2, DGAT2L4, EPAS1, FGFR2, GALNT9, GNA12, GRAP2, GUCY2D, IL21R, IRF7, ITGA4, ITGA6, KAT2A, KLRK1, MKNK2, MTHFR, NAGLU, OR14A16, OR1G1, OR2B2, OR2C1, OR5M8, OR6C4, ORC6L, PDXP, PIK3R5, PLK1, PLXNB2, POLD1, RFNG, RFXANK, SKP1, TP73, ATP6V1B2
Negative_5150	IL15, NGEF, TUBAL3
Negative_5151	F8, ANAPC7, CRHR1, KLRK1, MTMR6, OR10H3, OR1A2, OR5H6, PFKFB1, PIK3CB, SLC2A4, TUBA3E
Negative_5152	RFK, ALAD, APOC3, BHMT, CAMK4, HSD3B1, LAMC3, LTBP1, UROS
Negative_5153	FUK, ALG3, APPL1, ATP5I, CDS1, CLDN1, COL4A4, CXCL11, FSHB, GALNS, GATM, HMGCS2, ITGB8, NKX3-1, NLGN3, NTF3, OR1A1, OR1F1, RAC2, RFX5, STAT1, UGT8, UNC5C
Negative_5154	BTK, CRH, CSK, CTH, GK, MSN, ADRA1A, AKR1C4, ARSB, ATP5J2, BPGM, CCR5, CD44, CDC25C, CKS1B, DDX52, EDNRA, EPHA7, EPHB4, FGF18, FRAT2, HTR6, IL18R1, KIR3DL3, MAP4K1, NLGN3, OR13C5, OR1Q1, OR2D3, OR56A1, OR5F1, OR5P3, OR7C2, OR8I2, OR9I1, PDE1C, PLCD4, POLR3A, PSEN1, RGS3, TYK2, UGT2B11, TNFRSF6B
Negative_5155	ADA, ZYX, ACAD9, ACOX3, ATP5I, BAI1, CCR4, DAG1, EGFR, ERO1L, GCNT4, HSD3B1, LAMB3, LRDD, MAD2L2, NCR1, OR4F6, PDE8A, PDHB, PGM1, PLEKHA8, RAB23, SGMS1, SH2D1B, SLC27A4, SMPD3, SSH3, ST8SIA5, TLR3, ULK2, VAV2, VCAM1, ZIC2, GABARAPL2
Negative_5156	GP5, LYN, AARS2, ADH4, C1QB, CHST11, CYP3A5, DEGS2, EFNA4, EFNB2, IPMK, MAOB, MAPK14, MASP1, OR10G3, OR2F1, PLAT, PLCG1, PON1, RAC1, SCIN, SPCS1, TXNDC12, TYRP1, CSNK1A1L
Negative_5157	CEL, ACY1, ADH1A, CDC42, CHRM3, CSH1, DUSP10, GGT1, HLA-DMA, HLA-G, IMPDH2, INHBE, MAPK10, NUMBL, OR6K2, OR7D4, ORC2L, PARD3, PDE7A, RASGRP1, MAPK8IP2, SERPINE1, TNFRSF13B
Negative_5158	HGF, ACP6, ACPT, AGMAT, ALDH2, APOA2, ASIP, CDK6, CES7, CLDN22, CMPK1, DARS, FMO2, GAD1, GCLC, GLO1, GNG13, HAGHL, JAG2, KRAS, NEFM, NLGN1, OR1K1, OR2M5, OR4A16, OR51T1, OR52L1, OR5A2, P4HA2, PDE10A, PDE1A, PDE5A, PDE7A, PLAU, PRKCI, PTK2, RBX1, SDHD, SPRY1, SUCLG2, TAS2R41, TAS2R48, TUBA1A, TUBB2B, VAV2
Negative_5159	AK3, AK5, CD6, CGN, F10, IL2, APOA1, ATP6V1A, B4GALT3, CD27, CD59, CHUK, COX15, CRY2, CSNK1G1, CTPS, CXCL1, CYP26B1, FGF3, GADD45A, GSTA5, HNF1B, LDHAL6B, MAD1L1, MCM3, MYLK2, NAT6, NRXN3, NT5C3, NTF4, OR1K1, OR3A1, OR9Q2, PCK1, PIK3C3, PLA2G5, POLR1C, PYCR1, RBL2, SEMA4B, SOCS4, STK3, STK36, TNFRSF8, TNFSF8, WARS2, XCL1
Negative_5160	HK3, INS, LSS, ACO1, ALG12, APOA5, BMP7, CFLAR, CYP2U1, DDX54, DHDDS, DPAGT1, HPSE, IAPP, IKBKE, IL10, ISYNA1, MYH10, OR51L1, OR52I1, P2RX3, PDE8A, PIK3CG, PRKAA1, PYCR2, SCD5, SPRY3, TAS1R2, TKTL1, TKTL2, TYMS, USE1, VEGFA, WNT2, PRICKLE2, TNFRSF18
Negative_5161	BUB1B, CAMK2G, CLTA, DAPK2, FGF5, GALNT1, GNA12, H6PD, IARS2, KLRC3, LCMT1, OCRL, OR10G9, OR4D2, OR7G3, OR8K3, PCCB, PPP2R2B, PRKCZ, RPE65, SULT1A2, TLR2, WNT16

Negative_5162	ABL1,ALPPL2,ARNT,BAAT,E2F1,FMO2,GALNTL1,GALNTL4,GLI3,GNAL,GP1BB,GRM1,HYAL1,ITGB7,LRDD,NRAS,NTF3,OR13D1,OR4S1,OR51B2,ORC1L,PPP3R1,SAT1,SLC27A5,SMAD6,SOAT1,SOS1,TBL1Y,TLR4
Negative_5163	AANAT,ACSM5,ALG10B,APOA1,ARPC2,ATP12A,CHKA,CLCA2,CREB3,CSNK2A2,CYP2F1,DERL1,ECSIT,EHMT2,GRHPR,GRIA2,IPMK,ITGB7,KLRC4,MBL2,MMP2,MTFMT,NKX6-1,OR10A4,OR6S1,PDCD1,SEMA3C,SNAP25,SULT1A2,TPM3,TSNARE1,UBE2L6,ZMAT3,ATP6V1G3,SLC25A31,TNFRSF14
Negative_5164	LHB,PGF,SCD,ADAM10,ASRGL1,ATP6V0C,CACNG4,CDK7,CHEK2,CHST13,CNTNAP2,CXCL6,CYP7A1,DDIT3,GAD2,LTB4R2,MAML1,NPAS2,NUMBL,OLAH,OR1J2,OR2T12,OR4D5,OR4N4,OR6C70,PAK1,PDE9A,PIGW,PIK3R1,PLA2G5,PMM1,RAF1,RBL1,UBA1,VAMP3,ATP6V0A2
Negative_5165	CD6,F11,HK2,IHH,ANAPC10,CACNG3,COL3A1,CTSL1,DUSP2,FGFR4,FMO3,GALNT11,GPX5,GRIN2C,GSTO2,IFNA10,LAMC3,LARS2,LRRC4C,MAPT,OCRL,OR10X1,OR13C9,OR1M1,OR2G6,OR51Q1,OR52I1,OR6C74,PI4KA,PIP5K1B,POLR2C,PVRL3,RALBP1,RARS2,SEMA4C,SOS2,SPRED1,SUFU,ATP6V0D2,HSD17B12,TNFRSF13C
Negative_5166	CD4,RFK,TNN,ACAD9,AGMAT,AMY2A,ATP13A2,B3GALT1,B3GAT2,C3AR1,CCR2,CDC20,CHD8,CHEK1,CTSL1,CYBA,FABP3,FARSA,GALM,GNAI3,GRIN2B,GSTA5,HES1,IARS2,IL2RB,ITGA2,LAMB2,LGMN,NRG4,NUDT8,OR10AD1,OR2T5,OR5AC2,OR6C3,OR8D4,PAK7,PLXNB3,PVRL3,ST3GAL4,TNFSF10,MAPKAPK2
Negative_5167	BMPR2,CD226,CPOX,CYP4A11,DCTD,MDH1,ODC1,OR56B4,PFKP,RAC3
Negative_5168	KHK,ACVR1C,AKR1B10,ALDOB,DLL1,GALNT5,IQGAP1,MCAT,OR11L1,OR14J1,OR2AG2,OR4D6,OR4K17,OR52I2,OR5AC2,PCK2,PFKFB4,PLA2G1B,PTGFR,RBL1,SNAP25,TCF7,TUBB4,PPP1R12A
Negative_5169	ABO,ADRBK2,ALDH4A1,ALG9,AOX1,ATN1,CBLB,CCL1,CD3E,COQ6,DDX56,EPHX2,FLT3LG,GNAI1,GNAQ,GNRHR,HCCS,IGF1,IL25,ITGAE,LIAS,MAN1A2,NTN1,ONECUT1,OR13G1,OR4F5,OR4F6,PIGX,RPS6KA6,TNFRSF8,TRIP10,B3GALNT1
Negative_5170	C1S,GP6,HDC,B3GALT1,CCNA1,CRLF2,CXCL10,DFFB,FOSL1,GALK1,GLI1,ITPKA,MYH8,MYL7,OR51M1,PAX4,PIGO,PPP3R2,PPT1,SMURF1,WNT7B,PAFAH1B3
Negative_5171	AGL,ALDH1A1,BRAF,CASP6,CD99,CLTB,GMPR,OR10H2,OR13C4,SIAH1
Negative_5172	FST,MPI,ALDH1A2,ARG2,B3GAT3,CADM3,COQ3,DUSP1,DUSP14,FLT1,GLUD1,HSP90B1,HTR5A,ITPKA,MYH2,NAGLU,NEFL,NOD1,OR4A15,OR4A5,P2RX2,PARS2,PAX4,PDE1C,PDE6D,PPP1R3A,PSAT1,RDH12,RFXAP,SV2B,ULBP1,EIF4EBP1,HLA-DPA1
Negative_5173	AGK,BTC,MAG,ABCC8,ATP5I,CLTA,CSNK2B,FGF21,ITGA10,L1CAM,NCK2,OR10A5,OR10J5,OR2T11,RASA2,SLC2A4,VKORC1
Negative_5174	F8,LPL,MET,PGD,TNN,ACO1,ALDH3A2,ALOX12B,ANAPC7,ARSB,C1QC,CCR6,CD19,CHAT,CHRNA7,DDB2,DUSP4,EHHADH,ERBB3,GALNT10,GMPPA,GNAS,HIP1,HSD17B8,ITGA5,ITGA7,KCNQ1,KLRK1,LAMA4,LIPE,OR13H1,OR8K3,P2RX4,PCYT1B,PDGFB,PER2,PKMYT1,POLR2I,PTDSS1,PTK2B,RALB,RBL2,RPSA,SAT2,SLC9A1,TGFBR1,WASF3,ATP6V1E1,CTNNBIP1
Negative_5175	GPI,ACSM5,ALG13,ATP13A2,BUB3,CACNA1G,CACNG3,CASP8,CCNG1,CD80,CYP27A1,GCDH,GGT7,KCNQ1,NOTCH3,OR11H4,OR2L13,OR5M3,OR8K1,PLCB3,PPP1CC,PTPN5,RRAS,STAT6,TUBB3,UROD,MAPKAPK2
Negative_5176	ACSL4,CASK,CHEK1,ENPP3,IFNW1,OR2AG2,OR4C16,OR51F2,PER3,SPTLC2
Negative_5177	BTK,F12,PLN,ABL2,ACAD9,ACTG2,ADH1A,ASCC3,ATP5G2,CDK6,CLDN14,CTBP1,DVL1,GFPT1,GMPR2,GRIN2A,IGF1,MCEE,NRXN3,OR52E4,OR9K2,PAX6,PGK2,PIGO,PLCG1,PRKG1,PSME1,QARS,SAT1,SSH1,ATP6V0D2
Negative_5178	BDKRB1,CCNB2,OR10A7,OR5P3,PLA2G3,PTAFR,TACR1,HLA-DQA2

Negative_5179	IDS, IL6, A4GALT, BMPR1B, CASP10, CCNB2, CCND1, CCND2, CHRM2, COL5A2, CTTN, DDIT3, ENO1, ENTPD7, FUT5, GCH1, HHEX, KLRK1, MAP4K2, MKNK1, NUDT2, OR10X1, OR11H6, OR51G1, OR6N2, OR6Y1, PFKFB3, PPARG, RDH5, RIPK1, ROCK2, SSH2, SUOX, TAS2R9, TGDS, TP53I3, TNFRSF11B
Negative_5180	ILK, MOS, MSN, NPY, RDX, ACVR2B, AGXT2, ARAF, ATIC, BCAT1, CASP6, CD74, CDK6, CHST11, COL2A1, DGUOK, DVL2, E2F4, EGFR, F2RL1, FCGR2B, FIGF, IL10RA, IRAK4, MMP9, NFKBIE, NOS1, NOTCH1, NUDT2, OR10AG1, OR1B1, OR1D2, OR4A15, OR51F2, OR5L1, OR6N2, PDHA1, PIAS4, PRIM2, RDH8, RERE, SCP2, SUFU, TRAF6, TRPC1, TUBB, VAMP2, WNT9A, XCL1
Negative_5181	HGF, ADCY2, ALG6, AMPD1, BCL10, CSF1R, CXCL12, CYFIP2, DHCR7, IL28B, IPPK, ITGA1, LLGL1, NCK1, OR2H2, OR2Y1, PANK2, PLCD3, PLCE1, ROBO3, TLR3, TNFSF11, TYROBP, WASF3, YES1
Negative_5182	HGD, ALDH1A3, CSF1, E2F3, GPLD1, IL24, SC4MOL, TALDO1, UBA1
Negative_5183	CCL1, CYP2C9, HLA-DOA, MARS2, MECR, MYH10, OR4D6, TNFRSF17
Negative_5184	C8G, CRK, GGH, LAT, NLK, ALAS2, APBB1, APOA2, ARHGEF1, B3GNT4, CASP6, CAV3, CCR9, CDKN1C, CTSG, CX3CL1, CYP27A1, DPM2, FGF21, FLT3, GABARAP, GNA12, GRM5, HSD3B2, IL17A, INPP1, LIFR, MGST1, OR14A16, OR4Q3, OR52N2, PGM1, PPP3CC, SEMA4F, STK36, SUCLG2, THBS2, TIAM1, TJP3, TSHR, TUBA1B, TUBA3E, TUBB, WNT5B, ATP6V0A1
Negative_5185	LCK, WAS, ACMSD, ACSL5, ARAF, B4GALT5, CACNA1F, CCL20, CHUK, CMAS, CNTNAP2, DGKH, DMGDH, FGF12, FMO5, FSHB, FUT5, IFNA4, IL22, IL8RA, JAG1, MAP4K1, MASP2, NODAL, OR10H3, OR2C1, OR2M5, OR5AK2, OR7G3, OR9G1, P2RX2, PAICS, PIK3CB, PLCD1, PPARG, PTCH2, SELE, SH2D1B, SMAD2, SMAD6, SNAI2, SPHK1, STX16, TAS2R4, TBXAS1, TNFSF8, UGCG, WNT2B, HSP90AB1
Negative_5186	AMDHD2, CAV3, CCL13, CTSB, CTTN, ENTPD8, ESAM, FMO3, FZD5, GLYCTK, GPD1, GSTA2, GUCY1A2, HIP1, KLRC2, NADK, NEFH, OR10G3, OR2D2, PANK4, PDE11A, SEMA4D, STK11, TAS2R14, TAS2R48, TIAM1, TJP2, VAMP4, VANGL1, YWHAQ
Negative_5187	GP9, GSTM2, IL28RA, IL8RB, PDE4D, RPS6KA4, STT3B, MAPKAPK2
Negative_5188	EBP, GCK, ETNK1, GDE1, KIR2DL3, NT5C, OR1D2, OR52H1, PITX2, PRKAG3, RARA, THBS2, TUBB2B, PNLIPRP1
Negative_5189	C1S, INS, TYR, ABLIM1, ATP6V1A, BAAT, BIRC2, CCNE2, CD74, CD79A, CLDN9, DDIT3, DDX23, DHRS4, DPM2, EPAS1, EPHB6, HNF1B, HSD11B1, MTHFD1L, OR10G7, OR1S2, OR4F6, OR6B2, OR9A2, POLR2K, PRKAB2, RAF1, RND1, SELP, TAS1R2, TAS2R49, TJP2, UGT2B4, UPP1
Negative_5190	ABP1, ACADL, ACY3, APOC3, CAPN2, CFL2, CFTR, CNTNAP1, FCGR3B, FGF1, FUT3, GFPT1, MCM2, NEU4, OR2A25, OR4N2, ORC1L, PDE10A, POLR1E, PROS1, RALBP1, SUV39H2, UPP1, WBSCR22
Negative_5191	ADO, AMT, GLA, MPL, ACSS2, ARSA, B4GALT1, BBC3, CARS, CDC25A, CDH2, CHIA, CHKA, CNGB1, EFNB2, FGF11, IAPP, IDH2, MAN1C1, OR9G1, OXSM, PDE8B, PIGS, PTCH1, RFXAP, SHC2, SHC3
Negative_5192	AK1, BTD, KDR, RB1, SCD, ARPC2, CSNK1A1, CTNNA1, CYP1B1, DTX4, GARS, GP1BB, HTR2B, JUND, KRAS, LAMB3, NEGR1, NKD1, NPAS2, PRSS3, PVRL2, SEMA3G, TAS2R5, UPP2, PDCD1LG2
Negative_5193	MSN, PGD, ACVR1B, CALML6, CANT1, CHUK, CIITA, CNDP1, CNTN1, DUSP3, EFNB3, FGF18, FLT4, G6PC, GCLC, GLYCTK, GUCY1B3, HAAO, IRAK2, MYH10, NFATC4, NTN1, NUMB, OR10Q1, OR10V1, OR2T3, PRODH2, PTPRJ, RALGDS, RERE, SMURF2, TGFB3, UGT2A3, WNT8A
Negative_5194	HDC, BAIAP2, EHMT1, FRAT2, GALNT9, NRXN2, VDAC2, HLA-DRB3, PAFAH1B1
Negative_5195	AGXT2, CHEK2, CSF1R, HARS, PGAM4, PLXNC1
Negative_5196	B3GAT1, CYP1A1, FGF20, GALNT5, IPMK, PRKCZ, RASGRP4, TNFSF11
Negative_5197	BIRC2, CFTR, CLDN18, CYP7A1, DDB2, EDAR, GCLM, MADCAM1, NCR1, OR2A14, PON1, WNT1, ARHGEF12, ATP6V1G3
Negative_5198	CD40LG, DUSP9, GLUL, HLA-DOB

Negative_5199	PLG,ACSM5,ACTN4,ALDH1A3,APRT,ASIP,ATP5O,BCKDHA,CCKAR,CDKN1C,CSNK2A1,CTNNA1,CYP26A1,DDX47,DERA,EPB41L1,IARS,LCMT2,MAPK13,NMNAT2,NOTCH3,NR4A1,OR10J1,OR1L8,OR51E2,OR51S1,OR52R1,OR6F1,PAK3,PAK4,PIK3C2A,PPP2R1A,PRKCD,SMAD6,STAT1,THBS2,TRPM5,TSC1,PRICKLE1,SERPINA5
Negative_5200	AKR1B10,AREG,C7orf16,CDK5,CISH,COL6A1,CSF3,CX3CL1,FTMT,IFNW1,IL10RA,IL24,LRAT,NCF1,NRG2,NRXN1,OR1B1,PHKA2,PVRL3,TAS2R9
Negative_5201	AUH,GP9,MPL,ACADS,ACP5,ALAS1,ALDOA,APPL1,AVPR1A,BMP4,C5AR1,CAPN2,CCL28,CCL4,CD27,CHD8,CSNK2A1,CYFIP1,DARS2,DBF4,DDX4,DHRS9,FGF6,FUT6,GALNT2,GALNT7,GPD2,INSRR,ITGA7,LIPE,MAGI1,MYL9,NUMBL,OR10R2,OR51I1,OR8B4,PLXNB1,PPBP,PTPRJ,RASGRP1,SUFU,TFDP1,TUBA1C,WNT11,WNT7B,CSGALNACT2,PNLIPRP2
Negative_5202	ARD1A,ATP6AP1,CANT1,IFNA6,ITGA2B,NT5C1A,POLE4,PRKACB
Negative_5203	GP6,IHH,TH,CASP8,CCR6,CYP3A7,DERL1,EFNA2,EPHB2,FUT1,GALNT7,GMPPA,GNA11,IL10,IL23R,ITGA8,MAP3K3,MEF2C,MGAT4A,MTHFD2,OR51B4,PAK7,PGLS,PRKCB,PTPRZ1,RALA,RAPGEF4,UAP1,XCL2,HSD17B12
Negative_5204	CD6,GGH,LPO,AADAT,ACTG1,ALPL,BMP8A,CACNG8,CSF3,GADD45A,GAPDH,GCLM,GLS2,GSTZ1,HAO2,HSD17B4,IL22RA2,KIR3DL3,NQO1,OR4C16,OR6X1,PANK2,PCYT2,PFKFB1,PHKG2,PLA2G2A,PLA2G6,PTTG1,RAC1,RPS6KA6,SELPLG,SIAH1,SLIT2,SPCS1,SPCS3,SPRY2,STX10,TALDO1,TNFSF9,TUBA8,VNN1,GABARAPL2,PAFAH1B2
Negative_5205	PGP,SYK,ACTN4,AMPD1,CCL26,COL5A1,CYP3A43,CYP7A1,DDHD1,EPHA6,F2RL3,GAL3ST1,GALNT10,GSK3B,GSTP1,HRH1,IL12RB2,MAD2L2,MAOA,MAP3K11,MAP4K3,MGLL,NRXN2,OR2K2,OR2T8,OR6K6,OR6T1,PANK1,PDE6D,PIAS4,PPP1R3C,UGT2B11,VANGL1,TNFRSF10A
Negative_5206	AK7,C1S,GPT,HGD,ME2,ACACA,ADORA2B,ALOX12,ATP2A2,BMP5,BMPR2,C5AR1,CACNA1H,CAPN2,CCL17,CCR9,CER1,CHKA,CHRM1,CLTC,CX3CR1,CYP27A1,DHRS4,ECHS1,ENTPD1,FABP4,FGF3,GALK1,GALNT13,GRM5,GSTZ1,IL7R,ITGA2,L1CAM,OR1A1,OR1A2,OR51F2,OR5T3,PFKL,PLA2G10,PNMT,PRIM1,PSAT1,PSENEN,RBPJL,UGT2B7,SERPINA5
Negative_5207	AKR1A1,OR1S1,STX5
Negative_5208	BAD,F9,INS,ME3,ALCAM,AQP7,CREB3L1,CYP4A11,DDOST,DDX52,EFNA5,GNAS,GPD1L,HARS2,IL29,OR2F2,OR4A15,OR4D1,OR6X1,PDE4A,PLA2G2F,PMM2,POLR2I,PPP2R5B,PTPRR,RRM2,SELPLG,SGMS2,SSH1,ST8SIA5,TAS2R3,TGFB2,TSLP,VAMP3,YWHAE,PDCD1LG2,TNFRSF13C
Negative_5209	CHKA,EDNRB,HADH,MGLL,MTHFD2,NME2,RND1,ROBO2,SEMA4C
Negative_5210	ACAD8,ACSL1,ACVR1,CCL21,CHST12,DLAT,EDNRA,FADS2,NKX3-1,OR2M2,POLD1,POLD3,PTPRB,RBL2,SETD1A,TAS2R50,UROC1,XYLT1
Negative_5211	ALG8,ATP5C1,CXCL2,DDX51,FECH,GCNT1,HCST,ITGA9,KLK3,NCSTN,NFX1,NTNG1,OR11L1,OR52D1,OR8D2,RFNG,SDHC,TAS2R1
Negative_5212	MET,ACTG1,ALDH1A1,ARSB,ATP12A,C1QA,CAMK2B,CRLF2,CXCL13,CYCS,CYP2A7,DHRS3,EXOC7,FBXW11,GSTM1,HIF1A,INPP5E,LPAR1,NCR1,NRG1,OR13C9,OR1G1,OR2M2,OR52N2,OR5AR1,PHGDH,PI4KB,POLA1,PPM1A,PRKX,PSME2,RBPJ,RHOA,ROBO2,SAT1,SULT2B1,TAS2R16,WNT6,YWHAE,SERPINB5,ST6GALNAC5
Negative_5213	C2,IL7,ABLIM3,AVPR1A,CD55,CTBP2,CXCR6,FGF21,FIGF,FLAD1,GCDH,HSP90B1,HSPA2,IQGAP2,MCM2,NODAL,PNPLA3,PTGES2,STAT1,STX12,STX7,WASF3
Negative_5214	FYN,LPL,ACSL5,ACVR2B,AGRN,CYFIP1,EPHA5,FABP4,FGF13,FGF3,GLI1,HLA-C,IDI1,IL17RA,IL1A,LIPF,NAT5,NCK2,OR10A4,OR1S1,OR56A3,OR6M1,P2RX7,PDE1B,PIK3CD,PNPT1,POLR3H,PTPRB,RPIA,SOS2,SUV39H2,TYROBP,UROS

Negative_5215	CP,PGF,COL4A4,CXCL9,DGKG,EPHB3,HLA-F,KIR3DL1,MAGI2,NFYA,OR4F6,PGM2,PIP5K3,SCD5,TOMM40L,TNFRSF10C
Negative_5216	ATP6AP1,FASLG,FZD4,LDHD,NFYC,NRXN1,PDE8A,PPP1R3D,PTTG1,RPS6KA3,STX12,STX1B,TJP1,TUBB,ULK3,ST6GALNAC5
Negative_5217	ALDH5A1,ALG5,ALOX12,B3GAT1,BAIAP2,BET1,CACNB3,CASP8,CCR9,CES2,CHEK1,COX15,CSNK2B,FTH1,GAS1,GRHPR,NAT5,OR1J4,OR1Q1,P2RX2,PDE6G,PIAS2,PIGL,POLA2,PPAT,PPT1,PTGES2,RDH12,RPN1,TACR3,TAS1R3,TAS2R3,TNFRSF9,WNT2,ZNRD1
Negative_5218	OAT,PDC,ADSSL1,CCL17,CD80,GSTZ1,ICOSLG,IL17A,OR2A42,PLA2G2D,PLCE1,PPM1A,PRPS2,RARS2,WASF2,SERPINA1
Negative_5219	ATP6V1H,CAMK4,CBR1,CCKAR,CD47,CTNNB1,CTSB,DKK1,FOXA3,GCLM,GGT6,HTR4,ITGAX,MCCC2,MGAT1,MGST3,MYD88,NR1D1,OR10A3,OR4M1,PAK6,PDE4D,PPP1CA,PRKAB2,SERPIND1
Negative_5220	ADSL,CD244,DKK1,IQGAP3,OR5B12,RALA
Negative_5221	GALNT11,GFPT2,POLR3K,TCF7L1,YARS2
Negative_5222	PC,ACADL,AKR1B1,UROD,ATP6V1G3
Negative_5223	ACACA,ALDH1A3,AOX1,CCDC6,CD59,CD79A,CRHR1,DAAM2,DRD5,ELK4,FGFR3,FUT1,GFAP,GSTA3,IKBKG,LAMA3,LGMN,MAGI3,MTMR2,OR5AP2,PANK1,PLD2,PRKAG1,SARS2,TNFSF4,TNFSF9,YWHAH,CSNK1A1L,HSD17B10,KIR2DL5A
Negative_5224	BTC,FGA,FN1,GP9,JUP,MAX,SRF,ABLIM1,ACAT2,ACOX3,ALG13,ALG6,ANAPC2,CCL24,CD34,CDK4,CNGB1,COX15,CREB1,F11R,FBXW11,GRIN2B,NEU3,NME7,OR1B1,OR2L13,OR3A3,OR7C1,OXCT2,OXSM,PFKFB3,PIGU,PRKCQ,PRSS2,PRSS3,SPRED1,TAS2R60,THBS4,UGCGL1,B3GALNT1,TNFRSF12A
Negative_5225	TFG,ACTN2,ACVRL1,AGRN,ATP6V1D,CDKN1A,CLDN1,CTNNB1,CYP11B2,CYP2C9,CYP4A11,DGKE,DHX58,DPAGT1,EDAR,EPOR,F13B,GGT5,GSTK1,HTR2A,HTRA2,IL18RAP,INPPL1,KAT2A,LARS,NCOR2,OR11H4,OR13G1,OR5D13,OR7A5,PIGN,PNPO,POLR3D,PPOX,PRKAG3,PRKCQ,PSPH,SEMA3A,SH2D1A,SIPA1,TP73,TRAF6,TUBB2B,ATP6V1G3
Negative_5226	C4B,FH,TNC,ARAF,CLDN22,CSNK1D,CXCL10,CXCL11,DGAT2,DUSP9,FDFT1,FLT3LG,GMDS,GP1BA,IL22,IMPA1,ITGB8,LFNG,MAP3K4,OR4X1,OR6Q1,PIGM,PPID,PRKAR1A,TNFRSF9,TPH1
Negative_5227	F3,CCL16,CLTB,GALNT8,GNRH1,KLK3,OR10G3,OR10G9,OR2F2,SOCS5,UAP1
Negative_5228	TKT,TYR,ACAA1,CKMT2,CYBB,GAPDH,NFYA,NRXN2,NTNG1,OR2B11,OR6C6,THBS4,VTI1B
Negative_5229	EIF4B,HNF4A,KYNU,MCM6,OR52D1,OR8G1,PCCA,PLXNA1,POLR2K
Negative_5230	C8G,TPO,ACTG1,AMDHD1,ATP5D,CDK5,EIF4E,FGF10,FGF23,HADH,IL20RA,IMPA1,KAT2A,KLRC1,LTA4H,MAOB,NCR2,NR1H3,NUDT8,OR14I1,OR8D4,OXSM,PIK3R1,PLXNC1,PRSS2,PTGS1,RAP1A,SUFU,TAS2R14,TSLP,TUBA3E
Negative_5231	FUK,LCT,ADRA1B,ALDH3B1,CARS2,CCL4,CCNB3,CDKN1B,COL1A2,CTSS,DAPK2,DERA,DPYD,ENTPD8,FARP2,GNPDA2,MAD2L2,MS4A2,MYH8,OCRL,ODC1,OR10AG1,OR10T2,OR2L8,OR51Q1,OR6C74,PGAP1,PLAU,PPP2R2C,PRKAA1,PTDSS1,PVRL2,RPN2,RPS6KA5,SOD1,ST3GAL1,TCF7L1,UGT2B7,VAMP4,VAV2,MAPKAPK3
Negative_5232	C4B,ALDH5A1,ATP6V1H,CCL2,CCR6,CDC27,FGF14,LAMC2,MTHFD2,P2RX5,PRPH,ROCK1,SIGLEC1,ST8SIA5,UCP1
Negative_5233	LEP,ACVR2A,ALOX12B,APRT,CCL24,CDH15,CDKN1A,COX15,EPHA8,GLI2,ITGB3,MCCC1,OR10P1,OR51B4,OR52E8,OR5I1,OR5L1,P4HA2,PIP5K1A,PTGFR,RCHY1,RPS6KA1,RRAS,UBA7,YWHAG

Negative_5234	NPL, PAH, UBC, ARHGEF4, CCDC6, CCL21, CCR5, CHEK1, CLTCL1, CNTN2, COQ6, CSNK1G3, DHRS9, DPM1, EDAR, EDNRA, EFNA1, EPHA5, G6PC2, GALNT10, GLUD1, GSTM4, HMGCS2, IL20RB, MAP3K2, MMP7, NRG1, OR1A2, OR1B1, OR2T12, OR51F1, OR51S1, OR5L2, PGS1, PLCD4, POLR2D, POLR3B, PRKAB2, PRKCG, PTGS1, ROBO1, RPE65, SLC2A1, SPHK1, THBD, TP53I3, TUBB4Q, UBA1
Negative_5235	EZR, GLA, NPL, MAD2L1, P2RX1, P2RX5, P2RX6, PAFAH2, POLA1, PPARD, STX1B, ULK3
Negative_5236	ME3, NGF, SRF, SYK, BBOX1, CHKA, FOXA2, OR1S1, PIK3R2, POLR3D, RDH5, SDHB, PPP1R12A
Negative_5237	AMH, JUP, ACAA1, ACOX1, ALDH1A3, ALG8, ARSE, ATP5D, BDKRB2, BMPR1A, CDH1, CTNNA1, CXCL2, DGUOK, E2F5, EHMT1, ENO1, ENO2, ENPP3, FGF13, FZD6, GFAP, GNAO1, HTRA2, IFNA6, IFNGR1, IL1R2, ITGA1, ITPK1, KLRC2, MBL2, MCM5, MGAT4A, MYH13, NFYB, NME7, OR4D2, OR51B6, OR52J3, OR8D2, POLR1B, PTPN6, SARS2, SETD7, ST3GAL5, TAS2R48, TLR4, TPH1, UXS1
Negative_5238	RDH16, SSH3, B3GALNT1
Negative_5239	BTC, CFH, SRF, ACACA, AMHR2, AOC2, ARHGAP5, ARNTL, ARRB2, CASP10, CES1, COQ5, COX15, CREB3L4, CXCL16, DAD1, DUSP5, E2F5, ERBB4, GAMT, GCLC, GRB2, IDI2, IL1RAP, KAT2B, MAPK11, NUDT8, OGDH, OR10K2, OR1I1, OR2T6, OR52H1, OR5R1, PDGFD, PFKP, POLR2B, POLR2K, RENBP, SMPD1, SOS2, STAT2, SULT1A2, TAS1R2, UGDH
Negative_5240	ADC, C1S, CRK, DBT, TK1, ANAPC5, BMPR2, CCL13, CHDH, CLDN2, CREB3L2, GPX5, HMGCL, ICOSLG, IL13, KCNB1, KLRK1, OR10G8, OR2AG2, PFKP, POLR2A, SRGAP1, SSH3, TGDS, TLR3, TPH2, TPI1
Negative_5241	FST, AASS, ALDH3B1, B4GALT1, BUB3, CD3E, CDC2, DLL4, DPYSL2, FUT9, IGF1, IL22RA2, IL4R, ITGB1, LDHD, OR10P1, OR2D3, OR5L2, PPP5C, RAB5A, RPSA, SEMA4F, SH3BP2, TAS2R13, WNT7A, ATP6V0A1
Negative_5242	C3, PGD, UBC, ADH1B, AKR1B10, BCKDHA, BUB1, CALML3, CCL20, CCNA2, CCND2, CD46, CD55, COMT, CPS1, CRB3, CYP3A5, DDX41, DUSP5, EHMT1, GAD2, GIT1, HCCS, IQGAP1, ITGA3, MAP3K7, NCR3, NFX1, OR4D6, OR6B2, OR6X1, PDE1B, PKLR, PLA2G1B, PLA2G3, PNLIP, PPP2R5A, PSME1, RALB, RAPGEF1, RND1, RPSA, RRM2B, SRGAP2, TAS2R13, TAS2R42, UBE2L3, VEGFB
Negative_5243	FAH, FH, CAMK2G, CHST12, CYP1A1, CYP3A5, DKK1, EIF4B, FDFT1, GALNTL1, IL15RA, JAG2, NFATC1, NRG4, NUDT9, OLR1, OR2A2, OR2AE1, OR4A16, OR4N5, OR9A2, PAK3, RHOQ, SARS, TM7SF2, ULK2
Negative_5244	ILK, LCK, ADIPOQ, ARAF, ASPA, BPNT1, CACNG3, CCL23, CCR4, CLDN5, CSNK1E, E2F2, GNG12, HSPA2, ITGB4, MAP3K13, MAPK3, MYL7, NKX2-2, OR2AT4, OR6C2, PPP2R5A, SGMS2, SLC2A1, SUV39H2, TSHR, WASF3
Negative_5245	CCL25, CFTR, CTNNA1, HSD17B3, LDHD, OR2T29, PER3, PTGS2
Negative_5246	CFH, DLD, F3, LAT, NLK, ACP6, ADCY5, ALOX12, ALOX12B, ARPC4, ATP13A2, CAMKK1, CFL2, COL3A1, COL4A6, CYP3A4, DDX23, DHRS9, FGD1, FGFR2, H6PD, LEFTY1, MAP4K4, MGAM, MGAT2, NFAT5, OR10C1, OR10S1, OR1J2, P2RX4, PDE3A, PDE6G, PHKA2, PIGB, PRKCE, PROC, PRODH, RALGDS, RUVBL1, SH2D1A, SORD, UAP1, HSD17B12
Negative_5247	ABCC8, ACAA2, CCL20, GDE1, GLUL, OR2L3, OR2T1, THBS4
Negative_5248	CTH, GP5, TFG, TNN, ACMSD, ACVR1, ADH1A, CCL15, CD226, CHST13, CHSY1, CREB3L4, CSNK2B, DAPK2, ENPP1, FGF9, GALK1, HAP1, IFIH1, IFNK, IL20RA, KLKB1, LEFTY1, OR1K1, OR4K1, OR51G1, OR8H2, PHKB, PPOX, SETD1A, SFRP1, TIAM2, TOMM40L, MAP3K7IP1, SUV420H2
Negative_5249	BPGM, CAMK2D, CD247, CLTA, CMAS, CYCS, CYP2A6, DGUOK, EFNB2, FMO1, GCDH, IFNAR1, IFNK, MAFA, MAPK10, MASP1, MMP9, NPAS2, NTNG1, OR6N1, PHKG2, PPA1, RCHY1, SETD1B, SIPA1, SPTLC2, TPI1, ARHGEF12, SERPINA1
Negative_5250	SCD, ACSM1, ARPC3, CD58, CISH, ITGAX, LRP5, NEFH, NOS3, NPR1, OR2M2, PFN1, PLA2G1B, PLCG2, PRKCQ, SCLY, SOCS3

Negative_5251	CKM, IL3, TH, ABI2, AKR1C2, CD244, DDX50, F2RL2, NFKB2, P2RX4, PLCB1, POLR3H, PRKACG, SSX2IP, TGFBR1
Negative_5252	CGN, CP, AKR1B10, ALG8, COX15, CTBP2, CTPS2, DERL1, ENTPD7, FGF6, FUT7, GSTP1, HSD17B4, NAT1, OR1A2, OR4C11, PPP2R5C, PTGES2, PTGFR, RASGRF2, SV2C, SLC25A31
Negative_5253	EDA, NNT, CAV1, FMO1, GMDS, ITGA3, KAT2A, KIR2DS4, NAT6, PIK3C2B, PLXNA3, SPRY4
Negative_5254	IL9, ARRB1, BLVRA, CCL16, DLL4, FGF20, PDGFD, PMAIP1, PTPN5
Negative_5255	F12, PC, ACOT12, ACSL4, AKR1C3, AMDHD1, ANAPC1, CD79A, CSF1R, CSNK1G1, DAAM1, GBA3, GSTM4, HBEGF, HLCS, IL22RA1, IMPDH2, LDHAL6A, NFYA, OR1A2, OR52A1, OR5D13, PDHA1, PI4KA, RPS6KA6, SDHB, ST8SIA5, STX4, TAS2R5, VAMP3, ATP6V1E2
Negative_5256	ADRA1D, ARHGEF6, B3GNT2, CLDN8, DLL3, DVL1, EDAR, FLT1, FZR1, HNMT, JAG2, KIR2DL1, OR4N4, PER3, PLAU, RDH11, SV2A, TBL1Y, THBS2, ATP6V0A1, HSP90AA1
Negative_5257	EPO, GH2, MVK, PGD, ADCY10, ATP5D, CREB3L2, CTLA4, ENPP3, FRAT1, HES1, MCAT, MFNG, MYH3, NAGS, OR11H6, OR2S2, PIGK, PIP5K1C, RALGDS, SETD7, STAT4, SV2C, TAS2R8, TNFSF8, UNC5D, WARS2, ATP6V0E1
Negative_5258	TFG, ADCY8, ATP5L, CAMK2D, GUCY2F, MAP2K3, OCLN, OR13D1, OR1N2, OR2T6, OR4C15, OR4F6, OR51G2, OR5P3, ORC4L, P2RX2, PVRL1, SLC27A4, VAMP4
Negative_5259	TNC, CLTC, EPB41, HDAC1, IMPA2, LEF1, OR2B6, PLAT, RAC1, SDC3, SKIV2L2, UBA1
Negative_5260	CD4, NNT, TNF, ACSL5, ACTB, ALG12, ALPL, HSD17B1, OR5AN1, PDE11A, PRODH, SYNJ2, CACNA2D3
Negative_5261	FSHR, OR4C6, RPS6KA5, SERPING1
Negative_5262	ACVRL1, ALDH6A1, APC2, DGKA, IFIH1, MGAT4B, POLE2, SEMA3E, STX5, TLN2, TUBB2A
Negative_5263	GADD45B, IL28B, LAP3, MDM4, OR4K2, SMAD6, ULBP3, VANGL2
Negative_5264	CLDN6, EPRS, PDE3B
Negative_5265	AGL, F2, LAT, ACP6, ANAPC2, AVPR1B, BMP2, BMP8A, CCKAR, CREB3L4, ELK4, GABARAP, GRIN2B, GSTA1, IFNW1, NKD1, OR1A2, OR2T12, OR4F17, P2RX6, SHC2, SMURF2, SRGAP1, TRDMT1, WNT9B, WWP2, CSGALNACT1, CTNNBIP1, HLA-DQB1
Negative_5266	BTC, BTK, IL4, ASIP, CIITA, GPT2, INSR, NUDT8, SFRP1, SNW1, PLA2G12A
Negative_5267	CR2, ACVR2B, ADAM10, BAI1, C1QC, C5AR1, CD274, CNGA4, CYP2E1, DDB2, EREG, FRAP1, GADD45A, GGT5, IL22RA2, IL5RA, KIR2DS4, LAMA3, LIMK1, MNX1, NCF4, NFATC4, OR10V1, OR52K1, PDE3A, PFKM, PIGN, PLIN, POLE2, PROC, PTPRM, PYCR2, RALA, RASGRP2, RRAS2, RRM1, SNAP23, SUCLG1, XCL2, ATP6V1B2, MAPKAPK3
Negative_5268	CTSG, IL18R1, KYNU, MAN1C1, OR52N2, PKMYT1, TRPC1
Negative_5269	CTPS, OR1N1, OR5K1
Negative_5270	C3, ABLIM3, AOC2, BIRC5, CDK6, CNTNAP2, COL5A2, CSNK1G3, CTNNA1, EDNRB, FGFR1, GUCA1C, HES1, INPP5A, IRF3, JAK1, LIFR, LRP5, NCKAP1, NFKBIE, OCLN, OR2B6, OR51M1, PIAS2, PPARD, RAPGEF4, SCD5, SCLY, SLC2A1, SRGAP3, ST6GAL1, TKTL2, YARS2, YWHAG
Negative_5271	ID3, IHH, BPNT1, CCKBR, COL5A3, CYP4F2, GNRH2, GYS2, IPMK, KLRC3, MCM7, MMP1, MMP14, NFASC, OR14J1, OR6K6, OR6S1, PIK3CB, PLA2G2F, ST8SIA1, TALDO1, TNFSF4, TUBA4A, YKT6, HLA-DQA2, HSD17B10, MAPKAPK5
Negative_5272	NOG, TFG, VTN, ABLIM2, AMPD3, AREG, CACNG3, CD8A, CHEK1, CSH1, CYP2C9, DAXX, ENPP7, GALNT2, GALNT5, LYPLA2, MLLT4, NKX2-2, NOS3, NOTCH3, NR1H3, OR6C76, PAK7, PDE4D, POLR2G, PPP2R5A, PTGDS, RXRB, SUFU, TBXAS1, UNC5A, HSP90AB1

Negative_5273	A2M,PAH,TH,ALG6,AMD1,ARPC1B,ATP5H,CD58,COL5A1,DDHD1,DTX3L,ITPA,MDH1,MYH3,NCK2,OR1F1,OR3A2,OR5F1,POLR1B,RALB,TFPI,TSC1,UCK1,WNT10A
Negative_5274	SCD,TAT,ASIP,ATP6AP1,CCL24,COQ3,EXT2,KAT2B,MAP2K2,OR11H6,OR2H2,OR4D1,PARD6A,STK3,XCL1
Negative_5275	F2R,KDR,ADCY7,CACNA1A,DGKI,ERBB3,IARS,ICOSLG,IL11RA,IL8RA,NFATC4,OR1F1,OR56A4,OR8J3,PFAS,PFN2,PISD,PRKAA1,RASGRP1,RFXANK,SPRY1,TAS2R4,VANGL2,MAPK8IP3,PRICKLE1
Negative_5276	C4B,GLS,PDC,RPE,ACADSB,BCAR1,CD8A,COX15,CXCL2,GSTA4,ITGA1,KNG1,LDHA,MAFA,MYD88,NPR1,OR4F15,OR5B21,OR6B2,PDIA4,PRDX6,PSME1,SREBF1,SV2C,TNFRSF9,TPH1,UGT2B7,VDAC3
Negative_5277	GCK,ACO2,AGXT,AKT1,ALDOC,AMY2A,BET1,BET1L,BPNT1,CACNB3,CFL2,CTPS,DHDH,DPYSL2,DRD2,EDN1,FABP3,HMGCL,HNF1A,LEF1,MAOB,MAP3K5,OR14I1,OR6A2,PFKFB2,PIGH,PRIM2,PRKAR1A,PTGS2,PYCR1,QPRT,RALGDS,SLC25A4,SUV39H2
Negative_5278	F9,ACADVL,ARHGEF1,BCAT2,CACNA1E,CCL7,CYP17A1,CYP2C8,DKK4,DUSP9,FLT4,G6PC2,INSRR,IRF9,MYL5,NAMPT,OGDHL,OR2T34,OR4K1,OR52B4,OR5A2,PPAT,PRKCH,RDH8,TAS1R1,TYK2,HSD17B12
Negative_5279	FYN,ID4,ILK,INS,ACSM2A,ALDH3B2,ALDH5A1,AMD1,BET1,CCND3,CHD8,CHUK,COX15,CSNK1A1,DDIT3,DDX19A,DERA,DVL1,EPB41L2,ETNK1,EXTL3,FGD3,FGF10,FGF21,FGF4,GAD2,GAMT,GRIN2C,IRS2,MCM7,NCSTN,NT5C,OR52B4,OR52N5,OR6S1,PLCD1,PMM2,PPM1B,PPP2R5D,PRPH,RALB,RFWD2,RUVBL1,SCP2,SULT1A1,UGT2B28,WNT1,WNT7A,YOD1
Negative_5280	FES,GSR,APRT,BDKRB2,CCL1,CDH3,CHST12,CNDP1,CSF1,CYP2A13,CYP2C19,DPYD,DTX1,DUSP8,EDA2R,ENPP7,EXTL3,GRHPR,HSD17B1,HTR2B,KAT2A,KRAS,LIMK2,MGLL,MGST2,NFKBIE,NKD1,NRCAM,OR10H4,OR10X1,OR13A1,OR1L1,OR1N1,OR2G2,OR2M4,OR52H1,OR56A4,OR5F1,OR5H6,OR5M3,PFN4,PIGP,PIP4K2B,RPS6KA2,SNAI1,SPAM1,ULK2,UROS
Negative_5281	AKR1B10,ALOX5,BST1,CNTNAP1,CRY1,LARS2,MCM3,OLAH,OR1E2,OR51A7,PLA2G10,TAS2R4,ARHGEF12,ATP6V1B2
Negative_5282	HK1,ITK,NGF,TH,AK3L1,ANAPC4,ASMT,CER1,CYP26A1,F11R,FGD3,IL1RAP,ITCH,LYPLA1,NFASC,OR7A17,PPP2CA,PRODH,HLA-DRB4
Negative_5283	CRLF2,CYSLTR1,PNPT1
Negative_5284	GK2,CTSG,DLL4,EXTL3,LLGL2,OCLN,TIAM1,ST6GALNAC6
Negative_5285	MAX,AADAC,ACP1,ADH1B,CYSLTR1,DHRS9,DKK2,DPYD,EPHA3,GAB1,GJA1,HCST,MAPK7,NFATC2,OR2M7,OR51A4,OR52I1,POLR1E,POLR3G,PPP2R1A,PRKAR2A,PTK2B,RFWD2,RHOQ,SOS2,TNXB,UCKL1,WNT9A,YWHAB
Negative_5286	FUK,BPNT1,BUB1,CXCL6,HAGHL,KLKB1,LLGL2,YWHAH
Negative_5287	OSM,TEC,CAMKK1,DPM1,FMO3,GRIN2A,ITGA9,LDHAL6A,MKNK1,PRODH2,TALDO1,VAMP2
Negative_5288	ACOT12,ACYP2,ALG12,CCKBR,CMPK1,CSNK2A2,DVL3,FCGR2B,GRIN2D,HNF4A,IFNGR2,ITGA2B,MAP4K3,NCKAP1L,OR1S2,PIAS2,PLD1,POLR2F,PVRL1,SFRP4,SMAD3,TLR3
Negative_5289	F11,F9,UBC,CHAD,COASY,EHHADH,MYLK2,NRP1,OR52I1,SHC1,TFPI
Negative_5290	IL7,TPR,ALDH3A1,APPL1,B3GNT2,CCL2,CCR2,CUL1,EHMT1,GFPT2,GPX7,HLA-F,HSD17B2,INPP5A,ITGAD,LIPE,LIPF,MAP3K4,MTMR6,NFKBIB,NKD2,OR9G4,PAX8,PDCD1,PSEN2,RUVBL1,SDC3,SELE,SFRP5,SMURF1,SOAT1,STX12,STX3,THBS4,TJP1,TPI1,WARS2,WASF1,WEE1,WNT7B,XYLB,SERPINB5
Negative_5291	BIRC5,DUSP10,IFNAR2
Negative_5292	CCL20,IDI2,PPP2R1B

Negative_5293	GNS,MVD,PDC,AASDH,ACO1,ALDH4A1,ALDH6A1,APAF1,ARSD,CCL4,CCL7,CSNK2A1,EPHA1,FBP2,HLCS,HRH1,IL1R1,LAMC3,MAP2K4,MLYCD,MTHFD2,MYH7B,OR10G8,OR10Q1,OR11H6,OR11L1,OR13H1,OR14A16,OR4S1,OR8D4,PI4KA,PIK3R2,PIM2,PLTP,PPP2R5D,PVRL2,PYCRL,SETMAR,TJP3,YES1,ZNRD1
Negative_5294	TK2,ADRA1B,ARPC4,CD19,FGF20,GMPR2,LCMT1,NCR2,OR5L1,PAX4,PDE1C,PPP1R3B,SMAD7,SULT1A1,TAS2R13,WASL
Negative_5295	HPD,AADAC,ACAD9,APOA5,BHMT,CCL5,CDC26,CES2,CLDN9,CTSS,CYP2D6,FLT4,GALNT13,GNA14,NCKAP1L,NCOA4,NMNAT2,OR10J1,OR4K13,OR52K1,OR56A3,OR6K6,PCK2,PDGFD,PGAM2,PPP1R3B,SKP1,SLC25A6,ST3GAL1,TALDO1,TNFSF12
Negative_5296	IDS,MYC,AANAT,ABL2,ACAA1,ACYP1,ALPI,B3GALT4,CACNG4,CALML5,CCND2,CXCR3,EXT1,GALNTL1,GMPPA,HTR2B,IGSF5,MAP3K14,NAE1,OR10C1,RENBP,RPS6KA3,SNAP25,TBL1XR1,VAMP3,VDAC3
Negative_5297	AR,FH,AGPAT3,ALG5,B4GALT4,BAIAP2,CACNA1E,CACNG4,CD46,CDKN1B,CHDH,CLDN7,CLTB,CMPK1,CNTNAP2,CTPS,CYP26C1,FZD5,IFNGR2,IL10,PCYT1A,RPE65,RPSA,STX4,STX7,VAMP4,CSNK1A1L,HLA-DPB1,PAFAH1B3
Negative_5298	CD6,F10,FES,ACO2,CLOCK,CLTCL1,IL22,ITGAD,NEU2,NTRK2,PDCD1,PDE11A,PERP,SETD1A,SIAH1,SULT1A2,PPP1R12A,SLC25A31
Negative_5299	C6,RFK,SHH,ALG13,ALPPL2,ANAPC4,ARPC1B,CACNB1,CD274,CSF1R,CTPS,CXCL11,CYP2B6,DHX58,ENO1,F13A1,FDPS,FMO1,GALNS,GARS,GCDH,GUCA1C,HLA-DOA,ICOS,ITPKA,LEFTY1,MARS,MCM6,MYL2,NCK1,NTRK1,NUDT12,OR4K15,PDE9A,PGS1,PLCE1,POLR2H,PPARD,PTPN1,RDH10,SFRP4,SMPD1,SPHK1,TYRP1,UROS,XIAP,PHOSPHO1
Negative_5300	CS,EGF,SP1,GP1BA,OR4F5,OR8B12,PER1,SEMA4C,TUBB2C,XYLB
Negative_5301	ASMT,CALML6,CAMK2A,CBR3,CSF2RB,DAPK3,FZD7,GDF6,IFNB1,MAGI2,MAGI3,MAPK8,MINPP1,MYH6,MYH7B,NRG2,OR4M1,PPP2R5D,RPS6KB1,SAT1,STK3,TBXA2R,ATP6V0A4
Negative_5302	ABLIM1,ACSS2,ALOX5,CDC2,CYBA,FGF18,GDF5,IL21,LCAT,LIPT1,MAN1B1,OR1L8,OR2B6,OR51Q1,TPH2,TPK1,TUBA3E,TUBB,ATP6V1G1,PAFAH1B3
Negative_5303	F10,JUP,MUT,B4GALT5,CD274,CD72,CSF3R,CYP4F2,DDX56,DERL1,DVL1,EGFR,EGLN1,F2RL3,HAO2,IL13RA2,MAP3K5,NRXN2,NTSR1,OR1I1,OR2AT4,OR4K17,OR7A5,OR7D4,PDXK,PLA2G6,PLCB1,RPS6KA5,SDHA,SRD5A1,TGFA,TYK2,UGCGL2,VAV3
Negative_5304	HPD,ALCAM,ATF2,ATP5E,CREB3L4,CRKL,CTPS2,CYP3A43,HLA-DOA,HSD3B1,MAN2A1,MDH1,NRCAM,OR2T3,OR52E6,PLCB3,TBXA2R,WASL
Negative_5305	ACAD8,ACAD9,ALG3,ARD1A,ASIP,ATP5E,B4GALT4,BAI1,CASP3,CCR9,CD58,CDC20,CFTR,CHST12,ENPP2,EPHA7,EVI1,HSD17B3,NFE2L2,OR3A2,PARVA,PTGS2,RXRA,SARDH,SMPD2,TACR3,VNN1,TNFRSF10C
Negative_5306	BTK,GK,HGD,ATG7,CCL11,CCR6,CLTCL1,CREBBP,CTSG,FOXO1,GALNT1,HCCS,HPSE2,IFIH1,MAML3,MAP2K2,MTHFD2,NTF3,NTF4,OR14C36,OR5T3,PIP4K2A,PPARA,PTTG1,QPRT,SFRP1,SGMS1,SH2D2A,SHMT2,TRMT11,VANGL1,VDAC2,YWHAH,CSGALNACT1
Negative_5307	CGN,CR2,FOS,GSR,ADCY2,ALDH7A1,B3GAT3,CCL13,CLDN16,CYP2C8,DGKH,GRIN1,MAP3K11,NTSR1,OR1D2,OR2G6,OR52K2,OR5K1,POLE3,SMAD4,SMARCA2,THBD,TLR3,TNFSF4,TPK1,YWHAB
Negative_5308	EZR,TH,ACAD9,ACVR1,ADSS,AMD1,AOX1,CASP9,CD19,CRKL,CYP3A7,FASN,FLT4,GNB1,GSTM2,HNMT,IKBKE,IL28A,METTL6,MYLK,NCK2,NMNAT2,OR10G2,OR2F1,PRPS1,PTPN1,SH2D1A,UPP1,C1GALT1C1,MAP3K7IP1,MAP3K7IP2

Negative_5309	KIT,LHB,MUT,PTS,ABLIM3,ADORA2A,ARHGEF6,ATP6V1D,BAIAP2,BCL2,CALML6,CHKA,CHUK,DVL3,EXOC7,GALNT13,GARS,GJA1,HARS,IPMK,IRAK4,MAP2K5,MTHFD1L,OR10V1,OR11H6,OR13J1,OR4A5,P2RX7,PIGH,PPAT,PPP2R2A,RARA,RPS6KB1,SGMS2,SLIT2,SMC1B,THBS4,TNFSF9,ULBP2,VDAC2,ZMAT3,MAP3K7IP2
Negative_5310	ADSL,B4GALT5,IL15RA,OR5A1,PRODH2
Negative_5311	LCT,PVR,SMS,ACOT12,ACY3,B3GALT6,B3GAT1,B4GALT4,BMP6,CASP3,CXCR5,CYP2A13,DDIT3,DUSP1,EIF4B,HSD11B1,LEF1,OR1A2,OR1M1,OR4K5,PIGS,PKLR,PRKAR1B,RFWD2,ST8SIA5,STX8,TBL1X
Negative_5312	ACADL,APLP1,CCR8,CDC25C,CHRNA7,CKS1B,CTSL1,CYP11B2,FBXW11,FGF10,FSHB,ITGA7,MCM5,MYLK3,NRXN2,OR2B2,OR4C12,PIGO,PLA2G5,PLD2,PRSS2,SORBS1,SYNJ2,TLR3,WARS2,SERPINB5
Negative_5313	SYK,ACADS,ACAT2,ALG5,ARPC1B,ATP6V0B,COL1A2,FLT4,HAO1,IDH2,ITGA1,PGK1,PNMT,PPP1CB,SMC1B,ULK2,YES1,TNFRSF10A
Negative_5314	PLG,UBC,CACNB4,DGAT2,FLT3LG,GFPT1,GMPPB,GSTA3,LDHD,MAPT,NFKBIE,OR10G2,OR52N5,OR6C70,PMVK,TAS2R4,YARS2,PPARGC1A
Negative_5315	ASL,CFH,MPL,AKR1C3,ATF2,CAMK2A,CCL20,CD226,CD47,CLDN3,DDB2,DPM1,DUSP10,EARS2,ERBB4,HSD17B8,ICOSLG,ITGB2,KLRD1,OR1Q1,PDGFRA,PLA2G1B,POLR3A,PPAT,PRPS2,PVRL1,RAPGEF1,RETSAT,SFRP4,SOCS3,TGFBR1,TLR3,TNFRSF4,TSLP,ARHGEF12
Negative_5316	ADK,CD4,GAA,MPL,UBB,ACTA2,ACVR1C,ADH6,ASMT,ATP5J2,BDKRB2,CALML3,CCKAR,CLDN9,CNTF,DGKA,ELK1,EPHA3,EXT2,FUT9,GLG1,GOSR2,HMGCS1,HTR5A,IL23R,IMPA1,IRF5,IRF7,ITGA8,LIMK1,MADCAM1,MITF,MYH14,OR8H2,PANK4,PFKFB4,PGK1,POLE3,PPP2CA,PRODH,ST8SIA1,ST8SIA5,TBL1Y,TGFB3,TP73,UCP1,TNFRSF18
Negative_5317	NNT,ACP6,ALDOC,ANGPTL4,ARG2,BCL2,CD226,CDC42,CUL1,EPHB3,EXT2,GRIN2A,GSTK1,IARS,IFNA2,IL18,IL28B,MAP4K2,NR1H3,NT5C3,OR5B17,POLR2E,PPP2R2D,PRKAR2B,PRKX,RDH12,RDH5,ROBO3,RUNX1T1,SARDH,SKIV2L2,TGFBR1,TRHR,TUBA3C,TYRP1,ULBP3
Negative_5318	CBL,FOS,HGF,LAT,RDX,TPR,ABI2,ACSL1,BACE1,C4BPB,CCL21,CD79A,CDC7,CHPT1,CKS1B,DLAT,EFNA1,F13A1,FGF8,GNAQ,GSTM3,ITGB4,LILRB3,MAFA,MAPK11,MYH13,NKD2,OR2T8,OR4K17,OR5A2,OR5B3,OR8H3,PARD6G,PPP3CA,PRKACB,PRKAR2B,RENBP,SLC25A6,TOLLIP,TRDMT1,UGCG,WNT5B
Negative_5319	BAD,CP,FST,GSS,KMO,SDS,ADCY6,AHCYL2,ARNT2,ATP12A,B4GALT7,CAMK2A,CFTR,CRLS1,CTNNB1,CYP2A6,DKK1,EPHA2,EPHA3,ETS1,HAO1,HDAC2,IL22RA2,IQGAP3,MAPK11,MGAT4B,MYL2,MYL5,NRXN1,OR11H6,OR51Q1,PDCD1,PIK3C2B,PIP4K2B,PTCH2,PTPRZ1,RHOA,SETD8,SV2B,TNFSF4,TP53I3,WWP1,YES1
Negative_5320	ATIC,CTF1,DKK4,FGF23,OR5AU1,OR5B3,PIGX,PIP5K1B,PKMYT1
Negative_5321	CBL,MVD,NLK,ALDH9A1,BCL10,CACNG3,CNTN2,CYP17A1,DDX41,FABP1,IL12RB2,ITGA11,MYH2,MYL2,NCR3,NEGR1,OR2H1,OR4C6,OR52H1,OR52R1,OR9G1,OXSM,PRKAR1B,PTGES2,TNXB,VDAC3,WNT3A,CSGALNACT2,TNFRSF10A
Negative_5322	CCL21,OR2T11,OR51A4,OR6C2
Negative_5323	GK2,KHK,MET,SDS,TK2,APAF1,CAV2,CD79A,CHPF,CLDN16,CNGA3,CREB3L2,CXCL1,DARS,DGKA,DOT1L,DTYMK,ENPP1,F11R,FGF11,FUT9,GZMB,HTR5A,ICAM3,IMPDH1,INPP1,KCNB1,LTB4R2,MKNK2,NEFL,NEU2,NLGN3,OR1J4,OR2L2,OR4S2,OR52A5,OR5D18,OXSM,P2RX3,PARVA,PDIA4,PIGH,PIGK,PLCE1,SMURF2,ST3GAL2,TAS2R40,WHSC1

Negative_5324	CSK, F5, HK2, PTS, ARD1A, ARHGEF6, ATG7, CACNA1D, CASP7, COQ5, EIF4E, FLT3LG, G6PD, HEXB, IL1B, LIPF, LYPLA2, MAP2K4, NAGA, NLGN2, NOX3, OR4C11, OR4D2, OR4K2, OR52B4, OR7G1, PDE8B, PLIN, POLR2L, PRDX6, RALGDS, RPS6KA2, SMAD5, STX4, SULT1A1, TRAF5, TSC2, UPP1, VAMP3, WNT2, ATP6V1G1, CACNA2D1, TNFRSF11A, TNFRSF13B
Negative_5325	GBA, ALG12, AOX1, ARNTL, BCL10, CAMK2D, CD44, CD99, COL5A2, CREB3, CSH1, CSNK1D, ENPP7, FZD1, GDF6, HMGCS2, HSD17B2, IL21, INPP5B, MAP2K5, MYLK2, NRG2, OR1Q1, OR5V1, OR6M1, OR8B4, ORC1L, PAX8, PFKL, PGAP1, PIGF, PLOD1, PRIM2, PTPRM, RASSF1, SEMA4F, STAT2, SUV39H1, AASDHPPT, KIR2DL5A
Negative_5326	ACVR2B, CLTC, ESPL1, FRAP1, OR13J1, OR1A1, RASSF1, SETD7, TNFRSF8
Negative_5327	LSS, OTC, RDX, SRC, ALG12, C3AR1, CDC25A, CDH2, CEBPA, CNTF, CSNK2A2, CTNNA2, FSHR, GSTM4, GSTO2, IL24, LAMB1, LRP5, LRP6, MYH13, NFAT5, NLGN1, OR1I1, OR2H1, OR4C16, OR4K1, OR6C3, PLXNB3, PRKAG2, PRSS1, PYGL, SELE, SEMA3D, SESN2, SETD1A, SGPP2, SPP1, TRDMT1, HLA-DRB3, HSP90AB1
Negative_5328	ADK, ABAT, CHRNA7, DDOST, GFPT1, ITGAM, OR2L2, OR4S1, P2RX6, PDHA2, PDX1, PFKFB1, PMM2, SDC2, ST8SIA5, TKTL1, TP73, VASP, ATP6V1G1
Negative_5329	GSR, TNN, ACTC1, AKR1C1, BMPR1A, CALML5, MCM5, PLAUR, RPS6KB2, TJP2
Negative_5330	IHH, LIF, ADCY10, ARG2, CHRM5, CLDN6, DGUOK, GCLC, GLDC, GLI2, GNPNAT1, INPPL1, MAP3K1, OR51D1, P4HA1, PSEN2, TSHR, XYLB, TNFRSF13B
Negative_5331	IDS, ACTG2, CYCS, GZMA, HGSNAT, HSD17B7, OR10J3, PTGS1, UROC1
Negative_5332	NGF, AKR1C3, ARG1, AXIN1, BDKRB2, BIRC3, CCND3, CHRNA7, CTSG, FUT5, GNA12, HSD11B1, HSD17B8, IL8RB, ITGB1, LRDD, MGAT3, OR4A16, OR5B3, OR5D14, PAFAH2, PARVB, PDE4D, PLXNC1, POMC, SELP, SMAD9, UGCG, UPB1, SUV420H1
Negative_5333	C1S, CTH, GH1, RDX, ACSM3, AKT2, AKT3, ARD1A, BDKRB2, CD247, CDK4, CDKN1C, CHRM1, COL2A1, CYBA, CYP1A1, CYP2C9, EPHB4, F2RL2, GPD1, HARS, HSPA2, KIR2DS5, MAP3K14, MYLK, NAGA, NEFH, OR10A4, OR1N1, OXCT2, PI4KA, PLA2G2E, POLE4, PPP5C, RELA, SGPP2, SMPD3, SUCLG2, SUV39H1, TPM3, UGT2B10, MAP3K7IP1, MAPK8IP3
Negative_5334	ADH6, AXIN1, CCNE1, ENPP2, GJD2, ITPK1, MFNG, MPST, MYH2, OR5K1, PDXP, PEMT, RUNX1T1, SUOX, TGFB2
Negative_5335	ACTG1, ACVR2B, BACE1, CANT1, CCR5, EXT2, GALE, IL6ST, ITGA1, NTF4, OR4F6, OR51B2, OR51F1, OR52A5, PGAM1, PIGS, PIGV, PLCE1, PRKCI, SMC1B, TSHB, UBA7, WNT2, WNT8A
Negative_5336	DBT, F11, FN1, GNS, LPO, TKT, B4GALT1, CASK, CDH3, CHIT1, CLTC, CNTNAP2, EPHA7, FPGT, GABARAP, GCH1, HSD3B1, KLRC4, LILRB3, LTB4R2, NFYC, NOX1, OR13C5, OR51E2, OR5AS1, OR5D14, PANK3, PIGL, PON3, PPAP2B, PYCRL, RAPGEF3, RDH11, RDH8, SC5DL, TUBB2B, VAV1, WBSCR22, YWHAE
Negative_5337	CYP11A1, OR4S1, PLCG1
Negative_5338	DCC, F7, ACVR2B, ARHGEF6, ARPC4, ATP2A2, CBLC, CCL22, CDC6, CFL2, CREB3L1, CXCL11, GAS1, HCLS1, HTR2A, ITGAX, ITGB3, KLRD1, MAD2L1, MAPK8, MYH15, NAT1, NNMT, OR4N5, OR8K1, PGAM2, PPM1D, PRODH2, PRPS1, PTPRC, RASSF5, TSHB, ULBP1, XCL1
Negative_5339	BAX, CKB, ID2, SYK, AKR1A1, ANAPC7, APAF1, CALML6, CCR7, CHPF, DDX23, DGUOK, ETNK2, FGF5, GMPPA, GRIA2, HADHB, HMGCR, IARS2, ITGA7, MTHFD1, OR2G3, OR2T2, OR52B4, OR5F1, ORC1L, PAK2, PCYT1A, PDE6D, POLE, POLR2E, POLR2H, PRKACB, PRKCZ, SH2D2A, SNAI2, SRD5A2, TJP2, TUBB2C, UGT1A10, YKT6, PHOSPHO1, TNFRSF10B
Negative_5340	EHMT2, IKBKG, STX8, ULBP1

Negative_5341	ILK, LCT, RPE, ACP6, ADH5, ADRA1B, ADSL, ALDH9A1, CACNA1C, CACNB1, CASP3, CHIT1, CMPK1, CTSB, CXCL6, DOT1L, DPM1, EP300, EPHA2, GRIA3, GSTP1, GUCA1B, HARS, IL17A, IL3RA, LTC4S, MALT1, MGST2, MRAS, NTF3, ODC1, OR52K2, PAK3, PDE1B, PLCG2, POLD2, PPP1R3B, PRKCE, PTGER1, RPSA, SARS2, SEMA3G, TAS2R1, TGFBR2, UMPS, HLA-DRB4, HSP90AB1, PAFAH1B2
Negative_5342	CDKN2C, CSNK1E, DCTN1, GSTA3, IFNA6, LEFTY2, ONECUT1, OR9G4, PPOX, PRKACG, HLA-DRB3
Negative_5343	AK5, GCK, GSN, PVR, ACADVL, ADIPOQ, CCR2, CNGA3, CYP4F3, DDOST, EPB41, FGFR3, GNG12, HGSNAT, HSPA8, HTR2A, LRP5, LTBP1, MAP3K13, MAP4K1, MAT1A, MDM4, OR10J3, OR2M4, OR7G1, PCYT2, PDPK1, PIM1, PLAU, STX1A, TAS2R10, UGT2B28, YWHAB, TNFRSF10B
Negative_5344	BMP8B, EPHB6, LCAT, OR10J3, OR4E2, PLXNA3, POLE3, PPM1A, PSENEN, SLC33A1, SPRED1, THTPA, PNLIPRP2
Negative_5345	IVD, TPO, AASS, ACAA1, BRAF, DFFA, DHCR24, EREG, FGF23, FLAD1, FZD9, MCM5, ORC1L, P2RX4, PIK3C2G, PTDSS2, PTPN6, PYCR2, THBS3, TUBB4Q, ZFYVE9, HLA-DPA1, ST6GALNAC1
Negative_5346	BTC, FGG, ID1, OAT, SP1, ACSM3, AKR1C3, ALDH1A3, BMP7, CFTR, CNDP1, CRB3, EHHADH, GJA1, GRHPR, GUCY1A2, HHEX, HSD3B7, INMT, KIR2DL2, KITLG, MDH1, MKNK1, MYH3, NCK1, OR2S2, OR4K13, OR51E2, OR6Y1, OXTR, PGAM4, PIGL, PTPN11, TUBA3C
Negative_5347	C6, AHCYL2, ALDH6A1, ALPI, ATP6V1D, CAV3, CCL5, CCNG1, CD40LG, CD79B, CDKN2D, CPT1B, CYFIP2, CYP21A2, DHCR24, GALT, HAGH, HAO2, HDAC2, HSP90B1, IL20RA, IPPK, IQGAP3, KLRC4, MMP9, OR10J1, OR2F2, OR51B2, OR6C6, PER2, PPOX, PTPN7, RASGRP2, ROCK1, SELL, XYLT2, CACNA2D3
Negative_5348	AGL, LPO, RET, TNN, ACOX3, ACSL5, ALDH1A3, CKMT2, CMAS, CMPK2, COL4A4, CSNK1E, CYP2J2, DUSP6, E2F4, EPB41L1, FZD10, GADD45A, GMPPA, HAO2, IL11, LTBP1, NLGN2, OR10AD1, OR4S1, OR5K1, PGAM4, PKLR, POLR2E, PRKY, RASA1, ROBO3, STX8, SULT1A4, TNFSF14, UBE2K, ATP6V1G3, TNFRSF10A, TNFRSF10D
Negative_5349	AK3, CKB, LHB, CHST11, E2F1, EFNA3, FCER1G, HSD3B2, IL23R, INPP5A, LRAT, NR5A2, OR6C3, PPP2R2A, SFRP1, SIGLEC1, SOCS3, VAV1
Negative_5350	ATP5G3, CPT2, CXCL2, ENAH, VARS
Negative_5351	CBL, TH, ABI2, ACLY, ALDH9A1, ALPI, ARD1A, ASS1, ATP5O, CARD11, CCND1, CDH1, CHAT, CLDN3, CLTA, CREB3L3, CYFIP1, CYP17A1, CYP3A4, DIAPH1, HADH, IL1B, ITGB8, LAMC1, NKX3-1, OR10G2, OR1J2, OR2M3, OR4K14, OR4K2, OR52E2, OR5C1, OR6C2, OR6C4, PTPRM, RBPJ, RELB, RFXAP, STAT4, STEAP3, SUV39H2, TNFSF11, ATP6V1C2
Negative_5352	OTC, VCL, ALG10B, BCL10, CCL2, CCR2, CDH2, CREB3L3, CSNK1E, DUSP5, FGF22, FLT3, HMBS, HYAL1, IFNA16, IFNAR1, LTA4H, MAD2L2, MGAT5B, OR2T34, PFN3, PPP1R3C, PSME3, PSPH, SARDH, SH2D1B, TCF7L2, UBE2L3, UMPS, ATP6V1B1
Negative_5353	CS, ME2, ADH7, AMHR2, ARRB2, B3GAT1, BCAT1, CLDN22, EDNRB, EGLN1, EIF4E, FCER1G, FLOT2, GZMA, IL12RB2, KARS, LHCGR, MAP2K3, OR52E4, OR5V1, P2RX3, PARD3, PDGFB, PFAS, PNLIP, POLE3, POLR1C, PPP2CB, PRPH2, SHC1, SPHK1, TCF7L1, UAP1
Negative_5354	EBP, TPO, AKR1C4, AMY2A, BBC3, BCL2, CDH2, CLDN3, CLDN8, CRY2, DAD1, DHRS4, EFNB1, GNG12, HPSE2, HRH1, HSD17B3, IFNA5, IL12A, INADL, ITGA2B, KLRC2, MAP2K1, NNMT, NOTCH2, OR2W1, OR7A5, PDPK1, PER2, PIK3R4, PPP2R2D, PSME3, ROBO3, SPRED1, SQLE, STAT5A, SULT1A1, HSD17B12, PLA2G12A, ST6GALNAC6
Negative_5355	FH, STS, ACVR1, ADCY9, ANGPTL4, CCL20, CCND2, CXCL1, CXCL9, DTX3L, FDFT1, FGFR2, GNAI2, GRPR, HTR2B, INHBE, JAM2, MAPK3, NCK2, NCKAP1L, NRG2, OR4C11, OR4F17, PDE4A, PFKFB2, PIK3R2, PIK3R3, PSEN1, RRM2, SARS, SRGAP2, SSH3, TBL1X, TBL1XR1, TGFB3, TP73, TUBB4Q, UCKL1, ZIC2

Negative_5356	AMT, HAL, ABL2, FZD9, PIK3CG, PRPS2
Negative_5357	RPE, ASPA, ATP5E, CBR3, PTGFR
Negative_5358	ID4, SRC, ACSM5, CCL23, CDS1, DERA, DGKE, ENTPD2, EVI1, FARSA, FGF18, FUT2, IMPDH2, ISYNA1, ITGB5, MAP2K7, NCKAP1, OR2A4, OR2Z1, OR4D1, PDE3A, PGK1, POLR2F, PRKAB1, PRKAG1, SDC1, TJP1, TUBA3C, WNT7A, XYLT1, CSGALNACT2, SERPING1
Negative_5359	ADO, HK1, ME3, OAT, ACVR1C, AFMID, APOA2, ARNTL, ARPC3, CXCL2, DIAPH2, E2F1, ENTPD3, ENTPD6, EPHA6, FGFR2, GALNT5, GJA1, GRIN1, HTR5A, IL13RA1, IL4R, MAML1, MAPK11, MPZL1, OR10A6, OR10C1, OR2AE1, OR52H1, OR5M8, PGM1, PLK1, PLXNB3, POLE4, PSME1, PTPMT1, RETSAT, SDC1, SDC2, STX10, UGT2B28, UROD
Negative_5360	ID4, ARG1, CYP27A1, EPAS1, GNPAT, IFNA5, IL2RG, OR10T2, OR6K3, PIGH, PLA2G2A, PON2, STAM2, UPB1, ATP6V0D1
Negative_5361	FH, KMO, MAX, ATP5G2, CDC26, CDH1, FUT2, HSPA2, IRAK2, ITPKB, NCF2, NFYC, OR2C3, OR4K17, OXTR, POLE, PPP2CB, PPP3CA, PRKCZ, PTAFR, SPRY3, TRIP10
Negative_5362	C8A, CBS, FOS, A4GALT, ACADSB, ATP5G3, C1QB, CACNG6, CDH4, CTBS, DRD2, EPHA7, ETNK2, FBP2, FGF20, FLT1, FUT8, GALNT12, GNAI1, GNMT, GYS2, HLA-DRA, IARS2, IDH1, IL5RA, IRF3, ITGB8, NCOR2, OR52K1, P4HA2, PAX4, PDGFB, PDGFRA, PGAM4, PIP4K2B, PLD1, POLE4, POLR1C, PROS1, PYGL, RAF1, RRM2B, SQLE, THY1, TPI1, UNC5D, TNFRSF1B
Negative_5363	DAPK2, LEPR, OR4C12
Negative_5364	LAT, BCKDHA, CACNA1H, CCL28, GART, INPP4A, OSMR, WNT6
Negative_5365	AHCYL1, ALPPL2, BCAR1, CTSL1, CXCL12, EPHB2, FCER1G, HTR2B, IL21, MGAT3, OR2L2, OR2L3, OR4E2, PDXK, PLCD4, RCHY1, RELA, STAM, SUCLG1, TPMT, MAPK8IP2, SERPING1
Negative_5366	DBH, ID4, BUB1B, CCL28, CD74, CHAD, CLDN3, CREB3L4, E2F4, EVI1, FGF9, FPGT, HPRT1, IL1R1, INMT, MAN1A1, NFE2L2, OR10G4, OR2A5, OR5AU1, PDE11A, PLOD1, POLD1, PPP2CA, PSEN2, PTGES, SELL, SH3BP2, MAPKAPK5
Negative_5367	LEP, OSM, ACSS2, ADIPOQ, AKR1C1, ALPL, ATP5G3, CDS2, CES7, EFNA3, HTR6, IL24, KLK3, MAN1B1, OR14I1, OR1L8, OR6B3, OR6M1, PAPSS2, PNPT1, POLR3G, RASGRP4, SUCLG2, TAS2R1, TSLP, VASP, WARS2, WASL, WIF1, XYLT2, CSNK1A1L, TNFRSF11A
Negative_5368	BCR, SMS, ADCY7, C3orf10, CDC2, CDO1, CHKA, CLCF1, CYSLTR2, DDIT3, EDN1, FGF17, HAGH, HRH2, LYZL1, MAD2L1, MGAT4A, NCKAP1, OR52H1, PFN4, PIP4K2A, RPS6KB1, TNFSF9, WNT3A, WNT5B, WNT7B, MAPKAPK2
Negative_5369	BTK, OGT, SDS, ACSS2, ADCY9, ALDH5A1, ARRB1, BMP4, C1QA, COL4A4, EVI1, HIF1A, IFNA17, IRAK3, LDHA, LEPR, MGAT4A, MGLL, NFE2L2, NFKBIB, NFYA, OR1J1, OR2G2, OR7A5, PER1, PLCD4, PPP2R5B, QPRT, STAT4, STEAP3, SYNJ2, TALDO1, TRMT11, WNT10B, WNT2
Negative_5370	AMT, EPO, NPL, TNF, ACLY, ADCY10, ATP5D, CADM3, CLCA4, CSNK1G3, CXCL9, DDX54, DIAPH3, DOCK1, FBP1, GCNT4, GUCY2D, HIF1A, HSD17B1, ITPKA, ITPKB, MAOB, MOV10L1, NT5C1B, NTRK1, OR2L13, OR51V1, OR5B21, OR5H2, PLA2G10, RAPGEF3, RASGRP4, RFXANK, RPS6KA6, SLC25A5, TUBB1, UAP1, ULBP3, WNT2, PAFAH1B1, TNFRSF1B
Negative_5371	BAD, AACS, ADCY8, CACNB2, CDH4, CYP2A13, FZD5, HSPA2, ITGB7, MTFMT, NRG1, PDE9A, RELB, VTI1B
Negative_5372	CR1, COQ3, DDX4, IL22RA1, PRNP, TOMM40L
Negative_5373	HAL, SPN, CDC16, CDS1, ERO1L, ETS1, HPRT1, MTHFD1, OR5M3, PLTP, PPOX, SHMT1, TBL1X, UBA1, CSGALNACT1, TNFRSF1A
Negative_5374	AGK, IL9, ABL1, AKR1B1, CD58, CLDN15, COL11A1, ELK1, FABP3, ITGAM, KLRK1, MTHFR, MYH8, NME7, OR1J1, OR1Q1, OR51L1, SC5DL, SDC4, SEMA3B, STAT6, TBK1

Negative_5375	DLD,AGMAT,AGTR1,ALAS2,ALDH3A2,AMDHD1,APOA1,CCL25,DEGS1,DHRS9,FARP2,GCNT2,IFNA4,MDH2,NRG2,NRG3,OLAH,RASGRF2,STAT2,TBL1XR1,TUBA1B,ATP6V0A4
Negative_5376	CAMK2B,CAV3,CISH,GARS,NTN1,SEMA3C,SPTLC1,TOMM40
Negative_5377	F11,LPO,AADAT,ACTA2,B4GALT5,BCAR1,CANT1,CDKN2A,CHD8,COL5A2,CRY1,CYP27A1,EVI1,FGF18,GDF6,GNA15,HPSE2,IBSP,IDUA,IL28RA,INSRR,ITGA10,MAD1L1,NKD2,NME7,NR4A1,NT5C1B,OCRL,OR8B4,PLAT,POLR2I,PRKACG,SEMA3E,SMAD3,SSH3,TALDO1,TAS2R40,TUBA1C,UGT8,ULBP3,WBSCR17,TNFRSF18
Negative_5378	FMO4,FZD5,PIAS3
Negative_5379	AK5,GK2,SPN,ACOT12,ALPL,ALPP,CD226,CD34,CDH2,COQ3,COX15,GALNT13,GANAB,HAAO,KLRC3,LEFTY1,MAP3K6,MECR,NCR2,OR2L13,PDE4C,POLR2K,PPCS,PPP2R5C,SV2A,VAV3,TNFRSF6B
Negative_5380	CFD,DNMT1,MCCC1,PIK3C3,TNFRSF18
Negative_5381	JUP,NF1,PAH,ACSL5,AHCYL1,CACNA1H,CASP8,CNTFR,DAD1,DGKG,GCNT2,GGT6,ICAM3,ITGB5,OR5P3,PFN3,PHPT1,PLAT,PSME2
Negative_5382	C7,CAT,IL5,BAIAP2,CALML3,CD274,CHAD,CPS1,DHFR,DMGDH,DTX3L,GALNT1,HES1,IFNA16,IMPA2,ITGA2B,LTB4R2,OPLAH,OR8K5,POLR2L,PRKAB2,SFRP2,TAP2,TRADD,TUBA4A,WARS,WNT6,SERPINF2
Negative_5383	BAX,ASIP,CCNA1,CCNE1,COL4A2,CYBB,GPX5,MCM4,NEU3,QPRT,RAPGEF3,RCHY1,SETD8
Negative_5384	BCR,GNS,ACAD9,ARD1A,ARG2,ARPC1A,ATP5F1,B3GALT4,B4GALT2,CDH15,COL5A3,GALE,GALNTL1,GTSE1,HCCS,HSD17B8,HSPB1,IGFBP3,IL6R,LIPF,MAP2K7,MASP2,NEU4,NMNAT1,OR6C74,OR7A17,OR8G1,POLR1C,POLR2E,PRNP,RARS2,SEMA4B,SQLE,ST8SIA5,TAS2R3,TAS2R60,TIAM2,TYRP1,C1GALT1C1
Negative_5385	ADCY3,CDH3,CHEK1,GATM,GMPR2,IL1RAP,OR8J1,PGAM1,RENBP
Negative_5386	C4B,GNS,PTS,ACYP1,ASAH1,ETNK1,GSTA2,OR10T2,OR2T6,OR4C16,OR5C1,OR6B3,PDCD1,SPTLC2,ULK1,UROC1
Negative_5387	TKT,ACTG2,ATP5G3,BDH2,GAD1,HEMK1,HYAL1,MAP4K4,MNX1,P2RX1,MAPK8IP3
Negative_5388	SMO,ABP1,B4GALT1,CSNK2B,HBEGF,LIAS,NOS3,OR6K3,PDE1A,PPP2R1B,PRKCE,RAPGEF1,STX7,TAS2R3,UCP1,HSD17B12
Negative_5389	ACPP,ADCY10,ALDH6A1,ANAPC11,CACNB1,CCNA1,CD274,CD44,CDC25A,CDS2,CLDN19,CTNNA2,CYP2E1,EFNB1,FGF9,G6PC2,GOSR2,GSTA3,IFNA7,ITGA2,KIR2DL4,LAMB1,LARS2,MAP2K6,MAP3K2,MAP3K8,MDM2,MYH10,MYH13,NFYC,NPAS2,OR10AD1,OR2M3,OR8B12,PCYT1A,PLCG1,PLK1,PRKCH,RFX5,SNAI2
Negative_5390	TH,CES1,CHRM3,CMPK1,ECSIT,FGF22,FUT4,GRAP2,PDE6H,ST3GAL1
Negative_5391	F2R,IL7,BLNK,CCL22,E2F3,GALNTL1,KIR2DS4,MAPK11,MTHFS,OR13J1,OR4A15,PAICS,RASGRP3,RPS6KA4,SPRED2,STAT2,TGFB3,TNFSF10,TNFRSF10B
Negative_5392	C1S,GNS,ADAM17,AGPAT4,ALCAM,ARG2,BBC3,CD86,DGAT1,ENO3,FTCD,GALNT2,ITGA7,MTAP,MYL2,OR51M1,PRKAG2,PRKG2,RBPJ,SETDB2,TLN2,UGP2,XIAP,YES1,TNFRSF18
Negative_5393	DDC,LTB,OGT,ABLIM1,ATP5E,CCL15,CDH4,CDS2,CLTB,CNDP1,CSNK1E,DAAM2,EVI1,FARSA,FLT4,FUT3,GCNT1,HRAS,HRH2,IL23R,KIR2DS5,KLRD1,OR5D16,ORC5L,PRKAG1,PSME3,PTCH1,SMAD1,SPRY4,SUV39H1
Negative_5394	CD4,MPZ,NF1,ABCC8,AKT3,ASIP,ATG12,BACE2,COL5A2,COQ5,DDB2,DDX47,DLST,EPHA2,FGFR3,GCNT2,GGCX,GLUD2,GNRHR,GUK1,GUSB,IFNA4,IFNA7,IL12RB1,IRAK2,MAFA,MGAT1,OR13A1,OR4C46,OR52I2,OR5AK2,OR6C75,PDGFC,POLE2,PTDSS2,RARS2,SLC2A2,ULBP1,B4GALNT1
Negative_5395	ME2,ACSS3,ALOX15,ATP5G3,CBLC,LTB4R2,MAPK10,NAE1,OR2A5,PDE6D,STX1A,UROS

Negative_5396	CKM, AMY2B, CDS2
Negative_5397	TAT, ACPP, BUB1B, CDH3, CSNK1G3, ELK1, ENTPD8, FGF22, GBA3, GSTA5, MYLK, NCF2, NEU2, OR10G4, OR10K2, OR1L8, OR4K5, PCYT2, PTK2, SDC1, SDC3, SEMA4C, UXS1, VEGFB
Negative_5398	OTC, ASPA, ATP2A2, ATP5F1, B4GALT2, COQ3, EDN1, FGF10, IL23A, ITGA8, LEFTY1, MCAT, NFASC, OR11H1, OR2B11, OR52N1, OR5M8, OR7A10, PAFAH2, PDE3A, PDX1, SEMA3B, SHC2, SLIT1, SMAD9, TPH1, UGDH, TNFRSF10B
Negative_5399	ALDOC, CCL1, CDK5, CXCR6, DVL2, F2RL1, FGFR3, GBE1, MAP4K2, OXCT2, PEMT, RPE65, WNT16, ATP6V1B2, TNFRSF1A
Negative_5400	DBI, AKR1C4, BUB1, COL11A1, EDNRA, ETNK2, GALNT6, IL23R, LIPG, NFAT5, NRG3, OR4N4, PPP1CC, PRKCI, SLC12A2
Negative_5401	GGH, ADCY2, ALOX12B, ATP6V1D, BUB3, CCNE1, CCNH, CHKB, CYP2S1, ENTPD6, GALM, GAMT, GLYCTK, GYS2, IRS1, ITGAM, MAFA, MBL2, NAGLU, OR1A2, OR4K17, OR52N1, ORC1L, P4HA3, POLR1A, PVRL3, RAP1B, RBX1, TGFB3, HLA-DRB5
Negative_5402	AMT, EBP, TFG, ACPP, ACVR1C, AMPD1, BDH2, BLNK, CAMK2B, CCL19, CD86, CYP2C9, FADS2, FBP1, FBP2, GCAT, GCNT3, GNAO1, GRIN2D, GSTA5, GSTM1, HLA-C, IL6ST, KIR2DS5, MAP3K8, MGAT5B, ONECUT1, OR14C36, OR52H1, OR9K2, PDE11A, PHKA1, PTCH1, RHOD, ST6GAL1, STX1B, SULT1A4, THTPA, TRADD, TSC2, UGT2B28, WNT9A, ARHGEF12, PAFAH1B3
Negative_5403	ACVR1B, DGKD, EFNB1, FGF21, OR2K2, TGFB1
Negative_5404	AMH, GLS, OTC, ACACB, ACSM5, ARSA, ATP5O, CACNA1H, CD28, GSTA2, IL1RAP, INPP1, LRAT, OR10A4, PAK4, PDE8B, THY1, ST6GALNAC6
Negative_5405	GNS, TXK, ALDH3B1, BCL10, CACNA1E, COL6A1, COQ3, FUT6, GNG12, ITGAX, MARS, MDM4, MYL5, NGEF, OR4F15, OR8I2, PDE8A, PNPLA4, PYGB, RDH11, RPSA, VAMP4
Negative_5406	CTH, ACADS, ALOX12, AMDHD1, CYP2A6, CYP2C18, DDIT4, DDX56, ECSIT, HRAS, IFNA14, IGF1, LRDD, MTMR1, MTMR2, NUMB, OR10Q1, OR2D2, OR51I1, OR6C2, OR6F1, OR9Q2, PARVG, PDE4C, PRIM2, PRODH, PTEN, RALBP1, RXRG, SDC2, SETD2
Negative_5407	ATM, C5, F12, GNS, IL2, ACOT12, AHCY, ATP5J, BMP6, CLTB, CYP3A4, EPHA2, FOSL1, FZD10, GART, GCDH, LDHC, LFNG, NPR2, NUMB, OR4S2, OR51B5, OR51V1, OR5AK2, OR5I1, PAK2, PFKL, PHPT1, PPP3CC, SMAD9, TNFRSF9, TUBB2A, TUBB6, ATP6V1G2, CACNA2D2, TNFRSF13C
Negative_5408	LHB, C4BPA, CFLAR, CHRM5, CHST11, CISH, FECH, FRAT1, GPD2, ITGAD, LRP5, MGST3, OR5K1, OR8J3, PDE3B, PIK3C2G, POLR3K, PPP2R5E, TNFRSF4, TUBB2B, ULK2
Negative_5409	HSD17B8, IL17RA, MAP3K2, OR56A3, OR6C75, PKMYT1, SLC25A5, UAP1
Negative_5410	AMT, ADCY9, ALG10B, B3GALT2, B4GALT4, CD36, CD8A, CHRNA7, CLDN6, COQ6, CSF3R, DARS, DDX52, DFFA, DTYMK, FZD3, GALNT2, HTR2C, IL20RA, IL28B, IRAK1, ISYNA1, LILRB3, LRP5, MLLT4, MYL2, OR10G4, OR1A1, OR1G1, OR2A2, OR5M9, OR6N1, OR8J1, POLR2A, PRPS1, PTPRR, RAC3, SARDH, SETD1A, ST6GAL1, SUFU, TAS2R49, TSC2, VARS, XIAP, XYLT2, HLA-DPB1
Negative_5411	KHK, BDKRB1, CAMK4, MGST1, PIK3C3, PLA2G3, PPP2R1A, SNAI2
Negative_5412	HDC, CACNG8, EXTL1, GLO1, HYAL1, LIPC, MAFA, OR4D6, OR4F4, PCK2, PDE6H, TGFB1, WNT16, YARS, PPP1R12A
Negative_5413	ADIPOR1, AK3L1, BTRC, EPB41L3, GSTA4, HSPA8, PPP3R1, RAPGEF4
Negative_5414	F7, ANAPC4, CHST12, HCLS1, RPS6KA5, SHMT1
Negative_5415	EZR, PDC, RB1, TNN, TPO, ACSS2, APAF1, ARSB, CDKN2C, CRKL, DDX47, ECSIT, ETNK1, GZMA, HTRA2, IL17RA, IL1R2, IQGAP1, LIPC, MYD88, NTRK1, OR1E2, OR1S1, OR8A1, ORC1L, PTTG1, RPN2, SARS, SH2D2A, SIPA1, SNAP25, TAS2R16, TLR5, VAMP3
Negative_5416	GNAS, IKBKB, LRAT, MINPP1, PDE11A, PRPH2, RPS6KA5, TNFRSF11A

Negative_5417	ALDH3B2,BBC3,CLDN20,CXCL10,DLST,GFPT2,GGCX,GGT5,GNPAT,HTR4,IL18R1,MAP2K6,OR13D1,TP53
Negative_5418	AHCY,APBB1,CCL8,FBP1,GAS1,GBE1,OR10K2,OR6B2,OR6N1,POLE4,PTGIS
Negative_5419	AADAC,BUB1B,CUL1,DCTN1,GAD2,GNPDA2,IL13,OR5C1,POLR3A,SENP2,SETD2
Negative_5420	ALG10,CCND2,CSF2,DTX3L,IFNK,ITGA2B,LIPG,MAPK8,MYH14,OR7G3,OR8G1,PTPN5,ROCK1,TPK1,CSNK1A1L
Negative_5421	CCKBR,CSNK1D,ENPP3,GNAQ,IARS2,LYPLA2,NANS,OR4F15,OR4M1,OR5T2,OR6F1,OR9Q1,P4HA3,PPA1,PTPN5,SMAD1,TOMM40,UCK1
Negative_5422	EZR,GH2,TAT,ADRB3,ADSS,AGPAT6,ALDH9A1,ARHGEF6,ATG3,ATP6V0B,CLDN3,COMP,CTNNA1,CXCL16,DDB2,DNMT3L,E2F2,ENO1,F11R,FARSA,GNAI3,GSTZ1,ISYNA1,ITPKA,JAK3,KAT2B,MITF,NEU1,NFATC4,P2RX6,POLR2L,PPBP,PPP2CB,PPP5C,PTAFR,PTCRA,STX18,SV2A,TAS2R3,TRAF3,UBE2K,ATP6V1B1,HLA-DRB5
Negative_5423	ADA,MSN,B4GALT3,CCR1,CD48,CLDN4,DDX47,GALC,HLA-DRA,NRCAM,OR10P1,OR52I2,PDPK1,PLA2G2A,PLXNB1,PON2,PPOX,RAPGEF2,RPE65,SAT2,SC5DL,SEMA3B,PAFAH1B3,TNFRSF18,TNFRSF1A
Negative_5424	ADH1B,LTBR,OR13H1,OR2V2
Negative_5425	CACNA1B,ECSIT,LIPE,OR1L4,PRKAB1,HLA-DRB4
Negative_5426	AR,CD6,HAL,TNR,ACHE,ATP2A3,ATP6V1H,B3GNT2,C1QB,CCL28,CDKN1C,CSNK2B,CYBB,CYP1B1,DGUOK,DIAPH1,DLL4,EDNRB,FABP1,GLO1,IARS2,LHCGR,MAPK9,MEF2C,OR2T11,OR4K2,OR5T2,ORC6L,PKLR,PLA1A,PTGIS,PVRL2,RHOD,RPS6KA3,UCK1,UPB1,VDAC2,VEGFB,VKORC1
Negative_5427	FAH,NPL,AOC2,CDS2,CIITA,CYBA,CYP4A11,H6PD,JAK2,LLGL1,PPP2R5D,TAS2R4,ATP6V1E2
Negative_5428	HAL,MAG,MVK,ACY3,AOC3,ARHGEF4,BAIAP2,CCL7,CDC25A,CLDN16,CREB1,CX3CL1,DOT1L,DUSP9,EVI1,FGF20,FRAT1,FUT3,GALNT8,GNRH1,HCLS1,HPSE2,INPPL1,IRF3,ITGB8,MAP2K1,MGAT5B,OR10G8,OR5A1,PDE1C,PIK3CA,PPCS,PPP3CA,PPP3R2,PRKAB2,RAC3,RASGRP1,RBPJ,SFRP2,SNAP23,SORD,ST6GALNAC3
Negative_5429	LEP,HADHA,PRKAA2
Negative_5430	AR,ACTN1,AGXT,ALG3,ATP5D,CD74,CDH5,DNMT1,FUT6,HMOX1,NME7,NR4A1,PIGM,PRLR,PTGES2,PTPN5,SESN1,SLC2A1,YWHAE
Negative_5431	JUP,ACTG1,ADH7,AKR1C2,ALDOA,ALPL,ARG2,ARRB2,BUB3,CACNA1E,CALML6,CAMKK2,CLDN3,CYP27A1,DDX41,FADS2,FMO1,FUT3,GDE1,HNF4G,KIR2DL2,LIAS,LPAR1,MAGI3,MAP2K3,MMP2,MS4A2,MYH14,NCR1,OR13C5,OR6K3,OR7A5,PIK3C2B,PMM2,PNPO,PPP2CB,PPP2R5C,PRPH2,PRSS1,RASGRF2,SLC27A5,ATP6V1G1,TNFRSF1B
Negative_5432	AGPAT6,CALML5,CXCL3,EIF4B,GAB1,GADD45B,GIT1,GLUL,GNAI3,GSTO2,HYAL1,IQGAP3,ITGAV,ITGB7,LAMB3,NLGN3,OR10H5,PLOD1,PPP1R3D,PRKAG3,PRNP,RERE,SELE,TNFSF10
Negative_5433	IL6,CANT1,COMP,CYP3A5,DDX19A,EPHA2,GSTO2,IL20RB,MGAT5B,NCK1,OR5I1,PDCD1,PDHA2,PIK3C3,PON3,PRKAR2A,PRODH,PTGDS,SKP2,SUCLA2,SULT1A4,TAS2R50,VEGFA,HLA-DPB1,SUV420H1
Negative_5434	TNN,APPL1,CCR9,CDH1,FCGR3B,GGT5,GNAS,MAP3K5,MCM3,NKX2-2,OR2V2,PHKA1,PPP2R5E,PRKAG2,SRGAP1,ST3GAL5,TSTA3,PAFAH1B2
Negative_5435	PGD,ADSSL1,BDH1,CERK,CSF1,MAFA,MASP2,NTRK2,OR2A12,PYCR2,RRAS2,SLC33A1,SERPING1
Negative_5436	ADK,EDA,GHR,IL8,ABAT,ACADL,ATP6V1A,C9orf95,CD38,CDH15,CHAT,CXCL13,DCXR,DGKZ,FDPS,GMPS,HADH,HTR4,MGAT4A,MTAP,OR2A12,PLCG2,PPP3CB,PRKCQ,SUV39H1,TNFSF11,TP53I3,UGT2B7,TNFSF13B
Negative_5437	PAH,CHRM3,CSF3,DGKD,GSTM2,HHIP,IFNA21,IFNW1,LAMA4,LTB4R2,NARS2,OR7A5,PIPOX,PLAU,SUV39H2,TNFSF18

Negative_5438	GK2,ABLIM3,ACACB,ARPC1B,BCMO1,BUB3,CHSY3,CKMT2,CNTFR,CYP2C18,FLT4,FZR1,GUCY1A3,IDI2,ODC1,OR10X1,OR11H1,OR2G3,OR2T4,OR51I2,OR52N1,OR8J3,PVRL3,RIPK1,SH3BP2,STEAP3,TSTA3,SERPINC1,SERPINE1
Negative_5439	ADCY5,ALDH3B1,CYP2C18,ENPP7,GYS2,MAPK3,OR5B21,OR8J3,PRKACA,SCP2,SMPD1,TACR3,VAMP1
Negative_5440	CD79B,DTX3,GNAS,PTCH1
Negative_5441	ACTA2,ALDH9A1,ATP5J,ATP6V1A,B3GNT2,CCL16,CD19,CDIPT,CXCL1,F2RL1,HHEX,IFNA14,IL2RB,MAPK3,NGEF,PDE9A,PLCD3,RFXAP,ROBO1,UBE2L3,UGT2B17,VAMP4,TNFRSF10C
Negative_5442	ACY3,CDH4,CNTFR,FASLG,PANK1,TUBA4A
Negative_5443	GLS,PRL,AASDH,ACAD8,BACE2,CASP10,CHEK1,DAAM2,FABP1,HTR7,OR1L8,PARS2,PCCA,PCCB,PFKFB2,PPP5C,PVRL2,THBS2,TP53
Negative_5444	CAT,F2,F3,ACTN2,ALG6,AMHR2,ATP6V0C,BMP6,CCKBR,COMT,CXCR4,DDX54,DLL4,DNMT3L,EXT2,FADS2,FGF13,IFT57,KIR2DS5,KLRK1,KRAS,NT5M,OR7A5,PAK4,PDE8B,PDXK,PPP5C,PRSS1,PYCR1,RDH8,RPS6KB2,SLC25A5,SMURF2,SOCS7,STX1A,TALDO1,TYK2,WEE1,PLA2G12A,TNFRSF1B
Negative_5445	CR2,LYN,ACACA,ADRB2,ETNK1,EXTL1,GALNT1,LDHAL6A,OR1S1,OR2L8,PFN4,RPS6KA5,ST3GAL3,TAS2R9,TRPM5,ATP6V1G2,MAPKAPK2
Negative_5446	F10,SMS,ADCY1,AKR1C4,ALDH5A1,ASRGL1,B3GNT2,B4GALT2,CACNA1A,CANT1,CCL26,CCR4,CD3E,CDKN1C,CIITA,COMP,CPOX,CYP4A22,DHCR24,DLL3,E2F1,EFNA2,ETNK2,GALM,GLI1,GUCY1A2,HHEX,HMGCR,MGAT4A,NEU2,OR10S1,OR11H1,OR13C2,OR13D1,OR2D3,OR2M4,OR2T2,OR6C3,OR9G1,PFKFB4,PFN2,RHOA,RPN2,STX16,SULT1E1,TAS2R1,HSP90AA1
Negative_5447	ACSL6,B3GALT5,C1QC,CDH3,CXCL2,IL17A,NLGN1,PIGK,PPAP2C,SLC27A4,SSH1
Negative_5448	CR1,SHH,ADH7,ADORA2B,ATP5D,BNIP1,CACNB2,CDC23,DDX55,DTYMK,FGF12,GART,GUCY2D,GZMB,HARS,HMBS,HMGCL,HNF4G,IDH3A,IL11RA,IL12RB2,INSR,IRS2,LIPT1,ONECUT1,OR10A3,OR10H5,OR2F2,OR4X2,OR52E4,OR8K5,P2RX3,POLA1,POLR1C,POLR2A,RERE,SELPLG,SMURF1,UCK2
Negative_5449	CKB,DDC,F2,PDC,ACADS,ACOT11,ADCY5,ALDOA,ANAPC2,AOC2,CCL11,CCR6,DDB2,DRD5,E2F5,FBXW11,FSHR,FZD6,GP1BA,GPX5,IL23A,ITGA7,ITGB1,MAD2L2,NFATC1,NOX1,OR10H3,OR10J1,OR14A16,OR4D5,PDE7B,PLOD1,RARS2,RXRG,SEMA3D,SGMS2,STK36,SUOX,TAS2R41
Negative_5450	ADCY9,ATP5C1,BMP2,CD58,CLDN5,CNGB1,CYP3A43,DDX50,EPHA5,ERBB2,FLOT2,IDUA,NR4A1,OR10S1,OR4C11,PARS2,PFKFB4,RHOD,RPS6KA3,TBL1XR1,TOLLIP,WASF1,XYLT1,SUV420H1
Negative_5451	EBP,SRM,ADRA1D,AHCYL1,APOA2,BET1L,CD82,CDC2,CDKN2C,COMT,DRD2,F13A1,FDPS,FUT9,GALNT1,HSD11B1,IL5RA,IMPA2,INPP4B,LAMB4,MMP14,PAICS,PFKFB1,PFKFB2,PIP4K2B,POLR2J,POLR2L,POLR3D,PRPS2,RBKS,SMAD4,TLR5,ULK3,VDAC1,VDAC3,SERPIND1,TNFRSF11A,TNFRSF6B
Negative_5452	C4B,HK1,ACP6,BDNF,CXCL13,CXCR4,DDX52,DNMT3B,FGF21,GLB1,GNB1,HMOX2,INPPL1,LYZL1,MARS,MYH6,NAGS,OR4A16,OR4F6,OR52R1,PDGFA,PDGFC,PHGDH,PIP5K3,PRKCQ,PSME2,PTPRJ,TAS2R7,TXNDC12,PDCD1LG2
Negative_5453	AGPAT3,ALDOC,APLP1,BACE1,BCAT2,BDH2,CACNG5,CES7,CYP1B1,GRIN1,HPRT1,MDH2,NTRK2,OR13J1,PIGB,PRKCG,PRKG2,PTPRZ1,SARS,TOMM40L,TUBA3E,TUBAL3
Negative_5454	GP5,LPL,TK2,C3orf10,CDO1,CNTN1,CYP2U1,ERBB4,FLAD1,HTR4,HTR6,MTHFR,OR1J4,OR4F17,OR52E4,OR5B3,OR5M3,PARVG,PCYT1B,PDCD1,PDE3B,PDXP,PIGK,PIK3R5,PNPO,PPP2R1A,PRKCB,PRPS1,RAD51,RRM2B,SUFU,TAS2R4,TBXA2R

Negative_5455	DBH, F5, IL4, IL8, ATG3, CASP9, CD72, CSAD, ENTPD3, FGF12, GNAS, HNF1B, HSPA1B, IARS, IL22RA2, ODC1, OR4K13, SMAD6, STK36, TKTL1, WHSC1
Negative_5456	FUK, TAT, ACTN4, ASCC3, ATP5J2, DUSP10, GNMT, NFYB, OR8H3, PPP1CB, SETD2, TXNDC12, ZBTB16
Negative_5457	IRF5, PCYT1B, ATP6V1B2, PRICKLE2
Negative_5458	C5, AADAC, ALG10, AMY2A, ANAPC5, ARRB2, ASAH1, BMP2, CCNG1, CDH2, CHKB, DGAT1, DHDDS, EFNA3, EHMT2, EI24, ELK4, ESPL1, GAD1, GALNT14, GP1BA, GSTA4, GSTM1, GSTO2, GUCY1A2, IDH1, IRAK3, IRF9, ITGA10, LDHAL6B, LLGL2, MYH1, NEUROG3, OR2Y1, OR5D13, OR6C3, OSMR, POLE2, POLE4, POLR2K, STK4, TKTL2, VCAM1
Negative_5459	DCN, APPL1, B3GALT5, CACNG2, CERK, CRY1, HSD17B2, IL12RB1, IRF5, LLGL2, MAP3K3, MAT2B, MTMR2, OR1A2, OR1L3, OR2A4, OR2W1, OR6K6, OR8S1, P2RX6, POLA2, PRKACG, RALB, SOCS4, SYNJ1, TRHR, VEGFA
Negative_5460	CR1, EZR, ACTG1, C5AR1, CFLAR, CSDA, EPHX2, F2RL3, GSTM5, IL6R, LAMC1, NOS1, PFKL, PRSS2, RPS6KB2, STAM, WHSC1L1
Negative_5461	AADAC, ALPPL2, BCL2, CNTF, DDOST, ENTPD3, IL20RB, L1CAM, MAML3, NAGA, NMNAT1, OR1E1, OR2V2, POLR2F, SKP1, SULT2A1, UGCG, ULBP3, UNC5B
Negative_5462	ACTB, ACVR2A, CCL15, CLCF1, DDX23, DFFA, ENTPD8, FOXA3, GAL3ST1, GALC, HEMK1, HMBS, HRH2, IFIH1, INPP5A, IRS4, LGMN, MAPK8, NT5E, OR2L13, OR4M2, OR5K2, PDE4D, PIAS4, PKMYT1, POLR1D, RASA1, RND1, WNT11, YES1, ZIC2, TNFRSF18
Negative_5463	CGA, FGA, ADRA1A, CXCL12, CYP17A1, FRAT1, MAN2A1, MAT2B, OR2K2, OR2M5, PDK1, PGM1, PTDSS2, TLR3, TNFSF10, UMPS, MAP3K7IP1
Negative_5464	IL9, ARPC2, CCKAR, CD48, CHST11, COL3A1, CXCL2, DDB2, DLL3, EHMT2, FGFR3, GAD2, GALNTL2, GNPDA1, GUCY2C, IBSP, IFNG, INPP4A, ITGB8, JAK3, KIR2DS4, KLKB1, LIPE, LRP6, MAD2L1, OR10H3, OR10J5, PARD6A, PIGK, PIK3R5, RAC2, SGMS1, SLC25A5, SMURF2, UNC5C, AASDHPPT, ATP6V1B2, ATP6V1E2
Negative_5465	F3, AKR1B10, BMP8A, CAMKK2, CD44, CDH4, CSF3R, CTBP2, CYP2F1, DARS, ECSIT, FGF2, FMO4, GALNTL5, GMPPA, GNB1, GPX5, HPSE, HPSE2, IL1A, MAN2A1, MAP3K3, METTL6, NCR1, NNMT, OCLN, OR11H4, OR52I2, PDGFD, PGAM1, PPP1R3D, PTGES2, QPRT, RALA, RASSF5, RHOD, RUVBL1, STX1A, TAS2R1, TJP2, TUBB4, TXNDC12, VARS, ZFYVE16
Negative_5466	ACY1, CACNA1I, CDC27, ENTPD4, GCNT4, GNA14
Negative_5467	ADO, CASP10, CLDN15, CYP2F1, DDX51, DTX1, GSTM2, NFKB1, OR10A3, OR2AG1, OR4D1, OR51E2, OR5I1, RFWD2, SAT2, STX5
Negative_5468	C5, CD6, EBP, ABCC8, ACAD8, AQP7, COQ5, COQ6, CTNNA1, CYP3A4, DDX41, ENAH, EPB41L2, GANC, GGCX, GP1BA, HSD17B8, IFNG, IL1R2, IL9R, INPP4A, INSRR, ITGAV, ITGAX, LAMB1, LRAT, MAML1, MMP9, NOTCH1, OR14A16, OR1L4, OR1Q1, OR4D11, OR4F15, OR52K1, OR52K2, OR5AC2, PIP4K2C, POLR1E, SLC2A2, ST3GAL4, TBL1X, TUBB4, CACNA2D1, GABARAPL1, SUV420H2
Negative_5469	BID, IDS, ACOX2, ADORA2B, ALDH3A2, ALPL, ARG1, ARNTL, ASCC3, BCKDHB, CCL17, CMBL, DIAPH1, ERBB3, FUT8, GADD45G, GANC, HHEX, HMOX1, IL7R, INHBE, NR4A1, NRG2, OR11A1, OR51I1, OR5A2, OR5T2, PDHB, PER3, PPP2R2D, RARS, STX1B, TRPM5, UBE2L3, VANGL1, WWP2, HSD17B10, HSD17B12, SERPING1
Negative_5470	AK7, CACNG5, CUL1, FLOT2, LDHAL6A, OR2C3, PGAP1
Negative_5471	CAD, ATP6V1A, BCAT2, BMP7, CD8B, FGF4, FUT4, LAMB3, OR4F4, OR51E1, PMAIP1
Negative_5472	ADCY4, DNMT1, PFAS, PLA2G2D
Negative_5473	ABL2, ACAA2, ARPC5, B4GALT1, CDS1, DDX23, DEGS2, DHRS3, GALM, MGAT1, MGAT4A, NT5C3, OR2L13, OR2T4, OR52E4, OR5L1, PLA2G3, PPP2R5A, RND1, SEMA3E, SETD2, SGPP1, SHISA5, SOCS3, STAM, SV2A, SERPIND1
Negative_5474	CD27, IL1B, IL5RA, IRF9, MAML1, OR4K1, OR5R1, PFN3, PRICKLE2

Negative_5475	MYC, ADCY6, AGPAT6, ALCAM, CCNA1, CD244, CD38, CDC25A, CDH1, CHRNA7, CYP2J2, DDX47, EARS2, GMPR2, GNAI2, GOSR2, IL2RB, IL6ST, ITGB2, LCMT1, LHCGR, MYH1, OR10Z1, OR5C1, PFKFB3, POLR2H, POLR3D, PPP2CB, SPCS3, ST3GAL3, STEAP3, SUCLG2, SYNJ1, TBL1Y, TUBA8, WNT3, ZNRD1, HSD17B10, PPARGC1A
Negative_5476	CHRM3, DUSP1, GNAI2, HLA-F, HMGCS2, ITGB7, KIR2DL4, NTN1, OR2AG2, OR52B4, POLE, UGP2, B3GALNT1
Negative_5477	ABO, AASS, ADH1B, BLVRA, CAMK2D, CCL4, CX3CL1, CYP3A4, EGFR, EHHADH, GALNT5, GCNT1, IBSP, ICAM2, IPPK, LCP2, MAML3, MAOA, NPR2, NR1D1, OR1B1, OR51A2, OR51B2, OR51E2, OR52E8, OR9A2, P4HA1, PDE6H, PNPO, PPP2R5A, SDC4, SEMA3B, STX12, TAS2R3, TPH1
Negative_5478	KMO, ACTC1, ARHGEF7, ATP6AP1, CCNG2, CHKA, CLDN11, CPT1A, CYFIP1, DARS2, EHMT1, MAD2L1, MAP3K4, OR2M7, PIK3C2A, PRKCZ, RPS6KB1, SUV39H2
Negative_5479	BTD, ABL1, ACTG1, AKR1C2, ALG3, BET1L, CCR9, CYFIP2, DERA, DNMT1, FTH1, GGT5, GLI2, GUCY1A3, H6PD, HLA-DOA, MKNK2, NFYC, OR14C36, OR1C1, OR4D11, OR52H1, PARVB, PLA2G2A, PLAT, PON1, PSEN1, PTPN7, SHC4, STK3, TAS2R41, TNFRSF4, TRAF3, TYK2, UGCG, UGT2B11, PAFAH1B2, TNFRSF11A
Negative_5480	F12, MUT, ACADSB, BUB3, CACNA1I, CAMK2A, CD14, CLDN18, DLL4, FABP1, FDFT1, FGF21, GNAO1, HMOX1, IL13RA2, IRF9, LDHAL6A, LRP6, MS4A2, MYH7B, NADSYN1, NT5M, OR10T2, PANK4, PDE6H, PIGN, PLTP, PON3, PPP5C, PTPRF, SGPP2, SNAI1, ST8SIA1, SUFU, TSC2, UCKL1, ATP6V0D1
Negative_5481	DCN, JUN, PAH, AANAT, EPHA7, GLI1, GUCA1A, OR5C1, PDHB, PIM1, PRKCQ, SHC1, TPK1, VAMP3
Negative_5482	AARS2, ARPC3, DGUOK, DHRS4, DTX2, FCGR3B, HRAS, IFNAR1, IL15RA, IL17RB, MAGI1, MAP3K13, NR1H3, OR4K1, PIGF, PPARD, TCIRG1, TJP2, ZMAT3, PAFAH1B1
Negative_5483	JUP, LPL, CSF3R, CYP2B6, DAD1, EIF4E2, GLO1, HHEX, NCKAP1L, NRG4, OR8H1, TM7SF2, TNFSF14, UBA1
Negative_5484	ACSL1, ACTN4, ADH7, AGPAT3, ALDH1A1, ARPC4, ARRB1, B3GALT4, BPGM, CDC20, CFLAR, CHSY3, CLCF1, CSNK2A2, DDX52, FRAT2, GOSR2, HMGCR, HNF4A, ITGB5, ITGB8, MAGI3, MAOB, MFNG, MYL9, OR10C1, OR11A1, OR2L13, OR4L1, OR6K3, OR6T1, OR7G3, PAX6, POLR2B, PROS1, QPRT, SELE, SLC25A6, SPRY2, STEAP3, UBA1
Negative_5485	XDH, ACSL1, B3GALT5, CSNK1D, CYP2J2, EIF4B, ENTPD1, FDFT1, GSK3B, GSTM2, HSD3B7, IFNA17, IQGAP1, ITGA11, ITGB4, KIR2DS5, MASP2, MYLK3, OR10A6, OR2T11, OR4A16, OR52J3, OR6C1, PFKFB4, PLXNB2, RARA, RHOA, SFRP5, SUV39H1, TAS2R50, HLA-DRB3, SUV420H1
Negative_5486	DCT, DHH, ACAT2, ALG8, AMD1, CASK, CD19, GLDC, KIR3DL1, P2RX6, PRKAA1, STK11
Negative_5487	AUH, C7, CDA, VCL, ADAM17, ALOX5, AMDHD1, AVPR1B, BBC3, CACNA1D, CD55, CHKA, CLDN4, CTPS2, CYP3A5, DDX18, DHDH, FGF18, FGF22, FGF8, FIGF, FLT1, FPGT, HSD17B7, IFNA21, IFNAR1, IKBKG, LILRB3, LTBR, MAFA, NFATC2, NT5C1A, P2RX5, PAX6, PLTP, RASGRF2, RPS6, RRAS, SENP2, SORBS1, SPRY2, SULT1A1, TRAF1, TUBA4A, VDAC3, YWHAG
Negative_5488	ID4, MVD, ACADM, ACADVL, ACTN2, BLNK, BMP7, BUB1B, CRY1, FTMT, GLDC, GPLD1, MYH15, NEU4, NOTCH1, OR2L3, OR3A3, OR4X2, OR56A3, POLR2G, PPBP, RDH5, YWHAQ, TNFSF13B
Negative_5489	DCK, IL5, LCK, NOG, ADCY1, ALG3, ANAPC11, BACE2, C4BPB, CASP9, CD38, CHDH, CLCA2, CNTN2, DAAM1, DKK1, DRD1, EGFR, FSHR, GSTM4, HRH1, HSD11B1, IL3RA, IQGAP2, ITGA1, KYNU, MAP2K6, MAPK12, MCM7, NUDT5, OR10H1, OR10H5, OR11H1, OR1S2, OR4K1, PCNA, PMM1, PPP2R5A, PTGFR, PTPRF, TAS2R38, TYMS, VAMP4, WARS2, B4GALNT1, CACNA2D3, SERPINC1

Negative_5490	EBP,F5,ME2,OAT,PGD,PXN,B4GALT5,CACNG8,COL2A1,CYP2C9,DGKI,ENTPD4,ETNK2,EXOC7,GPD2,GUCY1A2,IARS,IFNA7,IFNW1,INMT,LIPC,MAP3K11,NOTCH1,OR1E2,OR4C16,OR9I1,PDE10A,PPP5C,SELL,SEMA4F,SHISA5,SQLE,SUFU,UBE2L3,UGT2B11,VASP,WBSCR22,WWP1,ATP6V0A1
Negative_5491	OR51L1,RFX5,TUBB2C
Negative_5492	ARHGEF6,BDH2,CDC14B,CTPS2,DGAT2,EDAR,GALC,GALNS,HCST,IMPDH2,NCOR1,NNMT,OR6K3,PGM2,PLA2G3,PPM1A,SMAD3,STAM2,TRAF2,TUBB2A
Negative_5493	NME2,OR2M2,PIGF,PIP4K2A
Negative_5494	AASS,AGPAT2,IRAK3,NFKB1,NKX6-1,NT5C1A,OR7C1,PIK3CB,ARHGEF12
Negative_5495	IL5,ACY3,ADCY8,C4BPA,CARS2,CBR3,CCL15,CCL2,CLDN10,CNDP1,CNTF,CUL1,FOXA3,FRAP1,GADD45B,GNAO1,GNRHR,GSTO1,HTR7,ICAM1,IL13RA2,ITGB8,KIR2DS5,LYPLA1,MINPP1,MMP7,MPP5,NCAM1,NCR1,NME2,OR14A16,OR51V1,OR52J3,OR5P3,OR5T2,PMAIP1,PNPLA3,PPP1CC,PSME2,RRM2,SOAT1,TAS2R13,TNFRSF9,TUBA1B,YARS2,CTNNBIP1,PPARGC1A
Negative_5496	SI,GLUL,ORC2L,PKMYT1,PORCN,SFRP5
Negative_5497	ASIP,CASP10,CHKA,CNDP1,CRY2,CTPS,CXXC4,CYP2S1,CYP2U1,DDX50,DGKI,EXTL3,FZD5,GALM,GNG3,HSD3B7,IL1R2,INPP1,KCNB1,LTA4H,MAN1C1,MGST1,MIOX,NAT6,NEUROG3,NGEF,NT5M,NUDT9,OR2T4,OR4N5,P2RX5,PANK1,PDE4B,PON3,RDH12,RPS6KB2,SUCLA2,TAS2R1,TAS2R38,TMLHE,SERPINA5,TNFRSF10A
Negative_5498	WAS,ACOT11,ALDH1A3,ALPPL2,APOA1,ATP5B,CADM1,CYP2J2,MAGI2,NOTCH1,NTNG1,OR5P3,PLOD3,SORD,TUBB6,ATP6V1C1
Negative_5499	KMO,ASMT,ATIC,EPHA2,FGF1,FZD3,KCNQ1,MGST1,PLEKHA8,SETD1B
Negative_5500	ADA,CD6,EBP,HK3,IHH,ABL2,ALG12,BHMT,CAV2,CDC26,CXCL12,DBF4,GNG12,GNMT,GRIA2,IDUA,KLRC4,MYH8,OR10A4,OR2T1,OR4C13,OR51B2,OR52N2,OR6Y1,PDIA4,PRKG2,PRKX,PTPN11,PTTG1,ST8SIA5,STX5,TPH2,UGCGL2,ATP6V0A1,CACNA2D2,CACNA2D3
Negative_5501	ADH5,FMO1,HIF1A,ITGA1,OR2G6,OR3A2,POLE4
Negative_5502	NPY,ATIC,CDC16,CEBPA,CYP2C8,FCGR3A,GSTM3,GUCY1B3,HLA-DOA,HSPA2,OR12D3,OR52A5,PDHB,PDX1,PLAT,PPP2R2A,PPP3CC,PVRL1,RHOD,SGPP1,TUBB4Q,UAP1
Negative_5503	LCT,ADRBK2,ATP5I,BBC3,CACNG5,CCL25,CYP2U1,EFNA5,GCNT1,GNPDA2,IL17B,MAT2B,OR2A2,PDE2A,SPTLC2,TAS2R3,ZFYVE9
Negative_5504	AK1,CD4,CR2,ME1,AARS2,ACMSD,ATP5G2,ATP6V1H,CCR1,CD34,CYP1A2,DGKI,EFNA2,FDFT1,FGF19,GAD1,IL7R,INHBC,IQGAP1,JAG1,NT5E,OR13C8,OR13G1,OR2A42,OR3A3,OR4K1,OR51A2,OR6T1,PHGDH,PIGV,PROC,PRPS1,PTPN7,PVRL2,PYGB,RPN2,UBA7
Negative_5505	DBI,FN1,GAA,ID3,ACVR1C,AHCYL2,ALDH2,ASPA,ATP12A,BIRC5,C1QC,CACNG7,CCNB3,CLDN20,COASY,CPT2,ENPP7,EPHA7,FDPS,FGF1,FUT5,GAPDH,HMGCS1,IL1R1,LIFR,MAN1A2,MAP2K6,NEFH,NUDT12,OGDH,OR10S1,OR1I1,OR2Y1,OR56A4,ORC3L,PGS1,PLD2,RASGRP4,RCHY1,RPS6KB2,TFPI,TGFA,TRADD,XCR1
Negative_5506	ALG9,CCR2,FGF19,OR13J1,OR2AE1,OR4K17,OR6C2
Negative_5507	OSM,SPN,CHST13,CYP2A13,DARS2,EFNB3,FGF22,GMPR,GNRH1,GSTA4,ICAM1,KLRC4,NEU3,OR10V1,OR4C46,OR5AS1,PAK2,PDK1,PNPT1,POLE2,PVRL3,RFXAP,RPS6,VEGFA,MAPK8IP1
Negative_5508	CGN,FER,TXK,ADRA1D,AGPAT4,APPL1,AXIN2,CCDC6,FTH1,ITGB2,NLGN2,NOD1,OR8J3,PDGFA,PRKCE,PYCR2,RDH10,SNAP25,SOD1,SUCLG2,TGFBR1,UCK2,ULBP3,CTNNBIP1
Negative_5509	OAT,PLG,SPN,ADSL,AKR1B1,AMPD3,APOA1,ATG7,C5AR1,CCL25,CLOCK,CPT2,EPHA8,EXT1,FARP2,FOXA2,FUT9,GADD45G,GLI2,GNA11,MAGI2,MTHFS,NAGLU,NPR1,NRG4,NTNG1,OR2D2,OR2G2,OR8D2,PDXK,PFKFB4,PTGFR,SETDB2,SQLE,SUCLG1,TRAF5,TXNDC12,UROS,XCR1

Negative_5510	AMY2A, CCL5, CD3E, CD40LG, CHRNA7, CPOX, CRY2, CXCR4, CYP2E1, CYP4A11, GNA11, IL10RA, KITLG, LIPF, NPR2, PTDSS1, SDC3, TRAF2, SUV420H1
Negative_5511	HAL, ACMSD, ASNS, CD46, CYP1B1, CYP2A6, DGKB, ENTPD4, EPHA3, GNPDA1, HAGH, HNMT, LAMA4, NFATC2, OR10P1, OR1A1, OR5I1, OR7D2, PCCB, PTPRF, SDHA, STAM, STX7, ATP6V1C1
Negative_5512	CAD, GK, IL5, NGF, PGD, ACP1, ADCY4, ALDOA, ATP5D, ATP5H, CAMK2B, CCNH, CDK6, CISH, CLDN15, CRLS1, DEGS2, DGKQ, EI24, EPHB6, ERBB2, EXT2, FCGR3A, FSHB, GALNT1, GMPR, GRIN1, HCLS1, MAGI2, MAP3K5, NAMPT, NOS1, NOTCH1, OR13C3, PDE5A, PIP5K1B, PLCB3, PLOD3, PLXNA3, ST3GAL4, ST3GAL5, TRAF5, TUBA1C, TUBA8, VEGFA, ST6GALNAC1
Negative_5513	A2M, CAD, HYI, CYP21A2, CYP4A22, CYP8B1, DDX51, FLT1
Negative_5514	GBA, NOG, ALAS1, ALOX12, ATP5O, ATP6AP1, B3GNT3, CDKN1A, CES1, CRLS1, DUSP3, GCAT, GLI1, GYS1, HBEGF, INPP1, KIR2DS5, LMO7, MAP4K2, MTHFD1, OR10G8, PSME1, RBL1, RELB, SEMA3E, STX16, SUCLG2, MAPK8IP2, SERPINC1
Negative_5515	IL9, KIT, ACPP, ARPC4, BMPR2, CCNG2, CYP11B2, CYP2A6, CYP3A4, DDB2, DTYMK, EFNA2, GP1BA, HMGCL, HMOX1, IL2RG, LCP2, LTBP1, MAP3K14, MAPK14, MFNG, MYH15, MYLK, OR2A25, OR4C12, OR51L1, OR5V1, OR6N2, PCYT2, PLCB3, PRIM1, PTPRC, QARS, RPS6, SENP2, UMPS, VAV1
Negative_5516	ACACB, ACSL1, ALDOB, GSTM4, IFNA7, IL10RA, MALT1, NUDT2, PCCB, PLA2G3, PLAU, PPP2R2A, PSME3, RXRA, SCIN, ZIC2
Negative_5517	BTD, CTH, ACAA1, ACVR2B, AGRP, ALDH2, CLDN19, CNTN2, CYP2D6, DGKD, DGUOK, DLAT, HEXA, HTR2A, INPPL1, NFASC, NME4, OR11H4, OR1L8, OR6C6, PAK6, PIK3C2A, PKM2, PPP2R5A, SLIT2, SMC1B, SPCS1, SV2B
Negative_5518	BAX, GLS, ANAPC1, B4GALT7, CDH1, COL2A1, COQ6, GNAI2, LRP5, MAGI1, MAPK14, NFATC4, NME4, OR4P4, RHOA, WWP1
Negative_5519	CS, ABLIM1, ALG1, ALPP, CAV3, CCL27, CLDN5, CYP8B1, DUSP10, E2F1, FZD9, GADD45B, GNAI1, GOT1, GPX5, HNF4A, IL9R, KCNQ1, LAMC2, MAP2K3, MAP4K4, MMP1, NANS, OR11A1, OR2T2, OR52B6, OR5K1, P2RX1, PDGFD, PIAS4, PLOD1, RDH10, RDH5, RPS6KA2, SEMA3E, SETDB1, SKP2, SOCS3, SRGAP3, VANGL1, WBSCR17, WEE1, WNT2B, WNT4, HLA-DRB5, PPARGC1A
Negative_5520	FOS, ATP5H, CLDN9, CLOCK, DAXX, DGKH, FTCD, GCLC, GUCY1A3, HNF1A, IKBKG, LYPLA1, MAN2A1, NADK, NEFH, OR51Q1, OR52K1, OR5C1, PDE9A, PHPT1, POLD4, RAB5A, SARS2, ST8SIA1, STEAP3, TPI1, WNT7B, ZFYVE9
Negative_5521	ME3, MVK, EPHA6, GALNT2, GNPDA1, HMBS, IGSF5, OR2M4, OR4A15, OR6C3, PCK2, PRKG1, PYCR1, TUBB1, B3GALNT1
Negative_5522	FST, ADCY9, ANAPC2, AVPR1B, CDS2, FGF3, GPX5, GUCY2C, HMGCR, HNF1A, IL17RB, INHBE, IRS4, MCCC2, MCEE, NAT6, OR12D3, PFN3, PLA2G2E, PTGES, PTPRF, RASSF1, RDH10, TRPC1, HLA-DQA2, PAFAH1B1
Negative_5523	FAS, ACVRL1, AMHR2, APRT, CD8A, CFLAR, CLDN15, DCTD, DGUOK, E2F5, EXTL2, GANC, GDE1, GRIA3, GUCA1B, HRAS, IL25, IPPK, KDSR, KLRC2, OR1N1, OR8I2, PFKFB2, POLR3K, PRKX, SFRP1, SIPA1, SULT1A2, TBL1Y, TNFSF4, UNC5B, MAPK8IP3
Negative_5524	C8B, CKM, JUN, MPL, ACSM4, BRAF, CCND3, CSF3, CSNK1G1, DEGS2, FGF17, FOXO3, FUT4, GYS2, HEXA, ITPKB, MAGI2, MTHFD1L, NKD1, OR1L4, OR6C2, OR6K6, OR8D1, P2RX4, PDE8B, PPP1CC, PSEN1, RASSF1, SNAI1, SULT1E1, THBS3, TOLLIP, TYK2, VEGFA, WASF3, HSD17B10
Negative_5525	CFH, MYC, ACOT12, AMD1, DDIT4, DUSP14, EHMT1, FSHB, HLA-DMA, MAT1A, NCR1, NFATC3, OR10Z1, OR5P2, PARD3, PIK3R5, TJP2, ATP6V0E1
Negative_5526	BCMO1, CACNB3, CES2, CHPF, CLDN4, DHODH, ECHS1, ENO3, FGF7, GGPS1, GLO1, IFNA14, IL1R1, INSR, IQGAP3, LAMA4, LDHA, MYH14, NKD1, NKX6-1, OR2T33, OR5R1, PIAS2, PLXNA1, PLXNA3, POLR1B, PPP2R5C, PTDSS1, RHOD, SLC33A1, SULT1A4, TUBB, MAP3K7IP1

Negative_5527	AK7,CD4,ANAPC2,ARSD,BPGM,CCL27,CDKN2A,CSNK1A1,CXCR6,CYP4A11,DAPK2,DDB2,DIAPH2,FTCD,FZD4,G6PD,GRM5,GSTM1,IFNA14,IL28B,IL4R,MITF,MOV10L1,NKD1,OR5A1,OR5D16,OR5M8,OR6K6,PANK2,PFKFB3,PGAM1,PPARD,PPM1A,PPP2R5D,PVRL2,RALBP1,RPS6,SMAD1,SOCS2,SSH1,UCP1,PRICKLE2,SERPINB5
Negative_5528	CYP3A7,DGKD,EPHA2,IFNA16,PCNA,PGK2,POLA2,TAP2
Negative_5529	MUT,AARS2,ATF4,CCL26,CLTCL1,COL4A1,CXCL11,CYP2A6,DDIT4,EGLN1,GMDS,GOT1,MAD2L1,MAOB,MAPK14,OR13F1,OR2A12,OR8B12,PKMYT1,POLR1D,RENBP,SELP,SPTLC2,STT3B,STX1B,TAS2R9,TRADD,UPP2
Negative_5530	GLA,ADCY3,CLDN1,INSR,LRP5,MAP3K8,OR7A17
Negative_5531	ACHE,BDNF,CHRM5,COMP,FDPS,GNAT3,GP1BA,GPAM,LEPR,MMP1,NCR2,OR4D1,OR4K5,OR52J3,PDE3B,POLR2K,POLR3G,PTCH2,SPHK1,STK11,VAV1,VAV3
Negative_5532	DBT,NPY,ABLIM3,ACSL3,AK3L1,ARNT,ATG12,CACNA1G,CCL26,CD28,CLOCK,CNGA4,FUT5,GALNTL1,GGPS1,IDI2,MAPK9,OR56B4,PIK3R5,PPM1B,PTGDS,PTGES2,PYCR2,RBL1,RCHY1,SETMAR,TACR1,TAS2R7,WNT3,YWHAQ,ATP6V0D2
Negative_5533	SMO,ARSA,BLVRA,CARD11,CLDN9,COL3A1,CYP2S1,EPHB6,FADD,FGF5,FGFR4,FZD9,GCAT,GLI3,GNG13,GRIA3,GSTA2,ITGAE,KNG1,LTA4H,OR13A1,OR1A2,OR2AG1,OR5B3,PGK1,PLA2G6,PLCB3,POLR1B,PTPRF,RUNX1T1,SLC27A5,SSH3,ATP6V1C1
Negative_5534	RB1,ADCY9,ADRB1,AHCYL1,AKT3,CCNA2,CD46,CHRM2,COQ6,FADS2,HMGCL,IDH3G,JAM2,MGST1,NFKBIB,OR10H3,OR2H1,OR2M2,OR52E6,OR5B17,OR8D1,PAPSS1,PDGFD,PHKG2,POLR3G,PPA1,PPP3R2,PRKCI,PTAFR,SPRY1,TAS1R2,SERPINF2
Negative_5535	C8G,HK1,MOS,ACLY,AGPAT4,ATP12A,BAAT,BDH1,BDKRB2,BMP5,CACNA1I,CACNG4,CACNG7,CACNG8,CAMK2B,CCL11,CCL26,GCNT2,GGT1,GNRH2,GUCA1B,IFNK,ITGA2B,LYZL1,MAP3K13,MDM2,MGAT5B,MGST3,MTHFD1,NADK,OR10J1,OR13J1,OR6K2,OXCT2,PLA2G2F,PRSS3,RPS6KA6,TFPI,TGFB3,TM7SF2,TYRP1,B4GALNT1,TNFRSF12A,TNFRSF6B
Negative_5536	FUK,AARS2,ADAM10,ADCY1,ADCY10,CBR1,CCL27,CDC2,CDH4,CDK6,CHUK,CYP2A7,DGAT2,EFNB3,EPHA3,FABP3,FIGF,FUT1,GRHPR,HLA-DOB,HTR5A,IFNA4,IL10,IL13RA2,IL2RA,LAMA4,LTB4R2,MDH1,MGAT1,OR1L4,OR2AE1,OR2T29,OR2W1,OR4C16,ORC6L,P2RX4,PDE4D,PERP,PIK3C2A,PIP4K2B,PPP2R5E,PRKAB2,PTPN11,SDC2,WNT7B,SERPINB5,TNFRSF12A
Negative_5537	BAD,CAD,KDR,SFN,AARS,ACSL4,BMPR2,BUB3,CD14,CD19,CD274,CLDN1,CREB3L3,CXCL10,CYP4A11,DDX51,E2F4,EIF4E,GADD45B,GLUL,GSTM1,IFNA4,IL12B,IRAK4,KYNU,MAP2K2,MAP3K2,MAPK13,NCAM2,OR10K1,OR13C3,OR2T11,OR51S1,OR6Q1,PHKG1,PIGN,POLD4,PRKAR1B,SESN3,SLC25A4,SPTAN1,STX10,TAS1R2
Negative_5538	AGK,DBH,ACO2,ACTC1,CASK,CD72,CFLAR,CXCL2,DEGS2,DFFA,DHDDS,EPHB6,EREG,GUCY1A3,HSPA1B,HTRA2,IBSP,IDH3B,IL13RA2,IPMK,MAD2L1,MTAP,MYH7,MYH7B,NAGS,NR1H3,OR10P1,OR2A12,OR51B6,OR8I2,OR8U1,PIK3C2A,PRDX6,SLC27A4,TCIRG1,UROS,CACNA2D4
Negative_5539	AK7,AACS,ARNTL,ATP2A1,B3GAT2,CDKN2B,CDS2,CLDN3,CTNNB1,CYP2U1,EPB41L3,GIT1,GNRHR,NT5C1A,OR10A7,PGK1,RCHY1,SFRP5,SPP1,UNC5C,ATP6V0A2
Negative_5540	GP6,GSR,TFG,ADRB3,AKR1D1,ATP5G2,BCMO1,CASP9,CD3E,CDH15,COQ3,CXCR6,DLL4,IARS2,MGAM,NOS1,NT5E,OR1L1,OR6K2,PER1,PLAT,POLR2K,WWP1

Negative_5541	DBH, GLA, GSR, OTC, ADIPOQ, BBC3, CALML3, CAV1, CD244, CRKL, DHFR, DLL3, DLST, GPX7, IL8RA, INPP1, INPPL1, IRF9, KYNU, LALBA, LHCGR, LIPF, LRRC4C, MADCAM1, MAN2A1, MASP1, MDM2, MDM4, MTHFD1, NEU4, NR1H3, NRXN2, NUDT9, OR1A2, OR52E8, OR6Q1, PANK2, PARD6G, PLA2G2A, PTGES2, PTPRJ, RAPGEF3, RENBP, SDHD, SHC3, SHISA5, SMAD9, TUBB6, UAP1
Negative_5542	FGG, ACSM5, BCL10, HEXA, MAN1B1, OR12D3, OR52K1, OR7D2, ORC4L, PCK1, SOCS1, TGFA, TSC1, ULBP1, WIF1
Negative_5543	F11, MYC, TNR, ABLIM1, ACLY, ADCY1, CCR6, CD82, FADD, HAAO, HARS, HTR6, IL13RA2, IL3RA, JUND, LAMA3, NTRK1, OR10G3, OR2L2, OR6C76, PAFAH2, PAK6, PPP2R2A, PTPMT1, RHOA, TAS2R5, WASL, SERPINF2
Negative_5544	GK, PVR, BIRC3, CD34, CPT2, CX3CR1, CYP1B1, FUT3, GALK2, GALM, HYAL1, JAK3, MTMR1, NUDT5, OR4F6, OR4K5, ORC5L, PDHA1, PIGU, PLA2G2D, PRKAR1B, STEAP3, SUV39H2, TNXB, PAFAH1B1, TNFRSF10B, TNFRSF11A, TNFRSF11B
Negative_5545	AK5, GK, PC, PML, ACVR1B, ALPI, ARPC5, B4GALT6, CLDN22, CMBL, CREBBP, CTLA4, DCTD, DLL4, DPYSL2, DUSP16, FGFR3, GMPPB, GUCY2C, HLA-DRA, HLA-G, IFNB1, LYPLA1, MAPK1, MYH7B, NAE1, NEU4, NPR1, OR2D2, OR4E2, OR5AK2, OR8I2, PDE7B, PGS1, PKM2, POLR1C, PRPS1, RDH8, RENBP, SETD1B, STX6, UROD, WNT4, TNFRSF17
Negative_5546	EPHA8, NTN1, OR2T3, OR7A5, PPM1D, SNAI2, STX3
Negative_5547	DDO, F3, PRL, ABP1, ACOX3, ACSL6, ATP5G1, BDKRB2, C1QB, CHSY1, CTSS, EPHA8, HLA-DRA, IL15RA, IL1B, IL23R, ITGA11, ITGB4, KIR2DL4, NKD1, OR4C12, OR4K13, ORC5L, P4HA2, PANK2, PCK1, PIGT, PIK3C2G, PIK3C3, PMM2, RDH11, SH2D1A, SMAD7, SORD, TPMT, TUBA4A, TUBB6, VAV3, WNT10B, AASDHPPT
Negative_5548	DDO, DLD, IL4, IL7, ADH1B, ATP6V1D, CER1, COL2A1, COL5A2, COQ5, CTNNA3, CYP26B1, CYP2A7, DHCR24, FZD10, GANAB, GNA11, GSTA5, HSD17B2, IL18, IL20RA, KLRC1, LAMB1, LAMC3, LDHD, NFASC, NFKBIA, NTSR1, OLAH, OR4C15, OR7A17, OR7G3, OR9A2, PFAS, PIK3C3, POLR1B, PPM1D, PPP2R2A, PRKAR1B, RHOQ, RRAS2, SUV39H1, TLN1, TNFRSF8, ULBP1, VAMP2, WNT9A, ARHGEF12, ST6GALNAC3
Negative_5549	C7, FUK, HGD, LCT, B3GNT3, CLCA4, CYP26C1, GNMT, GUCA1C, HTR2A, IPMK, KNG1, LAMC3, OR2AG2, OR2M2, OR4A15, OR4F4, OR6C2, OR8D4, PDE11A, PHKA2, RENBP, ST8SIA5, VAMP1, WNT7A, XYLB
Negative_5550	AGK, ADRA1A, COASY, DNMT1, DUSP7, ERO1L, GNMT, IMPA2, KCNB1, MAP3K6, MTFMT, OR12D3, OR5B17, ORC6L, PAX8, PLD1, PPP2R5A, PROS1, PTGER3, RIPK1, SOCS5, SOS2, TIAM2
Negative_5551	C7, ACAD9, ALG2, ASS1, BMP2, CD19, CFL1, CPT1A, CREB3L4, CRHR1, CSF2, CSF2RB, CXCL13, DAD1, DGAT2, DOCK1, EFNA3, ENPP2, GUCA1B, HEXB, IL11RA, LEFTY2, LIPT1, MIOX, MYLK2, OR10AG1, OR1J2, OR2B2, OR2G6, OR5AP2, OR5H2, OR5M8, OR6C3, P4HA2, PIP5K1B, PLCB2, PLCD1, PLCG2, PLXNA2, RASA2, RFX5, RPS6, SESN3, SETD2, STK3, SUOX, ATP6V1C2, MAPK8IP1, TNFRSF12A
Negative_5552	BTD, CR2, F5, IL7, B3GALT6, CACNA1S, CYP2C18, ENO2, GNAI1, GOT1, HADHB, HTRA2, IL2RA, ITGA3, ITGA4, LAMB1, MGAT4A, MMP7, MTHFD1L, NLGN3, PAK7, PSPH, SRD5A2, STX2, TUBB3, CSNK1A1L
Negative_5553	AKR1C3, C4BPB, CCNG2, CXCL2, EDNRB, ENPP3, EPHB4, FGF13, FOXA3, FUT9, GPD1, GSTA4, HHIP, LLGL2, OR10C1, OR2A4, OR2T33, OR51B6, PGS1, PLCE1, SH2D2A, STK3, TRDMT1, UBE2L3, ALDH18A1, HLA-DQB1, TNFSF13B
Negative_5554	GH2, GP6, IL4, MPI, B3GNT3, BACE1, BBOX1, BET1, CACNA1C, CDH4, DUSP10, EPHA4, ICAM2, MASP1, NFKBIB, NTRK2, OR51B4, OR51G1, OR52K1, PDE2A, PLCB2, PON2, PPBP, PPP3CB, RAC1, SMAD3, SOCS2, SOS2, TLN1, TNXB, TOLLIP, UGCGL1, VTI1A, CSNK1A1L, GABARAPL1, MAP3K7IP2

Negative_5555	RFK,ABLIM3,CCR4,EFNA2,ITGAL,KITLG,NUDT8,OR10Z1,PAX4,STX6,SERPIND1
Negative_5556	ADRA1A,CMAS,DTX4,ENAH,F13B,MDH1,OR4K14,OR8K3,PPP1R3A,PPP2R2D,RPN1,SMAD3,TYROBP
Negative_5557	IL8,ACADS,AGMAT,AMPD3,CACNG6,CSNK1E,ENPP1,GTSE1,HMOX1,IL21,LAMB2,LDHAL6B,MGAT4A,MGAT4B,NEFH,NKX3-1,OR2L13,OR52N2,OR56A1,OR5D14,PDGFRA,PHKB,PLCG2,PTPRB,PYCRL,RRAS,SDHB,STAT4,THBS4,TNFSF14,VTI1B,YARS2,PNLIPRP1
Negative_5558	DLD,F2,GH2,MVK,ADRA1A,ALG13,AMD1,ANAPC7,APAF1,ARG1,BDKRB1,CADM3,CD3E,CES2,CLTCL1,CMPK1,CYP26B1,DFFA,GALM,GRAP2,HHIP,HSD17B7,HTR5A,IDUA,IL13RA2,IL17A,IRS2,LCAT,NRXN2,NRXN3,NT5C3,OR10H3,OR2AE1,OR51I1,OR56A1,PLA2G4A,PPID,PRNP,PTGIS,RAP1B,SLC27A5,TAS2R60,TRDMT1,UGT2B28,ULK1,YKT6
Negative_5559	IL8,LCT,PGP,ALG11,ANAPC11,AQP7,B3GNT3,BCKDHB,CD22,CXCR3,CYP2C8,CYP4A22,DAAM2,DAD1,DGKA,DHDH,DHRS4,FGFR1,GARS,GNG13,HTR2B,IL10RB,IL12A,INPP4A,ITGA9,LTA4H,LYZL1,METTL2B,NCR2,NFX1,OR8B4,PDE8B,PRSS2,RPN1,TKTL2,TNFRSF4,VAMP2,CACNA2D2
Negative_5560	CD4,GSN,ITK,NGF,ACACB,ACP6,ADCY10,AGRP,ARG2,ARHGEF7,CSNK2A1,DGKA,DPM1,ELK1,FARP2,GFAP,IAPP,MGAT4A,NEFM,OPLAH,OR11G2,OR4N5,PER1,PGAP1,PIP5K1B,PRSS3,SH2D1B,SRD5A1,STX18,UGCG,WHSC1L1,ATP6V0E1
Negative_5561	AUH,ID3,ITK,KMO,NLK,SMS,ACSL3,AMY2B,APOA2,ATP5J,B3GALT2,BBC3,BPNT1,C3orf10,CNTFR,CXCL1,DDX23,DLAT,ELK4,ENO3,FZR1,G6PC2,GALT,HNF4A,IL2RA,LAMC1,MYH7B,NRG2,OR4C13,OR4E2,OR52L1,OR7D4,OR8I2,P2RX6,PIAS3,PIK3CD,PLAUR,PLXNA3,SFRP2,SMAD7,TCIRG1,WASL,PRICKLE2,TNFRSF17
Negative_5562	F7,FAH,TNN,ALDH6A1,B3GNT3,CDC45L,COL6A2,COQ5,CREB5,EXT2,GLI2,GLUL,IL17A,IL4R,JAK3,LIMK1,MAFA,OR13C5,OR2L8,PPP1CC,PRIM2,RDH11,RPS6KA5,SOAT1,SULT2B1
Negative_5563	GH1,MUT,ALDH3B1,CACNG7,CASP10,CYP2F1,DGKB,DOLPP1,GCLC,GNA12,MYL9,OR6F1,ORC2L,CSNK1A1L,PLA2G12B
Negative_5564	NOG,ACACB,CANT1,CD226,DUSP7,GALNT11,HIF1A,ITGA10,NAT6,OCRL,OGDHL,OLAH,OR2A25,RASGRP4,SPRY4,ST6GAL1,TNXB
Negative_5565	CAD,EPO,F2R,NNT,ATF2,OR52K1,PRKAR1A,ATP6V0D1,ST6GALNAC6
Negative_5566	C8B,EZR,IL2,RDX,VCL,ADCY5,ALDH4A1,ARPC1A,ATP4B,BPNT1,C1QC,CALML5,CAMKK1,CDC26,DERL1,DUSP3,EPHA5,KLKB1,LDHA,MAD1L1,NAGLU,OR11H6,OR1A1,OR51I2,PARD6B,PRKAA1,RALB,SDHA,SFRP5,SULT2B1,TAS1R3
Negative_5567	SP1,ADH7,ARPC5,CACNA1E,CDC14A,CLCA4,COMP,EXOC7,FUT1,GALNT8,HYAL1,ICAM2,IL1A,MAOB,MGAM,NEUROG3,OR2C3,OR4C11,OR51B5,PPP2CA,SC4MOL,SCIN,STAT4,TJP2,TNFSF10,TOMM40,TRHR,VAMP2,HLA-DRB3
Negative_5568	F8,APH1A,CACNB4,CHKA,DGKB,GMPS,HPSE2,NCK2,OR1L3,OR1L8,ORC5L,P2RX3,PCCA,PDE10A,POLR3H,TNFSF12
Negative_5569	AUH,DHH,FUK,LTB,MPI,OSM,SDS,AACS,ACVR2A,ALG6,CAV3,CDC25B,CNGB1,CYFIP1,FGF1,FMO5,GATM,HNMT,IFNA17,IL13RA1,IL2RG,KIR2DL1,MAPK14,MLYCD,NAT5,NCK2,NUMBL,OR10G3,OR11L1,OR4K15,OR51E1,OR8K1,PPARG,PPP1R3C,PRKCH,RAP1B,RERE,RPE65,SEMA3B,SHISA5,TNFSF11,VDAC2,WHSC1L1,WNT4,MAPK8IP2,TNFRSF11B
Negative_5570	BID,FAH,ADH5,ADORA2A,BLVRB,CACNA1A,CCL5,CDC26,CNTNAP2,COX10,CYP2A7,DUSP16,IRAK1,OR1N1,RFXANK,SPRED2,TPM3,MAP3K7IP1,SUV420H2
Negative_5571	MUT,ADCY10,ATP6V0B,BCMO1,BMP6,CDKN1C,DHFR,EFNA2,GDF6,GDF7,MAP2K6,OR4K5,OR6Y1,OR8D1,PPAT,RPS6KA1,ST3GAL6,TNFSF4

Negative_5572	LHB,AARS,ANAPC10,ARPC5,BIRC2,BNIP1,CCL16,CDC6,EIF4E,FARSB,GALK2,GRM5,HDAC2,IDI1,IFNA16,IPMK,NCAM2,NUDT9,OR1M1,OR6B3,OR8D1,PANK2,PCYT2,PLCB1,PLXNB1,PRKACB,PRSS3,SOCS4,TRAF6,HLA-DPB1,PLA2G12B
Negative_5573	BID,GP5,INS,ACAA1,ACHE,ASPA,CCR3,CHUK,DAAM1,EARS2,GGT5,GNAL,GPX5,GSTM4,GUCY2D,HADHB,IL1B,ITGAD,LCAT,OR11A1,RAPGEF3,SHC4,SRGAP1
Negative_5574	CR1,ATP5A1,BCL10,CCL8,DBF4,E2F4,EPB41L3,GGT5,GSTM5,HEXA,IL1RAP,LILRB3,MAPK1,OR4C11,OR4K13,PDE6G,PER2,SLIT1,STX18,TPH2
Negative_5575	ATM,DDC,IHH,ACSM1,ANAPC7,ASRGL1,ATP2A3,ATP5H,CDS1,CLTCL1,CXCR3,CYP7A1,DGUOK,EGFR,FGF8,GALNT11,GALNT13,GMPR,HAGH,IL6ST,KDSR,KIR2DL4,KITLG,LAMC2,MAP3K8,MCM4,MTFMT,NFAT5,NFX1,NTF4,OR4K13,OR9A2,OR9A4,OR9Q1,PCK1,PFKFB1,PGK1,PTGFR,PTPRB,SPRY1,TLR9,TUBA1C,WWP2,PLA2G12B
Negative_5576	ACVR1B,B4GALT7,CCNA2,CD226,CDKN2C,CXCL12,EPHB4,FCGR2B,GALK1,GP1BB,IKBKB,IL6R,OR5D18,OR8G1,ORC6L,PSEN2,PTEN,TNFRSF8,VEGFA
Negative_5577	AGRN,LEFTY1,PFKL,SIAH1,UXS1,WIF1
Negative_5578	EDA,LEP,SYK,ADRA1B,ASRGL1,BET1,CDC27,CHDH,CTSB,GCNT1,JAG1,LLGL1,MAPK13,OR2M3,OR2T34,OR51B5,OR8G1,PAK1,PIK3C2B,PKLR,PTPN11,SPAM1,TOMM40,UGT2A3,PAFAH1B3,ST6GALNAC1,TNFRSF10A
Negative_5579	ALG6,ATP6V0C,CDH2,CYSLTR1,DARS2,DDX55,ENTPD7,GALC,GNRH2,H6PD,IL18RAP,JUND,NTN1,OR2H2,OR8J1,ORC2L,PDGFB,PERP,PRLR,PRPH2,TYMS,UGCG
Negative_5580	CAD,CAT,CEL,TYR,ADH7,ALDH3B2,CAV3,CMAS,COL1A2,CYBB,DHDDS,EFNA4,GNAI2,GNAT3,MLLT4,OR2AE1,OR4F15,PGAM2,PGM2,PLOD2,PTGES,RAC1,SYNJ2,TRAF4,UROC1,UROS,WEE1,PDCD1LG2
Negative_5581	C1S,C7,HK1,ASRGL1,B3GALT6,COL6A1,CTF1,CXCL1,FOSL1,GGT5,GGT7,GNAI1,GNAZ,GRIA3,IQGAP2,IRF3,ITGA4,LAMA3,LAMC3,LIPT1,MAP2K3,MAPK8,METTL6,NCK2,OR13H1,OR1B1,OR2M2,OR51B2,PHKG1,PKMYT1,PLCE1,POLR3B,PPP2R1A,PRODH2,RUNX1T1,SETDB2,TJP3,ULBP2,ZNRD1,SUV420H1
Negative_5582	C4B,ACAA1,ATP2A1,BET1L,CLDN16,DARS2,ECHS1,EXT2,FGFR2,FGFR3,HAGH,IKBKB,NOS1,NT5C1A,OR8K1,RAPGEF3
Negative_5583	DCT,MTR,ABL1,ACPT,ADCY9,ALDH3A2,ATG7,ATP5B,BNIP1,CCR10,CDC7,CDK2,CDK4,CLDN19,CLDN22,COL3A1,CREB3L2,CTNNA2,GNAI2,HNF1A,HYAL1,INPP5A,IQGAP3,ITGA8,LCMT1,MAP2K6,MAP4K1,NFKBIB,NOX3,NRG1,OCRL,OR2A14,OR2M7,OR52A5,OR5AP2,OR5H2,OR5P3,OR9Q1,OXSM,PLXNB1,PPP1CB,PPP2R5A,PYGL,SEMA3F,ULBP2,UNC5C
Negative_5584	ABO,CD2,FN1,GNE,HK3,IDS,LPO,LSS,SFN,ACAA2,ALPI,APOA2,APOC3,BST1,CD274,CD46,CDC20,CHST12,CSF3R,CYP1B1,CYP2U1,DAAM1,DDIT4,DPM1,EDN1,ENTPD6,EPHX2,GMDS,HTR2A,IFNGR1,INSR,LAMC1,MYH1,OR4C46,OR4K15,OR4K2,PIGF,POLR1D,PPP2R2C,PRSS1,ROBO3,TAS2R1,TAS2R7,TUBB4,ATP6V0A4
Negative_5585	CBL,ADCY1,ANAPC7,B3GALT4,B3GALT6,CDC14A,CSNK1G2,DUSP2,OR6N2,TRAF3,TRMT11
Negative_5586	CREB3L1,HSP90B1,LAMB4,NTNG1,OR5B12,WHSC1
Negative_5587	ARD1A,ATP5E,BACE2,BCAT2,CAV2,CCNB1,CDKN2C,DGKZ,FZD4,HLA-DOB,IL23R,KLKB1,MAP2K1,NTF3,OR10H1,OR10H5,P2RX3,PPP3CB,PTGER1,SHMT2,SPCS3,UNC5B,WNT2B
Negative_5588	BTC,CCND2,CHKA,IRS1,MCAT,OR4N2,P2RX1,SQLE
Negative_5589	CCR4,IL1RAP,MAP3K4,PDK1,PTPRB,RFWD2,TCF7,TNFRSF9,UPB1,WARS2

Negative_5590	ID2,ACAA2,ACACA,BRAF,CCL17,CDC14A,CDC26,CDC7,DGKD,FSHB,GART,GIT1,GJA1,GUCA1C,HSD17B8,JAM2,KLRC1,LDHB,MAP3K7,NTF3,OR10J3,OR13C2,OR2T1,OR2T8,OR2W1,OR52A5,PCNA,PDE2A,PFKFB2,PIK3C2G,PIK3C3,PNMT,POLR1E,SETDB1,SMPD3,ST3GAL6,TPH2,WWP1,ALDH18A1,ATP6V1E2
Negative_5591	B3GNT5,CHSY3,CSNK1G2,DOLPP1,DOT1L,ENAH,GRIN2D,HSD3B2,IDI2,IFNB1,ITGAL,KCNQ1,KIR2DS5,MADCAM1,MEF2C,MTMR6,MYH8,OGDHL,OR11H6,OR14C36,OR1E2,PFKM,PIK3CB,PLCB2,PLCE1,PPP1CC,RPIA,TUBB1,UROD,WARS2
Negative_5592	C8B,CD2,NNT,ACAA2,ALG10B,ALPL,CDC14A,E2F2,ENPP1,GADD45A,GALNTL2,HSD17B3,IAPP,IKBKG,LDHB,MAT2B,MYL2,NUDT9,OR10G3,OR13C5,OR2A12,OR51V1,OR5D13,OR6B2,OR8J3,PNPLA3,POLR2E,POLR3K,PSEN2,RARS,SETD7,SOS2
Negative_5593	JUN,LPL,BDKRB2,DHDDS,GATM,GLG1,HTR4,IFNW1,IRF3,OR1Q1,OR4Q3,OR8U1,P2RX1,PANK4,POLR3B,PTGIS,RASGRP2,SETMAR,SNAI2,SOAT1,STX10,TSLP,UNC5D,XYLT2
Negative_5594	PXN,APC2,CHRNA7,CHSY3,CMAS,CPOX,CSNK2A1,CYP3A5,DDIT3,ENO3,FGF11,G6PC2,GPX5,H6PD,INADL,IQGAP3,LDHD,NPAS2,OR10Z1,OR4C13,OR52K1,PDE6D,PDGFB,PNPT1,PSME2,RERE,RRM2B,SDHA,SHC1,SPCS1,TNFSF12,TOLLIP,ULK1
Negative_5595	EBP,APOC3,ASCC3,CACNA1C,CAMK4,CHIT1,CLTCL1,DRD2,DTX3,EFNA3,FUT5,GRIN1,ITCH,ITGA1,NR5A2,OR10AG1,OR4D2,OR9A4,PDE4A,PIK3R2,PRKACA,PTK2B,PYCRL,SC5DL,ZIC2
Negative_5596	MPL,ACSL1,ALAS2,ARG2,AVPR1A,BCMO1,CD27,DGKI,GALNT6,MYL7,NME6,NR1D1,NT5C,ODC1,OR10A6,OR4A5,OR4N4,OR5I1,OR5M3,OR9A2,POLD3,PPP2CA,SMAD2,STAT2,UGT2B28,WARS2,HLA-DPA1,SERPINF2
Negative_5597	AMT,DBH,UBC,AARS2,ADH1B,ALDH1A2,ARSB,CNDP1,CX3CL1,CXCL11,DGKA,EVI1,FADD,FZD8,GALNT12,GDE1,HTR5A,IMPDH2,NFYB,NTSR1,OR10K1,OR8H1,PDE8B,PFN2,PLCZ1,PNPT1,PPP2R5D,RPS6KA6,RUVBL1,SIAH1,SOCS1,THBD,UBE2L6,VANGL1,WIF1,PAFAH1B1
Negative_5598	ARAF,OR2M5,QARS,TUBB2C
Negative_5599	TAT,ALG13,BBC3,GRM5,IL1R1,MCM7,OR7C2,RPN2
Negative_5600	VCL,ACP5,ACYP1,ALDH7A1,ALG6,ANAPC1,ATP5L,BUB1,CACNA1D,CCR10,CD247,CYP3A5,FSHR,GALT,HAGH,IL17B,KIR3DL1,LCMT2,LRAT,MAP3K14,ONECUT1,OR1J2,OR2L3,OR5B3,OR9A2,PIK3C2G,PPM1B,RARA,RASGRP1,SAT2,STK11,STX16,STX5,TACR1,TUBA3C
Negative_5601	CD38,CYCS,DDX23,DVL3,FSHR,OR13F1,PDIA4
Negative_5602	SMO,ABAT,ACSS2,ALOX12,AMDHD2,ATG12,ATP5B,CCL2,CCNB3,CERK,CLDN1,COMT,DPYD,EP300,GOT1,HMGCS2,HSD17B1,LAMC1,LDHD,MGAT3,NOX1,NUDT9,OR1E2,OR7A17,POLD1,POLR2C,RAP1B,RPS6,SAT2,SCD5,SELL,SMC1B,THBS1,MAPKAPK2
Negative_5603	IVD,SP1,TNN,ACTG2,AKT2,APOE,ATP4A,ATP5O,BBOX1,CCDC6,CCR7,COX15,CTBP1,ENO3,GCNT4,GDF5,IL6R,INPP5A,ITPKB,LTBP1,MYH4,NAT6,NR4A1,OR10Z1,OR11A1,OR1A1,OR2T33,OR2T4,OR4C46,OR6T1,OR7A17,OR8H3,OR9Q1,PCYT2,PLK1,PNPLA3,PON3,PPP2R1B,RBPJ,RRAS,SETD1B,SGMS1,ST3GAL1,TGFBR1,TRAF3,TRIP10,HSP90AB1
Negative_5604	F10,AKR1C4,ALG10B,B4GALT3,CDC6,CDKN2D,CXCL10,FGF17,FZD10,KLRC1,MAD2L1,MAN1B1,MMP9,MYL9,NADK,NADSYN1,OR10V1,OR13A1,OR2AG1,OR2T33,OR5K1,OR5L1,OR6B3,OR8A1,PDE4C,PGM1,PIGC,POLD4,PTEN,PTGDS,RALGDS,RARS2,SEMA3B,TCF7L2,TNFSF10,TOMM40L,WNT11,MAPKAPK5
Negative_5605	ACYP2,ALDH5A1,ATP5D,B3GALT4,CACNG7,CCNB1,CYP2A6,DDIT4,DDX55,GZMB,IFNA5,MAN1A1,MGST3,MYH15,NR1H3,OR4M1,PDHA2,STAT5A,THBS4,VTI1A

Negative_5606	MVK,CSNK1D,GNRHR,IL9R,LAMA4,OR10G4,OR10H3,RRM2
Negative_5607	FH,MET,AKR1B1,APLP1,CCNG1,CREB5,EFNB3,EPHB6,EXTL3,FARS2,FPGT,GCNT1,GGPS1,GNG13,HAAO,IDH3G,IL23A,IL28B,IL8RA,KCNJ11,MYH9,OR13C4,OR4D2,OR8I2,ORC4L,ORC6L,PHKB,PLXNA1,PRKCB,PTEN,QPRT,RELA,SH2D1B,SHMT1,SMC1B,SPRED1,SRD5A2,SRGAP1,TNXB,TP53I3,WWP2,ZFYVE9,ATP6V0A4,B4GALNT1
Negative_5608	DDO,OAT,OGT,UBC,ADH1A,CDH1,COX10,CXCL12,CYP21A2,DEGS2,DGAT1,EFNB1,ERBB4,FUT2,GAD2,GNA12,GPX7,GUCY2C,HES1,ITGB8,KIR3DL1,KLRC1,METTL2B,NRXN3,OR11L1,OR13C3,OR2L3,PLOD1,PPP1CC,STAT5B,TSLP,UBE2L3,ULK1,TNFRSF6B
Negative_5609	F2R,ABLIM3,CCR4,CYP2A6,DEGS2,EPHA4,FARSB,GCLC,IL1A,ITGAL,LAMB2,LYZL1,MAD2L2,MAP4K4,MTHFD1,OR51B5,ORC5L,PDGFRB,PIAS3,PLA2G10,POLE,POLR2B,POLR3B,PRKAB1,PRKACG,RELA,RPS6KA2,WWP2,MAPKAPK2,ST6GALNAC4
Negative_5610	C2,HAL,ADAM17,ALDH1A2,ALPL,ATIC,B4GALT4,C1QB,CLDN9,CLTC,FOXA2,GSTZ1,HYAL1,IDI2,IFNA4,IL11,ITGA5,LYPLA2,MYH11,OR1N1,OR56A4,OR5P3,OR6K6,PLCB4,PPP2R2C,SLC25A6,SLC2A1,SOCS1,TAS2R3,YWHAE,PDCD1LG2,TNFRSF18
Negative_5611	APOA5,CYP2A13,IL18R1,LHCGR,LIAS,NCOA4,NFAT5,PHKA1,PRKCZ,SLC25A6,TRPC1
Negative_5612	ATP6V1F,CYP2C9,GALNT13,NFATC4,POLR1D,ULBP1,MAPKAPK3
Negative_5613	EGF,IL4,NNT,AHCY,ARHGEF4,BCKDHB,BIRC3,DERL1,FZD8,GAD1,GALNT9,GDF6,GOSR2,MAP4K3,NFX1,OR10X1,OR14J1,OR52L1,PNPO,PPP2R1A,SOAT2,TAS2R1,TGFBR1,TKTL1,TNFSF9,TUBB6,UCP1,WARS,WNT5B
Negative_5614	CFI,ARSD,BDKRB2,CASK,CCL26,HTR2B,SEMA3A,UGT2B11
Negative_5615	AUH,KIT,ME1,TEC,APLP1,BHMT,CACNA1F,CCNG2,CCR3,COMP,DDX23,EVI1,FPGT,FUT4,HRH1,LCP2,LMO7,NAT2,OR2Y1,RPS6KB1,SEMA4G,SPTAN1,SRGAP3,TAS2R16,TICAM2,TLR2,UPP1,UROC1,TNFRSF14
Negative_5616	C6,DCT,DDC,ADH6,AKR1C2,AKR1D1,ALG6,ARSE,ASPA,BCL2L1,CCL3,CDH1,CLDN7,DHCR7,EPHB2,FGF5,FZD4,FZD7,HMBS,HMGCS2,HTR6,IL12B,INSR,KIR2DL4,LCP2,MFNG,MLYCD,NANP,NR1D1,OR2T8,OR8D1,PDX1,PIGX,POLA2,PTPN11,SDHA,ATP6V0A2,ATP6V0E1,TNFRSF10A
Negative_5617	LTB,ARRB2,FPGT,OR4A16,PIK3R5,POLR3G,CSGALNACT2,TNFRSF1B
Negative_5618	CAD,TPO,CD44,CDC6,CLCA4,ENPP3,FLT3LG,NT5E,OR4D5,OR9Q2,PEMT,RASGRF1,TAP2
Negative_5619	ILK,LTB,ME2,TKT,ALG2,ATP4A,CACNG8,CAMK2A,DGAT1,EARS2,FGF21,GJA1,GNA11,HCCS,IARS,IFNA21,LIFR,NEU4,NFKB1,NGEF,NPR2,OR10K1,OR13A1,OR2F2,OR51B4,PARD6B,PAX6,PHPT1,PIAS2,PIP5K1B,PLA2G5,PON3,PPAP2C,PVRL3,SEMA4G,SLIT2,ST3GAL1,TRAF6,ARHGEF12,TNFRSF14,TNFRSF1A
Negative_5620	CR2,DHH,ILK,BACE2,CACNA1B,CD8B,CDC25B,CRB3,CYFIP2,DGAT2L4,DUSP4,EPB41L2,GLDC,GUCY1A2,H6PD,HTR7,ITGA10,ITGB2,LIAS,MAP3K3,MGLL,NCK2,OR2T33,OR2Y1,OR51I1,PIGS,PIP4K2A,PITX2,PLEKHA8,PLXNB1,PPM1D,RALA,SCP2,SOD1,SPCS1,SV2B,TNFRSF17
Negative_5621	FOS,IL3,ALDH1A2,B3GAT1,B3GAT2,CASK,CDH1,CHIT1,CLDN8,COL4A4,CXCL16,DDX18,EFNA4,FUT5,HES1,HTR7,LDHA,MAML3,MAP4K1,MASP1,NFKBIA,NME4,OR14I1,OR1E2,OR1L6,OR2AG2,OR2S2,OR4C13,OR51B6,OXSM,PARS2,PDHA1,PER3,PHKG1,PIGO,PIGU,PIGW,PPP2R2C,PPP3CC,RALGDS,RPN1,SMAD1,SNW1,TAS2R7,TNFRSF9,TP53,UNC5B,VDAC3,PNLIPRP2
Negative_5622	IL2,MAG,ACADSB,ARPC5L,ARSD,CDO1,F2RL1,IDI1,IL13,LARS,OR2Y1,OR6C2,PCCB,PLCE1,PYGB,RBPJL,SLC27A1,TGDS,UGT2B28

Negative_5623	DDO, HDC, ACPP, ATP12A, BCMO1, BLVRB, CD244, CXCL5, CYP17A1, DFFA, DHRS3, FGFR3, FRAT1, G6PC2, G6PD, GMPR2, HSD17B8, IL19, MAN1C1, MBL2, MOV10L1, NCR2, OLAH, OR6M1, OR7D4, PANK4, POLR2L, PPAP2A, PSAT1, UNC5A, WARS2
Negative_5624	ADCY9, B4GALT5, CHEK1, CPT2, CTBP1, CTSB, DIAPH1, ECHS1, EFNA2, EFNA5, EHMT1, FZR1, GAS1, HMGCR, HSPB1, ITGA4, KAT2A, KCNQ1, KIR3DL1, KLRC2, LILRB3, MAML2, MAN1C1, MYH8, NAE1, NAT5, NODAL, OR51G1, OR8B12, OR8K1, OR8U1, PAX6, PER3, PI4KB, PPCS, PRKAR2B, PRODH2, RBL1, SETD2, ST3GAL4, STX1B, TBL1XR1, TGFBR2, TYMP, HLA-DPA1, TNFRSF10B, TNFRSF17
Negative_5625	AK1, GP6, GSR, VTN, AASDH, ACVR2B, AQP7, ARNT, ATP6V0C, AXIN1, CCNA1, CDK5, CDK6, CES1, COL5A2, CREBBP, DCXR, DDX19A, F2RL2, FGF18, FGF20, FUT3, GNG12, GNG3, GSTA2, IL2RA, IRAK2, KLRD1, MAN1B1, MAP3K8, OR10AD1, OR51B6, PCNA, PDHB, PIK3C2A, PLA2G6, PPP2R5C, RND1, SPP1, SULT2A1, TM7SF2, TRDMT1, TUBB, UGCGL1, VKORC1, ATP6V0A4
Negative_5626	ATP5E, EXTL2, GNAI2, IFNAR1, LAMC1, MAP3K2, OR1N2, OR2C3, OR6N2
Negative_5627	GSR, MAG, NOG, ADH1B, ADRB1, AKR1B1, ALDH1B1, ALDOC, APOA1, CACNA1B, CCL16, CD79A, CDK7, CHUK, CLDN5, DGKI, DNMT3L, EHMT2, ELK4, GRIA2, HNMT, IFNA13, INPP4B, ITGB2, LIPC, LIPF, LYZL1, MAML2, OCRL, OR1J4, OR2M4, OR2T29, OR56A3, OR8K1, OR9K2, PDE3B, PPP1R3B, PRODH2, RASGRP1, RPS6KA1, STEAP3, TKTL2, TRAF5, UCKL1, UGT2B28, XYLT2, ATP6V1B1
Negative_5628	IL10, OR3A2, OR4F4, PIGQ, SULT1E1
Negative_5629	GBA, ACADS, AMPD1, CACNG6, CLDN8, CPT1C, CXCL3, DAPK3, DGAT2, ENPP2, GATM, GNG13, GOT1, IKBKE, IKBKG, IL13, LEFTY1, NCK1, NOTCH1, NTRK1, OR4C15, OR51E2, OR56A1, PAK1, PGK2, PGM3, PIK3C2A, PRKG2, PTGER3, RDH16, SETD1A, STMN1, TAS2R16, TIAM1, TNFSF4, TUBA1A, YWHAG, ATP6V0D1
Negative_5630	BAX, BTK, CFI, TPR, ACLY, ACSM3, AMY2A, ATG12, BET1, CRY2, CYP11A1, CYP26B1, EPHB1, FGD3, FLOT1, GUCA1A, GUCA1C, HEMK1, IFNG, KLK3, OR2A25, OR2T1, OR4C16, OR52E6, OR9A2, PIAS2, POLE, PTCRA, RAC3, SIAH1, SSH2, STAT5A, STX8, TGFB2, WBSCR22, PAFAH1B3
Negative_5631	ITK, TFG, AARS2, ACOX1, ALDH1L1, ATP5C1, CD79A, CDC25A, CNGA4, DDX18, DUSP1, GLO1, HSD17B1, INPP4A, ITGA2B, MAP3K8, MPST, NAE1, NR5A2, OR10J3, OR2T1, OR3A3, OR51B5, OR9G1, PRSS3, PTPRC, ST3GAL6, PDCD1LG2
Negative_5632	ADH1B, BDNF, CACNG5, CASP7, CDC45L, CNTFR, DDX19A, IRS4, OR1N1, OR2Y1, OR7C1, PEMT, RAD51, RDH12, RPS6, TM7SF2, TRAF4, TYROBP, VDAC3, SERPINB5
Negative_5633	KMO, CALML3, CCR1, CHRNA7, CLDN9, CYP26B1, GP1BB, OR4Q3, OR52B4, SLC2A4, TCF7L2, SERPINA5
Negative_5634	EZR, NGF, CAMKK1, CD47, CYP1A1, DRD1, ENPP2, GART, IL19, MAP2K1, MARS2, MEF2C, PDGFC, ULK3, WNT3A
Negative_5635	C3, ATP4B, AVPR1B, BLNK, CDKN1A, E2F3, EPHB4, EXTL1, FTMT, GNMT, GRIA3, MAP2K3, MYH8, MYH9, OR14I1, OR1L6, OR4A5, PDE6D, PDK1, PIP4K2A, PNPO, PTPN1, RAPGEF1, RASGRP1, SMAD3, WNT10B
Negative_5636	ABL2, FZD6, HPSE, OR2D2, OR4X2, OR5AS1, PLD2, PTPN11, SH2D1B, TLN2, VANGL2, ATP6V0A2
Negative_5637	ACO1, COL5A3, GJA1, HAGHL, HNF1B, MTHFD1L, OR4D1, OR4S1, ROCK1, TAP1, TUBA3C, TUBA4A
Negative_5638	CDC25B, IL11RA, OR11L1, PLOD2
Negative_5639	F11, SMO, ALOX15, AMDHD1, APOE, CDH1, CNTNAP1, CSF2, CTLA4, CX3CR1, DAPK1, EFNB2, FZR1, GSTA5, HAP1, HES1, LTB4R2, NEU4, OR13C9, OR4E2, OR5P2, PIGH, PIK3R3, PIP5K1A, PLA2G2E, PRKX, SMAD9, SULT2A1, TAP2, TBL1Y, UGDH, UGT2B7, USE1, VCAM1

Negative_5640	AK1,AUH,CRH,ACTN1,ADAM17,ADCY2,APBB1,BUB1,C9orf95,CCL25,CPS1,CSF2,DHRS4L2,DLAT,EFNA2,ENTPD3,EPHX2,FARS2,GPAM,GUCA1A,MKNK2,OR2C3,OR8I2,PCYT1B,PDE3A,PIAS1,PIAS2,PIGQ,PIP5K1B,PLA2G6,PMAIP1,POLE,PPBP,PRKAG2,PTGES,RAB23,RAP1A,RARS2,RASGRP1,RBKS,RETSAT,RPS6KA1,SESN1,SLC12A2,SPHK1,SPTAN1,TCIRG1,TPI1,VAV1
Negative_5641	C2,GSN,IDS,PAH,TK1,APPL1,ATP5A1,C4BPA,CARS,CMBL,CPT2,ESPL1,FGF19,FZD1,GALNT2,GGT5,GPD2,HSD3B2,IDI1,IQGAP1,KIR3DL3,LGMN,LRAT,MAD1L1,NCR3,NEGR1,OR13C4,OR13G1,OR2AE1,OR2F2,PANK4,PDXP,POLR2A,PPP2R2A,PRKCQ,PRPH2,RAPGEF1,RHOD,RXRA,SARDH,SEMA4G,SLC2A1,SMURF2,SV2B,TFPI,TICAM1,HLA-DRB3
Negative_5642	GLA,MAX,NLK,SRF,ACO2,AKR1C4,APOE,ATP5F1,ATP5H,BMP8B,CACNA1I,CCL21,CDC7,CHDH,CHRM2,CHST13,COL5A1,EPHA6,F11R,FGF3,FMO3,GGT5,IFNG,IL15RA,KLRC4,MTHFD1L,MYH7,MYH8,OR13C4,OR13J1,OR1N1,OR4C12,OR4Q3,OR4S2,OR5R1,OR6K2,PDE4A,PFKFB1,RDH5,SEMA3C,TCIRG1,TM7SF2,TSTA3,WNT5B
Negative_5643	F3,GP5,BDH1,BLNK,CCL8,CCR7,CIITA,CLDN20,COL4A1,COQ7,CTSS,CYP19A1,ENTPD6,ERBB2,FIGF,GAB1,GSTM5,ICAM2,IRS1,LAMC2,MTHFS,NUMB,OR10G8,OR1I1,OR4C12,OR4D2,PAICS,PERP,PIP4K2A,POLE3,SLIT1,STK3,TNFSF4,UNC5A,WHSC1
Negative_5644	CKB,FH,GLA,ADH5,BTRC,CASP3,CRY1,DDHD1,DDX19A,DPYD,EPHA8,EPHX1,FGF17,HMOX2,HSPA8,HYAL2,IFNA14,ISYNA1,LALBA,LARS2,LIPT1,OR12D3,OR5AP2,OR5L1,OR6C1,OR6K2,OR6T1,OR8I2,OR8K5,PKMYT1,POLR2D,PPP3CA,RASSF5,STX12,SUV39H1,TNFSF4,MAP3K7IP2
Negative_5645	ALDH4A1,CHKB,CTBP1,CXCL12,DFFB,EPHB6,IDH1,OR1A1,OR7G1,RDH12,ST3GAL2,WNT3A,SERPINB5
Negative_5646	DBH,ABLIM3,CCND3,CLCA4,CYP2S1,EFNA1,GAB1,GALNT7,GPAM,GRM5,GSK3B,GUCY1B3,IL1R2,LIPG,OR2F2,OR6C6,OXTR,PDE8A,PFAS,PIGX,PLXNA3,PRKAR2A,PRKY,RCHY1,SESN1,SRGAP2,TBK1,ATP6V1G3
Negative_5647	JUN,CDC26,DLL4,GPD2,LAMB2
Negative_5648	F2,ALG3,ALG9,ARHGEF7,ATP5J2,BMP4,BMP7,CAPN2,CARD11,CCL23,CHAT,DARS2,DDX52,DGKD,EARS2,GFPT2,GSTA5,ICAM1,IL10RA,ITGA3,NAT6,NME7,NOTCH3,NRXN2,OR9A4,PDE3A,PTCH1,PTGES2,PTPMT1,SELL,ATP6V0E1
Negative_5649	BTK,CASP8,FGF10,ITGAV,OR8H1,RND1,UCK1,PRICKLE2
Negative_5650	HGF,AK3L1,ATP6V0C,BDH2,CCDC6,CCL16,COMP,DTX4,DUSP14,LARS,OR7D2,PGAM4,PLCD4,PLIN,RHEB
Negative_5651	GNE,HPD,SP1,BMP8B,CD226,CXCL3,FGD1,GALNT13,GNAL,HAGHL,IFNA8,IRAK3,MAP3K8,MGAT3,MIOX,NCAM2,NOS1,NT5C1B,OR6B2,PCNA,PIP5K1A,POLD4,PRODH2,SENP2,SLC12A2,SNAI1,YWHAQ
Negative_5652	CBL,ME1,ALDH1L1,ALG3,ATP5D,CDC6,CMAS,CPT1C,CX3CL1,EPHX2,FABP3,FZD7,GLO1,JAG2,MAP3K14,MYLK3,NKX6-1,OR10C1,OR1N2,PLEKHA8,PPA2,RBX1,SESN1,SYNJ2,TAS2R16,TAS2R4,TRAF1,TUBA4A,WARS
Negative_5653	OSM,SDS,ACTG2,AKR1C3,CADM3,DDIT4,FGF4,GGT1,HSD17B7,KIR3DL1,MAGI2,MCM6,MGAT2,NT5C,OR2T33,PER3,PERP,POLD3,POLE3,PRKCA,RAD51,RCHY1,SOAT1,TUBA3C
Negative_5654	CLCA4,CLDN9,COX10,DOLPP1,EVI1,GRIN2B,LRDD,MTMR1,NODAL,PFKL,PIP4K2B,PPP2CB,TLR3,VDAC1
Negative_5655	AANAT,ACSL3,ADH7,ALG13,CCL15,DDIT3,DGKI,DGUOK,DVL1,INHBC,OR6K6,OR7G3,PDE5A,PRKAG2,PRKG1,PTCRA,SETD1B,ST3GAL6,TBL1XR1,ZFYVE16,HLA-DQA2
Negative_5656	AK3,NGF,TNR,ALDH2,ARPC2,C5AR1,NME7,OR51A7,OR5A1,OR6K6,PAK4,STX1B,TPM3

Negative_5657	FAS, GLA, MVD, ALOX5, B3GAT2, BCL2, CCL1, CCL21, CDC45L, CPT2, FGF23, GGT5, HNMT, IL8RB, LAMB1, NAT2, NKD2, OR2T34, OR4C15, OR52B6, PGS1, PLCZ1, PPP1CA, RHOQ, ROBO3, SH2D1A, TDO2, TIRAP, HSD17B10
Negative_5658	C3, GPI, ABAT, CACNB4, CD244, CREB3L2, IL13RA2, ITGB4, LIPF, MCM4, NARS, NCF4, OR5M8, PIGK, RAC1, RUNX1, ST8SIA1
Negative_5659	F7, MIF, ACOX2, ALDH1A3, ANAPC2, CCR9, IL13RA1, IL1A, ITGA8, KIR2DL3, OLR1, OR4K5, OR5B12, PFKFB3, PLCD4, QARS, SESN2, STEAP3, STX10
Negative_5660	BMP5, CCNB1, CFTR, CYP11B1, GALNT2, MYH1, OR2A2, PDGFA, PDGFRA, PPAP2C, PRKAG1, SCLY, SETD8, STAT1
Negative_5661	ADA, GDA, LAT, ACVR1, ARD1A, ASNS, BUB1B, CNTF, CYFIP2, FGF6, GGT7, GJA1, GOT1, GSTA4, HSPA1B, IFNK, MAP3K8, MINPP1, MMP1, PGLS, PLCB1, RAC3, RFNG, SMPD3, STX18, TLR4, TNFSF9, UXS1
Negative_5662	NLK, LAMA4, MAP4K1, PPARD, PPP2R5C, STX3, TIAM1, WNT2
Negative_5663	FUK, GSS, IL4, MAX, ALG10, ALPPL2, B4GALT1, BCAT1, CACNA1F, CBR3, CDC14B, DHRS3, ENTPD6, FGF18, FZD5, IL25, IMPA1, ITGAX, NFATC2, OR2F1, OR4A15, P2RX2, PIGC, PTGER1, TAS2R9, TGFB1, TGFB3, UGT2B17, ATP6V0A1, MAP3K7IP2, TNFRSF12A
Negative_5664	CR2, ACTA1, ATP5L, CCNB3, CXCL13, DDX50, GAPDH, GLDC, GUCY1A3, IDH1, IL1A, MMP9, OR10H5, OR7C1, OR8H3, RHEB, UNC5B, ZFYVE9
Negative_5665	MOS, MVD, RDX, SMO, AGPAT2, ARPC4, CX3CL1, DERA, DUSP6, EXTL1, GFPT1, GNG13, HLA-C, IL12RB2, ITGA3, MAP2K2, MTAP, NOTCH1, NT5M, OR56A1, OR7G3, OR8K5, ORC4L, PLXNA2, PNLIP, RPS6KA4, SC4MOL, SLC27A5, VEGFB, ST6GALNAC3
Negative_5666	C5, DCT, MOS, NGF, TH, AADAC, ADH4, AKR1C4, ALG2, ARSD, CCR5, CHIT1, DTX2, GLUD2, GOT2, HNF4G, ITGA6, KCNJ11, KLRC2, MAN2A1, MAP2K6, MTFMT, NKX2-2, OR10C1, OR4N5, OR52M1, OR52R1, OR5D14, PAX8, PFKFB1, POLR2C, PVRL2, RFX5, RPS6KA2, SHISA5, SPRY4, SUCLG2, UGT2B17, SUV420H1
Negative_5667	ATM, CGA, ACOT11, ACSS3, ARSA, ARSD, CCDC6, CDH3, CYP2A13, DGKH, DUSP9, G6PC, MAPK11, NTNG1, OR10G7, OR52K1, OR5F1, PDGFRB, PPP2R2A, PTEN, RRM1, SIPA1, SPRY4
Negative_5668	GP6, NGF, AGPAT2, B3GALT5, BST1, C1QA, CD86, CDC45L, CLCF1, CNGA3, FUT7, GNPNAT1, HRAS, IL8RB, MAP3K1, MCCC1, NUDT9, PDE3B, PLXNA1, PRKAR2B, PTPRR, SDHD, SOS1, TNFSF14
Negative_5669	ATP5B, CD79B, CHAD, CLDN3, CX3CR1, DOLPP1, HPRT1, MAP3K7, OR10Q1, PFKFB1, PFKFB3, PIGS, TUBA3E
Negative_5670	MET, ABCC8, ACAD9, ACSS3, ALG13, ANPEP, BAAT, CACNG5, CCL5, CTNNA1, DPAGT1, ENTPD7, FRAT2, HSD17B1, IKBKE, INMT, IRF7, L1CAM, LDHAL6A, NFX1, NKD2, OR13H1, OR4K17, OR52N1, OR52N4, OR5J2, OR7G2, OR9G1, OR9Q2, PARD6A, PDHB, PGAM4, PKLR, PLXNA1, POLD1, PORCN, PRPS1, PTCH2, SMPD2, TAP2, TRPC1, WARS2
Negative_5671	GSN, MAX, NT5C, OR4B1, OR5T3, PCCB, PRKAB1, UXS1
Negative_5672	CFH, MUT, VCL, ACSL3, ADH5, AKR1C2, ALOX12B, BMP7, BRAF, CAMK2D, CARD11, CCR4, CD36, CD40, CHKB, COL5A2, CSDA, DAD1, DDIT3, F2RL3, GCNT2, HTR2A, IL22RA2, ITGA1, KIR2DS5, MAP3K1, MCCC1, OR13G1, OR2A12, OR4C12, OR8A1, OR9A4, PARD6B, PFKL, PFKP, PTPRB, PYGB, PYGL, SLC25A4, SMAD4, STK36, TDO2, ST6GALNAC5
Negative_5673	KMO, ABCC8, ACAA1, ACADL, ATP12A, CACNA1F, CD48, CDIPT, CYP2C8, CYP3A5, DGKG, DUSP1, EARS2, EFNB1, FZD10, FZD9, GYS2, GZMA, JAG1, MAP3K6, MCCC1, MTHFR, MYH1, NCKAP1L, NFKBIA, OGDH, OPLAH, OR10A3, OR1S1, OR2F1, OR4E2, OR5P3, PAK1, PDCD1, POLR3K, PPM1B, SHMT2, SLC27A1, STX4, ZBTB16, ATP6V1G2

Negative_5674	C5, CFH, IL8, MVD, PRL, ADH1B, ADSSL1, ALG1, ATP5G3, BIRC5, BMP7, CDK4, CDKN1A, CDS2, CLDN3, DARS2, ENTPD4, FGF6, IL10RB, ITGB1, MAP3K11, NPR1, OR1B1, OR52L1, OR52N2, PHPT1, PIGH, PNMT, PPARD, PRPH, PTAFR, SOCS4, TGFB2
Negative_5675	AMDHD2, ATP5A1, CACNB1, CHEK2, CRKL, HMGCS1, JAM2, KCNJ11, OR9A2, PDGFC, PLCE1, RPS6KB1, YWHAB
Negative_5676	EGF, IL8, SYK, VCL, CDC25A, CHDH, CSNK1A1, CTSG, EPHB4, GALNT14, HEXB, ITGA7, JAK1, LIAS, PLXNB2, PSEN1, PSPH, PTPRM, RBL1, SESN2, TUBB, VANGL1, ATP6V1E2
Negative_5677	GDA, MVK, ACADVL, ADRBK2, CHPT1, CREB3, CSF3, DVL1, EDA2R, GPAM, GSK3B, IL23R, ITGA3, LRP6, MAP3K3, MTHFS, NFX1, OGDHL, OR10H5, OR2A25, OR2L2, OR56A4, OR6C74, PECAM1, PLIN, PLXNA1, PPID, PPP1CB, PPP3R1, PTCRA, RBPJ, RXRG, SC4MOL, SDC1, SHISA5, SUCLG1, UPP2, YWHAB, AASDHPPT, ATP6V0A4
Negative_5678	FGA, GK2, UBB, UBC, AMD1, B4GALT7, CCL24, CNTN1, CRLF2, CXCL11, DDB2, DDX18, DNMT3L, HADH, HAGH, LAP3, LLGL1, OR1J2, OR1K1, OR4F4, PDHB, PIK3C2B, PIK3CG, PPP2R5A, ULBP3, XYLT1, ST6GALNAC5
Negative_5679	FGFR3, PIP5K1B, PTEN, QPRT
Negative_5680	ATF4, GAL3ST1, POLD4, RPS6KA6, THTPA, TYROBP
Negative_5681	ACP1, AMY2A, CLDN1, CSF2, CYP1A1, GNB1, HNF1B, MAGI1, NFAT5, OR4C11, PFN1, RFXANK, SCP2, WNT3A, CACNA2D1
Negative_5682	ACTN4, ADRBK2, CACNA1D, CDH5, GALNT13, MASP1, MASP2, OR51F1, OR5M9, PFKL, POLR3D, RUVBL1, SEMA4G, USE1
Negative_5683	GDA, GP9, UBB, ACSM5, ADCY1, ADIPOR2, ALPI, AMY2B, ARPC4, ATP4B, ATP5J, B3GAT3, CD55, CHDH, DGAT1, DLAT, DPM3, EPHB2, EPHB4, EPHB6, FBXW11, HSD3B2, IL20RB, NCKAP1L, NCR3, NT5C2, NT5C3, OR4X1, OR51I2, OR52E2, OR52N1, OR6K3, OR6N1, PDIA4, POLR1E, PORCN, PPBP, PPP5C, PRLR, PTPN5, RPS6KA3, SMAD6, TOMM40, TRPM5, ATP6V0A2, CSGALNACT1, TNFRSF17
Negative_5684	IL2, TK2, ABP1, ACADSB, AGXT2, AKR1C2, ALG11, CD3E, CD79A, CEBPA, CHPT1, DAAM2, DCTD, FRAP1, HNF4A, ITGA5, ITGAL, KIR3DL2, LAMB2, MCM5, MRAS, NCOR2, NQO1, OR10A7, OR10V1, OR12D3, OR2AT4, OR2T5, P2RX6, POMC, RRM2, TAP1, TKTL2, UPB1
Negative_5685	GP5, ACVR1, AMY2B, ANGPTL4, ATP4A, ATP5G3, BDKRB1, CHRM5, CLTCL1, CNTN2, CREB3, CRHR1, CXCR4, DAAM2, DERL1, DGKG, DHDDS, DLL3, DPM1, FOXA3, GLI1, GP1BB, HPRT1, HSPA2, IRS1, KIR3DL3, LIPA, MAD2L2, MAP3K6, MAT2B, MLLT4, MTHFD1L, MYLK, NCOR1, NEFL, OR1E2, OR51G1, OR51Q1, P2RX1, PCYT1B, PIAS3, PRKCD, RPE65, SESN2, SETD2, VAMP3, VAV3, ZNRD1, MAPKAPK2
Negative_5686	BBOX1, CYP2A7, G6PC2, GBE1, HCCS, IL29, SMAD9, SUV39H1, THY1
Negative_5687	GH2, ADCY4, CNTN1, CRY2, CSF3, CYP1B1, HIBADH, MAP2K2, NAT5, NPR1, OR8K3, PDGFD, PHPT1, RDH12, RPN2, STAT4, TLR4
Negative_5688	ABO, AADAC, ALG9, ARHGEF6, ARRB2, BACE1, CAMKK2, CBR1, CCL22, CDC45L, CLDN18, CMPK2, CX3CL1, CX3CR1, DBF4, DUSP5, ENTPD1, EPHA6, FGF3, GFPT2, GYS2, HGSNAT, IRS2, JAM3, MAD2L1, MAGI2, MMP7, MPP5, OR10AG1, OR11L1, OR2AE1, OR2G3, OR5B21, OR8D2, PIGN, PIM2, PKM2, PTGIS, PTPN6, RPS6KB1, RRM2B, SMARCA2, TAS2R8, TIRAP, TRAF6, WNT10B, TNFRSF11B, TNFRSF6B, TNFSF13B
Negative_5689	CTNND1, IL17B, UNC5B
Negative_5690	DBI, FAS, HPD, ME1, MUT, ACSM3, ADRB1, AGXT2, ATP12A, BIRC3, CCNE1, CXCL2, CYP1A2, DRD1, FDFT1, FGF11, GALT, GNAQ, GNAT3, IDH3G, INSRR, KDSR, MTFMT, NCOR2, OLR1, OR2F2, OR4C11, OR9I1, PIP5K1A, PITX2, PLIN, SPCS1, STAT4, TUBB4Q, VANGL2, WNT7B, XCL2, ARHGEF12, SERPIND1
Negative_5691	APC2, CACNA1S, CDS2, HADHA, HSD3B2, IARS, IL8RA, ITGB1, MAD2L2, PFN4, RALGDS, SYNJ1, TRIP10, YWHAB, PAFAH1B1

Negative_5692	ID3, IL5, ACADVL, CTNNA1, DEGS2, DHDH, HADH, IL8RA, ITGAX, NSD1, OR10A3, OR1S2, OR51B5, OR8H1, PDE3A, SETD7, SORD, TAS2R40, TSLP, YOD1, PDCD1LG2
Negative_5693	AK7, IHH, KHK, PLG, ACP1, ADCY9, APRT, ATP6V0C, BDH2, BUB1, CAMK2A, CDC45L, CYP4A22, CYP4F3, DGKB, EPHB2, FLT4, GALNTL4, GNAL, GP1BB, HLA-F, HTR6, IL8RB, KLRC1, MLLT4, OR4D5, OR6B3, OR6X1, PANK2, PGAM2, PGM3, POLR3A, PPP1R1A, PPP3CB, PSPH, RALA, RFWD2, SLC2A2, SPCS3, STAT4, TXNDC12, YWHAG
Negative_5694	HK3, MVK, STS, ACP5, ACTA1, ACTA2, AKR1B10, BCL10, CD72, CDC27, CHST12, DAPK2, DTX4, EGLN1, FECH, FGF9, GATM, HMGCR, INHBC, MPDZ, NAE1, NTRK1, OR10J3, OR1A2, OR6K2, PDE2A, PI4KA, SARS2, SETD1A, SV2A, THBS2, THBS4, TNFSF18
Negative_5695	ADSS, ALDH7A1, BLVRA, CDC6, CDKN2D, CFLAR, CNTN1, GPX5, PRKAG1, RAB5A, RAD51, SLC2A4, UROS, ST6GALNAC4
Negative_5696	AUH, ACP1, ADRB1, ARPC5, ARSA, B3GAT2, CACNG2, CARS, CD47, CDIPT, CLCA4, CYP4F2, DPM2, E2F3, ETNK1, FUT1, GLUD1, GNG3, GPD1L, GSTZ1, IL17A, JAK2, KIR2DS5, MAP2K1, MGAT4A, MYL5, NAGK, NFATC2, NOX1, NRG3, OR5AS1, OR5AU1, OR5M3, OR8K3, PANK1, PLAT, POLD1, POLR3G, STAT5B, TACR2, TAS2R9, TCF7L1, TGDS, TPI1, VAV1, WNT9A, ST6GALNAC3
Negative_5697	CD80, CLCA4, EPHA7, EPHX1, HES1, IBSP, KLRC1, KLRC4, LFNG, MAP3K7, OR5P3, SCP2, SFRP5, UBE2L6, UPP2, ZMAT3, ALDH18A1, ATP6V1G1
Negative_5698	BACE1, BCKDHA, CBLB, CDC6, CHAD, CHEK1, CLCF1, DUSP10, LIMK2, OR2L2, PTGES, SGPP1, SIGLEC1, TLR3, TNFSF14
Negative_5699	DCN, GK2, GLA, ABL1, ACMSD, ATP5E, B3GALT4, CLOCK, COQ7, CREB5, DUSP10, FGF2, GALC, GNAS, HHIP, HNF4A, IARS, IL8RA, INADL, LCMT2, LLGL1, NMNAT1, OR11H4, OR4F17, OR51B2, PAPSS2, PDE10A, PLCB1, PPP1R3C, RAPGEF2, RDH5, RFX5, RFXANK, RPRM, SPHK2, STX16, TAS2R60, TAS2R7, WNT1, YWHAG, KIR2DL5A, SERPINB5, SERPINF2
Negative_5700	C4B, CRK, ACOX3, ALOX15B, BLNK, CACNA1H, CACNG3, CES7, CLDN19, COL2A1, CYP2A7, EDNRB, FARSA, GALC, GNA11, GPX7, GSTM4, HAO1, INPP5B, ITPKA, LIPC, MAOB, MKNK2, NANP, NCAM1, NT5E, OR2G6, OR2H1, PCCB, PIGB, PIK3CD, PLCD3, PLD1, POLR3H, PPP2R2B, PPP2R5E, PRKAB2, PRKCQ, PTCH2, RBL2, SDC1, STX1B, SULT1A2, ATP6V0A4, CSGALNACT2, HLA-DPA1
Negative_5701	APP, FH, NGF, TNC, ARHGEF2, ATP6AP1, CDH2, COX15, CYP21A2, DAXX, EGLN3, IFNK, LIPT1, NADSYN1, NUDT12, PLD1, PLXNB1, PRKAA1, RENBP, SFRP2, SRD5A1
Negative_5702	INS, ITK, ACSM2A, ATP5L, CACNG5, CCL19, CCR9, CD48, CYP21A2, EFNB2, FARSA, FOXA3, GCNT1, HIP1, HMGCS1, HTR7, IL22RA1, ITGA1, JAK2, MAOA, METTL6, OR5AS1, OR5D14, OR6C1, PER1, PHPT1, PIGX, PIK3R1, RASA2, UCKL1, UROC1, PPP1R12A
Negative_5703	ADC, C7, GGH, ACVR2B, AMDHD2, BMPR1A, CACNA1E, CCNG2, CD74, CHRNA7, COL11A1, CXCL2, DAPK2, DNMT1, EFNA4, EPHB4, HYAL2, IL13, IL2RG, LDHAL6B, NCAM1, NCR1, OR13G1, OR4S1, OR5B3, OR5T1, OR8D1, PHKA1, PPP2R5D, PRSS1, PTEN, RAB5A, SDHC, SOCS7, STX8, TAS2R60, TOMM40, UGCG, UGT2B4, YES1, ATP6V1G1, HLA-DPA1
Negative_5704	UBC, ACP1, ACP6, AGMAT, ALPPL2, ASS1, BET1L, CHKA, CISH, CXCL11, DDB2, ENPP7, GFPT1, GMPS, GSTZ1, IDI2, KIR2DL3, LCP2, NEUROD1, NFATC3, NRXN3, OR1L4, OR2A14, OR2F2, OR4C46, OR6B2, PARD6B, PTAFR, RBX1, RDH8, RRM1, SELPLG, SIGLEC1, SMURF1, STAT6, STX3, TBL1X, UCK1, UNC5A, UROC1

Negative_5705	EGF, IL6, KMO, ACADVL, ADSL, ALG10B, ATP5I, ATP6V0B, CD44, CDH5, CHRD, CLDN5, COL4A4, CTSL1, CYP1B1, CYP2C8, DDX47, E2F1, EPB41L1, FGF3, GALE, GZMA, HBEGF, IARS2, LILRB3, MAP3K14, MBL2, NOTCH3, NT5M, NTN1, OR52I2, PDGFRA, POLE2, QARS, SARS2, SHISA5, SMARCA5, TAS2R38, TBL1XR1, TCF7L1, TNFSF14, YWHAH, ZAP70, ARHGEF12
Negative_5706	UBC, ACP6, ANAPC5, C1QC, GGT1, GUK1, HTR2A, IFNK, MGLL, NME7, NTF4, OR10V1, OR5B3, PFKFB4, PLCG2, PLK1, POLE, PORCN, SRGAP3, PPP1R12A
Negative_5707	GSR, ACVRL1, ACYP1, ACYP2, ADSL, AKR1C3, APPL1, CASP7, CLTB, CTNNA1, DHCR24, EDA2R, FABP1, FLT1, GALC, HLA-DOA, IGSF5, IL25, INSRR, ITGB6, KIR3DL1, LIFR, LMO7, OR10K2, OR10Z1, OR11H6, OR5L1, OR8K1, OXCT1, PDE6G, PDE6H, PPP1CC, PRKAR2B, RPS6KA5, RPS6KB1, SORBS1, TGFB1, TIAM1, TUBA4A, VASP, WNT8A, XYLT1, TNFRSF13C, TNFSF13B
Negative_5708	IL3, ACSS3, ALDH3B2, B3GALT1, CACNA1G, CACNG7, CASP6, CD80, CLDN8, CYP2A7, CYP2C8, DHODH, EIF4B, ETS1, GDF7, GMPR, GP1BA, HHEX, HMGCS2, IFNA21, IL1B, ISYNA1, ITGB8, JUND, MAP4K1, MGST2, MOV10L1, OGDHL, OR51B4, OR52W1, OR5B12, OR8A1, PDE5A, PTGIS, QPRT, SEMA4G, SLIT2, SPRED1, TAS1R1, TGFBR2, TUBA4A, TYRP1, ULK3, AASDHPPT, HLA-DRB5, PAFAH1B2
Negative_5709	NPL, ACTA1, ATG12, B4GALT1, CCL7, CXCL11, CYP4F3, DAXX, EDA2R, ENTPD3, GAS1, HSPA2, LCP2, MAML2, MYH7, NFKB2, OR13C3, OR52B6, OR52N2, PMM1, PMM2, POLR3K, PRPS1, SHISA5, SMARCA2, STX2, TUBAL3, VTI1B, ATP6V1B1
Negative_5710	ABLIM3, ACVR2A, ADRA1A, ALG9, ALOX12, ANAPC1, APOC3, ATP4A, B4GALT1, BIRC5, CACNG7, CAMK4, CCL5, CCNE2, CD19, CD22, DDX47, DDX56, ERO1L, FSHB, GADD45A, GGT1, GLUD1, HEXB, ICAM2, IL22RA1, IRS4, ITGA7, MGAT2, MGST3, MYLK2, NCF4, ONECUT1, OR10C1, OR1J1, PAK1, PARVA, PDE10A, PFAS, PIM1, POLD2, PTEN, PTPRB, RBKS, RFXAP, SSX2IP, TAS1R3, UGP2, PPP1R12A
Negative_5711	CTH, EDA, TPO, ABI2, ACSM3, ATP5G3, BAI1, BMPR1A, CACNG3, CCKBR, CHPT1, COQ6, CYP2F1, DUSP14, ERBB3, FASN, FGFR2, GBE1, GLO1, GNA15, GRIA1, IL11, IL20, IRAK2, KLRC1, NCK1, OR1L3, OR51I2, OR52W1, OR8J3, PFAS, PPP2R2B, RDH11, RHOQ, SELP, SELPLG, STX16, TDO2, TOLLIP, VAMP1, WNT7B, ATP6V1C1
Negative_5712	MVK, ACAA2, ACAT1, ACTA1, ALG8, BTRC, CD28, CDKN1B, CDKN1C, COL4A4, CTNNA2, EPB41L3, EPHA2, FMO3, GCNT2, GNRHR, GRAP2, HMGCS2, ITGA6, LAMB4, NUDT9, OR2AT4, PARVB, PDHB, PEMT, PGAP1, RPN2, SH2D1A, STK3, SUOX, TCF7L2, TLN1, TNFSF8, WASF1
Negative_5713	ADC, EDA, FH, IL9, ALDH5A1, ANAPC2, C4BPB, CACNA1I, CD3E, CHRD, CKMT2, CREB3L4, ENO1, FGF13, GALNTL4, GP1BA, GRAP2, IFT57, IKBKE, JAK2, JUND, LRP5, MCM3, MTHFD1, ONECUT1, OR10AG1, OR4M2, OR5B17, PLA1A, POLR2K, PRKACA, RDH12, SFRP2, ST3GAL4, STAT3, STX4, TAS2R10, ZNRD1
Negative_5714	FES, PVR, SCD, ACSM4, ACYP1, B3GALT6, CCND1, CD36, CDC45L, CLDN11, COL1A1, CREB3L2, CTSS, DAPK2, DNMT1, EPHB2, FARP2, FBP1, FLT1, GALNT6, IGSF5, IL10RB, IL3RA, KIR2DL1, LARS, MAP2K3, MGST2, MS4A2, MYH4, OR13C3, OR13F1, OR2AG1, OR7C1, P4HA2, PDE4D, PDGFC, PFAS, PIGW, PSME2, RARS2, SMPD2, STX18, UGDH, UROC1, YKT6, MAPKAPK3, PRICKLE1
Negative_5715	CTH, MVD, MYC, ARSD, ATP12A, ATP6V1H, COQ5, CYP2C8, CYP2F1, DAD1, DTX3, EFNA2, EHMT1, FZD7, GSTA3, HSPB1, IL24, INPPL1, LTB4R2, MECR, NME7, OCLN, OR10R2, OR13C4, OR2AG2, OR2D2, OR5T3, PDHA2, PIK3CB, PPCDC, PPP1CB, RRM2, SNAP25, SPRY4, TBL1Y, TP53I3, VEGFC, XCL1, MAPK8IP3
Negative_5716	HDC, XDH, ACOT12, APOC3, C1GALT1, CACNG7, CD226, CES2, CNTN2, EIF4E, HTR2A, IL6R, IRF9, MAP2K7, MAP3K12, MYH8, OR52K1, OR5T1, OR6C74, OR8J3, PAK1, PDGFRA, TBL1XR1, TGFB1, THY1, TLR2, TP73

Negative_5717	FST, ME2, FUT5, HMGCR, IL10RA, PIK3CD, PLA2G6, TBXAS1
Negative_5718	DBH, DLD, AGPAT2, ASPA, C1QB, CBLB, CD14, CFL2, CLDN4, CRLF2, CTSB, CYP2S1, DVL1, EPHA6, FABP1, FGF3, GALNT11, GCNT3, GOT2, GP1BB, HSD11B2, IL3RA, IPMK, MAPK8, NEU1, NME7, NUDT12, NUDT9, OR10G9, OR5AN1, POLR2J, STMN1, TAS2R41, TCF7L1, TOMM40L, WNT9B, ATP6V0A1
Negative_5719	ADO, CSK, PAH, ACOX3, ACTN4, APH1A, BDNF, BPNT1, CACNG2, CCL17, CDC45L, CDK5, CHD8, CLDN17, CNTNAP2, COL2A1, CSAD, CSH1, CTBS, EFNA4, GLYCTK, IL17RA, IL18, MAP4K3, MTHFD2, OR10P1, OR2L3, OR6T1, P2RX6, PRODH2, PRPS2, SLC2A4, TAS2R5, TOLLIP, TRADD, TRAF4, TRPM5, UGT1A10, SERPINF2, TNFRSF18
Negative_5720	AGK, BTK, AHCY, ALAD, ARPC4, ARSB, CCR2, CFL1, DDX19A, E2F4, FZD6, GALNTL5, GJD2, GNAO1, GSTA4, HLA-G, IL28RA, INPPL1, ITGAV, LAMB1, LAMB4, MTFMT, NCSTN, NME7, OR52E4, OR52E6, OR5F1, OR6K2, OR8H3, P2RX7, PKLR, RASGRP1, SORD, WBSCR22
Negative_5721	ACHE, AKR1A1, ALDH2, CACNB1, CALML3, CDS1, DKK2, FARSB, FGFR3, GRIN2A, HCST, HIF1A, IL18, IL1RAP, IL22RA2, INPP4B, ITPKB, JAG2, LEF1, MGST1, MTHFD1L, MYD88, OR1A1, OR2T12, OR5B3, PIGM, PIK3R5, PPP2R5E, PRKAG2, PRODH2, PTCH1, RND1, ROCK1, SEMA7A, STAM, TP73, TRHR, WARS, YARS
Negative_5722	ID1, AMY2B, CACNA1C, CCR9, CTLA4, MGAT5, MRAS, MYH8, SPRY1, STX6, SUCLG2, UNC5A, YWHAQ
Negative_5723	AK5, DBI, HDC, LTB, MPL, OSM, STS, ABLIM1, ACSM3, ACVR2A, CDC7, CLDN6, CTNNA3, CYBA, DGAT2, DHODH, FOXO3, GALNT1, GPD2, GSTM3, HLA-DOB, HMOX2, IL15, IMPA1, LCMT2, LEPR, METTL6, OR1F1, OR2T34, OR52M1, PDE8B, PIGW, POLR2G, PTCRA, PYCR1, RALA, RAP1A, RPS6KA5, SKP2, SPI1, TLR2, TUBA1C, UBE2K, PLA2G12A, PNLIPRP1, SERPINF2, ST6GALNAC4
Negative_5724	HK3, RET, SDS, AHCYL1, AKT2, ALS2, ATP5J2, CCNE2, CHIT1, CLDN10, DVL2, EPHA7, EPHA8, ESAM, FGF7, FGF9, FLT3, GART, GGT6, GNAZ, GUCY2D, IARS, IL9R, ITGB3, L1CAM, LYPLA1, LYZL1, MAPK7, OR10A7, OR10H5, OR1S2, OR52K2, OR6C70, PARVA, PHKG1, PRLR, PRNP, PTTG1, RRM2, SOS2, ST6GAL1, TAS2R41, PAFAH1B2
Negative_5725	TH, ACAD8, CAMK2G, CCR3, CYP26C1, DKK2, FARSA, GMPR, IL19, NCF2, NUDT2, OR4D1, PFN4, PIGA, PLXNA1, POMC, SMC1B, UBA7, UCP1
Negative_5726	F2, HAL, ACYP2, ADAM10, ADRB2, AGPAT1, AKT1, AOC2, ASNS, CCNA1, CLDN7, DAAM2, DHFR, IFNA16, IFNGR1, IKBKE, IL10RB, IL17A, IL23A, ITGB6, MAT2B, MMP2, MYH2, OR11H1, OR2V2, OR5AK2, OR5F1, ORC1L, PIP5K1C, PLA2G2A, PTDSS1, RASGRP2, SEMA4D, SETD2, SOCS4
Negative_5727	ARG2, ASRGL1, CREB3L3, CYP1A2, GMDS, GNPNAT1, HTR2A, IL21R, IL22RA2, IL6ST, IMPA2, LAMB2, LEFTY1, LLGL2, MAP4K3, NEUROG3, NTSR1, OR2A4, OR4A5, OXCT1, PAFAH2, PDGFA, PHKA1, PIK3R2, PRKAA1, PTPRR, PTTG1, SH3BP2, TSLP, TNFRSF1A
Negative_5728	DCC, GGH, IL6, NF1, ALDOB, ALG11, AOC2, ARG2, ATF2, BMP4, CD19, COL1A1, CTSB, DHCR7, DHRS9, DRD1, DVL3, EPHB2, FARS2, HYAL1, IL10RB, IRS4, LCMT2, LRAT, LTB4R2, OR52N4, ORC5L, PARVA, PIK3C2G, PLXNC1, PPP2R1A, PRPH2, ROBO2, WASF1
Negative_5729	EPO, GK2, AKT1, ARD1A, ASS1, CACNA1C, CCND2, CCR5, CLTA, COL2A1, CXCR4, EDNRA, EPB41L1, FOSL1, FUT9, GP1BB, IRS4, MEF2C, MS4A2, NAT1, NKX3-1, OR2G2, OR2T34, OR4M1, OR52L1, PIAS4, PLA1A, PMAIP1, PPP1CC, PTDSS2, PTGES, RAB23, RASA1, RDH10, RDH12, SLC25A4, SMPD1, SPTLC1, TAS1R2, TAS2R16, WNT1, YOD1, CACNA2D4
Negative_5730	FES, IDS, TXK, ACADSB, ADH1B, AGPAT6, ALDH1A3, ALDH3B2, ATF2, BACE2, BDKRB2, CDC16, CDC45L, CHRM2, CREBBP, DDIT3, DNMT3B, FCGR3B, GRIN2B, NUDT2, OR4K13, OR5AU1, P2RX2, PLA2G2E, POLA2, POLR2E, RXRA, SEMA3C, STX7, ATP6V1C1, SERPING1, ST6GALNAC1, TNFRSF10D

Negative_5731	ATP5D, ATP5J2, ATP5O, BAAT, BBC3, BCAR1, BDNF, CCDC6, CDK7, CHD8, CRY2, DHCR24, EHMT2, FBXW11, FGF18, GALNT1, GART, GUCY2C, HYAL2, IAPP, IFNAR1, IL20, IL29, L1CAM, LPAR1, MYLK2, NAT1, OR1F1, OR2A42, OR4F15, OR51G1, OR7G3, PAPSS1, PIK3C2B, PPCDC, PPP1R3A, PPP3CB, PSPH, PTGES2, RRAS2, SOS2, ST3GAL4, TNFSF14, TPH1, TPMT, TYROBP, ALDH18A1, SERPINE1
Negative_5732	AASDH, GDE1, MYH8
Negative_5733	IVD, AQP7, AREG, CCL28, COX10, DPM1, GRHPR, IL11, IL28A, IL8RA, IPPK, LARS, MKNK1, NT5C, OR10G7, OR10R2, OR8J1, OR8K1, PTPN11, TAS2R42, TNFRSF11B
Negative_5734	F11, IL5, BUB3, CCL7, CDH1, CNGA4, CRKL, DNMT1, DUSP6, EPHB3, FGF2, GDF7, GOT1, GRIN2D, HIF1A, HTR2A, IRF3, LDHAL6B, NMNAT3, OR10C1, OR2F1, OR2T1, OR3A3, OR51F1, OR6Q1, OR7D4, OR8D4, OR9A2, PFKM, PIGH, PISD, PLTP, SPI1, SPRED2, SRGAP3, ST3GAL6, STT3B, TBL1X, THBS4, TKTL1, TNFSF4, VEGFA, WBSCR17, SERPINE1
Negative_5735	CD4, MPZ, BDKRB2, CCND3, COL2A1, COQ7, DUSP9, FMO3, GAL3ST1, IL19, IL22RA2, MAOB, NAT6, NCK1, NFKB2, NFKBIE, OR10G7, OR10H2, OR1E2, OR2T4, OR4M1, OR6M1, P4HA2, PIK3R3, PIPOX, PKM2, PLCB2, POLR1B, PRKAR1A, PRKY, RBL2, RXRB, STX2, TPK1, UCK1, UNC5B
Negative_5736	CSAD, ENPP7, OR5AR1, OR6N2, ULBP2
Negative_5737	AASS, ACLY, CEBPA, CHAT, FTCD, GCNT1, GNAQ, GSTM1, IRS2, ITGB3, OR4F15, OXSM, PIK3R1, PLA2G5, POLR1C, RAP1B, ROCK2, SETDB1, SOCS7, SRGAP2, STAM2, UGT2B17
Negative_5738	CR2, ALPL, ASMT, CD8A, COL5A2, DHRS9, DPM1, LEFTY1, MASP1, MKNK2, MYH7B, PER2, PNMT, SGPP1, SMAD4, STX8, SV2B, TKTL1, TNFSF9, TUBB6, VAV2, PPARGC1A
Negative_5739	MUT, ACSL5, AKT3, APOA5, ATP5J, CALML5, CCL13, CCL14, CX3CR1, DDX41, DUSP16, FGF23, FMO2, FZD1, INPPL1, IQGAP3, MAPK9, MYL9, OR1A2, OR3A3, OR52I2, PDE1A, PIK3C2A, PLCG2, POLE3, POLR3K, PON1, RBKS, SARS2, SDHD, SKIV2L2, SULT1E1, TUBA8
Negative_5740	ADC, TKT, AOC3, ATIC, BNIP1, C1QA, CACNB4, CALML6, CCR3, CDC27, CDIPT, CYP1B1, CYP2E1, DLL4, GCAT, GCNT2, HPRT1, HPSE2, IFNA13, IL21R, LIAS, OR2G3, OR2V2, OR4N5, OR8B4, OR8H1, PGAM4, PHPT1, POLE, POLR2H, PPARG, PTPN11, RAPGEF3, SEMA3B, STAT6, SUFU, TACR3, TAS2R5, THBD, TP53I3, UNC5C, UPRT, VNN1, YWHAB, SERPINF2
Negative_5741	GCK, ADIPOQ, AKR1C3, CPT1B, FGF22, KLRC3, LILRB3, MCM6, SESN3, SOS1, TAS2R38, VAMP2
Negative_5742	ACTB, CACNB1, CTLA4, GLS2, IMPDH2, OR13C8, PIK3C2G, PLA1A, POLR2H, SETDB2
Negative_5743	NF1, ACVR1, AKT2, ALG8, BACE1, BCL2L1, DUSP10, GALK2, GCNT2, GSTM4, ITGAM, LIAS, MYH8, NT5C1B, OR1N1, OR2AT4, OR51V1, P2RX7, PRKAB1, PRKG2, PRKX, SENP2, SIGLEC1, SLC27A5, SMPD3, SMURF1
Negative_5744	IL3, ACVR1C, ADRA1A, AKR1C4, ANAPC2, CANT1, COQ5, CSF2RB, DDX50, DPM2, DTX3L, DUSP10, EDNRB, EFNA2, FCGR2B, FZD6, MBL2, NCF4, OR1E1, PHKA1, PIGF, PLCE1, POLD1, PPARD, PTPN1, RAB5A, RARA, RDH5, RHOQ, SETD1A, SPRED2, TAS2R16, TRADD, YWHAZ, HLA-DRB5
Negative_5745	TNF, ACAT2, ACTA1, ATP5G2, CACNA1C, CAMK2B, CBLC, CCL11, CD59, DARS, FRAT1, GART, GCNT4, GLG1, MAGI2, MGLL, NTRK1, OR4K14, PAX8, PLXNA1, RFXANK, TPK1, WARS
Negative_5746	CPOX, DFFB, ENPP1, FBXW11, FCGR2B, MCM4, NPR2, OR4A15, PIGQ, SULT1A2, TACR3

Negative_5747	BTK, CKB, GNS, ME1, ME2, SHH, UBC, ADSSL1, AMPD3, ATP13A2, B3GAT2, BMP5, C5AR1, CAV3, CCL15, CHST11, DKK4, FUT5, FUT7, HARS2, HLA-DRA, IDH1, IL23R, IMPA2, KLRD1, MAGI2, MINPP1, NT5C3, NT5M, OR2T34, OR52K2, P2RX1, PGK2, PLTP, PLXNA1, POLR2B, PRIM1, TBL1Y, TLR3, TRADD, UBA1, ATP6V1G1, ATP6V1G3, SERPINF2, TNFRSF17, TNFRSF1B
Negative_5748	C9, ACY1, ADRA1B, AHCYL2, ALDH3B2, APBB1, B3GNT4, CACNA1H, CDH15, COL11A1, CSF3, CYP2F1, DUSP6, EGLN1, FGF8, GGT6, HSD17B2, IRS2, MLLT4, NCOA4, NCOR1, OR2A25, OR2L2, OR2S2, OR4C15, OR4X1, OR6K2, PARS2, PTGES, RUNX1T1, STK11, TBL1X, THBS3
Negative_5749	C9, FGG, AGXT2, APAF1, B4GALT7, BMP5, BMP6, BUB1B, CLDN15, DHCR7, HARS, HMGCL, ICOSLG, NCK2, NTF4, OR13C5, OR13C8, OR2T5, OR4D5, OR8K3, PIAS4, PIGN, PPP1R1A, PRKCD, SCLY, TNFRSF10B
Negative_5750	ATP5G1, CXCL12, DLL3, KLRC3, NEU3, STX2, WEE1
Negative_5751	ALAD, ECSIT, ENPP2, FZD6, HLA-C, HTR6, OR9I1, PHKG1, PIP5K1B, PPAP2A, PPAT, PRKACB, PRKCZ, PRPS2, PTCRA, SLC25A4
Negative_5752	ACSM5, ATP5F1, CACNG2, CSDA, CYP2A6, DOT1L, EP300, HMOX1, IL28RA, LAMB3, PARD6G, PSME1, PYGM, SNAI1, STX8, SUV39H2, UGT2B4
Negative_5753	ACHE, FGD3, IFNA8, IFNW1, SULT2A1
Negative_5754	ATM, F11, ALG2, ATP5L, CALML6, CD244, CLDN17, DERA, DLL4, DUSP16, GFAP, GSTM4, IL13RA1, IL21, IQGAP1, IRF7, NFATC4, NFKB2, OR11H4, OR14I1, OR2V2, OR5L2, OR6N2, OR6T1, PHKA2, POLR3D, PRKAG1, PRKAG2, PTGDS, SHC3, ST3GAL4, UPP1, UROC1, HLA-DPB1
Negative_5755	ACAD8, ATP5G2, CHRM3, FRAT1, HSD3B1, NME6, NRG4, SELL, UPP1, TNFRSF11B
Negative_5756	HDC, IL7, AASS, ACADVL, ADORA2A, ALDH1A3, DAXX, EFNA3, FGD1, FUT4, GZMA, IL8RA, KAT2A, LAMB2, MTHFD2, OR2AG2, PIP4K2B, POLD3, PPT1, STX5, HLA-DPA1
Negative_5757	FOS, INS, PRL, PTS, ACTB, ACY3, AKR1C3, ALAS2, APRT, ATF2, CCL21, CCR4, CRLS1, CTLA4, CYP2E1, DGUOK, ENO2, FMO2, HLA-DOB, HPRT1, HSD3B2, ITGA7, ITGB5, JAK1, KLRK1, MAP2K5, MAPK13, NLGN2, OR2T2, OR52L1, OR5AK2, P2RX6, PDHB, POLR3K, PPP2R2A, PRKAR1A, PRKCA, RASSF1, RPS6KA2, SGMS2, SLC12A2, SMPD3, SMPD4, THBS2, THBS4, TSNARE1, WARS, WNT2
Negative_5758	HK2, ITK, ADCY5, BIRC3, C7orf16, CLTB, CTBP2, CYP2B6, DAAM2, ENO3, GMPS, IGSF5, MAP3K1, OR1E1, OR2AG2, OR6C74, THTPA, TYK2, YWHAQ, CACNA2D3
Negative_5759	CD4, ME3, MTR, TK1, TNF, VTN, ALDH9A1, ALPPL2, ASNS, ATP5C1, B3GAT2, C5AR1, CD40, CLDN2, DGKH, ESAM, GIT1, GOT1, GPAA1, GSK3B, ITGA5, KIR2DL1, KYNU, MTMR6, OR10H3, PIK3C2A, RETSAT, SEMA3A, SETD1B, SLC33A1, SPTLC2, TLN2, TRAF6, XIAP, ZNRD1
Negative_5760	HAL, IL9, PRL, SYK, ADSS, ALAD, ANAPC5, B3GALT2, CCR3, CHDH, CHIT1, CSF1, DGKA, DLAT, E2F2, FLOT1, FRAT1, FUT9, HMBS, IGF1R, IL11RA, LIPE, LRRC4C, MAP2K7, MAP3K11, MYH7, OR13J1, PGAM4, PGM2, PHGDH, PIK3C2G, PLCD3, POLR1D, PRKX, PTCRA, PYGL, SEMA7A, SLC2A1, SMPD1, PNLIPRP2, SLC25A31
Negative_5761	CR2, DCC, OTC, RFK, WAS, ALDH1A2, AMPD3, ARSB, BDNF, CAMKK2, CD40, CTNNA2, CXCL10, DOLPP1, EPAS1, FMO2, GALNT13, GCNT2, GGT7, GLG1, GNAI3, GNAQ, GYS1, HSPA8, IRAK2, ITGB5, MGAT4B, MYH9, NUDT9, OR1M1, P2RX4, PDE6D, PLCB2, PLXNB2, PNPLA4, POLR1D, POLR2I, RFXAP, RPE65, SENP2, SESN1, SH2D1A, ST3GAL2, VDAC2, WNT9A
Negative_5762	BLVRB, CD72, FOXO1, HMGCS1, LAMA4, RAPGEF4

Negative_5763	ILK, ACAA2, ARHGAP5, AXIN1, CACNA1F, CHST13, CTBP2, CTLA4, CYP4F3, DDX56, ECHS1, GALNT2, GDF6, GFPT2, GNRH2, IL10RA, IMPA2, LIPE, METTL2B, MIOX, MLYCD, NAGS, NLGN1, OR10H1, OR13D1, OR4N5, OR8K3, PDE1A, PDPK1, POLR2I, PON1, PPID, PRIM1, PTPRJ, RHOD, SENP2, SOCS2, WNT3, EIF4EBP1
Negative_5764	SMS, UBB, ARHGAP5, ARSE, B3GALT6, BCAR1, BLVRB, BST1, CD274, CD79A, CYP19A1, GDF5, GUSB, IPPK, MAML2, MAP3K13, NOX3, PCCA, PGAM2, PLEKHA8, RASGRP2, SNAP25, WNT7B, ATP6V0E1
Negative_5765	CDA, IL8, NF1, ATG3, ATG7, ATN1, ATP5H, ATP5O, BACE1, CBLB, COQ5, CSF1, CSNK1E, CUL1, CYP4F3, DAPK2, DDHD1, DDX56, DHDH, EPHA3, FMO4, GALC, GAS1, IDI1, IL15, LRRC4C, MTMR1, NANP, NR5A2, NRXN3, OR11H4, OR51I2, OR6F1, OR8J3, PANK3, PDE3B, PIGQ, PIP4K2A, PNPT1, PSEN1, SEMA3G, SETD1B, STX2, TNFSF18
Negative_5766	GNS, ACTN4, GUCA1B, IFNK, IL20RB, LEF1, MGAT5B, OR4B1, POLA2, POLR2H
Negative_5767	HYI, OAT, AASDH, ADH4, ATP5E, BCL2L1, CACNG7, CALML6, COASY, CTPS, DLAT, FGF22, FGF9, HIP1, ICOS, INSR, INSRR, MCM3, MYH11, NMNAT2, NOX3, OR10H4, OR14C36, OR2T8, OR51A2, OR5T3, OR6T1, OR8K5, PAICS, PAK1, PDE3A, POLR1E, PPA1, PPARA, ROBO2, ROCK2, RPN1, SARS, SLC2A2, SYNJ2, TAS2R5, TGFB3, TJP3, ATP6V0D1, HLA-DRB4
Negative_5768	CFD, CRK, EZR, NPY, ABI2, ACO1, ACSL6, ACSM5, ADCY8, AKR1C1, AVPR1A, CCR1, CDC27, CHEK2, CHST11, EPHA7, GLUL, HLA-DOA, IFNA4, IL13RA2, IL6R, INPP5A, LAMB3, MAP2K6, MAT1A, MITF, MTHFD2, NCSTN, OR51Q1, OR52K1, PAK6, PDCD1, PDE10A, PDE6D, PSEN2, PSENEN, PTGS2, RPS6KA6, SULT2A1, VDAC3, HSP90AB1, ST6GALNAC5
Negative_5769	CD4, SRC, AGRP, ALG12, B4GALT5, BMPR1A, C9orf95, CCND3, CXCL10, DGKQ, FLAD1, FZD10, GOSR2, GUK1, HSD17B4, HTR4, IL5RA, INHBB, LDHB, MAD2L1, NCAM1, OR13C5, OR4D5, OR51A2, OR5B17, ORC3L, PLA1A, PLA2G2F, POLR2I, SPAM1, SV2B, TAS2R16, TRADD
Negative_5770	ABO, MSN, ADRBK2, ARHGAP5, ARNTL, MAML1, PIGL, PLCD3
Negative_5771	ACACB, ACADS, ADSSL1, ATP5H, B4GALT5, CCL4, CLTA, CTF1, CXCL2, DLST, ECSIT, EGLN3, FGF10, GBGT1, GGCX, GNG13, GPD1L, HARS2, IL2RG, MYH7, NTRK1, OR6C1, P2RX7, PHKG2, PIGT, POLE, PON3, PPBP, PPCDC, PTPRF, WNT5B, XCR1
Negative_5772	ACP6, AKT3, ATP5G1, CLCF1, DGUOK, GADD45A, GAPDH, GLUD1, NFKBIB, OR13G1, OR2T33, PFKFB2, RAB23, RPS6KA3, RRAS, SDC4, STX12, MAPK8IP3
Negative_5773	ENPP1, GPX5, IRS4, ITGA2
Negative_5774	CR1, SCD, ABL2, ALG12, ALG6, ARSE, CD22, CSAD, CYP1A1, DUSP6, EPHA5, GSTM3, HADHB, HARS, IFNA4, IL20, MYH1, NCR1, OR10J3, OR1C1, OR2L3, OR4K5, OR6N1, OXCT1, P2RX4, PDE4C, PLXNA2, PRPH, PTPN5, RPN2, SMAD3, STAM2, STK36, STX18, SULT1A1, TAS2R13, UPP1, ZBTB16
Negative_5775	FGA, CDO1, IL28B, OR10G2, OR8A1
Negative_5776	GPAA1, IL10, LTB4R2
Negative_5777	ID4, PLG, SDS, SHH, CACNG6, CCL1, CDH2, CEBPA, CSNK1A1, CXCL2, CYP2B6, DHRS4, EFNA3, ENTPD5, FGD3, GGT5, GMPR2, GPAM, GRIN2A, HLA-F, LIMK2, MAP2K2, MRAS, OR2T10, OR51E1, OR5D16, OXTR, PCK1, PDHB, PIP5K3, PLXNC1, POLR1D, PTTG1, PYCRL, SAT2, SHMT2, SUCLG2, TAS2R49, TP73, TUBB2C, UNC5D, ATP6V0E1, ST6GALNAC1
Negative_5778	MAX, MET, MOS, ACY1, BBC3, BPGM, DGAT2, EPHA6, EPHB3, FLT3LG, GAMT, GMPPB, HSD17B7, MFNG, NFAT5, NOX1, OR4C13, OR7A10, PCYT2, PDGFC, PITX2, RASGRP4, SETDB1, SKP1, SULT1A4, WNT3, PRICKLE1, ST6GALNAC1, TNFRSF1B
Negative_5779	ABLIM2, AKR1B10, ALDH7A1, BUB1, DOT1L, HTR6, IKBKE, IRS4, KLRK1, LTC4S, MTHFD2, OR4Q3, PFN2, PIGU, RFNG, SARS, SCD5, STAM2, STX5, SUOX, SV2A, TBXA2R, VAMP3, GABARAPL1, GABARAPL2

Negative_5780	ACYP2, ASCC3, ASS1, CXCR4, CYBB, DEGS2, OR10K2, OR14A16, OR1N2, OR4X1, PLAUR, PPP1CA, RCHY1, UROD, B3GALNT1, SUV420H2
Negative_5781	ADSSL1, AFMID, AKT3, DERL1, LTB4R2, PFKL, RXRA, UBA7, VEGFC
Negative_5782	AGRN, CLDN20, FGFR4, GUSB, HHIP, LDHC, OR10C1, OR8H3, PIGM
Negative_5783	AK1, CTH, SFN, ACMSD, CCKAR, CDKN2B, CSNK1G1, CTSL1, GALNT5, GLI1, GNRH1, IDH3B, IL13, NOX3, OR4K17, PDE6D, TUBAL3, UGDH, WNT7B
Negative_5784	ATP12A, DAPK1, GADD45G
Negative_5785	ID4, ACADVL, ALG11, COL6A2, EHMT2, HSPB1, OR4A15, OR51E2, OR8K3, SPTLC1, TBK1, B4GALNT1, GABARAPL2
Negative_5786	F8, GSS, ACYP1, ADCY5, ADCY9, ALDH3B2, ALG1, AOC2, C1QA, CACNG1, CD74, CDC14B, CFL1, CTTN, CYP2U1, DDX51, DDX54, DGKZ, EPHA7, FGFR4, FUT6, GTSE1, HMGCR, HPRT1, ICAM2, IL21R, INPP1, MAP3K13, MAPK1, MYH11, NODAL, OR1S1, OR2AT4, OR3A3, PNMT, PTPN7, PTPRR, RIPK1, THBS4, UCK1, UNC5C, VEGFB, WASL, YKT6, ATP6V1G3
Negative_5787	EZR, ADRB3, CACNB3, CDH1, CDO1, CLDN18, FGF3, GADD45B, GALNT6, HNF4G, LAMB4, LGMN, NRG3, OR10A4, OR10S1, OR14A16, OR2A2, PDE10A, PLD1, PROS1, STAT2, TUBA1C
Negative_5788	IDS, ACSL3, ALDH1A2, ATP5G2, BLNK, CDC16, CDC25A, CDK5, EXT2, MLLT4, PFKFB1, PRKX, RRM2, TSTA3, UNC5B, GABARAPL1
Negative_5789	EZR, VTN, B3GALT1, CD36, CHIA, CNTF, CSNK1G2, CXCR5, EGLN1, FGD1, FLT4, FZD3, GCAT, GJA1, IMPA2, MYLK, PFN3, PLCB3, POLD1, RAC2, RARA, SESN2, SH3BP2, TBXAS1, TSTA3, UBE2L6, ULK1, WNT9B, ATP6V1B2, PLA2G12B
Negative_5790	EZR, AMY2B, CD55, CD99, CX3CL1, DIAPH3, EFNB1, FUT4, GALNT12, IL4R, MAML3, NARS2, NOTCH1, NRXN2, OCLN, OR13C8, OR2T8, PIP5K1C, PLXNC1, PTCRA, THBS4, UPP2, CACNA2D2, PDCD1LG2
Negative_5791	ALPI, BAI1, CDC7, CHKB, CLOCK, CPOX, CREB3L4, CXCL9, FGF1, FUT8, GCNT2, GMPPA, GYS2, OR6B2, OR8I2, PIGL, POLR1B, ST8SIA5, STAT6, TAS2R40, TNFSF18, TRAF1, ULBP1, WHSC1L1
Negative_5792	ADIPOR1, ADRA1B, CACNG2, CD226, GDF5, GNA15, LIPF, NOS3, NPR1, ORC2L, PLA1A, PLA2G6, POLA2, SH3BP2, PLA2G12A
Negative_5793	ASL, BTC, F2R, ACSM5, ALG1, ATP2A2, CACNG7, CD19, CD59, DCTN1, DGKQ, DHRS4, DUSP6, EIF4B, ESAM, FLOT1, FUT8, GJD2, HADHB, HIP1, HSPA8, LIPT1, MECR, NLGN3, OR8D2, PCCA, PDE10A, PFKM, PMM2, PPAP2B, PPARG, PRODH, SDC1, ST8SIA5, YWHAZ, ATP6V1B1, SERPINB5
Negative_5794	CEL, LEP, SMO, TXK, ADRA1B, ASMT, B4GALT5, CCNB1, CD86, CRHR1, CYP2A7, DVL2, EHMT2, ETNK1, GABARAP, GALNT5, GALNTL2, GART, GDE1, GNPNAT1, LDHAL6A, LTC4S, MTHFR, MTMR2, NUDT12, OR10C1, OR1L4, OR4D2, OR6K3, PDPK1, POLR3A, PPP2R5B, PRKAR1B, PRPH2, PRPS1, RAB23, SOX17, TBXAS1, TRHR, UGT2B28, ULBP1, SERPINF2
Negative_5795	NEUROD1, SULT1A2, TNXB
Negative_5796	BTD, INS, ADCY2, ADRBK2, AGMAT, ALDH1A3, BHMT, CD72, CLCF1, CSF2RB, CXCR6, CYP3A5, DAXX, E2F2, ENPP7, FZD6, GNAO1, GPLD1, HLA-DRA, HMOX2, IFNAR2, IFNB1, IMPA2, LALBA, MAD2L2, MAN1C1, MAPK7, MGAT5B, NCAM1, NOD1, OPLAH, OR13D1, PARD6G, PDHB, PHPT1, PI4KB, PIK3R5, PPP3R1, PRKCH, PTPN5, RIPK1, SOCS5, TAS2R38, TAS2R5, TKTL1, ST6GALNAC3
Negative_5797	A2M, FGB, FUK, ID2, ACSL6, ADCY10, ALPL, APPL1, ASS1, AXIN2, CASP9, CD55, CSNK1A1, CXCR4, CYP8B1, DTX2, GNA14, GPAM, GRIA2, GSTM5, IFNA6, IFNK, IRF5, NT5C2, OR4K17, OR51E2, OR5H6, OR5T2, OR5T3, OXTR, PMM1, PNPLA4, POLR3D, PTCH1, SARDH, SIGLEC1, SOX17, SULT1E1, TNFSF12, TUBA3E, ZBTB16, ATP6V1C2, CACNA2D1
Negative_5798	CKM, ATP6V1D, CCL5, ELK1, IKBKE, IQGAP3, METTL6, MGAT4A, NFATC2, OR2T2, OR4C15, OR51D1, OR52D1, PTDSS1, PVRL2, RCHY1, SMPD2, SNAI1, VKORC1, WNT9A

Negative_5799	F2, ACSS1, ACYP1, ADCY8, ALDH1A1, AMY2B, ARAF, ARNT2, CLDN16, EPHB1, FABP3, FDPS, GALNT11, GNG12, MCM6, NT5C1B, OR4D6, OR51B5, PAPSS2, PMAIP1, PMM2, PPOX, RAD51, SLC27A1, SMPD3, TIRAP, UCK1, UGT2B11, WWP2, XCL2
Negative_5800	FGG, SP1, ALDH1A1, ATP5G3, CD27, COL4A1, CSNK1A1, JAK3, MCCC1, NEFH, NQO1, OR2T1, PIAS4, PTAFR, TBL1Y, TNFRSF11A
Negative_5801	DAO, HK3, AGTR1, B3GNT5, COL4A6, CSDA, CTBP2, DDHD1, EVI1, GOSR2, HSD17B4, INADL, MAD1L1, MAP3K4, MDH2, NAT5, NEFH, OR4F6, OR51E2, PLA2G3, PMM2, PRODH, RAD51, RASA1, SEMA3C, SH2D1A, ST8SIA1, UPB1, YWHAH
Negative_5802	DBI, LEP, ACSS3, ALOX15, CNGB1, CRY1, CXCL16, DDX51, DDX55, DRD5, FUT3, GRM1, GYS2, IL10, INADL, ITGA5, LFNG, MCM6, OR4D5, PERP, PIM2, RXRG, TBXAS1, TKTL2, VAV2
Negative_5803	OAT, PC, AKR1C3, ALAS1, ARHGEF6, BCL2, CHAT, DHRS4, DPM1, FDPS, FMO3, FRAP1, HYAL2, ITGA8, MKNK2, NAGA, NFKBIA, PDHA1, PIK3R4, PLCB3, TUBA1B
Negative_5804	MVD, ACAT2, ACP5, AGPAT1, AKR1A1, ALG5, CACNA1A, CCNG2, CD47, CDS2, CTPS, DUSP6, FBXW11, GART, IDH3B, IL11, IL13RA2, JAK2, LAMA3, NCKAP1, NFKBIB, NRG3, OR1F1, OR1J2, OR1J4, OR2T8, OR5T2, OR6C4, PDE1C, PFN3, PGM3, PRPS2, RBKS, SCIN, ST6GAL1, STAT1, TAS2R10, TAS2R14, TAS2R48, THTPA, TUBA3E, ULK1, YARS, SERPING1
Negative_5805	DBH, TK2, ANPEP, ARPC5L, CACNA1I, CAV3, CD38, CDC2, CNTNAP2, COL4A1, GGPS1, GRHPR, IFNA10, IFNA14, IL2RB, KLRD1, NCSTN, NLGN2, NSD1, OR2F1, OR2H2, P2RX1, PCK1, PLA2G2A, PLA2G2E, HLA-DPB1
Negative_5806	ALAS1, CXCL6, CYP2C19, DGKA, FUT5, GBGT1, IL13RA2, LRRC4C, OR1B1, OR7G1, TUBA1A, YWHAQ
Negative_5807	APP, ALAS2, C1QA, CALML6, CCNA1, COASY, CTNNB1, DAPK2, DGKH, ENAH, FGFR4, FOXA2, GNAO1, GOSR2, GSTZ1, IL22RA2, IL8RB, LDHA, MAP4K2, MPST, MYL9, OR51L1, OR52K1, OR56B4, PIGX, PLA2G2A, SULT2B1, TUBB2B, VAMP2, WNT1, ST6GALNAC5, TNFRSF10C
Negative_5808	ABO, HAL, PC, SCD, ACACB, ADH4, AHCYL2, AKR1B10, CADM3, CAMK4, CHDH, CYP2J2, DAPK1, DGKQ, EPHB2, FGF3, GALNT2, IL18RAP, IMPA1, LAMA3, LYPLA1, MAN1A1, OCLN, OR13G1, OR6C3, PARVB, PLXNB2, RAB5A, RBL1, SOCS1, ST3GAL2, TAS2R40, TAS2R48, TBXA2R, TCF7L1, TICAM2, TOLLIP, UBA7, MAPKAPK3, SERPINA1
Negative_5809	CRY2, GALC, IL6ST, KYNU, LDHA, P2RX7, PIK3CD, POLR1B
Negative_5810	ADCY9, CACNG5, CDKN2C, CPOX, DAXX, FTH1, HSD17B1, HTR2A, MCM4, NAE1, OLAH, PPBP
Negative_5811	ADCY8, ADORA2A, AGXT2, ALDH3A1, BMP5, CACNA1H, CIITA, CNTN1, FMO4, GCLC, GUCY2F, INPPL1, KLRK1, LIMK1, NAGK, OR10G4, OR4N2, OR5K2, OR9G4, PIP5K1B, POLR3G, PPP5C, PTGFR, RARS2, ROBO2, SMPD2, TAS2R60, ULK2
Negative_5812	AK5, ATM, B3GNT5, CACNA1F, G6PC, GCNT1, GDF6, GNA11, HNF4A, IL10RA, IL4R, ITGA3, MCM4, MTMR6, MYH3, NFATC3, NLGN3, OR10AD1, OR13C2, OR51M1, OR52E2, OR8J3, PARD6G, PARS2, PGM1, POLR1D, PPP2R5E, PRPH2, RASGRF1, RBL2, RPS6KB1, RUNX1T1, SETD1B, SLC27A1, TAS2R50, THBS3, TNFSF14, TPMT, UGT2A3, YES1, ZMAT3, ATP6V0A2
Negative_5813	HAL, APAF1, CACNG1, CAMK2B, DTX3, GGT1, IL25, IL3RA, KLKB1, KNG1, MAML3, MAP4K2, MCM6, NEUROD1, NOS1, NRXN1, OR2A2, OR3A1, OR4P4, OR52D1, PPM1D, RPE65, SEMA3E, SUCLG1, TGDS, XCL1
Negative_5814	CD6, ACY1, AHCY, AKR1C4, ALG10, ALOX12, APOA5, DAG1, GALNT14, GSTM4, LAMC1, NMNAT1, OR1G1, OR56A4, PERP, TSLP, UNC5A

Negative_5815	HGF, MAG, SCD, TEC, ABLIM2, ACVR2B, AKR1C2, ALG1, B4GALT2, B4GALT5, BDKRB2, CHAT, CHST13, DAPK1, DNMT3B, EDA2R, EXTL3, GALNTL4, GRIN1, GRIN2A, GSTA4, GUCY2D, IFNA8, INPP4B, MINPP1, NEGR1, OR14A16, PDE3A, PLCB4, PPP1R3B, PRKAA1, PROC, PVRL4, RASSF1, RFXAP, RIPK1, RPS6KA4, SETD8, SLIT2, SMC1B, SPHK1, TLR2, TUBA8, UNC5B, PPARGC1A, SERPINF2
Negative_5816	ASL, GP6, MOS, SRC, ADH6, AGPAT4, ALPPL2, ANGPTL4, ATP2A2, BUB3, C9orf95, CLDN1, CXCL10, CYP26A1, DOT1L, DTX4, EGFR, F2RL3, GOT2, GUSB, HAAO, IDH3G, IGFBP3, IKBKG, IL13, IQGAP3, KLRC2, MAP3K2, NT5C1A, NTRK1, OR13C3, OR2M3, OR3A3, OR5AR1, ORC2L, PDGFA, PKLR, RAC2, RPS6KA4, RRAS2, SLC2A4, SNAI1, SYNJ1, TGFBR2, TNFSF4, USE1, XYLB, CACNA2D2
Negative_5817	C1S, CD2, JUP, MVD, AANAT, ALG3, CCNB3, CREB3L3, DIAPH1, EDA2R, IL10, LDHAL6A, MOV10L1, NANS, NRG4, OR2A5, OR2G2, OR6N1, PDE8B, PRKY, PTPN11, SPRY2, TJP3, ULBP1, WNT4, ZMAT3
Negative_5818	ATP6V0C, BCL2L1, BLVRB, CFLAR, CLDN20, DUSP10, ENTPD2, GALNTL4, HAO1, NUDT9, P4HA1, PRODH, PTDSS2, RIPK1, RRM1, SEMA4F, SOCS1, SREBF1, YES1
Negative_5819	ADK, ADO, AK5, GK2, TNF, ABAT, AMDHD1, B3GNT5, BCAR1, C3AR1, CCL20, CDH4, CLCA2, CXCL2, CYCS, EPB41L1, ETNK2, G6PC, GAD2, GRLF1, GSTM5, IL18, IL22, JAK2, KLRC3, KYNU, LEFTY2, MAT2B, MYH7, NANS, NOX3, NRXN3, OR1D2, OR3A2, OR4A16, OR52R1, OR5AR1, OR5B17, OR6C1, PTCH2, SC4MOL, SHISA5, SLC27A5, SPTLC1, TSNARE1, WWP1, SERPINC1, TNFRSF17
Negative_5820	TNN, APPL1, CDH1, CNGB1, CSAD, ETNK1, ETS1, GGCX, IL6R, JAK1, OR5K2, PFN4, PIGX, PIP4K2C, STAM, STX7, TDO2
Negative_5821	C7, DCK, F3, NGF, ACAA1, ALDH9A1, ALPP, BMP8A, CCNB1, CDH2, CNTF, CXCR5, DDX19A, DMGDH, E2F3, EARS2, ENTPD8, EPHA6, FLAD1, GRPR, HHIP, HSPB1, INMT, LCP2, LTA4H, MGAM, MMP9, MYH1, NFKB1, OR4P4, OR4S2, POLR1D, PPP3R1, PRKAG3, PRKCZ, RASGRF1, RHOA, SH2D2A, SNAP23, SPTAN1, SPTLC2, ST8SIA1, TPK1, YWHAZ, ATP6V0D1
Negative_5822	GH2, ALG10, AMDHD1, AMPD1, ASMT, ATG12, ECSIT, GLI2, GSTM1, HARS2, HSD17B3, IL12A, IL1RAP, KIR2DL1, NNMT, OR5AR1, OR6Y1, PHKA1, PPP3R2
Negative_5823	SDS, AANAT, ACSL5, AFMID, AGPAT1, AKR1A1, BCMO1, CD80, COL1A2, CRLF2, CTBP1, CXCL10, CYP11B2, DUSP8, EDNRA, FGD1, FGF11, HCLS1, HSD3B1, IL28B, IMPA2, JAK2, MAPK14, MTMR2, OR10G4, OR2F1, OR51B5, OR56A3, PDGFA, PDIA4, PFKM, POLE, POLR3D, PTK2, SEMA4C, SLC27A5, SPHK2, STAT2, TKTL2, UROD, UXS1, WNT3, TNFRSF6B
Negative_5824	TNN, ADCY6, AMY2B, EFNB1, ITGB5, MAPK12, MAPT, NFAT5, OR51G2, P2RX2, UGT2B4
Negative_5825	CKM, SCD, TAT, AARS, ADRB1, ALOX5, ATP5O, C7orf16, CD46, CDKN2D, CHEK1, CIITA, CSH1, DDX51, FBXW11, FUT9, GYS1, HSD17B8, ICOS, IL5RA, MYL5, NT5E, OR10G7, OR11G2, OR51B5, OR5M9, PIP4K2C, RASSF1, SNAI2, SOCS4, SPTLC1, SUV39H2, TCIRG1, PHOSPHO1
Negative_5826	ACADM, CCNB1, ITGB3, LIPT1, OR5AK2, PTEN
Negative_5827	CTH, ATP4A, CSNK1G3, CYP3A7, EPHA2, GLDC, IDI2, INPP4B, MAGI3, OR4C15, ATP6V1B1
Negative_5828	AGL, AUH, CGN, EDA, A4GALT, ACLY, AGPAT1, ARHGEF7, ASAH1, CHST11, CLOCK, DIAPH2, DMGDH, DTX3L, ENTPD2, FGF1, FUT2, GCDH, HTR5A, IL13RA2, IL8RA, ITGB5, ITGB8, L1CAM, LIPT1, LLGL1, MAPK14, OR1E2, OR2S2, OR7G2, OR9G1, PDE3B, PKMYT1, PPP2R5A, PRKCD, PTK2B, RPE65, RPS6KA3, SEMA3C, SETD1B, SPRED1, TPK1, UCP1, YKT6, SERPINA5
Negative_5829	BID, AFMID, BIRC3, CAV2, CDC2, CHAD, CNTN2, CYP1A1, CYP1B1, DHFR, EGLN3, FARP2, FZD2, GNG3, GRHPR, IKBKE, INHBC, LIMK2, MAOA, MEF2C, NSD1, PDE7B, PFKFB3, PMVK, PPAT, PPCDC, PPP2CA, SOCS7, UGCGL1, ZFYVE9, PHOSPHO1

Negative_5830	CEL,CGN,F10,KMO,LYN,RPE,STS,ALDH2,ATP5J,B3GAT2,B4GALT5,BBC3,C4BPB,CCL16,CCL27,COL5A1,CSH1,CXCR5,CYP3A4,EPAS1,FADD,GCNT2,KITLG,LAMB1,NAGA,OR10A3,OR1L8,OR1Q1,OR2T10,OR4X1,POLR3G,PPP1CB,PRKCG,RPIA,SLC12A2,SYNJ2,TNFRSF4,TUBAL3
Negative_5831	ADH7,CACNA1C,CHAT,CRLS1,DRD1,GALC,HLA-DOB,MAFA,MAP2K7,MPST,NCF1,OR5A2,PDK1,PGK1,PGS1,PPAT,STX12,TLR9,VKORC1,TNFRSF13C
Negative_5832	DHH,F8,MVK,NF1,ADCY4,ARPC2,C1QC,CCNH,DUSP8,GNA14,GRAP2,IFNA21,JAM2,LCP2,MGAT5,MIOX,OR1F1,OR2L13,OR4N5,OR6F1,PGLS,PPP2CA,RRAS2,SOD1,SV2A,UGCG,UPB1,CSGALNACT1
Negative_5833	LCT,LSS,MVK,ADCY3,AKR1C2,ALG1,ATP6AP1,BACE2,BMP7,CFTR,CNGA4,CYBB,DBF4,FUT3,GALNT13,GALNT6,H6PD,HIF1A,IFNW1,ITGA9,LAMC2,LTBP1,MCEE,METTL2B,NUDT2,OR10AG1,OR10J3,OR1G1,OR1I1,OR9Q1,P2RX4,PCYT2,PDE1C,PDHB,PFKFB2,PIK3C2B,PLCG2,PLXNA3,SDC3,SMC1B,SOCS3,SULT1A4,TNFRSF4,TSC1,UGT2B11,UROD,TNFRSF13C
Negative_5834	GP5,PRL,ACP5,ADCY7,ARPC5,CHRNA7,CMBL,DGKA,ECHS1,ENPP2,GALNTL4,GSTK1,GSTM4,HIF1A,HSD3B7,IFNB1,IL11,ITGAL,KLRC2,KLRC3,MAFA,NMNAT1,OR1J1,OR4Q3,OR9Q2,PER1,PIAS4,PNPLA4,POMC,PPA1,PRKAB1,PTPRB,SAT1,SMPD4,ST3GAL4,TLR2
Negative_5835	PXN,BCAT2,CCNA2,CDC14B,CHKB,DDB2,GSTM5,MYH7,NTN1,OR4M2,OR6Y1,OR9G4,PIAS2,PRSS2,PTPRC,WARS2,WASF3,ST6GALNAC1,TNFRSF10B
Negative_5836	AK5,FER,SRC,ACSL5,APRT,BCL2,BMPR1A,DPYS,GRPR,HTR6,KAT2A,KIR2DL1,MAGI1,MECR,NFATC1,OR2T34,OR6C70,PAK4,PARVG,PGK2,POLD2,POLR2G,RBPJ,SEMA3F,SEMA7A,SLC9A1,SMARCA5,SV2A,THBS3,TRPC1,ULBP3
Negative_5837	CKB,TYR,ALDH6A1,ATP5A1,CD72,CDC25A,COL1A2,CXCL3,CXXC4,CYP1A1,CYP2E1,EDNRB,ELK4,EPHA7,EPHX1,GALNT1,HMGCL,HRH2,ICOSLG,IL17RB,NFKB2,OR1E2,OR2C3,OR2T6,OR2V2,OR56B4,PIGU,PSAT1,PTCH2,PTGIS,RALA,ROCK2,SMPD4,VAMP2
Negative_5838	C2,ALDH1B1,CCL23,CCNG1,CD22,CHIT1,CLCA4,CMPK1,DOLPP1,ENO1,HHEX,IL1B,IL22,IL2RG,KLRC4,LIAS,MAGI3,MNX1,NAE1,OR10AG1,OR2B2,OR4N5,PIGL,PPP2R2C,RXRG,SLC2A2,TYRP1,ZIC2,SUV420H1,TNFRSF11A
Negative_5839	GDA,IL8,ADRBK2,AXIN2,CYP11B1,ETS1,EXT2,FARP2,GCNT4,GUK1,HNF1A,IL13RA1,LYZL1,MAP4K3,NEGR1,NOTCH1,NOX3,OR5D14,PARVB,PIK3C3,PLCB3,PLCD3,PLCG1,PPOX,PPP3CA,RAP1A,TCF7L2,TRAF2,VANGL1,WNT4
Negative_5840	CACNB2,CER1,FASN,GNA13,KITLG,MADCAM1,PLCG2,PMM2,PPT1,PRDX6,RHEB,SH2D1A,SPTLC2,SSH3,ALDH18A1,ATP6V0A1
Negative_5841	CSNK1E,IMPDH1,MAP2K3,OXSM,TRPM5
Negative_5842	ABO,IL5,TNC,ANAPC1,ATP5J2,BMP6,CD19,CDKN2A,CXCR6,CYP11B1,EIF4E,IRS1,LARS2,NAGLU,NKD2,NOS1,NRG1,OR1F1,OR2T11,OR4B1,OR8H1,PLAT,POLR3A,PRKAB1,RHOA,SCIN,SLC2A4,TAS2R10,UGCG,UGP2,VDAC1
Negative_5843	CKM,EBP,F3,ID3,OAT,RB1,AANAT,ADSS,ARHGEF4,ATP5C1,BCKDHB,BNIP1,BUB1B,C1QA,CACNA1G,CDH5,CNTNAP2,COASY,CREB3L1,DHX58,DLAT,EIF4E2,ENTPD2,EPHA7,EPHB2,FARSA,GFPT2,GNB3,ITGAD,LRRC4C,MMP1,NGEF,OR13C2,OR51D1,PDE4C,PIK3CB,PLCD3,POLR3B,PPAT,QPRT,SDHA,SMPD1,SOAT1,ST6GAL1,TFDP1,TUBAL3
Negative_5844	NNT,APOC3,C3orf10,EI24,FZD5,GALK2,GSTA2,INPP5B,NT5M,OR4C16,OR4D6,UGT2B11,VDAC2,MAP3K7IP2,TNFRSF6B
Negative_5845	CKB,MAT2B,OR7C1,PFKL,POLR3K,PPP2CA,STEAP3

Negative_5846	BTK,C7,SYK,ALOX15B,B3GALT5,B3GALT6,C5AR1,CACNB2,CHPF,COL4A4,CYP4A22,GNRHR,GSTT1,HSD17B1,IFNG,IL21R,IL7R,INHBB,INPPL1,MAP2K7,MYH7B,ODC1,OR5H2,P2RX3,PARVB,PIGA,PPAP2A,PSENEN,PSPH,RAPGEF1,RERE,RIPK1,RRM1,SORBS1,ATP6V0D1
Negative_5847	AK7,C3,EZR,ACSL3,ACTG2,ALG10,ALG9,BIRC2,BMPR1B,BUB1,CACNA1I,CD14,DHRS9,ENPP1,EPHA2,FCGR3B,FUT1,FUT2,GJA1,GSTA3,HTR2B,HTR7,LYZL1,MPP5,NCAM2,NCOA4,NEU2,OR10Q1,P4HA3,PLA2G2D,POLD1,POLE,POLR2E,PRKAG2,PTGFR,ROCK2,RPS6KA5,SEPHS1,SNAP25,SPTAN1,STK4,TRPC1,UGT2B4,VAMP3,WARS,EIF4EBP1,TNFRSF1B
Negative_5848	ACP1,DDX52,DUSP10,FPGT,GSTA1,IFNB1,LCAT,LDHA,MYD88,NFATC2,OR13G1,OR1L1,OR4K17,OR52N2,PDCD1
Negative_5849	BTD,DCC,F9,ABLIM3,ACVR1B,AKR1A1,ALDH1A2,APAF1,BACE1,BCL2,CCL4,CLDN2,CSDA,CSNK2A1,CXCL1,CXXC4,DARS,DHRS3,EPHB6,EPRS,ERBB4,GALT,GDF6,HSD11B1,IL21,LARS,OR13C2,OR2L8,OR4K13,OR8J3,PCYT1B,PFKFB1,PGS1,PIGL,PLA2G3,SETD7,SMAD9,STAT5B,TAS2R48,TICAM2,ATP6V1B2
Negative_5850	CR1,ACVR1,ADRB2,ALDH1A2,AMDHD1,ATP5J,CD244,ERBB3,GGCX,GGT5,GSTM5,IFNA21,IL12A,JAM2,KIR3DL2,LYPLA2,MYL2,MYLK3,OR1L3,OR3A2,OR52D1,OR6C4,PARD6G,PDE6G,PDGFRB,PFKP,PGAM4,PIAS1,PIGO,PIP4K2A,PISD,PPP5C,PTDSS2,PTPN1,PYCRL,RARA,RXRA,SESN1,ST3GAL4,UCK1,WWP1
Negative_5851	CYP1A1,DPM3,MAD2L1,ODC1,PIGP,PPP2R2A
Negative_5852	OTC,GNAI2,SRD5A1,TAS1R1
Negative_5853	GLA,ABLIM2,ADCY8,ATP5D,CCR3,CTNNA2,DHRS3,ERO1L,ITGB7,NCOR2,PDE4C,SMAD5,SMPD4,TAS2R8,HSD17B10,TNFRSF17
Negative_5854	DAO,AKR1C2,MDM2,OR4C15,OR51T1,OR6Q1,PDXK,RARS2,SEMA4A,TAS2R42,TUBB3,ATP6V0D1
Negative_5855	TNF,ALG6,CIITA,CMAS,FGF20,GGT5,HMGCR,MAPK12,MAT2B,OR10G4,OR1S2,OR4P4,PDE6D,PIK3CA,PPP2R2D,RAP1A,SELL,TBL1X
Negative_5856	CKM,ACAA1,ACPP,ADCY7,ADSSL1,ARPC3,ATP4A,ATP5B,B3GALT6,BMP6,CDC20,CYP2C9,CYP2D6,DARS2,EVI1,HIF1A,IBSP,LIPT1,MGLL,NFYA,NMNAT3,NR5A2,OR10G7,OR10S1,OR8D4,PANK4,PIAS2,PIAS3,PIGH,PIGL,PMAIP1,POLR3D,PPP2R1B,PTAFR,PTPRC,VEGFB,ATP6V1E1,ST6GALNAC4
Negative_5857	DAO,ASMT,CYSLTR2,GP1BA,GUCY2F,IL21R,MS4A2,NT5C,PLXNB1,POLD4,SLC25A6
Negative_5858	GH1,IVD,ACMSD,ANPEP,BACE2,CRLF2,CYP11B2,DDX55,DHODH,E2F1,FUT6,GNG12,HCCS,MAOA,MAP2K7,NRG4,OR2T6,OR5D13,PLCB3,PLCD3,PYGM,SPTAN1,TBXA2R,TUBB,ZIC2
Negative_5859	CFI,NLK,SCD,ACTG1,ADCY6,ATP5O,B3GNT2,CASP6,CASP8,CBR1,CDKN2A,CEBPA,CLCA4,CXCL16,ENPP2,ENTPD2,FCGR3A,FUT4,FUT8,FZD3,GRHPR,NCOR2,OR10J5,OR2A5,OR2T12,PER2,PKLR,PRKAR2A,RELA,RFXANK,ATP6V1C2
Negative_5860	ADH1B,ATP5J,CD8B,HRH2,ITGAE,LMO7,OR2D2,OR51E2,OR5H6,PIK3R3,PPP2R1A,SCIN,UGT2A1,ATP6V1G3
Negative_5861	ADC,LAT,TAT,ADCY2,CYSLTR1,DIAPH1,HSPB1,ICAM1,ISYNA1,LHCGR,LRAT,NOS1,ODC1,OR1N2,OR4N2,PRKAB2,PTPRZ1,STAT2,TFDP1,VAMP4,ATP6V1B1,HSP90AB1,ST6GALNAC3
Negative_5862	AADAC,ACTA1,ALDH3A1,ALG9,CMBL,DHDDS,GSTA1,LCAT,PLXNA2,PYGM,SMAD2

Negative_5863	FAH,FGA,IL8,ADH4,AKR1A1,ALDH2,ATP5H,B3GALT4,B4GALT4,CACNA1B,CADM1,CCL21,CCNA2,CCND1,CD14,CD40,CD47,CDK2,CLCA4,CLDN20,COL4A6,CXCR4,CYP2A6,DEGS2,EPHA1,EXTL1,FBP2,FUT3,GANAB,HTR2B,IL23A,IL2RA,IRS2,LIPC,NAMPT,NCAM2,NOD1,OR10AG1,OR1S1,OR5F1,OR7C2,OR9G4,PLCG2,PLOD2,POLR2I,PTK2,RXRG,SETD1B,TUBB1
Negative_5864	CD6,GPAA1,HYAL1,IL23A,PON3,PTPMT1
Negative_5865	LAT,ACVR1,B3GNT5,BCKDHA,CDC23,CFTR,EPHA1,F13B,GALNT6,GRB2,GSTM2,GSTM5,IFNA6,IL17RB,IL19,ITGB1,OCLN,OR2M4,POLR1E,PPP1CC,SETD2,SFRP1,SV2A,UPB1,YKT6
Negative_5866	CAD,TKT,ABAT,B4GALT3,CAV3,CDS1,CYP8B1,DDHD1,IL10RA,LRDD,MCM2,OR3A2,OR4D1,OR52E4,OR8H2,PPP1R3A,PTDSS2,RBKS,SLC27A4,SLC2A4,SMARCA5,YES1,C1GALT1C1,PNLIPRP2,TNFRSF13B
Negative_5867	SRM,CALML3,CREBBP
Negative_5868	CTH,EBP,NPY,ACSS2,ATP4B,CCR4,CDS2,COL6A2,CSH1,CTBP2,CXCL12,CXCR4,DGKD,DGKI,EGLN1,ELK1,F2RL2,GAL3ST1,HPSE2,IL17B,MCM2,MTAP,NARS,OR5K1,PLA2G2A,PNPLA3,PPP2R5B,RASGRF2,SETD8,SKP1,SLC27A5,SNAI1,TOLLIP,VANGL2,VEGFC
Negative_5869	C2,PXN,CDKN2A,CLDN18,DTX4,HLA-G,ITGA9,LPAR1,MPP5,OR10H1,PLA2G2F,WASF1
Negative_5870	DCK,KMO,ACLY,AMY2B,CCL17,CCL24,CCR9,CD44,CLTA,CYP11B1,DRD1,DUSP10,E2F3,EARS2,EDAR,FZD2,GSK3B,GSTO2,HPRT1,IDI2,IMPDH2,MAOA,MGST3,MS4A2,NME4,NOD1,OR10H4,OR7D2,OR8B4,PDGFA,POLD3,POLR2K,POLR3A,PRSS2,RBX1,RELA,RELB,SFRP2,SOCS4,SPAM1,SPRY1,ST8SIA1,TAS2R4,TFPI,THBS2,YWHAG
Negative_5871	CBS,SPN,TK1,ACADVL,ARHGEF4,CCL25,CD19,CD244,CER1,DHODH,EXTL2,GAMT,GBE1,GSTA3,GTSE1,HEMK1,INHBB,IRS2,ITGB1,KCNB1,MARS2,NFKBIB,NME7,OR2T4,OR5C1,OR6K6,PLA2G4A,POLR3K,PTPRF,SARDH,SESN2,SPRY1,TAS2R10,ULK2,VAMP2,XYLT2,MAPK8IP3,SERPINA1,SERPINB5
Negative_5872	MET,ACSM1,AGPAT6,AMD1,AMPD3,BET1,CAMKK1,COQ6,CTSL1,DCXR,DERL1,ELK4,EPHA3,GADD45A,GALNT2,GGCX,GNAL,GOSR2,GPAA1,HLA-G,IDI1,IFNK,IL18RAP,LAMA4,LIPA,OR2T34,OR5AK2,OR5K2,PGAM2,PON1,PPCS,PRSS1,PTTG1,SETMAR,SGPP1,SLC2A4,TGDS,TJP2,TPI1,TRAF3,TSC1,UGDH,UGT2B7,VARS,PHOSPHO1
Negative_5873	C7,EBP,FES,GPT,LEP,MVD,PLN,ABL2,B4GALT1,CCR3,CLDN20,CNTNAP2,CREB3,DCTD,DGKA,ENTPD4,EVI1,FGF1,GALNT13,GPAM,HNMT,ICOS,ITGAV,KIR3DL2,LDHA,NAGLU,NRG4,OR10G8,OR10R2,OR2H2,OR2K2,OR2M7,OR2T11,OR5D13,OR6A2,PDGFA,PIP5K1A,PLAU,PRKAR1B,RUVBL1,SMAD6,SORBS1,TNFSF11,WEE1,TNFRSF10D
Negative_5874	INS,ACOX1,ARPC4,BMP5,BMPR1B,CBLC,CDK4,CHDH,COL1A1,CX3CL1,DDOST,FCGR3A,FGF1,FGFR2,FUT8,HAO1,OR3A1,OR6B3,PDE5A,PGAM4,PIK3CD,PISD,PLCB4,RASGRF2,SEMA3G,TAS2R16,UROC1,PAFAH1B1,PLA2G12A
Negative_5875	RDX,ADSS,CCNB1,DKK2,DUSP2,DUSP6,LAMA4,OR1G1,RALA,RFXAP
Negative_5876	IDS,ARPC1A,CAMKK1,CCNE2,CXCL10,DRD1,FUT5,GATM,GNPDA1,HRAS,IBSP,LIMK1,NCF1,OR1B1,OR7A17,PANK3,PCCB,PGLS,PPAT,PRNP,TP53,VANGL1,WBSCR17,WNT16,B3GALNT1
Negative_5877	ACTG1,ARSE,ATP5O,B3GAT1,CCND1,CCNE2,CHST11,CTNND1,DDX23,FMO5,HMOX1,IL1R1,KCNJ11,MTHFD1,NTSR1,OR52M1,OR6C1,OR6X1,OR9Q2,PRKAG1,PTK2B,SOCS1
Negative_5878	ARG2,CXCR4,FLOT1,GGT6,GNAZ,OR13C5,POMC,TNFSF12

Negative_5879	EGF,GCK,OSM,STS,ALDH3B1,AMDHD2,APOC3,ARPC3,ASNS,BCL2L1,CAV1,CLDN14,CLDN18,CYCS,DMGDH,ELK1,FZD8,H6PD,LAMC2,LCMT2,LIPA,MAP3K14,MYH7,NARS2,NEUROD1,NOD1,OR1L6,OR2T29,OR4F5,OR51G1,OR51I1,OR51V1,OR52A1,OR7A5,PANK3,PHKB,PISD,PTGS1,RRM2,SIAH1,SNAP25,SV2C,TYMP,VAV1,WHSC1L1,WNT11,XIAP,CSNK1A1L,TNFRSF10D
Negative_5880	ACVR2B,AGXT2,ALOX15,CYP3A5,ECHS1,GALNTL2,GGT1,GUCY2C,IL11,LFNG,LPAR1,OR10J5,OR2A42,OR2H2,OR4C11,OR6K2,PANK4,PKM2,POLR1C,PPA1,QARS,RALBP1,RARA,STAM2,ZFYVE9,ATP6V0D2,MAPK8IP1
Negative_5881	ALDH3A1,AOC2,ATP5D,BDNF,BHMT,BUB1B,CCL24,CPT1C,CTPS,DHRS9,DKK1,DOLPP1,FBP2,FGF4,FUT4,GLG1,GRIN2D,HTR2A,IMPA1,ITGB3,KIR2DL2,MAGI2,MALT1,MYL5,NCK1,NTRK1,OR52E8,PDE3B,PDE9A,PLAUR,POLD3,PPP2R2D,PTPRZ1,PYCR1,RALBP1,RASGRF2,RRAS2,SPHK2,TRMT11,TUBB,UROC1,WNT2B,ZNRD1,CSGALNACT1
Negative_5882	CTPS2,CYP2A7,E2F3,FBXW11,GCH1,GMDS,GMPR2,GNA14,RDH16,STAT5B,TCF7L1,ARHGEF12,ATP6V1G2
Negative_5883	LPL,NF1,SI,ACTA2,CACNB4,CANT1,CD38,E2F4,EFNB3,ESPL1,FARP2,GMPR,HADHA,IL18RAP,NCOA4,OR10C1,OR2M5,OR4A16,PIGK,PIGS,POLR3B,SH2D1B,SMAD4,SNAP23,TPH2,ST6GALNAC3
Negative_5884	ABO,TH,ATP5H,BAIAP2,COQ7,CPT1B,FZD5,GALNT8,HLA-DRA,KLK3,LTBR,MPZL1,OR10Q1,P4HA1,SULT2B1,VANGL2,VAV3,PHOSPHO1
Negative_5885	TKT,ARSE,ASIP,CASP10,CCL22,CCND2,COQ6,CYCS,CYP3A43,DAAM2,DUSP5,E2F5,FBP1,FZD7,GMPPA,HRAS,HSD17B2,IL28RA,INPP4B,LAMC3,MALT1,NNMT,NODAL,PDIA4,PGS1,PLA2G2D,RRM2B,STK36,WASF2,WBSCR22,XYLT2,CACNA2D2
Negative_5886	ADIPOQ,DLST,MMP7,NFAT5
Negative_5887	ACP1,ALS2,ARAF,ARPC2,ATP5G1,BCKDHA,CLCA2,CLDN11,COL2A1,CYP17A1,CYP19A1,CYP4F3,DDX23,ENO3,GDF6,GRLF1,IL12RB2,ITGB1,KIR3DL2,KLK3,NOX3,NTRK1,OR1J4,OR2AG2,OR2F2,OR4F5,OR5P3,OR6N2,OR7C2,POLR3H,PPP1R3C,PPP2CA,PRKCI,ROCK2,RPS6KB1,SMAD7,STAT2,TAS2R14,TCIRG1,TRAF5,UGT8,VANGL1,ST6GALNAC1
Negative_5888	GBA,IL7,WAS,ABI2,AK3L1,AKR1C1,ATF4,ATIC,BPGM,CARS2,CHDH,CHEK1,CLDN14,CLTA,DFFA,EFNA4,FZD6,GALNS,GANAB,HMBS,IQGAP3,LMO7,MIOX,NFASC,NFKBIA,OR4C46,OR51I1,OR5AS1,OR9A2,PPP1CB,RALB,RDH16,SIPA1,SOAT2,TALDO1,TAS2R14,VEGFC,WNT8B,XYLT2
Negative_5889	ABLIM2,ACSL1,BLVRB,CDH4,FARP2,INPP5E,ITGAL,MAPT,OCLN,OR10A2,OR13G1,OR1A2,OR4B1,OR4C46,OR52K2,PAFAH2,PAK1,PNMT,POLE2,PTPRZ1,RALA,RETSAT,RPN1,SERPINC1
Negative_5890	AQP7,CACNA1B,F11R,GSTZ1,IL20,NKD2,POLR1B,PRKAR1A,PSME2,PVRL3,SCD5,SLC9A1,ULBP1,YWHAG
Negative_5891	ATP4B,CD44,CDKN1A,CHRM5,CLTB,COASY,COMP,CSAD,CYP19A1,DNMT3B,DPYS,ECSIT,ETNK1,FPGS,IL10RA,LAMB4,LDHAL6A,LIPG,MAD1L1,MAP2K6,MCM4,NFAT5,OCLN,OR10G2,OR10S1,OR11G2,OR2C1,OR7G1,PNPLA3,PPP2R1A,RDH16,SLC25A4,SRD5A1,ST3GAL3,VAMP1,WNT1,WNT9B
Negative_5892	GNE,HK1,OSM,ACSL5,AGMAT,AQP7,CCL22,CCNB2,CD70,CHAD,CLDN17,CRY1,GSTA5,GSTP1,GYS2,HCCS,IFNA4,INPP5B,IPMK,LAMC2,MAP3K3,MAP3K7,MCM2,NAT6,NT5C3,OR2B2,OR2T10,P2RX5,PMAIP1,POLD4,PPCDC,PPP2R5B,PTGER3,SEMA4F,SETD2,SGMS1,SMURF1,TAP2,TYMS,ZMAT3
Negative_5893	ALDOA,CCL21,OR13C5,PLXNB2,STX1A
Negative_5894	ITK,ASPA,CRY1,ENPP1,GRB2,LIAS,OR51S1,OR5AN1,OR8K5,STAM,TUBA1A,WNT9B
Negative_5895	ATP2A2,FGF20,GALNT12,ITGB7,NRXN1

Negative_5896	GP5, CNTF, CYP26B1, DAAM1, FTCD, FZR1, GARS, GLUD2, HSPA2, HTR7, INHBC, INPP5B, LARS, NCF4, NTNG1, OR4D2, PGK1, PLD2, PPP1CB, RRAS, SARDH, SLC2A1, ST3GAL1, TLR4
Negative_5897	LPL, B4GALT3, FBP2, LAMA3, LTB4R2, OR2W1
Negative_5898	GNE, ALG10, ATP5L, BDKRB1, CAMK2G, CCND2, CREB3, DUSP1, FGF19, FMO1, GALNTL2, HRAS, KLRC1, KRAS, LALBA, LRAT, NOTCH1, NR1H3, OR10A7, OR10H3, OR51F1, PIGW, PLOD2, PVRL1, SEMA4A, SEMA4B
Negative_5899	IVD, PGP, ABP1, BACE2, C5AR1, CACNG6, CHDH, CHKB, DNMT1, EFNA3, ENO2, FDPS, FGD3, HSD17B8, IL15RA, ITPKA, MAP2K3, MAP3K3, MAPK11, OR10H1, OR8S1, PPP1R3D, SFRP4, UXS1
Negative_5900	AKR1B1, CLDN7, CYP3A5, EFNB2, IDH2, MAP3K11, OR10G8, OR6C4, OR7G3, PDE8B, PDHB, SMPD4, SPHK2, TUBA1A, TXNDC12, HSP90AB1
Negative_5901	IDS, CASP10, CD226, CDK5, CFL2, CHRM5, CISH, CRY1, DDIT3, GNRH1, HSD3B2, IQGAP3, LMO7, MLLT4, MTMR6, OR4D6, OR8G1, PNLIP, PPBP, WHSC1L1, WNT9A, MAPKAPK3
Negative_5902	CSF2RB, EPRS, GNRHR, GOT1, ITGAD, MYH10
Negative_5903	AK2, GDA, JUP, ZYX, ABLIM1, AGXT, CD55, CDH3, CHKB, CXCL10, CXCL12, CYP2S1, DPYD, ESAM, FGF21, GNA13, GRIN2C, H6PD, HMBS, HNF1A, HSP90B1, KYNU, LCMT1, MLLT4, NEU1, NMNAT3, OR51A7, OR5D16, PARVA, PARVB, PGM2, PLCD4, PYGB, RUNX1T1, SMAD2, TNFSF8, TXNDC12, UCK2, ULK2, WASF3
Negative_5904	PC, ASIP, B4GALT3, CACNA1A, CHRM3, CNTN1, FIGF, HBEGF, IL25, JAK2, MCAT, MGLL, NCAM2, OR2AG1, OR2D2, OR2T8, OR8I2, PRKAA2, SETD7, ST8SIA5, STAT6, STEAP3, MAPKAPK3
Negative_5905	GLA, ACHE, AKR1A1, APLP1, ARPC5, ATP6V1H, CCL7, CLDN8, CYP3A7, DDX55, FGD1, GBE1, GPAM, IL18R1, LIPF, LYPLA2, MLLT4, OR8U1, STAT4, SUCLA2, CTNNBIP1, HLA-DRB4
Negative_5906	FH, PVR, ACY1, ANGPTL4, ARPC5L, ARSE, ATP5H, B3GNT2, CASP10, CCL3, CDK7, CES7, CLDN16, CLDN9, CNGA4, GCLM, GNAI1, GNAI3, GSTA1, IDH3B, IFNA13, IL1R2, IL22RA1, IL29, IL9R, IPMK, LEFTY1, NRXN3, OR13G1, OR51E2, OR52N1, OR8U1, PAX6, PCK2, PGK2, PHPT1, PIM1, PRKAB2, RASGRP1, SEMA4A, STAM, TAS2R5, TLR3, TRIP10, ULK2, MAP3K7IP2, TNFRSF11A
Negative_5907	TAT, UBC, ZYX, ACVR1B, ALG10B, ATP5J, CFLAR, CHIA, CHKB, COL5A2, CYP26A1, FGF9, HPRT1, MAML3, OR51T1, OR5P3, PFKFB1, PLEKHA8, PNPT1, SDHB, SEMA3D, TNXB, CSNK1A1L
Negative_5908	ACVRL1, ATP6V1F, CCNA2, CSNK1G1, FZD4, IRAK2, KLRC3, MAP3K3, PLCD4, RAC3
Negative_5909	VTN, ATP6V0C, CDC20, CHIA, COL5A3, CTBP1, CTNNA2, DUSP9, ESAM, FGF1, GALNT7, ITGAD, NCR3, NKD2, OR2C1, OR2T4, OR5AR1, PIK3R5, PMM1, PRIM2, SARDH, SETD8, SOCS7
Negative_5910	ADC, AK7, FOS, ID1, IL5, ALOX12, ATP2A3, B3GAT1, CD79B, CDKN2D, CHPT1, DOLPP1, FUT2, FZR1, GCNT1, GLDC, HIP1, IFNB1, ITPKB, KCNB1, KLKB1, LDHAL6A, LRDD, MDM4, MKNK1, OR1D5, OR52N4, OR56A4, OR5A1, OR5B21, ORC1L, PDPK1, PLEKHA8, PLK1, POLA2, POLD3, POLR2L, PPP1R3B, PRKX, PTGES, RXRB, SETMAR, SOCS2, ST3GAL1, STK4, SULT1A2, TLN2, TOMM40
Negative_5911	DCK, MAX, AANAT, ACOX3, ADCY1, AMPD3, APOA1, BAIAP2, CARD11, CD274, CSF3, CYP2C19, GALK1, GUK1, HSP90B1, IL4R, LIPC, LRP6, NARS2, NCF4, NPR1, OR10G9, OR4F15, OR4S1, OR51B2, OR56A1, OR5D18, PLD1, PLOD3, PRKAA2, RAP1A, RARS2, RELA, SCD5, SLC2A1, SMAD2, SOS1, TAS1R1, TPI1, CSNK1A1L
Negative_5912	AR, CAT, F11, FOS, ILK, LSS, MET, TPO, ACVR1, AKR1D1, ANAPC10, AXIN1, CCL16, CD36, CYP1B1, CYP4A22, DIAPH1, DKK2, FGF12, FUT8, FZR1, GCLM, GNA15, INADL, LAMB1, MAOB, MPZL1, NEU1, NOS3, NRG4, P4HA2, PPCDC, PRIM2, SPRY2, SPRY3, TOMM40, UCK2, YES1, SERPINF2, ST6GALNAC1
Negative_5913	CNTNAP1, STX18, WNT7A

Negative_5914	AGL, ACHE, ATP2A1, BACE1, CCR4, CISH, GNA11, HGSNAT, MCCC1, MCM3, MGAT2, NTSR1, OCLN, OR13H1, OR1G1, OR2A14, OR8K3, PIGM, SDHB, TP73, ATP6V0E1
Negative_5915	TPR, ACOX2, AGRN, BAI1, BTRC, CDKN1B, CLCA4, CREB1, CXCL9, CYP1A2, DBF4, FCGR3A, GALNTL5, HARS, MAP2K6, OR5H6, OR8K3, PNMT, PRKACG, PTPRM, RBKS, TUBB3, UGT2B4, VEGFA, ZFYVE16, CSGALNACT2
Negative_5916	C4B, GNE, BDKRB1, CACNB4, CASP6, CCR5, CHUK, FCGR3A, FGFR2, GSTP1, GUCY2C, IL19, ITGA10, MGLL, MRAS, OR5D14, OR8A1, PAK1, PCYT1A, SIGLEC1, TAS1R3, TBXA2R, TKTL2, B4GALNT1, HSD17B10, PAFAH1B1, ST6GALNAC3
Negative_5917	APLP1, ATN1, ATP6V1D, B3GAT2, BCL2, BDKRB1, CACNA1D, CD36, COL4A2, CYP26B1, DNMT3L, FGF19, FTMT, FZD10, GCNT3, IRF9, NFATC4, NFKBIE, OR10Z1, OR4N5, OR5B3, PAK3, PDE7A, PER2, PLD1, PTK2, RASGRP1, RRAS2, SAT1, SEMA3A, SMAD4, TAS2R49, UGT8, VNN1, VTI1B, WHSC1L1, ZAP70
Negative_5918	CS, ID3, MYC, SMS, TPR, ADCY3, ALDH2, ATP5G1, C1GALT1, CACNG1, CDKN1A, CNTNAP1, COL6A1, CXCR4, CYP4F3, DIAPH2, ENO2, F2RL3, FGF6, G6PC2, GALNT10, HADHB, IFNA8, ITGAE, JAG2, LDHD, NADSYN1, OR2C1, P4HA3, PPP3R2, PRKAR1A, PRKCA, RUNX1, SEPHS1, ST3GAL4, THBD, TNFRSF4, UBA1, VAMP1, WWP2
Negative_5919	HK3, NF1, RDX, ALG13, AMHR2, APAF1, ATP6V1A, C3AR1, C3orf10, CCNE2, CFL1, CYSLTR2, DLAT, ENO2, ENPP3, EPHA3, EPHX1, GADD45A, GPD1, HSD11B2, IL11, KDSR, KYNU, MGLL, MYH7B, NLGN2, OR51B4, OR51I1, OR51L1, OR5P3, OR5V1, OR8A1, PGS1, PTK2, RASGRF2, RBL1, SETD2, SIAH1, SKIV2L2, SRGAP3, STAT1, UCK2, UNC5D, WNT2, XYLT1, ALDH18A1, ATP6V1G3
Negative_5920	GNS, PLG, PRL, TK2, ACOX2, ACY3, ADAM17, ADH5, ALDH1A1, ATP5O, CERK, CHDH, CNTNAP1, CREBBP, CTF1, DAAM2, DDX41, DDX52, EPHA2, GALNT7, GPLD1, GRIN2A, HTRA2, IKBKB, IL15, IL1R2, INPP5E, LAMB4, LFNG, MAOB, MDH1, NME7, OR2A12, OR52L1, P2RX4, PAK2, PANK1, PDE7B, PLXNB2, POLR3D, PPAT, PPM1A, PRIM2, SEMA3C, STAT5B, TNFRSF4, TOMM40, UBA7
Negative_5921	LYN, CHST11, CYSLTR2, FARP2, FGF8, GUCY2C, LMO7, PKM2, STX5, TNFRSF9, YOD1, TNFRSF6B
Negative_5922	F2R, HGF, BBC3, CBLB, CCNB1, CLDN16, GUCY2F, IL2RB, IRAK3, LIPT1, MGAT5, MYH9, NFATC4, OR10A5, OR52E6, PLA2G1B, POLD4, TAS2R3, TNFSF18, TPI1, SERPING1
Negative_5923	TPO, AVPR1B, EXTL1, PFAS, RAF1, TUBB2C
Negative_5924	ILK, ACTN2, E2F2, EPRS, EXT2, GOT2, GPD2, HTR7, IARS, IRAK3, MAOB, MAP2K3, NT5C1A, PDGFC, STAT2, TRAF3, TUBB2C, UPP2, ATP6V1G3
Negative_5925	DDC, F12, FER, FGA, ACADVL, ACSM2A, ADRBK2, ANAPC5, CDC6, CLDN8, COL6A1, CRKL, CXCL16, ERO1L, EXTL1, FMO4, FRAP1, HEMK1, HIBADH, HSD17B2, IBSP, IFNA10, ITGB1, MAGI3, NFKB1, NFKBIA, NOX1, NTNG1, OR10A3, OR10H2, OR13C4, OR2A42, OR6K6, PDE8A, PIGH, POLR2G, POLR3H, PRKAA2, PSENEN, PTDSS1, RASSF5, THBS1, TNFSF8, CSGALNACT1, TNFRSF12A, TNFRSF1A
Negative_5926	ALAS1, EDA2R, MINPP1, NAE1, SETD1A, TYROBP
Negative_5927	DBT, ARPC3, BMP2, CLDN20, CRKL, DAAM1, FGF3, HNF1B, ICOSLG, OR52N1, PIK3C2B, PTPRR
Negative_5928	CTH, FOS, LSS, ADCY10, ALS2, BMP5, CCKAR, CHSY3, CLDN3, DCXR, DGKZ, ENTPD8, FUT3, GCH1, IFNGR1, IMPA2, LAMB4, MCM3, MYL2, OR2M5, OR51F2, PDE3B, SETMAR, SHISA5, SLC2A4, TRADD
Negative_5929	CKB, FN1, KIT, SFN, ACAD9, ACOT11, ADH7, ARSD, BDNF, BMP8A, DGKI, DOT1L, DPM1, EXTL3, FABP4, FPGS, GALNT10, GNAI3, GRIN2A, IL22, KLRC2, MMP1, MMP14, MYH10, OR1L3, OR7D4, PIGK, PROC, SOX17, SPTAN1, ST6GAL1, STK36, TAP1, TRADD, TUBA1C, WARS2, GABARAPL2
Negative_5930	PC, ATP5D, ATP5G1, CACNG6, CXCL9, HMBS, HTR5A, JUND, MYH11, NME6, OR11H1, OR52K1, PLEKHA8, POLD3, PRKAB1, RALA, SKP2, SNAP23, TJP3, VAMP3

Negative_5931	TKT, ABCC8, ACACB, B3GNT4, CHPT1, COQ3, CSNK1G2, EPOR, FASLG, FGF9, IL10RA, IL1B, ITGAX, OR2G2, OR4A16, OR5L2, PRKCH, SGMS1, SPRED2, STX1B, SUFU, TUBB6, VANGL2, ZAP70
Negative_5932	EPHA5, IL12RB1, OR8D2
Negative_5933	CDK5, CPT2, CYP27A1, IL18RAP, NMNAT3, SERPINC1
Negative_5934	ADA, ADH7, ARSB, DRD1, GSTM4, IGFBP3, NUDT5, OR6C75, PRSS3, RERE, ROBO2, SAT1, SH2D1A, TAS2R48, TNFRSF10B
Negative_5935	GAA, IL8, TYR, ACSL4, C3orf10, CACNA1S, CSNK1E, DDX55, EXOC7, ICAM1, IL23A, LAMB4, LTC4S, MECR, OR1F1, OR1S1, OR6A2, OR6M1, PLXNA1, POLR2L, PSEN2, ST3GAL6, VDAC3, WNT6, YWHAE
Negative_5936	C5, PLG, DAG1, FPGS, GLI1, NTRK2, OR51M1, PIGS, PIM1, SPHK1, STX10
Negative_5937	AGL, ATP5F1, CAMK2D, CCNB3, CCNG2, CCR9, CD99, CSF2RA, CYFIP2, EGLN1, GNAI2, GOT1, GSTA4, IFNK, MAP4K4, MTMR6, OR11G2, OR1L8, OR2C3, OR4C46, PFKFB2, PLA2G2E, STAT6, YWHAQ, PHOSPHO1
Negative_5938	AK3, HK1, OGT, VTN, ACP5, ADCY3, AMPD3, ASCC3, CCL25, DAG1, ERBB2, FGF21, FOXO1, GBGT1, INPP1, ITGA2, ITGAL, LAMB3, LILRB3, MAP3K2, METTL6, NFKB2, NOTCH1, OR2W1, OR4K2, PHKA1, PRKX, PRNP, PTGES, RFXANK, SRD5A1, ST3GAL6, XYLB
Negative_5939	GP6, SRM, ACAT2, ACVR1C, AKR1B10, AREG, ATF4, CACNA1C, CALML6, CLDN14, DCXR, EPB41, EPHA4, FGF8, FZD10, GJD2, GNB1, ICAM1, IFNAR1, ISYNA1, ITGAD, ITPA, LEF1, MAOA, MGST3, MTHFD1L, OR10H4, OR1C1, OR2F1, OR51B4, PHKB, PSME1, PYCR1, SGMS1, STMN1, TM7SF2, TRMT11, UBE2K, VANGL2, XYLT1
Negative_5940	SMS, CALML5, CASK, CCR2, CDKN2C, CYP2B6, GYS2, IFNA14, KYNU, MDH2, MTMR1, MYH2, NFKB2, NFYA, PFKL, PLA2G3, PLA2G6, POMC, SEMA4B, SLC33A1, TAS2R4, TAS2R9, THBS3, TIRAP
Negative_5941	AADAT, CYP26A1, IL18, IRS2, OR2D2, OR4K2, P2RX6, SETD1A, TSNARE1
Negative_5942	AGPAT6, BTRC, CNGA4, DGAT1, GALNTL2, GP1BA, HTR4, IL15, IRAK1, OR1M1, SMAD6, TCF7L2, WASF2, HLA-DRB4, SUV420H2
Negative_5943	CSNK1G2, CTPS2, FZD4, HBEGF, TNFSF12, WNT2B, PAFAH1B1
Negative_5944	PTS, AMHR2, BET1L, CLDN14, KCNQ1, KIR2DS5, PCNA, RAPGEF3, STAT6
Negative_5945	DHH, HPD, MPI, ACTG1, ADCY1, AMDHD1, CASP7, CHRM2, CTLA4, CYP3A43, ENO2, FARP2, GGCX, GGT7, HTR4, IL2RG, IRS1, MAP2K4, MGAT4B, MYH1, NANP, OR10A7, OR14C36, OR2T34, PCNA, PDE4B, PIGH, PIP4K2A, PRKAR2A, PRKCA, PRKCI, SESN3, SETD8, SOCS1, SPTAN1, SRD5A1, TKTL1, TNFSF8, VAMP3, WIF1
Negative_5946	LEP, AMY2A, ARAF, EVI1, GP1BA, IL1R1, JUND, MASP1, NEUROG3, OR2Y1, SLC25A4, TSHR
Negative_5947	ATP4A, CD47, FUT7, GMPPA, GSTA5, HPSE, IL8RA, INPP1, MAP3K3, NEUROD1, OR4K13, OR8G1, UCKL1
Negative_5948	A2M, BTD, IHH, LCT, MVD, RET, AASDH, ACYP1, BPGM, CDC2, CDKN2B, CX3CR1, DERL1, DRD2, DUSP7, ENTPD5, ETS1, FGF11, FTMT, GGCX, GJA1, IL1A, IMPA1, INMT, JUND, MGAT5, NAGLU, NMNAT3, OR10J1, OR10X1, OR11L1, OR13J1, OR1L1, OR4E2, OR5L2, OR7A5, PDE1B, PFN4, PPAP2C, PPP2CB, RALBP1, RASSF5, ROCK1, RPRM, SDC1, SETMAR, STX12, SYNJ1, SERPINA5
Negative_5949	DBI, VCL, ACHE, ALG12, ATP5L, CACNB3, CHSY1, CMPK2, CPS1, CXCL6, CYP1B1, DGKI, DUSP14, DUSP5, FARSA, FPGT, FUT8, GMPPB, GNAZ, HLCS, HSD17B2, ITGAD, LAMC2, MAPK13, NANS, NT5C2, OLAH, OR14A16, POLR3K, PRKACG, PRPS2, RALBP1, RAP1A, RHOD, RIPK1, SFRP4, TAP1, UGDH, UGT2B4, ULBP3, TNFRSF10C

Negative_5950	BID, BTC, C2, CKM, DBH, GGH, GNS, ACSS1, ACSS2, ACVR1, ADAM17, AKR1C2, ALG10B, ARG1, ARRB1, BCAR1, CCR5, CHRNA7, CMPK1, CSNK1G3, DGKA, DHCR24, ERBB3, GAD1, GMPR, HMGCL, HMOX1, HNF1B, HSD3B1, JAK1, JAM3, KCNQ1, LAMC1, LLGL1, OR2M7, OR8I2, OR9G1, P4HA1, PCCB, PIK3CG, POLA2, PPP1CC, TAS2R42, TKTL2, TUBB3, UBA1, YKT6
Negative_5951	ATP5A1, IDI2, ITGAV, NFX1, QARS
Negative_5952	MVD, TK1, ALG5, APAF1, ATP5I, AXIN1, BAAT, CALML3, CBR1, CD22, CD70, CLCF1, CYP2C9, CYP4F3, DFFB, DGAT1, DIAPH2, FGF2, GAD2, HSPB1, IRF3, ITPA, LARS, OR13F1, OR2L8, OR51A4, OR51I1, OR6S1, PAK7, PDHA1, PDHA2, PGK1, PLK1, POLR1A, PON2, PVRL3, RDH12, SSH1, STT3B, TSLP, UCKL1, ULK1, YARS2
Negative_5953	DHH, ALDH3B1, ASS1, BMP5, CD247, DAXX, DLAT, DUSP9, FZD3, GATM, IL17RA, ITGA10, MAP2K4, MGAT3, OR10AG1, OR51G2, P4HA2, PDE4B, PDGFC, PIGP, PIK3CD, POLD3, PPBP, PPP2R5D, PRKCZ, PTEN, SARS, SLC25A4, SLC27A4, SRGAP1, CSGALNACT1, HLA-DRB3, SUV420H2, TNFRSF10B
Negative_5954	CBL, ILK, ALOX15B, BHMT, CACNG7, CALML3, CCKAR, CNTNAP2, FABP1, FUT2, GNAT3, HAGH, HRH1, HSD17B8, IL12A, IL13RA2, MAD2L2, NFKBIA, NR4A1, NRG3, OR1N2, OR5H2, OR6C70, PIM1, POLR2J
Negative_5955	DCK, TFG, ALDOC, ATG7, ATP6V0C, B3GNT3, CAV3, CCR3, CD22, CDO1, CTPS2, DNMT3B, FADD, FGF5, FOXA3, FTH1, GALK2, GNAI1, GSTP1, HGSNAT, HYAL1, ITPKB, MCCC2, MCEE, NFATC4, NUMBL, OGDHL, OR52E2, PDHB, PPP1R3C, QPRT, RBX1, SDHB, SLC33A1, SNW1, XIAP, CACNA2D3
Negative_5956	LSS, ACTN4, ALG10B, ARHGAP5, ASMT, BDH2, CACNA1A, CSNK1G1, ECHS1, FADD, FGF14, GRM1, HADHB, HAGHL, HEXA, HSD17B8, KAT2A, MAP2K5, MCCC1, MYH11, NRG4, OR10H4, OR6C76, PKMYT1, PRKAB2, PSAT1, STAT4, TLN2, UGT2B4, UPRT, TNFRSF12A
Negative_5957	BCR, CD4, ADCY10, ATP6V0B, CCND3, CRKL, DUSP14, EDAR, ENPP1, EXTL2, GNA14, KLRK1, LLGL2, LTBR, MAP2K2, NADSYN1, NEUROD1, OR10G3, OR13C8, OR2M3, OR2M5, OR9G4, PMVK, PRKAR2B, RAB5A, WNT8B
Negative_5958	C1S, OGT, SYK, TAT, ADCY8, ADRB3, ANAPC4, BMP7, CCR4, CD46, CDH4, DHDDS, EGLN1, EPOR, GOT1, GSTT1, IFNG, MGAT1, NADSYN1, OR10A3, OR10K2, OR4D9, OR52E6, OR8S1, PCK1, PLCB2, PTPN11, ROCK1, SARS2, SLC12A2, TBL1XR1, THTPA, UNC5A, ZMAT3, HLA-DRB3
Negative_5959	LPL, UBB, CCL8, CFLAR, GUCA1C, IRAK4, MGAT1, NTF3, PGM2, UBE2L6
Negative_5960	LYN, ACSS3, ALG6, ALPI, AOC3, CAV1, CCL16, CDC7, CHEK1, CLDN2, CYP19A1, CYP4F3, CYSLTR1, FGF14, FGF9, GADD45B, GALNTL5, HAGH, HLA-DMA, HSD11B1, IKBKG, IL13RA2, IL1RAP, IL2RA, IL7R, ITPKB, NKD2, OR10T2, OR5AC2, OR6K2, OR7G3, PFKFB4, PPP2R2B, PSPH, RAPGEF4, RFX5, SCD5, SHC3, SRGAP1, STK3, STK36, STX7, TRMT11
Negative_5961	NNT, PC, ACAD9, ACVR1B, BCL10, CCL24, CDC25C, DGKH, DPYD, DTX1, DUSP7, E2F5, ERO1L, FOXA2, GALNT11, HAGHL, HIBADH, INSRR, ITGA2B, LEFTY2, MIOX, NFYA, NOTCH2, OGDH, OR4K13, OR4K2, OR52N2, OR8I2, OR8K3, PDPK1, PIK3R2, PLAU, PRKAB1, SAT1, SDHB, STAT5B, TAS2R7, TBK1, TNFSF10, VTI1B, WNT6, HLA-DRB4, MAPK8IP1
Negative_5962	ADC, CCL26, CPT1A, CPT1B, DEGS1, DHFR, EXOC7, FUT9, GALE, GCH1, GNA15, HAP1, HMGCR, IAPP, IL10RA, ITGAM, KARS, KNG1, MMP7, NPAS2, NQO1, OR10A6, OR1A1, OR1S2, OR51L1, OR7D4, PAK6, PLOD1, POLR2E, PPP1R3A, RAP1B, RFXANK, SDHA, THY1, TIAM2, SERPINA5
Negative_5963	GP5, ACADS, BMP2, CCR4, COX15, F2RL3, FOXO3, GSTM4, GUCY1B3, IGSF5, ITPA, PGAM2, PLCG1, POLA2, SUCLG1, SERPINE1

Negative_5964	C1S, C9, DDO, LSS, APOA1, ASPA, ATF4, ATP6V1A, B4GALT3, BCL2, BHMT, CACNG2, CLDN5, CYP26C1, DHRS9, DPAGT1, FDFT1, FIGF, GPAM, HSD3B2, ITGA5, LCP2, LMO7, MCM2, MMP2, NPR2, NR4A1, OR11H1, OR13C4, OR1J2, OR2AG2, OR5AS1, OR6C4, OR7C1, POLR2D, POLR3A, PON1, PRPH2, PTPRM, PYCRL, SDC4, SENP2, SLC2A2, SMC1B, TYROBP
Negative_5965	CACNA1S, CACNG6, CNTN2, DHDDS, DPM1, DUSP4, EPHA8, FASN, GSTO1, MOV10L1, NCR3, OR11L1, OR2AG2, OR4C15, OR4F6, PDPK1, PLA2G4A, PRKCZ, SFRP5, SH2D2A, TRMT11
Negative_5966	LIF, PGF, ACOT12, AHCY, CDH1, CHIA, CLDN4, CSF1R, DGKZ, GADD45A, IDH2, IL1RAP, ITGA2B, ITGB1, KCNB1, MGAT5B, NSD1, OR2V2, OR51A4, P2RX1, PDE2A, PDXP, PGAM4, SH2D2A, SMAD6, SMURF2, SPTLC2, VKORC1, YWHAH, HSP90AA1, MAPKAPK5, SERPINB5
Negative_5967	ADC, ID3, NGF, ACVR1B, APOC3, CACNA1B, CDC14A, CDKN2C, CNGA3, COMP, EHMT2, ENAH, FBXW11, FRAP1, GDF6, HTR5A, LFNG, MYLK2, OR2F1, OR56A3, OR5P2, PAICS, PDHB, PHKG2, PIP5K1A, POLR1D, SESN1, SHMT1, SLC2A1, STAT6, TNFSF18, TOMM40, WHSC1L1, YWHAE, TNFRSF10A
Negative_5968	ADK, FGG, GP5, ACP1, ACY3, ADRB2, AGXT2, BDH1, CALML5, CCL24, COL5A2, CREB3L3, CXCR5, FGF12, FMO5, HMGCS1, ITGB8, NCAM2, NCOR1, NR4A1, PDE7A, PIK3C2B, PLA2G3, RARS, SFRP4, TAS2R40, VAV3
Negative_5969	DCN, DLD, FN1, KHK, ALPL, AOX1, ATP6AP1, C3orf10, CACNG6, CANT1, CBLB, CIITA, CLDN3, CREB1, CTLA4, DGAT1, EGLN1, EPHB2, GBE1, GNA14, GRLF1, GSTA5, GSTP1, HMGCS2, ICAM1, IFNA10, IGSF5, IL28B, ITGB1, LEFTY2, LIAS, MMP2, NCR2, NEFM, NMNAT1, OR2G2, OR4X1, PAK4, PDE7A, POLA1, POLR3G, RAP1B, SNAP23, SSX2IP, TUBB2B, WARS2
Negative_5970	FUK, TFG, TKT, ALAS2, B3GNT2, B4GALT7, CCR5, CHKA, CLDN9, CNTNAP2, DDX23, DFFA, FGFR2, GABARAP, GGPS1, GLI1, GNPDA1, IL23R, INMT, ITGA10, MYH2, NCOA4, OR11H4, OR12D3, OR1L8, OR2AG2, OR2K2, OR4S2, PLD1, PMM1, POLR1B, PRLR, PVRL4, ROCK1, RRM2B, TRAF4, YARS, CSGALNACT2, HSD17B12
Negative_5971	NGF, ACSL1, AMY2B, AREG, CDC2, EXTL1, GAB1, IL19, PKM2, PPP2CA, TAS2R48, TCF7L2, WIF1
Negative_5972	AKR1B1, ATP6V0C, BPNT1, CRY1, DGKB, ENTPD5, GALNT1, GUCY1A3, HSD3B7, ITGA9, KIR2DL1, MTMR1, NFKBIB, P2RX2, PCYT1A, PIGU, PPAP2A, TAS2R16, ZFYVE16
Negative_5973	CTH, INS, ACVR2A, ADH1B, ADRB1, ALOX5, CD46, CNTFR, CSNK1E, CTBP1, EPHA1, FGF14, FSHB, G6PD, GART, HLA-DOA, IL18R1, IRAK4, LIMK2, NAMPT, OR10G3, OR51B6, OR52I2, OR8D2, PDE9A, PHPT1, PLA2G5, PLCZ1, PPAP2B, PPP2CB, RALA, RPS6KA6, SETD1A, SH2D1B, SHMT2, SLC2A2, SULT1A4, SUOX, TAS2R5, TGFBR2, TNFSF4, TRAF4, UCP1, UGT2B4, WNT8A, YWHAQ, KIR2DL5A, MAPKAPK5
Negative_5974	C8A, DAO, GNE, RPE, WAS, ABP1, AKT3, CD8A, CDC26, CXCR6, ENPP1, HDAC2, LAMA4, MAP3K12, MS4A2, NRAS, OR2A4, OR8K3, PIK3C2B
Negative_5975	HYI, AGPAT3, AMY2B, DHDDS, F13A1, FMO2, GLG1, GLUD2, HAGHL, HSPA8, INSRR, LDHAL6B, OR5AN1, OR8D4, ORC1L, PAK1, PDE4C, PRKCE, TBL1Y, TJP2, SLC25A31
Negative_5976	ADO, ACP1, APOE, CACNA1S, CD40LG, CYP4F3, DGKD, FGF22, FMO3, GARS, GDE1, GNPDA1, HMGCL, IFNA2, IRF3, MAN1A1, NFATC2, NFKB1, OR1K1, OR4C15, OR52N1, OR5B21, OR6C4, P2RX3, PIAS4, PIGW, POLR2C, SPHK1, TAS2R3, TKTL2, HSP90AA1
Negative_5977	DDO, SDS, CD79A, CHDH, CHPT1, CYP2C19, CYP3A5, ENTPD3, FLT3LG, GANC, GLG1, IL28RA, IPPK, ITGA9, NRAS, NSDHL, OR1D2, OR51A7, OR51I2, PFKM, PRPH2, PRSS2, PTPN5, ATP6V1G3
Negative_5978	NNT, IL2RA, MYL9, PRODH, SRD5A1, TAP2

Negative_5979	ACP6,B3GAT1,BDKRB1,ERBB2,GNPAT,IFT57,IL21,INSR,MAN1A2,OR13D1,PIP4K2C,POLR2A,PPCDC,TICAM2,TRPC1,UBA7,ST6GALNAC3
Negative_5980	ADO,PLG,SMS,ADRA1D,B3GALT2,B3GAT2,CCR9,CHRM2,CHSY3,CYP26C1,CYP3A4,FASN,GGPS1,GMPR,IFNAR1,LYPLA1,OR2L3,OR5L2,PARD6A,PDGFC,PLA1A,POLE,RAPGEF2,ROBO1,SCLY,SNAI2,SOAT1,SULT2B1,TP73,WNT2,HLA-DQA2,ST6GALNAC6,SUV420H2
Negative_5981	ATM,GK2,PXN,SP1,AARS,BCL2,CCL14,CCR9,CER1,CLCF1,DPYSL2,ECSIT,EDAR,EHMT2,FUT4,GCNT3,GJA1,GOT1,IFNG,IQGAP2,MAGI3,NCOR2,NKX6-1,OPLAH,OR10K1,OR10T2,OR1S2,OR2T34,OR51B6,PARD6G,PIP5K1B,PISD,PLA2G1B,RBPJ,SOAT1,TSNARE1,WARS,B4GALNT1
Negative_5982	AADAC,ABLIM2,ACSM2A,AK3L1,ANAPC4,CANT1,CD27,CDH1,CREB3L2,CYP11B2,DAXX,DGAT1,EFNA5,EGFR,ENTPD5,F11R,FADS2,GOT2,GPAM,HNMT,IQGAP1,LYZL1,MAPK14,MDH2,MOV10L1,NUDT5,OR10S1,OR4K15,ORC6L,PIGW,PLA2G2D,PTPRR,RBPJ,SARDH,SPRY1,STK4,TLR4,TPH1,TRAF3,VAV2,ATP6V0E1,TNFRSF12A
Negative_5983	AXIN1,CDH3,CXCR5,ESPL1,FGF11,GDF6,HSD17B8,ITGAX,OR2T3,OR4F5,SMARCA2,TSHR
Negative_5984	AK2,CR1,GSR,PLN,TPO,ACPT,ACYP2,CCR6,CD79B,CRY1,CUL1,CYP17A1,DBF4,ENTPD8,FTCD,GALNT9,GUCA1A,HMOX1,IFNA8,IFNK,IL12RB1,IL25,LCMT1,MAPK11,MCM3,MGAT4B,NCK2,NUDT5,OR10J3,OR4K17,OR51A2,OR52B6,OR5B17,OR6Y1,PANK4,PARVG,PDE1B,PLXNB2,PPP1R1A,PRKCE,STX1B,TP53,TRDMT1,TRIP10,WASL,WNT2,YKT6,ZMAT3,TNFRSF14
Negative_5985	MUT,ADSS,ALDOA,CALML6,CDC23,CKS1B,FGFR1,GCDH,HNMT,PLOD1,SKIV2L2,UGT2B11
Negative_5986	LPL,NF1,CREB1,IL13RA1,PIGK,PIP5K1B,POLE3,ROBO1,SEMA4B
Negative_5987	CR2,HAL,PDC,ACADM,ADRA1A,ARRB1,BACE1,BUB1,CASP7,CCL28,CES2,CHAD,CRKL,CSNK2B,CYP11B2,DNMT1,E2F5,FZD1,GRIN1,HIP1,IL15,IL20,INADL,LFNG,LIMK2,MAP2K6,MASP1,MDM4,NCR1,NRP1,NT5C,OR10X1,OR13C5,OR4D9,OR4K5,OR5M3,OR5P2,OR9K2,POLR2I,PRKCI,SMC1A,SOCS1,TIAM1,ATP6V1B1,PPARGC1A,TNFRSF14
Negative_5988	FGG,ACADVL,B4GALT7,BDNF,CACNG8,CFTR,FECH,GNAT3,GNRH1,IL24,MYH9,NMNAT3,OR2T34,OR5M9,PDE3B,PDGFRB,PPP2R5D,PRKCA,RGS3,ROCK1,SETDB1,STAT4,TPI1,UGT2A3
Negative_5989	AMY2B,CYP2D6,HNF4A,IFNA6,OR2T10,ORC2L,EIF4EBP1,TNFRSF11B
Negative_5990	CBL,TNC,ACOT12,ADCY4,ALG6,ARPC5,C1QC,CCL1,CCL4,CD40LG,CPT2,DHRS4,E2F5,ENTPD7,EPAS1,GADD45B,GRM1,HNF1A,ITGA6,LRP6,MDH2,MYH10,NLGN3,NR1H3,NSD1,OR10J3,OR4K5,PANK2,PDE3A,PGK2,PMM2,PPM1D,PPP1R3B,PRKAB1,PRPH,RASA2,UCK1
Negative_5991	LIF,TNC,ADAM17,AMPD3,ASPA,BIRC2,COL2A1,CYP26A1,CYP2A6,IL12A,INSR,ITPA,KLKB1,MAP3K1,MRAS,NLGN2,NUDT8,NUDT9,OR1M1,OR2F2,OR4F17,OR4K13,OR52W1,OR5L1,PGM1,PIAS2,RARS,RDH5,SPRY1,TAP2,TMLHE,EIF4EBP1
Negative_5992	HAGHL,OR2T3,OR5R1,WASF3,WWP2
Negative_5993	MAX,ARNTL,ASIP,CCR3,EARS2,GSTM5,LALBA,NAE1,OR1A1,OR4N5,PIPOX,TICAM2
Negative_5994	CXCL11,GALNTL4,GNAI2,OR4D5,OR5L1,PIGH,PTPRB,VAMP1
Negative_5995	ACO1,C1GALT1,DGUOK,IL10RB,IL20RB,NFKB1,PGS1,RBPJ,VDAC2
Negative_5996	B4GALT3,CCNB2,CHPF,CHRD,CLDN1,CSNK2A2,FZD9,GART,GGT6,GLI1,GNMT,OR56A1,POLE,PPP2R2C,RASSF5,SGMS2,STX7,TSC1

Negative_5997	PVR, AACS, ALG1, ALG11, B3GALT1, BPNT1, CCNB1, CCNH, CCR7, CD3E, CD82, COL1A1, CXCL3, DUSP3, DVL3, EPHB1, GLI1, HNMT, HSD3B2, IFNA7, NCR3, NKX3-1, NOX1, OR5AS1, OR5T1, PANK3, PANK4, PLCZ1, RFXANK, RPS6KA6, RXRB, SELE, SETDB2, SMARCA5, SMURF1, STX2, TAP2, TNFSF12, TRAF4, ATP6V1B1, CACNA2D1
Negative_5998	APP, CAT, CBS, JUN, ACHE, ACP1, ANAPC1, B3GNT2, CCR5, CCR6, CDC23, CSNK1G3, CTNNA1, CYP3A7, DDX18, DDX47, EPHA5, FGF21, FUT8, GGPS1, GMDS, GNAI2, HLA-DOB, HSPA8, HTR5A, IRF7, NTNG1, OGDH, OR1L6, OR2V2, OR52D1, OR6C76, OR6Y1, ORC6L, PLAU, POLD1, PRKY, RALA, RHOA, SDHC, SEMA3E, SLC25A6, TGFB1, TNXB, UNC5D, WBSCR17, CTNNBIP1, PNLIPRP2
Negative_5999	AK5, BCR, FGA, FYN, MPL, AASDH, ADRA1B, BACE2, BCAT1, CCL27, DFFA, DGKG, DUSP7, ETNK2, HMGCS2, HSD17B2, HTR7, IL11RA, ITGA5, MAN1A1, NFATC2, OR10AG1, OR13C8, OR1B1, OR1J1, PARVA, PDE2A, PFKFB2, PIK3R3, PLA2G10, POLD1, PRKCD, SDHC, SHISA5, SLC2A4, SLIT1, SULT1A2, TNFSF9, PRICKLE1
Negative_6000	ACOX1, AFMID, ALOX12, ANAPC1, ARSD, CD28, CDKN1A, COQ3, CYP11B2, DDX50, DUSP4, FLT1, FOXA3, FUT5, GUCY1B3, HIBADH, HSD17B7, MDH1, NFX1, NLGN1, OR2L2, OR4K17, OR4N2, OR51A4, OR6K3, PKM2, TBXAS1, TUBA1B, WNT11
Negative_6001	LPO, AGTR1, ATP6AP1, CNDP1, CTNNA3, CYP21A2, FLT3LG, GPAM, IL11, IL2RB, INHBE, OR2A5, OR5B3, RFNG, SSX2IP, TSHR, TUBB6
Negative_6002	B3GNT5, CDS1, OR5R1
Negative_6003	C4B, INS, ADIPOR2, ARG2, ARHGEF4, B4GALT1, BAI1, BIRC5, CACNA1S, CD70, CFL1, CYP11B1, DOLPP1, EPHB2, JAG2, LAMA3, MCM4, OR4A5, OR51Q1, OSMR, PIK3C3, PRKCH, RBL2, SHC4, SMAD2, STX5, WNT5A, HSD17B12, HSP90AA1
Negative_6004	MPZ, AMPD1, AMY2B, APOA5, ARSE, B3GAT3, CLDN8, CYP21A2, CYP2A7, DHRS9, DUSP16, EPHB6, EPRS, FUT1, GALK1, GCH1, HAP1, IL20, IL28A, KDSR, LRRC4C, OR10A2, OR10P1, OR13F1, PAX8, PDE11A, PLAUR, PNPLA4, PTGES, RXRB, SEMA3G, SKP1, SPRED2, SULT1A4, WWP1, YWHAB, ZFYVE16, CTNNBIP1, MAPK8IP1, TNFRSF11B
Negative_6005	FH, MSN, ATP5O, HNF4A, INMT, PDE10A, PTPRF, TNFSF12
Negative_6006	CGA, CKB, CR2, FGA, AASS, AKR1C1, ATIC, CADM1, CCL16, CCNG2, CD226, COQ5, CYFIP1, DGKE, DHRS4L2, EIF4B, FUT7, GSTO1, HSPA2, MAFA, MAP3K13, MASP2, MGAT4B, MYH3, MYL5, MYLK, MYLK2, OR2M3, OR2T2, OR4C6, OR51I2, PCYT1A, PDGFC, PER1, PFKP, PHKB, PIGF, PLCB1, POLE3, PRKAA2, PTGIS, RALB, RND1, STK3, UGT2B17, VAV2
Negative_6007	ABL2, ALAD, AMPD2, ASMT, B3GAT1, CBLB, DDX51, ENTPD2, FZD4, GADD45A, GALM, GCH1, GLI3, GYS2, HCST, MAPK1, OR4D9, PDE5A, SNAI1, SRGAP3, TACR3, TMLHE
Negative_6008	GLA, ID1, PLG, TEC, ANAPC7, ATP2A1, C1GALT1, CCKAR, CSF2RA, CUL1, EPHA8, GNA11, LIPC, LPAR1, MBL2, NSD1, OR51F2, OR56A3, OR5L2, OR8K5, PARVA, PAX4, PLCD1, PMVK, POLA1, PPAP2A, PRKACA, PTPRF, RAC3, RASA2, RCHY1, SARDH, SMPD2, TIAM1, TLN2, TRAF4, WNT1, WNT2B, ST6GALNAC4, TNFRSF6B
Negative_6009	TFG, ADORA2A, ATP5F1, EGFR, TAS2R5, VAMP2, B3GALNT1
Negative_6010	ZYX, ACADL, ALDOA, EPB41L2, GSTA3, ITGAX, MTHFR, OGDHL, OR51B2, OR52B4, OR8B4, POLR1D, SMAD4, TLR3, TSC2, UGT2B4

Negative_6011	GSN,ACVRL1,ALG10,ALG13,ASRGL1,BDH1,CCL24,CDC25B,CLDN18,CSNK1A1,EXTL3,GALNT3,GIT1,GNPAT,HSD17B3,IFNA5,IPMK,ISYNA1,ITGA9,KNG1,LCP2,MAP4K2,MGAT5,NFKB2,NMNAT2,NRP1,OR13J1,OR51A7,OR5L2,OR5P3,OR8H1,PAK4,PDGFRA,PEMT,PIK3R4,PLAU,PTGFR,RASGRP1,RIPK1,STX3,TAS1R1,UPB1,WNT3A,YWHAQ
Negative_6012	ATP5I,CFL1,CRKL,DIAPH2,FGF14,GCNT3,MAP2K4,NEFH,SELP
Negative_6013	FGB,MNX1,NR5A2,PSME1,RAC2,SEMA4A,STAT5A,TPI1,VAMP1
Negative_6014	MET,ACSS2,ADCY6,ARNT,CCL13,CD36,COL5A3,CRB3,CSNK1D,EFNB3,ENPP3,GNA12,HLA-DOA,HSD3B1,MIOX,NUDT12,OCLN,OR1L6,OR2AT4,OR56A1,OR5D13,OR5H6,PAPSS1,PIAS1,PIGL,PIM1,PLCG1,PON2,PPM1D,SPTAN1,TBL1XR1,UGP2
Negative_6015	NPL,CREBBP,CYCS,F2RL3,GSTO2,IL7R,MGAT5B,NCAM2,OR2B2,OR5I1,PDE5A,PHKA1,PIK3CB,PPP2R2C,WASF3
Negative_6016	C8G,IL4,CHST12,CNGB1,FGF4,GMDS,GUCY1A2,ITGA1,KIR3DL3,MMP14,MYH9,NAT6,OR10G2,OR10J1,OR4C16,OR8K3,PDE6D,PIAS3,PIK3C2G,POLR2K,SEMA3D,SMAD4,TPK1,TSTA3
Negative_6017	ACTN4,ADRB1,BDKRB2,CRHR1,DOCK1,EPHB6,GTSE1,ITGA7,PNMT,PPP1CC,RAC1,SEMA4B,WNT7B
Negative_6018	DDO,NOG,PTS,CD22,CNDP1,COQ6,CREB3L3,CTSG,DDIT4,DGAT2,GUCY2F,IL1A,IL1RAP,IL22,KLRC1,LMO7,MCCC2,METTL2B,NTN1,OR2G3,OR4K5,OR51B6,OR7G1,OR8S1,PGLS,PIGT,SELPLG,SMARCA2,SOX17,SRGAP3,STEAP3,MAPKAPK5
Negative_6019	JUP,ALDH3B1,ALOX5,CYP11A1,EGFR,FCGR3A,FGF9,GCLM,GLI3,LARS,LTBR,MAPK13,OR7G2,PAK6,PLXNA2,PNPT1,POLR1D,PPOX,PTPRZ1,RPN2,SARDH,SOCS4,ST3GAL4,WIF1
Negative_6020	AASS,ACSM3,APOA2,ATP13A2,CNTN2,DDHD1,GP1BA,HADHB,HDAC1,ITGA2,MAPK1,MAPK9,MARS,MEF2C,MYH10,NTF4,OR2K2,PPM1D,STK3,TSNARE1,UPP1,HLA-DRB5,TNFRSF1B
Negative_6021	ID3,NOG,GPT2,GRIA3,HTR5A,NAGLU,OR10H1,OR52L1,OR5D16,OR5L2,PHKG2,PIK3C2G,POLR2H,SNAP23,SOCS4,SV2A,TMLHE,TSC2,UMPS
Negative_6022	ADK,GGH,PAH,TNC,ACSS1,ADH1A,BDKRB1,CDC23,DBF4,DDHD1,EPHA7,FUT3,GCH1,HRH2,ICOS,IFNA6,ITGAV,LIPG,MAP4K1,MECR,MTMR6,ODC1,OR1F1,OR1N1,OR2H2,OR2M4,OR4A5,OR6C4,PDGFC,PLCZ1,PLEKHA8,POLR2A,PROC,RDH12,RXRB,SKP2,STX16,SUCLA2,TFDP1,TRPM5,UBA7,WNT10A
Negative_6023	BNIP1,COMP,EGLN3,FUT4,ITPK1,NEU1,NFKB1,OR1A2,PDE6D,PDGFRA,STX1A,SUV39H2,UBE2L3,ULK3,YKT6,MAP3K7IP1
Negative_6024	AK2,GSR,CAMK2B,CLTC,GCLM,GNAI2,HMGCS2,LAMB1,MTFMT,NFX1,NME2,PGM2,PIAS2,POLA2,RDH8,RRM2B,ST8SIA1,VAMP4,VKORC1,WNT8B,XIAP,ATP6V1B2,HSD17B10
Negative_6025	CGN,CCL17,EPHB6,PON2,SFRP2
Negative_6026	PGD,ABLIM1,ARSB,ATP5B,ATP5F1,ATP5G3,CCL1,CCL21,CERK,CTF1,CTLA4,ENAH,EXTL3,FBP1,FGFR4,GCNT2,GGT1,GGT7,HMOX2,MAP2K7,MYH2,NME6,OLAH,OR10A3,OR10T2,OR11H1,OR2A5,OR4M1,OR51G1,OR52H1,OR8H3,PARS2,PAX8,PIP4K2A,PRKCE,RAC1,SPHK2,TALDO1,TUBB4,SERPINC1
Negative_6027	HPD,CDC26,CHPF,CHRM1,CSF3R,CYP11B2,ENPP6,LTBP1,NKX3-1,OR4A15,OR4A5,OR6K6,PRDX6,RRM2B,SMAD1,SMAD4,TAS2R41,YOD1,PLA2G12B
Negative_6028	AADAC,ADH1B,ATP5D,BDH1,BPGM,CYBB,EGFR,FGD1,GALNT6,GLS2,HSD17B1,MAP4K3,NANP,NOS1,OR10H1,OR1G1,OR2A12,OR6K3,RENBP,STAT3,KIR2DL5A

Negative_6029	MET, ACSS2, ADIPOR1, AGXT, ALCAM, B3GALT4, CPT1B, ETNK1, GNB3, IL12RB1, IL4R, OR4C13, OR52E4, SDHD
Negative_6030	CEL, KMO, ANAPC5, ATG12, B3GALT6, BACE1, BMP8B, CBR1, CREB3L3, DDX23, ELK1, ENTPD1, FASLG, HSD11B1, INPP5E, LEF1, NT5C, OR4K2, OR51B2, PIM1, PVRL1, STX2, SV2A, WASL, TNFRSF11A
Negative_6031	C4B, ALDH4A1, CTSL1, OR9Q2, PER2, PIGV, PLCD3, PRKCD, PRODH, RAB23, UCKL1, VAV3
Negative_6032	NLK, SRM, STS, ADH7, ALPP, ARPC5, CACNB3, CBR3, COL4A6, CSNK2B, CX3CR1, DAXX, EDNRB, GDF6, GSTM5, HLA-F, HYAL1, IRF9, ITGA11, LIPG, NQO1, NTRK1, NUDT9, OR11A1, OR5AU1, OR9G4, OR9I1, PDE11A, PDGFD, RIPK1, RRAS2, SGPP1, SOX17, TNFSF14, MAPK8IP1, ST6GALNAC5
Negative_6033	DAO, GLA, ID4, ILK, MAX, ACSL5, AKR1D1, ATG12, B3GALT6, BCKDHB, CARD11, CCL3, CCR1, CD70, CDH3, CES1, CRY1, DOT1L, DRD5, GALNT6, IAPP, IFNA10, IL18, IL18R1, KIR2DS4, MPST, NANP, NFX1, OR10H4, OR13D1, OR2A42, OR2L13, OR4B1, OR5AS1, OR6C76, RFXANK, SC4MOL, SEMA4B, SEMA4G, SMPD3, SPTLC1, SUCLG2, SULT1A4, YWHAZ, ATP6V1C1, GABARAPL1
Negative_6034	BID, CTH, DCC, GBA, ID1, UBC, ADRB2, AMPD3, BMPR2, CD72, CMPK1, CTPS, CXCL2, CYP4A11, CYP4F2, DFFA, DUSP14, EDAR, ERBB2, EXTL1, FDFT1, FZD8, GSTO1, LYZL1, NRG3, OR1B1, OR3A1, OR5P2, OR6C4, OR7G2, P2RX3, PDE8B, PIGU, PIK3C2B, PORCN, PRIM2, PRPH, PSAT1, RDH11, RPS6KB2, TAS2R14, TDO2, TLR5, VARS, YWHAB, SUV420H1
Negative_6035	IVD, CNGA3, DDIT3, GFAP, NFKB2
Negative_6036	ACACB, APOC3, ARPC1B, CASP7, CCL13, COL6A1, CYP3A5, FSHR, LAMA4, PDE4D, SPP1, SPTLC1, TBL1XR1
Negative_6037	DDO, MAX, ADCY8, ADORA2A, AGPAT6, ANAPC7, B4GALT4, BDKRB2, CACNA1I, CCR7, CDC14B, CX3CL1, DAAM2, FGF9, FMO3, FPGS, GALNT10, HLCS, OR1S2, OR4A16, OR51T1, OR52I1, OR5R1, ORC2L, PDE8B, PIAS1, PIGO, PIGV, POLR2I, PSEN1, RRM1, SKIV2L2, SLC25A4, SMURF1, SORBS1, STK36, SULT1E1, TALDO1, TP73, TPM3, UGP2, XYLT2, MAPK8IP2, PLA2G12A, TNFRSF17
Negative_6038	AASS, AGPAT3, BLNK, CAMK2A, CD72, DDX4, EHHADH, FABP3, GCLM, GLG1, GSTM2, IBSP, IL9R, IRF3, ITGA1, ITGA6, NFATC3, OR10T2, OR52N1, PDHB, PIGC, PLXNA1, SARDH, SHMT1, STAT5B, TNFRSF18
Negative_6039	GPI, OGT, WAS, ACSM1, CACNG4, CCL17, CDC25C, CES1, CHRM3, DUSP6, FRAT1, GANC, GART, GNAS, GNRH2, GSTT1, GYS2, IL21, MAD2L1, METTL6, NCR3, NPR2, OR1L1, OR1L6, OR5AN1, OR6K6, OR8H2, PDE3A, PDGFRB, PIGC, PIK3C2G, PPP2R5A, PTPN1, PYGM, SESN3, SFRP5, SLC2A4, SMAD4, STAT5B, TGDS, TGFB3, TRAF3, ULBP2, UPB1, WNT5B
Negative_6040	HYI, LAT, CDC6, CREB3L4, DDX55, DTX4, EFNA5, ESAM, FBXW11, FMO4, GALC, GAPDH, GSTA1, GTSE1, GUCY1A2, HYAL1, INHBE, ITCH, LGMN, MKNK1, NEUROD1, OR4B1, OR4F6, PFKFB3, PNPLA3, POLD4, RARA, SETD8, STAT3, TICAM1, TRADD
Negative_6041	ADCY5, ADCY9, APPL1, ATP5J2, BMP6, CACNG7, COL2A1, EIF4E, EPHA7, FARS2, FGF12, FGF18, GALNT14, GALNTL1, HSD17B7, INHBB, ITGB5, LAMA3, NTRK1, OR2M3, OR4C13, OR51L1, OR9K2, PDE7A, PNPLA3, SESN2, SOS2, VASP, TNFRSF13B, TNFRSF13C
Negative_6042	AANAT, B4GALT1, CACNA1D, CSF1R, FADD, HADHB, HSPA8, IL1B, NCR2, OR1K1, OR5P2, PGS1, PIK3CD, PLOD3, ST3GAL1, STK11, TAS2R48
Negative_6043	B3GALT6, CCL14, CCL22, CDC14A, CLDN3, CREB3L2, CRKL, CTSB, CXCL12, EP300, FZD8, GLUD2, GNA15, GNAI1, GNAZ, HSD17B7, HSPA2, ITGA3, L1CAM, NCKAP1L, NSDHL, NT5C1A, OR10C1, OR11A1, OR11H4, OR13C5, OR4C11, OR52R1, OR6K6, PIGA, PPP2R5C, PPP2R5D, PPP3CC, PRODH2, PTGS2, PTK2B, PTPRF, RAF1, SMC1A, ST6GAL1, UMPS, WNT9B

Negative_6044	PVR,ACACA,ACVR2A,ALDH4A1,AMDHD2,ANAPC1,BBC3,BCAR1,CDC25A,CHUK,CLDN3,CNGA4,CYP4A11,DGAT1,DLAT,DTX2,DUSP4,EP300,F13B,FADD,FBP1,FPGT,GGPS1,GPD2,HLA-DOA,LARS2,OR51E1,OR51I1,OR51S1,OR7A10,OR8D1,OXSM,P2RX5,PAK4,PDGFA,PGAM2,PIGW,PMM2,PON2,PPP3CC,PRNP,RASSF1,RPN1,RXRA,STAT5A,SYNJ1,TACR2,TPK1,ATP6V1G2
Negative_6045	FGA,NOG,ADH1A,CLTC,DGKE,EHMT1,GALC,HSD17B2,HSD17B3,OR2F1
Negative_6046	CAD,TPO,ABL2,ACOT12,ACP5,ACVR1C,ACY1,ADAM10,ALDH7A1,APAF1,ARG1,ATP5A1,ATP5J,CBR1,CD14,CRY1,CTNNB1,CYP2A6,DGAT1,DGKI,E2F2,EIF4E,HMGCS1,HSD17B8,IL13RA1,IL17A,ITPKA,LMO7,MDM2,NAGA,NUDT9,OR10G7,OR10K2,OR14J1,OR2T3,OR3A1,OR4B1,OR6K6,PDE6G,PTDSS1,TLN2
Negative_6047	CGA,GLS,KIT,LEP,ATIC,CADM3,CD244,CNTN1,CSNK1G2,CYP17A1,CYP4A11,DDX23,FDPS,HCCS,MAP4K4,NTF4,OR51F1,OR5P3,PAX8,PDE4D,POLR1D,RPS6KA3,SDHB,SH2D2A,SUCLG2,TYRP1,VTI1B,AASDHPPT
Negative_6048	ACPT,ADSL,C7orf16,CD8B,CXCL6,EPHA3,ERBB2,KIR2DS5,LAMB2,MTMR2,SOCS1
Negative_6049	ACTN4,AXIN1,ENTPD5,GNA12,MAP4K2,OR4D2,OR51G2,OR52E2,PIGP,SETDB1,SYNJ2,TLR9,TSLP,ATP6V0A2,ATP6V0D2
Negative_6050	GP9,ABL1,ABLIM1,AKT3,ATP5O,CD72,CDC23,EHMT1,ENTPD2,IL5RA,INPP4A,ITGA11,NFX1,OR2A42,OR5B21,OR7G1,PAK2,PAPSS1,PIK3R2,SHMT2,YWHAG,ATP6V0A1,SERPINA1,SLC25A31
Negative_6051	CR1,C1QB,CSNK1A1,DFFA,TBXAS1
Negative_6052	ALDH2,CHRD,CMPK1,CSNK1D,CUL1,CYBA,FGD3,MCM5,NEGR1,OR4C46,OR52W1,PDE6D,PLA2G2F,SKIV2L2,SYNJ1,ATP6V0D2
Negative_6053	HTR2A,IL12A,OR51L1,PLOD3
Negative_6054	TNC,ACHE,CACNG5,CADM3,CLDN3,GALNTL1,GUCA1C,MAPK9,NANP,NKD1,OR4P4,OR5H6,PDXK,PER1,PRKACA,RDH12,TNFRSF4,TSLP,UGCG,VNN1
Negative_6055	AASDH,ACTN4,ADIPOR1,ATP13A2,CACNA1C,CCL8,CDS1,CREB3L3,DGKG,DUSP2,E2F2,ELK4,FPGS,GPD1,GSTO2,HLA-G,HLCS,IL12A,LYZL1,MYH7,NOS1,OR1Q1,OR2B11,OR52B6,PIAS3,PLOD1,PLXNB1,POMC,PPP2R1B,RPS6KA1,SHC3,ST6GAL1,STAT4,TLN1,TNFSF12,TRAF1,TNFRSF10A
Negative_6056	STS,ACSL3,ADRBK2,CCL20,CSNK1A1,EHMT2,KRAS,LALBA,MTHFD2,OR10K1,OR2AE1,PER2,SMAD6,SULT1A1,TAS2R48
Negative_6057	DCK,F10,F9,JUP,SPN,ABL2,ACSS3,ALDOA,AMY2A,ATG12,ATP6V1H,BCL2L1,CADM3,CAMK2A,CANT1,CBLB,EFNA5,ETS1,EXT2,F2RL3,HHEX,HTR2A,IDH2,IFNA6,IL1B,NQO1,NR1D1,NRXN3,NT5C2,OR2T5,OR51S1,PDE2A,POLR3K,PRLR,PRSS1,RARS2,RASA2,RDH8,RPS6KA3,SIPA1,SMPD4,SPAM1,TRDMT1,TUBB,VEGFA,ATP6V1E1,PAFAH1B1,PPARGC1A
Negative_6058	CSK,DBH,GNS,ADCY6,ATP5G1,C3AR1,CHUK,CYP3A7,EGFR,FGD1,GOT1,IDH1,ITGA4,L1CAM,MPDZ,NMNAT3,NSDHL,OR6K3,PCCB,PCYT1A,PDE6G,PIK3R3,PPP1CB,RPS6KA1,STK11,STX4,SYNJ2,UNC5D
Negative_6059	CS,ABLIM1,ACTG2,CLTC,CPT2,ENTPD8,H6PD,OR4K5,PIGS,PPP3R1,RFWD2,RGS3,SCP2,SGPP2,SLC25A5,UNC5C
Negative_6060	INS,OTC,CCL23,CIITA,CSNK2A1,DIAPH2,DLL3,EPHB2,MAP3K6,NPR1,NUDT12,OR10A3,OR4M2,OR9G1,POLR1E,RBPJL,TAS2R48
Negative_6061	CEL,FYN,B4GALT5,CFTR,CHEK2,GLDC,GNAI3,GNAZ,HAGH,IL15RA,INADL,LGMN,MFNG,NANP,OR8H1,PDE7A,PGS1,PIGA,PIK3C2A,SKP2,SMAD7,ST6GAL1
Negative_6062	ADK,C9,F12,FYN,HAL,MSN,ACAA2,ACVRL1,ALG11,CCL22,DAD1,DAPK3,DGKA,FGD3,FTCD,GSTK1,HTRA2,IFNA6,IL20,MAML1,MASP2,NRXN2,OR14J1,OR4K14,OR4X1,OR5R1,OR5T2,OR6C70,PHKB,PLA2G2F,RIPK1,RPS6KA4,TP53,TRIP10,VAMP4,VNN1

Negative_6063	CD2,FGB,TH,AQP7,ARSA,BDH2,BMP5,CLDN1,CNTFR,CYFIP1,CYP2S1,DAAM1,DOLPP1,FGF13,FUT1,INADL,MCM2,NCF4,OR51I2,PFAS,PFN2,PIK3R5,PLA2G2D,SDHD,SGMS1,TAS2R10,TM7SF2,TMLHE,SUV420H1,TNFRSF10B
Negative_6064	AOC2,CMAS,CSAD,DAAM1,LRAT,SNAI2,SOS1,HLA-DPA1
Negative_6065	CBS,ZYX,ACY1,AHCY,ARHGEF4,CACNA1C,CCNG1,CCR8,CD226,CDS2,CHIT1,CLDN3,CXCL2,DGUOK,DHDH,FBP2,GPT2,GRM1,HTR2A,HTR6,IL1B,IL8RA,INHBB,ITGA8,KNG1,MARS2,MBL2,MDH1,MGAT2,NODAL,NR5A2,NTNG1,OR10K1,OR52W1,OR7D4,PDPK1,PLAUR,PMAIP1,PRKCE,RALA,RASSF1,SEMA3E,SHMT2,TAS2R3,TAS2R41,VAMP2,ZAP70,ZFYVE16,ST6GALNAC4
Negative_6066	A2M,C9,FST,ME3,ACACB,ADCY3,ANPEP,B4GALT6,CACNA1I,CAMK2A,CASP10,CDK7,CDKN2C,CNGA4,CRKL,CYP2S1,EFNA5,EIF4B,F2RL1,GCLM,GRAP2,ICAM2,IFNB1,LRP6,MAP3K3,MDH2,MMP9,OR10R2,OR13C4,OR1G1,OR52I2,OR5B12,OR6N1,PPOX,PRKAB1,RPN1,SORD,STT3B,TBL1XR1,HLA-DRB3
Negative_6067	ACADSB,ADH1B,ANGPTL4,APOA1,CARD11,CFLAR,CHST13,DDX19A,GCLM,HSD17B3,LDHB,OR4K1,OR56A4,OR8K1,PLA2G5,POLE,PPARA,TUBA3C,UBE2L6,WARS2,HSD17B12,HSP90AA1
Negative_6068	DDC,GAA,ADSS,AHCYL2,ANAPC1,ARG2,ATF4,CCL8,CD226,CDC23,CDH15,CDS1,CLDN17,COL6A2,COQ7,DHRS4L2,DIAPH2,EHMT1,FADS2,GMPS,HPSE,IL12RB2,IL28RA,IL3RA,LDHA,MAFA,MAN1B1,MCM6,NCK2,OR14A16,OR2T8,OR8H3,ORC6L,PARVA,PCK2,PIP4K2A,PLA2G2A,PPM1A,PTGFR,QARS,SETD7,SNAI1,STAT1,STX2,TRMT11,TUBA3E,TUBA4A,WNT3
Negative_6069	IL9,KHK,OAT,ACADVL,APRT,ARSD,ATP5G3,AVPR1A,BNIP1,CYP2A13,ESPL1,EXOC7,EXTL3,GALNT8,GLS2,HEXB,HIBADH,KLKB1,MEF2C,MYH7B,NFYB,NT5C3,OR4C12,OR8H3,PRPH2,PTPRZ1,RAD51,SCIN,THBS4,ATP6V0D2,MAPKAPK5,PDCD1LG2
Negative_6070	F2R,PVR,ABL2,ADSSL1,BST1,CCL16,CD80,CDS2,COL4A6,CTSS,DGAT2L4,GALNT5,IL18RAP,IL7R,NCAM1,OR10H5,OR13C8,OR6C3,OR6C74,PIGN,PLXNC1,ROBO1,SLC2A4,STAT5B,TAS2R13,VASP
Negative_6071	BHMT,BLNK,CNTN2,COL4A1,CYP11A1,DVL2,FBP2,GALK1,MCCC2,OR1G1,OR4S2,OR7C1,ZMAT3
Negative_6072	GH1,ADCY2,ASAH1,ATP6AP1,CCL16,CDS2,DOT1L,ELK4,GRIA1,GSTM3,IGF1R,MAP2K7,MPZL1,NT5C1B,OR51B4,ORC3L,PDGFA,PNPT1,PTGER1,PYCR1,RASGRP1,SCLY,SLC27A1,UPP2,B4GALNT1,CTNNBIP1,PPARGC1A
Negative_6073	F5,ATP4A,CDKN1B,CDKN2B,CXCL16,DAG1,ERBB2,GJA1,GNA11,GYS1,LDHA,MAFA,MAPK10,NAT1,NCF1,OR11H6,OR1L8,OR5K1,PDGFRA,PFKFB1,PRKACG,PRKY,PSPH,SEMA3F,SEMA4B,SLIT1,SPRY4,ATP6V1B2,CSNK1A1L,TNFRSF13C
Negative_6074	FST,AKR1D1,ASCC3,CX3CL1,CYP19A1,DDX41,ETS1,FARS2,GNG3,IL22RA2,MAPK11,NFASC,OR5C1,OR9A4,SESN2,SH3BP2,SULT1E1,TGFA,TRAF6
Negative_6075	A4GALT,B3GALT4,CACNA1I,CHRM2,CPT1C,CYFIP2,CYP3A4,EPHA4,HTR2A,LIMK2,NAGA,NKD1,NOD1,NR5A2,OR10A3,OR5B12,RUVBL1,TPI1,ZIC2
Negative_6076	ALAS1,CCL4,CNTN1,CREB3,CTBP1,CYFIP2,EPHA4,FUT6,HLA-F,ITPKB,NCF2,NR5A2,OR1S1,OR52A5,PFN2,POLR2E,PTDSS2,RASSF1,SETMAR,SLC27A5,SSH1,TAS2R13,WNT4,PAFAH1B2
Negative_6077	MAG,PAH,CD19,COL4A6,COL5A2,DNMT3A,EHMT2,ENO3,F2RL1,FGF18,GLUD2,GUCY1B3,HDAC1,IL17RB,IL18R1,MAP2K2,MAPK8,MCCC1,MMP1,NFKBIB,NGEF,NMNAT3,NQO1,NTNG1,OR10G4,OR2A14,OR52I1,P4HA1,PAX8,PER1,PLA2G1B,RAPGEF2,SEMA3E,SLC2A1,SOCS3,SRGAP3,STX18,TACR3,TRAF3,XIAP

Negative_6078	OSM, ALAS2, ASPA, ATP2A1, B3GALT2, BRAF, C5AR1, CD86, CDC20, CNDP1, CNGA4, CX3CL1, CYP4A11, DUSP6, FDFT1, FOXO1, GMPPA, GNA15, GNMT, IFNA10, IL15RA, IRS1, LRDD, LRRC4C, MAN1B1, MAP4K2, MAPK12, NAGA, NANP, NMNAT3, OR10A7, OR4C11, OR52A1, OR56A1, OR5B3, PFN4, PLCD3, PVRL4, RRM2B, SC4MOL, SMARCA5, SORBS1, STX4, SUV39H1, TAS1R3, TUBA1A, UGT2B17, CTNNBIP1, TNFRSF12A
Negative_6079	BTK, F9, GNS, MIF, MSN, RB1, ADAM17, AKR1C3, AQP7, ARRB2, B3GAT3, BAIAP2, CMPK1, COL4A1, CSF1R, DAD1, DMGDH, ECHS1, EFNA2, EREG, FGF20, FOSL1, GAS1, GFPT2, GLO1, GZMB, IL7R, INHBC, IPMK, LAP3, LFNG, MKNK2, NEFH, NRP1, NRXN2, OR11H4, OR1E2, OR4K13, PAFAH2, PIK3R3, PLOD1, PNLIP, RBL2, RERE, RPIA, SOCS3, TIAM2, TUBAL3, ALDH18A1
Negative_6080	AGK, DDO, EZR, FUK, ME1, TNC, AKT2, ASS1, CAMKK1, CASP7, CDC42, CTSL1, CYP2B6, CYP3A43, DAPK3, DCTD, DFFA, EFNA1, EPHA1, FECH, FGD1, GCH1, GCNT4, HARS2, HIP1, ICAM2, LIPG, METTL2B, MPZL1, MRAS, OR10A4, OR2Z1, OR4Q3, OR5T1, OR8S1, PLAT, PPP2R5A, PPP3CC, RBPJ, SARS, SDHB, SLC27A4, SLIT1, TGFB2, TRAF2, VAMP3, WARS, YARS
Negative_6081	C2, F11, MPL, ACADVL, ACY3, ADSS, AMY2B, APOE, ARNT2, CCL21, CD34, CSF1R, DAAM2, GALE, IL10RB, IRF9, ITGA9, ITGB3, KIR2DL3, KLK3, MFNG, MYH4, NME7, NMNAT2, OGDHL, OR3A3, OR52N2, OR5C1, OR6C70, OSMR, PDGFC, PDGFRB, PGS1, PI4KA, PIP4K2B, PPP2R5A, PYCR1, RGS3, SMURF1, SOAT1, SSH2, STAT3, TAS2R8, TCF7L1, TNFSF10, TSC2, TSNARE1, WNT7A, HSD17B12
Negative_6082	ACTA1, DDX18, DUSP2, EGFR, GAL3ST1, IGSF5, IL1A, IRAK4, ITGAV, MAGI3, PRSS2, RPIA, RPS6KA1, SMAD2, WASF2
Negative_6083	HGD, ME3, WAS, AKR1D1, ALCAM, ALOX12, ARHGEF6, ATP12A, CCNG2, CDKN2D, EFNA4, EGLN3, FGFR3, FZD8, GNPNAT1, GUCA1A, IL1RAP, INSRR, JAM2, KIR2DS4, LYPLA1, MAP3K4, MMP14, OR10H1, OR2A25, OR2T10, OR5AU1, OR8B12, PAK4, PDE6D, PGM1, PGM2, PPP3R2, PSAT1, RFNG, RGS3, ROCK1, RPS6KA5, SARS2, SDC1, SRGAP1, SULT2B1, UBE2L3, VCAM1
Negative_6084	C4B, CFI, AASS, ABL2, ALDH7A1, ALS2, ATP2A3, CAMK4, CEBPA, CHST13, CLDN9, CNTFR, CSNK2A1, DDX55, EDA2R, FZD10, GADD45A, GLO1, GPD1, H6PD, IFIH1, IL15RA, IL6ST, MAML2, NCK1, NQO1, OR10AD1, OR10C1, OR10Q1, OR2T2, OR4D1, OR4K17, OR51D1, OR5F1, PPCDC, PRDX6, PTPN7, RPRM, SUV39H1, SUV39H2, TAS2R14, TAS2R3, TRADD, UNC5C
Negative_6085	TNC, BMPR1A, CACNG2, GSK3B, KIR2DS5, OR4S1, SMARCA2, TNFRSF10D
Negative_6086	CDK2, COL6A2, OR2A14, TUBB1
Negative_6087	F11, ILK, PVR, AADAT, ATF2, ATP5O, B3GALT5, BMP5, CLDN5, COL1A2, CREB3L2, CYP8B1, DOLPP1, ENTPD8, ESAM, F13A1, FGF21, FLT4, FZD10, GALM, GUCY1A2, GZMA, HTR4, IL1B, KCNJ11, OGDH, OR10G3, OR4X1, PDE5A, SFRP2, SLIT1, TAS2R5, VCAM1, WARS, WNT5A, ATP6V1G3
Negative_6088	E2F5, ERBB3, OR52B6, RBPJ
Negative_6089	DCK, IL3, AGPAT4, ALOX5, ATP5J, CACNG2, CXXC4, E2F2, FZD5, GSTM2, HADHA, HTRA2, OR4A5, OR8B8, RIPK1, SDHD, ST8SIA5, TNFSF4, AASDHPPT
Negative_6090	CCR3, CD27, FGF4, IL10RA, OR13C4, OR14A16, P2RX4, SNAP23, VTI1A
Negative_6091	TPO, ALOX12, AMDHD1, ARHGEF4, CHPT1, DDB2, DHX58, FUT8, GBGT1, GCNT2, GNA13, GRHPR, LEPR, NAT2, NEFL, NOS1, NRG2, OR4D6, OXTR, PDE8A, PIP5K1B, PRKCZ, SEMA4C, SNAP25, ST3GAL1, TBK1
Negative_6092	ALPI, ATIC, BAI1, SIGLEC1
Negative_6093	F8, FGB, HK2, IHH, ALAS1, BMP8B, CCND3, CD47, CDC27, CDH4, CDKN2C, CPOX, DDHD1, DKK4, DOT1L, EDAR, EIF4E2, ENO1, GCLC, GRHPR, GSK3B, HADHB, HAGHL, HAO1, IAPP, LIPT1, MINPP1, NOD1, OR11H4, OR2Z1, PTPN5, RASA2, SLC27A5, SPRY3, STAT4, SUV39H2, VAMP4, WNT7A, TNFRSF17
Negative_6094	SI, CAMK2D, CASP8, CHPT1, CISH, CXCL3, DGAT2L4, GUCA1C, IDH3A, MAP2K2, MINPP1, OR4A5, OR52R1, OR5V1, PER3, PPP1CB, PPP1CC, PTCH2, SLC33A1, SPRY3, SSH3, TNFRSF18

Negative_6095	FST, CD55, CYP17A1, DCXR, DGKE, EXT1, GDF5, HIF1A, KIR2DL2, OR6N2, POLA1, PPP1R3C, RUNX1, STAT5B
Negative_6096	C1S, GNE, AMPD3, APC2, CACNA1S, CDC16, CES1, CHSY3, CNGB1, COL3A1, EFNA3, ETS1, GRIN2A, GSTM4, ICOSLG, IFNW1, IMPDH1, ITGB5, KIR3DL1, LTB4R2, OR52E2, OR5AK2, PIK3C2B, POMC, PPP1CB, PRODH2, RAPGEF2, STAT4, TAS2R50, TGFBR2, TIAM2, WWP2, PNLIPRP1
Negative_6097	DDO, F10, FGB, CCL13, GLS2, GPAA1, IQGAP2, OR5T2, PLA2G2E, PLCE1
Negative_6098	AK7, DCT, FYN, GNE, IL2, JUP, ACAD8, ACMSD, ACSL5, ACTG1, ATP6V1A, BLVRB, CFTR, CYP19A1, DKK4, ENPP3, GALNT12, GFAP, GGT6, HARS2, HSD11B2, MAPT, NEU1, NTNG1, OR2A14, OR4Q3, OR9A4, OSMR, PARVB, PFKP, PNPLA4, PNPO, PPARA, PRKAB2, PTGS1, RHOA, SNAP25, SULT1A1, UBE2L3, UGT2B17, CACNA2D3, PLA2G12A, PPARGC1A, PPP1R12A, SERPINA1, SERPINE1
Negative_6099	EDA2R, GMPR2, MYH14, PIGA, TAS2R8
Negative_6100	EPO, ADH6, ALDH2, CLTC, DAPK3, EFNA3, MAPK13, NCOR1, PLOD3, PRKAB2, PTPRF, RPS6KA2, SETD1B
Negative_6101	CAT, MVK, SI, SMO, ACOX1, AKT1, ATP5G2, CHPT1, CLDN17, CYCS, CYP2C19, DOCK1, FGF22, G6PC2, GLUD1, HTR2C, ITGB7, MAP2K1, MYH13, NKD2, OR13G1, OR2K2, OR3A2, OR5B17, P4HA3, PARD6A, PRKCB, PRODH2, SEMA3C, SETD1A, STK36, TLR2, TLR9, TUBB2B, WNT4, XYLT1, XYLT2, ZFYVE9, HSP90AB1, ST6GALNAC3
Negative_6102	CSK, FLT1, GART, IL18, MITF, OR4K17, OR6Q1, SH2D1B, TLR2
Negative_6103	C4B, RPE, ACTN1, ACYP1, ALDH1A1, ALG9, COQ3, CYP8B1, EPAS1, FMO5, GALK2, GGCX, GPD1, HAO1, JAG1, KNG1, LARS, LCP2, NRXN3, PIK3R3, SETD1A, SLC2A4, TCF7L2, THBS2, UGT2A1, WNT2B
Negative_6104	DHH, CACNA1I, FASN, FGF5, GFPT1, GRM5, LILRB3, MAPK7, PFKFB3, RHOD, ST8SIA1, WHSC1L1
Negative_6105	GSR, HK2, ABLIM2, APOA2, ATP6AP1, CADM3, CCL19, CFL2, CLDN15, DOCK1, DRD5, EARS2, EPB41L1, GALC, GAS1, GGT6, GUCY2F, HCCS, HSPB1, KYNU, LYZL1, MYLK, NFATC1, OR2T4, OR4K5, OXCT1, OXSM, PFAS, POLR1E, RDH8, SMAD2, SOAT2, TBL1Y, TNFSF12, TP53, UPRT, VDAC1, YWHAQ, HSP90AB1, PNLIPRP1
Negative_6106	ASMT, ATP2A1, CACNA1S, CARS, COL1A1, DHCR7, IPMK, ITGAX, LIMK2, OR1S2, OR4N5, PCK2, PTPRF, SAT1, SGPP2, SPI1, TJP1, TPM3, UNC5B, YWHAB, PAFAH1B1, TNFRSF10D
Negative_6107	AMH, LYN, MPI, ACOT12, ACVR2B, B3GAT2, C3orf10, CLDN3, E2F2, GLUL, KLKB1, OR1F1, PLCB1, PPP2R5E, PTPRR, TAS2R8, THBS4, WNT10B, MAPKAPK5
Negative_6108	AR, C9, DCT, FH, GK, SRC, TXK, ACAT2, ATP2A2, ATP6V1D, BCAT1, BTRC, CHRM2, CXCR4, DHRS4L2, DPYS, FGF17, FUT2, GALNTL4, GNPDA2, GUCY2D, HLA-G, IL12RB2, LTA4H, MYH11, MYH7B, OR1E1, OR2A42, OR4C12, P4HA1, PDE7A, PTGER1, PYCRL, RAPGEF2, SDHC, SETD2, SOCS4, SQLE, TAS2R42, TCF7L2, TGDS, THBS2, UGT2A3, MAPK8IP2
Negative_6109	CCL2, EPOR, FIGF, GRIA1, MTFMT, MYH11, PAFAH2, POLR1C, PTGDS, WEE1
Negative_6110	AGRP, F2RL1, OR4B1
Negative_6111	AK1, ALOX15, ASCC3, CCR2, CXXC4, DARS2, GALNTL4, GRPR, HAO1, HPSE, HSD17B2, HTRA2, INMT, LAMA3, LCMT2, MAP3K4, MPP5, NCR2, NPR1, NTN1, NUDT2, OR2T2, OR2V2, OR4K2, PDE4A, PDGFRA, PERP, PRKY, PTPN7, RENBP, TBK1, TKTL1, TSTA3, UBA1
Negative_6112	IL3, ACP5, ACSL1, ACSS3, ALDH3A2, ASCC3, BAI1, CACNG3, CALML6, CD34, CLDN22, CRHR1, DCXR, E2F2, EDNRB, ELK1, FABP4, FZD3, GNG3, HCST, HSPA1B, IDUA, IL18, IRAK2, KLRK1, LLGL2, LRAT, MAN1A2, MCM7, NAE1, OR52N2, PDE1C, PDE8A, PLA2G6, POLR2D, PPCDC, RARS2, RXRG, SCLY, SEMA4A, SETDB2, SOS2, ST8SIA5, STX1B, TLR2
Negative_6113	MUT, ACSM2A, CTSS, CYP2E1, DDX56, ERBB4, GPAA1, HAGH, IL13RA1, ITGB6, NCR2, NEFM, OR4S2, PDGFD, SETD1B, SMURF2, SPRY3, VASP, WNT8B

Negative_6114	C4B, IL2, ACY1, AMHR2, BDKRB1, CACNB3, CD14, CD44, CDO1, CFLAR, CHAD, CHEK1, COL4A6, CREB3L2, ENTPD4, ERBB3, ERBB4, FGF18, FMO2, FZR1, GALNTL1, GNA14, HAO2, IFNA14, IKBKB, ITPKA, KIR2DL1, LDHD, LYPLA2, MAPK1, MYL9, NCOA4, NKD2, NOD1, OR10A3, OR10G8, OR2M2, OR52K1, PDE5A, PLAU, PNMT, PPA1, RAP1A, RDH11, SEMA4C, THBS1, UCK1, ULK3, HLA-DRB3
Negative_6115	CBL, TFG, ACVR1, AGMAT, C5AR1, CALML6, CAMK4, CDC42, COL4A6, DDX18, EDNRB, FZD6, GALNS, GALNT10, GALNT3, GCLM, GMPS, GNG3, IKBKG, MAOB, MAPK7, MPDZ, MPP5, NARS, NCR1, ONECUT1, OR10A3, OR5P3, OR7A10, PKM2, POLR2K, SETD1B, SMURF2, STK3, STK4, TICAM1, TNFSF8, TRHR, AASDHPPT, SLC25A31
Negative_6116	BTC, CFI, OSM, RFK, ABP1, ACOX1, AHCYL2, BBC3, BMPR2, C1GALT1, COQ7, CX3CR1, CYBB, DTX3, FUT5, FZD2, GNRH2, IRAK1, LFNG, NCKAP1, OPLAH, OR52E6, OR8I2, PDPK1, PFN3, PIGS, PIGT, RRAS, SDHA, SLC9A1, SMC1A, SUCLG1, UCK1, ZFYVE9, TNFRSF1A
Negative_6117	A2M, DDC, LTB, MAG, PLG, TNR, ALAS1, APOA1, APOA5, ARHGEF6, CD8A, CHEK1, CHPF, CSH1, CXCL10, DPAGT1, DPM2, DVL1, EPB41L2, ESAM, INPP5B, NCAM1, OR52E8, PLCB4, PPP2CB, PTPRB, RRAS2, SLC27A4, SOCS4, SOCS5, TLR4, TYK2, WNT16
Negative_6118	BTK, ASS1, CTNNA1, DAG1, DDIT3, DDX51, DNMT3B, F13A1, HIP1, INHBE, IRF7, IRS4, ITGAX, LAMB4, LIPG, MAP3K13, OLR1, OR12D3, OR1C1, OR1L4, OR2AG1, OR2C1, OR6C76, OR6Y1, PIK3C2G, PIK3R4, PLA2G5, PLCZ1, POLE2, PPP2R2B, PSPH, RHOQ, SEMA4D, SFRP4, SMPD3, SOAT1, ST8SIA1, STAT2, THBS4, TLR3, TUBA3C, TYROBP, VANGL1
Negative_6119	CDA, FUK, HYI, ADSS, AGMAT, AK3L1, ALDH1A3, ALG12, APBB1, BACE1, CLDN4, CLTA, CNGB1, DGKB, FBP1, FOXO1, HES1, IGF1R, IL24, ISYNA1, ITCH, MYH11, NCAM1, NFE2L2, NQO1, OR10A6, OR1L6, OR2A4, OR2M7, PKMYT1, PLD1, RAPGEF1, RAPGEF4, RASA1, RASGRP1, SMURF1, SV2A, UGT2B28, VKORC1, ATP6V1B2
Negative_6120	ID4, ATP13A2, LAP3, OR2M3, PLCE1, UPP1
Negative_6121	CS, MET, ABL1, ATP5G1, CLDN20, CSF1, CTBP2, CYP2E1, CYP2J2, EFNA5, ENTPD5, EPHB4, IFNGR2, IL24, LAMC1, LTB4R2, NME7, NRXN1, OR14J1, OR1C1, OR4C46, OR6C70, OR9G1, PDPK1, PFKFB4, SC4MOL, SEMA3B, SMAD6, SOCS7, STK36, TAS2R40, TGFB2, VANGL1, XIAP, XYLT1
Negative_6122	DCK, HAL, HGF, LEP, MAG, MET, MPZ, ACY1, ADSS, CCL5, CFLAR, CSDA, CYSLTR2, DAPK1, DBF4, ECSIT, F2RL2, FMO3, G6PD, GALNTL2, GCDH, GSTM1, HTR6, IL13RA1, LTBP1, MAFA, MAP2K4, MAP3K12, MCCC2, MCM5, MTHFD1, NCF1, OR2A25, OR2D2, OR8B4, PI4KA, PIK3R1, QPRT, RAPGEF3, SMARCA5, ST8SIA5, STK3, TUBA1B, CSGALNACT1, SUV420H2
Negative_6123	BTK, C5, GLA, SPN, ACOX1, ACTA2, CD274, CERK, COL5A2, CTBP2, FGF13, GDF6, GNRHR, GUCY2F, HGSNAT, HTR7, IDH1, KAT2A, LEFTY1, OR2M7, PDE1B, PGS1, PMAIP1, POLD4, PPP1R1A, PPP3CA, SEMA4D
Negative_6124	AMPD2, CD86, CYP17A1, DGKD, DHRS9, FMO4, KLRD1, NNMT, OR1D5, OR52W1, STEAP3, TSHB, VCAM1
Negative_6125	PGP, CYCS, MADCAM1, OR1F1, PIGO, SRGAP2, SV2C, TGDS
Negative_6126	C2, ACADM, B4GALT2, DTYMK, EFNA2, GALNT10, GSTA4, ITGA4, KITLG, MAN2A1, NAE1, NAT5, OR2A4, PGAM2, PPP2R2A, ULBP3
Negative_6127	CACNA1D, ENO1, GLS2, HTR7, NAGS, OR6Q1, POLR3A, RENBP, SFRP5, SORBS1, TICAM2
Negative_6128	HYI, OAT, ZYX, ALG2, ASRGL1, CACNA1S, CCDC6, HAGH, IL13RA2, IL15, ISYNA1, LALBA, NAT2, OR7D4, PDE4B, PRKAB2, PYGB, SETMAR, TOMM40L, TUBB4, TNFRSF10A
Negative_6129	AREG, GDF7, KITLG, PON3, RDH12

Negative_6130	ATM, DCN, ID2, ADCY8, AGTR1, ALG13, ANAPC10, ATP5I, CEBPA, CYP4A22, DVL1, E2F1, ENPP6, FOXA2, GALNTL1, GLS2, GUCY1A2, HTR4, IFNA14, IL4R, JAM2, KCNB1, LIPF, MAN1A2, MAPK7, MPST, MYH1, MYH9, MYL7, OR10A7, OR1K1, OR4C12, OR4M1, OR4N5, OR51B5, OR9Q1, PGLS, PIM2, PRKCA, PYGM, RDH10, RND1, STK4, UBA7, UCK2, VANGL2
Negative_6131	ACTN1, ACVR1, ALOX15, BDH1, BUB3, CAMK4, CAV1, CHDH, CTNNA3, CYP2C8, ESAM, GUCY1B3, HLA-DMA, IDH1, ITGA4, MKNK1, NEGR1, NOX3, NUDT5, OR1N1, OR56B4, OR5T3, OR8B4, PIGS, PPCDC, PSAT1, RPS6KA6, SLC9A1, SMAD6, SMPD4, TIAM1, TJP2, TUBB2B, VNN1, YWHAQ, MAPKAPK3
Negative_6132	MAX, ZYX, AASDH, ADCY5, BMP8B, CLDN6, CSDA, DPYS, EPB41L3, F2RL1, LIMK2, LTB4R2, LYPLA2, OR6M1, OR9A4, OR9Q2, PTPRB, RAC1, STMN1, VARS
Negative_6133	ADC, DDC, HYI, SP1, ALDH5A1, ARG1, ENTPD5, EPB41, OR1L8, ORC4L, PTCRA, TPH1
Negative_6134	HK2, PML, ACSM2A, ACYP1, ADCY5, CDC26, COL5A1, COL5A2, CREB3, DERL1, F2RL2, IFNA17, IL25, IL2RB, KCNB1, OR10A6, OR10G7, OR2M7, PFKFB1, PLCD4, PRSS2, PSEN2, SOAT2, TGFB3, TRAF5, UGT2B10, YWHAE, SERPIND1
Negative_6135	C4B, DUT, ACTN2, B3GALT2, CDC25A, CDC45L, CDS1, FADD, GRM1, HIF1A, IL1R1, KLRD1, MAP2K1, OR10A2, OR10C1, OR9K2, PDE3A, PLAUR, TUBB4, UNC5D, YES1, PRICKLE1
Negative_6136	F10, F8, FES, KDR, NLK, SFN, ACTB, AMPD1, ANAPC5, CCNG1, CSNK2A2, CTBP2, CTLA4, DTX3L, EXT1, FGF22, GALNTL1, IFNA13, NRG1, OR10C1, OR4C11, P2RX2, PLAUR, RAD51, SMAD4, MAP3K7IP1
Negative_6137	FST, GLA, MVD, ACAT2, AGTR1, BDKRB1, CAV2, CD36, CYP8B1, ENTPD8, FGF5, FIGF, GLG1, HDAC1, HLA-DOB, KLK3, METTL6, NNMT, OR10G8, OR1C1, OR2AE1, OR9A2, PDE6G, PGK1, PIM2, PRKCI, SHMT1, ST3GAL6, STK36, SULT1A4, VAMP1, ATP6V1B2, SERPINC1
Negative_6138	GCK, MVK, ADCY2, ATP5J2, CADM3, DGAT2L4, GALNTL1, HTR5A, IFNGR2, KIR2DL3, LCP2, MAD2L2, OR5T2, PCNA, PHKA2, PIGC, PLA1A, PLA2G2E, PORCN, PRKCZ, RPS6KA1, CTNNBIP1
Negative_6139	MPZ, TKT, C3AR1, CD80, CES7, DHDDS, HAGHL, HAO1, IPMK, IRF5, LRP6, MAML1, OPLAH, OR4A16, OR5B3, PCK1, PROS1, PTPRF, SYNJ2, TACR1, ATP6V0A2
Negative_6140	FH, PVR, CAMKK2, CASP3, CCL16, CCL28, CD19, CD79A, CFL1, CHST12, CNTN2, CX3CR1, EPHX2, FMO5, GUCA1B, INPPL1, IRF5, KAT2A, MAN1A2, MAP3K3, MAP3K5, MYLK2, OR11A1, OR2A42, OR52N2, OR52N4, PLA2G2A, PLD2, PYCRL, SEMA4B, SETDB2, STAT6, SUFU, TAS2R9, TOMM40, WWP2, XYLT2, YES1, ATP6V1C2
Negative_6141	BTC, OTC, ADCY9, ADIPOR2, ALAS1, ALDH3B1, BNIP1, CD44, CDC16, CFLAR, CISH, CLDN3, CYP11A1, FBP2, FECH, FGF3, FLT4, FRAT2, G6PC, GALE, GCNT4, GOT1, GPX5, GRIN2A, IFNB1, MPDZ, OR1S1, OR2M7, OR4Q3, OR51E1, OR51F2, OR8J1, OR9I1, PLA2G2D, PRKAB1, RASGRP4, SDC2, STAT5B, STK3, SUCLA2, TRMT11, VEGFC, VTI1B, WNT7A, CSGALNACT1, SERPINC1
Negative_6142	PDC, TNF, CD59, DLST, METTL2B, NANS, NEGR1, NLGN3, OR51Q1, PGM3, STX12, TAS2R14, TRAF6
Negative_6143	DDC, CACNA1E, CAMKK1, CCL25, CD22, CD8B, CNGA3, CNGA4, CSF2, CSNK1G1, DDX51, DGKE, FGF23, GCAT, OR10H3, OR1I1, PITX2, PRKACG, RXRB, SETD2, SHISA5, SMPD1, TAS1R3
Negative_6144	NNT, PVR, SMO, TPO, ACTG1, ALDH1A3, ATP5J, B4GALT5, B4GALT6, BBC3, BIRC2, BMPR2, CACNG4, CCL22, COX15, CSNK2B, CYP4F3, ENTPD5, FGF12, FSHB, GAL3ST1, GCDH, GP1BB, HMGCL, MAP2K4, NEU1, NFYB, OGDH, OR51B6, PANK4, PON2, SESN1, SPAM1, TAS2R60, THY1, TJP3, TKTL1

Negative_6145	TNR,ARHGEF4,BUB1,DDX52,F13B,FZD4,GARS,GLI3,HLA-DOB,IFNAR1,LLGL2,MPP5,MTMR6,OR2M7,PDE4A,PRPS1,PTK2B,SETD2,SULT2A1
Negative_6146	IL8,OSM,SPN,ACHE,ANGPTL4,APLP1,ATP5E,BAAT,CCL7,CDC14A,DDX52,GNAI2,GNAI3,HTR6,IL17RB,IL21,MAGI2,MARS,NEU4,NLGN3,NT5C3,OR13F1,OR2K2,OR51D1,OR56A4,OR5F1,PDE1C,PHKB,PI4KB,PLTP,POLR2A,PPAP2C,PTPRM,RPS6,SDHA,SDHC,SMAD5,STX7,WASF2,WNT10A,YARS,B3GALNT1,GABARAPL1,GABARAPL2
Negative_6147	C4B,CD6,EGF,SRF,ABL2,ADIPOR2,ALDH9A1,CNTN2,CYFIP1,EPHX1,FGF9,FTCD,FUT8,GDE1,GNAI3,GNB3,ITGA8,KIR3DL3,MAP3K14,OR10G7,OR13C2,OR13C8,OR2AG2,OR4K1,PKLR,PLA2G4A,SMAD6,TLN1,XYLT1
Negative_6148	CR2,ACTA2,ATP6V1H,CCNB1,CD70,CHAD,FGF4,NTF4,OR2AG1,P4HA2,PLCG1,PMAIP1,POLR2E,PRKAR1A,PTPN11,PTPRF,STAT4
Negative_6149	ALG3,ATP6AP1,B3GAT3,CD8B,CHAD,CHIA,CNTNAP1,EGLN3,ETS1,FBP1,FGF6,GNMT,GNPDA1,GUCA1C,ITGA7,KIR2DL3,OR2T29,PIGT,PPM1D,RBKS,SENP2,SHMT2,SMPD2,SPAM1,STMN1,CTNNBIP1
Negative_6150	MPI,CAMKK1,CXXC4,ETS1,OR10H4,OR2L3,STK36,UGT2A1
Negative_6151	CD6,HPD,RFK,SDS,ADCY10,ADRBK2,ALPPL2,ASIP,CSNK1G3,CTNND1,CXCL13,DHRS4,EFNB1,ENPP2,FBXW11,FLT3,FLT3LG,GGT7,GPX5,GRPR,HCST,HDAC2,HEXB,HTR5A,IL22RA2,IL8RA,MCM6,NOTCH3,OR13F1,OR2T34,OR51I2,OR5A1,OR5T2,OR6B3,OR9Q2,P4HA3,PLCD4,PPP3R1,RRM2B,SORBS1,SPP1,TBL1Y,WNT10B,WNT3,TNFRSF17
Negative_6152	FH,ABLIM3,ACP1,BIRC2,BLVRB,BNIP1,CCL26,CCNG2,CERK,COL1A2,CREB3L2,CYP3A43,DOCK1,GUCY1A3,IL10,IL17B,MAML2,MGLL,NCKAP1L,P2RX2,PLD2,SELPLG
Negative_6153	CR1,GPI,KMO,RDX,ACOT12,ACTA2,BHMT,CDH3,CNDP1,ENO1,FOXA3,MTHFR,PDE4D,PFKP,POLR2A,PPOX,PPP1R3A,PTPN7,PTPRJ,SESN1,SGMS2,SHISA5,SLC9A1,TIAM1,TRHR,VNN1,WARS2,HLA-DQB1
Negative_6154	AGL,AK3,BID,DCC,ILK,OGT,TK1,ABLIM3,ARHGEF4,ASIP,CAMK2G,CD14,CD274,CD55,CHIA,CHRD,CSNK2A1,CX3CR1,DIAPH3,ESPL1,FASN,FMO4,FTH1,IFNAR1,IL15RA,LMO7,MCCC2,MMP2,NFKBIA,OR2L3,OR4P4,OR5T3,OR8J3,PAK7,PFN1,PON1,PPA2,PVRL3,RPN1,SARS2,SNAP23,STK11,TAS2R38,TIAM1,UGT2B7,CACNA2D4,PAFAH1B1
Negative_6155	MSN,CD28,NT5C,POLR1A,STK36,TJP1,WNT7A
Negative_6156	FOS,GPI,LHB,SP1,CD44,CDK5,COL6A1,EARS2,FPGT,GANC,GOT1,GSTT1,GUCY1B3,LAP3,MAGI1,NFKB2,OR2V2,OR5AC2,OR5P2,PLCE1,POLA2,POLR3D,RDH16,STAM,TGFB2,TPH1,TUBB4Q,TYK2
Negative_6157	ASL,CS,IL5,ADCY10,ALDH1A3,AREG,ASRGL1,CBR1,CCKBR,CMPK2,CSF2RB,GALE,GALK1,GRIN2A,GZMB,HLA-F,HTR6,IMPDH1,NNMT,NT5C1A,OR5D14,OR6X1,OR8S1,PDX1,PLK1,PPM1A,SELE,SRD5A1,STX18,TGFB2,VTI1B,WNT2B
Negative_6158	AK2,APP,F12,GK2,ALDH3B2,BET1,CD247,CPT1A,CSNK1A1,CYFIP1,CYP2A7,DDX55,DOCK1,EGLN3,EIF4B,ENPP7,EREG,FGF21,FZD4,GRLF1,HPSE,IGFBP3,IL12A,IL15,INSRR,ISYNA1,KIR2DS5,LAMB2,NANS,NCR3,NR4A1,OR10G3,OR10S1,OR51F1,OR52N1,OR5T1,ORC3L,PLCB3,PNPLA4,PRKAG2,PRKAR2A,ROBO1,SPHK1,STK4,YWHAB,ZNRD1,ATP6V0E1,HLA-DRB4
Negative_6159	C8G,MUT,SHH,ACTG2,ADCY8,ADORA2A,ALAS1,ALDH3A2,BCAR1,BCL2L1,C4BPB,CYP1A1,CYP3A43,DDX55,EFNA3,GAD2,IARS2,LIPT1,MAP2K6,MAPT,MCM2,NR1D1,NT5C1A,OR4C11,OR4C16,OR9G4,PDHA2,PISD,PPAP2B,PVRL1,SMPD2,SOCS3,SUCLG1,TACR3,TUBB4Q,VAV1,VTI1B,SLC25A31
Negative_6160	AK7,IL4,CFL2,DGAT1,EFNB1,EPB41L2,GLO1
Negative_6161	CTH,CDC25B,CLDN18,CSNK2A2,EFNA3,ELK4,GBGT1,HMGCS1,HYAL2,INPP5B,KIR3DL2,NRAS,OCRL,ODC1,OR1L6,OR2AE1,OR52K1,OR52R1,OR8H1,PDE5A,PPP2CB,PRKAR2A,RHOQ,SOCS1,TRAF1

Negative_6162	ACLY,ADAM10,ADH5,ASNS,CARS,CBR3,CD59,CNGA4,CREBBP,DPM3,DUSP7,DUSP8,GRIN2C,IFNA21,ITGAL,KYNU,NTNG1,OR4P4,OR6B3,OXCT2,POLD2,SEMA3G,ST8SIA1,STAT2,TICAM2,TNFRSF10D
Negative_6163	GDA,HK1,SHH,ACSL5,AMPD2,CD247,CES7,CNTNAP1,CSF2RA,CTPS,CYP2U1,DFFA,DLST,ELK4,FGFR3,FZD1,GMPR,GP1BA,GPT2,GSTZ1,HSPA8,IFNAR1,IL13,IL6R,MGST1,OGDH,OR2A12,OR2M2,OR6C3,P2RX1,PCK2,PGAM2,PLAT,PLCD3,RFNG,RXRG,SH2D1B,SMAD1,SRGAP2,TSHB,TUBB4,UROD,SUV420H1
Negative_6164	C7orf16,CACNA1G,DPAGT1,FGD1,GAPDH,MAP4K1,OR10A4,OR2H2,OR52E6,OR7A17,PDE10A,PDGFD,POLR1E,PPP3R2,MAPKAPK2
Negative_6165	FN1,CHDH,CYP2C8,GSTM4,PDE4B,PIM1,PPP1R1A
Negative_6166	ACAT2,ALG10B,ALG9,ASS1,BMPR2,CAV1,CCNB1,CD72,CSNK1G3,DDOST,DTX3,OR13C4,OR6Q1,POLR2F,POLR2G,SMAD5,STX18,WNT2B
Negative_6167	CDKN2B,CXCL10,CYP4A22,EPAS1,EXTL1,F2RL3,FECH,NR4A1,OR1S1,OR52E4,TBXAS1,TSNARE1
Negative_6168	AR,C2,JUN,PLN,SMS,ACAA2,ADSSL1,AHCY,ALG5,ARRB1,ASS1,ATP6V1D,B3GNT2,CLCF1,CLDN16,CLDN22,CREB3,CTSB,DHRS3,DNMT3L,HARS,HSP90B1,HYAL2,IARS2,MADCAM1,MMP14,MPDZ,OR10AD1,OR2A42,OR2T11,OR5AP2,OR5C1,ORC6L,PDGFB,PIP4K2A,PLA2G10,RXRB,SMPD4,STX4,THBS3,TUBB2A,UCK2,VASP,SERPINB5
Negative_6169	FH,CERK,SHC1
Negative_6170	ADK,AUH,GSR,IL6,SCD,ANAPC2,AXIN2,B3GAT3,CACNB2,CACNG8,CCL28,CYP4A11,KRAS,MYH11,NRCAM,OR10AG1,OR4A15,OR4K1,OR52N4,PAX6,PEMT,PIGS,SETD2,PNLIPRP2
Negative_6171	KMO,ALOX15B,BAAT,CDC42,CDS1,CSNK2A1,FUT6,HNF4A,MTHFD1L,NUMB,SETMAR,SLC25A5,STX7,TMLHE,TSC2,WNT1
Negative_6172	SYK,ASPA,CSDA,E2F1,FABP4,FGD3,LAMA4,MTMR2,OR6C70,PIGU,RAPGEF2,ST3GAL6
Negative_6173	C7,F10,GHR,GLA,ME3,NPL,ADH1B,AKT1,ATP6V1A,BCL2L1,CARD11,CFL1,COX15,CSDA,DKK2,DKK4,DLL1,DUSP10,E2F4,EFNA5,EXTL2,FGF14,IL20,NRCAM,NTRK2,NUDT8,OR10A5,OR13C9,OR4D6,OR51B2,OR5B21,OR6Q1,PER2,PIGQ,PIK3CA,PIK3R2,PRKAB1,PTGES,SGPP1,TSLP,WNT11,YWHAQ,TNFSF13B
Negative_6174	C9,ME3,MSN,SMO,CD86,CHPT1,E2F3,ERO1L,ETNK1,FLOT1,GMDS,GTSE1,HTR2C,IL13RA2,INSR,JAK1,LIPF,MAN1C1,MAT1A,MTHFD1L,NODAL,NT5C,NT5C1A,OR10A4,OR1S2,OR51E1,OR51F1,OR6Q1,PAFAH2,PDX1,PER1,PHKG2,PYGM,RASGRP1,SC4MOL,SDC2,SEMA3B,SHISA5,SSH3,STX10,WNT6,YOD1,AASDHPPT,TNFRSF10B
Negative_6175	DBH,FOS,GSR,HDC,MTR,RET,ABL1,ALS2,CAMK2B,CD28,COASY,CYP26C1,DTX2,EPB41L3,FGF1,GLS2,IL20RB,JUND,MAP3K3,MITF,OR12D3,OR51A7,OR7D4,PISD,PLXNA3,POLR3G,PPP1R3D,PPP2R2C,SUOX,TFPI,TLR4,UROC1
Negative_6176	GSTO2,HYAL2,WNT3A
Negative_6177	GP6,ACOX3,ALDH5A1,APLP1,ATIC,CASP8,CD72,CMBL,EGFR,EIF4B,ETS1,GJA1,GOT2,HSPA1B,IFNAR2,ITGB7,MAPK10,MTFMT,MYH4,NCSTN,OR2T4,OR8B12,PLCB2,PLCB3,POLR3A,PTK2,PTPRB,PTPRM,SHISA5,SMURF1,TAS1R1,TIAM2,TNFRSF11B,TNFRSF13B
Negative_6178	BTD,CS,FAS,MVK,ADRBK2,CANT1,CDH5,CTNND1,CXCL6,CYP2J2,DGKD,FSHR,FUT3,HYAL2,ITGAE,KLRC3,LALBA,LRDD,MRAS,OR2D2,OR4A5,OR56A4,P2RX5,POLR2A,PPP2R2C,PRKAG1,PVRL4,RASGRP3,RBL1,TAS2R13,THY1,WNT4,B4GALNT1,ST6GALNAC5
Negative_6179	CFI,HAL,ANAPC4,APC2,CFTR,COMT,DGAT2,GUCY2C,GUCY2F,ICOSLG,INHBE,KLRC1,MAPK11,MYLK,OR2H2,OR2M2,ROCK1,SHC4,TPK1,VKORC1,C1GALT1C1

Negative_6180	FH, KMO, ACP1, ADAM17, ALG5, ANAPC11, CHUK, COL4A6, CYP11A1, CYP2J2, DUSP8, ERBB3, GMPR, GNG3, GRIN2A, IL28B, MAP3K4, MAPT, NT5E, OR2AG1, OR4N5, OR52J3, OR5AK2, OR5T1, PCYT1A, PHGDH, PIGB, PVRL3, PYCRL, RAPGEF1, RBX1, TAS2R16, TCF7L2, TGDS, TNFRSF10B
Negative_6181	CSK, HK1, TFG, ALDH7A1, ALOX5, C3orf10, CREB3L1, CSNK1E, CTNND1, DDX50, FABP1, FUT6, GLO1, GNPAT, GNPDA2, IL2RB, INHBB, MARS, MYLK2, NRG3, OR10A5, OR2W1, OR51G1, OR6C74, OR7D2, OR8B12, PAK2, PPP5C, PYCR2, SLC27A5, SPRY4, TFPI, TGFA, TUBB2C, UGCGL2, ALDH18A1, PAFAH1B3
Negative_6182	VTN, CYFIP1, INPP4B, OR10A7, OR9G4, PAK4, POLR2I, PTDSS2, TKTL2
Negative_6183	CFD, GP9, ADCY8, ADIPOR1, AKR1A1, ALDOA, ALG6, CDS1, CRKL, CSDA, CYP11B2, CYP3A5, CYP4A22, DDHD1, GALC, GSTO2, IL18, NANP, OR4K2, PON1, PPA1, PPP2R5D, PRODH, SAT1
Negative_6184	PDC, SCD, ACTN1, ACVRL1, B3GALT2, CACNA1S, CACNB1, CCL5, CCR8, CSNK1G3, CTSG, CYBA, CYP2B6, DDX23, DDX47, DDX54, HCLS1, IKBKB, IL19, IL21R, IL6ST, INPP4A, IPMK, KDSR, MAPK3, MGAT3, NAGK, NCAM1, NFATC4, OR10Q1, OR13D1, OR56A1, OR8K3, PIP5K1B, PRODH2, PTPMT1, RAPGEF4, RARA, SMAD6, SNW1, STX7, TAS2R38, TAS2R5, TBXAS1, THBS1, TUBAL3, TUBB2B, UGT2B4
Negative_6185	AK2, ARPC5L, CASP3, CCL8, CDKN2B, CYP2U1, DKK4, ENTPD5, FGFR2, GSTZ1, ITGAM, OR2T8, OR5H2, SUCLA2, TNFRSF17
Negative_6186	STS, APOC3, CALML3, COMT, CTNND1, CYFIP1, CYP17A1, EHMT1, ERO1L, FBP2, HLA-DOA, IL6ST, OR1D2, PDE3A, PDGFA, PDK1, PPARD, SLC2A4, SMAD5, STX1A, VAV2, VEGFB, WHSC1, WNT16
Negative_6187	ID3, AKR1C3, FGF12, GANAB, GZMB, KCNB1, MYH13, PTGDS, RALB, SOAT2, STAT3, TNFRSF8, ULBP2, VKORC1
Negative_6188	SMO, ABP1, ACP1, ALPP, AMY2A, ATP2A3, B3GNT5, C1QB, CD274, CD38, CSF3R, CXCL13, CYP11A1, ENAH, EPHB3, FASN, FLT1, GCNT4, GDF6, HLA-DOA, KLRC4, KYNU, LIPG, MGAT4B, NFE2L2, OR5D14, PLK1, PPAP2C, PYCRL, RAC3, SEMA7A, SLC27A4, TBL1Y, TGFB3
Negative_6189	EPHA6, JAK2, PIAS4, YARS
Negative_6190	ACTG1, ALOX15, ATP6V1H, DRD2, GDF7, GSK3B, LIMK1, MCM2, MCM3, MDM4, MGAT5, MYLK, OR2T1, SUFU
Negative_6191	ACSL3, ACYP2, ADCY8, BPNT1, CNTN2, CTBS, CXCL10, CYBB, CYP2A6, DUSP9, ENPP2, FGF2, GRIN1, GSTM2, IKBKB, IL15RA, IMPDH2, ITPK1, KLRC2, LTC4S, MAT2B, MGAT3, MYD88, NKD1, OR13C8, OR1D5, OR2H1, OR8D1, PITX2, PNPT1, POLR2D, PRKY, PTDSS2, SOD1, TBL1X, TOMM40, UCKL1, VTI1A, ATP6V0A1
Negative_6192	AK3, MSN, PGP, ACADM, AGRP, ALDH3B1, APH1A, ARHGAP5, ATP6V1F, CD99, CLTC, CX3CR1, CXCL10, EXTL2, FARP2, GADD45G, GALNT13, GLI3, HSD3B1, LTC4S, MAOA, MAOB, MGAT4B, NOTCH3, OR51D1, OR8U1, PGAP1, PLCB4, POLR1E, POLR2K, PRKAR1A, PRKY, PTPN6, RBX1, SLC27A5, SLC2A4, SSH1, ST3GAL5, TRAF2, TSNARE1, TUBA3C, PAFAH1B2
Negative_6193	ALDH4A1, ALPL, FGF19, FMO4, HHEX, NFYC, ATP6V1B1
Negative_6194	SYK, ACSL1, ACSS2, ACTA2, ADAM10, ADRA1A, AGPAT2, ALDH1A1, ATP2A1, ATP5C1, CCNB1, CHRM1, CYP3A5, DMGDH, DOCK1, DRD2, E2F5, F13B, FZD9, G6PC, G6PC2, GAD1, GALNT6, GOSR2, GPT2, HIP1, HPSE, NKX3-1, OR52N2, PIK3C2A, RAP1A, RDH8, TAS2R13, TIAM2, TJP3, TXNDC12, USE1, ATP6V1G1, SERPINB5
Negative_6195	APP, AGXT, ALG5, ATP4A, ATP5D, ATP5J2, BMPR2, CCL17, CD72, DDX55, FARP2, FGF11, FLT3LG, G6PD, HSD17B8, HYAL2, IARS2, IFNK, JAG2, MAP2K4, MAP3K4, MAPK11, MYH15, NRCAM, OR10G4, OR11L1, OR1B1, OR2AG1, OR2L3, OR2Z1, OR4X2, PAFAH2, PLA2G5, RAP1A, SEMA3F, SENP2, SMARCA5, SOX17, TYROBP

Negative_6196	FH,MET,MPZ,PAH,TNR,ACAA2,ACADL,ACSS2,ALOX15B,ARG1,ATP5G1,CACNA1H,CBLB,CD28,COASY,DARS2,DOLPP1,E2F4,EI24,ERBB2,FCER1G,GALNT7,GAMT,GCAT,GRM5,GSTZ1,GUCY2F,HYAL1,IRAK1,JUND,LEF1,MAN1C1,MGAT5,MMP7,MTFMT,OR10X1,OR4D6,PDXK,PIGT,PIK3R1,PRKG2,RBX1,ROCK2,SEMA3D,SORD,STK36,STX2,TAS2R8,YWHAH
Negative_6197	EBP,SFN,ACP6,BST1,CCL4,CCR5,CD247,CDC45L,CPT2,CREB3L3,CTNNA1,CTPS2,DFFA,DHCR24,EHMT1,EIF4E2,EPHA3,ERBB4,GART,GLI2,IFNA13,IL21R,INSRR,IPPK,MDM2,MTHFS,NT5C1A,OR2T4,OR4D1,OR4D11,OR51D1,OR6Q1,PAK1,PIAS2,PRSS2,SDHA,SLC27A1,CSGALNACT2
Negative_6198	CAD,F2,FYN,LPO,LTB,MPI,PLG,A4GALT,ACOX3,ALPI,CCL17,CCL25,COL6A2,CRY2,FABP3,FGD3,FGF8,GPLD1,GSTM2,HHIP,IFNW1,IL10,IL23A,ITGB3,KIR2DS5,MAD1L1,MAN1B1,MCM5,MMP7,MOV10L1,MYLK2,NFASC,OR1G1,OR1J2,OR2AT4,OR2B6,OR2F2,OR4K17,OR5B17,POLE2,SMPD3,SPHK2,SRGAP2,SYNJ2,TACR1,TAS1R2,XYLT2,ZIC2,PLA2G12B
Negative_6199	CBL,F2,ABLIM1,ALDH3B2,CACNA1C,DHRS9,FASN,GLS2,ICAM2,LALBA,MTFMT,NFYA,NNMT,OR4A16,OR4C12,OR6K2,PERP,PLAU,PTTG1,SC5DL,SEMA4F,TAS2R40,TKTL1
Negative_6200	HYI,AADAC,AGXT2,APAF1,CHRM1,CLDN17,CYFIP2,CYP4F3,FOXA2,GCLC,GRM1,IL2RG,LIMK1,NANS,OR4Q3,OR5AC2,PDCD1,PISD,SDC2,SNW1,TAP2,TLR9,TYK2,XCR1
Negative_6201	MIF,AKR1C1,ARSB,DAPK2,EXTL3,GALT,HYAL2,IFIH1,IFNA13,IL29,KCNQ1,OR8B12,PAK2,PI4KB,PLA2G2A,PPM1B,TIAM2,TNFRSF9
Negative_6202	LCT,ATP5G2,GRM1,MOV10L1,PCCA
Negative_6203	C2,HAL,HK1,PLN,ABCC8,CACNG3,DPAGT1,DTX2,DUSP8,EPHB4,H6PD,IFNA10,IL1R1,KCNQ1,MECR,NUMB,OR5H6,P2RX1,PI4KA,PLA2G5,PNMT,PPAP2A,PPP3CB,PRKG1,PSEN2,SEMA7A,SENP2,TCF7L2,UAP1,ATP6V0D1,GABARAPL2
Negative_6204	IDS,TH,ACP2,AFMID,ALG9,AMPD2,ASAH1,CCDC6,COL11A1,CTSG,CXCL5,CXCL6,DNMT3B,DPYD,ENPP2,EPHB2,FGF14,GPAM,H6PD,HAP1,HPSE2,IL23A,IL24,JAG1,LDHA,MYH14,MYH4,MYL5,MYLK,MYLK2,NADK,NFYB,NOS1,OLAH,OR11H1,RPS6KB1,RPS6KB2,SNAI2,TNFSF9,VEGFB
Negative_6205	C2,DDC,GP6,ALG8,BUB1B,CDC45L,CPT1B,CREB1,FRAT2,GRB2,HHIP,IFNW1,INPP4A,LAMC1,MYH2,NME2,OCRL,OR4A16,OR4C15,OR5P2,OR6T1,PARVG,PGAM2,PIK3CD,PTGER3,RHEB,SC4MOL,SLC27A4,SMAD6,TAS2R3,TCF7L1,THBD,TNFSF9
Negative_6206	DDO,MIF,ACAT2,ACSL3,B3GNT2,CLDN3,CTNNB1,CXCL2,DGKA,DUSP3,DUSP5,GALNT10,GCLM,GNAL,GRIN2B,IDI2,IRAK4,KRAS,KYNU,MAPK13,MYH1,OR13C4,OR4D5,PPAP2C,PPP2R1B,PPP2R2C,PRKCZ,PTTG1,RAC2,STK3,STX18,UGT2B28,VANGL2,WASF1,ZIC2
Negative_6207	C8B,HK2,SPN,ACYP2,ADRB3,AREG,BDH2,CDS2,DHDH,DPYS,DTX3L,EDN1,ENPP3,FGF17,FGF8,FLOT1,FRAP1,HEXA,IL11,IRAK4,IRS1,KYNU,NADK,NCR3,OR2A25,OR5D18,OR6C75,PCCA,PDK1,PLCE1,PLTP,POLR3A,POLR3B,PPP2R5D,RXRA,SEMA4C,SLC27A4,TAS2R5,TAS2R50,TCF7L1,TNFSF11,WNT1,CSNK1A1L
Negative_6208	CCR6,GAS1,GGCX,IFNA16,MGAT5,NFASC,SMAD2
Negative_6209	AR,F7,BHMT,CACNB3,CCL22,CDIPT,DVL2,EVI1,EXTL1,FGFR4,HLA-DOB,MTMR1,OR6K6,PNPO,PRIM2,UGCG,UGT2B28,VAMP4,WARS,YWHAB,ZNRD1
Negative_6210	CMPK1,PIM2,PLCB1,TIRAP
Negative_6211	AK1,MPZ,SRC,TXK,ACADL,ACYP1,ALAS1,ALG1,ALOX12,ATP6V0C,CCKBR,CCL15,CYP2E1,DEGS2,DLAT,FASN,GGT6,GYS2,HSD3B2,IL18RAP,ITGA5,LIPG,MGLL,MOV10L1,MYH4,MYH7B,NTN1,ODC1,OR10G7,OR10X1,OR2T29,PER3,PKLR,PLA2G3,POLR1E,PRKY,PTPRJ,RAF1,SEMA4B,SOCS1,SUCLA2,TGDS,TGFBR1,HLA-DPA1,MAPK8IP3

Negative_6212	SRM,ADH1A,ADORA2B,ASCC3,ATP5L,CACNB1,CAV1,CDC25C,CX3CL1,DPM1,HRH1,HSD17B2,LAMB3,NARS,NLGN2,OR2G2,OR7D2,PLCG1,PPP1R3B,PRKAB1,PTGER1,PTPN1,SPRY4,SULT1A2,TIAM2,WNT5B,CACNA2D1
Negative_6213	ARPC5,CTBS,CTSS,DGKZ,FIGF,GMPS,INSR,IRAK1,ITPK1,NCSTN,OR1S2,OR4D5,PIK3CG,RASSF1,SEMA7A,TOLLIP,TPM3
Negative_6214	BTC,DHH,OGT,SFN,ADORA2B,CACNG5,CARS2,CCL7,CDC42,CSNK1G1,CTLA4,FGFR2,FSHR,GALK2,GLG1,GMPR2,GRLF1,HSP90B1,IFNA4,MGAT4B,MINPP1,MMP1,OR1D5,OR4F5,OR4P4,OR52L1,OR7A10,OR8I2,POLD2,PPP3CB,RBL1,RFNG,TRAF5
Negative_6215	CFH,GK,RFK,TNF,AGPAT2,ALCAM,ALG11,ANPEP,APBB1,ARAF,AVPR1A,GNPDA1,HEMK1,IDUA,IFNA2,IL12B,ITGA5,ITPKB,NCKAP1,OR2T4,OR8B8,RRM2B,SESN1,SLC9A1,SUCLG1
Negative_6216	AK2,ME2,NPL,TFG,AACS,ADSS,ALG12,ATP6AP1,CACNA1G,CACNG7,CDH3,CLDN10,CXCL5,DDB2,F2RL1,GFAP,HARS,HNF4G,MAN2A1,MAP2K1,MCAT,NCKAP1,OR2AG1,OR52A5,OR52B6,OR52W1,PHPT1,PIP5K1C,PRKACG,RRM2B,SEMA3D,SOCS7,SPTLC2,TPMT,TYMP,WEE1,PHOSPHO1,ST6GALNAC6
Negative_6217	GH2,PXN,A4GALT,ADH1A,ALOX12B,BPNT1,C3orf10,CAMK2A,CLDN15,COL1A2,COQ7,DPM3,FDFT1,GAS1,GGT1,IFNK,ITGA2,NCF4,NFYB,NOS3,NRG4,OR1D2,OR2H1,OR6N1,PDE4B,PDGFC,PIK3C2B,PMAIP1,POLA2,POLR1E,POLR3K,PPAT,PRPS1,SH3BP2,ST8SIA5,STK36,TCF7L1,TUBA1B,VAMP3
Negative_6218	HGD,LSS,ACAA2,CCND1,CDH1,CDKN1A,CYP1A1,DTX1,FGF9,FSHR,IL4R,OR4C15,OR4Q3,OR6T1,PPARD,PSME2,SLC27A5
Negative_6219	DDO,MIF,ACPP,ADCY5,ADORA2B,AMPD2,CCKAR,CTBP1,ENPP2,GSTA2,HSD3B7,IDH2,ISYNA1,MTMR2,OR6X1,PFKM,PRLR,RPS6KB1,STAM,ULK3
Negative_6220	CGN,ACY1,AGXT2,ARPC5,ATP6V1D,CACNB3,CCNH,CD74,EFNB2,ENO1,HIF1A,IFNA21,IL29,INPP4B,LDHB,MMP7,NT5C,ONECUT1,OPLAH,OR1G1,OR52L1,PRKACB,PRKCD,PYGM,RND1,SDC1,SEMA3C,SLC2A4,TRPM5,PLA2G12A
Negative_6221	ALG10,AQP7,COL4A2,CXCR4,CYBB,FGF6,FUT7,GLS2,LYPLA2,MARS,OR51F2,PGK1,PLA2G2E,PPP1R1A,PPP2R2C,PRKAR1B,SHC2,TAS1R1,UGT2B10
Negative_6222	TK1,AKT3,ANAPC1,BAIAP2,CANT1,CCR7,CD38,CLTCL1,COL4A6,CREB5,CTNND1,DGKB,ENPP2,FLOT1,GNAL,GNG13,GSTA4,GSTM5,GZMB,ICAM2,INHBB,ITGA6,LDHC,LIPA,LYPLA1,MOV10L1,NSD1,OGDH,OR10Z1,OR2G6,OR51B5,OR52N1,PON2,PPP2R2B,PTGES2,RPS6,RPS6KB2,SEMA3G,ATP6V0D1,MAPK8IP3
Negative_6223	CP,EDA,ABAT,ABLIM3,AKR1C1,ALDOB,ARHGEF4,ARPC2,CDC42,CDC6,DGKQ,GUSB,HRH1,IGFBP3,ITGA4,KIR2DL1,NMNAT1,NOTCH3,OR51V1,OR5K1,POMC,PSEN1,RALA,SESN3,TSC2,VDAC2,ST6GALNAC4
Negative_6224	ADCY4,ADRA1B,ASNS,B3GALT1,B4GALT1,CACNA1S,CDC26,CDC27,CLDN16,CLTC,CYP2A13,CYP2U1,EI24,EPB41L3,F11R,F2RL1,FADD,GALNTL1,GSTP1,IFNA8,IL13,IMPDH2,INHBB,ITGB6,KLRK1,MPZL1,MTHFS,NCF1,NEFL,NFKBIB,NODAL,OR4F17,OR51B4,OR5D18,PARVG,PDE4A,PFN2,PLK1,PPP1R3B,RALBP1,RPS6KA3,SCP2,SMAD9,ST8SIA1,STX12,UNC5B,PAFAH1B1
Negative_6225	ATF2,CNTFR,DUSP9,G6PC,MYL2,WNT2B
Negative_6226	C5AR1,CACNA1F,NSDHL,OR3A3,OR5H2,RPS6KA2,TOLLIP,TRAF5,XCR1,XYLT2,SERPINE1
Negative_6227	FER,SYK,TK2,ALDH1A3,ALDOB,ALG6,ALS2,ARRB1,CACNG6,CCL23,CCL27,CHSY1,CSNK1D,CX3CL1,EFNA4,ENTPD7,EPB41,GDF6,GGT5,GNG12,GNRH1,HSD3B1,IRS1,NLGN3,NUMBL,OR2T8,OR4F6,OR6C3,PAK2,PDHA2,PIK3CA,PLAUR,PLXNC1,POLR1A,PPP2R5C,PRKG2,RARS2,RDH5,SETMAR,SMAD7,SMURF2,TBL1XR1,UGT1A10,VNN1,ALDH18A1,ATP6V0D2,GABARAPL1

Negative_6228	CDKN2D,CER1,EFNA2,HTR5A,ITPA,OR5AN1,RHOA,SARS,ATP6V1E2,SERPINA1
Negative_6229	BLVRA,CALML5,ESAM,JAM3
Negative_6230	CCL27,CSF2RA,HEXB,IL17RB,NTF4,TNFRSF10D
Negative_6231	CLDN15,DDX19A,FSHB,IL2RG,IMPA2,LYPLA2,OR10H1,SH2D1A,ST3GAL5,HLA-DPA1
Negative_6232	ILK,ARPC3,BMPR1A,FGF2,OR5AS1,OR8G1,OXTR,PLXNA1
Negative_6233	F3,PTS,ACVR1C,B3GNT5,CSNK1A1,GFPT2,GRB2,OR13C9,OR1M1,OR2D2,OR4K15,OR4S2,OR56A3,OR9G1,POLR1A,RASGRP3,RPS6KA3,SETD7,WNT9B
Negative_6234	AUH,F7,GNS,IL4,AGPAT4,AKT3,ATP5G3,ATP5J2,CDKN2C,CHEK1,CLCF1,CLDN16,CTSB,ENTPD3,FGF19,FGF2,FLT3LG,GCNT4,HCLS1,HDAC2,IDH3A,INADL,ITGA6,LAMB2,MINPP1,OR12D3,OR4A5,OR52D1,OR6A2,OR7G1,OR8H2,OR8J3,PGAM1,POMC,PORCN,RAPGEF4,SMAD2,STT3B,SULT1E1,TSHB,VAMP3,PHOSPHO1,TNFRSF13B
Negative_6235	ARPC1B,ASPA,CXCL3,F2RL2,IL11RA,LAMC3,MAN1A1,MMP9,OR1A1,OR52D1,ORC1L,PPP3CA,WNT7B,SERPINC1
Negative_6236	CRK,AKR1C4,ATP6V0B,BTRC,CASP6,CASP8,DFFA,DGKZ,EPB41L2,FGD3,GARS,GLI3,GNPDA2,GRLF1,HSD17B4,IDH1,IFNA4,IL1R2,MAD2L1,MDH2,NFKBIA,OR1E2,OR6C1,PRIM2,SMAD1,SRGAP3,ULK3,MAPKAPK2,PPARGC1A
Negative_6237	FAH,ALG1,CCL20,CDC25A,CYP26B1,GLO1,GSTA1,GSTA4,HLA-DMA,KYNU,LAMB2,MEF2C,NTSR1,OR4F17,PIGN,RDH8,STX4,UBA1
Negative_6238	AMT,CFH,GCK,NGF,ABAT,ABL1,ADRA1D,ARRB1,C1QA,CBR1,CD28,CSDA,CXCL3,EXOC7,FCER1G,GUCY2D,IFNA6,IFNAR1,IRS4,LAMA3,MAP3K5,OR2L13,PAX4,PCCA,PIGA,PIGB,PLTP,RPE65,SLC25A4,TBL1X,TSNARE1,ULK2
Negative_6239	LTB,AGXT2,AMD1,APAF1,ARNTL,CASP10,CDK6,CHST11,CHST13,DGAT2,DGKI,DUSP6,ENTPD8,ETS1,FGF3,FUT8,FUT9,GALNT1,GALNT3,GLDC,GRIN2A,HGSNAT,HSPA1B,IRS1,ITPKB,MAP2K2,MAP3K4,MDH2,MS4A2,NKX2-2,NOTCH2,OR2L2,OR51B5,PFN1,PHGDH,PI4KA,PRIM2,PRODH2,PSAT1,PSME3,PYCR1,SGPP2,TNFSF4,ATP6V1B1,TNFRSF18,TNFRSF6B
Negative_6240	GSN,ITK,TYR,ACMSD,ADSL,AMD1,ARD1A,ASRGL1,BBC3,BMP4,CADM1,CCNG1,CDKN1C,CSNK2B,CTNNB1,DIAPH2,E2F3,ENTPD8,EPB41L2,GPD2,GRAP2,GRIA1,IL1B,IL6ST,ITGB7,LIPF,MAP4K1,MAPK3,MPZL1,NFKB2,NOS1,OR10G7,OR12D3,OR13D1,OR51T1,OR7A17,OR8S1,PGK1,PNPO,POLE2,PPBP,PTPRF,TAS2R16,UGT8,UROS,ATP6V1C2,CACNA2D1,PHOSPHO1
Negative_6241	F12,ABLIM2,ADRA1D,ATP5A1,AXIN1,B3GNT3,BBOX1,BMPR2,BUB3,CACNB1,CACNG3,CDC14B,CDKN2B,CLCA2,CLDN8,CTBP1,CTBP2,CTSL1,CYBB,CYP8B1,DLAT,EVI1,FMO2,FTH1,FUT8,GLI2,HSD3B1,IL2RG,LIMK1,MAP2K2,MAT1A,METTL6,MYD88,MYH7,OR2K2,PLAT,RALGDS,RBPJ,RPS6KB1,SAT1,SESN2,SMAD7,TLN2,ULK2
Negative_6242	IL2,ABAT,AGPAT3,ATIC,CCL28,CDC45L,CSAD,CYP19A1,CYP2A6,ENO3,FPGT,GALE,HIF1A,HSPA1B,IFNG,KITLG,NCR1,OR2A2,OR51E1,OR6F1,OR6Q1,OR8S1,PIK3CA,POLR2G,RAC3,RERE,SHC2,TAS2R9,TBXA2R,TUBB,TUBB6,UGCGL1,VNN1,WASF3
Negative_6243	HK3,JUN,AACS,ACAA2,ACSM2A,ADH1A,B3GAT2,CTF1,DHCR24,HEMK1,ITGA10,LTA4H,MYD88,MYL5,NMNAT2,NOS3,OR11H4,OR5P2,PGAM4,SFRP5,SLC27A4,TCF7,WBSCR17,TNFRSF10C
Negative_6244	CRK,F12,FAH,HYI,IL6,PGD,ABI2,ACVR1B,ASMT,CERK,DUSP5,FUT2,GDF6,GNA14,GNAI2,GOT1,GPD1L,GPX5,HMBS,MCEE,MYH10,NFYB,NT5C3,OR13C5,OR1N1,PER2,PLXNC1,PTGIS,RPS6KA6,SMAD1,SMC1A,SQLE,STX4,TGFB1,TPH2,TSHR,UNC5D,PHOSPHO1

Negative_6245	RPE, ALS2, C3AR1, CDIPT, CSNK2A1, DDX51, DUSP3, ENTPD7, FMO5, GLUL, ICOS, IRF7, JAM3, MAP3K8, MYH13, NEU4, OR51I2, OR52M1, P2RX3, PPID, SEMA3B, SORBS1
Negative_6246	MOS, STS, TXK, AANAT, ACAD9, ACSM2A, APC2, CACNG2, CCL26, FCGR3B, FGF10, GABARAP, GNMT, IL1RAP, MAPK9, MYH15, NADK, NCKAP1L, OR51T1, OR52B4, PDE5A, PIK3CA, PPP1R3A, RPS6KA4, SHC1, SLC27A5, WNT2B
Negative_6247	CDKN2C, GSTM5, PTCH1, TXNDC12, UGT2B7, SERPINB5
Negative_6248	LHB, ALDH3A2, CLDN16, COL6A1, CRKL, IFNA2, MTHFR, NAT1, OR1I1, OR2M2, ORC3L, P2RX1, PRKAG3, RARS, ROBO3, SHISA5, SOCS3, SERPIND1
Negative_6249	ADORA2A, CCNB2, CXCL3, EPHA6, GADD45B, GMPR, GNAI3, HNF1A, KDSR, LCMT1, MGAT3, OR5B12, PTPRR, UCK2, CSGALNACT2, SUV420H2
Negative_6250	F2, ID4, APOA1, CCND1, CHIT1, CMPK1, COL6A1, DLL3, DPM2, F2RL1, GAMT, GRIA3, KIR2DL1, LAMC3, LRP5, MARS2, MPST, OR2A2, PANK1, PIGB, RARS2, SH3BP2, TGFBR1
Negative_6251	CBL, DUT, F5, FER, GLA, LPO, NLK, ABLIM1, ACP2, ALG10, ALOX5, CD72, CFL2, DGKH, DUSP16, EFNA1, ESAM, FGF5, GALNT7, GPLD1, IMPDH2, KIR2DL1, NEU1, NME6, OR2T33, PCYT1B, PYCR2, SHC1, SMAD6, ST3GAL5, ST3GAL6, SYNJ2, TOLLIP, TUBB4, VANGL2, VTI1A, WNT5A, YWHAB
Negative_6252	GLA, LPL, TEC, ACSM2A, ACTG2, ACVR1C, AMPD1, ANGPTL4, CACNG2, COASY, COL4A6, CXCR5, DHDH, DLAT, FCGR3B, GALNT7, GALNT8, GCH1, GFPT1, HIBCH, HSP90B1, IFNAR2, IL20RB, ITGAL, LTBR, MAFA, MCM2, MGAT2, NEFH, NUDT2, OR10Z1, OR11H1, OR13G1, OR2A14, OR4K5, OR9Q1, P2RX1, PDE4C, PIGH, PLCB2, PON1, PPCS, RFWD2, SCP2, SOX17, STT3B, TCF7L2, TPM3, YWHAE
Negative_6253	IVD, MIF, ARNT, GUCY1A3, HYAL2, NFATC3, NRXN1, PHKG1, SFRP1, SRGAP3
Negative_6254	F9, BAAT, DPYS, GOT1, MLLT4, NSD1, NT5C, OR4F15, PTPRC
Negative_6255	IL6, ILK, BDKRB1, BET1, CAMKK2, CFL1, CNGA3, DGKH, EFNB2, IFIH1, IFNA10, ITGAX, KAT2A, NAGLU, NT5M, OR10C1, OR10R2, OR2T2, OR5AC2, POLA2, PTCH1, PTK2, SFRP2, TAS2R5, TSLP, YARS
Negative_6256	ACHE, ACTG1, ATP5E, DDX56, DPM3, GPD1, HEXB, HSD11B2, HSD17B2, KIR3DL3, OR6F1, OR7C1, OR7G3, OR8J1, PDE9A, PDK1, POLR2A, PPM1D, TSHR
Negative_6257	C8B, ACYP2, APOA2, CACNG5, CASP9, CAV3, CCNB3, CD40LG, CLTA, CNTF, CRKL, CSNK1A1, DAD1, DRD2, GANAB, GART, GCNT3, HAO1, HLA-DOB, HLA-DRA, HMGCL, HMOX2, IKBKB, ITGA3, OR51F2, OR51M1, OR5AS1, OR5K2, OR6X1, OR8G1, OR8H3, PCYT1A, PIP4K2A, POLR2J, PPP3R2, RBPJ, RERE, TRADD, VEGFC, WEE1, WNT9A
Negative_6258	CD55, DGKG, GANAB, IFNA16, OR5L2, SV2B, KIR2DL5A
Negative_6259	CFI, GHR, HGD, TEC, ACY1, ADRB1, ATP5J2, CASP3, CES7, CLOCK, CRLS1, CYBA, EXOC7, F13A1, HSD17B3, IQGAP3, ITGB2, NADK, OR13D1, OR2A25, OR5H6, PI4KA, PPAT, RAC2, RND1, RPIA, SHC2, TAS2R14, VAMP4, WIF1, WNT6, ST6GALNAC1
Negative_6260	F3, ACSM4, AKR1A1, ALDH3B1, ARRB1, CD80, CHSY1, DEGS2, DPM1, EFNA4, FARP2, FZD8, GUCA1B, GZMB, KDSR, MAML1, NUDT9, OR13C9, OR3A2, OR51B4, P4HA1, PIGC, PIP4K2B, POLR1A, PRKAR1A, RASGRP4, STX10, TAS2R42, UCK2, UGCGL2, WNT2
Negative_6261	GLA, AKR1C3, ATP6AP1, CACNA1B, GALK2, HYAL2, OR10AD1, PIGB, RASGRP3, RFXANK, SLC12A2
Negative_6262	ACHE, AGRN, ALDH6A1, ALOX12, B4GALT7, DOCK1, E2F1, GRIA3, GUCA1C, NR5A2, OR11H6, OR2A4, OR5P3, POLR3H, STAT4, STAT6, THBD
Negative_6263	F2, TXK, ACACB, ALDH1B1, APRT, ARHGEF6, ASRGL1, ATP5L, B3GNT4, CD19, CDC42, CRB3, CYP2C8, CYP2U1, GALNT7, GALNTL1, GSTA5, IFNAR2, ITPA, OR10G7, OR3A2, OR5B17, PFN3, PPM1A, PPP3CB, RPIA, SGMS1, TUBB3, ATP6V1E1, TNFRSF11B
Negative_6264	DDO, ARPC1A, CCL24, CD274, CPT1B, EPHB6, GPX5, HTR5A, NFATC1, OR52W1, PIAS4

Negative_6265	AK2, CBL, HSD17B4, MGAM, SEMA4C
Negative_6266	FUK, GHR, ACSM2A, ADRA1B, BCKDHA, CAMK4, CLDN7, CREB3L2, CYP2A6, EP300, F2RL2, FGF11, FMO1, GNB3, GNRH2, IL2RA, ITPA, KIR3DL1, LDHAL6A, MICA, MLLT4, NEU2, NEUROD1, OR10K1, OR10S1, OR2T11, OR2Z1, OR52N2, PLA2G2A, RAF1, SC4MOL, SFRP2, SGMS2, STX1B, TICAM2, ZIC2
Negative_6267	CD2, CGN, PRL, ALDH4A1, ALDH5A1, ALOX15B, ATP5O, ATP6V1D, CCL25, CER1, COQ3, CYP2E1, FADS2, GAD1, HTR5A, IRF5, MAPK11, MAPK3, MMP7, MPDZ, OR2T29, OR6C2, OR8B4, OR8D4, PAK2, PLXNB2, PRSS2, RDH11, ROCK2, SMPD3, ST3GAL1, ST8SIA1
Negative_6268	F11, IL4, LIF, MVK, PDC, ADCY3, APC2, CCL28, CPT2, DDOST, DDX51, EXTL3, FGF12, GAD1, GDF5, HIBCH, HMOX1, IL20RB, KCNQ1, KLRC2, LAMA4, MDH1, NT5C, NT5C2, OR14C36, OR2D3, OR5R1, PDE7B, PER2, PFKFB4, PPP2R5C, PRKCD, QARS, SAT1, SRD5A1, TAS1R3, TGFB1, VDAC3, WNT8B
Negative_6269	C9, FAH, GK, SMO, XDH, ACAT2, APOC3, CD27, CFL1, CHRM2, CHSY3, CSF2, DCTN1, DDX18, FGF10, FLT4, FOXO1, GALNT5, GGT5, IDI1, IFNB1, IL2RA, IL6R, LIMK1, LRP5, LRRC4C, MAP2K1, MPDZ, NT5C1A, OR14A16, OR14J1, PCK1, PPP2R2D, PRKAR2A, SEMA4C, SEPHS1, TNFRSF8, UBE2K, UGCG, UGT2B17, ZAP70, ZFYVE9, CSNK1A1L, PNLIPRP1, TNFRSF18
Negative_6270	CD2, ACTC1, BDKRB1, CLCA4, DDX4, DGKB, DNMT1, EDNRA, MAOB, NOTCH1, PFN4, SEMA7A, SOCS4
Negative_6271	CPT1A, GADD45A, NADK, NAMPT, NT5C1A, OR13A1, PLCD3, QARS, RBL1, SEMA3D, TAS2R50, YARS2, HSP90AA1
Negative_6272	F2R, F8, AADAT, ADCY6, ADCY7, AGPAT3, ALOX5, AMPD1, ASRGL1, B3GALT4, BACE2, CACNA1B, CACNB4, CIITA, CLDN8, CNGA3, DBF4, GALNT9, GRIN2C, GSTT1, IRAK4, IRF5, MTHFD1L, OR10G8, OR13C5, OR2K2, OR51G2, OR5M8, PARVA, PDE9A, PITX2, PPP3CB, PRKACG, SEMA4D, SETD1B, SETMAR, SUCLA2, SULT2A1, SV2B, TALDO1, TAS2R42, ATP6V1G2, CACNA2D4, ST6GALNAC4
Negative_6273	CAT, FST, TH, AADAC, ACMSD, AOC3, BIRC2, CDS1, COASY, COQ5, FGF9, FTCD, GALNT13, GANC, GFAP, HSD11B2, IDH1, LAMB2, LIPT1, MKNK1, NAT1, NLGN2, OR13F1, OR2T4, OR5F1, OR6B3, OR8B12, PPP2R2B, PRKG1, PTGFR, SAT2, SDC2, TAS2R40, WNT5B
Negative_6274	AK5, TFG, TNF, ACOX1, ACOX3, ACTC1, ACYP2, CACNA1A, CCL25, CERK, CLTB, DAPK2, DTX2, DUSP8, EPHB3, FOXO3, FUT3, G6PD, GABARAP, GPD1, GSTA5, IL13RA1, ITGA2, ITGAD, MYLK, NLGN1, OR10G3, OR14I1, OR2AT4, OR2C3, OR2L13, OR5K2, PDE3B, PNMT, POLR1D, POMC, RETSAT, SEMA3F, SLC12A2, SLC2A2, ST3GAL4, STX6, TAS2R1, TRIP10, UGT2A1, VNN1, WBSCR17, SERPINA1, TNFRSF12A
Negative_6275	CKB, FGA, TEC, ABLIM3, ACADVL, ALG2, ATP13A2, CCL21, CD99, CDKN1B, CEBPA, CSF2, CYP1B1, CYP4F3, DDX19A, FRAT1, FSHR, G6PC2, GALNTL2, GPD1L, GYS2, H6PD, HSD11B2, ICOSLG, IL1R2, IL6ST, KAT2B, LILRB3, MAP2K1, MAP2K2, MGLL, NLGN1, OR1M1, OR2D3, OR4C13, OR6Q1, PCK1, PDE8B, PFN3, PRKCB, PYCRL, SCLY, STMN1, TAS2R10, TNFSF8, TOMM40, VAMP4
Negative_6276	CEL, CTH, F11, MUT, AGMAT, AKT3, ASAH1, CD59, DUSP8, EPB41L1, EXTL3, FGF10, GPX5, IFNA6, IL11RA, IL1B, IL21, INPP1, ITGB4, MARS2, NKD1, NME2, NRXN2, ONECUT1, OR10G2, OR1J4, OR4C15, PAK2, PFKP, RARS, SHC2, EIF4EBP1, TNFRSF11A
Negative_6277	CD6, MTR, ACPP, AGXT, CACNA1F, CALML5, CLDN5, CNTF, CSF2RB, FLT3LG, FUT7, GLDC, GOT1, GUCY2F, HAGH, HBEGF, IL24, ITGAX, LIPA, NAGA, NTF4, OR10A6, OR10G2, OR13C8, OR2B2, OR52I2, OR5D18, OR5T2, PLA1A, PLCZ1, PPP2R2A, ROBO3, SEMA3G, SPRY3, STK4, VAV3, WNT8B, HLA-DPB1, PAFAH1B2
Negative_6278	CFD, CKB, UBB, ATP5B, C4BPB, CD86, CTSG, DVL1, FGF5, GNRHR, HADH, HTRA2, IL22RA2, IL8RB, MINPP1, MYL7, NLGN3, OR52L1, OR5B17, OXCT2, PIM1, PLXNB1, PRKACA, ROCK2, SEMA4A, CACNA2D1

Negative_6279	DUT,MPI,SYK,AARS2,ABAT,AKR1C3,ALOX15,BMPR1A,CACNB2,CCR9,CTBP2,DARS,EXOC7,FOXA2,GALNS,GCDH,ITGAV,KCNQ1,KIR2DL1,LTB4R2,MAPK14,MCCC2,MYLK3,NADSYN1,NOS3,NUDT2,OR5D18,OR5L2,OR6C70,OR6X1,PDE10A,PLTP,RASA1,RASGRP3,SEMA3C,SESN3,SETD8,SH2D1A,STAT5A,CSNK1A1L,HSP90AB1,SERPINC1
Negative_6280	HK2,CCL1,CTNND1,IL24,LIFR,OR1L3,OR4F5,OR5AS1,PIGB,PIGQ,STAT4,SUV39H2,UBE2K
Negative_6281	TK2,CCNH,CHKA,ENO3,FOXO3,HSPB1,IFNA10,KARS,KIR2DL1,MCM7,OR1A1,OR51A7,PAK3,PSME2,SEMA3F
Negative_6282	CDA,MAG,ME1,ARAF,CXCL6,DUSP2,DUSP7,GALK1,IAPP,ICAM3,MAOB,MYL9,OR14J1,OR2T3,OR4N4,OR5AP2,OR6C74,PAICS,PAPSS1,PDHB,PLOD3,PPP1R3A,PTDSS2,SLC9A1,UGT2B7,TNFRSF6B
Negative_6283	PML,AGMAT,CD46,CXCR3,DDOST,DOT1L,FGFR1,FZD2,HIF1A,IL10RA,IL2RG,LLGL2,MAD2L1,MAML1,NFYA,NQO1,OR14I1,OR6C1,PFKFB4,PRKCB,RPS6KB1,SNAP23,STX6,TCF7L1,TIAM2
Negative_6284	LTB,PLG,UBB,ALDH1A3,BMPR1B,OR2C3,PDHA1,PPP2CA,CACNA2D1
Negative_6285	F8,GLS,ACTN2,AGRP,AOC3,C1QA,CCNA2,COL2A1,CX3CR1,DEGS1,GSTA2,GSTM1,LTA4H,LYZL1,NUDT8,OR4K14,OR6C3,OR8K1,PCK1,PDHA2,PIP5K3,SAT2,SETDB1,ST8SIA5,TUBB2C,VAV3,YWHAH,CACNA2D3,PPP1R12A,TNFRSF10B
Negative_6286	CD4,PGP,TNF,ACVR1C,AKT2,ALDH1L1,ALPP,ASCC3,ASRGL1,CCNE1,CLDN8,CREB3L3,CXCL13,GSTM4,HAAO,HADH,HIP1,HLA-DOA,LIPG,MNX1,NKX3-1,NR4A1,OR4N4,PDE6D,PFKL,PHKG2,POLR2B,PPP3CC,PTCRA,PTPRM,RRAS2,TAS2R41,TCF7L1,TRAF1,VAMP4,PPP1R12A
Negative_6287	ARHGEF4,ATP12A,BAIAP2,CERK,CLDN8,DGKI,EVI1,GARS,HARS,IL18R1,JAM3,NADK,OR13F1,OR1L6,OR52A5,OR52L1,P2RX5,POLA1,CACNA2D3
Negative_6288	ARPC4,ATG7,ATP5G1,CTNNA3,CYP11B2,EDAR,FCGR2B,HIP1,KAT2B,LILRB3,MAP3K4,MGAM,NCOR2,OR51F2,PIK3R3,PNMT,POLR3A,POLR3K,RASSF1,SLC12A2,TNFSF14,TRPM5,TUBA3C,UCK1,WASF3
Negative_6289	ID3,ARPC5,B3GALT5,BCKDHB,CCR9,CDC14A,CLDN19,CRY2,CSNK2A2,CXCL10,DCXR,DDB2,DGKI,DKK4,FLT3LG,GALNT6,GDF5,GPX7,IL7R,LDHB,MIOX,NAMPT,OR2T29,OR51B4,PLA2G2F,PLCD3,PNPO,POLR2I,PRKACG,UPP1,WNT6,SERPINE1
Negative_6290	ABO,ACAD8,ACO1,ALDOB,AMDHD2,AVPR1B,C4BPB,CCND3,CCR10,CSF2RA,DMGDH,EPHA8,FCER1G,FLT1,GALNT13,GUCY1A2,GUCY2C,IFNA4,IL20RB,IRAK1,KIR3DL3,LMO7,LRP5,MKNK1,MMP7,NTRK1,ODC1,OR11H6,OR2T11,OR4D1,OR4D2,OR52E4,OR5K1,OR9A4,ORC2L,PDIA4,PLA2G5,PRKG2,PVRL2,PVRL3,RHOA,STMN1,UNC5D,WBSCR22,ATP6V0A1
Negative_6291	ACAA1,ACP5,ALG10,CHST12,DUSP16,ENPP1,GGT7,GRB2,IFNA13,ITGB2,JAM3,LAMC2,LTBR,MTHFR,MYH2,NEFL,NPR2,NRG1,OR10V1,OR2F2,OR9G1,PLA2G2A,PNLIP,POLR2B,PTGER1,RFWD2,SESN3,STAT3,TAS1R1,TRAF6,TUBB4Q,VDAC1,YES1,SUV420H2
Negative_6292	ADCY8,AGMAT,BPNT1,CCL3,CCNB1,CD74,CHRM1,DHDH,GAD2,GFPT1,GSTO1,NFATC1,RPS6KA1,ATP6V1E2,SERPINA5
Negative_6293	ADC,GH2,LHB,TKT,ACO2,AGRN,ARRB2,CACNA1F,CHIT1,CLDN9,COL4A4,E2F5,EDNRA,FGF7,FSHB,GADD45B,GSTA3,GSTP1,GUCA1B,HMBS,HMGCS2,HRAS,HRH2,HSPA8,HSPB1,IL6R,ITCH,KIR3DL1,MAP3K5,MAPK7,NEFL,OR1G1,OR2W1,OR4C12,OR56A3,OR8H3,PDHA1,PDIA4,PERP,PHKB,PNPT1,PSME2,PTPRC,RBL1,RPS6KA2,SNAI1,UROD,B3GALNT1,ST6GALNAC6
Negative_6294	OAT,AASS,ACP5,ALG10B,ATP5G3,CAMK2D,CTNNA2,EARS2,FGF5,GPAM,GSTP1,HSD11B1,MDM4,PDE3B,PGM2,PHKA1,PRKCA,PTPN11,SSH2,THBS1,UMPS,VAV1

Negative_6295	ID2, ACSL3, CAMK2D, CAMK4, CTSG, DBF4, FASLG, HLA-DRA, ICOSLG, IRS1, LCP2, LEF1, OR13G1, OR2G6, RASA2, RBL1, SPRED2, TAS2R1, THBS4, WNT3, MAPK8IP3
Negative_6296	NPY, CAPN2, GALNTL1, GSTM2, KARS
Negative_6297	ATP2A3, CLDN9, COL1A2, IL8RB, NCOR2, OR7A17
Negative_6298	CFD, FER, GP6, LSS, PTS, XDH, AOX1, BNIP1, CACNG6, CARS, CDH15, CDKN2D, CHST11, CLDN6, CRY2, CX3CL1, DDB2, DDOST, DEGS1, EIF4E2, EPB41L3, EPRS, EXTL3, FGF6, FZD7, GRB2, HTR4, ITGB2, LDHB, NCF1, NOTCH3, OR10H4, OR10Z1, OXTR, PIP5K1A, PPP3CC, PSPH, RAC3, RALB, SDC4, STX3, TGFB2, TNFSF4, TRADD, ATP6V0D2, SERPING1
Negative_6299	ENO1, GNPNAT1, HIF1A, TNFRSF13B
Negative_6300	BMP7, HAO1, IL17B, IL25, PRKAA1, PSME1, TAS2R50
Negative_6301	ANAPC1, BMPR1B, CDC16, DPM2, FGF18, G6PC2, GAL3ST1, GARS, GSTO2, IL28RA, NR1D1, OCRL, OR2B11, OR2L8, OR2T11, OR52L1, OR6S1, PLAU, PPP1R3D, TUBB3, ULBP1, HLA-DRB5
Negative_6302	FER, ACAD9, ADIPOR1, BMP8A, CASP7, CD38, CD47, CHST12, CLDN11, CYP2E1, DIAPH3, ESAM, FABP4, FGF18, FGF4, FLT4, GDE1, INHBE, KITLG, LCMT2, LLGL1, MGAT1, NAMPT, NARS, OR4F17, OR5R1, OR7A5, PER3, PRNP, ROCK1, RPSA, SCP2, STX6, SULT1A2, YKT6, HLA-DQB1, HSD17B12, PAFAH1B3
Negative_6303	PC, PTS, ABCC8, ANAPC1, B3GNT4, BACE1, BMPR2, CCL28, CDIPT, CES2, CHIA, COL11A1, CRHR1, DAPK1, DAPK3, DGKZ, DOT1L, DVL3, FSHR, GNA11, GYS2, HSD3B1, IAPP, IARS2, IMPDH2, MYH14, NT5M, OR1K1, PDE4B, PLCE1, PLXNA2, PTDSS2, RALGDS, RASA1, RBL1, SDHB, SENP2, SIPA1, SQLE, SRGAP2, STAT4, UGT2B11, WNT8A
Negative_6304	HPD, AMPD1, ANAPC5, APOE, DGKZ, GNRH2, GSTA5, HRH1, LDHAL6A, MARS2, PRKCD, SEMA3E, SFRP2, TYRP1
Negative_6305	BAX, CFH, EDA, GPI, KMO, LCT, ARPC2, CDC27, CPS1, CSF1, CSNK1G2, CYCS, DIAPH2, EPHB3, FGF23, GALNT12, ICAM2, IL17A, ITGAD, LCP2, MMP1, NLGN2, OLR1, OR2T5, OR52A5, OR6K2, OR7G2, PERP, PLCG2, PTK2, SCIN, SEMA3E, TAS2R40, TNXB, YES1, EIF4EBP1, SERPIND1
Negative_6306	DOLPP1, F11R, HLA-G, ITGA2B, LYPLA2, OR5A2, PGAM2, PLOD1, STX16, XYLB
Negative_6307	FUK, GSS, CMBL, IQGAP3, MYLK, PLA2G2D, PPCS, TUBB3
Negative_6308	C2, ALOX15B, APAF1, ARSD, ARSE, CASP9, CHIT1, CLCA2, CSF1, CSNK1G2, GNA15, GUCA1B, HES1, HSPA2, ITGA2B, ITGA4, NAMPT, NAT2, NLGN3, NMNAT1, NPR1, OR1L4, OR2G2, OR2M3, OR4D1, OR4N4, OR9Q2, OXTR, PHPT1, PPAP2C, RAPGEF1, RDH16, SGPP1, SKP1, SMAD1, TACR2, TAS1R1, UBE2L6, ATP6V1C2
Negative_6309	AR, GLA, MUT, TK1, VCL, CLDN11, CLDN2, CREB3, DHRS9, DHX58, FASLG, FDPS, FGF2, GALNT3, GNG12, GRIN2A, GUCY2C, IL28B, MAPK7, MAT2B, MINPP1, MPDZ, MTHFD1L, MYH7B, NPAS2, NPR1, NUDT5, OR1I1, OR1K1, OR9A4, PARD3, PHKG2, PNMT, PPM1D, PTGS1, SEMA3B, STAT2, TAS2R5, UGCG, UROC1, XIAP, YWHAQ, ARHGEF12
Negative_6310	CD4, GP9, TEC, AARS2, ACMSD, ALDH3B2, CARD11, CD70, CD80, CDC45L, COL6A1, CPT1A, CREB3, DEGS2, DOLPP1, DPM3, DRD5, DTX3, DUSP1, EPHX1, GSTM4, GYS1, KITLG, MAN2A1, NFASC, NMNAT3, OLR1, OPLAH, OR2L3, OR2L8, OR51M1, OR5I1, OR5P3, PARVA, PGLS, PIGW, PLAU, PSME3, PTK2, PTK2B, RAF1, SLC25A5, VDAC2
Negative_6311	ACACA, B3GALT2, CDKN2A, COL5A3, CPOX, CSDA, EP300, GDF5, MGST1, OR2H1, PRKCG, B3GALNT1, PRICKLE2
Negative_6312	ANAPC1, ARPC5, ENPP3, OR5AR1, ATP6V0A2
Negative_6313	CCR8, EHHADH, EPHA5, FMO5, ICOS, IL1R2, OR8K3

Negative_6314	F7,ACTB,ALDOB,ALOX12B,ASPA,B3GALT2,CACNG3,CD38,CYP3A43,CYP8B1,EPHA4,FABP4,GNB1,GSK3B,GSTO2,HIBADH,ICAM1,MAP3K12,MAPK7,NCK2,OR9G4,PARVA,PDE7B,PFKFB1,PFKM,PPP2R2B,PVRL1,RHOA,SDC2,SESN3,SULT1A4,TJP1,ULBP3,TNFRSF11A
Negative_6315	ADRA1D,EPHA8,GGT6,LEPR,MYLK2,NCR3,NFASC,OR4C12,PARVB,PGM1,PIK3R3,PTPRB,TYK2,UGT2B11,ULK3,VDAC1,YARS2
Negative_6316	RET,CD46,DCTD
Negative_6317	ADCY8,CHRM2,FRAT1,NME7,OR8G1,PDE7B,PRODH2,SMARCA2,UROD
Negative_6318	PXN,DTX3L,IRAK1,KNG1,MCCC2,OR9K2,PDX1,RRM2B
Negative_6319	C8G,ADORA2B,AGRP,ALDOC,APPL1,B4GALT2,CYP17A1,DUSP16,GNAI3,INHBC,LIPG,MAPK9,MYH1,NOD1,OR10A5,OR13C4,OR2W1,OR4D1,OR4F5,OR52N5,OR5AN1,OR9A2,PPCS,PVRL1,SMAD4,SPRY1,STX2,TAS2R48,TNFSF14,TP53,VAMP3,VARS
Negative_6320	CD4,MVK,ACO2,ACTG2,B3GALT2,CTF1,CXCR4,CYFIP2,DDIT4,DHRS9,IL10RB,KIR3DL1,MMP2,MYH3,NTRK2,OR2T11,OR2Y1,OR6M1,PDGFC,PPARD,PPP2R5E,PTPN5,PTPRJ,TAS2R1,TPMT,TRAF5,WASF3,TNFRSF14
Negative_6321	BTK,GP6,ACSL5,ADORA2B,ALAD,CACNA1A,CALML6,CD38,CD79A,CDH1,CDK5,CDKN2A,COL1A1,CRLS1,DGAT1,FGF8,GADD45B,GNA14,HAGHL,HBEGF,IFIH1,ITGB1,KDSR,MAPK10,MBL2,MICA,MTMR6,NCR2,OR10G8,OR5B12,OR6X1,PFKFB2,PHKB,PHKG1,PIGT,PIK3CD,PIM2,PKMYT1,POLR2I,RPS6KA5,SLC27A5,STK11,TAS2R49,THY1
Negative_6322	EZR,AMDHD2,ARPC1A,ASAH1,B3GALT1,CACNA1C,CDH5,CHAT,DDX47,DGUOK,ENO3,FGF9,FIGF,ICOS,IMPDH2,LDHAL6A,MYH4,MYLK3,OR11G2,OR1N1,OR4D9,ORC2L,PDK1,RND1,TAP2,ZFYVE9,CACNA2D2
Negative_6323	IL7,MAG,MIF,ACOT12,ADRA1B,ANPEP,APOE,ARHGEF2,ATP5G3,BAI1,C3orf10,CCL1,CSNK1D,DUSP5,HMGCS2,HSD17B4,IKBKG,IQGAP1,JAM3,LIPE,MPP5,NFATC4,NME6,OR10G8,OR11A1,OR4F6,TBL1X,TM7SF2,TUBA1B,ATP6V1B1,MAP3K7IP1
Negative_6324	EBP,GPT,ACO2,ACSM4,CMPK2,CTPS2,CXCL9,DHCR7,DIAPH3,GBA3,HMOX1,MCM2,NFKB1,NKD1,NPR2,NUMB,OR1K1,OR5K2,OR8K5,PDE1B,SNAI1
Negative_6325	AASDH,CMBL,EDA2R,GALK1,GPLD1,IL2RG,PLCB1,SEMA4F,SLC25A5,UGCG
Negative_6326	FGG,RB1,ABLIM3,CDC26,CDS2,EPHX1,EXTL2,GUCA1A,HCLS1,IKBKG,KLRC1,MAGI2,MYH7,NMNAT2,NUDT8,OR7G1,PIM2,PLA2G10,PPCDC,PTK2,STEAP3,UGT2B28
Negative_6327	GP9,IL4,LAT,PDC,UBB,ALG8,ASNS,CCNE2,CDK6,CTPS,CYP26A1,FARSA,GUCA1B,HMOX1,HSPA2,HYAL2,IL12RB2,NEU1,NFATC1,NLGN3,NRP1,OR51A2,OR8U1,PIP4K2C,PLCB2,PLCG1,PNPO,POLR2F,SPAM1,MAP3K7IP1,ST6GALNAC1
Negative_6328	F2,GAA,ABLIM1,ACADL,ACADS,ADIPOR2,C1QC,C7orf16,CACNA1G,CASK,CD244,CSNK1E,CYBA,DFFA,FRAP1,GAMT,GFPT2,GIT1,GLI2,GNPDA1,HAAO,HAGH,HSD17B1,IFT57,INSRR,MITF,OR10A7,OR1S1,OR5D16,OXCT2,PDK1,POLE2,POLR2E,PPP3CA,PRKG1,PRKG2,PRPH,RASGRF2,SH2D1B,SNAP25,SPRY1,STX10,TBL1X,ATP6V1B2,CSGALNACT2,TNFRSF13B
Negative_6329	PLN,SCD,ACSL3,ARPC3,CCL13,CDH3,EPHX1,LRP5,PNLIP,THBS1,TUBB2B
Negative_6330	CTH,DHH,GP6,ILK,LTB,MPL,ACVR2A,AMDHD1,ARPC3,CASP9,CD34,CEBPA,CHAT,COMT,FGF5,FZD8,GCDH,GCH1,GSTO2,HIBCH,HLA-DMA,IL22,ITCH,MAP2K3,NCOR1,OLAH,OR10S1,OR52L1,OR5D13,OR5F1,OR6Y1,PCNA,PDGFC,PKM2,POLA1,PRKAG3,PRSS2,PTGS1,RBL2,SHC4,STAM2,WASF2,ZAP70,ZFYVE9
Negative_6331	VTN,CFLAR,EPB41L1,FZR1,PDE8A
Negative_6332	ADRB1,BCL2L1,CCR6,CD247,CD40,DDX56,GNRH1,HCCS,HSP90B1,IL29,KARS,MLYCD,PDGFA,PEMT,RBX1,SC4MOL,STAT5A

Negative_6333	DDX4,EPB41L2,GLI2,GUCY2C,OR5P2,RDH12,UPRT
Negative_6334	ADIPOR2,BBC3,CDC6,CLDN3,HTR4,ICOSLG,MLLT4,MYH2,OR11L1,OR4N4,OR8B4,PI4KA,PPARG,PTPN7,WHSC1L1
Negative_6335	FGA,MSN,AKR1C1,CCL19,CCNA1,CD44,CDC42,CSH1,CYP2C18,DFFB,DPYS,ERBB3,GNPDA2,IDH3A,IDH3G,IFNA10,IFNAR2,KCNQ1,MKNK1,NODAL,OR11A1,OR2F2,OR4D1,OR52E4,P4HA3,PDE4D,PDE7B,PKMYT1,POMC,PPP1R3A,PPP2CA,PRKAB1,PTGDS,PYCRL,RAB5A,RAPGEF4,ROCK2,SEMA3B,SLC27A5,STX3,UCP1,MAP3K7IP1
Negative_6336	F12,ABLIM3,ATG12,B3GNT4,CADM1,EPB41L2,GLUD2,GNAI3,IL6ST,OR2T2,OR7C1,OR8I2,PFKFB2,PFN3,PIP4K2B,SMAD4,SNAP23,SRD5A2,SSH1,TNFSF10,ULBP1,YARS,CTNNBIP1,MAPK8IP1
Negative_6337	EZR,AHCYL2,B3GNT3,BCMO1,DPM1,NT5C1A,PAX6,PRKCH,HLA-DPA1
Negative_6338	FGD1,GRIN2D,PRDX6,SIGLEC1
Negative_6339	CFI,COX15,DUSP16,PANK2
Negative_6340	GH1,ACADM,ATG12,ATP6V1H,B3GNT2,DCTD,DGKE,ENO3,FRAT1,GBE1,GNRHR,GYS2,HTR6,IFNA16,IL7R,MAP4K4,NEUROG3,NMNAT1,NR5A2,OR2K2,OR5AS1,OR7D4,PHGDH,POLR2H,PRKAB1,PTPMT1,RXRG,SPAM1,SPRY3,UGT2B7,VANGL2,HLA-DQA2
Negative_6341	CCNG2,CISH,CNTN1,CYP11B2,IL22RA2,IL2RA,INSRR,NANP,OR10AG1,OR1E2,OR6K3,PCK2,PHKA2,PIK3C2G,PIP5K1A,PTPMT1,ROCK2,SFRP2,SPP1,TAS2R42,UPP2,SUV420H1
Negative_6342	APP,CD6,CDA,ADH1B,BUB1,CDC27,CTF1,CYP21A2,DDX4,DKK4,GCLC,GOT2,INADL,IRF9,LTBP1,MICA,NARS2,OR1M1,OR2B11,OR2H1,PDE6H,PFN2,RAP1B,SCIN,SDC1,SEPHS1,SOCS2,TAS2R42,UNC5B
Negative_6343	AGL,FST,KMO,LHB,TNR,AKR1C2,AOC2,APRT,CD48,CD79A,CDC16,CDC7,CIITA,CYP2C9,HMOX2,HNF4G,IL1R1,IL21,MAML2,MAP3K13,MCM7,MYLK2,NAGA,NT5M,NUDT5,OR10G3,OR4N2,PAK1,PFKFB3,PLA2G3,PLA2G5,PLCG2,PTPRM,PYCRL,RPRM,RPS6KA6,SGPP2,STAT3,THBS3,TRHR,UBE2L6,ULK1,UROC1,VEGFB,YARS,B4GALNT1,GABARAPL1,TNFRSF11B,TNFSF13B
Negative_6344	F10,ID4,ME3,AGPAT6,ARG2,ATP2A2,CD58,CYP2C18,DAAM2,EFNB3,FRAP1,HTR2B,MGST1,NAGA,NEFH,OR2B11,OR2D3,OR2L8,OR4N2,OR56A3,PLXNA1,PMVK,PROS1,RFNG,RPS6KA4,TIAM1,PPP1R12A
Negative_6345	CEL,ADAM10,ADRA1B,CHKA,ESPL1,FUT5,HADH,ITPKB,NCF4,NFYB,NRXN1,OR52A1,OR5K2,OR5T2,PDE6G,PKM2,PLA1A,PRSS1,PVRL2,RAF1,RPS6KA3,SETMAR,SMAD9,ST8SIA1,SV2B,WNT7B
Negative_6346	ALDH1B1,NFKBIE,PIK3C2A,PKM2,PTK2
Negative_6347	CAT,ITK,SYK,AGPAT3,ARNTL,ATP2A2,BMP8A,BNIP1,CAMKK2,CCNG2,DAPK1,GLDC,GNAT3,ITGAX,ITPKA,MTMR2,OR10G7,OR14A16,OR1E2,OR51A7,OR8B12,PANK4,PDE11A,PDXK,PIAS2,SPRED1,SULT2A1,TPH2,HLA-DQB1
Negative_6348	GSN,LHB,ARPC4,BCL10,CLTC,CYP8B1,FTH1,FUT5,HDAC2,ITGB6,NR5A2,OR5AK2,SHC3
Negative_6349	KDR,ATP6V1H,BMP5,CD58,CSNK1A1,CYBA,FTMT,GTSE1,IAPP,IL4R,MAPK9,MCAT,MCM6,NCR1,OR1N1,OR4A16,OR5AK2,OR5C1,OR6B2,OR6F1,PCYT1A,PDE3B,PDK1,PIGF,PIK3CD,PIM1,POLD4,PRKAA2,PRKAG3,ST3GAL4,ST3GAL5,UGCGL2,VAMP4,TNFRSF10B
Negative_6350	HPD,IL8,ACSM5,ADCY8,ALG6,ARPC5L,CD86,CHSY3,CYP2A13,CYP2A7,FCGR3B,FZD9,GP1BB,GSK3B,IFNA4,NGEF,OR4D5,PAFAH2,PARD3,PER1,PNPO,PPP2R5D,PRKCZ,ROBO1,SARS2,SLC25A6,SORD,UNC5A
Negative_6351	CAT,EGF,GNE,ME1,SI,ADH5,ALPI,AVPR1B,CACNG2,CCL13,CDC25B,CXCR4,CYSLTR2,DAG1,EARS2,EP300,EPB41,FASN,GDF5,HAGHL,HHEX,ITGA5,ITGAE,ITGB2,ITGB6,JAK1,LTC4S,NCKAP1L,OR13G1,OR51V1,OR5AR1,OR7C1,OR9K2,PARD3,PGAM2,PPAP2C,PSME1,PTCRA,RPN1,SFRP4,ST8SIA1,STK11,TUBA1C,UCK1,WNT4,XCR1,YKT6

Negative_6352	AK7,F5,MVK,ALDH1B1,ATP2A1,ATP5J,ATP6V1H,CBLB,CD27,CDC45L,CER1,CHPT1,CLCA4,CMPK2,CYP4F2,EIF4E,FGF10,GNA13,IFNAR1,IGSF5,ITGA4,LARS2,OR13C8,OR4A5,OR56A4,OR5J2,OR5R1,OR6C75,PLXNB1,PRPS2,PTPRR,RUVBL1,SDHD,STX1A,TNFSF18,UBE2K,VARS,YWHAG,B4GALNT1,HSD17B10
Negative_6353	ACPP,APOE,ATP6V1A,BACE1,COL2A1,FGF7,GALNT7,GSTO1,IL2RG,IL9R,OR2F1,OR4D2,OR8J3,PAK4,PFKFB4,PYCR1,SEMA3C,SHC4,SUV39H2,TICAM2,TPI1,VDAC3,XCL1
Negative_6354	UBB,AFMID,APOE,ARG2,ATP6V1A,B3GALT6,C9orf95,CALML3,CASK,CCL8,CLDN2,CRLS1,DDX4,DNMT3B,ENTPD8,FABP4,FGF3,GLI3,GSTM4,LAMC2,LYPLA2,MEF2C,NRG2,OR10H5,PARS2,PIK3R2,PNPLA4,POLR2J,PPP2CB,RPIA,RPS6,SMAD2,TIAM2,WIF1
Negative_6355	ABO,HDC,LTB,ACADSB,ACTN2,AKR1C2,AOX1,APH1A,ATP5E,ATP6V0B,B3GAT3,BCL2,CACNA1G,CCL22,CDC25A,COL1A1,COL5A2,CPT2,CREBBP,DEGS2,ESPL1,EXTL2,F2RL1,G6PC,GPD1,GSTA4,GSTP1,GUCY2F,GYS2,IFNGR1,KLRC1,MAOA,MBL2,NOTCH2,OR2B2,OR4D9,OR9Q2,ORC4L,PLA2G4A,RAD51,RASGRF2,SELE,SESN2,SKIV2L2,SMC1A,TAS2R42,UPP1,YKT6,PNLIPRP2
Negative_6356	AGK,DLD,HK1,MVK,ALDOA,ALG13,CD48,CPS1,DRD2,DVL1,FADD,GALNT1,GCNT2,GDF6,GNAT3,GUCY1B3,HCLS1,HMOX1,IL22RA2,MCM2,MGAT5B,MTHFD2,NRP1,NUDT9,OR52N1,OR52R1,OR5B3,OR5T1,PDE10A,PIAS2,PLA2G1B,PLD1,PLD2,POLR2B,RIPK1,SH3BP2,SLC25A5,XIAP,ZBTB16
Negative_6357	DDC,SRF,TK1,ACY1,BDH1,BDKRB2,BMP2,CCNE1,CYP2A7,DUSP7,EHMT1,FUT9,HSPA2,IL6R,INPP4A,LAMA4,MAP3K13,MKNK1,OR2T6,PDHA2,PLD2,PNPT1,PORCN,PRKG2,SORD,THY1,ATP6V1G3
Negative_6358	ACAD9,ARPC3,CLDN2,DDX4,FGF22,GALM,HTR6,MGST1,OR5AP2,PCYT1A,PGK2,SSH2,TLR4,TNFSF14,TSLP
Negative_6359	CREB3L2,ERO1L,HSPA8,IL17RB,NFATC1,NFX1,OR1Q1,OR2M5,PPM1D,PROC,PRSS3,PTCH2,WASF2
Negative_6360	FGG,GK2,LPO,MAX,PGD,PGF,AKR1C3,ALPP,AMY2A,ARNTL,ARSA,ATP6V1D,CCKBR,CCL4,CDH1,CHRM5,CYP4F3,DAG1,EPB41L3,GLDC,GRIN1,HSD3B2,HYAL2,IDH1,IL1RAP,IRS1,ITGA10,ITGA9,MAFA,MYH8,NCOA4,NFASC,NFKBIB,NSDHL,OR13C5,OR2D2,OR4N4,OR52E6,PIK3CG,PSPH,RASGRF1,RBL2,SGMS1,UGT2A3,VTI1B,WEE1,WWP2,PAFAH1B1
Negative_6361	BCR,ACADL,ACOX2,ADAM10,BAAT,CD226,CLDN7,COL4A4,CPT1B,CTNNA3,CYP3A7,DHX58,E2F5,ETNK1,FMO5,FUT3,GJA1,GMPR2,GRIN2A,GSK3B,HMOX2,HSPB1,IL12RB1,MAML1,MKNK1,NFATC2,NRG4,OR11H1,OR14A16,OR1E1,OR1I1,OR2D3,OR56A4,OR7C2,PLXNA2,PNPO,PPP2R2D,PRSS2,SPTLC2,STX10,STX18,TUBB4,ULK1,WNT10A,WNT2,YWHAQ
Negative_6362	CBS,ID2,MAG,ACTG1,ACTN2,AHCYL2,ALOX5,ATG3,CLTB,CTNNA1,DTX3,GRIN2A,HSD17B4,IGFBP3,IKBKB,IL1R1,ITGB8,MPZL1,NRCAM,OR5D13,OR5J2,OR5K2,PAX8,PER3,PPA1,PPP1R1A,PPP1R3D,PTPN7,RELB,RHEB,RPIA,SESN3,ARHGEF12,PAFAH1B1,TNFRSF18
Negative_6363	C1S,IL8,ACP5,ACSS1,CAMK2B,CAMKK1,CCKAR,CHKA,CLDN20,CYP3A43,DTX4,DUSP3,FGF6,GOT2,GRPR,GSTO1,HGSNAT,HSPA2,IARS,IL2RG,ITGA2,ITGA3,JAM3,LTBR,MAPT,MGAT2,NFASC,OR2M5,PAX6,PGLS,PPP5C,PRKACA,PRKAG3,SFRP4,TCF7L1,TP73,YWHAB,MAP3K7IP2,TNFRSF11A
Negative_6364	ACSL6,ACY3,APPL1,BBC3,CAMK4,CSNK2A1,DDX41,DPM3,FGF10,IL2RG,ITGA1,ITGB1,JUND,MAP3K12,NCAM2,NLGN2,OR52K1,SDC1,VAMP4,ZFYVE9,HSP90AB1
Negative_6365	ALDH1B1,ATG3,COL3A1,CXCL11,CXCL3,DUSP9,EXTL1,GNPDA1,HAO1,HEMK1,KIR2DL3,MAP3K11,MTHFD2,OR2B2,OR56A4,PANK4,PIK3R3,PLCG2,POLE4,POLR3D,PPCDC,TJP1,TRMT11,UGT2B17,UPB1,UROC1,VCAM1,YWHAG

Negative_6366	ACSL1,ADRBK2,CD19,CSF2RB,GSTO1,ITGA10,LTBP1
Negative_6367	GP9,ME1,PGF,C7orf16,CLDN10,CLDN20,FRAT2,GALK1,IAPP,ITPA,KCNJ11,MINPP1,MLLT4,MMP9,MYLK3,NUDT5,OR10H4,OR4A15,OR51G2,PLCB1,PPCDC,PRKAG3,RPS6KA6,TNXB
Negative_6368	ABAT,AMY2B,ARG1,B3GAT1,CD72,CDC16,CYP19A1,DHRS4,FLOT2,GALC,GAMT,GUCA1A,LHCGR,METTL2B,NFKB1,OR52K2,OR6K6,PDX1,PLIN,PPID,SMURF1,TCIRG1,YARS2
Negative_6369	ABO,AK7,IL4,LPO,AACS,ALG11,CACNG5,CASK,CCL13,CHKB,CYP1A2,CYP2S1,DAPK2,DHRS3,EPHA1,FZD8,GLB1,HRH1,IFNA17,IFNA7,IL3RA,IRF5,KAT2B,KLK3,MAP3K13,MGST3,MYH8,NFYC,NKD2,NMNAT1,NTF3,OR2AG1,OR2B6,OR2F1,OR52N1,OR5AK2,OR5B3,OR5D14,ORC2L,PDE6G,PLA2G5,PMM1,PRKCG,PSEN2,PTPRB,RELB,RRM1,TFDP1
Negative_6370	TK1,CACNA1E,CLOCK,CMAS,CRKL,DTX4,EREG,FGF1,GNG12,HCLS1,HRH2,IL23R,LAMC2,LCMT1,MLYCD,MRAS,OR8S1,SHC3,SSH2,STAM2
Negative_6371	C2,F2R,LEP,ACP5,ARRB1,CACNG6,CLTA,DIAPH3,GZMA,HAAO,HTR6,MAT2B,MYL9,OR6B2,PTPRJ,PVRL4,SH2D1B,THBS2,UGCGL1,UPP1,B4GALNT1
Negative_6372	CKM,ACSM1,AKT2,BCAT2,CD79B,COL11A1,CYP2B6,DEGS2,EP300,FPGS,GSTM1,HLA-C,MAT2B,NAMPT,OR10A6,OR1K1,PLEKHA8,POLR1B,PPP2R2C,STX1A,TACR1,TAS2R38,TAS2R7,ULK1,TNFSF13B
Negative_6373	GNE,ACSS2,ADRA1A,ALAS2,BPNT1,CALML3,CLTCL1,CSDA,CSNK1G3,CXCL2,CXCR5,DVL1,ENO1,ERBB3,ERO1L,GAD2,GNA13,IDH3G,ISYNA1,JAM2,MAP3K1,MAP4K2,NAGS,NAT1,OPLAH,P2RX1,PIGO,PMM2,SCLY,SEMA4D,UCK1,VDAC2,XCL1
Negative_6374	TNN,AKR1C3,ALDH6A1,ALOX12B,APRT,ATP5O,CCL2,CLDN9,CYP1B1,ETS1,FZD9,GGCX,GMPPB,HLCS,ITGB2,JAK1,MARS2,NUDT2,OR1K1,OR2T1,OR4K2,P2RX3,PDE1B,PDE3B,PRKAA1,PRKCB,PRKCI,PSME1,PVRL3,RPN1,RPS6,SARS,SFRP2,SOS1,SSH3,UNC5D,YWHAZ,TNFRSF10B
Negative_6375	IL9,ACYP2,DGKQ,EDNRA,NMNAT2,OR10V1,OR4B1,OR51L1
Negative_6376	C5,GP9,TK1,ACPT,ATP13A2,B4GALT3,BAAT,BMP6,CACNG3,CACNG8,CD22,CDH2,CRKL,CSDA,CSNK1G2,CYP17A1,CYSLTR2,E2F4,GLUL,HCCS,HLA-DRA,IL6R,KITLG,LAMC1,LCMT1,MAGI1,MAP2K4,MPDZ,NCF2,NODAL,OR2AG1,OR51I1,OR5B3,OR8D2,PIGP,PLA2G10,PSME1,RALA,RFX5,SETD8,SMARCA2,STX6,TAS2R14,VEGFC
Negative_6377	ASL,ACAD9,AK3L1,AKR1B10,CACNB3,GGT6,GNPDA1,HADH,HAGHL,IL22,IRS2,ITGB1,MYLK,NEUROG3,NPR2,OR7C1,PFKFB2,PIK3C2A,PNPT1,PTAFR,PTPMT1,SHMT1,TNFRSF4,TNFRSF10D
Negative_6378	DLD,F10,IL5,MPZ,MVD,TEC,AKT2,B3GAT1,BDKRB2,CCNB3,CLDN14,CLDN5,DEGS1,DGKD,FGFR3,FPGT,GNA14,GNPDA1,HDAC2,HES1,IL1RAP,IRAK3,MAP3K8,MKNK1,NAGK,NRXN2,OR1A1,OR1L1,OR2T8,OR51F1,OR6N2,PDXP,PER2,PIK3R4,PIPOX,PLCB1,PPP2R5D,PRKCD,SH2D1A,SMAD4,TAP1,TOMM40L,TUBB2C,TUBB6,UCK2,VKORC1,YWHAQ,ATP6V1B1,EIF4EBP1
Negative_6379	HDC,HK2,CACNG6,CCL16,CCR8,CDC14A,CKS1B,CLDN8,CPS1,DFFB,ESAM,FDFT1,G6PD,GRIA1,GUCY1B3,HBEGF,LDHC,NADK,OXSM,RFXANK,XYLT2,ZFYVE9
Negative_6380	SRF,CALML5,CFL1,DFFB,GART,MAPK10,OR10S1,OR51Q1,OR8A1,PRSS1,SSH1
Negative_6381	CSK,DBI,DCN,HGD,ACYP1,AKR1C2,ALAS2,ARHGEF7,B4GALT2,CSDA,CYP2C19,DBF4,DGAT2L4,EDN1,GMPR2,GNAS,GNAT3,GPD1,GSTM1,IL1R1,ITGAE,MYH1,NEU4,NRXN3,OR4F17,PARD3,PCYT1A,PLCZ1,PLOD2,POLE3,PPAP2A,PRKCZ,PYCRL,RASGRP3,RHOQ,RRAS2,SEMA4C,SMPD4,STX12,VKORC1,VTI1A,WNT5B,ZAP70

Negative_6382	C2, F9, IL2, LCK, SFN, SRF, TNF, UBC, ARG1, ATP2A1, C1GALT1, CD79A, CDH15, DNMT1, FUT1, GALNT14, GBGT1, GLYCTK, GNA14, KIR3DL2, MTMR1, OR2B11, OR2T33, OR4A15, OR4K17, OR5B3, ORC1L, PFAS, PFKFB1, POLR2H, POLR3D, PPP1CA, RAP1A, RELA, SPHK2, SRD5A2, TGFBR2, TIAM2, TNFSF18, USE1, ATP6V1G2, MAP3K7IP1, MAPK8IP1
Negative_6383	CKM, ALDH1L1, ARPC5, BPNT1, CAMK2G, CANT1, CLDN6, CYP11A1, DHRS4L2, GANC, GPD1, GUCY1A3, HLA-DMA, INHBC, INPP4B, ITGA2B, KIR2DS5, MAT1A, NCAM1, OR10J5, PANK2, PIAS3, PORCN, TACR1, TRAF6, VNN1, ARHGEF12, ATP6V0E1, MAPKAPK2
Negative_6384	AR, CSK, ACP6, ARSD, ATP5L, CDKN2B, DHCR7, EPHB6, HSD17B4, IFNGR2, JAK2, MPDZ, MYL5, NTRK1, OR2T1, PRDX6, PTK2, TIAM1, TICAM2
Negative_6385	LLGL2, RHOD, ARHGEF12
Negative_6386	DDO, ADSSL1, AGPAT1, AGPAT2, ATP5D, ATP6V0C, BUB3, CCNE1, CCR5, CDIPT, CREB5, DAPK2, DHDH, ENTPD5, FGFR2, GATM, ICAM3, IL22, IL28A, ITGA3, LALBA, LAMB3, LCAT, LIPA, LMO7, MAFA, MYLK2, OR10G9, OR2B11, OR6C6, OR9Q2, PARD3, PDE7A, PDE8B, PIP5K3, PLA2G2D, PPP3CA, PPT1, RUVBL1, SMAD4, SMARCA2, SMPD4, TRAF2
Negative_6387	DHH, ACOT11, ALOX15B, ARRB2, B3GALT5, C4BPA, CD19, CDS1, CHAD, CNTN2, COL6A1, DHODH, FUT1, GNAI2, GNAT3, HLA-C, IL20, MAD2L1, MTMR1, NNMT, OR10H2, OR56B4, OXCT1, OXCT2, PHKB, PIP5K1C, RDH16, ROCK2, RPS6KA6, TUBA3E, TNFRSF18
Negative_6388	C6, CD2, TK2, ACTB, ACTN4, ADCY10, AOX1, CER1, CLCF1, COX10, CPT1A, CTF1, CYFIP2, DGAT1, DLL1, E2F4, EPB41L1, FADD, FGF5, FGFR2, GNAO1, HLA-DMA, IL20RB, ITGAD, MAP2K6, MYH8, MYLK, NKX2-2, OR1N2, OR2A14, OR2G6, OR51G2, OR6N1, PARVA, PDE7B, PON3, PRKACG, PTPN5, SC4MOL, SDHB, SIGLEC1, STX5, TAP1, TGFB3, VAV1, VDAC3, WNT6, YWHAQ
Negative_6389	LRRC4C, NGEF, NT5C3, OR14J1, PIP5K1C, SFRP4, STK11
Negative_6390	F8, LCK, ARHGEF6, ATF4, B4GALT1, C4BPA, CXXC4, DHCR7, FDPS, FLT1, GBGT1, HRH1, IL11RA, ITGA9, ITGB6, LEFTY2, LYPLA1, MAP3K12, MCCC1, OR1N1, OR56B4, OR8D2, PANK4, PDK1, PIP4K2A, PLCB4, PPP1R3D, SFRP1, SGMS1, SKP2, ST3GAL5, VARS, MAPK8IP2
Negative_6391	TXK, ASS1, ATP5G2, CAV1, CHPT1, CYP2A13, DCTN1, ENTPD6, FGF19, GAB1, GCNT3, GRPR, MAD1L1, MTAP, NFX1, OR2A25, PLOD2, SDC4, SHMT1, TAS2R8, TGFBR2, ST6GALNAC1
Negative_6392	CRH, ALG12, ELK1, GOT1, NAT6, OR8S1, ORC1L, ORC3L, PDE9A, PYGB, SQLE, UGT2B17, VANGL1, VNN1, ATP6V1G1
Negative_6393	OSM, ARSD, CD59, CPT2, CYP11B1, DDX55, DHRS4, DIAPH1, EARS2, EFNA1, EGLN3, EPHB6, FLAD1, FRAP1, FTH1, GANAB, GCDH, GNRHR, HAAO, MITF, NLGN3, OR1E2, OR4A15, OR4C11, OR5D13, PLK1, PLTP, PPA1, PRKCE, SLIT1, TIRAP, TNFSF11, TPM3, PLA2G12A
Negative_6394	ADC, BCR, C2, CD6, CDA, ACSL3, ALDH5A1, CADM1, CCL23, CLDN3, CLDN8, CYP2D6, DDX18, DUSP3, DUSP5, FGFR2, GCNT4, INADL, INPP5A, KIR2DS5, NOTCH1, NT5C1B, NTRK2, OR10H4, OR2T2, OR4B1, OR5B12, PAK2, PLOD2, PNPLA3, POLR1B, POLR2A, POLR2J, PPP2R5E, PRPH, RASSF5, SSH3, TAS2R1, TLR9, TP73, TYK2, UGCG, VNN1, WARS
Negative_6395	CES7, DDX41, LDHB, LRDD, MAPK14, NKD1, OR13C5, OR2A4, OR2L2, OR8S1, PHKG2, SFRP4, UCP1, MAPKAPK2
Negative_6396	CAT, CFD, IL2, INS, VTN, XDH, AADAT, ADH1A, C1QA, CD46, CDC25B, CDC6, CSF3, CXCL9, EPHA7, EPRS, HSPB1, KLKB1, LLGL2, MAGI1, MGAT5B, PDE2A, PLA2G6, PRKAG2, PTGS2, RPE65, YWHAZ
Negative_6397	BTK, IL6, TPO, ACP6, B3GALT4, C4BPB, CCNG1, CD40, CDO1, CHST12, COL4A2, CREB1, DHCR7, EI24, GUCA1A, IQGAP3, IRAK4, ONECUT1, OR13D1, OR4N2, PIP5K1B, RALB, SETDB1, EIF4EBP1, PPP1R12A

Negative_6398	NPY, PLG, WAS, APBB1, ARSE, B3GALT1, BRAF, C1QA, CAV1, CBLB, CCNB1, CHD8, CHIT1, CNGA4, COL4A4, CXCL16, DARS, DFFB, EFNA5, ENO2, ENPP2, ENTPD6, ENTPD7, FGFR2, GCNT2, GLYCTK, IDH2, ITGB4, ITPA, KNG1, LCAT, NEFL, NOD1, OLAH, OR11A1, OR11G2, OR1E2, OR51G1, PAX6, PLK1, POMC, SOAT2, TAS1R2, TUBA8, UBA1, VASP, PRICKLE2, TNFRSF14
Negative_6399	ANAPC11, DFFA, FGF20, GUCY2F, KIR3DL2, OR52B6, SEMA4C, SLC25A5, TICAM2, ATP6V0D2
Negative_6400	ACSL4, ACSM5, ADH6, CCKAR, CCL16, CFL1, DGAT2, EHMT1, ELK4, EXTL2, GNG13, GOT1, GSTA3, GUSB, GYS2, IFNK, IL20RB, ITGA5, MAPK8, MCM6, NFYC, OR4D6, OR5K1, PDE4D, PIK3C2A, PMAIP1, PRKAR1B, PYCRL, RELA, RFWD2, RPSA, RXRB, SKIV2L2, SPHK2, TGFBR1, TRHR, TUBB3, TUBB4, WBSCR22, TNFRSF13B
Negative_6401	ADO, FAH, SFN, ACAD9, ALCAM, ATP2A2, CDC27, CDC7, CYP2S1, DHFR, E2F1, GALNTL4, GNA11, ICAM2, INMT, MCCC1, OR14I1, OR5D13, OR8I2, PLA2G5, PLCB3, PPP2R2D, PVRL2, RAC1, SCP2, TAS2R41, UNC5C, YWHAZ
Negative_6402	ABL2, ACP2, DUSP14, HMOX1, LAMC2, NCR2, OR4A15, OR4C6, PIGN, ATP6V1B2, TNFSF13B
Negative_6403	MIF, DOLPP1, OR10J1, OSMR, PRSS1
Negative_6404	CKM, ACAA1, ACTN4, ALG12, APC2, ARPC3, BCMO1, CCL26, COL4A2, COL4A4, COX10, CREB3, CRLS1, EFNA1, G6PC2, GALNT1, GCNT4, GDE1, HIBCH, HNF1B, IL19, INPP5B, NFAT5, OR10A7, OR4F5, OR6F1, OR9I1, PERP, PIGP, PIM1, PON2, PPP2R5C, PRKAG3, PROC, PTGS1, RHOQ, ROBO1, SEMA3C, SETD8, ULK3
Negative_6405	GSS, HAL, NOG, TNR, ADCY9, AHCYL2, AMD1, BMP2, CACNA1S, CD28, CHRM3, COASY, CREB3L4, DPM2, ENPP2, ERO1L, GCLM, GGT1, GPAA1, GUCY1A3, HADHB, ICOS, ITGAX, MAP2K3, MYLK2, NOS3, NPR1, NRG1, OR2D2, OR2M7, OR2T11, OR4D1, PPOX, PPP3CB, PTGIS, SEMA4D, STAM2, SV2A, TBXAS1, UPP1, ATP6V1C1, B4GALNT1, CSGALNACT1
Negative_6406	ALDOC, ATP5E, CASK, CCR4, COL4A1, E2F3, GPD2, POLR3A, PTPN7, RFWD2, TGDS, UNC5C, YWHAZ, HSP90AB1
Negative_6407	GK, CLDN16, CSNK2A2, HARS2, ITGB4, OR1E1, OR51S1, PDCD1, PDE5A, PPP1CC, PROS1, RPN1, SEMA3D, PRICKLE2
Negative_6408	GNS, LEP, MOS, TH, ADCY9, ALDH2, ARHGEF1, CACNA1D, CACNA1I, CACNG4, CD36, CD8B, CLDN2, CLDN20, CRLS1, CYP2A6, ENAH, FASLG, GP1BA, GPLD1, HHEX, MAT2B, OCLN, OR10J5, OR5R1, OR8J3, PLAU, POLR3B, PPP1CC, PVRL2, SMPD2, UPP1, VAMP3, TNFRSF18
Negative_6409	GDA, AADAC, AANAT, ABAT, ACLY, ACO2, AXIN2, CDC7, CDKN1A, CNTF, CRB3, CYFIP1, DGKQ, DTX3, EPHX2, FOXO3, FUT3, GOT2, GPX5, IDUA, IL9R, MAP3K3, MTHFD1L, MYH14, NCR1, OR11G2, OR4C16, OR51F2, OR51S1, OR52M1, PDE11A, PLA2G2A, PRKCA, PTPN6, SC4MOL, SETDB1, SLC27A1, SMPD2, SPTAN1, STK36, STX18, TRMT11, ULBP1, ULK3, VTI1B, WHSC1, ATP6V1G1
Negative_6410	DAO, ACTN2, AKT3, ALOX12, CAMKK1, CDK4, CYP3A5, DAPK2, DDX18, DHRS3, FGF3, FSHB, GNAI1, ITGB3, JAG1, KIR2DL3, LHCGR, OR4K14, OR6C1, P4HA2, PAFAH2, PIK3C2A, PTK2B, SNAP23, STX16, UGT2B4, ATP6V1G3
Negative_6411	BDH1, CREBBP, CYP4A22, DGAT1, EDAR, EGLN1, ELK1, MYH2, NTSR1, OR51Q1, POLD2, RERE, RGS3, SULT1E1, SERPINE1
Negative_6412	RB1, BUB1B, C1QB, C3AR1, CAMK4, CCND2, CD44, DNMT3A, EIF4E, EPHX1, GALC, GPD2, MDH2, MTMR1, NT5M, OGDHL, OR10X1, OR2T34, PLEKHA8, PRPS1, TAS2R10, TLR9
Negative_6413	NLK, PLN, SRM, AGPAT2, AGPAT4, BCL2, CACNB4, CFTR, DDX50, DFFA, EXT1, GRLF1, HLA-DOA, IKBKB, NR4A1, OR1L4, OR2B2, PI4KB, PLOD1, SETD1B, TGFB2

Negative_6414	FER, IVD, PC, RDX, ACO2, AGMAT, CAMK2A, CCL28, CD36, CHPF, CTSL1, DTX4, ENTPD7, GAB1, GNG3, GPLD1, HIP1, HLA-DRA, IL13, ITGAX, NRG1, NTN1, OR5M3, OR6Q1, PTTG1, PVRL1, RBPJ, RRAS2, SAT1, SMAD3, ST3GAL5, STAT3, TAS2R48, TAS2R5, TUBA3E, UGT2B17, VKORC1, WNT1, ZAP70, TNFRSF17, TNFRSF6B
Negative_6415	PML, TPR, TXK, ALDOC, CADM3, CD8A, CHST11, CSNK1G2, CTPS, E2F2, FMO1, GFPT2, GNPDA1, GZMB, HSD3B7, KCNQ1, NFYB, OR5B21, PPP3CA, SETDB1, WNT7A, MAPK8IP1
Negative_6416	BLVRB, FARP2, GRM5, HSD11B1, HTR6, NEFL, NFATC2, OR13H1, PDGFA, PIK3R5, PTPRB, ROCK2, STK11, UNC5C, CSGALNACT2
Negative_6417	GRM5, NOTCH1, OR10P1, OR2T3, PAK7, PIK3R4, SDHA, TCIRG1, ATP6V1G1
Negative_6418	CD4, FN1, IL8, ACOX1, FASN, IQGAP1, NCOR2, PDE4A, PLXNA2, PRPH, SUV39H1, TNFRSF9, HLA-DQB1
Negative_6419	AUH, CREB3L2, IMPDH1, OR9I1, RBKS
Negative_6420	AGL, OR6N1, SEMA4D
Negative_6421	FYN, ATG7, CCL11, CDC26, CXCL5, GRIN2C, MAT2B, MGLL, MYH3, OR1A1, PDCD1, PPM1B, PTAFR, SKP1, SOX17, TUBB4, WNT2, WNT5B
Negative_6422	ADRA1A, COASY, DKK1, G6PC2, GNRHR, GOT1, HLA-DMA, IDI2, IL1R2, MAP3K3, MECR, PPP1CC, PRKCA, PYCRL, TUBB2A, ULBP1, VAMP1
Negative_6423	GP5, ITK, OTC, TNN, ACOX3, ADCY2, ATF2, ATP5O, BDH1, CCNA1, CDC27, CDKN2D, CLCF1, CXCL16, GOT2, IGF1, IL12A, INADL, NPR2, NT5C1B, OCRL, OR13A1, OR5D14, OR8H1, OR8J1, PPP2R5E, PROC, PTDSS1, SGMS2, SIGLEC1, SMAD5, SMURF1, STK3, STK4, TCIRG1, TKTL1, TMLHE, TUBA1B, XCL2, ATP6V0E1, CSNK1A1L, PAFAH1B3
Negative_6424	ARPC3, CKMT2, DGKE, DPM1, EPHA8, FGD1, HYAL1, IL28A, IL28RA, NAE1, OR51A7, RASGRF2, SPRY2, TNFSF11, ARHGEF12, GABARAPL1, TNFRSF17
Negative_6425	FCGR3A, IFNGR1, INHBC, NFKBIB
Negative_6426	JUN, ME3, ANAPC10, CASP8, CYP26A1, DAPK1, GANC, IMPDH2, LAMB2, NCOA4, OR10Z1, PDPK1, PTDSS2, SETD2, SLC25A4, TNFRSF10D
Negative_6427	A2M, AARS2, ADCY3, ALDH1A2, ALDH7A1, ANAPC11, ATF4, B4GALT6, BIRC2, CACNB2, CDH5, CLDN9, CX3CR1, CXCL1, GCNT3, GNAQ, GRIN2C, HTR2A, IL22, ITPK1, NEFH, NRG4, OR10Z1, OR11H6, OR5A1, OR5T3, PAK6, PLXNC1, PMVK, PTCH2, SULT1E1, TAS2R13, TLN1, UBE2K, WARS, WNT10A, WNT4
Negative_6428	ANAPC7, ATG7, DHCR7, HRH1, SULT1A4
Negative_6429	ABCC8, ADCY2, ATP2A1, AXIN2, BDH1, BMP2, CACNA1D, CDC23, CFL2, CLDN19, CREB5, CXCL11, EARS2, EIF4E2, FGF3, FGFR3, FTH1, GALNTL1, GALNTL2, GMPS, HAP1, HLA-G, HSD17B3, IL2RG, LAMA3, MAPK8, NUDT9, OR4F6, OR52I1, OR5R1, PDGFC, PRKAG2, RASA2, RRAS, RRM1, SARS, SMPD1, SMPD4, TNXB, TUBAL3, TUBB2C, WNT11, YWHAE
Negative_6430	AMH, XDH, ADRA1D, ALDH1B1, ARPC2, CPS1, EARS2, EPHA6, FARP2, FTMT, GGT6, HYAL2, OR1N2, OR2A5, OR2H2, OR2V2, POLR1E, SMC1B, VAMP1, YWHAG
Negative_6431	DDO, FES, MPZ, ABP1, ACVR2B, ADCY9, AGRN, AK3L1, ATP13A2, CD40, CD82, CYP1A1, DLL3, DUSP1, FGD3, FGF14, GARS, GNA14, GNPDA1, IL29, INPP5A, ITGA9, NLGN1, NTRK1, OR10A2, OR2F2, OR5AK2, OR8K1, PDHA2, PRKAR2B, PRODH, PTCH1, ROBO3, SEMA3B, STX7, SYNJ1, TGFB3, UBE2L6, HLA-DQB1
Negative_6432	AGL, GK, AKR1D1, BMP4, CD226, FUT7, ITGA8, KLRC1, OR51F2, PSAT1, SMURF1
Negative_6433	RB1, ALAS1, CAPN2, CDKN2D, CLTA, CXCR5, CYCS, DGAT1, EDA2R, EPB41L1, GSTZ1, IRF9, LIAS, MEF2C, NFKBIE, OR51E1, OR8D2, PLA2G5, TCIRG1, TUBA1C, CSGALNACT2, TNFRSF12A

Negative_6434	A2M,C8G,CFD,AASDH,ACO2,ALG11,ALOX12,AQP7,C9orf95,CD22,CDC14A,CDC6,CDO1,CDS2,CTPS,CTSB,DUSP6,GNAI3,GSTA2,GSTM3,HCCS,IL28A,JAM2,KIR2DL1,LLGL2,MARS,NCK2,NFE2L2,NTRK1,OR10C1,OR1L8,OR4C13,OR4S1,OR7D2,OR8D2,OR8K3,PANK4,PLA1A,POLE,POLR1B,PPP2CA,PRKAB1,SEMA3B,STX6,TAS2R4,TCF7,WASF1,PLA2G12A
Negative_6435	CSK,DHH,FAH,JUN,MAG,C1GALT1,CACNG5,CALML5,CLDN22,CSNK1G1,CTTN,CXCL5,DOLPP1,ECHS1,ECSIT,ENTPD3,FBXW11,FZD2,HRAS,HSD3B2,ICAM3,IL17RA,IL18R1,ITPK1,MCCC1,MCEE,MGAT1,MTAP,NCF2,NFATC2,NPR1,OR4X1,PIGP,PRKCB,PRKCD,PTPRJ,PYCRL,RALGDS,RPRM,SDC3,SEMA7A,TAS2R60,TRAF4,UROC1,VAV1,WNT10A,WNT8B
Negative_6436	CKB,CSK,AACS,AGPAT6,ALG9,B4GALT5,CHPT1,CMPK1,COL4A6,DKK2,EI24,FGF5,HADHA,HBEGF,MAGI1,MAN1C1,MYH6,OR4K5,PAK6,PAX6,RPS6KA3,RUNX1T1,SOCS5,SRGAP3,SV2C,TNFSF10,WWP2
Negative_6437	AK3,C2,C9,ME1,ATP6V1H,BTRC,CD74,CSF2RB,DHRS3,EDNRA,ETNK2,FGF20,GNPDA1,HCCS,HPSE,HYAL2,IL23A,INHBE,IRF7,LIPF,MAPK10,MTMR2,NTF4,OR10H1,OR1J4,OR2F2,OR2G6,OR51G2,OR52E8,OR8I2,OR9G1,PARS2,PARVG,PIK3R2,PLA2G6,POLE3,PPP3CC,PRKCH,RASA2,SOCS2,TLR4,TNFSF4,TRDMT1,WNT3,XCL1,SUV420H2
Negative_6438	TPR,ACHE,CD46,CHSY1,COL2A1,CTNNA1,DHCR7,ENO1,FMO1,GALNT3,GLI3,GSTP1,HMGCR,IARS,IDH3A,IFNW1,IL21R,LGMN,MAN1A1,OR2A25,OR2L2,OR2M7,OR2V2,OR5T1,PARS2,PRIM2,PRKAA2,PRLR,PROC,PTPRR,SEMA3D,SLC33A1,SORD,ST3GAL5,SUCLA2,SUCLG1
Negative_6439	FAH,MAG,DHFR,ETNK2
Negative_6440	CEL,TEC,AMDHD1,BACE2,BET1,CHSY3,CREB3L1,CRLF2,CRLS1,CYP1A2,ECSIT,ELK4,FGF23,FGF4,FZD5,GALNTL4,GLDC,GRM5,LILRB3,MAP4K2,NOS3,OR13C4,P2RX1,PARD6A,PHKG1,SIPA1,SLIT2,TAS2R5,UGDH,WBSCR17,PNLIPRP2
Negative_6441	AK7,BCR,FOS,MTR,ALAS1,ARHGEF1,ATP5G3,BMP8A,BMPR1B,CD55,CLDN19,COL1A1,COL5A1,CYP2J2,DGKD,DIAPH3,DUSP6,EFNB1,ENTPD4,EP300,FGF11,FTCD,GCNT1,GPAA1,HSD11B2,ITPA,MAN2A1,OR1I1,OR6C75,PAK3,PIP4K2B,POLD2,PTAFR,RBPJ,TIAM2,PPP1R12A
Negative_6442	TXK,ACOT12,ACSM2A,BMP5,CCR2,CLDN4,CYCS,CYP2E1,DAD1,DHRS3,HMGCS1,IL2RG,KARS,OLR1,OR52K2,OR5M9,OR8A1,PFN4,PMM2,PVRL3,SH2D1B,SMURF1,TJP2,UGT2A1,WNT1
Negative_6443	DBH,HPD,IHH,VCL,ACTG1,ATP5E,B3GALT2,CES2,CNTNAP2,CREB3L3,CYP4F2,DDOST,EPHA2,FOXO1,GAD2,GALNT1,GMPS,HADH,HRH1,INPP5E,ITGA3,LDHB,MAOA,MGAM,NT5M,OR1L1,OR2B11,OR4A5,OR4X2,OR51A2,OR8A1,PFAS,PGM3,PLCB1,PON1,PRSS1,PTAFR,PTGER1,PTPN7,PVRL1,SCP2,SEMA3F,SIPA1,SOAT1,STAM,STMN1,TGFBR1,TOLLIP,ATP6V0D2
Negative_6444	CGN,F9,MPI,ATP5C1,AVPR1A,CALML3,CCL1,CTNND1,DERA,FLOT2,FUT9,ICAM1,IL29,LIPC,MAP2K5,MPST,MYH14,NAT2,NCKAP1,NRCAM,OR1J1,OR2T3,OR3A1,OR4K1,OR6B3,PI4KB,PLA2G4A,ST3GAL2,TBXA2R,TOMM40,TOMM40L,AASDHPPT
Negative_6445	CGA,SDS,ACSL5,ACSL6,ACTG1,B3GALT4,BRAF,CHRM1,EPHA5,ERBB3,FCER1G,FZD1,GPT2,GSK3B,IL1B,MAT2B,NFKB1,OR4C13,OR51B6,OXCT1,PLD1,ROCK2,SMPD1,VAMP2,VDAC1,WBSCR22,XYLB
Negative_6446	VTN,ZYX,ACSM3,ADCY9,AGPAT2,ALDH9A1,B3GAT3,CHRM1,GFPT2,HAAO,IL18R1,KIR2DL2,OR4C15,OR5AC2,OR5K2,OR5T1,RALGDS,SOAT1,TUBA1A,WNT6,YWHAZ,ST6GALNAC3
Negative_6447	DBI,FST,IL2,IL6,ME2,ABL1,CACNB2,COL1A2,CSAD,DDX51,DTX2,FCER1G,FGF10,FLT4,INPP5A,L1CAM,LAMB2,LDHAL6B,MAD1L1,MAP2K4,MAP3K4,MAPK1,NT5E,OR2T4,OR4K1,OR5F1,OR7C2,PEMT,PFN1,POLD1,PPAP2B,PPAT,PRKG1,RARA,RBL2,SHC4,SMAD7,SOD1,ST3GAL5,SULT2B1,TDO2,TRADD,UAP1,UNC5C,ZNRD1,PAFAH1B1,SUV420H1

Negative_6448	BAX, MOS, PGF, PLN, AGRN, ARNTL, CCL11, CDKN1A, GRIN2D, GSTA5, HMOX2, HPSE, KARS, LAMC3, MTFMT, MYL7, NCK2, NKD2, OR10A4, OR4C13, QPRT, SARDH, SEMA7A, SPCS3, SPTLC2, SUCLG1, TRAF6, WNT1, TNFRSF1A
Negative_6449	ADK, ALCAM, B4GALT1, BLNK, CACNG7, CKMT2, CSNK2A2, DHDH, FMO4, FZR1, GCH1, GRB2, HHEX, IL13RA1, LAP3, LILRB3, LLGL2, MPZL1, OR2L2, OR7A5, PER1, PFAS, PFKFB2, PGS1, PIK3C2B, PRPH2, RERE, RPS6, SHC1, SLC27A1, SMAD6, SPHK2, ST8SIA1, SUV39H1, EIF4EBP1, MAPKAPK3, TNFRSF10B
Negative_6450	DDC, HGF, ADCY9, ADORA2A, AFMID, ALS2, ASNS, B3GALT6, B3GAT1, BUB1B, CLCA4, CYP11A1, CYP17A1, FARS2, GLYCTK, HTR6, LAMC3, MADCAM1, MAP2K6, OR5H6, PIP5K1A, PLOD3, PTCRA, PTGDS, RALB, TP53I3, WEE1
Negative_6451	GBA, IL8, ACSS3, ATG12, B4GALT7, BMPR1B, CCL27, CCNH, CLTCL1, CTSB, DDOST, DEGS2, G6PD, GMPPA, IL13RA2, ITGAL, MTMR2, OR2A2, OR51I2, OR52B4, OR8B4, PDGFRB, PIGQ, TAS2R16, XCL1
Negative_6452	AMH, NF1, SRF, TFG, UBB, AMY2B, CDC23, COL4A2, DNMT3L, DRD2, GOSR2, ICAM1, IL3RA, LDHAL6B, MCEE, MYLK, NODAL, OR10A2, OR2T33, OR2T5, OR4F5, OR4M1, PAK6, PDE6H, PIK3CG, PPID, PTK2, PTPRR, TRPM5, ZFYVE9, HLA-DRB3
Negative_6453	F2R, AADAT, ALDH5A1, C9orf95, COMP, CSNK2B, CYP2A13, CYP3A7, DGKZ, DUSP14, EFNA4, EREG, GUCA1C, IDH3G, IL17RB, INPP5B, IPMK, LIMK2, MAP3K1, MECR, NEFH, NEU1, NEUROD1, OR51G1, PIP4K2B, PPP1R3A, PPP2R1B, PTCRA, RAD51, RASGRP1, SOAT2, STX2, SULT2B1, TRIP10, ULK3, VAMP4
Negative_6454	BCR, DLD, SP1, ACTG1, ACYP2, ALDOC, ATG7, CCL13, CLTA, CNTN1, CPT1B, EPHA2, EXOC7, FOXA2, GALE, GUCA1B, GUK1, HCLS1, HMGCL, HSD3B1, HSP90B1, IL19, KIR3DL3, MAP2K2, NKX2-2, OR10A3, OR10G7, OR13C2, OR2F2, OR51E1, OR6C3, OR8J3, PDE1C, POLD1, PRKAA2, PRPH2, RAC2, RFWD2, RHOD, RPSA, RRM1, TUBA1C, TUBB4Q, WASF3
Negative_6455	FN1, MVD, ABP1, ACACB, APPL1, BACE1, BDH2, CDC26, DGKB, EDAR, FUT4, GALNT13, GRIA2, HDAC1, IL21, IRAK1, LHCGR, LRAT, NKD1, NKX3-1, NRG1, NSD1, NUDT5, OR4C6, OR4P4, OR52B4, OR8B12, PAFAH2, PIK3R3, PPCDC, SARDH, SEMA4D, TIRAP, ULBP3, CACNA2D4
Negative_6456	AUH, CD2, LHB, LIF, UBB, ALDH1A2, ATP5G1, ATP5J2, B3GALT2, B3GNT5, CADM1, CAMK2G, CDC14A, COL4A1, EXT1, GFAP, GIT1, GPAA1, GPD2, GRAP2, HIBCH, IFNA8, IL23R, LDHD, MEF2C, ODC1, OR5AR1, PHKB, PPP5C, PRKAB2, PSME3, RBPJL, RFX5, SOCS5, SRGAP3, SUFU, TAS2R14, YARS
Negative_6457	ACAA1, ADH4, CDH15, CLDN19, DDX18, FECH, GALNT12, GNA14, IGF1, IRF3, NAT5, NCAM2, NPR1, OR2T3, OR2T33, OR4K14, PAPSS1, POLR2H, RAP1B, SOAT1, UPP1
Negative_6458	CKB, FES, ME3, NOG, ADORA2B, ALDH1A2, APAF1, ATG12, B3GAT1, CCR4, CSNK2B, CYP4F3, CYP8B1, DFFA, DHRS4, FGD1, FRAT1, GABARAP, GALT, GNA15, GRM1, HPRT1, ICOS, IFNAR1, INPP5E, KIR2DL2, LTBP1, MAPK14, MYH3, NADSYN1, NAT1, NAT6, NCAM1, NRG2, OR10H1, OR2A42, OR51B6, PAPSS2, PARS2, PIP5K3, PITX2, PRKCD, PVRL1, TAS2R14, THBS3, TYK2, TNFRSF10C
Negative_6459	C9, FH, IVD, OTC, SPN, WAS, ABI2, ATP4B, C3AR1, CCL20, CD19, CLDN2, CPT1C, CYP26B1, DGKD, DUSP4, GLYCTK, GSTZ1, HEXA, IBSP, IRAK4, IRF9, LIPT1, NFYB, OR10A5, OR13C8, OR2B2, OR2T4, OR5B17, OR5D14, ORC6L, PDE10A, PDE3A, PEMT, PIAS3, PIK3C2A, PLA1A, PRDX6, RAPGEF2, ROBO2, SMAD5, TRMT11, VEGFA, ATP6V0A4, CACNA2D4, KIR2DL5A
Negative_6460	C1S, CFD, F10, OGT, TFG, ABL1, ACAA1, ACOX2, ATG12, BDNF, BET1, CCKAR, CDKN1A, COL5A3, CXCL1, EI24, GLI2, GNA11, GNRH2, GSTM4, GYS2, HARS, HTR2B, INHBA, IQGAP1, MGAT3, MTAP, OR2A5, OR52N5, OR7D2, OR8H2, PIK3CA, PPA1, PPOX, PPP2CA, TUBB2A
Negative_6461	NF1, TK2, CASP10, CLDN17, CNTF, CSF3R, GNAO1

Negative_6462	GGH,ABLIM2,APAF1,ATP5D,B4GALT3,BET1,BIRC2,C1QC,CAMK2B,COL1A1,CXCL16,GNA14,GNB3,GNRH1,MAP2K2,OCLN,OR2Y1,PTCH1,ST3GAL1,SUV39H2,TNFSF9
Negative_6463	ATM,HPD,PTS,AADAC,ACTN1,ADRB1,AKT2,ANAPC1,BDKRB2,CDH1,CLCA4,CTPS2,CXCR6,DERL1,EPHA7,ESPL1,GLDC,GPAA1,GPX5,HIBCH,IRAK1,ITPKB,JUND,MAD2L2,MRAS,MYH4,NCOA4,NEFM,NEU1,NFKBIA,NKD1,OR10V1,OR14I1,OR2A25,OR5AC2,OXTR,PAK2,PPP1R3A,PRPH2,RFNG,SEMA4F,STMN1,STX5,TBK1,ULK1
Negative_6464	MYC,NOG,ATN1,BAI1,BMPR1B,CD8A,CEBPA,CNGA3,CPT2,CYP27A1,DVL1,EDA2R,ENTPD4,ESAM,FBP1,GTSE1,GUCY1A3,GZMA,IL25,LAMC3,MAGI1,MAPK3,NME4,OR2H1,OR4A15,OR5T1,OR8B4,ORC1L,ORC5L,PIK3CA,POLR3A,RBL2,SPTLC2,TRAF3,B3GALNT1
Negative_6465	AK1,AGXT,ALG12,CCL15,CXCL1,GJA1,GSTM5,ITGA9,NFATC1,OR4D6,PRKAA2,SLIT2,TGFA,TRMT11,TUBA4A,ULBP2
Negative_6466	ADO,ALDOC,ANAPC11,ARHGEF1,BMP5,CCL20,CDKN1C,CHST11,CMPK2,CYP2A7,EDNRB,GAPDH,HBEGF,IL6R,ITGB8,MCM2,NR5A2,OR10G7,OR51B4,OR6C6,PDE1A,PDE2A,PRPH,PSME1,SHMT2,TFDP1,TPH2,USE1,VTI1A
Negative_6467	F2R,CLDN8,OR3A3,PER3,PPP2R1A,RPS6KA6,SDC2,STX6,HLA-DRB5
Negative_6468	BCAR1,BCL10,CHRM5,GALNT12,GNRH2,OR10G7,TIAM1
Negative_6469	FGB,AANAT,ACOT12,CCL28,CYP2A7,ENPP2,ITGAM,NRG1,OR10K1,OR4P4,PLCE1,PPP2R1A
Negative_6470	AK2,GK,LPO,MUT,TPR,VCL,ABI2,ACY3,ATP5J2,ATP5O,BCL2,BUB1,CACNA1F,CANT1,CBLB,CBR1,CD19,DDX23,DHRS3,DOT1L,FLT4,GLI1,GNMT,HES1,HMGCS1,HTR2C,IDH1,IPMK,JAK3,JAM2,KNG1,NEFM,NEU3,NFATC2,OR1K1,OR2T1,OR5M3,OR5M9,PDE10A,PIGM,PLXNA2,SFRP5,TJP3,TOLLIP,TUBB2A,VEGFA
Negative_6471	AVPR1B,CASK,IL12RB2,NANP,OR1S1,POLR2G,POLR3D,PPP3CA
Negative_6472	BAD,GAA,ANAPC10,CCR1,CFTR,CPS1,DAPK2,DCTN1,DIAPH3,FABP3,GZMA,IDH3B,IL8RA,KIR3DL1,LDHAL6A,MCM5,NADSYN1,NCSTN,NT5C2,OR2T11,OR4C12,OR51E2,P2RX6,PIGQ,PPP1R3D,PRKAA1,PRLR,PTPN11,QPRT,SEMA3A,SPRED2,TKTL1,TYRP1,WNT9A,ST6GALNAC5,TNFRSF11A
Negative_6473	ADAM17,FGF17,GUCY2D,OR10A5,PPP1CB,RAB5A,TAS2R13
Negative_6474	BTC,DBH,PXN,AKR1C2,ALDH1A3,ALDH3A2,CD27,CD36,COMP,CYP2A13,CYP2C9,DNMT1,GALNT7,INPP4A,IRF5,IRS2,MAP3K11,MTHFR,MTHFS,MYH14,OR2AG1,OR4F17,OR51S1,OR5AR1,PAK4,PIK3C3,PLCD3,PLK1,PPM1B,PTPRF,SH3BP2,SMAD6,SMC1A,PAFAH1B3,SERPIND1
Negative_6475	C5,GCK,GHR,TK1,ALAD,ANAPC5,CACNA1H,CXCL16,CYP2E1,FRAT1,GNAI1,GRIA3,HHIP,ITGB3,NRG1,OR2AG2,OR4B1,OR6B3,OR6M1,PDE2A,PROS1,PRPS2,PTPMT1,RBPJ,SELE,TAS2R48,TCF7L1,ATP6V0A1,TNFRSF13B
Negative_6476	PML,ACP2,ADSS,ALAS1,CD247,CYP2D6,CYP3A7,DUSP3,GRB2,IDH3A,IDUA,ITGB5,OR11H6,OR13C2,OR14I1,OR5M3,OR8H1,PDGFRB,PER1,POLA2,PRKCA,PRKCD,RPSA,SRD5A1,TJP3,UBE2L6
Negative_6477	GCK,ADCY5,CAV1,CD19,DGKG,GNA13,GNAT3,IL13RA2,KAT2B,PSPH
Negative_6478	BTD,GHR,ID1,SFN,CTTN,CYFIP1,DPM3,GSTM4,HTR2C,OR2L13,SPRED1,TNXB,TYRP1,WNT2B,SLC25A31
Negative_6479	CFH,ACTA2,BET1,CARS2,CLDN8,CPS1,DHX58,FGF13,KIR2DL2,OR13C3,PARVG,POLR3A,SEMA4F,SMPD1,TJP3,TUBA8
Negative_6480	DLD,LSS,ALDH2,APH1A,CHD8,CLDN14,CYP2A7,CYP2U1,INPP5E,IPPK,LRAT,OR2B2,OR2T2,OR2Z1,OR4D11,OR51E2,OR5R1,PLCD4,PLD2,PNPLA3,TGFB1,TLN1,TPMT,YOD1,ATP6V0A1
Negative_6481	AK2,C4B,TNR,ADCY5,AKR1C1,ALG13,APC2,BIRC5,CASP10,CAV1,CD82,CMAS,COL5A2,DPYD,DUSP9,EPHA2,GCDH,HEMK1,KITLG,LPAR1,NLGN1,NUDT5,OR10Z1,OR7A10,PI4KB,PIK3R5,PPP1CA,PRDX6,RALB,SH2D1A,THBS2,TLR5,UBE2K,UNC5A,ATP6V1C1

Negative_6482	C3,F2R,RPE,UBB,ATP4B,CACNB4,CALML5,CDK6,DEGS2,ETNK1,GCNT4,HADHB,HEXA,IL20RA,IL4R,LTBR,NTRK2,OLR1,OR8U1,OSMR,PIGP,PNPT1,RETSAT,SEMA4A,SESN1,SPRED1,TNFRSF11B
Negative_6483	GCK,PGD,BACE2,CFL1,CHAD,CLDN2,COL6A2,CREB3,FGF5,FGFR4,GALNT13,HSD17B2,LDHAL6B,LDHC,MAP3K1,MYLK3,NANS,OR52E8,OR5B17,OR5B21,PARD6B,PIAS4,POLR2J,PPP2CA,PPP5C,RFWD2,STX12,TSC2,VANGL1
Negative_6484	PLN,TK2,AGPAT4,AKR1C1,ALDH3A1,ALG10,ALG10B,ARNT2,B4GALT6,CCL4,CCL8,CTPS,CYFIP2,DAD1,DAXX,DHRS4L2,EFNB1,FGF22,FMO4,FSHB,GANAB,GLB1,GPLD1,HAAO,ITGA10,MAPK12,MOV10L1,OR2A4,OR51B6,OR8K3,PDHA1,PISD,POLA2,PPP3R2,PRKACA,PRKAG3,PRKAR2B,RAPGEF1,RBX1,SARS2,SETD2,SKP2,SPTLC2,STK3,TAS2R38,TRPM5,HSD17B10
Negative_6485	CR2,ID3,ANAPC1,ASMT,CCR7,CDC25B,EDN1,EI24,GALNT12,GGT5,GNA11,GNG12,GPX5,GZMB,IFNA21,IL13RA2,ITGA1,OR8H3,PANK4,PIAS3,RPS6KB2,SPHK2,ATP6V1E1,MAP3K7IP2
Negative_6486	RFK,ACSL1,ACTB,ARG1,CADM3,CBR1,CD82,COL3A1,DKK1,DLL4,DTX4,IFNA10,ITGB2,OR2G2,PIGF,PIP5K1A,PRKAR1B,PRLR,PVRL4,TAS2R16,TRIP10,UGT2B10,MAPK8IP1
Negative_6487	AK2,CGA,DBI,MIF,ADH5,APOA2,CAMK2B,CAMK2G,CCL23,CISH,CLCA4,COASY,CSDA,CYP21A2,DGKE,DMGDH,FUT5,G6PC2,HAAO,HMGCL,IL15,INSR,ITGA7,JAK3,KLRC3,MCM5,NR4A1,OR10A6,PIGV,POLR2F,PPP3CA,RPS6,TGFBR2,TRMT11,VDAC1,WBSCR22,WWP2,ST6GALNAC6
Negative_6488	ABO,AMT,C3,CFH,ADCY8,ARPC1B,CDC6,DLL1,HSD17B3,IFNA7,IFNAR2,KCNQ1,OR10AG1,OR2T8,OR4K13,OR51T1,OR6C2,PIP5K1A,PRKY,RASGRF2,RASSF1,SGMS2,SKP2,STX2,TAS2R49,MAP3K7IP2
Negative_6489	F3,OGT,SRM,ALDH1A2,BDH2,CACNB1,CHPF,CYP26B1,DHODH,DKK2,ENO2,FCER1G,FGF14,FMO3,GBA3,GCDH,GYS2,H6PD,IFNA2,NRG3,OR2W1,P2RX7,PPP3R1,PTGFR,RPS6KB2,TBXA2R,TGFB1,TLR9,MAP3K7IP2,PRICKLE2
Negative_6490	AR,CSNK1A1,CYP3A43,DUSP5,GYS2,HAGHL,IFNA21,INPPL1,MYL5,OR2A42,OR2T34,PHOSPHO1
Negative_6491	CDC25A,COL4A6,CRHR1,EFNB1,EPRS,LTBR,MCM2,PAK2,PER2,VAMP2
Negative_6492	MPZ,ADCY10,ADCY7,ALG12,ATP5J2,B3GALT2,CASP10,COL1A2,CXCL12,CYP26C1,CYP3A5,CYSLTR1,DDX52,DNMT1,DUSP1,EDAR,EXTL3,FCER1G,FGFR3,GFPT2,GSTO1,MAGI1,MCAT,NAGLU,NQO1,OR1L4,OR2M2,OR3A2,OR56A4,PAICS,PCCB,PPP1CA,PRKAR2A,PTPN1,SNAI1,TIAM2,TPH2,ULBP3
Negative_6493	IL2,KIT,WAS,ADORA2B,CDK2,CHST12,CNTF,CXCL16,CYCS,CYP3A4,DTX1,HMGCS2,MDM2,MYH15,NR4A1,NT5E,OR52R1,PMM2,PROC,PSENEN,SETDB1,VKORC1,KIR2DL5A
Negative_6494	ADO,EGF,TH,AADAT,AMDHD1,ATF4,CHIT1,EPB41L1,GCH1,HLA-DOA,ITGA3,JAK1,LEPR,MYH11,NME2,OR10V1,OR51L1,OR6Y1,PDE6H,PDPK1,PIGL,PIGN,PLXNB1,PPARA,RPN1,SHC1
Negative_6495	ACSM2A,MAP2K3,NRCAM,OR2AE1
Negative_6496	GP9,HAL,MET,MVK,ABI2,ACYP2,APBB1,ARHGAP5,ATP6V0B,CACNG7,DAG1,DDX55,DUSP5,ERBB4,FDPS,FPGT,GALNT7,GGT5,GNPDA1,HPSE2,IL11RA,KIR2DL3,NAGK,NAT2,NMNAT3,OR10A6,OR2D3,OR4C15,OR51Q1,PHKB,POLE3,POLR2K,RAD51,RALA,SEMA4F,SESN3,SETDB1,TDO2,TRHR,ULK2,UNC5C,PLA2G12A
Negative_6497	AMH,HGD,ALG10B,CDC2,OR52B6,PRKCD,TOMM40,TPH2,PHOSPHO1,ST6GALNAC5
Negative_6498	SHH,AGXT,GPX7,MADCAM1,MGLL,NSDHL,OR56A4,OR5H2,OR8D2,ORC6L,PLA2G5,PNPLA3,PPP3CB,PTK2
Negative_6499	CSNK2B,HSD17B2,IL25,POLR2B

Negative_6500	AR,CD4,CGA,F2R,HK1,SI,TPO,ALG11,ALG3,AQP7,ARHGEF4,ARPC3,ATP5H,B3GAT3,BDH1,C5AR1,COASY,GNPAT,GSTA4,HIP1,ITGA2,ITGAL,JAK2,JUND,NAGLU,NAT5,NSDHL,NUMB,OR4D6,OR52E8,OR52N4,PDHA1,PGAM1,PLCD4,PLXNB2,RELA,SDC1,SEMA4G,SPRED2,TRPC1,VAV2,WBSCR17,WNT1,XCR1,ATP6V1C2
Negative_6501	ILK,AGPAT1,ALDOA,ATP2A2,CANT1,CBLC,CCL24,CDKN1B,CNTFR,CRHR1,CXCL3,CYP3A7,CYP4F3,DVL3,ENO1,FTCD,FUT5,FZD1,GALM,GYS2,HPSE2,KLKB1,MAGI3,NFE2L2,NOD1,NSDHL,NTSR1,OR51A2,OR5L2,OR8K1,PHKA2,PMAIP1,PPAP2B,PPP2R5D,PTPMT1,ROBO2,SAT2,SETDB2,SIGLEC1,SOCS3,TIAM1,UROD,PAFAH1B1,SERPIND1
Negative_6502	AK1,ASL,C4B,HYI,IL9,BMP6,CCL13,CCL17,CDC14B,CER1,CHKB,CREB3L4,CRLS1,CTNND1,DAAM1,EDAR,G6PC2,GALNT14,JAK2,OR10A3,OR10X1,OR2K2,OR2T34,OR51A7,OR51B5,OR5AC2,OR5M3,PHKA1,PNMT,PPP2R2D,RENBP,SEMA3F,SPRED1,SRD5A2,TAS2R10,VAV1,WNT7B
Negative_6503	F7,ACOX3,IRF7,ITGA2,NUMBL,OR2B6,OR8H3,RDH12,STEAP3,TGFB1,UGT2B28
Negative_6504	IVD,ACYP2,CCL16,IL18RAP,PLA2G2A,SHMT2
Negative_6505	AACS,ACPT,DOT1L,DUSP14,EGFR,GLB1,GSTO2,IL28B,OR2C3,OR5B17,PAFAH2,PCK1,PTGFR,RAPGEF4,SPRY2,TKTL2,TOMM40,XYLT1,ST6GALNAC5
Negative_6506	SI,ALPP,CXCL1,CYP8B1,DDX50,NRG1,PDIA4
Negative_6507	ID2,CALML6,CCNE2,CHEK2,CHKA,CLDN1,CREB3,CTNNA2,CXCL2,DGKG,DUSP4,FGF3,FLT1,GMPR2,GPD1L,GPD2,GSTA5,GUCA1A,HLA-C,HSPB1,IKBKE,IL28A,INPP4A,OR10G4,OR13C5,OR1J2,OR1L1,OR2T34,OR52E2,OR7A10,RFX5,SARS,SAT1,SOCS4,SOS1,TAS1R2,TAS2R13,TLR3,TP53,TUBB6,TXNDC12,ATP6V0A1,TNFRSF12A
Negative_6508	NGF,SRC,ACSS2,AKR1C1,CCL3,CD79B,DAXX,DPYD,EDAR,GSTA2,HCCS,HIF1A,MYH8,OR2A25,OR2H2,OR4K1,PAICS,SAT1,SEMA3E,SMPD2,SOCS2,SOS2,SV2B
Negative_6509	HK1,PRL,ACSM1,ARSE,CACNA1G,CD40,CHAD,CHAT,COX10,CPOX,CTSB,DDX56,DUSP16,FUT9,GSTM2,GSTM5,HMBS,IL20RA,KIR3DL1,LDHAL6B,MAP4K4,OR13J1,OR2A14,OR52I2,PMVK,PPARD,PRKAR1A,SORBS1,THBD,TIAM1,TUBB2B,ULBP1,ULBP3,PNLIPRP1
Negative_6510	AASDH,BUB3,CYP2C9,IL21,ITGB4,MS4A2,OR10K1
Negative_6511	MOS,CACNG1,CCL13,CHST11,CREB3L2,CSAD,CYP1A2,FGF18,GBGT1,LAMC1,MAP3K13,MYH13,NME4,OR14J1,OR51A7,OR7A10,PER2,PIGX,PTGER1,SAT2,SPCS1,SLC25A31,SUV420H1
Negative_6512	CD244,DUSP6,OR4K13
Negative_6513	CDA,ID2,AGRP,ALG12,C7orf16,CCNB1,CSNK1E,DAAM1,EFNB2,F13A1,FGF14,FGF22,GNPAT,GRHPR,GSTA4,HCLS1,HSD17B1,IRS4,KIR3DL3,KLRC4,KLRK1,MAP2K6,MAT2B,NOTCH2,NOX1,OR4N2,OR52N1,OR56A1,PAK7,PANK1,PCK2,PCYT1A,PFN3,PITX2,POMC,PTGS1,RIPK1,SUFU,TBL1Y,TMLHE,WNT10A,YKT6,TNFRSF11B,TNFRSF6B
Negative_6514	F2R,ACAD8,ALAS1,MAP3K14,MYH15,NANS,NCR1,OR3A3,OR6S1,PRKACB,SPP1,TCF7L2,TRPM5,YARS2
Negative_6515	GP6,ACVR1C,AMPD2,EDA2R,GLI2,GSTA1,MAP3K12,METTL2B,NEU4,OR11H1,PDE5A,SHMT1,TRPC1,VEGFA
Negative_6516	GH2,GK,GLS,RPE,TK2,ACVR2B,AKR1B10,AKT2,CASP3,CD40,CD46,CD82,CHIA,CHKA,COL1A1,CTNNA2,DHCR24,ENTPD8,ETNK2,FLT4,GALM,GRIN2A,IL20RA,IL28RA,KLRC4,MAN1C1,MDH2,NMNAT3,OR1J4,OR2M4,OR5AR1,OR8B4,PCK1,PFKFB3,PLCD1,PRKG1,STK36,TALDO1,TM7SF2,TYROBP,VASP,WASL,MAP3K7IP1

Negative_6517	C6, C1QA, CACNB1, CLCA4, CRLF2, CTNNB1, DHDH, DPM3, EARS2, FGF18, GGT7, IARS2, LFNG, MRAS, NR5A2, OCLN, OR2T1, OR4C12, OR4D2, OR4F17, OXCT1, PDGFD, PDHA1, PIGN, PLAT, POLR1A, POLR2F, PPP2R1A, PTGES, RASA2, RPN1, SEMA3G, TGFBR1, THBS2, THBS4, VARS, YARS2
Negative_6518	CS, FH, AACS, ACADVL, ACPP, ADCY6, AGPAT4, AKR1C1, BAAT, CCL17, CD40, CD47, CDH1, CDS1, COMP, GNA15, HIBCH, LARS, LIMK1, LPAR1, MAP2K3, NLGN2, OR10C1, OR5M9, POLA1, POLR3H, RDH5, RGS3, ST3GAL2, TNFRSF8, TYMS, UBA1, YWHAB, ST6GALNAC5
Negative_6519	ACADSB, ACVR1, CREB3L4, DGKB, EPHA5, LAMA3, PRKAR1B, PTK2B, PTPN11, WASF3
Negative_6520	CD4, DCN, ACTA2, BCL2, CDC20, CHRNA7, CYP1A2, FCGR3A, IFT57, IL25, IRF7, MECR, NLGN2, OR4E2, ULBP2
Negative_6521	UBC, DGKH, HDAC2, HLA-DOA, IL7R, KIR2DL2, MGST2, MMP14, PTPN1, SLC2A4, TAS2R13, PNLIPRP1
Negative_6522	AHCYL1, ALAS1, APOA2, ATP6V0C, CAV2, CCNE2, CFL1, CHUK, CLCA2, CPOX, CYP4F2, CYP8B1, DDX19A, EDAR, ETNK2, FGF8, FUT1, GNA12, GNAS, ICOS, ITGA4, LAMB3, MMP9, MYH9, NLGN1, NT5C3, OR13C2, OR2A14, OR2M7, OR6B2, OR9G4, PGM2, PRODH2, PTK2B, SCP2, SORBS1, TAS2R42, TAS2R5, VTI1B, YOD1, PRICKLE2, TNFRSF18
Negative_6523	ABO, AMT, MAG, TPR, ADORA2B, ADRA1D, ALDH4A1, ATP6V1D, CCR8, CLDN2, COMT, CTBP2, DKK2, ECHS1, F11R, FADD, GAMT, GRIN2B, HMOX1, IL8RA, JAG1, LAMC2, LLGL2, NFE2L2, NTNG1, OCLN, OR5D18, OR6B2, OR9I1, PLA2G2F, PLCD3, POLR3G, PPP2R2C, RASGRP2, WNT2, ATP6V1E2, PAFAH1B1, SERPING1
Negative_6524	C2, IL4, LCT, RB1, ADCY2, CD34, CTPS2, CXCL13, CYP7A1, DUSP14, ICAM1, IRAK1, JUND, LCAT, LTBR, MAP3K4, MS4A2, OR2A4, PDE1A, PLA2G2F, PLXNA3, THBS3, UGT1A10, VAMP3, VDAC2, ATP6V1E2
Negative_6525	GLA, IL7, ACP1, ADAM17, AKT2, ALOX15B, APPL1, ARRB2, AVPR1B, BCAT2, CARS2, CD14, CD34, CHIT1, CKS1B, CXCL2, FMO4, GNPAT, HLA-DOB, HMOX2, MYLK3, OR5R1, OR8S1, PARD3, PIAS2, PPP1R3B, PPP2R5C, PRKCG, RAF1, SOCS4, STX1B, UCK1, UGT1A10, UNC5A, VNN1
Negative_6526	EZR, CCNB2, CD226, CLDN16, CXCL13, EXT1, FOXO3, FZD7, GALNT13, KIR2DL2, LCP2, MDH2, NEU2, OR14A16, OR1M1, OR2A2, PDE4B, RRAS, SIPA1, TBXAS1, PDCD1LG2, PHOSPHO1, SUV420H1, TNFRSF6B
Negative_6527	BAX, C9, GSR, GSS, IVD, ME3, ALDH1A1, AMY2A, ATF4, CCNH, CDC42, CHIT1, CSF3, CYBA, DTX4, EPHA2, FGF4, FMO5, GNAZ, GSTA4, GSTM5, HLCS, HNMT, MAD2L2, NOD1, OR52E4, OR8D1, PER2, PFN3, PPP2R5A, PRKCQ, PRPH2, PTPN5, RFWD2, ROCK1, SENP2, SH2D2A, SLC27A4, SRD5A1, TRHR, WNT16
Negative_6528	ACY3, AREG, B4GALT7, BDH1, CDH2, CLCF1, COL2A1, CXCL12, DCTN1, FASLG, FASN, FGF18, GNPDA1, HMOX2, HSD3B7, IGSF5, MAP3K12, MYH7, NEU1, NODAL, OR51V1, P2RX1, PIM1, PLEKHA8, PLXNB3, PPP2R5B, RASA1, SFRP1, SHC4, STAT6, STX4, ULK3, UNC5A, UNC5B, YOD1, SUV420H1
Negative_6529	CGA, ACSM1, ALDH1A1, ALG9, CCNB2, CCND2, CDH4, CDK2, DRD1, IDH3B, MAML3, NEGR1, NR1D1, NTF3, OR2A42, RALBP1, TOMM40L
Negative_6530	BTC, ABLIM1, ACSL6, ATP5D, BDH2, C1QB, CD274, CYP11B2, GNRH1, KAT2B, MKNK1, MTMR6, OR4F15, OR5F1, PDE7B, PFN1, PHKA1, PLCG1, PTGES2, STEAP3, TBL1X, VTI1A, WNT10B, WNT11
Negative_6531	GNE, ME2, TKT, ACTC1, CDC2, COL1A2, EHMT1, FZD6, HLA-F, HLCS, HSPB1, ICOS, MAP2K4, NCKAP1L, OR51G2, PDE1B, PGLS, PLD1, SPRY2, ST8SIA1, TKTL1, WHSC1L1, MAPKAPK5
Negative_6532	DCN, IL6, ALG2, ARSD, ATP2A1, CCL20, CDC45L, CIITA, DHRS4, FGF12, FIGF, GSK3B, HSD17B8, HSPA1B, INPP5B, IRF9, LAMA3, MAPK13, MTMR2, NAE1, NFYB, NMNAT1, OR1A2, OR4K1, OR8G1, PIK3C2G, POLR2I, PRKG1, RALA, RARS2, RPS6KA3, SC4MOL, WASF2, MAPKAPK2
Negative_6533	ME1, B3GALT4, EFNB3, OR2T6, PIGL

Negative_6534	AKR1A1,AKT2,C1QA,CYP2B6,DHODH,DHRS4L2,DPAGT1,DRD1,DVL3,ETNK1,GALNT11,GNB1,IMPA1,OR51E1,OR52B6,OR5AP2,OR9G4,PIM2,PIP5K3,PTGS1,TUBA1C,TUBB,TNFRSF10D
Negative_6535	RET,SCD,TFG,AADAT,ALDH3A1,ALG2,ARHGEF7,ATP2A3,ATP6V1F,B3GALT6,CACNA1I,CAMKK1,CASK,CDK6,CHKB,CISH,CNTF,COL4A1,CSNK2A2,CYCS,GANAB,GP1BB,IL22,INHBA,JAM2,MAP3K8,MYL7,NFE2L2,OR10G7,OR13C5,OR14I1,OR3A1,OR4D5,OR52E6,OR6N2,OR6Y1,POLR1E,PPM1A,PRKCE,PRKX,PSME2,RDH16,TAP2,TAS2R4,TP73,UROC1,XIAP,ATP6V1E2
Negative_6536	SPN,ALG10B,CDC25A,CHEK2,IMPDH1,MPZL1,OR51A7,OR52I2,PIK3R5,PPAP2A,ATP6V1E1
Negative_6537	IL8,ABAT,CYP4A11,ENAH,GNA13,NRXN1,OR2AT4,OR52D1,OR9K2,TNFSF13B
Negative_6538	GH2,HDC,ACAA2,ACTN2,ALDH3B1,CLDN19,CSH1,CYP3A43,DHFR,FGFR4,GRM1,GUCY2C,HLA-DOB,MAD1L1,MAGI2,MITF,NFYC,OR10G7,OR13A1,OR2Y1,PGAM2,RBPJ,SCLY,SIPA1,THTPA,UNC5A,WNT8B,ZFYVE9,ATP6V1E2,B3GALNT1,TNFRSF10C
Negative_6539	GNS,HK1,IL4,MTR,OAT,ACO1,ADIPOR2,AKT3,ALOX15B,ALPI,AOC3,ARNT,ARPC1A,ATP2A2,BBOX1,BIRC2,BPNT1,C1GALT1,CCKAR,CLOCK,ENAH,FUT4,GNG3,GUK1,JAK1,MAPK8,MYL7,NANS,OR11H4,OR51G1,OR6C74,PGK2,PGM2,PHKA2,PIK3R3,PLCZ1,PLXNA2,PRPH2,PTDSS1,SLC2A2,SLIT2,TLN2,TSLP,VAMP1
Negative_6540	FES,HK2,MAX,ACOT12,ACSM4,AKR1D1,ARSE,CACNA1A,CBLC,CCNG1,CDH3,CDKN2D,COL5A3,COX15,CTLA4,DNMT3B,DRD1,ENTPD5,GNA11,GPAM,HSPB1,LEF1,MAPK7,OR10A7,OR51Q1,PDE7A,PLA2G2E,PLXNA2,PLXNA3,PPP1R3A,PYGL,RPS6KA3,SEMA3E,SMURF1,SORBS1,STK3,STX7,TBXAS1,TLN1,TNFRSF9,TRPC1,VAV1,WNT10A,CSGALNACT1
Negative_6541	JUN,PAH,RFK,TEC,VCL,ACTC1,ADSS,ARD1A,BACE1,BMP8B,C1QC,CDC25A,COL2A1,COL3A1,CYP3A43,FMO2,HMGCL,HSD11B2,HSD17B1,ITPA,KLRD1,LRAT,MAML1,MTHFD1L,NCF1,OR11H1,OR2F2,OR2M2,OR56B4,OR5B3,OR5T3,PDE1A,PERP,POLR2A,POLR3B,PRKCE,RDH5,SESN2,STAM2,TAS2R42,TPH2,ULK2,PAFAH1B1,TNFRSF10A
Negative_6542	DCK,NGF,PDC,ALDH3A1,CALML3,CHRD,CNTNAP1,DIAPH3,ERBB3,FOXO3,GSTM5,HBEGF,IARS2,MADCAM1,MARS2,NCAM2,NFKBIB,OR2A12,OR4L1,OR51B5,OR8J1,PTCH1,RARS,STAT5B,TGFB2
Negative_6543	F7,ADH4,ARPC2,CAMK4,CD247,CD27,DFFA,DUSP4,FMO3,FMO5,FOXO3,GADD45B,GPT2,GRIA1,GUK1,GZMB,HIBADH,IL24,JAK3,LARS,LTBR,MAP2K6,MYLK2,NLGN2,OR1J4,OR1L3,OR2A4,OR2F2,OR4S2,OR52N5,OR9G4,ORC4L,PCK2,PDE6H,PIGC,PIGH,PIK3C2B,POMC,PTGDS,PTGS2,SPRY2,TYRP1,YES1,C1GALT1C1,CSGALNACT1,SERPINE1
Negative_6544	PGD,CAMK2D,CSF1,GSK3B,HMBS
Negative_6545	MPZ,WAS,ABL1,ADCY10,ADRBK2,ALDOA,ARNTL,AXIN1,BACE2,C1QC,CACNG1,CARD11,CCL11,CD19,CKMT2,COL4A4,CSF2,CYP2C9,CYP2U1,DUSP1,EI24,ENO2,FSHR,GLG1,IDH3A,ITGA3,LCMT1,MAPT,NEUROD1,NPAS2,OR5R1,PDK1,PDPK1,POLR1D,POLR2K,PPP2R2B,PRKY,SDHD,SEMA7A,SFRP1,SLC33A1,STX1B,SUCLA2,TAS1R1,TFPI,TGDS,TPM3,UNC5A,UNC5B
Negative_6546	TFG,ACHE,CCNB2,CYP1A1,DRD1,DTX3,GALM,GSTK1,IKBKB,INHBB,ITGA11,JAK1,SETD1B,TACR1,TIRAP,TLR2,VEGFA,PPP1R12A
Negative_6547	SRM,ECSIT,MGAT4A,OR2A25,RAP1B,YKT6
Negative_6548	ALG8,CACNA1A,CHD8,COL6A1,DFFA,DUSP4,FDFT1,MGST1,MYH9,NRCAM,OR2C3,OR2L8,PDGFRB,PPP2R5E,SH2D2A,SPTAN1,SRD5A1,TM7SF2,TYMS,UGT8,B4GALNT1

Negative_6549	C6,ITK,NF1,ADIPOQ,AKR1C2,BBC3,CCL25,CDC20,CDKN2D,CDS1,EIF4E2,EPB41,HMBS,IAPP,IFNGR1,NFASC,NKX3-1,OR6T1,OR9A4,PLAUR,ATP6V1C2,ATP6V1G1,TNFRSF13B
Negative_6550	CISH,LCMT2,ONECUT1,OR10AG1,OR5B21,UGT2B28
Negative_6551	GHR,NGF,FGF4,GALNT12,IFNA21,LDHD,OR52I1,OR7C1,P4HA3,PAK1,PARS2,PCYT1B,PDHA2,PIGH,PKMYT1,PLA2G2D,PTGIS,SHC4,HLA-DRB3,TNFRSF13B
Negative_6552	ADIPOQ,EGFR,FGF5,GALNT9,LIPT1,MYL2,NME2,OR1F1,OR1L6,OR2M5,PIK3C2A,PPARA,PROC,STX7,TPI1,VANGL1
Negative_6553	CTH,IL8,ADRA1A,CCL22,CTSB,CXCL13,DAAM2,HLA-DMA,OR2G6,PFKFB2,PKLR,PLA2G3,PLCG1,PLIN,STAT3,SULT1E1,TMLHE,TYK2
Negative_6554	CXCL6,LAMB2,TP53,WBSCR22
Negative_6555	KDR,CSNK1D,CTBP2,DNMT3L,FGF4,GLUL,IARS2,KCNB1,MCAT,NUDT2,OR2H1,OXCT2,POLD3,PSENEN,RPS6KA5,SMAD4,TNXB
Negative_6556	CBL,RDX,SDS,ABLIM3,ADRB3,ANAPC4,ATG7,ATP5A1,CCR10,CD14,CD27,CSF2RA,EFNA3,HSPA8,IPMK,KAT2B,NCAM2,OR9G4,PAICS,PDE5A,PIK3R1,PRKY,PTK2,PVRL1,RASSF5,SMURF2,WNT6
Negative_6557	ATP5F1,BIRC2,CACNA1A,CDKN2A,CYP3A43,HCLS1,HEMK1,IARS2,ITGB2,MPST,MYL5,NT5C,OR13F1,OR4K2,OR52B4,OR6K3,OR6T1,PAK1,PAX8,PIGM,PLD2,PRKX,RXRG,SELPLG,SMAD4,SPRY2,YWHAQ,GABARAPL2,TNFRSF1A
Negative_6558	KIT,ACVR1,ADRA1A,ADSS,ALDH3A2,ARNT2,ATP6AP1,CD3E,CHST13,CTBP2,CXCR6,GALE,ITGA6,OPLAH,OR6B3,PCCB,PIP4K2C,PRKCG,RPN2,WBSCR22
Negative_6559	ATP6V1H,FARSA,OR4K1
Negative_6560	CTSB,CYP2C8,DKK1,ITGA6,NKD1,OR5H2,THTPA
Negative_6561	LRDD,OR52I1,PERP,SPRY4,STX18,TPI1,TYK2,AASDHPPT,MAPKAPK5
Negative_6562	FUK,GBA,NGF,ALG11,APOA2,CCL23,CFL1,CKMT2,CLDN7,CREB3L3,CYP2C19,EPHA6,FUT1,GP1BB,HTR7,JAM3,OGDH,OR1L4,OR6K3,PIK3R1,PNPO,PPAP2C,SH2D2A,SULT2B1,SYNJ1,TAS2R3,TOMM40L,TUBAL3,PAFAH1B2,TNFRSF13C
Negative_6563	CBS,MUT,PC,PGP,SCD,SHH,ACAD9,ACVR2B,ADCY7,AKR1A1,CCR1,CHRM5,CLDN11,CREB3L2,CYP4A11,FTH1,GLI3,MAN2A1,MAP3K14,ORC5L,P2RX4,PAPSS1,PPP2R5E,SDHA,SOS1,UBE2L3,ATP6V0E1,PAFAH1B2
Negative_6564	GPT,B3GNT4,BCL2L1,CASP6,CCR6,EDNRB,FZD8,GNA15,GSTM1,ITPKA,MECR,OR9I1,SOS2,SV2C
Negative_6565	ABO,F7,ACOX3,AKR1A1,APBB1,BCAT2,BCMO1,CACNA1B,CASP3,CDC14A,FMO2,GNAL,HNF4A,IFNA13,IL4R,INHBC,LAMB1,LEPR,MGAT2,MMP2,NT5C1A,OGDH,OR10H1,OR10J3,OR1L6,OR2S2,OR51I2,OR8I2,PANK2,PDE7A,PIGF,RXRA,SEMA4G,STK36,TOMM40,VDAC1,YWHAQ,HLA-DPA1,PHOSPHO1,TNFRSF10A
Negative_6566	AK2,ATM,GH1,MOS,MPZ,SI,ABCC8,ADIPOQ,ALDH1A2,ANAPC4,C1GALT1,CCDC6,CCKBR,CDS2,DHX58,GABARAP,MITF,OR2A42,OR2T29,OR5D18,OR7G2,OR8S1,PLTP,POLR1B,ROBO3,SMARCA5,SOCS7,SPRY2,HLA-DPA1
Negative_6567	DBI,F7,TH,ADCY7,BMPR1B,IL2RB,KLRC4,MCM3,MPZL1,NFKBIB,OR51S1,OR6N2,PSME2
Negative_6568	CDA,GH1,UBB,ADCY8,BCAT1,CDC16,CDK4,CDKN2B,CISH,DHRS4L2,ENPP2,EPHB2,EPHB4,FABP4,FUT8,GGT6,GNAI1,HSD11B1,IL12A,INPPL1,INSR,LAMB4,MYH13,NANP,NOX1,NTNG1,NUMBL,OR1L3,PARD3,PDGFRA,PIGC,PKMYT1,PLA2G1B,PLD2,PLTP,PLXNA1,PRIM2,PSAT1,SARS,SETD1B,SV2B,TCF7L1,ULK2,WHSC1L1,MAPKAPK3,SERPINA1

Negative_6569	BTK, DAO, NLK, PLG, SDS, ALDH1L1, C1QC, CCKAR, CDKN2D, DDX4, DERA, DHDDS, FGF9, FGFR1, GCAT, GPD2, HSD17B1, ICOSLG, LARS, LCMT1, NAT6, OR1Q1, OR4K1, OR5AK2, PIK3R4, PLOD3, PTCRA, RAPGEF1, SLIT2, SMARCA2, TUBB1, TYMP
Negative_6570	AGK, ALDH3B1, ATG12, ATP2A3, EPHA7, GSTO1, SDHB
Negative_6571	CFH, XDH, ACVRL1, ALDH3A2, APOA2, CDC7, DGKB, EXTL1, GDE1, GUCY2F, HADHB, HES1, HSD3B2, IL29, MAML1, OGDH, OR6N1, PFN1, PTPN1, RENBP, SESN1, SFRP4, SUV39H1, TRAF6
Negative_6572	ADK, AK3, C1S, ME3, RFK, ACLY, ACSL5, ACTA1, ACTA2, ACYP1, AHCYL1, ALDH3A2, AMPD2, AOX1, B3GAT3, C3AR1, CAV1, CCNA1, CD244, CDH2, DHFR, EDNRB, ENPP6, FCGR2B, FGF7, GBA3, HYAL1, IL13, IL18R1, MCCC2, MYH1, NCR3, NFE2L2, OR10X1, OR51E2, OR5K1, OR7C2, OR9I1, PDE4A, PDK1, PER3, SAT1, SMPD1, SNAP23, SSH2, STT3B, SYNJ2, CSGALNACT1, EIF4EBP1
Negative_6573	ADRB1, APPL1, ARPC4, COL5A2, FASN, G6PD, HLA-DMA, MAPK13, PPP2R2B, THY1, VARS
Negative_6574	DLL1, DUSP8, ITPA
Negative_6575	DCT, APOA2, B3GNT4, DLAT, HMGCR, HTR6, MICA, MTAP, OR10J1, OR4K1, PARD3, PIP5K1C, POLR1D, POLR2C, UGT2A1, UROS, TNFRSF13C
Negative_6576	CGN, F3, FN1, LPL, OGT, AADAT, ACSL4, ACSM4, ADRB3, ALDOB, B4GALT5, CCR4, CLTC, DGUOK, DPYSL2, FECH, FGD3, GBE1, GNG3, GPAA1, HPSE, KLRK1, NFATC3, NPR1, OR4K14, PDXK, RALGDS, SRD5A2, STK4, ULBP2, WNT8B, YWHAG, AASDHPPT, ATP6V0A1, HLA-DQA2
Negative_6577	APP, CGN, GK2, LEP, ACOX3, ALG2, APAF1, ARNTL, CLDN15, DARS2, E2F5, FGF8, GNAO1, GNB1, HEMK1, HSD17B3, IL13RA2, IL17A, INHBE, ITGAV, OCRL, OR10G4, OR10S1, OR13F1, OR4C12, OXCT2, PGM1, PIAS2, POLD3, PPM1D, PPP3CC, PRKAR2B, PYGM, RXRB, SELE, SEMA3C, SQLE, ST8SIA5, STX1A, TAS2R1, TAS2R16, TAS2R4, TRHR, TUBB, UMPS, ST6GALNAC6
Negative_6578	HAL, ACYP2, B4GALT7, CD19, CDC6, DRD5, ENTPD1, HTR6, HTRA2, IL23R, LIAS, NFATC2, OR1I1, PAICS, PIGN, POLR1B, POLR1C, SMPD1, TRHR, UPP1, UXS1
Negative_6579	C1GALT1, CD19, CD44, INPP1, NTN1, PIAS2, PMM2, PPAT, PRKACB, RCHY1, SETD1B, SORBS1, SOS1, SULT2B1
Negative_6580	CAD, CBL, GGH, TNR, ALPPL2, ANAPC11, BDH2, CACNA1G, CTSL1, DDX47, FGF22, FGF23, FGF5, GABARAP, GANAB, GGT1, GLYCTK, INPP1, MAP3K5, NEUROG3, NFKB1, OR4P4, PHKA2, PIK3R1, PLTP, PPCS, RBKS, SETMAR, STAM2, STX2, TAS2R38, TUBB4Q, WBSCR17, PNLIPRP1
Negative_6581	LYN, ATP5C1, CCL13, CYP26C1, DOT1L, DTX4, EFNB1, ENAH, GALNT10, NOTCH3, NUDT9, OR4X2, OR51A2, OR7A17, RUNX1T1, SMAD1, TYK2
Negative_6582	FYN, ACADVL, ACOX3, ARPC5, ATP5J, ATP5O, B4GALT7, CD28, CHRD, CRHR1, DAAM1, DDX56, DPYD, FADD, FARSA, GLUL, GNRH2, HEXB, HTR7, ITGB4, MAGI2, MMP2, NKD1, NOTCH1, OR10A5, OR1L8, PIK3CB, POLA2, POLR2F, SREBF1, SUV39H2, TAS2R10, TUBB2B, WWP1, HSP90AA1
Negative_6583	TNF, VCL, ADCY4, CADM1, CKMT2, CTBP2, FASLG, GLUL, IMPDH1, MECR, OR51A2, PAX8, PCYT1A, RALA, SETMAR, SV2B
Negative_6584	EBP, ITK, LPL, PC, ACVR1, ALDH7A1, ARPC1B, CHRM2, CNTF, ENPP7, FLAD1, GALNT8, GDE1, GSTM1, HADH, IL12B, IL28B, LRP5, MAGI2, MAP2K2, MFNG, NFKB1, OR10K2, OR4K2, OR52L1, OR6C70, OR6Y1, OR8B12, P4HA2, PHKG2, PIGX, POLR3K, PPAP2C, PPP3CA, PSME3, PTGIS, RBPJ, SEMA4B, STAM2, ULBP3, VEGFA, WBSCR17

Negative_6585	BAD, PRL, ACP5, ALDH3B2, APOC3, AQP7, BST1, C4BPB, CARD11, CDC25C, CD C45L, CDH1, COQ7, CPS1, CUL1, CYP2U1, E2F3, EHMT1, GNAT3, ICOS, ITGAX , JAK1, JAM2, LRP6, MMP7, NFYC, NKX2-2, OR2B2, OR4K5, OR51F2, OR5D18, PER1, PLXNC1, PORCN, PPP2R2D, PPT1, PTPN7, RAF1, RAPGEF1, RASA2, SAT2, ST6GAL1, TP53I3, TRAF6, WNT11, YW HAZ, ZBTB16, B3GALNT1
Negative_6586	KHK, MYC, OSM, AACS, ACYP2, ATG3, ATP5H, BMP6, CRB3, CSNK2A2, CYP2A7, HNMT, IL11, MARS, MAT1A, MFNG, NCR1, NOD1, NUDT9, OR13C3, PKM2, POLR3 G, PVRL1, SORBS1, TGDS, TJP3, TRHR, UBA7, UGT2A1, YWHAB, YWHAG
Negative_6587	AHCYL2, CHIA, CXCL2, ENO3, HBEGF, IPPK, OR13J1, PRKACA, STX12, TPMT
Negative_6588	FOS, NLK, SYK, AKR1C2, B3GALT2, CACNA1E, CD14, CD59, CHIA, CLDN7, DNM T3A, EIF4E, EREG, FRAP1, GMPR, GNAQ, HSD17B1, OR2T10, PFN2, RPS6KA4, SLC27A4, ST8SIA1, TNFRSF9, YWHAB, ATP6V0A1, ST6GALNAC4
Negative_6589	TPR, ACADL, CCL11, DGAT2, DHDH, FGF22, GSTM3, OR8K5, OXTR, RRAS, WNT7 B
Negative_6590	DCC, OR2T3, PLOD1, UROS, CSGALNACT1
Negative_6591	ACSS1, CACNA1B, CCR6, CD40LG, CD44, CPT1C, EDA2R, EFNA5, EPHA5, GUCA 1A, HPSE2, ICAM2, ICAM3, IL12B, IL21R, IRF5, ITGB8, L1CAM, LIPT1, MAP K11, NEU4, OR10A6, OR10H1, OR51A2, OR6N1, OR9A2, P2RX2, PDGFC, PLA2G 10, PTAFR, SDC2, SLC25A5, STX2, TAS1R3, TAS2R7, TNFRSF8, WNT9A
Negative_6592	EGF, F2R, SCD, ACACA, ACAT2, CPT1B, DDX47, EGLN1, FGF10, G6PD, GNPNAT 1, HPRT1, HTR6, IBSP, ITGAV, LYPLA2, MAPK14, NAT2, NRG3, NRXN1, OR10A 6, OR10H5, OR52N1, OR5L2, OR7G3, ORC4L, PDE4A, PDXP, PITX2, POLR1C, Q PRT, RAD51, SARS, SH3BP2, TFPI, UPB1, PPARGC1A
Negative_6593	AGPAT3, CCL3, HHEX, PIK3C3, PPP1R3D, HLA-DRB4
Negative_6594	AUH, ADCY8, ALAS1, APBB1, ATP6V0C, BCKDHB, C9orf95, CASP9, CCL7, CD8 6, DLST, F2RL3, HRAS, ICOS, IL17RA, IL23R, LAMB1, NTRK2, OR10G8, OR51 A7, OR5AN1, OR6S1, RHOD, SLC12A2, TKTL2, TUBB6
Negative_6595	GPT, CPT1B, CSF2RB, CYP2C18, DAXX, DHRS4L2, IDUA, OR2F2, RHOA, TUBA8
Negative_6596	DBH, DDC, ACYP1, APAF1, AREG, ATIC, ATP5E, B4GALT3, BDKRB2, BMP7, C1Q B, CADM3, CES1, CLDN11, CMPK1, CPT1A, FSHB, GALC, GALNT8, HSD17B7, IL 2RG, ITGA2B, LAP3, MAT1A, MTHFS, NTNG1, OR10A7, OR2D2, OR2M5, OR4D6, OR4S1, OR6X1, OR9A2, PDGFRA, PLCB2, PLCD3, PPP1CC, PPP1R3B, PRKCG, S LC2A1, STAT5B, TACR2, ULK1, WBSCR22, MAPK8IP1
Negative_6597	JUP, CFL1, CTSB, GNA12, ITGAE, MAPK13, NADSYN1, OR51S1, PLXNC1, PPP1 CA, SETD7, SLC9A1, TALDO1, VNN1
Negative_6598	CBS, NGF, ABP1, HSD11B1, PDE3A, PI4KA, PLA1A, PLD2, POLR1A, POLR3A, S TAT1, TOLLIP, ULBP3
Negative_6599	C4B, ME2, SCD, ACVR2B, ADRA1D, ALDH1B1, ALDH1L1, ARHGEF4, AVPR1A, CC L26, CDH1, CDK2, CDKN2C, CREBBP, G6PD, GUK1, IQGAP2, ITGA1, MYH7B, NF ATC4, NTF4, OR10H2, OR52D1, OR5L2, PDE1A, PMM1, PNPO, PPP2R5B, PSME2 , RARS2, SIPA1, SKP2, STAT5A, TAS1R1, UROC1, WNT3, WNT5B, ZNRD1
Negative_6600	GPT, ARSD, CCL24, CD8B, COQ5, FARP2, GYS2, H6PD, MTAP, NCR3, NEU1, NLG N2, NUMB, PGAM1, PPP2R5C, PSPH, PYGM, SEMA3C, TFPI, TRPM5, HSP90AA1
Negative_6601	CFI, GAA, MVD, BLVRA, CACNG2, CCNH, CD44, CIITA, CREB3L4, CSF3, CUL1, CYP8B1, DHFR, DPYD, EIF4B, GALT, GSTZ1, HSD11B2, IFNA7, IFNK, IGSF5, IL23A, LIAS, MCEE, NEU4, OR52E2, OR52N2, OR6K3, P2RX1, PIK3C2G, PRKG 2, RAB5A, RCHY1, SC5DL, SFRP4, TAS2R13, TUBB4, WBSCR22
Negative_6602	IVD, MOS, ACPP, ALPPL2, ATP6AP1, CDS2, COASY, EHMT2, FABP4, H6PD, MYD 88, OR2S2, OR7C1, PLA2G3, SDHA, ST8SIA5

Negative_6603	GDA, ACSM1, ALPPL2, ANAPC7, C1QC, CASP9, CCL25, CSF3R, DKK4, DRD1, FABP1, FLT3, GMPS, GNAQ, GRIA1, GSTZ1, HNMT, IFNA21, MAP3K3, MTMR1, NFYB, OR13D1, OR6S1, OXSM, P2RX3, PCCB, PER1, PIP5K1A, POLR2E, PRIM1, PRKAR1B, PRPS1, RAPGEF1, SESN1, TAS2R5, TBXAS1, TSLP, VDAC3, YOD1, GABARAPL1, TNFRSF13C
Negative_6604	CBS, ACVR1, ALG12, BCKDHB, CD22, CLTCL1, GDF6, HTR2B, ITGA5, LIPT1, MYLK2, NSDHL, OR10G3, OR4C46, OR5L2, SFRP5, SKIV2L2
Negative_6605	B4GALT1, EPHB6, FGF22, GNA12, LDHB, PLD2, SETDB1, TAS2R60, ULK2, MAPKAPK2
Negative_6606	BTD, CGA, KMO, ADAM17, ARPC3, B3GAT2, CAMK4, CXCL12, EIF4B, FMO2, GNPDA1, HSP90B1, IL11, IL28A, ITGA7, LRP5, MAP3K6, MARS, NAMPT, NLGN2, NOS1, OPLAH, OR1N1, OR2T3, OR4F15, OR52B4, OR6K2, PARS2, PDE11A, PDGFRB, PMM2, PPAP2B, PPP2R1A, PSENEN, PSPH, RPS6KA3, SPTLC1, TAS2R14, THTPA, TSC2, TUBB6, UXS1, CSGALNACT2, HSD17B12
Negative_6607	GP5, ABI2, ACAA2, AKR1C3, AMPD2, ARPC5L, ARSE, ASIP, ATP5I, CCNE1, CHIA, CTNNA1, CTSG, CYP2J2, DGKG, DIAPH2, EPAS1, EPHA8, FGF20, FMO2, HEXA, HIP1, IL17A, MGAT5B, NAGS, NFATC1, NUDT2, OR10G4, OR10H1, OR13F1, OR2F2, OR52A1, ORC6L, PI4KA, RDH5, RRM1, SETD1A, SKP1, SPTLC1, SSH3, TAS2R16, TUBB3
Negative_6608	KMO, ME1, TNF, AGMAT, BMP8B, C3AR1, CD48, COL4A4, CPT1C, CTF1, EPHA4, FECH, FGF1, FGF4, FZD6, HCLS1, HLA-DRA, HSD3B7, HSPA8, ICOSLG, IL10RA, MYH11, NCOR1, NUDT8, OCRL, OGDHL, OR1J1, OR2T10, OR51E1, OR5F1, PDE1A, PIK3C3, POLE3, SNAP25, TUBA1B, WWP2, CSGALNACT2
Negative_6609	CKB, CRK, F2, RPE, SRC, AANAT, ADCY8, ALDH3A1, ASMT, BDKRB2, CACNA1D, CASP7, CCR2, CHEK2, CLDN2, DAAM1, DGAT1, E2F3, EPOR, GALNT6, GDF6, IDH3B, IDUA, INPPL1, MAN1C1, NEFM, NFYA, OR1L4, OR4K13, OR4N2, OR4Q3, OR51I1, OR51I2, OR5K2, PCCB, PHKA2, PHKB, PKMYT1, POLR3B, PPP2CA, PSME2, PYGM, SEMA7A, SHC4, SMAD1, SULT1E1, TAS2R42, TYROBP, ATP6V1B1
Negative_6610	AR, ADH1A, AKR1C4, ALG3, ANAPC5, ARPC5L, BRAF, CCNA1, CD79B, EFNA1, ENPP3, FGFR1, GMPPB, IL22RA2, IMPDH1, ITGA9, LYPLA1, MAD2L1, MAPK1, MGAT5B, NTRK2, OR10G7, OR10H1, OR2AT4, OR52J3, PAX4, PCNA, PIGB, PPP1CB, PRIM2, PRNP, RPS6, RRM2B, SDC4, SETD1B, SLC9A1, TUBB2B, TYMP, WNT9A, XCL2, CACNA2D3
Negative_6611	GSS, ABCC8, ADH4, AGPAT4, ASNS, ATP5C1, CHRD, CKMT2, CLCF1, CLDN2, CXCL5, DDX23, DFFA, EP300, EPHA1, FUT9, IL17RA, KIR2DL2, LARS2, LIPE, LIPF, MAN1C1, MGAM, NCF4, NME6, NRCAM, OR1J4, OR2AG2, OR4A15, OR4F4, OR52W1, OR5AK2, PDGFC, PGAM1, PIK3R2, PLOD2, PTCH1, PYCR1, TGFBR1, TIAM1, TJP2, TRIP10, UMPS, TNFRSF10D
Negative_6612	AR, MAX, ACAT2, ADRB3, ASS1, BET1L, CACNG7, COL4A2, CRHR1, CRY1, FUT2, GJD2, GNA12, MDH1, MYL9, NEUROD1, OR1G1, POLR2B, PPP2R2C, PPP3CA, SV2B, UGT2A1, UPP1, WIF1, TNFRSF10C
Negative_6613	ALDOB, BMP5, BMP8B, CD55, CSF2, CYP2C18, F13A1, GPX7, GTSE1, OR2A42, OR52J3, OR52L1, OR5T1, PLA1A, PPCS, PPP2CA, PTPN5, RBL1, TSHB, PLA2G12A, SUV420H2, TNFRSF11A
Negative_6614	BTD, ABCC8, AHCYL1, CNTF, CREB1, CXCL13, G6PD, GSTA2, HSD3B1, IGSF5, LTB4R2, LYPLA1, MYL5, NTF4, OR2T8, OR4C46, PGLS, PHKG2, PTPRF, SUCLG2, TFDP1, TMLHE, TNFRSF17
Negative_6615	BTD, ADH1B, AMDHD1, AQP7, CDKN1B, CLTC, CNTN2, CXCL12, DARS2, DOLPP1, DUSP8, EPHA5, FUT5, GANAB, GYS2, HPRT1, HSD3B7, HTR2B, IFNGR1, IL13RA2, IL15RA, IL1RAP, ITGA4, KIR3DL3, MCM5, MGLL, NKD1, NRG1, OLR1, OR11A1, OR13D1, OR6M1, OR8H2, PDE6H, PIAS2, PIM1, PLA2G2A, POLR2B, PRKACA, RDH10, SCD5, STX5, TAS1R3, THTPA, TYROBP, WARS2, WIF1, WNT9B, YARS

Negative_6616	EPO,FUK,BBOX1,CCND2,DAPK3,FZD1,GGT1,GP1BA,LAMB1,OR51B2,PMM1,PRKCI,PTPN5,SPCS1,PDCD1LG2
Negative_6617	GH1,GSN,PRL,TK2,ACO2,ALG5,ARSB,BCKDHB,CCND2,CD82,CDH4,CER1,CLCF1,CLDN10,COL5A2,CYP27A1,DERL1,DGKI,DHDDS,EFNA4,ENTPD2,EREG,GARS,HTR2C,IFT57,IMPDH1,LDHC,MAP3K1,NOS3,NUDT12,OR10C1,OR2AG1,OR4S1,POLR2G,PTDSS2,PTPMT1,RDH12,RND1,RPN1,SAT2,SHISA5,TAS2R10,HSP90AA1,MAPK8IP1
Negative_6618	MOS,AGTR1,CPT1A,DUSP5,EPHA7,LDHAL6A,OR4K17,OR52K1,PANK4,PIK3R4,PTPN7,RPS6KA5,TPH2
Negative_6619	AMT,IL8,NOG,TEC,AADAC,ABL1,ACP5,AGRP,AKT1,ALDH3B2,B3GAT1,C1QA,CCL11,CLDN22,DIAPH1,EPHX2,FPGS,GLI2,GNA14,GNG3,GPX7,GSTM4,HES1,IKBKG,MAPK10,NFAT5,NRCAM,OR13C2,OR7G3,PIGX,POLR3B,PRKAG1,PTPN11,SELP,TPK1,HLA-DQB1,MAP3K7IP2,SERPIND1
Negative_6620	ADH1B,AMY2A,ASS1,ATP6V1D,CCL2,CD70,IRAK4,MAP4K3,MGST2,PPA1,PRKAR1A,PRKCQ,SUFU,YARS2,ST6GALNAC3
Negative_6621	ADK,AK2,AADAC,AGTR1,ASRGL1,CD34,DDX50,EHMT2,FMO5,GSTA4,IDUA,IFNA17,IL13,IL29,LAMA4,LHCGR,OR1E1,OR2T3,OR4X1,OR52N1,OR7D4,PNPO,POLR2K,PSME1,PYGL,SFRP2,UGCGL2,VANGL1,ZIC2,HLA-DRB3
Negative_6622	C7,TNN,ABLIM2,AGTR1,AK3L1,ALDH7A1,C1QC,CD59,CSF3,CYP2A6,FCGR3B,FLT3,ICOS,IL13RA2,NRXN1,OR2T1,OR4K14,OR6K2,PCK1,PIGS,PRKAA1,RALGDS,TRPC1,TUBA3E,TUBA4A,VEGFA,YARS2,SERPINB5
Negative_6623	KDR,AARS2,ADCY3,ADH1B,B3GNT4,B4GALT7,DHRS4,EPHX2,GALNT7,ICAM3,IL7R,INHBA,KAT2B,NPR2,OR9G4,PARVB,PDE6D,PPP1R1A,RARA,SFRP1,HLA-DPA1,KIR2DL5A,TNFRSF10C
Negative_6624	GGH,NPY,CBR1,CYP7A1,HSD17B4,MAP2K5,OR5T2,PDE4A,RAC2
Negative_6625	C1S,ID3,AMPD3,BCL2L1,CACNA1D,CADM3,CCND2,COQ3,COX15,CREB3L1,CTPS2,CYP1A2,CYP2A7,DKK1,EFNB2,EIF4B,FABP1,FMO4,FTMT,HSD17B4,IL2RG,NFKBIB,NFX1,NFYB,OR4E2,PDHB,PER2,PFKFB2,PIP5K1A,PLA2G10,POLE,PPCS,SHC3,SLC27A4,STX8,TAS2R49,UNC5A,HLA-DQA2,ST6GALNAC5
Negative_6626	DBH,OR4F5,RPS6KB2
Negative_6627	LCT,CDC45L,COX10,KIR2DS4,LCMT1,MAP3K14,OR14J1,TXNDC12
Negative_6628	ADC,ACPT,ACVRL1,ALG9,BCAT2,BMPR1B,CACNA1G,CHSY1,DDIT4,EIF4E,ELK4,FLT4,FUT7,GAD2,GUCY1A3,IL15,IL22RA1,KARS,KIR3DL1,LIPT1,MYLK2,NEGR1,NFX1,OR52I1,OR52J3,OR5A1,OR6T1,PDE1B,PECAM1,PI4KA,PIGP,PKM2,PTPMT1,RENBP,RFX5,RRM2B,SETMAR,SLIT2,ST8SIA5,TCIRG1,UBE2L6,VEGFC,MAPK8IP2,PAFAH1B2,SERPINA5
Negative_6629	GSR,CX3CR1,FARS2,LDHA,MMP2,OR2F2,PPP1CB,RFXAP,TSHB
Negative_6630	HYI,TAT,CD48,IFT57,MGAT5,OR13H1,OR51B4,OR8K5,PAK1,PNPLA3,PPA1,SNAI2,SRGAP3,TYROBP,AASDHPPT
Negative_6631	C7,DLD,F11,F12,ACSM2A,ACY3,AKR1C3,ATP6V1D,B4GALT1,BBC3,CCL16,CCL23,CSH1,CYP17A1,F2RL1,G6PD,GNPDA2,HSD17B1,IL11RA,IMPDH2,INADL,LDHA,MYH8,NARS,NEUROG3,NRXN1,NTF4,OR2F2,OR5J2,RARA,STX2
Negative_6632	ADC,GPT,CD19,DDX4,IKBKB,MPDZ,PARVA,SCD5
Negative_6633	POLR2L,SGPP1,TBXA2R,WNT9A
Negative_6634	ACADVL,ARHGEF1,CACNB1,CARS2,CYP1A2,DDX18,GAL3ST1,GALNTL1,MAPK8,OR4D2,OR52L1,SYNJ1,TLR9
Negative_6635	GBA,MAX,NLK,ADH1B,AGPAT1,ALPI,APBB1,B4GALT2,CLDN3,COL1A1,CTNNB1,CTSB,CYP1B1,DAG1,DUSP2,G6PD,HTR4,ITGB6,MAGI3,MCM3,MYL5,NFASC,OR10H2,OR14A16,OR2A2,OR2M2,OR4B1,OR5F1,OR6B3,P2RX7,PAK1,PAK7,PIP4K2B,PPP3CA,PRKCG,RENBP,RETSAT,RPS6,SESN3,SH2D2A,SHC4,WNT7A,ATP6V0D1,MAPKAPK2

Negative_6636	CTH, GLS, IVD, CSF3, IL15RA, INPP5A, OR14I1, OR56A3, PIK3R5, PON1, RXRG, TPI1
Negative_6637	GK2, MIF, ADCY5, ADRA1D, ALG1, ATN1, ATP6V1A, CCL21, CD99, CDC25C, CLDN18, CXCL9, CYP2B6, DDX55, EHHADH, FGF3, GADD45A, GBGT1, LAMB3, NOX1, NRAS, OR4S2, OR6Q1, PDE11A, PFKFB4, PGK1, PLCD4, PLD2, PTPRF, RAD51, SMPD4, SOAT2, ST3GAL1, SV2B, SYNJ1, TAS2R13, TUBA3C, TUBB3, UGT2B7, ATP6V1B2, SUV420H1, TNFRSF1B
Negative_6638	GP6, GPI, MVK, APAF1, ATP2A1, ATP5L, BCAT1, BST1, C4BPA, CDC7, CDH15, DGAT1, EPHA4, FPGT, IARS, IQGAP1, MAP3K12, METTL6, MGST2, MYH1, MYH14, NCOR1, OR4F4, OR52A5, OR6M1, OR9Q2, OXCT2, PI4KB, POLR3B, PPP2CB, PSPH, RGS3, SSH1, TAP2, THBS3, TLR9, TOLLIP, TYMP, UBA1, SERPINF2
Negative_6639	AK1, CTH, MIF, XDH, ACO1, ACSL3, ADAM17, ALDH1A2, CCL3, CCR5, CREB3L2, DEGS2, DOCK1, DPM2, GGCX, GGT1, GNG13, GNPDA2, JAM2, MAP2K4, MAP2K5, MTFMT, OR1C1, OR1N2, OR2K2, OR6K6, PARVB, PDXP, PMAIP1, PPAP2A, SFRP4, SMURF2, SPRED1, TCF7, TNFSF18, TNFSF4, UCK1, WBSCR17
Negative_6640	MAX, CACNG2, GOSR2, IDH3G, IL9R, MGST3, SAT1, TAS1R3
Negative_6641	ABCC8, AGPAT2, BIRC5, CYP11A1, ENO1, FLT3, GALNT5, INPP5A, LDHD, LEFTY2, OR10K2, OR4C12, OR6A2, OR7G2, PDE1C, PLA2G2E, PPP1R3B, SUCLG2, WNT3A
Negative_6642	APOA1, CACNA1D, CACNB4, CERK, CRB3, ESAM, F2RL1, MYH1, NFKBIB, OR2T33, PANK1, PARS2, TRPM5
Negative_6643	HK3, JUP, ME3, SP1, ACTN2, BNIP1, CANT1, CCL8, CHSY1, CLDN5, CYP26B1, FABP1, G6PC, GALNTL2, GLG1, GNA15, GP1BB, HTRA2, IDUA, KARS, LAMA3, LAMC3, MAP3K11, NAT1, NCF1, NMNAT2, NPR2, OR2C1, OR2T2, OR4K13, OR6C74, PIK3C2A, PIPOX, POLR2D, RASGRP2, SGMS1, SIGLEC1, SNAP25, SOAT2, STAT5A, TUBA3E, UNC5C
Negative_6644	B4GALT2, DVL1, EXTL1, NEU3, RASSF1, SETD7, HLA-DPA1, TNFRSF10B
Negative_6645	F2R, HDC, CCR6, CD36, CDK6, CYP4A11, EPHX2, FGF23, FTMT, GABARAP, GUCY1B3, IDI1, IFIH1, KAT2B, MAP3K11, MCM6, MYH14, NFKBIB, OR4X1, OR5AS1, OR8G1, PPARG, PPP1CA, PRIM1, PSME3, STX6, SUOX, TALDO1, TRAF6, UGDH, SERPINA5, TNFRSF13B
Negative_6646	ACO2, ARPC3, IPPK, OR5H6, OR8G1, PYGM, VTI1B, SERPINA5
Negative_6647	ALDH1A2, GCLM, INHBA, OR10J3, PIGH, PRKACB, RAPGEF1, RFWD2, SHC1
Negative_6648	FYN, HYI, ADH7, AKR1B1, CACNA1C, COL4A1, CXCL2, CXCL9, LALBA, LFNG, NAT1, OR4K1, OR51F1, OR5A1, PNPLA3, POLD1, POLE4, RAB5A, UGCGL2, UXS1
Negative_6649	MAX, ADRB3, AOX1, ATP5D, BDKRB2, CD79A, CDC14B, CNGB1, CTSL1, CYP26A1, DAG1, DDX23, DDX54, DGKH, EIF4E, FGD3, GAL3ST1, GMPS, GNAT3, GPT2, JAM2, LFNG, MLLT4, MYH13, NCR1, NOD1, NT5C3, OR10G8, OR5A2, POLE3, RAB5A, ROCK2, SULT1A1, TCF7L2, TKTL1, TSHR, TUBB3, UGT1A10, VASP, VAV2, WASF3
Negative_6650	AKT2, ASRGL1, ATN1, ATP5C1, CACNG7, DCTD, DKK2, F13B, GPD1L, IL1RAP, ITGB2, JAK1, MRAS, NAE1, NEFM, OR10J3, OR1G1, PIGL, PIP5K1C, PTGS1, RPE65, TIRAP
Negative_6651	C9orf95, CD27, DDX47, HSD17B7
Negative_6652	AK3, ZYX, ACAA1, CCL25, CCNH, CES1, COL6A2, CXCL16, CYP11B1, CYP1B1, DTX2, HSD3B2, IL28B, KLK3, PDE8B, SGPP2, TGFB1, ST6GALNAC1
Negative_6653	MDM2, OR2A12, OR5AS1, P2RX5, PLXNA2, SLC25A4, SPRED1
Negative_6654	C8B, CGN, DCC, FER, STS, ACYP2, DLL3, DUSP16, E2F4, ESAM, FARP2, FMO2, FRAP1, GALE, GLI1, GPAA1, HNF4A, LAMC2, MAPK12, NSD1, OR10A5, OR14J1, OR2G6, OR51I1, OR52I2, OR52M1, PDE1C, PDGFB, PFAS, PIGQ, PIGX, PLEKHA8, POLR2D, PPP2R5B, SETD1B, SPTAN1, STX18, TAS2R8, TBL1X, TFPI, ULBP1, ATP6V0A4
Negative_6655	GAA, PC, CDK7, COL4A2, ITGAM, LAMC1, LRRC4C, OR4S1, OR51B4, PIAS3, POLR3G, SHC2

Negative_6656	FYN, RDX, UBC, ACPT, ALG10B, CCL17, CCNB1, DGAT2L4, DUSP6, GNG12, KRAS, NTNG1, OR13F1, PORCN, RBL1, VARS, SUV420H2, TNFRSF18
Negative_6657	ASL, CACNG6, FGF7, GUCA1B, NFATC1, NOTCH3, OR10K2, OR13C2, OR4A5, OR4D11, PRKCH, PRKCZ, PPP1R12A
Negative_6658	C8G, F5, IL8, ZYX, ACADS, AKR1A1, ALDH3A2, AMPD1, AOC2, APLP1, B3GAT1, BLNK, CCND2, CHEK1, CHSY1, COL3A1, CXCR3, DOCK1, DUSP2, EPRS, GALNT13, GNG12, HRH2, IMPDH2, ITGA11, METTL6, MYLK3, NRP1, NUDT9, ORC3L, PARD3, PDE6G, PMVK, PON3, PPID, PTPN6, RENBP, SPRED2, SUCLG2, TRAF6, VAMP4, SERPING1, ST6GALNAC4, TNFRSF1B
Negative_6659	AK5, CACNG8, CCR9, COL6A2, DPYS, FARSB, GALT, GARS, ITGA9, LTBP1, OR51E1, PLCD4, PNMT, SELP, ALDH18A1
Negative_6660	ASCC3, KCNJ11, PIGU, PRIM2
Negative_6661	NLK, ACADS, ACSM5, AGTR1, APPL1, CLDN3, COL5A1, DNMT1, EPHB6, HSPA8, MDH1, MYH9, NADK, OR2T34, OR4X1, OR5A2, PAX8, PIGV, PRKAA1, PRKAR1A, PRKCA, PYGB, SETD7, SH2D1A, SH2D1B, WBSCR17, SERPIND1
Negative_6662	BTD, ILK, LIF, RDX, TPO, ACOX3, ANAPC7, ATP5G3, BCAT2, BMP2, BUB3, CACNB1, CCNB3, CD38, CDKN2B, DDX18, DVL2, FMO5, GALNTL5, GNRHR, GSTP1, HRAS, IL1A, MEF2C, MGAT3, NME2, OR6C3, OR7G2, PAX4, PDE6G, PIK3R5, PNMT, POLR2A, POLR3G, PPBP, PRKAB2, RBPJ, RPSA, SULT2A1, TRAF4, WNT10B
Negative_6663	ZYX, CDKN2B, CNTN1, ENO1, GMPR2, HCCS, HSD3B2, IL18RAP, IRAK2, OR4D9, OR51G1, OR9I1, PGM1, PIK3CD, PRKAG1, RALA, SOCS3, SOCS4, SPCS3, ULK2, UNC5A, WWP2, XCR1
Negative_6664	CYP4A22, E2F3, PIK3R3, WBSCR22
Negative_6665	HGD, ME3, ARG1, BMP8A, DIAPH2, GUCA1C, HADHB, HSD17B8, KLRD1, MAP3K12, MCM7, MNX1, OR14J1, OR51F2, POLR3G, RPRM, SMURF1, TAS2R48, TRAF3, TSC1
Negative_6666	MIF, MYC, ALG1, CAMKK1, CFLAR, CHRD, DHRS3, DPM3, GALM, RAP1B, RRAS, SSH1, TSC2, VAMP4
Negative_6667	AADAT, ACSL3, ADH7, BCAT2, BLVRB, CFL1, CHRM2, CLDN6, CLTC, COMP, CSF1, EPHA5, EXTL3, GATM, IL23R, IL3RA, KIR3DL1, NLGN1, OR2T34, PFKFB2, PPP3R1, SETMAR, SUFU, TACR2, TNFSF4
Negative_6668	A2M, ITK, RB1, XDH, ACSL3, AGPAT6, ALAS1, ALDH6A1, CACNA1S, CAV1, CDC14A, CRY2, CYBB, FLOT1, FMO5, FPGT, GALK1, HTR2B, IL20, ITGB5, KLRC1, MCEE, NGEF, OR13F1, OR2T11, OR2T8, OR5A1, P2RX3, PNMT, POLD1, POLR2I, ROBO2, SCLY, SELE, SRD5A2, TAS2R49, TUBB, UBA7, WNT3A, ZAP70, AASDHPPT, ATP6V1C2, PAFAH1B3, TNFRSF1A
Negative_6669	C3, CBS, DDC, GK2, LHB, ADSL, ALPP, APH1A, ATP6V0C, CACNG7, CHRM3, CTSL1, DDOST, DLL1, DUSP14, FBP2, FRAT2, FZD6, GALNT3, GAS1, GRB2, KIR2DS5, MAP3K13, MYH10, MYH9, NAE1, OR10S1, OR51V1, OR9G4, PIAS4, PIGA, PKLR, PRPH, PTGDS, SFRP4, SOD1, TIAM1, TSHB, TUBA1B, TUBA3C, TUBA3E, TUBB2B, XYLT2, ZMAT3, SERPING1
Negative_6670	BIRC3, E2F4, FGF18, FLAD1, FRAP1, GRIA3, HHEX, INHBB, NAT5, OR2L8, OR5P2, P4HA3, PDHB, PIK3CB, PRKAR1A, PRKAR2B, PTCRA, RBPJ, RDH16, RPS6KB1, SEMA3G, SEPHS1, ST8SIA1, TDO2, TIAM2, CACNA2D3, SERPINA1, TNFRSF10A
Negative_6671	AMT, F8, GNS, IL2, MAG, PC, PLN, APH1A, APOA5, CCL19, CD74, CLCA2, GNPDA1, GPT2, HCST, ICAM3, INPP5E, MAP2K2, MAP3K5, MTHFD1L, OR13H1, OR2S2, OR4E2, OR5AU1, ORC1L, OXCT2, PCK1, PDIA4, PON3, PPP2R2A, RARS2, RHOQ, RRAS, SPRY4, TOMM40, TRAF6, UGCG, ULK3, TNFRSF10A
Negative_6672	ID3, ACSM1, CCL25, CREB3L3, CXCR3, DCTN1, GALK2, GBGT1, GOSR2, IARS2, ICAM2, IL15, INPP1, INPP5A, ITGB5, NODAL, OR1J4, OR52W1, OR5B17, OR6C1, OR6N2, OR8D4, PGM2, PHKA2, PNLIP, PTGER3, RAF1, RASGRP3, SRGAP1, ST3GAL6, SULT2A1, UMPS

Negative_6673	AASS,ACSS3,ADH5,ANAPC7,BPGM,CACNG8,GLI1,GNA13,IGF1R,IL1A,IL29,IRF3,MAPK9,MKNK1,MLYCD,MTHFR,OLAH,OR14C36,OR4X2,OR5T3,OR8I2,ORC6L,PDCD1,PIGL,PLXNB3,POLR1E,RAB23,WASL,YARS2,TNFRSF10D
Negative_6674	EGF,CALML3,CDH1,CFLAR,CLDN16,CXCL11,FMO2,FSHB,GCH1,GMPPB,MCM3,NCF4,OR51B4,OR51V1,ORC2L,PVRL2,SENP2,SULT1A1
Negative_6675	C4B,DDC,F3,HK2,ACVRL1,AKR1A1,B3GALT6,BCAT1,CACNA1A,CLCA2,CLDN15,CLDN19,CLTB,COX10,CSF2RB,CYP26B1,ECSIT,FPGT,G6PD,GALNT2,GCLC,GJD2,GSK3B,GUCY2F,HTR2B,IRF3,ITGAL,NCF1,OR10S1,OR4A5,P2RX2,PARS2,PFN3,PSAT1,SCP2,SETDB1,SFRP2,SMAD1,TNFSF10,TRMT11,TUBB1,HSD17B10,PLA2G12A
Negative_6676	BTK,FES,ADCY8,ALDH1A1,BCKDHB,CCL22,CHDH,CMPK2,COMT,CSNK1E,EHMT1,ENPP1,GGT5,GOSR2,GUCY1B3,IRF7,LARS,LRP5,MCM3,NT5C3,OR2A4,OR2K2,OR51B6,PIP5K3,POLD3,PRDX6,PRKAR1B,PSENEN,PTCRA,TSTA3,TNFRSF14,TNFRSF1A
Negative_6677	HTRA2,MITF,SOCS7
Negative_6678	GDA,NGF,ABLIM1,ACVR1B,ADSL,APOA5,BCAR1,CLDN16,COL1A1,COL4A6,DGKA,GNAO1,GUCY1A3,HADH,IFNA21,IL22RA1,INMT,IQGAP1,MAP3K8,MYH4,NCF2,NKX2-2,NOTCH3,NT5C2,OR11H1,OR13F1,OR2A42,OR4D5,OR6C76,PIGB,PLCB1,POLR1E,PRKCQ,PRKG1,PTGS1,RELA,SPTLC1,SULT1A1,TBL1Y,TDO2,TNFSF10,TSNARE1
Negative_6679	BTD,F11,ME1,ACSL3,ACYP2,AFMID,AGPAT4,B4GALT6,BCL2,CAMK2B,CAMK4,CCNH,CCR3,CCR7,CD47,DARS,ENPP2,ETS1,FOXO3,FRAT1,GGCX,IL12RB1,INHBA,JAG1,KLKB1,MAFA,MTAP,NUMB,OR2F1,OR4N4,OR51A4,OR8K5,P2RX3,PDE9A,PFKFB1,PFKFB2,PFKM,PIAS3,PIGM,PSME2,RXRG,SLC2A4,ST3GAL1,TSC2,TUBA3C
Negative_6680	F8,ATP2A1,BCMO1,CD27,IRF9,MMP2,OR4D11,PARVA,STX5,TNFRSF11B,TNFRSF14
Negative_6681	LCK,MIF,MPL,TNC,AADAT,ACAA2,ALG5,ATF4,BCAT2,CACNA1I,CBR3,CCL5,CCND3,CD40LG,CD59,CDK2,CRB3,CSNK1D,CYP7A1,FGF13,FGF20,GALNT13,INPPL1,IPMK,MAGI3,MYH3,NRXN2,OR4D5,OR51G2,OR56B4,OR6K2,PLXNA2,POLR2C,PTDSS2,PYGB,RFXANK,SEMA4F,SETD7,SFRP1,SMURF2,SOCS5,SUCLG1,TLR4,UAP1,UPP2
Negative_6682	KMO,BDKRB1,CDK2,CYP1A2,FLAD1,GFPT1,HLA-DMA,HMOX1,IDI2,IL17B,L1CAM,MDH2,OR1Q1,ORC3L,SULT1A4,TOMM40
Negative_6683	ACADSB,AGMAT,ALG10B,BCKDHB,BLNK,CHUK,EHMT1,ENAH,GNPNAT1,GUCY1A2,HTR5A,IL1R1,ITGB3,JAK3,MAOA,MECR,MYH7,NCAM2,OR2L2,OR52N2,PGAP1,SIPA1,SNAP23,SPRY2,SQLE,XCL2,GABARAPL1,MAPK8IP1,TNFRSF1B
Negative_6684	LCK,CACNG5,DHX58,GANAB,GNRHR,IL3RA,OR2T5,OR4N5,OR5M9,OR6C74,PDE2A,PERP,SMARCA2,TAS2R5,HSD17B10,PNLIPRP1,TNFRSF12A
Negative_6685	ADA,C8G,F5,GH1,PC,ALDH6A1,ARSB,CASP9,CCR1,CDH2,CYP26B1,CYP3A7,DHRS4,DUSP3,ECSIT,EFNB1,EXT2,F11R,FGF9,GSTK1,GSTO2,HSD17B1,HSPB1,ICAM2,MAGI1,MAPK13,NAGLU,NAT1,NAT6,NFKBIE,NRXN1,OR10C1,OR13C9,OR2B6,OR6K2,OR7G1,PLK1,PPP5C,PRSS3,PTPRB,RENBP,SLC27A5,SNAP23,TCF7,ATP6V1B2,HLA-DPA1,PPP1R12A,ST6GALNAC3
Negative_6686	CRK,PDC,AKR1A1,APC2,APOA1,ATP5E,BHMT,CANT1,CCR8,CDC16,CTF1,CYP2D6,DMGDH,DOT1L,HYAL2,INHBB,ITGB4,ITGB6,JAK1,MAN1C1,MAPK8,NEU4,ODC1,OR5C1,OR5D18,OR6C3,PDGFC,PRKAG2,SIPA1,SLC25A5,SOS1,SUCLG1,TAS2R60,TP53,WASL

Negative_6687	FGG, AGTR1, ALG2, ALS2, ATP5D, CD40, CDC16, CDK2, CLDN20, CMPK1, COX10, CTPS, DIAPH2, DUSP1, E2F5, EIF4E2, ESPL1, EXTL3, FABP4, FTCD, GCNT2, GJD2, HSPA1B, ISYNA1, KLRC3, KYNU, LILRB3, NEU2, NUDT2, OR10H4, OR7A10, PFKFB2, PGAP1, PLXNA2, PRKG2, RPS6KB2, SETD2, STEAP3, TCF7L2, TNFSF11, TYK2, UGCGL1, ULK1, XCL2, ATP6V1C1, ATP6V1G2
Negative_6688	GPT, ITK, ALS2, CCL22, CYP4F3, MKNK2, NEU1, OR14A16, OR2T10, ORC1L, PTGER3, SPRY2, ATP6V0A4
Negative_6689	LYN, PXN, SHH, AHCY, ALS2, ATF4, ATP4B, B4GALT5, BAAT, BCKDHB, C7orf16, CADM1, CCR4, CLDN15, CTNNA3, CYP11A1, CYP2S1, DGKQ, DHRS4, EIF4B, FABP3, HIBADH, IFNA6, ITGA9, MGST1, OR10T2, OR1J4, OR2B11, OR51B5, OR5M3, PAFAH2, PDE2A, PDGFC, PPP2R1B, ROBO2, SEMA4F, THTPA, TNFRSF8, TUBA8, ST6GALNAC5
Negative_6690	CFH, B3GAT2, CAMK2G, CISH, DEGS2, FUT9, GALNTL5, GYS1, IRAK3, ITGB7, MAP3K3, MGLL, ORC6L, RAB23, STK11
Negative_6691	DCK, PXN, ACTN2, ADCY6, APAF1, CDIPT, CTBP1, CXCL11, DGKB, DHDH, DLL1, ENTPD6, FGFR2, FOSL1, GAL3ST1, GALT, HSD11B1, IARS, IL12RB1, LTA4H, MGAT5B, NME6, NRG4, NUDT12, OR1S1, OR52R1, OR6T1, OR8U1, PECAM1, PRIM1, STX12, TGFB3, TNFRSF4, TNXB, TOMM40, TUBB2B
Negative_6692	HK1, NF1, ALG3, BLVRA, CALML6, CYSLTR1, DUSP6, EI24, EPB41L1, EPHX2, GLI2, HAGH, ISYNA1, KLRC3, KLRK1, LLGL1, MYLK2, OR1L3, OR52M1, OR6K2, OR7C1, PDXK, PPP1R3B, PTPRZ1, RASGRP1, RPS6KA2, SETDB1, SORD, SPP1, STT3B, TLR5, ATP6V0D2
Negative_6693	PML, CHKB, CHSY3, CTPS, CTPS2, ECSIT, GCLM, GNAQ, GPD1, GSTM1, IL13RA2, ITGA1, LAMC3, LIMK1, MCM7, MYH14, SELE, SMAD3, TNFSF10, UGT2B17
Negative_6694	AK5, ATM, CD2, GNE, GNS, MPL, AARS, ATG12, BIRC2, C1GALT1, CLDN18, EGLN1, EVI1, FGFR1, FMO2, FTH1, IL9R, KAT2A, LIPG, MAGI1, MASP1, MCM7, NEU1, NFYA, OLAH, OR2AE1, OR2T1, OR51M1, PITX2, PNMT, PRPH, PTPN7, RDH16, SULT1A1, TAP1, TLR3, WBSCR17, CACNA2D2, HLA-DPB1, SUV420H2
Negative_6695	C3, NF1, SDS, AQP7, ATP12A, ATP6AP1, B3GNT2, BST1, CDS2, CTLA4, CXCR6, ENO2, FMO3, ITGA5, LDHD, MYH4, OR13C9, OR1I1, PLCE1, PRKCA, SMPD1
Negative_6696	ID4, TK2, TPO, ATG12, CACNA1C, CERK, CLCA4, DGKI, DPYS, FRAT1, FZD7, GCNT1, GRHPR, IRAK1, ITGA1, LEFTY2, LLGL2, LRP6, MGLL, OR2S2, OR2T4, OR5B17, OR5T3, PDGFRB, PLK1, POLR1B, RARS2, RFXAP, SEMA4G, SMAD6, SMURF1, SSH1, TACR1, VASP, SERPINE1, TNFRSF10C
Negative_6697	CKB, F7, ID3, IL7, LHB, AGPAT2, ASRGL1, CACNB3, CD3E, CNTNAP1, CSH1, CTLA4, DUSP5, EFNA1, EGLN3, ETNK2, GATM, GPD1L, HLA-C, HTR2B, HTR6, IL1R1, INSRR, LAMC2, MAGI2, MTHFD2, NLGN2, NSDHL, OCRL, OR2C1, OR5C1, OR6B3, OR6K3, OR7A17, OR9G4, OXSM, PAK3, PCCA, PRPH, RDH16, SNAP25, STK4, TAS2R42, TPMT, TSHB
Negative_6698	AK5, CBL, IDS, ME3, OSM, ACOX3, ACTG1, ADCY10, ADRB3, ALDOB, CD19, CHKA, COMT, CXCL11, DGKG, EHMT1, ENPP6, FCER1G, FUT4, GANAB, GBE1, GRIA3, KIR2DL2, MIOX, OR1A2, OR2M4, OR2T4, OR4N5, PDGFRB, PFN3, PRKCI, PRPH2, PTTG1, RAPGEF3, RASA1, TPMT, TSNARE1, ULBP2, UPP1, WNT2B, PDCD1LG2
Negative_6699	PLG, ACO2, ACTN4, AGPAT1, ATP4B, ATP5G3, C4BPA, CD40LG, CLTC, DAPK1, DMGDH, E2F5, ECHS1, EPAS1, EPHB2, EPHB4, FGF7, FUT9, HIBCH, HTRA2, ICAM2, IGF1R, KYNU, NAGS, NEU4, OR10A4, OR2A14, OR2A42, OR4C12, OR51A7, OR5C1, OR7A5, OR9I1, P2RX1, PARVG, PER2, PIGB, PIGS, PRKACG, PTPRB, RAPGEF1, SNW1, STAT5A, ATP6V1E1, HSP90AB1
Negative_6700	FYN, IHH, ADCY5, APC2, ARPC5, BET1L, CACNA1F, CCL1, CDKN1B, CLDN9, COL1A1, CYP2S1, DDX52, DIAPH3, DKK4, DLST, EXT2, GANAB, GLDC, GLI1, HDAC2, HPSE, IGF1R, IRS4, LCP2, MCM7, OLR1, OR2F1, OR4D1, ORC6L, PER3, PLA2G3, PPP3CA, PRKCB, PRKG2, PTGES2, PTGS2, SOCS1, ST3GAL6, TUBB6, WNT10A, HLA-DQA2, TNFRSF13B

Negative_6701	GGH, IL7, KMO, ACP1, ADCY10, ARD1A, BCAR1, C7orf16, CCL8, CDH3, CLTB, CYP19A1, EXTL2, FGFR1, FIGF, GLDC, GNRH1, GSTK1, HEMK1, IL19, IL20, KIR2DL3, MARS2, MITF, MTAP, MYLK, OR11G2, OR1B1, OR2B2, OR51A7, OR56B4, OR8J3, PISD, SETD7, HSP90AA1, MAPKAPK3, TNFRSF10D
Negative_6702	DDO, SRC, TAT, TEC, ACSL3, ACYP1, ALDH3B2, AOC3, ATP12A, C1QB, CLDN15, CLDN4, DGKA, DPAGT1, EFNA1, GALNT5, GLUD1, GNAQ, GUCY2D, IDI1, IGF1R, IGSF5, KCNB1, LIAS, MASP1, METTL2B, NT5C1A, OR2D2, OR2H1, OR2T11, OR9A2, PFAS, PIP4K2B, PKLR, PLIN, PPP2CB, PVRL2, RBL1, STX2, TLR3, TYROBP, TYRP1, VAMP4, VAV1, WNT8B, CSNK1A1L, HLA-DRB3, PDCD1LG2
Negative_6703	CFI, F8, IHH, SRC, UBC, ATP4B, CACNA1F, CCNG2, CDH1, CLDN10, CYP7A1, DDX41, DHRS3, FUT2, HSD17B1, IL11RA, IL28RA, ITGAX, MCM4, MYH2, NOTCH2, OR14I1, OR8D2, PSME2, SPRED1, SPRY3
Negative_6704	GHR, AADAT, ARG2, ATP6V0C, CCR2, CDKN2B, CSH1, CXCL1, FMO2, FOXO1, FUT6, GARS, HTR4, IL22, INHBA, INHBE, JAG2, NFKBIA, NRG1, OR1A2, OR2F2, OR4N5, OR51A2, OR5AN1, P2RX3, P2RX7, PITX2, POLD4, POLR3K, PTEN, PYCR1, ROBO3, SETD1B, STX1A, TNFSF8, UGT2A1, WARS, AASDHPPT, CACNA2D3
Negative_6705	RFK, SFN, ACTA1, ADCY7, AKR1C2, ALAS1, ANAPC11, BMP2, CACNA1I, CHKB, COL2A1, CYP2C9, DDX4, EPHA1, GALNTL4, GNPNAT1, GPAM, HTR6, IARS2, IL20RA, LAMB4, MAP2K1, NME4, OR10J5, OR2AG1, OR52R1, OR5M8, PHKB, PIGP, PLA2G5, PLK1, POLR1E, RELA, RELB, RPS6KA1, SLC9A1, SOX17, STAT5B, UNC5C, PAFAH1B1, SERPINA5
Negative_6706	ALG11, B3GAT2, BPNT1, CCL17, CHKB, CHRM1, CTPS, CYP2E1, DDX18, GDE1, NRG4, OR10A5, OR2AE1, OR2T2, OR5B17, PDE5A, SPI1, STAM2
Negative_6707	AK2, GSS, NPY, PAH, ACOX2, AKR1B1, ALG3, ATP6V0B, C5AR1, C9orf95, CCL17, CCNA2, CSF2RB, CXCL12, CYP11B1, CYP4F3, DCTD, ECSIT, FGF12, FGF9, G6PC, GNRHR, IL1A, LAMC2, MAN1A1, MYLK, NARS, NCKAP1, NEFH, NT5C2, NUDT12, OR10A2, OR10H3, OR10Q1, PAK3, PPA1, RCHY1, SIAH1, SMAD3, SRGAP3, STAT5A, SYNJ2, TAS2R41, TYRP1, UNC5D, WHSC1L1, WNT6
Negative_6708	GK2, IL8, ALDH6A1, ALOX12, ALPI, BCL2, BLVRA, CDC25A, CDIPT, CLDN7, CYP2F1, DLAT, DUSP5, EPOR, GNAS, GNRHR, HMGCS1, HSPA8, IL17B, IL17RB, IMPA2, ISYNA1, LIMK1, NAE1, OR52B4, PGK1, PIK3CA, PTPRC, SDC1, SPRY1, ST6GAL1, STAT1, SULT1E1, WNT9A, SUV420H2
Negative_6709	LPL, ALOX12B, GNG3, ICAM3, IMPA2, KLRC1, TOLLIP
Negative_6710	CNGB1, GALNS, HSP90B1, NCAM1, STAT5B, TAS2R4, ST6GALNAC5
Negative_6711	MPL, LIPF, PGLS, PPP2R2D, QARS, TSC2
Negative_6712	FGG, MPL, MPZ, ACOT11, ADH1B, B3GNT4, BMP5, CHIT1, COMT, CYBB, DDOST, FGFR1, INMT, KLK3, MAP2K2, MKNK2, MNX1, NCF2, NUDT5, OR11G2, OR1K1, PANK1, PDXP, PIP4K2C, PPP2CB, PTGFR, SMURF2, ST8SIA1, STX16, STX1A
Negative_6713	CTH, F5, SDS, AGXT, AKR1C4, ANGPTL4, CAMK2G, CD14, CLTB, DDX23, DGKZ, FGFR3, FOXA3, HSPB1, IARS, IFNA2, IL19, IL22, IL23A, IL6R, ITPKA, MYH7B, NCSTN, NFATC3, NUDT12, OR2S2, OR2T5, OR4P4, OR4Q3, OR6X1, OR7D4, PIGA, POLR2D, PSME1, SDHB, SLC25A6, SPTLC1, TLR3, TUBB4Q, ULBP1, ULBP3
Negative_6714	AK5, ABLIM1, ACAT2, ACSS1, ALAS1, AOC2, BLVRB, CCL8, CHEK1, CRHR1, CSH1, CYP2S1, DAG1, DTX4, EFNB3, GGT7, GPX5, GZMA, HLA-G, HMOX2, IFNA17, IL1B, KIR3DL2, NT5C, NTF4, ONECUT1, OR2M4, OR2Z1, OR4C12, OR4L1, OR52E4, OR5P3, PCK2, SETMAR, SRD5A1, WNT6, HLA-DQA2, SERPINA5
Negative_6715	NPL, CACNA1E, CACNG7, COL3A1, CTSL1, EP300, IL13RA2, KLRC3, LIMK1, NPAS2, OR6B2, PRKCZ, RALA, SOCS1, TOMM40L
Negative_6716	DCN, EDA, IL9, ACP5, ADH1A, ATP5G2, BIRC2, CDS2, CHKB, CX3CR1, FECH, HMGCL, IL18, ITGA2B, LYZL1, MAP3K13, OR2G3, OR3A2, OR51A4, OR56A1, OR5L2, OR7A5, OR9K2, OXSM, PERP, PLCG1, PRKAB2, PRKCE, RPRM, STAT1, STMN1, TICAM2, TLR5, HSD17B12

Negative_6717	B3GNT2, CDIPT, GRIN2A, HSD17B3
Negative_6718	HGD, NPY, OAT, ACTN1, ADCY4, ALDH3A1, B3GALT4, BCAT1, BMP5, CDC20, CTNNA2, CYP1A2, DUSP14, GLI3, GNB1, GSTO2, HNMT, INPP5A, MAGI3, MYH2, MYH6, NMNAT3, NTF3, OR10P1, OR13C3, OR1C1, OR2M2, OR2T33, OR5A1, OR5AR1, P2RX3, PDHA2, PHKB, PIGS, PPP2R2D, PRKAR2A, PTDSS2, PTPRR, RENBP, STAT5A, STAT6, TAS2R10, TAS2R7, TGFB2, WIF1, WNT1, TNFRSF10D
Negative_6719	FER, GNS, HK1, ID3, LTB, NPL, ARSD, CCND2, CCR2, CD59, CYP2A6, DGKB, DGKQ, DHFR, EHHADH, ETNK1, GCLC, GOSR2, HADH, IFNAR2, INSRR, ITGAL, KAT2B, MAML3, NMNAT1, NTRK1, OR10K2, OR2A42, OR7G1, OR8H3, PDCD1, PDGFRA, PLXNB1, PNLIP, PRKCA, ROBO1, SHC2, STX1A, TICAM1, TNFRSF17
Negative_6720	ADCY2, AHCYL2, ATP6V1F, B4GALT3, CACNA1G, CRKL, HYAL1, KRAS, MAP4K3, MGAT5B, MPZL1, OR2A25, OR2A42, OR51G1, OR51S1, RRAS, RUVBL1, TAS2R4, UXS1, WNT7A
Negative_6721	ANAPC4, AVPR1A, ENPP2, INPP5E, OR2T2, PLCG1
Negative_6722	DHH, GLS, ENTPD4, EXTL2, FLOT2, FTMT, HPRT1, KIR2DS5, OR2Z1, PDHB, POLD2, PRKG1, RALB, ATP6V0A1
Negative_6723	F2, ME2, PXN, AKR1C1, ATP5A1, ATP5O, BCAR1, BLVRA, BMP2, CACNA1F, CACNA1H, ENTPD1, EPHA1, FABP1, GTSE1, HIF1A, IFNA17, INPP5E, NANP, OR10H1, OR11L1, OR2K2, OR4C11, OR6A2, PTPMT1, RERE, RHOA, SETD1A, SSH1, TAS2R10, TNFSF10, ULBP3, VARS, SERPINA5
Negative_6724	GHR, MTR, OAT, AKT2, ALG3, CAV2, CCL27, CCR6, CLDN17, COL3A1, EVI1, FTMT, FUT6, GPX5, GSTA1, GUSB, LAMA4, MAP3K7, MCM6, MDM2, MGAT4B, OR10AG1, OR7G1, P2RX1, PAFAH2, PDE1B, PTPRM, STK36
Negative_6725	AADAC, CCR10, CTNND1, CYP7A1, HMGCL, LRDD, MAP2K6, OR13C4, OR5K1, OR9A4, PDPK1, PDX1, PLIN, POLR2E, RRAS, TMLHE
Negative_6726	C7, IVD, ADRA1D, BDKRB1, CIITA, FGF20, MAP2K7, MCAT, OR4N2, OR5C1, OR9G4, OSMR, RAPGEF4, STK36, MAP3K7IP1, ST6GALNAC3
Negative_6727	MGAT5B, OR13A1, OR4D11, OR7G3, PGS1
Negative_6728	AMH, AR, CRK, IL4, PML, AARS, ACTN1, ADRA1A, ALAS2, BMP4, CACNG1, CBLC, CXCL2, CXCL9, DGKG, DHRS3, DUSP5, GGT7, IL29, IRF9, LPAR1, MAN1C1, MYH7, NCAM1, NCK2, NPR2, OR1A2, OR4K17, OR5D14, OR8B12, OR8K3, PEMT, PFKP, PNLIP, PPARG, PPP2R1B, PRKAA2, RAPGEF1, RASGRF1, THBS4, UNC5B, ATP6V0D2, CACNA2D3, ST6GALNAC4
Negative_6729	DHH, ACADL, ADH1B, CERK, DAPK3, DUSP8, ITGAL, NAGLU, OR13C8, OR13C9, OR4A15, PRKCZ, RERE, RHEB, SULT1A4
Negative_6730	PAH, ACVRL1, ADH6, CXCL11, FGF4, FLOT1, OR1D2, PIGF, PORCN, RFNG, SEMA4F, TAS1R1, HSD17B12
Negative_6731	MAG, RPE, ACPT, ANAPC2, CREB5, DLAT, ERO1L, FGFR1, GLI1, HRAS, OR1Q1, OR51G1, OR52N1
Negative_6732	BAD, ADCY10, ALG9, COL1A2, CSNK1D, FGF1, FZR1, GSTM2, LEFTY2, MAP3K3, NME6, OR1E1, OR2A42, PPP2R5D, SEMA4B, ST8SIA5, ULBP2, UNC5D, VCAM1, CSGALNACT2, ST6GALNAC6
Negative_6733	FGB, LIF, ARG2, CARS, DAXX, HRH1, IRF3, OR4C16, PLD1, PPP2R2A, WNT9A, MAPKAPK3
Negative_6734	F8, FER, MAX, AADAT, ADAM10, ADH1A, ADIPOR1, ADORA2A, CCL22, CCL25, CD55, CD70, CHPF, CNTNAP1, CYP2B6, DDB2, DHRS4, DHRS4L2, DPAGT1, ENTPD7, FARP2, FGFR1, GALNT1, GRIN1, ICAM1, IL12B, KIR2DS4, NRXN2, OR10H5, OR1L8, OR2F1, OR4K14, OR51F2, OR9G4, PECAM1, PFAS, PLCD1, POLR2B, PROC, RFXAP, SMAD3, SOX17, TGFB3, TLR9, TPH2, TSHB, UGT2A1, XCL2
Negative_6735	DAO, F5, PC, CCNA2, CD72, CDH1, CFTR, CLDN7, COQ5, CYP26B1, DHODH, DPYS, ENAH, FGF22, GRIN2C, GRPR, IL6ST, KIR2DL2, MAP3K12, NLGN1, OR1C1, OR4S2, PDHA1, PIAS1, PPARD, PPP1R3A, PPP2R5C, RBL2, TNFSF12, UCP1

Negative_6736	GGH,GP6,HK3,IL9,NLK,VCL,ACAA2,ADH5,ADRB3,ALG2,AOC3,APOA5,ARRB1,B4GALT6,BIRC5,CACNB1,CNGB1,COMT,CSNK1G2,FGF22,FIGF,HTR5A,ITGA4,LIFR,NEFH,NEUROD1,NT5C,OR10A3,OR52N4,OR5AC2,OR6X1,OR8B8,OR8D4,OR9Q2,PFKFB3,PIAS1,PPP1R1A,PRKAR2B,TCF7,TGFB2,CACNA2D3,HSP90AA1,SERPING1
Negative_6737	ACACB,ADCY5,CDK2,CLDN14,EDNRA,INHBB,NUMB,OXTR,PRKCH,RPS6KB2,STAT1,TM7SF2,TOMM40L,TRPM5,VEGFB,HLA-DPA1,PHOSPHO1
Negative_6738	GADD45G,HADH,POLR2H
Negative_6739	ILK,MPL,B3GAT2,BAAT,CARS,CCR3,GGT5,GLI1,GYS1,HPSE2,HSP90B1,IGSF5,IL12RB1,LCMT2,NOTCH3,OR2D2,OR6X1,PNPLA4,PRKCB,RRAS,TGFA,TLN1,TRIP10,TYK2,ATP6V1G3
Negative_6740	CBS,CFI,PRL,CD36,CD8B,CNDP1,CRY2,GALNT10,HNF4G,HTR7,ICAM3,PISD,POLA1,PPA1,PRKX,SCP2,SDHB,SESN1,STX8,TAS2R7,THY1,ST6GALNAC5
Negative_6741	INS,CDK7,FZD1,HEXA,NRXN3,NT5C1B,SRD5A2
Negative_6742	ADA,ADCY4,ADIPOR1,ALG8,AMY2A,BCMO1,CD244,CDC45L,COL11A1,CTSG,CYCS,EI24,GLO1,HSP90B1,IFNA21,IGF1,IKBKG,JAK2,MAD2L2,OR1D2,OR2T12,OR51E2,OR6B3,POLD2,PPCS,SLC27A4,SPRY4,TCIRG1,VANGL1,ST6GALNAC3
Negative_6743	A2M,GH2,GK2,INS,SMS,ACSS3,BIRC2,CACNG7,CD40,CSF1,DDX54,DNMT3A,E2F5,ENTPD3,EPHB3,ETNK2,FOXO1,GMPR2,ITGA2,LDHD,MAP4K3,NEFL,NRCAM,OR1E1,OR2H2,OR2M3,OR52E2,OR52I2,OR52N1,OR6C70,OR7D4,ORC3L,PDGFD,POLD4,RAPGEF3,SNAI2,TAS2R42,VANGL1
Negative_6744	AGL,DCC,LCT,ACADVL,ARHGEF6,AXIN1,B3GNT3,B4GALT1,CAMK2G,CARS2,CD99,CYP2S1,FMO4,IFNA4,IRAK4,LTA4H,NCR1,NFKB2,NRG2,OCLN,OR1G1,OR4C12,OR52B6,PLXNB3,PPM1A,PRKAG3,RPIA,SEMA4D,SIPA1,UCP1,WASF1,WIF1,WNT11
Negative_6745	ADSL,CDO1,CNGA4,COQ5,CREB5,FRAT2,GADD45B,GUK1,HAO1,KRAS,PDK1,PERP,PRODH2,PLA2G12A,TNFRSF10A
Negative_6746	KMO,TK1,ABLIM3,ADRA1A,AKR1A1,ATF2,BMP8A,C1QC,CALML5,CASP8,CCL23,CCR4,CHUK,DPM2,ENTPD6,EPHA5,GNA14,GRIA2,INSR,LRP5,NCF1,OR10A7,OR2A4,OR4D1,OR8K3,PLAT,PRPH,RASGRF2,TLR5,UROD,ATP6V1C2,KIR2DL5A
Negative_6747	AMT,C9,IL5,INS,LAT,MPL,ABL2,ARNT,CCDC6,CCNG2,CD8A,CNGB1,DDB2,ENTPD2,ENTPD6,EPHX2,EXTL3,GALK2,GPAA1,GRIN2B,HSD11B2,IDH3A,IL1R1,IL23R,ITGA9,LRP6,MAPK8,MYH10,OR10A7,OR1D2,OR52B4,OR8K1,PI4KA,PSME1,RAP1B,RRAS2,SETD2,SHMT2,WBSCR22,XIAP,PHOSPHO1
Negative_6748	DCT,DHH,APLP1,ASPA,B4GALT2,CCR3,CD40LG,CES1,CSF3,CTBS,CTSL1,CYP2J2,DDX47,DGAT2,ENPP2,ERBB2,FGF14,FZD1,GALNT2,GFAP,GIT1,GRPR,GSTZ1,HMGCS1,HSPA8,LLGL2,LRP5,LRP6,NEU3,OR5H2,PDHB,PIGN,PIP4K2A,PPAP2A,PPP2R1B,PRSS2,RBKS,RFNG,SDC3,SMC1A,SRGAP3,ST3GAL5,TRIP10,VTI1A,WNT5B
Negative_6749	F7,ACADL,ADSSL1,ASMT,COQ5,FUT3,MAPK3,OR4E2,OR5AN1,PDE1B,PI4KA,PTEN,PTPN1,RPS6KA4,WNT10B,YWHAH
Negative_6750	BCR,IL5,AKT3,CPT2,IFNGR2,INPP4A,L1CAM,NKD1,OR2T2,POLR3A,PPP2R1B,PRKAG2,RAB5A,RFNG,RPRM,STAT2,TLR9
Negative_6751	C1S,CARD11,CHST11,CLDN7,EPHA4,OR51B5,TPH1
Negative_6752	C8A,DCT,ILK,JUN,SI,APBB1,ARHGEF1,ATP5H,CAMK2B,CARS,CBR1,CCNB1,CD82,CDC7,CXCL11,DPM2,FGF9,GIT1,IMPDH1,NOX1,OR14J1,OR2S2,PIK3R4,PNPO,PRODH2,SCD5,SHMT1,THTPA,TNFSF18,TOMM40L,UCKL1,WARS2,MAPK8IP1
Negative_6753	AKR1D1,CDK6,PRKAG3,STAM

Negative_6754	ADO, AK1, AK2, C4B, ACSL5, BCAT2, BHMT, HSD17B7, ITGA8, KLRC1, NCR2, NFASC, OR1B1, OR1E1, OR1S2, OR2C1, OR51A4, OR52L1, OR5B12, OR5T3, PDCD1, PDE4A, PMM1, PNPLA3, POLE4, PTCH2, RERE, SDHC, SEMA3F, SUCLA2, UCP1, UGCG, SERPINE1, TNFRSF11B
Negative_6755	ID2, ALPL, ATP4A, CD40, CLDN8, CPT1A, DKK4, DLST, DUSP6, EHMT1, EPHB4, ETNK1, GAB1, GUCY1A3, IRS4, NEU4, OR10AG1, OR1K1, OR1L6, PDE4A, SPTLC2, TBL1Y
Negative_6756	CD2, IL3, ADCY10, ANAPC7, ARHGEF7, B4GALT2, CSNK2A2, DEGS1, DUSP6, GGT7, HSD3B7, MAP4K3, MTHFR, OR2T12, OR6A2, PDE3B, PDIA4, PPM1D, PTTG1, SCLY, SEMA4A, SMPD3, SPTLC2, TAS2R10, WWP1, TNFRSF13B
Negative_6757	IL7, ACSL1, CAV1, CD74, CSNK1G1, DDIT3, EGLN3, EXTL3, GSTA5, GSTT1, HDAC2, LILRB3, NAT6, OR4F17, OR8B12, OXTR, P2RX2, PIGN, PIK3CG, PRKY, RAC2, RETSAT, RPS6KB1, SDC1, TNFSF4
Negative_6758	PRL, ACPP, ACSS3, ACTB, ADCY1, ARPC4, BCAT2, BMP8B, BPNT1, CCR3, CCR4, CLDN6, CYP26A1, EDNRB, EFNB1, GLUD2, GNAO1, KLK3, LHCGR, MCM5, NFATC2, NODAL, NTRK1, OR10J3, OR2B11, OR2M2, OR51B4, OR51F2, OR52E4, OR5D16, OR5T2, PIK3CA, POLR2F, PRKACG, PTPRJ, RAPGEF3, SDHB, SKP2, SLC2A4, TRAF2, UCP1, WNT8A, YWHAZ, ALDH18A1, PPP1R12A, TNFRSF11B
Negative_6759	TNC, TPO, AKR1C3, AMPD1, CBR3, CCKAR, CNGB1, CSDA, EFNA2, ERBB4, ETS1, F13B, FGF9, GYS1, HRH2, HTR2B, IPPK, KRAS, MAP4K4, MYLK3, NOTCH1, OR8D1, PDE7A, PFKFB3, PIP5K3, PRKCQ, QARS, RPS6KA1, TFPI, UBE2L3, UGT2B4, VANGL2, XCR1
Negative_6760	IL5, SP1, TH, ABCC8, ASAH1, B3GNT3, CDC23, CHST13, CRY1, CTTN, DPM3, GNA13, GNAI2, GTSE1, HAGH, ICAM1, KIR3DL3, MAP2K6, MCCC2, OR10A7, OR10J5, OR4D9, OR4N5, OR5H6, PAK4, PDE3B, PIGC, PLAT, POLE, RAB5A, RPRM, SEMA4D, THTPA, UNC5B, ATP6V0D1
Negative_6761	OR52M1, PPP1R3A, TUBB4Q
Negative_6762	F7, TPO, ACSM3, AGRN, AMPD1, BMP8B, CAPN2, CLCA4, CLDN1, CSF3, DDIT4, DDX54, FARP2, FRAT2, GNB3, GPT2, HNF4A, INPP4B, LDHC, NKD1, OR10T2, OR2G2, OR2H2, OR4B1, OR51G1, OR5B17, OR5D14, OR9G4, PAK7, PARVB, PER1, PTPN5, RRM2B, SORD, SPHK1, ST3GAL1, STX10, THTPA, TSC1, YKT6
Negative_6763	CRH, F7, PAH, ABLIM2, ALOX15B, AMDHD1, AOX1, CASP10, CPS1, CYP2C19, DUSP16, F13B, FARP2, IL23R, IRF7, NCKAP1L, NFE2L2, NT5C2, OR4F4, P2RX2, PARVA, PTPN6, RPN1, SDC4, STAM2, TM7SF2, KIR2DL5A, SERPINF2
Negative_6764	LYN, BRAF, DUSP10, FCGR3B, JAK3, MGAT4A, OR52D1, OR6C74, PHKG1, PLXNB2, POLD1, PRKCA, SAT2, SOCS7, STAM, TNFRSF1A
Negative_6765	CAT, FYN, NNT, PML, AADAT, ACTB, AKT2, ANAPC10, ATF2, BTRC, CALML6, DTYMK, EHMT2, ENPP6, FGF7, FUT7, GJA1, GNA14, H6PD, HPSE, HSPA8, IL29, IL7R, LDHA, MCM7, MTMR1, OR10A4, OR13C8, PFKFB4, POLR1B, PPM1B, PRPH2, RBL2, RPS6KA6, SH2D1A, TPH1, UMPS, VAV1
Negative_6766	APP, CKM, IL7, TH, ATP6V1H, BET1, C1GALT1, CCL23, CYP2C9, EHHADH, FUT7, FZD3, FZD4, GSTO2, HCCS, HSD11B1, HSD17B8, HSP90B1, IL4R, LAMC1, LEFTY2, LTBR, MAP3K12, NCR1, OR4K14, OR5B17, OR6C74, PTDSS2, SGMS1, SPI1, TAS2R60, TRAF6, ULBP3, VNN1, CSGALNACT2
Negative_6767	IHH, KIT, OTC, ACP1, ARD1A, ATF2, ATP2A3, ATP5H, CAMK2B, CLDN1, CLDN6, COL5A1, CTPS, CXCL1, CYP3A4, DGKZ, EFNA5, ENTPD5, GRIA2, HRAS, IL15RA, MAML1, NCK2, NT5C3, OR1N1, OR2D3, OR3A2, OR4C46, PHKG2, POLR2D, RPN2, RXRA, SARS, SDHD, SEMA4A, SLC12A2, SUV39H1, WASL, YES1
Negative_6768	C7, TK1, PNPLA4, USE1
Negative_6769	ASPA, CKS1B, DNMT3B, IKBKG, OR13A1, TALDO1, TRMT11, TYMS
Negative_6770	BNIP1, CTNNA1, FGF8, IFNA13, MDM4, MIOX, RDH5, RGS3, SCD5, SKP2, XYLT1

Negative_6771	C4B, FAH, ID3, ACAA2, C7orf16, CAV1, DAD1, DHX58, DOLPP1, DVL3, ENTPD1, GALNT14, IFNAR2, IRS1, MAP3K7, MGST3, OR1J2, OR2A14, OR2G3, PIK3R4, PRKAR2A, SV2B, TRPM5, ZIC2
Negative_6772	ALDH3A2, BCKDHB, CACNA1I, CCNB3, CDC16, CXCL16, GADD45G, GUK1, MTHFS, PIM2, PLCB2, POLR3K, RAF1, UGT2B11
Negative_6773	FH, SRF, ADCY5, DDX19A, EXTL2, FGF17, GNAZ, ITGB5, JAG2, KIR2DL2, LEFTY2, MCM7, OR1A2, OR2Z1, OR6C70, PDE10A, POLR2F, PTDSS2, SMURF2, TRDMT1, ZMAT3, PLA2G12A
Negative_6774	A2M, CDA, HGF, TNN, ACOX1, ACSS3, ACTN1, CAMK2G, CFTR, COL4A6, CTSG, DCXR, DGUOK, EPHA7, FBXW11, ITGB8, MYH11, OR5L2, OR8H2, P2RX5, PNPO, PROC, RHOD, TP53, PLA2G12A, TNFRSF14
Negative_6775	F11, ID1, ATP5E, B3GALT5, CACNA1D, CLDN15, CLDN18, CMPK1, CYP2A13, DKK2, FABP3, FGFR2, FOXO3, HBEGF, HRAS, IL10RB, LGMN, MGLL, NCOR2, NEFL, NUDT5, OR13C4, OR1S1, OR6K2, PARD6B, PFKFB4, POLR3H, PPOX, PTPN5, PTTG1, RAPGEF3, RFX5, RPS6KB1, SETD7, SLC12A2, SLC27A5, SMARCA2, SOCS7, TUBB, VAV3, WNT2, WNT5A, PAFAH1B3, PDCD1LG2
Negative_6776	CBS, ACVR2B, ADH7, AHCY, ASAH1, BST1, CDC45L, CNGB1, DPYS, DRD5, EPB41L2, GRIA3, ICOS, MAPK9, OR14J1, OR2A25, OR2T2, OR4S1, OR8B8, PCCB, PFKFB4, PMM1, RASA2, SRD5A1, TPI1
Negative_6777	SI, AKR1D1, ALPI, CSNK1D, CTSL1, NMNAT2, P4HA1, PRPH2, SPCS3, SREBF1, TOMM40
Negative_6778	F5, AANAT, ACTA2, ARHGEF4, ATP5E, B3GNT3, CNTN2, COL11A1, CSDA, CYP8B1, DUSP8, FUT5, GMPPA, GRHPR, ITGB6, LCAT, LTBP1, MPST, NKX3-1, OR10A6, OR11H4, OR2M5, OR2T1, OR8S1, PARD6A, PER1, RDH11, SELE, SPRY2, STAM2, STX7, TAS2R40, TFDP1, UROC1, ATP6V0D2
Negative_6779	ADC, IL8, AKR1B10, ARPC1B, B3GNT5, CACNA1G, CHRM3, COQ6, DAXX, DTX1, EPHA8, HEXB, IL1R1, IL22RA2, IL28B, IRS2, MAML1, MAP3K1, MTHFD1L, MYLK2, NAE1, NAGA, OR4K15, OR4M2, OR51A2, OR52W1, OR5A1, OR8H1, ORC5L, PLD1, PPP1R3A, SMARCA2, SULT1A1, TBXA2R, TICAM1, ULK1
Negative_6780	GK2, KDR, AARS2, ACADVL, AGXT2, ALG6, ALPPL2, ATP4A, ATP5J2, AVPR1B, CBR1, CDC25A, CHST12, CSF1, DGAT2L4, DIAPH1, DKK4, FASLG, GADD45G, GALE, GIT1, GMPPA, GZMA, HMGCS1, LDHD, LIAS, MGAT4B, NEFH, NOX1, OR1D5, OR1G1, OR2G3, OR52N1, OR5B17, OR5M3, OR5T1, PLA1A, POLR3K, PPP2CB, PPP3CB, PRDX6, PTDSS2, PTPRB, PTTG1, XYLT2, TNFRSF14
Negative_6781	C7, CFI, DBH, GP6, CACNG3, CCNE1, DIAPH3, GALNT2, GALNTL1, GBE1, GNB1, GRIN2A, HADH, HLA-F, IARS2, IL1RAP, KIR3DL1, MAP3K14, MCCC1, NCF4, NME4, OR10Z1, OR1D5, OR1J1, OR1J4, OR6C76, PRKY, SRGAP3, SULT2B1, UGP2, ULK3, UPRT, WNT3A, YOD1
Negative_6782	CSK, ILK, LTB, ALDH1L1, ASPA, EFNB2, EPHA1, GALNT8, NCOR2, NOX3, OR5D18
Negative_6783	HK3, LAT, ADORA2A, BMPR1A, CASP3, CNTFR, CSDA, CTF1, DGKB, DHRS9, FZD8, GNB1, HEXB, IAPP, NT5C, OR6C4, OR6C6, PANK2, PAPSS1, PIGK, PRKAB1, SYNJ2, TM7SF2, TP73
Negative_6784	C8G, AOC2, BAIAP2, CAMKK1, CDC16, EPAS1, ESAM, GNAZ, JUND, MGST1, OR10Z1, OR2A5, OR3A3, OR51G2, SDHD, SKP2, SMAD2, TMLHE, UNC5D
Negative_6785	DCC, FER, ACSS3, CBLB, CCR5, CLDN18, DLL1, FGF8, GALNT2, GALT, GMPPA, IFNB1, LARS, NAGLU, NME6, OLR1, OR5H2, PANK1, PIGC, PLXNA1, PON2, PRKCZ, RFX5, RXRG, SPCS1, SULT1A4, TICAM2, TOMM40L
Negative_6786	OAT, OTC, ACACB, ACVR2B, ALDOC, BDNF, CHRD, CLDN11, CNGA3, CYP2A6, FUT7, GALNTL2, GUCA1A, IDH3B, IFNK, IL9R, MAPK12, NCSTN, NEGR1, OR2F1, OR52B6, PAPSS1, PDGFA, RXRG, TPH2, UBE2K, UBE2L3, VAMP1, XCR1, YWHAG, ATP6V1C2, HLA-DRB3

Negative_6787	APP,C8B,CGN,CKM,DCT,PGP,TPO,CASP8,DAG1,DGKG,ETS1,FMO1,HAGHL,LIAS,LRRC4C,MCM4,MYL9,NAGLU,NCK1,NCK2,NNMT,NRCAM,OR4C15,OR4D9,OR7G3,OXTR,PHKA1,PLXNC1,POLR2I,PPP2R5E,PTGER3,STEAP3,STX16,TACR3,TCF7L2,THBS1,ULK1,UPB1,VAV1,WNT10B,TNFRSF14
Negative_6788	DAO,PDC,ARHGAP5,ATG3,BMPR1B,CBR3,CCL1,CDC2,CREBBP,CXCR3,DIAPH3,ENPP1,FGFR4,GRIA3,IL17A,INPP5E,ITPKB,KIR3DL2,KLRC3,MAD1L1,NGEF,NNMT,NUDT5,OR10G2,OR10T2,OR5AU1,PAX8,PCCA,PIGN,RAPGEF1,SDC1,SPRY2,STK3,TAS2R10,ATP6V1E2,TNFRSF10A,TNFRSF13C
Negative_6789	AVPR1B,CTNNB1,CTSG,DGAT1,GSTA1,KCNQ1,LMO7,PDGFB,PIGL,PMVK,PRKAA1
Negative_6790	ADK,AK7,IL3,PGD,ACSL6,AGPAT2,B3GALT2,BACE2,CD79A,CDC27,CDKN2B,CDO1,CLTC,COASY,CRY1,CYP1A1,EHMT2,EPB41L1,FLT3LG,ICAM2,KLKB1,LIPF,MAML3,NAT6,OR10R2,OR1Q1,OR51A2,OR51B5,OR52N1,OR6F1,PDE9A,PFKFB3,PFN3,PIGK,PIGN,PLCZ1,PLXNC1,PRPS1,PSEN1,RELB,SARS2,SHC2,SLC25A5,SORD,TAS2R48,TUBA3C,UCKL1,UGT2A1,ULBP3
Negative_6791	AGTR1,ANPEP,ARPC2,BRAF,CYP2D6,DPM3,H6PD,MAGI2,NFKB2,NT5C1A,OR10X1,OR1J1,OR56A3,OR6C4,PLA2G4A,PPOX,RASSF1
Negative_6792	ACADL,ACOT11,ACSL6,ADRB1,AGMAT,ALDH4A1,AMPD1,ARHGEF2,ARPC2,CCL22,CD74,CFL2,COL11A1,COL5A3,CSDA,CYP7A1,DKK1,EPHA4,GCNT4,GUSB,HDAC1,HTR2A,ICAM1,IKBKE,ITCH,LARS,NCKAP1,OR10G3,OR10X1,OR2T11,OR4C11,OR52L1,OR6B3,PPM1B,RASSF5,SETD8,SHC4,SNW1,SPHK1,STX18,SYNJ1,TAS2R50,TP53,TXNDC12,UGT2B11,XCL2,AASDHPPT,CACNA2D1
Negative_6793	CKM,AMPD1,CARD11,CREB3,CTBS,OR1E2,OR2T4,TIAM1,TPI1
Negative_6794	F8,GPT,HK1,ABL2,ACSL1,ARRB1,ATP6V0C,AXIN2,C7orf16,CACNB3,CMBL,CYP1A1,FLT4,IL7R,KIR2DS4,MGAT5,MYH11,OR10H1,OR1D5,OR5M9,OR9G4,PDE2A,PDGFRA,PLA2G6,PLXNB2,PRKAR2A,PRKX,SOCS3,TYROBP,UGT2B7,WNT7B
Negative_6795	HK3,ACTN1,AGXT2,CALML5,COQ6,CXCR6,DNMT3B,GMPPA,GSTM3,PPP2R2D,SKP1,VAMP1
Negative_6796	PTS,ACMSD,ALDH3A1,ALG1,ALOX12B,ALPL,ATP13A2,B3GALT1,BMP8A,CCL11,CD44,CD8A,CDIPT,COL4A2,COQ7,DHRS4L2,DHRS9,FBXW11,HRH2,IFNA4,LEF1,MCCC1,OR1N1,OR2A2,OR7A17,PHKA2,PLCB4,POLE3,POLE4,PRKCB,RFXAP,SHC3,TNXB,VKORC1,YARS
Negative_6797	LAT,GCH1,GNA12,HMOX1,IL1B,NKD1,PRKY,TNFRSF4
Negative_6798	ABO,BCR,MUT,ACSM5,AKR1C1,ATP13A2,CDC26,CDO1,CLDN6,COMT,CSNK1G3,DGKB,FCGR3B,GALNT10,GRHPR,HLA-G,IGSF5,IL11,IL13RA1,IL17RA,L1CAM,LYPLA2,MTHFS,NME2,NUDT2,OR10G8,OR2A25,OR2M5,OR4C6,OR4M1,OR51D1,OR52E4,OR5F1,POLR1A,POLR1E,PRKAB1,PRKACG,PYCRL,RDH5,RUNX1T1,SMPD3,SULT1A2,TAS2R1,TNFRSF9,TRAF1,HLA-DRB5
Negative_6799	C3,IL8,MOS,CCL16,CDO1,CHKA,DPM3,GALNTL4,HNF1A,IL6ST,MAP4K4,NCKAP1L,OR5L2,PLCB2,SLC2A1
Negative_6800	DLD,DUT,PML,ACSL1,CARD11,CYP1A2,CYP4F3,DPYD,F2RL3,FZD4,GNAQ,GNAZ,JAK3,NT5C2,OR1M1,OR2D2,OR2M3,OR6Q1,OR6T1,PIK3R2,PLA2G6,PRKAG3,RBL1,RFXAP,UROD,WASF1,PAFAH1B2,PNLIPRP1,ST6GALNAC5
Negative_6801	ACACA,APAF1,ASMT,FUT1,OR11G2,SESN1,PPP1R12A
Negative_6802	VCL,VTN,ACSM4,AGRP,AHCY,ARHGEF1,BAIAP2,CDKN1C,CHD8,CTSS,CYP2F1,DCXR,DHRS4L2,GNA12,HSD17B7,IL28A,INPP1,IRF3,LAMB3,MAP3K3,MFNG,MYLK2,OR14I1,OR4K5,OR52K2,OR8K3,OR8U1,PAK2,PDE9A,PHKA1,SDHA,SMPD4,UGCGL2,WHSC1,WNT4

Negative_6803	AUH,CBS,LPO,SP1,ACSM2A,ADSL,ANAPC10,C1GALT1,C5AR1,CBR3,CDC20,CDKN1B,CTTN,DIAPH2,FGF10,GALE,GNAS,GNG3,HLA-DRA,IL19,IL5RA,ITGB2,LAMB2,LILRB3,OR13C4,OR1J4,OR2C3,OR2S2,OR6C2,OR8J1,PCCB,PGK2,PIGA,PIGU,PON1,SETMAR,STAT3,THY1,TPMT,TUBB3,UCK1,TNFRSF11B
Negative_6804	AHCY,ARG2,CIITA,GRIN2A,HAGHL,RALGDS,RENBP,SNW1
Negative_6805	EDA,GH1,IVD,LCT,RB1,ABAT,ALDH1B1,ALG11,CCL15,CLDN16,CYP2C19,CYP4F3,EXOC7,FMO4,FZD4,GALNS,GALNTL2,GNB3,HEXB,HTRA2,KIR3DL3,LIPC,MAP3K13,MGAT2,NFYC,NR1H3,OR1J1,OR8K5,P2RX6,PHGDH,PLCB3,POLA2,PPARG,PRKACB,PTCRA,RRAS2,SLC2A1,TAP2,UPP2,PDCD1LG2
Negative_6806	IVD,DNMT3L,FLOT2,GALNTL1,GTSE1,MAP2K3,OR52B4,PCYT1A,PGAP1,PRKG1,SCD5
Negative_6807	ESPL1,GCNT2,OR5A2,UMPS
Negative_6808	ID4,MTR,PTS,ALG8,CLDN4,CLOCK,CYP2C18,DHRS4,E2F4,GAD1,GP1BA,GPAA1,GRIN2C,GSTA3,HAO2,HMGCS1,IFNA2,IL13RA1,ITGA9,MAD2L1,MTFMT,MYLK3,NAT6,NOTCH2,PARD6A,PIAS4,PPP2R5A,PRKG1,PTPMT1,SHC3,STX4,SUCLG1,TAS2R4
Negative_6809	AK3,CAD,F2R,GCK,VCL,ACSM5,AGPAT1,CACNA1D,CSNK2B,FUT3,GNG3,HSPA2,IL17RA,MLYCD,NCF1,OR2C3,OR6C4,OXCT2,PIGS,RAPGEF2,SMAD4,TYK2,UGT2B11
Negative_6810	ATM,EPO,GH1,SPN,ACP2,C3orf10,CCNA2,CDKN2C,COL1A2,CSNK2A1,CXCL3,DGKZ,EARS2,ECHS1,F2RL1,FGF19,GSK3B,IFNA13,ITGAD,MAPK12,NFE2L2,NRG4,NRXN1,NTN1,OCRL,OR10J5,OR4K5,OR52H1,PIGA,POLR2F,RALGDS,TOLLIP,UBE2L3,ARHGEF12,ATP6V1E1
Negative_6811	FGF6,HIBCH,HNF1A,IL11,OR52I2,SKP1,SMAD9,TAS2R50,USE1
Negative_6812	GH1,ID1,ILK,NOG,ALDH3A2,ASPA,ASRGL1,ATP5L,B3GALT2,BDNF,CDIPT,CHST12,CXCL11,CYP3A4,DHODH,DOT1L,DPYSL2,DVL2,EPHA5,GART,GSTO2,GUCY1B3,IL8RB,ITGAX,ITPKA,NCK2,NT5C1B,OR10K1,OR1J1,P2RX1,PLXNA3,POLD2,PON2,RALGDS,SHISA5,SOCS2,SRGAP1,STK11,STX3,TPK1,UGT2B4,VTI1B,YKT6,ATP6V0D2
Negative_6813	C7,HGF,MPZ,AHCYL2,AXIN2,CASP10,CDC14B,CDC25C,CLTB,COL4A6,CYP3A4,DAAM1,DKK2,FGF22,FGF4,GANC,GNAO1,GNAS,HMGCR,IARS,IFNK,IL13RA2,IL17RB,IL21,IRS2,ISYNA1,KCNJ11,KLRC1,LDHC,MAP2K6,MAP3K11,MAPK9,OR13A1,OR1S1,OR4C11,OR4S1,OR5H6,OR5P2,PAPSS2,PIK3C2A,POLR3K,PPAP2A,PPAP2B,PTAFR,PTGFR,VKORC1,ATP6V1C2
Negative_6814	AK3,SYK,TNC,ACMSD,ARPC3,ATP6V0B,CHPF,CREB3L1,CTTN,DPYS,G6PC2,GCNT4,GJD2,HHEX,ITGA1,LMO7,MAP2K1,MYL2,OR14A16,OR1E1,OR2AT4,OR51I1,P2RX3,ROBO2,RPS6KA6,SARS,SELL,SLC2A2,SV2A,B3GALNT1,PAFAH1B1
Negative_6815	ATM,FER,TPR,ACACA,BCAT2,CPT1B,CTSG,DAAM2,DNMT1,EIF4B,EXT1,FGF6,FGFR2,GOT1,HCLS1,IARS2,ICOS,IL4R,ITGAE,LAMC1,NCR2,NFE2L2,NGEF,NRG4,NTRK2,OR4N2,PER2,PPP2CA,PRKAR2B,PTDSS2,SSH2,ST3GAL2,ST3GAL6,STEAP3,SV2B,TCF7,TYROBP,ATP6V1E1,TNFRSF13B,TNFRSF13C,TNFRSF1B
Negative_6816	ANAPC2,BUB1B,CISH,CYP2U1,ENTPD5,FTCD,HARS2,HTR5A,IL13RA1,ITGB1,MTMR6,ODC1,OR4C15,P2RX5,PIK3C2B,PLA2G2F,PPP3CB,RAB5A,RBPJL,RFWD2,SEMA3D,SMPD1,TRAF5,TRDMT1,UGT2A3
Negative_6817	ACYP1,ATP5L,DPAGT1,FRAP1,IL13,IMPDH1,MAP3K14,NCSTN,SSX2IP,SULT1A2,WNT4
Negative_6818	CHUK,COL4A1,DARS,DPYD,GLB1,GNAL,GNAS,GRM5,INPP4B,JAK3,NEU1,PER3,PI4KA,POLR2L,PPP2R5B,PVRL4,SFRP1,STX16,YWHAB

Negative_6819	TPR,A4GALT,ACHE,ACY3,ADAM17,AGTR1,ALDH3B2,AREG,ARSB,ATG3,C5AR1,CDH15,CFL1,CMBL,DUSP9,ECHS1,ENAH,EXT2,FARP2,FGF18,FGF4,GLS2,MAP3K4,MINPP1,OR11G2,OR2Z1,PCNA,PDE4C,PDE6H,PIGN,PITX2,PRKAA1,PRKG2,PSEN2,RAPGEF2,RDH16,STK3,TRAF1,WNT7A,WNT8B,WNT9A,HLA-DRB4,TNFRSF11A
Negative_6820	CRK,OGT,ANGPTL4,ASS1,ATP5J2,CHD8,FGF8,IARS2,MYL7,MYL9,OR10A6,OR10G8,OR7G3,TSTA3,UPB1,TNFRSF12A
Negative_6821	PGD,ARPC4,CACNA1G,CAPN2,COL6A1,CRHR1,CTSG,DGKB,EXTL3,FZD6,GAMT,HPRT1,ITGA4,OR2A12,OR2G3,OR2T12,OR4N2,ORC3L,PRIM2,RAD51,SEMA4C,SMAD9,STX12,TUBB,WASL
Negative_6822	LPO,AGPAT1,AGPAT2,AOC2,CACNG5,CYP3A4,DLL1,ENO1,ERO1L,GGT1,HARS2,OR6K6,PLA2G3,POLR2B,SOD1,SUOX
Negative_6823	DCN,ID4,LCT,NGF,ABLIM1,B4GALT7,BIRC2,CCR4,CTTN,CYFIP1,CYP3A7,DOLPP1,GALNTL4,GNA11,LYPLA2,MDM4,NOTCH2,OR11L1,OR1N1,OR2L2,OR7A10,PLCB1,PON3,PPP3R2,PSAT1,PTK2,RAF1,SEMA4B,VANGL1,WBSCR22
Negative_6824	C2,EZR,TAT,ALDH2,ALDH9A1,ATN1,BDKRB2,CAV1,CCL22,CCL4,CD8A,CDH2,CLDN4,CLDN5,CYCS,DGAT1,DPM2,DUSP7,ENAH,F2RL1,FADS2,FLT3,FMO5,GDE1,ITGAE,MAP2K6,MASP2,NTRK1,OR2T6,OR4D5,OR51L1,OR52D1,P2RX6,POLR1A,PRKACB,PRKY,RUVBL1,ST8SIA5,TJP2
Negative_6825	FST,ID1,ATP6V1F,BMP7,CES1,CTPS2,FMO2,GALNT6,GRHPR,IGSF5,MAOA,MECR,NRG4,NT5C,OR11H4,OR52J3,OR52W1,PDE9A,PFN3,PLAT,POLR1B,POLR2F,PPAP2C,PRSS1,RPS6KA5,STAM,XYLB,B3GALNT1,HLA-DPA1
Negative_6826	ANAPC1,ARPC5,ATP4A,BMP7,CXCL16,CYP4A22,IL2RG,MTHFD2,OCLN,OR2L2,OR4D6,OR4K15,SV2A,WWP1
Negative_6827	FER,MVD,ARPC1A,B3GAT3,BMPR2,CCL20,CDO1,DDHD1,DLAT,DPM2,EIF4B,FZD5,GNAZ,IDI2,IL1RAP,IRF7,MAOA,MAP4K3,NMNAT3,OGDHL,OR1C1,OR2AT4,OR51G2,ORC5L,OSMR,PARD3,PDGFRA,PDK1,PER2,PGAM1,PON3,RALA,SEMA3G,SSX2IP,STMN1,SULT1E1,SULT2B1,TOMM40L,PDCD1LG2,SERPINF2
Negative_6828	CREB1,EREG,OR6C75,OR8H1,PPP3R2,RPS6
Negative_6829	DHH,F12,FYN,TFG,TPO,ACSM5,AHCY,ALPP,BLNK,BMP8A,CCNE2,CHSY1,CKS1B,CLDN10,CNDP1,CTNNA3,CYP21A2,ENTPD6,FGF21,GNA14,HADH,HAGH,HPSE2,MPZL1,NFKBIB,NOTCH2,OR4P4,OR8H3,OR8U1,PDHA2,PIM1,PLA2G10,PPM1D,PSEN1,PVRL3,RBKS,RCHY1,RXRG,SEMA7A,SNW1,TBXA2R,TJP1,VCAM1,GABARAPL1
Negative_6830	F5,IVD,ACADS,ATP5J,B3GNT2,C1QB,CADM3,CASP3,CCNG2,CHRNA7,DIAPH1,DPAGT1,E2F4,FGF19,GADD45G,GALNT9,GARS,GIT1,GMPPB,HADHB,IL11,IL17RB,KLRC3,MAD2L1,MCM6,MTAP,OR4A16,PFKFB1,PIAS4,PIK3R3,POLR3K,PSME1,PTDSS1,SPTLC1,STX1B,TNFSF18,UMPS,XYLT2,CACNA2D3,HLA-DRB3,TNFRSF17
Negative_6831	AASDH,CD99,DLAT,GUCA1A,IKBKE,OR13H1,PARD6G,PDHA1,PRKAR2A,PTPN1,PTPRR,RASGRP3
Negative_6832	INS,TK1,AASDH,CARS,GALNTL5,GALT,GSTM5,OR4D9,PIK3CB,PORCN,RFWD2,RGS3,CTNNBIP1
Negative_6833	ADH1B,ALS2,DDX52,ENTPD3,INHBC,OR2B11,PKM2,UCK2
Negative_6834	C2,JUP,CCL8,CHDH,CX3CR1,GRIN2B,IRF3,MAN1A1,NAMPT,OR10AD1,PMM1,SESN3,TLN1,TUBB2A,YWHAZ,ZIC2
Negative_6835	AMT,IHH,PAH,AGXT2,BPGM,CNGA4,COL5A3,DDIT3,FGF6,GGCX,L1CAM,OR56A3,PRPH,RASGRP2,RETSAT,ST3GAL5,TAS1R1,YWHAZ
Negative_6836	AXIN1,BNIP1,CXCR3,EPHA8,GUSB,IL15RA,IL9R,INHBC,PPAP2A,TBK1,TJP3

Negative_6837	ACTC1,ADRB1,ALG11,ATP6AP1,B4GALT5,BUB1,CES2,CHEK2,CHSY3,CNTN1,COL1A1,CXCL1,CYP11B1,ERBB2,FLT3,GLG1,MYH11,MYL7,OR8J1,PAK6,PGLS,PHKA2,PLA2G10,PSME2,RAP1A,RCHY1,STX3,TCF7L1
Negative_6838	ADAM17,BMP5,CALML5,CCKAR,CMBL,CNTNAP1,DCTD,DLAT,DUSP9,ECSIT,GMDS,IL17B,IL1R1,IQGAP2,LRP6,MAPT,MBL2,NTSR1,OR2B6,OR51E1,OR8B4,OXSM,PIGP,PRDX6,TYRP1,UNC5D,WNT7A,YWHAE,ARHGEF12
Negative_6839	FGB,ABL1,ACTN2,ALPP,ARPC1A,ATP5F1,B3GALT6,CX3CR1,FGF2,GP1BB,IL10,MAP2K6,NT5M,OR5B17,STK3,TGFA,UNC5B,ATP6V0A1,PRICKLE2
Negative_6840	C4B,ME2,AGPAT3,APOE,ATP6V0B,B4GALT1,CCNG1,CKS1B,CSH1,CTLA4,DDIT3,E2F2,FZD8,GANAB,GNA15,LYPLA2,MCM6,OR10G8,OR14C36,OR7G1,RAPGEF4,RBX1,SCLY,SREBF1,SRGAP2,TUBA1A,UGT2B4,YWHAE,HLA-DRB5,TNFRSF1B
Negative_6841	ACAA1,CTNNB1,OR14J1,PRODH,RASSF1
Negative_6842	BTK,F11,GNE,ME2,ACAA2,ALCAM,ALOX5,ANPEP,APOA1,ATN1,B3GAT3,B3GNT5,CDC7,CDH2,CRKL,GSTP1,HLA-F,HMGCR,IL1RAP,ITGA7,KAT2A,LIPA,LRP6,LTA4H,MAPT,NRG2,NSDHL,OR2AG2,OR4S1,OR51A4,OR7A10,OR7A17,PARD6B,POMC,PRKCQ,RFXANK,SLIT2,SORD,STAT5B,THBD,UGP2,ALDH18A1
Negative_6843	BTC,BTD,C1S,IL3,IL7,LPL,APOC3,ARRB1,CCL24,CHEK1,CHSY3,COL5A1,EREG,FDPS,FGF5,GNPDA2,GPAM,GRAP2,GRIA2,HEXA,IDH3G,IL1R2,ITGAL,MAML2,MAN2A1,MGAM,MGAT2,NFYA,OR4D11,PIP4K2C,POLD4,PPAP2C,PPP2R1A,RASA1,SETD7,SKP2,SQLE,TYRP1,ZIC2,ATP6V1B2
Negative_6844	AOX1,CD40LG,CDC27,CKMT2,DLAT,GALNT11,HEMK1,INHBE,KNG1,NOTCH1,PFKFB3,PRODH,SYNJ2,TAS2R40,WNT1
Negative_6845	FST,TNN,A4GALT,ALDH1A1,APAF1,ATP6V1A,B4GALT4,CCKBR,CD99,CKS1B,CYP1A1,CYP26B1,DDB2,DGAT2L4,DUSP3,EFNA4,GALNT5,GNAZ,GNMT,HSPB1,IFNA8,IL24,METTL6,MFNG,NFKB1,OR2C1,OR2L2,OR4A16,OR4N2,OR51M1,OR52B4,OR52I1,OR5J2,OR5R1,PAK2,PDE6G,PDGFRA,PIGS,PIP5K1B,PLD1,PTPN6,RASGRP3,SARS2,SETD8,SGPP2,SRGAP3,ATP6V0E1
Negative_6846	CGA,ACSS3,ANPEP,APOA2,ARG2,CAMK2A,CLCF1,CNTN2,DDHD1,DDX19A,FGFR3,FOXO3,IGFBP3,MAP2K1,MAP4K3,MCEE,OR10H2,OR2F2,PERP,PRKAR1A,PRODH,TRAF6,TSNARE1,WNT11
Negative_6847	A4GALT,ADH1A,ADRB3,ATP12A,ATP13A2,ATP6V1D,BCAT2,BMP5,CREB3L2,CXCL13,CYP2C19,DDX4,ECHS1,FGF9,GMPS,HYAL2,IFNGR1,IL8RA,KIR3DL1,LAMB1,LTB4R2,LYZL1,MASP2,MYH4,NTRK1,OR10A4,OR10P1,OR2H2,OR2T34,PFAS,PIGH,PNPLA3,PNPO,POLR2A,PRKAR2A,PROS1,PTDSS2,PTGER3,PTK2,SC4MOL,SDHD,TNFSF4,TUBB2C
Negative_6848	LIF,ABCC8,ACSS2,ADH7,CHST12,CMPK1,COQ5,CPOX,CSF2RB,CTPS2,CYBB,CYP21A2,DDX52,DGKG,E2F5,FZD4,GNAO1,IKBKB,JAM2,NEU3,NFKBIA,NKD2,TSNARE1,TUBA1A,HLA-DPA1,PLA2G12A,TNFRSF10A
Negative_6849	ACSL6,ACSM3,ATG3,B3GALT2,CCL27,CD80,COL6A1,EFNB1,EREG,PDX1,PFKFB4,PLCD4,PRIM2,RASSF1,SARS,TUBB4Q,UPRT,ATP6V1G2,HLA-DRB4
Negative_6850	DCN,IL8,ACTC1,ADH6,ATP5F1,CREB5,GRIN2D,IARS,ITPK1,NKX3-1,PIGQ,PIK3CG,PRPS1,RARS,SCLY,SDHC,SFRP2,SMPD4,TGFBR2
Negative_6851	FGG,CD99,CSDA,CYP11B1,EP300,FLT3LG,HADH,HEXA,ICAM1,NCKAP1,OR4D5,OR5L1,OR7A10,PFKFB3,PLCB1,PYGM,TCIRG1,WHSC1
Negative_6852	OSM,ARSA,ASIP,CCL8,CDKN2A,CYP7A1,DARS,DGAT2,E2F2,ESPL1,FGF18,GLUL,GRIN2D,HPRT1,IFNGR2,INMT,IQGAP2,ITGAD,MAP2K1,NFATC2,OR10A3,OR52D1,OR5M3,PFKP,PLOD2,PPM1A,PRKAB2,PRKG2,PROS1,PRPS2,SMAD7,STX4,TAS2R9,TGFBR2,TOMM40L,TP73,WNT3,YWHAE,PLA2G12B,PNLIPRP2

Negative_6853	IDS,ACLY,ALDH1A2,ANGPTL4,CDC25C,CDS2,CTF1,CTLA4,G6PC,GBGT1,GRM5,GSTM4,HDAC2,HMBS,MCCC1,NME2,NME4,OR2L8,OR2Z1,PLCG2,PTAFR,QPRT,RASA1,SGPP2,SH2D1B,SHC2,TAS1R1,TAS2R60,TCIRG1,TDO2,TKTL2,UGT1A10,WNT4,ARHGEF12
Negative_6854	BID,FGG,GK,MSN,ADCY10,ADSS,ARSB,ASMT,ASNS,B3GAT2,BUB1,CSNK1G1,CTPS2,CXCL11,DUSP3,GPLD1,GUCY2C,GUCY2F,INHBE,ISYNA1,MYL2,OR10G8,OR1L8,OR2M2,OR52E2,OR52E8,OR7D4,PHKG1,PIPOX,RDH12,RDH16,SLIT2,SOAT1,SYNJ1,TUBA4A,TUBB3,PNLIPRP1,SERPINE1,TNFRSF11B
Negative_6855	MET,ACSL3,ACYP2,APAF1,B3GNT3,CD58,CHAD,DDX18,FZD4,GCNT2,GSTA3,HHIP,HTR2C,IL6ST,ITGAD,LAMB4,MAOB,MYL9,NUDT5,OR6M1,PDXK,PSPH,SAT1,TICAM2,UBE2L3,VCAM1
Negative_6856	BCR,DCN,ACTA2,ADCY3,AOC3,C4BPA,CCNB1,CHUK,CYP2E1,DCTN1,DDOST,DIAPH3,EFNB3,EPHA6,FABP3,FOXO1,FUT1,GANC,GSTO2,HLA-C,KIR3DL3,KLK3,MAN2A1,MCM7,MTFMT,MYL5,NOX3,OR1D5,OR2D2,OR51Q1,PAK1,PDE1B,PDGFD,PLA2G2A,PLEKHA8,POLR1D,PRKAA1,PTGDS,PTGS1,PTTG1,STK36,TLN1,ULBP3,ATP6V0A4,MAPK8IP3
Negative_6857	KMO,FLAD1,HMBS,MAPK7,NGEF,NKX2-2,PDE4D,PFKFB2,PIP5K1C,PLXNA2,PRKY,RASA2,SHC4,ULK1,CTNNBIP1
Negative_6858	DCT,RFK,ACAD9,CREBBP,CTNNA1,CXCL6,F2RL3,GBGT1,HNF4G,IL20,LGMN,OR2A5,PFKM,SLC2A4,SOCS5,ULK3
Negative_6859	CD6,PGF,SRF,AMDHD2,BAI1,BMPR2,BRAF,CACNG3,CNTN1,CPT1B,CXCR4,CYP4A11,DPYS,ERBB2,F2RL2,FBXW11,FPGS,FTCD,GOSR2,GYS2,H6PD,IDI2,INMT,IRAK4,ITGA3,LCMT1,LGMN,NKD1,NPAS2,OR2AG1,OR4D11,OR4K2,OR5AS1,PFKFB4,PIP4K2B,PKM2,PLA2G10,POLE,RXRG,SARS,SEMA3C,SMPD1,TRHR,TRMT11,TSHR,VASP,WASL,WNT3A,HSP90AB1
Negative_6860	C7,CDA,F9,ACVR2B,AHCYL1,ALG3,ALG6,ATF2,ATP5C1,CD99,CDC45L,DDX19A,DGKD,DUSP10,FLOT1,GABARAP,GLI3,GOSR2,IKBKG,IL18,INPP4B,ITGAX,NCAM2,NRCAM,OR10Q1,OR2L13,OR4D6,OR5C1,OR6N1,OR9Q2,PDGFC,PDHB,PIP5K1B,PLA1A,PPCDC,PPID,PPP1R3C,PPP3R1,PYGL,RAC1,RBL2,SNAP23,ST3GAL2,TACR3,TAS2R42,TNFRSF4,UNC5C,ATP6V1B1
Negative_6861	C7,JUN,ABAT,APPL1,CKS1B,CSF3R,CYP4F3,DKK4,DPM1,EPHB3,FASN,FGD3,GAS1,GGT1,GRHPR,HLA-F,HSPA1B,HTR4,IL1B,IRAK1,ITGB7,LALBA,LDHC,LTB4R2,MAP4K2,MAPK1,MMP7,MYH13,OR4C16,PDXP,PLK1,RAC3,RASSF5,RHOQ,SOCS5,STX2,UGT8,UMPS,CACNA2D2
Negative_6862	C4B,ID2,ANAPC4,CACNA1B,CDKN2B,CLDN15,CLDN18,CNGB1,CSF2,CXCR6,DHODH,DKK4,EPHB1,FGF8,GOT1,GRM5,HIF1A,JAM2,LAMC3,MAGI3,MTHFS,MYH2,NFAT5,NLGN2,OR1Q1,PAICS,PDE5A,RHOQ,SGMS1,SMARCA5,SPRY3,THBS2,TSNARE1,HLA-DQA2,TNFRSF12A
Negative_6863	IHH,NF1,ATP5C1,ENTPD4,GLI3,LTC4S,NOS3,SMURF1
Negative_6864	CRH,ARHGEF4,ATF2,AVPR1A,CACNA1H,CACNG5,CCL4,COL1A2,COL4A6,CYP2U1,DHRS3,E2F4,FRAT1,FUT1,G6PC,GARS,GGT1,GMPR,ITGAX,LTBR,MAP3K11,MYH2,NCF1,NEU1,OR10G4,OR10H4,OR10J1,OR10Q1,OR13C3,OR13C5,OR7D4,PDK1,PLTP,SEMA3B,SIPA1,STAT2,THY1,TOLLIP,TRAF6,KIR2DL5A
Negative_6865	TK2,TNR,C7orf16,CCNB1,CDIPT,DDHD1,DPAGT1,EHHADH,FGF17,GALC,GALNT9,HADH,HEXB,IFNG,KIR3DL1,MAP3K7,NNMT,NRG4,OR10Z1,OR4K5,OR51G2,OR6C75,OR6S1,PDE10A,PGS1,PIK3C2A,PIP4K2C,PRKCD,PRKCH,PTPRB,PTPRJ,PVRL2,SHC2,TLR4,UGT2B11,ULK3,ARHGEF12,ST6GALNAC4
Negative_6866	ATG7,CYP11A1,ENTPD6,FZR1,GALT,IFNA7,MYL5,NCF4,NCSTN,OR13C2,OXCT1,PLA2G4A,SEMA3E,WNT10B

Negative_6867	AMT,KMO,SFN,SYK,ALDOA,CHRD,CYP3A7,DTX3,DUSP8,EP300,FGF12,GALNT11,GGT5,GMPPB,GRHPR,HMOX2,IFNAR1,IFT57,IL8RA,ITGA4,MGAT3,NTN1,OR10A3,PAK2,PHKG2,PIAS4,PIK3R3,POLR2D,PPID,RHOD,SEMA4D,YARS2,ATP6V0E1
Negative_6868	EDA,ALAS2,ARSB,BMP6,C1QB,CCNG2,CD58,CLDN10,FZR1,OR51B5,PON1,RBL2,SMC1A,THBS4
Negative_6869	ALDH1A2,CCKBR,CCNB3,CHKB,CYP2C8,E2F2,ENO1,GRM1,MTFMT,OR4N4,OR5AS1,PARD6G,PPAT,SPAM1,SLC25A31
Negative_6870	ACAT1,ADCY1,CCL3,CD226,FLOT1,OR11L1,PPP1R1A,PVRL4
Negative_6871	AK2,APLP1,CHST12,DPYSL2,FUT2,GALE,GFPT2,GNAI2,MAP3K7,OR10X1,OR4C16,OR4X1,OR52D1,PHKA2,SEMA3B,STK3,SUOX,WNT10B,YWHAZ
Negative_6872	ACTG1,CCR8,HLCS
Negative_6873	GNS,WAS,ANAPC7,BRAF,CTNNA3,CYP11B2,DAAM2,DBF4,EFNA3,EPRS,FCGR3A,FLT3LG,GNRH2,H6PD,INSR,ITGA3,OR10C1,OR2AE1,OR4K14,OR51M1,OR5B3,OR8D1,PIGS,PLXNB2,POLR3A,PRSS3,RND1,SAT1,SMAD4,TAS2R1,TFPI,TLR3,ULK1,WBSCR22,MAPK8IP2
Negative_6874	GK2,IVD,OSM,ACYP2,ATP5C1,BACE2,CHIT1,CTSL1,CXCL12,CYP1B1,CYP2B6,ETNK1,FCGR3A,GLI1,IDUA,IL28B,IMPDH2,MDH1,OR52B6,OR5AR1,PDHB,RARS2,SLC25A6,SMURF1,STX12,SUOX,TAP2,TAS2R41,TIRAP,TM7SF2,TP53,VEGFC,WNT6,WNT8A,MAP3K7IP2
Negative_6875	CCL26,CNTFR,DUSP7,GRAP2,IL10RB,NFASC,NT5E,OGDHL,OR4D5,OR52N5,OR56A3,TSC2
Negative_6876	ABLIM2,ACAA1,ACSL4,AKR1B10,ALDH1A3,BCKDHB,BMPR1A,BMPR1B,BUB1B,CACNB4,CARS2,CCL23,CCNB3,CDC25C,CDC45L,CREB3,CTPS,CYP7A1,CYP8B1,DAXX,EPHA8,FCGR3A,HADHA,ITGA9,JAM3,MCM2,MGAT5,MYH14,NMNAT3,OR10R2,OR10V1,OR1C1,OR2G6,OR5T1,OR6C74,OR8J1,P4HA2,PAFAH2,PDE7A,POLR2I,PPP2R2B,PRKAR1A,PTGER3,SH2D2A
Negative_6877	F8,CCNG1,CLTCL1,CTSB,CYBB,ENTPD6,NAE1,NANS,OR2G2,PYGL
Negative_6878	GAA,ACTN4,ADAM17,CACNB1,CAMK2D,CASP6,CDC23,CDKN2B,CNTN2,CSDA,EP300,EPHA7,FBP2,GPAA1,GSK3B,HMGCL,HSD17B7,HSD17B8,IFNGR1,IL6R,INPP5A,LMO7,MAOA,MAPK9,MCEE,MPP5,MYH3,MYH6,NCAM2,NOX3,OR1A1,OR4N5,OR6C75,OXCT1,PFN2,PLD2,RAD51,SPAM1,THBD,VTI1B,SERPINB5
Negative_6879	EIF4B,GALNT2,TUBA3C
Negative_6880	AR,ADAM17,APH1A,ATP12A,ATP5H,BET1,BIRC5,CDH4,CHST11,CNDP1,CTPS,DDX19A,DHFR,EPRS,GP1BB,IDH1,IFT57,IL12B,MS4A2,OR13F1,OR2A14,OR4C6,OR4P4,OR51G1,PANK4,PAPSS1,PDE9A,SETD1B,SLC25A6,SPP1,STAM2,SUV39H2,TIRAP
Negative_6881	PLN,ATP5B,ATP5E,BCAT2,DARS2,EGLN1,FGF23,FZD3,IL1R2,OPLAH,OR4D11,OR51S1,PRNP,RPS6KB1,SH2D2A,TAP2
Negative_6882	GP6,ADIPOR1,ARPC5L,CD46,EGLN3,FCGR2B,NEFM,NFE2L2,NKX6-1,OR8H1,PRKAR2B,PTGS2,SC4MOL,SMC1B,SUV39H2,WASF2,YWHAQ
Negative_6883	CACNG3,CYBA,DNMT1,FGF6,GALNTL5,GNB3,MAFA,OLAH,OR2L8,PKLR,PLEKHA8,PRKY,RPN2,TAS1R1
Negative_6884	MPI,ARNT,DUSP7,GNAI1,GNAI3,IL8RA,LRRC4C,MGAM,NCKAP1L,OR1B1,OR56A1,RBX1,RFWD2
Negative_6885	CTH,GAA,ACSS2,AMY2A,APAF1,CAMK4,CASP10,DDX52,EPAS1,FGF1,GFPT1,HAAO,IDI2,IFNA17,ITGA5,JUND,L1CAM,LAP3,LIMK1,LTBR,MICA,MYH10,NCAM2,OR10G9,OR1J4,OR1L4,OR2T12,OR4S2,PCYT1A,PIK3C2B,PNPLA3,PPP2R1B,PRKY,RAC2,TJP1,TRAF6,TRHR,TUBAL3,WWP1

Negative_6886	CP, SP1, AASDH, ABLIM2, ACAT2, ACYP1, ADSSL1, ARPC1A, ATIC, ATN1, CLDN20, COL6A1, DPYSL2, ELK4, FARSA, FRAP1, GALNTL4, GNA12, GRHPR, GSTO2, HNF4A, ICOS, ITGAX, MYH10, NMNAT1, OR10G3, OR10P1, OR4C12, OR4F17, OR51L1, PDHA2, PIGV, POLD3, RBL2, RPN1, SETD2, SHC3, SORBS1, ST3GAL2, STAT5A
Negative_6887	ADCY8, ADIPOQ, APLP1, CCL5, CTNNA3, EFNB2, FGF2, FGFR3, HMBS, IDH3A, MAP3K11, OR1D2, OXSM, ROBO1, SLC27A5, SORBS1, STX8, TPH1, UGCGL2, WHSC1L1, YWHAH
Negative_6888	GGH, LCK, ACACB, ALDH1A1, CACNA1H, CAMK2B, DLL1, DNMT3B, EPHA1, EPHB1, FBP2, FMO2, GNRH2, IDH3A, IL13RA2, IL2RB, KAT2B, KDSR, MGST1, NFX1, PDHB, PLCB1, PMM2, POLR2A, PRPH, SAT2, SPHK2, TBXA2R, TRAF5, TRPM5, TUBA3E, VAMP4, VANGL1
Negative_6889	ACADM, CAMK2A, CLDN4, DDX19A, MEF2C, NAGS, PIGA, PPOX, RAD51, TRADD, ATP6V1C2
Negative_6890	GLA, ARD1A, CASP9, CBR3, CHST11, CYP4A11, DAAM2, DCTD, DOT1L, EDAR, EHMT1, EPHB6, F13A1, FGF6, FGFR2, GNA12, HPSE2, IRAK3, JAK2, MAP2K7, MBL2, MTHFD1L, MYH7B, NRG4, OR10P1, OR2K2, OR4K13, PGK2, PIGP, PIK3C2G, PLOD2, POLR1E, PPP2R2B, PSEN2, PTPRM, SCD5, SIPA1, SLC27A4, STK4, SULT1A1, TRADD, TSHR, TUBA1A, VANGL1, KIR2DL5A, PAFAH1B1
Negative_6891	ADA, SI, AMY2A, ANPEP, BCKDHA, CCR4, CD40LG, CDKN2A, CNGB1, CTBP2, FZD9, GUCY2F, INPPL1, MGST1, OR4B1, OR52L1, OR6C74, OR8B4, PER3, POLE2, UGP2
Negative_6892	EPO, PLG, SRC, ARRB1, CCR8, CTSL1, CUL1, CYP19A1, EDN1, EPHB2, EXTL1, FLT3LG, IL1A, IL8RA, KLRC4, MAP3K5, MAPK10, MGST3, NANS, OR10AD1, OR51B5, OR5A1, OR5AU1, OXTR, PDE5A, PIK3R1, PLA2G6, PPP1R1A, SRGAP2, SULT1A4, TJP3, ZFYVE9, HLA-DRB4, MAP3K7IP2
Negative_6893	CRK, GPT, JUN, RDX, ACADL, ADH1B, ATP5G1, CACNG4, CCNB3, CMPK1, CTBP2, CYP27A1, DAAM1, DGKI, FARSB, FMO3, GADD45A, GALK2, GSTK1, LCMT1, MAP2K6, MGST2, NAGLU, NMNAT2, NPR2, OR10H5, OR51B6, OR5M3, PDE6H, PIK3CG, PLA2G2A, PLXNA1, POLR3A, PRKAR1B, PTPN7, SCLY, SH2D2A, TSC1, XCR1
Negative_6894	ACAD8, ATP2A2, BBOX1, C1QA, CALML6, CCL14, FABP1, ITCH, ITGA4, ITGB4, MALT1, OR5B21, PAK6, PPM1A, PRPS1
Negative_6895	HGD, AKT1, ANPEP, CDK4, CXCR3, DAAM2, DBF4, EHMT1, GADD45B, GMDS, GPD2, HSD17B3, IL22, NT5C1A, OR5AC2, OR5K2, P4HA2, PAICS, PDE1C, PRIM1, PSAT1, RAB23, RPS6KA3, SRGAP3, ST8SIA5, TNFRSF1A
Negative_6896	NAT5, RBPJL, SETD1A
Negative_6897	AACS, ADH4, ALDH3A1, CNTNAP2, CXCL10, CYP8B1, DNMT3L, FECH, FGD3, HNF4A, IKBKB, ITGA3, KIR2DL1, MTMR2, NR4A1, OR11H4, OR2T29, OR4C12, OR4C15, OR5D13, PARS2, PLXNB2, TJP1, VNN1, WNT11
Negative_6898	F7, AHCY, CASP10, DBF4, EFNA1, EPHX1, FGF22, GLO1, GZMB, HARS, LIPT1, OR4S2, PDE4D, PLA2G4A, PPARD, RAP1B, SEMA3C, SPRY2, TAS2R16, ULK2, ST6GALNAC3
Negative_6899	EPO, FST, GCK, TNC, TNN, ANAPC4, BCL10, CD99, CNGA3, DAPK3, DDX23, FZR1, GAPDH, GOT2, GSTM3, GUCY1A2, KARS, KDSR, MAP3K5, NME7, OR1B1, OR4N5, OR52M1, OR7D4, P2RX5, PHPT1, PIP5K1A, RETSAT, SOCS1, SRD5A2, TDO2, UGP2, WASF3, B4GALNT1
Negative_6900	IL5, LYN, UBC, ACTN4, ARSD, BDH1, BMP2, CSAD, CTBS, EDN1, GARS, GRIA3, HIBADH, IGFBP3, IL15, ITGA3, NRG4, NT5C, OR2G2, OR5T2, RHOA, RIPK1, RPS6KA1, SCLY, ZAP70, HLA-DPB1, MAP3K7IP1

Negative_6901	C2,PTS,SP1,ADCY1,ALDH7A1,ANAPC5,CACNG3,CLDN10,CYP1B1,CYP2C8,EGFR,EPHA3,FECH,GANAB,GSTA5,GUCA1B,IL15RA,MAP3K5,MPP5,MTHFD1,NAT1,NCF2,OLR1,OR1L1,OR2B2,OR6C76,OR7D4,PCK1,PITX2,PPP1CA,RAB5A,RDH10,SCP2,SOS2,STK11,STK4,TLR2,TUBB2C,TYMS,UBA1,VEGFA,XIAP,GABARAPL2,PAFAH1B1,TNFRSF13C
Negative_6902	ATP5B,GALNT8,MTFMT,NCOR2,NFE2L2,PKLR,PLCG2,ZFYVE9
Negative_6903	ACACA,OR1C1,PON1
Negative_6904	IL7,IL8,ACOX1,ARNTL,CAMKK2,CD46,CD79B,CDC25A,CREB3L1,CTSS,DHDH,EXTL1,FOXA3,FZD3,GALM,GALNT7,GNPNAT1,GUK1,IL12RB1,IL15RA,MYH3,NRXN1,OR13D1,OR1I1,PIGW,PLXNA3,SPP1,TNFRSF10B
Negative_6905	BAD,GPI,HYI,SDS,TK1,ADCY1,CD8A,FGFR1,FGFR2,GUCA1B,HSD3B2,IL15,JAG2,LIPC,MCAT,NUDT8,OR2M7,OR52B6,OR5T1,P4HA3,PCNA,PFN2,PRKCB,PSAT1,SEMA7A,SUFU,TBK1,TGFBR2,TUBA1A,UGT2A1
Negative_6906	AK3,DCT,GHR,ID4,ACSM4,C3AR1,CBLC,CHPT1,CTNNB1,ESAM,FOXA3,GSTA2,IL3RA,ITCH,MAP4K2,MTHFD1L,OR10R2,OR13H1,OR6C75,PIGK,POLR2G,PSEN1,RDH5,TAS2R38,TNFSF10,HSD17B12
Negative_6907	AMT,CGN,MYC,ATP6V1F,BAI1,BCL10,CCNB1,COX15,CSNK1A1,DTX3,EFNB1,FGFR1,G6PD,GNPDA1,GOSR2,HAO1,HAP1,IL22RA2,KLRC2,LARS,LEF1,MAP3K2,MARS,MGAT5B,MYH10,NARS,NOX3,OR10H1,OR1J2,OR2Z1,OR5T2,OR8U1,OR9A4,PGK2,PIK3C2A,PLAUR,ROCK1,RPRM,RPS6KA6,SETDB1,SPRY1,TAS2R1,TICAM1,VDAC2,YKT6,ATP6V1C1,PAFAH1B1,SERPING1
Negative_6908	PML,ARSA,CD47,CLDN15,CLDN8,COQ3,CYP2J2,DDX41,EPB41,EREG,GMDS,GNRHR,GPD1,GSTM5,HAO1,IRF3,ITGA6,LAMB1,MAML3,MAP4K3,MTHFD2,NRG2,OR10K2,POLD1,PRODH,THBS3,UCKL1,WNT9A,YWHAG
Negative_6909	GHR,ACTA2,AGPAT2,CCNB1,CHPT1,E2F3,FZD4,IFNG,IL11,PFKFB3,SNW1,SOS1,TNFSF4,TRAF3,B3GALNT1
Negative_6910	DDC,ACTC1,ATG3,CCR9,COMP,ECHS1,FOXA3,FUT1,GALNT14,GNPNAT1,IL13RA2,KIR2DL2,MAP3K14,MAT2B,MGAT5B,NUDT5,OR14I1,OR51Q1,PAFAH2,PIK3CB,PPM1B,PPP3R2,RASSF5,RPE65,SMURF2,PLA2G12A
Negative_6911	BTC,ME1,MET,AACS,C7orf16,C9orf95,DLAT,GSTM2,GUCY2C,IL1B,KLRC4,NTNG1,OR5AP2,PRKCE,TAS2R16,THY1
Negative_6912	F2,F2R,GSR,LPO,MPL,TPO,ATP6V0C,CASP6,CNGA3,CNTF,CSF2,CTSG,DDX51,DOLPP1,ENO3,FMO1,FSHR,GANAB,GLO1,GP1BA,GRIN2D,LIMK1,MAN1C1,NFKB2,NT5E,NUDT5,OR1L1,PFKL,PKMYT1,POLR3K,PPP1R3B,PPP5C,PRSS2,RAC1,RAF1,RCHY1,RPS6KA3,SARS2,STAT2,TNFSF14,TUBB6,XYLB,AASDHPPT,ATP6V0A4
Negative_6913	EBP,EPO,AGPAT3,ALG10,ARPC1B,CACNG7,CCNB2,CD40,CES2,CKS1B,CSNK1A1,DERL1,DUSP6,EGLN3,FUT7,GCDH,HRH1,ITGB3,MMP1,NMNAT2,NT5C,OR1I1,OR2L3,PANK3,PDE11A,PLA2G2D,PPP2R2B,PROS1,PRPS1,PSEN2,PTPN11,RAP1B,RPS6KB1,SDC1,TAS2R49,XCL1,TNFRSF10A
Negative_6914	PAH,ABLIM3,ANAPC1,B3GALT2,BDKRB2,BET1,CD38,CDH5,CREB1,CSNK1E,CXCL3,EI24,EPHA6,FGF1,FGF23,FUT1,GADD45G,GALNT14,GNA15,GSTM4,GUCY2F,IL10RB,IL20,IL20RB,INSRR,NT5C1B,NTN1,OR4F17,OR6K2,OR6S1,P4HA1,PHKB,PLOD3,PNPT1,POLR2L,PPP2R2A,SETDB2,SNAI1,SRGAP3,TPK1,ATP6V0D1,HLA-DPB1,ST6GALNAC6,TNFSF13B
Negative_6915	ADK,FYN,TYR,ABCC8,ADCY9,AGPAT4,APLP1,ARPC4,CAPN2,CD70,CHST13,CRB3,CYBB,DFFB,ENTPD4,GMPS,GNAO1,GNAZ,INPP5E,IRAK2,ITGA6,MCM7,PFKFB2,PIK3CB,POLD3,PPP2R5C,PPT1,RDH11,SCLY,SEMA3D,SHISA5,SKP1,SSH2,STEAP3,TAS2R38,TUBA1C,PLA2G12A
Negative_6916	BTK,JUP,LSS,MYC,RFK,AANAT,ABLIM3,ANAPC5,BMP5,C4BPB,CHAD,CLCF1,CLTC,CNTFR,CYP1A1,DUSP2,EFNA5,FECH,FRAT2,FTCD,FUT1,FUT7,GCLM,GIT1,GRIA3,GZMA,LIMK1,MAOB,NCR3,NR5A2,OR10T2,PDE11A,PER2,PIK3C3,PKLR,PPT1,WNT11,ZFYVE16,TNFRSF10A
Negative_6917	GNE,LYN,CYP2F1,GGT6,HSD11B2,ITGA8,OR2C3,RASA1,WHSC1

Negative_6918	ABO,AUH,CTH,HK3,ME3,ACSL3,ADCY6,AGPAT6,ATG7,ATP5G2,BAIAP2,CD247,CLDN4,COMT,DDX18,DVL1,EFNA4,EPHX2,F2RL3,GALNT1,GALNT13,GLI2,HAAO,HNF4G,HPSE,LIPA,NCR1,NEFH,NOX3,NUDT12,NUMBL,OR10G2,OR2T33,OR7C1,PDE4D,PGAM2,PLOD2,PLOD3,PVRL4,RDH8,RRM1,SUV39H2,TAS1R1,ATP6V1G3,TNFRSF1B
Negative_6919	ABCC8,ALOX12,APOE,CD46,DDX19A,IMPDH2,IRF9,ITGB7,NT5M,PAX8,PLCD1,PLOD1,SLC27A1,TCF7,TOMM40
Negative_6920	AR,ACAT2,ADCY9,ALDH3A2,ARHGAP5,AXIN1,BET1L,CASP6,CD244,ELK4,GALNS,LAMC3,OR2T12,OR52A5,TSNARE1,TUBAL3,TUBB4Q,TNFRSF17
Negative_6921	AARS,BMP4,IL18R1,NEFL,NR1D1,OR1C1,PIK3R3
Negative_6922	ID3,INS,PRL,ACAA1,ACMSD,ACY3,ATP5A1,B3GALT4,B3GNT3,BAAT,CACNA1H,CCND2,CHKB,COL2A1,CYP26B1,CYP2A7,DGUOK,DLL1,FGFR3,GBGT1,GNPDA2,HLA-F,ISYNA1,LEFTY2,MAFA,MCCC1,MLYCD,NOTCH3,OR2L3,OR56A4,OR5H6,OR6K2,PCCA,PIGC,RALBP1,RXRG,SMAD1,TGFB2
Negative_6923	IL2,KHK,RB1,B3GALT4,BDNF,BNIP1,CARD11,CDC27,EPHA4,GANAB,GNPNAT1,GSTA2,HMOX2,IBSP,ICAM2,IDH3G,IL1RAP,INHBE,KIR2DS5,KNG1,MAPK10,NFYC,NUDT5,OR7A17,PAICS,PER1,PIP5K3,PRKCQ,RARS2,STAM2,WNT4,CSGALNACT2
Negative_6924	CFD,AANAT,ATP6V1D,GLYCTK,HLA-DOB,UGCGL2,ULK3
Negative_6925	FES,ID1,JUP,ACSL1,APOA5,ARNT,BET1L,CAV1,CDC6,CFL1,CHST13,CMPK2,COL4A4,CXCL10,DHCR24,DHRS3,DLL1,DLL4,DOCK1,DUSP1,DUSP14,EPB41L3,EVI1,FCGR2B,FZR1,HAO2,IL29,LCAT,MCM7,MYH13,OR13F1,OR4D11,OR4F6,OR51B5,OR8B4,POLR2A,PRKCZ,PSEN2,PTPN7,RETSAT,SOCS7,SSH1,ST3GAL4,TAS2R10,TCIRG1,TUBB2C,UGP2
Negative_6926	AGPAT3,ALDOB,CACNB3,CD58,CDH5,FDFT1,GNAI1,GOT1,HPSE2,INHBC,ITGA1,MAP3K2,MYH11,OR4A16,OR6M1,OR8B8,OR9G1,OXCT2,PAPSS2,PLA2G2A,POLR2H,PPP1CA,RBL2,TLR5
Negative_6927	ADSL,AFMID,APOA2,CLTC,CYP4A22,DTX1,EPHA4,GALM,GGT7,GLUD2,IARS,MAN2A1,OR13J1,OR2G3,OR3A1,OR4K5,OR5V1,OR9I1,PDE4A,PGK2,PHPT1,PPP1CB,RAC1,SETD7,STX10,SULT1A2,WASF1,WNT2B,PRICKLE1
Negative_6928	F3,C4BPB,CD55,DDHD1,DHDDS,GALNT9,GUK1,HSD3B2,IMPDH2,IRS1,NCSTN,OR2T2,OR9G1,P4HA3,PARVB,PPAP2B,TUBA3C
Negative_6929	C1S,ACHE,AKR1A1,ATP5I,CSNK2B,DGAT1,DUSP16,ECSIT,EDN1,FGF6,GALM,ITCH,LRAT,MKNK2,MYH9,OR4E2,OR5AC2,SMPD4,TAS1R3,WNT10B,WNT7B
Negative_6930	AK7,DDC,ACADL,AFMID,AMPD2,CCL27,CLDN9,CYP2B6,DIAPH2,EPHB3,EPHX1,FGFR1,FLAD1,GNG13,GSTO2,GUCY1A2,IL13,IL20RA,MAN1A1,MCM7,MRAS,NAT2,NSD1,OR13D1,OR51M1,OR51Q1,P2RX3,PAK3,PDE1C,PIGS,PIK3C2A,PLCB3,PMVK,POLR3A,PRIM1,PRLR,PTPN1,RBL1,SESN2,SGPP2,SHC3,SULT1A1,SULT1A2,THBD,UBA1,YARS,CACNA2D2,PHOSPHO1
Negative_6931	CKB,ME1,MTR,ACP2,ADAM10,ARPC1A,CCR9,CD99,CDC14A,CHKB,CLDN14,COL5A1,CYP2A13,DHDH,FABP3,GFPT1,GGPS1,ICAM1,ITGA3,LCAT,MGAT3,MTMR6,NT5C1A,OR10H4,OR4F4,P2RX6,PAPSS1,PDXK,PTPRZ1,RPN2,SLC27A1,SV2A,TNFSF14,UGT8,PNLIPRP1
Negative_6932	SPN,TK2,BUB1,CCKAR,COL5A2,EXOC7,GCNT3,GZMB,OR6C1,PAK4,PDE2A,PPP1R1A,PSEN2,SEMA4D,SPTAN1,TAS2R13
Negative_6933	ATM,A4GALT,CES1,F13B,OGDHL,OR4A15,OR52N5,PLA2G2D,HLA-DRB3
Negative_6934	IVD,A4GALT,ADSL,ANAPC4,BLVRA,CD19,CTNNA1,CYP1A1,EHMT1,FZD4,KRAS,MAPK13,NFKBIA,OGDHL,OR11L1,OR1J4,OR4X1,OR6M1,PDE6H,PDE9A,PLD2,PNPLA3,SGPP2,TCF7L1,TIAM2,UGCGL2
Negative_6935	CACNB1,CLCA2,GAB1,IFNA13,PLK1,PPP1CB

Negative_6936	DCK,ARHGAP5,CAV1,CLTB,CTSG,DGKE,ENO3,FARS2,GALNT13,GJD2,INPPL1,NAT5,NSDHL,OR2A12,OR2A5,OR2L8,OR52L1,OR5AN1,OR8B12,PARD6B,PPP2R2D,RALBP1,SMARCA2
Negative_6937	ALOX12,AREG,DGKE,GSTO1,MAML1,MCM2,OR4K17,PRKCA,ST6GALNAC1
Negative_6938	C5,F2,IL6,JUN,PGD,TXK,ADSSL1,ALG10B,BNIP1,CNDP1,COQ3,DCTN1,DGAT2,ENTPD2,GNAS,IL13RA1,ITGB3,MKNK2,OR10A3,OR10J3,OR4D1,PANK4,PHKG2,PLCG1,PPP1R3A,PPT1,PTEN,PVRL4,PYGM,SHC3,STX18,SUCLA2,TUBB2C
Negative_6939	SMO,SP1,ACTA1,AGXT2,BAIAP2,BCL10,BLNK,CCL4,CCNH,CDH5,CDIPT,CER1,CNTF,CREB3L2,CREB3L3,DGKI,DHX58,FMO1,FPGT,ICAM3,IL1RAP,IL2RG,IL7R,INMT,IQGAP2,ITGAV,LAMB3,MIOX,MYLK2,OR5A1,PDE7A,PIGP,PNPO,POLD2,PSEN2,SMURF2,TAS2R41,TPI1,TPK1,TRADD,TRDMT1,UGT2B17,ULBP1
Negative_6940	HGD,AKR1B1,C3orf10,CACNB4,CD80,CREB3L4,CXCL12,CYP2J2,DTX3L,EPHX1,GGCX,HAO2,IFNA13,JAG2,OR5I1,OR8B8,ORC1L,PCYT2,PIGQ,PVRL3,RRAS2,SGMS2,STAT4,TACR1,TYMP,ULBP2,WNT16
Negative_6941	IL2,ADIPOR1,BCKDHB,CCL1,CXCL12,CYP2C18,DPM1,FARS2,FGF2,FRAT1,LAMC3,MAN1B1,MGST1,NTRK2,OR1E2,OR2A2,OR4N2,OR5AN1,OR7C1,PARD3,PHKA2,PLA2G2D,PLCB3,PPP5C,RBX1,SETD8,ATP6V1E2
Negative_6942	ADA,ALDOC,CCL24,CREB3L2,DGKH,ENPP6,FUT8,FZD1,HSD3B1,HSPA2,IFNA7,MAP2K4,MAP3K2,MYH4,MYLK,OR51S1,PIP5K1A,PPCS,SEMA3F,SLC27A4,WASF1
Negative_6943	ACAD8,ARPC4,CD244,EPHX2,FABP4,FCGR3A,GCDH,GUCY2F,MGAT5,OR4C12,PER1,PLCG1,PON2,PPP2R1A,RFXAP,SAT2,TUBB2A,VAMP1,TNFRSF10D
Negative_6944	JUP,ACOT11,BMP8B,CTPS2,FGF19,FGF23,G6PC,ITGB4,NTF4,OR1L4,OR6B3,PPM1A,RBL2
Negative_6945	CAMK2B,IL5RA,IL8RA,LLGL1,MYH14,MYLK2,NME7,NR4A1,OR13C2,PDGFB,PTAFR,SMPD1
Negative_6946	GCLM,GNA14,NEU4,ST3GAL1
Negative_6947	DCC,GGH,ALG3,ATG12,BIRC5,CSF2,DFFA,DIAPH2,DNMT1,FUT9,HSD17B7,IL12B,MYL9,PAX6,PDE4A,PTGS1,SOCS3,WNT9B
Negative_6948	CSF1,LGMN,MAP4K1,PRKX,SORD
Negative_6949	C2,C4B,DBI,FUK,IL9,LIF,RET,AARS2,ACPT,AGRN,ALG13,ARPC1B,COL1A2,COL4A2,COQ3,CPT1A,DGKD,DGKE,GALE,GALNT2,GLDC,HAO1,IDI2,IRF3,ITGAE,KIR3DL1,MAOA,MKNK1,MYH8,NFASC,NRXN2,OR2L2,OR51A7,OR52E8,OR5AU1,OR6F1,OR6K2,OR7A5,PANK4,PIP5K1A,PSME2,RASSF1,RBPJ,TUBB2C,VKORC1
Negative_6950	GSS,AASS,ACSL1,ACSL4,ACVR1,ADH5,ANAPC7,BLNK,CAV1,CCR3,CD244,DPM3,DUSP7,ENTPD6,FABP3,FDFT1,FGF12,GCH1,GPD2,GRM1,HMGCS2,HRH1,MGST2,NGEF,NMNAT2,OR2M4,OR5A2,P2RX1,PFAS,POLR3H,PRKAR2A,PSEN1,PSME3,PTPRM,RAC3,RGS3,SOCS7,SPTLC1,TOMM40,UGT2B17,YWHAE
Negative_6951	FBP1,FGF18,IGSF5,SHMT2
Negative_6952	C2,C6,DHH,LEP,AGTR1,ALDH5A1,ALOX12,ARPC2,ARSB,ATP12A,AXIN1,BDH1,C1QA,CDC26,CDC42,CUL1,CYCS,CYP27A1,CYP8B1,DAPK2,EPHA7,GCLM,IRAK4,LAMA4,LAP3,LIPA,MAP3K12,NAMPT,NME4,OR11H6,OR1S1,OR4P4,OR5D16,PCYT1A,PIP4K2B,PRKACB,RELB,RPE65,RPN2,SETD1A,SHISA5,SRD5A1,TGDS,UAP1,UNC5D,VAMP2,ZBTB16
Negative_6953	GH1,ID2,IL4,SCD,AASS,ACAD9,ACLY,ADIPOR2,AMPD3,ARPC5,BCL10,CLTC,DAPK1,DUSP2,ENPP6,FGD1,GALNT5,GRIA1,HLA-DMA,MASP1,MGST1,MYLK2,NOS3,NUDT12,OR52H1,OR56A3,OR5F1,OR6S1,OR9G4,PLD2,PPARD,PPCDC,PRPH2,PRPS1,PTK2,RAC3,SSX2IP,STAT2,STEAP3,TAP1,TCF7,UNC5C,ZFYVE9,SLC25A31

Negative_6954	F2,FST,KMO,MIF,ADCY5,ASNS,ATP6AP1,CCR5,CMBL,COASY,CREB5,CSF2RA,EPB41L3,FLT1,GNRH2,GSTM1,HAP1,IKBKG,LAMC1,LIPA,MAGI2,MAP3K1,MYH13,OR10G4,OR1E2,OR51A7,ORC3L,PARD3,PSEN2,RAPGEF4,STX2,STX6,THTPA,TNFSF9
Negative_6955	PRL,ATN1,BAI1,COL2A1,FGF19,GRIA1,IL12A,NUDT8,OR52E2,PIK3CA,PLA2G2E,STK36,ATP6V0A1
Negative_6956	MPI,ADCY8,AGXT2,CCDC6,CLDN11,HNMT,IFNGR2,ITPK1,JAM3,MAP3K4,NR4A1,OR1B1,OR1J4,OR6N2,OR6Q1,OXCT1,PIGM,PRKAR1B,PYGM,SHMT1,SORBS1,TGFA,TUBB1,TUBB3,ULK1
Negative_6957	F11,AKT1,MYH7B
Negative_6958	F3,GK2,GNS,IL4,AMHR2,ANAPC7,BHMT,CCKAR,CCNA1,CDC45L,CERK,CIITA,CXCL9,CYP17A1,CYP4F2,DDIT3,DDX19A,FADS2,FGFR4,FUT2,GSK3B,HLA-F,HMGCR,HYAL2,IL12A,ITGB6,KLKB1,OR13J1,OR1S2,OR2F1,OR2T33,OR52K1,OR6Q1,OR7G2,PDE7A,PTPRF,PTPRJ,RUNX1T1,SAT2,SULT2A1,TAS2R60,THBD,TPH1,TUBA1B,WASF1,XYLB,SERPINA1
Negative_6959	ADRB2,ALOX12,CACNB3,CCR10,CHIT1,CRHR1,CXCL3,CYP2C9,DHFR,FMO5,FOXO1,GLYCTK,ICAM1,IL18RAP,IRF3,IRF9,KIR2DS4,KLRD1,LAP3,LCAT,MAN1A1,MAP2K6,MCM5,NUDT12,OR10Z1,OR1B1,OR51B6,OR5L1,ORC5L,PFKM,PIGK,POMC,PTGDS,RBPJL,TLR2,UGT2B11
Negative_6960	AK5,CAD,TNN,AASDH,C4BPB,CACNG2,CAV3,CCNH,CD244,CD36,CLDN16,CREB3L3,CTPS2,DDX19A,FGF7,GSTZ1,HBEGF,IL17RB,NT5C2,ONECUT1,OR10J5,OR2A14,P2RX6,PANK1,PFN3,PLCD4,PTDSS2,QARS,SDHD,SMAD7,STX16,TKTL1,VAV1,ATP6V0D1
Negative_6961	NOG,TPO,ALDH2,BET1L,CISH,CNGA3,DHRS3,ESPL1,ITGA10,LDHD,MAPK14,OR5A1,OR6Q1,OR7A5,PDIA4,PIPOX,POLE,SRD5A1,ARHGEF12
Negative_6962	ASL,DDC,GLA,PVR,SDS,TPR,ADSL,CASK,CCKBR,CCL17,CCL7,CCR3,CCR9,CD72,CDC23,CDC7,CLDN19,CNGA4,DIAPH2,GMPPB,HSPB1,IDH3B,IFIH1,INPP4A,IQGAP3,NEFH,NPR1,OR6C3,OR8J3,PIK3CB,PRODH2,SETD7,SHC2,THBS2,TRAF5
Negative_6963	AGK,F8,GCK,RPE,AGRN,ALPL,CCL7,CDK7,CDKN2C,CYP17A1,CYP2A6,CYP2E1,DERA,DUSP4,EDA2R,FASN,FBP1,FGF19,FZD9,IL25,INPP5E,KIR3DL1,MAGI2,MYH11,NARS2,NPR1,OR2AG2,OR2AT4,OR2T8,OR4N2,ORC4L,PDHA1,PHKA1,POLR3K,PRPS2,PTPRZ1,RAB5A,RFXANK,SEMA4A,SMPD3,SOCS2,SYNJ2,TNFSF11,TYMP,UGCG,VANGL1,VKORC1
Negative_6964	AASS,ACAT2,ALDH3A1,ANAPC7,ATP6AP1,ATP6V0B,BET1,CHAT,DDX51,DGKZ,DHX58,ELK4,GCNT3,GDF7,GRIA1,GRM5,HLA-G,HYAL1,L1CAM,MAPT,MYH2,OR51I1,PDE4A,PTPN11,RASGRP2,ST3GAL2,STAT1,TNFSF14,WNT9B,MAPKAPK2,TNFRSF10A,TNFRSF1A
Negative_6965	GGH,AASDH,ABAT,ADRBK2,ALOX5,ATP6V1F,BAAT,CHPF,CSNK1G1,CYP3A43,DFFB,GGCX,GYS1,HBEGF,HMGCS2,HRH2,ITGAX,NFATC3,OR5I1,OR7A10,PIGN,POLE2,SIGLEC1,UGP2,YWHAB,CACNA2D2,TNFRSF10C
Negative_6966	ADC,C8B,TEC,ABL2,ACTA1,ALG2,CD58,CDC45L,CDH2,CLTB,CREB3L1,CTNNB1,CYP3A43,DDIT3,DUSP3,ENTPD1,FGF23,GCDH,HDAC1,HTR4,INADL,MAP3K11,MGST2,MICA,MYLK3,NAGK,OR10A7,OR1L6,OR2F1,OR7D4,PAFAH2,SMAD3,TAS1R1,TAS2R40,TYMS
Negative_6967	ADC,NNT,ADORA2B,C3AR1,CASP8,CES2,DGKH,E2F1,GALNT13,ICAM1,KIR3DL1,NR5A2,OR12D3,OR4F4,PAX4,PLA2G10,PMAIP1,PNPT1,VEGFA,ATP6V1B2,SERPINE1
Negative_6968	CR2,DCC,PDC,ALPL,ARPC5L,ATP5C1,CYP26A1,CYP7A1,DDX47,HCST,IL22RA1,JAK3,KARS,MGLL,MKNK2,MLLT4,MTHFD1L,OR5M3,PIGH,PRKCE,RAC2,RFXANK,WNT16,CSGALNACT1,SERPINF2

Negative_6969	CAT, CBL, HK2, MPI, ACP6, ADRB2, ALG11, CACNA1F, CD44, COL11A1, CSNK2B, DDX54, ERBB3, GAMT, GNA15, GNG3, GSTK1, HYAL2, NAGLU, NANS, NR1D1, NT5C2, OR51D1, OR52D1, PER3, PHKA1, PIP5K1B, TIRAP, TRIP10, TSHB, UGT2A1, SLC25A31, TNFRSF11A
Negative_6970	ALAS2, B4GALT5, CAMK2A, CCL16, DHRS4L2, FMO5, MCCC1, PTPRR
Negative_6971	CD4, EGF, IL2, STS, XDH, ABI2, ACAT1, ACSM3, AKR1C2, C5AR1, CBLB, CCL16, CCR4, CHD8, CX3CL1, DIAPH2, DOCK1, EPAS1, FMO3, FZD3, GBA3, HLA-F, ICOSLG, ITGAD, NAMPT, NFKB2, NKX6-1, OR10AD1, OR10J3, OR1C1, OR2W1, OR4F6, OR52A5, OR52I1, PLA2G4A, PNLIP, PPT1, RAPGEF2, RDH5, TRAF3, UBE2L6
Negative_6972	CR2, DDO, NLK, VTN, ACAD8, ACADL, ADCY7, AOX1, APPL1, BACE2, CASP10, CCL7, CLDN18, CTBS, FMO5, MGLL, MKNK1, NFYA, OR4K2, OR51I2, PDE8B, PFN3, POLR2J, PRKG1, SETD7, SNAI2, UBE2K, UGT2B4, ZAP70, HSD17B12
Negative_6973	AXIN2, CAMK2B, CDC23, FOXA3, GLUD1, LLGL1, MCAT, NAT5, OR1B1, PPM1A, SLC33A1, PRICKLE2
Negative_6974	F5, GBA, ID1, PXN, TEC, AADAC, ADH7, ALDH5A1, BCMO1, BDH1, CACNB1, CDH1, DLAT, ENTPD1, GGT7, HAGH, KIR2DS5, LCMT1, LHCGR, MAPK14, NUDT5, OLR1, OR14C36, OR2A4, OR51E2, OR52E6, OR52E8, OR52H1, P2RX1, PANK2, PFKFB3, PIGU, POLR2H, PORCN, PPCS, PTTG1, RELB, SMARCA2, TAS2R50, TNFRSF9, TYK2, ATP6V0D1, SERPINA5
Negative_6975	NGF, TPR, ALAS2, ALDH4A1, ATP2A3, AXIN2, CLDN9, CNGA4, CYP2U1, DTX3, GAB1, GALK2, GCNT2, GGT7, GLUD2, GOT1, GSTA5, HARS, IRS1, ITGA2B, LILRB3, MASP2, MNX1, OR10P1, OR1A2, OR5AS1, OR5K2, PLA2G4A, PMAIP1, PNPO, SDC2, SHC1, THTPA, TPMT, UGT2B17, PNLIPRP2
Negative_6976	ARHGEF1, BLVRA, CCL5, GRPR, LEF1, MAP3K6, MYH8, OCRL, OR2T6, RPN1, TAS2R8, TLR4, TSHR, UPRT, CACNA2D2
Negative_6977	CEL, GLS, IHH, ACAD8, ADRA1B, ASPA, B3GALT4, BACE2, BMPR2, CCL4, CDC25A, FRAT2, GALNT7, GUCA1B, HNF1A, HSD17B2, IFNA10, IKBKB, IL28A, KAT2B, LDHD, MYH14, NCKAP1, NTF4, OR1L4, OR6B3, OR9I1, PAPSS2, PFKFB3, PIK3CD, PTPRB, PVRL2, RPE65, TLR5, TSHR, WNT11, WNT7B, MAPK8IP3
Negative_6978	CTH, ACHE, CARS2, CASP10, CXCL1, DPYD, FZD10, FZD6, G6PD, GAPDH, GNRH1, GRIN2A, IDH3G, IL25, INHBE, INPP4A, IRAK4, ITGA5, KYNU, MTHFS, NKX6-1, OR13C3, OR13F1, OR4C11, OR52A5, ORC4L, PGS1, PLAU, PLXNB2, PPCDC, PRKCD, SETMAR, SMARCA5, SMC1A, SMC1B, TAS2R38, TLN2, WASF1, WASF3, TNFRSF17
Negative_6979	DBH, CXCL2, GLS2, MTMR6, OR11H1, OR13C8, PFAS, PIGU, PLA2G3, SRGAP2
Negative_6980	ADK, CAT, TK1, ACAT2, ACSL1, AKR1A1, ALDH1A3, B4GALT5, CCL8, CCR7, CHEK2, CKMT2, CSF3, DDX55, E2F5, EDNRB, FGF12, FGF2, GCDH, HSD17B8, IL29, IMPA1, ITGA7, ITGAX, LLGL1, LTB4R2, LYPLA2, NR5A2, OR1A1, OR56A1, OR7A5, OR7G1, PRODH, RPSA, SKIV2L2, SLIT1, SOCS5, TOLLIP, TPI1, TUBB6, UCKL1, UROD, WWP2, ATP6V0A1
Negative_6981	F8, ABP1, ANAPC1, ARPC5L, B4GALT3, BBOX1, COL4A6, DAPK2, DHRS9, ERBB3, IRF9, LHCGR, OR10A2, OR4B1, OR5D16, PLCB3, PTEN, RDH12, SMPD4, TRIP10, YWHAB
Negative_6982	CTH, F3, FYN, IL3, TYR, ABLIM2, ACVR1C, ACVR2B, ARRB1, CD22, CHPF, COASY, CXCL6, CYP3A43, DERL1, GCLC, GNAI1, GNAI3, GNAZ, GPD2, GPLD1, IGF1R, IMPDH1, IRAK1, ITGA4, ITGB8, LTC4S, MAPK9, MYH6, NCR2, OR10J3, OR2A42, OR52N2, OR52N5, OR5K2, PAICS, PANK4, PFKFB2, PIP4K2A, PLA2G6, POLR3K, PON1, PVRL4, SLC2A4, TGFB1, UGCGL1, PNLIPRP1, SERPINB5, SUV420H1
Negative_6983	AFMID, B4GALT2, BMPR1B, BUB1, CBLB, F13A1, FGF17, MAGI3, MOV10L1, NME2, NOTCH3, NUMBL, OR10T2, TAS2R9, TRPM5, ALDH18A1, PDCD1LG2

Negative_6984	NF1, ACAD8, BMPR1B, CALML5, CDKN1A, FGF18, IL18R1, MAP4K3, MGAM, NR1H3, OR2AG2, OR52I2, PANK3, PPCS, PPP2R5C, RAB23, SKIV2L2, STX3, TSTA3, MAP3K7IP1
Negative_6985	ADA, FGA, ID1, ME3, MET, ALCAM, ALS2, ANAPC11, ARHGEF7, ARSD, BAAT, CAMK2D, CDC14B, CDC16, CDC45L, CYSLTR2, EI24, HNF4G, IARS2, IL12B, INADL, IRF7, ITGA7, LDHA, MAP3K3, MAPK10, NFKBIE, OGDHL, OR10G2, OR10S1, OR1E2, OR1S2, OR2T11, OR4S2, OR4X2, OR52I1, PDGFRB, PLCG2, PPP2CB, PPP3CA, RPS6KA4, SHC4, SLIT1, SORD, SPRY4, THTPA, UGT2B11, WNT7B
Negative_6986	DBH, F9, TNF, ACOT11, B4GALT2, BDH1, CDKN2B, CNTFR, CSNK1A1, CYP2A6, FMO3, IL17A, IRAK3, LIPF, MDH1, MTMR2, MYH4, NCR1, NEFH, NOX1, OR13G1, OR1B1, OR52I1, OR6C1, PDE6G, PIP5K3, PPA2, PPP2R5C, PRKAA1, PRKAA2, RBX1, SCLY, SETDB1, SHMT1, SPRY4, TUBA3C, UGT8, VEGFA, XYLB, ST6GALNAC4
Negative_6987	AK3, CBL, GP5, ALS2, B3GALT1, CCL7, CDK5, CREB3L1, CTSG, CX3CR1, DAD1, DPAGT1, DUSP2, EDNRB, FGF10, GOT2, HSD17B2, HSP90B1, IL12A, IL7R, MAD2L2, MARS, MLYCD, OR10P1, OR51B6, OR52H1, PLA2G2A, POLE, POLR1A, PRODH2, SARS2, SULT1A4, TBL1Y, UGCGL1, PLA2G12B, TNFRSF10B
Negative_6988	GCK, TYR, CACNB3, CNTF, CYP11A1, FGF13, IL10RA, KLRC1, LTBP1, MMP2, OR52K2, STK36, YKT6
Negative_6989	ACTN2, ALG10B, ANAPC2, CDS1, CLDN1, FGF22, GART, IL22RA2, MBL2, MMP2, MPST, NADSYN1, OR10H1, OR6M1, OR7D2, OR8D4, POLR3B, RALBP1, RASGRF1, SULT2A1, TMLHE, VCAM1, ZNRD1
Negative_6990	MVD, PGD, ACSS2, APLP1, ATP6V1H, CCL26, COX15, ESPL1, GSTM3, GYS1, HARS, ITGB4, MAP3K11, OR10G8, OR4C13, OR51G2, PPA2, PRDX6, RRM2B, SEMA3G, SMPD3, TAS2R41, TGDS, TJP1, UBA7, WNT5B, MAP3K7IP1
Negative_6991	AK5, CFI, AKR1C3, CACNG2, CD8B, COQ3, CYP27A1, EPB41L3, FOXA3, GNPDA1, GSTA5, HCST, ITGA7, KIR2DL3, LTA4H, MS4A2, OR1L8, OR4F6, OR5AN1, PER2, PNPLA3, RHOQ, SOCS3, THBS1, TNFRSF10A
Negative_6992	LPL, BUB1, CCL16, CCL2, CLDN10, CLDN15, DDIT4, ENTPD3, IMPDH1, ITGA4, KCNQ1, MGAT5, PHKA1, TAS2R3, TNFSF4
Negative_6993	SYK, CPT2, OR1J1, PPARA, ATP6V0E1
Negative_6994	DBH, EGF, SDS, ACADSB, ACVR2B, ADSL, APBB1, ARHGEF4, ARPC3, ARRB2, CASP6, CDK7, CHAT, CREBBP, CYP1B1, CYP2B6, DDX4, EFNB3, ENPP2, F2RL2, GJA1, GLO1, HNF4G, ICAM2, KLRC4, MTHFD1L, NME7, NR1D1, OR13C2, OR4C12, OR51A4, OR5B17, OR6F1, PCYT1B, PDE9A, PGAM1, PLXNA3, POLR2E, SFRP5, ST3GAL6, STX10, SULT1A4, VNN1, MAPK8IP1
Negative_6995	DAO, LPO, VCL, ACTN4, ADCY2, APH1A, ARRB2, C4BPA, CCNG1, DOT1L, DVL3, F11R, FOXA3, GALNT8, HSD17B2, OR52B4, OR52D1, PDHA1, PPP2R2A, SSH2, TBL1Y
Negative_6996	ACTN4, CACNA1G, COQ7, F13A1, HAAO, IRF7, KLRC4, MAPK12, OR1A2, OR2T5, OR4D9, OR51I2, OR5P3, PNMT, PON1, RPS6, TAS1R1, TPM3, TXNDC12, UPP1, XCL1
Negative_6997	CFH, CKM, GSR, OTC, ACP1, ACVRL1, BNIP1, CDC20, CRY2, CSF2RA, CTSG, GAD1, HNMT, IDH3B, IL7R, ITCH, OR10H4, OR1A1, POLR3A, PPP3R2, STX3, TSNARE1, VCAM1, WNT2B
Negative_6998	AK7, LPO, TNF, ADCY6, APRT, ASIP, CYP3A5, IL10RB, LAMB1, OR51B4, OR52N2, P4HA1, PCCA, WASF2, WBSCR17
Negative_6999	FGA, BIRC2, CCR4, CDC42, CTLA4, DCTN1, DPAGT1, FUT7, GNG12, GSTM2, MGST2, NEFH, OR10A3, OR10T2, OR1E1, OR51A2, PLA2G4A, PLK1, PNLIP, POLR3A, PTGS2, ROCK1, SEMA4F, SPTLC2, TGFB3, TRAF4, UGT1A10, YES1
Negative_7000	ALDH4A1, FGF12, FGF21, LHCGR, PCCB, RBKS, SEMA4C, UCKL1
Negative_7001	CLDN9, IKBKB, TBK1, UBE2L3, WNT5B

Negative_7002	ASL,ALDH2,ANAPC5,ASIP,BDKRB1,CACNB2,CCL28,DUSP3,EI24,ENTPD8,EPHA1,INHBE,ITGB4,LIPF,NFATC4,NLGN2,NR4A1,OR10G3,OR10H3,OR10H5,OR13C9,OR4E2,OR4N5,OR5AU1,OR5D18,PGM3,PTEN,RDH5,ROBO2,SGPP1,SMURF2,TGFBR1,TRAF5,VDAC2,WBSCR22,WHSC1L1,WNT2B,XCL1,ATP6V1G3,HLA-DPB1
Negative_7003	HK1,ATG12,B3GNT3,CADM3,CLDN11,IKBKB,IRAK3,LAMA4,MDH2,MGAT3,NFAT5,OR4C12,PRKCB,RAP1B,SMPD2,YARS
Negative_7004	C8A,ACSL4,ADIPOR2,BDNF,CACNG5,CDH3,CFL1,CLDN2,CYP2S1,DGUOK,DLL4,EFNA5,FMO2,GART,GCNT1,HRH1,HSPA1B,IRAK2,ITGB6,NFKBIA,NODAL,OR1J1,OR2C1,OR2F2,OR2H1,OR2L2,OR51F1,OR51G1,OXTR,PKM2,PLXNA3,PROC,RARS2,RDH12,RPE65,SMURF1,SPHK1,TAS2R50,TUBA3C,UPP1,WNT2,WNT4,PDCD1LG2,ST6GALNAC6
Negative_7005	GH1,AKR1D1,ALOX15B,FGF21,IGF1R,OR4B1,PDGFRA,PTPN7
Negative_7006	CD2,CRH,ACSL6,AMHR2,ATG3,DDX51,HPSE,HPSE2,HSPB1,HYAL2,IL22RA1,NKD2,OR6A2,PGAP1,SNAI1,TUBB4,VAMP4,VAV3
Negative_7007	EGF,FYN,GGH,HK1,ACO1,AKR1A1,AKT2,ALDOC,CASP8,CCL28,F2RL2,G6PC2,HPSE,HRH2,IKBKE,IMPA1,OR1M1,OR2AT4,OR56A1,PDE8B,PIK3C2G,PSEN1,SGMS2,SMAD4,TAP1,UROD,ATP6V0E1,HSD17B10,PLA2G12B,TNFRSF6B
Negative_7008	CKB,DHH,MPZ,A4GALT,AGXT,ALDH1A1,ALDOC,ARSE,BACE1,C4BPA,CACNB3,CACNG1,CAMK2B,DAPK2,DGKI,ENTPD8,FABP3,FCGR3A,FDFT1,FLOT2,GANC,GNAI1,GNMT,IL18R1,MAP2K6,MAPK8,MGST2,NARS,NEU3,OR2A42,OR2G6,OR2T10,OR51F2,PTPN6,RELB,SFRP5,SORBS1,TCF7L2,VAMP1,WNT10A
Negative_7009	ATM,ATG12,B3GALT1,CLDN15,CYP3A5,EGFR,EPB41,GPX7,HMGCS1,LIPC,MAT2B,MMP1,NRG4,OR11H1,OR5M9,OR6C6,PFN3,PHKA1,PI4KB,PRKAB2,SOD1,TNFRSF11A
Negative_7010	AK3,LAT,TNC,AKR1B1,ALDH1B1,ALDH6A1,BMP5,CCND3,CDC25C,CHST12,CNGA4,FCGR3A,FLOT2,FZD5,GCNT2,GUCY2D,IFNA10,IL4R,KLKB1,LIMK1,MAP2K2,MIOX,MPDZ,NAT2,NOTCH3,NRG1,NTF3,NTF4,OR1D2,OR2D2,OR4C6,OR4M2,OR8A1,PAK6,PDE1C,PDE4C,PIGX,POLR3A,RARS2,SEMA4A,STK3,TAS2R9,TNFRSF9,TSC1,UGT2B28,VTI1A,PNLIPRP2,SERPINE1
Negative_7011	EBP,TPR,ATP5D,COL3A1,DAPK2,DKK2,EPHB3,ICOS,OR4K17,PDGFRB,PSME2,SPRY2,TIAM1
Negative_7012	FRAP1,MAP3K5,OR5H2,OR8B8,OR8I2,PAFAH2,ST3GAL3,TCF7
Negative_7013	MVD,ACADL,ALOX5,ASAH1,BUB1,CCL23,CCR5,CD79A,CHRM2,COL5A3,CPT2,DAAM2,DPM2,EXOC7,FMO2,FZD2,GCNT1,GTSE1,HLA-G,HSD17B1,INPP4A,IQGAP3,MTAP,NFKB1,NT5C1A,OR1J1,OR9Q1,PPARG,PPP5C,PRODH,PTPRZ1,RASGRF2,RERE,SEMA4D,TGFB2,TNFSF11,WASF1,ATP6V1B2,ATP6V1G3,SERPINA5
Negative_7014	GPI,IDS,PGF,ALG10,ATG3,ATP6V1H,BET1L,CADM1,CCND1,CHKA,DCTD,ENTPD4,ERBB3,GNG13,GRM1,IL19,IMPA2,ITGA6,ITGAL,NEUROD1,NLGN2,OR2T3,OR52L1,OR7A5,PNLIP,POLR2H,RAD51,RBKS,SELE,STAT1,SUFU,TAS2R50,TLR2,TP53,VTI1B,GABARAPL2,SERPINA5
Negative_7015	MAP2K6,OXTR,SESN1,SETMAR,SFRP4
Negative_7016	AMT,ASL,C9,CP,PGD,ACACB,AGRP,ALOX15,ARNT,BMPR2,C7orf16,CARS2,CASP10,CHD8,CNTN1,DRD1,FCGR2B,FGFR3,GSTA1,GSTK1,IL10RA,JAK2,MLLT4,MYH6,NPAS2,NQO1,OR4D9,OR8B8,PCCB,PDPK1,PFKFB2,PIGM,PIGS,POLR3B,PPP5C,PTGS1,RDH16,SEMA3C,SUV39H1,TIRAP,TPK1,TRAF6,VANGL1,WNT7B
Negative_7017	ARAF,BMP5,CACNG6,CLDN2,GNA12,MGST3,OR2AG1,OR4K14,OR51L1,OR52J3,PFKFB1,PGAM4,PGS1,PLOD3,PLTP,PPCS,SOS2,ULK3,WNT9B

Negative_7018	ABLIM2,ACVR2B,ADCY7,ADORA2A,AKT3,BIRC3,CACNA1C,CD34,CD58,CDK7,CLDN4,COL6A1,COMT,CXCL1,CXCL11,CYP21A2,CYP2E1,E2F1,EPHA2,HEMK1,HRH1,IDH2,IL28A,INPP4B,MAGI1,OR10A6,OR13F1,OR8S1,PARD6A,PIK3CG,PRKAG2,RRM2B,STAM,TAS2R49,TPI1,UCK2
Negative_7019	COQ5,CXCL2,DBF4,OR14I1,OR2L3,PDE4C,PLCB4,RALB,SHMT2
Negative_7020	ARHGAP5,CACNA1A,CD36,CFL1,COL5A1,CPT1A,DAXX,DUSP5,EDA2R,FGF14,GNPDA2,GP1BA,IL17B,LAMB3,NNMT,OR11G2,OR1D2,OR2L13,OR51B2,OR51M1,OR6X1,OR8K3,OXTR,PAFAH2,PGLS,POLR2B,PPP2CA,SAT2,SMAD2,TGFB1,ST6GALNAC4
Negative_7021	EBP,FAS,LIF,LTB,SYK,ACAD9,ALDH1L1,ALDH5A1,ALDH9A1,CCL15,CD46,CDC25A,CDC45L,CTBP1,CXCL11,DVL3,EVI1,F2RL3,FLOT1,FZD7,GSTO2,ICOS,MAPK14,OR2AG1,PFKFB4,PLCG1,PPP2R5A,PVRL1,RAC3,RFNG,SEMA3A,SGMS2,SHC2,SPHK2,TBK1,ATP6V0A2
Negative_7022	FN1,HAL,ACSL4,ACY3,ASMT,CAMK4,CASK,COMT,CTF1,CTSB,DHCR7,E2F5,ENPP1,FGF18,GBGT1,GRIN2A,HYAL1,IL28B,IQGAP3,ITGAV,MAP4K2,OR1L4,P2RX3,PLCD1,PLCZ1,PSEN1,RAB23,SDC4,SETD2
Negative_7023	BET1,CCNG1,HSD11B1,HSD3B7,LIPF,NKD1,NUDT9,OR2V2,PANK1,PTPRZ1,SEMA3B,SMPD1,TNFRSF10A
Negative_7024	ACACB,APAF1,CACNG4,CARS2,CHDH,DHODH,ETNK2,FUT6,GLB1,HNMT,IPPK,KIR2DL4,NCOR2,OR10G9,OR14I1,OR52H1,PRPH2,TGFBR1,WNT9A,TNFRSF17
Negative_7025	CAPN2,CD274,CD40LG,DDB2,DDX51,ENTPD1,EPHA5,GMPS,IL28RA,IL7R,ITGA10,MAP2K7,MKNK2,NEFL,OR1L8,OR2T6,OR51G1,OR8J1,OXCT2,PCNA,RASGRF1,TM7SF2,TRADD,TSHB,WWP2,YWHAQ
Negative_7026	CRH,ID2,IL3,LYN,ACAD9,ALPL,BET1,CSF3,EPHA5,EXT1,EXTL3,GLI1,ICAM2,IFNGR1,JAM3,JUND,MAN1C1,MAN2A1,MTMR2,MYH8,NEFL,OR51I1,POLR2I,RFXAP,SPP1,TNFRSF8,WNT4,CSNK1A1L
Negative_7027	CKB,F10,F2,IL7,ACSM4,ACSM5,ADRB2,APOA5,B3GAT2,CARS2,CASP10,CD38,CSDA,CXCL5,DKK4,EPHB3,ERBB4,FUT8,HADH,IKBKG,LALBA,MAGI2,MAPK13,NFKB2,OGDH,OR13C8,OR51A4,OR52D1,OR5A2,OR5D16,PIAS2,PLA2G2A,PLAU,PLCB4,PRKACB,SAT1,SDC1,SHMT1,SLC25A6,TLR5,TRPC1,VDAC2,YWHAE
Negative_7028	VTN,ACTA2,CACNG6,CLDN2,CNDP1,DPYD,DUSP14,DUSP9,GALK2,GDE1,HPSE,HTRA2,LDHAL6B,OR2V2,OR4X2,OR51A4,OR52N1,OR6K2,PDE3B,PGAP1,PORCN,PPARG,RALBP1,RASGRF1
Negative_7029	ATP2A1,BRAF,CDC6,CFTR,CXCL9,DDX56,FARP2,GNPDA2,GPD1,OR1D5,PGM1,PIGF,PIGP,PNMT,SELL,SEMA3C
Negative_7030	DAO,FAS,ILK,PGF,PLG,AADAC,ACADL,ADIPOR2,ALDH1A2,ARNT2,B4GALT5,BMPR1A,BUB3,CCNA2,CCNG2,CPT1B,CYP3A5,DAXX,GPX5,ITPKA,LYPLA1,MAPK1,NT5C1A,OR4L1,OR4N4,OR5B17,OR6C1,OR9I1,PDX1,PKLR,PLA2G6,PPP2R2D,PTPMT1,PTTG1,ROCK2,TKTL1,VEGFB,ZFYVE16
Negative_7031	ADORA2B,BMP4,FUT1,GSTA1,IFNA5,LAMA3,MAPK10,OR8J3,SDHD,SESN1,SMPD3,UGT2B28
Negative_7032	ME1,OGT,AKT1,ALDH3A2,ATG12,CCKAR,CCL3,CD247,EPHB3,GCNT1,IKBKE,IL1R1,LLGL2,MARS2,MMP7,OR10T2,OR1A1,ORC1L,PDE6H,PIGM,RALBP1,SC4MOL,SDC1,SELP,TYMS,ULK2,UROS,PNLIPRP1,TNFRSF10A
Negative_7033	AMT,IL6,ACHE,AFMID,BMPR1B,CACNG5,CDS1,CHSY1,CMBL,COL4A2,CTF1,CYP2F1,DTX2,ESAM,FABP1,FGF1,FOXA3,GALNT2,GNAL,GRB2,GRIN2D,GSTO2,GUCY2C,GUSB,HLCS,HSD17B2,IL9R,IRF7,NPR2,OR10S1,OR2L2,OXTR,P4HA1,PDE4A,PDE6H,PDE7B,PIAS4,PTPRF,SETD7,SGMS1,SLC2A2,ST3GAL5,TKTL1,TRHR,TRPC1,TUBAL3,WNT2B,HLA-DQA2

Negative_7034	ME3,PTS,ACSM3,AGTR1,ANPEP,ATP5J2,BET1L,BMPR1A,BMPR2,CACNA1B,CD244,COL4A6,COQ7,COX15,FTH1,GALM,GNG3,HCLS1,HTR2A,JAM3,MAPK14,MDH1,METTL2B,NKX2-2,NT5C,OR2A12,OR2D3,OR52A5,OR52K2,OR5AR1,OR8B4,OR8D4,ORC3L,PLK1,PLXNB2,POLR2F,PRNP,RETSAT,SDC2,SELE,SEMA3A,SLC27A1,THTPA,TUBB2C,UPB1,VEGFA,XCL2,YWHAZ
Negative_7035	ADA,CD4,MPL,ACMSD,ACTC1,ANAPC7,CHRM1,DHRS9,FABP3,GSTM4,IL12RB1,LCMT1,NME2,OLR1,OR10G3,PFKP,PIGH,RASGRF1,ST3GAL1,SYNJ2
Negative_7036	AK1,RPE,ZYX,AHCY,ALDH3A1,APBB1,APH1A,APOA2,CALML3,CD44,CDC25A,CHSY3,DDX41,DPM3,ENPP6,IL8RB,ITGB7,LIFR,MAPK7,NOX1,NR1D1,NUDT9,OR4N4,P2RX1,PFKM,PON1,RETSAT,SMAD6,SYNJ2,UMPS
Negative_7037	IL5,SRM,ADH7,ALDH1A2,ANAPC7,ATG12,C1QC,CAMK2G,CSNK2B,CXCL2,EFNA3,GALNT14,GNA15,HSD17B1,IL24,INHBA,NARS2,NTN1,RFNG,SQLE,STAT4,TLR4,TLR9,TNFRSF8,VANGL2
Negative_7038	GCK,ID3,MPZ,ABCC8,CASK,CBLC,CCR3,CDC25A,CDC45L,CREB3L4,CRLS1,CYSLTR1,DHDDS,DMGDH,EPOR,FRAT2,GSTM5,HNMT,HSD3B2,IFNGR2,IL1A,IL2RB,ITGA1,ITGAD,METTL6,NCF2,NFATC4,NOX3,OR4F4,OR4F6,OR4N4,OR51F1,OR5J2,OXCT2,PCCB,PIK3C3,POLR2A,POLR2J,RBPJ,RDH10,RGS3,RPS6KB1,SFRP5,SMAD2,SPHK2,STEAP3,VASP,ATP6V0A2,HLA-DRB5
Negative_7039	FN1,LIF,CHEK2,IFT57,OR5M3,PFN4,PPAP2A,RAPGEF1,SDC2
Negative_7040	BAX,GK,HK1,ALDH3A1,APOE,B3GAT2,BLNK,C3AR1,CADM3,CCL27,CDC27,CFL1,CSAD,CXCL10,DAAM2,FGF4,IFNA2,IFNA21,LAMA3,LPAR1,LTC4S,MDH2,MYH3,NEFH,NME4,OR10P1,OR4K15,OR7D4,OR8J1,PFKFB4,PPCDC,PPID,PRKAA2,RRM1,SMPD4,SREBF1,SYNJ1,TBL1Y,TPI1,UBA1,ATP6V1B1,ATP6V1E1,MAPKAPK5,SERPIND1
Negative_7041	AASS,ACTB,B4GALT4,BAI1,CD19,GPT2,IFNAR1,MARS,OR5R1,ORC2L,PCYT2,RUNX1T1
Negative_7042	DCK,MIF,BST1,CXCR6,ENTPD5,HAO1,INPP1,ITGB3,OR10G3,OR4C46,OR5C1,UGP2
Negative_7043	F2R,ACAA1,ACVR1C,ASPA,BCKDHA,CAMK2G,CD244,CLTC,CMPK1,CSDA,CSNK2A1,CYP2D6,EPB41L2,ESPL1,FZD9,GMDS,GPAA1,GSTT1,HHEX,KIR2DS5,LALBA,LIPA,MASP2,NFE2L2,NME4,NRG3,OR5T3,PNPLA3,PPP1R3A,PTGER1,RBL2,RXRA,SKP1,SLC12A2,TP53I3,TUBB1,ATP6V0D2
Negative_7044	NPY,OGT,PAH,AASDH,CD247,CSF2RA,DHCR7,DUSP6,FSHB,FZD9,HCST,HTR2C,IFNA8,LCAT,LEF1,NODAL,NSDHL,OR4C16,OR52N1,OR5M9,OXCT1,PFN4,PPP2R1A,SPRY4,WNT9A
Negative_7045	PDC,TKT,ARSD,ATP2A3,B4GALT7,CCL22,CCL27,CCNA2,CCNH,CD79B,CLTCL1,CPS1,DAAM1,DEGS1,DUSP16,EXOC7,FABP4,FCER1G,FGF4,GALK1,GNAT3,HES1,HMGCL,LILRB3,LMO7,LRP5,MGST2,MTHFD2,NAMPT,NME2,NQO1,ODC1,OR2L13,OR51F1,PAK2,PGM3,PTGES,PTPRF,RASSF5,RERE,RPE65,SETD1A,SKP1,SOCS3,TAS2R3,TSC1,TSTA3,HSP90AA1,PPARGC1A
Negative_7046	ACHE,ACO2,ACP2,ACTN2,ANAPC5,ARRB1,B3GAT2,CDK4,IL18,MAPK3,MCM7,MGST3,OR10K1,OR2W1,P4HA3,PHKA1,PLA2G6,POLR2G,PRSS2,PYGB,SOX17,ST3GAL1,UNC5A,SERPINB5
Negative_7047	TNF,ABI2,ADCY8,ADIPOR1,AGPAT3,AGRP,ALG5,ALS2,ATP2A3,BDKRB1,C5AR1,CDO1,CHAD,CLTA,CNTNAP1,DGKD,DGKE,DUSP4,EFNA4,FLT4,HADHA,HEXB,HIP1,HRAS,LRP5,LTA4H,MAP3K8,OR2A4,OR4C46,OR4K17,OR6C75,PCYT1B,PDX1,PIGP,PRLR,PSENEN,RFNG,SHC3,SMARCA5,STX4,TACR1,TDO2,TGFB3,YKT6,SERPINA5

Negative_7048	FGG, HGD, OSM, UBB, ACADS, AGXT, B3GALT2, CALML6, CCKBR, CD80, CLDN17, CMPK1, COMT, CTSG, CXCL12, DHRS4, F13A1, GNAI2, IDH1, ITGB1, NAGK, NFAT5, NLGN1, OR4F15, OR51A2, OR6B3, P2RX6, PFN4, PHKA2, PLA2G1B, PLA2G2F, PLCB2, PRKG1, RASGRP2, SESN1, SH2D1A, SOAT2, TAP2, TCF7L1, TNFSF4, ARHGEF12, ATP6V1G1, GABARAPL2, TNFRSF12A
Negative_7049	AMH, GK2, IL9, MIF, XDH, ACSM1, ACSM2A, CCNH, CD36, CD3E, CLTCL1, CSNK2A2, DPM1, DPYS, FUT2, LDHAL6A, MAP3K7, MAPK12, NPR2, NT5C3, NTF3, OR1L6, OR52M1, PPAP2A, PRKAB1, PRPH, RPN2, SDC2, SLC27A4, SOCS4, TBL1Y, TSC1, UAP1, VAMP4
Negative_7050	JUN, TK1, ACAT1, CDK7, CHDH, CLOCK, COL4A6, CYP2B6, DPYSL2, ELK1, ERBB3, FGF19, GNA13, HARS2, HIP1, IL1B, LTB4R2, MAD2L1, MCM6, MNX1, MTMR6, NFYC, OR10J3, OR13C9, OR6N2, ORC2L, PHKG1, PLA2G4A, POLR2F, PRKAG2, SOAT1, ST3GAL2, SUCLG1, SV2B, TICAM2, UAP1, MAPKAPK2
Negative_7051	CTH, DDC, B3GNT3, COQ6, IL23A, OR13A1
Negative_7052	LPL, MVK, NOG, ACVRL1, ACYP2, ADSL, AGTR1, ALPI, AOC3, BMPR2, CHEK1, CHKA, CSF2RA, DAPK2, DHCR24, EDN1, EHMT1, EPHX1, FZD2, GNA15, GRB2, GRIN2B, HTR4, IL21R, IQGAP3, ITPKB, MCAT, MYLK3, NPR1, NR4A1, OR4Q3, OR6C75, PDGFRB, PDK1, PIP4K2A, PLOD3, PRKAR1A, PRSS3, RASSF1, RFNG, RPSA, SELE, TAS2R16, TXNDC12, ULK2, WASL
Negative_7053	BTK, CBL, LPL, OAT, TEC, AANAT, ACVR2B, ADRBK2, ALG10B, CACNA1E, CHST12, COL1A1, CYFIP2, DDX23, DGKD, FGF13, GRIN1, IFNG, KITLG, OR11G2, OR13C9, OR4X2, OR51S1, OR5M3, RAB23, RAF1, TLN2, TNFRSF13B
Negative_7054	RB1, VCL, ADH6, ARHGEF4, BPNT1, CACNB4, CASP10, CDC20, DGKB, DNMT3B, EXT1, FARS2, GNRH1, HRH1, HSP90B1, ITGAM, MAPK13, MIOX, MYD88, NPR1, OCLN, PAK2, PLA2G2A, POLR2E, SFRP5, SMAD1, SMARCA5, SSH2, STX18, TAS2R7, TOMM40, TUBB2B, YWHAH
Negative_7055	CD4, ID1, MOS, ACP5, ANAPC7, ARPC5L, ARRB1, BAIAP2, BCAR1, CD82, CHPF, CTF1, CXXC4, CYP26A1, EDNRB, F2RL2, FTH1, FZD2, GOSR2, HARS2, ITPA, KIR2DL4, MCM5, MTHFS, MYH14, OR10G3, OR51B2, OR56A1, P2RX5, PIK3C3, PLA2G2A, POLR2F, PPP2CA, PRPH2, RAPGEF3, RASGRP4, RDH11, RHOA, SLIT1, SMURF1, SSH2, TAS2R13, TIAM1, TJP1, TJP3, UGCGL1, VAV2
Negative_7056	LAT, OSM, ADRA1B, ASMT, COX15, CREB3L3, CREB5, CYP4F3, DTX1, EFNB2, HSD17B4, MKNK1, OR4K13, PPM1B, PTEN, RXRA, UBE2L3, UGT2B28, WNT10B
Negative_7057	CFI, DBI, DHH, PLG, TKT, ADSL, AMHR2, BLVRA, CD22, CLDN22, CMAS, ENPP3, FZD5, FZR1, GBGT1, GNAL, GNAS, GNB3, IRAK3, MYH7, NUDT5, OR14C36, OR1D2, OR1E1, PDGFD, PLAU, PNPLA3, PPA2, PPP3CB, PVRL3, RUNX1, SFRP4, SUOX, TAS2R1, TGFB2, TJP1, UGCG, XYLB, XYLT1
Negative_7058	AGL, BAX, F12, LEP, ACAD9, ACTG2, ADRA1B, ADRA1D, CYP17A1, CYP2E1, CYP2F1, FGFR4, GCNT2, IL17RA, MAP4K3, NEFH, OR1L6, PDE7A, RASGRP1, RRM2, SGPP1, PLA2G12A, PRICKLE2
Negative_7059	TNR, ATN1, EXT2, LDHC, PDCD1, RFWD2, SDHD, SMAD1, TMLHE, PLA2G12B
Negative_7060	CBL, APAF1, APOA1, CYFIP1, FLT3LG, GADD45B, HCLS1, INHBA, KLRK1, OR10G4, OR2F2, PAK2, POLE2, TRPC1, TUBA3C, WBSCR22
Negative_7061	HYI, ABLIM1, ACSL3, ADORA2B, AKR1B1, BCKDHA, CBLC, CD40, CYP2J2, GNG3, GUCY1A2, NOTCH3, RBX1, RRM2B, SH2D1A, TMLHE
Negative_7062	A2M, C2, LTB, AADAT, ACSM2A, ACSS2, AMY2B, CACNG1, CCL15, CCL3, CD40LG, DBF4, DGKH, GGT6, GRIA3, HADHA, HTR2A, IFNA10, IL22, NFASC, NT5M, OCLN, OR11G2, OR4F17, P2RX4, PARD6G, PDE7B, PPCS, RBX1, SRD5A1, TCIRG1, TP53I3, UCK2, YWHAZ, SERPINC1
Negative_7063	EPHA2, NAT5, SFRP4
Negative_7064	SRF, ALOX12B, APPL1, BMP5, CACNA1S, CDH5, CTSS, EXT1, FCGR2B, FLAD1, IL17B, IL24, MAN2A1, MGAT5, NFKBIB, OR2B6, OR2G6, OR52I2, PI4KB, PLCD4, PTPRB, PVRL2, TGFB3, TPI1, UPP1, MAP3K7IP2, TNFRSF12A

Negative_7065	C5,FN1,HK1,RET,SCD,ACADVL,ADAM10,AMHR2,CAMK4,CHST12,COASY,CTTN,CYP2C19,DIAPH1,DTX4,GRIN2B,HHIP,MAT2B,MECR,MMP14,MTMR2,NNMT,NT5C1A,NTF3,NUDT8,OR14A16,OR1N1,OR4D9,OR52K2,PDE4C,PIAS2,PLA2G10,PLXNA2,POLR1B,PTK2B,PYGL,PYGM,QARS,RPN1,RPS6KA2,RUVBL1,SEMA3F,UBE2L3,UBE2L6,VTI1A,WASL,ARHGEF12,HLA-DPB1,TNFRSF10B
Negative_7066	CFI,GPT,MAX,ACY3,ARAF,BCAT1,BDKRB2,BMP5,DDHD1,EXTL3,FCER1G,HMOX2,IL1R2,LPAR1,OR10A4,OR11H1,P4HA3,PIM2,PNPLA4,POLR2G,RND1,TAS2R10,TAS2R4,TPH1
Negative_7067	HGD,ID1,PGD,ADH7,AFMID,ALOX15B,ALPP,ATP5B,CACNG5,CD3E,DGAT2L4,DPYS,FDFT1,FUT5,FZD5,GLUD2,HSPA2,IGSF5,IL20,KCNJ11,LRP5,MGST1,NLGN1,OR10J5,OR13C4,OR4D9,OR51G2,OR52I2,POLR1A,PPP1CC,PRKCD,RPS6KA4,RUVBL1,SGMS1,SLC25A4,STT3B,SULT2A1,VNN1,MAPKAPK5,PHOSPHO1
Negative_7068	ADO,ME2,ADH7,ALAS2,ALDH3B1,C4BPA,CCR6,CREBBP,DEGS1,EXT2,FZD8,GLDC,IFNG,IFNK,JAM3,MAP3K3,MCM5,OR1N2,OR3A1,OR4D1,OR51F2,OR5T2,OR7D2,OR7G3,OR8G1,OR9Q2,PANK1,PITX2,PPA1,RAB5A,RAPGEF1,RARS,RASGRF2,SEMA3A,SESN3,SOD1,ST3GAL6,STMN1,STX12,SULT1A2,TAS2R13,TGFB2,TSHR,KIR2DL5A,TNFRSF12A
Negative_7069	C9,PGD,ACPP,APPL1,CLDN3,CTSG,CYP2E1,FABP4,GNA12,MAD2L1,MDH1,NAGLU,OR2G2,OR2K2,OR6B3,PARVG,PFKL,PGK2,PRIM1,PRPS1,PTPRZ1,SCIN,SLC27A4,THTPA,TRADD,VAV1,XIAP
Negative_7070	ADO,HK3,PML,PVR,TAT,ACSM2A,AMPD2,ATP5I,CCL16,CD55,CES7,CLDN10,CYP27A1,DGKB,FUT9,FZD8,HTR2C,IL21R,MAML1,OR2V2,OR52I2,OR5AS1,P4HA1,PIGX,RAP1A,RFX5,ROBO3,SEMA4B,SH3BP2,SMARCA5,STX6,ZIC2,ATP6V1C1,B3GALNT1
Negative_7071	F9,CRLF2,GOT2,GRM1,GZMB,IRF9,OR2T1,OR5D18,PEMT,PGAM2,RAPGEF2,SRD5A2,TBXAS1,ST6GALNAC3
Negative_7072	DDC,AHCYL2,ANAPC2,ARD1A,ARPC1A,B3GAT3,BCKDHB,BIRC5,CFTR,COASY,CTLA4,DDX52,EHHADH,F2RL2,HCST,IDI2,IL21,INPP4B,IRAK4,KARS,KIR3DL1,L1CAM,LRRC4C,LYPLA2,MAP3K13,MDH2,NEU4,NFATC1,NRXN3,OR2M2,OR2Y1,OR51V1,OR5AP2,P4HA2,PAK7,PFKFB3,PGS1,PIGS,PLCB4,PRKCQ,PROC,PYGL,STMN1,SUCLG1,SUV39H1,WWP1
Negative_7073	CD46,HTR7,LYZL1,OR5T2,PDE6H,SGPP2
Negative_7074	LSS,ACP6,AKR1C3,ALDH1B1,ATP5E,B3GALT5,CD28,COL3A1,EP300,FZD4,GCNT2,GNAS,GNG13,HBEGF,LEPR,MYH11,NADSYN1,NCOA4,NFYA,NTN1,OR11L1,OR1G1,OR5AU1,OSMR,PIP4K2A,POLR3B,RASSF5,RPS6KB1,RPS6KB2,TUBB6,UGT2B17,HLA-DQA2,SERPINB5
Negative_7075	B3GALT4,CYP2A7,GUCA1A,MPDZ,NCF2,OR10T2,POLR1A,PRPH2,SH2D1A
Negative_7076	ASCC3,CD82,COL4A4,FGF19,TAS2R49
Negative_7077	FST,OTC,ABLIM2,ACAD8,AGPAT6,ALG12,BAAT,CCNA1,CD40,CDC25B,CTSL1,CYFIP2,ENPP2,ENTPD1,FZD1,GALM,GBE1,GMPPA,GRM5,HSD17B1,IDH1,IL21,IPMK,NANP,OR1J4,OR2AG1,OR4N2,OR51F1,OR52N5,OR9Q2,P2RX6,PLAUR,PLCD3,POLD2,PRPH2,RBL1,SEMA3G,SGMS2,SPRY4,ULK2,VAV2,CACNA2D3
Negative_7078	FER,SRM,AQP7,ERO1L,GDF6,GRM1,GSTM2,IL9R,NEFM,NEU1,NOTCH1,OR2T5,OR6K6,OR9Q2,PNLIP,PNPO,PPP2CA,PTPRC,SSX2IP,STT3B,TUBA3C
Negative_7079	IL8,MVK,ACY3,APLP1,ASS1,CD226,CD8A,CES2,CHDH,CLTC,COMT,CRB3,DEGS2,ECHS1,GNAI3,MITF,MYH9,MYL2,NEUROG3,OCLN,OR51L1,OR5C1,OR7A10,PARVA,PIP5K1B,PLCG1,PPP3CC,PRKAB2,RASGRP1,SDHC,SLC12A2,SMAD2,SMPD2,SNAP23,UPRT

Negative_7080	AK1,AMH,GLS,KHK,ABL1,ACOX2,ALDH1B1,ARHGEF7,ASCC3,BCKDHA,BDH2,BHMT,BMP6,CCL2,CCR7,CXXC4,CYP26C1,DGKE,EDA2R,FGF17,FTCD,FZD10,GBE1,GPD2,HEXB,HIBADH,HLA-DRA,KIR2DL1,LTC4S,MCM5,NCF1,NUDT9,OR2M7,OR5AU1,PANK1,PFKL,PPP2R5C,RAPGEF4,SHISA5,SYNJ1,TAS2R41,TSHR,TYROBP,ZNRD1
Negative_7081	CGN,ADH5,AGPAT2,ARG2,BMP5,CPT1B,GOT1,IL18RAP,MTFMT,OR51A2,PLA2G2A,SMPD4,SULT2B1,TCF7L1,TNXB,SERPINB5,ST6GALNAC3
Negative_7082	AGL,DAO,EBP,FER,IL4,ACAD9,CLCA4,CRKL,FASLG,GDE1,HNF1A,IFNB1,KNG1,MCCC2,MDH2,OR10K2,OR1A1,PHKG2,POLD2,PRKACB,PRKCA,SMAD1,UBA7,ATP6V1B1
Negative_7083	CBS,ID2,ACADM,ALDH1L1,ALDOC,ATF4,CCL26,CD22,CFTR,CYSLTR2,FECH,GDE1,GPD1L,GYS1,INPP4A,KIR3DL3,NRG4,OR10A5,OR5F1,OR7G3,PAK1,PFKFB3,POLR2J,POLR3D,PRKACB,PROS1,QPRT,SLIT1,SPRY4,SSH1,TSHR,SUV420H2,TNFRSF13C
Negative_7084	GRHPR,HMGCS1,OR4D2,PPP2R5C,PPP3R2,TRAF1,TUBB2C
Negative_7085	DUT,PGP,SFN,ADIPOR1,CACNA1H,CACNG7,CAMK4,ESAM,EXOC7,GUCY1A3,IL28B,ITCH,OR6M1,PHKG2,TAS2R5,WNT2B,WNT3A
Negative_7086	NNT,PLG,ACADSB,AKR1C2,ALDH7A1,APC2,BNIP1,CD40,COL4A2,CYP21A2,EDNRA,IARS,IL9R,LYZL1,MAN1A2,MAP3K8,NANS,NKD2,NLGN2,OR10J5,OR2A12,OR7D4,OR9Q1,PARVA,PPOX,PPP2R5B,PRKAB2,RAP1A,SETDB2,SHC4,SNW1,ST3GAL5,TCIRG1,PRICKLE1,TNFRSF12A
Negative_7087	STS,AARS,ANAPC11,ARRB1,B4GALT2,EPB41,GPT2,NLGN3,OR7D4,PNPT1,PPP2CA,PRKAR1A,PRPS1,PTDSS1,PTPMT1,TUBA4A
Negative_7088	AK3,BCR,ACOX2,ADRB3,ARG1,ASPA,CD36,COL5A3,CSF3R,GNAS,HEMK1,IKBKE,IL22,INPP5A,MAN1B1,MAP2K6,MDM4,OR2AG1,OR6K6,PANK4,PGM3,PISD,PLCB2,PLXNB2,PMM2,PPARD,PPP2R5A,PRLR,RAPGEF2,RASSF1,RBX1,RGS3,TAS2R14,VAMP3,YWHAQ,ARHGEF12,SERPINA5
Negative_7089	GH2,AARS2,ACTN4,ACVR1B,ACYP2,ALG10,CD58,CDC6,CDKN1B,CDO1,CHRM5,CNGB1,COL5A2,CYP2C19,CYP2E1,DAAM1,DGKQ,EARS2,FGF4,FPGT,GALNT7,GLO1,GMPS,HEMK1,HRH1,IFIH1,IL2RA,ITGB2,MCM4,OCRL,OR14J1,OR2T4,OR4C15,OR8J3,PGS1,PMAIP1,PPAT,RUVBL1,SNAP23,SRGAP3,STEAP3,TYMP,UGT2A3,UGT2B28,UGT2B7
Negative_7090	BTK,ILK,PTS,ABLIM1,AGPAT6,ARNTL,ATP5A1,CCL26,CCR3,CD19,CD3E,CSNK1G3,CYP11A1,E2F1,ELK4,EPHB1,EXOC7,F13B,FOXA2,FPGS,HIF1A,IRAK4,ITGA6,MTHFD2,MYH6,NQO1,OR1L6,OR1N1,OR4C12,OR4K14,OR7A5,PAK1,PDE5A,POLR2L,PRKCA,PTCH2,RAP1B,RERE,RHOA,RPIA,SDHC,SGPP1,SLC33A1,SPP1,SSH3,STX10,TKTL2,XYLT2,ST6GALNAC4
Negative_7091	MPI,ARNTL,CBLB,IL4R,LCAT,NKX6-1,NPR2,PARVA,PLCG1,PPP3CC,STX1A,TNFSF8,UGCG
Negative_7092	FER,AKT2,CALML3,CDH4,CDKN1A,CPS1,CSNK1G1,CUL1,EDAR,ENTPD7,HADH,MAPK8,OR13C4,OR5H2,OR8D1
Negative_7093	MIF,ACSL5,CARS,ESAM,H6PD,IRS1,KIR2DS4,OR2D2,PFKP
Negative_7094	ABAT,ACVR1,AKT3,ALDH3A1,CCL13,CD48,CER1,EI24,GALNT9,GSTM5,IFNB1,IFNGR2,IL12RB2,OR10X1,OR5A2,OR6C75,PIK3C2G,PLIN,RPN1,TLR9,TNFSF14,UPP2,VAMP3
Negative_7095	AK1,C5,ADIPOR1,APC2,ATP4A,ATP5G1,AXIN2,CACNB3,CCL19,CCND2,CD40,CDC23,CDKN2C,CXCL16,DPYS,EPHA3,FLT3,FRAP1,FUT4,GADD45A,HEXB,IDI2,ITGAX,MBL2,NCAM1,NEGR1,OR10S1,OR1N1,OR4L1,OR9G4,PDHA1,POLR2A,PRKAB1,PTPN11,ROCK1,SARS,ST8SIA5,STAT5B,SULT1A2,TAS2R7,TSTA3,PAFAH1B1
Negative_7096	AADAC,ACSL1,ALS2,C1QA,CCL11,CLDN1,CSNK1E,E2F5,EHMT1,HBEGF,IL11RA,MCM5,MTMR1,NANS,OLAH,PGS1,PISD,PPID,PTPRC,RASA2,RASGRF2,RRAS,ST3GAL4,SUV39H2,TNFSF9,UCKL1

Negative_7097	DUT, F9, GPT, IL2, ACAA1, ATF4, CCL23, CHRM5, DPM2, FGF12, FRAT2, GRIA1, HRAS, IL17RB, LILRB3, LTB4R2, MDH2, NARS, NRCAM, OR2A4, OR2F1, OR5A1, OR6C2, PIAS3, PPP3CB, STAT1, TAS1R3, UGCGL2
Negative_7098	C8A, ACSL4, ALDH1A3, CACNA1F, CACNB2, CDC25C, CDC45L, DNMT3L, DPM3, FGFR1, GNB3, HIP1, HLA-DOA, IL3RA, NCOA4, NRAS, OR2T12, PAK2, PDE1A, PRKAA2, SDHD, SESN2, STX4, TALDO1, UBE2K
Negative_7099	A2M, CR2, IL6, ACSL4, APOE, ARPC5, B4GALT6, BCL10, CACNG6, CHPF, CLDN6, CLTB, CMPK1, CYP11A1, CYP2A13, DDX56, DERL1, ENTPD8, FMO2, GAMT, GSTM3, IRF5, LGMN, MAOB, MKNK1, OGDHL, OR56A1, OR7G3, P4HA2, PLXNB1, PPAP2A, PTPRF, SDHA, UPRT, WASL, WNT2, XCL1
Negative_7100	FER, AVPR1B, B3GAT1, BCAR1, DHCR24, FTCD, GMPS, HNMT, HRH2, LEFTY2, PAK6, PARD6G, PLAU, SMC1B, SPCS1, TUBB4
Negative_7101	IDS, KHK, XDH, ACOX1, ASRGL1, CBLB, CCL4, CDKN2B, COQ3, CTF1, DGUOK, DTX2, EPHX1, INPP5E, KDSR, MIOX, MYH11, NR1H3, OR2T2, OR4F15, OR4X1, OR51A7, P2RX2, PDE8B, PLD1, PVRL1, SMURF1, SPTLC1, TNFSF11, VDAC1
Negative_7102	C8A, HAL, RB1, SRF, AHCYL1, AMDHD2, AMHR2, ARNTL, CD247, CD274, CES2, COL11A1, COL4A4, CSNK1G2, CYP2C8, FARS2, GNB1, HDAC2, HEXB, HLA-DRA, HSPB1, IARS2, IL13RA1, IL17A, IL17RB, IRF5, LCMT1, LYPLA2, MAGI2, MAP3K8, MARS2, MGST2, MTHFD2, OR2T33, OR4N4, OR6K2, P4HA1, PDE8B, PPP3CB, RASA2, RBL1, SDHC, SHISA5, SMARCA5, TRMT11, ULBP3, UNC5B, CACNA2D3, SERPING1
Negative_7103	BTC, RB1, VTN, ABCC8, ACSL5, ACTB, CANT1, CCR8, CDC42, CDKN1A, CNDP1, CPT1B, CYP2A7, DKK2, EPHB4, FDFT1, FZD6, GALNS, GSTZ1, IKBKB, IL12B, IL20RB, ITPKB, MAD2L1, MAML2, MAPK1, OR1M1, OR2L2, OR56A4, OR5H6, OR6C4, OR6C74, PDE9A, PIK3CA, POLR1E, POLR3D, PPP2CA, PTPRJ, RASGRF2, RPN1, UGCG, XCR1, ATP6V1B1, CSGALNACT2
Negative_7104	IL4, ADRA1D, ADSL, ALDH3A1, ASIP, ATP6V0C, B4GALT4, CAMKK2, CD27, CDKN2A, CNTNAP1, CXCL10, DDX51, DNMT3L, EI24, ERO1L, GLI1, GLI3, IARS2, IL28B, IL29, IL5RA, INSR, IRS2, NKD2, NRG3, NUDT8, OR1L1, OR2M3, OR4D11, OR52D1, PARD3, PDE1B, PER3, PFKM, POLD1, PTK2B, PYGB, RDH5, SPRED1, SRGAP2, TGFBR1, THBS3, TRMT11, TUBA4A, WNT2
Negative_7105	AKR1B10, ATP2A3, BTRC, IL17A, NRG1, PAK2, PTPN1, SPHK2, PPP1R12A
Negative_7106	ASL, C2, FAH, FGG, MVD, PLN, ACVR2A, AGTR1, BLVRA, BMPR2, DGKG, DHDDS, DTX3, EPOR, GAPDH, GDF5, GLO1, GNPNAT1, GPD2, GUCA1B, IFNAR1, IFT57, IL20RB, LAMA4, OR10G9, OR10T2, OR2A2, OR4P4, OR51V1, OR5D14, OR6C2, OR8B8, P4HA1, PAICS, PDE3A, PFAS, PLAU, PPP2CA, PPP2R2D, PSPH, PTK2B, RASA1, RFNG, SSX2IP, TACR3, ULBP3, VEGFB
Negative_7107	CBL, CD40LG, EPHX2, GNG3, LRDD, OR5K2, PIK3C2A, ATP6V0A4
Negative_7108	DAO, ACVR1B, AGPAT2, ANGPTL4, CASP3, CLDN4, COQ6, CXCR4, ENO3, ENTPD8, FOXA3, GUCY2D, HTR7, IL17B, IL17RA, NKD2, NUDT9, OR13A1, OR1E1, OR1L4, OR52J3, OR6T1, OR9I1, PIGP, PON1, PSPH, PYGM, QPRT, SLC27A4, SMAD5, TLR9, TPK1, UCP1, UGCG, WNT10B, ATP6V1C2, B3GALNT1
Negative_7109	AK1, DHH, GH1, ACSL3, ADH1A, C3orf10, CACNG1, CD80, CNTNAP1, EHMT1, EPHA3, FARS2, FGF12, ISYNA1, ITPKB, MAP2K4, MYH2, MYH3, OR1A1, OR1S1, OR2T34, PHKA1, PLCD4, PRKCZ, PTPN7, SC5DL, TAS2R3, TBXAS1, TIAM1, TYROBP, VAMP2
Negative_7110	DDO, NGF, OGT, ACOX1, ADH4, ADORA2A, AKT2, B3GAT3, CYP11B1, DRD5, HTR4, IARS, NCKAP1L, OR2A25, OR4K1, OR5L1, OR8J3, PGM3, PLD2, PTGES, RAPGEF2, SDHA, SOX17, SULT1A4, TJP1, TLR9, TNFSF11, HLA-DPA1
Negative_7111	MOS, MAPK8, PTGER3, PTTG1, TAS2R8, TCIRG1
Negative_7112	LPL, AGMAT, AMPD3, CD82, CHUK, CSNK2B, ENAH, FCGR3A, GALNT11, HSD11B1, LGMN, MGAT4B, NFYB, OR10G7, PDE7B, SEMA4F, SMPD3, SOCS4, ATP6V1B1, CSNK1A1L, TNFRSF1B

Negative_7113	GP9, AARS2, ACADVL, AHCYL2, ATP6AP1, C1QA, CD74, COL5A3, CYP4A11, ENPP1, FMO2, GLYCTK, IMPDH2, LAMB4, NCR1, NRG3, OR1L8, OR4K5, OR6N1, P2RX6, PHKB, PLAUR, POLA2, PVRL1, SOAT2, TAP2, TICAM2, TP73, TSLP, PLA2G12A, PLA2G12B, ST6GALNAC4
Negative_7114	CD4, ACSM2A, ACTN4, ALDH7A1, ARSB, ATP4B, ATP6V0C, BIRC2, BTRC, CCR5, CDK5, CHPT1, GNMT, ICAM3, IMPDH2, INPP4B, MTMR2, NCF2, NCK2, OR1E2, OR51L1, OR56A3, OR8U1, ORC1L, ORC6L, PNMT, POLR3B, RALBP1, RASSF5, RCHY1, RRM1, SHC4, SHMT1, SUFU, TAS2R16, TUBB2A, UGT2A3, UPB1, VAMP4, WASF3, ZBTB16, ATP6V1G1, HLA-DQB1
Negative_7115	PTS, CDK4, CTF1, GGT1, PHOSPHO1
Negative_7116	MVK, NGF, PTS, ACSL4, ADAM17, ADSL, ANAPC10, BDKRB1, BDNF, BRAF, CCND1, CDC14A, CYP2C18, DUSP2, FCER1G, GBE1, GNRH1, HHEX, HMBS, IFNA2, IL6R, MYH11, NNMT, OR2Y1, ORC4L, PAPSS1, PARD6B, RAPGEF2, SDC1, SELE, STX5, TIAM1, TRPC1, TSC2, TUBB1, MAP3K7IP2, MAPK8IP1
Negative_7117	GK2, IVD, UBB, ABL2, AKT2, ALDH5A1, APOE, AQP7, ARHGEF7, ARSA, BLVRB, C4BPA, CDC42, CHPF, DGKA, DGUOK, DHRS9, DPM1, EXOC7, FGF10, FUT5, GALNT10, GGT7, GP1BA, GRIA1, HPSE2, IL20RA, ITGA6, ITGAM, LEF1, OR5R1, ORC2L, PAPSS2, PARD6A, PDE1C, POLR3G, PRPS1, PVRL3, RHOQ, SETDB1, SMAD2, TAS2R38, TAS2R42, UGDH, WNT2B, WNT3, CACNA2D1, TNFRSF1B
Negative_7118	ACSL5, ARRB1, ATP4B, CCL2, CYP19A1, CYP1A1, HARS, IL10, IL21, IL8RA, OCLN, OR6C1, RARA, UCP1, WNT4, ATP6V0A4, TNFRSF14
Negative_7119	ACO1, ACSL5, BBC3, BCL10, CAMK2A, GBGT1, GNA15, IL5RA, ITGB6, LYZL1, OR51S1, PGK1, PI4KB, PTGES, PTPN1, RFX5, SH3BP2, TBL1Y
Negative_7120	C1S, NGF, AKR1B1, B3GNT2, BLNK, CALML6, CD79B, CHPT1, CKMT2, CTSB, CTTN, CXCR4, CYP1A1, DMGDH, DPM3, ENTPD5, EP300, EXTL3, FECH, GLB1, HCST, HSD11B2, IFNW1, ITCH, ITPKA, LIMK2, MCM2, METTL6, MYL7, NANS, OR14A16, PAK4, PDE3A, PDXK, PIGK, PLCB4, PNPLA3, POLR1C, PPP2R1A, SOCS4, STX8, UGT2B4, ULBP3, PPP1R12A
Negative_7121	APLP1, G6PC, HSD17B4, OR2AE1, OR52N1, SIAH1, SMPD2
Negative_7122	DHH, KIT, ADCY7, B4GALT1, CACNA1E, CCL20, CLTB, CPS1, CSH1, CYP26C1, DFFA, DPYSL2, DUSP16, EPHA2, FGF6, GGT5, GOT1, HLA-DOB, IL2RG, KLRC4, LALBA, MAT1A, MCCC2, NAMPT, NCKAP1, NT5C2, OR4F4, P2RX4, PPP1R3B, RPS6KA4, SEMA3D, SYNJ2, TAS1R1, TPH1
Negative_7123	AK1, CRH, ADAM10, ADH6, ALAD, BCAR1, CCL8, CDKN2D, CER1, CFL1, CHPF, CHPT1, CHRNA7, CTNNA3, CYP26C1, CYP2C19, ERBB3, FUT1, GNPDA2, HIBADH, IDI1, LDHAL6B, MYLK3, OR1S1, OR2Y1, OR9K2, ORC4L, PARD6G, PIK3R3, PPP1CC, PPP2CA, PPP2R5C, PRKX, ROBO2, RRAS2, SETD7, SYNJ1, TAS2R10, ULBP1, WBSCR22, ST6GALNAC1
Negative_7124	C9, HK1, TNC, ACTG1, B3GAT3, BCAT1, CADM1, CCR5, CD59, CSF2RB, CSNK1G2, DLL4, ENO1, HTR4, IKBKE, MYH15, NCOR2, OR4K14, OR5B17, OR5D14, OR5V1, PDX1, PIK3C2G, SETD2, SPRY3, ST3GAL2, TAS2R3, TGDS, TP53, TRADD, PHOSPHO1, ST6GALNAC6
Negative_7125	IL6, MTR, ACVR1, ADRB3, ALDH1A2, ALDH4A1, ANPEP, ATP5B, CYP2B6, DLAT, DUSP3, IL5RA, IMPDH1, INADL, MCM2, NCOR2, NRP1, OR14J1, OR51D1, PIAS1, SNAI2, VARS, VEGFA
Negative_7126	ALAD, AOC2, APC2, ATG3, BCKDHA, CACNA1E, CD79B, CD80, COL5A1, CSNK2B, CTPS2, DDOST, DDX50, FGFR3, FLOT2, FUT5, GALNT3, GNPDA2, IFNB1, IL1RAP, INPP5A, MCM7, MKNK2, MYLK2, NCOR2, OCRL, OR1Q1, OR2A5, OR4D5, OR4K13, OR52E6, OR56A3, OR9Q2, PTPRZ1, PVRL4, RASA2, RASGRP1, RGS3, SNAP25, TAS1R3, TGDS, TGFB1, THBS2, VEGFB, TNFRSF10A
Negative_7127	PPP2R5D, RAB23, HLA-DRB4

Negative_7128	ACACA, CD36, CD82, CKS1B, CTNND1, DRD2, DVL3, EFNA5, FTMT, GABARAP, GLB1, ICOSLG, IFNGR1, IL8RA, ITGAL, ITGB3, KAT2B, LAMC2, MYH7B, MYLK, NFKB2, NR1H3, OR1N2, OR2M5, OR8K5, PARD6G, PLXNA1, POLD4, POLR2J, PPT1, RHOA, RXRB, SIPA1, STX1B, TJP1, TNFSF14, TNXB, WNT3, CACNA2D3, MAPK8IP3, MAPKAPK2, TNFRSF11A
Negative_7129	AMT, FGA, ACP1, ACTA2, ATP5F1, BCL10, CACNG6, CCL7, CDH2, CKMT2, COMP, CYP3A5, DHCR24, EFNB3, ETS1, EVI1, FGF23, FUT9, GANC, GJD2, GNAZ, GRM5, HTR6, IL2RB, ITGA3, NCOR2, NRP1, NT5C3, OR10C1, OR5AK2, OR5M3, OR8K1, PDE11A, PLCD3, PPP2R5B, PTGS1, RAC2, RASA2, RASSF1, SPCS3, STK3, SUFU, TCIRG1, TNFSF18, AASDHPPT
Negative_7130	C8A, GH2, GLA, ASIP, ATF4, CLDN11, CTNND1, DTX3, FGFR3, GALNT14, GMPR, GNA15, IL11RA, IL12RB1, INPP5B, IRS1, LALBA, MAT1A, MIOX, NEU1, OR2F1, OR4C16, OR5AS1, OR5H6, OXCT2, PGS1, PIGA, POLE2, PRDX6, PRODH2, SLC27A4, SMAD3, SMAD5, SNW1, UBE2L6, ATP6V1C2, CTNNBIP1, PAFAH1B2
Negative_7131	DHH, F12, ADCY1, AOC2, B3GALT1, BUB1, CNTFR, CSNK1D, DDX54, FLT4, GALNT12, GALNTL4, HRAS, NRAS, NRG2, OR10J5, OR8I2, P2RX5, PIGS, PLXNB1, PRKAA1, SCP2, SREBF1, YWHAZ
Negative_7132	BTK, HK3, ID2, SI, ANAPC10, AQP7, CMPK2, DKK2, DUSP3, GNAI1, H6PD, ITPK1, MAP3K7, MAP3K8, MAP4K2, NCF2, NFKB1, OR2A42, OR5AU1, PLCD1, SC4MOL, SLIT2, TNFRSF8, TNFSF4
Negative_7133	ADH1B, AOC3, APRT, BCAR1, CD59, CHRM5, COMT, CTNNA1, FGF22, GMPPA, IRF5, KIR2DS5, OR5B21, OR5D16, PDE6D, SEMA4D, SMAD7, TAS1R1, TBL1X, TGFA, TNFRSF9, UBE2L3, UGCG, UROS, ATP6V1G1, ATP6V1G3, PLA2G12A
Negative_7134	ID2, BUB1B, CBLB, CCL25, CDC14B, CHKB, CLTA, FGF17, GANC, GNRHR, GUCY2D, MAPK1, MYH10, OR10G8, OR14A16, OR2T1, OR2T29, OR52N4, OR6F1, PIK3C3, PRKAA1, RBKS, TAS2R50, TUBA1C, XYLB, ALDH18A1
Negative_7135	AK3, BAD, BID, CTH, KDR, MTR, AASDH, ADAM17, AKR1C1, AKT2, APOA2, ARPC4, ATP6AP1, C9orf95, CCND2, COL11A1, CYP2F1, DDX56, DGAT2, GNAO1, GRIA3, ITGA5, LCP2, MAPK7, NCKAP1, NFASC, NGEF, OR2D2, PDE6H, PIK3CA, PKM2, PPP2R5A, SUFU, PDCD1LG2
Negative_7136	LSS, ALG8, ATP5L, BMP4, BTRC, CPT1C, CRLF2, CSDA, CTNNA1, CTNND1, DGKA, EARS2, GOT1, GSTO2, HLA-DMA, LCAT, MGAM, MYH7, OR2T11, PRKAR1A, PSEN2, PVRL4, STX8, TDO2, HLA-DPB1
Negative_7137	ADO, FN1, IL8, VTN, XDH, ACSL4, ADSSL1, ALOX15, ANGPTL4, ASCC3, CACNA1D, CFL1, CSNK1D, CTF1, DDX56, DPYD, ERBB4, FDPS, GARS, IMPDH1, NADK, NEU4, NFATC2, NSDHL, OR1M1, OR51B6, OR6A2, PKM2, SH2D1A, SHMT2, SUV39H2, TNFRSF17
Negative_7138	CYP7A1, FUT8, FZD10, IL3RA, OR10J5, OR4C15, OR5B3, P2RX2, RPS6KA3, SELPLG, SHC4, SLC27A4, TAS2R41, UPB1
Negative_7139	B3GALT5, EHMT2, OR8J3
Negative_7140	ALAD, ALDOC, APOC3, ATP4A, CCL16, CNTN2, CNTNAP1, COMT, ENTPD2, GSTT1, IRS2, LEFTY1, LPAR1, NOD1, OR51L1, PCCA, ZNRD1
Negative_7141	DGKB, YWHAB, MAPKAPK2
Negative_7142	C8B, PML, ACMSD, ADIPOR1, ALOX15, ANAPC4, CCL17, CD28, CDS2, CHRM1, COL4A6, CXCL1, CYFIP2, DDX18, DGKQ, DHCR24, FGF3, FSHR, FUT1, HAGH, LYPLA2, METTL6, MRAS, NTRK1, OR10AD1, OR1E2, OR2T1, OR2T12, OR51L1, OR52A1, PDE8B, PIK3CA, PLAUR, PLXNA3, PPM1B, RASGRP3, STX12, TGFA, UGT2B7
Negative_7143	CKM, PTS, ACSM1, ACVR2B, ADSSL1, CACNG3, CISH, COL6A2, CYP27A1, DIAPH2, DPYS, EPB41, G6PD, GSTA3, IL17RA, IL6R, KIR2DS4, MGAT5, NCAM2, NCK2, NRXN3, OR14A16, OR2G6, OR6B3, PIK3CG, PIP4K2C, POLR2I, PRPS1, RAC1, SLC27A1, SOCS3, TALDO1, PAFAH1B1, TNFRSF10B

Negative_7144	TFG, XDH, AGTR1, ALOX15, B3GALT1, BDKRB1, CHRD, EPHX2, GRM1, MAPK14, MCCC2, MYH7, NCK1, NOS3, OR13A1, OR1N1, OR5J2, PARVG, PDE4D, PIAS1, PIK3R5, PTCRA, PYGM, RPS6KA3, SC4MOL, SORD, TNFRSF4, TUBB3, UNC5D, YARS2
Negative_7145	AK2, CFI, DDC, MYC, ALG2, BCL2, CACNG3, CCL19, CCNE1, CHRM1, CHRNA7, CHUK, CLDN7, CSDA, CXCL9, DIAPH1, ENTPD8, FGF4, FLOT1, GALK1, GALNTL1, GGPS1, GPD1L, HSPA1B, HTR4, INPP4A, ITGB7, MAN2A1, MAP2K4, NAE1, NEU4, OGDH, OR2F1, OR2L13, OR52A1, PCYT2, PFKM, PFN2, PGAM4, PIK3R4, PLXNC1, POLR2B, TNFSF10, ATP6V0D2, TNFRSF10C
Negative_7146	CAT, DHH, FST, AOC3, BIRC3, CDH5, CDKN2D, CMBL, CXCL1, DDHD1, DDX50, DRD5, DUSP1, ECHS1, FMO4, HAGH, HTR2B, IRF3, NKX6-1, NOD1, OR2B11, OR52E8, OR5H6, OR5R1, PIGT, PLA2G5, PLAU, SDC3, TBL1XR1, TPMT
Negative_7147	TH, TNN, ADCY1, CD244, CD79B, GNAI3, GUCY2F, HEXA, IKBKG, IL15, JAK1, OR4F4, PLCZ1, PPP2R2B, PRKAB1, RHOA, ZBTB16
Negative_7148	OR10G4, PCNA, PGS1, PIAS3, PTGER1, STX4, HSP90AB1
Negative_7149	CDA, PGF, CLDN8, CYP2C9, FLT3LG, IL20, IL20RB, INPP1, NFYB, OR56A3, RAC1, RBL2, SERPING1
Negative_7150	CAT, CP, KMO, TH, ADSSL1, ALDH3A1, BCMO1, BLVRA, BPNT1, CARS2, CCND2, CDC23, CDIPT, CNDP1, CYP1B1, DUSP3, EFNA1, EVI1, FGF20, FZD6, GDF6, GMPPB, GNAO1, GNAZ, HMGCS2, IL8RA, LRDD, NADSYN1, NKX2-2, OR2H1, OR4K2, PLCZ1, PRKCZ, RBKS, SARS, SPCS1, STAT5A, UAP1, UBE2L3, UCK2, HLA-DRB3, SUV420H1
Negative_7151	CFD, IL4, NGF, ALG1, B3GAT2, CACNG7, CNTN2, DIAPH2, DUSP1, DUSP4, EPAS1, FECH, HNMT, ITGAE, MAML1, MTHFD1L, NEFL, NMNAT2, NUMB, PIM2, RPSA, TNFSF8, YWHAE, MAPK8IP2, PAFAH1B2
Negative_7152	BAIAP2, CTSL1, FCER1G, KNG1, VTI1A
Negative_7153	IL3, PVR, ALDH1A3, ALDOC, ANAPC11, ATP5O, ATP6V0B, B3GAT2, BIRC2, BIRC3, BMP6, CCL5, CYP26C1, DPYS, DTYMK, EIF4B, EPHA1, EPHA4, FGD3, FGFR3, FOXO3, GALNTL4, GCNT4, GRM1, INPP5B, IQGAP1, LILRB3, MKNK1, MYL9, NFATC4, OR10A4, OR4N2, OR7D4, PIGK, PLXNB1, POLR2A, PRSS3, TDO2, UROC1, UXS1, HLA-DQA2, HLA-DQB1, PAFAH1B1
Negative_7154	C6, LCK, AGPAT4, ALDH3B1, ARPC1B, ASCC3, BBOX1, BMP5, CACNA1S, CD74, CISH, CLDN3, CTBP2, CTSG, F2RL2, FCER1G, FGF7, GALE, GALK1, GRHPR, GUCA1C, HLA-G, IFNB1, IGSF5, IL13RA1, INPP5A, KCNB1, NEGR1, NUDT9, OR4C6, OR4S2, OR5B17, ORC4L, PCYT2, PDE7A, PYCR1, RXRA, SORBS1, STAT4, TLR3, UGCGL2, GABARAPL2
Negative_7155	PER3, PLCD4, POLD4, SFRP2, PPP1R12A
Negative_7156	B3GALT4, DCXR, ENPP2, OR2W1, OR5A2
Negative_7157	ADH6, CFLAR, EPHA8, FCGR3B, GRIN2C, HEXB, MAD2L2
Negative_7158	EPAS1, HPRT1, OR5I1, P2RX4, WNT11
Negative_7159	HDC, SRC, BCAT1, EDA2R, ENTPD2, ENTPD7, FZD5, IL12RB1, KLK3, PDE8B, PI4KA, PIGU, PTPRJ, RPS6KA4, SPRY3, TRDMT1, CTNNBIP1
Negative_7160	F2, FGA, HPD, NOG, ATP5B, CDO1, CLDN17, CRHR1, CTPS, ERBB2, GRM5, GSTZ1, MAD1L1, MDM4, OR10K1, POLR1C, PORCN, PPP2R1A, PPT1, SPAM1, TOMM40, UGT2B28, CACNA2D3
Negative_7161	LPL, ATP5G1, B4GALT2, B4GALT6, CAMKK1, CCNA2, CXCL9, E2F3, E2F5, EPHB2, ETS1, EXT2, FECH, GALNT3, GSTA4, KIR3DL1, MYH10, NT5C1B, OR10G3, OR1Q1, OR51I2, PARVG, RPN1, SOS2, UCKL1, UGT1A10, UGT2B7, VEGFC, WASF1, WHSC1, YWHAZ, PPARGC1A

Negative_7162	OAT, RET, AGPAT3, BAAT, BET1L, CACNB1, CALML3, CDKN1B, CHRM5, CMPK1, CSF1, CTNNA3, DGKD, DTX1, EPB41L1, FDFT1, FGF5, FGFR1, FGFR2, FLOT2, GADD45B, GAL3ST1, GNAS, GRIN2C, HARS, IL1R1, MAP2K1, OR6C74, OR6X1, PIK3CG, PPARD, PRIM2, RBL2, RPN1, ST8SIA5, UCP1, UROC1, HSD17B10, SERPINE1
Negative_7163	CCR9, GNA14, MGAT5, MYH2, RASGRF2, TM7SF2, ULK1
Negative_7164	F2R, GP6, TNF, ACACB, ALS2, ANAPC7, BUB1B, CCNB3, CFL1, DCTN1, EFNA5, ENTPD5, GNG13, HDAC2, HTR6, LAMC2, LIPG, MCM4, MPST, OR4P4, OR51F2, OR51V1, OR5M3, PERP, PIK3R3, POLD1, POLR2G, PPAP2C, PPP1R3D, PRKAG1, PRKCZ, PRKG2, RGS3, SDC3, SFRP1, SOCS5, SULT1A4, THBS4, TRMT11, TSTA3, WNT4, YWHAG, HLA-DRB3, MAPK8IP1
Negative_7165	FST, GRIN2A, HTR4, IFNA14, NFYA, NRXN3, OR4C15, OR8H3, SESN3, SPP1, ZIC2
Negative_7166	SI, ACVR1, AKR1C2, APLP1, AREG, CACNA1H, CDS2, CHSY1, CHUK, CLDN20, DVL3, G6PC, GFPT1, HSD17B1, ITCH, ITGA3, MAD1L1, OR1J2, OR2A4, OR2L2, OR4N5, OR52N2, PTPN7, RAPGEF4, RPS6, TAS2R38, UBE2K, WNT7A
Negative_7167	DUT, ALDOB, ATF2, CLDN17, CPT1C, DGAT2L4, DUSP1, EPHB6, GMPR, MECR, NKX3-1, NT5C3, OR1Q1, OR2M7, OR5M3, OR8B12, PGK2, RHOD, SHC3, TRIP10, ATP6V0A2
Negative_7168	APP, MVD, AASS, ACVR1B, APOA1, AQP7, ARSE, ATP6V1A, AXIN1, CASP6, CD86, DGKH, DTX3L, E2F3, EPHA6, FOXA3, GLI2, GNA12, IL13RA1, INPPL1, LIFR, MTFMT, OR10A5, OR10A6, OR4M2, OR5P2, PCYT1A, PLA2G2E, PNLIP, PPCS, RERE, RXRA, SMURF1, STAT4, TGFBR2, HSP90AA1, SERPING1
Negative_7169	CFH, F8, ACTC1, ADAM10, ATP5E, CD48, CDS2, CLCA2, CLOCK, DFFA, FDPS, INPPL1, MAPK9, ODC1, OR1E1, OR2T5, ST3GAL3, STAT3, TFDP1, YWHAH
Negative_7170	CGA, PAH, ACVR2B, ADCY9, ALG13, ARSD, ATP5G2, CACNA1H, CASP8, CCL13, DGAT1, DGKE, FLT3, GATM, HNF1A, HTRA2, IRF9, ITGA7, ITGAL, MMP7, MYL7, OR6C74, PCCB, PKM2, PLCD3, POLR1E, POLR2F, RCHY1, SESN3, THBS2, TOMM40, TPMT, TSNARE1, ZIC2, HLA-DPB1, PDCD1LG2
Negative_7171	HK1, ACTA2, ACVR1C, BUB1B, CCR2, CD40LG, DPM1, ETNK1, GPD1, HADHA, IFNA17, IL17RA, IL29, IRAK1, MYH9, NFATC2, OR2M7, OR5T2, PCYT2, PHKG1, PLA2G10, PTPN5, SETD2, SRD5A2, ST3GAL2, TAS2R1, TRAF4, VEGFB, WNT2, SERPIND1
Negative_7172	CRK, DUT, FAS, PRL, ADORA2A, ALG8, ANAPC11, B4GALT4, BIRC5, CACNA1C, CDKN1C, CEBPA, CLDN6, CTSL1, EFNA2, ENTPD1, GALNT1, GNAZ, IL22RA2, LAMB4, LLGL1, OR13C5, OR4A16, OR4K14, OR51S1, OR52N5, OR56B4, OR5AU1, OXSM, P4HA3, POLR2K, PPP1R3D, QPRT, RASA1, SLC33A1, SUOX, TDO2, TLR9, TP53, TRPM5
Negative_7173	SMO, IL24, NUDT5, OR4P4, OR9Q1, PHPT1, POLD4, PRKACB, RDH16, TMLHE, VARS, ZNRD1, TNFRSF13B
Negative_7174	C8G, AMPD1, CCR2, CD3E, CLDN19, COL4A2, DUSP16, ELK1, GMPR, GYS1, HTR2B, LRAT, MGAT1, NOTCH1, OR13C8, OR4D6, OR56B4, OR5T2, P2RX2, PCNA, PRNP, RDH12, SIPA1, SOCS2, SOS1, STK36, TGFBR1, UBA1, UMPS
Negative_7175	ABO, LTB, SCD, SHH, UBB, AKR1C3, ASRGL1, CAMK4, CHUK, CYP26B1, CYP2A13, EDNRA, GMDS, GPAA1, IFIH1, IFNA21, IL1B, LIMK1, MAPK3, NAGK, NFASC, NKX3-1, NMNAT1, NRG3, NTRK2, OR11A1, PDE1A, PLXNA2, SEMA3B, STT3B, UPP2, TNFRSF1A
Negative_7176	DDO, F11, TFG, ADRA1B, ADRBK2, AGTR1, AHCYL2, ALOX15B, ATP6V0C, CACNB4, CAMKK1, CFLAR, CRY1, CSNK2B, CYP27A1, DKK1, DUSP16, EPHB6, GNA15, GSTA4, HIF1A, HSD17B4, IDH3G, LCMT1, OR10Z1, OR2T8, OR5D14, OR6C76, PAK4, PANK3, PGM1, PLOD1, PMM1, PPP3CA, SETD1A, SGMS2, SPRED1, STK4, TAS2R10, TAS2R48, TRAF6, TSLP, ULK3, YARS2, YWHAH

Negative_7177	C8A, F2R, ACLY, ACVR2B, ADSSL1, BLVRA, CYP3A4, DDIT4, KLRD1, LEFTY2, MAD2L2, OR13D1, OR5K1, PIK3R1, PISD, PLCE1, PRKACA, PSENEN, ROBO1
Negative_7178	CACNG7, CASP8, CSF1R, DAXX, LAMC2, MTHFD2, OR6K6, PAPSS1, TUBA3C
Negative_7179	APP, C6, PRL, AHCYL2, ALG13, ATP2A3, CCL28, CD274, CDC42, CDH15, CXCL5, DHODH, EFNA4, FGFR3, GLI3, IL2RG, ITGA5, OR4K1, P2RX1, RCHY1, RPS6, SMURF1, TGFB3, CACNA2D2
Negative_7180	F7, TEC, ACAA2, ADSS, ATP13A2, BMP7, BPNT1, CACNG2, CCL23, CD48, CDKN1C, FMO4, FUT7, GALM, GARS, GCNT2, H6PD, HSD11B2, ICAM2, IL13RA1, IL17RA, IL1RAP, MAPT, MIOX, NADSYN1, OR10H2, OR2B2, OR2F2, OR4C46, OR4N5, OR6C70, PDHB, PDPK1, PIGN, PNPO, PORCN, RHOA, SARS2, SELP, SFRP5, UGT2B10, ATP6V1E2
Negative_7181	GALNTL2, MBL2, MGST3, OR8H2, PARD6B, PDE11A, RARA, SPRED1, ATP6V1E2, CSGALNACT1
Negative_7182	BTD, NNT, RET, CCL2, CFL2, CYP1B1, CYSLTR1, DPYD, MYH11, NLGN1, OR10G2, OR51D1, OR9I1, PRKCB, YWHAZ, ST6GALNAC3, TNFRSF11A
Negative_7183	F7, IL2, MIF, ACAT2, AKR1C3, APH1A, CASP3, CASP7, CDC20, COL5A1, CSF1R, CSF3, ENPP1, FUT3, GFPT1, GMPS, GNAO1, GRIA2, HCCS, HSD17B1, IL17RB, IL28RA, ITGB7, LIMK1, NLGN1, NR1H3, OR10V1, OR2K2, OR4F4, OR4N4, OR6C76, OR6K3, OR7D4, OR9G1, PAX6, PDE5A, PDE8A, PPP3CA, PTAFR, PTPRB, RAPGEF2, TYMS, PAFAH1B1
Negative_7184	FH, HGD, NLK, AACS, ACADSB, ALDOC, ALPPL2, CD47, CD48, CRLF2, CXCL11, DDX52, DGKB, FLT1, FUT7, FZD4, G6PC2, GADD45B, IFNAR2, IRF3, ITGA4, LILRB3, LRRC4C, MCM4, MINPP1, NAT5, NFX1, OR13C3, P4HA1, PAX6, PLAT, PLIN, PRKACB, SYNJ2, TBL1Y, WNT5A, ATP6V0E1, B3GALNT1, CSGALNACT2
Negative_7185	AK7, ZYX, ENO1, FLOT1, GCNT3, GLG1, HRAS, HSPA8, MGLL, RRAS, SUCLG2, TRADD, PNLIPRP1
Negative_7186	BCR, F8, GSN, ACVR1, C1QA, C7orf16, EPHB4, GNAL, GTSE1, GYS2, LAMA3, LAMB2, NME2, NSDHL, OR10G3, OR13C9, OR2G3, OR5A1, OR6N2, PDE4A, PDGFD, PIGH, PRKX, RPS6KB2, SNW1, TUBB2A, UCP1, PRICKLE1
Negative_7187	LYN, AASDH, ACOX2, AKR1B1, ATP2A1, CCR5, CDKN1B, CXCL2, GZMA, IFNA8, MMP1, MTMR2, NANP, NFX1, OR13C9, PHKA2, POLE, POLE2, PRDX6, SUFU
Negative_7188	GALNT11, OR8B12, PTGER3
Negative_7189	B4GALT3, DGKD, GANAB, GNB1, GRIA1, GYS1, MADCAM1, OR2F1, OR6C6, OR8H3, P4HA1, WNT3A
Negative_7190	LSS, PRL, ALDH6A1, ALS2, ARD1A, BAIAP2, BLVRB, CDKN2A, CES1, CNTNAP1, COQ6, CSH1, DGUOK, DKK1, EDNRB, ENTPD7, FLT4, GAMT, GJD2, GRAP2, HSD17B4, IQGAP1, MAP3K4, NCR3, OR51B4, OR51F1, OR6C74, PFAS, SEMA3D, SETD2, TACR2, UGCG, ATP6V1G2
Negative_7191	ADA, INS, ACAD9, ADCY3, AOC3, ATP5C1, ATP5G3, CACNG5, CACNG8, CNTNAP2, CPT1A, CX3CR1, FGF9, GNPNAT1, ICAM3, IFNK, IL20, IL6R, LEPR, LIPE, MDH2, NCF1, OR10R2, OR13G1, OR51T1, OR6N1, OR7G1, PDE4C, PKM2, PPP2R5B, PYCR1, SMAD3, STAT6, TIRAP, TLR5, TP53I3, UGT1A10
Negative_7192	SCD, CMAS, EPHB6
Negative_7193	AGL, ALOX5, ANPEP, APPL1, AVPR1B, CLDN4, CREB3L2, CXCL13, F2RL1, FPGT, FUT2, IFIH1, IRAK2, LCMT2, NCOR2, NT5C3, OR10H3, PDE3B, PDE8B, PTGES, ROBO1, SEMA3B, SFRP5, SLC2A1, STX1A, TPH1, PHOSPHO1
Negative_7194	ADSSL1, BMP8A, CD44, CTBP1, DGAT1, HTR4, MAP2K5, MAP3K4, OR2H1, PLA2G2E, ST6GALNAC4
Negative_7195	CDA, CKB, ACP6, ASNS, CAPN2, CASP10, CBR3, CDKN2D, CLTC, DHFR, GNMT, ICOSLG, IL12RB2, LIPE, MCM7, NEFM, OR6C3, PECAM1, PIAS1, PLA2G4A, PTPMT1, RASSF5, SETD1B, TRADD, WIF1, HSP90AA1
Negative_7196	MSN, BIRC5, FABP4, TLR4
Negative_7197	HK1, ACSM2A, ACTG1, CSNK2A2, DNMT3L, FMO4, GBA3, HTR2C, IFNA14, IL19, INPP5A, OR2T2, OR4C11, PLXNA2, POLR3G, PPP1CA, RDH5

Negative_7198	AR, BID, CD2, DCN, ACAD9, ADH1A, AVPR1A, B3GALT2, B4GALT2, CACNA1S, CACNG5, CAMKK2, CBR3, CCL5, CCR10, CD74, CDKN2B, CHIT1, CSNK1A1, CXCL11, DIAPH3, GDE1, HDAC1, HSD17B3, IFNA8, IL8RB, IRAK2, NFKBIE, NT5M, OR10H3, OR4K2, OR9K2, PDPK1, RAC2, SEMA3A, SPAM1, STMN1, TGFB2, WNT6, XCL2, MAPK8IP2
Negative_7199	AMT, PDC, ACSL5, ALOX5, ATP2A1, ATP5G1, BCAR1, CACNA1H, CACNA1S, CARS2, CLDN8, COL4A1, CSH1, CXCR3, DHRS4L2, DKK4, DLL4, DNMT1, FBXW11, FCGR3B, FGF21, GADD45G, GNAS, GNB1, HSD3B1, IDUA, KIR2DS4, LYPLA2, MASP1, NCAM2, NSD1, OR10A6, PPARD, SEMA3G, SLC25A6, STX3, TSHR, CACNA2D3, GABARAPL1
Negative_7200	ADCY2, CBLC, EXOC7, FSHR, NAGK, OR10A3, OR1D5, OR51S1, PGLS, SEMA4A, SLC9A1, TAS2R38
Negative_7201	DCC, NPY, PML, APOA2, ATP5H, CCL2, CCR7, CDC23, DLL1, ERO1L, GCNT2, GUSB, IL1R2, ITGA8, MAP3K3, NCF2, NSDHL, OR51A2, PIGK, PLA1A, PYGB, UCKL1
Negative_7202	LYN, NNT, PXN, ABLIM1, AMPD1, APOA2, BBC3, BMP8B, C4BPB, CD19, CDKN1B, CLDN7, CRY2, CSF3R, DDX52, DUSP8, FABP1, GAD2, GATM, GNAO1, GNPNAT1, NMNAT1, PDE4B, PDXK, PROC, PVRL2, RASGRF1, SLC12A2, SPCS3, SPTLC1, UGT2B10
Negative_7203	ACHE, AGRN, ARPC5L, BMPR1B, CCDC6, CD55, CD82, CDIPT, CLDN10, CLDN5, CTNND1, DNMT3L, DUSP7, EPHX1, GPD2, HLCS, HSD17B7, IFNAR2, JAM2, KYNU, L1CAM, LCP2, PDE6H, PLOD2, PNPLA3, QARS, RPS6KA1, SETD2, SMAD9, TBL1XR1, TOMM40L, UGCGL2, ULBP2, VAV1, YWHAH, YWHAQ
Negative_7204	C1S, C8A, GDA, LHB, SMO, ADSS, ALG6, ALOX12B, APBB1, CBLC, CD86, CDH3, CRLS1, CSNK2B, ENTPD3, F11R, GCNT2, GRIN2B, HLA-F, HMGCR, LRAT, LRRC4C, MAFA, NT5C1B, NUDT12, OR2M5, OR5C1, OR5M3, OR6T1, OR7C2, PARD6G, PCYT2, PFKFB2, POLE, PPP1R3C, RPS6KA4, SCD5, TRAF6, UGDH, WNT3, YWHAG, CSGALNACT2, HLA-DQB1, SERPINF2
Negative_7205	ADCY7, CHRM5, EP300, GALNT10, IL17A, OR13C5, OR13D1, OR2M4, PLCB1, UROS, USE1, MAPK8IP1
Negative_7206	C8A, F2R, PGF, ADRB3, AKR1C3, BLVRA, CALML3, CCL21, FZD3, HSD3B7, ITGA3, MAGI1, MAN1A1, MAP2K6, MMP1, NLGN3, OR13F1, OR4F6, OR5C1, OR5F1, PIP5K1B, PLD2, SRGAP1, STX10, STX12, UBA1, TNFRSF13C
Negative_7207	CGN, CTH, GBA, TNF, BST1, CCKAR, CCL28, CD40, COL1A1, CXCL6, DDOST, DFFA, EXT2, FGF21, GNPAT, GNPNAT1, IDI2, ITGA8, KIR2DS5, MCCC1, MCM2, NCR1, NMNAT2, NTSR1, OR2T6, OR51D1, PDE8B, PIGF, PPP3R1, PTPRF, RASGRP4, SELL, SEMA3G, SHMT1, TAP2, TP53I3, VTI1B, XYLT1, CTNNBIP1, HLA-DPB1, SERPIND1
Negative_7208	DUT, CPT1C, DDX54, EDNRA, NRP1, PDGFA, PLOD1, SELP, SUV420H2
Negative_7209	DCC, ACSS1, ACTN1, ASRGL1, CASP8, CBLB, CLDN4, DHFR, DTX3L, ENPP2, ENPP3, EPB41L3, FMO3, GALNT9, GCNT1, GRM1, GYS2, HMGCS2, IDH3B, IL12B, IL13, ITPK1, LAMB2, MAOB, NPR1, OR4M2, OR52K1, OR5D18, OR7A17, PDGFRB, PDIA4, PHGDH, PIK3CB, PPAP2A, RALB, SCD5, SH2D1A, SPCS3, THTPA, ULBP2, WEE1, HLA-DQA2
Negative_7210	AR, NOG, ARPC3, ARPC5L, CACNA1I, CACNB3, CSH1, CTTN, CXCL3, CYP2C18, FGF13, FUT5, GNG3, GUCA1A, IL11RA, IRF7, ITGB5, KDSR, MAP3K12, MTHFS, MYH6, NSDHL, NT5M, OR51M1, OR52K1, OR52K2, P2RX7, P4HA3, PRKCI, RPS6KA3, SDC1, SPRY2, SSH1, SUV39H2, UPRT
Negative_7211	C4B, ADCY10, AGPAT4, APOA1, ATP2A3, ATP5E, ATP5I, B4GALT7, BBOX1, CCKAR, CCR8, CLDN17, CNTN1, GLYCTK, MAPK14, MASP2, NPR1, OR52E4, OR9Q1, PDGFA, PIGP, PLA2G4A, PLXNC1, RAC2, SUFU, SUOX, TBXA2R, UGT2B10, TNFRSF11B
Negative_7212	LTB, ATP4A, CSNK1A1, CYP3A7, DGKB, FUT6, GYS1, LAMB1, NRG2, OR10AD1, PGAM4, PIGN, PLCB2, PYCRL, SMC1A, STAT4, SV2A, THBS1, TNFSF11

Negative_7213	STS, ACP6, ATP5G2, ATP5J2, B4GALT2, CDC25B, COL6A1, CREBBP, CXCR6, CYP2E1, DUSP10, ENPP7, EPHA7, F13B, FLAD1, FOSL1, GRIN2B, INHBE, INPP1, LDHAL6A, MCM5, MYL7, OCLN, OR2F2, OR4M1, OR6A2, OR9A4, PLIN, POLR2C, PPAP2B, PTPN1, PVRL2, RAC3, RERE, RFXAP, SNAI1, ZAP70, PNLIPRP1
Negative_7214	B3GAT1, GAD2, MAD2L1, POLR2L
Negative_7215	C9, ADRB1, AGMAT, AMD1, ATN1, ATP6V1F, BCL2, C1QC, CISH, DTX3, F2RL1, F2RL3, FGF19, FGF3, GANC, GNAT3, GSTO1, GUCA1B, IKBKB, IKBKE, IL13RA2, IL21, LCP2, LDHAL6A, LIPE, NARS, OR1J4, OR4D2, OR52N1, OR8D4, OR8H3, P2RX1, P4HA1, PLCG2, PRKCG, PVRL2, PVRL3, SEMA3G, SNAI1, SPRY3, STX16, STX2, TNFRSF8, UROS
Negative_7216	ADAM10, ALG11, ASCC3, DGKQ, HMGCL, ITGA2B, OR13C2, STAM, TLN2, TNFSF13B
Negative_7217	F11, GSR, IL2, SMS, CACNB4, DFFA, FGF12, GDE1, GSTZ1, HPSE, IL20RB, MYH7B, MYLK, OR11H6, OR4C13, PANK3, PDE2A, POLR2G, PPAT, PRKY, RBL2, TAS2R14
Negative_7218	EBP, TEC, DCXR, DRD5, DUSP8, GALNT11, GALNT6, KYNU, OR10AG1, PDE2A, PLAUR, SOS2, SPAM1, TAS2R49, ATP6V0E1
Negative_7219	CD2, APH1A, B3GNT5, CAPN2, DNMT3L, EPHB1, GALE, HMGCS1, IL5RA, NEGR1, NLGN2, NT5C1A, OR10Q1, OR10S1, OR2A4, PIP5K1B, PISD, RARS, RRAS2, SEMA4C, SHC4, TM7SF2, TYK2, UGT2B17, WWP1, XCL1
Negative_7220	ACVR1C, AMY2A, ASIP, ATP5A1, ATP5J2, B3GAT1, BAIAP2, BET1L, BUB1B, CACNA1D, CAMKK1, CSF2RB, CSNK2A2, CYP27A1, DUSP10, HIBADH, IFNK, IL13RA1, MAML2, QARS, SFRP4, SULT1A4, TGFBR2, VAV1
Negative_7221	BST1, CASP9, COL11A1, COQ3, DAPK2, DRD1, GCDH, GSTK1, IL22, LAMB1, LAMB4, OR10G3, OR10G4, OR8K1, PLCB3, POLR2J, PRODH, PTPN1, RBPJL, STX3, TKTL1, TNFSF18, UBA7, TNFRSF10B, TNFRSF11A
Negative_7222	CGA, GSR, LCT, SI, AHCY, CFL1, DNMT3B, EPHA6, FCGR2B, HSD17B2, MTHFS, NEFM, OR2D2, PARD6B, TIAM2, TJP2, CACNA2D1, MAP3K7IP1
Negative_7223	TFG, ACP5, BDNF, CD19, DUSP2, IGF1, KIR2DS4, NCKAP1, NT5C3, OR10K2, OR3A2, OR5V1, OR6C1, PDE3B, PLA2G2A, VAMP2, PRICKLE1
Negative_7224	GP5, DUSP9, DVL1, ITGAL, OR2B6, OR8J3, PTGFR
Negative_7225	MSN, ATP5J, BPNT1, CCL1, CDC14A, CXCL6, IL13, LAMA3, OR2V2, OR4D5, OR4X1, RAC2, UGT2B28
Negative_7226	DLD, EBP, ID4, ANAPC10, DKK2, PLA2G4A, SELL, SMC1B, ZIC2
Negative_7227	DUT, OTC, RFK, ABP1, ADH5, ADH6, ALS2, ANGPTL4, ASMT, B3GALT5, CACNB1, CCNG1, CNGA4, CXXC4, CYP2A7, CYP2B6, DGKI, DHRS4L2, DLST, ELK1, FGF21, G6PC2, GSTM1, IL12A, IRS2, LDHB, NEUROD1, NFKBIA, NQO1, OR13D1, OR4N5, OR5A1, OR9G1, PLXNB3, PRKACB, PSME2, RARA, RPS6KA3, SPHK2, SRGAP2, STK4, TPI1, WNT16, WNT7A, YWHAZ, HLA-DQA2
Negative_7228	AMH, APP, ADCY10, ALG2, B3GALT2, BAAT, CDH4, CDH5, COMT, FBXW11, GFPT1, GNA12, HARS, IFNGR1, IRAK2, ITGA6, ITGB8, LILRB3, LIPC, LTA4H, MAD2L2, MAGI2, MTHFS, NCR2, NT5E, OGDHL, OR2W1, OR4E2, OR51T1, OR5AC2, OR8I2, OR8K5, PNPO, PRPS1, RDH10, RXRA, SOCS3, TNFSF4, UNC5A, WNT10B, ATP6V0E1, HLA-DPA1
Negative_7229	DFFA, DUSP5, HDAC2, IRF5, LEFTY1, OR10A6, RPS6KA3
Negative_7230	CEL, EPO, ID3, NOG, TNR, VCL, ADRB3, AMHR2, ANAPC11, ATP5G1, ATP6V1D, BCL10, BDNF, CDC14A, CMPK1, CNGA3, CTBS, DVL1, ERBB3, FGF18, GAPDH, GCAT, GGT7, GSTO1, HCCS, HSPB1, IL22RA1, LIPC, MAPK11, MYL5, NFX1, OR10C1, OR4S2, OR8B8, OR9I1, PIGP, PLK1, PPP3CB, PTCH2, PTPN5, RALB, SDHB, SMAD5, TAS2R7, TICAM1, UGT2B7, WEE1, SERPINF2, TNFRSF18
Negative_7231	F3, GSN, AGPAT4, CAMK2G, CD3E, CDC20, CLCA4, CYP2J2, CYP3A43, F2RL2, GART, MPDZ, OR10R2, OR2T4, PLXNA2, PPA1, PPCS

Negative_7232	AUH,LHB,MPI,ACAD9,ALOX12B,BBC3,CACNB4,CCND2,CD22,CDC14A,CKS1B,CMPK2,ECSIT,GLS2,HHIP,IL17RA,LHCGR,MPST,NEU4,NFATC2,NODAL,OR14I1,OR1A1,OR51B2,OR51M1,OR5D18,OR8G1,PIGB,PTDSS1,PTPRF,PTPRZ1,RFNG,SEMA3A,SFRP2,STAM2,STK11,VEGFA,YWHAB,ATP6V1E1
Negative_7233	HK2,AASDH,ASRGL1,CDC25C,CDK6,COL4A6,CYP3A43,DAPK3,DUSP7,FOXO3,GALNT8,IL25,MAOA,MAP2K7,MLYCD,ODC1,OR10A2,OR5H6,RXRG,SHC2,STX4,TIRAP,WNT4
Negative_7234	CTH,DDO,FGA,OSM,UBB,ADORA2A,ALG10B,ALG5,ALOX15B,CD99,DVL3,FZD2,GCH1,GJA1,HAGHL,IBSP,IQGAP2,KIR2DS4,KLK3,MAP3K3,NANP,NEFM,NEU2,OR10K1,OR11G2,OR56B4,OR5M3,OR8D4,P2RX3,PISD,RND1,PAFAH1B2
Negative_7235	CKB,ATP6V1F,BPGM,NMNAT2,TAS2R13
Negative_7236	AGXT2,CCND1,CMPK1,CSNK1D,CYP26C1,DPM2,FTCD,GMPS,HSD17B2,IL1A,IL22RA2,NFKBIA,NUMB,OR14J1,OR4X1,OR8H1,PDE3B,SLC27A4,TKTL1,TYK2
Negative_7237	C5,F7,PLG,ACAD9,ALG9,AXIN2,CBLB,CDK5,CFL1,CHPT1,CHSY3,COASY,CRY2,DMGDH,FCGR2B,GNA15,GP1BA,GSK3B,HARS2,HIBADH,HMOX2,HSD17B8,HSD3B1,INPPL1,MAPK12,OR11H1,OR8J3,P4HA1,PAK2,PDE4C,PGAP1,PHKB,PLCE1,RAPGEF1,SKP2,SREBF1,ST3GAL3,TCF7L2,TUBA3E,HSP90AB1,PDCD1LG2
Negative_7238	C5,ME3,ADH4,CANT1,CCL16,CD19,CLDN19,CTTN,CYP2S1,DGAT1,EFNB3,FDFT1,FGF8,GCH1,GGCX,GRIA2,IL1A,LIFR,LLGL1,MAPK14,NCOR1,NOX1,NUDT9,OR1G1,OR51G1,PAPSS2,PRPS2,PTCH1,SCIN,SMAD5,TNFSF14,CACNA2D3
Negative_7239	F11,AASDH,BCMO1,CD38,DRD2,MAT1A,OR2V2,ORC2L,PCYT1B,PDGFB,PLCG1,PSME3,SKIV2L2,SMPD4,SMURF2,STX18,VCAM1
Negative_7240	MVK,RDX,ACP1,ACTN2,CDC45L,DPM3,GPX5,IARS,ITGA4,KIR2DS4,LLGL2,OR4D1,OR5L1,PRKCD,TGFB3,UPB1
Negative_7241	AMH,ACAT2,ACTA2,AGPAT3,AXIN2,CD274,CHRM5,DGKD,EPHA1,FZD4,GAD2,GALNT12,GAS1,GFAP,HTR2B,KAT2B,MAT1A,NAE1,NCKAP1L,OR2H2,OR6C3,PDE11A,PPP3R1,PRKAB1,PRKCB,PTPRR,RASSF1,RFNG,RPIA,TIAM2,TUBA1B
Negative_7242	C2,DHH,F11,ABLIM1,AQP7,ATP5C1,CBR1,CRLF2,DDX4,FGFR2,GNPAT,HEXA,INADL,ITGA4,ITGB4,LGMN,MAP3K5,MAPK9,MLYCD,NAT5,NTRK1,OR52E6,OR8B4,PFN1,PLOD2,POLR3K,PSME1,PSME3,TAP2,TPK1,USE1,VANGL2,VAV2,YWHAQ
Negative_7243	ME2,CLCF1,FZD3,GABARAP,GNA11,IRF9,MCM7,NRAS,PFKFB3,RRM2B,SEMA3A,SREBF1,STX4,TYROBP,VDAC1
Negative_7244	CGA,FES,PGD,ACY3,ADCY3,ALG10,CACNB3,CCL17,CLDN15,CPT1A,CXCL6,DUSP6,ETS1,FGF19,ITGAD,LARS2,MAN1A2,MMP9,MYD88,MYL7,OR10A2,OR2A25,OR5T2,POLR2E,PRKAG2,PYGM,RPS6KA6,SEMA3F,SHC4,SOCS2,TAS2R16,TICAM1
Negative_7245	ALDH1A1,ALG10B,ALG8,C4BPA,CACNG3,COL1A2,CXCL16,DERL1,GLI2,HADHB,IL21,INADL,KCNJ11,MAP2K7,NEU4,NFATC2,OR10G8,OR10R2,OR2A25,OR5T2,OR6N1,OR8B4,POLD3,RASGRF2,SMPD2,STAT6,TRADD,TUBB2C,XCL1,MAP3K7IP2,MAPK8IP2
Negative_7246	CEL,IL5,RFK,ACSL3,ACSM3,ALDH6A1,ALOX12,AOX1,APH1A,ARNT2,ASCC3,ATP6V1D,CCKAR,CCL13,CD22,CDK7,COL5A1,COX15,DUSP1,EDNRB,GAPDH,GYS1,INADL,IRAK4,IRS2,LRAT,MAP3K8,MASP2,MDM2,METTL2B,NRAS,NTF3,OR4C13,PARS2,PCK2,PHPT1,PIGW,PMM2,PRPH,PYGM,SOCS2,ST6GAL1,TAS2R40,TNFSF9,UBA7,SERPINF2
Negative_7247	LIF,B3GAT2,CLDN11,CRB3,DAAM1,GAL3ST1,KCNQ1,LCP2,NT5C1B,OR10AD1,OR2C3,PFKFB1,PIP5K1C,PLXNB2,PRKAA2,TAS2R38,UGT2B28

Negative_7248	FER, ME2, UBB, AASS, ADH7, ANAPC1, ARPC5L, ASCC3, ASS1, ATP4B, BCL2L1, CCL1, CCL21, CCL27, CCND1, CFTR, CHRNA7, CPT2, DHDH, GUSB, HRH2, HSD11B2, ICOS, IRF5, ITGAL, ITGB7, MAP3K7, MYL7, NT5C1A, OR2A42, OR4C11, PIK3R4, POLR2J, PRKCD, PSEN1, PTPRJ, PTTG1, RFXAP, SMURF2, SOCS2, SPRY1, TJP2
Negative_7249	FER, FOS, SYK, ABLIM1, ACOT11, ACSL5, ADCY2, AREG, CBLC, CCNG2, DHCR24, DHDH, ETNK1, PARVG, PDGFB, PTGS1, ROBO1, SDC3, SHISA5, TNFRSF4
Negative_7250	DDC, ADCY4, ADH1A, ADSSL1, C4BPB, CD47, CDKN2D, EPHA6, FGF9, GRIA2, NMNAT2, OR7A5, P2RX7, PLXNB2, POLR2B, PTGS1, SEMA3C, TAS2R16
Negative_7251	FER, MVK, PML, SMS, TKT, ACADS, AVPR1B, BMP8B, BMPR1A, CCNA1, CDKN1C, CNTNAP2, FGF11, GALNT10, GMDS, GMPS, GNAQ, GPD1L, GRIN2C, GUCY1B3, INPP1, JAM2, KIR3DL3, LCP2, MAP4K1, MMP14, MTHFD1L, NFKBIE, OR1S1, PDK1, PIK3C2B, POLA1, PRSS2, RASSF1, SH3BP2, SLC27A5, SMAD2, SMARCA2, TRAF2, ULK3, VDAC1, YES1, PHOSPHO1
Negative_7252	EBP, F8, ABP1, AKR1A1, ALDH1A3, CDC7, CLDN16, CYP2S1, DPM1, EPHX1, EXTL1, GALNT8, OR4K13, OR4N2, OR51D1, OR51I2, OR52E6, OR6C75, ROBO3, SUCLA2, THBS3
Negative_7253	MAX, ADCY8, AGRP, AKR1A1, ASIP, CCR3, CLDN19, CYP2A13, EPHB1, ERO1L, ETNK1, FARSB, GBE1, GCLC, GRLF1, GRM5, HAO2, IFNA14, IL17A, INPP5E, KNG1, NME2, NRG4, OR2A25, OR4F15, OR4M2, OR5K1, OR6X1, PARD6G, PDGFA, PIGV, PPP1CB, PPP1R3C, RDH5, RUNX1T1, SPP1, TBL1XR1, UGT2B4, VARS, WWP1, PPP1R12A, SUV420H1
Negative_7254	FST, ME2, PC, ACADSB, AMDHD2, ARRB2, ATP5C1, ATP6V0C, CHEK2, CYP2C18, DBF4, DHDH, FBXW11, FZD4, GATM, GLUD1, KCNB1, KLRD1, OR2G6, OR5AU1, OR5T3, OXCT2, PIM1, QARS, ST3GAL3, TLR9, TPH2, UGT2B11
Negative_7255	ASMT, CCND3, CMAS, CYP4F3, DEGS1, DGKG, GUCY2F, IL23A, ITGAM, MASP2, MYLK2, NAE1, PTDSS1, TLN2, TSC2, TNFRSF10A
Negative_7256	ACSS3, B3GALT4, CACNA1G, CDC14B, CYP26A1, DTX2, ITGAD, LARS, MGST2, MITF, NFX1, NMNAT1, NTSR1, PANK4, PIK3C3, PLCG2, TUBB4, UROC1
Negative_7257	EPO, FGG, MPL, NNT, SMS, ACOX2, ALPI, BCMO1, BMP4, BMP5, CASP3, CDC45L, CYP4A22, DFFB, DHODH, DUSP1, ENTPD1, FSHR, FTMT, FZD1, GUCA1C, IDH3G, MTFMT, NOS3, OR10P1, OR1A1, OR2G2, OR4E2, OR8H1, ORC4L, PANK2, PDHA1, PPCDC, PPP2CB, RAF1, SMAD6, SPTLC2, TACR1, TBL1XR1, TCIRG1, TP53I3, TUBA1C, UGCG, VASP
Negative_7258	ALG10, LYPLA2, PTCH1, UAP1
Negative_7259	A2M, HGD, ABP1, ACAD9, ADRB1, ARHGEF6, ATP13A2, ATP5G1, CDC7, CNTN2, CRY2, DAG1, DEGS2, DHRS4L2, DHX58, DUSP6, EPB41L3, EPHA7, EPHB2, FGF2, GNAI1, GPAM, HSD11B2, LIPE, MMP1, OR1G1, OR5D18, OR5M8, OR8I2, PDE8B, PGM1, PLA2G4A, PLCD1, POLR2F, POLR3H, PORCN, PRIM1, PTPRZ1, SMPD3, UGCGL2, UMPS, WNT8A, AASDHPPT, CTNNBIP1, HSP90AB1
Negative_7260	CRK, DCK, CYP2B6, EPHB2, GPX5, KLRC1, MAML1, OR6B2, TSTA3
Negative_7261	ASL, GK, GSS, IL5, AQP7, ASCC3, ATP12A, CCL23, CCNE1, CCR1, CDH5, CHRD, CSNK2B, CYP11B1, CYP2D6, CYP3A5, CYSLTR1, EHMT2, FLT1, GMPPA, GRIN2C, IL23A, ITGA10, KIR2DL1, LAMA3, LIFR, LIMK1, MAOA, NUDT8, OR10V1, OR1N2, OR7D2, PFN1, PGLS, PSME2, PTGES2, PTPRB, RPS6KA2, SHC1, UGCGL1, YES1
Negative_7262	FER, IL3, ABCC8, ABLIM1, ADRA1A, ATP2A2, AVPR1B, B3GALT4, CDH15, CKS1B, CLDN1, CMPK2, CYBA, DCXR, DOCK1, EIF4E, FGF12, FGF13, FLOT2, GALNTL4, GAS1, GNA11, IDH3B, ITPKA, KIR2DS4, LAMC3, MTHFD2, NODAL, OCLN, OR10A4, OR12D3, OR1A1, OR4S2, OR6K6, OR8S1, PDE4C, PGS1, PHKA1, PPARA, PPAT, PPP1R3C, RASSF5, RRM1, SMAD6, SOAT2, TAS1R1, WNT8B, ATP6V1E1, PAFAH1B2
Negative_7263	MYH9, PKLR, PPP3R1, SH3BP2, TICAM2

Negative_7264	GP5,ACY3,ARG2,BCAT1,CKS1B,CLDN3,CYP4F3,FGFR2,GDE1,HLA-DRA,HPSE,IL12RB2,MMP7,NCK2,NRXN2,OR52K2,OR56A1,OR5AC2,PGM3,POLA2,RPSA,SCP2,SMAD7,TRADD,TRAF3,TSHR,TNFRSF10A
Negative_7265	CR1,CRH,PRL,ADIPOR2,CASP3,CBR1,CCNB3,CDC6,CLCA4,E2F4,ERBB4,EXOC7,FGF20,GFPT2,GNG3,GSTA3,GUK1,IFNA5,IL23A,IRAK3,MAPK13,NMNAT2,NQO1,NTNG1,ONECUT1,OR10H5,OR51E1,OR52I2,OR6C2,PCCB,PIGL,PIK3C2B,PLOD2,PPP2R2B,PTK2,SMPD2,STMN1,TCF7L2,VEGFA,YARS2
Negative_7266	DDO,ALAD,CDC14B,CER1,FBP2,GALNT11,GYS1,IFNA8,MMP1,TNFRSF1B
Negative_7267	DHH,ME2,NLK,TK1,ADH7,AOC3,ARNT2,CAMKK2,CD99,CHST11,CTBS,CYP2J2,DERA,DGKG,DUSP3,EFNA1,ITGA7,LIAS,MPDZ,NAGA,NMNAT2,OR10H5,OR2G6,OR52I1,OR6C4,OR8H1,P2RX4,PFKP,PSME1,PSME2,PTGS2,RELB,RPS6KA6,SORBS1,TAS2R41,TLR2,TUBB1,YKT6,ATP6V0E1,ATP6V1B1,HSP90AA1
Negative_7268	CSF1,CTSS,CYP2A6,DLL3,GBE1,IL20RA,ITGA4,OR52D1,PFKFB2,PIGB,PPARA,RBL1
Negative_7269	ACACA,ATP5H,CANT1,CAV2,CD55,DIAPH1,FZD6,GNB3,IGF1,ITGAM,MAML3,NT5C1B,OR6X1,RASGRF2,SOS2,TFDP1,YES1
Negative_7270	CKB,INS,ACAD9,ATP6V0B,C4BPA,CASP6,CCNH,CNTN2,CTNNA1,ENO3,FGF6,FOXA2,GCNT2,HCLS1,IL18R1,INHBC,KIR2DS4,LIPC,MAP4K4,MGST3,NAT1,OR10H1,OR3A1,OR5D18,ORC4L,P2RX7,PER1,PIGV,PTGER3,PTPRZ1,SHC2,SV2C,TPH2,WNT3,TNFRSF14
Negative_7271	ME1,SHH,ACAA1,ACOX2,AKR1C3,ALDH1A1,AQP7,ASCC3,CCL8,CNTN1,CRLS1,CTSS,CYP1A1,DDX56,FDFT1,FZD6,GALC,GALNT14,HADH,HSD17B2,IFIH1,IFNB1,MAFA,MAP3K2,MAPK8,MASP2,MYL5,OR10G8,OR13C8,OR1A1,OR2AG2,OR52H1,PDXK,PIP5K1C,PYGM,SEMA7A,SRGAP3,STK3,TPH2,UCKL1,WNT10A,YWHAE,ARHGEF12,PRICKLE1
Negative_7272	CD4,DDC,HGF,ACSL1,ACSL6,ACTA2,ALCAM,ARG1,ATP5J,BCKDHB,CACNG3,CDC25B,CDC6,CSDA,DDX47,EPB41,EPHA3,GNAL,LAP3,MPZL1,MTMR6,MYD88,NADK,NARS2,OR10G3,OR10G4,OR1S1,OR4K2,OR56A3,OR5AP2,OR8H3,PLAU,RAD51,RBKS,RPS6KB2,ST8SIA1,WASF3,YWHAG
Negative_7273	GRIN1,OCRL,RBL1
Negative_7274	CD6,ATP6V1F,CASP10,CBR3,CCKAR,FADS2,FARSB,GSTA5,HMGCS2,NMNAT1,PLK1,RDH11,TSHR,UROC1
Negative_7275	CAD,EBP,TFG,TPO,ADH5,AGPAT4,AHCYL2,B3GALT5,CADM1,CAMK2B,CBLC,CCL17,EPHA2,FGF18,FMO1,LRDD,OR2Y1,OR5P3,OR8B8,OR8K3,PARVA,PGS1,PIGW,PLOD2,PNLIP,PPP1CB,PPP2R5C,PRKACG,PYGL,RARS2,SETD8,SGPP2,STX8,TACR3,TNFRSF4,TRAF4,VAV3
Negative_7276	AGRN,CACNA1D,CD86,CDC27,CHRM5,CLCA2,CLOCK,CTTN,DAPK3,DIAPH3,DLAT,FRAT2,G6PC2,GUCY1B3,HIF1A,HSPA2,INHBA,ITPKB,MGST3,NARS2,NKD1,NOS1,NUMB,OR4Q3,PER3,PTGES,SPCS1,ST3GAL3,THBS4,TRAF3,SERPINA1,SERPING1
Negative_7277	SHH,VTN,AGRP,ARG2,ATP4A,AVPR1A,BLVRB,CACNA1D,CASP8,CD48,CREB3L4,DUSP4,DUSP9,FPGS,GMPPB,GRIA2,HCST,HMGCS2,IL17RB,IL6ST,KARS,KIR3DL2,MYH15,OR10A2,OR2C3,OR2M5,OR4D5,OR5D16,OR6A2,PAK4,PCCB,PDE1C,PDE4A,PGK1,PIGH,PLA2G4A,PLCD3,PLCD4,POLR2C,RDH8,SH2D1A,SKP2,ST8SIA5,SUCLG2,TFPI,VANGL2,WNT11,WNT7B,WNT9A
Negative_7278	NPY,AVPR1B,CCNG2,MAN2A1,NFASC,NFKB2,RCHY1,SESN3
Negative_7279	F2,ALG12,BCAR1,CCL25,CD86,CDH15,CHRNA7,CTPS2,CTSL1,CXCR3,DMGDH,EDN1,ENPP6,ENPP7,EPOR,FGFR4,FMO1,GAMT,GJA1,HEXA,HSD11B2,ICOSLG,INHBC,IQGAP1,MAD2L1,MAP3K2,MAPK1,MAPK11,NCOA4,NRG1,OR10G7,OR2T5,OR4F5,P2RX2,PIGT,PLA2G2E,PLA2G6,SV2A,TGFB3,TPI1,TPK1,TRAF4,TUBA3C,UGT2A3,ATP6V0A2,B3GALNT1,HLA-DRB5,SUV420H2

Negative_7280	AANAT,ALOX5,DDX56,DUSP10,ICAM3,IFNW1,LAMA4,NCAM1,NUMB,OR4N2,PARD6B,PDXK,PIGF,SARS2,THBD
Negative_7281	ACAD9,ANAPC7,CDH1,DNMT3L,EI24,FPGT,HEXB,IFNA7,MFNG,MYH15,NRG1,PEMT,PPARG,PRKAR2A,RRAS,TUBA1C,UPP2
Negative_7282	EPO,LAT,ADCY5,ALG2,APOA2,CACNG4,CAMKK2,CNGB1,CTBS,CYP2C19,DAXX,DLL4,EPHA3,FADD,FLT1,GALNT10,GALNT13,GGPS1,HEMK1,HYAL2,IDI2,INHBA,LEFTY1,MYH7,OR2G6,OR2T2,OR51V1,OR52D1,OR52N5,OR6C2,PIK3C2G,PIK3CB,PLA2G2E,POLE,PVRL4,RPS6KA5,RRM2,SSH2,STK4,SUFU,TRIP10,UBE2L6,UGCG,UGP2,HSP90AB1
Negative_7283	PC,ACP6,ADAM17,AHCY,AKR1C4,ATP5I,BAI1,BPGM,BTRC,CACNB2,CCL25,COL4A4,CSH1,CYCS,DDHD1,DUSP16,FGF23,FUT3,GDF7,GRIA2,H6PD,HARS2,IFIH1,IPMK,KCNJ11,LIMK1,MAP2K5,OR13G1,OR1D2,OR2A5,OR4C46,OR4K17,PCCB,POLD2,PRKAR1B,PTCH1,PTGDS,SETD7,SRGAP3,TACR2,TAS2R1,TAS2R40,WNT6
Negative_7284	PRL,CD82,CDS2,CREB3L2,CYP2A7,DDX55,FZD6,IL23A,KAT2A,LTA4H,NAT2,OR10J3,OR10S1,PNPLA3,TNFRSF9,ZNRD1
Negative_7285	ABL1,ACVR1,DDIT4,HRAS,IGFBP3,PDE4C,SKIV2L2
Negative_7286	APP,BAD,CKB,DDO,OAT,AGPAT3,ASMT,ATP5L,BDH1,C4BPA,CCL3,CLDN16,COMP,COX10,CYP26B1,DFFA,FZD1,GBE1,GNAL,GNAQ,GRIN2A,GUCA1A,IL2RA,IRAK1,LAMC1,MGAT2,MTFMT,NCK1,OLAH,PANK2,PFN1,PIGO,PTPRR,SFRP5,SMAD3,SMAD5,SULT1A4,TSTA3,UGT8,UNC5B,YARS,HLA-DQB1,PAFAH1B2
Negative_7287	BID,EGF,NGF,SP1,ALDH1L1,ARHGEF1,ARHGEF2,ATP5G3,CCNA2,CD247,CMPK2,CNDP1,CNTN1,CPS1,CYP3A4,CYP4A11,DHX58,DPYS,GIT1,GJA1,GOSR2,GRM1,GRPR,HAO1,HPRT1,IL11RA,IL18R1,ITGB3,LYPLA1,NCKAP1L,NFKBIE,NLGN2,NSD1,OR10Z1,OR1A2,OR5I1,RCHY1,RFWD2,RIPK1,SAT2,SLC12A2,TNFRSF4,ULK2,XCL2,ATP6V0A1
Negative_7288	IL8,ALG13,ARSE,ATP6V0C,B4GALT2,CFL2,COL5A2,CSNK2B,DIAPH1,FASLG,FUT6,GSK3B,HAO2,IL11RA,IL7R,MAGI3,NCKAP1,OR1L3,OR1Q1,OR2AE1,OR2G6,OR4L1,PIGP,PIK3CA,POLA2,RIPK1,SMAD4,TAS2R42,TUBA3C,TUBB3,VANGL1,YWHAQ
Negative_7289	DBI,AARS2,ABP1,ADSL,APRT,ATP5I,C1QC,CLDN19,COL4A1,COL5A3,CPS1,CSNK1G2,CYP4F2,E2F4,FSHR,G6PD,GADD45A,GANC,GNAQ,IL8RB,ITGA10,ITGA11,ITGB4,OR10P1,OR2L3,OR52K1,OR52N1,OR6B2,PANK3,PAX6,PDE5A,PER1,PITX2,POLR1D,PPAP2A,PPP2R2C,PPP2R2D,PSPH,SCP2,SEMA3F,SORD,TAS2R8,TFPI,TNFRSF12A
Negative_7290	BCR,F9,ALG9,ANPEP,ATG12,B4GALT7,BMP8A,CAMKK2,CASP8,CBLB,CREB3,CSNK2B,DAG1,DFFA,DHCR7,FABP1,FGF3,GSTK1,GZMB,HLA-C,HPSE2,IDH2,IFNAR1,IL12RB2,IL15RA,KAT2B,KLK3,LAMC1,MGAT4B,NAGA,NRXN1,OR7A17,ORC3L,PDCD1,PDE10A,PPP2CA,PRKACG,PRPS1,PTPN11,RXRA,SELPLG,STAM2,STX18,TCIRG1,THTPA,UGT2B7,WNT8B,SERPINA5
Negative_7291	GPI,ALDH3B2,BIRC5,BPNT1,CHPF,CYP4F3,DDX54,FRAT2,GSTP1,IFNA7,OR2AG1,OR2M5,PDXK,PIPOX,PLXNB2,POLE4,PTGER1,RPRM,SIAH1,TGFB1,TICAM1,YWHAE
Negative_7292	C8B,CD4,INS,ACP1,ATP6AP1,CAV3,CLDN3,COL6A2,LTC4S,MAPK1,MYH3,MYLK,NANP,OR14I1,OR56A1,PIK3R1,POLR2E,PYCR1,TAS2R14,TSTA3,WASF1
Negative_7293	AK5,CTH,DHH,CCL16,COL2A1,COQ5,NCKAP1L,WNT5B
Negative_7294	SFN,ARSE,BCAT1,CREB3L1,CYBB,DDX56,DUSP7,EPHA7,IMPDH1,LIPG,MTMR1,NCOR1,NEUROD1,OR13A1,PIK3CD,RDH5,TICAM2,TPI1,WNT16
Negative_7295	GK,IL8,ADCY6,ADIPOR2,ATP5I,CALML3,CAMK2A,CES7,EGFR,GMPR2,HEXA,HLA-DOB,HSPA8,MNX1,NCSTN,NRAS,PDHB,PIPOX,SCP2,SGMS2,ULBP2

Negative_7296	BTC, FOS, GH2, CACNA1S, CLDN14, GAS1, METTL2B, PDCD1, PDIA4, PIK3R5, TNFSF11, SERPINB5
Negative_7297	PLN, COL2A1, FUT2, ITPKB, LTC4S, POLR2H, PTEN, STX16
Negative_7298	CD6, F3, MUT, MYC, ALG2, C3orf10, CBLC, CHAT, CYP26C1, DDX18, DNMT3B, ICAM3, IMPDH1, KIR2DL3, MGST1, MYH1, MYLK, NAE1, NEUROD1, NPR1, OR10P1, OR1I1, OR2A4, OR52B4, OR56A4, OR8D1, OR8H2, OXCT2, PISD, PLOD3, PTEN, ROCK1, SLIT1, TAS1R3
Negative_7299	C4B, FYN, GPT, HK2, APLP1, BBC3, CACNB1, CCKAR, CDK5, CDO1, CES1, CLDN22, CXCL10, CYBA, EPB41L3, FOXA2, FRAP1, GAB1, GALC, GSTA5, GTSE1, HYAL2, ITGA5, ITGB2, NEFM, OR10A3, PDE9A, SIGLEC1, SORBS1, TGFA, TNFSF18, ATP6V1C1, CSNK1A1L
Negative_7300	LIF, ADIPOQ, ATP6V1A, BMP6, CCL26, CHST13, CLCA2, FUT6, GSK3B, GSTA4, IL18, IL22, INSRR, ITGB8, KAT2B, LEFTY1, MGST3, MLYCD, OR2AT4, OR2C1, OR2K2, OR2L2, PCK1, PIGM, PIGO, PLIN, PRKX, SLC25A6, STX3
Negative_7301	AMT, CRK, HGD, HK1, ITK, JUP, ADRA1D, AOC2, ARSE, ATG7, ATP5C1, BLVRB, CADM3, CAMKK1, CASP10, CCL4, CCR1, CD55, CX3CR1, DPYS, FGF5, FPGS, GDF6, HNF1B, HSD11B2, IFNAR1, IL12B, IL18, KAT2A, KIR3DL2, MAP4K4, MAT1A, MGAT4A, MTHFD2, MYH9, NRCAM, OR2L2, PIK3CB, PPARD, PPP2R1A, RENBP, SFRP5, SOCS2, ST8SIA5, TP73, WNT11, ATP6V1E1, MAPKAPK3
Negative_7302	SFN, ACYP1, ARHGEF6, ARSB, CCL5, CHPF, DEGS1, DHDH, ENTPD1, FGD3, FGFR2, HDAC2, IL29, IL2RA, ITGB6, KYNU, LALBA, LAMC1, MRAS, NAGS, NRG1, OR1L1, OR51A7, OR51B6, OR52I2, OR6C76, PCYT1A, RIPK1, TKTL1, TNXB
Negative_7303	CBS, FGA, FGB, GP9, ACP5, ACVR1C, BACE1, BLVRA, CD44, CIITA, COL4A6, CRB3, CTSB, DFFB, ETS1, FBP1, FGF20, GALNTL4, GALT, GSTA5, INPP5A, IRF5, ITGA1, MAML2, MAN1C1, MBL2, MIOX, MYH4, OR2T8, OR3A3, OR4C13, OR51A2, OR52R1, ORC5L, PPP2R2D, RAD51, RFX5, RHOD, SCIN, SGPP2, SMC1B, TLR4, VEGFB, SERPINC1, TNFRSF10D
Negative_7304	C9, CD4, DUT, PRL, SDS, ACSM3, ARPC3, CCNG1, CD47, CD8A, CNTFR, COL2A1, CYP1A1, CYP2C8, DDX41, DNMT3L, EPHX1, EVI1, FCGR2B, FMO3, FUT3, FZD2, GDF6, HNF4G, IKBKG, IL12RB1, IRAK4, MAGI1, MDM4, NFYB, NT5E, OR14J1, PANK3, PIGA, PNPT1, PRKAR2A, PTPRR, RPS6KA1, STAM2, STX16, TCF7, UGT2B28, VTI1B, HSP90AA1
Negative_7305	CAD, SRM, ACVR1, ARNTL, DGKE, FCGR2B, GAB1, HNF1A, KLRC3, OR52B6, PDE1A, POLE3, SMAD5, TMLHE
Negative_7306	KIT, ACVR2B, ARHGEF1, CPT1A, CYP11B2, MTFMT, OR13G1, OR5H2, QPRT, SMARCA2, SUV420H2
Negative_7307	PGP, ALDH1A2, B4GALT2, CD226, CD34, CX3CL1, EPHA6, HGSNAT, IL8RB, KIR2DL1, LDHB, MAP4K1, MNX1, MYH8, OR11G2, PRKG2, SETD1A, STX12, WARS, PDCD1LG2
Negative_7308	AMH, C4B, LPL, ACO1, ACO2, ACY3, ALCAM, ALG8, ALOX12B, CCR9, CD99, ENPP1, EVI1, GALT, GJA1, GNPDA1, GRAP2, IL29, MAP3K2, OR2Z1, OR4D5, PCK2, PLCD4, PLTP, POLR2I, PPP1R3C, RAC3, RFXAP, RPRM, SIGLEC1, SKP2, SPHK1, SULT2A1, THBS4, TLN1, TLR3
Negative_7309	GSN, INS, ACADM, ACOT11, AVPR1A, BACE2, CD226, CDC45L, CYP7A1, IDH3G, IGFBP3, INSRR, PDCD1, PFKFB2, PIGO, PRIM1, PRKG1, SDHA, SGPP2, TBL1X, THBS1, UBA7, TNFRSF1A
Negative_7310	INS, ACOT12, CCNB2, CCND3, GNAT3, GP1BA, IL21, MKNK1, MTHFD2, PAK1, PIK3CB, SELL
Negative_7311	C9, DBH, ME2, APOA2, ATP13A2, BAIAP2, BCAT2, C1QA, C3orf10, CASP9, COQ7, CXCR3, CYP2A7, CYP3A5, DKK1, DUSP14, ENO1, GANAB, GUCY2D, HEMK1, IDI1, IMPDH2, MASP1, NT5C1B, OR13F1, OR2A4, OR2T1, OR2T34, OR7A5, ORC3L, PGS1, PIP5K1B, PPBP, PSME2, RAB23, RASSF1, RPSA, SESN2, SETMAR, STEAP3, TAS2R3, TGDS

Negative_7312	UBB, CD40, CYSLTR1, GNAI1, HLA-DOB, MOV10L1, OR2T33, PPAT, PPP2R5D, QPRT, ST3GAL4, SERPINC1
Negative_7313	ID2, UBC, FZD4, NCOA4, PPT1, ST8SIA1, TUBA1A, HLA-DQB1
Negative_7314	E2F1, GMPPA, PLCD4, RPS6KB1, SMAD7
Negative_7315	AGK, ACTC1, ACYP2, ADH5, ARSD, GFPT2, GPLD1, GRIA1, IL23R, MGAM, OR1S1, PTPN5, RUNX1, TNFSF18, TOMM40, UNC5C, ATP6V0A1, TNFRSF13B
Negative_7316	ME1, ACP1, ALAD, OR10X1, OR4D2
Negative_7317	ALDH1A2, ALG9, BCAT2, CYP3A4, FMO1, GPD1L, GPLD1, GRIA2, IL12RB2, INADL, ITGA2, MLYCD, NODAL, OR10Q1, OR4A5, OR7G2, PARD3, PNPLA4, PORCN, PPA2, RDH12, STX1A, TP53, TRAF2
Negative_7318	CYP2U1, GRB2, ZBTB16, SERPINC1
Negative_7319	ALDH3A1, GAS1, GCDH, GUCY1A2, HAGH, INPPL1, INSR, MYH11, NKX2-2, PLAT, PLIN, TP73, TNFRSF6B
Negative_7320	AGK, HAL, LAT, ACTG1, ATG7, CDH4, CHRM5, CTBS, DDOST, DGKB, GALNT8, GNPDA1, HNF1B, ITPA, LPAR1, MAP3K8, MGST2, OR1E2, OR2AG2, OR52L1, OR6C3, OR9Q1, PDCD1, PDGFB, PDK1, PER2, PPP2R5E, PRKAR2B, SORD, TAS2R14, UPP2, XCL2, TNFRSF11B
Negative_7321	CAD, ACY3, AGPAT2, BMP8B, CACNA1G, CACNG8, CAPN2, CRKL, DKK2, FGF3, GDF7, GMPPB, GNPNAT1, GSK3B, GSTO1, IFNAR1, IFNGR2, IL11, IL8RA, MALT1, MAP4K3, NOTCH3, OR10H5, OR6C75, PDE6G, PDPK1, PFKM, PHPT1, PTPN5, RPN1, SDHA, SEMA4C, SFRP1, TAS2R48, UGT8, WARS2, ZFYVE16, ATP6V1B2, ATP6V1C2
Negative_7322	ALG9, CDC16, DTYMK, IFNK, ITGA7, METTL2B, MTMR1, OR3A2, OR4N2, OR8K3, PAK3, SV2A, TNFRSF9
Negative_7323	FAS, ACSS3, ADRB2, AOC3, CACNG8, CUL1, DNMT1, IL1R2, LAP3, OR8G1, PIK3R1, SEMA4G, SIGLEC1
Negative_7324	CAT, MPI, PC, ACVR2B, AGRN, ATP6V0B, CACNB3, CCND1, CDC27, CHKA, CSNK1D, CYP19A1, DEGS1, DUSP16, GCDH, GFAP, GMPPB, GP1BA, IL21R, MAPK9, NME6, OR2A14, OR52L1, OR6X1, OR8H2, PDE4C, PIP5K1C, PLAU, POLR3A, PTGER3, SFRP1, SMAD6, TLR2
Negative_7325	APP, DHH, GSS, MPL, MVK, SDS, ADAM10, ATP6V1A, B3GALT4, BUB1, C9orf95, CAPN2, CCNE2, CD99, CERK, CFL2, CMBL, CSF1, CYP2E1, DIAPH3, DTX3L, ENTPD4, FIGF, IFNA13, IL8RA, INSR, IRF5, LARS2, MDM4, MYH2, OR51E2, ORC2L, PCK1, PIGH, POLR1B, PPA1, PTDSS2, PTGER3, SAT1, SOCS1, STAT3, SUV39H2, SV2B, TP73, TUBA1A, TYK2, VTI1A
Negative_7326	B3GALT4, GALK2, HIBADH, ST6GALNAC3
Negative_7327	GLA, MET, ACOX1, ACSL6, ACVR1C, ALAD, AMY2B, BAI1, BCL10, CCNE2, CDC6, CHD8, COL6A2, DGAT1, DUSP10, ESAM, FIGF, FPGT, GALK1, GALNT1, GPD2, HLA-DRA, HPSE2, JAM2, KIR3DL1, MAN2A1, MCM6, MGAT1, MINPP1, MLYCD, NLGN1, OR1K1, OR4C16, OR51T1, OR52W1, OR6C4, PCYT2, PLD2, PRIM2, PRKAR2A, PRPH2, PTGS1, RPS6KA4, SNAP23, SSH1, TGFBR2, CSNK1A1L, EIF4EBP1
Negative_7328	GH2, IL3, RB1, SCD, SRF, XDH, CCL14, CLCF1, CLDN9, COL6A1, CREBBP, HAAO, POLR2G, PROC, PTK2, RHOQ, SC4MOL
Negative_7329	CBL, CFH, MPL, AKR1C4, AMPD3, CAMK2G, FABP1, IFNA10, IL28RA, ITGA3, KYNU, MYH4, OR1C1, OR2A12, OR5T1, PDX1, PIGA, PLXNB2, PTPRF, PTPRJ, SMAD1, SV2C, TGDS, TKTL1, TUBA1C, UCP1, VDAC2, MAP3K7IP1
Negative_7330	DMGDH, HARS2, HEMK1, HNF4G, IFNA2, IL2RG, ITGB1, LCMT1, MINPP1, NLGN3, PER1, SOS1, UCKL1, ATP6V1G2
Negative_7331	AMT, GK2, ATP5D, B4GALT7, BAI1, BCAT1, CAMK2G, CDKN1C, CYP11A1, CYP4F2, DAAM1, DFFB, EFNA5, EPHA6, FGF4, GADD45A, GALNT12, GBGT1, GCDH, IMPA2, INPP5A, IQGAP2, ISYNA1, KIR2DL4, KITLG, KLKB1, LLGL1, MYH7B, NSDHL, OR14A16, OR56A1, OR6N2, PDE10A, PDE4A, PIGA, PPA1, PPP1CC, PTGER3, SEMA7A, SMC1A, TUBA1B

Negative_7332	GP9,RET,ZYX,AMPD3,ATP2A2,CASK,CD274,CLDN20,CLDN4,CSNK1E,CTLA4,CYP1B1,DAD1,DFFB,DHX58,FUT5,GNA13,GSK3B,HNF1A,IL1RAP,MASP2,MLYCD,NAMPT,NRP1,OR1A1,PFN1,POLR1E,PPP2R2D,RAF1,SGMS2,UPRT
Negative_7333	ACVR2A,CAPN2,DPM2,IL1B,LCP2,OR10H2,OR10Z1,PPP1R1A
Negative_7334	AK5,CFI,F8,ACYP2,C4BPA,CAMK2A,CCL26,EHMT1,EREG,GRIN2A,HLA-DRA,HSD17B2,HSPA1B,IFNA6,LAP3,MAP3K8,MARS,MLYCD,NAGA,NAGLU,NME2,PIGV,PRPS2,PTEN,PYCR1,SETDB1,SHC1,SLC2A1,SORBS1,SRD5A2,TRDMT1,TSC1
Negative_7335	AASS,ACADSB,ADCY10,AKR1A1,CACNA1E,CACNA1H,CBR1,CCNA1,CD38,CNTNAP1,CYP8B1,DHRS4,EFNA4,ENAH,FGF12,HDAC2,HIBADH,IFNW1,IGF1R,ITGAD,KYNU,MARS,MYL2,NFATC4,NR1H3,OR10G9,OR14J1,OR2AT4,OR3A3,OR4F6,OR4K15,OR51B4,OR5F1,PIGB,PIGN,POMC,PRKAA2,SDHC,SEMA4F,SETD1B,SETD8,SLC27A4,UROC1,VAMP4,ATP6V1C2,PLA2G12A
Negative_7336	CCL21,CCR8,CD72,CREB3L3,CXCL16,ETS1,F13A1,FRAT1,GUCY2C,ICAM2,IFNA8,KLRC1,OR52N5,ORC6L,PLXNA1,POLR1B,PRKAG3,RASGRF1,SORBS1,STX7,TYMP,HSP90AB1
Negative_7337	C4B,C8B,FUK,VCL,ABCC8,ALG2,C1QC,CAMK2G,CLDN20,COL6A1,CREB3L1,CXCL12,CYP2C9,DLL4,DNMT3B,GMDS,IFNGR2,LAMB1,NARS,OR10H1,OR1L3,OR4S2,OR51G2,OR52M1,PDE6D,PDHA2,PITX2,PLAUR,PLXNB1,PRKG2,PTPN1,SPI1,SPRY2,TNFSF18,TUBA4A,PNLIPRP1
Negative_7338	CD36,CFL2,GUCY1B3
Negative_7339	ACOX3,APBB1,GMPR,PDE6H,ROBO3
Negative_7340	CBS,CS,MSN,ADCY6,ADH5,AMPD2,ATF4,BDH1,BDH2,CCR10,CD55,CER1,CLDN14,CSNK1G1,DDX55,DTX3,EDN1,ENTPD7,GNB1,ITGA9,ITPKA,MAGI3,MALT1,MDM2,NCK2,NEU3,OR1E2,OR2G6,OR2T33,OR51M1,OR5A1,OR6Q1,OR6Y1,OR8H3,OR8K1,ORC1L,PIGQ,PIK3C2B,POLR1B,PPP2R5A,PRKAG2,PVRL1,SH2D1B,TICAM1,TRPM5,WHSC1
Negative_7341	AGL,CGA,ALG3,C4BPA,CHAD,CHDH,DRD5,FUT9,GRAP2,GRPR,IBSP,IL9R,IQGAP2,JAG1,MS4A2,NCOR1,OR4N2,OR52R1,OR6C2,PAK1,PARD6G,POLE4,PRKCB,SDC1,SNW1,ST3GAL2,ST3GAL3,TBXAS1,VARS
Negative_7342	HDC,NPY,ACADSB,ACSS1,ALG1,ANAPC10,CCL23,CCNG1,CCR3,CDC45L,CEBPA,CNGA3,CTLA4,CYP1A2,DDX56,DGAT2L4,E2F2,EHMT1,FGF4,FMO1,FRAT2,GALNT14,GDF6,IL13,MPZL1,NEFH,OR10A5,OR10A7,OR2L3,OR51L1,PMVK,POLD2,SMAD1,STAT3,SUCLA2,TAS2R7,ST6GALNAC6
Negative_7343	IL3,MPI,PGP,UBB,ACTN2,ALG10B,B3GALT2,B4GALT5,CAV2,CCR3,CSNK1G3,CTPS,DPM2,GSTO1,HSD17B2,IL13,MITF,MYL9,MYLK3,NFKB2,OR4A15,PGAM2,PIAS3,PRKCZ,PTK2B,RAPGEF2,SLC25A4,SMAD1,TRADD
Negative_7344	ACAT2,ACVR1C,AFMID,ATP13A2,CACNA1S,CCNG1,CD36,CHRM5,CYP26A1,CYP2F1,EXTL1,FBXW11,FGD3,GSTM1,HLA-G,IL12B,IL1R2,MAOA,MGST1,OR14J1,OR2Y1,OR4D5,OR4F17,OR5D13,PDE8A,SFRP1,TSHB,TUBB6,UGT2A1,ATP6V0A4,EIF4EBP1
Negative_7345	CDA,DDO,AARS2,ABL2,CCL8,GNAL,KYNU,NARS2,NEUROD1,TSC2,WASL,PLA2G12A
Negative_7346	B4GALT2,CDKN2D,CXCL9,CYP2J2,HADHB,LAMA3,PTAFR
Negative_7347	VTN,AMPD3,ARHGEF4,ATP5J,CYP26C1,EXT1,ITGB4,NCR1,OR1D5,OR1L1,OR2F1,PAICS,PLA2G3,PMVK,PRKAA2,SDC1,SEMA4C,ATP6V0A4
Negative_7348	ADO,AANAT,APOA1,CD27,DUSP1,IDH2
Negative_7349	AASDH,NTF3,OR2M3,RAPGEF1,TCF7L1
Negative_7350	EDA,JUN,ARHGEF4,CCNB2,CYP2C18,CYP4F3,DDX4,OR13C3,SYNJ1,TACR2
Negative_7351	ABL1,ADCY4,BLVRB,DPM3,OR1S2,OR8B12,PLXNB2,TNFSF14,WASF2,B4GALNT1,TNFRSF11B

Negative_7352	GK, IL7, LIF, AKR1B10, ALAS2, ALPP, ATP2A1, C1QC, CACNG3, CCL7, CD36, CDKN2C, CHKB, CSF1R, DGKQ, FCGR3A, GALNT8, GNA11, LHCGR, LIPC, LIPF, MYH7B, OR7D2, OR7G1, RASGRP3, SNW1, TAS2R14, THBD, TLN1, CACNA2D4
Negative_7353	SI, CYP2E1, FCGR3A, GAB1, GALK2, GAPDH, IDH1, MAT1A, MINPP1, NOTCH2, TAP2, TYMP, WNT2, YWHAE
Negative_7354	ABO, INS, ADIPOQ, AGPAT4, AQP7, ARD1A, ARPC1A, ARPC5, CCNA2, CD244, CXCL6, DTYMK, FGF4, FSHB, GALNT3, HADH, HHIP, HLA-DMA, ITGB1, KAT2B, KYNU, LIMK2, MAP3K1, MAP3K3, MMP9, MTMR6, NGEF, OR10A7, OR13C4, OR2Z1, OR3A3, OR5B3, OR6C1, PDE5A, PIAS3, PLOD3, POLR2D, PPP5C, PSEN1, RAPGEF3, RASGRP3, RPN1, SELP, TGFBR2, TLR4, TUBB4, PHOSPHO1, TNFRSF14
Negative_7355	ID4, DIAPH3, HAGHL, PARD6A, PFAS, PON1, PRKAB1, PTPN6, RXRG, SHMT1, WNT9B
Negative_7356	A2M, CD4, CDA, HK3, MPI, ALDH4A1, ANAPC4, APOA1, APOA5, AVPR1B, BMP8B, CACNA1I, CCL3, DPYS, GANAB, HAGHL, HIP1, IMPA1, MAPK13, MLLT4, MRAS, NUDT5, OR2A4, OR8G1, PAPSS1, PAPSS2, PDHA1, PIGU, TAS2R5, THBD, UNC5D, CACNA2D2, HSP90AA1, SERPINB5
Negative_7357	ATP5J, HAAO, JAG1, MMP2
Negative_7358	HCLS1, IRF9, POLD2, PPP1CC
Negative_7359	DCN, DCT, AASS, ALPL, ASIP, ATP6V0C, BTRC, CCL11, CD244, CDC14A, CYP26B1, CYP2A7, HLA-G, HMGCS1, IFNA17, IL1B, LIAS, MTHFS, NFYB, OR10Q1, OR4F6, OR4S2, OR52B4, OR5K1, OR7G1, PIP5K1B, PLXNB2, RPN1, TBL1Y, TBXAS1, WIF1, MAPK8IP1
Negative_7360	AHCYL2, ALDH1B1, CD3E, MYH14, MYL7, OR4K2, SNAI1, PRICKLE1
Negative_7361	CR2, GCK, IL9, PXN, RDX, ANPEP, ARSE, BCAR1, CD58, CDC23, FGF23, GCLC, GPAM, GUK1, IDH3G, ITGA2, ITGB3, MGAT2, OR2B2, OR2B6, OR2Y1, OR52K2, OR56A3, OR5D13, OR5P3, P2RX1, PIGV, POLR2J, PSPH, PTGS1, RPS6KA2, SKP2, SLC25A6, ULK1, UMPS
Negative_7362	GSN, CARS, CD72, CDC27, CDK4, CFL1, CXCL5, FMO4, GMPR, IRF5, MAP4K4, NEU1, PDCD1, POLE4, SDHC, SPTAN1, STX18, TAS1R1
Negative_7363	F2, RET, ACAT2, AQP7, ATP5A1, CACNG2, CSAD, DTYMK, FTMT, IL11, IL6ST, MGAT4B, NCK1, OR6C6, PDE5A, PIGM, POMC, RND1, SQLE, B4GALNT1
Negative_7364	LPL, ABLIM2, ARPC1A, ASNS, GALNT6, GRPR, ITGA9, MAPK8, MKNK2, NCSTN, OR10J5, OR2AE1, PIK3C2G, SDHC, SOS2, WNT8A, YWHAG, SUV420H1
Negative_7365	C8A, CGA, SMS, ACSL3, ADH4, ASCC3, ATP6AP1, CASP8, CDH5, CXCL12, FASLG, HLCS, JAM2, MRAS, NQO1, OR10J3, OR4C46, PIK3R1, PPP1CB, UGT2B4
Negative_7366	GSN, IL5, LSS, STS, VCL, ALDOB, ARG1, ATF2, B3GALT1, BST1, C1QA, CCNG2, CYP2F1, FGF1, GSTA3, IL2RG, ITGA1, MASP2, MGST3, MPST, OR4E2, OR51A7, OR51F2, PIGV, PIP4K2B, PLCB2, PRNP, TAP1, SERPINA5
Negative_7367	PLN, RPE, ACADM, ADSS, CD47, CNTNAP1, FZD2, GARS, GLB1, GNG12, GUCY1A3, HAGHL, IFNA2, ITGB3, MMP2, OR2C1, OR2H2, OR4S1, OR51E1, OR9A2, PARD6G, PERP, PISD, QPRT, SMARCA2, SMURF2, STAT1, SUV39H1, TCF7L1
Negative_7368	IL2, ACP6, ADAM17, FTCD, HEXB, MDM2, MDM4, MRAS, OR52E2, PDE6G, TICAM2, WNT10A
Negative_7369	BAX, BID, CFH, GDA, GLS, NLK, ALG13, CD274, CYP11A1, CYP4A11, DBF4, DIAPH2, DKK2, DLAT, FGF19, GALC, GCNT4, INPP1, MAGI2, MAP4K1, MYH11, MYH9, NOX3, NT5M, OLR1, OR2M3, OR5J2, P2RX2, P2RX7, PPP2R1A, PVRL4, RAPGEF4, RASSF5, SETD8, SHC2, TACR3, TAS2R1, TNFSF9, UGT2B17, VAV2, VNN1

Negative_7370	CFI,ATP6V1F,CAMKK2,CDH4,CES1,CHST13,CLDN9,CSF2RB,CSNK2A2,CYP21A2,CYP3A5,DGAT2,DGKQ,DHCR7,G6PC,IL10RA,IL20,IL28A,IRAK2,ITPA,LDHAL6A,MAML3,MTHFD2,NCF4,NEU1,OR2F1,OR2T34,OR4F15,OR51F2,OR7C1,OXSM,P2RX5,PNPT1,PPP3R2,PRKAR1A,PRKY,PTPMT1,SEMA3E,SOCS4,SPCS1,STX12,TAP1,THY1,VAMP1,VDAC3,WWP2,YKT6,MAPKAPK5,SERPINA5
Negative_7371	FH,CACNA1D,CLDN14,EXOC7,GLI3,IL19,LAMC1,OR2K2,POLR3H,SETD2,SLC27A1,SOCS5,SULT1E1
Negative_7372	DHH,ADSS,ATP6AP1,CACNA1I,CERK,COL4A6,COL6A2,CRKL,CXCL6,CYFIP1,GJD2,GPX5,GSTO2,HLA-DRA,IL17RB,IL6R,INSR,MRAS,MYH6,NFKB2,OR2A42,OR4C11,P2RX5,PAX4,PHKA1,PIK3C2G,PLCB3,PLD2,RAP1B,SMAD2,STAM2,TAS2R1,TNFSF4
Negative_7373	ADK,CKM,ID1,RFK,ACACA,ACADVL,ACLY,AK3L1,ASCC3,CCL22,CYP2A7,E2F1,EIF4B,EPHA5,FDFT1,GALM,GRM1,ICAM2,IFNA13,IL13RA2,ITGA2,ITGAM,JAK3,KIR3DL3,MAPT,MYD88,MYLK3,NSD1,ODC1,OR10H3,OR1D2,OR2D2,OR2T10,OR2T4,OR8K5,PER3,PIP5K1A,PPM1A,PPP5C,PTGFR,RUNX1T1,SDC4,SHC4,SOX17,TRPC1,UGT2B17,WNT9A
Negative_7374	DBT,ADRA1A,ATP2A2,ATP5D,CD44,CRY1,DAAM1,EFNA1,EGFR,ENTPD1,ENTPD3,FRAT2,FUT4,FZR1,G6PC,IDH1,LAMC2,LEF1,MTMR1,NOS1,NOX1,NPAS2,OR2C3,OR52L1,PCCB,PGAM4,PLCG2,PTPRB,SOS1,SRD5A1,TNFRSF4,TUBB2B,VCAM1,XIAP,C1GALT1C1,ST6GALNAC1,TNFRSF10D
Negative_7375	C2,IHH,PML,RPE,ACAA2,ADORA2B,ADRB3,AMHR2,B4GALT6,COL11A1,CYP3A7,DAPK2,FGFR3,FIGF,GMPR,GP1BA,GUCA1B,IL22RA1,IL28RA,IQGAP3,JAK3,LTBR,MEF2C,NCOA4,NFKBIA,OGDH,OR13C4,OR2S2,OR2T6,OR4D1,POLR2L,PRKAR1A,PRKG2,PSME3,RAPGEF4,SEMA3G,SHMT1,STX4,TRAF6,TSHB,UBA7,UNC5B
Negative_7376	CS,AFMID,ALDH3A1,AMPD3,ATIC,CDH1,DMGDH,EIF4E,GMDS,GNPDA1,HEXA,MDH2,MTHFD1L,NCOR2,OR13G1,OR4C6,PERP,ST8SIA5
Negative_7377	APOA2,ATP2A2,C1QC,CD59,FGF4,HIBADH,PIGU,RDH12,TUBA1A,UGT8
Negative_7378	AK3,AGMAT,AGPAT6,APOC3,CCR1,CHPT1,CYP26A1,GALNTL1,GCDH,GNA11,GRM5,HSPB1,IAPP,IFNA4,IGFBP3,KLK3,LRRC4C,MCCC1,MTHFR,OR4K13,OR52E8,ORC6L,PNMT,PTPMT1,PTPRZ1,SPI1,TPM3,B4GALNT1
Negative_7379	C2,CFH,ACSM1,ATP6V0B,BCL2L1,BDNF,C5AR1,CHPT1,CHRM1,CRB3,CSF1R,DAG1,DGKB,DPYSL2,FABP4,GALNT6,GNA13,IRAK1,KLRC4,NFYB,OR2D3,OR3A1,PAPSS1,PDE1B,PDE4B,POLR1D,RBX1,ROBO3,SKIV2L2,TPM3,VAMP3,WNT6,YWHAZ,ZNRD1,CACNA2D1
Negative_7380	DCK,LCK,LPO,ACADVL,ALDH3B1,APLP1,CD58,CNGB1,CYP3A5,DAAM2,EDAR,ENPP3,FUT5,FZD2,FZD4,GCH1,GRAP2,HEMK1,HSD17B8,IFNA7,KITLG,LALBA,MAPK14,MAPK8,NADK,NOD1,OR10H1,OR10H3,OR1A2,OR52N5,ORC2L,OXCT2,PLCB1,PPP2CA,SDHC,SESN1,SH3BP2,TGFA,TNFSF8,TNFSF9,UNC5B,HSP90AB1
Negative_7381	F10,FST,GNE,HAL,MYC,AKR1D1,B3GALT1,BMPR2,CACNG7,CCL20,CES1,CTSG,CYBA,CYP8B1,DUSP9,ENAH,F13B,FGF6,FLAD1,GAD2,GALC,GSTM5,HTR6,HTR7,IPPK,ITGB2,LAMB4,MBL2,MMP2,MYL9,NANS,NFKB2,NUMBL,ONECUT1,OR13C3,OR2AG1,OR5H2,OR5I1,OR6B2,P2RX4,PHKA1,PIP5K3,PLCZ1,PLXNB2,POLE,PRKCI,PSPH,RDH10,SOCS5
Negative_7382	ADCY5,ANAPC1,COL1A2,CYP21A2,GRIN1,IFNA8,MAP3K14,OR9G1,POLR3D,STX8
Negative_7383	ATP2A2,FUT3,OR5V1,OR6C70,OR6T1
Negative_7384	ABCC8,AKR1B10,ALDH1A2,CACNB2,CARS,DEGS1,DKK1,EPHX2,FGF3,NOX3,OR51Q1,OR56A3,OR5T3,PAX6,PIGB,PMM1,SEMA3B,SEMA3F,TJP2,SERPINF2

Negative_7385	F2R,FGB,GPI,SMO,TEC,TKT,ACOT11,ADRB1,APOA5,ASMT,BHMT,CCL22,CKMT2,CLDN17,CLTB,CSNK2A2,DARS,DDB2,DFFA,EXT1,F13B,GGPS1,GIT1,GNRH2,GUCY1B3,HAO2,HRH1,IFNA10,IL17RB,MAP4K1,MYL2,OR12D3,OR3A1,OR56B4,OR8D4,PDGFC,PLAU,POLE,PRKY,RAB23,SPTLC2,SV2C,SYNJ2,TCIRG1,TMLHE,TNFSF12,UNC5A,ZAP70,ZNRD1
Negative_7386	CLDN5,CLOCK,CSDA,EPRS,GSTA1,GSTA2,HDAC2,HRAS,IL23A,MLYCD,MMP14,NPR2,OR5D16,OR5K1,PAX6,PIK3R2,PLXNB2,PPP2R5E,PRIM2,SELP,UGT2B4,UNC5D
Negative_7387	AK1,HGD,TK2,ACVR1C,ADRBK2,ALG11,BUB3,CDH15,DBF4,DIAPH3,EDAR,FRAT1,GAL3ST1,GYS1,IL9R,IRS2,LDHAL6A,LLGL2,LMO7,NFAT5,OR10G3,OR1S2,OR5AR1,OR5D16,OR5H6,OR6Q1,PDE5A,PIK3R3,PISD,PLOD3,PRKCI,TRAF5,UCKL1,ULBP1
Negative_7388	TPR,AADAC,ADRB1,AQP7,ARPC2,ARPC5L,BET1,CAPN2,CREB1,GNMT,HSD17B1,LRP6,MDH2,MDM4,MYLK3,NLGN2,NR5A2,OR10H1,OR4Q3,OR7A10,PIGF,PLXNB3,PPP2CB,PPP3R2,PRKCA,RASSF5,STT3B,TAS1R1
Negative_7389	GNE,LHB,RFK,SRC,AGMAT,AGRP,B4GALT5,CFTR,CHST13,CLDN6,CYP1A2,FUT3,GRIA1,GSTP1,IL10RA,IL22RA1,ITGAV,LTBP1,MAP2K2,OR2V2,OXTR,PDGFB,PEMT,SETDB2,TNFRSF10C
Negative_7390	AKT2,BBC3,CNGA3,DHDH,MAML3,MAP2K3,MAP3K7,OR51A7,OR8K1,PCYT1A,PDX1,SORD,HLA-DRB3
Negative_7391	RDX,TNC,ADCY1,AKR1C3,ARRB2,ATP5G1,BLVRA,BPGM,CDC7,CXCR6,CYP27A1,DGKG,DUSP8,EPHA7,GARS,GMPS,GNAI2,ITPK1,ITPKB,NCF2,NTF3,NTRK1,PLXNC1,POLR2K,SMPD4,THBS3,ST6GALNAC4
Negative_7392	AGL,ADH7,AKT1,ALCAM,AMDHD1,APC2,ARRB2,CCL11,CCND3,CHDH,CX3CR1,CYP2C9,ESPL1,FOSL1,GALM,GMPR2,GP1BA,GPX5,HPRT1,KIR2DL3,LEFTY2,MAN1B1,NEGR1,NKX6-1,NTF4,NTRK2,OR10J1,OR2M3,OR2M5,OR51I1,OR6B3,OR8H2,PDE1B,PDE7B,PDK1,PGK2,PIK3CA,PPAT,PTPN6,PYCRL,SGMS2,SLC27A4,SPTLC1,STAT4,SUV39H2,UGT1A10,VAV2,TNFRSF11A
Negative_7393	GNE,ZYX,A4GALT,ATP2A3,ATP6V0C,CD70,CDH2,CLDN10,DARS2,DGAT2L4,DHDDS,E2F1,GJA1,GNA11,GNA15,GNAL,GNMT,HARS2,IFNA21,IL17A,ITGB8,MAP4K3,MDM4,OR10H1,OR5F1,PCK1,PIPOX,PLA2G10,PLAUR,PPARD,PPP2R1B,PRKAG2,RCHY1,RXRA,SMPD2
Negative_7394	BTK,GNE,PGP,ALDH3B1,ALPP,AMPD2,ATF2,CACNA1D,CACNG4,CASK,CD80,CHRNA7,COL6A2,ENTPD3,EPB41,FARSB,HMGCR,IL23R,LEF1,MAML3,MDM2,MKNK2,MMP1,MS4A2,NANS,NEUROG3,NFATC3,NKX3-1,NLGN2,NMNAT2,NTF4,OR51E2,OR6N2,OR8D2,PIAS1,PIAS2,PIK3CA,PIK3R3,PIP4K2C,PLXNB2,POLR3A,PPM1D,RAF1,STX1A,TUBA8,VDAC2,YWHAQ,ZIC2,ATP6V0E1
Negative_7395	IL5,KMO,PC,VTN,ANAPC2,APPL1,CD74,CHDH,CYP2S1,DMGDH,DPM2,EDA2R,ENAH,GSTA1,HSD17B2,JAM2,LIMK1,PAK3,PDCD1,POLR1E,RRM2,SARS2,SRGAP1,STAT1
Negative_7396	ATM,PML,TKT,ADCY7,B3GALT5,CCNB1,CD22,CDKN1A,CFTR,CRB3,CSNK2B,CXCL2,DHRS4L2,EPHX2,FTCD,GLG1,GNRH2,HLA-DRA,IFNB1,IL24,KAT2A,OR10G2,OR2A25,OR4B1,OR51D1,OR6S1,PIK3CB,PIP4K2A,SOAT2,SPHK2,SPP1,STAT4,SUCLG2,TPK1,TSHR,TUBB2C,ST6GALNAC3
Negative_7397	ADCY6,CDK6,CLDN6,CXCR3,CYP4F3,FTMT,IDI1,MMP2,MPZL1,NFE2L2,OR2M2,OR8U1,SAT2,TBL1X,TUBA1C,HSD17B12
Negative_7398	CEL,ACY1,COL4A4,MYH2,MYH7B,OR2T5,SMAD9,SPRED2,TPK1,TUBB4,SUV420H1
Negative_7399	HK2,ACOT11,ACSL5,ATP5D,CCL19,EARS2,ERBB3,FGF19,IFNA6,JAK3,KIR2DL1,OR10V1,OR2T29,POLA2,PPP1CA,PRKAR2A,PVRL2,SMURF2,STX1A,TICAM2,TYK2,WNT5B

Negative_7400	ID3, LPO, ME1, MPL, AADAC, ADIPOR2, AGRP, AKR1C4, B3GALT2, BCAR1, BDNF, BUB3, CACNA1H, CCL13, CLDN20, CXCL2, DAD1, FASN, FGF2, FGF6, GNAQ, HADHA, HAO1, HDAC2, IFNA14, IL22RA1, MAP2K6, MAPK14, NARS2, NEU2, NRAS, OR10G3, PON3, PPP2R2A, PRKAG3, PRPH, PTK2B, PTPN5, PVRL1, RAF1, ROCK1, RUNX1, SDHC, SOCS2, TNFRSF14, TNFRSF6B
Negative_7401	ALOX15B, CSF1R, IFNA17, OR2M4, PIGF, SDC3
Negative_7402	ACOT11, ATP4A, CCL11, CCL5, CLDN5, CYP8B1, DARS, DDX41, DGKQ, GADD45G, GUCY2C, HNF4A, IKBKG, LAMA4, LEF1, LTB4R2, NAGA, NRP1, NTF4, OR10G7, OR1N1, OR2L2, OR6K2, OR8A1, OR8K1, P2RX6, PDGFD, PDHA2, PIK3R1, PRLR, PTPRB, QARS, ROBO3, RPS6KA3, SEPHS1, SPHK2, SRGAP3, ST6GAL1, STX1B, SUOX, TAS2R4, TM7SF2, TRHR, TUBB, UGCG, UGT2B17, UGT2B28, ATP6V1C1
Negative_7403	EDA, F11, HK3, ALPL, AMY2B, B3GALT1, CACNA1E, CCNB3, CSNK1E, CYP2F1, DGKA, EARS2, EPHX1, GUSB, HLCS, IDH1, IGF1, IRS2, KDSR, LAMC1, METTL6, MGAT5B, NAGA, NANS, NOX1, OGDHL, OR4F15, OR5D13, OR5P2, OR6B3, OR6K6, PDE1B, PLA2G2F, PLCD3, POLR2B, PPAP2C, SOS2, SPCS1, TCF7L1, TNXB, UGT2B7, ATP6V1E1, TNFRSF18
Negative_7404	ALDOA, ATP5E, BET1, CALML6, CDC20, CDC45L, CERK, CLDN6, CYP11B1, CYP2B6, DUSP8, ENPP2, FGF21, FZD6, GALK1, GALNT11, GATM, HIBADH, MMP2, MMP9, NT5M, OR2L3, OR4P4, OR51E2, OR5AS1, OR6K2, PDGFA, PLEKHA8, POLR2F, POLR3G, PRKCQ, PRPH, PSME1, RDH10, RPS6KA1, SELE, SMARCA5, SOCS2, TAS2R7, TICAM2, TNFRSF10C
Negative_7405	C1S, TXK, ARG2, CBR3, EDNRA, GRM1, IL22RA2, OR4C13, OR7G2, PIGV, PRKAR2B, SAT2, SELP, TUBA1A, UNC5C
Negative_7406	FGG, KDR, LCK, A4GALT, ADORA2B, BACE2, BBC3, CAMK4, COL5A1, CYP3A43, EPHA1, GNAZ, HAAO, HMGCS1, IDUA, ITGB5, LRP5, MAT1A, MCM4, MGST3, MRAS, NCF2, NCOR1, NKX3-1, OR1M1, OR51B6, OR52M1, PON2, PPOX, PTDSS2, ROBO3, SESN1, SH2D2A, TAS2R8, TAS2R9, TLR5, VAV1, XIAP, ATP6V0A2, CACNA2D3
Negative_7407	ATM, CRH, ACSL3, AGPAT6, ALDH1A1, ALPPL2, ARHGAP5, C5AR1, CASP9, CHEK1, CRY1, EXT2, FCGR2B, FGF13, FUT9, GALNTL2, GSTM4, IL6ST, LDHAL6B, MCM7, MTMR6, OR1L4, OR5D18, OR6B2, OR9K2, PDCD1, PHGDH, PIK3R3, PLA2G2F, PPP2R1B, PRKCG, PYCR2, PYCRL, RBPJL, SESN1, SH2D2A, STAM, TJP2, ATP6V0A1, TNFRSF13B
Negative_7408	GBA, SDS, ACSS2, ADCY5, ADH1B, AREG, CCNB1, CYP26C1, DAPK1, FGF7, GSTM2, GSTO1, HSPA8, IMPA2, INPP4A, MMP1, OR51T1, PLA2G2A, PTTG1, RHOA, SGPP2, SMURF1, SYNJ1, UGCG, WWP1
Negative_7409	GH2, MPL, SYK, ADRB1, B3GALT4, BMPR1B, CASP6, CDH15, ENTPD7, EP300, FCGR2B, FRAP1, GGCX, IFNA8, MALT1, OR13C2, OR2AG1, P2RX4, P4HA2, PDE4D, PMVK, PSPH, SHC2, STAT4, UBE2L6, WWP2
Negative_7410	F2, FAH, SRF, AGTR1, ALDH3A1, ATP5E, BUB1, C3orf10, C4BPB, CCL19, E2F4, ECHS1, FGF12, GALNT6, GIT1, GSTA4, HMBS, IL15RA, MTHFD1, NAT2, OR14J1, OR1E2, OR1F1, OR5A2, OR8I2, POLR2J, PON3, PPP2R2B, RHEB, SEMA3D, TACR1, TLN2, TUBAL3, ULBP2, UNC5A, UPP1, WASF1, WASL, ATP6V0D2
Negative_7411	F2, TK2, ABAT, ACP1, ADCY5, CCL2, CSF2, CSNK1G3, CYP2E1, CYP2F1, DAAM1, DLL3, GUCY2D, HSPA2, IL12A, ITGA4, LAMC3, LPAR1, OR10A2, OR4A5, OR4C13, OR4K14, PCYT1A, PPBP, ROCK1, STX6, TCF7L2, WNT3A, HLA-DPA1
Negative_7412	PGD, TH, ABLIM2, ACTN1, ANAPC11, ASIP, CACNB2, CCL24, CDH2, CHRNA7, COQ7, CREB3L4, CSNK1G1, DDHD1, DDX56, DGUOK, EPHA6, GRM5, IFNA16, ISYNA1, MPP5, MYL2, OR2H1, OR4A16, OR6Q1, ORC4L, PLCB2, PRPH2, SARS, WNT9B
Negative_7413	MVD, SP1, ACSL4, AGPAT6, CYP2A13, DTYMK, EXTL2, GLYCTK, KIR2DS5, NCF4, OR52A1, OR52I2, PHPT1, RPN2
Negative_7414	ACYP1, AGXT, CSNK2A2, CTBS, IRAK4, KDSR, ZFYVE9, ATP6V1G1

Negative_7415	GSTT1,NANP,SMAD3
Negative_7416	MUT,CMPK2,COX15,ENTPD7,OR1D5,PDE3A,RPN2,RPS6KA5,SIAH1
Negative_7417	F9,NGF,ACAD8,ALS2,ANAPC7,AREG,BDH1,CDC14B,CYP4A11,EIF4E2,EPHX1,FGF19,GNPNAT1,GRIA2,HIP1,HTR7,IL13RA2,INSRR,NCK1,NEGR1,NFAT5,NLGN1,NRP1,OR1L4,OR2G2,OR2T11,OR4K1,OR4S2,OR52A5,PLA2G2F,SELPLG,SESN2,SMURF1,SPCS3,ST3GAL4,TAS2R13,THTPA,XCL2,CSGALNACT2
Negative_7418	ADK,ATP6V0B,BMP8B,CDH2,CDK2,CLDN10,CLDN2,FGF13,MCM2,NAT6,NFAT5,NT5C,OR5M3,PTPRJ,RAC3,TNFRSF18
Negative_7419	ADA,C8G,KMO,SHH,VTN,ADAM10,ALCAM,ALDH9A1,ALOX15,APC2,ATP6V1H,BHMT,CHST11,CNTN1,CSNK1G2,DRD2,GNPNAT1,GPX7,KLRC4,LCAT,LIFR,MAPT,MDM4,MYH2,NARS2,OR4D1,OR4D11,OR52N1,OR56B4,OR8D2,P2RX2,PDE2A,PLCG2,PSENEN,ROCK1,SKIV2L2,SMAD3,SORBS1,SPTAN1,STK4,YWHAB,KIR2DL5A,PPARGC1A,PRICKLE2,ST6GALNAC5
Negative_7420	HDC,IL2,SI,TFG,ACOX3,COMT,DEGS1,ELK4,EPHA6,EXT1,GALK1,GCLM,KLRC3,LAMB3,MAPK9,NAGA,NMNAT1,OR2T6,OR8J3,PTPRC,RUNX1,UBE2K,VAMP3,PDCD1LG2
Negative_7421	KIT,PLG,AACS,ADSS,AGMAT,AGPAT1,ARNT,C9orf95,CCL17,CDC2,CDH1,COL2A1,CSNK1G2,EPB41L2,EPHB3,FZD8,GNG13,GSTT1,IFNA16,ITGA1,LAP3,NAGLU,NME2,OR10K1,OR14I1,OR1E2,PDHA2,PIAS4,PNPLA4,PPP2R2B,PTGFR,PTPRJ,SAT1,SCP2,ST8SIA5,STX8,TAS2R14
Negative_7422	IL9,ALPI,ATP6V1A,CACNA1I,CHEK1,CLDN5,COL6A2,DAXX,DPM3,FOXO1,FZD2,GALNT2,GALNT8,GGCX,GNRH2,INSR,KAT2A,MAP3K13,MPST,MYL9,NCSTN,OR1L1,OR7G3,PANK1,PIP4K2A,PRKCZ,PVRL3,PYCR2,RAB23,ROCK1,SFRP1,SLC25A4,SMPD2,SV2C,TAS2R9,YKT6,CACNA2D4
Negative_7423	DBH,ALG2,CCL20,COL5A1,CUL1,EPHA7,EVI1,GNA14,GRPR,HLA-DRA,IFNA7,ITGA1,LRAT,MNX1,NOTCH3,OXSM,PANK3,PDE3A,PSME2,PTPRM,RBL1,SLC2A1,SMURF1,SUV39H1,TBL1X,UGT2A3,UMPS,UROS
Negative_7424	ABO,DCC,EDA,F12,IL5,ATP6V1F,CSF2RA,DVL3,FOXA3,GPD2,HDAC1,IFNAR1,INPP5B,ITGAV,KCNQ1,MCM2,NEFH,NLGN2,OR4X2,PARD3,PFN1,POLR2K,PVRL3,SPHK2,TOMM40,TXNDC12,WWP2
Negative_7425	CP,TK2,ACAA2,ATP5J2,B4GALT4,BUB1,CCR1,FDFT1,FZD10,GNG12,IFIH1,IRF9,MBL2,NEUROD1,OR10A5,OR10H5,OR1N1,OR7D2,PARD6A,PDE1C,PIGT,PLA2G2A,POLR3A,PPP1R3A,SETMAR,SIGLEC1,SMARCA2,MAPKAPK2
Negative_7426	TNC,ALOX15B,ATP6AP1,BCKDHA,CRHR1,EARS2,GALC,GMPPB,GPD2,HAGH,INPP4A,LAMC2,MAP2K2,NCR2,NT5C1B,PHKA2,PSAT1,PTPRZ1,ULK3,WASF3
Negative_7427	ASL,ADIPOR2,ALPPL2,AMDHD2,ARPC4,ATP6V0B,BDH1,C4BPB,COX10,CYP2B6,DDX18,ICOS,IL24,LAMC3,MAP3K8,MAP4K2,MNX1,NCKAP1L,NEU2,NFATC4,NFKBIB,NTRK2,OR2F1,OR2Z1,OR52B4,PLCB4,POLR2A,POLR2H,PTCH1,PTGS2,RAPGEF2,RBL1,RBPJL,RDH5,RPN2,SDC1,TNFSF9,UROS,VAMP2,VDAC1,WNT2B,EIF4EBP1,SERPINC1
Negative_7428	CAT,FAH,GP6,MOS,NNT,BAIAP2,CCL24,HRH1,NRXN1,OR4N2,OR51B6,OSMR,PDE6G,PIK3CA,PRKACG,SDHA,SEMA3F,TYMP
Negative_7429	APRT,CD86,CDC6,CPT1A,CSNK2A1,DEGS1,HLA-DMA,HSP90B1,IL3RA,OR56A4,PLAUR,PTDSS1,RPS6KB1,SIAH1,ST3GAL1,STX6,ST6GALNAC6
Negative_7430	AK1,MTR,AMDHD1,ARNTL,EPHX1,GALNT11,GUCY1B3,IL1B,LAMC2,MAD2L1,NFKB2,NOTCH1,OR2L2,OR4C15,OR52E2,PIK3R3,PRKACA,PRKCD,RPRM,STX12,TPMT,TNFRSF11B

Negative_7431	ABO, AUH, DDC, SRF, SRM, ACSL5, ACTG2, ADCY4, ALG1, ATF4, ATP5D, CARS2, CAV3, CCR3, CLCA2, CYP19A1, DFFA, DGKA, DTX2, FGF5, GUCY1B3, IL12RB2, MPDZ, NME2, NNMT, OR2M7, OR2W1, PDGFB, PEMT, PLD1, PLK1, POLR2K, PPA2, PPP1CC, RXRA, SMAD3, SULT1A1, TCIRG1, UGT2B4, UNC5B, WNT6
Negative_7432	GNS, BTRC, FZD7, HGSNAT, INPP4A, MAP3K1, MYH15, OR13C2, SH2D1A, SLC2A1, UGP2, WARS, PNLIPRP2, TNFRSF13C
Negative_7433	CRH, CSK, IL5, PLG, AADAC, AANAT, ACTN1, ALAD, ALAS1, ALG5, ARPC1A, CCR4, CNGA4, CXCL10, ERBB2, GDF7, GJA1, HSD17B1, MAP3K14, MYH8, NAT2, NCR1, OR51A2, OR6C1, OR7D4, OR8J1, PAK1, PDE6H, PDE7B, PGAM4, PPM1D, PPP1R3B, RAP1A, SFRP1, TRADD, WNT7B, ZIC2
Negative_7434	ACSL1, B4GALT4, BIRC3, C4BPB, CYP3A4, GNG13, IDI1, MAML3, MGAT4A, PIP4K2C, ZNRD1
Negative_7435	C8B, CRH, F2, FER, ME1, ADORA2A, BACE1, CACNG5, CD82, CREB3L2, CTSB, CYP2C9, DTX3L, FLOT1, GGT1, GMPPB, INPP4B, IPPK, KCNJ11, MAGI1, NARS2, OR52E4, PARD6B, PDE3B, POLR2C, PRKAB1, PTPN7, RERE, STX6
Negative_7436	BTC, C4B, F7, PML, TK1, ACMSD, AKR1A1, AKT3, BCL10, BDNF, CBR3, CD79B, CLDN3, DGUOK, GRLF1, HLA-DRA, HPRT1, ITCH, LIPF, OR1B1, OR2G3, OR4F4, OR52J3, OR5P2, OR8H1, OR8I2, PDE2A, PLA2G2A, PPOX, PPP2R5B, PPP2R5E, PPP3R1, PVRL2, SMAD7, TDO2, TUBB2A, UBA1
Negative_7437	ALG3, ASCC3, COQ5, CXCL1, HSD17B2, ITGA11, LIPG, MAPK1, NQO1, NUDT12, OR52L1, PIK3C2G, PLIN, PTPRC, PVRL1
Negative_7438	PTS, B3GALT1, CACNB4, CREB3L4, CYP11B1, HTR7, INPPL1, SCP2, SDHD
Negative_7439	CAD, CSK, DDO, DLD, SMS, ACTA1, AK3L1, AKR1C3, ALPPL2, CTPS, CYP2D6, DARS, DDX23, DOT1L, DPYS, FTCD, FUT3, GNA12, GSTM3, ICAM1, IFNW1, IGF1R, INPP5E, MAP4K2, MAPK13, NEFH, OGDHL, OR2AG2, OR2M5, OR2T5, OR4F6, OR4S1, OR9I1, PCK1, PDE6G, PGM1, PIAS1, PIK3CB, POLD3, PTPRC, RPN2, SDC3, SFRP1, SNAP23, TNFSF4, XYLT1
Negative_7440	SDS, BAIAP2, CCL21, CLDN14, COQ7, GPD2, ITPA, MNX1, NOX3, OR52N4, P2RX5, POLA1, UGT2B11
Negative_7441	OTC, ACTA2, ACTN1, CACNB2, CCR3, GALK1, GNAI3, HMGCS2, IL29, LAMB3, OR9I1, SELE, TFDP1, HLA-DQB1
Negative_7442	ARNTL, CSF1R, FZD5, IFNA14, MTMR1, OR11H4, PAPSS1, PRKCQ, STK3, B3GALNT1
Negative_7443	ACPP, ACTN2, ALDH4A1, CACNA1B, CADM1, CLDN5, CSNK1G2, CYP19A1, CYP2C18, ENTPD8, FGFR3, GALNT8, GUCA1B, HRH1, IL20RA, IL21, LHCGR, MAD2L2, MAPK14, NADK, NEFL, NRG4, OR10X1, OR2B2, OR3A3, OR8J1, POLE2, PPP5C, SFRP4, SMAD2, SRGAP2, STAM, STAT3, UGT2A1
Negative_7444	ADCY9, CCKBR, CNGB1, FGF8, GBA3, ITGA10, NOTCH3, OR2B11, OR52W1
Negative_7445	GDA, MTR, ABAT, ARNT, BCAT2, CDH3, CLCF1, COL4A2, DGKI, DTX3L, GALNTL5, HLA-G, IDH3B, INPPL1, MAPK14, MCM4, OR8H3, PHGDH, PRKCH, ATP6V1E2
Negative_7446	CRH, VTN, ADRA1D, ALDH3B1, CLDN4, CREB3L1, CRKL, CXCL5, DHFR, DKK4, DUSP3, ESAM, EXOC7, FZD5, GNA14, HAP1, HSD3B1, LDHC, LLGL1, NT5C1B, OR13C4, OR5T3, PIK3CG, PON1, PRNP, RAC2, SFRP1, SMPD2, YWHAZ, PAFAH1B1
Negative_7447	ME2, ACSM5, ADSS, CSDA, DAXX, DVL1, FASN, GALNTL1, ICAM2, IL21, IRF7, KLRK1, NME4, NOS3, OR2G6, OR8D1, OR8J1, OR9Q1, PAX6, PAX8, RUNX1T1, SFRP2, SSH2, TAS2R9, SUV420H1
Negative_7448	AMPD1, DUSP3, IL22RA1, MYH6, PAX4, PSEN1, RRM2B, TUBA8, WNT2B, GABARAPL2
Negative_7449	ACO2, CACNA1F, CYBA, DGAT2, ECHS1, EFNB3, IRS4, ITCH, JAK1, LAP3, MDH1, OR2F1, OR5I1, OR8H3, STX6, TAS2R13, TAS2R42, TAS2R48, TGFB3, UAP1
Negative_7450	MAX, XDH, CDC42, LAMC2, OR4B1, OR5P3, TNXB, WASL

Negative_7451	ABO,C8B,CAT,PRL,ACO1,ACVRL1,AMDHD2,APOE,B3GALT6,BCKDHB,CCL27,CCL4,CD247,CSAD,CYP27A1,CYP2S1,CYP8B1,DGKI,F13A1,F2RL3,FABP3,GRPR,GSTZ1,HAO1,HAO2,IFNA6,IL17A,LHCGR,MBL2,MMP14,MYL5,NFYB,NPR2,OR11A1,OR13G1,PDE7A,PMM1,POLR3B,PPARG,PTGFR,SEMA3A,SUCLG2,TAS1R3,TLR2,WASF1,WNT5A,SERPIND1
Negative_7452	F2,FN1,LEP,MSN,SFN,AARS,ACTG2,BCKDHB,BMP8B,CBLB,CCL8,CCND2,CCND3,CCR5,CD55,CDC25C,CDKN2A,CLCF1,DCTN1,DDX19A,DGUOK,DKK1,EGLN3,ENO2,FGF1,FZD4,GALNT8,GNMT,GNPAT,GNPNAT1,GZMB,HIBADH,IFT57,LAMB4,MAOA,MAP2K2,MAPK14,OR2K2,OR4D11,OR6C1,PHKB,POLR3G,RARS2,RDH12,SPTAN1,STAT2,TACR2,TRAF2,VAMP3
Negative_7453	FAS,ACTG2,CD40LG,CNTFR,FDPS,GOT1,IL25,ITGAX,OR10V1,PDE10A,PEMT,PLXNB1,PPP2R5D,SEMA4C,TGFBR1,TRADD,TYROBP,UPP1
Negative_7454	CS,ADRB2,CLCF1,CSF1,CSNK1G2,CXCR4,DUSP1,FDPS,FZD7,IL5RA,OR10J1,OR7A5,OR7G3,PDE8A,PDGFRA,PER1,PPP2R5D,SDHA,SMPD2,SUCLG2,TGFBR2,THBS4,TSLP,UGDH,YWHAE,HLA-DPB1,ST6GALNAC3
Negative_7455	ACADM,F2RL3,FGF8,PRKG2,MAP3K7IP1
Negative_7456	F2,GGH,ID1,ADIPOQ,AKT1,ALDH1A3,ARHGAP5,CARS,CDKN2A,CSAD,CXCL6,DTX1,ELK4,GLS2,GSTA2,GSTA3,IFNA14,IL12RB2,IL20RB,OLR1,OR10G3,OR11G2,OR14A16,OR2A4,OR51I2,OR7G3,PFKFB1,PNPLA3,PTPRF,SEMA4D,SH2D1B,SSH2,SV2B,TNFSF9,TSHB,WNT5B,XCL1,SERPINA5,TNFRSF10B,TNFRSF17
Negative_7457	TXK,CYP11A1,FGFR3,HLA-F,OR5A2,STX16
Negative_7458	ACVR1,AGTR1,CACNG8,CBLB,CCR4,CDK2,CLDN10,CLDN8,COL4A6,CPT1B,CTF1,CYFIP2,F2RL2,FARSB,FZD1,GALNT14,GMPS,GNRH1,GTSE1,IL15RA,IL17A,ITGB2,MAOB,OR2T4,OR52E2,OR7A5,PLXNA2,POLR2L,PPP2CA,PPP2R5B,SEMA3D,SMPD4,SPRY2,SPTLC1,SSH3,TAS2R1,TNFSF4,WASF3,XCL1
Negative_7459	EZR,IL3,LAT,NF1,ACOT11,ATP13A2,BCAR1,BET1,CCL7,CKS1B,IKBKB,LIPC,NCAM1,NSDHL,OR10H2,OR9A4,OR9Q2,PERP,PIGQ,PLA2G1B,POLR3B,PRKACB,PRSS1,RAPGEF3,RETSAT,RPE65,SEMA4B,TAS2R48,TPH2,TUBB
Negative_7460	DDO,GSS,CACNA1G,CBLC,CYP2C8,GCLM,MCM6,MGLL,MTFMT,NT5M,OR6C1,PIK3CB,PLCZ1,PPP2R5D,PSEN1,RASGRP1,TRHR,TSC2,HLA-DPA1,PNLIPRP1,SERPINF2
Negative_7461	DBH,KIT,MPL,AGPAT2,ALAD,ALPPL2,APLP1,C5AR1,CRY2,DAPK1,E2F5,GRB2,GSK3B,HMGCL,ICAM1,INADL,JAM2,MBL2,MRAS,OR13F1,OR1L4,OR5AU1,OR5L1,PDE9A,PGAP1,RBPJ,RFXAP,RPE65,SMAD3,SOCS1,ST3GAL6,UGT2B28,YARS,ALDH18A1,ATP6V1G2,HSD17B12
Negative_7462	ACTN1,AGRN,BHMT,CCL7,INSR,JUND,MDH1,NT5C1B,NUDT12,OR52I2,ORC2L,PCK1,POLR2K,RIPK1,ROBO2,SLC25A5,SOS1,TACR2,UPRT
Negative_7463	ATP5C1,BAIAP2,BLNK,CDH5,CXCL2,CYP2A6,DAG1,DIAPH2,E2F3,EPOR,HADHB,IRAK4,NAE1,NFYC,OGDHL,OR5AC2,PGAM1,PIAS3,PRKX,PRPH,RPE65,TAS2R3,TNFRSF8,TSHR,PLA2G12B
Negative_7464	CLDN14,CXCL2,CXCL9,HAAO,OR8J3,POLA2,WNT10B,XCR1
Negative_7465	C8B,GP6,LEP,NPL,RET,TNN,ADH4,ALG10,AXIN1,C5AR1,CD59,CHKA,CX3CL1,EFNA1,MCEE,MPZL1,NKX2-2,OR4B1,OR4K14,OR5D16,OR7G2,PANK4,PDE4C,PHPT1,RBL2,RFXANK,SHMT2,SMC1B
Negative_7466	FER,KMO,MIF,ACVR2B,ATP5D,BAAT,CACNG4,CCL23,COL4A4,CPT1C,CSNK2A2,DERA,DHCR24,EPB41L3,F13A1,FZD3,GSTA5,GYS1,HMBS,IL2RB,INSR,IRF5,ITGAV,MAP3K6,NPR1,NRG1,OR6C76,PDK1,PECAM1,PFN1,PPP2R1B,PRKAA1,RDH11,SMAD5,TAS2R8,TUBA1C
Negative_7467	B3GAT3,CDC2,CES2,CTNNB1,CXCL12,DGKH,EPHA4,FARSB,GBA3,NCOA4,OR52B4,OR52I2,OR52K1,PLEKHA8,STAM2,WNT2B,MAPKAPK3

Negative_7468	FH,ID2,CCL28,EDA2R,FADD,FUT2,LARS2,NFYA,OR6K2,OR7D2,PIAS1,PPP2R5E,PPP3CB,RAP1A,RASA1,SEMA4B,SLC9A1,SULT1A2,PLA2G12A
Negative_7469	AMY2B,CYP26A1,DDX52,DKK2,ELK1,GBE1,GMPPB,HMGCL,IARS2,MCAT,PDE1B,ST6GALNAC4
Negative_7470	HDC,UBB,ALDH7A1,ANAPC11,CCND3,CNTNAP2,CYFIP2,FGF7,FUT4,GNMT,GRIN2C,IDH1,IDH3B,NODAL,OR3A1,OR9K2,PAK7,PRKCD,PTAFR,PVRL2,RND1,SMAD1,SOCS2,SPCS1,SV2B,TKTL1,VAV1
Negative_7471	C2,SPN,TPO,ALG1,BMP4,CACNG6,CYP4A11,EDNRA,EPHA8,IKBKB,NCR2,PDE8A,PHKG2,PTGES2,SCLY
Negative_7472	KIT,APOA1,ATP5G3,CALML5,CYP26C1,CYSLTR1,DPM1,DUSP5,GNAO1,GSTK1,HSP90B1,LARS2,LRDD,LYPLA1,MAP4K4,MCM4,MDH2,MPDZ,NFKBIE,PAK6,PLXNB3,PRKCQ,RALGDS,TUBB2C,TYMS
Negative_7473	CLDN19,EPHA8,FMO1,KCNB1,MTMR6,PTAFR,RENBP,TRAF2
Negative_7474	CBL,ALDOC,ARSE,BCL2L1,CDC26,CLDN1,FGD3,GLYCTK,HADHB,HLA-C,IFNGR2,INHBB,LAMC3,NCF1,NKD2,OR12D3,OR2A42,OR4C11,POLR1D,PTPN6,SYNJ2,TOMM40L,UAP1,UGT2B7,ZIC2
Negative_7475	MAG,ADSSL1,ATP2A1,B3GALT2,CALML6,CCL13,CNTFR,CRKL,CXCR5,DTX2,EPHA1,GLDC,GRIA2,HLA-C,KLRC4,MAP2K1,NEUROG3,NRAS,OR10V1,OR2A2,OR52K2,PARD6G,PIGC,PIGX,PIK3CG,PLOD2,TYMS,WNT5B,ZFYVE9,CSGALNACT1,PDCD1LG2,SERPIND1,TNFRSF13B
Negative_7476	ADCY8,ADORA2A,BCAT1,CCL11,CCL19,CCL20,CCNB3,CREB3L1,CTNNA3,EFNA5,GCNT1,ITGB4,ITPK1,LDHB,MCCC2,OR2H2,OR6M1,OR6N2,PAPSS2,PFKFB2,PNPO,PPP2R1A,RAB5A,SIAH1,TPH1
Negative_7477	SDS,CCR1,CDS2,CHST12,ENTPD8,FABP4,FGF2,NCOR1,OR8B8,PPP1CC,RASA1,TGFBR2,TLN1,ULK1,UPB1,PPP1R12A
Negative_7478	CASP8,DGKZ,IFNGR1,MCM6,PPP1CB,RUNX1,SELP,UGT2A3
Negative_7479	BAD,B4GALT7,BDNF,COL3A1,COL5A2,EPHA2,GAS1,KLRC2,KLRC4,NCKAP1,OR11A1,PLA2G1B,PPP2R5B,SULT2B1
Negative_7480	IL8,ABL1,AGMAT,ALG10B,ANAPC2,BCKDHB,CDC25A,DRD1,FUT1,GABARAP,IFNGR2,IL1RAP,OR1A1,OR1A2,OR2G3,PIAS1,PRKAR2A,SHC1,SLIT2,STK3,TRAF2
Negative_7481	CFI,DLD,FGA,HPD,SRM,ACO2,ARPC1A,CACNA1H,CCL8,CCNH,CTPS2,DGKB,GSK3B,GSTT1,HARS2,MAP3K7,MMP9,MYL9,OR1G1,OR2M4,OR4D5,OR5AN1,PDE5A,SEMA3F,ULK3,SLC25A31
Negative_7482	APP,DUT,FST,ZYX,AMD1,ATP13A2,CACNB4,CADM3,CSNK1G2,DAPK2,FCGR2B,HRH2,IFT57,IRF5,MAP3K2,METTL2B,MTAP,NARS,NTRK1,OCRL,OLAH,PHPT1,PPP1R3A,PRKAG2,STAM2,SULT1E1,TAS2R5,TBL1Y,UGT2B4,WNT4
Negative_7483	ACP2,AKR1A1,CCL16,CD99,CDC7,CTNNA1,CTTN,DUSP16,ESPL1,HMOX1,ISYNA1,LIMK2,MAGI3,MNX1,NAT5,OR1N1,OR4K2,PCYT1A,PIGH,PLAT,ROCK1,UGCGL1
Negative_7484	APP,BTD,CP,PGP,ADRB3,ARPC5L,CAMK2A,CTPS2,DRD1,FSHB,FZD8,GCLC,GNB1,IL18,KDSR,KIR2DL2,MALT1,NLGN1,NME6,NOS3,OR2T33,OR4C13,OSMR,P2RX2,PCYT2,PDK1,PGM3,PIPOX,POLD2,PRKCA,PTPN1,RBPJ,RPN1,SKIV2L2,SMAD4,SV2C,C1GALT1C1,KIR2DL5A
Negative_7485	EBP,INS,ME2,AASDH,ACO1,ADORA2B,ANAPC5,ARHGEF7,B3GAT2,DDX56,FABP1,FBXW11,FMO1,ICOS,IL13RA2,IMPA1,LAP3,LTB4R2,MASP1,NAT2,NEGR1,NFYA,NOX1,OR4A16,OR4D6,OR51L1,OR8D2,PDE1A,PDE6D,PFKL,PGK2,PIGA,PLOD2,PPP2CB,RAPGEF3,RDH16,ROCK2,TAP2,TLN2,TNFRSF8,TRAF1,WNT11,WNT9A,YWHAE,EIF4EBP1

Negative_7486	AK5,GK2,LCT,MPI,SMS,A4GALT,ACHE,ARHGEF4,BMP6,CAPN2,CCR2,CDC14B,CDH15,CNTNAP2,DAG1,FGD1,GLDC,GNRH1,GP1BB,IL22RA1,IRAK3,IRAK4,ITGB7,NTN1,OR1N2,OR2H1,OR51I1,OR8I2,OR9I1,PAK1,PCYT2,PDX1,RETSAT,RUNX1T1,SEMA3G,SKIV2L2,TAS2R41,TCF7L1,TUBB6,VAMP3,WASL,ZMAT3
Negative_7487	ACP5,CDS2,CHRM5,GSTM1,IL11RA,OR5P2,OR8I2,ROCK1,THY1
Negative_7488	BTK,ID4,LCK,ACSL6,ADIPOR1,ADRBK2,FGF14,GSTO2,ITGA2,MAPK9,NFYA,OR10J5,OR1N2,OR2C1,OR5M9,PDE6H,PIGN,PIGS,PMAIP1,PTTG1,RHOQ,SIPA1,STX6,TRHR,TSHB,UBE2L6,WNT6
Negative_7489	GP6,LTB,PVR,RDX,SRC,ABL1,CCND3,CD34,CPOX,DCXR,DIAPH2,ELK4,ENPP7,FBP2,GNB1,IFNA17,IL20RA,IL2RA,MCM3,MMP2,MYH15,OR13C3,OR1C1,OR52I2,OR52N2,OR5AC2,ORC6L,PARS2,PIK3R2,POLR2K,PPP2R2C,PTGIS,RALGDS,SPHK2,ST8SIA1
Negative_7490	C5,FH,HYI,MPI,ACAT2,ACPP,ADRB2,ANPEP,CBR1,CHUK,COL2A1,CPT2,CSF2,CYP2B6,CYP3A7,EPOR,FGF12,GUCA1C,GUCY1A2,IL28RA,IL2RB,ITGA2B,LIPG,MAN1A2,MAP3K8,MARS2,NFKB1,OR2AE1,OR2D3,OR51A4,OR52J3,PIK3C2A,PLCB3,RRM2B,SARDH,SDHB,SEMA3C,SMAD9,SMPD4,STX12,PNLIPRP2,SLC25A31
Negative_7491	FN1,GPI,INS,MET,ALOX5,ASNS,BNIP1,CHST13,DDX41,DFFA,FGFR2,FLOT1,GALNT3,GALNT6,GNA12,ITGAM,ITGB2,MAD2L2,MYLK3,NCKAP1,NUDT2,OR10P1,OR4M2,OR8B12,PRKX,PRPH2,ULK1,CACNA2D4,HSD17B10
Negative_7492	OR2A4,OR2T3,OR51F2,UBA1
Negative_7493	ADIPOR1,AGXT,AKR1D1,AKT3,ALG10,CIITA,FGF4,GOT1,HES1,IRS1,NUDT12,OR1J1,OR2T34,OR52B6,OR8S1,PDGFRB,PIGH,PPP1R1A,TAS2R16,TSC2
Negative_7494	CAT,LSS,C1QB,CHUK,CYP1B1,F2RL2,GNMT,HLA-C,ITGA10,PPOX
Negative_7495	PXN,ABCC8,ALG12,CYP17A1,ELK4,PNMT,SEMA4F,SPTAN1,TAS2R13
Negative_7496	SRF,ADH1B,CCL4,CCR3,DARS,DGKE,FTCD,GP1BA,HLA-DMA,IARS,ITGAX,MPZL1,NFATC1,OR10AG1,OR1M1,OR5T2,PAK1,PCYT1A,PNPLA4,PSEN2,SETMAR,STT3B,MAPK8IP3
Negative_7497	IHH,INS,KMO,LHB,CCL15,CLDN5,DGKB,DKK2,FOXO3,GBA3,GCNT2,GPX7,HEXA,IL17RA,KIR2DL1,NTNG1,NUDT9,OR1F1,OR52E6,OR6C76,OR6M1,PCYT1A,PDGFD,PPP1CA,SDHA,TNFSF14,TYK2
Negative_7498	DCT,FST,GHR,HPD,ARNT2,BCKDHB,BMPR1B,C5AR1,CACNB4,CACNG8,CANT1,CCNA1,CLTC,CYP2A7,DAAM1,DOT1L,DPAGT1,DVL2,GALNT6,GGPS1,GNAI2,HLA-F,HNF4A,HRAS,ITGA11,LFNG,MAGI2,MGAT2,MTMR2,NQO1,NTRK2,OR1L1,OR4C12,PFKFB3,PRDX6,PRKAR2A,PTCH1,PTDSS2,RPN2,RPS6KA6,SEMA7A,TAS2R50,TJP1,USE1,MAP3K7IP2
Negative_7499	AK5,ACTN2,ANAPC2,ASRGL1,BET1L,BMPR1A,CNGA4,DGAT1,DOCK1,EPAS1,FGD3,KIR2DL1,MTHFD2,OR9A2,PTGS1,ROCK1,SLC25A5,SREBF1,TGFB3,TSC1,UGT2B28,TNFRSF13B
Negative_7500	C1S,LPO,RFK,AKT3,CHKA,CRY1,CSNK1G1,GNRH2,HPRT1,ITGA9,NCAM1,NFYC,NRG4,PDPK1,PPM1D,RARA,RGS3,SC4MOL,SUV39H2,TNFSF4,UAP1
Negative_7501	DBI,SCD,TAT,ACSM5,ADH1A,ARHGEF6,ARNT,ASIP,BCAT1,CBR1,GLYCTK,HSD3B7,INPP1,IQGAP2,NCAM1,QPRT,TAS1R3,WNT5A,YWHAE
Negative_7502	C1S,KMO,MAX,NGF,UBB,AARS2,ADRB2,ALS2,AMHR2,AMY2A,AOX1,ATP5A1,B3GNT3,CCR8,CD36,CDC25A,CLDN14,DHRS4L2,DPAGT1,F2RL1,FUT2,GRIA2,HAAO,IFNA14,IL23A,KLRC4,LDHA,MCM7,NARS,NOX3,OR11G2,OR2F1,OR51Q1,OR52K1,OR5M3,PAK3,PIP4K2C,PROS1,PTPN7,RDH11,RPSA,SHISA5,ST8SIA5,SULT1A1,TLR9,TM7SF2,TNFSF14,TRMT11,HLA-DRB4

Negative_7503	CAD, GP5, MAX, PGP, TXK, CACNG7, CDC6, CHRNA7, CLDN4, DDX56, EGLN3, ERBB2, FGF8, FGFR4, GNAI3, GNRH2, GRM5, HSD17B3, IFNA10, MAD1L1, MAP3K7, MGAT5, MS4A2, NMNAT2, OR4D1, POMC, PPP1CB, PRKG1, PTAFR, RDH10, RELA, SARDH, SEMA3B, SEMA4D, SLC2A4, TAS2R16, TYRP1, VCAM1, YARS
Negative_7504	GP6, GSR, IVD, PXN, SRC, ACPP, AKR1B1, ALG1, AREG, ATP5H, BCMO1, CACNA1G, CASP7, CCL24, CTSS, CXCR6, EIF4B, FARSA, FLT3, FOSL1, FSHR, GBGT1, ICAM2, IL1A, ITGA7, ITPA, METTL6, MTFMT, OR10AD1, OR1N2, OR2M5, POMC, PPP3CA, PRKACG, PRKAR1B, PTPRR, SPCS3, SPTLC1, ULBP1, ST6GALNAC4, SUV420H1, TNFRSF1B
Negative_7505	IL2, ATG7, CYP11B2, IARS2, OR10A3, PAX4, PLTP, PYCRL
Negative_7506	C6, LPL, ACPP, ALDH5A1, ALOX12, AMPD1, ASCC3, ATG12, ATP5I, CAMK2D, CAV1, CHKB, DUSP3, FLT1, GLI1, GNG12, GNPNAT1, GOT1, GPD2, GUCA1C, MAPK14, NCK2, OR10H1, OR14A16, OR5L2, OR7D2, PNPO, RASGRF1, SC4MOL, SELE, SEMA7A, SLC27A4, SMAD1, TJP2, TNFRSF8, TUBA1C, PLA2G12B, SUV420H1
Negative_7507	AK7, DCN, MET, SRM, ABLIM2, ACACA, AGRN, ARHGEF6, ATP13A2, B3GALT1, CACNG5, CAMKK2, CDC14B, CDK2, CES1, CXCR5, DLL4, DNMT3A, EPHA4, GNAS, GSTP1, HIF1A, HNMT, HSPA2, ICAM3, IDH3A, IL17RB, IL20, ITGA3, LIPA, LRP5, MAT2B, MYLK, NEFM, OGDH, OR4N4, OR5AP2, PANK3, PDE9A, PLTP, PYGM, STAT6, TBXA2R, TRPM5, TSC1, TUBB6, WHSC1, SERPIND1
Negative_7508	AANAT, ACSM2A, AKR1C3, ALG10, APAF1, CACNG8, CDKN2A, CHAT, CNTFR, CSF1R, DHRS4, DRD1, ERBB4, GRIN1, GSTZ1, ITGAL, ITGB4, OR2C3, OR4K14, OR52I1, OR5D13, RBPJL, TAS2R60, VARS, CSGALNACT1, CTNNBIP1
Negative_7509	CKM, GHR, ID4, MOS, AARS, ABCC8, ARNTL, ARSB, ATP5B, CDK6, CLDN2, CRLS1, DARS2, EXTL2, FBP2, FCGR3B, FGF6, FUT1, GNA11, HSD17B8, IKBKG, MCM4, NCK2, NT5E, OR2F2, OR2T4, OR4K2, OR52D1, PNMT, POLR1B, POLR2K, SHC4, SPHK2, VANGL1, WARS2
Negative_7510	CR1, GPI, SMO, ACAA2, ADH4, AGPAT1, AKR1C3, ALG8, BCKDHA, COQ5, CYP17A1, CYSLTR2, ENTPD1, EPB41L3, EREG, GBE1, GPAM, GRAP2, HSD17B3, HSPA2, HSPB1, INPP4A, LAMB2, MAD1L1, MAPK14, NANP, NTRK1, OR4L1, OR56A4, PLCD1, PPP3CC, RASGRP1, RPS6KA1, SRD5A1, SSH1, TGFBR1, VEGFB
Negative_7511	BCR, CDA, DCC, GGH, ADCY5, APOC3, ARHGAP5, BPNT1, CD27, CMAS, COQ5, CRKL, DAPK2, DHODH, HMOX2, HNF1B, HSPB1, IL17A, MFNG, NKX3-1, NTN1, OCRL, OR1S1, OR2L3, PAPSS2, PDXK, PFN4, PIGL, PON2, PRKAG2, SARS, SHC3, SSH1, TIAM1, TOMM40L, XIAP, TNFRSF10C
Negative_7512	SRC, AGRN, APLP1, ARSB, AXIN1, BUB1B, CSF1, CYP1A1, DFFA, GAD2, HLA-DMA, HRH1, HTR7, ITGA10, MAD2L1, MECR, NFASC, OR10H5, OR2D2, OR5D18, PGAM2, PIK3C2B, PPP1CB, PRKAB1, PTGFR, RASGRP3, VAMP4, WASF2, ATP6V1B1
Negative_7513	FST, VCL, CALML6, CASP8, CCR2, CLDN1, COL1A1, DHRS4L2, DRD1, DRD5, E2F1, GCLM, MAML3, MIOX, NAGLU, NRG2, OR2Z1, OR4N2, OR5A1, OR8D4, OXCT2, P2RX3, PAK4, PIP5K3, PISD, PLXNB3, PRKCA, RPS6KA1, SARS, STX1B, TSLP, TNFRSF11B
Negative_7514	GSS, HGD, TXK, AACS, ACAT2, ACOX2, ALDOC, APH1A, ARPC1A, CARS2, CCL25, GNA11, IAPP, INMT, JUND, NMNAT3, OR10G9, OR13C5, OR13J1, OR4A16, PIGF, PIK3R1, PIPOX, PLOD1, PRKCH, PRKCZ, ROBO3, RPS6KA5, SLC9A1, SPHK2, UNC5D, ATP6V1C2
Negative_7515	F5, TAT, ACOX1, ALDH3A2, APOA2, CBLB, CDIPT, CYP17A1, DDX18, DDX4, ENO2, GUSB, IRS4, KLRD1, LLGL2, LYZL1, NFYB, OR2H1, OR52H1, P2RX6, PIK3R5, PLXNC1, PTGS2, RHOQ, PLA2G12B
Negative_7516	C9, TXK, ACSM1, ADORA2A, COL4A1, OR1M1, TPH1
Negative_7517	MUT, CACNG7, CASK, CCL28, CCL8, CCNB3, CD44, CES2, CSAD, CSF3, HTR6, INHBA, NKX2-2, PIAS4, POLR1D, RHOD, ULK2, HSD17B10

Negative_7518	CSK,ALOX15B,ANAPC11,ARPC3,DLST,ENTPD5,GBGT1,GNG13,MAML3,MPZL1,NFKB2,NOTCH3,OR1F1,OR2L2,PAPSS1,PLD1,PPP1R1A,SC5DL,SETMAR,SLC27A5,ST3GAL2,TNFRSF9,TNFSF10,ZBTB16,TNFRSF12A
Negative_7519	DDC,IL2,ILK,ATP5H,CAV3,EPAS1,FBP2,GGT1,GNPNAT1,OR8B4,PARD6B,PDE11A,PPP2R2D,PRODH2,SPRY4
Negative_7520	ME2,ALOX12,ENPP7,FECH,GARS,GJD2,GRIN2C,HAGHL,IRAK2,ORC1L,PDE2A,PHKA1,PLCB1,PLCE1,PPARA,PPP2R5E,PTGES2,SEMA3C,TRAF5,VEGFB
Negative_7521	AGL,SMS,ACOX3,ADRA1D,AKR1C1,AOC2,ASCC3,CD247,CLDN10,CREBBP,DOT1L,ENPP1,ENTPD8,ERO1L,FGF7,FMO4,GAS1,GBA3,HRAS,HSPA1B,IFNA6,IL17RB,JUND,MAP3K11,MBL2,NKD1,OR4D2,OR7C2,ORC4L,PLOD1,PPP1R3A,PRKACB,PVRL4,RGS3,RRM2,SDHB,SGMS1,STX5,YWHAB,CTNNBIP1
Negative_7522	GGH,AMY2B,ANPEP,APBB1,ARAF,CLDN5,COL5A1,DGKH,EHMT2,ENPP6,EPB41L1,FABP3,FGF20,GALNT10,GNB3,HTR2A,IFNA21,MAPT,NEFH,OLR1,OR52I2,OR5K2,OR6K6,OR9Q2,PDCD1,PDGFRA,PPARD,PRKCG,RASGRF2,RDH11,RELA,RND1,SRGAP1,STAT5B,STX1A,TACR1,TBL1X,TNFRSF11B
Negative_7523	EPO,PLG,SHH,TNN,ANAPC1,ATP4B,CCL27,CHIA,CNTN1,DHDH,DPYD,EFNA4,FBXW11,GYS2,IFNGR1,MAD2L1,MAPK14,MYL2,NAT2,NEU2,OR13F1,OR2Z1,OR4C16,PIGT,PIK3C3,PRKAR2B,PRKCE,PRPH,PRSS1,SULT1A2,SULT1A4,TUBA1C,PNLIPRP2
Negative_7524	APP,C7,AASS,ACHE,ALDOA,ATP5H,CCDC6,CERK,CRY1,DOT1L,IFNA10,IFNAR2,IL1R1,JAG2,MYH8,OGDH,OR11H6,OR14I1,OR4N4,OR8H3,PARS2,PIM1,POLR2E,PPARA,PRNP,RDH8,STAT4,TNFSF8,TUBB4Q
Negative_7525	LYN,ASAH1,ATP5C1,CACNA1C,CAV2,CD79B,CTSB,CYP1B1,CYP2C18,DDIT4,DDX23,DGKA,DOLPP1,EGFR,ENO3,GNG13,GNPAT,GSTM2,LDHA,MDH1,MGAT3,MMP14,MYLK3,NFASC,OR1D5,PIGK,PIM1,PPP2CB,PSME2,RHOQ,SSH3,SULT1A2,TAP1,USE1,ZFYVE9,PLA2G12A
Negative_7526	FAS,LEP,ACSL6,ACSM2A,ADCY1,ARPC4,CCL21,CDH4,DFFA,DGKD,DOT1L,ERBB4,GNAI1,GPD2,HES1,HMGCS2,JUND,LIPA,MYH13,MYH6,NFATC2,OR11A1,OR1K1,OR2T10,OR6T1,OR8K5,PGK1,PGM3,PRKAG3,PTCH1,PTPRJ,SMAD9,SOCS4,TALDO1,TAS1R3,TNFSF11,UMPS,CACNA2D2,PNLIPRP2
Negative_7527	SPN,ALG11,DTX3,GSTP1,ITGAL,LDHA,MYH3,PRPH,SOS2,TAS2R40,TNXB,UGCGL2
Negative_7528	TNC,UBB,APOC3,B3GNT5,BMP4,CD226,CTSG,CYP19A1,DPYS,GUCY2F,HSD17B3,IPPK,MARS2,MBL2,NAGLU,OR4D5,OR5AP2,OR8H2,OR8J1,PIGA,PPP3R2,PSPH,RASGRF1,TGFB1
Negative_7529	ACADSB,ACSM4,BCL10,CACNA1B,DARS2,EDAR,EXOC7,FARSA,IFNA10,IL22RA2,LIPC,MITF,OR1J1,OR2Z1,PIK3CG,PNMT,PRKAR2A,PRKCD,PRNP,SKIV2L2,SPCS1,TBL1XR1,TNFRSF13B
Negative_7530	ADO,GAA,NF1,PGD,SRC,AGPAT3,ALDH4A1,APRT,BET1L,BST1,CACNA1S,CD44,CHD8,CTNNA3,CXCL11,CXCL12,DIAPH3,GMPS,GNRH1,GP1BB,HLA-C,KLRC1,LIFR,MAP4K2,NCSTN,NMNAT1,NMNAT2,NODAL,NOX1,NSDHL,NTNG1,PAK1,PAK4,PDE2A,POLR2E,PRPS2,PTK2,RAB5A,RASGRP2,RBKS,SDC3,SLIT1,SMAD7,ST8SIA1,TJP2,TPH2,YES1,ATP6V0A2
Negative_7531	C3,ALDH9A1,ANAPC5,APH1A,CD22,CLCA4,COL5A2,CYP2S1,DARS2,DDX56,EP300,FADS2,FDPS,FLT4,HTR5A,METTL2B,MPZL1,NLGN3,OR2V2,OR52E6,OR6C76,OR7G1,P4HA2,PDGFD,PIK3C3,PLCG1,POLR2J,PPP3R2,PTGER1,PVRL1,RELB,RFXAP,RRM2B,STX7,TUBB,WHSC1
Negative_7532	TXK,ADAM10,DGKI,EPAS1,FGF19,FSHB,FTMT,GMPR2,MGAM,PRKCE,ROBO1

Negative_7533	ABO,C3,CR1,GSN,SHH,ACPP,ALG3,ARHGEF1,ASNS,CCL7,CD247,CDH3,CFTR,CMBL,COX10,CPT1A,DTX3,EXTL3,FOXO3,GALNT14,GDF6,GNPAT,GPD1,HRH2,HSD17B8,ITGA1,MDH2,OR1S2,OR2D2,OR5P3,OR8D1,OR9G1,ORC4L,PAK3,PAK4,PAX8,PDE6G,PI4KA,PIK3CA,PRKCE,RFWD2,SDC2,ST3GAL6,STEAP3,STX10,TAS2R13,TSNARE1,TNFRSF12A
Negative_7534	BAD,BTD,IL3,INS,MSN,SYK,ACSL1,ACSM5,ADIPOR2,ATP4B,BMP6,CDC14B,CHSY1,CNGA3,EHMT1,GSTA3,GUCA1B,GZMA,IL4R,IL9R,IRF7,MITF,NARS,NAT2,NCF2,NCKAP1,OR1S2,OR6B2,P2RX5,PDE8B,PI4KA,RAC2,RAPGEF2,SLC27A5,STX16,STX8,TIAM2,WBSCR17,B4GALNT1,PNLIPRP1,TNFRSF10A
Negative_7535	CACNA1F,CHSY3,CRHR1,DTYMK,FASN,GALNT8
Negative_7536	BID,C9,ID4,APH1A,CCL22,CFLAR,ECHS1,EXT2,FGF18,FTH1,FTMT,GCAT,HMGCS1,HTR2B,IL17B,IL7R,KIR3DL2,MAD1L1,MAML2,MAP4K2,OR2G3,OR4D1,OR56A3,OR7A5,OXCT2,P2RX1,PIAS2,PPP2R2D,SCIN,STK36,STX10,TACR1,TUBA3C,VAMP2,TNFRSF17
Negative_7537	CD4,EBP,GHR,GSR,ADIPOR2,ATG12,B3GALT5,BHMT,CCR3,CD46,CLDN15,CLTC,CYP21A2,DUSP10,EPRS,FECH,FLT3LG,FUT2,FZD7,GLUL,GNRH2,IFNA13,IFNA6,IL10,ITGA4,KIR3DL3,LALBA,NCOA4,NRG1,OR4D5,OR52J3,OR9Q2,PLA2G1B,PLA2G2D,PRKACB,PRKCZ,PYGM,SARS,SHMT1,SMPD1,SNAP23,ST3GAL6,STX6,TAS2R5,WBSCR17
Negative_7538	HK1,SCD,AKR1B10,ARRB2,CALML6,CCR6,DHDDS,DUSP7,EPHA4,FRAT2,GALNTL4,GALNTL5,GRIN2C,ITGA6,JUND,LEPR,OR2T1,OR6C1,PLIN,POLR2K,RASSF1,RPRM,RRM2,SEMA4A,SERPINB5
Negative_7539	MPZ,DDX52,DHX58,IFNA14,OR10T2,SETD2,SSH1,STAT1,STX1B
Negative_7540	ADO,BTC,GSN,HK3,AK3L1,ALG5,BBOX1,BRAF,COL5A1,CYP2C9,EDNRB,ENTPD3,GADD45A,GLI2,HARS,MAN1A1,NFATC2,NMNAT1,PANK3,PDXK,PER2,PLCD1,PLXNA3,PRKAA1,RARA,RASGRP4,RBL1,SRGAP2,STEAP3,WWP1,YWHAE,YWHAZ
Negative_7541	BCL2L1,CCND1,CXCR4,EFNA2,PPP3CB,SDC1,TLR9,WIF1
Negative_7542	ACYP2,ALPI,ATIC,BCL2L1,BLVRB,C3AR1,CBLB,CCR9,CDC25A,CHAT,CNDP1,COL4A1,FSHB,IL18,OR1N1,OR3A2,OR6N2,SV2A,TAS1R2,ZAP70
Negative_7543	CGA,AHCYL2,CACNA1G,DGKQ,IFNA6,IFNB1,IL6ST,IRAK3,LIPA,MARS2,MMP14,NTNG1,OGDH,OR51A4,OR8B4,PRKAA1,PTGER1,TP73
Negative_7544	AGK,CTH,AASS,ACSS3,AKR1C1,ARRB1,ATP4A,BMPR1A,CARS,DIAPH3,DNMT3A,ENPP1,G6PC,HADHB,KDSR,MAP4K1,NMNAT2,NODAL,OR1E2,PANK4,PDE4A,PIGX,PLCG2,PPCDC,RBPJL,SHC3,ST3GAL3,TBK1,TP53,VAMP4,PPARGC1A
Negative_7545	ADO,C7,ID2,ACLY,ALS2,ATP6V1F,CAMKK2,CDH15,CYP2U1,DERA,EGLN1,EPB41,EPHB3,ESPL1,FBP2,FGF8,GTSE1,MAPK7,NEGR1,NT5C,OR2T1,OR56A4,PPP2R5E,RFX5,SENP2,SH2D1A,SLC2A2,SOCS5,TAP2,TAS1R3,TSC1,UNC5C,YOD1,GABARAPL2
Negative_7546	COQ3,CRLF2,CRLS1,GALNT9,GRLF1,IFNGR2,ITGA10,MAP2K3,NKX6-1,PRSS1,VAMP3,ARHGEF12,TNFRSF10C
Negative_7547	CEL,F2R,ADH6,ADRA1A,APRT,ARPC5L,B3GALT6,CNGA3,CSNK1G2,E2F3,F13B,GALC,IFT57,MAPK8,OR3A2,OR5T2,PARD3,PGS1,PIGW,PLA2G5,PRKAG2,RPS6KA2,SDC1,SMPD2,SOAT2,STX2,TAS2R50,ATP6V0A2
Negative_7548	ADA,F12,SMS,TEC,ABLIM3,ARPC3,BMP2,CHEK2,COASY,CRY1,DAD1,ETS1,EXOC7,GALNTL1,GGT5,GPT2,HHIP,HPSE,IFNA14,LGMN,MAP4K4,MGST2,MTMR1,NAT2,NCOR2,NSD1,NUDT9,OR10T2,OR1K1,OR51B5,OR51V1,PAK6,PDE2A,POLR2D,PPP1R3A,PRKAA2,RARS,RDH16,SKIV2L2,SNAP23,TCIRG1,UGT2B4,VANGL1,VDAC2,WASL,TNFRSF1A
Negative_7549	CR2,EDA,ZYX,AKR1C3,AMPD1,BMP2,CALML3,CCL13,CXCL10,DDIT3,DKK2,DRD1,ENTPD5,FZD3,GMPS,NADSYN1,NT5C1A,OR4F4,PDGFRA,PPP1R1A,RDH10,RPRM,ST3GAL5,TRPM5

Negative_7550	C3,CTSL1,PTPRM
Negative_7551	MPL,ALOX5,LGMN,LIMK2
Negative_7552	ARD1A,ATP6AP1,BTRC,C1QA,CDK6,DUSP7,E2F2,E2F4,GALNT14,HLA-DOB,HTR5A,ICOS,ITGB3,MAD2L2,MAP3K1,MGST2,MMP1,MYH8,OPLAH,OR2Z1,OR4N2,OR9A4,POLR1C,RASGRP2,SCP2,TNFRSF1B
Negative_7553	SRC,ARPC5L,ARSD,ATP6V1D,CD40LG,CTLA4,CYP2S1,EARS2,IL12B,MAP3K5,MAP4K2,MCM2,OR10H3,OR2T8,OR4C13,PI4KB,RHEB,TLR2,ULBP3,WBSCR17,ZAP70
Negative_7554	ADSSL1,ALG1,ALPI,ATP6V0C,CCL16,ENPP6,IFNA17,OR4S2,PDE6D,PIGT,PLA2G2F,PPP2R1A,TMLHE,TRADD,UMPS
Negative_7555	IHH,KHK,XDH,ARHGEF4,BDNF,CD40LG,CHEK1,CXCL13,CXXC4,CYP27A1,CYP2A13,CYP7A1,DLST,DPYD,EPHB6,FDFT1,FZD9,GNPNAT1,HIP1,HTR2C,IL11,IL11RA,INPPL1,IRF5,KIR2DS4,KYNU,MARS,NAGA,NAGS,NEU3,ORC6L,PNPT1,PPP1R1A,PPP2CA,PTPMT1,RRM1,SESN3,SKIV2L2,SKP1,SMAD1,SUCLG1,TPH2,TPMT,UGDH,ATP6V1E2,SERPINF2
Negative_7556	ALG9,ASS1,BET1L,CBLB,CBR3,CCL24,CDS2,CHRM5,CSF2RB,CXCL1,CYP4F2,E2F1,EPB41L1,FSHR,FUT8,GALNT6,GTSE1,HMOX1,ICAM2,MAPK12,MTHFD1,NR1D1,OR13A1,OR1J4,OR4B1,OR4Q3,P2RX3,PDE8B,PGM2,PIGW,PLA1A,PLCB4,SC5DL,SNAI1,SOS1,TNFSF14,TSC2,TUBAL3,UBE2L6
Negative_7557	EGF,CNTN1,DTYMK,ECSIT,FGF9,IMPDH1,MADCAM1,OR10A5,PDE8B,POLD3,POLD4,PTCH1,PYCRL,RASA2,STK4,TFPI,VAMP2,CSGALNACT2
Negative_7558	ACVR1C,ADCY4,ADH4,AGRP,ALCAM,ALG2,ANAPC2,B3GNT5,CCL19,CCL2,CCR8,CNTNAP2,COL5A1,CYP26A1,DGKE,DOT1L,DTX4,EDNRA,ERBB2,FOXO3,IFNA4,IL4R,INHBB,ITCH,KCNJ11,KIR2DL1,MINPP1,MPST,NAGLU,NANP,OPLAH,OR1D2,OR2F1,OR51A4,PLXNA1,PPID,PRKAR2A,PTCH1,SEMA4D,SMAD6,TKTL2,TP53,TRHR,TSC2
Negative_7559	A2M,ACOX1,AKR1B1,ATN1,BAAT,CRY1,DAPK3,HHIP,HRH2,IARS,IFNB1,NOS1,OR10G2,OR10P1,OR2A2,OR6K6,SFRP4,TNFSF9,GABARAPL2
Negative_7560	ACAD8,ACTA1,ADCY3,ADSS,B4GALT1,CAMK2D,GALNTL5,GNAT3,HSD17B3,IRAK2,JAM2,MPST,NAE1,OR13C8,P4HA1,PGM3,PIGB,PRIM2,PTPMT1,RBL2,SARDH,SAT1,SIGLEC1,SLIT2,SMPD1,TAS2R14,TSLP,WNT11
Negative_7561	F3,GCK,GDA,KMO,PAH,ACSM2A,AKR1B1,ALDH3B2,ALG11,ATP6V1F,BMP7,CAMK4,CD8B,CHPT1,CLDN10,CYP3A4,DDHD1,DFFB,DHRS4,GRIN2C,GRIN2D,HNF1A,ITGA1,KCNQ1,LAMC3,MGAM,MYL2,OR1L3,OR2A42,OR7C1,OR7G1,OR8H2,PDE3A,PECAM1,PFN4,PLCD4,PLXNB2,PNPO,PPP3CB,PPT1,SEMA3F,STX8,TMLHE
Negative_7562	AKT1,ATP5L,CACNA1H,FZD7,IDI1,ITGA10,NFYA,OR2B11,OR4F15,OR4K14,OR6X1,OR9Q1,SULT1E1,TSHR
Negative_7563	DAPK1,DDX50,DEGS1,FARS2,FGD1,FZD7,IFNB1,KIR2DL1,MAP3K8,TFDP1
Negative_7564	CD2,IHH,PDC,RPE,SMS,AARS2,ALDH3A2,ALDH9A1,ANGPTL4,ARPC5L,BDH2,HARS2,HTR6,HYAL2,IDH3G,LGMN,MCEE,MGAT4B,MGST1,MOV10L1,MS4A2,NCAM2,NCF2,OR13C3,OR1D2,OR56B4,OR5AR1,PPA1,PTGES2,PYGM,RHOQ,SMARCA5,SPRY2,TAS1R1,TRAF1,TRMT11,UGT2A3,VAV1,ZBTB16,ATP6V1C2
Negative_7565	AGK,BAX,BTC,F3,ME1,TKT,BIRC3,CLDN8,COL4A1,EFNA3,EHHADH,GALNT1,HMOX2,KLRC3,OR4N5,OR5B12,OR5B21,OR8K3,OR9A2,P2RX3,PCK2,PFKM,PRKAR1A,SLC25A6,SMURF1,SUCLA2,TACR3,TP53,ULK3,ATP6V1B1,ATP6V1G3,HSP90AA1,PAFAH1B3
Negative_7566	ANAPC2,CHEK2,CSNK1A1,LDHAL6B,NT5C1A,POLR2G,PPM1B,SLC2A2,ATP6V0D1
Negative_7567	AKR1C3,CACNB2,CCL4,DCTD,EXT1,HHEX,IL23R,OR4D6,OR51B2,RBKS,TUBB1

Negative_7568	ALG10B, CALML3, CD58, CD74, CHSY3, DTX4, EXOC7, GSTA4, OR51B2, PDE4D, PIGO, PPA2, STAT5B, PLA2G12B
Negative_7569	FN1, ID2, IL7, PLG, SMO, ACSS2, APOC3, CD40LG, CYP3A5, DBF4, DDB2, DGKG, GCLC, HAGHL, HES1, INADL, LAMC3, LIMK2, MAP2K5, MAP3K3, NADK, NFKB2, OR10G9, OR10H3, OR13D1, OR4F5, OR52E6, OR52K2, OR8J3, PLA2G2D, PLCE1, SDHB, SPI1, TNFSF8, TP53, WNT8A, ATP6V0A4
Negative_7570	BCR, C1S, CR2, EZR, HGF, IL9, JUN, PC, AKT3, ATP2A2, BDNF, CASK, CCL21, CCNG1, CCR8, CDC27, CLDN2, CSNK1D, CSNK1G1, DDIT3, EP300, EPHB1, GALK1, HMGCS2, IKBKE, IL24, INPP4A, ITGA6, ITPKA, KLRC1, MAD1L1, MAD2L2, MCCC1, NARS2, NFE2L2, OR13F1, OR2T11, OR8H2, OR8J1, PLCB2, RBX1, SPRED1, TAS2R5, TBXA2R, TNFSF9, WASF3, GABARAPL1
Negative_7571	CGA, ID3, SRC, ACVR1C, ANAPC11, BIRC2, CDH5, CHRM3, COL1A1, CXCL3, CYP4A22, DTX3, GOSR2, GPD1L, HRAS, MAPK9, MGAT2, NCF4, NME4, OR4A16, OR8B4, OR9A2, PIGB, PSAT1, RAP1A, RPS6KB1, SETD7, SRGAP2, TALDO1, TNFSF11, TSNARE1, TUBB, TUBB2A, ULBP3, VANGL1, VNN1, PNLIPRP1, PPP1R12A, ST6GALNAC3
Negative_7572	B3GALT4, CACNA1D, CHKA, CMBL, CXCL5, CYP3A43, EGFR, HEXB, IL15RA, IRF3, MBL2, NCAM1, OR52M1, OR8H2, P2RX2, VNN1
Negative_7573	HGF, LIF, MTR, ALAS2, ALOX15B, ANAPC2, ASRGL1, CACNA1A, CARS, CD48, DTX1, DUSP8, GALNT8, GPT2, GSK3B, ITCH, KLRD1, MAP3K11, MAPK9, NAGA, NFKBIA, NQO1, OR2H2, OR51I1, OR8D2, OR9G1, PLA1A, PLOD1, RPS6KA4, SESN3, SOCS4, TRHR, UGT2B17, UROS, CACNA2D4
Negative_7574	F2, AMDHD1, ASRGL1, AXIN1, CACNA1E, CDC25B, CYFIP1, DDX52, GRAP2, IL1A, INPP4A, NCK1, NUDT5, OR10AG1, OR52A1, PAFAH2, PIK3C2B, PLXNB3, SC4MOL, ST8SIA1, STX10, TRPC1, VAMP1
Negative_7575	SCD, ALPP, ALPPL2, ATP5J2, C3AR1, CCL5, EPB41L3, GCNT2, GRIA2, IL25, KCNJ11, OR4C11, OR4S2, OR51A4, PPA2, TRIP10, UROD, B4GALNT1, ST6GALNAC4
Negative_7576	DBI, AMY2A, APC2, APOA5, ARPC1A, CASP3, CCKBR, CYSLTR2, GALNT9, GP1BB, HGSNAT, LARS, MAOB, OR10H5, OR1N2, OR52I1, OR5AC2, OR8D1, PGM2, PIGA, POLR2I, PRKAA2, SETDB1, STX8, WNT9B
Negative_7577	CD4, FGB, ACVR2A, B3GALT6, BAIAP2, CASP3, CBR1, CCL20, CCND3, CD274, CDC25C, CLTB, DLL4, EPHB6, GZMA, HTRA2, IL18R1, IL18RAP, LDHC, LTC4S, MGAM, NOTCH1, OR2C3, OR52D1, OR52N1, OR6N1, PAX6, PDGFB, PFAS, PHGDH, PIGQ, PLIN, SHC2
Negative_7578	CCL8, EPB41L1, GNA15, IDI1
Negative_7579	TKT, ADSSL1, AMPD1, APOA5, ARPC1B, OR8K1, PLAU, SSH3, TBXA2R, UMPS, ATP6V0E1, TNFRSF6B
Negative_7580	AMT, DLD, MAX, ACPP, AKR1A1, AKR1B10, B4GALT4, BBOX1, CCL1, CHRM1, COL5A1, CYP21A2, EPHA1, GLYCTK, GNRHR, GPT2, GRIA1, GUCY1B3, HRH1, ICOS, LIPA, MEF2C, MS4A2, NTNG1, OR4F15, OR51E1, OR7G1, OR9A4, PIGV, PLCB4, PNMT, PON2, PPP2R2D, PRODH2, PTPN5, RDH12, SETMAR, SHC2, VDAC2, WNT5B, YWHAE, CSGALNACT1, PLA2G12A
Negative_7581	C4B, GLS, ME1, SCD, BMP5, CAMK2G, CD79B, CDC45L, CXCL1, DBF4, GBA3, GP1BB, HGSNAT, MASP1, NFE2L2, OR1A1, OR51D1, OR51E1, OR5AR1, OR8I2, PECAM1, PGM3, POLR2L, POLR3B, SFRP4, SGPP1, SMAD2, TXNDC12, UROD, HLA-DQA2
Negative_7582	GPI, ME1, ALG6, ALPPL2, ATP6V1H, CCL16, CD79B, CRB3, CYP2J2, DUSP8, ENTPD6, FASLG, GALNT1, GNAI3, GNAS, ITGA4, NANS, OR1A2, OR1E2, OR1J2, OR4F15, OR7G2, POLR2D, SDC4, TAS1R3, WEE1, SERPINF2
Negative_7583	C7, ACP5, AGXT2, ASCC3, ATP6AP1, CD79A, COL5A1, CXCR3, DOLPP1, FUT5, GMPR2, INPP5B, INSR, NTF4, OR2L2, OR51E1, OR6C3, PKM2, PLCB4, PRKAG1, RUVBL1, TLR9, TPI1

Negative_7584	TYR,CDC7,CHPT1,EI24,EXTL1,HMGCR,MCCC2,MKNK1,OR2M3,PIK3R3,PSEN2,STK11,THY1,TRPM5,WIF1,YOD1
Negative_7585	CD2,ME1,MET,PLN,AGRN,C3orf10,CCL5,CD47,CDH3,EPOR,FARS2,FZD4,HDAC2,IDH1,IL15,INPP4A,ITGB3,LDHD,NCOA4,NEFH,NT5E,OR10J5,OR2T12,OR4M1,OR51F1,OR52R1,OR5M3,PDE3B,PERP,PNMT,POLE,PTCH2,RPS6KB2,SIAH1,TRIP10,ATP6V0E1,HLA-DQB1
Negative_7586	CFD,HYI,ME2,AK3L1,B4GALT3,BDH2,C1QB,CER1,CLDN16,COQ5,CPT2,CREB3L4,CXCL9,CYP27A1,DDX54,DGKQ,ENAH,EPB41L3,EPHX1,FADS2,FBXW11,FUT9,G6PC2,GAD1,GCAT,GP1BB,GPX7,HSD3B7,IL1B,MAML2,NRCAM,OR2T34,OR52H1,OR6N2,PDE3A,PPT1,PTK2,PYCR1,RPN2,SOCS3,SYNJ2,TUBA8,UGT2B11,YWHAB
Negative_7587	ACOX1,ACSL6,ARHGEF7,B4GALT6,CHPT1,CREB5,DDIT3,GLI3,HSD3B1,IFT57,IL10RB,MAP2K4,OR1K1,OR4A16,OR4K2,OR5T1,P2RX1,PLCB4,PPAP2C,PPP3R1,PRPH,RBPJL,RIPK1,TLR3,UGT2A1,UPP1,WNT11
Negative_7588	TYR,C1GALT1,CDK2,CLDN6,DAAM2,DLL3,EREG,KLRK1,NRAS,OR51L1,PIGP,POLD2
Negative_7589	DDC,FGA,ABL2,ACTN1,CACNG8,CCND1,CD8A,CDK6,CES1,CHDH,CXXC4,CYP26B1,CYP2U1,DDIT4,FGFR2,GFAP,HCLS1,ITGA8,NARS,NTRK2,OR10A2,OR14C36,OR4D1,OR51G2,OR5T1,PIK3R3,PLK1,RPS6KA4,SC5DL,SEMA4G,SHMT1,SLC2A2,STK36,TAS2R14,TAS2R48,TAS2R50,ULBP2,VNN1,CACNA2D4
Negative_7590	CPT1B,DHCR24,JAG2,PDE4A
Negative_7591	GNPDA1,IDI2,LTBR,OR2G6,OR51G2,RPN2
Negative_7592	CFI,FGB,ACADM,AKR1C1,APOE,GSTA1,JAK3,OR10G7,PIAS2,PIP5K1C,PPP3R1,UCK1
Negative_7593	CFD,HGF,ALG10B,CCL7,CCR7,CYP26B1,CYP2C19,ECSIT,GSTT1,IL10RB,ROCK1,SEMA3G
Negative_7594	AK7,F5,SMS,ACSM5,BIRC3,C4BPA,CD244,CHDH,CLDN15,CYP3A5,G6PC2,GAD2,GUCY2D,HLA-DMA,HSD17B7,IL17A,ITGA1,MADCAM1,NCAM2,OR10AG1,OR13C4,OR2L8,OR4N2,PDK1,PNLIP,PTGS1,SPTLC1,TCF7,TPMT,VDAC3
Negative_7595	ADC,C7,ACP6,ARHGEF1,CRLS1,DGKI,DGKZ,FRAT1,GDF6,GUCA1A,IL28B,KIR3DL3,KLRC2,MAPK14,MFNG,MGAT4B,NARS2,NRXN1,OR2AG2,OR6C74,OR6Q1,OR8B4,OR8H2,PDE8B,PLCG2,SDHD,SH3BP2,SOD1,TRAF5,PRICKLE1
Negative_7596	NF1,SDS,APLP1,BHMT,EPB41L2,FGF1,GCDH,HMGCR,ITPKA,LIPT1,MAPK11,NCF4,NEU4,OR10G3,OR1L3,OR6S1,TNFRSF13B
Negative_7597	AACS,AGPAT4,ASS1,B3GALT6,CAV3,CDH5,COL4A1,DVL2,EPHX2,ESAM,ETNK2,GNG13,GUCY2D,IL20,KCNJ11,LEFTY1,MMP14,MYH4,OPLAH,OR1D2,OR2A12,OR2C1,PDE4D,PIGO,PIK3C2G,PNLIP,PNPLA4,PPCDC,PRKACG,PYGB,RXRA,STAT4,STX8,SV2B,TUBAL3,TUBB2A,VEGFB,WARS,WBSCR22,YWHAQ,HSD17B10
Negative_7598	C4B,FGB,PC,PLG,PLN,ABAT,CACNA1H,CD72,GAS1,GUCY2D,KAT2A,MAP2K1,MPP5,NEFL,NTSR1,OR13J1,OR4K15,OR52A1,PIP5K1A,POLR2I,PPP3R2,TP73
Negative_7599	APRT,ARRB1,B4GALT5,CACNA1G,DAAM1,GCNT4,GFPT2,KIR2DL2,LAP3,LRAT,MARS,MCM4,MTHFD2,OR1L8,PPM1D,PPP2R2B,RAP1B,SFRP1,SH2D1B,TAS1R3,VAMP3
Negative_7600	FYN,NPL,SYK,AMD1,CD46,CD79B,CREB3,GRAP2,MAP4K4,MCM6,MGST1,MYL7,OR10A6,OR2D3,OR2T3,OR52L1,OR6C76,PAK2,PARVB,PGAM4,PLOD1,PPP1R3C,PRKAB1,RRM1,SPCS1,ST8SIA5,SUFU,TIRAP

Negative_7601	GBA,GLA,PLG,ADIPOQ,ADORA2B,ALCAM,ALDH1B1,AOX1,ARHGEF6,BET1L,CCR7,COQ6,CREB3L3,CYP2A13,ENTPD8,FMO4,FOXO1,GADD45B,HSD17B4,ICAM3,IFIH1,IFT57,NFX1,OR10A3,OR4D6,OR4M2,OR51A7,PANK3,PPAP2C,PPP5C,PSPH,PTAFR,SDHC,SETD7,SGMS2,SMAD7,TAS2R14,THBS4,TJP1,VAMP1,WASF1,ARHGEF12,HSP90AA1,SERPING1
Negative_7602	TYR,ATP6V0C,BCL2L1,CAMKK1,CD8A,CYP2C8,EP300,KARS,MAOA,MMP1,OR13H1,PNMT,PPM1B,PRKCZ,RETSAT,RGS3,SEMA3G,SLC2A4,SQLE,UGCG
Negative_7603	CKM,JUN,NGF,PTS,ACAT1,ADH1A,AGXT2,ARG2,ATP5G3,BCAR1,DNMT3L,FTMT,GBA3,HES1,HIP1,LAMB4,LIPF,MPST,OR10C1,OR4D5,PHKB,PNPLA4,POLD2,POMC,PPP5C,PSME1,RASGRF1,RBL2,SMAD1,SNAP23,SSH1,TAS2R48,UNC5A
Negative_7604	ATP5A1,PLAT,RARA
Negative_7605	ACSL4,BNIP1,FZD10
Negative_7606	MPZ,ACACA,AKR1C1,AKR1C3,AMPD1,APC2,ATP4A,CCL19,CDC2,CREB3L3,CTNNB1,DDX55,FGF18,GJA1,GP1BB,GRIA2,GUCY2C,IDH3B,IL20RB,INHBA,KARS,MAPT,NCR1,NFKBIE,NT5C1A,NT5C2,NT5M,OR4C16,OR4F4,OR7A17,OR8B4,OR8K3,ORC2L,PIK3C3,PLAUR,PON3,PPP1CB,RAC1,RDH16,RRM2B,SAT2,SEPHS1,XCL1,AASDHPPT,SERPINB5
Negative_7607	CGA,IL2,C4BPA,CCL13,CD44,CYP2C9,DAAM2,DPYD,GALNTL2,GCNT1,HTR7,LAMB1,NCSTN,OR14J1,OR6B2,OXCT2,PDHB,PHKG1,RASGRP3,TAP1,TICAM2
Negative_7608	SMO,CDH5,CLDN19,DHX58,LAMA3,MAOA,SLC12A2,SNAI1,ST3GAL6,THY1
Negative_7609	BAD,DLD,SMS,ACADS,AKR1B10,AOC2,ARHGEF1,ATG12,AXIN2,BMP6,CFTR,CLDN19,CXCL1,DARS2,DKK4,FARP2,GUCY1A2,HEXA,IFNA8,IKBKG,IQGAP2,KIR2DL1,LRP5,NCOR1,OR11H6,OR1D2,OR51A4,OR51A7,PPAT,PPID,PRKCG,PROS1,RFX5,SEMA4C,SHC1,SHMT1,SLC25A4,SLC25A6,SLC27A5,STX1B,TAS2R5,TJP2,TMLHE,TRAF4,WARS2,YES1,MAPK8IP3
Negative_7610	MPZ,ACAD9,ALDH3A1,ALDOA,AMY2B,CDKN2D,CTBP2,DDX47,EHMT2,GUCA1A,HNF1A,PFN4,SCIN,VAMP1
Negative_7611	CSF2,GADD45G,OGDH,PAPSS1,TBK1,TNFSF4
Negative_7612	GHR,AADAC,ADH1B,GALNTL2,INPP5E,MAP2K5,MPZL1,MYL9,OR6C75,POLA1,PRKCQ,UBE2K
Negative_7613	DUT,FAH,AARS2,ASNS,CAV3,CCR2,CD22,CLTC,COL4A1,COL5A3,CTBS,DEGS1,FBXW11,FLT3,FMO3,MGAT4A,MYD88,MYH11,NME2,NOD1,NRXN3,OR10H3,OR2M4,OR2T34,OR5D16,OR5I1,OR5T1,PDHB,PLA2G1B,PPP3CC,PTPRC,RBL1,RPS6KA1,SC4MOL,SLC27A1,STX6,STX8,TICAM2,TNFSF18,TYK2,VKORC1
Negative_7614	C4B,GP9,ADCY2,ARAF,CLDN10,CSNK1A1,CXCL2,DHCR24,ECSIT,GLI1,IL1RAP,LEPR,MTHFS,NRXN3,OR2A5,OR2G6,OR2L8,OR3A3,OR8H1,PDGFD,PLA2G4A,POLR2J,PPP2R5C,RPS6,RRAS,SELL,SOAT2,TAS2R50
Negative_7615	KIT,ADIPOQ,ARSE,BIRC2,CNTN1,CYP2A13,CYP2S1,DTX4,FGF13,GBA3,GNAI2,HMGCS1,HTR7,METTL6,NME2,NRCAM,OR2L13,OR4D11,RAC1,SETD8,UPP1,VAV3,XYLT2,MAPK8IP1
Negative_7616	AK1,C7,C8B,DBI,MVK,ACSM5,ALOX15,ATP6AP1,CCL20,CCNG1,CNTNAP2,COMP,CXCR5,CYP2S1,DHX58,EDA2R,HSP90B1,IFNA8,IRAK4,LFNG,MKNK2,NEU3,NOTCH3,OR1A1,OR1S2,OR4D6,PRKAR2A,PRKG2,PSENEN,SNAI1,SRGAP1,SRGAP2,WNT8A,YKT6,PAFAH1B3
Negative_7617	ID2,UBB,ACYP1,ALG9,BBOX1,CCND1,CSNK1D,CSNK2A1,DIAPH1,EPB41L1,EPRS,OR1D5,SEMA4C,TGFB3,PAFAH1B3
Negative_7618	ADAM17,CARS2,DLAT,DLL4,GDF5,MCCC2,NFATC4,NT5C1A,OR2A25,OR2D3,PDE8B,SEMA4C,SLIT2,TGFB3

Negative_7619	AK3, DHH, TFG, ADCY9, ALDH1B1, ATP5I, ATP6V0B, BMP2, CCL26, CXCL16, DARS2, DGKH, ENO1, ETNK2, FMO5, HEMK1, LAMB4, LAP3, LTC4S, MGAT4B, NFKBIE, NFYB, NKD1, OR10G2, OR10H5, OR10T2, OR13J1, OR2Y1, OR5AP2, OR5AR1, OR8K3, PDE4C, PDE9A, PLCD3, PLXNB1, POLE4, POLR2A, POLR2H, PRKAB2, PTCRA, SESN1, SHISA5, SOCS3, THBS3, TKTL1, ATP6V1G2, HLA-DRB3
Negative_7620	AKR1C3, CYP2U1, DFFB, DKK2, EPB41L3, FGF9, GNA12, GSTO1, HIBADH, HMGCR, HMGCS2, IDI2, NADSYN1, OR2W1, PEMT, PPP2R5B, RCHY1, TLR9, TSTA3
Negative_7621	IL4, ME2, ACSM1, B3GAT3, CCL15, FECH, LEPR, MNX1, OR56B4, OR6K3, PPA2, PRKCA, PRKG1, QARS, STAT3, WASL
Negative_7622	GBA, ACTN2, ADCY4, B3GAT3, CNTNAP2, GNAI2, KLRC2, MGAT4A, MYH11, NRCAM, OR2Z1, OR4F15, RFXANK, SH2D1A, UPP2
Negative_7623	DDO, TNC, ADRB2, AGMAT, CASP8, CDC45L, CHRM5, CSF1R, DGAT2L4, DTX4, FGF7, GSTM4, GUK1, ITGA10, LILRB3, LTBP1, MAN1A2, MGST2, NRXN2, OR10R2, OR5H6, PDHB, POLA1, PROC, PTAFR, PTGDS, RBPJL, SDHD, SPTAN1, WNT2, SUV420H1
Negative_7624	C4B, LAT, AKR1C3, AKR1C4, ATP4A, ATP6V1F, CACNA1I, CCR2, CES2, CFLAR, CYP3A5, DUSP10, DUSP7, FZD10, HES1, IL28RA, IL6ST, MAP3K14, MASP2, MMP2, NRP1, OR6A2, OR7G2, OXCT1, PDE6H, PI4KA, PIGB, PLA2G10, PRKCI, RFX5, RUNX1T1, XYLT2, ZIC2, TNFRSF10A
Negative_7625	SPN, ACY1, CYP1B1, GMPR2, ITGA3, OR2B11, OR6Y1, PNLIPRP2, TNFRSF10A
Negative_7626	ATM, PVR, TKT, ABI2, ADIPOR1, ALDH3A1, AOX1, ATP5F1, CADM3, CARS, CSDA, CX3CR1, FDFT1, FGD3, FTCD, INMT, ITGA2B, NCK2, NME7, NUMB, OR2A42, PHKA2, PLOD2, PPP2R5D, SCLY, SDC1, SLC27A4, SOAT2, TYROBP, ATP6V0D2
Negative_7627	EPHB3, HTR5A, PDE4D, PECAM1
Negative_7628	CACNG3, DTX1, ERO1L, HAGH, IAPP, OR52K1, OR6Y1, PRKCQ, UGT1A10, SERPING1
Negative_7629	CYFIP2, DLST, FTH1, GPT2, NFKBIE, NME6, PCK2, PFN2, PIK3R5, SMAD5
Negative_7630	CBS, GHR, SRF, AGPAT1, AGXT2, AKR1D1, ARG1, C1QB, CCL26, CHEK1, CHRM3, CYP4F2, DVL1, ERBB2, ESAM, FGF17, GADD45B, ITPKB, LALBA, LIFR, MAML1, MAN1A2, MDM4, MGAT2, MGST1, OGDHL, OR10R2, OR51D1, OR6M1, PTGS2, PTK2B, RALBP1, RFXANK, SV2C, UNC5B, CSNK1A1L, KIR2DL5A
Negative_7631	IL6, ACTN4, APOA1, CD14, CD19, CDC26, CRLF2, CYSLTR1, FCER1G, GNPDA1, MMP9, NSDHL, OR2M3, PDE8A, PDXP, PGS1, RBX1, YARS2
Negative_7632	GP5, ACACA, ACP1, ADIPOQ, ANAPC10, BCL10, CALML5, CCR9, CSAD, DIAPH1, FZD6, FZR1, GNRH2, ITGA3, LFNG, MCM3, NAT1, NAT5, NRCAM, OR2A4, OR2T8, OR7C1, P2RX2, POLR2K, PON2, PTPRF, PTPRM, RGS3, RRM2, SORBS1, TUBA8, WASF3
Negative_7633	SRM, STS, ACOX1, ANPEP, ASNS, BMP8A, CFLAR, CLDN20, FASLG, GP1BA, MAN1B1, MAP2K2, MAP2K3, NLGN1, NNMT, NT5C, OR1C1, PAX4, PCYT2, PGS1, TAS2R50, SERPINB5
Negative_7634	GSR, IRF9, OR4F15
Negative_7635	DDC, GGH, AKR1C1, CCNG1, DDB2, GCLC, ITGA2, KLRC2, NUDT12, PPAP2A, ROCK2, TOLLIP, WNT10A
Negative_7636	GP9, SMS, ATP5G1, BCKDHB, CAMK2D, CASK, CHRNA7, CTBP2, CXCR6, DNMT1, ENO2, ENPP1, IL20, KLK3, NAGS, NAT1, NFYA, NKX6-1, OR2T8, OR6N2, PDXK, PHKG1, PLXNA1, PTPN1, SUV420H1
Negative_7637	TAT, ADORA2A, ARHGEF7, ATF2, CASP9, CNTF, CREB3L3, CYCS, CYP3A5, FARS2, FZD5, GALNT7, GMPR, GNPAT, HNF4G, LTBP1, MDM4, MTMR6, OR2T8, OR51I1, PFKM, PIK3C2G, PLA2G4A, SESN2, SLC27A1, SMPD3, SPRY4, ST3GAL5, TAS2R8, TUBA1C, PRICKLE1
Negative_7638	DHH, MAX, ALOX12, APLP1, CACNG1, CD28, CTSS, DHDH, DPYS, ECHS1, FMO3, GCNT1, GGPS1, HIBCH, MCEE, MDH1, MGAT1, NEU3, NEUROG3, OR10AG1, OR4K14, OR7G2, P4HA2, RAP1A, SLC27A4, TYRP1

Negative_7639	DBI,LEP,CD274,CYP17A1,GSTK1,IMPA2,ITPA,MAD1L1,NT5C,OR4M1,PIGO,PIGU,POLD3,POLE,PSAT1,PTDSS2,RDH10,TJP3
Negative_7640	C3,INS,LEP,ABI2,ACSL1,ACSS3,ADRA1D,ALOX15,ATP6V1H,BST1,BUB3,CACNG4,CARD11,CD27,CD99,CYP2J2,DDX41,GSTZ1,IFNA10,IFNK,JAM2,MAP3K12,MAPK10,MAPK9,NEGR1,NRG1,OR52B6,ORC2L,P2RX3,PEMT,PPP2R2A,PPP3R2,PSME3,PTPN6,RFNG,SETD7,ST8SIA1,STX10,TNFRSF8,TSC2,UBE2L6,ZMAT3,KIR2DL5A,SUV420H2
Negative_7641	AMT,HGF,LCT,PGD,SMO,SMS,AKR1B10,ALDOA,ALOX12,AREG,ATG12,CACNA1F,COL3A1,DUSP1,GALC,GALNT13,GSTA4,IDH2,NOD1,NOTCH3,NR1D1,OR10A2,OR13C9,OR2D3,OR52L1,OR5AN1,P2RX1,PDCD1,PDHA1,PIPOX,PLA2G5,PLCB4,PPM1A,RALB,RPS6,SELE,SEMA3A,SH2D2A,TACR2,TCF7L1,TUBA1A,TUBB2A,VAMP1,MAPKAPK3,PAFAH1B2
Negative_7642	ALG8,APOA1,CTPS,FMO4,HIF1A,IFNW1,MFNG,POLR3A,SPTAN1,TAS2R7,ZIC2
Negative_7643	CAD,LCK,ASNS,CD70,HCCS,MARS
Negative_7644	KHK,ACSL3,ADAM10,AGPAT4,ALG13,ANAPC2,ARPC3,BCAT1,CALML6,CCNH,CDC14A,COL6A1,CSNK1G2,CYP1A2,DHCR24,DUSP7,FLT3LG,GAPDH,GCNT2,HLA-F,IFT57,INPP4B,ITPKB,LRDD,MAP3K1,OR10A6,OR1B1,OR1F1,OR2M2,PDE4B,PLCG1,POLA1,POLD3,PRKAG1,PSAT1,PVRL4,TPK1
Negative_7645	F11,LCT,ADCY1,B3GALT1,CCNB1,EDAR,FARSB,GAD2,HIBCH,HMOX2,INPPL1,KCNQ1,KIR2DL4,NCR2,PDE4D,SELP,SEMA3F,SSX2IP,UCK2
Negative_7646	ADC,ITK,PGP,APH1A,B4GALT4,BAI1,C4BPB,CDC27,CNTN2,CXCL2,DIAPH2,ERO1L,GNB1,HLCS,IL20RB,ITGB6,LARS2,OR4D9,PAX4,PGAM1,PLXNB1,POLR3A,PRKG2,RERE,SOCS5,ULBP3,C1GALT1C1,PNLIPRP1
Negative_7647	CGN,APBB1,ATP5L,FPGS,HSD11B2,NUDT2,OR51G2,OR51Q1,SHMT1,SMC1B,TYMS
Negative_7648	ITGA11,OR51M1,SKIV2L2
Negative_7649	APP,GP6,ACP6,B3GNT2,BUB3,DHDDS,ECSIT,F13A1,FGFR1,FMO3,FOSL1,GUCY2D,HRH2,IL2RA,KLKB1,MYH15,MYH4,MYLK3,OR11A1,OR13C8,OR13J1,OR1L3,OR4S2,OR51E2,OR52K2,OR5A1,OR5K2,PCK2,PHKA1,PIAS3,PLD2,POLR2L,PRKAB2,PRSS3,PTGES2,RXRB,SCIN,SMAD5,SPRED1,SULT1A2,SYNJ1,VAMP2,VASP
Negative_7650	AGL,IL8,CACNA1C,GSTM4,HIBADH,HSD17B3,IARS2,NFATC4,NGEF,OR2Z1,OR8I2,PAK1,PDE1A,PDE3B,RPN2,SDC3,TAS1R3,TOMM40,TYRP1,VEGFC,WWP1
Negative_7651	CGN,MIF,AGRP,APLP1,APRT,CACNG6,CHIT1,CYP2A7,FMO1,HTR5A,MAT1A,OR10S1,SMAD7,TIAM2
Negative_7652	EBP,IVD,ARRB1,CLTCL1,EPHA4,FLT4,SESN1,SLIT1,SUV39H1,TNFRSF6B
Negative_7653	C4B,F3,GLA,IL7,ATG3,C7orf16,CACNG1,CASK,CCL2,CLDN16,DTX3,EXT2,FGF3,GLYCTK,GNAT3,GRHPR,MTHFS,MTMR2,NME2,NODAL,NSD1,OR10H3,OR13C4,OR1J1,OR5B12,OR5V1,OR8A1,PARD6B,PDE8B,PLA2G5,POLR2C,PON1,RAP1A,TBL1XR1,VEGFB,HSD17B10
Negative_7654	CBS,CHRM2,ENTPD4,ESAM,FCGR3B,GLDC,MBL2,MKNK2,OR11G2,OR2Y1,PNPLA3,TJP3,VDAC2,ATP6V1C2
Negative_7655	NNT,OTC,HYAL2,KLRK1,PARVB,PFKM,PHKA2,STX5,TGFA,VTI1A,ATP6V0A1
Negative_7656	CR1,GBA,HK3,MVK,OSM,AKR1C1,APOE,AREG,ARPC3,ATP6V1D,CCR10,CD55,CD74,CYP3A5,DKK1,DUSP2,ENTPD4,FGD1,GALNT5,IFNA10,IGF1R,IGFBP3,IL17RB,MAFA,MGAM,MGAT3,MKNK1,MMP14,NODAL,OR4N5,OR56A3,PANK4,PDE3A,PPAP2C,PPP1R1A,PPP2R5D,PTPN7,RAPGEF2,RRAS,SMARCA2,TGFA,THBS4,TSHR,TUBB2C,ZBTB16

Negative_7657	BTD, IL3, LTB, SFN, ALOX15B, ATP2A3, ATP6V0B, CD40LG, CNGB1, DDOST, DUSP6, FZD1, GARS, GLDC, HNF4G, KIR2DL3, KLRC1, MCM5, MICA, MRAS, MTHFD1L, NCKAP1, OR56A3, PDIA4, PPP2R5C, PTPN1, SEMA3G, SPTAN1, STX2, TLR4, VDAC1, XIAP
Negative_7658	GLA, ASMT, CER1, CYP3A4, CYSLTR2, ENO3, EPB41L1, ITGAV, MGLL, SV2B, VKORC1
Negative_7659	FES, TNN, ACADL, AXIN2, CADM3, CDH4, COQ7, CSNK1G2, DAPK3, DTX4, DVL3, FRAP1, FZD5, HSPB1, IL12A, ITGA3, MGAT1, MTFMT, NCF2, NQO1, NT5M, OR10A2, OR14I1, OR1D2, OR4D6, OR51B2, OR51L1, OR5AR1, PDE10A, PHKA2, PLA2G2D, POLR2C, RAPGEF4, SEMA7A, TAS1R1, TSTA3, UNC5D
Negative_7660	AK1, GCK, ILK, ME1, NPL, SMS, ALDH7A1, ARNT, B4GALT4, BAAT, CACNB2, CCR1, CD3E, CD59, COL3A1, CPT2, CYP21A2, DGKB, GGT7, HSPB1, IL12RB1, LTC4S, NGEF, NOTCH1, NT5C1A, OR1N2, OR2V2, OR51A2, PPCDC, PRKAR1B, PTGES, PVRL4, TJP3, TRIP10, TSC2, YWHAB, ATP6V1E2
Negative_7661	CKM, MOS, OSM, AGPAT1, ATN1, B3GNT4, B4GALT3, CADM3, CSF3, DAAM2, EDNRA, FABP3, FBP1, GGPS1, GJD2, GNRHR, IL8RA, ITGA2B, MAN1A2, OR10G2, OR51A2, OR5I1, OR6B2, OR6K2, OR8D1, OR8H1, OR9K2, PANK4, PFKFB3, PGM3, PRKAB2, PTGDS, SESN2, SFRP5, SMAD7, SOX17, SSX2IP, TXNDC12, UBA1, ARHGEF12
Negative_7662	GLA, GLS, LCK, ACVR1C, ADCY2, ADSS, AKR1B10, ATP5B, BAIAP2, CD47, CDK4, CXCR5, CYP26B1, DDIT4, DDOST, DTX2, FGF13, GSTT1, IFNGR1, IL29, IL6R, ITGA5, ITGA8, LEFTY1, MMP1, MTFMT, NANS, NEU1, NTNG1, ODC1, OR12D3, OR51T1, OR5P2, PARD3, PIGM, PROC, RBL2, RIPK1, SOD1, STX12, TDO2, TNFSF4
Negative_7663	DHH, ACY3, ADRA1B, AGRN, AMY2B, APLP1, ATG7, CCKAR, CCR5, CPT1A, CXXC4, CYP2F1, DLL1, DUSP3, E2F2, FUT2, GALNT10, GSTM3, HNF1B, IL13RA1, IL22RA1, IL2RA, OGDHL, OR13C2, OR1J2, OR4A16, OR4K5, OR52E2, PIP4K2A, PIP5K1A, PIP5K1B, PLXNC1, PPM1D, PRNP, RAC3, RFNG, SDHA, SEMA4A, SKP1
Negative_7664	TNN, BMPR2, CYP2B6, DOT1L, EVI1, FZD5, GALNS, IDI2, LAMB1, MAPT, MTMR6, NAT1, OR4F4, PDGFA, SLC2A4, TCF7L1
Negative_7665	AR, F7, IVD, MET, ACVR1B, ACVRL1, AOC2, APOA1, ATP5O, B3GAT1, B3GAT2, CACNG3, CD27, CEBPA, CSF3R, DPAGT1, EREG, FGD3, FGF8, GALNTL1, GATM, GBA3, GCNT4, HLA-DOA, HTR7, ITGA5, JAK1, MS4A2, NUDT5, OR2M2, OR8B8, OR8K3, PAK6, PIGV, PIGW, PLXNB2, PMVK, POLR3D, RPN2, SETD1B, TACR2, TBXA2R, TDO2, TFDP1, TGFB1, TIAM2, TNFSF10, TOMM40L
Negative_7666	CRH, ADIPOQ, ADRB1, CCL8, DTYMK, EFNA1, FCGR3B, FGF10, FOXA2, HAP1, HIBCH, HMOX2, HRAS, MAT1A, NCKAP1L, OCRL, OR2Z1, OR5AP2, PFN1, PFN4, PIP5K1B, PLOD1, POLR2I, PPM1D, PSEN1, PSEN2, RASGRP1, RND1, SDHA, SESN1, SHMT2, SPCS1, WNT16
Negative_7667	GPI, MVD, NLK, NPL, ADIPOR2, AGPAT4, ALDOA, C1QA, CDC14A, CHEK2, CSNK1E, EFNB2, GALNT1, HSD3B2, IL13, ITGA11, MGST2, NTF3, OR13D1, OR9A2, PISD, PPCS, PRKY, PTPRZ1, RAP1A, SPP1, TCF7L1, TYMP, UGCGL2, ATP6V0A4, HLA-DPB1
Negative_7668	C1S, JUP, MSN, AADAC, ARPC2, CCL21, CLDN14, CXCL6, DARS, DTX3L, EFNA2, ENTPD6, FARS2, FECH, GNAI1, GRIA2, HARS2, IL17A, IMPDH2, INADL, MAGI2, MAN1A1, MAPK10, MTHFS, NANP, NRG4, OR10C1, OR2L8, P2RX4, PARVB, PER1, PHKA2, PPAP2B, PPP2R2D, RDH16, RERE, RHEB, SLC2A1, SOCS1, TNFSF14, TPM3, UNC5B, ST6GALNAC4, TNFRSF6B
Negative_7669	TXK, TYR, ADIPOR2, CREB3L1, CX3CR1, CYP27A1, EFNB1, HNF4A, KIR2DS4, MGST1, OR2F2, PANK3, PDGFD, PLCD4, PLCZ1, PLOD1, PPP1R1A, RFX5, SLC2A2, SV2B, UAP1, UGT2B11, HLA-DRB3

Negative_7670	C4B, DBI, GSS, MVD, OSM, PML, ACOX3, ALDH3A2, ANPEP, APH1A, BAIAP2, BLNK, BLVRA, CX3CR1, DDX55, DGKH, F11R, FGF18, FZD3, HSD3B1, IFNA17, ITGB7, MAP3K13, MEF2C, MYH7, NRG1, OR51G1, OR9Q1, PLD1, PLXNB3, POLR1C, POLR3G, PPA2, PPAP2C, PTGS1, SEMA4D, SETD8, SMAD3, TPM3, WNT8B, YES1, ZBTB16
Negative_7671	STS, ACYP1, AKR1C1, CTNND1, MAP2K3, MCCC1, NME7, OR5AU1, PDE7A, POMC, RIPK1, RPS6KA1, WEE1
Negative_7672	FES, LPO, ADH1B, ALDH5A1, ATG7, CCL3, CD34, CD59, CNTNAP2, CYCS, IFNA4, NRXN3, NUMBL, OR2C3, OR6K3, OR9Q1, P4HA2, PAK2, PDGFB, PGK2, PHPT1, PON3, SSX2IP, ST3GAL5, TLR2, VARS, VEGFC, WHSC1
Negative_7673	ID3, ID4, ALDH3A2, ARHGEF4, CDC20, CHST13, CTF1, CTNNA1, DCXR, DKK2, GLO1, HNF4G, IFNA17, MAP4K4, MYD88, NAE1, OR5AR1, PAX4, PECAM1, PLXNC1, PMM1, PRODH2, PTGDS, PTGES2, SEMA4G, SMC1A, SOAT2, TM7SF2, TRMT11, WHSC1, MAP3K7IP1
Negative_7674	DCC, GBA, KHK, TH, ACSM1, ALDH3B2, AMY2B, B3GALT4, BTRC, C3AR1, CD86, CDH3, CLDN19, CLDN6, DPM2, EGLN3, FGD3, FLAD1, GLI3, GYS1, HSD17B1, IFNAR2, ISYNA1, LMO7, MAPK9, NCF1, OR1M1, OR4L1, OR51A7, OR5K1, OR5K2, PARD6G, PPP1R3B, PRKAG1, PRKX, PSENEN, PTPN1, RENBP, SEPHS1, SORD, TAS2R49, TBL1XR1, TGFB3, ULK3, CACNA2D1
Negative_7675	AK3, DUT, TNN, ABCC8, ALOX12B, ARHGEF2, ATP6V1F, CADM3, CD55, CFL2, CFTR, CSNK1G3, EPHA7, FABP1, FZD8, GNG13, GSTA5, GSTO1, GZMB, HLA-DRA, HMOX2, ITGB5, KLK3, KLRC1, MAP3K6, METTL6, OR13H1, OR51E2, OR5I1, OR6C70, PISD, PLA2G5, PLXNB1, POLR3H, PRKCE, SDHB, SOX17, SSH1, STAM, TAS2R7, TAS2R8, TLR9, SERPIND1, SERPINE1
Negative_7676	CKM, IL7, MAG, ACPP, ACVR1B, AGMAT, AKR1C2, B4GALT1, BCL10, BET1, CDC2, CNDP1, CXCL11, DDX19A, EGLN3, EPRS, GBE1, GYS1, IFNGR1, IMPA2, KDSR, LLGL1, MYH1, NFX1, NPR1, NTRK2, OR10H1, OR52K1, PIGL, PIK3R5, POLR1D, PRKAA2, RALGDS, SEMA3D, SMAD2, SOAT1, SSH3, TCF7, TIRAP, ATP6V0D2
Negative_7677	CBS, BMP7, INPP5E, LIPG, OR10A4, PI4KB
Negative_7678	BTK, SRM, ACPT, ADIPOR2, AKR1C3, ALDH1A1, ALDH1L1, ALG13, ARSE, CACNG7, EFNA1, ENTPD7, FGF9, GALK1, GALNT3, GCNT3, GLDC, MGST1, MTHFD2, MYH8, NCAM2, NOD1, OR2H2, OR2T4, OR51B4, OR9A4, PAPSS2, PARD6A, PDCD1, PIGK, POLR1E, RFXAP, SGPP1, SULT1A2, TSNARE1, TSTA3, WWP2
Negative_7679	RDX, BMP8B, CCL24, CCL7, CXCR3, EPHA2, FTH1, GGT5, IL17RB, NMNAT2, OR4C16, PHKG2, PHPT1, STT3B, UCK1, ATP6V1B1
Negative_7680	GHR, HGF, APOA1, BTRC, CRB3, EPHA1, FGF3, MDM2, NAGLU, NEUROD1, PLCE1, PNPLA3, SESN3, SLC27A5, TFPI, TOMM40, TP73, WARS, ST6GALNAC5
Negative_7681	CAD, KIT, CASP10, CHST12, CHST13, CRLS1, CYP3A4, G6PC, GSTA5, HPSE2, LEPR, MYH11, NCSTN, OR52R1, P2RX2, PLA2G4A, QPRT, SEMA4G, SESN3, SLIT1, SPRED1, UXS1, VDAC2, WASF3, HLA-DPB1, TNFRSF1B
Negative_7682	MIF, OAT, ADCY4, ADORA2A, AHCY, ARPC5, B3GALT5, C4BPB, CACNA1I, CMBL, CPT1A, CTNNA2, ENPP2, FGFR4, GLYCTK, GPX7, GRPR, IL20RA, IMPA2, INADL, ITGB4, LTBR, MARS, NME4, NME6, PDGFD, PRKAB2, PRKX, PTPRF, RAD51, RFWD2, RIPK1, STX18, VAV3, YWHAG, ATP6V1E1, CACNA2D2
Negative_7683	ARHGAP5, CHAT, FZD5, HSD17B3
Negative_7684	ABLIM2, ACMSD, CDC2, CPT1A, CSNK1G1, CSNK2A1, CXCL10, EFNA4, EPHA7, FZD5, GALNT7, GCNT4, GLS2, GOSR2, GPT2, GUCA1C, MAP3K14, NKX6-1, NRXN1, PCCB, PLXNA1, PRKG2, RDH8, RPS6, SLC33A1, SOS2, TPMT, TRAF3, UGCGL1, UGT1A10, ULBP2, UMPS, WNT5A, SERPING1
Negative_7685	MYC, SRM, ABL1, ARSD, FLAD1, GANC, IL23R, MGAT3, OR51A2, PRKCE, RBKS, ST6GALNAC1, TNFRSF1A
Negative_7686	CBLB, GART, GGT5, GRPR, IRAK1, LDHA, NAT2, OR10P1, PPP2R5C, PTPN5, TGFB1, TSTA3, PAFAH1B3

Negative_7687	CAD, OTC, ACVR2A, ADAM10, ALG5, CACNA1H, CCNB2, CDC25B, CDKN1A, CLDN4, CYP11A1, CYP2C18, DFFB, GSTZ1, GUCA1B, LLGL2, LYZL1, NFATC2, NMNAT3, NPAS2, NTRK1, OR10X1, OR13C8, OR2F2, OR2T11, OR51Q1, PIGT, PLCE1, PPAP2A, PPP2R2D, SEMA4A, SOCS7, UCKL1
Negative_7688	MOS, ACP2, CDKN2B, FGF6, SHMT2
Negative_7689	FGF7, OR2AG2, OR51Q1
Negative_7690	APP, C8B, FAH, ID2, PML, SRC, ABLIM1, AGPAT3, ALDH6A1, ASNS, CDH2, COQ5, CYCS, DCTD, DFFA, DNMT1, DVL2, EIF4B, EPRS, GGCX, GNAZ, GNMT, IRS2, LCMT2, NEUROD1, NFATC2, OCRL, OR2T12, OR51G2, OR7G3, OR9Q2, PAX4, PIGK, PLA2G2F, PLCB2, PRKG2, PTPRZ1, SFRP2, SLC2A1, STK3, TLN1, TNXB, WNT2B
Negative_7691	CP, AADAC, ARPC1B, CASK, CCL17, EGLN3, ENTPD2, GADD45B, HLA-F, IL20RB, MMP9, NKD1, OR6C4, PIGT, RPN1, SELP, TGDS, TSC1
Negative_7692	DBI, ILK, JUP, AHCYL2, ATP5G3, CACNA1E, CHEK1, DBF4, DDX47, ENPP3, ERBB3, GALNT7, GRIA2, GYS1, INSRR, MAML3, MPZL1, MTMR6, NRXN1, NTF4, OR5B17, OR6S1, PIGW, PRKAR1B, RUNX1, SLC27A5, TRPC1, VDAC3, WNT8B, ZFYVE16
Negative_7693	JUN, PTS, SMO, TAT, ACAD9, ACLY, CDC16, COL4A6, FADS2, GALC, GAMT, GNMT, IL11, INPP5E, ITGB7, LTB4R2, NT5C3, OR10Z1, OR4F17, PAPSS1, PDE11A, PIGN, PIGP, PLOD3, PRKACB, PRSS1, TNFRSF6B
Negative_7694	APC2, BET1, CCR8, DGKE, FGF19, MYH10, NFKB2, POLR2D, SDHA, SEMA3E
Negative_7695	ADH4, ARHGEF2, ARSE, ATP5D, BIRC2, CAMKK1, CD226, FUT3, GNPAT, HHEX, ITGB8, KIR2DL4, LRAT, LRDD, MYD88, NCOR1, OR1E1, OR5M9, OR7C1, OXCT2, PDE6D, PIK3C2G, PPP2R5B, SC5DL, SEMA3A, SLC9A1, STX18, TNFSF10, USE1
Negative_7696	ADC, BTC, SPN, ADSSL1, APOE, ARPC5, CASP10, CBLC, CCR2, CNTNAP1, CRB3, DAXX, DHDDS, ECSIT, EFNB1, EIF4B, ENTPD5, EPOR, FARS2, FBP1, GNPAT, GRIN2C, HAGH, IDH2, IL21, IRAK4, KIR2DS5, MCAT, MCCC2, OR1L3, OR2A42, OR2M5, OR4A5, OR51Q1, OR5P3, OR5T1, P2RX4, PDGFRB, PNPLA4, RFNG, SELE, SMARCA2, ST3GAL4, TUBA1A, VEGFA, TNFRSF12A
Negative_7697	APPL1, ATP5J, COX15, GZMB, NT5C1B, OR56A1, PRKCI, TACR3, TUBB2B
Negative_7698	BCR, RPE, ACSS1, ACVRL1, CACNA1A, CACNA1G, CEBPA, CFLAR, CLDN17, CSNK2A1, CXCL5, CYP11B2, CYP2C8, EPB41, FGF18, GALT, HCCS, HIP1, IGF1R, IL12RB1, IL15RA, MAPK1, MLYCD, NCKAP1L, OR7C1, OR8G1, PPA2, RBX1, SETD1B, SLC25A4, SMARCA5, SOAT1, SRGAP2, TAS2R4, TIAM1, TNFRSF8, TPK1, CSGALNACT2, PAFAH1B2, PDCD1LG2
Negative_7699	EBP, ACSM2A, APAF1, DLL4, IFNAR2, ITPA, MGAT4A, PDE7A, SORBS1, MAPK8IP3
Negative_7700	SHH, ADCY4, AKR1A1, CCL19, OR5AU1
Negative_7701	C3AR1, CACNA1G, DHX58, GLO1, HNF1B, OR5D14, ROBO1
Negative_7702	APP, AACS, CBR3, GARS, HTR4, IL15RA, IMPA1, ITPKB, KLRK1, LIPG, MAFA, MCEE, OR51F2, RBX1, SEMA4D, SUCLA2, SV2A
Negative_7703	BUB3, CDK2, CYBB, FGF23, GSTM2, JUND, LAMC3, NMNAT1, PTCH2, RHOQ, SGPP2, SMC1B, SERPINC1
Negative_7704	EPO, LCT, UBC, AKT2, B3GNT5, CLDN20, GRHPR, INPP4B, JAM2, MAFA, MAP2K5, MAP2K6, NTF4, NUDT12, OR2L13, PLOD2, QARS, RARS2, VDAC3, PLA2G12A
Negative_7705	F8, IL3, TK2, AARS, AGRN, ALOX12B, ATP5D, CD55, CD79B, CDKN2D, DUSP14, EPHA1, GADD45B, IGSF5, KDSR, LCP2, LHCGR, MYH10, NCR3, NLGN1, OR1B1, OR1L8, OR2AG2, PCK2, PPP2R2D, PRKACG, PTGS1, SMAD5, THTPA, WNT5A, WNT5B, PRICKLE1
Negative_7706	FGF17, HAGHL, OR2B6, OR2T6, YWHAQ
Negative_7707	BTC, ME2, MPL, SCD, ADCY8, CACNA1G, CACNA1S, CHAD, CYP2S1, DCTN1, DPYS, EXT2, FZD3, GDE1, GFPT2, HTR2A, KITLG, LDHB, OR2M3, OR6Y1, PARD6G, STAT4, THBS2, TUBB3, WEE1, WNT7B, YWHAH, ST6GALNAC4

Negative_7708	GSR, MUT, AASDH, APH1A, B4GALT4, BDKRB1, BHMT, CALML3, CAMK4, CASP7, CHST11, COL1A1, DRD1, DRD5, ESPL1, EXTL2, GNRH1, GSTO1, IDUA, IQGAP1, ITGAV, LAMC3, LGMN, LIFR, NAMPT, NRP1, OR13C8, OR2H2, OR4D1, OR52I2, ORC3L, PAX6, PDGFRB, PSPH, PTK2, RDH5, SEMA4G, SKP2, SLC2A2, SMAD2, ST6GAL1, TOLLIP
Negative_7709	BIRC3, CD99, CLTCL1, DDOST, DFFA, DGKI, E2F3, EDNRB, ENPP3, GGPS1, HDAC2, IL11, IL28B, INHBC, IQGAP3, ITGA3, PHPT1, PLXNA3, POLR3A, SEMA4B, TUBB3, TYMS, TNFRSF13C
Negative_7710	ACP5, ATP5O, C5AR1, CD74, CDKN2B, CHRNA7, CXCL10, OR2A2, OR5H2, PIP4K2C, PLIN, PNPT1, SOX17
Negative_7711	CCR5, HAP1, ITGB8, OXCT2, PAX6
Negative_7712	ACAA1, ACACA, ANAPC4, APOE, ATF2, BACE2, CYP2F1, ELK1, GCAT, HLA-DOB, ITGB5, LAP3, MAGI1, MITF, NCR3, OR52W1, POLR1B, PVRL4, STK4, THY1, TNXB, TSNARE1, WASL, HLA-DQB1
Negative_7713	C7, CD4, GK, ATN1, CD28, CFL2, ETNK1, FCGR3B, GTSE1, IGF1, LEFTY2, NARS, OR4C11, OR5L2, PIP4K2A, PLAT, PPAP2A, PTK2, TNFSF18, VDAC3, YWHAH
Negative_7714	ME2, DNMT3L, ITGA3, NPR1, PRKCG
Negative_7715	DCC, FYN, GPT, PXN, SYK, ATP2A2, CARS, CCR4, CTNNA3, DGUOK, DKK4, E2F3, EFNA1, GGCX, HSD17B4, HTR7, IL17RB, MLYCD, NEU3, OR10J3, OR4D1, OR4K15, PDE9A, PDXK, PFKFB3, PGAM2, PIAS4, PIGQ, PIK3CA, PLA2G2F, PPP3CC, RPS6KA1, SETD7, STK4, TKTL2, TUBB2C, WASF3, WNT3, ZFYVE9
Negative_7716	ACVR1C, CDC26, CLDN22, CYP2A6, GNA12, GNB1, HAO2, IL22, INMT, KDSR, NSD1, P2RX7, RBL2, SARS, SOCS5, TAS2R13, WWP2, XYLT2
Negative_7717	DBH, F12, FOS, ACP1, AKR1C2, ATG3, ATP6V1D, BCAR1, CAMKK2, DRD1, FLOT1, GALT, GSTA3, ICOS, IMPDH1, INHBB, OR6C3, OXCT2, PARD6G, PDE11A, PIK3C2B, PLD2, PPP2CA, PPP2R5B, PRDX6, RPS6KA3, RUNX1, SGMS1, TMLHE, VASP, VDAC3, PDCD1LG2
Negative_7718	PGP, ASCC3, C1QA, CYBB, FSHR, LYPLA2, POLR2D, SIGLEC1
Negative_7719	AXIN2, CMPK2, PLCB1
Negative_7720	EDA, ACACA, ACADVL, ALDH5A1, ANGPTL4, ATG3, ATP6V0C, B3GNT5, CACNG2, COX10, CREB3L4, CSH1, DGAT2, EPHX2, FGF10, IL23A, MAPK11, MPST, NAGK, NEU4, NPAS2, OR10A5, OR10G9, OR4D1, OR56A4, ORC1L, PDCD1, PFKM, PIGV, PRKCH, RBPJ, RPRM, SHC3, SOAT2, SSH2, TACR3, TAS2R13, THBS1, TUBB2A, UCKL1, VEGFC, WEE1, ZAP70, GABARAPL1
Negative_7721	C7, SCD, ADCY10, ALG10B, AMPD1, ARSA, ARSE, BPGM, BUB1, CADM1, CALML3, CDKN1A, CES1, COL5A1, CSF3R, CTNNA2, DHRS9, ENTPD7, FUT2, GAB1, GAMT, GPX7, HPRT1, KIR3DL3, LIPG, MCAT, MDH2, MTAP, NEUROD1, NFATC4, OR11G2, OR13C8, OR1L3, OR5B3, OR6C70, PIGK, PRKAB2, PRKCE, PTGES2, SGPP1, TICAM2, TLN1, TLN2, WASF2, PAFAH1B2, PLA2G12A
Negative_7722	BAX, CD6, HPD, B3GNT5, BIRC3, CAV1, DVL1, MAD2L1, PLA2G1B, PRPS2, THBS1, UCK2
Negative_7723	C3, ACSL5, AHCY, ATP5A1, CACNG4, CAMKK1, CCL8, CD244, CHUK, CSF2RA, CTSB, DCTD, E2F3, FOXA3, HADHB, HAGH, IL21R, IQGAP1, MAP4K2, MYH8, OR2A25, OR7A17, PDE10A, PLCD3, POLE2, PYGL, RRAS, RRAS2, SLC33A1, ATP6V1C1, HLA-DQA2, MAPKAPK5, ST6GALNAC3
Negative_7724	AK3, AGXT, ATP2A1, COASY, E2F5, ESPL1, FZD1, GNRH1, GRM1, HHIP, IQGAP1, ITGB4, MAFA, MAOA, MMP1, NEFH, NRCAM, OR1D5, OR2AG1, OR2W1, OR56A3, OR8A1, PAPSS2, PDHB, PLCB1, TAS2R42, ATP6V1E2
Negative_7725	AK3, FAS, PRL, ACP1, ACSS1, ADCY5, ADRB2, AOC2, ATP6V0C, C4BPB, CER1, CHST12, CHSY1, CLDN1, CLDN18, CRKL, CYP19A1, CYP1A1, FZD10, GALNT3, GCNT2, HSPA2, IFNG, IL17RA, ITPKB, LTC4S, NCOR2, NPR1, OCLN, PDGFD, PGK1, PLA2G2F, PPA2, PRNP, PRODH2, PTGER1, PTPMT1, SETD1A, SPRY2, ST3GAL6, SUV39H2, TGFBR2, TLN1, TNXB, TUBB4Q, UBE2K, B4GALNT1, PLA2G12B, SLC25A31

Negative_7726	IL9, NPL, PRL, VTN, ADRB2, ALOX12B, BMP7, CDKN2A, DEGS1, OR10G7, OR1J4, PFN1, PLA1A
Negative_7727	DUT, ACADM, ACPT, ANAPC5, ASPA, BACE2, BCKDHB, C7orf16, CAMK2G, CARS, CXCL13, EDNRB, FASLG, FGF3, GP1BB, IFNA4, IL22RA1, NEU2, PDE1B, PGAM1, PGK1, PPAP2C, SV2A, USE1, PHOSPHO1, TNFRSF1A
Negative_7728	KDR, NPY, BET1L, CACNG8, CD8A, CRLS1, CSAD, ELK1, EPHA5, EPOR, FGF22, GALNT14, GCAT, HHIP, ITGB7, MAML1, NANS, NARS2, OR14C36, OR2H1, OR2L2, OR2M2, OR2M7, OR2T12, OR4D1, OR5T1, OR6T1, OXTR, PCK2, PDE4D, PLXNC1, PTPRZ1, QPRT, SAT1, SMARCA2, SMC1A, SOCS2, SRGAP2, HLA-DQA2, HSP90AA1
Negative_7729	A2M, ATF4, ATP6V0C, CXXC4, CYBB, FUT4, GCNT1, GUCA1C, HSD11B2, ITGB6, LYZL1, OR5K2, OR8H2, PIK3C2G, PLA1A, PLCG2, PPP1R3B, PTGIS, ROBO3, SEMA3B, TRAF4
Negative_7730	F12, F2, F2R, IL2, AARS2, ACADSB, AMDHD1, B3GALT4, BCL10, BLVRA, CCL3, CD14, CD59, CHRM1, ENO2, EPHB2, EPHB6, GNB3, ICAM1, KDSR, KIR2DL1, MMP7, MTAP, OCRL, OR10K1, PDE10A, PGAM2, PIK3C3, PNPLA3, PVRL2, RFXAP, ROBO3, RPN1, SEMA7A, STAM2, TJP2, WNT10B, ATP6V0A4
Negative_7731	LCT, ACADM, ACP1, ADIPOR2, ALDH1A2, BPNT1, C7orf16, CACNB1, CCNB1, CDH3, DIAPH3, FGFR1, FTMT, G6PD, GGT5, H6PD, HNMT, HRAS, IL28B, INPP5E, MAOA, MPDZ, NCF1, OR10J5, OR10T2, OR1I1, OR4C6, OR4D11, OR5AU1, POLA1, POLR2E, PTGFR, PYGM, RARS2, STX5, TKTL1, VDAC1
Negative_7732	ADO, RDX, BIRC5, C3orf10, CAV3, CHRM5, CYP2A7, CYP2E1, DAG1, GCDH, GNPDA1, GUCY2D, HNMT, IFNA17, LAMC2, LEFTY1, MCCC2, MMP14, MTFMT, NRG1, OR51Q1, OR56B4, PDE1B, PDE6G, PIPOX, PPP2CB, SKIV2L2, SRD5A1, WWP2, ATP6V1G3, SERPINB5
Negative_7733	GGH, OGT, SFN, ADCY3, ARSD, CTSL1, GRIA1, HBEGF, IDH3A, MAML3, MYH7B, NAE1, NOX1, OR4N5, OR52E2, PIK3C2G, PIP4K2A, PRNP, TNFSF14, CSGALNACT2
Negative_7734	B4GALT6, CDKN1C, DDX54, OR2F2
Negative_7735	CAD, ACADSB, ACSL6, ACTG2, AGPAT1, AVPR1A, CLCA4, CREB3L2, CYP2A6, GSTA1, GSTT1, IFNA4, IQGAP2, MYD88, NCR3, OR2K2, OR6S1, ORC5L, PARVG, PIGQ, SEMA3D, SHC4, TCF7, TLR3, UBA1, SUV420H1
Negative_7736	OAT, PAH, ACADSB, ADH6, AGPAT1, AGRN, ARNT, CACNB4, GALT, GPD2, HTR6, OR10A2, RRAS2, SDHD, TNFRSF10A
Negative_7737	AK1, C7, PTS, BACE1, CLDN2, CSNK1D, CX3CL1, DHODH, ENO3, FADD, GCNT4, GNB1, KAT2B, LAMC2, OR4K5, OR51D1, PCYT1B, PPA1, RPIA, SHC3, ZIC2
Negative_7738	ACTN2, ALOX15B, ATP5I, DLL1, EPHB4, FRAP1, HMOX2, IDH1, IL2RA, ITGA10, LDHAL6B, LHCGR, LRP5, MAP4K1, OR10K1, OR1G1, OR2C1, OR4C11, PGAM2, PRKAG2, RAP1A, SOCS5, TGFA
Negative_7739	ABAT, ACSS3, ADSSL1, ARPC5, B4GALT5, BMP8A, BMP8B, CCL16, CYP19A1, DNMT3A, ENO3, F13B, FGF10, FMO1, IKBKG, LIPA, NT5C3, OR2A25, OR5J2, OR6C4, OR9A4, PARS2, PPID, PRKCE, RPSA, SEMA4F, SOCS1, SUFU, TAS2R16, TDO2, VASP, YWHAB, SLC25A31
Negative_7740	ALG8, ATP6V0B, B3GNT4, CREB3, CYP11A1, DHX58, DUSP16, GALNT6, GAS1, GDF6, INPP5B, MAPK1, NKD2, OR10H3, OR5P2, OR6C3, PLTP, ST8SIA5, MAPK8IP2
Negative_7741	DCN, IL7, LSS, PXN, ACAD8, ADCY2, ALDH5A1, ALDH7A1, BCAR1, CDH5, CER1, CLDN2, EGLN3, FGF4, GNA11, GSTA2, GSTO2, ISYNA1, ITGB3, MALT1, MGAT1, MGAT4A, MPZL1, NARS, NCAM1, NPAS2, NT5C1A, OCLN, OR52L1, OR5A1, OR5L1, OR8B4, ORC5L, PGK2, PGM3, PIGW, PNPLA3, POLR2F, POLR2G, SLIT2, SPRY1, TAS2R7, TUBB, B3GALNT1, MAPKAPK2, PNLIPRP1

Negative_7742	IL6,MTR,ADAM10,ADORA2B,AMHR2,CACNA1A,CACNA1H,CCR10,CCR8,CDS2,CTSS,DHDH,GDF7,GJA1,HDAC2,ITGAV,MAPK11,NCKAP1,OR10H1,OR10P1,OR10R2,OR2L8,OR3A1,OR51S1,PDK1,PFN1,PIGF,PIPOX,PLA2G2E,PLA2G6,PON1,SETD1B,SHMT2,SNAI2,ST3GAL4,SUV39H1,WWP1,YWHAQ,PPP1R12A
Negative_7743	MAG,ATP12A,ATP5D,CXCL6,DRD1,EDAR,GALNT9,GYS1,HEXB,HHIP,IGF1,IL4R,LARS,LDHC,MAP3K2,MAT2B,NFAT5,NUDT9,OGDHL,OR1G1,OR6K2,PORCN,PPP3CA,PRSS3,PTPRJ,SMAD7,UAP1,UPP1,ALDH18A1
Negative_7744	AR,BTC,CGA,CRH,JUN,NF1,PLG,TKT,ARNT,COL5A1,CYP2B6,DDX52,DHODH,FRAP1,GGT1,GUCY1A3,ITPA,LDHD,MAP3K13,MAP3K6,NAT5,OR10A2,OR1A2,OR52H1,OR52J3,OR7G1,OXSM,PFKFB4,PIGO,PIP5K1A,PLCZ1,POLR1B,PRKCZ,PTGS1,RALGDS,RDH10,SDC4,TAP2,TRPM5,WNT10B,CTNNBIP1
Negative_7745	CAT,DLD,NNT,OAT,ABCC8,ANAPC7,AOC2,ARPC1B,ARPC5L,ATP4A,B4GALT5,CALML3,CASP8,CD55,CHSY3,CTSG,FZD6,GALNS,GOSR2,HNF4A,IFNB1,IL12A,IL2RG,IMPA1,IRAK4,LPAR1,MGAT2,OR10P1,OR6K3,PDGFC,PECAM1,PRKG2,PYGL,RASSF5,RPRM,SDC2,SETD2,SMPD4,SPAM1,TIRAP,TUBA8,UXS1,WARS2
Negative_7746	CKB,DLD,F3,APOA2,DUSP9,FMO2,MAPK14,PLAT,PTPRJ,RPS6KB2,TNFRSF13B
Negative_7747	ACO1,CDS1,CXCL10,DGKE,FLT3LG,IL22,PIGA,TGFBR2,TNFSF18,C1GALT1C1
Negative_7748	KIT,RPE,ACSL4,ACSM5,ATP5D,BACE1,BAI1,CHSY1,CSF2RB,DPYD,F2RL1,GLUD2,GSTP1,HLCS,IKBKB,MAD2L2,MAP4K3,MAT1A,OR2F2,OR4M1,OR51V1,PDGFC,PTPN1,RXRB,SELP,SEMA4A,ULK1,WBSCR17,ST6GALNAC4,TNFRSF1A
Negative_7749	RDX,AKT3,CCNB2,CMAS,CRB3,CTNNA1,DGKB,E2F2,FGFR2,HMGCS1,IFNB1,IGF1R,IGSF5,OR10K2,P2RX6,PAPSS1,PDE1B,TRPM5,UGT2B11
Negative_7750	CLOCK,GSTT1,HES1,NT5M,OR8H1,PSENEN,SMAD2
Negative_7751	ACOT12,ADCY10,CLDN8,CYP2E1,ETNK2,MAP3K5,OR52R1,PLCB1
Negative_7752	LIF,LPL,MPI,PGF,TYR,ACPT,AGPAT6,APLP1,B4GALT6,CDC26,EREG,F2RL3,FGFR2,GNAQ,GRM5,HLA-F,IMPDH1,ITGA4,OLAH,PAX6,PDGFA,POLD2,RDH5,SH2D1A,TNFSF12,TSNARE1,WNT3
Negative_7753	GSR,CHAT,CHSY3,CYP2A6,CYP2C19,DDX51,DERA,NUDT9,PAK6,PDE1A,PRKCE
Negative_7754	GP6,MAX,ADCY3,ADH7,AGPAT3,ANAPC11,ARPC1B,ATIC,CD58,CDC23,CX3CR1,CYCS,DGKH,EIF4B,FPGT,FRAT2,GGT7,GMPPB,GNAQ,IL17A,ITPA,MASP2,NAT5,NLGN2,OR6N2,OR8H2,PARVA,PIK3CG,PPT1,PVRL3,SH3BP2,SMC1A,STX1A,STX4,B4GALNT1
Negative_7755	AGK,BCR,ALDH3A2,CBLB,CCR7,CHIT1,OR8B4,POLR3H
Negative_7756	ADC,CRH,ADH6,AGPAT6,AKT2,ARPC5L,CCL3,CER1,CFL2,E2F5,FGF11,GNPNAT1,HARS,IFNG,IRS2,ITGAE,NCAM1,NME4,OR1M1,OR2AG2,OR4S1,PGM1,PIGV,POLR2K,PPP2CB,PTPN1,PTPN5,RDH5,RELA,SPP1,UGT2B11,WNT7B,PLA2G12B
Negative_7757	EDA,IL3,MET,UBC,ACSL6,BCAR1,BDNF,BUB3,CFL1,COL5A1,CPS1,CSF3,CTBP1,DUSP7,EPHA6,FMO3,GCLM,GLI2,GNRHR,GRIN2B,GZMB,HIBCH,IFNAR1,ISYNA1,ITGB4,LAMB4,LIPF,MGAM,MYD88,NAGS,OR13C3,OR1F1,OR2T4,OR4K5,OR52L1,OR5AP2,OR6M1,PDE1A,PLXNA3,SLC27A1,SPRED2,UGT2B4,VDAC2,ATP6V1E2
Negative_7758	IHH,TNR,ACSL6,ADCY4,CLTC,CYP2C8,E2F2,ENPP1,GANC,GNA15,GRIN2C,ITGB6,LCAT,LYPLA1,NOTCH1,PIP5K1B,POLD2,PVRL3,ULBP2,EIF4EBP1
Negative_7759	AGPAT2,CD48,HSPA1B,IDH2,OR2M7,OR4M2,OR6C1,TKTL2,WNT8B

Negative_7760	AK7,KDR,ACAD9,ACY3,AMDHD2,ATF4,CD14,CD274,CD48,CDK4,CFLAR,CLDN18,CPS1,DUSP5,ETNK1,FARSB,GALNT10,GALNTL4,GMPR2,GNAQ,IL1R1,ITGB2,MGAT2,NCF1,OR2A5,PIGM,PKLR,POLR1C,POLR3K,PPP1R3C,SKP1,SSX2IP,STX12,TNFSF14,UGCGL2,WNT2,MAPKAPK3
Negative_7761	NOG,ABLIM3,ATP5D,CCL16,CSNK2A2,CX3CL1,ENTPD2,GAD2,GALE,GNG13,IL10,MECR,OR1N2,OR51I1,OR51L1,PDXP,PYCR2,SMAD9,CSGALNACT2
Negative_7762	DCN,ACO1,ACVR1B,AMY2B,ARD1A,CACNA1G,CACNA1I,CAMK2A,CD28,CLDN6,CLTC,CYP11A1,CYP26B1,DGAT2,DKK1,EGFR,EP300,ETS1,FMO1,GNRHR,GSTA4,GUCA1B,IARS,IKBKG,ITGB8,MAP2K7,NADK,NAGA,NRG3,NRXN3,OR10A3,OR4Q3,OR6C76,PLD2,ROCK2,SV2A,TLR3,ATP6V1B2,SERPINB5
Negative_7763	PTS,ADAM17,BPNT1,CCL20,CES7,CSF3,DGAT2,DPYSL2,DTYMK,ENTPD1,GARS,GSTT1,HPSE,HSD17B7,IL11RA,INHBE,KDSR,MASP2,MYH15,NCSTN,NT5C1A,OLR1,OR11L1,OR2L3,OR6C76,OR6Y1,P2RX1,PNPLA4,POLR3H,PRIM1,RPS6KA6,TAS1R3,TAS2R3,TOMM40,UGT8,ZAP70,EIF4EBP1,TNFRSF12A
Negative_7764	ACVR1,BCL2,CXCR4,OR8J1,TRIP10,MAPKAPK3
Negative_7765	CGN,ADCY7,ALDOB,BMP7,CFLAR,EPB41,F13B,GUCA1A,IL2RB,MTMR2,NUDT9,OR2T12,OR9I1,SMURF1,VANGL2,ATP6V1B2,SERPINA5
Negative_7766	GLA,GSR,TNF,CACNB2,CNDP1,FZD7,NEGR1,OR10G7,PTCRA
Negative_7767	ATG3,ATP2A1,B3GNT3,CD27,GALNT9,HTRA2,OR5T3,SMARCA2
Negative_7768	OTC,ACAD8,ACSL1,ANAPC4,ARHGEF2,ARNTL,ASIP,CCL28,CCL3,CREB3L3,CTNNA2,CTSL1,EPHA1,FLOT1,GSTA4,GSTA5,HPSE2,IL17A,IL21R,IMPA2,MYL9,NKX3-1,NRXN2,OR10H2,OR2A14,OR2C3,OR3A3,OR4A16,OR9A2,PDE8A,PRKCE,RPS6KA1,SEMA3D,SLC27A1,SOCS2,VEGFA,HLA-DRB3
Negative_7769	HGF,NNT,ABLIM2,CCL20,CDC14A,CNTNAP1,COL4A1,CSF2RA,CSNK1G2,CYP21A2,ENAH,ENTPD5,FTH1,HLCS,HNMT,HSD17B4,HTR2B,IL13RA1,LTBP1,MAP4K4,MTHFD1L,OGDHL,OR10C1,OR10G3,OR2A2,OR2M2,OR4M2,OR4N4,OR5L1,OR6T1,OR8J1,ORC6L,PFKM,PHKA2,PIPOX,PLCD3,PRKAG3,PRKAR2B,PRKCH,RHOA,SEMA3D,SETD2,SOAT2,TJP1,SERPINB5,TNFRSF18
Negative_7770	C8B,SPN,ABAT,ATP2A2,CCR7,CHKB,CYP2U1,DOCK1,FGFR4,GUCY1A3,MGST2,NCR2,PECAM1,PRNP,SEMA3C,SGPP1,SPRED1,STX6,UNC5C,TNFRSF10A
Negative_7771	B3GAT1,BBC3,C1QC,CCL4,DVL2,GALNT11,GPD2,GUCY2D,IFNGR2,LDHAL6B,OR2D3,OR7G1,OXCT1,POLD1,PRKCA,RHOQ,RPS6KB2,SGMS1,TAP2,TNFSF4,TUBB6,VDAC2,GABARAPL2
Negative_7772	FUK,ME3,VTN,ALDH7A1,ARHGEF4,BCAR1,BUB1B,CCL17,CCR4,CD46,CHRM3,DNMT3L,DUSP7,ENPP6,GALC,IFNA16,IRAK2,LMO7,MAP4K3,OR10G4,OR10G8,OR8S1,PFKFB2,PIK3CA,PLA2G5,RARS2,RGS3,SFRP1,TRIP10,WNT9A,YWHAZ
Negative_7773	AMPD2,FASLG,HADHA,HSD3B2,INSRR,MAD2L2,OR13C9,OR51I2
Negative_7774	A2M,KHK,LCK,PRL,TNN,ALDH7A1,ALOX15B,ANAPC1,CCNB1,CLCA4,CYP2C19,DGAT2L4,DHDDS,FUT5,GDF7,HNF4G,IFNA5,LIPA,OR1S1,OR52A1,OR5T2,PANK4,POLR2C,PPP2R5D,PRLR,RASGRF1,RBL2,SARDH,SGPP1,SLC33A1,THBS4,SERPINE1,SUV420H1
Negative_7775	TNF,ACYP1,ATG7,CASP10,CLOCK,CNGB1,COQ5,COQ7,CREB1,CSH1,CTF1,CYBA,EIF4E,EPAS1,HCCS,IL22RA2,MTMR2,OR11L1,OR2T3,OR4K1,OR8K1,OXSM,PDHA1,PLCG1,RHOA,SDHB,SLC9A1,STX18,TJP3,TUBA3E,TNFRSF1B
Negative_7776	C1QC,CAV2,CDK4,CYP4F2,ITGA10,KLRC4,NTNG1,OR2T3
Negative_7777	BCKDHA,CFL2,CHRNA7,IMPA1,MALT1,NPR1,OR1D5,OR1K1,OR5D16,OR9A4,SPP1,TBK1

Negative_7778	CS, CDK2, CHRM2, CYP8B1, CYSLTR1, DHRS9, DTX2, F13B, FCGR3B, FZD7, GALM, GPD1L, GRLF1, GSTA3, ITGB3, KLKB1, LIPC, MAML3, MAP3K14, MAP4K3, MAPT, MCCC1, NCSTN, NUDT5, OR2T6, OR52W1, OR5A2, OR6A2, PCYT2, PDGFC, PI4KB, PRPS2, RAPGEF2, RDH12, RHEB, RHOA, SETDB1, TUBA4A, UCK2, ZBTB16, TNFSF13B
Negative_7779	ANPEP, CMBL, CYP7A1, ENTPD2, FTH1, GCNT2, HSD17B7, KYNU, NRCAM, OR1L1, OR4K15, OR56A1, PLD1, PRKAG2, PVRL3, STX4, SUV39H1, TRADD, MAPK8IP2
Negative_7780	BCR, GSR, PDC, RDX, CXCL9, DDOST, DGUOK, DHFR, DHX58, GALNTL4, HSD17B3, IL12RB2, IL22RA2, IRAK3, LYPLA2, MYH2, NEU4, OR5P2, PDE1C, PLXNC1, PROC, PTPRB, RPS6KB2, SETD8, TLR3, ATP6V1C1
Negative_7781	GAA, LHB, MOS, PC, TNN, ASIP, BCL2, C5AR1, CAV3, CCNE1, CD86, CLDN9, EHMT2, GSTK1, ITGAD, KIR2DL1, LDHAL6B, LLGL1, MMP7, OR2K2, OR6X1, OR8J3, P2RX4, PIGQ, RAB23, SARS, SEMA3E, SLC25A6, SNAI2, ST8SIA1, TAS2R41, THBS1, TOLLIP, VDAC3, WEE1, HLA-DPB1, SERPINF2
Negative_7782	AMHR2, ANGPTL4, CADM3, CCR8, GALNT10, JAK3, MAP4K3, OR4D9, P2RX7, PDGFRA
Negative_7783	GDA, HK3, JUN, STS, CCR1, CDK6, COL5A1, ESAM, GCNT4, HMGCR, KLKB1, KLRC4, LDHA, MAP3K2, OR2A4, OR4A15, OR4F6, OR5D18, PIK3C3, PLA2G2A, SHC3, SHC4, SMAD4, TAS2R5, TBXA2R, TLR3, UGT8, VARS, WNT8A, YOD1, ATP6V1C2, MAPK8IP3
Negative_7784	F8, PVR, ABLIM3, ALDOA, ANAPC1, AOC3, BCL10, CACNA1B, CCL23, DHRS9, EI24, FCER1G, FLT4, FZD5, GALNT9, GNG13, GRB2, HLA-DRA, IPMK, LCP2, PCK1, PLA2G4A, PLXNB3, RPIA, SMURF2, SPRY2, TAS2R7, UMPS
Negative_7785	CFH, CFI, FGB, TK2, ALOX15, ARSB, DGUOK, EXTL2, HCLS1, INSR, ITGB6, LHCGR, NFKB2, NRG2, OR52I1, OR52J3, POLR2A, PVRL4, SPRED1, UGT2B7
Negative_7786	C3, CEL, EPO, GGH, ID1, LPL, ACP1, ALDH9A1, AVPR1A, BLNK, CALML5, CHPF, CHRM5, CSNK1D, CYP26A1, EFNA3, EPB41L1, EPOR, EPRS, FGF17, GRIA3, GUCY1B3, HSD11B2, OR4F5, OR4M2, PARVB, PDX1, PIK3R4, POLR1D, POLR2F, PPID, PRSS2, RAB23, RBKS, RELA, SLC2A4, SPHK2, TP73, UGT2B28, VAMP4, WNT5B, ST6GALNAC1
Negative_7787	GHR, TK2, TXK, XDH, ACYP1, ADRBK2, ALG3, ALPI, APOE, ARNT, CDKN1A, DPYSL2, EDAR, FUT3, GDF5, GNPAT, GOT2, GYS1, HLA-G, IDH1, IPPK, KARS, NGEF, NSDHL, OR2T1, OR6C6, OR6Q1, PDE4D, PFKFB3, PIK3R3, PLXNB1, PRIM1, PRKAR1A, PRPH2, PYCR1, RALBP1, RASA1, SMPD2, TAS2R4, TAS2R48, TAS2R50, THY1, TRHR, WNT10A, WWP2, HSD17B12, MAPK8IP1, PAFAH1B2
Negative_7788	RFK, ABI2, ADCY3, ALDH3B1, ARNT, ATP6V1F, B3GALT4, CASK, CCNB1, CD38, CD40LG, CXCR4, EIF4E2, FLT1, FLT4, MAN1A1, MCM6, MYLK2, NAGK, NR1H3, OR2AG1, OR2M7, OR51F2, OR7A10, P2RX2, PITX2, POLR1D, PPA2, PRKACG, PTPRJ, RAB23, RAB5A, SMARCA2, STAM, STX8, SUFU, SULT1A1, SUV39H1, TAP1, TAS2R48, TGFA, VEGFA, WASF2, WIF1, KIR2DL5A
Negative_7789	BCR, ABI2, APH1A, APOA2, B3GNT4, BACE2, C4BPA, CPT1B, CXCL11, CYP2B6, DHCR24, EFNB3, G6PD, GMDS, GSTO1, IL23A, IMPDH2, ITGA7, MYL2, NT5C2, OR4A15, OR6C76, OR9G1, PLCZ1, POLE4, PRKAB2, PTGER3, QARS, RAC1, RAPGEF3, RHEB, SELP, STX8, TPH2, TXNDC12, UGT2B4
Negative_7790	GLA, UBC, ALPPL2, CACNA1H, CHST13, CTPS, DDX19A, DUSP3, EIF4B, FCGR2B, FOXA2, GCH1, GLDC, GNRH2, GNRHR, HNF4A, IL15RA, IL1RAP, INPP5B, KLRC3, LCMT2, LEPR, MAP3K3, MAPK12, PDX1, PER1, PTGS1, PTK2B, PTPN7, RASGRF1, RHOA, SMPD1, SPHK1, SSH2, TAS2R38, TGFBR2, TLR3, WNT9A, TNFRSF10D
Negative_7791	IHH, DDX50, FOXO1, OR5I1

Negative_7792	CDA, CRH, GCK, LSS, RDX, ACP1, CHDH, CTF1, DDX56, GDF5, HADH, MGAT5, OR4X1, OR8H3, PDGFA, PDGFC, PGM1, PI4KB, SLC33A1, SPTAN1, YWHAG, ATP6V1C2
Negative_7793	LCT, EPRS, GALNTL2, MGAT5B, OR5P3, PLXNB3
Negative_7794	AUH, CDA, F2R, GCK, ATG7, B3GALT4, CACNG3, CBLB, CD59, CYP2U1, DDOST, DKK1, ENO3, FUT9, GALNT11, GRIA2, HDAC2, HSPA2, IL22, IMPA1, IQGAP3, JAK3, LCMT1, MAP2K7, MYL2, NME6, NRG1, OR10H5, OR1N1, PANK3, PAX6, PDE7B, PHKG1, PIP5K3, PSME2, PTCRA, RASGRF1, RUNX1, SDC4, SEMA3B, SETD1A, SNAI1, ST6GAL1, TLR3, TP73, PDCD1LG2, PLA2G12A
Negative_7795	EGF, IL7, ACADVL, AKT2, CACNG7, CD244, CD36, CDKN2C, CES1, CLDN19, DERL1, ENPP6, EPHA3, FMO1, GABARAP, GLI3, HSPA8, IFNA17, IL20RB, IRS2, KLKB1, OR13H1, OR2D3, OR2L3, OR2T2, OR4D5, OR5A1, OR5C1, OR8I2, ORC1L, PIK3R3, POLR2C, PON3, PPP1R3C, PPP2R2D, PTPRM, RGS3, SEMA3E, TBXAS1, TM7SF2
Negative_7796	IHH, ABP1, ALG11, CLDN16, EPHA2, FRAP1, MAP3K5, NGEF, NUDT12, OR10G3, OR7A5, TUBAL3, VDAC2
Negative_7797	CBL, NNT, NPL, PLN, ACAA2, ATP4B, ATP6V1D, CD74, CDS2, COL4A4, CYP11A1, DDX18, DHDDS, FGF14, GP1BB, MFNG, OR11L1, OR5AS1, PLAU, POLR2K, PPARD, PROC, PTPN6, RUNX1T1, SEMA4C, SLC25A5, SLC9A1, SULT1E1, TGFBR1, TPI1, UNC5A, ZFYVE16, MAPKAPK2, PRICKLE1
Negative_7798	ASNS, CACNA1B, CACNG8, CD80, CXCL10, IL28A, MAP2K4, MTMR1, OR13F1, OR52W1, PRPS2, RAC3, STX2, UBE2L3, YWHAG, ATP6V1G3, C1GALT1C1, HLA-DRB4
Negative_7799	DBT, TPR, CAMK4, CHDH, DHRS3, DPM2, DTX4, ITGB1, ITGB8, LDHC, MLLT4, OR6A2, PARS2, POLA2, RPS6KA4, SLC2A4, TP53, ZNRD1
Negative_7800	GNE, GPI, PLG, ACSL4, ACTA1, ADCY10, ADCY7, ADIPOR1, AKR1B1, AKR1C4, AREG, B3GNT2, BET1, CFL1, DRD2, ENTPD3, EXTL1, GNAT3, HSPA8, IAPP, IRS1, ITGB7, OR10A4, OR4K13, OR52I1, OR5AS1, PPP2R2B, RELB, RUNX1T1, SEMA3F, SLIT2, STAM, STAM2, TAS2R4, TGFBR1, TRPC1
Negative_7801	LCT, MTR, FRAT2, GGCX, GP1BB, GRIA2, IFNW1, IL20, MYH8, OR1J1, OR56B4, OR5T1, PFKFB2, PIGO, PPP2R1A, PSEN1, PTGS1, SGMS1, SH2D2A, SSX2IP, ATP6V1G2, C1GALT1C1
Negative_7802	GK2, IDS, OGT, ACOT12, ACSS1, ACTB, ADSS, APOA1, ARPC1A, CACNB4, COL1A2, CXCL3, CYP2A6, CYP2C9, CYP4F3, DGKE, ENPP6, FCER1G, FOXA3, GMPR2, ITCH, MCCC1, MTMR6, NR4A1, OCRL, OR10AD1, OR1E2, OR1G1, OR5D16, OR7G1, OR8B12, PANK1, PCCB, PFAS, POLD3, PPA1, SCD5, SEPHS1, SFRP4, SUCLA2, TAS2R42, TJP3, TRHR, TUBA1B, UGT2B10
Negative_7803	ID4, IL8, ACTA1, ADRA1A, BDH1, CD22, CYP26C1, HAAO, IL3RA, ITGA9, LAMB3, MAD2L2, MGAT5B, OR51B2, PARD6G, PDE1C, PRKAA1, SPCS3
Negative_7804	AK5, MOS, TFG, ATF2, CPT1C, EI24, MITF, OR6C74, SULT1E1, MAPKAPK5, PAFAH1B3
Negative_7805	AK7, IDS, B3GALT5, CREB3L3, CXCL10, EDA2R, GALNT10, GNAS, GNAT3, GNRHR, GSTA4, INPP5A, JUND, MCM5, MOV10L1, OR3A3, OR4D11, PLA2G5, POLD1, RBKS, SKP1, ATP6V1G2, TNFRSF10B
Negative_7806	FGG, HK2, MTR, ADH1A, ADSS, ARSB, BRAF, CSF3, FLT3, FOXO1, GSTM2, GSTM4, HMGCS2, HTR2A, LDHAL6B, MAP3K6, OR1L4, OR51B4, OR52B4, OR5D16, RDH16, SEMA3A, SUCLA2, TAS1R1, VTI1A, WNT10A, XCL1
Negative_7807	AR, HK3, MTR, SCD, SP1, ANAPC11, APH1A, ARHGEF6, CACNB4, CCNG1, CD47, CD48, F2RL2, GUCA1A, HLA-DOB, HTR2C, INPP1, ITPK1, MITF, OR2L3, OR4N4, OR51S1, OR8H1, PDGFA, PECAM1, PIK3R3, PLA2G4A, PLA2G6, PPP1CC, RASA2, SSH3, STAT4, TAS2R7, TPH2, UGT1A10, WNT3, TNFRSF10C
Negative_7808	ADSL, CYP26C1, HMGCS1, LTB4R2, MCAT, NME7, OR7C2, PAFAH2, RETSAT, TRAF1

Negative_7809	ABO, ACOT11, BMPR1B, BTRC, CES7, CYP26B1, EP300, GNPDA2, HADHB, IMPA1, KIR2DS4, OR7A5, PDE3B, PGAM2, RALB, SCLY, TSHB
Negative_7810	ATP5D, BMP8B, CPT2, GALNT12, GMPS, GNG3, MYH10, NMNAT3, OR2AT4, OR4A15, PPP3R2, TUBAL3, UBE2L3, C1GALT1C1
Negative_7811	CSK, DCC, F10, GK, ACOX3, ADCY7, APOC3, CMAS, CRKL, CYP2C19, DGAT2L4, FIGF, FOSL1, LCMT1, NAT2, NCF4, OR10S1, OR1L3, OR51B6, OR52W1, OR6T1, PFAS, PLA2G5, PPT1, PRLR, PRPS2, PSEN2, SENP2, SKP2, SERPINB5
Negative_7812	C4B, MUT, AKR1D1, ARG1, CD99, CLTA, GFPT1, GUCA1B, HSD3B7, IL22, LPAR1, NEFM, OR4A16, OR5AS1, OR5D16, PARVB, PDE4A, PRSS1, RAP1A, RAPGEF4, TAS2R13, TRPM5, GABARAPL1, HLA-DPA1, TNFRSF6B
Negative_7813	CGN, F9, HPD, VTN, ACSM1, ADCY10, ADH5, ALPI, B4GALT1, BCL10, BIRC3, CDC45L, CDKN2A, CHIT1, CLDN10, CLDN20, DDX4, DUSP3, FADD, FUT9, GLG1, GSTP1, HEXA, HRH1, HSPA1B, ICAM1, IRF5, IRS1, ITPKA, LCP2, MGAT4B, OR10H5, OR12D3, OR2A5, OR2Y1, OR51B6, OR5AK2, OR8H1, PRKACB, PRKAG1, RALBP1, SEPHS1, SHMT2, ST3GAL1, TAS2R49, TUBA3C
Negative_7814	ASPA, CCNB2, EPHB3, EXTL3, OR4A5, OR5M9, OR6C4, PARS2, HLA-DRB3
Negative_7815	AGK, AK2, IL7, AMDHD1, CCL16, CD48, CXCL10, CYP26A1, FMO5, FUT7, HMGCR, IFIH1, MAML2, MS4A2, MYH7B, OR14C36, OR1S1, OR51D1, PLA2G4A, POLA1, RFX5, RFXANK, RPS6KA3, SFRP4, SOCS3
Negative_7816	BIRC5, BMPR1A, CCND2, DDB2, ERBB2, EXT2, FGF2, HMGCR, ITGA2B, MTHFS, OR10C1, OR10K2, OR2G3, OR4A5, PPP2R5C, PTCH1, RDH10, ST8SIA1, STX1B, TGFB2, TNFSF4, TNFSF9, ST6GALNAC1
Negative_7817	DCK, ACAT1, ACHE, ANAPC2, ARSA, ATP5H, BPGM, C3AR1, CACNG7, CCL22, CCNH, CD14, CD44, CHDH, CKMT2, COL5A3, CSF2RA, CYBA, EPOR, FGF13, GNAO1, IL5RA, ITGB8, MAP2K4, MAPK3, MYH1, OLR1, OR10H1, OR5P3, OR8K3, PDXK, PIAS1, PLD1, POLR2I, PPM1B, PPP2R5E, PRIM1, RPSA, SLC2A2, SPTLC1, ST3GAL1, VAV3, WNT7B
Negative_7818	ACVR2A, CHAD, IDUA, RRM2, TPM3, TRPC1, TUBA1A, WNT2, ZNRD1
Negative_7819	A2M, PGF, PVR, ALG10B, CYP1B1, DDX4, LRP5, MYH11, POLR2K, PTDSS2, RXRG, TKTL2, TSLP, WASF2
Negative_7820	FER, FST, GBA, IL4, ALDH1A2, ANAPC10, ARSA, BTRC, CAMKK2, CHRNA7, CMAS, CREB3L4, CTNNB1, CXCL6, GAPDH, GOT1, ITGA9, LIAS, OR13H1, OR2L3, OR2T29, OR52I2, OR52N2, OR56B4, PAX4, PRODH2, PYCRL, RPS6KA5, SELE, SELP, SPRED1, VTI1B, XIAP, XYLT2, ARHGEF12, ATP6V1G1
Negative_7821	GNE, ACACB, ADORA2B, CDC16, CDC25C, CDKN2B, CPT2, CXCL5, GAB1, GALNT9, GSTA1, ITPK1, MAOB, MYH3, NCF4, OR13C9, OR51B4, OR51L1, OR6T1, PDE11A, PFN2, PGK2, PIP4K2C, RFWD2, ZIC2
Negative_7822	CTH, LCK, NPY, CYSLTR2, ELK1, LDHAL6B, OR52E8, OR5H2, PIK3R1, POLE4
Negative_7823	MTR, TXK, ALCAM, CACNA1D, CACNA1G, CHKB, CHSY1, FECH, FGFR2, FSHB, GALNT13, HNF4A, HTR7, ITGB5, ITPKA, KAT2A, KAT2B, LARS, MAD1L1, MAP3K2, MYH7B, NEUROG3, NOS1, NUDT12, OR1N1, OR51D1, OR56B4, OR7C1, PIPOX, PLXNB2, PRKY, PTEN, SMPD2, SOCS3, SUV39H2, TAP1, TICAM2, UBE2L3, VNN1, CACNA2D4, SERPINF2
Negative_7824	CNTNAP1, GALNT11, GGPS1, KIR2DL3, MMP14, PMAIP1, RAPGEF4, STAT6, TACR2
Negative_7825	GP6, ID3, PML, AGRN, APOC3, BTRC, ESAM, HLA-DOB, ODC1, OR52N4, OR7G1, PIGU, PIK3R2, PLXNA2, PTPRC, TYK2, UBA1, EIF4EBP1, TNFRSF11B
Negative_7826	ABO, CR2, ALAD, ALDH3B1, ATP5J2, GMPR, GP1BB, GSTP1, HHEX, INPPL1, MAP2K3, MCEE, MYH4, NAGLU, NFYB, NOTCH3, NQO1, OR10H3, OR5AU1, OR8B4, PARVA, PCNA, PFKFB1, PMM1, STAM2

Negative_7827	CBL,FH,FYN,IHH,OTC,UBB,ALDOA,B3GAT1,CCKBR,CCL1,CDC27,CERK,CTSL1,CYBB,CYP4A22,DCXR,DPYS,GANAB,GDF7,GNA12,GRM5,HRH2,HSP90B1,IL13RA2,KCNB1,NEU1,NOD1,NOS3,NRG2,OR2V2,OR4C16,OR4D11,OSMR,PIK3CB,PLTP,PPT1,PRKAA1,PSME3,SDC3,SEMA3B,SEMA4G,UGT2B7,CSGALNACT2
Negative_7828	APLP1,ATP2A2,AVPR1B,E2F3,GALK2,HIBCH,RAPGEF1,TYRP1,YWHAZ
Negative_7829	ACYP1,ALPI,ATP5C1,BACE1,BCL10,BCMO1,CD14,CDC6,COQ3,CREB3L4,CSNK2A1,CSNK2A2,DDX4,DNMT1,FCGR2B,GCNT1,HMGCR,IFNK,IL28B,IL5RA,LILRB3,MAP2K1,NR5A2,OGDH,OR11G2,OR4K17,OR9K2,ORC2L,PARD3,PIGU,PLA2G1B,PLA2G6,PLK1,RAD51,RIPK1,SOAT1,SOAT2,ST3GAL5,STMN1,UGP2,UNC5A,XIAP,YWHAZ,HSP90AB1,SUV420H2
Negative_7830	TPO,AKR1C1,APPL1,ATP4A,BDKRB1,BPGM,CCKBR,CDC6,CHST12,CRLS1,DVL1,ECHS1,EPHA2,FBP1,FPGS,GALT,GFPT1,GMPPB,GP1BB,GSTM3,HAP1,MAD1L1,MAML1,MCM5,MYLK3,OR1A2,OR4P4,OR51F1,OR51S1,OR5AC2,OR5T1,OR7C2,P4HA2,PFN4,POLE,POLR3D,PTGER3,SMAD4,SOCS5,TAS1R3,UBE2K,VAMP3,WASF1,WNT9A,YWHAH,HLA-DQB1,HSP90AB1,ST6GALNAC4
Negative_7831	EGF,C5AR1,PARD3,PRODH
Negative_7832	TAT,CXCL6,ICAM3,IQGAP3,MYH4,SUCLG2,TAS2R42,WNT7A,SERPINA1
Negative_7833	CAT,FOS,TPR,ACSS2,ALDH1A1,AMPD3,ATP5A1,BMP2,CCNB3,CLDN20,CSNK1E,CYP2C18,DGKB,EPHX2,ESAM,FBP1,FIGF,GART,IL11,INADL,MAPK12,MYL7,NMNAT1,NOTCH1,OR14I1,OR4X1,OR7A17,PCK2,PFKFB4,PIK3R2,PON1,RHOD,RRAS,SEMA3F,SPRY3,SULT1A1,ARHGEF12,PPARGC1A
Negative_7834	AGK,F8,HK3,SRM,ACOT11,ACY3,AGPAT2,ALDH7A1,CCR1,CD79A,CRB3,DNMT3A,DUSP16,GUCY2D,IFNA10,IL15RA,OR2T4,OR4F6,OR51E1,OR52N2,OR5B17,PRKACB,PTPMT1,RRAS,SLC25A4,SNAI1,UGT2B17,UXS1
Negative_7835	SMO,DGKB,DPAGT1,DTX3L,EIF4B,FIGF,FUT4,IL10RB,IL23A,IL25,MYL5,OR4F4,OR5L2,POLR2A,THTPA
Negative_7836	DCT,EBP,SPN,ABL2,ACSS3,APLP1,DDX47,FARP2,GRPR,IRS1,MICA,NRP1,OR8H1,OR8J3,PITX2,PNMT,SMAD2,SPTAN1,SSH1,STX12,TKTL2
Negative_7837	EZR,ACTN4,ALDOC,MAML1,MAT1A,NCK1,NRP1,OR1J2,P2RX4,PPID,PYCR2,RIPK1,SDHC,SSH2
Negative_7838	KMO,ARPC1B,CACNA1A,CAMK2D,CSNK2B,DDX55,DPM2,E2F3,GABARAP,GOT2,LEPR,MPST,MTHFD2,NEU2,OR4M1,PDGFRA,PLA1A,PRKCI,TOLLIP,TNFRSF10B
Negative_7839	ACADS,ACOT11,CDC14B,COL6A2,CRB3,CYP3A4,F2RL1,FMO3,LTA4H,SEMA3F,SHC4
Negative_7840	ID3,OGT,RET,ABLIM3,ACADS,ACTG2,ACVR1B,AGPAT1,ALG1,B3GAT1,BCL2L1,BDKRB1,BIRC2,BMP4,CARD11,CD247,COL4A6,DLL3,EHMT2,EPOR,GNA12,HARS2,HIBADH,IRF3,LAMA4,MAP2K5,OR13A1,OR1B1,OR51G2,OR6Y1,PARD6B,PARVG,PIGS,PPP2R2D,RFX5,SGMS1,SLC2A2,SMPD4,TACR1,TAS2R8
Negative_7841	FES,MET,MPL,A4GALT,ADCY6,ADRB2,ALDH3B1,CD22,CD244,DKK1,EHMT1,F11R,G6PC2,GBE1,GCAT,GMPR,GPX5,IL22RA2,MAN1A2,MAN1B1,MYD88,OR2T33,PARD6B,PIGV,SEMA3B,SLC9A1,SOCS3,ST3GAL5,TSLP,VDAC2,WNT10B
Negative_7842	COMP,CTPS,PIM1,PTPRC,TP73
Negative_7843	CS,F11,AFMID,ALAS2,CALML3,CLOCK,COX15,CREB3L1,CSF3R,CYP2S1,DAPK1,DEGS1,DGKE,DRD2,ENTPD8,FGFR3,GDF7,GOSR2,HAGHL,HEXB,ICOS,OR2T2,OR51B4,PTGES,SETD1B,WARS2,WASF3
Negative_7844	C4B,ACSL3,APH1A,ATP6V1F,BCL2,CCL3,CNTN1,CYBA,DDX47,DDX55,FRAT2,GOT2,GZMA,IGF1,ITGA11,KRAS,MAP3K12,MAP4K1,NCK1,OR5AN1,OR6B3,PLA2G10,PPP1R3A,RPRM,SENP2,SLC27A5,TICAM1,TRPC1,UCK2,HSD17B12,ST6GALNAC6

Negative_7845	ABL1,CUL1,MRAS
Negative_7846	EPO,NLK,TNF,ASIP,ATP5E,B3GALT6,BAI1,CNGA4,CREB5,DLL4,EPHB1,GALNTL2,GBGT1,IFNA4,IGF1,IL18RAP,IL2RA,IRS1,ISYNA1,ITGA8,KAT2B,KLRC1,LAMC2,NEU2,OR11A1,OR5AU1,OR5H2,OXTR,PARD6B,PIGK,PLCB4,PLXNB3,PPID,PRKCZ,PRPH,PTGS1,RAC3,SDC3,SLC27A5,SMPD2,SOCS2,SRGAP2,ST3GAL6,STAT6,WNT7A,XCR1,ATP6V1E1,TNFRSF14
Negative_7847	CFI,PML,AASDH,AHCYL1,ANGPTL4,APOA2,ATP5H,B3GNT2,BMP7,BMPR1A,CASP10,CD48,CREB3L3,DVL2,FUT3,GALNT5,GMDS,ICAM2,IL18R1,IL6R,IRS4,LCMT2,MAPK12,MAPK9,MPDZ,NEUROD1,NR1H3,OGDHL,OR10K1,OR2T2,OR51V1,OR5M3,PARD6G,PDX1,PGLS,PTGER1,RPRM,RRM2B,SNAP25,TACR2,TAP2,TRPC1,TYROBP,UBE2L3,WWP2,MAP3K7IP1,MAPK8IP3
Negative_7848	AR,HK1,AKT3,ALDH6A1,ATP6AP1,B3GNT2,B4GALT7,BCL2L1,CACNB1,CAPN2,CCL16,CCR9,CMAS,COX15,CYP3A5,DPYS,DRD5,DUSP1,EDA2R,GP1BB,GYS2,IL21R,IL28A,LALBA,MAOB,MYH6,NTN1,OR10S1,OR13C9,OR2D2,OR2T11,OR3A3,OR51F2,OR9Q2,PARVA,PDE5A,PIP5K3,PNPLA3,PPBP,PRKAA2,RPS6KA1,SEMA4D,SFRP1,STAT6,STEAP3,VNN1,WNT1,B3GALNT1,ST6GALNAC1
Negative_7849	C7,HPD,OTC,ACY3,ADH1A,ASMT,CD38,CD8A,CNTF,CUL1,DDX18,DTX3L,EPRS,FMO1,GCLM,HDAC2,IFNK,IGSF5,IKBKE,LEFTY2,MAP3K2,MTFMT,NCAM1,OR2T8,OR4C46,OR4F6,OR5T3,OR6C2,PARVG,POLA1,PTPN11,RARA,TAS1R1,TCF7L1,THTPA,UGT2A3,WARS,WNT9A,MAPK8IP3,TNFRSF10B
Negative_7850	DCK,ARSE,ATF2,C1GALT1,DNMT1,OR12D3,SEMA3D,XYLT2
Negative_7851	CCNE2,DUSP1,FTMT,KRAS,MYH10,NEFM,WARS2,CSGALNACT1,TNFRSF17
Negative_7852	MAG,ACVR1B,CNTNAP1,DDX55,E2F3,EHMT2,JAK2,MAT1A,SDHA,TAP2,TPH2,XYLB,MAPKAPK5
Negative_7853	AGK,F8,MVD,TNR,ADORA2A,BMP2,BTRC,CCL16,CD48,CXCR6,DGUOK,FARSB,HMGCR,IFNAR2,IL10,ITGA2B,JAM3,LCMT1,MAP4K1,OR1L3,OR5AR1,OR5L1,OR6M1,OR6T1,OR8B4,PFN4,PON2,PPBP,SV2A,TAS2R42,TFDP1,TYROBP,VANGL1
Negative_7854	FN1,AGXT2,BET1,CYP3A5,ETS1,GALNTL4,HSD3B7,LYZL1,MTFMT,OR4F5,OR52N5,OR8K3,PGK1,PGM1,TAS2R14,UMPS,WNT10A
Negative_7855	NOG,ALDOA,ARHGAP5,BLVRA,CHIA,CLTA,CYP2U1,DEGS1,DHX58,FZR1,MGAT5B,NARS,NPAS2,OR2G6,PIGF,PLCD3,STX8,TACR1,TP73,TRAF3
Negative_7856	HYI,CD38,LAMB3,MYL9,NFYC,OR51V1,PGLS,POLR3A,SNAI2
Negative_7857	CS,F11,ITK,ALDH1A2,B3GALT2,BCL10,BMPR1B,CD36,CDKN2D,CHST13,CLDN22,CSF2RB,CTPS,CXCL16,CXCR3,DAPK2,EPHX1,F2RL3,GNPNAT1,GUCA1B,HAO2,IL24,ITGA2,ITPK1,MGAT3,MGAT5B,OR2L2,PAPSS1,PLA2G6,PRKAG1,PTGS2,PYGM,TAS2R48,TIAM1
Negative_7858	C9,ME1,ACVR1,ALDOC,ALG10B,APOA5,B3GNT4,CACNA1E,CDKN2D,DCXR,ECHS1,GADD45A,GALK1,GARS,GGCX,GLYCTK,GUCY2C,IFNGR1,IL22RA2,INPP4A,JAK1,LIMK1,LIPC,LMO7,MAML3,MAP3K4,MAPK1,OCLN,OR2A12,OR6K6,PLCD4,PPP3R1,TFDP1
Negative_7859	OAT,ARNTL,B4GALT4,CHRD,ERBB2,GRB2,GRIN1,KCNB1,MYH4,NGEF,OR14C36,PAX6,PPP1R1A,SGMS1,STK4
Negative_7860	FAH,MVK,ACMSD,ACSL4,ACTA1,BMPR1A,CCKAR,CCL4,CDC2,CDC27,CDKN1A,CHPF,CMPK1,DUSP6,DUSP8,ENO1,ENTPD3,EPHA8,FZD2,GGCX,HSD11B1,LIPT1,NCR2,OR51V1,OR56A4,PAICS,PTCH2,RBL1,RXRB,ST3GAL6,ST8SIA1,ST8SIA5,STEAP3,TNFRSF11A
Negative_7861	CFH,F7,SRF,TK2,ADIPOR1,ALDH6A1,BUB1B,CCNA1,CD79A,CSH1,DCXR,DGKH,DHRS9,ELK4,FGF2,FZD7,GDF6,GRIN2A,IL22RA1,IL5RA,LIPF,MAPK1,MGAT5B,MRAS,MTFMT,OR10R2,OR4S1,PLAU,PLCD1,RPE65,SNAP23,SPTLC1,STT3B,TACR2,TBL1XR1,TUBB,TNFRSF11B
Negative_7862	CFH,F5,GSR,TK2,CD27,CRKL,DIAPH3,FGF1,KIR2DL4,MAP3K7,OR5D18,PLXNA2,POLR2F,RAD51,SDC1,SQLE,TSC1,UBE2L3

Negative_7863	DHH,ACPP,ACTN2,ANAPC11,CRHR1,E2F3,ECSIT,GNG3,GSTK1,HTR2C,MAP2K1,NRG2,NRG4,OR1B1,OR2T12,OR3A1,OR7G3,OR8B12,OR9Q1,ORC2L,PDXK,RAB5A,RAC3,SETD1A,ST3GAL3,TGFA,WBSCR22,GABARAPL1
Negative_7864	C8B,GNE,ADRBK2,ALG6,APH1A,ATG7,BCL2,BMP6,CAV2,CD28,CDC14B,CES2,COL4A1,CTBP1,CYP2C8,DTX4,E2F2,ERO1L,FGF9,FMO4,GANAB,GNB3,IFNB1,ITGA9,MAPK9,NRXN2,OR10A3,OR10V1,OR13H1,OR1J4,OR1L6,OR2L2,OR5C1,OR6B3,PARD6B,PCCA,POLR1A,SMAD1,TDO2,THBS4,UBA1,VANGL2,VAV3,ATP6V1G2
Negative_7865	C7,FAH,GGH,LEP,LPO,SMO,ALPPL2,ATP4B,CANT1,CCNB2,CCNG2,CDC14A,CMAS,COQ5,CYFIP1,CYP27A1,CYP3A5,DEGS1,DGKQ,DHODH,DRD2,DTX1,E2F1,EP300,F13B,FTCD,GANAB,GNMT,IRAK2,MAD2L2,MAPK13,MYH3,NFATC3,NKD2,NT5C3,OR1S1,OR2T1,OR8K3,PDE7A,PDXP,STX5,TAS2R49,TAS2R60,TAS2R7,TOMM40,TRAF1,TRAF3,TRHR
Negative_7866	C2,HPD,PXN,AADAC,ADSS,ATP2A3,CCL27,CCNA1,CDKN1B,DHRS4L2,EXTL3,F2RL3,GRIN2D,IL12A,ITGA7,OR1L6,PORCN,PPARD,SNAP23,STX2,CSNK1A1L
Negative_7867	ACO2,APOA1,B3GALT5,B3GNT5,BCAT2,CACNB1,CCL13,CDIPT,CLDN15,CSF1,CSNK2A2,DNMT1,DTYMK,DUSP5,EFNA2,FLT3,FRAT2,G6PD,GUCY1A3,ICOSLG,IL29,IPMK,MCCC1,MDM4,NRG1,OCRL,OR52K1,OR8J1,OR8U1,POMC,PPP2CB,PPP3CA,PRDX6,PRIM1,PROS1,RAD51,SELPLG,SGMS1,SHISA5,SNAI1,VKORC1,WBSCR17,ATP6V1B1,CACNA2D4
Negative_7868	NNT,VTN,ACVR1B,B3GNT2,BMP7,CCL21,CXCL2,CXCR4,CYSLTR1,GBA3,GNB3,HPRT1,IQGAP2,MAP3K3,NCK1,OR10J1,OR14I1,OR4K17,OR4M1,OR52K2,PDPK1,PNPO,PRKAA2,PSME3,PYGM,SARDH,SETMAR,STX8,TAS2R38,TGFB1,WNT3,SERPIND1
Negative_7869	ADA,CRH,ACPP,ADH4,AGPAT2,ALG1,ATP6AP1,AXIN2,B3GALT6,B3GNT4,BCKDHA,CISH,CLDN14,CLDN4,CREB1,CRLS1,DHRS4L2,DPYD,FCER1G,GNB1,HES1,HLA-DMA,HMBS,IL21R,IL29,ITGAD,KIR2DS5,LDHC,LIMK1,OR2T8,OR5B17,OR9A2,PITX2,PLA2G4A,PNPLA4,POLD2,POLR2I,PSAT1,ROBO2,RPS6KA2,RPS6KB2,SEMA3E,SEMA3F,SQLE,SULT1A4,PRICKLE1
Negative_7870	CSK,ARPC5L,ATG3,CADM1,ENTPD3,HTRA2,IFNG,IQGAP3,ISYNA1,MALT1,MAOA,MYH8,NFATC2,NKX6-1,OR13C4,POLR3H,PTGIS,SMAD9,SNW1,STK11,TAS1R1,TAS2R41,KIR2DL5A
Negative_7871	FAS,GP5,SRM,STS,ACADS,ACOX1,ACOX3,ALAS2,CCL4,CD86,CDC27,CDH15,CDK4,CHRM2,DHDH,F2RL1,FOXA2,GMPS,IL20RB,IQGAP2,LIMK2,MAP3K1,MAP3K13,MAPK12,NCAM2,NTSR1,OR10H4,OR14I1,OR51F2,OR51T1,OR52E8,OR6K2,PAPSS2,POLR3A,POLR3G,PTCH1,PVRL1,RAC2,RBL2,RRM2,SH3BP2,SPTLC2,STMN1,TLR9,TOMM40L,ATP6V1B2,PRICKLE2
Negative_7872	KMO,NGF,ALG3,B3GAT3,FZD1,HYAL2,INPP4B,IRS1,OR10A5,OR4F6,PLCD4
Negative_7873	LAT,MUT,AASS,CASP3,CCL1,CD3E,CREB3L1,GBA3,GUCA1C,LAMB2,NODAL,NPR2,OR4D9,OR5H2,PPP1CB,PROS1,RASA2,STAT2,TAS2R4
Negative_7874	GLS,TAT,ABI2,ALDH6A1,ALS2,CDO1,CRLS1,CUL1,DDX52,DEGS1,EDN1,ERBB3,FABP4,FBXW11,FTMT,FZD8,GNAT3,GPX5,HPSE2,HSPB1,JAG1,LCAT,METTL2B,NCOA4,NCR3,OR11H1,OR14A16,OR4N2,OR52K2,OR9A2,PIK3C3,PMM1,RBL2,SEMA3B,SIAH1,TAS2R8,TBL1Y,TGFA,TIAM2,TJP2,TLR2,TUBB1,UAP1,WNT9B,HLA-DQB1
Negative_7875	ADO,C2,GSN,LSS,TNR,TYR,VCL,AKT2,ALDH1A1,BMP8B,CACNB1,CLCF1,EXT2,FADD,GMPPB,GZMB,IFNA16,IL22,IRS1,MYLK,NEFH,OR10J3,OR10P1,OR2M2,OR51G1,OR5K2,OR8J3,PDGFC,PRKCB,PSAT1,RASSF5,SDC1,SH2D1A,SMAD3,SNW1,TAS2R9,TBL1Y,TCF7L2,TICAM1,TUBB3,HLA-DRB5

Negative_7876	AMT,ATM,CBS,FAS,GSN,PC,AARS2,ALS2,ASMT,ATP6V0B,CD27,CD70,CDC45L,CHD8,CHKB,DDX55,FGF8,GAPDH,GBGT1,GUCY2D,IFNAR2,IL13RA2,ITGB7,LIPF,MAP3K3,MAPK7,MCM5,NOS3,NOTCH1,OR10G4,OR10J1,OR5K1,PAX6,PI4KA,PIGT,PLCD1,PLCZ1,SH2D2A,SORBS1,SPRED1,TNFSF4,TRAF2,TSNARE1,VKORC1,WNT6
Negative_7877	A2M,GPT,MUT,SYK,VCL,AASS,ALDH1B1,ATP4A,CACNA1C,CNGB1,CYP27A1,CYP2B6,CYP2F1,DLL1,ELK1,FCER1G,GLUD1,HMGCS1,ICOS,KLRD1,MAP3K2,MCM7,MGAM,NUDT12,OR2B2,OR52E6,PDE10A,POLR2A,PON2,TAS2R1,UAP1,UBE2L6,ZIC2,ATP6V1B2
Negative_7878	AMT,CRK,GAA,MUT,PRL,TFG,UBB,ACYP1,ADCY1,ADIPOR1,ADRB2,APOA2,BACE1,CASP7,COQ3,DARS,DNMT1,ELK1,ENPP2,GSTA3,GSTO2,IL29,JAG2,L1CAM,LRRC4C,NT5C,OR10C1,OR11H6,OR2B11,OR2L2,OR2Y1,OR4C6,OR52D1,PAX6,PI4KA,PIK3R4,PLAUR,PPAT,SEMA4C,TNFSF14,YARS,ARHGEF12,PRICKLE2
Negative_7879	F2R,NPY,SRM,ALDH3A2,ALDH4A1,ARAF,ARHGAP5,ARPC2,ATP5D,CD79B,CDH3,CFL2,CXCR5,EGLN1,FLOT2,FRAT2,GUCA1A,HES1,ITGAM,KLRC3,LEFTY1,LEFTY2,MAD2L1,MAP2K6,NRXN1,OR1G1,OR1S2,OR2A2,OR2B11,OR51T1,OR52H1,OR5A2,PIGC,PIGH,PITX2,PLEKHA8,ROBO2,SETDB2,SHC1,STK36,TYROBP,UCK1,WNT2B,HLA-DPA1
Negative_7880	CALML5,DPM1,NAGLU,OR2A14,PDCD1,PLXNA1,RALGDS,ATP6V0D1
Negative_7881	BID,MAX,ABAT,ARD1A,CACNG6,CREB5,DNMT1,GAL3ST1,GNA13,HLA-G,NAT5,NUDT5,OR51E2,OR5T3,OR6N2,PRNP,PTK2,ROBO3,RRM1
Negative_7882	CP,ID3,ABL2,ACY3,ADH1A,ARRB1,B4GALT2,CES7,EFNA2,EPB41L3,ETNK1,FPGS,FSHB,GLI2,HIP1,HTR5A,KLRC1,LIPG,PDE3A,PER1,PSME1,RAPGEF3,SCIN,SPRY3,SPTLC2,UGT2A1,UPP1,VAV1,HLA-DPB1
Negative_7883	AK5,FAH,GK,GNE,AACS,ACSL1,ADORA2B,ATP6V0B,B3GNT4,BCL2,CCNB1,CCR9,CDC7,CMPK2,CNDP1,CTTN,EDAR,F13A1,FARSB,FOXO1,FUT8,HTR7,IL22,IRAK2,MAOA,NPAS2,NRXN3,OR2T3,OR5H6,OR5T3,OR6C6,PDE8A,PDGFRB,PDHB,PHKA1,PPP1R3D,PPP2R2D,RASA1,RFNG,SLC2A1,SMAD1,ATP6V1E2,SLC25A31
Negative_7884	CD4,CP,AGPAT1,AQP7,ARPC5L,CACNA1I,CCR1,CD247,CDC20,CXCL11,DUSP10,E2F1,ENO2,GNA14,GSTM2,IFNB1,IL1B,IL29,IRAK2,ITGA10,ITGAV,LIPT1,LYZL1,MAP3K3,MTHFS,NKX6-1,OR4P4,OR51G1,OR5P3,OR6K3,PAPSS2,PIAS3,POLR3K,PTPRB,RDH8,TAS2R3,TBXA2R,TUBA3E,VAMP2,EIF4EBP1,MAPK8IP2,MAPK8IP3,TNFRSF10D
Negative_7885	AR,RB1,ALG12,CAMK2A,CASK,CD244,CD27,CD28,CD40LG,CRHR1,DRD2,ENO2,EPHA6,EPHB4,FDPS,KIR2DL4,NFATC3,OR1S2,OR2L2,OR2T33,OR52N4,OR5D16,OR5F1,OR6K2,OR8A1,OR8S1,ORC1L,PAK1,PDE3A,PIP4K2C,POLE4,PSAT1,PSEN2,RFXANK,SSX2IP,TRMT11,B3GALNT1
Negative_7886	CTSS,DHFR,GNG13,LAMB3,OR1L3,OR4E2,PLCB1,SETD8,UGP2
Negative_7887	GP6,KHK,NLK,PGP,B4GALT1,B4GALT2,BIRC3,CKMT2,CPOX,CYP11B2,HTRA2,IGSF5,IKBKB,INPPL1,LIPE,MYH2,NEFM,NGEF,OR11H1,OR1F1,PDHB,PFKFB2,PGM3,POLR1C,PRKAG2,PTGDS,PYGM,RALBP1,RELB,SAT2,SETD8,SKIV2L2,TUBB4Q
Negative_7888	F2R,IL7,LAT,AARS2,ACACA,ACP2,ADCY5,ALDH4A1,ALDH7A1,ALPI,CACNG8,CD44,CD74,CES2,CLCA2,COX15,CYP2A13,CYP2C18,DDHD1,DDX18,FABP1,GALE,GALNTL1,GCLC,GNA11,GNA12,HADHB,HARS2,HIF1A,HNF1B,METTL2B,METTL6,MYH7B,MYLK2,NKD1,NUMB,OR12D3,OR4D6,PARD6B,PPP2R1A,PRKAG3,PRLR,RALA,SCP2,SFRP1,THBS3,UROD,YWHAH,PRICKLE2
Negative_7889	GSR,SRC,TH,CD46,CLCF1,CLDN4,COL4A1,GNG3,HARS,HCCS,IMPDH1,ITGA7,NCR1,OR5L1,PFKFB3,PPP3CA,PYCR1,RARA,RASA1,SDC4,SETMAR,SMARCA5,SMURF1,THBS2,WNT8A,GABARAPL2

Negative_7890	PGD, ABAT, ALG10, AOC2, ARHGEF6, ARPC4, ASCC3, B3GNT2, CCL1, CCND2, CD19, CD46, CDC27, CEBPA, CER1, CLDN5, CPS1, DTX3L, GADD45A, GAS1, GIT1, H6PD, HLA-DRA, HSPA1B, IL28A, INHBB, LIMK2, MMP7, OR10T2, OR13C8, OR2D2, OR6C74, P2RX1, POMC, PRKAR2A, PSEN2, RAC3, RND1, ROBO3, RPS6KA6, SLC27A5, SLIT2, TIRAP, TNFSF18, TUBB1, ALDH18A1
Negative_7891	STS, TXK, ABLIM1, ACAA1, APOA2, CCL16, CLDN16, CLDN6, DHODH, ELK1, EPHB2, GALNT12, GCNT2, GLG1, GRIN2B, HSD3B2, LAMB2, LARS, MGST1, MIOX, NFATC3, NQO1, OLR1, OR2A14, OR4F15, OR5F1, P4HA1, PANK4, PFN4, PTPRF, SLC2A4, SMARCA2, TGFB3, THBS2, UNC5B
Negative_7892	GDA, SP1, B3GAT2, BET1, CBR1, COL4A2, DARS2, DNMT3B, E2F4, EFNB3, EPHB1, FADS2, GALE, GALNT12, GNAO1, HTR5A, MAP3K4, NAGS, NEFL, NTNG1, OR4K2, OR5H2, OR7A5, OXCT1, PLA2G6, PLCZ1, PYGB, RBKS, RHEB, SLC2A1, STAT5B, STX12, TJP3, TNFRSF9, CACNA2D2, PDCD1LG2
Negative_7893	ATP6V0C, CACNA1H, COL1A1, CYP26A1, DGKG, FUT7, GOT1, IPMK, ITGB7, NCF2, NTN1, NUDT9, OR14C36, OR4D5, OR4M1, ORC4L, PDE6G, PHGDH, PRODH, RAPGEF3, SH2D1B, SNW1, SPCS3, MAPK8IP3, SUV420H1
Negative_7894	CFI, FN1, TPR, ALAD, ALPI, ARHGEF4, B4GALT6, C1QC, CCL20, CD40LG, CDIPT, COQ7, DHCR24, EDNRB, F2RL1, GMPS, GNA13, GSTO2, IGSF5, LAMB3, LCMT1, MAGI1, MAP3K8, MYH7, NANS, NKX3-1, NOS3, NPR2, NQO1, OR14J1, OR2M7, OR7G2, PFKFB3, POLR2J, PTPRZ1, SDC3, SIPA1, TPH1, TSHB
Negative_7895	CAD, FGG, MPL, ACAD9, AGTR1, APOE, ARPC5L, CLDN15, FLT4, GCLC, OR2F1, PFN3, PIGC, PIK3R2, RHEB, STAT5A, TUBA8, WASF2, XYLB, MAP3K7IP2
Negative_7896	CSK, DCT, MOS, OAT, WAS, ACOX3, ACTG1, ARHGEF1, CFL2, CSAD, DUSP2, FGFR3, GPLD1, HLA-DOB, MAN1B1, MAP4K2, OCLN, OGDH, OR2D2, OR52W1, PON3, PPP2R5B, RPN2, SMC1A, TICAM2, UXS1, MAPKAPK2
Negative_7897	AMH, CSK, INS, OAT, AXIN1, CCL25, COL1A1, CSNK1A1, DTX3L, EHMT2, GCNT2, GMPPB, GRIN2B, HHIP, HRH1, IL20, ITGB4, KLK3, NEUROD1, NMNAT2, NOTCH1, OR10G7, OR1L1, OR52E2, OR5A2, OR5H2, OXCT1, PLCB3, PRKCE, PSEN1, PTDSS2, QARS, SKP2, SOCS4, TNFSF8
Negative_7898	CRH, OAT, PC, ADRBK2, AGRN, APOE, ARHGEF1, ARSD, ASMT, ATP6AP1, BUB1, CCL16, CDK6, CFTR, CHSY3, EHMT2, EIF4E, FMO2, GRIN2C, HARS2, HMBS, IL15, IL19, ITGB3, LTBP1, MAFA, MCCC2, NADSYN1, NAT5, NPR1, NRAS, OR10P1, OR4F6, OR8K3, OXCT1, PMM2, POLR1C, PPP1CA, PPP2R5B, PPP3R1, PYCR2, RFWD2, SMPD1, TAS2R3, TGFB2, MAPK8IP2
Negative_7899	CSK, IL3, TNR, ACAT1, ATN1, CBLB, CCKBR, CDKN2D, CXCL16, CYP2C8, CYP2J2, DDOST, FGF2, GUCY1B3, HEXA, IFIH1, MDH1, NFATC1, NR4A1, OR56A1, OR5D14, PDE1C, PPP2R5C, PRKAG3, SENP2, SNW1, WWP2, TNFRSF10D
Negative_7900	AGRN, ALOX12B, ARNT, BACE2, BMP8A, EIF4E2, GGT5, GPX5, LDHAL6B, MAP4K3, PLCB3, RHEB, SH3BP2, SLIT1, SQLE, SRD5A2, WWP2, ATP6V0D2, ST6GALNAC5
Negative_7901	CFH, AKR1C1, ALDOB, CCL24, CSAD, CYP1A2, CYP27A1, EI24, FMO2, FMO3, GALM, GNAI1, IL23R, NR1D1, PDGFA, POMC, PPP1R3B, PTGER1
Negative_7902	ADCY2, CD79B, LRAT, OR2F1, PCK1, PDHA2, PYCR2
Negative_7903	FGF7, KARS, NSDHL, POLD2, SEMA3D
Negative_7904	ID4, IL8, RET, ACVR1C, ALDOC, ARPC1A, ARPC2, AXIN1, BET1, CADM3, CCR3, CHPF, CLDN7, CYP2A7, CYP2C9, DDX19A, DOCK1, DRD1, ENO2, FTMT, FUT4, GALNT7, GCNT1, HHIP, IAPP, IRF5, ITGAE, MAOA, MCM5, MDH1, MDM4, MNX1, MOV10L1, NQO1, OR10G7, OR11L1, OR13F1, OR1J1, OR4L1, OR8K1, PPBP, PPCDC, PTGES2, PTGS2, SDC4, STK11, VAMP2, ZBTB16, TNFRSF12A
Negative_7905	CKB, IL7, UBC, COL6A1, CYP2S1, GPD1, KIR3DL2, MAN1C1, NFE2L2, OR4F15, OR4N5, SOAT2, WHSC1L1

Negative_7906	AK1,C8B,F8,GH1,GLA,IL5,IL6,ADCY6,AXIN1,B3GNT5,CAPN2,CCNB2,CCNE1,CD22,CDKN2B,CHRM5,CLTB,CSDA,CX3CL1,DGAT1,GNAI3,GPLD1,GRIA2,HAP1,HSD11B2,HTR7,ITGB4,KCNQ1,LRAT,NCKAP1,NMNAT2,ONECUT1,OR52B6,OR6A2,PAX6,PGAM1,PPP1R3B,PTGFR,PTK2,RAD51,RASGRP2,SARDH,SESN3,SPTLC1,SQLE,SUCLG1,ZMAT3,ATP6V0D1
Negative_7907	BTD,FOS,FST,PAH,RB1,TXK,ADCY9,ADH4,ADSS,AKR1B10,ALDH6A1,APC2,ARD1A,ATP13A2,ATP5D,B4GALT2,BACE2,CTNND1,DFFB,DLAT,EGFR,FGF4,GNG12,GRPR,IGFBP3,IL5RA,INPP5B,KIR3DL3,MCM2,MGAT1,NME6,NT5C1B,NUDT9,OR1A1,PDE8A,PDK1,PIM1,PLA1A,POLR2G,PTPRZ1,PYCR1,RPS6KA1,RXRG,SKP1,SLC2A1,SRD5A2,TNFSF14,TUBB3,TYRP1
Negative_7908	OAT,TEC,ACVR1,AMHR2,ARSA,ATP5I,ATP6V1A,CCL1,CHPF,CHRM2,CHST12,CHSY1,COL4A6,CSF2,CTSB,DAG1,EXOC7,FGF14,FZD1,GMPS,GRB2,GRIA3,MAPT,OR2A4,OR2F2,OR2T6,OR52I1,PAX4,PFAS,PI4KB,PIK3R1,PIP4K2C,POLR3D,POLR3K,RUVBL1,SEMA3G,SMAD1,SPCS1,SRGAP3,TGFB2,TRAF5
Negative_7909	BMP2,FOXO3,MAP3K13,NTF4,SETD8,SV2C,TUBA8,ULBP1
Negative_7910	GH2,NNT,UBB,BCAT2,CCL22,CD79B,CDK7,CPT1B,CSF3,CYP1A2,DRD2,EFNA1,FOXO1,IL8RA,LAP3,MMP14,MYH3,NEUROD1,OR10G8,OR14C36,OR51B6,OR56A1,PORCN,RASGRF1,SEMA4G,SH2D1B,SPRY4,STK36,TNFSF4,TUBA3C,TNFRSF18
Negative_7911	CHRD,CLDN5,HARS,KLK3,OR1S1,RAD51,SPTLC1,VAMP1
Negative_7912	SP1,AASS,ALDH1A1,ALOX15,ANGPTL4,CDKN2B,CNDP1,CNTFR,EFNB3,ERBB3,F2RL3,GSTM2,HMOX1,LIPT1,MAPK7,OR6C70,PDGFB,PFN4,PIP4K2A,PRKACB,SLC27A5,STX18,TCIRG1
Negative_7913	ADC,AGK,ALDH1A1,B3GNT5,CCL22,CCNB1,CLDN10,COL6A2,CSNK1E,DERL1,GSTA1,NRXN3,OGDHL,OR4C11,OR6C75,PDE1A,PIGC,PRKAG2,RDH12,SLC25A5,WNT10A,TNFRSF12A
Negative_7914	B3GALT6,FUT5,NSD1,THBS2
Negative_7915	DLD,FOS,NGF,TFG,AADAC,ALDH3A2,ALG12,AMPD3,AREG,ATP2A3,CACNB2,CCL8,CCNA1,CCND3,CMBL,COQ6,DAAM1,DHODH,EI24,FGFR3,GMPR2,IL12RB1,LCAT,MAPK14,NCF2,NT5M,OR14I1,OR4X2,OR8A1,P2RX6,PAFAH2,PANK2,PLA1A,POLA2,POLD1,PRPH,PTPN1,RPS6KA3,SRD5A1,TRIP10,ULK1,VTI1B,ARHGEF12,ST6GALNAC4
Negative_7916	CD2,OGT,TFG,ABLIM2,ACADVL,ARHGEF2,ATP5O,C7orf16,CACNG5,CCNB1,CDKN1A,CES1,COL4A2,CYP2J2,DHODH,FGF20,GALC,GGCX,GLO1,GLS2,GMPR2,GRIA1,GSTA5,GTSE1,HIBADH,IL12A,IL5RA,ITGAE,KIR2DS5,KLKB1,LIPF,MAP4K4,NEFH,OR4N4,OR52N4,OR6C76,OR7A5,OR7D2,PLAUR,POLR1B,PROS1,PTAFR,SDHD,SETD8,TACR1,TGFBR2,UNC5C
Negative_7917	BTK,ID3,ADCY9,B3GALT5,BCAT2,CTSL1,CYP2J2,DDX23,DHCR24,DHRS4,FZD1,GPD1,HEXA,HSD3B2,ICOS,IL12RB1,OR10A5,OR2B2,OR4K14,OR52H1,OR6X1,OR9Q1,POLE,POLR1D,POLR3D,PORCN,PVRL2,RPN2,STX1B,STX4,TM7SF2,TSC1,TYMS,UBE2K
Negative_7918	IL3,IL4,ATP6AP1,CDC27,EDA2R,GATM,MAPK12,MAT2B,MCM4,MYH15,NEUROD1,NRG4,NT5C,OR4F17,OR51B5,OR52E6,PNPLA3,TFDP1,TLR4,MAPKAPK5
Negative_7919	OSM,PTS,SMO,ALG1,ATG3,ATP5B,B3GAT1,CACNG7,CADM1,CYP21A2,CYP3A43,DGKZ,DGUOK,EXT2,F2RL2,FLOT2,GALK1,GALNT3,GALNT6,GJA1,IL28B,INADL,INPP4A,MTHFD2,NAMPT,NAT1,OR52B6,OR5AR1,OR6N2,PIGF,SIPA1,SOX17,SRGAP3,TGFBR1,TUBB1
Negative_7920	MYC,ATP6V1D,B4GALT5,DHRS9,FGF11,FGF22,FOXA2,GSTM2,MAP2K5,MAPK13,OLAH,OR2V2,OR4D1,PIK3R1,SMPD3,SPHK2,STMN1,TKTL1
Negative_7921	MAX,ACAT1,BCKDHA,CCL21,CHIA,COX15,DHCR24,FCGR3A,GNPAT,IL12A,MAPK9,OR10AG1,P2RX5,PDGFC,PRKAR1B,RAP1A,RARA,SMARCA5,ST3GAL4

Negative_7922	PLN, AGTR1, ATP5I, CCL4, CLDN22, GLI2, HIF1A, IL1R2, LFNG, LIMK2, MAML1, MAP2K1, NOTCH2, OR5AC2, OR6B3, PIAS2, SEMA4A, ST8SIA5, TACR2, TAS2R41, TRAF6, ULBP1, WNT3, YOD1, ST6GALNAC4
Negative_7923	ALCAM, ALOX5, CACNG4, CAMK2B, CAMKK1, CDC23, CDKN2C, CLOCK, COMP, HCCS, IL17B, ITCH, MAML2, MTAP, MYH7, NFASC, NFE2L2, OR1J2, OR2T2, OR5B21, PAPSS1, PIK3C2B, PLAU, PSME3, PTGES, RFXANK, SARS, SV2B, TAS2R42, TM7SF2, ZMAT3
Negative_7924	AMHR2, BDH1, BUB1B, CACNG1, CHRM2, COX10, CREB3L3, EDN1, FASLG, FGF21, FGF9, FMO5, GALNT5, GARS, GSTP1, HRAS, ITGAV, LFNG, MASP2, MGAT5, NADK, OR2V2, OR4C12, OR51I2, OR5I1, OR8K3, PDE10A, PDE1C, PTPMT1, SIPA1, SREBF1, SUV39H2, TMLHE, TPM3, TRMT11, TUBB2C, TUBB4, USE1
Negative_7925	MSN, XDH, COL5A3, CSNK2A1, EHHADH, ETNK2, KIR2DL4, KIR3DL1, NR5A2, OR13C9, OR5P2, PPARA, SIAH1, UROS, ST6GALNAC3
Negative_7926	ADRB3, ATP5L, CDS1, CNTN1, CYP3A4, GBGT1, GJD2, ITGA2B, MDM4, OR5AK2, STX1B, UCP1, VAMP2
Negative_7927	ACACA, CSNK1A1, HAP1, MAN2A1, NFASC, OR10G3, PAK6, PIK3C2A, RBKS, TKTL2, TOMM40, TPMT
Negative_7928	HGD, CALML5, ERO1L, GADD45A, GSTA2, MAPT, OR4C16, OR56A3, PORCN, RPN1
Negative_7929	IL7, ACP5, ACSS1, ACY1, AGPAT4, CAMK2D, CDKN2D, COL5A1, CYP27A1, E2F4, FASLG, GRHPR, GRIN2A, HCST, JAM3, LRAT, MAN1B1, MAP2K7, MGAM, MYH3, NOTCH1, NRAS, NTSR1, OR7A5, PLOD2, POLR3B, PPP1R3B, PRKAR1B, PTDSS1, PTEN, RAPGEF2, RRAS2, TFPI, TLR3, UGCGL2, VAMP1, WNT10B, YARS
Negative_7930	FER, ADIPOR1, ATP2A2, ATP5J, CAMK2G, DGKI, EPHA3, GALK1, GCNT2, LTBP1, OR2K2, POLE3, RUNX1T1, TNFSF9
Negative_7931	CKB, FYN, AARS2, ABLIM1, ABLIM3, ADRBK2, ALCAM, ARPC5L, BIRC2, CCL15, CDK7, CLTC, CPT1B, CPT1C, CYP2C8, DAAM1, DHRS3, DPAGT1, EXTL2, FCGR3B, GCH1, GLYCTK, GNPNAT1, GPX7, HLA-DOA, IFNA5, IL8RA, KIR2DS5, LCMT2, LEPR, MAP2K5, MGAT3, MMP2, NADK, OR2L3, PERP, POLE2, POLE4, PRLR, PTGER1, UGT2B11, GABARAPL1
Negative_7932	C8G, F7, HDC, IVD, ATP5I, CLTC, DAAM2, FGF20, GCLC, HRH1, LAMC3, LCP2, ODC1, OR1B1, OR1L6, OR6F1, PTGES, TPH2
Negative_7933	DIAPH1, OR2T11, PTGFR
Negative_7934	C9, F10, ITK, PAH, ALG1, AQP7, ASS1, BDKRB2, CACNG2, CCL17, CHRD, CIITA, CYP11B1, DGKB, EDA2R, FPGT, FZD2, GALNT1, GSTA3, GUCA1C, HADHA, IDI1, MAP3K13, MAP3K7, MAP4K2, MPDZ, NAGA, OR4N4, OR51I2, OR52B4, OR52B6, OR52K2, OR5M3, PFN1, PGM3, PIP5K1B, PLD2, PVRL4, RBX1, SEMA3G, SH3BP2, TICAM2, TUBB1
Negative_7935	FUK, ACSL5, ACVR2B, AGRN, ASRGL1, ATP6AP1, BMP2, CD38, CES1, CLDN18, ENTPD2, FGF21, GFPT2, GTSE1, IRF9, ITGAD, KLRC4, MARS, MCM3, MTMR2, MYL2, NAT5, NPR2, OR52E4, OR5AK2, OXCT1, PFN3, PLA1A, PPP1CB, RBKS, SPRY4, TAS1R2, TAS2R3, TRPC1, VAMP3, ALDH18A1
Negative_7936	EBP, MAX, ACOX1, ADRA1D, ARHGEF6, ARPC2, BDNF, C1QC, CACNB2, CDC25B, CDK4, CDKN2B, CLDN1, EGLN3, EPHB1, GP1BB, HAGH, HMBS, HSD3B2, KIR2DL4, LCMT1, MAPK8, NANP, NCR2, OR11L1, OR4C46, OR51B6, OR51D1, OR5D13, OR5F1, PCK2, PHKB, PIK3C3, PIK3CA, PLXNB1, PPP2R2C, PTDSS2, PTTG1, QARS, RRM2, SETD1B, SOAT2, ST3GAL4, TICAM1, TSNARE1, GABARAPL1
Negative_7937	IL8, ACSM4, ACYP1, ADRB2, BIRC3, CALML3, CASP10, FMO2, GALE, GSTO2, LIPE, MGAT5B, NFKBIA, OR2A2, OR5T1, OR8K1, ORC5L, PAX8, PFN1, PKLR, PLOD3, SLC25A6, SV2C, TJP1, CSGALNACT1
Negative_7938	GHR, TPR, ACTA1, ATP13A2, CACNB4, CD8A, CISH, CLDN3, CMAS, CYP3A43, CYP8B1, DAAM1, E2F3, E2F4, ERBB4, GRIA2, HTRA2, IL22RA1, ITGA4, KYNU, LARS, LTBP1, MAP2K6, NFAT5, NOD1, OR9I1, PTGES, RDH16, RPS6KA5, SCD5, SV2A, TBL1Y, TPK1, WASF3, WHSC1, WNT3, YES1, MAPK8IP1

Negative_7939	MUT, ALDH1A2, AXIN2, BMPR1B, C1QC, CDS2, ETNK2, FABP1, FMO3, HLA-DRA, IDH3A, NARS, P4HA2, THY1, WNT2B
Negative_7940	GP6, ATG3, BDKRB2, BLNK, CYP2S1, EIF4E, ENPP2, HTR2C, IDI2, IFNAR1, MAP2K5, OR4L1, OR8I2, PAK6, PRPS2, PSAT1, SLC27A1, STX7, ATP6V0D1
Negative_7941	INHBE, OR52N4, PPT1, TGFB1, TNFSF11
Negative_7942	GLA, ME1, SMS, ACSM3, ADH5, ADIPOR1, ADSS, AKT2, AMD1, ARPC5, CCL17, CCL27, CMAS, CRKL, CSNK1D, CYFIP2, DDHD1, GJA1, GLUD1, HTR2B, NANP, NCAM2, NFATC4, OR1C1, OR2S2, OR51T1, OR52A5, OR9G1, PIK3CA, POLR2H, POLR3D, PRSS1, RPSA, SEMA3D, SEMA3F, SEMA4C, SETD7, SH2D1A, SPRED2, SUCLG1, TNFRSF8, TUBAL3, UGCGL1, WASF3, ATP6V1E2, PNLIPRP2, TNFRSF11B
Negative_7943	CD4, IL6, ITK, OTC, BCKDHB, CADM1, CARS, CCNE2, CCR5, CLDN5, CSF2RB, CSNK1G1, CXCL13, ETS1, GPX7, GRIN1, ICAM3, MAP4K1, MYH13, PIGQ, PIK3C2A, POLR3G, PPARG, PPP2R1A, SETD1B, HLA-DRB4
Negative_7944	CGA, BMPR1B, BTRC, CASP8, CSF3R, DHRS4L2, FOXO3, IQGAP2, MAP3K6, NAMPT, NOX1, OR5K1, OR8K3, POLR2E, PPP2R5E, RELB, SESN3, SOS1
Negative_7945	NGF, ACY3, HADHB, HIBCH, HMOX2, ITPA, LAMB4, POLE3
Negative_7946	ACSL3, APOA1, B3GAT3, CD86, CHPT1, CPT1B, DGKG, FGF19, GALNT7, GNB3, ITGA5, KIR2DL3, MAP2K3, MTHFD2, NAE1, OR4S1, OR51I2, OR52I2, OR52N5, OR6K3, OR9A4, P2RX1, PIAS2, PPP3R1, RERE, STAT5B, TRAF4, TYMP, WIF1, SLC25A31
Negative_7947	IDS, LEP, NF1, SHH, TYR, ALAD, CDH5, COL4A1, CRHR1, DDX18, DDX56, DHCR7, ECHS1, EPHA6, EPHB6, F2RL2, FADD, FZR1, GNAZ, GP1BA, HHIP, IFNA21, IL13RA2, INHBB, IPMK, LCAT, MAP2K5, MRAS, OR13C9, OR51L1, OR52D1, OR8K3, PMVK, POLD2, PTGER1, RAB23, SULT1E1, SV2B, TAP2, TM7SF2, TP53, TYMS, VAMP3, WNT16, ZIC2
Negative_7948	FGG, ARHGEF6, CCR3, CDC2, CDKN2D, CLDN19, EGLN3, EPHX2, FGF7, GATM, HTR5A, INSRR, MAP3K5, MAP4K3, MGAT3, MYH7, NPAS2, OR2T3, OR4D1, OR4L1, OR51Q1, OR52R1, PIK3CA, PIK3R5, PMVK, POLR2K, PPP2R1A, PPP2R5C, PTGIS, SETD1A, SMARCA2, STAT5B, TAS1R2, TGFB1
Negative_7949	C2, F12, APPL1, CES2, COX10, CYP3A7, CYP8B1, GAL3ST1, GAS1, GCNT3, IDH1, ITGA7, MAN1C1, MMP2, MTAP, OR13C2, PRKAG1, RGS3, RXRA, SOS1, SPI1, TUBB2B, XYLT2, YES1
Negative_7950	ID3, STS, ABAT, ACSM1, CAMK2A, CPS1, FZD7, IGSF5, ITPA, KLKB1, PIP5K3, POLR2C, PPA1, RHEB, STX6, TUBA4A
Negative_7951	EPO, FGA, FUK, PTS, SFN, ABL2, ACTA2, APAF1, APOA1, BIRC5, CD46, CD80, CDH4, CHPF, CLDN2, CSNK2B, CXCL9, E2F2, EPHB1, FSHR, FTH1, HTR5A, IDH1, IFNA4, IGF1R, ITGAD, LAMB2, MAPK7, METTL6, MGST1, MYH14, NCR2, NEU2, OR2A42, OR2L8, OR2M4, OR56A3, OR5B12, PDXP, PFKFB2, PPP2R2D, RFWD2, SPTLC2, SSH2, TAS2R14, TAS2R8, AASDHPPT
Negative_7952	GGH, LAT, SRM, ACSL6, ACTG2, ALG2, B3GALT6, BBOX1, CCL8, CNGB1, CSF3, CTBS, GNAZ, ICOS, IL13RA2, ITPA, KCNQ1, LAMA3, LCP2, MGLL, NEU1, NODAL, NOS3, NRCAM, OR4K1, OR51I1, PAK4, PIGW, PLOD2, PPARG, PTPRB, STAT2, ULBP1, SERPING1
Negative_7953	ALPPL2, CDKN1A, CKS1B, CYP17A1, ENTPD2, GALNT7, GNAI1, GSTP1, IMPDH2, MAP3K11, NEU1, OR8S1, POLR2J, PRKG2, PVRL3, TAS2R3, TUBA8, SLC25A31
Negative_7954	ADCY1, ARSE, BRAF, CCR7, CNTN2, DDHD1, DDX23, DRD2, ESAM, FGF1, GALE, GCNT2, GGCX, HCLS1, HSD17B8, IMPA1, LCMT1, MAP3K5, NCF1, OR2T3, PPAT, PPOX, PTGIS, RPS6KA5, SELP, SLIT2, SMAD7, TPI1
Negative_7955	CFD, ABAT, B3GALT5, BAAT, BUB1, CAMK2D, CAV3, CCL11, CDS1, CNDP1, EXTL3, KIR2DL3, LCMT1, LCMT2, LEFTY1, LGMN, MAP3K1, NOX1, OR11H4, OR2D3, OR2T11, OR6N2, OR8G1, P4HA2, PIPOX, PPP1R3B, PTCH2, SHC4, SPHK1, STAT1, TNFSF12, ZMAT3

Negative_7956	CKM,CHAT,EARS2,ENPP3,EPB41L3,FMO3,GUCA1C,IL4R,ITGA9,OR2S2,OR4K13,OR5AP2,OR6K6,PLXNB2,RPSA,SGPP2,TAS2R9,TUBA1A,PNLIPRP1
Negative_7957	F9,MUT,ACAA2,AHCYL2,ALS2,BACE1,CHSY3,DDB2,DKK4,DPM1,DTX4,EGFR,EPHA2,FRAP1,GCAT,GJD2,GPX7,HTRA2,MAD2L1,MAPK1,MGST3,NEUROG3,OR11H1,OR2A42,OR2F1,OR51A2,OR5K1,PDE7A,POLE2,POLR2K,PRKAA1,PSPH,RAC2,SSH3,SUCLG1,TGFBR2,TUBAL3,UCK1
Negative_7958	PVR,RB1,AANAT,AARS2,ARAF,BDKRB2,C1QA,CLDN20,COASY,COMP,CYP2C19,DCXR,GCNT1,GSK3B,HIBADH,IL10,ITGAD,NEU1,OR5AR1,PLA2G2D,PTK2B,SDC4,STAT6,TP53I3,TUBB2B,UBE2L6,VNN1,ALDH18A1,HSP90AB1
Negative_7959	PLN,AOC2,APOE,ATP5F1,COQ6,GRIN1,H6PD,ITGAD,LEPR,NEU1,OR4N2,PNLIP,PRPH,PTCH2,SENP2,UCP1,WHSC1,AASDHPPT
Negative_7960	BAX,AASDH,ALG10B,CDK6,KARS,KNG1,LIPE,LLGL1,MTMR1,NARS,PER1,PIK3C2B,POLE,PPM1B,PPP1R3B,PTPRJ,RAPGEF2,SRD5A1
Negative_7961	BTC,C3,PLN,ALG8,APOA5,B3GALT4,CACNA1H,CCKBR,CCNE1,CD3E,CTNNA1,CYP26C1,DMGDH,DUSP16,ENAH,FARS2,GDF5,GNA13,HAAO,HSD11B2,IGF1,IL12A,IL13,LIPA,MAOA,MKNK1,NME4,OR4D6,OR51A2,OR6C3,OR6K6,P2RX5,PANK4,PDE11A,PRKACA,PRKCD,PRKCQ,RAPGEF2,RASA2,SHC3,STK11,TLR4,HLA-DQB1,PRICKLE1
Negative_7962	ATM,SRC,ACADM,ACVRL1,ALG10B,B3GALT5,BPGM,CACNA1B,CMPK2,CXCL5,CYP11B1,CYP26B1,CYP4A11,DCXR,DPM1,DTX3,FABP3,FMO5,GUCA1A,HCST,HES1,HEXA,HSD3B2,IFNA5,MAPK14,MKNK2,MTAP,NEU3,OR2A14,OR52E4,OR52I1,OR6C76,PHPT1,PIGH,PLXNA2,PNPLA3,PPP3CC,PRKCZ,PRKY,PRLR,PTPRF,PVRL2,SELL,SLC25A6,SPCS3,TJP2,YKT6
Negative_7963	ADH6,ALDH3A2,B3GALT5,CDC23,CHIA,CLDN5,DUSP7,FGF8,GLUD2,GMPR2,GNA13,GP1BB,HCLS1,HLA-DOA,HMBS,HMGCR,HTR2C,IDH3A,IL2RG,ITGAL,LPAR1,MYH14,NMNAT3,OR1L1,PANK1,PDE4B,PFKL,PNPLA4,PPM1D,PRKAA1,PROS1,SLC33A1,ST8SIA5,SUV39H2,VANGL1,ZNRD1,C1GALT1C1,MAPK8IP2,PLA2G12B,ST6GALNAC4
Negative_7964	ACAT1,CHRD,ECSIT,EIF4E,ERBB4,GNRH2,GSTT1,NRAS,OR5P2,OR6K2,PARD6G,PDGFC,PDHB,RPIA,SH2D1B,TSNARE1,WASF3,WNT2,CACNA2D3
Negative_7965	DHH,MSN,RDX,RET,TH,ALG12,CDKN2A,CYFIP2,E2F2,ERO1L,FBP1,FZD7,GADD45B,GART,GGCX,GRM5,GRPR,HCCS,HMGCL,HMGCS1,HTR2A,INADL,LARS,MGST1,MKNK2,MYH1,NCAM1,NMNAT1,OR6S1,PHKG1,PLXNC1,POMC,RENBP,ROCK1,SESN2,SIPA1,SMAD5,TGFB1,TLR9,TSC1,WNT11,WWP1,YARS,HLA-DPA1,ST6GALNAC1
Negative_7966	HK2,TNN,ARRB1,ATP5J,BAAT,CACNG1,CARS,CBR3,CD74,CPT1A,FGFR1,GMPS,HMOX2,IL6ST,ITGB6,MAGI1,NFASC,OR1K1,OR6C4,OR8J1,ORC2L,P2RX3,PDE9A,PIK3R2,SEMA4G,SMAD2,SSH1,STX5,STX8,TAP1,TCIRG1,VKORC1,MAPK8IP2,PHOSPHO1,SERPINC1,SLC25A31,TNFRSF14
Negative_7967	CKM,MET,ADIPOR1,AGMAT,AREG,ASIP,ATP5G2,ATP6V1D,CCL7,CD40,CTNNA3,DMGDH,ENO2,FGF4,FLOT1,GGT6,GNRH1,GP1BB,HPSE,IL10RB,IRAK1,MGST3,MIOX,MYH6,NARS2,NEUROD1,NFX1,NOTCH2,OR10R2,OR2A4,OR2A5,OR2T12,OR3A3,OR5C1,OR5M3,OR9Q2,P2RX1,POLR2G,SDHA,TOLLIP,WNT9B
Negative_7968	GNMT,JAM2,OR10G4,OR5B21,TJP3
Negative_7969	ACAD8,GADD45A,IDH3G,MAP3K2,MYH13,OR13C2,OR1D5,OR5P3,OR6K3,PAK3,SDC1,TJP3,ATP6V0E1
Negative_7970	PML,TEC,ALDH1A1,BMP2,CDKN2A,CTNNA3,EDN1,FIGF,GGT6,GLO1,NR4A1,NRP1,OR1L6,OR2T4,PARS2,PLK1,PPP1CA,PYGL,TAS2R3,THTPA,TIAM2,TNFSF14,ULK3,TNFRSF1A
Negative_7971	ID2,ADRBK2,EXTL3,HSD11B1,PIK3CD,PPP2R5E

Negative_7972	DLD, PLG, TPR, ACOT12, ACVR2A, ARPC3, B3GAT1, B4GALT5, CCR5, CD247, CDK4, CDK5, CHRM1, CYP17A1, DNMT3L, GAD2, GALE, GALNT2, GPX5, GRIN1, GUCY1A2, IFNA17, IFNA7, INSRR, ITCH, ITGAL, MAML2, MCM5, MTMR1, NFKBIB, NOX1, NT5M, OR10AG1, OR1L8, PLCB3, PLTP, POLR1D, PPP1CB, PRKCZ, PTK2, RASGRF1, SARDH, SDC2, SLC27A4, STT3B, TKTL2, ALDH18A1, CSNK1A1L
Negative_7973	EPO, GSN, ACY1, ADRB3, ATF2, BAAT, C4BPA, CLTCL1, CSNK2A2, CXCL2, DEGS2, DGUOK, ENTPD1, ENTPD5, FGD1, FLAD1, FUT1, GFAP, HAO1, HLCS, HTR4, IL15, INPP5A, KLRC3, MAPK11, MAPK9, NLGN2, OR13C5, OR8I2, RPN1, TGFB1, TUBB, SLC25A31
Negative_7974	CTH, DCK, FH, IL7, MPZ, CBR1, CCND2, CCNE2, CCR2, CHD8, CHIA, DHX58, DIAPH1, DVL1, EFNA3, ERBB2, GSTA1, HAGH, ITGAD, LYPLA2, MAGI2, MAP4K3, NCAM1, OR8K3, PHKB, POLR2D, PRPS2, PTCH1, PTPN7, SEMA3C, ZBTB16
Negative_7975	ADA, DCT, LTB, TAT, ADCY8, ADCY9, AGMAT, AOX1, BLNK, BPNT1, CAMK2G, CAMKK1, CCL13, CD99, CDC25B, CSNK1D, CYP2J2, CYP7A1, DNMT3B, EPHB1, FGF2, FOXO3, GALNT2, GALNTL5, HARS, HHEX, IDI2, IFNA6, LCMT2, LIPA, MAPK10, MYH8, NARS, NAT2, OR2T1, OR4C16, PARVG, PCCB, PKMYT1, PPP3R2, SELE, SPTAN1, STX18, SV2B, UXS1, YWHAB, B3GALNT1, CACNA2D4, TNFRSF18
Negative_7976	DAO, FAH, ME2, ACTA1, BMP5, CCL22, CCNG1, CHEK1, CRY1, CSNK1A1, DLL1, FASN, FUT8, GNAI2, GUCY2F, HHEX, IL18RAP, KLRD1, LDHC, LTA4H, MYL5, OR2G6, OR2S2, OR4E2, OR51I1, PIGB, PMAIP1, SELE, SPRY4, STX7, TAS2R8, TCF7L2, WNT1, C1GALT1C1, HLA-DQA2
Negative_7977	CACNB1, CDC14A, CDH3, DVL3, ELK4, FZD5, GSTK1, HTR6, MTHFD1, PIGB, PPAT, PPP1R3C, SHMT1, TAS2R7, TM7SF2, TRAF4
Negative_7978	MPZ, NNT, ACADVL, ALDH9A1, AVPR1A, BACE2, CHEK2, CYP2A6, CYP2U1, DAG1, DDX23, FZD4, G6PD, GALK2, GATM, GNAL, GNPNAT1, GSTA1, IDI2, JAK1, MAPK3, MGAT5, NPAS2, NUDT12, OR5AK2, PAPSS2, PARD6G, PCCA, PFN2, PIM2, PPOX, PPP2CB, RARS2, SCD5, STX12, WHSC1L1
Negative_7979	DHH, MTR, ADCY10, ADCY5, ATP5O, CFL2, CHKA, CLDN8, COL3A1, CXCL1, CYP19A1, E2F2, GDF7, IL17B, MAML1, MAP3K1, MTMR6, OR2B11, OR52E8, OR8B8, PDHA2, PFKL, PLXNC1, PMVK, POLR2D, PTGS2, RPN2, SETMAR, SPAM1, TAS2R3, UROS, HLA-DPA1
Negative_7980	CRH, NLK, OR51B6, OR8A1, TAS2R14
Negative_7981	ASIP, CYBB, OR8S1, PAK4, RASGRF2, SSH1, TGFB3
Negative_7982	C5, MYC, SRM, AREG, CACNA1A, CD47, CDKN2B, DGKE, FGF12, FZD5, GALNT2, GUCY2C, GUK1, HTR2A, IKBKG, IL20, L1CAM, LIPC, MAOA, MAP2K2, MITF, NAMPT, OR2D2, OR5F1, OR8B8, PIGN, PPP2R5D, RPS6KA3, SEMA3A, TAP1, TGFB3, TUBB2A, UBE2L3, UGT2B4
Negative_7983	NPL, SFN, B3GNT4, CTF1, CYP2B6, DPYD, ERBB3, GPLD1, IFNA7, IL2RB, MMP2, OR2AG2, PDE5A, PIP5K1B, PPP2R5A, SLIT1, TAS2R5
Negative_7984	IL9, JUN, ATP5G3, B3GALT5, CCL21, CDC7, CES1, CHIA, HDAC1, HSD17B1, PAICS, PRKACB, RPS6KA2, STMN1, TNFSF18, SERPINC1
Negative_7985	BTD, BCL10, CDH1, CDKN1C, COQ5, DLL3, EPHX2, GALNT5, OR2T1, OR52N2, RDH8, SGMS1, TRAF2, VEGFA
Negative_7986	CD6, DBI, AGPAT4, CACNB2, FOXO3, FRAT1, HSD17B8, MTFMT, OR7D2, RDH8, TIAM2
Negative_7987	GUCY2D, IPMK, LIMK2, MECR, MYD88
Negative_7988	F11, ACLY, ALG1, ARNTL, CCL20, CDC26, CHST13, CHSY1, COL6A2, E2F5, EGLN3, FGF19, FZD3, G6PC2, GALNT2, GMPPA, GSTM2, IL2RB, IRS1, ITPKA, OR11H6, PPT1, PTEN, TNXB, UNC5C, UROD, WASF3
Negative_7989	BTD, CP, AKR1D1, AMY2A, CCNG2, CCR7, COL4A2, COX10, CYP3A5, GAL3ST1, GALNT6, HMGCR, IGF1, MAP3K12, OR10A6, OR10K2, OR4K2, PDGFB, POLE4, PPAP2B, PPP3CB, PRKAA1, SLC2A2, SMAD4, STEAP3, TCF7L1, GABARAPL1

Negative_7990	ALDOC,C4BPA,CCR10,CTBP2,CYP4F2,DDX18,DEGS2,DLL3,DNMT1,DPM3,ELK1,EPHA7,IRAK1,KLKB1,LRAT,MGAT3,NCF2,NFATC1,NSD1,OR2W1,OR5C1,OR5R1,OR9A2,OXSM,PDE3A,POLR3D,RARS2,SLC25A6,SSX2IP,SV2B,WHSC1,WNT7A,ATP6V1G3
Negative_7991	CKM,DBI,FAH,SYK,ACAD9,ACSL6,CCR9,COL5A2,DERL1,DHDDS,ECSIT,EPHX2,FASN,GALE,GUCY1B3,HRH1,ICAM2,INPP4B,MECR,NCR2,OR52A5,OR5C1,OR8D1,PAK3,PDHB,PIK3C3,PNPT1,PTPRB,RBX1,UGCGL2,TNFRSF18
Negative_7992	ABO,DTYMK,ENTPD3,KIR2DL2,LDHB
Negative_7993	UBC,CLDN11,CYP2D6,GFPT1,MTMR2,OXCT1,PDE1A,PLXNA2,PPP2R5A,PRKY,RALBP1,TPH2,TNFRSF1B
Negative_7994	AGK,F2,GP5,MAG,ACTB,ACTG2,ADORA2B,AFMID,APAF1,APRT,ARPC2,ATP5I,ATP6V0C,B3GALT2,B4GALT1,BCMO1,CDH15,CXCL2,EFNB1,EPHA3,FARSA,FMO3,GRAP2,IFNA8,IRS2,LCMT2,MAN2A1,OR2T4,OR2W1,OR6N1,OR9Q1,ORC6L,PIK3R3,PPA2,PRKAA2,PRKACB,PRSS3,PSME3,PYGL,RPS6KA2,SRGAP2,TCF7L2,TDO2,TIAM2,CACNA2D3
Negative_7995	ASL,CFD,F10,ADRA1A,ALDH5A1,CACNA1A,FGF8,GNG12,IL11RA,IQGAP1,MARS,OR1S1,RARA,SMPD4,TPH1
Negative_7996	AVPR1B,GNPNAT1,OR6N2,PLXNC1,VNN1
Negative_7997	F12,VCL,ATP2A2,BMP7,C4BPA,CACNB3,CDC6,CMBL,CPS1,CREB3,CRHR1,CXCL5,ENPP3,ENTPD4,FGF19,FZD1,GIT1,GNPAT,GNPNAT1,HLA- DMA,HSD3B2,IFNAR2,IL22RA2,INHBB,ITPK1,JAM3,KCNQ1,LAMB2,LIMK2,LRAT,MGAT5B,MMP9,MYH6,NFAT5,NOD1,NPR2,ONECUT1,OR4K2,OR5V1,OR6A2,PDHA2,SKIV2L2,SMAD4,STX12,THBS1,TNXB
Negative_7998	IDS,TPR,ACTN1,ACVRL1,ANAPC1,APBB1,ARRB2,ATP5L,BDKRB2,BUB1B,CADM3,CARS2,CBR3,CDO1,CLDN11,CNDP1,CREB3L3,CTSS,DARS2,DGKQ,DPM3,DPYD,ENPP2,ENTPD6,EPHB2,FLT3LG,GUCA1A,GUCY1A2,HYAL2,IL20RA,ITGA2B,KLK3,MGAT4A,MTMR6,ODC1,OR51E1,OR52N5,OXCT1,PKM2,TAS2R41,TBK1,TLN2,TRHR,PPP1R12A
Negative_7999	C5,CR1,CRH,SYK,AARS2,ADH4,ADH5,ALCAM,ALG11,ARPC5L,ASMT,B3GNT2,BCL2,BDKRB1,CCL16,CLCA4,COL6A1,CYP2C9,CYP2F1,DPYS,DTX2,EPHA3,GLG1,HADH,HAGHL,HLA- DOA,IDH1,IL18R1,IL22,INPP5A,IRF7,JAM3,NFYB,OR13C8,OR2A2,OR9A4,PANK3,PGK2,PI4KA,PLA1A,PNMT,PPP3CB,TDO2,TLR3,ULK3,SERPINC1
Negative_8000	AR,C8B,AMHR2,ANAPC2,BLVRB,CACNA1C,CCL27,CHEK2,CLTC,CXCL3,CYP2C18,DAAM1,DDX52,DHRS4L2,EXT2,FLAD1,G6PC2,G6PD,GUCY2F,KLKB1,OR10H3,OR1J4,OR52J3,OR5L1,PARD6A,PDIA4,PNMT,PPP3R2,PRKAR2B,PRKX,RHOQ,RXRA,SYNJ2,UCK1,ST6GALNAC3
Negative_8001	AK2,ADIPOQ,FGFR3,HLA- DRA,HRH1,IFNB1,LTB4R2,NKD1,OR4K13,PIP4K2B,POLR2F,RALBP1,WASF1
Negative_8002	IVD,ADRBK2,ALOX15,ATP5L,CIITA,CLDN10,CXCR3,DPM3,FGF21,IFNA14,MAT2B,MINPP1,NLGN3,NRXN3,NT5C1B,OR2A2,OR2F2,OR6A2,POLR3D,TBL1XR1,UCK2,YWHAB
Negative_8003	AK3,CD6,GNE,NF1,NGF,SI,TKT,ALDH1L1,ATP5F1,CCL16,CD34,CDK2,CYP2D6,DDX50,EDN1,FRAT2,HHIP,IDH1,KLRC3,MAP3K14,MCM5,MGAT2,MGAT4A,OR1S2,OR4F4,OR5AN1,OR7D2,PDE10A,PDXK,PDXP,PLTP,POLE,PPP2R5C,TOMM40,WNT6,ATP6V0D1
Negative_8004	C3,EGF,HK1,ATF4,ATIC,CADM3,CCNH,CYP2B6,DVL2,GNMT,GNPNAT1,GZMB,HSD3B1,IL22,LCMT2,LEF1,MARS2,OR13C2,OR1J2,OR1S2,OR4C16,OR51A2,OR6C70,P2RX7,PFKFB3,PGK2,PTDSS2,ST3GAL5,UGDH,WNT3,XCR1,SERPINE1,TNFRSF1A
Negative_8005	SMS,CDC20,GDF7,GMPR2,PHKG2,PIP5K3,VANGL1,WNT1,WNT10B,WNT7B

Negative_8006	C9,CD6,CRH,PGF,APAF1,ATP6V1F,CCL2,CD48,CLCA4,CNTNAP1,CPT1C,CSF1,CTBP1,CXCL2,CYP2E1,DHRS9,DIAPH2,EFNB2,EPHA2,FZD1,GALNT10,GTSE1,HMGCS1,HNF1B,HSD11B1,HTR2A,IL10,KLKB1,LCMT1,NCR1,NFX1,NR5A2,OR1J1,OR4D2,OR4S1,OR51E1,OR8S1,PGK2,PIGX,PLCB4,POLD1,PPP2R5E,PSAT1,SDC3,TALDO1,TPI1,XYLB,YKT6,ZBTB16
Negative_8007	EBP,BMP2,EPHA2,MFNG,OR4D11
Negative_8008	ACTN4,CAMKK2,CDC20,FGF2,IDH3B,NCAM1,NFATC1,OR12D3,OR4C6,OR5B12,OR7C1,PIK3CG,SPRED1,TSC2,XCL2,XYLT2
Negative_8009	OR51Q1,UPB1,YES1,TNFRSF17
Negative_8010	F3,GK2,AOC2,APRT,BACE1,CDC45L,CDKN1C,CLDN11,DARS2,DVL2,EFNA5,GNPAT,GYS2,HES1,INHBA,MAGI3,NARS2,NAT2,NFYA,OR52H1,PDHB,PIK3C2A,PITX2,PPP2R5C,PTK2,PTPRR,RCHY1,SHC3,SUV39H2,WNT10A,TNFSF13B
Negative_8011	C4B,C6,FAS,FH,HGF,IL2,TAT,ALDH4A1,CACNA1H,CCKBR,CD244,CDC26,CDO1,CYP3A5,DARS,DCXR,FGF2,GALNT8,GNAT3,IFNA10,IKBKB,IL11RA,IL17RB,IL23A,ITGAD,KYNU,MAT1A,MECR,MYH2,NPAS2,NRG1,OR13C8,OR1L3,OR52E6,OR5AK2,OR5B3,PDX1,PIP5K3,PLD2,PNPT1,SMARCA2,UBE2L3,VASP,YWHAB,C1GALT1C1,GABARAPL1,HLA-DQA2,SUV420H1
Negative_8012	DCK,ADH7,ATP5C1,CLDN16,FRAT1,HTR2C,OR13C5,OR2L8,OR5M8,OR8D4,PCCA,PPA1,PTPRB,RASA2,TNFRSF9,TSHR
Negative_8013	PTS,WAS,CDC20,CDO1,COL5A2,CYCS,DNMT3A,ITGAX,NKX6-1,TAS2R1,ST6GALNAC1
Negative_8014	C9,MIF,NLK,BDH2,COL3A1,DUSP5,EXT1,F2RL2,GUSB,HAO2,HLA-G,IL20RB,NRXN2,NT5C1A,OR1J4,OR4K14,PIK3C2B,PIP5K1B,PLCB4,PPP1R3A,PRKG2,PYCR1,UAP1,VANGL1,ATP6V1E2
Negative_8015	CD80,GALNT6,IL13,RXRA,SH2D1A,SMPD4,VAV2
Negative_8016	BTK,F8,OTC,TPR,ACLY,ACPP,ALDH9A1,ALG1,ATP5C1,CDC14B,CDC26,CHAT,CLTC,CRB3,CXCL9,DDX51,FGF11,GALNT3,GDF5,GSTA4,HGSNAT,HTR4,INHBC,ITGA11,KDSR,NCOR1,NME7,OCRL,OR13C4,OR3A3,OR51B5,OR56A3,OR8B4,PDGFRB,PIK3CD,PLOD2,POLR2I,RAD51,RHOD,SHC4,SPI1,SUCLG1,UGCG,WASL,SERPING1
Negative_8017	RDX,ADRB1,ALDH9A1,ALOX15B,ARHGAP5,CCL26,CHKA,CLCF1,CLDN22,COQ6,DGUOK,FARP2,FARS2,G6PD,GDF6,GUCY2C,ICOS,ITGB6,LCP2,MARS2,NME2,OR10G7,OR10H5,P2RX4,PDE4D,PLCG1,PNPLA3,PPP2R2C,PSAT1,SC5DL,SEMA3E,SEMA3G,SIAH1,SMAD3,STEAP3,SYNJ2,UNC5B,ATP6V1B1,KIR2DL5A,SERPINC1
Negative_8018	GP6,TNC,CLDN3,DGKA,GALNT5,GLI2,HLA-DOB,HNF4G,IFNK,ITGA7,ITGAE,JUND,KDSR,LMO7,LRP5,MYH14,OR10G8,OR11G2,OR6C75,PAX6,PRKAG1,PSME1,SORD,SOS1,TSC1,TUBA8,MAP3K7IP2
Negative_8019	CFH,IL6,LCK,ALOX15B,APH1A,ARHGEF1,CACNA1S,CDK4,DDX47,DUSP7,ENTPD7,GNAI2,GNRH1,GSTK1,HCLS1,IFNA8,JAG2,MAP2K4,NT5C3,OR10J3,OR4X1,PFKP,PIGT,PLA2G4A,PNPT1,PSME1,RALBP1,RHEB,ROCK1,SDC2,SFRP1,SORD,TGFBR1,VARS,PPP1R12A,SUV420H1
Negative_8020	AK7,DAO,EGF,ZYX,ACAT1,ALDH2,ALOX12B,ATP13A2,BUB1,CLDN14,ENPP1,ENPP6,GGCX,GNG12,GSTK1,GSTM2,HAGHL,IL1R2,MAP4K1,NEU1,NUMB,OLAH,OR1G1,OR3A1,OR4K17,OR51G2,OR6B2,OR8U1,P4HA1,PARVB,PHGDH,PIK3CB,PLA2G4A,POLA1,PON1,PRKACA,PTK2,RAF1,RAPGEF4,SRD5A1,SSH3,SUFU,MAP3K7IP2,PRICKLE1
Negative_8021	APBB1,ATP5G2,BAI1,CCL5,DUSP10,F13B,FBP2,GCH1,GDF7,GP1BA,IDH2,IL1A,MAOA,NKD1,NT5C2,OR11G2,OR1L8,OR2T10,OR6T1,PAK3,PDE1C,PLCB1,SOAT2,SORBS1,SUCLG1,WNT7A

Negative_8022	C4B,FGA,FGB,NNT,TPR,A4GALT,BCKDHB,CD58,DHRS4,DRD5,E2F2,ESAM,F13B,FGF6,FPGS,FRAP1,FUT4,GALNT14,GRIN2C,IDI2,IGF1,IL2RB,MBL2,MTMR1,OR10H4,OR2AG1,OR4C11,OR8A1,OR9I1,PDHB,PIP4K2B,POLR2B,RFXAP,SGPP1,SPRY2,STAT3,TLN1,VDAC2
Negative_8023	ACSL6,AKR1B1,CLDN5,COL4A2,COL6A2,CSAD,CYBA,FZD9,GADD45A,IDH1,LAMB1,LDHC,MAP3K11,MAP3K4,OR4N2,OXSM,PTK2,RAF1,VAMP4,CACNA2D1
Negative_8024	ADRA1B,AGPAT4,ATF2,BUB3,CASK,CCR5,COL4A2,DHRS4L2,DLST,EXT2,FGD3,FUT9,GRIN2D,GSK3B,HCCS,HLA-C,HSPA2,IGSF5,ITGB5,MAP4K3,MAPK3,MRAS,MYD88,NTRK2,OR13C3,OR13D1,OR2M4,OR51B2,OR56A3,OR8K3,PDE6G,PDX1,PGAM4,PLCB2,PMM1,RDH12,SCIN,UCK1,VTI1A
Negative_8025	LPO,PGP,PVR,TYR,ABL2,ACOT11,ADCY4,ALG2,AMDHD1,AOC3,ARPC5,BMP6,CCNA1,CDKN1C,CHDH,CHRM3,EXT1,FABP3,FCGR3B,FGF7,GCNT2,GUCY1B3,HCST,HLA-G,HLCS,IKBKG,IL18RAP,KLRC3,LAMA4,MYH8,NFYA,OR1K1,OR4E2,OR4F5,OR4K15,OR52N1,PFKFB3,PIK3CB,SEMA3E,TJP1,TLR5,VAMP2,WNT6,ZBTB16,CACNA2D1
Negative_8026	ADCY6,ADH1A,ARRB1,B4GALT4,CACNA1D,CCR4,FZD6,MYH10,NOS1,OR2T6,OR5T1,OR6M1,PRKACB,PTPRM,SGPP1,YWHAE
Negative_8027	ADC,AK3,GSS,HDC,ABL2,AMD1,APOA2,B3GAT1,BCKDHB,BMPR1A,CCL5,COL4A6,CSF2RA,CYP26B1,CYP2J2,DDX19A,DDX41,DEGS1,DPYS,FRAP1,GALNT8,GCDH,GLI2,GSTO2,GUCY2F,IFNA17,IL2RA,KAT2A,KIR3DL2,NCOR1,NGEF,OR10G8,OR2H1,OR51M1,OR52N1,OR5B17,PDE7B,PRKCE,PTK2B,PTPN6,RAD51,RAP1B,ST3GAL6,TSLP,UGT2A1,PNLIPRP1,ST6GALNAC4,ST6GALNAC5,TNFRSF13B
Negative_8028	FN1,ABL2,ACAD9,ACMSD,ACSL3,ARSB,C4BPB,CMPK1,COL11A1,CTSG,CYP2A13,EPHX1,IAPP,JAM3,MAP3K6,MGST2,NMNAT1,PITX2,POLR1D,SMAD4,THBS2,TJP3,WNT9B,CACNA2D1,PHOSPHO1,TNFRSF1B
Negative_8029	C3,CFI,TYR,ALG9,ATP2A3,BAI1,BCAT1,C1QC,CCL26,CDC27,CRLS1,CSF3R,DCTD,F13B,FGF10,GBGT1,GRB2,IDH2,ITGAE,MAP4K4,MTFMT,NRXN3,OR1B1,OR2AE1,OR2M3,PAPSS1,PITX2,PPAP2A,PRKAG2,RALB,RPS6KA2,SIGLEC1,SMURF1,SNAP23,SULT2A1,TRMT11,TYMS,UGT1A10,VAMP3,WASL,ATP6V1E1,B3GALNT1,PNLIPRP2
Negative_8030	ACSM5,CDC16,CFLAR,GNG12,MFNG,MGST1,MMP9
Negative_8031	DCC,ID4,ADAM17,AQP7,ASMT,CHRM5,CNTNAP1,CSF1R,CYP2S1,DGKZ,DUSP2,EP300,IFNG,LAMA3,LTA4H,MAN2A1,MAP2K7,NAT2,NCF2,NMNAT3,OR5K2,OR9G1,PLA2G2F,PLXNB1,PRKG2,RPS6KA3,UAP1,UBA1,UBE2L3,VAV3,WASF1,WNT5A,KIR2DL5A
Negative_8032	CDC23,FCGR2B,GLG1,HNMT,LIPC,MAN1B1,MAN2A1,MYL2,PLCD3,RND1
Negative_8033	BACE1,CCL2,CD79A,GDF5,HTR2B,ITGB7,OR3A2,PLA2G6,TLN1,TLR2
Negative_8034	MUT,ACSL3,AKR1C4,ARAF,BMPR1A,CDC14B,CDKN1B,CNTN1,CRB3,DAPK2,DGUOK,ECHS1,FGF7,FZD1,GNAI3,GNG13,GSTM3,HSPA1B,IL2RB,KLRC2,LIPT1,NPR1,OR10P1,OR13J1,OR2T29,OSMR,PARVB,PAX6,PIAS2,RALGDS,RARS2,SC4MOL,TNFRSF9,TPK1,TUBA3C
Negative_8035	GLA,SYK,ANAPC5,APBB1,BET1L,C3orf10,CCL11,CDC42,CLDN11,CMPK2,CXCL13,IRAK3,NMNAT2,OR2B11,OR4X2,OR9G4,PLCD3,PRKACB,PRKAR2A,PYGM,SCP2,SH2D1A,SOCS4,TAS2R14,TP53,TRAF3
Negative_8036	HGD,ACSM1,ACVR1B,ALDH1B1,ARSB,CCL25,CLDN6,CYP8B1,FGF11,GALNT10,HAO1,IFIH1,IFNA8,IL1B,ITGB7,NPAS2,OR5D18,PIK3C2G,SOAT1,WNT10B

Negative_8037	BAX, C5, C6, CBL, GGH, ACVR2A, AKT1, ALDH1B1, ATP5G1, BCKDHA, CAV3, CCL5, CDC25B, DKK1, DPM3, DUSP5, FGF17, G6PC2, GALNT12, GSTT1, ITGA9, KDSR, OR10C1, OR13J1, OR5M9, OR8K1, P4HA1, PIGF, PLOD1, PPAT, PSEN1, SEMA3B, SMAD2, SNAI1, SPTLC1, ST6GAL1, TBL1X, TP53, TPI1, YARS, HLA-DRB4, PRICKLE2
Negative_8038	ALG12, CCNB3, CDKN1C, CHKB, DTX3L, DTX4, GAS1, LAP3, MYH15, OR10K1, OR2M7, OR51B5, PDGFRA, PLXNA1, POLR2F, POLR3D, PPP2R5E, PRLR, PTGFR, RASGRF1, RDH11, SSH2
Negative_8039	DHH, EBP, UBC, ACADM, ARG1, ATP13A2, ATP6V1D, CDC14A, CES2, CLTC, COL5A2, CTSB, DPM1, DVL3, EFNB3, GGT7, GSTP1, OR10A3, PCK1, PLA2G2E, PLK1, PPA2, RAB5A, SELP, TOMM40L, TPMT, HLA-DPA1
Negative_8040	ID2, PAH, ALDH7A1, ASNS, BIRC2, CCR8, ENAH, NEFM, PARS2, PCNA, POLR2L, PPP1R3B, PPP2R5A, VAV3
Negative_8041	TNN, ACSM5, ARAF, ARHGEF6, C3AR1, CACNG4, CD226, CDC25C, CTSG, CYBA, DDX47, DLST, EPHA8, FABP4, FGF22, GALNT1, GANAB, GNB3, HMGCR, IL18RAP, IL20, IQGAP2, ITCH, LRAT, MAN1A2, MIOX, NQO1, OR2B11, OR52N4, OR5AR1, OR5I1, PARD3, PLAU, PMVK, POLR1E, PTGER1, PTPN5, RAB5A, RAPGEF2, SMC1B, TAS2R38, UGT2A1, WBSCR22, WHSC1, SERPINC1
Negative_8042	CHUK, ITGAM, KYNU
Negative_8043	IDS, ADH4, CCNE1, CD74, CYP2A6, EFNA2, F2RL1, FGF7, FUT4, GRPR, IDH2, IFT57, IL15RA, LLGL2, MTMR2, NEU2, OR2G6, OR51L1, OR52M1, OR7D4, PDCD1, PKMYT1, PLTP, PNPLA3, PPARD, PTPRB, RPE65, SETMAR, SMPD4, SORBS1, TAS2R3, THBS4, WNT10B
Negative_8044	EZR, ACTG2, ADIPOR2, ALOX5, AMD1, ATP6V0B, B3GAT1, CDC27, CDC42, CRB3, CYP1A2, DDX19A, F2RL1, GALNT14, GALNT8, GFAP, MTMR1, NCSTN, NEFL, OR2T6, OR4A15, OR8H3, OXSM, P2RX6, PAPSS2, PDE1A, PDE2A, PDIA4, PFN2, PLCB2, PLOD3, PMVK, PON1, PPM1A, PPP2R5A, PRKACA, PRKCB, PTGDS, RASSF5, RBPJL, SLC27A4, SPTLC1, SYNJ1, TSHR, UGT2A3, SUV420H2
Negative_8045	ACP2, ADH4, ARPC2, ATP13A2, B4GALT3, BAIAP2, BMP8B, BMPR1B, CCL1, CCR7, CLDN9, ECHS1, ENTPD8, FMO2, GUCA1B, IFNA10, IL11, IL1R1, IRS4, MLLT4, NFX1, OR4A15, OR4K14, OR5D14, OR8D4, PGM1, PLOD1, PPAP2B, PPARA, ROCK1, SPRY2, TJP2, UBE2L3
Negative_8046	CD6, ANPEP, ARD1A, BDKRB1, CCL24, CCR8, CHPF, CHSY3, CLDN2, DIAPH1, EXT1, IL10RA, MCCC2, MOV10L1, NAT5, NUDT5, NUDT8, OR10K1, OR1A1, OR2T10, OR56A3, OR56B4, P2RX4, PIGM, PKMYT1, PLA2G3, POLR3B, PPAP2A, PPBP, PRDX6, PRODH, PTCH2, TOLLIP, TRAF4, TYK2, CACNA2D1, HLA-DRB4
Negative_8047	CTPS2, FGF19, FUT7, OR1L1, OR1N1, OR2T6, OR52N2, VARS
Negative_8048	ILK, ADCY4, ADSL, ATP5G2, BUB3, C1QB, CDH3, CHPF, CNGB1, COL4A6, COL5A3, CSF3R, DAPK3, DDX56, DERL1, DOCK1, DUSP7, EFNA1, FASLG, FCGR3B, GABARAP, GGPS1, GLUL, HPRT1, ICAM2, IDH3A, OR6C4, OXTR, P2RX3, PDE5A, PLK1, PLXNB2, PPAT, RAC3, RPS6KA4, RPS6KA6, SDHB, SMARCA2, TJP3, VCAM1, WNT4, XYLB, YWHAZ, ZBTB16, GABARAPL2, SERPINB5
Negative_8049	CR1, GNS, ACVRL1, AKT1, BIRC2, CACNG8, CTSL1, DCXR, DGKB, EPAS1, EPHA2, EPRS, FMO3, GNB3, GNRH2, NTSR1, OLR1, OR6S1, OR9A2, PHKB, PPP1CC, PRKACG, PRSS1, RXRB, TYMS, ATP6V0A2
Negative_8050	AUH, OSM, ALG6, ATP5D, CHPT1, CNTF, DDX52, EDNRA, FGF1, GSTM3, HSPB1, KIR2DL4, MCM2, NRG2, POLD3, PRKCZ, QPRT, SULT1A1, PNLIPRP2
Negative_8051	GDA, KIT, ACSL1, ADCY3, ADCY4, AGRN, CADM3, CD72, CDKN2D, CRY1, DUSP8, GNAQ, IL18, IRF5, MCM3, NSDHL, OR4M1, PGM3, POLR1D, RDH8, SMARCA2, SORD, THBS1, TIRAP, TNFRSF14
Negative_8052	LAT, SI, ALG3, CCNE2, CDIPT, CXCR4, DTX3, E2F2, IFNA16, KIR2DL3, LARS, MCM4, NCK2, OR2L13, OR2S2, OR56A1, OR6K3, OR8A1, PER1, PPP3CA, SRD5A2, TAS1R1, TP73, UGT2A1, CACNA2D3

Negative_8053	GCK,AADAT,AGPAT3,AMHR2,ATP6AP1,CD72,CLCA2,CTNNA1,JAG2,MYH15,POLD3,PON3,PSME3,SLC12A2,SMURF1,TAS1R1
Negative_8054	ACADM,ALDH1A3,ALDH2,ALG2,AMPD2,ANAPC11,BCL10,CCL19,CCL2,CYP21A2,E2F5,EHMT2,F2RL3,GFPT1,HEMK1,HMGCR,MAN1B1,MMP14,NCOR1,NFASC,NGEF,NKX6-1,OLAH,OR2AE1,OR2M7,OR52L1,OR6X1,P2RX4,PFN2,PIP4K2B,PPARD,PRLR,PTGDS,SESN2,SRGAP1,STX1B,SV2C,TAS2R48,UCP1,UGT2A3,WNT5A,WNT9B
Negative_8055	ALPP,AMDHD1,CACNA1A,CLDN15,CLDN17,CYP2F1,FBP2,IL10,OR51T1,OR6B3,OR6K2,ORC2L,PDGFD,PFKL,PROC,RDH12,SETD8,UNC5A,SERPINB5
Negative_8056	ITK,ZYX,ACP1,COL3A1,FBP2,GLDC,GLO1,GUCY2D,IDH1,LIAS,MAP3K14,NT5C,OR2T4,OR6C75,OXTR,POLR1C,PSME2,PTPN5,SFRP2,SNAP25,ST3GAL1,TICAM2,TRAF5,WEE1,SERPING1
Negative_8057	ACAA2,AGRN,ATP5I,CCL16,CHKA,FMO4,FOXA2,GNB3,HYAL2,IARS,MAGI2,MGAT1,NARS,OR4X1,PI4KB,POLR2L,RAD51,SPRY2,TAS2R9,THBS3
Negative_8058	HGF,ITK,ADORA2A,AKR1C3,ALDH1B1,ARPC4,B3GAT3,BMP7,CD38,CD47,CHIA,CHPT1,CLCA2,CREB1,CSDA,CTTN,DUSP7,FGD3,GLUD2,GNPDA2,GSTM2,INADL,INHBB,MGAT5B,OR5AR1,OR6N2,PARVB,PDE1B,PIGB,POLA2,PPAP2C,PRIM1,PRKAG1,SPHK2,SULT2B1,TCF7,TUBB1,TNFRSF10B
Negative_8059	C9,CD59,DARS2,GALK1,GRIN2B,HNF1A,IL13,OR1D2,OR2Y1,OR5AR1,OR8H2,STEAP3,TNFRSF4,YWHAH,GABARAPL2,HSP90AB1,TNFRSF17
Negative_8060	ME3,STS,ADIPOR1,AGXT,ALG9,CAV3,CD34,CD72,CES2,CHAD,CLOCK,DEGS1,DGKD,DGKZ,DUSP6,ENPP3,EPHA6,HIBADH,IARS2,ITGB2,JAM2,KNG1,LIMK1,MGAM,MMP1,NAGK,NGEF,NTF4,OR10A3,OR10AD1,OR11H6,OR2V2,PDE7A,PON3,PPP2R5B,PSEN2,RDH5,SPTLC1,SPTLC2,SSH1,TP73,B4GALNT1,MAPK8IP2,SERPING1,TNFRSF18
Negative_8061	ALG13,DNMT3A,FGFR4,HTR2B,MYL9,NFASC,NPAS2,OR10A5,OR2L2,OR2T11,OR4D2,OR5AK2,RPRM,TNFRSF1B
Negative_8062	CS,TEC,TK2,TPR,ATP6V1F,BUB1,CACNA1G,CMPK2,COQ6,CSAD,CSNK2A2,E2F5,FASLG,HAO1,HIF1A,IFNA16,LGMN,MFNG,NT5C1A,ODC1,OR13F1,PIP5K1B,PNPT1,PTDSS1,RASGRP4,RBX1,SEMA4C,SOCS3,SPTLC1,ST8SIA5,TGFBR1,WASF1,ARHGEF12
Negative_8063	ACSL5,ADRA1A,CDK6,CHRNA7,CSNK2A1,CYP2B6,DUSP8,EXOC7,FASN,GALNT1,HPRT1,HSPB1,HTR2C,KDSR,LDHAL6B,LIPA,MYH11,OR10J3,OR14J1,OR4C46,OR4F6,OR5K2,OR6K3,OR7G2,P4HA2,PARD6A,PDGFA,PDGFRA,PLCD3,POLR2J,RAD51,SLC25A4,SPRY4,SRGAP3,STX18,SUV39H2,TDO2,TRAF3,WNT16
Negative_8064	TH,ACHE,ACY1,ADCY5,ATF4,ATP5B,CLDN6,CYP21A2,EFNA1,EXOC7,F2RL3,FASLG,MMP1,OR6K6,OR8D1,OR9A4,PDE2A,SPTAN1,TAS2R5,THBD,HSD17B12,SERPINA1
Negative_8065	HGD,HYI,MOS,TPR,ABL2,ALG10,CLDN7,CNTNAP1,COL3A1,CSNK1E,CYP2B6,DCTN1,GMPPB,GUK1,IL20,IL22,MAN2A1,MPZL1,NTSR1,OR52E2,OR52H1,OR5M3,PDE3B,PGK2,POLD3,PPP5C,PRKAA1,PTPMT1,RFX5,TUBA3C,WNT11,ATP6V1B1,HSD17B12,PAFAH1B3
Negative_8066	ABO,CGN,ACVR1C,CACNA1D,GSTA5,IPPK,OR10H4,OR4P4,OR8B4,PIP5K1C
Negative_8067	FGG,MIF,ACTN1,ATP6V1H,CLDN18,CPS1,CSF2,CYP2C19,DDX19A,E2F1,ENO3,GSK3B,IFNB1,IL28RA,KLRC4,NANS,NCSTN,NFYC,OR11H4,OR1D2,OR52D1,PIK3R2,PIK3R5,PLA2G2E,PPA1,PPP1R3C,PSME1,SEMA4G,SPAM1,TMLHE,TP53I3,TUBA3C,UPP1,USE1,CSGALNACT1
Negative_8068	AK7,GSS,SDS,ACP5,AGPAT3,BDKRB2,BMP8B,CTNNA2,CTNNB1,DHODH,DUSP16,FOXA2,GGCX,GSK3B,ICAM1,IL15RA,IL4R,IMPA2,MAT2B,NCF1,NME7,OR2B2,OR56B4,ORC4L,PLCE1,PRSS1,PTCH2,PTGER1,RPS6KA2,RPS6KA3,RXRG,TACR3,TRAF5,TUBA4A,TUBB4Q,UBA7

Negative_8069	ATP5B,NTRK2,RAC2,RAC3
Negative_8070	AK5,OTC,ACOT12,ACTG1,ACVRL1,ARSE,ASCC3,B4GALT4,C1QA,CACNA1F,CHRNA7,CLDN20,CLDN7,CPS1,CYP11B1,CYP2U1,DBF4,DLL1,EI24,EIF4E,FLOT2,GJA1,KAT2B,MDM4,NAT1,NCR3,NR5A2,OR10G3,OR1N2,OR2T8,OR5D18,OR6T1,OR8B4,PAK2,PDE2A,PGAM4,PPP3CA,PROS1,PSEN1,RPS6KA5,SEMA4D,SHISA5,SLC2A2,STMN1,THTPA,TLR2,TOMM40,TYROBP,ST6GALNAC4
Negative_8071	C9,FGG,HYI,IHH,UBC,WAS,ACSM4,ADAM17,ADCY5,ADRB3,ALCAM,AXIN2,C9orf95,CACNA1G,CD36,CD79B,CHD8,CHRM5,CXCR3,CYP26A1,CYP26B1,EPB41L3,FARSA,FGF13,FZR1,GALK1,GALNT12,HSD3B7,HSPA8,IL28RA,IQGAP3,LIFR,NR4A1,OR10AG1,OR14J1,OR1A1,OR51G1,ORC6L,PAX6,PER3,PIGK,PIP4K2B,RXRB,SCIN,SEMA3F,SYNJ1,THBS3,TSC1,PRICKLE2
Negative_8072	VCL,AKT3,CHIA,DDX52,DGKD,ENPP2,FGF17,FZD7,GALNT5,LAMA4,MAP2K5,NEFH,OCLN,OR8U1,PPAP2A,SH2D1B,SOCS3,SOCS7,HSP90AA1
Negative_8073	ABO,ADC,CP,DCK,MPZ,ADRA1D,B3GALT6,B4GALT1,BMP8B,C1GALT1,CCDC6,CCND3,CD34,CDKN2B,CTNNA3,CX3CL1,CXCR3,DERA,DGKQ,FGF8,FZD8,GLUL,GNPDA2,GPAM,HDAC1,IDH3A,IPMK,ITGAE,LIMK2,MAGI2,NANP,NTN1,OR10Z1,OR1S2,OR52N2,PARD3,PAX6,POLR3H,PTPN5,PVRL1,SEMA3G,TGFA,UNC5C,XCR1
Negative_8074	BAX,TEC,ACADS,ACP2,ALDH9A1,BUB1B,CACNG8,CISH,COL4A4,CREB3,DPM3,DPYS,EFNB3,GALNTL2,GANAB,GFAP,GGCX,GUCA1C,HARS2,HCLS1,HIBCH,IDH2,IFNA5,KRAS,LHCGR,MYH10,NFKB1,OR10A7,OR10K2,OR52K1,PGM2,PIGF,PIM1,PPP2R1B,PRKG1,PTTG1,QPRT,RBX1,ST6GAL1,STAM2,SULT1A1,SULT2B1,TRPM5,WNT8A,TNFRSF10B
Negative_8075	CFI,MAX,AKT2,ALDH3A2,ANAPC10,CACNG2,CHSY1,CLDN11,CREB5,CTBP1,CXCL9,CYP11A1,CYP2A6,EPHB4,GADD45G,GALNT10,GBE1,GJA1,HSD17B3,HTR5A,IL13RA1,IL8RB,INPP4A,LLGL2,MARS,MTHFD2,NCF4,NCOR1,NEFM,OR1A1,OR2A12,OR4X2,OR52A5,OR6F1,OR7A10,OR8U1,ORC4L,PAX4,PRKAR2B,PSME3,PTCH2,RASGRF2,SLC2A2,STEAP3,STX1B,TAP1,TNFSF4,TRAF1
Negative_8076	LPO,MVK,ADRA1B,AKR1C2,CLDN16,CPT1C,CX3CL1,ENTPD8,F2RL3,FGF8,FGFR3,IL20,IL22RA2,ITPKA,MLLT4,OR4F15,OR56A3,OR5P3,OR8D1,P2RX6,PIK3C3,PPAT,PPP1R3D,PRKAB2,PRKACG,PSME3,ROBO2,SELP,SMAD5,TBL1Y,UBE2L6,WNT4,YOD1,ZFYVE9,ALDH18A1
Negative_8077	C2,GHR,GP5,ME3,ABAT,APOA1,CAMK4,CCL13,CD80,CLTB,COL4A4,CYP2E1,DAAM1,DDIT4,DGKH,ECHS1,GADD45B,HEMK1,HSD17B7,INSRR,ITGA6,JAK3,LAP3,NKX3-1,OR8D1,PAK1,PAK2,PLCB1,PPM1D,PRKCZ,PVRL2,RAC3,SLC25A4,SRGAP1,TBL1Y,VAMP3,WASF1,YARS2,PDCD1LG2
Negative_8078	CDA,CR1,EPO,MPZ,SCD,TNR,ACVR1,AMPD3,CACNB3,CSNK2A1,DGKA,ENTPD1,GBGT1,GUCA1C,IL13,IL15RA,MAP3K12,MECR,NANP,NCOR2,ONECUT1,OR52B6,PAX4,PRODH,RPS6,SMAD5,STK4,TAS2R10,ULK1,MAPKAPK5,PNLIPRP2
Negative_8079	STS,ALOX12,COL5A1,FCGR3B,FGF3,FZD2,IRAK3,MYH6,RAP1A,RBL1,SLC27A5,THBS4,TRAF6
Negative_8080	AMD1,ARSB,BCL2,EPB41L2,ERBB4,FGF1,HSPB1,HTR4,ITPKB,MAOB,NKX2-2,OR13A1,OR6N1,SMPD2,ZFYVE16
Negative_8081	CFI,ALG10,BMP8B,CACNA1D,CERK,DDX18,G6PC2,GALNT5,HSPA8,LAMB4,OR2T33,OR4F5,OR52W1,OR5B21,RBKS,SPRY2,SPTLC2,TUBA1C

Negative_8082	CBL, DUT, RFK, RPE, ZYX, AASDH, B3GALT4, BIRC3, BMP6, CASP9, CAV3, CCL2, CCL4, CDC25C, CHAT, CYP2C19, DHCR7, ENPP6, FGF20, FZD5, GALT, GFAP, GSTZ1, HLA-DRA, IL13, MAP3K6, MAPK9, OR13A1, OR2A42, OR2L13, OR51A2, PAX8, PDGFRA, PIGB, PIGL, PLOD2, PMM2, POLR2F, PPP2R5A, PRSS2, ROCK2, SETD1B, SLIT2, SPRY4, STAM, TRHR, XIAP, ATP6V0D2, PNLIPRP1
Negative_8083	ABLIM2, ACLY, ATF2, B3GALT5, CD44, CSNK1G3, DHX58, DOCK1, ENTPD2, EPHA7, FLT3LG, GBE1, GPD2, GSTM3, HSD3B2, IL12RB1, IMPA2, IRF5, ITGA2, ITGB4, LMO7, MAP4K4, MPZL1, NEFH, OGDH, PISD, PLEKHA8, PTGDS, RELB
Negative_8084	CHKB, CREB3L1, GNAI3, GNB1, MTHFR, PRKAR1B, PRODH, RDH5, SORBS1, HLA-DRB3
Negative_8085	PVR, TFG, AKR1A1, ASIP, CASP3, CDC26, CLDN5, COMT, CYP27A1, GUCA1C, MMP9, OR4F6, OR5F1, OR5R1, OR8J3, PAK4, PLA2G3, ST8SIA1, STAM, TFPI
Negative_8086	EDA, LCT, RET, ALDH1A1, CACNA1B, CASP7, CHDH, CLDN19, COL5A2, DTX3L, ETNK2, GALNT6, GUCY2C, IL22RA2, LAMB3, NCR1, NR4A1, NRXN2, OR4C12, PGAP1, PIGQ, PITX2, PLOD1, QPRT, SAT1, SPTLC1, TRAF2, UGP2, YARS, MAP3K7IP2
Negative_8087	ADC, DCC, MSN, PGP, ACY1, AK3L1, BAIAP2, CACNG7, CCL19, CCNE2, CLCF1, CLDN11, COMT, DUSP14, ECSIT, EFNB1, FASLG, FRAT2, GAPDH, HMGCL, HMGCS2, KLRC4, MAP3K8, NCK2, NMNAT2, NR5A2, NTF3, OR1G1, OR1L6, OR2A2, OR2B6, OR2C1, OR4C13, OR5P2, OR7C1, PDE1A, PLXNC1, RAF1, RETSAT, SHC1, TAS2R4, THBS1, TYMS, WNT10A, WWP1
Negative_8088	F2R, ATP6V1D, B4GALT5, BHMT, C1QC, CHST12, COL2A1, COMT, CYP3A4, E2F3, GALK2, GALT, GBGT1, GLI2, GPD2, GYS1, HSP90B1, JAK3, OR4A15, OR52A1, OR52B4, OR5P2, PIGH, POLR2G, RASSF1, RXRA, SENP2, SULT1E1, SV2C, VARS
Negative_8089	ALG10, ALOX12, ESPL1, G6PC2, GNAI3, IFNA6, NUDT12, PLTP, PYGM, SPAM1, UGCGL2
Negative_8090	ATM, GP9, AOC2, ATG12, ATP5G1, B3GALT5, B3GALT6, B3GAT2, CASP9, CCL5, CCNB3, CDC2, CSNK1G3, CXCL1, DNMT3A, FGF3, FZD7, GLUD1, GPT2, HNF4A, HTR6, LIPA, MGST1, NCK1, NME6, NT5E, OR2M2, OR5M8, OXCT1, PHKG2, PLCB3, PLXNA1, PTGFR, SH2D1B, STAT2, TPMT, TRAF1, TYROBP, ULBP2, WNT2B, ZMAT3, HLA-DQB1
Negative_8091	C8G, F11, F8, CASP3, CD8B, CDK4, CHUK, CRLS1, CSF3R, CYP4F2, EDAR, GALNT11, IL22, INSRR, LARS2, OR2AT4, OR4B1, OR4N4, OR6X1, OXCT2, PLXNA1, PMVK, RBPJL, RFX5, SDHC, SEMA4D, TYMS
Negative_8092	ADA, CBS, ACP5, ACP6, ACPP, ACSL3, ACTC1, ADCY4, CCL22, CCNG2, CTBP2, DARS, F13B, GSTM1, HPSE2, IAPP, IL29, MCCC1, NTRK2, OR1D5, OR2AG2, OR51G2, OR52I1, P2RX5, PIGU, PIK3C2B, PLEKHA8, PLIN, PNLIP, PNPLA3, PTCRA, PTPRM, PVRL2, SGMS2, TALDO1, VANGL1, HLA-DPB1, PAFAH1B3
Negative_8093	AGK, ACTG1, ADORA2B, AMD1, ARG2, AVPR1B, BCAT1, CDKN1C, CISH, CLDN8, CNTNAP2, COL4A1, CRHR1, DGKB, DHODH, FMO5, FZD3, GRPR, GZMB, HLA-DOB, LIFR, MKNK1, NEFL, OR10G2, OR13H1, OR4K15, OR5AU1, OR6C6, ORC5L, PDGFC, PLCB3, PMM2, PYCRL, TKTL2, TRPM5, WNT9A, ATP6V0D2, HSD17B10
Negative_8094	IVD, LAT, ATP5G1, BHMT, CDKN2C, CREB3L3, CXCL2, EIF4B, ENTPD2, FUT3, FUT5, GALNTL2, GCDH, GNRHR, HYAL1, IDH3A, IL11RA, INPP5B, IRF9, ITPKA, KLRK1, MGAM, NCAM2, OR10X1, OR2M4, OR2M5, OR4B1, OR4N2, OR5AR1, OR9A4, PLOD3, POLD4, POLR2E, PON2, RBL2, SELPLG, SEMA4B, SHISA5, TLR5
Negative_8095	ACAA2, DDB2, PGAM2, POLE2
Negative_8096	MIF, PGF, ABP1, ALDH6A1, ATIC, ATP6V0B, BUB1B, CACNG2, CCR5, CD99, CLCF1, CSNK1E, DAPK2, DHX58, DUSP16, IL22, KYNU, MEF2C, OR1N1, OR2S2, OR7C1, PECAM1, PFKP, PLA2G5, PNPLA4, SHMT2, TBL1X, TOMM40L, TUBB2A, KIR2DL5A, MAP3K7IP1

Negative_8097	CAD,CLTCL1,E2F5,GGT7,HLA-F,INPP5A,LAMB1,NT5M,PAPSS2,PIP4K2A,PLK1,RBKS,SIGLEC1,SLC12A2,TACR1,TRPM5,VDAC2,HLA-DPB1,PDCD1LG2
Negative_8098	FST,ABI2,ADH7,AKR1C1,ALG3,ARNT,BCAT2,CCL7,DAG1,DPYD,E2F3,ITGB2,JAM3,KCNJ11,KDSR,KLKB1,LDHC,MAD2L2,METTL2B,NOTCH1,OR13C5,OR1N2,OR5A2,OR5AC2,PFN3,PTK2,SLC33A1,SPRED1,TNFRSF8,ZFYVE9,CSNK1A1L,HLA-DPB1
Negative_8099	AK7,BTC,ACO1,ACTA1,ADAM17,ALAS1,ALDH3A1,ATP5F1,CBLB,CCL21,CRHR1,EFNA1,FCGR3A,GNRH1,GSTM5,ICOS,ITGAE,MALT1,MAPK12,NRG1,OR10A5,OR8H3,PYGL,RDH10,RFXAP,SLC2A4,VEGFC,WWP2
Negative_8100	GLA,CALML5,CDH4,CTLA4,FLT4,ATP6V1B2
Negative_8101	BID,FST,ACACA,ANAPC4,APOA1,BBOX1,CCNA2,CD19,CNGA3,EFNA5,FUT4,GALNT13,IDI2,LAMB4,LIPT1,MGAT4B,NEUROG3,NME6,NMNAT1,OR10A5,OR10AG1,OR2F1,OR2M7,OR4F15,OR9G1,PDHB,PFN3,PRODH,RASGRP1,RRM1,SDHD,SETD2,SOCS7,TFDP1,HSP90AA1
Negative_8102	C8G,ALDH1A3,APOC3,B3GALT4,B3GALT6,B3GNT2,CADM3,CCND2,CDH4,CYP11B2,DHCR24,DPM3,EPOR,FGF22,ICOSLG,MAOB,NOS3,OR2F1,OR52M1,OR5T3,PIK3C2A,POLE3,PRKACA,SAT2,SH2D1B,STAM,TAS2R7,UGCGL1,VNN1,ZFYVE9
Negative_8103	AUH,MPZ,TNR,ACAT2,AKR1B10,ANGPTL4,ARHGEF1,B4GALT5,CD82,CDC2,CSF1,DLAT,FECH,FZD6,NCKAP1,NRG3,OR2L2,OR2Z1,PLCB4,PPP2R5C,RAF1,SESN1,SHISA5
Negative_8104	F7,PLG,AADAT,ARHGEF4,BUB1,CHEK2,CHIA,CHRM2,CIITA,CTSS,FGF3,GBE1,GLG1,GSTM4,GUCY1A2,IFNGR1,IFT57,IL12A,ITGB4,NCF2,NR1H3,PDHA2,PGAM1,PIM2,PRPS1,RFWD2,RRAS,SMARCA2,VTI1A,ATP6V1B2
Negative_8105	ATP5A1,OR2B11,RUNX1T1,STT3B,HLA-DRB5
Negative_8106	IL9,MIF,TPR,AADAC,ADH7,ATP5O,AXIN1,BCL10,CCL22,CCR3,CCR8,COQ7,CYP2C8,EGLN1,ENO2,FCGR3B,FGF10,GRM5,GSTM5,GUCY2C,H6PD,HAGHL,IL28RA,ITGAE,KLRC1,LDHA,MAGI2,MCM3,NFYA,NOTCH2,NUDT2,OR1A1,OR2V2,OR4D1,OR4K13,OR51E2,OR5AR1,OR6K6,PCK1,PDE4B,PLA2G3,SDHD,SFRP2,SHC2,SLC2A2,TUBA3E,TUBB4,UGT2B7,GABARAPL2
Negative_8107	ACOX3,ADRA1D,CCND3,FARP2,NAGK,PDE10A,PPARG,PTPRZ1
Negative_8108	NLK,PLG,SYK,AASDH,ABLIM1,ACADS,ADAM17,AKR1C2,ATP6V1D,AVPR1B,C1QB,CACNG1,CACNG3,CAMK2B,CCL23,CCND2,CDKN1C,CDS1,CRY2,CYP4A11,DAPK3,DHDDS,GNAO1,GRIA3,GSK3B,HCLS1,HES1,HIBADH,IFNA2,IMPDH2,JAM2,NAT2,NFYA,NNMT,OR1G1,OR4X2,OR51A4,OR51A7,PLEKHA8,RHOA,SETD2,SOCS5,VARS,VKORC1,MAP3K7IP1
Negative_8109	FER,GDA,VTN,ACAA1,ACADM,ADRBK2,AFMID,AGPAT6,BCMO1,CASP3,CCL26,CCL28,CDK6,COMT,EGFR,FUT4,GARS,HSPA8,IDI2,IL22RA2,IMPA2,INPP1,ITGA1,KLRC3,KNG1,MAP4K4,MARS2,MCCC2,NCK2,NUDT8,OCRL,OR10C1,OR2M5,OR4A16,OR4C16,OR4K2,OR52K1,OR7D2,ORC5L,PTCH2,RASGRP4,RFXAP,TAS2R8,TPMT,TYK2
Negative_8110	MPZ,SFN,ALDH3B2,CAMKK1,CDKN1A,CTPS,DLAT,ENTPD8,GUK1,HLA-DRA,NME2,OR2A4,OR5F1,SSX2IP,PAFAH1B2,ST6GALNAC5
Negative_8111	FES,FGA,CHEK2,DUSP9,FGD1,FGF10,GLUD1,IBSP,IDI1,OR6X1,TAS2R8,WNT1
Negative_8112	DBI,BCKDHA,GUCA1C,OR2H2,OR56A3,PARD6B,RPRM
Negative_8113	CDH4,DAG1,GART,GOT2,HIBADH,HTR6,NPR1,OR1G1,OR6C1,PDE11A,RAC3,SREBF1,SULT2B1,ATP6V0D2,ATP6V1G2
Negative_8114	SMS,SPN,CDC14B,CER1,GFPT1,GNMT,HEXA,LEFTY1,MGST1,OR10G4,OR2T34,OR4N5,OR4S2,SERPINA5,ST6GALNAC4
Negative_8115	AMH,GNS,FGFR3,GANAB,GPAA1,MAPK7,MCM2,NEFH,PANK1,PDE5A,TUBA8,WEE1,KIR2DL5A,SLC25A31
Negative_8116	GYS1,HMOX2,LDHAL6B,PSEN2,SEMA4G

Negative_8117	ADCY3,ALAS2,BACE1,BCKDHA,CACNB4,CD47,CD79B,CDS1,CHPT1,EPHA3,FTCD,GALNT1,HYAL2,ITGAM,OGDHL,OR10H4,ORC2L,P2RX6,PLD2,PPP2R2C,PPP2R5A,PTAFR,PTPN7,SDC2,SDHD,SLC27A1,SPP1,THY1
Negative_8118	HK2,ACP6,ADCY1,AKR1A1,AMHR2,AMY2B,APBB1,B3GNT5,BET1L,CCR2,CCR7,CLDN18,CLDN2,CNTNAP2,CYP2C18,DDX41,EGLN3,FOXO3,GALE,HNF4A,IL23A,MAP2K1,MKNK1,NTNG1,OR51G2,OR52J3,OR6C1,OXSM,PDGFA,PDGFC,RAP1B,RASGRP1,RUVBL1,SNAI1,STAT6,SUV39H1,WNT7A,ZMAT3,ATP6V0A1
Negative_8119	AK1,C1S,GPI,TH,ALG2,APOA5,CYP2C8,DGKD,DKK4,FMO5,GALNT14,HHIP,LTB4R2,NFKBIA,NME2,NPAS2,OR2S2,ORC2L,PDGFA,SNAP25,TOMM40
Negative_8120	PML,PVR,SHH,ADH6,AKR1C3,ALAS1,ALOX12B,CBLB,CHST12,CLDN10,CPT1C,CTNNB1,CX3CR1,EDN1,FRAP1,GPD1L,HMGCR,HSD17B2,IL12B,IMPDH1,INHBA,IPPK,IRF7,IRS4,MGST3,NFX1,NFYA,NLGN2,NTN1,OR7C1,OR9K2,PFN3,PFN4,PNMT,PRKAG1,PSME1,PTK2B,SEMA4D,SHMT1,STAT5B,TNFRSF8,TSNARE1,UNC5C,WNT11,YWHAQ,HSD17B10,PPARGC1A
Negative_8121	CKB,LTB,ACLY,AKT1,ATF2,CD59,CDK6,CDS2,CHRNA7,COL4A4,CXXC4,CYP26C1,DHRS3,E2F2,ENPP3,EPHA4,FGF3,GAD2,GMPR,IFNA21,IRS4,ITGB7,LCAT,MAP3K14,MARS2,MCM7,OR1J4,OR2W1,OR52A5,OR52E8,OR7G2,PLCE1,PNLIP,PRKCZ,PTGFR,RAPGEF3,TLR2,HLA-DPB1,TNFRSF17
Negative_8122	LIF,AMY2A,ANPEP,BDNF,CADM1,COL5A3,COQ6,EGLN3,ENO1,FBXW11,GRIN2A,IL20RA,NEUROG3,NFKBIE,NRG2,NT5C1A,OR2C1,OR52E6,PFN1,PGAM4,PIGH,PIK3C2G,PPAP2A,PPAT,PRKAR1A,RARS2,RFNG,SMAD1,TRIP10,WNT10B,ALDH18A1,ATP6V0D1,CTNNBIP1,TNFRSF17
Negative_8123	CXXC4,FMO2,MGLL,PRKACA,RGS3,SAT2,TAS2R5,TSHR
Negative_8124	CBS,CR1,EGF,IVD,NNT,ACACB,ADH1B,ALDH1L1,APPL1,B4GALT6,CCL22,CDKN2A,FGF4,FGF9,FIGF,FZD8,GCNT2,GPT2,HADHB,HSP90B1,IARS2,IBSP,KLKB1,KLRK1,MCCC2,MTMR6,MYH3,NAT6,NKX3-1,OR2F1,PAPSS1,PIK3C2B,PTGES,SEMA3B,SEMA4B,SKIV2L2,SQLE,STX4,TLN2,ZMAT3,ATP6V1G2
Negative_8125	ME1,NOG,VCL,ALG1,CACNG4,CDK2,CLDN1,CLDN3,CYP2U1,FGF5,GALNT3,GLUD2,HARS,HMGCS2,IRF7,LRP6,NME4,OR14A16,OR2V2,OR4X2,UCKL1
Negative_8126	HAL,RDX,ACVR1B,ALG3,APOA5,ARD1A,B4GALT3,CACNG6,CCL27,CDC2,CHRM2,CMPK2,COASY,CXCL9,CYP4F3,DDIT4,ENPP1,FCGR2B,GFAP,GJD2,ICAM1,IL1R2,IQGAP3,MAPK13,MTMR2,MYLK,NEUROG3,OR1L3,OR51B4,OR52E4,OR52R1,OR5M3,PDE9A,PDGFRB,PGAM4,PLTP,PRKAG1,PVRL1,RRAS2,SGPP1,SLC2A1,SSX2IP,TSHB,TUBA4A,VAV3
Negative_8127	AKR1B1,ALPL,CLDN14,CLDN9,IL6ST,NLGN3,OR10V1,OR4K13
Negative_8128	PLN,ACADSB,CHSY1,CX3CL1,CYP3A5,DDX4,DHCR24,DUSP16,ENTPD3,EXT1,GNAT3,GSK3B,HSPA1B,HYAL1,MAD1L1,MADCAM1,MAN1C1,MYH2,OR6X1,OR7D2,PARD6A,PIK3C2A,PTGDS,SEMA3E,SPRED1,TAS2R9,TNFSF14,TNXB,TSNARE1
Negative_8129	DUT,F2R,GPI,HAL,RFK,ACAA1,ALDH9A1,ATP6V0C,B3GNT5,BET1L,CSNK1G2,EFNA2,FTMT,GNAI3,GUCY1B3,ITPKA,JAG2,KCNQ1,OR13D1,OR2H1,OR5A1,OXTR,PDK1,PIGP,PRKAG1,PRKCE,PTPRB,RAB5A,SDHD,SSX2IP,TGFB3,UPP1,CSGALNACT2,HLA-DQB1,TNFRSF1B
Negative_8130	FES,IVD,ACADSB,ACAT2,ARHGAP5,CAV1,CDH4,CMAS,CYP2C8,DGKA,EPHB3,FGFR2,FOSL1,FZD10,IDH3G,IL20RA,MYL5,OR5V1,OR8I2,PPP2R5C,SEMA3B,SIGLEC1,SLIT2,SUFU
Negative_8131	ACTN1,ATP2A3,C4BPA,CDC7,DTX2,GPAA1,IFIH1,INPP5E,LAMB3,OR1I1,OR51F2,OR51I2,PRKX,SPTLC1

Negative_8132	C4B,FES,GLS,ACTB,AGRN,ALDOB,ARHGEF2,ATP5H,ATP6V0C,CAV3,CCL26,CD58,CHAD,CLCF1,CXCL2,CXCL5,DPM2,DPYS,DUSP9,ENO3,EPHX1,FGF11,FTMT,GPAA1,INPP1,MAP2K1,MAP3K12,MCEE,NKX3-1,NPR2,OR10H2,OR2T3,OR4N5,OR5T3,PER2,PIK3C2A,PPP2R5E,SPHK2,SQLE,SUV39H1,UCKL1,VEGFA,WWP2,XIAP,XYLT1,PRICKLE1
Negative_8133	EZR,ME1,XDH,AKR1B1,AMPD1,CALML5,COL5A3,CTNNA2,ENO3,FGFR2,FUT6,HLA-DOB,IAPP,IL13RA1,IL17A,MAP2K1,MYH14,NAGA,NLGN1,OR10G3,OR14I1,OR14J1,OR4K2,ORC4L,PCNA,PDE2A,PIM1,PIP4K2B,PPA2,PPAP2B,PPM1A,PRNP,SDC2,TAS2R48,UROD,VEGFA,WASF2,WHSC1,HLA-DQA2,PNLIPRP1,PPP1R12A,PRICKLE2
Negative_8134	AR,FGB,GK,ATP5L,BRAF,CCL22,CNTFR,CTNND1,DCXR,EGLN1,EIF4E,GALNT14,HIBADH,IL17RB,INPP5A,ITGAL,MAP2K1,MMP14,NANP,NEFM,NTF4,OR10G9,OR12D3,OR13C9,OR13F1,OR14I1,OR1N1,OR4B1,OR6N1,OR9K2,ORC6L,PARD3,PDCD1,PIAS3,PNPLA4,RGS3,RHOQ,SLC33A1,SUV39H1,UGCGL1,UGT2A1,PHOSPHO1
Negative_8135	PVR,TPR,ACOX1,ALAS1,APPL1,CLCA2,DPM1,DRD5,DUSP3,FLT3,GALNT12,GPAM,GUCY2D,HAP1,IL29,IRF7,LAMC1,MYH7,NKD1,OR8J3,PCCB,PIGU,RDH8,SAT2,SRGAP1,TAS2R1,HLA-DRB5
Negative_8136	FYN,KDR,SPN,CD58,CDKN1B,CDO1,CNTNAP1,DHRS4,EFNA5,ENTPD2,FGFR2,FZR1,HHEX,IMPDH1,ITGB8,NEU1,OR10X1,OR1C1,OR5P3,PAPSS2,PIGB,POLR3D,PTPN11,ROBO3,SNAP25,STX4,TSLP,WNT6,PPP1R12A,ST6GALNAC1
Negative_8137	A2M,CGA,FGA,PLG,ACAA2,ACADS,ASMT,B3GNT2,CCL15,CCND2,CKS1B,CSNK1G1,CYP3A5,DGAT2L4,ENTPD7,FADD,GALE,IL11RA,KAT2B,KLK3,MAN1A2,MAP4K1,MCCC2,OR1A1,OR1N1,OR51Q1,OR51T1,OR5F1,PCK2,PIM2,POLE3,PPARD,RALGDS,RIPK1,SC5DL,SDC3,TOMM40,XCR1,MAP3K7IP1
Negative_8138	ADK,C5,ID2,LCK,TAT,UBC,ACVR1,ADH7,ALDH1A3,CREB3L2,CYP21A2,EFNA4,GALM,HSP90B1,IFNA6,INPP1,IRS4,LTBP1,MAP2K3,MYL5,NLGN2,NRG3,OR10A4,OR2S2,OR52N5,OR5K2,OR7G3,PGAP1,PIK3R4,PLEKHA8,POLR3H,RFXANK,SENP2,SYNJ1,TLR2,TUBA3E,TUBAL3,MAPK8IP3
Negative_8139	TFG,AASS,ABCC8,ACOT12,ACTC1,AKR1C3,ATP6AP1,BHMT,CDK5,CMPK2,COL5A3,GMPR2,GYS2,IFNA13,IL1A,ITGAE,LYPLA1,MAP4K1,MYL5,OR10K1,OR4K2,OR8K5,ORC1L,PIGA,POLR2K,PTGDS,PYGL,SARS,SLC2A2,SPTLC1,ATP6V1E2,SUV420H2
Negative_8140	C9,ALG2,CHRM2,DOLPP1,FLT3LG,FUT2,FZD8,HGSNAT,ICOSLG,IDH3A,ITGA6,KARS,KLRC1,LCP2,LGMN,LTBP1,LTBR,MAP2K2,MYH10,MYH11,NEUROG3,OR10S1,OR10V1,OR6C6,P2RX5,PIAS1,PNPLA4,PPP1R1A,ROBO3,RPS6KA6,SRD5A1,TBK1,TSHB
Negative_8141	TXK,ALPL,AMPD2,CKMT2,DGAT1,DIAPH3,IRS2,JAG1,MAP2K6,NARS2,OR13C9,PYGB,SLC27A4,SRGAP1,YWHAZ,PAFAH1B1
Negative_8142	C7,FGA,PVR,TKT,TPR,ADCY6,BMPR2,CD48,CDC27,CPT1A,CX3CL1,CYP2S1,CYP3A5,DAD1,DPYSL2,DRD2,DUSP3,EDNRA,EIF4E2,EPB41,EPHB6,FADS2,FGF23,GALK2,GNA11,HLA-DOA,HTR6,IL10RA,IL15,IL28B,ITGB6,MAP3K14,NEU1,NMNAT1,OR2C1,OR5H6,PGAM2,PIAS3,PIGN,SAT1,SETD1A,SMAD2,SPRY4,TUBA1A,TUBA1B,UROS,CACNA2D3,HLA-DRB4
Negative_8143	MVD,ACOT12,ADRA1B,ALG9,CCL28,CD80,CDH3,CSF2,CXCL3,DTX2,E2F4,EFNA4,ENTPD5,FGF10,GALNT14,GCNT1,GNA13,GNA15,GSTA2,MAN1A1,MAP2K7,MGST3,NCK1,NEFL,NR4A1,NRP1,OR11L1,OR2A25,OR4K13,OR5AK2,PARVG,PGAM2,PHKA2,PIGT,PIP5K1C,PNMT,POLE4,PPM1A,PRPS1,RBX1,TUBB2A,UGCGL1,YWHAE,ATP6V1G1,SERPINC1
Negative_8144	CPT1A,CRHR1,DHCR24,EGLN3,ENO1,LALBA,OR52D1,PDHA2,RPS6KB1,SMPD1,SERPINF2

Negative_8145	AGK,C4B,ATP5E,FUT5,JUND,OR5B12,OR5H2,SNW1,UGT2A1
Negative_8146	BCR,CR1,VTN,ADIPOR1,AOC2,ARNTL,BUB1,CACNB3,CFLAR,CHRM2,CSF2RB,CXCR4,DDX50,DNMT3B,EXTL3,FGF3,FGFR1,HAAO,HSD17B7,IFNA14,IKBKG,IL21,MAT1A,NARS,NFASC,OR10J3,OR14A16,OR4D1,OR4M2,OR5T3,PDE9A,PITX2,POLR2H,PRKCE,PRLR,RAP1A,SELPLG,SMAD9,SULT2A1,THBS3,TP53I3,UBE2L6,CACNA2D3
Negative_8147	GHR,GLS,MIF,MVD,TNN,AACS,ACSL1,ACSM1,ACTB,ALAS2,AOC3,CACNA1F,CDKN1B,CHIA,ECHS1,GALE,GALNT9,GMPR,GNRH2,HEXB,IDI1,IL20RA,INADL,ITPKB,KLRD1,MPST,MYH10,NFASC,OR10G3,OR10G8,OR11H4,OR4A16,OR4S2,OR56A1,OR5L2,PIGA,RGS3,RPSA,SLC33A1,SMAD9,STX1B,SUOX,TFPI,UMPS,WASL,GABARAPL2,MAP3K7IP1
Negative_8148	AK5,GP5,LEP,ACVR2B,ADCY3,AKT2,ALG9,CNGA3,CREB5,CRLF2,DDX51,ENTPD8,FBP1,FCGR3A,FGF20,FOXA2,FUT3,GATM,HLA-DMA,IFNA13,IL23A,ITGAX,MDH1,MDM2,NCAM2,OR11H1,OR4E2,OR8J1,OXSM,PDE8B,PTPMT1,PYGB,SDHD,SMAD9,SMARCA2,TSTA3,ATP6V1C1,PPARGC1A
Negative_8149	AR,AGPAT2,AGPAT4,CACNA1G,CD38,EVI1,EXTL2,FZD6,FZR1,GALNT5,GBA3,GLI1,GRIN2A,GRIN2D,JAK1,MCCC1,NOD1,OR9Q2,PIK3C2G,PLAT,PPP1R3B,PPP2R5C,SMURF2,ST8SIA1,STAT5B,SULT1A2,SULT1E1,TYMS,WNT10A,YWHAB,YWHAG,SERPINC1
Negative_8150	C7,HYI,LPL,TH,ACOT11,ACP2,ADAM17,AGPAT2,APOE,ARSD,CDK5,ERBB3,FGF23,FGFR3,FZD3,GNAI1,GRIN2B,IL1A,ITGA3,ITGAX,LAMB2,MTAP,OR10A5,OR10C1,OR1E1,OR4A15,OR4K13,OR5D16,OR6B3,OR6C76,OR6M1,PLIN,PPT1,RASGRF1,RDH10,TACR1,XYLT2,PDCD1LG2,SERPINB5,SERPING1
Negative_8151	GNS,OGT,PLN,ARHGEF2,ARNT,ASMT,BCMO1,CD47,CHSY3,CYP7A1,DDOST,DHRS4L2,EPHB2,FCGR2B,FRAT1,GSTA2,ICOS,INHBC,MAPT,OR4K15,OR6C4,RAPGEF3,RFNG,SIAH1,TICAM1,TJP2
Negative_8152	IL4,AGRP,ASCC3,CAMKK1,CHST12,F11R,GNB3,INPP5B,MGLL,NFKB1,OR11H6,PPARD,RPS6KA3,TMLHE,TUBB2B,YARS,TNFRSF10C
Negative_8153	AK3,C5,DBI,F7,FAS,FOS,ACSS2,ADCY3,CCR10,CCR8,CD274,DDX51,DRD1,FGFR2,GUCY1A2,HAAO,IL3RA,KIR3DL1,LDHAL6B,MADCAM1,NCKAP1,NEU4,OR4N5,OXCT1,PITX2,PMM2,PMVK,POLR2I,PPP1CC,ROBO2,SH3BP2,SPTAN1,STK4,WNT10B,HSD17B10,PDCD1LG2
Negative_8154	ACSS1,ATP4A,BCAR1,CYP2A6,FGF9,FLT3,GDF7,GLI1,GSTO1,HARS,IFNG,LGMN,MDM2,NME2,PAX8,PGS1,PHKG1,PLAU,PLCG2,RARS,SAT1,SHMT1,TRPC1,MAP3K7IP2
Negative_8155	TPR,CRY1,IL1RAP,KYNU,MPP5,POLR1E
Negative_8156	VTN,FDPS,GUCA1A,HSD17B7,INSRR,NFKBIE,PLAU,RDH10
Negative_8157	SDS,CAMKK2,FOSL1,GADD45A,GNA13,GUCY2D,IFNA13,OR5B3,PLOD1,PPP2R2A,RALA,STX18,TLR9
Negative_8158	ACYP2,ARPC1A,ARSB,ATP5F1,BBC3,CD28,CD55,CDKN1C,CES2,CHD8,CHST13,CREB3L1,CXCR6,CYP26B1,EDAR,FGF17,GLDC,GSK3B,HEMK1,HNF4G,HTRA2,IL15RA,JAK2,JUND,KIR2DS4,LDHD,NT5C1B,OR10R2,OR2G6,OR4D5,OR56A3,OR5D18,OR5K1,OR6C6,OR7G1,OR9I1,PDE2A,PLAU,RHOD,SMAD7,SPP1,STAT6,TBL1XR1,TNFSF12,TUBA8,WASF3
Negative_8159	KHK,AQP7,CALML6,CMPK1,CSF2RA,CXXC4,DDX56,GCNT4,HAP1,HSD17B1,ITGA8,LAMC3,OR13A1,OR14J1,POLA1,PRKCQ,PTPRB,RASSF1,SIAH1,TOMM40L,WIF1,PDCD1LG2
Negative_8160	HK2,IL5,NNT,AADAT,ACTN4,B3GNT2,CD40,DOLPP1,EDA2R,FABP1,GNRHR,HNMT,IFNA7,IRF3,LIMK1,MAP2K4,NUMBL,OR10V1,OR52E2,OR5AP2,ORC5L,SLIT2,TACR2,UGDH
Negative_8161	RDX,C1QB,CACNB4,CFTR,FADS2,MAP2K4,MAT2B,OR2F1,OR52A1,POLR1E,PPP2CB,RAPGEF2,SHISA5,TNFRSF8,TSTA3,WNT6

Negative_8162	COL5A3,DNMT3L,EDNRA,GJA1,GSTZ1,GUCY2F,PERP,TAS2R5,TAS2R7,XIAP,SERPINC1,TNFRSF10D
Negative_8163	ME1,NPY,AMPD3,BHMT,CACNA1I,CASP10,CMBL,CTBP2,CYP2D6,DGKE,INMT,MYH7,OR5K2,PFN3,POLE4,PYCR2,ROCK1,SHISA5,SOAT1,ST8SIA5,SUCLG2
Negative_8164	FER,FUK,ACO2,ALDH3A1,CCL7,CCL8,CEBPA,E2F1,EPHA7,EPHA8,FTMT,FZD9,G6PC,GUSB,HARS,HSD3B1,ICAM1,IL28RA,IL7R,ITGA3,OR52B6,PDE1A,PDE1B,PDE4C,PDGFRB,PIP5K3,PLOD1,PNPT1,RENBP,SHC4,SMAD1,STX10,TNFSF12,TSC1,UNC5A
Negative_8165	CCR9,CLDN18,CLDN6,HNF1A,OR6X1,SMAD7
Negative_8166	ATM,FES,UBC,AGPAT2,AKR1B10,B4GALT6,CCL3,GALNT3,OR10K1,OR1E2,OR1K1,OR2T29,OR4X2,RDH12,RFX5,RUNX1,SESN3,SSH1
Negative_8167	ACSM4,BLVRB,CCL5,CD44,CD82,COQ5,DVL2,EPB41L1,FMO3,INSR,MTAP,NEGR1,PIGC,VAV1
Negative_8168	CFI,ID3,LSS,ALOX12,CLCA4,PGK1
Negative_8169	FH,MET,NOG,VCL,ACADVL,ALOX5,ATIC,CACNA1B,CCL17,CD247,CES2,COX15,DLL3,ELK1,GALNT12,GDF5,GSTK1,HCST,HNF1B,HTR4,HYAL2,LAMB4,LYPLA2,MAPK11,MARS2,NEFL,NUDT2,OR4X1,OR52N4,OR5AK2,OR5J2,PARD3,PDE8B,PKM2,PLCZ1,RDH12,RFXANK,RXRA,SMPD2,TAS2R7,THBS1,UBA7,VTI1B,ATP6V1E1,TNFRSF17
Negative_8170	JUP,LIF,MUT,AKR1B1,ALG8,ANAPC10,ATF4,CDKN1B,CES2,CRKL,CTTN,CYP8B1,DPM2,GNG13,HDAC2,INMT,ITGA1,LMO7,MALT1,OR2T5,OR4D9,OR5D16,OR6C1,PARVA,PDE6D,PROC,PVRL3,SHC4,SULT1A1,TCF7L1,TRAF3
Negative_8171	C9,DHH,GPI,ACP6,AGPAT3,AKT2,ANAPC2,BAIAP2,BCMO1,CCL26,CCR3,CD44,CLDN3,CTF1,DUSP1,F13A1,FABP1,GDF6,HIBCH,IL11,IL21R,IL4R,LPAR1,NADSYN1,NFATC4,NKX6-1,OR1G1,OR51A7,OR5B17,OR5D14,PAPSS2,PDE4D,PIK3C2A,PLA2G3,POLR1D,RAPGEF1,RPIA,STX1B,SULT1E1,TAS2R13
Negative_8172	FUK,ME1,UBB,AREG,CLDN14,DHDDS,GLUD2,HSD11B1,LIPT1,MLYCD,MYD88,NLGN3,OR2M7,OR51A7,OR52N4,PDGFB,PIGS,PRNP,PTGS2,SEMA4B,TIRAP,WBSCR17,YARS,ZIC2
Negative_8173	DCK,ADIPOR1,ASMT,COL5A3,ECHS1,GABARAP,HSPB1,ICAM3,NFKB2,OR1A1,OR4P4,OR5D18,P2RX1,PIM1,PPARA,PRDX6,TRMT11,PNLIPRP2
Negative_8174	CACNB1,CALML5,FUT9,GGT1,HHEX,IL1R2,OR8K1
Negative_8175	GGH,ARSB,BCMO1,CDKN2A,CXCL3,CYP2A6,FCGR3B,ITGB1,JAK1,MAP4K4,MAPK10,OR6C70,OR7C1,PDE6H,POLE4,PROC,PTGER3,RCHY1,ST3GAL1,ST8SIA1,TBXAS1,UBA1,UGCGL2
Negative_8176	CD2,CRK,IDS,PTS,ABL1,ACADSB,ADORA2B,ALAD,ALG6,ATP5I,BET1L,CD244,CD40,CFL1,CXXC4,CYP3A4,EDNRB,ESPL1,EXTL3,FIGF,G6PC,GALC,IFNW1,INPP1,LDHA,MAPK13,NAGK,NSDHL,ORC1L,PANK2,PGS1,POLE2,PTGFR,PTPRC,RPIA,TAS2R40,TYK2
Negative_8177	CTH,NPY,MAD2L2,OLAH,PDE1B,RRM2B,SMARCA2
Negative_8178	STS,ANAPC10,COL3A1,GABARAP,GLYCTK,LTBR,OR14J1,PDE4B,RPS6,RPS6KA6,TPI1,HSD17B10
Negative_8179	CBS,F2,ACHE,ANAPC10,CHKA,DOLPP1,EPHX2,OR1A2,STK11
Negative_8180	AR,CBS,ATP5E,CCR5,CDC42,CTTN,ETNK1,GSTM5,H6PD,HAO1,ITGAX,MAP3K12,MYH7,NARS,POLR2D,PRKAR1A,PRKG2,SEMA3G,TAS2R49,PPP1R12A
Negative_8181	ANPEP,APBB1,ATG7,BACE2,BMPR2,CPT2,CTNNB1,DPM3,EPOR,ERBB3,GANAB,GNG12,INPP5A,IQGAP3,MAD1L1,MAP3K14,NAGA,NCF4,OR2L8,OR3A2,OR4M1,OR5K1,OR8B12,PIGP,POLE3,PON2,PON3,PPP1R1A,PRKCI,RCHY1,SELP,SQLE,TAS2R50,TJP3,TSNARE1,UCK1,VANGL2,WASF2,WNT7B
Negative_8182	ADRA1D,EDNRA,IFNA21,OR51M1,SELL,ST3GAL3

Negative_8183	F12, AMDHD2, DEGS1, FGF12, IL18, MTMR1, OR4F5, SDC1
Negative_8184	AK7, BCR, GK2, ID4, IL5, SI, TK1, AGXT2, ALDH4A1, ARSB, ATP5E, CCL24, COL4A4, CX3CR1, DDX50, ENPP1, GALNT7, GIT1, GOSR2, HTR5A, IL1R2, IL20, INSR, ITGB7, KIR2DL3, MAPK1, NEU1, NMNAT2, NT5M, OR10J1, OR52A1, OR52R1, OR5K2, OR7D2, P2RX2, PIK3C3, PLCD1, PLXNA2, PNPLA4, PPBP, PRDX6, PRPH, PTAFR, PTGS1, PYCR2, SGMS2, SPCS1, TFDP1, TRDMT1
Negative_8185	GGH, GPT, MPZ, ADCY6, AKT1, APPL1, CALML6, CHIT1, CSF2RA, DCXR, DGAT2L4, EPRS, ETNK2, EVI1, FGF2, FGFR1, GALNTL2, GPAM, HAGHL, LDHB, MAD2L2, NOTCH2, OR51G1, PER3, PIK3R4, PRKCD, PSENEN, RPS6KB2, SNW1, SPCS3, THTPA, ZIC2, ATP6V1B1, ATP6V1B2, CACNA2D2
Negative_8186	DCT, FYN, HGF, ALDH3B2, ASCC3, ASIP, ATP5G3, AXIN2, CDKN2B, CHST11, CXCL12, CXCR5, DDX19A, ETNK2, EXT2, GNG13, GOSR2, GRB2, HSD3B1, ICAM1, IFNA10, IGSF5, IL10RA, IL8RB, KIR2DL2, KYNU, MMP9, NFATC1, NFKBIA, NODAL, OR4F4, OR4K1, OR8K1, PCCA, PGAM4, PI4KB, PNPO, POLR2F, PRKCA, ULBP2
Negative_8187	DCT, EZR, AACS, ACTG1, ADCY7, ARSE, B3GALT5, CASP3, CASP7, CLCA2, CRHR1, CYP4A11, DDX55, EHMT1, ERBB3, FGF10, FRAT2, GALK2, GDF5, GNA11, IGF1R, IKBKE, LDHB, NKD2, OR2T1, ORC4L, PIGS, RHOD, RPRM, SEMA3B, MAPK8IP1
Negative_8188	ACVR1C, ADRA1A, ARHGEF2, CD28, CHEK2, COL6A2, COQ7, DAXX, DERA, GPAM, GRHPR, IL6ST, ITGA2B, ITGB5, LHCGR, MAGI2, MAPK3, OR51S1, PAK1, POLE2, SOCS2, SPP1
Negative_8189	CRK, HK2, IL9, KDR, NLK, AMY2A, ARPC1B, B3GALT4, CBLC, CCL8, CCNE1, CDK6, CFTR, CLCF1, CLTC, DERL1, DGKD, EDA2R, FABP1, FUT6, GLYCTK, GNA15, GOT2, IL21R, IMPDH1, LAMB4, MBL2, MCEE, MTHFD2, MYL5, NT5M, OR10K2, OR1J4, OR52I2, PNPT1
Negative_8190	AADAC, CASP6, CDC14B, CYP2J2, DDIT3, FOXO1, FZD8, HPRT1, KLRC2, MCCC2, OR4F17, OR52N1, OR5L1, PAK2, POLE2, POLE3, TNFSF11, UMPS
Negative_8191	SYK, AHCY, AMPD3, CAMK4, CLDN17, CSF1, DGKE, DLAT, GALNT1, LALBA, NME4, PER2, RENBP, STX7
Negative_8192	AK7, ACP6, ASIP, BDH1, GPAA1, KCNB1, KIR3DL1, NCF1, OR2M3, OR8D4, SMAD4, TLR2, HSD17B10
Negative_8193	BTC, ID4, MAX, ADAM17, ATP5J, DTX2, EPAS1, EPHX2, ESPL1, GALNT7, GCLC, GLYCTK, LEFTY1, NR5A2, OR2A4, OR3A3, PARVA, ROBO2, SLC27A4, SOCS7, SSX2IP, WNT5B
Negative_8194	ACVR1C, NUMBL, ULBP2
Negative_8195	BTD, DBI, LCT, PGP, TFG, ADORA2B, CD46, CSAD, EIF4E, FRAT2, GMPS, IDUA, IFNA21, IL10RA, MAD2L2, NFAT5, OCLN, ONECUT1, OR10G7, OR2T12, OR2T33, OR4C15, P2RX5, PDK1, PLA2G3, POLR2L, PPP2R1A, SOX17, ST3GAL1, ST3GAL5, TAS1R1, TAS1R2, TJP3, TUBA1C, TUBA3C, YKT6, ATP6V1E1, PPARGC1A
Negative_8196	DBT, PML, SYK, NRAS, HLA-DPB1
Negative_8197	CADM3, CASP6, OR2H2, OR4K14, OR51B2
Negative_8198	CSK, GP9, AKR1D1, BLVRA, CACNA1I, CCL16, CCNG2, CD40LG, CDC45L, COL4A6, CPOX, CXCL3, DDOST, F11R, F13B, FZD6, GRIN2B, GSTT1, HRAS, HSP90B1, ITGB8, KDSR, MAGI2, OR2T11, OR6F1, PFN4, PGAM1, PIGO, PLCD3, PRKAB1, PRKAB2, PYGB, RENBP, STT3B, TNFSF10, UGCGL2, UNC5C, UPB1, PAFAH1B1
Negative_8199	DAXX, MAD1L1, OR2T33
Negative_8200	ADSS, ATP2A1, ATP5A1, CACNA1E, COL5A1, CYCS, DDOST, G6PD, GMPPB, IFNA10, IFNGR1, KAT2B, MAP2K7, MCM5, OR10S1, OR2K2, PAX8, POLR3H, RDH8, SAT1, SEMA3B, TM7SF2, VAMP4, WASF1

Negative_8201	ADRB1, ALG8, BLNK, CYP2A6, DAD1, FLOT2, GADD45G, GZMB, HYAL1, MAGI1, MKNK1, NTN1, OR2G2, OR4F4, PARVG, PLXNA1, PRKAB1, RAC3, RFXAP, TICAM2, TPH2, YWHAE, PHOSPHO1
Negative_8202	SRF, CPT2, CTBS, FLT3, G6PD, H6PD, IFNA14, ITPA, KIR3DL3, MECR, NRCAM, OR11L1, OR1A2, OR1S2, OR9Q2, PCK2, SNAI1, XYLB, SERPINB5
Negative_8203	CFH, ZYX, ATP5G3, ATP6V1D, DUSP1, FTH1, IL5RA, IMPA1, PLCB2, SULT2A1, TGDS, ATP6V0D1
Negative_8204	ACAA2, ALG10B, CPT1C, GRIN2A, HDAC1, PAFAH2, PHPT1, PIGK, RDH11, RFWD2, STAT6, TNFSF10, XYLT1, TNFRSF10B
Negative_8205	CRH, ERBB2, NCAM2, UPRT
Negative_8206	F9, ARSB, ATP6V1A, BCAT1, BIRC2, CLDN2, DBF4, DFFA, DRD1, FGF6, GALT, GUCY1A3, HTR2A, IQGAP3, MAML3, MAP3K3, MAP3K4, NCF1, NRXN1, OR2AG1, OR2H2, OR4A16, OR51T1, OR7C2, PYGB, RFX5, RRM1, SMURF1, ST8SIA1, STX16, TSNARE1, VTI1B, WIF1, MAPKAPK3
Negative_8207	FYN, ID3, MVK, NLK, PML, ACSL6, ADCY1, ALPL, BLVRA, BMP8A, CACNB2, CD34, CDH15, CLDN7, DFFA, DRD1, DTX2, ENTPD6, FGF2, FLT4, FMO2, GALNTL4, GSTM3, HSD17B1, IDH2, INADL, IRAK1, KLRC3, MITF, MYL7, OR13D1, OXCT2, PLTP, PRKAR1B, SMPD3, SPRY4, SRD5A2, SRGAP2, VAMP4, ATP6V0D1
Negative_8208	AK3, DCC, MPZ, PC, RET, CCDC6, CCL5, CD34, CTBP1, CYP1A1, DERL1, DGAT2L4, DGKB, GBGT1, IL3RA, IL9R, ITGA1, KLRK1, NEFM, NMNAT2, OR2H2, OR2T33, OR4F6, OR51B2, OR52E4, OR6C2, OXSM, PAK7, PIAS2, PIAS3, PLCB3, PSME3, SMAD5, STK11, TDO2, TICAM2
Negative_8209	PLG, PLN, RFK, ACP1, ALDH3B1, CD47, DGKD, DKK4, DVL3, EPHA7, GALNTL5, GBGT1, GNA14, HMGCS2, IL1R2, IL6R, MASP2, NRXN2, NT5C, OR8J1, POLR2B, PPP1R3C, PTPN7, TAS1R3, TYMS, WNT2B, PPP1R12A, TNFRSF10A
Negative_8210	AKR1D1, CACNA1A, CDK7, CLCA4, COL6A1, GNRHR, MICA, OR1F1, OR2G2, PLCD3, UPB1, WNT7A
Negative_8211	ADO, HGD, LTB, MPI, AMPD2, CCDC6, CCR5, CDC25B, CHIT1, CYP4A22, DHDH, ENAH, FGF11, GALNS, GGT7, GLO1, GNA11, GRHPR, GSTA4, GSTM4, GUK1, HAAO, MTHFR, NCF2, NMNAT3, OR10G9, OR10J5, OR4K1, OR4L1, PLA2G10, POLD4, PRKCH, RFXANK, SDHB, SLC27A4, SNAI1, SRGAP1, SUCLA2, YWHAB, ATP6V1E2, GABARAPL1
Negative_8212	ACMSD, ALG8, APOA1, ARNTL, ARSE, ATP5A1, ATP6V0B, CACNB4, CASP9, CCL17, CDC25A, GNA14, HDAC2, IKBKG, IRS4, JAG2, OR10H5, OR10K2, OR1K1, OR2AT4, OR5D13, OR5K1, OR6C70, OR7A5, PNPLA4, POLR3B, RHOA, SULT1E1, TAS2R48, UGT2A3, UPP1, WNT11
Negative_8213	MVK, NNT, B3GALT5, CBLB, HSD17B3, HSD17B8, MAFA, NEFH, NFKBIB, OR2D3, SPCS3, SPRY3
Negative_8214	ADC, APP, ACPP, ATP12A, BMPR2, CHRM1, CNTFR, COX10, CYP19A1, CYP2J2, DVL2, FADS2, FDFT1, FMO2, GRIA1, IL28B, INPPL1, NAE1, OR1L8, OR2D3, OR2T34, OR52E4, OR52J3, P2RX4, PDHA1, PYCRL, RPS6KA4, SPRY4, SULT1A4
Negative_8215	C5, LEP, AOX1, ATP5G2, CD28, CD38, EARS2, FGF12, FGF18, FMO5, GALNT7, GNPDA2, IRF5, MGAT3, OR51F2, POLE4, PTPRF, SETD7, SLC2A1, TLR4, TNFRSF9, HLA-DRB4
Negative_8216	ADORA2A, ADORA2B, ATP6V0C, CES7, CHST11, CNTF, CPS1, CXCL5, CXXC4, FOXA2, HPSE, IFNAR2, IFT57, KCNQ1, KRAS, LDHB, LIAS, MAOB, MGAT5, NR4A1, OR13C8, PIGL, PLD2, POLR2J, PTGS2, QARS, RASGRP4, SEMA4D, TACR1, TAS1R1, TGFB1
Negative_8217	CCR3, CD244, LEF1, MCM2, OR2T1, TCF7L2
Negative_8218	EFNA1, GP1BB, IL12B, IL23A, ROBO2
Negative_8219	PLN, ACAD8, ACP5, CCNG1, CYP17A1, DLL1, ENTPD7, FABP1, IFIH1, IL21, IL24, NARS, NKX3-1, NRG2, OR3A2, OR4K15, QPRT, SEMA3C, WHSC1

Negative_8220	ACTN4,AKR1C4,ATP2A1,BCL10,CACNA1F,CACNG3,CAMKK2,CCL19,CCNA2,CDC25B,CDK2,CLDN10,CYP3A4,DCTD,DCXR,DVL1,EFNB3,ENTPD1,GGCX,HYAL2,ICOS,IL22RA1,IL23A,ITGA4,MAT2B,MKNK2,NRG2,NUDT5,OR2AG1,OR4A5,OR4D9,OR4F5,OR52N4,ORC6L,PDGFRB,PLOD3,POMC,ROCK1,TUBB3,UGT2B28,VAMP3,XYLB,AASDHPPT
Negative_8221	AGPAT1,ATP5G1,ATP5G3,BBOX1,CD36,CDS1,DFFA,ELK4,FGF22,HSPA8,KLRC2,OR4D2,PIK3CA,PLA2G3,PNPLA3,STT3B,UNC5A
Negative_8222	FGG,TK1,UBB,ARSA,CACNA1S,CYP4F3,FABP4,GALNT2,HIBCH,MAP2K6,MAP3K8,MAPK12,NUMBL,OR10G2,OR2H1,OR5K1,OR7A10,PDGFD,PIGU,PPP2CB,RBKS,RRM2,TJP2,TSLP,UGT2B17
Negative_8223	FN1,HK1,ANAPC2,CACNG1,CLDN16,CYP3A43,DDX4,DLL4,ECHS1,ENTPD6,FGD1,FMO1,FUT5,GMPS,GNAI1,HADHB,KLRD1,MAGI1,OR13C4,OR1M1,OR2M3,OR2T10,PHGDH,PLXNB2,POLR2G,PRKAB1,SLC27A5,SLC9A1,TRAF1
Negative_8224	IL4,ATP6V0C,C9orf95,CD34,CDS1,CLDN11,CLDN3,COL2A1,ENPP2,EXTL3,FGF7,IDH3A,IMPA2,INHBE,MYH4,NCKAP1,NMNAT2,OR2L8,OR4D1,OR5H6,PDE1B,PDGFRA,PIK3CD,PNPT1,POLR2I,RFXAP,SLC2A2,SMC1A,TP53,VNN1,HLA-DQB1
Negative_8225	DDIT4,OR6C76,PTK2,TNFRSF11B
Negative_8226	ADO,GNS,ALPP,APH1A,CD99,CYP11B2,EPB41L1,FBP1,FTMT,GSTM2,IL25,ITGA11,KIR2DS4,OR10G7,OR5A1,PARD6B,PHKG2,PIK3R3,SEMA4F,SUV420H1,TNFRSF11A
Negative_8227	ALS2,ANAPC4,BDKRB2,CRKL,ENO3,ENTPD1,GALNT9,HPSE,HSD17B8,IRF3,LIMK2,NCKAP1,PDXP,PIK3CD,SMAD4
Negative_8228	FGG,SP1,TKT,AGPAT6,ATP6V1H,B4GALT1,C1QA,CDC20,CSF1,DHDH,FZD7,GGT7,GRIN1,GUCA1A,HSPA8,IFNA8,IGF1R,LAMC1,MAGI1,MDH1,NFKBIA,OR2C3,OR56A4,PGK1,PLD1,PPP1CC,RPSA,SYNJ1,UBE2K,YARS2
Negative_8229	ACADS,CAV2,FGF21,GNMT,IARS,ICOS,ITGB7,MGLL,OR1E2,OR1I1,PDE4D,PLA2G2E,RXRA,SMARCA5,ALDH18A1
Negative_8230	AGPAT3,ELK1,GADD45G,NOTCH3,UMPS
Negative_8231	ABO,BTK,F12,MPL,NGF,TK2,AANAT,ACOT11,ADH1B,ALDH6A1,ARHGEF4,BCKDHB,BUB1B,CACNG4,CFTR,CLCA4,CYP2D6,G6PC2,GNRH1,GUCY2D,IRAK2,IRAK3,LDHAL6A,LDHD,MAP4K4,MAPK3,MYH14,NCSTN,NKX3-1,OR2A14,OR51A7,PIGH,ROCK2,SORBS1,SPRED1,TPH1,WNT3,ATP6V1E2,ST6GALNAC3
Negative_8232	DCK,F8,FES,ACOX3,ACSL1,AGPAT6,ALG6,AMPD3,BBOX1,BNIP1,CACNA1G,CASP9,CCR9,CDH3,CYBA,DHODH,DHRS4,DUSP2,EDNRB,FGF21,INHBB,LAMC2,MAT1A,MGST2,NKX3-1,OR10J1,OR2D3,OR4X1,OR51I1,OR5B12,OR6B2,PRKAR2A,PRKCZ,PTPRC,SDC1,SELE,SEMA4B,SOCS7,TRAF5,XYLT1,PAFAH1B3
Negative_8233	ABO,CAT,ABL2,ACPP,AMY2B,BMPR2,CLDN15,CLDN5,DTX3,EPHA7,GSK3B,NAGK,OR2AG2,OR2V2,OR51I1,P2RX1,PARD6B,PARS2,PDE11A,PDHB,PPP1R3A,TRAF3,TSHR
Negative_8234	BTK,EZR,SHH,ACYP2,AKR1B1,AKR1C1,ALDH3A2,ALDOB,AMDHD2,CAV2,CCR3,CES7,CXCL12,CXCR6,GCNT1,GSTA4,IFNGR2,IRS2,KDSR,MAPK7,MTAP,NEU3,NRG2,OR13F1,OR4M2,OR52D1,ORC2L,PDE9A,PDHA1,PLCE1,PMM1,PNLIP,PRKCI,RBPJ,SEMA3A,SLC25A6,SPRY4,TCF7L1,TYROBP,PRICKLE2
Negative_8235	DCK,F5,GNS,ME2,PGF,ACTN4,ADRA1B,B3GAT1,B4GALT3,BBOX1,C3orf10,CCR4,CDS2,CTNND1,DPM1,ENAH,ENTPD4,GABARAP,GALNT7,GNRH2,GOSR2,LCMT1,MADCAM1,MGAM,OR10A7,OR1A2,OR2A14,OR4N2,OR56A4,OR56B4,PIM1,POLE3,PRKAG3,PRSS1,RALB,SC5DL,SLC2A1,SPRY1,TAS2R50,TLR2,TUBB,ULBP2
Negative_8236	ADORA2A,ALDH5A1,HAAO,KNG1,MAP4K3,MGLL,NEU1,OR51B4,OR51E2,OR6M1,PFN2,RARS

Negative_8237	F8,HPD,LCK,ACSL3,ACSL5,AKR1B1,AKR1C2,ASPA,CCL17,CCNA1,CDC2,CDC6,CYSLTR2,DVL1,G6PD,GAPDH,GCLM,HPRT1,HTR7,IL12RB1,ITGB8,LDHB,MAPK10,MTAP,NCAM2,NOTCH1,OR51A2,OR5B12,OR5M8,OR9K2,OR9Q2,PDIA4,PHKB,PIK3R2,RARS,RASGRF1,RASGRP3,STX18,THBS3,TNFSF4,WNT6,XCL2
Negative_8238	B4GALT5,CAV1,CHKA,CHRM1,CXCL9,GPD1L,IFNW1,MAP2K6,MTHFS,NCR1,OR52N1,OR5J2,OR5K1,PRPH2,PTEN,ROBO2,SLC2A4,STX2,TBL1Y,ATP6V0A1,PAFAH1B3,TNFRSF11B
Negative_8239	BCR,C3,ACAD9,ACTA1,AKR1D1,ALCAM,ASRGL1,CACNG8,CD244,CD28,CD47,CDC25B,CSF2,CXCL3,CYFIP2,DDIT4,DHX58,ELK1,ENPP7,FUT4,GFAP,HSD17B4,ITGB1,NFATC3,NLGN3,NOX3,OGDHL,OR11H6,OR2AG2,OR5R1,OR8I2,OXTR,PLCD4,PLEKHA8,PRKAG2,RASA2,RBL1,RELA,RELB,SEMA7A,SETD1A,ST3GAL1,TSLP,TUBB1,TYRP1,XYLT2,GABARAPL2,MAP3K7IP1,MAPK8IP3
Negative_8240	GLA,MPL,MPZ,RB1,APOA2,ATP6V1H,CES2,CIITA,COL1A2,CTSS,CXCR4,DRD5,DUSP6,FTH1,GAPDH,GCNT3,HARS,HNMT,IL22,ITGAD,KITLG,NSD1,NTNG1,OR2K2,OR2S2,OR52J3,PIK3CA,PLXNB3,PPOX,PTPMT1,RPRM,RUNX1,SCLY,SEMA3A,SULT1A1,TAS2R38,TP53,WNT9B,YWHAG,MAPK8IP1
Negative_8241	C1S,F2R,ACOX3,AMHR2,ARD1A,ASRGL1,EPB41L1,HTR5A,IL23R,LARS,MAPK13,OR1Q1,OR1S2,OR2K2,OR9A2,OXCT2,PPP2R5D,PYCRL,SETDB1,SMC1B,TLR2,YES1,ZIC2,PLA2G12A,ST6GALNAC6
Negative_8242	FUT6,GRIN2D,MGLL,OR6F1,PDE6H,PIAS4,PMM2,PPM1A,PPP2R2C,PRKAB2,PRKACG,TPI1
Negative_8243	CR2,F8,MOS,ZYX,ADCY6,ADRB3,CACNA1G,CD74,CERK,CRY2,CTBP2,CXCL9,CYCS,DOT1L,DUSP5,ECHS1,FGF5,GALNT9,GLUD1,HAGH,HSD3B2,IFNA16,INMT,IQGAP3,MAP3K6,NTN1,NUMBL,OR10G9,OR13H1,OR2V2,OR4K13,OR8K5,OR9G4,PDGFRB,PHKA1,PLCG2,PVRL1,RPS6KA3,SMC1B,SPRED2,STAT5B,STX16,VAV3,HLA-DPB1,MAPKAPK3
Negative_8244	NNT,NPL,RDX,SRC,ACACB,ATP5J,CHAD,CXCL16,DAD1,DAPK3,DDIT3,DEGS1,DMGDH,ENTPD1,ENTPD8,GCNT3,GMPR,GNRH1,GOT1,HSD11B2,KCNJ11,MAOB,NT5C2,OR2Y1,OR51Q1,OR5D16,OR6K2,ORC1L,PARVB,PPP2R2A,PYCR2,SETDB1,SMAD7,SRGAP3,ST3GAL2,TP53I3,UGT2B11
Negative_8245	AASS,ACSM2A,ARRB2,CCR10,CLDN9,CYSLTR2,DDX55,DPM1,DUSP8,EXTL2,FABP4,FLT4,GMPPA,GNG3,IFNA6,INPP5A,ITGA4,NCOR2,NGEF,NLGN1,OR10S1,OR4M2,OR51Q1,PDIA4,POLR1C,PPP2CB,PRKCG,RRM1,SHMT1,STAT3,TCF7,UCKL1
Negative_8246	DAO,INS,KIT,AASDH,AKR1B10,ASRGL1,ATP5E,CCR6,CD47,CD59,CPS1,CTLA4,CXCL6,DGAT1,DUSP6,EIF4E2,ENTPD2,EPB41L1,ETNK1,HTR2C,IFNAR2,OR1E2,OR2T5,OR4L1,OR52N2,OR5T2,OR6K3,OR8K5,PCNA,PGAM1,PGM3,PISD,PRKAR2A,PTPRM,RDH16,RPS6KA3,SGMS1,ST3GAL3,TGFBR2,TSHR,TUBAL3,TUBB,USE1,WNT1,CACNA2D2,HLA-DRB4
Negative_8247	ADC,GCK,ABP1,ACACA,BIRC2,CD40,CPS1,DAXX,DIAPH1,EPB41L2,GUCY2F,ICOS,KLRC4,LDHC,MGAM,NCAM1,NFATC3,NKX3-1,NOS1,NRXN1,OR10A5,OR10G8,OR2A2,OR2G6,OR4B1,OR4N5,PIK3C2G,PLA2G4A,POLR3G,PRKAB1,PVRL4,RAP1B,RPRM,ST3GAL6,STX12,TGFA,TKTL1,AASDHPPT
Negative_8248	AR,ACVRL1,BCAT1,CNTNAP1,CTSL1,DVL1,ENO2,GLUD2,HSPB1,INHBB,NKX2-2,NRP1,OR2AG2,OR52L1,PLCE1,POLR2B,RAP1B
Negative_8249	ARHGAP5,B3GAT1,CCR5,CD40LG,CDK5,CYP2S1,DDX56,FGFR1,FRAT1,OR6B3,PGAP1,SLC33A1
Negative_8250	CHRNA7,CPT1B,CSNK1E,FTCD,GSK3B,OR1J1,SMURF1,STK4,TPH2,TUBB,MAPK8IP3
Negative_8251	ALOX12,ANAPC7,CRY1,FBP1,GOT1,GRIN2B,GSTA5,IL1B,LCMT2,OR13F1,OR51B6,OR5C1,PPAP2B,RETSAT,TACR1,TGFBR1,TMLHE,YWHAQ

Negative_8252	AK5,ME2,CACNA1B,CACNG3,CX3CR1,DNMT1,DTX3,ERBB4,FOXO3,IFT57,PFKM,SNAP25,SORD,UGDH,ZAP70
Negative_8253	BTC,C2,C8G,FST,LHB,OAT,UBC,ACSL3,ALDH1A1,ALDH3B1,ALOX15,BACE1,C5AR1,CD28,CDKN2D,CTPS,CXCL12,HTR6,IDH3B,IKBKB,IRS2,ITGAM,MAP4K4,MARS,NFAT5,NTRK2,OR4C46,OR51B6,OR5M8,OR6X1,PPAP2B,RDH5,RXRG,SLC2A1,SMURF2,TBK1,MAPK8IP2,MAPK8IP3,PRICKLE2
Negative_8254	ADA,IL5,MPL,PAH,ANAPC5,BCL10,CCNA1,CHKB,CTNNA3,DVL3,GALC,GPAM,IL8RB,LLGL1,MAT1A,MECR,MPP5,NOTCH1,OR4A5,P2RX5,PARVB,PDE5A,PIAS1,SFRP5,SHMT1,STAT1,STX4,TJP3
Negative_8255	C6,IL5,ACMSD,ACYP2,AHCY,ALDOA,APOE,ARPC3,ARSB,CSAD,CXCL3,DFFB,EHMT2,ENPP3,FLOT2,GNAO1,GSTP1,GUCY1A2,IFNA5,IGF1,INMT,ITGA10,LARS,MYLK,NCKAP1,NLGN3,NME6,NOTCH1,OR10P1,OR1C1,OR1J2,OR2F2,OR4E2,OR5AK2,OR8B4,OR8K5,PAPSS2,PPID,PTK2B,SEMA3C,SGPP1,SPRY2,TJP3,ATP6V0D1,PRICKLE2
Negative_8256	C1S,ABCC8,ATP6V1A,BIRC3,EPHA5,GART,IL2RB,PDE8A,PIM1,PLA2G4A,TRAF2,PAFAH1B3
Negative_8257	EDA,GHR,TAT,TNR,ALDH1A1,ANGPTL4,CXCR5,DARS2,DDX41,FMO5,GAS1,GNPNAT1,HLA-DOA,HTR2C,ICAM2,IFNA2,IL17RA,INHBB,INPP5E,IRAK4,MAP4K4,NFAT5,NKX6-1,NUMBL,OR10K1,OR2T4,OR7G3,SLC9A1,SMPD3,SOCS3,SULT2A1,XIAP
Negative_8258	FGA,NNT,ARHGEF6,BBOX1,GCLC,HTR5A,IL15,NOX3,OR2A5,OR51A7,SELL,VCAM1
Negative_8259	CSK,ACSL3,AMHR2,ASIP,ASNS,B3GAT3,BET1,CACNG6,CAMK2A,CCNG2,CNDP1,DGKQ,GALNT1,GPD1,GRM5,GSTM5,HLA-F,HMOX2,ITGA5,LMO7,MECR,MTHFD1L,NCOR1,NFAT5,OR10A3,OR2L3,OR4S1,OR52B4,P4HA2,PFKM,PPP1R3A,PTEN,PTPRB,RRAS,SAT1,SDC4,SFRP4,SHC4,SHISA5,SPCS3,SRD5A1,TBK1,TSNARE1,TUBAL3,TUBB2A,VASP
Negative_8260	INS,LCK,A4GALT,AACS,ALDOA,CD226,CHST11,DEGS2,DPYD,FASN,FGFR2,GMPPB,GNPNAT1,GPLD1,GRM1,GZMA,HTR5A,IFNGR1,ITGAM,MYL2,NCF4,NCR2,NUDT2,OR10G3,OR1A2,OR2L3,OR2T8,OR4K17,OR52J3,OR6F1,P4HA1,PLA2G3,PPP2R1B,PTGS1,RPS6KA2,STEAP3,TYROBP,UGCGL1,ATP6V1C2
Negative_8261	FES,OGT,AMD1,B4GALT1,CACNB2,COX15,F2RL2,GCLC,GGT6,GNB3,ITGB7,MAN1C1,MGAT4B,PVRL3,RETSAT,SMARCA2,VEGFA,MAPK8IP3
Negative_8262	AK5,CGN,DDC,EZR,LCK,ABAT,ARG2,CACNG3,CASP7,CHRM1,CNTN2,CXCL1,CYBA,FOSL1,GALNT7,GCH1,GIT1,IL18R1,IQGAP1,ITGB1,LDHB,LTBR,MAFA,NCF2,OR6C74,OR6X1,PARVA,PHKA1,PLCB1,PPP1R3D,PRKAR1A,PRKAR2A,PTCRA,PTEN,RAPGEF4,RETSAT,TNXB,XYLT1
Negative_8263	ITK,SRF,TKT,ALOX5,BCAR1,CTBP2,CTLA4,DDX51,DGKE,DHRS4,DUSP1,EFNA2,EPB41L1,FARSB,FIGF,GALNT12,GPD2,GSTM1,HYAL1,IFNAR2,IL20RA,IL24,LAP3,LPAR1,MAP3K3,MYH9,NAGA,NAT5,NFKBIB,NKX2-2,OR2H2,OR4C12,OR4D2,PGM3,PISD,PLCD4,PRPH2,PTPRJ,RAC2,RASGRF1,SUV39H1,TRPC1,UGT2B7
Negative_8264	XDH,DKK4,FMO1,GLO1,GRIN2C,HLA-DOB,IARS,ITGA3,KLKB1,MAP2K4,MYLK3,OR1L8,OR2AG2,OR4D9,OR4S1,OR5AC2,PFN3,PHKB,SSH3,STT3B,ATP6V1G1
Negative_8265	NLK,AASDH,ACPP,ARPC2,B4GALT7,BRAF,C4BPB,CXCL1,CYP2F1,FGFR2,GPD1L,GZMB,HSD17B2,HSD17B3,IDUA,IRAK3,ITGAX,LAMA3,PDE6H,PIGM,PNPT1,PPP3R1,RPN1,SGPP2,SSH2,TAS2R50,TGFB2,TLR9,UGP2,WNT2
Negative_8266	GPT,ID3,CTSB,E2F1,GMDS,HLA-DOA,MYD88,OR11A1,OR6C2,PFAS,PLD1,PRODH2

Negative_8267	EZR,GDA,ALG1,ALOX12B,ATP5J2,CHEK1,DHCR24,DLL4,EFNA3,FIGF,IL3RA,MAML1,MAP4K4,NKD2,OR8J1,POLD3,PPP2R2D,PRKCG,PSAT1,SHMT1,SMARCA2,SNW1,STT3B,ATP6V1C1,KIR2DL5A
Negative_8268	EDA,FES,LPL,PLN,B3GALT4,CNTFR,CRLS1,CYP11B1,DOCK1,ENAH,GALNTL4,JAM2,NME2,NRP1,OR2AT4,OR2K2,OR2M3,PAFAH2,PAK3,PPA1,PRKCD,RAPGEF2,SMAD3,SNAI2,PPP1R12A,TNFRSF13C
Negative_8269	LSS,CHKB,CYBA,MCCC2,MCM7,NSD1,PNPT1,RAPGEF2,RXRB,YWHAB
Negative_8270	GP9,ANAPC11,ARHGAP5,CNDP1,COQ7,GRB2,GUCA1B,IGSF5,MAP2K6,OR10V1,OR52E8,PHKA1,PIK3CB,PSEN2,PTPN11,ROBO2,TAS2R49,TSHB,WNT2B
Negative_8271	AMY2A,NANP,PARD6G,SPAM1
Negative_8272	F10,TNR,ALOX5,BACE2,BUB1B,CCL26,CDC2,CTNND1,CXCL10,DDX56,DUSP5,ETNK2,FZD2,GGT7,GNRH2,GPD1L,HSD11B2,IGSF5,INHBB,MYLK3,OR2A2,OR2B11,OR8B4,ORC2L,PDHA1,PRKAR1A,PTPN5,SDHB,SEMA3E,SLC25A4,TLN1,WNT3,YWHAQ
Negative_8273	CTBP2,PRKAB1,TIRAP,VDAC2
Negative_8274	AK7,ILK,ABLIM2,ACADM,ACAT2,ADH7,ADIPOR1,AHCY,ALCAM,ALG2,BLNK,CACNG1,CCR5,CHEK2,CNGA4,CRKL,CTNNA2,CYP3A4,DAPK3,DPM2,EDA2R,GALM,GALNT9,GNPNAT1,HCLS1,IMPA2,MAD1L1,NEU4,NNMT,OR10A2,OR1A1,OR2A14,OR2T10,OR4D1,OR51I1,OR7D2,PGM1,POLE,RAP1B,RASA1,RBX1,ROBO1,SOCS7,VAMP2,ARHGEF12,C1GALT1C1,HLA-DQA2,SUV420H1
Negative_8275	DBH,HDC,OSM,BIRC3,COL3A1,FLT1,IL1R2,JAK1,LAMA4,NQO1,OR11H4,OR2Z1,PAK6,PGM1,PLA2G3,RAP1B,TSC2,UGT2B28
Negative_8276	DKK2,GADD45B,GNPDA1,INPP1,NEU2,OR51V1,PPID
Negative_8277	EGF,JUN,MPL,TFG,ACTB,ANGPTL4,CHKB,CXCL9,EPB41L3,EPHA4,FGF3,FTMT,GRB2,HEXA,ICAM1,ITGB5,KAT2A,LAP3,NEUROD1,OR10X1,OR1B1,OR4C15,PDGFB,PIAS1,POLR2E,PTEN,RDH12,SSH2,ST6GAL1,TNFSF12,UGCGL2
Negative_8278	CFI,IL7,ALAS1,B3GAT1,CDC14B,EIF4B,GYS1,HCST,NOS1,PPOX,SEMA4D,TAS2R48,TUBA1C,WNT7B,ATP6V1B2,PLA2G12A,TNFRSF12A
Negative_8279	FGB,ACTN2,AHCYL2,ALDH3A2,ANAPC1,BRAF,CCR4,CD274,CDC25C,CHEK2,CXCL16,CYBA,CYP27A1,DGKG,GGCX,GSTM3,IL1RAP,IRS1,MAN1B1,MCAT,NUDT9,OR52E6,OR7C1,OR8K5,P2RX1,PLOD3,PON2,PTEN,SEMA4F,SLC27A5,SMC1B,SNAP25,TAS2R8,TBL1XR1,TCF7L1,ST6GALNAC3
Negative_8280	BID,DCT,FGB,GSS,NNT,SPN,AGRN,ALDH9A1,ALPI,ANAPC7,ARNT,BMPR2,C3AR1,CASP6,CD70,CDC6,CLDN17,COL1A1,CTPS2,CYP19A1,DIAPH2,DKK2,FABP3,HDAC2,LDHAL6A,NAMPT,OR12D3,OR1S2,OR2H1,OR4F15,OR52N4,OR5M8,OR6Y1,PFN4,PIK3C2A,PIK3R3,PMM2,PMVK,PPP1R3A,PPP2R2C,PSAT1,PTGES,QPRT,SKP2,SMPD2,TLR2,UNC5A,HSD17B10
Negative_8281	ADK,RET,FGF20,OR5D18,PMVK,SARS2,SEMA4G,TRAF4
Negative_8282	DCN,FER,FGB,AARS2,ABP1,ACAD9,ALG13,ARG2,BMPR1A,CD46,CDIPT,CHRM3,CRLS1,CSDA,DDX18,EGLN3,EIF4B,FGF13,FGF17,HEXB,INPP5E,LCP2,MGST3,NAT5,NEU2,NKX3-1,NOTCH2,NOTCH3,NPAS2,OR13C3,OR1M1,OR2T2,OR56A4,OR6A2,PDXP,PGM1,PHKB,PHKG2,PLXNA1,POLR1A,SEMA4G,SLIT1,SMAD4,STK36,TAS2R4,TGFB1,KIR2DL5A
Negative_8283	F5,TNC,TNR,ACSS3,ADCY1,ARHGAP5,BNIP1,CDC7,CDIPT,CSNK1E,IAPP,IGF1R,INPP1,ITGB1,MAGI2,MAP2K7,NRXN1,OR2T12,OR4X2,OR8H3,PIK3CB,POLR1B,POLR1C,POLR2D,PON2,SC5DL,SETDB2,SOCS7,STAM2,STAT1,UGT2B4,UXS1,VEGFB
Negative_8284	PGD,ATF2,CASP8,CTBP2,DCTN1,EI24,FLT3LG,FUT5,FUT7,MAP2K2,NCR2,OR52E2,OR7G3,PRKCB,PTPN11,SMPD4,TLR9
Negative_8285	COX15,GALNT9,OR1A2,OR4M2,OR51T1,P2RX3,PAX8,PTPRF

Negative_8286	EZR, ABLIM1, APPL1, BET1L, CCL8, CD72, CSF1, CTSB, CXCL16, CYP19A1, FCER1G, GALNT11, IKBKE, IRAK3, NRG1, OR13C4, OR1L1, OR3A3, OR5AP2, OR6C3, OR6C6, P4HA1, PEMT, PLCB1, PPP3R2, ROCK1, SOAT2, EIF4EBP1
Negative_8287	EGF, GNE, PLN, ABLIM1, ADAM17, AGPAT1, ASCC3, ATP5I, ATP6V1D, CCNG2, CCR10, CD34, CDKN2D, CLDN19, CREB3L1, GLDC, GMDS, GSTM5, IL5RA, IMPDH1, LIAS, LIPA, MAGI1, NOX3, NT5C, OR4D6, P2RX3, P4HA1, PANK4, PKLR, PLOD1, PRKAG1, RAB5A, RAPGEF2, RHOA, SEMA4B, SEMA4D, SOCS7, TAP1, TP53I3, UAP1, USE1
Negative_8288	C7, LCT, OGT, PAH, PVR, ACOX3, ALDH4A1, AOC3, B3GAT2, C7orf16, CCNG2, COQ3, CRB3, DIAPH2, DTX2, FGF23, GBA3, IL17RB, INPP5E, KLKB1, LCP2, NAE1, NAGS, OR10S1, OR2T33, OR52N1, OR5B17, OR6T1, OR7G3, PDE9A, PIGO, PIK3CB, PLA2G4A, PNPO, PON1, PPARA, PPM1D, PRKAR2A, PTGES2, PYGB, SOS2, SQLE, ST3GAL2, VAMP4
Negative_8289	NF1, ACADL, ACSL4, ANGPTL4, ARRB2, ASAH1, ATN1, ATP5A1, ATP6V1F, ATP6V1H, CASP7, CAV2, CDC42, CHRD, CISH, COL2A1, COL5A2, COX15, DAPK1, DUSP5, EDAR, GFPT1, GLI2, GLI3, GMPPB, GMPR, GNRH1, HGSNAT, IL2RA, ITGA3, MYLK3, NFYC, NT5M, OR4K17, PLCG1, RAPGEF2, SEMA3C, SEMA3E, SGMS1, SYNJ1, TGDS, TNFRSF4, WNT7A, ATP6V0A2
Negative_8290	CFD, EHHADH, KIR2DL1, PHKA1, TBXA2R
Negative_8291	AR, PAH, ADIPOR1, APOA5, CBLC, DEGS1, EIF4B, EPHA5, EXTL3, FGF1, GNA12, GNPAT, HNF4G, LCMT2, NOS1, NR1H3, OPLAH, OR10T2, OR4K1, PER2, PITX2, POLE, PPP2CA, PPP3CB, PRKCQ, PROS1, QARS, SGMS1, ST6GAL1, SYNJ2, TUBB2B, WNT2
Negative_8292	DAO, GAA, GNE, HK1, LAT, OGT, AANAT, ACSL3, ACVR2B, ADORA2A, C1QB, C9orf95, CCR3, CDK7, CDKN2D, CSNK2A1, ENPP7, FGF22, FRAP1, GALNT10, GCNT2, GNA15, GRIN1, IGSF5, IL28RA, INMT, KAT2B, KCNQ1, LAMB3, LEFTY1, MASP2, MS4A2, NARS, NLGN1, OR4F4, OR6N2, OR9Q2, PARVA, PIK3C2B, SELE, SEMA4F, SHC4, TAS2R49, TFPI, TUBB4, VTI1A, VTI1B, WNT5A
Negative_8293	ASNS, CARS, CRB3, CREB3L3, FMO1, GAD1, GLUL, GPAM, IL3RA, IRS1, MADCAM1, MARS, METTL6, MGAM, NRAS, OR10H1, OR2L13, OR51B2, OR5J2, OR7G2, OR8K5, PDGFD, PIGA, PLA2G4A, POLR2A, PRKACB, PTPN1, PVRL1, SULT1A1, ULBP1, VDAC1, WWP2, ZAP70
Negative_8294	FYN, ACPP, ALAS2, BCL2, BNIP1, CDC25C, IFNA4, IL5RA, MKNK2, NUMB, RAPGEF4, TNFRSF6B
Negative_8295	GP6, NPL, ARPC1A, CMPK1, CRY2, HARS, IL18, PRKX, PYGM, RBX1, SYNJ1, TGFB3, TUBB2A
Negative_8296	FGB, GP6, MYC, TEC, ACSL5, ADAM10, ATP5H, BMP6, CD82, CRLS1, CXCR5, EPB41L3, FBXW11, GAMT, HAGHL, HHIP, HNF1A, IKBKB, ITGAM, KIR2DS4, KRAS, MYL2, OR2W1, OR5P2, OR6C1, PIGO, PLA2G2A, SQLE, ATP6V1G1
Negative_8297	CGA, FGB, ARPC1A, CAMK2G, FZD5, ISYNA1, KIR2DS4, NRCAM, OR10C1, OR52K1, OR5A2, PCK2, PLD1, PPP2R1B, TNFSF8, TUBB
Negative_8298	CD6, FGG, JUP, SRC, ACACB, ADRB3, AKR1C3, ARPC1B, ATP13A2, COL1A1, CXCL6, GALNT6, GNA15, GNAT3, GSTA1, HNMT, IL1R1, LEFTY2, NFKBIA, NME6, NR4A1, OR1L3, ORC5L, ORC6L, PDXP, POLR3B, PRDX6, RARS, TACR2, TYROBP, TNFRSF10C
Negative_8299	ADO, CCNB3, COQ6, HLA-DRA, OR1E2, P4HA2, PAK1, RAPGEF2, SRD5A2, VAMP3
Negative_8300	ADCY1, ADH4, ALG12, ALPI, ATP5E, CACNA1I, CHPF, DAD1, EIF4B, ENPP6, GALNT8, HSD3B2, IKBKE, MAT1A, NSDHL, NUDT12, OR3A1, OR5A1, OR5B17, OR6M1, PANK2, PCYT2, PERP, PIPOX, PLCD1, PPP3R1, RFXANK, STX1B, VNN1, PAFAH1B1, SERPINA1
Negative_8301	FH, ABAT, AKR1C1, EPHA2, ERBB3, MICA, OR4L1, OR8J1, PAX8, PRKAR2B, SUCLA2

Negative_8302	ABL1,BCAT2,CDH15,DGKH,FARS2,FLT1,IL20RB,MYH14,OR5AN1,OR5D18,PFKFB4,PIGA,SUCLA2
Negative_8303	F5,FGA,ACADS,ACSL5,ADCY6,ADSL,ARAF,ARSB,ATP5G2,C1QB,C3AR1,CCNE2,CLTB,CXCR5,CYP2F1,DGKA,DHCR7,FGF18,FUT7,HARS,IFNA10,IL12B,IL1RAP,IRAK3,JAK3,KARS,LAMC2,LCAT,LTC4S,MAPK14,MGST3,NFKBIE,OR1C1,OR1F1,OR1L6,OR6T1,PDE1C,PDPK1,PRIM1,PRKAR1A,SMC1A,SNAI2,SPRED1,TNFSF4,UBA7,UPB1,XYLT1,ATP6V0E1
Negative_8304	MAX,MPZ,OAT,SYK,ADIPOR1,NLGN1,OR9Q2,P4HA3,PIK3R1,SMARCA5,TLR2,ZMAT3
Negative_8305	ADO,IL5,TFG,B3GNT4,BAAT,C4BPA,CCL14,CD79A,CD8A,CYP3A5,DDIT3,DGKQ,DUSP6,ENPP2,F13A1,FCGR3B,FLT1,GCAT,GLUD2,HAAO,HAO2,ITGB1,MAD2L1,MGAT3,OR10G2,OR13C8,OR13H1,OR1D5,OR2AT4,OR2T10,OR51I1,PAK6,PGAM1,PIP4K2C,PLCZ1,PPP3CC,RAD51,ROCK1,SRD5A1,SUFU,TAS2R48,TIAM2,TM7SF2,TPMT,TUBAL3,UNC5B,WNT10B,HLA-DQA2
Negative_8306	GK2,TK2,CCNE2
Negative_8307	CRH,F8,LEP,NPY,ATP5G2,C1QA,CCL13,CD14,CD38,FIGF,LRDD,NCKAP1L,NUDT8,OR1E2,PTPMT1,RBPJ,SC5DL,ST3GAL5,TIRAP,TLR3,TUBA8,UBA1
Negative_8308	GLI3,PDGFD,WNT16
Negative_8309	DDC,AGPAT3,CADM3,CHRM5,CSNK1E,ENPP6,FGF8,GLG1,HTR2A,IRAK2,NFAT5,NTN1,OR1C1,PAX4,PDE6D,PLAT,POLR3B,PPM1B,SPRY2,TACR3,TJP3,EIF4EBP1
Negative_8310	FAH,ID3,PDC,SP1,AMY2A,ARNT,ATP6V0B,AXIN2,BCAT1,CACNG7,CD226,CD79A,CFL2,DAAM2,DDX23,EIF4E2,F13A1,GADD45G,ICOS,IFNA13,LDHC,LIPG,MYLK3,NPAS2,NRXN3,OR10J5,OR10P1,OR1D5,OR1I1,OR2T1,OR2W1,OR8A1,OR8H1,PCYT1B,PLXNA2,PNPLA3,PTCRA,PTPRM,RBX1,RPSA,SETMAR,SSH3,STX5,TCIRG1,TUBB4Q,ULK3,C1GALT1C1,PNLIPRP2
Negative_8311	HAGH,OR2L8,PHKG2,SETMAR,TAS2R1
Negative_8312	BLVRA,CCR2,COL6A2,CXCR6,DNMT3B,HNF1B,NEUROG3,PDCD1,PGK1,ST6GALNAC5
Negative_8313	C6,C7,IL6,PTS,SHH,TK2,AGMAT,ALDH4A1,ARSA,BCL10,CCL16,CD19,CHDH,COQ3,CRY1,CSDA,DLL3,DVL1,EGLN3,EPHA1,GALNT6,GCNT1,GP1BA,HLA-DOB,IFNA10,INHBC,LARS,MGLL,NNMT,NRXN3,NTN1,OR2A25,OR2F1,OR8K5,PDE6G,PIGW,PLA2G1B,POLR2A,POLR2G,PPID,SMAD6,STAM2,TOMM40,TRHR,TYMP,VARS,VDAC2,VNN1
Negative_8314	GSR,ABLIM2,AFMID,CYP11A1,FUT7,GNB3,METTL6,MYH14,OR52A5,POLD2,SHMT1,TLR9
Negative_8315	ARSA,CDS2,CLDN6,CLDN9,TRMT11
Negative_8316	AUH,GBA,SCD,ATN1,ATP13A2,CACNB1,CLDN7,ENO2,EXTL2,FGF18,FGF3,FOSL1,GOT2,GPAA1,GSTA2,HADHA,HIF1A,HMGCS1,IDH3B,IL18R1,IL20RB,IL8RB,IL9R,ITGA7,LHCGR,MALT1,MCCC1,NME6,OR10A6,OR10AG1,OR2D3,OR4C15,OR6A2,OSMR,PDE6D,PFN4,PPARG,PTPRM,SLC27A5,SPAM1,SPHK2,TAS2R40,TRAF2,VTI1B,PRICKLE2,ST6GALNAC3
Negative_8317	CKB,CTH,F8,HK1,ID3,KHK,LYN,MET,ACADVL,ADSS,ARPC1A,ATP6V0C,B4GALT2,BCKDHB,BDNF,BLNK,CASP9,CCL14,CD274,CEBPA,CHAD,CTBP2,DGKD,DHX58,EPHB2,EPHB6,FZD6,GALT,GNPAT,HBEGF,LDHAL6A,OR1G1,OR1S2,OR4F17,OR4F6,OR51B4,OR5B21,ORC1L,PLIN,PLXNA3,PPP2R2D,SESN1,SLC2A2,SMAD2,CACNA2D4
Negative_8318	CDH2,EPB41L3,OR52E4,TRADD
Negative_8319	GSS,PRL,ACTC1,ANAPC5,ARHGAP5,ATP13A2,AVPR1A,CCL17,CCL4,CD82,CYP4F2,ETS1,FGF21,GALM,HBEGF,LARS2,MCM4,MGLL,OR4A15,OR4S2,PAK2,PDE7A,PGLS,PLCD1,PLD1,PPP2R1A,PRKAB2,PRKCQ,SEMA4F,STAT1,STAT5A,STK3,SULT2B1,TGDS,VEGFC,TNFRSF10D

Negative_8320	F9, GNS, SFN, CASP8, CLDN19, COMT, CPT2, CXCL10, CXCL6, CYFIP1, CYP2A7, G6PC2, GAB1, GALNT11, GDE1, GNAO1, HHEX, OR13C3, OR2M7, OR2Z1, OR8B8, OXTR, PDGFD, PECAM1, PERP, PLAUR, PRKCD, PRKCG, RFNG, SEMA4D, SETD1A, SLC2A4, SV2B, TAS2R10, TICAM1, UGT2B10
Negative_8321	GSS, PGP, AARS2, ACSS3, CDKN2B, CSNK2B, DGKB, EFNA4, EGFR, FGF9, FIGF, GNRHR, ITGAM, LIPG, OR10A2, PDXP, PON3, SARDH, SELP, SFRP1, THY1, TOMM40, ATP6V0A4
Negative_8322	C3, INS, CACNG7, CALML6, DNMT3A, FGF22, G6PC, MAP3K5, OR5P2, OR6K3, PDX1, PIK3C2B, PLCG2, PLD1, RXRB, SKIV2L2, SUOX, TRADD, UNC5C, MAPKAPK2
Negative_8323	FAH, A4GALT, AGPAT3, CACNA1A, CASP8, CD40, CYFIP1, ENTPD2, FMO4, GATM, GSTO2, GUCY2D, INPP5B, LTB4R2, MADCAM1, MAML1, MAP3K5, MOV10L1, NCR3, NOTCH2, OR51L1, PCYT1A, PDE8A, PDGFA, PERP, PIK3C2G, POLE, RPS6KB1, CACNA2D1
Negative_8324	ADC, FES, ALDH2, ATF4, B4GALT3, CCL7, CDKN1A, CYFIP1, CYFIP2, CYSLTR1, DCXR, ECSIT, EPAS1, FABP3, GGT1, HSP90B1, IDI2, IL18R1, JAG2, MAP3K6, MAP4K2, NT5E, NUDT5, OR11H1, OR1J1, OR2AG2, OR8K5, OR9K2, PAICS, PCYT1A, PCYT1B, PGK1, PPP1CC, PRKAG3, PRKCZ, PRKY, SREBF1, SSH3, TLR3, TNFSF10, UGDH, WNT10B, C1GALT1C1
Negative_8325	FST, ADRA1A, B3GALT2, BMP2, CAMK4, CASP10, CHRM3, CTBP1, CYP2B6, GGCX, GNRH1, ITCH, KLRC4, LARS, MAPK3, MFNG, NLGN3, NUDT8, OR10A7, OR4F6, OR8H2, PLAU, POMC, RPN2, SAT2, SETDB2, SQLE, VAV1, WHSC1, YES1
Negative_8326	CBL, MUT, OTC, CALML6, CAMKK1, CHRNA7, DAPK2, GAMT, GCNT4, IARS, KNG1, MAP3K8, OR1D5, OR1L4, OR2AG2, OR2M3, OR52I2, OXSM, PGM2, PHKB, POLR2E, POLR2J, PPAT, SESN3, ST3GAL4, STAT4, TIAM2, TRAF6, WNT1
Negative_8327	BTC, AKR1C1, ALPPL2, CLTCL1, FOXO1, HDAC2, IGFBP3, MGLL, OR10A7, OR4E2, OR9G4, PTGS2, RAPGEF1, RPN2, SHC3, TNFRSF8, WNT16, TNFRSF6B
Negative_8328	ACAD8, ANPEP, ASMT, ENTPD3, FGD1, FUT5, GALNT5, GALNT8, GGT5, GNAQ, HIP1, IL9R, MMP9, MYH11, MYL2, NNMT, OR10H1, OR2AG1, OR4F17, OR6F1, PIK3C2B, POLR3D, PTPRF, SEMA4C, SNW1, TCF7, UROD, WNT16, ZAP70, B3GALNT1
Negative_8329	CD2, NNT, ACOX1, ACP5, ACVR1B, AMPD3, ASMT, ATP5F1, ATP6V1A, BST1, CDC25A, COL2A1, COQ3, CREB3L1, CREBBP, DARS2, DHODH, DRD2, DUSP16, GGT5, GRM1, HLA-DOA, HRH2, ICAM1, IMPDH1, ITGB3, MAP2K2, MITF, MYH7B, NFE2L2, NME2, OR14J1, OR2A4, OR4A15, OR51S1, OR5H2, OR6C2, OR6Q1, PIM1, PPP1CA, SDHC, SEPHS1, TP53, TRAF4, ATP6V0D1, SERPINB5, TNFRSF10D
Negative_8330	INS, JUN, NGF, PDC, AGXT, ALG6, ALPL, ASAH1, CBLB, CD34, CD48, CFLAR, CLDN18, CREBBP, DAG1, EHHADH, EXOC7, FECH, FUT8, FZD1, GALNT1, GALNT14, GNAZ, GYS2, IBSP, IL29, LDHB, LRRC4C, MAP2K6, MYL7, OR52N2, OR5B17, OR9G1, PIK3C3, POLR1D, POLR2J, RASA1, SIPA1, SLC2A4, STX3, TAS2R14, TGFB1, TRAF6, UCK2, XYLT1, HSP90AA1
Negative_8331	MET, ADCY4, CYP27A1, CYP2B6, CYP3A7, DGUOK, DUSP3, EIF4E, ENTPD4, ENTPD8, FMO5, GALNTL2, GDF5, GMPPA, GNPDA2, IL12A, IL28RA, IL2RB, IRS1, MGAT1, NTRK2, OR10K1, OR2T12, OR4K13, OR4S2, OR9G1, PDGFC, PEMT, PER1, PRKAB2, PRNP, RAPGEF2, RASSF1, RPIA, RPS6KA4, SV2B, VANGL2, WWP1, YWHAH, ZAP70, PAFAH1B2
Negative_8332	CLCF1, FGF14, IQGAP3, NR1D1, SEMA3A, ATP6V0D2, TNFRSF6B
Negative_8333	DCT, DDC, F11, F3, A4GALT, ADRB1, ALOX12B, APPL1, ATG7, CTNNB1, CXCL1, DDX52, FGF17, FMO1, GART, GBE1, GNAT3, GP1BA, IL21, MAP3K5, MCEE, NOS1, OR11L1, OR1L1, PAK6, PCYT1A, PIK3C2B, PLA2G2A, POLR3G, PRPS1, QPRT, RAB23, RPS6KA6, SARDH, SDHB, SNAP23, STAT3, SULT1A1, TACR3, TAS1R3, UGCG

Negative_8334	GBA,BDKRB1,CCL3,CLDN17,DAG1,DKK2,HSP90B1,IFNA7,OR4P4,PLXNB3,SMAD4,TRIP10,VNN1
Negative_8335	HAL,NPL,ACAT2,BIRC3,C5AR1,CCL20,CD79A,CLDN7,DARS,E2F1,EIF4B,EXOC7,FGF20,FPGS,GAMT,GNA12,GNB1,GPD1L,GUCY1A2,HHIP,HSD17B3,IBSP,NAMPT,NLGN3,OR10A5,OR2AG2,OR5A1,OR6C76,OR8D4,OXCT2,PIAS4,PLXNB3,PRKAR2A,PRSS1,PTGFR,RAPGEF4,RDH8,SEMA3G,STK36,TAS1R2,TUBA1B,VDAC2,WNT1,WNT10B
Negative_8336	AGK,BAX,ACOX3,B3GALT4,CD47,GNAI2,LIPG,OR1S1,PLCB1,RPS6KB2
Negative_8337	ADCY1,DGKD,GAD1,GGT6,IL25,MYH10,OR2A14,PIGU,SDHC,STX1B,TM7SF2,EIF4EBP1
Negative_8338	C4B,CD2,MET,OTC,SRC,ADIPOR2,ADSL,ALDH4A1,B4GALT7,CACNA1C,CACNB1,CAPN2,CASP7,CES1,CNDP1,CPT1B,DGAT2L4,EDN1,GALNT9,GRAP2,HLA-DOA,HMBS,IFNAR1,IFT57,LEFTY1,LIPF,MYLK2,NAGK,NCK2,NUDT5,OR10A2,OR10S1,OR3A2,OR5AU1,OR7G2,PDE9A,PIK3C2B,PRIM2,PRKCB,SEMA3C,SLC2A4,THBS3,UPP1,ATP6V1B2
Negative_8339	B3GALT6,FSHB,GPD1L,IL15RA,PHKG1
Negative_8340	ARHGAP5,CACNG1,EPHB1,GNAL,UBE2K
Negative_8341	ASL,C5,ASAH1,BDKRB2,CDKN2B,DFFA,EDAR,GFPT2,GGPS1,GSTZ1,ITGB1,MKNK2,OR4K14,PIP4K2B,PSME2,PTGIS,RDH5,RENBP,SYNJ1
Negative_8342	FER,TEC,FOSL1,G6PC2,GNPDA2,NSDHL,OSMR,UGCGL2,SERPINC1
Negative_8343	ZYX,ACOT11,ATP2A1,ATP5G2,CLDN1,CRB3,CSDA,DIAPH2,DUSP6,EXOC7,EXTL2,EXTL3,FASLG,GABARAP,GALC,HCST,HDAC2,IL22RA1,LAMB2,MGST3,MYH3,NARS2,NCOR1,NEFL,NUMB,OGDH,OR1Q1,OR2Z1,OR52J3,OR6C75,OR7A17,PAX6,PDGFRA,PIGQ,PIK3R1,PMVK,POLR2B,PRKAG3,RAP1B,SEMA3B,SEMA4C,TNFSF14,ULK1,UROS
Negative_8344	CACNG2,CCNE2,CYP2A13,EXTL1,GALE,MAP3K13,MCCC1,OR2F1,OR3A2,OR5D13,OR8U1,POLR3K,PRKACA,PTPN7,TNFSF9,TRPM5,UGCGL1
Negative_8345	C8G,FUK,ADCY10,ADH7,ALCAM,CCR2,CD38,E2F5,FZD5,GALNT2,GMPR,GNA12,GSTM5,IFNW1,NPAS2,OR10J5,PAK2,PLOD3,POLR2F,PRODH2,PTPRM,RASGRF1,SESN2,TCF7L2,TJP3,TSC1,CACNA2D4
Negative_8346	CBL,IL3,AMDHD1,BMP8A,CACNB1,CCL8,COL3A1,CTSL1,ENTPD3,FGD1,GRIN2D,HLA-DMA,ICOSLG,ITGAX,KLRK1,MCCC1,NCF2,OR4C13,OR5P3,PIAS2,PLA2G2D,POLR2C,PRKX,PTPRR,RALB,TAS2R3,TIRAP
Negative_8347	B4GALT2,IL8RA,ITCH,LTA4H,NCAM2,PTGS1
Negative_8348	CD4,CS,DBT,ID3,KMO,MSN,ACSM2A,ARHGAP5,BNIP1,C7orf16,CLOCK,DEGS1,FGD3,GABARAP,GATM,GGT5,GTSE1,HSPB1,IFNA6,IL1RAP,KARS,KLRC4,MS4A2,MYH9,NT5C2,OR2T33,OR2Z1,OR4Q3,OR5B12,OR5H2,PAPSS2,PCNA,PDE3B,PDE8B,PI4KB,PPOX,PRKAB2,RASGRP2,SLC2A2,ST3GAL4,STX3,TBXA2R,TSTA3,TUBB3,TXNDC12,UNC5C,ZAP70
Negative_8349	CD6,PRL,ALG2,B4GALT2,CDH1,CLDN15,CLDN20,CTLA4,FGF1,FPGS,G6PD,GATM,GJA1,GNAI1,HEXA,IFNA13,IKBKE,IL11RA,IL4R,L1CAM,MCM3,NANP,PHKB,POLR3B,RPS6KB1,SLC27A1,TNFSF10,UCK1,ULK2,UPP2
Negative_8350	ACYP1,CDC25A,COQ6,CXCR6,CXXC4,DARS,FMO4,FTMT,HTR2A,MRAS,OR2C3,OR52B4,PRKCD,PRKCZ
Negative_8351	ACSS3,CYP19A1,EIF4E,FABP4,GSTP1,HADHB,HLA-F,ITGAV,LYPLA2,NEFL,NR4A1,OR6C75,PGM3,PLXNA3,PPP2R2C,PRKAB2,PRKCE,PTDSS1,RAPGEF1,RENBP,ROCK2,SMAD6,ST3GAL3,ST8SIA5,TNFSF9
Negative_8352	MYC,SYK,ATG7,B3GALT5,CCL13,CDC27,CLDN10,IL15RA,LCMT2,MAD2L2,OR1E2,OR2H1,OR4K13,OR4X2,PAK7,PPP3CC,PRKX,PTEN,RAPGEF4,ROBO3,TAS2R3,ULBP3

Negative_8353	FAS, ACTA2, AGPAT6, CCL7, CD226, CLCA4, FADS2, GFPT1, GRM1, GSTK1, MAP2K7, MYH8, NEFL, OR2H1, OR2L8, PLAT, PSME2, RAB5A, SORD, VARS
Negative_8354	ARG1, CCR3, CD80, CSF2, CYFIP1, JUND, KRAS, OR5B3, PRKACG, PSME2, SCLY
Negative_8355	MYC, NPL, ADAM10, ARPC1A, ARPC5L, ASAH1, ATP5H, BCL2, C5AR1, CADM3, CCL13, CCL24, CCL27, CD86, EXTL1, GNAI1, IL20RA, KARS, LDHD, LIPF, MAP3K5, MAPK12, NFX1, NPAS2, OR2K2, OR2L13, OR52I2, OR52J3, PAPSS1, PIK3R1, PKLR, RHEB, SPTLC1, THBS2, TSHR, UGT2B28, WNT11, PNLIPRP1, TNFRSF14
Negative_8356	BID, F11, FH, GNS, ACP5, ALPP, ATG3, CYBB, GALNT8, GOSR2, IFNA21, MLLT4, OR13C9, OR52A1, PYCR1, RBKS, RPN1, SLC2A2, ST8SIA1, STAT6, SERPINA5
Negative_8357	A2M, IL2, ADCY1, CAMK2D, CASP9, CNGA3, CNGA4, COASY, DGKG, GALE, HLA-C, IL12A, MAN1A2, NKD1, OR1A1, OR51B4, OR51T1, OR8U1, PCCB, PDGFB, PIGT, PRKAB2, SERPINE1
Negative_8358	ALAD, ARNTL, ELK1, INPP1, MASP2, NAGLU, OR2F1, SH3BP2, SSH1, UBE2L3, PAFAH1B2
Negative_8359	NF1, ALCAM, APOA1, BAIAP2, BMP8B, BPGM, CAV2, CCR4, CLDN1, GNAI1, GOSR2, IDI2, JAG2, LDHB, LRDD, PIK3C2A, PSME1, SH2D1A, SHC3, TNFSF12, VANGL1
Negative_8360	C9, CP, ADCY6, ANAPC5, ATP4B, BNIP1, C4BPA, CACNB1, CD14, CNTN2, CREB5, CXCL11, FTMT, GUK1, HYAL2, IFNA10, NEFH, NFATC3, NMNAT3, OGDHL, OR2H1, OR2M3, OR51Q1, OR8J3, P2RX2, PFN1, PMM1, PPCDC, PPP3R2, RPS6KA2, SEMA4C, SESN3, ST3GAL3, SUFU, SULT1E1, TP53, UROS, VEGFB, WNT3, TNFRSF10A
Negative_8361	INS, ACSL1, ATP5H, COL2A1, HNMT, NFAT5
Negative_8362	ACOX3, CDC25B, EIF4E2, MAN1A2, MAPK1, NRXN1, OR2A5, PLAUR, PLOD1, POLR2K, SIPA1, WEE1, WNT10A
Negative_8363	CEL, F12, AADAC, ALDH1L1, ATG12, CACNG5, CD38, CLDN2, COL5A2, CSF1, CSNK1E, CXCL5, CYP1A2, CYP2E1, DUSP1, EARS2, GNG13, HTR2B, ICOSLG, IFNG, IL1RAP, OPLAH, OR10A2, OR4P4, PKLR, POLE2, PON2, PPP1CA, PTPN7, RAC1, RALGDS, RPS6KA3, SEMA4A, SH2D2A, THY1, TRHR, WNT2B, WNT5B, ZFYVE16, TNFRSF11A
Negative_8364	CD2, EBP, FH, JUP, KDR, TK1, TYR, ACP2, C1QB, CCND2, CXCR3, DLL1, GNAI1, HTR4, MAGI3, MAPK10, MLYCD, MPDZ, MTMR6, MYLK3, OR7A17, PDE6H, PDE7B, PRPS1, PYGB, STX2, SUCLG2, TCIRG1, TICAM2, VASP, HSP90AA1, TNFSF13B
Negative_8365	CD4, ACYP2, ALDH2, CCL3, CHRM3, CNTN1, CNTNAP1, COL5A3, GNA11, GNB1, GRPR, IRAK1, MTHFD1L, MTHFS, MYH11, OR10Q1, OR13H1, OR5AC2, PGAP1, PPP2R2A, PPP2R5A, PRKACA, RXRG, SLC2A4, SULT1A4, TFPI, TUBA1B, WNT16, TNFRSF14
Negative_8366	AARS, ALG8, CAMK2D, FARSB, GALNT8, IMPA2, MLYCD, NOTCH1, OR10G8, OR51A2, PRODH, ST3GAL4, TKTL1, HLA-DRB3
Negative_8367	C8A, CP, OGT, B3GALT5, CAV1, CD226, CSF2, CXCL9, DGKQ, FGD1, MEF2C, MRAS, OR4S1, OR52N1, PDGFA, PIGK, PIP5K1C, PPP1CA, PTGIS, RDH12, RPN2, SDHA, STK3, SULT1A2, SUOX, XCR1
Negative_8368	CD6, DUT, GLS, GP9, ALG10, ATIC, B4GALT5, CCDC6, CD34, CSF2, CYP4F3, ESAM, FARS2, FOSL1, GANC, GPX5, HADHA, HLA-G, IL18R1, IL22RA2, ITGB8, LDHA, OR5AU1, OR8G1, ORC2L, P2RX5, PGAP1, PLA2G2D, PRKCI, RDH5, ST3GAL4, STAT6, TAS2R41, TPK1, TUBA3E, WASF1
Negative_8369	GHR, ACSS1, APLP1, B3GALT4, BCL2L1, BIRC2, CLDN3, CNTNAP2, CTNNA1, FADD, FOXA2, GCLC, HSD11B1, IL23A, IL4R, MLYCD, OR13C8, OR6B2, OR8K1, PLA2G2F, POLR2B, PON2, RAPGEF2, TCF7L1, WNT4

Negative_8370	BTK,ALDH1A2,ALG10B,BET1,CCND2,CDC27,CFTR,CHRNA7,CSH1,CYP7A1,ENO3,EPRS,GPAM,GSTT1,KRAS,MAML3,MYL7,OR1Q1,OR2A42,OR2T34,OR51E1,OR7C2,PLCB3,POLR3B,PRKAB1,PRLR,PTTG1,RBL1,MAP3K7IP2
Negative_8371	GHR,AVPR1A,CHEK2,CYP3A43,IL2RG,ITGAL,ITGB4,MADCAM1,NKX3-1,PRKCZ,SIPA1
Negative_8372	ATM,PVR,RDX,ADRA1B,CCL22,CCL5,DGKA,DMGDH,DPAGT1,FCGR3A,GAL3ST1,GALNT7,GNPNAT1,HDAC1,MTMR6,MYL2,OR7D2,OR9K2,POLE2,PRKAG3,STX4,TDO2,TNFRSF4,TRAF3,TUBA1C,VDAC1,B4GALNT1
Negative_8373	FH,CLCA2,FABP3,ITGB7,MAD2L2,NFYB,NRG4,OR10A2,OR6Y1,OR8J3,PRSS1,SC4MOL,UGT2B10
Negative_8374	AMH,CSK,SRF,BDH1,BRAF,CAV3,CERK,COL6A1,DKK1,EPAS1,FOXO1,HSD17B7,NCAM1,OR4B1,OR52E2,PDE11A,PFAS,PGAP1,RFNG,SFRP1,SLC2A2,SMPD1
Negative_8375	HGF,ME2,MUT,ALPI,B4GALT6,BAIAP2,CER1,DKK4,FGF22,FLT3,HMOX1,IFNAR2,IL2RA,OR10K1,OR2L3,PDE4A,PRPH,STX1A,THBS2,UMPS
Negative_8376	DCT,ABI2,ALDH6A1,ALG3,ARSB,ASCC3,BMP4,CYP2D6,GZMB,MARS2,MITF,OR14I1,OR5D14,OR5K1,OR6C2,STT3B,SULT2B1,HLA-DQB1,TNFRSF17
Negative_8377	ADA,ACADSB,ACP2,AREG,ARPC3,CD226,EFNA5,JAK2,KAT2B,NT5C1A,OGDHL,OR1L3,OR51Q1,PRKCA
Negative_8378	ILK,TNN,ADH5,ALDH3B2,BMPR1B,CARD11,CPT2,CSF2RB,CTLA4,CTNNA2,CX3CL1,CYP2A7,DEGS1,DLL1,DPM3,FGF20,GNRH2,HMGCS2,LEPR,MTHFD2,NKD2,NKX6-1,OR10A7,OR10G2,OR8D4,OR9A4,PDPK1,PFN2,PSEN1,SGPP1,STAT3,TPI1,UGDH,ATP6V1B1,KIR2DL5A,ST6GALNAC5
Negative_8379	DDC,HK1,ACVR1,AGPAT6,ARHGEF4,ARPC1B,ATP6V0C,ATP6V1D,CACNA1F,CDC7,CXCL1,DGUOK,DRD2,GANC,GNPAT,GNPDA2,GRM1,HNF1B,HSD3B2,IDH1,IRAK3,NCSTN,NT5C3,OR5M9,OR8D4,PAPSS1,PKMYT1,PPARA,SHC3,SKIV2L2,TAS2R14,TRPC1,WNT10A,ATP6V0A1,PNLIPRP1
Negative_8380	DDO,GGH,MTR,NLK,RET,ACLY,BMP8A,C1QC,CACNB1,CARS2,CCNB1,CYP2E1,DAXX,ETNK1,FGD1,GCNT1,GLO1,GNAO1,GP1BA,IL1B,IL22RA1,IPMK,IRS4,JAK2,MAN1C1,MRAS,MYH1,MYL7,NRG2,OLAH,OR5AS1,OR5M8,OR7A17,OR8B4,ORC4L,PDXK,PIP5K3,PLA2G5,PMVK,POLA2,PTEN,SFRP2,SOCS2,SREBF1,STX10,WASF1,WNT10A,PPARGC1A
Negative_8381	APP,CBS,OSM,ALOX12B,ALS2,APPL1,CASP10,CPT1C,CXCL12,DGKB,EPHX1,FUT9,IRS1,ITGB5,MAP3K12,MAP3K13,MCM5,MGST3,MITF,MPST,NME2,OR10S1,OR10X1,OR2L8,OR5M9,OR9G4,PDK1,POLR3D,PPT1,PTTG1,RAD51,SEMA3C,SETDB2,SH2D2A,SKIV2L2,TOMM40
Negative_8382	ME2,ACAA1,AKR1C3,BMP4,CCL13,CHDH,F2RL1,F2RL2,GMPPB,MAP3K1,MCM2,MGAT4B,NAT1,OR4K17,OR5B17,OR8B8,ORC2L,PANK1,PLTP,POMC,RPS6KA5,SHC4,SLC9A1,SNAI2,TRDMT1,ULK2,ATP6V0A1
Negative_8383	MUT,OSM,GNB3,HRAS,OR52L1
Negative_8384	ITK,ALDH1A1,ALG1,ATP5E,BET1,CACNA1E,CCR3,CD36,CLCA2,CXCL11,DHFR,EPHA2,ETNK1,EXOC7,FGF23,GALNT3,GALNT9,GNAZ,GNRH2,HSD3B1,HSPB1,IL4R,KIR2DS5,KLRC3,LHCGR,NOX1,NRG1,NTSR1,OR10AG1,OR2L2,OR2M2,OR5AS1,OR5C1,OR5D14,OR5M3,OR6N1,RCHY1,SAT2,STX5,TNFSF14,ZFYVE9,HLA-DRB3,TNFSF13B
Negative_8385	IL5,TEC,CCL28,CSAD,HAGHL,HSD3B1,MITF,OR10T2,OR2A14,PAPSS1,PLCB3,RAC3,SOAT2,STMN1,SUOX,TCIRG1
Negative_8386	ATP5A1,CCND2,CDIPT,CLDN6,IL24,OR13C9,OR13H1,OR2B2,OR2G6,OR56A4,OR7D4,PPP1R3A,PPP1R3D,PTK2,PYCRL,SLC2A2,WNT4

Negative_8387	CR2, JUP, MAX, SCD, TYR, XDH, ACADVL, ACTN4, AHCY, AKT1, B4GALT3, B4GALT6, C4BPB, CACNG4, CD27, CLTC, DHRS3, FUT7, GLUL, HLA-C, ICOS, IGF1R, IL23A, IL28B, LAMB4, LDHB, LILRB3, LRP5, MKNK2, NCOA4, NFX1, NR1H3, OR10Q1, OR2T10, PITX2, POLD1, RCHY1, RHOQ, RXRG, STX18, TOMM40, TUBB2C
Negative_8388	AASDH, ACAA1, ADRA1B, ALDH9A1, ALOX12B, ANAPC7, AOC2, ARNTL, ATP5L, CCKBR, CCND1, CCR9, CDC25A, CHAD, EGFR, ENAH, EPHA8, GALNT14, GARS, GFPT1, GLI3, GRLF1, HSP90B1, HTR2C, IFNA17, IL20RA, ITGA10, ITGA7, MAML2, NUDT5, OR10AG1, OR12D3, OR1J4, OR2B6, OR51D1, OR8J3, PIK3CG, PITX2, POLR2E, PPP5C, PTAFR, RASSF1, RPS6KA4, RRM2B, TAS1R2, TAS2R4, TDO2
Negative_8389	XDH, CHAT, FTH1, KAT2B, LLGL2, ODC1, OR10A4, OR5L2, PIK3R1, PIK3R2, PLEKHA8, PORCN, STMN1, THTPA, TICAM1, UNC5D
Negative_8390	DBT, CACNG3, CLOCK, HLA-DRA, IL3RA, MAD1L1, OR4D2, PGS1, PLOD3, PPARA, PPP1R1A, SPRED2
Negative_8391	ITK, ME2, ACY3, AKR1C3, ALG9, ARPC4, B3GAT3, CARS, CPT1B, CRB3, DDX54, GCNT1, GDE1, GLI2, GNPDA2, GUCY1B3, HNF1A, HSPA8, INMT, IRS4, LEF1, LEPR, MTFMT, NEFL, OR10R2, OR1G1, OR1J4, OR4N4, PTPRJ, RASA1, UGT1A10, XYLB, TNFRSF11B
Negative_8392	A2M, CFH, GPI, HYI, KHK, ACAT1, ALG10, AOX1, ARPC1B, ATP2A3, BMP7, C7orf16, CCL5, CLDN22, CLDN4, CTNNA3, CXCL3, DHDDS, EFNA5, ETNK2, GNA11, GNAZ, HHIP, KIR3DL2, MAPK13, NME4, NRCAM, OR14J1, OR52D1, OR8A1, PAFAH2, PDCD1, PFKM, PIGO, PLXNA2, PTGIS, PTPRB, RRM2, SPCS3, SQLE, USE1, MAPK8IP2
Negative_8393	CFH, SCD, SI, TKT, AADAT, ARNTL, ARPC1A, ATP6AP1, CACNA1B, COL4A4, DUSP16, DUSP2, ERBB3, EXT1, EXTL2, HNF4G, IFT57, IQGAP3, MAOB, MASP2, MCM3, NTRK2, OR10G3, OR4P4, OR6X1, POLR2B, POLR2I, SEMA3B, SETD1B, SLC33A1, SRGAP3, TAP1, TUBA8, UCKL1
Negative_8394	MUT, ALAS2, ATP6V0B, CBLC, CDC16, CDC2, CFTR, CHSY1, CREB3, FUT3, GLG1, GP1BA, GZMA, ICOS, IL13, MAP2K2, MCM4, MGAM, MMP14, MTMR2, NEU4, OR1A2, OR1J1, OR2AT4, OR51M1, PDE6H, PDPK1, PFKFB4, PLAUR, ZNRD1, CACNA2D1, HLA-DPB1
Negative_8395	ALDH6A1, CYP4F2, GDE1, GRIN2C, NKX3-1, OR13G1, OR2T3, PIGB, PIK3CA, SRD5A1
Negative_8396	MAG, MPL, PLG, RB1, SMO, ACLY, ALCAM, AREG, BBOX1, CLDN9, CPT1A, CYP27A1, FUT3, GPLD1, HCLS1, INHBE, OR13H1, ORC2L, PAK6, PAX8, PDXP, PHKG1, PLCB2, PPP1CA, PPP2R2B, SHC4
Negative_8397	JUP, ALG11, ALG5, ATP5G2, BDH1, CCNG1, DTYMK, HLA-DRA, HSD17B3, HSD3B1, HSPA8, LIMK1, NOTCH3, NT5C2, OR10G2, OR4K15, OR51A4, OR6C70, PARD6G, PARS2, PIGP, PRPS2, SPHK2, TOLLIP, VCAM1
Negative_8398	TXK, CCL17, CD274, CDH3, CHUK, CTLA4, DERL1, DNMT1, GNAZ, HSD17B8, IL9R, KIR3DL3, LAMA3, MAT1A, MGST3, OR1L6, OR51B2, OR5C1, RRM2, SNAP25, TNFSF8
Negative_8399	HDC, JUP, ACLY, C1QB, CAMK2D, CCNB3, CDH15, CSF3, DIAPH1, EGLN3, GRIN2B, HSPA1B, HYAL1, ITGA3, LRP6, ODC1, OR4A15, OR52I1, PIK3CB, PITX2, PLA2G2F, POLE, PTDSS2, SETDB2, SUV39H1, TAS2R5
Negative_8400	DCK, HK1, PXN, ACSS1, ACYP1, ARNT2, C1QC, CAMK2A, CDC25B, COASY, CYP19A1, DKK2, GAS1, GNPNAT1, GNRH1, HIBCH, IL21R, LAMB3, MAGI1, MGLL, NFKB1, OR10K1, OR11L1, OR1D5, OR2T34, OR52K2, OR6X1, PDGFRA, PHGDH, PIP5K1A, PPP2R5C, PYGM, RBL2, ROBO3, SETMAR, ST8SIA1, TLR5, UGT1A10, WHSC1, ZNRD1, ST6GALNAC3

Negative_8401	BCR,MAG,MYC,ALDOC,ASRGL1,BCAT1,CLDN19,CSNK2B,DIAPH2,DUSP5,EDN1,EFNB1,F2RL2,FTMT,FZD6,HAP1,HTR4,HTRA2,IFNA6,NCSTN,OR10K2,OR2AE1,OR7A10,OXTR,PDE10A,PLXNA3,RPS6KA3,SLC12A2,SREBF1,TOMM40,WNT11,WNT5B
Negative_8402	ALG2,ASCC3,CASP6,CES7,CHEK1,CHRM5,GOT2,IFNA6,IFNGR2,NFKBIA,OR1L4,OR4K14,OR51I2,OR52E4,PARD6B,PDCD1,PIM2,POLR2I,PYCR2,RPS6KB1,SAT2,SESN1,TGFBR2,TP53,WARS2,KIR2DL5A,PNLIPRP1
Negative_8403	C1S,C8G,ACSM2A,ALDH9A1,ATP5F1,C1GALT1,CNTNAP2,FGF14,FSHB,GNA12,ISYNA1,PAX6,PFKFB1,PIK3C2B,PPA1,QARS,SPCS1,STT3B,SERPINA1
Negative_8404	C6,ALDH3B2,EHMT2,GSTO2,MAML3,MAP3K6,MYH6,ODC1,OR52N1,PYGM,SMURF1,SV2B,TDO2
Negative_8405	ACP1,BDKRB2,FRAP1
Negative_8406	ACP2,BMP5,CTNNA1,CYP4F2,GNPDA2,MAP2K7,OR4D1,RASGRP3,TRAF4
Negative_8407	B3GNT3,CALML3,CLTC,GALNTL4,GLG1,GMPS,HES1,HSD17B1,MCCC2,MDM2,OLAH,OR4L1,PAX4,PLA2G2A,TLN1,TRAF5
Negative_8408	ID2,MAX,CCR6,DKK1,EPAS1,EPB41L2,GRLF1,LIPE,MCCC2,MTFMT,MYH13,OR2B2,OR2T29,OR4K2,OR5AU1,OR5C1,OR5M9,RALGDS,TUBB4
Negative_8409	ACOT12,CDIPT,CYP3A43,FGD3,GCLM,GTSE1,HSPA2,OR1L4,OR5C1,PYCR1,SENP2,SMC1B,SOCS7,STX16,TGFBR1,ARHGEF12
Negative_8410	CFI,EPO,GP6,MVD,ACTB,ADAM17,ALDH1A3,ARSE,ATP5D,CACNA1H,CARS,CASP3,CCR8,CDH15,CFTR,CTBS,DKK1,GALNT1,GCLM,GNAI2,GSTA5,IL13,IL1R1,ITGAD,MAN1B1,MCM2,NT5C1B,OR11L1,OR1C1,OR2AE1,OR4C11,OR52I1,OR5M8,P4HA3,PFKL,PPAP2C,PPP3CB,PRSS3,RAB5A,STEAP3,TAS2R8,TNXB,UGT2B7,VDAC1,AASDHPPT,HSP90AB1,PRICKLE2,ST6GALNAC4,TNFRSF14
Negative_8411	C3,CFI,CKB,HK2,HPD,AKR1B10,ALS2,AXIN1,BBC3,COL4A6,CSNK1A1,DNMT3B,DOLPP1,DUSP5,ENO3,GOT1,GRIN1,GTSE1,GUCY2D,HCCS,IL20RA,INMT,NCK1,OGDHL,ONECUT1,OR10V1,OR56B4,OR6Y1,OR9K2,PFN3,PMM1,PON3,PPAP2A,PTCH2,SMAD3,SOCS7,STAT6,SULT1A4,TAS1R3,THY1,UBE2K,UBE2L3,YWHAZ,PNLIPRP2,TNFRSF10A
Negative_8412	ALPPL2,ATP5I,CD58,CDKN1C,CNGA3,ECSIT,ENTPD4,EPHB2,FMO3,FUT6,GNAT3,HSP90B1,ITGA6,LAMB4,MAPK3,OR11A1,PANK4,PCCB,PDGFRA,PLA2G3,POLE4,POLR2I,PPP3R2,PPP5C,SENP2,SH2D2A,STMN1
Negative_8413	MSN,ACAA2,ALDH3B2,ALPI,B3GALT6,B4GALT3,C4BPB,CTLA4,CXCL6,CYP2J2,FARP2,FTMT,FUT5,GRIA1,IRAK1,LCMT1,LILRB3,MAPK11,NUDT5,OR1J4,OR2T11,OR51L1,PAK4,PARVB,PFKFB4,POLR3D,PON3,PPP2R2A,PPP3CA,SMARCA2,TIAM2,TYK2
Negative_8414	ADRBK2,ARPC5L,ASAH1,CD244,CLDN11,CREB3L2,DGKE,FABP3,FADS2,IL22RA1,IL6R,LTA4H,OR1L1,OR2T2,PDE4A,PLXNB3,PNPT1,PRKAB1,PTPN7,SV2A
Negative_8415	ACACB,ADH7,ALDOB,BACE2,CACNA1C,CCNB3,CHAT,CLTB,GCLM,GGCX,GNAL,HLA-DMA,KARS,PLOD3,PRKCQ,RARS,SMARCA5,SYNJ1
Negative_8416	FH,CACNA1D,CYBA,GLUD1,MNX1,PDGFD
Negative_8417	GCK,ACVR1,ARG2,ATG12,CLCF1,CSNK1G2,ENTPD4,GALNT10,GALNT12,GALNT9,H6PD,KLRC3,LAP3,OR52H1,OR6K2,OR8B4,OR8K1,PDE3B,PIAS1,PMM2,STK11,TLR9,TYMS
Negative_8418	ACSL3,AGPAT6,C3AR1,CCR6,CTBS,FGF6,GALNTL5,GNAZ,LGMN,MGLL,MYH10,PDGFRA,PHKA2,PIGU,PIK3CG,PLCB4,POLR2G,RARS2,SEPHS1,TNFRSF8,UBE2L3
Negative_8419	ACY3,ARPC1B,CACNG3,CYP17A1,ERBB4,ETNK2,ITGA9,MASP1,MNX1,OR5AP2,PGAM2,PPP1R3A,PRNP,ST3GAL2,WNT7A

Negative_8420	CFD, PRL, ABCC8, ADRB3, AGPAT2, ARHGEF2, ATP5I, ATP5J2, CCL4, CCND3, CDS2, CLOCK, CTBS, CYP4A22, GALK1, GNAT3, IL22RA2, ITGA7, LRP5, MDH2, MTHFD2, OR8K3, PIGC, PSEN1, PYGM, RPS6KA3, SDHC, SEMA4B, TKTL2, ST6GALNAC5
Negative_8421	ELK4, HLA-G, MAD1L1, MYH7, OR2F2, SPTAN1, WNT10B, HSP90AB1
Negative_8422	F3, HGF, ARPC1B, B3GALT5, BDKRB2, EFNA3, ENTPD3, EPHX2, HNF4G, LCMT2, OR2H1, OR6C76, PAPSS1, PLA2G1B, PRPH2, SPRY4, SREBF1, HLA-DPA1, SUV420H1
Negative_8423	MYC, CHSY3, COL3A1, CPT1B, CREB3L2, EVI1, FGF17, HDAC1, IL13, IL5RA, OR51V1, PKM2, PLA2G2E, PTDSS1, RAPGEF1, SEMA7A, SENP2, SLC25A5, STK3, UPP1, SERPINC1
Negative_8424	CDA, CEL, DLD, ILK, AASS, ANAPC5, ATP5J2, AVPR1A, CCNA2, CLDN17, CYP2A6, EPB41L1, FGF18, FZD2, GNAI1, HCST, IL21, INADL, ISYNA1, ITPA, KAT2B, LGMN, MTMR1, NFAT5, NRG3, OGDH, OR13C5, OR1A1, OR51E2, OR52E6, OR5AS1, OR6B2, P4HA3, PARD6G, PDE3B, PGK1, PIGF, PLA1A, PMM2, PRODH, PTPN1, STX5, STX7, SYNJ1, TUBA3E, UGP2, YWHAE, HSD17B12, SLC25A31
Negative_8425	BAX, ID3, LCK, ACMSD, ACOX2, CCNH, CDIPT, DGUOK, DHX58, EHMT2, EIF4E2, EXTL2, GDF7, HAGH, HIF1A, IDUA, IL3RA, IMPA2, INHBB, ITCH, ITGAE, KLRK1, LIMK2, MAP2K4, MASP2, MGST3, OR1G1, OR51M1, OR52K1, OR8B4, PHKA2, PIGH, PLA2G2A, PRLR, RAB23, RPN2, SDC1, SULT2A1, TBXA2R, TRAF5, TSHR, YWHAQ, ZFYVE9, GABARAPL2, PAFAH1B3, TNFRSF13C
Negative_8426	ILK, ACTN4, ARPC1B, CACNA1E, CYP17A1, DDX4, EPHA1, IL13RA2, NKX2-2, PDCD1, RALA, RERE, SRGAP2, ATP6V0D2, HSD17B12
Negative_8427	PML, SRC, TAT, TKT, ADSSL1, ATP6V0C, BAAT, CAMK2A, CASK, CCL25, CCR10, CHST11, CKS1B, DDX23, EDAR, FCGR3B, FGF13, FTCD, GRM1, IDUA, IL12RB2, LIAS, MOV10L1, NFKBIA, NFYA, NSDHL, OR2A25, OR5AK2, OR5D14, OR8G1, PDE4D, PDHA1, PLOD1, POLR2I, RASGRF1, SGMS2, SH3BP2, SLC25A4, SMPD2, TSHR, TUBB3, ULK1, VAV2, WNT11, TNFRSF12A, TNFRSF13B
Negative_8428	DAO, DCN, FER, HAL, ASCC3, ATP5G2, C1QC, C9orf95, CALML5, CDS1, COL6A2, CXCL10, DGKQ, ENAH, FGF21, GNAS, GRIN2B, GSTM5, HADHB, HNF4G, ICOS, IL17RB, IL18, ITGAM, KCNB1, LCMT1, MAP3K11, MKNK1, MRAS, NARS2, NLGN3, NMNAT3, OR14J1, OR1E1, OR1J4, OR51T1, OR5J2, PLK1, PNMT, PPAP2B, PSPH, PTEN, RPSA, SEMA3E, STX3, TSHR, PLA2G12A, SERPINA5
Negative_8429	FGB, TK1, ABCC8, ACVR1C, CD58, CHST12, CYP11B1, DGAT2, DLL1, FASLG, HNF4G, IFNA7, LCMT1, OR10AD1, OR10C1, OR4A16, OR4C46, PLCZ1, PTGS2, RAPGEF4, RENBP, SEMA3A, SMAD2, TCF7L2, UCK1, VANGL1
Negative_8430	ABO, JUN, PC, A4GALT, ACP1, ACVR1C, ANAPC2, AOC2, CD274, CHRNA7, CYP1A2, DDIT3, EDNRA, FUT5, FZD4, G6PD, GNPDA1, GPLD1, GSTO1, HNMT, ITPA, MYH2, NAT2, OR10G7, OR4A15, OR51G2, OR5L1, OR8B4, PDE1B, PHKA1, PKM2, PLCG2, PLXNA1, PON2, RPE65, TDO2, TNFSF9, TPMT, VEGFC, WEE1, WNT6, WNT8A, TNFRSF10D
Negative_8431	ADCY2, CCL19, CD8A, HLA-DRA, INPP5E, NFATC2, PGAM2, SETD2
Negative_8432	GSS, ADH6, ATP4A, BUB3, CD80, CDH4, CER1, CLDN18, CLOCK, CXCL5, DDX19A, DDX55, DRD2, DUSP14, DUSP3, ENO3, EPHX1, ETS1, FBP2, FGF23, FGFR3, GAL3ST1, GCAT, HNF4A, ITCH, KLRD1, NFE2L2, NTSR1, NUMB, OR1L1, OR2K2, OR2T2, OR8G1, PDE8A, PGAM4, PLOD1, QPRT, RASGRF2, RHOD, RRM2, TRIP10, TUBB4Q, VEGFB, YOD1, ZAP70, SLC25A31, SUV420H1
Negative_8433	EGF, CACNG1, CHSY1, CYP27A1, DERL1, EXTL2, FCER1G, G6PD, GALNTL4, GDF6, GNG13, GUCA1A, HCCS, INPP5B, IQGAP2, ITGA3, LCMT1, MALT1, MAP2K7, MGAT4A, NCK1, NOS3, NRXN2, OR2K2, OR51D1, PER1, PIK3R2, PIP5K3, PRKAG1, SEMA3D, SPRY2, SPTLC2, ST3GAL2, ST3GAL4, STK11, TACR1, TAS2R4

Negative_8434	DHH, IL3, IL8, NLK, BCAT1, CDH2, CLDN11, CLDN17, COL6A1, CYP2C18, CYP3A7, GCDH, IL1R1, NCF2, NFAT5, NKX6-1, NPR2, OR2AG2, OR52E8, OR9K2, PPP2R5E, PYCRL, RAC3, RALBP1, RRM2, SPAM1, SUV39H2, TAS2R3, WEE1
Negative_8435	FUK, GDA, ADRA1D, CYSLTR2, DEGS1, EXTL1, IL7R, ITGAL, MAP3K2, MYLK3, NME4, NRCAM, OR51B6, OR5M9, PPP2R1B, RPRM, SCLY, SLC27A5, THY1
Negative_8436	LSS, B3GALT5, CACNA1G, GDE1, GNA15, LAMB1, NRG1, NSD1, OR2D3, OR4A5, OR4D5, PDE4D, POLR2G, PRKAR2B, SELP, SLC2A2, ARHGEF12
Negative_8437	GDA, ALDH2, ALG3, ALG6, APAF1, ATG7, C3orf10, CD38, CSF2, CSF2RA, DGKG, EPB41L2, FGFR1, FOXO1, FZD9, GARS, GCNT1, ITGB3, KIR2DL3, LIMK1, LYPLA2, MAN1A2, NR1H3, OR52E8, PIAS3, PIK3CA, PIP4K2A, PLAUR, PNMT, PPAT, PRKCG, SULT2A1, TUBA4A
Negative_8438	FST, ABAT, C4BPB, CCR9, CTBP1, CXCL11, DUSP4, ENPP7, FADS2, FASN, FMO5, FPGT, IDH3B, IL28A, INPP5A, JAG2, LDHB, MAD2L1, MAP3K1, NADSYN1, OR10G2, OR2D3, OR2Z1, OR5K1, OR6N2, PARD3, PDE3B, PER2, PFKFB1, PLCG1, SRD5A2, STAT4
Negative_8439	CBL, F5, MOS, RPE, ALPP, C4BPB, CDK4, CDKN1B, CLTB, CYP19A1, FUT3, ICAM2, ITGA6, ITGAE, NAGLU, ONECUT1, OR51F1, PIGL, POLR2K, PPP2R2B, RASGRF2, RASGRP1, RDH11, SHC2, SMPD3, UBA7, UROS, CSGALNACT2, PLA2G12A, PPARGC1A, SERPINF2, TNFRSF1B
Negative_8440	DDC, GP5, ABI2, BMP5, BRAF, CACNG1, CHRNA7, COL5A1, COMP, DDIT4, DHCR7, ECSIT, ENTPD4, EPHA3, GAB1, HLA-DRA, HSPA8, NAGA, OCLN, OR2D2, PDE8A, PGM2, POLR1C, PON1, PPP2R2B, PRKCQ, PSEN2, PTGER3, RASA1, STX8
Negative_8441	SI, TK1, AADAT, ACYP2, ADIPOQ, AMHR2, ARSE, BPNT1, CACNG4, CLDN15, DDX4, DPYSL2, EDNRA, FBP1, GNAT3, GSTM4, HAO1, HMOX2, HTR5A, IFNW1, IL9R, LRP5, MGAM, MMP14, NCR1, OGDHL, OR13D1, OR3A1, OR4F15, PDE6H, PFN4, PIGH, PLA2G2D, PLCD4, PSME3, PYCR2, RRAS2, SDC1, SEMA3C, SGMS2, SH2D1A, SLC2A1, SUCLA2, TRDMT1, TSC2, TUBA1C
Negative_8442	GSS, SRF, ACSM3, ADRB2, AKT1, CCL14, CCNB3, CYP11B1, EPB41L2, GALNT12, IL13RA2, NR1D1, OR5J2, OR6K3, PDE4D, POLR2D, PTGS2
Negative_8443	C1S, DAO, F10, FN1, PAH, RET, AARS2, ACADL, ADIPOQ, ARHGEF7, CLDN17, CXCR4, CYP8B1, DDX52, FSHB, GNB1, GOSR2, HTRA2, IL20RB, INHBB, ITGA3, ITGB8, JAM3, MKNK1, MYH1, OR56A4, OR5B21, OR5P3, OR6T1, OXTR, PGAM1, PIK3C2A, PPM1D, PRKCQ, PRKX, QARS, RAP1A, RAPGEF2, RPE65, SELPLG, SETD1A, TGFB3, TIRAP, TOMM40, UGCG, USE1, WHSC1L1, YARS, HSD17B10
Negative_8444	ASIP, CTPS, SEMA4D, SPAM1, UROD
Negative_8445	AK7, GPT, KDR, OTC, TK1, ACVR2B, ACVRL1, C1QB, CARD11, CAV3, CCR5, CCR9, CHEK1, CXCL9, CYP1A2, CYP4A22, DDX51, DGKZ, ENTPD3, ETNK2, FARS2, FOXO3, GOT1, GZMA, HHEX, HMBS, IGF1R, MAN2A1, MAP2K4, MYH15, MYLK, OR2H1, OR2M2, OR2T10, OR2T5, OR7D4, PERP, PIM2, PRIM1, PSEN1, RASGRF2, SAT1, SV2C, TAS2R40, UNC5A, VAMP3, ALDH18A1
Negative_8446	C5, DLD, PAH, ACSL4, AMD1, BCMO1, BPGM, CD22, CES1, CHSY3, CLDN7, DDX51, EHMT1, EIF4E2, ERBB4, F2RL2, FLT3, FOXO1, GRB2, HSD17B1, IRS4, LILRB3, LRP6, LYPLA1, MCCC2, NAT1, NGEF, OR2Z1, OR8B8, PDE3A, PIGC, PIGP, PIP5K1A, PRKAR2A, RBL1, RDH8, RPS6KA6, SDHD, SEMA3D, SRGAP2, ULK3, WNT4
Negative_8447	DUT, HK3, PAH, ADCY2, ADRA1B, ADRB2, BET1L, BIRC5, CLTCL1, CSDA, CSNK2B, CTNNB1, CYP1A1, E2F3, FECH, GLI2, GNG13, IGF1, ITGAE, OR13H1, OR52H1, OR9K2, PPT1, PRLR, ST8SIA1, TAS1R3, UCKL1, ULBP3, HSP90AB1
Negative_8448	AHCY, CCNG1, GART, GSTO2, ICAM2, IFIH1, IFNA2, IL22RA1, IRF5, ITGA3, NME4, NTF4, OR4K1, POLR2C, PTGDS, RASSF5, TJP2, UCK1, PPARGC1A

Negative_8449	BAD,FUK,IL5,AGPAT1,ALDH3B1,B3GALT6,CD55,DAAM2,DERL1,DUSP5,EXOC7,HTR7,HTRA2,INHBB,ITPA,NTN1,OR1C1,OR2F1,OR5M3,OR8D4,PLXNB2,RASGRF1,SENP2
Negative_8450	AGK,DBI,ACSM3,ALPL,ATG3,ATP5G2,CAMKK1,CCNB2,CXXC4,CYSLTR2,DHX58,DUSP8,EGLN1,ESPL1,FGF14,HNF4G,ICOSLG,IL1B,IL20RA,IMPDH1,INPP1,IQGAP1,IRF7,JAK2,LEFTY2,LIPC,MITF,MTAP,MYH7,NSD1,NUMB,OR2W1,OR7G3,OR8S1,PIGB,PIGT,PLA1A,PLOD1,PON2,PRDX6,PTK2,RPS6KB1,TAS2R16,THBS1,UGT2B17,PAFAH1B3
Negative_8451	C6,ACACB,ALAD,ALDH1A2,ARPC4,ATP5O,CAMK2A,CAMKK1,CCL17,CDC7,CHIA,CYP2S1,CYP7A1,DDX47,DDX51,DHRS4L2,EVI1,FTMT,GP1BA,IDH2,IL22RA2,INHBB,INHBE,ITGB4,LDHC,MAP3K4,MTHFD2,MYH7B,NAGA,NCKAP1L,NRG4,OR10G3,OR2L2,OR2L3,OR52B4,PAX4,PPP1R3A,RXRG,SARS2,SETD1A,TAS2R38,TAS2R41,TBK1,PLA2G12B
Negative_8452	CGN,F7,GH2,GSN,IL4,ACOT12,ACSL4,ADH1A,ANAPC10,COL6A1,DDOST,DIAPH3,ENO2,EXTL3,GRB2,GUCA1B,GYS1,HSD17B7,IL6R,IMPDH1,L1CAM,NMNAT1,OR13C4,OR1F1,OR51B5,PIGM,PLOD1,POLD4,PRKAR2B,RXRG,TACR3,ULK2,ATP6V1G1
Negative_8453	CD6,HAL,HK1,ME2,ACP5,ALG6,ANAPC1,ARD1A,ATP5E,C1GALT1,CCR5,CCR9,CDH1,CHSY3,CYP11B1,CYP26B1,DCXR,DDX19A,DHDDS,ENPP6,FLAD1,GGCX,HNF1A,IMPDH1,ITGA4,JAK3,LAP3,MCCC2,NT5C3,OR1B1,OR4D11,OR4K5,OR5D18,OR8B12,PANK1,PARS2,PLAT,PPP1CC,TIAM2,TUBB6,UCK2,UGCG
Negative_8454	AKT3,CNTN2,CTNNA1,DTX1,GALNT1,GPX5,STX18
Negative_8455	AGL,ITK,ACOT12,ALDH3A1,ALG8,ALPI,ARSB,CCL4,CHRNA7,CNTFR,CTNNA3,DDX50,GAPDH,GNPDA2,ICAM3,ITGB8,MBL2,OR1G1,OR2M4,OR5H2,PDCD1,PIGP,PPARG,PSENEN,SHMT2,STX4,SYNJ1,UCP1,UGT2B17,YKT6
Negative_8456	ADRB2,LYPLA1,PLCB2,PRKAG2
Negative_8457	FES,HYI,MVD,MYC,ZYX,AADAT,AKR1C1,AKT2,ALDH1A2,ALPL,ARHGEF1,ASPA,ATP5B,CXCL5,E2F4,ELK1,GGT1,GZMB,IL28RA,IMPA1,IMPDH2,INPP4B,KNG1,LRP5,MMP1,MYH3,OR2AG2,OR4S1,OR5AU1,PIGH,PLA2G4A,PPP2R5D,PRDX6,PRKAB1,SSH3,TCF7L2,THBS1,UGT2A3,VAMP4
Negative_8458	HK1,AKR1C2,ARHGEF7,ATP5J,CACNA1I,CCL11,CTNNA2,DPYSL2,FADS2,FOXA3,GGPS1,HCST,IDI1,IL1R2,NAGLU,OCRL,OR1L3,OR2AT4,OR8J3,POLD1,PRKACB,PTPRB,RARS2,SEMA3F,SMURF1,ST3GAL5,SUCLA2,TSC2,WHSC1L1,WNT1
Negative_8459	GH1,TPR,ALDH3A1,ATP6V1H,COQ5,CRHR1,CXCL6,EIF4E2,ENPP6,HPSE,IL13RA2,INPP5B,OR2T10,OR8J1,RHEB,SARDH,TGFB2,TICAM1,TNXB
Negative_8460	HAL,CCL23,CSNK2A1,EFNB2,NFKBIA,PLXNB3,PPP5C,TSNARE1
Negative_8461	BID,C1S,KDR,ABCC8,ACY1,ADSS,ALDOA,CAMK2A,CD22,CYP2J2,CYP3A5,EFNB2,FGF4,FLOT2,GCNT4,GUCY2F,HSD17B3,IL20RB,IRAK3,IRS1,MGST2,MGST3,NUDT2,OR10A4,OR2A5,OR2V2,P2RX7,PHKA1,PIP4K2B,PPA2,PPP2R2C,PTGES2,SHMT2,SMPD3,SOAT2,SQLE,TNFSF18,WBSCR17,WNT3,WNT8B
Negative_8462	HGD,HK1,ALDH1L1,ASMT,BMP7,CDC27,CLDN15,CREB1,CTSS,GADD45G,OR10J3,OR1J4,PMM2,POLR2J,PPP2R2C,PTPRB,SLC27A4,SMAD7,TPH1,CSGALNACT1
Negative_8463	CBS,ACOT12,ALPI,CCL27,EFNA2,EIF4B,FGF13,FZD10,GALE,GLB1,GNAO1,GSTK1,IFNA5,IL8RA,INHBE,INPP4A,ITGA6,MYH7,MYL2,OR1A1,PDE10A,PON2,PPAT,PYCRL,SHC1,SLC2A2,SYNJ1,TAS2R16,TSTA3,CSNK1A1L,PDCD1LG2,TNFRSF13C
Negative_8464	HK3,CHAT,GP1BB,IRF9,MYH9,OR13J1,OR6A2,OR7G1,POLR2E,SESN3,SRGAP3

Negative_8465	MVK, SDS, SMO, ACAT1, ACMSD, BCKDHB, BUB3, CANT1, CRKL, DGKG, DUSP7, EPHA8, EPHX1, FMO3, FZD1, GARS, GMPR, HMGCR, L1CAM, MALT1, MAP2K4, MYH6, OR4S1, ORC5L, OSMR, OXSM, PDE7B, RAP1A, RASGRP4, RRAS, THY1
Negative_8466	BID, RDX, ACAT2, AKR1C4, AMY2A, AVPR1B, CCKAR, CCR6, CD99, CFLAR, CMPK2, CRHR1, CTSL1, EIF4B, G6PC, GGT7, GRIA3, HSPB1, IDI1, IMPA1, LILRB3, LTBR, MAP3K13, NARS2, OR2F1, OR52E6, OR6B2, ORC3L, PIGL, PPCDC, PRKG1, PSME3, PTGFR, RBKS, ROCK2, SHC1, SMAD5, TUBAL3, TUBB4, WNT3
Negative_8467	ACSM1, ADCY2, ALG2, AMD1, CASP7, CCL4, CLCF1, CYP11B2, DHRS4, FGF2, FSHR, HADH, HIBCH, HLA-G, MAP3K1, NT5M, OR2A14, OR2A42, OR52I1, PER2, PRKAB2, PRKACB, SAT1, MAPK8IP3
Negative_8468	DCT, F12, INS, ANAPC5, ATP6V1D, BMP6, CACNG2, CCND3, CHRM1, COMP, CREB1, CYFIP1, CYP1B1, DGUOK, DUSP7, DVL3, FDFT1, GNA15, HMGCR, HSD17B2, HYAL2, IARS, IFIH1, IL1B, IL6R, NCSTN, OR8I2, PMM2, POLR2H, PPP1R3D, PRODH, SDC3, SETD1A, SH2D1B, SREBF1, ZMAT3, TNFRSF11B
Negative_8469	F10, GH1, GSS, HAL, ALG2, C1GALT1, CAMK2A, CDC2, CLDN5, CLOCK, DHRS3, ENAH, ENTPD5, FZD2, GCLM, GLO1, GPD1, GSTM2, LAMA4, LCAT, MAN1B1, MPP5, OR5AK2, OR5B17, PRKG2, RASSF1, SELL, STMN1, TSC1, TSHR, WNT11
Negative_8470	ID2, SFN, TK1, WAS, ALG13, APOC3, C4BPA, CBLC, CBR1, COL4A1, CRLS1, CSH1, CYP2C8, DUSP7, E2F4, GALK1, GALM, GAMT, HAAO, IGFBP3, IL17RB, IL1A, INPP4B, MAP3K7, NTF4, NTNG1, NUDT2, OR2AG2, OR2C1, OR52A1, OR52J3, OR5D18, OR5V1, PDGFB, PGLS, PLAU, POLA2, PPA2, PRKACB, RBKS, SEMA4F, SLC33A1, SPP1, TNXB, TRADD, TUBB6, ATP6V0A1
Negative_8471	F7, OAT, ACVR1C, AGRP, AKR1D1, ALDOA, C1QB, CD58, CDC42, CHKA, CSNK2A1, DCTD, DPYSL2, ENO3, FGF22, FOXA3, GRM1, IL12A, KLRC4, MASP2, OR2A4, OR5AC2, OR5AR1, PER1, PLA1A, PLA2G2A, PLCB4, POLR3B, PRKAR2B, PTPRZ1, RDH16, SDC2, SPCS1, ST6GAL1, STX6, TNFRSF13B, TNFRSF1B
Negative_8472	BTC, C3, CRK, HK3, ALOX15, ARSA, CACNG4, CD40LG, HEMK1, MGAT4A, OR4D5, OR51I1, OR6F1, PIM1, PIP4K2A, SULT1A2, TNFSF14, TRMT11, UBE2L6, UGDH
Negative_8473	EGF, ITK, ZYX, ACADL, BAAT, CD99, CDC45L, CHEK1, CXCL6, DEGS1, E2F4, FGFR2, GMPR2, GP1BA, KLRK1, LTA4H, MTHFD1L, OR10Z1, OR3A3, PON2, PPAP2B, PPP1CA, SETD8, SHMT2, SMAD4, STX8, SV2A
Negative_8474	AKR1B1, B3GAT3, CACNA1S, CD59, CDC26, CYP2A6, FCER1G, FIGF, ITGA5, OR6C4, OR9Q2, OSMR, PFKM, PNPO, POMC, SEMA4F
Negative_8475	IL6, CAMK2B, CBLB, CLOCK, CTPS, EFNA1, FRAP1, GDF7, INHBB, ITGA2B, MAP2K2, NOX1, OLR1, OR14J1, OR1A2, OR5K1, PLA2G2D, PLAT, RDH10, RELB, SESN2, USE1, VNN1, HSP90AA1
Negative_8476	C8A, ME3, OGT, ATN1, BCL2L1, CD40, CDH1, CHST12, FUT2, GRIA3, HCCS, IQGAP2, LARS, LDHAL6A, OR14A16, OR4K5, OR4X1, P2RX2, PIAS1, PPP1R1A, PSAT1, SESN3, TNFRSF18
Negative_8477	BAX, CCKAR, CCL23, DTX1, EPB41L3, OR52N2, OR7C2, PDHA1, SPHK1, TGFBR1, CACNA2D1, PPARGC1A
Negative_8478	ADK, GP9, NF1, CHST13, CSF2RB, CSNK1D, DLST, EREG, FGF14, FTCD, GRIN2A, GSTK1, IARS2, IFNA8, OR10R2, OR13G1, OR1N2, OR8D1, PER1, PLOD1, PLTP, PPP2R1A, SEMA4G, SETDB1, SRD5A2, SSX2IP, TSHR, TUBB1
Negative_8479	AKR1C2, BMP8B, CD19, CRHR1, JAK3, LTB4R2, PFKFB4, PPP1CC, TBK1, TLN1, SERPINF2
Negative_8480	AK5, C3, GCK, ACAA1, AMDHD2, BUB1, CYP21A2, ENO1, FPGT, GALNT14, GNA13, JAM2, KLK3, MAD2L1, MPST, OR13C3, OR5D13, OR8A1, OR8H2, PDE1C, PIGS, PIP4K2B, PRKACA, PRKG1, RASGRF1, STX1B, PNLIPRP2

Negative_8481	IL7, ACO1, ACTG1, ADCY1, ALDH1A3, ALDH2, ARAF, ASIP, ATIC, ATP6V0B, CACNA1F, CES1, CHEK1, CLDN7, CREB3L1, CSF2RA, CYP2A7, DOLPP1, E2F2, ELK1, ENPP7, EPB41L3, FBP2, FLOT2, IL10RA, ITCH, ITGB1, KIR2DL4, MAP3K12, MGAT3, MRAS, NAT2, OR1A2, OR8A1, PFAS, PGAM1, PGK2, PPOX, PVRL1, RAP1A, RPS6KA2, SDC3, SLIT2, SPRY2, ST6GAL1, SULT1A1, VAMP1
Negative_8482	C9, CRH, EDA, IL4, ACP5, ACSM2A, ADRA1A, AVPR1B, BET1, BMPR1A, CCL27, CDC6, CES1, CX3CL1, DHCR7, DLST, EPAS1, FTCD, IL13RA1, INPP5E, IRAK3, IRS4, ITGAX, MAFA, MTHFD1, MYH8, NT5C2, NTN1, OR11H1, OR1E2, OR1J4, OR5C1, PDE3B, PGAM1, POLA2, PRKY, PTAFR, RPIA, RXRB, SEMA7A, TAS1R3, TKTL2, HSD17B12
Negative_8483	C1S, LCT, ACAT1, B3GALT2, BUB3, CHAT, F11R, GALNT8, IL13, IL1RAP, IRAK2, LCP2, MGAT4A, NME7, NT5C, OR14J1, OR6C4, OR8D4, PER2, PIGA, PPBP, PRPS2, TAS2R38, YARS
Negative_8484	CSK, ADRA1B, CTBP2, FLT4, GRB2, MAN2A1, MAPT, OR2S2, SMARCA2
Negative_8485	AK3, BCR, CGA, FYN, BAIAP2, CD99, CLTC, CPT1B, CYP3A4, DDIT3, DHRS4L2, EXTL3, GJA1, LDHB, MAP2K2, MAP2K4, MCM7, MYL5, NLGN3, OR5B21, OR6A2, OR6X1, PPP5C
Negative_8486	ADC, BTD, GLS, SMO, AOC3, CACNA1H, CALML5, CHRNA7, CSNK2B, CXCR6, CYFIP1, DAD1, DHCR24, EI24, ELK1, FARSB, FUT1, FUT9, GNAI2, IFNAR1, IKBKB, IL18, IMPDH2, LYZL1, MAOB, NFKB2, OR10G2, OR10J3, OR5B3, OR5P3, OR7A10, P2RX5, PAK2, PDE8A, PIP4K2C, POLD4, PPA2, PPP3R1, PRKACA, PTGFR, RFXANK, RND1, SHISA5, TICAM2, TSLP, TUBB3, VCAM1, TNFRSF11A
Negative_8487	PXN, ACVRL1, ADCY1, CCL21, CLOCK, CPT1C, CREB3L2, EHHADH, FTCD, HTR6, IL23A, LMO7, MMP7, NFKB1, OR7D2, PIGO, PSME2, WNT7A
Negative_8488	LTB, MSN, PLN, ACAA1, ANAPC10, AXIN1, CAV2, CXCL5, DDX23, DNMT3L, FGF22, FMO3, GALNTL4, GBE1, GCNT4, GJA1, GOT1, GSTZ1, HLA-G, HSPB1, ICAM1, IL23A, IRAK3, ITGB2, KDSR, LAMB4, MASP1, NCOR2, NEU3, OR2M2, PDE4C, PFN3, PHPT1, PIP5K3, PLOD1, PPP3CB, PRKAA2, SCP2, SPRY3, STAM, TOLLIP, TUBA1B, TUBB2A, WASL, WNT8B, YOD1
Negative_8489	AK1, C8B, LHB, PVR, TYR, BDH1, CBLB, CCR9, CDK7, CLDN9, COQ6, CREB3L4, CRKL, CTPS2, CXCL5, DMGDH, EFNA2, EPHA4, GALNT1, GSTO1, HIF1A, IFNGR1, JAK2, OR52I1, OR52K2, OR6C3, PIK3C3, PNLIP, PPID, PPP1R1A, PSME1, RASA1, RPS6KA3, SIGLEC1, SQLE, TICAM2, TRADD, TYROBP, ATP6V1B1, HSD17B10, TNFRSF10D
Negative_8490	SFN, AKR1C4, ALS2, ARSD, ATP5E, BLVRB, CD38, EPHB1, GPD1L, HHIP, HTR7, ITGA1, NAT2, NAT6, NRP1, OR13D1, OR4X1, OR8B4, OR8G1, OR9Q1, PDIA4, PSEN1, TBL1X
Negative_8491	PML, CAMK2G, DEGS2, F13B, LAP3, LEPR, NTSR1, OR52K1, PDE4C, RBPJL, ALDH18A1
Negative_8492	PDC, SI, CD47, CD80, DHRS4, FBP1, NCK2, NEU4, OR4D6, PIGH, SGMS2, B4GALNT1
Negative_8493	F7, MAX, B3GALT4, CREB5, GALNT8, GRIA2, HMOX1, OR13C8, POLE2, RELA
Negative_8494	ABO, ABAT, AGMAT, ASMT, BMP5, CACNA1S, CTBP2, CXCL2, CYP4A11, EDN1, FBP2, GALNT14, GP1BA, GRB2, IDH2, IL15, IL8RA, IRF5, KAT2A, LYPLA1, MTMR2, NCR3, NT5C3, NTSR1, OR10X1, OR1A1, PLA2G6, PPP1CC, PTGES, SMAD9, SOCS4, VTI1B, WNT2, WNT2B, YWHAQ, CACNA2D2, PNLIPRP1, SERPING1
Negative_8495	DUT, INS, KIT, SFN, ACSS2, APH1A, ARAF, ARHGEF2, ARHGEF4, BDH1, BDNF, CACNA1C, CCR3, CD86, CDK6, COQ7, CTNNB1, DLL1, EREG, FUT6, HTRA2, IFT57, LIMK2, MDH1, OR5B21, OR8H3, P2RX6, PARVB, PPP2CB, PRKCH, PYGL, RAF1, RASGRP4, ST3GAL2, VDAC1, WWP1, YWHAQ, B4GALNT1
Negative_8496	F5, LYN, ARSE, B3GAT2, BCMO1, CCR7, COL1A2, DDX56, FTH1, INPP1, LIPF, NCR3, OR10H5, OR2T2, OR6M1, PTCRA, RHOQ, SEMA3B, TCF7

Negative_8497	GCK, ACAA1, ACAD9, ACTC1, ACVR1, ALDOB, CCL20, CCL8, CCNA2, CD34, CDKN1A, CNTFR, CYP2A6, DHODH, ECSIT, ENO2, ERBB3, G6PC2, GALNT7, GATM, GNAS, GSTA3, HEXB, HTR4, HTR7, IAPP, MAP3K5, OR10G4, OR13C3, OR14I1, OR1D5, OR6C4, PAK1, PIGL, PIGS, PTPRZ1, RASGRP1, RRAS, SMAD3, SQLE, SV2B, TGFB1, TUBA1C, UBE2L6, VANGL1
Negative_8498	CR2, BDH1, CAV1, CCL24, CYP4A22, DOLPP1, DRD1, HYAL2, ITGAD, OR10J1, OR1D2, OR2AG1, PPP2R5C, PTPN6, TRAF1, TUBB4, ARHGEF12
Negative_8499	ADH1B, ADSSL1, CD70, CDKN1A, IAPP, KLRC2, OR51G1, SHC4
Negative_8500	A2M, AK3L1, DDX52, ECHS1, FGF14, FGFR3, GCLM, GRM1, IAPP, IMPDH1, KIR2DL1, LAMA3, OR10T2, OR2C1, OR4K1, PTPRC, SUV420H2
Negative_8501	F2, KHK, ALAS1, ALDH2, ALDH3B1, APH1A, C1QB, CAPN2, CCR6, CCR9, CD55, CDH2, COL3A1, CTSG, CYP2E1, FGF17, FUT1, HDAC2, IL23R, INHBB, ITGA7, MCM7, OR10A3, OR52M1, OR6M1, OR6N2, PLOD2, ROBO3, SHC1, SRGAP2, SULT1A4, TNFRSF4
Negative_8502	CGA, GBA, IL6, ABP1, ADH1A, ADRA1B, ADSSL1, ALDOA, ATP5E, ATP5J2, B3GALT5, BCAR1, CBLB, CD46, CDC14B, CSDA, CYP2S1, EARS2, ECHS1, GARS, GCDH, GGT6, GMPPA, GNRH1, GYS1, IQGAP1, ITGA1, LFNG, MAN1B1, MAN1C1, OR1A2, OR52N2, ORC4L, PIAS2, POLD3, PPP2R5D, PTPRR, RDH16, SKIV2L2, SOS1, WNT9A, ZMAT3, ATP6V1C2, PRICKLE1
Negative_8503	ALPP, APH1A, CDK4, GCLM, HIBCH, IL18R1, ITCH, ITGB5, MAN1A1, MTHFR, NAGLU, NR5A2, OR6T1, PIGK, POLR2A, STAT2, ZFYVE9, TNFSF13B
Negative_8504	HK3, ME2, AKT2, APPL1, BMPR1A, CD247, CYP2D6, CYP3A5, DAPK1, DPYSL2, EVI1, GCLC, HDAC1, IFNA16, IKBKB, IL13RA2, INPP4A, ITGB7, LAP3, MAOB, MAP4K2, MECR, NCAM2, NCOA4, NLGN3, OR52D1, OR7D2, P2RX2, PFKFB1, POLR3K, SETMAR, TALDO1, TRIP10, VTI1B
Negative_8505	F10, ADORA2B, ANAPC5, ARG1, ARSD, C7orf16, CACNA1F, CCR5, CFLAR, CLDN8, CSDA, CSNK2B, CYP1B1, DDX19A, DNMT1, DTX2, GALNT8, GDF5, HMBS, IFIH1, IGSF5, MAFA, MPST, NR4A1, OR13C9, OR1N2, OR2G2, OR2M4, OR4F17, OR5AN1, OXTR, PDGFC, PIAS4, PRKG2, PTEN, PTPN6, STAT3, UGT8
Negative_8506	CAMK2G, FRAT1, IMPDH1, ITGA1, OR2AE1, OR4M1, OR8I2, TOMM40, PLA2G12A
Negative_8507	ACOT11, ACTN2, AFMID, ATP12A, B4GALT3, BMPR2, CARS2, DGAT2, DKK1, GGT6, GMPPA, IL17A, ITGA10, MAP4K2, OR4C46, OR5F1, OR8B12, PLA2G3, PTDSS2, QARS, RARS, SMAD4, SUCLG1, TNFRSF8
Negative_8508	FGG, GNS, ARD1A, ATP13A2, B3GNT2, CACNG5, FZD10, GALNT7, IMPDH1, KLRD1, OR10G4, OR10H5, OR13C9, PSPH, RDH12, RPE65, SELE, SETD1B, SGMS2
Negative_8509	C2, F7, ADCY9, AFMID, BIRC2, C5AR1, CACNA1E, GALNT9, INPP5B, LHCGR, MGAT3, NQO1, OGDH, OLAH, OR10H5, OR8B12, PDGFC, PLCD4, PPID, PTGER3, XYLB, MAPK8IP3, PAFAH1B2
Negative_8510	CLDN18, CLDN22, IDH1, IFNAR1, IL15, OR1L1, PDGFRB, SARDH, ST6GALNAC1, TNFRSF10B
Negative_8511	DCK, ACAT1, ADIPOR2, CCND3, CDKN1A, CNTN1, COMT, GGT1, OR1L3, OR52B6, OR5V1, PIGN, RPN1, SARDH, SULT2B1, SUV39H1, TUBB1
Negative_8512	TPO, ABLIM2, ACAT2, AGPAT1, APPL1, B3GAT3, BCL2, CD27, COL4A2, CXCL16, GCH1, IKBKE, IL18RAP, LYPLA2, MYD88, OR4K17, PDGFRB, POLD2, PPP1CA, PTGES2, PTPN7, RAPGEF3, SNAP25, STMN1, TAS1R2, VDAC2, WBSCR17
Negative_8513	ASMT, NEUROD1, OR2M7
Negative_8514	AANAT, NUDT9, OR13C8, RRM2
Negative_8515	CAT, CSK, HGF, IL8, MPZ, SRF, ACOX1, ADCY6, ADRA1D, ALCAM, CCL11, CD86, CDS2, COX10, DCTD, E2F3, GATM, GRM5, LIPF, MAD2L2, OR10P1, PDE7A, SEMA4A, SHMT1, TNFSF9, ATP6V1E2

Negative_8516	IL7, SDS, SHH, ACADS, ALAS1, ASIP, AVPR1B, B4GALT6, BCMO1, CCR8, CCR9, CLDN17, CLDN19, CLDN3, CNTNAP1, CREB3L4, CYP2S1, DCTD, DHRS4L2, DVL2, FMO1, IFNA2, IL1R1, INADL, LTBP1, MALT1, NKX3-1, NQO1, ODC1, OR1E1, OR4N5, OR8A1, OR8J1, PFKFB3, PIK3C2A, PIK3C2G, PISD, POLR1C, PRKACA, RASGRP1, SPHK1, TAP1, TUBB, ULBP1, VAV3, ATP6V0E1, ST6GALNAC4
Negative_8517	NGF, AMPD3, CHSY3, DDOST, DGKB, DPYSL2, FUT9, GADD45B, GSTM3, GUCY2C, IL17B, IL8RA, INHBB, ITPA, MGAT3, OR4F15, OR6B3, OR6K3, P4HA3, PHPT1, PIGM, PIK3CA, RPS6KA2, SAT2, TAS2R48
Negative_8518	CR1, SMO, ARNT2, FTMT, NCF1, OR13C8, PLD2, RPS6KA4
Negative_8519	AGL, GNS, IL8, ACTA2, ALDH6A1, AMDHD1, ARRB2, BMP7, CASP8, CDC14B, CSNK2A2, CYP26A1, CYP2A7, DAAM1, FGF1, GARS, GFAP, GPD1L, HYAL1, INHBC, INPP1, IRF3, ITGB2, KIR2DL4, MAP3K8, NANP, NCOR2, OR5F1, OR8H2, PI4KA, PLAT, PNPT1, POMC, PTK2, PVRL3, RARA, SESN2, TKTL1, TPK1, TRMT11, TUBA1C, TNFRSF13B, TNFRSF18
Negative_8520	COL4A2, ITGAE, KCNQ1, NANP, NCAM1, OR3A2, SOCS3, STX7
Negative_8521	LTB, AGPAT1, AKT2, ALDH2, AMDHD2, ARNT, ASS1, B4GALT6, BCL2, CACNB3, CD38, CD80, CDH3, CDK2, CDK7, CHEK1, CHRM3, CYP17A1, FTCD, FZD10, HARS2, IFNA14, IRS4, MYH14, OR10R2, OR3A3, OR51A7, OR51S1, OR52B4, ORC4L, PAPSS1, PAX8, PIAS4, PIGX, SH2D2A, SLC12A2, UGT1A10, UGT2B17, UGT2B7
Negative_8522	ATP2A1, ATP4B, C9orf95, CD86, CLDN9, CYP4A22, GALNTL1, ITGA5, ITGAV, ITGB8, LALBA, OR11H4, OR2T6, OR4D6, OR6Y1, PIGQ, PLA2G2F, RELA, SIGLEC1, SLC2A1, SMC1A, SOCS2, TALDO1, TFPI, YWHAH, TNFRSF10B
Negative_8523	ADK, DCC, AGRN, APOA5, ATG3, ATP6V1F, B3GNT5, CDH3, CLDN14, GALNS, GRM5, IARS, INMT, LHCGR, SEMA4F, TCF7L1, YWHAE, ST6GALNAC1, TNFRSF11A
Negative_8524	CDA, GPT, SMS, ADCY7, CASP9, CDKN1C, CHAT, CHIA, CHRM5, DAPK1, FMO1, GUSB, GYS1, HEXB, IKBKE, NFATC1, OR10A4, OR4C6, OR6C76, PLA2G2F, PLOD1, PRDX6, SELPLG, UGT2A1, WIF1, YWHAE, CACNA2D3, ST6GALNAC1
Negative_8525	CAT, CBS, GHR, GSS, LHB, CD34, GNA14, H6PD, IQGAP3, OCLN, OR4K1, OR6F1, OR6K2, PDE7A, PDPK1, SULT1A2, TAS2R8, ATP6V0A1
Negative_8526	A2M, ADH1A, ADORA2B, ALDH1A1, ALG11, ARNTL, C1QC, DPM3, DUSP1, DUSP6, EARS2, EDN1, GALK1, GGPS1, HEXA, HSD17B7, IDH1, LEF1, MAP2K4, MCM5, NTRK1, OR14A16, OR1M1, OR2F1, OR4F6, OR6C3, PCYT1B, PLOD3, PPAT, PVRL1, RARA, TSTA3, TUBA1B, UGDH, VAV1, YES1, ATP6V1G1
Negative_8527	F8, TXK, ACADS, ACOX3, ACP2, AKR1C1, BIRC3, BPNT1, CHKA, CREB1, CXCL12, CYBA, CYP11B2, CYP2S1, EPHA6, GLG1, IL2RB, KLKB1, LIMK2, MS4A2, NUMB, OR1M1, OR1S2, OR2AG2, OR2T4, OR6C1, OR7D2, OR8B12, PAICS, PIAS1, PIPOX, SC5DL, VAMP3, VCAM1, ATP6V0A2, ATP6V1G2, PPARGC1A
Negative_8528	JUP, ADRB3, ATIC, BIRC3, CCR9, CDC14A, DDX55, FOXO1, GNAI3, GRHPR, IL24, MYH3, OR5A1, PFKM, POLR1C, PTGFR, SESN3, TPH2, WNT9B
Negative_8529	CD6, CXCL2, CXCR6, HAO1, HGSNAT, POLR1B, KIR2DL5A
Negative_8530	DDO, ACADM, ACP6, BCL2L1, CACNG7, DARS2, DHRS3, ENPP6, GABARAP, GNMT, GSTK1, HRH1, KITLG, LAMB3, LDHD, MAT2B, MINPP1, NCF1, NLGN1, OR6F1, OR7A10, OR7G1, PMAIP1, RASSF5, RDH11, ST3GAL6, TSLP
Negative_8531	ANAPC1, CCKAR, CSNK1A1, EPHB2, GCNT2, GSTM2, HAAO, KAT2B, KIR2DL1, LTA4H, ORC2L, PIM1, PIPOX, PLCB1, POLE4, PRKCH, RPIA, SARDH, SEMA4A
Negative_8532	CAT, BMP2, CDC20, DDIT3, EXTL1, GCNT4, IGF1, INPP4A, ITCH, LCMT2, MCAT, NFAT5, OR10G4, OR7A17, PLCD1, SOCS7, THY1, TICAM1, TICAM2, WNT8B
Negative_8533	ADA, CRK, IL5, TNC, ADCY6, CD22, CLDN6, CNTN1, CTF1, GNPAT, ITGB2, OR10Z1, PFKFB3, PRKG1, SMC1B, SSH1, SULT1A1, TRAF2, WHSC1, HSP90AA1
Negative_8534	FH, GSR, ACVRL1, ALDH1L1, GGCX, ITGA5, MYL5, NMNAT2, OR10H1, OR11A1, OR51F1, OR52H1, PIK3CG, PPARG, PTPMT1, RPS6KA4, TYROBP

Negative_8535	ANAPC2,APOE,DLAT,FARSA,FZD3,IL18,ITGA1,KIR3DL2,MAP2K4,OR1B1,OR4K13,PLXNC1,POLE,PPP3R1,PRPS1,RPS6KA4
Negative_8536	LCK,PDC,TNR,ADCY4,ALG9,ATP5A1,CDC25B,LDHB,OR10AD1,PIGL,PTDSS2,SSH2,UGT2B17,ALDH18A1,PRICKLE1
Negative_8537	GCK,ATP5I,FRAT2,IBSP,IGF1R,LIMK2,MAPT,OR10G2,OR4K15,SLC12A2,TICAM1,VAMP3,CTNNBIP1
Negative_8538	EZR,TNF,VTN,ATP5G2,BMP7,C9orf95,CACNA1B,CACNB2,CCKBR,CCNA2,CDC16,CDC27,CDK5,DRD2,DUSP9,GGT5,GRLF1,IGF1R,MYH8,NCK1,OR2V2,OR9Q2,PANK3,PIGN,PRLR,PSENEN,SEMA4G,SNAI2,TOMM40,WARS
Negative_8539	ACSS2,CACNG5,MRAS,NT5C1A,OR7G3,PSPH,SLC33A1,TAS2R8,TAS2R9,TPH2,UCKL1,WBSCR17
Negative_8540	AGL,EGF,F5,FUK,LIF,MET,ADRBK2,AKR1D1,ALOX15,ATF2,CACNG1,CAMK2B,CASP7,CASP8,CCL26,CER1,DTX2,FABP4,FRAT2,FUT2,GCNT1,GNPAT,GRIN1,HNF1A,HPSE2,IAPP,IL13RA1,LTB4R2,MCCC1,MYLK,NEFM,OR1N2,OR2C3,OR51E1,OR7D2,OR8H1,ORC6L,PDGFRA,PGAP1,PLA2G2D,PRDX6,RRM2B,SLC25A4,SORBS1,SSH2,TAP1
Negative_8541	ARAF,C3orf10,HTR4,SLC27A5,SLC9A1
Negative_8542	XDH,AARS2,ATP5A1,BMP6,CCR1,CD59,CDIPT,CHEK1,CHKA,CLDN17,CTBP1,CYP2E1,DAPK1,DDX52,GRHPR,GRIN2A,GSTA4,IL21R,IL22,ITGA7,MKNK1,NFYB,NOTCH1,NOTCH2,NT5C1A,OLAH,OR6C76,OR6N2,OR8D4,OXSM,PLXNA3,PON1,PPARG,PYCRL,SAT2,SDHA,ST3GAL2,TUBA1A,YOD1,ATP6V0E1,ATP6V1G2,MAPKAPK5
Negative_8543	ADC,LPO,ACAA1,ACSS2,ACTN4,ALG5,ASNS,ATP2A2,B3GALT2,BMP8A,BST1,CAV3,CCL21,CCND1,CD14,CHEK1,CHUK,CXCR5,EPHX1,FCGR3A,FLT1,FZD7,GUCA1B,HMGCR,HPRT1,ITGA7,OLR1,OR2A42,OR2D2,OR51F1,OR52N1,OR56A4,P2RX3,PIGF,PSPH,SELP,SIAH1,ST8SIA1,TAS2R4,HLA-DPB1,MAP3K7IP1
Negative_8544	GSS,KDR,NNT,NPL,TNN,ACADM,ADRB2,ATG7,CHKA,CLCA2,CLDN7,CPS1,CXCL2,CXCR3,CYP17A1,CYP4F3,DARS2,E2F2,E2F3,EPHB2,EPHX2,FADS2,FMO1,GUK1,IL2RG,ITCH,MAP2K3,NCSTN,OR2A5,OR2D3,OR2M3,OR51B2,OR6Q1,OR7C1,PDE9A,PIGX,PLXNB1,PPARD,PSPH,PTAFR,SEMA3G,SH2D1A,SLC27A1,TALDO1,TNXB,TRPC1,TXNDC12,VEGFC
Negative_8545	TAT,CEBPA,DHDH,GNAO1,OR6C70,PRKAA2,ST6GAL1,TGFB1
Negative_8546	FST,GPI,IHH,ALG12,ARNTL,ATP5C1,COL2A1,GALE,GALNT9,GBA3,GCNT1,GSTO1,KIR3DL3,OR1L8,OR8D4,PANK3,PLEKHA8,PYCR2,SDC2,SGPP2,SULT1E1,TBXAS1,TNFRSF9,TNFRSF13C
Negative_8547	AR,GPI,LCK,LCT,TFG,ACO1,AHCYL2,ASCC3,B3GALT6,B4GALT6,BMP4,BPGM,CALML3,CREB3,CRY2,CXCL3,DOT1L,DPM2,DTX3,DUSP5,DVL1,GFPT1,GLUD1,GOT1,GRIA1,GSTM5,INPP5A,ITGB3,LRAT,MAT2B,MMP9,OR9G4,POLR1D,POLR2A,PRPS1,SFRP4,ST3GAL6,SUCLG1,TRAF6,TRDMT1,UCKL1,WNT1,WWP2,ALDH18A1,ATP6V1G3
Negative_8548	GCDH,IFNA5,MYH6,PNPT1,PON3
Negative_8549	ABO,AK7,DLD,FGA,LPO,SYK,WAS,ACHE,ADCY6,ADSS,AHCYL1,B4GALT6,BCKDHB,CASK,CCKAR,CD59,CMBL,CSNK2B,DDX55,ENO1,FARSB,FOXO3,GZMB,IL28B,KIR2DL3,LTB4R2,MAP3K8,NLGN1,OR10A3,OR13H1,OR2D3,OR4X1,OR7G2,PANK4,PARVA,PIAS4,PIK3C2A,POLE4,PTPRZ1,RASSF1,RBL2,SLIT2,TAS1R1,THTPA,TKTL1,TPM3,TUBA3E,VANGL2,ATP6V1C1
Negative_8550	CD6,HGF,IL7,SPN,AKR1A1,ALAD,BET1L,BMP6,CACNA1C,CCDC6,CHRM5,CLDN17,DPYSL2,FMO4,GNG12,GSTT1,LILRB3,MAD2L1,MIOX,MTHFS,MYH6,OR1L1,OR4L1,PKMYT1,PNMT,PSENEN,PTGES2,SPRY4,STT3B,TPK1,TUBB1,TYROBP,VAV2,MAPKAPK3

Negative_8551	SCD, AOC3, B4GALT6, CACNA1A, CLDN5, CYP2C19, CYP3A5, EFNA5, EFNB1, FUT4, GALNT7, HMGCL, IL9R, MAGI1, MAN1A1, MAPK10, MGAT4B, MYLK, NAGLU, NEUROD1, OR10A3, OR11L1, OR2G3, POLR1B, PSPH, TUBB1, TYROBP, ZMAT3, EIF4EBP1
Negative_8552	CP, DAO, ALDH1A2, AMY2B, CCL22, CHAD, DDB2, HIBADH, IL18R1, INPP5E, LAMB2, NME6, OR1N1, OR2AG2, OR51A2, PIP5K1C, PLCB2, QPRT, RPE65, SETDB1, SLC25A6, UNC5D, VAMP1, CSNK1A1L
Negative_8553	C2, FUK, MET, ACYP1, ARHGEF7, ATP5J, BLVRB, CACNB1, CCDC6, CCNB2, CHEK1, CHSY1, CLDN16, CSNK1G1, EGLN3, FGF7, FUT8, GART, GMPPB, HTR2C, INPP5A, KLRK1, LTC4S, MBL2, METTL6, NR1D1, OR4S1, OR6Y1, PDIA4, PRNP, PRPH2, PVRL4, PYCRL, PYGB, SHMT1, STAM, STK4, TAS2R4, THY1, TPMT, VASP, ZAP70, PNLIPRP2, TNFRSF1A
Negative_8554	CSK, MUT, PGD, ADRB2, AKR1A1, AMY2A, ARHGEF1, ASS1, CHPT1, CSF1, EPHA5, EPOR, FARP2, GRHPR, GZMB, IGF1, ITGA11, KDSR, LIMK2, MAGI1, MAP4K2, OR2Z1, OR51T1, OR56A1, PDGFD, PFKL, PIGH, PLXNB1, POLR1A, POLR2E, PRKCA, RDH11, RPS6KA4, SC4MOL, STX7, TNFSF9, TUBA3E
Negative_8555	CBL, ACPP, ALDH1L1, AXIN1, CANT1, CTNNA2, DDIT3, EPHB2, ETNK2, FBP2, GALC, GRHPR, GUCA1A, HPSE, IDH3G, IL13, IL1R1, JAG1, LCMT2, MAP2K4, MAPK8, MYLK3, NCK1, OR1E1, PDXK, PIAS4, PIK3R1, PIP4K2B, PLA1A, PLOD1, RRM2B, SPHK2, STAT4, UPP2, WNT1
Negative_8556	MPL, MPZ, SRM, ACSL3, ASCC3, ATP12A, ATP6V0B, C1GALT1, CERK, CXCL12, DDOST, EDNRB, EPHA6, GADD45B, GNAQ, GSTA5, GUCY1B3, INPP4B, KIR2DS5, KITLG, LGMN, NFATC3, NT5C1B, OR14J1, OR51G1, OR52N4, OXCT2, PRDX6, PRKACG, PRODH2, PSAT1, PTPN7, RAPGEF3, SESN2, SKP2, SLIT2, SYNJ1, TGFB3, TIAM1, GABARAPL1, PNLIPRP1
Negative_8557	CHKB, EI24, FGF20, IGF1, PDE4C, PRPH, SOCS7, TACR1, MAPKAPK5
Negative_8558	ID2, ME3, ACVR1, ALOX12B, ASPA, BCAR1, C1QC, CCL26, CD80, CSNK1G2, CXCR6, CYP2U1, MAOA, MYH2, NCR3, NT5M, OR10J3, OR5D14, PIP4K2C, PRKACG, PRKCA, RCHY1, STT3B, TAS2R13, TBL1Y
Negative_8559	ADA, GH1, STS, B3GALT5, CD48, CYBA, GALT, GLI3, HHEX, IFNG, MITF, OR51A2, RDH10, THTPA, TPK1
Negative_8560	COMP, GALNT1, HDAC2, IFNA8, ITGB7, TACR2
Negative_8561	ADCY8, B4GALT2, DBF4, GNA11, GRPR, IL1B, MAN2A1, MAP3K8, METTL2B, MGST3, NUDT5, OR13C3, OR7D4, OR9Q1, OR9Q2, PKLR, RASGRP1, SETDB2, SPTAN1, STX4, ST6GALNAC4
Negative_8562	IDS, ALAD, CCR10, HSD3B2, IGF1, MCCC1, MYH6, NR5A2, PTCH2
Negative_8563	ACO1, ACSM1, C4BPA, CTBP2, DDX18
Negative_8564	F8, IDS, MVK, ACP5, BCAT1, CACNA1B, CBR3, CCNA2, CD14, COL4A4, CXCL16, DDX41, ENPP1, EXT1, GUCY2F, IL6R, INHBA, IRF5, LAMA4, MAML2, MGAT2, OR2T8, OR4M2, PAICS, PIK3R5, POLD1, POLR2H, PPP2R2D, PTPRZ1, RETSAT, RPS6, SHMT2, ULBP2, YARS2, MAPKAPK5, TNFRSF14
Negative_8565	CHSY1, CLCA4, RPS6, MAPKAPK3
Negative_8566	NPL, PGP, CHRM5, DGAT2L4, OR4C12, PDE11A, RDH12, RRAS, SFRP1, SLC25A5, SLIT2, ATP6V1G1, MAPKAPK2, SUV420H2
Negative_8567	IL2, AARS, ALG5, B4GALT6, CACNB2, CLDN8, GABARAP, GNPAT, H6PD, HSPB1, IARS, MAGI1, NPR2, NUMB, OR10Q1, OR2A5, OR51A7, PLAUR, POLR3A, PPP3CA, PRKAG2, PTGFR, PTPRM, WHSC1L1, XYLB
Negative_8568	AK1, TNN, AGRP, ALDH1L1, ALG2, ATP13A2, CACNA1F, CLDN11, COL1A2, ENO2, EXTL1, FLAD1, FUT5, GSTA3, GZMA, HIF1A, IMPDH2, LRAT, NAGLU, OR2M2, OR52I1, OR7C1, PDE1C, PDE9A, PFN4, PLCB3, POLA2, PPP1R3B, SPCS1, TJP3, XCL2

Negative_8569	BID,ADIPOQ,ATP5I,CCND3,CDC25C,CDK2,CHKB,CMPK2,CREBBP,CYP2A13,DCXR,DGKZ,DUSP14,EPB41L1,EPB41L3,FGF4,HIF1A,IDI2,IMPA1,MAP3K12,MASP1,MYH9,NKX2-2,OR2A4,OR4D11,PRODH2,PVRL4,SELP,SLIT1,SRD5A2,TUBA4A,TNFRSF13C
Negative_8570	GK2,GLA,AGPAT2,ALOX15,COMT,FABP1,FPGT,GNA12,GSTA1,IRAK1,KCNB1,MEF2C,PFKP,PRKCH,RAPGEF4,STK4,TBL1Y,THBS4,UAP1,ULK2,YARS,YWHAG
Negative_8571	LCK,ZYX,ARPC4,ATP5C1,CCL11,CLTC,DGKE,HSD17B7,IL23A,MYLK3,NAGS,OR2D3,OR51M1,OR52E4,OR6N2,PDE3B,PGM2,PIGH,PPAT,SEMA7A
Negative_8572	DBH,IL5,ACTA1,CACNB2,CAMK2A,CAMKK1,CCR6,CDC16,CDK7,CHSY3,CLOCK,CRY2,CYP2S1,CYSLTR2,EVI1,F13B,FOXO3,FZD3,GALNT1,GNG13,HEXA,HMGCR,ITGAV,KNG1,LIAS,MYH14,NCKAP1,OR5P2,P2RX6,PDHA2,PLA2G5,PLAT,PPP2R2D,PSENEN,RASA1,RBL2,SDC1,SMPD1,SNAP25,THTPA,TKTL2,TNFSF14,TRAF6,PRICKLE2,TNFRSF1B
Negative_8573	DLD,KDR,ABAT,ALDH6A1,ARHGAP5,CDC16,CDC23,CDC26,CEBPA,CHPF,CHST11,ENAH,GALNT1,GSTO2,GUCY2F,IL19,IL8RB,LEF1,MAPK9,NCK2,OR11L1,OR2M4,OR51G1,OR51I1,PIK3R2,PLCD3,PPP2CA,PRKAG1,PSME3,PTPRB,PTPRF,RELA,SETD2,SPRED2,SV2C,TAS2R42,TKTL2,TLR2,TLR5,TYRP1,WNT1,ST6GALNAC1
Negative_8574	A4GALT,ABI2,BACE1,DPM2,ITGA11,L1CAM,OR1J2,OR6A2,TCF7
Negative_8575	IHH,ADSL,ALDH3B2,CCL19,CCL24,CCL7,CCNB1,CD59,CD72,DERA,DKK2,EIF4E2,GJD2,HNF4A,HNF4G,IL21,INSR,KIR2DL4,MGST2,NT5M,OR13G1,OR4F4,PLCB2,PLXNA3,PNPLA3,SHC3,SUFU,TBL1Y,TUBAL3,UGCG,UGT2A3,UPB1,VARS,WNT8A,YWHAH
Negative_8576	CEL,CTH,NLK,SPN,ACY1,ATP5G3,CACNB3,CYP2A7,GAS1,MMP1,OR8H1,PIGM,PIK3CD,PLCB4,PLOD1,STAM,TACR2
Negative_8577	LHB,UBC,ANAPC2,COL5A2,GUCY1A2,HAAO,HAGHL,IKBKB,MAGI2,OR9Q1,OSMR,PGM3,RPS6KA3,SMAD3,SPRY4,UGT2B7,WHSC1L1,PPARGC1A
Negative_8578	CD6,ID3,ALG12,C1QB,GUCY2D,IFNGR2,IL6R,IRF7,OR2B6,PIGW,PIK3C3,STX4,ATP6V0A1,CSNK1A1L,PAFAH1B1,TNFSF13B
Negative_8579	ITK,BMP5,CCL16,CPT1A,EPB41L1,GSTA5,HRAS,OR13F1,OR2D3,OR4X2,PCK2,PFKM,PGM2,SNW1,SOCS4,PPP1R12A
Negative_8580	HBEGF,PARD3,PSAT1,TAS2R50
Negative_8581	F5,HGF,CAV3,CCR5,CDC14A,CNGA4,FMO2,HAGHL,IL1RAP,IL21R,KIR2DL3,KIR2DL4,LARS2,MAP3K5,MAP4K4,OR2G2,PRKAB1,PRODH2,PTGES,WBSCR17,YWHAE,MAPK8IP2
Negative_8582	CFH,PC,ALG9,ATP5B,CD72,DUSP7,EPHA4,FBP2,FGD3,FGF8,FZD6,GNAI1,GSTO1,GUK1,HTR7,IDH3A,IL10RA,KCNQ1,NEFH,NFKB1,NRG1,OR2AT4,OR2F2,OR2T34,OR52K2,OR5F1,ORC1L,PDE4C,PKM2,PLA2G3,PPARA,PPP3R1,PRKAR1A,RAPGEF4,RBKS,SDHA,SLIT2,SOD1,ST3GAL5,TCIRG1,UNC5C,ATP6V1E1,HLA-DPB1
Negative_8583	NGF,NNT,CCNB2,MGAT2,OR2M2,OR2T2,PGK2,PIGV,PRKAG2,PRPS1,SULT1E1,CSNK1A1L
Negative_8584	CD6,CP,EDA,AARS,ACLY,ACVRL1,AGPAT4,AKT1,ANAPC1,ANAPC10,BIRC3,CACNA1S,CADM3,CD19,CD274,CDC14B,CSF2RA,DLST,EPHA4,FGF14,GRAP2,IFNA21,IFNB1,IFNK,IL22RA1,ITGAL,MAP3K14,OR2G2,OR52B6,OR5K1,PFKL,RUNX1T1,ST6GAL1,STX12,SULT1A4,TGFA,XYLB,PLA2G12B
Negative_8585	LCT,ACTG1,ACYP1,AOX1,B3GNT3,CAMKK2,CCNB2,CDC25C,CYP2S1,ENTPD1,EXOC7,F2RL1,GNPDA2,GOSR2,ITPK1,MAML1,NRCAM,OR52N4,PCYT1B,PLXNA2,PYCR2,RDH10,RPSA,SOAT2,STK36,HSD17B12,PDCD1LG2

Negative_8586	DCN, KHK, MIF, MVD, NF1, ABLIM1, ATG12, B3GALT6, CD70, CLCA4, CYP1B1, CYP2C8, DHX58, DIAPH3, EPHB4, EPHX2, GRHPR, GSTK1, GSTT1, IL13RA2, ITGAL, JAM2, MGAT1, MYH14, NAT6, OR10A2, OR1E2, PFKP, POLA2, POLR2C, POLR2L, PPT1, PRKCD, PSAT1, PYCR2, RALB, RASA1, SMAD5, SRGAP3, STK11, STX1A, TAS2R42, TAS2R5, UGT8, ULBP1, CACNA2D3, ST6GALNAC4
Negative_8587	HYI, ACOX1, AGXT, AKT1, ALDH1A1, ALDH6A1, ATP5J2, CDK2, CNGB1, CREB1, CRHR1, CXCL10, CYP26A1, DOLPP1, FOSL1, GALNT12, HGSNAT, HLA-DMA, HSD17B8, ITPA, MAOA, NFATC4, NFKB1, NUDT9, OR10A2, OR5P3, OR6B3, OR7A5, PGLS, PI4KA, PRKACB, PRKCE, PRNP, RARS, SHC1, SREBF1, STAT4, STAT6, TLN2, TUBB4Q, WNT3, XCL1
Negative_8588	AASS, GNRH1, MGAM, SNW1
Negative_8589	GNA15, GNG12, NFX1
Negative_8590	FABP4, OR4D9, OR5L1, STT3B, STX10
Negative_8591	DCT, GPI, KDR, PLN, ABAT, ATP5B, ATP6V0C, ATP6V1F, BCMO1, CD47, COL1A1, DEGS2, G6PD, GNRHR, HARS, IL22, IRAK2, ITGA2B, MYH14, NTF4, OR10H2, OR13J1, OR4M1, OR6F1, VANGL2, CACNA2D2
Negative_8592	F11, ID4, ALPPL2, ATP5O, CDH1, ITPK1, KLRC4, LDHC, MMP2, NCK2, OR51G2, OR5M9, OR5T3, PDGFA, POLD2, PPP2R5C, PPP3CC, ATP6V0D2
Negative_8593	AK3, LYN, NOG, SDS, ACO2, ACSL6, ACTG2, ACY1, ALCAM, ALDH1A3, ATP4B, C5AR1, CD40, CLDN3, CNTFR, DMGDH, DNMT3B, DUSP10, ENPP2, ETNK2, HADHA, LDHB, LTA4H, MDM2, MECR, MITF, NCAM2, OR10G2, OR13C4, OR13G1, OXCT1, PMM1, TNFRSF9, WNT3A
Negative_8594	MPI, CFTR, CMBL, CNDP1, CYP1B1, DHODH, DUSP3, ENAH, GNRHR, HHEX, IQGAP3, KNG1, LALBA, LAMA3, LDHAL6A, MAGI3, OR13G1, OR1A1, OR2G6, OR5K2, PDCD1, PTDSS2, RPS6KA2, SGPP1, TIRAP, TSHR, MAPKAPK2
Negative_8595	DUT, GLA, IVD, PRL, ACY3, ADRB2, ATP6V0B, CANT1, CCR9, CDC25A, CHAD, CLCA4, CLDN8, CXCL9, CYP3A7, E2F4, EPHB2, FADS2, FUT8, GCAT, GMPR, GSTZ1, IL20, LIFR, MCM3, NFAT5, NRP1, OR1N1, OR5A2, P2RX7, PAK2, PFKP, PGS1, PLAT, PPP2R1B, PRKCD, PTGIS, RPN1, SDC1, SGMS1, SHISA5, SHMT2, SOCS4, STMN1, TFDP1, TRHR, UNC5A, YWHAG
Negative_8596	NLK, PGF, ACADSB, ADRB3, ALAS1, ALOX15B, CCR4, IL22RA2, ITGB6, LHCGR, OR5M9, PARD6B
Negative_8597	BIRC3, CSF3R, PPP1R3D, SELL
Negative_8598	SRM, ANGPTL4, CERK, GSTA2, MCM3, MMP7, NGEF, OR4K15, OR4X1, PLTP, RASGRP2, THY1, TSHB
Negative_8599	SMS, ACP6, AGXT2, ATF4, BMP8B, CARS2, CNTFR, GUCA1A, MCM6, MYH7B, NRCAM, OR8B12, PAK4, PIGT, STAT3, UROC1
Negative_8600	ACTB, CACNA1G, DHDH, FUT8, NCR1, NRG4, OR10Q1, OR3A3, OR5T3, RHOQ, ROBO2, STX10, UCK1, ARHGEF12, B4GALNT1
Negative_8601	MAX, TXK, ACVRL1, AGRN, COQ3, CYP17A1, CYP2D6, CYP3A43, ECHS1, FARSA, FGF18, GALNTL2, GMDS, IL2RB, ITPKA, NKD1, OR2AT4, OR2T4, OR6C3, PHKB, POLD3, PRKCD, SLIT1, SPTLC1, ST3GAL3, TLR3, VDAC3, WNT3A, CACNA2D3, KIR2DL5A
Negative_8602	DCC, MSN, ACAA2, ACAT1, ACSM1, ACSS1, ADCY1, ADCY7, ALG12, BET1, BLVRB, BUB1, CADM1, CCND1, CCNH, CDC14A, CDC16, CDH1, CISH, CYP7A1, FGFR1, FZD9, GALNTL5, GNAO1, GSTA4, HPRT1, INPP5B, INSRR, LCAT, MALT1, MAP3K5, MCM3, OR13J1, OR14I1, OR1F1, OR2A42, OR4F6, OR4N5, OR6A2, PIK3C2A, PIK3CD, POLR3K, SHC3, SV2C, UROD, VANGL1, VEGFA, VEGFC, PRICKLE2
Negative_8603	ALDH9A1, ENPP3, NUDT8
Negative_8604	DBI, IL5, CD274, CDC25C, COL5A3, EPRS, FARSA, FGF14, GFPT1, OR7C1, ORC5L, PAPSS2, PFKFB1, PPA2, TXNDC12, UMPS, UPP2, WWP2, ATP6V1G1

Negative_8605	GK, IVD, ADCY3, ADRBK2, AHCY, ALDH1A1, CAMK2G, CAV1, CDO1, CHRM5, EDNRB, EFNB3, ESPL1, FLOT1, GALNT13, GALNT14, GALNT5, GBA3, GRIN2A, HMOX2, IL1A, ITGA10, NADK, NOS3, OR52A5, OR8A1, PAX6, PIK3C2A, PRKG2, RAB5A, RASSF5, UGDH, WHSC1L1, PDCD1LG2
Negative_8606	RET, ACOT12, CIITA, CMPK1, EPHA5, FZD8, MDM2, NQO1, OR2M7, STX10, TMLHE
Negative_8607	TPO, ACP1, ADIPOR2, ADRA1A, ATP4A, BMPR1B, CACNA1I, CADM1, CCL11, CDH5, CFLAR, CLDN17, CSNK2A1, FARS2, FGF23, FMO1, GALK2, GNAI2, HMOX1, HSD17B2, MAP3K2, MCCC2, MS4A2, NFKBIA, NFYB, OR2D2, OR5T3, P2RX3, PHPT1, PLA2G4A, PNPT1, PRKCZ, PTCH1, RAB23, RDH11, ROBO3, SMPD3, UNC5A, VASP, TNFRSF1B
Negative_8608	F7, ADCY6, CCR1, CDC25B, EFNB2, FSHB, FUT9, GGT6, GUCY1A3, IFNA13, IRF9, LAMC1, LRAT, MAOB, OR13C5, OR2A42, PDE3A, PGS1, PRKY, SELL, SMURF2, SPRY3, C1GALT1C1, TNFRSF10C
Negative_8609	AGL, IVD, NGF, PXN, ATG3, ATP5A1, CACNA1B, CAV1, CCL1, CLCA2, DGKI, DUSP1, HAP1, HIBADH, IQGAP1, LCMT1, NCOR1, NCOR2, NFATC3, NR1D1, NR5A2, NTF3, OR11A1, OR11H1, OR11H6, OR2T10, OR2T4, OR6C6, ORC1L, PCYT1B, PIAS3, PIGX, PRKAR1B, RAC2, SPRED1, STK4, UROS, KIR2DL5A, TNFRSF11B, TNFRSF1A
Negative_8610	BCR, BLVRB, CACNG1, CAMKK2, CCNE1, CLCA2, DPAGT1, NAGS, OR4F4, PLOD2, POLA2, POLR2G, POLR2H, PTGES, STAT4, PPP1R12A
Negative_8611	ACOX3, ADCY8, ARRB2, CBLB, CCKAR, DPAGT1, DTX4, IGSF5, OR52B6, PIGV, SFRP4, TNFSF9, CACNA2D4
Negative_8612	IVD, ACAD9, ALPP, AREG, BMP8B, CALML3, CAPN2, CCNB2, CDKN1C, CYP26B1, DARS, DHRS9, DUSP10, DUSP4, EPAS1, FLT1, GALT, HYAL2, IFNW1, IL1B, KLRD1, MMP2, NRG1, OR2AE1, OR2T29, OR5B12, OR8K3, OR9K2, ORC3L, PFKFB1, PPP1R3C, RELA, RERE, RFXANK, SEMA3A, SEMA3B, STAM, HLA-DRB3, SERPINE1, ST6GALNAC3
Negative_8613	SMO, VTN, ABCC8, CASP3, CYBB, DDX52, DPM3, DTX3, FBXW11, FGF6, G6PC2, GADD45A, HTR2C, IFNA4, IFNAR2, ITGA4, LAMA4, MMP1, OR13D1, OR2M3, OR5C1, PHGDH, PIK3C2A, RASGRP1, SEMA3B, STAM2
Negative_8614	AARS, ADSSL1, AGPAT3, ALG3, AOC3, BTRC, COASY, CYP2A7, EGLN1, FZD4, GABARAP, GALNT2, GUCY2F, HLA-DOA, MAP3K6, NOX3, OR14J1, OR4N4, OR5AN1, OR7C1, PDE6G, PLA2G2F, PPP2R5B, PPP3CC, PRKCI, PTCRA, SEMA3E
Negative_8615	AGL, CTH, GCK, LPO, ADAM10, ALCAM, ALG6, ARPC2, ATP6V1F, B3GAT3, BHMT, CADM1, CCDC6, CCNH, COL5A1, DPM1, FUT8, GDF6, GUCY1A3, IDH3G, IGSF5, KLKB1, KLRC4, KRAS, MAD1L1, MGAT5B, MTHFD2, MYH7B, NCKAP1, NFYB, NPR1, OR2B6, OR4C46, OR52A1, OR6X1, OR8D2, PCNA, PIK3C2A, PIK3CB, PIK3R2, POLR2J, RFX5, SDHA, SH3BP2, SKIV2L2, TLR3
Negative_8616	ASL, TAT, CTBP2, EHMT2, NAMPT, PGK2, PTGFR, RFX5
Negative_8617	IHH, TK2, ABL2, ACSM5, ACTG2, ADORA2B, B3GAT3, CACNA1E, CDC25A, CYP2J2, DHX58, ECHS1, ENO3, GSTM3, HNF4A, IL1A, ITGB7, LAMA4, LFNG, NANS, OR10H4, OR2D2, OR2H1, PIK3R2, POLE, PTEN, SARS, VANGL2
Negative_8618	TNC, ADCY8, ADSS, C3orf10, FGFR3, FUT2, HAO2, HSD17B8, ITGA5, KAT2B, MAP3K14, MAP3K7, MGAT5B, NFATC3, NOTCH3, NT5E, OR10H3, OR2AG1, OR5B21, OR5V1, PCNA, PHGDH, PIGA, PLXNA1, PNPT1, POLR2B, PPP2R5A, RHEB, SH2D1A, TACR2, TAS2R4, TNFSF10
Negative_8619	DCT, FADS2, PVRL1, RELA, SOCS5
Negative_8620	MPL, BDKRB1, CD82, CDC20, CDKN2A, CHRM5, GRIN2C, KIR2DS4, MAP3K5, MYL7, NR5A2, OR52N4, OR5B17, OR5L2, PCK1, PLCZ1, SORBS1, SSH2, YWHAB, PHOSPHO1

Negative_8621	GP6,PLG,ATN1,CD14,CD247,CLDN9,DPM3,E2F3,HMGCL,HTR2B,KIR3DL1,LEF1,MCAT,NLGN3,OR10G2,OR51G2,OR52N5,OR56A4,OR6B3,OR8U1,OR9A2,PIP4K2A,PIP5K1A,PRSS3,SDC1,SETDB2,TNXB
Negative_8622	LPO,MIF,ABAT,AGRN,ALDH4A1,CTPS2,DGAT1,DHDH,DOCK1,EFNA4,FZD9,GNAZ,GRIN1,GSTM5,IL6ST,JUND,MYH10,NOTCH2,NTF3,OR1S1,OR7G1,PCK1,PECAM1,STK36,TIAM1
Negative_8623	C4B,F3,ID1,AACS,DGKQ,FBXW11,GLI1,GRIA2,GUCY1B3,ITGA7,MAP3K3,NLGN1,OR51B6,POLR2L,PPBP,SEMA3E,ZIC2
Negative_8624	ADCY9,ALG8,ASS1,BAAT,CCNG1,CYP17A1,GNAI1,HTR5A,KIR2DS4,RERE,SSH3
Negative_8625	F3,ADCY9,CRB3,CRLS1,CX3CR1,DDX55,DERA,GNG12,MPST,NCR2,NOS1,PDK1,PSME2,SMURF2,SOAT2,STAT5B,TSHR,TNFRSF6B
Negative_8626	AFMID,ALG1,CD34,DERL1,EPHX1,IL12RB1,IPMK,NKX3-1,OR13C8,PPARG,SMAD9
Negative_8627	GH1,CASP6,COL6A2,DDHD1,DDIT4,DHRS9,DUSP2,ENTPD2,FABP4,GPLD1,HNF1B,IL15,INSR,NCF2,OR5A1,PDGFRA,PLA2G3,SELL,SETD1A,WASL,ATP6V0A1,ATP6V1G2
Negative_8628	CP,HDC,KHK,TNF,TPO,ADCY8,CCL16,CDO1,COL5A3,DIAPH2,EFNB2,ENPP6,GALE,GALNTL4,GDF5,GGPS1,GSTP1,ICOS,IL13,IL5RA,KIR3DL3,KLKB1,MAP3K3,MTMR2,NADSYN1,NEFL,OR11A1,OR3A2,OR4K15,PDE8A,PLCD4,PPP1R1A,STAT4,SUCLG1,PNLIPRP2,ST6GALNAC3
Negative_8629	GLS,IL8,BDKRB2,CHPF,EPHA8,FADD,GALNT12,GPAM,IRF7,OR51A2,OR8B4,UGCGL2
Negative_8630	PTS,ACSM5,BLNK,CASP7,CD274,CHRM3,COL6A1,CXCL13,CYSLTR1,EI24,ERBB3,FGF10,FOSL1,FOXO1,HSD11B1,INHBB,ITPKB,OR4C13,OR5B17,PDE6G,PERP,POLR2G,PTPN6,RDH5,SELL,SEMA3B,SEPHS1,TGFBR1,UAP1,ULBP1,WEE1,WNT2B,ATP6V0A4,HLA-DRB5,PRICKLE2,TNFRSF14
Negative_8631	DCK,MPL,NOG,AARS,ACOT11,ADSL,ALOX15B,ARG1,CACNG7,CALML6,CASP3,CCNB1,CD44,CMBL,CRHR1,CTPS,CYP11A1,CYP3A7,DGKA,FGFR2,GADD45A,GPD1L,GUK1,GUSB,IFNA13,IL13RA2,IRF9,KIR2DL4,MADCAM1,MAGI1,MAPK3,MCCC2,MGAM,MGAT3,MKNK2,NR4A1,P2RX2,PAICS,PIGK,PLA2G6,SULT2A1,TAS2R10,TRAF6,TSNARE1,TUBB1,UGT1A10,ULK1,ULK3,TNFRSF13B
Negative_8632	ALG6,CD99,COL5A1,CREB3L2,E2F3,GRIN2B,GRPR,IFNAR1,IKBKE,IL17RA,KIR2DL1,LCP2,NANS,OGDHL,OR1N1,OR4L1,PHKA1,PTPRM,TLR4,VTI1A,WNT3,ZAP70
Negative_8633	TEC,ABP1,ALDH3B1,CYP4A11,DAD1,DPYSL2,EPHA6,GDF7,LRRC4C,MAN1A1,OR1B1,OR6Q1,PPP2R2B,RDH5,SLC12A2,SMC1A,TYRP1,WNT4,SERPINF2
Negative_8634	TYR,ADCY6,CXCL16,MAN2A1,MCM7,NCAM2,NR5A2,OR10A3,OR51Q1
Negative_8635	ADC,AR,FAS,FH,GLA,KIT,SI,AASDH,ANGPTL4,B4GALT6,CAV1,CDKN2D,CHD8,CMBL,CSF2,CTBS,CXCR4,DGAT1,DUSP6,ECHS1,EPHA8,GALNT7,GCNT4,GNRH2,IRF5,LTC4S,MYLK3,NUDT9,OR2T5,OR8D2,PARVA,PMVK,PRKAG2,PRKCH,PRPS2,PTPRR,RENBP,SCLY,SORBS1,SQLE,STEAP3,TRDMT1,WNT5A,PAFAH1B1
Negative_8636	ACADS,CHEK1,COL2A1,EFNA4,ITGB8,NFKBIA,NMNAT3,NRCAM,OR10P1,OR6C4,WBSCR17
Negative_8637	GP5,SFN,ABLIM1,ADH7,AFMID,ALAD,BBC3,CTBP2,DGKE,ENPP7,FARP2,LDHAL6B,LRDD,MAP2K1,MLYCD,NFATC3,OR13C5,P4HA3,POLR2C,WNT3A,WNT6
Negative_8638	ADK,ASL,TH,ACTA2,ADCY7,ADRB3,AHCYL2,ASPA,ATP5I,CCL4,CD19,CD27,CDC16,CPOX,CREB3,EFNB1,EXT2,ICAM2,MYD88,MYLK2,OR4F6,PIGB,PSAT1,RARS,TACR2,KIR2DL5A

Negative_8639	KIT,LEP,CAMK2A,GPAM,HEMK1,IGSF5,LTBP1,MTMR2,NTN1,OR2M5,PSME3,XCR1,PHOSPHO1
Negative_8640	ABI2,CCL5,CLDN7,CRY1,OXTR
Negative_8641	DCC,B4GALT3,BIRC5,FGF17,JAK3,MBL2,OR4N5,OR51D1,OR5AU1,PLD1,POLE4,POLR3K,ZIC2
Negative_8642	ALDH3A2,AMPD1,CD19,CREB3L2,DIAPH1,ELK1,FZD2,IL28A,MAOA,MYH1,NKD1,NOS3,OR10Z1,OR1M1,ORC2L,PRPH2,SHC1,SHC4,TALDO1,TUBA1B,VAMP2,CACNA2D4
Negative_8643	C9,FGB,SFN,ADRBK2,ATP4B,BIRC5,BMP7,CRY1,FADD,FCGR3A,HRH1,IPMK,IQGAP2,METTL2B,NR1D1,OR10H2,OR51A4,PDE4D,PIK3R3,RARS2,SDC1,SEMA3B,TNFRSF18
Negative_8644	FGG,HGD,ABAT,APOA1,BMP7,CLDN6,CXCL16,CXCR5,CYFIP1,CYP2C8,DDX41,FARS2,GALK1,MPST,NEGR1,OR10AG1,OR1C1,ORC6L,PDE6D,PER3,PIK3R3,PPAT,PPP2R5D,TBL1X,TNFRSF9,TNFRSF1B
Negative_8645	MVD,MVK,RET,TK1,ACADS,ACTN1,ATP5J2,BACE2,CNTFR,COQ3,CTBP1,CTPS,CX3CR1,CYP2C18,DARS,FGF5,FMO5,GAS1,IFNA21,IKBKE,KAT2B,KCNQ1,LCMT1,NEU4,NFYC,OR1J1,PARD3,RBX1,RXRB,VDAC1,MAP3K7IP1
Negative_8646	DDC,IVD,AGRN,CALML6,CCL23,CD8A,CHAD,CLTCL1,EPOR,FARS2,GPD1,ISYNA1,JAK2,LAMA4,NEFH,OR14A16,OR4A15,OR4D11,OR5F1,OXCT1,PGM1,PIGL,PIP5K3,PTPRR,PYCRL,QARS,SRGAP1,STK3,STX16,TALDO1,TCF7L2,VAV2
Negative_8647	ADH1B,B4GALT5,CLDN7,CPT1C,CYP1A1,DKK4,EPHX2,FZD4,GDF6,HLA-DRA,LEFTY1,MAP4K1,NT5M,OR4D11,P2RX7,PANK1,PIGN,PIGO,POLR2K,PPARD,RASGRP4,RBPJ,TNFRSF13C
Negative_8648	OSM,SMO,BST1,CAV1,CLCF1,CMPK2,CNGA3,CYP17A1,CYP2F1,DGKQ,DNMT1,HADHB,IFIH1,KIR2DL3,MAP3K5,MAPT,MTMR1,NCSTN,NEUROD1,NLGN1,NOTCH3,NTSR1,OR2AG2,OR2T1,OR5B12,P2RX7,PDE6H,PDGFD,PFKFB1,PLAU,POLD2,POLR2F,PPAP2A,PRKCH,PTDSS2,RAP1B,TAS2R38,TUBA3C,UGT2B7,UGT8,ULK1,XCL2
Negative_8649	AR,FOS,GGH,ITK,ABL1,ALG2,BET1,BIRC3,CD46,CD70,CDKN2D,CLTCL1,CNDP1,DUSP8,EPHB6,GABARAP,GPT2,HSPA2,IL15,ITGA7,KCNQ1,KLKB1,LIPT1,MMP9,NEU2,OR10C1,OR52A1,OR8J3,ORC4L,PDE4C,PLTP,PNMT,PTTG1,RAP1B,RDH16,SHISA5,SPRED2,TLN1,TOMM40L,TUBB3,UNC5D,ATP6V1B1,CSGALNACT2
Negative_8650	ABAT,ALG6,BLNK,CDH2,JAK1,METTL6
Negative_8651	IL13RA1,IL29,MAML2,NNMT,OR13C2,OR1J1,PAICS,PLAU,TRAF4
Negative_8652	EXT2,MAP4K2,ORC5L
Negative_8653	ALDH1B1,C9orf95,CX3CR1,FPGS,GBGT1,INPP4B,LRP5,OR4C12,PFKL,PIP4K2C,RDH11,TRAF1
Negative_8654	ALCAM,ALG5,APOC3,BAIAP2,DCXR,GSK3B,LIFR,NT5M,OR8B8,PLD2,POLR3G,PPP2R1B,PTGFR,RERE,SMPD3,TUBB2A
Negative_8655	ADRA1D,ALDOA,CCNB1,CLDN17,DHX58,DNMT1,DPM3,EVI1,FTH1,GNAZ,IFNAR2,INPPL1,MARS,NCR2,OR4N2,OR4X2,OR51G1,OR52E4,PAK6,PAX4,PIGM,POLE4,POLR1C,PPP1R3D,PRSS3,TPMT,ALDH18A1,CACNA2D1,TNFRSF1B
Negative_8656	GBA,ACOX2,DERA,HSD17B3,NCOR2,OR8J1,SOCS3
Negative_8657	MIF,RPE,ALPP,BCKDHB,CACNB4,CCND3,CKMT2,CTBS,FGF13,GALNT14,HLA-DMA,IARS,IL19,NME2,NUDT9,OR3A1,OR52K2,OR5B12,OR9A2,PCYT1A,PGM3,PIGA,RAC3,SFRP4,SNAP23,STAM2,UGT2B7,ULK2,UNC5B,SERPINE1
Negative_8658	DHFR,DVL2,GMPPA,NAMPT,PAPSS2,POLA2,RAPGEF3,SEMA3D,SPAM1,UGT2B11

Negative_8659	VTN,ADORA2B,ANGPTL4,BMP2,CARS2,CLDN16,CRLS1,CXCL12,CYCS,FPGS,FTH1,HCST,HSD17B4,KCNJ11,KIR3DL2,KITLG,LAP3,MAN1A1,NFKBIA,NNMT,NOX1,OR1E2,OR2Z1,OR4F4,ORC5L,P2RX3,P2RX4,PIK3R4,PIP4K2C,PRPS2,PRSS3,RAPGEF4,RCHY1,TAS1R3,TJP1,TYMP,TNFRSF6B
Negative_8660	KMO,TK1,ACVR2A,AGXT2,ALOX12,ANAPC10,ATIC,CSNK1E,EDNRB,FZD9,GUCY1B3,HADH,ITGB2,MTHFS,NR1H3,OR10AG1,OR1L1,OR2AG1,OR4P4,OR5P3,OR6C76,OR7G3,PAK3,PDE7A,PDE9A,PDHB,PDIA4,PIK3R5,PMM2,RXRG,SIAH1,SUV39H1,CSGALNACT1
Negative_8661	ABL2,ALDH2,CCNE2,CPT1B,DRD1,ENTPD6,FGF3,GLUL,GPAM,IPMK,KIR2DS5,NFYA,NLGN2,OR52B4,PIGN,PIK3C3,PIK3R3,PTPRJ,RPE65,RPS6KB1,SDC1,TAS2R13,TP73,TRADD,TSC1
Negative_8662	FN1,BCL10,CHD8,CNTN2,FUT9,LAMC2,MMP9,NFKB1,SOS1,SERPINB5
Negative_8663	TH,B3GALT4,BMP6,CACNB2,DDX50,EIF4E2,ERBB2,EXOC7,F11R,GALK1,IFNG,IPPK,ITGA5,MAPK13,OLR1,OR4K5,PPP1CB,PPP3CB,PPP3CC,PRKCE,SRGAP1,TGFBR1,TSC2,UNC5B
Negative_8664	AMH,CRH,DAO,AARS2,ACMSD,ACVR2A,ANAPC10,ARRB1,BMPR1B,CBR1,CCL15,CHST13,CPT1A,EXT2,FZR1,GRIN2B,HMGCS2,IL12B,NCSTN,NFATC2,NME4,OR2T10,OR51I1,OR51L1,PDE3B,RALB,SEMA4B,SUCLG1,VEGFA,GABARAPL2,HSD17B10,ST6GALNAC1
Negative_8665	ACOX2,AGXT,ARNT2,CAMK2B,CDH3,IFT57,OR5B17,OR5K1,SHC4
Negative_8666	AHCYL2,ARNTL,CLTB,CRLS1,KAT2A,MAP2K2,OR10H3,OR13H1,OR2T2,TBL1X,TNFRSF1A
Negative_8667	NLK,ALPI,ARNT,CACNA1I,CAPN2,CYP26A1,E2F2,GSTM4,GYS1,MCM5,PIAS2,PSEN2,SORBS1,TGFBR2,TNXB,XIAP
Negative_8668	GH1,IL8,IL9,LTB,MIF,SRC,ALDH3B1,AOC3,B3GAT2,CCL26,CEBPA,CLDN17,CNGA4,COL2A1,ENPP3,FGF9,G6PC2,GALNT7,HAGHL,HES1,IL17B,MAOA,MAOB,MAPK8,OR13C2,OR1Q1,OR2M7,OR6F1,OR8D4,PARS2,PDHA1,PVRL1,RFNG,SHC4,SPCS3,SUCLG1,TP73,VAMP3,TNFRSF13C
Negative_8669	PDE8B,PPARG,RPS6KA4,SDHB,SOS1
Negative_8670	MAX,ALDH6A1,BACE2,BDH2,CDC25A,CTNNA1,CXCL12,CYP2C19,DDX23,ECHS1,FOXA2,GADD45A,GGT5,HSD17B4,IFNA16,IL2RA,KIR2DL3,LPAR1,MDH1,OR2G2,OR5T1,PAK2,PIP5K3,PTTG1,RIPK1,TPM3,TUBB2A,PNLIPRP1,SERPINC1
Negative_8671	IFNA4,IL20,TAP1,TYRP1
Negative_8672	OR7A5,PIP5K1B,TUBA4A
Negative_8673	ALG12,BCAT1,BNIP1,CAMKK2,CCL27,CHIA,CTBP1,CUL1,EGFR,FGF18,GAD2,GAPDH,GCLC,GLS2,GNB3,IL20RB,KIR3DL2,NCOR2,NT5C1A,OR4F5,OR6B3,PIGS,PLCB3,POLR3H,PYCR1,RFWD2,RUNX1,SEMA4G,ST8SIA5,STK3,TSC1,UGT2B4,VNN1,WHSC1L1,YARS2,TNFRSF1B
Negative_8674	AGPAT1,AHCY,CYP2U1,CYP4A11,HSD3B1,IDUA,OR13C9,PARD6G,PIK3C2A,PRKAR1A,TAS1R3,UXS1,VAV2
Negative_8675	EBP,SP1,ADCY8,CES2,CFL1,CLDN3,CNDP1,DLAT,DRD2,EPHA2,HIP1,HLA-C,HNF4G,KCNJ11,MAN1B1,MAPK10,MAT2B,OR51D1,OR51Q1,PDX1,PLXNB1,POLR1E,PTK2,RRM2B,SPRED1,ST3GAL1,TGFBR2,TRHR,TUBA1A,UPB1,UPRT,YWHAH,ZNRD1,HLA-DQA2,HSP90AA1
Negative_8676	IVD,TYR,ALOX15,ATG7,BBC3,BPNT1,CCR5,CHRNA7,CNTN2,ENTPD3,EPHA5,FTCD,GADD45G,ICAM3,INHBB,INPP1,IRAK2,KLRC1,LHCGR,MAP3K12,NEGR1,NFAT5,OR4S2,OR56A4,OR9A2,PTGFR,TPMT,YARS2
Negative_8677	GPT,NF1,NNT,B4GALT7,BCL2L1,CDC42,CREB3L4,CYP2A6,DGKZ,DHCR24,DPYS,EPHB3,EPHX2,GATM,GLYCTK,GNB3,HHEX,HSPA8,INPP4A,LDHAL6B,MTHFD1L,MTMR1,MYLK2,OR13J1,OR5AU1,OR7G1,OR9G1,PIAS1,PIK3C2G,PLCD3,PTGS2,RPS6KB2,RRM1,SARS,SELE,SETD7,TPH2,VARS

Negative_8678	CD47, CNGA3, CYP26C1, DUSP7, ENTPD8, FLT1, FLT3, FSHR, GSTK1, NAT2, NEU3, OR3A1, PCYT1A, PKLR, SPRY4, THTPA, ATP6V1C2, SERPINE1
Negative_8679	LCT, FDPS, NADK, NKD1, OR52E2, OR6Q1, RIPK1, RPS6KA3, TLR2, PDCD1LG2
Negative_8680	GSN, TK2, ALG5, ARD1A, ATP6V0C, CARS, CASK, CCNE2, CD72, CHRNA7, CRHR1, EP300, ETS1, FMO3, GGT5, GNRH1, GRB2, ITGAM, KIR2DL2, NCKAP1L, NPAS2, NR5A2, OR51B6, PER3, PTCRA, PTPRZ1, PVRL4, RALBP1, SORD, ST8SIA5, STX8, TBXAS1, TNFRSF9, TOMM40L, VAV2, WARS, YWHAB, YWHAG, YWHAZ, CACNA2D4, PNLIPRP2
Negative_8681	F10, MOS, TXK, ACAA1, ALAD, ALOX12, ARPC3, ATF2, ATG3, BPNT1, CCL11, COL4A4, DGAT2L4, ENTPD7, EXTL1, FGF19, FUT7, GAD1, GDF7, GFPT2, GMDS, GNA15, IDH3A, IFNAR2, IL29, ITGAM, KLK3, LALBA, LAMB4, LCP2, MAP2K5, NCF2, NFATC1, NME7, OR2C3, OR52D1, OR52K2, OR5L2, P2RX3, PDE3B, PFAS, POLR3H, PPP1CA, RFXANK, TUBAL3, UPP1, WNT11, CACNA2D2, PRICKLE2
Negative_8682	AK1, GGH, TXK, ACLY, ACSS3, ATP5J, CD27, CDC2, CDC7, CYP17A1, CYP3A43, DDX52, DHCR24, DUSP9, FGF3, GLS2, GMPR2, GNB3, GSTM5, IL10RB, IL21R, ITGAD, KIR2DS4, KLK3, NCF4, OR2D2, OR2T5, OR4X2, OR51B2, PANK4, PCYT2, PVRL3, SC4MOL, SMAD7, TGFB1, ULBP1, WNT2, ZIC2
Negative_8683	TYR, ABLIM3, ANPEP, ATP6V1A, B3GNT3, CCL23, CCR9, CLDN4, CNTN2, CRY2, DFFA, DTX1, GMPR, IPMK, MMP14, MRAS, OR4C13, OR52N1, OXSM, PDE4B, PNPLA4, POLR2L, PRKAA2, PTGER3, PYCR2, SUFU, TGDS, TNFRSF14
Negative_8684	ANAPC11, AOX1, CCL2, CTNNA2, EARS2, EHMT2, EPHA2, GGT7, GZMA, HAO2, HCST, INPPL1, KIR2DS5, NLGN1, NTRK2, NUDT2, OR9Q2, PLAU, PLCB2, PLXNA2, PMM1, PSPH, PTPRF, PYGL, RASGRP1, RDH16, RENBP, RPS6KA5, SETDB1, SHC2, UGT2B17, UROC1, WNT2B, YKT6, ATP6V0A4, HLA-DPA1
Negative_8685	IL3, TNC, ATP6V0C, CSF2RB, EPB41L3, HTR4, IKBKE, IL8RB, INPP5A, MAP4K3, NT5C, PIP4K2C, SETD1A
Negative_8686	CCL4, GRIA1, RFNG, SDC2
Negative_8687	BID, CR1, CS, DCN, PLG, ASPA, ATP5G1, ATP5J2, CCL20, CDC42, CDKN1C, CHAT, DAPK1, DUSP10, E2F1, FGD3, GALNT3, HAAO, HEXB, IRF9, NANS, NRG2, NRXN2, OR10S1, OR4F17, OR52B6, OR6C70, OR9I1, PARVG, PDE4A, RFX5, RPS6KA3, SGMS2, SMAD4, SORD, SOX17, TCIRG1, CACNA2D3, TNFRSF6B
Negative_8688	ACOT12, DMGDH, NEFH, OR2M7, SMAD2, UAP1
Negative_8689	CDA, CRH, DAO, TEC, TNC, ACAA2, ACPT, AGPAT1, ALOX15B, ARHGEF7, BIRC5, C1QC, CCL20, CCNE2, CLDN19, CXCR5, CYBB, E2F5, FUT8, GSTA4, HIF1A, HSPA2, IQGAP3, MAN1C1, MAPK1, MCM3, NRCAM, OR13H1, OR52R1, OR5T3, OR7C1, OR8B8, PKM2, PLAUR, PTCH1, SCLY, SEMA7A, SFRP1, TUBA3C, UAP1, ATP6V1C2, SERPINB5, SERPINE1
Negative_8690	AMT, APP, BID, C6, CAD, FST, IL7, ADH5, ANPEP, BPNT1, CD226, CFL1, COL4A2, CSF1, CYP2C18, EPHA7, GNAS, HCLS1, HLA-F, HSD3B7, ITGB5, MAP2K5, MAP3K12, MARS, MCM5, MPDZ, OR2AE1, OR4N2, OR51B4, OR56A4, OR8B8, OSMR, P2RX7, PAX4, PGLS, PGM2, PIP5K1A, PLXNA1, PPAP2C, PRKAA2, SESN1, TOMM40L, UAP1, VAMP4, WNT10A, ZMAT3
Negative_8691	TNR, ACTG2, ALPPL2, CDC26, FGF3, GSTA3, MAPK13, PCCB, TAS2R14, TPK1
Negative_8692	OTC, ALDH6A1, ANAPC11, ATP5H, CAMKK1, DKK2, EI24, FMO1, GLS2, GUCA1C, IL9R, IRS1, ITGB8, KIR3DL2, KLRC4, OR2H1, OR5L1, PDGFC, PIP5K1C, PLCB3, PNMT, PTGES2, SOCS5, STX4, SULT2B1, TGFB3, TICAM1, TNFRSF4, TRDMT1, TUBB1, UAP1
Negative_8693	ASL, ID2, IL3, ACACA, ACOT11, ALG2, APLP1, ARAF, ATP5D, CNTNAP2, DARS2, DDB2, ENTPD1, FABP1, FOXO1, FPGS, GCNT3, HNF4G, HTR5A, KRAS, LGMN, LTC4S, MYH13, NAT2, NRAS, NTRK1, OR5R1, PGM1, PHPT1, PIK3R2, PPP3CB, QPRT, RRM2, SMAD9, STX3, TOMM40, ULBP3, VEGFC, ZIC2
Negative_8694	MYC, CDC23, DIAPH2, EPRS
Negative_8695	ACOX1, ACVR1, CCL26, IGF1R, IL13RA1, KLKB1, LHCGR, MAPK10, MCM6, NEFH, OR7C1, PAK1, PIM1, PRKX, PYGM, SPP1, ULK1, GABARAPL2, MAPK8IP2

Negative_8696	NPL, ACTA1, ACTB, AKT3, ARNT, ATP5G2, CAV2, CBR3, CHRD, COL4A4, CTLA4, DDX54, EPHA6, FUT1, GADD45B, GDF5, GNAT3, IDH2, IQGAP2, ITGA9, MGAT3, OGDHL, OR10G3, PLA2G6, RUNX1T1, STAT5A, ULBP1, VTI1A
Negative_8697	AK2, ACTA2, ALOX15, ATP5J, B3GALT1, CAMKK1, CDH4, CHST13, DNMT3B, EPHX2, FGF10, FUT1, GALNT3, GPX5, GRAP2, HLA-DOA, IL4R, MAFA, MAN1C1, OR13C4, OR1G1, OR56B4, OXCT2, PLXNB2, PORCN, PPP3CA, PTTG1, SEMA4B, STAT1, TLN2, TUBB3, VAMP2, XYLT2
Negative_8698	F9, PTS, ACADS, ANAPC10, AREG, BLVRA, BMP7, CALML3, CCDC6, CCNE2, CLDN15, CRHR1, CXCL2, CYBA, GSTM1, HLA-DOA, IDH2, ITGB6, MCM5, OR4Q3, OR51B5, OR5M3, PIGF, PLA2G2A, PLXNA3, PNLIP, RBPJ, RHOQ, TNFSF12, TPI1, TUBB2C, UGP2, PRICKLE2
Negative_8699	EHHADH, IL18R1, IL22, NFATC4
Negative_8700	GH2, GSR, MOS, TKT, ACADM, ACOX3, ACSM2A, ACY3, AKR1C1, ALAS2, B3GAT2, C1GALT1, CCL25, CD38, CDC14A, CHD8, CHSY1, CYP2S1, DDIT3, DHRS3, DPM2, EPHA8, GAL3ST1, HYAL2, LTBP1, MAN1A2, MICA, MOV10L1, NCAM1, NRG1, OR10H2, OR7D2, PGAM4, POLR2I, SEMA3C, SHISA5, SIGLEC1, SULT1A1, TRHR, UGCGL1, WNT2, C1GALT1C1, PRICKLE1, TNFRSF13C
Negative_8701	AR, PGP, SRM, TXK, ABP1, ACACB, ACTG2, ADRBK2, ANGPTL4, APH1A, ARG2, ARPC5L, B4GALT7, CASP9, CAV3, CCL3, COL5A3, CSNK1G3, CYP2A6, DAPK1, DIAPH3, DTX1, ECSIT, EPHA4, FADD, FZD5, HBEGF, ITGA2B, KITLG, LRP6, NFATC1, NME4, NPR2, NQO1, NUDT2, PLA2G1B, RAP1A, RASA2, RBPJL, RDH12, RENBP, SEMA3C, SMARCA2, STX5, TUBA3C, UBE2L3, ULK1
Negative_8702	ADSSL1, ALDOC, ATP5C1, ATP6V1D, CD58, CLDN9, GALNTL4, GNAI1, GRB2, GSTK1, GTSE1, HMGCL, LYPLA2, MITF, OR1L8, OR2B6, OR2M5, OR4M2, PIK3CA, PRPH, SNAP23
Negative_8703	ARHGEF7, CACNA1S, CD19, GRAP2, IFNA16, IRF3, NEFL, PFAS, WNT10B
Negative_8704	C3, AKT1, ATF2, CTSL1, FBXW11, HPSE2, IL13RA2, LMO7, MAD2L2, MAP3K6, MAPK13, MECR, MYLK3, NAGA, NCK1, NSD1, OR1D2, OR2A2, OR6X1, OR6Y1, PIP4K2C, QARS, RAPGEF2, RRM1, STEAP3, TRIP10
Negative_8705	DDO, PVR, CACNB3, CCNB1, CHRM5, ENTPD8, IRF7, MYH4, OR1C1, OR52W1, PTDSS1, RDH5, RELA, SLC12A2, TGFBR2, CACNA2D3
Negative_8706	AR, FUK, GHR, SMS, ZYX, ADAM10, ARNTL, CAMK2A, CES1, CPS1, CTNNA3, DARS, DDX56, GPLD1, ICAM2, KLRC4, OR12D3, OR4N5, OR52L1, OR5R1, OXCT2, PARD6B, PLA2G4A, POLE2, PRKAR1A, QPRT, SETD2, SLC27A5, SPRY1, ST8SIA5, STAT2, TUBB2B, HSP90AA1, PNLIPRP2
Negative_8707	CAD, CAT, CKM, IVD, MSN, MVK, ACTG1, ADIPOQ, BMP6, CREB3L3, CSNK2A2, DCTD, DNMT3L, GNAO1, GPD2, IFNA21, IL4R, KIR3DL2, KLRC4, MECR, NAE1, NFATC2, OR1D2, POLE, PPA1, PYCR1, RDH11, TNFSF9
Negative_8708	LEP, SRM, AKT1, ASNS, BMP6, CDH4, CTNND1, ENPP2, FABP1, FADS2, FASN, FLOT1, GATM, GP1BA, GPD2, GSTO1, IFNA5, MGAT4B, MYLK2, OR2A14, PLA2G4A, SETD8, SUFU, TAS2R42, TSHR, VCAM1, PAFAH1B3
Negative_8709	APP, CD2, ATP5D, B3GNT3, BAI1, C5AR1, CDC25C, CDKN2A, CES2, CHRM2, CHST13, DDX41, DPM1, ELK1, EXTL1, FLOT1, HIBADH, HSD17B1, LIPT1, MCM7, NTSR1, OR10Q1, OR5A2, OR5I1, OR5K1, PDE1C, PIK3R4, POLR2H, PPA1, SETMAR, SMURF2, UROD, YWHAH, TNFRSF10B
Negative_8710	CRH, ACOX3, CCL28, CD80, DUSP3, FGF11, GYS2, IAPP, IMPDH1, INPP1, LAMB3, MAN2A1, NKX6-1, OR4K5, SAT1, SPRY4, TAS2R7
Negative_8711	BCAR1, BET1, E2F4, FBXW11, IKBKB, LRP6, MDM4, MMP9, NCF1, NPAS2, OR5M3, PCYT2, PMM2, TGFBR1, THBS1, TSC1, ULBP3
Negative_8712	MIF, ARSE, B4GALT2, DNMT3A, FUT6, GPD1, IL11RA, OR4N2, OR8K3, RPIA

Negative_8713	FGG, ADH4, ADIPOR1, ALG10B, ATP4B, BIRC5, BPGM, CACNA1F, CCND3, CLTA, CNGB1, COL5A1, CREB3L4, CYFIP1, DDIT4, DTX2, FGF1, GNA13, GRHPR, IFNA7, KCNQ1, MAPK9, MPZL1, MYH15, OR1K1, OR2D3, OR4C16, OR4D11, OR5T2, P2RX5, PARVA, PTDSS1, RASSF1, RPN2, SESN1, STMN1, TACR1, TAS2R1, TAS2R3, MAPK8IP1, MAPKAPK5, PLA2G12A
Negative_8714	C8A, GP9, GSN, ARHGEF1, ARHGEF2, CACNA1E, CAPN2, CNDP1, COL4A1, CTNNA1, DGAT2L4, EPB41L2, EVI1, GAL3ST1, GDF7, GNA12, GPAA1, GPAM, HEXA, IL15, LEFTY2, NR1D1, OR10H3, OR52R1, OSMR, OXCT2, PLD2, PTGS1, RGS3, STT3B, TAS1R2, TAS2R49, TAS2R50, TGFB2, UAP1, XCL1, PAFAH1B2, PNLIPRP1, TNFRSF11A
Negative_8715	ADO, FH, ARHGAP5, ATP5E, NRXN2, OR3A2, OR6C4, PDCD1, PGK2, RXRA, SRGAP3, ST3GAL4, TAP1
Negative_8716	DHH, FGA, HGF, IL9, AGPAT1, AGPAT3, ANAPC7, APOA1, CDK2, CHIA, COL4A2, CTLA4, DFFA, DGKG, FGF5, FMO3, FZD9, GALNTL2, GAPDH, GNMT, GUK1, IL12RB1, ITGAM, ITGB6, MYH2, NADSYN1, NOS1, NOX3, OR11A1, OR1A1, OR2B2, OR2D2, OR2L2, OR2L3, OR4D5, OR51S1, OR5V1, PDE1B, POLE3, PPAP2A, PPM1D, PTAFR, RPS6KB1, STX8, SULT1A1, VKORC1, WWP1, PAFAH1B3, ST6GALNAC3
Negative_8717	CRK, GNS, ACP5, ACTG1, ASPA, BIRC5, CCNG1, CSF3R, CYP2A7, DAD1, DGAT1, DHRS3, DPYS, DVL3, EPHA8, FABP4, GALNT5, GNA14, GNAS, LTBP1, NQO1, NUMBL, OR10J5, OR10Z1, OR2L2, OR51M1, OR5B3, OR5P2, OR7D2, PDE4A, PDIA4, POLR2L, PTGER3, PTPN6, RAC3, RARA, RASSF1, RPIA, SUFU, UROD, ATP6V1G3, MAPK8IP3, TNFRSF13B
Negative_8718	MOS, AVPR1A, DCTN1, NME4, OR2M4, PTTG1
Negative_8719	PDC, CXCL11, FZD5, GRIA2, GRIN2C, GSTM1, GUCY1A2, ISYNA1, ITGB4, MALT1, MAN1B1, MASP1, OR2B6, PGK1, PISD, PLXNB1, PRNP, PTGIS, WARS, TNFRSF10C
Negative_8720	FAH, ACTG1, ANAPC2, ATP5J2, CD72, CD99, CHAD, CYP1A1, EXTL1, GALT, GLO1, GNAT3, GNPDA1, HIBADH, INMT, MAP2K3, MYH14, OR4E2, OR5D14, PGAP1, RASGRP1, RPS6KA1, TAP2
Negative_8721	LYN, MVK, ACSM1, B3GALT2, B4GALT5, CACNG6, CD19, CDH1, CHKB, CISH, COL4A6, CYP2C8, ENAH, G6PC2, GGCX, HSP90B1, IL10RA, IL12A, MASP1, MGAT4B, NFATC4, NMNAT1, NOX1, OCRL, OR2AE1, OR56A1, P2RX4, PDE1B, PLA2G2F, PTK2B, RXRA, RXRB, SFRP2, SPHK2, SSH2, STAT1, UGT2B17, WASL, WBSCR22, XYLT2, YWHAQ, HLA-DPB1
Negative_8722	RB1, ADRB1, CDC23, EREG, FRAT1, GSK3B, GSTM2, IL12RB1, NAE1, OR4N4, OR5T2, PHKB, PRKCE, ST3GAL5, SULT1A4, HLA-DPA1
Negative_8723	ACVR2B, ALG1, ATP5O, BCAR1, CHSY1, GJA1, GRIA2, IFNA6, KLRC2, LTBR, MCEE, NADK, NFKBIB, TCIRG1, UBE2L6
Negative_8724	ADAM17, ASPA, CCR5, GSTO1, UGT2B4
Negative_8725	CFD, ADIPOR1, GPAA1, ITGB5, LEPR, OR11H6, PRICKLE1
Negative_8726	LCK, NNT, NOG, TEC, TXK, ADCY4, AMY2B, B4GALT1, CCR7, COMT, CSNK2A1, CYP2A13, DAPK1, EIF4E, FGF3, IRF7, NFATC4, OR10S1, OR1L1, OR2T33, OR51M1, OR5B3, OSMR, P4HA2, PAK3, POLR3D, PTDSS1, PTGES, SGMS1, STX16, SV2A, WASF2, ZBTB16
Negative_8727	CAT, OSM, ACADVL, AKT3, ALOX5, ARPC5, AVPR1B, CACNA1A, CD74, CHKB, CHST13, CYBB, DUSP14, E2F1, EFNB1, EGLN3, FGF10, FMO3, GRHPR, GSTO1, HARS2, HLA-DOB, HLA-DRA, HSPB1, ITGB2, KAT2A, LTBP1, MALT1, MAP3K4, MTHFS, NCF1, NMNAT2, NOTCH2, OR10G4, OR2G3, OR4F6, RFNG, SPTLC2, TPMT, ULBP1, ATP6V1B1
Negative_8728	ALG6, ARG1, CDKN1B, COQ7, GMPR2, GPD1L, HEMK1, IDI1, IFNA17, IL20RA, MAP4K4, MAPK1, OR2T3, OR52D1, PNPO, PRPS2, RPS6KA4, SEPHS1, SETD7, TLR2, TUBA1C, UCP1, MAPKAPK5

Negative_8729	JUP,LSS,ACSL3,ACSS3,AK3L1,ATP5H,B4GALT3,C1QA,CDC14A,CHRNA7,CHST13,CHUK,COL4A1,CSF1,CYFIP1,DIAPH1,DTX3L,DUSP14,EFNA2,EPHA1,EPHA2,GSTO2,IL29,INHBE,ITCH,LDHAL6A,LIMK1,MAN1A1,MAPK12,NANP,NUDT5,OR2F2,PCYT2,PDE10A,PGAP1,PGM3,PIGS,PIGU,PLCD1,PLK1,PRKAR2A,RARS2,SNAI1,SPP1,SSH3,TM7SF2,CACNA2D2,HSD17B10
Negative_8730	CAD,HGF,ARSE,CAV2,CLTA,LRAT,OR2A4,OR8K1,PDGFC,RARS2
Negative_8731	IHH,MYC,ADCY9,ADRA1B,ARPC5,CDKN1C,DDHD1,DNMT1,EDAR,F11R,HBEGF,HSD17B7,MAGI2,MAT2B,MYH15,MYLK3,OR11A1,OR2B11,P2RX4,PFKFB3,PLXNA3,PRKY,SHISA5,SMAD4,STAT2,SULT2B1,TLN1,UPB1,TNFRSF11A
Negative_8732	F3,GNS,ACTA2,ADRA1D,ARRB1,ATP5H,B3GALT2,CTSG,DGKG,EARS2,ENTPD6,ERBB2,FCGR3A,FPGT,GGCX,GJD2,GNPDA2,GPD1,GUK1,HSP90B1,HSPB1,KLKB1,LDHC,LRP5,MAN1C1,OR1J1,OR2T5,OR4X1,PIGF,POLE2,POLR2G,PRKAA2,SMARCA2,TAS2R8,TDO2,TKTL1,TNFRSF4,TNFSF8,TUBB,UGCG,XYLB,EIF4EBP1,SLC25A31,TNFRSF18
Negative_8733	AGTR1,ALDH1L1,B3GAT2,CALML5,CASP6,EGFR,GSTM4,GUCA1C,NANS,NUDT5,PEMT,RAD51,ST8SIA1
Negative_8734	ACO2,BLNK,CD79A,FOXO1,MARS,MNX1,PDE1B,SYNJ1,TNFRSF10A
Negative_8735	F2,ZYX,AHCYL1,ALPI,ARHGEF7,C1QC,CACNB1,CYP3A4,F13B,GNMT,HSD17B2,ICOSLG,IL3RA,MKNK1,NEU4,OR5K1,PPARD,PTPN5,RAP1B,RAPGEF3,RHEB,SLC2A4,WNT11,SUV420H1
Negative_8736	EZR,ACSS1,ACSS2,ACVR2A,BDH1,CD36,CDK2,CLDN15,CTSL1,DDX19A,DERL1,IL4R,IRF3,ITGB7,MAPK8,OR1E1,OR52M1,PEMT,PFN2,PLAUR,PLXNC1,PTGER1,PTGES2,SLC12A2,SERPINF2
Negative_8737	CD55,CHRNA7,CMPK2,COL4A6,CREB1,CYP3A43,CYP8B1,DDX50,DLST,EFNA2,G6PC2,GNAS,IL18RAP,NAGS,NRAS,NUDT5,OR52L1,OR9G1,PLA2G3,PPP2R2D,SMAD2,STX6,TUBA8,TYROBP,HSP90AB1
Negative_8738	CFH,KDR,NPY,ALDH4A1,ARHGEF4,BACE1,COMP,DDX51,IFNA6,IFNGR2,MYH7B,NODAL,OR51E2,OR52A1,OR5F1,PDGFD,PGLS,PPOX,PTPRJ,RFWD2,RHOD,THBD,TMLHE,TRHR,TUBA1A,TUBB2B,UPB1,ATP6V1G3,PLA2G12B,SERPINF2
Negative_8739	GP6,SRC,ALOX12B,ANAPC11,ARPC5,ATP5E,CD247,CD36,CHPT1,CLDN6,DERL1,EFNA2,FMO3,FUT8,GPAM,GUK1,HIBCH,HLA-DMA,ICAM1,IQGAP3,LALBA,LTBP1,MDH1,NARS,NUMB,OR11G2,OR4P4,OR51G2,OR5P3,OR8K5,PANK2,PDE1B,PHPT1,POLE2,PPT1,PRKAR1B,PRKAR2A,SLC25A4,THBS4,ATP6V1E2,ST6GALNAC4
Negative_8740	F10,HK2,BHMT,CCNG1,CLDN7,COASY,CREB5,DDX54,E2F4,GDE1,LAMA3,MEF2C,NOTCH1,NRXN1,OR13G1,OR2M2,OR8I2,PGAM4,PIGB,POLR2C,POLR2G,SYNJ2,UAP1,XIAP
Negative_8741	ACP6,AFMID,AKR1D1,ALOX12B,ARSA,C7orf16,NUMBL,OR10H2,OR4S1,OR56A4,SC5DL,SSX2IP,STX4,WEE1,HLA-DPB1,HSP90AA1
Negative_8742	ADRB3,ATP5C1,CXCR3,CYP19A1,EPRS,GSTO2,OXSM,PPP3CA,PRKCH,SUCLG1,TJP3,WWP2,XYLB
Negative_8743	C8B,PVR,C5AR1,CACNA1G,CACNG4,CAMKK1,CD70,CSF1,DARS2,E2F1,EPHA2,GAMT,GATM,GLYCTK,GNPAT,IFNA14,IL1R1,IL20,IL22RA1,IRS1,KLRC3,KLRD1,MYLK3,NCOA4,NTSR1,OR1A2,OR1F1,OR1L4,OR5C1,OR6C4,ORC1L,P2RX2,PIGV,POLR3G,PPAT,PRKCA,PRKCH,PYCR1,RARS2,RDH5,SDC4,SELPLG,SEMA4D,SLC2A1,SPRY4,UAP1,VAMP2,VANGL2,ATP6V0D2
Negative_8744	OR5R1,OR8H1,PNPLA3,PPCS,VAV1
Negative_8745	ME2,ALAS2,CHRD,FABP3,GALNT12,PHPT1
Negative_8746	CSH1,CYP2U1,DGAT1,EPHX1,FARSB,FGF17,MMP9,OR10T2,OR6M1,PHKG1,PLCG1,SOAT1,TM7SF2

Negative_8747	ACYP2,ANAPC7,BMP8A,CBR1,CTNNB1,GADD45B,HRH1,IFNAR1,LAMB2,METTL2B,MYL9,NOX3,OR2AG2,OR4K14,PGK1,PPOX,PPP1CA,PTPRR,C1GALT1C1,SERPING1
Negative_8748	BTK,ITK,ACAA2,ACVR1C,ADCY10,ALS2,CACNG4,DTX3,FMO1,HCST,INPPL1,JAK3,LDHA,NOTCH3,NT5C1B,OR10G9,PAFAH2,PHKG2,PNPT1,PPP2R2B,PPP2R5B,PTPN1,RAPGEF4,TLR2,TNFSF4,UAP1,PPARGC1A
Negative_8749	AMPD1,CCR8,CYP7A1,DCTD,EVI1,HCLS1,HSPA2,KRAS,MAPT,MMP2,ODC1,OR52D1,PIP4K2C,POLE2,PTGDS,SETD7,THBS3,GABARAPL2,PPP1R12A
Negative_8750	HGD,HK2,STS,AADAC,ADCY3,AHCY,ARSE,CD72,CD8B,CDC25B,CDC42,CDK4,COL2A1,CREB3L4,CYP3A43,DHRS3,ENTPD3,FPGT,GAD2,GALNT7,GNAI2,HHIP,HMGCS1,IL10RA,IL15,JAK1,LDHAL6A,MYL9,OR5F1,OR9G4,PIK3R1,PNPT1,PTDSS2,PVRL3,RALA,RELA,STAM,TBL1Y,THY1,UBE2K,UCKL1,UGT2B4,UGT2B7,ATP6V0A1
Negative_8751	ADC,C7,FST,GK2,AADAC,ADAM10,ADORA2B,ALDH9A1,ALG5,CCND1,CLDN17,COL5A1,DUSP3,ENTPD7,ERBB4,FZD3,ITGB1,LAMB4,MAPK14,MS4A2,NFAT5,OR2F2,OR4N2,OR51A7,RAB23,RPE65,SC5DL,SMC1A,SPCS1,STX2,SV2C,VAMP1
Negative_8752	ABL2,BPGM,CACNG3,CD247,CD72,CHRD,CLTC,FGF3,IL23R,LPAR1,NADK,OR4A16,OR4F17,POLR2I,RAB23,RPSA,SHC4,SLC25A5,TNFSF4,VEGFC,WARS,MAP3K7IP1,SERPIND1
Negative_8753	FYN,HYI,TFG,ACACA,FGFR4,GCNT4,GRIN2C,LFNG,LRP5,LTC4S,MAPK1,MCEE,MMP9,NT5C3,OR10Q1,OR11H6,OR13H1,OR2B2,OR2H1,OR4C11,OR4S2,PCCB,RAB5A,SGMS2,TAS2R42,TGFBR2,TPM3,TRAF6,TUBA3E,VAMP4,XCL1,ZMAT3,CACNA2D3
Negative_8754	ATP5D,CACNA1B,CCR7,NT5M,OR6F1,PGAM1
Negative_8755	HK2,ARPC3,NFYB,OR1E1,RPN1
Negative_8756	CS,EDA,SI,AKR1B1,ALDH1A2,CHST13,CNTNAP1,CYP2E1,DGKG,FUT1,FUT8,GALNT1,GALT,GLYCTK,GSTP1,HSPA8,HTR4,IFNAR2,IL11RA,ITGAV,MTMR2,MYLK2,OR4S2,OR52K1,PFKFB2,PIP4K2A,RPS6,SOAT2,YOD1,ATP6V0A1,ATP6V1C1,HSP90AB1
Negative_8757	ACO2,ADRA1D,APRT,BMP6,CSF2RB,CTNNA3,DDX50,FABP4,GPLD1,HSD3B2,IL19,IL23R,MTMR2,NT5C1A,OR1L4,OR8K3,PAK6,PLA2G2D,PRKAR2A,SDHC,SEMA4A,ATP6V1C2,SLC25A31,TNFRSF6B
Negative_8758	CAT,AACS,ACSM3,APOA2,CNDP1,CRHR1,CSNK2B,CXCR6,DCXR,GPX5,HMBS,HPSE2,IL13RA2,KAT2A,LIPC,NGEF,NT5C1A,OR13D1,OR6C2,PLA2G2A,POLR1C,PRPS1,PSME3,PYGM,RALBP1,RIPK1,STAT4,TNFSF8,CACNA2D2
Negative_8759	CFD,HK1,AFMID,AGPAT2,ATN1,CCND2,CD14,CYP2F1,FRAP1,GUCY2C,HSPA8,IPPK,ITGAX,MAGI1,MAGI3,MCAT,NOTCH3,OR10AG1,OR1K1,OR6T1,OR9Q2,PDE4C,PIGM,PLK1,POLR2D,PPP1CC,RDH10,SC4MOL,SENP2,SPHK1,STX2,TAS2R7,SUV420H2
Negative_8760	CS,CDC26,CTBS,DAAM1,GART,IFNG,IGF1R,MAP3K14,OR2L3,OR2T29,OR52N1,PDHB,PNPLA3,SETD1B,TAS2R50,UGT2A1,WNT3A
Negative_8761	AR,EPO,AASS,AXIN1,CES7,CPOX,CYBA,DDX51,DOT1L,F2RL3,FTH1,FZD3,IL10RB,IL6ST,KIR2DL2,MGAT4B,NTNG1,ODC1,OR3A1,OR4D11,OR51B2,OR6C4,PLCB3,PLIN,PPP3CC,SEMA3G,SOD1,SRGAP1,SYNJ1,TALDO1,UGT1A10,WNT5B
Negative_8762	AK7,GNS,HGD,ALG3,ALPP,APOE,ATN1,CCL23,COL5A3,CXCL9,DFFA,DTX2,FMO4,FZD9,GP1BA,GPT2,HRH2,IL2RA,INPP4B,ITPKA,NEGR1,NEU1,NFX1,NRG1,OCRL,OR5H2,OR5R1,PDK1,POLR2D,PORCN,PTPRZ1,PTTG1,RASGRP2,SOCS3,STX7,VDAC3,WASF2,ZFYVE16,ZNRD1,HLA-DRB5,TNFRSF10B
Negative_8763	COQ7,FMO3,GOT2,PRKAB1

Negative_8764	GHR,HPD,MSN,ALDH3B2,AMDHD1,BCAR1,CD74,CHPT1,CYP2S1,ENTPD8,FASN,FGF10,GOT1,GUCY1A2,HEXA,IGFBP3,IL13RA1,IL20RB,IL6R,ITPA,LIPE,MCM3,NOX3,NPAS2,OR10H5,OR2S2,PARVA,PIP5K3,PISD,PLCE1,PLD2,PPAT,RPS6KA5,SOAT1,TPI1,TRMT11,TSLP,ULBP2,UROD,WNT7A
Negative_8765	IL5,ACACB,ALG9,ATP6V1D,AVPR1B,BUB3,CCR2,CLTB,CSF1,CYP19A1,DHRS3,F2RL2,FLT1,FLT3,GOT1,HCST,HNF1A,HSD17B3,HSPA2,IFNA13,IFNAR2,IL1RAP,IQGAP2,KIR3DL2,MAP3K5,MCM5,MGST3,MKNK2,NFE2L2,OR10A4,OR1D5,OR51E1,OR51I1,PAPSS1,PDK1,PLA2G2E,PRKAG1,SARDH,SDHA,SKP1,SSH3,TAS2R50,TLN2,ULK1,UPP2,WASF2,MAPK8IP2
Negative_8766	AMY2A,CCNB1,GALC,GSTA1,NFAT5,PGS1,PIAS3,PTGES,SLC9A1,TAS2R5,TPH2,VAV1
Negative_8767	AMT,FYN,HDC,ACSL1,ADCY8,CACNA1I,CACNG1,CBR3,CDH5,COL5A1,CXCL10,FGF7,FZD8,IKBKG,IL20,IL24,KCNB1,LLGL2,NCR1,OR52N2,OR8H1,P2RX3,POLE,PRKAG1,PPARGC1A
Negative_8768	HAL,ACVR1,DGKQ,ITGAV,OR10H3
Negative_8769	C8A,CTH,EGF,EZR,F9,MUT,NF1,OGT,OTC,AKT1,ASAH1,ATP5J2,CCND2,CLOCK,E2F5,FRAT1,GALNT1,GCNT1,GPAM,GRIA2,GRIN2D,HLA-DMA,HSP90B1,IL11RA,ISYNA1,LDHB,MAT1A,MGAT4A,NLGN1,NNMT,NRP1,OR10K1,OR1J1,OR2B6,PAICS,PDE6D,POLR1D,RPRM,SETD2,SPRED1,ST6GALNAC3
Negative_8770	CD4,SDHB,TNFSF4
Negative_8771	AADAC,GLG1,NANS,NEU4,NPAS2,OR13F1,OR56A3,RBPJ,ST3GAL3
Negative_8772	GLS,VCL,ABL1,CACNG8,CD8A,CDK4,CXCR5,DUSP16,FCGR3A,FGF11,IMPDH1,ITGAL,NCR1,OR10H4,OR4B1,PER1,POLR3H,PRIM2,PTCH1,SHC4,SMAD5,SMURF1,UGCGL2
Negative_8773	LEP,AACS,ASNS,B3GALT4,CACNA1C,COASY,CXCL2,DGKG,DGKZ,E2F5,FGF2,GJA1,GJD2,GLI1,GUSB,H6PD,HTR2B,IPPK,KLKB1,LTA4H,LTB4R2,MAPK10,OR4M2,OR51L1,OR52A1,OR5AK2,OR5B12,OR8S1,OXCT2,PFKL,PLXNA3,POLD3,POLE2,PRSS1,RARS2,RHOQ,RPRM,SDC3,SRGAP3,TP53,TYMS,WNT6,ATP6V0E1,CSGALNACT1,PHOSPHO1
Negative_8774	FES,UBB,ACP2,APPL1,ENTPD8,GRIA3,IL12B,IRF3,KLKB1,NFX1,OGDH,OR5B21,PDGFB,PIP4K2B,SPCS3,ST8SIA1,STAM,TP53,TSC2
Negative_8775	ITK,LIF,RB1,ABI2,ACP2,ADRB1,APAF1,ARPC1B,ATP2A2,CCL28,CDK5,CSF1,DNMT3A,DNMT3L,DVL2,EFNA3,FRAP1,GALNT14,GNPNAT1,HTR6,LAMA3,MAML3,MAP3K11,MAPT,MYD88,OR4C13,OR51G2,OR52E2,OR52M1,OR5H6,OR9Q2,PIAS2,PIK3R5,PLCB3,PPM1A,PRKACA,PROC,PSME3,PTK2,RRM2B,SELPLG,STAT2,SV2A,SYNJ1,TFDP1,TUBA8,ATP6V1C2,SERPINE1
Negative_8776	BTC,GUCA1B,NFYB,OR6C76
Negative_8777	CHAT,HLA-DMA,IL20,ITGAX,MMP9,NUDT12,OR4B1,PTGFR,SORD,STX3,STX4,ST6GALNAC5
Negative_8778	FGA,MPI,SP1,ACAT2,ACSL5,ADIPOQ,AGTR1,ARHGEF2,CAV1,CD59,COL2A1,COL5A1,DARS,GADD45G,GALNT2,GRIN2C,IGF1,INHBA,OR51G1,PDE4A,PFN1,PIK3R4,PPAP2C,PVRL2,QARS,SAT2,ST6GAL1,STX8,TGDS,UBA7
Negative_8779	AK2,HDC,ID3,ADCY8,APRT,BMP2,BMP4,CADM3,CXCL3,CYP26A1,GYS1,GZMB,HHIP,IL1R2,NME4,OR4C6,OR8J1,OR8S1,PARD3,PIP5K1A,PNPLA3,PNPO,SEMA3F,SPHK2,ST3GAL2,UGT2B28,PNLIPRP2
Negative_8780	BIRC2,CACNA1D,OR13D1
Negative_8781	CSK,F2R,JUN,NNT,TNN,AGPAT2,AMPD2,APOA2,ARNT,ATP5L,BMPR2,CDH15,CDH4,CLDN10,CSNK1A1,DKK2,EGLN3,EPHA5,GRIN2B,GSTO2,KIR3DL2,MGAT4B,MINPP1,MYLK2,NAT5,NPAS2,OR10A6,OR10AD1,OR2T5,OR4F15,OR4X2,OR8B8,OR8D1,PDE5A,PDE8A,POLR3H,SDC4,SH2D2A,SNAP23,SPRY3,SRGAP3,ST3GAL6,SYNJ1,TYMS

Negative_8782	CYP26A1,GANAB,GRLF1,LIFR,PKLR,PTPN1,RAB5A,UMPS,VEGFC,WWP2,ATP6V1B1
Negative_8783	ACTB,ACTN4,AKR1C2,BCL2,GDF7,IFNA8,IL21R,TAS1R3,ZFYVE16
Negative_8784	AK3,FGG,GHR,PTS,TFG,ACTN1,CACNG1,CAMKK1,CHD8,CHST12,CLDN11,CSDA,CXCR5,CYP4F3,DAPK3,DEGS2,FCGR3B,FMO2,G6PC2,GAD2,GNA14,GTSE1,GZMB,HRH1,HSD11B2,IL21R,IRF9,KIR2DL1,MAN1B1,MAP3K13,MCM3,MTHFD1L,MYH4,NARS,OR10A7,OR4A15,PIK3R4,PPAP2C,RASGRP4,RPS6KA3,SEMA3G,SLC33A1,STAT3,TAS2R16,TBXAS1,THBD,TUBA4A
Negative_8785	ARD1A,ATP5G3,BLVRA,SETMAR,UMPS
Negative_8786	KMO,LCK,LYN,XDH,ADRB2,ADRB3,AHCYL1,ALAS1,ARNT,CDK2,CYP4A22,DAPK3,DCTD,FDFT1,FGF22,FTH1,HADH,HMOX1,HMOX2,HPSE,HSD11B1,HSPA2,IFNG,IL2RB,INADL,OR4F15,OR4L1,PDE1B,PLA2G10,PON1,QPRT,RFNG,RRAS,SPP1,SRGAP2
Negative_8787	CKM,PGF,CCR6,CPOX,DCTN1,EPHB2,EXTL2,FGF20,NEGR1,PYCR1,TP53I3,TPM3,ULK3,UNC5D
Negative_8788	FGF17,HSD11B2,OR10G2,SH2D1A,THY1,VANGL1
Negative_8789	AK3,DBI,GCK,ACADS,ACO1,ACTN2,AMD1,ANPEP,ATN1,CCL27,CDC20,CLTA,CSNK1A1,CXCL10,DDOST,DIAPH1,EFNB2,FZD6,GGT6,GSTZ1,HSD17B2,HSD3B2,IDH3G,IL4R,IRAK1,ITGAL,KIR2DL1,MTMR1,OR10K2,OR11A1,OR2AT4,OR2M2,OR51B6,P4HA2,PAICS,PDE6H,PIK3C2G,PLCG1,PTGFR,RPSA,SCLY,SDC1,UGT8,UPP2,WASF3,WHSC1,WNT8B
Negative_8790	ADH6,CCL21,CTPS,CXCR5,GABARAP,GMPS,HSPA8,MAOB,MGST1,PPP2R1B,PRIM2,RHOA,SUCLG1,TP73,UCK1,XCR1
Negative_8791	AMH,BAX,KIT,TPO,ACADL,ADH6,BST1,CACNG5,CD80,COASY,CUL1,DDX54,DNMT3A,EHHADH,EPAS1,EVI1,FGF19,HMOX2,IBSP,IL20,LDHB,LIPE,MAP4K3,MCAT,MECR,NFKBIE,NUDT2,OR10J1,OR1S2,OR2D2,OR4F4,OR4P4,OR4S2,PCCA,PDHB,PLA2G2E,PLXNB1,PRKG2,SOX17,SPCS1,HLA-DRB5,MAP3K7IP1
Negative_8792	CBS,HGD,ACADVL,CD58,CSNK2A2,ITGA4,TAS2R10
Negative_8793	BTC,CD4,CRK,TNF,AADAC,CARS,CD38,CDKN2B,CTNND1,DHRS9,FCGR2B,FDPS,HIP1,HRH2,IL23A,ITGAV,KLRK1,LARS,NKX3-1,OR4D11,OR51G1,RDH5,SETD7,TAS1R2,UAP1,YARS,CTNNBIP1,TNFRSF11A
Negative_8794	ACTC1,ACYP2,AMY2B,ASS1,AXIN1,CAMK2D,CD44,CD55,CDC25B,CHRM3,CLTCL1,CREB3L4,CSNK2A2,CYP1A2,CYP21A2,DGUOK,DTX1,E2F4,EPHA4,FCGR3B,FGF7,GALNT11,GALNT8,IPMK,MAN1B1,MDM4,MGAT1,NR4A1,OR1L4,OR5A2,PGAM1,PTAFR,PTGER3,SELPLG,ST3GAL1,STAT5A,SUFU,SV2B,TAS2R48,TCIRG1,ATP6V0A4
Negative_8795	CBL,CS,F9,ACVR1B,ALDH3A2,CCR6,CDKN1B,CFLAR,CTSB,CXCL5,FARSB,FTH1,GALNTL5,GLO1,GPLD1,GRM5,GSTZ1,HTR6,IFNG,JAK2,LIMK1,LRP6,NCR2,OR13C8,OR4C12,OR51A2,PPM1A,PTPRM,SETDB1,SORBS1,ST8SIA5,WARS,WNT10A,XYLB
Negative_8796	HPD,ACVRL1,ADAM17,AGPAT4,AMD1,AMHR2,ARHGEF6,BAI1,CCL25,CHAD,CHST13,CMPK2,CTSS,DERA,DHCR7,EPB41L1,FTCD,GALNT2,GALNTL2,IQGAP2,ITPK1,LAMC2,MMP9,NFATC2,NKX6-1,NPR2,NT5C1A,OR51D1,OR52I1,OR7G2,OR8B8,POLD4,PRKAR1B,PRKX,PTGFR,RDH5,SORBS1,SQLE,ULK2,VAV3,WNT5A,YOD1,ZFYVE9
Negative_8797	AK2,ASL,C9,GH1,HGD,LCT,SCD,ACSL4,ADIPOR1,ALDH1A2,CARS2,CCR7,CDC2,CYP17A1,DHDDS,EPB41L2,FOSL1,FZD2,GALNT11,GAMT,GUCA1B,GUCY1A3,IL15,ITGA2B,JAG2,LALBA,LIMK1,LTA4H,MTMR2,OR2T10,OR5T3,PIK3C2A,SH2D1A,SLC25A6,UGCGL1
Negative_8798	DDC,ACTG1,ACTN2,AXIN1,CCNA1,CCND3,CLCA4,CMAS,HMOX1,OR4D1,OR52B4,PRPS2,SOCS1,ST8SIA5,TUBA1C

Negative_8799	GGH, NLK, ACACA, ACPT, ADRBK2, ATP5G2, CCNB3, COL3A1, CYFIP1, DHX58, F13A1, FBP1, GALNT3, GRIN2B, GUCA1C, HAGHL, HSD11B2, IL29, INHBC, ITGA8, ITPA, MAFA, MGAT1, NRXN3, NT5C2, OR52A5, P2RX3, PAK6, PDE1C, POLD3, RAPGEF1, TNFSF10, TUBA4A
Negative_8800	CTH, AARS, BMP2, C5AR1, CNTNAP2, CXCL10, DUSP6, EPB41L1, FZR1, GPLD1, ICOSLG, IL15RA, ITGA1, LARS2, MECR, OR1S2, OR2T33, OR5C1, ORC6L, PIGC, PPP3CC, RASSF1, SPRY1, STMN1, UCK2, TNFRSF10A
Negative_8801	FES, GLS, AGTR1, ARSD, B4GALT7, CCL5, CCR2, CD44, CFTR, CLDN3, CLTB, CNTNAP2, COL1A1, CSF2, DLL1, ENTPD4, EPAS1, EPHB6, GMPR, GRIN1, HYAL2, IDI1, INPP4A, ITGB6, ITPA, LPAR1, LYPLA1, MAML3, OR1G1, OR51E1, OR8K5, OR9G4, PDE4D, PGAP1, PIP5K1B, PPP2R1B, PPP2R2A, RPS6KA3, SRD5A1, STX10, TKTL2, TUBB4, TUBB6, VANGL1, HLA-DRB5
Negative_8802	ACVR1, ARPC4, BACE2, DDIT4, GIT1, HHIP, IRAK1, LAMA4, LDHA, NCAM1, NFYC, TPM3, PPP1R12A
Negative_8803	GP6, TK2, ZYX, C5AR1, COL3A1, COX10, GUCA1A, ITPKA, OR8B8, THTPA, TPK1, MAPK8IP3
Negative_8804	ACY3, FOXA3, FZD7, GALNT13, GNRH2, HCST, ITGAE, NPR2, OR5AU1, TACR2, TBXA2R, UGDH
Negative_8805	NPY, SRF, TK2, ADCY4, BDKRB2, CHUK, CYP2C8, DAPK1, DNMT3A, ENO1, EPHA4, FZD5, GLUD1, IL10RA, IL17RA, IL21, MKNK1, PIK3R5, PIP4K2B, PPARG, PRPS2, RPE65, SETD1B, TGFBR1, THBS3, TSLP, TNFRSF18
Negative_8806	ID4, MAG, AHCYL1, APH1A, CCNB1, CD226, DKK1, MLYCD, OR13F1, OR2T5, PFKFB1, PLA2G4A, TSTA3, ATP6V0D2
Negative_8807	IL8, ACSM2A, ANAPC7, BHMT, C1GALT1, CAPN2, CCR4, CEBPA, CSNK1G2, DAAM1, EDN1, EPHA6, EPHB2, EPHB4, GUCY1B3, IL11RA, L1CAM, MAFA, MAN1A2, MAPK8, MCCC1, MCM5, MDM4, MTAP, NANP, NODAL, OR10A3, OR11G2, OR52K2, OR5AK2, PGM1, PLAU, PLD1, POLD4, POLR2F, PPP1R1A, PTK2B, PYCR1, PYCRL, RASGRP4, SCD5, SPRED2, SV2A, TNFRSF11B
Negative_8808	ATP2A2, DUSP3, FOXA3, IL17RA, SUCLA2
Negative_8809	DHH, FER, ATG3, CYP2C8, LAMB4, MAPT, OR11G2, ORC5L, PDXP, PLA2G2F, ATP6V1G3
Negative_8810	ID1, CACNA1C, CASK, CD274, COQ7, CSF3, CTTN, CYCS, CYP3A43, DAAM2, DLL3, FGF17, FMO3, GNAI2, HMOX2, OLAH, OR51A4, SRGAP3, UGP2, VTI1B
Negative_8811	ACTB, ARNT, CCL2, CRKL, CYBA, ECHS1, EXTL2, GNAI1, GRB2, MYH4, OR13C2, OR13C8, OR51L1, OR52K1, PLXNC1, PRKAA1, RBPJL, RRM2
Negative_8812	ACADVL, ACLY, ADIPOR1, CASP7, CCDC6, CHRD, COQ3, CSNK2B, CYP2A13, CYP3A5, DHRS3, DLST, HSD17B2, IL1R1, KLRC1, MDM2, MTHFD1, NFATC4, NT5C2, OR10K2, OR9K2, PTDSS1, RPN2, SOCS2, THTPA, XYLT1, ATP6V0A4
Negative_8813	C2, CCR5, DGAT2L4, DHODH, GZMA, KIR2DL3, MAP3K14, OR2V2, OR51B4, POLR2I, TPH2
Negative_8814	CD8B, DGKB, GNG3, GP1BA, GPD1L, HADH, HTRA2, ITPA, LRAT, MAOB, NAMPT, NKD1, OR51B4, OR52E8, OR52J3, PYGM, SDC3, TPI1, VAMP4, PPARGC1A
Negative_8815	DCK, EBP, ADCY6, AMHR2, ARSE, C1GALT1, CALML6, CASP9, CD36, CREB5, CYCS, ENO2, FGF8, GGT6, GLI1, MCCC1, MYH1, OR52D1, OR5I1, PAPSS2, PCCB, PRKAB1, PRKACA, PSME2, RBPJ, SLC27A5, SNAP23, UBE2L3, VKORC1, SERPINA1
Negative_8816	F7, PVR, ACP6, ADRA1D, APRT, ARPC5L, CNTNAP2, CREB3L3, CSH1, CYP3A7, DVL1, EHMT1, ENO3, EVI1, EXTL3, FGF13, FZD3, HEMK1, IL17B, LEPR, MDM2, MGAT3, MTMR2, OR2G6, POLR2F, PTCRA, TAS1R1, WNT9A
Negative_8817	CD55, CDS1, HMOX1, POLR2L, PAFAH1B2
Negative_8818	ME2, AARS, AGRP, AKR1C1, CHPT1, COL4A2, COQ6, EIF4B, GALNT2, GRM1, IDH3G, MCM3, OR10H5, OR5D13, OR5I1, OR7G2, POLD2, PPP3R1, PRKCH, PYGM, RAPGEF3, SMURF1

Negative_8819	ID4,CHIT1,CYP17A1,DGKZ,FSHB,FSHR,GJA1,ITGB5,MAP3K11,MLLT4,OR5M3,PAX8,POLE4,PRKAB2,PTPRF,RAC3,SGMS2,SLC33A1,SNAP25,ST3GAL2,ST8SIA5,SUCLG1,SYNJ2,TNFRSF11A
Negative_8820	AMH,GK,ADCY2,ADRA1B,AGRN,BCAR1,CAMKK1,CDH5,CISH,CYP11B1,DUSP16,FABP1,GALE,MAPK1,MAPT,NAT5,NCK2,NCKAP1,NUDT9,OR51I2,OR52M1,OR9A2,PLXNC1,RFXAP,SOX17,ST3GAL1
Negative_8821	F2R,RFK,TNC,ACOX3,ADAM17,CHST12,DHFR,FASN,FECH,FUT3,GGT7,GNG3,IFNK,IL29,JAK3,MAPK14,MCM4,OR52E4,OR5H2,OR9G4,PHKB,POLR1C,RARA,RBKS,SV2C,TAS1R3,TAS2R8,WARS,XCL1,ZFYVE9
Negative_8822	CEL,F8,AKR1C4,AMY2A,CLCA2,CYP2B6,MCCC2,PARVA,PNMT,SLC12A2
Negative_8823	ALG9,ALOX12B,FABP4,MPP5,STX1A,UGT2A1,XYLT2
Negative_8824	GSS,IHH,AFMID,ATP6AP1,CCKBR,CDC26,CREB3L2,CSNK1G2,DARS,ECSIT,EFNA4,ENPP2,EXT2,FBP2,FLOT2,FZD8,GMDS,GNMT,GPD1L,GRM5,GSTA2,GSTK1,HLA-DMA,IL7R,LRRC4C,MAML2,MAOB,MYD88,OR10K2,OR2M3,OR2V2,OR6T1,PPP1R1A,PPP2R1A,PPP3CC,PTTG1,SMARCA5,STAT5B,TRPC1,TUBA1C,WNT1,ZFYVE9,SERPIND1
Negative_8825	B3GNT5,IL2RA,OR6C6
Negative_8826	CSK,NLK,ARNT,ARPC2,ATG7,B4GALT4,BCAR1,CHSY3,EIF4B,EPHX1,ESPL1,FUT4,GNAI3,IRF3,ISYNA1,MCEE,MYD88,NSD1,NTNG1,OR4K5,OXTR,P4HA3,PFN3,PKMYT1,POLR1A,PPP3CB,PSME1,RRM2,SMAD9,TRAF3,WNT1,TNFRSF6B
Negative_8827	DDC,MPL,PDC,AASS,ADAM17,AGMAT,ALDH3B1,C4BPB,CCL8,CDH5,CYP2A7,EFNB3,ENPP3,FDFT1,HMGCS2,IL12RB1,IL7R,KDSR,LCMT1,NEFH,OR2D2,OR52E8,OR5L2,RHOA,SESN3,SFRP1,SIAH1,TJP1,WNT9B,MAP3K7IP1
Negative_8828	C8B,OGT,ACP1,ALG6,ASRGL1,ATP6V1A,C1QC,CCND3,CNTNAP2,CREB3L3,CYP11A1,DARS,EPHA2,EXTL2,GSTO1,HAGH,HSD3B2,HSPA1B,IKBKB,IL1RAP,IL8RB,LRP6,MAP3K1,NEFM,OGDH,OR10X1,PGM1,PLCG1,PRKCI,TAS2R5,WNT10B,WNT8B
Negative_8829	ALDH1A3,ALG8,AOX1,CNTF,DDX4,DHCR7,GFPT1,IFNB1,MDM4,OR10AG1,PPAP2B,RALB,TAS2R50
Negative_8830	KDR,SDS,ACP1,ATP2A3,CCL23,CDC25B,CYP2C19,DPYSL2,GDE1,HIBADH,MAPK11,NFASC,NRXN2,OR2AG1,OR2AT4,OR5T3,PRKCD,RDH12,RPN2,SOCS4,STX3,TAS2R38,TLN1
Negative_8831	AKR1A1,C3AR1,CCL24,CXCL6,EDN1,FASN,HHEX,ITGB5,KIR2DL2,LDHAL6A,MAP2K3,OR10J5,OR1A1,OR4K2,PAK3,PIK3C2G,PPP2R2D,SLC12A2,SLC33A1,SULT2A1,TAS2R13
Negative_8832	ACADL,ACADVL,ACSS3,ALG13,APOA1,ATP5F1,C3orf10,CCNE1,COL5A3,CSF2,ERO1L,F2RL1,FMO3,GTSE1,HMGCL,KAT2B,MAP4K4,MFNG,NCF4,NCOR1,OLAH,OR1F1,OR1L1,OR2A42,OR4L1,OR4M2,OR5F1,OR8B12,OR9A4,OXCT2,PDE1A,PIGX,PLA2G3,PTPRB,RAF1,RBL1,SLC27A1,SOS2,TAS2R38,THBD
Negative_8833	ACSM2A,APOA2,CPS1,DHDDS,DPAGT1,GJA1,GRIN2A,HTR7,IL20RA,MTAP,MYH15,NOX1,OR13A1,OR2D2,OR6C2,PNPO,PPP3CA,RBL2,SDC2,SELL,SEMA3G,SFRP1,UGP2
Negative_8834	DDC,CBR1,SPRY3,STX3,TUBA1C,UROC1
Negative_8835	F8,RFK,AACS,ADRB2,CCNG1,CDH2,CHST12,CHUK,DDX50,DUSP9,ENPP3,ETNK1,FARS2,FTCD,GSTM4,MAP2K1,MAP2K4,MCCC1,MYD88,NRCAM,NTSR1,OPLAH,OR4N2,OR52N2,OR56A1,OR8D1,PAX8,PTGER1,RELA,RPRM,ST8SIA1,VAMP2,WNT9A
Negative_8836	ALDOA,BMP2,GALNT11,IQGAP3,LDHD,LTBP1,NANS,NOS1,OR10V1,OR5D14,OR6A2,PARVG,PDXP,PTPRJ,SDHB,SHMT1,STX10,TAS2R16,TNFSF14
Negative_8837	MVD,ITGB8,LLGL2,NME2,OR2D3,OR52B6,WNT8B

Negative_8838	TYR, BIRC3, C4BPB, CDK2, CNGB1, CYP2A13, DIAPH3, HMOX1, HRAS, NCR2, OR1J2, OR6Q1, PLA2G2D, PTEN, TAP2, UPP1
Negative_8839	AGL, APP, DHH, ACOX3, ACTG2, ADCY6, ALDH4A1, ALG9, CAPN2, CASK, CASP7, ENPP3, ENTPD7, EPB41L2, FLT3, GALNT12, GNG13, IFNA14, IL7R, KIR3DL1, MALT1, MAP4K1, MDH1, MYL2, NOTCH2, OR11H4, OR2A14, OR2T3, OXCT2, PCYT2, PFKFB4, PIP5K1A, PLCB3, PLOD1, PPP2R1B, PSPH, SETD1A, TAP2, TAS2R1, TAS2R40, XIAP, YWHAB, ATP6V1G3, TNFRSF6B
Negative_8840	FAH, IL4, ABLIM2, ACSS2, APAF1, B4GALT2, CAMK2G, CBLC, CHIT1, COL4A6, CXCL11, DGKB, EPHB6, FGF13, FRAP1, IFNB1, IL21R, KCNQ1, LAMB1, LDHC, MDM2, NAE1, NARS2, OR13C9, PIK3CD, PLA2G4A, PLA2G5, PLOD3, POLR2C, PORCN, PRLR, PRODH, SFRP1, STX3, TP53, UBE2L6, WARS, ZMAT3, MAPK8IP2, SERPINB5, SERPINC1
Negative_8841	C3, LHB, MVK, NLK, TNF, ACSM5, ALDH5A1, ALPL, CAMK2G, CD70, CNTNAP1, CREB3, DBF4, DGAT1, EDN1, ENPP1, GPD2, HMGCR, IFNG, ITGB8, KLRC2, MASP2, METTL6, MYH3, NME2, OR10A6, OR1K1, OR2T10, OR4A15, OR51I1, OR9G1, PDK1, PECAM1, PPA2, TACR2, TPH1, UGT1A10, UGT8, WNT6, ATP6V0E1, HSP90AB1
Negative_8842	C2, CR1, CYP2D6, DHDH, FASLG, GLUD2, GNPDA1, IFNA13, MAP2K7, MAP3K3, NOX3, NSD1, OR2B11, OR51D1, OR51S1, OR52A5, PANK1, PDE5A, POLR2E, SLC2A1, SNAI2, VAV2, C1GALT1C1
Negative_8843	B3GALT1, CPT1A, CRY1, CXCR4, FECH, FLOT2, FTCD, HMOX1, KIR3DL1, LAP3, PRKG1, TALDO1, VEGFC, PDCD1LG2
Negative_8844	IL4, NPY, AKR1B1, APBB1, BHMT, CACNG2, CAMK2G, CDKN2C, CNDP1, CTTN, CXCL10, CYBB, CYP3A5, DDX55, DLL3, FZD1, GALT, GIT1, HAP1, INMT, LAMA3, LAMA4, LIPA, LYZL1, MAP3K13, METTL6, NEU2, NFATC2, NODAL, OR2A2, OR2B11, OR4K13, OR9G4, PGAP1, PLXNB2, PNPLA4, PRKCG, PYCRL, SARS, SEMA7A, SH2D1B, SLC27A1, SMPD4, TGFBR2, VANGL2, HLA-DRB5
Negative_8845	GPT, IL5, IVD, MET, PXN, AADAC, ADCY10, AKR1A1, BCL2L1, CARS, CCL3, CCL5, CLDN1, CSF2RA, CTF1, EPB41L1, FGF14, FLAD1, G6PD, IFNA4, IL13RA1, IL2RG, LAMC1, MAN2A1, MNX1, NFKBIA, OR2A4, POLR2E, POLR2L, PON1, RAP1B, RGS3, SPRY4, TAS2R49, CACNA2D2
Negative_8846	OGT, AGTR1, GRIN2A, PDCD1
Negative_8847	MIF, PML, AANAT, ABL1, ALG5, ATP2A2, C1QA, CD19, CLCF1, CLDN4, CNTN2, COL4A6, COL5A1, CTTN, FASLG, GLG1, HLA-G, IL12RB2, IRS1, JAK3, MLLT4, MOV10L1, NRXN2, NT5C2, OR10K2, OR1S2, PIGF, PPP1CA, PRSS2, PTPN1, PYCR1, RHEB, RRAS2, SLC27A4, SUCLG1, UGCGL1, VAMP3, WIF1, HLA-DQA2, PDCD1LG2, TNFRSF18
Negative_8848	ACVR1B, ECSIT, GSTA1, GSTA3, JAM3, KIR2DL4, OR10H5, SETDB1, ULK1, ARHGEF12
Negative_8849	DAO, IL3, LAT, TAT, ACTN1, ATP5O, BCKDHB, CCL15, CCR10, CCR9, COQ5, CXCL12, CYBB, CYP17A1, DDIT3, DDX18, DTX4, EIF4E, EPHA3, FARS2, FECH, FGF13, FGF4, GSTP1, HEXB, IBSP, IFNA4, IRS4, MAP3K2, MTHFD1, MYH8, NFKB2, OR10K2, OR1F1, OR2T6, OR4Q3, OR5L1, OR7A17, POLR1D, PRKCB, SMAD1, ST3GAL3, STK3, SYNJ1, TGFBR1, TM7SF2, UNC5D
Negative_8850	FZR1, OR3A2, OR52N4, OR5A2, OR5J2, PANK1, PTGER1, SREBF1, TNFRSF10A
Negative_8851	CDA, DKK2, MDM4, OR1B1, PMM1, PSME3, RAC2, SLC27A4, SMAD2, TAS2R4, TNFSF10
Negative_8852	HAL, PGD, CMBL, GNA13, GUCA1A, HMGCS2, ITGA7, OR51B6, PARVA, PIK3CA, RXRA, ZBTB16
Negative_8853	JUP, NPL, SRF, ACVRL1, ATP13A2, CTBP1, CYP1A2, CYP2D6, CYP2S1, DAAM2, DNMT3B, DRD1, GNMT, GSTT1, GUCY2D, HSD11B1, INPP1, ITGA7, KITLG, MAML1, MAP3K13, MTHFD1L, NANP, NCR1, NTF3, NTSR1, OR2C1, OR4M2, ORC6L, PARD6G, PDE2A, PFKFB4, PIGO, PLA2G2F, PTDSS2, PTGES, SCIN, SRGAP2, ST3GAL1, STK36, UCK1, SERPINA1, SUV420H2

Negative_8854	AGL,GH2,GNE,SYK,ABLIM1,ACADSB,ARD1A,CCL11,CLDN4,CYBB,DOT1L,EXTL2,GIT1,IFNB1,ITGB3,MAN1C1,MGAT3,MMP2,MTMR2,NEGR1,OR2B2,OR4K13,OR52N4,PAX4,PDGFD,PKLR,PPP3R1,PRKAA2,PTGS1,SHMT1,SOX17,HLA-DRB5
Negative_8855	CS,EGF,FER,ADCY3,ALG1,ALG12,ARSB,BCAT2,BNIP1,CACNB1,CDC42,CDK2,CLDN15,CLTC,CYP2U1,DCXR,DVL3,EHMT2,F2RL1,FDPS,FLOT2,HCST,IL21R,ITGA4,ITGB2,KIR2DL1,MNX1,NAE1,NAGS,NMNAT3,NPAS2,OR2L2,OR4B1,OR4L1,OR5T3,P2RX1,PHGDH,POLR1A,PPP2R5C,PTPMT1,RUNX1,SPRED2,SULT1A4,TJP1,UBE2L3,XIAP,ATP6V1G2,PAFAH1B1,PNLIPRP2
Negative_8856	EZR,RFK,ASAH1,ATP5A1,B3GALT5,CHRM5,COQ7,GANC,GOSR2,HEMK1,HSP90B1,IL13RA1,ITGAX,MAP3K13,MAPK1,MCM5,MYH2,MYH4,MYH9,PIP4K2C,PPP1R3A,SDC2,SIGLEC1,TAS1R2,TGFB1,TUBB6
Negative_8857	AGK,CFH,DHH,MOS,STS,ADCY6,ADORA2A,ALAS2,ANAPC7,B3GNT5,CDC26,CRY2,ECHS1,EPB41L2,FGF1,FUT6,FZD8,GGPS1,GNB3,GRIN1,HCST,KITLG,LDHAL6B,MFNG,MYLK,OR1G1,OR4A5,OR51E2,OR5B3,OR5T1,OR6F1,OR8D4,PANK3,PDK1,PLA2G6,PLCD3,POLR3K,PPP3CC,SCIN,UGDH,VEGFA,MAPKAPK3
Negative_8858	NPL,ACTB,ACVR1,ARSD,CCNB3,CCR7,CD226,CDK6,CHPF,CLDN18,COQ6,CTBP2,CYP1A2,EFNA3,EXT2,F2RL3,FGF9,FZD2,GPX7,GSTT1,GUCA1A,HEXA,IFNG,IL13RA2,IL24,OR2H2,OR2T2,OR6C1,PDIA4,POLD1,PPAT,RPSA,SFRP2,SH3BP2,SMPD3,TAS2R38,TYMP,UGT2B17,WNT6,ATP6V1G2,TNFRSF6B
Negative_8859	MAG,CACNG8,CEBPA,FABP3,FMO1,GLG1,GNAO1,GSTA3,HSPA2,LLGL1,MKNK1,NAMPT,NT5C1A,NTN1,OR1L4,OR52R1,OR8J1,POLR2J,STX12,SULT1E1,TRDMT1,XYLT1,YES1
Negative_8860	HK2,ITK,LIF,ADRBK2,ATP5H,BDH1,CBLB,CDH3,DDX51,DLL4,FECH,FMO5,HTR4,IRF3,LARS,MGAT4B,MMP14,MYH13,NEUROD1,RALGDS,RARS,RND1,ROBO1,RPN1,STAT4,STK4,SUCLG2,TLN1,TYRP1,WNT9B,CSGALNACT2
Negative_8861	RDX,AARS,BMPR2,DCXR,DDX51,GNAI3,GPX5,INPP4B,MDH2,MICA,ONECUT1,OR4B1,OR7D4,POLR1A,RETSAT,SEMA4B
Negative_8862	C6,F7,ALG10,CLDN2,DDX4,INPP5A
Negative_8863	GAA,ATP5I,ATP6V0B,CD8A,CSF3R,CYP26C1,DDIT4,DKK4,FMO1,HLA-DOB,MAD1L1,PPAP2C,PPP3CC,SC4MOL
Negative_8864	ID3,DDX23,ENTPD7
Negative_8865	GGH,ACADL,ATP5H,CDC25A,EPHB1,GALNT14,GPT2,IPMK,OR14A16,OR4D6,OR56B4,OXCT1,PANK2,PDE8A,PDGFRA,PIP5K1C,PNLIP,RAC1,TIRAP
Negative_8866	SCD,ARPC5L,ENO3,GALNT2,GALNTL1,GFPT1,GMPPA,OR2M5,P4HA3,PIK3C3,RAP1B,SC4MOL
Negative_8867	MPI,ACYP2,AGMAT,CCL11,CYP3A7,FGF9,IL22,LMO7,NCOR2,OR6Q1,PIK3R5,PKLR,PTPRB,RARA,SUCLG1
Negative_8868	CRK,BCKDHB,CREB3L4,GPLD1,GUCY1A3,HSP90B1,MAPK9,NEU4,NRAS,WWP1
Negative_8869	ID3,SPN,AADAT,ADRB1,ANAPC1,APH1A,AXIN2,B3GALT1,BPGM,CANT1,CDO1,CHEK1,CLDN9,CNTF,CSDA,CXCL12,DERL1,DGKI,FGFR1,GAD1,HEMK1,IFNA7,IFNA8,IL10RA,IL5RA,INHBC,JUND,LDHA,LTA4H,MAP2K6,MAP3K3,MCCC1,MIOX,NFATC1,NOX3,NT5C1B,OR1D5,OR2T3,OR8D2,PDGFC,PIK3R5,PLA2G10,PRKAR1A,SMAD7,SNW1,THBD,WNT3A,TNFRSF10C
Negative_8870	GH2,IL2,TNN,ACP2,ALDH2,ALDH3B2,ANAPC11,APPL1,ATF2,BCAR1,BET1L,BNIP1,CACNB1,CCL17,CDKN1C,CISH,CNTN2,CTPS,DARS2,ENAH,EVI1,FCGR3A,HIBCH,KCNQ1,KLRD1,LPAR1,NFX1,NTRK2,OR13F1,OR1A2,OR51Q1,OR6B3,PCCB,PDHA1,PDPK1,PRKCD,PVRL1,QPRT,SORD,SULT1A1,VDAC1

Negative_8871	DCT, GDA, ME2, ACOT11, ALPI, AMPD2, ARHGEF7, ARRB2, CCL23, CLDN4, CNGA4, DLST, F11R, FOXO3, FPGT, FZD8, GNAT3, GSTM3, HTR5A, IL3RA, KARS, MCAT, OR13D1, OR4S1, OR51S1, OR5AN1, OR5M9, OR5T3, OXCT1, PLXNB2, POLR1E, PPP2R1B, PRKG2, PVRL3, SARS, SLC25A5, SNAI2, UGT2B11, C1GALT1C1
Negative_8872	DDC, MVD, PML, ALG9, CCL2, CCND3, CNTNAP2, CSNK1E, EXT2, FASN, FGF20, IRS1, ITGAE, MIOX, NFKBIA, NPR2, OR10T2, OR13C4, OR14J1, OR1K1, OR51G2, PAPSS2, PCCB, PI4KB, PLA2G3, PRIM1, PTPRB, RND1, STX1B, TSTA3, WNT2B, ATP6V1E1
Negative_8873	DEGS1, IFNA8, SKP2
Negative_8874	FGB, BLVRB, CXCL5, CYBA, CYP2C9, HGSNAT, MYLK2, NME4, NPAS2, OR5H6, POLR3H, PRKACA, SMAD1, SUFU, THBD, TPH1, UGP2, TNFRSF10A
Negative_8875	DCK, LSS, ACACB, CCND3, CDC25C, CYP2A13, DARS2, DCXR, DDX55, ETNK1, GARS, LIAS, LYPLA1, MINPP1, NRAS, OR10A7, OR5V1, P2RX2, PLIN, PYCRL, SMAD4, SOCS1, WBSCR17
Negative_8876	AK1, MVK, PVR, ACOT11, ALAS2, APOC3, ARHGEF4, ARRB2, ARSD, AXIN1, CCNA1, CHRM3, CSF2RB, DKK4, ENO1, FOSL1, GGPS1, HMBS, HRH2, IL28RA, IQGAP2, LRP5, LTBP1, MAOB, MTFMT, NFAT5, NFKB2, OR1L3, OR4A5, OR4K14, PAK3, PDE7B, PIP5K1A, PORCN, PSEN2, PSME1, PTGS1, ST3GAL6, TMLHE, UPRT, VAV2, ZIC2, HLA-DRB4, MAPK8IP2, SERPINF2
Negative_8877	ZYX, CARS, LAP3, OR1A2, PDXP, PLA2G1B, WNT16, ATP6V1E1, TNFRSF12A
Negative_8878	C4B, LIF, ATP5E, CD247, IFNA13, MAD2L2, NUDT9, RAPGEF1, SEMA4B, HLA-DRB3
Negative_8879	BAD, RFK, ASAH1, ATP5O, DKK1, DUSP7, ENPP7, FGF13, GLB1, IL25, LDHA, MGST2, NRG1, OR12D3, PTGER1, PTPN1, TGDS, TUBB2C, TNFRSF14
Negative_8880	PGF, ASAH1, DAPK3, IGSF5, PYGM, TRHR
Negative_8881	C2, ILK, A4GALT, BCL10, C1QC, CD22, CLDN15, CRKL, CXCR4, DGKI, E2F1, EPAS1, FADS2, GNPDA2, ICAM3, MYH8, OR13A1, OR4B1, OR4N4, OR8G1, OR8I2, P2RX5, PLXNA3, POLR2E, PPBP, RAP1B, RFXANK, RPIA, SOX17, TAS2R40, UAP1, UROS
Negative_8882	ME1, UBC, ACTN4, ASMT, BCKDHB, BCL2L1, COL2A1, COMT, CYP2C9, FUT5, HCST, HRH1, IL10, MAOA, MAP2K7, NCKAP1, OR10K1, OR1J4, OR2T34, OR51A2, OR5D16, OR6N1, PLCG2, POLR1B, RBPJL, SLC27A5, SMURF1, TAS1R2, B3GALNT1, ST6GALNAC3
Negative_8883	EGF, FAS, GP5, LCT, SRF, ALG10, ALOX12B, ATG12, CACNA1I, CD19, CD274, CDC6, CYSLTR1, DDX41, ERBB3, ERBB4, GAPDH, GRIN2B, HCST, HMOX1, HPSE2, IDH3B, IFT57, IRAK1, ITGB1, KLRK1, MDM4, MS4A2, NME6, OR1N1, OR5V1, ORC5L, PCYT1A, PDE3A, PDE4A, PIK3C2G, PIK3R3, PLAUR, POLR2J, PRIM1, PRODH, RBPJL, SGMS1, SMPD2, TLN1, TPI1, TPK1, WASL
Negative_8884	FH, ARPC1B, CCND1, DNMT3B, FGF14, FUT4, GADD45G, HAO2, LIMK2, NADK, NKX3-1, OR13C9, PNPT1, PTGES2, SELL, SV2B, TYMP, SERPINA1
Negative_8885	ALDH1A2, BLNK, EFNA3, EPB41, GALNT2, WNT4, YWHAB, ZNRD1, TNFRSF10B
Negative_8886	SMS, CDH1, FMO4, FOSL1, GAD2, MAP4K3, NAT1, NT5C2, OR1K1, OR51I1, PARD6A, PRDX6, PTDSS1, PTGS2, RPS6KA4, SEMA3E, SNAI1, TYMS, UGT2B10, UROD, YWHAG
Negative_8887	JUP, AARS2, ACP2, ALDH6A1, BCKDHB, BMP6, CDC27, DERA, FECH, NAMPT, NOTCH3, NT5C2, OR13C2, OR6N1, OR8D1, PDCD1, PDHB, PIGA, PTCH2, RFX5, RPS6KB1, TMLHE, UROS, ZFYVE9, PPP1R12A
Negative_8888	ACAA1, CCR9, CDKN2A, CHST12, FGFR1, IFNA14, IRAK4, KIR2DS4, NADK, NEU2, OR4D11, OR6C76, PFKFB3, PFKL, POLA1, PRKACA, RDH8, SELE, STX7, TAS2R49, TPM3, TRPC1, UBE2K, UPRT
Negative_8889	CISH, EFNB3, FGF4, FMO4, GFPT2, GRIN1, GSTT1, IL13, IL20RA, IL23R, ITGAM, MAP3K3, OR1E2, SARS, TNFSF12, WBSCR22

Negative_8890	C8B,GGH,LEP,ADCY4,ALAS2,AMY2A,ANAPC11,CD59,CD8A,CDC27,CLDN10,EHMT1,FADD,IDH3B,IL10RB,IL28A,IL5RA,NLGN3,NNMT,OR10A2,OR2A5,OR4C16,OR4D11,OR6Y1,OR7D4,PAPSS2,PFKFB3,RALBP1,SGMS1,SLC33A1,UCK2,UPP1
Negative_8891	CTH,ACVR2A,ALG5,B4GALT1,CARS2,FUT8,MTHFD1,MYLK2,SETDB1,SLC25A4,STX18
Negative_8892	DDO,SRF,VTN,ACP6,ACPP,APOE,ARNT,CACNA1H,CHIA,CSNK2A2,DKK1,EPHA3,FECH,GLUD2,MAP3K1,MDM4,PRKCE,PRPS2,PYCR1,SELE,TGFA,TXNDC12,WNT7B
Negative_8893	ADH6,DHFR,IDH1,IL15,MAFA,OR13C2,OR2L8,OR51T1,PHKA1,RAC2
Negative_8894	CTH,DCT,ALOX12,CASP10,CLDN6,DDIT4,GUSB,LDHAL6B,LIPF,NPAS2,OR14A16,OR1E1,OR56A4,OR6N2,RASA2,RDH11,SFRP5,SUV39H1,TRAF4,UBE2L3,SERPINC1
Negative_8895	AMH,CKB,FH,NPY,STS,ABAT,ACY1,ADH1B,ANPEP,CLDN1,CYP8B1,NANP,NRP1,OR10G8,OR2A2,OR5B12,PAPSS1,PDE8B,PIGV,SYNJ2,TACR2,TUBB1,WNT4,TNFRSF13C
Negative_8896	EBP,KDR,SRM,ABL2,AKT1,BCL10,CCL16,CCL28,CCNA2,CHIT1,CLTB,CREB3L2,CSDA,E2F4,FLT3,GPD1L,HEXA,MGST1,OR10T2,PIGM,RASGRP2,ROBO2,TICAM1,VANGL2,PLA2G12A
Negative_8897	ACADS,CD3E,CREB3L2,DKK4,HYAL1,OR4C16,PDHB,TPK1,UGT2B11,CSNK1A1L
Negative_8898	FES,ADSSL1,AMDHD2,ANAPC1,AOC3,AQP7,ATP5L,BLNK,BST1,CASP8,CCR10,CD8A,CDS2,CPS1,CYP3A7,DGKQ,EFNA1,EFNB2,HSP90B1,INPP1,ITGB2,KRAS,LRP5,MDM2,NCK1,NLGN2,NPR1,NUDT8,OR1L4,OR5J2,ORC4L,PFKFB3,PFKL,POLR3B,PRKCA,PTPN7,SOD1,SUFU,TAP1,TAS1R1,TAS2R1,WEE1,WNT11,B3GALNT1
Negative_8899	MIF,PML,ALAS2,ALDH1A3,ASS1,BDH1,BMPR1B,C1GALT1,CD99,CYSLTR2,DEGS2,EFNA2,GCH1,GRM5,GSTM1,ITGB5,LCP2,MAGI2,MAOA,MAP2K3,MLLT4,MTMR1,OR10G4,OR2G2,OR4C11,OR7A17,OR9A4,P2RX3,PRKACG,PRKCI,RUVBL1,SDC1,SEMA3G,SETD2,STAT4,SUV39H2,TAS2R38,TLN2,TUBB4,VARS
Negative_8900	ACMSD,AGRP,AMPD2,ATP5J2,BST1,CCL26,CNDP1,GALNT11,HLCS,IFNA4,IFNA6,IL12A,OR52L1,OR9G4,OXCT2,PKLR,PRKCZ,PRLR,TGFB1,TGFB3,CSNK1A1L,TNFRSF10C
Negative_8901	ARG2,CBR1,CCNB1,ENTPD8,HTR7,IFNA4,IL18RAP,MTHFS,NAGA,NRXN2,OR1D2,OR4C13,OR8G1,PCCB,PDE3B,PPP2R5C,TDO2,UGDH,SERPINE1
Negative_8902	AUH,HK3,KIT,RB1,APRT,CD244,CD44,CLDN20,COQ6,CYP1B1,CYP2U1,GUCA1A,LAMC1,LIPE,MAP2K5,OR52D1,OR7G1,PRKAG3,TICAM1
Negative_8903	BTK,CAT,STS,AKR1C2,ALPL,ATP5D,BDH1,CACNA1A,CD8A,CHSY1,CSNK1A1,DDX51,F2RL3,FGD3,FLT1,GNPDA2,GRIA1,IL12B,IL22RA2,JAM2,LILRB3,MAPK7,NRG4,NT5E,NTF3,OR10C1,OR13C8,OR1S2,OR56B4,PARD3,PDE6H,PLCG1,PLIN,PPP1CB,ROBO3,SAT1,SLC25A4,TSHB,TUBB3,ULBP3,CACNA2D2,GABARAPL1
Negative_8904	GH1,PVR,RET,ADH1A,AMPD2,ATP5G1,CCL5,CCNA1,CCR5,CDC16,COQ7,CSF2RB,DGKQ,FGF1,GALNS,GALNTL4,GCNT3,GNPAT,GSTM1,MAPK3,MASP1,OR1J1,OR2T34,OR51V1,OR6M1,OR8B4,PFKM,RPS6KB2,TLN2,TLR3,UGT2B10,HLA-DQB1
Negative_8905	C2,DLD,FAH,ABLIM1,ACAT1,ARHGEF2,CTSL1,CYP4A11,DDX4,EPHX1,FLT4,GBGT1,HCLS1,IL23R,IL2RG,INHBA,JAK1,MAP2K2,MAP3K8,NEUROG3,OR2F1,OR2M5,OR51S1,PIK3C2G,PLTP,POLR2G,PPP2CB,PRKAG2,PTK2,SSH1,WNT2B
Negative_8906	AMH,GAA,GDA,AACS,ACSM3,ARRB2,COL11A1,CX3CL1,CYP4A22,FPGT,ICAM3,KARS,MAP3K6,MAT1A,OR13A1,PARS2,PLCB2,SCP2,SIPA1,UGCGL2,WWP1,ATP6V0D1

Negative_8907	ACADL,AKR1C2,AMDHD1,C3orf10,CACNA1E,CBR3,HADHB,HNF4A,HTR7,JAG1,LARS2,MASP1,OR10G3,OR4F17,PAK7,PIGP,PIGU,PIK3R5,PPID,PVRL4,SEMA4G,TAS2R48,WARS2,WNT1,SERPINC1
Negative_8908	SRM,SYK,AACS,ACVR1,ACY3,ADSS,ALDH1A1,B4GALT6,BST1,CDC23,CHKA,CLDN8,CREB3,CREB3L4,CTSL1,DPM1,ENTPD8,EPOR,ETNK2,GSTO1,HDAC2,ITGA2B,LCP2,MAD2L1,MECR,NAE1,NEGR1,NKX6-1,NR1H3,NUDT8,OR13C8,OR1F1,OR51E2,OR5H6,OR8J3,OXTR,P2RX6,PLD2,RALBP1,RPS6KB2,SV2B,TUBA1A,UGT2B28,MAPK8IP2,SLC25A31
Negative_8909	C3,ILK,MSN,SRF,ACTN2,BHMT,BUB1,C5AR1,COX15,CXCL10,CXCL9,EDNRB,G6PC,GABARAP,GALNT3,GNAI3,IFNGR1,KIR3DL1,LIMK2,NRP1,NSD1,PDX1,POLR3G,PON1,PRPS1,RAF1,RPS6KB1,SLC2A2,SOAT2,TAS2R38,TKTL2,UROD,HLA-DRB4,MAPK8IP3
Negative_8910	HGD,MPL,TXK,AANAT,AOX1,ARSD,ATF4,C4BPA,CCNE2,CHDH,CYP2C8,DDX19A,DKK2,E2F2,EXTL2,FGF20,GAD1,GGT7,GOSR2,ITGB7,NFATC4,NTSR1,OR14C36,OR2H2,OR2T5,OR4Q3,OR52E4,PDGFRB,PRKAA1,RASGRF1,RFXAP,SEMA4A,SPCS1,TAS2R49,TPH2,TRAF2,XCL1
Negative_8911	GCK,ACAT2,ADIPOQ,CDO1,CLDN16,CNTFR,DAAM2,DPM1,EIF4E,EPHA5,FGD3,FOXO1,GLUD1,GNAI3,HTRA2,LHCGR,NMNAT2,OR51V1,OR52I2,OR5AS1,OR6K6,OR7G3,PDE11A,PHKA1,SLC27A4,TNFRSF11B
Negative_8912	CS,FOS,CDKN1B,CMPK1,GALNT10,HPSE,ISYNA1,MGAM,MMP9,OR14C36,OR2B6,OR4K15,OR5AK2,PAPSS2,PCNA,PRKCG,RHOA,RXRG,ATP6V0A2
Negative_8913	NNT,ACSL3,BAAT,C1QC,CD8A,COQ6,COX15,CSNK2B,CXCL13,CYP19A1,DKK2,ENTPD8,FGF6,GSTT1,IL10RB,ITGB3,MLLT4,MPP5,NANS,NCKAP1L,NOTCH2,PCK2,PDCD1,PSPH,SEMA3C,TRAF4
Negative_8914	A2M,ADA,CFH,F9,SP1,ACADM,ACSS2,ADRA1D,B4GALT3,BMP5,BMP6,BMPR1A,CASP9,CCR4,CCR5,CFL2,CHD8,CRHR1,DNMT3A,FADS2,FCGR2B,FIGF,FLT1,FLT3LG,GSTK1,IDH1,IL13RA2,IMPA1,LLGL1,MYH6,OR4F17,OR51V1,OR6X1,OR8H3,OR8U1,PHKG1,PVRL1,PYCR2,RALB,RPS6KA1,RPSA,SMAD4,STMN1,TICAM2,TYK2
Negative_8915	ABO,BAX,CFH,FGB,ME1,MPI,ADH1B,C4BPA,CCNE2,CD38,CISH,CLTCL1,EPHA5,EVI1,EXOC7,GALNT3,HCST,HGSNAT,KRAS,MDM4,MITF,MYL7,NAMPT,NANS,NKX2-2,OGDHL,OR5AN1,OR7C1,P2RX2,PANK1,PDE4A,PER2,PGAM4,PIAS4,PITX2,RARS2,SMURF1,SPHK1,SPI1,TSNARE1,TUBB3,UCK2,WASF2
Negative_8916	NPL,PML,ACOX2,ACSM3,AOC2,CCR2,FMO1,GNPNAT1,GUK1,IDH3G,IL2RA,ISYNA1,OR2Z1,OR4K2,PDE4A,PDE6D,PPP2R5A,PTGES2,PTGS2,PYCR2,RRAS,SGPP1,SHC4,SUOX,TAS2R42,VAMP2
Negative_8917	GGH,CXCL12,FMO2,GFAP,HSD17B8,NCF4,NOTCH1,OR11H4,OR2AG2,OR4D6,OR5L2,PLA2G6,PLCB2,SULT2B1
Negative_8918	ACY1,CHPF,CTF1,ITGA6,NT5C1A,TNFSF11
Negative_8919	BID,C8G,ACLY,AQP7,CDK2,CSNK2A1,CXCL12,DHX58,FGF7,HHIP,KLK3,OR51T1,RUVBL1,SULT1E1
Negative_8920	AGL,ATM,FAH,KHK,LCT,A4GALT,ADSL,AKT3,CCL3,CTSG,CXCR5,CYFIP1,CYP26B1,DHRS4L2,HIF1A,KRAS,LTBP1,MTHFD2,OR4D9,OR51V1,PAK6,PGAM2,PPAP2B,PRKAA1,TICAM2
Negative_8921	ACVR2B,AKT1,ARPC3,BMP8B,CACNA1S,CD47,CPT1B,EFNA2,FIGF,GNB1,IDH2,LDHAL6B,NFKBIB,OR2T12,OR4K1,PSEN1,ZAP70
Negative_8922	RET,SRF,ACAD9,ATP2A3,BAI1,CD46,DUSP9,EPHA3,FABP1,HTR4,LDHC,NME7,OR6C76,PGAM2,SULT1A2,UCP1
Negative_8923	ADH5,PLCD4,TRAF2
Negative_8924	OSM,SPN,AANAT,ACMSD,BMPR1A,BUB1B,CAMKK1,CHIA,DARS2,ENTPD2,FCGR3B,FRAT1,HAGH,HIBADH,IKBKE,LAMA3,MAP3K13,NFATC3,NKX2-2,OR10A6,OR2C3,PCYT1A,PPP2R2B,PTGIS,SMARCA5,SPHK1,TNFRSF4,TUBA8,VDAC1,VNN1,PAFAH1B1,SERPINA5

Negative_8925	CBS,LPO,AADAC,ACOX1,ACSS3,ADCY1,ASAH1,ASMT,CADM3,CDC2,CLTCL1,ENTPD4,FGD3,FGF8,FZD7,GNRH2,HIBCH,ITPK1,KYNU,MLYCD,MYL5,OR2Y1,OR2Z1,OR5AR1,OR8I2,PARD6A,PGAM1,PIK3CB,PLCB4,POLR2D,SDHC,ST3GAL2,STEAP3,MAPKAPK2
Negative_8926	ANPEP,BACE1,EARS2,HTR2B,OR51A2,PLCD4,PRKAB1,TGFB2,PHOSPHO1
Negative_8927	ACSM5,ALPPL2,G6PD,OR10A6,OR2Z1
Negative_8928	C7,CBL,DCT,MPZ,ABAT,ABL2,ALDH1A1,ALOX12,ARD1A,CSNK1G3,CXCR4,ENPP1,IDI2,LRP5,NMNAT1,OR1Q1,OR4C12,OR4F4,OR5AC2,OR8H2,PCK1,RND1,SDC4,SMPD1,SPCS3,STK3,ATP6V0D1,ST6GALNAC1
Negative_8929	GCK,ACTN1,ADRBK2,AKR1C1,ALDH9A1,ARHGEF2,CDC14B,CHPT1,COX15,CRB3,CTBP2,CXCL9,ENTPD3,FABP4,FECH,FGF10,FZD10,GALNTL1,GPAA1,GZMA,IRF7,KIR2DL3,MGST2,NLGN2,OR10G2,OR52I2,OR52K2,OR9Q1,PGAM2,PIP5K1C,PLAUR,PLCB2,PNLIP,POLR3B,POLR3K,PPAP2A,PPM1A,PPM1D,RALGDS,RFX5,SC4MOL,SEMA3D,SOCS3,ULBP1,CTNNBIP1
Negative_8930	ADC,CBL,CSK,GSS,ARHGEF2,ATG12,ATP5L,B3GALT1,BDH1,BLNK,CACNG5,CASP10,CCKBR,CD55,CHKB,CHRM5,COQ3,DDX19A,DGKA,DKK4,DUSP1,FGF8,GALNTL2,IL21,KIR2DS4,MAN1A1,MEF2C,MYH13,MYH8,NME7,NOD1,NUDT5,OR10J3,OR2F2,OR51B6,OR5B17,OR5B21,P4HA3,PLA2G1B,PLD2,PON2,RHOQ,SUFU,TFPI,TNFRSF4,TUBA1A,UGT2B28,MAPKAPK5
Negative_8931	FES,PGP,ACLY,ALAD,CACNA1G,GCAT,NFE2L2,OLR1,OR4K15,OR4M1,P2RX5,PANK1,PIK3R2,RXRA
Negative_8932	AR,ASL,DBI,DCC,F9,AASS,ACVR1C,ARHGEF7,ARSD,ASAH1,ATP6V1F,B4GALT3,BACE1,BMP5,CLDN9,CYP26A1,CYP2A6,DDX55,DTX1,ENPP1,FCGR2B,GLI1,HAO1,IL11RA,IL20RB,IMPDH2,KIR2DL2,KIR3DL1,LDHAL6B,LILRB3,MAP3K11,NOS1,OR1S1,OR2D3,OR52A1,OR52E2,OR7G3,ORC6L,PER3,POLR1A,PRPS2,RUVBL1,SETD7,TXNDC12,PPARGC1A
Negative_8933	ASL,SRC,AASS,ABL1,ACTA1,ACVRL1,ALG12,AMDHD2,BPGM,BST1,CLDN14,DGKD,DGKH,EPHX1,FABP1,FBP2,GLG1,IDH1,KAT2A,OR2T29,OR51I1,OR5K2,OR5L2,OR5V1,OR9A2,PFKM,STAT1,TFDP1,TOMM40L,TRAF6,TUBA1C,TYRP1,VANGL2,ARHGEF12,ATP6V1C2
Negative_8934	AGXT2,ALDH1L1,ARPC2,ARPC5L,ATP5G3,CD58,CHKA,CSNK2A2,DDX4,FUT6,GNA11,HMGCS2,IFNG,ITGA1,MAP3K8,NCF2,NMNAT3,NT5C1B,OLR1,OR11H4,OR51B2,OR7A5,PLA2G2A,POLR2C,PRKCZ,PRPH,SESN1,SOS1,STAT5A,UCK2,PRICKLE2
Negative_8935	ADRB3,ALDH3B2,ALG2,ALPP,ATP2A2,ATP5J2,B3GAT2,CANT1,CHKA,CHRNA7,CRY1,EPOR,GNPDA1,GSTM5,HEMK1,HSD17B7,IL17B,IL18RAP,IRAK4,LDHAL6A,LYPLA2,MCCC1,MYLK3,OR4F5,OR4K1,OR51B6,OR52I1,OR6A2,PIGA,PMM1,PNLIP,PSENEN,SC4MOL,SMAD2,TPMT,UCK2,WNT10A
Negative_8936	GNE,AACS,ACSL4,ACTN4,MAPT,NFKBIB,NNMT,NT5C2,OR2L8,OR51G2,PPP3R1,RPN2,SPRY4,TSC2,YKT6,YWHAH,GABARAPL1
Negative_8937	B4GALT3,BIRC5,CCR2,CHRM5,DUSP6,GNG3,GRLF1,OR1N2,OR4S2,PAK2,POLR1C,SCIN,UBA7
Negative_8938	FGB,FN1,PLN,AASDH,CHPT1,CMBL,CNDP1,CTNNA3,CYP2S1,ECHS1,FZD3,HCST,HMGCL,IGF1,INPP1,LAMB1,MCAT,MYH7,OR2G2,OR5M9,OR7G3,PCYT1A,PDE8A,PGM2,POLR2B,RAPGEF2,SPTLC1,TM7SF2,UGT2A1,USE1
Negative_8939	GNE,HAL,RB1,SRM,ACVRL1,ADCY6,ADH1A,ASNS,CACNA1S,CYP2C8,DAPK3,EPHB4,EXTL1,GLI3,LEF1,MAP4K3,MGAT5B,MYL9,NCR2,OR51A2,OR9K2,PANK1,PCYT2,PHKG2,PIK3R4,PIK3R5,PPP2R2C,RPS6KA4,TAS2R16,TLR2,VDAC3,CTNNBIP1
Negative_8940	C9,ILK,ATP13A2,CSH1,DGKE,DHRS4,LIPE,MAPK12,OR1J4,OR6A2,PARD6B,TCF7

Negative_8941	AK7,GLS,GSN,SRF,ACTC1,ADCY1,ADRB1,ASNS,ATG3,B3GALT4,BUB1B,CDK6,CPS1,CREB3,CTLA4,CYP2C18,F11R,FSHR,GARS,GCLM,GLUD1,MTAP,NKD1,OR2AG2,OR2C1,OR51B6,OR51E2,OR5B21,OR6A2,PNMT,POLR1A,SARS,SETD7,SHC2,TLR5,WBSCR17,HLA-DRB4,MAPK8IP2,ST6GALNAC5
Negative_8942	AR,FES,TPR,ALOX15B,BCL2L1,CCR6,CDH5,CTPS2,CYP2J2,DLAT,EPAS1,ESAM,FGFR2,ISYNA1,LAMC3,LEPR,MAGI2,MYH13,OR10G7,OR2T5,OR4D11,OR51B4,OR9I1,PAICS,PIK3C2B,PLIN,PTGS2,RHOA,SETD7,SULT1A4,TCF7,TJP1,ST6GALNAC5
Negative_8943	SP1,CD8A,CRHR1,RALA,SLC27A1,HLA-DRB4
Negative_8944	AFMID,ALDH2,C7orf16,CCNB3,CD22,EFNA1,FOXO1,G6PC,GALE,GAS1,GLUD1,GRLF1,HHEX,MGAT5B,OR10G9,OR13J1,OR8J3,PIK3R1,PISD,POLR3G,PPP2R2B,SELL,TGFBR2
Negative_8945	AR,F7,GLS,LAT,MAG,PAH,ACACB,AMY2A,AOC3,CRY2,DPM2,GSTM2,MMP14,OR2A5,OR4C16,OR7A5,PDE4B,PMVK,POLR3A,RRAS2,SMAD9,SPCS1,SRGAP2,TLN1,UMPS,YES1
Negative_8946	GLA,HPD,TEC,VCL,ABI2,AKR1C1,AMDHD1,C5AR1,CACNG2,CSF2RB,FPGT,GCH1,IKBKE,IRAK4,MGLL,OR2G3,OR2H2,OR4P4,OR51B4,OR5AU1,OR7D4,PCK2,PDGFRB,PFKFB2,PGM2,PIGV,POLR3A,PPOX,RAP1A,RDH5,ROBO1,RXRA,SDHD,SGPP1,SH2D1B,SYNJ1,TAS1R1,TJP1,YARS2,YWHAH,TNFRSF6B
Negative_8947	GH1,MVD,CACNG3,F2RL3,IRS2,OR2Z1,ORC1L,PANK1,SRGAP1
Negative_8948	AGK,ADRBK2,CD40,CDS1,CLDN16,CSF1R,CSF2RA,CSH1,DEGS2,DIAPH3,EPB41L2,GNAI2,GSTK1,HADHA,HNF1A,LAMA4,MCM6,MGAT5,NSD1,NT5C1B,OR1G1,OR4D1,OR51I1,OR8D4,PIGQ,PLA2G3,PPOX,PRODH,PTPN5,PTPN7,PYGL,SMAD7,SMPD1,SUV39H2,TCF7L1,UBA1,UROC1,VNN1,WNT8B,HSD17B10
Negative_8949	F2,CDKN1A,CNGA4,CXCL2,DHCR24,DHODH,GNRHR,IL15RA,P2RX6,PLOD3,PMM2,RALB,ST3GAL2,CACNA2D2
Negative_8950	SHH,NPR1,OR2T11
Negative_8951	APAF1,APBB1,ASRGL1,B3GNT5,B4GALT1,BIRC5,C7orf16,CD14,CD79A,CHRM3,COL5A2,CSNK1A1,DPYS,DRD2,HRAS,IAPP,IL17RA,LAP3,LRAT,MAP2K6,NODAL,OR2F1,OR2M3,OR4E2,OR52B4,OR5K1,OR5T1,OXSM,PAK2,PARD6B,PIGH,PLA2G3,SIGLEC1,STX1B,TPH2,TSC2,VDAC3,YARS
Negative_8952	DDC,GH1,GSR,ADORA2A,ALOX12,CDS2,CNTN1,CX3CR1,CYP11B2,HLA-C,LDHC,NFATC4,OR4K14,PFN1,PI4KB,PLD1,PPP2R5A,SELPLG,SETDB1,TACR3,WNT3
Negative_8953	C2,PGD,ABL2,ALG1,AMY2B,ARSE,BLVRA,CCL8,CCNB2,CSNK2B,CTLA4,CTNNA1,CYP2B6,DERA,EPHA6,HYAL2,IDI1,MASP1,NAGA,NKX2-2,OLAH,OR10A4,OR5L2,POLD1,RXRB,SDC4,SGPP2,TPH1
Negative_8954	ABO,ACVR2A,ALDH6A1,APOA2,CACNA1C,CALML3,CTSB,DPAGT1,EGFR,EPB41L2,FGF13,FOXO3,GNRH2,GPD1L,MAPK10,MCM3,MYL5,NCKAP1L,NRXN3,OR3A2,OR51E2,OR5B17,PIGK,PRKAB1,PRKAG2,SDC1,SSX2IP,TACR3,TICAM1,TP53,WNT10B,WWP2,SERPINA1
Negative_8955	LPL,MUT,ABL2,ACVR2B,ACYP2,ATP2A3,CACNG8,CBR3,CLDN5,COL5A3,CYP2C18,EGFR,ENPP3,F2RL1,GNA13,GSTZ1,IDH3A,IFT57,IGF1,IKBKG,IRAK2,IRF3,KYNU,LIPT1,MARS2,NAT5,NCAM1,NFATC2,NOX1,OR10AD1,OR13G1,OR5B21,OR7A5,OR8I2,PFKFB2,PGK1,PLA2G2E,PPM1D,RARS,RUNX1T1,SFRP2,SGMS2,SGPP2,SMURF1,SNAI1,UBE2L6,YWHAG
Negative_8956	C5,EBP,F5,LTB,RET,ALOX5,ALS2,APOE,B4GALT7,BMPR2,CALML5,CASP8,CCL5,CRHR1,CXCL10,DAAM2,DPM2,E2F2,ERBB3,FRAP1,FSHR,G6PC2,GGCX,GJA1,GLI3,HLA-F,ITGA6,ITGAE,MGLL,MOV10L1,NAT2,NEUROG3,NKX6-1,NMNAT3,OR10R2,OR14I1,OR2K2,OR9K2,PFKFB1,PHPT1,PLCD1,PPOX,PPP3CA,RPE65,UAP1,ZAP70,HSD17B12,PPARGC1A,SERPINA1

Negative_8957	DBI, HGF, ALOX15B, APC2, CREB3, FZD2, FZD7, GLB1, INPPL1, MAPK7, OR1L8, PARD6B, PTCH2, SMPD2, WNT2, YKT6
Negative_8958	GCK, TNF, ACSL5, AGTR1, ARPC2, CHRNA7, FTCD, GALNTL1, GCLM, ICOSLG, LRP6, NAT5, NOTCH2, OR2A2, OR2C1, OR4K2, OR5T2, PAK1, PDGFRB, PNPT1, POLA2, PTPN6, RALA, RETSAT, SDHA, SETD7, SETMAR, SLC12A2, TYMP, WARS
Negative_8959	F12, ITGA11, KLRD1, MASP1, MTMR1, OR4F17, OR6T1, OR8D4, SULT2B1, TRMT11, VAMP4, ATP6V1C2
Negative_8960	AR, ATM, BAD, C1S, CD6, CKB, CR2, ID2, AARS, ABLIM3, AHCYL1, ATP6V1D, BMP7, CTNNA2, E2F5, EHMT2, EPB41L1, FGF11, FLT3LG, G6PD, GALNT1, HNF1B, HRH1, IL17RA, INMT, LEPR, MAPK7, MYH6, NEUROD1, NRG2, OR10J5, OR4K14, P2RX3, PAX6, PI4KB, PPP2R5B, PTPRJ, SIPA1, SLC27A5, SPP1, TRAF3, TYMP, WIF1, ZBTB16, ATP6V1G2, PAFAH1B2, PAFAH1B3, TNFRSF6B
Negative_8961	ADO, DBH, FN1, NLK, PRL, STS, ACACA, ANPEP, B3GNT5, CACNB4, CD58, CDC14B, CDK6, CDKN2B, CX3CR1, CYP2A7, DGKQ, DVL3, EPB41, FTH1, GAD2, GALNTL4, IL28A, MAGI3, MALT1, MAPK1, NFKB1, OR51T1, OR6T1, PFKFB4, PPAP2A, PPP3CB, RXRA, TP53I3, YWHAQ
Negative_8962	CKB, DBT, DCN, GNE, GSS, B4GALT2, CACNG5, CTSB, ENAH, FGF6, HSD17B1, NAGS, NEU3, OR13H1, PGS1, POLD3, RUVBL1, SHISA5, TNFSF18, UGT2B11, PLA2G12A
Negative_8963	FRAT2, ITGA3, MAPK7, MTMR2, OR2T3, TCIRG1, YKT6
Negative_8964	ATM, DCN, OSM, ADRA1D, BCKDHA, CACNA1F, CFL1, CYCS, FGF13, GDE1, GDF5, IL18, IL23R, IL28RA, IL9R, MAD2L2, MAN1A2, MAP4K1, MDH2, NPAS2, OR10A4, OR13C3, PDE4B, PFKFB2, RAPGEF1, SUFU
Negative_8965	LCT, ACPP, HRH1, IFNA8, KLRC4, LIPA, NCR2, NUDT9, OR10G8, OR6K2, PIP5K1A, PYCR2, RASA2, SLIT1, SSH2, TNFRSF10B
Negative_8966	CR2, F7, LYN, PGD, TNR, BLVRB, CCNB1, CNTFR, CSDA, DARS2, DGKZ, DHCR24, DLAT, FTH1, GALE, GANAB, HTR2B, IL28B, LAMC1, OR13C5, OR52N4, OR5T1, PIGH, PIK3CD, PIM1, PPP2R2B, PSME2, PTK2B, RASGRP2, TUBB2A
Negative_8967	ACAA1, ADRB3, CLDN2, DHRS4, FGF3, FZR1, G6PC2, GALE, GCNT3, GSTM1, HPSE2, IL5RA, ITGA9, ITGAM, LRAT, MAP3K11, NNMT, NR1H3, OR4S2, OR5T1, OR9K2, PFKM, PLEKHA8, POLR3B, RASGRP4, SPP1, TNFRSF11A
Negative_8968	CCL28, DNMT3B, EPHA5, GALNT13, MAPK3, NAMPT, OR13C3, OR51F1, OR5V1, RELB, SOCS2, TPI1, UCK2, YOD1
Negative_8969	ANAPC5, ETNK2, LCMT2, OR9G4
Negative_8970	CACNB1, GSTA3, HLA-G, NEUROD1, NTRK1, PDCD1, PNPO, RALGDS, WNT7A
Negative_8971	C5, F7, ACO2, ACSS2, ADH7, AGTR1, AOX1, APRT, ATP5G2, BCL10, CCL20, CDC7, CLDN11, CNGB1, CYCS, CYP26A1, CYP2S1, FGF1, GNA11, GSTA3, GUK1, IL3RA, ITGB8, LIPE, MRAS, MYD88, NODAL, OR13H1, OR2T6, OR4D11, OR6N1, OR7D2, PFKL, PLXNB1, POLR2G, PPP3CC, PRLR, RBL2, ROCK1, RPS6KA3, SPRED2, STAT3, TGDS, TNFSF18, WIF1, YWHAE
Negative_8972	ME3, ACOT12, EPHB2, GSTA1
Negative_8973	AK5, ACY3, APPL1, B3GALT1, B3GAT2, CACNG4, CD8A, CDC14A, COL4A1, DGKH, EDNRB, EFNA2, EXTL1, FCGR2B, GSTM1, IFNA14, IFNGR1, IKBKE, IL24, MAP2K3, NOTCH1, OR51F1, OR7A10, RCHY1, SDHB, STT3B, TAS2R49, TRHR, WNT4, WNT5A, XYLT2, ATP6V0A4, CACNA2D1
Negative_8974	AARS2, ACSL4, BUB3, CAV3, DFFA, DHDDS, GCNT1, GJA1, GLUD2, GNA13, LAMA3, MAN2A1, MAP2K4, MTHFR, NOTCH3, PSAT1, RAC3, SAT2, STK4, TGFB1, UGT2B10, SERPIND1, ST6GALNAC6
Negative_8975	ASL, AUH, F7, ID1, MAG, TH, AARS, ADCY7, AGXT2, ALDH1B1, AQP7, ARHGEF6, ATP2A2, CACNA1A, CDKN2B, CSNK2A1, DVL2, EDNRB, EFNA2, EFNB2, FBP2, GALNT8, HSD17B8, INPP5E, INPPL1, LCAT, LTC4S, MAD1L1, MAGI1, MGAT3, MTHFS, OR1D2, OR1L3, OR4C16, OR4F17, OR4M2, OR7A10, OR8B8, PDE8A, PKM2, PTGS1, RUNX1, STAT5A, TAS2R50, YARS, EIF4EBP1, SERPINC1

Negative_8976	APBB1,ARPC4,BST1,CACNA1H,CD48,CDC45L,DDX52,EIF4E,EPB41L3,GCLM,HCST,IFNA2,ITGAX,L1CAM,LDHAL6B,MNX1,NOD1,OR2AG1,OR2L2,OR4F5,OR52A1,OR5AN1,OR5I1,OR5V1,OR6K6,OR6Y1,OR8B12,PDE7B,PIK3R3,PLD1,PNPT1,PPM1D,PPP3R1,PRKCE,RDH12,RELB,SMURF1,TCF7L2,TNFSF10,TPM3,TRADD,TRAF5,TRPM5,UPRT,WNT5B
Negative_8977	AK1,ACAT2,ADCY10,AGPAT1,COQ6,FBP1,FLAD1,HSD11B2,INHBE,MGAT5B,NODAL,OR1N2,OR8A1,PFKL,PGK1,SH2D1A,SH2D2A,SLC2A2,TALDO1,TUBA8,VAMP2,WARS2
Negative_8978	ACYP1,CYP8B1,DDX55,GNAI1,HSD17B8,ITGA7,PHKG1,PRKCD,PSME2,TAS2R4,ATP6V1G2
Negative_8979	C8B,F3,AGPAT4,FGF11,HSPA2,NAGS,NFAT5,OR10Z1,OR2A5,OR5T2,PTPRZ1,TAS2R10,TAS2R40,TBXA2R,TCIRG1,ZIC2,SERPINA1
Negative_8980	FH,NOG,ADIPOR1,ANAPC5,ENTPD7,EXTL1,FSHB,IL24,KIR3DL2,OR2A2,OR52E4,OR5K1,OR5T2,OR6Y1,PAK3,PLAUR,PLXNB3,POLD3,PPP1CC
Negative_8981	CR2,DDC,EDA,GH2,SDS,SHH,ACO2,AKR1C3,ALDH3A1,ARNTL,ATP2A2,CBLB,CCDC6,CSNK2A1,CYP2A6,DVL2,ENTPD6,EPAS1,EPHA5,GALNT3,IARS2,IFNA2,IL17A,KCNJ11,MAML1,MAT1A,OR10C1,OR10J5,OR1L6,OR5B3,PDE9A,PDGFRB,POLR2D,RASGRF1,RRAS2,SORD,STAT1,STMN1,UGT2A1
Negative_8982	MVD,A4GALT,BDKRB2,DHDH,DHRS3,ENPP2,EPHA7,MTMR6,OR2F1,OR2L8,OR51S1,OR52N2,OR6C76,PRKCQ,RAB5A,SMAD7,TGFB3,SERPINB5,TNFRSF1B
Negative_8983	AUH,ADCY2,ALDH4A1,CHEK2,CRB3,EPRS,FOSL1,IL22,PON3,SGPP1,SRGAP1
Negative_8984	F2RL2,SOAT2,TNFRSF10C
Negative_8985	HYI,LPO,AOC3,ATP6V1F,B3GALT1,B3GALT2,CACNB1,CACNB2,CPOX,CSNK1A1,CYP4A11,DOT1L,DVL3,EDA2R,FOXA2,FZD10,GJD2,GLO1,GLUD1,GRM5,GSTK1,HNMT,IDH3A,ITGA7,ITGA9,MDH1,MYLK3,OR1L4,OR2T3,OR2T34,OR4M1,OR52I2,PDE11A,PPP1R3A,PRKAG1,PRKG1,PTDSS2,RELA,SHC2,SHC3,SOCS7,TAS2R4,TNFSF11,TOMM40L,TPM3,UGCG,WASL,EIF4EBP1,TNFRSF6B
Negative_8986	ACADM,ALDH4A1,CREB3L4,GSTM1,HTR5A,ICAM3,OR52L1,PPM1B,RARS2,TPH1
Negative_8987	AGK,CD4,FST,PC,VCL,ACSL3,ALG9,APPL1,ATP6V0B,CCR4,CUL1,CX3CL1,CXCR4,CYP11B1,DGKB,GCAT,IL18RAP,ITGB1,KLRD1,MIOX,NCK2,NEUROD1,NUDT9,OR1S2,OR4M2,OR5L1,PAX8,PHPT1,PPAT,PRKCA,PRKCD,PYGB,SEMA3D,TUBB2C,VAMP1,CSGALNACT2,PNLIPRP1,SERPINC1
Negative_8988	ILK,OSM,ACSM1,ACVR1C,ADRB3,ALDH7A1,APBB1,ATF4,CDC14A,CER1,CHDH,CTBS,CTPS2,ECSIT,EPHA8,FADD,FGF22,FGF4,G6PC,GALNT13,GALNT5,GART,GIT1,HADHB,IL22RA2,MDH2,NANS,NFYA,OR2C1,OR52H1,OR5B21,PRKAR1A,PSEN1,RELB,RFWD2,STAT4,TRPM5
Negative_8989	A2M,HGF,MTR,ADIPOQ,ALAS1,ALAS2,ALOX5,ARNT,CACNA1I,CHPT1,CLDN6,CMPK1,CPT1C,DGKB,EGFR,GLUD2,HARS,ICAM2,IDH3A,IL9R,KCNJ11,KITLG,LALBA,LCMT2,LIPE,MYH6,NME2,NOTCH2,OR2A5,OR2V2,OR8S1,P4HA2,PDE5A,PGK1,PIK3C2B,PPP2R1B,PRKCE,PVRL2,RASGRP1,RASGRP4,TJP3,ULK2
Negative_8990	C3,ALDH3A1,APLP1,ATP5A1,BMP8B,CDH4,CRY2,CYP17A1,DPM2,DRD2,G6PC,GAB1,GALNT10,IFNA16,IL19,IL8RB,MAP3K4,MAPK10,NFATC1,OR1A2,OR1L1,OR52E8,PFKL,PHKA1,PIGL,PLCD3,PLD1,PTPRM,RFXAP,TRAF3,TSTA3,HLA-DQA2,TNFSF13B
Negative_8991	FER,NPY,RPE,TYR,AADAT,AASDH,ACSS2,AKR1A1,BUB1B,CASK,CCR9,CNTFR,CYP2C8,GANC,GOSR2,GZMB,HSD17B1,IFNA7,JAK1,LAMA4,MAGI1,NME6,OR1D2,OR2AG1,OR2F1,OR2G6,OR2M4,OR8H2,PIAS3,PLD1,PPP2R5D,PTK2,RASGRP4,RGS3,RPS6KA5,SEMA3F,SEMA4D,SMAD3,SMAD6,TSTA3,TUBB4Q

Negative_8992	AGK,ME1,APAF1,CD19,DTX3L,GLG1,GNPNAT1,KITLG,NME2,OR5H6,RAC2,SPRED1,UGDH,ZIC2
Negative_8993	BID,DCN,GP6,SCD,AARS2,AKT1,ANPEP,BDKRB1,BTRC,CD19,CNDP1,CRKL,DDX51,DHDDS,ENO3,FSHB,GFPT2,GRPR,ITGB8,MAGI2,MYD88,NT5C,OR10K2,OR9A4,PFKFB1,PTPMT1,RFWD2,SC4MOL,SKIV2L2,SOX17,SPHK1,SPRED2,STK4,TAS2R9,TSNARE1,TUBA3C,WNT2,CACNA2D2
Negative_8994	APP,CAD,PDC,ZYX,CACNA1B,CSF2RB,CXCL5,DHRS3,FABP1,GLS2,LMO7,NPR1,OLAH,OR10T2,OR11G2,OR52B6,OR5K2,PAPSS2,PCCA,PPARD,PRKCB,SELE,SPCS3,STX1B,TGFB3,TGFBR1,TRAF6,UGT2B10,VAMP3,HLA-DQB1
Negative_8995	FST,RPE,AARS2,AGPAT4,ATP6AP1,B3GNT3,BIRC3,CD27,CD36,CHPT1,CKMT2,CNGA3,CNGA4,CSNK2A1,CTLA4,CYP1A1,CYP2D6,DKK1,DNMT3A,GALNT14,GPD1,GRIN1,HGSNAT,KIR2DL4,LALBA,MAN1A2,MAP3K14,MTMR6,NMNAT3,NSDHL,OR10J5,OR52I1,PITX2,POLR2J,SELL,SNAP23,SPRY1,STAM,TCIRG1,THTPA,UGT8,VEGFB
Negative_8996	DDO,ABLIM2,ALOX15B,CD58,DERL1,ETS1,PLCB1,PTAFR,SERPINB5
Negative_8997	CFI,STS,DHDH,FGF6,OR1D2,PIK3CD,SLC25A4,YWHAZ
Negative_8998	EDA,ALOX12,ASRGL1,CACNB4,CCND2,CMBL,CYP2C19,F11R,GANC,GCLC,GSTP1,IL25,MAP3K5,NFAT5,PLCD1,SAT1,SEMA3C,SNAP25,TPH1,PHOSPHO1
Negative_8999	CKM,AXIN1,GSTA5,PRSS3,PSEN2
Negative_9000	GLS,ABI2,ACACB,ATP2A2,GPD1,IL17RA,OR2B2,OR5T1,POLR2H,TP53I3,TRAF2
Negative_9001	GNAZ,INPP1,OR52R1
Negative_9002	TKT,BRAF,CD48,EFNA4,GPD2,NR1D1,RDH8,RFXAP,SMPD4,HSD17B12,MAP3K7IP2
Negative_9003	DUT,FER,FST,GAA,ACSM4,ARHGEF7,CDH4,CHRM3,COL5A1,CTPS2,CYP3A4,DGKQ,DNMT3A,F13A1,GALNTL5,GNA14,MGST2,MYLK3,NANP,NCK2,NFX1,ONECUT1,OR1E2,PAK4,PPARG,PPCS,PPP5C,RERE,SETD1B,SH3BP2,TAS2R4,TIAM2,TNFSF10
Negative_9004	LTB,ALOX12,DDX41,ESPL1,GNG3,NMNAT2,ORC3L,PNPO,SOX17,HSP90AA1
Negative_9005	CADM1,CCL8,CDC25A,CLDN6,CSNK2A1,CYP3A4,DRD2,ECSIT,GADD45B,GRIN2B,HLA-DOA,HSD17B1,HSD17B2,MTHFR,NUDT2,PIGQ,PTPN1,SGPP1,SLC25A4
Negative_9006	DCN,CSNK2B,CTSS,DDX19A,EFNA1,EVI1,FGF23,FOXO3,GALNTL5,INHBB,MPST,PLXNA2,PPP1R3B,RPS6,STX10,WNT2,CSGALNACT1,PAFAH1B2,TNFRSF10D
Negative_9007	DHH,ME3,ACAA2,ADRB2,ALDH1A3,CAV2,CCR10,CLTA,DHCR24,DTX3,FGF19,GALNT10,GLI2,HARS2,HIF1A,IL15,MYL5,NNMT,OR10H2,OR2G3,OR4L1,OR5L2,PIK3C3,PLCZ1,PPP3CB,RRM2B,STAT4,STX1A,TBL1XR1,WNT3,WNT8B,B4GALNT1,HLA-DRB5
Negative_9008	C7,EGF,GPI,AOC2,CCL26,CDH4,CXCL11,DHRS9,DNMT3A,EDNRA,MAN1A1,NADSYN1,OR13J1,PGAM1,PTPRB,RALBP1,SGMS2,TJP1
Negative_9009	C8A,ALDH6A1,CACNG3,CCND3,CYFIP1,CYP1A1,FASN,FGF11,FZD9,OR2T11,RND1,ATP6V1G3
Negative_9010	FAS,BUB3,CAV3,CD40,CLDN1,CSF2RB,CXCL10,EDA2R,EFNB2,ENPP7,IBSP,IL12B,IRF5,MAML3,MTHFD1,NMNAT1,OR10C1,OR2A14,P2RX4,PNPLA3,PPM1B,SDHB,TAS2R60
Negative_9011	CTH,GK2,ACTG2,ANGPTL4,COL4A2,CSDA,FGF11,GRHPR,IL20RA,LTBP1,MYLK,OR2L8,PLA2G6,SDHD,SNAP25,SPHK2,YWHAE
Negative_9012	LSS,ACSL4,AKR1C2,ARHGEF4,CD8B,CYBB,FGF11,KAT2B,KIR2DL1,MAGI3,PGAM2,PLD2,RFNG,SLC25A4

Negative_9013	ADA,B4GALT2,BCKDHB,CCL17,CCL27,CPT1A,GLUD2,GSTM2,MCM2,NODAL,PFKP,PLA2G2E
Negative_9014	CEL,ENTPD6,TAS2R42
Negative_9015	DCN,ATP6AP1,BIRC3,BUB3,CCR7,CDC25A,GGT5,GSTA5,ITGA5,LAMC3,MINPP1,MTAP,OR5B17,PIK3C2B,PIK3C3,SEMA4G,SETD1B,SMC1A,ST3GAL1,TLR9,TYROBP,PAFAH1B1
Negative_9016	CR1,LAT,ACTB,BCAT2,CD40,CHAD,CTTN,CYP11A1,HTR4,MICA,OR6K2,PPCS,PPP2R2D,TP53I3,TYMP,VAV3,WEE1
Negative_9017	AKR1B1,GMPPB,IFNK,NCR1,RHEB,SDHB,SNAI2,TFPI
Negative_9018	FN1,LSS,AKR1C2,FGF19,HTR7,NANP,PPP2R1B,PVRL3,SARS,SOCS3,SPTAN1
Negative_9019	BTK,ADCY10,ANPEP,APOE,ARSD,BDKRB1,CADM3,CNDP1,DDX55,DUSP9,ENTPD3,EPB41,FLT3,GRPR,GSTM2,IL18R1,MAFA,MAP2K3,MCM6,MGST1,OCLN,OR10H2,OR11G2,OR2AG1,OR4C6,OR51V1,OR52D1,PIGS,POLR2D,TAS2R7,TPH2,WASL
Negative_9020	BET1,OR52B4,STAT2
Negative_9021	B3GNT4,CD34,F13B,GNAI2,INADL,NUDT12,POLD1,TAS2R5,UBE2L3
Negative_9022	HGF,KMO,ACSM5,ADH1B,ARPC5,CAMK4,CBR1,CCKAR,CCR7,CD79B,CREBBP,CRY1,E2F2,FADD,GNAQ,IGF1R,ITGAV,LIPG,MAGI1,MGAT4A,NR1H3,NTSR1,OR11H6,OR1G1,OR51D1,OR51E1,OR9K2,SETD7,XYLB,GABARAPL1,PNLIPRP1,TNFRSF13C
Negative_9023	ADC,HGF,ALPP,APOA1,AREG,BLVRA,CCR2,CNTF,COL4A1,DUSP8,FOXA3,GBE1,GGT6,GLS2,GLUD1,GNG3,HTRA2,IFNA5,IL12RB1,IL8RB,ITGA6,MAPK8,NR1H3,OR1D5,OR4D2,PFKM,PLXNB3,POLR3G,PPAP2A,PPP1R3D,PTGES,SESN2,SETD7,SPRED2,SUCLA2,SULT1A2,TAS2R48,UNC5B,SERPINE1,TNFRSF17
Negative_9024	MUT,ADH4,AK3L1,CAPN2,CD99,CDC23,COQ7,CYP3A5,GABARAP,GCLC,GNRH1,GSTA4,HEXA,INHBB,MADCAM1,MCEE,MTHFD2,NANP,NR1D1,OR51S1,OR8D4,ORC5L,SLIT1,TAS2R48,TYROBP,WEE1,MAPKAPK5
Negative_9025	ACSL3,B3GAT1,BMP8A,CACNB1,CCR1,CD48,CDS2,DCXR,FRAT1,GSTM3,IL12RB1,IL12RB2,NFKB1,NOTCH3,OR2A2,P2RX2,PEMT,PIP5K3,PKM2,PPP2R5C,SFRP4,SHC3,SNAI2,TICAM1
Negative_9026	RFK,ADRB1,ALOX15B,ASNS,B3GNT5,BMP7,CCL8,CCNA2,CDC20,CES1,CLDN5,CTNNA2,CXCL11,CXCL16,CYP3A4,DNMT1,EPHA4,FGF12,FTH1,FZD1,GUCA1A,GUCY1B3,HTR2C,LEFTY2,MAGI1,MAP3K6,MAPK1,MITF,MYLK2,NT5C3,NUDT5,OR2T11,OR51B4,OR52L1,OR6T1,PDIA4,PHKG2,PIGC,PPOX,PTGFR,PYGL,SLIT1,STX6,TJP2,TRIP10,UGT8
Negative_9027	ILK,ACACB,GLUD2,LIMK2,OR14J1,SRD5A2,SULT1A4,TBXAS1,MAP3K7IP2
Negative_9028	CFH,MVK,NPL,PC,TNF,ACO2,ACSM4,ADH1A,ALDOC,B3GNT3,CBR1,CD55,CREB5,CYP3A7,CYP4F3,DDX23,DTX4,EPHA7,ERBB3,HAAO,HMGCR,IL8RB,JAK3,JUND,NEGR1,NFKB2,OR13C4,OR14I1,OR4N4,OR5AN1,PAK6,PIGA,PIK3CD,PPT1,PRKG2,RASSF5,SMPD2,SOS1,SPTAN1,STMN1,UGDH
Negative_9029	CBL,CD4,CR1,IL3,RFK,RPE,SPN,ALPPL2,BCL10,C5AR1,CDKN1B,DUSP7,EPHX1,GADD45A,ICAM1,IL2RG,IL7R,INPP4B,MYL9,NANP,OR5AC2,OR6C75,OR9K2,PDE11A,PIK3C2B,POLR3B,RBL1,RPS6KA5,SEMA3C,TM7SF2,TUBB6,VARS
Negative_9030	AK3,INS,AHCYL1,ATP6V0C,B4GALT1,CLDN9,DGKZ,FZD9,PDE6G
Negative_9031	ADO,AMT,C8B,FGG,MPZ,AASS,ACAT1,ACSL6,ALCAM,ALDOB,ALG11,ALG3,CHST12,CYBB,CYP2E1,DAAM2,DEGS1,EPHA7,ERBB3,FGF21,GYS1,IFNAR1,IL17RA,IL1R2,LEF1,MGAT3,MYH2,NANS,NFX1,ONECUT1,OR8J1,OR8J3,PCNA,PPP2R2D,SARS,SFRP2,STAT1,STX2,ULBP3,YWHAZ

Negative_9032	CR2, SCD, ACACB, ACVR2A, ALPPL2, BAAT, CD19, COL4A2, CSNK2A1, CYP2J2, DGKA, FUT2, FUT7, GALNT9, HSD17B1, IL2RA, KLRC1, LIPT1, LRRC4C, LTC4S, MAGI3, MAN1B1, MAP2K3, MAP3K6, OR13C2, OR1B1, OR4K14, OR4S2, P2RX1, PAK1, PDGFD, PGK2, PHKB, PIGB, PLA1A, PLCG2, POLR3A, PYGB, RPE65, SEMA3D, SEMA4B, SSH1, STX18, VASP, WARS2, HSD17B12, SLC25A31
Negative_9033	APLP1, BMPR2, CISH, IFNA21, MAN1A2, OR2H2, OR2L2, OR52M1, PHKB, PPP2R5B, RASGRP2, RPS6KA4, SKP1, ULBP3
Negative_9034	CGN, CSK, ME3, ANAPC5, ARD1A, B4GALT1, CRHR1, EDA2R, FLOT2, IARS, IL19, LCMT1, MAP4K2, OR2G3, OR51E2, OR51L1, OR52N4, PFN4, PIK3C3, PLA2G4A, QPRT, RAF1, RND1, SERPINE1
Negative_9035	B3GALT6, CYP26B1, DDX19A, FGF21, GLB1, IL10RA, IL18, LIPE, MAD2L1, OR1E1, PDE1A, PDE7A, PIK3CG, PLA2G2D, PPP1CC, RPS6KA2, SRGAP1, TAS2R49, TYMS, ULK3, YWHAG, PAFAH1B2
Negative_9036	MVK, SMO, ACP1, ADIPOR2, ATP2A1, ATP5E, ATP5G1, ATP5J2, BMP8B, BRAF, CAV3, CBR3, CDC20, CDC25C, GAL3ST1, GBA3, HTRA2, IL12A, IL23A, MAP3K6, OR1A2, OR1L3, OR2T29, OR3A1, OR56A1, OR6N2, PIGH, PKLR, PLA2G1B, POLD4, PPP2R5C, PYGM, RHOA, RUVBL1, SIAH1, SPRY2, SRGAP3, VASP, VEGFB, ST6GALNAC3
Negative_9037	GGH, SYK, ACY3, AMPD1, ARPC3, CSDA, FGF6, GCH1, LDHAL6B, MAP3K3, OR8D2, ORC1L, P4HA3, PEMT, RPS6KA3
Negative_9038	HAL, LIMK1, RASA2, UPB1
Negative_9039	MET, TNN, CKMT2, COL5A1, DERA, GP1BB, HPSE2, INPP5E, MTHFS, OR7G1, PFKFB1, PHOSPHO1
Negative_9040	F8, KMO, SCD, SRF, AACS, AKT2, ALPP, ANAPC2, ASPA, BUB1, CCL1, CCND1, CCR5, CLDN4, CXCR3, DDX52, DVL1, EFNA5, ETS1, FOXO3, FUT3, FUT5, GCH1, GIT1, GNPDA1, GRIN2D, HCCS, HIBADH, KDSR, LAMB3, MYH7, NADK, OR13C3, OR13G1, OR52E6, OR7G3, PIP5K1C, PRKAB1, RBKS, STAT4, TAS2R49, TAS2R9, TCF7, TICAM2, TNFRSF8, VTI1A, ATP6V1G1
Negative_9041	FAS, CCNH, CES7, CLTC, CPT1A, DKK4, ENPP7, EPHA5, GALE, GLS2, IKBKB, IL17A, JUND, NME4, OR13C9, OR1L3, ORC2L, PER2, PMAIP1, PRSS2, SKP1, SUV39H1, UGT8, WHSC1, WNT5A
Negative_9042	F12, ALG10B, BMP7, CCL17, CNTNAP1, CTSB, CYP26A1, EREG, IBSP, MAPK1, PPAP2B, PRKCH, PTPRR
Negative_9043	GSN, SPN, AKT1, CCNG1, CLDN2, DDHD1, EPHB1, GLI1, GNG13, HAGH, IGFBP3, IL18, IRS1, ITGA3, MAP3K4, MAT1A, NAGLU, NOTCH2, OR4Q3, OR7A5, ORC6L, PGM3, PHKB, PPP1CA, PTGS2, SEMA4C, SETMAR, SFRP1, SRGAP3, TGDS, TRAF3, YKT6, PNLIPRP1
Negative_9044	HPD, ABL2, APH1A, ARNTL, B4GALT1, CAMK2A, CAV3, CDC7, CHDH, CLDN20, CYFIP1, DDX19A, DVL1, EXTL3, FZD9, GALNT7, GGT6, GMPR, MAPT, MMP9, NFASC, NKX2-2, OR2M4, OR6N1, OR6X1, PARD6A, PTCRA, PTPRJ, RBL1, SDHA, TCF7L1, UGDH, UNC5D, YWHAZ
Negative_9045	ABO, GCK, LSS, BLVRB, CCND3, CXCR3, DLL1, DUSP2, GALNS, GALNT10, LIMK1, OR2M7, OR2T33, RPS6KA1
Negative_9046	IVD, SI, ACAA1, ACO1, ACY1, ADH1A, ADH5, AGMAT, AKR1A1, ALG1, ARNT, BDNF, CCL2, CD19, CDC23, CER1, CLDN17, CLTB, CREB5, EIF4B, F13B, FGF6, FLT4, GGT5, IDH3A, IL1R1, JAG1, KLK3, MADCAM1, METTL6, NKX2-2, OR1E1, PIK3C3, PIP4K2B, PKM2, PPP2R1A, PRPS1, PSEN2, PTPN1, RPRM, SEMA3E, SH2D1A, SLIT2, SMAD4, SUCLG2, TPK1, TRIP10, ULK2, VCAM1
Negative_9047	NRXN3, OLR1, SH2D2A, TUBA1C
Negative_9048	CD99, PVRL1, SLIT2, TAS2R48, TUBB4
Negative_9049	IARS, MAP2K4, SESN2, SULT1E1, TRAF3

Negative_9050	GCK,NGF,NPY,ABL2,ACADM,ACSL5,ACVR2B,ADRA1D,ASIP,ATP5J,CARD11,CCR1,CDC2,CHKB,CSNK2A2,CTNNB1,CTPS,CYP1A1,GAMT,GGT5,GNMT,HAO2,INPP5E,LTBP1,MKNK2,OR10AD1,OR11L1,OR14J1,OR2T1,OXSM,P4HA3,PIAS3,POLR2H,PPP3CC,PTGFR,RELB,RFNG,SOS1,SPP1,SPTLC1,TAS2R16,UBA7,UCK1,WNT4,ALDH18A1,GABARAPL2
Negative_9051	A2M,ALDH1A3,ALDH3A2,APH1A,B3GAT2,CTNNA1,DAD1,EPHA8,FUT5,FUT9,GNB3,HBEGF,HHEX,HNF4A,IFNA13,INMT,LDHAL6B,MCAT,MNX1,NAGA,NKX6-1,NRG2,OR8B8,PDGFC,PLXNB3,PPAP2A,PPP2R2C,PRPH2,PTGFR,PTPN7,SH2D2A,SNAP25,SOCS7,SOS2,SULT1A4,TAS1R2,TNFRSF4,TRAF3,UCK1,WBSCR17
Negative_9052	LIF,NLK,ACADVL,ADAM10,APBB1,ARRB1,ATP4B,CASP9,CLDN10,FUT5,GRIN2C,GSTM4,IARS2,ITPA,KIR2DL4,LILRB3,MAPK14,NCR1,OR10AG1,OR51E2,OR6C74,OR8K5,PDE1C,PDE3B,PIK3CB,POLR2D,POLR2F,PSEN1,PTPRB,RPS6KA1,SDC2,SEMA4A,SPP1,TCIRG1
Negative_9053	GK2,KIT,SDS,TNF,ACAA1,ALDH5A1,ALG10B,ALOX12,APC2,ARHGEF7,ATG3,BAIAP2,CFL2,CLTA,DDHD1,DIAPH2,DPM1,ENPP7,FPGT,IGFBP3,IL22,JAK3,MCM3,MGAT3,NEU1,OR10A4,OR6F1,PANK2,PER2,PHPT1,PIK3C3,PLCD1,PNMT,POLE4,POLR2K,PPP2R2A,PRKACA,SHC1,TSTA3,UGCGL1,VDAC2,B4GALNT1
Negative_9054	CGN,ACHE,ALDH9A1,AMD1,CCNB1,CD86,CSF3,CYP2A7,DCTN1,DERL1,EGLN3,HMOX1,JAK1,KCNQ1,KIR3DL2,MYH6,MYLK,NAMPT,NCAM1,OR2B2,OR5A2,OR8I2,PAK1,PCCA,PITX2,PTGDS,RDH16,SEMA3F,ST8SIA5,TIRAP,YARS,ALDH18A1,HLA-DRB5
Negative_9055	AMH,CTH,TNC,TXK,ACTB,AGPAT3,CACNA1F,CASP10,CCL16,CD14,CHDH,COQ5,CTNND1,CYP3A5,EI24,ENTPD8,EXTL3,FZD9,GGT5,GLYCTK,GNAI1,GNAO1,GSTT1,GUSB,ITPK1,NFATC4,NODAL,NQO1,OR1A2,OR2A12,OR2W1,OR52D1,OR6N2,OR6X1,OR9Q2,PARD6G,PDK1,PNPLA3,POLR3D,PRKCA,PVRL1,PVRL3,RASGRP3,SEMA7A,TJP1,ATP6V0A4,B4GALNT1
Negative_9056	INS,ANAPC10,CAV2,GALNT13,GNB3,MECR,PGS1,SORBS1
Negative_9057	GCK,KHK,CD8B,CLDN15,CNGB1,DGKE,DLAT,IFNGR1,LRAT,OR8K3,PER1,PPP2R1A,PYGL,ZFYVE9,TNFRSF1A
Negative_9058	GH1,ANGPTL4,ASRGL1,ATIC,CDKN1A,CLDN7,CRY1,DKK2,FLT3,GALNTL4,GRIN2D,GSTO1,MAPK11,NANS,OR10J3,OR1L6,OR2C1,PPBP,RASGRP3,RPSA,ZMAT3,HSP90AB1,PRICKLE2
Negative_9059	GSR,ACVR2B,ACYP1,AGTR1,ALDOC,AMHR2,CCR8,CLDN8,COL11A1,CSNK2A2,CYCS,CYP21A2,CYP2J2,DARS,DNMT3B,DUSP14,ECSIT,FRAT2,GMPR,IFNA5,LDHD,LHCGR,MAOB,MAP3K11,MINPP1,MPP5,OR4K2,OR51G2,PFKFB1,PGM1,POLR2G,PPARD,PTPRF,RAPGEF3,WNT1
Negative_9060	PC,UBB,AGPAT3,APOA1,B4GALT4,B4GALT6,CD28,CD74,CDH3,CNTNAP1,CTPS,CYP19A1,CYP26A1,DDB2,DPM1,ERBB4,FCER1G,GALNT1,GCLM,GDE1,GGT6,GNG12,GSTA2,IL20,MCCC2,MCM5,NEFH,NPAS2,OR8B12,PDX1,PSME2,PTPRM,RHOD,RUNX1,TAS2R4,TLR4,VAMP1,YWHAE,ALDH18A1
Negative_9061	EBP,GAA,IL5,ACP6,ARHGEF1,ARHGEF6,ATP5D,BIRC2,BTRC,CDC2,CLDN9,CMPK2,CSNK1D,DAPK1,DVL2,EPOR,FDFT1,FZD1,GANC,HMGCL,IMPA1,MDM4,NR5A2,NRP1,NTSR1,OR10X1,OR4K17,OR5L1,OR9I1,PIGU,PPAT,PTGS1,RRAS2,SDC1,SHC1,SLC27A4,TACR1,TYK2,VARS
Negative_9062	BID,F10,MUT,ABL1,ACTC1,ATP13A2,ATP6V1D,B4GALT6,BMP8A,CACNB2,CACNB3,CD82,CDC23,CDC25C,CIITA,CRLF2,CXCL6,DARS,DHRS4L2,GNA12,LMO7,OR2M3,OR2S2,OR6C76,PPP3R2,PRKAA2,PTTG1,SCD5,SDHD,SFRP1,SLC27A1,SPRY1,SRGAP1,SRGAP2,TAS2R9,WNT1,TNFRSF10A
Negative_9063	AMH,CFD,CSF2RB,HAGHL,NUDT9

Negative_9064	ADCY7,ADIPOR2,CD226,CD3E,CYP21A2,IRF9,ITGB4,LEPR,MCEE,OR2M5,P2RX1,PDE5A,PFKFB1,PI4KA,PIM1,PPARG,PPP2R5D,RHEB,SELL,SETDB2,SMPD2,SOCS2,SORD,SOS2,TAS2R8,TPM3,UNC5D
Negative_9065	ACSS3,DAPK1,PRKX,TUBA1B,TUBB2C
Negative_9066	C2,FAH,IL7,TFG,ACADS,AGPAT3,BCAT2,BCL2L1,BIRC3,BUB3,CMBL,COL4A6,CREB3L1,CTSL1,DCXR,DHFR,DKK1,FDFT1,FGF7,FOSL1,HSD17B8,IL23R,KAT2B,KITLG,MAP3K8,MARS,MDM4,OR10G7,OR10K1,OR5I1,PDE6D,PGK2,PLA2G2D,PPARG,PRKACB,RALA,SOAT1,SPHK2,TAS2R38,WARS2,KIR2DL5A
Negative_9067	DBI,F9,AARS2,AKR1C4,ALG13,ASAH1,CACNA1B,CASP9,CD46,CES7,CLDN2,CLTA,CTBS,CYP2D6,CYP2E1,DPYS,E2F4,FMO1,GRIA2,HTR7,IDH3A,IMPA1,ITGA8,KIR2DL4,LALBA,LCMT1,MAPK9,NEU3,NTF3,OSMR,P4HA3,PIGF,PITX2,PLD1,PPOX,PPP2R5C,PRKACB,RCHY1,RDH12,RELB,RENBP,SESN3,SMAD7,TAS2R1,TSC1,UROC1,UROD,ZAP70
Negative_9068	APP,HK3,TNR,ACADSB,CCL17,EDNRB,LDHC,ZMAT3
Negative_9069	BTC,GNE,ABAT,ACTN2,ADAM17,ALDH3A2,ALDH7A1,CCR9,CHST11,DDX54,DUSP7,FLOT2,FLT3LG,GGT7,KIR2DL2,NCOR1,NEUROG3,NOS3,OR10AD1,OR2L8,OR8J3,OR9A4,PAICS,PANK3,PON2,PORCN,PTPN5,RALBP1,SMC1A,TAS2R3,TICAM1,TRADD,WNT3A,WNT9B,YOD1,MAPK8IP1,SERPINC1
Negative_9070	AK2,CSK,ACADVL,APOC3,BST1,GMPS,LDHC,LPAR1,NFYB,PDE9A,RASGRP4,RPIA,TNFSF11,ZMAT3
Negative_9071	OAT,CSNK2A2,GLS2,GSTO2,LDHAL6B,OR10V1,HLA-DQB1,PLA2G12B
Negative_9072	BTK,TYR,ACMSD,AHCYL1,ALOX5,ATP5J2,CREB3L3,CYP1B1,DEGS2,DGAT1,DNMT3A,DPM3,EXTL1,FOXA3,GALNTL1,HSD11B1,HSPB1,HTR2B,LIPG,MMP9,OR10S1,OR1J4,PIGS,POLR2E,PPP3R2,PRKG1,PSAT1,RHEB,SEMA3A,TAS2R9,TUBA8,TNFRSF10C
Negative_9073	OR5AK2,TUBA1B,GABARAPL2
Negative_9074	AK3,DUSP2,HLA-DMA,LIMK1,NFAT5,OR2Z1,OR5A2,OR5T1,P2RX3,PDE11A,PYCRL,SH3BP2
Negative_9075	PRL,AASDH,ADH7,CCL3,CDC25C,COQ3,GCH1,IRAK1,OR10H2,OR4F17,THBS1
Negative_9076	PGD,TKT,CDKN2D,MMP14,OR13C5,OR2H1
Negative_9077	C6,F8,GPI,MPL,ADIPOR1,ALG12,ARPC5,BCL2L1,CDIPT,FBP2,FOXA2,GP1BB,IDH2,IL22RA2,KIR2DL4,NCSTN,NFASC,OR1S2,OR2H1,OR2T4,PAK4,PLXNB1,POLR3G,PPP3R1,PVRL3,SLC25A6,TUBB4Q,ATP6V0E1,ATP6V1C2,SERPINE1
Negative_9078	CR2,DCK,F7,ATG7,ATN1,CBLC,DGAT1,DOCK1,DUSP7,EHMT2,FMO1,GOSR2,GPD1L,IFNAR2,MYL7,NAT6,OR52R1,OR6Y1,OR8B8,ORC5L,PDE9A,PEMT,PIGW,POLE3,PPP2R5D,PTGS1,SKP2,SPHK2,STX16
Negative_9079	CD6,ACSM4,CDS1,DGKD,GDF5,GUCA1C,IL1R1,ITGA4,JAK2,LEPR,PNLIP,SORD,STX4
Negative_9080	TK2,ALPL,ARPC3,B3GNT5,CFTR,CHSY1,CNTFR,DHDDS,DPM2,EPHA5,EPHB1,FLOT2,GALT,GGT1,GNAI3,GPT2,GUSB,HSD17B7,IFNGR2,IL29,ITGAV,MAML2,NEU2,NTSR1,OR2D2,OR51G1,OR5AS1,OR8J1,PDPK1,PFN3,PIK3C2A,PIK3CA,PIK3R2,PITX2,PPARG,PRKAB1,PRKG1,RDH10,SEMA3G,TACR1,TRADD,UGT2B17,WIF1,CACNA2D4,HSD17B10,TNFRSF10C
Negative_9081	FGB,GPT,HGD,RB1,SMS,ACAD9,ATP5L,BET1L,CAPN2,CHAT,CMBL,CNTF,CYP2S1,F13A1,GALNT13,HMBS,IAPP,IGFBP3,IL18RAP,MGST2,MPDZ,MYH9,OR13H1,OR1A2,OR51F1,PDE1B,PIP5K3,PNPO,PPARA,PPCDC,SMC1A,UGT2B4,VASP,VTI1B,ATP6V1C1,HSD17B12
Negative_9082	HK2,AMDHD1,CACNG8,EPHB6,FGF9,GAB1,GGT6,GLYCTK,IMPDH1,ITGA7,LIPG,OR2G6,OR5H6,PDHB,RDH8,RPRM,RRM1,SETD1A,SH2D2A,SPRED1

Negative_9083	UBB,UBC,ADCY8,ADCY9,ADRB1,ATP5G2,BCAR1,CDC6,DPM1,EXT2,FGFR1,FUT1,FZD8,GCAT,GRM5,JAM2,LRRC4C,NT5C3,P2RX6,PDE8B,RCHY1,RIPK1,SULT2B1,TAS2R4,VAV1,HSD17B12,HSP90AA1
Negative_9084	ATIC,F13B,GOT1,GPD1,ICAM1
Negative_9085	ALDH1L1,DAG1,KLRC1,MAP3K5
Negative_9086	INS,LIF,ARNT2,ATP6V0C,BLVRB,CBLC,CD99,ENPP3,FGF1,MAOA,NLGN2,OR8B4,PGM3,PLAUR,POLR2C,PRKCI,PTPN11,PVRL2,UNC5D,ZMAT3
Negative_9087	EBP,HK1,IHH,APBB1,ARNT2,CLDN8,DHCR7,DPAGT1,ENTPD2,GNAT3,HSD17B4,IL6ST,MAML1,MYH10,PIGB,PLCE1,PSME3,THBD,WHSC1L1,TNFRSF11B
Negative_9088	LPL,ALDH1L1,ALOX15,CDC26,CDKN2D,DHRS4L2,FARSB,INPP1,LDHAL6B,MTHFS,MYLK,NFKBIB,NGEF,OR5B17,OR8G1,OR9A4,PFN2,PRKAB1,RASGRP3,RPS6,SC4MOL,SCD5,SCIN,SSH3,UGCGL2,WNT1,ATP6V1G2,TNFRSF10D
Negative_9089	DCC,GPI,HGF,AKR1A1,ALOX15,CHST13,CLDN10,CRY2,CXCL10,DRD5,EPOR,GRPR,LCAT,OR4F4,OR6C4,OR6S1,PIGQ,PLD1,PON1,PVRL4,SETD1B,SPP1,TNFSF12
Negative_9090	CAD,F11,HPD,ILK,LEP,ACVR2B,AGXT,ATP6V0B,BACE1,BNIP1,CCL1,CD14,CLDN19,CPT1A,CX3CL1,CYP3A7,DARS2,DHRS9,DUSP4,FGF2,GLS2,GTSE1,HHIP,HMGCS1,KAT2A,MGAT5,NTRK1,OR10S1,PDE1A,PIK3C2A,PLXNA1,PON3,PPP2R5D,PRKAR1B,PRKCA,RBPJL,SESN2,SMARCA5,TUBB1,UGT2A3,UNC5C,WIF1,XYLT2
Negative_9091	IL5,ABLIM2,ACVR1C,ARSE,BCMO1,BPGM,CES2,CFL1,COMT,CXCL13,CYP2F1,EFNA2,FZD10,GSTA2,GSTK1,HSD17B4,IFNA5,IFNAR2,IRS2,LRP6,MAPK13,MCM6,MFNG,MPDZ,NFYB,NTNG1,OR1E2,OSMR,PON1,PORCN,PRKCE,PTCH2,PTGER1,PTGS2,SAT1,SDHC
Negative_9092	GNE,KIT,ZYX,ACVR1,ASIP,BAI1,C7orf16,CCL1,CCL13,CCL21,CYSLTR2,GSTA4,PDE1A,PDGFC,POLE4,PON2,SHC3,ST3GAL2,SULT1A1,UPP1
Negative_9093	FES,HGF,LCK,LTB,TH,AASS,ABLIM2,ACO1,ACPP,ACSM5,ADRB3,AOC2,ATP6V1F,CD58,GOT1,HTR2C,IDH3A,IFNA10,IL11,LYZL1,MBL2,NOX1,NTF3,OGDH,OR10H3,OR6B2,PANK1,PHKA2,PMM1,PRKAG2,SETD1A,SUCLA2,TNFRSF8,TUBB4,CACNA2D3,PLA2G12A
Negative_9094	ALG10B,ARSD,BDH1,BUB3,CACNB2,CCL13,CNDP1,E2F5,EPHA2,FGF8,GNAO1,HTR2A,HTR6,NCOR2,PRNP,RPS6KA6,TNFSF4
Negative_9095	CNTFR,HIBCH,NR1H3,SMAD3
Negative_9096	CARS,GNA14,GRIN2B,LIPA,OR8G1,PRKAR2A
Negative_9097	COL4A6,CRY1,OR1D2,SLC27A1,STAT6
Negative_9098	ACSM3,AKR1C1,ARG1,ATP5L,CACNG3,CDH1,CSF2,CTNNA2,DFFA,EFNA4,F2RL3,GFPT1,HTRA2,IFNG,IKBKE,JUND,MYH6,NEGR1,NKD1,NT5C2,OR10S1,OR1C1,OR1Q1,OR2L3,OR56A4,OR5B17,OR8U1,ORC4L,P2RX4,PVRL2,RPS6KA2,ST6GAL1,TPH2,WNT10A,PLA2G12A
Negative_9099	FH,ILK,ADRA1D,AKT2,ARHGEF7,ATP2A1,ATP5G3,CD14,CHRM5,COL4A2,CYP3A4,CYP3A7,DGAT2,ELK1,ENO1,EPAS1,FGF17,GFAP,GNAI2,IL24,IRAK4,MAT2B,OR13D1,OR3A2,OR8K3,PIP5K1C,PLOD2,RHOQ,SETMAR,TBXA2R,TNFRSF4,TNFSF14,ATP6V0A2
Negative_9100	ADIPOR1,AGRP,ATF2,B3GNT4,B4GALT2,DHRS4,EIF4E,ENPP6,FASLG,GMPS,HSD17B7,LAMB1,NFKBIE,NOS3,OR52A5,OR52E4,OR5D14,PLIN,PPP3R1,PSEN1,STAT4,TMLHE,MAPKAPK5
Negative_9101	RDX,ACSM1,AGRN,ANAPC10,ARPC3,ARSA,ATP5G1,ATP6V0C,BCKDHA,BCL2,C3orf10,CCL20,CCR9,CD40LG,DOT1L,FCGR2B,FUT1,GJD2,GLYCTK,GUCY2D,IL1B,IRS1,LIFR,MPP5,NCSTN,NR1H3,NRG1,OR10H3,OR13A1,OR1L6,OR4S1,OR51L1,PAK2,PGAM1,PHKA2,PON2,PRSS1,PTGIS,RRAS2,SELP,SETD8,SETMAR,SHISA5,SMC1A,SREBF1,SV2A,MAP3K7IP1,PRICKLE1
Negative_9102	JUP,ALDH1A1,CACNA1H,CASP3,GADD45G,NCF4,PAK4

Negative_9103	ERBB4, IKBKB, OR10A6, TAS2R50
Negative_9104	FGA, CYP2F1, HSD17B3, IL3RA, OR5T3, OR8H1, POLE4
Negative_9105	KMO, MSN, CALML3, CPT2, CYP2C9, EPHB1, ERBB3, IFNGR2, IMPDH2, LLGL2, MAPK7, MTMR6, ONECUT1, OR8A1, PON2, PPP1CB, PRKCA, RASGRP2, SYNJ2, MAPK8IP2
Negative_9106	ARSD, FZD7, MAGI2, NOS1
Negative_9107	GGH, IL8, MPI, SDS, SRM, ABLIM3, ALDH1A3, BCL2, CACNG5, CCNB2, CNTNAP2, CYP2A7, DCXR, EDNRB, EPOR, FARSB, FBP2, FGF7, HRAS, IL15RA, INHBB, KLRD1, KYNU, LIMK1, LIPG, MAP2K2, NAMPT, NEUROD1, OR13A1, OR2G6, OR2T3, OR51A2, OR5AP2, OR6C4, OR6K3, ORC1L, PARVG, PDE4C, PDE8A, POLR3D, PRKAG1, PTGFR, SUFU, TNFSF10, VEGFB, WASF3
Negative_9108	F3, AGPAT1, APOE, CCNB1, COQ5, CPT1A, CSF2, DDOST, EFNA1, EIF4E2, FUT4, MPST, MYL5, NCOA4, OR11H6, OR1I1, OR5J2, PAK4, PIGK, PRKCE, PTPN7, TYK2
Negative_9109	ALDH7A1, ALG9, ARSE, BACE1, BUB1, CASP10, CCND3, CD48, CD70, CDC2, CHRD, CYP2F1, EPHX2, GIT1, HDAC1, IFNA6, IL11, ITCH, ITGB3, JAG2, LEF1, NCK1, NCK2, PHKA1, PIK3C3, PLA2G2F, PVRL3, PYCR2, RAP1B, RASGRP3, SOS2, ST3GAL1, SUCLA2, TAS2R40, UGT8, WNT3A, PAFAH1B3
Negative_9110	BID, C3, LAT, APOA2, BCAT2, CACNG4, CCL15, CD34, CD8A, COL2A1, COL5A1, COL6A2, CYP1A2, DAD1, DUSP9, EPHA4, FGD3, FZD1, GANAB, GUCA1B, HSD3B2, IFNA10, IQGAP2, KIR2DS4, LDHA, LTBR, MAP3K8, OR52R1, OR5B3, OR7G3, PLA1A, PLD1, PLOD3, PPP1R3B, RAB23, SDHA
Negative_9111	CRK, EZR, KMO, PLN, RB1, ACADL, AHCYL1, CCL3, CLCF1, CNTFR, CSDA, ECSIT, G6PC, GRIN2D, GUCA1B, HLA-F, HNF1B, HSD11B1, ICAM1, MCEE, OR10AD1, OR2G3, OR2W1, OR51V1, PDK1, PFKL, PPM1D, PPP1CC, PPP2R1B, PRKAR2A, PTPRF, RAPGEF1, RHOD, SPHK1, STAT4, SUCLG1, TRPC1, TYROBP, ATP6V0E1
Negative_9112	ACOX3, CD86, RASGRF2
Negative_9113	C2, FGB, IHH, ME2, ACSL5, ADAM17, ADORA2A, AOC3, ATP5I, AXIN2, BTRC, C1GALT1, CDC25C, DDX51, DHDDS, G6PD, GLI1, IL15RA, IL9R, ITGAD, LALBA, MAP3K13, NAT6, NFYB, OR13F1, OR1I1, OR1N1, OR2B2, OR4D6, OR52E4, P4HA2, PAX4, PDGFRB, PGK1, PPP3R1, PRKCQ, PYCRL, RXRA, SEMA3C, SH3BP2, WNT8B, SLC25A31, TNFRSF11B
Negative_9114	ADC, KDR, SCD, ACVR2B, ADCY4, ALAD, ARHGEF6, BMP2, C4BPA, CCL16, CLDN3, CTSG, CXCR6, DHCR24, FARS2, FARSB, FMO5, GALNTL5, GCDH, HSD17B4, ICAM1, IMPDH1, KIR3DL3, LTC4S, MGAT4B, MMP1, MPZL1, MYH7B, NEU3, NKX3-1, NR4A1, NUDT12, OR10K1, OR4K17, P2RX3, PAPSS1, PFKFB1, PLAT, PON1, PPP3R2, RDH10, RELB, SUCLA2, TPI1, TRAF2, TRMT11, UGCG, XYLT2
Negative_9115	BTC, DBH, GCK, ADCY7, AGMAT, ATIC, BCL2L1, BIRC2, C1QA, CDK6, CLTCL1, CMAS, CXCR3, DLAT, DNMT3L, EDNRA, EFNA4, ETS1, FOXO3, GALC, GGPS1, GNAI2, GNMT, HMOX1, HTR2C, HTR4, IL12B, IMPA1, ITGAX, LDHD, MADCAM1, MMP14, MYLK2, NRG1, OR10R2, OR13F1, OR1D2, OR1M1, OR52E8, OR6C74, OR6K2, P2RX7, P4HA1, PRKAR2B, PTK2B, RAP1B, SEMA3C, WBSCR22, PLA2G12A
Negative_9116	CD4, DCC, TAT, ACAD8, ATP6V0B, ATP6V0C, ATP6V1A, BNIP1, CDH2, CDK7, CTBP1, DFFB, ENPP2, ENTPD8, ESPL1, GNPDA1, GSK3B, GSTM5, HMGCR, IL10, IL18RAP, KLRK1, MAT2B, MITF, PIGT, PNPO, PTGES2, PTTG1, RAP1B, SEMA3D, SMURF2, SOCS4, STX2, TAS2R3, TAS2R50, THBD, UGCGL1, VAMP4, WARS2, XYLB, CTNNBIP1
Negative_9117	MPL, VCL, AASDH, ABCC8, ACOX2, ADORA2B, ALDH2, CALML3, CDS1, CLDN1, DHCR7, EHMT1, ENTPD5, FMO5, FUT8, FZD2, GART, GFPT1, GJA1, HEXA, IDH3B, IFNAR1, IFT57, IL12RB1, JAK2, KITLG, MTAP, NKD1, NOS3, NUDT5, OR2G2, OR4A16, ORC2L, PDE7A, PIAS1, PLXNC1, RUNX1T1, ST3GAL5, TPK1, UGT8

Negative_9118	ACVR1, BCAT2, IFNA13, IRAK3, MCM3, OR2M5, OR4P4, P2RX4, PLD2, PPP2R5B, SLC27A4, TSTA3
Negative_9119	ACSM5, ARNT, ARNTL, ARSE, BMP6, CACNA1E, CACNG4, CDH3, CDK5, CTPS2, CXCL1, DHFR, FMO1, GCLC, GDF6, GRIN1, IFNA10, IL5RA, KIR2DL3, MAP2K1, NFE2L2, NFX1, NSD1, OR14C36, OR1D5, OR2F1, OR6Q1, OXCT2, PGK2, PMM1, PPP2R2A, PRKCZ, SHMT1, STX18, TBL1XR1, TRAF2, VANGL1, WNT7A
Negative_9120	ALG6, CES1, COQ6, EPHA6, FMO3, GALE, GNAL, IL15RA, ITGA1, ITGA2, LDHC, LTA4H, NARS
Negative_9121	CD6, PXN, ALG12, ATP5J, B4GALT4, C1GALT1, CARS2, CD38, CDC23, CREB3L2, EGFR, ELK1, EPHA3, FABP1, IFNB1, NPR1, OR1K1, OR4D6, OR4M1, OR5T1, OR8U1, ORC5L, POLE2, SC4MOL, TBXA2R
Negative_9122	DBH, FGG, ALG9, BAAT, BIRC2, CCL16, CCR5, CDC45L, CSF1R, DGUOK, ENO2, FLT1, GNPDA2, GSTA4, HTR5A, IFNGR2, LCAT, LYPLA1, MAML2, OR13C2, OR6K6, PLAT, RELA, SEMA3F
Negative_9123	ATP6V0C, CCR6, CD48, CYP2A6, DTYMK, FGF4, GGT6, PDCD1, PIGN, PON1, RBPJL, TAP2
Negative_9124	SPN, ACP2, AMY2B, B3GALT5, CD80, CYFIP2, FUT2, IL28RA, OR52L1, PANK4, PLOD1, RAC2, UGDH, XYLT2
Negative_9125	FST, RET, APPL1, GALM, OR6B3, PPP2R5A
Negative_9126	AGK, APP, BTK, F3, ADCY10, AKR1C1, ALAD, BAIAP2, CNTF, DCTD, ENTPD8, FGF17, GALNT12, GNA15, HCLS1, IFNA14, IL15RA, IL18R1, NARS2, OR2C3, OR2L3, P2RX1, PDE1C, PLA2G1B, PLAUR, PNPO, POLR1C, PYCR2, RAC2, RBL1, RFWD2, SUCLG2, YWHAH, PLA2G12B
Negative_9127	GSS, MIF, AGPAT1, AGRN, ALDH6A1, AMDHD1, B4GALT4, BCMO1, CACNG6, COL1A2, ENPP6, GARS, GYS2, HTR4, MRAS, NANS, OR11H4, OR51A2, OR51D1, PDE9A, PIGM, PLCB2, PON1, SCLY, ST6GAL1
Negative_9128	ACOX1, ALG12, AMPD1, BIRC5, CLDN6, COQ3, COQ5, CRLS1, MAPK1, MECR, MGAT5B, NLGN2, NQO1, OLAH, PLA2G2D, PLCB3, PRKAG3, RPS6KA4
Negative_9129	AR, MOS, ACOX3, ACSM3, ADCY8, ARHGEF4, BBC3, CADM1, CDC16, CDH2, DDX4, DGKQ, DOT1L, FGFR1, GADD45A, GRIA3, GUCY2D, HSD11B1, IFNA16, IL1R1, KARS, KLK3, MAP3K12, MLYCD, MYH2, MYL5, NT5C1A, NT5C3, OR52N1, OR52N2, OR5A1, OR6C2, PDGFRA, SIPA1, STX16, KIR2DL5A, ST6GALNAC1
Negative_9130	ALDH1A1, APOA5, ARPC1B, ATF2, BPNT1, C1GALT1, CACNB3, CLDN14, CXXC4, CYP2A7, DDX41, FLT3, GALM, IFNA21, ITGAD, MGST2, NFKBIE, NPR1, OR10G4, OR10J3, OR1K1, OR2H1, PDE7A, PIGN, PPA1, TUBA1C, WEE1, PAFAH1B1
Negative_9131	CD6, AKR1A1, ASNS, BBC3, CNTN2, CSF1R, DGAT2, DTX2, DTYMK, GPLD1, HADHA, HSPA1B, ITGA8, LIPT1, MYL9, NFATC2, OR52B4, ORC3L, PDPK1, SETDB1, SIAH1, ST3GAL2, MAPK8IP2
Negative_9132	GNS, LHB, CLDN14, FOXO1, GSTA1, HAO2, NRAS, OR1G1, PDE11A, PTPMT1, SFRP4, SLC25A6, TP53I3, UGT2A3
Negative_9133	IL4, ACOX2, ACSM4, ALCAM, CCL15, CCL23, CDC14A, CDS2, CLDN1, CREB1, CTBS, CX3CR1, FTMT, FZD9, GCLM, HEXB, IMPDH1, OR2S2, OR4C12, OR52W1, PDE11A, PIGP, PPT1, PTPN6, SRGAP3, TIRAP, TUBB, VNN1, WIF1, WNT11, CSNK1A1L, PLA2G12A
Negative_9134	ADCY5, B4GALT4, CDS2, CTNND1, EIF4B, HTR5A, INMT, MINPP1, MYLK, OR51G1, SEPHS1, WASF1
Negative_9135	TNR, AKR1C4, CEBPA, CPT1A, EDN1, ENPP7, GALNTL2, IL5RA, MCM5, NCAM2, NNMT, OR2G2, PHKG2, PIGC, PPP2R5E, RPN1, SEMA4A, TAS2R42, TCF7L2
Negative_9136	DBT, ACAA2, APOE, B4GALT3, CD82, CES1, CLTB, CYP4F2, DNMT1, GALNT5, GFPT2, GGT1, GRM1, HMOX1, IL11RA, LIPC, MAML2, MYLK, NLGN3, OR2L13, OR51B4, OR5B17, OR5F1, RFXANK, ST3GAL1, STAM, TAS2R8, UGT2B4, VAMP4, ST6GALNAC3

Negative_9137	AK3,CD6,PML,AARS,ACADL,ACO1,AKR1D1,ATP6AP1,AXIN1,CDC27,CNTF,CYP4F2,DBF4,DHRS3,FZD5,GAD2,GALNT14,KRAS,MAPK8,NCAM1,OR4C6,OR52H1,OR6Y1,PFAS,PIGK,PIK3CD,PTCRA,SEMA7A,SMAD2,SNAI2,TALDO1,TAS2R38,TYROBP,ZFYVE9
Negative_9138	PTS,AXIN2,CBLC,CCL3,CDKN2A,CHEK1,CPT1A,CYP3A7,FCGR2B,GCNT3,GRIA3,GSTO2,HDAC1,HLA-C,MAN1C1,MAP3K5,MMP9,OR2A12,OR6C4,OR6K6,PPP2R5B,PVRL4,SLC27A4,ST8SIA1,ATP6V0A4
Negative_9139	LCT,AHCYL2,AMHR2,ASCC3,CCR3,CLDN7,COL5A2,GCNT2,IMPA1,LCMT1,OR4D1,OR4K14,PCNA,POLE3,PPOX,TOMM40L,WEE1,YARS
Negative_9140	ADIPOR1,INHBC,MYH6,OR1L4,SPRY4,STAT5A,SERPINC1
Negative_9141	CD6,CKB,DAO,FYN,GK2,PGF,TEC,ACAT2,ADCY1,ADORA2A,ATP6V0B,AVPR1A,CACNA1B,CHIA,E2F5,ENTPD8,EPB41L2,FMO3,GALNT7,GALNT8,GNA11,HNF1A,MAP2K7,MGAT2,NCF1,NEU2,NFATC4,OR5D14,OXCT2,PFKL,PGAM2,PIGL,PIK3CB,PLXNA1,POLD3,RCHY1,ROCK2,SHC4,SMAD9,TAP2,THBS1,THBS2
Negative_9142	DCN,HPD,NGF,ACSL4,ADRA1A,ATF4,C1QB,CCR9,CXCL5,CXCL6,CYP3A43,DDX18,GYS2,HHIP,HIP1,MYH2,NARS2,NCR2,OR13C5,OR2M7,OR4K13,OR56B4,PARD3,PLXNA3,POLE,PPP1CC,PPP3CC,PRSS1,PSME3,SHMT2,SKIV2L2,SNW1,TGFB1,UMPS,VAMP2,VANGL1,KIR2DL5A
Negative_9143	AR,ARHGEF4,CIITA,CNDP1,CTNNA2,CTPS,CYP3A43,DTX3L,FASN,GTSE1,HSD17B8,JAK1,MAN1B1,MEF2C,MTHFD1,MYD88,NUDT2,OR2M3,OR8J3,PDE7B,PIGX,PPP1CC,PTK2,RPS6KA1,SCD5,SEMA4F,SMC1B,SMPD4,CSGALNACT2
Negative_9144	ILK,MPI,SCD,ACACA,ACSM4,AKR1B1,C3orf10,CDH3,CTBP1,DHX58,DLST,EFNA1,FGFR3,FOXO3,GALNTL5,GCLM,HSD3B2,HSPA2,IL10RA,LAMB3,LDHAL6B,LDHC,LEF1,MAFA,MGAT4B,MITF,MMP2,NARS2,NR5A2,OR10H5,OR1C1,P2RX4,PAICS,PLK1,POLE3,POLR3G,PON3,SETD1B,SFRP2,STX16,TUBAL3,TNFRSF12A
Negative_9145	MTR,RB1,ADCY8,CAPN2,CDIPT,CHD8,COX10,CYP2A7,CYP7A1,EIF4B,FLAD1,GAB1,GLB1,GOT2,MGAM,OR5AR1,PDE1C,PDGFRB,PIGS,SELE,SPP1,ST3GAL5,SUV39H1,SERPINC1
Negative_9146	AGPAT6,APBB1,AREG,CDK4,CER1,CHST13,CREB3L1,CTSS,DMGDH,FGF13,GDF5,GGPS1,GRPR,GUCY2F,HSD17B2,ITPA,MS4A2,NME6,NMNAT2,OR4C11,PANK1,PDX1,RBPJL,STAM2,TGFBR2,TNFRSF9
Negative_9147	CPT1A,HAAO,HCCS,PKLR,SARDH,SLC25A4,SV2C,MAPK8IP1
Negative_9148	C1S,ID3,NF1,PGD,ACVR2B,ALDH1L1,ALG13,APOA2,ASNS,CCL13,CDC16,CHRNA7,CLDN4,COX10,CYP1A2,DDOST,DUSP5,EFNB2,FARS2,FZD2,GNAO1,GOT2,HNF1B,IKBKB,IL1R1,ITGAE,KCNB1,MAOB,MGAT1,MKNK2,NAT2,OR1J1,OR2AT4,OR2L13,OR52D1,OR52M1,PDE3B,PIGF,PIGM,PIK3R4,PPP1R3D,PRKAG1,QARS,RETSAT,SDHB,SLIT1,SPRY3,WWP2,TNFRSF10B
Negative_9149	BMP4,BNIP1,COL1A1,DDX18,GANAB,HSPA8,ICAM3,IRF9,PCK2,PDE10A,PIPOX,TACR2,TICAM1,UGCGL2,WNT5B
Negative_9150	PDX1,SOCS1,STX18,PRICKLE1
Negative_9151	CEL,DBH,GLA,ME2,PGP,ACTB,ADRB2,ASIP,ATP5L,ATP6V0C,BMPR1A,CCNH,CD8A,CEBPA,CLDN22,CMPK1,CSF1R,CSNK1G2,CSNK2A1,CYP2U1,DEGS1,FZD10,GRM1,HES1,IL4R,IPPK,LYZL1,MAP4K3,OR1C1,OR1E1,OR2F1,OR51B4,OR6C75,OR8J3,PAK7,POLR3H,PPA2,PPP2R5B,PRPH2,PRSS3,RPS6KA4,RRAS2,SGMS2,SMAD3,STX16,TM7SF2,TRPM5,SERPING1,SUV420H1
Negative_9152	DUT,GLA,ID3,ANAPC2,ATP5I,CLDN1,EDAR,FSHB,ITGA5,OCRL,OR56A1,OR8S1,PRPS2,RASGRP3,RPN2,RPS6KB1,SRGAP3,HLA-DRB3
Negative_9153	AK2,CDA,AGPAT3,EARS2,GRPR,JAK2,NCSTN,NTF3,OR1C1,OR4L1,OR9K2,P4HA1,PIP5K1B,TGDS

Negative_9154	ATM,CKB,F8,ACSM1,AREG,CHST13,CLTCL1,DAD1,DDX56,FGFR1,GOSR2,GRIA1,IGF1R,ITGB4,JAK3,OR1S2,PLCG2,RAP1A
Negative_9155	AMH,ME2,ALOX15,ATP4B,ATP5J,CACNA1B,CACNA1H,CD47,CD59,CD79A,COL4A1,CSF2,CX3CR1,CYP19A1,CYP2C9,DDX4,F11R,GAD2,GALNS,GALNTL4,GNAQ,LHCGR,MGST2,MYH6,NPR2,NRP1,OR2A5,OR2G6,OR4C6,OR56A3,OR6M1,OR9I1,PCNA,PPT1,ST3GAL6,STEAP3,TAS1R3,WNT6,XYLB
Negative_9156	GSS,ARHGEF6,BCL10,DDX19A,DLL3,FLT3,GOT2,HSP90B1,IGFBP3,INSRR,IRS2,LAMB4,LIPA,MLLT4,NKX3-1,NRG3,ONECUT1,OR4D1,OR4Q3,OR51B4,OR8A1,PPP2R1B,PRKCD,SHISA5,TSLP
Negative_9157	F10,GLA,ABP1,ACOX1,ATP4A,CCL20,CD226,CHAD,CLTCL1,FPGS,GPAA1,GUCY1A2,HCLS1,HHIP,IDH1,IL2RA,IQGAP1,MAGI1,MAP3K6,MAP3K7,NME6,OR10A7,OR10R2,OR51A7,OR51G2,OR5A2,PAX8,PPA2,PPP5C,STEAP3,TAS2R16,TAS2R41,TSTA3,PAFAH1B2
Negative_9158	UBB,ACPP,ADAM17,ARPC5L,ASNS,BMP5,CARD11,CCR9,CDH3,CHPT1,DRD1,GLI1,IFNA5,INSRR,IRS4,ITPKA,MYH3,NCF1,NEUROD1,ODC1,OGDH,OR56A3,OR5K2,SMPD1
Negative_9159	F3,ACACA,CAPN2,CDH5,CDS1,CKS1B,CLDN20,CLDN5,CYP4A22,DUSP7,ERBB4,GSTM5,HTR4,MAD1L1,OR13C8,OR1N1,OR4C6,OR5AS1,OR6K2,PRKAG1,RASSF5,TGDS,UMPS,WNT10B,YWHAE,ZFYVE16,KIR2DL5A
Negative_9160	CBS,LTB,ADH4,ADORA2B,APH1A,ATP5G1,CDH5,CRY1,CTBP2,CYP2A6,CYP2S1,EFNB1,GLG1,IKBKG,KCNB1,LAMC2,LCP2,LDHA,MTHFD1L,NCF1,NCK1,NFE2L2,NFKB2,OR4A16,OR56B4,P4HA2,PI4KB,PLOD1,POLR3A,PTPN1,RDH8,STMN1,TAS2R9,UCK2,ULBP3,VEGFB
Negative_9161	B3GNT4,CASP8,PPP2R1A,QPRT,SEMA7A,ST8SIA5,PHOSPHO1
Negative_9162	TPR,VCL,ACAA1,ACY3,AFMID,ALCAM,AMPD3,ARG2,CACNA1F,CD82,CHRM5,COASY,COL4A1,CSNK1D,EDA2R,ENAH,FMO1,FUT5,GABARAP,GMDS,ICOSLG,IDH3G,IFNA17,MYH9,NEFL,NT5E,OR4K17,OR4K2,OR56A4,PLA2G6,PNPLA3,PRKAB2,RND1,XIAP
Negative_9163	ADK,BTD,F8,LYN,ALAD,ANAPC5,ARHGEF2,CSNK1D,DEGS1,GGT5,LFNG,MAPK8,OR51E2,OR8H1,P4HA2,RPN1,RPS6KA1,SULT1A1,TLN2,GABARAPL1
Negative_9164	C2,F12,LCK,AKR1B10,ATP5B,ATP5G2,CAMK2D,CCNG2,CDH4,CHSY3,COL6A2,COQ5,CRKL,CYP2F1,DOCK1,ENO1,FGF4,FGF7,GANC,GNAT3,IL6R,LTC4S,OR10H1,OR11G2,OR2B11,OR4C46,OR4Q3,PARS2,PDE1C,PGAM1,PIK3C3,PLCD4,PLOD3,POLR2G,PRDX6,RPS6KA3,SEMA3D,SEMA4B,SNAP25,TACR2,TJP3,TYMP,UGT2B28
Negative_9165	INS,MET,RPE,TK1,ABLIM1,ADCY7,AKR1C4,ALG11,APBB1,ASS1,B3GNT4,B4GALT5,BAIAP2,BPGM,EDNRA,ESPL1,FLT4,FRAP1,G6PC2,GNAS,HAAO,HCLS1,HLCS,IL18R1,IL2RG,ITGA2,MAN1A2,MTFMT,MTHFD1,NFYC,NUMB,OR2T4,OR51G1,OR52J3,OR8H3,PDE4C,POLR2B,SCP2,SETDB2,SLC27A5,TAS2R48,WEE1,ATP6V0D1,ATP6V1G2
Negative_9166	IL6ST,OR14I1,OR5A1,PANK4,PDE1B
Negative_9167	TH,ACAD9,ACY1,ANGPTL4,ATP5C1,B3GALT2,B4GALT1,CD27,CFL2,CLDN11,CLDN17,CTBP1,DVL3,EARS2,ERBB2,EXTL3,FGF23,FUT6,GCNT4,HAGH,IL18,IQGAP1,IRAK3,LCMT2,LDHC,NLGN1,OPLAH,OR2L8,OR4A5,OR5AP2,PARD3,PFN1,PPAT,PSME2,PTK2B,RALBP1,SARDH,SETDB1,SFRP4,SLIT1,SMPD1,SNW1,STEAP3,UPB1,VANGL1,VARS,SERPINB5
Negative_9168	C9,DCN,ACVR2B,ALOX12B,ARRB2,BAIAP2,CACNA1C,CCL7,CHDH,DTX1,ENAH,FZD9,GALNTL4,GNA12,GNB1,GUCY2C,IRF7,ITGA7,ITGB6,NRG1,OR2M5,OR7C1,PIK3C3,PRKAG2,ST3GAL6,TNFRSF9,WNT8A,MAPK8IP1

Negative_9169	PRL, RDX, ACTC1, ACTN1, ADCY4, ADIPOQ, ATG7, B3GALT4, BMPR1B, CCNG1, CD44, CDS1, CHPF, CNGB1, COMP, CYP19A1, DIAPH3, DVL3, EARS2, ENPP3, ENTPD3, GCDH, GNAZ, HHIP, IL1B, IL28B, IL8RB, MASP2, OPLAH, OR1Q1, OR56A4, OR5L1, PIGL, PIGU, PIK3CG, PIP4K2A, PON2, PTPRJ, RAB23, RDH12, RRM2, SMAD5, TPMT, TRPM5, UGCGL2, VDAC2, WHSC1L1, XYLT2, ST6GALNAC4
Negative_9170	ALDH3B1, DRD2, MCCC2, OR7D2, SEMA7A
Negative_9171	DCN, NLK, PGD, CFTR, KITLG, LIMK2, OR2A4, PHGDH, PLA2G1B, SMAD5, SOCS3, TICAM2, WNT7B, HLA-DPB1
Negative_9172	C7, AVPR1A, BACE2, FGF2, IL21R, MGAT4A, NMNAT1, NTN1, OR51B2, P4HA2, PDE10A, PDE7B, RERE
Negative_9173	EIF4E, IFNAR2, NRG2, PHKA2, PRPH2
Negative_9174	BTK, F2, ALDH7A1, ATP12A, ATP6V1F, CASP9, CD38, CHPT1, CHST12, CSNK1E, DCXR, FGF11, GUCY1B3, HLA-DOA, HSD17B4, HSPA2, IFNAR2, IKBKG, IL1A, KIR2DL3, MBL2, MIOX, NODAL, NSDHL, OR13H1, OR2G3, OR4C15, OR5I1, OR5J2, OR7A10, OR8I2, PIP5K1B, PLA2G2F, PSENEN, PSME3, RHOA, ST3GAL4, ST6GAL1, SUCLA2, SULT1E1, TAS2R49, UBE2L3, HLA-DRB5
Negative_9175	SPN, ACSM3, ADH4, ALDH4A1, ALPL, CCND1, CFL1, CFLAR, CLDN17, CTLA4, CYP2F1, DPM2, ENAH, ENO3, GAPDH, GCLC, GLUD1, GMPPA, HLA-C, IL24, MAP3K8, MAPK14, NCAM2, NUDT9, OR10V1, OR13C8, OR2T2, OR4N2, OR56A4, OR9G4, OR9Q1, PRSS2, RDH5, SELL, UPP2, ZFYVE16
Negative_9176	TYR, CAMK2B, CEBPA, GYS2, P2RX2
Negative_9177	NPY, FGF12, GADD45B, GLUL, LDHD, OR4M1, PIGA, VARS, TNFRSF17
Negative_9178	LCT, ADH7, CCL15, CCL5, CCNA1, DCXR, DUSP2, EIF4B, FZD2, GNRH1, GPAA1, MTHFS, NLGN2, NOTCH3, OR51M1, OR5K1, OR7A5, PDE6G, PDK1, PPCDC, SHC3, TRAF1
Negative_9179	XDH, EREG, FGF8, NR4A1, OR1L8, PRDX6, RASA2, SOCS7, WWP1, PAFAH1B3
Negative_9180	CRH, GLS, GP9, ACVR2A, CSNK1E, CXCL13, CYP11B1, DAPK1, ETNK2, FGFR3, FLT4, IDI1, IQGAP3, MAN2A1, MAPK9, MTHFD2, NMNAT3, NQO1, NRG4, OR1F1, OR6C70, PGAP1, PLCB3, PRODH, PRPH2, RPS6KA3, SOAT2, THBD, TSC2, TSTA3, UGT2B11, WARS, HSP90AA1
Negative_9181	C1S, OAT, ACLY, ARSA, C3AR1, CD70, CDK5, CERK, CHD8, CNTN2, CYP1B1, CYP8B1, DGKB, DUSP3, GCNT2, GSTM1, HSD11B2, IFNA21, IL25, KLRC1, LYPLA2, MAN1B1, MFNG, OR13C5, OR2A5, OR2D2, OR51B4, OR5F1, OR7D4, P2RX5, PAK3, PIP4K2B, SPCS1, TUBB3, VANGL1, WWP1
Negative_9182	FGG, FYN, PC, ALDH1L1, ALDH5A1, APC2, ARSB, BMP6, DAXX, DDX54, FOXA2, FUT2, GMPR2, IDI2, LARS2, OLR1, OR1A2, OR2T33, OR52B6, OR52R1, PIP5K1C, SPHK2, ST8SIA5, TKTL1, TNXB, YARS
Negative_9183	ID4, ME3, ADH5, ALG10B, AREG, ARHGEF1, B3GALT2, COL6A2, CTTN, CYP4F2, ENAH, FZD8, GABARAP, GALNT11, GLI1, GNG13, GRPR, GZMB, HTR2C, JAK3, LAMC2, MAPK3, MDM4, MYL9, NEUROG3, NOS3, NRP1, OR51A7, OR5D13, PAK3, PMVK, PNLIP, SLC2A4, TKTL2, UGT2B10, VKORC1, TNFRSF11A
Negative_9184	GHR, LYN, MAG, ME1, ACP5, ALDH5A1, C4BPA, CLTCL1, COMT, COX10, CYP3A7, DLL1, FUT4, GLO1, GNA14, GPAM, GPD1, GSK3B, GSTZ1, HAO2, IFNA4, IL8RA, INSRR, ITGB1, LEF1, METTL6, NFKBIB, NKX3-1, OR10T2, OR2T33, OR51F2, OR52K1, OR52N1, OR56A1, PARVG, PPA1, PPP3CC, PVRL3, STX1B, TUBB6, UNC5D, SERPINF2
Negative_9185	ACACA, DDX19A, EREG, F2RL2, HADHB, INPP1, LALBA, LAMB2, PLXNA2, SC5DL, SPHK2, UNC5C, WNT7B
Negative_9186	F12, ID1, ACO2, ACTN2, ACVRL1, C1QA, CACNA1S, CD46, CDC45L, CDH3, COL5A1, CYP1B1, DDX52, DIAPH2, EIF4B, FARSA, FDPS, GSTZ1, GUCY2C, HDAC2, HNF4G, INPP4A, LAMB2, LAMC1, LAMC2, LDHC, MAD2L1, MAGI1, MMP14, MYH4, NEUROD1, OR2T29, OR3A3, OR51D1, OR52A1, OR7A17, PAK7, PNPO, PPM1B, PRKCQ, PYGL, RBPJ, RDH8, SOCS4, HLA-DQB1

Negative_9187	BID, KHK, ADAM17, AKR1C1, APOA1, ATF4, ATP5E, CAMK4, CPT1C, CRLF2, DDX47, DHRS4, FGF9, GGT7, GRIN2A, GSTM1, HNF4A, ICOSLG, IL9R, INHBB, ITGAM, JUND, LGMN, MADCAM1, MAPK13, MYH7B, NR5A2, OR1N1, OR2M2, OR2M7, OR2T10, OR4M2, PER1, PPP1R3B, RASGRP4, RPS6KA4, TAS2R42, THBD, TLN1, TSTA3, WASF1, ZFYVE9
Negative_9188	PGF, NAGS, NTN1, PON3
Negative_9189	AK2, ID3, LIF, NNT, PVR, ALG1, APC2, C3orf10, CCL11, CMPK2, COQ7, DAG1, DGKQ, EFNB3, ENTPD1, G6PC2, GGT5, GLO1, GNRH2, IL10RB, KIR2DL4, LAMA4, MOV10L1, NARS, NEU1, NME6, NPR1, NT5M, OR13C9, OR13D1, OR2T12, OR4D6, OR51B5, OR52L1, OR5D18, P4HA1, PIK3R5, PLCB1, PLOD3, PNPLA4, POLE3, PRKAG1, PSME1, PSME2, QARS, SMAD7, SSH1, STX16, UROD
Negative_9190	C6, ALG1, CD48, CLDN11, CNGB1, CSNK1G3, DCTN1, EI24, GGT5, GUCA1A, GUSB, IFNB1, OR1E2, OR2Z1, OR5M9, PANK3, PARVG, PFN1, PHKB, PIK3C2G, PPA1, PPP2R2A, SHC4, SLC33A1
Negative_9191	AOX1, CDK4, GPAM, ITGB8, OR51T1, PFN4, RBPJL
Negative_9192	GRAP2, GRM1, HPSE
Negative_9193	TH, ACTA2, AKR1B1, ANAPC2, ASCC3, B3GAT1, CCL16, CDC20, CSNK2B, CYP11B2, DIAPH2, GNAS, GUK1, ICOS, IMPDH1, LAMB3, OR10H4, OR2B11, OR2M7, OXCT2, PIK3R4, PLXNC1, PTGIS, RAB23, SCD5, SFRP5, SH3BP2, SMURF1, STX8, HSD17B10, ST6GALNAC6
Negative_9194	AASDH, CAMK2A, CCL16, CDKN1B, DGKQ, ENTPD6, FARP2, FUT1, HCLS1, HSPA8, IDH2, LARS, OR14I1, OR2C1, OR2D2, OR4K2, OR8B4, PDE10A, PIM2, PVRL4, SENP2, SETD8
Negative_9195	AK3, SI, AADAT, ALPPL2, ARPC2, CCL15, CHST13, CPT2, ETS1, FMO1, GSTA4, HSPA8, ITGA1, KLRC3, MAP3K3, MAPK1, MYD88, MYL9, NFKBIA, NUDT8, OR13J1, OR1J4, OR5B17, PIGQ, PKMYT1, STX3, TAS2R42, WNT5A
Negative_9196	LCT, ATG3, BDH1, CBLB, CREB3L2, FUT1, MYLK3, NPR1, OR4N4, OR6C6, PFKFB2, PPBP, PPP3CA, PRKCD, PRKCE, SH3BP2, ST3GAL2, STK4, VTI1B, B4GALNT1, GABARAPL2, HSD17B12
Negative_9197	IL8, MET, TAT, ACTN4, ASRGL1, CCND1, CCND2, CNTN2, CTPS, DUSP3, ENO1, GUCA1A, HSD17B4, LTB4R2, MAML2, NOTCH1, OR10J1, OR1D5, OR2A14, OR4C13, OR4D2, OXCT1, PPP1R1A, PRKG2, SMURF2, TBK1, YWHAG, HLA-DQA2, TNFSF13B
Negative_9198	GH2, KMO, TPO, ADCY8, ALDH1A1, CYP2C9, F2RL2, FZD3, KLK3, PLD1, POLR2J, PPP1R3A, SLC2A2, SMAD9
Negative_9199	NUDT9, PDE11A, PIGP
Negative_9200	IL6, RFK, ACSS2, ATP6V1D, BIRC2, CACNA1H, CASP8, CHEK2, CREB3L2, CXCL12, CXCL13, EPHB6, FUT2, GALNTL1, NEFL, NFX1, OR2V2, OR4M2, OR51B4, OR56A1, OR5A2, PLD1, PPT1, SARS2, SHC3, ULK3, UNC5C, WNT2B, WNT3, HSP90AB1, PRICKLE1
Negative_9201	HPD, A4GALT, ADH4, ADIPOQ, APOA5, ARSA, ASAH1, ATP12A, ATP2A2, CACNA1F, CCDC6, CDS2, CLDN19, DHX58, EFNB1, EPHB1, F2RL1, FARSA, FASN, GALNT8, GGT7, IFNA8, IL22RA1, IL29, INPP5A, MMP14, NEFH, NEU3, NOS1, NT5M, OR10A6, OR10G2, OR10P1, OR12D3, OR1L1, OR2M4, OR2Z1, OR4C11, OR52I1, OR5B3, PDE3B, PI4KB, PPP2CA, PPP5C, SUCLG2, TUBB2C, SLC25A31, ST6GALNAC3, TNFRSF1B
Negative_9202	F2, FH, XDH, ACAT2, AKR1A1, CCNA2, CCND1, CDK5, COL3A1, CPT1A, CYP11A1, CYP4A22, EFNA2, FECH, GLUD2, GSTM1, IDH3A, IFNA5, IL8RB, KIR2DL4, NAT6, OR10S1, OR2A5, OR52I1, OR6F1, OR7D4, PIGP, PLCD4, RXRB, SMPD4, TKTL1, TUBA1C, YWHAH, B3GALNT1
Negative_9203	TK2, ACAA1, ACSL6, ACVR1, ALDH1A1, B4GALT1, CYP21A2, EPHA4, FLT4, GPD1, IRF5, MAOB, MCM6, NTRK2, OR51G2, OR51S1, OR5D13, OR6X1, PDGFC, POLE3, PRKCB, PTCRA, RHOQ, RRAS, SEMA4A, SLC25A4, SLC2A2, ST3GAL6, TRAF1, XCL1

Negative_9204	C7,SPN,ACADL,ADSL,ALDH1B1,ALG10,ALOX12,ATP6V1A,BLVRA,CASP7,CLCF1,COQ7,CXXC4,EGLN1,ENTPD1,FOXO3,GNPAT,GNRH1,HDAC2,ICAM3,IKBKG,IL21R,IL2RA,LARS2,MAN1B1,MAPK13,NEU4,OR1B1,OR1J2,OR2AE1,OR52N1,OR5D13,OR8H2,PAX4,PDE9A,POLE4,POLR2F,RIPK1,SELPLG,SHC2,TUBB6,XIAP,ZFYVE16
Negative_9205	F8,GALNTL5,SEMA3A
Negative_9206	CD4,ACSM3,ACVR2A,ALG12,ATP6V1F,C3orf10,CD47,CDC42,DEGS2,FGF2,FGF8,GALNS,KDSR,OR10C1,OR2L2,OR5T2,PDE5A,SARDH,SPRY1
Negative_9207	HGF,NGF,NOG,OGT,SMS,ACVR1,ALDH1A2,ASPA,CALML6,CCR2,CDS1,COASY,CYP11A1,DGAT2,DUSP5,GALNTL2,INHBE,ITGA7,KAT2A,MAP4K2,MMP2,NEU2,NPAS2,NRCAM,NUDT5,OR2T6,OR51L1,PI4KB,PIK3R1,PLCE1,PMM1,RFX5,SELPLG,TAS1R1,TAS2R50,TFPI,WNT4,XYLT2,PAFAH1B2
Negative_9208	LPL,PGP,SYK,ACOX3,APC2,CLDN20,CRB3,DKK2,ENTPD4,GALNT8,GATM,GCNT2,GSTM1,IL13RA1,IL2RB,MAPK3,MGST3,NANS,NFATC4,NTNG1,OPLAH,OR52I1,OR52K2,ORC5L,PDXP,PFN1,PTPRZ1,RAP1B,TAS2R5,TNFSF10,TYMP,UGT1A10,ATP6V0A2,B3GALNT1,HSP90AA1
Negative_9209	ARPC1B,CCL16,CCL22,CLDN4,CXCR4,EPB41L2,HMGCR,MMP9,MYH3,NEU2,PPCDC,PTGES,RASGRP1,STX12,TBXAS1,CSNK1A1L
Negative_9210	ABO,MOS,MVD,XDH,ALDH9A1,ATIC,CHST11,COL4A2,CSF2,GRIA3,GSTT1,HRH2,ISYNA1,NTRK2,OR2T33,OR4F15,OR6C1,OR6Q1,PAK6,PIGU,PIP5K3,PPP1CA,PSENEN,RALB,SPRY2,STX2,TBL1XR1,TBXA2R,TP53I3,TRAF4,TUBA3C,UCK2,ATP6V1B1
Negative_9211	DLAT,GUCY1A3,MGLL,OR4N2,PMM1,PRNP,PSME3
Negative_9212	RET,ASCC3,ATG12,AVPR1A,CACNG5,CALML3,CEBPA,CLDN8,CNGA3,COL5A3,COX10,CPS1,CXCR3,CYP7A1,DHDDS,DIAPH1,FARP2,GSTO2,INHBA,IRAK2,ITGB7,MINPP1,MLYCD,MOV10L1,NME6,NTF3,OR10A5,OR10G8,OR10H4,OR1A2,OR1D2,OR4B1,OR4D6,OR51A7,OR52E2,OR6M1,PARD3,POLR3D,PPA1,PPP1CB,PSENEN,RPS6KA5,RUNX1T1,STX12,TAS2R3,TAS2R41,TNFSF10,VKORC1
Negative_9213	MSN,PXN,SCD,AK3L1,ALDH1A1,ALG2,ALS2,BCAT2,CCL4,CHRD,CLDN18,CYP4F3,EHMT1,FCER1G,GPX5,INPP4A,ITPKA,LAMB4,LDHC,NTSR1,OR2A12,OR4N4,OR5D13,OR6N1,PDX1,PHKG1,PIGU,PISD,PPP2R2D,PPP2R5E,SPHK2,SPRY3,WNT9A,XYLT1,CSGALNACT1,HLA-DPA1,MAPK8IP1,ST6GALNAC3
Negative_9214	IL2,JUP,PVR,AMPD3,BBC3,C7orf16,CDKN1B,CNTNAP1,CYSLTR2,EDN1,EGFR,FGF10,GALM,HSD17B4,INPPL1,LDHAL6A,OR2A25,OR2L2,OR2T6,OR51V1,OR52E6,OR52M1,PANK3,PNPLA4,POLR1C,PPP1CB,PPP1CC,RPN1,MAPK8IP1
Negative_9215	HK3,ACTN1,ADCY3,AGPAT4,ARPC1B,BMP4,CAMK2B,CCL15,CCNE2,CCNG2,EPHA5,FMO4,GCAT,GDE1,GLO1,HDAC1,HDAC2,ITCH,LTB4R2,MAP2K2,MYH6,OR2M5,OR52I1,OR5L1,PAK3,PDE5A,PDE7A,PIP5K1A,PPAP2B,SEMA4C,STX8,TNFSF8
Negative_9216	NOG,ACAD9,ALG11,BCAT1,DUSP3,EFNB2,FABP4,GOSR2,IL1R1,POLR2I,SDC2,STAM,UGT2A1
Negative_9217	ATIC,CRY2,DAXX,IL8RA,ITPA,LAMA3,MYH10,NLGN3,OR4F15,OR6A2,PLA2G12B
Negative_9218	B4GALT7,CAMK2B,CSNK1G1,DARS2,ERBB4,HMOX1,MYH7,PIK3C2A,PLOD3,SPRY1,TUBA3C
Negative_9219	CP,F5,TAT,AOC3,B3GAT1,CNTN2,EDA2R,ESAM,FGF6,FGF7,GBGT1,GGT6,GNA15,HLA-F,HSD17B8,IDH2,IL12A,IMPA1,LRAT,LTA4H,MAP2K4,NAT5,NUDT8,OR10G9,OR14A16,OR1D5,OR2AE1,OR4D1,OR52J3,P2RX1,PGK2,PIK3CD,PLA2G2A,PPID,PPOX,PRKACB,PRPS1,SELE,SEMA4B,SHC4,SUCLA2,SYNJ2,ATP6V1G3,MAPKAPK5

Negative_9220	ASL, SRC, ACY1, ADCY6, ADRA1D, B3GALT5, BCKDHA, CD244, CD40, CHSY3, DHFR, FGD1, FGF12, FGF13, FUT1, FUT8, IDUA, ITGA10, ITPKB, LAMA4, LGMN, MTFMT, MYH11, NR4A1, OR4D6, OR4F17, OR4K13, OR51A7, OR51E2, OR6Q1, PDE3A, PIM1, PIPOX, PNLIP, PNPLA3, RPS6KB1, SLC25A5, SLIT2, SPHK1, SPTLC2, SV2B, TGFBR2, THBS2, UCP1, UGT2B11, WNT8B, ATP6V1C1, MAPKAPK2, TNFRSF10D
Negative_9221	NPL, SYK, TFG, CDK6, HIF1A, LTBR, PIGU, SAT1
Negative_9222	ADO, AMDHD2, CAPN2, CCL7, CES1, CMAS, CMPK1, CSF1, GGCX, IDH2, MAP2K7, MTHFD1, MTMR1, PLA2G1B, PNMT, PRKCE, TAS2R48, TGFBR1, TPM3, WHSC1
Negative_9223	IDS, ALDH3B1, CARD11, CRKL, CYP2U1, DAAM2, FGF19, FGF2, GMPR, INPP4A, ITGA5, MAP3K5, MGAT5, NEUROG3, NGEF, OR14I1, PDGFD, PTGDS, PTGFR, RRM2, TUBA8, VAMP3, HLA-DPB1
Negative_9224	CS, GBA, AADAT, ALAD, CACNG3, CADM3, CANT1, CD72, CXCL10, CYP2C9, DRD5, DUSP4, EGFR, FCER1G, GAB1, IFNA6, IL18RAP, IL28RA, ITGA3, NRG3, OR11A1, OR14A16, OR2F2, OR2T29, OR8D1, POLD4, POLR2E, PTPMT1, RARS2, RPSA, SEMA3B, SLC9A1, THBD, TLN2, ULK3, MAPK8IP3
Negative_9225	FUK, TNF, APBB1, ATG7, BMP8A, CCNA1, CER1, CES7, CLCF1, CXCL3, FMO3, FRAP1, GUCA1B, IL15RA, IL20RB, LAMB2, MGAT3, NODAL, PLK1, PLXNB2, SIPA1, SLC12A2, SLC25A4, SMPD2, SUV39H1, TMLHE, UNC5D
Negative_9226	F2R, GLS, ID2, SHH, SPN, TXK, ACOT12, AVPR1A, B3GAT2, BST1, CANT1, CASP3, CCL22, CCNB1, CHKA, CREB3L4, CX3CL1, CYP2E1, CYP3A43, DGKB, EPRS, ERBB3, FUT6, IBSP, INPP4B, KLKB1, LIPG, MAOB, MITF, NME4, NOTCH1, OR2D2, OR2T5, OR51G2, OR5M9, PPOX, PTPN5, RASGRP2, RHEB, RPS6KA6, SEMA4B, SLC33A1, SPRY1, TALDO1, TRDMT1, XIAP, GABARAPL1, PPP1R12A, SERPINA1
Negative_9227	ADK, FH, GPI, ME2, ACADSB, ACSM1, ACVR1B, ACYP1, CCL19, CD8B, CHPF, CLDN10, CLTB, CSNK2A1, CYP1B1, CYP3A7, CYP4A11, EPOR, FZD9, GAMT, GLI1, ICAM3, IL21, IL29, IRS2, MGST2, MYL5, NT5M, OR10G9, OR11L1, OR4E2, OR6C4, PDE3A, PEMT, PRPS2, RFXANK, TAS2R38, TRPM5, YWHAZ, CSGALNACT1, GABARAPL2, MAPK8IP2
Negative_9228	EDA, HK2, LEP, ACTA1, ALPPL2, ATP6V1H, CASP10, CCL24, FZD6, IL13RA2, ITGAX, KIR3DL3, OR4F17, PRKAA2, RPRM, SELE, SGMS2, SH3BP2, TAS2R3, TNFRSF4, TNFSF8, UCK2, UPP1, VEGFB, HSD17B12
Negative_9229	GK, MOS, MPI, PVR, ALCAM, ALDH1A1, ATP5C1, ATP5G2, CD19, CDH4, CLDN2, CREB3L2, GART, GJA1, GJD2, GUCY1B3, HYAL1, IQGAP1, LEFTY2, MAP3K13, MYL7, NOTCH3, OR4F4, OR4L1, OR51M1, OR51S1, P2RX7, PDGFRB, PGAM4, PIK3R3, PKM2, PLA2G2D, PRSS3, RASGRF1, SELPLG, SIAH1, ST8SIA1, STAM2
Negative_9230	BAD, ID2, ARSB, CSF1, DDX47, EPB41L2, FCGR3B, FGFR3, IL11RA, INSRR, ITGAL, ITGB4, LDHA, NTSR1, OR2T33, PIM2, PLCD1, PROC, PYGM, RASGRP3, RBX1, SYNJ2, TIAM2
Negative_9231	AK1, ACACB, ALDH1L1, ALG13, APBB1, BACE2, BMP4, BMPR1B, CD55, CDC27, CIITA, CLOCK, COL4A1, CSF2, CYSLTR1, DDOST, DDX18, EPHA7, EPHX1, FUT1, GRM5, HIBADH, IFNA8, KLKB1, MAP4K4, NR5A2, OR13J1, OR14J1, PFKM, PLA2G2F, POLR3A, PPP1R3D, PTGIS, RALGDS, ST3GAL5, STX7, TGFB3
Negative_9232	C1S, IL9, MYC, PDC, ACP2, ACTN1, ADH1A, ALOX12B, APC2, CACNA1G, CD19, CKS1B, CTSS, CX3CR1, CXCL12, CYP17A1, DPYSL2, DTX4, ENPP2, GUCA1A, HMGCS1, IFNA17, JAM2, MAP3K14, MCM2, MYH15, NRXN2, OR1S1, OR52N4, PCCB, PDE1B, PROS1, STAT4, STX10, TNFSF9, WASL
Negative_9233	BID, CDA, DBI, GLS, ABAT, ACSL1, ADCY4, ANAPC5, AOC3, APC2, BLVRA, CANT1, DGKE, DUSP10, DUSP3, GALNT10, GOT1, GRLF1, IAPP, IDH3A, INPP4B, KIR2DL2, MYH7, NEU4, NFYB, OR10A7, OR10G8, OR11H4, OR12D3, OR13G1, OR2T8, OR4E2, OR51G2, OR52N4, OR5AC2, OR6B2, OR6X1, P2RX3, PKM2, POLR2A, SLC27A4, SUV39H2, TAS2R16, HLA-DRB5

Negative_9234	DBT,ME3,VTN,ADH7,AKR1C1,ALDH3A1,CCL28,CCL3,CLDN19,CTTN,DPM2,DTX1,DVL2,EDA2R,F11R,FGF20,FOXO1,GALC,GOSR2,GRIN2B,ICOS,IL2RB,IQGAP1,MAPK9,MGAT4B,MKNK2,MPP5,NANP,OR10G2,OR1A2,OR1Q1,OR52I2,OR52R1,ORC2L,POLR1A,POLR3A,PTCRA,PTGS1,RALBP1,SELP,TKTL1,VANGL1,XYLB,SERPINC1
Negative_9235	CP,PC,AKR1D1,CHKB,CHST11,CSNK1A1,CTSS,DPM1,FGF14,GAD1,GALC,GAPDH,GMPPA,GRIA3,JAK1,KIR2DS5,MALT1,NANS,NFKBIB,NPR1,OR14C36,OR51T1,OR52N2,OR8H2,PHKA2,PPP2R5C,PTCRA,SDC4,SSH1,TNXB,ATP6V1G1,GABARAPL2
Negative_9236	ATG3,BCMO1,BDH1,BPGM,CCND3,CES2,DPYSL2,EPHB6,INHBB,KIR2DL2,MIOX,OR4K5,OR51L1,P2RX1,PAX4,PDE6D,POLR1D,POLR2K,PTPN11,RDH11,SDHC,TAS2R9,YWHAZ
Negative_9237	C6,CFD,KDR,SCD,ADCY3,AKR1C1,ALOX5,AMDHD2,BIRC2,BLVRA,CACNA1S,CNTNAP1,DMGDH,EDNRB,EFNA5,FDPS,FGF10,FRAT1,GALNT14,GALNTL4,GP1BB,HES1,MARS2,NT5E,OR2B6,OR2W1,OR51T1,P4HA2,PCYT1A,PECAM1,PGLS,PLA2G2F,POMC,PPBP,PPP2CA,PPP3R2,SIGLEC1,TPK1,UPRT,MAPKAPK5
Negative_9238	EDA,F9,GSR,RET,ACMSD,ALDH2,ATP6V1D,C1QA,CD27,CDK6,COL6A1,CRHR1,CX3CL1,CYP1A1,CYP2S1,EPOR,ETS1,F13B,GCLM,GSTA3,HES1,HTR5A,IL10RB,IRAK3,MCCC2,NRG1,ONECUT1,OR2C3,OR2T3,OR5AU1,PAFAH2,PHGDH,PHKA2,PIGT,PLCD3,POLE3,POLR1C,PPP3R1,PVRL4,SEMA4A,SLIT1,STAM2,TMLHE
Negative_9239	BCR,HAL,IHH,AADAC,AADAT,ACVR1,ADCY1,AKR1C1,ALCAM,C1QB,CERK,EDAR,EDNRB,FUT8,GCLC,GNAL,HMGCL,ICOS,MCM4,MTMR1,NOTCH3,OR4D1,PPP5C,PRPS2,SDC2,SIAH1,TAS2R3,TBL1Y,WHSC1L1,WNT7A
Negative_9240	BAX,OAT,ABLIM3,B4GALT5,CREB3,CYP2D6,ESPL1,GLUD2,GSTA4,GZMA,HTR5A,IL12A,MMP9,NCOA4,NUDT12,OR3A3,OR5B3,OR6N1,OR8B4,OR9A4,PFKFB3,PIAS4,PLA2G5,SMAD7,TACR2,TMLHE,TSHR,TUBA3C,TUBA3E,XCL1
Negative_9241	DCN,B4GALT7,CFL2,EPHA2,MAPK12,NR1H3,PDHA1,PRKACG,TPI1,UNC5B,UROD,WBSCR22,YKT6
Negative_9242	NGF,PC,SHH,ANPEP,BLVRB,CCL1,CCL27,CD247,CHKA,CHKB,CNGB1,CSDA,CYP2D6,DDX51,DERL1,ETS1,GNG12,GOT2,HAP1,IL9R,JAG1,LIMK1,MPST,OR2A12,OR52K2,PAFAH2,PAPSS1,PPP3R2,PPT1,PRKG2,PVRL3,QPRT,RALGDS,RRM2,SEPHS1,SORD,SOX17,TUBA3E,VTI1B,C1GALT1C1
Negative_9243	C3,C4B,C8G,PC,PDC,ADH6,AGPAT1,BCKDHA,CPT1C,CXCR3,EGLN1,GUCY1A3,GZMB,HEXA,HTR2B,ICOS,IL22RA2,ITGA10,LLGL2,MAP3K3,MAP3K5,MAP4K3,MCAT,MDH2,NEFH,NT5M,OR1N1,OR1Q1,OR2M2,OR3A3,OR8K3,OXCT2,PARD6G,PDE2A,PERP,PPOX,RASGRP3,SOAT2,STK36,TNFRSF8,TUBB1,ST6GALNAC3,TNFRSF1A
Negative_9244	EBP,IL2,ALOX12B,BAIAP2,CCL2,CCR3,COL11A1,CRB3,DUSP2,EDA2R,ENTPD8,EPHA7,FASN,GJD2,HAO1,IDI1,IGF1R,ITGA3,ITGB1,METTL2B,MMP14,MMP9,NFKB2,OR10A6,OXTR,PAPSS2,PDE2A,PLA2G2A,PLK1,PTCRA,PYGL,RFXANK,RRM2,TACR2,TNFSF8,TUBB2B
Negative_9245	LHB,B3GALT1,CD55,CXCL13,GFAP,NT5M,OR4K2,POLR2A,PPOX,SHC3
Negative_9246	CRK,JAK2,OR5AK2,OR5R1,PCK2
Negative_9247	C2,CAT,DBI,DCN,FES,ACTC1,B4GALT1,CARS2,CASK,CBLB,CDC14A,DGUOK,DTX1,FGFR3,GALNT8,GUCA1C,HSPA2,IL22,LAMA4,LYPLA2,MAGI2,MAN2A1,MAPK7,OR10G2,OR10G9,OR6K3,PDGFA,PFN2,PIGC,PLCG1,POLR1B,POLR3D,PRKCH,PRSS2,PYGM,SH3BP2,SYNJ2,TAS2R14,TPK1,UMPS,VDAC3,WBSCR22,C1GALT1C1,SERPINB5

Negative_9248	AGK, F9, PTS, SMS, ACPP, ACSL4, AGRN, ARPC3, C1QA, CACNA1F, CPOX, CXCL9, DGKQ, ENPP2, FARS2, FUT1, GNAO1, IL12B, IQGAP3, JAK3, LAMB4, MTMR2, MYL7, OR10C1, OR1A2, OR1J2, OR6K3, PIAS4, PIGB, PIK3CD, PIP5K1B, PON3, PYGL, RBPJL, SEMA4C, SPRED2, SV2C, TAS2R16, THBS2, TSHR, VASP, WASF2, HLA-DQA2, TNFRSF10B
Negative_9249	BAX, NPL, ADCY2, CPOX, DHCR24, FGF18, GALNT13, GSTA2, GSTM1, IRS2, ITGA8, OR10J1, OR10X1, OR2Y1, OR5H6, SCP2, STK11, STT3B, TAS2R14, UBA1, TNFRSF18
Negative_9250	HDC, ID4, AADAC, ACSM3, BNIP1, CMAS, COQ5, CSNK1G2, CTPS2, GALNT13, IBSP, IL13, LLGL2, MADCAM1, OR4N4, OR51A7, OR7D4, PDE2A, PPM1D, TAS2R42, XYLT2, SERPINB5
Negative_9251	ABLIM2, ACP1, ATP5A1, CPT2, EPHA4, GFPT1, GYS1, HLA-DOA, IL15RA, INSRR, MAFA, OR10S1, OR13C9, POLR1C, POLR2I, PRODH2, RAC2, RPS6KA2, ST8SIA1, USE1, MAP3K7IP2
Negative_9252	CS, ADAM17, ADH7, BAAT, CACNG2, CYP4A22, CYSLTR1, DDX41, DRD1, EPHB6, FBP1, FGF11, INMT, ITPK1, MGST2, OR5T3, PIAS1, POLR3D, PRKAR2B, RPSA, SDHA, SSX2IP, UBA7, WNT5B, HLA-DQB1
Negative_9253	AK3, CBL, F3, HK2, ACSM3, AMPD3, B3GALT4, CALML6, CCND1, CCNE1, CYP3A43, DDX23, FOXO3, GPD2, IDH3B, MCM2, OR10P1, OR1J1, OR1Q1, OR8K1, RAC2, TFDP1, UGT2B7, TNFRSF12A
Negative_9254	AK2, GAB1, GANAB
Negative_9255	IDS, OAT, ABLIM2, AKT3, ALG6, AREG, ASS1, CAV2, CCND3, CD3E, CD58, CLOCK, CREB3, CREB5, CTF1, CXXC4, DFFB, DGKZ, FUT5, GAD1, GADD45B, GRM1, GUCY2F, IFNG, IQGAP1, MAN1A2, MCAT, MDM4, OR13C2, OR4F5, OXSM, PLD1, PPP2R1B, PTGS1, PTPRR, RAC3, SORD, SPTLC1, TAS2R10, TLN2, TUBAL3, UGT2B7, ATP6V1C2, PNLIPRP2, SUV420H2
Negative_9256	F9, DHODH, GSTO2, MAP3K4, SGPP1, STMN1, TICAM2, UGT2B10, VAMP2, HLA-DQA2
Negative_9257	F11, FGA, FGG, AARS2, ACYP1, ADCY2, B3GNT4, CCR10, CDKN2B, COL1A2, CSF2, DDX51, DUSP1, HAGH, HDAC2, HMOX1, INHBC, INMT, L1CAM, MAP2K5, MGAT2, MMP1, MYH8, OR10K1, OR1L4, OR2M3, OR51B2, OXTR, PGAM1, POLR1A, POLR3B, PON1, PRPS2, PSPH, SDC2, SHC3, STAM2, TAS2R38, TSLP
Negative_9258	ALDH7A1, CDH15, OR52A1, PLCB4, RALA, RETSAT, RRM1, ST6GALNAC6
Negative_9259	BAD, ADRB1, ARSE, ATP5F1, ATP5L, CCL3, CD40LG, CYP2U1, DHCR7, DOLPP1, EPHA4, FGFR2, GRIA1, HBEGF, HRH1, IFNA5, IL20RA, INPP5B, MAP2K7, MAPK12, NFATC3, NFE2L2, NR1D1, OR5D13, PIK3CA, PPP1CC, SRGAP3, SULT1E1, SV2C, VAMP3, HSD17B12
Negative_9260	GLS, SPN, CALML6, CD55, CDKN1C, CISH, CRLS1, CYFIP2, CYP4A22, EXOC7, EXTL3, GALNTL4, IRS1, MAT1A, NFYC, OR10K2, OR2L13, OR51G2, OR7C2, OR7G2, PLA2G2F, PLA2G3, PLA2G5, PPP3CC, SNW1, SPP1, THBS1, TNFSF14, ST6GALNAC5
Negative_9261	ACACA, CTPS2, GALNT1, GGPS1, GYS2, IDI2, OR10J3, OR5J2
Negative_9262	AASS, ACACB, ACADSB, ACVR2A, ALG12, BMP5, CCR3, CNTF, CXCL10, DDX51, DGKD, DGKQ, DHDH, DKK2, EHHADH, ENO3, ESPL1, GART, GCLM, IRS1, ITGA2, ITPKA, MCCC2, MGAT5, NRG4, OR10G4, OR2D2, OR6C1, P4HA1, PI4KB, POLA2, SARS, SEMA3F, SLC2A1, STX2, STX5, WNT4, WWP1, PAFAH1B1
Negative_9263	GLS, ACADS, BCKDHA, DBF4, DVL2, F11R, GALNT2, GNAS, HSD3B7, LIPA, LPAR1, MAML3, MAN2A1, MYH6, NCKAP1, OR10A7, OR6A2, PIGP, PLCE1, POLA2, SFRP1, TAS2R3, ATP6V1B2, TNFRSF13C
Negative_9264	RB1, ACTG2, ADORA2B, ALDH2, CD8A, CDC25B, CDC26, CYP4F3, DAPK1, GBA3, GFAP, IGSF5, IL22, INPP5B, ITPK1, MITF, MYH8, NT5C1B, OR5T3, PPP1CB, PPP2R2B, PRPS1, SAT2, SEMA3B, STAM2, TAS2R4, WIF1, ATP6V0A1

Negative_9265	HAL, PAH, ACADM, ATP6V1A, CBLB, CREB3L1, CSNK1G2, EPHA8, GALNT7, GALNTL5, KAT2B, LTC4S, NUMBL, OR14J1, PGM2, PLA2G2D, PPP2R2D, PYCR2, RUNX1T1, TAS2R4, UPRT
Negative_9266	AGL, AK2, ARNTL, ATP5H, AXIN1, CCR5, CHDH, CLDN19, DAAM1, EPHA8, FSHB, GALNTL4, GSTP1, INHBB, MAP2K7, MAPK13, MINPP1, MYH13, OR10C1, OR2C1, OR2K2, OR2T2, OR4K15, PARD6B, PDE11A, PFN3, PPARA, PRKACA, SOS1, THBS3, UGDH, PNLIPRP1
Negative_9267	AGK, BTD, C2, CR1, CSK, F8, INS, PC, ACHE, ACY3, AKT3, ALDH1A1, ALDH7A1, C1GALT1, CCL20, CDKN2B, CUL1, CXCR5, CYP4A11, HTR5A, IDI2, IL18, KDSR, LYZL1, MAML1, OR2B11, OR51V1, PLCD3, PPAP2A, PPCS, PRIM1, PRIM2, RPS6KA5, SHC3, SMAD2, TMLHE, UGT2B10, MAPK8IP2
Negative_9268	MYC, ABLIM3, ACTN4, ALG10B, AMPD3, ARPC4, CCNG1, CDC23, CSNK1D, CYP26B1, DAAM1, DHCR24, EHMT1, FOXO3, GABARAP, GPAA1, HAGHL, HNMT, IL1B, IL9R, MAP3K13, NT5C, OR10A3, OR14J1, OR1D5, OR2H2, OR4N2, PGM3, PIK3R2, POLE2, RAPGEF2, SH2D1B, STAT5B, TAS2R48, VKORC1, WARS2, PNLIPRP2
Negative_9269	DUT, SFN, ACADSB, ACLY, ACSM4, ADCY10, AGXT, ARG2, ATP5O, B3GAT1, CACNG1, CD19, CLCF1, COL6A2, E2F4, FCGR3A, FGD1, GMPS, GUSB, HSP90B1, IL5RA, ITPKA, KAT2B, MAP2K2, MRAS, NOX1, OR1L4, OR4M1, OR56B4, PCYT1B, PFN1, PIGH, POLA1, RHOD, SDC3, SSX2IP, STX10, SUFU, WNT16
Negative_9270	ALG1, ANAPC4, CD40, CTSG, GNG13, IMPDH2, NOTCH3, P2RX2, PDE1B, C1GALT1C1
Negative_9271	KITLG, NFKBIE, RBPJL
Negative_9272	FUK, ATN1, CES7, IFNA13, IL22, LILRB3, OR2T11, OR52A1, PHKG1, PIGB, TPI1, UGCGL1, UNC5C
Negative_9273	C8B, DCK, KDR, ACOX3, ACTN1, ADH7, BHMT, CACNA1B, CAPN2, CCND3, CES7, CHKB, DCTN1, ENO1, EPHA4, GALNTL1, HARS2, HSD17B3, IL17A, KIR2DS4, MAP4K4, METTL2B, NRG1, NTRK2, NUDT5, OR10G8, OR2A42, OR51A4, OR5A2, PCYT1B, PIK3C2G, PLAU, PROC, RRAS2, SEMA4F, SLIT1, SPTAN1, TKTL2, TSNARE1, WNT4, CACNA2D1
Negative_9274	AK5, EBP, FH, LPL, VCL, ADH4, ALDH5A1, B3GNT5, BAAT, BDNF, CHPF, DHDDS, DNMT3B, DUSP1, EXT1, GAB1, GALNTL4, GLG1, GLUL, GPD2, HARS2, MTHFD1, MTHFD2, NCOA4, NR4A1, OR2T10, OR5M8, ORC1L, P2RX2, PDE3B, PIGT, PLA2G4A, POLA1, POLR2E, PPP2R5C, PRPS2, RALB, SPTLC1, VTI1A, WNT1, HLA-DQA2, PNLIPRP2, PPARGC1A
Negative_9275	ADORA2B, BMP8A, OR1I1, PIP5K1C
Negative_9276	DBT, GLA, GSS, AKR1B10, AKT3, ALG13, ALPL, CASP8, CCR1, CD3E, CFL2, CHPT1, CLTA, DLST, DVL1, EP300, FADS2, FTCD, GGT5, HAO2, HLCS, IL11, INSRR, IRF5, LAMC1, LYPLA1, MAP3K11, NAGLU, NFKB1, NT5M, OR13C4, OR2C1, OR4Q3, OR6M1, PFAS, PGAM2, PIP4K2A, PLEKHA8, PPP2CA, PTPN1, PTPN6, SESN3, SH3BP2, SLC25A6, SPRED2, STX8, TAS2R38, TAS2R5
Negative_9277	INS, SRC, AARS, ACAT1, ALG9, ATP2A1, CLTC, CNGB1, COQ3, DGAT2, DGKZ, DRD5, ENO3, FZD2, GDF7, GRIN1, IFNA21, KNG1, KYNU, MKNK1, NLGN1, OR13C4, OR8D1, OR9G1, P2RX2, PDE3B, PIP4K2C, POLR2L, PTEN, RHOA, ATP6V0A1, HSP90AA1, PNLIPRP2, PPARGC1A
Negative_9278	C2, TAT, BRAF, CTNNA2, FUT5, IRF5, OR4F5, OR51I1, PIP4K2A, PLA2G4A, SESN1, XCL2
Negative_9279	AMT, GLS, ACHE, ACSL6, ARSA, BDNF, CD79B, CLTA, CXCL6, ENTPD7, GAL3ST1, GGT5, GNA15, GP1BB, GPAA1, HIBCH, IFNAR2, IKBKB, IL1R2, ITPA, LLGL2, MAP2K2, MCEE, NAGK, OPLAH, OR10G7, OR2T8, OR52I2, PDXK, PERP, PFKFB1, PGK1, PRKAG1, PRKCB, PSPH, RAD51, RENBP, SFRP2, STAT5B, STAT6, TAS2R5, THBS2, THTPA, ST6GALNAC5

Negative_9280	DCK,F9,IVD,NGF,ADCY7,ALDH1L1,ALDH4A1,ALDOB,APPL1,ATP5H,CD244,CDC14B,COL5A3,CREB1,CSNK2A1,CTLA4,FUT1,GALNTL4,GLI3,GNPDA2,GRM5,GSTK1,LHCGR,MAP2K5,MAP3K12,MTHFD2,NOX1,OR10H4,OR1M1,OR2AE1,OR4N2,OR51B6,OR52K2,OR52W1,OR5B17,OR6C1,PSAT1,RRAS,STX16,TCF7L2,TUBA3E,TNFSF13B
Negative_9281	ABO,AK7,FST,ID1,ME3,ACYP2,ADCY5,AKR1C4,ALS2,APPL1,ARPC1A,CCND3,CRLS1,DDX50,GLUD1,GRB2,IFNB1,IL13RA2,LRAT,NRG3,OR10G8,OR4C15,OR9Q2,PIK3CD,PIK3R4,PROC,RAPGEF2,RBL2,SDC2,STX6,YWHAH
Negative_9282	NF1,ACTG1,ANGPTL4,CEBPA,CXCR3,CYP1B1,HSD17B1,OR10A4,OR1L1,PAK7,PLXNB3,TAS2R38
Negative_9283	ACP1,ANAPC5,ATP13A2,CHRD,CRY1,DFFB,FZD7,GZMA,IFNA5,IFNA8,LAMB1,MAP2K7,OR6N1,PGAM4,PIAS4,PIGS,PIM2,PLD2,PNPLA3,PRPS1,RDH5,SMURF2,SPRED2,TAP1,UBE2L3,UPP2,XCR1,ALDH18A1
Negative_9284	BCAR1,CXCL6,HAGH,OR10A4,OR6M1,PRKY,SUCLG2,SERPING1
Negative_9285	CFD,LHB,PGP,ACSL1,ACVRL1,ADH7,ANAPC5,BMP7,CLDN20,ENTPD2,FLT4,HSD11B2,HSD17B1,IL8RA,ITGAL,MYH4,MYH7B,OR10H1,OR1K1,OR4K1,OR6M1,PDE1C,PPAT,PPCDC,SDHA,SH2D1A,STAT5A,ATP6V1C2
Negative_9286	ADC,CFI,F12,HDC,LTB,PXN,SDS,ACSS2,ADCY2,ATP6V0C,CCND3,CCR10,COL11A1,CYP26C1,CYP2J2,DPYS,DRD5,EPB41,FARSA,FLT1,GNAO1,MAP3K1,MASP2,MBL2,MYH15,NUDT12,OR13F1,OR2AG2,OR51L1,OR8J1,P2RX1,PLA2G3,PPP2R2B,PRDX6,PTCH1,RPS6,SH2D1B,STAT2,STX16,TLN1,TUBB4,YWHAE
Negative_9287	ACP1,ADSL,ALG5,APPL1,C9orf95,CCR7,CDO1,CHEK1,CLDN14,CXCL12,DIAPH3,DUSP14,DUSP9,EP300,GRIN2C,GUCY2F,HHIP,IFNA17,INMT,ITGAV,LARS2,LIPC,NCSTN,OCRL,OR10Z1,OR1A2,OR1I1,OR52H1,OR5AK2,PDE1C,PIK3CB,RUVBL1,SH3BP2,SPI1,SPRY3,SULT1E1,TBXA2R,TNFRSF9,VANGL2,B3GALNT1,TNFRSF13C
Negative_9288	MAX,MPZ,ACTB,ATP12A,ATP5E,B4GALT2,CACNA1H,CCL25,CCL5,CLDN16,COL11A1,CYP17A1,CYP3A4,DHRS3,FGFR4,GNG12,LIPA,LTB4R2,MCM5,MGAT4B,MPZL1,OPLAH,OR4D9,PCNA,PGM1,PIAS1,PIK3C3,PNLIP,POLD2,PRSS3,RPIA,SHC3,TAS2R7,TNFSF8,UMPS,VCAM1,ATP6V1E1
Negative_9289	CGA,F5,FOS,ADCY9,B4GALT4,C1GALT1,C9orf95,CACNB1,CACNG4,CCL13,CDC6,CLDN4,CSF2RB,CSNK1G3,CTLA4,FZD10,G6PC2,IPPK,KYNU,MAML1,NEU1,OR10AD1,OR4C11,PAK7,PDE4B,PFN2,POLD2,RELA,SOD1,TGFA,ATP6V1C2
Negative_9290	GCK,ABP1,ALDOC,ANAPC5,ATP2A1,AXIN2,B3GAT1,CLOCK,CPT2,CYCS,CYP2C9,ENPP3,EPHB3,GALNT2,GGCX,NADK,NKD1,OR10J5,OR1G1,P2RX6,PIAS1,PRKCD,PSME1,PSME3,THY1,UPRT,WNT8B,ATP6V1E2,TNFRSF10C
Negative_9291	TYR,AKR1D1,APC2,CXCL5,DKK1,F13B,FARS2,FZD8,MMP9,PARD6A
Negative_9292	ACSM1,ANAPC11,AOC3,ARSB,AXIN2,CAMKK1,CD99,CLDN18,COL4A4,DTX2,FZD8,GALNT14,GGT1,GPLD1,HTR2A,IFNA7,INSR,IRF7,KYNU,MGST2,MYH7B,NCR1,NCR2,NKX3-1,OR2L8,OR51I2,OR6C75,PARD6A,PIGU,SELL,SMARCA2,SRD5A2,TAS2R5,TSLP,ZBTB16,HSD17B12
Negative_9293	CD6,ADAM10,ADIPOR1,CLDN10,CNTNAP1,CRB3,CYP2F1,FZD7,ICAM3,IL20RB,MAOA,OR2G2,STAT5B,STX18,VCAM1
Negative_9294	FYN,INS,ACADL,ACTN1,ATG12,BMP6,BMP8A,CHKB,COQ5,ENTPD3,F2RL3,FSHB,GUCY2D,KARS,MNX1,PCK1,PIGH,PIK3CB,POLR2B,RND1,ST8SIA5,TRDMT1
Negative_9295	IL4,ACAT2,ADIPOQ,CCL4,CCL7,COQ7,CSF2RB,CYP11A1,CYP2E1,DDX19A,FGF13,FGF21,GALE,GALNTL4,GLUD1,GMPR,GNAI2,GPLD1,GSTM2,IL8RB,INPP5E,ITGA10,LIAS,LRDD,MICA,NCAM1,NEU1,OR51L1,OR5C1,OR5D16,OR5T2,OXSM,PDE3B,PTGIS,TCF7L2,WHSC1

Negative_9296	UBC,ADAM17,ALG3,APH1A,CHEK2,IL4R,MTHFR,MYH1,OPLAH,OR2B6,OR2M2,PANK2,SETD7,SORD,SPRED2,TACR3,TBXA2R
Negative_9297	FGB,FST,GSN,HK2,IDS,LPL,TPR,ABL1,ACADL,ADH5,ARPC5,B3GALT4,BIRC2,CACNG4,CCL26,CHRNA7,CLDN2,CSNK1G2,CSNK2A2,CTBP2,CXCL5,DDHD1,DRD2,EFNA3,FCER1G,FUT1,GADD45A,GAL3ST1,GALNT1,HSD3B2,IL15RA,MAP3K4,NOS3,NTN1,OR10H1,OR1N1,OR2H2,OR51B4,OR52N1,OR5H6,ROCK1,RPN2,SETD7,SGMS1,SH2D1A,STAT5A,SV2B,UGT2B11,HLA-DQA2
Negative_9298	ADK,DUT,TXK,AGPAT4,ALDH2,BET1,CAV3,CCNA1,CD72,CFL2,CYP1A1,FGF6,FSHR,FUT1,GART,GAS1,GFPT2,GSTM4,GZMA,IRF7,LRP6,MADCAM1,MYH9,NCR3,NR5A2,OR13C2,OR13C9,OR2S2,OR2T1,OR2T10,OR4M1,OR4N5,OR8D2,PLXNB1,PRKAA1,PRKACG,RAF1,RND1,SESN3,TLR2,VKORC1,HLA-DQA2
Negative_9299	HYI,ITK,VCL,ABL2,AGPAT4,ALDH1B1,ALOX15,APH1A,APOA1,AREG,ASNS,B3GALT4,BMPR2,C1QC,CD48,CDC14A,CDC25B,CDK5,CHRM1,COL4A1,DPM2,ENPP1,FGF20,G6PD,GAMT,ITGB1,KNG1,LFNG,MASP1,MGAT4A,OR10Q1,OR13H1,OR1M1,OR52W1,OR5K2,OR6Y1,OR8H2,PHKG1,PIAS3,PLXNC1,STAT3,TAS2R50,THY1,TKTL1,TOMM40L,TUBB6,TNFRSF10D
Negative_9300	TYR,AMD1,CD48,CKMT2,DHDDS,NFE2L2,OR52H1,OR7G2,PDGFB,PDGFC,RHOA,RUNX1T1,TNFSF11,XYLB,CACNA2D3
Negative_9301	CGN,COQ5,HTR5A,IFNW1,MARS2,NLGN3,NR4A1,OR2L2,OR2L8,PLA2G10,PPP1R1A,TLR3
Negative_9302	EPO,ADORA2A,ALDH2,AMPD2,BST1,CLTC,DVL1,ENTPD2,GRM1,KCNJ11,NCF2,NEU2,NPAS2,OR1I1,SEMA3E,STT3B,TUBA3C
Negative_9303	BTC,GGH,INS,AKR1C1,AKR1C2,B4GALT7,CD40LG,EPHA1,FMO2,GALNT12,GP1BB,GSTA3,GSTO2,IRF3,LDHAL6A,MYL5,NSD1,OR10AD1,OR1A1,OR1G1,OR1J4,OR4F5,OR51A2,PI4KB,PKMYT1,PLCB4,POLR3H,PRKACB,RENBP,SETMAR,SLIT1,TRAF2,UGT2B11,ULBP3,VAV1,YARS2,CACNA2D4
Negative_9304	FER,OAT,SI,AGRP,AMDHD2,AOC2,APC2,APOE,ASCC3,ATP5G2,AXIN1,CACNA1G,CNGA3,DDX56,DUSP16,FRAP1,GPD1L,GUCY1B3,GUCY2D,HBEGF,IARS2,IL12RB2,IL22,KIR2DL4,LIPG,MTHFD1L,MYL7,OR2T2,OR4B1,PRKCD,RPS6KA5,STAT1,TUBAL3,TYMS,UGCGL2,ULBP2,ATP6V1G2,CACNA2D2,TNFRSF14,TNFRSF6B
Negative_9305	CFH,SI,AANAT,ACSM2A,ADIPOR2,AKR1D1,BDNF,CCNA2,CD47,CSNK2A2,DDOST,GFPT1,GPT2,GRIA3,GRIN2D,HRH1,HSD3B2,MYLK2,NFAT5,OR2T6,OR6K2,OR7A10,OR9Q1,OSMR,PAK4,POLR2L,PTAFR,PTPRC,RDH10,SEMA4C,SLIT1,ST3GAL5,SUOX,TPK1,VEGFA,SERPIND1
Negative_9306	F2R,SRF,ACSL5,ARRB2,CD44,CHST12,CMBL,CTNND1,CYP27A1,DAPK2,DMGDH,DTX3,DUSP10,FABP3,HIBADH,IL1R1,MYH7B,OR2L2,OR2L8,OR2V2,PLA1A,PPP3CB,PTPN1,PTPRZ1,RASA1
Negative_9307	LTB,AGRN,ALDH3A2,ATG3,CCR4,CNGA4,CNTNAP2,COL4A4,FARP2,LDHA,LIPG,LTBR,MAPT,MGST2,OR56B4,P2RX4,PIP4K2B,PLXNA1,POMC,PTGES,ST3GAL4
Negative_9308	PGD,TPO,AASS,ARNT,BCMO1,FLAD1,HAO2,NCAM2,OR14C36,PAK6,PAX4,PLCB1,PLCD1,PPAP2C,PYCR2,RENBP,TSHB,PLA2G12B,SUV420H1
Negative_9309	ADK,BTD,CR1,ME2,ACP1,ALOX15B,APPL1,ASNS,CCL1,CCL5,CD22,CD82,CERK,CHIT1,COL2A1,CUL1,ETS1,GALNS,GLI3,GNB3,GP1BA,IL12RB1,IL13RA2,IL2RG,JAG1,KAT2B,LYPLA1,LYPLA2,NAMPT,NAT1,OR1L3,OR4K15,PDE6G,PIGO,PLD1,TP53,TYMP,UBA1,UGT2B11,VASP,WNT11,XCL1
Negative_9310	OTC,PGP,ABI2,ARD1A,ARPC2,BUB1,CCR7,CHRM1,CMBL,DARS2,EPHB4,ETNK1,HADH,HNF1B,KIR2DL4,LFNG,MAP3K14,NFX1,NPR1,OR10G7,OR10J5,OR6B2,POLR3H,PRKAA1,PTGS1,RRAS2,SEMA3D,SMAD4,SOCS7,TAS1R2,TAS2R8,UGT2B4

Negative_9311	AADAC,ATP2A2,ATP5J,BUB3,CD8B,ITGAL,MAPK1,NCK2,NEFL,NFAT5,OR2T3,PDX1,PGM2,PISD,SCD5,SULT2A1,TNFRSF14
Negative_9312	MSN,A4GALT,ABLIM3,ACACB,ADH6,ADRB3,B3GAT2,CACNB2,CADM3,CMAS,CSNK1G2,CYP4F3,DBF4,EDA2R,HLA-G,IL18,ITGAX,LRDD,NOTCH2,OR10A6,OR52I2,OR6C75,RAP1B,RRAS,ST8SIA1,XCR1
Negative_9313	F3,GP9,PXN,TPR,ZYX,ABL2,ADRBK2,AMPD2,B3GNT3,BCL2,CCR8,CDK4,CLDN19,CPT1B,DLAT,ELK4,EXTL1,FGF10,GPD2,IL21R,OR10V1,OR13C9,PDGFC,PIK3C2G,POLR3G,PPCDC,SEPHS1,SULT1A1,ULK3,TNFRSF17
Negative_9314	ACTG2,ATP2A1,CCL1,DTX3,EPHA2,FADD,FARP2,GMDS,GPX5,GRLF1,HLA-G,ITGB3,NCF4,NOD1,NOX1,OR5T3,PDGFC,PLA2G4A,PRKAA1,PRKAG3,PRKAR2B,SMARCA2
Negative_9315	AK5,F8,FGA,ACLY,AGRN,AHCY,ALOX12B,APRT,B3GNT2,CSNK2B,DARS2,ENPP7,ENTPD6,FZD8,GRAP2,GSTM5,HTR2B,IL18,MAD2L2,MAP2K5,NR1D1,OR5M8,PDK1,PTCH2,SEMA7A,SRD5A2,TUBA3E,WNT5A
Negative_9316	AMT,C2,ME1,TPR,AARS,AGPAT2,ALAS2,ATP5E,BCAT2,CCR7,CDO1,CREB3L1,CSAD,CXCL12,CYSLTR2,F2RL1,FDFT1,GALNTL4,GDF6,GMPR2,GNAO1,GRIA3,GRM5,HTR2A,INPPL1,ITGA10,LYPLA1,OR2A12,OR2A14,OR2T3,OR51A7,OR6C6,OR8J3,PARS2,PDGFD,PDK1,PGK2,PMAIP1,PPP2R5A,PRKCG,PTGS2,RPS6KA5,SDHB,SULT1A4,TAS2R48,TSHB,TSNARE1,UGDH,ZFYVE9
Negative_9317	FAS,ABLIM2,ATP6V1F,CDC20,CHAT,CNTNAP2,CTBS,E2F4,ELK1,GADD45B,GLI2,IDUA,IL23A,INPP5B,IRF3,IRS1,ITGA10,OR10T2,PLOD2,PPAP2A,RPS6KA2,SULT1A2,VDAC1,ST6GALNAC6
Negative_9318	BTK,PXN,TK1,XDH,ANGPTL4,BNIP1,CHRNA7,CLDN14,COMP,CTSG,DTX2,GALNT5,GCLM,GIT1,GSTO1,HMBS,HNF1B,IDI1,IFIH1,KRAS,KYNU,LMO7,LRAT,NCOA4,NFATC3,NFKB2,OR52N1,OR6B3,OR7C1,OR9Q2,PPP2R2D,PTPRJ,SKP1,SMPD4,SPP1,STX5,TAP1,TGDS,TUBB2B
Negative_9319	DLD,TK1,APH1A,C1GALT1,CAMK2G,COL1A2,CXCL13,FABP4,GAS1,GSTM4,HRAS,IL4R,L1CAM,OR2G6,OR4M1,OR9G4,SETD8,SLC25A4,SSH1,UGT2B4,ATP6V0D1
Negative_9320	PAH,ADH6,AXIN2,BCL10,EPHB6,FGF18,FMO5,GSK3B,GSTM5,HEMK1,HTR4,IL24,NRXN1,OR3A3,OR5L2,PCNA,PEMT,PIP5K1C,PLCB4,PLXNB1,PNPLA3,PTPRM,PTPRZ1,RAB23,SH2D1B,ST8SIA5,HLA-DRB4,MAPK8IP2,MAPKAPK5,SERPINA5
Negative_9321	CD4,FYN,OAT,AKR1B1,AKR1B10,AKT2,ALG2,ATP4B,C1QC,CDC23,DNMT1,EARS2,EREG,GALNT5,GDF5,GLI3,GUCA1A,IDH3G,IKBKB,IRS2,ITCH,NAT5,OLR1,OR10H1,OR2W1,OR56A1,OR5P3,PANK2,PIGB,PLD1,PRODH2,QPRT,SOAT2,SUOX,TAS1R3,THBS1,TRMT11,TUBB6,ARHGEF12,ATP6V1C2
Negative_9322	ADC,LYN,NNT,ABL1,AHCYL1,ALCAM,B3GNT2,BUB3,CD46,CSF1,EPHX1,FGF9,FUT9,GARS,GPAA1,GUCY2C,HIBADH,IL17RB,ITGAV,LARS,LRP5,MCM6,NAGLU,NEU2,NTSR1,OR4K1,OR51B5,OR8K1,OXCT2,PIGX,PLAU,PLCG2,PRKCA,SEMA3D,TAS2R60,TGFBR1,TNFSF13B
Negative_9323	C8G,IL9,KHK,ACP2,AKR1C2,ATP6V0C,CDKN1B,CLDN6,COL5A2,CXCL2,CYP1A2,GADD45B,GALT,IL17RB,ITGA8,ITGB2,MAP3K3,MDM4,MGAT5B,MYH13,NAT2,OR11H6,OR4N2,OR4X2,OR52B4,OR9Q1,PHKG2,PIGN,PIGS,PRKG2,PYGM,RAF1,SLC9A1,TLN2,ULK2,WWP2,PLA2G12B
Negative_9324	APP,DCC,GSR,ALDH1A1,CCR1,CREB1,CYP2F1,FBXW11,GNAL,GRIN1,IFNA7,MAP3K12,MYH13,NFYB,OSMR,XIAP
Negative_9325	ADCY3,ADSSL1,APOA1,ARPC5,BMP7,CDKN2B,CHPT1,CHSY3,DRD5,FTMT,GCNT1,GLUD2,GSTM4,HAO1,HEXA,INPP1,LAMC3,LARS,LDHD,MYH11,NARS2,NFATC3,NOTCH3,OR5D14,OR6Y1,OR7G1,PIP5K1B,PLA2G4A,PPA1,PPARG,QARS,RASGRF1,SFRP1,SMAD9,STX12,TAS2R4

Negative_9326	HYI,CCL2,EHMT1,FSHR,GNB3,OR1L3
Negative_9327	BTC,FGA,ATP5G2,BMPR1A,CAMK2D,CAV3,CD3E,CDKN1C,CHSY3,CREB3,DGKA,EARS2,EPB41L1,GTSE1,IGF1R,IL3RA,IPPK,LDHB,MYH7B,NAMPT,NMNAT1,ODC1,OR2B2,OR4F15,OR9Q1,PAX6,PDE6H,PGM1,PIK3CD,POLR2B,PRODH,RGS3,SEMA4D,SMAD5,SOCS3,STK36,TCF7L1,TLR3,PRICKLE2
Negative_9328	CFH,FH,ACACA,BIRC3,CD34,DHDDS,EPHA3,ITGA11,OR52E4,PLAT,POLR1D,POMC,TACR1,TAS2R10,TYRP1,UPP2
Negative_9329	ITK,CYP4F3,NUDT2,RHOQ,SMARCA2,TGFA
Negative_9330	FER,CHIA,CHST12,CRLF2,CSNK1A1,GNPNAT1,HARS,ITGB6,MAP4K1,NOS1,OR2Z1,OR5B3,OR6C70,PLA2G10,POLR2B,SGPP1,SHC3,SOCS3,SULT1A1,ULBP3,WNT11
Negative_9331	AK7,F5,FGA,ID3,ACOX1,ACSS2,AKT2,ALDH1B1,AMPD3,AOC2,ATG7,ATP5B,BCAR1,BDNF,CACNA1S,CCL15,CCNA1,CLTA,CLTCL1,COL4A1,COL5A3,CPOX,CREB3,CXCL6,CYP3A7,CYP4F3,DLST,DUSP14,DUSP8,E2F5,G6PC,GLB1,GSTA1,HAO1,IFNA5,IGSF5,IL5RA,MAGI1,OR13G1,OR2M3,OR2T10,PAK1,PDGFC,PIK3CB,VTI1B
Negative_9332	ACSL5,AGPAT2,BST1,COL5A3,GPLD1,HLCS,IDUA,IFNA14,ITGA1,MTFMT,OR1L3,OR6B2,OR6X1,PNLIP,SKIV2L2,YARS
Negative_9333	C7,ACSM1,CHST13,CPT1B,FCGR2B,FLAD1,OR52M1,OR8J3,PDE3B,SOCS3,STK36,SYNJ1,TUBB3
Negative_9334	AMT,RFK,VTN,ADRA1B,COL2A1,CTPS2,GGCX,MGAT5B,PPP1CA,PSEN1,SFRP5,SNAP25,UBA7,VANGL1,WHSC1
Negative_9335	ALDH3B2,CDH2,COL11A1,CREB3L2,CXCL1,FGD3,GALK2,GOSR2,HAO2,HARS,HDAC1,MTHFD2,NPR1,OR10C1,OR10Z1,OSMR,UROD,YWHAG
Negative_9336	ABO,FH,HDC,PGD,AGRP,ARHGAP5,ASS1,BPGM,CASP10,CD47,CDH15,CEBPA,CPT2,CRHR1,DGUOK,EFNA1,FMO2,FOXO1,FUT2,FZR1,IL12RB1,IQGAP1,MYH8,NEU4,NTF3,OR4C13,OR4F17,OR52A5,PCCB,PDE5A,PDE8A,POMC,PPP3CB,PRKCZ,PTDSS1,SARS,SKP2,STAT2,SUV39H1,TNFSF4,UGT2B4
Negative_9337	ADA,BTK,F12,F9,GHR,SMO,AADAC,AASDH,ACLY,ADCY7,AMY2A,B4GALT6,BCKDHA,C1GALT1,CLDN2,CSNK1G2,DERA,ENO2,ETS1,FGD3,FGF6,GCNT3,GSTM5,IL13,LHCGR,LTA4H,NARS2,NT5M,OR4K2,OR51A7,OR51B4,OR8B12,OR9Q1,PGAM4,PLA2G4A,PLTP,PPCS,PTGES2,PYCR2,SEMA4F,SETD8,SHC1,SMAD5,STX18,TAS1R3,TNFRSF18
Negative_9338	HK1,MAG,MUT,RPE,ACLY,ADH7,AXIN1,CAMK2B,CCR6,CD82,CFL1,CREB3,EXT1,FARP2,FECH,FGF11,FGF23,GALNT13,GAPDH,GOT2,ITGAD,LDHD,MGAT4A,NRG4,OR11A1,OR6C2,OR7C2,PANK2,PDK1,PIP4K2B,PPCDC,PPP2R2D,PRKAG2,SELL,SMAD5,STX1B,TJP2,TPH2,TUBA4A,AASDHPPT,HLA-DRB4
Negative_9339	ADC,SRF,VTN,AGMAT,APH1A,CACNA1H,MAD1L1,MDM4,OR7C1,OR9G1,PIAS2,PIM2,PROS1,SAT2,SGPP2,SOCS2,TFPI,PNLIPRP2,TNFRSF17
Negative_9340	CAMK2B,DHRS9,EFNA3,FLOT1,KIR2DS5,OR4D9,OR5P2,PLA2G1B,WNT8A
Negative_9341	AOC2,CD34,CSF3R,CXCL11,JUND,ORC1L,PPARA,RFXAP
Negative_9342	FER,GLS,ACADL,CCKAR,CD44,CTNND1,DHDDS,EFNA2,FZD7,GALNTL5,GGT1,GSTA4,INHBB,IQGAP3,KLRC3,LPAR1,NCK2,NFYB,NTSR1,OR10H4,OR14C36,OR4N5,OR5AR1,OR5T3,PANK1,RCHY1,RIPK1,RPSA,SH2D1A,STAT5B,TAS2R60,THBS1,TSTA3,TYK2,ATP6V0D1,ST6GALNAC3
Negative_9343	C8B,CD6,CFD,RDX,ACP1,ARHGEF4,C4BPA,CACNA1H,CASP8,CD82,CLDN10,CTF1,CYP11B1,DUSP3,FCGR3A,FGF2,FGF22,GCLC,GZMB,HIBADH,IL12A,KIR3DL3,LIPC,MAP3K4,MTAP,MTMR1,MYL9,MYLK3,NEGR1,OR51L1,PLA2G2D,PPP2R5B,PTPRR,PVRL2,RFXANK,RPS6KA4,SHISA5,SLC25A5,SMAD2,TAS2R7,UNC5C

Negative_9344	GPT,ALDH1A2,ALPL,BMPR2,CCNE2,CRLS1,CTSB,CXXC4,CYP2U1,DLST,F2RL2,FGF5,FLT4,GJD2,IL17B,LTBP1,MALT1,MAPK13,MASP2,NOX1,OR11G2,OR52B4,OR5D18,OR5V1,PFKFB4,PFKP,PFN1,PLA1A,PRKG1,SAT2,SCD5,TUBAL3,UNC5A
Negative_9345	BTD,KMO,NGF,ACLY,AMY2B,BST1,CCDC6,CDKN2C,COL2A1,DAAM2,EXT2,FGF22,GAD2,GAMT,GNA14,GNAZ,GUSB,HGSNAT,IL25,ITCH,MAP3K8,MOV10L1,NUDT12,OR13C3,OR51B5,OR5L1,P2RX7,PDK1,PHKG1,PIK3CD,PLA2G2F,PLXNA1,PSME1,RPSA,SELL,SH2D1B,STAT3,VAV3,VEGFB,CACNA2D2
Negative_9346	GNE,ACVR1B,BBC3,BMP4,BMPR2,CAMKK1,CHUK,CLDN3,CTNNA3,EFNA2,FSHR,GNAT3,IL1B,IL22,NR5A2,NRG1,OR10H4,PFKFB4,PIK3CB,PLXNA3,PNPLA3,PPBP,PPP5C,PRKAB1,SMPD1,TAS2R13,TNFSF14,TNFRSF18
Negative_9347	BAD,OAT,RB1,ACP6,ATP2A3,CACNG6,CCL13,CD247,CFL1,CYP1A1,DRD1,DVL1,GARS,GDF5,GFPT1,GPD1,HMGCS1,IL12B,IL8RA,LAMB3,NAGLU,NFATC3,NKX3-1,OR1K1,OR2AG2,OR52E4,OR5L1,PDE8A,PFKFB3,PGAP1,PHKA2,PLOD3,PLXNA2,PPBP,PRLR,PTGS1,SEMA3A,SESN3,SIPA1,SMAD9,SV2B,TAS2R48,VAV1,VEGFA,HSD17B10,TNFRSF1A
Negative_9348	CAV3,FADD,NRP1,OR1Q1,PAK6,HSD17B12
Negative_9349	DLD,AKT2,ALG10,APRT,B3GNT3,CHST11,CLDN7,CMPK1,GALNS,GGT1,GNAO1,GYS2,HSD17B3,IL10RA,MAP2K7,OR10G3,OR4K1,OR9G1,PGAM4,PIK3R2,PLD1,PROC,RELA,RFXAP,RHOD,RUVBL1,SFRP4,SLC2A1,TLR3,UROD,YARS2,MAPKAPK5
Negative_9350	CKB,KHK,ACTC1,ALG10B,AXIN2,CXCL5,CYP3A43,IDH1,ITGB4,LMO7,NAT6,NOS1,NR1H3,NSD1,OR10A5,OR51A4,OR5A2,PDPK1,PPM1A,PTPRB,RFNG,RRM2B,SYNJ2,TLR4,UPB1,VTI1B
Negative_9351	LCT,OTC,TKT,TPO,ABLIM3,ACACA,ACYP2,AGPAT1,ALDOA,ANAPC2,ATP6V1D,BTRC,CCL5,CCNG1,CD34,CDC20,CES7,CHUK,CTTN,FABP3,FGF9,HMGCS2,IGFBP3,ITGA11,LRP6,OR1J1,OR51T1,POLE,POLR3G,RASA1,XYLB,TNFRSF10A
Negative_9352	CLDN22,OR10G8,SPHK1
Negative_9353	BTD,C4B,FES,INS,ME1,MVD,OAT,ADCY9,ADIPOR1,AGXT2,CLDN11,CLDN16,CXCL9,DKK4,DUSP6,EHHADH,ERBB3,FGF12,FGF21,FGF4,GALNT14,GPD1L,IFNAR2,IL22,MS4A2,NEU4,OR1L6,OR2M2,OR5P2,OR9A2,PDE5A,PFKFB2,POLE,PPP1R3D,PPP3R1,SOD1,TAS2R50,WARS2
Negative_9354	BID,LIMK1,NARS,NT5C3,PNPT1,SESN2,SMAD7
Negative_9355	ACAA2,ADSSL1,ARG1,CHPF,CHRNA7,COL4A1,F2RL2,FGF20,HCCS,IL22RA2,KIR2DL3,MAD1L1,MYL9,NOX3,OR10A6,ORC3L,PIP4K2C,PLXNB3,PTPRF,RAD51,SETDB1,TRMT11
Negative_9356	AGK,FGB,ID4,TEC,AANAT,ALDH9A1,B3GALT1,CASP6,CCL11,CD46,CDC23,CDH3,CLDN7,COL3A1,CXCR3,FLOT1,FUT8,IL2RG,IL6R,MAOA,MAPT,MARS,OR10J1,OR1A1,OR2A2,OR5K1,PDE6D,PGAM1,PIGX,POLD4,PPP2R1A,RIPK1,SMAD9,SOX17,TNFRSF8,TYK2,ULBP3,UPB1,ATP6V1B1,HLA-DPA1
Negative_9357	IL6,NNT,ACYP2,ADH1A,ADH5,ALDH9A1,CACNG4,CCL24,CLDN9,CXCR3,DUSP5,EDN1,EFNA1,FGF20,GCNT4,GSTM5,OR13C4,OR4C6,OR4K2,OR4M2,OR5I1,OR5L1,OR5V1,OR9G4,PCK2,PLAUR,PLCG2,PNMT,STX5,TLN2,TNFSF11,TRAF3,VKORC1,MAPKAPK3,PPARGC1A
Negative_9358	IL3,IL5,PLN,PVR,RPE,ZYX,ADIPOR2,AGMAT,ALDH4A1,ARG2,ASPA,ATP6V1F,BCL10,BDKRB2,CDC27,CDK2,CREBBP,CTSS,CYP2A7,DAAM2,DDX23,DEGS2,DTX1,FGF6,GNRH1,HLA-F,IL19,NT5C3,NTRK1,NUDT2,OR10Q1,OR2T33,OR8B12,OSMR,PIGP,PLCB4,POLA2,PPID,PRNP,PRPH,RDH5,RPS6KA6,SFRP1,UGDH,ATP6V0D2,PLA2G12B,TNFRSF10A

Negative_9359	CDA, LCT, MUT, ACADM, ATP6V0B, CD38, CYP3A43, DDX52, DGAT2, EPHA2, EPHB6, F13A1, GSTA4, IFNG, MAP3K14, MCM7, NTSR1, OR7A5, PTGER3, SSH1, SSX2IP, UROD, WNT2
Negative_9360	PRL, TNC, ALOX15, ANAPC5, ANAPC7, ARPC4, BLVRB, BPGM, CACNG5, CAMK2D, CCNB2, CD48, CFLAR, CHRM1, CKMT2, DPYS, ENPP3, GNA11, GNA14, GSTT1, IL1R2, ITGAX, KIR2DS4, LAMC3, MYH10, OR5C1, PAK1, PLA2G2A, PLCZ1, PLXNB3, POLR2C, PPID, PRKAB2, PTPRC, RRAS, SGPP1, SHC4, SMAD4, TSHR, ZAP70, GABARAPL2, PPP1R12A
Negative_9361	ASNS, ATP2A1, ATP5I, CAV3, CCKAR, CDKN1B, CREB3L3, DDX4, ENPP2, EPB41L1, FOXA2, GADD45A, GCH1, GSTM5, HAGHL, ICOSLG, LIAS, MAP4K3, PANK3, PHKA1, PLAUR, PRKAR1B, SUV39H1, TALDO1, TAS2R60, TFPI, TLR4, WNT10A
Negative_9362	IL5, ACP1, CCR8, CDC45L, CES2, COX10, CUL1, DPYS, EARS2, FIGF, GNRH2, GRIN2A, HCST, HNF1A, HSPA8, IL1A, INHBE, KIR2DL2, MAP2K7, MTFMT, OLAH, OPLAH, OR4K2, OR5AR1, PDE1C, PI4KA, PIGL, POMC, PTPRR, ULBP3, WHSC1, ST6GALNAC1
Negative_9363	ADCY4, ADORA2B, BPNT1, COL4A1, EXOC7, GAB1, GALNTL2, GSTP1, ITGAD, OR5B17, PFN1, PIGW, PPP2R1A
Negative_9364	NPL, ACP1, AKR1A1, AKR1C1, ALG11, ANAPC4, B3GAT2, CALML6, CCL19, CLDN7, GLB1, GYS2, HAGHL, HIF1A, IFIH1, IL20RB, OR11G2, OR4A5, OR51B5, PARVG, PDE4A, PDE5A, PLA2G5, PPP2R5B, PPP2R5E, PPT1, RASGRP3, RIPK1, RPS6KB1, RPSA, SMURF2, SOAT2, SULT1A2, TSC1, PDCD1LG2
Negative_9365	C1S, CSK, DDO, EDA, NPY, SFN, TPR, AGPAT1, ANAPC2, ARSE, CACNA1C, CCNB3, CDH1, CES7, CLTB, CSNK1G3, CYP11A1, DDIT4, DMGDH, EREG, GADD45B, GP1BA, GSTM2, HSPA2, INPP5E, ITGB4, JUND, KLRC3, LDHC, MAPK1, MASP2, MMP1, ODC1, OR10T2, OR11L1, OR52D1, OR52W1, OR5AR1, ORC3L, PER2, POLE3, PYGL, SDHB, SGMS2, SGPP2, TAS2R1, TUBB6, B3GALNT1
Negative_9366	CR2, NPL, ACACB, ACP1, ACPT, B4GALT1, BAAT, CCL11, DCTD, DPYD, DUSP1, DUSP4, FGF17, GRIA3, ITGA5, LAMC3, MTMR2, MYLK2, NFX1, OR13C2, OR2L3, OR2M2, OR5AR1, OR7C1, PARVA, PIP4K2B, PLCB3, PPAP2A, RAP1B, TKTL2, UBE2K, VEGFA
Negative_9367	EZR, UBC, ARD1A, CDC6, GALNT1, GNAO1, IRS4, LIPC, MYLK3, OR2F2, OR5P3, PIP5K3, SHMT1
Negative_9368	APP, FES, IL3, MSN, WAS, ATP5O, B3GNT4, BCAR1, BET1L, BST1, CACNA1S, CD226, CISH, CNTF, CREB5, DGKI, ENTPD3, EPOR, F13A1, GBE1, GSK3B, HLA-F, IFNAR1, IL29, IL9R, IQGAP2, NUDT12, OR14J1, OR2L8, OR5B12, OR7G1, PLAU, PLCB3, PPA1, RASGRF2, SARDH, SH3BP2, SHMT1, ST3GAL6, SUCLG2, TAS2R49, TAS2R9, TNFRSF13C
Negative_9369	ADCY6, AKR1D1, EPHA8, OR2T4, PIGO, RHOD, SUCLG1, UGT2B28
Negative_9370	ID3, ME1, PGP, PVR, PXN, ACVR2B, ARD1A, BUB1B, CACNB3, DPM1, FGD3, FRAT2, GAD1, GALNTL1, GMDS, OR1D5, OR2T10, SENP2
Negative_9371	ID4, MVK, PVR, XDH, AOC2, ATF2, CBLC, CCNA1, CDC16, CDK7, CDS2, CLDN22, CLTC, DGKA, EGLN1, EPHA6, FGF13, GSTM5, GUCA1A, IDUA, IFNK, IL12B, IL18, INPP5A, KCNQ1, LCMT1, MYL2, NFE2L2, NFKBIA, OR10J1, OR13C3, OR13H1, OR52K1, OR52R1, PAX4, PIK3CA, PLA2G2E, PLXNB1, PRKX, RDH16, SPAM1, STAM, SUV39H1, TPMT, TRPC1, UROD, TNFRSF10A
Negative_9372	MVD, CHST13, NKD1, OR4P4, PNPO, PPP2R5C, TAS2R42, TFPI
Negative_9373	CP, CD70, CFL2, EPHB1, INHBC, JAK3, PDIA4, PIGU, PKMYT1, PLAT, SMPD2, STT3B, TRAF5, TSLP, TNFRSF12A, TNFRSF1B
Negative_9374	CBS, F7, NPL, AASDH, ARHGAP5, CXCR3, GSTO2, IFNGR1, ITGA5, JAG2, LRDD, MAGI1, MAN1A2, MAPT, MYH7B, NANP, NUDT8, OR1C1, OR2V2, OR51I1, OR6K2, PIAS3, PRKCH, RUNX1, SETD1A, SETMAR, SLC27A4, SMAD9, TPK1, XYLT1, PNLIPRP2, TNFRSF10B

Negative_9375	GK,ADH4,C4BPA,CACNG5,CPT1C,CRY1,DVL2,EHMT1,FABP3,GCDH,GMDS,GNPAT,GUCY1A2,HLA-F,IL3RA,LCP2,LDHC,NRXN3,OR10G8,OR10R2,OR2G2,OR52A1,P2RX3,PNPO,PRIM2,PRKCE,PRKCQ,PTEN,SEMA3D,SEMA4D,TCF7L2,UROD
Negative_9376	GP6,NF1,ACTC1,ALDOB,AXIN1,BIRC5,CLTCL1,CPT2,CSNK1G1,CYP19A1,DAPK3,DERA,DLL3,EPHB2,GALK2,GALNT13,HYAL1,NT5C,OR10R2,OSMR,RAC1,SETD7,TGFB2,WASF2,WHSC1L1,SERPINE1,ST6GALNAC1
Negative_9377	DCT,ILK,ACSL3,ADCY5,ADRB1,ARHGEF7,ATP5H,BACE1,CCL20,CDK5,CDKN1A,DAPK3,DDX56,EPHA8,EPRS,GDF7,GNB3,GNG13,ITGB6,MAGI3,MAP3K12,MDM2,OR11A1,OR2M2,OR4F6,OR52E6,PIGT,PITX2,PRSS3,SEMA4C,SETD7,UGCGL1
Negative_9378	ALDH1A2,ALOX15,CHDH,CXCL2,ENTPD6,GALNT1,GALT,PDE8B,RPS6KA5,TAS2R1
Negative_9379	BAX,TK2,ADAM10,CDC7,CXCL9,GLI3,GSTA5,IGF1,IL13RA1,NLGN2,NMNAT3,OR2T4,OR5V1,OR6N1,PFKFB1,PIK3R5,PPARA,PTGS1,RAPGEF2,SEMA4B,SLIT2,STX8,TGFBR1,TUBA3E,UGP2,UGT1A10,XYLT2,KIR2DL5A
Negative_9380	SRM,BMP8A,CHEK1,CHIT1,CLCA4,COASY,CREBBP,CYP11A1,DPYD,EXTL3,FPGS,HRH2,ITGAX,LTBP1,NCKAP1L,OR51S1,PTDSS2,SREBF1,STX16,TJP2,TRADD,UGCGL2,ST6GALNAC5
Negative_9381	TKT,UBB,VCL,ALDH1A1,ATP4A,B3GALT5,DLL1,FMO3,HHEX,HSP90B1,HTR2C,KIR2DS4,MARS2,NCR1,NRG1,OR6C6,PGS1,PLXNB2,PPM1B,RIPK1,TP73,WEE1
Negative_9382	ABL2,ALPP,ATP5G2,BCAR1,CADM3,CD48,CNTN2,CSF2RA,CXCL2,CXCL9,DAAM1,FCER1G,GGPS1,GPD2,IFNA7,IFNAR2,INMT,IRAK2,IRF9,JAK3,LAMB2,LAMC1,MYL2,NME4,OR10H3,OR10Q1,OR14J1,OR3A1,OR52B6,OR52E8,ORC6L,POLD1,POLD4,PRKCB,RELB,RPS6KA6,SETD1A,SFRP4,SHMT2,SMPD4,STX3,TPH2,CACNA2D2,SUV420H1
Negative_9383	CP,FOS,JUN,MSN,AARS,ABL1,ABLIM1,ACY3,BBC3,CAV1,CCL23,CYP1A1,DBF4,DCXR,DDX18,DHRS3,EFNA4,ENTPD8,FGFR3,IARS,IL11,IL20RA,ITPA,KITLG,KRAS,MAN1B1,MGAT4B,MIOX,NCF2,NMNAT3,NOS3,OR51A4,OR52N2,OR5K2,PARD6B,PAX6,PHGDH,PIAS3,PIPOX,PTPRF,SHC3,SOCS2,TCF7,ULK2,VDAC2,VDAC3,WASL,ATP6V0E1
Negative_9384	A2M,MPL,ACP2,ALDH9A1,BCKDHA,BMPR1B,C7orf16,CACNG1,CCL24,CCR5,CDKN2D,CFTR,CISH,COQ5,CXCR4,DGKB,EDN1,ENTPD6,GGPS1,GNAS,GNPAT,GSTK1,HCLS1,IRAK2,LFNG,MS4A2,NEFM,NKX6-1,OPLAH,OR4D2,OR51Q1,PLCB2,PLOD2,RRM2,SPTLC2,SUCLG1,TRPC1,WNT10B,WNT2B,YES1,ZAP70
Negative_9385	FER,ILK,ACOX3,ACTA2,ALOX15B,AMPD3,BCAT2,CBR1,CCL19,CCR6,CD27,CD8B,CER1,CYP3A7,DARS,GCNT2,GNB1,GRLF1,HLCS,HRH2,KCNQ1,MAFA,NCSTN,OR2L3,OR4X1,PFN2,POLR2C,PON3,PRLR,STAM,WNT3,CSGALNACT1
Negative_9386	C9,CD2,EZR,IDS,NPL,PXN,UBC,APOC3,ARHGEF7,BCL2L1,CCL23,CCR5,CLDN5,CTBS,CXCL6,DPM2,DUSP9,ENTPD5,FGFR2,FMO4,FZD6,HTR2B,IDI2,IL22,MAN2A1,MAP4K2,MCM5,MFNG,MS4A2,MYL9,OR13A1,OR1E1,OR2AE1,OR4K5,OR9K2,PAICS,PHKG1,POLA1,POLD1,PRODH,TGFB1,TUBA1A,WNT7B,MAPK8IP3,SUV420H2
Negative_9387	GP6,LSS,NPY,ACTC1,APOA5,AVPR1A,B3GNT2,BAIAP2,BUB1,CADM3,CASP10,CASP9,CDC27,DVL3,EPB41L2,EPHA3,FTCD,FZD10,GLI1,GRIN1,GRIN2B,GUCY2F,HSD3B2,IL1R2,IL28A,IMPA2,ITGAE,LAMC3,MBL2,MCM4,NAGA,NUDT2,OR4D11,OR5K1,PDHA1,POLA1,PRKCA,PTGFR,SLC9A1,SUV39H2,YARS,ATP6V0A4

Negative_9388	A2M, MVK, SPN, TH, AKT2, ALDOC, CACNA1F, CAV1, CHST13, DDX18, DOLPP1, DUSP1, DUSP4, DVL3, EHMT1, EIF4E2, FECH, FGD1, G6PC2, HMGCS1, IL1RAP, ITGA2, ITGAV, KIR2DS4, NADK, NCF4, ODC1, OR10AD1, OR2T3, OR51A7, OR5T2, OR8U1, PIGO, PLCE1, SOCS1, SPRY2, B3GALNT1, GABARAPL2
Negative_9389	NNT, TNC, CHSY1, FBP2, FBXW11, GATM, OLR1, P2RX2, PDE5A, PIGQ, PIK3C2G, PTCH1, RASGRP3, SMC1B, TRAF6
Negative_9390	FGB, RB1, ACOT11, BIRC5, CYSLTR1, HMOX2, HSD11B1, LAMB1, OR1J4, OR4F5, OR6C3, SPTLC1, UCKL1, SERPINA5
Negative_9391	BAD, C4B, F2R, HPD, MTR, ACACB, ACMSD, ACSS1, ATP5I, BCMO1, CAV2, CCL20, CDC25B, CNTFR, CTLA4, DAPK3, DDX18, DLL1, DRD1, DRD5, FTCD, ICOSLG, IL19, JUND, KIR2DL3, MAP3K14, MCM6, MDH1, NMNAT3, NOX1, OR2AG2, OR4B1, OR4L1, OR4N4, OR5C1, OR7C2, POLR2J, POLR3K, PPBP, PPP1CA, PTPRJ, RXRB, STK4, TAS2R13, TRMT11, ATP6V0A1, TNFRSF6B
Negative_9392	AMH, C4B, SFN, ACSM4, AKT3, BBOX1, CD80, CD82, CD8A, CDC27, COL6A2, DAPK2, DDB2, DDX47, DUSP2, FOXO3, GAS1, GBA3, GNG12, GUCY2C, HTR2C, LIPF, MASP2, MMP2, NCK1, NCOR2, NT5C, OR1B1, OR4K14, OR5B17, OR6A2, OR6Q1, RASA2, SHISA5, ST3GAL3, MAPKAPK3
Negative_9393	TH, ATP6V1A, BCKDHB, C1GALT1, CDIPT, CNTF, DHODH, OR10A5, OR3A3, OR51G1, PAK7, PLOD1, SLC2A1, SUCLG1, SULT1E1, TUBA8, B4GALNT1
Negative_9394	ZYX, ACAA1, ACAT1, ACP5, CACNA1G, CACNA1H, COL5A3, CRKL, CSNK1G2, CSNK1G3, DGAT2L4, DRD5, FGF19, FGF4, GNAI1, GNAT3, H6PD, INHBE, OCRL, OR11G2, OR4Q3, PDCD1, PDE3B, PIAS3, PIK3R2, PRKCE, PRODH, PRPH, PTPN1, SEMA4G, SLC25A4, TBL1X, CSNK1A1L, HLA-DQA2, PAFAH1B1
Negative_9395	DAO, ASIP, ATP5J2, CACNG8, CNDP1, CYP19A1, CYSLTR1, EREG, ETNK2, FGF10, GAB1, GNAI3, GPAA1, IL20RB, LAMA3, MAP3K6, MAPK12, MPST, NTNG1, OR10S1, OR2AT4, OR2C3, OR2K2, OR4D6, OR52H1, PIGL, PIK3C2G, PRKX, PSEN1, QARS, RBL1, SC4MOL, SEMA4G, SRD5A2, TACR3, TBXAS1, UCP1, WIF1
Negative_9396	MPZ, ARPC4, ATP4B, CCR5, DHRS4L2, FBP1, ROCK1, EIF4EBP1
Negative_9397	FAH, GLA, A4GALT, ACSM1, ACTN4, ALDH1L1, AMY2A, CACNB3, CBR1, CCL1, CDC23, CER1, COL4A1, CXCR3, DLL3, DPYSL2, ENTPD6, G6PC, GALNT6, GSTM5, HSPA2, IFNA21, IL9R, IRAK2, LDHB, NR5A2, OR1K1, OR51I2, PIM2, POLR1C, PPP1R3B, PPP2CB, RETSAT, SELL, SMARCA5, TAS2R14, TPK1, TYRP1, ATP6V0A1, CSNK1A1L, HLA-DRB4
Negative_9398	ABO, ADO, F11, ADRA1D, ARG1, GLI3, HBEGF, HLA-DMA, HSD17B1, IFNG, LALBA, RASA1, SMAD3, UGT2A3, CACNA2D2, GABARAPL1
Negative_9399	MUT, ACSL5, BIRC5, DDX54, HAAO, OR3A1, PIK3CD, PLTP, RXRB
Negative_9400	LHB, MIF, RFK, ALDH3A1, BAI1, BCL10, BIRC5, CCL21, CDC42, CDH5, CLDN16, CSNK2A1, CXCL1, CYP11B2, DDB2, GMPPA, GNAO1, GSTM2, HAP1, HEXB, IL28A, INPP5A, INPP5B, ITPKB, KAT2B, MAD1L1, NRCAM, OR5I1, OR7C2, PDE5A, PFKFB1, PGLS, PIGQ, PIK3CB, POLD1, PPP1R1A, PPP2R5C, PRKX, PTCRA, PTEN, PYCRL, RASSF1, RND1, RPS6KA2, SFRP2, STAT6, WBSCR22, XCL1, TNFRSF14
Negative_9401	ADA, CAT, HYI, IL7, NF1, PDC, ABLIM1, ATP5I, BCKDHA, BDH2, CDH1, CHUK, CYP19A1, CYP3A43, DFFA, FDPS, GALNS, HNMT, IMPA1, INPPL1, IRS1, KLRK1, LIPE, MGAT3, NCOA4, OR1B1, PDE6D, PIP4K2B, PPP2R2B, PVRL2, RARS, RBKS, SDC4, SESN3, SNAP23, XYLT2
Negative_9402	ACAA1, ADRB3, ALDOA, ARNTL, CD226, CDO1, CHUK, CKMT2, COL1A1, CYP2C18, CYP4F2, DTX3, EFNA1, ENAH, ENO1, ERBB4, GALNT3, HSD17B2, IL20RB, IRAK2, ITGB6, MDH2, NMNAT1, NOTCH1, NPR1, OR10A4, OR2C1, OR2L8, OR4F4, PIGO, PRKAR2A, PRPS1, PTPN6, SNAP23, SV2B, TAS2R9, TFDP1, YWHAQ, HLA-DRB5

Negative_9403	DHH, GP6, ABLIM2, ATP5G2, AVPR1A, CD14, CD8A, CFL1, CHRM1, EFNA3, FTMT, HBEGF, IDH1, IFNA6, ITGAE, MCM6, NCAM2, NFATC1, OR10Z1, OR1J1, OR4D6, PGLS, PLCG1, RPIA, SDC2, SPTAN1, TLR5, TSNARE1, VDAC1, ATP6V1B2, MAP3K7IP1
Negative_9404	CCNE1, HIBADH, NR1H3, PIAS4, PPA1
Negative_9405	AK2, CS, DCK, PLN, VCL, ADIPOR1, ARSE, B4GALT5, BACE2, CACNG2, CLDN5, CLDN9, CREB3L4, DDX56, DVL2, EFNA3, F13B, GALC, GALNT12, GRB2, HPSE, HSD17B8, IFNAR2, IRF9, ITGAV, ITPA, KIR2DS4, KITLG, LDHAL6A, MAPK11, MAPK9, NFATC1, NFYC, NT5C, OCRL, OGDHL, OR52A1, PDE2A, PGM1, PGS1, PHKG2, PIAS3, PLCG2, POLR2K, PRKACA, PTPRZ1, ROCK1, STX1A, TBL1X
Negative_9406	NNT, TH, AARS2, ACTA2, ASMT, CCL1, CCNB3, CD82, CLDN3, CYP17A1, DDX41, GNB3, IARS, IL17A, LYPLA2, MAPK1, MGLL, NME4, NMNAT3, NUDT9, OR1B1, OR4K5, OR5C1, PIK3R1, PYCR1, RAPGEF4, RASSF1, SEMA4A, SETDB1, STAT3
Negative_9407	C3, CDA, EDA, LPL, MUT, ACO1, ACYP1, ADRB2, APOC3, BLNK, BST1, CACNA1D, CD44, CDH2, CNTNAP1, COL5A3, FOXO1, FZD3, GABARAP, GLS2, GOT1, GP1BB, HBEGF, HEXB, ICAM3, IL6ST, MCM5, MYLK3, NCOR2, NCR2, OR4S1, OR6C75, PAX6, PDGFRA, PGM1, PIGW, PIK3C3, PNPO, POLD1, PTGER1, PTTG1, SREBF1, STAM, STX3, XCL2, ALDH18A1, GABARAPL1, TNFRSF10C
Negative_9408	ADRB2, AGMAT, BST1, CDC7, COMT, DUSP16, EGLN3, F13B, F2RL1, FGF9, LIAS, NAE1, OR52H1, P2RX3, PIGN, SETMAR, TACR2, TBK1, WARS2, WNT10A, MAPKAPK5
Negative_9409	CFD, ABLIM1, ADCY1, ADCY9, AGXT2, ALDH3A2, AMPD2, ARHGEF1, BPNT1, CLTCL1, FSHB, GALK2, GUCY2F, GYS1, HRH1, IL17RB, LAMC1, LIMK2, MYL7, NFYA, NTRK1, OR11G2, OR52M1, OR5B21, PFKFB3, PPP2R2B, PRKACA, PTCRA, PYGB, SLC25A5, SPI1, TAS2R40, TAS2R9, TNXB, TRPC1, TYROBP, ULBP2, WNT10B, ST6GALNAC6
Negative_9410	MPZ, OTC, ADCY9, ARHGEF1, ARHGEF7, B3GALT2, CBLB, CD244, COL2A1, DHRS9, E2F1, EREG, FOXA3, GALNT3, GDF5, GNA15, GNRHR, IL10, MGAM, MTHFD1L, MYLK2, OR10H2, OR13F1, OR13J1, OR1N2, OR2F2, OR4F17, OR5B3, OR5I1, PHKG1, PIGF, QPRT, RCHY1, SEMA3G, SUFU, SULT2B1, SV2C, TAS2R49, TJP2, TNFSF4, WASF3, WNT4, ATP6V0D1, B4GALNT1
Negative_9411	ABL1, AGPAT1, C4BPA, EPHX2, GABARAP, ITGA4, MCM3, OR4D2, TP53
Negative_9412	CEL, GLA, LPL, CLTCL1, CMBL, DIAPH2, DLL3, GFAP, GLUL, IL20RB, TNFSF8
Negative_9413	CSK, ALDH5A1, CAV2, CD70, CXCR4, HSD17B1, ITGA3, LIPE, NAT1, NEFL, PIM2, SHMT1, TAS2R16, TJP2
Negative_9414	CBL, CTH, FES, GLA, TFG, ACOX3, ADCY5, ADSSL1, AGPAT3, AKR1C1, ALPP, BBOX1, CD58, CXCL10, DTX2, FGFR3, GCLC, GNAL, GZMA, HARS, IFNA16, ITGB5, KARS, KITLG, L1CAM, MAFA, MAP3K3, MAP3K6, MDH1, MGAM, MOV10L1, MTMR2, OR14A16, OR7G1, OR9G4, PECAM1, PIK3C3, PIK3R4, PMM2, PON2, PTCH1, PTDSS1, RPS6KA2, SLC27A5, ST3GAL2, THBD, ZNRD1, HSD17B12
Negative_9415	ACO1, CD55, CHKA, CKS1B, COL1A1, CREB3L3, CXCL2, EARS2, ECSIT, ENPP6, FLAD1, FOXA3, FTCD, GCNT2, GNPDA1, HPRT1, HSPA1B, HTR6, INMT, MYL7, NMNAT1, NR5A2, OR11G2, PLA2G1B, PLCD1, PLOD1, RAPGEF1, SOCS3, TAS2R3, TSTA3, UNC5B, WBSCR22, XCL1, ATP6V0A2
Negative_9416	F3, NPY, AFMID, CPT1A, GRIA1, HMBS, HSPA1B, NPR2, OR11H4, OR5M9, PPP3CA, RGS3, THBS3, TSNARE1
Negative_9417	CS, FYN, GDA, GK2, SFN, ACAT1, ACSM5, ANAPC11, ASCC3, B3GNT3, BET1L, CCND2, CDK5, CTPS, DHRS9, EFNB2, FGF20, GRLF1, GSTA1, GUCY2C, HPSE, HTR4, IFNA16, IFNGR2, IKBKG, IL22RA1, ITGAL, ITGAX, MAP2K5, NLGN3, OR10A4, OR13J1, PAK7, PDCD1, PHKB, PIK3CD, PIP5K1B, POLR2E, PRKCZ, PRKG2, PTPRB, PVRL4, RARS, RHEB, SMARCA2, TBL1X, UBE2L6

Negative_9418	ABO,FUK,NOG,AARS,AKT2,ALDH9A1,B3GAT3,CACNB2,CDC27,COL4A6,CSNK2A2,CTPS,CXXC4,CYP3A5,FGD1,GALNT12,GALNT2,GLO1,GNPDA1,IL17B,ITPA,JAM2,MFNG,MTMR6,NCKAP1,NCOR1,NEFL,OR10G4,OR10K1,OR2L3,OR2T10,OR2T12,OR3A2,OR51B5,OR51F1,OR52I2,OR5K2,OR7G3,PDGFRA,PIK3C3,PPP1R3C,PRKAR1A,PTGS1,SEMA3C,SIGLEC1,WARS2,TNFRSF14
Negative_9419	IL7,ACTN4,ADIPOR2,CD38,CDKN1A,CDKN2C,DCTD,GNPNAT1,MGAT4A,MOV10L1,NRXN2,NT5C2,OR1J4,PHKA1,PIK3C2A,PISD,PPAP2A,PPP2R5B,SLC25A5,TGFB1,TNFSF9,SUV420H1
Negative_9420	LAT,AOX1,CBR3,CCL26,DAD1,ENTPD8,EPRS,GADD45B,IDI2,IFIH1,IL12B,MAD1L1,NRCAM,OR4S1,OR5V1,OR6C1,PNMT,POLR1A,PPA2,PRKX,SGPP1,UGT2B7,B4GALNT1
Negative_9421	AFMID,AGPAT6,ALPPL2,ARAF,CACNG5,CARS,CXCL11,DGAT2L4,ENTPD4,GATM,GUCY1B3,OR2M5,OR51Q1,POLR1B,RAB5A,STAM2,STAT6
Negative_9422	GK,ANAPC7,DOCK1,MAPK1,RAP1B,RDH16
Negative_9423	AGK,CTH,GPT,HK2,B4GALT6,CAMK2A,DLL3,ENPP1,ENPP3,FECH,FGF2,FGFR1,FLOT1,GLDC,GSTM4,HLA-C,MAP2K2,MARS2,MGAT5,MLYCD,OR10A7,OR1Q1,POLR2B,RHEB,SETD1A,SPRED1,TAS1R2,TAS1R3,TNFSF14
Negative_9424	CD6,GK,NPY,ADH4,AOC3,CD55,CDKN1A,GPLD1,OR13C2,OR7C2,PIPOX,RAF1,SMPD3,WBSCR22,XYLB
Negative_9425	CGA,IHH,PRL,ABAT,ACHE,ADCY5,ALDOC,ASAH1,ASNS,CARS2,CCR3,CDC7,CLDN22,CTNND1,CYP26B1,EPRS,FECH,FGFR4,FIGF,GALNTL4,GFAP,HADHA,ICAM1,IFNA6,ITGA8,MAP4K2,ORC2L,PRKG2,SOS2,TOMM40,MAP3K7IP2
Negative_9426	CKM,ADIPOQ,B3GNT5,CDH2,DGUOK,FUT3,GAS1,HAO1,ICOSLG,IMPA2,NKD1,PAK7,PDIA4,STX10,TPH2,TRAF2,YARS2
Negative_9427	GAA,LPL,ACVR1,AKT1,ANAPC7,B4GALT5,C7orf16,CACNG7,CCNH,CD44,DHX58,GALNT5,GNRH1,GSTO2,GUCY2F,HLA-G,HTR6,KARS,LDHAL6B,MMP7,NFYC,NTN1,OR1C1,OR1G1,OR4C6,OR4F15,OR6F1,POLR1B,POLR2E,PRKCA,PRKX,PTPN1,PTPRM,SORBS1,STEAP3,TALDO1,TP73,TRAF6,WNT6,ATP6V0A1
Negative_9428	SP1,BIRC5,CD72,CHSY3,CNTF,CYP21A2,DBF4,DDOST,ENPP7,HARS,IFT57,INADL,KNG1,ORC1L,PORCN,SMAD2,SRGAP1,STX7
Negative_9429	GP9,PC,ATP5J2,B3GALT5,COX10,CRLF2,CYP26B1,DDX41,FARS2,FGD3,GFPT1,GRPR,IL10RA,LHCGR,OR10G4,OR4F5,PCYT2,PPP2R2A,SMPD2,STAT4,TICAM1,TNFRSF8,ZFYVE16
Negative_9430	CGA,FOS,GPT,AFMID,AKT2,B3GNT3,CCR2,CKMT2,COL4A6,FGF19,GSTK1,GUCY2C,HNF1A,HRH2,HSPA1B,HSPA2,IFNGR2,IGF1,KYNU,LMO7,NAMPT,OLR1,OR1D5,OR1K1,OR2T33,RHEB,SEMA3D
Negative_9431	C8G,MET,ADCY2,ASMT,ATP12A,BDNF,CDC25C,CIITA,CLTCL1,DNMT3A,DPAGT1,FARSA,FGF10,FGFR4,FOSL1,GGT1,GNG3,GNMT,HIBCH,IL1RAP,INSRR,KIR2DS5,LCMT1,LEF1,NEUROD1,NFASC,OR2C1,OR4L1,OR51B5,PKMYT1,POLD1,PYCRL,RAF1,SMAD6,SOCS5,STX3,TGFA,TIAM2,TUBB3,TYMP,ATP6V0A2,MAP3K7IP2
Negative_9432	AUH,GBA,HDC,TKT,ACACB,ACSM5,ALPPL2,ASRGL1,CDH1,CFLAR,CXCL1,DUSP9,HARS,HSD17B3,MGAT5B,OR2T2,OR8J3,PARD6B,PON3,TACR2,TLN1,TNFRSF4,UGT2A1
Negative_9433	NPY,BUB1B,CCR8,CPOX,FGF23,FPGS,GAL3ST1,GANAB,GRIA3,ITGAX,LILRB3,MYH2,NEU1,NTF3,OR10G7,OR6F1,PLCG1,PMM1,PSME2,RFXANK,SHMT1,SRGAP1

Negative_9434	ID3,MOS,ACP6,ARSE,B3GAT3,CCNE2,CD86,CHRD,CYP2A13,DDX52,DDX56,DUSP14,DUSP6,DVL3,FGF4,GUCY2C,HMGCS2,KDSR,KLKB1,LAMC1,MAPK9,MMP2,NANS,OR4A5,OR4K5,OR52E8,OR5L1,OR9Q1,PGS1,PLCB3,PPP2R5A,PPT1,PTPRC,SOD1,STX6,TJP3,TLN2,ZFYVE9,SERPIND1,SLC25A31
Negative_9435	CD244,CXCL12,E2F2,ISYNA1,NRXN1,OR5AP2,PEMT,TRAF2
Negative_9436	C8B,HYI,ALG2,DNMT1,EFNB1,EGFR,GFPT2,IFNA13,IL28A,MTFMT,MYL5,OCLN,OR3A2,OR6N1,OXSM,PANK2,PPP2R5C,PRKCZ,RAP1A,RND1,ROBO3,SHC2,SLC27A1,TICAM2,UXS1
Negative_9437	C5,HK2,MPI,PC,ABI2,ACOX3,ACTN1,AGRP,B3GNT3,BUB3,CACNG7,CAPN2,CHIT1,COL4A6,CREB3L1,CYP2F1,DDX19A,GALNTL2,GDF7,GLI1,HYAL1,ITGAM,LDHD,LGMN,NANP,NCK1,NFKB1,NR4A1,OR2D2,OR5K2,PDE10A,POLR2E,PTTG1,SLC9A1,SMAD5,STAT1,YKT6
Negative_9438	DLD,HDC,PGP,SI,ALOX12,ATP6V1A,CDIPT,CMPK1,DCTD,DDX23,FGF13,HDAC1,IL1R1,IL2RA,LFNG,LRAT,LTC4S,MGAM,MINPP1,NCK1,NRXN3,OR6B2,P2RX1,PORCN,PSME1,SOCS2,SPP1,TSTA3,VTI1A
Negative_9439	AK5,CAD,AK3L1,BMPR2,CACNG2,CCKBR,CCL1,CDH15,CDK6,CHPT1,CRKL,CYP2C18,E2F5,ENTPD2,FBP2,GANC,HPSE,IFNA17,ITGB6,JAM3,KLRC1,LDHD,MAP3K2,MLLT4,NGEF,NR1D1,OLAH,OR13C2,OR51E1,OR5B21,OR6C76,PGK1,PLA2G2A,PPP2R2B,PRPS1,RALBP1,RHOD,RRM1,SCIN,SESN1,SRGAP2,SUV39H2,TAS1R1,TAS2R38,TUBB2A,TUBB4
Negative_9440	ARAF,B3GALT2,CDH3,CHKB,CHRM5,CIITA,CLDN1,COL4A1,CYFIP1,GNAO1,HDAC2,HSPA8,ICAM3,IL18R1,ITGA4,ITGAD,MYH2,OR7A10,PPP3CA,PTEN,PVRL1,RELB,TAS2R16,MAPKAPK3
Negative_9441	AKR1D1,GALNT14,MAP2K4,PDE4D,SEMA4B,TYRP1
Negative_9442	CAD,ARG1,B4GALT5,CCR4,DCTD,ENTPD4,EPOR,FZD8,G6PC,GNAO1,INPP5E,NAGLU,NCKAP1,NTF4,OCRL,OR10G9,RASGRF1,SIAH1,TSC1,UNC5A
Negative_9443	TNR,AMY2B,DFFB,DNMT3L,DPYSL2,EHMT1,EPB41,MYH2,OXCT2,PAPSS2,PLXNB2,SGPP1,SLC25A4,WNT2
Negative_9444	ACPT,CSNK2A1,OR51E1,POLR1E,RAB5A,TNFSF9
Negative_9445	TNN,AANAT,ACSS2,ADRB1,CADM3,CCL1,CCL8,CCR1,CYP11A1,DLST,FZD8,GGT5,GRM1,IDH3B,ITGB6,ITPA,KNG1,NOS3,OR11H1,OR2G3,OR51T1,OR5B12,OR5P3,ORC6L,PCNA,PDE1C,PHGDH,PIAS4,PIP5K1A,PRKAR2A,PTGIS,RAB23,RAF1,SCLY,ST3GAL6,STX12,TAS2R42,HLA-DPB1
Negative_9446	IL6,ACTG1,ASS1,C1QA,CACNA1D,CAMKK1,CHST12,COL11A1,CSNK2A2,DDOST,FUT1,GADD45G,LGMN,MAN2A1,MTHFD2,NEUROD1,OR1S2,OR5A1,PDE1C,PIGQ,PTPRC,WHSC1,PAFAH1B1
Negative_9447	C5,ADIPOQ,ASIP,BMP4,CDC25C,CDK2,CLDN15,DDX23,IL8RA,NCOR1,OR1J1,OR2A14,OR2T10,PAICS,SULT1A2
Negative_9448	AKR1C3,BLVRB,CCR4,CHDH,DIAPH1,FZD9,HRH1,IL17RB,LHCGR,OR10A5,OR4Q3,PIGF,PPM1B,PYCRL,SMAD4,SOAT2,TLN2,CACNA2D4
Negative_9449	AGL,C1S,PTS,UBB,ADRBK2,ALG2,CCL27,CCNG2,CD59,CNTNAP2,CYP27A1,DDX54,DHRS9,EPB41L1,FGF10,FZD10,G6PC2,GNA11,GSTM2,GUCA1B,HSD17B7,IARS2,INPP1,JAM2,LAMA3,LDHC,MAP2K3,MMP7,MYH10,NFATC4,OR4C11,OR51I2,OR5P2,PARVG,PLXNA3,PPP1R3D,PRKCI,PTPN1,RBL1,TJP2,ZIC2
Negative_9450	CP,GSR,SFN,ACSM3,ACTN2,ACVR1C,B3GNT5,B4GALT7,CSF2,F11R,GOT2,HYAL2,IL1A,LIPE,MAGI1,MCCC2,NSD1,OR11H6,OR4K15,OR5T2,PKM2,PRKAR1B,RALB,SMURF2,ST3GAL2,TAS1R1,WNT6,XYLT1,YWHAE
Negative_9451	ATM,IL8,TNN,ACSL6,ACTN4,ACY3,ASAH1,ATP5G3,B4GALT7,BET1L,COQ5,CSDA,FLOT1,FZD8,GABARAP,GNAQ,GNRHR,GSTT1,HEXA,ICAM3,IGF1R,IL25,ITGB4,LLGL2,MGAT4A,MYL7,NLGN1,ONECUT1,OR1L8,OR4X2,OR52E8,OR8I2,ORC1L,PIGP,ROBO2,SCD5,SMARCA5,SYNJ2,TBL1Y,TNFSF8,WARS,YWHAZ,SLC25A31

Negative_9452	GP6,GPT,BCL2,CHIT1,CTSB,GDF5,GUCY2F,HLA-DOA,ITGA6,KLRC2,LIPT1,LYPLA1,MAFA,OR2T29,PHKG2,TLR4,VTI1A,XYLB,TNFRSF10A
Negative_9453	ATP5O,CD70,CTPS2,CYP11B1,LIPT1,LRP6,OR1C1,OR8B12,SQLE,TUBB6,HLA-DRB4
Negative_9454	ADCY4,CAMK2D,CNTFR,DGKH,FCGR3A,FGD3,GNA14,HHEX,OR10A7,OR52N5,ORC6L,PHKA2,PIAS4,SLC27A1,VAMP3,YWHAB,YWHAE,HLA-DQA2
Negative_9455	ADIPOQ,ATP6V1F,CASP7,CBR1,CRB3,GNRHR,NTNG1,ORC1L,PCYT1B,PPP2R5C,PRKAA2,UGT1A10,MAPK8IP3
Negative_9456	ABLIM3,ARG1,CBLC,FLAD1,GRIN2A,GSTM1,ITGA9,ITPA,POLR3K,RDH5,SDHC,TSC1,UNC5A
Negative_9457	ABL1,ACAT2,ACSL1,ACTB,AKT2,ASMT,CACNA1G,CCL20,COL5A1,CPT2,CX3CR1,CXCL11,ENTPD7,FCGR2B,IBSP,IFNA7,IL10RB,IPMK,ITGB8,MPP5,NARS2,NUDT2,OLR1,OR10H2,OR4F17,OR4K17,OR51B6,PARVB,PON2,PPP2CB,RAPGEF4,RBPJ,RPN1,TLR4,WEE1,ATP6V1B2
Negative_9458	IL7,LYN,ADRA1D,AHCYL2,AKT3,ATP2A3,ATP5L,BIRC3,BPNT1,CAMK2A,CFLAR,DCTD,DKK2,EFNA3,FTCD,GCNT3,GMPPA,GNAT3,HSPA1B,KARS,MAN1A1,MAP3K3,MAP4K3,MMP2,OR4D5,OR4P4,OR5B12,POLR3K,PPARG,PPP3CA,PRKX,RPRM,SMURF2,SNAI1,SOAT1,THBD,YWHAG,HLA-DRB3,TNFRSF1A
Negative_9459	ABO,ADIPOR1,ARPC3,CD55,DAD1,FGF13,GSTA4,HHIP,IFNK,ITGAX,OGDHL,OR14A16,OR52D1,PHKG2,PIK3R5,PRKACA,PTGIS,SGPP1
Negative_9460	NOG,ALDH6A1,B3GALT4,BBOX1,CCL2,CDK2,CHKA,CNTN1,CRY1,ITGB5,OR13C3,PIP5K1B,PNPLA4
Negative_9461	COL6A2,CPT2,GSTM3,GSTO2,IDH3A,LIPF,MAP2K4,NMNAT2,OR7C2,PCK1,PGAM2,PIP5K1C,PRKAG1,RAP1B,UGT8,XCL1
Negative_9462	BIRC5,CTBP2,DAXX,GGPS1,GSTA4,IL20RA,IL2RG,MGST2,PPP2R2B,B4GALNT1
Negative_9463	ABCC8,APLP1,BACE2,DGUOK,DHRS4,GLDC,HPSE2,IMPDH2,MAP4K2,NT5C1B
Negative_9464	MPI,TK1,CACNA1A,CCL7,CDC14B,CTNNA2,CYP7A1,EDN1,EDNRB,GGPS1,GNMT,GRIN2A,HHEX,HIF1A,IAPP,IMPA2,KCNQ1,LRRC4C,MAPK12,NOS3,OGDHL,OR2T12,OR52N4,OR5M3,OXTR,PPP1R1A,PPP5C,PYGL,RXRG,SMURF1,XYLT1
Negative_9465	ATP6V1H,CLDN10,FZR1,GUK1,HLCS,IL28B,JUND,OR6F1,PIK3R4,SESN2
Negative_9466	AK5,KIT,RPE,ACP6,ACYP1,ADCY5,B3GALT1,B3GNT2,CACNG8,CD27,COL6A1,DAAM1,DGKD,FGF6,GCNT4,IFNA6,INADL,IRS2,ITCH,MAP3K1,NAGA,NRG1,OR14C36,OR2T12,OXCT2,PFAS,PHKB,POLR2B,PTGS1,TAS1R1,CSNK1A1L,SLC25A31,TNFRSF1A
Negative_9467	CKM,DBT,IVD,VCL,ABL1,ACAD9,ALDOC,ALG5,BCAT1,BPGM,CDH4,CDKN1B,CDS2,CHEK2,CYBB,CYP2C8,DHRS4L2,FGF23,GZMA,IFNG,IRAK3,KIR2DS5,LLGL2,MAOA,MMP7,MYH7,OR1C1,OR5B12,PANK4,PCCA,PDGFD,PLCE1,POLR2K,PPA1,PPP2CA,PVRL3,PVRL4,RBKS,RDH5,RND1,SLC12A2,TRDMT1,WASL,WHSC1L1,YWHAB,HSD17B12,MAPKAPK2,ST6GALNAC1,ST6GALNAC6
Negative_9468	LPO,ANAPC5,ATG3,ATP4B,ATP5G1,CDS1,COASY,CXCL12,FGF22,GDF5,IGF1R,KAT2B,LDHA,NME2,NOX1,OR4D5,OR56A4,OR8I2,PDGFRB,PNPO,POLR2L,RRM1,SETD1B,SULT2B1,TAP2,VANGL2,WNT3
Negative_9469	IDS,JUP,LHB,ACTG2,ADRA1D,BMPR2,CD36,CDC25A,COL4A2,CYFIP1,FDPS,HYAL1,IFNA14,IL17B,IMPDH1,NFKBIE,NFYA,OR10AG1,OR2D2,OR2D3,OR4K1,OR4M2,OR5A1,RBKS,SELP,WNT7B,PLA2G12A,ST6GALNAC1,SUV420H1

Negative_9470	GLS, JUN, RDX, VCL, ACO2, ACVR2A, ADAM17, ARPC4, CCL23, COQ3, CPT1B, CSF2RA, EFNB2, FECH, FGF20, GP1BB, HSD17B1, LEF1, MAP4K4, MOV10L1, OR2T34, PMVK, POLR2F, PPARA, RPIA, SMPD3, SPRY3, TNFSF18, TSLP, TUBB2B, UPRT, WNT11
Negative_9471	BMPR2, COX15, CPT1B, DGKA, GSTT1, OR4C46, PPP1R3B, TNFRSF1A
Negative_9472	AADAT, AK3L1, BMPR1B, CES7, COQ3, CRLF2, CYP3A5, DUSP9, GALE, GALNT10, GSTA1, IL18, NAT2, NME6, OR4K13, OR52A5, ORC2L, PARVB, PNPLA3, PRKAA1, RDH16, RUVBL1, TBL1X, TP53, WARS2, WNT5B
Negative_9473	DCT, PGD, PXN, ABI2, ACOT12, ADCY5, ADH1A, ADH6, ALDH1B1, ALG8, ARSD, ATP2A3, AXIN2, CACNB2, CCL11, CHRM2, CKMT2, DARS, DHDDS, HHIP, IDI1, IFNA21, IPPK, LAMB4, LTA4H, MALT1, MCM4, OR1L1, OR5H6, PDCD1, PDE9A, PFKL, PGK1, PIK3C2A, PITX2, PLA2G3, PLCB1, PLCZ1, PPP3R2, PRKAR2B, PSME2, PTGES2, SV2A, TGFB1, TRAF4, TSTA3, WNT9B
Negative_9474	C1S, CD2, FAS, HGD, MAG, ACMSD, CD247, CDKN2D, CKS1B, COL4A2, CSNK2A1, DAG1, DERA, DUSP7, FCGR2B, GALNT6, GANAB, GDF6, GYS1, HEXA, HTR2B, IL23A, IL6R, MAP3K1, MAP3K11, MAP3K8, MDH2, MPP5, OR10AD1, OR2AE1, OR2AT4, PAK1, PON1, PROC, PTPRJ, RASA1, RPS6KB2, SFRP1, SHC4, SLIT2, SNAI2, SOAT1, SV2A, TPH1, TRAF5, ULBP2, HSD17B12, KIR2DL5A
Negative_9475	CFH, ASAH1, ATP6AP1, BMP6, CAMKK2, CLDN10, CSF2, EPHX1, FGD1, FGD3, GALNT7, GNRHR, HMGCS1, IRAK4, MGAT4B, MTFMT, NADSYN1, OR10J1, OR1S2, OR4K15, PARVG, PDE9A, PDGFRB, PIGN, PLAU, POLD3, PRPH, PYCRL, RASGRP1, SEMA4F, SMPD3, SULT1A1, ZFYVE16, PAFAH1B1
Negative_9476	AGTR1, GALM, THBS4
Negative_9477	ADC, ALDH9A1, ALS2, AMPD3, CUL1, CXCR6, HCCS, MAPT, MTAP, NFE2L2, OR52K2, TOLLIP
Negative_9478	ATM, LAT, NPL, ATP6V1A, BPGM, CRHR1, EPHA3, ETNK2, FSHR, GUSB, IL12RB2, IL13RA1, PCCA, PDHB, PIK3R4, PLXNA2, PPT1, PRKG2, PTPRZ1, RPS6KA5, SDC1, SKIV2L2, SMPD2, STAT5B, TAS2R16, TLR2
Negative_9479	ATM, NNT, ABL1, ACP5, ALAS1, CCL21, CCND3, CREB3L3, CYP2C8, CYP4A11, DHFR, DLL3, EDAR, ENTPD6, GAB1, GBA3, HARS, IDI1, LYPLA2, MAN2A1, MAP3K8, MGAT5B, MKNK1, NMNAT1, NTN1, OGDHL, OR1A2, OR2T6, OR2Z1, OR52B4, OR52E6, OR52E8, OR52J3, PCK1, PFN2, PHKA2, PLCE1, PNPLA3, PRKAA2, PTPMT1, PTPN7, RELA, RRAS2, RXRA, TAS2R48, TDO2, VTI1B, WWP1, TNFRSF6B
Negative_9480	ACMSD, AMDHD2, DKK4, OR1J4, SEMA3D, SOAT1, YWHAG
Negative_9481	F2, CCR7, F13A1, ITGA11, MASP1, MS4A2, SLC25A5, WNT5A
Negative_9482	C3AR1, C7orf16, CCL25, CDC45L, CDKN1C, CYP3A7, DKK2, EVI1, FUT3, FZR1, GNPDA2, GSTP1, GUSB, KLRD1, OR2G3, PDIA4, PPA2, PTPRZ1, RPS6KA5, RPS6KB1, SMARCA5, SPHK1, SULT1E1, VEGFA, CACNA2D4
Negative_9483	ADC, CD47, CDC20, GMPR, GUCY2C, IL13RA2, LCMT2, MYH4, NCK2, OR51M1, PDE4B, RALA, RAP1B, SCLY, UROC1
Negative_9484	ATP13A2, JAG2, MMP9, NAMPT, NCKAP1L
Negative_9485	ACVR2A, ATP4B, COL4A2, CYP2A6, DPM2, DUSP6, DUSP7, EIF4E2, FMO2, IL12B, LILRB3, MALT1, NLGN1, NRG1, OR14J1, RARS, STAT5B, STEAP3, UPB1
Negative_9486	BAX, CS, FAH, ARPC3, B3GNT2, BMP4, CSNK2B, DVL2, EDNRB, EPHA8, ETNK1, GBE1, IL18R1, INPP1, ITGAL, MTHFD2, OR1D5, OR52N2, ORC2L, OSMR, PGK2, PYCR2, ST8SIA1, STX5, TOMM40L
Negative_9487	ACSL4, CAMK4, OR5AC2, OR5H6, PIGB, PPA2, SPRY1
Negative_9488	BCR, C8G, IL3, PC, ACVR2A, ADH6, AGPAT3, ARRB2, BRAF, CD247, CDC25A, DAPK1, DARS, DDHD1, DEGS2, DUSP10, GAS1, GGT1, IL18, KAT2A, KAT2B, KLK3, LAMB2, LDHAL6B, NT5C1B, OR2G6, OR4K2, OR5T2, OR8K3, P2RX1, P2RX3, PIK3R1, PLCD1, POLR2E, PRPH2, RCHY1, RDH8, RPIA, SFRP1, TAP2, UGT2B17, ST6GALNAC3

Negative_9489	CBS,CD2,GPI,ID3,IL6,NOG,PDC,AGMAT,AHCYL1,CAV2,DHRS4,DOCK1,EFNB2,EXT1,GABARAP,GNG12,IFT57,IL21R,MAN1C1,NME7,OGDHL,OR2T34,OR8H1,PGAM2,SETD7,SOAT1,TDO2
Negative_9490	C4B,ACVR2B,ADIPOR1,COL4A2,CYP2C9,DDX55,GGT5,IL29,OR51I2,PIP5K3,STK4,VAV3
Negative_9491	ACTB,ALDH3A2,DDX55,E2F3,EHMT2,FGF19,FSHB,MALT1,PERP,PRKY,SDC4,THTPA,TRAF5,TUBB,ZFYVE9
Negative_9492	BUB3,OR4C46,PTDSS2
Negative_9493	GSTT1,PTPRC,TAS2R7,TYROBP
Negative_9494	CKM,F9,HAL,IL6,TH,ABL2,ADRB1,AKR1C2,ASIP,ATP5J2,BNIP1,CHAT,DBF4,DDX54,DERA,DHRS3,FABP3,FASLG,FGF2,FGF20,FPGT,GCNT2,IGF1,IL1R2,ITGA11,KDSR,LAMB3,MAP4K1,MYLK3,OGDH,OR10T2,OR2A12,OR4K17,OR51F2,P2RX4,PFKM,SCP2,SHMT2,TIAM1,TRAF1,CSGALNACT1,TNFRSF10A
Negative_9495	ACOT12,ADCY5,ADSSL1,B3GAT1,CLDN2,EIF4E2,F13A1,FDPS,FRAT1,GNAZ,GSTA3,GSTM5,HNF1A,KIR2DS4,MGAT4B,NR5A2,OR1L6,OR2C3,OR51D1,OR52M1,OR52R1,OR9Q1,PANK3,PAPSS2,PARVA,PCCA,PER2,PFAS,PIK3C2G,PRKAB2,RARS,SDHB,SEMA4G,SETD7,SYNJ1,TAS2R48,TRAF5,TSC1,WNT10A,SERPIND1
Negative_9496	IDS,MPZ,VTN,ACADM,ACTC1,ADAM10,ALG10B,AMHR2,AOX1,B3GALT1,CCR6,CD80,ELK4,ENO2,GPAA1,HMGCR,HYAL2,IL12B,IL3RA,NUDT5,NUMBL,OR10AG1,OR2AT4,OR4B1,OR5R1,OR5T2,OR8S1,PDCD1,PDE8B,PIGS,RDH10
Negative_9497	AREG,BMPR1A,C5AR1,CASP6,CYP7A1,DPM1,IFNW1,NFYC,PRNP,STX3,THBS2
Negative_9498	FES,ACVR2A,ADIPOQ,ANPEP,ARSA,CYP2C8,FMO1,FOXA2,GNAI1,GRM5,HNF1B,HSD17B4,IFNA8,JAG2,MCEE,MYH11,OR1C1,OR8J1,PLXNC1,PRKY,PRSS1,RAB23,TAS2R42
Negative_9499	IL5,JUN,TNR,AGTR1,ATP5G2,B4GALT1,BIRC3,C3orf10,CCL25,CCR1,CMBL,CTPS,CXCR3,DAPK3,DTX3,EIF4E2,FGD1,FGF2,GBA3,GPD2,IL20RA,LIAS,MTFMT,NR5A2,OR1A1,PPT1,PRIM1,PRIM2,PSPH,PTCRA,PTGIS,PTPN1,SSH2,SUCLA2,TSLP,TUBB4Q,ULBP2,VANGL1,YWHAQ,ATP6V1E1
Negative_9500	CS,LCT,CREBBP,DHX58,EPB41,FUT3,KIR3DL2,OR12D3,OR13C2,PFKFB1,TNFSF14,VKORC1
Negative_9501	IL4,ACADS,ACP2,ADORA2A,AKT1,ALDOA,CAMK2D,CD19,CLDN8,CNGA3,COL4A1,COL4A6,COX10,DHFR,DLST,F11R,FIGF,GNA11,GNPAT,GRM5,HES1,IFNB1,IL19,IL1R2,MAP4K2,NLGN1,OR10P1,OR51A4,OR5L2,OR6S1,OR9Q2,OXTR,PAICS,PTCH2,SPAM1,SUCLA2,TUBB1,WIF1,MAPKAPK2,TNFRSF10D
Negative_9502	ILK,ATG12,AVPR1A,AXIN2,B3GALT6,CASP6,CD8A,CDKN2C,CHEK2,CHRM3,CRLF2,DHCR24,DLL1,EFNB3,GLO1,JAK1,MAPK14,NFATC4,OR51A2,OR5B21,PDPK1,PIK3CG,PLEKHA8,PPARD,PRKX,PRPH2,PSPH,PTPN7,ST3GAL6,TAS2R7,UGT8,YOD1,ZIC2,TNFRSF11A
Negative_9503	SCD,ACOX3,ACY1,ALDH2,ATP5F1,BLVRB,C3orf10,CADM3,CHPF,CLDN5,CLTCL1,CTNNA2,CXCR6,CYP11B2,DERL1,DHFR,DVL2,EGLN1,ENTPD3,ERO1L,GCNT4,HADHA,HNF1B,HSD17B1,HSD17B8,IRS1,ITGAV,KLRK1,MIOX,MPST,NAGS,NEUROG3,NFATC1,OR10A2,OR11A1,OR13C9,OR2T29,P2RX1,PCYT1B,PDE4C,PDHB,PLCB3,SOX17,TGFA,TRAF4,WNT10A,MAPK8IP3,SERPIND1
Negative_9504	F11,UBB,ADCY2,AHCYL1,CACNA1I,CADM1,CD59,CYP2A13,F11R,FABP3,GSTO1,HTR6,IL3RA,L1CAM,NEU1,NOX1,OR4B1,SLC27A1,SSH2,YOD1,SERPINC1
Negative_9505	ASL,CCR7,CD79A,CHD8,CLDN2,DDX41,GDF7,MAPT,RASSF1,SPCS1

Negative_9506	C8A, IL3, SP1, AMD1, AMDHD2, ATG7, CAMK2B, EGFR, HAO2, IFIH1, LEF1, LIPA, MAOB, MPDZ, NKD2, OR11A1, OR6C75, P4HA1, PTCH2, SFRP2, TAS1R2, TPM3, TRDMT1, WHSC1
Negative_9507	RB1, AADAC, ACADM, B3GNT3, CACNB2, CYFIP2, DUSP1, HLA-DMA, HSPB1, IMPA2, MGST2, MYH3, NEUROG3, NTF3, OR12D3, OR14C36, OR2AG1, OR5L1, OR6B3, PDE7B, PLCB1, PRKCI, RBL1, RPS6KA6, SULT2B1, HSP90AB1
Negative_9508	PRL, BDKRB2, CDC7, CRLS1, CTNNA2, CTNND1, DAD1, GADD45B, GAL3ST1, LIPT1, NADSYN1, OR10P1, OR5D14, SUCLA2, TBL1XR1
Negative_9509	CS, MOS, ADAM17, BCMO1, CD79A, CHPT1, DGKI, FOXO3, G6PD, GZMB, HCLS1, MAPK14, NAT1, OR10J5, OR4N2, OR5B21, OR5T2, OR7G3, PGM1, SHC4, SMPD4
Negative_9510	CEL, AMY2B, ANAPC2, CRKL, E2F5, EPB41L2, FZD3, GADD45A, GALM, GSTM5, HAAO, HSD3B1, IFNA21, LEPR, NAE1, PAK6, PIGM, PIP4K2A, POLR2A, PPAT, PTPN1, PTTG1, RBPJ, RELB, SNAI1, TAS2R14, TUBB4
Negative_9511	APP, BTC, GSN, IL8, AGPAT4, APRT, B3GALT2, BCAT2, C4BPB, CDO1, CTF1, CXCR5, DAG1, DUSP1, DUSP14, FABP4, FTMT, GFPT1, GPD1, IDH1, NR5A2, NT5E, OR13H1, OR56A3, OR5AC2, OR5AR1, OR5M8, OR8G1, PIK3R2, PPAP2B, PTCH1, RASSF5, SYNJ1, TNFSF14, UGT2B10, ULBP2
Negative_9512	F12, RPE, TK1, ANPEP, CBLC, CCL27, COMP, DRD2, ENTPD8, IL7R, ITGA4, LCAT, NCKAP1, NCR1, OR10A2, OR10K2, OR52N5, OR5R1, TBL1XR1, TLR9
Negative_9513	GSN, APOE, CLDN20, GALNT12, GNAT3, ITGB7, LDHAL6B, LLGL1, MAN2A1, MDH1, OR10A3, PTGS1, STX18, TGFBR2
Negative_9514	GDA, NLK, RDX, RET, ARHGAP5, CALML3, CD3E, CDC14B, CDK2, CX3CL1, DAPK1, DGKQ, E2F3, FARS2, GAB1, GPD2, GTSE1, IDUA, ITGA8, JAG1, OR10G9, OR10H3, OR2B6, ORC6L, PGK1, POLR2E, PTGDS, RPS6KA4, SOCS2
Negative_9515	LPO, OAT, ASMT, CYP17A1, GGCX, GRIA1, GSTA4, GSTM1, GZMA, IFNA16, IL15RA, LFNG, MAP3K14, PAK4, PDE6H, PTGES2, QARS, SMPD4, SPP1, WBSCR22, PLA2G12B
Negative_9516	LCT, ADCY4, ARNTL, CXCL12, CYP2C9, G6PC, HDAC2, IFNA16, IFNG, MAML3, MAP2K1, MGAM, ODC1, OR2T12, OR51T1, OR9G4, PDXK, PIGF, PROC, RAF1, SEMA3G, SGMS1, THBS3, ULK2, USE1, YWHAQ, SLC25A31
Negative_9517	DFFB, GPX5, LAMC3, OR2A5, OR51B2, PCNA, PTGDS, ULK1
Negative_9518	FN1, CDC16, COL5A3, FTH1, GALNT1, GPD2, GSTA1, IDH2, ITGA6, KLK3, MADCAM1, OR3A2, PANK3, PCYT1B, SSH2, VAV2
Negative_9519	UBB, ATP6V0B, B4GALT7, CD8B, DDX51, DUSP3, E2F4, EXTL2, GAL3ST1, GANC, GLI3, GNB3, JAM2, LIPE, MAFA, MCCC2, MGST2, MYH10, OR10AD1, OR2T2, OR6C1, OR6N2, SPP1, TAS2R7
Negative_9520	FN1, BUB1, CHST13, CSF1R, CSF2RB, CTLA4, EGLN1, GALNTL1, GJA1, HTRA2, IRAK2, MAN2A1, OR10A7, PDE1A, PDE6G, TACR2, TBL1X, TCF7L1, TJP1, UMPS
Negative_9521	FGA, ASPA, ATIC, BTRC, C4BPB, CDKN1B, COL6A2, GADD45B, GALNT8, GMDS, GMPPB, GSTM1, IL6ST, MAP3K4, METTL6, OR5AC2, RENBP, SEMA4G, STAT4
Negative_9522	C8B, FGA, PAH, TEC, UBC, ABAT, ACP6, ACTB, ALAS2, ATP5H, CCR7, CDS1, CHRM3, DGAT1, DVL1, FMO5, GALK1, HSD17B4, IL23R, LARS, MYH2, NFATC1, NLGN3, OR13C8, OR1G1, OR5P3, PAK7, PDGFRA, PFKL, PHGDH, PLA2G2D, RAC1, RFXAP, SMPD3, SRGAP1, ULK3, VEGFB, WIF1
Negative_9523	AK7, F9, ILK, LPL, ARHGEF1, CCL27, CDC16, CHUK, CIITA, CXCL9, EDA2R, EFNA1, ENO2, FGF9, MAPK14, NEU1, NRXN2, OR10H5, OR2B11, OR4X2, OR56A4, OR5K1, P2RX1, PRPS1, PTCRA, PTPN5, TNFSF4, TUBA3C, WNT16, MAPK8IP3, PLA2G12A
Negative_9524	C8A, AMD1, AXIN1, CCNE2, CDIPT, CDO1, CLTC, EPRS, GBA3, GPT2, GSTM3, IPMK, NCOA4, OR2AT4, PFKL, PGK1, PLOD3, PON2, PTPRZ1, SPRY4, TPH2, WEE1, SERPINF2

Negative_9525	CP, GH1, ACOX1, AGTR1, CCL17, CTF1, DGKQ, EIF4B, ICOS, KIR3DL3, LRDD, LTC4S, MYH8, OR2D3, OR4X1, OR6K3, PIP5K1B, PRPS1, RUNX1T1, SLC12A2, SLIT1, STT3B, TLR5, TRAF4, SERPINB5, SUV420H2
Negative_9526	RPE, TAT, ACADS, AKR1B1, APC2, CACNA1S, CCNA2, CDC2, CHRM2, CRLF2, CTSS, DTX4, DUSP2, F2RL3, FABP3, FABP4, GPD2, HEXA, HSD17B3, IGSF5, IMPA2, IPPK, ITPKB, MYL7, OGDH, OR5T1, PARD6B, PCCB, PFKP, PIK3CD, PRKCH, PRKG1, SESN2, SESN3, TAS2R48, TPH2, UCK2, UCP1, UNC5D, WNT1, CACNA2D2, SERPINA1
Negative_9527	GNE, CACNA1E, CCL17, CLDN11, DAD1, DDIT3, GNPDA1, GSTA2, MYH15, NCF4, NCKAP1, PECAM1, SPRY3, TAS2R50, PLA2G12B
Negative_9528	HK3, LCT, AGXT, ARNT, ARSD, CACNB4, CD8A, COL5A2, CRY1, DGUOK, EHMT2, EI24, EP300, GADD45B, GNA13, GNG13, IKBKE, IL1R2, IL23A, ITGAL, KLRC2, MAGI1, MCM4, MTHFR, MYL7, NFKB1, NRAS, OR1D2, P4HA3, PDE5A, PIK3CD, PNPLA4, POLE, PPAP2B, PPARA, PYCR1, SLC33A1, SLC9A1, SPRY2, SV2B, UGT2A1, GABARAPL1, HLA-DRB4, MAPKAPK3
Negative_9529	C8A, BCL10, CASP10, CCL17, CD58, CLDN6, CXCR4, EGLN3, GALNT11, IRAK2, MAP4K1, MAPK9, MYH1, NFATC3, NKD1, NODAL, OR1L3, OR2F1, OR4A15, OR5A1, PAX8, PLA2G4A, PPP1R3A, PRKCI, RASSF1, SMAD3, STAM2, STX2, WNT5A, ATP6V1G3
Negative_9530	BID, EDA, SMO, AANAT, ACADVL, APC2, BUB1B, CAMK2G, CCL25, CLCA4, CLDN17, EDN1, GDF6, HAAO, HARS, IDH1, ITGAE, MTMR2, MYH6, NFATC4, OR51Q1, OR6C70, OR6K2, PAX8, PDE1B, PDE6D, PER1, PFKM, PIPOX, PPP2R2B, PPP2R5D, PTDSS2, RBL2, RPS6KB2, CTNNBIP1
Negative_9531	ADC, AK3, BID, DBH, F9, GSR, MSN, TK2, UBB, ACP1, AMPD2, ASNS, ASS1, CASP7, CD8B, CES7, CLDN9, COL1A2, CPT2, CREB3, CREB3L3, CYP27A1, DPM3, FGF23, GALM, GNA12, GRIN2B, GRPR, HMGCS2, IL28B, IL5RA, MCM7, NEU3, NME7, OR52A1, OR9K2, PNPO, POLR3D, PRKAG3, RFXAP, SFRP5, TDO2, TNFSF18, WARS2, CACNA2D1, PPARGC1A
Negative_9532	B3GAT1, CADM3, CAPN2, CYP7A1, EPHA5, ERBB4, MGST3, PERP, STX12
Negative_9533	NPY, TPR, ACSM3, ADCY2, ANGPTL4, ARHGEF1, ARHGEF4, ATP5D, BAIAP2, CACNG3, CCL23, COQ3, CSF1R, CYP2A7, DUSP5, EDA2R, FLT1, FUT8, GAD1, GDF5, GUCA1C, HAGH, HSP90B1, IGFBP3, MAP3K3, MYH10, OR1L8, OR1N1, OR4F15, OR51A4, ORC2L, PDE2A, PDE5A, PDGFA, PLA2G2D, PTDSS2, PTGER1, RPS6KA5, SH2D1B, STMN1
Negative_9534	AK7, AMT, IL7, SHH, BET1L, CNTFR, FCGR3B, FGFR1, FRAP1, HADHA, HDAC1, HLA-<br>DOB, IL17RA, MYH10, NCR1, OR10K2, OR5K1, PDE6D, PTDSS2, PVRL3, RAD51, TDO2, ATP6V0A1
Negative_9535	AMH, F9, HK2, IL2, MAG, RPE, AGPAT4, BDH1, CCR7, CDC7, CDKN2D, CHKA, CRKL, DGAT2, DPYS, EP300, FCGR3A, FDFT1, IDI1, IFNA16, NAGK, NFAT5, NKD1, NUDT9, OR13C9, OR4E2, OR4N5, OR56A3, OR9Q2, PAK3, PDGFA, PFN1, PGAM4, PPP1CC, RAC3, RERE, RXRA, SFRP4, SOX17
Negative_9536	AK1, DCT, GPT, IL9, LPO, MPI, RDX, AGPAT1, ARAF, BMPR1A, CCL22, CD8A, CFLAR, CHD8, CLDN6, COL11A1, CYP3A43, DGKB, IL18R1, IL1RAP, ITGA7, ITGB6, MAPK10, MDM4, OR2D3, OR2G2, P4HA1, PPP5C, RALB, SHC1, SORBS1, TICAM2, UGT2B28, PDCD1LG2
Negative_9537	ACSS3, ADRBK2, ATP6V0B, ATP6V1D, BAAT, BET1, BIRC5, BLVRB, BRAF, C3AR1, CCKAR, CLOCK, CSF2RA, FTH1, GLDC, GMPPB, GRIA1, GUCA1B, IDI2, ITGB4, JAG2, LCP2, MAP3K2, OR14I1, OR2M3, OR2Y1, PCCB, PPAT, PRKAA2, PTPN1, THTPA, UBE2L6, WNT5B, XCL1, GABARAPL2

Negative_9538	C1S, DCN, F2, OAT, PLG, ACTN4, AKR1D1, CACNA1D, CCL5, CES2, CHKA, CNDP1, CRLS1, DDX18, DDX56, DOCK1, ENTPD2, FRAP1, GNAI2, GUCY1A3, H6PD, HADH, HDAC2, HSD17B1, IARS, IL5RA, KLK3, MAP3K12, MMP7, MYH2, MYL2, NAGK, NCR1, NRAS, OR1G1, P2RX1, PGM1, POLD2, PON1, PRKAR2B, SEMA4A, ST8SIA1, SUV39H2, TAP2, TAS2R14, THBS1, UGT2B28, SERPINA5
Negative_9539	ADA, CBL, EZR, F8, FGG, IL8, LIF, ACOX3, ADCY4, ARRB2, CALML5, CCNG1, CCR7, CISH, CTBP2, CYP26A1, DRD1, GSTM2, IFNA2, IL11RA, MAP3K1, OR4F4, OR52D1, OR8K5, PPP2R2B, PRKCB, RALGDS, RAP1A, ST6GAL1, TIAM2, UBA1, ALDH18A1, MAPK8IP1
Negative_9540	ACSM5, ALDOA, APRT, ARNT, CANT1, CASP3, CCL24, CDH4, CLTA, FLAD1, FUT4, GALE, KIR2DS4, MAP3K8, NFX1, OR10K1, OR4C11, OR52A1, PAX6, PDCD1, PDE3A, PFN2, PIP4K2B, PLEKHA8, POLR2H, RPS6KA5, SAT1, SDHA, SNW1, STMN1, TYMS, VAMP3
Negative_9541	TFG, ANAPC2, ATP5B, CNTF, COL4A1, CYP2C8, CYP4A22, DAPK2, DGAT1, EGLN1, FGF7, FZD3, GBE1, IL2RA, MDM2, MTHFR, NARS2, NFYC, OR2T29, OR52N5, PDE8A, PGK2, PPP2R5A, PRPS1, PTPRM, RCHY1, SEMA4F, TAS2R5, TRAF6, TRPC1, VEGFA, WARS, TNFRSF1A
Negative_9542	CP, FES, NLK, SYK, ADRA1D, ARNT, BDH1, CCR6, DCTD, EDA2R, FCGR3A, FGF19, GALNT13, GALNT2, GNPNAT1, GPD2, GRPR, HCCS, HYAL1, IRAK3, ITGAE, MAP3K3, NT5C2, PANK1, PPARD, PPP2R1B, PTPN11, RENBP, SESN1, SMURF2, TKTL1, TXNDC12, TNFRSF13B
Negative_9543	AR, LPL, SMS, ADRB2, ATP5F1, BLVRA, C3AR1, CARD11, CCL17, CER1, CYCS, CYP11B1, CYSLTR1, EPHA8, GLO1, GMDS, GRM1, NAT5, NCF2, OR10G4, OR2T10, OR4B1, OR56A3, PIGN, PTPRR, STAT5A
Negative_9544	GK, KDR, A4GALT, C9orf95, CD86, CHUK, HTRA2, ST3GAL1, SYNJ1, TP53I3, YOD1
Negative_9545	DAO, HK2, KDR, SMS, ACVR1, ALPPL2, AMDHD1, BCKDHA, C7orf16, CCNB1, CLDN3, DDIT3, DKK4, EIF4E2, ENPP3, ERBB2, GSTO2, HAGHL, IDUA, IFNAR1, ITGA10, LARS, MAPK1, NCR1, NLGN2, NOTCH1, OR4X1, OR51S1, OR52L1, OR5H2, OR6B3, OR9G4, PDGFD, PIGN, PNMT, PNPLA4, PPP2R5A, PRPS1, SARS2, SLC2A1, TGFBR1, TNFSF12, ULBP3, WNT4, WWP1, PAFAH1B2
Negative_9546	CFD, CACNA1D, COL5A3, GPAM, ITGB7, MADCAM1, MAN1C1, OR2K2, OR4D2, PIK3C2G, PLA2G2D
Negative_9547	GP5, ID1, APC2, APOA1, ATP6V1A, CREB3L2, DKK2, DLST, G6PC, HEXB, ICAM3, IKBKG, LIPC, LIPE, LTB4R2, OR10S1, OR1L4, OXSM, PCNA, STX16, TYRP1, UNC5D
Negative_9548	AK5, IL4, MPZ, AANAT, ADRB1, ARPC5, ATP2A1, ATP6AP1, CACNA1A, CAMK2G, CD244, CHD8, CPT1C, EPHA8, FRAT2, GCNT4, GSTO2, GTSE1, HMBS, IQGAP3, ITGB4, JAK1, LAP3, METTL6, MPZL1, OR1S1, OR2T29, OR2T6, OR4D6, OR6S1, OR7G2, PAPSS1, PAPSS2, PIPOX, PLTP, POLD1, PPP1R3B, PRDX6, RBX1, SH2D1A, ST3GAL1, TAP2, UCK1, UPB1, WNT11, TNFSF13B
Negative_9549	AK7, ACTA1, ADRBK2, ASNS, BIRC3, CARS, CCL5, DVL1, EHMT2, HLA-DOB, HMGCL, HSPA1B, LEFTY1, LIPT1, MAPK1, MAPK10, OR10T2, OR2A42, OR4D2, P4HA1, PDGFB, PSEN2, RFXANK, SSH3, TRIP10, UBE2L3, YWHAE, HSP90AA1
Negative_9550	C9, ADCY1, CHRM3, CLTC, ELK4, EPHA6, FZD5, GAMT, OR10A7, OR6N1, UGT2A3
Negative_9551	BMP5, DGKD, DHCR24, FGF10, FZD7, GALNT1, GNMT, HSD11B1, IFNA8, IRAK3, LIPA, PVRL2
Negative_9552	ABLIM3, ATP5I, CDKN1C, DUSP14, GATM, GNB3, IL12RB1, IQGAP3, ITGAL, KIR2DL4, LIPG, OR10T2, OR2F2, OR51F2, PPP3R2, RASA1, RASGRF1, WARS, MAPK8IP2, SUV420H2

Negative_9553	CAD,HDC,ABP1,ACO1,ASRGL1,CCL16,CD79A,CDH5,EHHADH,GAD2,GDF5,GMPPA,GOT1,INSRR,KCNB1,LCAT,NRG2,OR5T3,PAPSS2,PLA1A,PLXNC1,PRKCB,PTPRZ1,STAM2,UGT2B7,UNC5D,PRICKLE1
Negative_9554	CFD,GK,PGF,ABL2,ACP1,BAI1,CCL5,CREBBP,EPB41,F2RL2,FMO1,GALNT13,GRIN1,GSTM1,GTSE1,IL13RA2,IL18RAP,LEFTY2,MCM3,METTL6,MINPP1,NANP,OR10P1,OR2M4,OR56A4,OR5P3,OR7G1,PCYT1A,PEMT,PFKFB4,PKMYT1,PPOX,PRKY,PTCH2,ROBO3,SDC1,SOCS7,SOX17,SRGAP1,SSX2IP,TAS2R3,TMLHE,VDAC3,SERPING1
Negative_9555	DCK,TAT,ADCY7,AKR1C1,AVPR1B,DNMT3A,EXT2,GAMT,HSD17B4,IRS2,ITGB2,LAP3,MGST3,NAGK,OR10A5,PMAIP1,PSPH,PTDSS2,RPN1,RPS6,SOCS3,TACR1,TPI1,CSGALNACT1
Negative_9556	IFNA5,UGP2,YWHAG
Negative_9557	ME1,AREG,BACE1,BMP8B,CER1,CHSY1,CLTCL1,DGAT2L4,DRD1,FABP1,FGFR1,FOXA2,FUT7,GCLM,IL13RA2,IL15,ITGA2B,LIFR,LRDD,MPZL1,MRAS,NRXN2,OR10K1,OR9G4,PAK4,SLC2A2,SULT2A1,TBL1X,TSC2,WARS,CACNA2D4,HLA-DPA1
Negative_9558	BID,C8G,DDO,PLN,TAT,VTN,XDH,ALDH2,ALDOC,AMPD2,ATP5D,CANT1,CBR1,COL1A2,CREB1,CXCL1,DUSP14,DUSP16,EIF4E,FUT5,GALNT1,HEXA,HNF4G,IRF7,LDHAL6A,NCAM2,OR4B1,OR4K5,OR5AP2,PFKFB1,PTGES,RUNX1T1,SOS1,STAT6,TNFRSF8,TRADD,TRMT11
Negative_9559	ALDH6A1,BAIAP2,CACNB3,CCNA1,CD3E,CDH15,COQ6,CPT1C,CYP2C8,DAAM2,GALNS,GALNT5,GDF6,GNPDA1,GOT2,IAPP,ITCH,JAK2,LCP2,MAD2L1,MIOX,MITF,MTMR2,NUDT8,OR2H1,OR2L3,OR52R1,OR5B3,OR7D4,OR8K5,PTAFR,SEMA3C,SGMS1,TGFBR1,TUBB2B,USE1,WASF1
Negative_9560	AGK,CKMT2,CLDN17,DIAPH3,FGF23,FLOT1,HNF1A,INPP5E,NKD1,PLA2G4A,POMC,PRKCH,TLR9,TRHR,MAPKAPK2
Negative_9561	ADA,MPI,PAH,SMS,ADCY3,APH1A,ARHGEF6,ASS1,CD55,CNGB1,CSF3,DAG1,DDX18,DEGS1,FGF23,HEXB,HSD11B2,IL10,IL17RB,IL1B,INPP5A,INPP5B,MAPK12,MAPK13,NEGR1,OR10G4,OR2S2,OR52N1,OXTR,PGS1,PLOD3,SETMAR,SRGAP3,STAT1,TGFBR1,TKTL1,TLR3,TOMM40L,TUBB,ZMAT3
Negative_9562	CRH,FER,HAL,ADH1B,ADORA2A,ASIP,BPGM,BTRC,CALML3,CCL14,CCNB3,CHUK,CSNK2A2,CTBP2,DDIT4,DIAPH3,DPAGT1,EPB41,EXT2,FUT1,GCNT2,GMPR2,HIF1A,IL12RB2,INPP4B,JAK1,LDHC,MCM5,MYH1,MYL5,OR52H1,OR5A1,PDGFB,PLA2G1B,PMM2,PTGFR,RAB23,RARS2,ROCK2,RPS6,RUNX1,SDHD,TP73,TUBB,UGT2B7
Negative_9563	C8G,ABL1,AHCYL2,ALDH1L1,ARAF,ATP5G3,BPNT1,CD70,CDC16,CDC26,CTLA4,CYP2B6,DDX18,DIAPH2,FCGR3B,FGF22,GALNTL5,GNG12,GUSB,MAPK9,NEFM,OR2A2,OR51I2,OR5AN1,OR6F1,PHKG2,PIGN,POLR2A,SH2D1B,SPTAN1,STAT3,TACR1,UCP1,ULBP1,YKT6,SERPINB5,TNFRSF11B
Negative_9564	ADO,C8B,GNAI2,ITGB7,PLCB3,RASGRF1,SULT1E1
Negative_9565	AGK,GSR,ALDH3A1,BDH2,CD8A,CDH5,CHRM5,CLDN18,COL6A1,EXTL2,GALNT2,GLI1,IFNK,IL18R1,IL6ST,NCKAP1,OR4D11,SOCS1,SRGAP1,TAS2R60
Negative_9566	CP,ACTG2,ACVR2B,AGRP,BACE1,BCAR1,CLDN22,CSAD,CXCL13,EHMT1,FBP1,FGFR3,GALNT11,GGT7,GNAI3,HMOX2,INADL,NANS,NR5A2,NTRK1,OR2AG1,OR51T1,OR52E2,PIK3R2,POLR1C,POMC,PPAP2A,PSAT1,PVRL3,RASGRF1,RDH10,RPS6KB1,RRM2B,SLIT1,SMAD4,SUCLG1,SV2B,TYK2,UGT2B7,UPP2
Negative_9567	ABO,C2,EPO,F3,PRL,ACOX2,AGMAT,ALG6,ATP5A1,CD72,CHPT1,CPT1C,EXOC7,FGF12,FTCD,GADD45A,GART,GMPPA,GMPR2,IGFBP3,IL1R2,LIMK1,MMP9,OR10G8,OR10X1,OR51E2,OR8I2,P4HA3,PDE4B,RARA,RPIA,SKP2,SOX17,TPK1,ZFYVE16
Negative_9568	ADCY8,ASNS,AVPR1B,CACNB3,COL3A1,DAAM2,FUT4,MTAP,OR10A2,OR2L13,OR52I2,PYGB,TRHR,WASF3,ATP6V1G3

Negative_9569	FGF4, IL13, ITCH, MDM4, OR52E6, ORC5L, RASGRP2
Negative_9570	AK3, F5, MYC, OGT, PC, BMP2, CACNA1D, CACNA1E, CD48, CDC20, CTSG, CUL1, CYP2D6, DCXR, GNMT, ICOS, IDH3B, IL11RA, KLRC2, NAT2, NFKB1, NFKBIE, NME4, OR10A4, OR2A4, OR51B6, OR6X1, PDE7B, PI4KB, PRDX6, PRSS2, RUVBL1, RXRB, SLC25A5, SLC2A1, SUFU, TFDP1, WASF1, PRICKLE1
Negative_9571	PRL, ATP5H, BCKDHA, CHRD, COQ5, DGKB, DOT1L, DPM3, DUSP6, FARS2, FLAD1, GPD1L, IMPDH1, KLRK1, MAOB, NCAM1, OR13F1, OR2W1, OR3A1, PLOD3, PLXNB3, PRPS2, TAS1R2, UGCGL2, UROD, YWHAB, TNFSF13B
Negative_9572	CCR10, OR8B8, TPH1
Negative_9573	BAX, FGFR1, HIP1, MAP3K2, YARS
Negative_9574	PC, ADCY3, ADH6, C3AR1, CAMK2A, CXCL9, CYP1B1, HNMT, IFNB1, IL4R, ITGA4, LLGL1, MAP4K3, OR51G1, PLIN, SELPLG, SEMA7A, SETD8, SLIT2, TGFB2, SERPINE1
Negative_9575	OGT, ZYX, FRAP1, OR10A6, OR52B4, RENBP, SEMA7A
Negative_9576	ANAPC10, CDC26, CREB3L2, LLGL2, MTHFD2, OR1A2, OR51E2, RRM2B, SPTLC1, TBL1XR1
Negative_9577	CEL, CRH, EPO, PC, ABLIM2, ACACA, ACLY, ACVR1C, ARPC5, CASK, CD47, CDC14A, CHIA, CREB5, CXCR4, CYP2A6, DUSP14, ECHS1, EDNRB, FGF18, FPGT, GALNT12, GDE1, GLUD2, GRIA3, GSTA5, IFNB1, JAK2, MAP2K6, MAP3K6, NFASC, NMNAT3, OR10A6, OR4F6, OR4X1, OR52E2, PIM1, PLCE1, RENBP, RXRA, RXRG, SELL, ST3GAL4, TGFB3, THY1, TXNDC12, EIF4EBP1, PRICKLE1
Negative_9578	PTS, AGXT, CAMKK1, CCNE1, COL5A3, CSF3, FGF6, HSPA2, HTR2B, ICAM1, IL21R, KLRD1, MGAT4A, OR1C1, OR5V1, ORC3L, RAPGEF2, RASGRP2, SUOX, UNC5B, PDCD1LG2
Negative_9579	MPZ, AKR1C1, ATP5E, B4GALT4, BUB3, C1QA, CCL27, CDC7, COQ5, DDX54, DGAT1, EPHA6, FGF9, FLOT2, FZD9, GALNT1, GLUD1, HCST, HSPA1B, KLRC2, LEPR, MADCAM1, MLLT4, MLYCD, MYH11, MYH14, NAMPT, NRXN2, NTRK1, OR10H2, OR51A2, PIGW, PIK3C3, POLR1C, POLR2H, PPA2, PTGIS, RDH16, ROBO1, SESN1, SMURF2, SULT1E1, TGDS, ATP6V1B1, HLA-DPA1, TNFRSF12A
Negative_9580	ADA, SMO, ARHGEF2, ATP5L, C1QB, CDC25C, DDX50, DPYS, E2F1, FGF12, FGF13, IFNA5, IL17B, IL28A, ITGAE, MGST3, NT5C, OR1A1, OR1Q1, OR2A4, PFAS, PGAM1, PLAT, PNLIP, POLR2D, PPP2R5D, SOCS4, SSH1, STX16, TALDO1, TSLP, WNT2
Negative_9581	FOS, PGD, AADAC, AHCYL2, ALDOC, ATP2A3, C1GALT1, CCL17, CD28, CD79A, CYP3A4, CYP3A7, GALNT1, GGT5, GGT6, GNA15, GUCY1A3, HCST, HSD3B7, IL11RA, IL1A, KIR2DS5, MGST3, OR10AD1, OR1Q1, OR4D9, OR51B4, OR5AC2, PDXK, PIGU, PPAP2A, PTAFR, RBX1, RFWD2, RRM2B, TAS2R49, TKTL2, TRMT11, UNC5B, VNN1, YWHAE, HLA-DPB1, SERPINC1
Negative_9582	BAD, FOS, GNS, TEC, AADAT, ARHGEF1, CACNA1F, CSF2RA, CXCL13, CYP26B1, DDX55, DERL1, ENTPD2, FUT9, GOSR2, HCCS, KARS, LCMT2, MAOA, MGAM, NSD1, OR10A4, OR5D16, P4HA3, PDE3B, PIAS4, PTPRZ1, SEMA4A, SORBS1, TAS2R4, TNFSF12, TOMM40, TYRP1
Negative_9583	CRK, DCT, IL8, CCNB3, EGLN3, F13A1, GAD1, GPD1, GRIN1, GRM1, GUCA1B, IFNA10, INHBA, ITGA7, KDSR, LCAT, OR51B5, PIP5K1B, PRKCD, SUV39H2, TLN2
Negative_9584	JUP, OGT, ACADSB, AKT2, BHMT, CCL2, CDC45L, CHEK1, COMT, CYP3A4, GDE1, HAGHL, NOD1, OR10H4, OR2A25, OR6B2, OR9A4, POLA2, PPCDC, SLC9A1, TNXB
Negative_9585	CCND1, CXCL5, CXCR6, DGKE, IL11, OR13C4, TRAF6, VAMP3, WWP1
Negative_9586	DUT, GHR, MIF, AGXT, APOC3, BUB1B, C1QA, C5AR1, CCL23, CCL28, CCNE1, CCNE2, CD48, CHPT1, CSH1, DHRS4L2, DVL1, GGCX, GGT7, GMPR2, HCLS1, HNF1A, HYAL1, ITGB3, JUND, KDSR, LEF1, MAP3K6, NAE1, NARS, OR10A2, OR10K2, PLCG1, PVRL4, RARA, SPAM1, SPRY3, TACR3, TAS2R42, THBS3, UGDH, WBSCR17, XYLT1

Negative_9587	IL5, ACVR1, CDC7, CRLS1, CTTN, DRD5, INPP5E, JAM3, NCAM2, OR51B2, OR7G2, OR9I1, PIGH, PTK2, SH2D1B, ZAP70
Negative_9588	FGB, HDC, HK2, HK3, OAT, TPO, ACOX2, ALAD, CACNB1, CCNG1, CSNK1G3, CYP11B2, DHDDS, EFNA1, FABP3, FADS2, FZD5, GNA13, IL6ST, IL8RA, ITGB4, KIR2DL4, LRP6, MGST1, NFATC1, NOTCH1, NOTCH3, NTNG1, OR51L1, OR52L1, OR8J3, P2RX5, PARS2, PLCB3, PSENEN, VANGL1, XCR1
Negative_9589	AK1, DCT, TK1, AASS, ACTA1, AMD1, ATP5I, CACNB2, CNGB1, CRLF2, DLST, EP300, ESAM, FGF19, FGF9, GSTK1, HNF1A, HSD17B4, IFNG, IL2RG, ISYNA1, ITGB7, LCAT, LCMT2, MAPT, MTHFD1, NCF2, NCK1, NCR3, NFKBIB, NTN1, OR4B1, OR4D9, OR5J2, PIAS1, PLXNB2, POLR2D, PPP2R1A, ROCK1, RRAS2, SH3BP2, SNAI2, STMN1, TRMT11, TUBAL3, UCKL1, WNT3A, WNT9B
Negative_9590	ADH1B, CCND2, DKK2, E2F5, GALC, GBA3, LCAT, OR51V1, PTPRC, RAP1B, SETD2, TNFSF12, CACNA2D3
Negative_9591	F7, HAL, HGD, NOG, PDC, ACOT11, ACSM4, ACSS3, ADCY6, ALOX15, ARPC3, ATP6V1A, C5AR1, CACNB1, CCL8, CHRM1, CSNK1E, ECSIT, EPHA1, FGF7, GALNT2, GSTM1, HEXB, IL21, KYNU, MMP7, MYH9, NAT2, OR2A4, OR4C16, OR6Y1, OR9A2, PAX6, PGM1, PLOD3, PPP1R1A, PPP2R5E, PRKX, RARS, RASGRF2, SSH2, TNFSF12, UCP1, ATP6V1C1, ATP6V1E1
Negative_9592	C8B, ME3, MOS, RPE, ACSL5, ALDH1A3, ALG8, ALPPL2, AMY2B, APOA5, BRAF, CCKAR, CCL5, CDK5, COL6A2, COQ3, CPT1C, CXCL13, DUSP10, ENTPD5, FOXA3, GLB1, GSTM2, GSTT1, GUSB, HYAL2, IFNB1, IL12B, IL9R, INADL, ITGA2B, LIPC, LMO7, MCM3, MGST2, NADK, NMNAT2, OCRL, OR4L1, OR5T1, OXTR, PGM3, PHKA1, PIGW, PON3, TCF7L1, TUBA4A, VAMP1
Negative_9593	AGRP, AQP7, CCL24, CYP2A13, CYP4F2, DTX3L, GDE1, INSR, OR5A1, OR8I2, ORC2L, TGFB1
Negative_9594	DHH, GBA, ID4, OSM, SP1, ACMSD, AMPD1, CRB3, CYP11B1, DDHD1, FCGR3A, GLI3, GNAT3, HCCS, IL2RG, LIMK1, MAN1A1, MGAT4B, OR10A7, OR2H2, OR2V2, PIGF, PIP4K2A, PLOD2, POLR2K, PPARG, PPP2CB, RPS6KA1, SEMA4D, SPAM1, TUBB1, VEGFC, WHSC1L1, GABARAPL1
Negative_9595	BBOX1, BMP7, CCL24, CD40LG, CHAT, DPM3, GDF7, GJD2, GLO1, GNRH1, IMPA1, JAK1, OR6X1, OR7G2, OR8D1, ORC6L, PRKCD, PRKG2, PRNP, PYCR1, SARS, TAS1R3, TRAF3, TUBB4, UGT1A10, VAMP4, WNT3
Negative_9596	F12, IL9, ABLIM3, ACTC1, AXIN1, CASP10, CHST13, CISH, COQ6, CRY1, EPHA7, ETNK1, FGF19, FTMT, GALNS, HAGH, HARS2, HIBADH, IFNA17, IFNGR1, IL8RB, IMPDH2, IRS4, MGLL, NOS1, OR1J4, OR51B5, OR7G3, ORC6L, PIGQ, RALGDS, SHISA5, SLC25A6, SMAD5, TSC2, TNFRSF13B
Negative_9597	CRK, ALG11, DERA, MGAM, PGAM2, STT3B, SYNJ1
Negative_9598	CLDN19, FZD2, NTF4
Negative_9599	COX15, CXCL16, GNAS, IL28RA, LCP2, OR2M5
Negative_9600	F3, ITK, PAH, CDKN2D, GNA14, GNMT, MAGI1, OR51F1, OR8H2, CACNA2D4
Negative_9601	AR, AUH, NF1, OTC, ACAA1, ADSSL1, ALG11, ASPA, CBR1, CCR2, CDO1, CYP2D6, DAXX, EHMT2, ELK1, F13A1, GJA1, IFNA21, ITGB6, LIPF, NCKAP1, NCOR2, NSD1, OR51B5, OR51F1, OR6B3, P4HA1, PPOX, PRPS1, RASGRP1, RDH12, RRAS2, SLC33A1, SOS2, SPHK2, STK36, ZIC2
Negative_9602	BTC, CDA, F3, ME2, ACOT11, AXIN2, BST1, CACNA1G, CLDN16, CRLF2, CYP11A1, ENTPD3, EPRS, GAD2, GJA1, GRM1, GSTM1, HTR7, INPP5E, MGAT2, NFKB1, NRP1, OR6K3, OR8A1, ORC3L, PCYT1B, PIAS3, PITX2, PPARD, PPOX, RAPGEF3, RASGRP2, SMURF2, SSH3, TAS2R5, TBXAS1, ATP6V1E1, PNLIPRP2
Negative_9603	MAX, MVD, SCD, ARPC1A, AXIN2, FARSA, FOXO3, HEMK1, IFNAR2, ITCH, MAML2, MKNK2, MYH8, NR1D1, PER1, POLR2F, RFNG, SPP1, SPRY1, TLR2
Negative_9604	ADA, GLA, HK1, SP1, ADRA1D, ASPA, CDC16, CES7, COL11A1, COL4A4, CYP3A43, FZD1, GALE, GNA11, GRIA2, IL28B, KAT2B, NOTCH3, OR52H1, OR5T2, OR7C1, PDE8A, PPP1R3A, RUNX1, SLC27A1, TJP2, ATP6V1B1, TNFRSF6B

Negative_9605	PTS, AXIN1, CHPF, CSF2RB, CYBB, DIAPH1, HCCS, OSMR, SDHC, SIPA1, SMARCA2, TNFSF9, WNT10A, PPP1R12A
Negative_9606	ABO, CBR3, DUSP2, FUT1, HMGCR, IFNA13, OR7A17, PAX4, PIGP, PYGB, RAB5A, TP53I3, YWHAB
Negative_9607	ACSL6, ALAD, ALG2, BCL2L1, BDKRB1, C1QB, CCR3, CD70, COL6A2, EFNA4, ELK1, EXTL3, FCER1G, FSHR, GLO1, GMPR, GUCA1C, HTR2C, IFNA17, IL22RA1, LAMA4, MDM2, MTHFS, NCOR1, NOS1, NRG4, ODC1, OR10G3, OR2A4, PANK4, PDE4D, PER3, PTCH1, RASGRF2, STX2, TAS2R13, TRAF3, ZFYVE9
Negative_9608	BID, F3, ZYX, AGPAT3, CCL4, F13A1, FGD1, FMO3, GCH1, MAGI3, OR10H5, POLR2H, SLC2A4, SPI1, WNT1
Negative_9609	ITK, PRL, CAMK2A, CDH1, CLDN9, CXCL13, DDX56, FABP4, GNG13, GPAA1, IDH3B, LIFR, LMO7, MAP2K4, MAPK1, MOV10L1, OR2G3, PLIN, TCF7L1, UGT2B4, VAV1, HSP90AA1
Negative_9610	FES, MVD, ACOX2, CCR4, DBF4, DFFA, EDA2R, ENPP1, ENPP3, FGF3, GGT1, GJD2, MAD1L1, MAP2K1, MAPK7, MMP9, NFYB, NGEF, OR13D1, PGS1, PRSS2, PTPRR, RPIA, SELL, SEMA3A, TLN2, ULBP1, UMPS, WNT2B
Negative_9611	ENO1, OR4A15, RETSAT
Negative_9612	BAX, DCK, DUT, F11, PML, TPR, AMHR2, ATG7, CALML3, CISH, CLDN4, CTNNA2, CYP1A2, DAD1, DDX41, DHFR, DNMT1, EREG, FRAT1, FZD2, GNAL, HSD3B7, IRAK1, IRAK3, KLRC3, LAMA3, LDHC, LEF1, MAP3K13, NARS2, OR10R2, OR2F1, OR2S2, PDGFA, PNPLA3, POLR2C, PRKAG1, RELA, SEMA3F, UPP2, PAFAH1B3
Negative_9613	AK2, FUK, GK, ABLIM1, ACPT, AGPAT2, AKR1B10, ALG9, ASPA, ATP6V1F, B3GALT4, BBOX1, CASK, CCL1, CCR10, CDK2, CHEK1, CTBS, DBF4, DHODH, ESPL1, FSHB, FZD4, G6PD, GNPDA2, GSTO1, LARS2, LTBR, MCM5, MYH7, NRCAM, OR4B1, OR4F5, OR52L1, OR56A3, PAK7, PCCA, PFKM, PIM1, PPP2R2B, PRIM2, PTCH2, RPS6KA2, RUVBL1, SPTAN1, SUOX, SV2C, TAS1R1, TUBA1C
Negative_9614	FH, IL9, ACTG1, ACVR1, AK3L1, AMPD2, APOA5, ARHGEF6, ATP6V0C, BDH1, CLDN16, CTSB, FZD5, GSTM3, LCMT1, NFYB, NT5C1A, OR10G9, OR52A1, PDE7B, PRKCE, PTCH1
Negative_9615	FN1, GSR, RB1, UBC, ADCY4, ADCY5, BMP4, CAV3, CCR10, FMO5, GRIN2B, IL20RA, IL28RA, LAMB3, NUDT5, OR10A6, OR56A4, OR8B8, ORC6L, PPM1A, PRKCE, PTPRZ1, SKIV2L2, TAS2R10, TNFSF14, UCKL1, UGT2A1, UGT2A3, VASP
Negative_9616	GK2, SRF, AACS, APH1A, CBR1, CCL28, FZD5, GALNT3, GALNT5, HADHA, INSR, OR10J3, OR4A5, OR5V1, OR9Q1, PARD6A, RELB, THY1, AASDHPPT, ATP6V0D1, MAPK8IP2
Negative_9617	MAX, TK1, ALG6, ALPL, ANAPC4, ANAPC7, ASPA, BHMT, CCDC6, CSNK1D, DARS2, FGF11, FGF3, FSHR, GANAB, IARS, IL20, LARS, NARS, NPR1, OR1B1, OR1C1, PAK1, PDXP, PIGN, PLXNA2, SEMA3F, SLC2A4, STAT5A, SULT1A1, TUBB2A, ATP6V1B2
Negative_9618	ABO, CKM, GGH, CACNA1D, CCL25, CDC7, CPT1B, ENTPD4, FCER1G, GALNT10, GUCA1B, GUCY1B3, HEXB, ITPK1, MAPK12, MTMR2, NFX1, NNMT, OR52W1, OR6C76, PAX8, POLR2I, PPP1R3B, SRGAP3, UPB1, VTI1B, AASDHPPT
Negative_9619	BTD, EPO, FOS, ACY1, ALDH3A1, APOA5, CD3E, CDC20, CER1, CPT1A, CPT1C, EFNB3, FOXA2, GALC, GALNT12, IMPDH2, ITGB8, MAP3K2, MGAT5B, MGLL, MRAS, NAGA, OR10G4, OR2L3, OR4C6, OR5C1, PCCB, PEMT, PLA2G3, POLR3B, PSME2, RGS3, RPN2, SSH1, TGFB1, UGT8, VDAC3, SERPINB5
Negative_9620	DDX50, FGD1, HSPA1B, JAM2, MYH4, NCOA4, NRXN3, OR10P1, OR5J2, POLD3, PSME1, TNFRSF13C
Negative_9621	F10, B3GALT6, CACNG3, CDIPT, CPS1, ENPP2, EXT2, FARSB, FLT3LG, FZD6, GALNT12, HSPA8, LEF1, MAGI3, MAP4K4, MYH7B, NKD1, OR52D1, PAX6, PLOD2, PRKCG, PYCR2, SH2D2A, SOCS4, SPHK2, STEAP3, TKTL2, TNFRSF8
Negative_9622	ATP6V1A, CCL19, CCL8, DPM3, FGF20, GNPDA1, HCCS, KARS, KAT2B, LEF1, MAT1A, OR5F1, PAX8, PLXNA1, RXRA, ST3GAL1, MAPKAPK5

Negative_9623	PGP, ADH7, AGTR1, ATP6V0B, CACNA1E, CAMK2B, CD99, MGST1, OR4C15, PAK3, SUOX, YWHAQ, HLA-DQB1, HLA-DRB4
Negative_9624	DDO, BCKDHB, CSF1, CYP17A1, DTYMK, IFNA6, IL13RA1, ISYNA1, OR4C11, PLXNA2, RPE65, SHMT1, TNFRSF8, TUBAL3
Negative_9625	TYR, ACVR1, CACNA1I, CALML5, CCL28, CCR3, CHRNA7, CHUK, CMPK1, COL11A1, DDIT4, DIAPH2, E2F5, EPB41L2, FECH, FOSL1, FPGS, GCLM, GLI2, IKBKB, IMPA1, ITGAX, LAMC2, LARS2, MAP3K13, MGLL, NOX3, OR1L1, OR2S2, OR4E2, OR52E2, OR56B4, PDCD1, PDE5A, PDGFRB, PFAS, PPAP2C, RBX1, SENP2, SOCS2, SSX2IP, TRHR, UGT2B28, XYLB, CSGALNACT2, HLA-DPA1
Negative_9626	KDR, ESAM, IFNW1, OR2L2, RPS6KA2, SHC4, TAS2R7
Negative_9627	ASL, DHH, ACSM4, CHIA, CHRM1, ENO1, GNAZ, LPAR1, MCM7, MPST, OR6C70, PIGL, PTPRR, RRAS, STEAP3, TM7SF2, TUBA3C, WNT2B
Negative_9628	ADO, RFK, TKT, AKR1B10, ATP5G2, CBLC, CCL11, CCND3, CYP4F2, DEGS2, EIF4E2, EP300, GRHPR, IMPDH2, LIAS, MGLL, NAGK, OR10G9, OR5H6, PIGH, PRKAA1, PRKAR1B, PSAT1, RFX5, SHC3, SLC2A1, SMPD4, THTPA, TNFSF18, UBE2K
Negative_9629	IL3, ABLIM2, AMDHD2, ANAPC2, ANGPTL4, B4GALT1, BDNF, CCNH, CDC23, CYBB, EREG, FRAT1, HSD17B1, ICOSLG, ITGB2, MKNK1, MYLK2, OR4D6, OR51E1, OR51I1, OR52N2, OR8H3, PDE1A, PECAM1, PFKL, ROCK2, RRM2, TNFSF14, UROD, VAV1
Negative_9630	GH2, TNC, AKR1B10, AMD1, APAF1, BHMT, CCL21, CD8B, CDS1, CREB1, FZD4, GADD45B, IL1R1, MYH8, NCOR1, NUDT9, PFN1, PISD, PLCE1, SMPD1, SRGAP2, TKTL1
Negative_9631	DCTN1, ERBB4, PIGU, PRSS1, PVRL1, TALDO1, USE1
Negative_9632	C9, F12, KIT, ACOX1, ACTG1, AMPD2, APLP1, ATIC, B3GNT3, BCAT2, BUB1B, CYP2C8, FASN, GABARAP, HEXB, HMBS, ITGB6, MASP1, OR13D1, OR2L8, PAK4, PCYT2, PLA2G4A, PPP1R3B, RGS3, STAT3, STX7, TFDP1, THBD, VAV1, VTI1B
Negative_9633	CCR9, DDX41, HMGCS2, MARS2, PDE6H, PDE7A, TNFSF9, TRMT11, GABARAPL1
Negative_9634	GP6, PTS, ALDH1A2, APPL1, ARSD, B3GALT2, B3GALT6, CAMK2A, EFNA4, EFNB2, FRAP1, FRAT1, GALNT8, GRIN2D, GSK3B, GSTM4, HYAL2, IFNW1, LYPLA1, MPDZ, NAMPT, NRG4, OR7A10, PDE11A, PIGB, PIK3C2A, QPRT, TJP2, TLR9, TRAF1, TRMT11, WASF3
Negative_9635	RET, ANAPC1, B4GALT2, C3orf10, CALML3, CCND3, CDC14A, CLDN11, CYP4F2, DGAT2, DGKZ, ENPP1, FGF1, GNAZ, IL10RB, IL2RB, IL8RA, ITGAL, MCM4, MGAT5B, NPR2, OR5T2, OR8J1, PDE4A, PIK3CD, PNPO, PRKCB, PTCRA, SAT1, STX8, TICAM2, WNT7B
Negative_9636	IDS, AACS, ACVR2B, ALG1, BAAT, BDKRB1, BLVRB, CDH1, CPT1C, CYP2A6, EIF4E, FTCD, GLS2, GMPS, GSTA4, H6PD, HMGCL, IFNA17, ITGA8, NCF1, NLGN2, NRXN2, OR2AG2, OR2H2, OR52A5, OR52N2, OR56A4, PRKAR2A, PTGES, RRM2B, SDC4, SEMA3D, SORBS1, TAS2R41, TFDP1, TUBB2A, TYROBP, UBE2K, UCP1, ZBTB16, SERPINB5
Negative_9637	CEL, GLS, GSR, TH, AADAT, ALDH2, ALG10, ATP5G2, BCKDHB, CACNA1H, CASP7, CCDC6, CXCR6, CYP2U1, DDOST, DRD1, EPHA8, FUT4, GALNT13, GALNTL4, GMPPA, IL17B, KLKB1, LAMA4, MAP2K5, OR11H6, OR2A12, OR2M5, OR51A7, OR8B4, PANK2, PECAM1, PLCG2, PPP5C, PRKACG, SNAP25, SORBS1, TCF7, TLR4, TRPC1, ULK1, VCAM1
Negative_9638	BTK, SHH, AGTR1, GALM, GSK3B, ITGA9, OR1L8, P4HA2, PCCB, PLA2G6, PPP3CC, RRM2, TYMS, VDAC1, WEE1, HSP90AB1
Negative_9639	CBL, FES, GPT, HK2, TNC, ADCY6, ALOX12, CARD11, CARS, CCR10, CDKN2A, CES1, CHIA, COQ6, CTSG, DHCR7, F2RL1, FARSA, FTCD, IFNB1, IL2RG, INPP5A, NFASC, OR13C2, OR1C1, OR1I1, OR2T29, OR4D1, OR4X2, OR52E8, OR52N1, OR7G1, PGAM2, PIGA, PPP2R5E, PPP3CA, RASA2, SC5DL, SMAD5, TLR5, TSHR, UNC5A, UPB1, YES1, YWHAZ, CACNA2D3

Negative_9640	ATM, FYN, ID3, TNC, BDKRB2, CALML3, CD274, CD40LG, CD8A, CREB5, DKK1, FDPS, FGF5, GALNTL5, GLUL, HCLS1, ICAM2, IL20, IL28B, IL5RA, IQGAP2, MAPT, MBL2, NKD1, OR13A1, OR6C1, OR6S1, OR7A5, PLAU, PLEKHA8, PRIM2, PSME3, SMPD3, SREBF1, TJP2, TLN2, AASDHPPT, HLA-DQA2, TNFRSF11B
Negative_9641	AOC3, BBOX1, CCR8, DDIT3, DGKE, IAPP, LPAR1, NCAM2, OR11A1, OR2Y1, OR8S1, PDGFRB, SHMT1, STX8, UGT2A3, SUV420H1
Negative_9642	CFI, DLD, ME1, SRC, ACSL5, ADH7, AKR1B10, ALDH1B1, ALDOC, CD55, EI24, GSTP1, H6PD, IL1RAP, ITGAM, MARS2, NADK, OR2T1, OR51A2, OR7G1, PANK3, PCCB, PCNA, PDE6D, PFKM, PLCD1, PRKAG1, PTPRZ1, RHEB, SELL, TBL1X, SERPING1
Negative_9643	ASL, CSK, CTH, GBA, CACNB1, CDS1, DHDDS, DVL1, ECHS1, EFNA5, EFNB2, GNRH2, GRPR, HRH2, NAMPT, NANS, OR2AT4, OR5D18, PAFAH2, PRKCI, RERE, SARDH, TAS2R8, TSC2, TYMS, VEGFA
Negative_9644	BTD, KMO, AASS, ALDH3A1, ARNT, BMP7, CADM3, CNTN1, DDX55, FGF14, GATM, GPT2, GUCY1A3, HAO1, HGSNAT, ICOS, IDI2, IKBKE, MAP4K4, NCKAP1L, NCR2, OR10H1, PDX1, PPM1B, SOCS2, SV2B, TGFB2, TRPM5, VDAC2
Negative_9645	A2M, ADO, CAD, SDS, ACO1, CACNB1, CCL3, CD99, CDKN2A, CHDH, CLDN19, CSNK1D, CYP1A1, CYP26C1, DHDH, EPHB4, LDHD, MYLK3, OR2T1, OR51A2, OR7A10, OR9G4, PERP, PGM1, PLXNB3, PPBP, SEMA4G, UGCGL1, VDAC1, WNT5B
Negative_9646	CR2, DLD, FAS, FOS, TFG, AGMAT, ALPP, APAF1, BNIP1, CACNB2, CDK6, CISH, CYFIP1, CYP2F1, EGFR, G6PC, HLA-G, IFNA10, IL3RA, NQO1, OR5R1, P4HA1, PDGFA, PGAM2, PI4KB, PIGN, PLA2G2E, POLD2, SELP, SUCLG1, SULT1A2, TIAM2, TUBB6, VEGFC, KIR2DL5A
Negative_9647	SP1, WAS, ABP1, ALG10, ALPI, APC2, ATP6AP1, CACNA1S, CACNG4, CCND1, CSF2RB, FGF22, GLI2, GRIN2A, GSTM2, KNG1, OR10K1, OR10R2, OR2D3, OR4P4, OR52L1, PDE9A, PGLS, PPOX, PPP2R1B, PRKCQ, RPS6KA4, TUBB1
Negative_9648	ABCC8, ADORA2A, AMY2B, CTBS, CTSG, GOSR2, HCLS1, KLKB1, LAMB4, OR52D1, PKLR, POLD4, STAT3
Negative_9649	CARD11, CCNB1, CLDN14, DRD5, GNAO1, GNG3, HSPA2, MAP2K2, MAP3K7, MTHFS, NT5M, OR2B2, OR51Q1, OR6M1, OR8J3, PIAS1, PLA2G2F, PRPS1, RHOQ, TAS2R7, UGCGL1
Negative_9650	TFG, ACTN1, ALDOB, ARHGAP5, BTRC, CD40, CD58, DDOST, ENO3, EPHX1, FZD1, IGF1R, IL15, IL1A, ITGA1, ITGAE, JAK1, MAT1A, MRAS, OR10S1, OR5L2, PIGU, POLE4, POLR2I, PPP3CC, STK36, TLR2
Negative_9651	GCLM, OR4N2, STX2
Negative_9652	ENAH, NRXN3, OLAH, OR4A15, PLCG2, VEGFC, MAPKAPK5
Negative_9653	SRM, ALG5, ANPEP, B3GNT3, CAMKK1, CD46, CHKA, DLL4, FMO4, GNRHR, HSD17B8, NAGLU, OR2T8, OR52N5, OR5I1, PIK3R3, PKM2, PLCG2, PROS1, RIPK1, SHMT1, XCL2
Negative_9654	C7, DCK, ACHE, ARPC1A, ATP5E, B3GALT4, CCL27, CXCL2, DGKH, DKK2, DLL3, DUSP6, ENTPD3, GAS1, GRB2, IL22RA1, MAP2K3, MGAT5, NEUROD1, NME2, OR11H1, OR13C2, OR2G3, OR2T4, OR4K2, OR52E4, OR7G3, PDGFB, PDHA1, RFWD2, SENP2, SIPA1, UCK2, ZFYVE16, MAPKAPK3
Negative_9655	GCK, ID2, AK3L1, ASMT, CCL15, CCNB2, CDKN1A, DPM2, HEXA, HLA-G, HMGCR, HSD3B1, IL1R1, IPPK, ITGA1, MAP2K5, MAPT, MYH4, MYH9, NFE2L2, NFX1, OR10Z1, OR11G2, OR1J2, OR56A1, PAK1, PDE7A, PDX1, PIAS1, PIGO, PMVK, POLR3B, SEMA4F, SSH2, STEAP3, STX18, SULT1A4, SUV39H1, TPM3, WBSCR22, YWHAZ, ALDH18A1, TNFRSF10B
Negative_9656	AGL, CRH, F7, JUP, ACTN4, ALPP, ATP2A2, CBLB, CD34, CPT1B, DUSP14, GALNT5, GNAO1, GNPAT, GUCA1A, MAN1A1, NRCAM, OR3A3, P4HA3, PDE5A, PGM3, PPT1, ROBO1, SULT1A2, THBS3, TLN2, TNFRSF8, XYLT2, YARS2, PAFAH1B1, TNFRSF1B
Negative_9657	COL4A2, ERBB3, TUBB

Negative_9658	FES, MVD, PVR, ADCY6, ADIPOQ, AKT1, ALDH1A3, BAI1, BDH2, CARS2, CDC42, EPHA8, EXTL3, FGF22, GDF7, HHIP, ICAM3, IDUA, IGF1R, IL11RA, IL12B, ITGA11, MAP3K12, MYL5, MYLK3, NOD1, OR10H4, OR1L3, OR2C3, OR2T11, OR51M1, PDX1, PGS1, RDH10, RDH12, SIAH1, SLC25A6, SOAT2, STAT1, STX10, VASP, WNT9B, ZMAT3, ATP6V1E1
Negative_9659	ADAM10, CYFIP1, IFNA13, RRAS2, SMURF2
Negative_9660	C4B, ALDH1A1, ALDH3A2, ANAPC4, CYP3A43, DGKE, DUSP10, DUSP4, EFNA5, ENTPD8, FCGR3B, FLT3, GALNT10, GCDH, GNRH2, GRIA1, GSTP1, IFNA2, KCNQ1, MAPK13, MYH11, NEFL, OR14A16, OR52B6, OR9G4, OSMR, OXCT1, PDHA2, PHKG1, PIK3R4, POLR2B, PPAP2B, PTPRM, PTPRR, RELA, ROBO3, SPRY3, TICAM1, WARS, WNT1, TNFSF13B
Negative_9661	TK2, XDH, ABAT, ACADVL, DGAT1, DIAPH1, GCNT1, HAO2, HLA-DOB, IL18, MYH2, NCK1, NEU2, OR6N2, P2RX3, PLA2G10, PYCRL, RDH12, RFWD2, TNFSF11, ZNRD1
Negative_9662	ANAPC1, CDO1, DLL4, HLA-G, OR4D5, P2RX5, SPTLC1
Negative_9663	ABLIM3, ADAM10, AKR1C2, ARPC5L, CACNA1E, CAMKK2, CD38, CXCL9, CYP11B2, FGF1, GLUD2, GSTK1, IL19, IL1R1, LCAT, MYLK3, NFATC3, NKX6-1, NRXN3, OR10P1, OR8I2, P4HA2, PPM1B, PPP2R5B, SPI1, UGDH, MAP3K7IP2
Negative_9664	F7, CASP6, F13A1, ITGA3, MAP3K1, OR5AK2, PYGL, UGT2A3
Negative_9665	A2M, CBL, GBA, IL4, NF1, PML, SYK, TNR, ADH5, AGMAT, ALDH1A2, ARHGEF1, ARNT, ATP5D, ATP5G3, B4GALT2, CES7, CLDN22, CMPK1, COL6A1, CYP2E1, DUSP10, ERBB4, FECH, FGF20, HMGCS1, NAT2, NCAM2, NGEF, NOS1, NRG1, OR5D16, OXSM, PANK1, PERP, PIK3R3, POLD4, PRKG2, RALB, RELA, SDHA, ST3GAL5, TAS2R48, TRIP10, WARS, TNFRSF10A, TNFRSF13B
Negative_9666	ATP6V1D, BHMT, CLCF1, GALNT13, LCMT2, SMAD5
Negative_9667	BMP4, DPM3, LAMA4, OR13C2, TUBA1A
Negative_9668	TH, AMHR2, ATP6V1F, CYP4A11, DGKD, MAP2K3, SEMA3F, TAS2R16
Negative_9669	IVD, CCL25, FPGT, GSK3B, HCLS1, HIP1, IAPP, IMPA1, NFATC3, OR51M1, SDHB
Negative_9670	C5, ABL1, ADCY6, ALDH9A1, APBB1, ATG12, C4BPA, CD79B, CYP2A6, DLL1, F13A1, FSHR, G6PD, GBE1, GMPS, GNA12, GNPNAT1, IKBKB, INHBB, LCMT1, MRAS, OR2C1, OR4B1, PAK7, PPID, PPP2R2B, PRKG1, PTGES, RERE, SAT1, SKP1, SLC27A1, SNAI2, SULT2B1, GABARAPL2
Negative_9671	AK2, IL8, MUT, NOG, ACO1, CASP3, CAV3, CD47, CDH5, GLYCTK, GPT2, IDI1, MPP5, MYL9, NEU3, NUDT2, OR2C1, PCYT2, PDE6H, PHKB, PLA2G2D, PYCR2, SETD1B, SFRP1, SOS1, SSH1, TCF7L1, TNFSF10, WASL
Negative_9672	CSK, EDA, JUN, CCNG1, CLDN20, COX15, CRLS1, CSF2RB, CYP21A2, CYP26A1, HAGHL, MAP4K2, MGAT2, MYLK2, NEU4, OR51D1, OR7D2, PDE10A, PDXP, PMAIP1, POLR3H, PRKAR2B, RHOQ, UGCGL1, UGCGL2, UGT2A1, UGT2B11
Negative_9673	ACSS2, ALG9, ANAPC2, ATP5G2, B4GALT7, CDKN1C, CXCL6, CYP17A1, DFFA, DMGDH, DTX2, FGFR3, HMGCL, LTBR, MYH7B, NARS, NLGN2, NTF4, NTN1, PCCA, PDE6D, PDGFB, PFKFB4, PFN4, PTTG1, RAC1, RALBP1, RASSF5, RPS6KA6, RRAS2, SEMA4C, SMAD1, SSH3, STAM2, STK3, THBS1, ATP6V0D1, B4GALNT1, CSNK1A1L, TNFRSF13B
Negative_9674	GHR, ABLIM3, ACHE, ADCY9, AHCYL2, ALDOA, ALOX12, ATP6V0C, BCAT2, CAMK2A, CARS, CCL22, CD19, CTLA4, DKK4, ENPP2, EVI1, F2RL2, FGF17, LEF1, LMO7, MYH10, MYH7, NCK2, NRG3, OR1N2, OR5K2, P4HA2, PARD6G, PIGS, POLR2J, PPP2R2A, RHEB, SFRP4, SKIV2L2, HSD17B10, SUV420H2, TNFRSF1A
Negative_9675	MPL, MVK, SMS, XDH, ACP5, ACSL5, ACSS2, ACYP2, ADH1A, ALG2, APPL1, CACNG4, CCL19, CCR4, CD27, CDH1, CLTC, CXCL1, DNMT3L, ELK1, FPGS, GMPS, HMGCS2, IL18R1, INPP1, ITGA10, ITGB2, KARS, LIFR, MAPK3, MTHFR, OR11H4, OR2T29, OR6M1, PAX6, PFN3, PRODH2, RAPGEF1, SPHK1, STX4, STX6, TP53, UXS1, SUV420H2

Negative_9676	DLD,FST,LCK,ALDH1B1,APH1A,BACE1,CCNA2,CD99,CES7,COQ7,CSF2,CTSS,CXCL9,DFFB,DGKB,EFNA2,ESAM,HTR4,MAML3,NRXN1,NT5C1A,OR4K15,OXTR,P4HA2,PAK4,PFAS,PIK3R3,PLA2G4A,PON3,RUNX1T1,SARS,STAT4,TAS1R1,TUBAL3
Negative_9677	ABI2,ACTB,CASK,CBLB,CDKN1A,CHEK2,CREB1,CRLF2,CTNNA1,CYP2C18,DUSP8,FGF17,GALNT1,GLDC,GLYCTK,INPP1,LAMB2,LTBR,MAP2K5,MAPK12,MGAT5,MYH1,MYH10,OR1F1,OR1L8,OR2B6,P2RX1,PPP3R1,PRKAA2,PTPRC,RBX1,SLC25A4,SQLE,XYLT1,TNFRSF11B
Negative_9678	CBS,MTR,TNC,ADRB1,AOX1,B3GNT5,DHCR24,DUSP10,EFNA1,HSD17B4,MAML2,MGAT4B,MYL7,OR10H3,PAK2,PIGH,PPP3R1,RND1,SOCS1,TYROBP,TNFRSF6B
Negative_9679	DBH,CACNA1C,IRF7
Negative_9680	GH1,DUSP2,OR4E2,TJP2
Negative_9681	C4B,F9,GBA,MPL,B3GAT3,BCMO1,CCR8,CHST11,COL6A1,CREB1,CYP4F2,DUSP8,ETS1,EXT1,GADD45G,GNA13,HMGCS1,IRAK4,LRDD,MAP3K11,NADSYN1,NAGK,OR14C36,OR2L13,OR2M3,OR8J1,OXCT2,PIP5K1C,PPP3CC,PTGS2,ST3GAL5,STK4,TAS2R4,TNFRSF4
Negative_9682	AK7,BTK,CRH,HPD,ID1,ABLIM2,AGRP,ARPC1A,ATP13A2,CASP3,CD28,CDKN2D,CPT1B,CXCL9,CYP27A1,DDX50,DHRS4,ENO3,FGFR4,FZD2,GCNT1,GSTM3,HAP1,KDSR,LAMA3,LCP2,MADCAM1,MAN1B1,MAP2K1,MCM5,MITF,NCOR2,OR13C4,OR4N4,OR8H3,PDGFD,PHKA1,SELL,SETDB2,SMPD3,SULT1A4,TUBB,UAP1,YARS2,YWHAB,HSD17B12
Negative_9683	ACAT2,ACP2,ALDH3A2,ARPC1B,ATN1,C3orf10,CACNA1A,CDH15,CLDN5,CNTF,COL5A1,COQ5,COX15,E2F4,ELK4,ENPP3,EPHB1,FECH,FZD2,GALC,GNPDA2,HHEX,HLA-DOB,KLRK1,LGMN,MYH9,NAT5,NCK2,NEU1,NFAT5,NNMT,NSD1,OR2F1,OR51F2,OR8G1,PDE8A,PFN2,POLR2D,PPCS,PPP5C,PROC,PTPRC,RASA2,SDHD,SEMA4G,TFDP1,TNXB
Negative_9684	LEP,LHB,LPL,ACOX2,ALAS2,BCKDHB,CASP8,CD82,CDH15,CDO1,CNTFR,DPM3,ENTPD3,ENTPD7,EPHX2,GANAB,GJA1,HCCS,HSPA1B,ICOS,ITGA7,ITGAX,LAMC2,LAP3,NPAS2,NTF3,OR51F2,PDGFB,PGLS,PLA2G2E,PPAP2A,PRKAR2A,PTPRC,SEPHS1,SLC25A4,SLC2A2,ST3GAL6,TIAM1,TNFSF9,TUBB3,PAFAH1B2,ST6GALNAC5
Negative_9685	F9,FES,MPL,RPE,AVPR1A,CAV1,DUSP10,LEF1,MDH2,MTFMT,MYH2,OR5B3,PRKCH,PSME3,RBKS,SRD5A1,ST3GAL2,TPI1,UROC1
Negative_9686	AKR1A1,ALG12,ATP6V0C,BCAR1,CACNA1A,CLDN6,COASY,DKK1,HSPA8,KAT2B,LARS,NEFL,NFATC4,OR10H1,OR13A1,OR1M1,OR2B2,OR4A16,OR4Q3,OR51D1,OR52N4,OR6M1,POLR3D,POLR3K,PPP2R5B,PRSS3,VAMP3,ZMAT3
Negative_9687	A2M,ADH1B,ALDH2,AMY2B,ASMT,CCR8,CD74,CHD8,CHPF,CHRM1,COL5A3,CUL1,DHRS4,DTX1,EFNB1,FOXA2,FTMT,GNA14,GNPDA2,GRB2,GSTO2,IDI2,IFIH1,IL17RA,IL25,KLRC1,LCMT2,LFNG,MALT1,MAP3K14,MAPK10,MAPK9,NUDT2,OPLAH,OR13C2,OR1A2,OR2M3,OR51A2,OR8S1,PDHA1,POLR3B,PPA2,PTGIS,STX8,PHOSPHO1,TNFRSF1B
Negative_9688	ALPI,CLDN4,DDX19A,EXTL1,MAN1A1,OR6C6,PDE4D,PDGFC,RALA,TAS2R16
Negative_9689	VTN,ACACA,ACAD9,ADH6,ARNTL,ARSD,ATP2A2,C1QB,CDS2,EFNA5,GMPR,HPSE,MGAT5,OR52R1,PNLIP,PTCH2,RASGRP2,RFXANK,SCP2,SFRP1,SHMT2,PPP1R12A
Negative_9690	BCMO1,OR56A4,RUNX1

Negative_9691	CBS,CD4,FGA,ME3,MYC,AGMAT,ALDH1B1,ALOX12,ARHGAP5,CACNG1,CCL17,CEBPA,COASY,CPOX,DDX56,DOCK1,EPB41,FGF18,GALE,GCH1,GGT7,GUCA1B,HARS,IL4R,IRF5,NCAM1,NRG3,OR10H1,OR2B6,OR51A2,OR56A4,PANK4,PDE10A,PRKCA,RDH16,RRAS,SLIT1,SPTAN1,TKTL1,TSLP,VARS,WNT1,XYLB,ATP6V0A2,SLC25A31
Negative_9692	GCK,C1QA,IL17RB,MAN1A2,VASP,YOD1
Negative_9693	DAO,GSS,TKT,ARPC3,BCAT2,BMP4,CDS1,CES7,GBE1,NUMBL,OR4K1,OR52R1,P2RX4,PNMT,POLR2C,POMC,PRKAR1B,ZAP70,MAPKAPK3
Negative_9694	XDH,ABP1,APLP1,BBOX1,CCL17,CLDN22,FGF8,FZD3,IDH2,ITGAX,PDGFRB,PLEKHA8,RAB5A,SFRP5,UGT8,SERPIND1
Negative_9695	FGB,OSM,ADH1A,AOX1,BIRC2,CASP7,CBR1,CDC27,CHRM2,CLDN16,CLTB,CNDP1,DGAT2,EXT1,GNAQ,INMT,IPMK,ITGB7,ITGB8,ITPKB,L1CAM,MAP3K11,MGAT4A,NOTCH2,OR11H4,OR6K6,PCCA,PDIA4,PLCB3,PSME3,PTCRA,RBPJ,RELA,SHC3,SOCS2,SRD5A2,ST6GALNAC5
Negative_9696	IHH,ACAT2,ACTN2,ALDOA,ALPL,ARHGEF7,ATP5E,C5AR1,CCNB1,CDC6,CDKN1A,CSH1,DEGS1,EI24,FTMT,HTR4,IFT57,IL17RB,IL1A,KARS,LFNG,MAN2A1,MGLL,OR10H3,OR2A4,OR2AE1,OR4K13,OR4N5,OR52B4,PDE3B,PDE6H,POLR2J,PTTG1,RASGRP2,RRAS2,RUNX1,SOD1,SSH3,STX5,THBS3,TUBA1A,UBE2K,VDAC1
Negative_9697	CACNG3,FGF9,GLI3,NFKBIE,OR52E4,PDE3A,POLR1D,SNAI2
Negative_9698	ADK,F10,F12,ANAPC2,ASCC3,BCL2L1,C3AR1,CACNA1I,CCL3,CDC6,DLL3,ETS1,FGF13,GDF6,IRAK1,JAK3,MCM2,NEU2,NEU3,NFX1,NRG2,OR1N1,OR2H2,OR4K1,OR52K1,OR7A17,OR8K3,PFN1,PTCH1,SMARCA2,SOS2,TAP1,EIF4EBP1
Negative_9699	ATP2A2,CACNG2,CTBS,OR10J5,OR5M3,SPRY3,SSH3
Negative_9700	AGPAT2,DGKI,DRD5,ENTPD8,GP1BA,NRXN1,PHKB,RARA,TGFBR2,TOMM40L,UROC1
Negative_9701	IL4,ALPL,CACNA1H,G6PC,SELL,SV2A
Negative_9702	LYN,ACTA1,APPL1,CCL11,CYP3A43,GALNT11,GALNT6,ITGA11,NTN1,OR1J4,OR4L1,PPAP2A,PPP1R3C,PRIM2,SOCS7
Negative_9703	BTK,KMO,XDH,ATP2A3,DERL1,DLL1,DUSP10,E2F1,GANAB,HYAL1,IFNA13,ITGAV,NFX1,NME4,NT5C3,OLAH,OR13C9,OR4K17,OR8K1,PDE8A,PIGX,SLC25A6,SORD,TRAF5,WEE1,WNT1
Negative_9704	GDA,AADAT,ALG3,ARHGEF2,CCKBR,CD82,CDC25C,CNTN2,COX15,CREB5,CYP11B1,DPM2,E2F4,EPB41L1,GALNT12,GCH1,GGT5,GSTP1,HSD11B2,LDHB,LRP5,NTSR1,OR10A7,OR10J5,OR1K1,OR5B12,OR5B21,OR5P2,OR6N2,OR8K5,PAFAH2,PAK2,PPP2CB,PPP2R5E,PRODH2,SELPLG,SGMS2,STX16,STX4,TAS2R4,TBL1X,VTI1B
Negative_9705	LCK,ALDH1L1,B3GALT1,CCKBR,CCL2,CCNH,CD47,DDIT4,FGF5,GALNT7,GCNT4,GSTM1,IFT57,ITPKA,LYPLA2,OR10A6,OR2B11,PANK1,PLAT,PYGB,RPS6,UPP1,TNFRSF13B
Negative_9706	GPT,ATP5F1,B3GNT2,CDK7,CHRM2,CLTC,CYP3A5,DAPK3,DNMT3A,EFNB1,F2RL3,GALNT12,GCDH,GOSR2,IL18R1,IL20RA,IMPDH1,ITGAD,MAN2A1,MAOA,MAP2K4,MAP3K8,MAPK1,MAPK3,MDH2,NEFH,NFYC,OR1S1,PFKM,PIK3CA,PLA2G5,PPP3R2,PROC,PRPH2,PYCR2,RAB5A,RASSF1,TNFRSF4,TUBB1,VAMP4,VCAM1
Negative_9707	DBI,GNE,MAG,MVK,ACAA1,ACVR1B,ADSSL1,ATF2,AXIN2,CLDN11,CSNK2A1,CYP26B1,FZD7,GADD45A,IL17A,JAK2,MASP1,MLLT4,OR10AD1,OR1E2,OR2L2,OR52N2,OR52N5,ORC3L,PAK3,PLIN,RASGRF1,RUNX1T1,SEMA3B,SMURF1,STAT5B,UGT8,VASP
Negative_9708	CTH,ACTG2,ADIPOQ,BIRC3,CCL21,CCL4,CD34,CD58,CDH1,ENPP2,GADD45A,GSK3B,HMOX2,HTR5A,HTR7,IL22RA1,NUDT2,OR1S1,OR2T1,OR2T4,PTCH1,SOCS4,SPRY2,SRD5A1,STK3,SV2B,TACR1,TAS2R4,TOMM40,VDAC1,WASF2,ST6GALNAC4

Negative_9709	BCR, BDNF, CSNK1G1, CTSB, GIT1, HMGCR, KLRC2, MCM2, MICA, NAT2, NT5C, OR2A14, PTPRB, UGT2B4, UGT8, VTI1B
Negative_9710	ME3, AVPR1A, CCND2, CDKN2D, CHRD, CYP19A1, DTYMK, ENPP1, FTH1, GPX7, GRIN1, GUCA1B, HNMT, HPRT1, ITGAL, LIPE, LYZL1, MAD2L2, MAN1A2, MCAT, NCKAP1, OR1G1, OR1N2, OR5A2, OR5L1, OR6K6, PRKG2, PSAT1, SESN3, TCF7L1, TDO2, TIAM1, VANGL2, HSP90AA1, PPP1R12A
Negative_9711	ABLIM1, ALAS1, C9orf95, CDKN2A, FARSB, GGPS1, GNAZ, GNG12, HAGHL, MGAT4A, NT5C, OR13C2, OR1L4, PDE1B, PIGF, PLXNB2, RDH10, RRM2B, SMC1B, TAP1, TAS2R4, TGFB3, UGT2B28, UPB1, VAMP2, YWHAQ, CACNA2D2, TNFRSF10B
Negative_9712	ITK, ACO1, BUB1, CAPN2, CCL20, CD226, CPT1A, CYBB, ENTPD2, FADS2, FCER1G, GUCY1B3, IFNA14, ITGA5, NCKAP1, OR1L1, OR2F1, RDH5, ST3GAL5
Negative_9713	C8B, EPO, PRL, ATP5D, B3GAT3, B4GALT5, CCL20, CHSY3, FZD9, GCLC, GSTM3, NCAM1, NUDT5, OR10AD1, OR10C1, OR2M5, PIK3CA, PLCG1, SETD8, TACR1, TSNARE1, UCP1, VAMP3, ZIC2, ATP6V0E1, ST6GALNAC5
Negative_9714	ATP6V0C, CCR8, CFLAR, IL12B, KIR2DS5, OR2A2, PIP5K3, PLOD3, POLR3D, RDH11, RPS6KA2, SC5DL
Negative_9715	CAD, F2R, FST, IL5, KDR, PGD, ACSL3, ARRB2, CCNB3, CD247, CYP2A7, DUSP14, GSTA1, GSTK1, KLRC1, OPLAH, OR10G9, OR10Z1, OR1D5, OR5B17, OR5M3, PAPSS1, PDCD1, PGLS, PIGB, PPP1CB, PPP2R2B, RASA2, SETD1A, SPRY1, HLA-DPB1
Negative_9716	CFI, CS, FOS, GH2, HK1, AGRP, ALAD, ALDH9A1, APRT, ATP5B, BCAT1, BDH1, BIRC3, CCL16, CCL23, CCL7, CD27, CLDN22, CTLA4, CYP3A7, DGAT2L4, DHCR7, DHX58, FARP2, GDF5, GRIN2C, HMGCS1, HPSE, IFNA8, IKBKB, IL24, ITGB6, KCNJ11, LDHAL6B, MAP3K6, MNX1, OCLN, OR10H1, OR51E2, OR5D18, PIGM, SORD, THY1, TMLHE, TNFSF8, UGT2B28, WNT6, XYLT1, ZNRD1
Negative_9717	FGG, GDA, MVD, ALDOB, CLCA4, EPHX1, ITPKA, LIFR, MINPP1, OR4D5, OR5C1, PIK3CG, RASGRF1, SRGAP2, TAS2R49
Negative_9718	F5, AXIN2, CHIT1, FZD1, GSTT1, GUCA1C, HLA-F, IGSF5, IL17B, LAMB4, NFE2L2, OR4K15, OR51B2, OR5AN1, PDE4C, PER3, PLA2G4A, PRKACG, RBL1, B4GALNT1
Negative_9719	CAD, ACOX1, BET1L, CYP4A22, IL12B, KDSR, NOX1, OR2Y1, OR4F17, OR5M8, TGFB1
Negative_9720	DUT, GSN, RB1, ALDOB, BLVRB, CRLS1, DARS, DDIT3, ENPP6, EXOC7, FARSA, FBP1, FGF2, FGF9, GUCA1A, GYS1, HLA-DMA, IGFBP3, IL9R, IRAK1, MAPK3, NFATC1, NPAS2, NRXN2, OR2AT4, OR4X1, OR5B3, PKLR, PLXNA1, POLE, PPP2R2A, PPP2R2D, PTGES2, RALB, TKTL1, TLR2, CACNA2D3
Negative_9721	CEL, OGT, ACAT1, ACTB, ATP5C1, ATP5G3, BAAT, BMP8B, C4BPB, CNTFR, CNTNAP1, CRLS1, CTBS, CYP11A1, DBF4, ENTPD1, GSTO1, HBEGF, IDH3B, IL22RA2, IRAK2, ITGA5, KLKB1, LCMT1, MAP4K3, MKNK2, MTAP, NCF4, NLGN1, OR1A2, OR5R1, PFKFB1, PHKG2, PIGU, POLR2G, PRDX6, PTGS2, SEMA4C, SMAD9, SOS2, SPRED2, TAS2R13, TUBB3
Negative_9722	MVD, ACADSB, ADCY5, ALDH2, ATP5C1, CBLC, CCL22, CSNK2A2, ESPL1, FGF2, GALNT3, OR10V1, OR11H1, PDE3B, PDGFA, PFKP, PLA2G3, PPM1D, PPP3R2, SEMA3G, SENP2, SH2D2A, TAS1R3, WHSC1L1, TNFRSF14
Negative_9723	ADC, FUK, ADSS, ALDH5A1, ATP5F1, CD40LG, CLDN16, CYP11B2, CYP2J2, DGKD, FGF12, GNPDA1, IARS, OR10J1, PIM1, PLCB2, UNC5A, WASF3
Negative_9724	ADSS, BACE2, CACNA1D, CCNG2, CD3E, CHEK1, FGF9, HNF1A, ITGA2B, OR52M1, PAK2, PFN1, PLA2G2D, RARS, SOCS5, TALDO1, TAS2R40, UAP1
Negative_9725	ACSL1, AXIN1, BCKDHA, CADM3, CAMK2B, GAB1, GMPPB, HLCS, MCCC2, NFATC4, OR1N2, OR2A42, OR5B21, PAX8, PLA2G1B, PLA2G3, RFXANK, SEMA3C, SEMA3F, SEMA4C, SOCS1, TJP2, HSD17B10

Negative_9726	F11,GK2,KMO,MTR,ABP1,ADH7,AGRN,AMD1,ATF2,CASP3,CD27,CD48,DHCR7,FGF6,GALNT10,GLB1,GMDS,HIBADH,HSD3B2,ITGB7,LIPA,MAP3K4,MAPK9,MCM4,MYL5,NR1D1,OR10A4,OR10G8,OR4C11,OR56A1,PAK2,PAK6,PIP4K2C,PLA2G2A,PNPLA4,PPP3CB,PTGS2,RPN1,SHC2,SLC2A2,ST3GAL5,TP73,UCK2,UNC5D,VDAC2,YWHAZ,ZNRD1,PRICKLE2
Negative_9727	EPO,RDX,ADRA1D,ALG10B,ATP4B,CCR7,CDK7,CDKN2B,COL4A2,CTNND1,CX3CL1,CYCS,CYP2B6,CYP2J2,DKK2,EIF4B,ENPP2,GLDC,GSTK1,HSD17B4,JAK3,LEFTY2,MAP2K3,MMP7,MYD88,OLR1,OR51I1,OR6F1,PCNA,PER2,PFN4,PIGB,PRDX6,RAD51,RDH12,SRD5A2,TAP2,TAS2R50,TNFSF8,TNFRSF1B
Negative_9728	AK7,GH2,GSR,SCD,CCL28,HCCS,MAN1A1,NFATC3,SCP2,TAS2R16
Negative_9729	DCT,LEP,AKR1C3,AQP7,BMP8A,CES2,COL11A1,CYP2B6,FABP1,HCST,HEXB,IL22RA2,ITGB2,OR2L2,P2RX6,PANK3,PPP2R5A,PTPN7,RXRA,SETD1A,SLC25A5,SLC2A2,TSC1,TSNARE1,XYLT2,YARS
Negative_9730	AUH,ABLIM1,ADCY5,AGPAT4,AGRP,ARG2,BBC3,CACNA1C,CARS2,CASP8,CCL5,CDK7,CDKN2A,CLDN16,COQ5,CREB3,CXCL6,CYP2A7,CYSLTR1,EP300,EPHA6,F11R,GUCY2C,IARS,IL23R,ITGA9,MAD2L1,NME4,OLAH,OR1D2,OR2L13,OR6N1,OR8B8,PARD6A,PFKFB1,PLA2G4A,RND1,SH2D2A,TAS2R16,TBK1,THBS1,VASP
Negative_9731	BCR,GGH,RDX,SCD,ACTA2,ACTG2,ADCY6,ADH1B,ALDH7A1,APOE,ARPC1B,BNIP1,C3orf10,CACNG5,CCL17,CDKN2D,CFTR,CXCR5,DDX54,E2F4,FABP4,FUT7,GCNT3,GMPR2,GNA13,GSTZ1,IL17B,IL19,LAMB1,MYLK,OR1S1,OR4C13,OR5AC2,P2RX6,PARD3,PLCZ1,POLR3K,PPCDC,PRKACG,RBX1,SOCS1,TNXB,TUBA1A,UNC5D,UROC1,MAPKAPK3
Negative_9732	AK7,AANAT,ATF4,ATP5G2,CACNA1H,COL1A2,CSF1R,FADS2,FGF6,GNA13,GUCY1A2,IDI1,IGF1,IKBKG,IL1R2,IL6R,IMPA2,ITGB5,MAN2A1,NKX2-2,NPR2,NUMBL,OR12D3,OR4C15,STX12,STX3,VAMP1,WNT5A,YWHAB,TNFSF13B
Negative_9733	CSK,F10,F5,MAG,ACOT11,ARPC5,C3AR1,FDFT1,FGFR2,GCAT,GNRH1,HEXB,HLA-DOA,IL5RA,INPP4B,ITGB7,KAT2B,KIR2DL3,LDHAL6B,LILRB3,MTMR2,MYLK,NME4,OR2A12,OR2T11,OR6C4,PGM2,POLR2B,PPARD,PRKAG2,PTPN7,ROCK1,RPS6KB1,RRM2B,SEMA3G,SESN2,SOD1,UCKL1,WASF2,WNT2B,WWP2,YWHAQ
Negative_9734	ACSM3,CACNG1,CCL17,CCL4,CHKA,CLDN19,CYP2E1,DHDH,DPM3,EVI1,GANC,LILRB3,MGST1,NEU4,NFYA,OR8H3,PAK1,PDE11A,PDE2A,PDGFC,PRKACA,RASGRP4,RDH10,TGFBR2,TMLHE
Negative_9735	CGA,CRK,GH2,IVD,B3GALT2,B3GALT5,BNIP1,CD8B,CDC23,CHUK,CLTA,CSNK1G1,CTBP1,DCXR,FABP1,FGF17,FLOT1,GOT1,GSTM4,HNMT,HSD3B2,IKBKE,IL1R1,LAMC2,MAN1A2,MCCC2,MECR,OGDH,OR5H2,OR6K3,OR7G3,P2RX3,PFKFB3,PIP4K2C,PLA2G6,PNLIP,PPP2CB,SFRP4,TUBB3,PAFAH1B2,TNFRSF14
Negative_9736	DCC,ACAD8,ALAD,ALAS1,AOC2,ARPC3,ARSD,CDKN1C,CHEK1,DFFB,EPHB1,FASN,FZR1,IARS2,MAML2,MCM5,NCR3,ODC1,OR14J1,PHKA2,PRKCE,PRPS2,PYGM,ROBO1,SLC25A5,SPRED1,STMN1,TUBA1C,XCL1
Negative_9737	BID,DBI,AMY2A,CNTNAP1,COMP,CX3CR1,CYP2B6,DGKE,GSTP1,HEXA,HPSE2,MAPK13,OR2A12,OR9K2,PCK1,PDGFC,PIP5K1B,PLOD1,PSPH,RFNG,ROBO2,SUFU,TAS1R3
Negative_9738	DBT,SCD,ACAD9,ADORA2B,ARNT2,B3GAT1,BIRC2,CCL14,CDH15,CDK6,EXTL1,FMO5,FTH1,GNMT,HCST,HIBCH,HRAS,IFNA14,IL12A,MAOA,NEUROD1,OR2A4,OR6C6,PAK2,PDE3A,PPP1R3B,PPT1,PRKAB1,TSHR,ULBP2
Negative_9739	AKR1D1,ALPP,ATP2A1,E2F2,EDNRB,GMPR,TRAF4,VEGFC
Negative_9740	CACNA1D,GRIA3,SLC33A1,SOCS4,TCIRG1,VAV3

Negative_9741	ATP6AP1,NUDT2,SRD5A1
Negative_9742	DBI,F9,FOS,ACADS,ACSM3,ARRB2,CACNG1,COQ5,EXTL2,IL20RB,JAG2,MAD2L2,MAPK7,PCK1,PRKAG1,PRKCA,PTPN6,SMAD6,ST8SIA5
Negative_9743	RPE,UBC,ACOX3,AGRP,ALDH9A1,ATN1,CASP9,CAV3,CD82,CREB3L4,F13A1,ITGAM,JAK3,KIR2DL3,MAFA,MAP2K4,MITF,NCK2,NFKB1,OR5V1,OR6A2,PDE4B,PFKFB2,SHC1,TJP2,TUBA1C,TUBB6,UAP1,UPP2,WWP2,HSD17B10,PNLIPRP2
Negative_9744	GCAT,NADK,SLIT1,SMURF1,TAS2R1,TCF7L1
Negative_9745	MAX,OAT,ACP1,ACPT,ANPEP,BMPR1A,CACNA1E,CSNK2A1,DIAPH3,DPM2,FLT3,FSHB,GRHPR,IARS,NOD1,OR10H4,OR10J1,OR2H2,OR6C1,PFN3,PLIN,TNFRSF8,SLC25A31,TNFRSF10A
Negative_9746	ILK,ALDH6A1,ALG6,ATP6V1A,CLDN8,LTBP1,NAGA,PCYT1A,POLR2J,VAMP4
Negative_9747	FER,ASIP,CASP8,GDF5,GP1BA,LEFTY2,OR2Y1,PRKACB
Negative_9748	C7,APAF1,CHUK,IBSP,IFNA4,PFN3,SOS1
Negative_9749	PGF,ACPT,CD59,CLDN7,GUCY2F,OR5AC2,OR6K3,PDE1B,PDE4A,RASA2,RDH16,RPS6KB2,SGMS2,SQLE,STAT3,C1GALT1C1,CACNA2D2,MAPKAPK2
Negative_9750	GP6,ACP2,AGXT2,ATP5F1,ATP5H,C7orf16,CAPN2,CCL8,CCND3,CD40,CD79A,CDH3,CNDP1,CSF2RB,CYP17A1,CYP26C1,DNMT3L,FABP3,FCER1G,GRIN2B,HNF4A,HSD17B7,ITGB3,MCM3,NCSTN,NUMB,OR10G3,OR2D3,OR9A4,OR9Q1,P2RX2,PDE7B,PTTG1,RXRB,UGT2B10,PAFAH1B3
Negative_9751	SDS,TKT,APC2,ATP4B,BCKDHA,CTSL1,CXCL10,IFNA5,LRRC4C,OR10G3,OR10G4,PTPN6
Negative_9752	DUT,EFNB1,GNAZ,NCR3,P2RX6,SEMA3D,TSHB
Negative_9753	RB1,ACP2,ADCY6,ALAS1,ARNTL,B4GALT6,BMP7,CDH4,CHAT,CSF3R,FLT3,GABARAP,GUCA1B,IQGAP3,MGAT2,OR11G2,OR2T34,OR4A16,OR4C6,OR52I1,OR6X1,PTPN7,TAS2R60,UGT2B4
Negative_9754	AADAT,ACTG2,ARG1,ASMT,GLUL,INHBE,PFKFB3,PIK3C2B,PLA2G2E,PRKACG,PSEN1,SMARCA5,TICAM1,TUBAL3
Negative_9755	ABLIM2,ALAD,ALOX15B,COMT,CXCL11,CYP27A1,EI24,IL12A,OCRL,OR51Q1,POLD3,RASSF5,SPRY4
Negative_9756	CBL,CKM,CRK,RB1,A4GALT,ABCC8,ABL1,ADCY7,AFMID,AOX1,ARHGEF6,CAMK2G,CARS2,CD36,DKK4,EPHA3,EPHB1,F13A1,FMO3,GCNT1,GGT1,LAMC1,LCMT1,MITF,NLGN1,NT5C1A,NT5C1B,PDX1,PKM2,PRKAG2,PRKY,PSENEN,RRM2,SEMA4F,SPCS1,STAT4,WNT16
Negative_9757	CAT,CBL,PXN,RFK,AMPD2,ARD1A,ATG3,B3GALT6,BDH2,CSF2,CTBS,DBF4,EPRS,FGD1,FLOT2,GALNT8,GRM1,HSD17B7,HTR7,IL6ST,KDSR,MAPK3,MYD88,NFAT5,NFYC,NRXN3,OR2M3,OR8D2,OR9G1,OR9I1,PARVB,PDHA1,PIGL,PISD,PLOD3,POLE4,PPCS,PVRL2,RALGDS,SENP2,SETD7,SSH1,TAS2R8,THBS4,UROC1,UROD,ZFYVE16,MAP3K7IP2
Negative_9758	DBT,OTC,PGD,ALCAM,APOE,B4GALT7,BCL2L1,CASP10,CBR3,CCL13,CCNB2,CIITA,CLDN14,COL2A1,CTNNB1,CYBB,CYP26A1,DDIT4,DPYS,EIF4E,EPB41L2,FGD3,G6PD,GALNT9,HIBCH,INPP4B,ITGAM,MTHFD1,NFATC1,OR51F1,OR6C74,OR7A10,OR7D4,PAK2,PAK3,PFKL,PIGQ,PIK3C3,PRKAR1B,PTPMT1,SAT2,SH2D1B,SLC25A5,UBE2L6
Negative_9759	AMDHD2,GNRHR,POLR3A,SPHK1,UGT2B17
Negative_9760	ACPT,APBB1,ATF4,B3GNT5,CAMK2B,COL5A3,ERO1L,KLRC1,OR4D1,PDCD1,PSME2,SHC2,TP53,WWP2,YKT6,TNFRSF13B
Negative_9761	ACTN2,CD86,CXCL1,GALK1,ITGA8,KRAS,MAPK13,PLA2G2F,ULBP3,HLA-DPA1
Negative_9762	GLA,HAO2,IL18R1,NFE2L2,OR1D2

Negative_9763	ADA, CGN, FGG, LHB, PGF, ALDH1A3, ALG13, APOA2, B3GALT1, BST1, CACNA1H, CCNG1, CDC14A, CNTNAP1, DUSP2, ENO3, EPHB1, GALK1, GLUL, HLA-G, IL7R, ITGAX, ITGB3, MAOA, NFASC, OR1J4, OR2H1, OR2M2, OR52H1, OR5AS1, PGLS, PHPT1, PPP1R1A, PRKACG, PYGM, SDHB, SEMA4B, SMARCA2, SOD1, TNFRSF4, TNFSF11, UCK2, UMPS, WNT7B, CTNNBIP1
Negative_9764	ARNT2, BACE1, CD244, CD40LG, CDKN2A, CPS1, CREBBP, CXCL11, EP300, FUT3, GALNTL2, HCLS1, HYAL2, IFNA4, LRP6, MCM4, NOD1, NTNG1, OR2M3, OR5B21, OR5D14, OR6C76, OR8G1, OR8K3, PCCA, PDCD1, PIGW, PPP2R5D, PSEN1, SMARCA5, UMPS, WASF2
Negative_9765	ADO, FER, ACADSB, ADAM17, ALG12, ARG1, CFLAR, DHRS3, ECSIT, FBP1, GSTP1, GUCY1A3, ITPA, MAOB, NADK, OR10R2, OR10S1, OR7G2, POLD4, RPSA, SUOX, YOD1, EIF4EBP1
Negative_9766	ILK, MPL, PGP, COL4A6, LCMT2, LTB4R2, MIOX, PDHB, POLR2G, PPP5C, PRKAR2B, SUCLA2
Negative_9767	A2M, AK3, BCR, CSK, EZR, ID3, AARS2, BDH1, CACNG8, CASK, CLCA2, CNGB1, CSF2RA, CYP11B1, CYP8B1, CYSLTR2, E2F4, EIF4E2, FMO1, FPGT, G6PC, GNRHR, GSK3B, IL5RA, IL6R, ITGAL, LCP2, MAOA, MAP3K8, MLLT4, OR1I1, OR2T12, OR6F1, OR9G1, PRKCA, RPSA, SH2D2A, SYNJ1, UBE2L6, VCAM1, YWHAB, CACNA2D4, MAP3K7IP2
Negative_9768	FGB, GBA, ILK, ARHGEF4, B3GALT4, BCL2L1, CHAT, CTBP2, CXCL13, CXXC4, DDIT4, EPB41L1, F13B, GSTM5, GUCY2F, MTFMT, OR2K2, OR2M5, OR4C11, PPP2R1A, SAT2, SHMT2, TAS2R48, TGFB2, TNFSF12, YWHAZ
Negative_9769	C4B, C8A, AGTR1, AKT1, ALG3, CDC7, CDKN2D, DAPK3, DGKQ, ECSIT, FGFR2, FSHR, FUT3, GOSR2, OR2AT4, PDE2A, PDE6H, PDE8A, PGK2, PHKA1, PRKG1, RDH16, RUNX1, SDC1, SMAD9, SOCS4, TCF7L1, WASF2, WNT9B, HSD17B10, PDCD1LG2, TNFRSF17
Negative_9770	APOC3, CACNB1, CD46, COL3A1, HTR2C, MYH7B, NFASC, NFX1, OR51V1, PARD6B, POLD4, TNFSF18
Negative_9771	BCR, CRH, MUT, NPY, SI, ACAA2, ACADVL, AGXT2, AKT2, ANAPC10, ASCC3, B4GALT5, CCNH, CD55, CDS2, CLDN14, DEGS1, DHRS3, DUSP9, FLOT2, GALNT2, IL20RA, IRF5, NFX1, OR2T8, OR5AS1, PHGDH, PKMYT1, PLXNC1, PRNP, SHC3, TAS1R3, TOMM40, TSC1, UCK1, PAFAH1B2
Negative_9772	ADO, C6, EBP, RFK, ACY3, ADCY5, AK3L1, ALOX12, BACE1, BMP2, DBF4, DGAT2L4, EHMT2, FOXO1, GRM1, HEXB, HPRT1, HSPA8, ICAM3, IL22, ITGB8, LIPF, MCM2, NTF4, OR14A16, OR1I1, PGAM2, PGM2, PIK3R2, PLA2G2F, PMM2, POLR2K, PPM1B, PPP2R2B, RELB, RPS6KA1, SC5DL, SEPHS1, SKP2, SPCS3, SPP1, THBS1, TYMS, VAMP1, WNT11
Negative_9773	DRD1, GPD2, IARS
Negative_9774	ANAPC10, CXCL1, GALNT12, HEXA, GABARAPL2, TNFRSF10B
Negative_9775	RDX, XDH, AHCYL2, AXIN2, CCL28, CD28, DHODH, ENAH, GRLF1, HDAC2, HRH1, HTRA2, OR10AD1, OR11L1, OR13C3, OR52B4, OR56A1, OR5T3, OR7A17, OSMR, PANK3, RXRB, SMAD1, TICAM2, MAPKAPK3
Negative_9776	ACPP, ACSM4, ACY3, CBR1, CCL15, CD19, CHRM3, CREB3L4, CSNK1E, CXCL11, DAPK3, DLL4, E2F4, FGF6, FTH1, GADD45B, GALNTL4, GCLM, IL18RAP, IL21, IL6ST, IRF5, LHCGR, LRP6, OR5M9, PAX4, PDPK1, POLR3H, PPOX, PPP1CB, QARS, RALBP1, RDH11, SELPLG, SESN1, TGDS, UNC5C, VDAC1
Negative_9777	AHCYL1, DPM2, ENTPD1, HNF1B, LYPLA2, MAP2K3, P2RX1, PAPSS2, PPP2R1B, TBXAS1
Negative_9778	C1S, ADORA2A, F2RL3, FSHR, OR1E1, OR5M9, VAMP1
Negative_9779	MVD, ABL1, B4GALT3, BCAT2, BCL2L1, CCL7, CREB3, CYP4F2, FTCD, HLA-DMA, IDH3B, IFNGR2, IMPA1, NUDT2, OR4M2, PDCD1, RPS6KB1, SESN1, SHC1
Negative_9780	EZR, ALG8, CNGA3, UPB1

Negative_9781	SDS, ALAD, ATF4, BCL2, CACNA1B, CDKN1A, CHRNA7, CSNK1D, CTNND1, CXCL2, DAG1, DDX23, DLST, DPYSL2, DTX1, FZD6, GAL3ST1, HDAC2, ITGAV, LHCGR, LTBR, MAPK11, NKX2-2, OR10A6, OR4P4, OR7D2, P2RX7, PAK7, PARD3, PDHA1, PIGL, PIK3C2G, PIK3R4, PLD2, PLIN, POLR2J, ROBO3, SMPD4, SPCS1, TUBB3, UGP2, VDAC2, PNLIPRP1
Negative_9782	ADH1B, CCL5, CMAS, CYP26C1, DDHD1, IL12RB1, NOD1, SIGLEC1, UAP1
Negative_9783	GGT5, GRB2, PDE2A, RAF1, STK36, SULT2A1
Negative_9784	FAS, MET, BDKRB2, BPNT1, CACNG7, CCL5, CCR8, CYP2B6, CYP3A5, CYP8B1, FABP1, FGF4, GNRH1, GZMA, HMGCL, LYPLA2, MCM7, NODAL, OR10AG1, OR1L4, OR2AG2, PFN3, PIK3R1, RASGRP1, SELPLG, SNW1, SOX17, STK11, WASF3
Negative_9785	PDC, TPR, EPRS, IDH1, IL20, OLR1, OR4K17, P2RX2, PIGS, PLXNA2, RDH11, TRPM5, UCK1, UPRT
Negative_9786	C1S, ASIP, CCL28, CCR8, CD40LG, CDH4, CLCA4, COX15, CTTN, DUSP7, FLT3LG, FUT7, GALNT11, GCAT, HNF1A, HPSE2, HSPA8, HTR2A, IL21R, IMPDH1, ITPKA, KDSR, OCRL, OR10A3, OXTR, PFN3, PLCD3, PLIN, POLR2E, PYGM, SGPP1, SPTLC1, SSH3, ST3GAL5, TAS2R3, WNT10B
Negative_9787	CGN, PVR, ALDH1A3, ASMT, CDC26, DGAT1, IL13, INPP5A, OR2M3, PPP1CC, PPP3CC, SRGAP2, STAT4, TCF7L2, TYROBP, WNT8A
Negative_9788	ACAD9, BLVRA, BMP8A, CACNG5, CALML5, CCL4, CDC26, CTTN, CYP3A7, DAPK3, EPHB3, EXT2, FABP1, GALNT6, GRIN2D, IRF7, IRS2, KNG1, LIPT1, MAT2B, METTL2B, MYH2, NAE1, OLR1, OR10AG1, OR1S1, OR2B11, OR2B2, OR4K2, OR5K1, PGS1, PHPT1, PPP2R2B, PRKX, PTPRZ1, RBKS, SEMA3E, SEMA4D, SFRP2, TAS2R4, TNFSF4, TRPC1, USE1
Negative_9789	ACP2, CCR6, CDK2, COL6A1, GALNT3, HDAC1, PAK2, PAPSS1
Negative_9790	DCN, PXN, ACTN4, B3GALT1, BPGM, BRAF, CTBP1, DAPK1, EXTL2, FGF13, GFPT2, GGCX, GSK3B, HAO1, HMGCS2, IFNA13, MAP4K1, NCF2, NCOA4, NT5C2, OR2M5, OR4D11, OR4S1, P2RX3, PER2, PER3, PISD, PRKAR1A, RRM1, SEMA4F, STX12, TAS2R49, TLR9, HSD17B12, TNFRSF17
Negative_9791	COQ6, CYP1B1, CYP4F2, DUSP6, ENO1, IAPP, IL2RA, IL2RB, MMP2, NAGK, NFX1, OR12D3, OR2H2, OR4F4, OR51V1, PARVG, PIGT, PPP2R5B, PTPRR, SMAD1, SRD5A1, UGT2B10, VEGFC, EIF4EBP1
Negative_9792	GDA, GLA, ACTG1, BDKRB1, CHKB, DGAT2, EHMT2, FGF17, GMDS, GRIA1, GRIN1, GSTK1, HEXA, HMGCS2, IL4R, NEU2, NLGN1, OR11G2, OR2B11, OR52N4, OR5I1, OR5K1, OR8G1, OR8S1, OSMR, PIGT, PLA2G4A, POLR1A, PRKACB, RAPGEF3, RFX5, RUVBL1, ST3GAL1, STAT1, TNFSF4, TUBB2C, VDAC2, WNT10A, C1GALT1C1
Negative_9793	ARNT, LAMC1, OR4D11, OR56A3, PHPT1, PIP5K1B, PLCD4, SPRY3, SYNJ1, UGT2B7, ULBP1, ZFYVE16
Negative_9794	IHH, TNC, AARS2, ACMSD, ACP2, ALDH1B1, ALG9, ALOX5, BDH2, CNGA4, CREB3L2, CXCR3, CYP3A7, EPHB6, EVI1, GGT6, GNAT3, GRM5, IL1A, IL21, IL5RA, NME6, OR10H3, OR2A4, OR51I2, OR6K3, OR8D2, PFKL, PKLR, PLIN, PLXNA1, POLD4, PTCH2, ROCK1, RPS6KA6, SDHB, SETDB2, STK36, TBXA2R, ULK2, YOD1
Negative_9795	CRK, F3, PRL, AKR1C2, AKT1, ALDH1A2, CDK7, DRD5, ENPP6, HTRA2, IL2RG, MCM2, OR2Z1, OR6C70, PAX6, PORCN, PRPS1, PTPMT1, RDH5, ATP6V0D1
Negative_9796	RET, C5AR1, EDNRB, FABP4, PSENEN, STK36
Negative_9797	DARS, IL23R, KLRC2, PNMT, RDH8, SELPLG, ST3GAL6
Negative_9798	ALDOC, CD8A, CDC42, CDH5, COL1A1, DDX50, DUSP8, FCGR3B, FGF8, GALC, GNPDA1, PERP, PLA2G2A, SEMA4B, STAT4, THY1, HLA-DRB3
Negative_9799	BTD, ABLIM3, ADCY9, ADRA1D, CLDN2, CLTCL1, CXXC4, DDB2, ECHS1, ENPP6, ENTPD4, FGF6, FZD4, GUCA1A, IFNA17, IL22, IL6ST, LDHAL6B, NCR1, NCSTN, NKD1, OR1F1, OR5K2, PDE6H, PI4KA, PIGL, PLAT, PLXNB2, PPOX, PPP3CC, PRIM1, PRKCB, SC5DL, SETMAR, STAT5B, SUV420H1

Negative_9800	CDK5,CHRM2,EGLN3,EIF4B,EPHA6,FASLG,IL11,JAG1,MAP3K5,MBL2,NAGK,OR5P3,OR6C1,OR6T1,OR8H1,P2RX5,PIP4K2C,PPP2R5D,PTPRR,WNT3,PRICKLE2
Negative_9801	C5,DUT,GAA,TK2,ACO1,APOA5,B3GAT3,COX15,CTBS,FLT1,FUT7,GALC,HAP1,HMOX2,MGST1,OR2A25,OR4D5,OR5H2,OR6F1,PDHB,SRD5A1,TUBB2A,MAPKAPK3
Negative_9802	CAD,FH,SRM,ACAA1,CAMKK2,CASP7,CLCA4,CYP1A1,DUSP14,EXTL2,HTR2B,ITGA2B,MNX1,NARS,NFX1,OR1Q1,OR2T2,OR51F2,OR5A1,OR5L1,PDIA4,PLOD3,POLE4,PTGER3,RBX1,RDH12,RPN2,TRPM5,VDAC2,WASF3
Negative_9803	C9,CACNA1E,CACNG5,CD44,CDC14A,CLDN2,COQ5,DHRS4,DOLPP1,FABP3,GCLC,IL22RA2,IRF3,MNX1,MTFMT,OXCT2,PLA2G1B,PNMT,PRKACA,RHEB,RIPK1,SETD8,SLC25A5,SOS2,TNFRSF9,TPK1,VTI1B,WNT2,PPP1R12A
Negative_9804	DAO,DCN,ABLIM2,ABP1,ACAT1,ACSL1,AHCYL1,ALAS2,ATP5I,B4GALT7,CD48,CER1,CES1,CSF1,E2F2,EPHB2,FCGR3A,H6PD,ICAM2,LTB4R2,NCAM2,OR2Z1,OR4D9,OR4K15,OR51V1,PCYT1A,PGK1,PIGU,PIK3CD,PIP4K2C,PLXNB1,PPM1D,SDHC,STK11,TAS2R60,TRMT11,TUBB2C,UPP1,SERPINA5,TNFRSF13B
Negative_9805	CFH,ADCY3,AGPAT2,B3GNT5,BCKDHB,C3AR1,CCDC6,CCL21,CFL1,CFLAR,CHD8,CSH1,CYBA,DCTN1,ENPP6,ENTPD6,FGF7,FRAT1,GDF7,HSD17B3,HSPA2,IL2RG,KRAS,MGAT3,MYH15,NCR1,NFATC2,NKX3-1,NTF4,OR6N2,PARVA,PDE1B,PDE8B,PIGP,PLOD2,PRKCQ,RAPGEF3,RBKS,SFRP2,SSH2,STAM,SUV39H2,TACR2,TCF7L2,TXNDC12,CACNA2D1
Negative_9806	CDA,MAG,ARSB,ARSD,ATN1,EPHA2,FOXA2,MAGI2,NT5C1A,OR51V1,PTAFR,ATP6V0A1
Negative_9807	ACLY,AHCY,CDK6,DHRS4L2,FDPS,ITGB2,MAP2K1,NAT6,OXCT1,PTGER1,SFRP5,STK4,TCF7L2,TUBB4Q
Negative_9808	GP5,ACSL3,ADCY1,ALDH1A2,AXIN2,CCNH,GALNTL5,GGT1,IGF1,MAPK1,OR8H3,PLOD3,PSAT1,UGT2A1
Negative_9809	ABLIM1,ATP2A2,FGFR1,GPD1,OR51B4,OR52D1,PCCA,POLR1E,ROBO1,SDHB
Negative_9810	AGRN,BRAF,CER1,CYP11B2,DAD1,DDX51,ENPP2,GMPS,IKBKB,IL21,KIR3DL3,KLRC3,MYH3,NT5C1A,OR10A5,OR2G2,OR4C12,OR4F4,OR5L1,OR5M3,OR6C1,OR8I2,PCCA,PDHB,PIK3CG,PIK3R2,PNPLA3,RPS6KA2,SELL,SHC2,ST6GAL1,TAS1R3,TJP1
Negative_9811	C8G,GALM,PLCB4
Negative_9812	IL3,OGT,PC,ALDH1A3,ALDH6A1,ATP5L,BDNF,C3AR1,CD99,CDH1,CRLF2,CSF2,CSNK1G3,CYP4F2,DCTD,EARS2,ENAH,ENPP2,FARSA,GOT2,IFNA17,OR1S1,PRKAB1,PRSS2,PTAFR,RBPJ,SFRP2,SMAD2,SOD1,SRGAP3,TAS2R7,TCF7L2,TICAM1,WASF1,HLA-DPB1,PRICKLE1
Negative_9813	GRM1,LIMK2,P4HA3,PIAS3
Negative_9814	DCC,GCK,GNS,HPD,KDR,SYK,AACS,ALAS2,ATP6AP1,CACNA1H,CACNB3,CBLB,CCND1,CLOCK,COQ3,CSNK1G3,CYBA,CYP11A1,CYP11B1,DGKZ,DHX58,DTX3L,ENO2,GLDC,HMOX1,HPSE,MAOB,MAP2K5,MAP3K12,MS4A2,NCOR1,NFKB1,NNMT,OR1J2,OR6T1,OR8H1,PGLS,POLD3,PPP3CB,SSH1,STX10,STX7,YWHAE,CSNK1A1L
Negative_9815	ACACB,ALOX5,B3GALT6,CCL8,CD40,CDKN1C,CNTFR,CRY2,DKK1,DLL1,DPM3,DPYSL2,EFNB1,FMO2,GALNT8,GSTA2,KLRD1,PPP1R3B,RPE65,TYROBP,WNT9B
Negative_9816	CD4,LIF,AADAC,AQP7,ARNT,BTRC,C4BPA,CACNA1E,CACNB2,CHSY1,CSNK2A1,CTNNA2,DUSP5,EFNA5,ENO3,ENPP7,GALNTL5,GCNT3,GLI3,HSD3B1,KLRK1,L1CAM,LIPG,LLGL2,MYH3,NRXN3,NUDT2,OR13C9,OR2V2,OR51B2,OR8D4,PFKP,PIP4K2A,PLA2G4A,PRNP,ROCK2,SLC27A1,SPI1,STAT6,SYNJ1,TAS2R38,TKTL2,WNT6,TNFRSF13B

Negative_9817	JUP,ARSA,B3GNT5,CHSY3,CXCL10,EHHADH,GGT6,HDAC2,IMPDH1,OR5B12,PFN1,PIP5K3
Negative_9818	APBB1,MAP3K2,OR7D4,SSH1,TAP1,TUBB4
Negative_9819	ACOX2,AHCYL1,ARHGEF6,B4GALT5,CCND3,CD59,CLDN10,CNTNAP1,COQ3,CREB3L1,EIF4E2,FMO3,FUT7,GYS1,HLA-DRA,IL12RB1,ITGA11,MAOA,OR2T33,OR6C6,PARD6B,PAX6,PDE2A,PRPS2,RRAS2,SARS2,SELE,SETD1A,SPCS3,SSX2IP,STX1B,TLN1,WNT9B,TNFRSF12A
Negative_9820	AK2,EBP,ALPPL2,ATP6V0C,C7orf16,CCL28,CSF2,FGF17,GCNT1,GSTM4,HAO2,IFNA21,LCAT,MS4A2,OR10R2,OR2D2,OR2H1,RASGRF1,RPS6KA2,SESN1
Negative_9821	BCR,CD6,CKM,XDH,ABL1,ADRA1B,ALOX12,BMP5,CTBS,CTSL1,ETNK1,GADD45A,GNAI2,LMO7,LRDD,MICA,NRCAM,OGDH,OR1L4,OR52R1,PIK3R1,PON2,PPP2CA,PRKG2,RASGRF1,SEMA3B,SEMA3G,TBXA2R,TRADD,ZBTB16,B3GALNT1,SERPING1
Negative_9822	CGA,LCT,CACNA1H,CD14,CHST12,CRB3,CYP2C18,DUSP1,HADHA,IL6R,MCM3,OR6N1,PRLR,RALB,RIPK1,SESN3,SNAP25,STAT5A,TICAM2,TNFSF10,TYMP
Negative_9823	CR1,LHB,ASNS,CD70,CNTFR,COL5A1,COQ3,CREB3L3,CSF3R,CYCS,CYP2D6,DHCR24,DHDH,FUT6,FZD1,GALM,GATM,GMPR,GNPAT,GNPDA2,IFNA16,IKBKG,INHBA,MYH8,NKD2,NMNAT2,NQO1,NT5C,OR4C15,OR51S1,OR52I2,OR8B12,PDE10A,PLCD1,POLD4,PPP2R2D,PRKAG1,PRKCZ,PRKY,PRSS2,SH2D2A,TACR3,TUBA1A,YWHAQ,ZFYVE16,ATP6V0A2,ST6GALNAC3
Negative_9824	F2R,ACAD9,ACADM,ACTG1,ADH5,ARD1A,ARSD,ATP5G3,BCAT1,CAMK2A,CYP11B2,DDX55,FLT3,GGT7,HSP90B1,KRAS,NOX1,OR1L6,OR56B4,OR5D14,OR5T3,PAPSS1,PARD6G,PDHA2,PGAP1,PIGQ,PIK3R4,PVRL1,RAD51,SYNJ1,THBS1,XCL1,TNFRSF18
Negative_9825	CD40,CD8A,CLDN2,COL1A1,COQ5,F2RL2,HLA-DMA,IFNA17,IL1RAP,IL2RG,KCNB1,KDSR,LEPR,LILRB3,MAP2K3,OGDH,OR51B2,RALBP1,RCHY1,SMARCA2,TM7SF2,MAP3K7IP2,TNFRSF1B
Negative_9826	VCL,ALG6,AOX1,ARHGEF7,ARSD,BCL10,CACNB2,CLTB,COL1A2,ESAM,FGF22,HNF1B,HSD17B8,IL28B,INSRR,ITPK1,KIR3DL2,KYNU,LDHB,MADCAM1,MTHFS,NCKAP1,NRCAM,NT5C3,OLR1,OR2M7,PAFAH2,PGM3,PNPT1,RDH8,SETD7,SLC27A1,TBK1,TPMT,TXNDC12,WNT10B,YOD1,YWHAG,ATP6V1B2
Negative_9827	FGG,HPD,MAX,ACP2,ALDH1B1,ANAPC10,ARPC1A,ATP5D,B4GALT3,BMP5,CASP7,CD22,CD8A,CREB3L1,DAPK3,DTX2,ESPL1,GLUD2,GNPNAT1,HLA-DOB,INADL,IQGAP2,IRAK3,LTBP1,LYPLA1,MAPK10,NME7,OGDH,OR1L1,OR2B2,OR2H1,OR51Q1,OR52E6,PARVB,PDHA1,PPAP2C,PRSS3,PTPN5,PYGB,SARS2,SEMA4D,SPRY2,TGFB3,THBD,CTNNBIP1
Negative_9828	CAMK2D,CCR1,CES2,CSNK1G1,FGF3,IRF7,MAP2K5,MYH13,OR7C1,PLOD1,SMAD2,WNT6
Negative_9829	ALDH3A1,C1QB,CAMK4,CD27,CD47,CD72,CDH1,CPT1A,CSDA,CXCL11,CYFIP1,ENO2,IL7R,LIPG,MAP2K2,MYLK,NFYB,NRXN1,OR10T2,OR2AT4,OR4K15,OR51A4,OR5D16,OR6C1,PDE9A,PER2,PHPT1,RAPGEF4,TAS2R48,TICAM2,YKT6,ATP6V1E2,MAPKAPK2,SUV420H2
Negative_9830	ADA,DLD,EDA,LPO,APPL1,BAI1,CCNA1,CHRM2,CHST11,EDN1,GFPT1,GUSB,LDHC,MECR,OR10J1,PDXK,PPOX,RALA,RDH11
Negative_9831	CFI,EZR,IL6,ADCY8,AGRP,AKR1D1,AKT1,ATP5D,BUB1,CACNA1G,CDC25A,CISH,CLDN2,CX3CL1,DKK4,EARS2,G6PC2,GLUD2,GMPR2,GMPS,HAGHL,HIP1,IFNK,IKBKB,IL11,IL6R,LEF1,MAP2K7,MYD88,OGDHL,OR2C1,OR2M4,OR4A15,OR4E2,OR4L1,OR7G1,POLR3B,PRKACG,PRKY,PTPMT1,PYCR2,RAC2,SLC27A4,WNT10B,PHOSPHO1,SERPIND1

Negative_9832	PXN, SCD, SYK, ARNTL, BDKRB1, CAMK2D, CDC16, CDH5, CDKN2A, CREBBP, DDHD1, DERL1, DFFA, EPHA5, GPD2, HNMT, IRAK3, NOTCH3, OR2M4, OR5M3, PARD6G, PLAU, PNLIP, PRKAR1A, RAB23, SH2D1B, VAMP3, YOD1
Negative_9833	EGF, AGPAT4, CD86, CNDP1, FASN, FGF19, GNRHR, IL28A, MYH9, OR2T12, OR8I2, PAX4, POLR2I, POLR2K, PTGFR, RPE65, SNAP23, TAP2, WHSC1L1
Negative_9834	SMS, ADSL, BCMO1, BIRC3, BMP5, CACNG1, CCNA2, CERK, CHDH, DHODH, DUSP2, DVL1, FARS2, FGF18, FGFR3, GLYCTK, GZMB, HIP1, IBSP, IDH3B, IFNG, MYL5, NUMBL, OR7D2, P4HA1, PCYT1A, PCYT2, PIGO, PRKACB, PTGIS, SMARCA5, SMC1A, SORD, STK3, TAS1R2, UGCGL1, WNT8B, HSD17B10
Negative_9835	ATP6V1D, CCR1, CHD8, OR10K1, PIK3C3, RARS2, SYNJ2, ARHGEF12
Negative_9836	ACSL6, CXCL5, CYP3A43, FGD1, FMO4, HBEGF, LAMB3, MAPK13, OR10H1, OR8I2, PERP, PFN3, SSH3, STAT3
Negative_9837	BTK, INS, MAG, MIF, ACP1, ACP6, AFMID, AK3L1, ALG1, ATP12A, BMPR1A, CACNB4, CAV3, CCL22, CRLF2, DDX56, EIF4B, FGF18, FIGF, GCNT2, GNPDA1, IL15RA, IL28A, ITGA7, OR10K2, OR13G1, OR2L13, OR9A4, PARD6G, PIP5K1B, RAC2, SLC12A2
Negative_9838	HK3, RET, SHH, AHCY, ALAS1, ANAPC11, ASPA, CAMKK2, CASP8, CBR1, CDK7, CHUK, COX10, CRB3, DGUOK, DTX4, FZD1, GALT, GBA3, GUSB, HAGH, IFNA5, IL2RB, IPMK, ISYNA1, MAD2L2, MGAT4B, MKNK1, OR10AG1, OR2B11, OR2L13, OR2M2, OR51E2, OR8H3, OXCT2, PAX4, PIK3C2B, PIK3C2G, PSME2, PTPRJ, SETD1B, SGPP2, STX7, TLN1, TLR9, UNC5C, YWHAB, PRICKLE2
Negative_9839	ARG1, CACNG4, DDOST, DUSP6, EPHB3, GALNT1, GCLM, HNF1A, LAMC3, MAP3K13, OR10A3, PDE6H, PLA2G1B, RBKS, ST3GAL4, SERPINB5, TNFRSF1A
Negative_9840	ATM, C1S, C3, ACPT, ATP6V1A, BMP4, CDC20, CXXC4, CYFIP1, DLL4, ELK4, EPHA3, GSTP1, HMBS, IDH3B, IFNGR2, INSRR, ITGA5, MOV10L1, NLGN2, OR10G7, OR51A7, OR51I1, PDE1C, PLTP, PON2, PPP2R1B, PRDX6, PRKAR1B, PTGFR, PTPRB, SMAD1, SRGAP2, TFDP1, TP73, WHSC1L1, ZBTB16, ATP6V1G2
Negative_9841	ADO, EGF, F8, LPO, SMS, TPR, ADRB2, ALS2, ARAF, BMP2, C7orf16, CACNG6, CAMKK2, CCNG1, CHD8, CLDN9, CNDP1, EGLN3, FECH, FGD3, GABARAP, GRB2, GRIA1, GSTA2, ITGB2, LILRB3, LLGL1, MDM4, MYL5, NRXN3, OR10J1, OR10V1, OR4F4, OR51B6, OR52E2, OR5AK2, OR6X1, OR7C1, OXSM, PIGA, POMC, SMC1A, STAT5A, TACR3, TLR2, TSC2, XYLB
Negative_9842	TFG, ACOX3, ARSB, CD247, CD38, COX10, CSF3R, CXCL3, DAPK2, HNF1A, IRS1, JAM3, LHCGR, LIFR, LLGL1, NCK2, OR4C46, PARD6B, PRIM2, PSME2, PVRL2, RASGRP1, RPRM, SEMA3F, SHC3, ST3GAL2, TNFSF18, UBE2L3, UGT2B7, MAP3K7IP1
Negative_9843	AK2, SPN, ACP1, ACVR2A, ALDOB, ALOX5, APOA2, CACNA1G, CNTN2, CXCL11, CYP1A1, DDX41, EXT2, FPGS, GALNT7, GGT5, GSTA5, JUND, LTBP1, MYLK, NPR1, OR2L2, OR2T5, OR4K13, OR6C74, OR7A10, OR8D2, PARD6A, PDGFB, PDGFRA, PIK3R2, PNMT, PTDSS1, PYCRL, RDH8, RELB, SPRED1, SRGAP2, ST6GAL1, TLR5, TSTA3, VNN1, VTI1B, ATP6V0A2
Negative_9844	DDO, ZYX, ABI2, ALAS1, ALDH2, BLVRB, CACNA1S, CDK7, CKMT2, COL3A1, CPS1, DDX18, DDX52, DTX3, DUSP14, EPHX2, GALNT1, GSTA4, GUSB, IL13RA1, LAMC2, LFNG, MAN1A1, MCM4, MYH15, OR11G2, OR2H2, OR5P2, OR6X1, OR8J1, PAK3, PDCD1, PPP2R2B, PRKAB1, PTPRJ, PTPRZ1, TAS2R1, TUBA1B, UGT2A3, UPRT, WWP2, ZFYVE9, ATP6V1E2
Negative_9845	BMP4, CADM1, MMP7, NKD1, OR2AG2, OR8B8, POLD1, PPP1R3A, PRKAA2, TNFRSF8, VTI1B
Negative_9846	ILK, ADSSL1, ATG3, CSDA, GRLF1, LDHD, MAN1B1, MASP2, OR2T5, OR6N1, PISD, PITX2, PPP2R2A, RPE65, WNT1, CACNA2D3

Negative_9847	ADO, ACP2, ACSS1, ACTN4, BTRC, CDKN1B, CYP21A2, ENTPD6, GALNT11, GALNT3, GDF6, GFAP, GNB3, GRAP2, IKBKG, IL28RA, IQGAP3, KIR2DL3, LIPA, MAP3K2, MYH10, MYH7B, NCAM1, NCK1, NFKBIE, NRCAM, OR56A1, OXSM, PFAS, PIGL, PRNP, RBKS, SH2D1B, SH3BP2, STAT4, SULT2A1, SV2C, TYROBP, WNT7B, YKT6
Negative_9848	EDA, ARRB1, EDNRA, ESPL1, OR5AC2, PGAM2, STX1A, WBSCR22, WHSC1, MAPK8IP1
Negative_9849	CKM, FAH, FER, ABL2, ADRB2, ANGPTL4, ATP13A2, BLNK, CACNG2, CD72, CHIT1, CTPS2, DAD1, FGFR3, FUT6, GNA14, GNAI2, GNG12, GNMT, GPD1L, HSD3B7, INPP5A, LAMB4, LTB4R2, MCEE, MOV10L1, MTHFD2, MYL5, NFKBIA, OR10A7, OR10P1, OR2AT4, OR4A16, OR4C11, OR4C16, OR51D1, OR52E6, OR8J1, PIGM, PIGN, PITX2, SEMA3C, SETDB2, TAS2R1, TUBAL3, UGT2A1, UGT2B4, WNT2, HLA-DRB3
Negative_9850	GHR, MIF, ABLIM2, ADCY6, ALPPL2, ATP5C1, AVPR1A, BPNT1, CASP7, CDC25C, CDIPT, CPT1A, CSF1R, CSNK1G1, DCTN1, DUSP16, EFNA2, FARS2, FGF20, FZD5, GLO1, GMDS, GNA13, HHEX, HHIP, HIP1, HSD17B7, INHBC, NCR2, NT5E, OR10V1, OR4C11, OR5I1, PAK1, PCCB, PFKFB4, PPBP, PROC, PTPRR, SEMA3C, SIGLEC1, TFDP1, TUBB6, WARS2, YKT6
Negative_9851	AMPD2, CLCA4, CYCS, NCF4, OR10Q1
Negative_9852	ASL, BTC, C7, DDO, IL6, MVK, ADORA2B, ALG5, ATG3, AVPR1B, C7orf16, CDKN1C, CES2, CLDN11, CPT1C, CXCL6, CYP4F3, GCLC, GNB3, HAGH, HTR7, IDI2, IL1A, INPP5E, LDHB, MAD2L2, NRXN3, OR10H1, OR2V2, OR52E4, PHKA1, PHKG2, PIP5K1C, PYGM, RAC3, RDH16, SETDB1, SHISA5, SKP2, SLC25A6, ST3GAL2, YKT6, ALDH18A1, HSP90AA1
Negative_9853	LPO, ACAA2, AGPAT6, AMDHD1, ARRB2, BCL2L1, CCL21, CDC20, CDC45L, FUT6, GSTM3, GUCY1A2, IL7R, ITPK1, MYLK2, NCF4, TAS2R13, TYRP1
Negative_9854	NNT, AKR1C3, ALG10, ANAPC7, C5AR1, CD86, CLCA2, OR1J4, OR1K1, PARVG, PDE11A, PIPOX, PPP1CC, PRDX6, TAS1R3, VCAM1, WNT8A
Negative_9855	LEP, SMO, AARS2, AKR1C2, AKT3, ALG12, ALPP, ANAPC1, APBB1, ARHGEF2, ATP5G3, CADM3, CCL1, CCL15, CCNB2, CD40LG, CLDN5, CXCL10, DPM2, DUSP14, EPHX1, FIGF, GSTP1, IFNK, INMT, ITGA5, LIPC, LYZL1, MCCC1, NOTCH3, OR13C5, OR1I1, OR56A1, PFKP, PIGN, POLR3D, PRPS2, PSENEN, PTGER3, SSH3, SULT1A1, VAMP2
Negative_9856	C8A, UBB, AGPAT1, B4GALT5, BMPR1B, CACNG8, CBR3, CYP21A2, DEGS2, EPHB2, FCGR3A, FGF19, GNA12, GP1BB, GRPR, IARS2, IFNW1, IL20, KARS, MAP3K3, MYH6, NUDT2, OR2G3, OR3A3, OR4S2, OR51F1, OR7G1, PER1, PLA2G2D, PLCD3, PPARA, PPP1CB, PRKACB, SPI1, TAS2R48, UGT2B4, XCR1, XIAP
Negative_9857	ACAT1, ATP5H, BBOX1, COL4A1, EHMT1, FMO4, HPSE2, IL20, IMPA1, ITGAD, JAK2, KLRC4, LAMC2, NEU1, NOS1, OR1M1, OR2AT4, OR2V2, OR5AK2, PDE7A, PFKFB2, PIK3R5, RELB, SCP2, TRHR, ULBP1
Negative_9858	AK5, CGN, ME3, ACACB, ACSS1, AGPAT2, ALOX5, ASIP, B4GALT1, BCMO1, CFL2, CHST13, CISH, CLCA2, CLDN22, CLDN6, COL6A1, CXXC4, FZD4, GMPR2, GPAM, HPSE, IFNAR2, IFT57, IMPDH1, ISYNA1, JAM2, MAP3K11, MAPK10, MCM3, MGAT1, MITF, OR2T11, PDE4D, PIK3C3, PLA2G5, PNPLA4, PPA2, PPP2CB, RARS, SOAT1, SUV39H2, TKTL1, TRAF5, UGCGL2, UGT2B28, WNT9B, HSD17B12
Negative_9859	CAD, MAG, PAH, RDX, AADAC, ANAPC7, AQP7, ARAF, ARHGEF7, CCL4, CREB3L2, CREBBP, DAG1, DARS, DERL1, DGUOK, FABP4, GADD45B, GDF5, GLG1, GLI2, GPLD1, HSPB1, NCAM1, NOTCH1, OR52A1, OR5H2, PIK3C2G, PTPN7, RAC2, SEMA4D, SPHK1, SRGAP1, STX10, TRAF1, TXNDC12, VNN1, WNT10B, XIAP, MAPK8IP2
Negative_9860	ADA, CD4, ACSS3, CACNA1D, CACNG8, CTTN, DRD5, DUSP7, EHHADH, HAP1, INPP1, LYPLA2, MAD1L1, NOTCH3, NT5E, OR2T1, OR4F4, PAFAH2, PDHB, PRKACA, RASGRP2, RPS6KA2, TLR3, UBA7, ALDH18A1, GABARAPL2

Negative_9861	FES, TNR, ACACA, AGPAT1, AKR1B10, ALOX15B, ARHGEF7, CARS, CCL3, CRLS1, CXCL3, DDIT4, ENTPD7, ETNK2, GALM, HAGHL, ICOSLG, IL3RA, IMPA1, IRF3, LGMN, LLGL2, MAP3K1, OR13C4, OR14A16, OR2W1, OR2Y1, OR5AK2, OR6B3, OR6C74, PDPK1, PIK3CA, PLAUR, PTK2, RAPGEF2, RPS6KB1, TAS2R3, WNT1, SERPINA5
Negative_9862	GP9, MPI, PGD, PXN, ACSM1, ALDH3A1, CCKBR, CDS2, CYP3A43, DNMT3B, ERBB2, EVI1, FASLG, GCDH, GMPPA, HSPA2, IFNA17, IQGAP2, LLGL2, MS4A2, MTHFS, NARS, NCSTN, OR2G6, OR8K3, OXCT1, PAX8, PFN2, PHPT1, PIGM, PLXNB1, POLA1, SARS2, SLC25A4, UGT2B7, ULBP2, WNT10A, WNT2B, ATP6V1E2
Negative_9863	FH, GBA, B4GALT1, CFLAR, CYP2C8, GALNT3, GSTA4, MAN1A2, OR4N4, OR7C2, PARVB, PPAP2C, TJP1, XYLB
Negative_9864	BTD, LPL, ME2, MVK, AKR1C1, AMPD3, CDC6, CLDN6, DERL1, DHX58, EI24, FMO5, IL17RB, NEFH, NT5E, OR2H1, OR2M7, PLA2G4A, POLR2F, PPM1D, PPP3CB, PSME1, PYGM, RPS6KA1, TFDP1, TNFSF4, TP73, TRPC1, TYK2, GABARAPL1
Negative_9865	AKT1, CD247, NTRK1, PAPSS1, PIPOX, TPM3
Negative_9866	ADK, CBS, DAO, AADAC, AGXT2, BAIAP2, CCR3, CDC7, CES7, CNGA4, FGF2, FMO2, GALT, GSTM3, HLCS, IFNGR2, IL1R2, IL22RA2, INMT, ITGB7, LARS2, LRP6, MAN2A1, MAT1A, MEF2C, NCKAP1L, OR13A1, OR2H2, OR2T6, OR52E2, OR5AN1, OR6F1, PPCDC, PPP1R3D, SEMA4C, SRD5A1, SRGAP3, ST3GAL5, TRPM5, WNT9A
Negative_9867	CD4, B3GALT4, CAMK2D, CCL20, CCL26, CCND2, CTSG, EP300, EPHB3, FOXA3, LIFR, LTBP1, MAD1L1, NAT6, NCAM2, NFATC4, NOTCH2, OR10K1, OR2V2, SDC3, SEPHS1, SH2D2A, SLC2A2, WASF3, WNT11
Negative_9868	PGP, ASRGL1, CACNG8, CMBL, CREB3L1, FGF19, FGFR4, IL28RA, MYH2, NAT1, OR51A2, ORC3L, SORBS1, TPI1, ULBP3
Negative_9869	B4GALT7, CASP10, ENPP1, GFPT2, IBSP, INPP4A, LGMN, MYL7, PIP5K1B, PRKG2, XIAP
Negative_9870	C4B, ATP5G3, KIR3DL1, OR7C1, PCCB, PDE6H, RASA2, UROC1, ATP6V1C1
Negative_9871	F12, KDR, ABP1, AKT1, ALG10, ARNT2, CCL23, CES1, COL3A1, DDB2, DGUOK, DMGDH, EFNA5, FOSL1, GMPPB, HIBCH, HYAL2, IKBKG, IL12RB1, IL25, ITGA9, ITGAM, KCNB1, NCR3, NEFL, OR52A5, OR5R1, OR6B3, RAB23, RASGRF2, RFX5, SMC1A, SUV39H2, UGT2B10, VAMP1, WARS2, TNFRSF11A
Negative_9872	ALG5, ASPA, BAI1, CALML3, CCL23, CNTNAP2, CYP2C8, DDX50, EARS2, EDNRA, EIF4E2, FARP2, FBXW11, GCNT3, GLYCTK, GNG12, GTSE1, HARS, IPMK, LAMC3, LTBR, MAN1C1, MICA, OR2AG1, OR4K2, PDE3A, PLXNA1, PPM1A, PPP1R3B, PRKCI, PRODH2, PRSS2, SOAT2, TKTL2, WNT6
Negative_9873	LYN, MAX, AARS2, ACSS1, ADCY6, ALG11, ALG3, ALOX12, BCAT1, CACNB3, CCNA1, CDK5, CDS1, CSDA, CXCL16, DGAT2, GNA12, GPD1L, GSTA1, HTR4, JAK1, LCMT1, LDHB, LIPE, MAP3K2, MBL2, MGST1, NAT5, NCR1, NFASC, NRXN1, OR1D5, OR6B3, PEMT, RRM1, SETMAR, TIRAP, XIAP, ARHGEF12
Negative_9874	F8, ACY1, APC2, BAAT, C1QB, CCNG2, DUSP10, GAPDH, GNB1, HEMK1, IL6R, INMT, ITPA, MBL2, MCM6, NCOR1, OR52N2, OR52N4, PPARA, PRKAB1, SEMA3B, SRGAP1, SUV39H2, WEE1, CACNA2D2, HSP90AA1
Negative_9875	CCR5, CYSLTR1, GFAP, MAPK1, P2RX4, SC4MOL
Negative_9876	DBH, APC2, ARD1A, CCNB3, DAAM2, EPHB4, EREG, NEU4, PLD1, TNFSF9
Negative_9877	ARPC5, CACNA1B, ETNK2, LYPLA2, PAK2, POLR2D, SCP2, TUBAL3
Negative_9878	BID, FUK, ACP2, AKR1B10, ARG1, CACNB4, ENO1, FCGR2B, FGF18, GNB3, GP1BB, GPAA1, HLA-C, INHBC, ITGAL, OR10A4, OR13C8, OR4N2, OR6C75, PLA2G2F, SFRP4, SLC12A2, ST3GAL3, TAS2R48, TPI1, WNT8A

Negative_9879	ADA,AK5,BID,LCK,ADRA1A,ALDH3B1,ATP6V0C,B4GALT5,BLNK,C3AR1,CACNA1A,CCL13,CD79B,CDC27,CLTCL1,CSNK1G3,DRD2,DVL2,FMO2,IGF1R,MOV10L1,NRG3,OR10G3,OR2A5,OR2T34,OR2Y1,OR4D5,OR4F15,OR4K14,PIP5K3,PLXNB3,POLA2,POLE4,PON3,PORCN,SDHC,STAT1,STX6,TOLLIP,UGCGL2,YWHAZ,ST6GALNAC3,TNFRSF13B
Negative_9880	DAO,F10,ACADSB,ADH4,DHDH,DUSP6,GBGT1,GUCA1B,IFIH1,IL12A,LIPA,OR13C5,OR52R1,PLXNB1,PROC,RAB23,ST8SIA5,TFPI
Negative_9881	CCND2,ESPL1,MAFA,OR52M1,PRPH,TBXAS1,TNFRSF13B
Negative_9882	ID2,ALDH2,CHRM5,OR2M2
Negative_9883	C8G,SRM,ACVR2B,ATG3,B3GNT3,CACNG6,CCNA2,CLTC,IDH3B,IL12RB2,NANS,OR5H6,OR5K2,PIGQ
Negative_9884	CBL,CP,DDC,MOS,TXK,ABLIM3,ALDH1L1,AXIN1,BCL10,CHDH,COQ5,CREB3L4,CSF2RA,CSNK2A2,CYP19A1,DDB2,EGFR,GCNT4,IKBKB,LMO7,MDM4,NQO1,OR2T6,PLA2G2A,PLXNA2,SAT2,SETD8,SETDB1,STAT2,STX8,TACR1,TBL1XR1,TUBA1B,PNLIPRP1
Negative_9885	BTD,MAX,ALG10,ANAPC5,CDC20,CSNK1G3,CYP2A6,DAAM1,GPLD1,HIBCH,PDE4D,PI4KA,SOAT1,STAM2,SERPINA1,SUV420H1
Negative_9886	CD6,TNN,ACSL6,ATIC,ATP5D,C3AR1,COL2A1,COL4A4,CREB5,CYP2C19,ENPP2,EPHA5,FGFR2,GRIA2,LTC4S,MAP3K2,MAPK7,OR10A7,OR14C36,OR2A4,PDE5A,PIP4K2C,PPP3CA,PYGB,SRGAP1,STX7
Negative_9887	AMH,TNC,ADORA2A,BBC3,DIAPH2,GRIA2,HLCS,IL1A,IL7R,LDHC,OR2T1,PIM2,SLC27A4,SYNJ2,UCP1
Negative_9888	DAPK2,GANC,GSTM2,MAP2K4,TAS2R4
Negative_9889	DBI,RET,ENPP2,ENTPD8,GALK2,GRIN1,GRIN2C,HHEX,HIBADH,ICOS,JAG2,LRDD,MAP4K1,OR1J4,OR6C76,PDE4C,PLA2G3,SMAD3,SRD5A1,TPMT,VAMP2
Negative_9890	ID4,DGAT1,GAS1,LTC4S,MYH10,PARVG,TUBB2C,HSD17B12
Negative_9891	SRM,ARSE,BAIAP2,CACNG6,CACNG7,CYP2F1,DOT1L,EARS2,GSTO1,IARS,IQGAP2,MARS,MGAT4B,OR2T6,OR7C1,PDE10A,PER3,PIGK,ROBO1,RRAS,SGMS1,SHC1,STAT5A,STX6,TUBA1C,WNT16
Negative_9892	AR,F3,GK2,HGF,IL2,PC,ABLIM2,ACAA1,AGPAT3,AHCY,AMDHD2,ARRB2,BACE2,CACNB4,CBLB,CBR3,CCNA1,CCND3,CCR3,CCR8,CD244,CDC25A,CFL1,CNTF,CRY1,CTNND1,CXCL1,DTX1,EGLN3,IL28A,KIR3DL2,NME4,NRXN2,OR14J1,OR4P4,OR5M8,OR6F1,PDE4A,POLA1,POLR1D,PRKACA,PTPRB,SGPP2,SLC27A4,SPTLC2,TLR5,WNT1,WNT10B,TNFRSF6B
Negative_9893	DHH,GSR,PRL,ACP2,AGPAT4,ALPP,ARD1A,ATP4B,BRAF,CCL11,CD46,DLAT,FUT1,GADD45A,GALE,GPD1L,GRIN1,GSTM2,GUK1,KARS,KAT2B,MAPK7,MMP14,MMP9,OR4F17,P4HA1,PAK2,PLCD4,PPP3CB,PPP3CC,PRKAR1B,TLR3,TMLHE,UROD
Negative_9894	ME1,MPI,ACACB,ACMSD,ANAPC11,CACNG5,CADM3,CLOCK,CYP26B1,CYSLTR1,DRD1,F13A1,FARSA,HMGCR,ITGA2B,ITGB4,KIR3DL1,NCKAP1,OR13C3,OR1N2,OR52E6,OR9K2,PIP5K1B,PLCB2,PRKACB,PVRL2,RASA1,RBPJL,WHSC1,WHSC1L1,C1GALT1C1,CSGALNACT2
Negative_9895	C2,FH,GK2,GP6,ACTB,ALDH4A1,AOX1,APAF1,B3GAT1,CD244,CHRD,GOSR2,H6PD,HMGCR,IRF9,LAMA3,LIPF,OR1E2,OR4F6,OR51L1,OR52E2,PDHA1,PIGN,PIGV,PIM2,PLA2G5,PTAFR,SDC1,SDC2,SH2D2A,TBL1Y,WNT5A,HSD17B12
Negative_9896	IL6,AKR1C4,ALG9,ARHGEF4,CCDC6,FBP2,FGF19,GOT2,HHEX,IPPK,MAN1A2,MGAT5,NARS2,PDHB,PLA2G3,PPAT,PTEN,RIPK1,RPE65,SELE,SPAM1,SYNJ2,TLN2,TYMP,YWHAB,YWHAE
Negative_9897	LCT,ACSL3,OR4D6,OR6C3
Negative_9898	CSNK1E,EPB41L1,FASN,MYH10,MYH8,NFKB1,OR52K1,PPP2R5A,RPS6KA3,SETD2,ST3GAL3,TAS2R9

Negative_9899	CKB, ACAA2, AQP7, ASCC3, ATP5G2, CEBPA, FABP4, GALNT14, GALNT7, GUCY2D, HDAC2, HLA-DRA, ITCH, LTC4S, MAD2L1, MAP3K4, MAP4K4, MDM2, NTRK2, OR10A5, OR2G6, OR2H2, OR51E2, OR5T3, PDE1B, PISD, POLE3, PPP2R1A, PYGM, SKIV2L2, SLC27A1, SOCS2, TNFSF12, TUBA1A, ULK2, VAMP4, XYLT2, PHOSPHO1
Negative_9900	FER, HGD, PGP, TXK, ACOT12, ADCY4, ANAPC11, BCKDHA, CACNA1F, CDC7, CHRD, CHRM2, CTBP2, ERBB2, F11R, FLT1, GGPS1, GSTZ1, HPSE2, HRH2, IL15RA, ITGAX, LDHD, LIAS, OR1L4, OR2M5, OR6A2, PCYT2, PDE4A, PIAS1, PIK3R4, POLR3A, PTPRR, RALBP1, SETDB1, SFRP1, STK11, STX8, USE1, VAV1, ST6GALNAC5, TNFRSF6B
Negative_9901	BTK, LCT, ACVR1C, AGPAT3, AKR1C2, ALDH6A1, ANAPC7, ATIC, ATP2A3, BCL10, CCR6, CKS1B, CYP19A1, DARS, DRD1, EPB41L3, F11R, FMO3, GOT1, IFIH1, IFNK, IL2RA, IQGAP1, ITGB6, LRAT, NPAS2, NT5C3, OR2T34, OR52E2, OR52M1, OXTR, PDXK, PFKL, PLA2G1B, PPOX, PPP3R1, PSME2, RPS6KB1, SETDB1, SFRP5, SLC27A1, SQLE, UBA7, YWHAE, TNFRSF17
Negative_9902	A2M, CEL, CTH, ACVR1, ALDH3A2, BDH2, BST1, CD38, CERK, CLTA, CNGB1, COL1A2, CPT1C, CSF2RB, CYFIP2, CYP3A43, DCTD, DDX18, DHRS9, DPM1, EGLN3, FLT3, FOXA3, FTMT, GALC, GNAI2, HLA-F, IDH1, IL11, INPP4A, KNG1, LAMC2, MAML2, NSDHL, OR4D2, OR4K1, OR51E1, PHKG1, PIAS3, PLXNA3, PPCDC, RELB, SCLY, SLC33A1, SV2A, UGCGL2, UROS, ZNRD1
Negative_9903	AK7, ASL, C9, CD4, FAH, MPI, PRL, ACP1, ALG10B, AMDHD2, ANAPC5, ARAF, CACNG3, CACNG8, CAV3, CCL24, EDN1, FUT4, GALNT14, GNA11, GNAI1, GRHPR, HNF1B, IFNA4, IL29, ITGA2B, ITGB6, KNG1, MDM4, OR10H2, OR11H1, OR1A2, OR2F1, OR5I1, OR8A1, PDGFD, PIK3C2G, PLXNC1, RPS6KB1, SLC25A4, SOAT2, TACR3, TBL1X, TCF7L2, TPI1, UCKL1, UGT2B7, WARS, ATP6V0A1
Negative_9904	DHH, LYN, ACSL3, ACTG1, ANGPTL4, CES1, CHST12, CNGA4, COL4A4, CYCS, DHDH, DNMT1, DPYS, E2F3, EFNB1, FGF11, GMPS, GSK3B, GUCY1A2, MAPK12, MITF, MLYCD, OR10H5, OR4F17, OR4P4, OR7A17, ORC2L, PIGP, PLA2G2E, PLCG1, PLCG2, PLXNA2, POLR1E, PTGES2, RDH11, SEPHS1, SNAI2, UBA1
Negative_9905	ACTN1, COMP, NRG3, OLR1, OR2L8, OR2M4, PAX6, PTPRZ1, RHOQ, TLR3, TNFRSF14
Negative_9906	ACY1, ARHGEF7, CMBL, FABP4, L1CAM, NFKBIA, OR10R2, PDE8A, PVRL3, RETSAT, SUCLG1
Negative_9907	CFD, RFK, TH, ATP5A1, B3GALT4, B4GALT5, CALML3, CDS1, CSNK2A1, DUSP4, EXTL2, FGFR1, FTCD, HAO2, HGSNAT, HRH2, HSD17B8, KLKB1, LHCGR, MAGI3, MAN1B1, MAOB, NCR1, OR2T8, PAK4, PDE3B, PIGO, PIGP, PRKCZ, PSAT1, PSENEN, PTGS2, STX2, SUCLA2, SULT2A1, TLR5, AASDHPPT, ST6GALNAC5, TNFRSF14
Negative_9908	CFI, GP6, LTB, OTC, PDC, ACOX3, ALDH4A1, ATP13A2, CCL20, CD244, CD47, COL1A2, CXCR3, FIGF, GALNT1, GCH1, GGT6, GMPR, GNPDA2, GUCY1A2, IARS, IDUA, MLLT4, NSD1, OR10J3, OR1L3, OR5P2, OR6Y1, OR9G4, PLA2G4A, PPCS, PSPH, PTGIS, SOCS7, SQLE, ST3GAL4, STX8, UGCGL1, ULBP3, VAV2, ATP6V1B2
Negative_9909	GPT, NLK, AARS2, CALML5, CYP26B1, GANAB, GGCX, GPAA1, ITGA10, LHCGR, MTMR2, NEU2, POLE3, POLR2I, PTK2, PTPRC, SELPLG, SSH2
Negative_9910	F2, PC, ACSS3, ALDH3A2, CHIA, GALNT13, GP1BA, GSTA5, IL21R, LCAT, NME7, NMNAT1, OR10H1, OR1F1, OR4S2, OR6T1, P4HA2, PGLS, PNMT, POLR3H, PTGS1, RFXAP, SLC12A2, SLC9A1, SPI1, SPP1, ST8SIA1, UCK2
Negative_9911	CXCL16, FGF22, FGFR1, H6PD, IFNGR1, ITPKA, NRG2, NRXN1, OR4C13, OR4K1, PFKM, PLCG1, POLR1C, POLR1D, PTPRM, RETSAT, ST3GAL1, ZAP70, ATP6V1B1
Negative_9912	BMP2, GSTP1, INPP5A, JAM2, MTMR6, NAMPT, OR5M8
Negative_9913	NNT, TK2, CLDN6, CTTN, ERBB2, GP1BA, NCF2, NR1D1, ORC2L, PPP1CA

Negative_9914	C2, FGB, FN1, LAT, ACOT12, ANAPC5, B4GALT6, BCAT1, BLVRA, CD38, CDC25B, CXCL13, CYP1A2, CYP2U1, EFNB2, GALNT14, GGT7, GPX5, GSTO1, HLA-C, HNF1B, HSD17B7, ITGB1, L1CAM, METTL2B, MMP14, OR13C4, OR1L6, OR4A16, OR51I1, OR6T1, PGM1, PIK3CA, PPP2R5C, PRKAG2, PRKCQ, ROCK1, SHC3, SRGAP3, STX12, TJP3, TMLHE, TPI1, TRAF3, UPP1, VAV1, CACNA2D1
Negative_9915	ERBB3, GCDH, MICA, PAX6
Negative_9916	LEP, AASS, ADSSL1, AKR1C3, ALDH3B2, CPOX, CYP26C1, E2F3, ENPP2, GLUD2, HSD3B1, IFNB1, ITGB5, MAPK11, NEUROD1, NFX1, PCCA, PSENEN, SLC2A4, STX16, STX18, UPP1
Negative_9917	AKR1D1, ARHGEF2, CARS2, CCL16, CTNND1, FTMT, IL21, NANP, NCAM1, NMNAT2, NRXN2, OR2T2, PFKFB3, PLAU, PPCS, PRSS1, SRD5A2, PAFAH1B3
Negative_9918	CARD11, CCNB3, CDC42, CDIPT, CHAT, CNGA4, CYSLTR1, DAPK1, FLT4, HDAC1, HTR6, IL12A, IL29, NFKB2, NFYC, NT5M, NUDT8, OR10A3, OR10X1, OR2M2, OR2T2, SLC27A4, SPRED1, TOLLIP, ULBP2
Negative_9919	GNS, ADAM10, ARSB, B3GAT1, CAV1, CCL24, CHD8, CHIT1, EGFR, GAB1, GAS1, GNA13, IFNGR2, IMPDH2, LIPA, NME2, OR5AU1, PAX4, PPAP2C, PTCRA, PVRL3, RASGRP2, TSHB, TUBA1B, UBA1, UROS, VASP
Negative_9920	ADRB1, HSD11B2, ITGA3, OR10V1, OR51F2, SMAD7, XCL2, XYLT2, ZNRD1, HLA-DRB4
Negative_9921	AMT, GCK, GLS, PRL, AGXT2, AKT1, ALAD, ANAPC4, CACNB3, CACNB4, CCND1, CHD8, CLTCL1, CYSLTR1, DHDH, FASLG, GCNT4, GNMT, HBEGF, HSD17B3, HTR2A, IFNW1, IL20, KIR2DL3, MAD2L2, MCCC1, MYH3, NANP, NFATC2, NTRK2, OR5R1, OR9I1, PDE5A, PGS1, PI4KA, PIK3R4, PNPO, PPP2R2A, PRKCQ, SEPHS1, SLC25A5, SLC27A4, STK4, TAS2R5, TCF7, UBE2K, WNT7B
Negative_9922	CDA, F5, FES, ACSL3, ADH1A, ATP5H, B3GNT5, BCAT2, CIITA, CRLS1, ENTPD3, EPB41L1, GOSR2, GRIN2D, IKBKE, ITGA10, LLGL1, MAP4K2, MGAT3, MYL5, OR10G4, OR8H2, ORC5L, PDX1, PER1, PHPT1, PMM2, RFX5, SDHA, STX8, CACNA2D3, PAFAH1B1, TNFRSF1B
Negative_9923	CLDN7, DDX4, MCEE, OR5AN1, RHOQ, SEMA4C, TCIRG1
Negative_9924	CGA, GPT, B3GNT4, CDC23, COQ3, CRKL, CTBP1, CTTN, CXCL6, CYP2J2, DPYS, FBP1, FGF6, GSTM3, GSTO1, GSTO2, H6PD, HSD17B7, INPP5E, MYH13, NFATC4, OR1S1, OR52W1, PAK3, PDE3B, POMC, PROC, RARS, SDC3, TACR3, TUBA3E, TNFRSF17
Negative_9925	HAL, ITK, MTR, AMY2A, CCNG1, CYP4F3, DAG1, EHMT1, FRAT2, GAB1, GALNT7, GATM, GSTA5, IL18RAP, MAP3K1, OR2AG2, OR7A10, PPP2R2A, PPP2R5C, PTGFR, RARA, TUBA1A, UROS, WNT1, HSD17B12, PAFAH1B1
Negative_9926	CKB, F10, LCT, LIF, TAT, ADORA2B, ALDH7A1, BACE2, BCMO1, BIRC5, CCL20, EPHB1, EXTL2, FRAT1, FZD8, GALNTL1, GPD1, GRIA3, IL4R, IPPK, ITGA6, KCNQ1, MKNK2, NFKBIE, NRG2, NTNG1, OR1L4, OR52B6, OR6C4, OR8B8, PIGF, PIGU, PIK3C2G, PRKAG3, PRODH, PTAFR, RBL1, RCHY1, ST8SIA5, STAT5A, TKTL1, TNFSF9, UGT2B11, UNC5B, VEGFA, WNT4, ZMAT3, CACNA2D4, PDCD1LG2
Negative_9927	CDA, HAL, TH, ADH6, AKT3, ATP5G3, B3GALT6, BMPR1B, CACNA1E, DHODH, FBP1, GALK2, GAMT, GLDC, HEMK1, HLA-DOA, HNF4G, IFNA8, IRF3, ITCH, ITGA4, LRRC4C, NCR1, NKD2, NLGN1, ODC1, OR12D3, OR2T2, ORC3L, P4HA1, PDPK1, PPP1R1A, SGPP1, STAT4, TACR2, TAS2R10, TAS2R7, TBXA2R, PAFAH1B1
Negative_9928	C7, CKB, HAL, LHB, ALOX5, BCL10, BMP2, CCKBR, CCL23, CCNG2, CD244, CD274, COX10, DUSP3, HTR2C, IL18R1, LTBR, OR10J3, OR1S1, PDE1B, PISD, RAC2, RELA, TAS2R10, UPRT

Negative_9929	IL8, LYN, NNT, TPO, AASS, ARAF, BET1L, BMP7, C7orf16, CACNA1H, CCNA1, CLCA4, CLDN10, CXCL12, DDIT3, DUSP8, DVL1, GAL3ST1, GDE1, GNAI2, GZMA, IFNA6, LIPG, NRG4, NT5C2, OR10H2, OR1K1, OR4K17, OR52W1, OR6C4, OR7A10, OSMR, PAX8, PFN3, PLXNA1, PPAP2A, PPID, PRDX6, PROC, SEMA4D, SOAT2, ST3GAL4, ST3GAL5, STK36, THBS4, TNFSF4, UMPS, WASL
Negative_9930	ATP6V1D, GAD1, HLA-DOB, OR2AE1, UPRT
Negative_9931	C9, GK, ACY3, ADCY10, ADCY9, ARD1A, BMP8B, CREB3L3, FLOT1, FSHR, GFPT1, GSTM5, HLA-DMA, HSP90B1, HTR5A, ICOS, NFKB1, NGEF, NPR1, OR2T34, OR2T4, PLAU, POLD2, PRKAG1, RXRA, SDHD, SEPHS1, TNFSF12, PRICKLE2
Negative_9932	B3GALT1, CCL8, CDS2, GSTA4, HNF4G, OR2C3, ST8SIA1
Negative_9933	ZYX, CTSL1, DIAPH2, FARS2, GNPNAT1, WEE1
Negative_9934	C8A, TK2, ACTG1, ALAS2, ALG1, ANAPC10, ATP2A1, BET1, CREB3L1, DKK1, DNMT3B, EP300, F11R, FDPS, GYS2, HAGH, IL20, IL5RA, IRS1, ITGAD, LAMC2, LDHB, MCAT, MTMR2, NKX3-1, NPAS2, OR1A1, OR4L1, PLCE1, POLE4, TGFA, WEE1, XYLT1, TNFRSF11B
Negative_9935	ME3, AKR1D1, ALDOC, ARNT2, ATP13A2, BDKRB2, CAMK4, CDC25A, CHRD, CTBP2, CYP17A1, CYP2D6, CYP4A22, DKK4, GALNTL5, GNA12, MTFMT, MTHFS, NADK, NOTCH2, OR10A7, OR13C3, OR1A2, OR2AE1, OR2T11, OR5D13, PARS2, PRKCG, PRKX, PTDSS2, RAPGEF4, RPS6KA3, SKIV2L2, SLC27A5, SMAD7, SPRY1, SSH2, ST3GAL1, STAT4, SYNJ2, TAS2R7, TUBA1A, XCL2, ATP6V0E1, HSP90AA1
Negative_9936	C8A, IL2, TNN, ALDH3B1, ALOX12B, ARSB, CD28, COL4A6, CYP4A11, DHDH, E2F3, FGF19, GALK1, GLI3, HADHB, HRH2, IL6R, ITGA4, KAT2A, MAD2L2, MECR, METTL2B, MTMR2, NCR1, NEFL, NR4A1, OR6C70, OR6T1, PHGDH, POLD1, PPP1R1A, PROC, RCHY1, RXRB, SOCS3, SUCLA2, UGT2B4, UROD, VDAC1, PAFAH1B3
Negative_9937	AGK, BCAT1, E2F5, GALNT11, GNA12, MAP2K2, OR56A3, OR7G1, OR9Q1, PDIA4, PI4KB, SESN3, SMAD7, SQLE
Negative_9938	ACVRL1, AGPAT4, CDKN2B, CLTCL1, CNDP1, COL1A1, DKK2, DOT1L, EGFR, ENO2, ERBB2, GBGT1, GCNT2, GLI1, GNPAT, GPX5, HSP90B1, IL20RA, MAP4K1, OR10Z1, POLR3B, RBX1, RPS6KA4, SH2D1B, SNAI2, STMN1, TIAM2
Negative_9939	CKB, GPI, ACLY, ACPP, ATP5D, CACNA1S, CD8A, CREB1, FBP1, FTMT, GADD45A, GNA15, IL2RA, IRF5, MYH6, OR4P4, OR51A2, PARVG, PIK3C3, PIK3R4, PTPN11, SH2D1A, SHC2, STK11, WHSC1, ATP6V0A4
Negative_9940	GGH, MVK, PVR, TK2, ACOT11, ACOX1, AMDHD2, ANPEP, CCL19, CCL5, CD48, CDC45L, CLOCK, EGFR, GAD1, GBE1, GCDH, GGT6, GRM5, GUCY2D, IKBKB, INPP5E, IRF3, LCP2, OR10H2, OR2T3, OR52I1, OR5R1, OR6B3, OR7C1, P4HA2, PANK2, PARD3, PDE4B, PEMT, PTCH1, RPN1, SMAD5, UXS1, WNT8B
Negative_9941	BLNK, CANT1, CTSB, DLL3, OR10K1, OR51E1, TBL1X, TIRAP, WARS2, YARS2, MAPK8IP3
Negative_9942	C4B, DLD, F7, HGF, ID3, SPN, ACTG2, ALOX5, ANPEP, AREG, ATIC, CCR6, GSTM4, HSD11B2, IMPA2, NCR3, OR13D1, ORC6L, RRAS2, UBE2L6, WNT6
Negative_9943	ADO, GAA, BNIP1, CACNA1H, CDK7, CLOCK, DAAM1, OR2F2, PMVK, PVRL3, SDHA, SELPLG, SENP2
Negative_9944	IDS, INS, SYK, ACO1, ADH4, BCL2, CADM3, CCL3, CTBP2, CYP21A2, ELK4, EPHB1, F13A1, FRAP1, ITGB6, LRDD, OR10G8, OR1G1, OR2A4, OR2T4, OR5J2, PIGL, PISD, RASGRP1, RHOD, SHC3, TAS2R3, TCF7, UBE2K, YWHAB
Negative_9945	GK2, PAH, PLG, TK2, ADH4, ADH7, ALAD, CAMKK2, CCR8, CKMT2, CSF3, CXCL9, DDX54, DGKH, FBP2, FRAT1, GFPT1, GSTA5, GZMA, HTR2B, IFNGR2, IL21R, IL22, KIR2DS4, LEFTY2, MAPK9, MDM2, MGAT2, NNMT, NRG4, OR2S2, OR51I2, OR5B12, P2RX1, PDXP, PI4KA, PIGS, PNLIP, PPP3R1, RPS6KB2, SH2D1B, TRAF4, TSLP, ULBP3, VASP, ZMAT3

Negative_9946	AMT, C5, CBS, RDX, ACSM3, ACSM4, ALDOC, CYP2C18, DNMT1, ENPP2, ETNK2, EXOC7, FARSB, FECH, FGF12, GNA12, GNAI2, HAO1, IFNA13, IFNAR2, IL19, IL20RB, IL21, LIPF, LYPLA2, MAN1B1, MTFMT, OR10Z1, OR1L6, OR1N2, OR51D1, OR8S1, PFKM, PIK3R1, PSEN1, PSME2, PTGFR, SDC1, SPRED2, SV2C, TNFSF10, UBA1, WEE1, CSGALNACT1
Negative_9947	KIT, CTBP2, CXCL12, DUSP6, IL15RA, PDE7A, PECAM1, PGM2, PNLIP, POLR3H, SRD5A1
Negative_9948	OAT, GLDC, HLA-C, OR2T3, TNFSF14, UGCG
Negative_9949	SFN, ACACB, APOA5, APOC3, CBLC, CSF2RA, DOCK1, FGFR3, LALBA, LIPF, PLA1A, PRIM2, UCKL1, WHSC1L1, TNFRSF1B
Negative_9950	CD4, PARD6G, RRAS2
Negative_9951	EGF, MPL, APOA5, B3GALT4, CCKAR, CCL14, CD8A, CYP11B1, DLL4, DOCK1, EPHA2, EREG, GNAZ, MAGI3, MGAM, MTHFD2, NOX3, OR10J5, OR1A1, OR4A15, OR4K5, OR51E1, OR5K2, PLIN, PPCS, PRKCB, SAT2, SCP2, SPAM1, SRD5A1, TDO2, UNC5C, WNT5A, AASDHPPT, CACNA2D4, HLA-DPB1
Negative_9952	CTH, PML, RDX, BLVRA, CACNA1E, CARS, DTX1, ENO1, GNMT, IL1R1, OR10H5, PCNA, RPRM, TGFBR2, TLR2, TNFSF4
Negative_9953	CKB, GK2, JUP, CCR5, CD3E, CDS1, COL4A6, CREB5, EXTL1, F11R, GALNT8, GCLC, GGCX, GJA1, GNPAT, GUCY2F, IFNAR1, IL20, MAML1, MAPK3, MGAT4B, MPDZ, MPP5, OR2AE1, OR4C11, OR4S1, OR9G4, PARVB, PAX4, PDE11A, PIGX, PPP1CC, PRKAG1, SEPHS1, STAT3, SULT2B1, TRAF4, WNT10B, ATP6V1C1, TNFRSF10B
Negative_9954	GNS, HDC, PXN, ADCY9, ADH4, ALDH1A2, ATP2A1, CALML3, CCNB2, CD27, COL4A2, CSNK1G3, DLAT, DRD2, DTX1, G6PC2, GLUD1, GNAI1, GUSB, HLA-DRA, HRH1, HSD17B3, IL10RA, IL18R1, IL6ST, MINPP1, NFX1, NOX1, NUDT9, OR13H1, OR2L2, OR4C16, ORC4L, OXCT1, PGM1, PIK3C2B, POLR2A, POLR2B, PPAP2A, PPARA, PTCH1, RETSAT, SLC2A4, TAP1, TYROBP, VEGFC, WNT5B
Negative_9955	AASS, ALDH1B1, ARPC3, CACNG3, CCL19, CD58, CDC25A, CLOCK, CRLF2, CSF3, CYSLTR1, E2F2, EFNA1, HNMT, IRS4, ITGA1, MITF, NEU4, NKD1, NKX3-1, NLGN3, OR6Y1, OR8D1, OR9Q1, PDE5A, PDGFRB, PMAIP1, POLA1, RALGDS, RHEB, SEMA3E, SMAD7, TAS2R16, TM7SF2, UGCGL2, VAMP1, HLA-DPA1, SERPINA5, SUV420H2
Negative_9956	ACP1, ACPP, APOA5, ATP5G3, CCND1, CDK5, CTBP1, CYP26C1, ELK1, EPHX2, ESPL1, EXTL1, GLDC, GNRH1, IGFBP3, IL21, LYZL1, NOTCH3, OR10H4, OR2C1, OXCT2, PARVA, PI4KB, PRKAR2A, SEMA3G, SULT2B1, SUV39H2, TAS2R1, TP73, TXNDC12, TYK2, UNC5A, TNFRSF13C
Negative_9957	C4B, EPO, HK2, TKT, ADRB1, ATG12, ATG3, BUB1B, CACNA1D, CACNB1, CTSL1, DAD1, EPHA1, FGF5, FLT1, GMPS, HLA-DOB, HLA-G, IDH3G, IRAK3, ITGA9, ITGB4, JAK3, LDHA, MKNK1, MYH3, NAE1, OR4A16, PIK3R4, PRIM2, PRKG1, SARS, UCP1, VKORC1, SERPINA5, ST6GALNAC4
Negative_9958	RPE, SRF, ACAD9, ACLY, ACOX3, AGRP, ALDH3B2, ALDH7A1, ALG8, AMHR2, C4BPB, CACNA1A, CCL11, CHKA, CNTNAP1, CREB3, CYP4F2, DDIT4, ELK4, FARSB, FGF19, FGF4, GCLM, HHEX, HMOX2, KIR2DL2, LAMC1, LEFTY2, LMO7, MCCC1, MICA, MYL9, OR10V1, OR4B1, OR5M9, P2RX1, P4HA2, PTGES, PYGM, RAB23, RAC3, SMURF2, TALDO1, UPB1
Negative_9959	ILK, TNC, TPO, ACADL, AHCY, BIRC3, CTSS, DGKQ, DMGDH, FUT9, GALNT7, HNF1B, HSD17B8, IL23R, LRRC4C, LYZL1, MAP2K1, MGAT1, NCR1, OR2D3, OR2V2, OXCT2, PHKA2, PIAS4, PIP4K2B, PISD, PPP1R3B, PTGS2, PYCRL, RAC1, RPN2, TAS2R38, TAS2R48, TBL1Y, TDO2, TGFB3, THBS2, TLR5, XYLT2, SLC25A31
Negative_9960	AMH, CEL, ACLY, CREB1, DOLPP1, GBE1, OR4D1, OR52H1, PIGT, PPBP, ROCK1, ST3GAL4, TNFSF11, SUV420H1

Negative_9961	GP9, TEC, XDH, ACVRL1, ALS2, ARHGEF4, ARNT2, ATP6V1A, CAMK2B, CARD11, CD19, CDC16, CYP3A5, FGF3, FLOT2, FSHR, FZD9, GALNTL2, GIT1, GNG12, GNRHR, KIR3DL1, L1CAM, LCAT, MAN1A2, MAP2K1, NKD1, OR10G3, OR1C1, OR2F1, OR5T3, OR6C1, PARVB, PDE1B, PFKFB1, PRKACA, PRKACB, RFNG, SEMA3F, SMAD7, TAS2R7, TCF7L2, TNFSF9, TUBB3, WASF2, WNT10A, WNT6
Negative_9962	SCD, TNR, ACSM3, AMD1, ANAPC1, CCL19, CCND2, CD70, CLDN20, COMP, CYBB, DLL3, EHHADH, EPHB3, ESAM, FGF11, GALNT3, IDH3G, LTBR, MAOB, MYH10, OR10G7, OR10Z1, OR13D1, OR1J4, OR2T3, OR5D18, PDHA1, PIGB, PIK3R2, PLA2G3, POLE3, PPP1CB, PPP2R5A, PPP2R5E, PPP3CC, PTK2B, RBPJ, RDH12, SCIN, SDC3, STX2, TOLLIP, TUBB, TYRP1, UBE2K, XYLB, ATP6V0A2
Negative_9963	DLD, NEU4, PDE6D, PDE6H, PLA2G2A, RDH11, STAT3, STX5, HLA-DRB4
Negative_9964	ZYX, AMDHD1, B3GALT2, COL6A1, CXCL3, DDX19A, DGKB, DUSP5, GNAZ, GP1BA, HMGCS2, HSD17B3, ICAM3, IL23A, IRF5, KLKB1, MCCC2, MFNG, NCF4, ODC1, OR4C15, OR4F17, P2RX6, P4HA3, PIGF, PTGER3, RAPGEF2, SLC27A1, SMAD9, TKTL1, TNXB, TRADD, TUBA3E, VAV1, TNFRSF13B, TNFRSF6B
Negative_9965	BTC, ID4, MET, ACY1, ALDH4A1, BBOX1, C7orf16, CDK4, CLDN20, CSNK1G2, CX3CR1, CYP11A1, DGKH, EPHB3, EPRS, FLT1, GAL3ST1, HMBS, NRCAM, OR10P1, OR13A1, OR4C16, OR52B6, PGK2, PLXNA2, POLR3D, TOMM40L, TPM3, XYLT1, C1GALT1C1, CACNA2D1, HLA-DPA1
Negative_9966	EPO, NPY, AACS, CACNG2, CD72, CLCA2, FCGR3A, GALT, INHBA, P2RX5, P4HA2, PDE1A, PDE7B, PLOD3, RASSF5, TBL1Y, TBXA2R, UGDH, WBSCR22
Negative_9967	TXK, ADCY8, ADSSL1, ALG6, ALPL, APOC3, CCR2, CERK, EXT1, GALNT10, GBE1, HEXA, IL2RG, IL8RA, IMPDH1, ITGAE, ITGAV, OR5V1, PLOD1, PLXNB2, PPARA, PPM1A, PTGES, SELL, SESN2, SETD1B, WARS2
Negative_9968	DAO, AKR1B1, CD58, CTNNA2, DHCR7, DUSP6, GFPT2, HES1, IL17B, MYH15, NCKAP1L, OR51B5, ORC1L, PCK2, RASGRF2, RRM1, VDAC3, XIAP, SERPINA1
Negative_9969	LAT, TNC, ALDH2, ANAPC11, APPL1, B4GALT3, CMPK1, COL6A2, CSH1, CXCL12, DHCR24, DHFR, EPRS, ETNK2, G6PD, HSD3B7, IMPDH1, KCNQ1, MGAM, MYH11, OR2AT4, OR52R1, OR5J2, ORC1L, ORC2L, PGAM2, PITX2, SESN1, SHISA5, SORD, TSC1
Negative_9970	KDR, NF1, AXIN2, CCL25, CLDN17, DDX50, DPAGT1, GRM5, ITGB8, KIR3DL2, MAP3K6, MAPK9, MPZL1, NFKBIB, NKD1, RXRG, SMAD2, TCF7, VAV1, WARS, WASL
Negative_9971	ALG2, ARPC5L, CADM3, CCR4, CISH, CNTN1, CYP3A5, DPYSL2, DUSP3, FGF1, FGF13, GLB1, LIPG, MCM5, PIGS, RAF1, STAT5B, STX5, TPMT
Negative_9972	AK1, AR, DCT, LTB, TK1, AACS, ALG9, ALOX12B, ARPC5L, B3GAT3, BPGM, CHRM5, CHST12, CISH, CNTN1, CXCL6, DPYSL2, EDNRA, FGF8, FGFR2, G6PC, GDF6, GRB2, GSTO2, GUCY1B3, HES1, HNMT, INPP5A, JAK1, MECR, MGAM, MYH6, NUMBL, OR1F1, OR2B6, OR4M2, OR51M1, PFAS, PLA2G4A, POLA1, PPP1R3C, RAPGEF2, SLC12A2, SPCS1, TAS2R40, TGFBR1, SERPING1
Negative_9973	F2, SCD, ACP2, ACTA2, DIAPH1, EGLN1, ETNK1, FGF14, FGF21, GDE1, GGT7, GPD1L, GYS1, HIBADH, HSD17B2, IL23R, ITGAV, MYH11, NFYA, OR1N2, OR2T5, OR9G1, P2RX2, PDE3A, PGS1, PMAIP1, PVRL3, SLC27A1, SNAP25, SOAT1, STX3, TNFSF11, WBSCR17
Negative_9974	AR, BUB3, CCL16, CHRM5, CYP1A1, CYP2A7, DRD5, FSHR, GMPPB, GNAI1, GSTT1, IL10RB, NUMB, OR10H3, PAK7, SDC3, STX7
Negative_9975	DBT, NF1, DOT1L, ITGA3, METTL2B, OR5B21, RXRA, TNFSF4
Negative_9976	AK3, ACSL4, ANAPC2, CACNA1C, CALML6, CCNB1, CDK7, FADS2, GNB1, GSTK1, HTR2C, KAT2B, LAMC3, NME6, NMNAT1, OR1N2, OR2AG2, OR51E1, OR52B6, OR52N2, ORC4L, PLA2G4A, PPP2R2C, PPP2R5B, PRKAA2, PRKG2, SOCS2, SUFU, THBD, VDAC1, ATP6V1E2, HLA-DRB4, SERPINA5
Negative_9977	SMO, CCDC6, CD22, CER1, CYP2C8, MAP3K2, PGS1, PIK3CD, RXRG, STX1B, ATP6V1C1, ST6GALNAC5

Negative_9978	BID,HK1,AGPAT4,ANAPC1,ARHGEF2,BACE1,BST1,BTRC,CCND3,CLDN16,CSF2RB,CSNK1A1,CTNND1,CXCR3,DARS,DCTN1,DERA,DUSP7,FDPS,FGF23,FGF9,ISYNA1,ITGAD,ITPKA,LLGL1,MTHFD1,NCKAP1L,NCR1,OCLN,OR2T34,OR7G3,PER3,PIGH,POLA1,POLD3,POLR1B,PPBP,PTPRM,RAB23,RAB5A,SELP,SGMS1,WNT4,CSGALNACT1
Negative_9979	IL6,B4GALT1,DNMT3B,GPD1L,LIPA,MGAT3,OR52B6,OR6C4,PPP2CB,TRAF1
Negative_9980	ADC,BAX,PAH,ACTG1,ADH5,APPL1,AREG,ATIC,CAMK2D,CARD11,CD36,CD40,CD8A,CLDN6,DOT1L,EI24,EXTL1,FGF1,FOSL1,GBGT1,GUCA1A,HSD3B2,IFNA16,KIR2DL4,LPAR1,MAP2K7,MECR,NFATC3,NFKB1,OR10H4,OR1D5,OR1N1,OR51D1,OR6C6,PAK6,PCYT2,PECAM1,PLCD3,PPP1R3A,PRSS2,PTPRC,RPS6KB1,RPS6KB2,TICAM2,ULBP2,UXS1,VKORC1,ZNRD1,MAP3K7IP2
Negative_9981	SDS,ALCAM,ALDOC,C4BPB,CAMK4,CCL2,DDB2,DUSP16,GNAI3,INPPL1,ITGAE,MYL7,NMNAT1,OR4C6,OR5T1,PDHB,PRKACG,RALB,SOD1,TPI1,ULBP1,TNFRSF6B
Negative_9982	GSN,PAH,SRF,TAT,TXK,AACS,AADAC,ACAT1,ACVRL1,BCAT1,BST1,CD244,COL4A1,CRKL,CTSB,GADD45A,GJD2,GRB2,GRHPR,GSTA1,IL29,KAT2B,KNG1,LIPC,MGAT4B,OR4C15,OR52A5,OR5AR1,OR5B21,PCYT1B,POLE4,POLR1B,PTTG1,RHOA,RPS6KA4,STX7,SYNJ2,WIF1,XYLT2,YWHAG,CACNA2D4,SERPINA1,ST6GALNAC5,TNFRSF14
Negative_9983	CR1,DDC,ALG3,AMPD3,APOC3,ARNT,CCND1,CCND3,CD58,CNDP1,CRY2,DDX50,DUSP14,EPHB3,GLDC,GLS2,GUCY2F,HADHA,HTR7,HYAL2,IGF1R,IL5RA,IMPA2,INADL,ISYNA1,KLRC4,LTA4H,MTHFD2,MTHFS,MYL7,NAT5,NUDT9,OR2F2,OR9G1,PFKFB3,PI4KB,PRPH2,PTDSS1,PTGES2,SEMA3E,SHMT1,TBXA2R,THTPA,TOMM40,TUBA3C,UGCG
Negative_9984	DCT,GGH,HAL,CALML3,CCL24,CCNA1,CREB1,CYP2A6,DLST,DTX1,FRAP1,HLA-DRA,HNF1A,IFNA13,ITCH,KAT2B,LAMC3,LIPF,MAT1A,MCEE,MGST3,MYL7,OR4K1,PAX8,PPP3R1,PSME3,SDC2,SEMA3B,SOCS2,SULT2B1,TPK1,WARS2,WWP2,ZFYVE16,B3GALNT1,HLA-DRB5,TNFRSF17
Negative_9985	SMS,ADCY10,CALML3,COL11A1,DCTD,ERBB3,EXTL1,GLUL,GRM1,GYS1,IRF3,ITGA2,ITPA,MAP3K6,MTHFR,NOTCH3,OR1D5,PAX6,PFKFB4,POMC,TAS2R7
Negative_9986	AKR1C2,DHRS4,GDF5,OR5A2,OR6C4
Negative_9987	C3orf10,DIAPH2,EFNB3,FLT4,HAAO,MCM3,NODAL,PDIA4,PTCH2,SELE,STAT2,TCF7,THBS3
Negative_9988	LCT,SI,TEC,TH,AARS2,ARSB,ATG3,CASP8,CDO1,CLTA,CLTB,DLAT,DUSP14,ECHS1,GLUD1,HSD3B2,NCKAP1L,NCR2,OR2M5,OR52E6,OR52N5,OR5B12,PIPOX,POLR3G,PROS1,PTPRB,RPN1,SARS2,SRGAP3,SYNJ1,UGT2B11,HLA-DQB1
Negative_9989	IL8,SRF,ACTC1,ACTN2,ACVRL1,CACNA1C,CACNG7,CAMK2G,CCL2,CD79B,CSDA,CSF1R,CXCL16,CYP26B1,CYP2F1,DGUOK,DIAPH2,DUSP8,EPHA8,EPHB4,GALNT1,GALNT2,GALNT6,GAMT,GFPT2,GLUD2,HAP1,MCM7,MTAP,NAT1,NAT2,NEUROG3,OR1J2,OR3A2,OR51B2,OR8B8,PLOD3,POLR2H,PRKCI,PSAT1,PTCH1,PTGS2,SHC1,SULT2A1,TRAF6,UNC5A,WHSC1,CTNNBIP1
Negative_9990	CFH,F2R,AACS,ACP5,AKT3,BMP7,C1GALT1,CYP2E1,DVL2,EXT2,FGF21,GDF7,GGT5,L1CAM,MAP3K4,NME6,OR2A4,OR2V2,OR4C11,OR4F17,OR5B17,OR9A4,POLR2I,RPS6,THBS1,TNFRSF8,TYK2,VNN1
Negative_9991	PLG,ATF2,CCR1,CDC16,CYP19A1,FOXA3,GZMA,HYAL1,IARS2,OR1F1,P2RX3,PPARG,TGFA,TSHR,CACNA2D1

Negative_9992	PML,AACS,AGRN,APC2,CCL1,CDS2,CKMT2,CXCR6,DGAT2,DUSP4,FLT4,GANC,IL22,KLRC4,MTHFD2,MYL7,OR1L1,OR4A15,PERP,PGAM4,PKMYT1,PTGER3,PVRL1,RIPK1,RRM1,SARDH,SLC27A5,SMAD5,TNFRSF10C
Negative_9993	IL12RB1,LIMK1,MYH11
Negative_9994	FOS,ID2,A4GALT,ADCY10,AMDHD2,APAF1,CACNG1,CALML3,CSF3,CXCR3,CXCR4,CYP2A7,DOLPP1,EARS2,F13A1,GARS,GLO1,GRIN2A,GRIN2C,HLCS,IAPP,IL23A,ITGA2B,LAMA3,MAP3K14,MCM3,NCAM2,OR2C1,OR52H1,PLXNB1,POLR3H,PRLR,RBL1,RPS6KA3,RUNX1,RUNX1T1,SELPLG,SFRP2,SSH3,TNFRSF18
Negative_9995	AR,TNF,ALDH1L1,AMPD2,ARRB1,CCL27,ENPP1,EXTL2,FMO3,G6PC,GNMT,GRIA1,HADHA,HLA-G,IL8RA,LRDD,MAFA,MYH7B,OR10A4,OR2T3,OR4Q3,OR52E6,OR6N2,PIM1,PIP5K1C,PLXNA3,POLR2J,SEMA3C,SEMA3D,SEMA3G,TAS2R40,TCF7,TOLLIP,TRDMT1,UNC5A,YWHAE
Negative_9996	AK3,CAD,TNF,ATP5L,C4BPB,COL2A1,DAPK1,DPAGT1,HNF4G,MAGI3,OR1E1,PARD6B,PDE1A,RDH10,UBE2K
Negative_9997	C4B,NPL,ABLIM1,AGPAT6,ATG12,CHIT1,CREB3L2,CYP2F1,FGD3,IL12RB2,LYZL1,MAP3K7,MTMR1,OR5P2,OR7G1,OR8J1,PARS2,PKLR,PNMT,POLR3D,PPP1R3D,WNT7A
Negative_9998	AHCYL2,ANAPC10,ARHGEF6,ATG7,B4GALT3,BCL2,BMPR1A,CACNA1E,CAMK4,CDK4,CES2,CHSY1,CREB5,CXCL10,CYP2E1,DTX2,GAB1,GPD1,HPSE2,IFIH1,IFNGR2,IKBKB,IL17B,IL1R2,MAP3K13,MAT2B,NCOR1,NR4A1,NRAS,OR13J1,OR4D1,OR51B6,OR5C1,OR6X1,OR7A17,PFN2,PLOD2,PPP3R2,PTCH2,RALGDS,RFWD2,ROBO2,SPI1,TPI1,UBE2L3,VAMP4,TNFRSF6B
Negative_9999	AGK,DCK,HPD,PGD,ACTN1,ALDH1A2,ATP5O,BACE2,CNTN2,COL5A2,DUSP10,FMO2,GRLF1,HHIP,HLA-DOA,KCNJ11,LTB4R2,ODC1,OR10J1,PAK2,PFKM,PNPT1,PRPH2,SCP2,SDHC,SPCS1,STX5,TAS2R50,TBXAS1,TFDP1,UMPS,ATP6V1G1
Negative_10000	EDA,PLN,SP1,TKT,AADAT,ACLY,ADCY8,ALDH3A1,ARPC1B,B3GNT2,C4BPB,CCR7,CDKN2A,CYP2E1,CYP3A7,DGKE,DOT1L,ENPP6,GNPAT,ITGAM,NRCAM,OR10X1,OR13C8,OR4D1,OR56A3,OR5J2,OR8H2,PANK4,PDE6H,PDHA2,PON2,PTK2,RPS6KA4,SFRP1,SLC27A5,SMC1A,SOCS5,UBE2K,WEE1,CSGALNACT1,PDCD1LG2,PPARGC1A
Negative_10001	AK5,C9,CR2,TXK,ACP2,AMY2B,ANAPC2,C3AR1,CCNE1,CFTR,CTNND1,DGKE,DTX1,ERBB3,FBXW11,GCNT4,GGT6,GNB1,GUCY2C,HNF4G,IFNB1,IPMK,IQGAP2,ITGAE,KIR3DL1,LEPR,MAP2K2,MAP3K5,NUDT5,OR10A6,OR2M7,OR4A16,OR4D11,OR7D2,PIK3C3,PRKACA,RARS2,RPIA,SCP2,SEMA3D,SLC2A4,STX7,SULT1A2,TRAF3,TRAF4,TRDMT1,TXNDC12,ZFYVE16,TNFRSF6B
Negative_10002	APP,C8A,EDA,ACSS1,ALOX15,APOA1,APOE,B3GNT4,BACE2,BPGM,CACNG6,CCL5,CCNB1,CD28,CD86,ETS1,FABP1,FABP4,GCLM,GSTA4,IDH2,IFNA16,IL10,LAMA3,MTHFD1L,NMNAT2,NNMT,OR1N2,OR8G1,PKMYT1,PLK1,PLXNB2,PPBP,PPP1CB,PRKAR2A,PRKCZ,PRKG2,PRNP,RASA2,RERE,RHOA,SCIN,SCLY,TLR9,WBSCR22,PRICKLE2,TNFRSF10C,TNFRSF10D
Negative_10003	ADA,DCN,FAH,FYN,RDX,ACOX2,B3GNT3,CBLB,CCL21,CTPS2,IL1RAP,MFNG,MMP2,NTSR1,OR10K2,OR5AK2,OR6C4,OSMR,P2RX4,PDE4B,PPARA,UGT2B7,VKORC1
Negative_10004	BTD,C7,CBL,F3,ACAT2,ADORA2B,BMP8A,CACNG8,CD79A,CDK5,CHRD,CYP2E1,DEGS1,DGKD,EFNA1,EFNB3,ENTPD2,EPHA3,GBGT1,GCNT3,GLI2,IKBKB,IL24,IL25,IMPA1,KLRC2,KRAS,LEF1,NFKBIE,PDXK,PFKFB1,PGM3,PON2,PYGL,SOCS5,SRD5A2,TAP2,UNC5C,UROS,MAPKAPK2,TNFRSF14
Negative_10005	ACSM5,CYP26A1,GALNTL1,GRLF1,ITGA9,JAM2,LYZL1,POLR3A,PPOX
Negative_10006	AUH,IL6,MSN,AKT3,BST1,CYP4A22,DAAM2,DARS,DAXX,MTMR2,OR9Q2,PLD2,SGMS2

Negative_10007	TXK, ACYP2, ADCY4, CRB3, DGUOK, DHCR7, DHODH, E2F3, EPB41L2, GLI1, HLCS, IL2RB, LIPF, MAGI2, MAP3K1, OR1E1, OR4F15, OR52N2, OXCT1, PHKG2, PLXNA2, PVRL1, QARS, SAT1, TYROBP, ATP6V1G3
Negative_10008	DCC, DCK, GDA, GLA, GLS, GSR, GSS, IL6, KHK, MPI, PLG, AGPAT1, CCNA1, CCNG2, CHPT1, CPT1A, DGKH, ENTPD1, GPLD1, IDH3G, IFNA10, LAMB2, MAN2A1, MAT2B, MDM4, OR10X1, OR2S2, OR51S1, OR52E2, OR8D1, PCK1, PIK3C2G, PIK3CB, PPAT, PRKAG2, RBKS, SIPA1, TAS2R41, UBE2K, VEGFA, WBSCR17, WWP1, YES1, PNLIPRP1, ST6GALNAC1, TNFSF13B
Negative_10009	ATM, CRH, OGT, ARSB, CCL17, CCNE1, CSAD, CTNNA3, DHRS4, GALNT14, GPD1, GSTA3, HSD3B7, HTR5A, LHCGR, NAT1, OR2V2, PIPOX, SLC33A1, WASF3
Negative_10010	GSTM1, NFATC2, OR2T1, TNFSF8, SERPIND1
Negative_10011	AMT, AUH, FH, TNF, ABL1, ADCY8, ATG12, B3GALT4, BHMT, CCL26, CMPK1, CXCR4, DDX56, DUSP3, EPHA2, FECH, FGF3, GCNT3, GDF5, GMPS, HARS2, IGSF5, IRF7, MYH1, NCR1, OR10S1, OR4K2, OR6C70, OR8K3, P2RX5, PLA2G2E, PPARA, PPP3R2, PRSS3, PTCRA, RIPK1, SC4MOL, SPTAN1, TCF7, TLR9, CACNA2D2
Negative_10012	ABLIM2, ACO2, ADIPOR1, BCMO1, BUB1, CACNA1E, CD47, CDH5, CNGA4, DMGDH, FGF13, FGFR2, GLI2, LYPLA1, NFKBIB, OR51G1, PMVK, PNPLA3, RFNG, RPRM, SCD5, SREBF1, TLR2, TNFRSF9
Negative_10013	FAH, GK2, AANAT, ACAA1, ADCY9, BPNT1, C1QA, CAMKK2, CCR1, CDC25A, CLTC, COX10, CYP4F2, DCXR, DEGS1, DEGS2, DHRS3, EPB41, EXTL3, FCER1G, FGF5, GFAP, GNA14, GSTM3, HGSNAT, HSP90B1, INHBB, IPPK, LARS, MTHFD2, MYH2, OR4D1, PIGP, PKLR, PLA2G6, PLXNA2, POLR3H, VTI1B, YARS, TNFRSF13C
Negative_10014	AK2, FER, OGT, ACMSD, AMPD2, ATF2, B3GALT1, CHRM5, CLCA2, COX15, GJA1, HTR4, IL18RAP, IPPK, MYLK3, NMNAT3, NUMBL, OR10G3, OR10H1, OR1A1, OR9Q2, PDE8A, POLR3G, RFNG, SMAD2, TBXAS1, TUBB1, TYMP, ULK3, WNT3
Negative_10015	EZR, JUP, ABLIM3, ACSS2, ANAPC4, ARNTL, ASRGL1, B3GAT3, CACNG7, CAV3, CLCA4, CLOCK, DEGS2, DTX3, DUSP14, FABP4, GCLM, GLUD2, GMPPB, GMPS, HSPB1, IKBKE, LRP5, MAP3K11, MCM6, NCAM1, NKD2, OR2W1, OR5L1, P2RX7, PDE1B, PIK3C2B, PNPLA4, POLR2K, PPAP2A, PPP3CB, PRIM2, RBKS, SESN3, SUCLG2, TP73, TPH1, TSC1, WNT5A, XCL1, XCR1, CTNNBIP1, PHOSPHO1
Negative_10016	ASL, JUN, WAS, ADCY10, ANAPC1, CAMK2G, CDC25B, CDC45L, FGF7, GALNT3, GPAA1, IGF1, MCAT, NKD2, OR1F1, OR2B11, PAFAH2, PHPT1, PLCB3, PTGDS, RHOQ, RXRG, UBE2K, YWHAG
Negative_10017	BTD, ID1, IL8, PGF, TK1, AGPAT6, ALPL, BMP8B, CADM1, CAMK2B, CCL17, CFLAR, CFTR, CLDN10, CLTB, COL5A1, CSNK2A1, CXCR3, DEGS1, DUSP7, EARS2, FBP1, FGFR1, HIF1A, HYAL2, IGF1, IL18RAP, ITPKB, MARS2, MYLK, NARS2, NCKAP1L, NEFH, NOX3, OR1L3, OR8J1, OR9A4, PPP3CC, PRKCG, ST3GAL3, STX1B, VEGFB, GABARAPL1, MAPKAPK5, SERPING1
Negative_10018	DBT, FGB, ASIP, CACNG3, CAMKK2, CCNE2, CSF3R, DRD5, EFNB2, FMO3, FOXO3, GLI2, GSTT1, GUCA1A, ICAM1, ITPKB, KIR2DL2, NCK1, NKD2, NTNG1, OR2A25, OR6A2, OR7A5, PAK4, PON3, PPP1CC, PPP1R3D, ROBO3, STK36, TLR5, UBE2L6, YARS2, ZNRD1, MAPK8IP2
Negative_10019	FCGR2B, MPZL1, NEU4, OR4D5, OR5D16, OR6T1, OR7C1, PAK6, PAX4, PECAM1, PIK3R1, PLA1A, POLR3K, PTGIS, SLC2A4, SOCS3
Negative_10020	BTK, RB1, SFN, ABP1, ACP1, ACTN4, APOA2, CACNA1D, CCL7, CDKN2B, CLTC, COMP, COQ5, DGUOK, ESPL1, GNAT3, GUCY1A3, LIPT1, MGAT4B, NCF1, NODAL, NPR2, NR1D1, OR4C12, OR8J3, OR9K2, PDE1C, PFKP, PIK3CG, PPP2CB, PPP2R2C, PTEN, RAB23, SLC33A1, SMAD2, TRHR, TSTA3, WHSC1L1, WWP1, YWHAE, MAP3K7IP2, TNFRSF1A

Negative_10021	GBA,A4GALT,ACVR2A,ADCY2,ASMT,ATP4B,BUB3,CCR9,CD72,CSNK1G2,GALNT1,GIT1,GLUD1,IFNGR1,IL12RB2,OR10K1,OR11H6,OR11L1,OR4K13,OR4Q3,OR51F1,OR6C6,PCYT1A,PRKAR2B,SOD1,ST8SIA5,SUCLG1,TBL1X,TKTL1,ATP6V1G2,ST6GALNAC6
Negative_10022	IVD,DPM3,DTX4,FGF20,GNB1,GUSB,IMPA2,ITGA10,MAP2K7,MAT1A,OR4C16,OR5B17,OR9Q1,P4HA2,PCNA,PDE4B,PFAS,PPP2R2C,RASGRF2,RETSAT,SIGLEC1,STX4,TP53,WNT3A
Negative_10023	KHK,LPL,PGP,CAMKK2,CDC45L,CDC7,CES7,CLTB,CYFIP1,CYP11A1,DAG1,DGKD,ENPP1,GNAZ,HLA-F,HSPA2,IDH3G,IFNA16,KLRC2,NEFL,NTSR1,OR1G1,OR1J4,OR4B1,OR4C15,OR5AN1,RPE65,UNC5A,WNT4
Negative_10024	GLA,ADCY7,ALDH7A1,CER1,CYFIP1,FOXA2,GPT2,GRIN2D,HARS,IL20,LHCGR,OR10X1,OR13C5,OR2T8,OR52L1,OR6C76,ORC1L,PAK4,PIP4K2B,PLAU,PPAP2A
Negative_10025	B4GALT4,FZD8,MAPK3,PER3,SOCS5,TBXA2R
Negative_10026	NLK,SYK,ACSS3,ACVR1B,ALAS1,ALAS2,ALDH1B1,C1QC,CD80,CDKN2A,CSAD,CYP1B1,CYSLTR1,DDX55,DNMT1,GAL3ST1,GLUL,GNRH1,HADHA,IFNGR2,LAP3,MGAT2,NEUROD1,OR10G4,OR5V1,PARVA,PFKP,RAF1,RFNG,SEMA4A,SEMA4F,SPHK2,STK36,TNFSF8,VARS,WBSCR22,PLA2G12B
Negative_10027	B4GALT4,BMPR1A,CD28,CLDN3,IBSP,IFNA4,MAPK1,MCM6,MYH14,NCF1,OR52N4,OR7C2,SYNJ2,XYLT1
Negative_10028	CKB,IL7,SRM,ATP5O,B3GAT2,BET1,CCNG1,CD3E,CDIPT,CREB5,CXCL3,CYP27A1,DAXX,DGAT2,DGKQ,EDN1,EGLN3,ENPP3,ENTPD3,FGF13,GAL3ST1,GIT1,GRIN2A,ITGB5,KDSR,MTMR6,OR1F1,OR1Q1,OR4N4,OR56A1,OR56A3,PFN4,PIGH,PIP4K2C,PNPO,POLR2C,PRKCQ,RARS,SOCS3,VEGFC,B3GALNT1
Negative_10029	MTR,RPE,ADRB1,CNTFR,CYP2C19,DTX3L,ENO3,ESPL1,FLT1,GALC,HMGCS1,IL15,MGAT4A,OR2C1,OR2H2,OR5AR1,PFN4,PRLR,PYGM,RPS6KB1,WNT4
Negative_10030	ADCY10,CMAS,CSNK1G2,CYP4F2,EHHADH,FGD3,GSTM3,HEXB,IFNAR1,MAP3K13,MASP1,OR2AE1,OR8S1,PDGFA,PIK3CA,PIM1,TNFRSF4,TYK2,YWHAE,B4GALNT1,MAP3K7IP1
Negative_10031	CGN,CD28,CYBA,EFNA4,MADCAM1,OR1L1,PNPT1,PPP1R3B,PRPH2,SORBS1,TNFRSF8,TNFRSF12A
Negative_10032	C1S,EPO,HDC,ACY1,AHCYL1,ATG3,BAIAP2,CAMKK1,CNTFR,CTSB,CXCL5,DGAT2,DIAPH1,DNMT3L,EPHB3,FGF13,FUT3,GGT7,GNG13,HDAC2,IL13RA1,IL15RA,ITCH,MAP3K6,MYH2,NCOR1,NCSTN,OR13C9,OR3A3,OR5M3,OR7C2,PGLS,PTTG1,RBPJL,SCP2,SV2A,TUBA4A,VAMP4,HSP90AA1,MAPKAPK2
Negative_10033	SYK,ACVR1C,ALG9,ALOX15,ANAPC5,ARSE,B3GALT5,BAIAP2,BBOX1,CCR9,CFL1,COMT,CTSG,DARS2,ERBB2,HMGCS1,IDI2,IMPA2,ITGAX,MYH1,OR2A14,OR2W1,OR4S1,OR5M9,OR7A17,P2RX1,PAK4,PFKFB1,PIK3CA,PLXNA1,PPM1A,PRKCE,PRKCI,RARS,SETMAR,SMARCA2,SPRY4,TAP1,TICAM1,TYROBP,MAPKAPK5,TNFRSF17
Negative_10034	CD2,ACOT11,ADCY4,APOE,C1GALT1,CCR4,CD36,CHEK2,CREB3L1,DHODH,GALNT6,GNAL,GNAO1,IDI1,NEU2,NFYB,NRXN3,PAFAH2,PIP4K2A,PLCG1,PLOD3,PTPMT1,SPRY4,TAS2R5,TNFSF11,UGT2B17,WNT4,ATP6V0D2,PAFAH1B1,TNFRSF10D
Negative_10035	APP,HYI,ITK,MOS,AMDHD1,APBB1,ATP6V1F,BMPR1B,C1QB,CNTNAP2,CSH1,CSNK1G2,CTTN,DDX54,FUT8,GATM,IDH3A,IQGAP1,LYPLA2,MAP2K3,MMP1,NTNG1,OR2Y1,OR4C6,ORC2L,ORC4L,PCNA,PDE5A,PDGFD,POLD2,PON2,PPP2R5E,RASGRF2,RRM2B,SEMA3B,SHC1,SOCS3,TJP3,TKTL1,TUBA1C,UGCGL2,HSD17B12,PAFAH1B2,TNFRSF10C,TNFRSF1A

Negative_10036	GRM5, HADHA, IL12RB2, MASP2, OR2B2, OR52E8, OR52J3, OR5P3, PIGQ, PIK3R4, PRSS3, SESN1, TJP3
Negative_10037	CAD, AKR1C1, ALG11, AMDHD2, ARHGEF7, ASS1, BMP7, BMP8B, CACNG4, CAMK2G, CLDN20, DTX1, FGF9, GABARAP, GBGT1, GMPR2, MCM2, NT5C, OR10A7, OR10G4, OR10G7, OR13C9, OR2L3, OR5AK2, OR5V1, PDE3B, PIK3R1, PRLR, PTPMT1, RASSF5, STX3, TAS2R49, TLR5, TUBAL3, UBA7, UGT2B17, YES1, ATP6V1G1, B4GALNT1, ST6GALNAC6
Negative_10038	BAD, HGD, IL9, SP1, ABLIM1, ACTN1, ACVR2B, AKT3, AMPD3, ARPC1A, ARSA, CANT1, CCR5, CDK2, CDKN1A, CFTR, COL4A6, CREBBP, CRHR1, CTPS, DCTN1, DHDH, DLL1, EFNB3, EPHX2, EXOC7, FUT8, GNG12, GRB2, GSTT1, GUCY1A2, IL2RA, JAK1, MALT1, NEGR1, NFATC4, OR10R2, OR5B12, OR5P2, OR8G1, POLR2I, RALB, RXRA, SCIN, SH2D1B, B4GALNT1
Negative_10039	EGF, LIF, ACHE, ACTB, AHCYL1, AKR1A1, AKT2, ALG2, CCL4, CCR5, CDK5, CHRM2, CLDN6, COL5A2, CYP1A2, DMGDH, FPGT, GALNT8, HCST, IMPDH2, INPP4A, KLRC3, MAT1A, NT5C1A, OR10G2, OR52A1, OR52E6, OR5D14, OR6Q1, OR9G1, OR9Q2, PDE1C, PLCD1, PPID, PPP2R5A, PRKG1, PRKX, PTDSS2, PVRL2, PYGM, SHC4, SPAM1, SREBF1, TMLHE, HLA-DRB5, PAFAH1B3
Negative_10040	AK5, ADCY10, APPL1, ARSE, ATP5J2, CACNA1C, CES1, CHSY1, CYP4A11, EXT2, NGEF, OR10J1, STX1B, TUBB1, TNFRSF14
Negative_10041	UBB, ACVR2B, ALOX15, ATP5H, CCR9, CD80, CHKA, CHRM2, COL1A1, COX10, DHCR7, DUSP5, ECHS1, EHMT2, EPHA7, EPHB1, EXTL2, FUT2, FUT7, GNG13, GYS2, IL15RA, INPP4A, LIMK2, MADCAM1, MAML1, MAP3K7, NPAS2, NPR1, NUMBL, OR5AC2, PARVA, PDE6H, POLR2H, PPP2R5E, PPP5C, PTGER3, RPN1, SNAP23, SOCS4, TAS1R3, TGFB1, UCKL1, GABARAPL1, SERPIND1
Negative_10042	CEL, LCT, TXK, ACTB, ADH5, AGTR1, ALDH3A2, AVPR1B, B4GALT3, CASP10, CASP8, CCR8, GALNT1, GBGT1, GNRHR, GPX7, HARS2, HSD11B2, IL13, KIR2DL3, LIFR, MMP2, MTHFD1L, OR2W1, OR6T1, OXCT1, P2RX7, PRKAR1B, PTPRC, SLC27A1, SLC27A4, STAT5A, SV2C, UBA7, C1GALT1C1
Negative_10043	GBA, MET, ACO1, CALML6, CCL25, CDKN2D, DDX56, ENTPD8, EPHA8, FZD7, GALNT10, GMDS, GNPNAT1, HSP90B1, HYAL1, IL1RAP, IMPDH2, ITGA9, ITGB4, LGMN, LIPC, OR2T2, OR4M1, OR5B12, OR9K2, PFN3, PLAUR, PRKAG1, PSPH, PYCRL, SMAD6, SPCS3, SPRED2, TNFRSF9, WHSC1L1
Negative_10044	OAT, BIRC5, DARS2, ENPP1, FUT1, FZD10, GRIN1, KLRC4, LTC4S, NKX6-1, OR1E1, OR6C2, OR6S1, PLA2G6, PON2, RALGDS, WNT9A
Negative_10045	CRH, F5, A4GALT, ADCY1, ALG10B, ALPP, ATP5G3, CCL1, CDC6, CHUK, CRLS1, CSNK1A1, CTNNA1, CTNNA2, DRD2, ENTPD4, G6PC2, GLI2, HMGCS1, HYAL2, ICOSLG, ISYNA1, MCM2, MPP5, NRG2, NTN1, OR12D3, OR6B3, OR6X1, OR8H2, OR8K3, PPP2R2D, PYGL, RPS6KA1, SLC2A4, SUCLA2
Negative_10046	ADO, CFI, EZR, TNF, ACSL3, ADH7, AKT2, ANAPC7, B4GALT6, CAMK2G, CBR1, CCL19, CCNB1, CCNE1, CTNNA2, CXCL3, EFNA2, EI24, FUT8, GALNTL2, GNPAT, HEXA, HMOX2, INSRR, LILRB3, METTL6, MGAT5B, MYLK3, NOS1, NUDT2, OR1L3, OR2M4, OR2W1, PIK3R3, PLA2G2E, PPCDC, PRODH2, SMAD2, SOS2, STK3, TFDP1, TRMT11, TYROBP, WNT9B, TNFRSF13B
Negative_10047	CR1, F7, GH1, AGMAT, ALDH3A1, CCL15, CCL19, CHST11, CPS1, CSF3, CYP2F1, ENTPD1, FGD1, FGFR3, FUT2, FZD10, INHBB, MAD2L2, MASP2, MYH7B, NANP, NFATC3, NTF4, NUDT8, OR14I1, OR1M1, OR2L3, OR2W1, OR3A3, OR5L1, OR6T1, PDXP, PFN2, PLA2G10, PPM1D, PRKAG3, PTPN7, PYGL, SC4MOL, SDHB, SEMA3D, SETD2, SLC27A4, SMC1A, SMPD3, TAS2R40, UNC5C, WNT2
Negative_10048	RPE, TPR, ACAD9, ACTC1, ADCY10, APPL1, ATP6V0B, BST1, BTRC, CHPF, CISH, CYP11B2, DHRS9, EPHA3, FUT9, G6PC, GATM, GNG12, IMPDH2, MAPK8, MGAT4B, MLLT4, MTMR2, NCSTN, NFKB2, NUDT2, OCRL, OR10A6, OR6K2, PECAM1, PIK3CB, PLCB1, PLCB4, PLD1, PLXNA2, PRLR, PTCRA, SFRP4, TFPI, TKTL1, ULK1, WNT3, ZMAT3

Negative_10049	DHH,ABAT,ADH1B,ALS2,AMY2B,CACNA1H,CACNG2,CHKA,CYP3A5,DCXR,EDA2R,FZD2,OR1L1,OR51F2,OR52A5,ORC1L,PPP2R5B,SDC1,TFDP1,TRIP10,YWHAQ,MAPK8IP1,SERPINA1
Negative_10050	AKR1B1,B4GALT1,CNTN1,COL1A2,DOCK1,FTMT,GSTA4,HMOX1,IFNW1,LHCGR,MLYCD,MPZL1,NAGLU,NLGN1,OR10H3,OR1I1,OR51B6,OR6C74,OR8S1,POLR2A,PPP1CA,PPP3R2,RAPGEF3,STAT2,TDO2,YWHAG,CACNA2D1,CACNA2D4
Negative_10051	AGXT2,ALAS2,C1QC,CDC25B,DTX3L,DVL2,GLI2,OR14J1,OR51T1,PRSS1,SH2D1B
Negative_10052	ACOX3,ESPL1,FADD,FARSB,WASF2
Negative_10053	ABCC8,DNMT3A,HTR5A,IDH2,MTFMT,OR5L2
Negative_10054	EBP,CACNG6,COQ6,DNMT1,DNMT3A,GSTT1,IL15,KDSR,MDM4,METTL2B,OR1N2,PDK1,PPP3R2,RAPGEF2,RASGRP1,VEGFC,PAFAH1B2
Negative_10055	ACP1,ATG12,CCNE1,CHST13,CREB3L4,CRHR1,CXCL9,CYP2D6,CYP4F3,DDX55,DIAPH3,FGF20,FGF8,GAS1,GRIN2A,HSPA1B,IARS2,IMPA1,IPPK,IRAK3,ITGA2B,ITGB8,NT5M,OR12D3,OR4M2,PLXNC1,PPCDC,PTPN1,RALA,RDH16,RRM2B,SEPHS1,SGPP2,SPHK2,SULT1A4,YES1
Negative_10056	MAG,SRC,LIMK2,LLGL1,MAP2K2,TAP1
Negative_10057	CCL11,CPT1C,CRKL,GALNT13,HAO2,IGSF5,NAGA,PGLS,PLA2G4A,TYROBP
Negative_10058	ANPEP,CCL14,EDAR,OR4F6,PLEKHA8,POLR2A,XCR1,KIR2DL5A
Negative_10059	C4B,FGA,CACNG7,CREB1,CREBBP,CTBS,CTNNA1,CXCR3,GGT7,HNF4A,ITGB3,NPR2,NSDHL,OR11H6,OR2A14,OR2H1,OR4F5,OR8J1,PAK6,SMPD1,SMPD3,STX12,SUV39H1,SV2B,TAS2R16,TCF7,THBS4,TLN2,UNC5B,VDAC2,WASF2,YWHAH,MAPKAPK3
Negative_10060	FYN,CACNG2,CDK6,CLDN20,DEGS2,DPM3,GABARAP,HCLS1,LIPC,MYL7,NTRK2,PDE6H,PFKFB3,POLR3B,PPP1R3C,RXRB,STAT5B,TCF7L2,TRAF1,UBE2K,SERPINB5
Negative_10061	ACO1,CD46,ERO1L,LEFTY1,METTL2B,OR2A2,OR9I1,TMLHE,TSHR
Negative_10062	F8,ADH7,AKR1D1,ARPC1A,ARPC1B,ATP6V0C,BCMO1,CAV1,CD22,ESPL1,HNMT,IL18RAP,MAN2A1,NFATC1,OR2A5,OR2F1,OR5K1,ORC6L,PKM2,POLA1,PTGES,RERE,VDAC3
Negative_10063	AMH,RFK,ABAT,AHCYL1,AMDHD1,CDKN2C,GALNT5,GANAB,GMPPB,MAPK7,MGAT4B,NARS,OR1J2,PLIN,PLXNC1,PPP2CA,TAS2R7
Negative_10064	ACACB,ADCY8,APOA2,ATP5G3,CFLAR,CREB3,DGKZ,FOXA3,GRIA2,HARS2,HLA-DMA,HTRA2,INHBA,JAG2,KLRD1,MAP2K5,NADSYN1,NLGN2,OR10G8,OR1D2,OR4D1,OR4D2,OR51D1,PGM3,PIGV,PKMYT1,PMAIP1,RDH11,SFRP2,ULK1,HSD17B10
Negative_10065	DDC,ACAA2,AMHR2,CCL1,CD274,CSF3R,CSH1,CTNNA3,CYCS,HYAL1,NLGN3,OR10AG1,OR10G4,OR9I1,PGAM4,PIGS,PIK3R2,PPP2R5B,PTPN11,SDC2,SDHC,SPRY3,STX10,STX1A,STX1B,SUV39H2,YES1
Negative_10066	ADCY4,ARPC5L,ATP5E,CYP26B1,DGKI,GALNT2,H6PD,JUND,NODAL,OR11G2,OR1L6,RIPK1,TP53I3,VDAC2,YWHAH
Negative_10067	ADH5,CDC6,CTSG,FARSB,HCLS1,LAMC2,MCM3,NPR2,NT5C1A,PDPK1,PLXNB2,PTPRJ,TBXA2R,TUBB2C
Negative_10068	HGF,LALBA,PEMT
Negative_10069	IL4,MVD,UBB,CCR1,CSF2,CSF3,CXCR5,ETNK2,FBP2,FGF1,FMO4,HLA-DOA,IL23R,LAMB3,MMP1,NOTCH3,PRKCD,SMAD1,SMPD2,TACR3,TSTA3,UGT2B4,XYLT2,YWHAE,MAP3K7IP2
Negative_10070	PGD,ABLIM1,ACVR1B,ADCY7,ALDH1A1,ANAPC5,ATP5I,ATP5J,BST1,BUB1,CDC25A,CXXC4,E2F2,FUT7,GRB2,HMBS,IRF7,MOV10L1,MYH4,NFKBIA,NPR1,NT5E,PNPLA3,PON2,PSPH,PTPRJ,TBL1XR1,VANGL2,ZBTB16,CACNA2D2,HSP90AA1

Negative_10071	ACSM2A,ACVRL1,ADH6,ADRB1,ATP5H,BDNF,CD70,COMP,CPT1B,FRAT1,GCAT,IL25,ITGB5,LCP2,MCM5,MGAT5,MGST3,MYH7,NR5A2,OR1D5,OR1L4,OR4A15,OR56A1,PIM1,PIP5K1C,PKLR,PRKAR2B,STAT5B,SUCLG1,YWHAH
Negative_10072	BAD,HGF,ACADVL,APC2,ATP2A2,BCAT1,CCL14,DGKI,FADD,FOXO3,FZD1,GLI1,GRLF1,HTR2C,IFNGR1,ITGA5,MAT1A,MNX1,NFATC4,OR1S1,OR5H6,P2RX5,PRKAB1,PRKAR2A,RRM2,SHC1,SMAD2,WNT2,SERPINB5
Negative_10073	HAL,AASS,ACHE,AKR1C3,ATP5A1,BPNT1,CACNA1D,CCR8,CDS2,CLCA2,CNTN2,CTBP2,CXCL1,CYP2B6,DRD5,FCGR3B,GSTM4,IL23A,JUND,KIR2DL2,LRAT,MALT1,MAPK8,MFNG,NRP1,OR13C9,OR13F1,PDHA2,PFKP,POLR2L,PPARD,SPTLC1,TBK1,TNFSF4,TUBA8,YES1,ATP6V1G1,PNLIPRP2,SERPINB5
Negative_10074	DLD,FER,ALDH3A1,AXIN1,CAMKK2,CCKAR,CD79B,CKMT2,CLDN1,CNGB1,CSF1,CYP2A7,EPOR,GARS,GRIN1,HLA-DRA,IFNA7,IL15,IL1A,IL1R1,INPP5B,ITGB3,MAPK13,MDH1,PDXK,PHKA2,PPOX,TAS1R1,UGT8,WWP1
Negative_10075	A2M,IVD,ACSS3,ALDH3A2,ALOX15B,ATN1,ATP2A3,CLCA4,CREB5,DCTD,DDX54,DRD2,FUT1,GRB2,GRIN1,IFNA2,INHBA,KIR3DL3,LCP2,MAN1A1,NAT2,OR1L1,OR2AG1,OR4K2,OR51L1,P2RX3,PARVA,PGS1,PMM1,PTDSS1,SARS,SCIN,TACR1,TGFA,WASF2,XYLT2,ST6GALNAC3
Negative_10076	C1S,EZR,AHCY,ANAPC4,BET1L,CCR1,CDIPT,CYP26B1,FGFR1,GATM,GUCY2D,IBSP,ITGA2B,NSDHL,OR1B1,OR52M1,POLR1A,SKIV2L2,SORD,TAS2R38,TRDMT1,WNT16,CACNA2D1
Negative_10077	CCNH,CD28,CDS2,CPT1B,EPHX2,GP1BA,GRIA2,MAD1L1,PFKP,PLA2G6,PNPLA4,SHISA5,TAP1,YWHAZ
Negative_10078	AGK,C6,DBI,GSR,PDC,ACTG2,APOA5,ARD1A,BDH1,CLDN11,CRLF2,CYFIP1,DDIT3,DEGS1,DRD1,EDAR,FUT3,GPX7,GSTM4,HCLS1,HSD3B1,HYAL1,ITGAM,KAT2B,LGMN,OR10H4,OR10P1,OR10Z1,OR2T12,OR4B1,OR52E4,PLCD4,PRSS1,PTK2,RFNG,SCD5,UGT2B7,TNFRSF10D
Negative_10079	GAS1,MCEE,NAGA,NT5C1A
Negative_10080	C6,SP1,B3GNT4,BHMT,BPNT1,C9orf95,DRD5,EFNB2,FABP1,FTCD,FZD5,GAS1,GNG3,GUCY1A3,IL8RB,LCMT2,MPP5,MYD88,OR2V2,OR51Q1,RDH16,SMAD9,TNFSF11
Negative_10081	CCR3,MAP3K6,NAT6,NCF1,OR2D3
Negative_10082	PML,ACSM1,ATP5C1,ATP6V1D,AXIN1,CCNB3,CSNK1E,FTH1,FZD2,GABARAP,GGT5,HAO1,INPPL1,LEF1,OR3A1,OR51L1,OR52W1,OR6K6,OR8U1,OR9Q2,PLCD3,POLE2,POLR1C,PRKACB,RALB,SFRP2,SPAM1,TICAM1,TKTL2,ZMAT3,TNFRSF18
Negative_10083	BAX,CD6,PAH,ACTG1,ADCY3,AGXT,ATG7,B3GNT5,BMP5,CCR10,CD3E,CD79B,CD82,CXCR3,DDX4,GNA14,HLA-DOA,IL6R,INPP5A,NAGS,OR4D1,PDE1C,PDE3B,PIK3CG,PTDSS2,SEMA4G,TFPI,TNFSF18,TUBAL3,VDAC3,ALDH18A1
Negative_10084	ARPC3,CYSLTR1,NRAS,PCYT2,RPS6KA2,XYLT1
Negative_10085	AMH,ID4,ABP1,ADCY1,ADRBK2,AGPAT6,AK3L1,AKR1B1,B3GNT3,C1GALT1,CCR9,CLDN9,CPT1A,CTBS,CTNNA3,CTTN,DCTD,DLL4,GCDH,GLB1,GRAP2,GSTM1,GSTM3,HADHA,IBSP,IL21R,KIR2DL4,LAMC1,LEFTY2,NT5C1A,OR13C8,OR4N2,OR51F1,OR51S1,OR7C2,PRKAG3,RASGRF1,SENP2,SOX17,SUOX,TBK1,UGCG,VASP,SERPINA1,TNFRSF1A
Negative_10086	ID4,IL6,ACVRL1,ALDOC,B3GAT3,BDH2,CTNNA1,CYP2B6,DLL3,DTX4,EFNA3,FLOT1,INPP5E,IRF7,MAP3K13,MYH11,MYL5,NOTCH2,NR1H3,OR10Z1,OR56B4,OR5AP2,OR6C2,OR9I1,PIP5K1A,PLCZ1,PPP2R5E,PPP3R1,PRKAB1,RALBP1,RASGRP3,RFWD2,SHISA5,SLC25A6,STAT4,WNT4,YKT6,EIF4EBP1

Negative_10087	ANAPC1,BLNK,CASP8,CDC14B,DGKZ,FARP2,FZD10,HARS,INSRR,LDHA,NOD1,NPR1,NRG4,OR10G8,OR52I1,OR5M9,ORC2L,P2RX2,PARS2,PIPOX,PRKCB,PTPRB,RCHY1,SUCLA2,TGDS,TLN2,TPI1,VAV3,WNT5B,PAFAH1B1
Negative_10088	C4B,ATP5A1,GUK1,ITGB7,MGST1,PDE6G,PRIM1
Negative_10089	AK3,CSK,DAO,AHCY,CD274,CHKA,CNTNAP2,CRHR1,CSF3R,CYP19A1,CYP8B1,DIAPH3,GALNT5,GFPT1,GGT6,GNA15,HYAL2,IL18,INHBE,ITGA1,ITGA10,NEUROG3,NFKBIA,NOS1,OR6M1,OR8S1,PFN2,PIP4K2B,PLCG2,PNPT1,PRIM1,RUVBL1,SV2B,TAS2R7,TRAF4,SUV420H2,TNFSF13B
Negative_10090	ARHGEF2,DNMT1,MEF2C,NPAS2,OR4X1,OR9Q1,PIGT,POLR2G,RPIA,UGDH,TNFSF13B
Negative_10091	APP,CBL,EZR,F2R,GH1,ACP5,ANAPC5,BAAT,CBLB,CHPF,CSNK1A1,CTF1,DCTD,DHODH,FLOT1,FSHB,HNF1A,IL7R,KLRD1,LAMB2,LIPG,MCAT,MPZL1,OR10A5,OR3A2,OR7G1,PIAS3,PIGP,PNMT,PPCS,RPS6KA4,SFRP4,SMC1A,TUBB,WHSC1,MAPK8IP3,SERPING1
Negative_10092	C1S,MPZ,MVD,AARS2,ALDH6A1,CAMK2G,CCL28,CDKN1B,COQ6,CTNNA2,F2RL2,FRAP1,GALNT12,GLO1,GNAI1,HARS,HES1,IL17RA,L1CAM,MADCAM1,MAP3K2,MAPK14,MGAT4A,MKNK2,NFASC,NKX6-1,NPR1,NTF3,NUDT8,OR4F17,OR8S1,P2RX7,PAK6,PGAM1,PTPN11,RARS2,SEMA3G,SKP1,ST3GAL5,STAM2,TRAF1,TSHB,ULBP1,VEGFB,MAPKAPK3,SLC25A31,TNFRSF10C
Negative_10093	ADRB1,ALG2,ATP5F1,CCL25,CCNA2,DHRS4,EDNRA,FUT7,GGT5,GNAZ,KNG1,MAP3K6,MCM7,MEF2C,MTHFD2,NARS2,NTRK1,OR4D9,OR6C4,PCYT1A,PRNP,SMAD4,SOCS3,STAM,STX16
Negative_10094	BAX,EGF,GAA,STS,ACO1,AKR1A1,AKR1B1,AMY2A,APH1A,ARHGEF2,ATF2,ATP4A,ATP5F1,B3GAT3,B4GALT5,CACNA1I,CD55,CLTB,EPB41L1,GALNT2,HLA-G,INHBB,KIR3DL1,LIPC,NEU1,OR8I2,PIGA,POLR2G,PRSS3,PTGS2,PYCR1,SRD5A2,ST3GAL5,STT3B,TAS2R14,TNFSF10,TUBA1A,WEE1,MAPK8IP3,SERPINC1
Negative_10095	FAS,GALNT14,IL22,OR13H1,PIGF
Negative_10096	A2M,GLA,ACVR1,ADIPOR2,CLDN11,CLDN9,CNDP1,CSF1,CX3CR1,CXCR5,EXTL1,FBXW11,GSTP1,IFNA13,IRAK4,ITGAX,LYPLA2,MAP3K6,OR10G8,OR2B6,PDPK1,PIK3R2,PKLR,PYGB,RASA2,SMAD1,ALDH18A1
Negative_10097	INPP4B,OR4D11,P2RX2,PLAT,SLC12A2,STAT3,TM7SF2,SLC25A31
Negative_10098	LPO,MSN,ACACA,AQP7,ASMT,CCR6,CER1,CHIA,CYP1B1,FGF4,GGCX,IL17RB,IL20RA,ITGAL,MAP3K6,OR5C1,OR5H2,PRSS2,RASA1,SEMA3D,SEMA4F,SLC27A4
Negative_10099	FST,MET,BDNF,CCNE2,CDKN1B,COL4A1,COL4A4,CUL1,CXCL3,EPHB6,GALNT10,GNAS,MAP2K7,MAP3K4,MDH1,MYH13,MYL7,OR2A42,PIGM,POLE2,POMC,PRKCZ,PTPRM,RPSA,SETD1A,SHMT2,SRD5A1,SSH2,SULT1A4,TSNARE1
Negative_10100	MS4A2,OR4M2,OR8I2,OR9Q1
Negative_10101	GLS,BIRC3,C7orf16,CD247,DPYD,EFNA2,EPHX1,FASN,GALK2,IL6ST,MAP3K11,MCM5,NANP,NT5M,OR6Q1,PDE5A,PDE6D,POLD1,SARDH,SHC2,SUOX,TOLLIP
Negative_10102	ACTN2,ALDH7A1,AMY2B,ARHGEF6,CCND1,CYSLTR1,DPM2,GALNT6,IL6ST,POLR1A,PPP1CC,TP53
Negative_10103	EBP,ME2,RB1,ALS2,CD80,CES1,ESPL1,FARS2,GDF5,GPT2,GRAP2,IGF1R,IL1R1,IL8RA,KDSR,KIR2DL2,MAD1L1,NFASC,NR4A1,OR1A1,OR2AT4,OR52I1,OR52N5,OR7D2,PAK3,PDE5A,PHKA1,PHKG2,PPP2R5B,PSME3,PYCR1,SLIT2,SPTLC2,VTI1A,TNFRSF18
Negative_10104	CP,ABL1,ATF4,GOT1,IFNA2,IGF1
Negative_10105	ATG3,CADM3,DNMT3A,EPHA7,FASN,FOXA2,HMOX1,IL11,MDM2,OR1J2,STX12,UGT2B4,HLA-DPA1,HLA-DRB3

Negative_10106	TNF, ADCY4, ADH6, AQP7, BMP6, CAMK2A, CDH2, CXCL5, DHRS9, DUSP6, EARS2, EDA2R, HEXB, IL19, KDSR, OR10J1, OR2F1, OR2H2, OXCT1, PLA2G2F, PPP2R5D, PTPRB, RAC3, SEMA4G, ST6GAL1, VARS, MAPKAPK3
Negative_10107	F11, TNC, A4GALT, ACAT2, ACTN2, ADSS, AGMAT, AGPAT4, CASP9, CNTN2, COL1A2, CXCL13, CYCS, CYP2A7, CYP3A43, DAPK3, FGF3, IFNAR1, ITGA3, LAMC2, LIPF, MAP2K4, MGLL, MYH14, MYL5, NFATC3, NLGN1, NLGN3, NPAS2, OR51F2, OR5AS1, OR5B3, OR6F1, PHKA1, PIP5K3, PLA1A, POLR1D, POLR2D, PTPN5, PVRL1, ROBO2, ROCK2, RPSA, SRGAP1, ST8SIA1, TAS2R49, TNXB
Negative_10108	GK, ACADSB, ACTB, ALS2, ANPEP, APRT, ATP5D, BIRC3, BMP8A, CD244, CDKN1C, CES1, CHSY1, CLTA, CTSB, CYP17A1, DVL3, F11R, GABARAP, GLI2, GRLF1, GUCA1B, HSD3B7, ICAM1, IFNA5, IL10RB, IL20, IL6ST, KIR2DL4, LRDD, MAPK13, MGAT2, NCAM1, NFYC, OR5AK2, PARD6B, PCCB, PEMT, PER1, PPM1D, PROC, RPS6KA1, SEMA4A, SLC12A2, SNAI1
Negative_10109	CSK, IL2, KDR, LEP, TNF, UBB, ACADSB, ACY3, ALG10, ANAPC5, ARHGEF1, CAMK2B, DGKA, DGKE, DPM1, DUSP1, FADD, FGF3, INADL, INHBE, IRAK4, IRF7, ITPKB, LEPR, LIPA, LIPT1, MAPK1, NFKBIA, NGEF, NRXN2, OR52A5, OXSM, PDE5A, PDE6G, RAF1, WNT7B
Negative_10110	F9, WAS, ACTC1, AMY2B, B3GALT6, CHD8, CREB3L4, DPYSL2, EIF4E, GNAI2, HTR5A, IGF1, KIR2DL1, KIR2DS4, OGDHL, OR4F15, OR4K17, OR51Q1, OR8J3, OR9G1, PDE8A, PDXP, PIGO, PLA2G10, PLA2G3, PPP2R5A, PRKAR1B, PTPRC, SELP, TNXB, VTI1B, SERPINA5
Negative_10111	AK7, DBT, ME2, ME3, PLG, ALDH1A3, AMY2A, ARPC3, BDH2, BMP6, CACNA1B, CAMK2B, CCNB1, CCNE2, CD226, CFLAR, CXCL12, CYP2S1, CYP4F3, DGKG, ECSIT, GCDH, HTR5A, IDI2, IFNGR1, LARS2, LYPLA2, MBL2, MCM2, MRAS, OR14I1, OR1K1, OR2T6, OR56B4, OR5M9, OR5T3, PDE2A, PDGFC, PERP, PRKCG, RALGDS, RASGRF2, SIPA1, TAS2R1, TBL1X, TGFBR2, ULK1, YARS2, TNFRSF17
Negative_10112	CBL, EGF, JUP, ASCC3, BET1L, CHIA, CYP4F3, EPHA2, FCGR3A, MAN2A1, NRXN3, NT5M, OR6N2, OR8U1, PCCB, PCK2, PIGV, PLCE1, PPP3CA, STX4, TUBA8, WNT3, TNFRSF10A
Negative_10113	AMH, IL5, ALG11, ARHGEF7, ATG7, CLDN18, CREB1, CREB3L3, DAXX, DHRS4L2, FLAD1, GALK2, GART, GGT7, GRIN1, GUCY2D, HADHA, IFNA8, ITGB7, MAN1A1, MAP4K2, MNX1, NARS, NME2, ONECUT1, OR13C4, OR2M4, OR4C12, PERP, PIK3C2A, POLR1E, PPP3CB, SOCS7, STAT4, TAS2R8, WIF1, CACNA2D4, PNLIPRP2
Negative_10114	CBS, DCC, ALG12, BMP8A, E2F1, EIF4E2, GSTO1, HTR7, IMPDH2, LRP5, NGEF, OR7D4, PARD6G, PDE3B, PIGW, PPP1CA, RELA
Negative_10115	ACOX3, GATM, GCNT4, GNA13, IRAK1, XCL1, CSNK1A1L
Negative_10116	GLS, BMP4, GSTT1, LAMB4, MGST3, OR10P1, PIGV, SYNJ2, THBS1
Negative_10117	BTD, CD6, GH2, LTB, RFK, ATG3, ATP5H, ATP5J, BRAF, CFLAR, CNDP1, COL11A1, CTSS, DDX51, DTX1, EDNRB, GALNT3, HMBS, INHBE, MCM3, MCM7, MKNK1, MYH15, OGDH, OR10H1, OR4C15, OR4F4, PECAM1, PIGL, PLAUR, POLR2J, PROC, RFXAP, SKP1, ST3GAL3, UBE2L3, UGT2B4, MAPK8IP3, TNFRSF6B
Negative_10118	LTB, ACO1, ALDOC, AOC3, C3AR1, CDS2, DEGS2, GANC, IFNAR1, IL6ST, ITGA4, LIMK2, NAGK, NFATC3, NSD1, OPLAH, OR10A6, OR10Q1, PCK1, PMM2, PRIM1, PTK2B, SETD1A, TPH2, WNT2
Negative_10119	ADA, CFI, HDC, LYN, TNC, CAV2, CCL24, CCL26, CCND2, CCR10, FZD2, HMBS, IL29, ITPKB, LEF1, MAP2K2, MYL7, OR13C5, OR2M4, OR5I1, OR8K5, PIK3C3, PMM2, POLR2D, PRDX6, STX12, TAS2R8, UPB1, WEE1, YWHAE, PLA2G12A
Negative_10120	C5, FGG, JUP, LSS, AACS, ACOX2, AMDHD1, APAF1, ATP13A2, BIRC3, CERK, CLDN9, CYP11B2, DFFB, EFNA4, GAD2, GNAI2, GNRH2, GSTK1, IL17RA, IRAK3, OR10A6, OR10H3, OR2T33, OR6C3, ORC3L, P2RX5, PAK6, PIGL, TPM3, UBA7, YWHAE

Negative_10121	BTC, FUK, NOG, NPY, RDX, SCD, ACOT12, AKR1D1, ALPL, ANAPC11, CACNB2, CLDN6, CNTF, CYP7A1, GALNT13, GLI3, GUCY2F, HTR7, IBSP, LYZL1, MLLT4, OR2T3, OR52J3, OR5AP2, OR5V1, PER1, PHKA1, PIAS2, PLA2G5, PRKAA2, PSME1, PYCRL, SMPD4, TAS2R48, TGFB2, TLR2, TP53I3, TYRP1, WNT7A, XCL2, PAFAH1B3, PPARGC1A
Negative_10122	BAD, BCR, MAX, SDS, AADAT, ACADM, ANAPC4, ARPC5, ATP5E, B4GALT1, BMP6, CDKN2B, CYP1A2, DHRS4L2, DUSP16, FMO3, FUT5, GZMA, HSD3B1, HTR7, IGF1, IL10, ITGA2B, MAN2A1, MAP3K2, MAT2B, MTMR6, NADSYN1, NLGN1, NLGN3, OR10G7, OR2C1, OR4D1, OR4F17, OR5J2, OR5L2, OR6K2, PAK1, PCYT1A, PIP5K1B, PTGER1, PVRL3, SENP2, SLC33A1, SNW1, WASF3, GABARAPL1
Negative_10123	GNS, ACSM2A, ACTN2, B4GALT7, CACNG3, CD19, CEBPA, CMPK1, CTPS, DGKG, DGKZ, DUSP10, EARS2, ERBB3, FTH1, GSTA1, ITGA1, KLRK1, MMP14, NTSR1, OR10Q1, OR4A15, OR51I2, PKMYT1, PSAT1, TAS2R60, THTPA, WNT1, B3GALNT1
Negative_10124	GH2, ABI2, ABL1, AKR1C4, ARPC5, B3GNT4, BACE2, BMP6, CDH2, CDKN1A, CLCA2, CSDA, CSF1R, DDOST, DUSP8, FGD1, GLUD2, GMPR, GRAP2, HGSNAT, IFNA21, LAMB1, NFE2L2, NOTCH1, NOTCH2, NT5C, OR4B1, PARVA, PFKL, PIP5K1C, PLK1, PPA2, PRKY, PTEN, RALB, SC4MOL, SDHB, SULT1A1, TGDS, WHSC1L1, TNFRSF10B
Negative_10125	CDKN2C, FOXO3, FUT6, GARS, MAP3K8, TRAF1
Negative_10126	ESPL1, GALC, IDH1, LHCGR, MAN1A2
Negative_10127	ADH1B, AREG, ARHGEF4, B4GALT2, CAMK2D, CDKN2A, CES7, DAAM1, NEU2, OR11H4, OR1K1, OR52N4, PDE4A, POLR1E, SMAD9, VTI1A
Negative_10128	DCT, ALDH6A1, ATP5D, CDH1, COQ3, GLYCTK, IL2RA, MAPK7, OR13A1, OR4D2, P2RX5, PGS1, PKMYT1, PSME1, SDC1, SOX17, TCF7, TNXB
Negative_10129	ARSE, GALNTL5, IFNA21, NAE1, PFKFB2, SNAI2
Negative_10130	CDH5, DKK2, EPB41L3, GPT2, KDSR, NCOR1, NEU3, SRGAP2, PNLIPRP2
Negative_10131	FES, GDA, GHR, MVK, AARS, ACY1, ADIPOQ, ADRB1, BCKDHB, BDKRB1, CCNB3, COL3A1, FDFT1, GGT5, GPX5, GSTA2, HES1, HYAL1, NADK, OR13A1, P2RX1, PARVA, PDE4D, PLA2G1B, PTGER3, PTPN11, TAS2R40, TUBA8, UNC5C, YWHAB, ATP6V1B1, MAPK8IP3
Negative_10132	CRH, RPE, ACSM2A, ADH4, BIRC3, CCNG2, CHEK1, COL4A6, CTNNA3, CXCR5, DDX19A, DDX52, DGKQ, DHRS9, EHMT1, FGF13, FGF18, GRM5, IFNA21, MALT1, NFAT5, NQO1, NSD1, NTF4, OR10J1, OR2T4, OR4K15, OR4X1, OR51B6, P4HA1, PDE6G, POLR2F, PPP1R3D, PTTG1, RXRG, SAT2, TP73, HLA-DRB3, PPP1R12A
Negative_10133	ACSM4, CHRM1, FSHR, KIR2DL2, MTMR1, NCOA4, NR1D1, OR5I1, OR5V1, PLA2G6, POLE4, POLR3A, PSAT1, PSME3, SUFU, WNT10A
Negative_10134	C7, ACSL6, ADH1B, ALG5, APRT, ARRB1, C9orf95, CAV1, CCL1, CXCL16, EFNB3, EPAS1, EPHA5, GALNT10, GLYCTK, HAP1, IL18RAP, KIR2DS5, MAP4K2, MDH2, OR10H3, OR2C3, OR4P4, OR52A1, PERP, POLR3K, PPP1R3B, PRKAB2, PTPRC, ST3GAL2, SV2C, TNFSF8, UGDH, UGT2B28
Negative_10135	KMO, ADCY1, ALOX12, AMY2B, APOA1, CCNH, GNAZ, LIPF, NLGN2, OR1J1, OR1L4, OR51D1, POMC, PPARG, TAS2R48, ZFYVE9, B4GALNT1
Negative_10136	CDC25C, CNGB1, GLYCTK, OR10A3, OR5P2, PEMT, PRKCB, PVRL1, PVRL2
Negative_10137	IHH, PDC, CSNK1A1, IQGAP1, ITGA5, LDHAL6B, MFNG, MS4A2, OR4M1, PGK1, POLR2J
Negative_10138	ABCC8, ADSSL1, ALOX12, ATP5J, BHMT, CACNA1E, CCL23, CCR10, CDC25B, CHRM3, CHUK, CSNK2A2, CYP4A11, ENTPD6, FCGR3B, FLAD1, FPGS, GLG1, IFNB1, LDHA, LILRB3, MITF, NARS2, NT5C, NUDT8, OR51B4, OR5H2, OR7A10, PDE1C, PDE8B, PIK3C2B, PNMT, POLA2, PRKAR1B, PTPN11, SOCS2, SSH3, STAM2, TAS2R10, TBXA2R, UGCGL2, UGDH, UXS1, TNFRSF12A
Negative_10139	ADCY7, INMT, SEMA3C

Negative_10140	GPI, ALOX12B, ANAPC7, CCL21, CTSL1, CXCL9, DUSP4, FZD10, GALK2, HADH, HSD3B2, INPP4B, NOTCH1, NT5C3, OR51L1, PFN3, ROCK1, SDC1, UBE2L3
Negative_10141	C5, F5, GH2, ACOT11, ACTN2, ACY3, CLTB, CYP1B1, EPHA4, EPHB4, FUT9, HSD17B3, HYAL2, IL20RB, IMPA1, ITPK1, KLRC4, OR2A42, OR4X1, PGM2, PLA2G3, PTCH1, PYGB, SMAD6, SPHK1, STK36, STX1B, TCF7L2, UPP1, VKORC1, ATP6V0E1, HLA-DRB4, PNLIPRP2
Negative_10142	ACVR1C, ACY3, ARSA, CHST11, CXCL11, DPM1, GBE1, GDF7, GNA12, JAK3, LAP3, LRP5, NMNAT1, OR1F1, OR1K1, OR2M4, PGK2, PIP5K1B, PLXNB1, POLR2F, PRKACB, PTPRJ, QARS, RAP1A, RASGRP4, ROBO3, SGPP1, SHC3, SKP1, STAM, TGFB1, TRPM5, TUBB6, UGT2B11, UROS, ATP6V0D1, PHOSPHO1
Negative_10143	AMH, F10, FOS, SI, ACTN2, ALS2, ARAF, AREG, B3GAT3, BPGM, CXCR4, CYFIP2, DLST, GALNT8, GFPT1, GUCA1C, IFNAR2, IL18R1, IL28A, IL28RA, IPPK, ITGA6, MAN1A2, MAP3K11, NCK2, NEFM, OR10A7, OR10H1, OR2F1, OR4D2, OR4X1, OR8K1, PANK2, PANK3, PDHB, PIGT, PLXNA3, POLR2I, PRKACB, PRKACG, PSAT1, PTGIS, SEMA3B, SEMA3E, STAT6, SV2A, TBXAS1, UCK2, WARS2
Negative_10144	ACAD8, ACMSD, FUT1, NOS1
Negative_10145	CKB, F10, GGH, GSS, TNF, AASDH, ABL1, ABL2, ADCY10, ALDH1B1, APLP1, ATP6V1H, BHMT, CHST11, CLCA2, CLDN16, CLDN5, CREBBP, DEGS1, DUSP7, DUSP9, FGF1, GLYCTK, HMGCS2, IL8RB, KCNB1, KYNU, LILRB3, MAP3K14, MGAT1, NAE1, NAGK, NR4A1, OR2B6, OR5D16, OR5L2, PAPSS2, POLA2, PPID, PPP2R1A, PTPRR, SLC25A5, SORBS1, SORD, STK36, UXS1, SERPINA5
Negative_10146	LPL, APRT, CACNG7, COL1A1, CYP3A7, DEGS2, DHRS3, ENTPD5, EPHB4, GALNT12, HTR6, IDI2, IL29, LIFR, MAGI2, NTRK1, PDGFA, PFN1, PISD, PPID, RASGRP3, TJP1, UCK2, UNC5C, HLA-DRB4
Negative_10147	GP9, ADSL, AGPAT4, ALG1, ALPL, ARD1A, ASPA, BCKDHB, BDKRB1, BMPR1B, CACNB4, CBLC, CDKN2D, CHSY1, CRLS1, CYFIP2, FARS2, HAP1, IL19, IRAK3, ITGB4, ITGB6, JAM2, LIPT1, MTHFD1L, OR10Q1, OR4K17, OR4S1, PGS1, PRKACG, RARS, RDH12, RPSA, SHISA5, UBE2L3, WASF3
Negative_10148	MYH10, TAS1R3, YWHAB, MAPK8IP1
Negative_10149	DBT, ACACB, ARHGEF2, B3GALT5, CCNA1, CCNE1, DDX18, DTX1, DVL1, E2F3, EGLN1, HCST, HPSE, IDH1, IL10, IRAK3, KLKB1, MAP4K4, MTHFD1L, MTMR1, MYL7, NCKAP1, NEUROG3, NME7, OR10AD1, OR2A25, OR2T10, OR2T6, OR52E8, OR56A4, OR5B3, PCNA, PLCD3, PPCDC, PPOX, PSENEN, PYCR1, RAB5A, RENBP, RFXAP, SLC2A4, SREBF1, SULT1A2, THBD, TKTL2
Negative_10150	CRK, ACOX1, AGPAT4, ANAPC7, ARPC3, BRAF, CACNG3, CHIT1, CLDN19, CTNNA1, CYP2A7, DPM2, EFNA5, EIF4E2, FCGR2B, FGF10, HRH1, IFNA7, IL12B, IL19, KCNB1, KITLG, MALT1, MAN1C1, MCCC1, MRAS, MTHFR, NPR1, OR1S1, OR2B6, OR4C46, OR4K5, OR51Q1, OR5I1, PCYT1A, PDE3B, POLR2D, PPAP2C, PPP2R2A, PTGIS, RHOA, RPS6KA1, SDHA, SHC1, SLC2A1, SMAD2, SNAP25, SOX17, PRICKLE2
Negative_10151	EBP, GAA, IL7, MVK, TNC, ABCC8, ACPT, ADRA1A, ATP5G2, CCR10, CD226, CES1, CHIT1, CMAS, COL2A1, DDX55, FBP2, GAB1, GNAQ, IDH2, IL28RA, MYLK, NCR3, NOX3, NUMB, OR2AT4, OR52A1, ORC3L, PDE4A, PIK3R2, PMVK, PORCN, PRKCG, PYGM, RAPGEF2, RPIA
Negative_10152	CD6, ACPT, COMP, DLL4, FZD2, GGT6, GNRH2, IFT57, ITGAM, JAK2, MARS, OR13H1, PANK3, PHGDH
Negative_10153	AGK, DAO, DHH, EBP, GK, HK1, IL6, NF1, RPE, ADORA2B, AKR1B10, ARHGEF1, ATP5G3, BUB3, CACNA1G, CAV1, CYBA, HIBADH, IDH3B, IDUA, IGSF5, IL17RA, IL29, INPP4B, NME6, OR13C5, OR6C75, PKMYT1, POLR1D, PORCN, PRKCD, PRPS1, VAV1, WASF3, CACNA2D3
Negative_10154	ACAD9, ADH4, ADH5, ADRA1A, AMPD2, APAF1, CCL23, CCL3, CNTN2, F13A1, FUT7, GMDS, GRLF1, GZMA, IFNA13, IMPDH1, KIR3DL3, LMO7, MAPK13, NFE2L2, NMNAT2, OR11H1, OR52B6, OR8D1, PLCB4, PNPO, PSPH, SMPD4, SOCS3, SOS1, TLR5, UBE2K, WARS, WARS2, ZBTB16

Negative_10155	BCR, DDC, F10, GPI, PGP, RB1, ACAD9, ACADVL, APAF1, CCL28, CD79B, CDH4, CHEK1, DUSP8, ENPP7, ENTPD3, EPHB4, ETNK2, FLOT1, FMO5, GGT7, IL21R, INADL, MAN1A2, NCK2, NNMT, NT5C3, OR1I1, OR52A5, OR52B4, PDHA1, PRIM2, PRKACA, PRKAR1B, PSEN1, PTDSS1, PTPRB, SETMAR, SRD5A2, ST3GAL5, TBXA2R, TGFBR2
Negative_10156	ID2, PDC, TNC, AADAC, ABLIM3, AKR1B10, ALOX15B, CD72, COMT, CPS1, CTSL1, DUSP3, EPHB6, GALK2, GUCY1A3, HIBCH, IL20RB, IL2RG, INHBA, LCAT, LIPF, LRRC4C, LYZL1, MAN1A2, MAP4K2, MAPK8, NR1D1, NTF4, NTSR1, OR2L3, OR4K1, ORC2L, PAK4, PCCB, PFN3, PIAS4, PIK3C3, PIM1, PRKAB2, PTPN7, RAPGEF1, TAS2R14, ULK1, VAMP1, XYLB, ATP6V1B2, TNFRSF14
Negative_10157	ADA, C8B, AASDH, ALDH1A2, BTRC, C5AR1, CACNA1H, CARD11, CCR6, CD247, CYP2S1, DFFB, DHODH, DTX3L, FGF17, GUCY1A3, HAGHL, JAG2, MGAT5B, MYH2, NUDT9, OR10H2, OR6Q1, PANK3, PDXP, POLR3K, PPARG, PPP1R3B, PRKAR1B, PRKG1, PTCRA, SOS1, TAS2R60, TJP1, WNT7B, WNT9B
Negative_10158	SFN, SPN, ACADS, ADH6, ALDH2, APOA1, BMP4, CCL13, CD38, CDC16, CHSY1, CPT1C, CYP27A1, CYP2C9, CYP2E1, DCTN1, DOCK1, GGT5, IL1A, IRS4, JAG2, KITLG, LTA4H, LTB4R2, MDM2, OR5AP2, PIGQ, PIP4K2B, PRDX6, PRKCQ, RETSAT, SMARCA2, STK36, THY1, TPH2, WASL, WHSC1L1, MAPKAPK5
Negative_10159	A2M, C7, FUK, IL5, IL7, SCD, ACSS1, ACVR1B, ADIPOQ, ADORA2B, ALDH2, APOE, CASK, CD38, CRB3, CYP4A11, ENO1, GALNT7, GALNT9, GCDH, HMOX1, IL10RA, IL17B, ITGAD, KLRK1, MAD2L2, MMP2, NARS, NAT1, NOD1, OR51G2, OR7A5, OR8B12, PAFAH2, PLXNC1, RARA, RARS2, RHOQ, SETDB1, SLC33A1, TCF7L2, THBD, TP53, UMPS
Negative_10160	ADK, BTD, FAH, JUP, ACVR2A, AGRP, AMD1, BDKRB1, CBLC, CCL26, CCNB3, CD99, CHRM3, DHFR, GNA11, GRIN2D, GYS1, HNF1A, MDH1, POLD2, RFXAP, SEMA7A, TSLP, TYRP1, PPARGC1A
Negative_10161	EPO, PGP, PLN, SI, ACTA1, ANAPC2, CLCA2, CTBS, DHRS4, ELK1, GOSR2, GPAA1, IL4R, MMP1, RXRB, STX16, STX1B, YWHAQ
Negative_10162	PLN, ARSA, CD36, CES7, DHODH, FMO5, GALNT3, GJD2, GOSR2, GSTA3, GYS1, IL20RB, LRAT, OR9G1, PLD2, PPARD, RAPGEF3, SDHC, TUBA3C, TUBB, ZIC2, ATP6V1B1, C1GALT1C1, GABARAPL2, TNFRSF18
Negative_10163	CD2, ARHGAP5, CARS, CCNE1, CPT1C, CYP2F1, DHDDS, DUSP16, FGF22, FZD9, GALNT9, GBA3, GLO1, OR10A2, OR52E6, OR52L1, PLA2G2F, PPP2R1A, PPP2R2A, SMC1A, TNFSF4
Negative_10164	CLDN8, CXCR5, E2F1, EPHX1, GALNT10, MAN1C1, NEFL, OLR1, OR4M1, OR7D2, PFKFB1, TRPC1, YWHAE, SLC25A31
Negative_10165	AGK, RPE, AGPAT4, ATP2A2, JAK1, LAMC2, MARS2, MITF, MKNK2, MTFMT, OR4K17, OR56A3, OR5D14, PAK7, PDXK, PER3, PRDX6, PRKG2, TNFSF8, UBE2L3, VAV3, GABARAPL2
Negative_10166	CTH, AGTR1, ASAH1, DRD1, IFNA2, MAPK1, OR2M4, OR3A2, OR4X1, OR7C1, OR7D2, OR8J3, PAICS, PSAT1, SUCLA2, VEGFC, XYLB, CSGALNACT1
Negative_10167	OR4K1, OR5L2, PTPRR
Negative_10168	CRH, ADRB3, AKR1C2, AVPR1B, CACNA1G, CD55, CD79A, CTLA4, DGKQ, ENPP2, FARSB, FGF7, GJD2, HEMK1, HNF1A, ITGB6, MAPK8, NOTCH3, OR2H1, OR2T33, OR51B6, PAFAH2, PANK3, POLR2C, RRM2B, SGPP1, ST8SIA1, TAS2R7, TLR9, ZFYVE16, ATP6V1E1
Negative_10169	CAD, GLA, ADSS, ARD1A, CD34, COL1A1, ETNK1, LIPA, NEUROG3, P4HA1, PANK2, PECAM1, PIP4K2A, POLE, PRKACB, SESN1, TRAF4
Negative_10170	GSN, AADAT, ACACA, ACADVL, ACOT11, AKT1, B3GALT1, BIRC3, CCR8, CREB5, CXCL2, CYCS, DHFR, DLAT, DMGDH, GZMA, HAO1, MYLK2, NCF4, NR4A1, OR52N1, ORC5L, PDHA2, PI4KA, PTPRM, PYGM, TUBA3E, XYLT1, YWHAG, HLA-DQB1

Negative_10171	DCK, GSR, ADCY6, ARPC5L, ATP5G1, CHIA, DDIT3, DGKH, DUSP5, FGFR3, G6PD, GPX7, GUCY2D, HSD17B2, IL6R, KLRC1, KNG1, LIPG, NADK, NRG2, OR2A42, OR2M4, OR6M1, ORC5L, PRDX6, PSEN1, RUNX1, SHC4, SKP2, SMPD3, SOCS7, SPHK1, TAS2R8, TLR2, ULK3
Negative_10172	LIF, SFN, BTRC, CACNG2, CCDC6, CD8A, DGKQ, DHDH, DLAT, E2F2, EHMT2, ENPP3, ETNK2, FASLG, FDFT1, GCH1, HYAL2, IRAK4, IRF3, OR10Q1, OR1D2, OR52N5, PER3, PLCE1, PNPT1, PRKCA, RARS2, RDH11, RELA, SDC1, SOCS2, SREBF1, VAMP2, CSNK1A1L
Negative_10173	FST, BCL10, BIRC3, COL4A1, CSDA, DAPK2, DPM2, ENPP2, GALNT2, GSTM4, LMO7, OR51E2, OR51M1, OR52E2, OR5L1, PAK7, PIP4K2B, PLA2G2A, RARA, RFX5, RHOD, SOAT1, SUOX, TCF7L2, UGCGL1, TNFRSF10A
Negative_10174	F10, JUP, ACOX2, ADCY6, ASAH1, ATP13A2, CYP19A1, DAPK2, DPAGT1, FGFR4, GALNT8, GALNTL4, GJA1, MASP2, MGAT5, OR10X1, OR1B1, OR1N2, OR2L8, OR2T11, OR4C6, OR4M1, OR51Q1, PER1, POLR2H, PRSS1, SMAD7, TNFSF9, VAMP1, ATP6V1C2
Negative_10175	BTC, CFH, FOS, LPL, OGT, SCD, XDH, ARD1A, B4GALT5, BCL10, BTRC, CASP7, CES1, CLDN2, CLTC, FLAD1, FOXO3, GUCA1A, IFNA2, NAT5, NRAS, OR11H1, OR2C1, OR2T1, OR5M3, PARD3, PRODH, RALGDS, RRM2, SMAD2, SPHK1, SRD5A2, STX1B, TALDO1, TGFBR1, TJP1, WNT9A, HSD17B12, TNFRSF14
Negative_10176	FAS, FER, LTB, ALDOC, BDH2, CYP3A7, CYP8B1, DGAT2, DUSP1, EFNA3, FOXA3, FRAP1, FUT1, GNA11, HSD3B1, HSPA2, IL28B, IQGAP3, NFKB2, OR10A3, OR10H3, OR2B6, OR51A7, OR51E1, OR52N1, OR5AN1, PDGFRB, PIAS2, PPP2R1A, PRKCQ, PTCRA, PTPRZ1, RRAS, SMARCA2, VEGFB, PNLIPRP2
Negative_10177	EPO, LTB, PGP, ADH1B, AGPAT6, CCL2, CD80, CDC26, CLDN7, GALNS, GANC, GLUD1, HDAC1, HMOX2, IFNB1, MYH13, OR10AG1, OR1J1, OR2AG1, OR2B6, OR6C3, PDE5A, PLCD3, PPBP, PRODH, PTCRA, SEMA3G, SEMA4A, ZMAT3, ATP6V0D1
Negative_10178	EPO, PDC, AKR1C2, B3GALT5, CCNB1, COMT, CYP8B1, DGKA, FLOT2, FTMT, GLI2, INADL, ITGB7, ITGB8, MAD1L1, MAP3K5, NAGLU, NCKAP1L, NOTCH3, NT5C1A, OR10AG1, OR10G3, OR2A2, OR3A1, OR51I1, OR6K6, P2RX5, PKMYT1, PPAP2A, PPP3R1, PVRL3, SHC3, SLC27A5, THBS2, VTI1A, CSGALNACT2
Negative_10179	ACSL4, ADRA1B, AGRP, CACNA1S, CDC14B, CDH5, GALM, GMPPB, GSTA2, MARS, NCKAP1, OR52I2, OR52N5, POLR3K, TAS2R10
Negative_10180	DCC, FAH, ACVR2B, BAIAP2, DDX52, DGKZ, EHMT2, ENPP1, FLT3LG, HLA-DOB, HRAS, IDH1, INHBC, LARS2, LRP5, NKX2-2, OR10A2, OR6C1, PAICS, PDE10A, PDGFRA, PIPOX, PRODH, RBL2, RRM2B, SCD5, SETDB1, VAMP1, WBSCR17
Negative_10181	AR, ANAPC7, ATP2A3, CCL16, DDIT4, IMPDH1, ITGB1, MAP4K3, OR2S2, OR5J2, PDE9A, TUBA1C, YARS
Negative_10182	C8G, TNC, ACTB, CCR8, CRY1, PLOD2, RENBP, SETD7, SORBS1, PPARGC1A
Negative_10183	MIF, ACADS, ALDH1A3, ANAPC5, AREG, ATF2, B3GALT2, BET1, BPNT1, CACNA1A, CD44, CDKN2B, CPOX, DKK4, DPYSL2, ENTPD1, G6PC, GATM, GRIA2, HMGCL, JUND, MCAT, NCF1, OR13C2, OR13C5, OR13C9, OR4F4, OXCT1, PDE1B, PDE8B, PLXNC1, PPP1R3C, ST8SIA5, STX3, VEGFC, YWHAB, YWHAG, KIR2DL5A
Negative_10184	AGK, ADH1B, ATF2, BCL10, CCNE1, CD27, CDIPT, CNGA4, CSNK1G3, EHHADH, HAAO, HTR2A, NNMT, OR4C15, OR52N4, OR6K2, PGK1, PLCG1, PPA2, PPP2R2B, PTTG1, SDC3, TALDO1, TBL1Y, VTI1B, YWHAZ
Negative_10185	HK1, ALDH3A2, ARG2, CACNG4, CCND1, CES1, CRLS1, CTNNA1, DUSP4, IARS2, IL2RA, KLRC3, LHCGR, NLGN2, NT5C1B, OR6N2, OR9A2, PDE9A, PTPN7, RPN2, RXRA, SEMA4C, STAT2, STX3, TSLP, TUBA1A
Negative_10186	AGTR1, DLL3, IGSF5, MDH1, NFASC, UGT2A1, VDAC1, SERPINA1

Negative_10187	DBH,GGH,MET,AKR1C1,ALOX12B,ALOX15,AQP7,CBR3,CD244,CHRM3,CXCL13,CYP2C19,DDX41,DHCR24,ENTPD3,FECH,FZD6,GAS1,GFPT1,GNG13,GTSE1,HADHA,HLA-DOA,IFNA2,ITGAE,KIR3DL1,MAML3,MYD88,MYH11,OR2T12,OR2W1,OR4K17,OR5B3,PGAP1,PIGA,PIK3R3,POMC,PPP2R5E,RND1,ROBO3,SARDH,SETD2,SLC2A2,SLIT2,SPHK2,SPRY3,STX7,TM7SF2,ZNRD1
Negative_10188	CBL,SMS,SP1,ARPC2,ARRB2,BAIAP2,CACNG8,CYP11B2,CYP4F3,DKK1,EDNRA,FABP3,GCNT1,GNAO1,IL12RB1,IQGAP2,IQGAP3,MAP3K2,OR14A16,POLR1C,PPA2,SMC1B,SULT1A4,TIAM1,TUBB2A,WNT4,ATP6V1G1,TNFRSF11A
Negative_10189	ACSM5,ACVR1C,ADH5,BET1,C9orf95,CDC14B,CLDN5,COX15,DGKH,DLL1,DTX1,GAD1,GPX7,GRIN2C,HSP90B1,IL2RB,LIFR,MADCAM1,MAP4K4,MPST,OLAH,OR10P1,OR2T5,PDXP,PIGP,PLA2G2F,PLCD4,POLD4,POLR1D,PRKG1,PSEN1,SMPD4,SPAM1,SPHK1,ST8SIA1,TAS2R38,TCIRG1,TRADD,TRAF5,WNT8B,WNT9B,ZFYVE9,SERPINB5
Negative_10190	ATM,AVPR1B,CFL2,CHAT,EDAR,FGF20,MMP7,POLR2J,TBXA2R,VDAC1
Negative_10191	BAX,ACO2,ADSSL1,AGPAT6,APAF1,ASRGL1,CHRM5,CISH,CPT2,CSAD,CTPS,DAPK2,DCTD,DGKZ,EARS2,EPHB3,FDPS,FLT3LG,FTCD,HHEX,HIP1,IQGAP2,IRAK3,ITGB1,KLK3,LEF1,MAP3K7,MTMR6,NFAT5,NR5A2,OR13D1,OR5D18,OR6C74,PDE4A,PLAT,POLR2E,PPP1CB,PRPH,PYGB,RELB,RENBP,SARS2,SDHB,SPTAN1,STAT5B,TIRAP,TPM3
Negative_10192	MAG,DLL3,FGF12,NRXN2,OR2C3,PTPRJ
Negative_10193	HK1,ASIP,CD46,CES1,CNGA3,COX15,DRD1,ENPP7,GALNT12,GNPNAT1,GRIA2,IFIH1,IL12RB2,IL19,LDHAL6B,LIPA,LLGL2,LRDD,MAN1A2,MAPK10,MYH1,MYLK2,NEGR1,OR5AR1,PRKACB,PTPRB,PYGL,SPRY4,STAT1,TAS2R16,TGFB3,WBSCR22
Negative_10194	C4B,C7,C8B,DCK,ID3,MAX,ACSL5,ACSS1,ADH5,ARNTL,ATP6V1D,CACNB2,CD46,CD59,CD82,CNGB1,EPB41L2,GNAI3,GSTZ1,HAO2,ITPK1,LCAT,LDHD,METTL6,MKNK1,MLYCD,NCK1,NSDHL,OR10R2,OR2S2,OR6C6,PDE4B,PDE7B,PHPT1,PNPO,PRKAG3,RPS6KA4,RRM2B,SESN1,SREBF1,SRGAP2,TAS2R41,TAS2R5,TBXA2R,TGFBR2,THTPA,TOLLIP,XCR1
Negative_10195	CANT1,DDIT4,DGKI,GABARAP,GNRH1,GPAM,GRLF1,GRM5,HSD17B4,IPMK,NFASC,NQO1,PDE1C,PTCRA,RFX5,SC5DL,ST3GAL1,TAS2R60,TUBB4Q,UGCGL2,ATP6V1B1
Negative_10196	HK1,NPY,ALDH4A1,ATG12,BCAT2,CD8A,DLL3,ETNK1,HLA-F,IDUA,MCAT,MCM3,OR13G1,OR4C46,SNW1,TNFSF9,ZIC2
Negative_10197	GNE,LCT,AGXT,ALPP,AMD1,AOC3,B4GALT6,BHMT,BMPR1A,CCR7,CDO1,COL4A2,CSNK1E,CXCR3,CYP1B1,EHHADH,FCGR2B,GP1BA,HLA-C,IFNA13,ITGA10,MAP3K3,MAPK1,NEU2,NOS3,NR5A2,OR13C8,OR5B3,OR5T2,OR6C6,PAX6,PMAIP1,PPP2R2A,PRODH2,PROS1,SNAI2,SUOX,TFDP1,ULBP3
Negative_10198	DCTN1,MYH13,OCLN,OR10H4,PPARD,THBD,MAPKAPK2
Negative_10199	BTD,CFH,CR2,DCN,GP6,KDR,SI,ALG2,APOC3,ARSA,ATP4B,BDKRB2,CD34,CLDN11,CREB5,CSNK2A1,CTPS2,CYP1A1,DHODH,FGF17,FGF18,GANAB,GFAP,GMPR2,IFNA16,IFNK,LCMT2,MAPK3,NFATC4,NOS1,OR4X1,OR6B3,ORC2L,OXSM,PIGX,PLA2G5,PPP1R3B,PPP2R5D,SGMS1,STAM2
Negative_10200	AMDHD2,COMP,EVI1,GANC,IL6R,MAN1C1,SSH1
Negative_10201	ACOX3,CCL2,EPHB3,GALM,IKBKB,IL4R,OR10P1,OR2L3,PAX6,PDE7A,PPM1D,QARS,RCHY1,SHMT2,STX8,TNFSF14,UBE2K,WEE1
Negative_10202	ACVR2A,ADAM17,ALS2,AOX1,B3GNT4,CCKBR,CHSY3,CLDN1,CXCR3,DHDDS,FGF1,GCLM,HMOX1,IKBKB,IL17RB,MAP4K4,OR10G4,OR10S1,OR11L1,OR2T4,OR51E1,OR5B21,PON2,PPAP2B,PTPRC,RHEB,SMPD2,STEAP3,STX7,UBA7,VAMP1,PLA2G12A,SERPINE1

Negative_10203	CTH,GAA,GH1,SI,ADH7,ARPC5L,B3GALT4,CCL26,EI24,ELK4,GFPT2,HLA-DOA,IARS,IL18RAP,IPMK,IRF5,NUDT2,OR52B4,OR52B6,OR8D4,OR8I2,OR9G1,PHKA2,PIK3C3,PPA1,PSPH,RPSA,RUNX1,STAT1,SULT1A4,TAS2R48,TP73,UGCGL2,UNC5C,HSD17B10
Negative_10204	CTH,LPL,NNT,RET,SPN,ACADS,APRT,CDC25C,CSNK1G1,FADS2,FGFR2,GALNT9,GYS1,HTR2A,IL22,IMPA2,ITGA3,ITGAV,MAP2K3,NFKB1,OR10H3,OR14A16,OR1E2,OR2B11,OR2G6,OR4C46,PCYT2,PDGFA,PRKCD,PRKCE,PYCRL,RARA,RFX5,SPRY1,TUBB2A,B3GALNT1
Negative_10205	ANAPC7,B4GALT5,C1QB,CAMK2B,CDC25B,CLDN20,GUCY2C,HSD11B2,IL22RA2,KLRC4,PLA2G2D,PLA2G2E,PPA2,RAC2,RASGRF1,TACR3,TKTL1,TNFSF4,UGCGL1,UPP2,MAPKAPK3
Negative_10206	CCL24,CLTA,CRKL,GZMA,IL23A,PARD6B,PLCD4,UPP1,PLA2G12A
Negative_10207	BID,DHH,MPZ,ACTA1,ADH5,ARPC5L,BCKDHB,CACNA1B,CASP9,CDS1,CFL1,CFLAR,CKMT2,COASY,CSNK1G1,CTNNA3,DGKZ,DPM1,FRAT2,GJA1,GSTM4,HDAC1,IDH1,IFNA10,MAP3K14,MTHFD2,NOX3,OR14I1,OR2H1,OR5AU1,OR6C1,PDE8B,PIGN,PLIN,POLD3,PPP2R2D,PRKAR2A,RFWD2,SEMA4B,SEMA4G,ST3GAL1,STX4,TAS1R2,TJP3,TRAF4,TRDMT1,YWHAQ
Negative_10208	C8G,F3,FGA,PLG,SMO,STS,ABCC8,ACTN4,AKR1B1,B3GAT1,CACNG6,CFTR,CKS1B,CLDN10,ENTPD8,FLT3LG,FUT2,GALM,GSTA2,IDH2,IRAK1,LALBA,LIAS,LIPG,MAP4K3,NAE1,OR2T1,OR4Q3,OR52A5,PIGL,POLR2K,PRKCH,PTDSS2,RALGDS,RIPK1,RRM2B,SDHA,SNAI2,SNAP25,TAS2R16,TBXAS1,WBSCR17,XYLB,YWHAZ,ST6GALNAC1
Negative_10209	ATM,C8G,ATP5F1,CARS2,CDC23,CLOCK,COQ6,CPT1A,DHFR,FBP1,GALM,IFNB1,IFT57,IQGAP1,IRF7,MAP4K4,MPP5,MYL5,NR4A1,OLAH,OR10C1,OR10Q1,OR13J1,OR2G6,OR2M3,OR6C75,PDGFC,PGM1,PIK3R1,PLCD3,PRKY,RAB23,STAM2,SULT2B1,TAP2,UCP1,WHSC1L1,XYLT1,TNFRSF17
Negative_10210	IL3,ARG1,ARSD,ATP6V0C,B4GALT6,C1QB,DGKE,DGKZ,DUSP8,ERBB4,ETNK1,GADD45G,HPSE2,IL20RA,IL6ST,INHBE,IRS2,MINPP1,NAGS,NFAT5,OR1I1,OR1J2,PARD6B,PLCB3,POLD2,POLR2G,RARS2,SKIV2L2,SPRY1,TAS2R41,UNC5D,ARHGEF12,ATP6V0A1,PNLIPRP1,TNFRSF13B
Negative_10211	ACVR1,APOE,C4BPB,CACNG5,CER1,CHAD,CYP2C9,DBF4,DDX4,EDAR,FADD,HAO1,IGSF5,IL13RA2,IL9R,ITGB8,LMO7,MAPK9,OR11G2,OR4X2,OR51A4,PCYT1B,PIK3R1,PITX2,PPP3R1,PSPH,RAF1,SOCS3,STEAP3,TAS2R10,TKTL1,TYRP1,AASDHPPT,C1GALT1C1
Negative_10212	F2R,GP9,SHH,ACSS2,APOA1,CACNA1E,CCR6,CDH15,CRHR1,CXCL6,ECHS1,EFNB1,FOXO3,IFNA10,IL5RA,IMPA2,INHBA,IRS1,ITPKB,LARS2,MMP7,NAT2,NCR1,OR52A5,OR52N2,OR8D1,PIP5K3,PNLIP,PON3,PPA1,PRKAB1,PTPRM,RHOQ,YARS2,AASDHPPT,HSP90AB1
Negative_10213	CS,DDC,F9,RET,ACADSB,ADCY7,ANAPC11,APPL1,ARSB,ATP6V0C,CD14,CYBB,CYP26C1,CYP4F3,DAPK3,DARS2,FABP4,GDE1,GLS2,GMDS,GPLD1,GSTT1,GSTZ1,GZMB,HNF4G,ICAM2,KCNQ1,KIR2DS5,MAP2K3,MAP3K13,MCCC1,MCCC2,MYH7B,NADK,NFX1,OR10H1,OR11A1,OR51A2,OR6C6,OR7D4,OR7G2,PAPSS1,PAX6,PGK1,SFRP1,SLC2A4,TNFSF14,TRMT11
Negative_10214	ABAT,ADCY9,AOC2,CCL13,CCL3,CHRM1,CLOCK,FARP2,FZR1,GSTT1,HLA-F,HSD11B1,HSD17B1,IL20,LIPC,MASP1,OR5AP2,PAFAH2,PDE7B,PDHA1,PDK1,PER1,RAPGEF2,SDHD,SMAD7,SOCS5,TSTA3,AASDHPPT
Negative_10215	DCN,APOE,CADM1,DDX4,MDM4,PLCB2,SOCS7,SPTLC2,TSTA3,UROC1
Negative_10216	AK2,MSN,RPE,TNR,ACTA2,AGPAT3,ALG6,ARSB,BAAT,BCAT1,CACNA1A,CALML5,CBLB,CCL8,CDO1,CYP3A43,DERA,DGUOK,DLAT,DPM3,EPB41L3,FBP2,FUT4,GAS1,GRIN2A,HADH,HSD17B3,IPPK,KAT2B,LAMC1,OR13D1,OR2T8,OR5AC2,OR8J1,PLCB1,PLXNA1,PTDSS2,SDC1,SPI1,ST3GAL3,TUBA1C,TXNDC12,UCKL1,VKORC1,C1GALT1C1

Negative_10217	AMH,C1S,F10,FGA,MAG,ACTN4,ACVRL1,APOA5,ARD1A,AXIN1,CACNA1D,CACNA1I,CD19,CHPF,CYP8B1,DDOST,DNMT3B,DRD1,EDAR,EFNA3,EPHA4,GDF6,HSD3B1,IL29,IL4R,IPPK,IRAK4,LCAT,LLGL1,NCK2,NFKBIA,OR10J3,OR13C9,OR4F15,OR7A17,OR9G1,ORC3L,PIK3R5,PPAT,SPRY3,VAV3,EIF4EBP1
Negative_10218	EZR,F3,GPI,GPT,HGD,LPL,OTC,PGP,CD27,CHAT,CHUK,CYP11B1,CYP2A7,DGKZ,DPYD,GALNTL4,GLS2,HAGHL,HNF4A,HSD17B4,INPP4B,INPPL1,MMP14,NUDT8,OR9Q2,PDE11A,PDGFC,PIGV,PKMYT1,PRLR,RPIA,RXRG,SETDB1,ST8SIA5,TCIRG1,TUBB4Q,UGDH
Negative_10219	ABL1,CACNG3,DHRS4L2,GALNT9,HMGCL,OR2K2,OR4C11,PGLS,SQLE
Negative_10220	ACSL4,BCKDHB,CDC25A,CLDN1,COL5A3,CXCL5,MAD1L1,MAN1A1,NAGK,PIK3R3,SETDB2,VNN1,YOD1,ZMAT3,ST6GALNAC4
Negative_10221	IL7,LIF,TEC,ADCY9,AHCYL2,ASIP,DDX56,FSHB,IPMK,OR1G1,OR5K2,POLR3D,POMC,PPA1,SHC4,TOMM40,YOD1,TNFRSF11B
Negative_10222	CMPK2,HLCS,KIR2DL3,NCAM2,PRKCB
Negative_10223	AGL,IL9,ME2,CD19,CDKN1B,CNTF,COQ3,CSF1,CSF1R,CYP2E1,FGF8,FSHB,GALNT14,GAPDH,GNG12,GSTA2,HMOX2,IL17A,IQGAP2,LAP3,LTBR,OR2AT4,PER2,PON2,PROS1,SCP2,SOX17,SUCLG2,TLR4,TNFRSF10A
Negative_10224	IL5,RPE,ADRA1A,BIRC2,BUB1B,CCL15,CD247,CD79B,CLDN15,CLDN5,DEGS2,DOLPP1,EXT1,MCM3,OXCT1,PFKL,PIGN,PPP2R5D,RRM1,UBA7,WARS2
Negative_10225	DCT,LTB,MIF,PTS,ATP6V0B,B4GALT5,CAV1,CD247,CLDN8,DUSP9,FGF10,FGFR4,IDH3B,MAP3K4,MDM2,NEU3,NFKB1,NME2,OR2M3,PARD6G,PFN3,PPP1R1A,PRKAR2B,RAPGEF1,SELPLG,TLR5,UGT8,WHSC1
Negative_10226	MET,ACACB,AKR1C2,C1GALT1,CCL22,CER1,CFL1,CNGA4,CREB3L4,CSF2RA,CSNK2A1,CXCR6,CYP2E1,DDX50,DUSP9,EIF4B,ENO1,ENPP1,ENPP2,FGF13,FMO4,HMOX2,IL12RB1,IL21,IMPA2,INADL,LALBA,LIPF,MYH14,NRG2,OR1J2,OR2T11,OR5D18,PTPRF,PTPRZ1,RAPGEF1,RASSF5,SARS,SH2D2A,SLC25A5,SMAD9,ST3GAL1,STAT2,TAS2R1,TUBB6,VDAC3,WASF2,YWHAG,HSD17B12
Negative_10227	BTD,GSN,APPL1,BAI1,CDH1,CHRM2,CNTNAP2,CYP1A2,DDX54,DDX56,DVL2,E2F1,EFNA5,ENTPD5,FCER1G,FCGR2B,GALNT8,GALNTL5,GGT5,GRIA3,GRIN2A,HEMK1,HRH2,HSPA2,IL13,NCF4,OR10H3,OR56A4,OR5B21,PDXK,PFKFB1,PIGQ,PLXNA3,POLR1A,PPAP2B,PPP2R2B,PRKAG3,PVRL4,RALBP1,RBPJ,TGFB2,TRAF4,VAV1,WWP2
Negative_10228	ID3,PGF,APC2,ATP2A1,B3GALT5,B4GALT1,CCR2,CPS1,CRLF2,DDB2,EHMT2,EIF4B,GLI1,GNRH2,IFNW1,ITGA7,LAP3,MICA,NFAT5,NOS3,NUDT9,OR11H6,OR1K1,OR2M2,OR5AN1,OR5K1,PCYT1A,PDHA1,PER3,PFKFB2,PPARG,PTDSS1,PTPN7,RARS,SLC25A4,SRGAP1,TGFB1,TRAF4,WWP2
Negative_10229	ADCY6,CPS1,OR7D2
Negative_10230	DAO,GSN,HPD,SHH,ACP6,ADRBK2,AQP7,BCL10,CBR3,CCNA1,CCNB1,CCND3,CCR3,CLDN7,CXCL9,DLL3,EFNA5,GALC,HAGH,IL28B,JAM2,MAP4K1,MDM4,MPDZ,OR13C8,OR13G1,OR2B11,OR4M1,OR4S2,OR52N1,P4HA1,PGAP1,PON2,PPP3CB,PRKCQ,PTDSS2,RPIA,RPS6KB1,SDC2,SHC3,SMARCA5,TLR9,PPARGC1A
Negative_10231	EPO,KIT,MUT,ACTN4,ATP12A,CACNG8,CYP2C19,DDHD1,DUSP14,E2F5,GCNT1,GNG13,HTR5A,IFNA14,IFNA4,NCKAP1L,OR6B3,PDE4A,PFKFB2,PFN3,PLAT,POLD2,PPP1R3A,PRKX,PTPRF,RFXANK,SMAD5,YOD1
Negative_10232	TNC,AARS,ACTA2,ADCY2,CASK,CCL2,CCNG2,CDK7,CLDN7,CLTB,CNGA4,CSNK2A2,CYP2U1,EXTL2,FZD1,GLB1,GLYCTK,GSK3B,IL1RAP,IL25,INHBC,LIMK1,MAP3K6,MAP4K4,MCEE,NTNG1,OR10H5,OR13A1,OR52B4,OR9G4,PGAM4,PHKG1,PLCB1,PLXNC1,POLD3,POLR1C,PPM1B,RRM2B,SETDB1,SPCS3,SUV39H1,TLN2,TP53,TRAF6,TUBA3C,XYLT1,PLA2G12B

Negative_10233	APP,C8A,IL9,TH,AARS2,ACPT,AMY2A,APBB1,ATF4,C4BPA,CNGA4,CSDA,CYP2A7,CYP2J2,FGF9,FLOT2,FLT3LG,HRAS,IL18,IRF7,LAMA3,LDHAL6A,MTHFR,OR2A4,OR2L13,OR4Q3,OR51E1,PRKG1,PRKY,RDH12,ROCK1,STAT5B,UGT2B7,WARS2,WBSCR22,WWP1,YWHAB,MAPK8IP2
Negative_10234	ADO,MIF,APAF1,ATG7,C3AR1,CACNA1D,CACNB3,CDC27,CDKN1B,CDKN2A,CLDN5,COL1A2,ENPP1,FLOT1,GAD2,GUCA1A,IARS,IL17B,IL22RA1,KCNJ11,LAMB3,LIPE,LLGL1,NCF2,NKX2-2,NR1H3,NRAS,OR2B6,OR51A2,OR9A2,P4HA1,PFKFB1,POLR1A,PTGDS,SARS,SEMA3D,SGPP1,SLIT1,SRD5A1,SERPINA5
Negative_10235	MPZ,ACTG2,DUSP7,FGF9,GALNT1,OR4C46,CACNA2D2
Negative_10236	DBT,DLD,AMDHD1,BMPR2,DARS,DPYS,DVL1,GPD1L,GSTM3,INHBE,ITGB1,LRP5,MYLK,NCK1,NRG2,NRP1,OR10AD1,OR5AR1,OR6C6,RUNX1,SKP2,YOD1
Negative_10237	GNS,IVD,MYC,OTC,ADIPOR1,ADRB2,AGRP,ANPEP,ARG1,CACNG8,CDH1,CHPF,COQ5,CSNK1E,CYP2A13,CYP4F3,EFNA1,FMO4,GBGT1,IL12A,IL15RA,IL17B,IL1R1,IL3RA,INPP4B,LGMN,LYPLA2,NAGLU,NTF4,NUDT8,OR14A16,OR2G2,OR56A1,PDGFA,PKM2,PLEKHA8,PNPLA4,PPP1R3A,PTGS1,RDH8,SPTAN1,SULT1A4,ULBP1,VANGL1,WNT3A,WNT7A
Negative_10238	HAL,LIF,MPI,OSM,AADAC,ADH1B,ARRB2,ASS1,B4GALT4,BAI1,BCL2L1,BMP7,CACNA1H,CCND2,CDC25A,CTNNA1,ENTPD6,EPHA7,FGF14,FGF4,GAL3ST1,GCNT1,GJD2,GRIN2B,HTR6,ITGB4,MDH1,MGAT5,MYL2,NR1H3,NTF4,OR14J1,PANK1,PCYT1B,PFKFB4,PGK2,PLA2G2D,PLCZ1,PLXNA3,PNLIP,PPM1B,RPS6KA5,SOCS2,SREBF1
Negative_10239	FUK,GLS,NLK,AGPAT6,CCNE2,COL2A1,DDIT3,DOLPP1,EDNRA,EPHA3,EPHA7,FGF10,FZD2,GCNT2,GNAZ,HEMK1,HSD17B2,IMPA2,LARS,NEU1,OR10Z1,OR1K1,OR4D1,OR51A2,OR5D16,OR6C70,PCYT1A,PDE8B,PDE9A,POLR1B,POLR2D,PTGER1,RAC2,RALBP1,SMAD1,SULT1A2,TAS2R60,TP53,YKT6,YWHAH
Negative_10240	ILK,CDH1,CMPK1,GSTT1,HARS,IFNAR1,IKBKB,OR1N1,OR51A7,PLXNB2,PRKACB,RASGRP3,SETD1B,TSHB,VANGL1
Negative_10241	ADCY5,ANAPC10,ANAPC2,ATP6V1H,BLNK,BUB1,CES2,CSH1,CYP2C18,FGF20,FGFR1,FUT7,GUCY2D,HGSNAT,IFNA21,LIPC,OR52R1,OR6K3,OR9Q1,PIGA,PIGB,PPM1A,SMAD3,SOCS4,UAP1,ATP6V1E2,TNFRSF11B,TNFRSF14
Negative_10242	ADO,SYK,ADCY1,AK3L1,ALDH1L1,ALG10B,APRT,ARPC1B,ASPA,ATP5E,B3GALT6,B4GALT1,CAMKK2,CCL4,CFL2,CLTB,CYP1A1,DIAPH3,ENO2,EPB41L2,FCGR3B,GAD2,HLA-DOA,HNF4A,IFNA17,IRAK3,LIPT1,MDM4,NMNAT3,OR10H1,OR1E1,OR4C11,OR6N2,OR8D4,PER1,PIK3CA,PLCD3,PLXNB3,PYCRL,RHOQ,SPHK1,SUV39H1,TAS2R14,THBD,TPH1,UGP2,CACNA2D3,MAPKAPK5
Negative_10243	FST,IFNA13,IFNA17,P4HA3
Negative_10244	AK2,ACOX2,CCL11,CDKN2C,CIITA,FUT5,G6PC,HPSE2,OR10C1,OR1S1,SPRY4,ST6GALNAC6
Negative_10245	CAD,CD4,F3,KMO,ACAD8,ADRA1B,AKR1C4,B3GALT2,CD40,CES1,DUSP1,EPHB1,FGFR4,FLT4,FUT4,GATM,GCDH,GGCX,GGPS1,GSTM4,GUCA1B,HCLS1,HMGCS2,ICAM2,IGSF5,MAP2K6,MGAT5,MYL9,NOTCH1,NPR1,OR56A4,PER1,PFKM,PIGM,PLAUR,PSENEN,PSME2,RASA2,RBPJL,SDC4,SETD2,SMAD5,SOS2,TRAF2,XIAP,CACNA2D1
Negative_10246	B3GNT2,CDK4,CLDN7,DMGDH,MASP1,PARD3,PRNP,SEMA3C,SETDB1,CACNA2D4,ST6GALNAC1
Negative_10247	BMP4,CDC25C,CYP2C8,CYP4A11,FGF18,FZD6,GDE1,GDF6,ITPKB,MAP2K1,MMP7,MYH8,NTRK2,OR2S2,OR4E2,OR52A1,PAPSS2,PDE10A,PDE1A,TUBB2A

Negative_10248	AGK, CBS, F10, GCK, PGP, RPE, ACSS3, ADIPOQ, AMPD1, AOC3, ATP5G2, B3GALT2, BDKRB2, CLCA2, FDFT1, GDF5, GNRH2, GRIN2B, IFNA4, INSRR, ITGA7, MTHFS, MYL7, NOS1, OR11H6, OR1L1, OR2A5, OR4A15, OR5R1, OR8K1, PFN3, PRKAR1A, RASGRF2, SELPLG, TAS2R14, TFDP1, TP73, TRAF2, WNT10B, WWP2, MAPKAPK3, TNFSF13B
Negative_10249	CGN, F3, GP6, ACTN1, ACVR2A, ACVR2B, ADRB1, ALS2, ANGPTL4, ASMT, ATP5B, ATP6V0B, AVPR1A, CCL14, CHAT, CYP2B6, DGKH, DVL1, EPB41L1, EXTL3, GFAP, IDUA, IL15RA, IL22RA2, ITGA10, JUND, KIR2DS4, LDHA, LRP6, MYL5, NSD1, NUDT9, OR13A1, OR2T1, OR2T10, PROC, PTDSS2, RFXAP, SOX17, SSH2, STX6, TALDO1, TOMM40L, VAMP3, TNFRSF1A, TNFRSF6B
Negative_10250	IL2, NF1, RET, VTN, ADH5, AGTR1, CCL11, CD19, CFL2, COX15, CYP3A43, DCXR, DDIT4, DRD5, EFNB1, ENPP1, FGF10, HARS2, HLCS, IGFBP3, IL17RA, IL1A, INPP5E, LIMK2, MYD88, NRG2, OCRL, OPLAH, OR10S1, OR1L8, OR2V2, PARD6G, PIGT, PIK3C3, POLA2, PYGL, RARA, RPS6KA1, SARS, SEMA3D, SPCS1, TALDO1, TICAM1, TSHB, ULBP1, VEGFC, WNT7A, YWHAH
Negative_10251	HPD, ACSM3, ASRGL1, ATP12A, CASK, CLDN11, DAXX, DIAPH2, ESPL1, FECH, FUT5, GALK2, HADH, IL28B, INSR, MGAT5B, MYH7B, NEGR1, OR1J1, OR2H2, OR2T10, PDHA1, PI4KA, PIGC, POLE, POLR2A, PPARG, PRKCG, RAB23, SARS2, SC5DL, SNW1, TAS2R3, TICAM2
Negative_10252	F8, BAAT, C1QC, CCL27, FDFT1, GRIN1, PLAT, WNT3, ATP6V0D1
Negative_10253	UBB, AGPAT1, EGLN1, ITGB1, KIR2DL1, OR4D2, PDGFB, PGK2, RASA1, SRD5A2
Negative_10254	CD4, FH, GH2, HK3, ID4, IL2, INS, KDR, MOS, ASCC3, AVPR1B, BUB1, C9orf95, CACNB4, CAMK2B, CCL2, CLTCL1, COX15, CSH1, CTNNA3, CYP11A1, DDX52, DFFA, DRD1, IL17B, KLRD1, MECR, OR1E1, OR1L4, OR4F4, OR51F2, OR5F1, OR6K2, PDE6D, POLR2J, PPP2R1B, PTTG1, RASGRP4, SOD1, SUCLG2, SULT2B1, THBS4, TOMM40, TPM3, VAMP4
Negative_10255	MSN, ATP6V0B, CD99, DHDH, EPB41, GALNT3, NSDHL, OR1A2, OR5H2, OR6T1, PARD6G, PFKFB2, STAM2, TAS1R3, TUBB4Q, UNC5C
Negative_10256	FST, RET, ACPP, AGPAT3, AGTR1, ARNT2, B4GALT7, BCAT2, CD44, CYP26A1, CYP26C1, DUSP9, EPB41L1, FOSL1, HADHA, HSD17B8, IDUA, MGAT3, MIOX, MKNK1, NKD1, NODAL, OR6K6, PGM3, PIGL, RASGRP1, RDH8, ROCK2, RRAS, SPRY1, STAT3, STX18, TP53I3, TPH2, VAV2, HLA-DRB4
Negative_10257	BTD, GH1, ID1, ACSL5, AGPAT1, ANAPC1, CDC45L, COL4A1, COMT, CUL1, CYCS, CYP7A1, DAXX, FADS2, GADD45A, GNG13, GPAA1, GUCA1A, H6PD, HNF4A, HSD17B3, IGF1R, IMPDH2, IRS1, ITGA1, MAML2, NRCAM, NTN1, OR10G3, OR10P1, OR2T2, OR4D11, OR52I2, OR5AP2, OR6C2, PFKL, PLAUR, RAD51, RPS6KA2, SAT1, SEMA3D, ST3GAL4, TAS1R1, TAS2R60, UNC5B, ARHGEF12, TNFRSF18
Negative_10258	CDA, CR1, F10, FES, LCK, ZYX, ANAPC4, ASPA, CALML3, CCL13, CD72, CLDN20, CNTFR, CYP2A13, DNMT1, ENTPD1, F13B, HAGH, HPSE2, IFNAR2, KLRC4, MGAT4A, NADK, NLGN2, NSDHL, OR13C5, OR4E2, PIGM, PPAP2B, PRPH2, RASGRF1, TCF7L1, TPK1, WNT5A
Negative_10259	FAS, ID1, PDC, AKR1C2, ARNTL, ATP13A2, ATP5G2, DGKA, IL1R1, LAMB1, LIPF, OR51F2, POLR2I, PTGS1, ROBO1, SEMA3E, TRADD, TRAF5, UGT2B7, UROD
Negative_10260	GPI, ARAF, CNDP1, CSF2RB, HPSE, INHBB, MINPP1, OR52N5, OR7C2, PTGER1, SMAD1, UCP1
Negative_10261	MPL, ACTA1, ACTC1, AMY2B, BDKRB2, BIRC3, BMPR1B, CSDA, EP300, EPHB3, HES1, IFNA10, IPPK, ITGA7, KARS, LAP3, OR13F1, OR2M5, OR4N4, OR52N5, PDE1B, PIK3C3, PRDX6, SFRP1, SGMS2, SHMT1, SIPA1, THBS2, THTPA, TM7SF2, TSHR, TSNARE1, TUBA8, YWHAZ, ZMAT3, SUV420H1

Negative_10262	C8G, CTH, DDC, CACNA1C, CFLAR, CLDN1, CMBL, CYP3A7, EPHB6, ETNK2, FGFR2, GRIN2A, GSTK1, HTR6, INSR, ITGA2, MAPK1, NUMBL, OR2A5, OR2G3, OR51B6, PLCD3, POLR2L, PRKAA1, RAP1A, SAT1, SESN2, SLC27A4, TAS2R3, TICAM1, TNFSF4, TSTA3, WBSCR17, CACNA2D2, PNLIPRP2
Negative_10263	ADO, ADCY4, ALDH1A2, ANAPC10, CADM3, CBLB, CDH5, COL5A1, HAO1, IL1R2, IL1RAP, LIFR, MFNG, MTHFR, NMNAT1, OR2T29, OR4F4, OR5J2, PDE6H, PPP2R5B, PTPRF, RALGDS, STX12, ZNRD1, SERPINA5
Negative_10264	FUK, ID4, PTS, CDH5, EPAS1, EPHA2, GUCY2F, MAP3K12, NCAM2, NEFM, NOTCH1, OR8B8, PFKFB3
Negative_10265	GSR, ME3, NF1, ALAS2, CCL11, CCL23, CD70, CDK7, CNGA4, COL1A2, DGKZ, EPHX2, FMO3, ITGA1, KLRC4, LDHAL6B, LTA4H, MPP5, NRCAM, OR11L1, OR2AT4, OR5H6, OR8B12, PLCD4, PORCN, PPP2R2A, PRODH, PSPH, PYCR2, SEMA4A, TSHR, TSNARE1
Negative_10266	GCK, ACAT2, ADAM10, ADCY1, AFMID, BLVRA, CACNG2, CCND2, CDK6, CLOCK, DHODH, FOXO1, HES1, IL1A, KLRC4, MGAT5B, OR10H5, OR51B4, ROBO3, SDC1, SFRP5, SLC27A5, SOAT1, TALDO1, TGFBR1, WARS, CACNA2D2, CACNA2D4
Negative_10267	ACTN4, APRT, ASS1, CDC14B, GALNT10, HADHB, HTR7, MAP3K4, MMP1, OR8H1, SDC2, STMN1, TPK1, YWHAG, PLA2G12A
Negative_10268	MUT, ADH7, ARNT, ATP5J, CACNB1, CALML3, CALML6, CCND1, CLCF1, CYP2F1, DOLPP1, FGFR1, GLUD2, HAO1, HTR7, MCM3, NAGLU, OR6Q1, RPS6KA6, SC4MOL, SDHD, SLC33A1, SOAT2, SPCS3, SREBF1, TP73, WASF1, MAP3K7IP1
Negative_10269	FGF11, GCNT4, HMBS, IL13RA2, MAPK3, MLYCD, MMP7, NOS1, OR52E4, PIK3R4, SOS2, TLN1
Negative_10270	ACAD9, ASIP, FZR1
Negative_10271	AK5, ACOX2, ASMT, BPNT1, CCL25, FUT9, GALC, GART, LRDD, MDH1, NCSTN, NFATC3, OR10A7, OR10AG1, OR13A1, OR52E6, OR5AC2, OR5P2, PDE1C, PFKL, PFKM, PTK2B, PTPMT1, RALB, TNFRSF4, ULK3, UROS, VAMP1, XYLT2, GABARAPL2
Negative_10272	ANGPTL4, C1QB, CYP2C19, H6PD, HSPA2, NCR2, OR11H1, OR7G3, PI4KB, PIGM, SERPINE1
Negative_10273	ASL, FES, NPL, ADH4, BNIP1, CDKN2A, CYP3A5, DTX4, FASLG, GDF5, GLDC, HSPB1, IL20, NCKAP1L, OR1F1, OR1L4, OR2A12, OR51B5, OR52H1, PDCD1, PIK3CD, PTGER3, TGFB3, UROS, ATP6V1G2, HSP90AA1, TNFRSF11B
Negative_10274	AADAC, ALDH9A1, ATP5O, BCAR1, CHAD, CLDN16, ENTPD8, FGD1, FUT8, GAD1, HEMK1, HTR2B, MMP9, NKX6-1, NRXN3, OR1L6, OR2T11, OR9G4, PDE9A, PIAS2, PNPLA4, PRKCQ, PTK2, SCP2, SELL, UCK2, UPB1, VTI1B
Negative_10275	F7, GSR, COL4A2, CTPS, CXCL2, DVL3, FASLG, FZD9, IL21, INHBE, KIR2DL4, MCAT, NUDT2, NUDT5, OR10G8, OR4C13, OR6C1, PKM2, PLCB4, PRKAB1, RDH8, SAT2, SPRED2, TP53I3, HLA-DPA1, TNFRSF10C
Negative_10276	F2, ASMT, BCL10, CD28, COL4A4, CYP2C8, DDB2, GPAA1, GRIA2, GRIN2D, HAGH, IRF3, MIOX, NAGK, NEU3, ODC1, OR6K2, PECAM1, RPE65, SARS, UPP2, VDAC1
Negative_10277	CKB, FAH, GH2, IDS, IL3, UBC, ADRA1D, ALDH3B1, ALDH5A1, ARNT, ATP13A2, ATP4B, ATP6V1F, BMP2, CAV3, CCR4, CD8B, CLDN7, COL1A2, DDX54, DHRS9, ENTPD5, FGF9, FRAP1, GANC, GCNT3, IL28A, KLRC1, LRP5, NFATC2, NTN1, OR10G3, OR11L1, OR52N4, OR6F1, ORC3L, ORC5L, PERP, PLXNB2, PSEN2, SMAD2, SMPD3, STX1B, TAS2R40, TJP3, UGT2B17, ULBP1
Negative_10278	DHH, ID4, IL4, PC, PML, ACTN4, ATF2, ATG7, ATP4A, ATP5A1, CDH1, CYP2F1, EIF4E2, EPB41L1, FUT4, FZR1, GALNT1, GALNTL1, GNRH1, GNRHR, HCST, HIP1, HSPA8, IARS, MAP4K4, NAGA, OR4D5, OR4F4, OR5P3, PARD6G, PIAS4, PNLIP, PRKCZ, RASSF5, TUBB2B, UGT2B7, WHSC1L1, WNT2B, HLA-DQA2, TNFRSF10A
Negative_10279	F5, NOG, ENO2, ITPKB, MAP3K7, OR2T5, PIGX, PPP2R2B, SOD1

Negative_10280	ACTN1,KARS,SYNJ2
Negative_10281	BTC,CD86,CXCL13,DAAM1,DKK2,GDF6,GSTA4,HTR2A,IDH3B,PPA2,UPRT,YWHAG
Negative_10282	C8B,F12,LYN,AK3L1,ALG2,ARHGEF2,CASP9,CDH15,COL4A6,EPHB2,FRAT2,GNPDA1,GRB2,HLA-C,IL9R,MARS,NCF1,NFKBIA,NR4A1,OR1J1,ORC4L,PAFAH2,PARVG,PGAP1,PGM3,PLIN,PPP2R5B,SH2D2A,STX4,TUBB2B,WBSCR17,WNT3A,ATP6V0A1,B3GALNT1
Negative_10283	ADRBK2,ALDH2,APOE,BST1,CLCA2,GNPNAT1,MASP2,RELA,SHC2,TLR3,VANGL2
Negative_10284	ACAT1,ALPPL2,CCR2,CD86,CLDN8,CNTNAP1,CPOX,CYP2A7,DAPK2,EFNA2,F13A1,FGF19,GNAL,OR52L1,OR56A1,P2RX4,PDE10A,PRKAG1,RAF1,ST3GAL5,STAT1,SYNJ1,TAS1R1,THBS3,WNT7A
Negative_10285	ANAPC2,CALML6,CCL8,CD244,ENPP6,GRIA2,IGF1R,NODAL,OR51B4,PEMT,PRPH,SESN1,THBS3,TNFRSF6B
Negative_10286	SPN,ADAM17,ALOX15,ATP2A1,ATP5J,B3GNT4,EFNB2,FGF11,FGF9,FLT3,GRPR,GSTA1,LLGL1,MDH2,OR1G1,POLR1D,PRKCB,RPS6KB2,TBL1XR1,SERPINF2
Negative_10287	ABLIM3,AMDHD2,ATG3,CD274,COQ3,DUSP14,EPHA1,ETS1,FARS2,LEFTY2,LRP6,MBL2,MTFMT,OR12D3,OR4F6,PAK6,PFKL,SEMA3D
Negative_10288	FES,UBC,ACAA1,ACYP2,ALDH1L1,ALDH3A1,ARD1A,CER1,CREB5,DGAT2L4,EPRS,FCGR3B,FGF11,FMO1,LPAR1,MARS,NTRK2,OR2L8,OR52L1,PDE9A,PFKM,SETD2,SLIT2,XCL1
Negative_10289	ACOX1,CLDN15,CYP4A22,MAP3K1,OR6F1,PIP5K1C,STAT1
Negative_10290	BAX,NOG,ACADSB,ACSL6,ACSM3,AGPAT3,AKR1D1,AKT3,ANAPC5,BCL2L1,CHST13,DAAM1,EPHA4,FTH1,FUT7,GLI3,GSTZ1,HAO2,HLA-DOA,IKBKB,IRAK3,ITGA8,ITPK1,JAK2,MAGI2,MAP2K3,MCAT,MCM7,MDM4,METTL2B,MOV10L1,MYH7,MYH9,NNMT,OR4X1,OR6M1,PGAM4,PGS1,PISD,POLD1,PPP1R3B,SNAP23,TUBA1B,TUBB6,VANGL1,WASL,PLA2G12A
Negative_10291	ADCY7,CLDN20,GAPDH,GJA1,HPRT1,OR2H2,PARVA,PIK3R3,SDC2,SERPIND1
Negative_10292	CAD,DBH,TAT,FUT7,IDH3A,PIK3C2B,PLCG1,PON1,SOCS2
Negative_10293	BCR,F9,GK2,CDC27,CYP17A1,DDOST,FLOT2,FOXO1,GALNTL1,GPD1,HIP1,HLA-C,IL28A,IL28RA,LALBA,MAOB,NARS,NEUROD1,OR52L1,P2RX6,PDX1,PLXNB1,STEAP3,ZBTB16
Negative_10294	ABO,AGL,GP6,TNC,ALDH3A2,BST1,BUB3,CAMK2B,CDH15,COX10,DDX51,DHRS3,EDA2R,FBP1,FGF2,FSHB,GALNT9,GLDC,GRIN2D,IL11RA,LALBA,LCP2,LIPA,MAP2K2,OR2A2,OR52K1,OR5D13,OR5H6,OR5L1,P4HA3,PIGV,PLCB2,PON1,PRPS2,SDC3,SEMA4B,SOS2,STX8,TBL1Y
Negative_10295	ACTG2,ALCAM,ASS1,CASP7,GALNT3,GLB1,GLYCTK,HAGH,MAN1A2,MPZL1,NTNG1,OR5AS1,PECAM1,PRKCE,RERE,SHC2,SHC4,TFDP1,CSGALNACT1,ST6GALNAC6
Negative_10296	C7,FH,ATP5J2,CASP6,GDF5,NOTCH2,OR51I2,PLXNA2,PRDX6,PRIM1
Negative_10297	F8,ACPP,ACY1,AGMAT,AGXT2,ATF2,BMP4,BUB3,CCNE2,CCNG2,CHST11,CLDN2,CYP27A1,DAAM1,DHX58,EPHA7,FTCD,FUT4,GAD2,GDE1,GUK1,IDUA,MAP2K3,MAP4K3,MCM2,MCM3,NAT5,NNMT,NT5C3,OR5D13,POLR1D,TJP1,UCP1,WNT9A
Negative_10298	GP6,ACADSB,ACOX2,ACP5,AHCYL1,C1QC,CCL27,CCL5,CHIT1,COQ6,DRD2,EDNRB,FASLG,FCGR3B,FIGF,FMO4,GLUD2,GNPDA1,GRHPR,IL1B,LYZL1,MCEE,NADK,OR11H1,OR5AS1,OR6C6,PHPT1,POLR2E,PPID,PPP1CC,PPP2CA,PRKAR2A,PRKG2,SEMA4B,TPI1,TRAF4,YARS2,PHOSPHO1
Negative_10299	GK,COL1A2,ENPP2,OR2D3,OR2L8,OR4N4,OR5AU1,OR6S1,PPP1R3B,PTAFR

Negative_10300	GSS, PLN, ALCAM, EPHX2, GNAQ, OR4S1, OR5AP2, PPARA, XYLT1
Negative_10301	C6, CBS, KHK, LAT, ATF2, CHRD, CLTC, CYP3A5, DHFR, FGD3, GLUD2, GNG13, ITGA2, ITGA9, MCM5, OR10A2, OR11G2, OR8K3, POLR2I, PRSS1, TSHB
Negative_10302	AK1, AK2, AR, C8B, CAT, CRH, FES, NPY, ACHE, ACVR2B, AGPAT2, ATP6AP1, CARS2, CYP26B1, DUSP14, DUSP4, ENPP2, ERBB4, G6PD, GALK1, GSTM2, IDH3B, IMPA2, INPP1, NEU4, NSD1, OR10A3, OR10H3, OR13C4, OR1D5, OR4F6, OR4X1, PIK3CA, PLXNA3, PRODH, SMAD6, TFPI, TLR4, UGCGL1, ATP6V1G2
Negative_10303	ACAA1, APBB1, CSF2RA, CTSG, DGKG, DOLPP1, FLT3LG, GPD2, HADHB, KAT2A, OR1J4, PIAS1, PRKACB, PAFAH1B1
Negative_10304	CKB, DUT, LIF, ACTN4, ATP5J, COL1A1, GALM, GUSB, IL18RAP, INPPL1, KDSR, NADK, NUDT8, OR10A3, OR1D5, OR4D11, PLA2G10, PPP1CB, XIAP, YWHAZ
Negative_10305	GP6, ADCY10, AKR1C4, BACE2, BLVRA, CCL25, CCR2, CDK6, CHRM3, CTPS2, EPHA7, GATM, IL18RAP, IL20RA, LAMB3, MAML2, NCAM2, NME6, NUDT5, OR10C1, OR10H5, OR1L4, OR4D1, OR4N4, ORC1L, PECAM1, PIGA, POLR2H, RARS, RBPJ, SPHK1, STAT4, TAS2R40, TNFSF18
Negative_10306	C6, F2R, AHCYL1, ALDH1A1, ALOX15, ALS2, C9orf95, CBR3, CCL14, CDC25B, CEBPA, CMPK1, F13A1, FASLG, GADD45G, GARS, GRM1, ITGA4, ITGAE, LYPLA1, MAN1A1, MYLK2, NAE1, OR6N1, OR9Q2, RASGRP3, RDH8, SQLE, SUCLA2
Negative_10307	ADA, FOS, ALDH3A2, ATP5J2, BCL2, CCNE1, CD86, CDK2, CHEK1, CPS1, DGKB, FARP2, FOSL1, GNAT3, IFNAR1, IL18, INADL, LAMC1, LYZL1, MCM6, NANS, NKX2-2, NOX1, OR4F5, OR51A2, OR7C1, PCNA, POLR2A, PRKACB, RBL2, RFXANK, SOS2, SV2C, TGFB1, UGT2B10, ULK3, WNT3A, WNT7A, MAPKAPK5, ST6GALNAC3, TNFRSF1A
Negative_10308	ATP5J, CACNB4, COQ7, DKK2, DTX1, F13A1, GARS, GCNT4, GUCY1A3, GUCY2C, PCK1, POLR3D, ROCK1, UGDH, EIF4EBP1
Negative_10309	CCNE2, EGFR, GNRH2, PMM1, VCAM1
Negative_10310	BUB3, CD27, CDH4, FDFT1, GALNT9, GNPNAT1, HSD17B1, HSD17B2, MAPK8, PIP4K2A, STX12, SUCLA2
Negative_10311	CGN, ME2, ALDH6A1, ARAF, B3GAT3, CD74, CDC42, CX3CL1, DFFB, GGT6, GNA14, GYS1, HSD17B7, IFNA8, MCM4, OR13C2, ORC2L, P4HA3, PIAS4, PNPLA3, SPP1, STX16, TBL1XR1, TUBB1, WBSCR17, WNT2, HLA-DRB3
Negative_10312	F7, AK3L1, B4GALT4, CDK7, CX3CR1, DAPK3, ETS1, GALNTL2, IFNA8, IL28B, OR4K13, OR56B4, SEMA4A, ST3GAL5, THBD, UROC1
Negative_10313	AK5, C5, ADRB2, ALG1, ANAPC1, CDC45L, CSNK2B, CYP8B1, ECHS1, EPHB3, FGD1, FLT3, FTCD, G6PC, GZMB, HEMK1, IDH2, IFNA5, LAMB3, MAOA, MGST3, NEFL, OCRL, OR14I1, OR5F1, OR5K1, OXCT1, P2RX5, PDXP, PHKA1, PLIN, PLXNB2, RAPGEF1, SC5DL, SIGLEC1, SMARCA2, TAP2, TLR4, TNXB, TRAF5, VAMP1, WASF2, WNT2
Negative_10314	IVD, ACOX1, CCL16, CCL22, GGCX, HEMK1, IFNA14, MAML3, MAP4K2, MGAT3, OR8I2, PGK1, POLR3H, PRPS2, SULT2B1, WNT16
Negative_10315	ATM, PGD, ACAA2, CHD8, CSNK1G1, FZD3, GALT, GGT7, HAO2, IL17RA, IL20RA, NSD1, OR13H1, OR2A2, PAK1, PFKFB2, SFRP5, ST3GAL1, TNFSF4, UGP2, HSP90AB1
Negative_10316	FGG, GH1, LAT, NLK, OSM, ACSL3, ATP2A2, B3GAT3, B4GALT1, BCKDHA, CCL3, CHD8, DUSP8, ITGA2B, KLRC2, OR1B1, OR2T33, OR52E2, OR6Q1, OR9Q1, PCYT1A, PDGFRB, RBPJ, SPHK2, STAM2, SUV39H1
Negative_10317	AADAT, B3GNT3, BMPR1A, CCL21, CCNG1, CTF1, GNA15, GNAI2, KNG1, LALBA, MYH15, OR10AD1, OR11L1, POLE, RDH16, TNFRSF10D
Negative_10318	GPI, HGD, ADRA1A, CD46, CDKN1A, IL20, NFYA, NPR2, OR51S1, PGK1, PLIN, PTPN6, TAS2R60, UGT2A1, WASF3

Negative_10319	AGK, HAL, LTB, PGF, AASS, ADIPOQ, ALG1, ALPP, B3GALT6, CDK5, CHRM1, CSNK1D, DNMT1, DUSP3, EPHA6, G6PC2, GBGT1, GNAO1, GNPAT, IDUA, IFNA5, IL22, MAP3K13, MBL2, MYH3, MYH6, NAE1, NRXN2, OR1L8, OR56B4, PARVB, PDE2A, PGM3, PITX2, POLR2G, PPP2R1A, PPP2R2B, RPS6KA1, SDHC, SFRP5, SKP2, SPRED2, SPTLC1, TCF7L1, TRADD, TRAF2, WIF1, TNFRSF13B
Negative_10320	GGH, MSN, RPE, TXK, ARPC4, BCL2, CSDA, DDX18, EXT2, GPAA1, GRHPR, IL17B, MAN1A1, MCCC2, MCM2, MYLK2, NCF1, OR10G4, OR4S2, PGK2, PLOD2, RXRG, SHC4, TRPM5, WNT2, MAPKAPK5, SERPINE1, SLC25A31, SUV420H1
Negative_10321	C7, CGA, F3, ABL2, ACSL5, ACSM5, ADCY1, ARNTL, B3GALT5, CCNA1, CDH4, CLTCL1, COASY, CRLF2, DPM1, ENTPD6, FLOT1, GADD45A, GPX5, HEXA, MAGI3, MAP3K12, MAP3K2, METTL6, MYLK3, NAT2, NPR2, NUDT12, OR13C2, OR4K5, OR52N1, OR52R1, PER1, PLA2G2E, PLOD1, RDH8, SNAI2, SPRY3, STAM2, STX1A, STX6, TUBB, UGT2B4, B3GALNT1, GABARAPL2
Negative_10322	CCL19, DOLPP1, E2F5, FUT3, IRF5, ITGB1, LCAT, NLGN1, SPI1, VEGFA
Negative_10323	ACTN1, ASS1, BDH2, DHODH, EXT2, FZD2, GOT2, NPR1, OR5T3, SOCS2, TRPC1, WNT4
Negative_10324	FAH, GGH, MUT, AKR1C2, ASAH1, CD70, CLDN15, CRY2, CSF3R, CYBA, DNMT1, FABP4, FGFR1, FRAT1, LTA4H, MARS, NRG1, PSME3, PVRL4, RASGRF1, ROBO2, SHC2, STX3, XCR1, YWHAE, MAPK8IP1
Negative_10325	ID2, B4GALT7, CFL2, ELK4, OR11H4, PDE6D, PIP5K3, TSNARE1, XCR1
Negative_10326	ARD1A, ATP5L, CD14, CLDN16, DHCR24, DHODH, FGF6, FLT4, GALNS, HAGH, HMGCS2, HSPA1B, IL25, MAT1A, NAGS, NRXN3, ONECUT1, OR11L1, OR1L6, OR4Q3, OR52W1, OR5B17, PRKX, PTEN, ROBO1, ROCK2, RPN1, STK11, TBXAS1, UNC5C, SERPINC1
Negative_10327	CAD, CDA, F12, IDS, ABLIM1, ACAA2, ACADSB, ACMSD, APOA1, ARD1A, CACNA1G, CACNG6, CD36, DOT1L, DPM3, DUSP10, GALNT3, GLG1, GSTA4, HMOX2, IL10, IL1B, IRS4, ITGA6, MIOX, NCAM1, NME4, NRXN3, NTRK1, OR2T8, OR5B12, OR6Q1, PANK1, PDE11A, PIGL, PIGW, PNPT1, PPCS, PROS1, PTGDS, SGMS2, SSH3, TIRAP, WNT9A, YWHAE, B4GALNT1, CACNA2D4, SLC25A31
Negative_10328	FYN, RET, TEC, APOE, ARPC4, ARSD, ATF4, ATP6V1D, C4BPB, CACNA1G, CBLC, CD22, CTBP2, CTF1, DGKQ, FUT5, GGT5, GPD1L, IFIH1, IL18, IL3RA, ITGB1, MASP2, NFKB1, OR10H1, OR1E1, OR2B2, OR4C6, OR4D6, OR52R1, OR5T3, PDGFRA, PRPH2, PTPRF, RRAS, SESN1, SIGLEC1, TNFRSF13C
Negative_10329	OGT, FDFT1, KIR2DL1, OR52K2, OR5P2, PDE1A, PI4KA, PTPN1, SDC4, WARS2, YWHAG
Negative_10330	ARG2, CARS2, CDC25A, COL6A1, DUSP16, IFIH1, IL28RA, LPAR1, PORCN, PSAT1, PTPN1, PTPRM, SELL, SUV39H2, SV2A, TUBA1A, VAV2
Negative_10331	AK7, ATM, ATF2, CSNK1G3, CYP27A1, DAD1, FGF19, FZD2, GALNS, GBE1, HARS, MAP3K11, NPR2, OR14A16, PDXK, PHKB, PLCZ1, PTPRB, UGT8, UNC5D
Negative_10332	APP, CRK, APLP1, ATP6AP1, CD46, IL22RA2, MGAM, OR5T3, PROS1, RPS6KA1, SV2B, XYLT2
Negative_10333	ACOX1, PRKAB1, TNFRSF12A
Negative_10334	C7, ATP13A2, CDH4, CTSL1, CYP7A1, DERA, ENAH, EPB41L1, EXTL1, FZD7, GPD2, HSP90B1, KIR2DL2, MAP2K5, NME4, NRCAM, OR2K2, OR51V1, OR5T3, PFKFB3, PRKAG2, PSEN2, RRM2, TLN1, WNT6
Negative_10335	ATN1, GLYCTK, MAT2B, SELPLG
Negative_10336	TH, CPT2, TNFRSF13B
Negative_10337	CD6, F7, ATG12, CACNG1, CALML6, CNGB1, COL6A2, COQ3, DCXR, DDIT3, DHCR7, EIF4B, ESPL1, FMO1, GRIN2B, HSD17B1, KIR2DL4, LCMT1, LIPT1, MITF, MYLK2, NFKB1, NOX3, ODC1, OR2A2, OR7G1, PI4KB, PIGA, PLXNB3, POLD1, POLR3B, RAC3, RARS, RASGRP1, RPS6KA6, SV2A, TGFB3, UCP1, UGT1A10
Negative_10338	HBEGF, METTL6, PFN4, UGT2B28, VANGL1

Negative_10339	AMH,GCK,HGD,TK1,TNC,ABAT,ACVR1,ALDH6A1,ATP6V0B,CCR10,CDKN2B,EFNA4,EXT2,FOXO1,GADD45B,GP1BA,GUCY1A3,IGF1R,IL1B,IL24,IRS1,KIR3DL1,MYH9,MYL9,NCKAP1,NFATC2,ONECUT1,OR2W1,OR7G3,PPP1R3B,PPP2CA,RAC1,RASGRP3,SEMA3D,SLC9A1,SRGAP1,ST3GAL6,UNC5C,WBSCR22,WNT3
Negative_10340	HGD,MAG,SRF,ACSM1,ACSM2A,ALDOA,APAF1,CCL13,CCNB3,DNMT3A,DTX1,ECSIT,ENAH,GGCX,IL17A,MAGI3,MAP3K3,NEFL,NKD2,OR13H1,OR52K2,P4HA2,PDE11A,PDE5A,PIPOX,PTPRB,ROBO1,SIPA1,SLC25A4,WEE1
Negative_10341	CYP11B1,CYP2A6,DDX56,FMO2,GNPAT,PANK1,SARDH,SIGLEC1,SLC2A2,SMPD3,UGT2B4
Negative_10342	C9,GPI,RPE,ABLIM2,B3GALT4,CHD8,CHEK1,CYP4A22,DCTD,DGKA,FGF2,IFNA2,IL21,OR52K1,PCCA,PER1,POLR2I,PRDX6,SRGAP3,TAS2R16,WARS
Negative_10343	GHR,WAS,ACPP,ATP2A3,BLNK,CD40LG,CDH15,CRKL,CYP1B1,DCTD,E2F2,FADS2,FASLG,FPGT,FUT8,GGT5,HGSNAT,IFNAR2,IL13RA1,IL19,IL7R,KLRC1,MAPK7,MYH8,NOX3,OR10P1,OR5AR1,PPP2R5D,SETD7,SLC27A1,SMAD6,ATP6V1G1,TNFRSF1B
Negative_10344	APP,F3,PML,ACVRL1,CACNG7,CD59,CHRNA7,CPT2,CSNK2A2,DARS,DGUOK,DVL2,FGF13,FOXO1,GDF5,GNA12,GPAA1,HHEX,MAD1L1,OR10G4,PNLIP,RPS6KA4,SKP1,SLIT2,SRD5A2,SYNJ1,TOMM40L,UCK1,ULK2,ATP6V0A2,PDCD1LG2
Negative_10345	ADK,AGK,FAS,IL7,PRL,SI,TK2,ACTC1,AGPAT1,AHCYL1,ALG1,AOC2,CACNG4,CCL19,CCL20,CCL21,CHSY3,CTNND1,DLST,DPYSL2,ENO2,FGD3,FGFR4,FUT5,GAD1,GNAL,HMGCS2,IFNAR2,IFNB1,IL1R2,IL6ST,MYH4,NTN1,PDE3B,PLOD3,POLR3H,PPM1A,PPP2R1B,RDH8,SPRY4,SUCLG2,TPM3,TRDMT1,SERPINC1
Negative_10346	ADK,AMT,FGG,ACMSD,ADCY7,APAF1,CCL7,CSAD,DTYMK,GALK1,IFNA5,IFT57,MAP3K7,NMNAT2,NOS1,NRG3,NTRK2,OR13C2,OR14A16,PAX4,PPP3CA,PPP3R2,PTPRB,SAT2,SHC4,TUBA1A,XCL2,XYLB,XYLT2,HSP90AB1
Negative_10347	CD2,MUT,ALDH7A1,ALPPL2,ASIP,ATG12,ATG7,CCNG1,CNTN1,CYP2C19,DRD1,EDA2R,F13A1,FGFR2,GALC,GALNT1,GJA1,GUCY1A2,IL4R,IL7R,ITGB7,MAPK13,METTL6,MYH15,ODC1,OGDH,OR2D2,OR5A1,OR8K5,P2RX6,PIGC,PIP4K2A,PPBP,PPP2R5A,RDH11,RDH8,SEMA4B,STX10,TAS2R41,THBS2,TLN2,TNFRSF4,TSC1,KIR2DL5A,ST6GALNAC5,TNFRSF1A
Negative_10348	GLS,ABL1,ABL2,AGTR1,ALG13,B3GALT5,GAD2,GNA15,LIMK2,MAPK13,MARS2,PAK3,PCYT1B,RHOD
Negative_10349	ADA,GSS,OSM,ACSL1,ANPEP,ATG7,ATP5G3,ATP5L,CCL3,CLDN18,DDX18,DIAPH1,DVL1,ENPP6,EPOR,FGFR3,FRAT2,HSD3B2,HYAL1,INPP4B,KDSR,LCAT,LIAS,MAP3K11,MAP3K5,NARS2,NR4A1,NTRK1,NUDT9,OR4S1,OR8G1,PAK7,PAX4,PLCB4,PPP2R2A,PRKCZ,PRNP,PROS1,PTGIS,SLC12A2,TBL1Y,TRAF5,WNT11,ZAP70,TNFRSF6B
Negative_10350	FGB,FGG,TNF,ACTG1,ADCY1,AGPAT3,BET1,C4BPA,CCL16,CYP4A22,EPB41L2,FGF18,FUT4,GGCX,GPD1,GRM5,IFNA2,ITGA2,JAM2,OR4B1,PAPSS1,PDE5A,PFN2,PI4KB,PIAS4,PIGH,PIP4K2C,PLOD2,PRKG2,PROS1,SETDB2,SNW1,TRDMT1,TUBB3,UCKL1,YKT6,PLA2G12B
Negative_10351	PXN,ARPC4,ATIC,AXIN2,C3AR1,CLDN1,COL6A1,COQ3,CSF2RA,ERBB4,FARSA,FGF11,GGCX,IL2RG,IL6R,ITGA9,KIR2DS5,MAGI1,OR10A4,OR14J1,OR2T5,PARD3,PAX6,PNMT,PPAP2B,PRKCG,PRPS2,SFRP1,VAMP3,WARS,MAPKAPK3
Negative_10352	ATP6V1A,CEBPA,CSNK2A1,FLT3LG,GMPPA,GNAS,IL21,OR2M2,OR4C13,OR4S2,PFKFB1,SULT2B1,TBL1X,TNFRSF10D
Negative_10353	DDO,LCK,ACSL4,ACYP1,CALML3,CCNH,CHRM1,COL4A1,DUSP2,FPGS,OR10J5,OR2T2,PAK7,PHGDH,RPS6,SETDB1,SGMS2,SPRY4,SRGAP1,UGT8,UMPS,ZIC2,SERPINA1,ST6GALNAC4

Negative_10354	CD6,IL5,KDR,ARNT2,B4GALT3,BMP5,DDOST,DDX50,FCGR3A,FDPS,FGF13,GATM,GNRHR,IFNA14,KLRC3,MAFA,MYL9,NAGLU,NFKBIE,NKX3-1,OR5AK2,OR6K3,PIGL,PIK3CB,PKMYT1,PRIM1,RDH12,SC4MOL,SV2A,UGT1A10,YWHAH,PDCD1LG2
Negative_10355	NF1,OAT,ACVR1,ALDH1B1,ALDOB,BST1,CACNB2,CACNG7,CDC14A,CDIPT,CHIA,CYP2B6,CYP4A22,CYP4F3,DKK2,DPM2,DUSP16,FBXW11,FZD10,GALNTL4,GMPPB,IFNGR2,NT5C1A,NTNG1,OR51I2,OR5B12,PARD6G,PLA2G3,PNPLA4,PPAT,PRKCB,PRKG2,PTDSS1,PYCR1,RETSAT,ROCK1,RPN2,SESN1,SRGAP3,TAS2R42,THBS1,TMLHE,VAV3
Negative_10356	AADAC,OR1I1,PLA2G2F
Negative_10357	CBS,CGA,DAO,ADCY8,AGPAT1,AMY2A,AOC2,ARNT,B4GALT4,CCND2,CFL2,EPHB1,FGFR1,G6PD,HSD17B3,JAG2,JUND,KIR2DL4,LIFR,MAP3K13,MYH2,OR11G2,OR1J4,OR4F15,P2RX6,P4HA1,PDX1,POLR2A,RAB23,RAPGEF2,RRAS2,SHC3,SMAD5,SULT1A4,WASF2,WNT2B,WNT5B,B3GALNT1
Negative_10358	IL3,ACAT1,ACP2,ALDH3B2,AMDHD1,ARHGEF7,BDKRB2,CHRNA7,DAAM1,DDIT4,DDX55,FBP1,GLI3,GPLD1,HADHB,HHEX,JAK1,MAP2K5,NAT2,NTF4,OR10P1,OR51S1,OR56A3,OR5H2,OR5K2,OR8B4,OR8J3,OR9G4,PIM2,PLEKHA8,POLR2E,PPP1CB,PRKAR1A,PTTG1,RALB,RAPGEF1,RASSF5,RELB,RHEB,RPS6KA4,SARS2,SESN1,SETD8,SUV39H1,TIRAP,VNN1,WIF1
Negative_10359	HK2,CCNA2,DAAM1,HEMK1,HHEX,LDHAL6B,MYH11,MYH13,OR4S2,PFN3,PIGB,SARS,STAT6,VAV3,MAPKAPK2
Negative_10360	CR1,CSNK1E,CXCL11,ITGA1,OR4F5,PHKG1,PIGF,PLCB4,PPP2R2C
Negative_10361	C7,PVR,GLI1,HSD17B7,IL1R2,OGDH,OR1I1
Negative_10362	CRK,RET,TK2,ACSL1,BMPR2,CCNE1,CD244,CD247,CKS1B,DNMT3A,GGT1,GLG1,GNAO1,GPD1,GPT2,GRIN2B,HAO2,HMOX1,IL6ST,IL9R,INPP4A,KIR2DL3,MAP2K7,MGAT1,MYH6,NFKB1,OR10AD1,OR14I1,OR1D5,OR2T3,OR52E2,OR8A1,OR8I2,ORC6L,PDE7A,PHKG2,PIGH,PPA2,PTPRM,PVRL1,SMAD2,SMPD3,SMURF1,TYMS,UGT2B11,ARHGEF12,ATP6V1B1,SERPINA5
Negative_10363	EPO,NPY,ALDH1L1,ATP5D,CLTA,EPHB3,LTA4H,OR1A1,OR52E4,SCP2,SORD,VTI1A
Negative_10364	ACP5,ALOX12,APLP1,BIRC5,BMPR2,C4BPA,CD274,COX15,EDA2R,FMO2,GFPT1,HSPB1,IL22RA1,MINPP1,MYLK2,NCOA4,OR2G6,OR2H2,OR2M3,OR52E2,OR5AS1,OR6Y1,OR7C1,OR8A1,RUVBL1,ULBP2,PLA2G12B,SUV420H2
Negative_10365	DDO,ALG10,ARHGAP5,ARHGEF6,BLVRA,CHRD,CLDN1,CYP21A2,CYP2A7,DFFB,FMO2,GRIN2A,GSTA2,HTR5A,IL23R,INPPL1,NFKB2,OR10H4,OR10P1,OR51G1,OR7G3,OR9Q1,PDHA2,PPARG,PROC,PTPRF,SLC2A4,SPCS3,CACNA2D1,CACNA2D3,SLC25A31
Negative_10366	C8A,DCK,ALG13,CCKBR,CNTN1,CSF2,CXXC4,DPM1,FGF19,HGSNAT,IAPP,IFNAR1,LDHAL6A,LRP6,MAP4K2,MGST3,MYH10,NLGN2,OR14C36,OR2B11,PFKFB1,RGS3,TAS2R16,TGFA,ATP6V1C1
Negative_10367	C9,F8,ATP6V1A,CALML5,CCR2,CHIA,CNTN2,CREB3L1,ERO1L,GALNT11,GLUD1,GRIN1,GRM5,IL15RA,KITLG,NEGR1,OR1L1,OR5AS1,PEMT,PER2,PLA1A,PPM1B,PRNP,PSEN1,ROCK2,RPRM,SMAD4,ST6GAL1,TNFSF12,VNN1,WNT3A,WNT9A
Negative_10368	AKT2,ASNS,BTRC,CCR9,CDC6,CLDN3,CREBBP,CYP26B1,EIF4E2,GRIN2D,HEXA,MAP2K4,MYL9,NOS3,OR1F1,PIK3CD,PPP1R1A,CACNA2D4
Negative_10369	AARS,ACPP,AOC2,BMP7,CCL14,CHKA,COX10,CXCL13,CXCL2,DEGS1,FADS2,FIGF,GAB1,GIT1,GJD2,GLB1,GLI1,HMOX2,ITGA6,JAG2,KLK3,LDHC,MAP3K2,NCK1,NCOR2,NRG3,OR4K1,OR51B4,OR5D16,PHKB,PI4KB,PLA2G2E,PPAP2B,PPCDC,PPM1B,PPM1D,PPP3R1,PYCR1,RBX1,RRAS,SETMAR,TIRAP,TNFRSF9,TYMP,WNT2,YES1,YWHAE,ATP6V1G3

Negative_10370	BTD, TXK, ADIPOR1, CCL17, CCNB2, CKMT2, CLDN8, COL4A2, CYP26B1, CYP3A4, DAPK1, FCGR3A, GRM1, HRAS, HSD17B4, IFIH1, IL2RG, MTMR1, OR14A16, OR4C46, OR4K5, OR51B5, OR51D1, ORC2L, PARS2, PHKA2, PIGN, PLCD4, PPP2R1B, PRKAG3, RHEB, SLC27A5, STAT6, TAS2R4, TGDS, TNFSF10
Negative_10371	CCL14, CFL2, CHEK2, CKMT2, CTBS, CTPS2, EPHB2, HADH, ITGAM, ITPKA, LRP6, OR13C9, POLR1A, PRKAR1A, PRKCB, ST3GAL1, VEGFB
Negative_10372	ADC, GNS, ABL1, ACSM5, ALG10B, ALG6, ARNTL, ATIC, ATP5E, BLVRB, CACNA1H, CDKN1B, CNTF, COL6A2, CTSG, DGKQ, ENTPD5, FECH, FGF2, GAB1, GGCX, GRPR, GSTK1, GZMA, HHIP, IL20RA, IL2RB, IL3RA, LAMC3, MYH15, NEFH, NPR2, NT5C1A, OR10H2, OR4C46, OR6B2, PAK7, PANK4, PIM1, POLD3, PTGER3, SCLY, TUBB2B, VANGL1
Negative_10373	DLD, ACAD8, ACSM1, ATP5L, BRAF, CPT1B, IDI1, IL8RB, ITGA2B, KIR2DL3, LRP5, MADCAM1, OR1G1, POLE, PHOSPHO1
Negative_10374	ADIPOR1, ALAS1, ALG11, ALG13, BUB3, CD74, COASY, DCTN1, EPHA8, GPX5, HPSE2, ITPA, LAMA4, LTC4S, MGAT4B, NMNAT3, OR13C3, OR1S1, OR2A5, P2RX1, PARD6B, PER1, PLAU, PPP2R1B, PTPMT1, PTPRR, STK4, ARHGEF12, HLA-DPB1
Negative_10375	TFG, ATP5B, CNGB1, GRAP2, KLRC4, PDGFA, PIP4K2B, SLC9A1, TAS2R7, VDAC1
Negative_10376	AK2, ID3, MPI, AMD1, ATP6V1H, CACNB3, CFTR, COX15, CTSB, CTSL1, CXCL6, DKK2, DPAGT1, FGD3, FSHB, GATM, GNAQ, HLA-G, HPRT1, IL17RA, IL7R, IMPA2, JAG1, JAK3, MAP3K12, MOV10L1, MYH8, NCF4, NOS3, OR2T3, OR4C46, OR4L1, OR7G3, PECAM1, PLCD4, PLCE1, POLD2, PRKAB2, PTPN6, SLC2A1, SOCS4, SORD, SPP1, TBXAS1, CACNA2D3, HSP90AB1
Negative_10377	DUSP2, GABARAP, GALNT7, LEPR, MNX1
Negative_10378	STS, ARPC2, ASAH1, BIRC5, CLTA, COL5A3, FGFR3, GNPDA1, NCR2, NT5C2, QARS, SMARCA2, SPCS1, TNFRSF13B
Negative_10379	LSS, ALG3, BMP6, CACNG3, CD72, CHRM1, CHRM2, FGFR4, HMGCL, IFNW1, IL11RA, NAT1, NODAL, NOX1, OR10C1, OR2M3, OR5H6, PFAS, PIGB, PPP3CB, RAB23, RBL2, SETD1A, SETDB2, SOAT1, TAS2R16, TUBB4, ULK1, WNT3A, EIF4EBP1
Negative_10380	AOC2, AVPR1A, BMP4, ETNK1, SOCS5
Negative_10381	CD2, ACMSD, ARPC1B, ATG12, BNIP1, BUB3, CACNG2, CCL17, CCND2, CDS1, CDS2, CYP2A13, FGF13, GALNT9, HNF4A, IL17RB, IL8RA, IRAK3, ITGAM, MTHFD2, NCF1, NR1H3, OR11H6, OR4K13, OR4S1, PDCD1, PDE11A, POLR1E, POLR2C, RCHY1, SH2D2A, SLC25A4, SPRY4, SRD5A1, ULBP3, VAV1
Negative_10382	F7, GHR, ACAT2, ACO1, APPL1, ARSE, CD46, CHRD, GNRH2, GP1BA, HTR2A, IFNA2, IFNAR1, IL11, IPMK, NEFH, OR10G7, OR13F1, OR4C15, PLCB2, POLE3, SARS2, SC4MOL, SPHK2, TSC2, UBE2K, VASP, XCL2
Negative_10383	BMPR1A, CDK2, NEUROG3, OR5J2, PANK3, SEMA3A, SPTAN1, SULT1A1
Negative_10384	ADA, ADK, PLG, SHH, ACAA1, BCAT2, CDKN1B, FABP3, FLOT1, GALNT6, GMPR, IL12A, IL13RA1, NCF2, NFAT5, NFATC2, OR10A2, OR10A3, OR1D2, OR52K2, OR7C1, PER3, PFKFB3, PGAM1, PRDX6, WNT4, PLA2G12A, TNFRSF12A
Negative_10385	HGD, ADORA2A, BCL2L1, GSTM1, MAP3K7, NTF3, OGDHL, OR8G1, PDGFA, PPP2R5B, PTGFR, RASGRP4, SIAH1, VASP
Negative_10386	APAF1, DUSP9, GALNT10, GNA12, MAP4K2, OR1S2
Negative_10387	CAT, AHCYL1, AMPD2, DAAM1, GUCA1C, HLA-DRA, MYLK, NAGA, PIGO, RBL1, SH2D1B, YARS2
Negative_10388	DLD, F2R, SDS, TNF, APLP1, BIRC2, CCR8, CDH4, CSF3, CYP2C18, FGF10, FIGF, GALK1, GYS1, IMPDH1, KIR2DS5, MAN2A1, MAP3K4, MAT2B, MLLT4, NEFH, OR10S1, OR2B11, OR2T29, OR2T3, OR4S2, OR4X2, OR52K1, OR7C1, ORC1L, PDE1A, PDE6H, PTGFR, RBPJL, SETMAR, SORBS1, TFPI, THBD, TNFSF14, UBA7, YWHAQ, ZMAT3, MAP3K7IP2, MAPK8IP1

Negative_10389	EDA,RFK,BET1,C3AR1,CCL15,CCL2,CD28,CD40LG,COX15,DBF4,DPYSL2,DUSP6,DUSP7,ENTPD2,FLOT1,FZD4,GCH1,GLI1,HMBS,MDH1,NT5C1B,OR13C5,OR1S2,OR3A2,OR5AN1,OR5C1,OR6T1,ORC4L,P2RX6,PARD6B,PCK2,PIM1,PIPOX,PKLR,PRODH2,PTCH2,PTPRB,SNAI2,SOCS3,SSH3,TFPI,ZNRD1,PNLIPRP2,TNFRSF1A
Negative_10390	ABO,WAS,CCNE2,ENTPD1,KIR3DL1,MYH7,OR1I1,PGAM1,PSEN1,PSME2,SH2D1B,VAMP3
Negative_10391	OTC,ACYP2,AMDHD1,CDKN2A,CSF2,FGD3,LRRC4C,MYH8,OR4C12,OR6Y1,OSMR,PDXP,PECAM1,PIGQ,RHOA,SEPHS1,SFRP5,SGPP2,SOCS4,STX1B,ST6GALNAC6
Negative_10392	ASCC3,IL8RA,KLRC2,NARS,NMNAT2,OR13C8,HLA-DPB1,PLA2G12A
Negative_10393	C8B,PTS,AHCY,APBB1,B3GALT5,CD226,CES7,DOLPP1,EIF4E,ELK4,GPX5,IKBKG,IL13,IL1R2,INHBA,ITPKB,KIR2DS4,MGAT1,NAT5,NCKAP1L,OCLN,OR10R2,OR1I1,OR1S2,OR5T1,PARVA,PAX6,PIGC,PPP2CB,PPP3CC,THBD,THBS4,WASL,WHSC1L1,ZFYVE16,MAPK8IP3,SERPINC1
Negative_10394	HPD,BBC3,FLOT1,FRAT2,GALNT3,NPR1,OR8J3,PLCB3,PTGER1,TNFRSF6B
Negative_10395	F2R,TXK,ACTB,ADAM17,ALOX12,NT5C,RAC2,XCL2
Negative_10396	ALG10,CYFIP1,IARS2,ATP6V1C1
Negative_10397	GBA,AGPAT3,FGF14,ITGAL,OR52E8,PKM2,VDAC3
Negative_10398	F9,B4GALT3,BDH2,CCNB1,ETS1,FBP2,GALNT12,HEXB,NT5E,OR1L3,OR5T2,SV2B,TM7SF2,UGT2B10
Negative_10399	ADK,AK5,INS,MAG,ABAT,ACADL,ACSM2A,ACSS2,ACTN1,ALDH1B1,B3GNT5,CBR3,CD247,CD40LG,CDK2,CLDN1,CNGA4,CSNK1D,CTSB,CXCL10,EFNA5,FDPS,GLG1,GOSR2,GPT2,GYS2,HADH,KRAS,NKD2,OR10A6,OR6Q1,PDE5A,PDGFRA,PECAM1,POLD2,PRKACA,RUNX1,SLIT2,SOCS2,TAS2R49,HLA-DQB1,HLA-DRB4,MAPKAPK2
Negative_10400	MET,ACVR1C,ADRBK2,ATP5C1,CLDN3,CX3CL1,DAD1,DDX23,DGKA,FUT5,G6PD,GMPPB,GNAO1,GUCA1C,GUCY1A2,HMGCL,ITGA2,LLGL2,MAP3K5,NRXN2,OR10H1,OR5T2,OR9Q2,POLE4,SPHK1,TAS2R49,TCIRG1,TFDP1,TPM3,CSNK1A1L
Negative_10401	AMY2A,CHPT1,CYCS,FOSL1,NFASC,NOTCH3,NUDT12,OR1S2,OR2T12,PAX6,POLR1D,POLR3K,RETSAT
Negative_10402	C5,DBI,ACYP1,ADH1B,AKT2,CACNB2,CCR3,CMBL,COMP,CTPS,CX3CL1,CXCL6,EREG,FUT8,GAD2,GGT1,HEXB,HSPA8,MGLL,NFYB,OR10H3,OR1A1,OR1A2,OR2H2,OR5J2,ORC6L,PGM2,PIK3C2A,PIK3R3,PLCB2,PON1,PPP2R5D,RDH16,RPIA,SEMA4F,SESN1,SULT1A2,TRPC1,UGT2A1,UMPS,WNT2,ZIC2
Negative_10403	ACTN4,AKR1B1,APOC3,GALNT14,PISD,PRSS1
Negative_10404	SRF,AMPD1,ARPC1B,ARSE,ASPA,CHRNA7,CYCS,DGKZ,DHDDS,ENTPD5,HLCS,IDUA,NAGK,PDE3A,PISD,PRKACA
Negative_10405	AK3,FST,GK,NOG,AGPAT3,B4GALT6,CAMK2G,CCNB3,CHAT,CHIT1,COASY,CTNNB1,CYBA,DDX47,FGF6,GALNT5,GCAT,GRIN2D,IL22RA2,INHBB,ITCH,KLK3,KYNU,LIPA,MAML2,MBL2,MYH6,NCK1,OR10AG1,OR1M1,OR4M1,OR51G2,OR5C1,OR6Q1,OR8I2,PGAP1,PIK3C3,PPP2R5B,PTPN11,QPRT,SEMA3A,SEMA7A,WNT16,PAFAH1B2
Negative_10406	ITK,MYC,ACADVL,APOA2,ARPC1A,ATP5J,BLVRA,CCL14,COX10,CYP4A11,DERL1,ETNK2,FGF18,FTMT,GALNT11,GLB1,GZMA,IL15RA,IL29,IRAK2,KLRC3,MAP2K3,MDH2,METTL2B,MGST1,MYL9,OR5M8,OR6B2,P4HA2,PAICS,PIGA,PON2,PPP1CB,PTPRF,PTPRR,SDC4,SKP2,STAT2,STX1B,TBL1Y,TRADD,TRAF4,TRHR,WNT3,ATP6V0A2

Negative_10407	AMT, C2, EDA, IL9, PTS, SMO, B3GALT4, BUB1, CASP9, CLDN2, CNTNAP2, CYP26C1, DAAM1, DDB2, DDX56, EPHB3, EPRS, GSTA4, HRH1, IL21R, ITGAM, KNG1, MAOB, MAP3K7, MDM4, NCOR1, NCR1, NME7, NOS1, OR10Q1, OR52B4, OR5L1, PIP5K1B, PPM1A, PRSS2, SELE, SH3BP2, SIPA1, TP53, UGCG, VEGFB, WWP1
Negative_10408	ACTN4, B3GALT6, CCL14, CCL15, CCNG2, CD8A, CDK4, CDKN1C, CTSS, DPYD, G6PC, IL8RB, IRF5, LPAR1, MAN1A1, MASP1, NFKB1, NUMBL, OR13F1, OR1M1, OR4B1, OR5P3, PANK3, PON2, ROCK2, RPN1, SMC1A, SPP1, SPTAN1, UBE2L3, YARS, MAP3K7IP1
Negative_10409	DCN, EBP, TK1, B3GALT2, C1QC, CADM3, CTTN, CYP17A1, DGKH, DTYMK, GALK2, GNPDA2, HLA-F, IRS1, LTB4R2, MAPK11, MICA, OR10T2, OR2AE1, OR2H1, OR4E2, OR5B12, OR5D18, PDE6G, PFKM, PLD2, PPP1CC, PRKAR1B, PRKCE, RASSF1, STK36, TIAM1, TLN1, TP53, TRAF4, UGCGL1, UROC1, WNT9A, ST6GALNAC5
Negative_10410	BTD, FUK, IDS, SPN, ACPP, ADCY9, ADRA1B, ALPI, ASRGL1, BNIP1, CAV2, CDC2, COMT, EXTL3, GCNT2, GLUL, GMDS, HAO2, IGF1R, INHBA, ITGA10, MAP4K3, MEF2C, MYH4, NADK, OR10A2, OR1A2, OR7C1, PAK3, PARD3, PDE4B, PER2, PNPLA3, PTGER3, PTPRM, RDH5, SARS2, SLC9A1, SYNJ2, TNFSF10, VDAC1, WBSCR22, PLA2G12B, ST6GALNAC4
Negative_10411	DDO, STS, ABAT, ACOT11, ADCY3, BUB3, CDC16, CHDH, CNGA3, DUSP7, MGAT5B, NTRK1, OR10A6, OR1G1, OR2T11, OR8K5, OSMR, PPCDC, PTCH1, RERE, ROCK2, SMARCA5, TALDO1, THY1, ZFYVE16, ATP6V1B1
Negative_10412	ALG8, HDAC1, PDGFC, PRPS1, RHOQ
Negative_10413	CBS, CP, IL5, PTS, SI, ADH1A, ADRA1B, ALOX15, APOC3, ASCC3, CASP3, DUSP5, EGLN1, FGFR4, GALNT10, GALNT14, GDF6, GLUL, GNPDA2, HLA-DOB, HLA-G, HRH1, IGSF5, IL18R1, ITPA, MADCAM1, MAML3, MKNK2, NT5E, OCRL, OR5H2, OR5L1, PHKG2, PLA2G5, PLAUR, PMM1, PPP2R2B, RARS2, RRM2B, STX1A, SV2C, TLN2, TUBA1B, UGCGL2, WNT5B
Negative_10414	PLG, ADCY6, ATP6V1A, CES7, CPS1, E2F5, FGF23, GALK1, IL4R, LYPLA1, MAD2L1, MAP2K4, NCK2, NMNAT1, OR10A3, OR10J3, OR1A1, OR6N1, SLC27A1, SNW1, STK11, STK3
Negative_10415	ITK, PGF, EPHA6, NEU1, OR5AS1, QPRT, WNT6
Negative_10416	FYN, KMO, PXN, AASDH, ACSS2, ALCAM, ALG5, CADM1, CD247, CDC27, CPT1C, DGAT1, DPYSL2, EFNA4, EREG, GOT2, HSPA1B, HTR6, IFNA14, IFNG, IGSF5, IL8RA, KNG1, LFNG, MCEE, NOS1, NRXN1, OR13G1, OR4F17, OR51V1, P4HA2, PDE1A, PDE1C, PDE3B, PIP4K2B, PLOD1, PRKCB, RASGRP4, RERE, RRAS2, TACR1, TAS2R10, TAS2R9, TCF7L1, TP53I3, TRIP10, PAFAH1B2, PNLIPRP1
Negative_10417	CAD, DDO, F2R, FLAD1, HPRT1, HTR2B, IL18R1, IL4R, NT5C3, OR1F1, OR1Q1, PANK3, PGM1, PLXNA2, SH3BP2, SHC4, TLR3, TNFSF9, USE1, WNT8B, YWHAZ, TNFRSF13C
Negative_10418	GLA, GPT, PRL, TPR, ADH4, ANAPC11, APAF1, ARD1A, ARHGEF1, CCR7, CEBPA, EPHA8, FOXA3, GARS, GJA1, GNPDA1, GRIN2A, IFNA7, IL23R, INHBE, IRAK3, ITGAL, MAP4K3, MOV10L1, NT5C2, OR2T33, OR51Q1, OR6C1, PROC, SULT2B1, TAS2R49
Negative_10419	IL1B, PNLIP, PYCRL
Negative_10420	CKB, F10, AMY2A, ARG1, B4GALT5, BACE1, CHPT1, CNTNAP2, COQ3, CSNK2B, CUL1, CYBA, DHRS3, EFNB1, EFNB2, HIP1, IL11, IL6R, MAML1, MAP3K2, OR1S2, OR2AE1, OR8K1, PDE2A, PLAUR
Negative_10421	PXN, CAMK4, CREB1, DAD1, ENTPD1, EPB41L2, EPHX2, GLG1, IRAK1, MAT1A, MTMR2, OR52J3, OR5B12, TAS2R49, WARS2
Negative_10422	EI24, OR6C76, SEMA4A, SLC2A2, TYMP

Negative_10423	ACAD8, ADSSL1, AOX1, APC2, ATP5H, CACNA1F, CD22, FUT6, GFPT1, GYS2, HPRT1, HRAS, HSPA8, IDH3B, LDHC, MYH6, NOX1, OR11L1, OR52I2, OR52L1, OR56A4, PIGH, PLA2G2D, PRKCH, PYGB, RIPK1, SMAD4, SMAD6, SPI1, STX8, TUBB2A, UGT2A3, VKORC1
Negative_10424	CACNA1H, CACNB4, FDFT1, FZD9, GSTA1, GSTA4, ITGA5, NAMPT, NAT5, NR1H3, OR2D2, P2RX7, PARD6A, PIGU, PRIM2, RXRA, TAS2R9, TNFSF18, ULK2
Negative_10425	PTS, TXK, ACACA, ACP5, ANAPC7, CES7, COMP, CSF2RB, ENPP2, ESPL1, EXOC7, GADD45B, GFAP, GNAI1, GUCA1A, HAAO, IL2RG, NAT2, OR2H2, OR51T1, OR5J2, OR6K3, P2RX3, PDE6D, PDE8B, PIGA, PLXNB2, PPP1CB, PRIM1, PTPRZ1, UGT2B4, AASDHPPT, SERPINE1
Negative_10426	AUH, ALG6, FGF7, GOT2, GUCY1A3, IRS4, OCLN, OR10G8, OR2D2, OR5K2, PKM2, WASL
Negative_10427	C8A, CSK, EDA, IVD, VTN, APAF1, APOA1, CLTCL1, CYSLTR2, DUSP3, EPHB1, GALNT14, GALT, GUCA1A, LRRC4C, MARS, OR2M2, OR51F2, PAX4, PGM2, PSME3, PTPN11, PYCR1, RASGRP4, SESN1, SETD1B, TYMS, TYRP1, ARHGEF12
Negative_10428	HK1, MOS, OGT, PLN, SFN, ARHGAP5, ATP5J, BTRC, CASP7, CKS1B, CPT1B, DUSP2, EI24, ENTPD6, FMO1, GALT, GNAI1, IL2RA, LAP3, MGAT2, MYH10, NAGA, OR13C8, OR2W1, OR4C46, OR6N2, OR8H1, PDGFC, PIAS4, PIK3CD, PLOD1, PNLIP, POLR2B, PTCH1, SESN3, SIGLEC1, SOS2, SPP1, STAT3, TACR3, TAS2R48, TRMT11, TSHR, TUBAL3, ULK3, UPRT, YWHAG
Negative_10429	ADCY10, ALG10, ATG3, CASP6, CD27, EHHADH, HADH, LRDD, PARS2, PIK3C2B, SPCS3, SRGAP1, STK36, TNXB, ST6GALNAC5
Negative_10430	ATP5E, CD34, CD48, CDC14A, CDO1, NKD1, OGDHL, PLOD3, SDC1, SMARCA2, TPK1
Negative_10431	INS, IVD, ACP1, BIRC3, CDS2, CLTC, DGKI, EPHB4, F2RL2, GNA12, GRIN2D, LCMT2, MAP4K4, OR2S2, PLCG2, ST3GAL1, STX3, TAS2R49, THBS4, AASDHPPT, SERPINA5
Negative_10432	AMH, AKR1C4, ALDH3B1, ATP6V1D, CLTCL1, CREB3L2, CTNND1, DHFR, DUSP14, DVL3, GSTM4, IL21R, IL23A, KIR2DL1, MAGI2, MAP3K6, NLGN1, NRAS, OPLAH, OR2M4, PIGH, PLCD4, PMM1, PRSS3, PTAFR, TRHR, VAMP4
Negative_10433	MAP3K8, OR5AN1, PFKP
Negative_10434	PGD, ACTA2, ALDH1L1, CACNA1B, CCKBR, CDC20, DPYSL2, F2RL3, FUT5, GSTM4, HHEX, HTRA2, IFNA6, IFNG, IL28RA, NLGN1, PCYT1B, PIP5K3, PLA2G10, PLA2G2F, PTTG1, RBPJ, RRM2B, SLC2A4, ZIC2, ATP6V0E1, MAP3K7IP1
Negative_10435	ADIPOR1, AGXT2, B3GALT5, BAAT, CTF1, DIAPH2, ELK4, EXT2, GAMT, GSTA3, HNF4G, IL29, ITGB1, KLK3, MAGI2, MAP2K5, MYLK3, NLGN3, OR2M7, OR51M1, OR5P3, OXSM, PIGV, PPP1R3C, PRKCB, PRPS1, RAP1A, SLIT1, SRGAP3, STX2, TNFRSF9, TUBAL3, SERPINF2
Negative_10436	AMDHD2, CCL4, CDKN2D, COQ6, EPHA7, GMPR2, HTR2A, ITGA2B, LARS, MLLT4, MYH7, NME7, OR10G3, OR5H6, SAT2, SFRP2, STK3, TAS1R3, UGT2A1
Negative_10437	CTH, EGF, ID3, ATN1, ATP6V1H, CCDC6, CD74, CDH1, CHRM3, CLTC, CSF1R, CTBP2, CYP3A7, DGKZ, DUSP8, DVL2, EGLN3, FCGR3B, FUT6, FZD4, JAM3, KAT2B, MAP3K5, MOV10L1, PLA2G10, POLR2H, RFX5, SH3BP2, SMPD3, SNAI1, SOAT1, TALDO1, TLN2, VAV1, MAPK8IP1
Negative_10438	ANAPC5, CCNB1, CDS1, IDH1, MLLT4, PDGFB, RHOD, SFRP1, SPRED2, TAS2R4
Negative_10439	ABL1, ACADVL, CCL24, CD8B, DCTN1, GSTM1, ICOSLG, IFNA7, OR4D5, OR4K17, OR5B3, PARD3, PAX8, PFKP, PIGL, PMAIP1, SETD2, TRPM5, ATP6V0D1, SERPINB5
Negative_10440	DHH, CCNB1, CSF1R, IDH2, IFNA10, IL28RA, KIR2DL1, OR10A5, OR2C3, OR52B4, PAK2, PIK3CB, PLA2G5, PPOX, SEMA4D, ST3GAL1, TUBAL3, VDAC3, GABARAPL1

Negative_10441	AMH, CSK, F8, ACP6, ADCY6, AMY2B, ANAPC4, ATP6AP1, C7orf16, CCL3, CMPK1, EPAS1, FGF19, GALK1, HSD3B7, IL4R, IL7R, IPPK, LDHAL6B, NFATC2, NR1D1, OR10H1, PIGN, PPP2CB, PPP2R2D, PPP2R5A, PPT1, PRDX6, PTPRM, SEMA4F, TAS2R13, TIAM1, TLR4
Negative_10442	ITK, LCT, ADCY3, AGRN, APOA2, ARHGEF4, ATG12, ATP6V1F, CFLAR, CLDN7, CSF2, CSNK1A1, EHMT2, FGF10, G6PC2, HRH1, HSPB1, INHBE, MAD2L1, MAP2K2, NCAM2, NCOA4, OR4A15, OR4F17, OR51Q1, OR5R1, OR8U1, PDE2A, PLCD3, PLCG1, PRPS1, PVRL3, RPS6KA6, ST3GAL3, TLR2, TUBB3, UGT2A1, UNC5C, ATP6V0A1, CTNNBIP1
Negative_10443	GSN, AARS2, BBC3, CTBS, GALNT2, IL13RA1, MMP7, NRCAM, PFKP, PIGN, PLCD4, SLC2A1, TSC1, ST6GALNAC6
Negative_10444	FH, FYN, APLP1, CDK5, CLCA4, CSF2, GNAQ, HLA-DOA, OR4N5, POLE4, QARS, SOCS5, B4GALNT1, HLA-DQA2, TNFRSF12A
Negative_10445	IL5, ADSL, ATP2A2, B3GALT6, C9orf95, COL4A2, CPT1A, CSAD, CXXC4, GABARAP, IDH3G, IL1A, OR2AG1, OR51B5, OR8H1, PFKL, POLA2, PTCRA, RHEB, SLC12A2, SLIT2, TKTL1, CACNA2D2, ST6GALNAC6, TNFRSF6B
Negative_10446	ADH5, CARS, ENPP3, GCNT2, PARS2, SAT1
Negative_10447	DCT, MOS, ALS2, ANAPC1, APH1A, CAMK2G, CD79A, EPHA3, HMGCL, LAMB3, MAP3K1, NCK2, OR12D3, OR2A2, OR2B2, OR4C15, OR4C6, OR51T1, OR8B12, RPS6KB1, SMPD3, ST3GAL4, TM7SF2, TPK1, HLA-DQA2, ST6GALNAC6
Negative_10448	CD4, CFH, GP9, PXN, APRT, CALML6, CDH2, DARS, DLL4, FARS2, HLA-DOA, IDH2, IL7R, IRS1, ITGB5, NKD2, NUDT12, OR10P1, OR52K2, OR6C75, OR9Q2, P2RX3, PDE2A, PDGFRA, PLCB1, PRLR, RCHY1, ROCK2, ST3GAL6, XCL1, GABARAPL2, HLA-DPA1, SUV420H2
Negative_10449	AK5, FES, ZYX, A4GALT, ALCAM, APBB1, ASAH1, ATF4, ATN1, CFL2, CXCR3, DPYSL2, ENPP7, ENTPD7, EPHB1, ETS1, FUT1, FUT3, GPT2, GRHPR, GSTA3, GZMB, HAGH, IL19, IL1A, LAMB4, LTA4H, MAOB, MAP4K2, MTFMT, OR2L13, OR52K1, OR8H2, PPT1, SIGLEC1, SUCLG1, TAS2R5, UCK2
Negative_10450	DDO, GK2, ALDH5A1, B3GAT3, CCKAR, CHIA, DVL3, ENAH, FLT1, GMPPA, IFNA16, KIR2DS4, MICA, MYH1, NCKAP1L, NFATC2, OR10A6, OR1G1, OR1N2, OR3A2, OR8J1, PIGU, PIK3R2, PIP4K2B, PLA2G5, POLD4, PTDSS2, TUBAL3, ATP6V1G1, CTNNBIP1, TNFSF13B
Negative_10451	HYI, FCGR3A, OR4K2, PCCB, PDGFD
Negative_10452	BAD, BID, EGF, IL6, ME3, ACHE, ACSL4, ALOX12B, ARNT, BDNF, BUB3, CREB3L2, CSNK1G2, CTLA4, DHRS4L2, FOXA2, GFPT1, GNRHR, IL12A, IRS1, LAMC1, LCAT, LTBR, NPR1, OR11G2, OR13C4, OR2T34, OR52E4, OR6K2, OR8A1, PAK3, PARD6G, PAX4, PLA2G2A, PLK1, POMC, PRSS1, PYGM, RRM2B, SEMA4B, SV2B, VAV1, XCR1, XYLT2
Negative_10453	ADC, FST, ABAT, ACADL, ARRB2, C4BPB, CCNE1, CDH15, CYP17A1, FLT3, IL23R, IRAK1, NARS, OR6N2, PAK7, POLA1, SAT1, SKP1, SMC1A, TNFSF10, TPH1
Negative_10454	FUK, PAH, WAS, ACSL3, ADAM10, APOA1, CD36, CLDN2, CPT1C, CTPS2, CYP19A1, EXTL3, FUT9, GALNT13, GMPPB, GNPDA2, IARS2, IFIH1, IKBKG, LAMB2, LILRB3, MINPP1, MMP14, MMP9, OR2A5, OR2T3, OR6T1, OR7G1, OR8K3, OR9I1, PFN2, PLCZ1, POLR2E, PRKG2, PSME2, PSPH, RASGRP2, RDH12, SHMT1, SNAP25, SPRY3, SRGAP2, TLR2, TLR3, TMLHE, ATP6V1B2, TNFRSF11B, TNFRSF1A
Negative_10455	CD4, LAT, ALDH2, ALG10, ARHGEF1, B3GALT2, DHRS3, DUSP5, IL13RA1, LEPR, MYH6, NANS, NCF1, NFKBIB, NFX1, NUMB, RARS, SDHD, TAS2R10, WNT7A
Negative_10456	APP, F11, LEP, ACP2, ALDOB, CACNA1G, CD27, DTX4, GALNTL2, GNG13, IL5RA, NGEF, NODAL, OR2A2, OR4C12, OR51S1, OR8B12, PLOD1, PRKCB, ST3GAL1, TNFSF8
Negative_10457	APP, DDO, FAH, ACVR2A, ATP2A2, C3AR1, CD40, CHRNA7, EPHB4, GADD45G, GALNT7, GRIA2, LIPF, OR52M1, OR52R1, OR6C2, PAX8, PIAS4, PLXNB2, QPRT, SEMA3D, TAS2R14, TIAM1, ZMAT3, MAP3K7IP1

Negative_10458	C8A,APAF1,CACNB3,FGFR1,GPAA1,GRIN2B,IFNA2,OR13C8,OR2B11,OR2K2,SMARCA2,SPRED2
Negative_10459	HAL,PDC,ACOX3,ALG1,BDH2,CACNB1,CDC25A,DHRS3,EI24,GADD45B,IL6R,MAML2,NPAS2,OR1J4,OR8B12,PER3,PLA2G6,SIPA1,VDAC2,SUV420H2
Negative_10460	CFH,FOS,ACSS3,BDNF,C9orf95,CACNB2,CASP10,CHRM3,COL5A1,CSF1,CYP2C19,ELK4,EPHA4,FOXO3,GALNT6,IL10RA,IL10RB,INPP5A,MAP2K6,NCOA4,NEGR1,NLGN2,NUDT12,OR2H2,OR8D1,OR8H2,PIGW,PNMT,POLA2,POLR2D,PPP2R5A,PRPH2,RASGRP4,SHISA5,SOCS7,SUCLG2,TOLLIP,UGT2A3,WNT1,ATP6V1G2,ATP6V1G3
Negative_10461	ATP6V1D,GADD45B,MYH6,OR10C1,OR7G3,STK3,TAS2R1,TGFB2,TNFRSF10D
Negative_10462	VTN,ALAD,APRT,CDKN2C,CYP4A22,DEGS1,DGKE,EPHA4,EPHX1,FCGR3A,GNA12,GSTA4,GSTO1,HIF1A,IFNA8,LRP6,LTA4H,MEF2C,OR10G8,OR10P1,OR1G1,OR1L3,OR2A12,OR2M2,OR52L1,OR5AP2,PGLS,PLA2G3,PTGES,SDC4,ST3GAL6,SUCLG2,WNT10A,WNT10B,ZFYVE16,CSGALNACT1
Negative_10463	B4GALT2,CDKN2B,DHX58,GUCY1A3,HPRT1,NEUROG3,NPR2,OR6B3,OR6F1,PDPK1,PLA2G2F,TUBB2A
Negative_10464	CD4,HDC,ID2,ADRA1A,CDKN1A,CLDN4,DOLPP1,ENTPD7,FGFR4,HMGCR,IDH3A,IRF7,MYH6,OR14I1,OR2W1,P4HA3,PHKB,PIPOX,PLD1,SEMA7A,SLC2A4,STAM2,HSD17B10,PDCD1LG2
Negative_10465	ACOX1,ACYP2,ADRA1B,ALPL,ARSB,ATP5L,CCL1,CCR7,CHPF,CLTCL1,COL3A1,COL6A1,CYP1A1,DARS2,DDB2,DGKA,DPYSL2,ELK4,FGF23,HAAO,ITGA11,LRAT,LYPLA1,MAP3K2,MAP3K6,MCCC2,MECR,OR11L1,OR4D5,OR5P3,PIAS1,POLR2I,POMC,PPP2R5B,PTPN5,SEMA7A,SLC25A4,TFPI,UGT2B10,UGT2B28
Negative_10466	ITK,SHH,AOC3,ARPC1B,CLDN5,CSNK1A1,EGLN1,GARS,GPD1L,HAO2,ITGA1,LEFTY1,MAFA,MCAT,NRG2,OR2A12,PER1,PIK3CA,POLR3G,PTPMT1,RDH5,RPSA,SH3BP2,SLC27A1,SMARCA2,ST6GAL1,WARS2
Negative_10467	ACACA,CADM3,CD22,COASY,CYP2U1,DDIT4,GRM1,NFKB2,OR10G8,OR4M2,OR56A4,OR5AC2,OR5AK2,PHGDH,PHKG2,PVRL2,SORD,TAS2R50,PPP1R12A
Negative_10468	PLG,C1QB,GALNT8,PGAM4,STX10,SUV39H1,WHSC1L1
Negative_10469	FAH,FUK,MAG,ACVR1C,ADSL,ARG2,CD226,CDC20,CDKN1B,CHDH,CYFIP1,DKK2,DLL4,DMGDH,DUSP5,FGF1,GAL3ST1,HMGCL,ITGA6,KYNU,MAP3K7,MAT1A,MGAT3,MPP5,NOX3,NPR2,OR2T8,OR52J3,OR52N4,OR52W1,OR5AR1,PDE9A,PDPK1,PLAT,PPARD,PPP2CB,PRSS1,RAP1A,RAPGEF3,RRM2B,SLIT1,SMAD7,TFDP1,TKTL2,UGCG,KIR2DL5A
Negative_10470	CD2,MPL,ACSM1,ACSM3,ALDH1A3,ALDOC,B3GALT6,BMP2,BPGM,CD14,CD55,CNDP1,COL4A6,CRB3,DARS,F2RL1,GARS,GDE1,HYAL2,IFNGR1,IL17RB,IL21R,MAP4K4,MKNK1,NEU4,NFAT5,OR14A16,OR2A2,OR2T11,PPARA,PPP1R3C,PRSS1,PTDSS1,PTPRZ1,RELB,SHMT1,TAS1R1,TAS2R7,THTPA,TLR2,VCAM1
Negative_10471	CAD,GP6,ACSL4,ACTB,AKR1D1,ARPC1A,CACNA1I,CCL20,CDC6,CDKN2C,CLDN17,CXCR3,CYP26A1,FGF4,FGFR2,GNAS,HADH,HLA-DRA,IBSP,KIR2DL4,MAP3K2,MAPK1,MASP2,MFNG,NEFH,NRXN3,OR2L8,OR4Q3,PIP5K1A,PLXNB1,PON1,PTPRC,SHC1,SLC25A4,SOAT2,VTI1A,WNT1,ATP6V1G1,TNFRSF6B
Negative_10472	ITK,SMS,AASS,ALDH7A1,ALDH9A1,ARPC3,CCND2,CD3E,CLDN8,CXCL3,EPHB1,EXT1,FADS2,GCAT,GSTM1,HDAC2,HHIP,IAPP,IFNGR1,ITGB7,LCMT1,LIPA,MAPK13,MDM4,MECR,MTMR6,NEU1,NFYC,OR10A2,OR1N2,OR2AG2,OR4K13,OR6N1,PCYT1A,PDK1,PLA2G10,PRKAG3,QARS,RPRM,SC5DL,SEMA3A,STK3,TNFSF11,TUBB2C

Negative_10473	CR1,NF1,ACTB,ADCY1,ALDH6A1,ALG6,AOX1,ATP12A,BACE1,BMP8B,CASP8,CDKN2B,COL1A1,CTNNB1,DNMT3B,EIF4E2,FDFT1,GNPAT,HLA-DRA,HRAS,HSD3B2,ITGB6,ITGB8,KLRC1,MADCAM1,MAML3,MCM2,MYH15,NT5C1B,OCRL,OR10Z1,OR4D6,OR5K2,OR7D2,OR8U1,PDE1A,PEMT,PFAS,PFN4,PIP4K2C,POLA2,PRKAR2A,PYCR1,RALBP1,SENP2,SIGLEC1,SPTLC1,TRMT11,TNFRSF1A
Negative_10474	BID,TNN,TPR,ABAT,AGPAT3,ASRGL1,BRAF,COX10,CRB3,EDA2R,ENO1,GALNT11,HSD3B2,LARS,LCMT1,LFNG,NCOR1,NCOR2,OR1M1,OR2AT4,OR52J3,OR52N2,OR5D16,PDE6H,POMC,RRM2B,TMLHE,TUBB3,WNT9A
Negative_10475	FER,ACSS1,ADH6,ALPPL2,CACNG4,CD247,CIITA,COL11A1,FLAD1,FRAT2,GAD1,GADD45G,HHIP,IL17A,JAM2,MAP4K3,MAPK13,MDM2,NAMPT,NCF2,NCF4,OR13D1,OR1D5,OR1I1,OR2T11,OR4B1,OR4D5,PIGV,PLCD3,PLOD2,PTGER3,PYGM,RAC2,SRD5A2,TNFSF11,TNFSF8,SLC25A31
Negative_10476	ATM,FST,ACAT1,CACNG1,CD99,CLDN18,DGKH,ITGB1,MTMR2,NADSYN1,OR4P4,PPP1R12A
Negative_10477	DCT,MET,AASS,ACOX1,ADH4,ALDH1L1,APOA1,ARNT2,ATIC,ATP5G1,CALML6,CD99,CDC42,CHUK,CSNK1A1,DERA,DUSP14,EDNRA,ERBB4,FUT4,GFPT2,HDAC2,HES1,KIR3DL3,LDHAL6B,NEGR1,NFKBIE,NOX3,NTF3,OR2T8,P2RX4,PAFAH2,PAK2,PON2,PPP2R5C,PTGS2,PYGM,RALB,ST8SIA1
Negative_10478	ALOX15,BBC3,CD34,CD46,DARS,HLA-DOB,KCNQ1,MAGI3,OR1N1,PDE6G,PIGS,PRKCE,RPE65,STX10,SUCLG1,UCP1,WARS2
Negative_10479	ACSS2,AHCYL1,CMAS,FARSA,GRIN1,GUCY2D,OR2B2,PIGF
Negative_10480	XDH,ACTA2,APH1A,BBOX1,CLDN16,FOSL1,NOX3,NUDT2,OR11G2,OR52B4,OR52K2,SETDB1,VANGL1
Negative_10481	BAAT,CARS2,CHST12,FASLG,IL20RB,MYH3
Negative_10482	GLA,PGP,ACP1,AHCY,ARPC5L,B4GALT6,BAIAP2,BET1,CD72,CDK5,CDO1,CYP2E1,FLT4,IFIH1,IL20RA,INMT,IQGAP2,MYH7,OR10A7,OR11L1,OR4C13,OR52H1,OR5I1,POLR2C,QPRT,RAF1,SEMA4F,ULK1,WNT3,PNLIPRP2,TNFRSF1B
Negative_10483	GSR,ACTC1,ADH4,ADH7,ALG8,APBB1,CXCL9,CYSLTR2,DIAPH1,ERO1L,EXT1,HAAO,IL13RA1,IMPDH1,IQGAP2,MAP2K7,NT5E,OR1L6,OR8J3,P2RX2,PIGF,PLCB2,PPP3CA,RETSAT,TKTL1,VAMP2,WASF2,WNT5B,ATP6V0E1,HLA-DRB4
Negative_10484	ILK,LHB,NPL,ACADL,ACVR1C,ALAS1,B3GAT3,CDK7,CLTA,CSF2RA,CSH1,DLST,FABP1,FGF17,FRAT1,GAS1,GJD2,GLI1,HNMT,IDI2,NCKAP1L,OR1K1,OR2T11,OR6K6,OR6N1,OR6T1,OR7G3,PFN3,PIK3R4,RFXANK,SH3BP2,ST8SIA5,TCF7L1,TGFB2,UROD
Negative_10485	EI24,GANC,GART,IFNA21,MASP1,NFYC,OR1F1,PRKG1,TAS1R3,TLR5,ATP6V0D1
Negative_10486	ARSA,BLNK,CD74,CDH5,CES1,CNGA3,COL5A3,CYP2U1,DLL4,DNMT3L,GCLM,GLI3,GPAA1,HLA-DRA,LRP6,MAP3K6,MDM2,OR13C2,PIGT,PIK3R1,PPP2R5D,PTGES2,SEMA4A,STAT5B,TLN2,TNFRSF9,PLA2G12B,SERPIND1
Negative_10487	F9,FUK,AANAT,ADRB2,ANPEP,CEBPA,CRHR1,DVL1,EFNB1,ENO1,EPHA6,FBP1,FGF8,GANAB,GLYCTK,IFNB1,ITPKA,MAN2A1,MAP2K2,OR10G9,PARD6B,PDE3B,PPCDC,SEMA4B,SFRP2,SHC4,SMAD4,SPAM1,SSH3,STX8,VDAC3,WASL,WNT8A,MAPK8IP3
Negative_10488	C8G,GK,ACACB,ACO1,ALDH1A3,ALOX12,ARAF,ATP2A3,BMP6,CAPN2,COX10,CYP2S1,DDX47,DNMT1,GRIN2D,HTR2A,IL17RB,KYNU,MCAT,MTMR6,NFATC1,NOD1,NPAS2,OR10H2,OR5D13,OR6C75,PARS2,PIK3C2A,PLAUR,RETSAT,SEMA4A,SEMA4B,SLC12A2,ULBP3,YES1,ATP6V0D2,PNLIPRP2,PPARGC1A,TNFRSF17

Negative_10489	AGRN, AMY2A, ANAPC5, ARPC4, ATP5J2, CCR6, CHSY3, CLTCL1, CNTN2, FMO5, GADD45A, GLI3, GMPR2, GNPDA1, HAO1, IMPDH1, MTHFR, NFATC4, NME4, NRXN2, NT5C2, OR1S1, OR2D2, PLK1, PPCDC, PRKG1, TBK1, TUBA8, UAP1, ULK3, WBSCR22, WNT3A, YARS2
Negative_10490	ID3, KIT, PC, ALG10B, ARG2, ARPC5L, CCNA1, CD28, CTF1, CYP4F3, DKK2, DLL3, GSTA3, HTR2A, INMT, ITGA4, KCNB1, MYLK2, NADK, NEU1, NOD1, OR13C2, OR14C36, OR2B6, OR52E2, OR5AR1, OR8A1, PDE11A, POLD4, PRPS2, PTGIS, PYCRL, ST6GAL1, VEGFA, WNT11, ATP6V0D1, CSGALNACT2
Negative_10491	GSR, LSS, ACTG1, ALG12, ARRB2, CACNA1C, CSNK2A2, CYP4A22, DDX51, DPYSL2, ERO1L, HTR6, IRAK1, MBL2, MECR, NARS2, NT5M, OR10G8, OR13A1, OR2A25, OR4K1, ORC2L, P2RX6, PIP4K2A, PLAU, STAM, TACR3, TGFB3, UGT2B4, UROC1, ATP6V1B1
Negative_10492	SMS, ASIP, GLI2, IL2RB, LEFTY2, TAS2R7, TNFSF14
Negative_10493	CAD, GBA, AANAT, ALG8, ANAPC5, APOC3, ASCC3, CHD8, GSK3B, ITGA8, LCMT2, LIPE, MPDZ, MYLK2, OR51V1, OR52J3, OR5B17, OR8K1, OR8K5, ORC1L, PLAU, PON3, PRKCQ, RALB, RARA, RUNX1, SOX17, STX7, TAS2R14, UGT2B10, TNFRSF10C
Negative_10494	ARHGEF4, CADM1, DUSP1, EPOR, MAP3K1, NEFL, RDH16, TLN1, WNT7A, MAPKAPK2
Negative_10495	CP, ALDOC, B4GALT7, BACE1, CADM1, CDC25C, EXOC7, HBEGF, ICOSLG, IL18, IL1R1, NME7, OPLAH, OR13C3, OR2M7, OR2T11, OR2V2, OR4C16, PIAS3, PIP4K2C, PNMT, PNPT1, PPP2R5C, SH2D1A, SLC2A2, SOCS2, PNLIPRP2
Negative_10496	AGL, FH, GNS, SRC, SYK, ABI2, ACTN4, ALAS2, ALPP, AMD1, ATP2A3, CACNG1, CCL19, CCL4, CDH1, COL5A3, CSNK1G1, CYP3A5, EVI1, GAD2, GNAZ, GSTA5, HSPA8, KITLG, MCM4, NUDT5, OR10P1, OR2T10, OR5B3, OR5T3, OR8A1, PANK2, POLD4, STX18, TBL1XR1, UBE2L6, UNC5C
Negative_10497	C9, CHD8, ECSIT, FPGS, HSPB1, ORC1L, POLD4, POLR1C, POLR2G, TP53I3
Negative_10498	DAO, LHB, MAX, ACTA1, BET1L, C7orf16, CCL21, CDC14B, COL5A2, CPT1A, DAAM2, FADD, GOT2, GUCA1B, HAAO, HSD17B2, IFNA8, L1CAM, LIPA, MDH1, NAGLU, NT5C3, OR13C2, OR2A25, OR4C12, P2RX4, PCCB, PCYT1A, PDE8B, PI4KA, PIGN, PIP5K3, PLCB1, PLD1, RALB, RCHY1, TRADD, TUBA3E, WNT4, SERPINA1, SERPINF2
Negative_10499	DUT, GLA, MPZ, ALDH3A2, ALOX15B, DUSP9, EPHB4, ERBB2, HLCS, IFNA5, IL24, LTC4S, MYH8, NARS2, OR4K5, OR5B3, RDH11, SETD2, TACR2, THBS4, TMLHE
Negative_10500	NF1, ALDH1A3, BMP5, CAMKK2, CHD8, CISH, CLDN15, COQ7, DHFR, EPB41L3, FABP4, FZD1, GNA12, HMOX2, NFKBIB, OR1K1, OR2AE1, OR52W1, OR5A1, PGM1, PPARG, PPP1CB, PRKAB1, PRODH, QPRT, SESN3, VAMP1, VDAC2, C1GALT1C1, HLA-DQA2
Negative_10501	CS, EGF, SYK, ACP2, ACTG2, ADH5, AKT3, AMY2B, BHMT, BTRC, CAV3, CCL28, CES1, CYP8B1, DGKD, DPM2, EFNB2, ERBB3, IAPP, IPPK, JAG2, NPAS2, OR51F1, OR6M1, OR9I1, PPP2R1A, PRKAB1, PTCH2, RRAS2, SKIV2L2, SMC1B, UAP1, YARS2, MAPKAPK5
Negative_10502	TAT, ADSL, CLDN20, CTBS, CX3CR1, CYP11B2, CYP19A1, DGKD, DHX58, EGLN3, GNA13, GTSE1, GYS2, ITGA6, KRAS, LAMB1, MPDZ, NUDT9, OR4K1, OR5C1, OXCT2, PPAP2C, PTGDS, PYGL, RPS6KA5, SDHA, SENP2, ST3GAL3, TNFSF9, ZIC2
Negative_10503	SRC, ABCC8, ALDH1A1, ANGPTL4, ARHGEF1, ARHGEF6, BMPR2, BST1, CACNB4, CNGB1, EPB41L2, FGF11, FGFR1, HSD17B7, HSD3B1, HTR7, IL17RB, IL3RA, ISYNA1, LAMB4, LRP6, MAGI3, MAPK10, NADSYN1, NCKAP1L, OR6N2, OR8H3, OR8K1, POLR2E, PPP1R3B, PRKAB2, PRKCG, PRLR, RASA2, RIPK1, RPS6, SCIN, SLC25A4, SV2A, TLN1, TNFRSF1B
Negative_10504	ATM, FUK, PXN, CACNG1, CDS2, CSNK1D, CYP27A1, FLT1, GALT, GAPDH, GNPNAT1, HMOX2, MPDZ, NAMPT, NPR2, OR51Q1, SQLE, TAS2R7, TLR4

Negative_10505	GAA, IL9, INS, JUP, AGPAT2, ARNT2, ATP5A1, CCL26, CDK5, CXCL9, CYP27A1, FASN, FGF12, FZD1, GMPR, GUSB, HSD17B8, IL8RA, INSR, ITGA3, LEFTY2, LRDD, LTC4S, MAGI1, MCEE, NARS, NCAM2, OR10G3, OR2M3, OR4D2, OR4K15, OR51E1, OR52E2, PDGFRA, PERP, PGK2, PLCG2, PPP2R5A, PRKCZ, PSME3, PTPN7, RBKS, SMARCA5, SNAI1, SPRY1, TRADD, VTI1A, ATP6V0A2, TNFRSF12A
Negative_10506	ATM, CGA, F3, ALOX12B, AVPR1A, B3GAT2, CASP8, CDC7, CFL2, CHST11, CSF2RA, CYP26A1, DPM3, DPYSL2, ECHS1, GPD1, GPT2, HIF1A, HMGCR, INPP5E, KIR3DL3, MAP2K7, MLLT4, NME4, NUDT9, OR10H2, OR2A14, OR2T11, OR4C46, OR6T1, PHKA2, PLA2G4A, PPP2CB, PRKCZ, RALBP1, SDHC, SPTLC1, STX4, TAS2R49, PNLIPRP1
Negative_10507	DAO, AANAT, ACACA, ALDOC, ALS2, APBB1, CASP7, CASP9, CCL19, CMBL, CSNK1A1, CTBP2, DGKI, EDN1, GALK1, GNB3, IFNA16, IL19, JAK1, KLRC3, MAPK12, NUDT2, OR10A3, OR10J5, OR13C5, OR1Q1, OR2T34, OR2Y1, OR52J3, OR7C1, PGS1, PHKA1, PIP5K1C, PLD1, PPP1R1A, PPP2CA, PPP3R1, PRKAR2A, PRKCD, ROCK2, SMAD4, TBL1Y, TGFB1, UGCG, UNC5C, WASL, WNT6
Negative_10508	ACO1, AHCYL1, ALDOB, APOA1, CHEK2, CLDN20, CPT1B, CYP21A2, DHX58, E2F4, EPHA2, FGF3, GALNT12, GGT7, IFNGR1, MASP1, NFATC3, NRAS, OR11H4, OR13G1, OR14I1, OR8S1, P2RX5, PFKFB2, PPAP2B, PRKAB1, SEPHS1, ST8SIA1, STK11, TRHR, UPP1, USE1, PRICKLE1
Negative_10509	ASL, ACSS2, CADM3, DUSP6, ENTPD2, FLT3, HAGHL, IMPA1, LDHA, MAPK14, NRP1, OR1D2, OR2A4, OR2AG1, OR5AN1, PPP3R1, PRPS1, PTPMT1, SDC1, SFRP4, TRAF3, YWHAB
Negative_10510	DLL3, MAP3K13, MGST1, OR10AG1, OR14C36, PPAP2C, PPP1CA, PTPN5
Negative_10511	AK3, C8B, BCL10, CCND2, CDIPT, CTNNB1, EXTL3, FOXO1, ISYNA1, MAML1, MCM6, OR10T2, OR52N2, PDE3B, PER2, PLA2G2D, PRLR, PTPRB, RAPGEF3, SEMA3B, SEMA4A, TAS2R8, UROD, WNT8A, CTNNBIP1
Negative_10512	MSN, ACSM5, ACTA1, AGRN, FGF1, GALNTL5, KCNQ1, MAD1L1, MFNG, PIAS4, UROC1, ATP6V1E2
Negative_10513	GPT, MTR, NPY, ACAD9, ACHE, ARHGAP5, CASP3, CAV1, CCR10, CD38, ENTPD1, GJD2, GMPR2, GUCY2D, HSD3B1, IL17B, MCCC1, NAE1, OR2M4, PAICS, PGLS, PIGV, PPP2R1A, PSEN1, RPS6KA3, SOAT1, SPRY1, YOD1
Negative_10514	GDA, GGH, GHR, GP9, MYC, VTN, ADSL, AKR1B1, ALG12, ARNTL, B3GALT2, BIRC2, BIRC5, BMP6, C7orf16, CCR4, CCR8, CNTFR, CSNK2A1, DDX56, DUSP14, ENTPD2, ENTPD4, EPHA5, FGF9, GALNT2, HCLS1, ITGA9, MAGI2, MCM3, MPDZ, MYH2, OR2T10, OR4A16, OR4K2, PCK2, PIGW, PLIN, TGFBR2, TNFSF11, UBA7, PNLIPRP1, PPP1R12A
Negative_10515	ACTA1, ATP5D, ATP6V1H, CLDN1, CXCR6, DDX41, DDX50, FBP2, GFAP, GNAO1, GNB3, MNX1, NMNAT2, OR2A25, OR3A2, OR7G3, PRKAG3, RASGRP3, TAS2R3, TGFBR1
Negative_10516	HDC, TNF, AMY2B, CHSY3, DARS, GART, HSD3B7, KIR2DL3, NMNAT3, NOTCH3, PANK2, PIGT, STAT6, MAPK8IP3
Negative_10517	LTB, GALNT13, GUSB, HIBCH, ITGB4, LAMB2, MAPK11, OR8J1, PSENEN
Negative_10518	AK3, OAT, ACAD9, B4GALT1, CHPT1, CXCR4, ENTPD7, FGF13, GNAZ, GSK3B, GUCY2C, IL17A, LIMK1, MAP3K1, OR10A3, OR2H1, OR5M3, PDHA1, POLR3B, SGMS2, TNFSF4, TPI1, UXS1
Negative_10519	BTC, GHR, ARHGEF7, AXIN1, CCL7, DGKB, ENO1, PDE4C, PPAP2B, UGT1A10, VARS, VDAC3
Negative_10520	ADRA1B, ALG9, GBA3, GGT5, MAP3K2, MLYCD, MYH1, NOTCH2, OR1C1, PIGK, SHC4, SLC27A1, UGT2B11

Negative_10521	C5, FUK, ITK, PXN, ACTA2, ACTN2, ALG12, B3GNT2, B4GALT6, BMPR1A, COL4A6, CRLF2, CTSG, CYP3A5, CYP3A7, DDX52, DUSP6, FGF23, FZD8, GCNT1, GLO1, GRAP2, MAD2L1, MAGI2, MCM5, OR1N1, OR4E2, OR4Q3, OR5AU1, OR8S1, PANK4, PIM1, PPAP2A, PPAP2C, PRKCB, STX1A, TAS2R16, TOMM40L, ULBP3, USE1, XCL2, YARS2, TNFRSF13B
Negative_10522	FST, DDX23, HBEGF, P4HA3, SLC27A1
Negative_10523	RDX, SI, SYK, ARD1A, B3GNT2, CLDN15, CNTF, CYP26B1, CYP2A13, EXT1, ITGB2, MPP5, OR4C6, OR51A2, PFAS, PLA2G2E, POLR2E, PPP2R2D, PTPN6, RHOQ, SGMS1, SMPD1, CSNK1A1L
Negative_10524	IHH, ALG5, CACNG4, LCP2, OR2A2
Negative_10525	CFD, CP, PXN, ACP6, ARNT, ARNTL, CCL7, CD38, CDKN1A, CLDN14, COL2A1, COMP, DPM3, GUCY1A2, IL21, ITGA9, MAP3K13, MASP2, MCM5, NCKAP1, NR1H3, OR2AG1, OR4N5, OR8B4, PI4KB, PIPOX, PLA2G4A, PLXNA1, PPP2R1B, RRM2B, STK36, TKTL2, B3GALNT1
Negative_10526	C1S, LAT, LYN, OSM, ADRB1, BMPR1A, DDX19A, DGKZ, DHRS9, E2F2, EHHADH, ERBB2, FGF9, FZD7, GBE1, GNAI3, GNPDA1, GRIA1, GSTA5, HIBCH, IAPP, IL15, LTBP1, NAMPT, NLGN3, NOTCH2, NR5A2, OR10G2, OR1I1, OR52E8, OR5B17, PER2, PIK3R2, PIK3R4, PLEKHA8, PTGIS, SCIN, SSX2IP, ST3GAL2, TNFRSF8, TP53, WNT9A, CTNNBIP1, HLA-DPA1, PLA2G12A
Negative_10527	CR1, LEP, AARS2, ACVR1C, CALML5, CCL3, CDKN2A, CYP21A2, DBF4, DGKE, EPB41L2, EPHA8, FGFR1, GP1BA, GRIN2B, HTRA2, IFT57, KLRK1, MAOB, MIOX, MNX1, OR10J5, OR10K2, OR5AK2, OR6C74, PGAM1, PLA1A, PLCD3, PNLIP, SH2D2A, TAS2R48, TAS2R7, TNFSF4, TPM3, TUBB4, YARS2, ZFYVE9, ALDH18A1, ATP6V1B2, SERPINC1, TNFRSF13B
Negative_10528	AK7, CFD, OSM, TXK, ADH5, ALG2, ALG9, APLP1, BDKRB2, BET1, CDKN1A, CFLAR, CHRM1, CSNK2A1, DDIT3, FGF8, GCNT3, GPD1L, GUCY2D, MAGI2, MAPK3, NODAL, POLR2H, RRAS, SETD7, SLC2A2, VAMP4
Negative_10529	FGB, KHK, PTS, TYR, ADH6, AK3L1, ATN1, CACNB4, CACNG8, CEBPA, CHKA, CSNK2A2, CTNNA2, DDX47, F2RL3, FGF1, FOXA2, GFAP, GRAP2, HADHA, HNF4G, HSD17B2, HYAL1, IL12B, IL22RA1, IRAK4, MAN2A1, MAP4K1, MASP2, MGST3, NKX2-2, OR2M7, OR4L1, OR51F2, OR7G3, PAK1, PAK4, PDE2A, PPP2R5C, PRKAA2, SGPP2, TCF7, TGFB2, TUBA1B, UGCGL2, ZBTB16, ATP6V1E1
Negative_10530	FOS, ALDH1A2, ARSA, ARSD, CASK, CHPT1, CLDN8, DUSP5, EXT1, GLS2, GMPS, GSTM1, HLA-DOA, HSPA1B, IL11, IL9R, ITGB1, ITGB4, MAP3K7, MPST, NCKAP1L, NCOR2, OR10A2, OR10C1, OR1N1, OR5AR1, SNW1, SULT1A1, VNN1, ATP6V1B1
Negative_10531	ALDH1A1, ARPC3, CLOCK, HNF4G, KLRK1, PDE4A, SOCS2, TAS2R14, TFDP1
Negative_10532	CGA, CRK, FES, NPY, AADAT, ACADS, ACPT, ADRB2, ANGPTL4, B3GALT4, CXCL12, DDHD1, DUSP3, EGLN1, EPOR, ERBB2, IL12RB2, IL24, LAMB2, LHCGR, MAP4K1, MAP4K4, NCK2, OR2L13, OR8I2, OXCT1, OXTR, PDE6G, PIK3R3, PIK3R4, PLCB4, PRKAG3, PTPRM, RHOA, RPS6KA4, TJP3, VAV1, YWHAZ, ATP6V1G1, SERPINA5
Negative_10533	FN1, LEP, SYK, ADRB3, ATP5J2, CCL11, CD247, CD86, CLTB, CMBL, CYBB, DDX52, FCGR3B, HIBCH, HNF1A, HSD3B2, IL1R1, IL21R, NRP1, OR4K14, OR51E2, PIK3R2, PIK3R3, PKM2, SORBS1, THTPA, WNT1, MAPK8IP1, PNLIPRP1
Negative_10534	UBC, IL28B, OR10A3, TCF7L2
Negative_10535	EPHA6, EPHA8, KYNU, MAML3, OR1L3, PAK7, PVRL4
Negative_10536	C5AR1, GALNT10, GUCA1C, OPLAH, PEMT, RRM1
Negative_10537	AK1, MSN, ABLIM1, ACP1, ACVR1B, AKR1C2, BDH2, BPNT1, CD244, CSNK1A1, DDIT3, DDX41, DLL4, FZD5, GALNTL2, GMPR, GNAS, GRIN2B, GUCY1A2, HMGCL, IAPP, IDI2, INMT, KAT2B, LARS, LEF1, NEFH, NFKBIB, NRAS, NT5C3, OR10G3, OR2AG2, PLK1, PON3, PRSS1, PTDSS1, PTGES2, RFX5, SOX17, SRGAP3, THBS2, UPP2, WNT10A, HLA-DRB4, TNFRSF11B

Negative_10538	B3GALT2, C1GALT1, CALML3, CANT1, CCL28, CHEK1, CTBS, CXCL11, DDX54, EPHB3, FABP3, FGF22, GNB1, GZMA, HEXA, HPSE, IL2RA, KLRD1, MCM3, MOV10L1, MRAS, MYH15, NCSTN, NOX3, OR2A5, ORC5L, PAX8, PLCD3, PMAIP1, PPP2CA, PTPRF, RDH8, UCK2, WNT5B, PAFAH1B2, TNFRSF13B
Negative_10539	MPZ, AARS, CCNB2, CLDN16, DFFA, MNX1, OR11G2, RGS3, RPS6KB1
Negative_10540	IL4, AKR1B1, CTBS, DAPK1, LTB4R2, LTBR, MICA, OR10G7, TRADD, SERPINB5
Negative_10541	C1S, AASDH, CLDN18, CTSG, CYP7A1, DRD2, FUT5, GLI3, GSTK1, GUCY2F, HLA-G, MAML1, MGAM, MYH15, MYH8, NFYA, OR2G3, OR5AN1, PDE8B, PIAS1, PPP2CA, PVRL1, RASSF5, RPE65, SUFU, TUBAL3
Negative_10542	ATM, PDC, B3GNT5, CAMKK1, CHPF, CYP17A1, DGKZ, ERBB2, FLOT2, FUT4, GAD1, GALNT6, GMDS, GSTM3, GTSE1, KARS, MALT1, OR10H2, PAX4, PDHB, PIGL, TJP1, TSHB
Negative_10543	MSN, GNAL, HLA-F, HTRA2, NFE2L2, OR4F4, SPTLC1
Negative_10544	CSK, GNS, ALG5, ATP5A1, B3GNT4, CSF1, FSHB, FZD1, IDH1, NKX3-1, OR1B1, OR1J2, OR1N1, OR4X1, PDE3B, PRKAR2A, ARHGEF12, ATP6V0A4, HLA-DRB5
[truncated: 474,952 more chars]
