# Supplementary material for: Analysis of Protein Pathway Networks Using Hybrid Properties
Source: Molecules. 2010 Nov 12;15(11):8177–92. doi: 10.3390/molecules15118177 (PMC6259184; doi:10.3390/molecules15118177)
Supplement: Supplementary File 5 [file molecules-15-08177-s005.pdf]

Online Supporting Information S5: Accuracy in IFS (incremental feature selection)

Index	The last added feature	Accuracy of positive pathway	Accuracy of negative pathway	Total accuracy
1	out_local_density_0.6_mean	100.00%	0.00%	0.99%
2	polarity_composition_P_max	42.60%	95.88%	95.35%
3	in_local_density_0.7_max	54.44%	98.91%	98.47%
4	in_local_density_0.5_mean	58.58%	99.27%	98.87%
5	topological_change_0.6_0.7	69.23%	99.48%	99.18%
6	topological_change_0.1_0.2	76.33%	99.57%	99.34%
7	in_degree_variance	73.37%	99.59%	99.33%
8	topological_change_0.7_0.8	75.74%	99.62%	99.38%
9	out_local_density_0.8_mean	73.96%	99.62%	99.37%
10	secondary_structure_distribution_P-1.0_mean	75.74%	99.66%	99.43%

11	topological_change_0.2_0.3	75.15%	99.68%	99.44%
12	in_local_density_0.6_mean	74.56%	99.65%	99.40%
13	out_degree_variance	75.15%	99.63%	99.39%
14	secondary_structure_distribution_P-1.0_max	74.56%	99.66%	99.41%
15	out_local_density_0.5_mean	75.74%	99.65%	99.41%
16	out_degree_correlation_max	77.51%	99.66%	99.44%
17	out_local_density_0.7_max	78.11%	99.65%	99.44%
18	polarizability_distribution_N-1.0_max	76.92%	99.66%	99.44%
19	in_local_density_0.7_mean	78.11%	99.67%	99.46%
20	AA_composition_C_mean	79.88%	99.69%	99.49%
21	in_degree_correlation_variance	79.88%	99.69%	99.49%

22	secondary_structure_distribution_P-0.0_max	79.88%	99.69%	99.49%
23	out_local_density_0.7_mean	77.51%	99.66%	99.44%
24	AA_composition_S_mean	77.51%	99.70%	99.48%
25	out_degree_correlation_variance	77.51%	99.70%	99.48%
26	VanDerWaal_distribution_P-1.0_max	77.51%	99.70%	99.48%
27	polarity_distribution_P-1.0_max	77.51%	99.71%	99.49%
28	in_local_density_0.8_mean	74.56%	99.67%	99.42%
29	AA_composition_K_mean	73.96%	99.66%	99.41%
30	polarity_distribution_H-1.0_max	73.96%	99.67%	99.42%
31	polarizability_transition_PH_mean	74.56%	99.68%	99.43%
32	in_clustering_max	76.33%	99.63%	99.40%

33	hydrophobicity_distribution_N-1.0_max	76.33%	99.66%	99.43%
34	solvent_accessibility_composition_H_max	76.33%	99.68%	99.45%
35	out_local_density_0.9_mean	76.92%	99.63%	99.40%
36	VanDerWaal_distribution_N-1.0_max	76.92%	99.65%	99.42%
37	secondary_structure_transition_PH_mean	74.56%	99.67%	99.42%
38	in_local_density_0.3_mean	77.51%	99.67%	99.46%
39	hydrophobicity_distribution_H-1.0_max	76.92%	99.69%	99.46%
40	in_local_density_0.9_mean	77.51%	99.66%	99.44%
41	out_local_density_0_max	77.51%	99.64%	99.42%
42	solvent_accessibility_distribution_H-1.0_mean	76.92%	99.61%	99.38%

43	in_local_density_0.6_max	76.33%	99.64%	99.41%
44	AA_composition_V_mean	76.33%	99.61%	99.38%
45	VanDerWaal_distribution_H-1.0_max	76.33%	99.63%	99.40%
46	polarizability_distribution_P-1.0_max	75.74%	99.67%	99.43%
47	out_local_density_0.6_max	75.15%	99.66%	99.41%
48	hydrophobicity_distribution_P-1.0_max	75.15%	99.66%	99.42%
49	solvent_accessibility_distribution_H-0.5_mean	75.74%	99.66%	99.43%
50	secondary_structure_distribution_H-0.0_mean	75.74%	99.64%	99.40%
51	in_local_density_0_max	75.15%	99.64%	99.40%
52	solvent_accessibility_composition_H_mean	74.56%	99.65%	99.40%

53	out_degree_correlation_mean	75.15%	99.67%	99.43%
54	out_out_topological_variance	75.74%	99.64%	99.40%
55	AA_composition_W_mean	73.37%	99.60%	99.34%
56	out_local_density_0.8_max	71.60%	99.60%	99.33%
57	out_local_density_0.1_max	71.60%	99.60%	99.33%
58	hydrophobicity_transition_PH_mean	71.60%	99.63%	99.35%
59	polarity_distribution_N-1.0_max	71.60%	99.63%	99.35%
60	in_local_density_0.8_max	71.60%	99.62%	99.34%
61	VanDerWaal_transition_PN_mean	72.19%	99.60%	99.33%
62	out_clustering_max	72.78%	99.62%	99.36%
63	AA_composition_L_mean	72.78%	99.61%	99.34%
64	in_local_density_0.1_max	72.78%	99.60%	99.33%

65	secondary_structure_distribution_P-0.0_mean	72.78%	99.60%	99.33%
66	out_local_density_0.3_mean	71.60%	99.60%	99.32%
67	AA_composition_A_mean	71.01%	99.62%	99.33%
68	AA_composition_H_mean	71.60%	99.57%	99.29%
69	polarizability_distribution_H-1.0_max	71.60%	99.57%	99.29%
70	VanDerWaal_distribution_H-0.5_mean	72.19%	99.57%	99.30%
71	in_in_topological_variance	71.01%	99.57%	99.29%
72	AA_composition_G_mean	71.60%	99.56%	99.28%
73	secondary_structure_distribution_P-0.75_mean	71.01%	99.54%	99.26%
74	in_degree_correlation_mean	71.01%	99.55%	99.27%

75	secondary_structure_distribution_N-1.0_max	71.01%	99.54%	99.26%
76	AA_composition_T_mean	72.19%	99.52%	99.25%
77	polarity_distribution_H-1.0_mean	71.01%	99.50%	99.22%
78	out_local_density_0.9_max	70.41%	99.51%	99.22%
79	secondary_structure_distribution_N-0.5_mean	70.41%	99.49%	99.20%
80	in_local_density_0.9_max	70.41%	99.48%	99.19%
81	in_local_density_0.4_mean	72.19%	99.50%	99.23%
82	secondary_structure_distribution_H-1.0_mean	72.19%	99.48%	99.21%
83	polarizability_distribution_N-0.75_mean	71.60%	99.47%	99.19%
84	solvent_accessibility_distribution_H-0.0_max	70.41%	99.49%	99.20%

85	solvent_accessibility_distribution_H-0.25_mean	72.19%	99.48%	99.21%
86	AA_composition_D_mean	71.60%	99.46%	99.18%
87	out_local_density_0.2_max	71.60%	99.46%	99.19%
88	polarizability_distribution_H-1.0_mean	71.01%	99.45%	99.17%
89	polarizability_distribution_P-1.0_mean	69.82%	99.44%	99.14%
90	in_degree_max	69.82%	99.45%	99.16%
91	weight_edge_mean(with_missing_edge)	70.41%	99.46%	99.17%
92	hydrophobicity_distribution_P-0.0_mean	70.41%	99.45%	99.16%
93	secondary_structure_transition_PN_mean	70.41%	99.44%	99.15%
94	out_clustering_mean	69.82%	99.44%	99.15%

95	polarizability_distribution_N-0.25_mean	71.01%	99.43%	99.15%
96	secondary_structure_distribution_H-0.5_mean	69.82%	99.43%	99.14%
97	AA_composition_Q_mean	69.23%	99.42%	99.12%
98	solvent_accessibility_distribution_H-0.75_mean	68.64%	99.40%	99.09%
99	AA_composition_P_mean	69.23%	99.40%	99.10%
100	secondary_structure_distribution_P-0.25_mean	68.64%	99.41%	99.10%
101	hydrophobicity_composition_H_max	70.41%	99.43%	99.14%
102	polarity_distribution_H-0.25_mean	71.01%	99.41%	99.13%
103	AA_composition_N_mean	71.01%	99.41%	99.13%

104	hydrophobicity_distribution_N-0.0_mean	69.82%	99.39%	99.10%
105	in_local_density_0.2_max	69.82%	99.38%	99.09%
106	AA_composition_R_mean	69.23%	99.37%	99.07%
107	out_local_density_0.4_mean	70.41%	99.40%	99.11%
108	VanDerWaal_distribution_P-0.75_mean	69.82%	99.39%	99.10%
109	out_degree_max	69.23%	99.41%	99.12%
110	polarizability_distribution_N-1.0_mean	69.82%	99.38%	99.09%
111	polarizability_distribution_H-0.25_mean	69.82%	99.37%	99.07%
112	hydrophobicity_distribution_N-1.0_mean	69.23%	99.36%	99.06%
113	VanDerWaal_transition_PH_mean	69.23%	99.36%	99.06%
114	polarity_distribution_H-0.75_mean	69.23%	99.39%	99.09%

115	in_clustering_mean	69.82%	99.40%	99.10%
116	polarity_distribution_P-1.0_mean	70.41%	99.37%	99.08%
117	secondary_structure_transition_NH_mean	70.41%	99.37%	99.08%
118	secondary_structure_distribution_H-0.25_mean	71.01%	99.39%	99.11%
119	AA_composition_Y_mean	71.60%	99.37%	99.10%
120	polarizability_distribution_P-0.25_mean	71.01%	99.37%	99.09%
121	in_degree_correlation_max	70.41%	99.38%	99.09%
122	polarizability_composition_N_mean	70.41%	99.35%	99.06%
123	VanDerWaal_distribution_H-0.75_mean	70.41%	99.35%	99.06%

124	solvent_accessibility_distribution_H-0.0_mean	71.01%	99.33%	99.05%
125	VanDerWaal_distribution_N-0.5_mean	71.01%	99.35%	99.07%
126	polarizability_transition_PN_mean	71.01%	99.34%	99.06%
127	secondary_structure_distribution_N-0.25_mean	71.01%	99.34%	99.06%
128	AA_composition_E_mean	71.01%	99.33%	99.05%
129	out_local_density_0.3_max	71.01%	99.33%	99.05%
130	VanDerWaal_distribution_P-1.0_mean	71.01%	99.33%	99.05%
131	hydrophobicity_distribution_P-1.0_mean	71.01%	99.33%	99.05%
132	hydrophobicity_distribution_H-0.75_mean	71.60%	99.31%	99.04%

133	secondary_structure_composition_P_mean	70.41%	99.30%	99.01%
134	secondary_structure_distribution_N-0.75_mean	70.41%	99.29%	99.00%
135	AA_composition_G_max	70.41%	99.29%	99.00%
136	secondary_structure_distribution_H-0.75_mean	70.41%	99.30%	99.02%
137	in_local_density_0.3_max	70.41%	99.30%	99.01%
138	AA_composition_F_mean	69.23%	99.29%	98.99%
139	VanDerWaal_distribution_N-1.0_mean	69.82%	99.29%	99.00%
140	polarity_transition_PH_mean	69.82%	99.28%	98.99%
141	hydrophobicity_distribution_H-0.25_mean	69.82%	99.27%	98.98%

142	secondary_structure_distribution_N-0.0_mean	69.82%	99.26%	98.97%
143	graph_size	69.82%	99.27%	98.97%
144	weight_edge_mean(without_missing_edge)	70.41%	99.29%	99.00%
145	polarizability_distribution_P-0.0_mean	69.82%	99.28%	98.99%
146	secondary_structure_distribution_P-0.5_mean	69.23%	99.30%	99.00%
147	secondary_structure_distribution_H-1.0_max	69.23%	99.30%	99.00%
148	polarity_distribution_H-0.5_mean	69.23%	99.29%	98.99%
149	polarizability_distribution_N-0.0_mean	68.64%	99.27%	98.96%
150	polarizability_distribution_N-0.5_mean	69.23%	99.25%	98.95%
151	topological_change_0.3_0.4	68.64%	99.23%	98.93%

152	secondary_structure_composition_N_mean	69.23%	99.23%	98.93%
153	polarizability_transition_NH_mean	69.23%	99.22%	98.92%
154	solvent_accessibility_distribution_H-1.0_max	69.82%	99.19%	98.90%
155	polarity_distribution_N-1.0_mean	69.82%	99.18%	98.89%
156	solvent_accessibility_transition_HE_mean	69.82%	99.18%	98.89%
157	polarizability_distribution_P-0.5_mean	69.82%	99.17%	98.88%
158	hydrophobicity_distribution_H-1.0_mean	69.82%	99.17%	98.88%
159	polarity_distribution_H-0.0_mean	69.82%	99.18%	98.89%
160	secondary_structure_distribution_N-1.0_mean	69.23%	99.17%	98.87%

161	hydrophobicity_distribution_N-0.75_mean	69.23%	99.15%	98.85%
162	out_in_topological_mean	69.23%	99.15%	98.86%
163	AA_composition_I_mean	69.23%	99.14%	98.85%
164	VanDerWaal_distribution_H-1.0_mean	69.23%	99.14%	98.85%
165	hydrophobicity_distribution_P-0.25_mean	69.82%	99.14%	98.85%
166	AA_composition_M_mean	69.82%	99.13%	98.84%
167	VanDerWaal_distribution_N-0.75_mean	69.82%	99.11%	98.82%
168	hydrophobicity_transition_PN_mean	69.82%	99.12%	98.83%
169	in_clustering_variance	69.23%	99.14%	98.85%
170	polarity_distribution_N-0.25_mean	68.05%	99.15%	98.84%

171	polarizability_composition_H_mean	68.05%	99.14%	98.83%
172	VanDerWaal_composition_N_mean	67.46%	99.14%	98.82%
173	hydrophobicity_transition_NH_max	67.46%	99.18%	98.86%
174	AA_composition_Q_max	68.05%	99.18%	98.88%
175	VanDerWaal_distribution_P-0.0_mean	68.05%	99.17%	98.86%
176	out_clustering_variance	67.46%	99.18%	98.86%
177	polarity_distribution_P-0.5_mean	67.46%	99.16%	98.85%
178	VanDerWaal_distribution_N-0.0_mean	67.46%	99.17%	98.86%
179	VanDerWaal_distribution_H-0.25_mean	67.46%	99.16%	98.85%
180	hydrophobicity_distribution_P-0.75_mean	68.05%	99.16%	98.85%

181	VanDerWaal_distribution_N-0.25_mean	68.05%	99.17%	98.86%
182	polarizability_distribution_P-0.75_mean	68.05%	99.17%	98.86%
183	secondary_structure_distribution_H-0.0_max	68.05%	99.18%	98.87%
184	polarizability_distribution_H-0.75_mean	67.46%	99.17%	98.86%
185	hydrophobicity_distribution_P-0.0_max	67.46%	99.16%	98.85%
186	polarizability_composition_P_mean	68.05%	99.17%	98.86%
187	in_out_topological_variance	68.05%	99.15%	98.84%
188	secondary_structure_composition_H_max	67.46%	99.14%	98.83%
189	polarity_transition_PN_mean	67.46%	99.14%	98.82%

190	in_local_density_0.4_max	67.46%	99.14%	98.83%
191	polarity_distribution_P-0.25_mean	67.46%	99.13%	98.82%
192	polarizability_distribution_H-0.5_mean	68.05%	99.12%	98.82%
193	secondary_structure_distribution_N-0.75_max	67.46%	99.15%	98.84%
194	in_in_topological_mean	67.46%	99.17%	98.85%
195	hydrophobicity_distribution_N-0.5_mean	67.46%	99.15%	98.84%
196	polarity_distribution_N-0.0_mean	66.86%	99.14%	98.82%
197	VanDerWaal_transition_NH_mean	66.86%	99.14%	98.82%
198	out_local_density_0.4_max	66.86%	99.14%	98.82%
199	polarity_distribution_P-0.75_mean	66.86%	99.15%	98.83%
200	in_out_topological_mean	66.27%	99.14%	98.81%

201	hydrophobicity_composition_P_mean	66.27%	99.14%	98.81%
202	out_in_topological_variance	66.27%	99.15%	98.82%
203	AA_composition_L_max	66.27%	99.14%	98.82%
204	hydrophobicity_distribution_P-0.5_mean	66.27%	99.14%	98.82%
205	hydrophobicity_composition_N_mean	66.27%	99.14%	98.81%
206	polarity_distribution_N-0.75_mean	66.27%	99.13%	98.80%
207	AA_composition_I_max	66.27%	99.14%	98.81%
208	secondary_structure_composition_H_mean	66.27%	99.13%	98.80%
209	VanDerWaal_distribution_P-0.25_mean	66.27%	99.14%	98.82%
210	out_out_topological_mean	66.27%	99.15%	98.82%
211	polarity_transition_PN_max	66.27%	99.15%	98.82%

212	VanDerWaal_composition_H_mean	66.27%	99.15%	98.82%
213	hydrophobicity_distribution_H-0.5_mean	66.27%	99.14%	98.82%
214	out_local_density_0.2_mean	66.27%	99.15%	98.82%
215	polarity_transition_NH_mean	66.27%	99.15%	98.82%
216	secondary_structure_composition_N_max	66.27%	99.14%	98.81%
217	secondary_structure_distribution_N-0.5_max	65.68%	99.14%	98.80%
218	hydrophobicity_distribution_H-0.0_mean	65.68%	99.14%	98.80%
219	AA_composition_H_max	65.68%	99.14%	98.81%
220	VanDerWaal_distribution_P-0.5_mean	65.68%	99.12%	98.79%
221	hydrophobicity_distribution_N-0.25_mean	65.09%	99.12%	98.78%

222	AA_composition_R_max	65.09%	99.11%	98.78%
223	first_singular_values	65.09%	99.12%	98.78%
224	AA_composition_W_max	65.09%	99.11%	98.78%
225	hydrophobicity_transition_NH_mean	65.09%	99.12%	98.79%
226	polarity_transition_NH_max	65.09%	99.11%	98.77%
227	secondary_structure_distribution_H-0.75_max	65.09%	99.12%	98.79%
228	polarity_composition_N_mean	65.09%	99.12%	98.79%
229	in_local_density_0.2_mean	65.09%	99.13%	98.79%
230	VanDerWaal_distribution_N-0.0_max	65.09%	99.14%	98.80%
231	solvent_accessibility_distribution_H-0.5_max	65.09%	99.12%	98.78%
232	secondary_structure_transition_PH_max	65.68%	99.11%	98.78%

233	polarity_composition_H_mean	65.68%	99.12%	98.79%
234	polarity_distribution_N-0.5_mean	65.68%	99.12%	98.79%
235	hydrophobicity_distribution_P-0.75_max	65.68%	99.14%	98.80%
236	AA_composition_S_max	65.68%	99.13%	98.80%
237	AA_composition_E_max	65.68%	99.12%	98.79%
238	VanDerWaal_distribution_P-0.25_max	65.68%	99.13%	98.80%
239	polarizability_distribution_N-0.75_max	65.68%	99.12%	98.79%
240	AA_composition_C_max	65.68%	99.09%	98.76%
241	AA_composition_Y_max	65.68%	99.09%	98.76%
242	AA_composition_T_max	65.68%	99.10%	98.77%
243	VanDerWaal_distribution_H-0.0_mean	65.68%	99.10%	98.77%
244	in_degree_median	65.68%	99.10%	98.77%
245	AA_composition_D_max	65.68%	99.10%	98.77%

246	solvent_accessibility_distribution_H-0.75_max	65.68%	99.09%	98.76%
247	VanDerWaal_composition_N_max	65.68%	99.08%	98.75%
248	hydrophobicity_composition_H_mean	66.27%	99.08%	98.76%
249	polarizability_composition_H_max	65.68%	99.09%	98.76%
250	VanDerWaal_composition_P_mean	65.68%	99.08%	98.75%
251	secondary_structure_transition_PN_max	66.27%	99.06%	98.73%
252	VanDerWaal_distribution_H-0.25_max	66.27%	99.05%	98.72%
253	AA_composition_K_max	66.27%	99.05%	98.72%
254	VanDerWaal_distribution_P-0.0_max	66.27%	99.05%	98.73%
255	AA_composition_F_max	66.27%	99.05%	98.72%

256	polarizability_distribution_P-0.5_max	66.27%	99.04%	98.72%
257	polarizability_distribution_N-0.25_max	66.27%	99.04%	98.72%
258	AA_composition_N_max	66.27%	99.04%	98.72%
259	out_degree_median	66.27%	99.04%	98.72%
260	polarizability_distribution_N-0.0_max	66.27%	99.04%	98.71%
261	secondary_structure_distribution_H-0.5_max	66.27%	99.04%	98.71%
262	AA_composition_A_max	66.27%	99.03%	98.71%
263	polarizability_distribution_P-0.75_max	65.68%	99.04%	98.71%
264	polarity_composition_P_mean	65.68%	99.04%	98.71%
265	polarizability_distribution_H-0.0_mean	65.68%	99.04%	98.71%
266	polarizability_composition_N_max	65.68%	99.04%	98.71%

267	polarity_distribution_N-0.25_max	65.68%	99.04%	98.71%
268	VanDerWaal_transition_PH_max	65.68%	99.04%	98.71%
269	VanDerWaal_distribution_H-0.5_max	65.68%	99.04%	98.71%
270	AA_composition_V_max	65.68%	99.03%	98.70%
271	polarizability_transition_NH_max	65.68%	99.03%	98.70%
272	polarity_distribution_H-0.75_max	65.68%	99.04%	98.71%
273	polarity_distribution_P-0.75_max	65.68%	99.04%	98.71%
274	secondary_structure_distribution_P-0.25_max	65.68%	99.03%	98.70%
275	VanDerWaal_distribution_N-0.5_max	65.68%	99.02%	98.69%
276	hydrophobicity_distribution_N-0.5_max	65.68%	99.02%	98.69%
277	out_degree_mean	65.68%	99.02%	98.69%

278	polarizability_transition_PH_max	65.68%	99.01%	98.68%
279	solvent_accessibility_distribution_H-0.25_max	65.68%	99.01%	98.68%
280	solvent_accessibility_transition_HE_max	65.68%	99.01%	98.68%
281	VanDerWaal_distribution_P-0.75_max	65.68%	99.01%	98.68%
282	AA_composition_M_max	65.68%	99.01%	98.68%
283	polarity_distribution_P-0.0_mean	65.68%	99.01%	98.68%
284	hydrophobicity_distribution_P-0.5_max	65.68%	99.00%	98.67%
285	VanDerWaal_transition_NH_max	65.09%	99.00%	98.66%
286	out_local_density_0.1_mean	65.09%	98.99%	98.66%
287	VanDerWaal_transition_PN_max	65.68%	99.00%	98.67%

288	hydrophobicity_distribution_H-0.5_max	65.68%	99.00%	98.67%
289	secondary_structure_transition_NH_max	65.68%	99.01%	98.68%
290	polarizability_distribution_N-0.5_max	65.68%	99.00%	98.67%
291	hydrophobicity_composition_P_max	65.68%	98.99%	98.66%
292	VanDerWaal_distribution_H-0.75_max	65.68%	98.98%	98.65%
293	hydrophobicity_transition_PN_max	65.68%	98.99%	98.66%
294	polarizability_distribution_P-0.0_max	65.68%	98.99%	98.66%
295	VanDerWaal_distribution_N-0.25_max	65.68%	98.99%	98.66%
296	polarity_transition_PH_max	65.68%	98.98%	98.65%
297	VanDerWaal_distribution_P-0.5_max	65.68%	98.98%	98.65%

298	in_local_density_0.1_mean	65.68%	98.98%	98.65%
299	hydrophobicity_distribution_N-0.25_max	65.09%	98.96%	98.63%
300	secondary_structure_distribution_N-0.25_max	65.09%	98.96%	98.62%
301	secondary_structure_composition_P_max	65.09%	98.96%	98.63%
302	hydrophobicity_distribution_H-0.25_max	65.09%	98.96%	98.62%
303	polarity_distribution_H-0.0_max	65.68%	98.96%	98.63%
304	secondary_structure_distribution_P-0.5_max	65.68%	98.97%	98.64%
305	secondary_structure_distribution_H-0.25_max	65.68%	98.96%	98.64%
306	in_degree_mean	65.68%	98.97%	98.64%
307	VanDerWaal_composition_H_max	65.68%	98.98%	98.65%

308	topological_change_0.4_0.5	65.09%	98.98%	98.64%
309	out_out_topological_max	65.09%	98.98%	98.64%
310	polarizability_distribution_H-0.25_max	65.68%	98.98%	98.65%
311	polarizability_distribution_P-0.25_max	65.68%	98.97%	98.64%
312	polarizability_composition_P_max	65.68%	98.96%	98.64%
313	secondary_structure_distribution_P-0.75_max	65.68%	98.98%	98.65%
314	polarity_distribution_H-0.5_max	65.68%	98.97%	98.64%
315	polarity_distribution_N-0.75_max	65.68%	98.97%	98.64%
316	hydrophobicity_distribution_N-0.0_max	65.68%	98.98%	98.65%
317	VanDerWaal_distribution_N-0.75_max	65.68%	98.98%	98.65%
318	in_in_topological_max	64.50%	98.99%	98.65%

319	hydrophobicity_distribution_P-0.25_max	64.50%	98.99%	98.65%
320	out_local_density_0_mean	64.50%	98.99%	98.65%
321	polarizability_distribution_H-0.5_max	64.50%	98.99%	98.65%
322	polarity_composition_H_max	64.50%	98.99%	98.65%
323	VanDerWaal_composition_P_max	64.50%	98.99%	98.65%
324	polarity_distribution_N-0.5_max	64.50%	99.00%	98.66%
325	polarity_distribution_P-0.25_max	64.50%	99.01%	98.66%
326	polarity_distribution_N-0.0_max	64.50%	99.01%	98.66%
327	hydrophobicity_transition_PH_max	64.50%	99.00%	98.66%
328	hydrophobicity_distribution_N-0.75_max	64.50%	98.99%	98.65%
329	secondary_structure_distribution_N-0.0_max	64.50%	98.99%	98.65%

330	polarizability_distribution_H-0.75_max	64.50%	98.98%	98.64%
331	in_local_density_0_mean	64.50%	98.99%	98.65%
332	AA_composition_P_max	64.50%	98.98%	98.64%
333	polarity_distribution_P-0.5_max	64.50%	98.98%	98.64%
334	topological_change_0.5_0.6	64.50%	98.96%	98.62%
335	polarity_distribution_P-0.0_max	64.50%	98.96%	98.62%
336	hydrophobicity_distribution_H-0.75_max	64.50%	98.97%	98.63%
337	polarity_distribution_H-0.25_max	64.50%	98.97%	98.63%
338	weight_edge_variance(with_missing_edge)	64.50%	98.96%	98.62%
339	hydrophobicity_composition_N_max	64.50%	98.96%	98.62%
340	polarizability_transition_PN_max	64.50%	98.96%	98.62%
341	in_out_topological_max	64.50%	98.97%	98.63%

342	out_local_density_0.5_max	64.50%	98.97%	98.63%
343	weight_edge_variance(without_missing_edge)	64.50%	98.96%	98.62%
344	hydrophobicity_distribution_H-0.0_max	63.31%	98.95%	98.60%
345	in_local_density_0.5_max	63.31%	98.96%	98.61%
346	polarity_composition_N_max	63.31%	98.96%	98.61%
347	polarizability_distribution_H-0.0_max	63.31%	98.97%	98.62%
348	VanDerWaal_distribution_H-0.0_max	63.31%	98.96%	98.61%
349	out_in_topological_max	63.31%	98.96%	98.61%
350	second_singular_values	63.31%	98.96%	98.61%
351	graph_density	63.31%	98.96%	98.61%
352	third_singular_values	63.31%	98.96%	98.61%
